# Integrative Analysis of Blood Transcriptomics and Metabolomics Reveals Molecular Regulation of Backfat Thickness in Qinchuan Cattle

**DOI:** 10.3390/ani13061060

**Published:** 2023-03-15

**Authors:** Hengwei Yu, Sayed Haidar Abbas Raza, Yueting Pan, Gong Cheng, Chugang Mei, Linsen Zan

**Affiliations:** 1 College of Animal Science and Technology, Northwest A&F University, Xianyang 712100, China; yuhengwei@nwafu.edu.cn (H.Y.); haiderraza110@nwafu.edu.cn (S.H.A.R.);; 2 Guangdong Provincial Key Laboratory of Food Quality and Safety/Nation-Local Joint Engineering Research Center for Machining and Safety of Livestock and Poultry Products, South China Agricultural University, Guangzhou 510642, China; 3 College of Grassland Agriculture, Northwest A&F University, Xianyang 712100, China; 4 National Beef Cattle Improvement Center, Xianyang 712100, China

**Keywords:** cattle, backfat thickness, transcriptomics, metabolomics, blood

## Abstract

**Simple Summary:**

Subcutaneous fat deposition in cattle has become the focus of breeders’ attention because excessive deposition is not conducive to efficient feed conversion. In the present study, based on the results of blood transcriptome sequencing and the detection of metabolites, bioinformatic analysis was used to explore the differential genes and metabolites associated with the subcutaneous fat depot phenotype of beef cattle. In conclusion, the functional genes *SMPD3* and *CERS1*, as well as the metabolite sphingosine 1-phosphate, were identified as an important metabolite and candidate genes to account for the differences in phenotype. These differential genes and the metabolite are thought to have an important reference value for effective breeding to improve beef performance.

**Abstract:**

A crucial goal of reducing backfat thickness (BFT) is to indirectly improve feed conversion efficiency. This phenotype has been reported in certain papers; however, the molecular mechanism has yet to be fully revealed. Two extreme BFT groups, consisting of four Qinchuan cattle, were chosen for this study. We performed metabolite and transcriptome analyses of blood from cattle with a high BFT (H-BFT with average = 1.19) and from those with a low BFT (L-BFT with average = 0.39). In total, 1106 differentially expressed genes (DEGs) and 86 differentially expressed metabolites (DEMs) were identified in the extreme trait. In addition, serum ceramide was strongly correlated with BFT and could be used as a potential biomarker. Moreover, the most notable finding was that the functional genes (*SMPD3* and *CERS1)* and metabolite (sphingosine 1-phosphate (S1P)) were filtered out and significantly enriched in the processes related to the sphingolipid metabolism. This investigation contributed to a better understanding of the subcutaneous fat depots in cattle. In general, our results indicated that the sphingolipid metabolism, involving major metabolites (serum ceramide and S1P) and key genes (*SMPD3* and *CERS1*), could regulate BFT through blood circulation.

## 1. Introduction

In many countries, beef is regarded as an edible meat of high quality. It has been dependably proven that breeding plays a decisive role in the improvement of meat quality for domestic livestock [1]. Generally, consumers will pay higher prices for better meat quality grades, which are determined by the longissimus thoracis area, rib thickness, cold left-side weight, and subcutaneous fat thickness [2]. Backfat thickness (BFT) has been a practical indirect predictor of whole-body fat content and can be measured by ultrasound on live animals without requiring slaughter [3]. This process has received much attention because BFT reduction can indirectly improve feed conversion efficiency [4]. Fat deposition in different parts of the animal body have variations in terms of the preference for metabolite utilization, such as propionate and glucose for intramuscular fat and acetate for subcutaneous fat [5]. Recently, an increasing number of studies conducted by multi-omics association analysis aimed to explore the potential molecular mechanisms involved in the melanogenesis pathway [6], fat formation [7], meat quality [6,8], meat discoloration [7], and in intramuscular fat [9]. Moreover, genome-wide testing of the gene expression in human peripheral blood cells revealed that approximately 80% of the genes expressed in the 9 key tissues are also expressed in blood cells [10]. Therefore, it is useful to clarify the relationship between blood metabolome and transcriptome analyses and phenotypes. For example, Samantha [11] showed that subcutaneous rib fat showed a negative correlation with dimethyl sulfone and a negative tendency with acetate and isobutyrate for blood metabolomes in Wagyu-crossbred steers. Another example is that certain genes that are strongly expressed in uterine tissue are also highly expressed in blood [12]. Cattle with a high ultimate pH showed higher levels of plasma cortisol, glucose, lactic acid, creatine kinase, and lactate dehydrogenase than cattle with a low ultimate pH during bloodletting [13]. These studies support a clearer elucidation of phenotypic differences through blood metabolomic and transcriptomic analyses.

China has the largest middle-income population and has become the world’s largest consumer of meat [14]. Qinchuan cattle, an important Chinese indigenous cattle breed, are named after the Guanzhong Plain in Shaanxi Province, and are characterized by good meat quality [15]. Consumers are known to recognize Qinchuan cattle as having delicious meat and are openly fond of it. Excessive subcutaneous fat deposition, however, greatly reduces the feed conversion efficiency and growth yield. Furthermore, the molecular mechanism of BFT in Qinchuan cattle remains unclear.

Therefore, we hypothesized that metabolite types, concentrations, and gene expression in the blood would be related to the deposition of subcutaneous fat in beef cattle. The current study used mRNA and metabolite sequencing to identify the differences in blood transcriptomes and metabolomes between cattle with a differing backfat thickness. Independently, a number of differentially expressed genes and metabolites were found in both high- and low-BFT individuals. Furthermore, a joint analysis of transcriptome and metabolome approaches was utilized to provide insights into the identification of biomarkers for BFT, as well as to understand the relationship of the traits for BFT, blood metabolite concentrations, and mRNA. The revelation of the molecular mechanism of subcutaneous fat deposition in Qinchuan cattle and the reduction in BFT was found to have significant value for the development and utilization of excellent Chinese local-breed cattle resources, which are required to improve feed conversion rates.

## 2. Materials and Methods

### 2.1. Animals and Phenotypes

The cattle included in the trial belonged to breeds of the Shaanxi province, northwest China. In this study, a total of 117 female cattle was used from the same conservation experiment farm of the National Beef Cattle Improvement Center (Xianyang, China). All cows were raised under the same feeding and management conditions, with free access to water. Cattle were measured by an ultrasonic device. The animal sampling and management protocol was implemented by the Technical Specification for Determination of Beef Cattle Production Performance (NY/T2660-2014) of the Ministry of Agriculture of the People’s Republic of China. The tested cattle were tied up and smeared with vegetable oil on ribs 12–13 on the left. They were vertically pressed with an ultrasonic probe at approximately 5 cm below the side of the cattle’s spine to be measured until a clear image appeared on the ultrasonic scanner host; the BFT was then calculated. Finally, two extreme groups of four cattle were selected through this process.

### 2.2. Blood Sample Collection and Preparation

Whole blood was collected from the jugular vein of eight cattle into a 5 mL EDTA anticoagulant tube and mixed, separately, upside down. The sample was then quickly transferred to a laboratory (within 2 h) where 300 mL of the blood was taken in by a pipette and then administered into a 2 mL centrifuge tube. Next, 700 µL of trizol was added. The samples were shaken and mixed for 30–60 s, cultured at room temperature for 5 min, then quickly frozen in liquid nitrogen and stored at −80 °C to extract the RNA. Similarly, 1 mL blood was inhaled into a 2 mL centrifuge tube and centrifuged at 4000× *g* concentration. Then, 200 μg of supernatant was loaded into centrifuge tube, rapidly frozen in liquid nitrogen, and stored at −80 °C to extract the metabolites.

### 2.3. RNA Extraction, Sequencing, and Transcriptome Data Analysis 

Total RNA extraction, RNA integrity detection, library construction, and RNA-seq were performed by Biomarker Technologies Co., Ltd. (Beijing, China). BMKCloud (www.biocloud.net) was used to analyze the RNA-seq data. Stringent quality control was applied to the raw data and low-quality reads were removed with the following standards: reads with adapters; low quality reads (including reads with an N ratio greater than 10%); and the number of bases with Q ≤ 10 quality value (which was greater than 50% of whole reads). In clean reads, Q30 (the proportion of bases with Phred quality values greater than 30 to total bases) was greater than 93.12%. Qualified reads were aligned against the bovine reference genome (https://bovinegenome.elsiklab.missouri.edu/downloads/ARS-UCD1.2, accessed on 13 October 2022) using the HISAT2 software package (http://www.ccb.jhu.edu/software/hisat2, accessed on 13 October 2022).

Fragments per kilobase of transcript per million fragments mapped (FPKM) was used to quantify the level of gene expression or transcript [16]. Gene expression analysis was performed using the DEseq2 package [17]. The differentially expressed genes (DEGs) were defined on the basis of |log2(fold change)| ≥ 1 and FDR < 0.05. Lastly, gene ontology (GO) enrichment analysis and Kyoto Encyclopedia of Genes and Genomes (KEGG) pathway enrichment analysis were performed using the clusterProfiler package and the topGO R package.

### 2.4. Metabolites Extraction 

For the BFT groups (four L-BFT blood serums and four H-BFT blood serums), the frozen blood serum samples stored at −80 °C were defrosted first. Metabolites extraction adhered to the following process: (a) 100 μL of a sample was weighed, adding 500 μL of the extraction solution containing the internal standard (the volume ratio of methanol to acetonitrile = 1:1; internal standard concentration 20 mg/L), and the sample was then vortexed and mixed for 30 s; (b) ultrasound was conducted for 10 min (ice water bath); (c) the sample was left to stand at −20 °C for one hour; (d) 4000× *g* of the sample was then centrifuged at 4 °C for 15 min; (e) the sample had 500 μL of the supernatant carefully removed into an EP tube; (f) the extract was then dried in a vacuum concentrator; (g) 160 μL of the extract solution was added (acetonitrile to water volume ratio: 1:1) to the dried metabolites to reconstitute; (h) this was vortexed for 30 s, then sonicated in an ice water bath for 10 min; (i) the sample was centrifuged at 4 °C and 4000× *g* for 15 min; and (g) 120 μL of the supernatant was carefully removed into a 2 mL injection bottle, and 10 μL of each sample was mixed into a quality-controlled sample and then subjected to LC-MS/MS analysis.

### 2.5. LC-MS/MS Analysis

Based on the online METLIN database of Progenesis QI software and the identification library built by Biomark, Progenesis QI processed the raw data detected by MassLynx V4.2, performed peak extraction, peak comparison, and other operations, as well as theoretical fragment identification and quality analysis (the deviations were all within 100 ppm).

First, a follow-up analysis was performed after normalizing raw peak area information with the total peak area. Principal component analysis and Spearman correlation analysis were used to estimate the repeatability of samples within a group and quantitative control samples. Subsequently, taxonomic and pathway information on the identified compounds were searched by the KEGG, HMDB, and Lipid mass spectrometry databases. Then, the difference multiplier and comparison data were calculated based on the grouping information, and the significance value of the difference of each compound was calculated by a *t*-test. OPLS-DA modeling was performed using the R language package ropls; furthermore, 200 permutation tests were performed to verify the reliability of the model. The VIP value of the model was calculated using multiple cross-validations. Differential metabolites were screened by combining the fold difference, *p* value, and VIP value of the OPLS-DA model. Screening criteria were |log2(fold change)| ≥ 1, *p*-value ≤ 0.05, and VIP ≥ 1. Finally, the differential metabolites of the KEGG pathway enrichment significance were calculated using the hypergeometric distribution test.

### 2.6. Joint Analysis of the Transcriptomic and Metabolomic Data 

To better explain transcriptional regulatory mechanisms in metabolic pathways, the correlations between all genes and metabolites were calculated based on the Pearson correlation. Then, the 9-quadrant graph was drawn based on the correlation coefficient (CC, |CC| > 0.80) and the *p*-value of the correlation (CCP < 0.05). A bubble map was drawn using the KEGG pathway, which was enriched by the significantly correlated combinations. Therefore, to visually reflect the differences in expression patterns of significantly different genes and significantly different metabolites, the heatmap R package was used for hierarchical clustering analysis.

### 2.7. Statistical Analyses

To test for the statistical differences for BFT in the two extreme groups, an unpaired *t*-test was used in the SPSS program. The level of significance was set at *p*-value < 0.05.

## 3. Results

### 3.1. Animal Phenotypic Divergence for BFT

**Figure 1:**
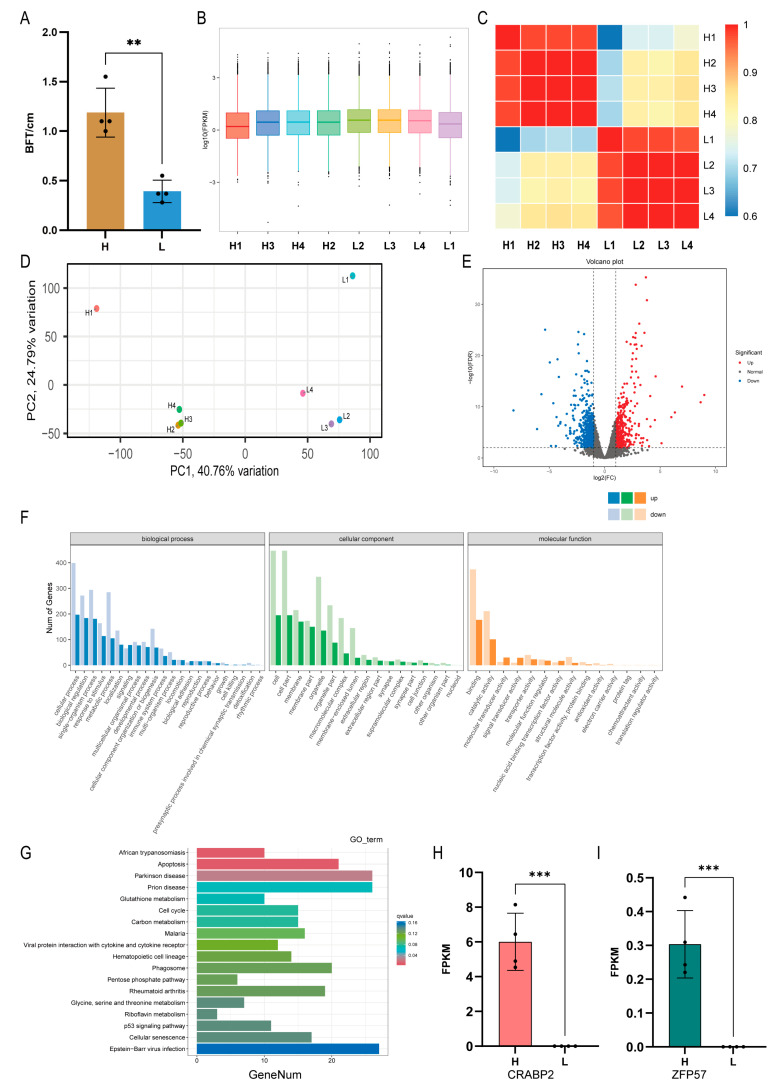
Basal analysis of the transcriptome and the different phenotypes profiles in the BFT (*n* = 8). (**A**) Comparison of the BFT phenotypes (** means *p*-value < 0.05). (**B**) The FPKM boxplot for each sample. (**C**) The heatmap of expression correlation between samples. (**D**) The principal component analysis (PCA). (**E**) Volcano plots of the DEGs in the BFT. (**F**) The GO annotation analysis histogram of DEGs for BFT. (**G**) The plot of the degree of KEGG pathway enrichment for DEGs. The vertical coordinates indicate the enriched pathway, and the horizontal coordinates indicate the value of the enrichment factor (ratio of the annotated DEGs to all genes in the enriched pathway). (**H**) FPKM of *CRABP2* in the H and L groups. (**I**) FPKM of *ZFP57* in the H and L groups (*** means *p*-value < 0.01).

In this study, a total of 117 Qinchuan cattle with an average age of 36 months (SD  = 7.71 month) was selected for the measurement of backfat thickness. According to BFT records, two groups were selected with a low or high backfat thickness (four H-BFT individuals and four L-BFT individuals, respectively). The average BFT of the cattle was 0.71 cm (SD  =  0.29 cm), ranging from 0.27 to 1.55 cm. As shown in Figure 1A, the histogram demonstrates a statistically significant difference between the groups’ H-BFT (with average_BFT = 1.19, SD = 0.21, *p*-value < 0.05) and L-BFT (with average_BFT = 0.39, SD = 0.10, *p*-value < 0.05). 

### 3.2. DEGs and Transcriptome Analysis

To compare the difference in blood mRNA between the H-BFT and L-BFT groups, next-generation sequencing was used. After removing the adaptors and low-quality reads, more than 93.95% of the Q30 was in each sample (Supplementary File S3: Table S1). Additionally, 91.78–93.64% of the clean reads were mapped to the bovine reference genome (Supplementary File S4: Table S2). As shown in Figure 1B, the boxplot shows that the distribution of gene expression levels was evenly distributed in each sample. Furthermore, it was also relatively centralized between the different samples. Interestingly, the correlation heatmap presented an obvious intergroup difference and high intragroup similarity (Figure 1C). Additionally, the PCA presented that the two BFT groups were clearly separated by the first principal component (Figure 1D). The volcano plot demonstrated that 1106 DEGs were identified in the BFT with 700 up-regulated genes and 406 down-regulated genes in the H group when compared with the L group (Figure 1E). The details of the DEGs are shown in Supplementary File S5: Table S3. Intriguingly, the significantly enriched genes, *CRABP2* and *ZFP57*, were mainly expressed in the H-BFT group (Figure 1H,I). Then, the GO annotation analysis showed that the DEGs were divided into 58 subcategories, including 23 biological process (BP) terms, 17 cellular component (CC) terms, and 18 molecular function (MF) terms. It was found that 53.89% (596 out of 1106) of the genes were related to the cellular process category for the BP category. In total, 58.05% of the genes (642 out of 1106) were annotated in regard to the cellular parts for the CC category. Furthermore, 49.82% of the genes (551 out of 1106) were located in the binding fraction for the MF category (Figure 1F and Supplementary File S6: Table S4). Furthermore, the enrichment analysis of the BP showed that the enriched terms were primarily involved in the granzyme-mediated apoptosis signaling pathway, megakaryocyte differentiation, neutrophil activation, the positive regulation of the ERK1 and ERK2 cascade, the arachidonic acid metabolic process, the long-chain fatty acid metabolic process, and the positive regulation of the establishment of protein localization to the telomeres (Supplementary File S1: Supplementary Figure S1A). The enrichment analysis of MF showed that the top enriched terms were mostly involved in GTP binding, GTPase activity, oxygen transporter activity, and 2′-5′-oligoadenylate synthetase activity (Supplementary File S1: Supplementary Figure S1B). Finally, KEGG pathway analysis indicated that the DEGs were involved in numerous signaling pathways, such as the p53 signaling pathway; the glycine, serine, and threonine metabolism pathways; the pentose phosphate pathway; glutathione metabolism pathway; and ribosome pathway (Figure 1G and Supplementary File S7: Table S5). Overall, these DEGs were involved in lipid metabolism, regulating systemic fat storage and utilization.

### 3.3. DEMs and Metabolome Analysis

**Figure 2:**
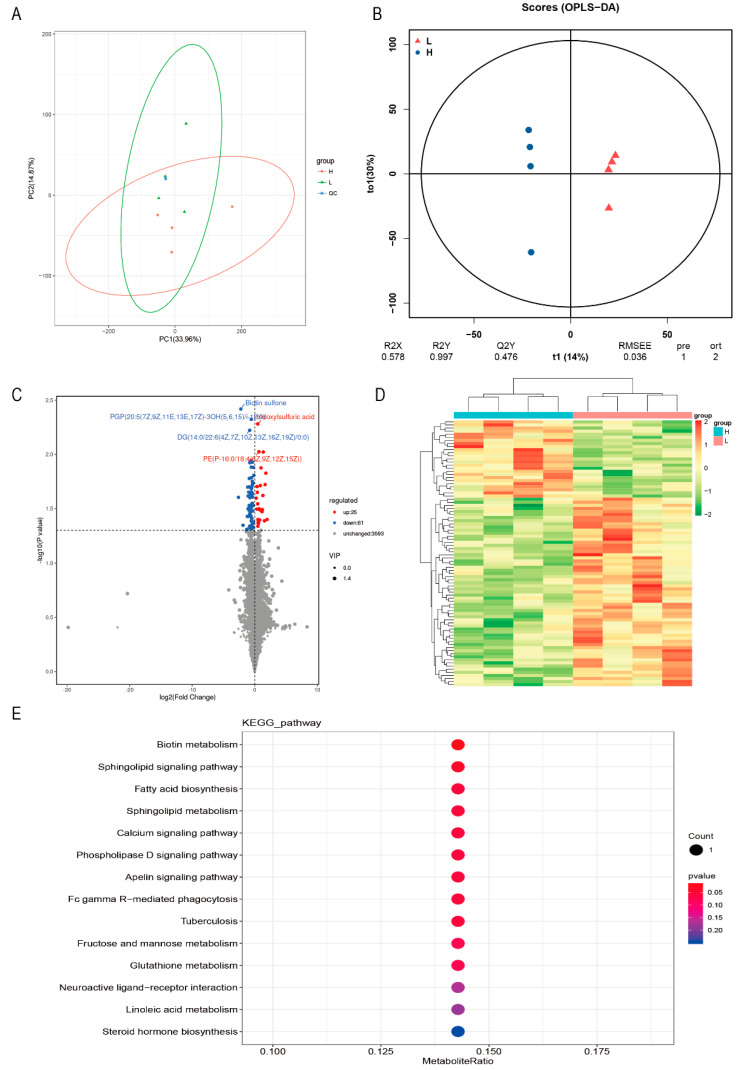
The separate metabolome data analysis of the BFT. (**A**) The principal component analysis (PCA). (**B**) The partial-least-squares-discriminant analysis (OPLS-DA) score plots. (**C**) The volcano plots of the DEMs. (**D**) The cluster heatmap of the DEMs. (**E**) The plot of the differential metabolite KEGG enrichment. The *x*-axis is the ratio of the number of DEMs in the corresponding pathway to the total number of metabolites that were detected and annotated in the pathway. The *y*-axis is the pathway name. The color of the dots represents the log (*p*-value), with more red indicating a more significant enrichment. The size of the dots represents the number of differentially enriched metabolites.

To evaluate the diversity of the blood metabolite composition in Qinchuan cattle, LC-MS/MS analysis was conducted with the L- and the H-BFT cattle groups. Collectively, a total of 3679 metabolites (1524 for negative ion mode and 2155 for positive ion mode) was identified (Supplementary File S8: Table S6). The PCA and PLS-DA score plots indicated that the quality-control samples were clustered together (Figure 2A,B). Based on the metabolome databases of KEGG, HMDB, and Lipidmaps, all metabolites were qualitatively analyzed. In total, 792 metabolites were identified in the KEGG database, including 85 amino acid metabolites, 64 biosyntheses of the other secondary metabolites, 42 carbohydrate metabolites, 8 glycan biosyntheses and metabolites, and 85 lipid metabolites (Supplementary File S2: Supplementary Figure S2A). In total, we identified 2709 metabolites in the HMDB database, including 906 lipids and lipid-like molecules, 475 organic acids and derivatives, 397 organic heterocyclic compounds, and 131 phenylpropanoids and polyketides (Supplementary File S2: Supplementary Figure S2B). We were able to identify a number of metabolites in total: 403 metabolites were identified in the database of the lipid maps, which included 208 fatty acyls, 9 glycerolipids, 79 glycerophospholipids, 22 polyketides, 20 prenollipids, 12 sphingolipids, and 53 sterol lipids (Supplementary File S2: Supplementary Figure S2C). These were used in the analysis. In addition, the criteria for the DEMs to be significant were |log2(fold change)| ≥ 1, VIP value ≥ 1 (variable importance in projection), and *p*-value ≤ 0.05. The volcano map displayed the DEMs related to the BFT (Figure 2C). A heatmap was created depicting the alignment of the metabolite levels that changed significantly in agreement consistently with the sample group (Figure 2D). In total, 25 up-regulated metabolites in the H-BFT group, including ceramide, beta-glucuronide, monacolin L acid, cornoside, vanilloyl glucose, etc., were higher than those found in the L-BFT. In contrast, 61 down-regulated metabolites in the H-BFT group, including biotin sulfone, phosphonic acid, propylene glycol stearate, dehydromakisterone, etc., were lower than those in the L-BFT (Supplementary File S9: Table S7). According to the KEGG annotation and enrichment results, the 86 DEM-relative metabolics were annotated into 14 pathways, such as sphingolipid metabolism, fatty acid biosynthesis, glutathione metabolism, the sphingolipid signaling pathway, biotin metabolism, the apelin signaling pathway, and steroid hormone biosynthesis (Figure 2E and Supplementary File S10: Table S8). In summary, these results indicated that the ceramide in the serum was closely related to backfat thickness and thus can be used as a potential biomarker.

### 3.4. Joint Analysis of the Transcriptome and Metabolome

**Figure 3:**
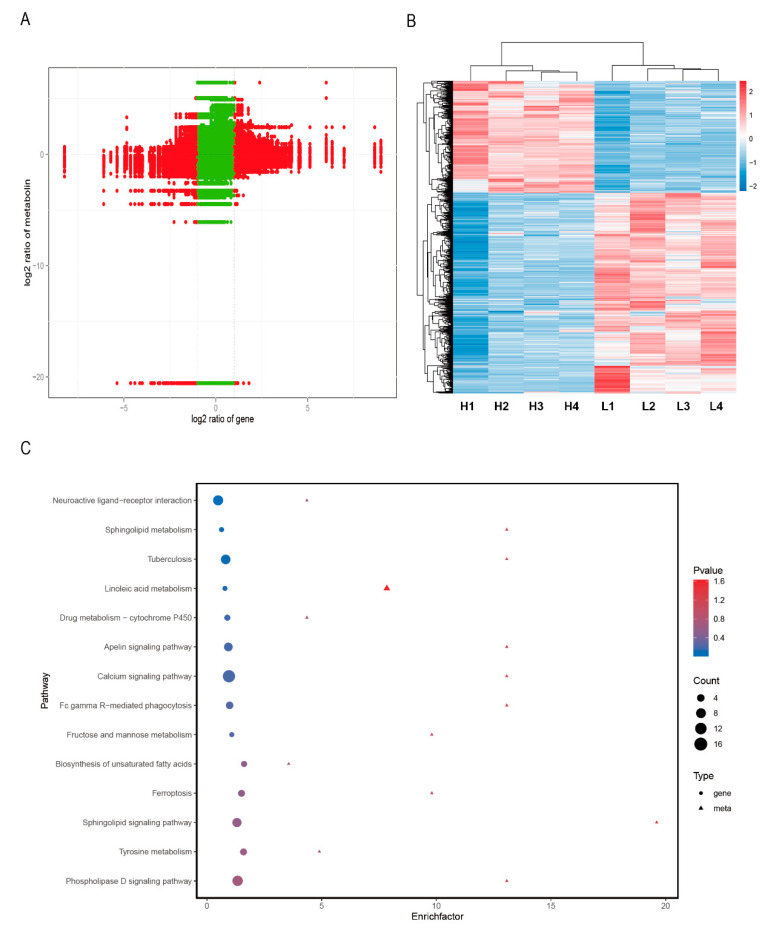
Combined metabolome and transcriptome analysis. (**A**) Nine-quadrant diagrams. The calculation of correlations between all genes and metabolites based on the Pearson correlation, with screening thresholds of |CC| > 0.80 and CCP < 0.05. Left-to-right and top-to-bottom indicates the order of quadrants 1 to 9. The 3rd and 7th quadrants represent the same trend of gene and metabolite expression, which indicate that the genes may be positively regulated metabolites. The 1st and 9th quadrants indicate that the genes and metabolites are negatively correlated. The 2nd and 8th represent not just the unchanged genes but also the up- and down-regulation of the metabolites. The 4th and 6th represent not just the unchanged metabolites but also the genes that were up-regulated. The 5th indicated no significant changes in both the metabolites and the genes. Only quadrants 1, 2, 3, 7, 8, and 9 were involved in this analysis. (**B**) The correlation analysis hierarchical clustering heatmap. Each row of the hierarchical clustering heatmap represented one DEG or DEM for each column of that differentially grouped sample. The clusters appear in the same cluster of significantly different metabolites or differential genes with similar expression patterns. (**C**) The differential gene/metabolite KEGG enrichment bubble plot. The enrich factor represents the enrichment factor of the pathway in different omics, and the ordinate represents the name of the KEGG pathway; the red-blue gradient represents the change in the significance of enrichment from high to low, represented by the *p*-value. The shapes of the bubbles represent the different omics, the circles represent the transcriptome, and the triangles represent the metabolome. The size of the bubbles stands for the number of DEMs or DEGs, with the larger the number the larger the dots.

To reveal the candidate genes involved in the BFT, we combined the analysis of the blood metabolome and transcriptome. The Pearson correlation coefficient (CC) between all genes and metabolites was calculated and screened according to |CC| > 0.80 and the *p*-value < 0.05 (Figure 3A and Supplementary File S11: Table S9). A hierarchical cluster analysis was used to visualize the differences in expression patterns of the DEGs and DEMs (Figure 3B). Among the genes, we presented those genes that have been reported in lipid metabolism as trait-related candidate genes (*APCDD1*, *ENHO*, *FBP1*, *FADS6*, and *KCTD15*). Further, we annotated the KEGG pathway with the important DEGs and with their strongly correlated DEMs (Figure 3C and Supplementary File S12: Table S10). It is worth noting that sphingosine 1-phosphate (S1P) was associated with several KEGG pathways, such as the phospholipase D signaling pathway, calcium signaling pathway, apelin signaling pathway, sphingolipid signaling pathway, and the sphingolipid metabolism. Moreover, S1P was significantly positively correlated with the expressions of *GABARAPL1*, *CXCL8*, *VDAC3*, *IL18*, *S1PR1*, and *ARPC3*, while cases were observed to the contrary for *TMIGD3*, *SMPD3*, *PLCB2*, *CAMK1*, and *CERS1* (|PCC| > 0.80) (Supplementary File S13: Table S11). Surprisingly, we observed that the sphingolipid metabolic pathway clustered two DEGs (SMPD3 and CERS1), both of which were remarkably up-regulated in the H-BFT group when compared with the L-BFT group. Together these results provided important insights into the blood transcriptomics and metabolomics in Qinchuan cattle for BFT.

## 4. Discussion

As an important indicator of meat quality, BFT is an essential guiding indicator of significance for breeding. A test hypothesis of this study was that the concentrations of blood metabolites and transcriptomes were correlated with the BFT traits of beef cattle. In regard to transcription, one interesting finding was that the candidate genes *CRABP2* and *ZFP57* were mainly expressed in the H-BFT group but not in the L group. *CRABP2* was the highest expression in the adipose tissue when compared with the different tissues of pigs [18]. In a high-fat diet mouse model, *CRABP2* can activate the RA/RAR pathway in adipocytes in order to inhibit the adipocyte differentiation [19]. Furthermore, *ZFP57* recognized its methylated site and played a pivotal role in the establishment of genomic imprints [20]. Moreover, it combines with its methylation site to maintain allele-specific gene repression [21]. Additionally, the mRNA expression of *ZFP57* in human adipose tissue was influenced by the genome-wide DNA methylation quantitative trait locus [22]. Moreover, its hypomethylation and mutations were associated with transient neonatal diabetes [23]. In addition, the DEGs and up-regulated and down-regulated KEGG pathways might be associated with fat metabolism and adipogenesis in beef cattle. Furthermore, *CRABP2* and *ZFP57* might be critical candidate genes related to BFT.

In our study, the blood metabolites may be potentially related to BFT. The ceramide and beta-glucuronide found in the H-BFT group were dramatically higher than those in the L-BFT group, whereas biotin sulfone and phosphonic acid were found to be in a negative correlation with BFT. These results are consistent with a previous study, which demonstrated that subcutaneous rib fat showed a negative correlation with dimethyl sulfone and a negative tendency with acetate and isobutyrate [11]. In metabolic syndrome, obesity, and type 2 diabetes (T2D), ceramide and S1P played an important role [24]. Additionally, ceramide/S1P metabolism and signaling were associated with adipose tissue dysfunction in the presence of excess dietary energy intake [25]. The importance of this metabolome cannot be overemphasized as it implies that it can be used for the early identification of cattle with a high propensity for BFT, thereby suggesting that the selection of cattle with a low BFT propensity for fattening may improve feed utilization. In contrast, further research is needed to investigate the potential biomarkers regarding this or for its metabolite application.

**Figure 4:**
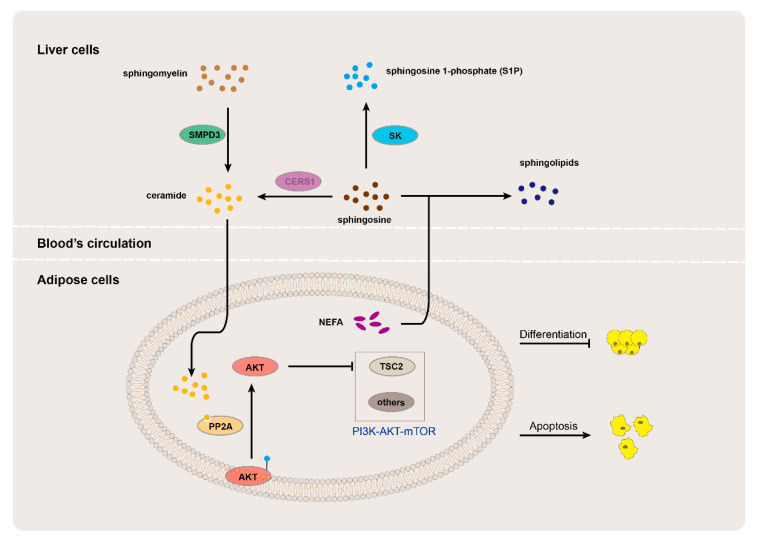
Correlation and pathway analysis of the DEGs and DEMs. The ceramide metabolic process was assigned with four central DEGs and DEMs of the KEGG-rich pathway.

Moreover, we focused on the integrative analysis of the transcriptomics and metabolomics for BFT. We identified several related genes, including *APCDD1*, *ENHO*, *FBP1*, *FADS6*, and *KCTD15.* The adenomatosis polyposis coli down-regulated 1 (*APCDD1*), a key regulator of adipogenic differentiation, was identified as an inhibitor of Wnt signaling. In addition, it positively regulated the adipogenic differentiation of subcutaneous adipose tissue during diet-induced obesity for the mouse [26]. Similar to this, *APCDD1* was significantly up-regulated in H-BFT individuals in our study. Adropin is a secreted protein that is encoded by an energy-homeostasis-associated gene (*ENHO*) that controls glucose and lipid homeostasis, as well as preventing the hepatic steatosis and hyperinsulinemia that are associated with obesity [27]. The glycoisomerase fructose-1,6-bisphosphatase 1 (FBP1) inhibits certain biological pathways, including cell proliferation, glycolysis, and pentose phosphate in a catalytic-activity-independent manner [28]. Polyunsaturated fatty acids perform critical physiological roles in human health, and Δ6 fatty acid desaturase (*FADS6*) is an enzyme that is essential in the polyunsaturated fatty acids production pathway [29]. Potassium-channel-tetramerization-domain-containing protein 15 (*KCTD15*), a member of the K+-channel-tetramerization-domain family, is an obesity-linked protein in humans and is implicated in the crucial physio-pathological processes that are involved in food uptake [30]. There is no doubt that these genes have direct or indirect effects on fat formation and degradation. In the current study, the most obvious finding to emerge from the analysis is that the expression levels of *SMPD3* and *CERS1* were higher in the H group than in the L group. This is in addition to the same trend being applicable for ceramide, while S1P showed the opposite trend. Ceramide is a core metabolite of the sphingolipid metabolic pathway, and it can promote insulin resistance [31]. Ceramide is bound to protein phosphatase 2A (PP2A) and mediates AKT dephosphorylation, thereby inhibiting glucose transport. In addition, PP2A is activated by ceramide [32,33,34]. Increased liver fat deposition in obese women is accompanied by high levels of ceramide in subcutaneous adipose tissue and in increased macrophage infiltration, thus suggesting that ceramide may also promote insulin resistance and chronic inflammation in adipose tissue [35]. S1P is a bioactive lipid and its level in cells is controlled by two factors: the sphingosine content and the catalytic activity of S1P metabolizing enzymes (such as sphingosine kinase (SK), S1P phosphatase, and S1P lyase [24]). Ceramide, sphingomyelin, and S1P were able to interconvert with each other. S1P, known as a “sphingolipid-variable blocker”, promoted the proliferation/survival pathway, while ceramide induced apoptosis/aging [36,37]. Additionally, research suggested that reducing intracellular ceramide levels may be an effective therapeutic strategy for the treatment of T2D and obesity [38]. It is of interest that the interaction between adipose tissue and the circulatory system is essential to maintain the homeostasis of systemic metabolism. The lipids secreted by adipose tissue may induce elevated levels of ceramide in the circulation of obese individuals. Furthermore, in metabolically active tissues, such as the liver and skeletal muscle, non-esterified fatty acid (NEFA) from dysregulated adipose tissue can be used for sphingolipid biosynthesis and thus can induce ceramide synthesis [39]. Furthermore, ceramides in the circulation may originate from adipose tissue [40], and the S1P secreted by adipose tissue in obese patients can also promote systemic inflammation. Therefore, we suggest that this is also a similar regulation process for BFT in cattle; specifically, *CERS1* and *SMPD3* were overexpressed by certain signaling molecules. CERS1 increases the rate of ceramide synthesis, thereby resulting in a decrease in sphingosine; in turn, the level of S1P was also decreased. Ceramide circulates through the blood system and enters into adipocytes by endocytosis. Then, the ceramide affects the *PP2A* to dephosphorylate AKT, thus preventing AKT from being transported to the cell membrane and also inhibiting the function of certain genes in the downstream pathways of AKT—which ultimately inhibits the adipocyte differentiation and promotes adipocyte apoptosis (Figure 4).

## 5. Conclusions

The findings of this study suggest that serum ceramide is closely related to backfat thickness and can be used as a potential biomarker. One of the most evident findings of this study, as determined by transcriptome- and metabolome-based analyses, was that the functional genes (*SMPD3* and *CERS1*) and metabolites (S1P and ceramide) were filtered and dramatically enriched in the processes related to sphingolipid metabolism. Overall, these results may contribute to a better understanding of the biological mechanisms of BFT, which has implications for both efficient farming and high-quality beef production. Considerably more work will need to be performed to determine the similarities and differences between blood and back subcutaneous adipose tissues for the purposes of transcriptomics and metabolomics.

## Data Availability

The RNA-Seq raw data have been deposited into the Genome Sequence Archive of the National Genomics Data Center, China National Center for Bioinformation/Beijing Institute of Genomics, Chinese Academy of Sciences, under accession number CRA009167 (https://ngdc.cncb.ac.cn/gsa/s/1T3O4cg0, accessed on 7 December 2023).

## Supplementary Materials

The following supporting information can be downloaded at https://www.mdpi.com/article/10.3390/ani13061060/s1. Figure S1: GO enrichment analysis. (A) Biological processes (BP) of DEGs. (B) Molecular functions (MF) of DEGs. Figure S2: All metabolites were qualitatively analyzed based on the metabolome database. (A) 792 metabolites were identified in the KEGG database. (B) 2709 metabolites were identified in the HMDB database. (C) 403 metabolites were identified in the Lipidmaps database. Table S1: Summary of sequencing data quality control. Table S2: Statistics on mapping ratio of sequencing data against reference genome. Table S3: Differentially expressed genes (DEGs) in BFT. Table S4: GO enrichment analysis for DEGs. Table S5: KEGG enrichment analysis for DEGs. Table S6: Identified full metabolites. Table S7: Differentially expressed metabolites (DEMs) in BFT. Table S8: KEGG enrichment analysis for DEMs. Table S9: Pearson correlations coefficient between all genes and metabolites. Table S10: KEGG enrichment analysis for DEGs and SEMs. Table S11: Significantly genes correlated with sphingosine 1-phosphate (S1P).

## References

[1] Berry D.P., Conroy S., Pabiou T., Cromie A.R. (2017). Animal breeding strategies can improve meat quality attributes within entire populations. Meat Sci..

[2] Polkinghorne R.J., Thompson J.M. (2010). Meat standards and grading: A world view. Meat Sci..

[3] Gol S., González-Prendes R., Bosch L., Tor M., Reixach J., Pena R.N., Estany J. (2019). Linoleic acid metabolic pathway allows for an efficient increase of intramuscular fat content in pigs. J. Anim. Sci. Biotechnol..

[4] Merks J.W.M., Mathur P.K., Knol E.F. (2012). New phenotypes for new breeding goals in pigs. Animal.

[5] Smith S.B., Crouse J.D. (1984). Relative contributions of acetate, lactate and glucose to lipogenesis in bovine intramuscular and subcutaneous adipose tissue. J. Nutr..

[6] Dou T., Yan S., Liu L., Wang K., Jian Z., Xu Z., Zhao J., Wang Q., Sun S., Talpur M.Z. (2022). Integrative analysis of transcriptomics and metabolomics to reveal the melanogenesis pathway of muscle and related meat characters in Wuliangshan black-boned chickens. BMC Genom..

[7] Zhan H., Xiong Y., Wang Z., Dong W., Zhou Q., Xie S., Li X., Zhao S., Ma Y. (2022). Integrative analysis of transcriptomic and metabolomic profiles reveal the complex molecular regulatory network of meat quality in Enshi black pigs. Meat Sci..

[8] Magalhães A.F.B., Schenkel F.S., Garcia D.A., Gordo D.G.M., Tonussi R.L., Espigolan R., Silva R.M.d.O., Braz C.U., Fernandes Júnior G.A., Baldi F. (2019). Genomic selection for meat quality traits in Nelore cattle. Meat Sci..

[9] Maciel F.C., Machado Neto O.R., Duarte M.S., Du M., Lage J.F., Teixeira P.D., Martins C.L., Domingues E.H.R., Fogaça L.A., Ladeira M.M. (2022). Effect of vitamin A injection at birth on intramuscular fat development and meat quality in beef cattle. Meat Sci..

[10] Iannaccone M., Ianni A., Contaldi F., Esposito S., Martino C., Bennato F., De Angelis E., Grotta L., Pomilio F., Giansante D. (2019). Whole blood transcriptome analysis in ewes fed with hemp seed supplemented diet. Sci. Rep..

[11] Connolly S., Dona A., Wilkinson-White L., Hamblin D., D’Occhio M., González L.A. (2019). Relationship of the blood metabolome to subsequent carcass traits at slaughter in feedlot Wagyu crossbred steers. Sci. Rep..

[12] Wojciechowicz B., Kołakowska J., Zglejc-Waszak K., Martyniak M., Kotwica G., Franczak A. (2019). The whole blood transcriptome at the time of maternal recognition of pregnancy in pigs reflects certain alterations in gene expression within the endometrium and the myometrium. Theriogenology.

[13] Lu X., Zhang Y., Qin L., Ma W., Zhu L., Luo X. (2018). Association of ultimate pH and stress-related blood variables in cattle. Meat Sci..

[14] Wang H.H. (2022). The perspective of meat and meat-alternative consumption in China. Meat Sci..

[15] Hengwei Y., Raza S.H.A., Wang S., Khan R., Ayari-Akkari A., El Moneim Ahmed D.A., Ahmad I., Shaoib M., Abd El-Aziz A.H., Rahman S.U. (2022). The growth curve determination and economic trait correlation for Qinchuan bull population. Anim. Biotechnol..

[16] Trapnell C., Williams B.A., Pertea G., Mortazavi A., Kwan G., van Baren M.J., Salzberg S.L., Wold B.J., Pachter L. (2010). Transcript assembly and quantification by RNA-Seq reveals unannotated transcripts and isoform switching during cell differentiation. Nat. Biotechnol..

[17] Love M.I., Huber W., Anders S. (2014). Moderated estimation of fold change and dispersion for RNA-seq data with DESeq2. Genome Biol..

[18] Li M., Chen L., Tian S., Lin Y., Tang Q., Zhou X., Li D., Yeung C.K.L., Che T., Jin L. (2017). Comprehensive variation discovery and recovery of missing sequence in the pig genome using multiple de novo assemblies. Genome Res..

[19] Noy N. (2013). The one-two punch: Retinoic acid suppresses obesity both by promoting energy expenditure and by inhibiting adipogenesis. Adipocyte.

[20] Quenneville S., Verde G., Corsinotti A., Kapopoulou A., Jakobsson J., Offner S., Baglivo I., Pedone P.V., Grimaldi G., Riccio A. (2011). In embryonic stem cells, ZFP57/KAP1 recognize a methylated hexanucleotide to affect chromatin and DNA methylation of imprinting control regions. Mol. Cell.

[21] Bina M. (2017). Imprinted control regions include composite DNA elements consisting of the ZFP57 binding site overlapping MLL1 morphemes. Genomics.

[22] Volkov P., Olsson A.H., Gillberg L., Jørgensen S.W., Brøns C., Eriksson K.-F., Groop L., Jansson P.-A., Nilsson E., Rönn T. (2016). A Genome-Wide mQTL Analysis in Human Adipose Tissue Identifies Genetic Variants Associated with DNA Methylation, Gene Expression and Metabolic Traits. PLoS ONE.

[23] Mackay D.J.G., Callaway J.L.A., Marks S.M., White H.E., Acerini C.L., Boonen S.E., Dayanikli P., Firth H.V., Goodship J.A., Haemers A.P. (2008). Hypomethylation of multiple imprinted loci in individuals with transient neonatal diabetes is associated with mutations in ZFP57. Nat. Genet..

[24] Fang Z., Pyne S., Pyne N.J. (2019). Ceramide and sphingosine 1-phosphate in adipose dysfunction. Prog. Lipid Res..

[25] Meikle P.J., Summers S.A. (2017). Sphingolipids and phospholipids in insulin resistance and related metabolic disorders. Nat. Rev. Endocrinol..

[26] Yiew N.K.H., Chatterjee T.K., Tang Y.L., Pellenberg R., Stansfield B.K., Bagi Z., Fulton D.J., Stepp D.W., Chen W., Patel V. (2017). A novel role for the Wnt inhibitor APCDD1 in adipocyte differentiation: Implications for diet-induced obesity. J. Biol. Chem..

[27] Kumar K.G., Trevaskis J.L., Lam D.D., Sutton G.M., Koza R.A., Chouljenko V.N., Kousoulas K.G., Rogers P.M., Kesterson R.A., Thearle M. (2008). Identification of adropin as a secreted factor linking dietary macronutrient intake with energy homeostasis and lipid metabolism. Cell Metab..

[28] Li B., Qiu B., Lee D.S.M., Walton Z.E., Ochocki J.D., Mathew L.K., Mancuso A., Gade T.P.F., Keith B., Nissim I. (2014). Fructose-1,6-bisphosphatase opposes renal carcinoma progression. Nature.

[29] Venegas-Calerón M., Sayanova O., Napier J.A. (2010). An alternative to fish oils: Metabolic engineering of oil-seed crops to produce omega-3 long chain polyunsaturated fatty acids. Prog. Lipid Res..

[30] Smaldone G., Pirone L., Capolupo A., Vitagliano L., Monti M.C., Di Gaetano S., Pedone E. (2018). The essential player in adipogenesis GRP78 is a novel KCTD15 interactor. Int. J. Biol. Macromol..

[31] Aburasayn H., Al Batran R., Ussher J.R. (2016). Targeting ceramide metabolism in obesity. Am. J. Physiol. Endocrinol. Metab..

[32] Chaurasia B., Summers S.A. (2015). Ceramides—Lipotoxic Inducers of Metabolic Disorders. Trends Endocrinol. Metab..

[33] Chavez J.A., Summers S.A. (2003). Characterizing the effects of saturated fatty acids on insulin signaling and ceramide and diacylglycerol accumulation in 3T3-L1 adipocytes and C2C12 myotubes. Arch. Biochem. Biophys..

[34] Blouin C.M., Prado C., Takane K.K., Lasnier F., Garcia-Ocana A., Ferré P., Dugail I., Hajduch E. (2010). Plasma membrane subdomain compartmentalization contributes to distinct mechanisms of ceramide action on insulin signaling. Diabetes.

[35] Kolak M., Westerbacka J., Velagapudi V.R., Wågsäter D., Yetukuri L., Makkonen J., Rissanen A., Häkkinen A.-M., Lindell M., Bergholm R. (2007). Adipose tissue inflammation and increased ceramide content characterize subjects with high liver fat content independent of obesity. Diabetes.

[36] Pyne S., Chapman J., Steele L., Pyne N.J. (1996). Sphingomyelin-derived lipids differentially regulate the extracellular signal-regulated kinase 2 (ERK-2) and c-Jun N-terminal kinase (JNK) signal cascades in airway smooth muscle. Eur. J. Biochem..

[37] Cuvillier O., Pirianov G., Kleuser B., Vanek P.G., Coso O.A., Gutkind S., Spiegel S. (1996). Suppression of ceramide-mediated programmed cell death by sphingosine-1-phosphate. Nature.

[38] Bellini L., Campana M., Mahfouz R., Carlier A., Véret J., Magnan C., Hajduch E., Le Stunff H. (2015). Targeting sphingolipid metabolism in the treatment of obesity/type 2 diabetes. Expert Opin. Ther. Targets.

[39] Lee Y.S., Li P., Huh J.Y., Hwang I.J., Lu M., Kim J.I., Ham M., Talukdar S., Chen A., Lu W.J. (2011). Inflammation is necessary for long-term but not short-term high-fat diet-induced insulin resistance. Diabetes.

[40] Hannun Y.A., Obeid L.M. (2018). Sphingolipids and their metabolism in physiology and disease. Nat. Rev. Mol. Cell Biol..

