# Supplementary material for: Integrative Analysis of Blood Transcriptomics and Metabolomics Reveals Molecular Regulation of Backfat Thickness in Qinchuan Cattle
Source: Animals (Basel). 2023 Mar 15;13(6):1060. doi: 10.3390/ani13061060 (PMC10044415; doi:10.3390/ani13061060)
Supplement: Supplementary file 1 [file animals-13-01060-s001.zip › Supplementary File S1 Supplementary Figure S1.pdf]

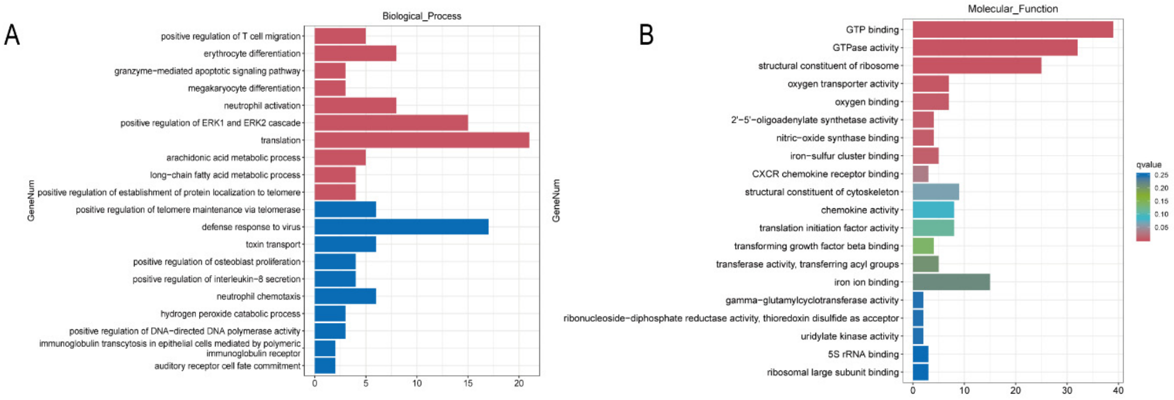

Supplementary Figure S1. GO enrichment analysis. (A) Biological processes (BP) of DEGs. (B) Molecular functions (MF) of DEGs.
