# Supplementary material for: Integrative Analysis of Blood Transcriptomics and Metabolomics Reveals Molecular Regulation of Backfat Thickness in Qinchuan Cattle
Source: Animals (Basel). 2023 Mar 15;13(6):1060. doi: 10.3390/ani13061060 (PMC10044415; doi:10.3390/ani13061060)
Supplement: Supplementary file 1 [file animals-13-01060-s001.zip › Supplementary File S10 Supplementary Table S8.pdf]

Table S8. KEGG enrichment analysis for DEMs.

#Pathway	ko_ID	Diff_Metabolites_in_Pathway
Tuberculosis	ko05152	1
Sphingolipid metabolism	ko00600	1
Fatty acid biosynthesis	ko00061	1
Glutathione metabolism	ko00480	1
Sphingolipid signaling pathway	ko04071	1
Fc gamma R-mediated phagocytosis	ko04666	1
Biotin metabolism	ko00780	1
Phospholipase D signaling pathway	ko04072	1
Linoleic acid metabolism	ko00591	1
Apelin signaling pathway	ko04371	1
Steroid hormone biosynthesis	ko00140	1
Calcium signaling pathway	ko04020	1
Neuroactive ligand-receptor interaction	ko04080	1
Fructose and mannose metabolism	ko00051	1

All_metabolites_in_Pathway	Diff_Mtabolites_in_All_pathways	All_Metabolites_in_All_pathways
3	7	353
3	7	353
3	7	353
4	7	353
2	7	353
3	7	353
1	7	353
3	7	353
10	7	353
3	7	353
14	7	353
3	7	353
9	7	353
4	7	353

Metabolite_id	KEGG_Orthology
pos_4929	C06124
pos_4929	C06124
pos_3312	C06424
neg_4693	C03170
pos_4929	C06124
pos_4929	C06124
pos_2804	C20387
pos_4929	C06124
neg_3626	C14833
pos_4929	C06124
neg_6246	C18041
pos_4929	C06124
pos_4929	C06124
pos_1270	C00507
