# Supplementary material for: Integrative Analysis of Blood Transcriptomics and Metabolomics Reveals Molecular Regulation of Backfat Thickness in Qinchuan Cattle
Source: Animals (Basel). 2023 Mar 15;13(6):1060. doi: 10.3390/ani13061060 (PMC10044415; doi:10.3390/ani13061060)
Supplement: Supplementary file 1 [file animals-13-01060-s001.zip › Supplementary File S11 Supplementary Table S9.pdf]

Table S9. Pearson correlations coefficient between all genes and metabolites.

Gene_ID	Gene_log2FC	Metabolite_ID	Metabolite_log2FC
gene-CRABP2	8.982505637	3-Thiacytidine	0.209387412
gene-CRABP2	8.982505637	LTB4-d4	0.226799982
gene-CRABP2	8.982505637	PE(20:0/18:1(12Z)-2OH(9,10))	0.438658253
gene-CRABP2	8.982505637	(-)-alpha-Terpineol	0.345716279
gene-CRABP2	8.982505637	Cyclosporin A	0.656529229
gene-CRABP2	8.982505637	Angiotensin A	0.247332017
gene-CRABP2	8.982505637	DG(18:0/LTE4/0:0)	0.681485773
gene-CRABP2	8.982505637	PC(17:0/PGJ2)	0.657689319
gene-CRABP2	8.982505637	PC(14:0/20:2(11Z,14Z))	1.110657378
gene-CRABP2	8.982505637	5-(2-Aminopropyl)-2-methylphenol	0.323231457
gene-CRABP2	8.982505637	Trimetazidine	0.438282534
gene-CRABP2	8.982505637	ingenol	0.942071652
gene-CRABP2	8.982505637	Armillane	0.52808635
gene-CRABP2	8.982505637	PC(P-18:1(11Z)/PGE2)	0.509503133
gene-CRABP2	8.982505637	(3R,4R)-3-Amino-1-hydroxy-4-methylpyrrol	0.471914506
gene-CRABP2	8.982505637	Isopropyl isothiocyanate	0.172385747
gene-CRABP2	8.982505637	(9Z)-Octadecenoic acid	0.142359556
gene-CRABP2	8.982505637	PC(18:1(9Z)/15:1(9Z))	0.534724682
gene-CRABP2	8.982505637	arachidyl amido cholanoic acid	1.24842952
gene-CRABP2	8.982505637	Nigroxanthin	0.705005059
gene-CRABP2	8.982505637	1-Octadecanoyl-2-(7Z,10Z,13Z,16Z-docosat	0.690496314
gene-CRABP2	8.982505637	PC(P-18:1(11Z)/PGJ2)	0.565243877
gene-CRABP2	8.982505637	PS(20:0/20:4(8Z,11Z,14Z,17Z)-2OH(5S,6R))	0.402595921
gene-CRABP2	8.982505637	CL(8:0/8:0/18:2(9Z,11Z)/20:0)	0.622988418
gene-ZFP57	8.653220864	3-Thiacytidine	0.209387412
gene-ZFP57	8.653220864	7(14)-Bisabolene-2,3,10,11-tetrol	2.446897974
gene-ZFP57	8.653220864	3-Deoxyestrone	0.282221709
gene-ZFP57	8.653220864	1-Oleoyl-sn-glycero-3-phosphocholine	0.18926952
gene-ZFP57	8.653220864	Psychosine	0.106475396
gene-ZFP57	8.653220864	(-)-alpha-Terpineol	0.345716279
gene-ZFP57	8.653220864	1,4-Undecadiene	0.462803973
gene-ZFP57	8.653220864	2-isopentyl-3,6-dimethyl pyrazine	0.710502562
gene-ZFP57	8.653220864	5-(2-Aminopropyl)-2-methylphenol	0.323231457
gene-ZFP57	8.653220864	4-Dimethylamino-L-phenylalanine	0.242110226
gene-ZFP57	8.653220864	Trimetazidine	0.438282534
gene-ZFP57	8.653220864	ingenol	0.942071652
gene-ZFP57	8.653220864	Armillane	0.52808635
gene-ZFP57	8.653220864	PC(P-18:1(11Z)/PGE2)	0.509503133
gene-ZFP57	8.653220864	(3R,4R)-3-Amino-1-hydroxy-4-methylpyrrol	0.471914506
gene-ZFP57	8.653220864	1-Palmitoylglycerol	0.084357408
gene-ZFP57	8.653220864	n-methyl-2-(4'-methylaminophenyl)-6-hydr	0.26655714
gene-ZFP57	8.653220864	Isopropyl isothiocyanate	0.172385747
gene-ZFP57	8.653220864	9-deoxy-9-methylene-16,16-dimethyl -PGE	0.606893884
gene-ZFP57	8.653220864	CL(8:0/8:0/18:2(9Z,11Z)/20:0)	0.622988418
Bos_taurus_newG	6.976293532	3-Thiacytidine	0.209387412
Bos_taurus_newG	6.976293532	D-Erythro-imidazole-glycerol-phosphate	0.355812431
Bos_taurus_newG	6.976293532	7(14)-Bisabolene-2,3,10,11-tetrol	2.446897974
Bos_taurus_newG	6.976293532	cis-p-Menth-2-en-1-ol	0.201017988

Bos_taurus_newG	6.976293532	(-)-alpha-Terpineol	0.345716279
Bos_taurus_newG	6.976293532	1,4-Undecadiene	0.462803973
Bos_taurus_newG	6.976293532	4-Dimethylamino-L-phenylalanine	0.242110226
Bos_taurus_newG	6.976293532	Armillane	0.52808635
Bos_taurus_newG	6.976293532	PC(P-18:1(11Z)/PGE2)	0.509503133
Bos_taurus_newG	6.976293532	(3R,4R)-3-Amino-1-hydroxy-4-methylpyrrol	0.471914506
Bos_taurus_newG	6.976293532	Sambutoxin	0.169786377
Bos_taurus_newG	6.976293532	Isopropyl isothiocyanate	0.172385747
Bos_taurus_newG	6.976293532	PS(20:0/20:4(8Z,11Z,14Z,17Z)-2OH(5S,6R))	0.402595921
gene-LOC540321	6.330528525	7(14)-Bisabolene-2,3,10,11-tetrol	2.446897974
gene-LOC540321	6.330528525	3-Deoxyestrone	0.282221709
gene-LOC540321	6.330528525	1-Oleoyl-sn-glycero-3-phosphocholine	0.18926952
gene-LOC540321	6.330528525	Lividamine	0.319679555
gene-LOC540321	6.330528525	Psychosine	0.106475396
gene-LOC540321	6.330528525	(-)-alpha-Terpineol	0.345716279
gene-LOC540321	6.330528525	1,4-Undecadiene	0.462803973
gene-LOC540321	6.330528525	2-isopentyl-3,6-dimethyl pyrazine	0.710502562
gene-LOC540321	6.330528525	4-cholesten-7伪,12伪,24-triol-3-one	0.097840451
gene-LOC540321	6.330528525	5-(2-Aminopropyl)-2-methylphenol	0.323231457
gene-LOC540321	6.330528525	4-Dimethylamino-L-phenylalanine	0.242110226
gene-LOC540321	6.330528525	Trimetazidine	0.438282534
gene-LOC540321	6.330528525	ingenol	0.942071652
gene-LOC540321	6.330528525	Armillane	0.52808635
gene-LOC540321	6.330528525	PC(P-18:1(11Z)/PGE2)	0.509503133
gene-LOC540321	6.330528525	2-(2-Aminopropanoylamino)bicyclo[3.1.0]he:	1.149807904
gene-LOC540321	6.330528525	(3R,4R)-3-Amino-1-hydroxy-4-methylpyrrol	0.471914506
gene-LOC540321	6.330528525	1-Palmitoylglycerol	0.084357408
gene-LOC540321	6.330528525	n-methyl-2-(4'-methylaminophenyl)-6-hydr	0.26655714
gene-LOC540321	6.330528525	Glutethimide	0.126237229
gene-LOC540321	6.330528525	Isopropyl isothiocyanate	0.172385747
gene-LOC540321	6.330528525	9-deoxy-9-methylene-16,16-dimethyl -PGE:	0.606893884
gene-LOC540321	6.330528525	CL(8:0/8:0/18:2(9Z,11Z)/20:0)	0.622988418
gene-MTUS2	6.010503498	5-Fluorouridine diphosphate	0.151761582
gene-MTUS2	6.010503498	L-Valine, N-(2-hydroxy-3-butenyl)-	0.253583227
gene-MTUS2	6.010503498	3-hydroxypristanic acid	0.548515835
gene-MTUS2	6.010503498	Canesceol	0.686312122
gene-MTUS2	6.010503498	Proclavaminic acid	0.060659628
gene-MTUS2	6.010503498	7(14)-Bisabolene-2,3,10,11-tetrol	2.446897974
gene-MTUS2	6.010503498	(3Z)-Phycoerythrobilin	1.456755874
gene-MTUS2	6.010503498	16-hydroxy hexadecanoic acid	0.309000958
gene-MTUS2	6.010503498	1,2-O-Isopropylidene-D-glucofuranose	0.080987667
gene-MTUS2	6.010503498	3-Deoxyestrone	0.282221709
gene-MTUS2	6.010503498	1-Oleoyl-sn-glycero-3-phosphocholine	0.18926952
gene-MTUS2	6.010503498	3,4-dihydroxy-5-all-trans-hexaprenylbenzoa	0.123615726
gene-MTUS2	6.010503498	Lividamine	0.319679555
gene-MTUS2	6.010503498	Psychosine	0.106475396
gene-MTUS2	6.010503498	Cyclotricuspidogenin C	0.440884085
gene-MTUS2	6.010503498	(-)-alpha-Terpineol	0.345716279
gene-MTUS2	6.010503498	Linoleamide	0.926003481
gene-MTUS2	6.010503498	DG(8:0/20:4(6Z,8E,10E,14Z)-2OH(5S,12R)/0:0	0.958378689
gene-MTUS2	6.010503498	Sapacitabine	0.213785561

gene-MTUS2	6.010503498	1,4-Undecadiene	0.462803973
gene-MTUS2	6.010503498	4-cholesten-7伪,12伪,24-triol-3-one	0.097840451
gene-MTUS2	6.010503498	PC(16:0/18:1(12Z)-2OH(9,10))	0.461843233
gene-MTUS2	6.010503498	4-Dimethylamino-L-phenylalanine	0.242110226
gene-MTUS2	6.010503498	Trimetazidine	0.438282534
gene-MTUS2	6.010503498	ingenol	0.942071652
gene-MTUS2	6.010503498	Armillane	0.52808635
gene-MTUS2	6.010503498	Dihydroethidium	5.075390639
gene-MTUS2	6.010503498	Tyrosyl-Arginine	6.462631098
gene-MTUS2	6.010503498	1,2-Benzisothiazol-3(2H)-one	0.055099967
gene-MTUS2	6.010503498	Misoprostol	1.349270093
gene-MTUS2	6.010503498	Pluviatolide	1.068135579
gene-MTUS2	6.010503498	Carboprost methyl	1.74923433
gene-MTUS2	6.010503498	11-Dehydro-2,3-dinor-txb2	0.307258797
gene-MTUS2	6.010503498	Val Trp Leu His	0.05980936
gene-MTUS2	6.010503498	2-(2-Aminopropanoylamino)bicyclo[3.1.0]he:	1.149807904
gene-MTUS2	6.010503498	1-Palmitoylglycerol	0.084357408
gene-MTUS2	6.010503498	N2-gamma-Glutamylglutamine	0.230065499
gene-MTUS2	6.010503498	n-methyl-2-(4'-methylaminophenyl)-6-hydr	0.26655714
gene-MTUS2	6.010503498	Corosin	0.175715347
gene-MTUS2	6.010503498	Permetin A	0.140869415
gene-MTUS2	6.010503498	Glutethimide	0.126237229
gene-MTUS2	6.010503498	Phosphorylcholine	0.087558558
gene-MTUS2	6.010503498	Isopropyl isothiocyanate	0.172385747
gene-MTUS2	6.010503498	L-Oleandrosyl-oleandolide	0.272998166
gene-MTUS2	6.010503498	9-deoxy-9-methylene-16,16-dimethyl -PGE:	0.606893884
gene-MTUS2	6.010503498	(E)-5-(3,4,5,6-Tetrahydro-3-pyridylidenemet	0.065093771
gene-MTUS2	6.010503498	1-Methylnicotinamide	0.203956241
gene-MTUS2	6.010503498	4-[(2,4-Dihydroxy-3,3-dimethylbutanoyl)ami	0.207947066
gene-MTUS2	6.010503498	CL(8:0/8:0/18:2(9Z,11Z)/20:0)	0.622988418
Bos_taurus_newG	5.124566706	3-Thiacytidine	0.209387412
Bos_taurus_newG	5.124566706	7(14)-Bisabolene-2,3,10,11-tetrol	2.446897974
Bos_taurus_newG	5.124566706	3-Deoxyestrone	0.282221709
Bos_taurus_newG	5.124566706	1-Oleoyl-sn-glycero-3-phosphocholine	0.18926952
Bos_taurus_newG	5.124566706	Psychosine	0.106475396
Bos_taurus_newG	5.124566706	(-)-alpha-Terpineol	0.345716279
Bos_taurus_newG	5.124566706	1,4-Undecadiene	0.462803973
Bos_taurus_newG	5.124566706	4-cholesten-7伪,12伪,24-triol-3-one	0.097840451
Bos_taurus_newG	5.124566706	5-(2-Aminopropyl)-2-methylphenol	0.323231457
Bos_taurus_newG	5.124566706	4-Dimethylamino-L-phenylalanine	0.242110226
Bos_taurus_newG	5.124566706	Trimetazidine	0.438282534
Bos_taurus_newG	5.124566706	ingenol	0.942071652
Bos_taurus_newG	5.124566706	Armillane	0.52808635
Bos_taurus_newG	5.124566706	PC(P-18:1(11Z)/PGE2)	0.509503133
Bos_taurus_newG	5.124566706	(3R,4R)-3-Amino-1-hydroxy-4-methylpyrrol	0.471914506
Bos_taurus_newG	5.124566706	1-Palmitoylglycerol	0.084357408
Bos_taurus_newG	5.124566706	n-methyl-2-(4'-methylaminophenyl)-6-hydr	0.26655714
Bos_taurus_newG	5.124566706	Isopropyl isothiocyanate	0.172385747
Bos_taurus_newG	5.124566706	9-deoxy-9-methylene-16,16-dimethyl -PGE:	0.606893884
Bos_taurus_newG	5.124566706	CL(8:0/8:0/18:2(9Z,11Z)/20:0)	0.622988418
gene-LOC52993(	4.59324894	3-Thiacytidine	0.209387412

gene-LOC52993(	4.59324894	(-)-alpha-Terpineol	0.345716279
gene-LOC52993(	4.59324894	1,4-Undecadiene	0.462803973
gene-LOC52993(	4.59324894	4-cholesten-7伪,12伪,24-triol-3-one	0.097840451
gene-LOC52993(	4.59324894	Cyclosporin A	0.656529229
gene-LOC52993(	4.59324894	5-(2-Aminopropyl)-2-methylphenol	0.323231457
gene-LOC52993(	4.59324894	ingenol	0.942071652
gene-LOC52993(	4.59324894	Armillane	0.52808635
gene-LOC52993(	4.59324894	PC(P-18:1(11Z)/PGE2)	0.509503133
gene-LOC52993(	4.59324894	PC(20:3(5Z,8Z,11Z)/24:0)	0.387959564
gene-LOC52993(	4.59324894	Isopropyl isothiocyanate	0.172385747
gene-LOC52993(	4.59324894	D-Fructose	0.167584616
gene-LOC52993(	4.59324894	CL(8:0/8:0/18:2(9Z,11Z)/20:0)	0.622988418
Bos_taurus_newG	4.523754331	3-Thiacytidine	0.209387412
Bos_taurus_newG	4.523754331	LTB4-d4	0.226799982
Bos_taurus_newG	4.523754331	(-)-alpha-Terpineol	0.345716279
Bos_taurus_newG	4.523754331	Cyclosporin A	0.656529229
Bos_taurus_newG	4.523754331	DG(18:0/LTE4/0:0)	0.681485773
Bos_taurus_newG	4.523754331	PC(17:0/PGJ2)	0.657689319
Bos_taurus_newG	4.523754331	PC(14:0/20:2(11Z,14Z))	1.110657378
Bos_taurus_newG	4.523754331	5-(2-Aminopropyl)-2-methylphenol	0.323231457
Bos_taurus_newG	4.523754331	ingenol	0.942071652
Bos_taurus_newG	4.523754331	beta-L-Dioxolane-cytidine	0.175916668
Bos_taurus_newG	4.523754331	PC(P-18:1(11Z)/PGE2)	0.509503133
Bos_taurus_newG	4.523754331	PC(20:3(5Z,8Z,11Z)/24:0)	0.387959564
Bos_taurus_newG	4.523754331	Isopropyl isothiocyanate	0.172385747
Bos_taurus_newG	4.523754331	arachidyl amido cholanoic acid	1.24842952
Bos_taurus_newG	4.523754331	Nigroxanthin	0.705005059
Bos_taurus_newG	4.523754331	D-Fructose	0.167584616
Bos_taurus_newG	4.523754331	CL(8:0/8:0/18:2(9Z,11Z)/20:0)	0.622988418
gene-PAMR1	4.127866288	3-Thiacytidine	0.209387412
gene-PAMR1	4.127866288	5-Hydroxy-2-oxo-4-ureido-2,5-dihydro-1H	0.122536033
gene-PAMR1	4.127866288	11-Maleimidoundecanoic acid	1.084942397
gene-PAMR1	4.127866288	13(S)-HpODE	0.163157753
gene-PAMR1	4.127866288	PE(20:0/18:1(12Z)-2OH(9,10))	0.438658253
gene-PAMR1	4.127866288	PE(22:2(13Z,16Z)/22:5(4Z,7Z,10Z,13Z,19Z)-O(	0.369745166
gene-PAMR1	4.127866288	Epomusenin A	0.766206049
gene-PAMR1	4.127866288	DG(18:0/LTE4/0:0)	0.681485773
gene-PAMR1	4.127866288	CDP-DG(PGF2alpha/16:0)	1.002512277
gene-PAMR1	4.127866288	Lamivudine	0.327402892
gene-PAMR1	4.127866288	1-beta-D-Arabinofuranosyl-5-fluorocytosine	0.152339645
gene-PAMR1	4.127866288	Cysteinyl-Glutamine	0.079050155
gene-PAMR1	4.127866288	15-keto-Prostaglandin E2	2.646629404
gene-PAMR1	4.127866288	Fluridone	0.127106682
gene-PAMR1	4.127866288	Norophthalmic acid	0.191432411
gene-PAMR1	4.127866288	5,6,7,8-Tetrahydromonapterin	0.724075531
gene-PAMR1	4.127866288	Guanidoacetic acid	0.542509265
gene-PAMR1	4.127866288	4-Oxo-9-cis-retinoyl-beta-glucuronide	1.611773742
gene-PAMR1	4.127866288	alpha-Terpineol formate	0.628238986
gene-PAMR1	4.127866288	Methionyl-Valine	0.502707745
gene-PAMR1	4.127866288	beta-L-Dioxolane-cytidine	0.175916668
gene-PAMR1	4.127866288	Ile His Leu Trp	0.124696937

gene-PAMR1	4.127866288	10-alpha-methoxy-9,10-dihydrolysergol	0.140851438
gene-PAMR1	4.127866288	PC(18:1(9Z)/15:1(9Z))	0.534724682
gene-PAMR1	4.127866288	Nigroxanthin	0.705005059
gene-PAMR1	4.127866288	PC(P-18:1(11Z)/PGJ2)	0.565243877
gene-PAMR1	4.127866288	PS(20:0/20:4(8Z,11Z,14Z,17Z)-2OH(5S,6R))	0.402595921
gene-HOXB6	4.106644497	3-Thiacytidine	0.209387412
gene-HOXB6	4.106644497	LTB4-d4	0.226799982
gene-HOXB6	4.106644497	PE(20:0/18:1(12Z)-2OH(9,10))	0.438658253
gene-HOXB6	4.106644497	Cyclosporin A	0.656529229
gene-HOXB6	4.106644497	DG(18:0/LTE4/0:0)	0.681485773
gene-HOXB6	4.106644497	PC(14:0/20:2(11Z,14Z))	1.110657378
gene-HOXB6	4.106644497	5-(2-Aminopropyl)-2-methylphenol	0.323231457
gene-HOXB6	4.106644497	beta-L-Dioxolane-cytidine	0.175916668
gene-HOXB6	4.106644497	PC(P-18:1(11Z)/PGE2)	0.509503133
gene-HOXB6	4.106644497	Isopropyl isothiocyanate	0.172385747
gene-HOXB6	4.106644497	PC(18:1(9Z)/15:1(9Z))	0.534724682
gene-HOXB6	4.106644497	arachidyl amido cholanoic acid	1.24842952
gene-HOXB6	4.106644497	Nigroxanthin	0.705005059
gene-HOXB6	4.106644497	PC(P-18:1(11Z)/PGJ2)	0.565243877
gene-HOXB6	4.106644497	PS(20:0/20:4(8Z,11Z,14Z,17Z)-2OH(5S,6R))	0.402595921
gene-HOXB6	4.106644497	D-Fructose	0.167584616
gene-HOXB6	4.106644497	CL(8:0/8:0/18:2(9Z,11Z)/20:0)	0.622988418
gene-APCDD1	3.999614213	5-(Ethylthio)-1H-tetrazole	0.29717344
gene-APCDD1	3.999614213	3-Thiacytidine	0.209387412
gene-APCDD1	3.999614213	Tsugarioside B	1.013262893
gene-APCDD1	3.999614213	Tetracosenoyl-CoA	0.187187233
gene-APCDD1	3.999614213	7(14)-Bisabolene-2,3,10,11-tetrol	2.446897974
gene-APCDD1	3.999614213	3-Deoxyestrone	0.282221709
gene-APCDD1	3.999614213	1-Oleoyl-sn-glycero-3-phosphocholine	0.18926952
gene-APCDD1	3.999614213	Psychosine	0.106475396
gene-APCDD1	3.999614213	2-isopentyl-3,6-dimethyl pyrazine	0.710502562
gene-APCDD1	3.999614213	PC(P-18:0/20:5(5Z,8Z,11Z,14Z,16E)-OH(18))	0.097499392
gene-APCDD1	3.999614213	PC(16:0/18:1(12Z)-2OH(9,10))	0.461843233
gene-APCDD1	3.999614213	5-(2-Aminopropyl)-2-methylphenol	0.323231457
gene-APCDD1	3.999614213	4-Dimethylamino-L-phenylalanine	0.242110226
gene-APCDD1	3.999614213	Trimetazidine	0.438282534
gene-APCDD1	3.999614213	Armillane	0.52808635
gene-APCDD1	3.999614213	5'-S-Methyl-5'-thioinosine	0.28772476
gene-APCDD1	3.999614213	(3R,4R)-3-Amino-1-hydroxy-4-methylpyrrol	0.471914506
gene-APCDD1	3.999614213	n-methyl-2-(4'-methylaminophenyl)-6-hydr	0.26655714
gene-APCDD1	3.999614213	PE(P-16:0/18:4(6Z,9Z,12Z,15Z))	0.546989864
gene-APCDD1	3.999614213	Isopropyl isothiocyanate	0.172385747
gene-APCDD1	3.999614213	(9Z)-Octadecenoic acid	0.142359556
gene-APCDD1	3.999614213	N-Myristoyl Glutamine	0.37856966
gene-APCDD1	3.999614213	9-deoxy-9-methylene-16,16-dimethyl -PGE	0.606893884
gene-APCDD1	3.999614213	2-Propenyl 2-aminobenzoate	0.128406829
gene-APCDD1	3.999614213	CL(8:0/8:0/18:2(9Z,11Z)/20:0)	0.622988418
gene-CELSR3	3.857834243	D-Erythro-imidazole-glycerol-phosphate	0.355812431
gene-CELSR3	3.857834243	7(14)-Bisabolene-2,3,10,11-tetrol	2.446897974
gene-CELSR3	3.857834243	cis-p-Menth-2-en-1-ol	0.201017988
gene-CELSR3	3.857834243	1,4-Undecadiene	0.462803973

gene-CELSR3	3.857834243	PG(20:1(11Z)/18:3(10,12,15)-OH(9))	0.626244347
gene-CELSR3	3.857834243	Armillane	0.52808635
gene-CELSR3	3.857834243	PC(P-18:1(11Z)/PGE2)	0.509503133
gene-CELSR3	3.857834243	(3R,4R)-3-Amino-1-hydroxy-4-methylpyrrol	0.471914506
gene-CELSR3	3.857834243	Isopropyl isothiocyanate	0.172385747
gene-CELSR3	3.857834243	PS(20:0/20:4(8Z,11Z,14Z,17Z)-2OH(5S,6R))	0.402595921
gene-PRG3	3.827782654	3-Thiacytidine	0.209387412
gene-PRG3	3.827782654	7(14)-Bisabolene-2,3,10,11-tetrol	2.446897974
gene-PRG3	3.827782654	3-Deoxyestrone	0.282221709
gene-PRG3	3.827782654	1-Oleoyl-sn-glycero-3-phosphocholine	0.18926952
gene-PRG3	3.827782654	(-)-alpha-Terpineol	0.345716279
gene-PRG3	3.827782654	1,4-Undecadiene	0.462803973
gene-PRG3	3.827782654	4-cholesten-7伪,12伪,24-triol-3-one	0.097840451
gene-PRG3	3.827782654	5-(2-Aminopropyl)-2-methylphenol	0.323231457
gene-PRG3	3.827782654	4-Dimethylamino-L-phenylalanine	0.242110226
gene-PRG3	3.827782654	ingenol	0.942071652
gene-PRG3	3.827782654	Armillane	0.52808635
gene-PRG3	3.827782654	PC(P-18:1(11Z)/PGE2)	0.509503133
gene-PRG3	3.827782654	(3R,4R)-3-Amino-1-hydroxy-4-methylpyrrol	0.471914506
gene-PRG3	3.827782654	1-Palmitoylglycerol	0.084357408
gene-PRG3	3.827782654	n-methyl-2-(4'-methylaminophenyl)-6-hydr	0.26655714
gene-PRG3	3.827782654	Isopropyl isothiocyanate	0.172385747
gene-PRG3	3.827782654	9-deoxy-9-methylene-16,16-dimethyl -PGE	0.606893884
gene-PRG3	3.827782654	CL(8:0/8:0/18:2(9Z,11Z)/20:0)	0.622988418
gene-PRKCG	3.807900385	3-Thiacytidine	0.209387412
gene-PRKCG	3.807900385	D-Erythro-imidazole-glycerol-phosphate	0.355812431
gene-PRKCG	3.807900385	7(14)-Bisabolene-2,3,10,11-tetrol	2.446897974
gene-PRKCG	3.807900385	(-)-alpha-Terpineol	0.345716279
gene-PRKCG	3.807900385	1,4-Undecadiene	0.462803973
gene-PRKCG	3.807900385	5-(2-Aminopropyl)-2-methylphenol	0.323231457
gene-PRKCG	3.807900385	4-Dimethylamino-L-phenylalanine	0.242110226
gene-PRKCG	3.807900385	Armillane	0.52808635
gene-PRKCG	3.807900385	PC(P-18:1(11Z)/PGE2)	0.509503133
gene-PRKCG	3.807900385	(3R,4R)-3-Amino-1-hydroxy-4-methylpyrrol	0.471914506
gene-PRKCG	3.807900385	1-Palmitoylglycerol	0.084357408
gene-PRKCG	3.807900385	n-methyl-2-(4'-methylaminophenyl)-6-hydr	0.26655714
gene-PRKCG	3.807900385	Isopropyl isothiocyanate	0.172385747
gene-PRKCG	3.807900385	PS(20:0/20:4(8Z,11Z,14Z,17Z)-2OH(5S,6R))	0.402595921
gene-OLIG2	3.805615593	3-Thiacytidine	0.209387412
gene-OLIG2	3.805615593	7(14)-Bisabolene-2,3,10,11-tetrol	2.446897974
gene-OLIG2	3.805615593	3-Deoxyestrone	0.282221709
gene-OLIG2	3.805615593	1-Oleoyl-sn-glycero-3-phosphocholine	0.18926952
gene-OLIG2	3.805615593	Psychosine	0.106475396
gene-OLIG2	3.805615593	(-)-alpha-Terpineol	0.345716279
gene-OLIG2	3.805615593	1,4-Undecadiene	0.462803973
gene-OLIG2	3.805615593	5-(2-Aminopropyl)-2-methylphenol	0.323231457
gene-OLIG2	3.805615593	4-Dimethylamino-L-phenylalanine	0.242110226
gene-OLIG2	3.805615593	Trimetazidine	0.438282534
gene-OLIG2	3.805615593	ingenol	0.942071652
gene-OLIG2	3.805615593	Armillane	0.52808635
gene-OLIG2	3.805615593	PC(P-18:1(11Z)/PGE2)	0.509503133

gene-OLIG2	3.805615593	(3R,4R)-3-Amino-1-hydroxy-4-methylpyrrol	0.471914506
gene-OLIG2	3.805615593	1-Palmitoylglycerol	0.084357408
gene-OLIG2	3.805615593	n-methyl-2-(4'-methylaminophenyl)-6-hydr	0.26655714
gene-OLIG2	3.805615593	Isopropyl isothiocyanate	0.172385747
gene-OLIG2	3.805615593	9-deoxy-9-methylene-16,16-dimethyl -PGE:	0.606893884
gene-OLIG2	3.805615593	CL(8:0/8:0/18:2(9Z,11Z)/20:0)	0.622988418
gene-ASB2	3.714413534	3-Thiacytidine	0.209387412
gene-ASB2	3.714413534	7(14)-Bisabolene-2,3,10,11-tetrol	2.446897974
gene-ASB2	3.714413534	3-Deoxyestrone	0.282221709
gene-ASB2	3.714413534	1-Oleoyl-sn-glycero-3-phosphocholine	0.18926952
gene-ASB2	3.714413534	Psychosine	0.106475396
gene-ASB2	3.714413534	(-)-alpha-Terpineol	0.345716279
gene-ASB2	3.714413534	1,4-Undecadiene	0.462803973
gene-ASB2	3.714413534	4-cholesten-7伪,12伪,24-triol-3-one	0.097840451
gene-ASB2	3.714413534	5-(2-Aminopropyl)-2-methylphenol	0.323231457
gene-ASB2	3.714413534	4-Dimethylamino-L-phenylalanine	0.242110226
gene-ASB2	3.714413534	Trimetazidine	0.438282534
gene-ASB2	3.714413534	ingenol	0.942071652
gene-ASB2	3.714413534	Armillane	0.52808635
gene-ASB2	3.714413534	PC(P-18:1(11Z)/PGE2)	0.509503133
gene-ASB2	3.714413534	(3R,4R)-3-Amino-1-hydroxy-4-methylpyrrol	0.471914506
gene-ASB2	3.714413534	1-Palmitoylglycerol	0.084357408
gene-ASB2	3.714413534	n-methyl-2-(4'-methylaminophenyl)-6-hydr	0.26655714
gene-ASB2	3.714413534	Isopropyl isothiocyanate	0.172385747
gene-ASB2	3.714413534	9-deoxy-9-methylene-16,16-dimethyl -PGE:	0.606893884
gene-ASB2	3.714413534	CL(8:0/8:0/18:2(9Z,11Z)/20:0)	0.622988418
gene-LTBP4	3.585204224	3-Thiacytidine	0.209387412
gene-LTBP4	3.585204224	7(14)-Bisabolene-2,3,10,11-tetrol	2.446897974
gene-LTBP4	3.585204224	3-Deoxyestrone	0.282221709
gene-LTBP4	3.585204224	1-Oleoyl-sn-glycero-3-phosphocholine	0.18926952
gene-LTBP4	3.585204224	Psychosine	0.106475396
gene-LTBP4	3.585204224	(-)-alpha-Terpineol	0.345716279
gene-LTBP4	3.585204224	1,4-Undecadiene	0.462803973
gene-LTBP4	3.585204224	5-(2-Aminopropyl)-2-methylphenol	0.323231457
gene-LTBP4	3.585204224	4-Dimethylamino-L-phenylalanine	0.242110226
gene-LTBP4	3.585204224	Trimetazidine	0.438282534
gene-LTBP4	3.585204224	ingenol	0.942071652
gene-LTBP4	3.585204224	Armillane	0.52808635
gene-LTBP4	3.585204224	PC(P-18:1(11Z)/PGE2)	0.509503133
gene-LTBP4	3.585204224	(3R,4R)-3-Amino-1-hydroxy-4-methylpyrrol	0.471914506
gene-LTBP4	3.585204224	1-Palmitoylglycerol	0.084357408
gene-LTBP4	3.585204224	n-methyl-2-(4'-methylaminophenyl)-6-hydr	0.26655714
gene-LTBP4	3.585204224	Isopropyl isothiocyanate	0.172385747
gene-LTBP4	3.585204224	(9Z)-Octadecenoic acid	0.142359556
gene-LTBP4	3.585204224	9-deoxy-9-methylene-16,16-dimethyl -PGE:	0.606893884
gene-LTBP4	3.585204224	CL(8:0/8:0/18:2(9Z,11Z)/20:0)	0.622988418
gene-SPOCD1	3.482465044	3-Thiacytidine	0.209387412
gene-SPOCD1	3.482465044	D-Erythro-imidazole-glycerol-phosphate	0.355812431
gene-SPOCD1	3.482465044	7(14)-Bisabolene-2,3,10,11-tetrol	2.446897974
gene-SPOCD1	3.482465044	1,4-Undecadiene	0.462803973
gene-SPOCD1	3.482465044	4-cholesten-7伪,12伪,24-triol-3-one	0.097840451

gene-SPOCD1	3.482465044	PE(22:2(13Z,16Z)/22:5(4Z,7Z,10Z,13Z,19Z)-O	0.369745166
gene-SPOCD1	3.482465044	PC(P-18:1(11Z)/PGE2)	0.509503133
gene-SPOCD1	3.482465044	Isopropyl isothiocyanate	0.172385747
gene-SPOCD1	3.482465044	PC(P-18:1(11Z)/PGJ2)	0.565243877
gene-SPOCD1	3.482465044	PS(20:0/20:4(8Z,11Z,14Z,17Z)-2OH(5S,6R))	0.402595921
gene-CELA2A	3.432009542	3-Thiacytidine	0.209387412
gene-CELA2A	3.432009542	D-Erythro-imidazole-glycerol-phosphate	0.355812431
gene-CELA2A	3.432009542	7(14)-Bisabolene-2,3,10,11-tetrol	2.446897974
gene-CELA2A	3.432009542	cis-p-Menth-2-en-1-ol	0.201017988
gene-CELA2A	3.432009542	(-)-alpha-Terpineol	0.345716279
gene-CELA2A	3.432009542	1,4-Undecadiene	0.462803973
gene-CELA2A	3.432009542	4-cholesten-7伪,12伪,24-triol-3-one	0.097840451
gene-CELA2A	3.432009542	Armillane	0.52808635
gene-CELA2A	3.432009542	2-Methyl-3-phenyl-2-propenal	0.407813975
gene-CELA2A	3.432009542	PC(P-18:1(11Z)/PGE2)	0.509503133
gene-CELA2A	3.432009542	(3R,4R)-3-Amino-1-hydroxy-4-methylpyrrol	0.471914506
gene-CELA2A	3.432009542	Isopropyl isothiocyanate	0.172385747
gene-CELA2A	3.432009542	PS(20:0/20:4(8Z,11Z,14Z,17Z)-2OH(5S,6R))	0.402595921
gene-LOC520402	3.400193769	Isomaltotriose	0.58434654
gene-LOC520402	3.400193769	3-hydroxypristanic acid	0.548515835
gene-LOC520402	3.400193769	(-)-alpha-Terpineol	0.345716279
gene-LOC520402	3.400193769	1,4-Undecadiene	0.462803973
gene-LOC520402	3.400193769	4-cholesten-7伪,12伪,24-triol-3-one	0.097840451
gene-LOC520402	3.400193769	N-[[3-Hydroxy-2-(2-pentenyl)cyclopentyl]ac	1.043930947
gene-LOC520402	3.400193769	PC(P-18:1(11Z)/PGE2)	0.509503133
gene-LOC520402	3.400193769	PC(20:3(5Z,8Z,11Z)/24:0)	0.387959564
gene-LOC520402	3.400193769	2-Amino-4-[carbamimidoyl(methyl)amino]bu	0.075703353
gene-LOC520402	3.400193769	D-Fructose	0.167584616
gene-SRMS	3.363094335	3-Thiacytidine	0.209387412
gene-SRMS	3.363094335	LTB4-d4	0.226799982
gene-SRMS	3.363094335	(-)-alpha-Terpineol	0.345716279
gene-SRMS	3.363094335	Cyclosporin A	0.656529229
gene-SRMS	3.363094335	5-(2-Aminopropyl)-2-methylphenol	0.323231457
gene-SRMS	3.363094335	Armillane	0.52808635
gene-SRMS	3.363094335	2-Methyl-3-phenyl-2-propenal	0.407813975
gene-SRMS	3.363094335	PC(P-18:1(11Z)/PGE2)	0.509503133
gene-SRMS	3.363094335	(3R,4R)-3-Amino-1-hydroxy-4-methylpyrrol	0.471914506
gene-SRMS	3.363094335	Isopropyl isothiocyanate	0.172385747
gene-SRMS	3.363094335	(9Z)-Octadecenoic acid	0.142359556
gene-SRMS	3.363094335	arachidyl amido cholanoic acid	1.24842952
gene-SRMS	3.363094335	Nigroxanthin	0.705005059
gene-SRMS	3.363094335	PC(P-18:1(11Z)/PGJ2)	0.565243877
gene-SRMS	3.363094335	PS(20:0/20:4(8Z,11Z,14Z,17Z)-2OH(5S,6R))	0.402595921
gene-SRMS	3.363094335	CL(8:0/8:0/18:2(9Z,11Z)/20:0)	0.622988418
Bos_taurus_newG	3.346994222	3-Thiacytidine	0.209387412
Bos_taurus_newG	3.346994222	3-Deoxyestrone	0.282221709
Bos_taurus_newG	3.346994222	1-Oleoyl-sn-glycero-3-phosphocholine	0.18926952
Bos_taurus_newG	3.346994222	LTB4-d4	0.226799982
Bos_taurus_newG	3.346994222	(-)-alpha-Terpineol	0.345716279
Bos_taurus_newG	3.346994222	1,4-Undecadiene	0.462803973
Bos_taurus_newG	3.346994222	4-cholesten-7伪,12伪,24-triol-3-one	0.097840451

Bos_taurus_newG	3.346994222	5-(2-Aminopropyl)-2-methylphenol	0.323231457
Bos_taurus_newG	3.346994222	4-Dimethylamino-L-phenylalanine	0.242110226
Bos_taurus_newG	3.346994222	Trimetazidine	0.438282534
Bos_taurus_newG	3.346994222	Armillane	0.52808635
Bos_taurus_newG	3.346994222	PC(P-18:1(11Z)/PGE2)	0.509503133
Bos_taurus_newG	3.346994222	(3R,4R)-3-Amino-1-hydroxy-4-methylpyrrol	0.471914506
Bos_taurus_newG	3.346994222	n-methyl-2-(4'-methylaminophenyl)-6-hydr	0.26655714
Bos_taurus_newG	3.346994222	Isopropyl isothiocyanate	0.172385747
Bos_taurus_newG	3.346994222	(9Z)-Octadecenoic acid	0.142359556
Bos_taurus_newG	3.346994222	9-deoxy-9-methylene-16,16-dimethyl -PGE:	0.606893884
Bos_taurus_newG	3.346994222	CL(8:0/8:0/18:2(9Z,11Z)/20:0)	0.622988418
gene-ENHO	3.328522186	3-Thiacytidine	0.209387412
gene-ENHO	3.328522186	3-Deoxyestrone	0.282221709
gene-ENHO	3.328522186	1-Oleoyl-sn-glycero-3-phosphocholine	0.18926952
gene-ENHO	3.328522186	Psychosine	0.106475396
gene-ENHO	3.328522186	(-)-alpha-Terpineol	0.345716279
gene-ENHO	3.328522186	1,4-Undecadiene	0.462803973
gene-ENHO	3.328522186	4-cholesten-7伪,12伪,24-triol-3-one	0.097840451
gene-ENHO	3.328522186	5-(2-Aminopropyl)-2-methylphenol	0.323231457
gene-ENHO	3.328522186	4-Dimethylamino-L-phenylalanine	0.242110226
gene-ENHO	3.328522186	Trimetazidine	0.438282534
gene-ENHO	3.328522186	ingenol	0.942071652
gene-ENHO	3.328522186	Armillane	0.52808635
gene-ENHO	3.328522186	PC(P-18:1(11Z)/PGE2)	0.509503133
gene-ENHO	3.328522186	(3R,4R)-3-Amino-1-hydroxy-4-methylpyrrol	0.471914506
gene-ENHO	3.328522186	1-Palmitoylglycerol	0.084357408
gene-ENHO	3.328522186	n-methyl-2-(4'-methylaminophenyl)-6-hydr	0.26655714
gene-ENHO	3.328522186	Isopropyl isothiocyanate	0.172385747
gene-ENHO	3.328522186	(9Z)-Octadecenoic acid	0.142359556
gene-ENHO	3.328522186	9-deoxy-9-methylene-16,16-dimethyl -PGE:	0.606893884
gene-ENHO	3.328522186	CL(8:0/8:0/18:2(9Z,11Z)/20:0)	0.622988418
gene-LOC101902	3.293272232	3-Thiacytidine	0.209387412
gene-LOC101902	3.293272232	11-Maleimidoundecanoic acid	1.084942397
gene-LOC101902	3.293272232	PE(20:0/18:1(12Z)-2OH(9,10))	0.438658253
gene-LOC101902	3.293272232	PE(22:2(13Z,16Z)/22:5(4Z,7Z,10Z,13Z,19Z)-O	0.369745166
gene-LOC101902	3.293272232	Cyclosporin A	0.656529229
gene-LOC101902	3.293272232	DG(18:0/LTE4/0:0)	0.681485773
gene-LOC101902	3.293272232	CDP-DG(PGF2alpha/16:0)	1.002512277
gene-LOC101902	3.293272232	Norophthalmic acid	0.191432411
gene-LOC101902	3.293272232	beta-L-Dioxolane-cytidine	0.175916668
gene-LOC101902	3.293272232	PC(P-18:1(11Z)/PGE2)	0.509503133
gene-LOC101902	3.293272232	PC(18:1(9Z)/15:1(9Z))	0.534724682
gene-LOC101902	3.293272232	Nigroxanthin	0.705005059
gene-LOC101902	3.293272232	PC(P-18:1(11Z)/PGJ2)	0.565243877
gene-LOC101902	3.293272232	PS(20:0/20:4(8Z,11Z,14Z,17Z)-2OH(5S,6R))	0.402595921
gene-LOC101905	3.217741113	3-Thiacytidine	0.209387412
gene-LOC101905	3.217741113	7(14)-Bisabolene-2,3,10,11-tetrol	2.446897974
gene-LOC101905	3.217741113	3-Deoxyestrone	0.282221709
gene-LOC101905	3.217741113	1-Oleoyl-sn-glycero-3-phosphocholine	0.18926952
gene-LOC101905	3.217741113	Psychosine	0.106475396
gene-LOC101905	3.217741113	(-)-alpha-Terpineol	0.345716279

gene-LOC10190€	3.217741113	1,4-Undecadiene	0.462803973
gene-LOC10190€	3.217741113	4-cholesten-7伪,12伪,24-triol-3-one	0.097840451
gene-LOC10190€	3.217741113	5-(2-Aminopropyl)-2-methylphenol	0.323231457
gene-LOC10190€	3.217741113	4-Dimethylamino-L-phenylalanine	0.242110226
gene-LOC10190€	3.217741113	Trimetazidine	0.438282534
gene-LOC10190€	3.217741113	Armillane	0.52808635
gene-LOC10190€	3.217741113	PC(P-18:1(11Z)/PGE2)	0.509503133
gene-LOC10190€	3.217741113	(3R,4R)-3-Amino-1-hydroxy-4-methylpyrrol	0.471914506
gene-LOC10190€	3.217741113	1-Palmitoylglycerol	0.084357408
gene-LOC10190€	3.217741113	n-methyl-2-(4'-methylaminophenyl)-6-hydr	0.26655714
gene-LOC10190€	3.217741113	Isopropyl isothiocyanate	0.172385747
gene-LOC10190€	3.217741113	9-deoxy-9-methylene-16,16-dimethyl -PGE	0.606893884
gene-LOC10190€	3.217741113	CL(8:0/8:0/18:2(9Z,11Z)/20:0)	0.622988418
Bos_taurus_newG	3.201186368	LysoPI(16:0/0:0)	0.379098336
Bos_taurus_newG	3.201186368	3-Thiacytidine	0.209387412
Bos_taurus_newG	3.201186368	cis-p-Menth-2-en-1-ol	0.201017988
Bos_taurus_newG	3.201186368	PE(20:0/18:1(12Z)-2OH(9,10))	0.438658253
Bos_taurus_newG	3.201186368	PE(22:2(13Z,16Z)/22:5(4Z,7Z,10Z,13Z,19Z)-O	0.369745166
Bos_taurus_newG	3.201186368	Lamivudine	0.327402892
Bos_taurus_newG	3.201186368	Norophthalmic acid	0.191432411
Bos_taurus_newG	3.201186368	4-Oxo-9-cis-retinoyl-beta-glucuronide	1.611773742
Bos_taurus_newG	3.201186368	alpha-Terpineol formate	0.628238986
Bos_taurus_newG	3.201186368	PC(P-18:1(11Z)/PGE2)	0.509503133
Bos_taurus_newG	3.201186368	Roxithromycin	0.268273077
Bos_taurus_newG	3.201186368	PC(18:1(9Z)/15:1(9Z))	0.534724682
Bos_taurus_newG	3.201186368	PC(P-18:1(11Z)/PGJ2)	0.565243877
Bos_taurus_newG	3.201186368	PS(20:0/20:4(8Z,11Z,14Z,17Z)-2OH(5S,6R))	0.402595921
gene-RAB44	3.117420423	3-Thiacytidine	0.209387412
gene-RAB44	3.117420423	(-)-alpha-Terpineol	0.345716279
gene-RAB44	3.117420423	1,4-Undecadiene	0.462803973
gene-RAB44	3.117420423	4-cholesten-7伪,12伪,24-triol-3-one	0.097840451
gene-RAB44	3.117420423	Cyclosporin A	0.656529229
gene-RAB44	3.117420423	5-(2-Aminopropyl)-2-methylphenol	0.323231457
gene-RAB44	3.117420423	beta-L-Dioxolane-cytidine	0.175916668
gene-RAB44	3.117420423	PC(P-18:1(11Z)/PGE2)	0.509503133
gene-RAB44	3.117420423	Isopropyl isothiocyanate	0.172385747
gene-RAB44	3.117420423	PC(P-18:1(11Z)/PGJ2)	0.565243877
gene-RAB44	3.117420423	PS(20:0/20:4(8Z,11Z,14Z,17Z)-2OH(5S,6R))	0.402595921
gene-RAB44	3.117420423	D-Fructose	0.167584616
gene-RAB44	3.117420423	CL(8:0/8:0/18:2(9Z,11Z)/20:0)	0.622988418
gene-GHSR	3.089945156	LysoPI(16:0/0:0)	0.379098336
gene-GHSR	3.089945156	3-Thiacytidine	0.209387412
gene-GHSR	3.089945156	cis-p-Menth-2-en-1-ol	0.201017988
gene-GHSR	3.089945156	PE(20:0/18:1(12Z)-2OH(9,10))	0.438658253
gene-GHSR	3.089945156	PE(22:2(13Z,16Z)/22:5(4Z,7Z,10Z,13Z,19Z)-O	0.369745166
gene-GHSR	3.089945156	Lamivudine	0.327402892
gene-GHSR	3.089945156	Norophthalmic acid	0.191432411
gene-GHSR	3.089945156	4-Oxo-9-cis-retinoyl-beta-glucuronide	1.611773742
gene-GHSR	3.089945156	PC(P-18:1(11Z)/PGE2)	0.509503133
gene-GHSR	3.089945156	PC(18:1(9Z)/15:1(9Z))	0.534724682
gene-GHSR	3.089945156	PC(P-18:1(11Z)/PGJ2)	0.565243877

gene-GHSR	3.089945156	PS(20:0/20:4(8Z,11Z,14Z,17Z)-2OH(5S,6R))	0.402595921
gene-MGAT3	3.076954518	Jasmolone glucoside	0.949312509
gene-MGAT3	3.076954518	7(14)-Bisabolene-2,3,10,11-tetrol	2.446897974
gene-MGAT3	3.076954518	3-Deoxyestrone	0.282221709
gene-MGAT3	3.076954518	1-Oleoyl-sn-glycero-3-phosphocholine	0.18926952
gene-MGAT3	3.076954518	Psychosine	0.106475396
gene-MGAT3	3.076954518	(-)-alpha-Terpineol	0.345716279
gene-MGAT3	3.076954518	1,4-Undecadiene	0.462803973
gene-MGAT3	3.076954518	4-cholesten-7伪,12伪,24-triol-3-one	0.097840451
gene-MGAT3	3.076954518	4-Dimethylamino-L-phenylalanine	0.242110226
gene-MGAT3	3.076954518	Armillane	0.52808635
gene-MGAT3	3.076954518	11-Dehydro-2,3-dinor-txb2	0.307258797
gene-MGAT3	3.076954518	2-(2-Aminopropanoylamino)bicyclo[3.1.0]he:	1.149807904
gene-MGAT3	3.076954518	Sambutoxin	0.169786377
gene-MGAT3	3.076954518	1-Palmitoylglycerol	0.084357408
gene-MGAT3	3.076954518	n-methyl-2-(4'-methylaminophenyl)-6-hydr	0.26655714
gene-MGAT3	3.076954518	Isopropyl isothiocyanate	0.172385747
gene-MGAT3	3.076954518	9-deoxy-9-methylene-16,16-dimethyl -PGE:	0.606893884
gene-LOC515276	3.071223421	3-Thiacytidine	0.209387412
gene-LOC515276	3.071223421	D-Erythro-imidazole-glycerol-phosphate	0.355812431
gene-LOC515276	3.071223421	LTB4-d4	0.226799982
gene-LOC515276	3.071223421	cis-p-Menth-2-en-1-ol	0.201017988
gene-LOC515276	3.071223421	(-)-alpha-Terpineol	0.345716279
gene-LOC515276	3.071223421	5-(2-Aminopropyl)-2-methylphenol	0.323231457
gene-LOC515276	3.071223421	4-Dimethylamino-L-phenylalanine	0.242110226
gene-LOC515276	3.071223421	Armillane	0.52808635
gene-LOC515276	3.071223421	2-Methyl-3-phenyl-2-propenal	0.407813975
gene-LOC515276	3.071223421	PC(P-18:1(11Z)/PGE2)	0.509503133
gene-LOC515276	3.071223421	(3R,4R)-3-Amino-1-hydroxy-4-methylpyrrol	0.471914506
gene-LOC515276	3.071223421	Isopropyl isothiocyanate	0.172385747
gene-LOC515276	3.071223421	(9Z)-Octadecenoic acid	0.142359556
gene-LOC515276	3.071223421	N-Myristoyl Glutamine	0.37856966
gene-LOC515276	3.071223421	PC(18:1(9Z)/15:1(9Z))	0.534724682
gene-LOC515276	3.071223421	arachidyl amido cholanoic acid	1.24842952
gene-LOC515276	3.071223421	Nigroxanthin	0.705005059
gene-LOC515276	3.071223421	PC(P-18:1(11Z)/PGJ2)	0.565243877
gene-LOC515276	3.071223421	PS(20:0/20:4(8Z,11Z,14Z,17Z)-2OH(5S,6R))	0.402595921
gene-LOC515276	3.071223421	CL(8:0/8:0/18:2(9Z,11Z)/20:0)	0.622988418
gene-COL7A1	3.02478168	3-Thiacytidine	0.209387412
gene-COL7A1	3.02478168	7(14)-Bisabolene-2,3,10,11-tetrol	2.446897974
gene-COL7A1	3.02478168	3-Deoxyestrone	0.282221709
gene-COL7A1	3.02478168	1-Oleoyl-sn-glycero-3-phosphocholine	0.18926952
gene-COL7A1	3.02478168	Psychosine	0.106475396
gene-COL7A1	3.02478168	(-)-alpha-Terpineol	0.345716279
gene-COL7A1	3.02478168	1,4-Undecadiene	0.462803973
gene-COL7A1	3.02478168	4-cholesten-7伪,12伪,24-triol-3-one	0.097840451
gene-COL7A1	3.02478168	5-(2-Aminopropyl)-2-methylphenol	0.323231457
gene-COL7A1	3.02478168	4-Dimethylamino-L-phenylalanine	0.242110226
gene-COL7A1	3.02478168	Trimetazidine	0.438282534
gene-COL7A1	3.02478168	ingenol	0.942071652
gene-COL7A1	3.02478168	Armillane	0.52808635

gene-COL7A1	3.02478168	PC(P-18:1(11Z)/PGE2)	0.509503133
gene-COL7A1	3.02478168	(3R,4R)-3-Amino-1-hydroxy-4-methylpyrrol	0.471914506
gene-COL7A1	3.02478168	1-Palmitoylglycerol	0.084357408
gene-COL7A1	3.02478168	n-methyl-2-(4'-methylaminophenyl)-6-hydr	0.26655714
gene-COL7A1	3.02478168	Isopropyl isothiocyanate	0.172385747
gene-COL7A1	3.02478168	9-deoxy-9-methylene-16,16-dimethyl -PGE	0.606893884
gene-COL7A1	3.02478168	CL(8:0/8:0/18:2(9Z,11Z)/20:0)	0.622988418
Bos_taurus_newG	3.023124336	Methylmalonate	0.241481249
Bos_taurus_newG	3.023124336	PE-NMe(18:0/18:3(9Z,12Z,15Z))	0.681884774
Bos_taurus_newG	3.023124336	CDP-DG(18:0/PGE2)	0.280573668
Bos_taurus_newG	3.023124336	3-Thiacytidine	0.209387412
Bos_taurus_newG	3.023124336	1-Phenylpiperazine	0.353584896
Bos_taurus_newG	3.023124336	5-Hydroxy-2-oxo-4-ureido-2,5-dihydro-1H	0.122536033
Bos_taurus_newG	3.023124336	PE(20:0/18:1(12Z)-2OH(9,10))	0.438658253
Bos_taurus_newG	3.023124336	PE(P-18:0/PGE1)	0.886597382
Bos_taurus_newG	3.023124336	PE(16:1(9Z)/18:0)	0.077115313
Bos_taurus_newG	3.023124336	PE(22:2(13Z,16Z)/22:5(4Z,7Z,10Z,13Z,19Z)-O	0.369745166
Bos_taurus_newG	3.023124336	Cyclosporin A	0.656529229
Bos_taurus_newG	3.023124336	Angiotensin A	0.247332017
Bos_taurus_newG	3.023124336	Epomusenin A	0.766206049
Bos_taurus_newG	3.023124336	PA(22:6(4Z,7Z,10Z,13Z,16Z,19Z)/16:0)	0.556532497
Bos_taurus_newG	3.023124336	DG(18:0/LTE4/0:0)	0.681485773
Bos_taurus_newG	3.023124336	PC(17:0/PGJ2)	0.657689319
Bos_taurus_newG	3.023124336	PC(14:0/20:2(11Z,14Z))	1.110657378
Bos_taurus_newG	3.023124336	CDP-DG(PGF2alpha/16:0)	1.002512277
Bos_taurus_newG	3.023124336	5-(2-Aminopropyl)-2-methylphenol	0.323231457
Bos_taurus_newG	3.023124336	1-beta-D-Arabinofuranosyl-5-fluorocytosine	0.152339645
Bos_taurus_newG	3.023124336	C20914	0.140383181
Bos_taurus_newG	3.023124336	S-(2-Hydroxyethyl)glutathione	0.269671835
Bos_taurus_newG	3.023124336	Norophthalmic acid	0.191432411
Bos_taurus_newG	3.023124336	6-hydroxymethyl-7,8-dihydropterin	0.190396346
Bos_taurus_newG	3.023124336	4-Oxo-9-cis-retinoyl-beta-glucuronide	1.611773742
Bos_taurus_newG	3.023124336	L-Histidinol	0.149249286
Bos_taurus_newG	3.023124336	beta-L-Dioxolane-cytidine	0.175916668
Bos_taurus_newG	3.023124336	DG(2:0/18:1(12Z)-O(9S,10R)/0:0)	0.033180989
Bos_taurus_newG	3.023124336	(4-Methylphenyl)acetaldehyde	0.208360133
Bos_taurus_newG	3.023124336	N-Palmitoyl Proline	0.470367693
Bos_taurus_newG	3.023124336	10-alpha-methoxy-9,10-dihydrolysergol	0.140851438
Bos_taurus_newG	3.023124336	17-Aminogeldanamycin	0.518144528
Bos_taurus_newG	3.023124336	MG(LTE4/0:0/0:0)	0.548257353
Bos_taurus_newG	3.023124336	PC(P-18:1(11Z)/PGE2)	0.509503133
Bos_taurus_newG	3.023124336	PC(18:1(9Z)/15:1(9Z))	0.534724682
Bos_taurus_newG	3.023124336	arachidyl amido cholanoic acid	1.24842952
Bos_taurus_newG	3.023124336	Nigroxanthin	0.705005059
Bos_taurus_newG	3.023124336	1-Octadecanoyl-2-(7Z,10Z,13Z,16Z-docosat	0.690496314
Bos_taurus_newG	3.023124336	PC(P-18:1(11Z)/PGJ2)	0.565243877
Bos_taurus_newG	3.023124336	PS(20:0/20:4(8Z,11Z,14Z,17Z)-2OH(5S,6R))	0.402595921
Bos_taurus_newG	3.023124336	DG(20:0/LTE4/0:0)	0.438074508
gene-B3GAT1	2.927236907	D-Erythro-imidazole-glycerol-phosphate	0.355812431
gene-B3GAT1	2.927236907	Tsugarioside B	1.013262893
gene-B3GAT1	2.927236907	7(14)-Bisabolene-2,3,10,11-tetrol	2.446897974

gene-B3GAT1	2.927236907	3-Deoxyestrone	0.282221709
gene-B3GAT1	2.927236907	1-Oleoyl-sn-glycero-3-phosphocholine	0.18926952
gene-B3GAT1	2.927236907	Psychosine	0.106475396
gene-B3GAT1	2.927236907	cis-p-Menth-2-en-1-ol	0.201017988
gene-B3GAT1	2.927236907	(-)-alpha-Terpineol	0.345716279
gene-B3GAT1	2.927236907	11-Oxahexadecanolide	2.378626329
gene-B3GAT1	2.927236907	1,4-Undecadiene	0.462803973
gene-B3GAT1	2.927236907	2-isopentyl-3,6-dimethyl pyrazine	0.710502562
gene-B3GAT1	2.927236907	PG(20:1(11Z)/18:3(10,12,15)-OH(9))	0.626244347
gene-B3GAT1	2.927236907	5-(2-Aminopropyl)-2-methylphenol	0.323231457
gene-B3GAT1	2.927236907	4-Dimethylamino-L-phenylalanine	0.242110226
gene-B3GAT1	2.927236907	Armillane	0.52808635
gene-B3GAT1	2.927236907	PC(P-18:1(11Z)/PGE2)	0.509503133
gene-B3GAT1	2.927236907	(3R,4R)-3-Amino-1-hydroxy-4-methylpyrrol	0.471914506
gene-B3GAT1	2.927236907	1-Palmitoylglycerol	0.084357408
gene-B3GAT1	2.927236907	n-methyl-2-(4'-methylaminophenyl)-6-hydr	0.26655714
gene-B3GAT1	2.927236907	Isopropyl isothiocyanate	0.172385747
gene-B3GAT1	2.927236907	9-deoxy-9-methylene-16,16-dimethyl -PGE	0.606893884
gene-FBP1	2.890556368	3-Thiacytidine	0.209387412
gene-FBP1	2.890556368	3-Deoxyestrone	0.282221709
gene-FBP1	2.890556368	1-Oleoyl-sn-glycero-3-phosphocholine	0.18926952
gene-FBP1	2.890556368	Psychosine	0.106475396
gene-FBP1	2.890556368	(-)-alpha-Terpineol	0.345716279
gene-FBP1	2.890556368	1,4-Undecadiene	0.462803973
gene-FBP1	2.890556368	4-cholesten-7伪,12伪,24-triol-3-one	0.097840451
gene-FBP1	2.890556368	5-(2-Aminopropyl)-2-methylphenol	0.323231457
gene-FBP1	2.890556368	4-Dimethylamino-L-phenylalanine	0.242110226
gene-FBP1	2.890556368	Trimetazidine	0.438282534
gene-FBP1	2.890556368	ingenol	0.942071652
gene-FBP1	2.890556368	Armillane	0.52808635
gene-FBP1	2.890556368	PC(P-18:1(11Z)/PGE2)	0.509503133
gene-FBP1	2.890556368	(3R,4R)-3-Amino-1-hydroxy-4-methylpyrrol	0.471914506
gene-FBP1	2.890556368	n-methyl-2-(4'-methylaminophenyl)-6-hydr	0.26655714
gene-FBP1	2.890556368	Isopropyl isothiocyanate	0.172385747
gene-FBP1	2.890556368	9-deoxy-9-methylene-16,16-dimethyl -PGE	0.606893884
gene-FBP1	2.890556368	PS(20:0/20:4(8Z,11Z,14Z,17Z)-2OH(5S,6R))	0.402595921
gene-FBP1	2.890556368	CL(8:0/8:0/18:2(9Z,11Z)/20:0)	0.622988418
gene-LOC11244	2.860384412	3-Thiacytidine	0.209387412
gene-LOC11244	2.860384412	D-Erythro-imidazole-glycerol-phosphate	0.355812431
gene-LOC11244	2.860384412	cis-p-Menth-2-en-1-ol	0.201017988
gene-LOC11244	2.860384412	PE(22:2(13Z,16Z)/22:5(4Z,7Z,10Z,13Z,19Z)-O	0.369745166
gene-LOC11244	2.860384412	PC(P-18:1(11Z)/PGE2)	0.509503133
gene-LOC11244	2.860384412	Roxithromycin	0.268273077
gene-LOC11244	2.860384412	PC(P-18:1(11Z)/PGJ2)	0.565243877
gene-LOC11244	2.860384412	PS(20:0/20:4(8Z,11Z,14Z,17Z)-2OH(5S,6R))	0.402595921
gene-CEBPE	2.842841126	3-Thiacytidine	0.209387412
gene-CEBPE	2.842841126	3-Deoxyestrone	0.282221709
gene-CEBPE	2.842841126	1-Oleoyl-sn-glycero-3-phosphocholine	0.18926952
gene-CEBPE	2.842841126	Psychosine	0.106475396
gene-CEBPE	2.842841126	LTB4-d4	0.226799982
gene-CEBPE	2.842841126	(-)-alpha-Terpineol	0.345716279

gene-CEBPE	2.842841126	1,4-Undecadiene	0.462803973
gene-CEBPE	2.842841126	5-(2-Aminopropyl)-2-methylphenol	0.323231457
gene-CEBPE	2.842841126	4-Dimethylamino-L-phenylalanine	0.242110226
gene-CEBPE	2.842841126	Trimetazidine	0.438282534
gene-CEBPE	2.842841126	ingenol	0.942071652
gene-CEBPE	2.842841126	Armillane	0.52808635
gene-CEBPE	2.842841126	PC(P-18:1(11Z)/PGE2)	0.509503133
gene-CEBPE	2.842841126	(3R,4R)-3-Amino-1-hydroxy-4-methylpyrrol	0.471914506
gene-CEBPE	2.842841126	n-methyl-2-(4'-methylaminophenyl)-6-hydr	0.26655714
gene-CEBPE	2.842841126	Isopropyl isothiocyanate	0.172385747
gene-CEBPE	2.842841126	(9Z)-Octadecenoic acid	0.142359556
gene-CEBPE	2.842841126	arachidyl amido cholanoic acid	1.24842952
gene-CEBPE	2.842841126	9-deoxy-9-methylene-16,16-dimethyl -PGE	0.606893884
gene-CEBPE	2.842841126	CL(8:0/8:0/18:2(9Z,11Z)/20:0)	0.622988418
gene-PADI3	2.835949139	3-Thiacytidine	0.209387412
gene-PADI3	2.835949139	3-Deoxyestrone	0.282221709
gene-PADI3	2.835949139	1-Oleoyl-sn-glycero-3-phosphocholine	0.18926952
gene-PADI3	2.835949139	Psychosine	0.106475396
gene-PADI3	2.835949139	(-)-alpha-Terpineol	0.345716279
gene-PADI3	2.835949139	1,4-Undecadiene	0.462803973
gene-PADI3	2.835949139	4-cholesten-7伪,12伪,24-triol-3-one	0.097840451
gene-PADI3	2.835949139	5-(2-Aminopropyl)-2-methylphenol	0.323231457
gene-PADI3	2.835949139	4-Dimethylamino-L-phenylalanine	0.242110226
gene-PADI3	2.835949139	Trimetazidine	0.438282534
gene-PADI3	2.835949139	ingenol	0.942071652
gene-PADI3	2.835949139	Armillane	0.52808635
gene-PADI3	2.835949139	PC(P-18:1(11Z)/PGE2)	0.509503133
gene-PADI3	2.835949139	(3R,4R)-3-Amino-1-hydroxy-4-methylpyrrol	0.471914506
gene-PADI3	2.835949139	n-methyl-2-(4'-methylaminophenyl)-6-hydr	0.26655714
gene-PADI3	2.835949139	Isopropyl isothiocyanate	0.172385747
gene-PADI3	2.835949139	9-deoxy-9-methylene-16,16-dimethyl -PGE	0.606893884
gene-PADI3	2.835949139	CL(8:0/8:0/18:2(9Z,11Z)/20:0)	0.622988418
Bos_taurus_newG	2.82943771	3-Thiacytidine	0.209387412
Bos_taurus_newG	2.82943771	cis-p-Menth-2-en-1-ol	0.201017988
Bos_taurus_newG	2.82943771	PE(20:0/18:1(12Z)-2OH(9,10))	0.438658253
Bos_taurus_newG	2.82943771	5-(2-Aminopropyl)-2-methylphenol	0.323231457
Bos_taurus_newG	2.82943771	Armillane	0.52808635
Bos_taurus_newG	2.82943771	PC(P-18:1(11Z)/PGE2)	0.509503133
Bos_taurus_newG	2.82943771	(3R,4R)-3-Amino-1-hydroxy-4-methylpyrrol	0.471914506
Bos_taurus_newG	2.82943771	Isopropyl isothiocyanate	0.172385747
Bos_taurus_newG	2.82943771	PC(18:1(9Z)/15:1(9Z))	0.534724682
Bos_taurus_newG	2.82943771	arachidyl amido cholanoic acid	1.24842952
Bos_taurus_newG	2.82943771	PC(P-18:1(11Z)/PGJ2)	0.565243877
Bos_taurus_newG	2.82943771	PS(20:0/20:4(8Z,11Z,14Z,17Z)-2OH(5S,6R))	0.402595921
Bos_taurus_newG	2.82943771	CL(8:0/8:0/18:2(9Z,11Z)/20:0)	0.622988418
gene-TDRD9	2.813890646	Methylmalonate	0.241481249
gene-TDRD9	2.813890646	PE-NMe(18:0/18:3(9Z,12Z,15Z))	0.681884774
gene-TDRD9	2.813890646	CDP-DG(18:0/PGE2)	0.280573668
gene-TDRD9	2.813890646	PG(18:1(11Z)/LTE4)	0.265299982
gene-TDRD9	2.813890646	LysoPI(16:0/0:0)	0.379098336
gene-TDRD9	2.813890646	3-Thiacytidine	0.209387412

gene-TDRD9	2.813890646	Methyl methacrylate	0.245645579
gene-TDRD9	2.813890646	1-Phenylpiperazine	0.353584896
gene-TDRD9	2.813890646	5-Hydroxyindoleacetaldehyde	0.448806354
gene-TDRD9	2.813890646	Azelaic acid	0.007180175
gene-TDRD9	2.813890646	Uridine 2',3'-cyclic phosphate	0.201589726
gene-TDRD9	2.813890646	5-Hydroxy-2-oxo-4-ureido-2,5-dihydro-1H	0.122536033
gene-TDRD9	2.813890646	Trihomomethionine	0.296210774
gene-TDRD9	2.813890646	Hygromycin B	0.930396619
gene-TDRD9	2.813890646	Metkephamid	1.159767003
gene-TDRD9	2.813890646	3,4-Dihydro-6-methoxy-2,2-dimethyl-2H-1	0.296379892
gene-TDRD9	2.813890646	2-Hexylbenzothiazole	0.396099297
gene-TDRD9	2.813890646	Valnemulin	0.408062514
gene-TDRD9	2.813890646	6-isobutyl-4-hydroxy-2-pyrone	0.473678412
gene-TDRD9	2.813890646	LTB4-d4	0.226799982
gene-TDRD9	2.813890646	cis-p-Menth-2-en-1-ol	0.201017988
gene-TDRD9	2.813890646	PE(20:0/18:1(12Z)-2OH(9,10))	0.438658253
gene-TDRD9	2.813890646	PE(18:0/18:1(9Z)-O(12,13))	1.009186776
gene-TDRD9	2.813890646	PE(P-18:0/PGE1)	0.886597382
gene-TDRD9	2.813890646	4,6-Heneicosanedione	0.113496143
gene-TDRD9	2.813890646	PC(P-16:0/18:1(12Z)-2OH(9,10))	1.231706326
gene-TDRD9	2.813890646	PE(22:2(13Z,16Z)/22:5(4Z,7Z,10Z,13Z,19Z)-O(	0.369745166
gene-TDRD9	2.813890646	DG(15:0/PGE1/0:0)	0.462019057
gene-TDRD9	2.813890646	Angiotensin A	0.247332017
gene-TDRD9	2.813890646	Epomusenin A	0.766206049
gene-TDRD9	2.813890646	PA(22:6(4Z,7Z,10Z,13Z,16Z,19Z)/16:0)	0.556532497
gene-TDRD9	2.813890646	DG(18:0/LTE4/0:0)	0.681485773
gene-TDRD9	2.813890646	PC(17:0/PGJ2)	0.657689319
gene-TDRD9	2.813890646	PC(14:0/20:2(11Z,14Z))	1.110657378
gene-TDRD9	2.813890646	CDP-DG(PGF2alpha/16:0)	1.002512277
gene-TDRD9	2.813890646	Lamivudine	0.327402892
gene-TDRD9	2.813890646	1-beta-D-Arabinofuranosyl-5-fluorocytosine	0.152339645
gene-TDRD9	2.813890646	Cysteinyl-Glutamine	0.079050155
gene-TDRD9	2.813890646	Pseudouridine 5'-phosphate	1.18431378
gene-TDRD9	2.813890646	15-keto-Prostaglandin E2	2.646629404
gene-TDRD9	2.813890646	Ribavirin monophosphate	1.091085027
gene-TDRD9	2.813890646	4-Hydroxyproline galactoside	0.148083825
gene-TDRD9	2.813890646	C20914	0.140383181
gene-TDRD9	2.813890646	S-(2-Hydroxyethyl)glutathione	0.269671835
gene-TDRD9	2.813890646	Fluridone	0.127106682
gene-TDRD9	2.813890646	Austdiol	0.046757893
gene-TDRD9	2.813890646	Norophthalmic acid	0.191432411
gene-TDRD9	2.813890646	4'-Thiothymidine	0.066948524
gene-TDRD9	2.813890646	Desmethylflumazenil (Ro 15-5528)	0.227256571
gene-TDRD9	2.813890646	6-Fluorohomovanillic acid	0.040947314
gene-TDRD9	2.813890646	kainic acid	0.836445456
gene-TDRD9	2.813890646	5,6,7,8-Tetrahydromonapterin	0.724075531
gene-TDRD9	2.813890646	Harmalol	0.364945111
gene-TDRD9	2.813890646	Guanidoacetic acid	0.542509265
gene-TDRD9	2.813890646	4-Oxo-9-cis-retinoyl-beta-glucuronide	1.611773742
gene-TDRD9	2.813890646	alpha-Terpineol formate	0.628238986
gene-TDRD9	2.813890646	Methionyl-Valine	0.502707745

gene-TDRD9	2.813890646	(3Z,6Z)-3,6-Nonadien-1-ol	0.185378696
gene-TDRD9	2.813890646	14alpha-Hydroxy-5beta-cholest-7-ene-3,6-	0.206315646
gene-TDRD9	2.813890646	DG(2:0/18:1(12Z)-O(9S,10R)/0:0)	0.033180989
gene-TDRD9	2.813890646	2-Phenylpropyl acetate	0.202302943
gene-TDRD9	2.813890646	LysoPE(0:0/22:5(4Z,7Z,10Z,13Z,16Z))	0.418671396
gene-TDRD9	2.813890646	Austalide L	0.4229014
gene-TDRD9	2.813890646	Spergualin	0.210690914
gene-TDRD9	2.813890646	2,3-Dihydroxypropyl octanoate	0.242447284
gene-TDRD9	2.813890646	N-Palmitoyl Proline	0.470367693
gene-TDRD9	2.813890646	10-alpha-methoxy-9,10-dihydrolysergol	0.140851438
gene-TDRD9	2.813890646	Ethyl salicylate	0.099640862
gene-TDRD9	2.813890646	Cichorioside J	0.191008611
gene-TDRD9	2.813890646	MG(LTE4/0:0/0:0)	0.548257353
gene-TDRD9	2.813890646	N-Stearoyl Glutamine	1.862695384
gene-TDRD9	2.813890646	Glutaminylphenylalanine	0.224405311
gene-TDRD9	2.813890646	N-Stearoyl Proline	0.469971646
gene-TDRD9	2.813890646	Galabiosylceramide (d18:1/20:0)	0.746737323
gene-TDRD9	2.813890646	LysoPI(0:0/18:0)	0.420441153
gene-TDRD9	2.813890646	Hypaconitine	0.176000431
gene-TDRD9	2.813890646	L-(-)Sorbose	0.168270222
gene-TDRD9	2.813890646	N-Palmitoyl Glutamic acid	0.250188721
gene-TDRD9	2.813890646	Cer(d18:2(4E,14Z)/TXB2)	0.236013333
gene-TDRD9	2.813890646	norerythromycin	0.401118778
gene-TDRD9	2.813890646	CDP-DG(a-25:0/PGF1alpha)	0.345292815
gene-TDRD9	2.813890646	N-Myristoyl Glutamine	0.37856966
gene-TDRD9	2.813890646	PC(18:1(9Z)/15:1(9Z))	0.534724682
gene-TDRD9	2.813890646	arachidyl amido cholanoic acid	1.24842952
gene-TDRD9	2.813890646	Nigroxanthin	0.705005059
gene-TDRD9	2.813890646	PE(P-18:0/20:5(5Z,8Z,11Z,14Z,16E)-OH(18))	0.339204508
gene-TDRD9	2.813890646	1-Octadecanoyl-2-(7Z,10Z,13Z,16Z-docosat	0.690496314
gene-TDRD9	2.813890646	PE(18:0/20:4(8Z,11Z,14Z,17Z)-2OH(5S,6R))	0.418208781
gene-TDRD9	2.813890646	CE(LTE4)	0.204616463
gene-TDRD9	2.813890646	PC(P-18:1(11Z)/PGJ2)	0.565243877
gene-TDRD9	2.813890646	PS(20:0/20:4(8Z,11Z,14Z,17Z)-2OH(5S,6R))	0.402595921
gene-TDRD9	2.813890646	PE-NMe(18:2(9Z,12Z)/18:2(9Z,12Z))[U]	0.694736593
gene-TDRD9	2.813890646	PS(16:1(9Z)/22:2(13Z,16Z))	0.659652661
gene-TDRD9	2.813890646	PC(P-16:0/20:3(8Z,11Z,14Z)-2OH(5,6))	0.174122169
gene-TDRD9	2.813890646	DG(20:0/LTE4/0:0)	0.438074508
gene-PKNOX2	2.812129619	3-Thiacytidine	0.209387412
gene-PKNOX2	2.812129619	Cyclosporin A	0.656529229
gene-PKNOX2	2.812129619	DG(18:0/LTE4/0:0)	0.681485773
gene-PKNOX2	2.812129619	PC(14:0/20:2(11Z,14Z))	1.110657378
gene-PKNOX2	2.812129619	5-(2-Aminopropyl)-2-methylphenol	0.323231457
gene-PKNOX2	2.812129619	ingenol	0.942071652
gene-PKNOX2	2.812129619	beta-L-Dioxolane-cytidine	0.175916668
gene-PKNOX2	2.812129619	PC(P-18:1(11Z)/PGE2)	0.509503133
gene-PKNOX2	2.812129619	Isopropyl isothiocyanate	0.172385747
gene-PKNOX2	2.812129619	Nigroxanthin	0.705005059
gene-PKNOX2	2.812129619	D-Fructose	0.167584616
gene-PKNOX2	2.812129619	CL(8:0/8:0/18:2(9Z,11Z)/20:0)	0.622988418
gene-C11H9orf5(	2.80209425	3-Thiacytidine	0.209387412

gene-C11H9orf5(	2.80209425	7(14)-Bisabolene-2,3,10,11-tetrol	2.446897974
gene-C11H9orf5(	2.80209425	4-cholesten-7伪,12伪,24-triol-3-one	0.097840451
gene-C11H9orf5(	2.80209425	Isopropyl isothiocyanate	0.172385747
gene-C11H9orf5(	2.80209425	PS(20:0/20:4(8Z,11Z,14Z,17Z)-2OH(5S,6R))	0.402595921
gene-ZBTB47	2.800822463	3-Thiacytidine	0.209387412
gene-ZBTB47	2.800822463	(-)-alpha-Terpineol	0.345716279
gene-ZBTB47	2.800822463	1,4-Undecadiene	0.462803973
gene-ZBTB47	2.800822463	5-(2-Aminopropyl)-2-methylphenol	0.323231457
gene-ZBTB47	2.800822463	Armillane	0.52808635
gene-ZBTB47	2.800822463	PC(P-18:1(11Z)/PGE2)	0.509503133
gene-ZBTB47	2.800822463	Isopropyl isothiocyanate	0.172385747
gene-ZBTB47	2.800822463	PC(P-18:1(11Z)/PGJ2)	0.565243877
gene-ZBTB47	2.800822463	PS(20:0/20:4(8Z,11Z,14Z,17Z)-2OH(5S,6R))	0.402595921
gene-ZBTB47	2.800822463	CL(8:0/8:0/18:2(9Z,11Z)/20:0)	0.622988418
gene-ALOX5	2.79889835	3-Thiacytidine	0.209387412
gene-ALOX5	2.79889835	LTB4-d4	0.226799982
gene-ALOX5	2.79889835	(-)-alpha-Terpineol	0.345716279
gene-ALOX5	2.79889835	1,4-Undecadiene	0.462803973
gene-ALOX5	2.79889835	5-(2-Aminopropyl)-2-methylphenol	0.323231457
gene-ALOX5	2.79889835	Armillane	0.52808635
gene-ALOX5	2.79889835	PC(P-18:1(11Z)/PGE2)	0.509503133
gene-ALOX5	2.79889835	(3R,4R)-3-Amino-1-hydroxy-4-methylpyrrol	0.471914506
gene-ALOX5	2.79889835	Isopropyl isothiocyanate	0.172385747
gene-ALOX5	2.79889835	PC(P-18:1(11Z)/PGJ2)	0.565243877
gene-ALOX5	2.79889835	PS(20:0/20:4(8Z,11Z,14Z,17Z)-2OH(5S,6R))	0.402595921
gene-ALOX5	2.79889835	CL(8:0/8:0/18:2(9Z,11Z)/20:0)	0.622988418
gene-MPO	2.760577841	3-Thiacytidine	0.209387412
gene-MPO	2.760577841	13(S)-HpODE	0.163157753
gene-MPO	2.760577841	LTB4-d4	0.226799982
gene-MPO	2.760577841	Cyclosporin A	0.656529229
gene-MPO	2.760577841	DG(18:0/LTE4/0:0)	0.681485773
gene-MPO	2.760577841	PC(14:0/20:2(11Z,14Z))	1.110657378
gene-MPO	2.760577841	CDP-DG(PGF2alpha/16:0)	1.002512277
gene-MPO	2.760577841	beta-L-Dioxolane-cytidine	0.175916668
gene-MPO	2.760577841	PC(P-18:1(11Z)/PGE2)	0.509503133
gene-MPO	2.760577841	PC(20:3(5Z,8Z,11Z)/24:0)	0.387959564
gene-MPO	2.760577841	arachidyl amido cholanoic acid	1.24842952
gene-MPO	2.760577841	Nigroxanthin	0.705005059
gene-MPO	2.760577841	PC(P-18:1(11Z)/PGJ2)	0.565243877
gene-MPO	2.760577841	PS(20:0/20:4(8Z,11Z,14Z,17Z)-2OH(5S,6R))	0.402595921
gene-MPO	2.760577841	D-Fructose	0.167584616
gene-MPO	2.760577841	CL(8:0/8:0/18:2(9Z,11Z)/20:0)	0.622988418
gene-LOC508441	2.747052937	3-Thiacytidine	0.209387412
gene-LOC508441	2.747052937	7(14)-Bisabolene-2,3,10,11-tetrol	2.446897974
gene-LOC508441	2.747052937	1-Oleoyl-sn-glycero-3-phosphocholine	0.18926952
gene-LOC508441	2.747052937	(-)-alpha-Terpineol	0.345716279
gene-LOC508441	2.747052937	1,4-Undecadiene	0.462803973
gene-LOC508441	2.747052937	4-cholesten-7伪,12伪,24-triol-3-one	0.097840451
gene-LOC508441	2.747052937	5-(2-Aminopropyl)-2-methylphenol	0.323231457
gene-LOC508441	2.747052937	4-Dimethylamino-L-phenylalanine	0.242110226
gene-LOC508441	2.747052937	Armillane	0.52808635

gene-LOC508441	2.747052937	PC(P-18:1(11Z)/PGE2)	0.509503133
gene-LOC508441	2.747052937	(3R,4R)-3-Amino-1-hydroxy-4-methylpyrrol	0.471914506
gene-LOC508441	2.747052937	n-methyl-2-(4'-methylaminophenyl)-6-hydr	0.26655714
gene-LOC508441	2.747052937	Isopropyl isothiocyanate	0.172385747
gene-LOC508441	2.747052937	PS(20:0/20:4(8Z,11Z,14Z,17Z)-2OH(5S,6R))	0.402595921
gene-LOC508441	2.747052937	CL(8:0/8:0/18:2(9Z,11Z)/20:0)	0.622988418
gene-PPP1R3C	2.735663895	Isomaltotriose	0.58434654
gene-PPP1R3C	2.735663895	1-(2-Furanyl)-1-pentanone	0.697147283
gene-PPP1R3C	2.735663895	2-Dehydro-3-deoxy-D-gluconate	0.65165299
gene-PPP1R3C	2.735663895	(-)-alpha-Terpineol	0.345716279
gene-PPP1R3C	2.735663895	1,4-Undecadiene	0.462803973
gene-PPP1R3C	2.735663895	4-cholesten-7伪,12伪,24-triol-3-one	0.097840451
gene-PPP1R3C	2.735663895	N-[[3-Hydroxy-2-(2-pentenyl)cyclopentyl]ac	1.043930947
gene-LOC100297	2.729207027	3-Thiacytidine	0.209387412
gene-LOC100297	2.729207027	3-Deoxyestrone	0.282221709
gene-LOC100297	2.729207027	1-Oleoyl-sn-glycero-3-phosphocholine	0.18926952
gene-LOC100297	2.729207027	Psychosine	0.106475396
gene-LOC100297	2.729207027	LTB4-d4	0.226799982
gene-LOC100297	2.729207027	(-)-alpha-Terpineol	0.345716279
gene-LOC100297	2.729207027	1,4-Undecadiene	0.462803973
gene-LOC100297	2.729207027	5-(2-Aminopropyl)-2-methylphenol	0.323231457
gene-LOC100297	2.729207027	4-Dimethylamino-L-phenylalanine	0.242110226
gene-LOC100297	2.729207027	Trimetazidine	0.438282534
gene-LOC100297	2.729207027	ingenol	0.942071652
gene-LOC100297	2.729207027	Armillane	0.52808635
gene-LOC100297	2.729207027	PC(P-18:1(11Z)/PGE2)	0.509503133
gene-LOC100297	2.729207027	(3R,4R)-3-Amino-1-hydroxy-4-methylpyrrol	0.471914506
gene-LOC100297	2.729207027	n-methyl-2-(4'-methylaminophenyl)-6-hydr	0.26655714
gene-LOC100297	2.729207027	Isopropyl isothiocyanate	0.172385747
gene-LOC100297	2.729207027	(9Z)-Octadecenoic acid	0.142359556
gene-LOC100297	2.729207027	9-deoxy-9-methylene-16,16-dimethyl -PGE	0.606893884
gene-LOC100297	2.729207027	CL(8:0/8:0/18:2(9Z,11Z)/20:0)	0.622988418
Bos_taurus_newG	2.718767341	3-Thiacytidine	0.209387412
Bos_taurus_newG	2.718767341	D-Erythro-imidazole-glycerol-phosphate	0.355812431
Bos_taurus_newG	2.718767341	13(S)-HpODE	0.163157753
Bos_taurus_newG	2.718767341	LTB4-d4	0.226799982
Bos_taurus_newG	2.718767341	cis-p-Menth-2-en-1-ol	0.201017988
Bos_taurus_newG	2.718767341	(-)-alpha-Terpineol	0.345716279
Bos_taurus_newG	2.718767341	4-Dimethylamino-L-phenylalanine	0.242110226
Bos_taurus_newG	2.718767341	2-Methyl-3-phenyl-2-propenal	0.407813975
Bos_taurus_newG	2.718767341	PC(P-18:1(11Z)/PGE2)	0.509503133
Bos_taurus_newG	2.718767341	(3R,4R)-3-Amino-1-hydroxy-4-methylpyrrol	0.471914506
Bos_taurus_newG	2.718767341	Isopropyl isothiocyanate	0.172385747
Bos_taurus_newG	2.718767341	(9Z)-Octadecenoic acid	0.142359556
Bos_taurus_newG	2.718767341	N-Myristoyl Glutamine	0.37856966
Bos_taurus_newG	2.718767341	PC(P-18:1(11Z)/PGJ2)	0.565243877
Bos_taurus_newG	2.718767341	PS(20:0/20:4(8Z,11Z,14Z,17Z)-2OH(5S,6R))	0.402595921
gene-CRTAC1	2.702109469	3-Thiacytidine	0.209387412
gene-CRTAC1	2.702109469	3-Deoxyestrone	0.282221709
gene-CRTAC1	2.702109469	1-Oleoyl-sn-glycero-3-phosphocholine	0.18926952
gene-CRTAC1	2.702109469	LTB4-d4	0.226799982

gene-CRTAC1	2.702109469	(-)-alpha-Terpineol	0.345716279
gene-CRTAC1	2.702109469	1,4-Undecadiene	0.462803973
gene-CRTAC1	2.702109469	5-(2-Aminopropyl)-2-methylphenol	0.323231457
gene-CRTAC1	2.702109469	4-Dimethylamino-L-phenylalanine	0.242110226
gene-CRTAC1	2.702109469	Trimetazidine	0.438282534
gene-CRTAC1	2.702109469	ingenol	0.942071652
gene-CRTAC1	2.702109469	Armillane	0.52808635
gene-CRTAC1	2.702109469	PC(P-18:1(11Z)/PGE2)	0.509503133
gene-CRTAC1	2.702109469	(3R,4R)-3-Amino-1-hydroxy-4-methylpyrrol	0.471914506
gene-CRTAC1	2.702109469	n-methyl-2-(4'-methylaminophenyl)-6-hydr	0.26655714
gene-CRTAC1	2.702109469	Isopropyl isothiocyanate	0.172385747
gene-CRTAC1	2.702109469	(9Z)-Octadecenoic acid	0.142359556
gene-CRTAC1	2.702109469	9-deoxy-9-methylene-16,16-dimethyl -PGE	0.606893884
gene-CRTAC1	2.702109469	PS(20:0/20:4(8Z,11Z,14Z,17Z)-2OH(5S,6R))	0.402595921
gene-CRTAC1	2.702109469	CL(8:0/8:0/18:2(9Z,11Z)/20:0)	0.622988418
Bos_taurus_newG	2.684814401	3-Thiacytidine	0.209387412
Bos_taurus_newG	2.684814401	11-Maleimidoundecanoic acid	1.084942397
Bos_taurus_newG	2.684814401	PE(22:2(13Z,16Z)/22:5(4Z,7Z,10Z,13Z,19Z)-O	0.369745166
Bos_taurus_newG	2.684814401	Cyclosporin A	0.656529229
Bos_taurus_newG	2.684814401	3b,6a-Dihydroxy-alpha-ionol 9-[apiosyl-(1-	0.045258177
Bos_taurus_newG	2.684814401	beta-L-Dioxolane-cytidine	0.175916668
Bos_taurus_newG	2.684814401	PC(P-18:1(11Z)/PGE2)	0.509503133
Bos_taurus_newG	2.684814401	Phorone A	0.079039095
Bos_taurus_newG	2.684814401	Nigroxanthin	0.705005059
Bos_taurus_newG	2.684814401	PC(P-18:1(11Z)/PGJ2)	0.565243877
Bos_taurus_newG	2.684814401	PS(20:0/20:4(8Z,11Z,14Z,17Z)-2OH(5S,6R))	0.402595921
Bos_taurus_newG	2.684814401	D-Fructose	0.167584616
gene-LOC618541	2.674229948	3-Thiacytidine	0.209387412
gene-LOC618541	2.674229948	D-Erythro-imidazole-glycerol-phosphate	0.355812431
gene-LOC618541	2.674229948	LTB4-d4	0.226799982
gene-LOC618541	2.674229948	(-)-alpha-Terpineol	0.345716279
gene-LOC618541	2.674229948	1,4-Undecadiene	0.462803973
gene-LOC618541	2.674229948	Cyclosporin A	0.656529229
gene-LOC618541	2.674229948	5-(2-Aminopropyl)-2-methylphenol	0.323231457
gene-LOC618541	2.674229948	4-Dimethylamino-L-phenylalanine	0.242110226
gene-LOC618541	2.674229948	Armillane	0.52808635
gene-LOC618541	2.674229948	PC(P-18:1(11Z)/PGE2)	0.509503133
gene-LOC618541	2.674229948	(3R,4R)-3-Amino-1-hydroxy-4-methylpyrrol	0.471914506
gene-LOC618541	2.674229948	Isopropyl isothiocyanate	0.172385747
gene-LOC618541	2.674229948	PC(P-18:1(11Z)/PGJ2)	0.565243877
gene-LOC618541	2.674229948	PS(20:0/20:4(8Z,11Z,14Z,17Z)-2OH(5S,6R))	0.402595921
gene-LOC618541	2.674229948	CL(8:0/8:0/18:2(9Z,11Z)/20:0)	0.622988418
gene-SFXN5	2.655262819	3-Thiacytidine	0.209387412
gene-SFXN5	2.655262819	milbemycin beta3	1.414447124
gene-SFXN5	2.655262819	3-Deoxyestrone	0.282221709
gene-SFXN5	2.655262819	1-Oleoyl-sn-glycero-3-phosphocholine	0.18926952
gene-SFXN5	2.655262819	Psychosine	0.106475396
gene-SFXN5	2.655262819	(-)-alpha-Terpineol	0.345716279
gene-SFXN5	2.655262819	5-(2-Aminopropyl)-2-methylphenol	0.323231457
gene-SFXN5	2.655262819	4-Dimethylamino-L-phenylalanine	0.242110226
gene-SFXN5	2.655262819	Trimetazidine	0.438282534

gene-SFXN5	2.655262819	ingenol	0.942071652
gene-SFXN5	2.655262819	Armillane	0.52808635
gene-SFXN5	2.655262819	PC(P-18:1(11Z)/PGE2)	0.509503133
gene-SFXN5	2.655262819	(3R,4R)-3-Amino-1-hydroxy-4-methylpyrrol	0.471914506
gene-SFXN5	2.655262819	n-methyl-2-(4'-methylaminophenyl)-6-hydr	0.26655714
gene-SFXN5	2.655262819	Isopropyl isothiocyanate	0.172385747
gene-SFXN5	2.655262819	(9Z)-Octadecenoic acid	0.142359556
gene-SFXN5	2.655262819	arachidyl amido cholanoic acid	1.24842952
gene-SFXN5	2.655262819	9-deoxy-9-methylene-16,16-dimethyl -PGE	0.606893884
gene-SFXN5	2.655262819	PS(20:0/20:4(8Z,11Z,14Z,17Z)-2OH(5S,6R))	0.402595921
gene-SFXN5	2.655262819	CL(8:0/8:0/18:2(9Z,11Z)/20:0)	0.622988418
gene-SYT8	2.617456529	Tsugarioside B	1.013262893
gene-SYT8	2.617456529	7(14)-Bisabolene-2,3,10,11-tetrol	2.446897974
gene-SYT8	2.617456529	3-Deoxyestrone	0.282221709
gene-SYT8	2.617456529	1-Oleoyl-sn-glycero-3-phosphocholine	0.18926952
gene-SYT8	2.617456529	3,4-dihydroxy-5-all-trans-hexaprenylbenzoa	0.123615726
gene-SYT8	2.617456529	Lividamine	0.319679555
gene-SYT8	2.617456529	Psychosine	0.106475396
gene-SYT8	2.617456529	Cyclotricuspidogenin C	0.440884085
gene-SYT8	2.617456529	(-)-alpha-Terpineol	0.345716279
gene-SYT8	2.617456529	1,4-Undecadiene	0.462803973
gene-SYT8	2.617456529	2-isopentyl-3,6-dimethyl pyrazine	0.710502562
gene-SYT8	2.617456529	5-(2-Aminopropyl)-2-methylphenol	0.323231457
gene-SYT8	2.617456529	4-Dimethylamino-L-phenylalanine	0.242110226
gene-SYT8	2.617456529	ingenol	0.942071652
gene-SYT8	2.617456529	Armillane	0.52808635
gene-SYT8	2.617456529	PC(P-18:1(11Z)/PGE2)	0.509503133
gene-SYT8	2.617456529	(3R,4R)-3-Amino-1-hydroxy-4-methylpyrrol	0.471914506
gene-SYT8	2.617456529	1-Palmitoylglycerol	0.084357408
gene-SYT8	2.617456529	n-methyl-2-(4'-methylaminophenyl)-6-hydr	0.26655714
gene-SYT8	2.617456529	Isopropyl isothiocyanate	0.172385747
gene-SYT8	2.617456529	9-deoxy-9-methylene-16,16-dimethyl -PGE	0.606893884
gene-SYT8	2.617456529	CL(8:0/8:0/18:2(9Z,11Z)/20:0)	0.622988418
gene-LOC50532	2.57917565	5-(Ethylthio)-1H-tetrazole	0.29717344
gene-LOC50532	2.57917565	3-Thiacytidine	0.209387412
gene-LOC50532	2.57917565	milbemycin beta3	1.414447124
gene-LOC50532	2.57917565	Tsugarioside B	1.013262893
gene-LOC50532	2.57917565	Tetracosenoyl-CoA	0.187187233
gene-LOC50532	2.57917565	1,2-O-Isopropylidene-D-glucofuranose	0.080987667
gene-LOC50532	2.57917565	3-Deoxyestrone	0.282221709
gene-LOC50532	2.57917565	1-Oleoyl-sn-glycero-3-phosphocholine	0.18926952
gene-LOC50532	2.57917565	3,4-dihydroxy-5-all-trans-hexaprenylbenzoa	0.123615726
gene-LOC50532	2.57917565	Psychosine	0.106475396
gene-LOC50532	2.57917565	Cyclotricuspidogenin C	0.440884085
gene-LOC50532	2.57917565	(-)-alpha-Terpineol	0.345716279
gene-LOC50532	2.57917565	2-isopentyl-3,6-dimethyl pyrazine	0.710502562
gene-LOC50532	2.57917565	PC(14:1(9Z)/P-18:1(11Z))	0.153578899
gene-LOC50532	2.57917565	PC(16:0/18:1(12Z)-2OH(9,10))	0.461843233
gene-LOC50532	2.57917565	5-(2-Aminopropyl)-2-methylphenol	0.323231457
gene-LOC50532	2.57917565	4-Dimethylamino-L-phenylalanine	0.242110226
gene-LOC50532	2.57917565	Trimetazidine	0.438282534

gene-LOC50532€	2.57917565	ingenol	0.942071652
gene-LOC50532€	2.57917565	Armillane	0.52808635
gene-LOC50532€	2.57917565	5'-S-Methyl-5'-thioinosine	0.28772476
gene-LOC50532€	2.57917565	PC(P-18:1(11Z)/PGE2)	0.509503133
gene-LOC50532€	2.57917565	(3R,4R)-3-Amino-1-hydroxy-4-methylpyrrol	0.471914506
gene-LOC50532€	2.57917565	n-methyl-2-(4'-methylaminophenyl)-6-hydr	0.26655714
gene-LOC50532€	2.57917565	PE(P-16:0/18:4(6Z,9Z,12Z,15Z))	0.546989864
gene-LOC50532€	2.57917565	Isopropyl isothiocyanate	0.172385747
gene-LOC50532€	2.57917565	(9Z)-Octadecenoic acid	0.142359556
gene-LOC50532€	2.57917565	arachidyl amido cholanoic acid	1.24842952
gene-LOC50532€	2.57917565	9-deoxy-9-methylene-16,16-dimethyl -PGE	0.606893884
gene-LOC50532€	2.57917565	1-Octadecanoyl-2-(7Z,10Z,13Z,16Z-docosat	0.690496314
gene-LOC50532€	2.57917565	2-Propenyl 2-aminobenzoate	0.128406829
gene-LOC50532€	2.57917565	CL(8:0/8:0/18:2(9Z,11Z)/20:0)	0.622988418
gene-CUEDC1	2.565084388	3-Thiacytidine	0.209387412
gene-CUEDC1	2.565084388	LTB4-d4	0.226799982
gene-CUEDC1	2.565084388	(-)-alpha-Terpineol	0.345716279
gene-CUEDC1	2.565084388	Cyclosporin A	0.656529229
gene-CUEDC1	2.565084388	DG(18:0/LTE4/0:0)	0.681485773
gene-CUEDC1	2.565084388	PC(14:0/20:2(11Z,14Z))	1.110657378
gene-CUEDC1	2.565084388	5-(2-Aminopropyl)-2-methylphenol	0.323231457
gene-CUEDC1	2.565084388	ingenol	0.942071652
gene-CUEDC1	2.565084388	Armillane	0.52808635
gene-CUEDC1	2.565084388	PC(P-18:1(11Z)/PGE2)	0.509503133
gene-CUEDC1	2.565084388	(3R,4R)-3-Amino-1-hydroxy-4-methylpyrrol	0.471914506
gene-CUEDC1	2.565084388	Isopropyl isothiocyanate	0.172385747
gene-CUEDC1	2.565084388	(9Z)-Octadecenoic acid	0.142359556
gene-CUEDC1	2.565084388	arachidyl amido cholanoic acid	1.24842952
gene-CUEDC1	2.565084388	Nigroxanthin	0.705005059
gene-CUEDC1	2.565084388	PC(P-18:1(11Z)/PGJ2)	0.565243877
gene-CUEDC1	2.565084388	PS(20:0/20:4(8Z,11Z,14Z,17Z)-2OH(5S,6R))	0.402595921
gene-CUEDC1	2.565084388	CL(8:0/8:0/18:2(9Z,11Z)/20:0)	0.622988418
Bos_taurus_newG	2.541881429	3-Thiacytidine	0.209387412
Bos_taurus_newG	2.541881429	7(14)-Bisabolene-2,3,10,11-tetrol	2.446897974
Bos_taurus_newG	2.541881429	(-)-alpha-Terpineol	0.345716279
Bos_taurus_newG	2.541881429	1,4-Undecadiene	0.462803973
Bos_taurus_newG	2.541881429	5-(2-Aminopropyl)-2-methylphenol	0.323231457
Bos_taurus_newG	2.541881429	4-Dimethylamino-L-phenylalanine	0.242110226
Bos_taurus_newG	2.541881429	Armillane	0.52808635
Bos_taurus_newG	2.541881429	PC(P-18:1(11Z)/PGE2)	0.509503133
Bos_taurus_newG	2.541881429	1-Palmitoylglycerol	0.084357408
Bos_taurus_newG	2.541881429	Isopropyl isothiocyanate	0.172385747
Bos_taurus_newG	2.541881429	PS(20:0/20:4(8Z,11Z,14Z,17Z)-2OH(5S,6R))	0.402595921
Bos_taurus_newG	2.541881429	CL(8:0/8:0/18:2(9Z,11Z)/20:0)	0.622988418
gene-CACNB3	2.539596587	3-Thiacytidine	0.209387412
gene-CACNB3	2.539596587	D-Erythro-imidazole-glycerol-phosphate	0.355812431
gene-CACNB3	2.539596587	7(14)-Bisabolene-2,3,10,11-tetrol	2.446897974
gene-CACNB3	2.539596587	1,4-Undecadiene	0.462803973
gene-CACNB3	2.539596587	Armillane	0.52808635
gene-CACNB3	2.539596587	PC(P-18:1(11Z)/PGE2)	0.509503133
gene-CACNB3	2.539596587	Isopropyl isothiocyanate	0.172385747

gene-CACNB3	2.539596587	PC(P-18:1(11Z)/PGJ2)	0.565243877
gene-CACNB3	2.539596587	PS(20:0/20:4(8Z,11Z,14Z,17Z)-2OH(5S,6R))	0.402595921
gene-GPR4	2.4938735	3-Thiacytidine	0.209387412
gene-GPR4	2.4938735	7(14)-Bisabolene-2,3,10,11-tetrol	2.446897974
gene-GPR4	2.4938735	3-Deoxyestrone	0.282221709
gene-GPR4	2.4938735	1-Oleoyl-sn-glycero-3-phosphocholine	0.18926952
gene-GPR4	2.4938735	(-)-alpha-Terpineol	0.345716279
gene-GPR4	2.4938735	1,4-Undecadiene	0.462803973
gene-GPR4	2.4938735	5-(2-Aminopropyl)-2-methylphenol	0.323231457
gene-GPR4	2.4938735	4-Dimethylamino-L-phenylalanine	0.242110226
gene-GPR4	2.4938735	Trimetazidine	0.438282534
gene-GPR4	2.4938735	ingenol	0.942071652
gene-GPR4	2.4938735	Armillane	0.52808635
gene-GPR4	2.4938735	PC(P-18:1(11Z)/PGE2)	0.509503133
gene-GPR4	2.4938735	(3R,4R)-3-Amino-1-hydroxy-4-methylpyrrol	0.471914506
gene-GPR4	2.4938735	1-Palmitoylglycerol	0.084357408
gene-GPR4	2.4938735	n-methyl-2-(4'-methylaminophenyl)-6-hydr	0.26655714
gene-GPR4	2.4938735	Isopropyl isothiocyanate	0.172385747
gene-GPR4	2.4938735	9-deoxy-9-methylene-16,16-dimethyl -PGE	0.606893884
gene-GPR4	2.4938735	CL(8:0/8:0/18:2(9Z,11Z)/20:0)	0.622988418
gene-KCTD15	2.468940288	3-Thiacytidine	0.209387412
gene-KCTD15	2.468940288	Tsugarioside B	1.013262893
gene-KCTD15	2.468940288	7(14)-Bisabolene-2,3,10,11-tetrol	2.446897974
gene-KCTD15	2.468940288	3-Deoxyestrone	0.282221709
gene-KCTD15	2.468940288	1-Oleoyl-sn-glycero-3-phosphocholine	0.18926952
gene-KCTD15	2.468940288	Psychosine	0.106475396
gene-KCTD15	2.468940288	(-)-alpha-Terpineol	0.345716279
gene-KCTD15	2.468940288	1,4-Undecadiene	0.462803973
gene-KCTD15	2.468940288	2-isopentyl-3,6-dimethyl pyrazine	0.710502562
gene-KCTD15	2.468940288	5-(2-Aminopropyl)-2-methylphenol	0.323231457
gene-KCTD15	2.468940288	4-Dimethylamino-L-phenylalanine	0.242110226
gene-KCTD15	2.468940288	Trimetazidine	0.438282534
gene-KCTD15	2.468940288	ingenol	0.942071652
gene-KCTD15	2.468940288	Armillane	0.52808635
gene-KCTD15	2.468940288	PC(P-18:1(11Z)/PGE2)	0.509503133
gene-KCTD15	2.468940288	(3R,4R)-3-Amino-1-hydroxy-4-methylpyrrol	0.471914506
gene-KCTD15	2.468940288	1-Palmitoylglycerol	0.084357408
gene-KCTD15	2.468940288	n-methyl-2-(4'-methylaminophenyl)-6-hydr	0.26655714
gene-KCTD15	2.468940288	Glutethimide	0.126237229
gene-KCTD15	2.468940288	Isopropyl isothiocyanate	0.172385747
gene-KCTD15	2.468940288	9-deoxy-9-methylene-16,16-dimethyl -PGE	0.606893884
gene-KCTD15	2.468940288	CL(8:0/8:0/18:2(9Z,11Z)/20:0)	0.622988418
gene-C6H4orf19	2.466577996	3-Thiacytidine	0.209387412
gene-C6H4orf19	2.466577996	D-Erythro-imidazole-glycerol-phosphate	0.355812431
gene-C6H4orf19	2.466577996	LTB4-d4	0.226799982
gene-C6H4orf19	2.466577996	cis-p-Menth-2-en-1-ol	0.201017988
gene-C6H4orf19	2.466577996	(-)-alpha-Terpineol	0.345716279
gene-C6H4orf19	2.466577996	5-(2-Aminopropyl)-2-methylphenol	0.323231457
gene-C6H4orf19	2.466577996	4-Dimethylamino-L-phenylalanine	0.242110226
gene-C6H4orf19	2.466577996	Armillane	0.52808635
gene-C6H4orf19	2.466577996	2-Methyl-3-phenyl-2-propenal	0.407813975

gene-C6H4orf19	2.466577996	PC(P-18:1(11Z)/PGE2)	0.509503133
gene-C6H4orf19	2.466577996	(3R,4R)-3-Amino-1-hydroxy-4-methylpyrrol	0.471914506
gene-C6H4orf19	2.466577996	Isopropyl isothiocyanate	0.172385747
gene-C6H4orf19	2.466577996	(9Z)-Octadecenoic acid	0.142359556
gene-C6H4orf19	2.466577996	N-Myristoyl Glutamine	0.37856966
gene-C6H4orf19	2.466577996	PC(18:1(9Z)/15:1(9Z))	0.534724682
gene-C6H4orf19	2.466577996	Nigroxanthin	0.705005059
gene-C6H4orf19	2.466577996	PC(P-18:1(11Z)/PGJ2)	0.565243877
gene-C6H4orf19	2.466577996	PS(20:0/20:4(8Z,11Z,14Z,17Z)-2OH(5S,6R))	0.402595921
gene-KRT42	2.432999816	5-(Ethylthio)-1H-tetrazole	0.29717344
gene-KRT42	2.432999816	Methylmalonate	0.241481249
gene-KRT42	2.432999816	PE-NMe(18:0/18:3(9Z,12Z,15Z))	0.681884774
gene-KRT42	2.432999816	3-Thiacytidine	0.209387412
gene-KRT42	2.432999816	1-Phenylpiperazine	0.353584896
gene-KRT42	2.432999816	Tetracosenoyl-CoA	0.187187233
gene-KRT42	2.432999816	1,2-O-Isopropylidene-D-glucofuranose	0.080987667
gene-KRT42	2.432999816	LTB4-d4	0.226799982
gene-KRT42	2.432999816	PE(18:0/18:1(9Z)-O(12,13))	1.009186776
gene-KRT42	2.432999816	PE(P-18:0/PGE1)	0.886597382
gene-KRT42	2.432999816	PC(P-16:0/18:1(12Z)-2OH(9,10))	1.231706326
gene-KRT42	2.432999816	Cyclosporin A	0.656529229
gene-KRT42	2.432999816	PA(22:6(4Z,7Z,10Z,13Z,16Z,19Z)/16:0)	0.556532497
gene-KRT42	2.432999816	DG(18:0/LTE4/0:0)	0.681485773
gene-KRT42	2.432999816	PC(17:0/PGJ2)	0.657689319
gene-KRT42	2.432999816	PC(14:1(9Z)/P-18:1(11Z))	0.153578899
gene-KRT42	2.432999816	PC(16:0/18:1(12Z)-2OH(9,10))	0.461843233
gene-KRT42	2.432999816	PC(14:0/20:2(11Z,14Z))	1.110657378
gene-KRT42	2.432999816	CDP-DG(PGF2alpha/16:0)	1.002512277
gene-KRT42	2.432999816	5-(2-Aminopropyl)-2-methylphenol	0.323231457
gene-KRT42	2.432999816	Trimetazidine	0.438282534
gene-KRT42	2.432999816	ingenol	0.942071652
gene-KRT42	2.432999816	Armillane	0.52808635
gene-KRT42	2.432999816	L-Histidinol	0.149249286
gene-KRT42	2.432999816	(4-Methylphenyl)acetaldehyde	0.208360133
gene-KRT42	2.432999816	17-Aminogeldanamycin	0.518144528
gene-KRT42	2.432999816	(3R,4R)-3-Amino-1-hydroxy-4-methylpyrrol	0.471914506
gene-KRT42	2.432999816	Gamithromycin	0.37168241
gene-KRT42	2.432999816	Cucurbitacin IIa	0.416650962
gene-KRT42	2.432999816	PE(P-16:0/18:4(6Z,9Z,12Z,15Z))	0.546989864
gene-KRT42	2.432999816	Isopropyl isothiocyanate	0.172385747
gene-KRT42	2.432999816	(9Z)-Octadecenoic acid	0.142359556
gene-KRT42	2.432999816	PS(O-20:0/14:1(9Z))	0.263612845
gene-KRT42	2.432999816	arachidyl amido cholanoic acid	1.24842952
gene-KRT42	2.432999816	AS 1-5	0.512683467
gene-KRT42	2.432999816	Nigroxanthin	0.705005059
gene-KRT42	2.432999816	9-deoxy-9-methylene-16,16-dimethyl -PGE:	0.606893884
gene-KRT42	2.432999816	3-Hydroxyheptadecanoylcarnitine	0.860642943
gene-KRT42	2.432999816	1-Octadecanoyl-2-(7Z,10Z,13Z,16Z-docosat	0.690496314
gene-KRT42	2.432999816	PC(16:0/20:5(5E,8E,11E,14E,17E))[U]	0.208077202
gene-KRT42	2.432999816	2-Propenyl 2-aminobenzoate	0.128406829
gene-KRT42	2.432999816	CL(8:0/8:0/18:2(9Z,11Z)/20:0)	0.622988418

gene-KRT42	2.432999816	PC(20:5(5Z,8Z,11Z,14Z,17Z)/P-16:0)	1.301095986
gene-KRT42	2.432999816	DG(20:0/LTE4/0:0)	0.438074508
gene-LOC10496ξ	2.431566331	3-Thiacytidine	0.209387412
gene-LOC10496ξ	2.431566331	LTB4-d4	0.226799982
gene-LOC10496ξ	2.431566331	PE(20:0/18:1(12Z)-2OH(9,10))	0.438658253
gene-LOC10496ξ	2.431566331	(-)-alpha-Terpineol	0.345716279
gene-LOC10496ξ	2.431566331	Cyclosporin A	0.656529229
gene-LOC10496ξ	2.431566331	DG(18:0/LTE4/0:0)	0.681485773
gene-LOC10496ξ	2.431566331	PC(14:0/20:2(11Z,14Z))	1.110657378
gene-LOC10496ξ	2.431566331	5-(2-Aminopropyl)-2-methylphenol	0.323231457
gene-LOC10496ξ	2.431566331	ingenol	0.942071652
gene-LOC10496ξ	2.431566331	Armillane	0.52808635
gene-LOC10496ξ	2.431566331	PC(P-18:1(11Z)/PGE2)	0.509503133
gene-LOC10496ξ	2.431566331	(3R,4R)-3-Amino-1-hydroxy-4-methylpyrrol	0.471914506
gene-LOC10496ξ	2.431566331	Isopropyl isothiocyanate	0.172385747
gene-LOC10496ξ	2.431566331	(9Z)-Octadecenoic acid	0.142359556
gene-LOC10496ξ	2.431566331	PC(18:1(9Z)/15:1(9Z))	0.534724682
gene-LOC10496ξ	2.431566331	arachidyl amido cholanoic acid	1.24842952
gene-LOC10496ξ	2.431566331	Nigroxanthin	0.705005059
gene-LOC10496ξ	2.431566331	1-Octadecanoyl-2-(7Z,10Z,13Z,16Z-docosat	0.690496314
gene-LOC10496ξ	2.431566331	PC(P-18:1(11Z)/PGJ2)	0.565243877
gene-LOC10496ξ	2.431566331	PS(20:0/20:4(8Z,11Z,14Z,17Z)-2OH(5S,6R))	0.402595921
gene-LOC10496ξ	2.431566331	CL(8:0/8:0/18:2(9Z,11Z)/20:0)	0.622988418
Bos_taurus_newG	2.418725189	Methylmalonate	0.241481249
Bos_taurus_newG	2.418725189	PE-NMe(18:0/18:3(9Z,12Z,15Z))	0.681884774
Bos_taurus_newG	2.418725189	CDP-DG(18:0/PGE2)	0.280573668
Bos_taurus_newG	2.418725189	PG(18:1(11Z)/LTE4)	0.265299982
Bos_taurus_newG	2.418725189	3-Thiacytidine	0.209387412
Bos_taurus_newG	2.418725189	1-Phenylpiperazine	0.353584896
Bos_taurus_newG	2.418725189	Coenzyme F420-1	0.507686022
Bos_taurus_newG	2.418725189	Azelaic acid	0.007180175
Bos_taurus_newG	2.418725189	milbemycin beta3	1.414447124
Bos_taurus_newG	2.418725189	Tetracosenoyl-CoA	0.187187233
Bos_taurus_newG	2.418725189	Isoplumbagin	0.127417868
Bos_taurus_newG	2.418725189	PC(24:0/20:3(8Z,11Z,14Z)-2OH(5,6))	0.43567608
Bos_taurus_newG	2.418725189	LTB4-d4	0.226799982
Bos_taurus_newG	2.418725189	DG(19:0/PGJ2/0:0)	0.415213526
Bos_taurus_newG	2.418725189	PE(20:0/18:1(12Z)-2OH(9,10))	0.438658253
Bos_taurus_newG	2.418725189	PE(18:0/18:1(9Z)-O(12,13))	1.009186776
Bos_taurus_newG	2.418725189	PE(P-18:0/PGE1)	0.886597382
Bos_taurus_newG	2.418725189	PC(P-16:0/18:1(12Z)-2OH(9,10))	1.231706326
Bos_taurus_newG	2.418725189	(2S,3'S)-alpha-Amino-2-carboxy-5-oxo-1-p	0.080821898
Bos_taurus_newG	2.418725189	PI(18:1(11Z)/PGF1alpha)	0.243592581
Bos_taurus_newG	2.418725189	Cyclosporin A	0.656529229
Bos_taurus_newG	2.418725189	Angiotensin A	0.247332017
Bos_taurus_newG	2.418725189	Epomusenin A	0.766206049
Bos_taurus_newG	2.418725189	PA(22:6(4Z,7Z,10Z,13Z,16Z,19Z)/16:0)	0.556532497
Bos_taurus_newG	2.418725189	DG(18:0/LTE4/0:0)	0.681485773
Bos_taurus_newG	2.418725189	PC(17:0/PGJ2)	0.657689319
Bos_taurus_newG	2.418725189	PC(14:1(9Z)/P-18:1(11Z))	0.153578899
Bos_taurus_newG	2.418725189	Alisporivir	0.430769197

Bos_taurus_newG	2.418725189	PC(14:0/20:2(11Z,14Z))	1.110657378
Bos_taurus_newG	2.418725189	CDP-DG(PGF2alpha/16:0)	1.002512277
Bos_taurus_newG	2.418725189	(1S,2R)-1-C-(indol-3-yl)glycerol 3-phosphat	0.374678916
Bos_taurus_newG	2.418725189	5-(2-Aminopropyl)-2-methylphenol	0.323231457
Bos_taurus_newG	2.418725189	1-beta-D-Arabinofuranosyl-5-fluorocytosine	0.152339645
Bos_taurus_newG	2.418725189	gamma-Glutamylcysteinylserine	0.098057967
Bos_taurus_newG	2.418725189	2-Methyl-5-nitroimidazol-1-ylacetic acid	0.701441397
Bos_taurus_newG	2.418725189	Fluoroazomycin arabinoside	0.590955233
Bos_taurus_newG	2.418725189	Cysteinyl-Tryptophan	0.586270687
Bos_taurus_newG	2.418725189	C20914	0.140383181
Bos_taurus_newG	2.418725189	S-(2-Hydroxyethyl)glutathione	0.269671835
Bos_taurus_newG	2.418725189	L-Histidinol	0.149249286
Bos_taurus_newG	2.418725189	14alpha-Hydroxy-5beta-cholest-7-ene-3,6-	0.206315646
Bos_taurus_newG	2.418725189	DG(2:0/18:1(12Z)-O(9S,10R)/0:0)	0.033180989
Bos_taurus_newG	2.418725189	2-Phenylpropyl acetate	0.202302943
Bos_taurus_newG	2.418725189	2,3-Dihydroxypropyl octanoate	0.242447284
Bos_taurus_newG	2.418725189	N-Palmitoyl Proline	0.470367693
Bos_taurus_newG	2.418725189	Atorvastatin	0.291646019
Bos_taurus_newG	2.418725189	17-Aminogeldanamycin	0.518144528
Bos_taurus_newG	2.418725189	MG(LTE4/0:0/0:0)	0.548257353
Bos_taurus_newG	2.418725189	7-Amino-4-hydroxy-2-naphthalenesulfonic	0.189964427
Bos_taurus_newG	2.418725189	N-Stearoyl Proline	0.469971646
Bos_taurus_newG	2.418725189	Gamithromycin	0.37168241
Bos_taurus_newG	2.418725189	PE(P-16:0/18:4(6Z,9Z,12Z,15Z))	0.546989864
Bos_taurus_newG	2.418725189	Isopropyl isothiocyanate	0.172385747
Bos_taurus_newG	2.418725189	N-Palmitoyl Glutamic acid	0.250188721
Bos_taurus_newG	2.418725189	(9Z)-Octadecenoic acid	0.142359556
Bos_taurus_newG	2.418725189	Cer(d18:2(4E,14Z)/TXB2)	0.236013333
Bos_taurus_newG	2.418725189	PS(O-20:0/14:1(9Z))	0.263612845
Bos_taurus_newG	2.418725189	norerythromycin	0.401118778
Bos_taurus_newG	2.418725189	PC(18:1(9Z)/15:1(9Z))	0.534724682
Bos_taurus_newG	2.418725189	PC(18:0/20:4(5Z,8Z,11Z,14Z)-OH(20))	0.318846141
Bos_taurus_newG	2.418725189	Ciprostene	0.108082983
Bos_taurus_newG	2.418725189	arachidyl amido cholanoic acid	1.24842952
Bos_taurus_newG	2.418725189	AS 1-5	0.512683467
Bos_taurus_newG	2.418725189	Nigroxanthin	0.705005059
Bos_taurus_newG	2.418725189	3-Hydroxyheptadecanoylcarnitine	0.860642943
Bos_taurus_newG	2.418725189	PE(P-18:0/20:5(5Z,8Z,11Z,14Z,16E)-OH(18))	0.339204508
Bos_taurus_newG	2.418725189	1-Octadecanoyl-2-(7Z,10Z,13Z,16Z-docosat	0.690496314
Bos_taurus_newG	2.418725189	PC(16:0/20:5(5E,8E,11E,14E,17E))[U]	0.208077202
Bos_taurus_newG	2.418725189	PE(18:0/20:4(8Z,11Z,14Z,17Z)-2OH(5S,6R))	0.418208781
Bos_taurus_newG	2.418725189	CE(LTE4)	0.204616463
Bos_taurus_newG	2.418725189	2-Propenyl 2-aminobenzoate	0.128406829
Bos_taurus_newG	2.418725189	CL(8:0/8:0/18:2(9Z,11Z)/20:0)	0.622988418
Bos_taurus_newG	2.418725189	PC(20:5(5Z,8Z,11Z,14Z,17Z)/P-16:0)	1.301095986
Bos_taurus_newG	2.418725189	PE-NMe(18:2(9Z,12Z)/18:2(9Z,12Z))[U]	0.694736593
Bos_taurus_newG	2.418725189	DG(20:0/LTE4/0:0)	0.438074508
Bos_taurus_newG	2.387813901	3-hydroxypristanic acid	0.548515835
Bos_taurus_newG	2.387813901	Canesceol	0.686312122
Bos_taurus_newG	2.387813901	3-Deoxyestrone	0.282221709
Bos_taurus_newG	2.387813901	1-Oleoyl-sn-glycero-3-phosphocholine	0.18926952

Bos_taurus_newG	2.387813901	Psychosine	0.106475396
Bos_taurus_newG	2.387813901	Cyclotricuspidogenin C	0.440884085
Bos_taurus_newG	2.387813901	(-)-alpha-Terpineol	0.345716279
Bos_taurus_newG	2.387813901	Linoleamide	0.926003481
Bos_taurus_newG	2.387813901	1,4-Undecadiene	0.462803973
Bos_taurus_newG	2.387813901	4-cholesten-7伪,12伪,24-triol-3-one	0.097840451
Bos_taurus_newG	2.387813901	Trimetazidine	0.438282534
Bos_taurus_newG	2.387813901	ingenol	0.942071652
Bos_taurus_newG	2.387813901	Tyrosyl-Arginine	6.462631098
Bos_taurus_newG	2.387813901	Misoprostol	1.349270093
Bos_taurus_newG	2.387813901	Pluviatolide	1.068135579
Bos_taurus_newG	2.387813901	Carboprost methyl	1.74923433
Bos_taurus_newG	2.387813901	PC(20:3(5Z,8Z,11Z)/24:0)	0.387959564
Bos_taurus_newG	2.387813901	1-Palmitoylglycerol	0.084357408
Bos_taurus_newG	2.387813901	N2-gamma-Glutamylglutamine	0.230065499
Bos_taurus_newG	2.387813901	n-methyl-2-(4'-methylaminophenyl)-6-hydr	0.26655714
Bos_taurus_newG	2.387813901	Isopropyl isothiocyanate	0.172385747
Bos_taurus_newG	2.387813901	D-Fructose	0.167584616
Bos_taurus_newG	2.387813901	1-Methylnicotinamide	0.203956241
Bos_taurus_newG	2.387813901	CL(8:0/8:0/18:2(9Z,11Z)/20:0)	0.622988418
Bos_taurus_newG	2.38728215	LysoPI(16:0/0:0)	0.379098336
Bos_taurus_newG	2.38728215	3-Thiacytidine	0.209387412
Bos_taurus_newG	2.38728215	Isopropyl isothiocyanate	0.172385747
Bos_taurus_newG	2.38728215	PC(18:1(9Z)/15:1(9Z))	0.534724682
Bos_taurus_newG	2.38728215	PC(P-18:1(11Z)/PGJ2)	0.565243877
Bos_taurus_newG	2.38728215	PS(20:0/20:4(8Z,11Z,14Z,17Z)-2OH(5S,6R))	0.402595921
gene-LOC101906	2.373096814	Glutamate carbon	0.671444516
gene-LOC101906	2.373096814	11-Maleimidoundecanoic acid	1.084942397
gene-LOC101906	2.373096814	Monacolin L acid	1.060562098
gene-LOC101906	2.373096814	cis-p-Menth-2-en-1-ol	0.201017988
gene-LOC101906	2.373096814	PE(22:2(13Z,16Z)/22:5(4Z,7Z,10Z,13Z,19Z)-O	0.369745166
gene-LOC101906	2.373096814	Norophthalmic acid	0.191432411
gene-LOC101906	2.373096814	(1R,6S)-6-Amino-5-oxocyclohex-2-ene-1-c	0.154751123
gene-LOC101906	2.373096814	alpha-Terpineol formate	0.628238986
gene-LOC101906	2.373096814	PC(P-18:1(11Z)/PGE2)	0.509503133
gene-LOC101906	2.373096814	Roxithromycin	0.268273077
gene-LOC101906	2.373096814	PC(P-18:1(11Z)/PGJ2)	0.565243877
gene-LOC101906	2.373096814	PS(20:0/20:4(8Z,11Z,14Z,17Z)-2OH(5S,6R))	0.402595921
gene-LOC101906	2.373096814	PE(20:5(5Z,8Z,11Z,14Z,17Z)/18:0)	0.427962506
gene-GSC2	2.372468863	3-Thiacytidine	0.209387412
gene-GSC2	2.372468863	7(14)-Bisabolene-2,3,10,11-tetrol	2.446897974
gene-GSC2	2.372468863	3-Deoxyestrone	0.282221709
gene-GSC2	2.372468863	1-Oleoyl-sn-glycero-3-phosphocholine	0.18926952
gene-GSC2	2.372468863	(-)-alpha-Terpineol	0.345716279
gene-GSC2	2.372468863	1,4-Undecadiene	0.462803973
gene-GSC2	2.372468863	4-cholesten-7伪,12伪,24-triol-3-one	0.097840451
gene-GSC2	2.372468863	5-(2-Aminopropyl)-2-methylphenol	0.323231457
gene-GSC2	2.372468863	4-Dimethylamino-L-phenylalanine	0.242110226
gene-GSC2	2.372468863	ingenol	0.942071652
gene-GSC2	2.372468863	Armillane	0.52808635
gene-GSC2	2.372468863	PC(P-18:1(11Z)/PGE2)	0.509503133

gene-GSC2	2.372468863	(3R,4R)-3-Amino-1-hydroxy-4-methylpyrrol	0.471914506
gene-GSC2	2.372468863	n-methyl-2-(4'-methylaminophenyl)-6-hydr	0.26655714
gene-GSC2	2.372468863	Isopropyl isothiocyanate	0.172385747
gene-GSC2	2.372468863	9-deoxy-9-methylene-16,16-dimethyl -PGE	0.606893884
gene-GSC2	2.372468863	PS(20:0/20:4(8Z,11Z,14Z,17Z)-2OH(5S,6R))	0.402595921
gene-GSC2	2.372468863	CL(8:0/8:0/18:2(9Z,11Z)/20:0)	0.622988418
gene-LOC10013{	2.365401325	3-Thiacytidine	0.209387412
gene-LOC10013{	2.365401325	1-Oleoyl-sn-glycero-3-phosphocholine	0.18926952
gene-LOC10013{	2.365401325	LTB4-d4	0.226799982
gene-LOC10013{	2.365401325	(-)-alpha-Terpineol	0.345716279
gene-LOC10013{	2.365401325	1,4-Undecadiene	0.462803973
gene-LOC10013{	2.365401325	Cyclosporin A	0.656529229
gene-LOC10013{	2.365401325	5-(2-Aminopropyl)-2-methylphenol	0.323231457
gene-LOC10013{	2.365401325	4-Dimethylamino-L-phenylalanine	0.242110226
gene-LOC10013{	2.365401325	Trimetazidine	0.438282534
gene-LOC10013{	2.365401325	ingenol	0.942071652
gene-LOC10013{	2.365401325	Armillane	0.52808635
gene-LOC10013{	2.365401325	PC(P-18:1(11Z)/PGE2)	0.509503133
gene-LOC10013{	2.365401325	(3R,4R)-3-Amino-1-hydroxy-4-methylpyrrol	0.471914506
gene-LOC10013{	2.365401325	n-methyl-2-(4'-methylaminophenyl)-6-hydr	0.26655714
gene-LOC10013{	2.365401325	Isopropyl isothiocyanate	0.172385747
gene-LOC10013{	2.365401325	(9Z)-Octadecenoic acid	0.142359556
gene-LOC10013{	2.365401325	Nigroxanthin	0.705005059
gene-LOC10013{	2.365401325	9-deoxy-9-methylene-16,16-dimethyl -PGE	0.606893884
gene-LOC10013{	2.365401325	PS(20:0/20:4(8Z,11Z,14Z,17Z)-2OH(5S,6R))	0.402595921
gene-LOC10013{	2.365401325	CL(8:0/8:0/18:2(9Z,11Z)/20:0)	0.622988418
gene-CD300LB	2.336108141	3-Thiacytidine	0.209387412
gene-CD300LB	2.336108141	LTB4-d4	0.226799982
gene-CD300LB	2.336108141	(-)-alpha-Terpineol	0.345716279
gene-CD300LB	2.336108141	Cyclosporin A	0.656529229
gene-CD300LB	2.336108141	5-(2-Aminopropyl)-2-methylphenol	0.323231457
gene-CD300LB	2.336108141	ingenol	0.942071652
gene-CD300LB	2.336108141	Armillane	0.52808635
gene-CD300LB	2.336108141	PC(P-18:1(11Z)/PGE2)	0.509503133
gene-CD300LB	2.336108141	(3R,4R)-3-Amino-1-hydroxy-4-methylpyrrol	0.471914506
gene-CD300LB	2.336108141	Isopropyl isothiocyanate	0.172385747
gene-CD300LB	2.336108141	Nigroxanthin	0.705005059
gene-CD300LB	2.336108141	PC(P-18:1(11Z)/PGJ2)	0.565243877
gene-CD300LB	2.336108141	PS(20:0/20:4(8Z,11Z,14Z,17Z)-2OH(5S,6R))	0.402595921
gene-CD300LB	2.336108141	CL(8:0/8:0/18:2(9Z,11Z)/20:0)	0.622988418
Bos_taurus_newG	2.32580551	3-Thiacytidine	0.209387412
Bos_taurus_newG	2.32580551	5-Hydroxy-2-oxo-4-ureido-2,5-dihydro-1H	0.122536033
Bos_taurus_newG	2.32580551	11-Maleimidoundecanoic acid	1.084942397
Bos_taurus_newG	2.32580551	cis-p-Menth-2-en-1-ol	0.201017988
Bos_taurus_newG	2.32580551	PE(20:0/18:1(12Z)-2OH(9,10))	0.438658253
Bos_taurus_newG	2.32580551	PE(22:2(13Z,16Z)/22:5(4Z,7Z,10Z,13Z,19Z)-O	0.369745166
Bos_taurus_newG	2.32580551	Norophthalmic acid	0.191432411
Bos_taurus_newG	2.32580551	Guanidoacetic acid	0.542509265
Bos_taurus_newG	2.32580551	4-Oxo-9-cis-retinoyl-beta-glucuronide	1.611773742
Bos_taurus_newG	2.32580551	alpha-Terpineol formate	0.628238986
Bos_taurus_newG	2.32580551	Methionyl-Valine	0.502707745

Bos_taurus_newG	2.32580551	PC(P-18:1(11Z)/PGE2)	0.509503133
Bos_taurus_newG	2.32580551	Roxithromycin	0.268273077
Bos_taurus_newG	2.32580551	Nigroxanthin	0.705005059
Bos_taurus_newG	2.32580551	PC(P-18:1(11Z)/PGJ2)	0.565243877
Bos_taurus_newG	2.32580551	PS(20:0/20:4(8Z,11Z,14Z,17Z)-2OH(5S,6R))	0.402595921
Bos_taurus_newG	2.32580551	PE(20:5(5Z,8Z,11Z,14Z,17Z)/18:0)	0.427962506
gene-TM4SF5	2.321028187	3-Thiacytidine	0.209387412
gene-TM4SF5	2.321028187	LTB4-d4	0.226799982
gene-TM4SF5	2.321028187	Cyclosporin A	0.656529229
gene-TM4SF5	2.321028187	DG(18:0/LTE4/0:0)	0.681485773
gene-TM4SF5	2.321028187	PC(14:0/20:2(11Z,14Z))	1.110657378
gene-TM4SF5	2.321028187	5-(2-Aminopropyl)-2-methylphenol	0.323231457
gene-TM4SF5	2.321028187	Trimetazidine	0.438282534
gene-TM4SF5	2.321028187	ingenol	0.942071652
gene-TM4SF5	2.321028187	Armillane	0.52808635
gene-TM4SF5	2.321028187	PC(P-18:1(11Z)/PGE2)	0.509503133
gene-TM4SF5	2.321028187	(3R,4R)-3-Amino-1-hydroxy-4-methylpyrrol	0.471914506
gene-TM4SF5	2.321028187	Isopropyl isothiocyanate	0.172385747
gene-TM4SF5	2.321028187	PC(18:1(9Z)/15:1(9Z))	0.534724682
gene-TM4SF5	2.321028187	arachidyl amido cholanoic acid	1.24842952
gene-TM4SF5	2.321028187	Nigroxanthin	0.705005059
gene-TM4SF5	2.321028187	9-deoxy-9-methylene-16,16-dimethyl -PGE	0.606893884
gene-TM4SF5	2.321028187	1-Octadecanoyl-2-(7Z,10Z,13Z,16Z-docosat	0.690496314
gene-TM4SF5	2.321028187	CL(8:0/8:0/18:2(9Z,11Z)/20:0)	0.622988418
gene-PLCXD1	2.31918465	5-Sulfosalicylic acid	1.31509494
gene-PLCXD1	2.31918465	4-Hydroxybenzoate	0.892336321
gene-PLCXD1	2.31918465	Ethyl nicotinate	1.024858052
gene-PLCXD1	2.31918465	Dopaquinone	1.087504518
gene-PLCXD1	2.31918465	Isomaltotriose	0.58434654
gene-PLCXD1	2.31918465	Glutamate carbon	0.671444516
gene-PLCXD1	2.31918465	1-(2-Furanyl)-1-pentanone	0.697147283
gene-PLCXD1	2.31918465	2-Dehydro-3-deoxy-D-gluconate	0.65165299
gene-PLCXD1	2.31918465	MG(0:0/20:4(5Z,8Z,11Z,14Z)/0:0)	0.488916341
gene-PLCXD1	2.31918465	(14S)-14,15-Dihydroxy-8(17),13(16)-labdadie	0.720697404
gene-PLCXD1	2.31918465	Deoxyshikonin	0.734877521
gene-PLCXD1	2.31918465	Serotonin	2.521565841
gene-PLCXD1	2.31918465	TETRAHYDROURIDINE	0.576545276
gene-PLCXD1	2.31918465	beta-Thujaplicin	0.681866194
gene-PLCXD1	2.31918465	Yucalexin P15	1.079168162
Bos_taurus_newG	2.30040615	3-Thiacytidine	0.209387412
Bos_taurus_newG	2.30040615	7(14)-Bisabolene-2,3,10,11-tetrol	2.446897974
Bos_taurus_newG	2.30040615	1-Oleoyl-sn-glycero-3-phosphocholine	0.18926952
Bos_taurus_newG	2.30040615	Psychosine	0.106475396
Bos_taurus_newG	2.30040615	1,4-Undecadiene	0.462803973
Bos_taurus_newG	2.30040615	5-(2-Aminopropyl)-2-methylphenol	0.323231457
Bos_taurus_newG	2.30040615	4-Dimethylamino-L-phenylalanine	0.242110226
Bos_taurus_newG	2.30040615	Trimetazidine	0.438282534
Bos_taurus_newG	2.30040615	ingenol	0.942071652
Bos_taurus_newG	2.30040615	Armillane	0.52808635
Bos_taurus_newG	2.30040615	PC(P-18:1(11Z)/PGE2)	0.509503133
Bos_taurus_newG	2.30040615	(3R,4R)-3-Amino-1-hydroxy-4-methylpyrrol	0.471914506

Bos_taurus_newG	2.30040615	n-methyl-2-(4'-methylaminophenyl)-6-hydr	0.26655714
Bos_taurus_newG	2.30040615	Isopropyl isothiocyanate	0.172385747
Bos_taurus_newG	2.30040615	9-deoxy-9-methylene-16,16-dimethyl -PGE	0.606893884
Bos_taurus_newG	2.30040615	PS(20:0/20:4(8Z,11Z,14Z,17Z)-2OH(5S,6R))	0.402595921
Bos_taurus_newG	2.30040615	CL(8:0/8:0/18:2(9Z,11Z)/20:0)	0.622988418
gene-LOC511713	2.260805718	3-Thiacytidine	0.209387412
gene-LOC511713	2.260805718	LTB4-d4	0.226799982
gene-LOC511713	2.260805718	(-)-alpha-Terpineol	0.345716279
gene-LOC511713	2.260805718	Cyclosporin A	0.656529229
gene-LOC511713	2.260805718	5-(2-Aminopropyl)-2-methylphenol	0.323231457
gene-LOC511713	2.260805718	Armillane	0.52808635
gene-LOC511713	2.260805718	PC(P-18:1(11Z)/PGE2)	0.509503133
gene-LOC511713	2.260805718	(3R,4R)-3-Amino-1-hydroxy-4-methylpyrrol	0.471914506
gene-LOC511713	2.260805718	Isopropyl isothiocyanate	0.172385747
gene-LOC511713	2.260805718	Nigroxanthin	0.705005059
gene-LOC511713	2.260805718	PC(P-18:1(11Z)/PGJ2)	0.565243877
gene-LOC511713	2.260805718	PS(20:0/20:4(8Z,11Z,14Z,17Z)-2OH(5S,6R))	0.402595921
gene-LOC511713	2.260805718	CL(8:0/8:0/18:2(9Z,11Z)/20:0)	0.622988418
gene-LOC407145	2.244607794	(-)-alpha-Terpineol	0.345716279
gene-LOC407145	2.244607794	Cyclosporin A	0.656529229
gene-LOC407145	2.244607794	DG(18:0/LTE4/0:0)	0.681485773
gene-LOC407145	2.244607794	beta-L-Dioxolane-cytidine	0.175916668
gene-LOC407145	2.244607794	PC(P-18:1(11Z)/PGE2)	0.509503133
gene-LOC407145	2.244607794	PC(20:3(5Z,8Z,11Z)/24:0)	0.387959564
gene-LOC407145	2.244607794	Nigroxanthin	0.705005059
gene-LOC407145	2.244607794	D-Fructose	0.167584616
gene-LOC407145	2.244607794	CL(8:0/8:0/18:2(9Z,11Z)/20:0)	0.622988418
gene-SCN2B	2.23913487	3-hydroxypristanic acid	0.548515835
gene-SCN2B	2.23913487	Canesceol	0.686312122
gene-SCN2B	2.23913487	Tetracosenoyl-CoA	0.187187233
gene-SCN2B	2.23913487	16-hydroxy hexadecanoic acid	0.309000958
gene-SCN2B	2.23913487	1,2-O-Isopropylidene-D-glucofuranose	0.080987667
gene-SCN2B	2.23913487	3-Deoxyestrone	0.282221709
gene-SCN2B	2.23913487	1-Oleoyl-sn-glycero-3-phosphocholine	0.18926952
gene-SCN2B	2.23913487	3,4-dihydroxy-5-all-trans-hexaprenylbenzoa	0.123615726
gene-SCN2B	2.23913487	Lividamine	0.319679555
gene-SCN2B	2.23913487	Psychosine	0.106475396
gene-SCN2B	2.23913487	Cyclotricuspidogenin C	0.440884085
gene-SCN2B	2.23913487	(-)-alpha-Terpineol	0.345716279
gene-SCN2B	2.23913487	Linoleamide	0.926003481
gene-SCN2B	2.23913487	1,4-Undecadiene	0.462803973
gene-SCN2B	2.23913487	PC(16:0/18:1(12Z)-2OH(9,10))	0.461843233
gene-SCN2B	2.23913487	5-(2-Aminopropyl)-2-methylphenol	0.323231457
gene-SCN2B	2.23913487	4-Dimethylamino-L-phenylalanine	0.242110226
gene-SCN2B	2.23913487	Trimetazidine	0.438282534
gene-SCN2B	2.23913487	ingenol	0.942071652
gene-SCN2B	2.23913487	Armillane	0.52808635
gene-SCN2B	2.23913487	(3R,4R)-3-Amino-1-hydroxy-4-methylpyrrol	0.471914506
gene-SCN2B	2.23913487	1-Palmitoylglycerol	0.084357408
gene-SCN2B	2.23913487	N2-gamma-Glutamylglutamine	0.230065499
gene-SCN2B	2.23913487	n-methyl-2-(4'-methylaminophenyl)-6-hydr	0.26655714

gene-SCN2B	2.23913487	Glutethimide	0.126237229
gene-SCN2B	2.23913487	Isopropyl isothiocyanate	0.172385747
gene-SCN2B	2.23913487	9-deoxy-9-methylene-16,16-dimethyl -PGE2	0.606893884
gene-SCN2B	2.23913487	CL(8:0/8:0/18:2(9Z,11Z)/20:0)	0.622988418
gene-FBLL1	2.237345327	3-Thiacytidine	0.209387412
gene-FBLL1	2.237345327	11-Maleimidoundecanoic acid	1.084942397
gene-FBLL1	2.237345327	13(S)-HpODE	0.163157753
gene-FBLL1	2.237345327	LTB4-d4	0.226799982
gene-FBLL1	2.237345327	cis-p-Menth-2-en-1-ol	0.201017988
gene-FBLL1	2.237345327	PE(20:0/18:1(12Z)-2OH(9,10))	0.438658253
gene-FBLL1	2.237345327	Cyclosporin A	0.656529229
gene-FBLL1	2.237345327	DG(18:0/LTE4/0:0)	0.681485773
gene-FBLL1	2.237345327	PC(14:0/20:2(11Z,14Z))	1.110657378
gene-FBLL1	2.237345327	CDP-DG(PGF2alpha/16:0)	1.002512277
gene-FBLL1	2.237345327	2-Methyl-3-phenyl-2-propenal	0.407813975
gene-FBLL1	2.237345327	beta-L-Dioxolane-cytidine	0.175916668
gene-FBLL1	2.237345327	PC(P-18:1(11Z)/PGE2)	0.509503133
gene-FBLL1	2.237345327	(9Z)-Octadecenoic acid	0.142359556
gene-FBLL1	2.237345327	PC(18:1(9Z)/15:1(9Z))	0.534724682
gene-FBLL1	2.237345327	arachidyl amido cholanoic acid	1.24842952
gene-FBLL1	2.237345327	Nigroxanthin	0.705005059
gene-FBLL1	2.237345327	PC(P-18:1(11Z)/PGJ2)	0.565243877
gene-FBLL1	2.237345327	PS(20:0/20:4(8Z,11Z,14Z,17Z)-2OH(5S,6R))	0.402595921
gene-FBLL1	2.237345327	DG(20:0/LTE4/0:0)	0.438074508
gene-FAM107A	2.221492044	3-Thiacytidine	0.209387412
gene-FAM107A	2.221492044	7(14)-Bisabolene-2,3,10,11-tetrol	2.446897974
gene-FAM107A	2.221492044	1,4-Undecadiene	0.462803973
gene-FAM107A	2.221492044	5-(2-Aminopropyl)-2-methylphenol	0.323231457
gene-FAM107A	2.221492044	Armillane	0.52808635
gene-FAM107A	2.221492044	PC(P-18:1(11Z)/PGE2)	0.509503133
gene-FAM107A	2.221492044	1-Palmitoylglycerol	0.084357408
gene-FAM107A	2.221492044	Isopropyl isothiocyanate	0.172385747
gene-FAM107A	2.221492044	PS(20:0/20:4(8Z,11Z,14Z,17Z)-2OH(5S,6R))	0.402595921
gene-FAM107A	2.221492044	CL(8:0/8:0/18:2(9Z,11Z)/20:0)	0.622988418
gene-CDKN2B	2.220773231	3-Thiacytidine	0.209387412
gene-CDKN2B	2.220773231	7(14)-Bisabolene-2,3,10,11-tetrol	2.446897974
gene-CDKN2B	2.220773231	3-Deoxyestrone	0.282221709
gene-CDKN2B	2.220773231	1-Oleoyl-sn-glycero-3-phosphocholine	0.18926952
gene-CDKN2B	2.220773231	(-)-alpha-Terpineol	0.345716279
gene-CDKN2B	2.220773231	1,4-Undecadiene	0.462803973
gene-CDKN2B	2.220773231	4-cholesten-7伪,12伪,24-triol-3-one	0.097840451
gene-CDKN2B	2.220773231	5-(2-Aminopropyl)-2-methylphenol	0.323231457
gene-CDKN2B	2.220773231	4-Dimethylamino-L-phenylalanine	0.242110226
gene-CDKN2B	2.220773231	Armillane	0.52808635
gene-CDKN2B	2.220773231	PC(P-18:1(11Z)/PGE2)	0.509503133
gene-CDKN2B	2.220773231	(3R,4R)-3-Amino-1-hydroxy-4-methylpyrrol	0.471914506
gene-CDKN2B	2.220773231	n-methyl-2-(4'-methylaminophenyl)-6-hydr	0.26655714
gene-CDKN2B	2.220773231	Isopropyl isothiocyanate	0.172385747
gene-CDKN2B	2.220773231	PS(20:0/20:4(8Z,11Z,14Z,17Z)-2OH(5S,6R))	0.402595921
gene-CDKN2B	2.220773231	CL(8:0/8:0/18:2(9Z,11Z)/20:0)	0.622988418
gene-JAG2	2.217558442	(-)-alpha-Terpineol	0.345716279

gene-JAG2	2.217558442	1,4-Undecadiene	0.462803973
gene-JAG2	2.217558442	4-cholesten-7伪,12伪,24-triol-3-one	0.097840451
gene-JAG2	2.217558442	Cyclosporin A	0.656529229
gene-JAG2	2.217558442	ingenol	0.942071652
gene-JAG2	2.217558442	N-[[3-Hydroxy-2-(2-pentenyl)cyclopentyl]ac	1.043930947
gene-JAG2	2.217558442	beta-L-Dioxolane-cytidine	0.175916668
gene-JAG2	2.217558442	PC(P-18:1(11Z)/PGE2)	0.509503133
gene-JAG2	2.217558442	PC(20:3(5Z,8Z,11Z)/24:0)	0.387959564
gene-JAG2	2.217558442	Isopropyl isothiocyanate	0.172385747
gene-JAG2	2.217558442	D-Fructose	0.167584616
gene-JAG2	2.217558442	CL(8:0/8:0/18:2(9Z,11Z)/20:0)	0.622988418
gene-PAQR7	2.212166775	Proclavaminic acid	0.060659628
gene-PAQR7	2.212166775	7(14)-Bisabolene-2,3,10,11-tetrol	2.446897974
gene-PAQR7	2.212166775	3-Deoxyestrone	0.282221709
gene-PAQR7	2.212166775	1-Oleoyl-sn-glycero-3-phosphocholine	0.18926952
gene-PAQR7	2.212166775	3,4-dihydroxy-5-all-trans-hexaprenylbenzoa	0.123615726
gene-PAQR7	2.212166775	Psychosine	0.106475396
gene-PAQR7	2.212166775	1,4-Undecadiene	0.462803973
gene-PAQR7	2.212166775	2-isopentyl-3,6-dimethyl pyrazine	0.710502562
gene-PAQR7	2.212166775	PC(16:0/18:1(12Z)-2OH(9,10))	0.461843233
gene-PAQR7	2.212166775	5-(2-Aminopropyl)-2-methylphenol	0.323231457
gene-PAQR7	2.212166775	4-Dimethylamino-L-phenylalanine	0.242110226
gene-PAQR7	2.212166775	Trimetazidine	0.438282534
gene-PAQR7	2.212166775	ingenol	0.942071652
gene-PAQR7	2.212166775	Armillane	0.52808635
gene-PAQR7	2.212166775	(3R,4R)-3-Amino-1-hydroxy-4-methylpyrrol	0.471914506
gene-PAQR7	2.212166775	1-Palmitoylglycerol	0.084357408
gene-PAQR7	2.212166775	n-methyl-2-(4'-methylaminophenyl)-6-hydr	0.26655714
gene-PAQR7	2.212166775	Glutethimide	0.126237229
gene-PAQR7	2.212166775	Isopropyl isothiocyanate	0.172385747
gene-PAQR7	2.212166775	9-deoxy-9-methylene-16,16-dimethyl -PGE	0.606893884
gene-PAQR7	2.212166775	CL(8:0/8:0/18:2(9Z,11Z)/20:0)	0.622988418
Bos_taurus_newG	2.207542374	3-Thiacytidine	0.209387412
Bos_taurus_newG	2.207542374	Isomaltotriose	0.58434654
Bos_taurus_newG	2.207542374	Cholic acid glucuronide	0.147398811
Bos_taurus_newG	2.207542374	11-Maleimidoundecanoic acid	1.084942397
Bos_taurus_newG	2.207542374	13(S)-HpODE	0.163157753
Bos_taurus_newG	2.207542374	PE(22:2(13Z,16Z)/22:5(4Z,7Z,10Z,13Z,19Z)-O	0.369745166
Bos_taurus_newG	2.207542374	3b,6a-Dihydroxy-alpha-ionol 9-[apiosyl-(1-	0.045258177
Bos_taurus_newG	2.207542374	3-(3-Methylbutylidene)-1(3H)-isobenzofurar	0.575422414
Bos_taurus_newG	2.207542374	beta-L-Dioxolane-cytidine	0.175916668
Bos_taurus_newG	2.207542374	PC(P-18:1(11Z)/PGE2)	0.509503133
Bos_taurus_newG	2.207542374	Phorone A	0.079039095
Bos_taurus_newG	2.207542374	Roxithromycin	0.268273077
Bos_taurus_newG	2.207542374	PC(P-18:1(11Z)/PGJ2)	0.565243877
Bos_taurus_newG	2.207542374	PS(20:0/20:4(8Z,11Z,14Z,17Z)-2OH(5S,6R))	0.402595921
gene-KCNK12	2.190315961	Cyclosporin A	0.656529229
gene-KCNK12	2.190315961	DG(18:0/LTE4/0:0)	0.681485773
gene-KCNK12	2.190315961	PC(16:0/18:1(12Z)-2OH(9,10))	0.461843233
gene-KCNK12	2.190315961	5-(2-Aminopropyl)-2-methylphenol	0.323231457
gene-KCNK12	2.190315961	L-Histidinol	0.149249286

gene-KCNK12	2.190315961	beta-L-Dioxolane-cytidine	0.175916668
gene-KCNK12	2.190315961	(4-Methylphenyl)acetaldehyde	0.208360133
gene-KCNK12	2.190315961	17-Aminogeldanamycin	0.518144528
gene-KCNK12	2.190315961	Isopropyl isothiocyanate	0.172385747
gene-KCNK12	2.190315961	1-Octadecanoyl-2-(7Z,10Z,13Z,16Z-docosat	0.690496314
gene-KCNK12	2.190315961	CL(8:0/8:0/18:2(9Z,11Z)/20:0)	0.622988418
Bos_taurus_newG	2.17347224	3-Thiacytidine	0.209387412
Bos_taurus_newG	2.17347224	D-Erythro-imidazole-glycerol-phosphate	0.355812431
Bos_taurus_newG	2.17347224	LTB4-d4	0.226799982
Bos_taurus_newG	2.17347224	cis-p-Menth-2-en-1-ol	0.201017988
Bos_taurus_newG	2.17347224	PE(20:0/18:1(12Z)-2OH(9,10))	0.438658253
Bos_taurus_newG	2.17347224	PE(22:2(13Z,16Z)/22:5(4Z,7Z,10Z,13Z,19Z)-O	0.369745166
Bos_taurus_newG	2.17347224	Cyclosporin A	0.656529229
Bos_taurus_newG	2.17347224	DG(18:0/LTE4/0:0)	0.681485773
Bos_taurus_newG	2.17347224	5-(2-Aminopropyl)-2-methylphenol	0.323231457
Bos_taurus_newG	2.17347224	2-Methyl-3-phenyl-2-propenal	0.407813975
Bos_taurus_newG	2.17347224	beta-L-Dioxolane-cytidine	0.175916668
Bos_taurus_newG	2.17347224	PC(P-18:1(11Z)/PGE2)	0.509503133
Bos_taurus_newG	2.17347224	Isopropyl isothiocyanate	0.172385747
Bos_taurus_newG	2.17347224	(9Z)-Octadecenoic acid	0.142359556
Bos_taurus_newG	2.17347224	PC(18:1(9Z)/15:1(9Z))	0.534724682
Bos_taurus_newG	2.17347224	arachidyl amido cholanoic acid	1.24842952
Bos_taurus_newG	2.17347224	Nigroxanthin	0.705005059
Bos_taurus_newG	2.17347224	PC(P-18:1(11Z)/PGJ2)	0.565243877
Bos_taurus_newG	2.17347224	PS(20:0/20:4(8Z,11Z,14Z,17Z)-2OH(5S,6R))	0.402595921
Bos_taurus_newG	2.17347224	CL(8:0/8:0/18:2(9Z,11Z)/20:0)	0.622988418
gene-EFNA2	2.166002867	3-Thiacytidine	0.209387412
gene-EFNA2	2.166002867	D-Erythro-imidazole-glycerol-phosphate	0.355812431
gene-EFNA2	2.166002867	7(14)-Bisabolene-2,3,10,11-tetrol	2.446897974
gene-EFNA2	2.166002867	13(S)-HpODE	0.163157753
gene-EFNA2	2.166002867	LTB4-d4	0.226799982
gene-EFNA2	2.166002867	cis-p-Menth-2-en-1-ol	0.201017988
gene-EFNA2	2.166002867	(-)-alpha-Terpineol	0.345716279
gene-EFNA2	2.166002867	PG(20:1(11Z)/18:3(10,12,15)-OH(9))	0.626244347
gene-EFNA2	2.166002867	2-Methyl-3-phenyl-2-propenal	0.407813975
gene-EFNA2	2.166002867	PC(P-18:1(11Z)/PGE2)	0.509503133
gene-EFNA2	2.166002867	(3R,4R)-3-Amino-1-hydroxy-4-methylpyrrol	0.471914506
gene-EFNA2	2.166002867	Isopropyl isothiocyanate	0.172385747
gene-EFNA2	2.166002867	PS(20:0/20:4(8Z,11Z,14Z,17Z)-2OH(5S,6R))	0.402595921
gene-LOC50885ε	2.162328796	3-Thiacytidine	0.209387412
gene-LOC50885ε	2.162328796	3-Deoxyestrone	0.282221709
gene-LOC50885ε	2.162328796	1-Oleoyl-sn-glycero-3-phosphocholine	0.18926952
gene-LOC50885ε	2.162328796	Psychosine	0.106475396
gene-LOC50885ε	2.162328796	LTB4-d4	0.226799982
gene-LOC50885ε	2.162328796	(-)-alpha-Terpineol	0.345716279
gene-LOC50885ε	2.162328796	1,4-Undecadiene	0.462803973
gene-LOC50885ε	2.162328796	4-cholesten-7伪,12伪,24-triol-3-one	0.097840451
gene-LOC50885ε	2.162328796	Angiotensin A	0.247332017
gene-LOC50885ε	2.162328796	PC(14:0/20:2(11Z,14Z))	1.110657378
gene-LOC50885ε	2.162328796	5-(2-Aminopropyl)-2-methylphenol	0.323231457
gene-LOC50885ε	2.162328796	Trimetazidine	0.438282534

gene-LOC50885ɛ	2.162328796	ingenol	0.942071652
gene-LOC50885ɛ	2.162328796	Armillane	0.52808635
gene-LOC50885ɛ	2.162328796	PC(P-18:1(11Z)/PGE2)	0.509503133
gene-LOC50885ɛ	2.162328796	PC(20:3(5Z,8Z,11Z)/24:0)	0.387959564
gene-LOC50885ɛ	2.162328796	(3R,4R)-3-Amino-1-hydroxy-4-methylpyrrol	0.471914506
gene-LOC50885ɛ	2.162328796	n-methyl-2-(4'-methylaminophenyl)-6-hydr	0.26655714
gene-LOC50885ɛ	2.162328796	Isopropyl isothiocyanate	0.172385747
gene-LOC50885ɛ	2.162328796	(9Z)-Octadecenoic acid	0.142359556
gene-LOC50885ɛ	2.162328796	arachidyl amido cholanoic acid	1.24842952
gene-LOC50885ɛ	2.162328796	CL(8:0/8:0/18:2(9Z,11Z)/20:0)	0.622988418
Bos_taurus_newG	2.129653913	3-hydroxypristanic acid	0.548515835
Bos_taurus_newG	2.129653913	7(14)-Bisabolene-2,3,10,11-tetrol	2.446897974
Bos_taurus_newG	2.129653913	(3Z)-Phycoerythrobilin	1.456755874
Bos_taurus_newG	2.129653913	3-Deoxyestrone	0.282221709
Bos_taurus_newG	2.129653913	1-Oleoyl-sn-glycero-3-phosphocholine	0.18926952
Bos_taurus_newG	2.129653913	Lividamine	0.319679555
Bos_taurus_newG	2.129653913	Psychosine	0.106475396
Bos_taurus_newG	2.129653913	Cyclotricuspidogenin C	0.440884085
Bos_taurus_newG	2.129653913	(-)-alpha-Terpineol	0.345716279
Bos_taurus_newG	2.129653913	Linoleamide	0.926003481
Bos_taurus_newG	2.129653913	1,4-Undecadiene	0.462803973
Bos_taurus_newG	2.129653913	4-cholesten-7伪,12伪,24-triol-3-one	0.097840451
Bos_taurus_newG	2.129653913	4-Dimethylamino-L-phenylalanine	0.242110226
Bos_taurus_newG	2.129653913	Trimetazidine	0.438282534
Bos_taurus_newG	2.129653913	ingenol	0.942071652
Bos_taurus_newG	2.129653913	Armillane	0.52808635
Bos_taurus_newG	2.129653913	N-[[3-Hydroxy-2-(2-pentenyl)cyclopentyl]ac	1.043930947
Bos_taurus_newG	2.129653913	11-Dehydro-2,3-dinor-txb2	0.307258797
Bos_taurus_newG	2.129653913	PC(20:3(5Z,8Z,11Z)/24:0)	0.387959564
Bos_taurus_newG	2.129653913	2-(2-Aminopropanoylamino)bicyclo[3.1.0]he:	1.149807904
Bos_taurus_newG	2.129653913	1-Palmitoylglycerol	0.084357408
Bos_taurus_newG	2.129653913	N2-gamma-Glutamylglutamine	0.230065499
Bos_taurus_newG	2.129653913	n-methyl-2-(4'-methylaminophenyl)-6-hydr	0.26655714
Bos_taurus_newG	2.129653913	Permetin A	0.140869415
Bos_taurus_newG	2.129653913	Glutethimide	0.126237229
Bos_taurus_newG	2.129653913	Isopropyl isothiocyanate	0.172385747
Bos_taurus_newG	2.129653913	9-deoxy-9-methylene-16,16-dimethyl -PGE:	0.606893884
Bos_taurus_newG	2.129653913	CL(8:0/8:0/18:2(9Z,11Z)/20:0)	0.622988418
gene-FADS6	2.107574658	3-hydroxypristanic acid	0.548515835
gene-FADS6	2.107574658	3-Deoxyestrone	0.282221709
gene-FADS6	2.107574658	1-Oleoyl-sn-glycero-3-phosphocholine	0.18926952
gene-FADS6	2.107574658	Psychosine	0.106475396
gene-FADS6	2.107574658	LTB4-d4	0.226799982
gene-FADS6	2.107574658	(-)-alpha-Terpineol	0.345716279
gene-FADS6	2.107574658	1,4-Undecadiene	0.462803973
gene-FADS6	2.107574658	4-Dimethylamino-L-phenylalanine	0.242110226
gene-FADS6	2.107574658	Trimetazidine	0.438282534
gene-FADS6	2.107574658	ingenol	0.942071652
gene-FADS6	2.107574658	N-[[3-Hydroxy-2-(2-pentenyl)cyclopentyl]ac	1.043930947
gene-FADS6	2.107574658	PC(P-18:1(11Z)/PGE2)	0.509503133
gene-FADS6	2.107574658	PC(20:3(5Z,8Z,11Z)/24:0)	0.387959564

gene-FADS6	2.107574658	(3R,4R)-3-Amino-1-hydroxy-4-methylpyrrol	0.471914506
gene-FADS6	2.107574658	n-methyl-2-(4'-methylaminophenyl)-6-hydr	0.26655714
gene-FADS6	2.107574658	Isopropyl isothiocyanate	0.172385747
gene-FADS6	2.107574658	(9Z)-Octadecenoic acid	0.142359556
gene-FADS6	2.107574658	9-deoxy-9-methylene-16,16-dimethyl -PGE	0.606893884
gene-FADS6	2.107574658	CL(8:0/8:0/18:2(9Z,11Z)/20:0)	0.622988418
gene-LOC100337	2.09868171	3-Thiacytidine	0.209387412
gene-LOC100337	2.09868171	cis-p-Menth-2-en-1-ol	0.201017988
gene-LOC100337	2.09868171	PE(22:2(13Z,16Z)/22:5(4Z,7Z,10Z,13Z,19Z)-O	0.369745166
gene-LOC100337	2.09868171	Armillane	0.52808635
gene-LOC100337	2.09868171	PC(P-18:1(11Z)/PGE2)	0.509503133
gene-LOC100337	2.09868171	Isopropyl isothiocyanate	0.172385747
gene-LOC100337	2.09868171	PC(18:1(9Z)/15:1(9Z))	0.534724682
gene-LOC100337	2.09868171	PC(P-18:1(11Z)/PGJ2)	0.565243877
gene-LOC100337	2.09868171	PS(20:0/20:4(8Z,11Z,14Z,17Z)-2OH(5S,6R))	0.402595921
Bos_taurus_newG	2.081292836	LysoPI(16:0/0:0)	0.379098336
Bos_taurus_newG	2.081292836	3-Thiacytidine	0.209387412
Bos_taurus_newG	2.081292836	11-Maleimidoundecanoic acid	1.084942397
Bos_taurus_newG	2.081292836	Monacolin L acid	1.060562098
Bos_taurus_newG	2.081292836	cis-p-Menth-2-en-1-ol	0.201017988
Bos_taurus_newG	2.081292836	PE(20:0/18:1(12Z)-2OH(9,10))	0.438658253
Bos_taurus_newG	2.081292836	PE(22:2(13Z,16Z)/22:5(4Z,7Z,10Z,13Z,19Z)-O	0.369745166
Bos_taurus_newG	2.081292836	Lamivudine	0.327402892
Bos_taurus_newG	2.081292836	Norophthalmic acid	0.191432411
Bos_taurus_newG	2.081292836	2-Methyl-3-phenyl-2-propenal	0.407813975
Bos_taurus_newG	2.081292836	Guanidoacetic acid	0.542509265
Bos_taurus_newG	2.081292836	4-Oxo-9-cis-retinoyl-beta-glucuronide	1.611773742
Bos_taurus_newG	2.081292836	alpha-Terpineol formate	0.628238986
Bos_taurus_newG	2.081292836	PC(P-18:1(11Z)/PGE2)	0.509503133
Bos_taurus_newG	2.081292836	Roxithromycin	0.268273077
Bos_taurus_newG	2.081292836	PC(18:1(9Z)/15:1(9Z))	0.534724682
Bos_taurus_newG	2.081292836	PC(P-18:1(11Z)/PGJ2)	0.565243877
Bos_taurus_newG	2.081292836	PS(20:0/20:4(8Z,11Z,14Z,17Z)-2OH(5S,6R))	0.402595921
gene-LOC101903	2.077010163	2-(alpha-Hydroxypropyl)thiamine diphospha	0.012996988
gene-LOC101903	2.077010163	3,3',5-Trihydroxy-4'-methoxy-6,7-methylene	0.137607639
gene-LOC101903	2.077010163	Benzoate	0.019595474
gene-LOC101903	2.077010163	Ascladiol	0.092884047
gene-LOC101903	2.077010163	1,5-Dibutyl methyl hydroxycitrate	0.108255051
gene-LOC101903	2.077010163	L-Citrulline	0.10853076
gene-LOC101903	2.077010163	Norophthalmic acid	0.191432411
gene-LOC101903	2.077010163	Ethylene brassylate	0.989156973
gene-LOC101903	2.077010163	Hercynine	0.111274419
gene-LOC101903	2.077010163	Imidazoline	0.232348181
gene-LOC101903	2.077010163	beta-L-Dioxolane-cytidine	0.175916668
gene-LOC101903	2.077010163	O-(17-Carboxyheptadecanoyl)carnitine	0.433790125
gene-LOC101903	2.077010163	Prostaglandin B2	0.552852305
gene-LOC101903	2.077010163	o-(pentafluorobenzyloxycarbonyl)-2,3,4,5-te	0.017661091
gene-LOC101903	2.077010163	Castanospermine	0.021364907
gene-LOC101903	2.077010163	5-Methoxytryptophan	0.079780961
gene-LOC101903	2.077010163	Azimexon	0.008689891
Bos_taurus_newG	2.06953157	3-Thiacytidine	0.209387412

Bos_taurus_newG	2.06953157	LTB4-d4	0.226799982
Bos_taurus_newG	2.06953157	Cyclosporin A	0.656529229
Bos_taurus_newG	2.06953157	PA(22:6(4Z,7Z,10Z,13Z,16Z,19Z)/16:0)	0.556532497
Bos_taurus_newG	2.06953157	DG(18:0/LTE4/0:0)	0.681485773
Bos_taurus_newG	2.06953157	PC(17:0/PGJ2)	0.657689319
Bos_taurus_newG	2.06953157	PC(14:0/20:2(11Z,14Z))	1.110657378
Bos_taurus_newG	2.06953157	CDP-DG(PGF2alpha/16:0)	1.002512277
Bos_taurus_newG	2.06953157	5-(2-Aminopropyl)-2-methylphenol	0.323231457
Bos_taurus_newG	2.06953157	beta-L-Dioxolane-cytidine	0.175916668
Bos_taurus_newG	2.06953157	PC(P-18:1(11Z)/PGE2)	0.509503133
Bos_taurus_newG	2.06953157	PC(20:3(5Z,8Z,11Z)/24:0)	0.387959564
Bos_taurus_newG	2.06953157	Isopropyl isothiocyanate	0.172385747
Bos_taurus_newG	2.06953157	arachidyl amido cholanoic acid	1.24842952
Bos_taurus_newG	2.06953157	Nigroxanthin	0.705005059
Bos_taurus_newG	2.06953157	1-Octadecanoyl-2-(7Z,10Z,13Z,16Z-docosat	0.690496314
Bos_taurus_newG	2.06953157	CL(8:0/8:0/18:2(9Z,11Z)/20:0)	0.622988418
Bos_taurus_newG	2.06953157	PC(20:5(5Z,8Z,11Z,14Z,17Z)/P-16:0)	1.301095986
Bos_taurus_newG	2.06953157	DG(20:0/LTE4/0:0)	0.438074508
gene-FRRS1	2.06147369	(-)-alpha-Terpineol	0.345716279
gene-FRRS1	2.06147369	3b,6a-Dihydroxy-alpha-ionol 9-[apiosyl-(1-:	0.045258177
gene-FRRS1	2.06147369	N-[[3-Hydroxy-2-(2-pentenyl)cyclopentyl]ac	1.043930947
gene-FRRS1	2.06147369	PC(P-18:1(11Z)/PGE2)	0.509503133
gene-FRRS1	2.06147369	Phorone A	0.079039095
gene-FRRS1	2.06147369	2-Amino-4-[carbamimidoyl(methyl)amino]bu	0.075703353
gene-FRRS1	2.06147369	Digalacturonate	0.473524431
gene-FRS3	2.031803569	LysoPI(16:0/0:0)	0.379098336
gene-FRS3	2.031803569	3-Thiacytidine	0.209387412
gene-FRS3	2.031803569	11-Maleimidoundecanoic acid	1.084942397
gene-FRS3	2.031803569	cis-p-Menth-2-en-1-ol	0.201017988
gene-FRS3	2.031803569	PE(20:0/18:1(12Z)-2OH(9,10))	0.438658253
gene-FRS3	2.031803569	PE(22:2(13Z,16Z)/22:5(4Z,7Z,10Z,13Z,19Z)-O	0.369745166
gene-FRS3	2.031803569	Lamivudine	0.327402892
gene-FRS3	2.031803569	Roxithromycin	0.268273077
gene-FRS3	2.031803569	PC(18:1(9Z)/15:1(9Z))	0.534724682
gene-FRS3	2.031803569	PC(P-18:1(11Z)/PGJ2)	0.565243877
gene-FRS3	2.031803569	PS(20:0/20:4(8Z,11Z,14Z,17Z)-2OH(5S,6R))	0.402595921
gene-TMIGD3	2.029112043	3-Thiacytidine	0.209387412
gene-TMIGD3	2.029112043	LTB4-d4	0.226799982
gene-TMIGD3	2.029112043	Cyclosporin A	0.656529229
gene-TMIGD3	2.029112043	beta-L-Dioxolane-cytidine	0.175916668
gene-TMIGD3	2.029112043	PC(P-18:1(11Z)/PGE2)	0.509503133
gene-TMIGD3	2.029112043	1-Heneicosanoyl-glycero-3-phosphoserine	0.214358093
gene-TMIGD3	2.029112043	Nigroxanthin	0.705005059
gene-TMIGD3	2.029112043	CL(8:0/8:0/18:2(9Z,11Z)/20:0)	0.622988418
gene-ADORA3	2.024496733	3-Thiacytidine	0.209387412
gene-ADORA3	2.024496733	3-Deoxyestrone	0.282221709
gene-ADORA3	2.024496733	1-Oleoyl-sn-glycero-3-phosphocholine	0.18926952
gene-ADORA3	2.024496733	Psychosine	0.106475396
gene-ADORA3	2.024496733	(-)-alpha-Terpineol	0.345716279
gene-ADORA3	2.024496733	1,4-Undecadiene	0.462803973
gene-ADORA3	2.024496733	4-cholesten-7伪,12伪,24-triol-3-one	0.097840451

gene-ADORA3	2.024496733	5-(2-Aminopropyl)-2-methylphenol	0.323231457
gene-ADORA3	2.024496733	4-Dimethylamino-L-phenylalanine	0.242110226
gene-ADORA3	2.024496733	ingenol	0.942071652
gene-ADORA3	2.024496733	Armillane	0.52808635
gene-ADORA3	2.024496733	PC(P-18:1(11Z)/PGE2)	0.509503133
gene-ADORA3	2.024496733	(3R,4R)-3-Amino-1-hydroxy-4-methylpyrrol	0.471914506
gene-ADORA3	2.024496733	n-methyl-2-(4'-methylaminophenyl)-6-hydr	0.26655714
gene-ADORA3	2.024496733	Isopropyl isothiocyanate	0.172385747
gene-ADORA3	2.024496733	(9Z)-Octadecenoic acid	0.142359556
gene-ADORA3	2.024496733	PS(20:0/20:4(8Z,11Z,14Z,17Z)-2OH(5S,6R))	0.402595921
gene-ADORA3	2.024496733	CL(8:0/8:0/18:2(9Z,11Z)/20:0)	0.622988418
gene-C2H2orf88	2.022932014	5-(3'-Carboxy-3'-oxopropenyl)-4,6-dihydro:	1.083232395
gene-C2H2orf88	2.022932014	D-Erythro-imidazole-glycerol-phosphate	0.355812431
gene-C2H2orf88	2.022932014	Erythronic acid	0.494007482
gene-C2H2orf88	2.022932014	Benzenesulfonamide, 2-(cyclohexylamino)-N	0.727905403
gene-C2H2orf88	2.022932014	5-Hydroxykynurenamine	0.870388402
gene-C2H2orf88	2.022932014	20-Carboxy-leukotriene B4	0.654456667
gene-C2H2orf88	2.022932014	Sonchuside C	1.385535636
gene-C2H2orf88	2.022932014	Undecanedioic acid	0.059955634
gene-C2H2orf88	2.022932014	(S)-10,16-Dihydroxyhexadecanoic acid	1.054006526
gene-C2H2orf88	2.022932014	Auxin b	1.052491354
gene-C2H2orf88	2.022932014	13(S)-HPOT	1.353059742
gene-C2H2orf88	2.022932014	13,14-Dihydro PGF-1a	0.390844466
gene-C2H2orf88	2.022932014	Cyclopentolate	0.486079401
gene-C2H2orf88	2.022932014	Monacolin L acid	1.060562098
gene-C2H2orf88	2.022932014	cis-p-Menth-2-en-1-ol	0.201017988
gene-C2H2orf88	2.022932014	PG(20:1(11Z)/18:3(10,12,15)-OH(9))	0.626244347
gene-C2H2orf88	2.022932014	Asparaginylcysteine	0.467538645
gene-C2H2orf88	2.022932014	(1R,6S)-6-Amino-5-oxocyclohex-2-ene-1-c	0.154751123
gene-C2H2orf88	2.022932014	3-Methoxytyramine	0.928926425
gene-C2H2orf88	2.022932014	L-histidinol-phosphate	0.865229875
gene-C2H2orf88	2.022932014	Hexanoylglutamine	0.593876112
gene-C2H2orf88	2.022932014	7-Aminomethyl-7-carbaguanine	0.474992724
gene-C2H2orf88	2.022932014	Nevirapine	0.820759262
gene-C2H2orf88	2.022932014	L-alpha-Acetyl-N,N-dinormethadol	1.567999001
gene-C2H2orf88	2.022932014	2-Methyl-3-phenyl-2-propenal	0.407813975
gene-C2H2orf88	2.022932014	2-(1-Adamantyl)-1,3-dioxetane	0.408916843
gene-C2H2orf88	2.022932014	Sequiterpene Lactone 326	1.141450991
gene-C2H2orf88	2.022932014	8-Methylthiooctanaldoxime	0.685273457
gene-C2H2orf88	2.022932014	Pitheduloside I	1.362662847
gene-C2H2orf88	2.022932014	Ajulemic acid	0.557832951
gene-C2H2orf88	2.022932014	PC(P-18:1(11Z)/PGE2)	0.509503133
gene-C2H2orf88	2.022932014	Roxithromycin	0.268273077
gene-C2H2orf88	2.022932014	PG(20:1(11Z)/18:3(9,11,15)-OH(13))	0.69829683
gene-MMP15	2.021335632	3-Thiacytidine	0.209387412
gene-MMP15	2.021335632	cis-p-Menth-2-en-1-ol	0.201017988
gene-MMP15	2.021335632	PE(22:2(13Z,16Z)/22:5(4Z,7Z,10Z,13Z,19Z)-O	0.369745166
gene-MMP15	2.021335632	Armillane	0.52808635
gene-MMP15	2.021335632	PC(P-18:1(11Z)/PGE2)	0.509503133
gene-MMP15	2.021335632	Isopropyl isothiocyanate	0.172385747
gene-MMP15	2.021335632	PC(P-18:1(11Z)/PGJ2)	0.565243877

gene-MMP15	2.021335632	PS(20:0/20:4(8Z,11Z,14Z,17Z)-2OH(5S,6R))	0.402595921
gene-CAPN5	2.01394984	3-Thiacytidine	0.209387412
gene-CAPN5	2.01394984	cis-p-Menth-2-en-1-ol	0.201017988
gene-CAPN5	2.01394984	PE(20:0/18:1(12Z)-2OH(9,10))	0.438658253
gene-CAPN5	2.01394984	PE(22:2(13Z,16Z)/22:5(4Z,7Z,10Z,13Z,19Z)-O	0.369745166
gene-CAPN5	2.01394984	Cyclosporin A	0.656529229
gene-CAPN5	2.01394984	5-(2-Aminopropyl)-2-methylphenol	0.323231457
gene-CAPN5	2.01394984	Armillane	0.52808635
gene-CAPN5	2.01394984	PC(P-18:1(11Z)/PGE2)	0.509503133
gene-CAPN5	2.01394984	Isopropyl isothiocyanate	0.172385747
gene-CAPN5	2.01394984	Nigroxanthin	0.705005059
gene-CAPN5	2.01394984	PC(P-18:1(11Z)/PGJ2)	0.565243877
gene-CAPN5	2.01394984	PS(20:0/20:4(8Z,11Z,14Z,17Z)-2OH(5S,6R))	0.402595921
gene-RAPSN	2.010701249	all-trans-Hexaprenyl diphosphate	0.277689133
gene-RAPSN	2.010701249	Stercobilinogen	0.144304212
gene-RAPSN	2.010701249	4-cholesten-7伪,12伪,24-triol-3-one	0.097840451
gene-RAPSN	2.010701249	5-(2-Aminopropyl)-2-methylphenol	0.323231457
gene-RAPSN	2.010701249	ingenol	0.942071652
gene-RAPSN	2.010701249	1-Palmitoylglycerol	0.084357408
gene-RAPSN	2.010701249	Isopropyl isothiocyanate	0.172385747
gene-RAPSN	2.010701249	D-Fructose	0.167584616
gene-RAPSN	2.010701249	CL(8:0/8:0/18:2(9Z,11Z)/20:0)	0.622988418
Bos_taurus_newG	2.007499957	Dextrorphan O-glucuronide	1.59476827
Bos_taurus_newG	2.007499957	LysoPI(16:0/0:0)	0.379098336
Bos_taurus_newG	2.007499957	3-Thiacytidine	0.209387412
Bos_taurus_newG	2.007499957	Methyl methacrylate	0.245645579
Bos_taurus_newG	2.007499957	5-amino-1-(5-phosphonato-D-ribosyl)imida	0.622612791
Bos_taurus_newG	2.007499957	8-Methoxykynurenate	0.424170332
Bos_taurus_newG	2.007499957	5-Hydroxyindoleacetaldehyde	0.448806354
Bos_taurus_newG	2.007499957	Azelaic acid	0.007180175
Bos_taurus_newG	2.007499957	5-Hydroxy-2-oxo-4-ureido-2,5-dihydro-1H	0.122536033
Bos_taurus_newG	2.007499957	p-Coumaroylputrescine	0.939330574
Bos_taurus_newG	2.007499957	Calicheamicinone	0.910932275
Bos_taurus_newG	2.007499957	Pterostilbene	0.899698314
Bos_taurus_newG	2.007499957	Phenylalanylhydroxyproline	1.183668928
Bos_taurus_newG	2.007499957	Trihomomethionine	0.296210774
Bos_taurus_newG	2.007499957	Arginine vasopressin 1-8	1.288601754
Bos_taurus_newG	2.007499957	Hygromycin B	0.930396619
Bos_taurus_newG	2.007499957	Netupitant	0.707108724
Bos_taurus_newG	2.007499957	Divinylprotochlorophyllide	0.629978387
Bos_taurus_newG	2.007499957	Metkephamid	1.159767003
Bos_taurus_newG	2.007499957	Tuberose lactone	1.590026295
Bos_taurus_newG	2.007499957	Leukotriene D4	0.6807158
Bos_taurus_newG	2.007499957	2-Ethyl-5-methyl-3,3-diphenyl-1-pyrroline	0.826978858
Bos_taurus_newG	2.007499957	11-Maleimidoundecanoic acid	1.084942397
Bos_taurus_newG	2.007499957	2-Hexylbenzothiazole	0.396099297
Bos_taurus_newG	2.007499957	Becocalcidiol	0.63162902
Bos_taurus_newG	2.007499957	Docosanamide	0.509975786
Bos_taurus_newG	2.007499957	Monacolin L acid	1.060562098
Bos_taurus_newG	2.007499957	cis-p-Menth-2-en-1-ol	0.201017988
Bos_taurus_newG	2.007499957	PE(20:0/18:1(12Z)-2OH(9,10))	0.438658253

Bos_taurus_newG	2.007499957	PE(22:2(13Z,16Z)/22:5(4Z,7Z,10Z,13Z,19Z)-O	0.369745166
Bos_taurus_newG	2.007499957	Epomusenin A	0.766206049
Bos_taurus_newG	2.007499957	Lamivudine	0.327402892
Bos_taurus_newG	2.007499957	1-beta-D-Arabinofuranosyl-5-fluorocytosine	0.152339645
Bos_taurus_newG	2.007499957	Pseudouridine 5'-phosphate	1.18431378
Bos_taurus_newG	2.007499957	15-keto-Prostaglandin E2	2.646629404
Bos_taurus_newG	2.007499957	S-Acetyldihydrolipoamide-E	0.429561283
Bos_taurus_newG	2.007499957	Ribavirin monophosphate	1.091085027
Bos_taurus_newG	2.007499957	4-Hydroxyproline galactoside	0.148083825
Bos_taurus_newG	2.007499957	Pro Gly Ser Ser	0.384303007
Bos_taurus_newG	2.007499957	Norophthalmic acid	0.191432411
Bos_taurus_newG	2.007499957	4'-Thiothymidine	0.066948524
Bos_taurus_newG	2.007499957	3'-N'-Acetylfusarochromanone	0.755683206
Bos_taurus_newG	2.007499957	cis,trans-5'-Hydroxythalidomide	0.589828342
Bos_taurus_newG	2.007499957	Indole-3-ethanol	0.096523128
Bos_taurus_newG	2.007499957	Ser Cys Ala Ala	0.603032447
Bos_taurus_newG	2.007499957	10-Hydroxycarbazepine	0.848001655
Bos_taurus_newG	2.007499957	Semilepidinoside A	0.527638644
Bos_taurus_newG	2.007499957	Arbutin	0.176184476
Bos_taurus_newG	2.007499957	Loganin	0.92782666
Bos_taurus_newG	2.007499957	7-Methylxanthosine	0.004641243
Bos_taurus_newG	2.007499957	3'-Deoxythymidine	0.791830758
Bos_taurus_newG	2.007499957	5-Acetylamino-6-amino-3-methyluracil	0.420050597
Bos_taurus_newG	2.007499957	Gly Asp Ala Ala	0.718656316
Bos_taurus_newG	2.007499957	N-Acetylhistidine	0.349369987
Bos_taurus_newG	2.007499957	ethyl 2-cyano-3-(1h-indol-3-yl)prop-2-eno	0.756861034
Bos_taurus_newG	2.007499957	1-{2-[(3-Ethylphenyl)amino]-2-oxoethyl}-6-	0.705711444
Bos_taurus_newG	2.007499957	kainic acid	0.836445456
Bos_taurus_newG	2.007499957	Zanamivir	0.839205907
Bos_taurus_newG	2.007499957	Tryptophyl-Glutamine	0.89722186
Bos_taurus_newG	2.007499957	Aminoglutethimide	0.652616628
Bos_taurus_newG	2.007499957	5-NITRO-2-PHENYLPROPYLAMINOBENZOI	0.713298779
Bos_taurus_newG	2.007499957	5,6,7,8-Tetrahydromonapterin	0.724075531
Bos_taurus_newG	2.007499957	Harmalol	0.364945111
Bos_taurus_newG	2.007499957	Guanidoacetic acid	0.542509265
Bos_taurus_newG	2.007499957	4-Oxo-9-cis-retinoyl-beta-glucuronide	1.611773742
Bos_taurus_newG	2.007499957	alpha-Terpineol formate	0.628238986
Bos_taurus_newG	2.007499957	4-Dihydroboldenone	0.692863202
Bos_taurus_newG	2.007499957	2'-Fluoro-2',3'-dideoxyinosine	1.214745496
Bos_taurus_newG	2.007499957	20-carboxy Arachidonic Acid	0.780988382
Bos_taurus_newG	2.007499957	17-beta-Estradiol glucuronide	0.827039442
Bos_taurus_newG	2.007499957	Nebramycin 5'	0.909280571
Bos_taurus_newG	2.007499957	Apronal	0.754033426
Bos_taurus_newG	2.007499957	Methionyl-Valine	0.502707745
Bos_taurus_newG	2.007499957	Argyrolobine	0.949134241
Bos_taurus_newG	2.007499957	3-{[(2E)-4-Amino-4-oxobut-2-enoyl]amino}	1.152542472
Bos_taurus_newG	2.007499957	Chitotriose	0.897504334
Bos_taurus_newG	2.007499957	4-Octylphenol	1.400657885
Bos_taurus_newG	2.007499957	Prolyl-Lysine	1.612991304
Bos_taurus_newG	2.007499957	Myricanol 5-[arabinosyl-(1->6)-glucoside]	0.744174399
Bos_taurus_newG	2.007499957	3-Pentadecylphenol	1.682258188

Bos_taurus_newG	2.007499957	MG(0:0/18:3(9Z,12Z,15Z)/0:0)	1.373784038
Bos_taurus_newG	2.007499957	Tetracycline	0.553304723
Bos_taurus_newG	2.007499957	N-Palmitoyl Proline	0.470367693
Bos_taurus_newG	2.007499957	10-alpha-methoxy-9,10-dihydrolysergol	0.140851438
Bos_taurus_newG	2.007499957	3beta-Hydroxyergosta-7,24(24(1))-dien-4alp	1.714710978
Bos_taurus_newG	2.007499957	MG(0:0/20:3(11Z,14Z,17Z)/0:0)	0.844949524
Bos_taurus_newG	2.007499957	N-Stearoyl Glutamine	1.862695384
Bos_taurus_newG	2.007499957	Palmitoylcarnitine	0.74779616
Bos_taurus_newG	2.007499957	Glutaminylphenylalanine	0.224405311
Bos_taurus_newG	2.007499957	Galabiosylceramide (d18:1/20:0)	0.746737323
Bos_taurus_newG	2.007499957	LysoPI(0:0/18:0)	0.420441153
Bos_taurus_newG	2.007499957	12-Hydroxyicosanoylcarnitine	1.730979067
Bos_taurus_newG	2.007499957	Milbemycin D	0.125637998
Bos_taurus_newG	2.007499957	Tragopogonsaponin A	0.169034237
Bos_taurus_newG	2.007499957	2,4-Undecadienal	0.228636005
Bos_taurus_newG	2.007499957	Sitosterol beta-D-glucoside	0.382944796
Bos_taurus_newG	2.007499957	PC(18:1(9Z)/15:1(9Z))	0.534724682
Bos_taurus_newG	2.007499957	Nigroxanthin	0.705005059
Bos_taurus_newG	2.007499957	PC(P-18:1(11Z)/PGJ2)	0.565243877
Bos_taurus_newG	2.007499957	PS(20:0/20:4(8Z,11Z,14Z,17Z)-2OH(5S,6R))	0.402595921
Bos_taurus_newG	2.007499957	PE(20:5(5Z,8Z,11Z,14Z,17Z)/18:0)	0.427962506
Bos_taurus_newG	2.007499957	PS(16:1(9Z)/22:2(13Z,16Z))	0.659652661
Bos_taurus_newG	2.007499957	PC(P-16:0/20:3(8Z,11Z,14Z)-2OH(5,6))	0.174122169
gene-TMEM205	2.003240964	3-Thiacytidine	0.209387412
gene-TMEM205	2.003240964	3-Deoxyestrone	0.282221709
gene-TMEM205	2.003240964	1-Oleoyl-sn-glycero-3-phosphocholine	0.18926952
gene-TMEM205	2.003240964	Psychosine	0.106475396
gene-TMEM205	2.003240964	(-)-alpha-Terpineol	0.345716279
gene-TMEM205	2.003240964	1,4-Undecadiene	0.462803973
gene-TMEM205	2.003240964	4-cholesten-7伪,12伪,24-triol-3-one	0.097840451
gene-TMEM205	2.003240964	5-(2-Aminopropyl)-2-methylphenol	0.323231457
gene-TMEM205	2.003240964	4-Dimethylamino-L-phenylalanine	0.242110226
gene-TMEM205	2.003240964	Trimetazidine	0.438282534
gene-TMEM205	2.003240964	ingenol	0.942071652
gene-TMEM205	2.003240964	Armillane	0.52808635
gene-TMEM205	2.003240964	PC(P-18:1(11Z)/PGE2)	0.509503133
gene-TMEM205	2.003240964	(3R,4R)-3-Amino-1-hydroxy-4-methylpyrrol	0.471914506
gene-TMEM205	2.003240964	1-Palmitoylglycerol	0.084357408
gene-TMEM205	2.003240964	n-methyl-2-(4'-methylaminophenyl)-6-hydr	0.26655714
gene-TMEM205	2.003240964	Isopropyl isothiocyanate	0.172385747
gene-TMEM205	2.003240964	9-deoxy-9-methylene-16,16-dimethyl -PGE	0.606893884
gene-TMEM205	2.003240964	CL(8:0/8:0/18:2(9Z,11Z)/20:0)	0.622988418
gene-CHAD	1.996291275	D-Erythro-imidazole-glycerol-phosphate	0.355812431
gene-CHAD	1.996291275	Isomaltotriose	0.58434654
gene-CHAD	1.996291275	2-Dehydro-3-deoxy-D-gluconate	0.65165299
gene-CHAD	1.996291275	(-)-alpha-Terpineol	0.345716279
gene-CHAD	1.996291275	N-[[3-Hydroxy-2-(2-pentenyl)cyclopentyl]ac	1.043930947
gene-CHAD	1.996291275	PC(P-18:1(11Z)/PGE2)	0.509503133
gene-CHAD	1.996291275	Roxithromycin	0.268273077
gene-CHAD	1.996291275	PC(P-18:1(11Z)/PGJ2)	0.565243877
gene-CHAD	1.996291275	PS(20:0/20:4(8Z,11Z,14Z,17Z)-2OH(5S,6R))	0.402595921

gene-LOC78612€	1.981568455	3-Thiacytidine	0.209387412
gene-LOC78612€	1.981568455	LTB4-d4	0.226799982
gene-LOC78612€	1.981568455	PE(20:0/18:1(12Z)-2OH(9,10))	0.438658253
gene-LOC78612€	1.981568455	(-)-alpha-Terpineol	0.345716279
gene-LOC78612€	1.981568455	Cyclosporin A	0.656529229
gene-LOC78612€	1.981568455	DG(18:0/LTE4/0:0)	0.681485773
gene-LOC78612€	1.981568455	PC(14:0/20:2(11Z,14Z))	1.110657378
gene-LOC78612€	1.981568455	5-(2-Aminopropyl)-2-methylphenol	0.323231457
gene-LOC78612€	1.981568455	Armillane	0.52808635
gene-LOC78612€	1.981568455	PC(P-18:1(11Z)/PGE2)	0.509503133
gene-LOC78612€	1.981568455	(3R,4R)-3-Amino-1-hydroxy-4-methylpyrrol	0.471914506
gene-LOC78612€	1.981568455	Isopropyl isothiocyanate	0.172385747
gene-LOC78612€	1.981568455	(9Z)-Octadecenoic acid	0.142359556
gene-LOC78612€	1.981568455	arachidyl amido cholanoic acid	1.24842952
gene-LOC78612€	1.981568455	Nigroxanthin	0.705005059
gene-LOC78612€	1.981568455	PC(P-18:1(11Z)/PGJ2)	0.565243877
gene-LOC78612€	1.981568455	PS(20:0/20:4(8Z,11Z,14Z,17Z)-2OH(5S,6R))	0.402595921
gene-LOC78612€	1.981568455	CL(8:0/8:0/18:2(9Z,11Z)/20:0)	0.622988418
gene-ALOX15	1.978948367	3-Thiacytidine	0.209387412
gene-ALOX15	1.978948367	cis-p-Menth-2-en-1-ol	0.201017988
gene-ALOX15	1.978948367	PE(22:2(13Z,16Z)/22:5(4Z,7Z,10Z,13Z,19Z)-O	0.369745166
gene-ALOX15	1.978948367	5-(2-Aminopropyl)-2-methylphenol	0.323231457
gene-ALOX15	1.978948367	Armillane	0.52808635
gene-ALOX15	1.978948367	PC(P-18:1(11Z)/PGE2)	0.509503133
gene-ALOX15	1.978948367	Isopropyl isothiocyanate	0.172385747
gene-ALOX15	1.978948367	PC(18:1(9Z)/15:1(9Z))	0.534724682
gene-ALOX15	1.978948367	Nigroxanthin	0.705005059
gene-ALOX15	1.978948367	PC(P-18:1(11Z)/PGJ2)	0.565243877
gene-ALOX15	1.978948367	PS(20:0/20:4(8Z,11Z,14Z,17Z)-2OH(5S,6R))	0.402595921
Bos_taurus_newG	1.974316749	D-Erythro-imidazole-glycerol-phosphate	0.355812431
Bos_taurus_newG	1.974316749	7(14)-Bisabolene-2,3,10,11-tetrol	2.446897974
Bos_taurus_newG	1.974316749	cis-p-Menth-2-en-1-ol	0.201017988
Bos_taurus_newG	1.974316749	1,4-Undecadiene	0.462803973
Bos_taurus_newG	1.974316749	Armillane	0.52808635
Bos_taurus_newG	1.974316749	PC(P-18:1(11Z)/PGE2)	0.509503133
Bos_taurus_newG	1.974316749	Sambutoxin	0.169786377
Bos_taurus_newG	1.974316749	Isopropyl isothiocyanate	0.172385747
Bos_taurus_newG	1.974316749	PS(20:0/20:4(8Z,11Z,14Z,17Z)-2OH(5S,6R))	0.402595921
Bos_taurus_newG	1.959801456	2(R)-HPOT	0.035267944
Bos_taurus_newG	1.959801456	13(S)-HpODE	0.163157753
Bos_taurus_newG	1.959801456	7(S),17(S)-dihydroxy-8(E),10(Z),13(Z),15(E),19	0.32147403
Bos_taurus_newG	1.959801456	LTB4-d4	0.226799982
Bos_taurus_newG	1.959801456	Cyclosporin A	0.656529229
Bos_taurus_newG	1.959801456	2-Methyl-3-phenyl-2-propenal	0.407813975
Bos_taurus_newG	1.959801456	beta-L-Dioxolane-cytidine	0.175916668
Bos_taurus_newG	1.959801456	PC(P-18:1(11Z)/PGE2)	0.509503133
Bos_taurus_newG	1.959801456	PC(P-18:1(11Z)/PGE1)	0.295520345
Bos_taurus_newG	1.959801456	1-Heneicosanoyl-glycero-3-phosphoserine	0.214358093
gene-LOC10713こ	1.954164085	LysoPI(16:0/0:0)	0.379098336
gene-LOC10713こ	1.954164085	2-[(3S)-3-[[(2S)-1-(Carboxymethoxy)-1-oxo	0.111577437
gene-LOC10713こ	1.954164085	4-Gingerol	0.222165012

gene-LOC10713	1.954164085	Monacolin L acid	1.060562098
gene-LOC10713	1.954164085	Norophthalmic acid	0.191432411
gene-LOC10713	1.954164085	7-Methylxanthosine	0.004641243
gene-LOC10713	1.954164085	Ethylene brassylate	0.989156973
gene-LOC10713	1.954164085	Terbutryn	1.186241428
gene-LOC10713	1.954164085	4-Oxo-9-cis-retinoyl-beta-glucuronide	1.611773742
gene-LOC10713	1.954164085	alpha-Terpineol formate	0.628238986
gene-LOC10713	1.954164085	4-Octylphenol	1.400657885
gene-LOC10713	1.954164085	Prolyl-Lysine	1.612991304
gene-LOC10713	1.954164085	10-alpha-methoxy-9,10-dihydrolysergol	0.140851438
gene-LOC10713	1.954164085	N-Stearoyl Glutamine	1.862695384
gene-LOC10713	1.954164085	Palmitoylcarnitine	0.74779616
gene-LOC10713	1.954164085	DL-Stearoylcarnitine	0.510965486
gene-LOC10713	1.954164085	12-Hydroxyicosanoylcarnitine	1.730979067
gene-LOC10713	1.954164085	Ascorbic acid 6-palmitate	0.201627014
gene-LOC10713	1.954164085	PE(24:1(15Z)/PGE1)	0.406225908
gene-LOC10713	1.954164085	PC(P-18:1(11Z)/PGJ2)	0.565243877
gene-LOC10713	1.954164085	PE(20:5(5Z,8Z,11Z,14Z,17Z)/18:0)	0.427962506
gene-LOC10713	1.954164085	PS(16:1(9Z)/22:2(13Z,16Z))	0.659652661
gene-LOC10713	1.954164085	PC(P-16:0/20:5(5Z,8Z,11Z,14Z,16E)-OH(18R)	0.26121581
gene-LOC78973	1.945680316	3-Thiacytidine	0.209387412
gene-LOC78973	1.945680316	LTB4-d4	0.226799982
gene-LOC78973	1.945680316	cis-p-Menth-2-en-1-ol	0.201017988
gene-LOC78973	1.945680316	PE(20:0/18:1(12Z)-2OH(9,10))	0.438658253
gene-LOC78973	1.945680316	PE(22:2(13Z,16Z)/22:5(4Z,7Z,10Z,13Z,19Z)-O	0.369745166
gene-LOC78973	1.945680316	5-(2-Aminopropyl)-2-methylphenol	0.323231457
gene-LOC78973	1.945680316	Armillane	0.52808635
gene-LOC78973	1.945680316	PC(P-18:1(11Z)/PGE2)	0.509503133
gene-LOC78973	1.945680316	(3R,4R)-3-Amino-1-hydroxy-4-methylpyrrol	0.471914506
gene-LOC78973	1.945680316	Isopropyl isothiocyanate	0.172385747
gene-LOC78973	1.945680316	(9Z)-Octadecenoic acid	0.142359556
gene-LOC78973	1.945680316	PC(18:1(9Z)/15:1(9Z))	0.534724682
gene-LOC78973	1.945680316	arachidyl amido cholanoic acid	1.24842952
gene-LOC78973	1.945680316	Nigroxanthin	0.705005059
gene-LOC78973	1.945680316	PC(P-18:1(11Z)/PGJ2)	0.565243877
gene-LOC78973	1.945680316	PS(20:0/20:4(8Z,11Z,14Z,17Z)-2OH(5S,6R))	0.402595921
gene-LOC10033	1.931530409	3-Thiacytidine	0.209387412
gene-LOC10033	1.931530409	D-Erythro-imidazole-glycerol-phosphate	0.355812431
gene-LOC10033	1.931530409	LTB4-d4	0.226799982
gene-LOC10033	1.931530409	cis-p-Menth-2-en-1-ol	0.201017988
gene-LOC10033	1.931530409	(-)-alpha-Terpineol	0.345716279
gene-LOC10033	1.931530409	Cyclosporin A	0.656529229
gene-LOC10033	1.931530409	5-(2-Aminopropyl)-2-methylphenol	0.323231457
gene-LOC10033	1.931530409	Armillane	0.52808635
gene-LOC10033	1.931530409	PC(P-18:1(11Z)/PGE2)	0.509503133
gene-LOC10033	1.931530409	(3R,4R)-3-Amino-1-hydroxy-4-methylpyrrol	0.471914506
gene-LOC10033	1.931530409	Isopropyl isothiocyanate	0.172385747
gene-LOC10033	1.931530409	Nigroxanthin	0.705005059
gene-LOC10033	1.931530409	PC(P-18:1(11Z)/PGJ2)	0.565243877
gene-LOC10033	1.931530409	PS(20:0/20:4(8Z,11Z,14Z,17Z)-2OH(5S,6R))	0.402595921
gene-LOC10033	1.931530409	CL(8:0/8:0/18:2(9Z,11Z)/20:0)	0.622988418

gene-IL5RA	1.921879109	3-Thiacytidine	0.209387412
gene-IL5RA	1.921879109	LTB4-d4	0.226799982
gene-IL5RA	1.921879109	cis-p-Menth-2-en-1-ol	0.201017988
gene-IL5RA	1.921879109	PE(20:0/18:1(12Z)-2OH(9,10))	0.438658253
gene-IL5RA	1.921879109	PE(22:2(13Z,16Z)/22:5(4Z,7Z,10Z,13Z,19Z)-O	0.369745166
gene-IL5RA	1.921879109	Cyclosporin A	0.656529229
gene-IL5RA	1.921879109	Epomusenin A	0.766206049
gene-IL5RA	1.921879109	PA(22:6(4Z,7Z,10Z,13Z,16Z,19Z)/16:0)	0.556532497
gene-IL5RA	1.921879109	DG(18:0/LTE4/0:0)	0.681485773
gene-IL5RA	1.921879109	PC(17:0/PGJ2)	0.657689319
gene-IL5RA	1.921879109	PC(14:0/20:2(11Z,14Z))	1.110657378
gene-IL5RA	1.921879109	CDP-DG(PGF2alpha/16:0)	1.002512277
gene-IL5RA	1.921879109	5-(2-Aminopropyl)-2-methylphenol	0.323231457
gene-IL5RA	1.921879109	beta-L-Dioxolane-cytidine	0.175916668
gene-IL5RA	1.921879109	PC(P-18:1(11Z)/PGE2)	0.509503133
gene-IL5RA	1.921879109	PC(18:1(9Z)/15:1(9Z))	0.534724682
gene-IL5RA	1.921879109	arachidyl amido cholanoic acid	1.24842952
gene-IL5RA	1.921879109	Nigroxanthin	0.705005059
gene-IL5RA	1.921879109	1-Octadecanoyl-2-(7Z,10Z,13Z,16Z-docosat	0.690496314
gene-IL5RA	1.921879109	PC(P-18:1(11Z)/PGJ2)	0.565243877
gene-IL5RA	1.921879109	PS(20:0/20:4(8Z,11Z,14Z,17Z)-2OH(5S,6R))	0.402595921
gene-IL5RA	1.921879109	CL(8:0/8:0/18:2(9Z,11Z)/20:0)	0.622988418
gene-IL5RA	1.921879109	DG(20:0/LTE4/0:0)	0.438074508
gene-OCSTAMP	1.921124979	3-Thiacytidine	0.209387412
gene-OCSTAMP	1.921124979	D-Erythro-imidazole-glycerol-phosphate	0.355812431
gene-OCSTAMP	1.921124979	11-Maleimidoundecanoic acid	1.084942397
gene-OCSTAMP	1.921124979	cis-p-Menth-2-en-1-ol	0.201017988
gene-OCSTAMP	1.921124979	PE(20:0/18:1(12Z)-2OH(9,10))	0.438658253
gene-OCSTAMP	1.921124979	PE(22:2(13Z,16Z)/22:5(4Z,7Z,10Z,13Z,19Z)-O	0.369745166
gene-OCSTAMP	1.921124979	PG(20:1(11Z)/18:3(10,12,15)-OH(9))	0.626244347
gene-OCSTAMP	1.921124979	PC(P-18:1(11Z)/PGE2)	0.509503133
gene-OCSTAMP	1.921124979	Roxithromycin	0.268273077
gene-OCSTAMP	1.921124979	PC(P-18:1(11Z)/PGJ2)	0.565243877
gene-OCSTAMP	1.921124979	PS(20:0/20:4(8Z,11Z,14Z,17Z)-2OH(5S,6R))	0.402595921
gene-RDH5	1.921010141	3-Thiacytidine	0.209387412
gene-RDH5	1.921010141	5-(2-Aminopropyl)-2-methylphenol	0.323231457
gene-RDH5	1.921010141	Armillane	0.52808635
gene-RDH5	1.921010141	PC(P-18:1(11Z)/PGE2)	0.509503133
gene-RDH5	1.921010141	Isopropyl isothiocyanate	0.172385747
gene-RDH5	1.921010141	PC(P-18:1(11Z)/PGJ2)	0.565243877
gene-RDH5	1.921010141	PS(20:0/20:4(8Z,11Z,14Z,17Z)-2OH(5S,6R))	0.402595921
gene-TMEM145	1.905992549	3-Thiacytidine	0.209387412
gene-TMEM145	1.905992549	cis-p-Menth-2-en-1-ol	0.201017988
gene-TMEM145	1.905992549	PE(20:0/18:1(12Z)-2OH(9,10))	0.438658253
gene-TMEM145	1.905992549	PE(22:2(13Z,16Z)/22:5(4Z,7Z,10Z,13Z,19Z)-O	0.369745166
gene-TMEM145	1.905992549	Cyclosporin A	0.656529229
gene-TMEM145	1.905992549	DG(18:0/LTE4/0:0)	0.681485773
gene-TMEM145	1.905992549	5-(2-Aminopropyl)-2-methylphenol	0.323231457
gene-TMEM145	1.905992549	Armillane	0.52808635
gene-TMEM145	1.905992549	PC(P-18:1(11Z)/PGE2)	0.509503133
gene-TMEM145	1.905992549	Isopropyl isothiocyanate	0.172385747

gene-TMEM145	1.905992549	PC(18:1(9Z)/15:1(9Z))	0.534724682
gene-TMEM145	1.905992549	Nigroxanthin	0.705005059
gene-TMEM145	1.905992549	PC(P-18:1(11Z)/PGJ2)	0.565243877
gene-TMEM145	1.905992549	PS(20:0/20:4(8Z,11Z,14Z,17Z)-2OH(5S,6R))	0.402595921
gene-SYNGR1	1.897766994	3-Thiacytidine	0.209387412
gene-SYNGR1	1.897766994	Hydroxyprolyl-Serine	0.519540341
gene-SYNGR1	1.897766994	PE(22:2(13Z,16Z)/22:5(4Z,7Z,10Z,13Z,19Z)-O	0.369745166
gene-SYNGR1	1.897766994	Cyclosporin A	0.656529229
gene-SYNGR1	1.897766994	Angiotensin A	0.247332017
gene-SYNGR1	1.897766994	DG(18:0/LTE4/0:0)	0.681485773
gene-SYNGR1	1.897766994	PC(14:0/20:2(11Z,14Z))	1.110657378
gene-SYNGR1	1.897766994	CDP-DG(PGF2alpha/16:0)	1.002512277
gene-SYNGR1	1.897766994	ingenol	0.942071652
gene-SYNGR1	1.897766994	beta-L-Dioxolane-cytidine	0.175916668
gene-SYNGR1	1.897766994	Nigroxanthin	0.705005059
gene-SYNGR1	1.897766994	PS(20:0/20:4(8Z,11Z,14Z,17Z)-2OH(5S,6R))	0.402595921
gene-SYNGR1	1.897766994	CL(8:0/8:0/18:2(9Z,11Z)/20:0)	0.622988418
gene-PDZD3	1.893506291	3-Thiacytidine	0.209387412
gene-PDZD3	1.893506291	D-Erythro-imidazole-glycerol-phosphate	0.355812431
gene-PDZD3	1.893506291	7(14)-Bisabolene-2,3,10,11-tetrol	2.446897974
gene-PDZD3	1.893506291	3-Deoxyestrone	0.282221709
gene-PDZD3	1.893506291	1-Oleoyl-sn-glycero-3-phosphocholine	0.18926952
gene-PDZD3	1.893506291	LTB4-d4	0.226799982
gene-PDZD3	1.893506291	(-)-alpha-Terpineol	0.345716279
gene-PDZD3	1.893506291	1,4-Undecadiene	0.462803973
gene-PDZD3	1.893506291	5-(2-Aminopropyl)-2-methylphenol	0.323231457
gene-PDZD3	1.893506291	4-Dimethylamino-L-phenylalanine	0.242110226
gene-PDZD3	1.893506291	Armillane	0.52808635
gene-PDZD3	1.893506291	PC(P-18:1(11Z)/PGE2)	0.509503133
gene-PDZD3	1.893506291	(3R,4R)-3-Amino-1-hydroxy-4-methylpyrrol	0.471914506
gene-PDZD3	1.893506291	n-methyl-2-(4'-methylaminophenyl)-6-hydr	0.26655714
gene-PDZD3	1.893506291	Isopropyl isothiocyanate	0.172385747
gene-PDZD3	1.893506291	9-deoxy-9-methylene-16,16-dimethyl -PGE	0.606893884
gene-PDZD3	1.893506291	CL(8:0/8:0/18:2(9Z,11Z)/20:0)	0.622988418
gene-PDE8A	1.887885593	Ascorbic acid 3-sulfate	0.300057469
gene-PDE8A	1.887885593	Methylmalonate	0.241481249
gene-PDE8A	1.887885593	N-Undecylbenzenesulfonic acid	0.139246407
gene-PDE8A	1.887885593	PE-NMe(18:0/18:3(9Z,12Z,15Z))	0.681884774
gene-PDE8A	1.887885593	DG(14:0/PGE1/0:0)	0.323973925
gene-PDE8A	1.887885593	Barbituric acid	0.164689286
gene-PDE8A	1.887885593	Hydroxyisonobilin	0.259767036
gene-PDE8A	1.887885593	PIP(20:1(11Z)/LTE4)	0.171016851
gene-PDE8A	1.887885593	CDP-DG(18:0/PGE2)	0.280573668
gene-PDE8A	1.887885593	2-Dodecylbenzenesulfonic acid	0.177256025
gene-PDE8A	1.887885593	CDP-DG(PGJ2/18:0)	0.287350234
gene-PDE8A	1.887885593	PE(16:0/18:1(11Z))	0.367326926
gene-PDE8A	1.887885593	Microcystin LR	0.167364914
gene-PDE8A	1.887885593	PG(18:1(11Z)/LTE4)	0.265299982
gene-PDE8A	1.887885593	[5-(Aminomethyl)furan-3-yl]methyl diphosp	0.138877972
gene-PDE8A	1.887885593	3-Thiacytidine	0.209387412
gene-PDE8A	1.887885593	1-Phenylpiperazine	0.353584896

gene-PDE8A	1.887885593	N-(2-(Methylamino)ethyl)-5-isoquinolinesulf	0.199224064
gene-PDE8A	1.887885593	p-coumaroyltriacetic acid lactone	0.057681273
gene-PDE8A	1.887885593	4-Aminophenyl 1-thio-尾-D-glucuronide	0.18194032
gene-PDE8A	1.887885593	3,3',5-Trihydroxy-4'-methoxy-6,7-methylene	0.137607639
gene-PDE8A	1.887885593	Asparaginyl-Methionine	0.228436756
gene-PDE8A	1.887885593	Coenzyme F420-1	0.507686022
gene-PDE8A	1.887885593	2-(Formamido)-N1-(5'-phosphoribosyl)aceta	0.35156894
gene-PDE8A	1.887885593	5-Hydroxyindoleacetaldehyde	0.448806354
gene-PDE8A	1.887885593	Azelaic acid	0.007180175
gene-PDE8A	1.887885593	2-(3-Carboxy-3-aminopropyl)-L-histidine	0.575765457
gene-PDE8A	1.887885593	Uridine 2',3'-cyclic phosphate	0.201589726
gene-PDE8A	1.887885593	Oxamyl	0.077223423
gene-PDE8A	1.887885593	5-[(Diaminomethylidene)amino]-2-(sulfanyln	0.076227549
gene-PDE8A	1.887885593	5-Hydroxy-2-oxo-4-ureido-2,5-dihydro-1H	0.122536033
gene-PDE8A	1.887885593	p-Coumaroylputrescine	0.939330574
gene-PDE8A	1.887885593	Pterostilbene	0.899698314
gene-PDE8A	1.887885593	Ancymidol	0.913827444
gene-PDE8A	1.887885593	Delgocitinib	1.051864373
gene-PDE8A	1.887885593	2H-1-Benzopyran-2-one, aminomethyl-	0.385162237
gene-PDE8A	1.887885593	Trihomomethionine	0.296210774
gene-PDE8A	1.887885593	2-[(2-Aminoethylcarbamoyl)methyl]-2-hydro	0.192497471
gene-PDE8A	1.887885593	Dihydroxy-(2-methoxyethoxy)-sulfanylidene-	0.124546591
gene-PDE8A	1.887885593	Hygromycin B	0.930396619
gene-PDE8A	1.887885593	Metkephamid	1.159767003
gene-PDE8A	1.887885593	Tuberose lactone	1.590026295
gene-PDE8A	1.887885593	PGP(20:2(11Z,14Z)/18:2(10E,12Z)+=O(9))	1.457698264
gene-PDE8A	1.887885593	Eicosanoyl-CoA	0.137091047
gene-PDE8A	1.887885593	5-Heptyltetrahydro-2-oxo-3-furancarboxylic	0.178798355
gene-PDE8A	1.887885593	Isoplumbagin	0.127417868
gene-PDE8A	1.887885593	L-2-Aminoethyl seryl phosphate	0.215980578
gene-PDE8A	1.887885593	PI(18:2(9Z,12Z)/PGF2alpha)	0.015701107
gene-PDE8A	1.887885593	2-Hexylbenzothiazole	0.396099297
gene-PDE8A	1.887885593	PC(24:0/20:3(8Z,11Z,14Z)-2OH(5,6))	0.43567608
gene-PDE8A	1.887885593	Valnemulin	0.408062514
gene-PDE8A	1.887885593	Testosterone isobutyrate	0.467664668
gene-PDE8A	1.887885593	6-isobutyl-4-hydroxy-2-pyrone	0.473678412
gene-PDE8A	1.887885593	LTB4-d4	0.226799982
gene-PDE8A	1.887885593	PE(20:0/18:1(12Z)-2OH(9,10))	0.438658253
gene-PDE8A	1.887885593	Molindone	0.353725645
gene-PDE8A	1.887885593	PE(18:0/18:1(9Z)-O(12,13))	1.009186776
gene-PDE8A	1.887885593	PE(P-18:0/PGE1)	0.886597382
gene-PDE8A	1.887885593	PC(P-16:0/18:1(12Z)-2OH(9,10))	1.231706326
gene-PDE8A	1.887885593	3-O-Sulfogalactosylceramide (d18:1/20:0)	0.255536938
gene-PDE8A	1.887885593	DG(17:0/20:3(8Z,11Z,14Z)-2OH(5,6)/0:0)	0.129288711
gene-PDE8A	1.887885593	PI(18:1(11Z)/PGF1alpha)	0.243592581
gene-PDE8A	1.887885593	[(3S,4S,5S,6R)-3,4,5-Trihydroxy-6-(hydroxym	0.008715229
gene-PDE8A	1.887885593	Cyclosporin A	0.656529229
gene-PDE8A	1.887885593	Angiotensin A	0.247332017
gene-PDE8A	1.887885593	Epomusenin A	0.766206049
gene-PDE8A	1.887885593	PA(22:6(4Z,7Z,10Z,13Z,16Z,19Z)/16:0)	0.556532497
gene-PDE8A	1.887885593	DG(18:0/LTE4/0:0)	0.681485773

gene-PDE8A	1.887885593	PC(17:0/PGJ2)	0.657689319
gene-PDE8A	1.887885593	Alisporivir	0.430769197
gene-PDE8A	1.887885593	PC(14:0/20:2(11Z,14Z))	1.110657378
gene-PDE8A	1.887885593	(4r,5s,6s,7r)-4,7-Dibenzyl-5,6-dihydroxy-1,3-	1.334600128
gene-PDE8A	1.887885593	CDP-DG(PGF2alpha/16:0)	1.002512277
gene-PDE8A	1.887885593	(1S,2R)-1-C-(indol-3-yl)glycerol 3-phosphat	0.374678916
gene-PDE8A	1.887885593	GDP-valienol	0.231837085
gene-PDE8A	1.887885593	Cyclohexa-1,2,4,5-tetraene	0.073496201
gene-PDE8A	1.887885593	1-beta-D-Arabinofuranosyl-5-fluorocytosine	0.152339645
gene-PDE8A	1.887885593	Cysteinyl-Glutamine	0.079050155
gene-PDE8A	1.887885593	gamma-Glutamylcysteinylserine	0.098057967
gene-PDE8A	1.887885593	D-Xylonate	0.386869454
gene-PDE8A	1.887885593	2-Methyl-5-nitroimidazol-1-ylacetic acid	0.701441397
gene-PDE8A	1.887885593	Fluoroazomycin arabinoside	0.590955233
gene-PDE8A	1.887885593	Cysteinyl-Tryptophan	0.586270687
gene-PDE8A	1.887885593	15-keto-Prostaglandin E2	2.646629404
gene-PDE8A	1.887885593	1,4-Benzothiazine-O-quinonimine	0.396873161
gene-PDE8A	1.887885593	4-Hydroxyproline galactoside	0.148083825
gene-PDE8A	1.887885593	C20914	0.140383181
gene-PDE8A	1.887885593	S-(2-Hydroxyethyl)glutathione	0.269671835
gene-PDE8A	1.887885593	Fluridone	0.127106682
gene-PDE8A	1.887885593	Austdiol	0.046757893
gene-PDE8A	1.887885593	3-O-fucopyranosyl-2-acetamido-2-deoxygl	2.892346598
gene-PDE8A	1.887885593	Norophthalmic acid	0.191432411
gene-PDE8A	1.887885593	4'-Thiothymidine	0.066948524
gene-PDE8A	1.887885593	Desmethylflumazenil (Ro 15-5528)	0.227256571
gene-PDE8A	1.887885593	5-Phosphoribosyl-4-carboxy-5-aminoimidaz	0.052809864
gene-PDE8A	1.887885593	6-Fluorohomovanillic acid	0.040947314
gene-PDE8A	1.887885593	L-Oxalylalbizziine	0.002304251
gene-PDE8A	1.887885593	cis-3,4-Phenanthrenedihydrodiol-4-carboxyl	1.374283937
gene-PDE8A	1.887885593	Met His Gly	3.037980823
gene-PDE8A	1.887885593	5,6,7,8-Tetrahydromonapterin	0.724075531
gene-PDE8A	1.887885593	Harmalol	0.364945111
gene-PDE8A	1.887885593	2,3-Epoxymenaquinone	2.040730224
gene-PDE8A	1.887885593	Etamicastat	0.052120936
gene-PDE8A	1.887885593	3-Hydroxypicolinic acid	0.088414928
gene-PDE8A	1.887885593	6-hydroxymethyl-7,8-dihydropterin	0.190396346
gene-PDE8A	1.887885593	1-[(2R,4S,5S)-5-[Difluoro(hydroxy)methyl]-4-	0.091474911
gene-PDE8A	1.887885593	Guanidoacetic acid	0.542509265
gene-PDE8A	1.887885593	Methionyl-Valine	0.502707745
gene-PDE8A	1.887885593	(5alpha,6beta,14alpha,20R,22R)-5,6,14,20,27-	1.296004093
gene-PDE8A	1.887885593	Macrocin	1.715200143
gene-PDE8A	1.887885593	9-Octadecenal	0.958949131
gene-PDE8A	1.887885593	Vulgarone A	0.102049407
gene-PDE8A	1.887885593	(3Z,6Z)-3,6-Nonadien-1-ol	0.185378696
gene-PDE8A	1.887885593	Zingiberenol	0.088860673
gene-PDE8A	1.887885593	14alpha-Hydroxy-5beta-cholest-7-ene-3,6-	0.206315646
gene-PDE8A	1.887885593	beta-L-Dioxolane-cytidine	0.175916668
gene-PDE8A	1.887885593	1,4,6-Trimethylnaphthalene	0.12150693
gene-PDE8A	1.887885593	DG(2:0/18:1(12Z)-O(9S,10R)/0:0)	0.033180989
gene-PDE8A	1.887885593	2-Phenylpropyl acetate	0.202302943

gene-PDE8A	1.887885593	MG(0:0/18:3(9Z,12Z,15Z)/0:0)	1.373784038
gene-PDE8A	1.887885593	2,3-Dihydroxypropyl octanoate	0.242447284
gene-PDE8A	1.887885593	N-Palmitoyl Proline	0.470367693
gene-PDE8A	1.887885593	Ile His Leu Trp	0.124696937
gene-PDE8A	1.887885593	Atorvastatin	0.291646019
gene-PDE8A	1.887885593	10-alpha-methoxy-9,10-dihydrolysergol	0.140851438
gene-PDE8A	1.887885593	Vipadenant	0.243429143
gene-PDE8A	1.887885593	17-Aminogeldanamycin	0.518144528
gene-PDE8A	1.887885593	Ethyl salicylate	0.099640862
gene-PDE8A	1.887885593	MG(LTE4/0:0/0:0)	0.548257353
gene-PDE8A	1.887885593	Dapdiamide B	0.07688703
gene-PDE8A	1.887885593	Iridal	0.699410612
gene-PDE8A	1.887885593	N-Stearoyl Proline	0.469971646
gene-PDE8A	1.887885593	LysoPI(0:0/18:0)	0.420441153
gene-PDE8A	1.887885593	1,3,5-Bisabolatrien-10-one	0.109736328
gene-PDE8A	1.887885593	Hypaconitine	0.176000431
gene-PDE8A	1.887885593	N-Stearoyl Valine	0.653773206
gene-PDE8A	1.887885593	PS(24:1(15Z)/24:1(15Z))	0.557296152
gene-PDE8A	1.887885593	L-(-)Sorbose	0.168270222
gene-PDE8A	1.887885593	N-Palmitoyl Glutamic acid	0.250188721
gene-PDE8A	1.887885593	Dopamine quinone	0.054568176
gene-PDE8A	1.887885593	(2E)-2-dodecen-1-ol	0.055772695
gene-PDE8A	1.887885593	1-Heneicosanoyl-glycero-3-phosphoserine	0.214358093
gene-PDE8A	1.887885593	DG(13:0/20:4(6E,8Z,11Z,14Z)+=O(5)/0:0)	0.124018655
gene-PDE8A	1.887885593	Cer(d18:2(4E,14Z)/TXB2)	0.236013333
gene-PDE8A	1.887885593	norerythromycin	0.401118778
gene-PDE8A	1.887885593	PC(18:1(9Z)/15:1(9Z))	0.534724682
gene-PDE8A	1.887885593	PC(18:0/20:4(5Z,8Z,11Z,14Z)-OH(20))	0.318846141
gene-PDE8A	1.887885593	arachidyl amido cholanoic acid	1.24842952
gene-PDE8A	1.887885593	Nigroxanthin	0.705005059
gene-PDE8A	1.887885593	3-Hydroxyheptadecanoylcarnitine	0.860642943
gene-PDE8A	1.887885593	PE(P-18:0/20:5(5Z,8Z,11Z,14Z,16E)-OH(18))	0.339204508
gene-PDE8A	1.887885593	1-Octadecanoyl-2-(7Z,10Z,13Z,16Z-docosat	0.690496314
gene-PDE8A	1.887885593	PE(18:0/20:4(8Z,11Z,14Z,17Z)-2OH(5S,6R))	0.418208781
gene-PDE8A	1.887885593	CE(LTE4)	0.204616463
gene-PDE8A	1.887885593	PC(P-18:1(11Z)/PGJ2)	0.565243877
gene-PDE8A	1.887885593	Azimexon	0.008689891
gene-PDE8A	1.887885593	PC(20:5(5Z,8Z,11Z,14Z,17Z)/P-16:0)	1.301095986
gene-PDE8A	1.887885593	PE-NMe(18:2(9Z,12Z)/18:2(9Z,12Z))[U]	0.694736593
gene-PDE8A	1.887885593	DG(20:0/LTE4/0:0)	0.438074508
gene-LOC10029(	1.865360006	D-Erythro-imidazole-glycerol-phosphate	0.355812431
gene-LOC10029(	1.865360006	7(14)-Bisabolene-2,3,10,11-tetrol	2.446897974
gene-LOC10029(	1.865360006	LTB4-d4	0.226799982
gene-LOC10029(	1.865360006	(-)-alpha-Terpineol	0.345716279
gene-LOC10029(	1.865360006	4-Dimethylamino-L-phenylalanine	0.242110226
gene-LOC10029(	1.865360006	Trimetazidine	0.438282534
gene-LOC10029(	1.865360006	Armillane	0.52808635
gene-LOC10029(	1.865360006	5'-S-Methyl-5'-thioinosine	0.28772476
gene-LOC10029(	1.865360006	(Z)-3-Oxo-2-(2-pentenyl)-1-cyclopenteneac	0.179805915
gene-LOC10029(	1.865360006	PC(P-18:1(11Z)/PGE2)	0.509503133
gene-LOC10029(	1.865360006	(3R,4R)-3-Amino-1-hydroxy-4-methylpyrrol	0.471914506

gene-LOC10029(	1.865360006	n-methyl-2-(4'-methylaminophenyl)-6-hydr	0.26655714
gene-LOC10029(	1.865360006	Isopropyl isothiocyanate	0.172385747
gene-LOC10029(	1.865360006	9-deoxy-9-methylene-16,16-dimethyl -PGE:	0.606893884
gene-LOC10029(	1.865360006	CL(8:0/8:0/18:2(9Z,11Z)/20:0)	0.622988418
gene-C11H2orf5(	1.854417509	D-Erythro-imidazole-glycerol-phosphate	0.355812431
gene-C11H2orf5(	1.854417509	Glutamate carbon	0.671444516
gene-C11H2orf5(	1.854417509	Urocortisol	0.926998516
gene-C11H2orf5(	1.854417509	11-Maleimidoundecanoic acid	1.084942397
gene-C11H2orf5(	1.854417509	13(S)-HpODE	0.163157753
gene-C11H2orf5(	1.854417509	7(S),17(S)-dihydroxy-8(E),10(Z),13(Z),15(E),19	0.32147403
gene-C11H2orf5(	1.854417509	LTB4-d4	0.226799982
gene-C11H2orf5(	1.854417509	(1R,6S)-6-Amino-5-oxocyclohex-2-ene-1-c	0.154751123
gene-C11H2orf5(	1.854417509	2-Methyl-3-phenyl-2-propenal	0.407813975
gene-C11H2orf5(	1.854417509	3-(3-Methylbutylidene)-1(3H)-isobenzofurar	0.575422414
gene-C11H2orf5(	1.854417509	[3-(2-Aminopropyl)-6-methylidenecyclohexa	0.422945997
gene-C11H2orf5(	1.854417509	3',4',5'-Trimethoxycinnamyl alcohol acetate	0.4221816
gene-C11H2orf5(	1.854417509	PC(P-18:1(11Z)/PGE2)	0.509503133
gene-C11H2orf5(	1.854417509	PC(P-18:1(11Z)/PGE1)	0.295520345
gene-C11H2orf5(	1.854417509	Roxithromycin	0.268273077
gene-C11H2orf5(	1.854417509	PC(P-18:1(11Z)/PGJ2)	0.565243877
Bos_taurus_newG	1.854166351	3-hydroxypristanic acid	0.548515835
Bos_taurus_newG	1.854166351	7(14)-Bisabolene-2,3,10,11-tetrol	2.446897974
Bos_taurus_newG	1.854166351	(3Z)-Phycoerythrobilin	1.456755874
Bos_taurus_newG	1.854166351	1,2-O-Isopropylidene-D-glucofuranose	0.080987667
Bos_taurus_newG	1.854166351	3-Deoxyestrone	0.282221709
Bos_taurus_newG	1.854166351	1-Oleoyl-sn-glycero-3-phosphocholine	0.18926952
Bos_taurus_newG	1.854166351	3,4-dihydroxy-5-all-trans-hexaprenylbenzoa	0.123615726
Bos_taurus_newG	1.854166351	Lividamine	0.319679555
Bos_taurus_newG	1.854166351	Psychosine	0.106475396
Bos_taurus_newG	1.854166351	Cyclotricuspidogenin C	0.440884085
Bos_taurus_newG	1.854166351	(-)-alpha-Terpineol	0.345716279
Bos_taurus_newG	1.854166351	1,4-Undecadiene	0.462803973
Bos_taurus_newG	1.854166351	2-isopentyl-3,6-dimethyl pyrazine	0.710502562
Bos_taurus_newG	1.854166351	5-(2-Aminopropyl)-2-methylphenol	0.323231457
Bos_taurus_newG	1.854166351	4-Dimethylamino-L-phenylalanine	0.242110226
Bos_taurus_newG	1.854166351	Trimetazidine	0.438282534
Bos_taurus_newG	1.854166351	ingenol	0.942071652
Bos_taurus_newG	1.854166351	Armillane	0.52808635
Bos_taurus_newG	1.854166351	PC(P-18:1(11Z)/PGE2)	0.509503133
Bos_taurus_newG	1.854166351	(3R,4R)-3-Amino-1-hydroxy-4-methylpyrrol	0.471914506
Bos_taurus_newG	1.854166351	1-Palmitoylglycerol	0.084357408
Bos_taurus_newG	1.854166351	N2-gamma-Glutamylglutamine	0.230065499
Bos_taurus_newG	1.854166351	n-methyl-2-(4'-methylaminophenyl)-6-hydr	0.26655714
Bos_taurus_newG	1.854166351	Isopropyl isothiocyanate	0.172385747
Bos_taurus_newG	1.854166351	9-deoxy-9-methylene-16,16-dimethyl -PGE:	0.606893884
Bos_taurus_newG	1.854166351	CL(8:0/8:0/18:2(9Z,11Z)/20:0)	0.622988418
gene-SLC7A11	1.851492544	3-Thiacytidine	0.209387412
gene-SLC7A11	1.851492544	milbemycin beta3	1.414447124
gene-SLC7A11	1.851492544	PE(20:0/18:1(12Z)-2OH(9,10))	0.438658253
gene-SLC7A11	1.851492544	PE(22:2(13Z,16Z)/22:5(4Z,7Z,10Z,13Z,19Z)-O(	0.369745166
gene-SLC7A11	1.851492544	Cyclosporin A	0.656529229

gene-SLC7A11	1.851492544	Angiotensin A	0.247332017
gene-SLC7A11	1.851492544	DG(18:0/LTE4/0:0)	0.681485773
gene-SLC7A11	1.851492544	PC(14:0/20:2(11Z,14Z))	1.110657378
gene-SLC7A11	1.851492544	CDP-DG(PGF2alpha/16:0)	1.002512277
gene-SLC7A11	1.851492544	PC(P-18:1(11Z)/PGE2)	0.509503133
gene-SLC7A11	1.851492544	Phorone A	0.079039095
gene-SLC7A11	1.851492544	Nigroxanthin	0.705005059
gene-SLC7A11	1.851492544	PC(P-18:1(11Z)/PGJ2)	0.565243877
gene-SLC7A11	1.851492544	PS(20:0/20:4(8Z,11Z,14Z,17Z)-2OH(5S,6R))	0.402595921
gene-MGLL	1.848556415	D-Erythro-imidazole-glycerol-phosphate	0.355812431
gene-MGLL	1.848556415	7(14)-Bisabolene-2,3,10,11-tetrol	2.446897974
gene-MGLL	1.848556415	(3Z)-Phycoerythrobilin	1.456755874
gene-MGLL	1.848556415	3-Deoxyestrone	0.282221709
gene-MGLL	1.848556415	1-Oleoyl-sn-glycero-3-phosphocholine	0.18926952
gene-MGLL	1.848556415	Lividamine	0.319679555
gene-MGLL	1.848556415	Psychosine	0.106475396
gene-MGLL	1.848556415	Cyclotricuspidogenin C	0.440884085
gene-MGLL	1.848556415	(-)-alpha-Terpineol	0.345716279
gene-MGLL	1.848556415	1,4-Undecadiene	0.462803973
gene-MGLL	1.848556415	2-isopentyl-3,6-dimethyl pyrazine	0.710502562
gene-MGLL	1.848556415	PG(20:1(11Z)/18:3(10,12,15)-OH(9))	0.626244347
gene-MGLL	1.848556415	5-(2-Aminopropyl)-2-methylphenol	0.323231457
gene-MGLL	1.848556415	4-Dimethylamino-L-phenylalanine	0.242110226
gene-MGLL	1.848556415	Trimetazidine	0.438282534
gene-MGLL	1.848556415	Armillane	0.52808635
gene-MGLL	1.848556415	PC(P-18:1(11Z)/PGE2)	0.509503133
gene-MGLL	1.848556415	(3R,4R)-3-Amino-1-hydroxy-4-methylpyrrol	0.471914506
gene-MGLL	1.848556415	n-methyl-2-(4'-methylaminophenyl)-6-hydr	0.26655714
gene-MGLL	1.848556415	Isopropyl isothiocyanate	0.172385747
gene-MGLL	1.848556415	(9Z)-Octadecenoic acid	0.142359556
gene-MGLL	1.848556415	9-deoxy-9-methylene-16,16-dimethyl -PGE	0.606893884
gene-MGLL	1.848556415	CL(8:0/8:0/18:2(9Z,11Z)/20:0)	0.622988418
gene-HSPG2	1.837274598	D-Erythro-imidazole-glycerol-phosphate	0.355812431
gene-HSPG2	1.837274598	Undecanedioic acid	0.059955634
gene-HSPG2	1.837274598	cis-p-Menth-2-en-1-ol	0.201017988
gene-HSPG2	1.837274598	(-)-alpha-Terpineol	0.345716279
gene-HSPG2	1.837274598	PE(22:2(13Z,16Z)/22:5(4Z,7Z,10Z,13Z,19Z)-O	0.369745166
gene-HSPG2	1.837274598	PG(20:1(11Z)/18:3(10,12,15)-OH(9))	0.626244347
gene-HSPG2	1.837274598	N-Eicosapentaenoyl Asparagine	0.371594025
gene-HSPG2	1.837274598	PC(P-18:1(11Z)/PGE2)	0.509503133
gene-HSPG2	1.837274598	PC(P-18:1(11Z)/PGJ2)	0.565243877
gene-HSPG2	1.837274598	PS(20:0/20:4(8Z,11Z,14Z,17Z)-2OH(5S,6R))	0.402595921
gene-HSPG2	1.837274598	PG(20:1(11Z)/18:3(9,11,15)-OH(13))	0.69829683
gene-EEPD1	1.836573841	3-Thiacytidine	0.209387412
gene-EEPD1	1.836573841	3-Deoxyestrone	0.282221709
gene-EEPD1	1.836573841	1-Oleoyl-sn-glycero-3-phosphocholine	0.18926952
gene-EEPD1	1.836573841	Psychosine	0.106475396
gene-EEPD1	1.836573841	LTB4-d4	0.226799982
gene-EEPD1	1.836573841	(-)-alpha-Terpineol	0.345716279
gene-EEPD1	1.836573841	1,4-Undecadiene	0.462803973
gene-EEPD1	1.836573841	5-(2-Aminopropyl)-2-methylphenol	0.323231457

gene-EEPD1	1.836573841	4-Dimethylamino-L-phenylalanine	0.242110226
gene-EEPD1	1.836573841	Trimetazidine	0.438282534
gene-EEPD1	1.836573841	ingenol	0.942071652
gene-EEPD1	1.836573841	Armillane	0.52808635
gene-EEPD1	1.836573841	PC(P-18:1(11Z)/PGE2)	0.509503133
gene-EEPD1	1.836573841	PC(20:3(5Z,8Z,11Z)/24:0)	0.387959564
gene-EEPD1	1.836573841	(3R,4R)-3-Amino-1-hydroxy-4-methylpyrrol	0.471914506
gene-EEPD1	1.836573841	n-methyl-2-(4'-methylaminophenyl)-6-hydr	0.26655714
gene-EEPD1	1.836573841	Isopropyl isothiocyanate	0.172385747
gene-EEPD1	1.836573841	(9Z)-Octadecenoic acid	0.142359556
gene-EEPD1	1.836573841	9-deoxy-9-methylene-16,16-dimethyl -PGE	0.606893884
gene-EEPD1	1.836573841	CL(8:0/8:0/18:2(9Z,11Z)/20:0)	0.622988418
gene-STARD10	1.833556348	3-Thiacytidine	0.209387412
gene-STARD10	1.833556348	(-)-alpha-Terpineol	0.345716279
gene-STARD10	1.833556348	1,4-Undecadiene	0.462803973
gene-STARD10	1.833556348	4-cholesten-7伪,12伪,24-triol-3-one	0.097840451
gene-STARD10	1.833556348	5-(2-Aminopropyl)-2-methylphenol	0.323231457
gene-STARD10	1.833556348	4-Dimethylamino-L-phenylalanine	0.242110226
gene-STARD10	1.833556348	Armillane	0.52808635
gene-STARD10	1.833556348	PC(P-18:1(11Z)/PGE2)	0.509503133
gene-STARD10	1.833556348	(3R,4R)-3-Amino-1-hydroxy-4-methylpyrrol	0.471914506
gene-STARD10	1.833556348	n-methyl-2-(4'-methylaminophenyl)-6-hydr	0.26655714
gene-STARD10	1.833556348	Isopropyl isothiocyanate	0.172385747
gene-STARD10	1.833556348	CL(8:0/8:0/18:2(9Z,11Z)/20:0)	0.622988418
Bos_taurus_newG	1.820187894	Isomaltotriose	0.58434654
Bos_taurus_newG	1.820187894	(-)-alpha-Terpineol	0.345716279
Bos_taurus_newG	1.820187894	4-cholesten-7伪,12伪,24-triol-3-one	0.097840451
Bos_taurus_newG	1.820187894	3b,6a-Dihydroxy-alpha-ionol 9-[apiosyl-(1-	0.045258177
Bos_taurus_newG	1.820187894	N-[[3-Hydroxy-2-(2-pentenyl)cyclopentyl]ac	1.043930947
Bos_taurus_newG	1.820187894	PC(P-18:1(11Z)/PGE2)	0.509503133
Bos_taurus_newG	1.820187894	PC(20:3(5Z,8Z,11Z)/24:0)	0.387959564
Bos_taurus_newG	1.820187894	Phorone A	0.079039095
Bos_taurus_newG	1.820187894	2-Amino-4-[carbamimidoyl(methyl)amino]bu	0.075703353
Bos_taurus_newG	1.820187894	Digalacturonate	0.473524431
Bos_taurus_newG	1.820187894	Phenethyl 6-galloylglucoside	0.069516774
Bos_taurus_newG	1.814063176	5-(3'-Carboxy-3'-oxopropenyl)-4,6-dihydro	1.083232395
Bos_taurus_newG	1.814063176	(+)-Bottrospicatol	0.68654221
Bos_taurus_newG	1.814063176	Hexahydro-4-methylphthalic anhydride	0.691665693
Bos_taurus_newG	1.814063176	Methionine sulfone	0.738197353
Bos_taurus_newG	1.814063176	7-Hydroxy-6-methyl-8-ribityllumazine	0.103672738
Bos_taurus_newG	1.814063176	Val-Cit	0.452308451
Bos_taurus_newG	1.814063176	3-(2,4-Dimethyl-5-(2-oxo-1,2-dihydroindol-	0.095524813
Bos_taurus_newG	1.814063176	3-carboxy-4-methyl-5-pentyl-2-furanpropa	0.375666997
Bos_taurus_newG	1.814063176	(S)-10,16-Dihydroxyhexadecanoic acid	1.054006526
Bos_taurus_newG	1.814063176	Auxin b	1.052491354
Bos_taurus_newG	1.814063176	Gemfibrozil	0.392694536
Bos_taurus_newG	1.814063176	4-Gingerol	0.222165012
Bos_taurus_newG	1.814063176	Aspartame	0.406719484
Bos_taurus_newG	1.814063176	Monacolin L acid	1.060562098
Bos_taurus_newG	1.814063176	Eicosapentaenoic acid	0.575884419
Bos_taurus_newG	1.814063176	3,4,3',4'-Tetrahydrospirilloxanthin	0.354666148

Bos_taurus_newG	1.814063176	(1R,6S)-6-Amino-5-oxocyclohex-2-ene-1-c	0.154751123
Bos_taurus_newG	1.814063176	7-Methylguanosine	1.215574844
Bos_taurus_newG	1.814063176	2-Hydroxyglutaric acid diethyl ester	0.799886767
Bos_taurus_newG	1.814063176	6-hydroxy-7E,9E-Octadecadiene-11,13,15,1	1.179129125
Bos_taurus_newG	1.814063176	7C-aglycone	1.015608521
Bos_taurus_newG	1.814063176	L-alpha-Acetyl-N,N-dinormethadol	1.567999001
Bos_taurus_newG	1.814063176	2-(1-Adamantyl)-1,3-dioxetane	0.408916843
Bos_taurus_newG	1.814063176	2-Oxo-10-methylthiodecanoic acid	0.695908469
Bos_taurus_newG	1.814063176	(-)-Huperzine A (HupA)	0.935198906
Bos_taurus_newG	1.814063176	Sequiterpene Lactone 326	1.141450991
Bos_taurus_newG	1.814063176	ent-16b,19-Kauranediol 19-acetate	0.400676827
Bos_taurus_newG	1.814063176	Pitheduloside I	1.362662847
Bos_taurus_newG	1.814063176	Ketosantalic acid	0.356663411
Bos_taurus_newG	1.814063176	(Z)-3-Methyl-3-decenoic acid	0.482455266
Bos_taurus_newG	1.814063176	Etazolate	0.149098864
Bos_taurus_newG	1.814063176	Roxithromycin	0.268273077
gene-MYO18B	1.808605222	D-Erythro-imidazole-glycerol-phosphate	0.355812431
gene-MYO18B	1.808605222	13(S)-HpODE	0.163157753
gene-MYO18B	1.808605222	LTB4-d4	0.226799982
gene-MYO18B	1.808605222	2-Methyl-3-phenyl-2-propenal	0.407813975
gene-MYO18B	1.808605222	PC(P-18:1(11Z)/PGE2)	0.509503133
gene-LOC51286	1.807148745	3-Thiacytidine	0.209387412
gene-LOC51286	1.807148745	D-Erythro-imidazole-glycerol-phosphate	0.355812431
gene-LOC51286	1.807148745	3-Deoxyestrone	0.282221709
gene-LOC51286	1.807148745	1-Oleoyl-sn-glycero-3-phosphocholine	0.18926952
gene-LOC51286	1.807148745	LTB4-d4	0.226799982
gene-LOC51286	1.807148745	(-)-alpha-Terpineol	0.345716279
gene-LOC51286	1.807148745	Cyclosporin A	0.656529229
gene-LOC51286	1.807148745	5-(2-Aminopropyl)-2-methylphenol	0.323231457
gene-LOC51286	1.807148745	4-Dimethylamino-L-phenylalanine	0.242110226
gene-LOC51286	1.807148745	Trimetazidine	0.438282534
gene-LOC51286	1.807148745	Armillane	0.52808635
gene-LOC51286	1.807148745	PC(P-18:1(11Z)/PGE2)	0.509503133
gene-LOC51286	1.807148745	(3R,4R)-3-Amino-1-hydroxy-4-methylpyrrol	0.471914506
gene-LOC51286	1.807148745	n-methyl-2-(4'-methylaminophenyl)-6-hydr	0.26655714
gene-LOC51286	1.807148745	Isopropyl isothiocyanate	0.172385747
gene-LOC51286	1.807148745	(9Z)-Octadecenoic acid	0.142359556
gene-LOC51286	1.807148745	9-deoxy-9-methylene-16,16-dimethyl -PGE	0.606893884
gene-LOC51286	1.807148745	CL(8:0/8:0/18:2(9Z,11Z)/20:0)	0.622988418
Bos_taurus_newG	1.797761813	LysoPI(16:0/0:0)	0.379098336
Bos_taurus_newG	1.797761813	3-Thiacytidine	0.209387412
Bos_taurus_newG	1.797761813	5-Hydroxy-2-oxo-4-ureido-2,5-dihydro-1H	0.122536033
Bos_taurus_newG	1.797761813	11-Maleimidoundecanoic acid	1.084942397
Bos_taurus_newG	1.797761813	Monacolin L acid	1.060562098
Bos_taurus_newG	1.797761813	cis-p-Menth-2-en-1-ol	0.201017988
Bos_taurus_newG	1.797761813	PE(20:0/18:1(12Z)-2OH(9,10))	0.438658253
Bos_taurus_newG	1.797761813	PE(22:2(13Z,16Z)/22:5(4Z,7Z,10Z,13Z,19Z)-O	0.369745166
Bos_taurus_newG	1.797761813	Lamivudine	0.327402892
Bos_taurus_newG	1.797761813	1-beta-D-Arabinofuranosyl-5-fluorocytosin	0.152339645
Bos_taurus_newG	1.797761813	15-keto-Prostaglandin E2	2.646629404
Bos_taurus_newG	1.797761813	S-Acetyldihydrolipoamide-E	0.429561283

Bos_taurus_newG	1.797761813	Cyclic Urea	0.1483082
Bos_taurus_newG	1.797761813	Fluridone	0.127106682
Bos_taurus_newG	1.797761813	Norophthalmic acid	0.191432411
Bos_taurus_newG	1.797761813	5,6,7,8-Tetrahydromonapterin	0.724075531
Bos_taurus_newG	1.797761813	Guanidoacetic acid	0.542509265
Bos_taurus_newG	1.797761813	4-Oxo-9-cis-retinoyl-beta-glucuronide	1.611773742
Bos_taurus_newG	1.797761813	alpha-Terpineol formate	0.628238986
Bos_taurus_newG	1.797761813	Ile His Leu Trp	0.124696937
Bos_taurus_newG	1.797761813	10-alpha-methoxy-9,10-dihydrolysergol	0.140851438
Bos_taurus_newG	1.797761813	MG(0:0/22:5(4Z,7Z,10Z,13Z,16Z)/0:0)	0.136549841
Bos_taurus_newG	1.797761813	PC(18:1(9Z)/15:1(9Z))	0.534724682
Bos_taurus_newG	1.797761813	Nigroxanthin	0.705005059
Bos_taurus_newG	1.797761813	PC(P-18:1(11Z)/PGJ2)	0.565243877
Bos_taurus_newG	1.797761813	PS(20:0/20:4(8Z,11Z,14Z,17Z)-2OH(5S,6R))	0.402595921
gene-ADAMDEC	1.79639996	3-Thiacytidine	0.209387412
gene-ADAMDEC	1.79639996	D-Erythro-imidazole-glycerol-phosphate	0.355812431
gene-ADAMDEC	1.79639996	11-Maleimidoundecanoic acid	1.084942397
gene-ADAMDEC	1.79639996	cis-p-Menth-2-en-1-ol	0.201017988
gene-ADAMDEC	1.79639996	PE(22:2(13Z,16Z)/22:5(4Z,7Z,10Z,13Z,19Z)-O	0.369745166
gene-ADAMDEC	1.79639996	Cyclosporin A	0.656529229
gene-ADAMDEC	1.79639996	beta-L-Dioxolane-cytidine	0.175916668
gene-ADAMDEC	1.79639996	PC(P-18:1(11Z)/PGE2)	0.509503133
gene-ADAMDEC	1.79639996	Roxithromycin	0.268273077
gene-ADAMDEC	1.79639996	Nigroxanthin	0.705005059
gene-ADAMDEC	1.79639996	PC(P-18:1(11Z)/PGJ2)	0.565243877
gene-ADAMDEC	1.79639996	PS(20:0/20:4(8Z,11Z,14Z,17Z)-2OH(5S,6R))	0.402595921
gene-ANKRD13D	1.789651616	3-Thiacytidine	0.209387412
gene-ANKRD13D	1.789651616	D-Erythro-imidazole-glycerol-phosphate	0.355812431
gene-ANKRD13D	1.789651616	3-Deoxyestrone	0.282221709
gene-ANKRD13D	1.789651616	1-Oleoyl-sn-glycero-3-phosphocholine	0.18926952
gene-ANKRD13D	1.789651616	LTB4-d4	0.226799982
gene-ANKRD13D	1.789651616	cis-p-Menth-2-en-1-ol	0.201017988
gene-ANKRD13D	1.789651616	(-)-alpha-Terpineol	0.345716279
gene-ANKRD13D	1.789651616	1,4-Undecadiene	0.462803973
gene-ANKRD13D	1.789651616	5-(2-Aminopropyl)-2-methylphenol	0.323231457
gene-ANKRD13D	1.789651616	4-Dimethylamino-L-phenylalanine	0.242110226
gene-ANKRD13D	1.789651616	Armillane	0.52808635
gene-ANKRD13D	1.789651616	2-Methyl-3-phenyl-2-propenal	0.407813975
gene-ANKRD13D	1.789651616	PC(P-18:1(11Z)/PGE2)	0.509503133
gene-ANKRD13D	1.789651616	(3R,4R)-3-Amino-1-hydroxy-4-methylpyrrol	0.471914506
gene-ANKRD13D	1.789651616	n-methyl-2-(4'-methylaminophenyl)-6-hydr	0.26655714
gene-ANKRD13D	1.789651616	Isopropyl isothiocyanate	0.172385747
gene-ANKRD13D	1.789651616	(9Z)-Octadecenoic acid	0.142359556
gene-ANKRD13D	1.789651616	9-deoxy-9-methylene-16,16-dimethyl -PGE	0.606893884
gene-ANKRD13D	1.789651616	PS(20:0/20:4(8Z,11Z,14Z,17Z)-2OH(5S,6R))	0.402595921
gene-ANKRD13D	1.789651616	CL(8:0/8:0/18:2(9Z,11Z)/20:0)	0.622988418
gene-BFSP2	1.782427588	3-Thiacytidine	0.209387412
gene-BFSP2	1.782427588	1-Oleoyl-sn-glycero-3-phosphocholine	0.18926952
gene-BFSP2	1.782427588	LTB4-d4	0.226799982
gene-BFSP2	1.782427588	(-)-alpha-Terpineol	0.345716279
gene-BFSP2	1.782427588	1,4-Undecadiene	0.462803973

gene-BFSP2	1.782427588	Cyclosporin A	0.656529229
gene-BFSP2	1.782427588	PC(14:0/20:2(11Z,14Z))	1.110657378
gene-BFSP2	1.782427588	5-(2-Aminopropyl)-2-methylphenol	0.323231457
gene-BFSP2	1.782427588	4-Dimethylamino-L-phenylalanine	0.242110226
gene-BFSP2	1.782427588	Trimetazidine	0.438282534
gene-BFSP2	1.782427588	ingenol	0.942071652
gene-BFSP2	1.782427588	Armillane	0.52808635
gene-BFSP2	1.782427588	PC(P-18:1(11Z)/PGE2)	0.509503133
gene-BFSP2	1.782427588	(3R,4R)-3-Amino-1-hydroxy-4-methylpyrrol	0.471914506
gene-BFSP2	1.782427588	n-methyl-2-(4'-methylaminophenyl)-6-hydr	0.26655714
gene-BFSP2	1.782427588	Isopropyl isothiocyanate	0.172385747
gene-BFSP2	1.782427588	arachidyl amido cholanoic acid	1.24842952
gene-BFSP2	1.782427588	9-deoxy-9-methylene-16,16-dimethyl -PGE	0.606893884
gene-BFSP2	1.782427588	CL(8:0/8:0/18:2(9Z,11Z)/20:0)	0.622988418
gene-LOC112443	1.781966175	3-Thiacytidine	0.209387412
gene-LOC112443	1.781966175	D-Erythro-imidazole-glycerol-phosphate	0.355812431
gene-LOC112443	1.781966175	cis-p-Menth-2-en-1-ol	0.201017988
gene-LOC112443	1.781966175	PE(22:2(13Z,16Z)/22:5(4Z,7Z,10Z,13Z,19Z)-O	0.369745166
gene-LOC112443	1.781966175	PG(20:1(11Z)/18:3(10,12,15)-OH(9))	0.626244347
gene-LOC112443	1.781966175	2-Methyl-3-phenyl-2-propenal	0.407813975
gene-LOC112443	1.781966175	PC(P-18:1(11Z)/PGE2)	0.509503133
gene-LOC112443	1.781966175	Roxithromycin	0.268273077
gene-LOC112443	1.781966175	Sitosterol beta-D-glucoside	0.382944796
gene-LOC112443	1.781966175	PC(P-18:1(11Z)/PGJ2)	0.565243877
gene-LOC112443	1.781966175	PS(20:0/20:4(8Z,11Z,14Z,17Z)-2OH(5S,6R))	0.402595921
gene-LOC518134	1.781949446	3-Thiacytidine	0.209387412
gene-LOC518134	1.781949446	LTB4-d4	0.226799982
gene-LOC518134	1.781949446	cis-p-Menth-2-en-1-ol	0.201017988
gene-LOC518134	1.781949446	PE(20:0/18:1(12Z)-2OH(9,10))	0.438658253
gene-LOC518134	1.781949446	5-(2-Aminopropyl)-2-methylphenol	0.323231457
gene-LOC518134	1.781949446	Armillane	0.52808635
gene-LOC518134	1.781949446	2-Methyl-3-phenyl-2-propenal	0.407813975
gene-LOC518134	1.781949446	PC(P-18:1(11Z)/PGE2)	0.509503133
gene-LOC518134	1.781949446	(3R,4R)-3-Amino-1-hydroxy-4-methylpyrrol	0.471914506
gene-LOC518134	1.781949446	Isopropyl isothiocyanate	0.172385747
gene-LOC518134	1.781949446	PC(18:1(9Z)/15:1(9Z))	0.534724682
gene-LOC518134	1.781949446	arachidyl amido cholanoic acid	1.24842952
gene-LOC518134	1.781949446	Nigroxanthin	0.705005059
gene-LOC518134	1.781949446	PC(P-18:1(11Z)/PGJ2)	0.565243877
gene-LOC518134	1.781949446	PS(20:0/20:4(8Z,11Z,14Z,17Z)-2OH(5S,6R))	0.402595921
gene-LOC518134	1.781949446	CL(8:0/8:0/18:2(9Z,11Z)/20:0)	0.622988418
gene-RAB17	1.778491514	3-Thiacytidine	0.209387412
gene-RAB17	1.778491514	D-Erythro-imidazole-glycerol-phosphate	0.355812431
gene-RAB17	1.778491514	LTB4-d4	0.226799982
gene-RAB17	1.778491514	cis-p-Menth-2-en-1-ol	0.201017988
gene-RAB17	1.778491514	PE(20:0/18:1(12Z)-2OH(9,10))	0.438658253
gene-RAB17	1.778491514	(-)-alpha-Terpineol	0.345716279
gene-RAB17	1.778491514	PG(20:1(11Z)/18:3(10,12,15)-OH(9))	0.626244347
gene-RAB17	1.778491514	5-(2-Aminopropyl)-2-methylphenol	0.323231457
gene-RAB17	1.778491514	Armillane	0.52808635
gene-RAB17	1.778491514	2-Methyl-3-phenyl-2-propenal	0.407813975

gene-RAB17	1.778491514	PC(P-18:1(11Z)/PGE2)	0.509503133
gene-RAB17	1.778491514	(3R,4R)-3-Amino-1-hydroxy-4-methylpyrrol	0.471914506
gene-RAB17	1.778491514	Isopropyl isothiocyanate	0.172385747
gene-RAB17	1.778491514	PC(18:1(9Z)/15:1(9Z))	0.534724682
gene-RAB17	1.778491514	PC(P-18:1(11Z)/PGJ2)	0.565243877
gene-RAB17	1.778491514	PS(20:0/20:4(8Z,11Z,14Z,17Z)-2OH(5S,6R))	0.402595921
gene-CATHL2	1.773080254	S-(Indolylmethylthiohydroximoyl)-L-cysteine	0.635042992
gene-CATHL2	1.773080254	PGP(20:1(11Z)/18:1(12Z)-2OH(9,10))	0.042651731
gene-CATHL2	1.773080254	LTB4-d4	0.226799982
gene-CATHL2	1.773080254	PE(18:0/18:1(9Z)-O(12,13))	1.009186776
gene-CATHL2	1.773080254	PC(P-16:0/18:1(12Z)-2OH(9,10))	1.231706326
gene-CATHL2	1.773080254	(2S,3'S)-alpha-Amino-2-carboxy-5-oxo-1-p	0.080821898
gene-CATHL2	1.773080254	Cyclosporin A	0.656529229
gene-CATHL2	1.773080254	DG(18:0/LTE4/0:0)	0.681485773
gene-CATHL2	1.773080254	PC(17:0/PGJ2)	0.657689319
gene-CATHL2	1.773080254	Alisporivir	0.430769197
gene-CATHL2	1.773080254	PC(14:0/20:2(11Z,14Z))	1.110657378
gene-CATHL2	1.773080254	CDP-DG(PGF2alpha/16:0)	1.002512277
gene-CATHL2	1.773080254	2-Methyl-5-nitroimidazol-1-ylacetic acid	0.701441397
gene-CATHL2	1.773080254	Trimetazidine	0.438282534
gene-CATHL2	1.773080254	(2R,3R)-3-Methylornithinyl-N6-lysine	1.014942199
gene-CATHL2	1.773080254	oleandomycin	0.996457692
gene-CATHL2	1.773080254	PC(20:3(5Z,8Z,11Z)/24:0)	0.387959564
gene-CATHL2	1.773080254	(9Z)-Octadecenoic acid	0.142359556
gene-CATHL2	1.773080254	PC(18:0/20:4(5Z,8Z,11Z,14Z)-OH(20))	0.318846141
gene-CATHL2	1.773080254	arachidyl amido cholanoic acid	1.24842952
gene-CATHL2	1.773080254	Nigroxanthin	0.705005059
gene-CATHL2	1.773080254	1-Octadecanoyl-2-(7Z,10Z,13Z,16Z-docosat	0.690496314
gene-CATHL2	1.773080254	2-Propenyl 2-aminobenzoate	0.128406829
gene-CATHL2	1.773080254	CL(8:0/8:0/18:2(9Z,11Z)/20:0)	0.622988418
gene-CATHL2	1.773080254	PC(20:5(5Z,8Z,11Z,14Z,17Z)/P-16:0)	1.301095986
gene-CATHL2	1.773080254	DG(20:0/LTE4/0:0)	0.438074508
gene-TUBA8	1.769817187	5-(3'-Carboxy-3'-oxopropenyl)-4,6-dihydro›	1.083232395
gene-TUBA8	1.769817187	2-Amino-6-methylpyrimidine-4-thiol	3.949216803
gene-TUBA8	1.769817187	D-Erythro-imidazole-glycerol-phosphate	0.355812431
gene-TUBA8	1.769817187	Benzoquinoneacetic acid	0.707114579
gene-TUBA8	1.769817187	4-Hydroxystyrene	2.854273107
gene-TUBA8	1.769817187	Dihydro-3-coumaric acid	1.950770185
gene-TUBA8	1.769817187	BYSSOCHLAMIC ACID	3.635460051
gene-TUBA8	1.769817187	Enterolactone 3'-glucuronide	1.086030454
gene-TUBA8	1.769817187	Methionine sulfone	0.738197353
gene-TUBA8	1.769817187	7-Hydroxy-6-methyl-8-ribityllumazine	0.103672738
gene-TUBA8	1.769817187	xi-2,3-Dihydro-3,5-dihydroxy-6-methyl-4H-	0.933518005
gene-TUBA8	1.769817187	(卤)-Enterolactone	0.766468338
gene-TUBA8	1.769817187	Enterolactone 3''-sulfate	2.060530283
gene-TUBA8	1.769817187	Val-Cit	0.452308451
gene-TUBA8	1.769817187	3-(2,4-Dimethyl-5-(2-oxo-1,2-dihydroindol-	0.095524813
gene-TUBA8	1.769817187	Fusidic Acid	1.929894931
gene-TUBA8	1.769817187	Docosanamide	0.509975786
gene-TUBA8	1.769817187	Monacolin L acid	1.060562098
gene-TUBA8	1.769817187	cis-p-Menth-2-en-1-ol	0.201017988

gene-TUBA8	1.769817187	11-Oxahexadecanolide	2.378626329
gene-TUBA8	1.769817187	L-Carnitine	0.2737116
gene-TUBA8	1.769817187	3,4,3',4'-Tetrahydrospirilloxanthin	0.354666148
gene-TUBA8	1.769817187	Indole-3-acetaldoxime N-oxide	2.727163281
gene-TUBA8	1.769817187	3-Methoxytyramine	0.928926425
gene-TUBA8	1.769817187	Hydroxyprolyl-Methionine	1.56890423
gene-TUBA8	1.769817187	2-Hydroxy-3,4,6-trimethoxydihydrochalcone	1.07715092
gene-TUBA8	1.769817187	7-Methylguanosine	1.215574844
gene-TUBA8	1.769817187	6-hydroxy-7E,9E-Octadecadiene-11,13,15,17	1.179129125
gene-TUBA8	1.769817187	7C-aglycone	1.015608521
gene-TUBA8	1.769817187	7-Aminomethyl-7-carbaguanine	0.474992724
gene-TUBA8	1.769817187	carbenicillin	1.429508875
gene-TUBA8	1.769817187	2-Methyl-3-phenyl-2-propenal	0.407813975
gene-TUBA8	1.769817187	(-)-Huperzine A (HupA)	0.935198906
gene-TUBA8	1.769817187	(1S)-3-[2-[(1R,7Ar)-7a-methyl-1-[(2R)-6-me	1.140894639
gene-TUBA8	1.769817187	4-Octylphenol	1.400657885
gene-TUBA8	1.769817187	Prolyl-Lysine	1.612991304
gene-TUBA8	1.769817187	Ajulemic acid	0.557832951
gene-TUBA8	1.769817187	Sorbitan palmitate	1.946147152
gene-TUBA8	1.769817187	Chalcone	4.157137778
gene-TUBA8	1.769817187	12-Hydroxyicosanoylcarnitine	1.730979067
gene-TUBA8	1.769817187	Milbemycin D	0.125637998
gene-TUBA8	1.769817187	Ascorbic acid 6-palmitate	0.201627014
gene-TUBA8	1.769817187	Sitosterol beta-D-glucoside	0.382944796
gene-ME3	1.763773147	3-Thiacytidine	0.209387412
gene-ME3	1.763773147	11-Maleimidoundecanoic acid	1.084942397
gene-ME3	1.763773147	cis-p-Menth-2-en-1-ol	0.201017988
gene-ME3	1.763773147	PE(20:0/18:1(12Z)-2OH(9,10))	0.438658253
gene-ME3	1.763773147	PE(22:2(13Z,16Z)/22:5(4Z,7Z,10Z,13Z,19Z)-O	0.369745166
gene-ME3	1.763773147	2-Methyl-3-phenyl-2-propenal	0.407813975
gene-ME3	1.763773147	PC(P-18:1(11Z)/PGE2)	0.509503133
gene-ME3	1.763773147	PC(18:1(9Z)/15:1(9Z))	0.534724682
gene-ME3	1.763773147	PC(P-18:1(11Z)/PGJ2)	0.565243877
gene-ME3	1.763773147	PS(20:0/20:4(8Z,11Z,14Z,17Z)-2OH(5S,6R))	0.402595921
gene-FZD8	1.761923783	3-Thiacytidine	0.209387412
gene-FZD8	1.761923783	11-Maleimidoundecanoic acid	1.084942397
gene-FZD8	1.761923783	PE(22:2(13Z,16Z)/22:5(4Z,7Z,10Z,13Z,19Z)-O	0.369745166
gene-FZD8	1.761923783	Norophthalmic acid	0.191432411
gene-FZD8	1.761923783	beta-L-Dioxolane-cytidine	0.175916668
gene-FZD8	1.761923783	PC(P-18:1(11Z)/PGJ2)	0.565243877
gene-FZD8	1.761923783	PS(20:0/20:4(8Z,11Z,14Z,17Z)-2OH(5S,6R))	0.402595921
Bos_taurus_newG	1.756251947	5-(3'-Carboxy-3'-oxopropenyl)-4,6-dihydro	1.083232395
Bos_taurus_newG	1.756251947	LysoPI(16:0/0:0)	0.379098336
Bos_taurus_newG	1.756251947	Val-Cit	0.452308451
Bos_taurus_newG	1.756251947	3-(2,4-Dimethyl-5-(2-oxo-1,2-dihydroindol-	0.095524813
Bos_taurus_newG	1.756251947	Docosanamide	0.509975786
Bos_taurus_newG	1.756251947	Monacolin L acid	1.060562098
Bos_taurus_newG	1.756251947	3,4,3',4'-Tetrahydrospirilloxanthin	0.354666148
Bos_taurus_newG	1.756251947	Lamivudine	0.327402892
Bos_taurus_newG	1.756251947	Pseudouridine 5'-phosphate	1.18431378
Bos_taurus_newG	1.756251947	15-keto-Prostaglandin E2	2.646629404

Bos_taurus_newG	1.756251947	S-Acetyldihydrolipoamide-E	0.429561283
Bos_taurus_newG	1.756251947	Ribavirin monophosphate	1.091085027
Bos_taurus_newG	1.756251947	Norophthalmic acid	0.191432411
Bos_taurus_newG	1.756251947	Indole-3-ethanol	0.096523128
Bos_taurus_newG	1.756251947	ent-16b,19-Kauranediol 19-acetate	0.400676827
Bos_taurus_newG	1.756251947	Palmitoylcarnitine	0.74779616
Bos_taurus_newG	1.756251947	MG(0:0/22:5(4Z,7Z,10Z,13Z,16Z)/0:0)	0.136549841
Bos_taurus_newG	1.756251947	Milbemycin D	0.125637998
Bos_taurus_newG	1.756251947	Ascorbic acid 6-palmitate	0.201627014
Bos_taurus_newG	1.756251947	PE(20:5(5Z,8Z,11Z,14Z,17Z)/18:0)	0.427962506
gene-PGBD5	1.74736545	3-Thiacytidine	0.209387412
gene-PGBD5	1.74736545	Stercobilinogen	0.144304212
gene-PGBD5	1.74736545	1,4-Undecadiene	0.462803973
gene-PGBD5	1.74736545	4-cholesten-7伪,12伪,24-triol-3-one	0.097840451
gene-PGBD5	1.74736545	5-(2-Aminopropyl)-2-methylphenol	0.323231457
gene-PGBD5	1.74736545	ingenol	0.942071652
gene-PGBD5	1.74736545	Armillane	0.52808635
gene-PGBD5	1.74736545	1-Palmitoylglycerol	0.084357408
gene-PGBD5	1.74736545	Isopropyl isothiocyanate	0.172385747
gene-PGBD5	1.74736545	PS(20:0/20:4(8Z,11Z,14Z,17Z)-2OH(5S,6R))	0.402595921
gene-KCTD14	1.747010779	Methylmalonate	0.241481249
gene-KCTD14	1.747010779	3-Thiacytidine	0.209387412
gene-KCTD14	1.747010779	1-Phenylpiperazine	0.353584896
gene-KCTD14	1.747010779	LTB4-d4	0.226799982
gene-KCTD14	1.747010779	PE(20:0/18:1(12Z)-2OH(9,10))	0.438658253
gene-KCTD14	1.747010779	PE(P-18:0/PGE1)	0.886597382
gene-KCTD14	1.747010779	PC(P-16:0/18:1(12Z)-2OH(9,10))	1.231706326
gene-KCTD14	1.747010779	Cyclosporin A	0.656529229
gene-KCTD14	1.747010779	Epomusenin A	0.766206049
gene-KCTD14	1.747010779	PA(22:6(4Z,7Z,10Z,13Z,16Z,19Z)/16:0)	0.556532497
gene-KCTD14	1.747010779	DG(18:0/LTE4/0:0)	0.681485773
gene-KCTD14	1.747010779	PC(17:0/PGJ2)	0.657689319
gene-KCTD14	1.747010779	PC(14:0/20:2(11Z,14Z))	1.110657378
gene-KCTD14	1.747010779	CDP-DG(PGF2alpha/16:0)	1.002512277
gene-KCTD14	1.747010779	5-(2-Aminopropyl)-2-methylphenol	0.323231457
gene-KCTD14	1.747010779	Armillane	0.52808635
gene-KCTD14	1.747010779	beta-L-Dioxolane-cytidine	0.175916668
gene-KCTD14	1.747010779	17-Aminogeldanamycin	0.518144528
gene-KCTD14	1.747010779	PC(P-18:1(11Z)/PGE2)	0.509503133
gene-KCTD14	1.747010779	PE(P-16:0/18:4(6Z,9Z,12Z,15Z))	0.546989864
gene-KCTD14	1.747010779	Isopropyl isothiocyanate	0.172385747
gene-KCTD14	1.747010779	PC(18:1(9Z)/15:1(9Z))	0.534724682
gene-KCTD14	1.747010779	arachidyl amido cholanoic acid	1.24842952
gene-KCTD14	1.747010779	Nigroxanthin	0.705005059
gene-KCTD14	1.747010779	3-Hydroxyheptadecanoylcarnitine	0.860642943
gene-KCTD14	1.747010779	1-Octadecanoyl-2-(7Z,10Z,13Z,16Z-docosat	0.690496314
gene-KCTD14	1.747010779	PC(P-18:1(11Z)/PGJ2)	0.565243877
gene-KCTD14	1.747010779	PS(20:0/20:4(8Z,11Z,14Z,17Z)-2OH(5S,6R))	0.402595921
gene-KCTD14	1.747010779	CL(8:0/8:0/18:2(9Z,11Z)/20:0)	0.622988418
gene-KCTD14	1.747010779	PC(20:5(5Z,8Z,11Z,14Z,17Z)/P-16:0)	1.301095986
gene-KCTD14	1.747010779	DG(20:0/LTE4/0:0)	0.438074508

gene-EBF4	1.738401509	3-Thiacytidine	0.209387412
gene-EBF4	1.738401509	D-Erythro-imidazole-glycerol-phosphate	0.355812431
gene-EBF4	1.738401509	7(14)-Bisabolene-2,3,10,11-tetrol	2.446897974
gene-EBF4	1.738401509	LTB4-d4	0.226799982
gene-EBF4	1.738401509	cis-p-Menth-2-en-1-ol	0.201017988
gene-EBF4	1.738401509	(-)-alpha-Terpineol	0.345716279
gene-EBF4	1.738401509	1,4-Undecadiene	0.462803973
gene-EBF4	1.738401509	2-Methyl-3-phenyl-2-propenal	0.407813975
gene-EBF4	1.738401509	PC(P-18:1(11Z)/PGE2)	0.509503133
gene-EBF4	1.738401509	(3R,4R)-3-Amino-1-hydroxy-4-methylpyrrol	0.471914506
gene-EBF4	1.738401509	Isopropyl isothiocyanate	0.172385747
gene-EBF4	1.738401509	PS(20:0/20:4(8Z,11Z,14Z,17Z)-2OH(5S,6R))	0.402595921
gene-TINAGL1	1.736705166	5-Hydroxy-2-oxo-4-ureido-2,5-dihydro-1H	0.122536033
gene-TINAGL1	1.736705166	19-hydroxyprostaglandin H2(1-)	0.116812012
gene-TINAGL1	1.736705166	Methyl (2E)-2-(10,13-dimethyl-11-oxo-3-py	0.478588158
gene-TINAGL1	1.736705166	PGP(20:1(11Z)/18:1(12Z)-2OH(9,10))	0.042651731
gene-TINAGL1	1.736705166	11-Maleimidoundecanoic acid	1.084942397
gene-TINAGL1	1.736705166	7(S),17(S)-dihydroxy-8(E),10(Z),13(Z),15(E),19	0.32147403
gene-TINAGL1	1.736705166	PC(24:0/22:6(4Z,7Z,10Z,12E,16Z,19Z)-OH(14)	0.188241061
gene-TINAGL1	1.736705166	Cyclosporin A	0.656529229
gene-TINAGL1	1.736705166	D-Xylonate	0.386869454
gene-TINAGL1	1.736705166	C20914	0.140383181
gene-TINAGL1	1.736705166	Fluridone	0.127106682
gene-TINAGL1	1.736705166	3b,6a-Dihydroxy-alpha-ionol 9-[apiosyl-(1-:	0.045258177
gene-TINAGL1	1.736705166	Asparaginylarginine	0.091306435
gene-TINAGL1	1.736705166	Guanidoacetic acid	0.542509265
gene-TINAGL1	1.736705166	Apronal	0.754033426
gene-TINAGL1	1.736705166	Methionyl-Valine	0.502707745
gene-TINAGL1	1.736705166	[3-(2-Aminopropyl)-6-methylidenecyclohexa	0.422945997
gene-TINAGL1	1.736705166	3尾-hydroxy-estra-5,7,9-trien-17-one	0.715223098
gene-TINAGL1	1.736705166	(22E)-3伪,7伪,12伪-Trihydroxy-5尾-chol-22-	0.522629215
gene-TINAGL1	1.736705166	3'-Hydroxyropivacaine	1.014215236
gene-TINAGL1	1.736705166	Vulgarone A	0.102049407
gene-TINAGL1	1.736705166	Sophoranol	0.282064826
gene-TINAGL1	1.736705166	L-Cysteine	0.214540754
gene-TINAGL1	1.736705166	1,4,6-Trimethylnaphthalene	0.12150693
gene-TINAGL1	1.736705166	Prostaglandin PGE2 1-glyceryl ester	0.007411313
gene-TINAGL1	1.736705166	7-Sulfocholic acid	0.039985455
gene-TINAGL1	1.736705166	1,8-Octanedithiol	0.133094504
gene-TINAGL1	1.736705166	Prostaglandin B2	0.552852305
gene-TINAGL1	1.736705166	PC(P-18:1(11Z)/PGE2)	0.509503133
gene-TINAGL1	1.736705166	PC(20:3(5Z,8Z,11Z)/24:0)	0.387959564
gene-TINAGL1	1.736705166	Phorone A	0.079039095
gene-TINAGL1	1.736705166	Nigroxanthin	0.705005059
Bos_taurus_newG	1.736009182	3-Thiacytidine	0.209387412
Bos_taurus_newG	1.736009182	3-Deoxyestrone	0.282221709
Bos_taurus_newG	1.736009182	1-Oleoyl-sn-glycero-3-phosphocholine	0.18926952
Bos_taurus_newG	1.736009182	Psychosine	0.106475396
Bos_taurus_newG	1.736009182	(-)-alpha-Terpineol	0.345716279
Bos_taurus_newG	1.736009182	1,4-Undecadiene	0.462803973
Bos_taurus_newG	1.736009182	4-cholesten-7伪,12伪,24-triol-3-one	0.097840451

Bos_taurus_newG	1.736009182	ingenol	0.942071652
Bos_taurus_newG	1.736009182	Armillane	0.52808635
Bos_taurus_newG	1.736009182	N-[[3-Hydroxy-2-(2-pentenyl)cyclopentyl]ac	1.043930947
Bos_taurus_newG	1.736009182	PC(P-18:1(11Z)/PGE2)	0.509503133
Bos_taurus_newG	1.736009182	PC(20:3(5Z,8Z,11Z)/24:0)	0.387959564
Bos_taurus_newG	1.736009182	(3R,4R)-3-Amino-1-hydroxy-4-methylpyrrol	0.471914506
Bos_taurus_newG	1.736009182	2-Amino-4-[carbamimidoyl(methyl)amino]bu	0.075703353
Bos_taurus_newG	1.736009182	n-methyl-2-(4'-methylaminophenyl)-6-hydr	0.26655714
Bos_taurus_newG	1.736009182	Isopropyl isothiocyanate	0.172385747
Bos_taurus_newG	1.736009182	(9Z)-Octadecenoic acid	0.142359556
Bos_taurus_newG	1.732398925	LysoPI(16:0/0:0)	0.379098336
Bos_taurus_newG	1.732398925	3-Thiacytidine	0.209387412
Bos_taurus_newG	1.732398925	milbemycin beta3	1.414447124
Bos_taurus_newG	1.732398925	cis-p-Menth-2-en-1-ol	0.201017988
Bos_taurus_newG	1.732398925	PE(20:0/18:1(12Z)-2OH(9,10))	0.438658253
Bos_taurus_newG	1.732398925	PE(22:2(13Z,16Z)/22:5(4Z,7Z,10Z,13Z,19Z)-O	0.369745166
Bos_taurus_newG	1.732398925	PG(20:1(11Z)/18:3(10,12,15)-OH(9))	0.626244347
Bos_taurus_newG	1.732398925	Lamivudine	0.327402892
Bos_taurus_newG	1.732398925	5-(2-Aminopropyl)-2-methylphenol	0.323231457
Bos_taurus_newG	1.732398925	Armillane	0.52808635
Bos_taurus_newG	1.732398925	PC(P-18:1(11Z)/PGE2)	0.509503133
Bos_taurus_newG	1.732398925	Sitosterol beta-D-glucoside	0.382944796
Bos_taurus_newG	1.732398925	Nigroxanthin	0.705005059
Bos_taurus_newG	1.732398925	PC(P-18:1(11Z)/PGJ2)	0.565243877
Bos_taurus_newG	1.732398925	PS(20:0/20:4(8Z,11Z,14Z,17Z)-2OH(5S,6R))	0.402595921
gene-IGFBP4	1.724733198	3-Thiacytidine	0.209387412
gene-IGFBP4	1.724733198	1-Oleoyl-sn-glycero-3-phosphocholine	0.18926952
gene-IGFBP4	1.724733198	LTB4-d4	0.226799982
gene-IGFBP4	1.724733198	(-)-alpha-Terpineol	0.345716279
gene-IGFBP4	1.724733198	1,4-Undecadiene	0.462803973
gene-IGFBP4	1.724733198	5-(2-Aminopropyl)-2-methylphenol	0.323231457
gene-IGFBP4	1.724733198	4-Dimethylamino-L-phenylalanine	0.242110226
gene-IGFBP4	1.724733198	Armillane	0.52808635
gene-IGFBP4	1.724733198	PC(P-18:1(11Z)/PGE2)	0.509503133
gene-IGFBP4	1.724733198	(3R,4R)-3-Amino-1-hydroxy-4-methylpyrrol	0.471914506
gene-IGFBP4	1.724733198	n-methyl-2-(4'-methylaminophenyl)-6-hydr	0.26655714
gene-IGFBP4	1.724733198	Isopropyl isothiocyanate	0.172385747
gene-IGFBP4	1.724733198	(9Z)-Octadecenoic acid	0.142359556
gene-IGFBP4	1.724733198	PS(20:0/20:4(8Z,11Z,14Z,17Z)-2OH(5S,6R))	0.402595921
gene-IGFBP4	1.724733198	CL(8:0/8:0/18:2(9Z,11Z)/20:0)	0.622988418
gene-EDA	1.713924328	D-Erythro-imidazole-glycerol-phosphate	0.355812431
gene-EDA	1.713924328	Glutamate carbon	0.671444516
gene-EDA	1.713924328	1-(2-Furanyl)-1-pentanone	0.697147283
gene-EDA	1.713924328	2-Dehydro-3-deoxy-D-gluconate	0.65165299
gene-EDA	1.713924328	16-Hydroxy-10-oxohexadecanoic acid	0.72302152
gene-EDA	1.713924328	11-Maleimidoundecanoic acid	1.084942397
gene-EDA	1.713924328	PE(22:2(13Z,16Z)/22:5(4Z,7Z,10Z,13Z,19Z)-O	0.369745166
gene-EDA	1.713924328	(1R,6S)-6-Amino-5-oxocyclohex-2-ene-1-c	0.154751123
gene-EDA	1.713924328	PC(P-18:1(11Z)/PGE2)	0.509503133
gene-EDA	1.713924328	Roxithromycin	0.268273077
gene-EDA	1.713924328	PC(P-18:1(11Z)/PGJ2)	0.565243877

gene-EDA	1.713924328	PS(20:0/20:4(8Z,11Z,14Z,17Z)-2OH(5S,6R))	0.402595921
gene-LOC100301	1.712897029	D-Erythro-imidazole-glycerol-phosphate	0.355812431
gene-LOC100301	1.712897029	Glutamate carbon	0.671444516
gene-LOC100301	1.712897029	Urocortisol	0.926998516
gene-LOC100301	1.712897029	11-Maleimidoundecanoic acid	1.084942397
gene-LOC100301	1.712897029	(1R,6S)-6-Amino-5-oxocyclohex-2-ene-1-c	0.154751123
gene-LOC100301	1.712897029	3-(3-Methylbutylidene)-1(3H)-isobenzofurar	0.575422414
gene-LOC100301	1.712897029	Chamissonin diacetate	0.324896191
gene-LOC100301	1.712897029	3',4',5'-Trimethoxycinnamyl alcohol acetate	0.4221816
gene-LOC100301	1.712897029	L-Anticapsin	0.426006862
gene-LOC100301	1.712897029	PC(P-18:1(11Z)/PGE2)	0.509503133
gene-LOC100301	1.712897029	Roxithromycin	0.268273077
gene-SMPD3	1.699160878	D-Erythro-imidazole-glycerol-phosphate	0.355812431
gene-SMPD3	1.699160878	7(14)-Bisabolene-2,3,10,11-tetrol	2.446897974
gene-SMPD3	1.699160878	3-Deoxyestrone	0.282221709
gene-SMPD3	1.699160878	1-Oleoyl-sn-glycero-3-phosphocholine	0.18926952
gene-SMPD3	1.699160878	(-)-alpha-Terpineol	0.345716279
gene-SMPD3	1.699160878	1,4-Undecadiene	0.462803973
gene-SMPD3	1.699160878	5-(2-Aminopropyl)-2-methylphenol	0.323231457
gene-SMPD3	1.699160878	4-Dimethylamino-L-phenylalanine	0.242110226
gene-SMPD3	1.699160878	Armillane	0.52808635
gene-SMPD3	1.699160878	PC(P-18:1(11Z)/PGE2)	0.509503133
gene-SMPD3	1.699160878	(3R,4R)-3-Amino-1-hydroxy-4-methylpyrrol	0.471914506
gene-SMPD3	1.699160878	1-Palmitoylglycerol	0.084357408
gene-SMPD3	1.699160878	n-methyl-2-(4'-methylaminophenyl)-6-hydr	0.26655714
gene-SMPD3	1.699160878	Isopropyl isothiocyanate	0.172385747
gene-SMPD3	1.699160878	9-deoxy-9-methylene-16,16-dimethyl -PGE	0.606893884
gene-SMPD3	1.699160878	CL(8:0/8:0/18:2(9Z,11Z)/20:0)	0.622988418
gene-LOC618787	1.695969633	D-Erythro-imidazole-glycerol-phosphate	0.355812431
gene-LOC618787	1.695969633	Tsugarioside B	1.013262893
gene-LOC618787	1.695969633	7(14)-Bisabolene-2,3,10,11-tetrol	2.446897974
gene-LOC618787	1.695969633	3-Deoxyestrone	0.282221709
gene-LOC618787	1.695969633	1-Oleoyl-sn-glycero-3-phosphocholine	0.18926952
gene-LOC618787	1.695969633	cis-p-Menth-2-en-1-ol	0.201017988
gene-LOC618787	1.695969633	2-isopentyl-3,6-dimethyl pyrazine	0.710502562
gene-LOC618787	1.695969633	PG(20:1(11Z)/18:3(10,12,15)-OH(9))	0.626244347
gene-LOC618787	1.695969633	4-Guanidinobutanoate	0.445426102
gene-LOC618787	1.695969633	Cornoside	0.781689516
gene-LOC618787	1.695969633	Armillane	0.52808635
gene-LOC618787	1.695969633	N-Eicosapentaenoyl Asparagine	0.371594025
gene-LOC618787	1.695969633	PC(P-18:1(11Z)/PGE2)	0.509503133
gene-LOC618787	1.695969633	(3R,4R)-3-Amino-1-hydroxy-4-methylpyrrol	0.471914506
gene-LOC618787	1.695969633	Isopropyl isothiocyanate	0.172385747
gene-LOC618787	1.695969633	Sitosterol beta-D-glucoside	0.382944796
gene-LOC618787	1.695969633	PS(20:0/20:4(8Z,11Z,14Z,17Z)-2OH(5S,6R))	0.402595921
gene-LOC618787	1.695969633	PG(20:1(11Z)/18:3(9,11,15)-OH(13))	0.69829683
gene-PADI4	1.687146126	3-Thiacytidine	0.209387412
gene-PADI4	1.687146126	D-Erythro-imidazole-glycerol-phosphate	0.355812431
gene-PADI4	1.687146126	3-Deoxyestrone	0.282221709
gene-PADI4	1.687146126	1-Oleoyl-sn-glycero-3-phosphocholine	0.18926952
gene-PADI4	1.687146126	LTB4-d4	0.226799982

gene-PADI4	1.687146126	(-)-alpha-Terpineol	0.345716279
gene-PADI4	1.687146126	1,4-Undecadiene	0.462803973
gene-PADI4	1.687146126	5-(2-Aminopropyl)-2-methylphenol	0.323231457
gene-PADI4	1.687146126	4-Dimethylamino-L-phenylalanine	0.242110226
gene-PADI4	1.687146126	Armillane	0.52808635
gene-PADI4	1.687146126	PC(P-18:1(11Z)/PGE2)	0.509503133
gene-PADI4	1.687146126	(3R,4R)-3-Amino-1-hydroxy-4-methylpyrrol	0.471914506
gene-PADI4	1.687146126	n-methyl-2-(4'-methylaminophenyl)-6-hydr	0.26655714
gene-PADI4	1.687146126	Isopropyl isothiocyanate	0.172385747
gene-PADI4	1.687146126	9-deoxy-9-methylene-16,16-dimethyl -PGE	0.606893884
gene-PADI4	1.687146126	PS(20:0/20:4(8Z,11Z,14Z,17Z)-2OH(5S,6R))	0.402595921
gene-PADI4	1.687146126	CL(8:0/8:0/18:2(9Z,11Z)/20:0)	0.622988418
gene-GFI1B	1.684235765	3-Thiacytidine	0.209387412
gene-GFI1B	1.684235765	D-Erythro-imidazole-glycerol-phosphate	0.355812431
gene-GFI1B	1.684235765	LTB4-d4	0.226799982
gene-GFI1B	1.684235765	cis-p-Menth-2-en-1-ol	0.201017988
gene-GFI1B	1.684235765	(-)-alpha-Terpineol	0.345716279
gene-GFI1B	1.684235765	PG(20:1(11Z)/18:3(10,12,15)-OH(9))	0.626244347
gene-GFI1B	1.684235765	5-(2-Aminopropyl)-2-methylphenol	0.323231457
gene-GFI1B	1.684235765	4-Dimethylamino-L-phenylalanine	0.242110226
gene-GFI1B	1.684235765	Armillane	0.52808635
gene-GFI1B	1.684235765	2-Methyl-3-phenyl-2-propenal	0.407813975
gene-GFI1B	1.684235765	PC(P-18:1(11Z)/PGE2)	0.509503133
gene-GFI1B	1.684235765	(3R,4R)-3-Amino-1-hydroxy-4-methylpyrrol	0.471914506
gene-GFI1B	1.684235765	n-methyl-2-(4'-methylaminophenyl)-6-hydr	0.26655714
gene-GFI1B	1.684235765	Isopropyl isothiocyanate	0.172385747
gene-GFI1B	1.684235765	(9Z)-Octadecenoic acid	0.142359556
gene-GFI1B	1.684235765	PC(P-18:1(11Z)/PGJ2)	0.565243877
gene-GFI1B	1.684235765	PS(20:0/20:4(8Z,11Z,14Z,17Z)-2OH(5S,6R))	0.402595921
gene-GFI1B	1.684235765	CL(8:0/8:0/18:2(9Z,11Z)/20:0)	0.622988418
gene-LY6G5B	1.68340844	D-Erythro-imidazole-glycerol-phosphate	0.355812431
gene-LY6G5B	1.68340844	7(14)-Bisabolene-2,3,10,11-tetrol	2.446897974
gene-LY6G5B	1.68340844	11-Oxahexadecanolide	2.378626329
gene-LY6G5B	1.68340844	Sambutoxin	0.169786377
gene-LY6G5B	1.68340844	Isopropyl isothiocyanate	0.172385747
Bos_taurus_newG	1.672941298	(-)-alpha-Terpineol	0.345716279
Bos_taurus_newG	1.672941298	1,4-Undecadiene	0.462803973
Bos_taurus_newG	1.672941298	4-cholesten-7伪,12伪,24-triol-3-one	0.097840451
Bos_taurus_newG	1.672941298	N-[[3-Hydroxy-2-(2-pentenyl)cyclopentyl]ac	1.043930947
Bos_taurus_newG	1.672941298	PC(P-18:1(11Z)/PGE2)	0.509503133
Bos_taurus_newG	1.672941298	PS(20:0/20:4(8Z,11Z,14Z,17Z)-2OH(5S,6R))	0.402595921
Bos_taurus_newG	1.65945079	3-Thiacytidine	0.209387412
Bos_taurus_newG	1.65945079	PE(20:0/18:1(12Z)-2OH(9,10))	0.438658253
Bos_taurus_newG	1.65945079	Cyclosporin A	0.656529229
Bos_taurus_newG	1.65945079	DG(18:0/LTE4/0:0)	0.681485773
Bos_taurus_newG	1.65945079	5-(2-Aminopropyl)-2-methylphenol	0.323231457
Bos_taurus_newG	1.65945079	Armillane	0.52808635
Bos_taurus_newG	1.65945079	beta-L-Dioxolane-cytidine	0.175916668
Bos_taurus_newG	1.65945079	PC(P-18:1(11Z)/PGE2)	0.509503133
Bos_taurus_newG	1.65945079	Isopropyl isothiocyanate	0.172385747
Bos_taurus_newG	1.65945079	Nigroxanthin	0.705005059

Bos_taurus_newG	1.65945079	PC(P-18:1(11Z)/PGJ2)	0.565243877
Bos_taurus_newG	1.65945079	PS(20:0/20:4(8Z,11Z,14Z,17Z)-2OH(5S,6R))	0.402595921
Bos_taurus_newG	1.65945079	CL(8:0/8:0/18:2(9Z,11Z)/20:0)	0.622988418
gene-CAMK1	1.658007734	3-Thiacytidine	0.209387412
gene-CAMK1	1.658007734	3-Deoxyestrone	0.282221709
gene-CAMK1	1.658007734	1-Oleoyl-sn-glycero-3-phosphocholine	0.18926952
gene-CAMK1	1.658007734	LTB4-d4	0.226799982
gene-CAMK1	1.658007734	(-)-alpha-Terpineol	0.345716279
gene-CAMK1	1.658007734	1,4-Undecadiene	0.462803973
gene-CAMK1	1.658007734	Cyclosporin A	0.656529229
gene-CAMK1	1.658007734	5-(2-Aminopropyl)-2-methylphenol	0.323231457
gene-CAMK1	1.658007734	4-Dimethylamino-L-phenylalanine	0.242110226
gene-CAMK1	1.658007734	ingenol	0.942071652
gene-CAMK1	1.658007734	Armillane	0.52808635
gene-CAMK1	1.658007734	PC(P-18:1(11Z)/PGE2)	0.509503133
gene-CAMK1	1.658007734	PC(20:3(5Z,8Z,11Z)/24:0)	0.387959564
gene-CAMK1	1.658007734	(3R,4R)-3-Amino-1-hydroxy-4-methylpyrrol	0.471914506
gene-CAMK1	1.658007734	n-methyl-2-(4'-methylaminophenyl)-6-hydr	0.26655714
gene-CAMK1	1.658007734	Isopropyl isothiocyanate	0.172385747
gene-CAMK1	1.658007734	(9Z)-Octadecenoic acid	0.142359556
gene-CAMK1	1.658007734	Nigroxanthin	0.705005059
gene-CAMK1	1.658007734	PS(20:0/20:4(8Z,11Z,14Z,17Z)-2OH(5S,6R))	0.402595921
gene-CAMK1	1.658007734	CL(8:0/8:0/18:2(9Z,11Z)/20:0)	0.622988418
gene-CPT1B	1.647381736	13(S)-HpODE	0.163157753
gene-CPT1B	1.647381736	LTB4-d4	0.226799982
gene-CPT1B	1.647381736	(-)-alpha-Terpineol	0.345716279
gene-CPT1B	1.647381736	Cyclosporin A	0.656529229
gene-CPT1B	1.647381736	beta-L-Dioxolane-cytidine	0.175916668
gene-CPT1B	1.647381736	PC(P-18:1(11Z)/PGE2)	0.509503133
gene-CPT1B	1.647381736	PC(20:3(5Z,8Z,11Z)/24:0)	0.387959564
gene-CPT1B	1.647381736	PC(P-18:1(11Z)/PGE1)	0.295520345
gene-CPT1B	1.647381736	D-Fructose	0.167584616
gene-CPT1B	1.647381736	CL(8:0/8:0/18:2(9Z,11Z)/20:0)	0.622988418
gene-RBM41	1.647181092	4-Hydroxybenzenesulfonic acid	0.748011467
gene-RBM41	1.647181092	D-Erythro-imidazole-glycerol-phosphate	0.355812431
gene-RBM41	1.647181092	Isomaltotriose	0.58434654
gene-RBM41	1.647181092	Glutamate carbon	0.671444516
gene-RBM41	1.647181092	1-(2-Furanyl)-1-pentanone	0.697147283
gene-RBM41	1.647181092	2-Dehydro-3-deoxy-D-gluconate	0.65165299
gene-RBM41	1.647181092	Undecanedioic acid	0.059955634
gene-RBM41	1.647181092	(-)-alpha-Terpineol	0.345716279
gene-RBM41	1.647181092	Threonylisoleucine	1.190547061
gene-RBM41	1.647181092	(1R,6S)-6-Amino-5-oxocyclohex-2-ene-1-c	0.154751123
gene-RBM41	1.647181092	3-(3-Methylbutylidene)-1(3H)-isobenzofurar	0.575422414
gene-RBM41	1.647181092	Chamissonin diacetate	0.324896191
gene-RBM41	1.647181092	3',4',5'-Trimethoxycinnamyl alcohol acetate	0.4221816
gene-RBM41	1.647181092	L-Anticapsin	0.426006862
gene-RBM41	1.647181092	PC(P-18:1(11Z)/PGE2)	0.509503133
gene-RBM41	1.647181092	Roxithromycin	0.268273077
Bos_taurus_newG	1.646273774	Glutamate carbon	0.671444516
Bos_taurus_newG	1.646273774	Urocortisol	0.926998516

Bos_taurus_newG	1.646273774	11-Maleimidoundecanoic acid	1.084942397
Bos_taurus_newG	1.646273774	PC(24:0/22:6(4Z,7Z,10Z,12E,16Z,19Z)-OH(14)	0.188241061
Bos_taurus_newG	1.646273774	PE(22:2(13Z,16Z)/22:5(4Z,7Z,10Z,13Z,19Z)-O	0.369745166
Bos_taurus_newG	1.646273774	Norophthalmic acid	0.191432411
Bos_taurus_newG	1.646273774	(1R,6S)-6-Amino-5-oxocyclohex-2-ene-1-c	0.154751123
Bos_taurus_newG	1.646273774	Guanidoacetic acid	0.542509265
Bos_taurus_newG	1.646273774	alpha-Terpineol formate	0.628238986
Bos_taurus_newG	1.646273774	3-(3-Methylbutylidene)-1(3H)-isobenzofurar	0.575422414
Bos_taurus_newG	1.646273774	[3-(2-Aminopropyl)-6-methylidenecyclohexa	0.422945997
Bos_taurus_newG	1.646273774	1,8-Octanedithiol	0.133094504
Bos_taurus_newG	1.646273774	Prostaglandin B2	0.552852305
Bos_taurus_newG	1.646273774	PC(P-18:1(11Z)/PGE2)	0.509503133
Bos_taurus_newG	1.646273774	Roxithromycin	0.268273077
Bos_taurus_newG	1.646273774	PC(P-18:1(11Z)/PGJ2)	0.565243877
Bos_taurus_newG	1.646273774	PS(20:0/20:4(8Z,11Z,14Z,17Z)-2OH(5S,6R))	0.402595921
gene-PTGDR2	1.645331746	3-Thiacytidine	0.209387412
gene-PTGDR2	1.645331746	(-)-alpha-Terpineol	0.345716279
gene-PTGDR2	1.645331746	1,4-Undecadiene	0.462803973
gene-PTGDR2	1.645331746	4-cholesten-7伪,12伪,24-triol-3-one	0.097840451
gene-PTGDR2	1.645331746	Cyclosporin A	0.656529229
gene-PTGDR2	1.645331746	5-(2-Aminopropyl)-2-methylphenol	0.323231457
gene-PTGDR2	1.645331746	ingenol	0.942071652
gene-PTGDR2	1.645331746	Armillane	0.52808635
gene-PTGDR2	1.645331746	PC(P-18:1(11Z)/PGE2)	0.509503133
gene-PTGDR2	1.645331746	Isopropyl isothiocyanate	0.172385747
gene-PTGDR2	1.645331746	PS(20:0/20:4(8Z,11Z,14Z,17Z)-2OH(5S,6R))	0.402595921
gene-PTGDR2	1.645331746	CL(8:0/8:0/18:2(9Z,11Z)/20:0)	0.622988418
gene-VIPR2	1.641259504	7(14)-Bisabolene-2,3,10,11-tetrol	2.446897974
gene-VIPR2	1.641259504	1,2-O-Isopropylidene-D-glucofuranose	0.080987667
gene-VIPR2	1.641259504	3-Deoxyestrone	0.282221709
gene-VIPR2	1.641259504	1-Oleoyl-sn-glycero-3-phosphocholine	0.18926952
gene-VIPR2	1.641259504	3,4-dihydroxy-5-all-trans-hexaprenylbenzoa	0.123615726
gene-VIPR2	1.641259504	Lividamine	0.319679555
gene-VIPR2	1.641259504	Psychosine	0.106475396
gene-VIPR2	1.641259504	Cyclotricuspidogenin C	0.440884085
gene-VIPR2	1.641259504	(-)-alpha-Terpineol	0.345716279
gene-VIPR2	1.641259504	1,4-Undecadiene	0.462803973
gene-VIPR2	1.641259504	2-isopentyl-3,6-dimethyl pyrazine	0.710502562
gene-VIPR2	1.641259504	PC(16:0/18:1(12Z)-2OH(9,10))	0.461843233
gene-VIPR2	1.641259504	5-(2-Aminopropyl)-2-methylphenol	0.323231457
gene-VIPR2	1.641259504	4-Dimethylamino-L-phenylalanine	0.242110226
gene-VIPR2	1.641259504	Trimetazidine	0.438282534
gene-VIPR2	1.641259504	ingenol	0.942071652
gene-VIPR2	1.641259504	Armillane	0.52808635
gene-VIPR2	1.641259504	(3R,4R)-3-Amino-1-hydroxy-4-methylpyrrol	0.471914506
gene-VIPR2	1.641259504	1-Palmitoylglycerol	0.084357408
gene-VIPR2	1.641259504	n-methyl-2-(4'-methylaminophenyl)-6-hydr	0.26655714
gene-VIPR2	1.641259504	Glutethimide	0.126237229
gene-VIPR2	1.641259504	Isopropyl isothiocyanate	0.172385747
gene-VIPR2	1.641259504	9-deoxy-9-methylene-16,16-dimethyl -PGE	0.606893884
gene-VIPR2	1.641259504	CL(8:0/8:0/18:2(9Z,11Z)/20:0)	0.622988418

gene-MGC13703	1.641092261	D-Erythro-imidazole-glycerol-phosphate	0.355812431
gene-MGC13703	1.641092261	Jasmolone glucoside	0.949312509
gene-MGC13703	1.641092261	3-hydroxypristanic acid	0.548515835
gene-MGC13703	1.641092261	7(14)-Bisabolene-2,3,10,11-tetrol	2.446897974
gene-MGC13703	1.641092261	24,25-Diacetylvulgaroside	0.920743312
gene-MGC13703	1.641092261	(3Z)-Phycoerythrobilin	1.456755874
gene-MGC13703	1.641092261	16-hydroxy hexadecanoic acid	0.309000958
gene-MGC13703	1.641092261	3-Deoxyestrone	0.282221709
gene-MGC13703	1.641092261	1-Oleoyl-sn-glycero-3-phosphocholine	0.18926952
gene-MGC13703	1.641092261	Lividamine	0.319679555
gene-MGC13703	1.641092261	Psychosine	0.106475396
gene-MGC13703	1.641092261	Cyclotricuspidogenin C	0.440884085
gene-MGC13703	1.641092261	(-)-alpha-Terpineol	0.345716279
gene-MGC13703	1.641092261	1,4-Undecadiene	0.462803973
gene-MGC13703	1.641092261	2-isopentyl-3,6-dimethyl pyrazine	0.710502562
gene-MGC13703	1.641092261	4-Dimethylamino-L-phenylalanine	0.242110226
gene-MGC13703	1.641092261	Trimetazidine	0.438282534
gene-MGC13703	1.641092261	Armillane	0.52808635
gene-MGC13703	1.641092261	N-Eicosapentaenoyl Asparagine	0.371594025
gene-MGC13703	1.641092261	PC(P-18:1(11Z)/PGE2)	0.509503133
gene-MGC13703	1.641092261	(3R,4R)-3-Amino-1-hydroxy-4-methylpyrrol	0.471914506
gene-MGC13703	1.641092261	Maraviroc	0.755297431
gene-MGC13703	1.641092261	N2-gamma-Glutamylglutamine	0.230065499
gene-MGC13703	1.641092261	Threoninyl-Tryptophan	0.69975167
gene-MGC13703	1.641092261	n-methyl-2-(4'-methylaminophenyl)-6-hydr	0.26655714
gene-MGC13703	1.641092261	Permetin A	0.140869415
gene-MGC13703	1.641092261	L-Oleandrosyl-oleandolide	0.272998166
gene-MGC13703	1.641092261	9-deoxy-9-methylene-16,16-dimethyl -PGE	0.606893884
gene-LRRC32	1.633474499	3-Thiacytidine	0.209387412
gene-LRRC32	1.633474499	D-Erythro-imidazole-glycerol-phosphate	0.355812431
gene-LRRC32	1.633474499	11-Maleimidoundecanoic acid	1.084942397
gene-LRRC32	1.633474499	LTB4-d4	0.226799982
gene-LRRC32	1.633474499	cis-p-Menth-2-en-1-ol	0.201017988
gene-LRRC32	1.633474499	PE(20:0/18:1(12Z)-2OH(9,10))	0.438658253
gene-LRRC32	1.633474499	PE(22:2(13Z,16Z)/22:5(4Z,7Z,10Z,13Z,19Z)-O	0.369745166
gene-LRRC32	1.633474499	PG(20:1(11Z)/18:3(10,12,15)-OH(9))	0.626244347
gene-LRRC32	1.633474499	2-Methyl-3-phenyl-2-propenal	0.407813975
gene-LRRC32	1.633474499	alpha-Terpineol formate	0.628238986
gene-LRRC32	1.633474499	10-alpha-methoxy-9,10-dihydrolysergol	0.140851438
gene-LRRC32	1.633474499	PC(P-18:1(11Z)/PGE2)	0.509503133
gene-LRRC32	1.633474499	N-Myristoyl Glutamine	0.37856966
gene-LRRC32	1.633474499	PC(18:1(9Z)/15:1(9Z))	0.534724682
gene-LRRC32	1.633474499	Nigroxanthin	0.705005059
gene-LRRC32	1.633474499	PC(P-18:1(11Z)/PGJ2)	0.565243877
gene-LRRC32	1.633474499	PS(20:0/20:4(8Z,11Z,14Z,17Z)-2OH(5S,6R))	0.402595921
gene-GGT1	1.633132657	3-Thiacytidine	0.209387412
gene-GGT1	1.633132657	D-Erythro-imidazole-glycerol-phosphate	0.355812431
gene-GGT1	1.633132657	11-Maleimidoundecanoic acid	1.084942397
gene-GGT1	1.633132657	LTB4-d4	0.226799982
gene-GGT1	1.633132657	cis-p-Menth-2-en-1-ol	0.201017988
gene-GGT1	1.633132657	PE(20:0/18:1(12Z)-2OH(9,10))	0.438658253

gene-GGT1	1.633132657	PE(22:2(13Z,16Z)/22:5(4Z,7Z,10Z,13Z,19Z)-O(	0.369745166
gene-GGT1	1.633132657	Cyclosporin A	0.656529229
gene-GGT1	1.633132657	Norophthalmic acid	0.191432411
gene-GGT1	1.633132657	2-Methyl-3-phenyl-2-propenal	0.407813975
gene-GGT1	1.633132657	Guanidoacetic acid	0.542509265
gene-GGT1	1.633132657	alpha-Terpineol formate	0.628238986
gene-GGT1	1.633132657	PC(P-18:1(11Z)/PGE2)	0.509503133
gene-GGT1	1.633132657	Roxithromycin	0.268273077
gene-GGT1	1.633132657	Nigroxanthin	0.705005059
gene-GGT1	1.633132657	PC(P-18:1(11Z)/PGJ2)	0.565243877
gene-GGT1	1.633132657	PS(20:0/20:4(8Z,11Z,14Z,17Z)-2OH(5S,6R))	0.402595921
gene-HID1	1.630123594	Methylmalonate	0.241481249
gene-HID1	1.630123594	PE-NMe(18:0/18:3(9Z,12Z,15Z))	0.681884774
gene-HID1	1.630123594	3-Thiacytidine	0.209387412
gene-HID1	1.630123594	Methyl methacrylate	0.245645579
gene-HID1	1.630123594	1-Phenylpiperazine	0.353584896
gene-HID1	1.630123594	Azelaic acid	0.007180175
gene-HID1	1.630123594	5-Hydroxy-2-oxo-4-ureido-2,5-dihydro-1H	0.122536033
gene-HID1	1.630123594	Trihomomethionine	0.296210774
gene-HID1	1.630123594	Hygromycin B	0.930396619
gene-HID1	1.630123594	Netupitant	0.707108724
gene-HID1	1.630123594	Divinylprotochlorophyllide	0.629978387
gene-HID1	1.630123594	Metkephamid	1.159767003
gene-HID1	1.630123594	11-Maleimidoundecanoic acid	1.084942397
gene-HID1	1.630123594	2-Hexylbenzothiazole	0.396099297
gene-HID1	1.630123594	Valnemulin	0.408062514
gene-HID1	1.630123594	LTB4-d4	0.226799982
gene-HID1	1.630123594	cis-p-Menth-2-en-1-ol	0.201017988
gene-HID1	1.630123594	PE(20:0/18:1(12Z)-2OH(9,10))	0.438658253
gene-HID1	1.630123594	PE(P-18:0/PGE1)	0.886597382
gene-HID1	1.630123594	PE(22:2(13Z,16Z)/22:5(4Z,7Z,10Z,13Z,19Z)-O(	0.369745166
gene-HID1	1.630123594	DG(15:0/PGE1/0:0)	0.462019057
gene-HID1	1.630123594	Cyclosporin A	0.656529229
gene-HID1	1.630123594	Epomusenin A	0.766206049
gene-HID1	1.630123594	PA(22:6(4Z,7Z,10Z,13Z,16Z,19Z)/16:0)	0.556532497
gene-HID1	1.630123594	DG(18:0/LTE4/0:0)	0.681485773
gene-HID1	1.630123594	PC(17:0/PGJ2)	0.657689319
gene-HID1	1.630123594	PC(14:0/20:2(11Z,14Z))	1.110657378
gene-HID1	1.630123594	CDP-DG(PGF2alpha/16:0)	1.002512277
gene-HID1	1.630123594	Lamivudine	0.327402892
gene-HID1	1.630123594	1-beta-D-Arabinofuranosyl-5-fluorocytosine	0.152339645
gene-HID1	1.630123594	Cysteinyl-Glutamine	0.079050155
gene-HID1	1.630123594	Pseudouridine 5'-phosphate	1.18431378
gene-HID1	1.630123594	15-keto-Prostaglandin E2	2.646629404
gene-HID1	1.630123594	Ribavirin monophosphate	1.091085027
gene-HID1	1.630123594	4-Hydroxyproline galactoside	0.148083825
gene-HID1	1.630123594	C20914	0.140383181
gene-HID1	1.630123594	S-(2-Hydroxyethyl)glutathione	0.269671835
gene-HID1	1.630123594	Fluridone	0.127106682
gene-HID1	1.630123594	Norophthalmic acid	0.191432411
gene-HID1	1.630123594	4'-Thiothymidine	0.066948524

gene-HID1	1.630123594	3'-N'-Acetylfusarochromanone	0.755683206
gene-HID1	1.630123594	Ser Cys Ala Ala	0.603032447
gene-HID1	1.630123594	3'-Deoxythymidine	0.791830758
gene-HID1	1.630123594	Gly Asp Ala Ala	0.718656316
gene-HID1	1.630123594	ethyl 2-cyano-3-(1h-indol-3-yl)prop-2-eno	0.756861034
gene-HID1	1.630123594	1-{2-[(3-Ethylphenyl)amino]-2-oxoethyl}-6-	0.705711444
gene-HID1	1.630123594	kainic acid	0.836445456
gene-HID1	1.630123594	Zanamivir	0.839205907
gene-HID1	1.630123594	Aminoglutethimide	0.652616628
gene-HID1	1.630123594	5,6,7,8-Tetrahydromonapterin	0.724075531
gene-HID1	1.630123594	Harmalol	0.364945111
gene-HID1	1.630123594	2-Methyl-3-phenyl-2-propenal	0.407813975
gene-HID1	1.630123594	Guanidoacetic acid	0.542509265
gene-HID1	1.630123594	4-Oxo-9-cis-retinoyl-beta-glucuronide	1.611773742
gene-HID1	1.630123594	alpha-Terpineol formate	0.628238986
gene-HID1	1.630123594	2'-Fluoro-2',3'-dideoxyinosine	1.214745496
gene-HID1	1.630123594	Methionyl-Valine	0.502707745
gene-HID1	1.630123594	Austalide L	0.4229014
gene-HID1	1.630123594	N-Palmitoyl Proline	0.470367693
gene-HID1	1.630123594	10-alpha-methoxy-9,10-dihydrolysergol	0.140851438
gene-HID1	1.630123594	MG(0:0/20:3(11Z,14Z,17Z)/0:0)	0.844949524
gene-HID1	1.630123594	N-Stearoyl Glutamine	1.862695384
gene-HID1	1.630123594	Iridal	0.699410612
gene-HID1	1.630123594	PC(P-18:1(11Z)/PGE2)	0.509503133
gene-HID1	1.630123594	Glutaminylphenylalanine	0.224405311
gene-HID1	1.630123594	N-Stearoyl Proline	0.469971646
gene-HID1	1.630123594	Galabiosylceramide (d18:1/20:0)	0.746737323
gene-HID1	1.630123594	LysoPI(0:0/18:0)	0.420441153
gene-HID1	1.630123594	12-Hydroxyicosanoylcarnitine	1.730979067
gene-HID1	1.630123594	Gamithromycin	0.37168241
gene-HID1	1.630123594	N-Palmitoyl Glutamic acid	0.250188721
gene-HID1	1.630123594	Cer(d18:2(4E,14Z)/TXB2)	0.236013333
gene-HID1	1.630123594	norerythromycin	0.401118778
gene-HID1	1.630123594	N-Myristoyl Glutamine	0.37856966
gene-HID1	1.630123594	PC(18:1(9Z)/15:1(9Z))	0.534724682
gene-HID1	1.630123594	arachidyl amido cholanoic acid	1.24842952
gene-HID1	1.630123594	Nigroxanthin	0.705005059
gene-HID1	1.630123594	1-Octadecanoyl-2-(7Z,10Z,13Z,16Z-docosat	0.690496314
gene-HID1	1.630123594	PE(18:0/20:4(8Z,11Z,14Z,17Z)-2OH(5S,6R))	0.418208781
gene-HID1	1.630123594	PC(P-18:1(11Z)/PGJ2)	0.565243877
gene-HID1	1.630123594	PS(20:0/20:4(8Z,11Z,14Z,17Z)-2OH(5S,6R))	0.402595921
gene-HID1	1.630123594	PE-NMe(18:2(9Z,12Z)/18:2(9Z,12Z))[U]	0.694736593
gene-HID1	1.630123594	PS(16:1(9Z)/22:2(13Z,16Z))	0.659652661
gene-HID1	1.630123594	PC(P-16:0/20:3(8Z,11Z,14Z)-2OH(5,6))	0.174122169
gene-HID1	1.630123594	DG(20:0/LTE4/0:0)	0.438074508
gene-MTCP1	1.629669253	3-Thiacytidine	0.209387412
gene-MTCP1	1.629669253	D-Erythro-imidazole-glycerol-phosphate	0.355812431
gene-MTCP1	1.629669253	LTB4-d4	0.226799982
gene-MTCP1	1.629669253	cis-p-Menth-2-en-1-ol	0.201017988
gene-MTCP1	1.629669253	PE(20:0/18:1(12Z)-2OH(9,10))	0.438658253
gene-MTCP1	1.629669253	DG(15:0/PGE1/0:0)	0.462019057

gene-MTCP1	1.629669253	5-(2-Aminopropyl)-2-methylphenol	0.323231457
gene-MTCP1	1.629669253	2-Methyl-3-phenyl-2-propenal	0.407813975
gene-MTCP1	1.629669253	PC(P-18:1(11Z)/PGE2)	0.509503133
gene-MTCP1	1.629669253	(3R,4R)-3-Amino-1-hydroxy-4-methylpyrrol	0.471914506
gene-MTCP1	1.629669253	Isopropyl isothiocyanate	0.172385747
gene-MTCP1	1.629669253	N-Myristoyl Glutamine	0.37856966
gene-MTCP1	1.629669253	PC(18:1(9Z)/15:1(9Z))	0.534724682
gene-MTCP1	1.629669253	PC(P-18:1(11Z)/PGJ2)	0.565243877
gene-MTCP1	1.629669253	PS(20:0/20:4(8Z,11Z,14Z,17Z)-2OH(5S,6R))	0.402595921
gene-ARHGAP5	1.628034043	5-Hydroxy-2-oxo-4-ureido-2,5-dihydro-1H	0.122536033
gene-ARHGAP5	1.628034043	Urocortisol	0.926998516
gene-ARHGAP5	1.628034043	Tuberose lactone	1.590026295
gene-ARHGAP5	1.628034043	Epothilone D	0.794619023
gene-ARHGAP5	1.628034043	Leukotriene D4	0.6807158
gene-ARHGAP5	1.628034043	2-Ethyl-5-methyl-3,3-diphenyl-1-pyrroline	0.826978858
gene-ARHGAP5	1.628034043	11-Hydroxyeicosatetraenoate glyceryl ester	1.226051047
gene-ARHGAP5	1.628034043	Azaspiracid 5	1.398898867
gene-ARHGAP5	1.628034043	PGP(20:2(11Z,14Z)/18:2(10E,12Z)+=O(9))	1.457698264
gene-ARHGAP5	1.628034043	Edetic Acid	0.028658217
gene-ARHGAP5	1.628034043	11-Maleimidoundecanoic acid	1.084942397
gene-ARHGAP5	1.628034043	PC(24:0/22:6(4Z,7Z,10Z,12E,16Z,19Z)-OH(14)	0.188241061
gene-ARHGAP5	1.628034043	Cyclic Urea	0.1483082
gene-ARHGAP5	1.628034043	Fluridone	0.127106682
gene-ARHGAP5	1.628034043	Norophthalmic acid	0.191432411
gene-ARHGAP5	1.628034043	kainic acid	0.836445456
gene-ARHGAP5	1.628034043	Ethylene brassylate	0.989156973
gene-ARHGAP5	1.628034043	Guanidoacetic acid	0.542509265
gene-ARHGAP5	1.628034043	alpha-Terpineol formate	0.628238986
gene-ARHGAP5	1.628034043	Apronal	0.754033426
gene-ARHGAP5	1.628034043	Methionyl-Valine	0.502707745
gene-ARHGAP5	1.628034043	[3-(2-Aminopropyl)-6-methylidenecyclohexa	0.422945997
gene-ARHGAP5	1.628034043	Argyrolobine	0.949134241
gene-ARHGAP5	1.628034043	3尾-hydroxy-estra-5,7,9-trien-17-one	0.715223098
gene-ARHGAP5	1.628034043	Cetamolol	0.770561315
gene-ARHGAP5	1.628034043	Eicosatetraynoic Acid	0.804823516
gene-ARHGAP5	1.628034043	(2E,4E)-5-[2-Methyl-2-(1,1,4,4-tetramethyl-	0.742652463
gene-ARHGAP5	1.628034043	3,7-Dihydroxy-12-oxocholanoic acid	0.832319882
gene-ARHGAP5	1.628034043	(22E)-3伪,7伪,12伪-Trihydroxy-5尾-chol-22-	0.522629215
gene-ARHGAP5	1.628034043	PGD2 ethanolamide	1.431143851
gene-ARHGAP5	1.628034043	(5alpha,6beta,14alpha,20R,22R)-5,6,14,20,27-	1.296004093
gene-ARHGAP5	1.628034043	SM(d19:1/PGE2)	0.725813678
gene-ARHGAP5	1.628034043	3-{[(2E)-4-Amino-4-oxobut-2-enoyl]amino}	1.152542472
gene-ARHGAP5	1.628034043	Leucylhydroxyproline	0.780056846
gene-ARHGAP5	1.628034043	3'-Hydroxyropivacaine	1.014215236
gene-ARHGAP5	1.628034043	3-Hydroxytetradecanoyl carnitine	1.314002596
gene-ARHGAP5	1.628034043	Chitotriose	0.897504334
gene-ARHGAP5	1.628034043	Macrocin	1.715200143
gene-ARHGAP5	1.628034043	Vulgarone A	0.102049407
gene-ARHGAP5	1.628034043	1,4,6-Trimethylnaphthalene	0.12150693
gene-ARHGAP5	1.628034043	1,8-Octanedithiol	0.133094504
gene-ARHGAP5	1.628034043	Ile His Leu Trp	0.124696937

gene-ARHGAP5	1.628034043	Prostaglandin B2	0.552852305
gene-ARHGAP5	1.628034043	MG(0:0/22:5(4Z,7Z,10Z,13Z,16Z)/0:0)	0.136549841
gene-ARHGAP5	1.628034043	PC(P-18:1(11Z)/PGJ2)	0.565243877
gene-SAG	1.624331794	3-Thiacytidine	0.209387412
gene-SAG	1.624331794	LTB4-d4	0.226799982
gene-SAG	1.624331794	cis-p-Menth-2-en-1-ol	0.201017988
gene-SAG	1.624331794	PE(20:0/18:1(12Z)-2OH(9,10))	0.438658253
gene-SAG	1.624331794	PE(22:2(13Z,16Z)/22:5(4Z,7Z,10Z,13Z,19Z)-O	0.369745166
gene-SAG	1.624331794	5-(2-Aminopropyl)-2-methylphenol	0.323231457
gene-SAG	1.624331794	Armillane	0.52808635
gene-SAG	1.624331794	2-Methyl-3-phenyl-2-propenal	0.407813975
gene-SAG	1.624331794	PC(P-18:1(11Z)/PGE2)	0.509503133
gene-SAG	1.624331794	Isopropyl isothiocyanate	0.172385747
gene-SAG	1.624331794	PC(18:1(9Z)/15:1(9Z))	0.534724682
gene-SAG	1.624331794	Nigroxanthin	0.705005059
gene-SAG	1.624331794	PC(P-18:1(11Z)/PGJ2)	0.565243877
gene-SAG	1.624331794	PS(20:0/20:4(8Z,11Z,14Z,17Z)-2OH(5S,6R))	0.402595921
gene-LOC11244	1.623873639	3-Thiacytidine	0.209387412
gene-LOC11244	1.623873639	D-Erythro-imidazole-glycerol-phosphate	0.355812431
gene-LOC11244	1.623873639	11-Maleimidoundecanoic acid	1.084942397
gene-LOC11244	1.623873639	13(S)-HpODE	0.163157753
gene-LOC11244	1.623873639	LTB4-d4	0.226799982
gene-LOC11244	1.623873639	cis-p-Menth-2-en-1-ol	0.201017988
gene-LOC11244	1.623873639	PE(20:0/18:1(12Z)-2OH(9,10))	0.438658253
gene-LOC11244	1.623873639	Cyclosporin A	0.656529229
gene-LOC11244	1.623873639	DG(18:0/LTE4/0:0)	0.681485773
gene-LOC11244	1.623873639	C20914	0.140383181
gene-LOC11244	1.623873639	2-Methyl-3-phenyl-2-propenal	0.407813975
gene-LOC11244	1.623873639	Guanidoacetic acid	0.542509265
gene-LOC11244	1.623873639	alpha-Terpineol formate	0.628238986
gene-LOC11244	1.623873639	Methionyl-Valine	0.502707745
gene-LOC11244	1.623873639	1,4,6-Trimethylnaphthalene	0.12150693
gene-LOC11244	1.623873639	Prostaglandin PGE2 1-glyceryl ester	0.007411313
gene-LOC11244	1.623873639	1,8-Octanedithiol	0.133094504
gene-LOC11244	1.623873639	PC(P-18:1(11Z)/PGE2)	0.509503133
gene-LOC11244	1.623873639	Nigroxanthin	0.705005059
gene-LOC11244	1.623873639	PC(P-18:1(11Z)/PGJ2)	0.565243877
gene-LOC11244	1.623873639	PS(20:0/20:4(8Z,11Z,14Z,17Z)-2OH(5S,6R))	0.402595921
gene-HIC1	1.623663274	3-Thiacytidine	0.209387412
gene-HIC1	1.623663274	1-Oleoyl-sn-glycero-3-phosphocholine	0.18926952
gene-HIC1	1.623663274	LTB4-d4	0.226799982
gene-HIC1	1.623663274	(-)-alpha-Terpineol	0.345716279
gene-HIC1	1.623663274	1,4-Undecadiene	0.462803973
gene-HIC1	1.623663274	Cyclosporin A	0.656529229
gene-HIC1	1.623663274	DG(18:0/LTE4/0:0)	0.681485773
gene-HIC1	1.623663274	PC(14:0/20:2(11Z,14Z))	1.110657378
gene-HIC1	1.623663274	5-(2-Aminopropyl)-2-methylphenol	0.323231457
gene-HIC1	1.623663274	4-Dimethylamino-L-phenylalanine	0.242110226
gene-HIC1	1.623663274	Trimetazidine	0.438282534
gene-HIC1	1.623663274	ingenol	0.942071652
gene-HIC1	1.623663274	Armillane	0.52808635

gene-HIC1	1.623663274	PC(P-18:1(11Z)/PGE2)	0.509503133
gene-HIC1	1.623663274	(3R,4R)-3-Amino-1-hydroxy-4-methylpyrrol	0.471914506
gene-HIC1	1.623663274	n-methyl-2-(4'-methylaminophenyl)-6-hydr	0.26655714
gene-HIC1	1.623663274	Isopropyl isothiocyanate	0.172385747
gene-HIC1	1.623663274	arachidyl amido cholanoic acid	1.24842952
gene-HIC1	1.623663274	Nigroxanthin	0.705005059
gene-HIC1	1.623663274	9-deoxy-9-methylene-16,16-dimethyl -PGE	0.606893884
gene-HIC1	1.623663274	CL(8:0/8:0/18:2(9Z,11Z)/20:0)	0.622988418
gene-LOC10190	1.618216748	5-(3'-Carboxy-3'-oxopropenyl)-4,6-dihydro	1.083232395
gene-LOC10190	1.618216748	Val-Cit	0.452308451
gene-LOC10190	1.618216748	11-Maleimidoundecanoic acid	1.084942397
gene-LOC10190	1.618216748	Monacolin L acid	1.060562098
gene-LOC10190	1.618216748	cis-p-Menth-2-en-1-ol	0.201017988
gene-LOC10190	1.618216748	PE(22:2(13Z,16Z)/22:5(4Z,7Z,10Z,13Z,19Z)-O	0.369745166
gene-LOC10190	1.618216748	3,4,3',4'-Tetrahydrospirilloxanthin	0.354666148
gene-LOC10190	1.618216748	Norophthalmic acid	0.191432411
gene-LOC10190	1.618216748	(1R,6S)-6-Amino-5-oxocyclohex-2-ene-1-c	0.154751123
gene-LOC10190	1.618216748	2-(1-Adamantyl)-1,3-dioxetane	0.408916843
gene-LOC10190	1.618216748	Milbemycin D	0.125637998
gene-LOC10190	1.618216748	Roxithromycin	0.268273077
gene-LOC10190	1.618216748	PC(P-18:1(11Z)/PGJ2)	0.565243877
gene-LOC10190	1.618216748	PS(20:0/20:4(8Z,11Z,14Z,17Z)-2OH(5S,6R))	0.402595921
Bos_taurus_newG	1.617150162	5-Hydroxy-2-oxo-4-ureido-2,5-dihydro-1H	0.122536033
Bos_taurus_newG	1.617150162	11-Maleimidoundecanoic acid	1.084942397
Bos_taurus_newG	1.617150162	PE(20:0/18:1(12Z)-2OH(9,10))	0.438658253
Bos_taurus_newG	1.617150162	Cyclosporin A	0.656529229
Bos_taurus_newG	1.617150162	Norophthalmic acid	0.191432411
Bos_taurus_newG	1.617150162	Ethylene brassylate	0.989156973
Bos_taurus_newG	1.617150162	Guanidoacetic acid	0.542509265
Bos_taurus_newG	1.617150162	4-Oxo-9-cis-retinoyl-beta-glucuronide	1.611773742
Bos_taurus_newG	1.617150162	alpha-Terpineol formate	0.628238986
Bos_taurus_newG	1.617150162	Methionyl-Valine	0.502707745
Bos_taurus_newG	1.617150162	beta-L-Dioxolane-cytidine	0.175916668
Bos_taurus_newG	1.617150162	PC(P-18:1(11Z)/PGE2)	0.509503133
Bos_taurus_newG	1.617150162	Nigroxanthin	0.705005059
Bos_taurus_newG	1.617150162	PC(P-18:1(11Z)/PGJ2)	0.565243877
Bos_taurus_newG	1.617150162	PS(20:0/20:4(8Z,11Z,14Z,17Z)-2OH(5S,6R))	0.402595921
gene-DSB	1.616947697	Urocortisol	0.926998516
gene-DSB	1.616947697	11-Maleimidoundecanoic acid	1.084942397
gene-DSB	1.616947697	13(S)-HpODE	0.163157753
gene-DSB	1.616947697	7(S),17(S)-dihydroxy-8(E),10(Z),13(Z),15(E),19	0.32147403
gene-DSB	1.616947697	Fluridone	0.127106682
gene-DSB	1.616947697	Norophthalmic acid	0.191432411
gene-DSB	1.616947697	Guanidoacetic acid	0.542509265
gene-DSB	1.616947697	alpha-Terpineol formate	0.628238986
gene-DSB	1.616947697	[3-(2-Aminopropyl)-6-methylidenecyclohexa	0.422945997
gene-DSB	1.616947697	Vulgarone A	0.102049407
gene-DSB	1.616947697	1-Heneicosanoyl-glycero-3-phosphoserine	0.214358093
gene-DSB	1.616947697	PC(P-18:1(11Z)/PGJ2)	0.565243877
gene-PLPP4	1.615860462	S-(Indolylmethylthiohydroximoyl)-L-cysteine	0.635042992
gene-PLPP4	1.615860462	1,2-O-Isopropylidene-D-glucofuranose	0.080987667

gene-PLPP4	1.615860462	3-Deoxyestrone	0.282221709
gene-PLPP4	1.615860462	1-Oleoyl-sn-glycero-3-phosphocholine	0.18926952
gene-PLPP4	1.615860462	LTB4-d4	0.226799982
gene-PLPP4	1.615860462	(-)-alpha-Terpineol	0.345716279
gene-PLPP4	1.615860462	Cyclosporin A	0.656529229
gene-PLPP4	1.615860462	PC(14:0/20:2(11Z,14Z))	1.110657378
gene-PLPP4	1.615860462	5-(2-Aminopropyl)-2-methylphenol	0.323231457
gene-PLPP4	1.615860462	4-Dimethylamino-L-phenylalanine	0.242110226
gene-PLPP4	1.615860462	Trimetazidine	0.438282534
gene-PLPP4	1.615860462	ingenol	0.942071652
gene-PLPP4	1.615860462	PC(P-18:1(11Z)/PGE2)	0.509503133
gene-PLPP4	1.615860462	PC(20:3(5Z,8Z,11Z)/24:0)	0.387959564
gene-PLPP4	1.615860462	(3R,4R)-3-Amino-1-hydroxy-4-methylpyrrol	0.471914506
gene-PLPP4	1.615860462	n-methyl-2-(4'-methylaminophenyl)-6-hydr	0.26655714
gene-PLPP4	1.615860462	(9Z)-Octadecenoic acid	0.142359556
gene-PLPP4	1.615860462	arachidyl amido cholanoic acid	1.24842952
gene-PLPP4	1.615860462	Nigroxanthin	0.705005059
gene-PLPP4	1.615860462	9-deoxy-9-methylene-16,16-dimethyl -PGE	0.606893884
gene-PLPP4	1.615860462	CL(8:0/8:0/18:2(9Z,11Z)/20:0)	0.622988418
gene-VWCE	1.614934138	3-Thiacytidine	0.209387412
gene-VWCE	1.614934138	D-Erythro-imidazole-glycerol-phosphate	0.355812431
gene-VWCE	1.614934138	11-Maleimidoundecanoic acid	1.084942397
gene-VWCE	1.614934138	LTB4-d4	0.226799982
gene-VWCE	1.614934138	cis-p-Menth-2-en-1-ol	0.201017988
gene-VWCE	1.614934138	PE(20:0/18:1(12Z)-2OH(9,10))	0.438658253
gene-VWCE	1.614934138	PE(22:2(13Z,16Z)/22:5(4Z,7Z,10Z,13Z,19Z)-O	0.369745166
gene-VWCE	1.614934138	PG(20:1(11Z)/18:3(10,12,15)-OH(9))	0.626244347
gene-VWCE	1.614934138	2-Methyl-3-phenyl-2-propenal	0.407813975
gene-VWCE	1.614934138	alpha-Terpineol formate	0.628238986
gene-VWCE	1.614934138	PC(P-18:1(11Z)/PGE2)	0.509503133
gene-VWCE	1.614934138	Roxithromycin	0.268273077
gene-VWCE	1.614934138	Sitosterol beta-D-glucoside	0.382944796
gene-VWCE	1.614934138	N-Myristoyl Glutamine	0.37856966
gene-VWCE	1.614934138	PC(18:1(9Z)/15:1(9Z))	0.534724682
gene-VWCE	1.614934138	PC(P-18:1(11Z)/PGJ2)	0.565243877
gene-VWCE	1.614934138	PS(20:0/20:4(8Z,11Z,14Z,17Z)-2OH(5S,6R))	0.402595921
gene-LOC789503	1.60962794	D-Erythro-imidazole-glycerol-phosphate	0.355812431
gene-LOC789503	1.60962794	13(S)-HpODE	0.163157753
gene-LOC789503	1.60962794	LTB4-d4	0.226799982
gene-LOC789503	1.60962794	cis-p-Menth-2-en-1-ol	0.201017988
gene-LOC789503	1.60962794	(-)-alpha-Terpineol	0.345716279
gene-LOC789503	1.60962794	PG(20:1(11Z)/18:3(10,12,15)-OH(9))	0.626244347
gene-LOC789503	1.60962794	2-Methyl-3-phenyl-2-propenal	0.407813975
gene-LOC789503	1.60962794	PC(P-18:1(11Z)/PGE2)	0.509503133
gene-LOC112445	1.608058481	3-Thiacytidine	0.209387412
gene-LOC112445	1.608058481	D-Erythro-imidazole-glycerol-phosphate	0.355812431
gene-LOC112445	1.608058481	LTB4-d4	0.226799982
gene-LOC112445	1.608058481	cis-p-Menth-2-en-1-ol	0.201017988
gene-LOC112445	1.608058481	(-)-alpha-Terpineol	0.345716279
gene-LOC112445	1.608058481	Cyclosporin A	0.656529229
gene-LOC112445	1.608058481	5-(2-Aminopropyl)-2-methylphenol	0.323231457

gene-LOC11244!	1.608058481	4-Dimethylamino-L-phenylalanine	0.242110226
gene-LOC11244!	1.608058481	Armillane	0.52808635
gene-LOC11244!	1.608058481	PC(P-18:1(11Z)/PGE2)	0.509503133
gene-LOC11244!	1.608058481	Nigroxanthin	0.705005059
gene-LOC11244!	1.608058481	PC(P-18:1(11Z)/PGJ2)	0.565243877
gene-LOC11244!	1.608058481	PS(20:0/20:4(8Z,11Z,14Z,17Z)-2OH(5S,6R))	0.402595921
gene-LOC11244!	1.608058481	CL(8:0/8:0/18:2(9Z,11Z)/20:0)	0.622988418
gene-GZMB	1.607952873	1,2-O-Isopropylidene-D-glucofuranose	0.080987667
gene-GZMB	1.607952873	3-Deoxyestrone	0.282221709
gene-GZMB	1.607952873	1-Oleoyl-sn-glycero-3-phosphocholine	0.18926952
gene-GZMB	1.607952873	LTB4-d4	0.226799982
gene-GZMB	1.607952873	(-)-alpha-Terpineol	0.345716279
gene-GZMB	1.607952873	Cyclosporin A	0.656529229
gene-GZMB	1.607952873	5-(2-Aminopropyl)-2-methylphenol	0.323231457
gene-GZMB	1.607952873	4-Dimethylamino-L-phenylalanine	0.242110226
gene-GZMB	1.607952873	Trimetazidine	0.438282534
gene-GZMB	1.607952873	ingenol	0.942071652
gene-GZMB	1.607952873	Armillane	0.52808635
gene-GZMB	1.607952873	PC(P-18:1(11Z)/PGE2)	0.509503133
gene-GZMB	1.607952873	(3R,4R)-3-Amino-1-hydroxy-4-methylpyrrol	0.471914506
gene-GZMB	1.607952873	n-methyl-2-(4'-methylaminophenyl)-6-hydr	0.26655714
gene-GZMB	1.607952873	Isopropyl isothiocyanate	0.172385747
gene-GZMB	1.607952873	(9Z)-Octadecenoic acid	0.142359556
gene-GZMB	1.607952873	9-deoxy-9-methylene-16,16-dimethyl -PGE	0.606893884
gene-GZMB	1.607952873	CL(8:0/8:0/18:2(9Z,11Z)/20:0)	0.622988418
Bos_taurus_newG	1.601909262	3-Deoxyestrone	0.282221709
Bos_taurus_newG	1.601909262	LTB4-d4	0.226799982
Bos_taurus_newG	1.601909262	(-)-alpha-Terpineol	0.345716279
Bos_taurus_newG	1.601909262	4-Dimethylamino-L-phenylalanine	0.242110226
Bos_taurus_newG	1.601909262	Trimetazidine	0.438282534
Bos_taurus_newG	1.601909262	5'-S-Methyl-5'-thioinosine	0.28772476
Bos_taurus_newG	1.601909262	(Z)-3-Oxo-2-(2-pentenyl)-1-cyclopenteneac	0.179805915
Bos_taurus_newG	1.601909262	2-Methyl-3-phenyl-2-propenal	0.407813975
Bos_taurus_newG	1.601909262	PC(P-18:1(11Z)/PGE2)	0.509503133
Bos_taurus_newG	1.601909262	PC(20:3(5Z,8Z,11Z)/24:0)	0.387959564
Bos_taurus_newG	1.601909262	(3R,4R)-3-Amino-1-hydroxy-4-methylpyrrol	0.471914506
Bos_taurus_newG	1.601909262	n-methyl-2-(4'-methylaminophenyl)-6-hydr	0.26655714
Bos_taurus_newG	1.601909262	Isopropyl isothiocyanate	0.172385747
Bos_taurus_newG	1.601909262	(9Z)-Octadecenoic acid	0.142359556
Bos_taurus_newG	1.601909262	9-deoxy-9-methylene-16,16-dimethyl -PGE	0.606893884
Bos_taurus_newG	1.601909262	2-Propenyl 2-aminobenzoate	0.128406829
Bos_taurus_newG	1.601909262	CL(8:0/8:0/18:2(9Z,11Z)/20:0)	0.622988418
Bos_taurus_newG	1.599933633	(2r,3r,4s,5r,6s)-2-(Hydroxymethyl)-6-Sulfanyl	0.392078794
Bos_taurus_newG	1.599933633	5-Sulfosalicylic acid	1.31509494
Bos_taurus_newG	1.599933633	4-Hydroxybenzoate	0.892336321
Bos_taurus_newG	1.599933633	4-Hydroxybenzenesulfonic acid	0.748011467
Bos_taurus_newG	1.599933633	Ethyl nicotinate	1.024858052
Bos_taurus_newG	1.599933633	Dopaquinone	1.087504518
Bos_taurus_newG	1.599933633	Isomaltotriose	0.58434654
Bos_taurus_newG	1.599933633	Lentialexin	0.257119065
Bos_taurus_newG	1.599933633	3-Acetyl-2,5-dimethylfuran	0.972578392

Bos_taurus_newG	1.599933633	Tyrosol 4-sulfate	0.918052785
Bos_taurus_newG	1.599933633	4-Ethylphenol	0.846486412
Bos_taurus_newG	1.599933633	4-Ethylphenylsulfate	0.685674676
Bos_taurus_newG	1.599933633	2,4-Dihydroxy-7,8-dimethoxy-2H-1,4-benzo	1.39970876
Bos_taurus_newG	1.599933633	Sulfite	0.908406967
Bos_taurus_newG	1.599933633	2,6,7-Trihydroxy-9-methylxanthen-3-one	0.49497168
Bos_taurus_newG	1.599933633	(E)-N-(3-(3-(4-Fluorophenoxy)phenyl)-1-(R,	0.26893724
Bos_taurus_newG	1.599933633	Glutamate carbon	0.671444516
Bos_taurus_newG	1.599933633	1-(2-Furanyl)-1-pentanone	0.697147283
Bos_taurus_newG	1.599933633	2-Dehydro-3-deoxy-D-gluconate	0.65165299
Bos_taurus_newG	1.599933633	(1(10)E,4a,5E)-1(10),5-Germacradiene-12-ac	0.476893826
Bos_taurus_newG	1.599933633	Crustecdysone	0.889062022
Bos_taurus_newG	1.599933633	(14S)-14,15-Dihydroxy-8(17),13(16)-labdadie	0.720697404
Bos_taurus_newG	1.599933633	Deoxyshikonin	0.734877521
Bos_taurus_newG	1.599933633	(R)-N1-((S)-3,3-Dimethyl-1-(methylamino)-1	0.382245196
Bos_taurus_newG	1.599933633	17alpha,20alpha-Dihydroxypregn-4-en-3-or	0.034933714
Bos_taurus_newG	1.599933633	3beta-7-Drimene-3,11-diol	0.176540144
Bos_taurus_newG	1.599933633	PE(18:1(12Z)-2OH(9,10)/P-18:0)	0.077744061
Bos_taurus_newG	1.599933633	(卤)-Menthyl acetate	0.218882699
Bos_taurus_newG	1.599933633	Valylarginine	0.627938493
Bos_taurus_newG	1.599933633	trans-Dodec-2-enoic acid	0.211807766
Bos_taurus_newG	1.599933633	3-O-alpha-Mycarosylerythronolide B	0.928450987
Bos_taurus_newG	1.599933633	Serotonin	2.521565841
Bos_taurus_newG	1.599933633	Threonylisoleucine	1.190547061
Bos_taurus_newG	1.599933633	5-Methoxyindoleacetate	0.597532846
Bos_taurus_newG	1.599933633	7-Amino-4-methylcoumarin	0.240088911
Bos_taurus_newG	1.599933633	TETRAHYDROURIDINE	0.576545276
Bos_taurus_newG	1.599933633	Sapidolide A	0.231383732
Bos_taurus_newG	1.599933633	Alongside	0.246470664
Bos_taurus_newG	1.599933633	2-Methyl-3'-hydroxyphenylpropionic acid	1.088489742
Bos_taurus_newG	1.599933633	N-Palmitoyl Cysteine	0.85339147
Bos_taurus_newG	1.599933633	Yucalexin P15	1.079168162
Bos_taurus_newG	1.599933633	(9R,10S,12Z)-9,10-Dihydroxy-8-oxo-12-octa	0.391102388
Bos_taurus_newG	1.599933633	Ascorbyl palmitate	0.376864555
Bos_taurus_newG	1.599933633	3-(3-Methylbutylidene)-1(3H)-isobenzofurar	0.575422414
Bos_taurus_newG	1.599933633	Chamissonin diacetate	0.324896191
Bos_taurus_newG	1.599933633	3',4',5'-Trimethoxycinnamyl alcohol acetate	0.4221816
Bos_taurus_newG	1.599933633	L-Anticapsin	0.426006862
Bos_taurus_newG	1.599933633	trans-trismethoxy Resveratrol-d4	0.731157657
Bos_taurus_newG	1.599933633	Sophoranol	0.282064826
Bos_taurus_newG	1.599933633	Abt-510	0.626815579
Bos_taurus_newG	1.599933633	PGF2伪 Alcohol methyl ether	0.167013539
Bos_taurus_newG	1.599933633	PGF2伪 1,11-lactone	0.041436917
Bos_taurus_newG	1.599933633	PA(PGE2/22:2(13Z,16Z))	0.24730102
Bos_taurus_newG	1.599933633	PS(20:5(5Z,8Z,11Z,14Z,16E)-OH(18R)/22:0)	0.090792733
Bos_taurus_newG	1.599933633	N-(2,3,4-Trihydroxybutyl)-L-valine	0.230879803
Bos_taurus_newG	1.599933633	N-Oleoyl Asparagine	0.060994498
gene-TMEM120B	1.599063603	5-(Ethylthio)-1H-tetrazole	0.29717344
gene-TMEM120B	1.599063603	S-(Indolylmethylthiohydroximoyl)-L-cysteine	0.635042992
gene-TMEM120B	1.599063603	3-Thiacytidine	0.209387412
gene-TMEM120B	1.599063603	LTB4-d4	0.226799982

gene-TMEM120B	1.599063603	(-)-alpha-Terpineol	0.345716279
gene-TMEM120B	1.599063603	DG(15:0/PGE1/0:0)	0.462019057
gene-TMEM120B	1.599063603	PC(14:0/20:2(11Z,14Z))	1.110657378
gene-TMEM120B	1.599063603	5-(2-Aminopropyl)-2-methylphenol	0.323231457
gene-TMEM120B	1.599063603	4-Dimethylamino-L-phenylalanine	0.242110226
gene-TMEM120B	1.599063603	Trimetazidine	0.438282534
gene-TMEM120B	1.599063603	Armillane	0.52808635
gene-TMEM120B	1.599063603	(2R,3R)-3-Methylornithinyl-N6-lysine	1.014942199
gene-TMEM120B	1.599063603	5'-S-Methyl-5'-thioinosine	0.28772476
gene-TMEM120B	1.599063603	(Z)-3-Oxo-2-(2-pentenyl)-1-cyclopenteneac	0.179805915
gene-TMEM120B	1.599063603	2-Methyl-3-phenyl-2-propenal	0.407813975
gene-TMEM120B	1.599063603	PC(P-18:1(11Z)/PGE2)	0.509503133
gene-TMEM120B	1.599063603	(3R,4R)-3-Amino-1-hydroxy-4-methylpyrrol	0.471914506
gene-TMEM120B	1.599063603	n-methyl-2-(4'-methylaminophenyl)-6-hydr	0.26655714
gene-TMEM120B	1.599063603	PE(P-16:0/18:4(6Z,9Z,12Z,15Z))	0.546989864
gene-TMEM120B	1.599063603	Isopropyl isothiocyanate	0.172385747
gene-TMEM120B	1.599063603	(9Z)-Octadecenoic acid	0.142359556
gene-TMEM120B	1.599063603	N-Myristoyl Glutamine	0.37856966
gene-TMEM120B	1.599063603	arachidyl amido cholanoic acid	1.24842952
gene-TMEM120B	1.599063603	Nigroxanthin	0.705005059
gene-TMEM120B	1.599063603	9-deoxy-9-methylene-16,16-dimethyl -PGE	0.606893884
gene-TMEM120B	1.599063603	1-Octadecanoyl-2-(7Z,10Z,13Z,16Z-docosat	0.690496314
gene-TMEM120B	1.599063603	2-Propenyl 2-aminobenzoate	0.128406829
gene-TMEM120B	1.599063603	CL(8:0/8:0/18:2(9Z,11Z)/20:0)	0.622988418
gene-TMEM120B	1.599063603	PC(20:5(5Z,8Z,11Z,14Z,17Z)/P-16:0)	1.301095986
gene-TMEM120B	1.599063603	DG(20:0/LTE4/0:0)	0.438074508
gene-C19H17orf	1.597180257	3-Thiacytidine	0.209387412
gene-C19H17orf	1.597180257	LTB4-d4	0.226799982
gene-C19H17orf	1.597180257	PE(20:0/18:1(12Z)-2OH(9,10))	0.438658253
gene-C19H17orf	1.597180257	(-)-alpha-Terpineol	0.345716279
gene-C19H17orf	1.597180257	Cyclosporin A	0.656529229
gene-C19H17orf	1.597180257	DG(18:0/LTE4/0:0)	0.681485773
gene-C19H17orf	1.597180257	PC(14:0/20:2(11Z,14Z))	1.110657378
gene-C19H17orf	1.597180257	5-(2-Aminopropyl)-2-methylphenol	0.323231457
gene-C19H17orf	1.597180257	Armillane	0.52808635
gene-C19H17orf	1.597180257	PC(P-18:1(11Z)/PGE2)	0.509503133
gene-C19H17orf	1.597180257	(3R,4R)-3-Amino-1-hydroxy-4-methylpyrrol	0.471914506
gene-C19H17orf	1.597180257	Isopropyl isothiocyanate	0.172385747
gene-C19H17orf	1.597180257	(9Z)-Octadecenoic acid	0.142359556
gene-C19H17orf	1.597180257	arachidyl amido cholanoic acid	1.24842952
gene-C19H17orf	1.597180257	Nigroxanthin	0.705005059
gene-C19H17orf	1.597180257	PC(P-18:1(11Z)/PGJ2)	0.565243877
gene-C19H17orf	1.597180257	PS(20:0/20:4(8Z,11Z,14Z,17Z)-2OH(5S,6R))	0.402595921
gene-C19H17orf	1.597180257	CL(8:0/8:0/18:2(9Z,11Z)/20:0)	0.622988418
gene-CRYM	1.594591581	Val-Cit	0.452308451
gene-CRYM	1.594591581	3-(2,4-Dimethyl-5-(2-oxo-1,2-dihydroindol-	0.095524813
gene-CRYM	1.594591581	Urocortisol	0.926998516
gene-CRYM	1.594591581	11-Maleimidoundecanoic acid	1.084942397
gene-CRYM	1.594591581	Gemfibrozil	0.392694536
gene-CRYM	1.594591581	Aspartame	0.406719484
gene-CRYM	1.594591581	13(S)-HpODE	0.163157753

gene-CRYM	1.594591581	Monacolin L acid	1.060562098
gene-CRYM	1.594591581	Norophthalmic acid	0.191432411
gene-CRYM	1.594591581	(1R,6S)-6-Amino-5-oxocyclohex-2-ene-1-c	0.154751123
gene-CRYM	1.594591581	3-(3-Methylbutylidene)-1(3H)-isobenzofurar	0.575422414
gene-CRYM	1.594591581	[3-(2-Aminopropyl)-6-methylidenecyclohexa	0.422945997
gene-CRYM	1.594591581	3',4',5'-Trimethoxycinnamyl alcohol acetate	0.4221816
gene-CRYM	1.594591581	ent-16b,19-Kauranediol 19-acetate	0.400676827
gene-CRYM	1.594591581	Ketosantalic acid	0.356663411
gene-CRYM	1.594591581	Isoamyl salicylate	0.393519337
gene-CRYM	1.594591581	Roxithromycin	0.268273077
gene-CHCHD10	1.59074733	3-hydroxypristanic acid	0.548515835
gene-CHCHD10	1.59074733	Canesceol	0.686312122
gene-CHCHD10	1.59074733	16-hydroxy hexadecanoic acid	0.309000958
gene-CHCHD10	1.59074733	1,2-O-Isopropylidene-D-glucofuranose	0.080987667
gene-CHCHD10	1.59074733	3-Deoxyestrone	0.282221709
gene-CHCHD10	1.59074733	1-Oleoyl-sn-glycero-3-phosphocholine	0.18926952
gene-CHCHD10	1.59074733	3,4-dihydroxy-5-all-trans-hexaprenylbenzoa	0.123615726
gene-CHCHD10	1.59074733	Lividamine	0.319679555
gene-CHCHD10	1.59074733	Psychosine	0.106475396
gene-CHCHD10	1.59074733	Cyclotricuspidogenin C	0.440884085
gene-CHCHD10	1.59074733	(-)-alpha-Terpineol	0.345716279
gene-CHCHD10	1.59074733	Linoleamide	0.926003481
gene-CHCHD10	1.59074733	DG(8:0/20:4(6Z,8E,10E,14Z)-2OH(5S,12R)/0:0	0.958378689
gene-CHCHD10	1.59074733	1,4-Undecadiene	0.462803973
gene-CHCHD10	1.59074733	4-cholesten-7伪,12伪,24-triol-3-one	0.097840451
gene-CHCHD10	1.59074733	4-Dimethylamino-L-phenylalanine	0.242110226
gene-CHCHD10	1.59074733	Trimetazidine	0.438282534
gene-CHCHD10	1.59074733	ingenol	0.942071652
gene-CHCHD10	1.59074733	Armillane	0.52808635
gene-CHCHD10	1.59074733	Misoprostol	1.349270093
gene-CHCHD10	1.59074733	Carboprost methyl	1.74923433
gene-CHCHD10	1.59074733	N-[[3-Hydroxy-2-(2-pentenyl)cyclopentyl]ac	1.043930947
gene-CHCHD10	1.59074733	11-Dehydro-2,3-dinor-txb2	0.307258797
gene-CHCHD10	1.59074733	Val Trp Leu His	0.05980936
gene-CHCHD10	1.59074733	PC(20:3(5Z,8Z,11Z)/24:0)	0.387959564
gene-CHCHD10	1.59074733	1-Palmitoylglycerol	0.084357408
gene-CHCHD10	1.59074733	N2-gamma-Glutamylglutamine	0.230065499
gene-CHCHD10	1.59074733	n-methyl-2-(4'-methylaminophenyl)-6-hydr	0.26655714
gene-CHCHD10	1.59074733	Permetin A	0.140869415
gene-CHCHD10	1.59074733	Glutethimide	0.126237229
gene-CHCHD10	1.59074733	Isopropyl isothiocyanate	0.172385747
gene-CHCHD10	1.59074733	9-deoxy-9-methylene-16,16-dimethyl -PGE:	0.606893884
gene-CHCHD10	1.59074733	1-Methylnicotinamide	0.203956241
gene-CHCHD10	1.59074733	CL(8:0/8:0/18:2(9Z,11Z)/20:0)	0.622988418
gene-PHF7	1.585081538	cis-p-Menth-2-en-1-ol	0.201017988
gene-PHF7	1.585081538	PE(20:0/18:1(12Z)-2OH(9,10))	0.438658253
gene-PHF7	1.585081538	PE(22:2(13Z,16Z)/22:5(4Z,7Z,10Z,13Z,19Z)-O	0.369745166
gene-PHF7	1.585081538	PG(20:1(11Z)/18:3(10,12,15)-OH(9))	0.626244347
gene-PHF7	1.585081538	5-Formiminotetrahydrofolate	0.118251287
gene-PHF7	1.585081538	Benzoyl glucuronide (Benzoic acid)	0.442844673
gene-PHF7	1.585081538	Pregnanediol	0.166857843

gene-PHF7	1.585081538	Milbemycin D	0.125637998
gene-PHF7	1.585081538	Sitosterol beta-D-glucoside	0.382944796
gene-PHF7	1.585081538	PC(P-18:1(11Z)/PGJ2)	0.565243877
gene-PHF7	1.585081538	PS(20:0/20:4(8Z,11Z,14Z,17Z)-2OH(5S,6R))	0.402595921
gene-PHF7	1.585081538	PG(20:1(11Z)/18:3(9,11,15)-OH(13))	0.69829683
gene-MAN1B1-2	1.582420462	D-Erythro-imidazole-glycerol-phosphate	0.355812431
gene-MAN1B1-2	1.582420462	milbemycin beta3	1.414447124
gene-MAN1B1-2	1.582420462	cis-p-Menth-2-en-1-ol	0.201017988
gene-MAN1B1-2	1.582420462	(-)-alpha-Terpineol	0.345716279
gene-MAN1B1-2	1.582420462	PE(22:2(13Z,16Z)/22:5(4Z,7Z,10Z,13Z,19Z)-O	0.369745166
gene-MAN1B1-2	1.582420462	PG(20:1(11Z)/18:3(10,12,15)-OH(9))	0.626244347
gene-MAN1B1-2	1.582420462	Armillane	0.52808635
gene-MAN1B1-2	1.582420462	PC(P-18:1(11Z)/PGE2)	0.509503133
gene-MAN1B1-2	1.582420462	PC(P-18:1(11Z)/PGJ2)	0.565243877
gene-MAN1B1-2	1.582420462	PS(20:0/20:4(8Z,11Z,14Z,17Z)-2OH(5S,6R))	0.402595921
gene-DGKG	1.57687909	D-Erythro-imidazole-glycerol-phosphate	0.355812431
gene-DGKG	1.57687909	Glutamate carbon	0.671444516
gene-DGKG	1.57687909	11-Maleimidoundecanoic acid	1.084942397
gene-DGKG	1.57687909	PE(22:2(13Z,16Z)/22:5(4Z,7Z,10Z,13Z,19Z)-O	0.369745166
gene-DGKG	1.57687909	Norophthalmic acid	0.191432411
gene-DGKG	1.57687909	(1R,6S)-6-Amino-5-oxocyclohex-2-ene-1-c	0.154751123
gene-DGKG	1.57687909	alpha-Terpineol formate	0.628238986
gene-DGKG	1.57687909	PC(P-18:1(11Z)/PGE2)	0.509503133
gene-DGKG	1.57687909	Roxithromycin	0.268273077
gene-DGKG	1.57687909	PC(P-18:1(11Z)/PGJ2)	0.565243877
gene-DGKG	1.57687909	PS(20:0/20:4(8Z,11Z,14Z,17Z)-2OH(5S,6R))	0.402595921
Bos_taurus_newG	1.575402582	D-Erythro-imidazole-glycerol-phosphate	0.355812431
Bos_taurus_newG	1.575402582	13(S)-HpODE	0.163157753
Bos_taurus_newG	1.575402582	LTB4-d4	0.226799982
Bos_taurus_newG	1.575402582	cis-p-Menth-2-en-1-ol	0.201017988
Bos_taurus_newG	1.575402582	(-)-alpha-Terpineol	0.345716279
Bos_taurus_newG	1.575402582	Cyclosporin A	0.656529229
Bos_taurus_newG	1.575402582	2-Methyl-3-phenyl-2-propenal	0.407813975
Bos_taurus_newG	1.575402582	PC(P-18:1(11Z)/PGE2)	0.509503133
Bos_taurus_newG	1.575402582	PC(P-18:1(11Z)/PGJ2)	0.565243877
gene-OLFML3	1.566838926	3-Thiacytidine	0.209387412
gene-OLFML3	1.566838926	PE(20:0/18:1(12Z)-2OH(9,10))	0.438658253
gene-OLFML3	1.566838926	PE(22:2(13Z,16Z)/22:5(4Z,7Z,10Z,13Z,19Z)-O	0.369745166
gene-OLFML3	1.566838926	Cyclosporin A	0.656529229
gene-OLFML3	1.566838926	DG(18:0/LTE4/0:0)	0.681485773
gene-OLFML3	1.566838926	PC(14:0/20:2(11Z,14Z))	1.110657378
gene-OLFML3	1.566838926	CDP-DG(PGF2alpha/16:0)	1.002512277
gene-OLFML3	1.566838926	beta-L-Dioxolane-cytidine	0.175916668
gene-OLFML3	1.566838926	PC(P-18:1(11Z)/PGE2)	0.509503133
gene-OLFML3	1.566838926	Nigroxanthin	0.705005059
gene-OLFML3	1.566838926	PC(P-18:1(11Z)/PGJ2)	0.565243877
gene-OLFML3	1.566838926	PS(20:0/20:4(8Z,11Z,14Z,17Z)-2OH(5S,6R))	0.402595921
gene-OLFML3	1.566838926	CL(8:0/8:0/18:2(9Z,11Z)/20:0)	0.622988418
gene-LOC100141	1.566320339	3-Thiacytidine	0.209387412
gene-LOC100141	1.566320339	(-)-alpha-Terpineol	0.345716279
gene-LOC100141	1.566320339	1,4-Undecadiene	0.462803973

gene-LOC100141	1.566320339	4-cholesten-7伪,12伪,24-triol-3-one	0.097840451
gene-LOC100141	1.566320339	5-(2-Aminopropyl)-2-methylphenol	0.323231457
gene-LOC100141	1.566320339	Armillane	0.52808635
gene-LOC100141	1.566320339	PC(P-18:1(11Z)/PGE2)	0.509503133
gene-LOC100141	1.566320339	(3R,4R)-3-Amino-1-hydroxy-4-methylpyrrol	0.471914506
gene-LOC100141	1.566320339	Isopropyl isothiocyanate	0.172385747
gene-LOC100141	1.566320339	PS(20:0/20:4(8Z,11Z,14Z,17Z)-2OH(5S,6R))	0.402595921
gene-LOC100141	1.566320339	CL(8:0/8:0/18:2(9Z,11Z)/20:0)	0.622988418
gene-ITGA9	1.558055654	3-Thiacytidine	0.209387412
gene-ITGA9	1.558055654	(-)-alpha-Terpineol	0.345716279
gene-ITGA9	1.558055654	1,4-Undecadiene	0.462803973
gene-ITGA9	1.558055654	4-cholesten-7伪,12伪,24-triol-3-one	0.097840451
gene-ITGA9	1.558055654	PE(22:2(13Z,16Z)/22:5(4Z,7Z,10Z,13Z,19Z)-O	0.369745166
gene-ITGA9	1.558055654	N-[[3-Hydroxy-2-(2-pentenyl)cyclopentyl]ac	1.043930947
gene-ITGA9	1.558055654	PC(P-18:1(11Z)/PGE2)	0.509503133
gene-ITGA9	1.558055654	Phorone A	0.079039095
gene-ITGA9	1.558055654	2-Amino-4-[carbamimidoyl(methyl)amino]bu	0.075703353
gene-ITGA9	1.558055654	PS(20:0/20:4(8Z,11Z,14Z,17Z)-2OH(5S,6R))	0.402595921
gene-NKIRAS1	1.557341706	7(14)-Bisabolene-2,3,10,11-tetrol	2.446897974
gene-NKIRAS1	1.557341706	1,4-Undecadiene	0.462803973
gene-NKIRAS1	1.557341706	PC(16:0/18:1(12Z)-2OH(9,10))	0.461843233
gene-NKIRAS1	1.557341706	5-(2-Aminopropyl)-2-methylphenol	0.323231457
gene-NKIRAS1	1.557341706	4-Dimethylamino-L-phenylalanine	0.242110226
gene-NKIRAS1	1.557341706	Armillane	0.52808635
gene-NKIRAS1	1.557341706	Sambutoxin	0.169786377
gene-NKIRAS1	1.557341706	1-Palmitoylglycerol	0.084357408
gene-NKIRAS1	1.557341706	Isopropyl isothiocyanate	0.172385747
gene-NKIRAS1	1.557341706	9-deoxy-9-methylene-16,16-dimethyl -PGE	0.606893884
gene-LOC615559	1.555310681	3-Thiacytidine	0.209387412
gene-LOC615559	1.555310681	D-Erythro-imidazole-glycerol-phosphate	0.355812431
gene-LOC615559	1.555310681	LTB4-d4	0.226799982
gene-LOC615559	1.555310681	cis-p-Menth-2-en-1-ol	0.201017988
gene-LOC615559	1.555310681	PE(20:0/18:1(12Z)-2OH(9,10))	0.438658253
gene-LOC615559	1.555310681	(-)-alpha-Terpineol	0.345716279
gene-LOC615559	1.555310681	5-(2-Aminopropyl)-2-methylphenol	0.323231457
gene-LOC615559	1.555310681	2-Methyl-3-phenyl-2-propenal	0.407813975
gene-LOC615559	1.555310681	PC(P-18:1(11Z)/PGE2)	0.509503133
gene-LOC615559	1.555310681	(3R,4R)-3-Amino-1-hydroxy-4-methylpyrrol	0.471914506
gene-LOC615559	1.555310681	Isopropyl isothiocyanate	0.172385747
gene-LOC615559	1.555310681	PC(18:1(9Z)/15:1(9Z))	0.534724682
gene-LOC615559	1.555310681	Nigroxanthin	0.705005059
gene-LOC615559	1.555310681	PC(P-18:1(11Z)/PGJ2)	0.565243877
gene-LOC615559	1.555310681	PS(20:0/20:4(8Z,11Z,14Z,17Z)-2OH(5S,6R))	0.402595921
gene-LOC615559	1.555310681	CL(8:0/8:0/18:2(9Z,11Z)/20:0)	0.622988418
gene-RHBDF1	1.553491675	Methylmalonate	0.241481249
gene-RHBDF1	1.553491675	Dextrorphan O-glucuronide	1.59476827
gene-RHBDF1	1.553491675	PE-NMe(18:0/18:3(9Z,12Z,15Z))	0.681884774
gene-RHBDF1	1.553491675	3-Thiacytidine	0.209387412
gene-RHBDF1	1.553491675	Methyl methacrylate	0.245645579
gene-RHBDF1	1.553491675	5-amino-1-(5-phosphonato-D-ribosyl)imida	0.622612791
gene-RHBDF1	1.553491675	8-Methoxykynurenate	0.424170332

gene-RHBDF1	1.553491675	5-Hydroxyindoleacetaldehyde	0.448806354
gene-RHBDF1	1.553491675	Azelaic acid	0.007180175
gene-RHBDF1	1.553491675	5-Hydroxy-2-oxo-4-ureido-2,5-dihydro-1H	0.122536033
gene-RHBDF1	1.553491675	p-Coumaroylputrescine	0.939330574
gene-RHBDF1	1.553491675	Calicheamicinone	0.910932275
gene-RHBDF1	1.553491675	Pterostilbene	0.899698314
gene-RHBDF1	1.553491675	Ancymidol	0.913827444
gene-RHBDF1	1.553491675	Phenylalanylhydroxyproline	1.183668928
gene-RHBDF1	1.553491675	Delgocitinib	1.051864373
gene-RHBDF1	1.553491675	Melatonin	1.134145229
gene-RHBDF1	1.553491675	2H-1-Benzopyran-2-one, aminomethyl-	0.385162237
gene-RHBDF1	1.553491675	Trihomomethionine	0.296210774
gene-RHBDF1	1.553491675	PIP(22:5(4Z,7Z,10Z,13Z,16Z)/PGJ2)	0.868452769
gene-RHBDF1	1.553491675	Arginine vasopressin 1-8	1.288601754
gene-RHBDF1	1.553491675	Hygromycin B	0.930396619
gene-RHBDF1	1.553491675	Netupitant	0.707108724
gene-RHBDF1	1.553491675	Divinylprotochlorophyllide	0.629978387
gene-RHBDF1	1.553491675	Benzoyl-fvr-pna	0.67370714
gene-RHBDF1	1.553491675	Metkephamid	1.159767003
gene-RHBDF1	1.553491675	Tuberose lactone	1.590026295
gene-RHBDF1	1.553491675	Epothilone D	0.794619023
gene-RHBDF1	1.553491675	Leukotriene D4	0.6807158
gene-RHBDF1	1.553491675	2-Ethyl-5-methyl-3,3-diphenyl-1-pyrroline	0.826978858
gene-RHBDF1	1.553491675	Azaspiracid 5	1.398898867
gene-RHBDF1	1.553491675	PGP(20:2(11Z,14Z)/18:2(10E,12Z)+=O(9))	1.457698264
gene-RHBDF1	1.553491675	3,4-Dihydro-6-methoxy-2,2-dimethyl-2H-1	0.296379892
gene-RHBDF1	1.553491675	2-Hexylbenzothiazole	0.396099297
gene-RHBDF1	1.553491675	Becocalcidiol	0.63162902
gene-RHBDF1	1.553491675	Docosanamide	0.509975786
gene-RHBDF1	1.553491675	Valnemulin	0.408062514
gene-RHBDF1	1.553491675	Oxyphencyclimine	0.098817805
gene-RHBDF1	1.553491675	Testosterone isobutyrate	0.467664668
gene-RHBDF1	1.553491675	6-isobutyl-4-hydroxy-2-pyrone	0.473678412
gene-RHBDF1	1.553491675	LTB4-d4	0.226799982
gene-RHBDF1	1.553491675	cis-p-Menth-2-en-1-ol	0.201017988
gene-RHBDF1	1.553491675	PE(20:0/18:1(12Z)-2OH(9,10))	0.438658253
gene-RHBDF1	1.553491675	4,6-Heneicosanedione	0.113496143
gene-RHBDF1	1.553491675	PE(22:2(13Z,16Z)/22:5(4Z,7Z,10Z,13Z,19Z)-O	0.369745166
gene-RHBDF1	1.553491675	Heptanoylcholine	0.218998318
gene-RHBDF1	1.553491675	DG(15:0/PGE1/0:0)	0.462019057
gene-RHBDF1	1.553491675	Epomusenin A	0.766206049
gene-RHBDF1	1.553491675	(4r,5s,6s,7r)-4,7-Dibenzyl-5,6-dihydroxy-1,3	1.334600128
gene-RHBDF1	1.553491675	CDP-DG(PGF2alpha/16:0)	1.002512277
gene-RHBDF1	1.553491675	Lamivudine	0.327402892
gene-RHBDF1	1.553491675	1-beta-D-Arabinofuranosyl-5-fluorocytosine	0.152339645
gene-RHBDF1	1.553491675	Cysteinyl-Glutamine	0.079050155
gene-RHBDF1	1.553491675	Pseudouridine 5'-phosphate	1.18431378
gene-RHBDF1	1.553491675	Aflatoxin P1	1.000661338
gene-RHBDF1	1.553491675	15-keto-Prostaglandin E2	2.646629404
gene-RHBDF1	1.553491675	S-Acetyldihydrolipoamide-E	0.429561283
gene-RHBDF1	1.553491675	Ribavirin monophosphate	1.091085027

gene-RHBDF1	1.553491675	4-Hydroxyproline galactoside	0.148083825
gene-RHBDF1	1.553491675	C20914	0.140383181
gene-RHBDF1	1.553491675	S-(2-Hydroxyethyl)glutathione	0.269671835
gene-RHBDF1	1.553491675	Fluridone	0.127106682
gene-RHBDF1	1.553491675	Austdiol	0.046757893
gene-RHBDF1	1.553491675	Norophthalmic acid	0.191432411
gene-RHBDF1	1.553491675	4'-Thiothymidine	0.066948524
gene-RHBDF1	1.553491675	3'-N'-Acetylfusarochromanone	0.755683206
gene-RHBDF1	1.553491675	Desmethylflumazenil (Ro 15-5528)	0.227256571
gene-RHBDF1	1.553491675	cis,trans-5'-Hydroxythalidomide	0.589828342
gene-RHBDF1	1.553491675	Ser Cys Ala Ala	0.603032447
gene-RHBDF1	1.553491675	6-Fluorohomovanillic acid	0.040947314
gene-RHBDF1	1.553491675	10-Hydroxycarbazepine	0.848001655
gene-RHBDF1	1.553491675	Semilepidinoside A	0.527638644
gene-RHBDF1	1.553491675	Arbutin	0.176184476
gene-RHBDF1	1.553491675	Loganin	0.92782666
gene-RHBDF1	1.553491675	3'-Deoxythymidine	0.791830758
gene-RHBDF1	1.553491675	5-Acetylamino-6-amino-3-methyluracil	0.420050597
gene-RHBDF1	1.553491675	Gly Asp Ala Ala	0.718656316
gene-RHBDF1	1.553491675	N-Acetylhistidine	0.349369987
gene-RHBDF1	1.553491675	ethyl 2-cyano-3-(1h-indol-3-yl)prop-2-eno	0.756861034
gene-RHBDF1	1.553491675	1-{2-[(3-Ethylphenyl)amino]-2-oxoethyl}-6-	0.705711444
gene-RHBDF1	1.553491675	kainic acid	0.836445456
gene-RHBDF1	1.553491675	Zanamivir	0.839205907
gene-RHBDF1	1.553491675	Tryptophyl-Glutamine	0.89722186
gene-RHBDF1	1.553491675	Aminoglutethimide	0.652616628
gene-RHBDF1	1.553491675	5-NITRO-2-PHENYLPROPYLAMINOBENZOI(	0.713298779
gene-RHBDF1	1.553491675	5,6,7,8-Tetrahydromonapterin	0.724075531
gene-RHBDF1	1.553491675	Harmalol	0.364945111
gene-RHBDF1	1.553491675	2,3-Epoxymenaquinone	2.040730224
gene-RHBDF1	1.553491675	Guanidoacetic acid	0.542509265
gene-RHBDF1	1.553491675	4-Oxo-9-cis-retinoyl-beta-glucuronide	1.611773742
gene-RHBDF1	1.553491675	alpha-Terpineol formate	0.628238986
gene-RHBDF1	1.553491675	4-Dihydroboldenone	0.692863202
gene-RHBDF1	1.553491675	2'-Fluoro-2',3'-dideoxyinosine	1.214745496
gene-RHBDF1	1.553491675	20-carboxy Arachidonic Acid	0.780988382
gene-RHBDF1	1.553491675	17-beta-Estradiol glucuronide	0.827039442
gene-RHBDF1	1.553491675	Nebramycin 5'	0.909280571
gene-RHBDF1	1.553491675	Apronal	0.754033426
gene-RHBDF1	1.553491675	Methionyl-Valine	0.502707745
gene-RHBDF1	1.553491675	Argyrolobine	0.949134241
gene-RHBDF1	1.553491675	Eicosatetraynoic Acid	0.804823516
gene-RHBDF1	1.553491675	(2E,4E)-5-[2-Methyl-2-(1,1,4,4-tetramethyl-	0.742652463
gene-RHBDF1	1.553491675	3,7-Dihydroxy-12-oxocholanoic acid	0.832319882
gene-RHBDF1	1.553491675	PGD2 ethanolamide	1.431143851
gene-RHBDF1	1.553491675	(5alpha,6beta,14alpha,20R,22R)-5,6,14,20,27-	1.296004093
gene-RHBDF1	1.553491675	3-{[(2E)-4-Amino-4-oxobut-2-enoyl]amino}	1.152542472
gene-RHBDF1	1.553491675	Leucylhydroxyproline	0.780056846
gene-RHBDF1	1.553491675	3-Hydroxytetradecanoyl carnitine	1.314002596
gene-RHBDF1	1.553491675	Chitotriose	0.897504334
gene-RHBDF1	1.553491675	Macrocin	1.715200143

gene-RHBDF1	1.553491675	Zingiberenol	0.088860673
gene-RHBDF1	1.553491675	Myricanol 5-[arabinosyl-(1->6)-glucoside]	0.744174399
gene-RHBDF1	1.553491675	MG(0:0/18:3(9Z,12Z,15Z)/0:0)	1.373784038
gene-RHBDF1	1.553491675	Austalide L	0.4229014
gene-RHBDF1	1.553491675	Tetracycline	0.553304723
gene-RHBDF1	1.553491675	Spergualin	0.210690914
gene-RHBDF1	1.553491675	N-Palmitoyl Proline	0.470367693
gene-RHBDF1	1.553491675	10-alpha-methoxy-9,10-dihydrolysergol	0.140851438
gene-RHBDF1	1.553491675	3beta-Hydroxyergosta-7,24(24(1))-dien-4alp	1.714710978
gene-RHBDF1	1.553491675	MG(0:0/20:3(11Z,14Z,17Z)/0:0)	0.844949524
gene-RHBDF1	1.553491675	Cichorioside J	0.191008611
gene-RHBDF1	1.553491675	N-Stearoyl Glutamine	1.862695384
gene-RHBDF1	1.553491675	Dapdiamide B	0.07688703
gene-RHBDF1	1.553491675	Iridal	0.699410612
gene-RHBDF1	1.553491675	Glutaminylphenylalanine	0.224405311
gene-RHBDF1	1.553491675	N-Stearoyl Proline	0.469971646
gene-RHBDF1	1.553491675	Galabiosylceramide (d18:1/20:0)	0.746737323
gene-RHBDF1	1.553491675	LysoPI(0:0/18:0)	0.420441153
gene-RHBDF1	1.553491675	N-Stearoyl Valine	0.653773206
gene-RHBDF1	1.553491675	12-Hydroxyicosanoylcarnitine	1.730979067
gene-RHBDF1	1.553491675	N-Palmitoyl Glutamic acid	0.250188721
gene-RHBDF1	1.553491675	Tragopogonsaponin A	0.169034237
gene-RHBDF1	1.553491675	Tylactone	0.340073797
gene-RHBDF1	1.553491675	Cer(d18:2(4E,14Z)/TXB2)	0.236013333
gene-RHBDF1	1.553491675	norerythromycin	0.401118778
gene-RHBDF1	1.553491675	2,4-Undecadienal	0.228636005
gene-RHBDF1	1.553491675	N-Myristoyl Glutamine	0.37856966
gene-RHBDF1	1.553491675	PC(18:1(9Z)/15:1(9Z))	0.534724682
gene-RHBDF1	1.553491675	arachidyl amido cholanoic acid	1.24842952
gene-RHBDF1	1.553491675	Ganglioside GM3 (d18:1/12:0)	0.163857715
gene-RHBDF1	1.553491675	Nigroxanthin	0.705005059
gene-RHBDF1	1.553491675	1-Octadecanoyl-2-(7Z,10Z,13Z,16Z-docosat	0.690496314
gene-RHBDF1	1.553491675	PE(18:0/20:4(8Z,11Z,14Z,17Z)-2OH(5S,6R))	0.418208781
gene-RHBDF1	1.553491675	PC(P-18:1(11Z)/PGJ2)	0.565243877
gene-RHBDF1	1.553491675	PS(20:0/20:4(8Z,11Z,14Z,17Z)-2OH(5S,6R))	0.402595921
gene-RHBDF1	1.553491675	PE-NMe(18:2(9Z,12Z)/18:2(9Z,12Z))[U]	0.694736593
gene-RHBDF1	1.553491675	PE(18:0/20:4(8Z,11Z,14Z,17Z))	0.344922006
gene-RHBDF1	1.553491675	PS(16:1(9Z)/22:2(13Z,16Z))	0.659652661
gene-RHBDF1	1.553491675	PC(P-16:0/20:3(8Z,11Z,14Z)-2OH(5,6))	0.174122169
gene-RHBDF1	1.553491675	DG(20:0/LTE4/0:0)	0.438074508
Bos_taurus_newG	1.550619303	LysoPI(16:0/0:0)	0.379098336
Bos_taurus_newG	1.550619303	3-Thiacytidine	0.209387412
Bos_taurus_newG	1.550619303	PE(20:0/18:1(12Z)-2OH(9,10))	0.438658253
Bos_taurus_newG	1.550619303	PE(22:2(13Z,16Z)/22:5(4Z,7Z,10Z,13Z,19Z)-O	0.369745166
Bos_taurus_newG	1.550619303	Cyclosporin A	0.656529229
Bos_taurus_newG	1.550619303	DG(18:0/LTE4/0:0)	0.681485773
Bos_taurus_newG	1.550619303	1-beta-D-Arabinofuranosyl-5-fluorocytosine	0.152339645
Bos_taurus_newG	1.550619303	Norophthalmic acid	0.191432411
Bos_taurus_newG	1.550619303	beta-L-Dioxolane-cytidine	0.175916668
Bos_taurus_newG	1.550619303	Hypaconitine	0.176000431
Bos_taurus_newG	1.550619303	Isopropyl isothiocyanate	0.172385747

Bos_taurus_newG	1.550619303	PC(18:1(9Z)/15:1(9Z))	0.534724682
Bos_taurus_newG	1.550619303	Nigroxanthin	0.705005059
Bos_taurus_newG	1.550619303	PC(P-18:1(11Z)/PGJ2)	0.565243877
Bos_taurus_newG	1.550619303	PS(20:0/20:4(8Z,11Z,14Z,17Z)-2OH(5S,6R))	0.402595921
gene-LOC61636	1.548765591	3-Thiacytidine	0.209387412
gene-LOC61636	1.548765591	3-Deoxyestrone	0.282221709
gene-LOC61636	1.548765591	1-Oleoyl-sn-glycero-3-phosphocholine	0.18926952
gene-LOC61636	1.548765591	Psychosine	0.106475396
gene-LOC61636	1.548765591	LTB4-d4	0.226799982
gene-LOC61636	1.548765591	(-)-alpha-Terpineol	0.345716279
gene-LOC61636	1.548765591	1,4-Undecadiene	0.462803973
gene-LOC61636	1.548765591	Cyclosporin A	0.656529229
gene-LOC61636	1.548765591	PC(14:0/20:2(11Z,14Z))	1.110657378
gene-LOC61636	1.548765591	5-(2-Aminopropyl)-2-methylphenol	0.323231457
gene-LOC61636	1.548765591	4-Dimethylamino-L-phenylalanine	0.242110226
gene-LOC61636	1.548765591	Trimetazidine	0.438282534
gene-LOC61636	1.548765591	ingenol	0.942071652
gene-LOC61636	1.548765591	Armillane	0.52808635
gene-LOC61636	1.548765591	PC(P-18:1(11Z)/PGE2)	0.509503133
gene-LOC61636	1.548765591	(3R,4R)-3-Amino-1-hydroxy-4-methylpyrrol	0.471914506
gene-LOC61636	1.548765591	n-methyl-2-(4'-methylaminophenyl)-6-hydr	0.26655714
gene-LOC61636	1.548765591	Isopropyl isothiocyanate	0.172385747
gene-LOC61636	1.548765591	(9Z)-Octadecenoic acid	0.142359556
gene-LOC61636	1.548765591	arachidyl amido cholanoic acid	1.24842952
gene-LOC61636	1.548765591	Nigroxanthin	0.705005059
gene-LOC61636	1.548765591	9-deoxy-9-methylene-16,16-dimethyl -PGE	0.606893884
gene-LOC61636	1.548765591	CL(8:0/8:0/18:2(9Z,11Z)/20:0)	0.622988418
gene-LOC100852	1.547938988	D-Erythro-imidazole-glycerol-phosphate	0.355812431
gene-LOC100852	1.547938988	cis-p-Menth-2-en-1-ol	0.201017988
gene-LOC100852	1.547938988	alpha-Terpineol formate	0.628238986
gene-LOC100852	1.547938988	PC(P-18:1(11Z)/PGE2)	0.509503133
gene-LOC100852	1.547938988	Roxithromycin	0.268273077
gene-LOC100852	1.547938988	PC(P-18:1(11Z)/PGJ2)	0.565243877
gene-LOC100852	1.547938988	PS(20:0/20:4(8Z,11Z,14Z,17Z)-2OH(5S,6R))	0.402595921
gene-SLC2A1	1.547422297	7(14)-Bisabolene-2,3,10,11-tetrol	2.446897974
gene-SLC2A1	1.547422297	3-Deoxyestrone	0.282221709
gene-SLC2A1	1.547422297	1-Oleoyl-sn-glycero-3-phosphocholine	0.18926952
gene-SLC2A1	1.547422297	Psychosine	0.106475396
gene-SLC2A1	1.547422297	(-)-alpha-Terpineol	0.345716279
gene-SLC2A1	1.547422297	1,4-Undecadiene	0.462803973
gene-SLC2A1	1.547422297	4-cholesten-7伪,12伪,24-triol-3-one	0.097840451
gene-SLC2A1	1.547422297	5-(2-Aminopropyl)-2-methylphenol	0.323231457
gene-SLC2A1	1.547422297	4-Dimethylamino-L-phenylalanine	0.242110226
gene-SLC2A1	1.547422297	Trimetazidine	0.438282534
gene-SLC2A1	1.547422297	ingenol	0.942071652
gene-SLC2A1	1.547422297	Armillane	0.52808635
gene-SLC2A1	1.547422297	PC(P-18:1(11Z)/PGE2)	0.509503133
gene-SLC2A1	1.547422297	(3R,4R)-3-Amino-1-hydroxy-4-methylpyrrol	0.471914506
gene-SLC2A1	1.547422297	1-Palmitoylglycerol	0.084357408
gene-SLC2A1	1.547422297	n-methyl-2-(4'-methylaminophenyl)-6-hydr	0.26655714
gene-SLC2A1	1.547422297	Glutethimide	0.126237229

gene-SLC2A1	1.547422297	Isopropyl isothiocyanate	0.172385747
gene-SLC2A1	1.547422297	9-deoxy-9-methylene-16,16-dimethyl -PGE	0.606893884
gene-SLC2A1	1.547422297	CL(8:0/8:0/18:2(9Z,11Z)/20:0)	0.622988418
gene-OVGP1	1.546726856	Methylmalonate	0.241481249
gene-OVGP1	1.546726856	LysoPI(18:2(9Z,12Z)/0:0)	0.358006727
gene-OVGP1	1.546726856	LysoPI(16:0/0:0)	0.379098336
gene-OVGP1	1.546726856	3-Thiacytidine	0.209387412
gene-OVGP1	1.546726856	2-[(3S)-3-[[(2S)-1-(Carboxymethoxy)-1-oxo-	0.111577437
gene-OVGP1	1.546726856	Azelaic acid	0.007180175
gene-OVGP1	1.546726856	PE(20:0/18:1(12Z)-2OH(9,10))	0.438658253
gene-OVGP1	1.546726856	2-hydroxyhexadecanoic acid	0.1755745
gene-OVGP1	1.546726856	5-Hexyltetrahydro-2-furanoctanoic acid	0.343510978
gene-OVGP1	1.546726856	DG(15:0/PGE1/0:0)	0.462019057
gene-OVGP1	1.546726856	Trifluoroacetamide	0.107717461
gene-OVGP1	1.546726856	5-(2-Aminopropyl)-2-methylphenol	0.323231457
gene-OVGP1	1.546726856	1-beta-D-Arabinofuranosyl-5-fluorocytosine	0.152339645
gene-OVGP1	1.546726856	4-Oxo-9-cis-retinoyl-beta-glucuronide	1.611773742
gene-OVGP1	1.546726856	L-Histidinol	0.149249286
gene-OVGP1	1.546726856	14alpha-Hydroxy-5beta-cholest-7-ene-3,6-	0.206315646
gene-OVGP1	1.546726856	(4-Methylphenyl)acetaldehyde	0.208360133
gene-OVGP1	1.546726856	10-alpha-methoxy-9,10-dihydrolysergol	0.140851438
gene-OVGP1	1.546726856	Hydrocinnamic acid	0.043440346
gene-OVGP1	1.546726856	Hypaconitine	0.176000431
gene-OVGP1	1.546726856	Gamithromycin	0.37168241
gene-OVGP1	1.546726856	Isopropyl isothiocyanate	0.172385747
gene-OVGP1	1.546726856	Neomycin B	0.395784943
gene-OVGP1	1.546726856	CDP-DG(a-25:0/PGF1alpha)	0.345292815
gene-OVGP1	1.546726856	3'-N-Acetyl-4'-O-(14-methylpentadecanoyl)	0.146786959
gene-OVGP1	1.546726856	N-Myristoyl Glutamine	0.37856966
gene-OVGP1	1.546726856	PC(18:1(9Z)/15:1(9Z))	0.534724682
gene-OVGP1	1.546726856	1-Octadecanoyl-2-(7Z,10Z,13Z,16Z-docosat	0.690496314
gene-OVGP1	1.546726856	CE(LTE4)	0.204616463
gene-OVGP1	1.546726856	PS(16:1(9Z)/22:2(13Z,16Z))	0.659652661
gene-LOC509881	1.543670391	3-Thiacytidine	0.209387412
gene-LOC509881	1.543670391	D-Erythro-imidazole-glycerol-phosphate	0.355812431
gene-LOC509881	1.543670391	3-Deoxyestrone	0.282221709
gene-LOC509881	1.543670391	1-Oleoyl-sn-glycero-3-phosphocholine	0.18926952
gene-LOC509881	1.543670391	LTB4-d4	0.226799982
gene-LOC509881	1.543670391	cis-p-Menth-2-en-1-ol	0.201017988
gene-LOC509881	1.543670391	(-)-alpha-Terpineol	0.345716279
gene-LOC509881	1.543670391	1,4-Undecadiene	0.462803973
gene-LOC509881	1.543670391	PG(20:1(11Z)/18:3(10,12,15)-OH(9))	0.626244347
gene-LOC509881	1.543670391	4-Dimethylamino-L-phenylalanine	0.242110226
gene-LOC509881	1.543670391	Armillane	0.52808635
gene-LOC509881	1.543670391	N-[[3-Hydroxy-2-(2-pentenyl)cyclopentyl]ac	1.043930947
gene-LOC509881	1.543670391	PC(P-18:1(11Z)/PGE2)	0.509503133
gene-LOC509881	1.543670391	(3R,4R)-3-Amino-1-hydroxy-4-methylpyrrol	0.471914506
gene-LOC509881	1.543670391	n-methyl-2-(4'-methylaminophenyl)-6-hydr	0.26655714
gene-LOC509881	1.543670391	Isopropyl isothiocyanate	0.172385747
gene-LOC509881	1.543670391	(9Z)-Octadecenoic acid	0.142359556
gene-LOC509881	1.543670391	PS(20:0/20:4(8Z,11Z,14Z,17Z)-2OH(5S,6R))	0.402595921

gene-LOC51508Ç	1.540925397	5-(3'-Carboxy-3'-oxopropenyl)-4,6-dihydro›	1.083232395
gene-LOC51508Ç	1.540925397	LysoPI(16:0/0:0)	0.379098336
gene-LOC51508Ç	1.540925397	Val-Cit	0.452308451
gene-LOC51508Ç	1.540925397	11-Maleimidoundecanoic acid	1.084942397
gene-LOC51508Ç	1.540925397	Monacolin L acid	1.060562098
gene-LOC51508Ç	1.540925397	PE(22:2(13Z,16Z)/22:5(4Z,7Z,10Z,13Z,19Z)-O	0.369745166
gene-LOC51508Ç	1.540925397	3,4,3',4'-Tetrahydrospirilloxanthin	0.354666148
gene-LOC51508Ç	1.540925397	Lamivudine	0.327402892
gene-LOC51508Ç	1.540925397	5-Formiminotetrahydrofolate	0.118251287
gene-LOC51508Ç	1.540925397	Pseudouridine 5'-phosphate	1.18431378
gene-LOC51508Ç	1.540925397	S-Acetyldihydrolipoamide-E	0.429561283
gene-LOC51508Ç	1.540925397	Ribavirin monophosphate	1.091085027
gene-LOC51508Ç	1.540925397	Norophthalmic acid	0.191432411
gene-LOC51508Ç	1.540925397	Indole-3-ethanol	0.096523128
gene-LOC51508Ç	1.540925397	Metaxalone	0.165579741
gene-LOC51508Ç	1.540925397	ent-16b,19-Kauranediol 19-acetate	0.400676827
gene-LOC51508Ç	1.540925397	Milbemycin D	0.125637998
gene-LOC51508Ç	1.540925397	Roxithromycin	0.268273077
gene-LOC51508Ç	1.540925397	Sitosterol beta-D-glucoside	0.382944796
gene-LOC51508Ç	1.540925397	PC(P-18:1(11Z)/PGJ2)	0.565243877
gene-LOC51508Ç	1.540925397	PS(20:0/20:4(8Z,11Z,14Z,17Z)-2OH(5S,6R))	0.402595921
gene-LOC51508Ç	1.540925397	PE(20:5(5Z,8Z,11Z,14Z,17Z)/18:0)	0.427962506
Bos_taurus_newG	1.539273744	3-Thiacytidine	0.209387412
Bos_taurus_newG	1.539273744	1,2-O-Isopropylidene-D-glucofuranose	0.080987667
Bos_taurus_newG	1.539273744	1-Oleoyl-sn-glycero-3-phosphocholine	0.18926952
Bos_taurus_newG	1.539273744	(-)-alpha-Terpineol	0.345716279
Bos_taurus_newG	1.539273744	Cyclosporin A	0.656529229
Bos_taurus_newG	1.539273744	PC(16:0/18:1(12Z)-2OH(9,10))	0.461843233
Bos_taurus_newG	1.539273744	5-(2-Aminopropyl)-2-methylphenol	0.323231457
Bos_taurus_newG	1.539273744	4-Dimethylamino-L-phenylalanine	0.242110226
Bos_taurus_newG	1.539273744	Trimetazidine	0.438282534
Bos_taurus_newG	1.539273744	ingenol	0.942071652
Bos_taurus_newG	1.539273744	Armillane	0.52808635
Bos_taurus_newG	1.539273744	PC(P-18:1(11Z)/PGE2)	0.509503133
Bos_taurus_newG	1.539273744	(3R,4R)-3-Amino-1-hydroxy-4-methylpyrrol	0.471914506
Bos_taurus_newG	1.539273744	n-methyl-2-(4'-methylaminophenyl)-6-hydr	0.26655714
Bos_taurus_newG	1.539273744	Isopropyl isothiocyanate	0.172385747
Bos_taurus_newG	1.539273744	arachidyl amido cholanoic acid	1.24842952
Bos_taurus_newG	1.539273744	9-deoxy-9-methylene-16,16-dimethyl -PGE	0.606893884
Bos_taurus_newG	1.539273744	CL(8:0/8:0/18:2(9Z,11Z)/20:0)	0.622988418
gene-LOC10030(	1.537521414	Hydroxyprolyl-Serine	0.519540341
gene-LOC10030(	1.537521414	Cyclosporin A	0.656529229
gene-LOC10030(	1.537521414	ingenol	0.942071652
gene-LOC10030(	1.537521414	beta-L-Dioxolane-cytidine	0.175916668
gene-LOC10030(	1.537521414	PC(20:3(5Z,8Z,11Z)/24:0)	0.387959564
gene-LOC10030(	1.537521414	Nigroxanthin	0.705005059
gene-LOC10030(	1.537521414	CL(8:0/8:0/18:2(9Z,11Z)/20:0)	0.622988418
gene-NECAB3	1.535661502	3-Thiacytidine	0.209387412
gene-NECAB3	1.535661502	LTB4-d4	0.226799982
gene-NECAB3	1.535661502	PE(20:0/18:1(12Z)-2OH(9,10))	0.438658253
gene-NECAB3	1.535661502	PE(22:2(13Z,16Z)/22:5(4Z,7Z,10Z,13Z,19Z)-O	0.369745166

gene-NECAB3	1.535661502	Cyclosporin A	0.656529229
gene-NECAB3	1.535661502	Angiotensin A	0.247332017
gene-NECAB3	1.535661502	DG(18:0/LTE4/0:0)	0.681485773
gene-NECAB3	1.535661502	PC(14:0/20:2(11Z,14Z))	1.110657378
gene-NECAB3	1.535661502	CDP-DG(PGF2alpha/16:0)	1.002512277
gene-NECAB3	1.535661502	5-(2-Aminopropyl)-2-methylphenol	0.323231457
gene-NECAB3	1.535661502	beta-L-Dioxolane-cytidine	0.175916668
gene-NECAB3	1.535661502	PC(P-18:1(11Z)/PGE2)	0.509503133
gene-NECAB3	1.535661502	Isopropyl isothiocyanate	0.172385747
gene-NECAB3	1.535661502	PC(18:1(9Z)/15:1(9Z))	0.534724682
gene-NECAB3	1.535661502	Nigroxanthin	0.705005059
gene-NECAB3	1.535661502	PC(P-18:1(11Z)/PGJ2)	0.565243877
gene-NECAB3	1.535661502	PS(20:0/20:4(8Z,11Z,14Z,17Z)-2OH(5S,6R))	0.402595921
gene-MYH7	1.535287912	3-hydroxypristanic acid	0.548515835
gene-MYH7	1.535287912	24,25-Diacetylvulgaroside	0.920743312
gene-MYH7	1.535287912	(3Z)-Phycoerythrobilin	1.456755874
gene-MYH7	1.535287912	16-hydroxy hexadecanoic acid	0.309000958
gene-MYH7	1.535287912	3-Deoxyestrone	0.282221709
gene-MYH7	1.535287912	1-Oleoyl-sn-glycero-3-phosphocholine	0.18926952
gene-MYH7	1.535287912	Lividamine	0.319679555
gene-MYH7	1.535287912	Psychosine	0.106475396
gene-MYH7	1.535287912	Cyclotricuspidogenin C	0.440884085
gene-MYH7	1.535287912	(-)-alpha-Terpineol	0.345716279
gene-MYH7	1.535287912	DG(8:0/20:4(6Z,8E,10E,14Z)-2OH(5S,12R)/0:0	0.958378689
gene-MYH7	1.535287912	1,4-Undecadiene	0.462803973
gene-MYH7	1.535287912	2-isopentyl-3,6-dimethyl pyrazine	0.710502562
gene-MYH7	1.535287912	4-Dimethylamino-L-phenylalanine	0.242110226
gene-MYH7	1.535287912	Trimetazidine	0.438282534
gene-MYH7	1.535287912	Armillane	0.52808635
gene-MYH7	1.535287912	N-[[3-Hydroxy-2-(2-pentenyl)cyclopentyl]ac	1.043930947
gene-MYH7	1.535287912	PC(P-18:1(11Z)/PGE2)	0.509503133
gene-MYH7	1.535287912	PC(20:3(5Z,8Z,11Z)/24:0)	0.387959564
gene-MYH7	1.535287912	(3R,4R)-3-Amino-1-hydroxy-4-methylpyrrol	0.471914506
gene-MYH7	1.535287912	2-Amino-4-[carbamimidoyl(methyl)amino]bu	0.075703353
gene-MYH7	1.535287912	N2-gamma-Glutamylglutamine	0.230065499
gene-MYH7	1.535287912	n-methyl-2-(4'-methylaminophenyl)-6-hydr	0.26655714
gene-MYH7	1.535287912	Permetin A	0.140869415
gene-MYH7	1.535287912	(9Z)-Octadecenoic acid	0.142359556
gene-MYH7	1.535287912	L-Oleandrosyl-oleandolide	0.272998166
gene-MYH7	1.535287912	9-deoxy-9-methylene-16,16-dimethyl -PGE	0.606893884
gene-MYH7	1.535287912	CL(8:0/8:0/18:2(9Z,11Z)/20:0)	0.622988418
gene-ANO9	1.529975807	D-Erythro-imidazole-glycerol-phosphate	0.355812431
gene-ANO9	1.529975807	7(14)-Bisabolene-2,3,10,11-tetrol	2.446897974
gene-ANO9	1.529975807	cis-p-Menth-2-en-1-ol	0.201017988
gene-ANO9	1.529975807	PG(20:1(11Z)/18:3(10,12,15)-OH(9))	0.626244347
gene-ANO9	1.529975807	2-Methyl-3-phenyl-2-propenal	0.407813975
gene-ANO9	1.529975807	Ajulemic acid	0.557832951
gene-ANO9	1.529975807	PC(P-18:1(11Z)/PGE2)	0.509503133
gene-ANO9	1.529975807	(3R,4R)-3-Amino-1-hydroxy-4-methylpyrrol	0.471914506
gene-ANO9	1.529975807	Sitosterol beta-D-glucoside	0.382944796
gene-ANO9	1.529975807	PC(P-18:1(11Z)/PGJ2)	0.565243877

gene-ANO9	1.529975807	PS(20:0/20:4(8Z,11Z,14Z,17Z)-2OH(5S,6R))	0.402595921
gene-DUSP4	1.522264687	PE(22:2(13Z,16Z)/22:5(4Z,7Z,10Z,13Z,19Z)-O(	0.369745166
gene-DUSP4	1.522264687	3,4,3',4'-Tetrahydrospirilloxanthin	0.354666148
gene-DUSP4	1.522264687	Asparaginylcysteine	0.467538645
gene-DUSP4	1.522264687	5-Formiminotetrahydrofolate	0.118251287
gene-DUSP4	1.522264687	Phenyllactate	0.55151911
gene-DUSP4	1.522264687	Erosone	0.527835387
gene-DUSP4	1.522264687	Indole-3-ethanol	0.096523128
gene-DUSP4	1.522264687	Metaxalone	0.165579741
gene-DUSP4	1.522264687	alpha-(Dimethylaminomethyl)-2-(3-ethyl-5-	0.712551948
gene-DUSP4	1.522264687	Milbemycin D	0.125637998
gene-DUSP4	1.522264687	Sitosterol beta-D-glucoside	0.382944796
gene-DUSP4	1.522264687	PS(20:0/20:4(8Z,11Z,14Z,17Z)-2OH(5S,6R))	0.402595921
gene-DUSP4	1.522264687	PG(20:1(11Z)/18:3(9,11,15)-OH(13))	0.69829683
gene-FFAR2	1.52075916	1,2-O-Isopropylidene-D-glucofuranose	0.080987667
gene-FFAR2	1.52075916	3-Deoxyestrone	0.282221709
gene-FFAR2	1.52075916	1-Oleoyl-sn-glycero-3-phosphocholine	0.18926952
gene-FFAR2	1.52075916	Lividamine	0.319679555
gene-FFAR2	1.52075916	Psychosine	0.106475396
gene-FFAR2	1.52075916	(-)-alpha-Terpineol	0.345716279
gene-FFAR2	1.52075916	1,4-Undecadiene	0.462803973
gene-FFAR2	1.52075916	5-(2-Aminopropyl)-2-methylphenol	0.323231457
gene-FFAR2	1.52075916	4-Dimethylamino-L-phenylalanine	0.242110226
gene-FFAR2	1.52075916	Trimetazidine	0.438282534
gene-FFAR2	1.52075916	ingenol	0.942071652
gene-FFAR2	1.52075916	Armillane	0.52808635
gene-FFAR2	1.52075916	PC(P-18:1(11Z)/PGE2)	0.509503133
gene-FFAR2	1.52075916	(3R,4R)-3-Amino-1-hydroxy-4-methylpyrrol	0.471914506
gene-FFAR2	1.52075916	n-methyl-2-(4'-methylaminophenyl)-6-hydr	0.26655714
gene-FFAR2	1.52075916	Isopropyl isothiocyanate	0.172385747
gene-FFAR2	1.52075916	9-deoxy-9-methylene-16,16-dimethyl -PGE:	0.606893884
gene-FFAR2	1.52075916	CL(8:0/8:0/18:2(9Z,11Z)/20:0)	0.622988418
gene-VWA5B2	1.518577941	3-Thiacytidine	0.209387412
gene-VWA5B2	1.518577941	PE(20:0/18:1(12Z)-2OH(9,10))	0.438658253
gene-VWA5B2	1.518577941	PE(22:2(13Z,16Z)/22:5(4Z,7Z,10Z,13Z,19Z)-O(	0.369745166
gene-VWA5B2	1.518577941	Lamivudine	0.327402892
gene-VWA5B2	1.518577941	5-Formiminotetrahydrofolate	0.118251287
gene-VWA5B2	1.518577941	Benzoyl glucuronide (Benzoic acid)	0.442844673
gene-VWA5B2	1.518577941	Pregnanediol	0.166857843
gene-VWA5B2	1.518577941	PC(P-18:1(11Z)/PGJ2)	0.565243877
gene-VWA5B2	1.518577941	PS(20:0/20:4(8Z,11Z,14Z,17Z)-2OH(5S,6R))	0.402595921
gene-CDC42EP1	1.516981701	3-Thiacytidine	0.209387412
gene-CDC42EP1	1.516981701	D-Erythro-imidazole-glycerol-phosphate	0.355812431
gene-CDC42EP1	1.516981701	LTB4-d4	0.226799982
gene-CDC42EP1	1.516981701	cis-p-Menth-2-en-1-ol	0.201017988
gene-CDC42EP1	1.516981701	PE(20:0/18:1(12Z)-2OH(9,10))	0.438658253
gene-CDC42EP1	1.516981701	DG(15:0/PGE1/0:0)	0.462019057
gene-CDC42EP1	1.516981701	PG(20:1(11Z)/18:3(10,12,15)-OH(9))	0.626244347
gene-CDC42EP1	1.516981701	2-Methyl-3-phenyl-2-propenal	0.407813975
gene-CDC42EP1	1.516981701	alpha-Terpineol formate	0.628238986
gene-CDC42EP1	1.516981701	PC(P-18:1(11Z)/PGE2)	0.509503133

gene-CDC42EP1	1.516981701	(3R,4R)-3-Amino-1-hydroxy-4-methylpyrrol	0.471914506
gene-CDC42EP1	1.516981701	(9Z)-Octadecenoic acid	0.142359556
gene-CDC42EP1	1.516981701	N-Myristoyl Glutamine	0.37856966
gene-CDC42EP1	1.516981701	PC(18:1(9Z)/15:1(9Z))	0.534724682
gene-CDC42EP1	1.516981701	Nigroxanthin	0.705005059
gene-CDC42EP1	1.516981701	PC(P-18:1(11Z)/PGJ2)	0.565243877
gene-CDC42EP1	1.516981701	PS(20:0/20:4(8Z,11Z,14Z,17Z)-2OH(5S,6R))	0.402595921
gene-COL6A3	1.510031056	D-Erythro-imidazole-glycerol-phosphate	0.355812431
gene-COL6A3	1.510031056	Sorbitan laurate	0.242315968
gene-COL6A3	1.510031056	4-Gingerol	0.222165012
gene-COL6A3	1.510031056	Monacolin L acid	1.060562098
gene-COL6A3	1.510031056	LTB4-d4	0.226799982
gene-COL6A3	1.510031056	cis-p-Menth-2-en-1-ol	0.201017988
gene-COL6A3	1.510031056	L-Carnitine	0.2737116
gene-COL6A3	1.510031056	DG(15:0/PGE1/0:0)	0.462019057
gene-COL6A3	1.510031056	Ser Cys Ala Ala	0.603032447
gene-COL6A3	1.510031056	2-Methyl-3-phenyl-2-propenal	0.407813975
gene-COL6A3	1.510031056	Guanidoacetic acid	0.542509265
gene-COL6A3	1.510031056	alpha-Terpineol formate	0.628238986
gene-COL6A3	1.510031056	4-Dihydroboldenone	0.692863202
gene-COL6A3	1.510031056	20-carboxy Arachidonic Acid	0.780988382
gene-COL6A3	1.510031056	4-Octylphenol	1.400657885
gene-COL6A3	1.510031056	3-Pentadecylphenol	1.682258188
gene-COL6A3	1.510031056	PC(P-18:1(11Z)/PGE2)	0.509503133
gene-COL6A3	1.510031056	12-Hydroxyicosanoylcarnitine	1.730979067
gene-COL6A3	1.510031056	N-Myristoyl Glutamine	0.37856966
gene-COL6A3	1.510031056	PC(18:1(9Z)/15:1(9Z))	0.534724682
gene-COL6A3	1.510031056	PS(16:1(9Z)/22:2(13Z,16Z))	0.659652661
gene-SNTA1	1.504223998	5-(3'-Carboxy-3'-oxopropenyl)-4,6-dihydro:	1.083232395
gene-SNTA1	1.504223998	3-Thiacytidine	0.209387412
gene-SNTA1	1.504223998	D-Erythro-imidazole-glycerol-phosphate	0.355812431
gene-SNTA1	1.504223998	11-Maleimidoundecanoic acid	1.084942397
gene-SNTA1	1.504223998	Monacolin L acid	1.060562098
gene-SNTA1	1.504223998	cis-p-Menth-2-en-1-ol	0.201017988
gene-SNTA1	1.504223998	PE(22:2(13Z,16Z)/22:5(4Z,7Z,10Z,13Z,19Z)-O	0.369745166
gene-SNTA1	1.504223998	(1R,6S)-6-Amino-5-oxocyclohex-2-ene-1-c	0.154751123
gene-SNTA1	1.504223998	PC(P-18:1(11Z)/PGE2)	0.509503133
gene-SNTA1	1.504223998	Milbemycin D	0.125637998
gene-SNTA1	1.504223998	Roxithromycin	0.268273077
gene-SNTA1	1.504223998	Sitosterol beta-D-glucoside	0.382944796
gene-SNTA1	1.504223998	PC(P-18:1(11Z)/PGJ2)	0.565243877
gene-SNTA1	1.504223998	PS(20:0/20:4(8Z,11Z,14Z,17Z)-2OH(5S,6R))	0.402595921
gene-ADAM11	1.502398886	3-hydroxypristanic acid	0.548515835
gene-ADAM11	1.502398886	3-Deoxyestrone	0.282221709
gene-ADAM11	1.502398886	1-Oleoyl-sn-glycero-3-phosphocholine	0.18926952
gene-ADAM11	1.502398886	Lividamine	0.319679555
gene-ADAM11	1.502398886	Psychosine	0.106475396
gene-ADAM11	1.502398886	Cyclotricuspidogenin C	0.440884085
gene-ADAM11	1.502398886	(-)-alpha-Terpineol	0.345716279
gene-ADAM11	1.502398886	1,4-Undecadiene	0.462803973
gene-ADAM11	1.502398886	4-cholesten-7伪,12伪,24-triol-3-one	0.097840451

gene-ADAM11	1.502398886	5-(2-Aminopropyl)-2-methylphenol	0.323231457
gene-ADAM11	1.502398886	4-Dimethylamino-L-phenylalanine	0.242110226
gene-ADAM11	1.502398886	Trimetazidine	0.438282534
gene-ADAM11	1.502398886	ingenol	0.942071652
gene-ADAM11	1.502398886	Armillane	0.52808635
gene-ADAM11	1.502398886	N-[[3-Hydroxy-2-(2-pentenyl)cyclopentyl]ac	1.043930947
gene-ADAM11	1.502398886	PC(P-18:1(11Z)/PGE2)	0.509503133
gene-ADAM11	1.502398886	PC(20:3(5Z,8Z,11Z)/24:0)	0.387959564
gene-ADAM11	1.502398886	(3R,4R)-3-Amino-1-hydroxy-4-methylpyrrol	0.471914506
gene-ADAM11	1.502398886	N2-gamma-Glutamylglutamine	0.230065499
gene-ADAM11	1.502398886	n-methyl-2-(4'-methylaminophenyl)-6-hydr	0.26655714
gene-ADAM11	1.502398886	Isopropyl isothiocyanate	0.172385747
gene-ADAM11	1.502398886	(9Z)-Octadecenoic acid	0.142359556
gene-ADAM11	1.502398886	L-Oleandrosyl-oleandolide	0.272998166
gene-ADAM11	1.502398886	9-deoxy-9-methylene-16,16-dimethyl -PGE	0.606893884
gene-ADAM11	1.502398886	CL(8:0/8:0/18:2(9Z,11Z)/20:0)	0.622988418
Bos_taurus_newG	1.501703681	11-Maleimidoundecanoic acid	1.084942397
Bos_taurus_newG	1.501703681	13(S)-HpODE	0.163157753
Bos_taurus_newG	1.501703681	Fluridone	0.127106682
Bos_taurus_newG	1.501703681	Norophthalmic acid	0.191432411
Bos_taurus_newG	1.501703681	(S)-Mandelic acid O-beta-D-Glucopyranosid	0.509637316
Bos_taurus_newG	1.501703681	Imidazoline	0.232348181
Bos_taurus_newG	1.501703681	3-(3-Methylbutylidene)-1(3H)-isobenzofurar	0.575422414
Bos_taurus_newG	1.501703681	beta-L-Dioxolane-cytidine	0.175916668
Bos_taurus_newG	1.501703681	Roxithromycin	0.268273077
Bos_taurus_newG	1.501703681	PC(P-18:1(11Z)/PGJ2)	0.565243877
Bos_taurus_newG	1.501703681	PS(20:0/20:4(8Z,11Z,14Z,17Z)-2OH(5S,6R))	0.402595921
Bos_taurus_newG	1.500270387	3-Thiacytidine	0.209387412
Bos_taurus_newG	1.500270387	D-Erythro-imidazole-glycerol-phosphate	0.355812431
Bos_taurus_newG	1.500270387	13(S)-HpODE	0.163157753
Bos_taurus_newG	1.500270387	LTB4-d4	0.226799982
Bos_taurus_newG	1.500270387	cis-p-Menth-2-en-1-ol	0.201017988
Bos_taurus_newG	1.500270387	(-)-alpha-Terpineol	0.345716279
Bos_taurus_newG	1.500270387	DG(15:0/PGE1/0:0)	0.462019057
Bos_taurus_newG	1.500270387	4-Dimethylamino-L-phenylalanine	0.242110226
Bos_taurus_newG	1.500270387	5'-S-Methyl-5'-thioinosine	0.28772476
Bos_taurus_newG	1.500270387	2-Methyl-3-phenyl-2-propenal	0.407813975
Bos_taurus_newG	1.500270387	PC(P-18:1(11Z)/PGE2)	0.509503133
Bos_taurus_newG	1.500270387	(3R,4R)-3-Amino-1-hydroxy-4-methylpyrrol	0.471914506
Bos_taurus_newG	1.500270387	Isopropyl isothiocyanate	0.172385747
Bos_taurus_newG	1.500270387	(9Z)-Octadecenoic acid	0.142359556
Bos_taurus_newG	1.500270387	N-Myristoyl Glutamine	0.37856966
Bos_taurus_newG	1.500270387	PC(18:1(9Z)/15:1(9Z))	0.534724682
Bos_taurus_newG	1.500270387	2-Propenyl 2-aminobenzoate	0.128406829
gene-HIST1H2AC	1.500142111	S-(Indolylmethylthiohydroximoyl)-L-cysteine	0.635042992
gene-HIST1H2AC	1.500142111	LTB4-d4	0.226799982
gene-HIST1H2AC	1.500142111	(-)-alpha-Terpineol	0.345716279
gene-HIST1H2AC	1.500142111	4-Dimethylamino-L-phenylalanine	0.242110226
gene-HIST1H2AC	1.500142111	Valylproline	1.009800636
gene-HIST1H2AC	1.500142111	Isoleucylproline	1.006580106
gene-HIST1H2AC	1.500142111	(Z)-3-Oxo-2-(2-pentenyl)-1-cyclopenteneac	0.179805915

gene-HIST1H2AC	1.500142111	2-Methyl-3-phenyl-2-propenal	0.407813975
gene-HIST1H2AC	1.500142111	PC(P-18:1(11Z)/PGE2)	0.509503133
gene-HIST1H2AC	1.500142111	(3R,4R)-3-Amino-1-hydroxy-4-methylpyrrol	0.471914506
gene-HIST1H2AC	1.500142111	(9Z)-Octadecenoic acid	0.142359556
gene-ADGRA2	1.497698244	7(14)-Bisabolene-2,3,10,11-tetrol	2.446897974
gene-ADGRA2	1.497698244	Stercobilinogen	0.144304212
gene-ADGRA2	1.497698244	1,4-Undecadiene	0.462803973
gene-ADGRA2	1.497698244	4-cholesten-7伪,12伪,24-triol-3-one	0.097840451
gene-ADGRA2	1.497698244	DG(2:0/20:3(8Z,11Z,14Z)-O(5,6)/0:0)	0.111978282
gene-ADGRA2	1.497698244	1-Palmitoylglycerol	0.084357408
gene-ADGRA2	1.497698244	Isopropyl isothiocyanate	0.172385747
gene-KLHDC9	1.496114905	5-Sulfosalicylic acid	1.31509494
gene-KLHDC9	1.496114905	4-Hydroxybenzoate	0.892336321
gene-KLHDC9	1.496114905	Ethyl nicotinate	1.024858052
gene-KLHDC9	1.496114905	Dopaquinone	1.087504518
gene-KLHDC9	1.496114905	Isomaltotriose	0.58434654
gene-KLHDC9	1.496114905	Glutamate carbon	0.671444516
gene-KLHDC9	1.496114905	1-(2-Furanyl)-1-pentanone	0.697147283
gene-KLHDC9	1.496114905	2-Dehydro-3-deoxy-D-gluconate	0.65165299
gene-KLHDC9	1.496114905	MG(0:0/20:4(5Z,8Z,11Z,14Z)/0:0)	0.488916341
gene-KLHDC9	1.496114905	Crustecdysone	0.889062022
gene-KLHDC9	1.496114905	Deoxyshikonin	0.734877521
gene-KLHDC9	1.496114905	Serotonin	2.521565841
gene-KLHDC9	1.496114905	Adrenosterone	0.050790129
gene-KLHDC9	1.496114905	5-Methoxyindoleacetate	0.597532846
gene-KLHDC9	1.496114905	TETRAHYDROURIDINE	0.576545276
gene-KLHDC9	1.496114905	beta-Thujaplicin	0.681866194
gene-KLHDC9	1.496114905	Crispolide	0.008726946
gene-KLHDC9	1.496114905	N-[[3-Hydroxy-2-(2-pentenyl)cyclopentyl]ac	1.043930947
gene-KLHDC9	1.496114905	Yucalexin P15	1.079168162
gene-KLHDC9	1.496114905	PGF2伪 1,11-lactone	0.041436917
gene-EEF2KMT	1.495705102	3-Thiacytidine	0.209387412
gene-EEF2KMT	1.495705102	11-Maleimidoundecanoic acid	1.084942397
gene-EEF2KMT	1.495705102	13(S)-HpODE	0.163157753
gene-EEF2KMT	1.495705102	LTB4-d4	0.226799982
gene-EEF2KMT	1.495705102	PE(20:0/18:1(12Z)-2OH(9,10))	0.438658253
gene-EEF2KMT	1.495705102	PE(22:2(13Z,16Z)/22:5(4Z,7Z,10Z,13Z,19Z)-O	0.369745166
gene-EEF2KMT	1.495705102	Cyclosporin A	0.656529229
gene-EEF2KMT	1.495705102	DG(18:0/LTE4/0:0)	0.681485773
gene-EEF2KMT	1.495705102	Norophthalmic acid	0.191432411
gene-EEF2KMT	1.495705102	Guanidoacetic acid	0.542509265
gene-EEF2KMT	1.495705102	beta-L-Dioxolane-cytidine	0.175916668
gene-EEF2KMT	1.495705102	PC(P-18:1(11Z)/PGE2)	0.509503133
gene-EEF2KMT	1.495705102	Nigroxanthin	0.705005059
gene-EEF2KMT	1.495705102	PC(P-18:1(11Z)/PGJ2)	0.565243877
gene-EEF2KMT	1.495705102	PS(20:0/20:4(8Z,11Z,14Z,17Z)-2OH(5S,6R))	0.402595921
Bos_taurus_newG	1.493359	3-Thiacytidine	0.209387412
Bos_taurus_newG	1.493359	3-Deoxyestrone	0.282221709
Bos_taurus_newG	1.493359	1-Oleoyl-sn-glycero-3-phosphocholine	0.18926952
Bos_taurus_newG	1.493359	Psychosine	0.106475396
Bos_taurus_newG	1.493359	(-)-alpha-Terpineol	0.345716279

Bos_taurus_newG	1.493359	1,4-Undecadiene	0.462803973
Bos_taurus_newG	1.493359	Cyclosporin A	0.656529229
Bos_taurus_newG	1.493359	PC(14:0/20:2(11Z,14Z))	1.110657378
Bos_taurus_newG	1.493359	5-(2-Aminopropyl)-2-methylphenol	0.323231457
Bos_taurus_newG	1.493359	4-Dimethylamino-L-phenylalanine	0.242110226
Bos_taurus_newG	1.493359	Trimetazidine	0.438282534
Bos_taurus_newG	1.493359	ingenol	0.942071652
Bos_taurus_newG	1.493359	Armillane	0.52808635
Bos_taurus_newG	1.493359	PC(P-18:1(11Z)/PGE2)	0.509503133
Bos_taurus_newG	1.493359	(3R,4R)-3-Amino-1-hydroxy-4-methylpyrrol	0.471914506
Bos_taurus_newG	1.493359	n-methyl-2-(4'-methylaminophenyl)-6-hydr	0.26655714
Bos_taurus_newG	1.493359	Isopropyl isothiocyanate	0.172385747
Bos_taurus_newG	1.493359	(9Z)-Octadecenoic acid	0.142359556
Bos_taurus_newG	1.493359	arachidyl amido cholanoic acid	1.24842952
Bos_taurus_newG	1.493359	Nigroxanthin	0.705005059
Bos_taurus_newG	1.493359	9-deoxy-9-methylene-16,16-dimethyl -PGE	0.606893884
Bos_taurus_newG	1.493359	PS(20:0/20:4(8Z,11Z,14Z,17Z)-2OH(5S,6R))	0.402595921
Bos_taurus_newG	1.493359	CL(8:0/8:0/18:2(9Z,11Z)/20:0)	0.622988418
gene-LOC10497	1.491315749	Cyclosporin A	0.656529229
gene-LOC10497	1.491315749	beta-Thujaplicin	0.681866194
gene-LOC10497	1.491315749	beta-L-Dioxolane-cytidine	0.175916668
gene-LOC10497	1.491315749	D-Fructose	0.167584616
gene-LOC10497	1.491315749	CL(8:0/8:0/18:2(9Z,11Z)/20:0)	0.622988418
Bos_taurus_newG	1.490377439	D-Erythro-imidazole-glycerol-phosphate	0.355812431
Bos_taurus_newG	1.490377439	Cyclosporin A	0.656529229
Bos_taurus_newG	1.490377439	PC(P-18:1(11Z)/PGE2)	0.509503133
Bos_taurus_newG	1.490377439	PC(P-18:1(11Z)/PGJ2)	0.565243877
Bos_taurus_newG	1.490377439	PS(20:0/20:4(8Z,11Z,14Z,17Z)-2OH(5S,6R))	0.402595921
Bos_taurus_newG	1.490377439	CL(8:0/8:0/18:2(9Z,11Z)/20:0)	0.622988418
Bos_taurus_newG	1.489879365	Norophthalmic acid	0.191432411
Bos_taurus_newG	1.489879365	Ethylene brassylate	0.989156973
Bos_taurus_newG	1.489879365	Imidazoline	0.232348181
Bos_taurus_newG	1.489879365	beta-L-Dioxolane-cytidine	0.175916668
Bos_taurus_newG	1.489879365	O-(17-Carboxyheptadecanoyl)carnitine	0.433790125
Bos_taurus_newG	1.489520655	5-(3'-Carboxy-3'-oxopropenyl)-4,6-dihydro:	1.083232395
Bos_taurus_newG	1.489520655	LysoPI(16:0/0:0)	0.379098336
Bos_taurus_newG	1.489520655	Val-Cit	0.452308451
Bos_taurus_newG	1.489520655	Docosanamide	0.509975786
Bos_taurus_newG	1.489520655	Monacolin L acid	1.060562098
Bos_taurus_newG	1.489520655	cis-p-Menth-2-en-1-ol	0.201017988
Bos_taurus_newG	1.489520655	PE(20:0/18:1(12Z)-2OH(9,10))	0.438658253
Bos_taurus_newG	1.489520655	1-(6-((3-Methoxyestra-1,3,5(10)-trien-17-yl)	0.042795602
Bos_taurus_newG	1.489520655	PE(22:2(13Z,16Z)/22:5(4Z,7Z,10Z,13Z,19Z)-O	0.369745166
Bos_taurus_newG	1.489520655	3,4,3',4'-Tetrahydrospirilloxanthin	0.354666148
Bos_taurus_newG	1.489520655	Dyphylline	0.664549753
Bos_taurus_newG	1.489520655	Lamivudine	0.327402892
Bos_taurus_newG	1.489520655	5-Formiminotetrahydrofolate	0.118251287
Bos_taurus_newG	1.489520655	Pseudouridine 5'-phosphate	1.18431378
Bos_taurus_newG	1.489520655	Benzoyl glucuronide (Benzoic acid)	0.442844673
Bos_taurus_newG	1.489520655	S-Acetyldihydrolipoamide-E	0.429561283
Bos_taurus_newG	1.489520655	Ribavirin monophosphate	1.091085027

Bos_taurus_newG	1.489520655	3'-N'-Acetylfusarochromanone	0.755683206
Bos_taurus_newG	1.489520655	Indole-3-ethanol	0.096523128
Bos_taurus_newG	1.489520655	n6-[2-(4-Aminophenyl)ethyl]adenosine	0.45059876
Bos_taurus_newG	1.489520655	7C-aglycone	1.015608521
Bos_taurus_newG	1.489520655	5-Acetylamino-6-amino-3-methyluracil	0.420050597
Bos_taurus_newG	1.489520655	Tryptophyl-Glutamine	0.89722186
Bos_taurus_newG	1.489520655	alpha-Terpineol formate	0.628238986
Bos_taurus_newG	1.489520655	4-Octylphenol	1.400657885
Bos_taurus_newG	1.489520655	Prolyl-Lysine	1.612991304
Bos_taurus_newG	1.489520655	12-Hydroxyicosanoylcarnitine	1.730979067
Bos_taurus_newG	1.489520655	Milbemycin D	0.125637998
Bos_taurus_newG	1.489520655	Sitosterol beta-D-glucoside	0.382944796
Bos_taurus_newG	1.489520655	PC(P-18:1(11Z)/PGJ2)	0.565243877
Bos_taurus_newG	1.489520655	PS(20:0/20:4(8Z,11Z,14Z,17Z)-2OH(5S,6R))	0.402595921
Bos_taurus_newG	1.489520655	Galactosylglycerol	0.738463143
Bos_taurus_newG	1.489520655	5-Hydroxy-2-acetamidofluorene	1.011174221
Bos_taurus_newG	1.489520655	PE(20:5(5Z,8Z,11Z,14Z,17Z)/18:0)	0.427962506
gene-RAB11FIP5	1.487467977	D-Erythro-imidazole-glycerol-phosphate	0.355812431
gene-RAB11FIP5	1.487467977	milbemycin beta3	1.414447124
gene-RAB11FIP5	1.487467977	Tsugarioside B	1.013262893
gene-RAB11FIP5	1.487467977	3-Deoxyestrone	0.282221709
gene-RAB11FIP5	1.487467977	1-Oleoyl-sn-glycero-3-phosphocholine	0.18926952
gene-RAB11FIP5	1.487467977	cis-p-Menth-2-en-1-ol	0.201017988
gene-RAB11FIP5	1.487467977	PE(20:0/18:1(12Z)-2OH(9,10))	0.438658253
gene-RAB11FIP5	1.487467977	(-)-alpha-Terpineol	0.345716279
gene-RAB11FIP5	1.487467977	PG(20:1(11Z)/18:3(10,12,15)-OH(9))	0.626244347
gene-RAB11FIP5	1.487467977	5-(2-Aminopropyl)-2-methylphenol	0.323231457
gene-RAB11FIP5	1.487467977	4-Dimethylamino-L-phenylalanine	0.242110226
gene-RAB11FIP5	1.487467977	Armillane	0.52808635
gene-RAB11FIP5	1.487467977	PC(P-18:1(11Z)/PGE2)	0.509503133
gene-RAB11FIP5	1.487467977	(3R,4R)-3-Amino-1-hydroxy-4-methylpyrrol	0.471914506
gene-RAB11FIP5	1.487467977	n-methyl-2-(4'-methylaminophenyl)-6-hydr	0.26655714
gene-RAB11FIP5	1.487467977	Isopropyl isothiocyanate	0.172385747
gene-RAB11FIP5	1.487467977	(9Z)-Octadecenoic acid	0.142359556
gene-RAB11FIP5	1.487467977	PC(P-18:1(11Z)/PGJ2)	0.565243877
gene-RAB11FIP5	1.487467977	PS(20:0/20:4(8Z,11Z,14Z,17Z)-2OH(5S,6R))	0.402595921
gene-RAB11FIP5	1.487467977	CL(8:0/8:0/18:2(9Z,11Z)/20:0)	0.622988418
gene-ITGB4	1.487325532	3-Thiacytidine	0.209387412
gene-ITGB4	1.487325532	D-Erythro-imidazole-glycerol-phosphate	0.355812431
gene-ITGB4	1.487325532	13(S)-HpODE	0.163157753
gene-ITGB4	1.487325532	(-)-alpha-Terpineol	0.345716279
gene-ITGB4	1.487325532	4-cholesten-7伪,12伪,24-triol-3-one	0.097840451
gene-ITGB4	1.487325532	N-[[3-Hydroxy-2-(2-pentenyl)cyclopentyl]ac	1.043930947
gene-ITGB4	1.487325532	PC(P-18:1(11Z)/PGE2)	0.509503133
gene-ITGB4	1.487325532	PC(P-18:1(11Z)/PGJ2)	0.565243877
gene-ITGB4	1.487325532	PS(20:0/20:4(8Z,11Z,14Z,17Z)-2OH(5S,6R))	0.402595921
gene-TMEM150E	1.479222435	3-Thiacytidine	0.209387412
gene-TMEM150E	1.479222435	1-Oleoyl-sn-glycero-3-phosphocholine	0.18926952
gene-TMEM150E	1.479222435	(-)-alpha-Terpineol	0.345716279
gene-TMEM150E	1.479222435	1,4-Undecadiene	0.462803973
gene-TMEM150E	1.479222435	Cyclosporin A	0.656529229

gene-TMEM150B	1.479222435	5-(2-Aminopropyl)-2-methylphenol	0.323231457
gene-TMEM150B	1.479222435	ingenol	0.942071652
gene-TMEM150B	1.479222435	Armillane	0.52808635
gene-TMEM150B	1.479222435	PC(P-18:1(11Z)/PGE2)	0.509503133
gene-TMEM150B	1.479222435	n-methyl-2-(4'-methylaminophenyl)-6-hydr	0.26655714
gene-TMEM150B	1.479222435	Isopropyl isothiocyanate	0.172385747
gene-TMEM150B	1.479222435	PS(20:0/20:4(8Z,11Z,14Z,17Z)-2OH(5S,6R))	0.402595921
gene-TMEM150B	1.479222435	CL(8:0/8:0/18:2(9Z,11Z)/20:0)	0.622988418
gene-SPRN	1.476792555	5-(3'-Carboxy-3'-oxopropenyl)-4,6-dihydro	1.083232395
gene-SPRN	1.476792555	(+)-Bottrospicatol	0.68654221
gene-SPRN	1.476792555	Val-Cit	0.452308451
gene-SPRN	1.476792555	3-(2,4-Dimethyl-5-(2-oxo-1,2-dihydroindol-	0.095524813
gene-SPRN	1.476792555	3-carboxy-4-methyl-5-pentyl-2-furanpropa	0.375666997
gene-SPRN	1.476792555	4-Gingerol	0.222165012
gene-SPRN	1.476792555	Monacolin L acid	1.060562098
gene-SPRN	1.476792555	3,4,3',4'-Tetrahydrospirilloxanthin	0.354666148
gene-SPRN	1.476792555	2-Oxo-10-methylthiodecanoic acid	0.695908469
gene-SPRN	1.476792555	(-)-Huperzine A (HupA)	0.935198906
gene-SPRN	1.476792555	ent-16b,19-Kauranediol 19-acetate	0.400676827
gene-SPRN	1.476792555	(Z)-3-Methyl-3-decenoic acid	0.482455266
gene-SPRN	1.476792555	Roxithromycin	0.268273077
gene-ELANE	1.474758876	3-Deoxyestrone	0.282221709
gene-ELANE	1.474758876	LTB4-d4	0.226799982
gene-ELANE	1.474758876	(-)-alpha-Terpineol	0.345716279
gene-ELANE	1.474758876	Cyclosporin A	0.656529229
gene-ELANE	1.474758876	5-(2-Aminopropyl)-2-methylphenol	0.323231457
gene-ELANE	1.474758876	4-Dimethylamino-L-phenylalanine	0.242110226
gene-ELANE	1.474758876	Trimetazidine	0.438282534
gene-ELANE	1.474758876	ingenol	0.942071652
gene-ELANE	1.474758876	Armillane	0.52808635
gene-ELANE	1.474758876	PC(P-18:1(11Z)/PGE2)	0.509503133
gene-ELANE	1.474758876	n-methyl-2-(4'-methylaminophenyl)-6-hydr	0.26655714
gene-ELANE	1.474758876	Isopropyl isothiocyanate	0.172385747
gene-ELANE	1.474758876	9-deoxy-9-methylene-16,16-dimethyl -PGE	0.606893884
gene-ELANE	1.474758876	CL(8:0/8:0/18:2(9Z,11Z)/20:0)	0.622988418
gene-SYTL1	1.474243992	3-Thiacytidine	0.209387412
gene-SYTL1	1.474243992	1-Oleoyl-sn-glycero-3-phosphocholine	0.18926952
gene-SYTL1	1.474243992	LTB4-d4	0.226799982
gene-SYTL1	1.474243992	(-)-alpha-Terpineol	0.345716279
gene-SYTL1	1.474243992	1,4-Undecadiene	0.462803973
gene-SYTL1	1.474243992	Cyclosporin A	0.656529229
gene-SYTL1	1.474243992	5-(2-Aminopropyl)-2-methylphenol	0.323231457
gene-SYTL1	1.474243992	4-Dimethylamino-L-phenylalanine	0.242110226
gene-SYTL1	1.474243992	Trimetazidine	0.438282534
gene-SYTL1	1.474243992	ingenol	0.942071652
gene-SYTL1	1.474243992	Armillane	0.52808635
gene-SYTL1	1.474243992	PC(P-18:1(11Z)/PGE2)	0.509503133
gene-SYTL1	1.474243992	(3R,4R)-3-Amino-1-hydroxy-4-methylpyrrol	0.471914506
gene-SYTL1	1.474243992	n-methyl-2-(4'-methylaminophenyl)-6-hydr	0.26655714
gene-SYTL1	1.474243992	Isopropyl isothiocyanate	0.172385747
gene-SYTL1	1.474243992	(9Z)-Octadecenoic acid	0.142359556

gene-SYTL1	1.474243992	Nigroxanthin	0.705005059
gene-SYTL1	1.474243992	9-deoxy-9-methylene-16,16-dimethyl -PGE	0.606893884
gene-SYTL1	1.474243992	PS(20:0/20:4(8Z,11Z,14Z,17Z)-2OH(5S,6R))	0.402595921
gene-SYTL1	1.474243992	CL(8:0/8:0/18:2(9Z,11Z)/20:0)	0.622988418
gene-SEPT5	1.473205559	3-Thiacytidine	0.209387412
gene-SEPT5	1.473205559	D-Erythro-imidazole-glycerol-phosphate	0.355812431
gene-SEPT5	1.473205559	LTB4-d4	0.226799982
gene-SEPT5	1.473205559	cis-p-Menth-2-en-1-ol	0.201017988
gene-SEPT5	1.473205559	(-)-alpha-Terpineol	0.345716279
gene-SEPT5	1.473205559	Cyclosporin A	0.656529229
gene-SEPT5	1.473205559	5-(2-Aminopropyl)-2-methylphenol	0.323231457
gene-SEPT5	1.473205559	Armillane	0.52808635
gene-SEPT5	1.473205559	PC(P-18:1(11Z)/PGE2)	0.509503133
gene-SEPT5	1.473205559	Isopropyl isothiocyanate	0.172385747
gene-SEPT5	1.473205559	Nigroxanthin	0.705005059
gene-SEPT5	1.473205559	PC(P-18:1(11Z)/PGJ2)	0.565243877
gene-SEPT5	1.473205559	PS(20:0/20:4(8Z,11Z,14Z,17Z)-2OH(5S,6R))	0.402595921
gene-SEPT5	1.473205559	CL(8:0/8:0/18:2(9Z,11Z)/20:0)	0.622988418
gene-OPLAH	1.473141443	Glutamate carbon	0.671444516
gene-OPLAH	1.473141443	2-Dehydro-3-deoxy-D-gluconate	0.65165299
gene-OPLAH	1.473141443	beta-Thujaplicin	0.681866194
gene-OPLAH	1.473141443	Yucalexin P15	1.079168162
gene-OPLAH	1.473141443	PC(P-18:1(11Z)/PGE2)	0.509503133
gene-HRH2	1.472937466	3-Thiacytidine	0.209387412
gene-HRH2	1.472937466	D-Erythro-imidazole-glycerol-phosphate	0.355812431
gene-HRH2	1.472937466	LTB4-d4	0.226799982
gene-HRH2	1.472937466	cis-p-Menth-2-en-1-ol	0.201017988
gene-HRH2	1.472937466	PE(20:0/18:1(12Z)-2OH(9,10))	0.438658253
gene-HRH2	1.472937466	PE(22:2(13Z,16Z)/22:5(4Z,7Z,10Z,13Z,19Z)-O	0.369745166
gene-HRH2	1.472937466	2-Methyl-3-phenyl-2-propenal	0.407813975
gene-HRH2	1.472937466	PC(P-18:1(11Z)/PGE2)	0.509503133
gene-HRH2	1.472937466	Roxithromycin	0.268273077
gene-HRH2	1.472937466	Nigroxanthin	0.705005059
gene-HRH2	1.472937466	PC(P-18:1(11Z)/PGJ2)	0.565243877
gene-HRH2	1.472937466	PS(20:0/20:4(8Z,11Z,14Z,17Z)-2OH(5S,6R))	0.402595921
gene-FHL3	1.47110397	3-Thiacytidine	0.209387412
gene-FHL3	1.47110397	D-Erythro-imidazole-glycerol-phosphate	0.355812431
gene-FHL3	1.47110397	LTB4-d4	0.226799982
gene-FHL3	1.47110397	cis-p-Menth-2-en-1-ol	0.201017988
gene-FHL3	1.47110397	(-)-alpha-Terpineol	0.345716279
gene-FHL3	1.47110397	5-(2-Aminopropyl)-2-methylphenol	0.323231457
gene-FHL3	1.47110397	4-Dimethylamino-L-phenylalanine	0.242110226
gene-FHL3	1.47110397	Armillane	0.52808635
gene-FHL3	1.47110397	2-Methyl-3-phenyl-2-propenal	0.407813975
gene-FHL3	1.47110397	PC(P-18:1(11Z)/PGE2)	0.509503133
gene-FHL3	1.47110397	(3R,4R)-3-Amino-1-hydroxy-4-methylpyrrol	0.471914506
gene-FHL3	1.47110397	Isopropyl isothiocyanate	0.172385747
gene-FHL3	1.47110397	PC(P-18:1(11Z)/PGJ2)	0.565243877
gene-FHL3	1.47110397	PS(20:0/20:4(8Z,11Z,14Z,17Z)-2OH(5S,6R))	0.402595921
gene-FHL3	1.47110397	CL(8:0/8:0/18:2(9Z,11Z)/20:0)	0.622988418
gene-NMUR1	1.471099474	Glutamate carbon	0.671444516

gene-NMUR1	1.471099474	11-Maleimidoundecanoic acid	1.084942397
gene-NMUR1	1.471099474	PE(22:2(13Z,16Z)/22:5(4Z,7Z,10Z,13Z,19Z)-O(	0.369745166
gene-NMUR1	1.471099474	Norophthalmic acid	0.191432411
gene-NMUR1	1.471099474	(1R,6S)-6-Amino-5-oxocyclohex-2-ene-1-ca	0.154751123
gene-NMUR1	1.471099474	(S)-Mandelic acid O-beta-D-Glucopyranosid	0.509637316
gene-NMUR1	1.471099474	Imidazoline	0.232348181
gene-NMUR1	1.471099474	3-(3-Methylbutylidene)-1(3H)-isobenzofurar	0.575422414
gene-NMUR1	1.471099474	O-(17-Carboxyheptadecanoyl)carnitine	0.433790125
gene-NMUR1	1.471099474	Roxithromycin	0.268273077
gene-NMUR1	1.471099474	PC(P-18:1(11Z)/PGJ2)	0.565243877
gene-NMUR1	1.471099474	PS(20:0/20:4(8Z,11Z,14Z,17Z)-2OH(5S,6R))	0.402595921
gene-LOC504773	1.464981303	3-Thiacytidine	0.209387412
gene-LOC504773	1.464981303	7(14)-Bisabolene-2,3,10,11-tetrol	2.446897974
gene-LOC504773	1.464981303	1-Oleoyl-sn-glycero-3-phosphocholine	0.18926952
gene-LOC504773	1.464981303	Psychosine	0.106475396
gene-LOC504773	1.464981303	(-)-alpha-Terpineol	0.345716279
gene-LOC504773	1.464981303	1,4-Undecadiene	0.462803973
gene-LOC504773	1.464981303	4-cholesten-7伪,12伪,24-triol-3-one	0.097840451
gene-LOC504773	1.464981303	5-(2-Aminopropyl)-2-methylphenol	0.323231457
gene-LOC504773	1.464981303	4-Dimethylamino-L-phenylalanine	0.242110226
gene-LOC504773	1.464981303	Trimetazidine	0.438282534
gene-LOC504773	1.464981303	ingenol	0.942071652
gene-LOC504773	1.464981303	Armillane	0.52808635
gene-LOC504773	1.464981303	PC(P-18:1(11Z)/PGE2)	0.509503133
gene-LOC504773	1.464981303	(3R,4R)-3-Amino-1-hydroxy-4-methylpyrrol	0.471914506
gene-LOC504773	1.464981303	n-methyl-2-(4'-methylaminophenyl)-6-hydr	0.26655714
gene-LOC504773	1.464981303	Isopropyl isothiocyanate	0.172385747
gene-LOC504773	1.464981303	PS(20:0/20:4(8Z,11Z,14Z,17Z)-2OH(5S,6R))	0.402595921
gene-LOC504773	1.464981303	CL(8:0/8:0/18:2(9Z,11Z)/20:0)	0.622988418
Bos_taurus_newG	1.464460369	3-Thiacytidine	0.209387412
Bos_taurus_newG	1.464460369	Metkephamid	1.159767003
Bos_taurus_newG	1.464460369	11-Maleimidoundecanoic acid	1.084942397
Bos_taurus_newG	1.464460369	Docosanamide	0.509975786
Bos_taurus_newG	1.464460369	cis-p-Menth-2-en-1-ol	0.201017988
Bos_taurus_newG	1.464460369	PE(20:0/18:1(12Z)-2OH(9,10))	0.438658253
Bos_taurus_newG	1.464460369	PE(22:2(13Z,16Z)/22:5(4Z,7Z,10Z,13Z,19Z)-O(	0.369745166
Bos_taurus_newG	1.464460369	PG(20:1(11Z)/18:3(10,12,15)-OH(9))	0.626244347
Bos_taurus_newG	1.464460369	Lamivudine	0.327402892
Bos_taurus_newG	1.464460369	5-Formiminotetrahydrofolate	0.118251287
Bos_taurus_newG	1.464460369	Pseudouridine 5'-phosphate	1.18431378
Bos_taurus_newG	1.464460369	Benzoyl glucuronide (Benzoic acid)	0.442844673
Bos_taurus_newG	1.464460369	S-Acetyldihydrolipoamide-E	0.429561283
Bos_taurus_newG	1.464460369	Ribavirin monophosphate	1.091085027
Bos_taurus_newG	1.464460369	Indole-3-ethanol	0.096523128
Bos_taurus_newG	1.464460369	Tryptophyl-Glutamine	0.89722186
Bos_taurus_newG	1.464460369	Guanidoacetic acid	0.542509265
Bos_taurus_newG	1.464460369	alpha-Terpineol formate	0.628238986
Bos_taurus_newG	1.464460369	2'-Fluoro-2',3'-dideoxyinosine	1.214745496
Bos_taurus_newG	1.464460369	PC(P-18:1(11Z)/PGE2)	0.509503133
Bos_taurus_newG	1.464460369	Galabiosylceramide (d18:1/20:0)	0.746737323
Bos_taurus_newG	1.464460369	Phorone A	0.079039095

Bos_taurus_newG	1.464460369	Milbemycin D	0.125637998
Bos_taurus_newG	1.464460369	Sitosterol beta-D-glucoside	0.382944796
Bos_taurus_newG	1.464460369	PC(18:1(9Z)/15:1(9Z))	0.534724682
Bos_taurus_newG	1.464460369	Nigroxanthin	0.705005059
Bos_taurus_newG	1.464460369	PC(P-18:1(11Z)/PGJ2)	0.565243877
Bos_taurus_newG	1.464460369	PS(20:0/20:4(8Z,11Z,14Z,17Z)-2OH(5S,6R))	0.402595921
Bos_taurus_newG	1.464460369	Galactosylglycerol	0.738463143
gene-RTKN	1.464051021	Cyclosporin A	0.656529229
gene-RTKN	1.464051021	beta-L-Dioxolane-cytidine	0.175916668
gene-RTKN	1.464051021	D-Fructose	0.167584616
gene-RTKN	1.464051021	CL(8:0/8:0/18:2(9Z,11Z)/20:0)	0.622988418
gene-GATA1	1.463783381	3-Thiacytidine	0.209387412
gene-GATA1	1.463783381	1-Oleoyl-sn-glycero-3-phosphocholine	0.18926952
gene-GATA1	1.463783381	LTB4-d4	0.226799982
gene-GATA1	1.463783381	(-)-alpha-Terpineol	0.345716279
gene-GATA1	1.463783381	1,4-Undecadiene	0.462803973
gene-GATA1	1.463783381	Cyclosporin A	0.656529229
gene-GATA1	1.463783381	5-(2-Aminopropyl)-2-methylphenol	0.323231457
gene-GATA1	1.463783381	4-Dimethylamino-L-phenylalanine	0.242110226
gene-GATA1	1.463783381	Trimetazidine	0.438282534
gene-GATA1	1.463783381	ingenol	0.942071652
gene-GATA1	1.463783381	Armillane	0.52808635
gene-GATA1	1.463783381	PC(P-18:1(11Z)/PGE2)	0.509503133
gene-GATA1	1.463783381	(3R,4R)-3-Amino-1-hydroxy-4-methylpyrrol	0.471914506
gene-GATA1	1.463783381	n-methyl-2-(4'-methylaminophenyl)-6-hydr	0.26655714
gene-GATA1	1.463783381	Isopropyl isothiocyanate	0.172385747
gene-GATA1	1.463783381	9-deoxy-9-methylene-16,16-dimethyl -PGE	0.606893884
gene-GATA1	1.463783381	CL(8:0/8:0/18:2(9Z,11Z)/20:0)	0.622988418
Bos_taurus_newG	1.460165017	D-Erythro-imidazole-glycerol-phosphate	0.355812431
Bos_taurus_newG	1.460165017	2(R)-HPOT	0.035267944
Bos_taurus_newG	1.460165017	13(S)-HpODE	0.163157753
Bos_taurus_newG	1.460165017	PE(18:1(12Z)-2OH(9,10)/P-18:0)	0.077744061
Bos_taurus_newG	1.460165017	7(S),17(S)-dihydroxy-8(E),10(Z),13(Z),15(E),19	0.32147403
Bos_taurus_newG	1.460165017	LTB4-d4	0.226799982
Bos_taurus_newG	1.460165017	2-Methyl-3-phenyl-2-propenal	0.407813975
Bos_taurus_newG	1.460165017	(4Z,7Z,10Z,13Z,16Z,19Z)-Docosahexaenoic a	0.434211486
Bos_taurus_newG	1.460165017	PC(P-18:1(11Z)/PGE2)	0.509503133
Bos_taurus_newG	1.460165017	9-OxoODE	0.111803831
Bos_taurus_newG	1.460165017	PC(P-18:1(11Z)/PGE1)	0.295520345
Bos_taurus_newG	1.458848788	D-Erythro-imidazole-glycerol-phosphate	0.355812431
Bos_taurus_newG	1.458848788	PC(P-18:1(11Z)/PGE2)	0.509503133
Bos_taurus_newG	1.458848788	Roxithromycin	0.268273077
Bos_taurus_newG	1.458848788	PC(P-18:1(11Z)/PGJ2)	0.565243877
Bos_taurus_newG	1.458848788	PS(20:0/20:4(8Z,11Z,14Z,17Z)-2OH(5S,6R))	0.402595921
Bos_taurus_newG	1.458543251	D-Erythro-imidazole-glycerol-phosphate	0.355812431
Bos_taurus_newG	1.458543251	Isomaltotriose	0.58434654
Bos_taurus_newG	1.458543251	11-Maleimidoundecanoic acid	1.084942397
Bos_taurus_newG	1.458543251	PE(22:2(13Z,16Z)/22:5(4Z,7Z,10Z,13Z,19Z)-O	0.369745166
Bos_taurus_newG	1.458543251	Cyclosporin A	0.656529229
Bos_taurus_newG	1.458543251	PC(P-18:1(11Z)/PGE2)	0.509503133
Bos_taurus_newG	1.458543251	Roxithromycin	0.268273077

Bos_taurus_newG	1.458543251	PC(P-18:1(11Z)/PGJ2)	0.565243877
Bos_taurus_newG	1.458543251	PS(20:0/20:4(8Z,11Z,14Z,17Z)-2OH(5S,6R))	0.402595921
gene-ZFPM1	1.456867895	3-Thiacytidine	0.209387412
gene-ZFPM1	1.456867895	Tsugarioside B	1.013262893
gene-ZFPM1	1.456867895	7(14)-Bisabolene-2,3,10,11-tetrol	2.446897974
gene-ZFPM1	1.456867895	3-Deoxyestrone	0.282221709
gene-ZFPM1	1.456867895	1-Oleoyl-sn-glycero-3-phosphocholine	0.18926952
gene-ZFPM1	1.456867895	Psychosine	0.106475396
gene-ZFPM1	1.456867895	(-)-alpha-Terpineol	0.345716279
gene-ZFPM1	1.456867895	1,4-Undecadiene	0.462803973
gene-ZFPM1	1.456867895	2-isopentyl-3,6-dimethyl pyrazine	0.710502562
gene-ZFPM1	1.456867895	5-(2-Aminopropyl)-2-methylphenol	0.323231457
gene-ZFPM1	1.456867895	4-Dimethylamino-L-phenylalanine	0.242110226
gene-ZFPM1	1.456867895	Trimetazidine	0.438282534
gene-ZFPM1	1.456867895	Armillane	0.52808635
gene-ZFPM1	1.456867895	PC(P-18:1(11Z)/PGE2)	0.509503133
gene-ZFPM1	1.456867895	(3R,4R)-3-Amino-1-hydroxy-4-methylpyrrol	0.471914506
gene-ZFPM1	1.456867895	n-methyl-2-(4'-methylaminophenyl)-6-hydr	0.26655714
gene-ZFPM1	1.456867895	Isopropyl isothiocyanate	0.172385747
gene-ZFPM1	1.456867895	9-deoxy-9-methylene-16,16-dimethyl -PGE	0.606893884
gene-ZFPM1	1.456867895	PS(20:0/20:4(8Z,11Z,14Z,17Z)-2OH(5S,6R))	0.402595921
gene-ZFPM1	1.456867895	CL(8:0/8:0/18:2(9Z,11Z)/20:0)	0.622988418
gene-CLEC7A	1.454978134	5-(3'-Carboxy-3'-oxopropenyl)-4,6-dihydro	1.083232395
gene-CLEC7A	1.454978134	Val-Cit	0.452308451
gene-CLEC7A	1.454978134	11-Maleimidoundecanoic acid	1.084942397
gene-CLEC7A	1.454978134	Monacolin L acid	1.060562098
gene-CLEC7A	1.454978134	3,4,3',4'-Tetrahydrospirilloxanthin	0.354666148
gene-CLEC7A	1.454978134	Lamivudine	0.327402892
gene-CLEC7A	1.454978134	S-Acetyldihydrolipoamide-E	0.429561283
gene-CLEC7A	1.454978134	Norophthalmic acid	0.191432411
gene-CLEC7A	1.454978134	(1R,6S)-6-Amino-5-oxocyclohex-2-ene-1-ca	0.154751123
gene-CLEC7A	1.454978134	3'-N'-Acetylfusarochromanone	0.755683206
gene-CLEC7A	1.454978134	Indole-3-ethanol	0.096523128
gene-CLEC7A	1.454978134	Tryptophyl-Glutamine	0.89722186
gene-CLEC7A	1.454978134	Guanidoacetic acid	0.542509265
gene-CLEC7A	1.454978134	alpha-Terpineol formate	0.628238986
gene-CLEC7A	1.454978134	ent-16b,19-Kauranediol 19-acetate	0.400676827
gene-CLEC7A	1.454978134	Milbemycin D	0.125637998
gene-CLEC7A	1.454978134	Roxithromycin	0.268273077
gene-CLEC7A	1.454978134	PC(P-18:1(11Z)/PGJ2)	0.565243877
gene-CLEC7A	1.454978134	2-OH-benzyl	0.05275011
gene-CLEC7A	1.454978134	PE(20:5(5Z,8Z,11Z,14Z,17Z)/18:0)	0.427962506
gene-PANX2	1.451674265	3-Thiacytidine	0.209387412
gene-PANX2	1.451674265	milbemycin beta3	1.414447124
gene-PANX2	1.451674265	Tetracosenoyl-CoA	0.187187233
gene-PANX2	1.451674265	1-Oleoyl-sn-glycero-3-phosphocholine	0.18926952
gene-PANX2	1.451674265	PE(20:0/18:1(12Z)-2OH(9,10))	0.438658253
gene-PANX2	1.451674265	(-)-alpha-Terpineol	0.345716279
gene-PANX2	1.451674265	Cyclosporin A	0.656529229
gene-PANX2	1.451674265	DG(18:0/LTE4/0:0)	0.681485773
gene-PANX2	1.451674265	PC(14:0/20:2(11Z,14Z))	1.110657378

gene-PANX2	1.451674265	5-(2-Aminopropyl)-2-methylphenol	0.323231457
gene-PANX2	1.451674265	ingenol	0.942071652
gene-PANX2	1.451674265	Armillane	0.52808635
gene-PANX2	1.451674265	PC(P-18:1(11Z)/PGE2)	0.509503133
gene-PANX2	1.451674265	(3R,4R)-3-Amino-1-hydroxy-4-methylpyrrol	0.471914506
gene-PANX2	1.451674265	Isopropyl isothiocyanate	0.172385747
gene-PANX2	1.451674265	(9Z)-Octadecenoic acid	0.142359556
gene-PANX2	1.451674265	arachidyl amido cholanoic acid	1.24842952
gene-PANX2	1.451674265	Nigroxanthin	0.705005059
gene-PANX2	1.451674265	PS(20:0/20:4(8Z,11Z,14Z,17Z)-2OH(5S,6R))	0.402595921
gene-PANX2	1.451674265	CL(8:0/8:0/18:2(9Z,11Z)/20:0)	0.622988418
gene-PRSS53	1.447562875	3-Thiacytidine	0.209387412
gene-PRSS53	1.447562875	D-Erythro-imidazole-glycerol-phosphate	0.355812431
gene-PRSS53	1.447562875	LTB4-d4	0.226799982
gene-PRSS53	1.447562875	cis-p-Menth-2-en-1-ol	0.201017988
gene-PRSS53	1.447562875	PE(20:0/18:1(12Z)-2OH(9,10))	0.438658253
gene-PRSS53	1.447562875	PE(22:2(13Z,16Z)/22:5(4Z,7Z,10Z,13Z,19Z)-O	0.369745166
gene-PRSS53	1.447562875	Cyclosporin A	0.656529229
gene-PRSS53	1.447562875	2-Methyl-3-phenyl-2-propenal	0.407813975
gene-PRSS53	1.447562875	beta-L-Dioxolane-cytidine	0.175916668
gene-PRSS53	1.447562875	PC(P-18:1(11Z)/PGE2)	0.509503133
gene-PRSS53	1.447562875	Isopropyl isothiocyanate	0.172385747
gene-PRSS53	1.447562875	PC(18:1(9Z)/15:1(9Z))	0.534724682
gene-PRSS53	1.447562875	Nigroxanthin	0.705005059
gene-PRSS53	1.447562875	PC(P-18:1(11Z)/PGJ2)	0.565243877
gene-PRSS53	1.447562875	PS(20:0/20:4(8Z,11Z,14Z,17Z)-2OH(5S,6R))	0.402595921
Bos_taurus_newG	1.446999719	3-Thiacytidine	0.209387412
Bos_taurus_newG	1.446999719	11-Maleimidoundecanoic acid	1.084942397
Bos_taurus_newG	1.446999719	Monacolin L acid	1.060562098
Bos_taurus_newG	1.446999719	cis-p-Menth-2-en-1-ol	0.201017988
Bos_taurus_newG	1.446999719	PE(20:0/18:1(12Z)-2OH(9,10))	0.438658253
Bos_taurus_newG	1.446999719	PE(22:2(13Z,16Z)/22:5(4Z,7Z,10Z,13Z,19Z)-O	0.369745166
Bos_taurus_newG	1.446999719	Lamivudine	0.327402892
Bos_taurus_newG	1.446999719	S-Acetyldihydrolipoamide-E	0.429561283
Bos_taurus_newG	1.446999719	Norophthalmic acid	0.191432411
Bos_taurus_newG	1.446999719	Indole-3-ethanol	0.096523128
Bos_taurus_newG	1.446999719	Guanidoacetic acid	0.542509265
Bos_taurus_newG	1.446999719	4-Oxo-9-cis-retinoyl-beta-glucuronide	1.611773742
Bos_taurus_newG	1.446999719	alpha-Terpineol formate	0.628238986
Bos_taurus_newG	1.446999719	PC(P-18:1(11Z)/PGE2)	0.509503133
Bos_taurus_newG	1.446999719	Roxithromycin	0.268273077
Bos_taurus_newG	1.446999719	PC(P-18:1(11Z)/PGJ2)	0.565243877
Bos_taurus_newG	1.446999719	PS(20:0/20:4(8Z,11Z,14Z,17Z)-2OH(5S,6R))	0.402595921
Bos_taurus_newG	1.446999719	PE(20:5(5Z,8Z,11Z,14Z,17Z)/18:0)	0.427962506
gene-RNF223	1.446457331	D-Erythro-imidazole-glycerol-phosphate	0.355812431
gene-RNF223	1.446457331	PC(P-18:1(11Z)/PGE2)	0.509503133
gene-LPIN1	1.440770589	3-Thiacytidine	0.209387412
gene-LPIN1	1.440770589	LTB4-d4	0.226799982
gene-LPIN1	1.440770589	Cyclosporin A	0.656529229
gene-LPIN1	1.440770589	5-(2-Aminopropyl)-2-methylphenol	0.323231457
gene-LPIN1	1.440770589	beta-L-Dioxolane-cytidine	0.175916668

gene-LPIN1	1.440770589	PC(P-18:1(11Z)/PGE2)	0.509503133
gene-LPIN1	1.440770589	Isopropyl isothiocyanate	0.172385747
gene-LPIN1	1.440770589	Nigroxanthin	0.705005059
gene-LPIN1	1.440770589	PC(P-18:1(11Z)/PGJ2)	0.565243877
gene-LPIN1	1.440770589	PS(20:0/20:4(8Z,11Z,14Z,17Z)-2OH(5S,6R))	0.402595921
gene-LPIN1	1.440770589	CL(8:0/8:0/18:2(9Z,11Z)/20:0)	0.622988418
gene-OPRL1	1.43936503	5-(Ethylthio)-1H-tetrazole	0.29717344
gene-OPRL1	1.43936503	3-hydroxypristanic acid	0.548515835
gene-OPRL1	1.43936503	(3Z)-Phycoerythrobilin	1.456755874
gene-OPRL1	1.43936503	16-hydroxy hexadecanoic acid	0.309000958
gene-OPRL1	1.43936503	3-Deoxyestrone	0.282221709
gene-OPRL1	1.43936503	1-Oleoyl-sn-glycero-3-phosphocholine	0.18926952
gene-OPRL1	1.43936503	3,4-dihydroxy-5-all-trans-hexaprenylbenzoa	0.123615726
gene-OPRL1	1.43936503	Lividamine	0.319679555
gene-OPRL1	1.43936503	Psychosine	0.106475396
gene-OPRL1	1.43936503	Cyclotricuspidogenin C	0.440884085
gene-OPRL1	1.43936503	(-)-alpha-Terpineol	0.345716279
gene-OPRL1	1.43936503	DG(8:0/20:4(6Z,8E,10E,14Z)-2OH(5S,12R)/0:0	0.958378689
gene-OPRL1	1.43936503	1,4-Undecadiene	0.462803973
gene-OPRL1	1.43936503	2-isopentyl-3,6-dimethyl pyrazine	0.710502562
gene-OPRL1	1.43936503	4-Dimethylamino-L-phenylalanine	0.242110226
gene-OPRL1	1.43936503	Trimetazidine	0.438282534
gene-OPRL1	1.43936503	ingenol	0.942071652
gene-OPRL1	1.43936503	Armillane	0.52808635
gene-OPRL1	1.43936503	Misoprostol	1.349270093
gene-OPRL1	1.43936503	Carboprost methyl	1.74923433
gene-OPRL1	1.43936503	PC(20:3(5Z,8Z,11Z)/24:0)	0.387959564
gene-OPRL1	1.43936503	(3R,4R)-3-Amino-1-hydroxy-4-methylpyrrol	0.471914506
gene-OPRL1	1.43936503	N2-gamma-Glutamylglutamine	0.230065499
gene-OPRL1	1.43936503	n-methyl-2-(4'-methylaminophenyl)-6-hydr	0.26655714
gene-OPRL1	1.43936503	Permetin A	0.140869415
gene-OPRL1	1.43936503	Glutethimide	0.126237229
gene-OPRL1	1.43936503	Phosphorylcholine	0.087558558
gene-OPRL1	1.43936503	Isopropyl isothiocyanate	0.172385747
gene-OPRL1	1.43936503	(9Z)-Octadecenoic acid	0.142359556
gene-OPRL1	1.43936503	L-Oleandrosyl-oleandolide	0.272998166
gene-OPRL1	1.43936503	9-deoxy-9-methylene-16,16-dimethyl -PGE	0.606893884
gene-OPRL1	1.43936503	2-Propenyl 2-aminobenzoate	0.128406829
gene-OPRL1	1.43936503	1-Methylnicotinamide	0.203956241
gene-OPRL1	1.43936503	CL(8:0/8:0/18:2(9Z,11Z)/20:0)	0.622988418
gene-PPEF2	1.438132467	5-(3'-Carboxy-3'-oxopropenyl)-4,6-dihydro	1.083232395
gene-PPEF2	1.438132467	2-Amino-6-methylpyrimidine-4-thiol	3.949216803
gene-PPEF2	1.438132467	Benzoquinoneacetic acid	0.707114579
gene-PPEF2	1.438132467	4-Hydroxystyrene	2.854273107
gene-PPEF2	1.438132467	4-Methylbenzyl alcohol	4.428526409
gene-PPEF2	1.438132467	Dihydro-3-coumaric acid	1.950770185
gene-PPEF2	1.438132467	BYSSOCHLAMIC ACID	3.635460051
gene-PPEF2	1.438132467	7-Hydroxy-2',4',5'-trimethoxyisoflavan	3.57282801
gene-PPEF2	1.438132467	(+)-Bottrospicatol	0.68654221
gene-PPEF2	1.438132467	xi-2,3-Dihydro-3,5-dihydroxy-6-methyl-4H-	0.933518005
gene-PPEF2	1.438132467	(卤)-Enterolactone	0.766468338

gene-PPEF2	1.438132467	Enterolactone 3''-sulfate	2.060530283
gene-PPEF2	1.438132467	Heptylmalonic acid	0.061016088
gene-PPEF2	1.438132467	Glutathionylspermine	0.87904089
gene-PPEF2	1.438132467	Docosanamide	0.509975786
gene-PPEF2	1.438132467	cis-p-Menth-2-en-1-ol	0.201017988
gene-PPEF2	1.438132467	11-Oxahexadecanolide	2.378626329
gene-PPEF2	1.438132467	L-Carnitine	0.2737116
gene-PPEF2	1.438132467	Dyphylline	0.664549753
gene-PPEF2	1.438132467	4-Hydroxyoctanedioylcarnitine	1.002008817
gene-PPEF2	1.438132467	Indole-3-acetaldoxime N-oxide	2.727163281
gene-PPEF2	1.438132467	4-Guanidinobutanoate	0.445426102
gene-PPEF2	1.438132467	Erosone	0.527835387
gene-PPEF2	1.438132467	7-Methylguanosine	1.215574844
gene-PPEF2	1.438132467	7a-Hydroxy-O-carbamoyl-deacetylcephalos	0.80981273
gene-PPEF2	1.438132467	Leu-Leu-Tyr	1.186798796
gene-PPEF2	1.438132467	7C-aglycone	1.015608521
gene-PPEF2	1.438132467	7-Aminomethyl-7-carbaguanine	0.474992724
gene-PPEF2	1.438132467	carbenicillin	1.429508875
gene-PPEF2	1.438132467	Humulinic acid A	0.602449819
gene-PPEF2	1.438132467	Suberenone	4.927547616
gene-PPEF2	1.438132467	Terbutryn	1.186241428
gene-PPEF2	1.438132467	(-)-Huperzine A (HupA)	0.935198906
gene-PPEF2	1.438132467	8-Methylthiooctanaldoxime	0.685273457
gene-PPEF2	1.438132467	(1S)-3-[2-[(1R,7Ar)-7a-methyl-1-[(2R)-6-me	1.140894639
gene-PPEF2	1.438132467	4-Octylphenol	1.400657885
gene-PPEF2	1.438132467	Prolyl-Lysine	1.612991304
gene-PPEF2	1.438132467	8-Acetylneosolaniol	4.201843387
gene-PPEF2	1.438132467	Ajulemic acid	0.557832951
gene-PPEF2	1.438132467	Sorbitan palmitate	1.946147152
gene-PPEF2	1.438132467	N-Linoleoyl Isoleucine	1.13160075
gene-PPEF2	1.438132467	11Z-Eicosenoic acid	0.170761996
gene-PPEF2	1.438132467	Chalcone	4.157137778
gene-PPEF2	1.438132467	12-Hydroxyicosanoylcarnitine	1.730979067
gene-PPEF2	1.438132467	Milbemycin D	0.125637998
gene-PPEF2	1.438132467	Ascorbic acid 6-palmitate	0.201627014
gene-PPEF2	1.438132467	Sitosterol beta-D-glucoside	0.382944796
gene-PPEF2	1.438132467	Lysyltryptophan	2.153119072
gene-PPEF2	1.438132467	Isopropyl hexadecanoate	0.803568925
gene-PPEF2	1.438132467	PS(22:0/18:1(12Z)-2OH(9,10))	0.158945417
gene-PPEF2	1.438132467	Galactosylglycerol	0.738463143
gene-PPEF2	1.438132467	PG(20:1(11Z)/18:3(9,11,15)-OH(13))	0.69829683
gene-FBXW9	1.438011432	3-Thiacytidine	0.209387412
gene-FBXW9	1.438011432	4-cholesten-7伪,12伪,24-triol-3-one	0.097840451
gene-FBXW9	1.438011432	Cyclosporin A	0.656529229
gene-FBXW9	1.438011432	PC(14:0/20:2(11Z,14Z))	1.110657378
gene-FBXW9	1.438011432	5-(2-Aminopropyl)-2-methylphenol	0.323231457
gene-FBXW9	1.438011432	ingenol	0.942071652
gene-FBXW9	1.438011432	Armillane	0.52808635
gene-FBXW9	1.438011432	beta-L-Dioxolane-cytidine	0.175916668
gene-FBXW9	1.438011432	Isopropyl isothiocyanate	0.172385747
gene-FBXW9	1.438011432	PS(20:0/20:4(8Z,11Z,14Z,17Z)-2OH(5S,6R))	0.402595921

gene-FBXW9	1.438011432	D-Fructose	0.167584616
gene-FBXW9	1.438011432	CL(8:0/8:0/18:2(9Z,11Z)/20:0)	0.622988418
gene-MBOAT7	1.432493645	3-Thiacytidine	0.209387412
gene-MBOAT7	1.432493645	D-Erythro-imidazole-glycerol-phosphate	0.355812431
gene-MBOAT7	1.432493645	LTB4-d4	0.226799982
gene-MBOAT7	1.432493645	cis-p-Menth-2-en-1-ol	0.201017988
gene-MBOAT7	1.432493645	(-)-alpha-Terpineol	0.345716279
gene-MBOAT7	1.432493645	PG(20:1(11Z)/18:3(10,12,15)-OH(9))	0.626244347
gene-MBOAT7	1.432493645	5-(2-Aminopropyl)-2-methylphenol	0.323231457
gene-MBOAT7	1.432493645	4-Dimethylamino-L-phenylalanine	0.242110226
gene-MBOAT7	1.432493645	Armillane	0.52808635
gene-MBOAT7	1.432493645	2-Methyl-3-phenyl-2-propenal	0.407813975
gene-MBOAT7	1.432493645	PC(P-18:1(11Z)/PGE2)	0.509503133
gene-MBOAT7	1.432493645	(3R,4R)-3-Amino-1-hydroxy-4-methylpyrrol	0.471914506
gene-MBOAT7	1.432493645	Isopropyl isothiocyanate	0.172385747
gene-MBOAT7	1.432493645	(9Z)-Octadecenoic acid	0.142359556
gene-MBOAT7	1.432493645	PC(P-18:1(11Z)/PGJ2)	0.565243877
gene-MBOAT7	1.432493645	PS(20:0/20:4(8Z,11Z,14Z,17Z)-2OH(5S,6R))	0.402595921
gene-MBOAT7	1.432493645	CL(8:0/8:0/18:2(9Z,11Z)/20:0)	0.622988418
gene-KIF12	1.427822204	4-Hydroxybenzoate	0.892336321
gene-KIF12	1.427822204	isochorismate	0.054781856
gene-KIF12	1.427822204	(14S)-14,15-Dihydroxy-8(17),13(16)-labdadi	0.720697404
gene-KIF12	1.427822204	Deoxyshikonin	0.734877521
gene-KIF12	1.427822204	5-Tetradecenoic acid	0.158247657
gene-KIF12	1.427822204	TETRAHYDROURIDINE	0.576545276
gene-KIF12	1.427822204	Imidazoline	0.232348181
gene-KIF12	1.427822204	beta-L-Dioxolane-cytidine	0.175916668
gene-KIF12	1.427822204	PS(20:5(5Z,8Z,11Z,14Z,16E)-OH(18R)/22:0)	0.090792733
gene-KIF12	1.427822204	D-Fructose	0.167584616
gene-MYBL1	1.427801758	Glutamate carbon	0.671444516
gene-MYBL1	1.427801758	2-Oxo-10-methylthiodecanoic acid	0.695908469
gene-MYBL1	1.427801758	Roxithromycin	0.268273077
gene-MYBL1	1.427801758	PC(P-18:1(11Z)/PGJ2)	0.565243877
gene-MYBL1	1.427801758	PS(20:0/20:4(8Z,11Z,14Z,17Z)-2OH(5S,6R))	0.402595921
gene-KREMEN2	1.424686434	5-Sulfosalicylic acid	1.31509494
gene-KREMEN2	1.424686434	4-Hydroxybenzoate	0.892336321
gene-KREMEN2	1.424686434	Gentisic acid	0.519724891
gene-KREMEN2	1.424686434	Ethyl nicotinate	1.024858052
gene-KREMEN2	1.424686434	Dopaquinone	1.087504518
gene-KREMEN2	1.424686434	Isomaltotriose	0.58434654
gene-KREMEN2	1.424686434	(1R,6R)-1,4,5,5a,6,9-Hexahydrophenazine-1,	0.26824502
gene-KREMEN2	1.424686434	Glutamate carbon	0.671444516
gene-KREMEN2	1.424686434	1-(2-Furanyl)-1-pentanone	0.697147283
gene-KREMEN2	1.424686434	2-Dehydro-3-deoxy-D-gluconate	0.65165299
gene-KREMEN2	1.424686434	Cholic acid glucuronide	0.147398811
gene-KREMEN2	1.424686434	16-Hydroxy-10-oxohexadecanoic acid	0.72302152
gene-KREMEN2	1.424686434	11-Maleimidoundecanoic acid	1.084942397
gene-KREMEN2	1.424686434	PE(22:2(13Z,16Z)/22:5(4Z,7Z,10Z,13Z,19Z)-O	0.369745166
gene-KREMEN2	1.424686434	Serotonin	2.521565841
gene-KREMEN2	1.424686434	Adrenosterone	0.050790129
gene-KREMEN2	1.424686434	(1R,6S)-6-Amino-5-oxocyclohex-2-ene-1-c	0.154751123

gene-KREMEN2	1.424686434	(S)-Mandelic acid O-beta-D-Glucopyranosid	0.509637316
gene-KREMEN2	1.424686434	2-(1-Adamantyl)-1,3-dioxetane	0.408916843
gene-KREMEN2	1.424686434	3-(3-Methylbutylidene)-1(3H)-isobenzofurar	0.575422414
gene-KREMEN2	1.424686434	Roxithromycin	0.268273077
gene-KREMEN2	1.424686434	PS(20:0/20:4(8Z,11Z,14Z,17Z)-2OH(5S,6R))	0.402595921
gene-GDPD3	1.423505643	1,2-O-Isopropylidene-D-glucofuranose	0.080987667
gene-GDPD3	1.423505643	1,4-Undecadiene	0.462803973
gene-GDPD3	1.423505643	PC(16:0/18:1(12Z)-2OH(9,10))	0.461843233
gene-GDPD3	1.423505643	5-(2-Aminopropyl)-2-methylphenol	0.323231457
gene-GDPD3	1.423505643	ingenol	0.942071652
gene-GDPD3	1.423505643	Armillane	0.52808635
gene-GDPD3	1.423505643	1-Palmitoylglycerol	0.084357408
gene-GDPD3	1.423505643	Isopropyl isothiocyanate	0.172385747
gene-GDPD3	1.423505643	9-deoxy-9-methylene-16,16-dimethyl -PGE:	0.606893884
gene-GDPD3	1.423505643	CL(8:0/8:0/18:2(9Z,11Z)/20:0)	0.622988418
gene-PDE4C	1.420825791	Hydroxyprolyl-Serine	0.519540341
gene-PDE4C	1.420825791	1,2-O-Isopropylidene-D-glucofuranose	0.080987667
gene-PDE4C	1.420825791	PC(16:0/18:1(12Z)-2OH(9,10))	0.461843233
gene-PDE4C	1.420825791	ingenol	0.942071652
gene-PDE4C	1.420825791	1-Palmitoylglycerol	0.084357408
gene-PDE4C	1.420825791	CL(8:0/8:0/18:2(9Z,11Z)/20:0)	0.622988418
gene-SSH3	1.420803007	3-Thiacytidine	0.209387412
gene-SSH3	1.420803007	D-Erythro-imidazole-glycerol-phosphate	0.355812431
gene-SSH3	1.420803007	cis-p-Menth-2-en-1-ol	0.201017988
gene-SSH3	1.420803007	5-(2-Aminopropyl)-2-methylphenol	0.323231457
gene-SSH3	1.420803007	Armillane	0.52808635
gene-SSH3	1.420803007	PC(P-18:1(11Z)/PGE2)	0.509503133
gene-SSH3	1.420803007	Isopropyl isothiocyanate	0.172385747
gene-SSH3	1.420803007	PC(P-18:1(11Z)/PGJ2)	0.565243877
gene-SSH3	1.420803007	PS(20:0/20:4(8Z,11Z,14Z,17Z)-2OH(5S,6R))	0.402595921
gene-SSH3	1.420803007	CL(8:0/8:0/18:2(9Z,11Z)/20:0)	0.622988418
gene-NOXO1	1.418524142	Tetracenomycin B3	0.744356245
gene-NOXO1	1.418524142	Jasmolone glucoside	0.949312509
gene-NOXO1	1.418524142	Lucyoside N	0.831231157
gene-NOXO1	1.418524142	Proclavaminic acid	0.060659628
gene-NOXO1	1.418524142	7(14)-Bisabolene-2,3,10,11-tetrol	2.446897974
gene-NOXO1	1.418524142	(3Z)-Phycoerythrobilin	1.456755874
gene-NOXO1	1.418524142	Lividamine	0.319679555
gene-NOXO1	1.418524142	11-Oxahexadecanolide	2.378626329
gene-NOXO1	1.418524142	1,4-Undecadiene	0.462803973
gene-NOXO1	1.418524142	2-isopentyl-3,6-dimethyl pyrazine	0.710502562
gene-NOXO1	1.418524142	4-Dimethylamino-L-phenylalanine	0.242110226
gene-NOXO1	1.418524142	Armillane	0.52808635
gene-NOXO1	1.418524142	N-Eicosapentaenoyl Asparagine	0.371594025
gene-NOXO1	1.418524142	Ajulemic acid	0.557832951
gene-NOXO1	1.418524142	LysoPE(0:0/16:0)	0.163938493
gene-NOXO1	1.418524142	2-(2-Aminopropanoylamino)bicyclo[3.1.0]he:	1.149807904
gene-NOXO1	1.418524142	Sambutoxin	0.169786377
gene-NOXO1	1.418524142	1-Palmitoylglycerol	0.084357408
gene-NOXO1	1.418524142	Threoninyl-Tryptophan	0.69975167
gene-NOXO1	1.418524142	Glycyl-Glutamine	0.129171527

gene-RASD1	1.416074941	3-Thiacytidine	0.209387412
gene-RASD1	1.416074941	7(14)-Bisabolene-2,3,10,11-tetrol	2.446897974
gene-RASD1	1.416074941	3-Deoxyestrone	0.282221709
gene-RASD1	1.416074941	1-Oleoyl-sn-glycero-3-phosphocholine	0.18926952
gene-RASD1	1.416074941	Psychosine	0.106475396
gene-RASD1	1.416074941	(-)-alpha-Terpineol	0.345716279
gene-RASD1	1.416074941	1,4-Undecadiene	0.462803973
gene-RASD1	1.416074941	5-(2-Aminopropyl)-2-methylphenol	0.323231457
gene-RASD1	1.416074941	4-Dimethylamino-L-phenylalanine	0.242110226
gene-RASD1	1.416074941	ingenol	0.942071652
gene-RASD1	1.416074941	Armillane	0.52808635
gene-RASD1	1.416074941	PC(P-18:1(11Z)/PGE2)	0.509503133
gene-RASD1	1.416074941	(3R,4R)-3-Amino-1-hydroxy-4-methylpyrrol	0.471914506
gene-RASD1	1.416074941	1-Palmitoylglycerol	0.084357408
gene-RASD1	1.416074941	n-methyl-2-(4'-methylaminophenyl)-6-hydr	0.26655714
gene-RASD1	1.416074941	Isopropyl isothiocyanate	0.172385747
gene-RASD1	1.416074941	9-deoxy-9-methylene-16,16-dimethyl -PGE	0.606893884
gene-RASD1	1.416074941	PS(20:0/20:4(8Z,11Z,14Z,17Z)-2OH(5S,6R))	0.402595921
gene-RASD1	1.416074941	CL(8:0/8:0/18:2(9Z,11Z)/20:0)	0.622988418
gene-DOC2G	1.407704593	3-Thiacytidine	0.209387412
gene-DOC2G	1.407704593	D-Erythro-imidazole-glycerol-phosphate	0.355812431
gene-DOC2G	1.407704593	LTB4-d4	0.226799982
gene-DOC2G	1.407704593	cis-p-Menth-2-en-1-ol	0.201017988
gene-DOC2G	1.407704593	(-)-alpha-Terpineol	0.345716279
gene-DOC2G	1.407704593	PG(20:1(11Z)/18:3(10,12,15)-OH(9))	0.626244347
gene-DOC2G	1.407704593	4-Dimethylamino-L-phenylalanine	0.242110226
gene-DOC2G	1.407704593	2-Methyl-3-phenyl-2-propenal	0.407813975
gene-DOC2G	1.407704593	PC(P-18:1(11Z)/PGE2)	0.509503133
gene-DOC2G	1.407704593	(3R,4R)-3-Amino-1-hydroxy-4-methylpyrrol	0.471914506
gene-DOC2G	1.407704593	PC(P-18:1(11Z)/PGJ2)	0.565243877
gene-DOC2G	1.407704593	PS(20:0/20:4(8Z,11Z,14Z,17Z)-2OH(5S,6R))	0.402595921
gene-DOC2G	1.407704593	CL(8:0/8:0/18:2(9Z,11Z)/20:0)	0.622988418
gene-DDR1	1.407223823	(-)-alpha-Terpineol	0.345716279
gene-DDR1	1.407223823	Cyclosporin A	0.656529229
gene-DDR1	1.407223823	DG(18:0/LTE4/0:0)	0.681485773
gene-DDR1	1.407223823	PC(14:0/20:2(11Z,14Z))	1.110657378
gene-DDR1	1.407223823	5-(2-Aminopropyl)-2-methylphenol	0.323231457
gene-DDR1	1.407223823	ingenol	0.942071652
gene-DDR1	1.407223823	beta-L-Dioxolane-cytidine	0.175916668
gene-DDR1	1.407223823	PC(P-18:1(11Z)/PGE2)	0.509503133
gene-DDR1	1.407223823	Isopropyl isothiocyanate	0.172385747
gene-DDR1	1.407223823	CL(8:0/8:0/18:2(9Z,11Z)/20:0)	0.622988418
gene-PTPN5	1.406726725	3-Thiacytidine	0.209387412
gene-PTPN5	1.406726725	LTB4-d4	0.226799982
gene-PTPN5	1.406726725	PE(20:0/18:1(12Z)-2OH(9,10))	0.438658253
gene-PTPN5	1.406726725	(-)-alpha-Terpineol	0.345716279
gene-PTPN5	1.406726725	Cyclosporin A	0.656529229
gene-PTPN5	1.406726725	DG(18:0/LTE4/0:0)	0.681485773
gene-PTPN5	1.406726725	PC(14:0/20:2(11Z,14Z))	1.110657378
gene-PTPN5	1.406726725	5-(2-Aminopropyl)-2-methylphenol	0.323231457
gene-PTPN5	1.406726725	4-Dimethylamino-L-phenylalanine	0.242110226

gene-PTPN5	1.406726725	Trimetazidine	0.438282534
gene-PTPN5	1.406726725	Armillane	0.52808635
gene-PTPN5	1.406726725	PC(P-18:1(11Z)/PGE2)	0.509503133
gene-PTPN5	1.406726725	(3R,4R)-3-Amino-1-hydroxy-4-methylpyrrol	0.471914506
gene-PTPN5	1.406726725	n-methyl-2-(4'-methylaminophenyl)-6-hydr	0.26655714
gene-PTPN5	1.406726725	Isopropyl isothiocyanate	0.172385747
gene-PTPN5	1.406726725	(9Z)-Octadecenoic acid	0.142359556
gene-PTPN5	1.406726725	arachidyl amido cholanoic acid	1.24842952
gene-PTPN5	1.406726725	Nigroxanthin	0.705005059
gene-PTPN5	1.406726725	9-deoxy-9-methylene-16,16-dimethyl -PGE	0.606893884
gene-PTPN5	1.406726725	1-Octadecanoyl-2-(7Z,10Z,13Z,16Z-docosat	0.690496314
gene-PTPN5	1.406726725	PC(P-18:1(11Z)/PGJ2)	0.565243877
gene-PTPN5	1.406726725	PS(20:0/20:4(8Z,11Z,14Z,17Z)-2OH(5S,6R))	0.402595921
gene-PTPN5	1.406726725	CL(8:0/8:0/18:2(9Z,11Z)/20:0)	0.622988418
Bos_taurus_newG	1.404116268	3-Thiacytidine	0.209387412
Bos_taurus_newG	1.404116268	11-Maleimidoundecanoic acid	1.084942397
Bos_taurus_newG	1.404116268	cis-p-Menth-2-en-1-ol	0.201017988
Bos_taurus_newG	1.404116268	PE(20:0/18:1(12Z)-2OH(9,10))	0.438658253
Bos_taurus_newG	1.404116268	PE(22:2(13Z,16Z)/22:5(4Z,7Z,10Z,13Z,19Z)-O	0.369745166
Bos_taurus_newG	1.404116268	Cyclosporin A	0.656529229
Bos_taurus_newG	1.404116268	Epomusenin A	0.766206049
Bos_taurus_newG	1.404116268	PA(22:6(4Z,7Z,10Z,13Z,16Z,19Z)/16:0)	0.556532497
Bos_taurus_newG	1.404116268	DG(18:0/LTE4/0:0)	0.681485773
Bos_taurus_newG	1.404116268	PC(14:0/20:2(11Z,14Z))	1.110657378
Bos_taurus_newG	1.404116268	CDP-DG(PGF2alpha/16:0)	1.002512277
Bos_taurus_newG	1.404116268	Norophthalmic acid	0.191432411
Bos_taurus_newG	1.404116268	Guanidoacetic acid	0.542509265
Bos_taurus_newG	1.404116268	beta-L-Dioxolane-cytidine	0.175916668
Bos_taurus_newG	1.404116268	PC(P-18:1(11Z)/PGE2)	0.509503133
Bos_taurus_newG	1.404116268	PC(18:1(9Z)/15:1(9Z))	0.534724682
Bos_taurus_newG	1.404116268	Nigroxanthin	0.705005059
Bos_taurus_newG	1.404116268	PC(P-18:1(11Z)/PGJ2)	0.565243877
Bos_taurus_newG	1.404116268	PS(20:0/20:4(8Z,11Z,14Z,17Z)-2OH(5S,6R))	0.402595921
gene-FOXO6	1.40358463	Isomaltotriose	0.58434654
gene-FOXO6	1.40358463	Cholic acid glucuronide	0.147398811
gene-FOXO6	1.40358463	Cyclosporin A	0.656529229
gene-FOXO6	1.40358463	beta-Thujaplicin	0.681866194
gene-FOXO6	1.40358463	Sophoranol	0.282064826
gene-FOXO6	1.40358463	L-Cysteine	0.214540754
gene-FOXO6	1.40358463	1,8-Octanedithiol	0.133094504
gene-FOXO6	1.40358463	Prostaglandin B2	0.552852305
gene-FOXO6	1.40358463	PC(P-18:1(11Z)/PGE2)	0.509503133
gene-FOXO6	1.40358463	Phorone A	0.079039095
Bos_taurus_newG	1.402062089	Dextrorphan O-glucuronide	1.59476827
Bos_taurus_newG	1.402062089	3,3',5-Trihydroxy-4'-methoxy-6,7-methylene	0.137607639
Bos_taurus_newG	1.402062089	5-amino-1-(5-phosphonato-D-ribosyl)imida	0.622612791
Bos_taurus_newG	1.402062089	5-Hydroxyindoleacetaldehyde	0.448806354
Bos_taurus_newG	1.402062089	(2S,4R)-4-(9H-Pyrido[3,4-b]indol-1-yl)-1,2,4	0.883685198
Bos_taurus_newG	1.402062089	Etimizol	0.852969435
Bos_taurus_newG	1.402062089	5-Hydroxy-2-oxo-4-ureido-2,5-dihydro-1H	0.122536033
Bos_taurus_newG	1.402062089	p-Coumaroylputrescine	0.939330574

Bos_taurus_newG	1.402062089	Calicheamicinone	0.910932275
Bos_taurus_newG	1.402062089	Pterostilbene	0.899698314
Bos_taurus_newG	1.402062089	Ancymidol	0.913827444
Bos_taurus_newG	1.402062089	Phenylalanylhydroxyproline	1.183668928
Bos_taurus_newG	1.402062089	Delgocitinib	1.051864373
Bos_taurus_newG	1.402062089	Melatonin	1.134145229
Bos_taurus_newG	1.402062089	2,5-Dihydroxy-1-octadec-9-enoyloxypyrrole	0.828088186
Bos_taurus_newG	1.402062089	Cyclo(glycylleucylvalylleucylprolylseryl)	0.824045744
Bos_taurus_newG	1.402062089	DG(10:0/20:5(7Z,9Z,11E,13E,17Z)-3OH(5,6,15	0.776712455
Bos_taurus_newG	1.402062089	Arginine vasopressin 1-8	1.288601754
Bos_taurus_newG	1.402062089	Hygromycin B	0.930396619
Bos_taurus_newG	1.402062089	Netupitant	0.707108724
Bos_taurus_newG	1.402062089	Divinylprotochlorophyllide	0.629978387
Bos_taurus_newG	1.402062089	Metkephamid	1.159767003
Bos_taurus_newG	1.402062089	Tuberose lactone	1.590026295
Bos_taurus_newG	1.402062089	Epothilone D	0.794619023
Bos_taurus_newG	1.402062089	Leukotriene D4	0.6807158
Bos_taurus_newG	1.402062089	2-Ethyl-5-methyl-3,3-diphenyl-1-pyrroline	0.826978858
Bos_taurus_newG	1.402062089	11-Hydroxyeicosatetraenoate glyceryl ester	1.226051047
Bos_taurus_newG	1.402062089	Azaspiracid 5	1.398898867
Bos_taurus_newG	1.402062089	PGP(20:2(11Z,14Z)/18:2(10E,12Z)+=O(9))	1.457698264
Bos_taurus_newG	1.402062089	Baccatin III	0.776246161
Bos_taurus_newG	1.402062089	Edetic Acid	0.028658217
Bos_taurus_newG	1.402062089	11-Maleimidoundecanoic acid	1.084942397
Bos_taurus_newG	1.402062089	Becocalcidiol	0.63162902
Bos_taurus_newG	1.402062089	Valnemulin	0.408062514
Bos_taurus_newG	1.402062089	Oxyphencyclimine	0.098817805
Bos_taurus_newG	1.402062089	(6Z,9Z,12Z)-Octadecatrienoic acid	0.266628199
Bos_taurus_newG	1.402062089	PC(24:0/22:6(4Z,7Z,10Z,12E,16Z,19Z)-OH(14)	0.188241061
Bos_taurus_newG	1.402062089	PE(20:0/18:1(12Z)-2OH(9,10))	0.438658253
Bos_taurus_newG	1.402062089	(3b,16b,20R)-Pregn-5-ene-3,16,20-triol 3-gl	0.870801075
Bos_taurus_newG	1.402062089	[(3S,4S,5S,6R)-3,4,5-Trihydroxy-6-(hydroxym	0.008715229
Bos_taurus_newG	1.402062089	Epomusenin A	0.766206049
Bos_taurus_newG	1.402062089	(4r,5s,6s,7r)-4,7-Dibenzyl-5,6-dihydroxy-1,3-	1.334600128
Bos_taurus_newG	1.402062089	Cysteinyl-Glutamine	0.079050155
Bos_taurus_newG	1.402062089	Pseudouridine 5'-phosphate	1.18431378
Bos_taurus_newG	1.402062089	Aflatoxin P1	1.000661338
Bos_taurus_newG	1.402062089	Benzoyl glucuronide (Benzoic acid)	0.442844673
Bos_taurus_newG	1.402062089	S-Acetyldihydrolipoamide-E	0.429561283
Bos_taurus_newG	1.402062089	C20914	0.140383181
Bos_taurus_newG	1.402062089	Fluridone	0.127106682
Bos_taurus_newG	1.402062089	Austdiol	0.046757893
Bos_taurus_newG	1.402062089	3-O-fucopyranosyl-2-acetamido-2-deoxygl	2.892346598
Bos_taurus_newG	1.402062089	Norophthalmic acid	0.191432411
Bos_taurus_newG	1.402062089	4'-Thiothymidine	0.066948524
Bos_taurus_newG	1.402062089	3'-N'-Acetylfusarochromanone	0.755683206
Bos_taurus_newG	1.402062089	Desmethylflumazenil (Ro 15-5528)	0.227256571
Bos_taurus_newG	1.402062089	cis,trans-5'-Hydroxythalidomide	0.589828342
Bos_taurus_newG	1.402062089	Ser Cys Ala Ala	0.603032447
Bos_taurus_newG	1.402062089	6-Fluorohomovanillic acid	0.040947314
Bos_taurus_newG	1.402062089	10-Hydroxycarbazepine	0.848001655

Bos_taurus_newG	1.402062089	Semilepidinoside A	0.527638644
Bos_taurus_newG	1.402062089	Arbutin	0.176184476
Bos_taurus_newG	1.402062089	Loganin	0.92782666
Bos_taurus_newG	1.402062089	3'-Deoxythymidine	0.791830758
Bos_taurus_newG	1.402062089	Gly Asp Ala Ala	0.718656316
Bos_taurus_newG	1.402062089	N-Acetylhistidine	0.349369987
Bos_taurus_newG	1.402062089	ethyl 2-cyano-3-(1h-indol-3-yl)prop-2-eno	0.756861034
Bos_taurus_newG	1.402062089	1-{2-[(3-Ethylphenyl)amino]-2-oxoethyl}-6-	0.705711444
Bos_taurus_newG	1.402062089	kainic acid	0.836445456
Bos_taurus_newG	1.402062089	Zanamivir	0.839205907
Bos_taurus_newG	1.402062089	Tryptophyl-Glutamine	0.89722186
Bos_taurus_newG	1.402062089	Aminoglutethimide	0.652616628
Bos_taurus_newG	1.402062089	5-NITRO-2-PHENYLPROPYLAMINOBENZOIC	0.713298779
Bos_taurus_newG	1.402062089	cis-3,4-Phenanthrenedihydrodiol-4-carboxyl	1.374283937
Bos_taurus_newG	1.402062089	Met His Gly	3.037980823
Bos_taurus_newG	1.402062089	5,6,7,8-Tetrahydromonapterin	0.724075531
Bos_taurus_newG	1.402062089	Harmalol	0.364945111
Bos_taurus_newG	1.402062089	Ethylene brassylate	0.989156973
Bos_taurus_newG	1.402062089	2,3-Epoxymenaquinone	2.040730224
Bos_taurus_newG	1.402062089	Guanidoacetic acid	0.542509265
Bos_taurus_newG	1.402062089	alpha-Terpineol formate	0.628238986
Bos_taurus_newG	1.402062089	Apronal	0.754033426
Bos_taurus_newG	1.402062089	Methionyl-Valine	0.502707745
Bos_taurus_newG	1.402062089	[3-(2-Aminopropyl)-6-methylidenecyclohexa	0.422945997
Bos_taurus_newG	1.402062089	Argyrolobine	0.949134241
Bos_taurus_newG	1.402062089	3尾-hydroxy-estra-5,7,9-trien-17-one	0.715223098
Bos_taurus_newG	1.402062089	Cetamolol	0.770561315
Bos_taurus_newG	1.402062089	Eicosatetraynoic Acid	0.804823516
Bos_taurus_newG	1.402062089	(2E,4E)-5-[2-Methyl-2-(1,1,4,4-tetramethyl-	0.742652463
Bos_taurus_newG	1.402062089	3,7-Dihydroxy-12-oxocholanoic acid	0.832319882
Bos_taurus_newG	1.402062089	(22E)-3伪,7伪,12伪-Trihydroxy-5尾-chol-22-	0.522629215
Bos_taurus_newG	1.402062089	PGD2 ethanolamide	1.431143851
Bos_taurus_newG	1.402062089	(5alpha,6beta,14alpha,20R,22R)-5,6,14,20,27-	1.296004093
Bos_taurus_newG	1.402062089	SM(d19:1/PGE2)	0.725813678
Bos_taurus_newG	1.402062089	3-{[(2E)-4-Amino-4-oxobut-2-enoyl]amino}	1.152542472
Bos_taurus_newG	1.402062089	Leucylhydroxyproline	0.780056846
Bos_taurus_newG	1.402062089	3'-Hydroxyropivacaine	1.014215236
Bos_taurus_newG	1.402062089	3-Hydroxytetradecanoyl carnitine	1.314002596
Bos_taurus_newG	1.402062089	Chitotriose	0.897504334
Bos_taurus_newG	1.402062089	Macrocin	1.715200143
Bos_taurus_newG	1.402062089	Vulgarone A	0.102049407
Bos_taurus_newG	1.402062089	Myricanol 5-[arabinosyl-(1->6)-glucoside]	0.744174399
Bos_taurus_newG	1.402062089	1,4,6-Trimethylnaphthalene	0.12150693
Bos_taurus_newG	1.402062089	Prostaglandin PGE2 1-glyceryl ester	0.007411313
Bos_taurus_newG	1.402062089	7-Sulfocholic acid	0.039985455
Bos_taurus_newG	1.402062089	1,8-Octanedithiol	0.133094504
Bos_taurus_newG	1.402062089	MG(0:0/18:3(9Z,12Z,15Z)/0:0)	1.373784038
Bos_taurus_newG	1.402062089	Alpha-Trisaccharide	0.928257808
Bos_taurus_newG	1.402062089	N-Palmitoyl Proline	0.470367693
Bos_taurus_newG	1.402062089	Dapdiamide B	0.07688703
Bos_taurus_newG	1.402062089	(1'R)-Nepetalic acid	0.499281338

Bos_taurus_newG	1.402062089	Iridal	0.699410612
Bos_taurus_newG	1.402062089	LysoPI(0:0/18:0)	0.420441153
Bos_taurus_newG	1.402062089	N-Stearoyl Valine	0.653773206
Bos_taurus_newG	1.402062089	Farnesyl acetone	0.222244889
Bos_taurus_newG	1.402062089	Undecylenic acid	0.352352175
Bos_taurus_newG	1.402062089	Nigroxanthin	0.705005059
Bos_taurus_newG	1.402062089	PC(P-18:1(11Z)/PGJ2)	0.565243877
Bos_taurus_newG	1.402062089	Azimexon	0.008689891
Bos_taurus_newG	1.402062089	PE-NMe(18:2(9Z,12Z)/18:2(9Z,12Z))[U]	0.694736593
Bos_taurus_newG	1.402062089	PC(P-16:0/20:3(8Z,11Z,14Z)-2OH(5,6))	0.174122169
gene-LOC112441	1.401201328	Tetracosenoyl-CoA	0.187187233
gene-LOC112441	1.401201328	1,2-O-Isopropylidene-D-glucofuranose	0.080987667
gene-LOC112441	1.401201328	3-Deoxyestrone	0.282221709
gene-LOC112441	1.401201328	1-Oleoyl-sn-glycero-3-phosphocholine	0.18926952
gene-LOC112441	1.401201328	Lividamine	0.319679555
gene-LOC112441	1.401201328	Cyclotricuspidogenin C	0.440884085
gene-LOC112441	1.401201328	(-)-alpha-Terpineol	0.345716279
gene-LOC112441	1.401201328	5-(2-Aminopropyl)-2-methylphenol	0.323231457
gene-LOC112441	1.401201328	4-Dimethylamino-L-phenylalanine	0.242110226
gene-LOC112441	1.401201328	Trimetazidine	0.438282534
gene-LOC112441	1.401201328	ingenol	0.942071652
gene-LOC112441	1.401201328	Armillane	0.52808635
gene-LOC112441	1.401201328	PC(P-18:1(11Z)/PGE2)	0.509503133
gene-LOC112441	1.401201328	n-methyl-2-(4'-methylaminophenyl)-6-hydr	0.26655714
gene-LOC112441	1.401201328	9-deoxy-9-methylene-16,16-dimethyl -PGE	0.606893884
gene-LOC112441	1.401201328	CL(8:0/8:0/18:2(9Z,11Z)/20:0)	0.622988418
gene-CDKN2A	1.395369461	3-Thiacytidine	0.209387412
gene-CDKN2A	1.395369461	1-Oleoyl-sn-glycero-3-phosphocholine	0.18926952
gene-CDKN2A	1.395369461	(-)-alpha-Terpineol	0.345716279
gene-CDKN2A	1.395369461	1,4-Undecadiene	0.462803973
gene-CDKN2A	1.395369461	4-cholesten-7伪,12伪,24-triol-3-one	0.097840451
gene-CDKN2A	1.395369461	Cyclosporin A	0.656529229
gene-CDKN2A	1.395369461	PC(14:0/20:2(11Z,14Z))	1.110657378
gene-CDKN2A	1.395369461	5-(2-Aminopropyl)-2-methylphenol	0.323231457
gene-CDKN2A	1.395369461	Trimetazidine	0.438282534
gene-CDKN2A	1.395369461	ingenol	0.942071652
gene-CDKN2A	1.395369461	Armillane	0.52808635
gene-CDKN2A	1.395369461	PC(P-18:1(11Z)/PGE2)	0.509503133
gene-CDKN2A	1.395369461	PC(20:3(5Z,8Z,11Z)/24:0)	0.387959564
gene-CDKN2A	1.395369461	n-methyl-2-(4'-methylaminophenyl)-6-hydr	0.26655714
gene-CDKN2A	1.395369461	Isopropyl isothiocyanate	0.172385747
gene-CDKN2A	1.395369461	(9Z)-Octadecenoic acid	0.142359556
gene-CDKN2A	1.395369461	arachidyl amido cholanoic acid	1.24842952
gene-CDKN2A	1.395369461	Nigroxanthin	0.705005059
gene-CDKN2A	1.395369461	D-Fructose	0.167584616
gene-CDKN2A	1.395369461	CL(8:0/8:0/18:2(9Z,11Z)/20:0)	0.622988418
gene-PRSS36	1.394331102	PE-NMe(18:0/18:3(9Z,12Z,15Z))	0.681884774
gene-PRSS36	1.394331102	Hydroxyisonobilin	0.259767036
gene-PRSS36	1.394331102	CDP-DG(18:0/PGE2)	0.280573668
gene-PRSS36	1.394331102	PE(16:0/18:1(11Z))	0.367326926
gene-PRSS36	1.394331102	3-Thiacytidine	0.209387412

gene-PRSS36	1.394331102	N-(2-(Methylamino)ethyl)-5-isoquinolinesulf	0.199224064
gene-PRSS36	1.394331102	p-coumaroyltriacetic acid lactone	0.057681273
gene-PRSS36	1.394331102	4-Aminophenyl 1-thio-尾-D-glucuronide	0.18194032
gene-PRSS36	1.394331102	Guanosine 3'-phosphate	0.147360452
gene-PRSS36	1.394331102	3,3',5-Trihydroxy-4'-methoxy-6,7-methylene	0.137607639
gene-PRSS36	1.394331102	2-(Formamido)-N1-(5'-phosphoribosyl)aceta	0.35156894
gene-PRSS36	1.394331102	5-amino-1-(5-phosphonato-D-ribosyl)imida	0.622612791
gene-PRSS36	1.394331102	8-Methoxykynurenate	0.424170332
gene-PRSS36	1.394331102	5-Hydroxyindoleacetaldehyde	0.448806354
gene-PRSS36	1.394331102	Vignatic acid B	0.136235161
gene-PRSS36	1.394331102	5-Hydroxy-2-oxo-4-ureido-2,5-dihydro-1H	0.122536033
gene-PRSS36	1.394331102	2-Hydroxymuconate	0.119367002
gene-PRSS36	1.394331102	2H-1-Benzopyran-2-one, aminomethyl-	0.385162237
gene-PRSS36	1.394331102	Trihomomethionine	0.296210774
gene-PRSS36	1.394331102	2-[(2-Aminoethylcarbamoyl)methyl]-2-hydro	0.192497471
gene-PRSS36	1.394331102	Cyclo(glycylleucylvalylleucylprolylseryl)	0.824045744
gene-PRSS36	1.394331102	Dihydroxy-(2-methoxyethoxy)-sulfanylidene-	0.124546591
gene-PRSS36	1.394331102	Metkephamid	1.159767003
gene-PRSS36	1.394331102	Tuberose lactone	1.590026295
gene-PRSS36	1.394331102	Epothilone D	0.794619023
gene-PRSS36	1.394331102	Leukotriene D4	0.6807158
gene-PRSS36	1.394331102	2-Ethyl-5-methyl-3,3-diphenyl-1-pyrroline	0.826978858
gene-PRSS36	1.394331102	11-Hydroxyeicosatetraenoate glyceryl ester	1.226051047
gene-PRSS36	1.394331102	Azaspiracid 5	1.398898867
gene-PRSS36	1.394331102	PGP(20:2(11Z,14Z)/18:2(10E,12Z)+=O(9))	1.457698264
gene-PRSS36	1.394331102	Eicosanoyl-CoA	0.137091047
gene-PRSS36	1.394331102	(15S)-15-Hydroxy-5,8,11-cis-13-trans-eicos	0.20506763
gene-PRSS36	1.394331102	11-Maleimidoundecanoic acid	1.084942397
gene-PRSS36	1.394331102	2-Hexylbenzothiazole	0.396099297
gene-PRSS36	1.394331102	PC(24:0/20:3(8Z,11Z,14Z)-2OH(5,6))	0.43567608
gene-PRSS36	1.394331102	Oxyphencyclimine	0.098817805
gene-PRSS36	1.394331102	6-isobutyl-4-hydroxy-2-pyrone	0.473678412
gene-PRSS36	1.394331102	PE(20:0/18:1(12Z)-2OH(9,10))	0.438658253
gene-PRSS36	1.394331102	PE(P-18:0/PGE1)	0.886597382
gene-PRSS36	1.394331102	4,6-Heneicosanedione	0.113496143
gene-PRSS36	1.394331102	3-O-Sulfogalactosylceramide (d18:1/20:0)	0.255536938
gene-PRSS36	1.394331102	medicagenate	1.084135703
gene-PRSS36	1.394331102	Petasalbin	0.667547079
gene-PRSS36	1.394331102	Isoniazid alpha-ketoglutaric acid	0.101041109
gene-PRSS36	1.394331102	Azetidinyl	0.131404372
gene-PRSS36	1.394331102	5-Hydroxyferuloyl-CoA	0.11702028
gene-PRSS36	1.394331102	N5-Citryl-D-ornithine	0.232123154
gene-PRSS36	1.394331102	PE(22:2(13Z,16Z)/22:5(4Z,7Z,10Z,13Z,19Z)-O	0.369745166
gene-PRSS36	1.394331102	Heptanoylcholine	0.218998318
gene-PRSS36	1.394331102	Angiotensin A	0.247332017
gene-PRSS36	1.394331102	Epomusenin A	0.766206049
gene-PRSS36	1.394331102	PA(22:6(4Z,7Z,10Z,13Z,16Z,19Z)/16:0)	0.556532497
gene-PRSS36	1.394331102	DG(18:0/LTE4/0:0)	0.681485773
gene-PRSS36	1.394331102	PC(17:0/PGJ2)	0.657689319
gene-PRSS36	1.394331102	CDP-DG(PGF2alpha/16:0)	1.002512277
gene-PRSS36	1.394331102	Isocolumbin	0.161623565

gene-PRSS36	1.394331102	1-beta-D-Arabinofuranosyl-5-fluorocytosine	0.152339645
gene-PRSS36	1.394331102	Cysteinyl-Glutamine	0.079050155
gene-PRSS36	1.394331102	5-Formiminotetrahydrofolate	0.118251287
gene-PRSS36	1.394331102	Aflatoxin P1	1.000661338
gene-PRSS36	1.394331102	15-keto-Prostaglandin E2	2.646629404
gene-PRSS36	1.394331102	Benzoyl glucuronide (Benzoic acid)	0.442844673
gene-PRSS36	1.394331102	S-Acetyldihydrolipoamide-E	0.429561283
gene-PRSS36	1.394331102	4-Hydroxyproline galactoside	0.148083825
gene-PRSS36	1.394331102	C20914	0.140383181
gene-PRSS36	1.394331102	S-(2-Hydroxyethyl)glutathione	0.269671835
gene-PRSS36	1.394331102	Fluridone	0.127106682
gene-PRSS36	1.394331102	Austdiol	0.046757893
gene-PRSS36	1.394331102	3-O-fucopyranosyl-2-acetamido-2-deoxygl	2.892346598
gene-PRSS36	1.394331102	4'-Thiothymidine	0.066948524
gene-PRSS36	1.394331102	Desmethylflumazenil (Ro 15-5528)	0.227256571
gene-PRSS36	1.394331102	cis-3,4-Phenanthrenedihydrodiol-4-carboxyl	1.374283937
gene-PRSS36	1.394331102	Met His Gly	3.037980823
gene-PRSS36	1.394331102	5,6,7,8-Tetrahydromonapterin	0.724075531
gene-PRSS36	1.394331102	Harmalol	0.364945111
gene-PRSS36	1.394331102	2,3-Epoxymenaquinone	2.040730224
gene-PRSS36	1.394331102	6-hydroxymethyl-7,8-dihydropterin	0.190396346
gene-PRSS36	1.394331102	Asparagine-betaxanthin	0.103826013
gene-PRSS36	1.394331102	1-[(2R,4S,5S)-5-[Difluoro(hydroxy)methyl]-4-	0.091474911
gene-PRSS36	1.394331102	Guanidoacetic acid	0.542509265
gene-PRSS36	1.394331102	Apronal	0.754033426
gene-PRSS36	1.394331102	Methionyl-Valine	0.502707745
gene-PRSS36	1.394331102	Argyrolobine	0.949134241
gene-PRSS36	1.394331102	3尾-hydroxy-estra-5,7,9-trien-17-one	0.715223098
gene-PRSS36	1.394331102	Eicosatetraynoic Acid	0.804823516
gene-PRSS36	1.394331102	(2E,4E)-5-[2-Methyl-2-(1,1,4,4-tetramethyl-1	0.742652463
gene-PRSS36	1.394331102	3,7-Dihydroxy-12-oxocholanoic acid	0.832319882
gene-PRSS36	1.394331102	(22E)-3伪,7伪,12伪-Trihydroxy-5尾-chol-22-	0.522629215
gene-PRSS36	1.394331102	PGD2 ethanolamide	1.431143851
gene-PRSS36	1.394331102	(5alpha,6beta,14alpha,20R,22R)-5,6,14,20,27-	1.296004093
gene-PRSS36	1.394331102	3-{[(2E)-4-Amino-4-oxobut-2-enoyl]amino}	1.152542472
gene-PRSS36	1.394331102	Leucylhydroxyproline	0.780056846
gene-PRSS36	1.394331102	3-Hydroxytetradecanoyl carnitine	1.314002596
gene-PRSS36	1.394331102	Chitotriose	0.897504334
gene-PRSS36	1.394331102	Macrocin	1.715200143
gene-PRSS36	1.394331102	Dicyclopentadiene	0.129868592
gene-PRSS36	1.394331102	DG(2:0/18:1(12Z)-O(9S,10R)/0:0)	0.033180989
gene-PRSS36	1.394331102	N-Palmitoyl Proline	0.470367693
gene-PRSS36	1.394331102	Phenol	0.097390283
gene-PRSS36	1.394331102	Cichorioside J	0.191008611
gene-PRSS36	1.394331102	MG(LTE4/0:0/0:0)	0.548257353
gene-PRSS36	1.394331102	Dapdiamide B	0.07688703
gene-PRSS36	1.394331102	N-Stearoyl Proline	0.469971646
gene-PRSS36	1.394331102	Phorone A	0.079039095
gene-PRSS36	1.394331102	N-Stearoyl Valine	0.653773206
gene-PRSS36	1.394331102	N-Palmitoyl Glutamic acid	0.250188721
gene-PRSS36	1.394331102	Nigroxanthin	0.705005059

gene-PRSS36	1.394331102	PC(P-18:1(11Z)/PGJ2)	0.565243877
gene-PRSS36	1.394331102	PS(20:0/20:4(8Z,11Z,14Z,17Z)-2OH(5S,6R))	0.402595921
gene-PRSS36	1.394331102	nona-4,6-dienoyl-CoA	0.07877806
gene-PRSS36	1.394331102	UDP-2-acetamido-2-deoxy-alpha-D-galactu	0.069033636
gene-PRSS36	1.394331102	L-Dopachrome	0.025660546
gene-PRSS36	1.394331102	Azimexon	0.008689891
gene-PRSS36	1.394331102	PE-NMe(18:2(9Z,12Z)/18:2(9Z,12Z))[U]	0.694736593
gene-GFRA3	1.392753727	1-Oleoyl Lysophosphatidic Acid (sodium salt)	0.375442922
gene-GFRA3	1.392753727	3-Thiacytidine	0.209387412
gene-GFRA3	1.392753727	Palmitoyl Ara-C	0.586890476
gene-GFRA3	1.392753727	9'-Carboxy-gamma-chromanol	0.068924993
gene-GFRA3	1.392753727	3-alpha-Androstanediol glucuronide	0.088637537
gene-GFRA3	1.392753727	Tsugarioside B	1.013262893
gene-GFRA3	1.392753727	Docosanamide	0.509975786
gene-GFRA3	1.392753727	LTB4-d4	0.226799982
gene-GFRA3	1.392753727	cis-p-Menth-2-en-1-ol	0.201017988
gene-GFRA3	1.392753727	PE(20:0/18:1(12Z)-2OH(9,10))	0.438658253
gene-GFRA3	1.392753727	DG(15:0/PGE1/0:0)	0.462019057
gene-GFRA3	1.392753727	PG(20:1(11Z)/18:3(10,12,15)-OH(9))	0.626244347
gene-GFRA3	1.392753727	5-(2-Aminopropyl)-2-methylphenol	0.323231457
gene-GFRA3	1.392753727	4-Guanidinobutanoate	0.445426102
gene-GFRA3	1.392753727	5'-S-Methyl-5'-thioinosine	0.28772476
gene-GFRA3	1.392753727	2-Methyl-3-phenyl-2-propenal	0.407813975
gene-GFRA3	1.392753727	2'-Fluoro-2',3'-dideoxyinosine	1.214745496
gene-GFRA3	1.392753727	Butanoic acid, [(diethoxyphosphinyl)oxy]meth	0.038991848
gene-GFRA3	1.392753727	Spergualin	0.210690914
gene-GFRA3	1.392753727	10-alpha-methoxy-9,10-dihydrolysergol	0.140851438
gene-GFRA3	1.392753727	PC(P-18:1(11Z)/PGE2)	0.509503133
gene-GFRA3	1.392753727	Galabiosylceramide (d18:1/20:0)	0.746737323
gene-GFRA3	1.392753727	(3R,4R)-3-Amino-1-hydroxy-4-methylpyrrol	0.471914506
gene-GFRA3	1.392753727	12-Hydroxyicosanoylcarnitine	1.730979067
gene-GFRA3	1.392753727	Gamithromycin	0.37168241
gene-GFRA3	1.392753727	PE(P-16:0/18:4(6Z,9Z,12Z,15Z))	0.546989864
gene-GFRA3	1.392753727	(9Z)-Octadecenoic acid	0.142359556
gene-GFRA3	1.392753727	Capryloylcholine	0.209692465
gene-GFRA3	1.392753727	norerythromycin	0.401118778
gene-GFRA3	1.392753727	3'-N-Acetyl-4'-O-(14-methylpentadecanoyl)	0.146786959
gene-GFRA3	1.392753727	N-Myristoyl Glutamine	0.37856966
gene-GFRA3	1.392753727	PC(18:1(9Z)/15:1(9Z))	0.534724682
gene-GFRA3	1.392753727	arachidyl amido cholanoic acid	1.24842952
gene-GFRA3	1.392753727	Nigroxanthin	0.705005059
gene-GFRA3	1.392753727	1-Octadecanoyl-2-(7Z,10Z,13Z,16Z-docosat	0.690496314
gene-GFRA3	1.392753727	PC(P-18:1(11Z)/PGJ2)	0.565243877
gene-GFRA3	1.392753727	2-Propenyl 2-aminobenzoate	0.128406829
gene-GFRA3	1.392753727	DG(20:0/LTE4/0:0)	0.438074508
Bos_taurus_newG	1.392589693	4-Amino-3-hydroxybutanoylcarnitine	0.089247351
Bos_taurus_newG	1.392589693	Stanolone enanthate	0.083202462
Bos_taurus_newG	1.392589693	Methyl 3b,24-dihydroxy-11,13(18)-oleanadie	0.056835859
Bos_taurus_newG	1.392589693	6.alpha.-Methylprednisolone	0.064521442
Bos_taurus_newG	1.392589693	trans-O-Methylgrandmarin	0.393786582
Bos_taurus_newG	1.392589693	Indanone	0.059392142

Bos_taurus_newG	1.392589693	5'-S-Methyl-5'-thioinosine	0.28772476
Bos_taurus_newG	1.392589693	Grifola frandosa Lectin	0.159622936
Bos_taurus_newG	1.392589693	(Z)-3-Oxo-2-(2-pentenyl)-1-cyclopenteneac	0.179805915
Bos_taurus_newG	1.392589693	O2'-4a-cyclic-tetrahydrobiopterin	0.005066256
Bos_taurus_newG	1.392589693	MG(0:0/18:1(9Z)/0:0)	0.361035605
Bos_taurus_newG	1.392589693	1,2,4-Nonadecanetriol	0.249655005
Bos_taurus_newG	1.392589693	N-[2-(3,4-Dihydroxyphenyl)ethyl]icosa-5,8,1	0.326021565
Bos_taurus_newG	1.392589693	Soyasapogenol A	0.016063148
gene-GAS6	1.388293331	3-Thiacytidine	0.209387412
gene-GAS6	1.388293331	D-Erythro-imidazole-glycerol-phosphate	0.355812431
gene-GAS6	1.388293331	5-(2-Aminopropyl)-2-methylphenol	0.323231457
gene-GAS6	1.388293331	Armillane	0.52808635
gene-GAS6	1.388293331	PC(P-18:1(11Z)/PGE2)	0.509503133
gene-GAS6	1.388293331	Isopropyl isothiocyanate	0.172385747
gene-GAS6	1.388293331	PC(P-18:1(11Z)/PGJ2)	0.565243877
gene-GAS6	1.388293331	PS(20:0/20:4(8Z,11Z,14Z,17Z)-2OH(5S,6R))	0.402595921
gene-GAS6	1.388293331	CL(8:0/8:0/18:2(9Z,11Z)/20:0)	0.622988418
gene-TGFBR3L	1.385909024	Cyclosporin A	0.656529229
gene-TGFBR3L	1.385909024	Imidazoline	0.232348181
gene-TGFBR3L	1.385909024	beta-L-Dioxolane-cytidine	0.175916668
gene-TGFBR3L	1.385909024	1-Heneicosanoyl-glycero-3-phosphoserine	0.214358093
gene-DNAH12	1.385523766	5-(3'-Carboxy-3'-oxopropenyl)-4,6-dihydro:	1.083232395
gene-DNAH12	1.385523766	Salicyluric acid	1.82393634
gene-DNAH12	1.385523766	4-Acetamidobenzenesulfonamide	0.620868172
gene-DNAH12	1.385523766	2-Amino-6-methylpyrimidine-4-thiol	3.949216803
gene-DNAH12	1.385523766	Benzoquinoneacetic acid	0.707114579
gene-DNAH12	1.385523766	Clusin	0.423362344
gene-DNAH12	1.385523766	4-Hydroxystyrene	2.854273107
gene-DNAH12	1.385523766	4-Methylbenzyl alcohol	4.428526409
gene-DNAH12	1.385523766	Dihydro-3-coumaric acid	1.950770185
gene-DNAH12	1.385523766	BYSSOCHLAMIC ACID	3.635460051
gene-DNAH12	1.385523766	Picroside I	1.144121381
gene-DNAH12	1.385523766	Erythronic acid	0.494007482
gene-DNAH12	1.385523766	Polyribophosphate	0.1080057
gene-DNAH12	1.385523766	7-Hydroxy-2',4',5'-trimethoxyisoflavan	3.57282801
gene-DNAH12	1.385523766	(+)-Bottrospicatol	0.68654221
gene-DNAH12	1.385523766	6-Methylnicotinamide	0.759498218
gene-DNAH12	1.385523766	Enterolactone 3'-glucuronide	1.086030454
gene-DNAH12	1.385523766	Methionine sulfone	0.738197353
gene-DNAH12	1.385523766	7-Hydroxy-6-methyl-8-ribityllumazine	0.103672738
gene-DNAH12	1.385523766	15-Epi-lipoxin B5	0.766800331
gene-DNAH12	1.385523766	xi-2,3-Dihydro-3,5-dihydroxy-6-methyl-4H-	0.933518005
gene-DNAH12	1.385523766	(卤)-Enterolactone	0.766468338
gene-DNAH12	1.385523766	Enterolactone 3''-sulfate	2.060530283
gene-DNAH12	1.385523766	DGAT-1 inhibitor 2	1.079931222
gene-DNAH12	1.385523766	Val-Cit	0.452308451
gene-DNAH12	1.385523766	3-(2,4-Dimethyl-5-(2-oxo-1,2-dihydroindol-	0.095524813
gene-DNAH12	1.385523766	Fusidic Acid	1.929894931
gene-DNAH12	1.385523766	(S)-10,16-Dihydroxyhexadecanoic acid	1.054006526
gene-DNAH12	1.385523766	Glutathionylspermine	0.87904089
gene-DNAH12	1.385523766	Docosanamide	0.509975786

gene-DNAH12	1.385523766	Monacolin L acid	1.060562098
gene-DNAH12	1.385523766	Methyl 8-[3-(2-hydroxyoctyl)oxiran-2-yl]octa	0.117374047
gene-DNAH12	1.385523766	cis-p-Menth-2-en-1-ol	0.201017988
gene-DNAH12	1.385523766	L-Carnitine	0.2737116
gene-DNAH12	1.385523766	3-Oxohexadecanoic acid	1.17619161
gene-DNAH12	1.385523766	Eicosapentaenoic acid	0.575884419
gene-DNAH12	1.385523766	3,4,3',4'-Tetrahydrospirilloxanthin	0.354666148
gene-DNAH12	1.385523766	Notoginsenoside T1	0.106462072
gene-DNAH12	1.385523766	PG(i-12:0/a-15:0)	0.182428802
gene-DNAH12	1.385523766	Dyphylline	0.664549753
gene-DNAH12	1.385523766	2-Hydroxy-6-keto-2,4-heptadienoate	2.23994791
gene-DNAH12	1.385523766	4-Hydroxyoctanedioylcarnitine	1.002008817
gene-DNAH12	1.385523766	Indole-3-acetaldoxime N-oxide	2.727163281
gene-DNAH12	1.385523766	Phenyllactate	0.55151911
gene-DNAH12	1.385523766	Erosone	0.527835387
gene-DNAH12	1.385523766	3-Methoxytyramine	0.928926425
gene-DNAH12	1.385523766	Hydroxyprolyl-Methionine	1.56890423
gene-DNAH12	1.385523766	2-Hydroxy-3,4,6-trimethoxydihydrochalcone	1.07715092
gene-DNAH12	1.385523766	7-Methylguanosine	1.215574844
gene-DNAH12	1.385523766	Eflornithine	0.287216354
gene-DNAH12	1.385523766	7a-Hydroxy-O-carbamoyl-deacetylcephalos	0.80981273
gene-DNAH12	1.385523766	Leu-Leu-Tyr	1.186798796
gene-DNAH12	1.385523766	6-hydroxy-7E,9E-Octadecadiene-11,13,15,17	1.179129125
gene-DNAH12	1.385523766	7C-aglycone	1.015608521
gene-DNAH12	1.385523766	7-Aminomethyl-7-carbaguanine	0.474992724
gene-DNAH12	1.385523766	Tryptophyl-Glutamine	0.89722186
gene-DNAH12	1.385523766	carbenicillin	1.429508875
gene-DNAH12	1.385523766	3,5,7-Trimethyl-2E,4E,6E,8E-decatetraene	1.105561224
gene-DNAH12	1.385523766	5a,6a-Epoxy-7E-megastigmene-3b,9e-diol 9	1.333480709
gene-DNAH12	1.385523766	Humulinic acid A	0.602449819
gene-DNAH12	1.385523766	Dodecatrienoic acid	0.134642109
gene-DNAH12	1.385523766	Suberenone	4.927547616
gene-DNAH12	1.385523766	6,7-dihydro-12-epi-LTB4	0.449117081
gene-DNAH12	1.385523766	Terbutryn	1.186241428
gene-DNAH12	1.385523766	(-)-Huperzine A (HupA)	0.935198906
gene-DNAH12	1.385523766	Sequiterpene Lactone 326	1.141450991
gene-DNAH12	1.385523766	8-Methylthiooctanaldoxime	0.685273457
gene-DNAH12	1.385523766	(1S)-3-[2-[(1R,7Ar)-7a-methyl-1-[(2R)-6-me	1.140894639
gene-DNAH12	1.385523766	ent-16b,19-Kauranediol 19-acetate	0.400676827
gene-DNAH12	1.385523766	4-Octylphenol	1.400657885
gene-DNAH12	1.385523766	Prolyl-Lysine	1.612991304
gene-DNAH12	1.385523766	Pitheduloside I	1.362662847
gene-DNAH12	1.385523766	8-Acetylneosolaniol	4.201843387
gene-DNAH12	1.385523766	3-Pentadecylphenol	1.682258188
gene-DNAH12	1.385523766	Ajulemic acid	0.557832951
gene-DNAH12	1.385523766	Sorbitan palmitate	1.946147152
gene-DNAH12	1.385523766	N-Linoleoyl Isoleucine	1.13160075
gene-DNAH12	1.385523766	QUININE ETHYL CARBONATE	0.116178312
gene-DNAH12	1.385523766	9,10-EOT	0.433129666
gene-DNAH12	1.385523766	Chalcone	4.157137778
gene-DNAH12	1.385523766	12-Hydroxyicosanoylcarnitine	1.730979067

gene-DNAH12	1.385523766	Milbemycin D	0.125637998
gene-DNAH12	1.385523766	Ascorbic acid 6-palmitate	0.201627014
gene-DNAH12	1.385523766	N-Palmitoyl tyrosine	0.089071561
gene-DNAH12	1.385523766	Sitosterol beta-D-glucoside	0.382944796
gene-DNAH12	1.385523766	DG(2:0/20:3(5Z,8Z,11Z)-O(14R,15S)/0:0)	0.046674703
gene-DNAH12	1.385523766	Lysyltryptophan	2.153119072
gene-DNAH12	1.385523766	PS(22:0/18:1(12Z)-2OH(9,10))	0.158945417
gene-DNAH12	1.385523766	Galactosylglycerol	0.738463143
gene-DNAH12	1.385523766	PG(20:1(11Z)/18:3(9,11,15)-OH(13))	0.69829683
gene-TMEM132A	1.384024724	D-Erythro-imidazole-glycerol-phosphate	0.355812431
gene-TMEM132A	1.384024724	cis-p-Menth-2-en-1-ol	0.201017988
gene-TMEM132A	1.384024724	5-(2-Aminopropyl)-2-methylphenol	0.323231457
gene-TMEM132A	1.384024724	PC(P-18:1(11Z)/PGE2)	0.509503133
gene-TMEM132A	1.384024724	PC(P-18:1(11Z)/PGJ2)	0.565243877
gene-TMEM132A	1.384024724	PS(20:0/20:4(8Z,11Z,14Z,17Z)-2OH(5S,6R))	0.402595921
gene-TMEM132A	1.384024724	CL(8:0/8:0/18:2(9Z,11Z)/20:0)	0.622988418
gene-LOC790181	1.383956124	D-Erythro-imidazole-glycerol-phosphate	0.355812431
gene-LOC790181	1.383956124	LTB4-d4	0.226799982
gene-LOC790181	1.383956124	cis-p-Menth-2-en-1-ol	0.201017988
gene-LOC790181	1.383956124	(-)-alpha-Terpineol	0.345716279
gene-LOC790181	1.383956124	PG(20:1(11Z)/18:3(10,12,15)-OH(9))	0.626244347
gene-LOC790181	1.383956124	5-(2-Aminopropyl)-2-methylphenol	0.323231457
gene-LOC790181	1.383956124	4-Dimethylamino-L-phenylalanine	0.242110226
gene-LOC790181	1.383956124	Armillane	0.52808635
gene-LOC790181	1.383956124	2-Methyl-3-phenyl-2-propenal	0.407813975
gene-LOC790181	1.383956124	PC(P-18:1(11Z)/PGE2)	0.509503133
gene-LOC790181	1.383956124	(3R,4R)-3-Amino-1-hydroxy-4-methylpyrrol	0.471914506
gene-LOC790181	1.383956124	n-methyl-2-(4'-methylaminophenyl)-6-hydr	0.26655714
gene-LOC790181	1.383956124	(9Z)-Octadecenoic acid	0.142359556
gene-LOC790181	1.383956124	CL(8:0/8:0/18:2(9Z,11Z)/20:0)	0.622988418
gene-SGSM1	1.382449488	3-Thiacytidine	0.209387412
gene-SGSM1	1.382449488	D-Erythro-imidazole-glycerol-phosphate	0.355812431
gene-SGSM1	1.382449488	11-Maleimidoundecanoic acid	1.084942397
gene-SGSM1	1.382449488	Monacolin L acid	1.060562098
gene-SGSM1	1.382449488	cis-p-Menth-2-en-1-ol	0.201017988
gene-SGSM1	1.382449488	PE(20:0/18:1(12Z)-2OH(9,10))	0.438658253
gene-SGSM1	1.382449488	PE(22:2(13Z,16Z)/22:5(4Z,7Z,10Z,13Z,19Z)-O	0.369745166
gene-SGSM1	1.382449488	Norophthalmic acid	0.191432411
gene-SGSM1	1.382449488	Guanidoacetic acid	0.542509265
gene-SGSM1	1.382449488	alpha-Terpineol formate	0.628238986
gene-SGSM1	1.382449488	PC(P-18:1(11Z)/PGE2)	0.509503133
gene-SGSM1	1.382449488	Roxithromycin	0.268273077
gene-SGSM1	1.382449488	PC(P-18:1(11Z)/PGJ2)	0.565243877
gene-SGSM1	1.382449488	PS(20:0/20:4(8Z,11Z,14Z,17Z)-2OH(5S,6R))	0.402595921
gene-FES	1.379077666	3-Thiacytidine	0.209387412
gene-FES	1.379077666	D-Erythro-imidazole-glycerol-phosphate	0.355812431
gene-FES	1.379077666	LTB4-d4	0.226799982
gene-FES	1.379077666	cis-p-Menth-2-en-1-ol	0.201017988
gene-FES	1.379077666	(-)-alpha-Terpineol	0.345716279
gene-FES	1.379077666	Cyclosporin A	0.656529229
gene-FES	1.379077666	5-(2-Aminopropyl)-2-methylphenol	0.323231457

gene-FES	1.379077666	Armillane	0.52808635
gene-FES	1.379077666	PC(P-18:1(11Z)/PGE2)	0.509503133
gene-FES	1.379077666	Isopropyl isothiocyanate	0.172385747
gene-FES	1.379077666	Nigroxanthin	0.705005059
gene-FES	1.379077666	PC(P-18:1(11Z)/PGJ2)	0.565243877
gene-FES	1.379077666	PS(20:0/20:4(8Z,11Z,14Z,17Z)-2OH(5S,6R))	0.402595921
gene-FES	1.379077666	CL(8:0/8:0/18:2(9Z,11Z)/20:0)	0.622988418
gene-ARVCF	1.375449772	5-(3'-Carboxy-3'-oxopropenyl)-4,6-dihydro:	1.083232395
gene-ARVCF	1.375449772	D-Erythro-imidazole-glycerol-phosphate	0.355812431
gene-ARVCF	1.375449772	(+)-Bottrospicatol	0.68654221
gene-ARVCF	1.375449772	Enterolactone 3'-glucuronide	1.086030454
gene-ARVCF	1.375449772	Methionine sulfone	0.738197353
gene-ARVCF	1.375449772	3-(2,4-Dimethyl-5-(2-oxo-1,2-dihydroindol-	0.095524813
gene-ARVCF	1.375449772	Fusidic Acid	1.929894931
gene-ARVCF	1.375449772	3-carboxy-4-methyl-5-pentyl-2-furanpropa	0.375666997
gene-ARVCF	1.375449772	(S)-10,16-Dihydroxyhexadecanoic acid	1.054006526
gene-ARVCF	1.375449772	Auxin b	1.052491354
gene-ARVCF	1.375449772	13,14-Dihydro PGF-1a	0.390844466
gene-ARVCF	1.375449772	Gemfibrozil	0.392694536
gene-ARVCF	1.375449772	Aspartame	0.406719484
gene-ARVCF	1.375449772	Monacolin L acid	1.060562098
gene-ARVCF	1.375449772	Eicosapentaenoic acid	0.575884419
gene-ARVCF	1.375449772	3,4,3',4'-Tetrahydrospirilloxanthin	0.354666148
gene-ARVCF	1.375449772	(1R,6S)-6-Amino-5-oxocyclohex-2-ene-1-c	0.154751123
gene-ARVCF	1.375449772	3-Methoxytyramine	0.928926425
gene-ARVCF	1.375449772	L-histidinol-phosphate	0.865229875
gene-ARVCF	1.375449772	Hexanoylglutamine	0.593876112
gene-ARVCF	1.375449772	Hydroxyprolyl-Methionine	1.56890423
gene-ARVCF	1.375449772	Eflornithine	0.287216354
gene-ARVCF	1.375449772	7a-Hydroxy-O-carbamoyl-deacetylcephalos	0.80981273
gene-ARVCF	1.375449772	6-hydroxy-7E,9E-Octadecadiene-11,13,15,1	1.179129125
gene-ARVCF	1.375449772	7C-aglycone	1.015608521
gene-ARVCF	1.375449772	loganate	0.253544733
gene-ARVCF	1.375449772	L-alpha-Acetyl-N,N-dinormethadol	1.567999001
gene-ARVCF	1.375449772	12R-hydroxy-5Z,8Z,12Z-eicosatrienoic acid	0.766411225
gene-ARVCF	1.375449772	2-Oxo-10-methylthiodecanoic acid	0.695908469
gene-ARVCF	1.375449772	(-)-Huperzine A (HupA)	0.935198906
gene-ARVCF	1.375449772	Sequiterpene Lactone 326	1.141450991
gene-ARVCF	1.375449772	2-(3-(Diisopropylamino)-1-phenylpropyl)-4-	0.497857255
gene-ARVCF	1.375449772	Pitheduloside I	1.362662847
gene-ARVCF	1.375449772	Ajulemic acid	0.557832951
gene-ARVCF	1.375449772	Ketosantalic acid	0.356663411
gene-ARVCF	1.375449772	(Z)-3-Methyl-3-decenoic acid	0.482455266
gene-ARVCF	1.375449772	Roxithromycin	0.268273077
gene-NUP210L	1.373345133	5-(3'-Carboxy-3'-oxopropenyl)-4,6-dihydro:	1.083232395
gene-NUP210L	1.373345133	LysoPI(16:0/0:0)	0.379098336
gene-NUP210L	1.373345133	11-Maleimidoundecanoic acid	1.084942397
gene-NUP210L	1.373345133	Monacolin L acid	1.060562098
gene-NUP210L	1.373345133	cis-p-Menth-2-en-1-ol	0.201017988
gene-NUP210L	1.373345133	PE(20:0/18:1(12Z)-2OH(9,10))	0.438658253
gene-NUP210L	1.373345133	PE(22:2(13Z,16Z)/22:5(4Z,7Z,10Z,13Z,19Z)-O	0.369745166

gene-NUP210L	1.373345133	Lamivudine	0.327402892
gene-NUP210L	1.373345133	Pseudouridine 5'-phosphate	1.18431378
gene-NUP210L	1.373345133	Ribavirin monophosphate	1.091085027
gene-NUP210L	1.373345133	Norophthalmic acid	0.191432411
gene-NUP210L	1.373345133	Guanidoacetic acid	0.542509265
gene-NUP210L	1.373345133	4-Oxo-9-cis-retinoyl-beta-glucuronide	1.611773742
gene-NUP210L	1.373345133	alpha-Terpineol formate	0.628238986
gene-NUP210L	1.373345133	4-Octylphenol	1.400657885
gene-NUP210L	1.373345133	Prolyl-Lysine	1.612991304
gene-NUP210L	1.373345133	PC(P-18:1(11Z)/PGE2)	0.509503133
gene-NUP210L	1.373345133	12-Hydroxyicosanoylcarnitine	1.730979067
gene-NUP210L	1.373345133	Milbemycin D	0.125637998
gene-NUP210L	1.373345133	Ascorbic acid 6-palmitate	0.201627014
gene-NUP210L	1.373345133	Roxithromycin	0.268273077
gene-NUP210L	1.373345133	Sitosterol beta-D-glucoside	0.382944796
gene-NUP210L	1.373345133	PC(P-18:1(11Z)/PGJ2)	0.565243877
gene-NUP210L	1.373345133	PS(20:0/20:4(8Z,11Z,14Z,17Z)-2OH(5S,6R))	0.402595921
gene-NUP210L	1.373345133	PE(20:5(5Z,8Z,11Z,14Z,17Z)/18:0)	0.427962506
gene-STX3	1.3728222	D-Erythro-imidazole-glycerol-phosphate	0.355812431
gene-STX3	1.3728222	cis-p-Menth-2-en-1-ol	0.201017988
gene-STX3	1.3728222	2-Methyl-3-phenyl-2-propenal	0.407813975
gene-STX3	1.3728222	PC(P-18:1(11Z)/PGE2)	0.509503133
gene-STX3	1.3728222	PC(P-18:1(11Z)/PGJ2)	0.565243877
gene-STX3	1.3728222	PS(20:0/20:4(8Z,11Z,14Z,17Z)-2OH(5S,6R))	0.402595921
gene-ANKRD61	1.370599467	Methylmalonate	0.241481249
gene-ANKRD61	1.370599467	3-Thiacytidine	0.209387412
gene-ANKRD61	1.370599467	LTB4-d4	0.226799982
gene-ANKRD61	1.370599467	cis-p-Menth-2-en-1-ol	0.201017988
gene-ANKRD61	1.370599467	PE(20:0/18:1(12Z)-2OH(9,10))	0.438658253
gene-ANKRD61	1.370599467	PE(22:2(13Z,16Z)/22:5(4Z,7Z,10Z,13Z,19Z)-O	0.369745166
gene-ANKRD61	1.370599467	Cyclosporin A	0.656529229
gene-ANKRD61	1.370599467	Epomusenin A	0.766206049
gene-ANKRD61	1.370599467	DG(18:0/LTE4/0:0)	0.681485773
gene-ANKRD61	1.370599467	CDP-DG(PGF2alpha/16:0)	1.002512277
gene-ANKRD61	1.370599467	5-(2-Aminopropyl)-2-methylphenol	0.323231457
gene-ANKRD61	1.370599467	Norophthalmic acid	0.191432411
gene-ANKRD61	1.370599467	Guanidoacetic acid	0.542509265
gene-ANKRD61	1.370599467	4-Oxo-9-cis-retinoyl-beta-glucuronide	1.611773742
gene-ANKRD61	1.370599467	alpha-Terpineol formate	0.628238986
gene-ANKRD61	1.370599467	10-alpha-methoxy-9,10-dihydrolysergol	0.140851438
gene-ANKRD61	1.370599467	PC(P-18:1(11Z)/PGE2)	0.509503133
gene-ANKRD61	1.370599467	LysoPI(0:0/18:0)	0.420441153
gene-ANKRD61	1.370599467	Gamithromycin	0.37168241
gene-ANKRD61	1.370599467	PC(18:1(9Z)/15:1(9Z))	0.534724682
gene-ANKRD61	1.370599467	arachidyl amido cholanoic acid	1.24842952
gene-ANKRD61	1.370599467	Nigroxanthin	0.705005059
gene-ANKRD61	1.370599467	1-Octadecanoyl-2-(7Z,10Z,13Z,16Z-docosat	0.690496314
gene-ANKRD61	1.370599467	PC(P-18:1(11Z)/PGJ2)	0.565243877
gene-ANKRD61	1.370599467	PS(20:0/20:4(8Z,11Z,14Z,17Z)-2OH(5S,6R))	0.402595921
gene-ANKRD61	1.370599467	PS(16:1(9Z)/22:2(13Z,16Z))	0.659652661
gene-ANKRD61	1.370599467	PC(P-16:0/20:3(8Z,11Z,14Z)-2OH(5,6))	0.174122169

gene-ERAS	1.370240813	Cyclosporin A	0.656529229
gene-ERAS	1.370240813	beta-Thujaplicin	0.681866194
gene-ERAS	1.370240813	Yucalexin P15	1.079168162
gene-ERAS	1.370240813	CL(8:0/8:0/18:2(9Z,11Z)/20:0)	0.622988418
gene-C7H19orf5	1.368289473	3-Deoxyestrone	0.282221709
gene-C7H19orf5	1.368289473	1-Oleoyl-sn-glycero-3-phosphocholine	0.18926952
gene-C7H19orf5	1.368289473	3,4-dihydroxy-5-all-trans-hexaprenylbenzoa	0.123615726
gene-C7H19orf5	1.368289473	Psychosine	0.106475396
gene-C7H19orf5	1.368289473	(-)-alpha-Terpineol	0.345716279
gene-C7H19orf5	1.368289473	1,4-Undecadiene	0.462803973
gene-C7H19orf5	1.368289473	5-(2-Aminopropyl)-2-methylphenol	0.323231457
gene-C7H19orf5	1.368289473	ingenol	0.942071652
gene-C7H19orf5	1.368289473	Armillane	0.52808635
gene-C7H19orf5	1.368289473	PC(P-18:1(11Z)/PGE2)	0.509503133
gene-C7H19orf5	1.368289473	1-Palmitoylglycerol	0.084357408
gene-C7H19orf5	1.368289473	n-methyl-2-(4'-methylaminophenyl)-6-hydr	0.26655714
gene-C7H19orf5	1.368289473	Isopropyl isothiocyanate	0.172385747
gene-C7H19orf5	1.368289473	9-deoxy-9-methylene-16,16-dimethyl -PGE	0.606893884
gene-C7H19orf5	1.368289473	PS(20:0/20:4(8Z,11Z,14Z,17Z)-2OH(5S,6R))	0.402595921
gene-C7H19orf5	1.368289473	CL(8:0/8:0/18:2(9Z,11Z)/20:0)	0.622988418
gene-DAGLA	1.367333349	3-Thiacytidine	0.209387412
gene-DAGLA	1.367333349	Tetracosenoyl-CoA	0.187187233
gene-DAGLA	1.367333349	1,2-O-Isopropylidene-D-glucofuranose	0.080987667
gene-DAGLA	1.367333349	3-Deoxyestrone	0.282221709
gene-DAGLA	1.367333349	1-Oleoyl-sn-glycero-3-phosphocholine	0.18926952
gene-DAGLA	1.367333349	Psychosine	0.106475396
gene-DAGLA	1.367333349	LTB4-d4	0.226799982
gene-DAGLA	1.367333349	(-)-alpha-Terpineol	0.345716279
gene-DAGLA	1.367333349	PC(16:0/18:1(12Z)-2OH(9,10))	0.461843233
gene-DAGLA	1.367333349	5-(2-Aminopropyl)-2-methylphenol	0.323231457
gene-DAGLA	1.367333349	4-Dimethylamino-L-phenylalanine	0.242110226
gene-DAGLA	1.367333349	Trimetazidine	0.438282534
gene-DAGLA	1.367333349	Armillane	0.52808635
gene-DAGLA	1.367333349	5'-S-Methyl-5'-thioinosine	0.28772476
gene-DAGLA	1.367333349	PC(P-18:1(11Z)/PGE2)	0.509503133
gene-DAGLA	1.367333349	(3R,4R)-3-Amino-1-hydroxy-4-methylpyrrol	0.471914506
gene-DAGLA	1.367333349	n-methyl-2-(4'-methylaminophenyl)-6-hydr	0.26655714
gene-DAGLA	1.367333349	PE(P-16:0/18:4(6Z,9Z,12Z,15Z))	0.546989864
gene-DAGLA	1.367333349	Isopropyl isothiocyanate	0.172385747
gene-DAGLA	1.367333349	(9Z)-Octadecenoic acid	0.142359556
gene-DAGLA	1.367333349	arachidyl amido cholanoic acid	1.24842952
gene-DAGLA	1.367333349	9-deoxy-9-methylene-16,16-dimethyl -PGE	0.606893884
gene-DAGLA	1.367333349	CL(8:0/8:0/18:2(9Z,11Z)/20:0)	0.622988418
gene-PRSS50	1.364644247	Methylmalonate	0.241481249
gene-PRSS50	1.364644247	3-Thiacytidine	0.209387412
gene-PRSS50	1.364644247	D-Erythro-imidazole-glycerol-phosphate	0.355812431
gene-PRSS50	1.364644247	LTB4-d4	0.226799982
gene-PRSS50	1.364644247	cis-p-Menth-2-en-1-ol	0.201017988
gene-PRSS50	1.364644247	PE(20:0/18:1(12Z)-2OH(9,10))	0.438658253
gene-PRSS50	1.364644247	PE(22:2(13Z,16Z)/22:5(4Z,7Z,10Z,13Z,19Z)-O	0.369745166
gene-PRSS50	1.364644247	DG(15:0/PGE1/0:0)	0.462019057

gene-PRSS50	1.364644247	DG(18:0/LTE4/0:0)	0.681485773
gene-PRSS50	1.364644247	PG(20:1(11Z)/18:3(10,12,15)-OH(9))	0.626244347
gene-PRSS50	1.364644247	5-(2-Aminopropyl)-2-methylphenol	0.323231457
gene-PRSS50	1.364644247	Armillane	0.52808635
gene-PRSS50	1.364644247	2-Methyl-3-phenyl-2-propenal	0.407813975
gene-PRSS50	1.364644247	10-alpha-methoxy-9,10-dihydrolysergol	0.140851438
gene-PRSS50	1.364644247	PC(P-18:1(11Z)/PGE2)	0.509503133
gene-PRSS50	1.364644247	(3R,4R)-3-Amino-1-hydroxy-4-methylpyrrol	0.471914506
gene-PRSS50	1.364644247	Gamithromycin	0.37168241
gene-PRSS50	1.364644247	PE(P-16:0/18:4(6Z,9Z,12Z,15Z))	0.546989864
gene-PRSS50	1.364644247	Isopropyl isothiocyanate	0.172385747
gene-PRSS50	1.364644247	(9Z)-Octadecenoic acid	0.142359556
gene-PRSS50	1.364644247	N-Myristoyl Glutamine	0.37856966
gene-PRSS50	1.364644247	PC(18:1(9Z)/15:1(9Z))	0.534724682
gene-PRSS50	1.364644247	arachidyl amido cholanoic acid	1.24842952
gene-PRSS50	1.364644247	Nigroxanthin	0.705005059
gene-PRSS50	1.364644247	1-Octadecanoyl-2-(7Z,10Z,13Z,16Z-docosat	0.690496314
gene-PRSS50	1.364644247	PC(P-18:1(11Z)/PGJ2)	0.565243877
gene-PRSS50	1.364644247	PS(20:0/20:4(8Z,11Z,14Z,17Z)-2OH(5S,6R))	0.402595921
gene-PRSS50	1.364644247	DG(20:0/LTE4/0:0)	0.438074508
gene-KCNC4	1.364370522	3-Thiacytidine	0.209387412
gene-KCNC4	1.364370522	3-Deoxyestrone	0.282221709
gene-KCNC4	1.364370522	LTB4-d4	0.226799982
gene-KCNC4	1.364370522	(-)-alpha-Terpineol	0.345716279
gene-KCNC4	1.364370522	4-cholesten-7伪,12伪,24-triol-3-one	0.097840451
gene-KCNC4	1.364370522	PG(20:1(11Z)/18:3(10,12,15)-OH(9))	0.626244347
gene-KCNC4	1.364370522	4-Dimethylamino-L-phenylalanine	0.242110226
gene-KCNC4	1.364370522	2-Methyl-3-phenyl-2-propenal	0.407813975
gene-KCNC4	1.364370522	N-[[3-Hydroxy-2-(2-pentenyl)cyclopentyl]ac	1.043930947
gene-KCNC4	1.364370522	PC(P-18:1(11Z)/PGE2)	0.509503133
gene-KCNC4	1.364370522	PC(20:3(5Z,8Z,11Z)/24:0)	0.387959564
gene-KCNC4	1.364370522	(3R,4R)-3-Amino-1-hydroxy-4-methylpyrrol	0.471914506
gene-KCNC4	1.364370522	n-methyl-2-(4'-methylaminophenyl)-6-hydr	0.26655714
gene-KCNC4	1.364370522	Isopropyl isothiocyanate	0.172385747
gene-KCNC4	1.364370522	(9Z)-Octadecenoic acid	0.142359556
Bos_taurus_newG	1.360680463	3-Thiacytidine	0.209387412
Bos_taurus_newG	1.360680463	D-Erythro-imidazole-glycerol-phosphate	0.355812431
Bos_taurus_newG	1.360680463	LTB4-d4	0.226799982
Bos_taurus_newG	1.360680463	cis-p-Menth-2-en-1-ol	0.201017988
Bos_taurus_newG	1.360680463	(-)-alpha-Terpineol	0.345716279
Bos_taurus_newG	1.360680463	4-cholesten-7伪,12伪,24-triol-3-one	0.097840451
Bos_taurus_newG	1.360680463	PG(20:1(11Z)/18:3(10,12,15)-OH(9))	0.626244347
Bos_taurus_newG	1.360680463	4-Dimethylamino-L-phenylalanine	0.242110226
Bos_taurus_newG	1.360680463	2-Methyl-3-phenyl-2-propenal	0.407813975
Bos_taurus_newG	1.360680463	N-[[3-Hydroxy-2-(2-pentenyl)cyclopentyl]ac	1.043930947
Bos_taurus_newG	1.360680463	PC(P-18:1(11Z)/PGE2)	0.509503133
Bos_taurus_newG	1.360680463	PC(20:3(5Z,8Z,11Z)/24:0)	0.387959564
Bos_taurus_newG	1.360680463	(3R,4R)-3-Amino-1-hydroxy-4-methylpyrrol	0.471914506
Bos_taurus_newG	1.360680463	PC(P-18:1(11Z)/PGE1)	0.295520345
Bos_taurus_newG	1.360680463	(9Z)-Octadecenoic acid	0.142359556
Bos_taurus_newG	1.357510261	D-Erythro-imidazole-glycerol-phosphate	0.355812431

Bos_taurus_newG	1.357510261	Sambutoxin	0.169786377
Bos_taurus_newG	1.354520616	(-)-alpha-Terpineol	0.345716279
Bos_taurus_newG	1.354520616	1,4-Undecadiene	0.462803973
Bos_taurus_newG	1.354520616	5-(2-Aminopropyl)-2-methylphenol	0.323231457
Bos_taurus_newG	1.354520616	4-Dimethylamino-L-phenylalanine	0.242110226
Bos_taurus_newG	1.354520616	Trimetazidine	0.438282534
Bos_taurus_newG	1.354520616	PC(P-18:1(11Z)/PGE2)	0.509503133
Bos_taurus_newG	1.354520616	n-methyl-2-(4'-methylaminophenyl)-6-hydr	0.26655714
Bos_taurus_newG	1.354520616	Isopropyl isothiocyanate	0.172385747
Bos_taurus_newG	1.354520616	9-deoxy-9-methylene-16,16-dimethyl -PGE	0.606893884
Bos_taurus_newG	1.354520616	CL(8:0/8:0/18:2(9Z,11Z)/20:0)	0.622988418
gene-PHETA2	1.352541112	3-Thiacytidine	0.209387412
gene-PHETA2	1.352541112	Isomaltotriose	0.58434654
gene-PHETA2	1.352541112	11-Maleimidoundecanoic acid	1.084942397
gene-PHETA2	1.352541112	13(S)-HpODE	0.163157753
gene-PHETA2	1.352541112	Cyclosporin A	0.656529229
gene-PHETA2	1.352541112	beta-L-Dioxolane-cytidine	0.175916668
gene-PHETA2	1.352541112	PC(P-18:1(11Z)/PGE2)	0.509503133
gene-PHETA2	1.352541112	Roxithromycin	0.268273077
gene-PHETA2	1.352541112	PC(P-18:1(11Z)/PGJ2)	0.565243877
gene-PHETA2	1.352541112	PS(20:0/20:4(8Z,11Z,14Z,17Z)-2OH(5S,6R))	0.402595921
gene-SLC38A8	1.351836939	D-Erythro-imidazole-glycerol-phosphate	0.355812431
gene-SLC38A8	1.351836939	Erythronic acid	0.494007482
gene-SLC38A8	1.351836939	Lucyoside N	0.831231157
gene-SLC38A8	1.351836939	Tsugarioside B	1.013262893
gene-SLC38A8	1.351836939	7(14)-Bisabolene-2,3,10,11-tetrol	2.446897974
gene-SLC38A8	1.351836939	24,25-Diacetylvulgaroside	0.920743312
gene-SLC38A8	1.351836939	(3Z)-Phycoerythrobilin	1.456755874
gene-SLC38A8	1.351836939	3-Deoxyestrone	0.282221709
gene-SLC38A8	1.351836939	1-Oleoyl-sn-glycero-3-phosphocholine	0.18926952
gene-SLC38A8	1.351836939	Lividamine	0.319679555
gene-SLC38A8	1.351836939	cis-p-Menth-2-en-1-ol	0.201017988
gene-SLC38A8	1.351836939	(-)-alpha-Terpineol	0.345716279
gene-SLC38A8	1.351836939	2-isopentyl-3,6-dimethyl pyrazine	0.710502562
gene-SLC38A8	1.351836939	PG(20:1(11Z)/18:3(10,12,15)-OH(9))	0.626244347
gene-SLC38A8	1.351836939	4-Dimethylamino-L-phenylalanine	0.242110226
gene-SLC38A8	1.351836939	Armillane	0.52808635
gene-SLC38A8	1.351836939	N-Eicosapentaenoyl Asparagine	0.371594025
gene-SLC38A8	1.351836939	Ajulemic acid	0.557832951
gene-SLC38A8	1.351836939	Siguazodan	0.338365222
gene-SLC38A8	1.351836939	PC(P-18:1(11Z)/PGE2)	0.509503133
gene-SLC38A8	1.351836939	(3R,4R)-3-Amino-1-hydroxy-4-methylpyrrol	0.471914506
gene-SLC38A8	1.351836939	MG(0:0/18:1(9Z)/0:0)	0.361035605
gene-SLC38A8	1.351836939	Threoninyl-Tryptophan	0.69975167
gene-SLC38A8	1.351836939	n-methyl-2-(4'-methylaminophenyl)-6-hydr	0.26655714
gene-SLC38A8	1.351836939	1,2,4-Nonadecanetriol	0.249655005
gene-SLC38A8	1.351836939	N-[2-(3,4-Dihydroxyphenyl)ethyl]icosa-5,8,1	0.326021565
gene-SLC38A8	1.351836939	2-Propenamide, 2-cyano-3-(4-hydroxy-3,5-	0.067779204
gene-SLC38A8	1.351836939	PG(20:1(11Z)/18:3(9,11,15)-OH(13))	0.69829683
Bos_taurus_newG	1.347060579	milbemycin beta3	1.414447124
Bos_taurus_newG	1.347060579	PE(22:2(13Z,16Z)/22:5(4Z,7Z,10Z,13Z,19Z)-O	0.369745166

Bos_taurus_newG	1.347060579	PG(20:1(11Z)/18:3(10,12,15)-OH(9))	0.626244347
Bos_taurus_newG	1.347060579	Benzoyl glucuronide (Benzoic acid)	0.442844673
Bos_taurus_newG	1.347060579	PC(P-18:1(11Z)/PGE2)	0.509503133
Bos_taurus_newG	1.347060579	Phorone A	0.079039095
Bos_taurus_newG	1.347060579	Sitosterol beta-D-glucoside	0.382944796
Bos_taurus_newG	1.347060579	PS(20:0/20:4(8Z,11Z,14Z,17Z)-2OH(5S,6R))	0.402595921
gene-ZDHHC14	1.345554777	3-Thiacytidine	0.209387412
gene-ZDHHC14	1.345554777	D-Erythro-imidazole-glycerol-phosphate	0.355812431
gene-ZDHHC14	1.345554777	3-Deoxyestrone	0.282221709
gene-ZDHHC14	1.345554777	1-Oleoyl-sn-glycero-3-phosphocholine	0.18926952
gene-ZDHHC14	1.345554777	LTB4-d4	0.226799982
gene-ZDHHC14	1.345554777	(-)-alpha-Terpineol	0.345716279
gene-ZDHHC14	1.345554777	Cyclosporin A	0.656529229
gene-ZDHHC14	1.345554777	5-(2-Aminopropyl)-2-methylphenol	0.323231457
gene-ZDHHC14	1.345554777	4-Dimethylamino-L-phenylalanine	0.242110226
gene-ZDHHC14	1.345554777	Armillane	0.52808635
gene-ZDHHC14	1.345554777	PC(P-18:1(11Z)/PGE2)	0.509503133
gene-ZDHHC14	1.345554777	PC(20:3(5Z,8Z,11Z)/24:0)	0.387959564
gene-ZDHHC14	1.345554777	(3R,4R)-3-Amino-1-hydroxy-4-methylpyrrol	0.471914506
gene-ZDHHC14	1.345554777	n-methyl-2-(4'-methylaminophenyl)-6-hydr	0.26655714
gene-ZDHHC14	1.345554777	Isopropyl isothiocyanate	0.172385747
gene-ZDHHC14	1.345554777	(9Z)-Octadecenoic acid	0.142359556
gene-ZDHHC14	1.345554777	Nigroxanthin	0.705005059
gene-ZDHHC14	1.345554777	CL(8:0/8:0/18:2(9Z,11Z)/20:0)	0.622988418
Bos_taurus_newG	1.344992942	D-Erythro-imidazole-glycerol-phosphate	0.355812431
Bos_taurus_newG	1.344992942	13(S)-HpODE	0.163157753
Bos_taurus_newG	1.344992942	7(S),17(S)-dihydroxy-8(E),10(Z),13(Z),15(E),19	0.32147403
Bos_taurus_newG	1.344992942	LTB4-d4	0.226799982
Bos_taurus_newG	1.344992942	(-)-alpha-Terpineol	0.345716279
Bos_taurus_newG	1.344992942	Cyclosporin A	0.656529229
Bos_taurus_newG	1.344992942	2-Methyl-3-phenyl-2-propenal	0.407813975
Bos_taurus_newG	1.344992942	Sophoranol	0.282064826
Bos_taurus_newG	1.344992942	PC(P-18:1(11Z)/PGE2)	0.509503133
Bos_taurus_newG	1.344992942	PC(20:3(5Z,8Z,11Z)/24:0)	0.387959564
Bos_taurus_newG	1.344992942	PC(P-18:1(11Z)/PGE1)	0.295520345
Bos_taurus_newG	1.344992942	(9Z)-Octadecenoic acid	0.142359556
Bos_taurus_newG	1.344992942	Nigroxanthin	0.705005059
gene-NLRX1	1.344776653	3-Thiacytidine	0.209387412
gene-NLRX1	1.344776653	D-Erythro-imidazole-glycerol-phosphate	0.355812431
gene-NLRX1	1.344776653	LTB4-d4	0.226799982
gene-NLRX1	1.344776653	cis-p-Menth-2-en-1-ol	0.201017988
gene-NLRX1	1.344776653	(-)-alpha-Terpineol	0.345716279
gene-NLRX1	1.344776653	PG(20:1(11Z)/18:3(10,12,15)-OH(9))	0.626244347
gene-NLRX1	1.344776653	4-Dimethylamino-L-phenylalanine	0.242110226
gene-NLRX1	1.344776653	Armillane	0.52808635
gene-NLRX1	1.344776653	2-Methyl-3-phenyl-2-propenal	0.407813975
gene-NLRX1	1.344776653	PC(P-18:1(11Z)/PGE2)	0.509503133
gene-NLRX1	1.344776653	(3R,4R)-3-Amino-1-hydroxy-4-methylpyrrol	0.471914506
gene-NLRX1	1.344776653	Isopropyl isothiocyanate	0.172385747
gene-NLRX1	1.344776653	PC(P-18:1(11Z)/PGJ2)	0.565243877
gene-NLRX1	1.344776653	PS(20:0/20:4(8Z,11Z,14Z,17Z)-2OH(5S,6R))	0.402595921

gene-NLRX1	1.344776653	CL(8:0/8:0/18:2(9Z,11Z)/20:0)	0.622988418
gene-RAPGEF3	1.344529161	3-Thiacytidine	0.209387412
gene-RAPGEF3	1.344529161	1-Oleoyl-sn-glycero-3-phosphocholine	0.18926952
gene-RAPGEF3	1.344529161	Psychosine	0.106475396
gene-RAPGEF3	1.344529161	LTB4-d4	0.226799982
gene-RAPGEF3	1.344529161	(-)-alpha-Terpineol	0.345716279
gene-RAPGEF3	1.344529161	PC(16:0/18:1(12Z)-2OH(9,10))	0.461843233
gene-RAPGEF3	1.344529161	5-(2-Aminopropyl)-2-methylphenol	0.323231457
gene-RAPGEF3	1.344529161	4-Dimethylamino-L-phenylalanine	0.242110226
gene-RAPGEF3	1.344529161	Trimetazidine	0.438282534
gene-RAPGEF3	1.344529161	ingenol	0.942071652
gene-RAPGEF3	1.344529161	Armillane	0.52808635
gene-RAPGEF3	1.344529161	5'-S-Methyl-5'-thioinosine	0.28772476
gene-RAPGEF3	1.344529161	PC(P-18:1(11Z)/PGE2)	0.509503133
gene-RAPGEF3	1.344529161	(3R,4R)-3-Amino-1-hydroxy-4-methylpyrrol	0.471914506
gene-RAPGEF3	1.344529161	n-methyl-2-(4'-methylaminophenyl)-6-hydr	0.26655714
gene-RAPGEF3	1.344529161	PE(P-16:0/18:4(6Z,9Z,12Z,15Z))	0.546989864
gene-RAPGEF3	1.344529161	Isopropyl isothiocyanate	0.172385747
gene-RAPGEF3	1.344529161	(9Z)-Octadecenoic acid	0.142359556
gene-RAPGEF3	1.344529161	arachidyl amido cholanoic acid	1.24842952
gene-RAPGEF3	1.344529161	9-deoxy-9-methylene-16,16-dimethyl -PGE	0.606893884
gene-RAPGEF3	1.344529161	1-Octadecanoyl-2-(7Z,10Z,13Z,16Z-docosat	0.690496314
gene-RAPGEF3	1.344529161	2-Propenyl 2-aminobenzoate	0.128406829
gene-RAPGEF3	1.344529161	CL(8:0/8:0/18:2(9Z,11Z)/20:0)	0.622988418
gene-CCDC30	1.343351783	(-)-alpha-Terpineol	0.345716279
gene-CCDC30	1.343351783	1,4-Undecadiene	0.462803973
gene-CCDC30	1.343351783	4-cholesten-7伪,12伪,24-triol-3-one	0.097840451
gene-CCDC30	1.343351783	Cyclosporin A	0.656529229
gene-CCDC30	1.343351783	ingenol	0.942071652
gene-CCDC30	1.343351783	N-[[3-Hydroxy-2-(2-pentenyl)cyclopentyl]ac	1.043930947
gene-CCDC30	1.343351783	beta-L-Dioxolane-cytidine	0.175916668
gene-CCDC30	1.343351783	PC(20:3(5Z,8Z,11Z)/24:0)	0.387959564
gene-CCDC30	1.343351783	D-Fructose	0.167584616
gene-CCDC30	1.343351783	CL(8:0/8:0/18:2(9Z,11Z)/20:0)	0.622988418
Bos_taurus_newG	1.339648773	3-Thiacytidine	0.209387412
Bos_taurus_newG	1.339648773	D-Erythro-imidazole-glycerol-phosphate	0.355812431
Bos_taurus_newG	1.339648773	11-Maleimidoundecanoic acid	1.084942397
Bos_taurus_newG	1.339648773	cis-p-Menth-2-en-1-ol	0.201017988
Bos_taurus_newG	1.339648773	PE(20:0/18:1(12Z)-2OH(9,10))	0.438658253
Bos_taurus_newG	1.339648773	PE(22:2(13Z,16Z)/22:5(4Z,7Z,10Z,13Z,19Z)-O	0.369745166
Bos_taurus_newG	1.339648773	Norophthalmic acid	0.191432411
Bos_taurus_newG	1.339648773	Guanidoacetic acid	0.542509265
Bos_taurus_newG	1.339648773	alpha-Terpineol formate	0.628238986
Bos_taurus_newG	1.339648773	PC(P-18:1(11Z)/PGE2)	0.509503133
Bos_taurus_newG	1.339648773	Roxithromycin	0.268273077
Bos_taurus_newG	1.339648773	Nigroxanthin	0.705005059
Bos_taurus_newG	1.339648773	PC(P-18:1(11Z)/PGJ2)	0.565243877
Bos_taurus_newG	1.339648773	PS(20:0/20:4(8Z,11Z,14Z,17Z)-2OH(5S,6R))	0.402595921
gene-WDR35	1.339449343	D-Erythro-imidazole-glycerol-phosphate	0.355812431
gene-WDR35	1.339449343	PC(P-18:1(11Z)/PGE2)	0.509503133
gene-WDR35	1.339449343	CL(8:0/8:0/18:2(9Z,11Z)/20:0)	0.622988418

gene-DNAH7	1.339377584	Norophthalmic acid	0.191432411
gene-DNAH7	1.339377584	Ethylene brassylate	0.989156973
gene-DNAH7	1.339377584	Imidazoline	0.232348181
gene-DNAH7	1.339377584	beta-L-Dioxolane-cytidine	0.175916668
Bos_taurus_newG	1.337857463	Glutamate carbon	0.671444516
Bos_taurus_newG	1.337857463	11-Maleimidoundecanoic acid	1.084942397
Bos_taurus_newG	1.337857463	PE(22:2(13Z,16Z)/22:5(4Z,7Z,10Z,13Z,19Z)-O	0.369745166
Bos_taurus_newG	1.337857463	Adrenosterone	0.050790129
Bos_taurus_newG	1.337857463	Norophthalmic acid	0.191432411
Bos_taurus_newG	1.337857463	beta-Thujaplicin	0.681866194
Bos_taurus_newG	1.337857463	Imidazoline	0.232348181
Bos_taurus_newG	1.337857463	beta-L-Dioxolane-cytidine	0.175916668
Bos_taurus_newG	1.337857463	Roxithromycin	0.268273077
Bos_taurus_newG	1.337857463	PC(P-18:1(11Z)/PGJ2)	0.565243877
Bos_taurus_newG	1.337857463	PS(20:0/20:4(8Z,11Z,14Z,17Z)-2OH(5S,6R))	0.402595921
gene-PLD3	1.336627591	7(14)-Bisabolene-2,3,10,11-tetrol	2.446897974
gene-PLD3	1.336627591	3-Deoxyestrone	0.282221709
gene-PLD3	1.336627591	1-Oleoyl-sn-glycero-3-phosphocholine	0.18926952
gene-PLD3	1.336627591	(-)-alpha-Terpineol	0.345716279
gene-PLD3	1.336627591	1,4-Undecadiene	0.462803973
gene-PLD3	1.336627591	4-cholesten-7伪,12伪,24-triol-3-one	0.097840451
gene-PLD3	1.336627591	4-Dimethylamino-L-phenylalanine	0.242110226
gene-PLD3	1.336627591	ingenol	0.942071652
gene-PLD3	1.336627591	Armillane	0.52808635
gene-PLD3	1.336627591	PC(P-18:1(11Z)/PGE2)	0.509503133
gene-PLD3	1.336627591	1-Palmitoylglycerol	0.084357408
gene-PLD3	1.336627591	n-methyl-2-(4'-methylaminophenyl)-6-hydr	0.26655714
gene-PLD3	1.336627591	Isopropyl isothiocyanate	0.172385747
gene-PLD3	1.336627591	PS(20:0/20:4(8Z,11Z,14Z,17Z)-2OH(5S,6R))	0.402595921
gene-PLD3	1.336627591	CL(8:0/8:0/18:2(9Z,11Z)/20:0)	0.622988418
gene-DKKL1	1.334673929	9'-Carboxy-gamma-chromanol	0.068924993
gene-DKKL1	1.334673929	Lucyoside N	0.831231157
gene-DKKL1	1.334673929	Tsugarioside B	1.013262893
gene-DKKL1	1.334673929	7(14)-Bisabolene-2,3,10,11-tetrol	2.446897974
gene-DKKL1	1.334673929	LTB4-d4	0.226799982
gene-DKKL1	1.334673929	cis-p-Menth-2-en-1-ol	0.201017988
gene-DKKL1	1.334673929	2-isopentyl-3,6-dimethyl pyrazine	0.710502562
gene-DKKL1	1.334673929	PC(P-18:0/20:5(5Z,8Z,11Z,14Z,16E)-OH(18))	0.097499392
gene-DKKL1	1.334673929	4-Dimethylamino-L-phenylalanine	0.242110226
gene-DKKL1	1.334673929	5'-S-Methyl-5'-thioinosine	0.28772476
gene-DKKL1	1.334673929	2-Methyl-3-phenyl-2-propenal	0.407813975
gene-DKKL1	1.334673929	(3R,4R)-3-Amino-1-hydroxy-4-methylpyrrol	0.471914506
gene-DKKL1	1.334673929	Isopropyl isothiocyanate	0.172385747
gene-DKKL1	1.334673929	Capryloylcholine	0.209692465
gene-DKKL1	1.334673929	N-Myristoyl Glutamine	0.37856966
gene-DKKL1	1.334673929	2-Propenyl 2-aminobenzoate	0.128406829
gene-LOC101906	1.330275398	D-Erythro-imidazole-glycerol-phosphate	0.355812431
gene-LOC101906	1.330275398	Hexahydro-4-methylphthalic anhydride	0.691665693
gene-LOC101906	1.330275398	Glutamate carbon	0.671444516
gene-LOC101906	1.330275398	1-(2-Furanyl)-1-pentanone	0.697147283
gene-LOC101906	1.330275398	2-Dehydro-3-deoxy-D-gluconate	0.65165299

gene-LOC10190€	1.330275398	16-Hydroxy-10-oxohexadecanoic acid	0.72302152
gene-LOC10190€	1.330275398	(1R,6S)-6-Amino-5-oxocyclohex-2-ene-1-c	0.154751123
gene-LOC10190€	1.330275398	2-Hydroxyglutaric acid diethyl ester	0.799886767
gene-LOC10190€	1.330275398	PC(P-18:1(11Z)/PGE2)	0.509503133
gene-LOC10190€	1.330275398	Roxithromycin	0.268273077
gene-LOC10190€	1.330275398	PS(20:0/20:4(8Z,11Z,14Z,17Z)-2OH(5S,6R))	0.402595921
gene-LOC100337	1.330208144	D-Erythro-imidazole-glycerol-phosphate	0.355812431
gene-LOC100337	1.330208144	11-Maleimidoundecanoic acid	1.084942397
gene-LOC100337	1.330208144	Monacolin L acid	1.060562098
gene-LOC100337	1.330208144	cis-p-Menth-2-en-1-ol	0.201017988
gene-LOC100337	1.330208144	PC(24:0/22:6(4Z,7Z,10Z,12E,16Z,19Z)-OH(14)	0.188241061
gene-LOC100337	1.330208144	PE(22:2(13Z,16Z)/22:5(4Z,7Z,10Z,13Z,19Z)-O	0.369745166
gene-LOC100337	1.330208144	Norophthalmic acid	0.191432411
gene-LOC100337	1.330208144	Tryptophyl-Glutamine	0.89722186
gene-LOC100337	1.330208144	Guanidoacetic acid	0.542509265
gene-LOC100337	1.330208144	alpha-Terpineol formate	0.628238986
gene-LOC100337	1.330208144	PC(P-18:1(11Z)/PGE2)	0.509503133
gene-LOC100337	1.330208144	Roxithromycin	0.268273077
gene-LOC100337	1.330208144	PC(P-18:1(11Z)/PGJ2)	0.565243877
gene-LOC100337	1.330208144	PS(20:0/20:4(8Z,11Z,14Z,17Z)-2OH(5S,6R))	0.402595921
gene-LOC100337	1.330208144	PE(20:5(5Z,8Z,11Z,14Z,17Z)/18:0)	0.427962506
gene-NOS1AP	1.326052357	D-Arabinono-1,4-lactone	0.1035711
gene-NOS1AP	1.326052357	Methylmalonate	0.241481249
gene-NOS1AP	1.326052357	3-Thiacytidine	0.209387412
gene-NOS1AP	1.326052357	1-Phenylpiperazine	0.353584896
gene-NOS1AP	1.326052357	2-[(3S)-3-[[(2S)-1-(Carboxymethoxy)-1-oxo-	0.111577437
gene-NOS1AP	1.326052357	Azelaic acid	0.007180175
gene-NOS1AP	1.326052357	4-Nitrophenol	0.147405743
gene-NOS1AP	1.326052357	Heptylmalonic acid	0.061016088
gene-NOS1AP	1.326052357	DG(19:0/PGJ2/0:0)	0.415213526
gene-NOS1AP	1.326052357	PE(20:0/18:1(12Z)-2OH(9,10))	0.438658253
gene-NOS1AP	1.326052357	5-Hexyltetrahydro-2-furanoctanoic acid	0.343510978
gene-NOS1AP	1.326052357	DG(15:0/PGE1/0:0)	0.462019057
gene-NOS1AP	1.326052357	PC(16:0/18:1(12Z)-2OH(9,10))	0.461843233
gene-NOS1AP	1.326052357	Trifluoroacetamide	0.107717461
gene-NOS1AP	1.326052357	5-(2-Aminopropyl)-2-methylphenol	0.323231457
gene-NOS1AP	1.326052357	4-Guanidinobutanoate	0.445426102
gene-NOS1AP	1.326052357	Armillane	0.52808635
gene-NOS1AP	1.326052357	5'-S-Methyl-5'-thioinosine	0.28772476
gene-NOS1AP	1.326052357	4-Oxo-9-cis-retinoyl-beta-glucuronide	1.611773742
gene-NOS1AP	1.326052357	L-Histidinol	0.149249286
gene-NOS1AP	1.326052357	(4-Methylphenyl)acetaldehyde	0.208360133
gene-NOS1AP	1.326052357	Spergualin	0.210690914
gene-NOS1AP	1.326052357	10-alpha-methoxy-9,10-dihydrolysergol	0.140851438
gene-NOS1AP	1.326052357	(3R,4R)-3-Amino-1-hydroxy-4-methylpyrrol	0.471914506
gene-NOS1AP	1.326052357	Butirosina	0.336730912
gene-NOS1AP	1.326052357	Hypaconitine	0.176000431
gene-NOS1AP	1.326052357	Dehydrodidemnin B	0.171013159
gene-NOS1AP	1.326052357	Gamithromycin	0.37168241
gene-NOS1AP	1.326052357	PE(P-16:0/18:4(6Z,9Z,12Z,15Z))	0.546989864
gene-NOS1AP	1.326052357	Isopropyl isothiocyanate	0.172385747

gene-NOS1AP	1.326052357	PS(O-20:0/14:1(9Z))	0.263612845
gene-NOS1AP	1.326052357	CDP-DG(a-25:0/PGF1alpha)	0.345292815
gene-NOS1AP	1.326052357	Avermectin B1a aglycone	0.60789276
gene-NOS1AP	1.326052357	3'-N-Acetyl-4'-O-(14-methylpentadecanoyl]	0.146786959
gene-NOS1AP	1.326052357	N-Myristoyl Glutamine	0.37856966
gene-NOS1AP	1.326052357	PC(18:1(9Z)/15:1(9Z))	0.534724682
gene-NOS1AP	1.326052357	arachidyl amido cholanoic acid	1.24842952
gene-NOS1AP	1.326052357	1-Octadecanoyl-2-(7Z,10Z,13Z,16Z-docosat	0.690496314
gene-NOS1AP	1.326052357	PC(16:0/20:5(5E,8E,11E,14E,17E))[U]	0.208077202
gene-NOS1AP	1.326052357	CE(LTE4)	0.204616463
gene-NOS1AP	1.326052357	PS(16:1(9Z)/22:2(13Z,16Z))	0.659652661
gene-DCBLD2	1.321759218	5-(3'-Carboxy-3'-oxopropenyl)-4,6-dihydro:	1.083232395
gene-DCBLD2	1.321759218	Sorbitan laurate	0.242315968
gene-DCBLD2	1.321759218	4-Gingerol	0.222165012
gene-DCBLD2	1.321759218	Docosanamide	0.509975786
gene-DCBLD2	1.321759218	Monacolin L acid	1.060562098
gene-DCBLD2	1.321759218	cis-p-Menth-2-en-1-ol	0.201017988
gene-DCBLD2	1.321759218	L-Carnitine	0.2737116
gene-DCBLD2	1.321759218	3,4,3',4'-Tetrahydrospirilloxanthin	0.354666148
gene-DCBLD2	1.321759218	Norophthalmic acid	0.191432411
gene-DCBLD2	1.321759218	7C-aglycone	1.015608521
gene-DCBLD2	1.321759218	Terbutryn	1.186241428
gene-DCBLD2	1.321759218	(-)-Huperzine A (HupA)	0.935198906
gene-DCBLD2	1.321759218	4-Oxo-9-cis-retinoyl-beta-glucuronide	1.611773742
gene-DCBLD2	1.321759218	alpha-Terpineol formate	0.628238986
gene-DCBLD2	1.321759218	(1S)-3-[2-[(1R,7Ar)-7a-methyl-1-[(2R)-6-me	1.140894639
gene-DCBLD2	1.321759218	4-Octylphenol	1.400657885
gene-DCBLD2	1.321759218	Prolyl-Lysine	1.612991304
gene-DCBLD2	1.321759218	3-Pentadecylphenol	1.682258188
gene-DCBLD2	1.321759218	(Z)-3-Methyl-3-decenoic acid	0.482455266
gene-DCBLD2	1.321759218	Palmitoylcarnitine	0.74779616
gene-DCBLD2	1.321759218	12-Hydroxyicosanoylcarnitine	1.730979067
gene-DCBLD2	1.321759218	Ascorbic acid 6-palmitate	0.201627014
gene-DCBLD2	1.321759218	PC(P-18:1(11Z)/PGJ2)	0.565243877
gene-DCBLD2	1.321759218	PE(20:5(5Z,8Z,11Z,14Z,17Z)/18:0)	0.427962506
gene-PODXL2	1.316397235	LTB4-d4	0.226799982
gene-PODXL2	1.316397235	(-)-alpha-Terpineol	0.345716279
gene-PODXL2	1.316397235	Cyclosporin A	0.656529229
gene-PODXL2	1.316397235	5-(2-Aminopropyl)-2-methylphenol	0.323231457
gene-PODXL2	1.316397235	4-Dimethylamino-L-phenylalanine	0.242110226
gene-PODXL2	1.316397235	Trimetazidine	0.438282534
gene-PODXL2	1.316397235	Armillane	0.52808635
gene-PODXL2	1.316397235	PC(P-18:1(11Z)/PGE2)	0.509503133
gene-PODXL2	1.316397235	(3R,4R)-3-Amino-1-hydroxy-4-methylpyrrol	0.471914506
gene-PODXL2	1.316397235	n-methyl-2-(4'-methylaminophenyl)-6-hydr	0.26655714
gene-PODXL2	1.316397235	Isopropyl isothiocyanate	0.172385747
gene-PODXL2	1.316397235	9-deoxy-9-methylene-16,16-dimethyl -PGE:	0.606893884
gene-PODXL2	1.316397235	CL(8:0/8:0/18:2(9Z,11Z)/20:0)	0.622988418
gene-DLGAP3	1.313677813	milbemycin beta3	1.414447124
gene-DLGAP3	1.313677813	Palmitoyl Ara-C	0.586890476
gene-DLGAP3	1.313677813	Tsugarioside B	1.013262893

gene-DLGAP3	1.313677813	cis-p-Menth-2-en-1-ol	0.201017988
gene-DLGAP3	1.313677813	2-isopentyl-3,6-dimethyl pyrazine	0.710502562
gene-DLGAP3	1.313677813	PG(20:1(11Z)/18:3(10,12,15)-OH(9))	0.626244347
gene-DLGAP3	1.313677813	4-Guanidinobutanoate	0.445426102
gene-DLGAP3	1.313677813	Cornoside	0.781689516
gene-DLGAP3	1.313677813	Armillane	0.52808635
gene-DLGAP3	1.313677813	PC(P-18:1(11Z)/PGE2)	0.509503133
gene-DLGAP3	1.313677813	(3R,4R)-3-Amino-1-hydroxy-4-methylpyrrol	0.471914506
gene-DLGAP3	1.313677813	11-Deoxycortisol	0.595462818
gene-DLGAP3	1.313677813	(9Z)-Octadecenoic acid	0.142359556
gene-DLGAP3	1.313677813	Sitosterol beta-D-glucoside	0.382944796
gene-DLGAP3	1.313677813	PG(20:1(11Z)/18:3(9,11,15)-OH(13))	0.69829683
gene-AHNAK2	1.312836046	milbemycin beta3	1.414447124
gene-AHNAK2	1.312836046	PE(20:0/18:1(12Z)-2OH(9,10))	0.438658253
gene-AHNAK2	1.312836046	5-(2-Aminopropyl)-2-methylphenol	0.323231457
gene-AHNAK2	1.312836046	Armillane	0.52808635
gene-AHNAK2	1.312836046	Pregnanediol	0.166857843
gene-AHNAK2	1.312836046	Val-Tyr-Leu-Arg	0.040793217
gene-AHNAK2	1.312836046	PS(20:0/20:4(8Z,11Z,14Z,17Z)-2OH(5S,6R))	0.402595921
gene-ZSCAN30	1.312460891	D-Erythro-imidazole-glycerol-phosphate	0.355812431
gene-ZSCAN30	1.312460891	9'-Carboxy-gamma-chromanol	0.068924993
gene-ZSCAN30	1.312460891	Tsugarioside B	1.013262893
gene-ZSCAN30	1.312460891	7(14)-Bisabolene-2,3,10,11-tetrol	2.446897974
gene-ZSCAN30	1.312460891	LTB4-d4	0.226799982
gene-ZSCAN30	1.312460891	cis-p-Menth-2-en-1-ol	0.201017988
gene-ZSCAN30	1.312460891	(-)-alpha-Terpineol	0.345716279
gene-ZSCAN30	1.312460891	2-isopentyl-3,6-dimethyl pyrazine	0.710502562
gene-ZSCAN30	1.312460891	DG(15:0/PGE1/0:0)	0.462019057
gene-ZSCAN30	1.312460891	PG(20:1(11Z)/18:3(10,12,15)-OH(9))	0.626244347
gene-ZSCAN30	1.312460891	4-Dimethylamino-L-phenylalanine	0.242110226
gene-ZSCAN30	1.312460891	Trimetazidine	0.438282534
gene-ZSCAN30	1.312460891	5'-S-Methyl-5'-thioinosine	0.28772476
gene-ZSCAN30	1.312460891	(Z)-3-Oxo-2-(2-pentenyl)-1-cyclopenteneac	0.179805915
gene-ZSCAN30	1.312460891	2-Methyl-3-phenyl-2-propenal	0.407813975
gene-ZSCAN30	1.312460891	PC(P-18:1(11Z)/PGE2)	0.509503133
gene-ZSCAN30	1.312460891	(3R,4R)-3-Amino-1-hydroxy-4-methylpyrrol	0.471914506
gene-ZSCAN30	1.312460891	MG(0:0/18:1(9Z)/0:0)	0.361035605
gene-ZSCAN30	1.312460891	1,2,4-Nonadecanetriol	0.249655005
gene-ZSCAN30	1.312460891	(9Z)-Octadecenoic acid	0.142359556
gene-ZSCAN30	1.312460891	N-Myristoyl Glutamine	0.37856966
gene-ZSCAN30	1.312460891	2-Propenyl 2-aminobenzoate	0.128406829
gene-TP53I13	1.309886859	3-Thiacytidine	0.209387412
gene-TP53I13	1.309886859	D-Erythro-imidazole-glycerol-phosphate	0.355812431
gene-TP53I13	1.309886859	cis-p-Menth-2-en-1-ol	0.201017988
gene-TP53I13	1.309886859	2-Methyl-3-phenyl-2-propenal	0.407813975
gene-TP53I13	1.309886859	PC(P-18:1(11Z)/PGE2)	0.509503133
gene-TP53I13	1.309886859	Roxithromycin	0.268273077
gene-TP53I13	1.309886859	PC(P-18:1(11Z)/PGJ2)	0.565243877
gene-TP53I13	1.309886859	PS(20:0/20:4(8Z,11Z,14Z,17Z)-2OH(5S,6R))	0.402595921
gene-WDR86	1.308996642	Methylmalonate	0.241481249
gene-WDR86	1.308996642	all-trans-Hexaprenyl diphosphate	0.277689133

gene-WDR86	1.308996642	1-Phenylpiperazine	0.353584896
gene-WDR86	1.308996642	1-Deoxy-D-glucitol	0.262280165
gene-WDR86	1.308996642	5-Acetamidopentanoate	0.163699446
gene-WDR86	1.308996642	4-Nitrophenol	0.147405743
gene-WDR86	1.308996642	Heptylmalonic acid	0.061016088
gene-WDR86	1.308996642	3-alpha-Androstanediol glucuronide	0.088637537
gene-WDR86	1.308996642	Tsugarioside B	1.013262893
gene-WDR86	1.308996642	Tetracosenoyl-CoA	0.187187233
gene-WDR86	1.308996642	Indinavir	0.008465584
gene-WDR86	1.308996642	Artemidinol	0.093675653
gene-WDR86	1.308996642	PC(P-18:0/20:5(5Z,8Z,11Z,14Z,16E)-OH(18))	0.097499392
gene-WDR86	1.308996642	PC(14:1(9Z)/P-18:1(11Z))	0.153578899
gene-WDR86	1.308996642	PC(16:0/18:1(12Z)-2OH(9,10))	0.461843233
gene-WDR86	1.308996642	Trifluoroacetamide	0.107717461
gene-WDR86	1.308996642	5-(2-Aminopropyl)-2-methylphenol	0.323231457
gene-WDR86	1.308996642	4-Guanidinobutanoate	0.445426102
gene-WDR86	1.308996642	Trimetazidine	0.438282534
gene-WDR86	1.308996642	Armillane	0.52808635
gene-WDR86	1.308996642	5'-S-Methyl-5'-thioinosine	0.28772476
gene-WDR86	1.308996642	Butanoic acid, [(diethoxyphosphinyl)oxy]meth	0.038991848
gene-WDR86	1.308996642	(3R,4R)-3-Amino-1-hydroxy-4-methylpyrrol	0.471914506
gene-WDR86	1.308996642	Butirosina	0.336730912
gene-WDR86	1.308996642	Gamithromycin	0.37168241
gene-WDR86	1.308996642	PE(P-16:0/18:4(6Z,9Z,12Z,15Z))	0.546989864
gene-WDR86	1.308996642	Isopropyl isothiocyanate	0.172385747
gene-WDR86	1.308996642	PS(O-20:0/14:1(9Z))	0.263612845
gene-WDR86	1.308996642	3'-N-Acetyl-4'-O-(14-methylpentadecanoyl)	0.146786959
gene-WDR86	1.308996642	9-deoxy-9-methylene-16,16-dimethyl -PGE	0.606893884
gene-WDR86	1.308996642	1-Octadecanoyl-2-(7Z,10Z,13Z,16Z-docosat	0.690496314
gene-WDR86	1.308996642	CE(LTE4)	0.204616463
gene-TTYH1	1.307630986	cis-p-Menth-2-en-1-ol	0.201017988
gene-TTYH1	1.307630986	PE(22:2(13Z,16Z)/22:5(4Z,7Z,10Z,13Z,19Z)-O	0.369745166
gene-TTYH1	1.307630986	PG(20:1(11Z)/18:3(10,12,15)-OH(9))	0.626244347
gene-TTYH1	1.307630986	PC(P-18:1(11Z)/PGE2)	0.509503133
gene-TTYH1	1.307630986	Sitosterol beta-D-glucoside	0.382944796
gene-TTYH1	1.307630986	PC(P-18:1(11Z)/PGJ2)	0.565243877
gene-TTYH1	1.307630986	PS(20:0/20:4(8Z,11Z,14Z,17Z)-2OH(5S,6R))	0.402595921
gene-LOC112445	1.303417602	3-Thiacytidine	0.209387412
gene-LOC112445	1.303417602	Cyclosporin A	0.656529229
gene-LOC112445	1.303417602	DG(18:0/LTE4/0:0)	0.681485773
gene-LOC112445	1.303417602	PC(14:0/20:2(11Z,14Z))	1.110657378
gene-LOC112445	1.303417602	beta-L-Dioxolane-cytidine	0.175916668
gene-LOC112445	1.303417602	Isopropyl isothiocyanate	0.172385747
gene-LOC112445	1.303417602	D-Fructose	0.167584616
gene-LOC112445	1.303417602	CL(8:0/8:0/18:2(9Z,11Z)/20:0)	0.622988418
gene-LOC788634	1.300796694	3-Thiacytidine	0.209387412
gene-LOC788634	1.300796694	4-cholesten-7伪,12伪,24-triol-3-one	0.097840451
gene-LOC788634	1.300796694	Isopropyl isothiocyanate	0.172385747
gene-LOC788634	1.300796694	PC(18:1(9Z)/15:1(9Z))	0.534724682
gene-LOC788634	1.300796694	PC(P-18:1(11Z)/PGJ2)	0.565243877
gene-LOC788634	1.300796694	PS(20:0/20:4(8Z,11Z,14Z,17Z)-2OH(5S,6R))	0.402595921

gene-LOC78863‹	1.300796694	D-Fructose	0.167584616
gene-KIAA1522	1.298827214	3-Thiacytidine	0.209387412
gene-KIAA1522	1.298827214	Cyclosporin A	0.656529229
gene-KIAA1522	1.298827214	DG(18:0/LTE4/0:0)	0.681485773
gene-KIAA1522	1.298827214	PC(14:0/20:2(11Z,14Z))	1.110657378
gene-KIAA1522	1.298827214	CDP-DG(PGF2alpha/16:0)	1.002512277
gene-KIAA1522	1.298827214	5-(2-Aminopropyl)-2-methylphenol	0.323231457
gene-KIAA1522	1.298827214	ingenol	0.942071652
gene-KIAA1522	1.298827214	Armillane	0.52808635
gene-KIAA1522	1.298827214	beta-L-Dioxolane-cytidine	0.175916668
gene-KIAA1522	1.298827214	17-Aminogeldanamycin	0.518144528
gene-KIAA1522	1.298827214	PC(P-18:1(11Z)/PGE2)	0.509503133
gene-KIAA1522	1.298827214	Isopropyl isothiocyanate	0.172385747
gene-KIAA1522	1.298827214	arachidyl amido cholanoic acid	1.24842952
gene-KIAA1522	1.298827214	Nigroxanthin	0.705005059
gene-KIAA1522	1.298827214	CL(8:0/8:0/18:2(9Z,11Z)/20:0)	0.622988418
gene-HK3	1.297698801	3-Thiacytidine	0.209387412
gene-HK3	1.297698801	D-Erythro-imidazole-glycerol-phosphate	0.355812431
gene-HK3	1.297698801	LTB4-d4	0.226799982
gene-HK3	1.297698801	cis-p-Menth-2-en-1-ol	0.201017988
gene-HK3	1.297698801	(-)-alpha-Terpineol	0.345716279
gene-HK3	1.297698801	Cyclosporin A	0.656529229
gene-HK3	1.297698801	5-(2-Aminopropyl)-2-methylphenol	0.323231457
gene-HK3	1.297698801	4-Dimethylamino-L-phenylalanine	0.242110226
gene-HK3	1.297698801	Armillane	0.52808635
gene-HK3	1.297698801	PC(P-18:1(11Z)/PGE2)	0.509503133
gene-HK3	1.297698801	(3R,4R)-3-Amino-1-hydroxy-4-methylpyrrol	0.471914506
gene-HK3	1.297698801	Isopropyl isothiocyanate	0.172385747
gene-HK3	1.297698801	Nigroxanthin	0.705005059
gene-HK3	1.297698801	PC(P-18:1(11Z)/PGJ2)	0.565243877
gene-HK3	1.297698801	PS(20:0/20:4(8Z,11Z,14Z,17Z)-2OH(5S,6R))	0.402595921
gene-HK3	1.297698801	CL(8:0/8:0/18:2(9Z,11Z)/20:0)	0.622988418
Bos_taurus_newG	1.295692624	3-Thiacytidine	0.209387412
Bos_taurus_newG	1.295692624	Hydroxyprolyl-Serine	0.519540341
Bos_taurus_newG	1.295692624	PE(22:2(13Z,16Z)/22:5(4Z,7Z,10Z,13Z,19Z)-O	0.369745166
Bos_taurus_newG	1.295692624	Angiotensin A	0.247332017
Bos_taurus_newG	1.295692624	ingenol	0.942071652
Bos_taurus_newG	1.295692624	PS(20:0/20:4(8Z,11Z,14Z,17Z)-2OH(5S,6R))	0.402595921
gene-TNFRSF18	1.294473273	3-Thiacytidine	0.209387412
gene-TNFRSF18	1.294473273	D-Erythro-imidazole-glycerol-phosphate	0.355812431
gene-TNFRSF18	1.294473273	13(S)-HpODE	0.163157753
gene-TNFRSF18	1.294473273	LTB4-d4	0.226799982
gene-TNFRSF18	1.294473273	cis-p-Menth-2-en-1-ol	0.201017988
gene-TNFRSF18	1.294473273	PE(20:0/18:1(12Z)-2OH(9,10))	0.438658253
gene-TNFRSF18	1.294473273	PE(22:2(13Z,16Z)/22:5(4Z,7Z,10Z,13Z,19Z)-O	0.369745166
gene-TNFRSF18	1.294473273	DG(15:0/PGE1/0:0)	0.462019057
gene-TNFRSF18	1.294473273	PG(20:1(11Z)/18:3(10,12,15)-OH(9))	0.626244347
gene-TNFRSF18	1.294473273	2-Methyl-3-phenyl-2-propenal	0.407813975
gene-TNFRSF18	1.294473273	PC(P-18:1(11Z)/PGE2)	0.509503133
gene-TNFRSF18	1.294473273	(3R,4R)-3-Amino-1-hydroxy-4-methylpyrrol	0.471914506
gene-TNFRSF18	1.294473273	(9Z)-Octadecenoic acid	0.142359556

gene-TNFRSF18	1.294473273	N-Myristoyl Glutamine	0.37856966
gene-TNFRSF18	1.294473273	PC(18:1(9Z)/15:1(9Z))	0.534724682
gene-TNFRSF18	1.294473273	arachidyl amido cholanoic acid	1.24842952
gene-TNFRSF18	1.294473273	Nigroxanthin	0.705005059
gene-TNFRSF18	1.294473273	PC(P-18:1(11Z)/PGJ2)	0.565243877
gene-TNFRSF18	1.294473273	PS(20:0/20:4(8Z,11Z,14Z,17Z)-2OH(5S,6R))	0.402595921
gene-BTN3A3	1.292809022	Glutamate carbon	0.671444516
gene-BTN3A3	1.292809022	11-Maleimidoundecanoic acid	1.084942397
gene-BTN3A3	1.292809022	PE(22:2(13Z,16Z)/22:5(4Z,7Z,10Z,13Z,19Z)-O	0.369745166
gene-BTN3A3	1.292809022	Cyclosporin A	0.656529229
gene-BTN3A3	1.292809022	Norophthalmic acid	0.191432411
gene-BTN3A3	1.292809022	alpha-Terpineol formate	0.628238986
gene-BTN3A3	1.292809022	beta-L-Dioxolane-cytidine	0.175916668
gene-BTN3A3	1.292809022	PC(P-18:1(11Z)/PGE2)	0.509503133
gene-BTN3A3	1.292809022	Roxithromycin	0.268273077
gene-BTN3A3	1.292809022	PC(P-18:1(11Z)/PGJ2)	0.565243877
gene-BTN3A3	1.292809022	PS(20:0/20:4(8Z,11Z,14Z,17Z)-2OH(5S,6R))	0.402595921
gene-MYADM	1.289966038	3-Thiacytidine	0.209387412
gene-MYADM	1.289966038	D-Erythro-imidazole-glycerol-phosphate	0.355812431
gene-MYADM	1.289966038	LTB4-d4	0.226799982
gene-MYADM	1.289966038	cis-p-Menth-2-en-1-ol	0.201017988
gene-MYADM	1.289966038	(-)-alpha-Terpineol	0.345716279
gene-MYADM	1.289966038	5-(2-Aminopropyl)-2-methylphenol	0.323231457
gene-MYADM	1.289966038	Armillane	0.52808635
gene-MYADM	1.289966038	PC(P-18:1(11Z)/PGE2)	0.509503133
gene-MYADM	1.289966038	(3R,4R)-3-Amino-1-hydroxy-4-methylpyrrol	0.471914506
gene-MYADM	1.289966038	Isopropyl isothiocyanate	0.172385747
gene-MYADM	1.289966038	PC(P-18:1(11Z)/PGJ2)	0.565243877
gene-MYADM	1.289966038	PS(20:0/20:4(8Z,11Z,14Z,17Z)-2OH(5S,6R))	0.402595921
gene-MYADM	1.289966038	CL(8:0/8:0/18:2(9Z,11Z)/20:0)	0.622988418
gene-EPHA2	1.288752541	alpha-Terpinyl pentanoate	0.098934683
gene-EPHA2	1.288752541	(15S)-15-Hydroxy-5,8,11-cis-13-trans-eicos	0.20506763
gene-EPHA2	1.288752541	PE(22:2(13Z,16Z)/22:5(4Z,7Z,10Z,13Z,19Z)-O	0.369745166
gene-EPHA2	1.288752541	5-Formiminotetrahydrofolate	0.118251287
gene-EPHA2	1.288752541	Benzoyl glucuronide (Benzoic acid)	0.442844673
gene-EPHA2	1.288752541	N-(3-oxo-octanoyl)-homoserine lactone	0.106580775
gene-EPHA2	1.288752541	Metaxalone	0.165579741
gene-EPHA2	1.288752541	Pregnanediol	0.166857843
gene-EPHA2	1.288752541	Phorone A	0.079039095
gene-NEURL2	1.286643235	1,2-O-Isopropylidene-D-glucofuranose	0.080987667
gene-NEURL2	1.286643235	1-Oleoyl-sn-glycero-3-phosphocholine	0.18926952
gene-NEURL2	1.286643235	PC(16:0/18:1(12Z)-2OH(9,10))	0.461843233
gene-NEURL2	1.286643235	5-(2-Aminopropyl)-2-methylphenol	0.323231457
gene-NEURL2	1.286643235	4-Dimethylamino-L-phenylalanine	0.242110226
gene-NEURL2	1.286643235	Trimetazidine	0.438282534
gene-NEURL2	1.286643235	Armillane	0.52808635
gene-NEURL2	1.286643235	5'-S-Methyl-5'-thioinosine	0.28772476
gene-NEURL2	1.286643235	PC(P-18:1(11Z)/PGE2)	0.509503133
gene-NEURL2	1.286643235	(3R,4R)-3-Amino-1-hydroxy-4-methylpyrrol	0.471914506
gene-NEURL2	1.286643235	n-methyl-2-(4'-methylaminophenyl)-6-hydr	0.26655714
gene-NEURL2	1.286643235	PE(P-16:0/18:4(6Z,9Z,12Z,15Z))	0.546989864

gene-NEURL2	1.286643235	Isopropyl isothiocyanate	0.172385747
gene-NEURL2	1.286643235	9-deoxy-9-methylene-16,16-dimethyl -PGE:	0.606893884
gene-NEURL2	1.286643235	1-Octadecanoyl-2-(7Z,10Z,13Z,16Z-docosat	0.690496314
gene-NEURL2	1.286643235	CL(8:0/8:0/18:2(9Z,11Z)/20:0)	0.622988418
Bos_taurus_newG	1.285716362	3-Thiacytidine	0.209387412
Bos_taurus_newG	1.285716362	D-Erythro-imidazole-glycerol-phosphate	0.355812431
Bos_taurus_newG	1.285716362	13(S)-HpODE	0.163157753
Bos_taurus_newG	1.285716362	LTB4-d4	0.226799982
Bos_taurus_newG	1.285716362	cis-p-Menth-2-en-1-ol	0.201017988
Bos_taurus_newG	1.285716362	(-)-alpha-Terpineol	0.345716279
Bos_taurus_newG	1.285716362	PG(20:1(11Z)/18:3(10,12,15)-OH(9))	0.626244347
Bos_taurus_newG	1.285716362	2-Methyl-3-phenyl-2-propenal	0.407813975
Bos_taurus_newG	1.285716362	PC(P-18:1(11Z)/PGE2)	0.509503133
Bos_taurus_newG	1.285716362	(3R,4R)-3-Amino-1-hydroxy-4-methylpyrrol	0.471914506
Bos_taurus_newG	1.285716362	(9Z)-Octadecenoic acid	0.142359556
Bos_taurus_newG	1.285716362	N-Myristoyl Glutamine	0.37856966
Bos_taurus_newG	1.285716362	PC(P-18:1(11Z)/PGJ2)	0.565243877
Bos_taurus_newG	1.285716362	PS(20:0/20:4(8Z,11Z,14Z,17Z)-2OH(5S,6R))	0.402595921
gene-COQ8A	1.284624449	3-Thiacytidine	0.209387412
gene-COQ8A	1.284624449	(-)-alpha-Terpineol	0.345716279
gene-COQ8A	1.284624449	Cyclosporin A	0.656529229
gene-COQ8A	1.284624449	DG(18:0/LTE4/0:0)	0.681485773
gene-COQ8A	1.284624449	5-(2-Aminopropyl)-2-methylphenol	0.323231457
gene-COQ8A	1.284624449	ingenol	0.942071652
gene-COQ8A	1.284624449	Armillane	0.52808635
gene-COQ8A	1.284624449	PC(P-18:1(11Z)/PGE2)	0.509503133
gene-COQ8A	1.284624449	(3R,4R)-3-Amino-1-hydroxy-4-methylpyrrol	0.471914506
gene-COQ8A	1.284624449	Isopropyl isothiocyanate	0.172385747
gene-COQ8A	1.284624449	Nigroxanthin	0.705005059
gene-COQ8A	1.284624449	PC(P-18:1(11Z)/PGJ2)	0.565243877
gene-COQ8A	1.284624449	PS(20:0/20:4(8Z,11Z,14Z,17Z)-2OH(5S,6R))	0.402595921
gene-COQ8A	1.284624449	CL(8:0/8:0/18:2(9Z,11Z)/20:0)	0.622988418
gene-PIGR	1.278643076	7(14)-Bisabolene-2,3,10,11-tetrol	2.446897974
gene-PIGR	1.278643076	1,4-Undecadiene	0.462803973
gene-PIGR	1.278643076	5-(2-Aminopropyl)-2-methylphenol	0.323231457
gene-PIGR	1.278643076	Armillane	0.52808635
gene-PIGR	1.278643076	PC(P-18:1(11Z)/PGE2)	0.509503133
gene-PIGR	1.278643076	1-Palmitoylglycerol	0.084357408
gene-PIGR	1.278643076	Isopropyl isothiocyanate	0.172385747
gene-PIGR	1.278643076	CL(8:0/8:0/18:2(9Z,11Z)/20:0)	0.622988418
gene-SRR	1.278365521	5-(3'-Carboxy-3'-oxopropenyl)-4,6-dihydro:	1.083232395
gene-SRR	1.278365521	Val-Cit	0.452308451
gene-SRR	1.278365521	(S)-10,16-Dihydroxyhexadecanoic acid	1.054006526
gene-SRR	1.278365521	Monacolin L acid	1.060562098
gene-SRR	1.278365521	PE(22:2(13Z,16Z)/22:5(4Z,7Z,10Z,13Z,19Z)-O	0.369745166
gene-SRR	1.278365521	3,4,3',4'-Tetrahydrospirilloxanthin	0.354666148
gene-SRR	1.278365521	Indole-3-ethanol	0.096523128
gene-SRR	1.278365521	tetranor-PGAM	0.584223916
gene-SRR	1.278365521	2-(1-Adamantyl)-1,3-dioxetane	0.408916843
gene-SRR	1.278365521	Milbemycin D	0.125637998
gene-SRR	1.278365521	Roxithromycin	0.268273077

gene-SRR	1.278365521	Sitosterol beta-D-glucoside	0.382944796
gene-SRR	1.278365521	PC(P-18:1(11Z)/PGJ2)	0.565243877
gene-SRR	1.278365521	PS(20:0/20:4(8Z,11Z,14Z,17Z)-2OH(5S,6R))	0.402595921
gene-SRR	1.278365521	PE(20:5(5Z,8Z,11Z,14Z,17Z)/18:0)	0.427962506
gene-SPNS2	1.278228124	D-Erythro-imidazole-glycerol-phosphate	0.355812431
gene-SPNS2	1.278228124	LTB4-d4	0.226799982
gene-SPNS2	1.278228124	cis-p-Menth-2-en-1-ol	0.201017988
gene-SPNS2	1.278228124	(-)-alpha-Terpineol	0.345716279
gene-SPNS2	1.278228124	PG(20:1(11Z)/18:3(10,12,15)-OH(9))	0.626244347
gene-SPNS2	1.278228124	4-Dimethylamino-L-phenylalanine	0.242110226
gene-SPNS2	1.278228124	2-Methyl-3-phenyl-2-propenal	0.407813975
gene-SPNS2	1.278228124	PC(P-18:1(11Z)/PGE2)	0.509503133
gene-SPNS2	1.278228124	(3R,4R)-3-Amino-1-hydroxy-4-methylpyrrol	0.471914506
gene-SPNS2	1.278228124	n-methyl-2-(4'-methylaminophenyl)-6-hydr	0.26655714
gene-SPNS2	1.278228124	(9Z)-Octadecenoic acid	0.142359556
gene-SPNS2	1.278228124	CL(8:0/8:0/18:2(9Z,11Z)/20:0)	0.622988418
Bos_taurus_newG	1.27458884	5-Fluorouridine diphosphate	0.151761582
Bos_taurus_newG	1.27458884	5-(Ethylthio)-1H-tetrazole	0.29717344
Bos_taurus_newG	1.27458884	3-hydroxypristanic acid	0.548515835
Bos_taurus_newG	1.27458884	Canesceol	0.686312122
Bos_taurus_newG	1.27458884	Tetracosenoyl-CoA	0.187187233
Bos_taurus_newG	1.27458884	16-hydroxy hexadecanoic acid	0.309000958
Bos_taurus_newG	1.27458884	1,2-O-Isopropylidene-D-glucofuranose	0.080987667
Bos_taurus_newG	1.27458884	DG(12:0/22:5(4Z,7Z,10Z,13Z,19Z)-O(16,17)/0	0.239819073
Bos_taurus_newG	1.27458884	3-Deoxyestrone	0.282221709
Bos_taurus_newG	1.27458884	1-Oleoyl-sn-glycero-3-phosphocholine	0.18926952
Bos_taurus_newG	1.27458884	3,4-dihydroxy-5-all-trans-hexaprenylbenzoa	0.123615726
Bos_taurus_newG	1.27458884	Lividamine	0.319679555
Bos_taurus_newG	1.27458884	Psychosine	0.106475396
Bos_taurus_newG	1.27458884	Cyclotricuspidogenin C	0.440884085
Bos_taurus_newG	1.27458884	(-)-alpha-Terpineol	0.345716279
Bos_taurus_newG	1.27458884	Linoleamide	0.926003481
Bos_taurus_newG	1.27458884	DG(8:0/20:4(6Z,8E,10E,14Z)-2OH(5S,12R)/0:0	0.958378689
Bos_taurus_newG	1.27458884	Sapacitabine	0.213785561
Bos_taurus_newG	1.27458884	PC(16:0/18:1(12Z)-2OH(9,10))	0.461843233
Bos_taurus_newG	1.27458884	5-(2-Aminopropyl)-2-methylphenol	0.323231457
Bos_taurus_newG	1.27458884	4-Dimethylamino-L-phenylalanine	0.242110226
Bos_taurus_newG	1.27458884	Trimetazidine	0.438282534
Bos_taurus_newG	1.27458884	ingenol	0.942071652
Bos_taurus_newG	1.27458884	Armillane	0.52808635
Bos_taurus_newG	1.27458884	Misoprostol	1.349270093
Bos_taurus_newG	1.27458884	(Z)-3-Oxo-2-(2-pentenyl)-1-cyclopenteneac	0.179805915
Bos_taurus_newG	1.27458884	Carboprost methyl	1.74923433
Bos_taurus_newG	1.27458884	(3R,4R)-3-Amino-1-hydroxy-4-methylpyrrol	0.471914506
Bos_taurus_newG	1.27458884	N2-gamma-Glutamylglutamine	0.230065499
Bos_taurus_newG	1.27458884	n-methyl-2-(4'-methylaminophenyl)-6-hydr	0.26655714
Bos_taurus_newG	1.27458884	Permetin A	0.140869415
Bos_taurus_newG	1.27458884	Glutethimide	0.126237229
Bos_taurus_newG	1.27458884	PE(P-16:0/18:4(6Z,9Z,12Z,15Z))	0.546989864
Bos_taurus_newG	1.27458884	Isopropyl isothiocyanate	0.172385747
Bos_taurus_newG	1.27458884	(9Z)-Octadecenoic acid	0.142359556

Bos_taurus_newG	1.27458884	L-Oleandrosyl-oleandolide	0.272998166
Bos_taurus_newG	1.27458884	9-deoxy-9-methylene-16,16-dimethyl -PGE2	0.606893884
Bos_taurus_newG	1.27458884	1-Methylnicotinamide	0.203956241
Bos_taurus_newG	1.27458884	CL(8:0/8:0/18:2(9Z,11Z)/20:0)	0.622988418
gene-C3H1orf22(	1.274210852	3-Thiacytidine	0.209387412
gene-C3H1orf22(	1.274210852	PE(22:2(13Z,16Z)/22:5(4Z,7Z,10Z,13Z,19Z)-O(	0.369745166
gene-C3H1orf22(	1.274210852	Cyclosporin A	0.656529229
gene-C3H1orf22(	1.274210852	PC(P-18:1(11Z)/PGE2)	0.509503133
gene-C3H1orf22(	1.274210852	Roxithromycin	0.268273077
gene-C3H1orf22(	1.274210852	Nigroxanthin	0.705005059
gene-C3H1orf22(	1.274210852	PC(P-18:1(11Z)/PGJ2)	0.565243877
gene-C3H1orf22(	1.274210852	PS(20:0/20:4(8Z,11Z,14Z,17Z)-2OH(5S,6R))	0.402595921
Bos_taurus_newG	1.273438808	milbemycin beta3	1.414447124
Bos_taurus_newG	1.273438808	3-Deoxyestrone	0.282221709
Bos_taurus_newG	1.273438808	1-Oleoyl-sn-glycero-3-phosphocholine	0.18926952
Bos_taurus_newG	1.273438808	3,4-dihydroxy-5-all-trans-hexaprenylbenzoa	0.123615726
Bos_taurus_newG	1.273438808	Psychosine	0.106475396
Bos_taurus_newG	1.273438808	Cyclotricuspidogenin C	0.440884085
Bos_taurus_newG	1.273438808	(-)-alpha-Terpineol	0.345716279
Bos_taurus_newG	1.273438808	PG(20:1(11Z)/18:3(10,12,15)-OH(9))	0.626244347
Bos_taurus_newG	1.273438808	Armillane	0.52808635
Bos_taurus_newG	1.273438808	n-methyl-2-(4'-methylaminophenyl)-6-hydr	0.26655714
gene-CPNE5	1.272116131	Colistin A	2.200819027
gene-CPNE5	1.272116131	D-Erythro-imidazole-glycerol-phosphate	0.355812431
gene-CPNE5	1.272116131	Dihydro-3-coumaric acid	1.950770185
gene-CPNE5	1.272116131	Lucyoside N	0.831231157
gene-CPNE5	1.272116131	Tsugarioside B	1.013262893
gene-CPNE5	1.272116131	7(14)-Bisabolene-2,3,10,11-tetrol	2.446897974
gene-CPNE5	1.272116131	cis-p-Menth-2-en-1-ol	0.201017988
gene-CPNE5	1.272116131	11-Oxahexadecanolide	2.378626329
gene-CPNE5	1.272116131	2-isopentyl-3,6-dimethyl pyrazine	0.710502562
gene-CPNE5	1.272116131	PG(20:1(11Z)/18:3(10,12,15)-OH(9))	0.626244347
gene-CPNE5	1.272116131	4-Dimethylamino-L-phenylalanine	0.242110226
gene-CPNE5	1.272116131	4-Guanidinobutanoate	0.445426102
gene-CPNE5	1.272116131	Cornoside	0.781689516
gene-CPNE5	1.272116131	Armillane	0.52808635
gene-CPNE5	1.272116131	5'-S-Methyl-5'-thioinosine	0.28772476
gene-CPNE5	1.272116131	7-Aminomethyl-7-carbaguanine	0.474992724
gene-CPNE5	1.272116131	Ajulemic acid	0.557832951
gene-CPNE5	1.272116131	(3R,4R)-3-Amino-1-hydroxy-4-methylpyrrol	0.471914506
gene-CPNE5	1.272116131	Sambutoxin	0.169786377
gene-CPNE5	1.272116131	Isopropyl isothiocyanate	0.172385747
gene-CPNE5	1.272116131	PG(20:1(11Z)/18:3(9,11,15)-OH(13))	0.69829683
gene-CNTN2	1.271845389	3-Thiacytidine	0.209387412
gene-CNTN2	1.271845389	(-)-alpha-Terpineol	0.345716279
gene-CNTN2	1.271845389	4-cholesten-7伪,12伪,24-triol-3-one	0.097840451
gene-CNTN2	1.271845389	N-[[3-Hydroxy-2-(2-pentenyl)cyclopentyl]ac	1.043930947
gene-CNTN2	1.271845389	PC(20:3(5Z,8Z,11Z)/24:0)	0.387959564
gene-CNTN2	1.271845389	PC(P-18:1(11Z)/PGE1)	0.295520345
gene-CNTN2	1.271845389	Isopropyl isothiocyanate	0.172385747
gene-CNTN2	1.271845389	D-Fructose	0.167584616

gene-ACE	1.271688925	Val-Cit	0.452308451
gene-ACE	1.271688925	11-Maleimidoundecanoic acid	1.084942397
gene-ACE	1.271688925	Monacolin L acid	1.060562098
gene-ACE	1.271688925	PE(22:2(13Z,16Z)/22:5(4Z,7Z,10Z,13Z,19Z)-O	0.369745166
gene-ACE	1.271688925	Lamivudine	0.327402892
gene-ACE	1.271688925	S-Acetyldihydrolipoamide-E	0.429561283
gene-ACE	1.271688925	Norophthalmic acid	0.191432411
gene-ACE	1.271688925	(1R,6S)-6-Amino-5-oxocyclohex-2-ene-1-c	0.154751123
gene-ACE	1.271688925	Indole-3-ethanol	0.096523128
gene-ACE	1.271688925	Tryptophyl-Glutamine	0.89722186
gene-ACE	1.271688925	Guanidoacetic acid	0.542509265
gene-ACE	1.271688925	alpha-Terpineol formate	0.628238986
gene-ACE	1.271688925	ent-16b,19-Kauranediol 19-acetate	0.400676827
gene-ACE	1.271688925	Roxithromycin	0.268273077
gene-ACE	1.271688925	PC(P-18:1(11Z)/PGJ2)	0.565243877
gene-ACE	1.271688925	PS(20:0/20:4(8Z,11Z,14Z,17Z)-2OH(5S,6R))	0.402595921
gene-ACE	1.271688925	PE(20:5(5Z,8Z,11Z,14Z,17Z)/18:0)	0.427962506
gene-C23H6orf1	1.27028734	D-Erythro-imidazole-glycerol-phosphate	0.355812431
gene-C23H6orf1	1.27028734	Palmitoyl Ara-C	0.586890476
gene-C23H6orf1	1.27028734	13(S)-HpODE	0.163157753
gene-C23H6orf1	1.27028734	LTB4-d4	0.226799982
gene-C23H6orf1	1.27028734	cis-p-Menth-2-en-1-ol	0.201017988
gene-C23H6orf1	1.27028734	PG(20:1(11Z)/18:3(10,12,15)-OH(9))	0.626244347
gene-C23H6orf1	1.27028734	2-Methyl-3-phenyl-2-propenal	0.407813975
gene-C23H6orf1	1.27028734	Guanidoacetic acid	0.542509265
gene-C23H6orf1	1.27028734	alpha-Terpineol formate	0.628238986
gene-C23H6orf1	1.27028734	Vulgarone A	0.102049407
gene-C23H6orf1	1.27028734	7-Sulfocholic acid	0.039985455
gene-C23H6orf1	1.27028734	PC(P-18:1(11Z)/PGE2)	0.509503133
gene-C23H6orf1	1.27028734	(9Z)-Octadecenoic acid	0.142359556
gene-C23H6orf1	1.27028734	N-Myristoyl Glutamine	0.37856966
gene-C23H6orf1	1.27028734	Nigroxanthin	0.705005059
gene-C23H6orf1	1.27028734	PC(P-18:1(11Z)/PGJ2)	0.565243877
gene-TBC1D16	1.269695623	LysoPI(16:0/0:0)	0.379098336
gene-TBC1D16	1.269695623	Norophthalmic acid	0.191432411
gene-TBC1D16	1.269695623	Imidazoline	0.232348181
gene-TBC1D16	1.269695623	PC(P-18:1(11Z)/PGJ2)	0.565243877
gene-TBC1D16	1.269695623	PS(20:0/20:4(8Z,11Z,14Z,17Z)-2OH(5S,6R))	0.402595921
gene-CREB5	1.267868236	Ascorbic acid 3-sulfate	0.300057469
gene-CREB5	1.267868236	Nona-4,6-dienoylcarnitine	0.567004808
gene-CREB5	1.267868236	Arachidonic Acid (peroxide free)	0.666664243
gene-CREB5	1.267868236	Dextrorphan O-glucuronide	1.59476827
gene-CREB5	1.267868236	CL(8:0/8:0/13:0/18:2(9Z,11Z))	0.088642926
gene-CREB5	1.267868236	3,3',5-Trihydroxy-4'-methoxy-6,7-methylene	0.137607639
gene-CREB5	1.267868236	5-amino-1-(5-phosphonato-D-ribosyl)imida	0.622612791
gene-CREB5	1.267868236	5-Hydroxyindoleacetaldehyde	0.448806354
gene-CREB5	1.267868236	(2S,4R)-4-(9H-Pyrido[3,4-b]indol-1-yl)-1,2,4	0.883685198
gene-CREB5	1.267868236	LysoPA(22:5(7Z,10Z,13Z,16Z,19Z)/0:0)	0.291568015
gene-CREB5	1.267868236	Bisbynin	0.993286894
gene-CREB5	1.267868236	Etimizol	0.852969435
gene-CREB5	1.267868236	Deoxyguanidinoproclavaminic acid	0.857672893

gene-CREB5	1.267868236	Azelaic acid	0.007180175
gene-CREB5	1.267868236	Pentahomomethionine	0.527487806
gene-CREB5	1.267868236	2-(3-Carboxy-3-aminopropyl)-L-histidine	0.575765457
gene-CREB5	1.267868236	N-[5-Methyl-8-(4-methylpiperazin-1-yl)-1,2	0.398723022
gene-CREB5	1.267868236	5-Hydroxy-2-oxo-4-ureido-2,5-dihydro-1H	0.122536033
gene-CREB5	1.267868236	p-Coumaroylputrescine	0.939330574
gene-CREB5	1.267868236	Calicheamicinone	0.910932275
gene-CREB5	1.267868236	Pterostilbene	0.899698314
gene-CREB5	1.267868236	Ancymidol	0.913827444
gene-CREB5	1.267868236	Phenylalanylhydroxyproline	1.183668928
gene-CREB5	1.267868236	Delgocitinib	1.051864373
gene-CREB5	1.267868236	Melatonin	1.134145229
gene-CREB5	1.267868236	Trihomomethionine	0.296210774
gene-CREB5	1.267868236	2,5-Dihydroxy-1-octadec-9-enoyloxypyrrole	0.828088186
gene-CREB5	1.267868236	Hygromycin B	0.930396619
gene-CREB5	1.267868236	Divinylprotochlorophyllide	0.629978387
gene-CREB5	1.267868236	Baccatin III	0.776246161
gene-CREB5	1.267868236	10-hydroxy capric acid	0.531721559
gene-CREB5	1.267868236	2-Hexylbenzothiazole	0.396099297
gene-CREB5	1.267868236	Valnemulin	0.408062514
gene-CREB5	1.267868236	6-isobutyl-4-hydroxy-2-pyrone	0.473678412
gene-CREB5	1.267868236	PE(20:0/18:1(12Z)-2OH(9,10))	0.438658253
gene-CREB5	1.267868236	Epomusenin A	0.766206049
gene-CREB5	1.267868236	DG(18:0/LTE4/0:0)	0.681485773
gene-CREB5	1.267868236	(4r,5s,6s,7r)-4,7-Dibenzyl-5,6-dihydroxy-1,3-	1.334600128
gene-CREB5	1.267868236	CDP-DG(PGF2alpha/16:0)	1.002512277
gene-CREB5	1.267868236	caffeoylshikimate	0.306700159
gene-CREB5	1.267868236	1-beta-D-Arabinofuranosyl-5-fluorocytosine	0.152339645
gene-CREB5	1.267868236	Pseudouridine 5'-phosphate	1.18431378
gene-CREB5	1.267868236	4-Hydroxy-6-methyl-3-(1H-pyrazol-3-yl)-2	0.006362816
gene-CREB5	1.267868236	15-keto-Prostaglandin E2	2.646629404
gene-CREB5	1.267868236	4-Hydroxyproline galactoside	0.148083825
gene-CREB5	1.267868236	S-(2-Hydroxyethyl)glutathione	0.269671835
gene-CREB5	1.267868236	Fluridone	0.127106682
gene-CREB5	1.267868236	Norophthalmic acid	0.191432411
gene-CREB5	1.267868236	4'-Thiothymidine	0.066948524
gene-CREB5	1.267868236	3'-N'-Acetylfusarochromanone	0.755683206
gene-CREB5	1.267868236	cis,trans-5'-Hydroxythalidomide	0.589828342
gene-CREB5	1.267868236	Ser Cys Ala Ala	0.603032447
gene-CREB5	1.267868236	Pantetheine	0.584830287
gene-CREB5	1.267868236	cis- and trans-Ethyl 2,4-dimethyl-1,3-dioxola	0.106321146
gene-CREB5	1.267868236	Pratenol A	0.886782548
gene-CREB5	1.267868236	6-Fluorohomovanillic acid	0.040947314
gene-CREB5	1.267868236	10-Hydroxycarbazepine	0.848001655
gene-CREB5	1.267868236	Semilepidinoside A	0.527638644
gene-CREB5	1.267868236	Arbutin	0.176184476
gene-CREB5	1.267868236	Loganin	0.92782666
gene-CREB5	1.267868236	7-Methylxanthosine	0.004641243
gene-CREB5	1.267868236	3'-Deoxythymidine	0.791830758
gene-CREB5	1.267868236	Gly Asp Ala Ala	0.718656316
gene-CREB5	1.267868236	ethyl 2-cyano-3-(1h-indol-3-yl)prop-2-eno	0.756861034

gene-CREB5	1.267868236	1-{2-[(3-Ethylphenyl)amino]-2-oxoethyl}-6-	0.705711444
gene-CREB5	1.267868236	kainic acid	0.836445456
gene-CREB5	1.267868236	Zanamivir	0.839205907
gene-CREB5	1.267868236	Aminoglutethimide	0.652616628
gene-CREB5	1.267868236	5-NITRO-2-PHENYLPROPYLAMINOBENZOIC	0.713298779
gene-CREB5	1.267868236	5,6,7,8-Tetrahydromonapterin	0.724075531
gene-CREB5	1.267868236	Harmalol	0.364945111
gene-CREB5	1.267868236	Ethylene brassylate	0.989156973
gene-CREB5	1.267868236	Kelampayoside A	0.744250453
gene-CREB5	1.267868236	2,3-Epoxymenaquinone	2.040730224
gene-CREB5	1.267868236	Guanidoacetic acid	0.542509265
gene-CREB5	1.267868236	4-Oxo-9-cis-retinoyl-beta-glucuronide	1.611773742
gene-CREB5	1.267868236	alpha-Terpineol formate	0.628238986
gene-CREB5	1.267868236	Methionyl-Valine	0.502707745
gene-CREB5	1.267868236	(S)-4-(2-(4-Amino-1,2,5-oxadiazol-3-yl)-1-	0.710706078
gene-CREB5	1.267868236	(Z)-11-Hexadecenal	0.208973533
gene-CREB5	1.267868236	Myricanol 5-[arabinosyl-(1->6)-glucoside]	0.744174399
gene-CREB5	1.267868236	MG(0:0/18:3(9Z,12Z,15Z)/0:0)	1.373784038
gene-CREB5	1.267868236	Alpha-Trisaccharide	0.928257808
gene-CREB5	1.267868236	LysoPE(0:0/22:5(4Z,7Z,10Z,13Z,16Z))	0.418671396
gene-CREB5	1.267868236	Austalide L	0.4229014
gene-CREB5	1.267868236	[(2S,4R,5R,6R,14S,16R)-14-Hydroxy-7,11-din	0.247987808
gene-CREB5	1.267868236	N-Palmitoyl Proline	0.470367693
gene-CREB5	1.267868236	Glu Cys Cys Tyr	0.269820719
gene-CREB5	1.267868236	10-alpha-methoxy-9,10-dihydrolysergol	0.140851438
gene-CREB5	1.267868236	3beta-Hydroxyergosta-7,24(24(1))-dien-4alp	1.714710978
gene-CREB5	1.267868236	MG(0:0/20:3(11Z,14Z,17Z)/0:0)	0.844949524
gene-CREB5	1.267868236	Bipindogulomethyloside	0.465702332
gene-CREB5	1.267868236	N-Stearoyl Glutamine	1.862695384
gene-CREB5	1.267868236	Palmitoylcarnitine	0.74779616
gene-CREB5	1.267868236	Glutaminylphenylalanine	0.224405311
gene-CREB5	1.267868236	LysoPI(0:0/18:0)	0.420441153
gene-CREB5	1.267868236	1,3,5-Bisabolatrien-10-one	0.109736328
gene-CREB5	1.267868236	MG(0:0/22:5(4Z,7Z,10Z,13Z,16Z)/0:0)	0.136549841
gene-CREB5	1.267868236	N1-(5-Phospho-a-D-ribosyl)-5,6-dimethylb	0.154251596
gene-CREB5	1.267868236	Tylactone	0.340073797
gene-CREB5	1.267868236	1-Heneicosanoyl-glycero-3-phosphoserine	0.214358093
gene-CREB5	1.267868236	3-Phenylpropylamine	0.220058937
gene-CREB5	1.267868236	2,4-Undecadienal	0.228636005
gene-CREB5	1.267868236	PC(18:1(9Z)/15:1(9Z))	0.534724682
gene-CREB5	1.267868236	Nigroxanthin	0.705005059
gene-CREB5	1.267868236	PE(18:0/20:4(8Z,11Z,14Z,17Z)-2OH(5S,6R))	0.418208781
gene-CREB5	1.267868236	PC(P-18:1(11Z)/PGJ2)	0.565243877
gene-CREB5	1.267868236	Azimexon	0.008689891
gene-CREB5	1.267868236	PE-NMe(18:2(9Z,12Z)/18:2(9Z,12Z))[U]	0.694736593
gene-CREB5	1.267868236	PE(20:5(5Z,8Z,11Z,14Z,17Z)/18:0)	0.427962506
gene-CREB5	1.267868236	PS(16:1(9Z)/22:2(13Z,16Z))	0.659652661
gene-CREB5	1.267868236	PC(P-16:0/20:5(5Z,8Z,11Z,14Z,16E)-OH(18R)	0.26121581
gene-LOC112444	1.263867097	3-Thiacytidine	0.209387412
gene-LOC112444	1.263867097	D-Erythro-imidazole-glycerol-phosphate	0.355812431
gene-LOC112444	1.263867097	11-Maleimidoundecanoic acid	1.084942397

gene-LOC112444	1.263867097	13(S)-HpODE	0.163157753
gene-LOC112444	1.263867097	LTB4-d4	0.226799982
gene-LOC112444	1.263867097	cis-p-Menth-2-en-1-ol	0.201017988
gene-LOC112444	1.263867097	PE(20:0/18:1(12Z)-2OH(9,10))	0.438658253
gene-LOC112444	1.263867097	PE(22:2(13Z,16Z)/22:5(4Z,7Z,10Z,13Z,19Z)-O	0.369745166
gene-LOC112444	1.263867097	Cyclosporin A	0.656529229
gene-LOC112444	1.263867097	DG(18:0/LTE4/0:0)	0.681485773
gene-LOC112444	1.263867097	PC(14:0/20:2(11Z,14Z))	1.110657378
gene-LOC112444	1.263867097	CDP-DG(PGF2alpha/16:0)	1.002512277
gene-LOC112444	1.263867097	5-(2-Aminopropyl)-2-methylphenol	0.323231457
gene-LOC112444	1.263867097	2-Methyl-3-phenyl-2-propenal	0.407813975
gene-LOC112444	1.263867097	beta-L-Dioxolane-cytidine	0.175916668
gene-LOC112444	1.263867097	PC(P-18:1(11Z)/PGE2)	0.509503133
gene-LOC112444	1.263867097	Isopropyl isothiocyanate	0.172385747
gene-LOC112444	1.263867097	N-Myristoyl Glutamine	0.37856966
gene-LOC112444	1.263867097	PC(18:1(9Z)/15:1(9Z))	0.534724682
gene-LOC112444	1.263867097	arachidyl amido cholanoic acid	1.24842952
gene-LOC112444	1.263867097	Nigroxanthin	0.705005059
gene-LOC112444	1.263867097	1-Octadecanoyl-2-(7Z,10Z,13Z,16Z-docosat	0.690496314
gene-LOC112444	1.263867097	PC(P-18:1(11Z)/PGJ2)	0.565243877
gene-LOC112444	1.263867097	PS(20:0/20:4(8Z,11Z,14Z,17Z)-2OH(5S,6R))	0.402595921
gene-LOC112444	1.263867097	DG(20:0/LTE4/0:0)	0.438074508
gene-UNC5A	1.26301304	PC(16:0/18:1(12Z)-2OH(9,10))	0.461843233
gene-UNC5A	1.26301304	CL(8:0/8:0/18:2(9Z,11Z)/20:0)	0.622988418
gene-IL9R	1.260845953	3-Thiacytidine	0.209387412
gene-IL9R	1.260845953	4-cholesten-7伪,12伪,24-triol-3-one	0.097840451
gene-IL9R	1.260845953	Cyclosporin A	0.656529229
gene-IL9R	1.260845953	5-(2-Aminopropyl)-2-methylphenol	0.323231457
gene-IL9R	1.260845953	beta-L-Dioxolane-cytidine	0.175916668
gene-IL9R	1.260845953	Isopropyl isothiocyanate	0.172385747
gene-IL9R	1.260845953	PC(P-18:1(11Z)/PGJ2)	0.565243877
gene-IL9R	1.260845953	PS(20:0/20:4(8Z,11Z,14Z,17Z)-2OH(5S,6R))	0.402595921
gene-IL9R	1.260845953	D-Fructose	0.167584616
gene-IL9R	1.260845953	CL(8:0/8:0/18:2(9Z,11Z)/20:0)	0.622988418
gene-B3GNTL1	1.257437888	3-Thiacytidine	0.209387412
gene-B3GNTL1	1.257437888	11-Maleimidoundecanoic acid	1.084942397
gene-B3GNTL1	1.257437888	PE(20:0/18:1(12Z)-2OH(9,10))	0.438658253
gene-B3GNTL1	1.257437888	PE(22:2(13Z,16Z)/22:5(4Z,7Z,10Z,13Z,19Z)-O	0.369745166
gene-B3GNTL1	1.257437888	S-Acetyldihydrolipoamide-E	0.429561283
gene-B3GNTL1	1.257437888	Norophthalmic acid	0.191432411
gene-B3GNTL1	1.257437888	Indole-3-ethanol	0.096523128
gene-B3GNTL1	1.257437888	Guanidoacetic acid	0.542509265
gene-B3GNTL1	1.257437888	alpha-Terpineol formate	0.628238986
gene-B3GNTL1	1.257437888	Roxithromycin	0.268273077
gene-B3GNTL1	1.257437888	PC(P-18:1(11Z)/PGJ2)	0.565243877
gene-B3GNTL1	1.257437888	PS(20:0/20:4(8Z,11Z,14Z,17Z)-2OH(5S,6R))	0.402595921
gene-ZMYND15	1.256529516	5-(3'-Carboxy-3'-oxopropenyl)-4,6-dihydro	1.083232395
gene-ZMYND15	1.256529516	Salicyluric acid	1.82393634
gene-ZMYND15	1.256529516	2-Amino-6-methylpyrimidine-4-thiol	3.949216803
gene-ZMYND15	1.256529516	Benzoquinoneacetic acid	0.707114579
gene-ZMYND15	1.256529516	4-Hydroxystyrene	2.854273107

gene-ZMYND15	1.256529516	4-Methylbenzyl alcohol	4.428526409
gene-ZMYND15	1.256529516	Dihydro-3-coumaric acid	1.950770185
gene-ZMYND15	1.256529516	BYSSOCHLAMIC ACID	3.635460051
gene-ZMYND15	1.256529516	Picroside I	1.144121381
gene-ZMYND15	1.256529516	Erythronic acid	0.494007482
gene-ZMYND15	1.256529516	7-Hydroxy-2',4',5'-trimethoxyisoflavan	3.57282801
gene-ZMYND15	1.256529516	Enterolactone 3'-glucuronide	1.086030454
gene-ZMYND15	1.256529516	Methionine sulfone	0.738197353
gene-ZMYND15	1.256529516	7-Hydroxy-6-methyl-8-ribityllumazine	0.103672738
gene-ZMYND15	1.256529516	15-Epi-lipoxin B5	0.766800331
gene-ZMYND15	1.256529516	xi-2,3-Dihydro-3,5-dihydroxy-6-methyl-4H-	0.933518005
gene-ZMYND15	1.256529516	(卤)-Enterolactone	0.766468338
gene-ZMYND15	1.256529516	Enterolactone 3''-sulfate	2.060530283
gene-ZMYND15	1.256529516	DGAT-1 inhibitor 2	1.079931222
gene-ZMYND15	1.256529516	3-(2,4-Dimethyl-5-(2-oxo-1,2-dihydroindol-	0.095524813
gene-ZMYND15	1.256529516	Fusidic Acid	1.929894931
gene-ZMYND15	1.256529516	3-carboxy-4-methyl-5-pentyl-2-furanpropa	0.375666997
gene-ZMYND15	1.256529516	Digoxigenin monodigitoxoside	0.07569922
gene-ZMYND15	1.256529516	Monacolin L acid	1.060562098
gene-ZMYND15	1.256529516	11-Oxahexadecanolide	2.378626329
gene-ZMYND15	1.256529516	L-Carnitine	0.2737116
gene-ZMYND15	1.256529516	Eicosapentaenoic acid	0.575884419
gene-ZMYND15	1.256529516	3,4,3',4'-Tetrahydrospirilloxanthin	0.354666148
gene-ZMYND15	1.256529516	2-Hydroxy-6-keto-2,4-heptadienoate	2.23994791
gene-ZMYND15	1.256529516	Indole-3-acetaldoxime N-oxide	2.727163281
gene-ZMYND15	1.256529516	3''-Hydroxy-geranylhydroquinone	0.459807452
gene-ZMYND15	1.256529516	N-(3-Aminopropyl)-N-methylcarbamic acid	0.313654734
gene-ZMYND15	1.256529516	3-Methoxytyramine	0.928926425
gene-ZMYND15	1.256529516	Hydroxyprolyl-Methionine	1.56890423
gene-ZMYND15	1.256529516	2-Hydroxy-3,4,6-trimethoxydihydrochalcone	1.07715092
gene-ZMYND15	1.256529516	7-Methylguanosine	1.215574844
gene-ZMYND15	1.256529516	6-hydroxy-7E,9E-Octadecadiene-11,13,15,17	1.179129125
gene-ZMYND15	1.256529516	7C-aglycone	1.015608521
gene-ZMYND15	1.256529516	7-Aminomethyl-7-carbaguanine	0.474992724
gene-ZMYND15	1.256529516	Nevirapine	0.820759262
gene-ZMYND15	1.256529516	carbenicillin	1.429508875
gene-ZMYND15	1.256529516	Suberenone	4.927547616
gene-ZMYND15	1.256529516	(-)-Huperzine A (HupA)	0.935198906
gene-ZMYND15	1.256529516	Sequiterpene Lactone 326	1.141450991
gene-ZMYND15	1.256529516	2-(3-(Diisopropylamino)-1-phenylpropyl)-4-	0.497857255
gene-ZMYND15	1.256529516	(1S)-3-[2-[(1R,7Ar)-7a-methyl-1-[(2R)-6-me	1.140894639
gene-ZMYND15	1.256529516	4-Octylphenol	1.400657885
gene-ZMYND15	1.256529516	Pitheduloside I	1.362662847
gene-ZMYND15	1.256529516	8-Acetylneosolaniol	4.201843387
gene-ZMYND15	1.256529516	Ajulemic acid	0.557832951
gene-ZMYND15	1.256529516	Sorbitan palmitate	1.946147152
gene-ZMYND15	1.256529516	QUININE ETHYL CARBONATE	0.116178312
gene-ZMYND15	1.256529516	Ketosantalic acid	0.356663411
gene-ZMYND15	1.256529516	Chalcone	4.157137778
gene-ZMYND15	1.256529516	Ascorbic acid 6-palmitate	0.201627014
gene-ZMYND15	1.256529516	DG(2:0/20:3(5Z,8Z,11Z)-O(14R,15S)/0:0)	0.046674703

gene-CLBA1	1.255569441	Cyclosporin A	0.656529229
gene-CLBA1	1.255569441	beta-Thujaplicin	0.681866194
gene-CLBA1	1.255569441	beta-L-Dioxolane-cytidine	0.175916668
gene-CLBA1	1.255569441	PC(P-18:1(11Z)/PGE2)	0.509503133
gene-CLBA1	1.255569441	Roxithromycin	0.268273077
gene-CLBA1	1.255569441	PC(P-18:1(11Z)/PGJ2)	0.565243877
gene-CLBA1	1.255569441	PS(20:0/20:4(8Z,11Z,14Z,17Z)-2OH(5S,6R))	0.402595921
gene-SPATA48	1.253190462	D-Glucono-1,5-lactone	0.240039573
gene-SPATA48	1.253190462	Glutamate carbon	0.671444516
gene-SPATA48	1.253190462	Urocortisol	0.926998516
gene-SPATA48	1.253190462	Deoxyshikonin	0.734877521
gene-SPATA48	1.253190462	13(S)-HpODE	0.163157753
gene-SPATA48	1.253190462	PE(18:1(12Z)-2OH(9,10)/P-18:0)	0.077744061
gene-SPATA48	1.253190462	Tetrofosmin	0.119741861
gene-SPATA48	1.253190462	7(S),17(S)-dihydroxy-8(E),10(Z),13(Z),15(E),19	0.32147403
gene-SPATA48	1.253190462	Valylarginine	0.627938493
gene-SPATA48	1.253190462	trans-Dodec-2-enoic acid	0.211807766
gene-SPATA48	1.253190462	3-O-alpha-Mycarosylerythronolide B	0.928450987
gene-SPATA48	1.253190462	(1R,6S)-6-Amino-5-oxocyclohex-2-ene-1-c	0.154751123
gene-SPATA48	1.253190462	(9R,10S,12Z)-9,10-Dihydroxy-8-oxo-12-octa	0.391102388
gene-SPATA48	1.253190462	3-(3-Methylbutylidene)-1(3H)-isobenzofurar	0.575422414
gene-SPATA48	1.253190462	Chamissonin diacetate	0.324896191
gene-SPATA48	1.253190462	[3-(2-Aminopropyl)-6-methylidenecyclohexa	0.422945997
gene-SPATA48	1.253190462	3',4',5'-Trimethoxycinnamyl alcohol acetate	0.4221816
gene-SPATA48	1.253190462	L-Anticapsin	0.426006862
gene-SPATA48	1.253190462	12-trans-Hydroxy juvenile hormone III	0.087265655
gene-SPATA48	1.253190462	Sophoranol	0.282064826
gene-SPATA48	1.253190462	L-Cysteine	0.214540754
gene-SPATA48	1.253190462	24,24-Dfhv	0.175278646
gene-SPATA48	1.253190462	7-Ethyl-3,6-dihydro-1,4-dimethylazulene	0.176621802
gene-SPATA48	1.253190462	Isoamyl salicylate	0.393519337
gene-SPATA48	1.253190462	9-OxoODE	0.111803831
gene-LOC617313	1.252940402	3-Thiacytidine	0.209387412
gene-LOC617313	1.252940402	LTB4-d4	0.226799982
gene-LOC617313	1.252940402	cis-p-Menth-2-en-1-ol	0.201017988
gene-LOC617313	1.252940402	PE(20:0/18:1(12Z)-2OH(9,10))	0.438658253
gene-LOC617313	1.252940402	(-)-alpha-Terpineol	0.345716279
gene-LOC617313	1.252940402	PE(22:2(13Z,16Z)/22:5(4Z,7Z,10Z,13Z,19Z)-O	0.369745166
gene-LOC617313	1.252940402	Cyclosporin A	0.656529229
gene-LOC617313	1.252940402	DG(18:0/LTE4/0:0)	0.681485773
gene-LOC617313	1.252940402	PC(14:0/20:2(11Z,14Z))	1.110657378
gene-LOC617313	1.252940402	5-(2-Aminopropyl)-2-methylphenol	0.323231457
gene-LOC617313	1.252940402	Armillane	0.52808635
gene-LOC617313	1.252940402	PC(P-18:1(11Z)/PGE2)	0.509503133
gene-LOC617313	1.252940402	Isopropyl isothiocyanate	0.172385747
gene-LOC617313	1.252940402	(9Z)-Octadecenoic acid	0.142359556
gene-LOC617313	1.252940402	arachidyl amido cholanoic acid	1.24842952
gene-LOC617313	1.252940402	Nigroxanthin	0.705005059
gene-LOC617313	1.252940402	PC(P-18:1(11Z)/PGJ2)	0.565243877
gene-LOC617313	1.252940402	PS(20:0/20:4(8Z,11Z,14Z,17Z)-2OH(5S,6R))	0.402595921
gene-LOC617313	1.252940402	CL(8:0/8:0/18:2(9Z,11Z)/20:0)	0.622988418

gene-ZNF175	1.251900647	N,N''-Sulfonylbisurea	0.308920258
gene-ZNF175	1.251900647	Imidazooxazole	0.20665788
gene-ZNF175	1.251900647	2,5-Dihydroxybenzenesulfonic Acid	0.220005737
gene-ZNF175	1.251900647	Pyrocatechol	0.215582237
gene-ZNF175	1.251900647	4-Hydroxybenzenesulfonic acid	0.748011467
gene-ZNF175	1.251900647	D-Erythro-imidazole-glycerol-phosphate	0.355812431
gene-ZNF175	1.251900647	Valeric acid	0.735658687
gene-ZNF175	1.251900647	O-methoxycatechol-O-sulphate	0.55600642
gene-ZNF175	1.251900647	Deoxyloganin	0.445304262
gene-ZNF175	1.251900647	Isomaltotriose	0.58434654
gene-ZNF175	1.251900647	Cymorcin monoglucoside	0.601123318
gene-ZNF175	1.251900647	2,6,7-Trihydroxy-9-methylxanthen-3-one	0.49497168
gene-ZNF175	1.251900647	1-(2-Furanyl)-1-pentanone	0.697147283
gene-ZNF175	1.251900647	2-Dehydro-3-deoxy-D-gluconate	0.65165299
gene-ZNF175	1.251900647	N1-Methyl-4-pyridone-5-carboxamide	0.751815577
gene-ZNF175	1.251900647	Jasmolone glucoside	0.949312509
gene-ZNF175	1.251900647	N-Docosahexaenoyl Lysine	0.392800451
gene-ZNF175	1.251900647	3beta-7-Drimene-3,11-diol	0.176540144
gene-ZNF175	1.251900647	PE(18:1(12Z)-2OH(9,10)/P-18:0)	0.077744061
gene-ZNF175	1.251900647	(-)-alpha-Terpineol	0.345716279
gene-ZNF175	1.251900647	Valylarginine	0.627938493
gene-ZNF175	1.251900647	3-O-alpha-Mycarosylerythronolide B	0.928450987
gene-ZNF175	1.251900647	4-Chloro-L-phenylalanine	0.256211739
gene-ZNF175	1.251900647	Cystine-glutamate	0.300382134
gene-ZNF175	1.251900647	1-(3-Fluoro-4-hydroxy-5-mercaptomethylte	0.992161192
gene-ZNF175	1.251900647	Valylproline	1.009800636
gene-ZNF175	1.251900647	Threonylisoleucine	1.190547061
gene-ZNF175	1.251900647	Isoleucylproline	1.006580106
gene-ZNF175	1.251900647	Pro-leu	0.931879628
gene-ZNF175	1.251900647	(1R,6S)-6-Amino-5-oxocyclohex-2-ene-1-c	0.154751123
gene-ZNF175	1.251900647	L,L-Cyclo(leucylprolyl)	0.634575464
gene-ZNF175	1.251900647	Hexanoylglutamine	0.593876112
gene-ZNF175	1.251900647	alatolide	0.26950377
gene-ZNF175	1.251900647	Ascorbyl palmitate	0.376864555
gene-ZNF175	1.251900647	Chamissonin diacetate	0.324896191
gene-ZNF175	1.251900647	3',4',5'-Trimethoxycinnamyl alcohol acetate	0.4221816
gene-ZNF175	1.251900647	L-Anticapsin	0.426006862
gene-ZNF175	1.251900647	(4Z,7Z,10Z,13Z,16Z,19Z)-Docosahexaenoic a	0.434211486
gene-ZNF175	1.251900647	PGF2伪 Alcohol methyl ether	0.167013539
gene-ZNF175	1.251900647	PC(P-18:1(11Z)/PGE2)	0.509503133
gene-ZNF175	1.251900647	(2S,3R)-2-Amino-4-octadecene-3-ol	0.308919473
gene-ZNF175	1.251900647	PA(PGE2/22:2(13Z,16Z))	0.24730102
gene-ZNF175	1.251900647	9-OxoODE	0.111803831
gene-ZNF175	1.251900647	Threoninyl-Tryptophan	0.69975167
gene-ZNF175	1.251900647	N-Oleoyl Asparagine	0.060994498
Bos_taurus_newG	1.249081743	PE(22:2(13Z,16Z)/22:5(4Z,7Z,10Z,13Z,19Z)-O	0.369745166
Bos_taurus_newG	1.249081743	beta-Thujaplicin	0.681866194
Bos_taurus_newG	1.249081743	PC(P-18:1(11Z)/PGE2)	0.509503133
Bos_taurus_newG	1.249081743	Roxithromycin	0.268273077
Bos_taurus_newG	1.249081743	PC(P-18:1(11Z)/PGJ2)	0.565243877
Bos_taurus_newG	1.249081743	PS(20:0/20:4(8Z,11Z,14Z,17Z)-2OH(5S,6R))	0.402595921

Bos_taurus_newG	1.246729443	5-(3'-Carboxy-3'-oxopropenyl)-4,6-dihydro:	1.083232395
Bos_taurus_newG	1.246729443	2-Amino-6-methylpyrimidine-4-thiol	3.949216803
Bos_taurus_newG	1.246729443	Picroside I	1.144121381
Bos_taurus_newG	1.246729443	Erythronic acid	0.494007482
Bos_taurus_newG	1.246729443	Benzenesulfonamide, 2-(cyclohexylamino)-N	0.727905403
Bos_taurus_newG	1.246729443	5-Hydroxykynurenamine	0.870388402
Bos_taurus_newG	1.246729443	(2R,4S,5R)-5-Ethyl-2-[(S)-hydroxy-(6-metho	0.385061056
Bos_taurus_newG	1.246729443	(+)-Bottrospicatol	0.68654221
Bos_taurus_newG	1.246729443	Enterolactone 3'-glucuronide	1.086030454
Bos_taurus_newG	1.246729443	15-Epi-lipoxin B5	0.766800331
Bos_taurus_newG	1.246729443	20-Carboxy-leukotriene B4	0.654456667
Bos_taurus_newG	1.246729443	13,14-dihydro-16,16-difluoro Prostaglandin	0.443708881
Bos_taurus_newG	1.246729443	DGAT-1 inhibitor 2	1.079931222
Bos_taurus_newG	1.246729443	Val-Cit	0.452308451
Bos_taurus_newG	1.246729443	Fusidic Acid	1.929894931
Bos_taurus_newG	1.246729443	(S)-10,16-Dihydroxyhexadecanoic acid	1.054006526
Bos_taurus_newG	1.246729443	Auxin b	1.052491354
Bos_taurus_newG	1.246729443	13,14-Dihydro PGF-1a	0.390844466
Bos_taurus_newG	1.246729443	8,8a-Deoxyoleandolide	0.305706087
Bos_taurus_newG	1.246729443	Cyclopentolate	0.486079401
Bos_taurus_newG	1.246729443	Monacolin L acid	1.060562098
Bos_taurus_newG	1.246729443	cis-p-Menth-2-en-1-ol	0.201017988
Bos_taurus_newG	1.246729443	3,4,3',4'-Tetrahydrospirilloxanthin	0.354666148
Bos_taurus_newG	1.246729443	PG(20:1(11Z)/18:3(10,12,15)-OH(9))	0.626244347
Bos_taurus_newG	1.246729443	Dyphylline	0.664549753
Bos_taurus_newG	1.246729443	Asparaginylcysteine	0.467538645
Bos_taurus_newG	1.246729443	4-Hydroxyoctanedioylcarnitine	1.002008817
Bos_taurus_newG	1.246729443	Phenyllactate	0.55151911
Bos_taurus_newG	1.246729443	Erosone	0.527835387
Bos_taurus_newG	1.246729443	3-Methoxytyramine	0.928926425
Bos_taurus_newG	1.246729443	L-histidinol-phosphate	0.865229875
Bos_taurus_newG	1.246729443	Hydroxyprolyl-Methionine	1.56890423
Bos_taurus_newG	1.246729443	2-Hydroxy-3,4,6-trimethoxydihydrochalcone	1.07715092
Bos_taurus_newG	1.246729443	7-Methylguanosine	1.215574844
Bos_taurus_newG	1.246729443	Eflornithine	0.287216354
Bos_taurus_newG	1.246729443	7a-Hydroxy-O-carbamoyl-deacetylcephalos	0.80981273
Bos_taurus_newG	1.246729443	Leu-Leu-Tyr	1.186798796
Bos_taurus_newG	1.246729443	6-hydroxy-7E,9E-Octadecadiene-11,13,15,1	1.179129125
Bos_taurus_newG	1.246729443	7C-aglycone	1.015608521
Bos_taurus_newG	1.246729443	7-Aminomethyl-7-carbaguanine	0.474992724
Bos_taurus_newG	1.246729443	Nevirapine	0.820759262
Bos_taurus_newG	1.246729443	alpha-(Dimethylaminomethyl)-2-(3-ethyl-5-	0.712551948
Bos_taurus_newG	1.246729443	3,5,7-Trimethyl-2E,4E,6E,8E-decatetraene	1.105561224
Bos_taurus_newG	1.246729443	5a,6a-Epoxy-7E-megastigmene-3b,9e-diol 9	1.333480709
Bos_taurus_newG	1.246729443	L-alpha-Acetyl-N,N-dinormethadol	1.567999001
Bos_taurus_newG	1.246729443	Humulinic acid A	0.602449819
Bos_taurus_newG	1.246729443	12R-hydroxy-5Z,8Z,12Z-eicosatrienoic acid	0.766411225
Bos_taurus_newG	1.246729443	2-(1-Adamantyl)-1,3-dioxetane	0.408916843
Bos_taurus_newG	1.246729443	(-)-Huperzine A (HupA)	0.935198906
Bos_taurus_newG	1.246729443	Sequiterpene Lactone 326	1.141450991
Bos_taurus_newG	1.246729443	8-Methylthiooctanaldoxime	0.685273457

Bos_taurus_newG	1.246729443	(1S)-3-[2-[(1R,7Ar)-7a-methyl-1-[(2R)-6-me	1.140894639
Bos_taurus_newG	1.246729443	4-Octylphenol	1.400657885
Bos_taurus_newG	1.246729443	Pitheduloside I	1.362662847
Bos_taurus_newG	1.246729443	Ajulemic acid	0.557832951
Bos_taurus_newG	1.246729443	Sorbitan palmitate	1.946147152
Bos_taurus_newG	1.246729443	Tetracosahexaenic acid	0.447398966
Bos_taurus_newG	1.246729443	QUININE ETHYL CARBONATE	0.116178312
Bos_taurus_newG	1.246729443	(3a,5b,7a,12a)-24-[(carboxymethyl)amino]-1	0.408427468
Bos_taurus_newG	1.246729443	N-[2-(3,4-Dihydroxyphenyl)ethyl]icosa-5,8,1	0.326021565
Bos_taurus_newG	1.246729443	Milbemycin D	0.125637998
Bos_taurus_newG	1.246729443	Sitosterol beta-D-glucoside	0.382944796
Bos_taurus_newG	1.246729443	(卤)-Ribaline	1.021609355
Bos_taurus_newG	1.246729443	Galactosylglycerol	0.738463143
Bos_taurus_newG	1.246729443	PG(20:1(11Z)/18:3(9,11,15)-OH(13))	0.69829683
gene-LOC101902	1.245460973	milbemycin beta3	1.414447124
gene-LOC101902	1.245460973	Hydroxyprolyl-Serine	0.519540341
gene-LOC101902	1.245460973	PE(22:2(13Z,16Z)/22:5(4Z,7Z,10Z,13Z,19Z)-O	0.369745166
gene-LOC101902	1.245460973	Cyclosporin A	0.656529229
gene-LOC101902	1.245460973	DG(18:0/LTE4/0:0)	0.681485773
gene-LOC101902	1.245460973	PC(14:0/20:2(11Z,14Z))	1.110657378
gene-LOC101902	1.245460973	CDP-DG(PGF2alpha/16:0)	1.002512277
gene-LOC101902	1.245460973	5-(2-Aminopropyl)-2-methylphenol	0.323231457
gene-LOC101902	1.245460973	ingenol	0.942071652
gene-LOC101902	1.245460973	Asparagine-betaxanthin	0.103826013
gene-LOC101902	1.245460973	DG(2:0/18:1(12Z)-O(9S,10R)/0:0)	0.033180989
gene-LOC101902	1.245460973	17-Aminogeldanamycin	0.518144528
gene-LOC101902	1.245460973	Nigroxanthin	0.705005059
gene-LOC101902	1.245460973	PS(20:0/20:4(8Z,11Z,14Z,17Z)-2OH(5S,6R))	0.402595921
gene-LOC101902	1.245460973	CL(8:0/8:0/18:2(9Z,11Z)/20:0)	0.622988418
gene-SDSL	1.243726896	3-Thiacytidine	0.209387412
gene-SDSL	1.243726896	D-Erythro-imidazole-glycerol-phosphate	0.355812431
gene-SDSL	1.243726896	(-)-alpha-Terpineol	0.345716279
gene-SDSL	1.243726896	Cyclosporin A	0.656529229
gene-SDSL	1.243726896	5-(2-Aminopropyl)-2-methylphenol	0.323231457
gene-SDSL	1.243726896	Armillane	0.52808635
gene-SDSL	1.243726896	PC(P-18:1(11Z)/PGE2)	0.509503133
gene-SDSL	1.243726896	Isopropyl isothiocyanate	0.172385747
gene-SDSL	1.243726896	Nigroxanthin	0.705005059
gene-SDSL	1.243726896	PC(P-18:1(11Z)/PGJ2)	0.565243877
gene-SDSL	1.243726896	PS(20:0/20:4(8Z,11Z,14Z,17Z)-2OH(5S,6R))	0.402595921
gene-SDSL	1.243726896	CL(8:0/8:0/18:2(9Z,11Z)/20:0)	0.622988418
gene-HIST1H1C	1.241653268	3-Thiacytidine	0.209387412
gene-HIST1H1C	1.241653268	D-Erythro-imidazole-glycerol-phosphate	0.355812431
gene-HIST1H1C	1.241653268	Armillane	0.52808635
gene-HIST1H1C	1.241653268	PC(P-18:1(11Z)/PGE2)	0.509503133
gene-HIST1H1C	1.241653268	Isopropyl isothiocyanate	0.172385747
gene-HIST1H1C	1.241653268	PC(P-18:1(11Z)/PGJ2)	0.565243877
gene-HIST1H1C	1.241653268	PS(20:0/20:4(8Z,11Z,14Z,17Z)-2OH(5S,6R))	0.402595921
gene-HIST1H1C	1.241653268	CL(8:0/8:0/18:2(9Z,11Z)/20:0)	0.622988418
gene-CACNB1	1.239303551	3-Thiacytidine	0.209387412
gene-CACNB1	1.239303551	11-Maleimidoundecanoic acid	1.084942397

gene-CACNB1	1.239303551	LTB4-d4	0.226799982
gene-CACNB1	1.239303551	cis-p-Menth-2-en-1-ol	0.201017988
gene-CACNB1	1.239303551	PE(20:0/18:1(12Z)-2OH(9,10))	0.438658253
gene-CACNB1	1.239303551	PE(22:2(13Z,16Z)/22:5(4Z,7Z,10Z,13Z,19Z)-O	0.369745166
gene-CACNB1	1.239303551	Cyclosporin A	0.656529229
gene-CACNB1	1.239303551	Epomusenin A	0.766206049
gene-CACNB1	1.239303551	PA(22:6(4Z,7Z,10Z,13Z,16Z,19Z)/16:0)	0.556532497
gene-CACNB1	1.239303551	DG(18:0/LTE4/0:0)	0.681485773
gene-CACNB1	1.239303551	PC(17:0/PGJ2)	0.657689319
gene-CACNB1	1.239303551	PC(14:0/20:2(11Z,14Z))	1.110657378
gene-CACNB1	1.239303551	CDP-DG(PGF2alpha/16:0)	1.002512277
gene-CACNB1	1.239303551	5-(2-Aminopropyl)-2-methylphenol	0.323231457
gene-CACNB1	1.239303551	1-beta-D-Arabinofuranosyl-5-fluorocytosine	0.152339645
gene-CACNB1	1.239303551	2-Methyl-3-phenyl-2-propenal	0.407813975
gene-CACNB1	1.239303551	Guanidoacetic acid	0.542509265
gene-CACNB1	1.239303551	10-alpha-methoxy-9,10-dihydrolysergol	0.140851438
gene-CACNB1	1.239303551	PC(P-18:1(11Z)/PGE2)	0.509503133
gene-CACNB1	1.239303551	(9Z)-Octadecenoic acid	0.142359556
gene-CACNB1	1.239303551	N-Myristoyl Glutamine	0.37856966
gene-CACNB1	1.239303551	PC(18:1(9Z)/15:1(9Z))	0.534724682
gene-CACNB1	1.239303551	arachidyl amido cholanoic acid	1.24842952
gene-CACNB1	1.239303551	Nigroxanthin	0.705005059
gene-CACNB1	1.239303551	1-Octadecanoyl-2-(7Z,10Z,13Z,16Z-docosat	0.690496314
gene-CACNB1	1.239303551	PC(P-18:1(11Z)/PGJ2)	0.565243877
gene-CACNB1	1.239303551	PS(20:0/20:4(8Z,11Z,14Z,17Z)-2OH(5S,6R))	0.402595921
gene-CACNB1	1.239303551	DG(20:0/LTE4/0:0)	0.438074508
Bos_taurus_newG	1.237297308	D-Erythro-imidazole-glycerol-phosphate	0.355812431
Bos_taurus_newG	1.237297308	Sorbitan laurate	0.242315968
Bos_taurus_newG	1.237297308	13(S)-HpODE	0.163157753
Bos_taurus_newG	1.237297308	Monacolin L acid	1.060562098
Bos_taurus_newG	1.237297308	LTB4-d4	0.226799982
Bos_taurus_newG	1.237297308	cis-p-Menth-2-en-1-ol	0.201017988
Bos_taurus_newG	1.237297308	PE(20:0/18:1(12Z)-2OH(9,10))	0.438658253
Bos_taurus_newG	1.237297308	DG(15:0/PGE1/0:0)	0.462019057
Bos_taurus_newG	1.237297308	3'-N'-Acetylfusarochromanone	0.755683206
Bos_taurus_newG	1.237297308	Ser Cys Ala Ala	0.603032447
Bos_taurus_newG	1.237297308	2-Methyl-3-phenyl-2-propenal	0.407813975
Bos_taurus_newG	1.237297308	Guanidoacetic acid	0.542509265
Bos_taurus_newG	1.237297308	alpha-Terpineol formate	0.628238986
Bos_taurus_newG	1.237297308	4-Dihydroboldenone	0.692863202
Bos_taurus_newG	1.237297308	2'-Fluoro-2',3'-dideoxyinosine	1.214745496
Bos_taurus_newG	1.237297308	20-carboxy Arachidonic Acid	0.780988382
Bos_taurus_newG	1.237297308	Nebramycin 5'	0.909280571
Bos_taurus_newG	1.237297308	4-Octylphenol	1.400657885
Bos_taurus_newG	1.237297308	3-Pentadecylphenol	1.682258188
Bos_taurus_newG	1.237297308	10-alpha-methoxy-9,10-dihydrolysergol	0.140851438
Bos_taurus_newG	1.237297308	PC(P-18:1(11Z)/PGE2)	0.509503133
Bos_taurus_newG	1.237297308	Galabiosylceramide (d18:1/20:0)	0.746737323
Bos_taurus_newG	1.237297308	12-Hydroxyicosanoylcarnitine	1.730979067
Bos_taurus_newG	1.237297308	N-Myristoyl Glutamine	0.37856966
Bos_taurus_newG	1.237297308	PC(18:1(9Z)/15:1(9Z))	0.534724682

Bos_taurus_newG	1.237297308	PC(P-18:1(11Z)/PGJ2)	0.565243877
Bos_taurus_newG	1.237297308	PS(16:1(9Z)/22:2(13Z,16Z))	0.659652661
gene-TPPP3	1.236546014	3-Thiacytidine	0.209387412
gene-TPPP3	1.236546014	D-Erythro-imidazole-glycerol-phosphate	0.355812431
gene-TPPP3	1.236546014	11-Maleimidoundecanoic acid	1.084942397
gene-TPPP3	1.236546014	13(S)-HpODE	0.163157753
gene-TPPP3	1.236546014	LTB4-d4	0.226799982
gene-TPPP3	1.236546014	cis-p-Menth-2-en-1-ol	0.201017988
gene-TPPP3	1.236546014	PE(20:0/18:1(12Z)-2OH(9,10))	0.438658253
gene-TPPP3	1.236546014	PE(22:2(13Z,16Z)/22:5(4Z,7Z,10Z,13Z,19Z)-O	0.369745166
gene-TPPP3	1.236546014	Cyclosporin A	0.656529229
gene-TPPP3	1.236546014	2-Methyl-3-phenyl-2-propenal	0.407813975
gene-TPPP3	1.236546014	beta-L-Dioxolane-cytidine	0.175916668
gene-TPPP3	1.236546014	PC(P-18:1(11Z)/PGE2)	0.509503133
gene-TPPP3	1.236546014	Roxithromycin	0.268273077
gene-TPPP3	1.236546014	PC(18:1(9Z)/15:1(9Z))	0.534724682
gene-TPPP3	1.236546014	Nigroxanthin	0.705005059
gene-TPPP3	1.236546014	PC(P-18:1(11Z)/PGJ2)	0.565243877
gene-TPPP3	1.236546014	PS(20:0/20:4(8Z,11Z,14Z,17Z)-2OH(5S,6R))	0.402595921
gene-PRR33	1.230761691	3-Thiacytidine	0.209387412
gene-PRR33	1.230761691	5-Hydroxy-2-oxo-4-ureido-2,5-dihydro-1H	0.122536033
gene-PRR33	1.230761691	11-Maleimidoundecanoic acid	1.084942397
gene-PRR33	1.230761691	PE(20:0/18:1(12Z)-2OH(9,10))	0.438658253
gene-PRR33	1.230761691	PE(22:2(13Z,16Z)/22:5(4Z,7Z,10Z,13Z,19Z)-O	0.369745166
gene-PRR33	1.230761691	Cyclosporin A	0.656529229
gene-PRR33	1.230761691	DG(18:0/LTE4/0:0)	0.681485773
gene-PRR33	1.230761691	CDP-DG(PGF2alpha/16:0)	1.002512277
gene-PRR33	1.230761691	C20914	0.140383181
gene-PRR33	1.230761691	Guanidoacetic acid	0.542509265
gene-PRR33	1.230761691	alpha-Terpineol formate	0.628238986
gene-PRR33	1.230761691	Methionyl-Valine	0.502707745
gene-PRR33	1.230761691	beta-L-Dioxolane-cytidine	0.175916668
gene-PRR33	1.230761691	1,4,6-Trimethylnaphthalene	0.12150693
gene-PRR33	1.230761691	PC(P-18:1(11Z)/PGE2)	0.509503133
gene-PRR33	1.230761691	Phorone A	0.079039095
gene-PRR33	1.230761691	Nigroxanthin	0.705005059
gene-PRR33	1.230761691	PC(P-18:1(11Z)/PGJ2)	0.565243877
gene-PRR33	1.230761691	PS(20:0/20:4(8Z,11Z,14Z,17Z)-2OH(5S,6R))	0.402595921
gene-B3GALT5	1.230522896	D-Erythro-imidazole-glycerol-phosphate	0.355812431
gene-B3GALT5	1.230522896	13(S)-HpODE	0.163157753
gene-B3GALT5	1.230522896	LTB4-d4	0.226799982
gene-B3GALT5	1.230522896	cis-p-Menth-2-en-1-ol	0.201017988
gene-B3GALT5	1.230522896	DG(15:0/PGE1/0:0)	0.462019057
gene-B3GALT5	1.230522896	Ser Cys Ala Ala	0.603032447
gene-B3GALT5	1.230522896	2-Methyl-3-phenyl-2-propenal	0.407813975
gene-B3GALT5	1.230522896	4-Dihydroboldenone	0.692863202
gene-B3GALT5	1.230522896	20-carboxy Arachidonic Acid	0.780988382
gene-B3GALT5	1.230522896	Vulgarone A	0.102049407
gene-B3GALT5	1.230522896	PC(P-18:1(11Z)/PGE2)	0.509503133
gene-B3GALT5	1.230522896	(9Z)-Octadecenoic acid	0.142359556
gene-B3GALT5	1.230522896	N-Myristoyl Glutamine	0.37856966

gene-CEP83	1.225685248	5-(3'-Carboxy-3'-oxopropenyl)-4,6-dihydro:	1.083232395
gene-CEP83	1.225685248	D-Erythro-imidazole-glycerol-phosphate	0.355812431
gene-CEP83	1.225685248	Dihydro-3-coumaric acid	1.950770185
gene-CEP83	1.225685248	Erythronic acid	0.494007482
gene-CEP83	1.225685248	Undecanedioic acid	0.059955634
gene-CEP83	1.225685248	(S)-10,16-Dihydroxyhexadecanoic acid	1.054006526
gene-CEP83	1.225685248	Auxin b	1.052491354
gene-CEP83	1.225685248	13(S)-HPOT	1.353059742
gene-CEP83	1.225685248	7(14)-Bisabolene-2,3,10,11-tetrol	2.446897974
gene-CEP83	1.225685248	Cyclopentolate	0.486079401
gene-CEP83	1.225685248	cis-p-Menth-2-en-1-ol	0.201017988
gene-CEP83	1.225685248	11-Oxahexadecanolide	2.378626329
gene-CEP83	1.225685248	PG(20:1(11Z)/18:3(10,12,15)-OH(9))	0.626244347
gene-CEP83	1.225685248	Asparaginylcysteine	0.467538645
gene-CEP83	1.225685248	3-Methoxytyramine	0.928926425
gene-CEP83	1.225685248	Hexanoylglutamine	0.593876112
gene-CEP83	1.225685248	7-Aminomethyl-7-carbaguanine	0.474992724
gene-CEP83	1.225685248	Nevirapine	0.820759262
gene-CEP83	1.225685248	N-Eicosapentaenoyl Asparagine	0.371594025
gene-CEP83	1.225685248	2-(3-(Diisopropylamino)-1-phenylpropyl)-4-	0.497857255
gene-CEP83	1.225685248	8-Methylthiooctanaldoxime	0.685273457
gene-CEP83	1.225685248	Ajulemic acid	0.557832951
gene-CEP83	1.225685248	PC(P-18:1(11Z)/PGE2)	0.509503133
gene-CEP83	1.225685248	LysoPE(0:0/16:0)	0.163938493
gene-CEP83	1.225685248	Sitosterol beta-D-glucoside	0.382944796
gene-CEP83	1.225685248	PG(20:1(11Z)/18:3(9,11,15)-OH(13))	0.69829683
gene-HIST1H1E	1.225026555	3-Thiacytidine	0.209387412
gene-HIST1H1E	1.225026555	D-Erythro-imidazole-glycerol-phosphate	0.355812431
gene-HIST1H1E	1.225026555	7(14)-Bisabolene-2,3,10,11-tetrol	2.446897974
gene-HIST1H1E	1.225026555	13(S)-HpODE	0.163157753
gene-HIST1H1E	1.225026555	cis-p-Menth-2-en-1-ol	0.201017988
gene-HIST1H1E	1.225026555	2-Methyl-3-phenyl-2-propenal	0.407813975
gene-HIST1H1E	1.225026555	PC(P-18:1(11Z)/PGE2)	0.509503133
gene-HIST1H1E	1.225026555	Isopropyl isothiocyanate	0.172385747
gene-HIST1H1E	1.225026555	PC(P-18:1(11Z)/PGJ2)	0.565243877
gene-HIST1H1E	1.225026555	PS(20:0/20:4(8Z,11Z,14Z,17Z)-2OH(5S,6R))	0.402595921
gene-NKD2	1.224441842	3-Thiacytidine	0.209387412
gene-NKD2	1.224441842	D-Erythro-imidazole-glycerol-phosphate	0.355812431
gene-NKD2	1.224441842	LTB4-d4	0.226799982
gene-NKD2	1.224441842	cis-p-Menth-2-en-1-ol	0.201017988
gene-NKD2	1.224441842	(-)-alpha-Terpineol	0.345716279
gene-NKD2	1.224441842	Cyclosporin A	0.656529229
gene-NKD2	1.224441842	5-(2-Aminopropyl)-2-methylphenol	0.323231457
gene-NKD2	1.224441842	4-Dimethylamino-L-phenylalanine	0.242110226
gene-NKD2	1.224441842	Trimetazidine	0.438282534
gene-NKD2	1.224441842	Armillane	0.52808635
gene-NKD2	1.224441842	PC(P-18:1(11Z)/PGE2)	0.509503133
gene-NKD2	1.224441842	(3R,4R)-3-Amino-1-hydroxy-4-methylpyrrol	0.471914506
gene-NKD2	1.224441842	Isopropyl isothiocyanate	0.172385747
gene-NKD2	1.224441842	(9Z)-Octadecenoic acid	0.142359556
gene-NKD2	1.224441842	arachidyl amido cholanoic acid	1.24842952

gene-NKD2	1.224441842	Nigroxanthin	0.705005059
gene-NKD2	1.224441842	9-deoxy-9-methylene-16,16-dimethyl -PGE2	0.606893884
gene-NKD2	1.224441842	1-Octadecanoyl-2-(7Z,10Z,13Z,16Z-docosat	0.690496314
gene-NKD2	1.224441842	CL(8:0/8:0/18:2(9Z,11Z)/20:0)	0.622988418
gene-BAIAP2	1.224249165	Glutamate carbon	0.671444516
gene-BAIAP2	1.224249165	Urocortisol	0.926998516
gene-BAIAP2	1.224249165	Deoxyshikonin	0.734877521
gene-BAIAP2	1.224249165	11-Maleimidoundecanoic acid	1.084942397
gene-BAIAP2	1.224249165	13(S)-HpODE	0.163157753
gene-BAIAP2	1.224249165	PE(18:1(12Z)-2OH(9,10)/P-18:0)	0.077744061
gene-BAIAP2	1.224249165	7(S),17(S)-dihydroxy-8(E),10(Z),13(Z),15(E),19	0.32147403
gene-BAIAP2	1.224249165	(1R,6S)-6-Amino-5-oxocyclohex-2-ene-1-ca	0.154751123
gene-BAIAP2	1.224249165	3-(3-Methylbutylidene)-1(3H)-isobenzofuran	0.575422414
gene-BAIAP2	1.224249165	[3-(2-Aminopropyl)-6-methylidenecyclohexa	0.422945997
gene-BAIAP2	1.224249165	3',4',5'-Trimethoxycinnamyl alcohol acetate	0.4221816
gene-BAIAP2	1.224249165	24,24-Dfhv	0.175278646
gene-BAIAP2	1.224249165	PC(P-18:1(11Z)/PGE2)	0.509503133
gene-BAIAP2	1.224249165	PC(P-18:1(11Z)/PGE1)	0.295520345
gene-BAIAP2	1.224249165	Roxithromycin	0.268273077
gene-BAIAP2	1.224249165	PC(P-18:1(11Z)/PGJ2)	0.565243877
gene-STAB1	1.219636596	3-Thiacytidine	0.209387412
gene-STAB1	1.219636596	D-Erythro-imidazole-glycerol-phosphate	0.355812431
gene-STAB1	1.219636596	cis-p-Menth-2-en-1-ol	0.201017988
gene-STAB1	1.219636596	PE(22:2(13Z,16Z)/22:5(4Z,7Z,10Z,13Z,19Z)-O	0.369745166
gene-STAB1	1.219636596	PG(20:1(11Z)/18:3(10,12,15)-OH(9))	0.626244347
gene-STAB1	1.219636596	PC(P-18:1(11Z)/PGE2)	0.509503133
gene-STAB1	1.219636596	Roxithromycin	0.268273077
gene-STAB1	1.219636596	PC(P-18:1(11Z)/PGJ2)	0.565243877
gene-STAB1	1.219636596	PS(20:0/20:4(8Z,11Z,14Z,17Z)-2OH(5S,6R))	0.402595921
Bos_taurus_newG	1.216410889	Hydroxyprolyl-Serine	0.519540341
Bos_taurus_newG	1.216410889	beta-Thujaplicin	0.681866194
Bos_taurus_newG	1.216410889	D-Fructose	0.167584616
gene-TKT	1.215664926	3-Thiacytidine	0.209387412
gene-TKT	1.215664926	D-Erythro-imidazole-glycerol-phosphate	0.355812431
gene-TKT	1.215664926	LTB4-d4	0.226799982
gene-TKT	1.215664926	cis-p-Menth-2-en-1-ol	0.201017988
gene-TKT	1.215664926	(-)-alpha-Terpineol	0.345716279
gene-TKT	1.215664926	Cyclosporin A	0.656529229
gene-TKT	1.215664926	5-(2-Aminopropyl)-2-methylphenol	0.323231457
gene-TKT	1.215664926	4-Dimethylamino-L-phenylalanine	0.242110226
gene-TKT	1.215664926	Armillane	0.52808635
gene-TKT	1.215664926	PC(P-18:1(11Z)/PGE2)	0.509503133
gene-TKT	1.215664926	(3R,4R)-3-Amino-1-hydroxy-4-methylpyrrol	0.471914506
gene-TKT	1.215664926	Isopropyl isothiocyanate	0.172385747
gene-TKT	1.215664926	Nigroxanthin	0.705005059
gene-TKT	1.215664926	PC(P-18:1(11Z)/PGJ2)	0.565243877
gene-TKT	1.215664926	PS(20:0/20:4(8Z,11Z,14Z,17Z)-2OH(5S,6R))	0.402595921
gene-TKT	1.215664926	CL(8:0/8:0/18:2(9Z,11Z)/20:0)	0.622988418
gene-RSPO4	1.211635183	4-Oxo-9-cis-retinoyl-beta-glucuronide	1.611773742
gene-RSPO4	1.211635183	Hydrocinnamic acid	0.043440346
gene-RSPO4	1.211635183	PS(16:1(9Z)/22:2(13Z,16Z))	0.659652661

Bos_taurus_newG	1.211622659	5-(3'-Carboxy-3'-oxopropenyl)-4,6-dihydro:	1.083232395
Bos_taurus_newG	1.211622659	Val-Cit	0.452308451
Bos_taurus_newG	1.211622659	Docosanamide	0.509975786
Bos_taurus_newG	1.211622659	cis-p-Menth-2-en-1-ol	0.201017988
Bos_taurus_newG	1.211622659	PE(22:2(13Z,16Z)/22:5(4Z,7Z,10Z,13Z,19Z)-O	0.369745166
Bos_taurus_newG	1.211622659	3,4,3',4'-Tetrahydrospirilloxanthin	0.354666148
Bos_taurus_newG	1.211622659	PG(20:1(11Z)/18:3(10,12,15)-OH(9))	0.626244347
Bos_taurus_newG	1.211622659	Lamivudine	0.327402892
Bos_taurus_newG	1.211622659	Asparaginylcysteine	0.467538645
Bos_taurus_newG	1.211622659	5-Formiminotetrahydrofolate	0.118251287
Bos_taurus_newG	1.211622659	Cornoside	0.781689516
Bos_taurus_newG	1.211622659	Phenyllactate	0.55151911
Bos_taurus_newG	1.211622659	Erosone	0.527835387
Bos_taurus_newG	1.211622659	7C-aglycone	1.015608521
Bos_taurus_newG	1.211622659	7-Aminomethyl-7-carbaguanine	0.474992724
Bos_taurus_newG	1.211622659	alpha-(Dimethylaminomethyl)-2-(3-ethyl-5-	0.712551948
Bos_taurus_newG	1.211622659	Milbemycin D	0.125637998
Bos_taurus_newG	1.211622659	Sitosterol beta-D-glucoside	0.382944796
Bos_taurus_newG	1.211622659	PS(20:0/20:4(8Z,11Z,14Z,17Z)-2OH(5S,6R))	0.402595921
Bos_taurus_newG	1.211622659	Galactosylglycerol	0.738463143
Bos_taurus_newG	1.211622659	PG(20:1(11Z)/18:3(9,11,15)-OH(13))	0.69829683
gene-LOC107131	1.210869092	3-Thiacytidine	0.209387412
gene-LOC107131	1.210869092	D-Erythro-imidazole-glycerol-phosphate	0.355812431
gene-LOC107131	1.210869092	Azelaic acid	0.007180175
gene-LOC107131	1.210869092	11-Maleimidoundecanoic acid	1.084942397
gene-LOC107131	1.210869092	Monacolin L acid	1.060562098
gene-LOC107131	1.210869092	LTB4-d4	0.226799982
gene-LOC107131	1.210869092	cis-p-Menth-2-en-1-ol	0.201017988
gene-LOC107131	1.210869092	PE(20:0/18:1(12Z)-2OH(9,10))	0.438658253
gene-LOC107131	1.210869092	DG(15:0/PGE1/0:0)	0.462019057
gene-LOC107131	1.210869092	Pseudouridine 5'-phosphate	1.18431378
gene-LOC107131	1.210869092	15-keto-Prostaglandin E2	2.646629404
gene-LOC107131	1.210869092	Norophthalmic acid	0.191432411
gene-LOC107131	1.210869092	3'-N'-Acetylfusarochromanone	0.755683206
gene-LOC107131	1.210869092	Ser Cys Ala Ala	0.603032447
gene-LOC107131	1.210869092	2-Methyl-3-phenyl-2-propenal	0.407813975
gene-LOC107131	1.210869092	Guanidoacetic acid	0.542509265
gene-LOC107131	1.210869092	4-Oxo-9-cis-retinoyl-beta-glucuronide	1.611773742
gene-LOC107131	1.210869092	alpha-Terpineol formate	0.628238986
gene-LOC107131	1.210869092	4-Octylphenol	1.400657885
gene-LOC107131	1.210869092	10-alpha-methoxy-9,10-dihydrolysergol	0.140851438
gene-LOC107131	1.210869092	N-Stearoyl Glutamine	1.862695384
gene-LOC107131	1.210869092	PC(P-18:1(11Z)/PGE2)	0.509503133
gene-LOC107131	1.210869092	Galabiosylceramide (d18:1/20:0)	0.746737323
gene-LOC107131	1.210869092	LysoPI(0:0/18:0)	0.420441153
gene-LOC107131	1.210869092	12-Hydroxyicosanoylcarnitine	1.730979067
gene-LOC107131	1.210869092	N-Myristoyl Glutamine	0.37856966
gene-LOC107131	1.210869092	PC(18:1(9Z)/15:1(9Z))	0.534724682
gene-LOC107131	1.210869092	Nigroxanthin	0.705005059
gene-LOC107131	1.210869092	PC(P-18:1(11Z)/PGJ2)	0.565243877
gene-LOC107131	1.210869092	PS(20:0/20:4(8Z,11Z,14Z,17Z)-2OH(5S,6R))	0.402595921

gene-LOC107131	1.210869092	PE(20:5(5Z,8Z,11Z,14Z,17Z)/18:0)	0.427962506
gene-LOC107131	1.210869092	PS(16:1(9Z)/22:2(13Z,16Z))	0.659652661
gene-ADAM8	1.209173353	3-Thiacytidine	0.209387412
gene-ADAM8	1.209173353	D-Erythro-imidazole-glycerol-phosphate	0.355812431
gene-ADAM8	1.209173353	LTB4-d4	0.226799982
gene-ADAM8	1.209173353	cis-p-Menth-2-en-1-ol	0.201017988
gene-ADAM8	1.209173353	(-)-alpha-Terpineol	0.345716279
gene-ADAM8	1.209173353	1,4-Undecadiene	0.462803973
gene-ADAM8	1.209173353	5-(2-Aminopropyl)-2-methylphenol	0.323231457
gene-ADAM8	1.209173353	4-Dimethylamino-L-phenylalanine	0.242110226
gene-ADAM8	1.209173353	Armillane	0.52808635
gene-ADAM8	1.209173353	PC(P-18:1(11Z)/PGE2)	0.509503133
gene-ADAM8	1.209173353	(3R,4R)-3-Amino-1-hydroxy-4-methylpyrrol	0.471914506
gene-ADAM8	1.209173353	n-methyl-2-(4'-methylaminophenyl)-6-hydr	0.26655714
gene-ADAM8	1.209173353	Isopropyl isothiocyanate	0.172385747
gene-ADAM8	1.209173353	PC(P-18:1(11Z)/PGJ2)	0.565243877
gene-ADAM8	1.209173353	PS(20:0/20:4(8Z,11Z,14Z,17Z)-2OH(5S,6R))	0.402595921
gene-ADAM8	1.209173353	CL(8:0/8:0/18:2(9Z,11Z)/20:0)	0.622988418
gene-CSRP1	1.2079514	D-Erythro-imidazole-glycerol-phosphate	0.355812431
gene-CSRP1	1.2079514	3-Deoxyestrone	0.282221709
gene-CSRP1	1.2079514	1-Oleoyl-sn-glycero-3-phosphocholine	0.18926952
gene-CSRP1	1.2079514	LTB4-d4	0.226799982
gene-CSRP1	1.2079514	(-)-alpha-Terpineol	0.345716279
gene-CSRP1	1.2079514	1,4-Undecadiene	0.462803973
gene-CSRP1	1.2079514	Cyclosporin A	0.656529229
gene-CSRP1	1.2079514	5-(2-Aminopropyl)-2-methylphenol	0.323231457
gene-CSRP1	1.2079514	4-Dimethylamino-L-phenylalanine	0.242110226
gene-CSRP1	1.2079514	Trimetazidine	0.438282534
gene-CSRP1	1.2079514	ingenol	0.942071652
gene-CSRP1	1.2079514	Armillane	0.52808635
gene-CSRP1	1.2079514	PC(P-18:1(11Z)/PGE2)	0.509503133
gene-CSRP1	1.2079514	PC(20:3(5Z,8Z,11Z)/24:0)	0.387959564
gene-CSRP1	1.2079514	(3R,4R)-3-Amino-1-hydroxy-4-methylpyrrol	0.471914506
gene-CSRP1	1.2079514	n-methyl-2-(4'-methylaminophenyl)-6-hydr	0.26655714
gene-CSRP1	1.2079514	Isopropyl isothiocyanate	0.172385747
gene-CSRP1	1.2079514	(9Z)-Octadecenoic acid	0.142359556
gene-CSRP1	1.2079514	9-deoxy-9-methylene-16,16-dimethyl -PGE	0.606893884
gene-CSRP1	1.2079514	CL(8:0/8:0/18:2(9Z,11Z)/20:0)	0.622988418
gene-ANKS3	1.206526356	3-Thiacytidine	0.209387412
gene-ANKS3	1.206526356	D-Erythro-imidazole-glycerol-phosphate	0.355812431
gene-ANKS3	1.206526356	11-Maleimidoundecanoic acid	1.084942397
gene-ANKS3	1.206526356	cis-p-Menth-2-en-1-ol	0.201017988
gene-ANKS3	1.206526356	PE(20:0/18:1(12Z)-2OH(9,10))	0.438658253
gene-ANKS3	1.206526356	PE(22:2(13Z,16Z)/22:5(4Z,7Z,10Z,13Z,19Z)-O	0.369745166
gene-ANKS3	1.206526356	PG(20:1(11Z)/18:3(10,12,15)-OH(9))	0.626244347
gene-ANKS3	1.206526356	2-Methyl-3-phenyl-2-propenal	0.407813975
gene-ANKS3	1.206526356	PC(P-18:1(11Z)/PGE2)	0.509503133
gene-ANKS3	1.206526356	Roxithromycin	0.268273077
gene-ANKS3	1.206526356	Sitosterol beta-D-glucoside	0.382944796
gene-ANKS3	1.206526356	PC(P-18:1(11Z)/PGJ2)	0.565243877
gene-ANKS3	1.206526356	PS(20:0/20:4(8Z,11Z,14Z,17Z)-2OH(5S,6R))	0.402595921

Bos_taurus_newG	1.206420165	5-(3'-Carboxy-3'-oxopropenyl)-4,6-dihydro:	1.083232395
Bos_taurus_newG	1.206420165	Dehypoxanthine futalosine	0.048862499
Bos_taurus_newG	1.206420165	Hexahydro-4-methylphthalic anhydride	0.691665693
Bos_taurus_newG	1.206420165	Glutamate carbon	0.671444516
Bos_taurus_newG	1.206420165	Val-Cit	0.452308451
Bos_taurus_newG	1.206420165	Monacolin L acid	1.060562098
Bos_taurus_newG	1.206420165	3,4,3',4'-Tetrahydrospirilloxanthin	0.354666148
Bos_taurus_newG	1.206420165	Norophthalmic acid	0.191432411
Bos_taurus_newG	1.206420165	(1R,6S)-6-Amino-5-oxocyclohex-2-ene-1-c	0.154751123
Bos_taurus_newG	1.206420165	2-Hydroxyglutaric acid diethyl ester	0.799886767
Bos_taurus_newG	1.206420165	(S)-Mandelic acid O-beta-D-Glucopyranosid	0.509637316
Bos_taurus_newG	1.206420165	tetranor-PGAM	0.584223916
Bos_taurus_newG	1.206420165	2-(1-Adamantyl)-1,3-dioxetane	0.408916843
Bos_taurus_newG	1.206420165	2-Oxo-10-methylthiodecanoic acid	0.695908469
Bos_taurus_newG	1.206420165	ent-16b,19-Kauranediol 19-acetate	0.400676827
Bos_taurus_newG	1.206420165	Etazolate	0.149098864
Bos_taurus_newG	1.206420165	Roxithromycin	0.268273077
gene-CDK3	1.206301877	5-(Ethylthio)-1H-tetrazole	0.29717344
gene-CDK3	1.206301877	3-Thiacytidine	0.209387412
gene-CDK3	1.206301877	LTB4-d4	0.226799982
gene-CDK3	1.206301877	DG(15:0/PGE1/0:0)	0.462019057
gene-CDK3	1.206301877	5-(2-Aminopropyl)-2-methylphenol	0.323231457
gene-CDK3	1.206301877	4-Dimethylamino-L-phenylalanine	0.242110226
gene-CDK3	1.206301877	Trimetazidine	0.438282534
gene-CDK3	1.206301877	Armillane	0.52808635
gene-CDK3	1.206301877	5'-S-Methyl-5'-thioinosine	0.28772476
gene-CDK3	1.206301877	(Z)-3-Oxo-2-(2-pentenyl)-1-cyclopenteneac	0.179805915
gene-CDK3	1.206301877	2-Methyl-3-phenyl-2-propenal	0.407813975
gene-CDK3	1.206301877	PC(P-18:1(11Z)/PGE2)	0.509503133
gene-CDK3	1.206301877	(3R,4R)-3-Amino-1-hydroxy-4-methylpyrrol	0.471914506
gene-CDK3	1.206301877	n-methyl-2-(4'-methylaminophenyl)-6-hydr	0.26655714
gene-CDK3	1.206301877	Gamithromycin	0.37168241
gene-CDK3	1.206301877	PE(P-16:0/18:4(6Z,9Z,12Z,15Z))	0.546989864
gene-CDK3	1.206301877	Isopropyl isothiocyanate	0.172385747
gene-CDK3	1.206301877	(9Z)-Octadecenoic acid	0.142359556
gene-CDK3	1.206301877	N-Myristoyl Glutamine	0.37856966
gene-CDK3	1.206301877	PC(18:1(9Z)/15:1(9Z))	0.534724682
gene-CDK3	1.206301877	arachidyl amido cholanoic acid	1.24842952
gene-CDK3	1.206301877	9-deoxy-9-methylene-16,16-dimethyl -PGE	0.606893884
gene-CDK3	1.206301877	1-Octadecanoyl-2-(7Z,10Z,13Z,16Z-docosat	0.690496314
gene-CDK3	1.206301877	2-Propenyl 2-aminobenzoate	0.128406829
gene-CDK3	1.206301877	CL(8:0/8:0/18:2(9Z,11Z)/20:0)	0.622988418
gene-CDK3	1.206301877	PC(20:5(5Z,8Z,11Z,14Z,17Z)/P-16:0)	1.301095986
gene-CDK3	1.206301877	DG(20:0/LTE4/0:0)	0.438074508
gene-CD63	1.206261182	3-Thiacytidine	0.209387412
gene-CD63	1.206261182	D-Erythro-imidazole-glycerol-phosphate	0.355812431
gene-CD63	1.206261182	LTB4-d4	0.226799982
gene-CD63	1.206261182	(-)-alpha-Terpineol	0.345716279
gene-CD63	1.206261182	Cyclosporin A	0.656529229
gene-CD63	1.206261182	5-(2-Aminopropyl)-2-methylphenol	0.323231457
gene-CD63	1.206261182	4-Dimethylamino-L-phenylalanine	0.242110226

gene-CD63	1.206261182	Armillane	0.52808635
gene-CD63	1.206261182	PC(P-18:1(11Z)/PGE2)	0.509503133
gene-CD63	1.206261182	(3R,4R)-3-Amino-1-hydroxy-4-methylpyrrol	0.471914506
gene-CD63	1.206261182	Isopropyl isothiocyanate	0.172385747
gene-CD63	1.206261182	Nigroxanthin	0.705005059
gene-CD63	1.206261182	PC(P-18:1(11Z)/PGJ2)	0.565243877
gene-CD63	1.206261182	PS(20:0/20:4(8Z,11Z,14Z,17Z)-2OH(5S,6R))	0.402595921
gene-CD63	1.206261182	CL(8:0/8:0/18:2(9Z,11Z)/20:0)	0.622988418
gene-DST	1.205345492	5-(3'-Carboxy-3'-oxopropenyl)-4,6-dihydro›	1.083232395
gene-DST	1.205345492	D-Erythro-imidazole-glycerol-phosphate	0.355812431
gene-DST	1.205345492	Glutamate carbon	0.671444516
gene-DST	1.205345492	Monacolin L acid	1.060562098
gene-DST	1.205345492	cis-p-Menth-2-en-1-ol	0.201017988
gene-DST	1.205345492	PE(22:2(13Z,16Z)/22:5(4Z,7Z,10Z,13Z,19Z)-O	0.369745166
gene-DST	1.205345492	Norophthalmic acid	0.191432411
gene-DST	1.205345492	(1R,6S)-6-Amino-5-oxocyclohex-2-ene-1-c	0.154751123
gene-DST	1.205345492	2-(1-Adamantyl)-1,3-dioxetane	0.408916843
gene-DST	1.205345492	alpha-Terpineol formate	0.628238986
gene-DST	1.205345492	4-Octylphenol	1.400657885
gene-DST	1.205345492	PC(P-18:1(11Z)/PGE2)	0.509503133
gene-DST	1.205345492	Roxithromycin	0.268273077
gene-DST	1.205345492	PC(P-18:1(11Z)/PGJ2)	0.565243877
gene-DST	1.205345492	PS(20:0/20:4(8Z,11Z,14Z,17Z)-2OH(5S,6R))	0.402595921
gene-LOC10496{	1.204967363	D-Erythro-imidazole-glycerol-phosphate	0.355812431
gene-LOC10496{	1.204967363	11-Maleimidoundecanoic acid	1.084942397
gene-LOC10496{	1.204967363	13(S)-HpODE	0.163157753
gene-LOC10496{	1.204967363	LTB4-d4	0.226799982
gene-LOC10496{	1.204967363	cis-p-Menth-2-en-1-ol	0.201017988
gene-LOC10496{	1.204967363	(1R,6S)-6-Amino-5-oxocyclohex-2-ene-1-c	0.154751123
gene-LOC10496{	1.204967363	2-Methyl-3-phenyl-2-propenal	0.407813975
gene-LOC10496{	1.204967363	3',4',5'-Trimethoxycinnamyl alcohol acetate	0.4221816
gene-LOC10496{	1.204967363	PC(P-18:1(11Z)/PGE2)	0.509503133
gene-LOC10496{	1.204967363	9-OxoODE	0.111803831
gene-LOC10496{	1.204967363	PC(P-18:1(11Z)/PGE1)	0.295520345
gene-LOC10496{	1.204967363	Roxithromycin	0.268273077
gene-LOC10496{	1.204967363	PC(P-18:1(11Z)/PGJ2)	0.565243877
gene-PCBP4	1.20496439	3-Thiacytidine	0.209387412
gene-PCBP4	1.20496439	D-Erythro-imidazole-glycerol-phosphate	0.355812431
gene-PCBP4	1.20496439	LTB4-d4	0.226799982
gene-PCBP4	1.20496439	cis-p-Menth-2-en-1-ol	0.201017988
gene-PCBP4	1.20496439	(-)-alpha-Terpineol	0.345716279
gene-PCBP4	1.20496439	PG(20:1(11Z)/18:3(10,12,15)-OH(9))	0.626244347
gene-PCBP4	1.20496439	5-(2-Aminopropyl)-2-methylphenol	0.323231457
gene-PCBP4	1.20496439	4-Dimethylamino-L-phenylalanine	0.242110226
gene-PCBP4	1.20496439	Armillane	0.52808635
gene-PCBP4	1.20496439	2-Methyl-3-phenyl-2-propenal	0.407813975
gene-PCBP4	1.20496439	PC(P-18:1(11Z)/PGE2)	0.509503133
gene-PCBP4	1.20496439	(3R,4R)-3-Amino-1-hydroxy-4-methylpyrrol	0.471914506
gene-PCBP4	1.20496439	Isopropyl isothiocyanate	0.172385747
gene-PCBP4	1.20496439	(9Z)-Octadecenoic acid	0.142359556
gene-PCBP4	1.20496439	PC(P-18:1(11Z)/PGJ2)	0.565243877

gene-PCBP4	1.20496439	PS(20:0/20:4(8Z,11Z,14Z,17Z)-2OH(5S,6R))	0.402595921
gene-PCBP4	1.20496439	CL(8:0/8:0/18:2(9Z,11Z)/20:0)	0.622988418
gene-CBX7	1.204715048	3-Thiacytidine	0.209387412
gene-CBX7	1.204715048	D-Erythro-imidazole-glycerol-phosphate	0.355812431
gene-CBX7	1.204715048	LTB4-d4	0.226799982
gene-CBX7	1.204715048	cis-p-Menth-2-en-1-ol	0.201017988
gene-CBX7	1.204715048	(-)-alpha-Terpineol	0.345716279
gene-CBX7	1.204715048	1,4-Undecadiene	0.462803973
gene-CBX7	1.204715048	PG(20:1(11Z)/18:3(10,12,15)-OH(9))	0.626244347
gene-CBX7	1.204715048	5-(2-Aminopropyl)-2-methylphenol	0.323231457
gene-CBX7	1.204715048	4-Dimethylamino-L-phenylalanine	0.242110226
gene-CBX7	1.204715048	Armillane	0.52808635
gene-CBX7	1.204715048	PC(P-18:1(11Z)/PGE2)	0.509503133
gene-CBX7	1.204715048	(3R,4R)-3-Amino-1-hydroxy-4-methylpyrrol	0.471914506
gene-CBX7	1.204715048	Isopropyl isothiocyanate	0.172385747
gene-CBX7	1.204715048	(9Z)-Octadecenoic acid	0.142359556
gene-CBX7	1.204715048	PC(P-18:1(11Z)/PGJ2)	0.565243877
gene-CBX7	1.204715048	PS(20:0/20:4(8Z,11Z,14Z,17Z)-2OH(5S,6R))	0.402595921
gene-CBX7	1.204715048	CL(8:0/8:0/18:2(9Z,11Z)/20:0)	0.622988418
gene-HRH1	1.203883889	D-Erythro-imidazole-glycerol-phosphate	0.355812431
gene-HRH1	1.203883889	2-Dehydro-3-deoxy-D-gluconate	0.65165299
gene-HRH1	1.203883889	11-Maleimidoundecanoic acid	1.084942397
gene-HRH1	1.203883889	13(S)-HpODE	0.163157753
gene-HRH1	1.203883889	(1R,6S)-6-Amino-5-oxocyclohex-2-ene-1-c	0.154751123
gene-HRH1	1.203883889	2-Methyl-3-phenyl-2-propenal	0.407813975
gene-HRH1	1.203883889	3-(3-Methylbutylidene)-1(3H)-isobenzofurar	0.575422414
gene-HRH1	1.203883889	3',4',5'-Trimethoxycinnamyl alcohol acetate	0.4221816
gene-HRH1	1.203883889	PC(P-18:1(11Z)/PGE2)	0.509503133
gene-HRH1	1.203883889	Roxithromycin	0.268273077
gene-HRH1	1.203883889	PC(P-18:1(11Z)/PGJ2)	0.565243877
gene-HRH1	1.203883889	PS(20:0/20:4(8Z,11Z,14Z,17Z)-2OH(5S,6R))	0.402595921
gene-TENM4	1.202287385	D-Erythro-imidazole-glycerol-phosphate	0.355812431
gene-TENM4	1.202287385	Glutamate carbon	0.671444516
gene-TENM4	1.202287385	1-(2-Furanyl)-1-pentanone	0.697147283
gene-TENM4	1.202287385	2-Dehydro-3-deoxy-D-gluconate	0.65165299
gene-TENM4	1.202287385	Undecanedioic acid	0.059955634
gene-TENM4	1.202287385	16-Hydroxy-10-oxohexadecanoic acid	0.72302152
gene-TENM4	1.202287385	(1R,6S)-6-Amino-5-oxocyclohex-2-ene-1-c	0.154751123
gene-TENM4	1.202287385	Hexanoylglutamine	0.593876112
gene-TENM4	1.202287385	2-(1-Adamantyl)-1,3-dioxetane	0.408916843
gene-TENM4	1.202287385	Chamissonin diacetate	0.324896191
gene-TENM4	1.202287385	L-Anticapsin	0.426006862
gene-TENM4	1.202287385	6-Hydroxypentadecanoylcarnitine	0.820747254
gene-TENM4	1.202287385	Roxithromycin	0.268273077
gene-BTBD8	1.200604639	Isomaltotriose	0.58434654
gene-BTBD8	1.200604639	Glutamate carbon	0.671444516
gene-BTBD8	1.200604639	Cholic acid glucuronide	0.147398811
gene-BTBD8	1.200604639	11-Maleimidoundecanoic acid	1.084942397
gene-BTBD8	1.200604639	PE(22:2(13Z,16Z)/22:5(4Z,7Z,10Z,13Z,19Z)-O	0.369745166
gene-BTBD8	1.200604639	(1R,6S)-6-Amino-5-oxocyclohex-2-ene-1-c	0.154751123
gene-BTBD8	1.200604639	beta-Thujaplicin	0.681866194

gene-BTBD8	1.200604639	3-(3-Methylbutylidene)-1(3H)-isobenzofurar	0.575422414
gene-BTBD8	1.200604639	PC(P-18:1(11Z)/PGE2)	0.509503133
gene-BTBD8	1.200604639	Roxithromycin	0.268273077
gene-BTBD8	1.200604639	PC(P-18:1(11Z)/PGJ2)	0.565243877
gene-BTBD8	1.200604639	PS(20:0/20:4(8Z,11Z,14Z,17Z)-2OH(5S,6R))	0.402595921
gene-AGER	1.199735097	3-Thiacytidine	0.209387412
gene-AGER	1.199735097	5-Hydroxy-2-oxo-4-ureido-2,5-dihydro-1H	0.122536033
gene-AGER	1.199735097	11-Maleimidoundecanoic acid	1.084942397
gene-AGER	1.199735097	LTB4-d4	0.226799982
gene-AGER	1.199735097	cis-p-Menth-2-en-1-ol	0.201017988
gene-AGER	1.199735097	PE(20:0/18:1(12Z)-2OH(9,10))	0.438658253
gene-AGER	1.199735097	PE(22:2(13Z,16Z)/22:5(4Z,7Z,10Z,13Z,19Z)-O	0.369745166
gene-AGER	1.199735097	Cyclosporin A	0.656529229
gene-AGER	1.199735097	Epomusenin A	0.766206049
gene-AGER	1.199735097	PA(22:6(4Z,7Z,10Z,13Z,16Z,19Z)/16:0)	0.556532497
gene-AGER	1.199735097	DG(18:0/LTE4/0:0)	0.681485773
gene-AGER	1.199735097	PC(17:0/PGJ2)	0.657689319
gene-AGER	1.199735097	PC(14:0/20:2(11Z,14Z))	1.110657378
gene-AGER	1.199735097	CDP-DG(PGF2alpha/16:0)	1.002512277
gene-AGER	1.199735097	C20914	0.140383181
gene-AGER	1.199735097	Guanidoacetic acid	0.542509265
gene-AGER	1.199735097	alpha-Terpineol formate	0.628238986
gene-AGER	1.199735097	Methionyl-Valine	0.502707745
gene-AGER	1.199735097	1,4,6-Trimethylnaphthalene	0.12150693
gene-AGER	1.199735097	N-Palmitoyl Proline	0.470367693
gene-AGER	1.199735097	10-alpha-methoxy-9,10-dihydrolysergol	0.140851438
gene-AGER	1.199735097	PC(P-18:1(11Z)/PGE2)	0.509503133
gene-AGER	1.199735097	Phorone A	0.079039095
gene-AGER	1.199735097	LysoPI(0:0/18:0)	0.420441153
gene-AGER	1.199735097	(9Z)-Octadecenoic acid	0.142359556
gene-AGER	1.199735097	PC(18:1(9Z)/15:1(9Z))	0.534724682
gene-AGER	1.199735097	arachidyl amido cholanoic acid	1.24842952
gene-AGER	1.199735097	Nigroxanthin	0.705005059
gene-AGER	1.199735097	1-Octadecanoyl-2-(7Z,10Z,13Z,16Z-docosat	0.690496314
gene-AGER	1.199735097	PC(P-18:1(11Z)/PGJ2)	0.565243877
gene-AGER	1.199735097	PS(20:0/20:4(8Z,11Z,14Z,17Z)-2OH(5S,6R))	0.402595921
gene-AGER	1.199735097	PS(16:1(9Z)/22:2(13Z,16Z))	0.659652661
gene-AGER	1.199735097	PC(P-16:0/20:3(8Z,11Z,14Z)-2OH(5,6))	0.174122169
gene-AGER	1.199735097	DG(20:0/LTE4/0:0)	0.438074508
gene-LOC61690:	1.198951222	D-Erythro-imidazole-glycerol-phosphate	0.355812431
gene-LOC61690:	1.198951222	7(14)-Bisabolene-2,3,10,11-tetrol	2.446897974
gene-LOC61690:	1.198951222	cis-p-Menth-2-en-1-ol	0.201017988
gene-LOC61690:	1.198951222	(-)-alpha-Terpineol	0.345716279
gene-LOC61690:	1.198951222	1,4-Undecadiene	0.462803973
gene-LOC61690:	1.198951222	PG(20:1(11Z)/18:3(10,12,15)-OH(9))	0.626244347
gene-LOC61690:	1.198951222	5-(2-Aminopropyl)-2-methylphenol	0.323231457
gene-LOC61690:	1.198951222	4-Dimethylamino-L-phenylalanine	0.242110226
gene-LOC61690:	1.198951222	Armillane	0.52808635
gene-LOC61690:	1.198951222	PC(P-18:1(11Z)/PGE2)	0.509503133
gene-LOC61690:	1.198951222	(3R,4R)-3-Amino-1-hydroxy-4-methylpyrrol	0.471914506
gene-LOC61690:	1.198951222	n-methyl-2-(4'-methylaminophenyl)-6-hydr	0.26655714

gene-LOC61690:	1.198951222	Isopropyl isothiocyanate	0.172385747
gene-LOC61690:	1.198951222	PS(20:0/20:4(8Z,11Z,14Z,17Z)-2OH(5S,6R))	0.402595921
gene-LOC10190:	1.198723395	Glutamate carbon	0.671444516
gene-LOC10190:	1.198723395	Adrenosterone	0.050790129
gene-LOC10190:	1.198723395	beta-Thujaplicin	0.681866194
gene-SAP25	1.197068967	milbemycin beta3	1.414447124
gene-SAP25	1.197068967	(-)-alpha-Terpineol	0.345716279
gene-SAP25	1.197068967	Cyclosporin A	0.656529229
gene-SAP25	1.197068967	PG(20:1(11Z)/18:3(10,12,15)-OH(9))	0.626244347
gene-SAP25	1.197068967	5-(2-Aminopropyl)-2-methylphenol	0.323231457
gene-SAP25	1.197068967	Armillane	0.52808635
gene-SAP25	1.197068967	PC(P-18:1(11Z)/PGE2)	0.509503133
gene-SAP25	1.197068967	Nigroxanthin	0.705005059
gene-SAP25	1.197068967	PC(P-18:1(11Z)/PGJ2)	0.565243877
gene-SAP25	1.197068967	PS(20:0/20:4(8Z,11Z,14Z,17Z)-2OH(5S,6R))	0.402595921
gene-SAP25	1.197068967	CL(8:0/8:0/18:2(9Z,11Z)/20:0)	0.622988418
gene-LOC78974ε	1.196276935	3-Thiacytidine	0.209387412
gene-LOC78974ε	1.196276935	3-Deoxyestrone	0.282221709
gene-LOC78974ε	1.196276935	1-Oleoyl-sn-glycero-3-phosphocholine	0.18926952
gene-LOC78974ε	1.196276935	LTB4-d4	0.226799982
gene-LOC78974ε	1.196276935	(-)-alpha-Terpineol	0.345716279
gene-LOC78974ε	1.196276935	1,4-Undecadiene	0.462803973
gene-LOC78974ε	1.196276935	Cyclosporin A	0.656529229
gene-LOC78974ε	1.196276935	5-(2-Aminopropyl)-2-methylphenol	0.323231457
gene-LOC78974ε	1.196276935	4-Dimethylamino-L-phenylalanine	0.242110226
gene-LOC78974ε	1.196276935	Trimetazidine	0.438282534
gene-LOC78974ε	1.196276935	ingenol	0.942071652
gene-LOC78974ε	1.196276935	Armillane	0.52808635
gene-LOC78974ε	1.196276935	PC(P-18:1(11Z)/PGE2)	0.509503133
gene-LOC78974ε	1.196276935	(3R,4R)-3-Amino-1-hydroxy-4-methylpyrrol	0.471914506
gene-LOC78974ε	1.196276935	n-methyl-2-(4'-methylaminophenyl)-6-hydr	0.26655714
gene-LOC78974ε	1.196276935	Isopropyl isothiocyanate	0.172385747
gene-LOC78974ε	1.196276935	9-deoxy-9-methylene-16,16-dimethyl -PGE:	0.606893884
gene-LOC78974ε	1.196276935	CL(8:0/8:0/18:2(9Z,11Z)/20:0)	0.622988418
Bos_taurus_newG	1.19500476	Hydroxytyrosol 1-O-glucoside	0.313284156
Bos_taurus_newG	1.19500476	Val-Tyr-Leu-Arg	0.040793217
gene-SCNN1D	1.193840919	D-Erythro-imidazole-glycerol-phosphate	0.355812431
gene-SCNN1D	1.193840919	Erythronic acid	0.494007482
gene-SCNN1D	1.193840919	(S)-10,16-Dihydroxyhexadecanoic acid	1.054006526
gene-SCNN1D	1.193840919	Auxin b	1.052491354
gene-SCNN1D	1.193840919	13(S)-HPOT	1.353059742
gene-SCNN1D	1.193840919	cis-p-Menth-2-en-1-ol	0.201017988
gene-SCNN1D	1.193840919	11-Oxahexadecanolide	2.378626329
gene-SCNN1D	1.193840919	PG(20:1(11Z)/18:3(10,12,15)-OH(9))	0.626244347
gene-SCNN1D	1.193840919	Asparaginylcysteine	0.467538645
gene-SCNN1D	1.193840919	(1R,6S)-6-Amino-5-oxocyclohex-2-ene-1-c	0.154751123
gene-SCNN1D	1.193840919	3-Methoxytyramine	0.928926425
gene-SCNN1D	1.193840919	Hexanoylglutamine	0.593876112
gene-SCNN1D	1.193840919	7-Aminomethyl-7-carbaguanine	0.474992724
gene-SCNN1D	1.193840919	Nevirapine	0.820759262
gene-SCNN1D	1.193840919	2-Methyl-3-phenyl-2-propenal	0.407813975

gene-SCNN1D	1.193840919	Ajulemic acid	0.557832951
gene-SCNN1D	1.193840919	PC(P-18:1(11Z)/PGE2)	0.509503133
gene-SCNN1D	1.193840919	PG(20:1(11Z)/18:3(9,11,15)-OH(13))	0.69829683
gene-PGD	1.19186774	3-Thiacytidine	0.209387412
gene-PGD	1.19186774	D-Erythro-imidazole-glycerol-phosphate	0.355812431
gene-PGD	1.19186774	LTB4-d4	0.226799982
gene-PGD	1.19186774	cis-p-Menth-2-en-1-ol	0.201017988
gene-PGD	1.19186774	(-)-alpha-Terpineol	0.345716279
gene-PGD	1.19186774	Cyclosporin A	0.656529229
gene-PGD	1.19186774	5-(2-Aminopropyl)-2-methylphenol	0.323231457
gene-PGD	1.19186774	Armillane	0.52808635
gene-PGD	1.19186774	PC(P-18:1(11Z)/PGE2)	0.509503133
gene-PGD	1.19186774	Isopropyl isothiocyanate	0.172385747
gene-PGD	1.19186774	Nigroxanthin	0.705005059
gene-PGD	1.19186774	PC(P-18:1(11Z)/PGJ2)	0.565243877
gene-PGD	1.19186774	PS(20:0/20:4(8Z,11Z,14Z,17Z)-2OH(5S,6R))	0.402595921
gene-PGD	1.19186774	CL(8:0/8:0/18:2(9Z,11Z)/20:0)	0.622988418
Bos_taurus_newG	1.191422004	D-Erythro-imidazole-glycerol-phosphate	0.355812431
Bos_taurus_newG	1.191422004	Urotensin-related peptide	0.9290699
Bos_taurus_newG	1.191422004	Netupitant	0.707108724
Bos_taurus_newG	1.191422004	Benzoyl-fvr-pna	0.67370714
Bos_taurus_newG	1.191422004	Metkephamid	1.159767003
Bos_taurus_newG	1.191422004	Sorbitan laurate	0.242315968
Bos_taurus_newG	1.191422004	Becocalcidiol	0.63162902
Bos_taurus_newG	1.191422004	4-Gingerol	0.222165012
Bos_taurus_newG	1.191422004	Polysorbate 20	0.727334778
Bos_taurus_newG	1.191422004	Monacolin L acid	1.060562098
Bos_taurus_newG	1.191422004	LTB4-d4	0.226799982
Bos_taurus_newG	1.191422004	cis-p-Menth-2-en-1-ol	0.201017988
Bos_taurus_newG	1.191422004	PE(20:0/18:1(12Z)-2OH(9,10))	0.438658253
Bos_taurus_newG	1.191422004	DG(15:0/PGE1/0:0)	0.462019057
Bos_taurus_newG	1.191422004	Pseudouridine 5'-phosphate	1.18431378
Bos_taurus_newG	1.191422004	Ribavirin monophosphate	1.091085027
Bos_taurus_newG	1.191422004	Norophthalmic acid	0.191432411
Bos_taurus_newG	1.191422004	3'-N'-Acetylfusarochromanone	0.755683206
Bos_taurus_newG	1.191422004	Ser Cys Ala Ala	0.603032447
Bos_taurus_newG	1.191422004	Loganin	0.92782666
Bos_taurus_newG	1.191422004	3'-Deoxythymidine	0.791830758
Bos_taurus_newG	1.191422004	kainic acid	0.836445456
Bos_taurus_newG	1.191422004	Tryptophyl-Glutamine	0.89722186
Bos_taurus_newG	1.191422004	2-Methyl-3-phenyl-2-propenal	0.407813975
Bos_taurus_newG	1.191422004	Guanidoacetic acid	0.542509265
Bos_taurus_newG	1.191422004	4-Oxo-9-cis-retinoyl-beta-glucuronide	1.611773742
Bos_taurus_newG	1.191422004	alpha-Terpineol formate	0.628238986
Bos_taurus_newG	1.191422004	4-Dihydroboldenone	0.692863202
Bos_taurus_newG	1.191422004	2'-Fluoro-2',3'-dideoxyinosine	1.214745496
Bos_taurus_newG	1.191422004	20-carboxy Arachidonic Acid	0.780988382
Bos_taurus_newG	1.191422004	Nebramycin 5'	0.909280571
Bos_taurus_newG	1.191422004	4-Octylphenol	1.400657885
Bos_taurus_newG	1.191422004	Prolyl-Lysine	1.612991304
Bos_taurus_newG	1.191422004	3-Pentadecylphenol	1.682258188

Bos_taurus_newG	1.191422004	7-Sulfocholic acid	0.039985455
Bos_taurus_newG	1.191422004	10-alpha-methoxy-9,10-dihydrolysergol	0.140851438
Bos_taurus_newG	1.191422004	N-Stearoyl Glutamine	1.862695384
Bos_taurus_newG	1.191422004	Palmitoylcarnitine	0.74779616
Bos_taurus_newG	1.191422004	PC(P-18:1(11Z)/PGE2)	0.509503133
Bos_taurus_newG	1.191422004	Galabiosylceramide (d18:1/20:0)	0.746737323
Bos_taurus_newG	1.191422004	LysoPI(0:0/18:0)	0.420441153
Bos_taurus_newG	1.191422004	12-Hydroxyicosanoylcarnitine	1.730979067
Bos_taurus_newG	1.191422004	N-Myristoyl Glutamine	0.37856966
Bos_taurus_newG	1.191422004	PC(P-18:1(11Z)/PGJ2)	0.565243877
Bos_taurus_newG	1.191422004	PE(18:0/20:4(8Z,11Z,14Z,17Z))	0.344922006
Bos_taurus_newG	1.191422004	PS(16:1(9Z)/22:2(13Z,16Z))	0.659652661
gene-KIF20B	1.188934625	2-[(3S)-3-[[(2S)-1-(Carboxymethoxy)-1-oxo	0.111577437
gene-KIF20B	1.188934625	Valyltryptophan	0.048149463
gene-LOC78785	1.188846419	L-Aspartate-semialdehyde	0.240059822
gene-LOC78785	1.188846419	Ascorbic acid 3-sulfate	0.300057469
gene-LOC78785	1.188846419	Methylmalonate	0.241481249
gene-LOC78785	1.188846419	Nona-4,6-dienoylcarnitine	0.567004808
gene-LOC78785	1.188846419	Arachidonic Acid (peroxide free)	0.666664243
gene-LOC78785	1.188846419	delta9-Tetrahydrocannabinol hemisuccinate	0.6166092
gene-LOC78785	1.188846419	Dextrorphan O-glucuronide	1.59476827
gene-LOC78785	1.188846419	Zucchini factor B	0.491282625
gene-LOC78785	1.188846419	Olsalazine-O-sulfate	0.134443146
gene-LOC78785	1.188846419	uric acid citrate	0.134413774
gene-LOC78785	1.188846419	PIP(20:0/18:1(12Z)-2OH(9,10))	0.044932711
gene-LOC78785	1.188846419	Niveusin C	0.161103545
gene-LOC78785	1.188846419	Diguanosine tetraphosphate	0.108790139
gene-LOC78785	1.188846419	N-Undecylbenzenesulfonic acid	0.139246407
gene-LOC78785	1.188846419	Furfural	0.12232573
gene-LOC78785	1.188846419	4-Carboxypyrazole	0.142560642
gene-LOC78785	1.188846419	sn-Glycerol 3-phosphate	0.129764323
gene-LOC78785	1.188846419	1-(3-Hydroxy-5-methyl-2-thienyl)ethanone	0.147518223
gene-LOC78785	1.188846419	thiol-maleimide	0.127080912
gene-LOC78785	1.188846419	Adenosine 3',5'-diphosphate	0.084396755
gene-LOC78785	1.188846419	Chelidonic acid	0.123550587
gene-LOC78785	1.188846419	D-Erythrose 4-phosphate	0.125002415
gene-LOC78785	1.188846419	Daucic acid	0.126034217
gene-LOC78785	1.188846419	dehydroascorbate (bicyclic form)	0.129137601
gene-LOC78785	1.188846419	Phosphogluconic acid	0.082715183
gene-LOC78785	1.188846419	(2-Carbamoyl-7-oxo-1,6-diazabicyclo[3.2.1]	0.105323986
gene-LOC78785	1.188846419	D-glycero-beta-D-manno-Heptose 1,7-bisp	0.122135244
gene-LOC78785	1.188846419	6-Methylthiopurine ribonucleoside	0.091885816
gene-LOC78785	1.188846419	2,3-Dihydroxy-2,4-cyclopentadien-1-one	0.158144253
gene-LOC78785	1.188846419	2-Hydroxy-2,4-pentadienoate	0.101082294
gene-LOC78785	1.188846419	4-Hydroxyphthalide	0.119380362
gene-LOC78785	1.188846419	cis-2-Methylaconitate	0.124396559
gene-LOC78785	1.188846419	Mercaptosuccinic acid	0.132992993
gene-LOC78785	1.188846419	1,5-Isoquinolinediol	0.088322271
gene-LOC78785	1.188846419	5-Hydroxyisourate	0.134630363
gene-LOC78785	1.188846419	D-Glyceraldehyde 3-phosphate	0.12399121
gene-LOC78785	1.188846419	Methazolamide	0.1226037

gene-LOC78785ℇ	1.188846419	Bergaptol	0.119008411
gene-LOC78785ℇ	1.188846419	4-Ketoniridazole	0.072360639
gene-LOC78785ℇ	1.188846419	1,7-Diphospho-1-epi-valienol	0.067989502
gene-LOC78785ℇ	1.188846419	Cysteic acid	0.090043703
gene-LOC78785ℇ	1.188846419	IDP	0.115710376
gene-LOC78785ℇ	1.188846419	PE-NMe(18:0/18:3(9Z,12Z,15Z))	0.681884774
gene-LOC78785ℇ	1.188846419	1-Cyclohexyl-N-{[1-(4-Methylphenyl)-1h-In	0.162668453
gene-LOC78785ℇ	1.188846419	Imidazotetrazine	0.117632006
gene-LOC78785ℇ	1.188846419	DG(14:0/PGE1/0:0)	0.323973925
gene-LOC78785ℇ	1.188846419	Barbituric acid	0.164689286
gene-LOC78785ℇ	1.188846419	CL(8:0/8:0/13:0/18:2(9Z,11Z))	0.088642926
gene-LOC78785ℇ	1.188846419	2-Hydroxybutyric acid	0.129292645
gene-LOC78785ℇ	1.188846419	Butylparaben	0.099047364
gene-LOC78785ℇ	1.188846419	Hydroxyisonobilin	0.259767036
gene-LOC78785ℇ	1.188846419	CDP-DG(18:0/PGE2)	0.280573668
gene-LOC78785ℇ	1.188846419	2-Dodecylbenzenesulfonic acid	0.177256025
gene-LOC78785ℇ	1.188846419	CDP-DG(PGJ2/18:0)	0.287350234
gene-LOC78785ℇ	1.188846419	Thiazole	0.144612903
gene-LOC78785ℇ	1.188846419	(1E)-4-Oxobut-1-ene-1,2,4-tricarboxylate	0.132863896
gene-LOC78785ℇ	1.188846419	7-Hydroxy-4-(trifluoromethyl)coumarin	0.126277898
gene-LOC78785ℇ	1.188846419	5,7-Dihydroxy-4H-1-benzopyran-4-one	0.122365814
gene-LOC78785ℇ	1.188846419	Microcystin LR	0.167364914
gene-LOC78785ℇ	1.188846419	Phosphonoacetate	0.124890095
gene-LOC78785ℇ	1.188846419	5-(Methoxycarbonyl)thiophene-2-carboxylic	0.083650045
gene-LOC78785ℇ	1.188846419	LysoPI(18:2(9Z,12Z)/0:0)	0.358006727
gene-LOC78785ℇ	1.188846419	PG(18:1(11Z)/LTE4)	0.265299982
gene-LOC78785ℇ	1.188846419	PI(16:0/22:4(10Z,13Z,16Z,19Z))	0.110181561
gene-LOC78785ℇ	1.188846419	[5-(Aminomethyl)furan-3-yl]methyl diphospl	0.138877972
gene-LOC78785ℇ	1.188846419	LysoPI(16:0/0:0)	0.379098336
gene-LOC78785ℇ	1.188846419	Hydroxyminaline	0.084122394
gene-LOC78785ℇ	1.188846419	3-Methyldioxyindole	0.113096293
gene-LOC78785ℇ	1.188846419	Theobromine	0.086592879
gene-LOC78785ℇ	1.188846419	Coumarin	0.111689981
gene-LOC78785ℇ	1.188846419	2-Chloro-L-phenylalanine	0.114608327
gene-LOC78785ℇ	1.188846419	2-Propenamide, 3-(3,4-dihydroxyphenyl)-	0.118389905
gene-LOC78785ℇ	1.188846419	GDP-D-mannuronate	0.164965956
gene-LOC78785ℇ	1.188846419	Degalloyltheaflavonin	0.303810853
gene-LOC78785ℇ	1.188846419	2-Amino-3-oxo-4-phosphonooxybutyrate	0.098315426
gene-LOC78785ℇ	1.188846419	2'-Deoxy-5-hydroxymethylcytidine-5'-triphc	0.146455502
gene-LOC78785ℇ	1.188846419	L-Adrenaline	0.226834671
gene-LOC78785ℇ	1.188846419	Methyl methacrylate	0.245645579
gene-LOC78785ℇ	1.188846419	1-Phenylpiperazine	0.353584896
gene-LOC78785ℇ	1.188846419	N-(2-(Methylamino)ethyl)-5-isoquinolinesulf	0.199224064
gene-LOC78785ℇ	1.188846419	p-coumaroyltriacetic acid lactone	0.057681273
gene-LOC78785ℇ	1.188846419	4-Hydroxy-3-methoxy-cinnamoylglycine	0.017759766
gene-LOC78785ℇ	1.188846419	4-Aminophenyl 1-thio-尾-D-glucuronide	0.18194032
gene-LOC78785ℇ	1.188846419	Guanosine 3'-phosphate	0.147360452
gene-LOC78785ℇ	1.188846419	3,3',5-Trihydroxy-4'-methoxy-6,7-methylene	0.137607639
gene-LOC78785ℇ	1.188846419	Asparaginyl-Methionine	0.228436756
gene-LOC78785ℇ	1.188846419	2-(Formamido)-N1-(5'-phosphoribosyl)aceta	0.35156894
gene-LOC78785ℇ	1.188846419	5-amino-1-(5-phosphonato-D-ribosyl)imida	0.622612791

gene-LOC78785ε	1.188846419	Belotecan	0.794871701
gene-LOC78785ε	1.188846419	8-Methoxykynurenate	0.424170332
gene-LOC78785ε	1.188846419	5-Hydroxyindoleacetaldehyde	0.448806354
gene-LOC78785ε	1.188846419	gamma-Butyrolactone	0.509389889
gene-LOC78785ε	1.188846419	(2S,4R)-4-(9H-Pyrido[3,4-b]indol-1-yl)-1,2,4	0.883685198
gene-LOC78785ε	1.188846419	Vignatic acid B	0.136235161
gene-LOC78785ε	1.188846419	LysoPA(22:5(7Z,10Z,13Z,16Z,19Z)/0:0)	0.291568015
gene-LOC78785ε	1.188846419	Bisbynin	0.993286894
gene-LOC78785ε	1.188846419	Etimizol	0.852969435
gene-LOC78785ε	1.188846419	Deoxyguanidinoproclavaminic acid	0.857672893
gene-LOC78785ε	1.188846419	Nicotine glucuronide	0.300940042
gene-LOC78785ε	1.188846419	Azelaic acid	0.007180175
gene-LOC78785ε	1.188846419	Pentahomomethionine	0.527487806
gene-LOC78785ε	1.188846419	Cinnavalininate	0.009509851
gene-LOC78785ε	1.188846419	2-(3-Carboxy-3-aminopropyl)-L-histidine	0.575765457
gene-LOC78785ε	1.188846419	3,4-Dimethyl-5-pentyl-2-furanpropanoic aci	0.896285742
gene-LOC78785ε	1.188846419	Uridine 2',3'-cyclic phosphate	0.201589726
gene-LOC78785ε	1.188846419	Oxamyl	0.077223423
gene-LOC78785ε	1.188846419	5-[(Diaminomethylidene)amino]-2-(sulfanylm	0.076227549
gene-LOC78785ε	1.188846419	N-[5-Methyl-8-(4-methylpiperazin-1-yl)-1,2	0.398723022
gene-LOC78785ε	1.188846419	5-Hydroxy-2-oxo-4-ureido-2,5-dihydro-1H	0.122536033
gene-LOC78785ε	1.188846419	p-Coumaroylputrescine	0.939330574
gene-LOC78785ε	1.188846419	Calicheamicinone	0.910932275
gene-LOC78785ε	1.188846419	Pterostilbene	0.899698314
gene-LOC78785ε	1.188846419	2-Hydroxymuconate	0.119367002
gene-LOC78785ε	1.188846419	Ancymidol	0.913827444
gene-LOC78785ε	1.188846419	Phenylalanylhydroxyproline	1.183668928
gene-LOC78785ε	1.188846419	Delgocitinib	1.051864373
gene-LOC78785ε	1.188846419	Melatonin	1.134145229
gene-LOC78785ε	1.188846419	2H-1-Benzopyran-2-one, aminomethyl-	0.385162237
gene-LOC78785ε	1.188846419	Trihomomethionine	0.296210774
gene-LOC78785ε	1.188846419	2,5-Dihydroxy-1-octadec-9-enoyloxypyrrole	0.828088186
gene-LOC78785ε	1.188846419	2-Maleylacetate	0.11603906
gene-LOC78785ε	1.188846419	2-[(2-Aminoethylcarbamoyl)methyl]-2-hydro	0.192497471
gene-LOC78785ε	1.188846419	Dehydroascorbate	0.130134414
gene-LOC78785ε	1.188846419	Alginic acid	0.103319393
gene-LOC78785ε	1.188846419	Cyclo(glycylleucylvalylleucylprolylseryl)	0.824045744
gene-LOC78785ε	1.188846419	Dihydroxy-(2-methoxyethoxy)-sulfanylidene-	0.124546591
gene-LOC78785ε	1.188846419	Hygromycin B	0.930396619
gene-LOC78785ε	1.188846419	Divinylprotochlorophyllide	0.629978387
gene-LOC78785ε	1.188846419	Metkephamid	1.159767003
gene-LOC78785ε	1.188846419	1-Hydroxyacorenone	0.352543272
gene-LOC78785ε	1.188846419	Tuberose lactone	1.590026295
gene-LOC78785ε	1.188846419	Leukotriene D4	0.6807158
gene-LOC78785ε	1.188846419	Azaspiracid 5	1.398898867
gene-LOC78785ε	1.188846419	PGP(20:2(11Z,14Z)/18:2(10E,12Z)+=O(9))	1.457698264
gene-LOC78785ε	1.188846419	2,3-Dihydro-6-methoxy-2,2-dimethyl-4H-1	0.145472538
gene-LOC78785ε	1.188846419	3,4-Dihydro-6-methoxy-2,2-dimethyl-2H-1	0.296379892
gene-LOC78785ε	1.188846419	Eicosanoyl-CoA	0.137091047
gene-LOC78785ε	1.188846419	3-Methylbutyl 3-oxobutanoate	0.188834236
gene-LOC78785ε	1.188846419	Brassilexin	0.175915609

gene-LOC78785‹	1.188846419	5-Heptyltetrahydro-2-oxo-3-furancarboxylic	0.178798355
gene-LOC78785‹	1.188846419	Baccatin III	0.776246161
gene-LOC78785‹	1.188846419	L-2-Aminoethyl seryl phosphate	0.215980578
gene-LOC78785‹	1.188846419	10-hydroxy capric acid	0.531721559
gene-LOC78785‹	1.188846419	PI(18:2(9Z,12Z)/PGF2alpha)	0.015701107
gene-LOC78785‹	1.188846419	3,4-Dihydroxy-9,10-secoandrosta-1,3,5(10)-	0.354025508
gene-LOC78785‹	1.188846419	(1'S)-Averantin	0.721766641
gene-LOC78785‹	1.188846419	2-Hexylbenzothiazole	0.396099297
gene-LOC78785‹	1.188846419	Heptyl 4-hydroxybenzoate	0.119601301
gene-LOC78785‹	1.188846419	9(S)-HpODE	0.09937774
gene-LOC78785‹	1.188846419	Cyclic GMP-AMP	0.023315703
gene-LOC78785‹	1.188846419	UDP-N-acetyl-3-(1-carboxyvinyl)-D-glucosa	0.097644027
gene-LOC78785‹	1.188846419	N-Lauroylglycine	0.266329671
gene-LOC78785‹	1.188846419	Peanut oil, hydrogenated	0.113412722
gene-LOC78785‹	1.188846419	I-Urobilinogen	0.120693099
gene-LOC78785‹	1.188846419	Fluoroacetate	0.09039162
gene-LOC78785‹	1.188846419	Valnemulin	0.408062514
gene-LOC78785‹	1.188846419	Ribavirin 5'-triphosphate	0.023732917
gene-LOC78785‹	1.188846419	1,1,2,2-Tetrahydroperfluoro-1-decanol	0.080705915
gene-LOC78785‹	1.188846419	Lotusine	0.129259623
gene-LOC78785‹	1.188846419	Testosterone isobutyrate	0.467664668
gene-LOC78785‹	1.188846419	6-isobutyl-4-hydroxy-2-pyrone	0.473678412
gene-LOC78785‹	1.188846419	Rotundine A	0.097713734
gene-LOC78785‹	1.188846419	DG(19:0/PGJ2/0:0)	0.415213526
gene-LOC78785‹	1.188846419	Ganoderic acid alpha	0.410281076
gene-LOC78785‹	1.188846419	PE(20:0/18:1(12Z)-2OH(9,10))	0.438658253
gene-LOC78785‹	1.188846419	CDP-DG(i-18:0/a-25:0)	0.24441782
gene-LOC78785‹	1.188846419	CPA(18:0/0:0)	0.085896823
gene-LOC78785‹	1.188846419	Avermectin A1b aglycone	0.34958727
gene-LOC78785‹	1.188846419	Fluoro 2,2-difluoroacetate	0.096767672
gene-LOC78785‹	1.188846419	PE(15:0/20:0)	0.306351064
gene-LOC78785‹	1.188846419	2-(3,7,11-Trimethyldodeca-2,6,10-trienylsulf	0.237778404
gene-LOC78785‹	1.188846419	meso-Tartaric acid	0.12328813
gene-LOC78785‹	1.188846419	PE(18:0/18:1(9Z)-O(12,13))	1.009186776
gene-LOC78785‹	1.188846419	PE(P-18:0/PGE1)	0.886597382
gene-LOC78785‹	1.188846419	Digitoxigenin bisdigitoxide	0.047257629
gene-LOC78785‹	1.188846419	2-Oxo-2,3-dihydrofuran-5-acetate	0.126701966
gene-LOC78785‹	1.188846419	2,2,2-Trifluoroethyl	0.180418313
gene-LOC78785‹	1.188846419	PE(20:0/20:3(8Z,11Z,14Z)-2OH(5,6))	0.154481111
gene-LOC78785‹	1.188846419	PE(14:1(9Z)/15:0)	0.042810582
gene-LOC78785‹	1.188846419	5-Hexyltetrahydro-2-furanoctanoic acid	0.343510978
gene-LOC78785‹	1.188846419	3-O-Sulfogalactosylceramide (d18:1/20:0)	0.255536938
gene-LOC78785‹	1.188846419	CDP-DG(20:4(5Z,8Z,11Z,14Z)/PGE2)	0.251717176
gene-LOC78785‹	1.188846419	PA(15:0/18:3(9Z,12Z,15Z))	0.137174289
gene-LOC78785‹	1.188846419	(S)-Ureidoglycolate	0.134540889
gene-LOC78785‹	1.188846419	PI(22:5(4Z,7Z,10Z,13Z,16Z)/PGE2)	0.104467297
gene-LOC78785‹	1.188846419	PE(16:1(9Z)/18:0)	0.077115313
gene-LOC78785‹	1.188846419	Methoxymalic acid	0.099765618
gene-LOC78785‹	1.188846419	Ceftizoxime	0.122210815
gene-LOC78785‹	1.188846419	Momordicin II	0.01234074
gene-LOC78785‹	1.188846419	Guanosine triphosphate adenosine	0.113994936

gene-LOC78785ɛ	1.188846419	Pinazepam	0.084413874
gene-LOC78785ɛ	1.188846419	(3S,7R)-16-Hydroxy-11-methoxy-6,8,19-trio	0.087126394
gene-LOC78785ɛ	1.188846419	Delphinidin 3-lathyroside 5-glucoside	0.213430556
gene-LOC78785ɛ	1.188846419	Carvone	0.069357978
gene-LOC78785ɛ	1.188846419	Alanylhydroxyproline	0.066041848
gene-LOC78785ɛ	1.188846419	Isoniazid alpha-ketoglutaric acid	0.101041109
gene-LOC78785ɛ	1.188846419	Calcein	0.072452031
gene-LOC78785ɛ	1.188846419	Amritoside	0.072777048
gene-LOC78785ɛ	1.188846419	Azetidinyl	0.131404372
gene-LOC78785ɛ	1.188846419	2,8-bis-Trifluoromethyl-4-quinoline carboxy	0.090998876
gene-LOC78785ɛ	1.188846419	(E)-2-Amino-5-phosphonopent-3-enoic aci	0.085518503
gene-LOC78785ɛ	1.188846419	Rutaretin 9-rutinoside	0.120184193
gene-LOC78785ɛ	1.188846419	5-Hydroxyferuloyl-CoA	0.11702028
gene-LOC78785ɛ	1.188846419	N5-Citryl-D-ornithine	0.232123154
gene-LOC78785ɛ	1.188846419	PI(18:1(11Z)/PGF1alpha)	0.243592581
gene-LOC78785ɛ	1.188846419	Heptanoylcholine	0.218998318
gene-LOC78785ɛ	1.188846419	1-(O-alpha-D-glucopyranosyl)-(1,3R,25R)-h	0.081253684
gene-LOC78785ɛ	1.188846419	Epomusenin A	0.766206049
gene-LOC78785ɛ	1.188846419	PA(22:6(4Z,7Z,10Z,13Z,16Z,19Z)/16:0)	0.556532497
gene-LOC78785ɛ	1.188846419	DG(18:0/LTE4/0:0)	0.681485773
gene-LOC78785ɛ	1.188846419	(4r,5s,6s,7r)-4,7-Dibenzyl-5,6-dihydroxy-1,3-	1.334600128
gene-LOC78785ɛ	1.188846419	CDP-DG(PGF2alpha/16:0)	1.002512277
gene-LOC78785ɛ	1.188846419	2-Amino-2-thiazoline-4-carboxylic acid	0.017174685
gene-LOC78785ɛ	1.188846419	Fluoromisonidazole	0.028528253
gene-LOC78785ɛ	1.188846419	L-quinate	0.038500771
gene-LOC78785ɛ	1.188846419	dTDP-alpha-D-glucose	0.099332103
gene-LOC78785ɛ	1.188846419	GDP-valienol	0.231837085
gene-LOC78785ɛ	1.188846419	Cyclohexa-1,2,4,5-tetraene	0.073496201
gene-LOC78785ɛ	1.188846419	Lamivudine	0.327402892
gene-LOC78785ɛ	1.188846419	caffeoylshikimate	0.306700159
gene-LOC78785ɛ	1.188846419	L-Citrulline	0.10853076
gene-LOC78785ɛ	1.188846419	1-beta-D-Arabinofuranosyl-5-fluorocytosine	0.152339645
gene-LOC78785ɛ	1.188846419	Cysteinyl-Glutamine	0.079050155
gene-LOC78785ɛ	1.188846419	Pseudouridine 5'-phosphate	1.18431378
gene-LOC78785ɛ	1.188846419	Aflatoxin P1	1.000661338
gene-LOC78785ɛ	1.188846419	4-Hydroxy-6-methyl-3-(1H-pyrazol-3-yl)-2	0.006362816
gene-LOC78785ɛ	1.188846419	15-keto-Prostaglandin E2	2.646629404
gene-LOC78785ɛ	1.188846419	S-Acetyldihydrolipoamide-E	0.429561283
gene-LOC78785ɛ	1.188846419	1,4-Benzothiazine-O-quinonimine	0.396873161
gene-LOC78785ɛ	1.188846419	Ribavirin monophosphate	1.091085027
gene-LOC78785ɛ	1.188846419	4-Hydroxyproline galactoside	0.148083825
gene-LOC78785ɛ	1.188846419	Pro Gly Ser Ser	0.384303007
gene-LOC78785ɛ	1.188846419	S-(2-Hydroxyethyl)glutathione	0.269671835
gene-LOC78785ɛ	1.188846419	Validamycin A	0.587705786
gene-LOC78785ɛ	1.188846419	Austdiol	0.046757893
gene-LOC78785ɛ	1.188846419	3-O-fucopyranosyl-2-acetamido-2-deoxygl	2.892346598
gene-LOC78785ɛ	1.188846419	Norophthalmic acid	0.191432411
gene-LOC78785ɛ	1.188846419	4'-Thiothymidine	0.066948524
gene-LOC78785ɛ	1.188846419	3'-N'-Acetylfusarochromanone	0.755683206
gene-LOC78785ɛ	1.188846419	Licoagrochalcone B	0.345948791
gene-LOC78785ɛ	1.188846419	Desmethylflumazenil (Ro 15-5528)	0.227256571

gene-LOC78785{	1.188846419	cis,trans-5'-Hydroxythalidomide	0.589828342
gene-LOC78785{	1.188846419	Ser Cys Ala Ala	0.603032447
gene-LOC78785{	1.188846419	Pantetheine	0.584830287
gene-LOC78785{	1.188846419	Tiaprost	0.249550474
gene-LOC78785{	1.188846419	cis- and trans-Ethyl 2,4-dimethyl-1,3-dioxol	0.106321146
gene-LOC78785{	1.188846419	6-Thioinosine	0.007981547
gene-LOC78785{	1.188846419	Pratenol A	0.886782548
gene-LOC78785{	1.188846419	5-Phosphoribosyl-4-carboxy-5-aminoimida:	0.052809864
gene-LOC78785{	1.188846419	Homovanillyl alcohol glucuronide	0.05686626
gene-LOC78785{	1.188846419	6-Fluorohomovanillic acid	0.040947314
gene-LOC78785{	1.188846419	L-Oxalylalbizziine	0.002304251
gene-LOC78785{	1.188846419	N-[[(2S,3S,4R,5R)-5-(6-Aminopurin-9-yl)-3,4	0.117601503
gene-LOC78785{	1.188846419	10-Hydroxycarbazepine	0.848001655
gene-LOC78785{	1.188846419	Semilepidinoside A	0.527638644
gene-LOC78785{	1.188846419	Arbutin	0.176184476
gene-LOC78785{	1.188846419	Loganin	0.92782666
gene-LOC78785{	1.188846419	7-Methylxanthosine	0.004641243
gene-LOC78785{	1.188846419	3'-Deoxythymidine	0.791830758
gene-LOC78785{	1.188846419	5-Acetylamino-6-amino-3-methyluracil	0.420050597
gene-LOC78785{	1.188846419	Gly Asp Ala Ala	0.718656316
gene-LOC78785{	1.188846419	ethyl 2-cyano-3-(1h-indol-3-yl)prop-2-eno	0.756861034
gene-LOC78785{	1.188846419	1-{2-[(3-Ethylphenyl)amino]-2-oxoethyl}-6-	0.705711444
gene-LOC78785{	1.188846419	kainic acid	0.836445456
gene-LOC78785{	1.188846419	Zanamivir	0.839205907
gene-LOC78785{	1.188846419	Aminoglutethimide	0.652616628
gene-LOC78785{	1.188846419	5-NITRO-2-PHENYLPROPYLAMINOBENZOI(	0.713298779
gene-LOC78785{	1.188846419	cis-3,4-Phenanthrenedihydrodiol-4-carboxyl	1.374283937
gene-LOC78785{	1.188846419	Met His Gly	3.037980823
gene-LOC78785{	1.188846419	5,6,7,8-Tetrahydromonapterin	0.724075531
gene-LOC78785{	1.188846419	Harmalol	0.364945111
gene-LOC78785{	1.188846419	5'-Methylthioadenosine	0.273413052
gene-LOC78785{	1.188846419	Ethylene brassylate	0.989156973
gene-LOC78785{	1.188846419	Kelampayoside A	0.744250453
gene-LOC78785{	1.188846419	2,3-Epoxymenaquinone	2.040730224
gene-LOC78785{	1.188846419	Arginylhydroxyproline	0.16320017
gene-LOC78785{	1.188846419	6-hydroxymethyl-7,8-dihydropterin	0.190396346
gene-LOC78785{	1.188846419	Calligonine	0.118287503
gene-LOC78785{	1.188846419	5-Formiminotetrahydrofolic acid	0.136750728
gene-LOC78785{	1.188846419	N1-(alpha-D-ribosyl)-5,6-dimethyl-benzimi(	0.446535514
gene-LOC78785{	1.188846419	1-[(2R,4S,5S)-5-[Difluoro(hydroxy)methyl]-4-	0.091474911
gene-LOC78785{	1.188846419	Guanidoacetic acid	0.542509265
gene-LOC78785{	1.188846419	4-Oxo-9-cis-retinoyl-beta-glucuronide	1.611773742
gene-LOC78785{	1.188846419	alpha-Terpineol formate	0.628238986
gene-LOC78785{	1.188846419	Methionyl-Valine	0.502707745
gene-LOC78785{	1.188846419	3-Hydroxytetradecanoyl carnitine	1.314002596
gene-LOC78785{	1.188846419	Macrocin	1.715200143
gene-LOC78785{	1.188846419	9-Octadecenal	0.958949131
gene-LOC78785{	1.188846419	Diisopropanolamine	0.110406861
gene-LOC78785{	1.188846419	Dicyclopentadiene	0.129868592
gene-LOC78785{	1.188846419	(3Z,6Z)-3,6-Nonadien-1-ol	0.185378696
gene-LOC78785{	1.188846419	(S)-4-(2-(4-Amino-1,2,5-oxadiazol-3-yl)-1-	0.710706078

gene-LOC78785{	1.188846419	Zingiberenol	0.088860673
gene-LOC78785{	1.188846419	L-Histidinol	0.149249286
gene-LOC78785{	1.188846419	(Z)-11-Hexadecenal	0.208973533
gene-LOC78785{	1.188846419	14alpha-Hydroxy-5beta-cholest-7-ene-3,6-	0.206315646
gene-LOC78785{	1.188846419	Myricanol 5-[arabinosyl-(1->6)-glucoside]	0.744174399
gene-LOC78785{	1.188846419	Apigenin	0.659004678
gene-LOC78785{	1.188846419	3-(4-Methylbenzylidene)camphor	0.301398724
gene-LOC78785{	1.188846419	DG(2:0/18:1(12Z)-O(9S,10R)/0:0)	0.033180989
gene-LOC78785{	1.188846419	2-Phenylpropyl acetate	0.202302943
gene-LOC78785{	1.188846419	MG(0:0/18:3(9Z,12Z,15Z)/0:0)	1.373784038
gene-LOC78785{	1.188846419	Alpha-Trisaccharide	0.928257808
gene-LOC78785{	1.188846419	LysoPE(0:0/22:5(4Z,7Z,10Z,13Z,16Z))	0.418671396
gene-LOC78785{	1.188846419	(4-Methylphenyl)acetaldehyde	0.208360133
gene-LOC78785{	1.188846419	8-Deoxy-11,13-dihydroxygrosheimin	0.017975582
gene-LOC78785{	1.188846419	Austalide L	0.4229014
gene-LOC78785{	1.188846419	[(2S,4R,5R,6R,14S,16R)-14-Hydroxy-7,11-dim	0.247987808
gene-LOC78785{	1.188846419	LysoPE(15:0/0:0)	0.20988633
gene-LOC78785{	1.188846419	Spergualin	0.210690914
gene-LOC78785{	1.188846419	2,3-Dihydroxypropyl octanoate	0.242447284
gene-LOC78785{	1.188846419	N-Palmitoyl Proline	0.470367693
gene-LOC78785{	1.188846419	(S)-Batatic acid	0.056098392
gene-LOC78785{	1.188846419	Dehydrooreadone	0.111045102
gene-LOC78785{	1.188846419	o-Xylene	0.036460942
gene-LOC78785{	1.188846419	Dihydrocaffeic acid 3-O-glucuronide	0.213679863
gene-LOC78785{	1.188846419	Eltrombopag	0.118484814
gene-LOC78785{	1.188846419	Imatinib	0.837527332
gene-LOC78785{	1.188846419	Deflazacort	0.118739616
gene-LOC78785{	1.188846419	Glu Cys Cys Tyr	0.269820719
gene-LOC78785{	1.188846419	10,12-Tetradecadiene-4,6-diynoic acid, (E,E)-	0.063733402
gene-LOC78785{	1.188846419	Atorvastatin	0.291646019
gene-LOC78785{	1.188846419	Tert-butyl (2-{7-[(2S)-3-(4-cyanophenoxy)-2	0.144488377
gene-LOC78785{	1.188846419	Lucidenic acid K	0.120873155
gene-LOC78785{	1.188846419	10-alpha-methoxy-9,10-dihydrolysergol	0.140851438
gene-LOC78785{	1.188846419	3beta-Hydroxyergosta-7,24(24(1))-dien-4alp	1.714710978
gene-LOC78785{	1.188846419	Vipadenant	0.243429143
gene-LOC78785{	1.188846419	MG(0:0/20:3(11Z,14Z,17Z)/0:0)	0.844949524
gene-LOC78785{	1.188846419	Ethyl salicylate	0.099640862
gene-LOC78785{	1.188846419	Phenol	0.097390283
gene-LOC78785{	1.188846419	Cichorioside J	0.191008611
gene-LOC78785{	1.188846419	MG(LTE4/0:0/0:0)	0.548257353
gene-LOC78785{	1.188846419	Octadecanol	0.1029866
gene-LOC78785{	1.188846419	Bipindogulomethyloside	0.465702332
gene-LOC78785{	1.188846419	3-oxo-2-(cis-2'-pentenyl)-cyclopentane-1-c	0.291387259
gene-LOC78785{	1.188846419	N-Stearoyl Glutamine	1.862695384
gene-LOC78785{	1.188846419	Imidazo(1,2-a)pyrazin-3(7H)-one, 2-methyl-	0.200597965
gene-LOC78785{	1.188846419	Dapdiamide B	0.07688703
gene-LOC78785{	1.188846419	(1'R)-Nepetalic acid	0.499281338
gene-LOC78785{	1.188846419	Iridal	0.699410612
gene-LOC78785{	1.188846419	Palmitoylcarnitine	0.74779616
gene-LOC78785{	1.188846419	(3b,5a,22x,25S)-Furostane-22-methoxy-3,26	0.208177061
gene-LOC78785{	1.188846419	PI(PGJ2/20:3(5Z,8Z,11Z))	0.235965446

gene-LOC78785{	1.188846419	Glutaminylphenylalanine	0.224405311
gene-LOC78785{	1.188846419	Biliverdin-IX-delta	0.006019056
gene-LOC78785{	1.188846419	N-Stearoyl Proline	0.469971646
gene-LOC78785{	1.188846419	Galabiosylceramide (d18:1/20:0)	0.746737323
gene-LOC78785{	1.188846419	Guanadrel	0.419227003
gene-LOC78785{	1.188846419	LysoPI(0:0/18:0)	0.420441153
gene-LOC78785{	1.188846419	Cerebronic acid	0.236898174
gene-LOC78785{	1.188846419	1,3,5-Bisabolatrien-10-one	0.109736328
gene-LOC78785{	1.188846419	24,25-Dihydrolanosterol	0.133512025
gene-LOC78785{	1.188846419	Mizoribine	0.254603976
gene-LOC78785{	1.188846419	3-Carboxy-2,3,4,9-tetrahydro-1H-pyrido[3,4	0.258570369
gene-LOC78785{	1.188846419	LysoPE(0:0/22:4(7Z,10Z,13Z,16Z))	0.23799354
gene-LOC78785{	1.188846419	Hypaconitine	0.176000431
gene-LOC78785{	1.188846419	Dehydrodidemnin B	0.171013159
gene-LOC78785{	1.188846419	Didemethylcitalopram	0.175767554
gene-LOC78785{	1.188846419	N-Stearoyl Valine	0.653773206
gene-LOC78785{	1.188846419	L-(-)Sorbose	0.168270222
gene-LOC78785{	1.188846419	Confertifoline	0.037624848
gene-LOC78785{	1.188846419	tetranor-12(S)-HETE	0.08903916
gene-LOC78785{	1.188846419	Gamithromycin	0.37168241
gene-LOC78785{	1.188846419	5-Nonyltetrahydro-2-oxo-3-furancarboxylic	0.038968516
gene-LOC78785{	1.188846419	Tyromycic acid	0.242165094
gene-LOC78785{	1.188846419	capsidiol	0.060941116
gene-LOC78785{	1.188846419	Prostaglandin F2alpha	0.247197962
gene-LOC78785{	1.188846419	N-Palmitoyl Glutamic acid	0.250188721
gene-LOC78785{	1.188846419	Morantel	0.099849467
gene-LOC78785{	1.188846419	Dopamine quinone	0.054568176
gene-LOC78785{	1.188846419	3-Hydrazinylpyridazine	0.029919446
gene-LOC78785{	1.188846419	Anofinic acid	0.064933209
gene-LOC78785{	1.188846419	(2E)-2-dodecen-1-ol	0.055772695
gene-LOC78785{	1.188846419	N1-(5-Phospho-a-D-ribosyl)-5,6-dimethylb	0.154251596
gene-LOC78785{	1.188846419	Tragopogonsaponin A	0.169034237
gene-LOC78785{	1.188846419	Tylactone	0.340073797
gene-LOC78785{	1.188846419	DG(13:0/20:4(6E,8Z,11Z,14Z)+=O(5)/0:0)	0.124018655
gene-LOC78785{	1.188846419	Nystatin	0.031925024
gene-LOC78785{	1.188846419	Lubiminol	0.122171707
gene-LOC78785{	1.188846419	3-Phenylpropylamine	0.220058937
gene-LOC78785{	1.188846419	Neomycin B	0.395784943
gene-LOC78785{	1.188846419	Cer(d18:2(4E,14Z)/TXB2)	0.236013333
gene-LOC78785{	1.188846419	O-Arachidonoyl Glycidol	0.163183179
gene-LOC78785{	1.188846419	norerythromycin	0.401118778
gene-LOC78785{	1.188846419	CDP-DG(a-25:0/PGF1alpha)	0.345292815
gene-LOC78785{	1.188846419	Dynorphin A 9-17	0.24833391
gene-LOC78785{	1.188846419	D-Pipecolic acid	0.23465295
gene-LOC78785{	1.188846419	2,5-Furandicarboxylic acid	0.091754756
gene-LOC78785{	1.188846419	Danielone	0.091899394
gene-LOC78785{	1.188846419	Osmundalin	0.061518589
gene-LOC78785{	1.188846419	1-(beta-D-Glucopyranosyloxy)-3-octanone	0.055505453
gene-LOC78785{	1.188846419	5-Heptyl-3,4-dimethyl-2-furanheptanoic aci	0.098397491
gene-LOC78785{	1.188846419	LacCer(d18:1/24:1(15Z))	0.285650466
gene-LOC78785{	1.188846419	Rocuronium	0.029736805

gene-LOC78785	1.188846419	2,4-Undecadienal	0.228636005
gene-LOC78785	1.188846419	Farnesyl acetone	0.222244889
gene-LOC78785	1.188846419	7-Dehydrodesmosterol	0.082527232
gene-LOC78785	1.188846419	12,13 diHOME-(d4)	0.012910854
gene-LOC78785	1.188846419	PC(18:1(9Z)/15:1(9Z))	0.534724682
gene-LOC78785	1.188846419	Cer(d18:2(4E,14Z)/5-iso PGF2VI)	0.035704229
gene-LOC78785	1.188846419	epsilon,gamma-Caroten-3-ol	0.098136867
gene-LOC78785	1.188846419	Ser Asn Ala	0.018912413
gene-LOC78785	1.188846419	Nigroxanthin	0.705005059
gene-LOC78785	1.188846419	PE(P-18:0/20:5(5Z,8Z,11Z,14Z,16E)-OH(18))	0.339204508
gene-LOC78785	1.188846419	1-Octadecanoyl-2-(7Z,10Z,13Z,16Z-docosat	0.690496314
gene-LOC78785	1.188846419	PC(16:0/20:5(5E,8E,11E,14E,17E))[U]	0.208077202
gene-LOC78785	1.188846419	PE(18:0/20:4(8Z,11Z,14Z,17Z)-2OH(5S,6R))	0.418208781
gene-LOC78785	1.188846419	CE(LTE4)	0.204616463
gene-LOC78785	1.188846419	PC(P-18:1(11Z)/PGJ2)	0.565243877
gene-LOC78785	1.188846419	UDP-2-acetamido-2-deoxy-alpha-D-galact	0.069033636
gene-LOC78785	1.188846419	Glutathione monoisopropyl ester	0.069078362
gene-LOC78785	1.188846419	Erdosteine	0.070965297
gene-LOC78785	1.188846419	PRIMA-1	0.159245247
gene-LOC78785	1.188846419	Azimexon	0.008689891
gene-LOC78785	1.188846419	2-Methoxyestriol	0.383594672
gene-LOC78785	1.188846419	PE-NMe(18:2(9Z,12Z)/18:2(9Z,12Z))[U]	0.694736593
gene-LOC78785	1.188846419	PE(20:5(5Z,8Z,11Z,14Z,17Z)/18:0)	0.427962506
gene-LOC78785	1.188846419	PS(16:1(9Z)/22:2(13Z,16Z))	0.659652661
gene-LOC78785	1.188846419	PC(P-16:0/20:5(5Z,8Z,11Z,14Z,16E)-OH(18R)	0.26121581
gene-LOC78785	1.188846419	PC(P-16:0/20:3(8Z,11Z,14Z)-2OH(5,6))	0.174122169
gene-LOC78785	1.188846419	DG(20:0/LTE4/0:0)	0.438074508
gene-LOC78785	1.188846419	PE(20:0/PGJ2)	0.198500208
gene-PACSIN3	1.185911637	5-(3'-Carboxy-3'-oxopropenyl)-4,6-dihydro	1.083232395
gene-PACSIN3	1.185911637	Salicyluric acid	1.82393634
gene-PACSIN3	1.185911637	2-Amino-6-methylpyrimidine-4-thiol	3.949216803
gene-PACSIN3	1.185911637	Benzoquinoneacetic acid	0.707114579
gene-PACSIN3	1.185911637	4-Hydroxystyrene	2.854273107
gene-PACSIN3	1.185911637	4-Methylbenzyl alcohol	4.428526409
gene-PACSIN3	1.185911637	Dihydro-3-coumaric acid	1.950770185
gene-PACSIN3	1.185911637	BYSSOCHLAMIC ACID	3.635460051
gene-PACSIN3	1.185911637	Picroside I	1.144121381
gene-PACSIN3	1.185911637	Erythronic acid	0.494007482
gene-PACSIN3	1.185911637	7-Hydroxy-2',4',5'-trimethoxyisoflavan	3.57282801
gene-PACSIN3	1.185911637	(+)-Bottrospicatol	0.68654221
gene-PACSIN3	1.185911637	6-Methylnicotinamide	0.759498218
gene-PACSIN3	1.185911637	Enterolactone 3'-glucuronide	1.086030454
gene-PACSIN3	1.185911637	15-Epi-lipoxin B5	0.766800331
gene-PACSIN3	1.185911637	xi-2,3-Dihydro-3,5-dihydroxy-6-methyl-4H-	0.933518005
gene-PACSIN3	1.185911637	(卤)-Enterolactone	0.766468338
gene-PACSIN3	1.185911637	13,14-dihydro-16,16-difluoro Prostaglandin	0.443708881
gene-PACSIN3	1.185911637	Enterolactone 3''-sulfate	2.060530283
gene-PACSIN3	1.185911637	DGAT-1 inhibitor 2	1.079931222
gene-PACSIN3	1.185911637	Fusidic Acid	1.929894931
gene-PACSIN3	1.185911637	(S)-10,16-Dihydroxyhexadecanoic acid	1.054006526
gene-PACSIN3	1.185911637	Auxin b	1.052491354

gene-PACSIN3	1.185911637	13(S)-HPOT	1.353059742
gene-PACSIN3	1.185911637	Cyclopentolate	0.486079401
gene-PACSIN3	1.185911637	11-Oxahexadecanolide	2.378626329
gene-PACSIN3	1.185911637	3,4,3',4'-Tetrahydrospirilloxanthin	0.354666148
gene-PACSIN3	1.185911637	Dyphylline	0.664549753
gene-PACSIN3	1.185911637	2-Hydroxy-6-keto-2,4-heptadienoate	2.23994791
gene-PACSIN3	1.185911637	Asparaginylcysteine	0.467538645
gene-PACSIN3	1.185911637	4-Hydroxyoctanedioylcarnitine	1.002008817
gene-PACSIN3	1.185911637	Indole-3-acetaldoxime N-oxide	2.727163281
gene-PACSIN3	1.185911637	Phenyllactate	0.55151911
gene-PACSIN3	1.185911637	Erosone	0.527835387
gene-PACSIN3	1.185911637	3-Methoxytyramine	0.928926425
gene-PACSIN3	1.185911637	Hydroxyprolyl-Methionine	1.56890423
gene-PACSIN3	1.185911637	2-Hydroxy-3,4,6-trimethoxydihydrochalcone	1.07715092
gene-PACSIN3	1.185911637	7-Methylguanosine	1.215574844
gene-PACSIN3	1.185911637	Eflornithine	0.287216354
gene-PACSIN3	1.185911637	7a-Hydroxy-O-carbamoyl-deacetylcephalos	0.80981273
gene-PACSIN3	1.185911637	Leu-Leu-Tyr	1.186798796
gene-PACSIN3	1.185911637	6-hydroxy-7E,9E-Octadecadiene-11,13,15,17	1.179129125
gene-PACSIN3	1.185911637	7C-aglycone	1.015608521
gene-PACSIN3	1.185911637	7-Aminomethyl-7-carbaguanine	0.474992724
gene-PACSIN3	1.185911637	Nevirapine	0.820759262
gene-PACSIN3	1.185911637	carbenicillin	1.429508875
gene-PACSIN3	1.185911637	3,5,7-Trimethyl-2E,4E,6E,8E-decatetraene	1.105561224
gene-PACSIN3	1.185911637	5a,6a-Epoxy-7E-megastigmene-3b,9e-diol 9	1.333480709
gene-PACSIN3	1.185911637	5-(3E-Pentenyl)tetrahydro-2-oxo-3-furanca	0.359344922
gene-PACSIN3	1.185911637	Suberenone	4.927547616
gene-PACSIN3	1.185911637	(-)-Huperzine A (HupA)	0.935198906
gene-PACSIN3	1.185911637	Sequiterpene Lactone 326	1.141450991
gene-PACSIN3	1.185911637	N-Eicosapentaenoyl Asparagine	0.371594025
gene-PACSIN3	1.185911637	8-Methylthiooctanaldoxime	0.685273457
gene-PACSIN3	1.185911637	8-Acetylneosolaniol	4.201843387
gene-PACSIN3	1.185911637	Ajulemic acid	0.557832951
gene-PACSIN3	1.185911637	Sorbitan palmitate	1.946147152
gene-PACSIN3	1.185911637	11Z-Eicosenoic acid	0.170761996
gene-PACSIN3	1.185911637	Chalcone	4.157137778
gene-PACSIN3	1.185911637	Milbemycin D	0.125637998
gene-PACSIN3	1.185911637	Sitosterol beta-D-glucoside	0.382944796
gene-PACSIN3	1.185911637	Lysyltryptophan	2.153119072
gene-PACSIN3	1.185911637	Isopropyl hexadecanoate	0.803568925
gene-PACSIN3	1.185911637	Galactosylglycerol	0.738463143
gene-PACSIN3	1.185911637	PG(20:1(11Z)/18:3(9,11,15)-OH(13))	0.69829683
Bos_taurus_newG	1.185345947	Urocortisol	0.926998516
Bos_taurus_newG	1.185345947	11-Maleimidoundecanoic acid	1.084942397
Bos_taurus_newG	1.185345947	13(S)-HpODE	0.163157753
Bos_taurus_newG	1.185345947	Tetrofosmin	0.119741861
Bos_taurus_newG	1.185345947	7(S),17(S)-dihydroxy-8(E),10(Z),13(Z),15(E),19	0.32147403
Bos_taurus_newG	1.185345947	Fluridone	0.127106682
Bos_taurus_newG	1.185345947	1,2,3,4-Tetrahydroisoquinoline-3-carboxylic	0.339248702
Bos_taurus_newG	1.185345947	Guanidoacetic acid	0.542509265
Bos_taurus_newG	1.185345947	1-[(5-Amino-5-carboxypentyl)amino]-1-dec	0.400321455

Bos_taurus_newG	1.185345947	3-(3-Methylbutylidene)-1(3H)-isobenzofurar	0.575422414
Bos_taurus_newG	1.185345947	[3-(2-Aminopropyl)-6-methylidenecyclohexa	0.422945997
Bos_taurus_newG	1.185345947	3',4',5'-Trimethoxycinnamyl alcohol acetate	0.4221816
Bos_taurus_newG	1.185345947	Vulgarone A	0.102049407
Bos_taurus_newG	1.185345947	Sophoranol	0.282064826
Bos_taurus_newG	1.185345947	L-Cysteine	0.214540754
Bos_taurus_newG	1.185345947	1,4,6-Trimethylnaphthalene	0.12150693
Bos_taurus_newG	1.185345947	24,24-Dfhv	0.175278646
Bos_taurus_newG	1.185345947	Prostaglandin PGE2 1-glyceryl ester	0.007411313
Bos_taurus_newG	1.185345947	1,8-Octanedithiol	0.133094504
Bos_taurus_newG	1.185345947	1-Heneicosanoyl-glycero-3-phosphoserine	0.214358093
gene-SV2B	1.183449645	D-Erythro-imidazole-glycerol-phosphate	0.355812431
gene-SV2B	1.183449645	7(14)-Bisabolene-2,3,10,11-tetrol	2.446897974
gene-SV2B	1.183449645	LTB4-d4	0.226799982
gene-SV2B	1.183449645	cis-p-Menth-2-en-1-ol	0.201017988
gene-SV2B	1.183449645	(-)-alpha-Terpineol	0.345716279
gene-SV2B	1.183449645	2-isopentyl-3,6-dimethyl pyrazine	0.710502562
gene-SV2B	1.183449645	4-Dimethylamino-L-phenylalanine	0.242110226
gene-SV2B	1.183449645	5'-S-Methyl-5'-thioinosine	0.28772476
gene-SV2B	1.183449645	2-Methyl-3-phenyl-2-propenal	0.407813975
gene-SV2B	1.183449645	PC(P-18:1(11Z)/PGE2)	0.509503133
gene-SV2B	1.183449645	(3R,4R)-3-Amino-1-hydroxy-4-methylpyrrol	0.471914506
gene-SV2B	1.183449645	Isopropyl isothiocyanate	0.172385747
gene-SV2B	1.183449645	N-Myristoyl Glutamine	0.37856966
gene-ZNF8	1.181429184	D-Erythro-imidazole-glycerol-phosphate	0.355812431
gene-ZNF8	1.181429184	3-Deoxyestrone	0.282221709
gene-ZNF8	1.181429184	1-Oleoyl-sn-glycero-3-phosphocholine	0.18926952
gene-ZNF8	1.181429184	Lividamine	0.319679555
gene-ZNF8	1.181429184	(-)-alpha-Terpineol	0.345716279
gene-ZNF8	1.181429184	1,4-Undecadiene	0.462803973
gene-ZNF8	1.181429184	PG(20:1(11Z)/18:3(10,12,15)-OH(9))	0.626244347
gene-ZNF8	1.181429184	5-(2-Aminopropyl)-2-methylphenol	0.323231457
gene-ZNF8	1.181429184	4-Dimethylamino-L-phenylalanine	0.242110226
gene-ZNF8	1.181429184	Armillane	0.52808635
gene-ZNF8	1.181429184	PC(P-18:1(11Z)/PGE2)	0.509503133
gene-ZNF8	1.181429184	(3R,4R)-3-Amino-1-hydroxy-4-methylpyrrol	0.471914506
gene-ZNF8	1.181429184	n-methyl-2-(4'-methylaminophenyl)-6-hydr	0.26655714
gene-ZNF8	1.181429184	Isopropyl isothiocyanate	0.172385747
gene-ZNF8	1.181429184	9-deoxy-9-methylene-16,16-dimethyl -PGE	0.606893884
gene-ZNF8	1.181429184	PS(20:0/20:4(8Z,11Z,14Z,17Z)-2OH(5S,6R))	0.402595921
gene-ZNF8	1.181429184	CL(8:0/8:0/18:2(9Z,11Z)/20:0)	0.622988418
gene-SLC25A29	1.179124411	3-Thiacytidine	0.209387412
gene-SLC25A29	1.179124411	D-Erythro-imidazole-glycerol-phosphate	0.355812431
gene-SLC25A29	1.179124411	LTB4-d4	0.226799982
gene-SLC25A29	1.179124411	(-)-alpha-Terpineol	0.345716279
gene-SLC25A29	1.179124411	Cyclosporin A	0.656529229
gene-SLC25A29	1.179124411	5-(2-Aminopropyl)-2-methylphenol	0.323231457
gene-SLC25A29	1.179124411	4-Dimethylamino-L-phenylalanine	0.242110226
gene-SLC25A29	1.179124411	Trimetazidine	0.438282534
gene-SLC25A29	1.179124411	Armillane	0.52808635
gene-SLC25A29	1.179124411	2-Methyl-3-phenyl-2-propenal	0.407813975

gene-SLC25A29	1.179124411	PC(P-18:1(11Z)/PGE2)	0.509503133
gene-SLC25A29	1.179124411	(3R,4R)-3-Amino-1-hydroxy-4-methylpyrrol	0.471914506
gene-SLC25A29	1.179124411	n-methyl-2-(4'-methylaminophenyl)-6-hydr	0.26655714
gene-SLC25A29	1.179124411	Isopropyl isothiocyanate	0.172385747
gene-SLC25A29	1.179124411	(9Z)-Octadecenoic acid	0.142359556
gene-SLC25A29	1.179124411	arachidyl amido cholanoic acid	1.24842952
gene-SLC25A29	1.179124411	Nigroxanthin	0.705005059
gene-SLC25A29	1.179124411	9-deoxy-9-methylene-16,16-dimethyl -PGE	0.606893884
gene-SLC25A29	1.179124411	CL(8:0/8:0/18:2(9Z,11Z)/20:0)	0.622988418
gene-TH	1.177372292	3-Thiacytidine	0.209387412
gene-TH	1.177372292	D-Erythro-imidazole-glycerol-phosphate	0.355812431
gene-TH	1.177372292	cis-p-Menth-2-en-1-ol	0.201017988
gene-TH	1.177372292	(-)-alpha-Terpineol	0.345716279
gene-TH	1.177372292	PG(20:1(11Z)/18:3(10,12,15)-OH(9))	0.626244347
gene-TH	1.177372292	Armillane	0.52808635
gene-TH	1.177372292	2-Methyl-3-phenyl-2-propenal	0.407813975
gene-TH	1.177372292	PC(P-18:1(11Z)/PGE2)	0.509503133
gene-TH	1.177372292	(3R,4R)-3-Amino-1-hydroxy-4-methylpyrrol	0.471914506
gene-TH	1.177372292	PC(P-18:1(11Z)/PGJ2)	0.565243877
gene-TH	1.177372292	PS(20:0/20:4(8Z,11Z,14Z,17Z)-2OH(5S,6R))	0.402595921
gene-CERS1	1.176056051	Cyclosporin A	0.656529229
gene-CERS1	1.176056051	ingenol	0.942071652
gene-CERS1	1.176056051	beta-L-Dioxolane-cytidine	0.175916668
gene-CERS1	1.176056051	CL(8:0/8:0/18:2(9Z,11Z)/20:0)	0.622988418
gene-KRTCAP3	1.175326509	D-Erythro-imidazole-glycerol-phosphate	0.355812431
gene-KRTCAP3	1.175326509	Sorbitan laurate	0.242315968
gene-KRTCAP3	1.175326509	cis-p-Menth-2-en-1-ol	0.201017988
gene-KRTCAP3	1.175326509	2-Methyl-3-phenyl-2-propenal	0.407813975
gene-KRTCAP3	1.175326509	PC(P-18:1(11Z)/PGE2)	0.509503133
Bos_taurus_newG	1.173952858	3-Thiacytidine	0.209387412
Bos_taurus_newG	1.173952858	D-Erythro-imidazole-glycerol-phosphate	0.355812431
Bos_taurus_newG	1.173952858	7(14)-Bisabolene-2,3,10,11-tetrol	2.446897974
Bos_taurus_newG	1.173952858	cis-p-Menth-2-en-1-ol	0.201017988
Bos_taurus_newG	1.173952858	2-Methyl-3-phenyl-2-propenal	0.407813975
Bos_taurus_newG	1.173952858	PC(P-18:1(11Z)/PGE2)	0.509503133
Bos_taurus_newG	1.173952858	Sambutoxin	0.169786377
Bos_taurus_newG	1.173952858	Isopropyl isothiocyanate	0.172385747
Bos_taurus_newG	1.173952858	PS(20:0/20:4(8Z,11Z,14Z,17Z)-2OH(5S,6R))	0.402595921
gene-S100A7	1.173801796	D-Erythro-imidazole-glycerol-phosphate	0.355812431
gene-S100A7	1.173801796	Gentisic acid	0.519724891
gene-S100A7	1.173801796	Valeric acid	0.735658687
gene-S100A7	1.173801796	O-methoxycatechol-O-sulphate	0.55600642
gene-S100A7	1.173801796	Deoxyloganin	0.445304262
gene-S100A7	1.173801796	6|A-Hydroxy-7|A-(thiomethyl)spirolactone	0.482613588
gene-S100A7	1.173801796	Hexahydro-4-methylphthalic anhydride	0.691665693
gene-S100A7	1.173801796	Butyl 3-hydroxy-2-methylidenebutanoate	0.644438067
gene-S100A7	1.173801796	Garcinone C	0.61676172
gene-S100A7	1.173801796	Glutamate carbon	0.671444516
gene-S100A7	1.173801796	Sonchuside C	1.385535636
gene-S100A7	1.173801796	1-(2-Furanyl)-1-pentanone	0.697147283
gene-S100A7	1.173801796	2-Dehydro-3-deoxy-D-gluconate	0.65165299

gene-S100A7	1.173801796	N1-Methyl-4-pyridone-5-carboxamide	0.751815577
gene-S100A7	1.173801796	Undecanedioic acid	0.059955634
gene-S100A7	1.173801796	7,8-Dihydro-3b,6a-dihydroxy-alpha-ionol 9-	0.274237338
gene-S100A7	1.173801796	Cholic acid glucuronide	0.147398811
gene-S100A7	1.173801796	16-Hydroxy-10-oxohexadecanoic acid	0.72302152
gene-S100A7	1.173801796	(10S)-Juvenile hormone III acid diol	0.351986626
gene-S100A7	1.173801796	(1R,6S)-6-Amino-5-oxocyclohex-2-ene-1-c	0.154751123
gene-S100A7	1.173801796	cis-trans-Nepetalactol	0.609089778
gene-S100A7	1.173801796	2-Hydroxyglutaric acid diethyl ester	0.799886767
gene-S100A7	1.173801796	L-alpha-Acetyl-N,N-dinormethadol	1.567999001
gene-S100A7	1.173801796	Alongside	0.246470664
gene-S100A7	1.173801796	2-(1-Adamantyl)-1,3-dioxetane	0.408916843
gene-S100A7	1.173801796	alatolide	0.26950377
gene-S100A7	1.173801796	3-(3-Methylbutylidene)-1(3H)-isobenzofurar	0.575422414
gene-S100A7	1.173801796	Chamissonin diacetate	0.324896191
gene-S100A7	1.173801796	3',4',5'-Trimethoxycinnamyl alcohol acetate	0.4221816
gene-S100A7	1.173801796	L-Anticapsin	0.426006862
gene-S100A7	1.173801796	6-Hydroxypentadecanoylcarnitine	0.820747254
gene-S100A7	1.173801796	Roxithromycin	0.268273077
gene-NACAD	1.167886325	D-Erythro-imidazole-glycerol-phosphate	0.355812431
gene-NACAD	1.167886325	Methyl (2E)-2-(10,13-dimethyl-11-oxo-3-py	0.478588158
gene-NACAD	1.167886325	11-Maleimidoundecanoic acid	1.084942397
gene-NACAD	1.167886325	13(S)-HpODE	0.163157753
gene-NACAD	1.167886325	7(S),17(S)-dihydroxy-8(E),10(Z),13(Z),15(E),19	0.32147403
gene-NACAD	1.167886325	LTB4-d4	0.226799982
gene-NACAD	1.167886325	cis-p-Menth-2-en-1-ol	0.201017988
gene-NACAD	1.167886325	Capillene	0.050385556
gene-NACAD	1.167886325	2-Methyl-3-phenyl-2-propenal	0.407813975
gene-NACAD	1.167886325	[3-(2-Aminopropyl)-6-methylidenecyclohexa	0.422945997
gene-NACAD	1.167886325	Vulgarone A	0.102049407
gene-NACAD	1.167886325	Sophoranol	0.282064826
gene-NACAD	1.167886325	PC(P-18:1(11Z)/PGE2)	0.509503133
gene-NACAD	1.167886325	9-OxoODE	0.111803831
gene-NACAD	1.167886325	PC(P-18:1(11Z)/PGE1)	0.295520345
gene-SCN1B	1.166783157	5-Hydroxy-2-oxo-4-ureido-2,5-dihydro-1H	0.122536033
gene-SCN1B	1.166783157	PGP(20:1(11Z)/18:1(12Z)-2OH(9,10))	0.042651731
gene-SCN1B	1.166783157	PC(24:0/22:6(4Z,7Z,10Z,12E,16Z,19Z)-OH(14)	0.188241061
gene-SCN1B	1.166783157	Cyclosporin A	0.656529229
gene-SCN1B	1.166783157	Epomusenin A	0.766206049
gene-SCN1B	1.166783157	DG(18:0/LTE4/0:0)	0.681485773
gene-SCN1B	1.166783157	CDP-DG(PGF2alpha/16:0)	1.002512277
gene-SCN1B	1.166783157	D-Xylonate	0.386869454
gene-SCN1B	1.166783157	Benzoyl glucuronide (Benzoic acid)	0.442844673
gene-SCN1B	1.166783157	C20914	0.140383181
gene-SCN1B	1.166783157	Guanidoacetic acid	0.542509265
gene-SCN1B	1.166783157	alpha-Terpineol formate	0.628238986
gene-SCN1B	1.166783157	Methionyl-Valine	0.502707745
gene-SCN1B	1.166783157	1,4,6-Trimethylnaphthalene	0.12150693
gene-SCN1B	1.166783157	Prostaglandin PGE2 1-glyceryl ester	0.007411313
gene-SCN1B	1.166783157	7-Sulfocholic acid	0.039985455
gene-SCN1B	1.166783157	1,8-Octanedithiol	0.133094504

gene-SCN1B	1.166783157	PC(P-18:1(11Z)/PGE2)	0.509503133
gene-SCN1B	1.166783157	Phorone A	0.079039095
gene-SCN1B	1.166783157	PS(24:1(15Z)/24:1(15Z))	0.557296152
gene-SCN1B	1.166783157	Nigroxanthin	0.705005059
gene-SCN1B	1.166783157	PC(P-18:1(11Z)/PGJ2)	0.565243877
gene-SCN1B	1.166783157	Azimexon	0.008689891
gene-C18H19orf3	1.166602248	S-(Indolylmethylthiohydroximoyl)-L-cysteine	0.635042992
gene-C18H19orf3	1.166602248	4-Trimethylammoniobutanoate	0.207169733
gene-C18H19orf3	1.166602248	(-)-alpha-Terpineol	0.345716279
gene-C18H19orf3	1.166602248	1-Benzazepine	0.130253707
gene-C18H19orf3	1.166602248	Trimetazidine	0.438282534
gene-C18H19orf3	1.166602248	oleandomycin	0.996457692
gene-C18H19orf3	1.166602248	(Z)-3-Oxo-2-(2-pentenyl)-1-cyclopenteneac	0.179805915
gene-C18H19orf3	1.166602248	(Z)-[3-(Methylsulfinyl)-1-propenyl] 2-proper	0.021537476
gene-C18H19orf3	1.166602248	Phantasmidine	0.044283869
gene-C18H19orf3	1.166602248	PC(20:3(5Z,8Z,11Z)/24:0)	0.387959564
gene-C18H19orf3	1.166602248	CL(8:0/8:0/18:2(9Z,11Z)/20:0)	0.622988418
gene-ELF3	1.165680006	N,N''-Sulfonylbisurea	0.308920258
gene-ELF3	1.165680006	D-Erythro-imidazole-glycerol-phosphate	0.355812431
gene-ELF3	1.165680006	8,8a-Deoxyoleandolide	0.305706087
gene-ELF3	1.165680006	13(S)-HpODE	0.163157753
gene-ELF3	1.165680006	LTB4-d4	0.226799982
gene-ELF3	1.165680006	cis-p-Menth-2-en-1-ol	0.201017988
gene-ELF3	1.165680006	(-)-alpha-Terpineol	0.345716279
gene-ELF3	1.165680006	PG(20:1(11Z)/18:3(10,12,15)-OH(9))	0.626244347
gene-ELF3	1.165680006	Isoleucylproline	1.006580106
gene-ELF3	1.165680006	Pro-leu	0.931879628
gene-ELF3	1.165680006	Hexanoylglutamine	0.593876112
gene-ELF3	1.165680006	2-Methyl-3-phenyl-2-propenal	0.407813975
gene-ELF3	1.165680006	L-Anticapsin	0.426006862
gene-ELF3	1.165680006	(4Z,7Z,10Z,13Z,16Z,19Z)-Docosahexaenoic ac	0.434211486
gene-ELF3	1.165680006	PC(P-18:1(11Z)/PGE2)	0.509503133
gene-ELF3	1.165680006	9-OxoODE	0.111803831
gene-ELF3	1.165680006	Threoninyl-Tryptophan	0.69975167
gene-RTN2	1.165209193	5-(Ethylthio)-1H-tetrazole	0.29717344
gene-RTN2	1.165209193	3-Deoxyestrone	0.282221709
gene-RTN2	1.165209193	1-Oleoyl-sn-glycero-3-phosphocholine	0.18926952
gene-RTN2	1.165209193	LTB4-d4	0.226799982
gene-RTN2	1.165209193	(-)-alpha-Terpineol	0.345716279
gene-RTN2	1.165209193	5-(2-Aminopropyl)-2-methylphenol	0.323231457
gene-RTN2	1.165209193	4-Dimethylamino-L-phenylalanine	0.242110226
gene-RTN2	1.165209193	Trimetazidine	0.438282534
gene-RTN2	1.165209193	Armillane	0.52808635
gene-RTN2	1.165209193	5'-S-Methyl-5'-thioinosine	0.28772476
gene-RTN2	1.165209193	(Z)-3-Oxo-2-(2-pentenyl)-1-cyclopenteneac	0.179805915
gene-RTN2	1.165209193	(Z)-[3-(Methylsulfinyl)-1-propenyl] 2-proper	0.021537476
gene-RTN2	1.165209193	PC(P-18:1(11Z)/PGE2)	0.509503133
gene-RTN2	1.165209193	(3R,4R)-3-Amino-1-hydroxy-4-methylpyrrol	0.471914506
gene-RTN2	1.165209193	n-methyl-2-(4'-methylaminophenyl)-6-hydr	0.26655714
gene-RTN2	1.165209193	Isopropyl isothiocyanate	0.172385747
gene-RTN2	1.165209193	(9Z)-Octadecenoic acid	0.142359556

gene-RTN2	1.165209193	9-deoxy-9-methylene-16,16-dimethyl -PGE:	0.606893884
gene-RTN2	1.165209193	2-Propenyl 2-aminobenzoate	0.128406829
gene-RTN2	1.165209193	CL(8:0/8:0/18:2(9Z,11Z)/20:0)	0.622988418
gene-ZNF775	1.16492904	3-Thiacytidine	0.209387412
gene-ZNF775	1.16492904	D-Erythro-imidazole-glycerol-phosphate	0.355812431
gene-ZNF775	1.16492904	LTB4-d4	0.226799982
gene-ZNF775	1.16492904	cis-p-Menth-2-en-1-ol	0.201017988
gene-ZNF775	1.16492904	PE(20:0/18:1(12Z)-2OH(9,10))	0.438658253
gene-ZNF775	1.16492904	Cyclosporin A	0.656529229
gene-ZNF775	1.16492904	2-Methyl-3-phenyl-2-propenal	0.407813975
gene-ZNF775	1.16492904	PC(P-18:1(11Z)/PGE2)	0.509503133
gene-ZNF775	1.16492904	N-Myristoyl Glutamine	0.37856966
gene-ZNF775	1.16492904	PC(18:1(9Z)/15:1(9Z))	0.534724682
gene-ZNF775	1.16492904	Nigroxanthin	0.705005059
gene-ZNF775	1.16492904	PC(P-18:1(11Z)/PGJ2)	0.565243877
gene-ZNF775	1.16492904	PS(20:0/20:4(8Z,11Z,14Z,17Z)-2OH(5S,6R))	0.402595921
gene-PACSIN1	1.164641518	3-Thiacytidine	0.209387412
gene-PACSIN1	1.164641518	D-Erythro-imidazole-glycerol-phosphate	0.355812431
gene-PACSIN1	1.164641518	LTB4-d4	0.226799982
gene-PACSIN1	1.164641518	cis-p-Menth-2-en-1-ol	0.201017988
gene-PACSIN1	1.164641518	(-)-alpha-Terpineol	0.345716279
gene-PACSIN1	1.164641518	PG(20:1(11Z)/18:3(10,12,15)-OH(9))	0.626244347
gene-PACSIN1	1.164641518	5-(2-Aminopropyl)-2-methylphenol	0.323231457
gene-PACSIN1	1.164641518	4-Dimethylamino-L-phenylalanine	0.242110226
gene-PACSIN1	1.164641518	Armillane	0.52808635
gene-PACSIN1	1.164641518	2-Methyl-3-phenyl-2-propenal	0.407813975
gene-PACSIN1	1.164641518	PC(P-18:1(11Z)/PGE2)	0.509503133
gene-PACSIN1	1.164641518	(3R,4R)-3-Amino-1-hydroxy-4-methylpyrrol	0.471914506
gene-PACSIN1	1.164641518	Isopropyl isothiocyanate	0.172385747
gene-PACSIN1	1.164641518	(9Z)-Octadecenoic acid	0.142359556
gene-PACSIN1	1.164641518	PC(P-18:1(11Z)/PGJ2)	0.565243877
gene-PACSIN1	1.164641518	PS(20:0/20:4(8Z,11Z,14Z,17Z)-2OH(5S,6R))	0.402595921
gene-PACSIN1	1.164641518	CL(8:0/8:0/18:2(9Z,11Z)/20:0)	0.622988418
gene-RABAC1	1.164177545	3-Thiacytidine	0.209387412
gene-RABAC1	1.164177545	D-Erythro-imidazole-glycerol-phosphate	0.355812431
gene-RABAC1	1.164177545	LTB4-d4	0.226799982
gene-RABAC1	1.164177545	cis-p-Menth-2-en-1-ol	0.201017988
gene-RABAC1	1.164177545	(-)-alpha-Terpineol	0.345716279
gene-RABAC1	1.164177545	PG(20:1(11Z)/18:3(10,12,15)-OH(9))	0.626244347
gene-RABAC1	1.164177545	5-(2-Aminopropyl)-2-methylphenol	0.323231457
gene-RABAC1	1.164177545	4-Dimethylamino-L-phenylalanine	0.242110226
gene-RABAC1	1.164177545	Armillane	0.52808635
gene-RABAC1	1.164177545	2-Methyl-3-phenyl-2-propenal	0.407813975
gene-RABAC1	1.164177545	PC(P-18:1(11Z)/PGE2)	0.509503133
gene-RABAC1	1.164177545	(3R,4R)-3-Amino-1-hydroxy-4-methylpyrrol	0.471914506
gene-RABAC1	1.164177545	Isopropyl isothiocyanate	0.172385747
gene-RABAC1	1.164177545	(9Z)-Octadecenoic acid	0.142359556
gene-RABAC1	1.164177545	PC(P-18:1(11Z)/PGJ2)	0.565243877
gene-RABAC1	1.164177545	PS(20:0/20:4(8Z,11Z,14Z,17Z)-2OH(5S,6R))	0.402595921
gene-RABAC1	1.164177545	CL(8:0/8:0/18:2(9Z,11Z)/20:0)	0.622988418
gene-LOC53900!	1.164172211	3-Thiacytidine	0.209387412

gene-LOC539009	1.164172211	11-Maleimidoundecanoic acid	1.084942397
gene-LOC539009	1.164172211	cis-p-Menth-2-en-1-ol	0.201017988
gene-LOC539009	1.164172211	PE(20:0/18:1(12Z)-2OH(9,10))	0.438658253
gene-LOC539009	1.164172211	PE(22:2(13Z,16Z)/22:5(4Z,7Z,10Z,13Z,19Z)-O	0.369745166
gene-LOC539009	1.164172211	PG(20:1(11Z)/18:3(10,12,15)-OH(9))	0.626244347
gene-LOC539009	1.164172211	alpha-Terpineol formate	0.628238986
gene-LOC539009	1.164172211	PC(P-18:1(11Z)/PGE2)	0.509503133
gene-LOC539009	1.164172211	Phorone A	0.079039095
gene-LOC539009	1.164172211	Roxithromycin	0.268273077
gene-LOC539009	1.164172211	Nigroxanthin	0.705005059
gene-LOC539009	1.164172211	PC(P-18:1(11Z)/PGJ2)	0.565243877
gene-LOC539009	1.164172211	PS(20:0/20:4(8Z,11Z,14Z,17Z)-2OH(5S,6R))	0.402595921
gene-MCAM	1.163285892	Methylmalonate	0.241481249
gene-MCAM	1.163285892	PE-NMe(18:0/18:3(9Z,12Z,15Z))	0.681884774
gene-MCAM	1.163285892	3-Thiacytidine	0.209387412
gene-MCAM	1.163285892	Methyl methacrylate	0.245645579
gene-MCAM	1.163285892	Metkephamid	1.159767003
gene-MCAM	1.163285892	11-Maleimidoundecanoic acid	1.084942397
gene-MCAM	1.163285892	LTB4-d4	0.226799982
gene-MCAM	1.163285892	cis-p-Menth-2-en-1-ol	0.201017988
gene-MCAM	1.163285892	PE(20:0/18:1(12Z)-2OH(9,10))	0.438658253
gene-MCAM	1.163285892	4,6-Heneicosanedione	0.113496143
gene-MCAM	1.163285892	PE(22:2(13Z,16Z)/22:5(4Z,7Z,10Z,13Z,19Z)-O	0.369745166
gene-MCAM	1.163285892	Angiotensin A	0.247332017
gene-MCAM	1.163285892	Epomusenin A	0.766206049
gene-MCAM	1.163285892	DG(18:0/LTE4/0:0)	0.681485773
gene-MCAM	1.163285892	CDP-DG(PGF2alpha/16:0)	1.002512277
gene-MCAM	1.163285892	Lamivudine	0.327402892
gene-MCAM	1.163285892	1-beta-D-Arabinofuranosyl-5-fluorocytosine	0.152339645
gene-MCAM	1.163285892	Cysteinyl-Glutamine	0.079050155
gene-MCAM	1.163285892	15-keto-Prostaglandin E2	2.646629404
gene-MCAM	1.163285892	2-Methyl-3-phenyl-2-propenal	0.407813975
gene-MCAM	1.163285892	10-alpha-methoxy-9,10-dihydrolysergol	0.140851438
gene-MCAM	1.163285892	PC(P-18:1(11Z)/PGE2)	0.509503133
gene-MCAM	1.163285892	N-Palmitoyl Glutamic acid	0.250188721
gene-MCAM	1.163285892	(9Z)-Octadecenoic acid	0.142359556
gene-MCAM	1.163285892	norerythromycin	0.401118778
gene-MCAM	1.163285892	N-Myristoyl Glutamine	0.37856966
gene-MCAM	1.163285892	PC(18:1(9Z)/15:1(9Z))	0.534724682
gene-MCAM	1.163285892	arachidyl amido cholanoic acid	1.24842952
gene-MCAM	1.163285892	Nigroxanthin	0.705005059
gene-MCAM	1.163285892	1-Octadecanoyl-2-(7Z,10Z,13Z,16Z-docosat	0.690496314
gene-MCAM	1.163285892	PC(P-18:1(11Z)/PGJ2)	0.565243877
gene-MCAM	1.163285892	PS(20:0/20:4(8Z,11Z,14Z,17Z)-2OH(5S,6R))	0.402595921
gene-MCAM	1.163285892	PE-NMe(18:2(9Z,12Z)/18:2(9Z,12Z))[U]	0.694736593
gene-MCAM	1.163285892	DG(20:0/LTE4/0:0)	0.438074508
gene-ADAMTSL4	1.161606807	3-Thiacytidine	0.209387412
gene-ADAMTSL4	1.161606807	D-Erythro-imidazole-glycerol-phosphate	0.355812431
gene-ADAMTSL4	1.161606807	LTB4-d4	0.226799982
gene-ADAMTSL4	1.161606807	cis-p-Menth-2-en-1-ol	0.201017988
gene-ADAMTSL4	1.161606807	PE(20:0/18:1(12Z)-2OH(9,10))	0.438658253

gene-ADAMTSL4	1.161606807	(-)-alpha-Terpineol	0.345716279
gene-ADAMTSL4	1.161606807	PE(22:2(13Z,16Z)/22:5(4Z,7Z,10Z,13Z,19Z)-O(	0.369745166
gene-ADAMTSL4	1.161606807	PG(20:1(11Z)/18:3(10,12,15)-OH(9))	0.626244347
gene-ADAMTSL4	1.161606807	2-Methyl-3-phenyl-2-propenal	0.407813975
gene-ADAMTSL4	1.161606807	PC(P-18:1(11Z)/PGE2)	0.509503133
gene-ADAMTSL4	1.161606807	Nigroxanthin	0.705005059
gene-ADAMTSL4	1.161606807	PC(P-18:1(11Z)/PGJ2)	0.565243877
gene-ADAMTSL4	1.161606807	PS(20:0/20:4(8Z,11Z,14Z,17Z)-2OH(5S,6R))	0.402595921
gene-DEPTOR	1.160007799	Val-Cit	0.452308451
gene-DEPTOR	1.160007799	DG(10:0/20:5(7Z,9Z,11E,13E,17Z)-3OH(5,6,15	0.776712455
gene-DEPTOR	1.160007799	11-Maleimidoundecanoic acid	1.084942397
gene-DEPTOR	1.160007799	Monacolin L acid	1.060562098
gene-DEPTOR	1.160007799	PE(22:2(13Z,16Z)/22:5(4Z,7Z,10Z,13Z,19Z)-O(	0.369745166
gene-DEPTOR	1.160007799	Isocolumbin	0.161623565
gene-DEPTOR	1.160007799	Lamivudine	0.327402892
gene-DEPTOR	1.160007799	Pseudouridine 5'-phosphate	1.18431378
gene-DEPTOR	1.160007799	S-Acetyldihydrolipoamide-E	0.429561283
gene-DEPTOR	1.160007799	Ribavirin monophosphate	1.091085027
gene-DEPTOR	1.160007799	Norophthalmic acid	0.191432411
gene-DEPTOR	1.160007799	3'-N'-Acetylfusarochromanone	0.755683206
gene-DEPTOR	1.160007799	Indole-3-ethanol	0.096523128
gene-DEPTOR	1.160007799	n6-[2-(4-Aminophenyl)ethyl]adenosine	0.45059876
gene-DEPTOR	1.160007799	Loganin	0.92782666
gene-DEPTOR	1.160007799	3'-Deoxythymidine	0.791830758
gene-DEPTOR	1.160007799	kainic acid	0.836445456
gene-DEPTOR	1.160007799	Tryptophyl-Glutamine	0.89722186
gene-DEPTOR	1.160007799	5,6,7,8-Tetrahydromonapterin	0.724075531
gene-DEPTOR	1.160007799	2-Methyl-3-(2-pentenyl)-2-cyclopenten-1-(	0.23873842
gene-DEPTOR	1.160007799	Guanidoacetic acid	0.542509265
gene-DEPTOR	1.160007799	alpha-Terpineol formate	0.628238986
gene-DEPTOR	1.160007799	Apronal	0.754033426
gene-DEPTOR	1.160007799	ent-16b,19-Kauranediol 19-acetate	0.400676827
gene-DEPTOR	1.160007799	Milbemycin D	0.125637998
gene-DEPTOR	1.160007799	PC(P-18:1(11Z)/PGJ2)	0.565243877
gene-DEPTOR	1.160007799	2-OH-benzyl	0.05275011
gene-DEPTOR	1.160007799	PE(20:5(5Z,8Z,11Z,14Z,17Z)/18:0)	0.427962506
gene-ZNF177	1.159692133	D-Erythro-imidazole-glycerol-phosphate	0.355812431
gene-ZNF177	1.159692133	1,4-Undecadiene	0.462803973
gene-ZNF177	1.159692133	PC(P-18:1(11Z)/PGE2)	0.509503133
gene-ZNF177	1.159692133	Isopropyl isothiocyanate	0.172385747
gene-ZNF177	1.159692133	PS(20:0/20:4(8Z,11Z,14Z,17Z)-2OH(5S,6R))	0.402595921
gene-ZNF177	1.159692133	CL(8:0/8:0/18:2(9Z,11Z)/20:0)	0.622988418
gene-RCOR2	1.159408536	2,6,7-Trihydroxy-9-methylxanthen-3-one	0.49497168
gene-RCOR2	1.159408536	D-Glucono-1,5-lactone	0.240039573
gene-RCOR2	1.159408536	1,3-Bis(4-nitrophenyl)urea	0.181755889
gene-RCOR2	1.159408536	2-Heptanone	0.764447226
gene-RCOR2	1.159408536	Aminohippuric acid	0.559220845
gene-RCOR2	1.159408536	19-hydroxyprostaglandin H2(1-)	0.116812012
gene-RCOR2	1.159408536	Methyl (2E)-2-(10,13-dimethyl-11-oxo-3-py	0.478588158
gene-RCOR2	1.159408536	Urocortisol	0.926998516
gene-RCOR2	1.159408536	Deoxyshikonin	0.734877521

gene-RCOR2	1.159408536	Cholic acid	0.073280104
gene-RCOR2	1.159408536	17alpha,20alpha-Dihydroxypregn-4-en-3-or	0.034933714
gene-RCOR2	1.159408536	2(R)-HPOT	0.035267944
gene-RCOR2	1.159408536	13(S)-HpODE	0.163157753
gene-RCOR2	1.159408536	PE(18:1(12Z)-2OH(9,10)/P-18:0)	0.077744061
gene-RCOR2	1.159408536	Tetrofosmin	0.119741861
gene-RCOR2	1.159408536	15(S)-Hydroxyeicosatrienoic acid	0.020035447
gene-RCOR2	1.159408536	7(S),17(S)-dihydroxy-8(E),10(Z),13(Z),15(E),19	0.32147403
gene-RCOR2	1.159408536	(卤)-Menthyl acetate	0.218882699
gene-RCOR2	1.159408536	Valylarginine	0.627938493
gene-RCOR2	1.159408536	trans-Dodec-2-enoic acid	0.211807766
gene-RCOR2	1.159408536	1,2,3,4-Tetrahydroisoquinoline-3-carboxylic	0.339248702
gene-RCOR2	1.159408536	(9R,10S,12Z)-9,10-Dihydroxy-8-oxo-12-octa	0.391102388
gene-RCOR2	1.159408536	1-[(5-Amino-5-carboxypentyl)amino]-1-dec	0.400321455
gene-RCOR2	1.159408536	[3-(2-Aminopropyl)-6-methylidenecyclohexa	0.422945997
gene-RCOR2	1.159408536	3',4',5'-Trimethoxycinnamyl alcohol acetate	0.4221816
gene-RCOR2	1.159408536	Vulgarone A	0.102049407
gene-RCOR2	1.159408536	12-trans-Hydroxy juvenile hormone III	0.087265655
gene-RCOR2	1.159408536	Sophoranol	0.282064826
gene-RCOR2	1.159408536	L-Cysteine	0.214540754
gene-RCOR2	1.159408536	(R)-2-Nitro-p-mentha-1,5-diene	0.145473521
gene-RCOR2	1.159408536	Chamazulene	0.145696377
gene-RCOR2	1.159408536	Isamoxole	0.121810485
gene-RCOR2	1.159408536	24,24-Dfhv	0.175278646
gene-RCOR2	1.159408536	PG(18:0/20:3(8Z,11Z,14Z)-2OH(5,6))	0.089406674
gene-RCOR2	1.159408536	7-Ethyl-3,6-dihydro-1,4-dimethylazulene	0.176621802
gene-RCOR2	1.159408536	9-OxoODE	0.111803831
gene-RCOR2	1.159408536	PC(P-18:1(11Z)/PGE1)	0.295520345
gene-RCOR2	1.159408536	PS(20:5(5Z,8Z,11Z,14Z,16E)-OH(18R)/22:0)	0.090792733
gene-RCOR2	1.159408536	(E,Z)-2,4-Dodecadiene	0.382588553
gene-RCOR2	1.159408536	1,3,5,11-Bisabolatetraen-10-one	0.319230011
gene-TNNI2	1.157996909	3-Thiacytidine	0.209387412
gene-TNNI2	1.157996909	cis-p-Menth-2-en-1-ol	0.201017988
gene-TNNI2	1.157996909	PE(20:0/18:1(12Z)-2OH(9,10))	0.438658253
gene-TNNI2	1.157996909	PE(22:2(13Z,16Z)/22:5(4Z,7Z,10Z,13Z,19Z)-O	0.369745166
gene-TNNI2	1.157996909	Armillane	0.52808635
gene-TNNI2	1.157996909	2-Methyl-3-phenyl-2-propenal	0.407813975
gene-TNNI2	1.157996909	PC(P-18:1(11Z)/PGE2)	0.509503133
gene-TNNI2	1.157996909	Isopropyl isothiocyanate	0.172385747
gene-TNNI2	1.157996909	PC(18:1(9Z)/15:1(9Z))	0.534724682
gene-TNNI2	1.157996909	PC(P-18:1(11Z)/PGJ2)	0.565243877
gene-TNNI2	1.157996909	PS(20:0/20:4(8Z,11Z,14Z,17Z)-2OH(5S,6R))	0.402595921
gene-LOC100847	1.155298302	D-Erythro-imidazole-glycerol-phosphate	0.355812431
gene-LOC100847	1.155298302	7(14)-Bisabolene-2,3,10,11-tetrol	2.446897974
gene-LOC100847	1.155298302	cis-p-Menth-2-en-1-ol	0.201017988
gene-LOC100847	1.155298302	PC(P-18:1(11Z)/PGE2)	0.509503133
gene-LOC100847	1.155298302	Roxithromycin	0.268273077
gene-LOC100847	1.155298302	PC(P-18:1(11Z)/PGJ2)	0.565243877
gene-LOC100847	1.155298302	PS(20:0/20:4(8Z,11Z,14Z,17Z)-2OH(5S,6R))	0.402595921
gene-SLC49A3	1.155261912	D-Erythro-imidazole-glycerol-phosphate	0.355812431
gene-SLC49A3	1.155261912	3-Deoxyestrone	0.282221709

gene-SLC49A3	1.155261912	1-Oleoyl-sn-glycero-3-phosphocholine	0.18926952
gene-SLC49A3	1.155261912	Lividamine	0.319679555
gene-SLC49A3	1.155261912	LTB4-d4	0.226799982
gene-SLC49A3	1.155261912	(-)-alpha-Terpineol	0.345716279
gene-SLC49A3	1.155261912	5-(2-Aminopropyl)-2-methylphenol	0.323231457
gene-SLC49A3	1.155261912	4-Dimethylamino-L-phenylalanine	0.242110226
gene-SLC49A3	1.155261912	Trimetazidine	0.438282534
gene-SLC49A3	1.155261912	Armillane	0.52808635
gene-SLC49A3	1.155261912	PC(P-18:1(11Z)/PGE2)	0.509503133
gene-SLC49A3	1.155261912	(3R,4R)-3-Amino-1-hydroxy-4-methylpyrrol	0.471914506
gene-SLC49A3	1.155261912	n-methyl-2-(4'-methylaminophenyl)-6-hydr	0.26655714
gene-SLC49A3	1.155261912	Isopropyl isothiocyanate	0.172385747
gene-SLC49A3	1.155261912	(9Z)-Octadecenoic acid	0.142359556
gene-SLC49A3	1.155261912	9-deoxy-9-methylene-16,16-dimethyl -PGE	0.606893884
gene-SLC49A3	1.155261912	CL(8:0/8:0/18:2(9Z,11Z)/20:0)	0.622988418
gene-ADGRB2	1.155166302	N,N''-Sulfonylbisurea	0.308920258
gene-ADGRB2	1.155166302	Imidazooxazole	0.20665788
gene-ADGRB2	1.155166302	2,5-Dihydroxybenzenesulfonic Acid	0.220005737
gene-ADGRB2	1.155166302	Pyrocatechol	0.215582237
gene-ADGRB2	1.155166302	D-Erythro-imidazole-glycerol-phosphate	0.355812431
gene-ADGRB2	1.155166302	N1-Methyl-4-pyridone-5-carboxamide	0.751815577
gene-ADGRB2	1.155166302	PI(22:3(10Z,13Z,16Z)/PGE2)	1.850545869
gene-ADGRB2	1.155166302	Glyuranolide	0.347923566
gene-ADGRB2	1.155166302	1-Octadecanoyl-sn-glycero-3-phosphoetha	0.318054581
gene-ADGRB2	1.155166302	Cholylserine	0.056568624
gene-ADGRB2	1.155166302	(9Z,12Z,15Z)-3-Hydroxyoctadecatrienoylcarr	0.280419534
gene-ADGRB2	1.155166302	(-)-alpha-Terpineol	0.345716279
gene-ADGRB2	1.155166302	4-Chloro-L-phenylalanine	0.256211739
gene-ADGRB2	1.155166302	Cystine-glutamate	0.300382134
gene-ADGRB2	1.155166302	Valylproline	1.009800636
gene-ADGRB2	1.155166302	Threonylisoleucine	1.190547061
gene-ADGRB2	1.155166302	Isoleucylproline	1.006580106
gene-ADGRB2	1.155166302	Pro-leu	0.931879628
gene-ADGRB2	1.155166302	L,L-Cyclo(leucylprolyl)	0.634575464
gene-ADGRB2	1.155166302	Hexanoylglutamine	0.593876112
gene-ADGRB2	1.155166302	2-Methyl-3-phenyl-2-propenal	0.407813975
gene-ADGRB2	1.155166302	N-[[3-Hydroxy-2-(2-pentenyl)cyclopentyl]ac	1.043930947
gene-ADGRB2	1.155166302	Ascorbyl palmitate	0.376864555
gene-ADGRB2	1.155166302	L-Anticapsin	0.426006862
gene-ADGRB2	1.155166302	4-Hydroxy-3-methyl-2-(2-propynyl)-2-cycl	0.120742395
gene-ADGRB2	1.155166302	(2R,4S)-1-(Tert-butoxycarbonyl)-4-phenylpy	0.192920298
gene-ADGRB2	1.155166302	Deoxycholic acid glycine conjugate	0.168887191
gene-ADGRB2	1.155166302	Siguazodan	0.338365222
gene-ADGRB2	1.155166302	(4Z,7Z,10Z,13Z,16Z,19Z)-Docosahexaenoic a	0.434211486
gene-ADGRB2	1.155166302	PC(P-18:1(11Z)/PGE2)	0.509503133
gene-ADGRB2	1.155166302	PA(PGE2/22:2(13Z,16Z))	0.24730102
gene-ADGRB2	1.155166302	9-OxoODE	0.111803831
gene-ADGRB2	1.155166302	PC(P-18:1(11Z)/PGE1)	0.295520345
gene-ADGRB2	1.155166302	2-Amino-4-[carbamimidoyl(methyl)amino]bu	0.075703353
gene-ADGRB2	1.155166302	Maraviroc	0.755297431
gene-ADGRB2	1.155166302	Saponin H	0.079671431

gene-EPS8L2	1.154841256	3-Thiacytidine	0.209387412
gene-EPS8L2	1.154841256	D-Erythro-imidazole-glycerol-phosphate	0.355812431
gene-EPS8L2	1.154841256	7(14)-Bisabolene-2,3,10,11-tetrol	2.446897974
gene-EPS8L2	1.154841256	LTB4-d4	0.226799982
gene-EPS8L2	1.154841256	cis-p-Menth-2-en-1-ol	0.201017988
gene-EPS8L2	1.154841256	PG(20:1(11Z)/18:3(10,12,15)-OH(9))	0.626244347
gene-EPS8L2	1.154841256	4-Dimethylamino-L-phenylalanine	0.242110226
gene-EPS8L2	1.154841256	Armillane	0.52808635
gene-EPS8L2	1.154841256	2-Methyl-3-phenyl-2-propenal	0.407813975
gene-EPS8L2	1.154841256	PC(P-18:1(11Z)/PGE2)	0.509503133
gene-EPS8L2	1.154841256	(3R,4R)-3-Amino-1-hydroxy-4-methylpyrrol	0.471914506
gene-EPS8L2	1.154841256	Isopropyl isothiocyanate	0.172385747
gene-EPS8L2	1.154841256	N-Myristoyl Glutamine	0.37856966
gene-EPS8L2	1.154841256	PC(P-18:1(11Z)/PGJ2)	0.565243877
gene-EPS8L2	1.154841256	PS(20:0/20:4(8Z,11Z,14Z,17Z)-2OH(5S,6R))	0.402595921
gene-TMEM53	1.154604831	2-[(3S)-3-[[(2S)-1-(Carboxymethoxy)-1-oxo	0.111577437
gene-TMEM53	1.154604831	PC(P-18:1(11Z)/PGJ2)	0.565243877
gene-TMEM53	1.154604831	PS(20:0/20:4(8Z,11Z,14Z,17Z)-2OH(5S,6R))	0.402595921
Bos_taurus_newG	1.151193745	Hydroxyprolyl-Serine	0.519540341
Bos_taurus_newG	1.151193745	Cyclosporin A	0.656529229
Bos_taurus_newG	1.151193745	DG(18:0/LTE4/0:0)	0.681485773
Bos_taurus_newG	1.151193745	PC(14:0/20:2(11Z,14Z))	1.110657378
Bos_taurus_newG	1.151193745	ingenol	0.942071652
Bos_taurus_newG	1.151193745	beta-L-Dioxolane-cytidine	0.175916668
Bos_taurus_newG	1.151193745	PC(20:3(5Z,8Z,11Z)/24:0)	0.387959564
Bos_taurus_newG	1.151193745	Nigroxanthin	0.705005059
Bos_taurus_newG	1.151193745	D-Fructose	0.167584616
Bos_taurus_newG	1.151193745	CL(8:0/8:0/18:2(9Z,11Z)/20:0)	0.622988418
gene-ZBED6CL	1.150669349	D-Erythro-imidazole-glycerol-phosphate	0.355812431
gene-ZBED6CL	1.150669349	LTB4-d4	0.226799982
gene-ZBED6CL	1.150669349	cis-p-Menth-2-en-1-ol	0.201017988
gene-ZBED6CL	1.150669349	PG(20:1(11Z)/18:3(10,12,15)-OH(9))	0.626244347
gene-ZBED6CL	1.150669349	2-Methyl-3-phenyl-2-propenal	0.407813975
gene-ZBED6CL	1.150669349	alpha-Terpineol formate	0.628238986
gene-ZBED6CL	1.150669349	PC(P-18:1(11Z)/PGE2)	0.509503133
gene-ZBED6CL	1.150669349	PC(P-18:1(11Z)/PGJ2)	0.565243877
gene-CCDC66	1.150117745	PE(22:2(13Z,16Z)/22:5(4Z,7Z,10Z,13Z,19Z)-O	0.369745166
gene-CCDC66	1.150117745	3,4,3',4'-Tetrahydrospirilloxanthin	0.354666148
gene-CCDC66	1.150117745	Lamivudine	0.327402892
gene-CCDC66	1.150117745	5-Formiminotetrahydrofolate	0.118251287
gene-CCDC66	1.150117745	S-Acetyldihydrolipoamide-E	0.429561283
gene-CCDC66	1.150117745	Erosone	0.527835387
gene-CCDC66	1.150117745	Indole-3-ethanol	0.096523128
gene-CCDC66	1.150117745	2-(1-Adamantyl)-1,3-dioxetane	0.408916843
gene-CCDC66	1.150117745	Milbemycin D	0.125637998
gene-CCDC66	1.150117745	Roxithromycin	0.268273077
gene-CCDC66	1.150117745	Sitosterol beta-D-glucoside	0.382944796
gene-CCDC66	1.150117745	PC(P-18:1(11Z)/PGJ2)	0.565243877
gene-CCDC66	1.150117745	PS(20:0/20:4(8Z,11Z,14Z,17Z)-2OH(5S,6R))	0.402595921
gene-CCDC66	1.150117745	PE(20:5(5Z,8Z,11Z,14Z,17Z)/18:0)	0.427962506
gene-KLK1	1.150004614	milbemycin beta3	1.414447124

gene-KLK1	1.150004614	1,2-O-Isopropylidene-D-glucofuranose	0.080987667
gene-KLK1	1.150004614	Lividamine	0.319679555
gene-KLK1	1.150004614	(-)-alpha-Terpineol	0.345716279
gene-KLK1	1.150004614	PC(P-18:1(11Z)/PGE2)	0.509503133
gene-KLK1	1.150004614	n-methyl-2-(4'-methylaminophenyl)-6-hydr	0.26655714
gene-KLK1	1.150004614	CL(8:0/8:0/18:2(9Z,11Z)/20:0)	0.622988418
gene-GAS7	1.149704022	3-Thiacytidine	0.209387412
gene-GAS7	1.149704022	D-Erythro-imidazole-glycerol-phosphate	0.355812431
gene-GAS7	1.149704022	LTB4-d4	0.226799982
gene-GAS7	1.149704022	cis-p-Menth-2-en-1-ol	0.201017988
gene-GAS7	1.149704022	PE(20:0/18:1(12Z)-2OH(9,10))	0.438658253
gene-GAS7	1.149704022	(-)-alpha-Terpineol	0.345716279
gene-GAS7	1.149704022	Cyclosporin A	0.656529229
gene-GAS7	1.149704022	PG(20:1(11Z)/18:3(10,12,15)-OH(9))	0.626244347
gene-GAS7	1.149704022	2-Methyl-3-phenyl-2-propenal	0.407813975
gene-GAS7	1.149704022	PC(P-18:1(11Z)/PGE2)	0.509503133
gene-GAS7	1.149704022	(3R,4R)-3-Amino-1-hydroxy-4-methylpyrrol	0.471914506
gene-GAS7	1.149704022	(9Z)-Octadecenoic acid	0.142359556
gene-GAS7	1.149704022	arachidyl amido cholanoic acid	1.24842952
gene-GAS7	1.149704022	Nigroxanthin	0.705005059
gene-GAS7	1.149704022	PC(P-18:1(11Z)/PGJ2)	0.565243877
gene-GAS7	1.149704022	PS(20:0/20:4(8Z,11Z,14Z,17Z)-2OH(5S,6R))	0.402595921
gene-GAS7	1.149704022	CL(8:0/8:0/18:2(9Z,11Z)/20:0)	0.622988418
Bos_taurus_newG	1.149207229	3-Thiacytidine	0.209387412
Bos_taurus_newG	1.149207229	D-Erythro-imidazole-glycerol-phosphate	0.355812431
Bos_taurus_newG	1.149207229	cis-p-Menth-2-en-1-ol	0.201017988
Bos_taurus_newG	1.149207229	PE(20:0/18:1(12Z)-2OH(9,10))	0.438658253
Bos_taurus_newG	1.149207229	(-)-alpha-Terpineol	0.345716279
Bos_taurus_newG	1.149207229	PE(22:2(13Z,16Z)/22:5(4Z,7Z,10Z,13Z,19Z)-O	0.369745166
Bos_taurus_newG	1.149207229	Cyclosporin A	0.656529229
Bos_taurus_newG	1.149207229	PG(20:1(11Z)/18:3(10,12,15)-OH(9))	0.626244347
Bos_taurus_newG	1.149207229	PC(P-18:1(11Z)/PGE2)	0.509503133
Bos_taurus_newG	1.149207229	Nigroxanthin	0.705005059
Bos_taurus_newG	1.149207229	PC(P-18:1(11Z)/PGJ2)	0.565243877
Bos_taurus_newG	1.149207229	PS(20:0/20:4(8Z,11Z,14Z,17Z)-2OH(5S,6R))	0.402595921
Bos_taurus_newG	1.149207229	CL(8:0/8:0/18:2(9Z,11Z)/20:0)	0.622988418
Bos_taurus_newG	1.14875135	3-Thiacytidine	0.209387412
Bos_taurus_newG	1.14875135	LTB4-d4	0.226799982
Bos_taurus_newG	1.14875135	Cyclosporin A	0.656529229
Bos_taurus_newG	1.14875135	DG(18:0/LTE4/0:0)	0.681485773
Bos_taurus_newG	1.14875135	PC(14:0/20:2(11Z,14Z))	1.110657378
Bos_taurus_newG	1.14875135	CDP-DG(PGF2alpha/16:0)	1.002512277
Bos_taurus_newG	1.14875135	5-(2-Aminopropyl)-2-methylphenol	0.323231457
Bos_taurus_newG	1.14875135	beta-L-Dioxolane-cytidine	0.175916668
Bos_taurus_newG	1.14875135	PC(P-18:1(11Z)/PGE2)	0.509503133
Bos_taurus_newG	1.14875135	arachidyl amido cholanoic acid	1.24842952
Bos_taurus_newG	1.14875135	Nigroxanthin	0.705005059
Bos_taurus_newG	1.14875135	PC(P-18:1(11Z)/PGJ2)	0.565243877
Bos_taurus_newG	1.14875135	PS(20:0/20:4(8Z,11Z,14Z,17Z)-2OH(5S,6R))	0.402595921
Bos_taurus_newG	1.14875135	CL(8:0/8:0/18:2(9Z,11Z)/20:0)	0.622988418
Bos_taurus_newG	1.148226719	LysoPI(18:2(9Z,12Z)/0:0)	0.358006727

Bos_taurus_newG	1.148226719	LysoPI(16:0/0:0)	0.379098336
Bos_taurus_newG	1.148226719	2-[(3S)-3-[[(2S)-1-(Carboxymethoxy)-1-oxo	0.111577437
Bos_taurus_newG	1.148226719	Trifluoroacetamide	0.107717461
Bos_taurus_newG	1.148226719	Neomycin B	0.395784943
Bos_taurus_newG	1.148226719	Ascorbic acid 6-palmitate	0.201627014
Bos_taurus_newG	1.148226719	3'-N-Acetyl-4'-O-(14-methylpentadecanoyl	0.146786959
Bos_taurus_newG	1.148226719	PC(18:1(9Z)/15:1(9Z))	0.534724682
gene-SULF2	1.146009917	D-Erythro-imidazole-glycerol-phosphate	0.355812431
gene-SULF2	1.146009917	Tsugarioside B	1.013262893
gene-SULF2	1.146009917	7(14)-Bisabolene-2,3,10,11-tetrol	2.446897974
gene-SULF2	1.146009917	24,25-Diacetylvulgaroside	0.920743312
gene-SULF2	1.146009917	(3Z)-Phycoerythrobilin	1.456755874
gene-SULF2	1.146009917	3-Deoxyestrone	0.282221709
gene-SULF2	1.146009917	1-Oleoyl-sn-glycero-3-phosphocholine	0.18926952
gene-SULF2	1.146009917	Lividamine	0.319679555
gene-SULF2	1.146009917	Psychosine	0.106475396
gene-SULF2	1.146009917	cis-p-Menth-2-en-1-ol	0.201017988
gene-SULF2	1.146009917	Cyclotricuspidogenin C	0.440884085
gene-SULF2	1.146009917	(-)-alpha-Terpineol	0.345716279
gene-SULF2	1.146009917	1,4-Undecadiene	0.462803973
gene-SULF2	1.146009917	2-isopentyl-3,6-dimethyl pyrazine	0.710502562
gene-SULF2	1.146009917	PG(20:1(11Z)/18:3(10,12,15)-OH(9))	0.626244347
gene-SULF2	1.146009917	Asparaginylcysteine	0.467538645
gene-SULF2	1.146009917	4-Dimethylamino-L-phenylalanine	0.242110226
gene-SULF2	1.146009917	Cornoside	0.781689516
gene-SULF2	1.146009917	Armillane	0.52808635
gene-SULF2	1.146009917	N-Eicosapentaenoyl Asparagine	0.371594025
gene-SULF2	1.146009917	Siguazodan	0.338365222
gene-SULF2	1.146009917	PC(P-18:1(11Z)/PGE2)	0.509503133
gene-SULF2	1.146009917	(3R,4R)-3-Amino-1-hydroxy-4-methylpyrrol	0.471914506
gene-SULF2	1.146009917	Threoninyl-Tryptophan	0.69975167
gene-SULF2	1.146009917	n-methyl-2-(4'-methylaminophenyl)-6-hydr	0.26655714
gene-SULF2	1.146009917	2-Propenamide, 2-cyano-3-(4-hydroxy-3,5-	0.067779204
gene-SULF2	1.146009917	PG(20:1(11Z)/18:3(9,11,15)-OH(13))	0.69829683
gene-FAM181B	1.145641308	D-Erythro-imidazole-glycerol-phosphate	0.355812431
gene-FAM181B	1.145641308	Tsugarioside B	1.013262893
gene-FAM181B	1.145641308	7(14)-Bisabolene-2,3,10,11-tetrol	2.446897974
gene-FAM181B	1.145641308	cis-p-Menth-2-en-1-ol	0.201017988
gene-FAM181B	1.145641308	11-Oxahexadecanolide	2.378626329
gene-FAM181B	1.145641308	2-isopentyl-3,6-dimethyl pyrazine	0.710502562
gene-FAM181B	1.145641308	PG(20:1(11Z)/18:3(10,12,15)-OH(9))	0.626244347
gene-FAM181B	1.145641308	Dyphylline	0.664549753
gene-FAM181B	1.145641308	Asparaginylcysteine	0.467538645
gene-FAM181B	1.145641308	Armillane	0.52808635
gene-FAM181B	1.145641308	7-Aminomethyl-7-carbaguanine	0.474992724
gene-FAM181B	1.145641308	N-Eicosapentaenoyl Asparagine	0.371594025
gene-FAM181B	1.145641308	Ajulemic acid	0.557832951
gene-FAM181B	1.145641308	PC(P-18:1(11Z)/PGE2)	0.509503133
gene-FAM181B	1.145641308	(3R,4R)-3-Amino-1-hydroxy-4-methylpyrrol	0.471914506
gene-FAM181B	1.145641308	2-Propenamide, 2-cyano-3-(4-hydroxy-3,5-	0.067779204
gene-FAM181B	1.145641308	Sitosterol beta-D-glucoside	0.382944796

gene-FAM181B	1.145641308	PG(20:1(11Z)/18:3(9,11,15)-OH(13))	0.69829683
gene-SGSM2	1.144952803	D-Erythro-imidazole-glycerol-phosphate	0.355812431
gene-SGSM2	1.144952803	(-)-alpha-Terpineol	0.345716279
gene-SGSM2	1.144952803	Cyclosporin A	0.656529229
gene-SGSM2	1.144952803	PC(P-18:1(11Z)/PGE2)	0.509503133
gene-SGSM2	1.144952803	PC(P-18:1(11Z)/PGJ2)	0.565243877
gene-SGSM2	1.144952803	PS(20:0/20:4(8Z,11Z,14Z,17Z)-2OH(5S,6R))	0.402595921
gene-SGSM2	1.144952803	CL(8:0/8:0/18:2(9Z,11Z)/20:0)	0.622988418
gene-SERPIND1	1.142786279	milbemycin beta3	1.414447124
gene-SERPIND1	1.142786279	3-hydroxypristanic acid	0.548515835
gene-SERPIND1	1.142786279	Tetracosenoyl-CoA	0.187187233
gene-SERPIND1	1.142786279	(3Z)-Phycoerythrobilin	1.456755874
gene-SERPIND1	1.142786279	1,2-O-Isopropylidene-D-glucofuranose	0.080987667
gene-SERPIND1	1.142786279	3-Deoxyestrone	0.282221709
gene-SERPIND1	1.142786279	1-Oleoyl-sn-glycero-3-phosphocholine	0.18926952
gene-SERPIND1	1.142786279	3,4-dihydroxy-5-all-trans-hexaprenylbenzoa	0.123615726
gene-SERPIND1	1.142786279	Lividamine	0.319679555
gene-SERPIND1	1.142786279	Psychosine	0.106475396
gene-SERPIND1	1.142786279	Cyclotricuspidogenin C	0.440884085
gene-SERPIND1	1.142786279	(-)-alpha-Terpineol	0.345716279
gene-SERPIND1	1.142786279	1,4-Undecadiene	0.462803973
gene-SERPIND1	1.142786279	PG(20:1(11Z)/18:3(10,12,15)-OH(9))	0.626244347
gene-SERPIND1	1.142786279	5-(2-Aminopropyl)-2-methylphenol	0.323231457
gene-SERPIND1	1.142786279	4-Dimethylamino-L-phenylalanine	0.242110226
gene-SERPIND1	1.142786279	Trimetazidine	0.438282534
gene-SERPIND1	1.142786279	ingenol	0.942071652
gene-SERPIND1	1.142786279	Armillane	0.52808635
gene-SERPIND1	1.142786279	PC(P-18:1(11Z)/PGE2)	0.509503133
gene-SERPIND1	1.142786279	(3R,4R)-3-Amino-1-hydroxy-4-methylpyrrol	0.471914506
gene-SERPIND1	1.142786279	N2-gamma-Glutamylglutamine	0.230065499
gene-SERPIND1	1.142786279	n-methyl-2-(4'-methylaminophenyl)-6-hydr	0.26655714
gene-SERPIND1	1.142786279	(9Z)-Octadecenoic acid	0.142359556
gene-SERPIND1	1.142786279	L-Oleandrosyl-oleandolide	0.272998166
gene-SERPIND1	1.142786279	9-deoxy-9-methylene-16,16-dimethyl -PGE	0.606893884
gene-SERPIND1	1.142786279	CL(8:0/8:0/18:2(9Z,11Z)/20:0)	0.622988418
Bos_taurus_newG	1.14158248	S-(Indolylmethylthiohydroximoyl)-L-cysteine	0.635042992
Bos_taurus_newG	1.14158248	2,6,7-Trihydroxy-9-methylxanthen-3-one	0.49497168
Bos_taurus_newG	1.14158248	1,3-Bis(4-nitrophenyl)urea	0.181755889
Bos_taurus_newG	1.14158248	2-Heptanone	0.764447226
Bos_taurus_newG	1.14158248	Aminohippuric acid	0.559220845
Bos_taurus_newG	1.14158248	Tetradecanedioic acid	0.382003831
Bos_taurus_newG	1.14158248	trans-Geranylgeranylbixin	0.220686938
Bos_taurus_newG	1.14158248	Milbemycin alpha7	0.494896921
Bos_taurus_newG	1.14158248	2(R)-HPOT	0.035267944
Bos_taurus_newG	1.14158248	13(S)-HpODE	0.163157753
Bos_taurus_newG	1.14158248	7(S),17(S)-dihydroxy-8(E),10(Z),13(Z),15(E),19	0.32147403
Bos_taurus_newG	1.14158248	4-Trimethylammoniobutanoate	0.207169733
Bos_taurus_newG	1.14158248	LTB4-d4	0.226799982
Bos_taurus_newG	1.14158248	Lucidenic acid A	0.05705234
Bos_taurus_newG	1.14158248	(-)-alpha-Terpineol	0.345716279
Bos_taurus_newG	1.14158248	(卤)-Menthyl acetate	0.218882699

Bos_taurus_newG	1.14158248	Valylarginine	0.627938493
Bos_taurus_newG	1.14158248	1-Benzazepine	0.130253707
Bos_taurus_newG	1.14158248	12-trans-Hydroxy juvenile hormone III	0.087265655
Bos_taurus_newG	1.14158248	Sophoranol	0.282064826
Bos_taurus_newG	1.14158248	PC(20:3(5Z,8Z,11Z)/24:0)	0.387959564
Bos_taurus_newG	1.14158248	PC(P-18:1(11Z)/PGE1)	0.295520345
Bos_taurus_newG	1.14158248	(E,Z)-2,4-Dodecadiene	0.382588553
Bos_taurus_newG	1.14158248	1,3,5,11-Bisabolatetraen-10-one	0.319230011
gene-KNTC1	1.141341672	3-Thiacytidine	0.209387412
gene-KNTC1	1.141341672	PE(22:2(13Z,16Z)/22:5(4Z,7Z,10Z,13Z,19Z)-O	0.369745166
gene-KNTC1	1.141341672	Cyclosporin A	0.656529229
gene-KNTC1	1.141341672	Angiotensin A	0.247332017
gene-KNTC1	1.141341672	PC(14:0/20:2(11Z,14Z))	1.110657378
gene-KNTC1	1.141341672	CDP-DG(PGF2alpha/16:0)	1.002512277
gene-KNTC1	1.141341672	3b,6a-Dihydroxy-alpha-ionol 9-[apiosyl-(1-	0.045258177
gene-KNTC1	1.141341672	beta-L-Dioxolane-cytidine	0.175916668
gene-KNTC1	1.141341672	PC(20:3(5Z,8Z,11Z)/24:0)	0.387959564
gene-KNTC1	1.141341672	Phorone A	0.079039095
gene-KNTC1	1.141341672	Nigroxanthin	0.705005059
gene-KNTC1	1.141341672	PS(20:0/20:4(8Z,11Z,14Z,17Z)-2OH(5S,6R))	0.402595921
gene-ALCAM	1.140136704	Glutamate carbon	0.671444516
gene-ALCAM	1.140136704	Urocortisol	0.926998516
gene-ALCAM	1.140136704	11-Maleimidoundecanoic acid	1.084942397
gene-ALCAM	1.140136704	Monacolin L acid	1.060562098
gene-ALCAM	1.140136704	Norophthalmic acid	0.191432411
gene-ALCAM	1.140136704	(1R,6S)-6-Amino-5-oxocyclohex-2-ene-1-c	0.154751123
gene-ALCAM	1.140136704	(S)-Mandelic acid O-beta-D-Glucopyranosid	0.509637316
gene-ALCAM	1.140136704	2-(1-Adamantyl)-1,3-dioxetane	0.408916843
gene-ALCAM	1.140136704	3-(3-Methylbutylidene)-1(3H)-isobenzofurar	0.575422414
gene-ALCAM	1.140136704	3',4',5'-Trimethoxycinnamyl alcohol acetate	0.4221816
gene-ALCAM	1.140136704	O-(17-Carboxyheptadecanoyl)carnitine	0.433790125
gene-ALCAM	1.140136704	Roxithromycin	0.268273077
gene-ALCAM	1.140136704	PC(P-18:1(11Z)/PGJ2)	0.565243877
gene-PALM	1.139553601	D-Erythro-imidazole-glycerol-phosphate	0.355812431
gene-PALM	1.139553601	3-Deoxyestrone	0.282221709
gene-PALM	1.139553601	1-Oleoyl-sn-glycero-3-phosphocholine	0.18926952
gene-PALM	1.139553601	LTB4-d4	0.226799982
gene-PALM	1.139553601	cis-p-Menth-2-en-1-ol	0.201017988
gene-PALM	1.139553601	(-)-alpha-Terpineol	0.345716279
gene-PALM	1.139553601	PG(20:1(11Z)/18:3(10,12,15)-OH(9))	0.626244347
gene-PALM	1.139553601	5-(2-Aminopropyl)-2-methylphenol	0.323231457
gene-PALM	1.139553601	4-Dimethylamino-L-phenylalanine	0.242110226
gene-PALM	1.139553601	Armillane	0.52808635
gene-PALM	1.139553601	PC(P-18:1(11Z)/PGE2)	0.509503133
gene-PALM	1.139553601	(3R,4R)-3-Amino-1-hydroxy-4-methylpyrrol	0.471914506
gene-PALM	1.139553601	n-methyl-2-(4'-methylaminophenyl)-6-hydr	0.26655714
gene-PALM	1.139553601	Isopropyl isothiocyanate	0.172385747
gene-PALM	1.139553601	(9Z)-Octadecenoic acid	0.142359556
gene-PALM	1.139553601	9-deoxy-9-methylene-16,16-dimethyl -PGE	0.606893884
gene-PALM	1.139553601	CL(8:0/8:0/18:2(9Z,11Z)/20:0)	0.622988418
gene-LOC100297	1.139395942	Sorbitan laurate	0.242315968

gene-LOC100297	1.139395942	4-Gingerol	0.222165012
gene-LOC100297	1.139395942	Monacolin L acid	1.060562098
gene-LOC100297	1.139395942	Norophthalmic acid	0.191432411
gene-LOC100297	1.139395942	Terbutryn	1.186241428
gene-LOC100297	1.139395942	Policapram	0.598028597
gene-LOC100297	1.139395942	alpha-Terpineol formate	0.628238986
gene-LOC100297	1.139395942	Isoamyl salicylate	0.393519337
gene-LOC100297	1.139395942	(Z)-3-Methyl-3-decenoic acid	0.482455266
gene-LOC100297	1.139395942	PE(20:5(5Z,8Z,11Z,14Z,17Z)/18:0)	0.427962506
gene-SCRN2	1.136303056	3-Thiacytidine	0.209387412
gene-SCRN2	1.136303056	11-Maleimidoundecanoic acid	1.084942397
gene-SCRN2	1.136303056	13(S)-HpODE	0.163157753
gene-SCRN2	1.136303056	4-cholesten-7伪,12伪,24-triol-3-one	0.097840451
gene-SCRN2	1.136303056	Cyclosporin A	0.656529229
gene-SCRN2	1.136303056	beta-L-Dioxolane-cytidine	0.175916668
gene-SCRN2	1.136303056	PC(P-18:1(11Z)/PGE2)	0.509503133
gene-SCRN2	1.136303056	PC(P-18:1(11Z)/PGJ2)	0.565243877
gene-SCRN2	1.136303056	PS(20:0/20:4(8Z,11Z,14Z,17Z)-2OH(5S,6R))	0.402595921
gene-SCRN2	1.136303056	D-Fructose	0.167584616
gene-DPM3	1.135471262	3-Thiacytidine	0.209387412
gene-DPM3	1.135471262	LTB4-d4	0.226799982
gene-DPM3	1.135471262	(-)-alpha-Terpineol	0.345716279
gene-DPM3	1.135471262	Cyclosporin A	0.656529229
gene-DPM3	1.135471262	Angiotensin A	0.247332017
gene-DPM3	1.135471262	DG(18:0/LTE4/0:0)	0.681485773
gene-DPM3	1.135471262	PC(17:0/PGJ2)	0.657689319
gene-DPM3	1.135471262	PC(14:0/20:2(11Z,14Z))	1.110657378
gene-DPM3	1.135471262	5-(2-Aminopropyl)-2-methylphenol	0.323231457
gene-DPM3	1.135471262	Trimetazidine	0.438282534
gene-DPM3	1.135471262	ingenol	0.942071652
gene-DPM3	1.135471262	Armillane	0.52808635
gene-DPM3	1.135471262	beta-L-Dioxolane-cytidine	0.175916668
gene-DPM3	1.135471262	PC(P-18:1(11Z)/PGE2)	0.509503133
gene-DPM3	1.135471262	PC(20:3(5Z,8Z,11Z)/24:0)	0.387959564
gene-DPM3	1.135471262	Isopropyl isothiocyanate	0.172385747
gene-DPM3	1.135471262	(9Z)-Octadecenoic acid	0.142359556
gene-DPM3	1.135471262	arachidyl amido cholanoic acid	1.24842952
gene-DPM3	1.135471262	Nigroxanthin	0.705005059
gene-DPM3	1.135471262	1-Octadecanoyl-2-(7Z,10Z,13Z,16Z-docosat	0.690496314
gene-DPM3	1.135471262	D-Fructose	0.167584616
gene-DPM3	1.135471262	CL(8:0/8:0/18:2(9Z,11Z)/20:0)	0.622988418
gene-DPM3	1.135471262	DG(20:0/LTE4/0:0)	0.438074508
gene-KCNT1	1.135076908	3-Thiacytidine	0.209387412
gene-KCNT1	1.135076908	D-Erythro-imidazole-glycerol-phosphate	0.355812431
gene-KCNT1	1.135076908	LTB4-d4	0.226799982
gene-KCNT1	1.135076908	cis-p-Menth-2-en-1-ol	0.201017988
gene-KCNT1	1.135076908	PE(20:0/18:1(12Z)-2OH(9,10))	0.438658253
gene-KCNT1	1.135076908	PE(22:2(13Z,16Z)/22:5(4Z,7Z,10Z,13Z,19Z)-O	0.369745166
gene-KCNT1	1.135076908	Cyclosporin A	0.656529229
gene-KCNT1	1.135076908	2-Methyl-3-phenyl-2-propenal	0.407813975
gene-KCNT1	1.135076908	PC(P-18:1(11Z)/PGE2)	0.509503133

gene-KCNT1	1.135076908	Nigroxanthin	0.705005059
gene-KCNT1	1.135076908	PC(P-18:1(11Z)/PGJ2)	0.565243877
gene-KCNT1	1.135076908	PS(20:0/20:4(8Z,11Z,14Z,17Z)-2OH(5S,6R))	0.402595921
gene-KCNT1	1.135076908	CL(8:0/8:0/18:2(9Z,11Z)/20:0)	0.622988418
Bos_taurus_newG	1.133449492	D-Erythro-imidazole-glycerol-phosphate	0.355812431
Bos_taurus_newG	1.133449492	LTB4-d4	0.226799982
Bos_taurus_newG	1.133449492	cis-p-Menth-2-en-1-ol	0.201017988
Bos_taurus_newG	1.133449492	PE(20:0/18:1(12Z)-2OH(9,10))	0.438658253
Bos_taurus_newG	1.133449492	PG(20:1(11Z)/18:3(10,12,15)-OH(9))	0.626244347
Bos_taurus_newG	1.133449492	2-Methyl-3-phenyl-2-propenal	0.407813975
Bos_taurus_newG	1.133449492	Guanidoacetic acid	0.542509265
Bos_taurus_newG	1.133449492	alpha-Terpineol formate	0.628238986
Bos_taurus_newG	1.133449492	PC(P-18:1(11Z)/PGE2)	0.509503133
Bos_taurus_newG	1.133449492	Nigroxanthin	0.705005059
Bos_taurus_newG	1.133449492	PC(P-18:1(11Z)/PGJ2)	0.565243877
Bos_taurus_newG	1.133449492	PS(20:0/20:4(8Z,11Z,14Z,17Z)-2OH(5S,6R))	0.402595921
gene-LOC52481(	1.129886406	3-Thiacytidine	0.209387412
gene-LOC52481(	1.129886406	D-Erythro-imidazole-glycerol-phosphate	0.355812431
gene-LOC52481(	1.129886406	7(14)-Bisabolene-2,3,10,11-tetrol	2.446897974
gene-LOC52481(	1.129886406	LTB4-d4	0.226799982
gene-LOC52481(	1.129886406	cis-p-Menth-2-en-1-ol	0.201017988
gene-LOC52481(	1.129886406	(-)-alpha-Terpineol	0.345716279
gene-LOC52481(	1.129886406	1,4-Undecadiene	0.462803973
gene-LOC52481(	1.129886406	PG(20:1(11Z)/18:3(10,12,15)-OH(9))	0.626244347
gene-LOC52481(	1.129886406	5-(2-Aminopropyl)-2-methylphenol	0.323231457
gene-LOC52481(	1.129886406	4-Dimethylamino-L-phenylalanine	0.242110226
gene-LOC52481(	1.129886406	Armillane	0.52808635
gene-LOC52481(	1.129886406	PC(P-18:1(11Z)/PGE2)	0.509503133
gene-LOC52481(	1.129886406	(3R,4R)-3-Amino-1-hydroxy-4-methylpyrrol	0.471914506
gene-LOC52481(	1.129886406	n-methyl-2-(4'-methylaminophenyl)-6-hydr	0.26655714
gene-LOC52481(	1.129886406	Isopropyl isothiocyanate	0.172385747
gene-LOC52481(	1.129886406	PC(P-18:1(11Z)/PGJ2)	0.565243877
gene-LOC52481(	1.129886406	PS(20:0/20:4(8Z,11Z,14Z,17Z)-2OH(5S,6R))	0.402595921
gene-LOC52481(	1.129886406	CL(8:0/8:0/18:2(9Z,11Z)/20:0)	0.622988418
gene-MEI1	1.128635658	3-Thiacytidine	0.209387412
gene-MEI1	1.128635658	Sambutoxin	0.169786377
gene-MEI1	1.128635658	Isopropyl isothiocyanate	0.172385747
gene-MEI1	1.128635658	PS(20:0/20:4(8Z,11Z,14Z,17Z)-2OH(5S,6R))	0.402595921
Bos_taurus_newG	1.128569596	3-Thiacytidine	0.209387412
Bos_taurus_newG	1.128569596	D-Erythro-imidazole-glycerol-phosphate	0.355812431
Bos_taurus_newG	1.128569596	7(14)-Bisabolene-2,3,10,11-tetrol	2.446897974
Bos_taurus_newG	1.128569596	1-Oleoyl-sn-glycero-3-phosphocholine	0.18926952
Bos_taurus_newG	1.128569596	LTB4-d4	0.226799982
Bos_taurus_newG	1.128569596	cis-p-Menth-2-en-1-ol	0.201017988
Bos_taurus_newG	1.128569596	(-)-alpha-Terpineol	0.345716279
Bos_taurus_newG	1.128569596	1,4-Undecadiene	0.462803973
Bos_taurus_newG	1.128569596	5-(2-Aminopropyl)-2-methylphenol	0.323231457
Bos_taurus_newG	1.128569596	4-Dimethylamino-L-phenylalanine	0.242110226
Bos_taurus_newG	1.128569596	Armillane	0.52808635
Bos_taurus_newG	1.128569596	2-Methyl-3-phenyl-2-propenal	0.407813975
Bos_taurus_newG	1.128569596	PC(P-18:1(11Z)/PGE2)	0.509503133

Bos_taurus_newG	1.128569596	(3R,4R)-3-Amino-1-hydroxy-4-methylpyrrol	0.471914506
Bos_taurus_newG	1.128569596	n-methyl-2-(4'-methylaminophenyl)-6-hydr	0.26655714
Bos_taurus_newG	1.128569596	Isopropyl isothiocyanate	0.172385747
Bos_taurus_newG	1.128569596	(9Z)-Octadecenoic acid	0.142359556
Bos_taurus_newG	1.128569596	PS(20:0/20:4(8Z,11Z,14Z,17Z)-2OH(5S,6R))	0.402595921
Bos_taurus_newG	1.128569596	CL(8:0/8:0/18:2(9Z,11Z)/20:0)	0.622988418
Bos_taurus_newG	1.128171959	5-(3'-Carboxy-3'-oxopropenyl)-4,6-dihydro	1.083232395
Bos_taurus_newG	1.128171959	D-Erythro-imidazole-glycerol-phosphate	0.355812431
Bos_taurus_newG	1.128171959	Monacolin L acid	1.060562098
Bos_taurus_newG	1.128171959	cis-p-Menth-2-en-1-ol	0.201017988
Bos_taurus_newG	1.128171959	PE(20:0/18:1(12Z)-2OH(9,10))	0.438658253
Bos_taurus_newG	1.128171959	Dyphylline	0.664549753
Bos_taurus_newG	1.128171959	Pseudouridine 5'-phosphate	1.18431378
Bos_taurus_newG	1.128171959	Ribavirin monophosphate	1.091085027
Bos_taurus_newG	1.128171959	Norophthalmic acid	0.191432411
Bos_taurus_newG	1.128171959	3'-N'-Acetylfusarochromanone	0.755683206
Bos_taurus_newG	1.128171959	Ser Cys Ala Ala	0.603032447
Bos_taurus_newG	1.128171959	Tryptophyl-Glutamine	0.89722186
Bos_taurus_newG	1.128171959	Terbutryn	1.186241428
Bos_taurus_newG	1.128171959	Guanidoacetic acid	0.542509265
Bos_taurus_newG	1.128171959	4-Oxo-9-cis-retinoyl-beta-glucuronide	1.611773742
Bos_taurus_newG	1.128171959	alpha-Terpineol formate	0.628238986
Bos_taurus_newG	1.128171959	20-carboxy Arachidonic Acid	0.780988382
Bos_taurus_newG	1.128171959	4-Octylphenol	1.400657885
Bos_taurus_newG	1.128171959	Prolyl-Lysine	1.612991304
Bos_taurus_newG	1.128171959	3-Pentadecylphenol	1.682258188
Bos_taurus_newG	1.128171959	PC(P-18:1(11Z)/PGE2)	0.509503133
Bos_taurus_newG	1.128171959	Galabiosylceramide (d18:1/20:0)	0.746737323
Bos_taurus_newG	1.128171959	12-Hydroxyicosanoylcarnitine	1.730979067
Bos_taurus_newG	1.128171959	Sitosterol beta-D-glucoside	0.382944796
Bos_taurus_newG	1.128171959	PC(P-18:1(11Z)/PGJ2)	0.565243877
Bos_taurus_newG	1.128171959	PS(20:0/20:4(8Z,11Z,14Z,17Z)-2OH(5S,6R))	0.402595921
Bos_taurus_newG	1.128171959	Galactosylglycerol	0.738463143
Bos_taurus_newG	1.128171959	PE(20:5(5Z,8Z,11Z,14Z,17Z)/18:0)	0.427962506
gene-DBNDD1	1.124576161	LysoPI(16:0/0:0)	0.379098336
gene-DBNDD1	1.124576161	3-Thiacytidine	0.209387412
gene-DBNDD1	1.124576161	11-Maleimidoundecanoic acid	1.084942397
gene-DBNDD1	1.124576161	PE(20:0/18:1(12Z)-2OH(9,10))	0.438658253
gene-DBNDD1	1.124576161	PE(22:2(13Z,16Z)/22:5(4Z,7Z,10Z,13Z,19Z)-O	0.369745166
gene-DBNDD1	1.124576161	Cyclosporin A	0.656529229
gene-DBNDD1	1.124576161	DG(18:0/LTE4/0:0)	0.681485773
gene-DBNDD1	1.124576161	CDP-DG(PGF2alpha/16:0)	1.002512277
gene-DBNDD1	1.124576161	1-beta-D-Arabinofuranosyl-5-fluorocytosine	0.152339645
gene-DBNDD1	1.124576161	Norophthalmic acid	0.191432411
gene-DBNDD1	1.124576161	beta-L-Dioxolane-cytidine	0.175916668
gene-DBNDD1	1.124576161	PC(18:1(9Z)/15:1(9Z))	0.534724682
gene-DBNDD1	1.124576161	Nigroxanthin	0.705005059
gene-DBNDD1	1.124576161	PC(P-18:1(11Z)/PGJ2)	0.565243877
gene-DBNDD1	1.124576161	PS(20:0/20:4(8Z,11Z,14Z,17Z)-2OH(5S,6R))	0.402595921
gene-SDK1	1.122629159	LysoPI(16:0/0:0)	0.379098336
gene-SDK1	1.122629159	2-[(3S)-3-[[(2S)-1-(Carboxymethoxy)-1-oxo-	0.111577437

gene-SDK1	1.122629159	5-(2-Aminopropyl)-2-methylphenol	0.323231457
gene-SDK1	1.122629159	Armillane	0.52808635
gene-SDK1	1.122629159	Isopropyl isothiocyanate	0.172385747
gene-SDK1	1.122629159	PS(20:0/20:4(8Z,11Z,14Z,17Z)-2OH(5S,6R))	0.402595921
gene-ZBTB48	1.122239013	PE(22:2(13Z,16Z)/22:5(4Z,7Z,10Z,13Z,19Z)-O	0.369745166
gene-ZBTB48	1.122239013	Cyclosporin A	0.656529229
gene-ZBTB48	1.122239013	PC(P-18:1(11Z)/PGE2)	0.509503133
gene-ZBTB48	1.122239013	PC(P-18:1(11Z)/PGJ2)	0.565243877
gene-ZBTB48	1.122239013	PS(20:0/20:4(8Z,11Z,14Z,17Z)-2OH(5S,6R))	0.402595921
gene-ZBTB48	1.122239013	CL(8:0/8:0/18:2(9Z,11Z)/20:0)	0.622988418
Bos_taurus_newG	1.119905735	3-Thiacytidine	0.209387412
Bos_taurus_newG	1.119905735	11-Maleimidoundecanoic acid	1.084942397
Bos_taurus_newG	1.119905735	cis-p-Menth-2-en-1-ol	0.201017988
Bos_taurus_newG	1.119905735	PC(24:0/22:6(4Z,7Z,10Z,12E,16Z,19Z)-OH(14)	0.188241061
Bos_taurus_newG	1.119905735	PE(20:0/18:1(12Z)-2OH(9,10))	0.438658253
Bos_taurus_newG	1.119905735	PE(22:2(13Z,16Z)/22:5(4Z,7Z,10Z,13Z,19Z)-O	0.369745166
Bos_taurus_newG	1.119905735	Benzoyl glucuronide (Benzoic acid)	0.442844673
Bos_taurus_newG	1.119905735	S-Acetyldihydrolipoamide-E	0.429561283
Bos_taurus_newG	1.119905735	Norophthalmic acid	0.191432411
Bos_taurus_newG	1.119905735	Guanidoacetic acid	0.542509265
Bos_taurus_newG	1.119905735	alpha-Terpineol formate	0.628238986
Bos_taurus_newG	1.119905735	Methionyl-Valine	0.502707745
Bos_taurus_newG	1.119905735	PC(P-18:1(11Z)/PGE2)	0.509503133
Bos_taurus_newG	1.119905735	Roxithromycin	0.268273077
Bos_taurus_newG	1.119905735	Nigroxanthin	0.705005059
Bos_taurus_newG	1.119905735	PC(P-18:1(11Z)/PGJ2)	0.565243877
Bos_taurus_newG	1.119905735	PS(20:0/20:4(8Z,11Z,14Z,17Z)-2OH(5S,6R))	0.402595921
gene-LOC52001	1.119483741	D-Erythro-imidazole-glycerol-phosphate	0.355812431
gene-LOC52001	1.119483741	Undecanedioic acid	0.059955634
gene-LOC52001	1.119483741	(S)-10,16-Dihydroxyhexadecanoic acid	1.054006526
gene-LOC52001	1.119483741	Auxin b	1.052491354
gene-LOC52001	1.119483741	cis-p-Menth-2-en-1-ol	0.201017988
gene-LOC52001	1.119483741	PG(20:1(11Z)/18:3(10,12,15)-OH(9))	0.626244347
gene-LOC52001	1.119483741	(1R,6S)-6-Amino-5-oxocyclohex-2-ene-1-c	0.154751123
gene-LOC52001	1.119483741	Hexanoylglutamine	0.593876112
gene-LOC52001	1.119483741	2-Methyl-3-phenyl-2-propenal	0.407813975
gene-LOC52001	1.119483741	2-(1-Adamantyl)-1,3-dioxetane	0.408916843
gene-LOC52001	1.119483741	Ajulemic acid	0.557832951
gene-LOC52001	1.119483741	PC(P-18:1(11Z)/PGE2)	0.509503133
gene-LOC52001	1.119483741	Roxithromycin	0.268273077
gene-LOC52001	1.119483741	PC(P-18:1(11Z)/PGJ2)	0.565243877
gene-LOC52001	1.119483741	PS(20:0/20:4(8Z,11Z,14Z,17Z)-2OH(5S,6R))	0.402595921
gene-TG	1.119309443	3-Thiacytidine	0.209387412
gene-TG	1.119309443	cis-p-Menth-2-en-1-ol	0.201017988
gene-TG	1.119309443	PE(20:0/18:1(12Z)-2OH(9,10))	0.438658253
gene-TG	1.119309443	5-(2-Aminopropyl)-2-methylphenol	0.323231457
gene-TG	1.119309443	Armillane	0.52808635
gene-TG	1.119309443	4-Oxo-9-cis-retinoyl-beta-glucuronide	1.611773742
gene-TG	1.119309443	PC(P-18:1(11Z)/PGE2)	0.509503133
gene-TG	1.119309443	Gamithromycin	0.37168241
gene-TG	1.119309443	Isopropyl isothiocyanate	0.172385747

gene-TG	1.119309443	PC(18:1(9Z)/15:1(9Z))	0.534724682
gene-TG	1.119309443	Nigroxanthin	0.705005059
gene-TG	1.119309443	PC(P-18:1(11Z)/PGJ2)	0.565243877
gene-TG	1.119309443	PS(20:0/20:4(8Z,11Z,14Z,17Z)-2OH(5S,6R))	0.402595921
gene-TG	1.119309443	CL(8:0/8:0/18:2(9Z,11Z)/20:0)	0.622988418
gene-SPATA20	1.118949054	D-Erythro-imidazole-glycerol-phosphate	0.355812431
gene-SPATA20	1.118949054	Palmitoyl Ara-C	0.586890476
gene-SPATA20	1.118949054	LTB4-d4	0.226799982
gene-SPATA20	1.118949054	cis-p-Menth-2-en-1-ol	0.201017988
gene-SPATA20	1.118949054	PE(20:0/18:1(12Z)-2OH(9,10))	0.438658253
gene-SPATA20	1.118949054	PG(20:1(11Z)/18:3(10,12,15)-OH(9))	0.626244347
gene-SPATA20	1.118949054	2-Methyl-3-phenyl-2-propenal	0.407813975
gene-SPATA20	1.118949054	PC(P-18:1(11Z)/PGE2)	0.509503133
gene-SPATA20	1.118949054	(3R,4R)-3-Amino-1-hydroxy-4-methylpyrrol	0.471914506
gene-SPATA20	1.118949054	11-Deoxycortisol	0.595462818
gene-SPATA20	1.118949054	(9Z)-Octadecenoic acid	0.142359556
gene-SPATA20	1.118949054	Nigroxanthin	0.705005059
gene-SPATA20	1.118949054	PC(P-18:1(11Z)/PGJ2)	0.565243877
Bos_taurus_newG	1.118568066	5-(3'-Carboxy-3'-oxopropenyl)-4,6-dihydro:	1.083232395
Bos_taurus_newG	1.118568066	D-Erythro-imidazole-glycerol-phosphate	0.355812431
Bos_taurus_newG	1.118568066	(+)-Bottrospicatol	0.68654221
Bos_taurus_newG	1.118568066	Undecanedioic acid	0.059955634
Bos_taurus_newG	1.118568066	Monacolin L acid	1.060562098
Bos_taurus_newG	1.118568066	cis-p-Menth-2-en-1-ol	0.201017988
Bos_taurus_newG	1.118568066	PE(22:2(13Z,16Z)/22:5(4Z,7Z,10Z,13Z,19Z)-O	0.369745166
Bos_taurus_newG	1.118568066	3,4,3',4'-Tetrahydrospirilloxanthin	0.354666148
Bos_taurus_newG	1.118568066	(1R,6S)-6-Amino-5-oxocyclohex-2-ene-1-c	0.154751123
Bos_taurus_newG	1.118568066	2-(1-Adamantyl)-1,3-dioxetane	0.408916843
Bos_taurus_newG	1.118568066	alpha-Terpineol formate	0.628238986
Bos_taurus_newG	1.118568066	4-Octylphenol	1.400657885
Bos_taurus_newG	1.118568066	PC(P-18:1(11Z)/PGE2)	0.509503133
Bos_taurus_newG	1.118568066	Milbemycin D	0.125637998
Bos_taurus_newG	1.118568066	Roxithromycin	0.268273077
Bos_taurus_newG	1.118568066	Sitosterol beta-D-glucoside	0.382944796
Bos_taurus_newG	1.118568066	PC(P-18:1(11Z)/PGJ2)	0.565243877
Bos_taurus_newG	1.118568066	PS(20:0/20:4(8Z,11Z,14Z,17Z)-2OH(5S,6R))	0.402595921
Bos_taurus_newG	1.118568066	PE(20:5(5Z,8Z,11Z,14Z,17Z)/18:0)	0.427962506
gene-AMPD3	1.11667558	3-Thiacytidine	0.209387412
gene-AMPD3	1.11667558	D-Erythro-imidazole-glycerol-phosphate	0.355812431
gene-AMPD3	1.11667558	7(14)-Bisabolene-2,3,10,11-tetrol	2.446897974
gene-AMPD3	1.11667558	cis-p-Menth-2-en-1-ol	0.201017988
gene-AMPD3	1.11667558	(-)-alpha-Terpineol	0.345716279
gene-AMPD3	1.11667558	1,4-Undecadiene	0.462803973
gene-AMPD3	1.11667558	5-(2-Aminopropyl)-2-methylphenol	0.323231457
gene-AMPD3	1.11667558	4-Dimethylamino-L-phenylalanine	0.242110226
gene-AMPD3	1.11667558	Armillane	0.52808635
gene-AMPD3	1.11667558	PC(P-18:1(11Z)/PGE2)	0.509503133
gene-AMPD3	1.11667558	(3R,4R)-3-Amino-1-hydroxy-4-methylpyrrol	0.471914506
gene-AMPD3	1.11667558	Isopropyl isothiocyanate	0.172385747
gene-AMPD3	1.11667558	PC(P-18:1(11Z)/PGJ2)	0.565243877
gene-AMPD3	1.11667558	PS(20:0/20:4(8Z,11Z,14Z,17Z)-2OH(5S,6R))	0.402595921

gene-AMPD3	1.11667558	CL(8:0/8:0/18:2(9Z,11Z)/20:0)	0.622988418
gene-SPTY2D1O!	1.11580336	5-(3'-Carboxy-3'-oxopropenyl)-4,6-dihydro›	1.083232395
gene-SPTY2D1O!	1.11580336	D-Erythro-imidazole-glycerol-phosphate	0.355812431
gene-SPTY2D1O!	1.11580336	(S)-10,16-Dihydroxyhexadecanoic acid	1.054006526
gene-SPTY2D1O!	1.11580336	Monacolin L acid	1.060562098
gene-SPTY2D1O!	1.11580336	cis-p-Menth-2-en-1-ol	0.201017988
gene-SPTY2D1O!	1.11580336	4-Octylphenol	1.400657885
gene-SPTY2D1O!	1.11580336	Ajulemic acid	0.557832951
gene-SPTY2D1O!	1.11580336	PC(P-18:1(11Z)/PGE2)	0.509503133
gene-SPTY2D1O!	1.11580336	Roxithromycin	0.268273077
gene-SPTY2D1O!	1.11580336	Sitosterol beta-D-glucoside	0.382944796
gene-SPTY2D1O!	1.11580336	PC(P-18:1(11Z)/PGJ2)	0.565243877
gene-SPTY2D1O!	1.11580336	PS(20:0/20:4(8Z,11Z,14Z,17Z)-2OH(5S,6R))	0.402595921
gene-LIN7B	1.115446974	Ascorbic acid 3-sulfate	0.300057469
gene-LIN7B	1.115446974	Azelaic acid	0.007180175
gene-LIN7B	1.115446974	5-Hydroxy-2-oxo-4-ureido-2,5-dihydro-1H	0.122536033
gene-LIN7B	1.115446974	Valnemulin	0.408062514
gene-LIN7B	1.115446974	PE(20:0/18:1(12Z)-2OH(9,10))	0.438658253
gene-LIN7B	1.115446974	PI(18:1(11Z)/PGF1alpha)	0.243592581
gene-LIN7B	1.115446974	Cyclosporin A	0.656529229
gene-LIN7B	1.115446974	Epomusenin A	0.766206049
gene-LIN7B	1.115446974	PA(22:6(4Z,7Z,10Z,13Z,16Z,19Z)/16:0)	0.556532497
gene-LIN7B	1.115446974	DG(18:0/LTE4/0:0)	0.681485773
gene-LIN7B	1.115446974	PC(17:0/PGJ2)	0.657689319
gene-LIN7B	1.115446974	CDP-DG(PGF2alpha/16:0)	1.002512277
gene-LIN7B	1.115446974	1-beta-D-Arabinofuranosyl-5-fluorocytosine	0.152339645
gene-LIN7B	1.115446974	4-Hydroxy-6-methyl-3-(1H-pyrazol-3-yl)-2	0.006362816
gene-LIN7B	1.115446974	C20914	0.140383181
gene-LIN7B	1.115446974	S-(2-Hydroxyethyl)glutathione	0.269671835
gene-LIN7B	1.115446974	Fluridone	0.127106682
gene-LIN7B	1.115446974	Norophthalmic acid	0.191432411
gene-LIN7B	1.115446974	5,6,7,8-Tetrahydromonapterin	0.724075531
gene-LIN7B	1.115446974	Harmalol	0.364945111
gene-LIN7B	1.115446974	Ethylene brassylate	0.989156973
gene-LIN7B	1.115446974	Guanidoacetic acid	0.542509265
gene-LIN7B	1.115446974	4-Oxo-9-cis-retinoyl-beta-glucuronide	1.611773742
gene-LIN7B	1.115446974	alpha-Terpineol formate	0.628238986
gene-LIN7B	1.115446974	Methionyl-Valine	0.502707745
gene-LIN7B	1.115446974	L-Histidinol	0.149249286
gene-LIN7B	1.115446974	beta-L-Dioxolane-cytidine	0.175916668
gene-LIN7B	1.115446974	1,4,6-Trimethylnaphthalene	0.12150693
gene-LIN7B	1.115446974	[(2S,4R,5R,6R,14S,16R)-14-Hydroxy-7,11-din	0.247987808
gene-LIN7B	1.115446974	N-Palmitoyl Proline	0.470367693
gene-LIN7B	1.115446974	10-alpha-methoxy-9,10-dihydrolysergol	0.140851438
gene-LIN7B	1.115446974	MG(LTE4/0:0/0:0)	0.548257353
gene-LIN7B	1.115446974	LysoPI(0:0/18:0)	0.420441153
gene-LIN7B	1.115446974	1,3,5-Bisabolatrien-10-one	0.109736328
gene-LIN7B	1.115446974	N1-(5-Phospho-a-D-ribosyl)-5,6-dimethylb	0.154251596
gene-LIN7B	1.115446974	1-Heneicosanoyl-glycero-3-phosphoserine	0.214358093
gene-LIN7B	1.115446974	Nigroxanthin	0.705005059
gene-LIN7B	1.115446974	1-Octadecanoyl-2-(7Z,10Z,13Z,16Z-docosat	0.690496314

gene-LIN7B	1.115446974	PE(18:0/20:4(8Z,11Z,14Z,17Z)-2OH(5S,6R))	0.418208781
gene-LIN7B	1.115446974	PC(P-18:1(11Z)/PGJ2)	0.565243877
gene-LIN7B	1.115446974	Azimexon	0.008689891
gene-LIN7B	1.115446974	PE-NMe(18:2(9Z,12Z)/18:2(9Z,12Z))[U]	0.694736593
gene-LIN7B	1.115446974	PS(16:1(9Z)/22:2(13Z,16Z))	0.659652661
gene-LIN7B	1.115446974	DG(20:0/LTE4/0:0)	0.438074508
gene-RFX2	1.115360952	D-Erythro-imidazole-glycerol-phosphate	0.355812431
gene-RFX2	1.115360952	Glutamate carbon	0.671444516
gene-RFX2	1.115360952	1-(2-Furanyl)-1-pentanone	0.697147283
gene-RFX2	1.115360952	2-Dehydro-3-deoxy-D-gluconate	0.65165299
gene-RFX2	1.115360952	11-Maleimidoundecanoic acid	1.084942397
gene-RFX2	1.115360952	13(S)-HpODE	0.163157753
gene-RFX2	1.115360952	cis-p-Menth-2-en-1-ol	0.201017988
gene-RFX2	1.115360952	PE(22:2(13Z,16Z)/22:5(4Z,7Z,10Z,13Z,19Z)-O	0.369745166
gene-RFX2	1.115360952	(1R,6S)-6-Amino-5-oxocyclohex-2-ene-1-c	0.154751123
gene-RFX2	1.115360952	2-Methyl-3-phenyl-2-propenal	0.407813975
gene-RFX2	1.115360952	2-(1-Adamantyl)-1,3-dioxetane	0.408916843
gene-RFX2	1.115360952	3-(3-Methylbutylidene)-1(3H)-isobenzofurar	0.575422414
gene-RFX2	1.115360952	3',4',5'-Trimethoxycinnamyl alcohol acetate	0.4221816
gene-RFX2	1.115360952	PC(P-18:1(11Z)/PGE2)	0.509503133
gene-RFX2	1.115360952	Roxithromycin	0.268273077
gene-RFX2	1.115360952	PC(P-18:1(11Z)/PGJ2)	0.565243877
gene-RFX2	1.115360952	PS(20:0/20:4(8Z,11Z,14Z,17Z)-2OH(5S,6R))	0.402595921
gene-SYNE1	1.114104386	D-Erythro-imidazole-glycerol-phosphate	0.355812431
gene-SYNE1	1.114104386	Isomaltotriose	0.58434654
gene-SYNE1	1.114104386	Glutamate carbon	0.671444516
gene-SYNE1	1.114104386	Cholic acid glucuronide	0.147398811
gene-SYNE1	1.114104386	Urocortisol	0.926998516
gene-SYNE1	1.114104386	Deoxyshikonin	0.734877521
gene-SYNE1	1.114104386	11-Maleimidoundecanoic acid	1.084942397
gene-SYNE1	1.114104386	7(S),17(S)-dihydroxy-8(E),10(Z),13(Z),15(E),19	0.32147403
gene-SYNE1	1.114104386	(1R,6S)-6-Amino-5-oxocyclohex-2-ene-1-c	0.154751123
gene-SYNE1	1.114104386	3-(3-Methylbutylidene)-1(3H)-isobenzofurar	0.575422414
gene-SYNE1	1.114104386	Chamissonin diacetate	0.324896191
gene-SYNE1	1.114104386	[3-(2-Aminopropyl)-6-methylidenecyclohexa	0.422945997
gene-SYNE1	1.114104386	3',4',5'-Trimethoxycinnamyl alcohol acetate	0.4221816
gene-SYNE1	1.114104386	L-Anticapsin	0.426006862
gene-SYNE1	1.114104386	Sophoranol	0.282064826
gene-SYNE1	1.114104386	L-Cysteine	0.214540754
gene-SYNE1	1.114104386	24,24-Dfhv	0.175278646
gene-SYNE1	1.114104386	1,8-Octanedithiol	0.133094504
gene-SYNE1	1.114104386	PC(P-18:1(11Z)/PGE2)	0.509503133
gene-SYNE1	1.114104386	Roxithromycin	0.268273077
gene-FCAR	1.112071823	LysoPI(16:0/0:0)	0.379098336
gene-FCAR	1.112071823	3-Thiacytidine	0.209387412
gene-FCAR	1.112071823	11-Maleimidoundecanoic acid	1.084942397
gene-FCAR	1.112071823	cis-p-Menth-2-en-1-ol	0.201017988
gene-FCAR	1.112071823	PE(20:0/18:1(12Z)-2OH(9,10))	0.438658253
gene-FCAR	1.112071823	PE(22:2(13Z,16Z)/22:5(4Z,7Z,10Z,13Z,19Z)-O	0.369745166
gene-FCAR	1.112071823	Lamivudine	0.327402892
gene-FCAR	1.112071823	5-Formiminotetrahydrofolate	0.118251287

gene-FCAR	1.112071823	Benzoyl glucuronide (Benzoic acid)	0.442844673
gene-FCAR	1.112071823	Pregnanediol	0.166857843
gene-FCAR	1.112071823	PC(P-18:1(11Z)/PGE2)	0.509503133
gene-FCAR	1.112071823	PC(18:1(9Z)/15:1(9Z))	0.534724682
gene-FCAR	1.112071823	Nigroxanthin	0.705005059
gene-FCAR	1.112071823	PC(P-18:1(11Z)/PGJ2)	0.565243877
gene-FCAR	1.112071823	PS(20:0/20:4(8Z,11Z,14Z,17Z)-2OH(5S,6R))	0.402595921
gene-ANG	1.111265617	3-Thiacytidine	0.209387412
gene-ANG	1.111265617	11-Maleimidoundecanoic acid	1.084942397
gene-ANG	1.111265617	PE(22:2(13Z,16Z)/22:5(4Z,7Z,10Z,13Z,19Z)-O	0.369745166
gene-ANG	1.111265617	Cyclosporin A	0.656529229
gene-ANG	1.111265617	DG(18:0/LTE4/0:0)	0.681485773
gene-ANG	1.111265617	beta-L-Dioxolane-cytidine	0.175916668
gene-ANG	1.111265617	PC(P-18:1(11Z)/PGE2)	0.509503133
gene-ANG	1.111265617	Nigroxanthin	0.705005059
gene-ANG	1.111265617	PC(P-18:1(11Z)/PGJ2)	0.565243877
gene-ANG	1.111265617	PS(20:0/20:4(8Z,11Z,14Z,17Z)-2OH(5S,6R))	0.402595921
gene-ANG	1.111265617	CL(8:0/8:0/18:2(9Z,11Z)/20:0)	0.622988418
Bos_taurus_newG	1.11026277	1,2-O-Isopropylidene-D-glucofuranose	0.080987667
Bos_taurus_newG	1.11026277	1-Oleoyl-sn-glycero-3-phosphocholine	0.18926952
Bos_taurus_newG	1.11026277	(-)-alpha-Terpineol	0.345716279
Bos_taurus_newG	1.11026277	Cyclosporin A	0.656529229
Bos_taurus_newG	1.11026277	PC(16:0/18:1(12Z)-2OH(9,10))	0.461843233
Bos_taurus_newG	1.11026277	5-(2-Aminopropyl)-2-methylphenol	0.323231457
Bos_taurus_newG	1.11026277	4-Dimethylamino-L-phenylalanine	0.242110226
Bos_taurus_newG	1.11026277	Trimetazidine	0.438282534
Bos_taurus_newG	1.11026277	ingenol	0.942071652
Bos_taurus_newG	1.11026277	n-methyl-2-(4'-methylaminophenyl)-6-hydr	0.26655714
Bos_taurus_newG	1.11026277	9-deoxy-9-methylene-16,16-dimethyl -PGE	0.606893884
Bos_taurus_newG	1.11026277	CL(8:0/8:0/18:2(9Z,11Z)/20:0)	0.622988418
Bos_taurus_newG	1.108810395	3-Thiacytidine	0.209387412
Bos_taurus_newG	1.108810395	5-Hydroxyindoleacetaldehyde	0.448806354
Bos_taurus_newG	1.108810395	5-Hydroxy-2-oxo-4-ureido-2,5-dihydro-1H	0.122536033
Bos_taurus_newG	1.108810395	11-Maleimidoundecanoic acid	1.084942397
Bos_taurus_newG	1.108810395	PC(24:0/22:6(4Z,7Z,10Z,12E,16Z,19Z)-OH(14)	0.188241061
Bos_taurus_newG	1.108810395	PE(20:0/18:1(12Z)-2OH(9,10))	0.438658253
Bos_taurus_newG	1.108810395	PE(22:2(13Z,16Z)/22:5(4Z,7Z,10Z,13Z,19Z)-O	0.369745166
Bos_taurus_newG	1.108810395	Epomusenin A	0.766206049
Bos_taurus_newG	1.108810395	CDP-DG(PGF2alpha/16:0)	1.002512277
Bos_taurus_newG	1.108810395	Lamivudine	0.327402892
Bos_taurus_newG	1.108810395	Pseudouridine 5'-phosphate	1.18431378
Bos_taurus_newG	1.108810395	Aflatoxin P1	1.000661338
Bos_taurus_newG	1.108810395	Benzoyl glucuronide (Benzoic acid)	0.442844673
Bos_taurus_newG	1.108810395	S-Acetyldihydrolipoamide-E	0.429561283
Bos_taurus_newG	1.108810395	Ribavirin monophosphate	1.091085027
Bos_taurus_newG	1.108810395	Norophthalmic acid	0.191432411
Bos_taurus_newG	1.108810395	Indole-3-ethanol	0.096523128
Bos_taurus_newG	1.108810395	kainic acid	0.836445456
Bos_taurus_newG	1.108810395	5,6,7,8-Tetrahydromonapterin	0.724075531
Bos_taurus_newG	1.108810395	Harmalol	0.364945111
Bos_taurus_newG	1.108810395	Guanidoacetic acid	0.542509265

Bos_taurus_newG	1.108810395	4-Oxo-9-cis-retinoyl-beta-glucuronide	1.611773742
Bos_taurus_newG	1.108810395	alpha-Terpineol formate	0.628238986
Bos_taurus_newG	1.108810395	Apronal	0.754033426
Bos_taurus_newG	1.108810395	Methionyl-Valine	0.502707745
Bos_taurus_newG	1.108810395	Argyrolobine	0.949134241
Bos_taurus_newG	1.108810395	Dicyclopentadiene	0.129868592
Bos_taurus_newG	1.108810395	Phenol	0.097390283
Bos_taurus_newG	1.108810395	Nigroxanthin	0.705005059
Bos_taurus_newG	1.108810395	PC(P-18:1(11Z)/PGJ2)	0.565243877
Bos_taurus_newG	1.108810395	PS(20:0/20:4(8Z,11Z,14Z,17Z)-2OH(5S,6R))	0.402595921
Bos_taurus_newG	1.108810395	Azimexon	0.008689891
Bos_taurus_newG	1.108810395	PE(20:5(5Z,8Z,11Z,14Z,17Z)/18:0)	0.427962506
Bos_taurus_newG	1.108810395	PC(P-16:0/20:3(8Z,11Z,14Z)-2OH(5,6))	0.174122169
gene-EIF4EBP1	1.107774296	3-Thiacytidine	0.209387412
gene-EIF4EBP1	1.107774296	D-Erythro-imidazole-glycerol-phosphate	0.355812431
gene-EIF4EBP1	1.107774296	11-Maleimidoundecanoic acid	1.084942397
gene-EIF4EBP1	1.107774296	LTB4-d4	0.226799982
gene-EIF4EBP1	1.107774296	cis-p-Menth-2-en-1-ol	0.201017988
gene-EIF4EBP1	1.107774296	PE(20:0/18:1(12Z)-2OH(9,10))	0.438658253
gene-EIF4EBP1	1.107774296	PE(22:2(13Z,16Z)/22:5(4Z,7Z,10Z,13Z,19Z)-O	0.369745166
gene-EIF4EBP1	1.107774296	Cyclosporin A	0.656529229
gene-EIF4EBP1	1.107774296	DG(18:0/LTE4/0:0)	0.681485773
gene-EIF4EBP1	1.107774296	PC(P-18:1(11Z)/PGE2)	0.509503133
gene-EIF4EBP1	1.107774296	Nigroxanthin	0.705005059
gene-EIF4EBP1	1.107774296	PC(P-18:1(11Z)/PGJ2)	0.565243877
gene-EIF4EBP1	1.107774296	PS(20:0/20:4(8Z,11Z,14Z,17Z)-2OH(5S,6R))	0.402595921
gene-METTL27	1.107102685	(Z)-[(4-hydroxyphenyl)acetaldehyde oxime]	0.158764133
gene-METTL27	1.107102685	Carindone	0.199333317
gene-METTL27	1.107102685	LysoPC(15:0/0:0)	0.069628101
gene-METTL27	1.107102685	Simulansine	1.719063191
gene-METTL27	1.107102685	4-cholesten-7伪,12伪,24-triol-3-one	0.097840451
gene-METTL27	1.107102685	desethyletomidate	0.192509641
Bos_taurus_newG	1.106600206	3-Thiacytidine	0.209387412
Bos_taurus_newG	1.106600206	D-Erythro-imidazole-glycerol-phosphate	0.355812431
Bos_taurus_newG	1.106600206	11-Maleimidoundecanoic acid	1.084942397
Bos_taurus_newG	1.106600206	cis-p-Menth-2-en-1-ol	0.201017988
Bos_taurus_newG	1.106600206	PE(20:0/18:1(12Z)-2OH(9,10))	0.438658253
Bos_taurus_newG	1.106600206	PE(22:2(13Z,16Z)/22:5(4Z,7Z,10Z,13Z,19Z)-O	0.369745166
Bos_taurus_newG	1.106600206	PG(20:1(11Z)/18:3(10,12,15)-OH(9))	0.626244347
Bos_taurus_newG	1.106600206	2-Methyl-3-phenyl-2-propenal	0.407813975
Bos_taurus_newG	1.106600206	alpha-Terpineol formate	0.628238986
Bos_taurus_newG	1.106600206	PC(P-18:1(11Z)/PGE2)	0.509503133
Bos_taurus_newG	1.106600206	Sitosterol beta-D-glucoside	0.382944796
Bos_taurus_newG	1.106600206	PC(18:1(9Z)/15:1(9Z))	0.534724682
Bos_taurus_newG	1.106600206	PC(P-18:1(11Z)/PGJ2)	0.565243877
Bos_taurus_newG	1.106600206	PS(20:0/20:4(8Z,11Z,14Z,17Z)-2OH(5S,6R))	0.402595921
gene-ARHGAP33	1.105001321	3-hydroxypristanic acid	0.548515835
gene-ARHGAP33	1.105001321	16-hydroxy hexadecanoic acid	0.309000958
gene-ARHGAP33	1.105001321	3-Deoxyestrone	0.282221709
gene-ARHGAP33	1.105001321	1-Oleoyl-sn-glycero-3-phosphocholine	0.18926952
gene-ARHGAP33	1.105001321	Psychosine	0.106475396

gene-ARHGAP33	1.105001321	Cyclotricuspidogenin C	0.440884085
gene-ARHGAP33	1.105001321	(-)-alpha-Terpineol	0.345716279
gene-ARHGAP33	1.105001321	DG(8:0/20:4(6Z,8E,10E,14Z)-2OH(5S,12R)/0:0	0.958378689
gene-ARHGAP33	1.105001321	1,4-Undecadiene	0.462803973
gene-ARHGAP33	1.105001321	4-cholesten-7伪,12伪,24-triol-3-one	0.097840451
gene-ARHGAP33	1.105001321	ingenol	0.942071652
gene-ARHGAP33	1.105001321	N-[[3-Hydroxy-2-(2-pentenyl)cyclopentyl]ac	1.043930947
gene-ARHGAP33	1.105001321	PC(20:3(5Z,8Z,11Z)/24:0)	0.387959564
gene-ARHGAP33	1.105001321	2-Amino-4-[carbamimidoyl(methyl)amino]bu	0.075703353
gene-ARHGAP33	1.105001321	N2-gamma-Glutamylglutamine	0.230065499
gene-ARHGAP33	1.105001321	n-methyl-2-(4'-methylaminophenyl)-6-hydr	0.26655714
gene-ARHGAP33	1.105001321	L-Oleandrosyl-oleandolide	0.272998166
gene-ARHGAP33	1.105001321	Digalacturonate	0.473524431
gene-ARHGAP33	1.105001321	Phenethyl 6-galloylglucoside	0.069516774
gene-ARHGAP33	1.105001321	1-Methylnicotinamide	0.203956241
gene-ARHGAP33	1.105001321	CL(8:0/8:0/18:2(9Z,11Z)/20:0)	0.622988418
gene-LRSAM1	1.104454954	3-Thiacytidine	0.209387412
gene-LRSAM1	1.104454954	D-Erythro-imidazole-glycerol-phosphate	0.355812431
gene-LRSAM1	1.104454954	LTB4-d4	0.226799982
gene-LRSAM1	1.104454954	cis-p-Menth-2-en-1-ol	0.201017988
gene-LRSAM1	1.104454954	PE(20:0/18:1(12Z)-2OH(9,10))	0.438658253
gene-LRSAM1	1.104454954	PG(20:1(11Z)/18:3(10,12,15)-OH(9))	0.626244347
gene-LRSAM1	1.104454954	2-Methyl-3-phenyl-2-propenal	0.407813975
gene-LRSAM1	1.104454954	alpha-Terpineol formate	0.628238986
gene-LRSAM1	1.104454954	PC(P-18:1(11Z)/PGE2)	0.509503133
gene-LRSAM1	1.104454954	(3R,4R)-3-Amino-1-hydroxy-4-methylpyrrol	0.471914506
gene-LRSAM1	1.104454954	N-Myristoyl Glutamine	0.37856966
gene-LRSAM1	1.104454954	PC(18:1(9Z)/15:1(9Z))	0.534724682
gene-LRSAM1	1.104454954	PC(P-18:1(11Z)/PGJ2)	0.565243877
gene-LRSAM1	1.104454954	PS(20:0/20:4(8Z,11Z,14Z,17Z)-2OH(5S,6R))	0.402595921
gene-USP20	1.10405382	3-Thiacytidine	0.209387412
gene-USP20	1.10405382	D-Erythro-imidazole-glycerol-phosphate	0.355812431
gene-USP20	1.10405382	LTB4-d4	0.226799982
gene-USP20	1.10405382	cis-p-Menth-2-en-1-ol	0.201017988
gene-USP20	1.10405382	(-)-alpha-Terpineol	0.345716279
gene-USP20	1.10405382	Cyclosporin A	0.656529229
gene-USP20	1.10405382	5-(2-Aminopropyl)-2-methylphenol	0.323231457
gene-USP20	1.10405382	PC(P-18:1(11Z)/PGE2)	0.509503133
gene-USP20	1.10405382	Nigroxanthin	0.705005059
gene-USP20	1.10405382	PC(P-18:1(11Z)/PGJ2)	0.565243877
gene-USP20	1.10405382	PS(20:0/20:4(8Z,11Z,14Z,17Z)-2OH(5S,6R))	0.402595921
gene-USP20	1.10405382	CL(8:0/8:0/18:2(9Z,11Z)/20:0)	0.622988418
gene-TTC31	1.103533762	11-Maleimidoundecanoic acid	1.084942397
gene-TTC31	1.103533762	PE(22:2(13Z,16Z)/22:5(4Z,7Z,10Z,13Z,19Z)-O	0.369745166
gene-TTC31	1.103533762	3,4,3',4'-Tetrahydrospirilloxanthin	0.354666148
gene-TTC31	1.103533762	S-Acetyldihydrolipoamide-E	0.429561283
gene-TTC31	1.103533762	Indole-3-ethanol	0.096523128
gene-TTC31	1.103533762	2-(1-Adamantyl)-1,3-dioxetane	0.408916843
gene-TTC31	1.103533762	Milbemycin D	0.125637998
gene-TTC31	1.103533762	Roxithromycin	0.268273077
gene-TTC31	1.103533762	Sitosterol beta-D-glucoside	0.382944796

gene-TTC31	1.103533762	PC(P-18:1(11Z)/PGJ2)	0.565243877
gene-TTC31	1.103533762	PS(20:0/20:4(8Z,11Z,14Z,17Z)-2OH(5S,6R))	0.402595921
gene-TTC31	1.103533762	PE(20:5(5Z,8Z,11Z,14Z,17Z)/18:0)	0.427962506
gene-TUBA1C	1.102789246	5-(Ethylthio)-1H-tetrazole	0.29717344
gene-TUBA1C	1.102789246	all-trans-Hexaprenyl diphosphate	0.277689133
gene-TUBA1C	1.102789246	Canesceol	0.686312122
gene-TUBA1C	1.102789246	Tetracosenoyl-CoA	0.187187233
gene-TUBA1C	1.102789246	1,2-O-Isopropylidene-D-glucofuranose	0.080987667
gene-TUBA1C	1.102789246	3-Deoxyestrone	0.282221709
gene-TUBA1C	1.102789246	1-Oleoyl-sn-glycero-3-phosphocholine	0.18926952
gene-TUBA1C	1.102789246	3,4-dihydroxy-5-all-trans-hexaprenylbenzoa	0.123615726
gene-TUBA1C	1.102789246	Psychosine	0.106475396
gene-TUBA1C	1.102789246	(-)-alpha-Terpineol	0.345716279
gene-TUBA1C	1.102789246	1,4-Undecadiene	0.462803973
gene-TUBA1C	1.102789246	PC(14:1(9Z)/P-18:1(11Z))	0.153578899
gene-TUBA1C	1.102789246	PC(16:0/18:1(12Z)-2OH(9,10))	0.461843233
gene-TUBA1C	1.102789246	PC(14:0/20:2(11Z,14Z))	1.110657378
gene-TUBA1C	1.102789246	5-(2-Aminopropyl)-2-methylphenol	0.323231457
gene-TUBA1C	1.102789246	4-Dimethylamino-L-phenylalanine	0.242110226
gene-TUBA1C	1.102789246	Trimetazidine	0.438282534
gene-TUBA1C	1.102789246	ingenol	0.942071652
gene-TUBA1C	1.102789246	Armillane	0.52808635
gene-TUBA1C	1.102789246	(3R,4R)-3-Amino-1-hydroxy-4-methylpyrrol	0.471914506
gene-TUBA1C	1.102789246	1-Palmitoylglycerol	0.084357408
gene-TUBA1C	1.102789246	n-methyl-2-(4'-methylaminophenyl)-6-hydr	0.26655714
gene-TUBA1C	1.102789246	Glutethimide	0.126237229
gene-TUBA1C	1.102789246	PE(P-16:0/18:4(6Z,9Z,12Z,15Z))	0.546989864
gene-TUBA1C	1.102789246	Isopropyl isothiocyanate	0.172385747
gene-TUBA1C	1.102789246	arachidyl amido cholanoic acid	1.24842952
gene-TUBA1C	1.102789246	AS 1-5	0.512683467
gene-TUBA1C	1.102789246	9-deoxy-9-methylene-16,16-dimethyl -PGE	0.606893884
gene-TUBA1C	1.102789246	CL(8:0/8:0/18:2(9Z,11Z)/20:0)	0.622988418
gene-LOC100848	1.101056324	D-Erythro-imidazole-glycerol-phosphate	0.355812431
gene-LOC100848	1.101056324	Forskolin	0.373721279
gene-LOC100848	1.101056324	cis-p-Menth-2-en-1-ol	0.201017988
gene-LOC100848	1.101056324	PG(20:1(11Z)/18:3(10,12,15)-OH(9))	0.626244347
gene-LOC100848	1.101056324	alpha-Terpineol formate	0.628238986
gene-LOC100848	1.101056324	PC(P-18:1(11Z)/PGE2)	0.509503133
gene-LOC100848	1.101056324	11-Deoxycortisol	0.595462818
gene-LOC100848	1.101056324	PC(P-18:1(11Z)/PGJ2)	0.565243877
Bos_taurus_newG	1.100186982	Isomaltotriose	0.58434654
Bos_taurus_newG	1.100186982	PE(22:2(13Z,16Z)/22:5(4Z,7Z,10Z,13Z,19Z)-O	0.369745166
Bos_taurus_newG	1.100186982	3b,6a-Dihydroxy-alpha-ionol 9-[apiosyl-(1-	0.045258177
Bos_taurus_newG	1.100186982	N-[[3-Hydroxy-2-(2-pentenyl)cyclopentyl]ac	1.043930947
Bos_taurus_newG	1.100186982	Phorone A	0.079039095
gene-LINGO3	1.099015635	Phorone A	0.079039095
gene-LINGO3	1.099015635	Digalacturonate	0.473524431
gene-MPV17L	1.098683106	PE(16:0/18:1(11Z))	0.367326926
gene-MPV17L	1.098683106	all-trans-Hexaprenyl diphosphate	0.277689133
gene-MPV17L	1.098683106	3-Thiacytidine	0.209387412
gene-MPV17L	1.098683106	LysoPC(15:0/0:0)	0.069628101

gene-MPV17L	1.098683106	2-(alpha-Hydroxyethyl)thiamine diphosphate	0.200020781
gene-MPV17L	1.098683106	Cyclosporin A	0.656529229
gene-MPV17L	1.098683106	Angiotensin A	0.247332017
gene-MPV17L	1.098683106	PA(22:6(4Z,7Z,10Z,13Z,16Z,19Z)/16:0)	0.556532497
gene-MPV17L	1.098683106	DG(18:0/LTE4/0:0)	0.681485773
gene-MPV17L	1.098683106	PC(17:0/PGJ2)	0.657689319
gene-MPV17L	1.098683106	PC(14:0/20:2(11Z,14Z))	1.110657378
gene-MPV17L	1.098683106	(1S,2R)-1-C-(indol-3-yl)glycerol 3-phosphat	0.374678916
gene-MPV17L	1.098683106	gamma-Glutamylcysteinylserine	0.098057967
gene-MPV17L	1.098683106	ingenol	0.942071652
gene-MPV17L	1.098683106	beta-L-Dioxolane-cytidine	0.175916668
gene-MPV17L	1.098683106	DG(2:0/18:1(12Z)-O(9S,10R)/0:0)	0.033180989
gene-MPV17L	1.098683106	17-Aminogeldanamycin	0.518144528
gene-MPV17L	1.098683106	Isopropyl isothiocyanate	0.172385747
gene-MPV17L	1.098683106	arachidyl amido cholanoic acid	1.24842952
gene-MPV17L	1.098683106	3-Hydroxyheptadecanoylcarnitine	0.860642943
gene-MPV17L	1.098683106	D-Fructose	0.167584616
gene-MTSS1L	1.097010198	Undecanedioic acid	0.059955634
gene-SCAP	1.096610602	3-Thiacytidine	0.209387412
gene-SCAP	1.096610602	D-Erythro-imidazole-glycerol-phosphate	0.355812431
gene-SCAP	1.096610602	3-Deoxyestrone	0.282221709
gene-SCAP	1.096610602	1-Oleoyl-sn-glycero-3-phosphocholine	0.18926952
gene-SCAP	1.096610602	LTB4-d4	0.226799982
gene-SCAP	1.096610602	cis-p-Menth-2-en-1-ol	0.201017988
gene-SCAP	1.096610602	(-)-alpha-Terpineol	0.345716279
gene-SCAP	1.096610602	1,4-Undecadiene	0.462803973
gene-SCAP	1.096610602	PG(20:1(11Z)/18:3(10,12,15)-OH(9))	0.626244347
gene-SCAP	1.096610602	5-(2-Aminopropyl)-2-methylphenol	0.323231457
gene-SCAP	1.096610602	4-Dimethylamino-L-phenylalanine	0.242110226
gene-SCAP	1.096610602	Armillane	0.52808635
gene-SCAP	1.096610602	PC(P-18:1(11Z)/PGE2)	0.509503133
gene-SCAP	1.096610602	(3R,4R)-3-Amino-1-hydroxy-4-methylpyrrol	0.471914506
gene-SCAP	1.096610602	n-methyl-2-(4'-methylaminophenyl)-6-hydr	0.26655714
gene-SCAP	1.096610602	Isopropyl isothiocyanate	0.172385747
gene-SCAP	1.096610602	(9Z)-Octadecenoic acid	0.142359556
gene-SCAP	1.096610602	PS(20:0/20:4(8Z,11Z,14Z,17Z)-2OH(5S,6R))	0.402595921
gene-SCAP	1.096610602	CL(8:0/8:0/18:2(9Z,11Z)/20:0)	0.622988418
Bos_taurus_newG	1.096464398	11-Maleimidoundecanoic acid	1.084942397
Bos_taurus_newG	1.096464398	PE(22:2(13Z,16Z)/22:5(4Z,7Z,10Z,13Z,19Z)-O	0.369745166
Bos_taurus_newG	1.096464398	Cyclosporin A	0.656529229
Bos_taurus_newG	1.096464398	Guanidoacetic acid	0.542509265
Bos_taurus_newG	1.096464398	beta-L-Dioxolane-cytidine	0.175916668
Bos_taurus_newG	1.096464398	PC(P-18:1(11Z)/PGE2)	0.509503133
Bos_taurus_newG	1.096464398	Phorone A	0.079039095
Bos_taurus_newG	1.096464398	Nigroxanthin	0.705005059
Bos_taurus_newG	1.096464398	PC(P-18:1(11Z)/PGJ2)	0.565243877
Bos_taurus_newG	1.096464398	PS(20:0/20:4(8Z,11Z,14Z,17Z)-2OH(5S,6R))	0.402595921
Bos_taurus_newG	1.094976329	5-(3'-Carboxy-3'-oxopropenyl)-4,6-dihydro	1.083232395
Bos_taurus_newG	1.094976329	D-Erythro-imidazole-glycerol-phosphate	0.355812431
Bos_taurus_newG	1.094976329	Docosanamide	0.509975786
Bos_taurus_newG	1.094976329	Monacolin L acid	1.060562098

Bos_taurus_newG	1.094976329	cis-p-Menth-2-en-1-ol	0.201017988
Bos_taurus_newG	1.094976329	PE(20:0/18:1(12Z)-2OH(9,10))	0.438658253
Bos_taurus_newG	1.094976329	PE(22:2(13Z,16Z)/22:5(4Z,7Z,10Z,13Z,19Z)-O(	0.369745166
Bos_taurus_newG	1.094976329	PG(20:1(11Z)/18:3(10,12,15)-OH(9))	0.626244347
Bos_taurus_newG	1.094976329	Dyphylline	0.664549753
Bos_taurus_newG	1.094976329	Lamivudine	0.327402892
Bos_taurus_newG	1.094976329	Pseudouridine 5'-phosphate	1.18431378
Bos_taurus_newG	1.094976329	Ribavirin monophosphate	1.091085027
Bos_taurus_newG	1.094976329	7C-aglycone	1.015608521
Bos_taurus_newG	1.094976329	Tryptophyl-Glutamine	0.89722186
Bos_taurus_newG	1.094976329	alpha-Terpineol formate	0.628238986
Bos_taurus_newG	1.094976329	(1S)-3-[2-[(1R,7Ar)-7a-methyl-1-[(2R)-6-me	1.140894639
Bos_taurus_newG	1.094976329	4-Octylphenol	1.400657885
Bos_taurus_newG	1.094976329	Prolyl-Lysine	1.612991304
Bos_taurus_newG	1.094976329	3-Pentadecylphenol	1.682258188
Bos_taurus_newG	1.094976329	PC(P-18:1(11Z)/PGE2)	0.509503133
Bos_taurus_newG	1.094976329	Galabiosylceramide (d18:1/20:0)	0.746737323
Bos_taurus_newG	1.094976329	12-Hydroxyicosanoylcarnitine	1.730979067
Bos_taurus_newG	1.094976329	Milbemycin D	0.125637998
Bos_taurus_newG	1.094976329	Ascorbic acid 6-palmitate	0.201627014
Bos_taurus_newG	1.094976329	Sitosterol beta-D-glucoside	0.382944796
Bos_taurus_newG	1.094976329	PC(P-18:1(11Z)/PGJ2)	0.565243877
Bos_taurus_newG	1.094976329	PS(20:0/20:4(8Z,11Z,14Z,17Z)-2OH(5S,6R))	0.402595921
Bos_taurus_newG	1.094976329	Galactosylglycerol	0.738463143
Bos_taurus_newG	1.094976329	PE(20:5(5Z,8Z,11Z,14Z,17Z)/18:0)	0.427962506
Bos_taurus_newG	1.094976329	PG(20:1(11Z)/18:3(9,11,15)-OH(13))	0.69829683
gene-SYCP2	1.093018493	5-Hydroxy-2-oxo-4-ureido-2,5-dihydro-1H	0.122536033
gene-SYCP2	1.093018493	19-hydroxyprostaglandin H2(1-)	0.116812012
gene-SYCP2	1.093018493	Methyl (2E)-2-(10,13-dimethyl-11-oxo-3-py	0.478588158
gene-SYCP2	1.093018493	Urocortisol	0.926998516
gene-SYCP2	1.093018493	PGP(20:1(11Z)/18:1(12Z)-2OH(9,10))	0.042651731
gene-SYCP2	1.093018493	11-Maleimidoundecanoic acid	1.084942397
gene-SYCP2	1.093018493	13(S)-HpODE	0.163157753
gene-SYCP2	1.093018493	7(S),17(S)-dihydroxy-8(E),10(Z),13(Z),15(E),19	0.32147403
gene-SYCP2	1.093018493	LTB4-d4	0.226799982
gene-SYCP2	1.093018493	PC(24:0/22:6(4Z,7Z,10Z,12E,16Z,19Z)-OH(14)	0.188241061
gene-SYCP2	1.093018493	Cyclosporin A	0.656529229
gene-SYCP2	1.093018493	C20914	0.140383181
gene-SYCP2	1.093018493	Fluridone	0.127106682
gene-SYCP2	1.093018493	1,2,3,4-Tetrahydroisoquinoline-3-carboxylic	0.339248702
gene-SYCP2	1.093018493	Guanidoacetic acid	0.542509265
gene-SYCP2	1.093018493	Methionyl-Valine	0.502707745
gene-SYCP2	1.093018493	[3-(2-Aminopropyl)-6-methylidenecyclohexa	0.422945997
gene-SYCP2	1.093018493	3'-Hydroxyropivacaine	1.014215236
gene-SYCP2	1.093018493	Vulgarone A	0.102049407
gene-SYCP2	1.093018493	Sophoranol	0.282064826
gene-SYCP2	1.093018493	L-Cysteine	0.214540754
gene-SYCP2	1.093018493	1,4,6-Trimethylnaphthalene	0.12150693
gene-SYCP2	1.093018493	24,24-Dfhv	0.175278646
gene-SYCP2	1.093018493	Prostaglandin PGE2 1-glyceryl ester	0.007411313
gene-SYCP2	1.093018493	1,8-Octanedithiol	0.133094504

gene-SYCP2	1.093018493	Prostaglandin B2	0.552852305
gene-SYCP2	1.093018493	PC(P-18:1(11Z)/PGE2)	0.509503133
gene-SYCP2	1.093018493	Phorone A	0.079039095
gene-SYCP2	1.093018493	PC(P-18:1(11Z)/PGE1)	0.295520345
gene-SYCP2	1.093018493	Nigroxanthin	0.705005059
gene-DDR2	1.09221076	LysoPI(18:2(9Z,12Z)/0:0)	0.358006727
gene-DDR2	1.09221076	2-[(3S)-3-[[(2S)-1-(Carboxymethoxy)-1-oxo-	0.111577437
gene-DDR2	1.09221076	2-hydroxyhexadecanoic acid	0.1755745
gene-DDR2	1.09221076	4-Oxo-9-cis-retinoyl-beta-glucuronide	1.611773742
gene-DDR2	1.09221076	(4-Methylphenyl)acetaldehyde	0.208360133
gene-DDR2	1.09221076	Hydrocinnamic acid	0.043440346
gene-DDR2	1.09221076	1,3,5-Bisabolatrien-10-one	0.109736328
gene-DDR2	1.09221076	1-Heneicosanoyl-glycero-3-phosphoserine	0.214358093
Bos_taurus_newG	1.08989206	PC(24:0/22:6(4Z,7Z,10Z,12E,16Z,19Z)-OH(14)	0.188241061
Bos_taurus_newG	1.08989206	Cyclosporin A	0.656529229
Bos_taurus_newG	1.08989206	1,4,6-Trimethylnaphthalene	0.12150693
Bos_taurus_newG	1.08989206	1,8-Octanedithiol	0.133094504
Bos_taurus_newG	1.08989206	Prostaglandin B2	0.552852305
Bos_taurus_newG	1.08989206	PC(P-18:1(11Z)/PGE2)	0.509503133
Bos_taurus_newG	1.08989206	Phorone A	0.079039095
Bos_taurus_newG	1.08989206	Nigroxanthin	0.705005059
Bos_taurus_newG	1.08989206	PC(P-18:1(11Z)/PGJ2)	0.565243877
gene-TMEM38A	1.088533024	Cyclosporin A	0.656529229
gene-TMEM38A	1.088533024	beta-Thujaplicin	0.681866194
gene-TMEM38A	1.088533024	beta-L-Dioxolane-cytidine	0.175916668
gene-TMEM38A	1.088533024	PC(20:3(5Z,8Z,11Z)/24:0)	0.387959564
gene-TMEM38A	1.088533024	Pyroglutamic acid	0.053168578
gene-GOLGB1	1.087601882	5-(3'-Carboxy-3'-oxopropenyl)-4,6-dihydro:	1.083232395
gene-GOLGB1	1.087601882	D-Erythro-imidazole-glycerol-phosphate	0.355812431
gene-GOLGB1	1.087601882	Glutamate carbon	0.671444516
gene-GOLGB1	1.087601882	11-Maleimidoundecanoic acid	1.084942397
gene-GOLGB1	1.087601882	Monacolin L acid	1.060562098
gene-GOLGB1	1.087601882	cis-p-Menth-2-en-1-ol	0.201017988
gene-GOLGB1	1.087601882	PE(22:2(13Z,16Z)/22:5(4Z,7Z,10Z,13Z,19Z)-O	0.369745166
gene-GOLGB1	1.087601882	Norophthalmic acid	0.191432411
gene-GOLGB1	1.087601882	(1R,6S)-6-Amino-5-oxocyclohex-2-ene-1-c	0.154751123
gene-GOLGB1	1.087601882	2-(1-Adamantyl)-1,3-dioxetane	0.408916843
gene-GOLGB1	1.087601882	Guanidoacetic acid	0.542509265
gene-GOLGB1	1.087601882	alpha-Terpineol formate	0.628238986
gene-GOLGB1	1.087601882	PC(P-18:1(11Z)/PGE2)	0.509503133
gene-GOLGB1	1.087601882	Milbemycin D	0.125637998
gene-GOLGB1	1.087601882	Roxithromycin	0.268273077
gene-GOLGB1	1.087601882	Sitosterol beta-D-glucoside	0.382944796
gene-GOLGB1	1.087601882	PC(P-18:1(11Z)/PGJ2)	0.565243877
gene-GOLGB1	1.087601882	PS(20:0/20:4(8Z,11Z,14Z,17Z)-2OH(5S,6R))	0.402595921
gene-GOLGB1	1.087601882	PE(20:5(5Z,8Z,11Z,14Z,17Z)/18:0)	0.427962506
Bos_taurus_newG	1.084139562	7(14)-Bisabolene-2,3,10,11-tetrol	2.446897974
Bos_taurus_newG	1.084139562	(3Z)-Phycoerythrobilin	1.456755874
Bos_taurus_newG	1.084139562	3-Deoxyestrone	0.282221709
Bos_taurus_newG	1.084139562	1-Oleoyl-sn-glycero-3-phosphocholine	0.18926952
Bos_taurus_newG	1.084139562	Lividamine	0.319679555

Bos_taurus_newG	1.084139562	Psychosine	0.106475396
Bos_taurus_newG	1.084139562	Cyclotricuspidogenin C	0.440884085
Bos_taurus_newG	1.084139562	(-)-alpha-Terpineol	0.345716279
Bos_taurus_newG	1.084139562	1,4-Undecadiene	0.462803973
Bos_taurus_newG	1.084139562	2-isopentyl-3,6-dimethyl pyrazine	0.710502562
Bos_taurus_newG	1.084139562	4-cholesten-7伪,12伪,24-triol-3-one	0.097840451
Bos_taurus_newG	1.084139562	5-(2-Aminopropyl)-2-methylphenol	0.323231457
Bos_taurus_newG	1.084139562	4-Dimethylamino-L-phenylalanine	0.242110226
Bos_taurus_newG	1.084139562	Trimetazidine	0.438282534
Bos_taurus_newG	1.084139562	ingenol	0.942071652
Bos_taurus_newG	1.084139562	Armillane	0.52808635
Bos_taurus_newG	1.084139562	PC(P-18:1(11Z)/PGE2)	0.509503133
Bos_taurus_newG	1.084139562	(3R,4R)-3-Amino-1-hydroxy-4-methylpyrrol	0.471914506
Bos_taurus_newG	1.084139562	1-Palmitoylglycerol	0.084357408
Bos_taurus_newG	1.084139562	N2-gamma-Glutamylglutamine	0.230065499
Bos_taurus_newG	1.084139562	n-methyl-2-(4'-methylaminophenyl)-6-hydr	0.26655714
Bos_taurus_newG	1.084139562	Glutethimide	0.126237229
Bos_taurus_newG	1.084139562	Isopropyl isothiocyanate	0.172385747
Bos_taurus_newG	1.084139562	9-deoxy-9-methylene-16,16-dimethyl -PGE	0.606893884
Bos_taurus_newG	1.084139562	CL(8:0/8:0/18:2(9Z,11Z)/20:0)	0.622988418
gene-CCDC17	1.083670675	D-Erythro-imidazole-glycerol-phosphate	0.355812431
gene-CCDC17	1.083670675	cis-p-Menth-2-en-1-ol	0.201017988
gene-CCDC17	1.083670675	PG(20:1(11Z)/18:3(10,12,15)-OH(9))	0.626244347
gene-CCDC17	1.083670675	2-Methyl-3-phenyl-2-propenal	0.407813975
gene-CCDC17	1.083670675	4-Octylphenol	1.400657885
gene-CCDC17	1.083670675	Ajulemic acid	0.557832951
gene-CCDC17	1.083670675	PC(P-18:1(11Z)/PGE2)	0.509503133
gene-CCDC17	1.083670675	MG(0:0/18:1(9Z)/0:0)	0.361035605
gene-ZCCHC13	1.083581527	3-Thiacytidine	0.209387412
gene-ZCCHC13	1.083581527	7(14)-Bisabolene-2,3,10,11-tetrol	2.446897974
gene-ZCCHC13	1.083581527	cis-p-Menth-2-en-1-ol	0.201017988
gene-ZCCHC13	1.083581527	5-(2-Aminopropyl)-2-methylphenol	0.323231457
gene-ZCCHC13	1.083581527	Armillane	0.52808635
gene-ZCCHC13	1.083581527	PC(P-18:1(11Z)/PGE2)	0.509503133
gene-ZCCHC13	1.083581527	(3R,4R)-3-Amino-1-hydroxy-4-methylpyrrol	0.471914506
gene-ZCCHC13	1.083581527	Isopropyl isothiocyanate	0.172385747
gene-ZCCHC13	1.083581527	N-Myristoyl Glutamine	0.37856966
gene-ZCCHC13	1.083581527	PC(18:1(9Z)/15:1(9Z))	0.534724682
gene-ZCCHC13	1.083581527	PC(P-18:1(11Z)/PGJ2)	0.565243877
gene-ZCCHC13	1.083581527	PS(20:0/20:4(8Z,11Z,14Z,17Z)-2OH(5S,6R))	0.402595921
gene-GCC2	1.081547165	5-(3'-Carboxy-3'-oxopropenyl)-4,6-dihydro	1.083232395
gene-GCC2	1.081547165	Glutamate carbon	0.671444516
gene-GCC2	1.081547165	Val-Cit	0.452308451
gene-GCC2	1.081547165	11-Maleimidoundecanoic acid	1.084942397
gene-GCC2	1.081547165	Monacolin L acid	1.060562098
gene-GCC2	1.081547165	3,4,3',4'-Tetrahydrospirilloxanthin	0.354666148
gene-GCC2	1.081547165	Norophthalmic acid	0.191432411
gene-GCC2	1.081547165	(1R,6S)-6-Amino-5-oxocyclohex-2-ene-1-c	0.154751123
gene-GCC2	1.081547165	2-(1-Adamantyl)-1,3-dioxetane	0.408916843
gene-GCC2	1.081547165	alpha-Terpineol formate	0.628238986
gene-GCC2	1.081547165	3-(3-Methylbutylidene)-1(3H)-isobenzofurar	0.575422414

gene-GCC2	1.081547165	3',4',5'-Trimethoxycinnamyl alcohol acetate	0.4221816
gene-GCC2	1.081547165	ent-16b,19-Kauranediol 19-acetate	0.400676827
gene-GCC2	1.081547165	Roxithromycin	0.268273077
gene-GCC2	1.081547165	PC(P-18:1(11Z)/PGJ2)	0.565243877
gene-ICAM3	1.078858163	3-Thiacytidine	0.209387412
gene-ICAM3	1.078858163	LTB4-d4	0.226799982
gene-ICAM3	1.078858163	(-)-alpha-Terpineol	0.345716279
gene-ICAM3	1.078858163	1,4-Undecadiene	0.462803973
gene-ICAM3	1.078858163	5-(2-Aminopropyl)-2-methylphenol	0.323231457
gene-ICAM3	1.078858163	4-Dimethylamino-L-phenylalanine	0.242110226
gene-ICAM3	1.078858163	Armillane	0.52808635
gene-ICAM3	1.078858163	PC(P-18:1(11Z)/PGE2)	0.509503133
gene-ICAM3	1.078858163	(3R,4R)-3-Amino-1-hydroxy-4-methylpyrrol	0.471914506
gene-ICAM3	1.078858163	n-methyl-2-(4'-methylaminophenyl)-6-hydr	0.26655714
gene-ICAM3	1.078858163	Isopropyl isothiocyanate	0.172385747
gene-ICAM3	1.078858163	PS(20:0/20:4(8Z,11Z,14Z,17Z)-2OH(5S,6R))	0.402595921
gene-ICAM3	1.078858163	CL(8:0/8:0/18:2(9Z,11Z)/20:0)	0.622988418
gene-IFNLR1	1.078851905	3-Thiacytidine	0.209387412
gene-IFNLR1	1.078851905	PE(20:0/18:1(12Z)-2OH(9,10))	0.438658253
gene-IFNLR1	1.078851905	PE(22:2(13Z,16Z)/22:5(4Z,7Z,10Z,13Z,19Z)-O	0.369745166
gene-IFNLR1	1.078851905	Cyclosporin A	0.656529229
gene-IFNLR1	1.078851905	DG(18:0/LTE4/0:0)	0.681485773
gene-IFNLR1	1.078851905	beta-L-Dioxolane-cytidine	0.175916668
gene-IFNLR1	1.078851905	PC(P-18:1(11Z)/PGE2)	0.509503133
gene-IFNLR1	1.078851905	Nigroxanthin	0.705005059
gene-IFNLR1	1.078851905	PC(P-18:1(11Z)/PGJ2)	0.565243877
gene-IFNLR1	1.078851905	PS(20:0/20:4(8Z,11Z,14Z,17Z)-2OH(5S,6R))	0.402595921
gene-IFNLR1	1.078851905	CL(8:0/8:0/18:2(9Z,11Z)/20:0)	0.622988418
gene-C1H21orf2	1.078475812	D-Erythro-imidazole-glycerol-phosphate	0.355812431
gene-C1H21orf2	1.078475812	Glutamate carbon	0.671444516
gene-C1H21orf2	1.078475812	1-(2-Furanyl)-1-pentanone	0.697147283
gene-C1H21orf2	1.078475812	2-Dehydro-3-deoxy-D-gluconate	0.65165299
gene-C1H21orf2	1.078475812	Undecanedioic acid	0.059955634
gene-C1H21orf2	1.078475812	(1R,6S)-6-Amino-5-oxocyclohex-2-ene-1-c	0.154751123
gene-C1H21orf2	1.078475812	PC(P-18:1(11Z)/PGE2)	0.509503133
gene-C1H21orf2	1.078475812	Roxithromycin	0.268273077
Bos_taurus_newG	1.078022427	Hydroxytyrosol 1-O-glucoside	0.313284156
Bos_taurus_newG	1.078022427	Tsugarioside B	1.013262893
Bos_taurus_newG	1.078022427	cis-p-Menth-2-en-1-ol	0.201017988
Bos_taurus_newG	1.078022427	PG(20:1(11Z)/18:3(10,12,15)-OH(9))	0.626244347
Bos_taurus_newG	1.078022427	Dyphylline	0.664549753
Bos_taurus_newG	1.078022427	4-Hydroxyoctanedioylcarnitine	1.002008817
Bos_taurus_newG	1.078022427	4-Guanidinobutanoate	0.445426102
Bos_taurus_newG	1.078022427	Erosone	0.527835387
Bos_taurus_newG	1.078022427	Leu-Leu-Tyr	1.186798796
Bos_taurus_newG	1.078022427	11-Deoxycortisol	0.595462818
Bos_taurus_newG	1.078022427	Milbemycin D	0.125637998
Bos_taurus_newG	1.078022427	Sitosterol beta-D-glucoside	0.382944796
Bos_taurus_newG	1.078022427	Galactosylglycerol	0.738463143
Bos_taurus_newG	1.078022427	PG(20:1(11Z)/18:3(9,11,15)-OH(13))	0.69829683
gene-ALMS1	1.077204748	11-Maleimidoundecanoic acid	1.084942397

gene-ALMS1	1.077204748	PE(20:0/18:1(12Z)-2OH(9,10))	0.438658253
gene-ALMS1	1.077204748	PE(22:2(13Z,16Z)/22:5(4Z,7Z,10Z,13Z,19Z)-O(	0.369745166
gene-ALMS1	1.077204748	Benzoyl glucuronide (Benzoic acid)	0.442844673
gene-ALMS1	1.077204748	S-Acetyldihydrolipoamide-E	0.429561283
gene-ALMS1	1.077204748	Indole-3-ethanol	0.096523128
gene-ALMS1	1.077204748	Guanidoacetic acid	0.542509265
gene-ALMS1	1.077204748	alpha-Terpineol formate	0.628238986
gene-ALMS1	1.077204748	PC(P-18:1(11Z)/PGE2)	0.509503133
gene-ALMS1	1.077204748	Sitosterol beta-D-glucoside	0.382944796
gene-ALMS1	1.077204748	PC(P-18:1(11Z)/PGJ2)	0.565243877
gene-ALMS1	1.077204748	PS(20:0/20:4(8Z,11Z,14Z,17Z)-2OH(5S,6R))	0.402595921
gene-ALMS1	1.077204748	PE(20:5(5Z,8Z,11Z,14Z,17Z)/18:0)	0.427962506
gene-GPR162	1.074996667	3-Thiacytidine	0.209387412
gene-GPR162	1.074996667	11-Maleimidoundecanoic acid	1.084942397
gene-GPR162	1.074996667	Cyclosporin A	0.656529229
gene-GPR162	1.074996667	DG(18:0/LTE4/0:0)	0.681485773
gene-GPR162	1.074996667	beta-L-Dioxolane-cytidine	0.175916668
gene-GPR162	1.074996667	PC(P-18:1(11Z)/PGE2)	0.509503133
gene-GPR162	1.074996667	Nigroxanthin	0.705005059
gene-GPR162	1.074996667	PC(P-18:1(11Z)/PGJ2)	0.565243877
gene-GPR162	1.074996667	PS(20:0/20:4(8Z,11Z,14Z,17Z)-2OH(5S,6R))	0.402595921
gene-ULBP13	1.074543705	5-(3'-Carboxy-3'-oxopropenyl)-4,6-dihydro:	1.083232395
gene-ULBP13	1.074543705	D-Erythro-imidazole-glycerol-phosphate	0.355812431
gene-ULBP13	1.074543705	(+)-Bottrospicatol	0.68654221
gene-ULBP13	1.074543705	Val-Cit	0.452308451
gene-ULBP13	1.074543705	Sonchuside C	1.385535636
gene-ULBP13	1.074543705	Undecanedioic acid	0.059955634
gene-ULBP13	1.074543705	3-carboxy-4-methyl-5-pentyl-2-furanpropa	0.375666997
gene-ULBP13	1.074543705	(S)-10,16-Dihydroxyhexadecanoic acid	1.054006526
gene-ULBP13	1.074543705	Auxin b	1.052491354
gene-ULBP13	1.074543705	13(S)-HPOT	1.353059742
gene-ULBP13	1.074543705	16-Hydroxy-10-oxohexadecanoic acid	0.72302152
gene-ULBP13	1.074543705	Monacolin L acid	1.060562098
gene-ULBP13	1.074543705	cis-p-Menth-2-en-1-ol	0.201017988
gene-ULBP13	1.074543705	3,4,3',4'-Tetrahydrospirilloxanthin	0.354666148
gene-ULBP13	1.074543705	(1R,6S)-6-Amino-5-oxocyclohex-2-ene-1-c	0.154751123
gene-ULBP13	1.074543705	3-Methoxytyramine	0.928926425
gene-ULBP13	1.074543705	7a-Hydroxy-O-carbamoyl-deacetylcephalos	0.80981273
gene-ULBP13	1.074543705	2-Hydroxyglutaric acid diethyl ester	0.799886767
gene-ULBP13	1.074543705	6-hydroxy-7E,9E-Octadecadiene-11,13,15,17	1.179129125
gene-ULBP13	1.074543705	7C-aglycone	1.015608521
gene-ULBP13	1.074543705	L-alpha-Acetyl-N,N-dinormethadol	1.567999001
gene-ULBP13	1.074543705	12R-hydroxy-5Z,8Z,12Z-eicosatrienoic acid	0.766411225
gene-ULBP13	1.074543705	2-(1-Adamantyl)-1,3-dioxetane	0.408916843
gene-ULBP13	1.074543705	2-Oxo-10-methylthiodecanoic acid	0.695908469
gene-ULBP13	1.074543705	(-)-Huperzine A (HupA)	0.935198906
gene-ULBP13	1.074543705	Sequiterpene Lactone 326	1.141450991
gene-ULBP13	1.074543705	8-Methylthiooctanaldoxime	0.685273457
gene-ULBP13	1.074543705	Ajulemic acid	0.557832951
gene-ULBP13	1.074543705	Roxithromycin	0.268273077
gene-PGA5	1.074362618	3-Thiacytidine	0.209387412

gene-PGA5	1.074362618	11-Maleimidoundecanoic acid	1.084942397
gene-PGA5	1.074362618	cis-p-Menth-2-en-1-ol	0.201017988
gene-PGA5	1.074362618	PE(20:0/18:1(12Z)-2OH(9,10))	0.438658253
gene-PGA5	1.074362618	PE(22:2(13Z,16Z)/22:5(4Z,7Z,10Z,13Z,19Z)-O	0.369745166
gene-PGA5	1.074362618	Norophthalmic acid	0.191432411
gene-PGA5	1.074362618	PC(P-18:1(11Z)/PGE2)	0.509503133
gene-PGA5	1.074362618	PC(18:1(9Z)/15:1(9Z))	0.534724682
gene-PGA5	1.074362618	Nigroxanthin	0.705005059
gene-PGA5	1.074362618	PC(P-18:1(11Z)/PGJ2)	0.565243877
gene-PGA5	1.074362618	PS(20:0/20:4(8Z,11Z,14Z,17Z)-2OH(5S,6R))	0.402595921
gene-GDF11	1.073694258	5-Sulfosalicylic acid	1.31509494
gene-GDF11	1.073694258	4-Hydroxybenzoate	0.892336321
gene-GDF11	1.073694258	Ethyl nicotinate	1.024858052
gene-GDF11	1.073694258	Dopaquinone	1.087504518
gene-GDF11	1.073694258	Isomaltotriose	0.58434654
gene-GDF11	1.073694258	Salbutamol	0.107762964
gene-GDF11	1.073694258	isochorismate	0.054781856
gene-GDF11	1.073694258	Methyl beta-D-galactoside	0.20810278
gene-GDF11	1.073694258	MG(0:0/20:4(5Z,8Z,11Z,14Z)/0:0)	0.488916341
gene-GDF11	1.073694258	Crustecdysone	0.889062022
gene-GDF11	1.073694258	(14S)-14,15-Dihydroxy-8(17),13(16)-labdadie	0.720697404
gene-GDF11	1.073694258	Deoxyshikonin	0.734877521
gene-GDF11	1.073694258	5-Tetradecenoic acid	0.158247657
gene-GDF11	1.073694258	3beta-7-Drimene-3,11-diol	0.176540144
gene-GDF11	1.073694258	PE(18:1(12Z)-2OH(9,10)/P-18:0)	0.077744061
gene-GDF11	1.073694258	Serotonin	2.521565841
gene-GDF11	1.073694258	4-(Methylnitrosamino)-1-(1-oxido-3-pyridin	0.158186549
gene-GDF11	1.073694258	5-Methoxyindoleacetate	0.597532846
gene-GDF11	1.073694258	(S)-Mandelic acid O-beta-D-Glucopyranosid	0.509637316
gene-GDF11	1.073694258	TETRAHYDROURIDINE	0.576545276
gene-GDF11	1.073694258	1-Tert-butyl 4-ethyl 5-oxoazepane-1,4-dica	0.248579928
gene-GDF11	1.073694258	Imidazoline	0.232348181
gene-GDF11	1.073694258	Citrusin I	2.390047936
gene-GDF11	1.073694258	1-[(5-Amino-5-carboxypentyl)amino]-1-dec	0.400321455
gene-GDF11	1.073694258	Homoarecoline	0.02475363
gene-GDF11	1.073694258	alantolactone	0.192300019
gene-GDF11	1.073694258	beta-L-Dioxolane-cytidine	0.175916668
gene-GDF11	1.073694258	PS(20:5(5Z,8Z,11Z,14Z,16E)-OH(18R)/22:0)	0.090792733
gene-GDF11	1.073694258	D-Fructose	0.167584616
Bos_taurus_newG	1.07262944	Methylmalonate	0.241481249
Bos_taurus_newG	1.07262944	3-Thiacytidine	0.209387412
Bos_taurus_newG	1.07262944	1-Phenylpiperazine	0.353584896
Bos_taurus_newG	1.07262944	milbemycin beta3	1.414447124
Bos_taurus_newG	1.07262944	cis-p-Menth-2-en-1-ol	0.201017988
Bos_taurus_newG	1.07262944	PE(20:0/18:1(12Z)-2OH(9,10))	0.438658253
Bos_taurus_newG	1.07262944	PE(22:2(13Z,16Z)/22:5(4Z,7Z,10Z,13Z,19Z)-O	0.369745166
Bos_taurus_newG	1.07262944	Cyclosporin A	0.656529229
Bos_taurus_newG	1.07262944	Epomusenin A	0.766206049
Bos_taurus_newG	1.07262944	DG(18:0/LTE4/0:0)	0.681485773
Bos_taurus_newG	1.07262944	CDP-DG(PGF2alpha/16:0)	1.002512277
Bos_taurus_newG	1.07262944	5-(2-Aminopropyl)-2-methylphenol	0.323231457

Bos_taurus_newG	1.07262944	Armillane	0.52808635
Bos_taurus_newG	1.07262944	4-Oxo-9-cis-retinoyl-beta-glucuronide	1.611773742
Bos_taurus_newG	1.07262944	alpha-Terpineol formate	0.628238986
Bos_taurus_newG	1.07262944	PC(P-18:1(11Z)/PGE2)	0.509503133
Bos_taurus_newG	1.07262944	PC(18:1(9Z)/15:1(9Z))	0.534724682
Bos_taurus_newG	1.07262944	arachidyl amido cholanoic acid	1.24842952
Bos_taurus_newG	1.07262944	Nigroxanthin	0.705005059
Bos_taurus_newG	1.07262944	1-Octadecanoyl-2-(7Z,10Z,13Z,16Z-docosat	0.690496314
Bos_taurus_newG	1.07262944	PC(P-18:1(11Z)/PGJ2)	0.565243877
Bos_taurus_newG	1.07262944	PS(20:0/20:4(8Z,11Z,14Z,17Z)-2OH(5S,6R))	0.402595921
Bos_taurus_newG	1.07262944	PC(P-16:0/20:3(8Z,11Z,14Z)-2OH(5,6))	0.174122169
gene-ITGAD	1.072354195	D-Erythro-imidazole-glycerol-phosphate	0.355812431
gene-ITGAD	1.072354195	cis-p-Menth-2-en-1-ol	0.201017988
gene-ITGAD	1.072354195	PC(P-18:1(11Z)/PGE2)	0.509503133
gene-ITGAD	1.072354195	PC(P-18:1(11Z)/PGJ2)	0.565243877
gene-ITGAD	1.072354195	PS(20:0/20:4(8Z,11Z,14Z,17Z)-2OH(5S,6R))	0.402595921
Bos_taurus_newG	1.06996792	Isovalerylglucuronide	0.507877754
Bos_taurus_newG	1.06996792	D-Lombricine	0.471458154
Bos_taurus_newG	1.06996792	Isomaltotriose	0.58434654
Bos_taurus_newG	1.06996792	Mesoporphyrin IX	0.426061873
Bos_taurus_newG	1.06996792	1,4-Undecadiene	0.462803973
Bos_taurus_newG	1.06996792	4-cholesten-7伪,12伪,24-triol-3-one	0.097840451
Bos_taurus_newG	1.06996792	Methyl helianthenoate F glucoside	0.282742357
Bos_taurus_newG	1.06996792	Illudin C2	0.309512507
Bos_taurus_newG	1.06996792	N-[[3-Hydroxy-2-(2-pentenyl)cyclopentyl]ac	1.043930947
Bos_taurus_newG	1.06996792	Casbene	0.36403781
Bos_taurus_newG	1.06996792	Abt-510	0.626815579
Bos_taurus_newG	1.06996792	PG(a-25:0/LTE4)	0.221651761
Bos_taurus_newG	1.06996792	2-Amino-4-[carbamimidoyl(methyl)amino]bu	0.075703353
Bos_taurus_newG	1.06996792	Pinolidoxin	0.047294647
Bos_taurus_newG	1.06996792	Tuliposide B	0.059657835
Bos_taurus_newG	1.064834364	milbemycin beta3	1.414447124
Bos_taurus_newG	1.064834364	PE(20:0/18:1(12Z)-2OH(9,10))	0.438658253
Bos_taurus_newG	1.064834364	Cyclosporin A	0.656529229
Bos_taurus_newG	1.064834364	DG(18:0/LTE4/0:0)	0.681485773
Bos_taurus_newG	1.064834364	CDP-DG(PGF2alpha/16:0)	1.002512277
Bos_taurus_newG	1.064834364	Benzoyl glucuronide (Benzoic acid)	0.442844673
Bos_taurus_newG	1.064834364	Guanidoacetic acid	0.542509265
Bos_taurus_newG	1.064834364	alpha-Terpineol formate	0.628238986
Bos_taurus_newG	1.064834364	PC(P-18:1(11Z)/PGE2)	0.509503133
Bos_taurus_newG	1.064834364	Nigroxanthin	0.705005059
Bos_taurus_newG	1.064834364	PC(P-18:1(11Z)/PGJ2)	0.565243877
Bos_taurus_newG	1.064834364	PS(20:0/20:4(8Z,11Z,14Z,17Z)-2OH(5S,6R))	0.402595921
Bos_taurus_newG	1.064834364	CL(8:0/8:0/18:2(9Z,11Z)/20:0)	0.622988418
Bos_taurus_newG	1.063986786	Yucalexin P15	1.079168162
Bos_taurus_newG	1.063986786	CL(8:0/8:0/18:2(9Z,11Z)/20:0)	0.622988418
gene-CCDC150	1.062817091	Dextrorphan O-glucuronide	1.59476827
gene-CCDC150	1.062817091	5-Hydroxy-2-oxo-4-ureido-2,5-dihydro-1H	0.122536033
gene-CCDC150	1.062817091	Calicheamicinone	0.910932275
gene-CCDC150	1.062817091	Pterostilbene	0.899698314
gene-CCDC150	1.062817091	Ancymidol	0.913827444

gene-CCDC150	1.062817091	Phenylalanylhydroxyproline	1.183668928
gene-CCDC150	1.062817091	Melatonin	1.134145229
gene-CCDC150	1.062817091	PIP(22:5(4Z,7Z,10Z,13Z,16Z)/PGJ2)	0.868452769
gene-CCDC150	1.062817091	Urotensin-related peptide	0.9290699
gene-CCDC150	1.062817091	Arginine vasopressin 1-8	1.288601754
gene-CCDC150	1.062817091	15-Keto-13,14-dihydroprostaglandin A2	0.447077042
gene-CCDC150	1.062817091	PA(2:0/18:1(12Z)-2OH(9,10))	0.512116195
gene-CCDC150	1.062817091	Hygromycin B	0.930396619
gene-CCDC150	1.062817091	Netupitant	0.707108724
gene-CCDC150	1.062817091	Divinylprotochlorophyllide	0.629978387
gene-CCDC150	1.062817091	Benzoyl-fvr-pna	0.67370714
gene-CCDC150	1.062817091	Metkephamid	1.159767003
gene-CCDC150	1.062817091	Methyl (2E)-2-(10,13-dimethyl-11-oxo-3-py	0.478588158
gene-CCDC150	1.062817091	Tuberose lactone	1.590026295
gene-CCDC150	1.062817091	Epothilone D	0.794619023
gene-CCDC150	1.062817091	Leukotriene D4	0.6807158
gene-CCDC150	1.062817091	2-Ethyl-5-methyl-3,3-diphenyl-1-pyrroline	0.826978858
gene-CCDC150	1.062817091	11-Hydroxyeicosatetraenoate glyceryl ester	1.226051047
gene-CCDC150	1.062817091	Azaspiracid 5	1.398898867
gene-CCDC150	1.062817091	PGP(20:2(11Z,14Z)/18:2(10E,12Z)+=O(9))	1.457698264
gene-CCDC150	1.062817091	PGP(20:1(11Z)/18:1(12Z)-2OH(9,10))	0.042651731
gene-CCDC150	1.062817091	11-Maleimidoundecanoic acid	1.084942397
gene-CCDC150	1.062817091	Becocalcidiol	0.63162902
gene-CCDC150	1.062817091	Valnemulin	0.408062514
gene-CCDC150	1.062817091	(6Z,9Z,12Z)-Octadecatrienoic acid	0.266628199
gene-CCDC150	1.062817091	LTB4-d4	0.226799982
gene-CCDC150	1.062817091	PC(24:0/22:6(4Z,7Z,10Z,12E,16Z,19Z)-OH(14)	0.188241061
gene-CCDC150	1.062817091	PE(20:0/18:1(12Z)-2OH(9,10))	0.438658253
gene-CCDC150	1.062817091	Physapubescin	0.679860022
gene-CCDC150	1.062817091	(3b,16b,20R)-Pregn-5-ene-3,16,20-triol 3-gl	0.870801075
gene-CCDC150	1.062817091	[(3S,4S,5S,6R)-3,4,5-Trihydroxy-6-(hydroxym	0.008715229
gene-CCDC150	1.062817091	Epomusenin A	0.766206049
gene-CCDC150	1.062817091	(4r,5s,6s,7r)-4,7-Dibenzyl-5,6-dihydroxy-1,3-	1.334600128
gene-CCDC150	1.062817091	Pseudouridine 5'-phosphate	1.18431378
gene-CCDC150	1.062817091	C20914	0.140383181
gene-CCDC150	1.062817091	Pro Gly Ser Ser	0.384303007
gene-CCDC150	1.062817091	Fluridone	0.127106682
gene-CCDC150	1.062817091	Norophthalmic acid	0.191432411
gene-CCDC150	1.062817091	4'-Thiothymidine	0.066948524
gene-CCDC150	1.062817091	3'-N'-Acetylfusarochromanone	0.755683206
gene-CCDC150	1.062817091	cis,trans-5'-Hydroxythalidomide	0.589828342
gene-CCDC150	1.062817091	Ser Cys Ala Ala	0.603032447
gene-CCDC150	1.062817091	10-Hydroxycarbazepine	0.848001655
gene-CCDC150	1.062817091	Semilepidinoside A	0.527638644
gene-CCDC150	1.062817091	Loganin	0.92782666
gene-CCDC150	1.062817091	3'-Deoxythymidine	0.791830758
gene-CCDC150	1.062817091	Gly Asp Ala Ala	0.718656316
gene-CCDC150	1.062817091	N-Acetylhistidine	0.349369987
gene-CCDC150	1.062817091	ethyl 2-cyano-3-(1h-indol-3-yl)prop-2-eno	0.756861034
gene-CCDC150	1.062817091	1-{2-[(3-Ethylphenyl)amino]-2-oxoethyl}-6-	0.705711444
gene-CCDC150	1.062817091	kainic acid	0.836445456

gene-CCDC150	1.062817091	Zanamivir	0.839205907
gene-CCDC150	1.062817091	Tryptophyl-Glutamine	0.89722186
gene-CCDC150	1.062817091	Aminoglutethimide	0.652616628
gene-CCDC150	1.062817091	5-NITRO-2-PHENYLPROPYLAMINOBENZOI(	0.713298779
gene-CCDC150	1.062817091	5,6,7,8-Tetrahydromonapterin	0.724075531
gene-CCDC150	1.062817091	Guanidoacetic acid	0.542509265
gene-CCDC150	1.062817091	alpha-Terpineol formate	0.628238986
gene-CCDC150	1.062817091	4-Dihydroboldenone	0.692863202
gene-CCDC150	1.062817091	2'-Fluoro-2',3'-dideoxyinosine	1.214745496
gene-CCDC150	1.062817091	20-carboxy Arachidonic Acid	0.780988382
gene-CCDC150	1.062817091	17-beta-Estradiol glucuronide	0.827039442
gene-CCDC150	1.062817091	Nebramycin 5'	0.909280571
gene-CCDC150	1.062817091	Apronal	0.754033426
gene-CCDC150	1.062817091	Methionyl-Valine	0.502707745
gene-CCDC150	1.062817091	[3-(2-Aminopropyl)-6-methylidenecyclohexa	0.422945997
gene-CCDC150	1.062817091	Argyrolobine	0.949134241
gene-CCDC150	1.062817091	3尾-hydroxy-estra-5,7,9-trien-17-one	0.715223098
gene-CCDC150	1.062817091	Cetamolol	0.770561315
gene-CCDC150	1.062817091	Eicosatetraynoic Acid	0.804823516
gene-CCDC150	1.062817091	(2E,4E)-5-[2-Methyl-2-(1,1,4,4-tetramethyl-:	0.742652463
gene-CCDC150	1.062817091	3,7-Dihydroxy-12-oxocholanoic acid	0.832319882
gene-CCDC150	1.062817091	(22E)-3伪,7伪,12伪-Trihydroxy-5尾-chol-22-	0.522629215
gene-CCDC150	1.062817091	PGD2 ethanolamide	1.431143851
gene-CCDC150	1.062817091	(5alpha,6beta,14alpha,20R,22R)-5,6,14,20,27-	1.296004093
gene-CCDC150	1.062817091	SM(d19:1/PGE2)	0.725813678
gene-CCDC150	1.062817091	3-{[(2E)-4-Amino-4-oxobut-2-enoyl]amino}	1.152542472
gene-CCDC150	1.062817091	Leucylhydroxyproline	0.780056846
gene-CCDC150	1.062817091	3'-Hydroxyropivacaine	1.014215236
gene-CCDC150	1.062817091	3-Hydroxytetradecanoyl carnitine	1.314002596
gene-CCDC150	1.062817091	Chitotriose	0.897504334
gene-CCDC150	1.062817091	Macrocin	1.715200143
gene-CCDC150	1.062817091	Vulgarone A	0.102049407
gene-CCDC150	1.062817091	Myricanol 5-[arabinosyl-(1->6)-glucoside]	0.744174399
gene-CCDC150	1.062817091	1,4,6-Trimethylnaphthalene	0.12150693
gene-CCDC150	1.062817091	Prostaglandin PGE2 1-glyceryl ester	0.007411313
gene-CCDC150	1.062817091	7-Sulfocholic acid	0.039985455
gene-CCDC150	1.062817091	1,8-Octanedithiol	0.133094504
gene-CCDC150	1.062817091	MG(0:0/18:3(9Z,12Z,15Z)/0:0)	1.373784038
gene-CCDC150	1.062817091	Tetracycline	0.553304723
gene-CCDC150	1.062817091	N-Palmitoyl Proline	0.470367693
gene-CCDC150	1.062817091	10-alpha-methoxy-9,10-dihydrolysergol	0.140851438
gene-CCDC150	1.062817091	(1'R)-Nepetalic acid	0.499281338
gene-CCDC150	1.062817091	Iridal	0.699410612
gene-CCDC150	1.062817091	Galabiosylceramide (d18:1/20:0)	0.746737323
gene-CCDC150	1.062817091	LysoPI(0:0/18:0)	0.420441153
gene-CCDC150	1.062817091	N-Stearoyl Valine	0.653773206
gene-CCDC150	1.062817091	Nigroxanthin	0.705005059
gene-CCDC150	1.062817091	PC(P-18:1(11Z)/PGJ2)	0.565243877
gene-CCDC150	1.062817091	PE-NMe(18:2(9Z,12Z)/18:2(9Z,12Z))[U]	0.694736593
gene-CCDC150	1.062817091	PE(18:0/20:4(8Z,11Z,14Z,17Z))	0.344922006
gene-CCDC150	1.062817091	PC(P-16:0/20:3(8Z,11Z,14Z)-2OH(5,6))	0.174122169

gene-CRACR2B	1.062539319	D-Erythro-imidazole-glycerol-phosphate	0.355812431
gene-CRACR2B	1.062539319	milbemycin beta3	1.414447124
gene-CRACR2B	1.062539319	Tsugarioside B	1.013262893
gene-CRACR2B	1.062539319	7(14)-Bisabolene-2,3,10,11-tetrol	2.446897974
gene-CRACR2B	1.062539319	cis-p-Menth-2-en-1-ol	0.201017988
gene-CRACR2B	1.062539319	2-isopentyl-3,6-dimethyl pyrazine	0.710502562
gene-CRACR2B	1.062539319	PG(20:1(11Z)/18:3(10,12,15)-OH(9))	0.626244347
gene-CRACR2B	1.062539319	Dyphylline	0.664549753
gene-CRACR2B	1.062539319	5-(2-Aminopropyl)-2-methylphenol	0.323231457
gene-CRACR2B	1.062539319	4-Dimethylamino-L-phenylalanine	0.242110226
gene-CRACR2B	1.062539319	4-Guanidinobutanoate	0.445426102
gene-CRACR2B	1.062539319	Armillane	0.52808635
gene-CRACR2B	1.062539319	N-Eicosapentaenoyl Asparagine	0.371594025
gene-CRACR2B	1.062539319	PC(P-18:1(11Z)/PGE2)	0.509503133
gene-CRACR2B	1.062539319	(3R,4R)-3-Amino-1-hydroxy-4-methylpyrrol	0.471914506
gene-CRACR2B	1.062539319	Isopropyl isothiocyanate	0.172385747
gene-CRACR2B	1.062539319	Sitosterol beta-D-glucoside	0.382944796
gene-CRACR2B	1.062539319	PS(20:0/20:4(8Z,11Z,14Z,17Z)-2OH(5S,6R))	0.402595921
gene-CRACR2B	1.062539319	PG(20:1(11Z)/18:3(9,11,15)-OH(13))	0.69829683
gene-TNFRSF4	1.061817581	3-Thiacytidine	0.209387412
gene-TNFRSF4	1.061817581	LTB4-d4	0.226799982
gene-TNFRSF4	1.061817581	cis-p-Menth-2-en-1-ol	0.201017988
gene-TNFRSF4	1.061817581	PE(20:0/18:1(12Z)-2OH(9,10))	0.438658253
gene-TNFRSF4	1.061817581	PE(22:2(13Z,16Z)/22:5(4Z,7Z,10Z,13Z,19Z)-O	0.369745166
gene-TNFRSF4	1.061817581	Cyclosporin A	0.656529229
gene-TNFRSF4	1.061817581	Epomusenin A	0.766206049
gene-TNFRSF4	1.061817581	DG(18:0/LTE4/0:0)	0.681485773
gene-TNFRSF4	1.061817581	PC(14:0/20:2(11Z,14Z))	1.110657378
gene-TNFRSF4	1.061817581	CDP-DG(PGF2alpha/16:0)	1.002512277
gene-TNFRSF4	1.061817581	5-(2-Aminopropyl)-2-methylphenol	0.323231457
gene-TNFRSF4	1.061817581	Guanidoacetic acid	0.542509265
gene-TNFRSF4	1.061817581	alpha-Terpineol formate	0.628238986
gene-TNFRSF4	1.061817581	PC(P-18:1(11Z)/PGE2)	0.509503133
gene-TNFRSF4	1.061817581	(9Z)-Octadecenoic acid	0.142359556
gene-TNFRSF4	1.061817581	PC(18:1(9Z)/15:1(9Z))	0.534724682
gene-TNFRSF4	1.061817581	arachidyl amido cholanoic acid	1.24842952
gene-TNFRSF4	1.061817581	Nigroxanthin	0.705005059
gene-TNFRSF4	1.061817581	1-Octadecanoyl-2-(7Z,10Z,13Z,16Z-docosat	0.690496314
gene-TNFRSF4	1.061817581	PC(P-18:1(11Z)/PGJ2)	0.565243877
gene-TNFRSF4	1.061817581	PS(20:0/20:4(8Z,11Z,14Z,17Z)-2OH(5S,6R))	0.402595921
gene-TNFRSF4	1.061817581	CL(8:0/8:0/18:2(9Z,11Z)/20:0)	0.622988418
gene-TNFRSF4	1.061817581	PS(16:1(9Z)/22:2(13Z,16Z))	0.659652661
gene-PAXX	1.061758742	3-Thiacytidine	0.209387412
gene-PAXX	1.061758742	D-Erythro-imidazole-glycerol-phosphate	0.355812431
gene-PAXX	1.061758742	LTB4-d4	0.226799982
gene-PAXX	1.061758742	(-)-alpha-Terpineol	0.345716279
gene-PAXX	1.061758742	Cyclosporin A	0.656529229
gene-PAXX	1.061758742	DG(18:0/LTE4/0:0)	0.681485773
gene-PAXX	1.061758742	PC(14:0/20:2(11Z,14Z))	1.110657378
gene-PAXX	1.061758742	5-(2-Aminopropyl)-2-methylphenol	0.323231457
gene-PAXX	1.061758742	4-Dimethylamino-L-phenylalanine	0.242110226

gene-PAXX	1.061758742	Trimetazidine	0.438282534
gene-PAXX	1.061758742	Armillane	0.52808635
gene-PAXX	1.061758742	2-Methyl-3-phenyl-2-propenal	0.407813975
gene-PAXX	1.061758742	PC(P-18:1(11Z)/PGE2)	0.509503133
gene-PAXX	1.061758742	(3R,4R)-3-Amino-1-hydroxy-4-methylpyrrol	0.471914506
gene-PAXX	1.061758742	n-methyl-2-(4'-methylaminophenyl)-6-hydr	0.26655714
gene-PAXX	1.061758742	Isopropyl isothiocyanate	0.172385747
gene-PAXX	1.061758742	(9Z)-Octadecenoic acid	0.142359556
gene-PAXX	1.061758742	arachidyl amido cholanoic acid	1.24842952
gene-PAXX	1.061758742	Nigroxanthin	0.705005059
gene-PAXX	1.061758742	9-deoxy-9-methylene-16,16-dimethyl -PGE	0.606893884
gene-PAXX	1.061758742	CL(8:0/8:0/18:2(9Z,11Z)/20:0)	0.622988418
gene-CCL3	1.061445066	Glutamate carbon	0.671444516
gene-CCL3	1.061445066	11-Maleimidoundecanoic acid	1.084942397
gene-CCL3	1.061445066	PE(22:2(13Z,16Z)/22:5(4Z,7Z,10Z,13Z,19Z)-O	0.369745166
gene-CCL3	1.061445066	Norophthalmic acid	0.191432411
gene-CCL3	1.061445066	(1R,6S)-6-Amino-5-oxocyclohex-2-ene-1-c	0.154751123
gene-CCL3	1.061445066	alpha-Terpineol formate	0.628238986
gene-CCL3	1.061445066	3-(3-Methylbutylidene)-1(3H)-isobenzofurar	0.575422414
gene-CCL3	1.061445066	O-(17-Carboxyheptadecanoyl)carnitine	0.433790125
gene-CCL3	1.061445066	PC(P-18:1(11Z)/PGE2)	0.509503133
gene-CCL3	1.061445066	Roxithromycin	0.268273077
gene-CCL3	1.061445066	PC(P-18:1(11Z)/PGJ2)	0.565243877
gene-CCL3	1.061445066	PS(20:0/20:4(8Z,11Z,14Z,17Z)-2OH(5S,6R))	0.402595921
gene-LOC61825(	1.060968593	5-Fluorouridine diphosphate	0.151761582
gene-LOC61825(	1.060968593	Phospho-anandamide	0.123015586
gene-LOC61825(	1.060968593	16-hydroxy hexadecanoic acid	0.309000958
gene-LOC61825(	1.060968593	4-Dimethylamino-L-phenylalanine	0.242110226
gene-LOC61825(	1.060968593	N-Acetylserotonin	0.136049296
gene-LOC61825(	1.060968593	Valylproline	1.009800636
gene-LOC61825(	1.060968593	1-Benzazepine	0.130253707
gene-LOC61825(	1.060968593	8-Isoquinoline methanamine (hydrochloride)	0.13476644
gene-LOC61825(	1.060968593	Trimetazidine	0.438282534
gene-LOC61825(	1.060968593	Harmalan	0.103514413
gene-LOC61825(	1.060968593	3-Furanmethanol glucoside	0.132831923
gene-LOC61825(	1.060968593	(Z)-3-Oxo-2-(2-pentenyl)-1-cyclopenteneac	0.179805915
gene-LOC61825(	1.060968593	Asp Tyr Asp Gly	0.575346771
gene-LOC61825(	1.060968593	(Z)-[3-(Methylsulfinyl)-1-propenyl] 2-proper	0.021537476
gene-LOC61825(	1.060968593	Phantasmidine	0.044283869
gene-PLXNB2	1.06013312	milbemycin beta3	1.414447124
gene-PLXNB2	1.06013312	cis-p-Menth-2-en-1-ol	0.201017988
gene-PLXNB2	1.06013312	PE(22:2(13Z,16Z)/22:5(4Z,7Z,10Z,13Z,19Z)-O	0.369745166
gene-PLXNB2	1.06013312	PG(20:1(11Z)/18:3(10,12,15)-OH(9))	0.626244347
gene-PLXNB2	1.06013312	Asparaginylcysteine	0.467538645
gene-PLXNB2	1.06013312	Cornoside	0.781689516
gene-PLXNB2	1.06013312	PC(P-18:1(11Z)/PGE2)	0.509503133
gene-PLXNB2	1.06013312	Sitosterol beta-D-glucoside	0.382944796
gene-PLXNB2	1.06013312	PS(20:0/20:4(8Z,11Z,14Z,17Z)-2OH(5S,6R))	0.402595921
gene-PLXNB2	1.06013312	PG(20:1(11Z)/18:3(9,11,15)-OH(13))	0.69829683
gene-AATK	1.059239014	Glutamate carbon	0.671444516
gene-AATK	1.059239014	Val-Cit	0.452308451

gene-AATK	1.059239014	Undecanedioic acid	0.059955634
gene-AATK	1.059239014	Cholic acid glucuronide	0.147398811
gene-AATK	1.059239014	11-Maleimidoundecanoic acid	1.084942397
gene-AATK	1.059239014	Monacolin L acid	1.060562098
gene-AATK	1.059239014	6-Ethylchenodeoxycholic acid	0.087193502
gene-AATK	1.059239014	PE(22:2(13Z,16Z)/22:5(4Z,7Z,10Z,13Z,19Z)-O(	0.369745166
gene-AATK	1.059239014	3,4,3',4'-Tetrahydrospirilloxanthin	0.354666148
gene-AATK	1.059239014	(1R,6S)-6-Amino-5-oxocyclohex-2-ene-1-ca	0.154751123
gene-AATK	1.059239014	Indole-3-ethanol	0.096523128
gene-AATK	1.059239014	tetranor-PGAM	0.584223916
gene-AATK	1.059239014	2-(1-Adamantyl)-1,3-dioxetane	0.408916843
gene-AATK	1.059239014	3-(3-Methylbutylidene)-1(3H)-isobenzofuran	0.575422414
gene-AATK	1.059239014	Roxithromycin	0.268273077
gene-ZFP41	1.057950143	D-Erythro-imidazole-glycerol-phosphate	0.355812431
gene-ZFP41	1.057950143	Glutamate carbon	0.671444516
gene-ZFP41	1.057950143	1-(2-Furanyl)-1-pentanone	0.697147283
gene-ZFP41	1.057950143	2-Dehydro-3-deoxy-D-gluconate	0.65165299
gene-ZFP41	1.057950143	N1-Methyl-4-pyridone-5-carboxamide	0.751815577
gene-ZFP41	1.057950143	Jasmolone glucoside	0.949312509
gene-ZFP41	1.057950143	Undecanedioic acid	0.059955634
gene-ZFP41	1.057950143	16-Hydroxy-10-oxohexadecanoic acid	0.72302152
gene-ZFP41	1.057950143	Hexanoylglutamine	0.593876112
gene-ZFP41	1.057950143	L-Anticapsin	0.426006862
gene-ZFP41	1.057950143	Threoninyl-Tryptophan	0.69975167
gene-KIAA0895L	1.057190305	Cholic acid glucuronide	0.147398811
gene-KIAA0895L	1.057190305	11-Maleimidoundecanoic acid	1.084942397
gene-KIAA0895L	1.057190305	PC(24:0/22:6(4Z,7Z,10Z,12E,16Z,19Z)-OH(14)	0.188241061
gene-KIAA0895L	1.057190305	Benzoyl glucuronide (Benzoic acid)	0.442844673
gene-KIAA0895L	1.057190305	3b,6a-Dihydroxy-alpha-ionol 9-[apiosyl-(1-:	0.045258177
gene-KIAA0895L	1.057190305	Asparaginylarginine	0.091306435
gene-KIAA0895L	1.057190305	1-Methylhistidine	0.750682149
gene-KIAA0895L	1.057190305	1,8-Octanedithiol	0.133094504
gene-KIAA0895L	1.057190305	10,11-Difluoro-8E,10E-dodecadien-1-ol	0.004391585
gene-KIAA0895L	1.057190305	Prostaglandin B2	0.552852305
gene-KIAA0895L	1.057190305	Phorone A	0.079039095
Bos_taurus_newG	1.056796001	D-Erythro-imidazole-glycerol-phosphate	0.355812431
Bos_taurus_newG	1.056796001	Tsugarioside B	1.013262893
Bos_taurus_newG	1.056796001	7(14)-Bisabolene-2,3,10,11-tetrol	2.446897974
Bos_taurus_newG	1.056796001	3-Deoxyestrone	0.282221709
Bos_taurus_newG	1.056796001	1-Oleoyl-sn-glycero-3-phosphocholine	0.18926952
Bos_taurus_newG	1.056796001	Lividamine	0.319679555
Bos_taurus_newG	1.056796001	LTB4-d4	0.226799982
Bos_taurus_newG	1.056796001	cis-p-Menth-2-en-1-ol	0.201017988
Bos_taurus_newG	1.056796001	(-)-alpha-Terpineol	0.345716279
Bos_taurus_newG	1.056796001	2-isopentyl-3,6-dimethyl pyrazine	0.710502562
Bos_taurus_newG	1.056796001	5-(2-Aminopropyl)-2-methylphenol	0.323231457
Bos_taurus_newG	1.056796001	4-Dimethylamino-L-phenylalanine	0.242110226
Bos_taurus_newG	1.056796001	Trimetazidine	0.438282534
Bos_taurus_newG	1.056796001	Armillane	0.52808635
Bos_taurus_newG	1.056796001	5'-S-Methyl-5'-thioinosine	0.28772476
Bos_taurus_newG	1.056796001	PC(P-18:1(11Z)/PGE2)	0.509503133

Bos_taurus_newG	1.056796001	(3R,4R)-3-Amino-1-hydroxy-4-methylpyrrol	0.471914506
Bos_taurus_newG	1.056796001	n-methyl-2-(4'-methylaminophenyl)-6-hydr	0.26655714
Bos_taurus_newG	1.056796001	Isopropyl isothiocyanate	0.172385747
Bos_taurus_newG	1.056796001	9-deoxy-9-methylene-16,16-dimethyl -PGE	0.606893884
Bos_taurus_newG	1.056796001	CL(8:0/8:0/18:2(9Z,11Z)/20:0)	0.622988418
gene-WDR31	1.055594072	Tetrahydroaldosterone-3-glucuronide	0.966478744
gene-WDR31	1.055594072	Undecaprenyl phosphate alpha-L-Ara4N	0.18214621
gene-WDR31	1.055594072	hydroxytetradecadienyl-l-carnitine	0.024526721
gene-WDR31	1.055594072	4-Hydroxyoctanedioylcarnitine	1.002008817
gene-WDR31	1.055594072	2-Propenamide, 2-cyano-3-(4-hydroxy-3,5-	0.067779204
gene-SLCO4A1	1.052461621	D-Erythro-imidazole-glycerol-phosphate	0.355812431
gene-SLCO4A1	1.052461621	Glutamate carbon	0.671444516
gene-SLCO4A1	1.052461621	2-Dehydro-3-deoxy-D-gluconate	0.65165299
gene-SLCO4A1	1.052461621	Undecanedioic acid	0.059955634
gene-SLCO4A1	1.052461621	(1R,6S)-6-Amino-5-oxocyclohex-2-ene-1-c	0.154751123
gene-SLCO4A1	1.052461621	3-(3-Methylbutylidene)-1(3H)-isobenzofurar	0.575422414
gene-SLCO4A1	1.052461621	3',4',5'-Trimethoxycinnamyl alcohol acetate	0.4221816
gene-SLCO4A1	1.052461621	L-Anticapsin	0.426006862
gene-SLCO4A1	1.052461621	PC(P-18:1(11Z)/PGE2)	0.509503133
gene-SLCO4A1	1.052461621	Roxithromycin	0.268273077
Bos_taurus_newG	1.051809119	D-Erythro-imidazole-glycerol-phosphate	0.355812431
Bos_taurus_newG	1.051809119	D-Glucono-1,5-lactone	0.240039573
Bos_taurus_newG	1.051809119	Methyl (2E)-2-(10,13-dimethyl-11-oxo-3-py	0.478588158
Bos_taurus_newG	1.051809119	Urocortisol	0.926998516
Bos_taurus_newG	1.051809119	13(S)-HpODE	0.163157753
Bos_taurus_newG	1.051809119	7(S),17(S)-dihydroxy-8(E),10(Z),13(Z),15(E),19	0.32147403
Bos_taurus_newG	1.051809119	9-Hydroxy-7-megastigmen-3-one glucoside	1.176625281
Bos_taurus_newG	1.051809119	1,2,3,4-Tetrahydroisoquinoline-3-carboxylic	0.339248702
Bos_taurus_newG	1.051809119	2-Methyl-3-phenyl-2-propenal	0.407813975
Bos_taurus_newG	1.051809119	[3-(2-Aminopropyl)-6-methylidenecyclohexa	0.422945997
Bos_taurus_newG	1.051809119	Vulgarone A	0.102049407
Bos_taurus_newG	1.051809119	7-Ketodeoxycholic acid	0.014650773
Bos_taurus_newG	1.051809119	Sophoranol	0.282064826
Bos_taurus_newG	1.051809119	L-Cysteine	0.214540754
Bos_taurus_newG	1.051809119	Methyl 4,7,10,13,16-docosapentaenoate	0.204170362
Bos_taurus_newG	1.051809119	24,24-Dfhv	0.175278646
Bos_taurus_newG	1.051809119	(4Z,7Z,10Z,13Z,16Z,19Z)-Docosahexaenoic a	0.434211486
Bos_taurus_newG	1.051809119	9-OxoODE	0.111803831
gene-PPL	1.051611817	Enprofylline	0.061479963
gene-PPL	1.051611817	1-Oleoyl Lysophosphatidic Acid (sodium salt)	0.375442922
gene-PPL	1.051611817	Sabine	1.8767788
gene-PPL	1.051611817	Sarcodon scabrosus Depsipeptide	1.433355295
gene-PPL	1.051611817	Thr His Phe Lys	2.161400139
gene-PPL	1.051611817	Gln Leu Glu Lys	1.069995273
gene-PPL	1.051611817	Fosinopril	1.857006593
gene-PPL	1.051611817	N-Arachidonoyl Glutamic acid	0.496582621
gene-PPL	1.051611817	N-Docosahexaenoyl Histidine	2.27720447
gene-PPL	1.051611817	(2S,3S,5S,8S,9S,10S,13S,14S,17S)-17-Acetyl-2	0.563570145
gene-PPL	1.051611817	Chlorophyllide	1.469089583
gene-PPL	1.051611817	Carmofur	0.625889788
gene-PPL	1.051611817	Apramycin	0.867064958

gene-PPL	1.051611817	Palmitoyl Ara-C	0.586890476
gene-PPL	1.051611817	Mupirocin	0.952072991
gene-PPL	1.051611817	PIP(22:5(4Z,7Z,10Z,13Z,16Z)/PGJ2)	0.868452769
gene-PPL	1.051611817	Urotensin-related peptide	0.9290699
gene-PPL	1.051611817	Arginine vasopressin 1-8	1.288601754
gene-PPL	1.051611817	Glucuronolactone	0.347424779
gene-PPL	1.051611817	15-Keto-13,14-dihydroprostaglandin A2	0.447077042
gene-PPL	1.051611817	PA(2:0/18:1(12Z)-2OH(9,10))	0.512116195
gene-PPL	1.051611817	Netupitant	0.707108724
gene-PPL	1.051611817	Benzoyl-fvr-pna	0.67370714
gene-PPL	1.051611817	Norfuraneol	0.526756614
gene-PPL	1.051611817	Metkephamid	1.159767003
gene-PPL	1.051611817	Leukotriene D4	0.6807158
gene-PPL	1.051611817	2-Ethyl-5-methyl-3,3-diphenyl-1-pyrroline	0.826978858
gene-PPL	1.051611817	PGP(20:1(11Z)/18:1(12Z)-2OH(9,10))	0.042651731
gene-PPL	1.051611817	Becocalcidiol	0.63162902
gene-PPL	1.051611817	His Leu Ser Lys	0.708465111
gene-PPL	1.051611817	(6Z,9Z,12Z)-Octadecatrienoic acid	0.266628199
gene-PPL	1.051611817	LTB4-d4	0.226799982
gene-PPL	1.051611817	Docosatrienoic acid	0.000323464
gene-PPL	1.051611817	4,6-Heneicosanedione	0.113496143
gene-PPL	1.051611817	Physapubescin	0.679860022
gene-PPL	1.051611817	C20914	0.140383181
gene-PPL	1.051611817	Ser Cys Ala Ala	0.603032447
gene-PPL	1.051611817	N-Acetylhistidine	0.349369987
gene-PPL	1.051611817	Lovastatin acid	0.456404915
gene-PPL	1.051611817	17-phenyl trinor Prostaglandin F2伪 cycloprc	1.374273176
gene-PPL	1.051611817	Dynorphin B (10-13)	0.692919909
gene-PPL	1.051611817	LysoPA(0:0/16:0)	1.30951833
gene-PPL	1.051611817	15H-11,12-EETA	1.71018828
gene-PPL	1.051611817	Cytochalasin B	1.792662701
gene-PPL	1.051611817	Taurocholate	2.056231295
gene-PPL	1.051611817	Cerulenin	1.323019945
gene-PPL	1.051611817	Perindopril	0.260541798
gene-PPL	1.051611817	Megestrol	0.703831777
gene-PPL	1.051611817	4-Dihydroboldenone	0.692863202
gene-PPL	1.051611817	2'-Fluoro-2',3'-dideoxyinosine	1.214745496
gene-PPL	1.051611817	20-carboxy Arachidonic Acid	0.780988382
gene-PPL	1.051611817	17-beta-Estradiol glucuronide	0.827039442
gene-PPL	1.051611817	Nebramycin 5'	0.909280571
gene-PPL	1.051611817	Argyrolobine	0.949134241
gene-PPL	1.051611817	3尾-hydroxy-estra-5,7,9-trien-17-one	0.715223098
gene-PPL	1.051611817	Eicosatetraynoic Acid	0.804823516
gene-PPL	1.051611817	(2E,4E)-5-[2-Methyl-2-(1,1,4,4-tetramethyl-1	0.742652463
gene-PPL	1.051611817	3,7-Dihydroxy-12-oxocholanoic acid	0.832319882
gene-PPL	1.051611817	SM(d19:1/PGE2)	0.725813678
gene-PPL	1.051611817	3-{[(2E)-4-Amino-4-oxobut-2-enoyl]amino}	1.152542472
gene-PPL	1.051611817	Leucylhydroxyproline	0.780056846
gene-PPL	1.051611817	3'-Hydroxyropivacaine	1.014215236
gene-PPL	1.051611817	Chitotriose	0.897504334
gene-PPL	1.051611817	Vulgarone A	0.102049407

gene-PPL	1.051611817	Prostaglandin PGE2 1-glyceryl ester	0.007411313
gene-PPL	1.051611817	7-Sulfocholic acid	0.039985455
gene-PPL	1.051611817	24,24-Difluoro-1alpha,25-dihydroxyvitamin I	1.131590894
gene-PPL	1.051611817	Valdiate	0.03448877
gene-PPL	1.051611817	Androsterone glucuronide	1.164397007
gene-PPL	1.051611817	Iridal	0.699410612
gene-PPL	1.051611817	Galabiosylceramide (d18:1/20:0)	0.746737323
gene-PPL	1.051611817	Phorone A	0.079039095
gene-PPL	1.051611817	(9Z)-Octadecenoic acid	0.142359556
gene-PPL	1.051611817	PE(18:0/20:4(8Z,11Z,14Z,17Z))	0.344922006
gene-TGM1	1.051554536	3-Quinolinecarboxylic acid, 7-(3-amino-1-py	0.614519401
gene-TGM1	1.051554536	Gentisic acid	0.519724891
gene-TGM1	1.051554536	Deoxyloganin	0.445304262
gene-TGM1	1.051554536	Isomaltotriose	0.58434654
gene-TGM1	1.051554536	Picrocrocin	0.381912689
gene-TGM1	1.051554536	6|A-Hydroxy-7|A-(thiomethyl)spirolactone	0.482613588
gene-TGM1	1.051554536	Cymorcin monoglucoside	0.601123318
gene-TGM1	1.051554536	1-Pyrimidinylpiperazine	0.320539209
gene-TGM1	1.051554536	Butyl 3-hydroxy-2-methylidenebutanoate	0.644438067
gene-TGM1	1.051554536	Garcinone C	0.61676172
gene-TGM1	1.051554536	Sebacic acid	0.461477247
gene-TGM1	1.051554536	Mevalonolactone	0.018076699
gene-TGM1	1.051554536	1-(2-Furanyl)-1-pentanone	0.697147283
gene-TGM1	1.051554536	2-Dehydro-3-deoxy-D-gluconate	0.65165299
gene-TGM1	1.051554536	20-Trihydroxy-leukotriene-B4	0.466763197
gene-TGM1	1.051554536	1-(2,6,6-Trimethyl-2-cyclohexen-1-yl)-1,6-h	0.473290341
gene-TGM1	1.051554536	13(S)-HPOT	1.353059742
gene-TGM1	1.051554536	Cholic acid glucuronide	0.147398811
gene-TGM1	1.051554536	16-Hydroxy-10-oxohexadecanoic acid	0.72302152
gene-TGM1	1.051554536	(10S)-Juvenile hormone III acid diol	0.351986626
gene-TGM1	1.051554536	Asparaginylcysteine	0.467538645
gene-TGM1	1.051554536	1-(5'-Phosphoribosyl)-5-amino-4-(N-succin	0.279811852
gene-TGM1	1.051554536	Val Gly His Thr	0.71344531
gene-TGM1	1.051554536	Citrinin	0.405093917
gene-TGM1	1.051554536	Illudin C2	0.309512507
gene-TGM1	1.051554536	2-(1-Adamantyl)-1,3-dioxetane	0.408916843
gene-TGM1	1.051554536	N-[[3-Hydroxy-2-(2-pentenyl)cyclopentyl]ac	1.043930947
gene-TGM1	1.051554536	Acutifolane A	0.095764856
gene-TGM1	1.051554536	Ascorbyl palmitate	0.376864555
gene-TGM1	1.051554536	6-Hydroxypentadecanoylcarnitine	0.820747254
gene-TGM1	1.051554536	Tetracosahexaenic acid	0.447398966
gene-TGM1	1.051554536	PA(PGE2/22:2(13Z,16Z))	0.24730102
gene-TGM1	1.051554536	Pinolidoxin	0.047294647
gene-SLC4A11	1.051103801	D-Erythro-imidazole-glycerol-phosphate	0.355812431
gene-SLC4A11	1.051103801	2-Dehydro-3-deoxy-D-gluconate	0.65165299
gene-SLC4A11	1.051103801	Undecanedioic acid	0.059955634
gene-SLC4A11	1.051103801	16-Hydroxy-10-oxohexadecanoic acid	0.72302152
gene-SLC4A11	1.051103801	7(14)-Bisabolene-2,3,10,11-tetrol	2.446897974
gene-SLC4A11	1.051103801	1,4-Undecadiene	0.462803973
gene-SLC4A11	1.051103801	PC(P-18:1(11Z)/PGE2)	0.509503133
gene-SLC4A11	1.051103801	Roxithromycin	0.268273077

gene-SLC4A11	1.051103801	PS(20:0/20:4(8Z,11Z,14Z,17Z)-2OH(5S,6R))	0.402595921
gene-LOC100297	1.050532539	Eudesmic acid	0.241222121
gene-LOC100297	1.050532539	Tetracosenoyl-CoA	0.187187233
gene-LOC100297	1.050532539	1,2-O-Isopropylidene-D-glucofuranose	0.080987667
gene-LOC100297	1.050532539	3-Deoxyestrone	0.282221709
gene-LOC100297	1.050532539	1-Oleoyl-sn-glycero-3-phosphocholine	0.18926952
gene-LOC100297	1.050532539	Lividamine	0.319679555
gene-LOC100297	1.050532539	Cyclotricuspidogenin C	0.440884085
gene-LOC100297	1.050532539	(-)-alpha-Terpineol	0.345716279
gene-LOC100297	1.050532539	4-Dimethylamino-L-phenylalanine	0.242110226
gene-LOC100297	1.050532539	Trimetazidine	0.438282534
gene-LOC100297	1.050532539	Armillane	0.52808635
gene-LOC100297	1.050532539	PC(P-18:1(11Z)/PGE2)	0.509503133
gene-LOC100297	1.050532539	(3R,4R)-3-Amino-1-hydroxy-4-methylpyrrol	0.471914506
gene-LOC100297	1.050532539	n-methyl-2-(4'-methylaminophenyl)-6-hydr	0.26655714
gene-LOC100297	1.050532539	9-deoxy-9-methylene-16,16-dimethyl -PGE	0.606893884
gene-LOC100297	1.050532539	CL(8:0/8:0/18:2(9Z,11Z)/20:0)	0.622988418
gene-IL3RA	1.05039077	D-Erythro-imidazole-glycerol-phosphate	0.355812431
gene-IL3RA	1.05039077	Jasmolone glucoside	0.949312509
gene-IL3RA	1.05039077	7(14)-Bisabolene-2,3,10,11-tetrol	2.446897974
gene-IL3RA	1.05039077	(3Z)-Phycoerythrobilin	1.456755874
gene-IL3RA	1.05039077	3-Deoxyestrone	0.282221709
gene-IL3RA	1.05039077	1-Oleoyl-sn-glycero-3-phosphocholine	0.18926952
gene-IL3RA	1.05039077	Lividamine	0.319679555
gene-IL3RA	1.05039077	(-)-alpha-Terpineol	0.345716279
gene-IL3RA	1.05039077	1,4-Undecadiene	0.462803973
gene-IL3RA	1.05039077	2-isopentyl-3,6-dimethyl pyrazine	0.710502562
gene-IL3RA	1.05039077	4-Dimethylamino-L-phenylalanine	0.242110226
gene-IL3RA	1.05039077	Armillane	0.52808635
gene-IL3RA	1.05039077	N-Eicosapentaenoyl Asparagine	0.371594025
gene-IL3RA	1.05039077	PC(P-18:1(11Z)/PGE2)	0.509503133
gene-IL3RA	1.05039077	(3R,4R)-3-Amino-1-hydroxy-4-methylpyrrol	0.471914506
gene-IL3RA	1.05039077	1-Palmitoylglycerol	0.084357408
gene-IL3RA	1.05039077	Threoninyl-Tryptophan	0.69975167
gene-IL3RA	1.05039077	n-methyl-2-(4'-methylaminophenyl)-6-hydr	0.26655714
gene-IL3RA	1.05039077	9-deoxy-9-methylene-16,16-dimethyl -PGE	0.606893884
gene-IL3RA	1.05039077	CL(8:0/8:0/18:2(9Z,11Z)/20:0)	0.622988418
Bos_taurus_newG	1.049338929	2-[(3S)-3-[[(2S)-1-(Carboxymethoxy)-1-oxo	0.111577437
Bos_taurus_newG	1.049338929	PE(22:2(13Z,16Z)/22:5(4Z,7Z,10Z,13Z,19Z)-O	0.369745166
Bos_taurus_newG	1.049338929	4-Oxo-9-cis-retinoyl-beta-glucuronide	1.611773742
Bos_taurus_newG	1.049338929	PC(P-18:1(11Z)/PGJ2)	0.565243877
Bos_taurus_newG	1.049338929	PS(20:0/20:4(8Z,11Z,14Z,17Z)-2OH(5S,6R))	0.402595921
gene-TELO2	1.049224053	D-Erythro-imidazole-glycerol-phosphate	0.355812431
gene-TELO2	1.049224053	LTB4-d4	0.226799982
gene-TELO2	1.049224053	cis-p-Menth-2-en-1-ol	0.201017988
gene-TELO2	1.049224053	(-)-alpha-Terpineol	0.345716279
gene-TELO2	1.049224053	4-Dimethylamino-L-phenylalanine	0.242110226
gene-TELO2	1.049224053	2-Methyl-3-phenyl-2-propenal	0.407813975
gene-TELO2	1.049224053	PC(P-18:1(11Z)/PGE2)	0.509503133
gene-TELO2	1.049224053	(3R,4R)-3-Amino-1-hydroxy-4-methylpyrrol	0.471914506
gene-TELO2	1.049224053	CL(8:0/8:0/18:2(9Z,11Z)/20:0)	0.622988418

gene-ROM1	1.048797565	3-Thiacytidine	0.209387412
gene-ROM1	1.048797565	D-Erythro-imidazole-glycerol-phosphate	0.355812431
gene-ROM1	1.048797565	(-)-alpha-Terpineol	0.345716279
gene-ROM1	1.048797565	5-(2-Aminopropyl)-2-methylphenol	0.323231457
gene-ROM1	1.048797565	Armillane	0.52808635
gene-ROM1	1.048797565	PC(P-18:1(11Z)/PGE2)	0.509503133
gene-ROM1	1.048797565	Isopropyl isothiocyanate	0.172385747
gene-ROM1	1.048797565	PC(P-18:1(11Z)/PGJ2)	0.565243877
gene-ROM1	1.048797565	PS(20:0/20:4(8Z,11Z,14Z,17Z)-2OH(5S,6R))	0.402595921
gene-ROM1	1.048797565	CL(8:0/8:0/18:2(9Z,11Z)/20:0)	0.622988418
gene-VXN	1.047726238	Glutamate carbon	0.671444516
gene-VXN	1.047726238	11-Maleimidoundecanoic acid	1.084942397
gene-VXN	1.047726238	PG(i-24:0/PGF1alpha)	0.443229376
gene-VXN	1.047726238	PE(22:2(13Z,16Z)/22:5(4Z,7Z,10Z,13Z,19Z)-O(	0.369745166
gene-VXN	1.047726238	Norophthalmic acid	0.191432411
gene-VXN	1.047726238	(S)-Mandelic acid O-beta-D-Glucopyranosid	0.509637316
gene-VXN	1.047726238	Imidazoline	0.232348181
gene-VXN	1.047726238	Guanidoacetic acid	0.542509265
gene-VXN	1.047726238	3-(3-Methylbutylidene)-1(3H)-isobenzofurar	0.575422414
gene-VXN	1.047726238	O-(17-Carboxyheptadecanoyl)carnitine	0.433790125
gene-VXN	1.047726238	Prostaglandin B2	0.552852305
gene-VXN	1.047726238	Roxithromycin	0.268273077
gene-VXN	1.047726238	PC(P-18:1(11Z)/PGJ2)	0.565243877
gene-VXN	1.047726238	PS(20:0/20:4(8Z,11Z,14Z,17Z)-2OH(5S,6R))	0.402595921
gene-VXN	1.047726238	Phenacetyl-carnitine	0.102314016
gene-VXN	1.047726238	5-Methoxytryptophan	0.079780961
gene-PPP1R26	1.046229594	D-Erythro-imidazole-glycerol-phosphate	0.355812431
gene-PPP1R26	1.046229594	Jasmolone glucoside	0.949312509
gene-PPP1R26	1.046229594	3-hydroxypristanic acid	0.548515835
gene-PPP1R26	1.046229594	7(14)-Bisabolene-2,3,10,11-tetrol	2.446897974
gene-PPP1R26	1.046229594	24,25-Diacetylvulgaroside	0.920743312
gene-PPP1R26	1.046229594	(3Z)-Phycoerythrobilin	1.456755874
gene-PPP1R26	1.046229594	3-Deoxyestrone	0.282221709
gene-PPP1R26	1.046229594	1-Oleoyl-sn-glycero-3-phosphocholine	0.18926952
gene-PPP1R26	1.046229594	Lividamine	0.319679555
gene-PPP1R26	1.046229594	Psychosine	0.106475396
gene-PPP1R26	1.046229594	Cyclotricuspidogenin C	0.440884085
gene-PPP1R26	1.046229594	(-)-alpha-Terpineol	0.345716279
gene-PPP1R26	1.046229594	1,4-Undecadiene	0.462803973
gene-PPP1R26	1.046229594	2-isopentyl-3,6-dimethyl pyrazine	0.710502562
gene-PPP1R26	1.046229594	PG(20:1(11Z)/18:3(10,12,15)-OH(9))	0.626244347
gene-PPP1R26	1.046229594	4-Dimethylamino-L-phenylalanine	0.242110226
gene-PPP1R26	1.046229594	Armillane	0.52808635
gene-PPP1R26	1.046229594	N-[[3-Hydroxy-2-(2-pentenyl)cyclopentyl]ac	1.043930947
gene-PPP1R26	1.046229594	N-Eicosapentaenoyl Asparagine	0.371594025
gene-PPP1R26	1.046229594	PC(P-18:1(11Z)/PGE2)	0.509503133
gene-PPP1R26	1.046229594	(3R,4R)-3-Amino-1-hydroxy-4-methylpyrrol	0.471914506
gene-PPP1R26	1.046229594	Maraviroc	0.755297431
gene-PPP1R26	1.046229594	Threoninyl-Tryptophan	0.69975167
gene-PPP1R26	1.046229594	n-methyl-2-(4'-methylaminophenyl)-6-hydr	0.26655714
gene-PPP1R26	1.046229594	Permetin A	0.140869415

gene-TAZ	1.046041437	3-Thiacytidine	0.209387412
gene-TAZ	1.046041437	D-Erythro-imidazole-glycerol-phosphate	0.355812431
gene-TAZ	1.046041437	cis-p-Menth-2-en-1-ol	0.201017988
gene-TAZ	1.046041437	(-)-alpha-Terpineol	0.345716279
gene-TAZ	1.046041437	PE(22:2(13Z,16Z)/22:5(4Z,7Z,10Z,13Z,19Z)-O(	0.369745166
gene-TAZ	1.046041437	PG(20:1(11Z)/18:3(10,12,15)-OH(9))	0.626244347
gene-TAZ	1.046041437	Armillane	0.52808635
gene-TAZ	1.046041437	PC(P-18:1(11Z)/PGE2)	0.509503133
gene-TAZ	1.046041437	PC(P-18:1(11Z)/PGJ2)	0.565243877
gene-TAZ	1.046041437	PS(20:0/20:4(8Z,11Z,14Z,17Z)-2OH(5S,6R))	0.402595921
gene-NINL	1.044820032	PE-NMe(18:0/18:3(9Z,12Z,15Z))	0.681884774
gene-NINL	1.044820032	3-Thiacytidine	0.209387412
gene-NINL	1.044820032	5-Hydroxy-2-oxo-4-ureido-2,5-dihydro-1H	0.122536033
gene-NINL	1.044820032	Netupitant	0.707108724
gene-NINL	1.044820032	Metkephamid	1.159767003
gene-NINL	1.044820032	Epothilone D	0.794619023
gene-NINL	1.044820032	Leukotriene D4	0.6807158
gene-NINL	1.044820032	2-Ethyl-5-methyl-3,3-diphenyl-1-pyrroline	0.826978858
gene-NINL	1.044820032	PGP(20:1(11Z)/18:1(12Z)-2OH(9,10))	0.042651731
gene-NINL	1.044820032	11-Maleimidoundecanoic acid	1.084942397
gene-NINL	1.044820032	2-Hexylbenzothiazole	0.396099297
gene-NINL	1.044820032	PC(24:0/20:3(8Z,11Z,14Z)-2OH(5,6))	0.43567608
gene-NINL	1.044820032	Valnemulin	0.408062514
gene-NINL	1.044820032	LTB4-d4	0.226799982
gene-NINL	1.044820032	cis-p-Menth-2-en-1-ol	0.201017988
gene-NINL	1.044820032	PE(20:0/18:1(12Z)-2OH(9,10))	0.438658253
gene-NINL	1.044820032	PE(18:0/18:1(9Z)-O(12,13))	1.009186776
gene-NINL	1.044820032	PE(P-18:0/PGE1)	0.886597382
gene-NINL	1.044820032	PC(P-16:0/18:1(12Z)-2OH(9,10))	1.231706326
gene-NINL	1.044820032	Cyclosporin A	0.656529229
gene-NINL	1.044820032	Epomusenin A	0.766206049
gene-NINL	1.044820032	PA(22:6(4Z,7Z,10Z,13Z,16Z,19Z)/16:0)	0.556532497
gene-NINL	1.044820032	DG(18:0/LTE4/0:0)	0.681485773
gene-NINL	1.044820032	PC(17:0/PGJ2)	0.657689319
gene-NINL	1.044820032	PC(14:0/20:2(11Z,14Z))	1.110657378
gene-NINL	1.044820032	CDP-DG(PGF2alpha/16:0)	1.002512277
gene-NINL	1.044820032	1-beta-D-Arabinofuranosyl-5-fluorocytosine	0.152339645
gene-NINL	1.044820032	Cysteinyl-Glutamine	0.079050155
gene-NINL	1.044820032	D-Xylonate	0.386869454
gene-NINL	1.044820032	2-Methyl-5-nitroimidazol-1-ylacetic acid	0.701441397
gene-NINL	1.044820032	Fluoroazomycin arabinoside	0.590955233
gene-NINL	1.044820032	Cysteinyl-Tryptophan	0.586270687
gene-NINL	1.044820032	Benzoyl glucuronide (Benzoic acid)	0.442844673
gene-NINL	1.044820032	C20914	0.140383181
gene-NINL	1.044820032	S-(2-Hydroxyethyl)glutathione	0.269671835
gene-NINL	1.044820032	Fluridone	0.127106682
gene-NINL	1.044820032	Harmalol	0.364945111
gene-NINL	1.044820032	Guanidoacetic acid	0.542509265
gene-NINL	1.044820032	alpha-Terpineol formate	0.628238986
gene-NINL	1.044820032	Apronal	0.754033426
gene-NINL	1.044820032	Methionyl-Valine	0.502707745

gene-NINL	1.044820032	Argyrolobine	0.949134241
gene-NINL	1.044820032	(5alpha,6beta,14alpha,20R,22R)-5,6,14,20,27-	1.296004093
gene-NINL	1.044820032	Leucylhydroxyproline	0.780056846
gene-NINL	1.044820032	Macrocin	1.715200143
gene-NINL	1.044820032	Vulgarone A	0.102049407
gene-NINL	1.044820032	1,4,6-Trimethylnaphthalene	0.12150693
gene-NINL	1.044820032	Prostaglandin PGE2 1-glyceryl ester	0.007411313
gene-NINL	1.044820032	7-Sulfocholic acid	0.039985455
gene-NINL	1.044820032	N-Palmitoyl Proline	0.470367693
gene-NINL	1.044820032	10-alpha-methoxy-9,10-dihydrolysergol	0.140851438
gene-NINL	1.044820032	Iridal	0.699410612
gene-NINL	1.044820032	PC(P-18:1(11Z)/PGE2)	0.509503133
gene-NINL	1.044820032	N-Stearoyl Proline	0.469971646
gene-NINL	1.044820032	Phorone A	0.079039095
gene-NINL	1.044820032	LysoPI(0:0/18:0)	0.420441153
gene-NINL	1.044820032	N-Stearoyl Valine	0.653773206
gene-NINL	1.044820032	PS(24:1(15Z)/24:1(15Z))	0.557296152
gene-NINL	1.044820032	N-Palmitoyl Glutamic acid	0.250188721
gene-NINL	1.044820032	(9Z)-Octadecenoic acid	0.142359556
gene-NINL	1.044820032	Cer(d18:2(4E,14Z)/TXB2)	0.236013333
gene-NINL	1.044820032	norerythromycin	0.401118778
gene-NINL	1.044820032	PC(18:1(9Z)/15:1(9Z))	0.534724682
gene-NINL	1.044820032	PC(18:0/20:4(5Z,8Z,11Z,14Z)-OH(20))	0.318846141
gene-NINL	1.044820032	arachidyl amido cholanoic acid	1.24842952
gene-NINL	1.044820032	Nigroxanthin	0.705005059
gene-NINL	1.044820032	1-Octadecanoyl-2-(7Z,10Z,13Z,16Z-docosat	0.690496314
gene-NINL	1.044820032	PE(18:0/20:4(8Z,11Z,14Z,17Z)-2OH(5S,6R))	0.418208781
gene-NINL	1.044820032	PC(P-18:1(11Z)/PGJ2)	0.565243877
gene-NINL	1.044820032	PS(20:0/20:4(8Z,11Z,14Z,17Z)-2OH(5S,6R))	0.402595921
gene-NINL	1.044820032	PC(20:5(5Z,8Z,11Z,14Z,17Z)/P-16:0)	1.301095986
gene-NINL	1.044820032	PE-NMe(18:2(9Z,12Z)/18:2(9Z,12Z))[U]	0.694736593
gene-NINL	1.044820032	PC(P-16:0/20:3(8Z,11Z,14Z)-2OH(5,6))	0.174122169
gene-NINL	1.044820032	DG(20:0/LTE4/0:0)	0.438074508
Bos_taurus_newG	1.043860104	3-Thiacytidine	0.209387412
Bos_taurus_newG	1.043860104	3,3',5-Trihydroxy-4'-methoxy-6,7-methylene	0.137607639
Bos_taurus_newG	1.043860104	5-Hydroxy-2-oxo-4-ureido-2,5-dihydro-1H	0.122536033
Bos_taurus_newG	1.043860104	11-Maleimidoundecanoic acid	1.084942397
Bos_taurus_newG	1.043860104	PE(20:0/18:1(12Z)-2OH(9,10))	0.438658253
Bos_taurus_newG	1.043860104	Cyclosporin A	0.656529229
Bos_taurus_newG	1.043860104	Epomusenin A	0.766206049
Bos_taurus_newG	1.043860104	PA(22:6(4Z,7Z,10Z,13Z,16Z,19Z)/16:0)	0.556532497
Bos_taurus_newG	1.043860104	DG(18:0/LTE4/0:0)	0.681485773
Bos_taurus_newG	1.043860104	CDP-DG(PGF2alpha/16:0)	1.002512277
Bos_taurus_newG	1.043860104	1-beta-D-Arabinofuranosyl-5-fluorocytosine	0.152339645
Bos_taurus_newG	1.043860104	Fluridone	0.127106682
Bos_taurus_newG	1.043860104	Norophthalmic acid	0.191432411
Bos_taurus_newG	1.043860104	5,6,7,8-Tetrahydromonapterin	0.724075531
Bos_taurus_newG	1.043860104	Harmalol	0.364945111
Bos_taurus_newG	1.043860104	Ethylene brassylate	0.989156973
Bos_taurus_newG	1.043860104	Imidazoline	0.232348181
Bos_taurus_newG	1.043860104	Guanidoacetic acid	0.542509265

Bos_taurus_newG	1.043860104	4-Oxo-9-cis-retinoyl-beta-glucuronide	1.611773742
Bos_taurus_newG	1.043860104	alpha-Terpineol formate	0.628238986
Bos_taurus_newG	1.043860104	Methionyl-Valine	0.502707745
Bos_taurus_newG	1.043860104	Dicyclopentadiene	0.129868592
Bos_taurus_newG	1.043860104	beta-L-Dioxolane-cytidine	0.175916668
Bos_taurus_newG	1.043860104	N-Palmitoyl Proline	0.470367693
Bos_taurus_newG	1.043860104	1,3,5-Bisabolatrien-10-one	0.109736328
Bos_taurus_newG	1.043860104	1-Heneicosanoyl-glycero-3-phosphoserine	0.214358093
Bos_taurus_newG	1.043860104	Nigroxanthin	0.705005059
Bos_taurus_newG	1.043860104	PC(P-18:1(11Z)/PGJ2)	0.565243877
Bos_taurus_newG	1.043860104	PS(20:0/20:4(8Z,11Z,14Z,17Z)-2OH(5S,6R))	0.402595921
Bos_taurus_newG	1.043860104	Azimexon	0.008689891
gene-RGS16	1.043780055	Glutamate carbon	0.671444516
gene-RGS16	1.043780055	Adrenosterone	0.050790129
gene-RGS16	1.043780055	beta-Thujaplicin	0.681866194
gene-RGS16	1.043780055	Imidazoline	0.232348181
gene-RGS16	1.043780055	O-(17-Carboxyheptadecanoyl)carnitine	0.433790125
gene-MST1	1.042433854	3-hydroxypristanic acid	0.548515835
gene-MST1	1.042433854	3-Deoxyestrone	0.282221709
gene-MST1	1.042433854	Lividamine	0.319679555
gene-MST1	1.042433854	Cyclotricuspidogenin C	0.440884085
gene-MST1	1.042433854	(-)-alpha-Terpineol	0.345716279
gene-MST1	1.042433854	1,4-Undecadiene	0.462803973
gene-MST1	1.042433854	N-[[3-Hydroxy-2-(2-pentenyl)cyclopentyl]ac	1.043930947
gene-MST1	1.042433854	Val Trp Leu His	0.05980936
gene-MST1	1.042433854	CL(8:0/8:0/18:2(9Z,11Z)/20:0)	0.622988418
gene-CEP112	1.040454203	S-(Indolylmethylthiohydroximoyl)-L-cysteine	0.635042992
gene-CEP112	1.040454203	Sarcodon scabrosus Depsipeptide	1.433355295
gene-CEP112	1.040454203	Thr His Phe Lys	2.161400139
gene-CEP112	1.040454203	N-Arachidonoyl Glutamic acid	0.496582621
gene-CEP112	1.040454203	N-Docosahexaenoyl Histidine	2.27720447
gene-CEP112	1.040454203	CL(10:0/10:0/10:0/11:0)	3.345075651
gene-CEP112	1.040454203	Cyclolinopeptide A	3.629188379
gene-CEP112	1.040454203	Ergocornine	1.065772597
gene-CEP112	1.040454203	Chlorophyllide	1.469089583
gene-CEP112	1.040454203	Carmofur	0.625889788
gene-CEP112	1.040454203	Apramycin	0.867064958
gene-CEP112	1.040454203	Palmitoyl Ara-C	0.586890476
gene-CEP112	1.040454203	Mupirocin	0.952072991
gene-CEP112	1.040454203	Methyl (2E)-2-(10,13-dimethyl-11-oxo-3-py	0.478588158
gene-CEP112	1.040454203	Tauroursodeoxycholic acid	1.430369356
gene-CEP112	1.040454203	2-Amino-N-[1-[[2-[[1-(2-hydroxyethylaminc	2.211846428
gene-CEP112	1.040454203	(3Z)-Phytochromobilin	0.67355307
gene-CEP112	1.040454203	PS(22:2(13Z,16Z)/LTE4)	2.431248895
gene-CEP112	1.040454203	PGP(20:1(11Z)/18:1(12Z)-2OH(9,10))	0.042651731
gene-CEP112	1.040454203	Glyuranolide	0.347923566
gene-CEP112	1.040454203	1-Octadecanoyl-sn-glycero-3-phosphoetha	0.318054581
gene-CEP112	1.040454203	Didemnin A	1.039530987
gene-CEP112	1.040454203	His Leu Ser Lys	0.708465111
gene-CEP112	1.040454203	13(S)-HpODE	0.163157753
gene-CEP112	1.040454203	LTB4-d4	0.226799982

gene-CEP112	1.040454203	(-)-alpha-Terpineol	0.345716279
gene-CEP112	1.040454203	PG(20:1(11Z)/18:3(10,12,15)-OH(9))	0.626244347
gene-CEP112	1.040454203	15H-11,12-EETA	1.71018828
gene-CEP112	1.040454203	Cytochalasin B	1.792662701
gene-CEP112	1.040454203	Taurocholate	2.056231295
gene-CEP112	1.040454203	2-Methyl-3-phenyl-2-propenal	0.407813975
gene-CEP112	1.040454203	Cerulenin	1.323019945
gene-CEP112	1.040454203	16-Ketoestradiol	0.560408527
gene-CEP112	1.040454203	Tetradecyl nicotinate	0.517405648
gene-CEP112	1.040454203	Docosahexaenoyl Ethanolamide	0.538090182
gene-CEP112	1.040454203	Antibiotic SB 202742	0.493908094
gene-CEP112	1.040454203	Glycocholic acid hydrate	0.476089117
gene-CEP112	1.040454203	Taurodeoxycholic acid	1.76790666
gene-CEP112	1.040454203	5尾-CHOLANIC ACID-3伪, 12伪-DIOL N-(2-	1.135007087
gene-CEP112	1.040454203	KIRENOL	0.568611097
gene-CEP112	1.040454203	24,24-Difluoro-1alpha,25-dihydroxyvitamin [	1.131590894
gene-CEP112	1.040454203	Sodium glycochenodeoxycholate	0.612043552
gene-CEP112	1.040454203	Androsterone glucuronide	1.164397007
gene-CEP112	1.040454203	PC(P-18:1(11Z)/PGE2)	0.509503133
gene-CEP112	1.040454203	Phorone A	0.079039095
gene-CEP112	1.040454203	PC(P-18:1(11Z)/PGE1)	0.295520345
gene-CEP112	1.040454203	(9Z)-Octadecenoic acid	0.142359556
Bos_taurus_newG	1.039612934	(-)-alpha-Terpineol	0.345716279
Bos_taurus_newG	1.039612934	Cyclosporin A	0.656529229
Bos_taurus_newG	1.039612934	PC(P-18:1(11Z)/PGE2)	0.509503133
Bos_taurus_newG	1.039612934	PC(20:3(5Z,8Z,11Z)/24:0)	0.387959564
Bos_taurus_newG	1.039612934	CL(8:0/8:0/18:2(9Z,11Z)/20:0)	0.622988418
gene-NRADD	1.039181606	D-Erythro-imidazole-glycerol-phosphate	0.355812431
gene-NRADD	1.039181606	LTB4-d4	0.226799982
gene-NRADD	1.039181606	cis-p-Menth-2-en-1-ol	0.201017988
gene-NRADD	1.039181606	Cyclosporin A	0.656529229
gene-NRADD	1.039181606	PC(P-18:1(11Z)/PGE2)	0.509503133
gene-NRADD	1.039181606	Nigroxanthin	0.705005059
gene-NRADD	1.039181606	PC(P-18:1(11Z)/PGJ2)	0.565243877
gene-NRADD	1.039181606	PS(20:0/20:4(8Z,11Z,14Z,17Z)-2OH(5S,6R))	0.402595921
gene-NRADD	1.039181606	CL(8:0/8:0/18:2(9Z,11Z)/20:0)	0.622988418
gene-PET100	1.037032096	5-Fluorouridine diphosphate	0.151761582
gene-PET100	1.037032096	5-(Ethylthio)-1H-tetrazole	0.29717344
gene-PET100	1.037032096	L-Valine, N-(2-hydroxy-3-butenyl)-	0.253583227
gene-PET100	1.037032096	all-trans-Hexaprenyl diphosphate	0.277689133
gene-PET100	1.037032096	[(1R,5R)-5-(6-Aminopurin-9-yl)cyclohex-3-e	0.021741756
gene-PET100	1.037032096	Tryptamine	0.044784028
gene-PET100	1.037032096	L-Tryptophan	0.063429384
gene-PET100	1.037032096	Thiophene-4,5-epoxide	0.018208639
gene-PET100	1.037032096	Canesceol	0.686312122
gene-PET100	1.037032096	16-hydroxy hexadecanoic acid	0.309000958
gene-PET100	1.037032096	Psychosine	0.106475396
gene-PET100	1.037032096	Linoleamide	0.926003481
gene-PET100	1.037032096	(R)-2-(4-(Tert-butoxycarbonyl)morpholin-3-	0.021193143
gene-PET100	1.037032096	9-(trans-4-Hydroxy-2-buten-1-yl)adenine	0.135307987
gene-PET100	1.037032096	2-Methoxy-4-vinylphenol	0.111453556

gene-PET100	1.037032096	5-[2H-Pyrrol-4-(3H)-ylidenemethyl]-2-furar	0.063836335
gene-PET100	1.037032096	Trp Gly Phe	0.392157812
gene-PET100	1.037032096	(1aalpha,2beta,3alpha,11calpha)-1a,2,3,11c-	0.12284867
gene-PET100	1.037032096	Trimetazidine	0.438282534
gene-PET100	1.037032096	ingenol	0.942071652
gene-PET100	1.037032096	Dihydroethidium	5.075390639
gene-PET100	1.037032096	Tyrosyl-Arginine	6.462631098
gene-PET100	1.037032096	Misoprostol	1.349270093
gene-PET100	1.037032096	Pluviatolide	1.068135579
gene-PET100	1.037032096	Carboprost methyl	1.74923433
gene-PET100	1.037032096	11-Dehydro-2,3-dinor-txb2	0.307258797
gene-PET100	1.037032096	Phantasmidine	0.044283869
gene-PET100	1.037032096	1-Palmitoylglycerol	0.084357408
gene-PET100	1.037032096	N2-gamma-Glutamylglutamine	0.230065499
gene-PET100	1.037032096	Glutethimide	0.126237229
gene-PET100	1.037032096	2-Amino-9-[4-hydroxy-2-(hydroxymethyl)bı	0.390342101
gene-PET100	1.037032096	5-Hydroxycapsanthin-5,6-epoxide	0.643327433
gene-PET100	1.037032096	PA(13:0/18:1(9Z)-O(12,13))	0.638080351
gene-PET100	1.037032096	B-Octylglucoside	0.163689453
gene-PET100	1.037032096	Leupeptin	0.307507603
gene-PET100	1.037032096	Antcin K	0.46373152
gene-PET100	1.037032096	Notoginsenoside R10	0.150329537
gene-PET100	1.037032096	(E)-5-(3,4,5,6-Tetrahydro-3-pyridylidenemet	0.065093771
gene-PET100	1.037032096	1-Methylnicotinamide	0.203956241
gene-PET100	1.037032096	4-[(2,4-Dihydroxy-3,3-dimethylbutanoyl)ami	0.207947066
gene-PET100	1.037032096	(2S)-1-[2-[[(2S)-Pyrrolidine-2-carbonyl]amir	0.147809994
gene-PET100	1.037032096	4-Amino-1-[(1S,2S,4S)-4,5-bis(hydroxymeth	0.109716854
gene-HAGHL	1.036833279	3-Thiacytidine	0.209387412
gene-HAGHL	1.036833279	Hygromycin B	0.930396619
gene-HAGHL	1.036833279	Metkephamid	1.159767003
gene-HAGHL	1.036833279	11-Maleimidoundecanoic acid	1.084942397
gene-HAGHL	1.036833279	4-Gingerol	0.222165012
gene-HAGHL	1.036833279	Docosanamide	0.509975786
gene-HAGHL	1.036833279	Monacolin L acid	1.060562098
gene-HAGHL	1.036833279	LTB4-d4	0.226799982
gene-HAGHL	1.036833279	cis-p-Menth-2-en-1-ol	0.201017988
gene-HAGHL	1.036833279	PE(20:0/18:1(12Z)-2OH(9,10))	0.438658253
gene-HAGHL	1.036833279	PE(22:2(13Z,16Z)/22:5(4Z,7Z,10Z,13Z,19Z)-O	0.369745166
gene-HAGHL	1.036833279	Lamivudine	0.327402892
gene-HAGHL	1.036833279	Pseudouridine 5'-phosphate	1.18431378
gene-HAGHL	1.036833279	15-keto-Prostaglandin E2	2.646629404
gene-HAGHL	1.036833279	Ribavirin monophosphate	1.091085027
gene-HAGHL	1.036833279	Norophthalmic acid	0.191432411
gene-HAGHL	1.036833279	3'-N'-Acetylfusarochromanone	0.755683206
gene-HAGHL	1.036833279	Ser Cys Ala Ala	0.603032447
gene-HAGHL	1.036833279	Loganin	0.92782666
gene-HAGHL	1.036833279	3'-Deoxythymidine	0.791830758
gene-HAGHL	1.036833279	Gly Asp Ala Ala	0.718656316
gene-HAGHL	1.036833279	ethyl 2-cyano-3-(1h-indol-3-yl)prop-2-eno	0.756861034
gene-HAGHL	1.036833279	kainic acid	0.836445456
gene-HAGHL	1.036833279	Zanamivir	0.839205907

gene-HAGHL	1.036833279	Tryptophyl-Glutamine	0.89722186
gene-HAGHL	1.036833279	Aminoglutethimide	0.652616628
gene-HAGHL	1.036833279	5,6,7,8-Tetrahydromonapterin	0.724075531
gene-HAGHL	1.036833279	2-Methyl-3-phenyl-2-propenal	0.407813975
gene-HAGHL	1.036833279	Guanidoacetic acid	0.542509265
gene-HAGHL	1.036833279	4-Oxo-9-cis-retinoyl-beta-glucuronide	1.611773742
gene-HAGHL	1.036833279	alpha-Terpineol formate	0.628238986
gene-HAGHL	1.036833279	4-Dihydroboldenone	0.692863202
gene-HAGHL	1.036833279	2'-Fluoro-2',3'-dideoxyinosine	1.214745496
gene-HAGHL	1.036833279	20-carboxy Arachidonic Acid	0.780988382
gene-HAGHL	1.036833279	Nebramycin 5'	0.909280571
gene-HAGHL	1.036833279	Apronal	0.754033426
gene-HAGHL	1.036833279	Methionyl-Valine	0.502707745
gene-HAGHL	1.036833279	4-Octylphenol	1.400657885
gene-HAGHL	1.036833279	Prolyl-Lysine	1.612991304
gene-HAGHL	1.036833279	3-Pentadecylphenol	1.682258188
gene-HAGHL	1.036833279	10-alpha-methoxy-9,10-dihydrolysergol	0.140851438
gene-HAGHL	1.036833279	MG(0:0/20:3(11Z,14Z,17Z)/0:0)	0.844949524
gene-HAGHL	1.036833279	N-Stearoyl Glutamine	1.862695384
gene-HAGHL	1.036833279	Palmitoylcarnitine	0.74779616
gene-HAGHL	1.036833279	PC(P-18:1(11Z)/PGE2)	0.509503133
gene-HAGHL	1.036833279	Galabiosylceramide (d18:1/20:0)	0.746737323
gene-HAGHL	1.036833279	LysoPI(0:0/18:0)	0.420441153
gene-HAGHL	1.036833279	12-Hydroxyicosanoylcarnitine	1.730979067
gene-HAGHL	1.036833279	N-Myristoyl Glutamine	0.37856966
gene-HAGHL	1.036833279	PC(18:1(9Z)/15:1(9Z))	0.534724682
gene-HAGHL	1.036833279	Nigroxanthin	0.705005059
gene-HAGHL	1.036833279	PC(P-18:1(11Z)/PGJ2)	0.565243877
gene-HAGHL	1.036833279	PS(20:0/20:4(8Z,11Z,14Z,17Z)-2OH(5S,6R))	0.402595921
gene-HAGHL	1.036833279	PE(20:5(5Z,8Z,11Z,14Z,17Z)/18:0)	0.427962506
gene-HAGHL	1.036833279	PS(16:1(9Z)/22:2(13Z,16Z))	0.659652661
gene-HAGHL	1.036833279	PC(P-16:0/20:3(8Z,11Z,14Z)-2OH(5,6))	0.174122169
Bos_taurus_newG	1.036823793	S-(2-(N,N-Diisopropylamino)ethyl)isothioure	0.479488177
Bos_taurus_newG	1.036823793	CL(10:0/10:0/10:0/11:0)	3.345075651
Bos_taurus_newG	1.036823793	Cyclolinopeptide A	3.629188379
Bos_taurus_newG	1.036823793	Matesaponin 3	2.228695487
Bos_taurus_newG	1.036823793	(3Z)-Phytochromobilin	0.67355307
Bos_taurus_newG	1.036823793	PS(22:2(13Z,16Z)/LTE4)	2.431248895
Bos_taurus_newG	1.036823793	Glyuranolide	0.347923566
Bos_taurus_newG	1.036823793	1-Octadecanoyl-sn-glycero-3-phosphoetha	0.318054581
Bos_taurus_newG	1.036823793	Didemnin A	1.039530987
Bos_taurus_newG	1.036823793	13(S)-HpODE	0.163157753
Bos_taurus_newG	1.036823793	LTB4-d4	0.226799982
Bos_taurus_newG	1.036823793	(-)-alpha-Terpineol	0.345716279
Bos_taurus_newG	1.036823793	2-Methyl-3-phenyl-2-propenal	0.407813975
Bos_taurus_newG	1.036823793	KIRENOL	0.568611097
Bos_taurus_newG	1.036823793	Sodium glycochenodeoxycholate	0.612043552
Bos_taurus_newG	1.036823793	4-Hydroxy-3-methyl-2-(2-propynyl)-2-cycl	0.120742395
Bos_taurus_newG	1.036823793	PC(P-18:1(11Z)/PGE2)	0.509503133
Bos_taurus_newG	1.036823793	(3R,4R)-3-Amino-1-hydroxy-4-methylpyrrol	0.471914506
Bos_taurus_newG	1.036823793	PC(P-18:1(11Z)/PGE1)	0.295520345

Bos_taurus_newG	1.036823793	(9Z)-Octadecenoic acid	0.142359556
Bos_taurus_newG	1.036823793	2-Propenyl 2-aminobenzoate	0.128406829
gene-VWA2	1.036045261	D-Erythro-imidazole-glycerol-phosphate	0.355812431
gene-VWA2	1.036045261	Erythronic acid	0.494007482
gene-VWA2	1.036045261	LTB4-d4	0.226799982
gene-VWA2	1.036045261	cis-p-Menth-2-en-1-ol	0.201017988
gene-VWA2	1.036045261	L-Carnitine	0.2737116
gene-VWA2	1.036045261	PG(20:1(11Z)/18:3(10,12,15)-OH(9))	0.626244347
gene-VWA2	1.036045261	2-Methyl-3-phenyl-2-propenal	0.407813975
gene-VWA2	1.036045261	4-Octylphenol	1.400657885
gene-VWA2	1.036045261	Ajulemic acid	0.557832951
gene-VWA2	1.036045261	PC(P-18:1(11Z)/PGE2)	0.509503133
gene-VWA2	1.036045261	(3R,4R)-3-Amino-1-hydroxy-4-methylpyrrol	0.471914506
gene-VWA2	1.036045261	MG(0:0/18:1(9Z)/0:0)	0.361035605
gene-VWA2	1.036045261	1,2,4-Nonadecanetriol	0.249655005
gene-VWA2	1.036045261	N-[2-(3,4-Dihydroxyphenyl)ethyl]icosa-5,8,1	0.326021565
gene-VWA2	1.036045261	N-Myristoyl Glutamine	0.37856966
gene-CYB561A3	1.034596007	D-Erythro-imidazole-glycerol-phosphate	0.355812431
gene-CYB561A3	1.034596007	3-Deoxyestrone	0.282221709
gene-CYB561A3	1.034596007	Lividamine	0.319679555
gene-CYB561A3	1.034596007	cis-p-Menth-2-en-1-ol	0.201017988
gene-CYB561A3	1.034596007	(-)-alpha-Terpineol	0.345716279
gene-CYB561A3	1.034596007	PG(20:1(11Z)/18:3(10,12,15)-OH(9))	0.626244347
gene-CYB561A3	1.034596007	4-Dimethylamino-L-phenylalanine	0.242110226
gene-CYB561A3	1.034596007	Armillane	0.52808635
gene-CYB561A3	1.034596007	PC(P-18:1(11Z)/PGE2)	0.509503133
gene-CYB561A3	1.034596007	(3R,4R)-3-Amino-1-hydroxy-4-methylpyrrol	0.471914506
gene-CYB561A3	1.034596007	n-methyl-2-(4'-methylaminophenyl)-6-hydr	0.26655714
gene-CYB561A3	1.034596007	9-deoxy-9-methylene-16,16-dimethyl -PGE	0.606893884
gene-CYB561A3	1.034596007	CL(8:0/8:0/18:2(9Z,11Z)/20:0)	0.622988418
gene-HOMER3	1.03451783	D-Erythro-imidazole-glycerol-phosphate	0.355812431
gene-HOMER3	1.03451783	Cyclosporin A	0.656529229
gene-HOMER3	1.03451783	PC(P-18:1(11Z)/PGE2)	0.509503133
gene-HOMER3	1.03451783	PC(P-18:1(11Z)/PGJ2)	0.565243877
gene-HOMER3	1.03451783	CL(8:0/8:0/18:2(9Z,11Z)/20:0)	0.622988418
gene-ACOT7	1.03438152	3-Thiacytidine	0.209387412
gene-ACOT7	1.03438152	D-Erythro-imidazole-glycerol-phosphate	0.355812431
gene-ACOT7	1.03438152	LTB4-d4	0.226799982
gene-ACOT7	1.03438152	cis-p-Menth-2-en-1-ol	0.201017988
gene-ACOT7	1.03438152	(-)-alpha-Terpineol	0.345716279
gene-ACOT7	1.03438152	Cyclosporin A	0.656529229
gene-ACOT7	1.03438152	5-(2-Aminopropyl)-2-methylphenol	0.323231457
gene-ACOT7	1.03438152	PC(P-18:1(11Z)/PGE2)	0.509503133
gene-ACOT7	1.03438152	(9Z)-Octadecenoic acid	0.142359556
gene-ACOT7	1.03438152	Nigroxanthin	0.705005059
gene-ACOT7	1.03438152	PC(P-18:1(11Z)/PGJ2)	0.565243877
gene-ACOT7	1.03438152	PS(20:0/20:4(8Z,11Z,14Z,17Z)-2OH(5S,6R))	0.402595921
gene-ACOT7	1.03438152	CL(8:0/8:0/18:2(9Z,11Z)/20:0)	0.622988418
gene-RIMS3	1.034081562	milbemycin beta3	1.414447124
gene-RIMS3	1.034081562	Tetracosenoyl-CoA	0.187187233
gene-RIMS3	1.034081562	Gentiobioside	0.022114549

gene-RIMS3	1.034081562	Val-Tyr-Leu-Arg	0.040793217
Bos_taurus_newG	1.031804398	Phytosphingosine	0.393831878
Bos_taurus_newG	1.031804398	Norophthalmic acid	0.191432411
Bos_taurus_newG	1.031804398	3'-N'-Acetylfusarochromanone	0.755683206
Bos_taurus_newG	1.031804398	Ser Cys Ala Ala	0.603032447
Bos_taurus_newG	1.031804398	Tryptophyl-Glutamine	0.89722186
Bos_taurus_newG	1.031804398	Guanidoacetic acid	0.542509265
Bos_taurus_newG	1.031804398	Policapram	0.598028597
Bos_taurus_newG	1.031804398	4-Oxo-9-cis-retinoyl-beta-glucuronide	1.611773742
Bos_taurus_newG	1.031804398	alpha-Terpineol formate	0.628238986
Bos_taurus_newG	1.031804398	4-Octylphenol	1.400657885
Bos_taurus_newG	1.031804398	PC(P-18:1(11Z)/PGE2)	0.509503133
Bos_taurus_newG	1.031804398	PC(P-18:1(11Z)/PGJ2)	0.565243877
Bos_taurus_newG	1.031804398	PE(20:5(5Z,8Z,11Z,14Z,17Z)/18:0)	0.427962506
Bos_taurus_newG	1.031804398	PS(16:1(9Z)/22:2(13Z,16Z))	0.659652661
Bos_taurus_newG	1.031804398	PC(P-16:0/20:5(5Z,8Z,11Z,14Z,16E)-OH(18R))	0.26121581
Bos_taurus_newG	1.031804398	PC(P-16:0/20:3(8Z,11Z,14Z)-2OH(5,6))	0.174122169
gene-SFI1	1.031135885	3-Thiacytidine	0.209387412
gene-SFI1	1.031135885	D-Erythro-imidazole-glycerol-phosphate	0.355812431
gene-SFI1	1.031135885	13(S)-HpODE	0.163157753
gene-SFI1	1.031135885	LTB4-d4	0.226799982
gene-SFI1	1.031135885	Cyclosporin A	0.656529229
gene-SFI1	1.031135885	beta-L-Dioxolane-cytidine	0.175916668
gene-SFI1	1.031135885	PC(P-18:1(11Z)/PGE2)	0.509503133
gene-SFI1	1.031135885	Nigroxanthin	0.705005059
gene-SFI1	1.031135885	PC(P-18:1(11Z)/PGJ2)	0.565243877
gene-SFI1	1.031135885	PS(20:0/20:4(8Z,11Z,14Z,17Z)-2OH(5S,6R))	0.402595921
gene-ARAP3	1.030759551	D-Erythro-imidazole-glycerol-phosphate	0.355812431
gene-ARAP3	1.030759551	3-Deoxyestrone	0.282221709
gene-ARAP3	1.030759551	cis-p-Menth-2-en-1-ol	0.201017988
gene-ARAP3	1.030759551	(-)-alpha-Terpineol	0.345716279
gene-ARAP3	1.030759551	PG(20:1(11Z)/18:3(10,12,15)-OH(9))	0.626244347
gene-ARAP3	1.030759551	Armillane	0.52808635
gene-ARAP3	1.030759551	PC(P-18:1(11Z)/PGE2)	0.509503133
gene-ARAP3	1.030759551	n-methyl-2-(4'-methylaminophenyl)-6-hydr	0.26655714
gene-ARAP3	1.030759551	PS(20:0/20:4(8Z,11Z,14Z,17Z)-2OH(5S,6R))	0.402595921
gene-ARAP3	1.030759551	CL(8:0/8:0/18:2(9Z,11Z)/20:0)	0.622988418
gene-SELENBP1	1.030666269	3-Thiacytidine	0.209387412
gene-SELENBP1	1.030666269	5-Hydroxy-2-oxo-4-ureido-2,5-dihydro-1H	0.122536033
gene-SELENBP1	1.030666269	milbemycin beta3	1.414447124
gene-SELENBP1	1.030666269	Palmitoyl Ara-C	0.586890476
gene-SELENBP1	1.030666269	PE(20:0/18:1(12Z)-2OH(9,10))	0.438658253
gene-SELENBP1	1.030666269	4,6-Heneicosanedione	0.113496143
gene-SELENBP1	1.030666269	PE(22:2(13Z,16Z)/22:5(4Z,7Z,10Z,13Z,19Z)-O	0.369745166
gene-SELENBP1	1.030666269	Epomusenin A	0.766206049
gene-SELENBP1	1.030666269	PG(20:1(11Z)/18:3(10,12,15)-OH(9))	0.626244347
gene-SELENBP1	1.030666269	CDP-DG(PGF2alpha/16:0)	1.002512277
gene-SELENBP1	1.030666269	5-Formiminotetrahydrofolate	0.118251287
gene-SELENBP1	1.030666269	Benzoyl glucuronide (Benzoic acid)	0.442844673
gene-SELENBP1	1.030666269	C20914	0.140383181
gene-SELENBP1	1.030666269	Asparagine-betaxanthin	0.103826013

gene-SELENBP1	1.030666269	Methionyl-Valine	0.502707745
gene-SELENBP1	1.030666269	Phenol	0.097390283
gene-SELENBP1	1.030666269	Phorone A	0.079039095
gene-SELENBP1	1.030666269	N-Palmitoyl Glutamic acid	0.250188721
gene-SELENBP1	1.030666269	(9Z)-Octadecenoic acid	0.142359556
gene-SELENBP1	1.030666269	Nigroxanthin	0.705005059
gene-SELENBP1	1.030666269	PC(P-18:1(11Z)/PGJ2)	0.565243877
gene-SELENBP1	1.030666269	PS(20:0/20:4(8Z,11Z,14Z,17Z)-2OH(5S,6R))	0.402595921
gene-HSF4	1.03000429	Dyphylline	0.664549753
gene-HSF4	1.03000429	4-Hydroxyoctanedioylcarnitine	1.002008817
gene-HSF4	1.03000429	Ribavirin monophosphate	1.091085027
gene-HSF4	1.03000429	Terbutryn	1.186241428
gene-HSF4	1.03000429	4-Oxo-9-cis-retinoyl-beta-glucuronide	1.611773742
gene-HSF4	1.03000429	alpha-Terpineol formate	0.628238986
gene-HSF4	1.03000429	4-Octylphenol	1.400657885
gene-HSF4	1.03000429	Milbemycin D	0.125637998
gene-HSF4	1.03000429	Ascorbic acid 6-palmitate	0.201627014
gene-HSF4	1.03000429	Sitosterol beta-D-glucoside	0.382944796
gene-HSF4	1.03000429	PC(P-18:1(11Z)/PGJ2)	0.565243877
gene-HSF4	1.03000429	PS(20:0/20:4(8Z,11Z,14Z,17Z)-2OH(5S,6R))	0.402595921
gene-HSF4	1.03000429	Galactosylglycerol	0.738463143
gene-HSF4	1.03000429	PE(20:5(5Z,8Z,11Z,14Z,17Z)/18:0)	0.427962506
gene-NMUR2	1.029550474	(Z)-[(4-hydroxyphenyl)acetaldehyde oxime]	0.158764133
gene-NMUR2	1.029550474	2-((3-Aminopyridin-2-yl)methylene)hydrazin	0.170153323
gene-NMUR2	1.029550474	Rumenic acid	0.306646116
gene-NOTCH1	1.029536367	D-Erythro-imidazole-glycerol-phosphate	0.355812431
gene-NOTCH1	1.029536367	cis-p-Menth-2-en-1-ol	0.201017988
gene-NOTCH1	1.029536367	(-)-alpha-Terpineol	0.345716279
gene-NOTCH1	1.029536367	PG(20:1(11Z)/18:3(10,12,15)-OH(9))	0.626244347
gene-NOTCH1	1.029536367	4-Dimethylamino-L-phenylalanine	0.242110226
gene-NOTCH1	1.029536367	PC(P-18:1(11Z)/PGE2)	0.509503133
gene-NOTCH1	1.029536367	CL(8:0/8:0/18:2(9Z,11Z)/20:0)	0.622988418
Bos_taurus_newG	1.029127488	5-(3'-Carboxy-3'-oxopropenyl)-4,6-dihydro›	1.083232395
Bos_taurus_newG	1.029127488	D-Erythro-imidazole-glycerol-phosphate	0.355812431
Bos_taurus_newG	1.029127488	Val-Cit	0.452308451
Bos_taurus_newG	1.029127488	11-Maleimidoundecanoic acid	1.084942397
Bos_taurus_newG	1.029127488	4-Gingerol	0.222165012
Bos_taurus_newG	1.029127488	Monacolin L acid	1.060562098
Bos_taurus_newG	1.029127488	cis-p-Menth-2-en-1-ol	0.201017988
Bos_taurus_newG	1.029127488	PE(20:0/18:1(12Z)-2OH(9,10))	0.438658253
Bos_taurus_newG	1.029127488	PE(22:2(13Z,16Z)/22:5(4Z,7Z,10Z,13Z,19Z)-O	0.369745166
Bos_taurus_newG	1.029127488	Lamivudine	0.327402892
Bos_taurus_newG	1.029127488	Norophthalmic acid	0.191432411
Bos_taurus_newG	1.029127488	(1R,6S)-6-Amino-5-oxocyclohex-2-ene-1-c	0.154751123
Bos_taurus_newG	1.029127488	3'-N'-Acetylfusarochromanone	0.755683206
Bos_taurus_newG	1.029127488	Tryptophyl-Glutamine	0.89722186
Bos_taurus_newG	1.029127488	2-Methyl-3-phenyl-2-propenal	0.407813975
Bos_taurus_newG	1.029127488	Guanidoacetic acid	0.542509265
Bos_taurus_newG	1.029127488	alpha-Terpineol formate	0.628238986
Bos_taurus_newG	1.029127488	4-Octylphenol	1.400657885
Bos_taurus_newG	1.029127488	Prolyl-Lysine	1.612991304

Bos_taurus_newG	1.029127488	PC(P-18:1(11Z)/PGE2)	0.509503133
Bos_taurus_newG	1.029127488	12-Hydroxyicosanoylcarnitine	1.730979067
Bos_taurus_newG	1.029127488	Roxithromycin	0.268273077
Bos_taurus_newG	1.029127488	PC(18:1(9Z)/15:1(9Z))	0.534724682
Bos_taurus_newG	1.029127488	PC(P-18:1(11Z)/PGJ2)	0.565243877
Bos_taurus_newG	1.029127488	PS(20:0/20:4(8Z,11Z,14Z,17Z)-2OH(5S,6R))	0.402595921
Bos_taurus_newG	1.029127488	PE(20:5(5Z,8Z,11Z,14Z,17Z)/18:0)	0.427962506
gene-PGLYRP4	1.027652333	N-Docosahexaenoyl Histidine	2.27720447
gene-PGLYRP4	1.027652333	Mupirocin	0.952072991
gene-PGLYRP4	1.027652333	Methyl (2E)-2-(10,13-dimethyl-11-oxo-3-py	0.478588158
gene-PGLYRP4	1.027652333	Tauroursodeoxycholic acid	1.430369356
gene-PGLYRP4	1.027652333	PGP(20:1(11Z)/18:1(12Z)-2OH(9,10))	0.042651731
gene-PGLYRP4	1.027652333	His Leu Ser Lys	0.708465111
gene-PGLYRP4	1.027652333	7(S),17(S)-dihydroxy-8(E),10(Z),13(Z),15(E),19	0.32147403
gene-PGLYRP4	1.027652333	LTB4-d4	0.226799982
gene-PGLYRP4	1.027652333	1,2,3,4-Tetrahydroisoquinoline-3-carboxylic	0.339248702
gene-PGLYRP4	1.027652333	Cerulenin	1.323019945
gene-PGLYRP4	1.027652333	Vulgarone A	0.102049407
gene-PGLYRP4	1.027652333	Sophoranol	0.282064826
gene-PGLYRP4	1.027652333	L-Cysteine	0.214540754
gene-PGLYRP4	1.027652333	Methyl 4,7,10,13,16-docosapentaenoate	0.204170362
gene-PGLYRP4	1.027652333	Prostaglandin PGE2 1-glyceryl ester	0.007411313
gene-PGLYRP4	1.027652333	7-Sulfocholic acid	0.039985455
gene-PGLYRP4	1.027652333	1,8-Octanedithiol	0.133094504
gene-PGLYRP4	1.027652333	24,24-Difluoro-1alpha,25-dihydroxyvitamin I	1.131590894
gene-PGLYRP4	1.027652333	Sodium glycochenodeoxycholate	0.612043552
gene-PGLYRP4	1.027652333	(4Z,7Z,10Z,13Z,16Z,19Z)-Docosahexaenoic a	0.434211486
gene-PGLYRP4	1.027652333	PC(P-18:1(11Z)/PGE2)	0.509503133
gene-PGLYRP4	1.027652333	TRIMOPROSTIL	0.000919109
gene-PGLYRP4	1.027652333	Phorone A	0.079039095
gene-GPAA1	1.027180897	3-Thiacytidine	0.209387412
gene-GPAA1	1.027180897	D-Erythro-imidazole-glycerol-phosphate	0.355812431
gene-GPAA1	1.027180897	LTB4-d4	0.226799982
gene-GPAA1	1.027180897	cis-p-Menth-2-en-1-ol	0.201017988
gene-GPAA1	1.027180897	(-)-alpha-Terpineol	0.345716279
gene-GPAA1	1.027180897	PG(20:1(11Z)/18:3(10,12,15)-OH(9))	0.626244347
gene-GPAA1	1.027180897	4-Dimethylamino-L-phenylalanine	0.242110226
gene-GPAA1	1.027180897	2-Methyl-3-phenyl-2-propenal	0.407813975
gene-GPAA1	1.027180897	PC(P-18:1(11Z)/PGE2)	0.509503133
gene-GPAA1	1.027180897	(3R,4R)-3-Amino-1-hydroxy-4-methylpyrrol	0.471914506
gene-GPAA1	1.027180897	PC(P-18:1(11Z)/PGJ2)	0.565243877
gene-GPAA1	1.027180897	PS(20:0/20:4(8Z,11Z,14Z,17Z)-2OH(5S,6R))	0.402595921
gene-GPAA1	1.027180897	CL(8:0/8:0/18:2(9Z,11Z)/20:0)	0.622988418
gene-GPI	1.026824101	3-Thiacytidine	0.209387412
gene-GPI	1.026824101	3-Deoxyestrone	0.282221709
gene-GPI	1.026824101	1-Oleoyl-sn-glycero-3-phosphocholine	0.18926952
gene-GPI	1.026824101	(-)-alpha-Terpineol	0.345716279
gene-GPI	1.026824101	1,4-Undecadiene	0.462803973
gene-GPI	1.026824101	4-cholesten-7伪,12伪,24-triol-3-one	0.097840451
gene-GPI	1.026824101	5-(2-Aminopropyl)-2-methylphenol	0.323231457
gene-GPI	1.026824101	4-Dimethylamino-L-phenylalanine	0.242110226

gene-GPI	1.026824101	ingenol	0.942071652
gene-GPI	1.026824101	Armillane	0.52808635
gene-GPI	1.026824101	PC(P-18:1(11Z)/PGE2)	0.509503133
gene-GPI	1.026824101	(3R,4R)-3-Amino-1-hydroxy-4-methylpyrrol	0.471914506
gene-GPI	1.026824101	n-methyl-2-(4'-methylaminophenyl)-6-hydr	0.26655714
gene-GPI	1.026824101	Isopropyl isothiocyanate	0.172385747
gene-GPI	1.026824101	9-deoxy-9-methylene-16,16-dimethyl -PGE	0.606893884
gene-GPI	1.026824101	PS(20:0/20:4(8Z,11Z,14Z,17Z)-2OH(5S,6R))	0.402595921
gene-GPI	1.026824101	CL(8:0/8:0/18:2(9Z,11Z)/20:0)	0.622988418
gene-MAN2B1	1.026198878	3-Thiacytidine	0.209387412
gene-MAN2B1	1.026198878	D-Erythro-imidazole-glycerol-phosphate	0.355812431
gene-MAN2B1	1.026198878	LTB4-d4	0.226799982
gene-MAN2B1	1.026198878	cis-p-Menth-2-en-1-ol	0.201017988
gene-MAN2B1	1.026198878	(-)-alpha-Terpineol	0.345716279
gene-MAN2B1	1.026198878	Cyclosporin A	0.656529229
gene-MAN2B1	1.026198878	5-(2-Aminopropyl)-2-methylphenol	0.323231457
gene-MAN2B1	1.026198878	Armillane	0.52808635
gene-MAN2B1	1.026198878	PC(P-18:1(11Z)/PGE2)	0.509503133
gene-MAN2B1	1.026198878	Isopropyl isothiocyanate	0.172385747
gene-MAN2B1	1.026198878	Nigroxanthin	0.705005059
gene-MAN2B1	1.026198878	PC(P-18:1(11Z)/PGJ2)	0.565243877
gene-MAN2B1	1.026198878	PS(20:0/20:4(8Z,11Z,14Z,17Z)-2OH(5S,6R))	0.402595921
gene-MAN2B1	1.026198878	CL(8:0/8:0/18:2(9Z,11Z)/20:0)	0.622988418
gene-NNAT	1.026115643	Cholic acid glucuronide	0.147398811
gene-NNAT	1.026115643	Methyl (2E)-2-(10,13-dimethyl-11-oxo-3-py	0.478588158
gene-NNAT	1.026115643	Urocortisol	0.926998516
gene-NNAT	1.026115643	Epothilone D	0.794619023
gene-NNAT	1.026115643	2-Ethyl-5-methyl-3,3-diphenyl-1-pyrroline	0.826978858
gene-NNAT	1.026115643	11-Maleimidoundecanoic acid	1.084942397
gene-NNAT	1.026115643	Becocalcidiol	0.63162902
gene-NNAT	1.026115643	Monacolin L acid	1.060562098
gene-NNAT	1.026115643	cis-p-Menth-2-en-1-ol	0.201017988
gene-NNAT	1.026115643	PC(24:0/22:6(4Z,7Z,10Z,12E,16Z,19Z)-OH(14)	0.188241061
gene-NNAT	1.026115643	S-Acetyldihydrolipoamide-E	0.429561283
gene-NNAT	1.026115643	3'-N'-Acetylfusarochromanone	0.755683206
gene-NNAT	1.026115643	Indole-3-ethanol	0.096523128
gene-NNAT	1.026115643	N-(3-oxo-octanoyl)-homoserine lactone	0.106580775
gene-NNAT	1.026115643	n6-[2-(4-Aminophenyl)ethyl]adenosine	0.45059876
gene-NNAT	1.026115643	Tryptophyl-Glutamine	0.89722186
gene-NNAT	1.026115643	Guanidoacetic acid	0.542509265
gene-NNAT	1.026115643	alpha-Terpineol formate	0.628238986
gene-NNAT	1.026115643	4-Dihydroboldenone	0.692863202
gene-NNAT	1.026115643	20-carboxy Arachidonic Acid	0.780988382
gene-NNAT	1.026115643	Apronal	0.754033426
gene-NNAT	1.026115643	Methionyl-Valine	0.502707745
gene-NNAT	1.026115643	[3-(2-Aminopropyl)-6-methylidenecyclohexa	0.422945997
gene-NNAT	1.026115643	Argyrolobine	0.949134241
gene-NNAT	1.026115643	3尾-hydroxy-estra-5,7,9-trien-17-one	0.715223098
gene-NNAT	1.026115643	Eicosatetraynoic Acid	0.804823516
gene-NNAT	1.026115643	(2E,4E)-5-[2-Methyl-2-(1,1,4,4-tetramethyl-	0.742652463
gene-NNAT	1.026115643	3,7-Dihydroxy-12-oxocholanoic acid	0.832319882

gene-NNAT	1.026115643	(22E)-3伪,7伪,12伪-Trihydroxy-5尾-chol-22-	0.522629215
gene-NNAT	1.026115643	SM(d19:1/PGE2)	0.725813678
gene-NNAT	1.026115643	3-{[(2E)-4-Amino-4-oxobut-2-enoyl]amino}	1.152542472
gene-NNAT	1.026115643	Leucylhydroxyproline	0.780056846
gene-NNAT	1.026115643	3'-Hydroxyropivacaine	1.014215236
gene-NNAT	1.026115643	Chitotriose	0.897504334
gene-NNAT	1.026115643	1,8-Octanedithiol	0.133094504
gene-NNAT	1.026115643	PC(P-18:1(11Z)/PGJ2)	0.565243877
gene-HS1BP3	1.02576432	3-Thiacytidine	0.209387412
gene-HS1BP3	1.02576432	Cyclosporin A	0.656529229
gene-HS1BP3	1.02576432	5-(2-Aminopropyl)-2-methylphenol	0.323231457
gene-HS1BP3	1.02576432	PC(P-18:1(11Z)/PGE2)	0.509503133
gene-HS1BP3	1.02576432	Isopropyl isothiocyanate	0.172385747
gene-HS1BP3	1.02576432	PC(P-18:1(11Z)/PGJ2)	0.565243877
gene-HS1BP3	1.02576432	PS(20:0/20:4(8Z,11Z,14Z,17Z)-2OH(5S,6R))	0.402595921
gene-HS1BP3	1.02576432	CL(8:0/8:0/18:2(9Z,11Z)/20:0)	0.622988418
gene-ASIC1	1.025500629	S-(Indolylmethylthiohydroximoyl)-L-cysteine	0.635042992
gene-ASIC1	1.025500629	milbemycin beta3	1.414447124
gene-ASIC1	1.025500629	Palmitoyl Ara-C	0.586890476
gene-ASIC1	1.025500629	PC(24:0/20:3(8Z,11Z,14Z)-2OH(5,6))	0.43567608
gene-ASIC1	1.025500629	LTB4-d4	0.226799982
gene-ASIC1	1.025500629	(2S,3'S)-alpha-Amino-2-carboxy-5-oxo-1-p	0.080821898
gene-ASIC1	1.025500629	Cyclosporin A	0.656529229
gene-ASIC1	1.025500629	Angiotensin A	0.247332017
gene-ASIC1	1.025500629	Epomusenin A	0.766206049
gene-ASIC1	1.025500629	PA(22:6(4Z,7Z,10Z,13Z,16Z,19Z)/16:0)	0.556532497
gene-ASIC1	1.025500629	DG(18:0/LTE4/0:0)	0.681485773
gene-ASIC1	1.025500629	PC(17:0/PGJ2)	0.657689319
gene-ASIC1	1.025500629	PC(14:0/20:2(11Z,14Z))	1.110657378
gene-ASIC1	1.025500629	CDP-DG(PGF2alpha/16:0)	1.002512277
gene-ASIC1	1.025500629	D-Xylonate	0.386869454
gene-ASIC1	1.025500629	2-Methyl-5-nitroimidazol-1-ylacetic acid	0.701441397
gene-ASIC1	1.025500629	C20914	0.140383181
gene-ASIC1	1.025500629	2-Phenylethyl propanoate	0.039485915
gene-ASIC1	1.025500629	PC(20:3(5Z,8Z,11Z)/24:0)	0.387959564
gene-ASIC1	1.025500629	Phorone A	0.079039095
gene-ASIC1	1.025500629	PS(24:1(15Z)/24:1(15Z))	0.557296152
gene-ASIC1	1.025500629	(9Z)-Octadecenoic acid	0.142359556
gene-ASIC1	1.025500629	arachidyl amido cholanoic acid	1.24842952
gene-ASIC1	1.025500629	Nigroxanthin	0.705005059
gene-ASIC1	1.025500629	CL(8:0/8:0/18:2(9Z,11Z)/20:0)	0.622988418
gene-ASIC1	1.025500629	PC(20:5(5Z,8Z,11Z,14Z,17Z)/P-16:0)	1.301095986
gene-ASIC1	1.025500629	DG(20:0/LTE4/0:0)	0.438074508
gene-DTX1	1.024678699	3-Thiacytidine	0.209387412
gene-DTX1	1.024678699	D-Erythro-imidazole-glycerol-phosphate	0.355812431
gene-DTX1	1.024678699	11-Maleimidoundecanoic acid	1.084942397
gene-DTX1	1.024678699	13(S)-HpODE	0.163157753
gene-DTX1	1.024678699	LTB4-d4	0.226799982
gene-DTX1	1.024678699	cis-p-Menth-2-en-1-ol	0.201017988
gene-DTX1	1.024678699	PE(20:0/18:1(12Z)-2OH(9,10))	0.438658253
gene-DTX1	1.024678699	(-)-alpha-Terpineol	0.345716279

gene-DTX1	1.024678699	PE(22:2(13Z,16Z)/22:5(4Z,7Z,10Z,13Z,19Z)-O(	0.369745166
gene-DTX1	1.024678699	PG(20:1(11Z)/18:3(10,12,15)-OH(9))	0.626244347
gene-DTX1	1.024678699	2-Methyl-3-phenyl-2-propenal	0.407813975
gene-DTX1	1.024678699	PC(P-18:1(11Z)/PGE2)	0.509503133
gene-DTX1	1.024678699	(3R,4R)-3-Amino-1-hydroxy-4-methylpyrrol	0.471914506
gene-DTX1	1.024678699	(9Z)-Octadecenoic acid	0.142359556
gene-DTX1	1.024678699	N-Myristoyl Glutamine	0.37856966
gene-DTX1	1.024678699	PC(18:1(9Z)/15:1(9Z))	0.534724682
gene-DTX1	1.024678699	Nigroxanthin	0.705005059
gene-DTX1	1.024678699	PC(P-18:1(11Z)/PGJ2)	0.565243877
gene-DTX1	1.024678699	PS(20:0/20:4(8Z,11Z,14Z,17Z)-2OH(5S,6R))	0.402595921
gene-SLC22A18	1.024541298	D-Erythro-imidazole-glycerol-phosphate	0.355812431
gene-SLC22A18	1.024541298	Undecanedioic acid	0.059955634
gene-SLC22A18	1.024541298	cis-p-Menth-2-en-1-ol	0.201017988
gene-SLC22A18	1.024541298	PG(20:1(11Z)/18:3(10,12,15)-OH(9))	0.626244347
gene-SLC22A18	1.024541298	alpha-Terpineol formate	0.628238986
gene-SLC22A18	1.024541298	4-Octylphenol	1.400657885
gene-SLC22A18	1.024541298	PC(P-18:1(11Z)/PGE2)	0.509503133
gene-SLC22A18	1.024541298	PC(P-18:1(11Z)/PGJ2)	0.565243877
gene-IL11RA	1.024119692	Hydroxyprolyl-Serine	0.519540341
gene-IL11RA	1.024119692	PE(16:1(9Z)/18:0)	0.077115313
gene-IL11RA	1.024119692	Cyclosporin A	0.656529229
gene-IL11RA	1.024119692	PA(22:6(4Z,7Z,10Z,13Z,16Z,19Z)/16:0)	0.556532497
gene-IL11RA	1.024119692	DG(18:0/LTE4/0:0)	0.681485773
gene-IL11RA	1.024119692	PC(17:0/PGJ2)	0.657689319
gene-IL11RA	1.024119692	PC(14:0/20:2(11Z,14Z))	1.110657378
gene-IL11RA	1.024119692	CDP-DG(PGF2alpha/16:0)	1.002512277
gene-IL11RA	1.024119692	5-(2-Aminopropyl)-2-methylphenol	0.323231457
gene-IL11RA	1.024119692	ingenol	0.942071652
gene-IL11RA	1.024119692	beta-L-Dioxolane-cytidine	0.175916668
gene-IL11RA	1.024119692	17-Aminogeldanamycin	0.518144528
gene-IL11RA	1.024119692	Nigroxanthin	0.705005059
gene-IL11RA	1.024119692	CL(8:0/8:0/18:2(9Z,11Z)/20:0)	0.622988418
gene-ADAMTS14	1.023056352	5-Sulfosalicylic acid	1.31509494
gene-ADAMTS14	1.023056352	4-Hydroxybenzoate	0.892336321
gene-ADAMTS14	1.023056352	Gentisic acid	0.519724891
gene-ADAMTS14	1.023056352	Ethyl nicotinate	1.024858052
gene-ADAMTS14	1.023056352	Dopaquinone	1.087504518
gene-ADAMTS14	1.023056352	Isomaltotriose	0.58434654
gene-ADAMTS14	1.023056352	Glutamate carbon	0.671444516
gene-ADAMTS14	1.023056352	Undecanedioic acid	0.059955634
gene-ADAMTS14	1.023056352	MG(0:0/20:4(5Z,8Z,11Z,14Z)/0:0)	0.488916341
gene-ADAMTS14	1.023056352	Cholic acid glucuronide	0.147398811
gene-ADAMTS14	1.023056352	16-Hydroxy-10-oxohexadecanoic acid	0.72302152
gene-ADAMTS14	1.023056352	Serotonin	2.521565841
gene-ADAMTS14	1.023056352	Adrenosterone	0.050790129
gene-ADAMTS14	1.023056352	5-Methoxyindoleacetate	0.597532846
gene-ADAMTS14	1.023056352	beta-Thujaplicin	0.681866194
gene-ADAMTS14	1.023056352	2-(1-Adamantyl)-1,3-dioxetane	0.408916843
gene-ADAMTS14	1.023056352	3-(3-Methylbutylidene)-1(3H)-isobenzofurar	0.575422414
gene-ADAMTS14	1.023056352	Roxithromycin	0.268273077

gene-LOC50864€	1.022009055	D-Erythro-imidazole-glycerol-phosphate	0.355812431
gene-LOC50864€	1.022009055	3-Deoxyestrone	0.282221709
gene-LOC50864€	1.022009055	1-Oleoyl-sn-glycero-3-phosphocholine	0.18926952
gene-LOC50864€	1.022009055	Lividamine	0.319679555
gene-LOC50864€	1.022009055	Psychosine	0.106475396
gene-LOC50864€	1.022009055	LTB4-d4	0.226799982
gene-LOC50864€	1.022009055	Cyclotricuspidogenin C	0.440884085
gene-LOC50864€	1.022009055	(-)-alpha-Terpineol	0.345716279
gene-LOC50864€	1.022009055	1,4-Undecadiene	0.462803973
gene-LOC50864€	1.022009055	2-isopentyl-3,6-dimethyl pyrazine	0.710502562
gene-LOC50864€	1.022009055	PG(20:1(11Z)/18:3(10,12,15)-OH(9))	0.626244347
gene-LOC50864€	1.022009055	5-(2-Aminopropyl)-2-methylphenol	0.323231457
gene-LOC50864€	1.022009055	4-Dimethylamino-L-phenylalanine	0.242110226
gene-LOC50864€	1.022009055	Trimetazidine	0.438282534
gene-LOC50864€	1.022009055	Armillane	0.52808635
gene-LOC50864€	1.022009055	5'-S-Methyl-5'-thioinosine	0.28772476
gene-LOC50864€	1.022009055	2-Methyl-3-phenyl-2-propenal	0.407813975
gene-LOC50864€	1.022009055	PC(P-18:1(11Z)/PGE2)	0.509503133
gene-LOC50864€	1.022009055	(3R,4R)-3-Amino-1-hydroxy-4-methylpyrrol	0.471914506
gene-LOC50864€	1.022009055	n-methyl-2-(4'-methylaminophenyl)-6-hydr	0.26655714
gene-LOC50864€	1.022009055	Isopropyl isothiocyanate	0.172385747
gene-LOC50864€	1.022009055	(9Z)-Octadecenoic acid	0.142359556
gene-LOC50864€	1.022009055	9-deoxy-9-methylene-16,16-dimethyl -PGE	0.606893884
gene-LOC50864€	1.022009055	2-Propenyl 2-aminobenzoate	0.128406829
gene-LOC50864€	1.022009055	CL(8:0/8:0/18:2(9Z,11Z)/20:0)	0.622988418
gene-MAST1	1.021807033	milbemycin beta3	1.414447124
gene-MAST1	1.021807033	Palmitoyl Ara-C	0.586890476
gene-MAST1	1.021807033	Tsugarioside B	1.013262893
gene-MAST1	1.021807033	Tetracosenoyl-CoA	0.187187233
gene-MAST1	1.021807033	3-Deoxyestrone	0.282221709
gene-MAST1	1.021807033	1-Oleoyl-sn-glycero-3-phosphocholine	0.18926952
gene-MAST1	1.021807033	3,4-dihydroxy-5-all-trans-hexaprenylbenzoa	0.123615726
gene-MAST1	1.021807033	Lividamine	0.319679555
gene-MAST1	1.021807033	Psychosine	0.106475396
gene-MAST1	1.021807033	Cyclotricuspidogenin C	0.440884085
gene-MAST1	1.021807033	(-)-alpha-Terpineol	0.345716279
gene-MAST1	1.021807033	1,4-Undecadiene	0.462803973
gene-MAST1	1.021807033	2-isopentyl-3,6-dimethyl pyrazine	0.710502562
gene-MAST1	1.021807033	PG(20:1(11Z)/18:3(10,12,15)-OH(9))	0.626244347
gene-MAST1	1.021807033	5-(2-Aminopropyl)-2-methylphenol	0.323231457
gene-MAST1	1.021807033	4-Dimethylamino-L-phenylalanine	0.242110226
gene-MAST1	1.021807033	Trimetazidine	0.438282534
gene-MAST1	1.021807033	ingenol	0.942071652
gene-MAST1	1.021807033	Armillane	0.52808635
gene-MAST1	1.021807033	PC(P-18:1(11Z)/PGE2)	0.509503133
gene-MAST1	1.021807033	(3R,4R)-3-Amino-1-hydroxy-4-methylpyrrol	0.471914506
gene-MAST1	1.021807033	n-methyl-2-(4'-methylaminophenyl)-6-hydr	0.26655714
gene-MAST1	1.021807033	Isopropyl isothiocyanate	0.172385747
gene-MAST1	1.021807033	(9Z)-Octadecenoic acid	0.142359556
gene-MAST1	1.021807033	L-Oleandrosyl-oleandolide	0.272998166
gene-MAST1	1.021807033	9-deoxy-9-methylene-16,16-dimethyl -PGE	0.606893884

gene-MAST1	1.021807033	CL(8:0/8:0/18:2(9Z,11Z)/20:0)	0.622988418
gene-SLC25A1	1.020920569	3-Thiacytidine	0.209387412
gene-SLC25A1	1.020920569	D-Erythro-imidazole-glycerol-phosphate	0.355812431
gene-SLC25A1	1.020920569	LTB4-d4	0.226799982
gene-SLC25A1	1.020920569	cis-p-Menth-2-en-1-ol	0.201017988
gene-SLC25A1	1.020920569	(-)-alpha-Terpineol	0.345716279
gene-SLC25A1	1.020920569	PG(20:1(11Z)/18:3(10,12,15)-OH(9))	0.626244347
gene-SLC25A1	1.020920569	5-(2-Aminopropyl)-2-methylphenol	0.323231457
gene-SLC25A1	1.020920569	4-Dimethylamino-L-phenylalanine	0.242110226
gene-SLC25A1	1.020920569	Armillane	0.52808635
gene-SLC25A1	1.020920569	2-Methyl-3-phenyl-2-propenal	0.407813975
gene-SLC25A1	1.020920569	PC(P-18:1(11Z)/PGE2)	0.509503133
gene-SLC25A1	1.020920569	(3R,4R)-3-Amino-1-hydroxy-4-methylpyrrol	0.471914506
gene-SLC25A1	1.020920569	Isopropyl isothiocyanate	0.172385747
gene-SLC25A1	1.020920569	(9Z)-Octadecenoic acid	0.142359556
gene-SLC25A1	1.020920569	PC(P-18:1(11Z)/PGJ2)	0.565243877
gene-SLC25A1	1.020920569	PS(20:0/20:4(8Z,11Z,14Z,17Z)-2OH(5S,6R))	0.402595921
gene-SLC25A1	1.020920569	CL(8:0/8:0/18:2(9Z,11Z)/20:0)	0.622988418
gene-GREB1L	1.020851496	Glutamate carbon	0.671444516
gene-GREB1L	1.020851496	Cyclosporin A	0.656529229
gene-GREB1L	1.020851496	Guanidoacetic acid	0.542509265
gene-GREB1L	1.020851496	alpha-Terpineol formate	0.628238986
gene-GREB1L	1.020851496	[3-(2-Aminopropyl)-6-methylidenecyclohexa	0.422945997
gene-GREB1L	1.020851496	Sophoranol	0.282064826
gene-GREB1L	1.020851496	L-Cysteine	0.214540754
gene-GREB1L	1.020851496	24,24-Dfhv	0.175278646
gene-GREB1L	1.020851496	1,8-Octanedithiol	0.133094504
gene-GREB1L	1.020851496	PC(P-18:1(11Z)/PGE2)	0.509503133
gene-GREB1L	1.020851496	Roxithromycin	0.268273077
gene-GREB1L	1.020851496	PC(P-18:1(11Z)/PGJ2)	0.565243877
Bos_taurus_newG	1.020602298	5-(3'-Carboxy-3'-oxopropenyl)-4,6-dihydro:	1.083232395
Bos_taurus_newG	1.020602298	Colistin A	2.200819027
Bos_taurus_newG	1.020602298	LysoPI(16:0/0:0)	0.379098336
Bos_taurus_newG	1.020602298	4-Acetamidobenzenesulfonamide	0.620868172
Bos_taurus_newG	1.020602298	4-Hydroxystyrene	2.854273107
Bos_taurus_newG	1.020602298	Dihydro-3-coumaric acid	1.950770185
Bos_taurus_newG	1.020602298	xi-2,3-Dihydro-3,5-dihydroxy-6-methyl-4H-	0.933518005
Bos_taurus_newG	1.020602298	(卤)-Enterolactone	0.766468338
Bos_taurus_newG	1.020602298	Glutathionylspermine	0.87904089
Bos_taurus_newG	1.020602298	Docosanamide	0.509975786
Bos_taurus_newG	1.020602298	2,8-Dibenzylcyclooctanone	0.368845452
Bos_taurus_newG	1.020602298	11-Oxahexadecanolide	2.378626329
Bos_taurus_newG	1.020602298	(-)-Huperzine A (HupA)	0.935198906
Bos_taurus_newG	1.020602298	Sorbitan palmitate	1.946147152
Bos_taurus_newG	1.020602298	Butanoic acid, [(diethoxyphosphinyl)oxy]meth	0.038991848
Bos_taurus_newG	1.020602298	DG(17:0/TXB2/0:0)	0.189148086
Bos_taurus_newG	1.020602298	Neomycin B	0.395784943
Bos_taurus_newG	1.020602298	Ascorbic acid 6-palmitate	0.201627014
gene-LTBP3	1.020386101	3-Thiacytidine	0.209387412
gene-LTBP3	1.020386101	D-Erythro-imidazole-glycerol-phosphate	0.355812431
gene-LTBP3	1.020386101	13(S)-HpODE	0.163157753

gene-LTBP3	1.020386101	2-Methyl-3-phenyl-2-propenal	0.407813975
gene-LTBP3	1.020386101	PC(P-18:1(11Z)/PGE2)	0.509503133
gene-LTBP3	1.020386101	Roxithromycin	0.268273077
gene-LTBP3	1.020386101	PC(P-18:1(11Z)/PGJ2)	0.565243877
gene-LTBP3	1.020386101	PS(20:0/20:4(8Z,11Z,14Z,17Z)-2OH(5S,6R))	0.402595921
gene-RNPEPL1	1.019847064	3-Thiacytidine	0.209387412
gene-RNPEPL1	1.019847064	D-Erythro-imidazole-glycerol-phosphate	0.355812431
gene-RNPEPL1	1.019847064	LTB4-d4	0.226799982
gene-RNPEPL1	1.019847064	cis-p-Menth-2-en-1-ol	0.201017988
gene-RNPEPL1	1.019847064	(-)-alpha-Terpineol	0.345716279
gene-RNPEPL1	1.019847064	PG(20:1(11Z)/18:3(10,12,15)-OH(9))	0.626244347
gene-RNPEPL1	1.019847064	4-Dimethylamino-L-phenylalanine	0.242110226
gene-RNPEPL1	1.019847064	Armillane	0.52808635
gene-RNPEPL1	1.019847064	2-Methyl-3-phenyl-2-propenal	0.407813975
gene-RNPEPL1	1.019847064	PC(P-18:1(11Z)/PGE2)	0.509503133
gene-RNPEPL1	1.019847064	(3R,4R)-3-Amino-1-hydroxy-4-methylpyrrol	0.471914506
gene-RNPEPL1	1.019847064	Isopropyl isothiocyanate	0.172385747
gene-RNPEPL1	1.019847064	PC(P-18:1(11Z)/PGJ2)	0.565243877
gene-RNPEPL1	1.019847064	PS(20:0/20:4(8Z,11Z,14Z,17Z)-2OH(5S,6R))	0.402595921
gene-RNPEPL1	1.019847064	CL(8:0/8:0/18:2(9Z,11Z)/20:0)	0.622988418
gene-RFNG	1.019612236	3-Thiacytidine	0.209387412
gene-RFNG	1.019612236	D-Erythro-imidazole-glycerol-phosphate	0.355812431
gene-RFNG	1.019612236	LTB4-d4	0.226799982
gene-RFNG	1.019612236	cis-p-Menth-2-en-1-ol	0.201017988
gene-RFNG	1.019612236	(-)-alpha-Terpineol	0.345716279
gene-RFNG	1.019612236	5-(2-Aminopropyl)-2-methylphenol	0.323231457
gene-RFNG	1.019612236	4-Dimethylamino-L-phenylalanine	0.242110226
gene-RFNG	1.019612236	Armillane	0.52808635
gene-RFNG	1.019612236	2-Methyl-3-phenyl-2-propenal	0.407813975
gene-RFNG	1.019612236	PC(P-18:1(11Z)/PGE2)	0.509503133
gene-RFNG	1.019612236	(3R,4R)-3-Amino-1-hydroxy-4-methylpyrrol	0.471914506
gene-RFNG	1.019612236	n-methyl-2-(4'-methylaminophenyl)-6-hydr	0.26655714
gene-RFNG	1.019612236	Isopropyl isothiocyanate	0.172385747
gene-RFNG	1.019612236	(9Z)-Octadecenoic acid	0.142359556
gene-RFNG	1.019612236	9-deoxy-9-methylene-16,16-dimethyl -PGE	0.606893884
gene-RFNG	1.019612236	PC(P-18:1(11Z)/PGJ2)	0.565243877
gene-RFNG	1.019612236	PS(20:0/20:4(8Z,11Z,14Z,17Z)-2OH(5S,6R))	0.402595921
gene-RFNG	1.019612236	CL(8:0/8:0/18:2(9Z,11Z)/20:0)	0.622988418
gene-ACHE	1.019214286	5-(3'-Carboxy-3'-oxopropenyl)-4,6-dihydro	1.083232395
gene-ACHE	1.019214286	D-Erythro-imidazole-glycerol-phosphate	0.355812431
gene-ACHE	1.019214286	Sorbitan laurate	0.242315968
gene-ACHE	1.019214286	4-Gingerol	0.222165012
gene-ACHE	1.019214286	Monacolin L acid	1.060562098
gene-ACHE	1.019214286	LTB4-d4	0.226799982
gene-ACHE	1.019214286	cis-p-Menth-2-en-1-ol	0.201017988
gene-ACHE	1.019214286	L-Carnitine	0.2737116
gene-ACHE	1.019214286	DG(15:0/PGE1/0:0)	0.462019057
gene-ACHE	1.019214286	2-Methyl-3-phenyl-2-propenal	0.407813975
gene-ACHE	1.019214286	4-Octylphenol	1.400657885
gene-ACHE	1.019214286	Prolyl-Lysine	1.612991304
gene-ACHE	1.019214286	3-Pentadecylphenol	1.682258188

gene-ACHE	1.019214286	PC(P-18:1(11Z)/PGE2)	0.509503133
gene-ACHE	1.019214286	12-Hydroxyicosanoylcarnitine	1.730979067
gene-ACHE	1.019214286	N-Myristoyl Glutamine	0.37856966
gene-ACHE	1.019214286	PC(18:1(9Z)/15:1(9Z))	0.534724682
gene-PLCB2	1.018877785	3-Thiacytidine	0.209387412
gene-PLCB2	1.018877785	D-Erythro-imidazole-glycerol-phosphate	0.355812431
gene-PLCB2	1.018877785	cis-p-Menth-2-en-1-ol	0.201017988
gene-PLCB2	1.018877785	PE(22:2(13Z,16Z)/22:5(4Z,7Z,10Z,13Z,19Z)-O	0.369745166
gene-PLCB2	1.018877785	PC(P-18:1(11Z)/PGE2)	0.509503133
gene-PLCB2	1.018877785	PC(P-18:1(11Z)/PGJ2)	0.565243877
gene-PLCB2	1.018877785	PS(20:0/20:4(8Z,11Z,14Z,17Z)-2OH(5S,6R))	0.402595921
gene-PLCB2	1.018877785	CL(8:0/8:0/18:2(9Z,11Z)/20:0)	0.622988418
gene-PLXNA1	1.01862832	D-Erythro-imidazole-glycerol-phosphate	0.355812431
gene-PLXNA1	1.01862832	milbemycin beta3	1.414447124
gene-PLXNA1	1.01862832	3-Deoxyestrone	0.282221709
gene-PLXNA1	1.01862832	1-Oleoyl-sn-glycero-3-phosphocholine	0.18926952
gene-PLXNA1	1.01862832	Lividamine	0.319679555
gene-PLXNA1	1.01862832	LTB4-d4	0.226799982
gene-PLXNA1	1.01862832	cis-p-Menth-2-en-1-ol	0.201017988
gene-PLXNA1	1.01862832	(-)-alpha-Terpineol	0.345716279
gene-PLXNA1	1.01862832	PG(20:1(11Z)/18:3(10,12,15)-OH(9))	0.626244347
gene-PLXNA1	1.01862832	5-(2-Aminopropyl)-2-methylphenol	0.323231457
gene-PLXNA1	1.01862832	4-Dimethylamino-L-phenylalanine	0.242110226
gene-PLXNA1	1.01862832	Armillane	0.52808635
gene-PLXNA1	1.01862832	PC(P-18:1(11Z)/PGE2)	0.509503133
gene-PLXNA1	1.01862832	(3R,4R)-3-Amino-1-hydroxy-4-methylpyrrol	0.471914506
gene-PLXNA1	1.01862832	n-methyl-2-(4'-methylaminophenyl)-6-hydr	0.26655714
gene-PLXNA1	1.01862832	(9Z)-Octadecenoic acid	0.142359556
gene-PLXNA1	1.01862832	9-deoxy-9-methylene-16,16-dimethyl -PGE	0.606893884
gene-PLXNA1	1.01862832	CL(8:0/8:0/18:2(9Z,11Z)/20:0)	0.622988418
gene-LOC51079	1.01848632	milbemycin beta3	1.414447124
gene-LOC51079	1.01848632	Tetracosenoyl-CoA	0.187187233
gene-LOC51079	1.01848632	1,2-O-Isopropylidene-D-glucofuranose	0.080987667
gene-LOC51079	1.01848632	3-Deoxyestrone	0.282221709
gene-LOC51079	1.01848632	1-Oleoyl-sn-glycero-3-phosphocholine	0.18926952
gene-LOC51079	1.01848632	3,4-dihydroxy-5-all-trans-hexaprenylbenzoa	0.123615726
gene-LOC51079	1.01848632	Lividamine	0.319679555
gene-LOC51079	1.01848632	Psychosine	0.106475396
gene-LOC51079	1.01848632	Cyclotricuspidogenin C	0.440884085
gene-LOC51079	1.01848632	(-)-alpha-Terpineol	0.345716279
gene-LOC51079	1.01848632	PC(16:0/18:1(12Z)-2OH(9,10))	0.461843233
gene-LOC51079	1.01848632	5-(2-Aminopropyl)-2-methylphenol	0.323231457
gene-LOC51079	1.01848632	4-Dimethylamino-L-phenylalanine	0.242110226
gene-LOC51079	1.01848632	Trimetazidine	0.438282534
gene-LOC51079	1.01848632	ingenol	0.942071652
gene-LOC51079	1.01848632	Armillane	0.52808635
gene-LOC51079	1.01848632	n-methyl-2-(4'-methylaminophenyl)-6-hydr	0.26655714
gene-LOC51079	1.01848632	Isopropyl isothiocyanate	0.172385747
gene-LOC51079	1.01848632	9-deoxy-9-methylene-16,16-dimethyl -PGE	0.606893884
gene-LOC51079	1.01848632	CL(8:0/8:0/18:2(9Z,11Z)/20:0)	0.622988418
Bos_taurus_newG	1.017471218	3-Thiacytidine	0.209387412

Bos_taurus_newG	1.017471218	(-)-alpha-Terpineol	0.345716279
Bos_taurus_newG	1.017471218	1,4-Undecadiene	0.462803973
Bos_taurus_newG	1.017471218	4-cholesten-7伪,12伪,24-triol-3-one	0.097840451
Bos_taurus_newG	1.017471218	5-(2-Aminopropyl)-2-methylphenol	0.323231457
Bos_taurus_newG	1.017471218	4-Dimethylamino-L-phenylalanine	0.242110226
Bos_taurus_newG	1.017471218	ingenol	0.942071652
Bos_taurus_newG	1.017471218	Armillane	0.52808635
Bos_taurus_newG	1.017471218	PC(P-18:1(11Z)/PGE2)	0.509503133
Bos_taurus_newG	1.017471218	1-Palmitoylglycerol	0.084357408
Bos_taurus_newG	1.017471218	n-methyl-2-(4'-methylaminophenyl)-6-hydr	0.26655714
Bos_taurus_newG	1.017471218	Isopropyl isothiocyanate	0.172385747
Bos_taurus_newG	1.017471218	D-Fructose	0.167584616
Bos_taurus_newG	1.017471218	CL(8:0/8:0/18:2(9Z,11Z)/20:0)	0.622988418
gene-LIPE	1.01655758	3-Thiacytidine	0.209387412
gene-LIPE	1.01655758	D-Erythro-imidazole-glycerol-phosphate	0.355812431
gene-LIPE	1.01655758	LTB4-d4	0.226799982
gene-LIPE	1.01655758	cis-p-Menth-2-en-1-ol	0.201017988
gene-LIPE	1.01655758	(-)-alpha-Terpineol	0.345716279
gene-LIPE	1.01655758	PG(20:1(11Z)/18:3(10,12,15)-OH(9))	0.626244347
gene-LIPE	1.01655758	4-Dimethylamino-L-phenylalanine	0.242110226
gene-LIPE	1.01655758	Armillane	0.52808635
gene-LIPE	1.01655758	2-Methyl-3-phenyl-2-propenal	0.407813975
gene-LIPE	1.01655758	PC(P-18:1(11Z)/PGE2)	0.509503133
gene-LIPE	1.01655758	(3R,4R)-3-Amino-1-hydroxy-4-methylpyrrol	0.471914506
gene-LIPE	1.01655758	Isopropyl isothiocyanate	0.172385747
gene-LIPE	1.01655758	(9Z)-Octadecenoic acid	0.142359556
gene-LIPE	1.01655758	PC(P-18:1(11Z)/PGJ2)	0.565243877
gene-LIPE	1.01655758	PS(20:0/20:4(8Z,11Z,14Z,17Z)-2OH(5S,6R))	0.402595921
gene-ZNF469	1.016327304	3-Thiacytidine	0.209387412
gene-ZNF469	1.016327304	3-Deoxyestrone	0.282221709
gene-ZNF469	1.016327304	(-)-alpha-Terpineol	0.345716279
gene-ZNF469	1.016327304	Cyclosporin A	0.656529229
gene-ZNF469	1.016327304	ingenol	0.942071652
gene-ZNF469	1.016327304	3b,6a-Dihydroxy-alpha-ionol 9-[apiosyl-(1-:	0.045258177
gene-ZNF469	1.016327304	N-[[3-Hydroxy-2-(2-pentenyl)cyclopentyl]ac	1.043930947
gene-ZNF469	1.016327304	PC(P-18:1(11Z)/PGE2)	0.509503133
gene-ZNF469	1.016327304	PC(20:3(5Z,8Z,11Z)/24:0)	0.387959564
gene-ZNF469	1.016327304	Phorone A	0.079039095
gene-ZNF469	1.016327304	2-Amino-4-[carbamimidoyl(methyl)amino]bu	0.075703353
gene-ZNF469	1.016327304	(9Z)-Octadecenoic acid	0.142359556
gene-ZNF469	1.016327304	Nigroxanthin	0.705005059
gene-ZNF469	1.016327304	CL(8:0/8:0/18:2(9Z,11Z)/20:0)	0.622988418
gene-AKAP9	1.016039126	Glutamate carbon	0.671444516
gene-AKAP9	1.016039126	11-Maleimidoundecanoic acid	1.084942397
gene-AKAP9	1.016039126	PC(24:0/22:6(4Z,7Z,10Z,12E,16Z,19Z)-OH(14)	0.188241061
gene-AKAP9	1.016039126	PE(22:2(13Z,16Z)/22:5(4Z,7Z,10Z,13Z,19Z)-O	0.369745166
gene-AKAP9	1.016039126	Norophthalmic acid	0.191432411
gene-AKAP9	1.016039126	Guanidoacetic acid	0.542509265
gene-AKAP9	1.016039126	alpha-Terpineol formate	0.628238986
gene-AKAP9	1.016039126	PC(P-18:1(11Z)/PGE2)	0.509503133
gene-AKAP9	1.016039126	Roxithromycin	0.268273077

gene-AKAP9	1.016039126	PC(P-18:1(11Z)/PGJ2)	0.565243877
gene-AKAP9	1.016039126	PS(20:0/20:4(8Z,11Z,14Z,17Z)-2OH(5S,6R))	0.402595921
gene-AKAP9	1.016039126	PE(20:5(5Z,8Z,11Z,14Z,17Z)/18:0)	0.427962506
gene-CLIC3	1.015618605	D-Erythro-imidazole-glycerol-phosphate	0.355812431
gene-CLIC3	1.015618605	Glutamate carbon	0.671444516
gene-CLIC3	1.015618605	Undecanedioic acid	0.059955634
gene-CLIC3	1.015618605	Cholic acid glucuronide	0.147398811
gene-CLIC3	1.015618605	11-Maleimidoundecanoic acid	1.084942397
gene-CLIC3	1.015618605	PC(24:0/22:6(4Z,7Z,10Z,12E,16Z,19Z)-OH(14)	0.188241061
gene-CLIC3	1.015618605	PE(22:2(13Z,16Z)/22:5(4Z,7Z,10Z,13Z,19Z)-O	0.369745166
gene-CLIC3	1.015618605	(1R,6S)-6-Amino-5-oxocyclohex-2-ene-1-c	0.154751123
gene-CLIC3	1.015618605	2-(1-Adamantyl)-1,3-dioxetane	0.408916843
gene-CLIC3	1.015618605	Guanidoacetic acid	0.542509265
gene-CLIC3	1.015618605	alpha-Terpineol formate	0.628238986
gene-CLIC3	1.015618605	3-(3-Methylbutylidene)-1(3H)-isobenzofurar	0.575422414
gene-CLIC3	1.015618605	PC(P-18:1(11Z)/PGE2)	0.509503133
gene-CLIC3	1.015618605	Roxithromycin	0.268273077
gene-CLIC3	1.015618605	PC(P-18:1(11Z)/PGJ2)	0.565243877
gene-CLIC3	1.015618605	PS(20:0/20:4(8Z,11Z,14Z,17Z)-2OH(5S,6R))	0.402595921
gene-PNPLA2	1.015305174	D-Erythro-imidazole-glycerol-phosphate	0.355812431
gene-PNPLA2	1.015305174	cis-p-Menth-2-en-1-ol	0.201017988
gene-PNPLA2	1.015305174	PG(20:1(11Z)/18:3(10,12,15)-OH(9))	0.626244347
gene-PNPLA2	1.015305174	Armillane	0.52808635
gene-PNPLA2	1.015305174	PC(P-18:1(11Z)/PGE2)	0.509503133
gene-PNPLA2	1.015305174	Sitosterol beta-D-glucoside	0.382944796
gene-PNPLA2	1.015305174	PC(P-18:1(11Z)/PGJ2)	0.565243877
gene-PNPLA2	1.015305174	PS(20:0/20:4(8Z,11Z,14Z,17Z)-2OH(5S,6R))	0.402595921
gene-PLXNB1	1.014187382	D-Erythro-imidazole-glycerol-phosphate	0.355812431
gene-PLXNB1	1.014187382	9'-Carboxy-gamma-chromanol	0.068924993
gene-PLXNB1	1.014187382	Tsugarioside B	1.013262893
gene-PLXNB1	1.014187382	7(14)-Bisabolene-2,3,10,11-tetrol	2.446897974
gene-PLXNB1	1.014187382	3-Deoxyestrone	0.282221709
gene-PLXNB1	1.014187382	1-Oleoyl-sn-glycero-3-phosphocholine	0.18926952
gene-PLXNB1	1.014187382	Psychosine	0.106475396
gene-PLXNB1	1.014187382	LTB4-d4	0.226799982
gene-PLXNB1	1.014187382	cis-p-Menth-2-en-1-ol	0.201017988
gene-PLXNB1	1.014187382	(-)-alpha-Terpineol	0.345716279
gene-PLXNB1	1.014187382	2-isopentyl-3,6-dimethyl pyrazine	0.710502562
gene-PLXNB1	1.014187382	DG(15:0/PGE1/0:0)	0.462019057
gene-PLXNB1	1.014187382	5-(2-Aminopropyl)-2-methylphenol	0.323231457
gene-PLXNB1	1.014187382	4-Dimethylamino-L-phenylalanine	0.242110226
gene-PLXNB1	1.014187382	Trimetazidine	0.438282534
gene-PLXNB1	1.014187382	Armillane	0.52808635
gene-PLXNB1	1.014187382	5'-S-Methyl-5'-thioinosine	0.28772476
gene-PLXNB1	1.014187382	(Z)-3-Oxo-2-(2-pentenyl)-1-cyclopenteneac	0.179805915
gene-PLXNB1	1.014187382	2-Methyl-3-phenyl-2-propenal	0.407813975
gene-PLXNB1	1.014187382	PC(P-18:1(11Z)/PGE2)	0.509503133
gene-PLXNB1	1.014187382	(3R,4R)-3-Amino-1-hydroxy-4-methylpyrrol	0.471914506
gene-PLXNB1	1.014187382	n-methyl-2-(4'-methylaminophenyl)-6-hydr	0.26655714
gene-PLXNB1	1.014187382	PE(P-16:0/18:4(6Z,9Z,12Z,15Z))	0.546989864
gene-PLXNB1	1.014187382	Isopropyl isothiocyanate	0.172385747

gene-PLXNB1	1.014187382	(9Z)-Octadecenoic acid	0.142359556
gene-PLXNB1	1.014187382	N-Myristoyl Glutamine	0.37856966
gene-PLXNB1	1.014187382	9-deoxy-9-methylene-16,16-dimethyl -PGE:	0.606893884
gene-PLXNB1	1.014187382	2-Propenyl 2-aminobenzoate	0.128406829
gene-WSCD1	1.011925152	5-(Ethylthio)-1H-tetrazole	0.29717344
gene-WSCD1	1.011925152	Tetracosenoyl-CoA	0.187187233
gene-WSCD1	1.011925152	16-hydroxy hexadecanoic acid	0.309000958
gene-WSCD1	1.011925152	1,2-O-Isopropylidene-D-glucofuranose	0.080987667
gene-WSCD1	1.011925152	3-Deoxyestrone	0.282221709
gene-WSCD1	1.011925152	1-Oleoyl-sn-glycero-3-phosphocholine	0.18926952
gene-WSCD1	1.011925152	3,4-dihydroxy-5-all-trans-hexaprenylbenzoa	0.123615726
gene-WSCD1	1.011925152	Lividamine	0.319679555
gene-WSCD1	1.011925152	Psychosine	0.106475396
gene-WSCD1	1.011925152	Cyclotricuspidogenin C	0.440884085
gene-WSCD1	1.011925152	(-)-alpha-Terpineol	0.345716279
gene-WSCD1	1.011925152	Linoleamide	0.926003481
gene-WSCD1	1.011925152	1,4-Undecadiene	0.462803973
gene-WSCD1	1.011925152	PC(16:0/18:1(12Z)-2OH(9,10))	0.461843233
gene-WSCD1	1.011925152	5-(2-Aminopropyl)-2-methylphenol	0.323231457
gene-WSCD1	1.011925152	4-Dimethylamino-L-phenylalanine	0.242110226
gene-WSCD1	1.011925152	Trimetazidine	0.438282534
gene-WSCD1	1.011925152	ingenol	0.942071652
gene-WSCD1	1.011925152	Armillane	0.52808635
gene-WSCD1	1.011925152	Misoprostol	1.349270093
gene-WSCD1	1.011925152	(Z)-3-Oxo-2-(2-pentenyl)-1-cyclopenteneac	0.179805915
gene-WSCD1	1.011925152	Carboprost methyl	1.74923433
gene-WSCD1	1.011925152	(3R,4R)-3-Amino-1-hydroxy-4-methylpyrrol	0.471914506
gene-WSCD1	1.011925152	1-Palmitoylglycerol	0.084357408
gene-WSCD1	1.011925152	N2-gamma-Glutamylglutamine	0.230065499
gene-WSCD1	1.011925152	n-methyl-2-(4'-methylaminophenyl)-6-hydr	0.26655714
gene-WSCD1	1.011925152	Isopropyl isothiocyanate	0.172385747
gene-WSCD1	1.011925152	9-deoxy-9-methylene-16,16-dimethyl -PGE:	0.606893884
gene-WSCD1	1.011925152	CL(8:0/8:0/18:2(9Z,11Z)/20:0)	0.622988418
gene-TESC	1.011774918	3-Thiacytidine	0.209387412
gene-TESC	1.011774918	LTB4-d4	0.226799982
gene-TESC	1.011774918	PE(20:0/18:1(12Z)-2OH(9,10))	0.438658253
gene-TESC	1.011774918	Cyclosporin A	0.656529229
gene-TESC	1.011774918	DG(18:0/LTE4/0:0)	0.681485773
gene-TESC	1.011774918	PC(14:0/20:2(11Z,14Z))	1.110657378
gene-TESC	1.011774918	5-(2-Aminopropyl)-2-methylphenol	0.323231457
gene-TESC	1.011774918	beta-L-Dioxolane-cytidine	0.175916668
gene-TESC	1.011774918	PC(P-18:1(11Z)/PGE2)	0.509503133
gene-TESC	1.011774918	PC(18:1(9Z)/15:1(9Z))	0.534724682
gene-TESC	1.011774918	arachidyl amido cholanoic acid	1.24842952
gene-TESC	1.011774918	Nigroxanthin	0.705005059
gene-TESC	1.011774918	1-Octadecanoyl-2-(7Z,10Z,13Z,16Z-docosat	0.690496314
gene-TESC	1.011774918	PC(P-18:1(11Z)/PGJ2)	0.565243877
gene-TESC	1.011774918	PS(20:0/20:4(8Z,11Z,14Z,17Z)-2OH(5S,6R))	0.402595921
gene-TESC	1.011774918	CL(8:0/8:0/18:2(9Z,11Z)/20:0)	0.622988418
gene-SCN4A	1.011069909	Methylmalonate	0.241481249
gene-SCN4A	1.011069909	CDP-DG(18:0/PGE2)	0.280573668

gene-SCN4A	1.011069909	1-Phenylpiperazine	0.353584896
gene-SCN4A	1.011069909	Coenzyme F420-1	0.507686022
gene-SCN4A	1.011069909	milbemycin beta3	1.414447124
gene-SCN4A	1.011069909	Tetracosenoyl-CoA	0.187187233
gene-SCN4A	1.011069909	PE(20:0/18:1(12Z)-2OH(9,10))	0.438658253
gene-SCN4A	1.011069909	Epomusenin A	0.766206049
gene-SCN4A	1.011069909	PA(22:6(4Z,7Z,10Z,13Z,16Z,19Z)/16:0)	0.556532497
gene-SCN4A	1.011069909	DG(18:0/LTE4/0:0)	0.681485773
gene-SCN4A	1.011069909	PC(14:0/20:2(11Z,14Z))	1.110657378
gene-SCN4A	1.011069909	CDP-DG(PGF2alpha/16:0)	1.002512277
gene-SCN4A	1.011069909	Benzoyl glucuronide (Benzoic acid)	0.442844673
gene-SCN4A	1.011069909	Gentiobioside	0.022114549
gene-SCN4A	1.011069909	6-hydroxymethyl-7,8-dihydropterin	0.190396346
gene-SCN4A	1.011069909	Calligonine	0.118287503
gene-SCN4A	1.011069909	5-Formiminotetrahydrofolic acid	0.136750728
gene-SCN4A	1.011069909	Nicotinate	0.119105893
gene-SCN4A	1.011069909	Asparagine-betaxanthin	0.103826013
gene-SCN4A	1.011069909	Pregnanediol	0.166857843
gene-SCN4A	1.011069909	L-Histidinol	0.149249286
gene-SCN4A	1.011069909	DG(2:0/18:1(12Z)-O(9S,10R)/0:0)	0.033180989
gene-SCN4A	1.011069909	3-Dehydrosphinganine	0.053002238
gene-SCN4A	1.011069909	17-Aminogeldanamycin	0.518144528
gene-SCN4A	1.011069909	MG(LTE4/0:0/0:0)	0.548257353
gene-SCN4A	1.011069909	Val-Tyr-Leu-Arg	0.040793217
gene-SCN4A	1.011069909	Didemethylcitalopram	0.175767554
gene-SCN4A	1.011069909	Lubiminol	0.122171707
gene-SCN4A	1.011069909	Cer(d18:2(4E,14Z)/TXB2)	0.236013333
gene-SCN4A	1.011069909	Nigroxanthin	0.705005059
gene-SCN4A	1.011069909	PC(16:0/20:5(5E,8E,11E,14E,17E))[U]	0.208077202
gene-SCN4A	1.011069909	PC(P-16:0/20:3(8Z,11Z,14Z)-2OH(5,6))	0.174122169
gene-TCAP	1.010637695	3-Thiacytidine	0.209387412
gene-TCAP	1.010637695	D-Erythro-imidazole-glycerol-phosphate	0.355812431
gene-TCAP	1.010637695	11-Maleimidoundecanoic acid	1.084942397
gene-TCAP	1.010637695	LTB4-d4	0.226799982
gene-TCAP	1.010637695	cis-p-Menth-2-en-1-ol	0.201017988
gene-TCAP	1.010637695	PE(20:0/18:1(12Z)-2OH(9,10))	0.438658253
gene-TCAP	1.010637695	PE(22:2(13Z,16Z)/22:5(4Z,7Z,10Z,13Z,19Z)-O(	0.369745166
gene-TCAP	1.010637695	Norophthalmic acid	0.191432411
gene-TCAP	1.010637695	2-Methyl-3-phenyl-2-propenal	0.407813975
gene-TCAP	1.010637695	Guanidoacetic acid	0.542509265
gene-TCAP	1.010637695	alpha-Terpineol formate	0.628238986
gene-TCAP	1.010637695	PC(P-18:1(11Z)/PGE2)	0.509503133
gene-TCAP	1.010637695	Nigroxanthin	0.705005059
gene-TCAP	1.010637695	PC(P-18:1(11Z)/PGJ2)	0.565243877
gene-TCAP	1.010637695	PS(20:0/20:4(8Z,11Z,14Z,17Z)-2OH(5S,6R))	0.402595921
gene-TCAP	1.010637695	PS(16:1(9Z)/22:2(13Z,16Z))	0.659652661
gene-CRIP1	1.007558769	5-(Ethylthio)-1H-tetrazole	0.29717344
gene-CRIP1	1.007558769	3-Thiacytidine	0.209387412
gene-CRIP1	1.007558769	Canesceol	0.686312122
gene-CRIP1	1.007558769	1-Oleoyl-sn-glycero-3-phosphocholine	0.18926952
gene-CRIP1	1.007558769	Psychosine	0.106475396

gene-CRIP1	1.007558769	(-)-alpha-Terpineol	0.345716279
gene-CRIP1	1.007558769	Angiotensin A	0.247332017
gene-CRIP1	1.007558769	PC(17:0/PGJ2)	0.657689319
gene-CRIP1	1.007558769	PC(14:1(9Z)/P-18:1(11Z))	0.153578899
gene-CRIP1	1.007558769	PC(14:0/20:2(11Z,14Z))	1.110657378
gene-CRIP1	1.007558769	5-(2-Aminopropyl)-2-methylphenol	0.323231457
gene-CRIP1	1.007558769	Trimetazidine	0.438282534
gene-CRIP1	1.007558769	ingenol	0.942071652
gene-CRIP1	1.007558769	Armillane	0.52808635
gene-CRIP1	1.007558769	oleandomycin	0.996457692
gene-CRIP1	1.007558769	PC(20:3(5Z,8Z,11Z)/24:0)	0.387959564
gene-CRIP1	1.007558769	(3R,4R)-3-Amino-1-hydroxy-4-methylpyrrol	0.471914506
gene-CRIP1	1.007558769	n-methyl-2-(4'-methylaminophenyl)-6-hydr	0.26655714
gene-CRIP1	1.007558769	Isopropyl isothiocyanate	0.172385747
gene-CRIP1	1.007558769	(9Z)-Octadecenoic acid	0.142359556
gene-CRIP1	1.007558769	arachidyl amido cholanoic acid	1.24842952
gene-CRIP1	1.007558769	AS 1-5	0.512683467
gene-CRIP1	1.007558769	9-deoxy-9-methylene-16,16-dimethyl -PGE	0.606893884
gene-CRIP1	1.007558769	2-Propenyl 2-aminobenzoate	0.128406829
gene-CRIP1	1.007558769	D-Fructose	0.167584616
gene-CRIP1	1.007558769	1-Methylnicotinamide	0.203956241
gene-CRIP1	1.007558769	CL(8:0/8:0/18:2(9Z,11Z)/20:0)	0.622988418
gene-C16H1orf1	1.006928397	3-hydroxypristanic acid	0.548515835
gene-C16H1orf1	1.006928397	3-Deoxyestrone	0.282221709
gene-C16H1orf1	1.006928397	Lividamine	0.319679555
gene-C16H1orf1	1.006928397	Cyclotricuspidogenin C	0.440884085
gene-C16H1orf1	1.006928397	(-)-alpha-Terpineol	0.345716279
gene-C16H1orf1	1.006928397	1,4-Undecadiene	0.462803973
gene-C16H1orf1	1.006928397	N-[[3-Hydroxy-2-(2-pentenyl)cyclopentyl]ac	1.043930947
gene-C16H1orf1	1.006928397	PC(P-18:1(11Z)/PGE2)	0.509503133
gene-C16H1orf1	1.006928397	n-methyl-2-(4'-methylaminophenyl)-6-hydr	0.26655714
gene-C16H1orf1	1.006928397	CL(8:0/8:0/18:2(9Z,11Z)/20:0)	0.622988418
gene-SLC4A3	1.006401498	Arachidonic Acid (peroxide free)	0.666664243
gene-SLC4A3	1.006401498	delta9-Tetrahydrocannabinol hemisuccinate	0.6166092
gene-SLC4A3	1.006401498	Dextrorphan O-glucuronide	1.59476827
gene-SLC4A3	1.006401498	Zucchini factor B	0.491282625
gene-SLC4A3	1.006401498	Belotecan	0.794871701
gene-SLC4A3	1.006401498	(2S,4R)-4-(9H-Pyrido[3,4-b]indol-1-yl)-1,2,4	0.883685198
gene-SLC4A3	1.006401498	LysoPA(22:5(7Z,10Z,13Z,16Z,19Z)/0:0)	0.291568015
gene-SLC4A3	1.006401498	Bisbynin	0.993286894
gene-SLC4A3	1.006401498	Etimizol	0.852969435
gene-SLC4A3	1.006401498	Deoxyguanidinoproclavaminic acid	0.857672893
gene-SLC4A3	1.006401498	Azelaic acid	0.007180175
gene-SLC4A3	1.006401498	2-(3-Carboxy-3-aminopropyl)-L-histidine	0.575765457
gene-SLC4A3	1.006401498	3,4-Dimethyl-5-pentyl-2-furanpropanoic aci	0.896285742
gene-SLC4A3	1.006401498	N-[5-Methyl-8-(4-methylpiperazin-1-yl)-1,2	0.398723022
gene-SLC4A3	1.006401498	5-Hydroxy-2-oxo-4-ureido-2,5-dihydro-1H	0.122536033
gene-SLC4A3	1.006401498	p-Coumaroylputrescine	0.939330574
gene-SLC4A3	1.006401498	Calicheamicinone	0.910932275
gene-SLC4A3	1.006401498	Pterostilbene	0.899698314
gene-SLC4A3	1.006401498	Ancymidol	0.913827444

gene-SLC4A3	1.006401498	Phenylalanylhydroxyproline	1.183668928
gene-SLC4A3	1.006401498	Delgocitinib	1.051864373
gene-SLC4A3	1.006401498	Melatonin	1.134145229
gene-SLC4A3	1.006401498	2,5-Dihydroxy-1-octadec-9-enoyloxypyrrole	0.828088186
gene-SLC4A3	1.006401498	Arginine vasopressin 1-8	1.288601754
gene-SLC4A3	1.006401498	Hygromycin B	0.930396619
gene-SLC4A3	1.006401498	Netupitant	0.707108724
gene-SLC4A3	1.006401498	Divinylprotochlorophyllide	0.629978387
gene-SLC4A3	1.006401498	Metkephamid	1.159767003
gene-SLC4A3	1.006401498	Tuberose lactone	1.590026295
gene-SLC4A3	1.006401498	Epothilone D	0.794619023
gene-SLC4A3	1.006401498	Leukotriene D4	0.6807158
gene-SLC4A3	1.006401498	2-Ethyl-5-methyl-3,3-diphenyl-1-pyrroline	0.826978858
gene-SLC4A3	1.006401498	11-Hydroxyeicosatetraenoate glyceryl ester	1.226051047
gene-SLC4A3	1.006401498	Azaspiracid 5	1.398898867
gene-SLC4A3	1.006401498	PGP(20:2(11Z,14Z)/18:2(10E,12Z)+=O(9))	1.457698264
gene-SLC4A3	1.006401498	Baccatin III	0.776246161
gene-SLC4A3	1.006401498	10-hydroxy capric acid	0.531721559
gene-SLC4A3	1.006401498	Becocalcidiol	0.63162902
gene-SLC4A3	1.006401498	Valnemulin	0.408062514
gene-SLC4A3	1.006401498	(6Z,9Z,12Z)-Octadecatrienoic acid	0.266628199
gene-SLC4A3	1.006401498	PE(20:0/18:1(12Z)-2OH(9,10))	0.438658253
gene-SLC4A3	1.006401498	Phytosphingosine	0.393831878
gene-SLC4A3	1.006401498	L-threo-sphinganine	0.388694728
gene-SLC4A3	1.006401498	(3b,16b,20R)-Pregn-5-ene-3,16,20-triol 3-gl	0.870801075
gene-SLC4A3	1.006401498	Epomusenin A	0.766206049
gene-SLC4A3	1.006401498	PE(P-18:0/20:4(5Z,8Z,11Z,14Z)-OH(20))	0.10814637
gene-SLC4A3	1.006401498	(4r,5s,6s,7r)-4,7-Dibenzyl-5,6-dihydroxy-1,3-	1.334600128
gene-SLC4A3	1.006401498	Pseudouridine 5'-phosphate	1.18431378
gene-SLC4A3	1.006401498	4-Hydroxy-6-methyl-3-(1H-pyrazol-3-yl)-2	0.006362816
gene-SLC4A3	1.006401498	C20914	0.140383181
gene-SLC4A3	1.006401498	7-Hydroxyenterolactone	0.232354714
gene-SLC4A3	1.006401498	Pro Gly Ser Ser	0.384303007
gene-SLC4A3	1.006401498	Validamycin A	0.587705786
gene-SLC4A3	1.006401498	Norophthalmic acid	0.191432411
gene-SLC4A3	1.006401498	3'-N'-Acetylfusarochromanone	0.755683206
gene-SLC4A3	1.006401498	N-trans-Feruloyloctopamine	0.301908137
gene-SLC4A3	1.006401498	Licoagrochalcone B	0.345948791
gene-SLC4A3	1.006401498	cis,trans-5'-Hydroxythalidomide	0.589828342
gene-SLC4A3	1.006401498	Ser Cys Ala Ala	0.603032447
gene-SLC4A3	1.006401498	Pantetheine	0.584830287
gene-SLC4A3	1.006401498	Pratenol A	0.886782548
gene-SLC4A3	1.006401498	10-Hydroxycarbazepine	0.848001655
gene-SLC4A3	1.006401498	Semilepidinoside A	0.527638644
gene-SLC4A3	1.006401498	Arbutin	0.176184476
gene-SLC4A3	1.006401498	Loganin	0.92782666
gene-SLC4A3	1.006401498	7-Methylxanthosine	0.004641243
gene-SLC4A3	1.006401498	3'-Deoxythymidine	0.791830758
gene-SLC4A3	1.006401498	Gly Asp Ala Ala	0.718656316
gene-SLC4A3	1.006401498	N-Acetylhistidine	0.349369987
gene-SLC4A3	1.006401498	ethyl 2-cyano-3-(1h-indol-3-yl)prop-2-eno	0.756861034

gene-SLC4A3	1.006401498	1-{2-[(3-Ethylphenyl)amino]-2-oxoethyl}-6-	0.705711444
gene-SLC4A3	1.006401498	kainic acid	0.836445456
gene-SLC4A3	1.006401498	Zanamivir	0.839205907
gene-SLC4A3	1.006401498	Aminoglutethimide	0.652616628
gene-SLC4A3	1.006401498	5-NITRO-2-PHENYLPROPYLAMINOBENZOI(	0.713298779
gene-SLC4A3	1.006401498	5,6,7,8-Tetrahydromonapterin	0.724075531
gene-SLC4A3	1.006401498	Harmalol	0.364945111
gene-SLC4A3	1.006401498	5'-Methylthioadenosine	0.273413052
gene-SLC4A3	1.006401498	Ethylene brassylate	0.989156973
gene-SLC4A3	1.006401498	Kelampayoside A	0.744250453
gene-SLC4A3	1.006401498	2,3-Epoxymenaquinone	2.040730224
gene-SLC4A3	1.006401498	Guanidoacetic acid	0.542509265
gene-SLC4A3	1.006401498	Policapram	0.598028597
gene-SLC4A3	1.006401498	4-Oxo-9-cis-retinoyl-beta-glucuronide	1.611773742
gene-SLC4A3	1.006401498	alpha-Terpineol formate	0.628238986
gene-SLC4A3	1.006401498	2'-Fluoro-2',3'-dideoxyinosine	1.214745496
gene-SLC4A3	1.006401498	Apronal	0.754033426
gene-SLC4A3	1.006401498	Methionyl-Valine	0.502707745
gene-SLC4A3	1.006401498	Argyrolobine	0.949134241
gene-SLC4A3	1.006401498	3尾-hydroxy-estra-5,7,9-trien-17-one	0.715223098
gene-SLC4A3	1.006401498	Cetamolol	0.770561315
gene-SLC4A3	1.006401498	Eicosatetraynoic Acid	0.804823516
gene-SLC4A3	1.006401498	(2E,4E)-5-[2-Methyl-2-(1,1,4,4-tetramethyl-:	0.742652463
gene-SLC4A3	1.006401498	3,7-Dihydroxy-12-oxocholanoic acid	0.832319882
gene-SLC4A3	1.006401498	PGD2 ethanolamide	1.431143851
gene-SLC4A3	1.006401498	(5alpha,6beta,14alpha,20R,22R)-5,6,14,20,27-	1.296004093
gene-SLC4A3	1.006401498	SM(d19:1/PGE2)	0.725813678
gene-SLC4A3	1.006401498	3-{[(2E)-4-Amino-4-oxobut-2-enoyl]amino}	1.152542472
gene-SLC4A3	1.006401498	Leucylhydroxyproline	0.780056846
gene-SLC4A3	1.006401498	3'-Hydroxyropivacaine	1.014215236
gene-SLC4A3	1.006401498	3-Hydroxytetradecanoyl carnitine	1.314002596
gene-SLC4A3	1.006401498	Chitotriose	0.897504334
gene-SLC4A3	1.006401498	Macrocin	1.715200143
gene-SLC4A3	1.006401498	Vulgarone A	0.102049407
gene-SLC4A3	1.006401498	(S)-4-(2-(4-Amino-1,2,5-oxadiazol-3-yl)-1-	0.710706078
gene-SLC4A3	1.006401498	Myricanol 5-[arabinosyl-(1->6)-glucoside]	0.744174399
gene-SLC4A3	1.006401498	Apigenin	0.659004678
gene-SLC4A3	1.006401498	1,4,6-Trimethylnaphthalene	0.12150693
gene-SLC4A3	1.006401498	Prostaglandin PGE2 1-glyceryl ester	0.007411313
gene-SLC4A3	1.006401498	7-Sulfocholic acid	0.039985455
gene-SLC4A3	1.006401498	MG(0:0/18:3(9Z,12Z,15Z)/0:0)	1.373784038
gene-SLC4A3	1.006401498	Alpha-Trisaccharide	0.928257808
gene-SLC4A3	1.006401498	Austalide L	0.4229014
gene-SLC4A3	1.006401498	Tetracycline	0.553304723
gene-SLC4A3	1.006401498	N-Palmitoyl Proline	0.470367693
gene-SLC4A3	1.006401498	Deflazacort	0.118739616
gene-SLC4A3	1.006401498	10-alpha-methoxy-9,10-dihydrolysergol	0.140851438
gene-SLC4A3	1.006401498	3beta-Hydroxyergosta-7,24(24(1))-dien-4alp	1.714710978
gene-SLC4A3	1.006401498	MG(0:0/20:3(11Z,14Z,17Z)/0:0)	0.844949524
gene-SLC4A3	1.006401498	N-Stearoyl Glutamine	1.862695384
gene-SLC4A3	1.006401498	(1'R)-Nepetalic acid	0.499281338

gene-SLC4A3	1.006401498	Iridal	0.699410612
gene-SLC4A3	1.006401498	N-Stearoyl Proline	0.469971646
gene-SLC4A3	1.006401498	Galabiosylceramide (d18:1/20:0)	0.746737323
gene-SLC4A3	1.006401498	LysoPI(0:0/18:0)	0.420441153
gene-SLC4A3	1.006401498	N-Stearoyl Valine	0.653773206
gene-SLC4A3	1.006401498	PS(24:1(15Z)/24:1(15Z))	0.557296152
gene-SLC4A3	1.006401498	Nystatin	0.031925024
gene-SLC4A3	1.006401498	2,4-Undecadienal	0.228636005
gene-SLC4A3	1.006401498	Farnesyl acetone	0.222244889
gene-SLC4A3	1.006401498	2-[(Tetrahydrofurfuryl)oxy]ethanol	0.07976822
gene-SLC4A3	1.006401498	Undecylenic acid	0.352352175
gene-SLC4A3	1.006401498	3,5,5-Trimethyl-2-cyclohexen-1-one	0.280596306
gene-SLC4A3	1.006401498	5-Ethyl-3-methyl-2E,4E,6E-nonatriene	0.147617686
gene-SLC4A3	1.006401498	9,12,15-Octadecatrien-1-ol	0.176982043
gene-SLC4A3	1.006401498	N-Myristoyl Aspartic acid	0.334387437
gene-SLC4A3	1.006401498	Nigroxanthin	0.705005059
gene-SLC4A3	1.006401498	PC(16:0/20:5(5E,8E,11E,14E,17E))[U]	0.208077202
gene-SLC4A3	1.006401498	PE(18:0/20:4(8Z,11Z,14Z,17Z)-2OH(5S,6R))	0.418208781
gene-SLC4A3	1.006401498	Azimexon	0.008689891
gene-SLC4A3	1.006401498	PE-NMe(18:2(9Z,12Z)/18:2(9Z,12Z))[U]	0.694736593
gene-SLC4A3	1.006401498	PE(18:0/20:4(8Z,11Z,14Z,17Z))	0.344922006
gene-SLC4A3	1.006401498	PS(16:1(9Z)/22:2(13Z,16Z))	0.659652661
gene-SLC4A3	1.006401498	PC(P-16:0/20:5(5Z,8Z,11Z,14Z,16E)-OH(18R)	0.26121581
gene-SLC4A3	1.006401498	PC(P-16:0/20:3(8Z,11Z,14Z)-2OH(5,6))	0.174122169
gene-SMAD6	1.006116689	5-Hydroxy-p-mentha-6,8-dien-2-one	0.363336951
gene-SMAD6	1.006116689	Gancaonin P	0.028135835
gene-HSPB2	1.00554143	Palmitoyl Ara-C	0.586890476
gene-HSPB2	1.00554143	Mupirocin	0.952072991
gene-HSPB2	1.00554143	(3Z)-Phytochromobilin	0.67355307
gene-HSPB2	1.00554143	(-)-alpha-Terpineol	0.345716279
gene-HSPB2	1.00554143	3b,6a-Dihydroxy-alpha-ionol 9-[apiosyl-(1-	0.045258177
gene-HSPB2	1.00554143	N-[[3-Hydroxy-2-(2-pentenyl)cyclopentyl]ac	1.043930947
gene-HSPB2	1.00554143	24,24-Difluoro-1alpha,25-dihydroxyvitamin I	1.131590894
gene-HSPB2	1.00554143	PC(P-18:1(11Z)/PGE2)	0.509503133
gene-HSPB2	1.00554143	PC(20:3(5Z,8Z,11Z)/24:0)	0.387959564
gene-HSPB2	1.00554143	Phorone A	0.079039095
gene-HSPB2	1.00554143	2-Amino-4-[carbamimidoyl(methyl)amino]bu	0.075703353
gene-HSPB2	1.00554143	(9Z)-Octadecenoic acid	0.142359556
gene-HSPB2	1.00554143	Digalacturonate	0.473524431
gene-HSPB2	1.00554143	Phenethyl 6-galloylglucoside	0.069516774
gene-FBN1	1.004283708	D-Erythro-imidazole-glycerol-phosphate	0.355812431
gene-FBN1	1.004283708	Undecanedioic acid	0.059955634
gene-FBN1	1.004283708	cis-p-Menth-2-en-1-ol	0.201017988
gene-FBN1	1.004283708	PG(20:1(11Z)/18:3(10,12,15)-OH(9))	0.626244347
gene-FBN1	1.004283708	N-Eicosapentaenoyl Asparagine	0.371594025
gene-FBN1	1.004283708	PC(P-18:1(11Z)/PGE2)	0.509503133
gene-FBN1	1.004283708	2-Propenamide, 2-cyano-3-(4-hydroxy-3,5-	0.067779204
gene-FBN1	1.004283708	PG(20:1(11Z)/18:3(9,11,15)-OH(13))	0.69829683
gene-PLEKHG3	1.003881454	3-Thiacytidine	0.209387412
gene-PLEKHG3	1.003881454	D-Erythro-imidazole-glycerol-phosphate	0.355812431
gene-PLEKHG3	1.003881454	LTB4-d4	0.226799982

gene-PLEKHG3	1.003881454	cis-p-Menth-2-en-1-ol	0.201017988
gene-PLEKHG3	1.003881454	(-)-alpha-Terpineol	0.345716279
gene-PLEKHG3	1.003881454	PG(20:1(11Z)/18:3(10,12,15)-OH(9))	0.626244347
gene-PLEKHG3	1.003881454	5-(2-Aminopropyl)-2-methylphenol	0.323231457
gene-PLEKHG3	1.003881454	4-Dimethylamino-L-phenylalanine	0.242110226
gene-PLEKHG3	1.003881454	Armillane	0.52808635
gene-PLEKHG3	1.003881454	2-Methyl-3-phenyl-2-propenal	0.407813975
gene-PLEKHG3	1.003881454	PC(P-18:1(11Z)/PGE2)	0.509503133
gene-PLEKHG3	1.003881454	(3R,4R)-3-Amino-1-hydroxy-4-methylpyrrol	0.471914506
gene-PLEKHG3	1.003881454	PC(P-18:1(11Z)/PGJ2)	0.565243877
gene-PLEKHG3	1.003881454	PS(20:0/20:4(8Z,11Z,14Z,17Z)-2OH(5S,6R))	0.402595921
gene-PLEKHG3	1.003881454	CL(8:0/8:0/18:2(9Z,11Z)/20:0)	0.622988418
gene-NLGN3	1.001375229	2-[(3S)-3-[[(2S)-1-(Carboxymethoxy)-1-oxo	0.111577437
gene-NLGN3	1.001375229	1,2-O-Isopropylidene-D-glucofuranose	0.080987667
gene-NLGN3	1.001375229	PC(16:0/18:1(12Z)-2OH(9,10))	0.461843233
gene-NLGN3	1.001375229	5-(2-Aminopropyl)-2-methylphenol	0.323231457
gene-NLGN3	1.001375229	Armillane	0.52808635
gene-NLGN3	1.001375229	L-Histidinol	0.149249286
gene-NLGN3	1.001375229	(4-Methylphenyl)acetaldehyde	0.208360133
gene-NLGN3	1.001375229	Valyltryptophan	0.048149463
gene-NLGN3	1.001375229	Isopropyl isothiocyanate	0.172385747
gene-NLGN3	1.001375229	CL(8:0/8:0/18:2(9Z,11Z)/20:0)	0.622988418
gene-APBB3	1.000575353	D-Erythro-imidazole-glycerol-phosphate	0.355812431
gene-APBB3	1.000575353	LTB4-d4	0.226799982
gene-APBB3	1.000575353	cis-p-Menth-2-en-1-ol	0.201017988
gene-APBB3	1.000575353	2-Methyl-3-phenyl-2-propenal	0.407813975
gene-APBB3	1.000575353	alpha-Terpineol formate	0.628238986
gene-APBB3	1.000575353	PC(P-18:1(11Z)/PGE2)	0.509503133
gene-APBB3	1.000575353	PC(P-18:1(11Z)/PGJ2)	0.565243877
gene-APBB3	1.000575353	PS(20:0/20:4(8Z,11Z,14Z,17Z)-2OH(5S,6R))	0.402595921
gene-CAMTA2	1.000537753	3-Thiacytidine	0.209387412
gene-CAMTA2	1.000537753	D-Erythro-imidazole-glycerol-phosphate	0.355812431
gene-CAMTA2	1.000537753	LTB4-d4	0.226799982
gene-CAMTA2	1.000537753	cis-p-Menth-2-en-1-ol	0.201017988
gene-CAMTA2	1.000537753	PE(20:0/18:1(12Z)-2OH(9,10))	0.438658253
gene-CAMTA2	1.000537753	(-)-alpha-Terpineol	0.345716279
gene-CAMTA2	1.000537753	PG(20:1(11Z)/18:3(10,12,15)-OH(9))	0.626244347
gene-CAMTA2	1.000537753	5-(2-Aminopropyl)-2-methylphenol	0.323231457
gene-CAMTA2	1.000537753	4-Dimethylamino-L-phenylalanine	0.242110226
gene-CAMTA2	1.000537753	Armillane	0.52808635
gene-CAMTA2	1.000537753	PC(P-18:1(11Z)/PGE2)	0.509503133
gene-CAMTA2	1.000537753	(3R,4R)-3-Amino-1-hydroxy-4-methylpyrrol	0.471914506
gene-CAMTA2	1.000537753	Isopropyl isothiocyanate	0.172385747
gene-CAMTA2	1.000537753	(9Z)-Octadecenoic acid	0.142359556
gene-CAMTA2	1.000537753	Nigroxanthin	0.705005059
gene-CAMTA2	1.000537753	PC(P-18:1(11Z)/PGJ2)	0.565243877
gene-CAMTA2	1.000537753	PS(20:0/20:4(8Z,11Z,14Z,17Z)-2OH(5S,6R))	0.402595921
gene-CAMTA2	1.000537753	CL(8:0/8:0/18:2(9Z,11Z)/20:0)	0.622988418

correlation_coefficient	correlation_coefficient_pvalue
0.930045605	0.000811552
0.83439368	0.00999099
0.822596729	0.012166794
0.800467074	0.017006737
0.831696332	0.010464675
0.807874978	0.01527279
0.842409194	0.008664369
0.801135838	0.016845383
0.847895503	0.00782455
0.911366522	0.001627073
0.80204618	0.01662729
0.810194075	0.014753838
0.868882596	0.005095714
0.852173209	0.007207138
0.832569063	0.01030989
0.928038061	0.000882083
0.826005399	0.011510156
0.838338315	0.009323127
0.860923767	0.006043136
0.856498301	0.006615438
0.822382748	0.012208785
0.81264478	0.014217637
0.837210715	0.00951105
0.861931443	0.005917426
0.854263484	0.006917132
0.846793056	0.007988943
0.817841172	0.013121684
0.843829811	0.008441667
0.828868091	0.010976374
0.843141496	0.00854911
0.844813287	0.008289648
0.800560057	0.016984245
0.88541013	0.003445776
0.853922009	0.006963989
0.830173552	0.010738261
0.801211894	0.016827094
0.919516921	0.001225923
0.843757212	0.008452958
0.883596778	0.003606849
0.80861938	0.015104983
0.844886601	0.008278386
0.951517105	0.000274649
0.839328706	0.009160031
0.831366241	0.0105236
0.811181366	0.014536322
0.841607511	0.008791677
0.894933224	0.002675901
0.809277296	0.014957641

0.807165325	0.015433848
0.851741195	0.007268026
0.815089703	0.013695088
0.846072316	0.008097597
0.866565108	0.00536094
0.824330091	0.011830006
0.814293981	0.013863805
0.88041234	0.003901309
0.829480827	0.010864201
0.89833051	0.002431049
0.866445243	0.005374893
0.883300304	0.003633636
0.807078481	0.015453631
0.871788502	0.004775252
0.864470243	0.005608165
0.909020007	0.001756558
0.830476284	0.010683515
0.811566114	0.014452105
0.851177275	0.007347998
0.892176986	0.002885864
0.839901865	0.009066478
0.819508731	0.012781659
0.920110524	0.001199546
0.801636875	0.016725128
0.805363476	0.015847565
0.869792223	0.004993962
0.881205976	0.003826526
0.889521301	0.003097975
0.830005705	0.010768691
0.943917334	0.000422646
0.879265606	0.004011016
0.831021726	0.010585324
0.825678408	0.01157215
0.863609493	0.005711826
0.868067861	0.005187974
0.898874819	0.002393225
0.871234357	0.004835333
0.813374877	0.014060304
0.84801662	0.007806623
0.907840192	0.001824116
0.853645623	0.007002063
0.935609877	0.000635602
0.953394175	0.000244318
0.900630176	0.002273849
0.857484639	0.00648501
0.954714715	0.000224359
0.908155441	0.001805902
0.864185989	0.005642263
0.93328774	0.000705619
0.849593043	0.007575664
0.857781768	0.006446044

0.917458713	0.001320301
0.80443722	0.016062919
0.815063894	0.01370054
0.889788568	0.003076187
0.892142653	0.002888544
0.90900296	0.001757522
0.868325889	0.00515864
0.819371819	0.012809364
0.858219266	0.006388943
0.81990093	0.012702504
0.912086248	0.001588642
0.844183564	0.008386784
0.917286456	0.001328408
0.910444498	0.001677184
0.846992493	0.007959042
0.851856589	0.007251731
0.930574	0.000793623
0.92921716	0.000840192
0.956340969	0.000201295
0.805060923	0.015917708
0.891878605	0.002909212
0.928111851	0.000879422
0.861624599	0.005955526
0.876011431	0.004333104
0.85668385	0.006590775
0.924358189	0.001021542
0.82225132	0.012234621
0.89855212	0.002415603
0.857371867	0.006499838
0.838769674	0.009251867
0.817324758	0.013228127
0.803622305	0.016253896
0.853900135	0.006966998
0.868388772	0.005151507
0.852656484	0.007139411
0.889229774	0.003121854
0.881928205	0.003759281
0.845756471	0.008145506
0.864812255	0.005567314
0.84408319	0.008402333
0.828956366	0.010960169
0.874729395	0.004464421
0.893165886	0.002809351
0.823859513	0.011920848
0.835498691	0.009800941
0.832769811	0.010274493
0.871213317	0.004837624
0.930812359	0.00078562
0.839401424	0.009148128
0.880189598	0.003922465
0.851151466	0.007351672

0.873230815	0.004621129
0.830331326	0.010709707
0.842496395	0.008650593
0.839415312	0.009145856
0.837924719	0.009391779
0.846711755	0.008001152
0.822710752	0.012144456
0.856281996	0.006644263
0.816442788	0.013411175
0.890089571	0.003051767
0.823070765	0.012074096
0.891396463	0.002947197
0.872300029	0.004720219
0.819342077	0.012815388
0.839928687	0.009062115
0.910696447	0.001663392
0.842544198	0.008643047
0.805530071	0.015809025
0.863983035	0.00566669
0.822523057	0.01218124
0.821619034	0.012359399
0.869041145	0.005077884
0.843342721	0.00851761
0.871349275	0.004822834
0.841489613	0.0088105
0.836107433	0.009697231
0.860758841	0.006063872
0.862569928	0.005838647
0.897977293	0.0024558
0.935255945	0.000645965
0.858471751	0.006356137
0.906655073	0.001893656
0.80537796	0.015844212
0.907889247	0.001821274
0.878108621	0.004123698
0.841553152	0.008800353
0.816102445	0.013482235
0.826219559	0.011469667
0.830301225	0.010715151
0.884609222	0.003516332
0.809654474	0.01487358
0.816700399	0.013357545
0.876926303	0.004240932
0.934010804	0.000683302
0.811306596	0.014508877
0.869290709	0.005049899
0.81900382	0.012884019
0.825501382	0.0116058
0.833657146	0.010118954
0.821719587	0.012339502
0.85506171	0.006808386

0.826350629	0.011444932
0.902044594	0.002180522
0.896335065	0.002573021
0.969403446	7.00E-05
0.919928491	0.001207595
0.939578652	0.000526769
0.842739642	0.008612237
0.813711405	0.013988154
0.87162143	0.004793316
0.832466483	0.010328007
0.825100243	0.01168228
0.822673798	0.012151693
0.845245957	0.008223326
0.888532937	0.003179413
0.85571903	0.006719662
0.824399352	0.011816673
0.819844663	0.012713841
0.877865732	0.004147609
0.866337657	0.005387437
0.87905401	0.004031474
0.810070813	0.014781137
0.821531653	0.012376705
0.800421953	0.017017658
0.810097814	0.014775155
0.866233587	0.005399589
0.807609677	0.015332876
0.824831665	0.011733664
0.828400612	0.011062447
0.883835316	0.003585389
0.865888774	0.005439977
0.86422205	0.00563793
0.804232657	0.016110725
0.830371678	0.010702412
0.943155706	0.000439844
0.883388817	0.003625626
0.915998936	0.001390029
0.965224862	0.000102412
0.873705268	0.004571139
0.937254131	0.000588885
0.8791731	0.004019951
0.868530154	0.005135494
0.960063756	0.000154505
0.811233342	0.014524927
0.800996065	0.016879027
0.907733321	0.001830318
-0.81480056	0.013756244
0.834238052	0.010017943
0.827498198	0.011229806
0.892321408	0.002874607
0.822172999	0.012250033
0.852676988	0.007136547

0.83471185	0.009936031
0.851440549	0.007310593
0.865269423	0.005513007
0.812259436	0.014301122
0.835289657	0.009836715
0.846794903	0.007988666
0.828080118	0.011121703
0.802450299	0.016531045
0.812393069	0.014272135
0.815663159	0.013574304
0.890047848	0.003055145
0.876586854	0.004274982
0.859596372	0.006211323
0.828577995	0.011029737
0.82989496	0.010788798
0.8292647	0.010903684
0.848223209	0.007776103
0.85098207	0.007375812
0.810935199	0.014590366
0.807859063	0.01527639
0.830365598	0.010703511
0.910810947	0.001657149
0.801306248	0.016804422
0.864805818	0.005568081
0.830193102	0.01073472
0.810818255	0.014616085
0.868313134	0.005160087
0.834661961	0.009944636
0.861797988	0.005933978
0.824751675	0.011748995
0.833078802	0.01022016
0.875579357	0.004377079
0.869652033	0.005009558
0.83471787	0.009934993
0.802918494	0.016419976
0.803791463	0.016214137
0.912600875	0.001561528
0.822364688	0.012212333
0.816977739	0.01329996
0.845987022	0.008110517
0.842044711	0.008722103
0.873679101	0.004573887
0.855519056	0.006746575
0.836996198	0.009547071
0.867290676	0.005276973
0.898694634	0.002405704
0.853629827	0.007004243
0.838767886	0.009252161
0.849504828	0.007588471
0.937493801	0.000582269
0.814806998	0.013754881

0.859071553	0.006278637
0.851201296	0.007344581
0.870634317	0.004900936
0.955724657	0.000209842
0.866401017	0.005380048
0.862825513	0.005807302
0.84740144	0.007897954
0.81489414	0.013736433
0.815746427	0.013556822
0.831843197	0.010438525
0.80928117	0.014956776
0.867045164	0.00530529
0.854469061	0.00688902
0.808970511	0.015026234
0.870810449	0.004881621
0.842433512	0.008660526
0.813171804	0.014103954
0.824520886	0.0117933
0.889837921	0.003072174
0.862731755	0.005818788
0.846040666	0.008102389
0.804105759	0.016140426
0.840936005	0.008899224
0.930342078	0.00080146
0.826640368	0.011390373
0.868888736	0.005095023
0.84732914	0.007908732
0.83212924	0.010387713
0.826483727	0.011419849
0.844522834	0.008334362
0.822862029	0.012114859
0.866535604	0.005364372
0.847431779	0.007893434
0.879260898	0.00401147
0.863320827	0.005746863
0.832393289	0.010340947
0.808091998	0.015223748
0.906107843	0.001926338
0.866793454	0.005334423
0.877848744	0.004149285
0.800202131	0.017070926
0.853143752	0.007071538
0.935136616	0.000649484
0.80304718	0.016389531
0.842886925	0.008589066
0.856763124	0.006580256
0.833535194	0.010140242
0.811130047	0.014547578
0.8337484	0.010103044
0.817111194	0.013272306
0.810375631	0.014713686

0.811309457	0.01450825
0.859590769	0.006212039
0.826587439	0.011400328
0.806053936	0.015688218
0.885548294	0.003433698
0.833581567	0.010132144
0.849456251	0.00759553
0.860515416	0.006094561
0.819578946	0.012767466
0.829927385	0.010782908
0.842404783	0.008665067
0.808477819	0.015136805
0.807766795	0.015297273
0.814896703	0.01373589
0.890751183	0.002998534
0.807662725	0.01532085
0.855354965	0.006768711
0.849966645	0.007521575
0.808059454	0.015231096
0.814708471	0.013775758
0.908367753	0.001793702
0.881329	0.003815017
0.900444508	0.002286289
0.906869113	0.001880971
0.829982162	0.010772963
0.816157877	0.013470645
0.833563685	0.010135266
0.807903826	0.015266265
0.897069991	0.002520125
0.848523974	0.007731808
0.85454458	0.006878712
0.833805323	0.010093127
0.849344254	0.00761182
0.829966784	0.010775755
0.8046031	0.016024218
0.918906987	0.001253416
0.820830107	0.012516211
0.876567304	0.004276948
0.819902182	0.012702252
0.803511024	0.016280085
0.835608661	0.009782154
0.830008268	0.010768226
0.850550771	0.007437502
0.859995425	0.00616045
0.827979565	0.011140335
0.814068615	0.013911825
0.815012038	0.013711498
0.83576709	0.009755128
0.910252631	0.001687736
0.842192829	0.008698612
0.82617569	0.011477954

0.829026163	0.010947366
0.870171189	0.004951959
0.838878155	0.009234
0.833331048	0.01017594
0.857193589	0.006523324
0.861399174	0.005983617
0.842159688	0.008703865
0.903133869	0.002110369
0.821907461	0.01230238
0.821612239	0.012360744
0.861797988	0.005933978
0.832063019	0.010399462
0.847790956	0.007840046
0.87211895	0.004739654
0.850765407	0.007406761
0.874743462	0.004462966
0.851347566	0.00732379
0.803956687	0.016175361
0.897696912	0.002475563
0.848876476	0.007680098
0.853768766	0.006985083
0.877294362	0.00420421
0.909809649	0.001712263
0.830664515	0.010649564
0.853780448	0.006983474
0.81641835	0.013416269
0.874734223	0.004463921
0.938608587	0.000552139
0.809085071	0.015000597
0.855934381	0.006690755
0.8988114	0.002397612
0.903354168	0.002096361
0.827012777	0.011320489
0.860085547	0.006148998
0.881723046	0.003778305
0.869372964	0.005040698
0.831419289	0.010514116
0.80240643	0.016541476
0.849109948	0.007645971
0.866487861	0.00536993
0.858311653	0.006376926
0.822809458	0.012125139
0.873256862	0.004618376
0.964444518	0.000109397
0.951394558	0.000276711
0.817321777	0.013228743
0.861776531	0.005936642
0.831946492	0.010420158
0.840171874	0.009022618
0.818744659	0.012936759
0.870617032	0.004902834

0.877426624	0.004191064
0.802391648	0.016544991
0.846543908	0.008026397
0.865870416	0.005442132
0.802583098	0.016499493
0.89529413	0.002649165
0.86838007	0.005152494
0.858084142	0.006406544
0.821480751	0.012386794
0.851581633	0.007290598
0.920387745	0.001187356
0.835788667	0.009751451
0.839660645	0.009105777
0.851876795	0.007248879
0.860956252	0.006039057
0.828105092	0.011117078
0.843341589	0.008517787
0.856358171	0.006634103
0.824623287	0.011773629
0.87665087	0.004268547
0.851694405	0.007274641
0.803277552	0.016335116
0.806523263	0.015580482
0.817047	0.013285603
0.860181272	0.006136849
0.948442578	0.000329509
0.935853481	0.000628532
0.866455615	0.005373685
0.832891762	0.010253027
0.808445573	0.015144059
0.824113429	0.011871777
0.843702972	0.008461401
0.81904614	0.01287542
0.817726374	0.013145299
0.870413125	0.004925262
0.877988636	0.004135498
0.803595543	0.016260192
0.838277459	0.009333208
0.817942739	0.013100813
0.86332798	0.005745993
0.852897704	0.007105759
0.916431308	0.001369132
0.862513304	0.005845607
0.866241395	0.005398677
0.871527493	0.004803491
0.821010649	0.012480215
0.8110708	0.01456058
0.802535295	0.016510846
0.84387058	0.00843533
0.891896665	0.002907795
0.938590765	0.000552613

0.951507211	0.000274815
0.853077114	0.007080796
0.912803888	0.001550915
0.8203668	0.012608884
0.818954647	0.012894016
0.806173503	0.015660727
0.847596884	0.007868864
0.935928702	0.00062636
0.842344999	0.00867452
0.859896541	0.006173031
0.847103536	0.007942425
0.816303074	0.013440317
0.817414045	0.013209684
0.830239832	0.01072626
0.893678188	0.002770234
0.838831782	0.009241635
0.896995902	0.002525425
0.828353286	0.011071184
0.905039668	0.001991184
0.823287606	0.012031842
0.882681429	0.003689966
0.818134785	0.013061406
0.84465909	0.008313367
0.840641856	0.008946596
0.807220459	0.015421297
0.816972673	0.01330101
0.857912779	0.006428911
0.905656934	0.001953542
0.851610601	0.007286497
0.888803542	0.00315698
0.824463189	0.011804392
0.81031549	0.014726979
0.825863063	0.011537115
0.810244739	0.014742626
0.808863699	0.015050161
0.807452917	0.01536845
0.819629431	0.012757266
0.825431824	0.011619039
0.811780453	0.014405322
0.82319653	0.012049578
0.838996232	0.009214579
0.841749847	0.008768988
0.817238867	0.013245884
0.900356352	0.002292211
0.876924872	0.004241075
0.824261785	0.011843165
0.84263587	0.008628587
0.868088067	0.005185673
0.818322182	0.013023025
0.822203398	0.01224405
0.870493293	0.004916437

0.868589342	0.0051288
0.84436357	0.008358944
0.813194931	0.014098979
0.860670984	0.006074937
0.909228921	0.001744767
0.83789134	0.009397334
0.876607239	0.004272932
0.851947725	0.007238877
0.818092942	0.013069985
0.821765602	0.012330403
0.932084203	0.000743813
0.854182184	0.00692827
0.812023938	0.014352294
0.928822398	0.000854069
0.819658697	0.012751356
0.815637171	0.013579763
0.862836242	0.005805988
0.904766262	0.002008007
0.808797836	0.015064928
0.890589535	0.003011484
0.878038406	0.004130601
0.931497097	0.000762926
0.852928817	0.007101426
0.8745206	0.004486047
0.916445851	0.001368433
0.844184935	0.008386572
0.811179698	0.014536688
0.804552317	0.016036059
0.800151169	0.017083291
0.808851242	0.015052954
0.806486428	0.015588921
0.821571529	0.012368806
0.831026852	0.010584404
0.862263262	0.005876401
-0.847488344	0.007885011
0.803536177	0.016274163
0.805192888	0.01588709
0.800795794	0.016927306
0.838605702	0.009278914
0.82293427	0.012100742
0.816310525	0.013438762
0.853297114	0.007050261
0.836681664	0.009600043
0.949185252	0.000315652
0.847667634	0.007858351
0.938219547	0.000562535
0.919658899	0.00121958
0.831515968	0.010496846
0.809300065	0.014952558
0.865250409	0.005515258
0.924104631	0.001031648

0.839231849	0.009175901
0.851827681	0.007255811
0.840488791	0.00897131
0.805284381	0.015865883
0.816561162	0.013386514
0.801685035	0.016713597
0.861450553	0.005977207
0.879889727	0.003951064
0.810014307	0.014793662
0.821028411	0.012476677
0.876193225	0.004314687
0.927124739	0.000915453
0.832658827	0.010294053
0.897428572	0.002494574
0.817160249	0.01326215
0.849465191	0.00759423
0.904970884	0.001995408
0.837465584	0.009468366
0.861442804	0.005978173
0.808258355	0.015186222
0.831491351	0.010501242
0.809500635	0.01490783
0.853564501	0.007013263
0.839531779	0.009126815
0.808965445	0.015027368
0.886299431	0.003368511
0.814172566	0.013889663
0.808608234	0.015107487
0.851157665	0.007350789
0.893984139	0.002747043
0.849428773	0.007599524
0.829859972	0.010795156
0.832537413	0.010315478
0.933744729	0.00069146
0.814725935	0.013772056
0.808003008	0.015243845
0.879116535	0.004025421
0.834596872	0.009955869
0.844512701	0.008335924
0.861433208	0.00597937
0.828062713	0.011124926
0.884154201	0.00355683
0.801585436	0.016737449
0.894426048	0.002713768
0.915802419	0.001399595
0.868088901	0.005185578
0.814788461	0.013758807
0.838236511	0.009339996
0.811653078	0.014433112
0.81732738	0.013227585
0.864255309	0.005633936

0.81339711	0.01405553
0.900508642	0.002281987
0.840125203	0.00903019
0.841104984	0.008872082
0.835577428	0.009787487
0.898323059	0.00243157
0.869176924	0.005062646
0.865906298	0.00543792
0.845188022	0.008232187
0.928804815	0.00085469
0.829451442	0.010869564
0.813827872	0.013963238
0.833311081	0.010179436
0.888536811	0.003179091
0.843500614	0.008492945
0.82083708	0.01251482
0.831460238	0.010506799
0.804486632	0.016051384
0.880016327	0.003938973
0.849537492	0.007583727
0.810982347	0.014580006
0.864629507	0.005589118
0.832806647	0.010268006
0.804646969	0.016013992
0.838099122	0.009362791
0.877630353	0.004170866
0.874737382	0.004463595
0.830787718	0.01062738
0.841646135	0.008785517
0.910932541	0.001650536
0.817224085	0.013248941
0.885789037	0.003412717
0.915687203	0.001405223
0.80436641	0.016079457
0.823412716	0.012007505
0.917145312	0.001335074
0.913304925	0.001524925
0.842652321	0.008625994
0.86301136	0.005784577
0.947921395	0.000339468
0.849030614	0.007657557
0.808937848	0.015033548
0.809676826	0.014868608
0.852497935	0.007161586
0.814607382	0.013797199
0.881106615	0.003835838
0.892716587	0.002843949
0.808725297	0.015081201
0.808557272	0.015118939
0.81856221	0.012973969
0.968265474	7.80E-05

0.875728965	0.00436182
0.86218977	0.005885471
0.831355572	0.010525508
-0.86048305	0.006098649
0.803520203	0.016277924
0.839920938	0.009063376
0.851035535	0.007368187
0.806493819	0.015587228
0.851685762	0.007275863
0.807002127	0.015471037
0.866827071	0.005330526
0.806270361	0.015638479
0.818091452	0.013070291
0.833736002	0.010105204
0.839922428	0.009063133
0.984130561	9.87E-06
0.819600642	0.012763082
0.856484592	0.006617263
0.82200104	0.012283916
0.813505292	0.014032316
0.842420936	0.008662514
0.815764666	0.013552994
0.823931634	0.011906897
0.922891796	0.00108089
0.855789721	0.006710164
0.883461237	0.00361908
0.83869803	0.009263678
0.827055097	0.011312565
0.889479756	0.00310137
0.876806319	0.004252947
0.917434633	0.001321432
0.856359243	0.00663396
0.837761819	0.009418907
0.897192061	0.002511408
0.824997783	0.011701866
0.851640761	0.007282229
0.833018959	0.010230668
0.856370211	0.006632498
0.82282573	0.012121957
0.836144924	0.009690867
0.84379524	0.008447043
0.83673656	0.009590784
0.836357594	0.009654815
0.822422862	0.012200906
0.806072176	0.015684023
0.831534863	0.010493473
0.850820661	0.00739886
0.836962759	0.009552694
0.85254395	0.007155146
0.809669018	0.014870345
0.822494328	0.012186877

0.803919196	0.016184155
0.812402964	0.01426999
-0.813794196	0.01397044
0.810672641	0.014648148
0.802742302	0.016461719
0.818959534	0.012893022
0.803144157	0.016366611
0.801560938	0.016743319
0.877070189	0.004226552
0.915039718	0.001437128
0.80819577	0.015200332
0.846372128	0.008052286
0.809714735	0.014860178
0.809846401	0.01483092
0.847526133	0.007879387
0.853652894	0.00700106
0.83455354	0.009963353
0.826363683	0.01144247
0.834342182	0.009999904
0.802494109	0.016520632
0.875692189	0.004365568
0.8237257	0.01194676
0.881754696	0.003775366
0.816719413	0.013353592
0.835045338	0.009878632
0.961915553	0.000134182
0.863275409	0.005752388
0.93547374	0.000639575
0.810694396	0.014643355
0.871488452	0.004807724
0.859672666	0.006201576
0.843725681	0.008457866
0.930330336	0.000801858
0.890099406	0.003050971
0.883791983	0.003589281
0.817272484	0.013238932
0.806449234	0.015597445
0.892695189	0.002845604
0.862920344	0.005795699
0.885047615	0.003477598
0.817928374	0.013103763
0.810872674	0.014604113
0.843980193	0.008418308
0.801048517	0.016866396
0.8621943	0.005884912
0.824841201	0.011731837
0.879943967	0.003945881
0.812289298	0.014294641
0.84104681	0.00888142
0.89390111	0.002753324
0.869030237	0.00507911

0.830542862	0.010671499
0.826328456	0.011449114
0.892849386	0.002833694
0.838789463	0.009248606
0.868317485	0.005159594
0.822880864	0.012111177
0.811190248	0.014534374
0.849949121	0.007524106
0.848395407	0.007750723
0.887341619	0.003279397
0.901744843	0.002200089
0.813086152	0.014122391
0.848728538	0.007701773
0.852738976	0.007127892
0.87824887	0.004109931
0.803104818	0.016375906
0.848178804	0.007782657
0.816656888	0.013366594
0.848089874	0.007795792
0.850257814	0.007479592
0.895279765	0.002650225
0.814724088	0.013772448
0.901261628	0.002231871
0.807607234	0.015333431
0.846485496	0.008035194
0.851447105	0.007309663
0.889960408	0.00306223
0.803089738	0.01637947
0.826051712	0.011501392
0.945469677	0.000388974
0.859498382	0.006223856
0.841488063	0.008810747
0.82379508	0.01193332
0.926209629	0.000949705
0.875187933	0.004417164
0.825272739	0.011649354
0.804288805	0.016097595
0.905382156	0.001970241
0.866108418	0.005414228
0.840108275	0.009032937
0.834309101	0.010005632
0.829619825	0.010838856
0.862885416	0.005799971
0.809829235	0.014834733
0.800744534	0.016939677
0.876447439	0.004289018
0.859367013	0.006240683
0.849144816	0.007640883
0.821476877	0.012387563
0.810054243	0.01478481
0.856409252	0.006627295

0.874413013	0.004497216
0.827320814	0.01126289
0.805736065	0.015761452
0.906060755	0.001929167
0.826978624	0.011326887
0.817329943	0.013227056
0.803089082	0.016379625
0.806220233	0.015649991
0.826893508	0.011342841
0.81102407	0.014570841
0.878035188	0.004130917
0.928978205	0.000848574
0.865653515	0.005467643
0.852561355	0.007152711
0.819241524	0.012835766
0.835566461	0.00978936
0.808697343	0.015087476
0.801915884	0.016658396
0.876700103	0.004263603
0.835472047	0.009805496
0.881391644	0.003809166
0.840554237	0.008960738
0.827048182	0.011313859
0.840020001	0.009047272
0.884803057	0.003499172
0.870111704	0.004958537
0.847267985	0.007917856
0.846190035	0.008079786
0.920989037	0.001161193
0.810698032	0.014642554
0.828899443	0.010970617
0.892260432	0.002879356
0.877548218	0.004179001
0.886167943	0.003379864
0.809660077	0.014872334
0.886937797	0.003313743
0.862313509	0.005870204
0.859289408	0.006250638
0.815048158	0.013703864
0.888884902	0.003150256
0.947528124	0.000347112
0.848634422	0.007715582
0.837050855	0.009537885
0.820149899	0.012652418
0.808197081	0.015200037
0.821697116	0.012343946
0.83271575	0.010284018
0.862212896	0.005882616
0.805458248	0.015825633
0.81986928	0.012708881
0.814444423	0.013831807

0.872611165	0.004686946
0.830324769	0.010710893
0.873456717	0.004597284
0.840638399	0.008947154
0.817745209	0.013141423
0.814936042	0.013727567
0.877966344	0.004137693
0.880492926	0.003893673
0.852475107	0.007164782
0.832895875	0.010252303
0.919862568	0.001210518
0.809110522	0.014994905
0.818733573	0.012939018
0.80019325	0.017073081
0.876518011	0.004281909
0.840663731	0.008943068
0.836463451	0.009636902
0.845781863	0.008141648
0.880308867	0.003911127
-0.810583711	0.014667752
0.881043434	0.003841766
0.879203916	0.004016973
0.80197376	0.016644575
0.834571064	0.009960326
0.869234264	0.00505622
0.887782276	0.003242181
0.810669839	0.014648766
0.854934514	0.006825641
0.817626774	0.01316581
0.812832713	0.014177032
0.855330288	0.006772044
0.804262042	0.016103852
0.805773139	0.0157529
0.847762585	0.007844255
0.816207647	0.013460244
0.851413369	0.007314449
0.921880603	0.001123098
0.821202099	0.012442116
0.874567032	0.004481232
0.814282775	0.01386619
0.841772318	0.00876541
0.869016051	0.005080704
0.876369059	0.004296921
-0.805549741	0.015804479
0.813903511	0.013947072
0.850921869	0.007384403
0.829801917	0.01080571
0.82467407	0.011763881
0.921686172	0.001131335
0.814981043	0.01371805
0.832787216	0.010271427

0.842567563	0.00863936
0.93264097	0.00072598
0.842644811	0.008627178
0.869589746	0.005016498
0.840062559	0.009040359
0.942379713	0.000457832
0.823624849	0.011966312
0.808707535	0.015085188
0.828243196	0.011091526
0.815774977	0.013550831
0.861911535	0.005919893
0.803253233	0.016340855
0.865295112	0.005509965
0.880693316	0.003874726
0.88391161	0.003578543
0.800982356	0.016882329
0.834917367	0.009900633
0.858818531	0.006311255
0.812576413	0.014232426
0.871464014	0.004810375
0.885852337	0.003407215
0.822573602	0.012171328
0.837655663	0.009436612
0.872343719	0.004715538
0.803505778	0.01628132
0.921946466	0.001120317
0.865204394	0.005520711
0.859874964	0.006175779
0.82698226	0.011326205
0.885158837	0.003467815
0.887764812	0.003243651
0.851122439	0.007355805
0.829890907	0.010789534
0.81276083	0.014192555
0.805079639	0.015913363
-0.849607527	0.007573562
0.831039786	0.010582083
0.869133532	0.005067513
0.853009462	0.007090203
0.868045807	0.005190486
0.912433743	0.001570301
0.828741789	0.010999587
0.88544172	0.003443012
0.809198618	0.014975214
0.834969819	0.009891612
0.82394129	0.01190503
0.809559286	0.014894767
0.831944644	0.010420486
0.947191298	0.000353749
0.876343548	0.004299496
0.920913756	0.001164448

0.863635182	0.005708714
0.956592262	0.000197877
0.81940335	0.01280298
0.803164661	0.016361767
0.916044354	0.001387824
0.907590032	0.001838654
0.872025907	0.00474966
0.930807292	0.00078579
0.856189191	0.006656655
0.835144818	0.009861551
0.907746315	0.001829563
0.803100705	0.016376878
-0.811421335	0.014483759
0.894736648	0.002690537
0.899199963	0.002370814
0.826151073	0.011482605
0.837201238	0.00951264
0.828586161	0.011028232
0.804794252	0.015979693
0.806195974	0.015655564
0.885807276	0.003411131
0.807590723	0.015337175
0.862923026	0.005795371
0.882790565	0.003679992
0.827972233	0.011141694
0.914082468	0.001485154
0.808689117	0.015089322
0.81724894	0.0132438
0.827493966	0.011230594
0.808482707	0.015135705
0.836329401	0.00965959
0.875820458	0.004352505
0.803765953	0.016220129
0.854552448	0.006877638
0.812668502	0.014212507
0.868365645	0.00515413
0.814353585	0.013851122
0.80515343	0.015896241
0.861091971	0.006022034
0.853971064	0.006957246
0.819256604	0.012832708
0.899306357	0.00236351
0.824611962	0.011775803
0.815033138	0.013707038
0.833228588	0.010193887
0.832325161	0.010353001
0.827933788	0.011148824
0.820871592	0.012507934
0.825026751	0.011696326
0.892221272	0.002882409
0.870414853	0.004925072

0.81369406	0.013991867
0.858573794	0.006342909
0.80754739	0.015347005
0.828025401	0.011131839
0.811425626	0.01448282
0.821275949	0.012427439
0.866529226	0.005365115
0.862630546	0.005831203
0.851604104	0.007287416
0.858581841	0.006341867
0.80625397	0.015642243
0.806264937	0.015639725
0.872031569	0.004749051
0.86845994	0.005143443
0.828732908	0.011001221
0.815706253	0.013565254
0.842843354	0.008595917
0.910360992	0.001681771
0.839205861	0.009180162
0.8806445	0.003879336
0.822909236	0.012105633
0.824180663	0.011858805
0.859734714	0.006193656
0.850073218	0.007506191
0.88294518	0.003665892
0.869783282	0.004994956
0.839923859	0.009062901
0.854972303	0.006820512
0.833703101	0.01011094
0.901587725	0.00221039
0.87410146	0.004529662
0.861668289	0.005950092
0.825811863	0.011546823
0.947398841	0.00034965
0.812724352	0.014200436
0.898711026	0.002404567
0.837911725	0.009393941
0.879005492	0.004036174
0.80941236	0.014927506
0.961202264	0.000141787
0.875974655	0.004336836
0.838307023	0.00932831
0.91528362	0.001425055
0.823029697	0.012082109
0.871888041	0.004764511
0.844557822	0.008328967
0.847287536	0.007914939
0.836611867	0.009611823
0.804153562	0.016129234
0.838080943	0.00936581
0.86139214	0.005984495

0.908466399	0.001788052
0.869953275	0.004976084
0.892427504	0.002866355
0.843770325	0.008450918
0.812814772	0.014180906
0.830279112	0.010719151
0.801866472	0.016670202
0.814093232	0.013906575
0.843959749	0.008421481
0.805478156	0.015821029
0.821548104	0.012373446
0.808892906	0.015043616
0.84155035	0.0088008
0.847324371	0.007909443
0.839153588	0.009188736
0.804312885	0.016091965
0.817744374	0.013141594
0.838979959	0.009217254
0.83654809	0.009622595
0.856302261	0.006641559
0.86113894	0.00601615
0.841354489	0.008832103
0.907510996	0.001843263
0.881759942	0.003774879
0.887649655	0.003253353
0.826499581	0.011416863
0.917079926	0.00133817
0.845958352	0.008114863
0.964437306	0.000109463
0.881615043	0.003788345
0.840331435	0.008996762
0.852795422	0.007120016
0.811693311	0.014424331
0.809696317	0.014864273
0.88922596	0.003122167
0.80546838	0.01582329
0.831568003	0.010487558
0.803070843	0.016383936
0.893973649	0.002747836
0.886087775	0.003386798
0.805785596	0.015750027
0.874210417	0.004518298
0.931895554	0.00074992
0.887775004	0.003242793
0.862306058	0.005871123
0.821017623	0.012478826
0.851880848	0.007248308
0.924294472	0.001024075
0.823712051	0.011949405
-0.820956588	0.012490987
0.937539816	0.000581005

0.866163254	0.005407811
0.870987654	0.004862237
0.896680057	0.002548102
0.813138902	0.014111035
0.817681193	0.013154601
0.803430438	0.016299066
0.82199204	0.012285691
0.820188224	0.012644718
0.808588386	0.015111947
0.913264811	0.001526995
0.805891395	0.015725639
0.890415728	0.003025449
0.880256176	0.003916133
0.834507585	0.009971293
0.915820479	0.001398714
0.801395059	0.0167831
0.808482409	0.015135772
0.82205689	0.012272905
0.835001647	0.00988614
0.801680684	0.016714639
0.825011015	0.011699335
0.850221336	0.007484843
0.878619254	0.004073718
0.874664545	0.00447113
0.889561296	0.003094708
0.803333402	0.016321941
0.835020602	0.009882882
0.909592807	0.001724354
0.902986765	0.002119756
0.833749771	0.010102805
-0.819710374	0.012740924
-0.819656014	0.012751898
0.801923871	0.016656489
0.8344962	0.00997326
0.860823572	0.006055728
0.855888307	0.006696933
0.805978715	0.015705529
0.894339621	0.002720255
0.898677111	0.002406919
0.902729034	0.002136268
0.907170713	0.001863191
0.818742573	0.012937184
0.812075257	0.014341132
0.877210557	0.004212553
0.886169076	0.003379766
0.900345027	0.002292972
0.912530065	0.001565241
0.951996803	0.000266675
0.938599229	0.000552388
0.843036592	0.008565561
0.841448367	0.00881709

0.959475756	0.000161358
0.92660135	0.000934942
0.819768608	0.012729175
0.92274636	0.001086896
0.810705602	0.014640887
0.823568404	0.011977264
0.850991309	0.007374494
0.805742681	0.015759926
0.81030935	0.014728336
0.840987265	0.008890985
0.825540185	0.011598419
0.808500171	0.015131778
0.805085242	0.015912062
-0.85900557	0.006287132
0.819570482	0.012769176
0.802249789	0.016578755
0.807669342	0.01531935
0.810549498	0.014675299
0.900537074	0.002280082
0.826283753	0.011457549
0.802247405	0.016579322
0.858320057	0.006375834
0.815872669	0.013530344
0.883512557	0.003614446
0.824026048	0.011888649
0.844923139	0.008272777
0.86638546	0.005381861
0.867713332	0.005228452
0.846802473	0.007987529
0.873248458	0.004619264
0.84993571	0.007526044
0.852282643	0.007191766
0.826106489	0.011491033
0.971509635	5.66E-05
0.865967691	0.005430716
0.947375178	0.000350115
0.879440367	0.00399417
0.815070271	0.013699192
0.939898491	0.000518575
0.838362157	0.009319179
0.845428586	0.008195433
0.84487474	0.008280208
-0.843447506	0.008501236
0.85495621	0.006822696
0.895243287	0.002652921
0.851638138	0.0072826
0.934255898	0.000675844
0.803269923	0.016336916
0.859680891	0.006200526
0.831851661	0.010437019
0.845526993	0.008180428

0.841309786	0.008839258
0.803832531	0.016204493
0.872867346	0.004659663
0.854473889	0.006888361
0.834706247	0.009936997
0.865117669	0.005530996
0.826757312	0.011368399
0.939036608	0.000540849
0.803872526	0.016195105
0.833456099	0.010154063
0.818306983	0.013026135
0.824211299	0.011852896
0.879826009	0.003957157
0.802499294	0.0165194
0.828417242	0.011059378
0.850313723	0.007471547
0.849676669	0.007563535
0.839196026	0.009181775
0.884891033	0.003491401
0.886357665	0.003363491
0.812174022	0.014319668
0.888196945	0.00320741
0.832157493	0.010382703
0.842991769	0.008572596
0.878225744	0.004112199
0.901277661	0.002230811
0.823695004	0.011952709
0.854654253	0.006863758
0.836266518	0.009670244
0.80516082	0.015894526
0.878832579	0.004052954
0.872063398	0.004745626
0.809464216	0.014915946
0.84060961	0.008951799
0.828963518	0.010958856
0.932696223	0.000724226
0.939294815	0.000534111
0.925849736	0.000963402
0.815732956	0.013559649
0.844218731	0.00838134
0.82590574	0.011529028
0.804535925	0.016039883
0.816656113	0.013366755
0.861941576	0.00591617
0.855324388	0.006772841
0.804571867	0.0160315
0.856666625	0.006593062
0.840472043	0.008974017
0.801442564	0.016771701
0.880839944	0.003860901
0.873658836	0.004576016

0.839941442	0.009060041
0.828764021	0.010995499
0.921492398	0.001139584
0.816384017	0.013423428
0.807374656	0.015386229
0.856265008	0.006646531
0.866267979	0.005395571
0.812041879	0.014348391
0.816867232	0.013322886
0.860138655	0.006142256
0.806455433	0.015596024
0.809687674	0.014866195
0.881668448	0.003783378
0.825801611	0.011548767
0.806011558	0.01569797
0.81289947	0.014162627
0.878646612	0.004071051
0.897537947	0.002486814
0.842859685	0.008593349
0.823881209	0.01191665
0.903771996	0.00206996
0.811778843	0.014405673
0.80324173	0.01634357
0.810584009	0.014667686
0.822492182	0.012187298
0.887268722	0.00328558
0.888075292	0.003217586
0.809977531	0.014801818
0.835748613	0.009758278
0.828732967	0.01100121
0.874796987	0.004457434
0.807304323	0.015402219
0.85412091	0.006936672
0.878725648	0.004063354
0.810773432	0.01462595
0.912415147	0.001571279
0.812039852	0.014348832
0.804344356	0.01608461
0.834315181	0.010004579
0.877121985	0.004221383
0.842220783	0.008694183
0.80654037	0.015576564
0.921593904	0.001135258
0.820998013	0.012482732
0.871949196	0.00475792
0.924471855	0.001017033
0.925551832	0.000974836
0.900076926	0.002311047
0.817804813	0.01312916
0.907878518	0.001821895
0.823013663	0.012085239

0.860176861	0.006137408
0.856315732	0.006639762
0.846501291	0.008032815
0.991892219	1.32E-06
0.951613665	0.000273032
0.810647607	0.014653665
0.891619623	0.002929576
0.820625365	0.012557111
0.831758499	0.010453601
0.828910649	0.01096856
0.83506906	0.009874557
0.919720173	0.001216849
0.834169865	0.010029766
0.821535945	0.012375855
0.863442719	0.005732051
0.822228134	0.012239182
0.822715104	0.012143604
0.941281497	0.000484106
0.814971745	0.013720016
0.85041213	0.007457402
0.817459404	0.013200322
0.80476135	0.015987351
0.825041711	0.011693466
0.894996166	0.002671226
0.86411041	0.005651352
0.875645578	0.004370321
0.842315614	0.008679169
0.855080009	0.006805906
0.865217566	0.005519149
0.872308016	0.004719363
0.806963742	0.015479792
0.814480305	0.013824182
0.80968684	0.014866381
0.803792059	0.016213997
0.839629471	0.009110863
0.848781645	0.007693988
0.846304536	0.008062487
0.891932368	0.002904996
0.868466616	0.005142687
0.881394625	0.003808887
0.808510005	0.015129566
0.830308914	0.01071376
0.816698849	0.013357867
0.808652341	0.01509758
0.910998344	0.001646965
0.800923467	0.016896518
0.808897197	0.015042655
0.816296518	0.013441685
0.933674753	0.000693615
0.822009206	0.012282306
0.861302137	0.005995735

0.815460205	0.013616974
0.958866358	0.000168669
0.81259346	0.014228737
0.828558326	0.011033361
0.823114157	0.012065633
0.882319212	0.003723195
0.821287274	0.012425189
0.828172743	0.011104557
0.835541248	0.009793667
0.87433219	0.004505619
0.845140815	0.008239411
0.887233198	0.003288596
0.809360623	0.014939045
0.90346086	0.002089599
0.813765705	0.013976534
0.810526669	0.014680335
0.829203188	0.010914938
0.883305788	0.00363314
0.831486046	0.010502189
0.927510917	0.000901244
0.800737083	0.016941475
0.87759161	0.004174702
0.888513625	0.003181018
0.84924984	0.007625569
0.838015556	0.009376674
0.800683975	0.016954299
0.895793498	0.002612457
0.80351913	0.016278176
0.817632079	0.013164717
0.830468893	0.010684849
0.810178757	0.014757229
0.875673294	0.004367494
0.911518931	0.001618885
0.930930197	0.000781684
0.862514377	0.005845475
0.830732882	0.01063725
0.907572627	0.001839668
0.873608887	0.004581266
0.888868511	0.00315161
0.806699574	0.01554013
0.881067812	0.003839478
0.815815866	0.013542254
0.875878751	0.004346577
0.863990724	0.005665764
0.870807528	0.00488194
0.932093799	0.000743504
0.912626982	0.001560161
0.823866904	0.011919418
0.859421968	0.00623364
0.834271908	0.010012075
0.930610955	0.000792379

0.824322939	0.011831383
0.902191997	0.002170941
0.895458519	0.002637044
0.921645999	0.001133042
0.934397817	0.00067155
0.817715943	0.013147446
0.849657476	0.007566318
0.913054109	0.0015379
0.824292064	0.011837331
0.858059049	0.006409816
0.893074632	0.002816356
0.855901301	0.006695191
0.833438694	0.010157106
0.827034175	0.011316482
0.854624152	0.00686786
0.847461998	0.007888933
0.925930083	0.000960333
0.824539602	0.011789703
0.846171439	0.008082598
0.851459086	0.007307964
0.927182376	0.000913323
0.900210261	0.002302046
0.865604997	0.00547336
0.81789428	0.013110768
0.818702221	0.012945408
0.816210747	0.013459597
0.814266384	0.013869679
0.864255846	0.005633871
0.876804352	0.004253145
0.819250286	0.012833989
0.809451044	0.014918882
0.919310153	0.001235198
0.828795791	0.010989658
0.836312771	0.009662407
0.838610351	0.009278146
0.833196938	0.010199435
0.81695044	0.013305621
0.820679605	0.012546268
0.86490649	0.005556092
0.865942061	0.005433723
0.812215984	0.014310555
0.833111644	0.010214396
0.825774252	0.011553957
0.883107245	0.003651149
0.884068966	0.003564449
0.833311558	0.010179353
0.824513257	0.011794766
0.902031541	0.002181372
0.834958792	0.009893508
0.824994922	0.011702413
0.845227778	0.008226105

0.848326325	0.007760899
0.892456234	0.002864123
0.820376515	0.012606936
0.800687909	0.016953349
0.836841941	0.009573027
0.819650471	0.012753017
0.815119743	0.013688744
0.804306865	0.016093372
0.805268884	0.015869474
0.869196951	0.005060401
0.836537778	0.009624337
0.803575754	0.016264848
0.909152329	0.001749084
0.844197929	0.00838456
0.88231504	0.003723579
0.816821694	0.013332341
0.868103564	0.005183908
0.8887344	0.003162702
0.838217258	0.009343188
0.840612113	0.008951395
0.869735539	0.005000265
0.888401687	0.003190331
0.855115592	0.006801085
0.815507114	0.013607104
0.935648441	0.000634479
0.850811779	0.00740013
0.914640069	0.001457054
0.887155056	0.003295236
0.845080137	0.008248704
0.953756452	0.00023873
0.912154317	0.001585038
0.828712463	0.011004981
0.810829997	0.014613501
0.854462206	0.006889956
0.801085532	0.016857487
0.908077359	0.001810402
0.812800288	0.014184033
0.872809708	0.004665792
-0.823744416	0.011943133
0.812671602	0.014211837
0.813782573	0.013972926
0.888143659	0.003211864
0.823863804	0.011920017
0.855310857	0.006774669
0.875236273	0.0044122
0.896043181	0.002594227
0.869278848	0.005051227
0.836157024	0.009688814
0.802641869	0.016485542
0.877371669	0.004196523
0.80974412	0.014853645

0.83020699	0.010732205
0.815010071	0.013711914
0.800789535	0.016928816
0.847263873	0.00791847
0.802443087	0.016532759
0.903636813	0.002078478
0.913014531	0.001539954
0.813922286	0.013943061
0.864685833	0.005582392
0.840486586	0.008971666
0.855361104	0.006767882
0.819708109	0.012741381
0.86826998	0.005164987
0.832811117	0.010267219
0.827425301	0.011243394
0.816430569	0.013413722
0.806841493	0.015507696
0.944610715	0.000407379
0.810760319	0.014628837
0.802256107	0.01657725
0.822056234	0.012273035
0.811860979	0.014387771
0.889843345	0.003071733
0.905152142	0.00198429
0.896839857	0.002536612
0.830858886	0.010614579
0.841184974	0.008859253
0.894915581	0.002677213
0.825794756	0.011550067
0.817788005	0.013132618
0.830418468	0.010693957
0.872488618	0.004700033
0.828041434	0.011128868
0.811273396	0.014516149
0.9079597	0.001817197
0.902004242	0.002183149
0.835980952	0.009718722
0.80411607	0.016138012
0.813834429	0.013961836
0.850768983	0.007406249
0.820478559	0.01258649
0.827769995	0.011179232
0.820537031	0.012574783
0.810770154	0.014626672
0.903460681	0.002089611
0.801612914	0.016730867
0.844038486	0.008409264
0.815911651	0.013522175
0.819315016	0.01282087
0.833506942	0.010145177
0.82329917	0.012029591

0.854463935	0.00688972
0.827542305	0.011221589
0.810859382	0.014607037
0.870051384	0.004965213
0.834209085	0.010022964
0.828509927	0.011042282
0.88450855	0.003525266
0.879339814	0.004003857
0.827422857	0.01124385
0.849333584	0.007613373
0.852760911	0.007124831
0.812327623	0.014286326
0.810028791	0.014790451
0.896302938	0.00257535
0.883046985	0.003656626
0.8270244	0.011318312
0.873699188	0.004571777
0.840349019	0.008993916
0.938118517	0.000565256
0.814929128	0.01372903
0.936251044	0.000617106
0.8985672	0.002414554
0.86312151	0.005771135
0.808180571	0.015203761
0.873620093	0.004580088
0.836995244	0.009547231
0.854194224	0.00692662
0.827667177	0.011198346
0.804433227	0.016063851
0.800116479	0.01709171
0.860862076	0.006050887
0.856249511	0.006648599
0.896463037	0.002563759
0.808885574	0.015045259
0.816137016	0.013475006
0.874499142	0.004488273
0.834737182	0.009931664
0.858123541	0.006401409
0.809461892	0.014916464
0.856320262	0.006639158
0.833510876	0.01014449
0.800727904	0.016943691
0.838160992	0.009352522
0.960715115	0.00014714
0.809751391	0.014852029
0.865879416	0.005441076
0.83505559	0.009876871
0.817373812	0.013217993
0.809602559	0.014885133
0.871074021	0.004852808
0.886261642	0.003371771

0.831833839	0.01044019
0.868406594	0.005149487
0.805188119	0.015888195
0.873683512	0.004573424
0.809998989	0.014797059
0.897530317	0.002487355
0.913831592	0.001497912
0.815230489	0.013665372
0.864904583	0.005556319
0.808178782	0.015204164
0.880555809	0.003887721
0.862599373	0.005835031
0.801524878	0.016751962
0.872959316	0.004649893
0.932292461	0.00073711
0.820749283	0.012532347
0.890905201	0.002986229
0.899352729	0.002360331
0.842228174	0.008693012
0.87049669	0.004916063
0.87286377	0.004660043
0.904334247	0.002034776
0.864631116	0.005588926
0.914383113	0.00146996
0.80291158	0.016421613
0.824949026	0.011711193
0.81444031	0.013832681
0.836236835	0.009675276
0.818864524	0.012912349
0.82979846	0.010806338
0.879702508	0.003968986
0.97087574	6.04E-05
0.94874686	0.000323784
-0.810979486	0.014580634
0.852255702	0.007195548
0.834579945	0.009958792
0.818566978	0.012972996
0.842173815	0.008701625
0.816514969	0.013396134
0.824627578	0.011772805
0.834070444	0.010047021
-0.835337639	0.009828496
0.908052146	0.001811857
0.827336073	0.011260041
0.837336421	0.009489982
0.832816124	0.010266337
-0.81997925	0.012686735
-0.802065134	0.016622768
-0.811266005	0.014517769
-0.849380314	0.007606572
0.864781916	0.00557093

0.852089107	0.007218966
0.947184086	0.000353892
0.812660336	0.014214273
0.899471939	0.002352172
0.849302888	0.007617842
0.895502746	0.002633789
0.845147967	0.008238317
0.852434516	0.007170467
0.880492091	0.003893752
0.851905882	0.007244777
0.823442817	0.012001655
0.808026493	0.01523854
0.880731642	0.003871109
0.902939558	0.002122774
0.833439708	0.010156929
0.923491895	0.001056338
0.80648607	0.015589003
0.825306177	0.011642978
0.84070605	0.008936244
-0.819539905	0.012775356
0.900821626	0.002261068
0.826756716	0.011368511
0.843713701	0.00845973
0.841399252	0.008824943
0.809362531	0.014938619
0.857762098	0.006448619
0.878885925	0.004047772
0.81629467	0.013442071
0.820503116	0.012581573
0.812392771	0.0142722
0.941810489	0.00047133
0.835111558	0.00986726
0.823011935	0.012085576
0.810619473	0.014659867
0.91937834	0.001232134
0.978625834	2.40E-05
0.814519644	0.013815825
0.841526926	0.00880454
0.894875944	0.002680161
0.858213842	0.006389649
0.846559167	0.0080241
0.82453841	0.011789932
0.809749246	0.014852506
0.882989228	0.003661881
0.877613842	0.004172501
0.807314694	0.01539986
0.825609744	0.011585195
0.810633004	0.014656884
0.858126163	0.006401067
0.838676393	0.009267247
0.818697035	0.012946465

0.870274425	0.004940556
0.8119995	0.01435761
0.823417485	0.012006578
0.892361283	0.002871504
0.857970059	0.006421429
0.847619355	0.007865524
0.822587907	0.012168523
0.931371629	0.000767052
0.80903101	0.015012691
0.823995352	0.01189458
0.840473413	0.008973795
0.813652575	0.01400075
0.932614625	0.000726818
0.85229218	0.007190427
0.817623973	0.013166387
0.823877215	0.011917422
0.802894533	0.016425649
0.800266743	0.017055258
-0.804518282	0.016043999
0.872999489	0.00464563
0.862499535	0.0058473
0.871441841	0.004812781
0.811255872	0.014519989
0.802760184	0.016457479
0.807534337	0.015349967
0.892059088	0.002895075
0.855188727	0.006791184
0.857733369	0.006452381
0.891977668	0.002901447
0.845108688	0.008244331
0.819720566	0.012738867
0.882579505	0.003699297
0.861039698	0.006028587
0.816345334	0.013431498
0.850442946	0.007452976
0.841307819	0.008839573
0.841053545	0.008880339
0.854220212	0.006923059
0.824762702	0.011746881
0.814892888	0.013736697
0.908445597	0.001789242
0.862418175	0.00585731
0.859130681	0.00627103
0.850399494	0.007459217
0.843039989	0.008565027
0.807769418	0.01529668
0.855895698	0.006695942
0.841435909	0.008819082
0.904231787	0.002041158
0.830760479	0.010632282
0.858136654	0.0063997

0.916588724	0.001361575
0.892525792	0.002858724
0.813071191	0.014125613
0.800773621	0.016932656
0.85394913	0.00696026
0.808995426	0.015020656
0.800050855	0.017107645
0.803800285	0.016212065
0.921777904	0.001127444
0.836119771	0.009695137
0.818416417	0.013003752
0.884837329	0.003496143
0.922445297	0.001099397
0.830629706	0.010655838
0.824919283	0.011716885
0.82045716	0.012590776
0.807541013	0.015348452
0.863686383	0.005702516
0.822999477	0.012088008
0.902281344	0.002165148
0.885824263	0.003409654
0.849962473	0.007522177
0.829239905	0.010908219
0.810974479	0.014581734
0.840632319	0.008948135
0.826036215	0.011504324
0.843801618	0.008446051
0.823154449	0.012057778
0.84248358	0.008652617
-0.811167657	0.014539328
0.805736125	0.015761438
0.803104281	0.016376033
0.827301264	0.01126654
0.806416214	0.015605015
0.828306794	0.011079772
0.803623378	0.016253643
0.806328893	0.015625044
0.856327116	0.006638244
0.837726355	0.009424819
0.835370779	0.009822822
0.892780662	0.002838998
0.816228509	0.013455886
0.81776154	0.013138062
0.815646172	0.013577872
0.826287687	0.011456806
0.810453892	0.014696399
0.813126981	0.014113601
0.870644391	0.00489983
0.865734875	0.005458065
0.878523767	0.004083034
0.94170624	0.00047383

0.840841889	0.008914364
0.820891023	0.012504059
0.925050616	0.000994274
0.815698564	0.013566869
0.930712581	0.000788964
0.922539949	0.001095457
0.881167531	0.003830127
0.923979223	0.00103667
0.838904619	0.009229645
0.805495679	0.015816976
0.92010498	0.001199791
0.841099262	0.008873001
0.923577964	0.001052847
0.85704869	0.006542453
0.817688882	0.013153017
0.864305317	0.005627933
0.843174219	0.008543982
0.834668994	0.009943422
0.849679291	0.007563155
0.888676226	0.003167522
0.82174921	0.012333644
0.878656447	0.004070093
0.840818346	0.008918154
0.853352547	0.007042581
0.804159582	0.016127825
0.864904106	0.005556375
0.848374546	0.007753795
0.89765507	0.002478521
0.859783053	0.006187491
0.892052472	0.002895592
0.866171479	0.005406849
0.840045094	0.009043195
0.898365378	0.002428615
0.81046623	0.014693675
0.914572775	0.001460427
0.89845556	0.002422325
0.926324487	0.000945361
0.809157193	0.014984471
0.878089428	0.004125584
0.85289669	0.007105901
0.805496037	0.015816894
0.84163624	0.008787095
0.833562791	0.010135422
0.852948964	0.007098621
0.810199499	0.014752637
0.804114699	0.016138333
0.808951974	0.015030385
0.858207464	0.006390479
0.852637589	0.007142052
0.805870831	0.015730378
0.839850187	0.009074888

0.81734699	0.013223533
0.827841222	0.011166002
0.84031713	0.008999078
0.916923523	0.001345594
0.825085819	0.011685036
0.860946953	0.006040224
0.890985012	0.002979865
0.860905826	0.006045389
0.839163363	0.009187132
0.906879425	0.001880361
0.818772972	0.012930991
0.898206353	0.002439731
0.805660903	0.0157788
0.820567727	0.01256864
0.818482637	0.012990219
0.809136987	0.014988988
0.892513692	0.002859663
0.817236066	0.013246463
0.934265077	0.000675565
0.864509761	0.005603435
0.885435104	0.003443591
0.815302074	0.013650279
0.827367842	0.011254113
0.806514382	0.015582517
0.854815185	0.006841854
0.85894084	0.006295474
0.832099855	0.010392926
0.905890703	0.001939407
0.87307781	0.004637325
0.822443426	0.012196868
0.853095949	0.007078179
0.857708454	0.006455644
0.82287401	0.012112517
0.855736732	0.006717283
0.876837432	0.00424983
0.863702595	0.005700555
0.834207118	0.010023305
0.809963584	0.014804912
0.87275064	0.004672079
0.901910305	0.002189274
0.840647876	0.008945625
0.897878826	0.002462729
0.847647071	0.007861405
0.818723738	0.012941022
0.812796116	0.014184934
0.825689793	0.011569988
0.802945852	0.016413501
0.931360126	0.000767431
0.849303782	0.007617712
0.813553452	0.014021989
0.848396659	0.007750539

0.889104784	0.003132128
0.834197104	0.010025042
0.80848223	0.015135813
0.834871411	0.009908541
0.845920324	0.008120629
0.816782653	0.01334045
0.806111813	0.015674907
0.878638923	0.0040718
0.853738964	0.00698919
0.913663447	0.001506502
0.819876075	0.012707512
0.878383458	0.004096748
0.815968156	0.013510338
0.81587261	0.013530356
0.848960221	0.007667846
0.836076736	0.009702444
0.853053391	0.007084094
0.883118629	0.003650115
0.907551289	0.001840912
0.814283192	0.013866101
0.82908684	0.010936244
0.811814308	0.014397942
0.80938375	0.014933886
0.902921021	0.00212396
0.867749572	0.005224305
0.807056606	0.015458616
0.801062286	0.016863082
0.877653301	0.004168595
0.913916469	0.001493588
0.840646982	0.008945769
0.891504109	0.002938689
0.801438928	0.016772573
0.832118332	0.010389648
0.830875993	0.010611503
0.856655002	0.006594606
0.820165455	0.012649292
0.834151924	0.010032879
0.842895329	0.008587745
0.817212343	0.01325137
0.94700712	0.000357413
0.831314027	0.01053294
0.839617848	0.00911276
0.803243339	0.01634319
0.826715171	0.011376314
0.82976979	0.010811553
0.803889275	0.016191175
0.90415895	0.002045704
0.848108172	0.007793088
0.822959423	0.012095829
-0.852437735	0.007170016
0.841663122	0.008782808

0.808965981	0.015027248
0.862724602	0.005819665
0.82108438	0.012465534
0.80363524	0.016250853
0.865158379	0.005526166
0.938612103	0.000552046
0.832915068	0.010248928
0.848999202	0.007662147
0.856176794	0.006658312
0.829524577	0.010856219
0.858909607	0.006299502
0.874991179	0.004437402
0.805012405	0.015928974
0.850815117	0.007399653
0.874779165	0.004459276
0.808532476	0.015124513
0.844758093	0.008298133
0.883217514	0.00364114
0.835938096	0.00972601
0.827320397	0.011262968
0.947465599	0.000348338
0.811539352	0.014457953
0.839279711	0.009168057
0.86710459	0.005298427
0.841178417	0.008860304
0.870203137	0.004948428
0.86648947	0.005369742
0.869825661	0.004990247
0.831307113	0.010534177
0.895714402	0.002618249
0.822710037	0.012144596
0.859604597	0.006210271
0.807426214	0.015374515
0.843269706	0.008529031
0.86291939	0.005795816
0.907508492	0.001843409
0.861415803	0.005981542
0.826346278	0.011445753
0.823454678	0.01199935
0.810806274	0.014618721
0.83442682	0.009985257
0.82396996	0.011899487
0.846313417	0.008061146
0.83670783	0.009595629
0.934527695	0.000667635
0.808529317	0.015125224
0.853903711	0.006966506
0.815312326	0.013648118
0.842260063	0.008687962
0.857359231	0.006501501
0.867170691	0.0052908

0.935333312	0.00064369
0.828725696	0.011002547
0.803119361	0.016372469
0.903155327	0.002109002
0.862860441	0.005803027
0.904260695	0.002039356
0.839530051	0.009127097
0.818621874	0.012961793
0.893292248	0.002799669
0.814982414	0.01371776
0.851870239	0.007249805
0.870396256	0.004927121
0.827610373	0.011208915
0.851435483	0.007311312
0.899545431	0.002347151
0.837879896	0.009399239
0.836020768	0.009711954
0.941560328	0.000477344
0.809792459	0.014842902
0.935295463	0.000644802
0.921690881	0.001131135
0.807905972	0.015265779
0.805959642	0.01570992
0.825094461	0.011683385
0.855609477	0.006734398
0.809474528	0.014913648
0.878348529	0.004100167
0.802681446	0.016476152
0.898649156	0.00240886
0.829729736	0.010818841
0.936128497	0.000620614
0.858728468	0.006322892
0.920527697	0.001181232
0.946819425	0.000361172
0.837577939	0.009449588
0.810829103	0.014613698
0.804167092	0.016126067
0.840404809	0.008984889
0.885323763	0.00345334
0.808915496	0.015038555
0.838374376	0.009317156
0.899820149	0.002328444
0.856202662	0.006654856
0.879934192	0.003946814
0.865978301	0.005429472
0.811942041	0.014370116
0.8076123	0.015332282
0.844164968	0.008389663
0.823412597	0.012007529
0.921481907	0.001140031
0.818625152	0.012961125

0.821783066	0.012326951
0.858176053	0.006394569
0.920901775	0.001164967
0.933073938	0.000712308
0.807437122	0.015372037
0.872849882	0.00466152
0.802531064	0.016511852
0.861181259	0.006010852
0.811519802	0.014462226
0.83001709	0.010766625
0.834666848	0.009943793
0.81699425	0.013296536
0.851545095	0.007295773
0.816086411	0.013485588
0.841013372	0.008886791
0.825130045	0.011676587
0.813218832	0.014093838
0.8295995	0.010842559
0.823813796	0.011929696
0.805765629	0.015754632
0.803940356	0.016179191
0.804099619	0.016141864
0.811731815	0.01441593
0.882564127	0.003700706
0.838800609	0.00924677
0.838280439	0.009332715
0.85468328	0.006859804
0.854524851	0.006881404
0.90846175	0.001788318
0.840428889	0.008980994
0.828888476	0.010972631
0.88576144	0.003415118
0.814345717	0.013852795
0.870496869	0.004916043
0.837327898	0.00949141
0.828665376	0.011013646
0.815256715	0.013659841
0.928835988	0.000853588
0.865513504	0.005484152
0.829008937	0.010950525
0.862916768	0.005796136
0.817521513	0.013187508
0.826548219	0.011407707
0.81097579	0.014581446
0.848002493	0.007808712
0.863191009	0.005762664
0.800863147	0.016911059
0.871894658	0.004763798
0.814362824	0.013849156
0.905314028	0.001974395
0.845351219	0.008207242

0.815167904	0.013678577
0.82663089	0.011392155
0.826052904	0.011501167
0.80092901	0.016895182
0.825713933	0.011565405
0.844533741	0.00833268
0.810748875	0.014631356
0.805039704	0.015922634
-0.856310785	0.006640422
0.801248133	0.016818384
0.868601382	0.005127439
0.842867315	0.008592149
0.827410042	0.01124624
0.91392678	0.001493063
0.804259658	0.01610441
0.803018272	0.016396367
0.807771862	0.015296126
0.812418759	0.014266567
0.867421627	0.005261909
0.87232697	0.004717332
0.81591785	0.013520876
0.803607285	0.016257429
0.805679023	0.015774617
0.821458697	0.012391167
0.804669678	0.016008701
0.803624511	0.016253377
0.838681638	0.009266382
0.826489329	0.011418794
0.811720431	0.014418413
0.813271761	0.014082458
0.807438374	0.015371753
0.881709874	0.003779528
0.864873409	0.00556003
0.867243946	0.005282355
0.825789809	0.011551006
0.849671543	0.007564278
0.819523215	0.012778731
0.894860089	0.00268134
0.864456058	0.005609864
0.900918305	0.002254632
0.914712846	0.001453412
0.897246897	0.002507498
0.852578998	0.007150243
0.809068918	0.01500421
0.829075933	0.010938243
0.811038852	0.014567595
0.878356814	0.004099356
0.86301595	0.005784016
0.947728932	0.000343195
0.932991087	0.000714911
0.94187516	0.000469784

0.930896282	0.000782816
0.886469603	0.003353855
0.909306288	0.001740414
0.837491989	0.009463951
0.952451169	0.000259264
0.839336932	0.009158684
0.8343243	0.010003
0.821833193	0.012317046
0.938123941	0.000565109
0.932335377	0.000735733
0.823854744	0.011921771
0.805730939	0.015762635
0.858356118	0.006371148
0.846419334	0.008045166
0.846215308	0.008075966
0.818207145	0.013046577
0.834110379	0.010040088
0.849842846	0.007539471
0.926311374	0.000945856
0.937199593	0.000590397
0.921286821	0.001148377
0.902944863	0.002122435
0.842518449	0.008647111
0.812975049	0.014146328
0.856546223	0.006609063
0.841529191	0.008804178
0.809707284	0.014861835
0.894468963	0.00271055
0.815246284	0.013662041
0.845920444	0.008120611
0.847304285	0.00791244
0.806583524	0.015566683
0.875280082	0.004407706
0.840691566	0.008938579
0.851146579	0.007352368
0.843145907	0.008548418
0.838673472	0.009267729
0.804007649	0.016163413
0.829049528	0.010943083
0.862269759	0.005875599
0.801184297	0.016833729
0.81694901	0.013305917
0.8236081	0.011969561
0.809124053	0.01499188
0.810284615	0.014733806
0.80470854	0.015999648
0.835544646	0.009793087
0.854123116	0.006936369
0.817192435	0.013255489
-0.827478886	0.011233405
0.842205822	0.008696553

0.804802775	0.015977709
0.839540184	0.009125442
0.904355466	0.002033456
0.810523748	0.01468098
0.805170715	0.015892231
0.849546731	0.007582386
0.80182606	0.016679862
0.815367937	0.013636401
0.827020407	0.01131906
0.841949165	0.008737278
0.825139105	0.011674857
0.81394881	0.013937396
0.912337005	0.001575393
0.852685392	0.007135373
0.800470173	0.017005987
0.820295632	0.012623157
0.859211326	0.006260664
0.830159605	0.010740787
0.807833672	0.015282135
0.883817792	0.003586963
0.821348488	0.012413033
0.839534223	0.009126416
0.843884945	0.008433098
0.847025454	0.007954107
0.837426722	0.009474866
0.889750898	0.003079251
0.879695654	0.003969644
0.909230769	0.001744663
0.903836071	0.002065931
0.966332614	9.30E-05
0.86559397	0.00547466
0.831617236	0.010478775
0.879136026	0.004023536
0.883448184	0.003620259
0.830277979	0.010719356
0.840240836	0.009011437
-0.806295872	0.015632623
0.866108239	0.005414249
0.941998363	0.000466847
0.948933244	0.00032031
0.845990777	0.008109948
0.834713936	0.009935671
0.801599801	0.016734008
0.8381688	0.009351226
0.931715012	0.000755795
0.853712678	0.006992814
0.801626861	0.016727526
0.843028903	0.008566767
0.818149805	0.013058327
0.822293818	0.012226263
0.865279913	0.005511764

0.840212047	0.009016104
0.834423602	0.009985813
0.879261434	0.004011418
0.828967154	0.010958189
0.843429804	0.008504001
0.805652082	0.015780837
0.824850142	0.011730124
0.891370237	0.002949272
0.910518289	0.001673137
0.820412636	0.012599696
0.803164124	0.016361894
0.841905355	0.008744242
0.831049263	0.010580382
0.864665508	0.005584818
0.811209857	0.014530075
0.861083865	0.00602305
0.898655653	0.002408409
0.801924944	0.016656232
0.843160987	0.008546055
0.8579548	0.006423421
0.808201969	0.015198934
0.849267662	0.007622973
0.829494357	0.010861732
0.840940058	0.008898572
0.915383101	0.00142015
0.915574789	0.001410729
0.823066175	0.012074992
0.88340807	0.003623885
0.881982565	0.003754251
0.849494576	0.00758996
0.911282718	0.001631587
0.886598706	0.003342763
0.835689962	0.009768279
0.845527291	0.008180383
0.932180047	0.000740723
0.867830634	0.005215037
0.829320371	0.010893505
0.908684909	0.001775578
0.851195931	0.007345344
0.880142868	0.003926913
0.840896487	0.008905579
0.815232813	0.013664882
0.936460793	0.000611133
0.878120124	0.004122567
0.91409868	0.001484332
0.889668643	0.003085951
0.847802162	0.007838385
-0.832499087	0.010322247
0.818096161	0.013069325
0.870711863	0.004892426
0.817798436	0.013130472

0.81010896	0.014772685
0.813342631	0.014067229
0.809785128	0.014844531
0.812496781	0.014249664
0.808472991	0.015137891
0.841002941	0.008888466
0.865792155	0.005451328
0.825335979	0.011637297
0.877028823	0.004230683
0.821453214	0.012392255
0.857412279	0.006494522
0.801516116	0.016754062
0.885439217	0.003443231
0.875380337	0.004397431
0.842514217	0.008647779
0.849662364	0.007565609
0.807472408	0.015364024
0.913446963	0.001517608
0.881777406	0.003773258
0.82622844	0.01146799
0.817006946	0.013293904
0.8185516	0.012976135
0.897933304	0.002458894
0.809390962	0.014932277
0.893974364	0.002747782
0.895582974	0.002627892
0.883180261	0.003644519
0.889206648	0.003123753
0.873340845	0.004609505
0.812097549	0.014336286
0.847824335	0.007835097
0.833010197	0.010232207
0.866911411	0.005320758
-0.807675719	0.015317905
0.849388778	0.007605341
0.804018199	0.01616094
0.823876917	0.01191748
0.883467793	0.003618487
0.807240665	0.015416699
0.931491613	0.000763106
0.804683924	0.016005382
0.867514729	0.005251216
0.803797781	0.016212653
0.820854187	0.012511406
0.845449567	0.008192232
0.839280784	0.009167881
0.805044949	0.015921416
0.814114273	0.013902088
0.917266846	0.001329333
0.83250767	0.010320731
0.838903308	0.009229861

0.84823823	0.007773887
0.806170642	0.015661384
0.82400316	0.011893071
0.854953468	0.006823068
0.908048272	0.00181208
0.806881249	0.015498618
0.841298342	0.00884109
0.856658459	0.006594147
0.861138642	0.006016188
0.841801763	0.008760722
0.812623441	0.014222252
0.891797125	0.002915609
0.838093996	0.009363643
0.878448129	0.004090424
0.85784483	0.006437793
0.843059957	0.008561895
0.816024721	0.013498496
0.81981504	0.012719813
0.827475965	0.011233949
0.872470915	0.004701926
0.801404655	0.016780797
0.802281141	0.016571289
0.896905005	0.002531938
0.85547322	0.006752754
0.895128429	0.002661418
0.84055829	0.008960083
0.818750441	0.012935581
-0.899623036	0.002341857
0.927518964	0.000900949
0.833230734	0.010193511
0.840572	0.00895787
0.852625966	0.007143676
0.887046158	0.003304504
0.807720721	0.015307709
-0.80283618	0.016439469
0.934630036	0.000664562
0.820038855	0.012674742
0.81058073	0.014668409
0.8024562	0.016529642
0.838831306	0.009241714
0.876425982	0.00429118
0.892009854	0.002898927
0.866268337	0.00539553
0.819657385	0.012751621
0.812483966	0.01425244
0.820977747	0.01248677
0.823352098	0.012019293
0.81540966	0.013627614
0.925023198	0.000995345
0.82620579	0.011472268
0.893274248	0.002801047

0.869230151	0.005056681
0.802456737	0.016529514
0.825279355	0.011648092
0.807224095	0.01542047
0.850331485	0.007468993
0.801131785	0.016846358
0.814603686	0.013797983
0.847387016	0.007900103
0.840005815	0.009049577
0.855338633	0.006770917
0.858990252	0.006289106
0.828623831	0.011021294
0.824778497	0.011743853
0.856325865	0.006638411
0.822724998	0.012141667
0.862404227	0.005859027
0.890181005	0.003044374
0.819300592	0.012823793
0.814903975	0.013734351
0.826467156	0.01142297
0.879086018	0.004028375
0.817110777	0.013272392
0.810080707	0.014778945
0.821600139	0.01236314
0.825424612	0.011620412
0.842127621	0.008708949
0.888676584	0.003167492
0.811497748	0.014467047
0.866217852	0.005401428
0.86315763	0.005766731
0.831128418	0.010566185
0.859901965	0.006172341
0.918684483	0.001263545
0.855730474	0.006718124
0.852108777	0.007216198
0.858481288	0.0063549
0.835418582	0.009814641
0.837339759	0.009489423
0.913186312	0.001531052
0.809671164	0.014869868
0.892842472	0.002834228
0.80219686	0.016591363
0.908784151	0.001769931
0.807255507	0.015413322
0.812024951	0.014352073
0.879553139	0.003983323
0.877847195	0.004149438
0.877135754	0.004220009
0.854520142	0.006882046
0.832197487	0.010375613
0.812312722	0.014289559

0.820328891	0.012616485
0.840704739	0.008936455
0.945419669	0.000390031
0.820321977	0.012617872
0.852907717	0.007104365
0.820510209	0.012580152
0.82607156	0.011497638
0.874156952	0.004523872
0.821708977	0.0123416
0.804503441	0.016047462
0.891939521	0.002904436
0.810781121	0.014624257
0.953640044	0.000240517
0.909996986	0.001701863
0.876024008	0.004331828
0.809247553	0.014964283
0.807882965	0.015270983
0.808054745	0.015232159
0.863363326	0.005741696
0.829814076	0.010803499
0.804209769	0.01611608
0.933757186	0.000691076
0.800379694	0.01702789
0.822461128	0.012193393
0.909748375	0.001715674
0.929506242	0.000830124
0.82610172	0.011491934
0.848548651	0.007728181
0.816004694	0.013502688
0.807611108	0.015332552
0.83623153	0.009676175
0.81019479	0.01475368
0.896970987	0.002527209
0.816915452	0.013312879
0.828280687	0.011084596
0.860547841	0.006090468
0.849321604	0.007615117
0.803711355	0.016232958
0.937986076	0.000568835
0.856177032	0.00665828
0.833568692	0.010134392
0.854299963	0.006912138
0.822705269	0.012145529
0.807147443	0.01543792
0.803616762	0.0162552
0.864391148	0.00561764
0.846414268	0.00804593
0.824738026	0.011751612
0.800872028	0.016908918
0.85487175	0.006834166
0.805483162	0.015819871

0.83108443	0.010574073
0.816522241	0.01339462
0.903239369	0.002103653
0.826382577	0.011438908
0.846263587	0.008068671
0.863017023	0.005783885
0.87456888	0.00448104
0.843429923	0.008503982
0.826280117	0.011458235
0.838800371	0.009246809
0.923144758	0.001070495
0.820890725	0.012504118
0.837978363	0.009382857
0.927154601	0.000914349
0.816116929	0.013479206
0.918840051	0.001256457
0.91849345	0.001272283
0.808063626	0.015230153
0.839314282	0.009162393
0.846783757	0.007990339
0.92611289	0.000953374
0.808540881	0.015122624
0.808575571	0.015114827
0.86306572	0.005777941
0.886853755	0.003320921
0.925102174	0.000992263
0.830778003	0.010629128
0.817465663	0.01319903
0.824123323	0.011869867
0.885765553	0.00341476
0.864144146	0.005647294
0.802528143	0.016512545
0.882469237	0.003709409
0.834607303	0.009954069
0.917894363	0.001299943
0.851511717	0.007300502
0.806925297	0.015488564
0.823728085	0.011946298
0.846319854	0.008060174
0.871743321	0.004780133
0.824129999	0.011868579
0.875494182	0.004385781
0.868691802	0.005117224
0.830693781	0.010644292
0.879027605	0.004034031
0.809156179	0.014984698
0.801585734	0.016737378
0.820912659	0.012499744
0.832969368	0.010239382
0.850228131	0.007483865
0.832427979	0.010334813

0.937928557	0.000570394
0.846010745	0.008106922
0.857127905	0.006531991
0.808238328	0.015190736
0.85732305	0.006506264
0.880845368	0.00386039
0.927050292	0.000918208
0.809333026	0.014945202
0.916330338	0.001373994
0.811919808	0.014374957
0.838745832	0.009255796
0.807903588	0.015266319
0.866718888	0.005343073
0.854580045	0.006873874
0.862965405	0.005790191
0.86398232	0.005666777
0.898413599	0.00242525
0.810682416	0.014645995
0.800081432	0.017100219
0.815549314	0.013598229
0.802453339	0.016530322
0.836718142	0.00959389
0.869292915	0.005049653
0.884436488	0.003531671
0.826359928	0.011443179
0.936644375	0.000605937
0.870540738	0.004911218
0.839028537	0.009209269
-0.864381611	0.005618783
0.872728229	0.004674466
0.916264534	0.001377168
0.871447504	0.004812167
0.940763593	0.000496831
0.801410317	0.016779438
0.85274893	0.007126502
0.808671594	0.015093257
0.808145404	0.015211694
0.826730311	0.01137347
0.801775575	0.016691934
0.802170575	0.016597627
0.846089542	0.008094989
0.805915773	0.015720023
0.835223496	0.009848055
0.81909579	0.012865336
0.812067091	0.014342908
0.810613573	0.014661168
0.832828283	0.010264197
0.819090486	0.012866413
0.831758916	0.010453526
0.906834245	0.001883034
0.889743328	0.003079868

0.815331876	0.013643998
0.833394587	0.010164821
0.84019208	0.009019341
0.821553111	0.012372454
0.82558614	0.011589681
0.81015569	0.014762336
0.81047368	0.01469203
0.848813534	0.007689315
0.811852753	0.014389563
0.883614719	0.003605232
0.808239102	0.015190561
0.816822827	0.013332105
0.822600484	0.012166058
0.857571959	0.006473543
0.857284546	0.006511335
0.865484953	0.005487522
0.829323709	0.010892895
0.862213731	0.005882513
0.834008574	0.010057769
0.809048653	0.015008744
0.825827181	0.011543918
0.806713402	0.015536968
0.854708374	0.006856387
0.816268623	0.013447509
0.923043311	0.001074656
0.846861124	0.00797873
0.863123178	0.005770932
0.832204998	0.010374282
0.928082645	0.000880474
0.806730032	0.015533165
0.890553415	0.003014383
0.820649326	0.01255232
0.922477722	0.001098046
0.969166875	7.16E-05
0.897833109	0.002465951
0.846348047	0.008055919
0.87175715	0.004778639
0.812216341	0.014310477
0.836108625	0.009697029
0.882957816	0.003664741
0.912205219	0.001582347
0.863345146	0.005743906
0.849999249	0.007516866
0.888446629	0.00318659
0.808811963	0.01506176
0.850413978	0.007457136
0.884214759	0.003551423
0.824192047	0.011856609
0.910490751	0.001674646
0.806251764	0.015642749
0.802271903	0.016573489

0.843997061	0.00841569
0.802817762	0.016443833
0.857727349	0.006453169
0.856909156	0.006560907
0.870386839	0.004928158
0.816730917	0.013351201
0.820523977	0.012577396
0.865375102	0.005500501
0.823584378	0.011974164
0.857766211	0.00644808
0.814001083	0.013926235
0.828273475	0.011085929
0.84577322	0.008142961
0.83797121	0.009384046
0.802192509	0.0165924
0.806706965	0.01553844
0.841061652	0.008879037
0.824609876	0.011776204
0.911271989	0.001632166
0.840137601	0.009028178
0.813527346	0.014027586
0.912635744	0.001559702
0.827640653	0.011203281
0.840570629	0.008958091
0.864553511	0.005598202
0.809843421	0.014831582
0.806388915	0.015611275
0.907327831	0.001853972
0.822073281	0.012269675
0.843750298	0.008454034
0.919513822	0.001226061
0.863212526	0.005760043
0.901164591	0.002238289
0.871632099	0.004792161
0.922192752	0.001109956
0.82226187	0.012232546
0.830589414	0.010663102
0.848893225	0.007677647
0.850704372	0.007415494
0.810086191	0.01477773
0.867277384	0.005278503
0.83883816	0.009240585
0.899390459	0.002357747
0.917034924	0.001340303
0.805228233	0.015878895
0.886102557	0.003385519
0.845890164	0.008125205
0.826904416	0.011340795
0.900970697	0.002251149
0.802792072	0.016449921
0.85263747	0.007142068

0.83998996	0.009052153
0.877206802	0.004212927
0.844103336	0.008399211
0.810608804	0.014662219
0.846653223	0.00800995
0.83884114	0.009240094
0.805202365	0.015884892
0.880211353	0.003920395
0.894240677	0.002727694
0.832294047	0.010358508
0.831197798	0.01055375
0.815990448	0.013505671
0.891530633	0.002936595
0.800154209	0.017082553
0.853560686	0.00701379
0.888170481	0.003209622
0.861250877	0.006002143
0.840488315	0.008971387
0.874676287	0.004469915
0.840509176	0.008968016
0.837949693	0.009387625
0.886722386	0.00333216
0.862092555	0.005897484
-0.863617957	0.0057108
-0.836529315	0.009625767
0.855314791	0.006774138
0.822163284	0.012251946
0.86660713	0.005356054
0.84091568	0.008902492
0.806558549	0.015572401
0.845552027	0.008176614
0.858835578	0.006309054
0.863518655	0.005722836
0.854760349	0.006849313
0.834877074	0.009907567
0.927437246	0.000903943
0.903586328	0.002081665
0.801954269	0.016649229
0.893661916	0.002771471
0.857628226	0.006466161
0.820400178	0.012602193
0.807646692	0.015324484
0.936443031	0.000611638
0.889056206	0.003136127
0.823866487	0.011919498
0.846251249	0.008070535
0.835318565	0.009831762
0.838422835	0.009309137
0.919216931	0.001239395
0.836847484	0.009572093
0.872406721	0.004708792

0.817497134	0.013192537
0.821955621	0.012292876
0.83599174	0.009716888
0.814770758	0.013762557
0.841708124	0.008775635
0.835674405	0.009770933
0.814427555	0.013835392
0.828446865	0.011053912
0.856663585	0.006593466
0.874561667	0.004481788
0.813241243	0.014089019
0.852885604	0.007107445
-0.825427294	0.011619902
0.857073784	0.006539137
0.90170306	0.002202825
0.895866752	0.0026071
0.823948681	0.011903601
0.820678473	0.012546494
0.821013391	0.012479669
0.834553599	0.009963342
0.84613359	0.008088323
0.867129743	0.005295524
0.801782012	0.016690394
0.898954868	0.002387695
0.932326674	0.000736012
0.878443182	0.004090907
0.80507499	0.015914442
0.810701072	0.014641884
0.861247361	0.006002582
0.825024545	0.011696748
0.865758598	0.005455274
0.821675956	0.012348133
0.875782788	0.004356339
0.886124969	0.00338358
0.847229302	0.007923631
0.813264072	0.014084111
0.91791743	0.001298871
0.806346595	0.015620983
0.82871443	0.011004619
0.853337467	0.00704467
0.811393917	0.014489759
0.824689031	0.01176101
0.809976399	0.014802069
0.827211678	0.011283276
0.80652225	0.015580714
0.830712974	0.010640835
0.834864676	0.009909701
0.869413793	0.005036134
0.850647807	0.007423594
0.932589889	0.000727605
0.840888619	0.008906844

0.888740957	0.003162159
0.804995716	0.015932851
0.947135031	0.000354865
0.822183013	0.012248062
0.813600302	0.014011948
0.889069855	0.003135003
0.900730371	0.002267154
0.833381712	0.010167073
0.89010942	0.003050161
0.807138145	0.015440038
0.812813878	0.014181099
0.925185382	0.000989023
0.814631701	0.013792039
0.837114394	0.009527213
0.812094331	0.014336986
0.807954311	0.01525485
0.877642632	0.004169651
0.874326229	0.004506239
0.813427687	0.014048966
0.857052803	0.006541909
0.849982381	0.007519302
0.887202203	0.003291229
0.813356221	0.01406431
0.806842506	0.015507465
0.834062338	0.010048429
0.880102456	0.003930762
0.819476843	0.012788109
0.861659706	0.005951159
0.800976336	0.016883779
0.891131639	0.002968196
0.840104163	0.009033604
0.802435935	0.01653446
0.804278076	0.016100103
0.867992461	0.005196565
0.85151583	0.007299919
0.915725708	0.001403341
0.810704589	0.01464111
0.83284229	0.010261731
0.893702209	0.002768409
0.816519678	0.013395153
0.851881027	0.007248282
0.870956898	0.004865598
0.805722594	0.01576456
0.849778831	0.007548735
0.821472049	0.01238852
0.85310781	0.007076531
0.836395383	0.009648418
0.818292081	0.013029185
0.805122197	0.015903486
0.810137808	0.014766296
0.807049036	0.015460342

0.870168149	0.004952295
0.826516628	0.011413654
0.854067087	0.006944057
0.846027493	0.008104385
0.869898319	0.00498218
0.912940979	0.001543776
0.842048347	0.008721526
0.829482317	0.010863929
0.885882914	0.003404559
0.81551528	0.013605386
0.816043913	0.01349448
0.874777555	0.004459442
0.853265226	0.007054682
0.850170493	0.007492167
0.854873121	0.006833979
0.828460038	0.011051482
0.881431401	0.003805455
0.802080989	0.016618986
0.835308552	0.009833478
0.84121567	0.008854333
0.843168437	0.008544888
0.821277261	0.012427179
0.944329143	0.000413535
0.855400383	0.00676258
0.810125768	0.014768962
0.855087578	0.006804881
0.832070589	0.010398119
0.805277884	0.015867388
0.824713171	0.011756379
0.857114255	0.006533793
0.807735503	0.01530436
0.858560383	0.006344647
0.838638484	0.009273502
0.842681289	0.008621429
0.808133185	0.015214451
0.85752058	0.006480288
0.858070493	0.006408324
0.893260717	0.002802083
0.867895961	0.005207575
0.86659801	0.005357114
0.840764165	0.008926879
0.863088489	0.005775163
0.807749331	0.015301228
0.87377429	0.004563896
0.822975934	0.012092605
0.849740565	0.007554276
0.812683105	0.01420935
0.807709157	0.015310328
0.913343549	0.001522933
0.829694629	0.010825231
0.852295339	0.007189984

0.869663298	0.005008304
0.877137423	0.004219843
0.880469859	0.003895857
0.847640455	0.007862389
0.811297596	0.014510848
0.816222787	0.013457082
0.816128194	0.01347685
0.888020515	0.003222174
0.805009782	0.015929583
0.83585006	0.009740994
0.860619485	0.006081429
0.816762567	0.013344623
0.833612859	0.010126681
0.852584422	0.007149484
0.901098371	0.002242675
0.807938457	0.015258434
0.821722507	0.012338924
0.830505431	0.010678253
0.871352375	0.004822497
0.830841959	0.010617623
0.817696989	0.013151348
0.802719176	0.016467203
0.904830337	0.002004056
0.849638343	0.007569092
0.823451817	0.011999906
0.837897897	0.009396243
0.869869232	0.004985408
0.85038352	0.007461513
0.880317748	0.003910284
0.841432333	0.008819653
0.857309163	0.006508092
0.815415502	0.013626384
0.808407784	0.015152564
0.806805253	0.015515974
0.856682956	0.006590894
0.862108052	0.005895568
0.833999932	0.010059271
0.879916012	0.003948551
0.890501916	0.003018519
-0.801879764	0.016667026
0.800683737	0.016954356
0.806914508	0.015491026
0.822707295	0.012145132
0.815697253	0.013567144
0.833923519	0.010072556
0.812972546	0.014146868
0.852747858	0.007126652
0.889258325	0.00311951
0.841110468	0.008871202
0.844560683	0.008328526
0.801511943	0.016755063

0.939320624	0.000533441
0.821950078	0.012293969
0.866755545	0.005338819
0.913840711	0.001497447
0.802152932	0.016601832
0.88921994	0.003122661
0.891541243	0.002935757
0.847419739	0.007895227
0.81662184	0.013373885
0.81461966	0.013794594
0.807011843	0.015468821
0.808369756	0.015161125
0.910406888	0.001679249
0.935345054	0.000643345
0.877347291	0.004198946
0.86648047	0.00537079
0.853338361	0.007044546
0.84226954	0.008686462
0.839129508	0.009192688
0.801213205	0.016826779
0.849131942	0.007642761
0.833580732	0.010132289
0.856033444	0.006677485
0.856649041	0.006595397
0.912327528	0.001575892
0.829572797	0.010847427
0.86046195	0.006101315
0.858106613	0.006403615
0.826644421	0.011389611
0.878438056	0.004091408
0.905044556	0.001990884
0.833598256	0.01012923
0.815672576	0.013572326
0.885594308	0.003429681
0.816159725	0.013470259
0.854764581	0.006848737
0.902164102	0.002172752
0.800764203	0.016934929
0.824649572	0.011768583
0.881789207	0.003772163
0.879013181	0.004035429
0.856239378	0.006649952
0.878359377	0.004099105
0.933405519	0.000701951
0.842742801	0.00861174
0.895768285	0.002614302
0.907465041	0.001845946
0.832692921	0.010288041
0.94385916	0.000423944
0.900688231	0.002269969
0.883799553	0.003588601

0.85211134	0.007215838
0.863754749	0.005694247
0.852104843	0.007216752
0.880472839	0.003895575
0.851793826	0.007260591
0.899016619	0.002383434
0.803366005	0.016314253
0.916422307	0.001369565
0.877046943	0.004228873
0.919662118	0.001219436
0.847696543	0.007854057
0.83707875	0.009533199
0.946702063	0.000363536
0.898527861	0.002417291
0.857148767	0.006529237
0.954676509	0.00022492
0.808200896	0.015199176
0.830283284	0.010718396
0.839805126	0.009082226
0.870390892	0.004927712
0.876348495	0.004298997
0.81650579	0.013398046
0.831212819	0.010551059
0.852603734	0.007146784
0.915931046	0.001393329
0.82013303	0.012655807
0.825633049	0.011580766
0.867374182	0.005267364
0.930811942	0.000785634
0.846644342	0.008011285
0.800872386	0.016908831
0.870929122	0.004868634
0.935836494	0.000629024
0.918808579	0.001257889
0.858496487	0.006352929
0.820215106	0.01263932
0.872147679	0.004736567
0.819063962	0.0128718
0.801874042	0.016668393
0.926917136	0.000923151
0.85664475	0.006595967
0.904142857	0.002046709
0.860958517	0.006038773
0.941486597	0.000479126
0.926162243	0.000951501
0.841099322	0.008872991
0.85480088	0.006843799
0.859086812	0.006276673
0.801927209	0.016655691
0.861780345	0.005936168
0.831347525	0.010526947

0.863063216	0.005778246
0.81460911	0.013796832
0.811086595	0.014557113
0.800160527	0.01708102
0.823101759	0.012068051
0.853092372	0.007078676
0.953795195	0.000238138
0.843551278	0.00848504
0.835857213	0.009739776
0.950382888	0.000294125
0.921212792	0.001151555
0.819453061	0.01279292
0.840044439	0.009043302
0.917744219	0.001306936
0.801523745	0.016752233
0.807233393	0.015418354
-0.867415011	0.005262669
0.851479828	0.007305023
0.812092304	0.014337426
0.827370286	0.011253657
0.821879148	0.01230797
0.803789258	0.016214655
0.867509127	0.005251859
0.805784523	0.015750274
0.831656158	0.010471835
0.842696846	0.008618978
0.869490743	0.00502754
0.869468093	0.005030069
0.973437309	4.59E-05
0.810289085	0.014732817
0.818717241	0.012942346
0.839812696	0.009080993
0.814071774	0.013911151
0.903450727	0.002090241
0.823705137	0.011950745
0.878506184	0.004084751
0.806477368	0.015590997
0.802531838	0.016511668
0.875599086	0.004375065
0.825277507	0.011648444
0.85765332	0.00646287
0.809344053	0.014942742
0.846254766	0.008070003
0.871307433	0.004827383
0.834492683	0.009973868
0.803449571	0.016294558
0.848652244	0.007712966
0.823493004	0.011991904
0.831278086	0.010539372
0.869802177	0.004992856
0.813060701	0.014127872

0.83395654	0.010066813
0.838236272	0.009340035
0.803093553	0.016378568
0.80982691	0.014835249
0.8136397	0.014003507
0.804694355	0.016002952
0.832042158	0.010403165
0.802099526	0.016614565
0.801015913	0.016874246
0.837763548	0.009418619
0.822189748	0.012246736
0.805495083	0.015817114
0.907648146	0.00183527
0.869803905	0.004992664
0.899523973	0.002348617
0.81927228	0.012829531
0.864246309	0.005635017
0.806037784	0.015691935
0.879428029	0.003995358
0.908877373	0.001764637
0.804580331	0.016029526
0.822932601	0.012101068
0.803904057	0.016187706
0.848331213	0.007760179
0.824843526	0.011731392
0.834316552	0.010004342
0.855463088	0.00675412
0.869108438	0.005070328
0.813527703	0.014027509
0.812583864	0.014230814
0.836216033	0.009678803
0.810121417	0.014769926
0.852775633	0.007122776
0.838296056	0.009330127
0.920796573	0.001169526
0.848595679	0.007721272
0.941666484	0.000474786
0.861719072	0.005943779
0.852731049	0.007128998
0.94638145	0.000370045
0.871937513	0.004759178
0.869955897	0.004975793
0.874598622	0.004477958
0.848544419	0.007728803
0.872842371	0.004662318
0.894982278	0.002672257
0.845359623	0.008205958
0.832722962	0.010282746
0.879640281	0.003974955
0.826758206	0.011368231
0.809862733	0.014827294

0.811322987	0.014505287
0.860546231	0.006090671
0.862011969	0.005907453
0.804354727	0.016082187
0.843560636	0.008483581
0.870533168	0.004912051
0.902862191	0.002127727
0.864191413	0.005641612
0.856726289	0.006585142
0.863491356	0.005726148
0.868695199	0.005116841
0.807710648	0.015309991
0.856189013	0.006656679
0.819084823	0.012867563
0.837412059	0.00947732
0.802183926	0.016594445
0.802983522	0.016404587
0.807239175	0.015417038
-0.808826149	0.015058579
0.915128767	0.001432712
0.829693794	0.010825383
0.805518329	0.015811739
0.905817688	0.001943815
0.933042347	0.000713299
0.819634974	0.012756147
0.832400084	0.010339746
0.942109227	0.000464214
0.885408282	0.003445938
0.91038692	0.001680346
0.892125785	0.002889862
0.915026546	0.001437782
0.858307481	0.006377469
0.865335166	0.005505225
0.829577684	0.010846536
0.869075477	0.005074028
0.851220846	0.0073418
0.850609362	0.007429102
0.863552034	0.005718789
0.852600992	0.007147167
0.824659705	0.011766638
0.832916021	0.01024876
0.844799101	0.008291829
0.850099504	0.0075024
0.895011008	0.002670124
0.827991724	0.011138081
0.842945814	0.008579813
0.834481299	0.009975836
0.8319363	0.010421969
0.861955464	0.00591445
0.860640407	0.006078791
0.801110208	0.016851549

0.805176318	0.015890932
0.822850108	0.01211719
0.856831849	0.006571146
0.925750375	0.000967206
0.815478563	0.013613111
0.827834606	0.01116723
0.811846673	0.014390888
0.831129551	0.010565981
0.818405032	0.013006079
0.812808156	0.014182334
0.811710417	0.014420598
0.907481611	0.001844978
0.87166059	0.004789078
0.824096262	0.01187509
0.82071805	0.012538586
0.875873387	0.004347122
0.911134422	0.001639595
0.814993739	0.013715366
0.814091206	0.013907007
0.825226068	0.011658256
0.800823808	0.016920547
0.883210838	0.003641745
0.820552707	0.012571646
0.83057934	0.010664919
0.881177306	0.003829211
0.81369108	0.013992505
0.802110374	0.016611978
0.808243275	0.015189621
0.870807648	0.004881927
0.860111773	0.006145668
0.801126242	0.016847692
0.837251842	0.009504154
0.802979589	0.016405518
0.803444266	0.016295808
0.946767747	0.000362212
0.903524876	0.002085549
0.892147541	0.002888163
0.814585924	0.013801753
0.856010854	0.00668051
0.813631833	0.014005193
0.800238431	0.017062123
0.854938328	0.006825123
0.805320501	0.015857516
0.838450611	0.009304542
0.800973475	0.016884468
0.811850965	0.014389953
0.893537581	0.002780935
0.813595772	0.014012919
0.822151303	0.012254305
0.853172123	0.007067599
0.869251847	0.005054251

0.861556649	0.005963985
0.817213356	0.01325116
0.826268375	0.011460451
0.913306594	0.001524838
0.811511874	0.014463959
0.80660969	0.015560693
0.818868637	0.012911512
0.918623269	0.00126634
0.852616787	0.007144959
0.813447237	0.01404477
0.869537771	0.005022293
0.878859818	0.004050308
0.834608614	0.009953842
0.873292446	0.004614616
0.8222363	0.012237576
0.851487815	0.00730389
0.864989221	0.005546251
0.801263928	0.016814588
0.805507302	0.015814289
0.93005234	0.000811322
0.912466109	0.001568599
0.91504854	0.00143669
0.806431174	0.015601585
0.863180995	0.005763884
0.808460295	0.015140747
0.842441857	0.008659208
0.934877217	0.000657176
0.812006831	0.014356015
0.902487576	0.002151813
0.82011807	0.012658814
0.899932444	0.002320826
0.821959436	0.012292123
0.840342402	0.008994987
0.806337059	0.015623171
0.841751158	0.008768779
0.938464642	0.000555971
0.84037286	0.008990057
0.853315413	0.007047726
0.849581122	0.007577393
0.833355606	0.010171642
0.823765397	0.011939069
0.837148845	0.00952143
0.89083302	0.002991992
0.861377299	0.005986347
0.828459144	0.011051647
0.862868905	0.005801991
0.804001033	0.016164964
0.832711458	0.010284774
0.827633083	0.011204689
0.815545261	0.013599081
0.828558624	0.011033306

0.803804934	0.016210973
0.805695772	0.01577075
0.835240483	0.009845142
0.876058519	0.004328329
0.854034722	0.006948501
0.815918505	0.013520738
0.844394386	0.008354184
0.831694663	0.010464972
0.840665877	0.008942722
0.813112378	0.014116744
0.878438056	0.004091408
0.826362431	0.011442706
0.802519858	0.016514514
0.842994332	0.008572193
0.842602611	0.008633831
0.830740988	0.010635791
0.813828945	0.013963009
0.826083601	0.011495361
0.955341518	0.000215274
0.855490983	0.006750359
0.911264658	0.001632561
0.818932533	0.012898513
0.826423287	0.011431235
0.964374542	0.000110038
0.843773007	0.008450501
0.866078734	0.005417703
0.886851847	0.003321084
0.883660316	0.003601125
0.805834115	0.01573884
0.949757934	0.000305233
0.808445692	0.015144032
0.844559193	0.008328756
0.81242007	0.014266283
0.819126606	0.01285908
0.912279248	0.001578438
0.916435122	0.001368949
0.915576339	0.001410653
0.800481021	0.017003362
0.804400146	0.016071576
0.813046694	0.014130889
0.881272376	0.003820312
0.817401469	0.013212281
0.83003211	0.0107639
0.973959863	4.33E-05
0.802673697	0.01647799
0.846643209	0.008011456
0.817328155	0.013227425
0.811760485	0.014409676
0.828375459	0.01106709
0.817429543	0.013206485
0.825314581	0.011641375

0.804750919	0.015989779
0.816302299	0.013440479
0.948940933	0.000320168
0.80179882	0.016686375
0.826050639	0.011501595
0.823217869	0.012045421
0.87790215	0.004144018
0.814683378	0.013781078
0.822033226	0.01227757
0.830444157	0.010689316
0.802525699	0.016513126
0.877476573	0.004186106
0.830825746	0.010620538
0.871318579	0.004826171
0.848598957	0.00772079
0.827600658	0.011210724
0.833962381	0.010065798
0.857936978	0.006425749
0.893404841	0.002791061
0.820474625	0.012587278
0.856076479	0.006671725
0.857134998	0.006531055
0.800830305	0.01691898
0.844607353	0.008321335
0.94012481	0.000512827
0.802515805	0.016515477
0.885011971	0.003480737
0.929341197	0.000835862
0.887059391	0.003303377
0.848896265	0.007677202
0.813435435	0.014047303
0.806503892	0.01558492
0.843966722	0.008420399
0.852575421	0.007150743
0.803426206	0.016300063
0.880227327	0.003918876
0.830272913	0.010720273
0.844089448	0.008401363
0.863092482	0.005774675
0.802791655	0.01645002
-0.815083206	0.01369646
0.825575709	0.011591664
0.822308242	0.012223427
0.880798995	0.003864758
0.879822254	0.003957517
0.926899433	0.000923809
0.851619959	0.007285172
0.86757654	0.005244124
0.925245225	0.000986697
0.805284679	0.015865814
0.897848129	0.002464892

0.898941875	0.002388592
0.864615083	0.005590842
0.837755084	0.00942003
0.859147549	0.006268861
0.855933845	0.006690827
0.844775856	0.008295402
0.804305732	0.016093637
0.839899659	0.009066837
0.924765408	0.001005447
0.927021325	0.000919282
0.823161006	0.0120565
0.833852828	0.010084856
0.813742995	0.013981393
0.9207654	0.00117088
0.811781824	0.014405023
0.806874573	0.015500142
0.866345525	0.005386519
0.905093312	0.001987894
0.802469969	0.016526369
0.830124795	0.010747095
0.90841651	0.001790908
0.889857531	0.003070581
0.853955448	0.006959392
0.869051635	0.005076706
0.815765321	0.013552857
0.814557672	0.01380775
0.906911373	0.001878473
0.812776506	0.014189169
0.803570271	0.016266138
0.858743429	0.006320958
0.856397808	0.00662882
0.888863325	0.003152038
0.834611773	0.009953297
0.801660538	0.016719462
0.842559159	0.008640686
0.908703685	0.001774508
0.853364825	0.007040881
0.880003512	0.003940196
0.828519464	0.011040523
0.807837665	0.015281231
0.818864286	0.012912398
0.884828329	0.003496938
0.865479231	0.005488197
0.847271323	0.007917358
0.826456249	0.011425024
0.84489584	0.008276968
-0.844932973	0.008271268
0.83141315	0.010515214
0.820220411	0.012638255
0.84109813	0.008873182
0.940268993	0.000509188

0.854436398	0.006893482
0.830128789	0.010746371
0.818828166	0.01291975
0.871546447	0.004801437
0.859009087	0.006286679
0.897142529	0.002514943
0.82007277	0.012667921
0.806124568	0.015671975
0.88057363	0.003886035
0.820412338	0.012599756
0.84735167	0.007905372
0.860684037	0.006073292
0.921507299	0.001138948
0.8163746	0.013425393
0.85039711	0.00745956
0.844289541	0.008370386
0.928299844	0.000872667
0.862778902	0.00581301
0.905850291	0.001941846
0.815226138	0.01366629
0.818802834	0.012924909
0.833991885	0.010060669
-0.911792934	0.001604232
0.855334163	0.006771521
0.845197976	0.008230664
0.823277652	0.01203378
0.889778912	0.003076972
0.829909921	0.01078608
0.811776876	0.014406102
0.822766483	0.012133547
0.861544728	0.005965469
0.831685781	0.010466555
0.818387926	0.013009577
0.882915199	0.003668623
0.847181141	0.007930825
0.907061577	0.001869612
0.805534065	0.015808102
0.876148582	0.004319205
0.805625975	0.015786865
0.822037697	0.012276689
0.857989252	0.006418923
0.840147793	0.009026524
0.852392495	0.007176356
0.891587377	0.002932118
0.842378676	0.008669194
0.829203606	0.010914861
0.887233734	0.00328855
0.891506433	0.002938505
0.818883479	0.012908492
0.804671586	0.016008256
0.801801085	0.016685833

0.908377051	0.001793169
0.830384791	0.010700042
0.901449859	0.002219455
0.867886364	0.005208671
0.835994601	0.009716401
0.859662592	0.006202862
0.871382177	0.004819259
0.800780773	0.01693093
0.840697706	0.008937589
0.843441844	0.00850212
0.871722996	0.004782329
0.81788075	0.013113548
0.846371472	0.008052384
0.839648068	0.009107829
0.901204646	0.002235638
0.900446951	0.002286125
0.81630528	0.013439856
0.837108314	0.009528234
0.880057812	0.003935017
0.861184955	0.00601039
0.920224309	0.001194533
0.870755672	0.004887622
0.814934373	0.01372792
0.90463233	0.002016281
0.843610406	0.008475821
0.868230283	0.005169496
0.854770184	0.006847975
0.918769062	0.001259688
0.83017683	0.010737667
0.834712505	0.009935918
0.82477355	0.011744801
0.808601201	0.015109067
0.843958139	0.008421731
0.80510056	0.015908507
0.819886148	0.012705482
0.866765201	0.005337699
0.879359365	0.004001972
0.920599818	0.001178085
0.807462156	0.015366352
0.810086489	0.014777664
0.859624386	0.006207743
0.877689123	0.004165051
0.834466815	0.00997834
0.918416798	0.0012758
0.814985275	0.013717156
0.809625328	0.014880066
0.932607353	0.000727049
0.873399436	0.004603323
-0.827598989	0.011211034
0.838975191	0.009218038
-0.812261701	0.01430063

0.827413321	0.011245629
0.881076217	0.003838689
0.829774439	0.010810707
0.892915785	0.002828576
0.808957696	0.015029103
0.809891641	0.014820876
-0.811679661	0.01442731
0.825063646	0.011689273
0.823759794	0.011940154
0.810653031	0.01465247
0.879334092	0.004004409
0.8172912	0.013235063
0.937727094	0.000575877
0.809743106	0.01485387
0.846723139	0.007999442
0.813012004	0.014138364
0.881136298	0.003833054
0.834177494	0.010028443
0.819053113	0.012874003
0.819924235	0.012697811
0.821709812	0.012341435
0.802450061	0.016531101
0.861232936	0.006004386
0.935075164	0.000651301
0.905212462	0.0019806
0.883144677	0.003647749
0.907014012	0.001872415
0.851095617	0.007359625
0.899842501	0.002326927
0.829715371	0.010821456
0.844789684	0.008293276
0.808078885	0.015226708
0.875716209	0.00436312
0.975708783	3.52E-05
0.81366986	0.013997048
0.868613362	0.005126085
0.816528261	0.013393366
0.820046067	0.012673291
0.944289267	0.000414411
0.881963789	0.003755988
0.836682796	0.009599852
0.848079085	0.007797387
0.910124481	0.001694808
0.85382688	0.006977079
0.903489769	0.00208777
0.897366643	0.002498975
0.88659215	0.003343326
0.849943638	0.007524899
0.876834929	0.00425008
0.858733058	0.006322299
0.840458274	0.008976243

0.849639237	0.007568962
0.822914004	0.012104701
0.834630787	0.009950015
0.867008209	0.005309561
0.804096043	0.016142702
0.908336103	0.001795517
0.829263747	0.010903858
0.825570405	0.011592673
0.801194906	0.016831178
0.851699412	0.007273933
0.807271779	0.01540962
0.842116535	0.008710707
0.833570898	0.010134007
0.86799109	0.005196722
0.854232728	0.006921344
0.824320197	0.011831911
0.805630207	0.015785888
0.849236131	0.007627567
0.880550742	0.0038882
0.835934281	0.009726659
0.812942505	0.014153345
0.858438253	0.006360483
0.815079629	0.013697216
0.89157021	0.002933472
0.813690305	0.013992671
0.882340312	0.003721254
0.958026052	0.000179104
0.848283052	0.007767277
0.882914126	0.003668721
0.870966554	0.004864542
0.850973964	0.007376968
0.862390935	0.005860664
0.803714216	0.016232285
0.809807539	0.014839552
0.80801779	0.015240506
0.843343139	0.008517544
0.952264547	0.000262291
0.807911336	0.015264566
0.830276489	0.010719626
0.803850293	0.016200324
0.811421752	0.014483668
0.87493521	0.004443169
0.882432103	0.003712818
0.80485791	0.015964883
0.813115299	0.014116115
0.848297954	0.00776508
0.845731735	0.008149266
0.900478601	0.002284001
0.869035304	0.00507854
0.846530795	0.008028371
0.832753897	0.010277296

0.859365821	0.006240836
-0.85283494	0.007114506
0.850589931	0.007431887
0.820220828	0.012638171
0.83009392	0.010752691
0.825040877	0.011693626
0.815362215	0.013637606
0.805610001	0.015790555
0.81101799	0.014572176
0.80769372	0.015313826
0.800292015	0.017049132
0.855153739	0.006795919
0.807862222	0.015275675
0.846059203	0.008099582
0.875505328	0.004384642
0.895465136	0.002636557
0.857123375	0.006532589
0.817281008	0.01323717
0.933327734	0.000704372
0.84781903	0.007835883
0.811023593	0.014570945
0.831396103	0.010518261
0.839232802	0.009175744
0.802287459	0.016569785
0.81970942	0.012741117
0.802999437	0.016400822
0.873606622	0.004581504
0.843218803	0.008536999
0.837581158	0.00944905
0.82775557	0.011181912
-0.802362263	0.016551982
0.800040603	0.017110135
0.819357514	0.012812261
0.830565751	0.01066737
0.878130198	0.004121578
0.974063516	4.28E-05
0.855784237	0.006710901
0.816146314	0.013473062
0.832678318	0.010290616
0.83547467	0.009805047
0.896062493	0.002592821
0.81758827	0.013173744
0.82303077	0.0120819
0.897685766	0.002476351
0.873880327	0.004552782
0.859889328	0.006173949
0.899837136	0.002327291
0.806460857	0.015594781
0.935600996	0.00063586
0.83012557	0.010746954
0.890283465	0.003036104

0.872033536	0.004748839
0.836151659	0.009689724
0.827744663	0.011183939
0.847944617	0.007817277
0.851707101	0.007272846
0.846458673	0.008039236
0.888438225	0.003187289
0.881787837	0.00377229
0.853364706	0.007040897
0.851065516	0.007363914
0.857747018	0.006450593
0.845942259	0.008117303
0.841020823	0.008885594
0.832574069	0.010309006
0.814570963	0.013804928
0.85852915	0.006348694
0.860104859	0.006146546
0.813905358	0.013946677
0.857383847	0.006498262
0.825974703	0.011515967
0.858917892	0.006298433
0.849393606	0.007604639
0.885860562	0.0034065
0.853595138	0.007009032
0.820296884	0.012622906
0.854415238	0.006896373
0.835833549	0.009743805
0.870543361	0.00491093
0.848712683	0.007704098
0.806814849	0.015513782
0.890025914	0.003056921
0.839935899	0.009060942
0.86454159	0.005599627
0.818892479	0.012906661
0.90198797	0.002184209
0.850788891	0.007403402
0.867125213	0.005296046
0.863358855	0.005742239
0.828341067	0.011073441
0.86881417	0.005103422
0.823734522	0.01194505
0.808486998	0.01513474
0.800587177	0.016977688
0.806165576	0.015662549
0.802798569	0.016448381
0.826862335	0.011348687
0.826051295	0.011501471
0.815237403	0.013663914
0.823865175	0.011919752
0.821897268	0.012304392
0.81698817	0.013297797

0.818496943	0.012987297
0.812384725	0.014273944
0.835281491	0.009838114
0.811402917	0.014487789
0.825865507	0.011536652
0.826896131	0.011342349
0.883916855	0.003578072
0.928527594	0.000864529
0.804227948	0.016111827
0.839667976	0.009104581
0.821062446	0.0124699
0.854494393	0.006885561
0.836572647	0.009618446
0.834721148	0.009934428
0.875506103	0.004384563
0.85172379	0.007270486
0.918646455	0.001265281
0.846775472	0.007991583
0.81429553	0.013863475
0.850975156	0.007376798
0.882196426	0.003734503
0.853037179	0.007086348
0.821680784	0.012347177
0.811150074	0.014543185
0.880200267	0.00392145
0.810703039	0.014641451
0.851103365	0.007358521
0.930337429	0.000801617
0.802673817	0.016477962
0.80506438	0.015916905
0.887721002	0.003247339
0.808376253	0.015159662
0.846895278	0.007973608
0.901628196	0.002207734
0.837581873	0.009448931
0.886171222	0.003379581
0.808095813	0.015222886
0.816814601	0.013333814
0.845031798	0.008256111
0.849547744	0.007582239
0.811476052	0.014471791
0.960602522	0.000148396
0.861911118	0.005919945
0.814807594	0.013754755
0.81234026	0.014283586
0.828792036	0.010990349
0.872010827	0.004751283
0.825183809	0.011666321
0.837631583	0.009440631
0.805498958	0.015816218
0.812885225	0.0141657

0.92162019	0.00113414
0.853566766	0.007012951
0.918833375	0.001256761
0.908114552	0.001808258
0.864117861	0.005650456
0.814737499	0.013769605
0.837068975	0.009534841
0.808258533	0.015186181
0.803912044	0.016185833
0.865218043	0.005519093
0.810440063	0.014699453
0.814859152	0.013743838
0.809572995	0.014891714
0.898177028	0.002441784
0.821607649	0.012361653
0.829348326	0.010888396
0.855438054	0.006757497
0.890385747	0.003027862
0.866502821	0.005368188
0.843189478	0.008541592
0.841744781	0.008769795
0.917307556	0.001327413
0.826794624	0.011361393
0.816151142	0.013472053
0.80799216	0.015246296
0.822396696	0.012206045
0.901888072	0.002190725
0.898427308	0.002424295
0.847238183	0.007922305
0.831723571	0.010459822
0.963212371	0.000121056
0.831343591	0.010527651
0.888802528	0.003157064
0.85082382	0.007398409
0.842938721	0.008580927
0.859167874	0.006266248
0.873940289	0.004546505
0.856758118	0.00658092
0.878412366	0.00409392
0.905223012	0.001979955
0.839127958	0.009192942
0.857075453	0.006538917
0.860719264	0.006068855
0.826356947	0.011443741
0.865260363	0.005514079
0.900543392	0.002279658
0.811989427	0.014359802
0.829493761	0.010861841
0.868880808	0.005095916
0.880348325	0.003907381
0.80002898	0.017112959

0.934268177	0.000675471
0.859427571	0.006232923
0.875787735	0.004355835
0.829535186	0.010854284
0.826558113	0.011405845
0.833610535	0.010127087
0.849569321	0.007579106
-0.841934264	0.008739646
-0.837009788	0.009544787
0.812068522	0.014342597
0.854002118	0.006952979
0.855660915	0.006727476
0.812465489	0.014256442
0.843366444	0.008513901
0.87023288	0.004945143
0.821741223	0.012335223
0.837984085	0.009381906
0.806879997	0.015498904
0.805618942	0.01578849
0.848999858	0.007662051
0.826850593	0.01135089
0.804700136	0.016001605
0.868525445	0.005136027
0.866657555	0.005350194
0.855100572	0.00680312
0.833399832	0.010163903
0.817732692	0.013143999
0.870150089	0.004954292
0.810087025	0.014777545
0.909680665	0.001719448
0.85348475	0.007024286
0.814903796	0.013734389
0.818180978	0.013051938
0.841392279	0.008826058
0.808771729	0.015070783
0.826908052	0.011340113
0.805902243	0.01572314
0.810014904	0.01479353
0.823748589	0.011942325
0.905388236	0.00196987
0.816808701	0.013335039
0.861020744	0.006030964
0.811273694	0.014516084
0.836135864	0.009692405
0.801512957	0.01675482
0.925219953	0.000987679
0.850341976	0.007467484
0.809809268	0.014839168
0.825407922	0.011623591
0.862643778	0.005829579
0.832851112	0.010260179

0.821278214	0.012426989
0.853081882	0.007080133
0.867536664	0.005248698
0.811235726	0.014524404
0.844619989	0.008319388
0.910271943	0.001686672
0.846768439	0.007992639
0.912319541	0.001576313
-0.83966881	0.009104445
0.82236737	0.012211806
0.915027022	0.001437758
0.820331156	0.012616031
0.860814393	0.006056882
0.941779196	0.00047208
-0.802457869	0.016529245
0.80362016	0.0162544
0.925086498	0.000992874
0.802583218	0.016499465
0.81514591	0.01368322
0.868714511	0.005114661
0.887188137	0.003292424
0.891796768	0.002915637
0.820502579	0.01258168
0.800109744	0.017093345
0.806926668	0.015488251
0.813966036	0.013933718
0.831992924	0.010411908
0.816176414	0.013466771
0.913418949	0.00151905
0.824163377	0.011862139
0.840955198	0.008896138
0.842424572	0.008661939
0.875602424	0.004374724
0.840642869	0.008946433
0.809264302	0.014960543
0.82697767	0.011327065
0.912310004	0.001576816
0.802659631	0.016481328
0.851584077	0.007290252
0.939550519	0.000527493
0.828491688	0.011045645
0.894731879	0.002690893
0.852504194	0.007160709
0.834891975	0.009905002
0.832553923	0.010312563
0.810301721	0.014730023
0.88273567	0.003685007
0.970731914	6.13E-05
0.804533064	0.01604055
0.85656774	0.006606201
0.854909778	0.006829

0.84327358	0.008528425
0.810033262	0.01478946
0.835278749	0.009838584
0.809770405	0.014847803
0.806708038	0.015538194
0.810366154	0.01471578
0.840676665	0.008940982
0.851644814	0.007281655
0.848250806	0.007772032
0.871416032	0.004815583
0.881585717	0.003791073
0.907882869	0.001821643
0.927396119	0.000905453
0.861199915	0.006008517
0.819725275	0.012737917
0.855655372	0.006728222
0.846545756	0.008026119
0.937591195	0.000579595
0.902877688	0.002126734
0.877832353	0.004150902
0.882321298	0.003723003
0.882439256	0.003712162
0.980925798	1.71E-05
0.80651319	0.01558279
0.855747104	0.006715889
0.808361888	0.015162896
0.913617671	0.001508846
0.842324018	0.008677839
0.821763158	0.012330886
0.832416713	0.010336805
0.846238732	0.008072426
0.82198894	0.012286303
0.880037487	0.003936955
0.802918375	0.016420005
0.855871975	0.006699124
0.925673962	0.000970138
0.823974311	0.011898646
0.886273146	0.003370779
0.878512144	0.004084169
0.841017187	0.008886178
0.828160346	0.011106851
0.92786777	0.000888243
0.819381475	0.012807409
0.924538016	0.001014414
0.868786097	0.005106587
0.84772402	0.007849978
0.82166183	0.012350928
0.924978614	0.000997087
0.808908939	0.015040024
0.823566854	0.011977565
0.832365394	0.010345881

0.866259873	0.005396518
0.896824241	0.002537734
0.810360909	0.014716939
0.859443009	0.006230945
-0.861990333	0.005910132
0.830551088	0.010670015
0.846291184	0.008064503
0.804776013	0.015983938
-0.816643417	0.013369396
0.832679152	0.010290469
0.921456218	0.001141128
0.890527606	0.003016455
0.840694964	0.008938031
0.866363585	0.005384413
0.838843226	0.009239751
0.857957065	0.006423126
0.863398433	0.00573743
0.832266688	0.010363353
0.815300584	0.013650593
0.888188243	0.003208137
0.834574044	0.009959811
0.861582041	0.005960823
0.891663611	0.002926111
0.849584818	0.007576857
0.831475139	0.010504137
0.874765635	0.004460674
0.875193238	0.004416619
0.837939084	0.00938939
0.892961383	0.002825064
0.86186707	0.005925406
0.885989189	0.003395337
0.934397161	0.000671569
0.808786809	0.015067401
0.805886507	0.015726765
0.983113647	1.19E-05
0.805081785	0.015912865
-0.847607791	0.007867243
0.810214221	0.014749379
-0.838604331	0.00927914
0.8924523	0.002864429
0.966516197	9.15E-05
0.845390618	0.008201227
0.909197032	0.001746564
0.930100024	0.000809693
0.923564136	0.001053407
0.849491358	0.007590428
0.834836185	0.009914606
0.830543458	0.010671391
0.898853183	0.002394721
0.813031912	0.014134074
0.965049088	0.000103959

0.831075251	0.01057572
0.830558419	0.010668692
0.868956447	0.005087404
0.825792968	0.011550407
0.837247491	0.009504884
0.862827837	0.005807017
0.812746882	0.014195568
0.906269968	0.001916617
0.853829086	0.006976775
0.811681151	0.014426984
0.828109741	0.011116217
0.893095851	0.002814726
0.914479375	0.001465116
0.815836847	0.013537854
0.878374279	0.004097647
0.858619928	0.006336935
0.909466445	0.001731425
0.801074922	0.01686004
0.849787772	0.007547441
0.810349464	0.014719468
0.829215586	0.010912669
0.860712171	0.006069748
0.819657266	0.012751645
0.871439278	0.004813059
0.844123423	0.008396098
0.89282161	0.002835837
0.800837755	0.016917183
0.802793086	0.01644968
0.816706598	0.013356256
0.801430106	0.01677469
0.830352485	0.010705881
0.834509194	0.009971015
0.819615424	0.012760096
0.881419063	0.003806606
0.913833141	0.001497833
0.805795133	0.015747827
0.867457151	0.005257827
0.822237372	0.012237365
0.904373646	0.002032325
0.836075604	0.009702637
0.91912514	0.001243537
0.952152729	0.000264116
0.801572323	0.016740591
0.904834092	0.002003825
0.901568294	0.002211666
0.811518788	0.014462447
0.885918379	0.00340148
0.854895651	0.006830919
0.934510767	0.000668145
0.871968389	0.004755852
0.834385157	0.009992465

0.82997185	0.010774835
0.855988681	0.006683479
0.836775184	0.009584273
0.880862474	0.003858779
0.875351071	0.004400428
0.82269311	0.01214791
0.826659381	0.011386798
0.830392301	0.010698685
0.847758114	0.007844918
0.816653728	0.013367251
0.903036773	0.002116562
0.874358177	0.004502916
0.852262735	0.007194561
0.802409708	0.016540696
0.835401714	0.009817527
0.872248113	0.004725786
0.936709344	0.000604105
0.858779132	0.006316344
0.80304116	0.016390954
0.808291733	0.015178699
0.840166986	0.009023411
0.839701533	0.009099108
0.849458039	0.00759527
0.869371533	0.005040858
0.811226189	0.014526495
0.853034556	0.007086713
0.82560575	0.011585954
0.841866255	0.00875046
0.903761744	0.002070606
0.827471018	0.011234871
0.945554256	0.000387192
0.871133089	0.004846365
0.852299631	0.007189382
0.812077761	0.014340588
0.849701822	0.007559889
0.849563837	0.007579902
0.930555403	0.000794249
0.932122231	0.000742586
0.890084922	0.003052143
0.801194608	0.016831249
0.859879732	0.006175171
0.878453076	0.00408994
0.803842604	0.016202128
-0.816322446	0.013436274
0.82672435	0.01137459
0.861667037	0.005950248
0.919704735	0.001217537
0.881444335	0.003804248
0.82547611	0.011610609
0.806323826	0.015626207
0.886698306	0.003334222

0.804208815	0.016116303
0.811047018	0.014565801
0.840643346	0.008946356
0.879213333	0.004016063
0.822322965	0.012220533
0.804182112	0.016122551
0.884608686	0.00351638
0.868086576	0.005185842
0.800113618	0.017092405
0.830306888	0.010714127
0.811356664	0.014497913
0.889902949	0.003066892
0.899441719	0.002354239
0.875022173	0.00443421
0.888621271	0.003172079
0.928675354	0.000859276
0.834440291	0.009982927
0.829716444	0.01082126
0.834195435	0.010025331
0.829926372	0.010783092
0.811208367	0.014530401
0.812811732	0.014181562
0.871359468	0.004821727
-0.807049215	0.015460301
0.826439917	0.011428101
0.853136003	0.007072614
0.88355428	0.003610681
0.812044144	0.014347899
0.82658565	0.011400664
0.808826625	0.015058472
0.834118724	0.01003864
0.897613943	0.002481431
0.876322985	0.004301572
0.821994305	0.012285245
0.823202014	0.01204851
0.835190237	0.009853758
0.851485491	0.00730422
0.895342469	0.002645597
0.820562065	0.012569774
0.819525123	0.012778345
0.810519099	0.014682005
0.880331695	0.00390896
0.917612493	0.001313091
0.896622419	0.002552254
0.947445691	0.000348729
0.914724827	0.001452813
0.851663113	0.007279066
0.820177734	0.012646826
0.838007331	0.009378041
0.805026293	0.015925749
0.866621614	0.00535437

0.811257601	0.01451961
0.819067419	0.012871098
0.801311135	0.016803248
0.889524102	0.003097746
0.81595856	0.013512348
0.81368798	0.013993168
0.855347574	0.006769709
0.887702465	0.003248901
0.877272964	0.004206339
0.831069171	0.01057681
0.842330217	0.008676859
0.899579763	0.002344808
0.821008861	0.012480571
0.801309109	0.016803735
0.809047282	0.01500905
0.816893101	0.013317517
0.807221055	0.015421162
0.904672325	0.002013808
0.908529401	0.001784449
0.829951227	0.010778579
0.912982166	0.001541635
0.814145684	0.013895392
0.853690743	0.006995839
0.806837797	0.01550854
0.842042506	0.008722453
0.908791482	0.001769514
0.87738204	0.004195492
0.860838652	0.006053832
0.832727253	0.01028199
0.876478612	0.004285877
0.837417364	0.009476432
0.93293947	0.000716536
0.836920381	0.009559823
0.872177243	0.004733392
0.823048711	0.012078399
0.806575656	0.015568484
0.834996521	0.009887021
0.86679095	0.005334713
0.866952837	0.005315964
0.833892047	0.010078031
0.828507185	0.011042787
0.809756935	0.014850797
0.894686222	0.0026943
0.816096067	0.013483569
0.830776274	0.010629439
0.921201169	0.001152054
-0.830460966	0.010686281
0.898465276	0.002421648
0.813760996	0.013977542
0.94060564	0.000500756
0.90656352	0.001899098

0.839742005	0.00909251
0.925283134	0.000985226
0.808128536	0.015215501
0.802082062	0.01661873
0.809155643	0.014984817
0.885281742	0.003457025
0.850177705	0.007491127
0.829587102	0.010844819
0.812654376	0.014215562
0.82796514	0.011143009
0.962151051	0.000131732
0.929745972	0.000821835
0.857409596	0.006494875
0.86213541	0.005892186
0.892829239	0.002835248
0.847278297	0.007916317
0.911794543	0.001604146
0.826246321	0.011464614
-0.837667704	0.009434602
0.801541924	0.016747876
0.801503956	0.016756978
0.808731675	0.01507977
0.859239817	0.006257004
0.802422881	0.016537563
0.805780351	0.015751236
0.87844497	0.004090732
0.842774093	0.008606814
0.814502537	0.013819459
0.888579369	0.003175557
0.92376709	0.001045202
0.857706368	0.006455918
0.811454713	0.014476458
0.807365835	0.015388234
0.812953591	0.014150955
0.803829908	0.016205109
0.835218132	0.009848974
0.807750165	0.015301039
0.814367712	0.013848117
0.834057748	0.010049226
0.804763734	0.015986796
0.847480714	0.007886146
0.814774692	0.013761724
0.801703155	0.01670926
0.929165423	0.000842002
0.822055042	0.012273269
0.851612628	0.00728621
0.811414719	0.014485207
0.80107224	0.016860686
0.852080405	0.00722019
0.827618539	0.011207396
0.840239763	0.009011611

0.845677376	0.008157532
0.855566442	0.006740192
0.843958318	0.008421703
0.86823386	0.00516909
0.811086059	0.014557231
0.864177763	0.005643252
0.810742497	0.014632761
0.910700798	0.001663155
0.895376503	0.002643087
0.800324082	0.017041361
0.819654226	0.012752259
0.807466447	0.015365378
0.814498007	0.013820421
0.816381752	0.013423901
0.877421677	0.004191555
0.862645268	0.005829396
0.890083373	0.003052269
0.81602484	0.013498471
0.822298288	0.012225384
0.897457182	0.002492543
0.849565387	0.007579677
0.802994907	0.016401894
0.811625421	0.014439151
0.891089857	0.002971518
0.835438788	0.009811184
0.82816422	0.011106134
0.847826302	0.007834805
0.803269684	0.016336972
0.801810741	0.016683524
0.810415506	0.014704876
0.905069649	0.001989345
0.836496532	0.009631308
0.824754298	0.011748492
0.822606862	0.012164808
0.933414578	0.00070167
0.866836727	0.005329407
0.816039741	0.013495353
0.815120697	0.013688543
0.854032993	0.006948738
0.802799821	0.016448084
0.804504454	0.016047225
0.888360381	0.003193772
0.826978624	0.011326887
0.809369206	0.01493713
0.812263191	0.014300306
0.886943102	0.003313291
0.89642024	0.002566854
0.848098218	0.007794559
0.82607013	0.011497908
0.906597912	0.001897052
0.812548161	0.01423854

0.80313617	0.016368498
0.815066993	0.013699885
0.824973524	0.011706506
0.885817111	0.003410276
0.83842206	0.009309265
0.841715097	0.008774524
0.81063199	0.014657107
0.828676522	0.011011595
0.818188071	0.013050485
0.816485226	0.01340233
0.820413709	0.012599481
0.817730546	0.01314444
0.951957941	0.000267315
0.80703038	0.015464595
0.814766765	0.013763404
0.85995239	0.006165923
0.869827628	0.004990028
0.849707901	0.007559008
0.81378895	0.013971562
0.808679879	0.015091396
0.823344052	0.012020858
0.822434723	0.012198577
0.801302731	0.016805267
0.856117249	0.006666272
0.870988548	0.004862139
0.80967927	0.014868065
0.860545397	0.006090776
0.813081384	0.014123418
0.824690282	0.01176077
0.811218023	0.014528285
0.94950825	0.000309748
0.802631378	0.016488032
0.807268023	0.015410475
0.916659951	0.001358165
0.90790832	0.00182017
0.840498149	0.008969798
0.878009319	0.004133463
0.84478265	0.008294357
0.844487131	0.008339868
0.833374083	0.010168408
0.814298391	0.013862866
0.809707999	0.014861676
0.810533881	0.014678744
0.821537793	0.012375489
0.956948459	0.000193098
0.816991866	0.013297031
0.819571793	0.012768911
0.842203259	0.008696959
0.844747245	0.008299802
0.841545045	0.008801647
0.899766147	0.002332114

0.891621411	0.002929435
0.846538842	0.00802716
0.865545332	0.005480396
0.830716372	0.010640223
0.832858801	0.010258826
0.827199459	0.011285559
0.835212767	0.009849894
0.812326729	0.01428652
0.944203079	0.00041631
0.90541935	0.001967975
0.888689697	0.003166405
0.884587884	0.003518225
0.801660657	0.016719433
0.819202721	0.012843635
0.811047137	0.014565775
0.837720215	0.009425843
0.831300735	0.010535319
0.820942879	0.012493719
0.880927145	0.003852693
0.809201598	0.014974548
0.811304986	0.014509229
0.821646035	0.012354054
0.895032704	0.002668514
0.823640943	0.011963191
0.853582919	0.00701072
0.81435132	0.013851603
0.955137253	0.000218207
0.801609397	0.016731709
0.828831434	0.010983108
0.847766697	0.007843645
0.804758549	0.015988003
0.844431818	0.008348404
0.812495708	0.014249897
0.915771008	0.001401128
0.919547796	0.001224541
0.941812277	0.000471287
0.864370108	0.005620162
0.885054052	0.003477031
-0.837908626	0.009394457
0.853823781	0.006977506
0.844804466	0.008291004
0.869915903	0.004980229
0.855719626	0.006719582
0.806109369	0.015675469
0.80616498	0.015662686
0.841142952	0.008865991
0.854751408	0.006850529
0.808606923	0.015107782
0.839025676	0.009209739
0.809451818	0.014918709
0.802851021	0.016435954

0.852846086	0.007112952
0.889389813	0.00310873
0.807392776	0.015382112
0.806128502	0.01567107
0.923529208	0.001054823
0.928261817	0.00087403
0.810537696	0.014677902
0.840871274	0.008909635
0.868107021	0.005183515
0.810959756	0.014584969
0.81828177	0.013031296
0.833375037	0.010168241
0.815012991	0.013711296
0.800680697	0.016955091
0.867799759	0.005218565
0.83031261	0.010713092
0.805123746	0.015903127
0.875408232	0.004394574
0.843491614	0.00849435
0.836169064	0.009686771
0.836667478	0.009602436
0.859541357	0.006218357
0.848668039	0.007710648
0.827576518	0.011215218
0.83664906	0.009605544
0.920989156	0.001161188
0.836247563	0.009673457
0.908703864	0.001774498
0.862553835	0.005840625
0.807991922	0.01524635
0.935375333	0.000642457
0.800319076	0.017042575
0.801187873	0.016832869
0.883154094	0.003646894
0.917554259	0.001315818
0.840552032	0.008961094
0.81825316	0.013037153
0.938353062	0.000558953
0.914302528	0.001474023
0.883072078	0.003654345
0.911121249	0.001640307
0.830447555	0.010688703
0.838206172	0.009345027
0.823519468	0.011986764
0.807592154	0.01533685
0.811480582	0.0144708
0.852667928	0.007137813
0.850432694	0.007454448
0.806927562	0.015488047
0.936888993	0.000599058
0.858896673	0.00630117

0.876795292	0.004254053
0.890145063	0.003047279
0.847733796	0.007848527
0.81385231	0.013958014
0.842219114	0.008694447
0.821967602	0.012290512
0.849491298	0.007590437
0.827630103	0.011205244
0.827864289	0.01116172
0.828695178	0.011008162
0.810943544	0.014588532
0.894221246	0.002729156
0.849935949	0.00752601
0.820554316	0.012571324
0.935497284	0.000638886
0.86036998	0.006112944
0.885905385	0.003402607
0.846732557	0.007998027
0.936696172	0.000604476
0.841828406	0.008756481
0.814411759	0.01383875
0.831090569	0.010572972
0.842119753	0.008710197
0.912192106	0.00158304
0.865592301	0.005474857
0.95156312	0.000273878
0.825307429	0.011642739
0.808418155	0.015150229
0.865769207	0.005454027
0.888302505	0.003198597
0.800732136	0.016942669
0.814127803	0.013899204
0.888293922	0.003199313
0.889759839	0.003078524
0.833692908	0.010112717
0.859265685	0.006253683
0.916587055	0.001361655
0.846201539	0.008078047
0.830162048	0.010740345
0.838203371	0.009345491
-0.837587714	0.009447955
0.883325756	0.003631332
0.863529325	0.005721542
-0.866191924	0.005404459
0.801189661	0.016832439
0.81439203	0.013842945
0.816789091	0.013339112
0.81671834	0.013353815
0.804505765	0.016046919
0.811141491	0.014545067
0.818494737	0.012987747

0.876548707	0.004278819
0.83606118	0.009705087
0.87944001	0.003994204
0.862097085	0.005896924
0.804524362	0.01604258
0.852276981	0.007192561
0.843159318	0.008546317
0.806517303	0.015581848
0.846007586	0.008107401
0.845153689	0.008237441
0.851010501	0.007371757
0.918197274	0.001285909
0.816195011	0.013462885
0.825874746	0.011534901
0.833186269	0.010201306
0.836280644	0.00966785
0.830969334	0.010594731
0.835959196	0.009722422
0.817556798	0.013180232
0.815006912	0.013712581
0.9156394	0.001407563
0.80951792	0.01490398
0.846073091	0.008097479
0.847571969	0.007872569
0.926017404	0.000957005
0.920397401	0.001186933
0.800976932	0.016883635
0.892634809	0.002850276
0.809688628	0.014865983
0.802263379	0.016575518
0.832887769	0.010253729
0.945045888	0.000397985
0.822944224	0.012098797
0.816470206	0.01340546
0.909655213	0.001720868
0.826362073	0.011442774
0.837239742	0.009506183
0.81459868	0.013799046
0.871077001	0.004852482
0.810266614	0.014737787
0.894112647	0.002737339
0.974826992	3.91E-05
0.964993477	0.000104451
0.848608613	0.007719372
0.830216527	0.010730478
0.823619604	0.01196733
0.871042371	0.004856262
0.805023253	0.015926455
0.861122668	0.006018188
0.824278891	0.011839869
0.825390637	0.011626883

0.900424838	0.002287609
0.84987092	0.00753541
0.818112552	0.013065964
0.850280941	0.007476264
0.854037404	0.006948132
0.862381637	0.005861809
0.851002812	0.007372853
0.825116992	0.01167908
0.822091699	0.012266046
0.878878593	0.004048484
0.929341257	0.00083586
0.919753432	0.001215368
0.800126493	0.017089279
0.826319218	0.011450857
0.9148404	0.001447043
0.882035196	0.003749385
0.94429785	0.000414222
0.821044445	0.012473484
0.813432693	0.014047891
0.818187237	0.013050656
0.902263105	0.00216633
0.902216315	0.002169364
0.822028458	0.01227851
0.832629859	0.010299162
0.840350568	0.008993665
0.832580745	0.010307828
0.820053399	0.012671816
0.900796771	0.002262725
0.859543085	0.006218136
0.930649996	0.000791066
0.823646843	0.011962046
0.813237965	0.014089724
0.805654347	0.015780314
0.817703187	0.013150072
0.907001734	0.001873139
0.891398311	0.002947051
0.858646452	0.006333501
-0.820800364	0.012522148
0.818326116	0.01302222
0.812110662	0.014333436
0.8646698	0.005584306
0.816095471	0.013483693
0.820429206	0.012596376
0.805330813	0.015855128
0.800924659	0.01689623
0.83471632	0.00993526
0.804127097	0.01613543
0.800744236	0.016939748
0.865774512	0.005453403
0.83379066	0.010095681
0.814346075	0.013852719

0.80411768	0.016137635
-0.800076008	0.017101536
0.829416573	0.010875929
0.837298036	0.009496412
0.835465074	0.009806688
0.84045136	0.00897736
0.8136338	0.014004771
0.944551706	0.000408664
0.896584928	0.002554957
0.810479105	0.014690833
0.818566501	0.012973093
0.809001684	0.015019255
0.838626444	0.00927549
0.851740837	0.007268077
0.859016895	0.006285674
0.857562363	0.006474802
0.800274134	0.017053467
0.813821971	0.0139645
0.802941322	0.016414573
0.803949177	0.016177122
0.803913713	0.016185441
0.869121671	0.005068844
0.815177321	0.01367659
0.812630832	0.014220653
0.889731646	0.003080819
0.848073602	0.007798197
0.810959876	0.014584943
0.823695481	0.011952616
0.829827726	0.010801017
0.822391033	0.012207157
0.834326982	0.010002535
0.819487214	0.012786011
0.826738596	0.011371914
0.855392277	0.006763674
0.874900281	0.004446771
0.943811953	0.000424999
0.823120415	0.012064413
0.806009114	0.015698532
0.860386372	0.00611087
0.911836803	0.001601894
0.857550144	0.006476406
0.901195705	0.002236229
0.835457563	0.009807973
0.826093316	0.011493524
0.804964483	0.015940107
0.854069829	0.006943681
0.831919074	0.010425031
0.821364284	0.012409898
0.817417204	0.013209032
0.923464835	0.001057437
0.838129818	0.009357695

0.852155387	0.007209643
0.834247649	0.010016279
0.855595112	0.006736331
0.854341388	0.00690647
0.835249245	0.00984364
0.851852775	0.007252269
0.843947053	0.008423452
0.822607517	0.012164679
0.84439081	0.008354736
0.822658241	0.01215474
0.858562291	0.0063444
0.80893147	0.015034977
0.946238577	0.00037297
0.850367427	0.007463826
0.85096997	0.007377538
0.812292099	0.014294033
0.833616316	0.010126078
0.855009317	0.00681549
0.839781046	0.009086148
0.824689925	0.011760839
0.817028344	0.013289469
0.812683165	0.014209337
0.873485982	0.0045942
0.835765481	0.009755402
0.834160328	0.01003142
0.841222644	0.008853215
0.900389969	0.002289951
0.873898447	0.004550885
0.895570338	0.00262882
0.83948189	0.009134968
0.851388991	0.007317909
0.899927557	0.002321157
0.843726039	0.00845781
0.848984241	0.007664334
0.81421566	0.013880481
0.834732533	0.009932465
0.82550019	0.011606027
0.848371744	0.007754208
0.861641705	0.005953398
0.929522693	0.000829554
0.801093578	0.016855551
0.847756088	0.007845219
0.827601552	0.011210557
0.833567142	0.010134662
0.854702234	0.006857223
0.824516416	0.011794159
0.838456035	0.009303645
0.812206447	0.014312626
0.814255476	0.013872002
-0.822421372	0.012201198
0.8341524	0.010032796

0.832174301	0.010379723
0.810422897	0.014703244
0.861966372	0.005913099
0.913099885	0.001535526
0.801850498	0.01667402
0.836450398	0.00963911
0.814621508	0.013794202
0.838066161	0.009368266
0.846335351	0.008057835
0.889396369	0.003108193
0.834423602	0.009985813
0.838528693	0.009291634
0.852636874	0.007142152
0.812068105	0.014342688
0.931902289	0.000749702
0.81609714	0.013483344
0.831964314	0.010416991
0.86555177	0.005479637
0.878246069	0.004110206
0.80267638	0.016477354
0.920900404	0.001165026
0.807744086	0.015302416
0.859468997	0.006227617
0.83216083	0.010382111
0.803175569	0.016359191
0.82771033	0.011190321
0.929308832	0.000836991
0.814255059	0.013872091
0.848717988	0.00770332
0.871154904	0.004843987
0.805382788	0.015843094
0.860840201	0.006053637
0.898143291	0.002444148
0.842733383	0.008613223
0.800036907	0.017111033
0.974996865	3.83E-05
0.815816164	0.013542191
0.828072071	0.011123193
0.904289544	0.002037559
0.869175375	0.00506282
0.863121152	0.005771179
0.830822229	0.010621171
0.832479239	0.010325754
0.858207405	0.006390487
0.8033638	0.016314773
0.870975614	0.004863552
0.80258131	0.016499918
0.895923257	0.002602973
0.858535707	0.006347844
0.841683626	0.00877954
0.82773602	0.011185546

0.808204532	0.015198356
0.842054248	0.00872059
0.828946114	0.01096205
0.840533674	0.008964059
0.814978182	0.013718655
0.851187646	0.007346523
0.877126515	0.004220931
0.851012468	0.007371476
0.835212171	0.009849996
0.817019522	0.013291298
0.901040018	0.002246545
0.86163044	0.0059548
0.82251507	0.012182807
0.827093601	0.011305358
0.844420731	0.008350116
0.911036551	0.001644893
0.824447036	0.011807499
0.81190908	0.014377293
0.851266325	0.007335333
0.810305417	0.014729206
0.910497308	0.001674287
0.855675936	0.006725456
0.873425364	0.004600589
0.844678044	0.008310449
0.838677704	0.009267031
0.825695693	0.011568868
0.839973032	0.009054905
0.975494742	3.61E-05
0.833016038	0.010231181
0.828194618	0.01110051
0.826613963	0.011395338
0.815942049	0.013515806
0.801029205	0.016871046
0.884898305	0.003490759
0.806955755	0.015481614
0.805664063	0.01577807
0.86084491	0.006053045
0.852478802	0.007164264
0.825705528	0.011567
0.809502602	0.014907392
0.860883653	0.006048175
0.956662118	0.000196933
0.871879697	0.004765411
0.856447756	0.006622167
0.816143155	0.013473723
0.827055216	0.011312542
0.839854896	0.009074122
0.830301166	0.010715162
0.820406318	0.012600962
0.907719016	0.001831149
0.814870059	0.013741529

0.8244102	0.011814585
0.870717585	0.004891798
0.906576872	0.001898304
0.851176739	0.007348075
0.80626899	0.015638794
0.878989756	0.004037699
0.835894048	0.009733505
0.848094463	0.007795114
0.850298464	0.007473742
0.811526239	0.014460819
0.819470644	0.012789363
0.811398029	0.014488859
0.811996818	0.014358194
0.905208528	0.00198084
0.944331586	0.000413481
0.834344268	0.009999543
0.805014968	0.015928379
0.914908886	0.001443631
0.914163828	0.001481032
0.870130777	0.004956427
0.83639276	0.009648862
0.802035451	0.01662985
0.868535876	0.005134847
0.809512794	0.014905121
0.855282784	0.006778463
0.821616828	0.012359835
0.800088286	0.017098555
0.843815088	0.008443956
0.871842861	0.004769385
0.852247953	0.007196636
0.923528552	0.00105485
0.966224015	9.39E-05
0.96288234	0.000124312
0.804349542	0.016083398
0.817281365	0.013237096
0.834293723	0.010008296
0.800364316	0.017031615
0.838379562	0.009316298
0.819169879	0.012850298
0.827453971	0.011238049
0.839570999	0.009120409
0.84540987	0.008198288
0.827734351	0.011185856
0.841379941	0.008828031
0.83414942	0.010033313
0.814061463	0.013913351
0.815326154	0.013645204
0.819400191	0.01280362
0.816888154	0.013318543
0.952525973	0.000258057
0.841089368	0.008874588

0.846769392	0.007992495
0.838229835	0.009341103
0.846258223	0.008069481
0.820682228	0.012545744
0.838312626	0.009327382
0.842383802	0.008668384
0.826998174	0.011323224
0.806607544	0.015561185
0.833211362	0.010196906
0.839054227	0.009205049
0.860854149	0.006051883
0.846297741	0.008063513
0.844653368	0.008314248
0.894585431	0.002701831
0.924092293	0.001032141
0.957343817	0.000187883
0.938050449	0.000567093
0.950630188	0.000289803
0.925728798	0.000968033
0.924867749	0.001001429
0.92696023	0.00092155
0.822525024	0.012180855
-0.818973482	0.012890186
0.85342598	0.007032415
0.900387824	0.002290096
0.852291107	0.007190578
-0.859710693	0.006196721
0.82258755	0.012168593
0.875386775	0.004396771
0.821744263	0.012334622
0.83107841	0.010575153
0.810150743	0.014763431
0.834572554	0.009960069
0.844826877	0.00828756
0.800636172	0.016965847
0.803128004	0.016370427
0.827523947	0.011225008
0.822355866	0.012214066
0.801546633	0.016746747
0.893523395	0.002782016
0.942351997	0.000458484
0.804771066	0.015985089
0.818228483	0.013042207
0.88680017	0.003325502
0.832139075	0.010385969
0.837692738	0.009430426
0.801460505	0.016767398
0.826136112	0.011485433
0.851413608	0.007314415
0.808727443	0.01508072
0.883844733	0.003584543

0.905426145	0.001967561
0.802733421	0.016463824
0.832378507	0.010343562
0.867520034	0.005250607
0.856754124	0.00658145
0.821100473	0.012462331
0.889853299	0.003070925
0.858220279	0.006388811
0.906257331	0.001917374
0.85775733	0.006449243
0.812625229	0.014221865
0.879136443	0.004023496
0.864590943	0.005593726
0.82462579	0.011773148
0.829155922	0.01092359
0.863855124	0.00568212
0.81908071	0.012868398
0.810205638	0.014751279
0.860420585	0.006106544
0.983109713	1.19E-05
0.910010159	0.001701133
0.978091776	2.59E-05
0.981898725	1.46E-05
0.883113325	0.003650596
0.95882231	0.000169206
0.956413627	0.000200302
0.928190947	0.000876576
0.9565835	0.000197995
0.950868309	0.000285682
0.952498376	0.000258502
0.942412317	0.000457067
0.951064587	0.000282314
0.93791461	0.000570773
0.92584306	0.000963657
0.943673491	0.000428103
0.95345819	0.000243324
0.947253704	0.000352513
0.939904571	0.00051842
0.946778893	0.000361987
0.940210164	0.000510671
0.900088429	0.002310269
0.829853296	0.010796369
0.948700547	0.000324651
0.88723892	0.00328811
0.852106452	0.007216525
0.875270605	0.004408678
0.827526748	0.011224486
0.818785012	0.012928538
0.848594904	0.007721385
0.810415745	0.014704824
0.842934489	0.008581592

0.826292157	0.011455963
0.829730451	0.010818711
0.821033597	0.012475645
0.819645107	0.0127541
0.823585093	0.011974025
0.850553572	0.0074371
0.838976979	0.009217744
-0.885421574	0.003444775
0.812514782	0.014245766
0.801515937	0.016754105
0.823670089	0.011957539
0.893689692	0.00276936
0.85922128	0.006259385
0.856967032	0.006553248
0.851117671	0.007356484
0.816997409	0.013295881
0.869269967	0.005052222
0.823823631	0.011927793
0.869996548	0.004971287
0.843313336	0.008522205
0.818642855	0.012957513
0.82222265	0.012240261
0.845748365	0.008146738
0.893974125	0.0027478
0.87845403	0.004089847
0.950276315	0.000296
0.841290414	0.008842359
0.811166227	0.014539642
0.887746811	0.003245166
0.812231183	0.014307255
0.845440209	0.00819366
0.838021994	0.009375604
0.834048748	0.010050789
0.846149564	0.008085907
0.805631161	0.015785668
0.883503079	0.003615301
0.820411623	0.012599899
0.817194045	0.013255156
0.871235073	0.004835256
0.818502247	0.012986213
0.886446118	0.003355875
0.821348906	0.012412951
0.814328432	0.013856473
0.800475419	0.017004717
0.835670471	0.009771604
0.900498033	0.002282698
0.823679447	0.011955724
0.842933476	0.008581751
0.811994314	0.014358739
0.822488308	0.012188058
0.858059406	0.00640977

0.854677379	0.006860608
0.838069081	0.009367781
0.826336801	0.01144754
0.825734615	0.011561479
0.827444196	0.011239871
0.804603994	0.016024009
0.82105124	0.012472131
0.851799667	0.007259766
0.894685984	0.002694317
0.819738209	0.012735308
0.948108554	0.000335869
0.805414557	0.015835742
0.835145295	0.009861469
0.843332708	0.008519176
0.806614101	0.015559684
0.82597059	0.011516745
0.820975006	0.012487317
0.832709908	0.010285047
0.861989558	0.005910228
0.833645582	0.010120972
0.816553891	0.013388029
0.834633112	0.009949614
0.853951991	0.006959867
0.852942109	0.007099576
0.851701915	0.007273579
0.810907543	0.014596446
0.851345122	0.007324137
0.810358107	0.014717558
0.833207548	0.010197575
0.812995493	0.014141922
-0.912990391	0.001541207
0.800857186	0.016912497
0.896317482	0.002574295
0.802952468	0.016411935
0.860697269	0.006071625
0.805299819	0.015862307
0.813835025	0.013961709
0.854152679	0.006932315
0.801874757	0.016668222
0.817556798	0.013180232
0.803434372	0.016298139
0.830309212	0.010713706
0.860255539	0.006127434
0.825395405	0.011625974
0.855322719	0.006773067
0.927619159	0.000897287
0.859180748	0.006264593
0.838585973	0.009282172
0.812685788	0.01420877
0.897501886	0.002489371
-0.843983829	0.008417744

0.817051589	0.013284652
0.835135341	0.009863177
-0.836153209	0.009689461
0.871133089	0.004846365
0.810219169	0.014748284
0.906604826	0.001896641
0.822891414	0.012109115
0.818701386	0.012945578
0.904093266	0.002049808
0.873608351	0.004581322
0.870503664	0.004915296
0.866004348	0.005426418
0.821273386	0.012427948
0.801701665	0.016709617
0.923378527	0.001060948
0.853973567	0.006956902
0.835921645	0.009728809
0.809129417	0.01499068
0.883497655	0.003615791
0.830284894	0.010718105
0.886210561	0.003376182
0.867427468	0.005261238
0.850714624	0.007414027
0.836001873	0.009715165
0.830922365	0.010603168
0.903638422	0.002078377
0.832129657	0.010387639
0.812006295	0.014356132
0.817665219	0.01315789
0.816853285	0.013325781
0.825596452	0.011587721
0.813829422	0.013962907
0.845474601	0.008188414
0.826080918	0.011495868
0.81117034	0.01453874
0.828484416	0.011046985
0.8507846	0.007404016
0.822984695	0.012090894
0.874306977	0.004508242
0.863250554	0.005755413
-0.8209306	0.012496167
0.846628845	0.008013616
0.916426957	0.001369342
0.863455951	0.005730445
0.814019978	0.013922203
0.813978374	0.013931083
0.863358557	0.005742275
0.828239918	0.011092132
0.822449088	0.012195756
0.867358685	0.005269146
0.913232803	0.001528648

0.856428146	0.006624778
0.828712821	0.011004915
0.812291741	0.014294111
0.833305538	0.010180407
0.874478936	0.00449037
-0.865639865	0.005469251
0.821807861	0.012322051
0.819205999	0.01284297
0.909609258	0.001723435
-0.817486823	0.013194664
-0.82128799	0.012425047
-0.848989129	0.00766362
0.817469716	0.013198194
0.813777924	0.01397392
0.908061683	0.001811306
0.934705913	0.000662289
0.91101408	0.001646111
0.893330812	0.002796719
0.843491733	0.008494331
0.825992703	0.011512559
0.822248995	0.012235078
0.863366544	0.005741304
0.88809067	0.003216298
0.800291002	0.017049378
0.825046301	0.011692589
0.812310755	0.014289985
0.826093137	0.011493557
0.845233262	0.008225267
0.829628825	0.010837216
0.900503576	0.002282327
0.811212301	0.014529539
0.815583408	0.013591061
0.837089121	0.009531457
0.889255524	0.00311974
0.870122373	0.004957357
0.860115707	0.006145169
0.823021412	0.012083726
0.945253432	0.000393555
0.923755825	0.001045656
0.85146755	0.007306764
0.82133466	0.012415779
0.830354691	0.010705482
0.811469913	0.014473134
-0.871253133	0.00483329
0.823592782	0.011972533
-0.826084971	0.011495102
0.913053393	0.001537937
0.828906119	0.010969391
-0.85135901	0.007322165
0.905902624	0.001938688
0.842561185	0.008640366

0.827229559	0.011279934
0.836878598	0.009566855
0.835832059	0.009744059
-0.821324408	0.012417814
0.931863844	0.00075095
0.927167535	0.000913871
0.878987134	0.004037953
0.80571878	0.015765441
0.851750851	0.007266662
0.809749663	0.014852413
0.842501402	0.008649802
0.829596579	0.010843092
0.877606153	0.004173262
0.894716322	0.002692053
0.806114554	0.015674277
0.826410651	0.011433616
0.842615366	0.00863182
0.899950087	0.00231963
0.855422199	0.006759636
0.939722657	0.000523069
0.851288199	0.007332224
0.977915645	2.65E-05
0.883623362	0.003604453
0.867785633	0.005220181
0.899643004	0.002340496
0.903601229	0.002080724
0.804584861	0.01602847
0.884748101	0.003504032
0.889800727	0.003075198
0.875361085	0.004399403
0.896139801	0.002587195
0.883640707	0.003602891
0.813636661	0.014004158
0.851594985	0.007288708
0.893945217	0.002749986
0.907427669	0.00184813
0.806599677	0.015562985
0.887636185	0.003254489
0.854791343	0.006845096
0.8312096	0.010551636
0.87785387	0.004148779
0.928409696	0.000868735
0.900936425	0.002253427
0.902160764	0.002172969
0.869484901	0.005028192
0.90372175	0.002073124
0.812950373	0.014151649
0.900238633	0.002300134
0.801257133	0.016816221
0.81754446	0.013182776
0.891020536	0.002977035

0.944338679	0.000413325
0.857731402	0.006452638
0.869182348	0.005062038
0.842059493	0.008719757
0.834439635	0.00998304
0.828760982	0.010996058
0.919613242	0.001221617
0.81934154	0.012815497
0.80978483	0.014844598
0.888713479	0.003164435
0.886325419	0.00336627
0.84651041	0.008031441
0.901098967	0.002242636
0.836821735	0.00957643
0.862065852	0.005900786
0.852991283	0.007092732
0.886466384	0.003354132
0.898769677	0.002400501
0.953470528	0.000243133
0.863139331	0.005768962
0.889663696	0.003086354
0.862600386	0.005834906
0.892914593	0.002828668
0.981785357	1.49E-05
0.879722297	0.003967089
0.80503124	0.0159246
0.897783756	0.002469431
0.865367591	0.005501389
0.859751344	0.006191535
0.880849719	0.00385998
0.834433734	0.009984061
0.894847095	0.002682307
0.858643889	0.006333833
0.813791752	0.013970962
0.946375191	0.000370173
0.82846868	0.011049888
0.841995299	0.008729949
0.960017085	0.000155042
0.819609106	0.012761372
0.913039505	0.001538657
0.880977273	0.003847981
0.83008498	0.010754312
0.894785941	0.002686862
0.861670792	0.005949781
0.882044375	0.003748536
0.919984818	0.001205101
0.81810689	0.013067125
0.864076257	0.005655462
0.884904087	0.003490249
0.905806303	0.001944503
0.865891159	0.005439697

0.921458602	0.001141026
0.878021777	0.004132237
0.848347425	0.00775779
0.912889421	0.001546458
0.821404517	0.012401914
0.859302759	0.006248925
0.874015272	0.004538664
0.865062892	0.005537498
0.838608921	0.009278383
0.854728103	0.006853701
0.826341629	0.011446629
0.807458222	0.015367245
0.925521314	0.000976013
0.854362011	0.00690365
0.839138269	0.00919125
0.824156046	0.011863553
0.893010616	0.002821276
0.84642154	0.008044833
0.856006742	0.00668106
0.831751108	0.010454917
0.841597319	0.008793304
0.80391258	0.016185707
0.852720261	0.007130504
0.815062523	0.013700829
0.800702155	0.016949908
0.955462158	0.000213553
0.851543546	0.007295992
0.801751733	0.016697637
0.810753286	0.014630385
0.840359926	0.00899215
0.81677562	0.013341911
0.818303406	0.013026867
0.901296377	0.002229575
0.826430202	0.011429932
0.858954132	0.006293761
0.837423146	0.009475465
0.88165468	0.003784658
0.89879179	0.00239897
0.873514354	0.004591212
0.904600501	0.002018251
0.894884765	0.002679504
0.883901954	0.003579409
0.972647548	5.01E-05
0.92713058	0.000915237
0.844034255	0.008409921
0.846670926	0.008007289
0.838801146	0.009246681
0.824032724	0.01188736
0.833770573	0.01009918
0.834824145	0.00991668
0.822173595	0.012249916

0.805156231	0.015895591
0.955726922	0.00020981
0.808003783	0.01524367
0.827167809	0.011291477
0.851441383	0.007310475
0.851098955	0.00735915
0.86470753	0.005579802
0.886195242	0.003377505
0.853601456	0.00700816
0.818289757	0.013029661
0.803446889	0.01629519
0.869538784	0.00502218
0.819469631	0.012789568
0.812602997	0.014226674
0.837446213	0.009471606
0.84131211	0.008838886
0.832482696	0.010325143
0.82363826	0.011963711
0.819382906	0.012807119
0.821160614	0.012450365
0.847979188	0.00781216
0.837929964	0.009390907
0.807329178	0.015396567
0.837544978	0.009455094
0.85879916	0.006313757
0.805566788	0.015800539
0.815249443	0.013661375
0.812351108	0.014281233
0.800951838	0.016889681
0.803945541	0.016177975
0.814371288	0.013847356
0.818050385	0.013078715
0.803092778	0.016378751
0.813124359	0.014114165
0.809263468	0.014960729
0.831666827	0.010469933
0.800327957	0.017040423
0.844368279	0.008358217
0.878761113	0.004059903
0.880750358	0.003869344
0.869086504	0.00507279
0.82917732	0.010919672
0.848162472	0.007785068
0.809952736	0.014807318
0.856257737	0.006647501
0.804126024	0.016135681
0.821039975	0.012474375
0.899065375	0.002380074
0.859276712	0.006252267
0.823407948	0.012008432
0.873276353	0.004616316

0.840349674	0.00899381
0.80101037	0.016875581
0.803740978	0.016225996
0.886889398	0.003317875
0.812365949	0.014278015
0.84663552	0.008012612
0.88704747	0.003304393
0.844344676	0.008361864
0.807545424	0.015347451
0.807963252	0.015252829
0.82088387	0.012505485
0.842325985	0.008677528
0.80836457	0.015162292
0.869809568	0.004992035
0.83847791	0.009300028
0.940400779	0.000505876
0.915845931	0.001397473
0.892467022	0.002863285
0.918063402	0.001292099
0.829449236	0.010869966
0.80824101	0.015190131
0.943803608	0.000425186
0.818743169	0.012937062
0.811953306	0.014367664
0.807742476	0.015302781
0.888675451	0.003167586
0.802820385	0.016443212
0.843858898	0.008437146
0.941818058	0.000471149
0.850400269	0.007459106
0.844638467	0.008316542
0.850296736	0.007473991
0.874457657	0.004492579
0.852033317	0.007226819
0.829649448	0.010833459
0.818164527	0.013055309
0.844727993	0.008302763
0.857873619	0.006434029
0.874493897	0.004488817
0.833022892	0.010229977
0.88313961	0.003648209
0.800282598	0.017051415
0.802866757	0.016432227
0.851913273	0.007243734
0.805670321	0.015776626
0.914796531	0.001449232
0.835149586	0.009860733
0.948926747	0.000320431
0.910596013	0.001668881
0.842510045	0.008648438
0.819805503	0.012721735

0.814910769	0.013732914
0.826661825	0.011386339
0.810608029	0.01466239
0.854257166	0.006917997
0.836145878	0.009690705
0.857459486	0.006488315
0.807069659	0.015455641
0.832315207	0.010354762
0.810745656	0.014632065
0.864647031	0.005587025
0.853220999	0.007060816
0.870171666	0.004951906
0.910954118	0.001649365
0.805609703	0.015790624
0.831321001	0.010531692
0.84068501	0.008939636
0.888046622	0.003219987
0.805370629	0.015845909
0.805493593	0.015817459
0.853004515	0.007090891
0.804450452	0.016059829
0.80382961	0.016205179
0.823932052	0.011906816
0.816999972	0.01329535
0.851235211	0.007339757
0.805371344	0.015845743
0.811628997	0.01443837
0.819779694	0.012726939
0.824758947	0.011747601
0.925518453	0.000976123
0.878104329	0.004124119
0.876895905	0.004243974
0.922006488	0.001117786
0.812590659	0.014229343
0.868013382	0.005194181
0.889802158	0.003075081
0.867561281	0.005245874
0.825662196	0.011575229
0.900974035	0.002250927
0.805763185	0.015755196
0.802968442	0.016408155
0.890721083	0.003000943
0.905350387	0.001972177
0.802351952	0.016554435
0.931120634	0.000775349
0.805250466	0.015873742
0.878486097	0.004086713
0.909851193	0.001709953
0.926957011	0.000921669
0.811576009	0.014449943
0.826895833	0.011342405

0.823560357	0.011978826
0.820434809	0.012595253
0.877536774	0.004180136
0.838410079	0.009311248
0.840054452	0.009041675
0.810542643	0.014676811
0.904592216	0.002018763
0.853009403	0.007090211
0.815770745	0.013551719
0.81001842	0.014792751
0.834114015	0.010039457
0.838427961	0.009308289
0.869177639	0.005062566
0.903880775	0.002063123
0.846194863	0.008079056
0.875115633	0.004424593
0.844321549	0.008365438
0.89436233	0.002718549
0.886225462	0.003374895
0.810639143	0.014655531
0.845090032	0.008247188
0.819720209	0.01273894
0.869237185	0.005055893
0.936210692	0.00061826
0.806998312	0.015471907
0.81703788	0.013287493
0.823090315	0.012070283
0.833643913	0.010121263
0.804363668	0.016080098
0.899704516	0.002336307
0.840415001	0.00898324
0.869191587	0.005061003
0.809581935	0.014889724
0.816824317	0.013331796
0.918213367	0.001285166
0.824301243	0.011835562
0.834248245	0.010016176
0.876741588	0.004259439
0.802246511	0.016579535
0.945645869	0.000385268
0.806280911	0.015636057
0.815118551	0.013688996
0.826099694	0.011492318
0.857848048	0.006437372
0.818179369	0.013052268
0.930618942	0.00079211
0.832660973	0.010293674
0.843985856	0.008417429
0.804084301	0.016145452
0.887429059	0.00327199
0.819318712	0.012820121

0.822518289	0.012182176
0.876502752	0.004283445
0.831626058	0.010477202
0.808622658	0.015104247
0.867987156	0.00519717
0.823286831	0.012031993
0.833930433	0.010071353
0.836122453	0.009694681
0.855079651	0.006805955
0.8458547	0.008130587
0.918407202	0.001276241
0.802829802	0.01644098
0.811309338	0.014508276
0.833653688	0.010119557
0.814981103	0.013718038
0.846546471	0.008026011
0.85715735	0.006528105
0.899977148	0.002317797
0.817685306	0.013153754
0.850180626	0.007490707
0.851550758	0.007294971
0.892552793	0.00285663
0.819534183	0.012776513
0.834008455	0.01005779
0.86246258	0.005851845
0.857829809	0.006439758
0.816223264	0.013456982
0.833308935	0.010179812
0.843258083	0.00853085
0.807248533	0.015414909
0.870092988	0.004960608
0.847075403	0.007946633
0.81424123	0.013875035
0.900913656	0.002254941
0.920455754	0.001184378
0.892695725	0.002845562
0.818515599	0.012983486
0.926840723	0.000925995
0.828292668	0.011082382
0.833194792	0.010199811
0.865269244	0.005513028
0.882253408	0.003729253
0.830479026	0.01068302
0.851964891	0.007236458
0.83017838	0.010737386
0.855986297	0.006683799
0.818316996	0.013024086
0.899508655	0.002349663
0.860378087	0.006111918
-0.828616142	0.01102271
0.894787252	0.002686764

0.891438365	0.002943883
0.808350146	0.015165541
0.830133319	0.01074555
0.826062977	0.011499261
0.830256462	0.01072325
0.864205658	0.0056399
0.812479436	0.014253421
0.809239864	0.014966
0.82984066	0.010798666
0.817852795	0.013119294
0.834213257	0.010022241
0.910404682	0.00167937
0.80911839	0.014993146
0.826353431	0.011444404
0.841769397	0.008765875
0.83527422	0.00983936
0.935467303	0.000639763
0.800199449	0.017071577
0.837256253	0.009503415
0.841137409	0.00886688
0.822376728	0.012209967
0.851580203	0.0072908
0.801179826	0.016834804
0.889746726	0.003079591
0.845215142	0.008228038
0.895461202	0.002636847
0.824882448	0.011723937
0.92651546	0.000938166
0.832724988	0.010282389
0.816842973	0.013327922
0.8730492	0.004640358
0.833641827	0.010121627
0.928341508	0.000871174
0.810445726	0.014698202
0.874805927	0.004456511
0.808434784	0.015146487
0.803563356	0.016267765
0.81451416	0.01381699
0.884242892	0.003548912
0.835265458	0.009840861
0.836861968	0.009569654
0.862949848	0.005792092
0.807769775	0.015296599
0.832451046	0.010330736
0.813026428	0.014135255
0.814580977	0.013802803
0.961066186	0.000143269
0.82459116	0.011779798
0.809678018	0.014868343
0.815246642	0.013661966
0.825867355	0.011536302

0.842664719	0.00862404
0.831357181	0.01052522
0.837725639	0.009424939
0.800503671	0.016997882
0.840765715	0.008926629
0.827184558	0.011288345
0.814960539	0.013722386
0.931111455	0.000775654
0.876579523	0.004275719
0.900301635	0.002295891
0.806528032	0.01557939
0.905168831	0.001983269
0.83963877	0.009109346
0.84436512	0.008358705
0.896048903	0.00259381
0.857271314	0.006513078
0.836419821	0.009644283
0.926851928	0.000925578
0.820407331	0.012600759
0.840545475	0.008962153
0.886052012	0.003389894
0.82001251	0.012680042
-0.806173265	0.015660782
0.907763183	0.001828583
0.82860291	0.011025147
0.805328667	0.015855625
0.820205986	0.012641151
0.855093896	0.006804024
0.871057391	0.004854622
0.830214977	0.010730759
0.814871073	0.013741314
0.852656841	0.007139362
0.834060192	0.010048802
0.905778646	0.001946174
0.832097173	0.010393401
0.830005586	0.010768712
0.862834752	0.005806171
0.873165429	0.004628046
0.851837695	0.007254397
0.9045555	0.002021037
0.838534951	0.0092906
0.834422886	0.009985937
0.878007233	0.004133668
0.926303387	0.000946158
0.864485919	0.005606289
0.812861741	0.014170767
0.966982067	8.78E-05
0.948828697	0.000322256
0.854043603	0.006947281
0.889931321	0.00306459
0.807280898	0.015407546

0.826466441	0.011423104
0.803441942	0.016296356
0.847514868	0.007881063
0.834333837	0.010001349
0.853749692	0.006987711
0.821146488	0.012453175
0.870438576	0.004922459
-0.805499017	0.015816205
0.811935723	0.014371492
0.847654462	0.007860307
0.82316649	0.012055432
0.80748862	0.015360343
0.898070872	0.002449227
0.898927927	0.002389555
0.914736986	0.001452205
0.810254931	0.014740372
0.823663414	0.011958833
0.815114975	0.013689751
0.868627131	0.005124529
0.884881556	0.003492237
0.819833219	0.012716148
0.803118646	0.016372638
0.807008862	0.015469501
0.853145182	0.00707134
0.877467453	0.004187011
0.801242709	0.016819687
0.822072864	0.012269757
0.856374443	0.006631934
0.821696162	0.012344135
0.840239108	0.009011718
-0.834664226	0.009944245
0.801065505	0.016862307
0.830818057	0.010621922
0.852817655	0.007116916
0.808731258	0.015079864
0.801143885	0.016843447
0.85749656	0.006483444
0.807123601	0.015443351
0.846936524	0.007967426
0.90042007	0.00228793
0.871139228	0.004845696
0.925884128	0.000962087
0.852558255	0.007153144
0.824923813	0.011716018
0.868076324	0.00518701
-0.807785392	0.015293063
0.853401303	0.00703583
0.807473898	0.015363686
0.812976182	0.014146084
0.863920808	0.005674193
0.880641282	0.00387964

0.825913608	0.011527537
0.872695923	0.004677908
0.801750839	0.016697851
0.846991599	0.007959176
0.902196288	0.002170663
0.816006899	0.013502227
0.801588178	0.016736792
0.86168468	0.005948054
0.842478752	0.008653379
0.842057824	0.008720022
0.854890227	0.006831655
0.854398608	0.006898646
0.824754238	0.011748503
0.817753971	0.01313962
0.883346498	0.003629454
0.936858892	0.000599901
0.871841073	0.004769577
0.87762022	0.004171869
0.90501678	0.001992589
0.857309043	0.006508108
0.831032991	0.010583302
0.813345671	0.014066576
0.857253015	0.006515489
0.8091048	0.014996184
0.82659936	0.011398085
-0.811802387	0.01440054
-0.83957231	0.009120195
0.856197894	0.006655493
-0.880500853	0.003892922
0.80847621	0.015137167
0.851871729	0.007249594
0.864684522	0.005582548
0.842839718	0.008596489
0.810495913	0.014687122
0.82718122	0.011288969
0.944312334	0.000413904
0.827533484	0.011223232
0.890812814	0.002993606
0.821156144	0.012451254
0.818661749	0.01295366
0.819125116	0.012859382
0.83490181	0.00990331
0.830050409	0.010760581
0.829507291	0.010859372
0.823451579	0.011999952
0.894900143	0.00267836
0.804269671	0.016102068
0.818090677	0.01307045
0.938323379	0.000559748
0.892893195	0.002830317
0.862963915	0.005790373

0.804574311	0.01603093
0.816595793	0.013379306
0.815601766	0.013587203
0.849237978	0.007627298
0.828717768	0.011004005
0.859555602	0.006216535
0.800777376	0.01693175
0.887899458	0.00323233
0.808265805	0.015184542
0.828603983	0.01102495
0.862495959	0.005847739
0.920546651	0.001180405
0.822194934	0.012245716
0.895858407	0.00260771
0.833301425	0.010181127
0.936484814	0.000610452
0.834171891	0.010029415
0.814388692	0.013843655
0.805773079	0.015752913
0.835540533	0.00979379
0.8338328	0.010088343
0.865080237	0.005535439
0.842780352	0.008605829
0.836305499	0.009663638
0.827985108	0.011139307
0.809587955	0.014888384
0.907306135	0.001855244
0.887467086	0.003268773
0.833591104	0.010130479
0.831010759	0.010587293
0.851052046	0.007365834
0.882644832	0.003693315
0.895104825	0.002663167
0.839615166	0.009113198
0.869207859	0.005059179
0.827977955	0.011140633
0.870768487	0.004886218
0.803826749	0.016205851
0.847567856	0.00787318
0.875452995	0.004389994
0.862609923	0.005833735
0.808236957	0.015191045
0.846486807	0.008034997
0.82210815	0.012262805
0.841709137	0.008775474
0.860751987	0.006064735
0.859578431	0.006213616
0.933692396	0.000693071
-0.862909496	0.005797026
-0.829372585	0.010883963
0.909918964	0.001706189

0.873343885	0.004609184
0.829840481	0.010798698
0.947592735	0.000345849
0.861087322	0.006022617
0.874958396	0.004440779
0.860755026	0.006064352
0.815199435	0.013671924
0.865771413	0.005453767
0.847515047	0.007881036
0.818095326	0.013069496
0.88528955	0.00345634
0.862533927	0.005843071
-0.885811508	0.003410763
0.817296267	0.013234016
0.830815852	0.010622318
0.838439345	0.009306406
-0.809843659	0.014831529
0.846576631	0.008021471
0.827537656	0.011222455
0.901750267	0.002199734
-0.828786671	0.010991335
0.840137303	0.009028226
0.805104673	0.015907553
0.894832373	0.002683403
0.892743409	0.002841876
0.804546535	0.016037408
0.870433927	0.004922971
0.836628497	0.009609015
0.83268708	0.010289071
0.893755078	0.002764394
0.813558757	0.014020852
0.935020924	0.000652907
0.824248493	0.011845727
0.922406018	0.001101035
0.85939157	0.006237536
0.959833741	0.000157162
0.806809664	0.015514967
0.836268425	0.009669921
0.849099994	0.007647424
0.918008208	0.001294657
-0.841701448	0.008776699
0.844968736	0.008265781
0.884096384	0.003561997
0.852411389	0.007173708
0.824043334	0.01188531
0.930746496	0.000787826
0.81653589	0.013391777
0.891517997	0.002937592
0.893633068	0.002773665
0.836863101	0.009569464
0.810208201	0.014750711

0.8067469	0.015529309
0.818002224	0.013088599
0.828962088	0.010959119
0.923099816	0.001072337
-0.846943259	0.007966417
-0.818400025	0.013007103
0.862020433	0.005906406
0.806540847	0.015576455
0.816542268	0.013390449
0.806655526	0.015550205
0.874997497	0.004436751
0.842845321	0.008595607
0.830668092	0.01064892
0.836226821	0.009676974
0.940727711	0.000497721
0.885999858	0.003394413
0.844058871	0.008406103
0.943189383	0.000439074
0.825611711	0.011584821
0.909994245	0.001702014
0.844336033	0.008363199
0.847538412	0.00787756
0.844844401	0.008284868
0.907104135	0.001867107
0.926393032	0.000942774
0.81384778	0.013958982
0.842795551	0.008603437
0.817017555	0.013291705
0.816003323	0.013502975
0.808835268	0.015056534
0.810798287	0.014620479
0.904618323	0.002017148
0.820971489	0.012488017
0.849501014	0.007589025
0.806065023	0.015685668
0.84529227	0.008216247
0.93489778	0.000656564
0.827611506	0.011208705
0.909445763	0.001732584
0.819998026	0.012682956
0.817857504	0.013118326
0.937394261	0.000585011
0.806367159	0.015616265
0.932403505	0.000733552
0.836415291	0.009645049
0.907137334	0.001865154
0.823128521	0.012062832
0.897348821	0.002500242
0.810761511	0.014628574
0.80770731	0.015310747
0.863170326	0.005765184

0.805067539	0.015916172
0.82589072	0.011531874
0.917676866	0.001310081
0.868117094	0.005182368
0.852062941	0.007222648
0.809191585	0.014976785
0.881655157	0.003784614
0.850943446	0.007381323
0.808739364	0.015078045
0.845066726	0.008250758
0.80167979	0.016714853
0.836873293	0.009567748
0.839709997	0.009097728
0.807124376	0.015443174
0.855052471	0.006809639
0.944943428	0.000400184
0.841313422	0.008838676
0.828529358	0.0110387
0.825068831	0.011688282
0.80294925	0.016412697
0.840252936	0.009009477
0.829938591	0.010780873
0.846429288	0.008043665
0.805926144	0.015717635
0.833526134	0.010141824
0.937476933	0.000582733
0.807938278	0.015258475
0.839981079	0.009053597
0.813905656	0.013946614
0.829900682	0.010787759
0.837908983	0.009394398
0.883615851	0.00360513
0.816898525	0.013316391
0.810978174	0.014580922
0.824491799	0.011798891
0.814929187	0.013729017
0.811555743	0.014454371
0.831607223	0.010480561
0.949290156	0.000313727
0.848330498	0.007760284
0.828944981	0.010962258
0.911302805	0.001630505
0.936286271	0.000616101
0.886752367	0.003329592
0.820781231	0.012525967
0.811300218	0.014510274
0.824768126	0.011745841
0.925427914	0.000979619
0.91625309	0.001377721
0.832047284	0.010402255
0.836756229	0.009587468

0.892528594	0.002858507
0.915477693	0.001415496
0.800116897	0.017091609
0.843712151	0.008459972
0.895142138	0.002660403
0.889775157	0.003077277
0.850393295	0.007460108
0.85683018	0.006571367
0.847774208	0.007842531
0.802461803	0.01652831
0.845791161	0.008140235
0.833220363	0.010195329
0.801415622	0.016778165
0.874135196	0.004526141
0.897416353	0.002495442
0.935615659	0.000635433
0.903790772	0.002068779
0.868589282	0.005128806
0.836934745	0.009557406
0.808548927	0.015120815
0.829845309	0.010797821
0.813579082	0.014016495
0.838772476	0.009251405
0.84344691	0.008501329
0.930194557	0.000806471
0.810324252	0.014725042
0.86208421	0.005898515
0.853925884	0.006963457
0.87844187	0.004091035
0.832640469	0.01029729
0.801833749	0.016678024
0.843755841	0.008453172
0.889177918	0.003126114
-0.880590856	0.003884406
0.860376775	0.006112084
0.841394842	0.008825648
-0.830952764	0.010597707
-0.85500437	0.006816161
0.821421921	0.012398461
0.801632047	0.016726284
0.822724938	0.012141678
0.86017859	0.006137189
0.936132252	0.000620506
0.857984543	0.006419538
0.879657745	0.003973279
0.915687978	0.001405186
0.814941049	0.013726508
0.800614655	0.016971047
0.859639466	0.006205816
0.839494109	0.009132971
0.816511095	0.013396941

0.937755346	0.000575106
0.894665778	0.002695826
0.802262962	0.016575618
0.898148239	0.002443801
0.85009855	0.007502537
0.921552241	0.001137032
0.850734651	0.007411161
0.914057434	0.001486424
0.922249854	0.001107563
0.859842896	0.006179863
0.884486914	0.003527188
0.943261385	0.000437431
0.966281593	9.34E-05
0.856029689	0.006677988
0.869033217	0.005078775
0.831469774	0.010505096
0.806333125	0.015624073
0.813741863	0.013981635
0.878377676	0.004097314
0.811172485	0.014538269
0.841142118	0.008866125
0.811398089	0.014488846
0.889487207	0.003100761
0.803890646	0.016190853
0.86540693	0.005496739
0.89090842	0.002985972
0.802031279	0.016630846
0.895335615	0.002646103
0.828264952	0.011087504
0.82971561	0.010821412
0.852148354	0.007210632
0.889662802	0.003086427
0.828180909	0.011103046
0.831345022	0.010527395
0.804157734	0.016128257
0.822682917	0.012149906
0.813675523	0.013995836
0.817959607	0.013097348
0.876597703	0.004273891
0.830990672	0.010590899
0.841427267	0.008820463
0.822990716	0.012089718
0.807486296	0.015360871
0.803672075	0.016242191
0.863061965	0.005778399
0.895125747	0.002661617
0.83936727	0.009153717
0.80857259	0.015115496
0.854558647	0.006876793
0.90118742	0.002236778
0.873550236	0.004587435

0.837518215	0.009459567
0.803915083	0.016185119
0.85737592	0.006499305
0.844736695	0.008301424
0.867735684	0.005225894
0.868206561	0.005172192
0.833529115	0.010141304
0.903604925	0.002080491
0.844241917	0.008377752
0.832126915	0.010388125
0.819167912	0.012850697
0.972285509	5.21E-05
0.834116757	0.010038981
0.800707817	0.016948541
0.861365199	0.005987858
0.84545517	0.008191378
0.807100773	0.015448551
0.887085199	0.00330118
0.825072765	0.01168753
0.82460475	0.011777188
0.860968769	0.006037486
0.911364138	0.001627202
0.899160504	0.002373526
0.813192666	0.014099466
0.922611654	0.001092478
0.942618072	0.000452257
0.859057903	0.006280393
0.841402888	0.008824361
0.917513371	0.001317736
0.891132593	0.002968121
0.888433635	0.003187671
0.842880964	0.008590003
0.833541512	0.010139138
0.829554439	0.010850774
0.826081932	0.011495676
0.847515404	0.007880983
0.909876585	0.001708542
0.958530724	0.000172788
0.893006384	0.002821602
0.860523343	0.00609356
0.898238122	0.002437507
0.804440081	0.016062251
0.886399388	0.003359897
0.860522985	0.006093605
0.867466688	0.005256732
0.960391998	0.000150764
0.806661069	0.015548937
0.813469291	0.014040038
0.801083565	0.01685796
0.836593091	0.009614993
0.828226209	0.011094667

0.842380881	0.008668845
0.937762022	0.000574924
0.865190089	0.005522406
0.91266191	0.001558333
0.802531779	0.016511682
0.898282826	0.002434381
0.805219412	0.01588094
0.894967735	0.002673337
0.803394794	0.016307466
0.803696156	0.01623653
0.910422564	0.001678388
0.935165465	0.000648632
-0.84695673	0.007964399
0.868153334	0.005178245
0.844614923	0.008320169
0.801608145	0.016732009
0.83746624	0.009468256
0.86145854	0.005976211
0.804965556	0.015939858
0.83180356	0.010445578
0.813782156	0.013973015
0.805940509	0.015714326
0.817587495	0.013173904
0.801726341	0.016703712
0.832004666	0.010409823
0.881609499	0.00378886
0.910227299	0.001689132
0.923351049	0.001062067
0.837616682	0.009443118
0.897706568	0.002474881
0.906479299	0.001904114
0.823968291	0.01189981
0.819434047	0.012796768
0.809396327	0.014931081
0.809239209	0.014966146
0.85810107	0.006404338
0.813383698	0.014058409
0.801984251	0.01664207
0.830518246	0.01067594
0.899086952	0.002378588
0.818689406	0.012948021
0.833496094	0.010147073
0.805395305	0.015840197
0.841134608	0.00886733
0.865764499	0.00545458
0.909976482	0.001702999
0.800671399	0.016957337
0.868334115	0.005157707
0.811189771	0.014534479
0.839831471	0.009077935
0.894837558	0.002683017

0.846452713	0.008040134
0.918051422	0.001292654
0.914830148	0.001447554
0.848973453	0.007665911
0.808118939	0.015217666
0.842437565	0.008659886
0.937941909	0.000570032
0.820402563	0.012601715
0.86147505	0.005974152
0.835074961	0.009873544
0.86386466	0.005680969
0.876744211	0.004259176
0.847057045	0.007949379
0.874584615	0.004479409
0.908446074	0.001789215
0.853145361	0.007071315
0.865816951	0.005448414
0.887081921	0.003301459
-0.81255877	0.014236244
0.825608313	0.011585467
0.945286334	0.000392856
0.895629644	0.002624465
0.84090358	0.008904438
0.900842845	0.002259654
0.817661166	0.013158725
0.815199018	0.013672012
0.809419036	0.014926017
0.808357179	0.015163957
0.958311439	0.000175514
0.863191068	0.005762657
0.816052139	0.013492759
0.827831447	0.011167817
0.8040483	0.016153886
0.809601426	0.014885385
0.836591125	0.009615325
0.80581677	0.015742839
0.870524526	0.004913001
0.875082374	0.004428014
0.834704697	0.009937264
0.808342636	0.015167232
0.814113259	0.013902304
0.823199809	0.012048939
0.830730855	0.010637615
0.838832676	0.009241488
0.810075164	0.014780173
0.817152441	0.013263766
0.823728621	0.011946194
0.846139491	0.00808743
0.820647955	0.012552595
0.830524564	0.0106748
0.814963341	0.013721794

-0.845189393	0.008231977
0.811279953	0.014514713
0.817542136	0.013183255
0.80907172	0.015003583
0.822641075	0.012158103
0.849829376	0.007541419
0.863518476	0.005722858
0.847267628	0.00791791
0.848596454	0.007721158
0.853120208	0.007074808
0.834709644	0.009936411
0.823922396	0.011908683
0.835104942	0.009868395
0.825610816	0.011584991
0.844992697	0.008262106
0.827456474	0.011237582
0.823895156	0.011913952
0.832550049	0.010313247
0.82190454	0.012302957
0.859197557	0.006262433
0.808046997	0.015233909
0.843669534	0.008466608
0.824638307	0.011770745
0.801619768	0.016729225
0.808635116	0.015101449
0.804715157	0.015998107
0.856939197	0.006556931
0.842839718	0.008596489
0.824457049	0.011805573
-0.904868901	0.002001681
0.851000667	0.007373159
0.84584868	0.008131501
0.820161462	0.012650094
0.813858151	0.013956765
0.961450219	0.000139112
0.825319886	0.011640364
0.834486365	0.00997496
0.843750954	0.008453932
0.814541757	0.013811129
0.822321117	0.012220896
-0.820535123	0.012575165
0.810339332	0.014721708
0.818368673	0.013013514
0.862836063	0.00580601
0.867044687	0.005305345
0.870200694	0.004948698
0.829098582	0.010934093
0.877616048	0.004172282
0.853621721	0.007005362
0.851468563	0.00730662
0.870537639	0.004911559

0.859385729	0.006238284
0.874551356	0.004482857
0.849226654	0.007628948
0.865636885	0.005469603
0.860553503	0.006089753
0.92385751	0.001041559
0.854743659	0.006851584
0.929772854	0.000820909
0.817849994	0.01311987
0.831388056	0.010519699
0.885411203	0.003445682
0.93533802	0.000643552
0.87693131	0.004240431
0.844566107	0.00832769
0.83442533	0.009985514
0.841567874	0.008798003
0.833813965	0.010091622
0.853638709	0.007003017
0.84361589	0.008474966
0.83721	0.00951117
0.852787137	0.007121172
0.834640384	0.009948359
0.878355324	0.004099502
0.807237208	0.015417486
0.88757813	0.003259388
0.863759518	0.00569367
0.867766082	0.005222416
0.8255862	0.01158967
0.860886633	0.006047801
0.801339924	0.016796335
0.800041318	0.017109962
0.870891869	0.004872708
0.887477994	0.00326785
0.801295936	0.016806899
0.805758417	0.015756296
0.805906355	0.015722193
0.822704434	0.012145693
0.829316378	0.010894235
0.883632362	0.003603642
0.814348578	0.013852187
0.81406945	0.013911647
0.820288599	0.012624568
0.874243975	0.004514801
-0.811949611	0.014368468
0.8048985	0.015955443
0.826936483	0.011334784
0.861844778	0.005928171
0.827227533	0.011280313
0.954959869	0.000220776
0.800907671	0.016900325
0.802497745	0.016519768

0.817376852	0.013217365
0.89053905	0.003015536
0.855087876	0.00680484
0.882605791	0.003696889
0.822011113	0.01228193
0.856210947	0.006653749
0.843735099	0.0084564
0.806443095	0.015598852
0.808932722	0.015034696
0.812074721	0.014341249
0.856439769	0.00662323
0.818129897	0.013062408
0.918647528	0.001265232
0.8085289	0.015125317
0.815064609	0.013700389
0.888898492	0.003149133
0.833600581	0.010128825
0.904250026	0.002040021
0.801360846	0.016791312
0.923325896	0.001063093
0.903651059	0.00207758
0.805888593	0.015726285
0.81556803	0.013594294
0.839625716	0.009111476
0.886420608	0.00335807
0.817195535	0.013254848
0.827918828	0.011151599
0.821912348	0.012301415
0.843523145	0.008489429
0.899075031	0.002379409
0.804786861	0.015981413
0.854982793	0.006819088
0.8570714	0.006539452
0.807912171	0.015264378
0.827943265	0.011147066
0.9089728	0.001759229
0.820384026	0.01260543
0.97213918	5.29E-05
0.802983403	0.016404615
0.889333904	0.003113311
0.810748398	0.014631461
0.805189788	0.015887808
0.802697301	0.016472391
0.865185142	0.005522993
0.966583848	9.10E-05
0.977729857	2.72E-05
0.917882621	0.001300489
0.848954737	0.007668648
0.855201244	0.00678949
0.864104688	0.00565204
0.871707857	0.004783966

0.855334699	0.006771448
0.871984065	0.004754164
0.862560868	0.005839761
0.862253189	0.005877643
0.817133605	0.013267665
0.85790205	0.006430313
0.858293176	0.006379329
0.945009172	0.000398772
0.865633309	0.005470024
0.839073718	0.009201847
0.876559675	0.004277716
0.856048048	0.00667553
0.871904373	0.00476275
0.800691962	0.01695237
0.897156298	0.00251396
0.886305869	0.003367956
0.825891554	0.011531716
0.853758276	0.006986529
0.84904021	0.007656155
0.847943366	0.007817463
0.852101147	0.007217272
0.857494891	0.006483663
0.803805351	0.016210875
0.85073626	0.00741093
0.876063526	0.004327821
0.805535495	0.015807771
0.808689713	0.015089188
0.896313429	0.002574589
0.877958298	0.004138486
0.874612927	0.004476475
0.880856276	0.003859363
0.865913153	0.005437115
0.887626171	0.003255333
0.822687864	0.012148938
0.810064971	0.014782432
0.875363946	0.004399109
0.860772848	0.006062109
0.856838882	0.006570214
0.805994451	0.015701907
0.865287304	0.005510889
0.881319761	0.003815881
0.805639327	0.015783782
0.887790024	0.003241529
0.856643796	0.006596094
0.893409967	0.00279067
0.883708119	0.003596822
0.848927855	0.00767258
0.828306615	0.011079805
0.860864341	0.006050602
0.807327628	0.015396919
0.844081581	0.008402583

0.820842862	0.012513666
0.807318509	0.015398993
0.819512665	0.012780864
0.845674574	0.008157958
0.802728295	0.01646504
0.845028222	0.008256659
0.876659811	0.004267649
0.844489753	0.008339464
0.829058409	0.010941455
0.863659143	0.005705813
0.864587665	0.005594118
0.800335884	0.017038502
0.828078866	0.011121934
0.818415642	0.01300391
0.855166793	0.006794152
0.831453621	0.010507981
0.833513319	0.010144063
0.839403391	0.009147806
0.802478433	0.016524357
0.801212728	0.016826893
0.85928309	0.006251449
0.8416816	0.008779863
0.810058653	0.014783832
0.901192725	0.002236427
0.849770486	0.007549943
0.838570893	0.009284662
0.842093527	0.008714357
0.820034921	0.012675533
0.898866057	0.002393831
0.805096447	0.015909462
0.823106945	0.01206704
0.810517788	0.014682295
0.89502275	0.002669253
0.809384406	0.01493374
0.80572933	0.015763006
0.841704071	0.008776281
0.804180086	0.016123026
0.819529712	0.012777417
0.84376204	0.008452207
0.826393962	0.011436762
0.80611825	0.015673427
0.870999932	0.004860896
0.851162076	0.007350162
0.913628519	0.00150829
0.8545084	0.006883649
0.81036526	0.014715978
0.804840803	0.015968862
0.866464674	0.00537263
0.86623764	0.005399115
0.888989151	0.003141653
0.857998729	0.006417686

0.896704733	0.002546325
0.892649651	0.002849127
0.870454371	0.00492072
0.867837846	0.005214212
0.86560303	0.005473592
0.872604847	0.00468762
0.837363183	0.009485501
0.838108063	0.009361307
0.80403024	0.016158118
0.852966666	0.007096157
0.843181074	0.008542908
0.80299902	0.016400921
0.903251946	0.002102854
0.909233868	0.001744489
0.889658332	0.003086791
0.803296208	0.016330714
0.820544779	0.012573233
0.800571382	0.016981507
0.84856683	0.00772551
0.84026444	0.009007613
0.809980094	0.014801249
0.813141942	0.01411038
0.857052147	0.006541996
0.844629526	0.008317919
0.806955636	0.015481641
0.802988827	0.016403332
0.909604788	0.001723684
0.841699719	0.008776975
0.866725266	0.005342332
0.818261743	0.013035396
0.833310425	0.010179551
0.858967721	0.006292009
0.849254966	0.007624822
0.894202709	0.002730552
0.867577016	0.00524407
0.816777527	0.013341515
0.932079613	0.000743962
0.869526088	0.005023596
0.817202985	0.013253306
0.842827559	0.008598401
0.80680424	0.015516206
0.887161314	0.003294704
0.87332654	0.004611015
0.841444016	0.008817786
-0.837367237	0.009484822
0.863876402	0.005679551
0.808335781	0.015168776
0.884079993	0.003563462
0.861266792	0.006000153
0.850600958	0.007430307
0.892969668	0.002824427

0.842364192	0.008671484
0.800598025	0.016975066
0.816974521	0.013300627
0.863934159	0.005672583
0.824813426	0.011737159
0.833637357	0.010122407
0.83394748	0.010068389
0.899548709	0.002346927
0.83592093	0.009728931
0.824361861	0.011823889
0.861053348	0.006026875
0.801533401	0.016749919
0.830914319	0.010604614
0.825444758	0.011616577
0.827852488	0.011163911
0.807667553	0.015319756
0.80570358	0.015768948
0.810618401	0.014660103
0.80839628	0.015155153
0.836384356	0.009650285
0.863846958	0.005683106
0.821368635	0.012409034
0.84311378	0.008553454
0.835761428	0.009756093
0.863973856	0.005667797
0.879139185	0.00402323
0.85240382	0.007174769
0.923873186	0.001040929
0.888509214	0.003181385
0.811017156	0.014572359
0.801517665	0.016753691
0.8286044	0.011024873
0.830383301	0.010700311
0.85561192	0.006734069
0.88761425	0.003256339
0.882389426	0.003716739
0.854398906	0.006898605
0.836245954	0.00967373
0.9003883	0.002290063
0.854675055	0.006860925
0.801862836	0.016671071
0.885190845	0.003465003
0.820878327	0.012506591
0.82809031	0.011119815
0.873398304	0.004603442
0.849931121	0.007526707
0.811519206	0.014462356
0.843109369	0.008554145
0.816147029	0.013472913
0.833226085	0.010194326
0.858978689	0.006290596

0.872091711	0.004742582
0.812021077	0.014352916
0.826469004	0.011422622
0.868402779	0.005149919
0.806801617	0.015516805
0.820469618	0.01258828
0.823857725	0.011921194
0.868022263	0.005193168
0.803495467	0.016283748
0.845327795	0.008210819
0.881064653	0.003839774
0.865474343	0.005488775
0.833538592	0.010139648
0.909947515	0.001704605
-0.828101575	0.011117729
0.882765651	0.003682268
0.80019027	0.017073803
0.830367386	0.010703187
0.829300463	0.010897144
0.8142941	0.013863779
0.814441681	0.013832389
0.812115848	0.014332308
0.847055793	0.007949567
0.805545509	0.015805457
-0.821178675	0.012446774
0.808928668	0.015035604
0.819446683	0.01279421
0.823875546	0.011917745
0.808122516	0.015216859
0.830528259	0.010674133
0.809297919	0.014953037
0.858034968	0.006412957
0.831050098	0.010580232
0.829030395	0.01094659
0.827781618	0.011177072
0.932439625	0.000732397
0.833854616	0.010084545
0.804278135	0.016100089
0.818673968	0.012951168
0.839438617	0.009142044
0.883639634	0.003602987
0.821104527	0.012461524
0.834632754	0.009949676
0.800138474	0.017086372
0.89807272	0.002449097
0.849103868	0.007646859
0.822072268	0.012269875
0.847342491	0.007906741
0.816653967	0.013367201
0.967412114	8.44E-05
0.814887822	0.01373777

0.879752278	0.003964217
0.870627165	0.004901721
0.934086263	0.000681
0.862201869	0.005883977
0.84864217	0.007714445
0.845604479	0.008168626
0.800023019	0.017114407
0.86953932	0.00502212
0.802802026	0.016447562
0.826568365	0.011403916
0.850431383	0.007454636
0.819266796	0.012830642
0.819338739	0.012816064
0.83461076	0.009953472
0.803154469	0.016364175
0.800871313	0.01690909
0.905135632	0.001985301
0.810642183	0.014654861
0.816536665	0.013391616
0.906601548	0.001896836
0.851408839	0.007315092
0.934106827	0.000680374
0.826681852	0.011382575
0.944794953	0.000403384
0.924641073	0.001010344
0.834203601	0.010023915
0.874210358	0.004518304
0.810060501	0.014783423
0.836722732	0.009593116
0.815444529	0.013620273
0.922943056	0.001078778
0.921140373	0.001154669
0.826773942	0.011365276
0.896117866	0.00258879
0.800068855	0.017103273
0.878967583	0.004039849
0.928525865	0.00086459
0.836428225	0.009642861
0.856494665	0.006615922
0.831647575	0.010473365
0.801004171	0.016877074
0.84568423	0.00815649
0.821234941	0.012435588
0.83788836	0.00939783
0.814093113	0.0139066
0.802616596	0.016491541
0.868179381	0.005175282
0.830183208	0.010736512
0.842507482	0.008648842
0.913893878	0.001494738
0.849152625	0.007639744

0.828706861	0.011006012
0.810134828	0.014766956
0.903138161	0.002110095
0.836339533	0.009657874
0.862879515	0.005800693
0.821928442	0.012298239
0.83268553	0.010289344
0.851971686	0.0072355
0.833875477	0.010080914
0.864287972	0.005630015
0.834721446	0.009934376
0.833818078	0.010090906
0.814686716	0.013780371
0.943393826	0.000434419
0.81206429	0.014343517
0.81575048	0.013555971
0.855863452	0.006700267
0.945017099	0.000398602
0.932207942	0.000739826
0.822735071	0.012139695
0.82954222	0.010853002
0.855348587	0.006769572
0.823297918	0.012029835
0.858110547	0.006403102
0.912477612	0.001567995
0.831815541	0.010443446
0.826262653	0.011461531
0.80689317	0.015495897
0.843911469	0.008428978
0.814707041	0.013776061
0.810836673	0.014612032
0.859916866	0.006170444
0.818213105	0.013045356
0.897265315	0.002506186
0.850808263	0.007400632
0.914251924	0.001476578
0.914242506	0.001477053
0.880352974	0.00390694
0.892039776	0.002896585
0.866934776	0.005318053
0.958502173	0.000173141
0.902100742	0.002176869
0.869153738	0.005065246
0.830217779	0.010730252
0.952080369	0.000265302
0.821314812	0.01241972
0.812836111	0.014176299
0.817867458	0.01311628
0.886471689	0.003353675
0.804458797	0.016057881
0.898574591	0.00241404

0.816784501	0.013340066
0.909797788	0.001712923
0.844146729	0.008392488
0.845390022	0.008201318
-0.847261071	0.007918888
0.873880863	0.004552726
0.851587296	0.007289796
0.823350668	0.012019571
0.855242968	0.006783846
0.807020962	0.015466742
0.869078338	0.005073707
0.881039083	0.003842175
0.849476695	0.007592559
0.827622473	0.011206663
0.838493705	0.009297417
0.847690821	0.007854907
0.853272557	0.007053666
0.8064062	0.015607311
0.82279098	0.012128754
0.803607345	0.016257415
0.856255591	0.006647788
0.913046002	0.00153832
0.846649766	0.00801047
0.805349052	0.015850904
0.84031558	0.008999329
0.876606584	0.004272998
0.874889314	0.004447903
0.861784279	0.00593568
0.800909817	0.016899808
0.804876566	0.015960544
0.84949249	0.007590263
0.867747724	0.005224516
0.894678652	0.002694865
0.825371802	0.011630471
0.828281224	0.011084497
0.871439934	0.004812988
0.803975165	0.016171028
0.840584397	0.008955868
0.870771766	0.004885859
0.80933702	0.014944311
0.809075356	0.01500277
0.809996724	0.014797561
0.871161401	0.004843279
0.841251075	0.00884866
0.822044849	0.012275279
0.82817018	0.011105031
0.918440342	0.001274719
0.847071946	0.00794715
0.818083525	0.013071917
0.805255532	0.015872568
0.814460278	0.013828437

0.806871653	0.015500809
0.822366059	0.012212063
0.801049173	0.016866238
0.898779988	0.002399787
0.812920451	0.014158101
0.866338372	0.005387354
0.819107413	0.012862976
0.899308503	0.002363363
0.924007058	0.001035554
0.801742613	0.016699819
0.850891769	0.007388701
0.854094207	0.006940335
0.896386325	0.002569309
0.831546664	0.010491366
0.899762928	0.002332333
0.821547627	0.01237354
0.824019432	0.011889927
0.819506168	0.012782178
0.830839574	0.010618051
0.821074486	0.012467503
0.813379824	0.014059241
0.893120646	0.002812822
0.890739262	0.002999488
0.846170187	0.008082787
0.830299556	0.010715453
0.955258191	0.000216467
0.83116281	0.01056002
0.904785097	0.002006845
0.911493599	0.001620244
0.833585143	0.010131519
0.871130109	0.00484669
0.816651464	0.013367722
0.876673698	0.004266254
0.806452274	0.015596748
0.812627256	0.014221427
0.80497551	0.015937545
0.836283505	0.009667365
0.80478543	0.015981746
0.86110127	0.006020869
0.800009251	0.017117752
0.861669719	0.005949914
0.915422261	0.001418222
0.824272573	0.011841086
0.886680245	0.00333577
0.805112898	0.015905644
0.830975533	0.010593618
0.861190557	0.006009688
0.857620716	0.006467146
0.813343585	0.014067024
0.90634793	0.001911954
0.817010164	0.013293237

0.825398922	0.011625305
0.830965519	0.010595416
0.833359241	0.010171005
0.857131302	0.006531542
0.857956171	0.006423242
0.842707813	0.00861725
0.841413677	0.008822636
0.842407644	0.008664614
0.867876291	0.005209821
-0.851655245	0.007280179
0.86097455	0.00603676
0.836858034	0.009570317
0.852164984	0.007208294
0.834986985	0.00988866
0.85573256	0.006717843
0.832322538	0.010353465
0.946491718	0.000367798
0.925971687	0.000958746
0.827130497	0.011298455
0.804774642	0.015984257
0.884267449	0.003546722
0.845594525	0.008170141
0.806917071	0.015490441
-0.819223881	0.012839343
0.806195378	0.015655701
0.819925845	0.012697486
0.837800384	0.00941248
0.881254256	0.003822007
0.900650203	0.00227251
0.825838029	0.011541861
0.805667639	0.015777245
0.807096779	0.015449461
0.892915308	0.002828613
0.830511153	0.010677221
0.841242373	0.008850054
0.822610199	0.012164153
0.892241359	0.002880843
0.873180389	0.004626463
0.924010992	0.001035396
0.834668815	0.009943453
0.862799346	0.005810506
0.816598117	0.013378822
0.863906741	0.00567589
0.816640437	0.013370016
0.809926569	0.014813124
0.845568538	0.008174099
0.827541053	0.011221822
0.878753304	0.004060662
0.847153425	0.007934966
0.940542996	0.000502318
0.852473259	0.007165041

0.802381039	0.016547515
0.864325166	0.005625552
0.831787288	0.010448475
0.838696241	0.009263973
0.810956836	0.014585611
0.809705496	0.014862232
0.86839962	0.005150278
0.810545921	0.014676087
0.83532989	0.009829823
0.835298002	0.009835285
0.855696261	0.006722723
0.934965372	0.000654555
0.834148169	0.01003353
0.815528929	0.013602516
0.856389523	0.006629924
0.803719342	0.016231081
0.833089411	0.010218297
0.861823976	0.005930752
0.800629437	0.016967474
0.837985158	0.009381727
0.829426229	0.010874166
0.851785898	0.007261711
0.924802601	0.001003986
0.800685763	0.016953867
0.809178472	0.014979715
0.8357988	0.009749725
0.873618186	0.004580288
0.837097108	0.009530116
0.803876102	0.016194266
0.830025136	0.010765165
0.912224233	0.001581342
0.81694901	0.013305917
0.852134287	0.00721261
0.86255002	0.005841094
0.838470459	0.00930126
0.854675591	0.006860852
0.832167983	0.010380843
0.914062679	0.001486158
0.849833012	0.007540893
0.879046857	0.004032166
0.859216213	0.006260036
0.92775625	0.000892293
0.812575579	0.014232606
0.909287274	0.001741483
0.885617554	0.003427653
0.847632706	0.00786354
0.949640691	0.000307348
0.845725298	0.008150245
0.8112638	0.014518252
0.912954807	0.001543057
0.939477026	0.00052939

0.829292595	0.010898583
0.823653996	0.011960659
0.810265541	0.014738025
0.852341056	0.007183569
0.860010922	0.00615848
0.862239063	0.005879386
0.856056094	0.006674453
0.818961263	0.01289267
0.849189579	0.007634353
0.819741964	0.01273455
0.825954139	0.01151986
0.802299201	0.016566989
0.841606736	0.008791801
0.848749816	0.007698653
0.867735207	0.005225948
0.848065913	0.007799334
0.920630515	0.001176747
0.825876832	0.011534506
0.801830769	0.016678736
0.896160364	0.0025857
0.870009124	0.004969894
0.909200668	0.001746359
0.874270618	0.004512027
0.920003116	0.001204291
0.854964793	0.006821531
0.920075774	0.00120108
0.832330287	0.010352093
0.809293926	0.014953929
0.818980336	0.012888793
0.922514856	0.0010965
0.816147923	0.013472726
0.836341441	0.009657551
0.8833161	0.003632206
0.869389415	0.005038859
0.847184181	0.00793037
0.879529059	0.003985638
0.806286335	0.015634812
0.874495447	0.004488656
0.86912787	0.005068148
0.858861268	0.006305738
-0.887104034	0.003299576
0.859446228	0.006230533
0.825115681	0.011679331
0.801136911	0.016845125
0.855541706	0.006743524
0.805108786	0.015906598
0.815841854	0.013536804
0.850398898	0.007459303
0.834685624	0.009940554
0.847908914	0.007822564
0.801386952	0.016785045

0.835012078	0.009884347
0.920005918	0.001204167
0.801730156	0.016702799
0.865258515	0.005514298
0.809246659	0.014964482
0.819099724	0.012864537
0.83786869	0.009401104
0.88484323	0.003495622
0.855149448	0.0067965
0.867728353	0.005226733
0.801818609	0.016681643
0.883347511	0.003629362
0.851559818	0.007293687
0.800504565	0.016997665
0.801510096	0.016755506
0.84216547	0.008702948
0.815220296	0.013667522
0.819234908	0.012837107
0.80295223	0.016411991
0.847706974	0.007852509
0.918441057	0.001274687
0.881515384	0.003797624
0.863015831	0.005784031
0.931800485	0.00075301
0.840989709	0.008890592
0.924548805	0.001013987
0.870243311	0.004943991
0.865064263	0.005537335
0.841827691	0.008756595
0.928336203	0.000871364
0.804041624	0.016155451
0.952136517	0.000264381
0.839623928	0.009111768
0.837875068	0.009400042
0.804670215	0.016008576
0.819780946	0.012726687
0.849307775	0.00761713
0.861241877	0.006003268
0.860933423	0.006041923
0.829360366	0.010886196
0.888383865	0.003191815
0.800936043	0.016893487
0.813544393	0.014023931
0.836781144	0.009583269
0.820881724	0.012505913
0.832862675	0.010258144
0.951717913	0.000271292
0.845616102	0.008166856
0.839439034	0.009141976
0.829318523	0.010893843
0.805786669	0.015749779

0.822329104	0.012219326
0.848262727	0.007770274
0.846448421	0.008040781
0.842496812	0.008650527
0.821494699	0.012384029
0.824710786	0.011756837
0.873595476	0.004582676
0.80109489	0.016855235
0.812785566	0.014187212
0.8181023	0.013068066
0.818174541	0.013053257
0.839888394	0.00906867
0.946261048	0.000372509
0.829576254	0.010846796
0.842204571	0.008696752
0.809709013	0.01486145
0.819682181	0.012746615
0.850638151	0.007424977
0.842607021	0.008633136
0.819975674	0.012687454
0.801452398	0.016769342
0.832410038	0.010337985
0.875331938	0.004402389
0.881663561	0.003783832
0.81548667	0.013611405
0.810010374	0.014794535
0.822431505	0.012199209
0.821071565	0.012468085
0.868873358	0.005096755
0.814773977	0.013761876
0.813903868	0.013946996
0.842009008	0.008727772
0.912516356	0.00156596
0.838204861	0.009345244
0.844653726	0.008314193
-0.934428453	0.000670625
0.851863503	0.007250755
0.855450034	0.006755881
0.804275572	0.016100689
0.865393937	0.005498275
0.808643878	0.015099481
0.818690538	0.01294779
0.809479296	0.014912585
0.912591577	0.001562015
0.821078837	0.012466637
0.900589049	0.002276601
0.812985003	0.014144183
0.894382656	0.002717024
0.846667051	0.008007871
0.815877497	0.013529332
0.817253411	0.013242876

0.863081336	0.005776035
0.844791412	0.008293011
0.93316263	0.000709528
0.879154086	0.004021789
0.870934367	0.00486806
0.899310589	0.00236322
0.84063828	0.008947173
0.804767609	0.015985894
0.867712915	0.0052285
0.844036758	0.008409532
0.936310291	0.000615415
0.838464439	0.009302256
0.880974352	0.003848255
0.8792485	0.004012667
0.821720421	0.012339337
0.892765045	0.002840204
0.814695299	0.013778551
0.840468943	0.008974518
0.883246303	0.003638529
0.857251108	0.006515741
0.876325369	0.004301331
0.86461395	0.005590977
0.840016425	0.009047853
0.807478249	0.015362698
0.807892561	0.015268812
0.800438464	0.017013661
0.910036802	0.001699657
0.804940581	0.015945661
0.800942063	0.016892036
0.811989009	0.014359893
0.834386289	0.009992269
0.85354203	0.007016368
0.960274041	0.000152101
0.828960836	0.010959349
0.932459772	0.000731753
0.881690025	0.003781372
0.805436552	0.015830652
0.810988486	0.014578657
0.836009145	0.009713929
0.880925894	0.003852811
0.838618457	0.009276808
0.842743158	0.008611684
0.823818684	0.01192875
0.805521727	0.015810954
0.828582406	0.011028924
0.800353885	0.017034141
0.845543623	0.008177894
0.861600816	0.005958486
0.927977502	0.00088427
0.838304341	0.009328754
0.803310394	0.016327368

0.827792346	0.011175079
0.826175392	0.01147801
0.829937637	0.010781047
-0.808779955	0.015068938
0.884247422	0.003548508
-0.831087232	0.01057357
0.81294632	0.014152522
0.800948679	0.016890442
0.82079196	0.012523825
0.828752279	0.010997658
0.833564878	0.010135058
0.92764765	0.000896248
0.813581705	0.014015933
0.810087681	0.0147774
0.819150865	0.012854156
0.867646813	0.005236069
0.823533535	0.011984033
0.80673331	0.015532416
0.821935833	0.01229678
0.809352696	0.014940813
0.867878258	0.005209596
0.805651069	0.015781071
0.827601552	0.011210557
0.883336425	0.003630366
0.844497681	0.008338241
0.830152154	0.010742137
0.832330048	0.010352136
0.866974175	0.005313496
0.885555625	0.003433058
0.828832567	0.0109829
0.844496429	0.008338434
0.889249444	0.003120239
0.840591908	0.008954656
0.92841953	0.000868384
0.833327353	0.010176587
0.836488008	0.009632749
0.965023279	0.000104187
0.867934585	0.005203167
0.819733441	0.01273627
0.806085408	0.015680979
0.818439126	0.01299911
0.923077404	0.001073257
0.802399218	0.016543191
0.895471454	0.002636092
0.841166854	0.008862158
0.83010149	0.010751319
0.829277277	0.010901384
0.883052766	0.0036561
0.838132024	0.009357329
0.801372468	0.016788522
0.885736704	0.003417271

0.909971416	0.00170328
0.895246267	0.0026527
0.851916134	0.007243331
0.894935489	0.002675733
0.803448796	0.016294741
0.816974282	0.013300676
0.80421114	0.016115759
0.812558711	0.014236257
0.830799401	0.010625278
0.842291355	0.008683008
0.824369729	0.011822374
0.918169796	0.001287178
0.869338036	0.005044604
0.826642096	0.011390048
0.862011254	0.005907542
0.883355916	0.003628602
0.843713939	0.008459693
0.822765231	0.012133792
0.800209463	0.017069148
0.802230418	0.016583368
0.821048021	0.012472772
0.823748112	0.011942417
0.855546832	0.006742833
0.806456029	0.015595888
0.833517015	0.010143417
0.829516828	0.010857633
0.853624642	0.007004959
0.829115629	0.01093097
0.886680901	0.003335714
0.834273577	0.010011786
0.839841902	0.009076237
0.807413459	0.015377413
0.807202399	0.015425408
0.810732484	0.014634966
0.884791255	0.003500215
0.925394475	0.000980912
0.842276752	0.00868532
0.809929013	0.014812582
0.810330153	0.014723737
0.868429065	0.00514694
0.815997481	0.013504198
0.902260542	0.002166496
0.802312851	0.01656374
0.947153449	0.0003545
0.867413342	0.005262861
0.842668653	0.00862342
0.889705658	0.003082935
0.850484371	0.007447028
0.883745193	0.003593487
0.827129483	0.011298644
0.851691544	0.007275045

0.801018059	0.01687373
0.823261142	0.012036994
0.812258363	0.014301354
0.80011797	0.017091348
0.834909737	0.009901946
0.867275178	0.005278757
0.813117087	0.014115731
0.880129576	0.003928178
0.822710454	0.012144514
0.840710819	0.008935475
0.832977533	0.010237947
0.859035075	0.006283333
-0.863232553	0.005757604
0.80465138	0.016012964
0.810182452	0.014756411
0.878885925	0.004047772
0.887810767	0.003239784
0.865381122	0.00549979
0.827925742	0.011150316
0.836752355	0.009588121
0.887396753	0.003274726
0.866130412	0.005411654
0.882493615	0.003707172
0.897304654	0.002503385
0.870336592	0.004933697
0.830123901	0.010747257
0.883628547	0.003603986
0.864871204	0.005560292
0.876936018	0.00423996
0.882534027	0.003703466
0.876878619	0.004245704
0.876185119	0.004315507
0.873313367	0.004612406
0.859805286	0.006184656
0.8736853	0.004573236
0.875555456	0.00437952
0.874111116	0.004528654
0.876560032	0.00427768
0.85592097	0.006692553
0.85392791	0.006963178
0.869385421	0.005039305
0.861973703	0.005912191
0.854631901	0.006866804
0.91264987	0.001558963
0.867762625	0.005222812
0.872944474	0.004651469
0.873970091	0.004543388
0.842725277	0.008614499
0.878610909	0.004074531
0.872375906	0.004712091
0.875095248	0.00442669

0.868479252	0.005141256
0.843047261	0.008563886
0.850400388	0.007459089
0.868626356	0.005124616
0.860808492	0.006057625
0.877303541	0.004203296
0.830196261	0.010734148
0.861100733	0.006020936
0.87127614	0.004830786
0.89040029	0.003026691
0.918854535	0.001255799
0.842811704	0.008600895
0.889033616	0.003137988
0.86567384	0.00546525
0.912992477	0.001541099
0.859659672	0.006203235
0.876412332	0.004292557
0.865876734	0.005441391
0.878076136	0.00412689
0.875388265	0.004396619
0.868151009	0.005178509
0.841362596	0.008830806
0.866217256	0.005401498
0.853063345	0.00708271
0.852309108	0.007188052
0.87876308	0.004059711
0.820087075	0.012665044
0.871566176	0.004799299
0.826332927	0.011448271
0.822314203	0.012222255
0.835590065	0.009785329
0.808015704	0.015240977
0.838929057	0.009225625
0.846579373	0.008021059
0.846905589	0.007972062
0.846559703	0.008024019
0.853420138	0.007033223
0.843270183	0.008528956
0.826073527	0.011497266
0.828362226	0.011069533
0.894749403	0.002689586
0.859713793	0.006196326
0.908002138	0.001814744
0.866788745	0.005334969
0.810263276	0.014738526
0.822389364	0.012207485
0.815145195	0.013683371
0.858093381	0.00640534
0.869596541	0.00501574
0.856240451	0.006649809
0.913393497	0.00152036

0.867608905	0.005240413
0.889520288	0.003098057
0.92123431	0.001150631
0.80533129	0.015855018
0.897462189	0.002492187
0.810420096	0.014703863
0.869357944	0.005042377
0.900303006	0.002295799
0.909224689	0.001745006
0.892701983	0.002845078
0.895424426	0.002639555
-0.904011488	0.002054926
0.847464561	0.007888551
0.801643133	0.016723629
0.908613384	0.001779655
0.876904726	0.004243091
0.874358833	0.004502848
0.862269223	0.005875665
0.859540284	0.006218494
0.844138443	0.008393771
0.838706017	0.009262361
0.922380149	0.001102115
0.926687956	0.000931699
0.921352744	0.001145553
0.85898596	0.006289659
0.91697216	0.001343282
0.899622381	0.002341902
0.906879365	0.001880365
0.893129468	0.002812145
0.894400358	0.002715695
0.941120446	0.00048804
0.906789839	0.001885662
0.805396914	0.015839825
0.852236331	0.007198269
0.800740123	0.016940741
-0.815752149	0.013555621
0.810634017	0.014656661
0.892081201	0.002893346
0.909946978	0.001704635
0.892561972	0.002855919
0.860837996	0.006053914
0.866947651	0.005316564
0.838520229	0.009293033
0.805190325	0.015887684
0.800232053	0.017063669
0.812477052	0.014253937
0.853323162	0.007046652
0.908697069	0.001774885
0.800850272	0.016914164
0.875843108	0.004350201
0.892139316	0.002888805

0.882154107	0.003738405
0.900075078	0.002311172
0.807708681	0.015310436
0.886179209	0.00337889
0.891057968	0.002974055
0.800324023	0.017041376
0.836761236	0.009586624
0.93110168	0.000775978
0.806606591	0.015561403
0.81413573	0.013897514
-0.848093629	0.007795237
-0.813575685	0.014017223
0.879424632	0.003995685
0.810854256	0.014608164
0.833311677	0.010179332
0.804240704	0.016108843
0.909949243	0.001704509
0.828813791	0.01098635
0.855021775	0.006813801
0.82837975	0.011066298
0.887865186	0.003235209
0.922066271	0.001115269
0.818915427	0.012901992
0.825180233	0.011667004
0.888547242	0.003178225
0.919954479	0.001206444
0.904855311	0.002002517
0.802025199	0.016632297
0.921176076	0.001153133
0.827425957	0.011243272
0.845949054	0.008116273
0.879483104	0.003990057
0.815797806	0.013546042
0.816281021	0.013444921
0.851372004	0.00732032
0.817769825	0.013136357
0.88017565	0.003923792
0.894593537	0.002701225
0.846089423	0.008095007
0.81437552	0.013846456
0.843460262	0.008499244
0.822967708	0.012094211
0.808049977	0.015233236
0.801318347	0.016801516
0.879866004	0.003953332
0.815614164	0.013584597
0.803664565	0.016243957
0.867390275	0.005265513
0.880416036	0.003900958
0.833072126	0.010221332
0.865522444	0.005483096

0.815670907	0.013572677
0.829553843	0.010850882
0.842622995	0.008630617
0.809720695	0.014858853
0.821940422	0.012295875
0.821258605	0.012430885
0.80198741	0.016641316
0.800389707	0.017025465
0.837135613	0.009523651
0.862166524	0.005888342
0.84322083	0.008536682
0.840268016	0.009007033
0.891316235	0.002953548
0.841767728	0.00876614
0.815205753	0.013670591
0.858826458	0.006310232
0.835726261	0.009762088
0.914314508	0.001473418
0.853815913	0.006978589
0.853758812	0.006986455
0.870205581	0.004948158
0.87165463	0.004789723
0.807230532	0.015419005
0.811557353	0.014454019
0.821117282	0.012458986
0.822893798	0.01210865
0.895668209	0.002621636
0.85167551	0.007277313
0.870553017	0.004909868
0.938336313	0.000559402
0.805234611	0.015877417
0.891265512	0.002957569
0.848064244	0.00779958
0.908619285	0.001779318
0.875081778	0.004428075
-0.908224583	0.001801923
0.942745328	0.000449298
0.802621603	0.016490352
0.873351395	0.004608391
0.888412654	0.003189418
0.935061991	0.000651691
0.814466417	0.013827133
0.905842066	0.001942342
0.847824275	0.007835106
0.854484797	0.006886871
0.851667166	0.007278493
0.907874048	0.001822154
0.895323634	0.002646987
0.867799699	0.005218572
0.864783585	0.005570731
0.880733311	0.003870952

0.914250016	0.001476674
0.805845857	0.015736133
0.905801117	0.001944816
0.858416438	0.006363315
-0.917946279	0.001297531
-0.87902391	0.004034389
0.891597509	0.002931319
0.858691931	0.006327617
0.83434236	0.009999873
0.882739842	0.003684626
0.873379588	0.004605416
0.857113302	0.006533919
0.913607359	0.001509374
0.902678668	0.002139504
0.935488999	0.000639129
0.858001709	0.006417297
0.93251133	0.000730107
0.896062791	0.002592799
0.856911004	0.006560662
0.906124294	0.00192535
0.910228729	0.001689053
0.918368161	0.001278036
0.912968993	0.001542319
0.899218202	0.002369561
0.909005702	0.001757367
0.910316885	0.001684197
0.882776797	0.00368125
0.858326793	0.006374959
0.969238997	7.11E-05
0.951860547	0.000268924
0.882757425	0.003683019
0.870365202	0.004930543
0.899539471	0.002347558
0.898128271	0.002445201
0.813025177	0.014135525
0.893835425	0.0027583
0.899964631	0.002318645
0.836454093	0.009638485
0.880910039	0.003854303
0.885878623	0.003404931
0.849435329	0.007598571
0.969174922	7.15E-05
0.838747442	0.009255531
0.851443529	0.00731017
0.80973959	0.014854652
0.820258081	0.012630693
0.902884245	0.002126314
0.803292513	0.016331586
0.896233618	0.002580379
0.914354146	0.001471419
0.901655138	0.002205966

0.83631891	0.009661367
0.8702479	0.004943484
0.942039192	0.000465876
0.900851607	0.002259071
0.907986403	0.001815654
0.882874668	0.003672318
0.809215546	0.014971432
-0.814714968	0.013774381
0.896876395	0.00253399
0.920885146	0.001165687
0.876122773	0.004321818
0.942396343	0.000457442
0.854183793	0.006928049
0.893011868	0.00282118
0.954845905	0.000222437
0.92659086	0.000935336
0.80650872	0.015583814
0.825462341	0.01161323
0.890720427	0.003000995
0.95353061	0.000242204
0.855981886	0.00668439
0.869301081	0.005048739
0.819573641	0.012768538
0.882819355	0.003677364
0.831947625	0.010419956
0.837547004	0.009454756
0.877970755	0.004137259
0.878127158	0.004121876
0.800111532	0.017092911
0.891156077	0.002966255
0.883509398	0.003614731
0.853947937	0.006960424
0.959276378	0.000163727
0.941949666	0.000468006
0.857144356	0.006529819
0.922457159	0.001098903
0.904356301	0.002033404
0.81019485	0.014753666
0.864200413	0.00564053
0.903404713	0.002093156
0.811328411	0.014504099
0.879822969	0.003957448
0.835830986	0.009744242
0.9232108	0.001067792
0.899932504	0.002320822
0.826329648	0.011448889
0.816000462	0.013503574
0.811129332	0.014547735
0.842907369	0.008585853
0.82714057	0.011296571
0.862606645	0.005834138

0.925210476	0.000988047
0.859227777	0.006258551
0.852357924	0.007181204
0.837774932	0.009416721
0.817046881	0.013285628
0.919889808	0.00120931
0.85074681	0.007409421
0.928654969	0.000859999
0.812363625	0.014278519
0.808777571	0.015069473
0.883637249	0.003603202
0.805450797	0.015827357
0.872488201	0.004700078
0.845502496	0.008184162
0.817675829	0.013155705
0.821979702	0.012288125
0.858547449	0.006346323
0.801816642	0.016682113
0.826257348	0.011462532
0.813199401	0.014098017
0.809922397	0.01481405
0.842129648	0.008708628
0.816922247	0.013311469
0.856726348	0.006585135
0.862138629	0.005891789
0.817670584	0.013156785
0.844218493	0.008381377
0.857784748	0.006445654
0.88138032	0.003810223
0.829400897	0.010878792
0.867437243	0.005260114
0.854112208	0.006937865
0.909493923	0.001729886
0.859068871	0.006278982
0.838491976	0.009297702
0.805867612	0.015731119
0.923593044	0.001052236
0.852748096	0.007126619
0.840599597	0.008953415
0.81178838	0.014403594
0.813325167	0.014070981
0.919483364	0.001227425
0.845925808	0.008119798
0.803668737	0.016242976
0.83196336	0.01041716
0.832122982	0.010388823
0.806037486	0.015692003
0.81045717	0.014695675
0.825941145	0.011522321
0.859445572	0.006230617
0.800226569	0.017064999

0.964413464	0.000109681
0.809593618	0.014887123
0.851310968	0.007328988
0.803425074	0.01630033
0.863584578	0.005714844
0.801022828	0.016872582
0.855879366	0.006698132
0.804533184	0.016040522
0.845518947	0.008181654
0.861230135	0.006004737
0.840137422	0.009028207
0.82621336	0.011470838
0.909154058	0.001748986
0.864504397	0.005604077
0.856750786	0.006581892
0.859644771	0.006205138
-0.819510698	0.012781261
0.841717482	0.008774144
-0.805570364	0.015799713
-0.827680349	0.011195896
0.850238919	0.007482312
0.874378443	0.004500809
0.83232826	0.010352452
0.888988078	0.003141741
0.866807818	0.005332757
0.881245255	0.003822849
0.830335319	0.010708985
0.874953747	0.004441258
0.832051992	0.01040142
0.849699616	0.007560209
0.860924959	0.006042986
0.847955525	0.007815663
0.855383694	0.006764832
0.847783387	0.007841169
0.904743075	0.002009438
0.852566004	0.00715206
0.852743328	0.007127284
0.81243819	0.014262356
0.846715569	0.008000579
0.861072838	0.006024432
0.84516871	0.008235142
0.850281775	0.007476143
0.838439286	0.009306416
0.846095324	0.008094114
0.833336234	0.010175032
0.801048875	0.01686631
0.849395692	0.007604335
0.856635392	0.00659721
0.839409947	0.009146733
0.88778013	0.003242361
0.866037965	0.005422478

0.815480769	0.013612647
0.807452083	0.01536864
0.902818382	0.002130534
0.837186754	0.009515069
0.86656034	0.005361495
0.853221118	0.0070608
0.875551522	0.004379922
0.895275354	0.002650551
0.86138624	0.005985231
0.881866097	0.003765034
0.885502696	0.003437681
0.832501292	0.010321857
0.842398226	0.008666103
0.848244071	0.007773026
0.841376483	0.008828584
0.809341609	0.014943287
0.860887706	0.006047666
0.898243129	0.002437157
0.854171395	0.006929749
0.842543185	0.008643207
0.912491381	0.001567272
0.823370218	0.012015769
0.854471028	0.006888752
0.867910504	0.005205915
0.863419473	0.005734874
0.838527322	0.00929186
0.845502257	0.008184198
0.839406133	0.009147358
0.801570535	0.01674102
0.801761687	0.016695256
0.922480404	0.001097935
0.854037046	0.006948181
0.880744874	0.003869861
0.859689236	0.00619946
0.843759775	0.00845256
0.855275571	0.006779438
0.848883808	0.007679025
0.905107617	0.001987018
0.821247339	0.012433124
0.815883875	0.013527995
0.806980669	0.015475931
0.93554014	0.000637635
0.873713136	0.004570313
0.828609943	0.011023852
0.845976591	0.008112098
0.829765379	0.010812355
0.921490014	0.001139685
0.829554915	0.010850687
0.802132487	0.016606706
0.84106034	0.008879248
0.805426717	0.015832928

0.808793604	0.015065877
0.904891968	0.002000261
0.807703197	0.015311679
0.865630805	0.005470319
0.823361635	0.012017438
0.822677851	0.012150899
0.852841675	0.007113567
0.885068834	0.00347573
0.827570498	0.011216338
0.877705276	0.004163454
0.804232538	0.016110753
0.901129842	0.00224059
0.85386157	0.006972304
0.857516825	0.006480782
0.828136861	0.011111197
0.837110102	0.009527934
0.824587166	0.011780565
0.897118747	0.002516641
0.874553561	0.004482628
0.900923908	0.002254259
0.85613817	0.006663474
0.905240119	0.001978909
0.822937727	0.012100066
0.8504439	0.007452839
0.80934	0.014943646
0.840606451	0.008952309
0.83425355	0.010015257
0.806649923	0.015551487
0.880695283	0.00387454
0.82216531	0.012251547
0.808998644	0.015019935
0.831186235	0.010555822
0.842236996	0.008691615
0.869308352	0.005047925
0.923251808	0.001066116
0.828021228	0.011132612
0.849456966	0.007595426
0.833385944	0.010166333
0.813040376	0.014132251
0.807330489	0.015396269
0.874495447	0.004488656
0.835894167	0.009733485
0.821334183	0.012415874
0.877399683	0.004193739
0.874277174	0.004511344
0.826818466	0.011356918
0.868367732	0.005153893
0.851442695	0.007310289
0.831319869	0.010531895
0.841469824	0.008813661
0.801143289	0.016843591

0.927186787	0.00091316
0.860398114	0.006109385
0.827004313	0.011322074
0.847251296	0.007920347
0.852040648	0.007225786
0.82877171	0.010994085
0.823575497	0.011975888
0.82036376	0.012609493
0.90750736	0.001843475
0.803658187	0.016245457
0.898470044	0.002421316
0.893679261	0.002770153
0.802839637	0.01643865
0.877204359	0.004213171
0.823662043	0.011959099
0.807622075	0.015330065
0.96086669	0.000145461
0.82516247	0.011670395
0.843592584	0.008478599
0.866210818	0.00540225
0.847732008	0.007848793
0.834051788	0.010050261
0.804640591	0.016015479
0.913042545	0.0015385
0.9246912	0.001008368
0.824123502	0.011869833
0.802254379	0.016577662
0.84862107	0.007717543
0.844278812	0.008372045
0.850651026	0.007423133
0.84857899	0.007723723
0.818884909	0.012908201
0.811930239	0.014372686
0.85171181	0.00727218
0.855280399	0.006778785
0.800705731	0.016949045
0.841002643	0.008888514
0.829037189	0.010945344
0.813306212	0.014075054
0.836100817	0.009698355
0.854241848	0.006920095
0.808102012	0.015221487
0.805172801	0.015891748
0.813869596	0.013954319
0.81487745	0.013739964
0.827127993	0.011298923
0.806665897	0.015547832
0.923464358	0.001057456
0.877575755	0.004176273
0.837249756	0.009504504
0.836926758	0.00955875

0.80364424	0.016248737
-0.911839306	0.001601761
0.825130522	0.011676496
0.8327595	0.010276309
0.864171624	0.00564399
0.851372004	0.00732032
0.928646028	0.000860317
0.85243237	0.007170768
0.803046584	0.016389672
0.829418719	0.010875538
0.833705425	0.010110535
0.933503091	0.000698923
0.836934686	0.009557416
0.895865262	0.002607209
0.851468503	0.007306628
0.869798839	0.004993227
0.863065124	0.005778013
0.867709458	0.005228895
0.951592863	0.00027338
0.850054681	0.007508865
0.838516772	0.009293604
0.803810596	0.016209643
0.9548406	0.000222514
0.851264954	0.007335528
0.948474646	0.000328902
0.819927216	0.01269721
0.82725203	0.011275735
0.935188413	0.000647955
0.802677214	0.016477156
0.8500278	0.007512745
0.808910131	0.015039757
0.896297157	0.002575769
0.829710066	0.010822421
0.892428279	0.002866295
0.838781059	0.009249991
0.816578865	0.013382829
0.821495831	0.012383805
0.883253753	0.003637854
0.811259091	0.014519284
0.837543845	0.009455283
0.840391934	0.008986971
0.842584372	0.008636708
0.816588759	0.01338077
0.873201907	0.004624187
0.851738751	0.007268372
0.820656419	0.012550902
0.846762657	0.007993507
0.889324784	0.003114058
0.859148979	0.006268677
0.848459065	0.007741354
0.832016587	0.010407706

0.834347904	0.009998913
0.834368706	0.009995312
0.82318604	0.012051622
0.904419839	0.002029454
0.80311203	0.016374201
-0.802046537	0.016627205
0.802827895	0.016441432
0.821326196	0.012417459
0.828562558	0.011032581
0.801834106	0.016677938
0.826931894	0.011335644
0.803218186	0.016349128
0.809103251	0.014996531
0.813536763	0.014025567
0.803212881	0.01635038
0.831556976	0.010489526
0.875938952	0.004340461
0.864547133	0.005598964
0.969958007	6.63E-05
0.810861826	0.014606499
0.804127157	0.016135416
0.816851616	0.013326128
0.824973285	0.011706552
0.84876442	0.007696512
0.821036339	0.012475099
0.836245537	0.009673801
0.813717067	0.013986942
0.850515902	0.007442504
0.881618381	0.003788034
0.846392751	0.008049175
0.836664438	0.009602949
0.926912546	0.000923322
0.857810199	0.006442323
0.819900513	0.012702588
0.806559622	0.015572155
0.815873682	0.013530132
0.820184171	0.012645533
0.8482005	0.007779454
0.896645963	0.002550557
0.818035603	0.013081748
0.920796454	0.001169531
0.908040941	0.001812504
0.800201833	0.017070999
0.839956343	0.009057618
0.853960752	0.006958663
0.804336488	0.016086449
0.805206239	0.015883994
0.877614558	0.00417243
0.869941056	0.004977439
0.85667026	0.006592579
0.814464033	0.013827639

0.879469693	0.003991347
-0.811115623	0.014550743
0.882406175	0.0037152
0.864706695	0.005579902
0.878313363	0.004103611
0.864583552	0.00559461
0.861222327	0.006005713
0.816287398	0.013443589
0.802601516	0.016495121
0.851268351	0.007335045
0.931923926	0.000749
0.813916564	0.013944283
0.834854782	0.009911404
0.814134777	0.013897717
0.88765645	0.00325278
0.852932513	0.007100912
0.847245276	0.007921246
0.895375729	0.002643144
0.866596997	0.005357232
0.887679517	0.003250835
0.845003128	0.008260506
0.853520751	0.007019309
0.805516362	0.015812194
0.818262041	0.013035335
0.818752646	0.012935131
0.850671649	0.007420179
0.972181201	5.27E-05
0.851699829	0.007273874
0.804449856	0.016059968
0.826981544	0.011326339
0.843368888	0.008513519
0.850308239	0.007472336
0.808627367	0.015103189
0.849615216	0.007572447
0.860764861	0.006063114
0.833494484	0.010147354
0.847042382	0.007951574
0.848426163	0.007746196
0.813564599	0.0140196
0.841144204	0.00886579
0.833623171	0.010124882
0.858517289	0.006350232
0.812883139	0.01416615
0.942825079	0.000447451
0.853360832	0.007041434
0.857545197	0.006477056
0.803616405	0.016255284
0.822982132	0.012091394
0.84565258	0.008161305
0.83938688	0.009150508
0.814981163	0.013718025

0.852762997	0.007124539
0.836918652	0.009560113
0.827200592	0.011285348
0.936722815	0.000603725
0.835932434	0.009726974
0.808622777	0.01510422
0.925397933	0.000980778
0.83889246	0.009231646
0.817761958	0.013137976
0.81918025	0.012848193
0.924132645	0.001030528
0.94339186	0.000434464
0.802487671	0.016522162
0.828147531	0.011109222
0.975461602	3.63E-05
0.803192377	0.016355221
0.805807948	0.015744872
0.806947172	0.015483572
0.882448256	0.003711335
0.89010793	0.003050282
0.948491931	0.000328576
0.868203998	0.005172484
0.852865577	0.007110235
0.830403626	0.010696638
0.860986471	0.006035264
0.821463048	0.012390304
0.826892495	0.011343031
0.828035116	0.011130039
0.859268844	0.006253277
0.80309701	0.016377751
0.820654333	0.01255132
0.857048213	0.006542516
0.83063072	0.010655655
0.842258155	0.008688264
0.823367238	0.012016348
0.846207321	0.008077173
0.850196898	0.007488363
0.859183311	0.006264263
0.937163293	0.000591405
0.852000296	0.007231469
0.909966886	0.001703531
0.809355974	0.014940082
0.904013097	0.002054825
0.890577555	0.003012446
0.826304376	0.011453657
0.850863099	0.007392796
0.843224704	0.008536075
0.863720775	0.005698355
0.831449389	0.010508737
0.869844973	0.004988102
0.811375678	0.014493751

0.818367839	0.013013685
0.840726137	0.008933006
0.851788461	0.007261349
0.811006784	0.014574637
0.980069995	1.95E-05
0.830322921	0.010711227
0.886348069	0.003364318
0.892360449	0.002871568
0.890376449	0.003028611
0.824611366	0.011775918
0.836608887	0.009612326
0.912301183	0.001577281
0.812158227	0.014323099
-0.805499852	0.015816012
0.861476481	0.005973974
0.859425128	0.006233236
0.937120676	0.00059259
0.851609528	0.007286649
0.894795358	0.00268616
0.855401456	0.006762435
0.949114084	0.000316963
0.813374281	0.014060432
0.82175976	0.012331558
0.805032253	0.015924364
0.82673943	0.011371757
0.914825022	0.00144781
0.82075876	0.012530454
0.802607417	0.01649372
0.847611606	0.007866676
0.862533629	0.005843108
0.828230739	0.01109383
0.892931461	0.002827368
0.870084584	0.004961538
0.830597341	0.010661673
-0.861871779	0.005924822
0.917002797	0.001341828
0.827703178	0.011191651
0.817388713	0.013214915
0.886821747	0.003323657
0.820991099	0.01248411
0.819808125	0.012721206
0.816065073	0.013490052
0.857031167	0.006544768
0.879680276	0.003971118
0.896802545	0.002539292
0.812490225	0.014251084
0.818092585	0.013070059
0.86134696	0.005990135
0.821764648	0.012330592
0.832878232	0.010255407
0.906938195	0.001876889

0.808498621	0.015132126
0.80872941	0.015080278
0.940327823	0.000507708
0.899092972	0.002378173
0.893474698	0.002785729
0.886054456	0.003389682
0.841893315	0.008746156
0.969650745	6.83E-05
0.803565443	0.016267274
0.88965559	0.003087015
0.822885036	0.012110362
0.819703996	0.012742211
0.807316303	0.015399494
0.875379801	0.004397485
0.832215071	0.010372497
0.830820978	0.010621396
0.814312458	0.013859872
0.838304937	0.009328656
0.917188346	0.001333039
0.852085233	0.007219511
0.810060441	0.014783436
0.82110548	0.012461334
0.831686616	0.010466406
0.925446928	0.000978884
0.815084159	0.013696259
0.813471854	0.014039488
0.818968654	0.012891168
0.930445552	0.000797957
0.812042296	0.0143483
0.825730681	0.011562225
0.816758692	0.013345428
0.931673646	0.000757146
0.864935338	0.005552659
0.835569501	0.009788841
0.861765504	0.005938011
0.814519048	0.013815952
0.814251423	0.013872865
0.871713519	0.004783354
0.865670323	0.005465664
0.864191711	0.005641576
0.88884604	0.003153467
0.93591398	0.000626785
0.904394627	0.002031021
0.826690733	0.011380906
0.830543339	0.010671413
0.9046067	0.002017867
0.837967336	0.009384691
0.810667455	0.014649291
0.847032189	0.007953099
0.844445407	0.008346307
0.832864106	0.010257892

0.828413367	0.011060093
0.811364174	0.014496269
0.819746196	0.012733696
0.884122074	0.0035597
0.841730654	0.008772045
0.87707454	0.004226117
0.810280442	0.014734729
0.862305403	0.005871203
0.932307422	0.00073663
0.857524395	0.006479787
0.857346058	0.006503235
0.833155155	0.010206762
0.813571692	0.014018079
0.84090358	0.008904438
0.889408946	0.003107164
0.898877144	0.002393064
0.878565907	0.004078921
0.882022798	0.00375053
0.876328111	0.004301054
0.839043796	0.009206762
0.810253441	0.014740701
-0.826518774	0.01141325
-0.855232	0.006785329
-0.821694314	0.0123445
-0.804213345	0.016115243
-0.805377126	0.015844405
0.921001911	0.001160637
0.831175029	0.01055783
0.814944744	0.013725727
0.841002226	0.008888581
0.875834465	0.00435108
0.859795094	0.006185956
0.849523306	0.007585787
0.81016767	0.014759683
0.80952704	0.014901948
0.805939913	0.015714463
0.847098291	0.007943209
0.837270677	0.009500997
0.826849818	0.011351035
-0.888059974	0.003218868
-0.81886667	0.012911913
-0.812172234	0.014320056
0.823618114	0.011967619
0.858740091	0.00632139
0.833178341	0.010202696
0.831157744	0.010560928
0.854747534	0.006851057
0.838149369	0.00935445
0.816423476	0.0134152
0.818923056	0.01290044
-0.841950238	0.008737108

0.815985322	0.013506744
0.917700231	0.001308989
0.812817395	0.014180339
0.815025389	0.013708676
0.892006218	0.002899212
0.801653564	0.016721132
0.805692554	0.015771493
0.809518933	0.014903754
0.851055026	0.007365409
0.922771871	0.001085841
0.835196197	0.009852736
0.814984918	0.013717231
0.814915299	0.013731955
0.826195478	0.011474215
0.834765673	0.009926753
-0.812042832	0.014348184
0.844806314	0.00829072
0.87530297	0.004405358
0.887612104	0.00325652
0.916247547	0.001377988
0.829289973	0.010899062
0.851014256	0.007371221
0.893655956	0.002771924
0.868243456	0.005168
0.863013148	0.005784359
0.809167981	0.01498206
0.833074689	0.010220882
0.898917317	0.002390288
0.943247437	0.000437749
0.857933939	0.006426146
0.931997538	0.000746615
0.89914614	0.002374514
0.858998597	0.006288031
0.826904416	0.011340795
0.976086557	3.36E-05
0.822345257	0.012216151
0.925445139	0.000978953
0.840283334	0.009004552
0.823148489	0.01205894
-0.802358031	0.016552988
0.828624129	0.01102124
0.802065432	0.016622697
0.859234035	0.006257747
0.801141858	0.016843935
0.925765872	0.000966612
0.819758117	0.012731291
0.886105597	0.003385256
0.826071024	0.011497739
0.897047162	0.002521757
0.815820456	0.013541291
-0.808774889	0.015070075

0.817216337	0.013250544
0.812049329	0.014346771
0.800999701	0.016878151
0.887606621	0.003256983
0.80303061	0.016393449
0.850932121	0.007382939
0.857499659	0.006483036
0.863648534	0.005707098
0.90641427	0.001907992
0.863736928	0.005696402
0.809625924	0.014879933
0.865773261	0.00545355
0.820267498	0.012628803
0.835665762	0.009772407
0.854715526	0.006855413
0.974892259	3.88E-05
0.834365129	0.009995931
0.875681221	0.004366686
0.808077633	0.015226991
0.854842782	0.006838102
0.837515414	0.009460035
0.825223029	0.011658836
0.832270503	0.010362677
0.823264122	0.012036414
0.812759221	0.014192902
0.812232316	0.014307009
0.803322911	0.016324415
0.805662692	0.015778387
0.818053305	0.013078116
0.843558729	0.008483878
0.800546169	0.016987603
0.955273211	0.000216252
0.843440354	0.008502353
0.872893512	0.004656882
0.875199914	0.004415933
0.839944601	0.009059527
0.849992573	0.00751783
0.826848865	0.011351214
0.936275959	0.000616395
0.859207153	0.0062612
0.834549725	0.009964012
0.802494109	0.016520632
0.818946958	0.012895579
0.878738165	0.004062135
0.891717613	0.00292186
0.813826025	0.013963633
0.878752053	0.004060784
0.837950349	0.009387516
0.807714939	0.015309018
0.907248259	0.001858638
0.817971766	0.013094852

0.887188613	0.003292383
-0.822612524	0.012163698
0.813955665	0.013935932
0.841513872	0.008806625
0.841885686	0.008747369
0.81518358	0.013675269
0.87231952	0.004718131
0.826092482	0.011493681
0.838387549	0.009314976
0.821435392	0.012395789
0.822027624	0.012278674
0.820473313	0.01258754
0.867806315	0.005217816
0.818840623	0.012917214
0.847083092	0.007945483
0.800769627	0.01693362
0.820272863	0.012627726
0.804426074	0.016065521
0.863305926	0.005748675
0.808022141	0.015239523
0.844313562	0.008366672
0.942589164	0.00045293
0.839720249	0.009096056
0.831824541	0.010441844
0.821846902	0.012314338
0.808805645	0.015063177
0.903897882	0.002062049
0.801432729	0.01677406
0.870492399	0.004916535
0.844155431	0.00839114
0.80660069	0.015562753
0.819918156	0.012699035
0.869747162	0.004998972
0.861328602	0.005992428
0.842498422	0.008650273
0.860474169	0.006099771
0.825830698	0.011543251
0.869173169	0.005063067
0.809712589	0.014860655
0.843137383	0.008549754
0.943710685	0.000427268
0.813760936	0.013977554
0.873709381	0.004570707
0.815168202	0.013678514
0.811131656	0.014547225
0.896969736	0.002527299
0.833198547	0.010199153
0.847822011	0.007835442
0.822487772	0.012188164
0.877290428	0.004204601
0.881203949	0.003826716

0.928105652	0.000879645
0.832615256	0.010301738
0.840117693	0.009031408
0.841947734	0.008737505
0.940776944	0.000496501
0.810250819	0.014741281
0.806608319	0.015561007
0.878343225	0.004100686
-0.83342129	0.01016015
0.804918945	0.01595069
0.8147645	0.013763883
0.803169668	0.016360585
0.851195335	0.007345429
0.923081815	0.001073076
0.909297884	0.001740887
0.824011028	0.011891551
0.880144775	0.003926731
0.869002938	0.005082177
0.881741226	0.003776617
0.916007102	0.001389633
0.833013892	0.010231558
0.812868536	0.014169301
0.810972989	0.014582062
0.879062772	0.004030625
0.81917578	0.0128491
0.828031063	0.01113079
0.877715468	0.004162446
0.868668914	0.005119809
0.847575903	0.007871984
0.803275466	0.016335608
0.919802308	0.001213195
0.835245728	0.009844243
0.807980955	0.015248829
0.857386827	0.00649787
0.87560302	0.004374663
0.822574079	0.012171234
0.899647832	0.002340167
0.835624695	0.009779416
0.834883571	0.009906449
0.83630383	0.009663921
0.843689382	0.008463517
0.85344851	0.007029298
0.820179403	0.01264649
0.89075762	0.002998019
0.805127501	0.015902256
0.821655214	0.012352237
0.885587573	0.003430269
0.806487858	0.015588593
0.83583039	0.009744343
0.829009414	0.010950438
0.841561377	0.00879904

0.859362543	0.006241257
0.833061099	0.010223268
0.818539917	0.01297852
0.839293897	0.009165732
0.860910833	0.00604476
0.923561156	0.001053528
0.861195624	0.006009054
0.895428061	0.002639287
0.810604632	0.014663139
0.813211799	0.014095351
0.868643165	0.005122717
0.819890022	0.012704702
0.814792633	0.013757923
0.81216228	0.014322219
-0.903081179	0.002113728
0.836960375	0.009553095
0.879071534	0.004029777
0.914102256	0.001484151
0.87814343	0.004120278
0.805173695	0.01589154
0.902157307	0.002173194
0.857714891	0.006454801
0.903502524	0.002086963
0.843503773	0.008492452
0.846463203	0.008038553
0.924811721	0.001003627
0.839314818	0.009162305
0.837347865	0.009488066
0.917841256	0.001302414
0.878642619	0.00407144
0.810837567	0.014611836
0.958786845	0.000169639
0.83827281	0.009333979
0.859686732	0.00619978
0.809267521	0.014959824
0.804825068	0.015972522
0.828785837	0.010991488
0.814850628	0.013745642
0.872475743	0.00470141
0.801077604	0.016859395
0.836399436	0.009647732
0.865658522	0.005467054
0.852176189	0.007206719
0.94144249	0.000480194
0.852067471	0.00722201
0.835381746	0.009820944
0.831832588	0.010440413
0.805308938	0.015860194
0.823662519	0.011959006
0.877670944	0.00416685
0.861660182	0.0059511

0.895862281	0.002607427
0.88212049	0.003741507
0.812402606	0.014270068
0.803430617	0.016299024
0.827345848	0.011258217
0.846210241	0.008076732
0.823057294	0.012076724
0.904090047	0.002050009
0.820048153	0.012672871
0.878099501	0.004124594
0.801386595	0.016785131
0.820142388	0.012653927
0.830129921	0.010746166
0.836043179	0.009708145
0.855703652	0.006721729
0.873723865	0.004569187
0.844896138	0.008276922
0.868820667	0.00510269
0.84813118	0.007789689
0.868744373	0.005111292
0.850362837	0.007464485
0.847300947	0.007912938
0.878073394	0.00412716
0.800646365	0.016963384
0.847866774	0.007828807
0.827549398	0.011220268
0.881188989	0.003828117
0.87767005	0.004166938
0.834311306	0.01000525
0.841707408	0.008775749
0.800220251	0.017066531
0.810454369	0.014696294
0.82547462	0.011610893
0.851335406	0.007325517
0.882943869	0.003666011
0.83462429	0.009951136
0.883543491	0.003611654
0.849287987	0.007620012
0.810432613	0.014701098
0.816800237	0.013336797
0.919026494	0.001247998
0.803127468	0.016370554
0.849559426	0.007580543
0.868884504	0.0050955
0.822760999	0.01213462
0.863923967	0.005673812
0.820786357	0.012524944
0.811295867	0.014511227
0.804897189	0.015955748
0.80374366	0.016225366
0.959160268	0.000165116

0.818704724	0.012944898
0.896957397	0.002528183
0.891517937	0.002937597
0.805144966	0.015898204
0.881000221	0.003845824
0.841189265	0.008858565
0.900804877	0.002262184
0.826813936	0.011357768
0.845317721	0.008212358
0.810725272	0.014636554
0.863107085	0.005772894
0.907856405	0.001823176
0.956690192	0.000196555
0.808824062	0.015059047
0.872452438	0.004703902
0.807283044	0.015407058
0.843743384	0.00845511
0.845704019	0.00815348
0.807500303	0.015357691
0.802152038	0.016602045
0.83413887	0.010035143
0.855307698	0.006775096
0.81416738	0.013890768
0.818085551	0.013071501
0.829744577	0.01081614
0.829042077	0.010944448
0.867305636	0.00527525
0.931972325	0.000747431
0.845762849	0.008144537
0.805536628	0.015807509
0.864845634	0.005563337
0.812877655	0.014167333
0.854145408	0.006933312
0.82428664	0.011838376
0.810661256	0.014650657
0.81911689	0.012861052
0.819951892	0.012692242
0.854642451	0.006865367
0.870573103	0.00490766
0.820376158	0.012607008
0.807265341	0.015411085
0.800023079	0.017114392
0.821042478	0.012473876
0.816053927	0.013492384
0.856974721	0.006552231
0.815899968	0.013524623
0.857945681	0.006424612
0.835024416	0.009882227
0.878082454	0.004126269
0.802007258	0.016636578
0.908833504	0.001767127

0.861854017	0.005927025
0.811858833	0.014388238
0.903308034	0.00209929
0.80682224	0.015512094
0.838258922	0.009336281
0.838599801	0.009279888
0.800000727	0.017119823
0.836903036	0.009562741
0.895604074	0.002626342
0.901985824	0.002184349
0.834406316	0.009988804
0.812289596	0.014294576
0.823764682	0.011939207
0.830091476	0.010753134
0.832161605	0.010381973
0.847379208	0.007901267
0.806036592	0.015692209
0.865829468	0.005446943
0.812399149	0.014270817
0.873347819	0.004608769
0.824446142	0.011807671
0.952774227	0.000254078
0.813047945	0.01413062
0.896930099	0.002530139
0.834611177	0.0099534
0.810852647	0.014608518
0.861904323	0.005920787
0.804352283	0.016082758
0.865458667	0.005490626
0.851275861	0.007333977
0.9001894	0.002303453
0.835155666	0.009859689
0.844248176	0.008376784
0.875780821	0.004356539
0.816825628	0.013331524
0.962762237	0.000125511
0.823698401	0.01195205
0.875321388	0.00440347
0.879988849	0.003941595
0.823899567	0.011913098
0.830769539	0.010630652
0.813205361	0.014096735
0.84990561	0.007530394
0.844455838	0.008344697
0.853273213	0.007053575
0.894171596	0.002732896
0.871561766	0.004799777
0.946686506	0.00036385
0.948558092	0.000327328
0.800642252	0.016964378
-0.81002593	0.014791086

0.829843521	0.010798146
0.85381341	0.006978934
0.859948158	0.006166462
0.859074473	0.006278261
0.813713074	0.013987797
0.834272683	0.010011941
0.928166509	0.000877454
0.861076117	0.006024021
0.874941826	0.004442487
0.831043303	0.010581452
0.806441069	0.015599317
0.946477056	0.000368096
0.826733887	0.011372798
0.840159297	0.009024658
0.839035034	0.009208202
0.940913439	0.000493127
0.805708528	0.015767807
0.802970111	0.01640776
0.886254132	0.003372419
0.896313906	0.002574555
0.897794902	0.002468645
0.820975065	0.012487305
0.875114202	0.00442474
0.880487144	0.00389422
0.818960726	0.01289278
0.972647011	5.01E-05
0.944198787	0.000416405
0.94627893	0.000372143
-0.847981632	0.007811799
0.818965137	0.012891883
0.800933242	0.016894162
0.888811171	0.003156349
0.841687918	0.008778856
0.879273534	0.004010251
0.827045023	0.01131445
0.827398837	0.01124833
0.839098454	0.009197786
0.829954028	0.01077807
0.928131044	0.000878731
0.905034959	0.001991473
0.819070816	0.012870408
0.836969137	0.009551621
0.854533434	0.006880232
0.822240233	0.012236802
0.852469504	0.007165566
0.878800869	0.004056036
0.838759959	0.009253468
0.800743163	0.016940007
0.871615112	0.004794
0.804216743	0.016114448
0.837224424	0.009508751

0.827507496	0.011228073
0.807243943	0.015415953
0.882797897	0.003679323
0.875545204	0.004380567
0.811886311	0.014382252
0.879709125	0.003968352
-0.816860199	0.013324346
0.874701381	0.004467318
0.906317234	0.00191379
0.82194382	0.012295204
0.904419661	0.002029465
0.826054871	0.011500795
0.965556741	9.95E-05
0.836832643	0.009574593
0.809952259	0.014807424
0.862923503	0.005795313
0.807521403	0.015352902
0.813328683	0.014070225
0.856364191	0.006633301
0.804246902	0.016107393
0.802227974	0.016583951
-0.826936483	0.011334784
0.890301228	0.003034671
0.824693561	0.011760141
0.834939659	0.009896798
0.841635287	0.008787247
0.80901742	0.015015733
0.873777747	0.004563533
0.848029852	0.007804665
0.803443372	0.016296019
0.806496143	0.015586695
0.837812483	0.009410465
0.923325896	0.001063093
0.83856976	0.009284849
0.865371287	0.005500952
0.857938886	0.0064255
0.849921107	0.007528154
0.831285536	0.010538039
0.846694112	0.008003804
0.81663996	0.013370115
0.827995539	0.011137374
0.811996222	0.014358324
0.804981887	0.015936063
0.820248604	0.012632595
0.804771423	0.015985006
0.859003842	0.006287355
0.919927239	0.00120765
0.814111412	0.013902698
0.859255731	0.006254961
0.827076554	0.011308548
0.868477643	0.005141438

0.807210505	0.015423563
0.858637989	0.006334597
0.886279285	0.003370249
0.812992096	0.014142654
0.834965348	0.00989238
0.878364623	0.004098592
0.812826395	0.014178396
0.819414616	0.0128007
0.859524667	0.006220492
0.868097365	0.005184614
0.829203188	0.010914938
0.853097677	0.007077938
0.869686544	0.005005716
0.805159152	0.015894913
-0.829956949	0.01077754
0.835952997	0.009723476
0.857891083	0.006431746
0.813911676	0.013945328
0.810796022	0.014620977
0.896046698	0.002593971
0.844711006	0.008305376
0.842477739	0.008653539
0.893721819	0.002766919
0.811530411	0.014459907
0.876145005	0.004319567
0.821998477	0.012284422
-0.850472391	0.007448748
0.812582254	0.014231162
0.834557235	0.009962714
0.820865035	0.012509242
0.88139081	0.003809244
0.814822614	0.013751574
0.806894004	0.015495706
0.829142928	0.01092597
0.853052795	0.007084177
0.84788996	0.007825371
0.821894228	0.012304992
0.826652944	0.011388009
0.812123656	0.014330611
0.875972867	0.004337017
0.801332235	0.016798181
0.804009676	0.016162938
0.85575372	0.006715
0.813033283	0.014133779
0.804199159	0.016118562
0.861427128	0.005980129
0.838737607	0.009257152
0.815823972	0.013540554
0.899608314	0.002342861
0.885732412	0.003417645
0.815978527	0.013508167

0.820845783	0.012513083
0.847696126	0.007854119
-0.821663082	0.01235068
0.818755507	0.012934549
0.856634617	0.006597313
0.824580252	0.011781893
0.882112026	0.003742288
0.825571775	0.011592412
0.810971379	0.014582415
0.825309455	0.011642353
0.869936049	0.004977994
0.832091451	0.010394417
0.815412581	0.013626999
0.805879533	0.015728372
0.926890552	0.00092414
0.816690743	0.013359553
0.809100628	0.014997117
0.855558515	0.006741259
0.83751595	0.009459945
0.891807079	0.002914827
0.938013554	0.000568091
0.841543496	0.008801894
0.855816782	0.006706531
0.845641315	0.008163019
0.801109612	0.016851692
0.86935544	0.005042657
0.831977963	0.010414566
0.849436104	0.007598458
0.816596985	0.013379058
0.807828665	0.015283268
0.814919412	0.013731085
0.847535849	0.007877941
0.893238127	0.002803813
0.814910352	0.013733002
0.820801497	0.012521921
0.887274206	0.003285115
0.838487506	0.009298441
0.865006268	0.005544225
0.850461125	0.007450365
0.870243013	0.004944024
0.80986762	0.014826209
0.875801504	0.004354434
0.853042483	0.00708561
0.831944942	0.010420433
0.908848703	0.001766264
0.816223681	0.013456895
0.818363905	0.01301449
0.904389083	0.002031365
0.973384917	4.62E-05
0.853069842	0.007081807
-0.823570549	0.011976848

0.80995971	0.014805771
-0.801333964	0.016797766
0.814326942	0.01385679
0.815890014	0.013526709
0.83780551	0.009411626
0.942240953	0.000461099
0.985188365	8.03E-06
0.871654272	0.004789761
0.808967113	0.015026994
0.818407476	0.01300558
0.894916594	0.002677137
0.805155516	0.015895757
0.877834976	0.004150643
0.909058273	0.001754394
0.856448829	0.006622024
0.914793134	0.001449401
0.899335921	0.002361483
0.801712036	0.016707135
0.871641278	0.004791167
0.808976769	0.015024832
0.841577232	0.008796509
0.810078681	0.014779394
0.821472168	0.012388496
0.814586341	0.013801664
0.836240172	0.00967471
0.860535741	0.006091995
0.852879047	0.007108358
0.858971953	0.006291464
0.870178342	0.004951168
0.984078407	9.97E-06
0.817893624	0.013110903
0.813165307	0.014105352
0.877936304	0.004140652
0.877036452	0.00422992
0.810781896	0.014624087
0.906970918	0.001874957
0.93359369	0.000696118
0.841042578	0.0088821
0.819155216	0.012853273
0.831227064	0.010548508
0.851073325	0.007362801
0.831224799	0.010548914
0.907309115	0.001855069
0.874738693	0.004463459
0.833921671	0.010072877
0.918180406	0.001286688
0.841269612	0.00884569
0.803876698	0.016194126
0.835764706	0.009755534
0.803283274	0.016333766
0.923134506	0.001070915

0.851622462	0.007284818
0.915273547	0.001425552
0.836408675	0.009646169
0.894078851	0.002739889
0.803613424	0.016255985
0.826549232	0.011407517
0.80315423	0.016364231
0.874789119	0.004458247
0.97072047	6.14E-05
0.930021346	0.000812381
-0.847308099	0.007911871
0.840454996	0.008976773
0.806587636	0.015565741
0.876110673	0.004323044
0.804840207	0.015969
0.816062033	0.013490688
0.822644114	0.012157507
0.837306917	0.009494924
0.853693306	0.006995485
0.865817964	0.005448295
0.864515305	0.005602772
0.811490774	0.014468572
0.955035448	0.000219679
0.888446987	0.00318656
0.925696909	0.000969257
0.916848302	0.001349174
0.806770265	0.015523969
-0.960695684	0.000147357
-0.80900687	0.015018094
-0.81471616	0.013774128
0.827765882	0.011179996
0.807755232	0.015299892
0.860618711	0.006081526
0.868732929	0.005112583
0.809888244	0.01482163
0.954214752	0.000231784
0.872285247	0.004721804
0.899059713	0.002380464
0.902286112	0.002164839
0.838957429	0.009220958
0.81535089	0.013639992
0.938613057	0.000552021
0.872992873	0.004646332
0.811500311	0.014466487
0.920407057	0.00118651
0.934977353	0.0006542
0.852358103	0.007181178
0.814692497	0.013779145
0.878174722	0.004117206
0.855184674	0.006791732
0.839089036	0.009199332

0.86735189	0.005269928
0.922998726	0.001076488
0.855166376	0.006794209
0.821946859	0.012294604
0.829910994	0.010785885
0.900962412	0.002251699
0.87959075	0.00397971
0.920094609	0.001200248
0.882904232	0.003669623
0.849749029	0.00755305
0.859884799	0.006174526
0.816473067	0.013404864
0.85565424	0.006728374
-0.80722338	0.015420633
0.832132518	0.010387131
0.859749317	0.006191793
0.826980412	0.011326551
0.949382842	0.000312032
0.871008277	0.004859984
0.959386468	0.000162416
0.816871643	0.01332197
0.877779067	0.004156162
0.908160925	0.001805586
0.806809843	0.015514926
0.972813368	4.92E-05
0.8019104	0.016659706
0.836051524	0.009706727
0.800029576	0.017112814
0.881375313	0.003810691
0.862519741	0.005844815
0.845906258	0.008122763
0.838421166	0.009309413
0.836885691	0.009565661
0.816698909	0.013357855
0.816243649	0.013452724
0.855605006	0.006734999
0.81080246	0.014619561
0.955659509	0.000210759
0.842687964	0.008620377
0.866959751	0.005315164
0.8583498	0.006371969
0.862636507	0.005830472
0.830283582	0.010718342
0.930037022	0.000811845
0.814979374	0.013718403
0.838505268	0.009295505
0.88245517	0.0037107
0.868173718	0.005175926
0.872645855	0.004683246
0.895932376	0.002602307
0.814151049	0.013894248

0.845290363	0.008216538
0.885297537	0.003455639
0.810253501	0.014740688
0.833489299	0.01014826
0.805058241	0.01591833
0.850999236	0.007373363
0.833382905	0.010166865
0.853306711	0.007048932
0.837768793	0.009417744
0.893634439	0.002773561
0.819616437	0.012759891
0.874760211	0.004461235
0.815004468	0.013713098
0.805174768	0.015891291
0.845637202	0.008163645
0.8674137	0.00526282
0.805666745	0.015777451
0.937714458	0.000576222
0.846784949	0.00799016
0.926941276	0.000922254
0.922441661	0.001099549
0.917552173	0.001315916
0.842003167	0.008728699
0.841363549	0.008830654
0.893684804	0.002769731
0.802278042	0.016572027
0.826952815	0.011331723
0.956172109	0.000203613
0.817152202	0.013263815
0.825432777	0.011618858
0.909867704	0.001709036
0.926506996	0.000938484
0.805105567	0.015907345
-0.832059562	0.010400076
0.813515306	0.014030168
0.818993807	0.012886055
0.855749786	0.006715529
0.902277529	0.002165395
0.8026613	0.016480932
0.811837912	0.014392797
0.827208161	0.011283933
0.813172042	0.014103903
-0.830497742	0.010679641
0.859537482	0.006218853
0.810272038	0.014736588
0.911993802	0.001593545
0.82604444	0.011502768
0.852268815	0.007193707
0.812809467	0.014182051
0.821893871	0.012305063
-0.88091886	0.003853473

0.80695796	0.015481111
0.841371715	0.008829347
0.879767537	0.003962755
0.813097656	0.014119914
0.832845032	0.010261249
0.857793927	0.006444453
0.885659516	0.003423995
0.804227769	0.016111869
0.823367536	0.01201629
0.822541356	0.012177651
0.861035526	0.00602911
-0.806480169	0.015590355
0.835028291	0.009881561
0.827644587	0.011202549
0.811971724	0.014363655
0.819926381	0.012697378
0.939176857	0.000537182
-0.853404582	0.007035376
0.821768403	0.012329849
0.831613541	0.010479434
0.834364414	0.009996055
0.821909428	0.012301992
0.814720035	0.013773307
0.804621577	0.01601991
0.905695915	0.00195118
0.832282901	0.010360482
0.817352176	0.013222462
0.818209887	0.013046016
0.851313114	0.007328683
0.855369627	0.006766731
0.934241474	0.000676281
0.850952446	0.007380038
0.840117335	0.009031466
0.848829448	0.007686984
0.828845084	0.0109806
0.813078344	0.014124072
0.852163196	0.007208545
0.823574722	0.011976038
0.83056134	0.010668165
0.885258555	0.003459058
0.804565549	0.016032973
0.811296284	0.014511135
0.920793712	0.00116965
0.871841967	0.004769481
0.883296907	0.003633944
0.872056603	0.004746357
0.848432422	0.007745274
0.848590672	0.007722007
0.834637702	0.009948822
0.867063224	0.005303203
0.874701858	0.004467269

0.95341152	0.000244048
0.851403952	0.007315785
0.85615629	0.006661052
0.882393539	0.003716361
0.885222733	0.003462202
0.846381843	0.00805082
0.820873141	0.012507625
0.837473452	0.00946705
0.829995871	0.010770475
0.824156284	0.011863507
0.857722878	0.006453755
0.807416558	0.015376708
0.903518319	0.002085963
0.849183321	0.007635266
0.811977923	0.014362306
0.854555368	0.00687724
0.804918706	0.015950746
0.871917188	0.004761369
0.89155817	0.002934422
0.812048376	0.014346978
0.955012381	0.000220013
0.879800558	0.003959593
0.879553378	0.0039833
0.900148153	0.002306236
0.832421243	0.010336004
0.832996607	0.010234595
0.813862562	0.013955823
0.825805783	0.011547976
0.884157717	0.003556515
0.834955275	0.009894113
0.873457193	0.004597234
0.862964511	0.0057903
0.878738761	0.004062077
0.935464025	0.000639859
0.83447808	0.009976392
0.827413201	0.011245651
0.818708718	0.012944084
0.803001165	0.016400413
0.856419623	0.006625914
0.82884866	0.010979943
0.84770745	0.007852438
0.806193233	0.015656194
0.907629967	0.001836328
0.8458305	0.008134261
0.905622602	0.001955623
0.90667665	0.001892374
0.879218519	0.004015562
0.904017448	0.002054552
0.884409249	0.003534093
0.89852953	0.002417175
0.907388568	0.001850417

0.812500715	0.014248812
0.848784506	0.007693568
0.827654421	0.011200719
0.825970709	0.011516723
0.829124987	0.010929255
0.803331435	0.016322405
-0.814440906	0.013832554
0.822431862	0.012199138
0.819550812	0.012773151
0.828139365	0.011110733
0.865891814	0.00543962
0.822894931	0.012108428
0.835427999	0.009813029
0.812013149	0.014354641
0.869345129	0.00504381
0.813663721	0.013998363
0.837652922	0.009437069
0.836071432	0.009703345
0.823273063	0.012034673
0.822511137	0.012183579
0.821239829	0.012434616
0.801664293	0.016718563
0.879390895	0.003998934
0.822897136	0.012107997
0.818487287	0.012989269
0.800218463	0.017066965
-0.823758304	0.011940443
0.811058164	0.014563354
0.810039222	0.014788139
0.8061046	0.015676566
0.848966181	0.007666974
0.879609704	0.00397789
0.837414145	0.009476971
0.867462158	0.005257252
0.829761863	0.010812995
0.82300365	0.012087193
0.853415608	0.00703385
0.807400823	0.015380283
0.882459819	0.003710274
0.842832386	0.008597642
0.810056865	0.014784228
0.933454871	0.000700419
0.811832607	0.014393953
0.905938745	0.001936511
0.886023581	0.003392357
0.84353441	0.008487672
0.857876778	0.006433616
0.803786159	0.016215383
0.891898811	0.002907627
0.895954192	0.002600715
0.839506865	0.009130886

0.874689162	0.004468583
0.820448816	0.012592447
0.909931779	0.001705478
0.910580575	0.001669726
0.80545783	0.01582573
0.818637609	0.012958583
0.843607724	0.008476239
0.905382991	0.00197019
0.852063239	0.007222606
0.847376287	0.007901702
0.89904213	0.002381676
0.830422342	0.010693257
0.860821366	0.006056005
0.863707364	0.005699978
0.809005678	0.015018361
0.911839843	0.001601732
0.808529854	0.015125103
0.91362685	0.001508376
0.887052774	0.003303941
0.878684282	0.004067381
0.981396616	1.59E-05
0.907874167	0.001822147
0.819101274	0.012864222
0.863242209	0.005756429
0.8272506	0.011276003
0.834507883	0.009971241
0.910399318	0.001679665
0.824173272	0.01186023
0.898487866	0.002420075
0.814316332	0.013859048
0.811436772	0.014480382
0.890021443	0.003057283
0.89611876	0.002588725
0.838868558	0.00923558
0.857852936	0.006436733
0.945942342	0.000379084
0.801944494	0.016651563
0.849053085	0.007654274
0.800834835	0.016917887
0.843003154	0.008570809
0.841251194	0.008848641
0.842555583	0.00864125
0.831810832	0.010444284
0.850786984	0.007403675
0.921402693	0.001143415
0.805332303	0.015854783
0.844876945	0.008279869
0.911211908	0.001635407
0.829913914	0.010785355
0.812792957	0.014185616
0.840366781	0.008991041

0.821488142	0.012385329
0.832248807	0.01036652
0.933384538	0.000702604
0.838765264	0.009252594
0.863105834	0.005773047
0.800611973	0.016971695
0.85621202	0.006653606
0.822684765	0.012149545
0.861481488	0.00597335
0.817540586	0.013183575
0.866685987	0.005346892
0.896383822	0.00256949
0.82627809	0.011458617
0.808993459	0.015021096
0.90282321	0.002130225
0.832693398	0.010287957
0.87045294	0.004920878
0.839458644	0.009138769
0.821215808	0.012439391
0.832583785	0.010307291
0.904369712	0.00203257
0.836912096	0.009561217
0.855562568	0.006740714
0.894929707	0.002676163
0.872749209	0.004672232
0.917598307	0.001313755
0.900174916	0.00230443
0.829532981	0.010854686
0.902467906	0.002153082
0.82540524	0.011624101
0.824232399	0.011848829
0.840541601	0.008962778
-0.841045737	0.008881593
0.821448267	0.012393236
0.918162584	0.001287511
0.875216365	0.004414244
0.859398961	0.006236588
0.802199662	0.016590696
0.80279696	0.016448762
0.851002991	0.007372828
0.915570736	0.001410928
0.890841305	0.00299133
0.859119594	0.006272455
0.824100614	0.01187425
0.806982458	0.015475523
0.850368202	0.007463714
0.873531699	0.004589386
0.964911342	0.000105182
0.915036738	0.001437276
0.890212953	0.003041794
0.805920959	0.015718829

0.849672914	0.007564079
0.953610539	0.000240971
0.884038091	0.003567211
0.829523206	0.010856469
0.810599506	0.014664269
0.813792646	0.013970771
0.849567354	0.007579392
0.805617809	0.015788752
0.812000155	0.014357468
0.92273885	0.001087206
0.938711762	0.000549404
0.800913215	0.016898989
0.809483767	0.014911589
0.825472176	0.011611358
0.934602737	0.000665381
0.804238617	0.016109331
0.923837066	0.001042382
0.873993397	0.004540951
0.860877216	0.006048984
0.883482158	0.00361719
0.849379241	0.007606728
0.902666867	0.002140263
0.846433103	0.00804309
0.839644849	0.009108354
0.82584691	0.011540177
0.804161906	0.016127281
-0.850438714	0.007453583
0.828254223	0.011089488
0.88726759	0.003285676
0.873011649	0.00464434
0.8191185	0.012860725
0.809899926	0.014819037
0.88918227	0.003125756
0.809055924	0.015007117
0.884769738	0.003502118
0.879056454	0.004031237
0.806737483	0.015531462
0.811153233	0.014542492
0.803986669	0.016168331
0.802293897	0.016568252
0.835563242	0.00978991
0.841167748	0.008862015
0.804730415	0.015994554
0.897723734	0.002473668
0.834202707	0.01002407
0.810459733	0.014695109
0.869350195	0.005043244
0.814639509	0.013790383
0.845881522	0.008126516
0.92932272	0.000836506
0.92091471	0.001164407

0.80411762	0.016137649
0.820713043	0.012539586
0.846304715	0.00806246
0.869222343	0.005057556
0.815746844	0.013556734
0.861395836	0.005984033
0.864505708	0.00560392
0.815963209	0.013511374
0.945816159	0.000381708
0.952446222	0.000259344
0.891053617	0.002974402
0.924166143	0.00102919
0.838766754	0.009252348
0.85204792	0.007224762
0.877544463	0.004179374
0.808946431	0.015031626
0.851653099	0.007280483
0.820848763	0.012512489
0.830579102	0.010664962
0.843667924	0.008466859
0.803591847	0.016261061
0.892098427	0.002891999
0.856259108	0.006647318
0.830088735	0.010753631
0.811914921	0.014376021
0.882847488	0.003674797
0.829866111	0.01079404
0.817181528	0.013257746
0.803947926	0.016177416
0.90206337	0.0021793
0.821972072	0.01228963
0.842705905	0.00861755
0.819766462	0.012729608
0.815677047	0.013571387
0.833921373	0.010072929
0.830797732	0.010625578
0.816881359	0.013319954
0.931516945	0.000762275
0.815691769	0.013568296
0.818410635	0.013004934
0.879376888	0.004000283
0.815904021	0.013523773
-0.8492226	0.007629539
0.843223691	0.008536234
0.93324995	0.000706798
0.850174606	0.007491574
0.807976425	0.015249852
0.836405814	0.009646653
0.856939435	0.006556899
0.835671902	0.00977136
0.867707253	0.005229148

0.819705248	0.012741959
0.80259037	0.016497767
0.816089392	0.013484965
0.878240108	0.00411079
0.822969496	0.012093862
0.812964439	0.014148616
0.895626247	0.002624714
0.828443527	0.011054528
0.906623781	0.001895515
0.899143338	0.002374707
0.810931265	0.014591231
0.894183815	0.002731975
0.867414832	0.00526269
0.933509588	0.000698722
0.855901599	0.006695151
0.844127178	0.008395517
0.821334481	0.012415814
0.806286752	0.015634716
0.816583037	0.013381961
0.855626106	0.006732159
0.842700303	0.008618433
0.85181278	0.007257914
0.801681817	0.016714368
0.807091236	0.015450724
0.946341932	0.000370853
0.820784032	0.012525408
0.829094172	0.010934901
0.806409299	0.0156066
0.855631888	0.006731381
-0.90489316	0.002000187
0.877163529	0.00421724
-0.82132107	0.012418477
0.849691331	0.00756141
0.845873654	0.00812771
0.825880527	0.011533805
0.809091449	0.01499917
0.800812066	0.01692338
0.810443699	0.01469865
0.833035886	0.010227695
0.895082593	0.002664815
0.882648826	0.00369295
0.858410239	0.00636412
0.82626909	0.011460316
0.806145251	0.01566722
0.819134355	0.012857507
0.816046715	0.013493893
0.833859622	0.010083673
0.884045124	0.003566582
0.818025529	0.013083815
0.802227557	0.01658405
0.802591145	0.016497583

0.806759417	0.015526448
0.804829836	0.015971413
0.854383707	0.006900683
0.857124686	0.006532416
0.882695198	0.003688707
0.850993931	0.00737412
0.840062082	0.009040436
0.861201942	0.006008264
0.885339916	0.003451925
0.848803759	0.007690747
0.869887948	0.004983331
0.911066055	0.001643295
0.804308653	0.016092954
0.872218788	0.004728932
0.909480691	0.001730627
0.907399774	0.001849761
0.917093396	0.001337532
0.860853136	0.006052011
0.860906422	0.006045314
0.870125771	0.004956981
0.811416805	0.01448475
0.820038319	0.012674849
0.92580235	0.000965215
0.846221745	0.008074993
0.879445314	0.003993694
0.816015482	0.01350043
0.859765708	0.006189702
0.840789855	0.008922741
0.829920173	0.010784218
0.832394958	0.010340652
0.803928137	0.016182057
0.806862056	0.015503
0.805778205	0.015751731
0.804767251	0.015985977
0.86517024	0.00552476
0.814286947	0.013865302
0.899303615	0.002363698
0.837637126	0.009439705
0.819193661	0.012845473
0.871369183	0.004820671
0.8125332	0.014241779
0.923093379	0.001072601
0.850605845	0.007429606
0.870885611	0.004873393
0.858261645	0.006383429
0.864544332	0.005599299
0.856070399	0.006672539
0.853170395	0.007067839
0.875100672	0.004426132
0.843275845	0.00852807
0.888656974	0.003169118

0.857403219	0.006495713
0.849770665	0.007549917
0.860568643	0.006087842
0.844361365	0.008359285
0.822049141	0.012274433
0.974258721	4.18E-05
0.932818353	0.000720358
0.873448551	0.004598144
0.850002527	0.007516393
0.863594651	0.005713624
0.855536938	0.006744166
0.860147715	0.006141106
0.938872814	0.000545151
0.93646872	0.000610908
0.877858996	0.004148273
0.921426237	0.001142409
0.914789081	0.001449603
0.875492096	0.004385995
0.91615653	0.001382389
0.908995032	0.001757971
0.907535255	0.001841847
0.886580169	0.003344354
0.889968216	0.003061597
0.897925556	0.002459439
0.892898858	0.00282988
0.90952754	0.001728004
0.914612949	0.001458412
0.930437088	0.000798243
0.884988904	0.003482769
0.906091928	0.001927294
0.869830072	0.004989757
0.922115922	0.001113181
0.800630867	0.016967129
0.922900498	0.001080531
0.890205324	0.00304241
0.900420189	0.002287922
0.848713517	0.007703976
0.806489527	0.015588211
0.80712992	0.015441911
0.830455124	0.010687336
0.805909514	0.015721465
0.858962417	0.006292693
0.853219688	0.007060998
0.842222214	0.008693956
0.847840488	0.007832702
0.805516899	0.01581207
0.84307766	0.008559118
0.830674767	0.010647717
0.823200345	0.012048835
0.853619158	0.007005716
0.819124103	0.012859588

0.818356216	0.013016062
-0.802982271	0.016404883
0.880127966	0.003928332
0.829877853	0.010791906
0.868672669	0.005119385
0.851849139	0.007252782
0.881681681	0.003782148
0.808467567	0.015139111
0.815660119	0.013574942
0.810123682	0.014769424
0.825108647	0.011680674
0.912932515	0.001544216
0.807005167	0.015470344
0.85332948	0.007045777
0.871335328	0.00482435
0.810921967	0.014593275
0.862768769	0.005814252
0.804765463	0.015986394
0.81967783	0.012747493
0.889281631	0.003117597
0.860809445	0.006057505
0.870123684	0.004957212
0.915681481	0.001405503
0.815369129	0.01363615
0.844967782	0.008265927
0.815801859	0.013545191
0.858352184	0.006371659
0.802082837	0.016618546
0.825513303	0.011603532
0.849241018	0.007626855
0.805834174	0.015738826
0.834811985	0.009918774
0.928872883	0.000852286
0.802292407	0.016568607
0.840718329	0.008934265
0.832989752	0.010235799
0.907950878	0.001817707
0.832763553	0.010275595
0.91656357	0.001362781
0.883833289	0.003585571
0.809998572	0.014797152
0.816416502	0.013416654
0.862740636	0.005817699
0.807783186	0.015293562
0.879642606	0.003974732
0.863914967	0.005674898
0.833783746	0.010096885
0.805671453	0.015776364
0.806656063	0.015550082
0.880862951	0.003858734
0.837991774	0.009380627

0.830688477	0.010645248
0.847802818	0.007838287
0.805892408	0.015725406
0.918057799	0.001292359
0.860587716	0.006085436
0.815386415	0.013632509
0.869025946	0.005079592
0.852017939	0.007228984
0.842810571	0.008601073
0.836004794	0.009714669
0.811726391	0.014417113
0.902162373	0.002172865
0.918601811	0.001267321
0.864954889	0.005550334
0.805020332	0.015927133
0.848244548	0.007772955
0.801761866	0.016695213
0.813409686	0.01405283
0.833926022	0.01007212
0.828312874	0.011078649
0.810810685	0.014617751
0.92788738	0.000887532
0.893244624	0.002803316
0.84922719	0.00762887
0.833177209	0.010202894
0.810951889	0.014586698
0.805706441	0.015768288
0.828347981	0.011072164
0.822388589	0.012207637
0.825610697	0.011585014
0.899115443	0.002376626
0.801667094	0.016717892
0.807971537	0.015250957
0.840132892	0.009028942
0.823763907	0.011939357
0.934707105	0.000662253
0.821350694	0.012412596
0.900330245	0.002293966
0.805020511	0.015927091
-0.823117673	0.012064948
0.839743197	0.009092316
0.878484011	0.004086917
0.9217242	0.001129721
0.808767796	0.015071666
0.814523697	0.013814964
0.840722561	0.008933583
0.879281402	0.004009491
0.864862382	0.005561343
0.852675617	0.007136739
0.857746422	0.006450671
0.801181257	0.01683446

-0.82185328	0.012313078
0.821167111	0.012449073
0.838884652	0.009232931
0.818362713	0.013014733
0.827296376	0.011267453
0.82464993	0.011768514
0.802523553	0.016513636
0.855690658	0.006723476
0.800656319	0.016960979
0.802637339	0.016486618
0.936963916	0.000596961
0.83837086	0.009317739
0.957439661	0.000186633
0.859449029	0.006230174
0.801521242	0.016752834
0.864474714	0.00560763
0.872926712	0.004653355
0.834856629	0.009911086
0.853644133	0.007002269
-0.910724998	0.001661834
0.812179267	0.014318529
0.859319568	0.006246768
0.83820951	0.009344473
0.895490229	0.00263471
0.832059026	0.010400171
0.805202782	0.015884795
0.856408179	0.006627438
0.818164229	0.013055371
-0.878281236	0.004106759
-0.842312455	0.008679669
0.821794391	0.012324713
0.822800756	0.012126842
-0.858831406	0.006309593
0.908459902	0.001788424
0.931236327	0.000771518
0.867099226	0.005299046
0.800489306	0.017001357
0.843079984	0.008558753
0.816718996	0.013353679
0.829956651	0.010777594
0.812624037	0.014222123
0.800532877	0.016990817
0.817060053	0.013282898
0.822384	0.012208539
0.845180213	0.008233381
0.853660464	0.007000015
0.915647149	0.001407184
0.843297601	0.008524666
0.893347621	0.002795434
0.834402084	0.009989536
0.885065317	0.003476039

0.902629316	0.002142679
0.860780537	0.006061142
0.856106818	0.006667667
0.885688722	0.00342145
0.837468028	0.009467957
-0.850715935	0.007413839
0.866864502	0.005326189
0.801264107	0.016814545
0.802323759	0.016561144
0.819378495	0.012808013
0.922293782	0.001105724
0.839122295	0.009193872
0.807615697	0.015331511
-0.816806138	0.013335572
0.854149818	0.006932707
0.82658875	0.011400081
0.823306262	0.012028211
0.825356126	0.011633458
0.93513298	0.000649591
0.876550496	0.00427864
0.818895042	0.012906139
0.823327959	0.012023989
0.925167561	0.000989717
0.849091768	0.007648625
0.817621529	0.01316689
0.839277685	0.009168389
0.817592621	0.013172847
0.838463485	0.009302413
0.819607258	0.012761745
0.871840835	0.004769603
0.808472514	0.015137998
0.86000669	0.006159018
0.849186301	0.007634832
0.837667286	0.009434672
0.843561947	0.008483376
0.82593751	0.01152301
0.879575253	0.003981199
0.83486551	0.009909557
0.858084381	0.006406513
0.866381168	0.005382362
-0.841198146	0.008857141
-0.838530064	0.009291407
-0.899989843	0.002316938
-0.802304208	0.016565797
-0.848132551	0.007789487
-0.929660857	0.000824772
-0.907800794	0.001826401
-0.820510983	0.012579997
-0.812005639	0.014356275
-0.82367903	0.011955805
-0.81298542	0.014144093

-0.947016418	0.000357227
-0.919417262	0.001230388
0.89333427	0.002796455
0.876939654	0.004239596
0.864918172	0.005554701
0.890424192	0.003024768
0.913547754	0.001512431
0.912441313	0.001569902
0.908549726	0.001783288
0.918076932	0.001291473
0.91782707	0.001303074
0.875683844	0.004366418
0.817195773	0.013254798
0.827134609	0.011297686
0.80578202	0.015750851
0.89681375	0.002538487
-0.939596117	0.000526319
0.875008166	0.004435652
0.803839266	0.016202912
0.910215378	0.00168979
0.803643763	0.016248849
0.896214604	0.002581759
0.812451422	0.014259489
0.801200151	0.016829917
0.818704486	0.012944946
-0.844534636	0.008332542
-0.869032502	0.005078855
-0.86612612	0.005412156
-0.863044143	0.005780574
-0.865945756	0.005433289
-0.823726833	0.01194654
-0.820682347	0.01254572
-0.925492465	0.000977126
-0.860670805	0.006074959
-0.802133501	0.016606464
0.897665262	0.002477801
0.877646983	0.00416922
0.870090127	0.004960924
0.941740096	0.000473017
0.891486168	0.002940105
0.823879838	0.011916915
0.812454224	0.014258882
0.812468231	0.014255848
0.813895047	0.01394888
0.807828963	0.015283201
0.821042895	0.012473793
0.815922618	0.013519877
0.825705767	0.011566955
0.828508496	0.011042545
0.80686897	0.015501422
0.806418359	0.015604523

0.806415915	0.015605083
0.861411631	0.005982062
-0.838043094	0.009372098
0.861469686	0.005974821
-0.883151412	0.003647138
0.84687674	0.007976388
0.820896327	0.012503001
0.803081751	0.016381358
0.845463693	0.008190078
0.810318649	0.01472628
0.86836493	0.005154211
0.910449862	0.001676889
0.887015522	0.003307115
0.891209424	0.002962018
0.82288003	0.012111341
0.870365798	0.004930477
0.87825042	0.004109779
0.830769658	0.01063063
0.892030954	0.002897276
0.81421119	0.013881433
0.851931512	0.007241162
0.859622896	0.006207933
0.834421337	0.009986205
0.834646046	0.009947382
0.804011106	0.016162603
0.857118905	0.006533179
0.842583299	0.008636877
0.821873009	0.012309182
0.888626754	0.003171624
0.817961812	0.013096895
0.804445803	0.016060915
0.814227998	0.013877853
0.863919675	0.00567433
0.832702696	0.010286318
0.918438256	0.001274815
0.824256957	0.011844095
0.826303482	0.011453826
0.830843449	0.010617355
0.800037265	0.017110946
0.807076156	0.015454161
0.806091368	0.015679609
0.869022012	0.005080034
-0.812454164	0.014258895
0.845376611	0.008203365
0.80487138	0.01596175
-0.824198961	0.011855276
0.82053411	0.012575368
0.827559888	0.011218314
0.814821661	0.013751776
0.87428838	0.004510178
0.859588802	0.00621229

0.858588338	0.006341025
-0.857202888	0.006522098
0.826813459	0.011357858
0.888702333	0.003165358
0.862409711	0.005858352
0.855335474	0.006771343
0.877022862	0.004231278
0.820548773	0.012572433
0.837175548	0.009516949
0.891408741	0.002946226
0.843223214	0.008536308
0.812593997	0.014228621
0.830939293	0.010600127
0.823118091	0.012064866
0.895915389	0.002603547
0.88581723	0.003410266
0.889467239	0.003102394
0.847875118	0.00782757
0.838876426	0.009234285
0.886999011	0.003308522
0.811628103	0.014438565
0.820912361	0.012499803
0.816049993	0.013493208
0.868176579	0.005175601
0.804709315	0.015999467
0.830723524	0.010638935
0.830493152	0.01068047
0.920806706	0.001169087
0.84144187	0.008818129
0.80229944	0.016566932
0.82383275	0.011926027
0.824398458	0.011816845
0.809119999	0.014992786
0.828820586	0.010985101
0.860352159	0.006115199
0.886793196	0.003326099
0.803086102	0.016380329
-0.803022921	0.016395267
0.807238758	0.015417133
0.823734403	0.011945073
0.88209343	0.003744005
0.887978196	0.003225722
0.904370546	0.002032518
0.844586492	0.008324549
0.821626782	0.012357865
0.833184838	0.010201556
0.845223248	0.008226798
0.822259128	0.012233085
0.9453215	0.00039211
0.815114558	0.013689839
0.844809413	0.008290244

0.843088567	0.008557407
0.804821253	0.01597341
0.820148766	0.012652645
0.829697251	0.010824754
0.844626963	0.008318314
0.917275488	0.001328925
0.853893161	0.006967957
0.839082658	0.009200379
0.87589848	0.004344572
0.846631408	0.008013231
0.810111403	0.014772144
0.926991284	0.000920397
0.827419162	0.011244539
0.880243719	0.003917318
0.91244936	0.001569479
0.856258571	0.00664739
0.871727645	0.004781827
0.810703158	0.014641425
0.804299474	0.0160951
0.833324492	0.010177088
0.833456814	0.010153938
0.868416309	0.005148386
0.902802169	0.002131574
0.873903394	0.004550367
-0.804341197	0.016085348
-0.801782727	0.016690223
0.818275273	0.013032626
0.853553951	0.007014721
0.802122831	0.016609008
0.880624592	0.003881217
0.821979284	0.012288207
0.85138309	0.007318746
0.894472182	0.002710309
0.85419023	0.006927167
0.878159702	0.00411868
0.834906399	0.00990252
0.826294363	0.011455546
0.910105765	0.001695842
0.912937522	0.001543955
0.821378529	0.012407071
0.844055533	0.008406621
0.870733738	0.004890027
0.840452433	0.008977187
0.823873162	0.011918207
0.860593975	0.006084646
0.873082638	0.004636814
0.825647473	0.011578026
0.801567018	0.016741862
0.831505597	0.010498698
0.86573863	0.005457623
0.80856508	0.015117184

0.802828312	0.016441333
0.878761947	0.004059821
0.868174195	0.005175872
0.81273818	0.014197448
0.828953147	0.010960759
0.866007864	0.005426006
0.800286353	0.017050505
0.959520638	0.000160828
0.851231337	0.007340308
0.886882424	0.003318471
0.833655119	0.010119308
0.895307183	0.002648201
0.913296819	0.001525343
0.823620915	0.011967075
0.85810113	0.00640433
0.803972244	0.016171713
0.800288618	0.017049956
0.817363799	0.013220061
0.817806542	0.013128805
0.816536367	0.013391678
0.804017723	0.016161052
0.810413003	0.014705429
0.838151634	0.009354074
0.887714148	0.003247917
0.805809915	0.015744419
0.933894694	0.000686854
0.821347356	0.012413258
0.820169747	0.01264843
0.82330507	0.012028443
0.870369494	0.00493007
0.92291671	0.001079863
0.858206093	0.006390658
0.902519584	0.002149748
0.857579112	0.006472604
0.864583671	0.005594596
0.913369179	0.001521612
0.813862681	0.013955797
0.818147242	0.013058852
0.833763659	0.010100385
0.835807502	0.009748242
0.805139959	0.015899365
0.806046307	0.015689974
0.804816663	0.015974478
0.935205996	0.000647436
0.841105103	0.008872063
0.882176816	0.003736311
0.804636061	0.016016534
0.905022562	0.001992234
0.848936856	0.007671263
0.8254565	0.011614341
0.902301669	0.002163831

0.814912915	0.01373246
0.800010026	0.017117564
0.806459665	0.015595054
0.802508116	0.016517304
0.841377974	0.008828346
0.880115271	0.003929541
0.822221577	0.012240472
0.831608295	0.01048037
0.860949934	0.00603985
0.820042551	0.012673998
0.827256083	0.011274978
0.850057125	0.007508513
0.874238372	0.004515385
0.835429549	0.009812764
0.839155138	0.009188482
0.832832396	0.010263473
0.837580502	0.00944916
0.851491332	0.007303392
0.825350106	0.011634605
0.826489687	0.011418726
0.844401181	0.008353135
0.841018915	0.0088859
0.825930893	0.011524263
0.88451308	0.003524864
0.981382489	1.59E-05
0.83507055	0.009874301
0.819022715	0.012880179
0.89722687	0.002508926
0.820021391	0.012678255
0.816779494	0.013341106
0.879220963	0.004015326
0.821644247	0.012354408
0.889148951	0.003128495
0.81108588	0.01455727
0.817546368	0.013182382
0.864483893	0.005606531
0.820924938	0.012497296
0.839703143	0.009098845
0.872209847	0.004729892
0.832850039	0.010260368
0.804822743	0.015973063
0.854548812	0.006878134
0.853242934	0.007057774
0.813817859	0.013965379
0.834389687	0.009991681
0.95243609	0.000259507
0.836223662	0.009677509
0.808126569	0.015215944
0.856928885	0.006558296
0.811595798	0.01444562
0.884414017	0.003533669

0.865528107	0.005482428
0.854219973	0.006923091
0.852291167	0.007190569
0.804135561	0.016133448
0.888920426	0.003147322
0.821522236	0.012378572
0.813989162	0.01392878
0.813007414	0.014139353
0.880392551	0.003903185
0.927696109	0.000894482
0.832891703	0.010253037
-0.847501636	0.007883032
0.867011964	0.005309126
-0.840840399	0.008914603
0.952458024	0.000259153
0.861211717	0.006007041
0.822172999	0.012250033
0.821043968	0.012473579
0.819219828	0.012840165
0.818921387	0.01290078
0.8140347	0.013919061
0.830531359	0.010673574
0.840691447	0.008938598
0.864056468	0.005657844
0.802093804	0.01661593
0.805039465	0.01592269
0.831974983	0.010415095
-0.830784142	0.010628024
-0.812008202	0.014355717
-0.816112995	0.013480029
-0.875157237	0.004420317
-0.837796152	0.009413185
-0.808434248	0.015146608
-0.832382441	0.010342866
-0.845397234	0.008200217
-0.845448494	0.008192396
-0.854922771	0.006827235
-0.878223062	0.004112462
-0.84646827	0.00803779
0.822526395	0.012180586
-0.840746224	0.008929769
-0.822051942	0.01227388
-0.841960549	0.008735469
-0.834412336	0.009987762
0.824943125	0.011712322
-0.851235151	0.007339765
-0.838867247	0.009235796
-0.868854403	0.005098889
-0.894411445	0.002714863
0.813438654	0.014046612
0.913778782	0.001500606

0.825784862	0.011551944
0.80080229	0.016925738
-0.837831914	0.009407228
-0.847821474	0.007835521
-0.846917748	0.00797024
0.864799559	0.005568827
-0.836562514	0.009620158
-0.817224085	0.013248941
-0.822043955	0.012275455
-0.829713523	0.010821792
-0.825128794	0.011676826
-0.813060403	0.014127936
-0.838324428	0.009325427
-0.810256004	0.014740134
-0.857315063	0.006507315
-0.875341475	0.004401412
-0.877418578	0.004191863
-0.822005272	0.012283082
0.850802839	0.007401408
0.827650785	0.011201395
0.827028573	0.011317531
0.920494735	0.001182673
0.803153276	0.016364456
0.882493556	0.003707178
0.827278316	0.011270825
0.834644616	0.009947629
0.881397724	0.003808598
0.81960386	0.012762431
0.801432908	0.016774018
0.810172439	0.014758628
0.828569412	0.011031318
0.946867347	0.00036021
0.830778837	0.010628978
0.843036234	0.008565617
0.817252576	0.013243048
0.850822091	0.007398656
0.848412216	0.007748249
0.839698911	0.009099535
0.968951344	7.31E-05
0.829338968	0.010890106
0.835085094	0.009871803
0.807104707	0.015447655
0.814000368	0.013926388
0.854865372	0.006835032
0.935242534	0.00064636
0.814898193	0.013735575
0.829810619	0.010804127
0.90982455	0.001711435
0.838624597	0.009275795
0.803258777	0.016339546
0.830905437	0.01060621

0.816895008	0.013317121
0.868548512	0.005133417
0.83784467	0.009405104
0.82895869	0.010959742
0.838776588	0.009250727
0.93749088	0.00058235
0.975153685	3.76E-05
0.921746016	0.001128796
0.882967472	0.003663861
0.939746976	0.000522446
0.859458506	0.006228961
0.819365442	0.012810656
0.811506331	0.014465171
0.80765754	0.015322025
0.845243633	0.008223681
0.848673046	0.007709913
0.938786328	0.000547432
0.944740295	0.000404567
0.886188388	0.003378097
0.889953554	0.003062786
0.913080513	0.00153653
0.881218195	0.003825382
0.916077137	0.001386234
0.947417855	0.000349276
0.952156961	0.000264047
0.907480717	0.00184503
0.902869225	0.002127276
0.801109552	0.016851707
0.802414417	0.016539576
0.841188371	0.008858708
0.825299203	0.011644307
0.806925654	0.015488482
0.804239631	0.016109094
0.877984047	0.00413595
0.801091969	0.016855938
0.904227912	0.0020414
0.904615462	0.002017325
0.801576436	0.016739606
0.825293899	0.011645319
0.868832171	0.005101394
0.837198555	0.00951309
0.864971697	0.005548335
0.897174776	0.002512641
0.896286309	0.002576556
0.880433798	0.003899274
0.845254838	0.008221968
0.827095449	0.011305012
0.802656531	0.016482063
0.814869344	0.01374168
0.841740608	0.00877046
0.809804142	0.014840307

0.877867758	0.004147409
0.808459818	0.015140854
0.829690516	0.01082598
0.80422157	0.016113319
0.929364145	0.000835063
0.870302856	0.004937419
0.964116931	0.000112421
0.814967752	0.013720861
0.838115215	0.009360119
0.852057934	0.007223353
0.818689883	0.012947923
0.812313497	0.014289391
0.831894755	0.010429355
0.877151728	0.004218416
0.836991668	0.009547833
0.829906404	0.010786719
0.865769386	0.005454006
0.81390518	0.013946716
0.843171716	0.008544374
0.825215936	0.01166019
0.845034778	0.008255654
0.869813561	0.004991591
0.810956657	0.01458565
0.885466754	0.003440823
0.805115819	0.015904966
0.844596207	0.008323052
0.814997137	0.013714648
0.933495581	0.000699156
0.844910264	0.008274754
0.862493873	0.005847996
0.842499435	0.008650113
0.812467277	0.014256054
-0.821134746	0.012455511
-0.817920089	0.013105465
-0.812610507	0.014225049
-0.806552768	0.015573725
-0.816253185	0.013450733
-0.812039077	0.014349
-0.890636861	0.003007689
-0.808417916	0.015150283
-0.846987545	0.007959783
0.851419389	0.007313595
0.910154343	0.001693158
0.883961499	0.00357407
0.903662562	0.002076854
-0.813203573	0.01409712
-0.834739029	0.009931345
-0.814297915	0.013862967
0.818517029	0.012983194
0.828870416	0.010975947
0.887796462	0.003240987

0.91468817	0.001454646
-0.826869071	0.011347424
0.972390354	5.15E-05
0.826320946	0.011450531
0.829057455	0.010941629
0.946020126	0.000377472
0.825739503	0.011560551
0.891106963	0.002970158
0.894881368	0.002679757
0.850255251	0.007479961
0.842187643	0.008699434
0.899562836	0.002345963
0.818064153	0.01307589
0.89284724	0.00283386
0.854460418	0.006890201
0.850890934	0.00738882
0.842542171	0.008643367
0.873829484	0.004558109
0.800277472	0.017052658
0.821536779	0.01237569
0.811030626	0.014569401
0.842978776	0.008574636
0.830896974	0.010607732
0.852972329	0.007095369
0.80632174	0.015626686
0.942772508	0.000448668
0.816612899	0.013375746
0.819536984	0.012775947
0.802502513	0.016518635
0.84645015	0.00804052
0.848058641	0.007800409
0.826509595	0.011414978
0.936437666	0.00061179
0.824718356	0.011755385
0.868248701	0.005167404
0.845705867	0.008153199
0.836333513	0.009658893
0.851014555	0.007371179
0.810808301	0.014618275
0.845021009	0.008257765
0.859644711	0.006205146
0.80316335	0.016362077
0.959532261	0.000160691
0.801235557	0.016821406
0.846458495	0.008039263
0.851220191	0.007341893
0.844133556	0.008394528
0.867923558	0.005204425
-0.953283489	0.000246042
0.846528053	0.008028784
0.831269145	0.010540973

0.894051194	0.002741977
0.806248188	0.015643571
0.818489432	0.012988831
0.839883089	0.009069533
0.841951132	0.008736965
0.829247952	0.010906747
0.907759905	0.001828774
0.850301147	0.007473356
0.823367596	0.012016279
0.971967697	5.39E-05
0.81128937	0.01451265
0.806788564	0.015519787
0.845071793	0.008249982
0.836422682	0.009643799
0.841318488	0.008837865
0.838190138	0.009347686
0.819721699	0.012738639
0.828168154	0.011105406
0.826088667	0.011494403
0.802036643	0.016629566
0.806114495	0.015674291
0.869235218	0.005056113
0.847785354	0.007840877
0.922360778	0.001102924
0.815561235	0.013595722
0.885564148	0.003432314
0.852184236	0.007205588
0.824932754	0.011714307
0.810634971	0.01465645
0.800350666	0.017034921
0.865879655	0.005441048
0.849711597	0.007558473
0.815788925	0.013547905
0.943436027	0.000433462
0.800114989	0.017092072
0.829556465	0.010850404
0.819452643	0.012793004
0.841921449	0.008741683
0.828697741	0.01100769
-0.830530047	0.010673811
-0.86384356	0.005683516
0.896796405	0.002539733
0.804223776	0.016112803
0.882678747	0.003690212
0.852252781	0.007195958
0.809307814	0.014950829
0.821807146	0.012322192
-0.803971827	0.016171811
0.894356072	0.002719019
0.839438975	0.009141985
0.820678532	0.012546482

0.814910233	0.013733027
0.825118661	0.011678762
0.911590397	0.001615055
0.800268054	0.01705494
0.823801875	0.011932005
0.888408959	0.003189725
0.841401041	0.008824657
0.852116883	0.007215058
0.8627882	0.005811871
0.821768939	0.012329743
0.807336748	0.015394846
0.807177722	0.015431025
0.822671413	0.01215216
0.815241873	0.013662971
0.820560277	0.012570131
0.837894976	0.009396729
0.805886805	0.015726697
0.879879892	0.003952004
0.802501261	0.016518932
0.808900476	0.01504192
0.888098121	0.003215674
0.891311169	0.00295395
0.81868279	0.01294937
0.899647713	0.002340175
-0.813208401	0.014096081
0.865887403	0.005440138
0.803534627	0.016274528
0.841989934	0.008730801
0.812279761	0.01429671
0.819615424	0.012760096
0.951028168	0.000282937
0.848001242	0.007808897
0.843414962	0.008506319
0.812093556	0.014337154
0.806463718	0.015594125
0.878550291	0.004080445
0.825569808	0.011592786
0.942939937	0.000444798
0.896440625	0.00256538
0.818551898	0.012976074
0.821772695	0.012329001
0.819558084	0.012771682
0.902299523	0.00216397
0.808150291	0.015210591
0.82453537	0.011790516
0.851489902	0.007303594
0.818398237	0.013007469
0.865712643	0.005460682
0.901598275	0.002209698
0.847837269	0.007833179
0.806693196	0.015541588

0.815566659	0.013594582
0.840553999	0.008960776
0.837294519	0.009497002
0.808920443	0.015037447
0.921272755	0.001148981
0.858872414	0.0063043
0.848741651	0.00769985
-0.824331999	0.011829639
-0.829077959	0.010937872
0.883325577	0.003631348
-0.815654695	0.013576082
0.837807715	0.009411259
-0.824784338	0.011742733
0.809285343	0.014955845
0.814555764	0.013808155
0.806433797	0.015600984
-0.85929662	0.006249712
0.806249619	0.015643242
0.897836983	0.002465677
0.852251351	0.007196159
0.802931547	0.016416886
0.844544351	0.008331044
0.800665319	0.016958805
0.805010319	0.015929459
-0.903141618	0.002109875
-0.800630033	0.01696733
0.804913402	0.015951979
0.841718256	0.008774021
0.812642157	0.014218204
0.805771351	0.015753312
0.806977332	0.015476692
0.900692761	0.002269666
0.831644416	0.010473929
0.86439389	0.005617311
0.842287123	0.008683678
0.839811742	0.009081148
0.807638884	0.015326254
0.819936335	0.012695374
0.975805283	3.48E-05
0.810724974	0.01463662
0.837808907	0.00941106
0.844499052	0.008338029
0.810459316	0.014695201
0.819719374	0.012739108
0.823451757	0.011999917
0.826860905	0.011348955
0.875411332	0.004394257
0.856349528	0.006635255
0.802781522	0.016452421
0.843727112	0.008457643
0.818415225	0.013003996

0.832726717	0.010282085
0.870313823	0.004936209
0.88526541	0.003458457
0.803311229	0.016327171
0.835900605	0.009732389
0.883542061	0.003611783
0.802807331	0.016446305
0.816265285	0.013448206
0.881145	0.003832239
0.862302423	0.005871571
0.829149067	0.010924845
0.812885284	0.014165687
0.822603226	0.01216552
0.823225796	0.012043877
0.818109393	0.013066612
0.834131539	0.010036416
0.830819786	0.010621611
0.940918505	0.000493002
0.839180946	0.009184248
0.817266762	0.013240115
0.859324813	0.006246095
0.877564549	0.004177383
0.849608719	0.007573389
0.817849278	0.013120017
0.863723636	0.005698009
0.813999295	0.013926617
0.805658817	0.015779281
0.800053298	0.017107052
0.884344101	0.003539892
0.818304062	0.013026733
0.863114059	0.005772044
0.81244874	0.01426007
0.933637381	0.000694769
0.844013453	0.008413147
0.803143263	0.016366822
0.826762557	0.011367414
0.854549408	0.006878053
0.843326926	0.00852008
0.899847746	0.002326571
0.900733352	0.002266955
0.904313862	0.002036045
0.814446747	0.013831313
0.807225645	0.015420117
0.879206896	0.004016685
0.816983342	0.013298798
0.864554763	0.005598052
0.838669062	0.009268457
0.844989896	0.008262536
0.850611269	0.007428829
0.831940353	0.010421249
0.816077173	0.013487521

0.861287236	0.005997597
0.855665863	0.006726811
0.800979018	0.016883133
0.830340922	0.010707972
0.893220246	0.002805183
0.813422561	0.014050066
0.822112262	0.012261994
0.814865589	0.013742475
0.824980915	0.011705092
0.837145627	0.00952197
0.852991402	0.007092715
0.866381407	0.005382334
0.827182531	0.011288724
0.857161462	0.006527562
0.84370333	0.008461345
0.860310495	0.006120473
0.866120458	0.005412819
-0.849470139	0.007593511
-0.846646786	0.008010918
0.846617043	0.008015391
0.812483609	0.014252517
0.840053201	0.009041879
0.826094031	0.011493388
0.851558745	0.007293839
0.807299793	0.015403249
0.807143033	0.015438925
0.846755028	0.007994652
0.849025905	0.007658245
0.896783352	0.002540671
0.840870917	0.008909692
0.843207538	0.008538763
0.832571924	0.010309385
0.834519863	0.009969171
0.815019011	0.013710024
0.885404229	0.003446293
0.842453778	0.008657324
0.803958893	0.016174844
0.923397124	0.001060191
0.891097605	0.002970902
0.891795576	0.002915731
0.819404602	0.012802727
0.801563323	0.016742748
0.811188817	0.014534688
0.887078524	0.003301748
0.894441664	0.002712597
0.804032624	0.01615756
0.810407937	0.014706548
0.888114333	0.003214318
0.917739689	0.001307147
0.82780236	0.011173219
0.821722865	0.012338853

0.830575049	0.010665693
0.870186687	0.004950246
0.893422544	0.002789709
0.979922235	1.99E-05
0.817470074	0.01319812
0.839017272	0.00921112
0.800853133	0.016913474
0.802115619	0.016610728
0.826868892	0.011347457
0.884704888	0.003507856
0.889285624	0.00311727
0.94332248	0.00043604
-0.808366776	0.015161796
0.894374192	0.002717659
0.821529865	0.01237706
0.812241316	0.014305055
0.823005259	0.012086879
0.928795576	0.000855017
0.818294168	0.013028758
0.8657552	0.005455674
0.816090107	0.013484816
0.93466413	0.00066354
0.818513215	0.012983973
0.887127459	0.003297583
0.819902241	0.01270224
0.87823832	0.004110966
0.84272927	0.00861387
0.848993599	0.007662966
0.880639374	0.00387982
0.877646923	0.004169226
0.843075216	0.008559501
0.853909314	0.006965735
0.924173534	0.001028895
0.831210732	0.010551433
0.841340482	0.008834344
0.811175168	0.014537681
0.859405816	0.00623571
0.869928122	0.004978874
0.894739509	0.002690324
0.898976803	0.002386181
-0.803752303	0.016223335
0.817471325	0.013197862
0.856501102	0.006615065
0.820482612	0.012585678
0.835095108	0.009870084
-0.88898629	0.003141889
0.83332181	0.010177558
0.867667377	0.005233714
0.830461502	0.010686184
0.846976221	0.007961479
0.911335707	0.001628732

0.81949532	0.012784371
0.880448699	0.003897862
0.865337312	0.005504971
0.824006796	0.011892369
0.833970666	0.010064358
0.872295737	0.004720679
0.800583959	0.016978466
0.947379947	0.000350022
0.802189648	0.016593082
0.81119889	0.014532479
0.810021996	0.014791958
0.817338407	0.013225307
0.918930769	0.001252336
0.870745361	0.004888753
0.846222281	0.008074912
0.812670887	0.014211992
0.813729703	0.013984237
0.900783777	0.002263591
0.919833601	0.001211804
0.895093977	0.002663971
0.832368255	0.010345375
0.908778012	0.00177028
0.851822019	0.00725661
-0.809351087	0.014941172
0.85813576	0.006399817
-0.861995757	0.00590946
-0.802313447	0.016563598
0.826134622	0.011485714
0.823454678	0.01199935
0.914554656	0.001461336
0.914007545	0.001488957
0.81525135	0.013660973
0.812909663	0.014160428
0.896868825	0.002534533
0.870690763	0.004894741
0.909476221	0.001730877
0.801811635	0.01668331
0.812120378	0.014331324
0.827260911	0.011274076
0.832591832	0.010305871
0.856805086	0.006574692
0.846037149	0.008102922
0.830395043	0.010698189
0.893395603	0.002791767
0.831527233	0.010494835
0.896122575	0.002588448
0.905225098	0.001979827
0.834771037	0.009925828
0.91175127	0.001606455
0.828616619	0.011022623
0.830453277	0.010687669

0.82331425	0.012026657
0.850470245	0.007449056
0.895398498	0.002641465
0.874527037	0.004485379
0.867192864	0.005288243
0.851202905	0.007344352
0.813022256	0.014136154
0.810414076	0.014705192
0.831041574	0.010581762
0.812851846	0.014172903
0.859541953	0.006218281
0.961237848	0.000141401
0.851146936	0.007352317
0.825761497	0.011556377
0.813207507	0.014096274
0.828568816	0.011031428
0.831679463	0.010467681
0.8231408	0.012060439
0.836788416	0.009582043
0.816989839	0.013297451
0.813456416	0.014042801
0.825169027	0.011669143
0.800558925	0.016984518
0.812775433	0.0141894
0.821881056	0.012307593
0.815260947	0.013658949
0.865654111	0.005467573
0.85242188	0.007172238
0.802307725	0.01656496
0.816846073	0.013327279
0.829119623	0.010930238
0.859722435	0.006195223
0.891863286	0.002910414
0.811326444	0.01450453
0.802715063	0.016468178
0.802447796	0.01653164
0.81885761	0.012913757
0.856997311	0.006549244
0.813054144	0.014129285
0.836125672	0.009694135
0.88827759	0.003200675
0.824225664	0.011850127
0.811740756	0.01441398
0.801018	0.016873744
0.808113873	0.01521881
0.82184732	0.012314256
0.80517894	0.015890324
0.881845295	0.003766961
0.811809301	0.014399033
0.861712992	0.005944535
0.852179646	0.007206233

0.829042256	0.010944416
0.90637958	0.001910063
0.826286435	0.011457042
0.843241751	0.008533406
0.836664498	0.009602939
0.819522738	0.012778827
0.885093272	0.00347358
0.82965225	0.010832949
0.850418448	0.007456494
0.84888196	0.007679296
0.822976291	0.012092535
0.818842173	0.012916899
0.815317929	0.013646937
0.812249005	0.014303386
0.964802146	0.000106158
0.820991695	0.012483991
0.802957654	0.016410708
0.83147049	0.010504968
0.843509734	0.008491522
0.830342233	0.010707735
0.856362641	0.006633507
0.841369629	0.008829681
0.832317531	0.010354351
0.825487316	0.011608477
0.852324784	0.007185852
0.859677017	0.00620102
0.844031632	0.008410327
0.871811271	0.004772794
0.806747198	0.015529241
0.923120499	0.001071489
0.861624658	0.005955519
0.81238097	0.014274758
0.886441052	0.003356311
0.803097844	0.016377554
0.806692719	0.015541697
0.804279983	0.016099657
0.83030653	0.010714191
0.853145719	0.007071265
0.815107405	0.013691349
0.903181791	0.002107317
0.855863273	0.006700291
0.856898069	0.006562375
0.873616517	0.004580464
0.814205408	0.013882665
0.890575707	0.003012594
0.844141603	0.008393282
0.827017605	0.011319585
0.897813797	0.002467312
0.861097038	0.006021399
0.807811618	0.015287126
0.809953332	0.014807186

0.813766122	0.013976445
0.834755659	0.009928479
0.871374846	0.004820056
0.80279386	0.016449497
0.800706446	0.016948872
0.850222051	0.00748474
0.8156569	0.013575618
0.805354953	0.015849538
0.939602315	0.00052616
0.870090008	0.004960938
0.884237647	0.00354938
0.803342462	0.016319805
0.850626409	0.007426659
-0.812266648	0.014299556
0.837645531	0.009438303
0.827798486	0.011173939
0.828786671	0.010991335
0.813691854	0.013992339
0.828164995	0.01110599
0.856044352	0.006676025
0.863744497	0.005695486
0.813662887	0.013998541
0.873563826	0.004586005
0.850526989	0.007440913
0.930838227	0.000784755
0.871549666	0.004801088
0.854860961	0.006835632
0.838496447	0.009296963
0.831224561	0.010548956
0.846906662	0.007971902
-0.829008698	0.010950569
0.892193198	0.002884599
0.943177462	0.000439347
0.856874406	0.006565508
0.889324009	0.003114122
0.843963563	0.008420889
0.814037025	0.013918565
0.832929969	0.010246308
0.832870901	0.010256697
0.808152437	0.015210107
0.878740549	0.004061903
0.891407132	0.002946353
0.828303516	0.011080378
0.860571802	0.006087444
0.904078841	0.00205071
0.883632898	0.003603594
0.900207281	0.002302247
0.822000206	0.012284081
0.900171757	0.002304643
0.968933403	7.32E-05
0.832845628	0.010261144

0.84730804	0.007911879
0.853182197	0.007066201
0.84036696	0.008991012
0.840912938	0.008902933
0.812624454	0.014222032
0.817280054	0.013237367
0.837463796	0.009468665
0.821240544	0.012434474
0.811379254	0.014492968
0.800817251	0.016922129
0.927363455	0.000906652
0.805375457	0.015844791
0.86619097	0.00540457
0.860878587	0.006048812
0.87039113	0.004927686
0.839477539	0.009135679
0.844656467	0.008313771
0.871370673	0.004820509
0.810156345	0.014762191
0.810701907	0.014641701
0.807432711	0.015373039
0.843767583	0.008451345
0.949178696	0.000315773
0.829389215	0.010880926
0.837663352	0.009435328
0.801279306	0.016810894
0.823712349	0.011949347
0.85484308	0.006838062
0.808851898	0.015052807
0.811696589	0.014423615
0.90728873	0.001856264
0.81458056	0.013802891
0.820625126	0.012557159
-0.806689918	0.015542338
0.838474274	0.009300629
0.873751998	0.004566234
0.892826736	0.002835442
0.832939267	0.010244673
0.821857154	0.012312313
0.872120798	0.004739455
0.819891214	0.012704461
0.844361663	0.008359239
0.805875838	0.015729224
0.882975578	0.003663123
0.804162085	0.016127239
0.887250841	0.003287098
0.865171909	0.005524562
0.873005807	0.00464496
0.911295831	0.00163088
0.877291799	0.004204465
0.894811869	0.00268493

0.948388398	0.000330535
0.909218848	0.001745335
0.802679241	0.016476675
0.80136621	0.016790024
0.833259404	0.010188487
-0.825106025	0.011681175
0.826482296	0.011420118
0.867121935	0.005296425
0.822454035	0.012194785
0.867823362	0.005215868
0.810782015	0.014624061
0.84921658	0.007630417
0.802280784	0.016571374
0.851936162	0.007240507
0.811806798	0.014399579
0.884992301	0.00348247
0.929789901	0.000820322
0.881987453	0.003753799
0.868894458	0.005094379
0.889000654	0.003140704
0.895147979	0.002659971
0.858610034	0.006338216
0.818800569	0.01292537
0.935601473	0.000635846
0.823776066	0.011937002
0.853811443	0.006979205
0.871312261	0.004826858
0.832343876	0.010349688
-0.800265908	0.017055461
0.87490052	0.004446747
0.848288298	0.007766504
0.8084355	0.015146326
0.8271088	0.011302514
0.811593413	0.014446141
0.851490974	0.007303442
0.826216042	0.011470331
0.822029114	0.012278381
0.906646132	0.001894187
0.805955946	0.015710771
0.831662178	0.010470762
0.928979933	0.000848513
0.889962912	0.003062027
0.87916553	0.004020683
0.943120956	0.00044064
0.832038999	0.010403726
0.850221276	0.007484852
0.815431118	0.013623096
0.978341401	2.50E-05
0.849951982	0.007523693
0.80300355	0.016399849
0.876843452	0.004249226

0.843549371	0.008485338
0.879758954	0.003963577
0.867820144	0.005216235
-0.853121936	0.007074568
0.821591914	0.012364768
0.808217347	0.015195467
0.83601433	0.009713048
0.884983242	0.003483268
0.868118107	0.005182253
0.896178126	0.002584409
0.80770874	0.015310423
0.827949524	0.011145905
0.856137335	0.006663586
0.809137166	0.014988948
0.857556522	0.006475569
0.806319594	0.015627178
0.804153621	0.01612922
0.87594831	0.00433951
0.878623247	0.004073329
0.924079359	0.001032658
0.944693148	0.000405589
0.858946204	0.006294783
0.870645702	0.004899686
0.822291672	0.012226685
0.801394284	0.016783285
0.816737592	0.013349813
0.851975322	0.007234988
0.823736787	0.011944611
0.802357018	0.016553229
0.930588305	0.000793141
0.875238538	0.004411968
0.953890085	0.000236691
0.946115792	0.000375496
0.868083417	0.005186202
0.842235863	0.008691794
0.828283966	0.01108399
0.89490962	0.002677656
0.852429867	0.007171119
0.803391695	0.016308197
0.845255494	0.008221868
0.827130198	0.011298511
0.911846638	0.00160137
0.80251354	0.016516015
0.811216772	0.014528559
0.875531197	0.004381998
0.807516277	0.015354066
0.880608439	0.003882744
0.845340192	0.008208926
0.874998689	0.004436628
0.802104831	0.0166133
0.838461995	0.00930266

0.843702912	0.00846141
0.800650358	0.016962419
-0.907884657	0.00182154
0.833636224	0.010122604
0.810183465	0.014756186
0.840434611	0.008980069
0.833829999	0.01008883
0.855523467	0.006745981
0.826410353	0.011433672
0.862483919	0.00584922
0.85209161	0.007218613
0.833014846	0.010231391
0.821532845	0.012376469
0.812359333	0.014279449
0.824605942	0.011776959
0.859930217	0.006168745
0.887299359	0.003282981
-0.805274129	0.015868259
0.814626992	0.013793038
-0.834125102	0.010037533
0.884633362	0.003514192
0.832545996	0.010313962
0.855351806	0.006769138
0.8460536	0.008100431
0.817575157	0.013176447
0.874738216	0.004463509
0.836958826	0.009553355
0.839385152	0.009150791
0.856644809	0.006595959
0.818625867	0.012960979
0.819273055	0.012829374
0.841863096	0.008750962
0.805379629	0.015843825
0.847499251	0.007883387
0.892155528	0.002887539
0.888835192	0.003154363
0.810869873	0.014604729
0.809130788	0.014990374
0.804709136	0.015999509
0.889453292	0.003103535
0.92445457	0.001017717
0.940157115	0.00051201
0.870383084	0.004928572
0.929990828	0.000813425
0.863665223	0.005705077
0.817583561	0.013174715
0.849765182	0.007550711
0.800235093	0.017062932
0.816534102	0.013392149
0.827199578	0.011285537
0.812942803	0.014153281

0.831052005	0.01057989
0.801698446	0.016710387
0.808300257	0.015176779
0.81985265	0.012712232
0.862644613	0.005829477
0.893759072	0.002764091
0.899228692	0.00236884
0.899619102	0.002342125
0.832202852	0.010374662
0.819484651	0.012786529
0.828863561	0.010977206
0.809066653	0.015004717
0.83518815	0.009854116
0.916276872	0.001376573
0.813285947	0.014079409
0.86803484	0.005191735
0.836143672	0.009691079
0.833900928	0.010076485
-0.809721231	0.014858733
0.802857697	0.016434372
0.805197656	0.015885984
0.912293792	0.001577671
0.820168316	0.012648717
0.84770745	0.007852438
0.85086298	0.007392813
0.814650893	0.013787968
0.888426542	0.003188261
0.842744112	0.008611533
0.855831146	0.006704603
0.863155544	0.005766986
0.820283055	0.012625681
0.825646341	0.011578241
0.853121877	0.007074576
0.819765627	0.012729777
0.85825181	0.006384708
0.830507278	0.01067792
0.802132726	0.016606649
0.838976622	0.009217802
0.812461019	0.01425741
0.810904562	0.014597101
0.829316139	0.010894278
0.834636271	0.009949069
-0.837415397	0.009476761
0.826205134	0.011472392
0.830502689	0.010678748
0.817586064	0.013174199
0.833764136	0.010100302
0.857719481	0.0064542
0.881899238	0.003761963
0.862450242	0.005853363
0.861329317	0.005992339

0.869875431	0.00498472
0.857461631	0.006488033
0.855471075	0.006753043
0.838923872	0.009226478
0.837216198	0.00951013
0.89432019	0.002721715
0.844817579	0.008288989
0.885332465	0.003452578
0.867667258	0.005233727
0.830804467	0.010624366
0.853642762	0.007002458
0.87755388	0.00417844
0.867452383	0.005258375
0.82078892	0.012524432
0.822992861	0.012089299
0.830270231	0.010720758
0.855032206	0.006812386
0.816182673	0.013465463
0.835847974	0.009741349
0.932367325	0.00073471
0.814545691	0.013810294
0.80878073	0.015068764
0.826722145	0.011375004
0.811758518	0.014410105
0.842062891	0.008719218
0.819734633	0.012736029
0.808235168	0.015191448
0.817940414	0.01310129
0.809684396	0.014866925
-0.864977837	0.005547605
0.83901751	0.009211081
0.802668929	0.016479121
0.861765206	0.005938048
0.817606986	0.013169887
0.81416285	0.013891733
0.874962628	0.004440343
0.836413741	0.009645311
0.823086441	0.012071038
0.850744307	0.007409779
0.926671743	0.000932305
0.844109595	0.008398241
0.830215156	0.010730727
0.88936466	0.00311079
0.908598542	0.001780501
0.84907192	0.007651523
0.859874904	0.006175786
0.844498992	0.008338039
0.886596382	0.003342963
0.897565246	0.00248488
0.85038811	0.007460853
0.90561986	0.001955789

0.892960787	0.00282511
0.891511202	0.002938128
0.894458354	0.002711345
0.888081789	0.003217042
0.892793536	0.002838004
0.860960603	0.006038511
0.805065155	0.015916725
0.825551629	0.011596243
0.830881596	0.010610496
0.832434058	0.010333738
0.830356181	0.010705213
0.932849407	0.000719377
0.866297424	0.005392133
0.828997552	0.010952613
0.92655772	0.000936579
0.800865889	0.016910398
0.864969492	0.005548597
0.900654495	0.002272223
0.858160257	0.006396626
0.838975251	0.009218028
0.840634048	0.008947856
0.836199045	0.009681684
0.841302514	0.008840422
0.844730675	0.00830235
0.833130658	0.010211059
0.849628031	0.007570588
0.803676307	0.016241196
0.83776933	0.009417655
0.846398175	0.008048357
0.801863909	0.016670815
0.841069758	0.008877736
0.850674927	0.00741971
0.82666862	0.011385062
0.846575499	0.008021642
0.868288696	0.005162862
0.867904186	0.005206636
0.811268926	0.014517129
0.883180499	0.003644497
0.83859098	0.009281345
0.912610471	0.001561025
0.861542821	0.005965707
0.889663219	0.003086393
0.804068089	0.01614925
0.811474741	0.014472078
0.886876106	0.003319011
0.851503015	0.007301736
0.838135004	0.009356834
0.832557321	0.010311963
0.864112318	0.005651123
0.815493703	0.013609925
0.909215093	0.001745546

0.882430553	0.003712961
0.801712394	0.016707049
0.82139039	0.012404717
0.940606117	0.000500744
0.83203733	0.010404023
0.801278353	0.016811123
0.874715328	0.004465876
0.807927489	0.015260914
0.877801776	0.00415392
0.802012801	0.016635255
0.865135074	0.005528931
0.852004111	0.007230932
0.82847178	0.011049316
0.856783867	0.006577505
0.886854053	0.003320895
0.816208839	0.013459995
0.807768762	0.015296828
0.831487715	0.010501891
-0.824196756	0.011855701
0.825192988	0.011664569
0.91530019	0.001424237
0.908609927	0.001779852
0.874263406	0.004512778
0.896863818	0.002534892
0.857584536	0.006471892
0.811040044	0.014567333
-0.849437773	0.007598216
-0.808790505	0.015066572
-0.831471384	0.010504808
0.8780334	0.004131093
-0.872566581	0.004691705
0.829545319	0.010852436
-0.841558754	0.008799458
0.834272802	0.01001192
0.904448032	0.002027703
0.918174803	0.001286947
0.857123911	0.006532518
0.900321186	0.002294576
0.874569833	0.004480941
-0.811948955	0.014368611
0.890125215	0.003048884
-0.875734389	0.004361267
0.835038424	0.00987982
0.892650604	0.002849053
0.831251025	0.010544217
0.925391078	0.000981043
0.8142609	0.013870847
0.811556697	0.014454163
0.818527281	0.0129811
0.882929504	0.00366732
0.84204942	0.008721356

0.855329752	0.006772116
0.828607321	0.011024335
0.818817854	0.01292185
0.80602628	0.015694582
0.806165457	0.015662576
0.805841506	0.015737136
0.810181141	0.014756701
0.967541933	8.34E-05
0.814605772	0.01379754
0.843022585	0.008567759
0.839614332	0.009113334
0.839729428	0.00909456
-0.808309019	0.015174805
0.816571653	0.01338433
0.887904048	0.003231945
0.885592699	0.003429822
0.852602065	0.007147017
0.820896864	0.012502894
0.813639104	0.014003635
0.801336586	0.016797136
0.806562066	0.015571596
0.843270659	0.008528882
0.899877787	0.002324532
0.806111991	0.015674866
0.858303249	0.006378019
0.804243028	0.016108299
0.85914433	0.006269274
0.924896061	0.001000319
0.874102712	0.004529531
0.807758987	0.015299042
0.822001576	0.012283811
0.842192054	0.008698735
0.82401371	0.011891033
0.841959834	0.008735583
0.801837802	0.016677055
0.830557525	0.010668853
0.830567598	0.010667036
0.841815054	0.008758606
0.809532225	0.014900793
0.847723603	0.00785004
0.950374842	0.000294266
0.83041811	0.010694021
0.806976557	0.015476869
0.812293649	0.014293697
0.811032891	0.014568903
0.831459165	0.010506991
0.839921951	0.009063211
0.858101845	0.006404237
