# Supplementary material for: Integrative Analysis of Blood Transcriptomics and Metabolomics Reveals Molecular Regulation of Backfat Thickness in Qinchuan Cattle
Source: Animals (Basel). 2023 Mar 15;13(6):1060. doi: 10.3390/ani13061060 (PMC10044415; doi:10.3390/ani13061060)
Supplement: Supplementary file 1 [file animals-13-01060-s001.zip › Supplementary File S12 Supplementary Table S10.pdf]

**Table S10. KEGG enrichment analysis for DEGs and SEMs.**

| #Pathway | ko_ID | DEG_in_Pathway\|DEM_in_Pathway | AllGene_in_Pathway\|AllMetabolite_in_Pathway |
|---|---|---|---|
| Tuberculos | ko05152 | 9\|1 | 219\|3 |
| Fc gamma | ko04666 | 5\|1 | 101\|3 |
| Biosynthes | ko01040 | 3\|1 | 37\|11 |
| Neuroacti | ko04080 | 10\|1 | 407\|9 |
| Phospholi | ko04072 | 11\|1 | 164\|3 |
| Fructose a | ko00051 | 2\|1 | 37\|4 |
| Drug meta | ko00982 | 3\|1 | 68\|9 |
| Linoleic ac | ko00591 | 2\|2 | 51\|10 |
| Calcium si | ko04020 | 16\|1 | 333\|3 |
| Tyrosine m | ko00350 | 4\|1 | 50\|8 |
| Ferroptosi | ko04216 | 4\|1 | 53\|4 |
| Apelin sig | ko04371 | 7\|1 | 150\|3 |
| Sphingolip | ko04071 | 8\|1 | 122\|2 |
| Sphingolip | ko00600 | 2\|1 | 63\|3 |

| DEG_in_All_pathways\|DEM_in_All_pathways | AllGene_in_All_pathways\|AllMetabolite_in_All_pathways |
|---|---|
| 533\|9 | 10647\|353 |
| 533\|9 | 10647\|353 |
| 533\|9 | 10647\|353 |
| 533\|9 | 10647\|353 |
| 533\|9 | 10647\|353 |
| 533\|9 | 10647\|353 |
| 533\|9 | 10647\|353 |
| 533\|9 | 10647\|353 |
| 533\|9 | 10647\|353 |
| 533\|9 | 10647\|353 |
| 533\|9 | 10647\|353 |
| 533\|9 | 10647\|353 |
| 533\|9 | 10647\|353 |
| 533\|9 | 10647\|353 |

| Gene_id | KEGG_Ortl | Metabolite | Compound |
|---|---|---|---|
| Bos_taurus | K06856+K | Sphingosir | C06124 |
| Bos_taurus | K06856+K | Sphingosir | C06124 |
| gene-ACC | K17360+K | Docosadie | C16533 |
| gene-F2R; | K03914+K | Sphingosir | C06124 |
| gene-F2R; | K03914+K | Sphingosir | C06124 |
| gene-HK3 | K00844+K | L-Rhamnc | C00507 |
| gene-ADH | K00121+K | 3-Carbam | C16591 |
| gene-ALO | K00460+K | 8(R)-HPOI | C14831+C04717 |
| gene-F2R; | K03914+K | Sphingosir | C06124 |
| gene-AOC | K00276+K | Hydroxyph | C05596 |
| gene-SLC4 | K14685+K | 1-Octadec | C21481 |
| gene-PLCI | K05858+K | Sphingosir | C06124 |
| gene-PLCI | K05858+K | Sphingosir | C06124 |
| gene-SMF | K12352+K | Sphingosir | C06124 |
