# Supplementary material for: Integrative Analysis of Blood Transcriptomics and Metabolomics Reveals Molecular Regulation of Backfat Thickness in Qinchuan Cattle
Source: Animals (Basel). 2023 Mar 15;13(6):1060. doi: 10.3390/ani13061060 (PMC10044415; doi:10.3390/ani13061060)
Supplement: Supplementary file 1 [file animals-13-01060-s001.zip › Supplementary File S13 Supplementary Table S11.pdf]

Table S11. Significantly genes correlated with sphingosine 1-phosphate (S1P).

geneName	metaName	PCC
GABARAPL1	Sphingosine 1-phosphate	0.89275223
CXCL8	Sphingosine 1-phosphate	0.886109293
VDAC3	Sphingosine 1-phosphate	0.876546144
IL18	Sphingosine 1-phosphate	0.840377629
S1PR1	Sphingosine 1-phosphate	0.828047931
ARPC3	Sphingosine 1-phosphate	0.818159521
TMIGD3	Sphingosine 1-phosphate	-0.833489478
SMPD3	Sphingosine 1-phosphate	-0.838780582
PLCB2	Sphingosine 1-phosphate	-0.841044962
CAMK1	Sphingosine 1-phosphate	-0.849737883
CERS1	Sphingosine 1-phosphate	-0.883688807
