# Supplementary material for: Integrative Analysis of Blood Transcriptomics and Metabolomics Reveals Molecular Regulation of Backfat Thickness in Qinchuan Cattle
Source: Animals (Basel). 2023 Mar 15;13(6):1060. doi: 10.3390/ani13061060 (PMC10044415; doi:10.3390/ani13061060)
Supplement: Supplementary file 1 [file animals-13-01060-s001.zip › Supplementary File S2 Supplementary Figure S2.pdf]

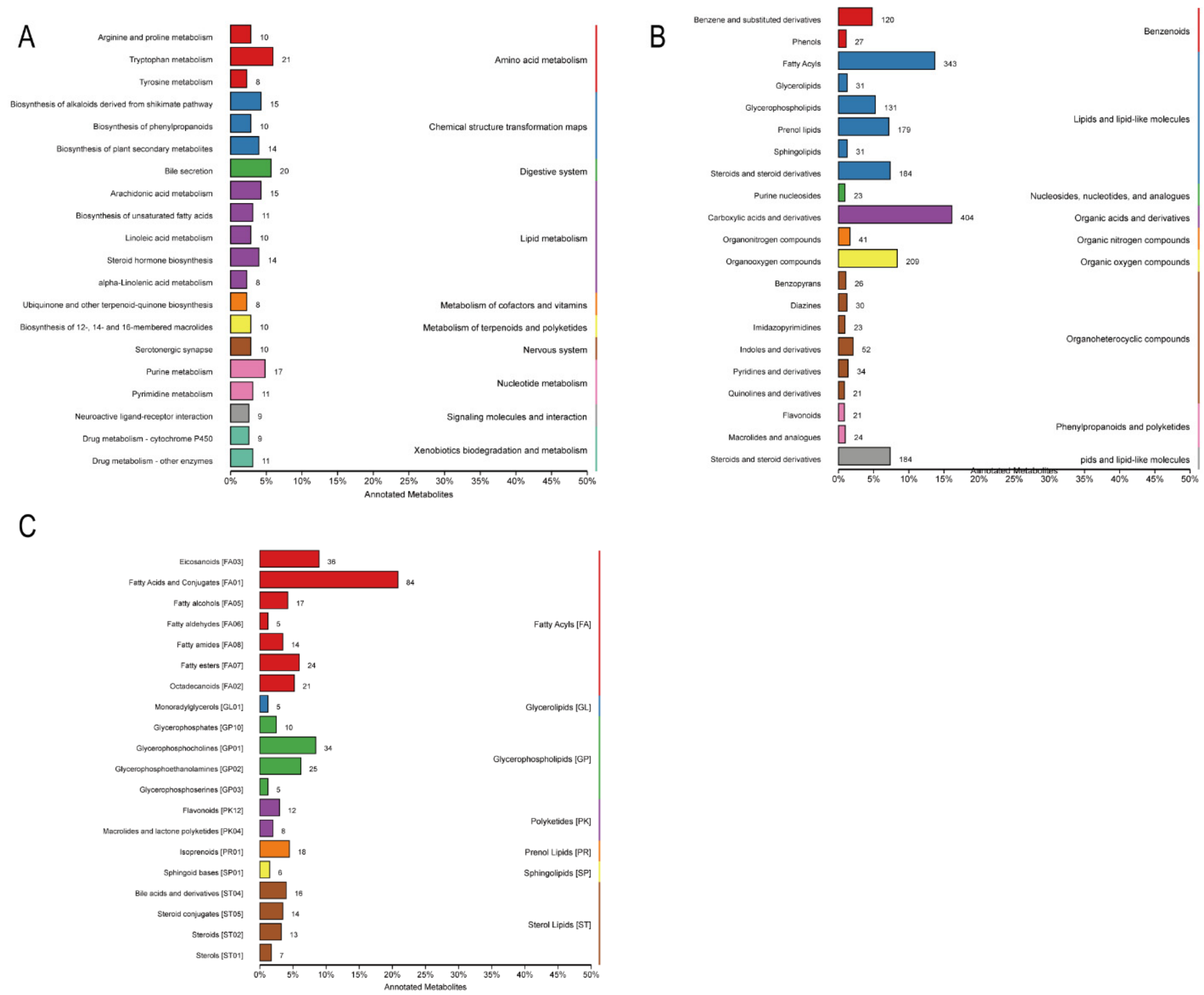

Supplementary Figure S2. All metabolites were qualitatively analyzed based on the metabolome database. (A) 792 metabolites were identified in the KEGG database. (B) 2709 metabolites were identified in the HMDB database. (C) 403 metabolites were identified in the Lipidmaps database.
