# Supplementary material for: Integrative Analysis of Blood Transcriptomics and Metabolomics Reveals Molecular Regulation of Backfat Thickness in Qinchuan Cattle
Source: Animals (Basel). 2023 Mar 15;13(6):1060. doi: 10.3390/ani13061060 (PMC10044415; doi:10.3390/ani13061060)
Supplement: Supplementary file 1 [file animals-13-01060-s001.zip › Supplementary File S3 Supplementary Table S1.pdf]

Table S1. Summary of sequencing data quality control.

#SampleID	ReadSum	BaseSum	GC(%)	N(%)	Q20(%)	Q30(%)
H1	25792726	7.71E+09	57.81	0	97.67	93.95
H2	21382719	6.4E+09	56.58	0	98.07	94.82
H3	20749636	6.21E+09	56.45	0	97.99	94.68
H4	20556578	6.16E+09	56.52	0	97.9	94.48
L1	23492013	7.03E+09	55.47	0	97.87	94.34
L2	20570129	6.16E+09	55.52	0	97.91	94.43
L3	20848874	6.24E+09	55.52	0	97.8	94.15
L4	20996424	6.28E+09	55.93	0	97.95	94.61

Note:
#SampleID Sample ID in BMK system. The correspondence table between BMKID and provided sample ID can be found in conclusive report.
ReadSum Number of total pair-end reads in clean data
BaseSum Total number of base in clean data
GC(%) GC content in clean dataClean N(%) Percentage of N in clean data.
Q20(%) Percentage of base with Q-score higher than 20 in clean data.
Q30(%) Percentage of base with Q-score higher than 20 in cleandata.
