# Supplementary material for: Integrative Analysis of Blood Transcriptomics and Metabolomics Reveals Molecular Regulation of Backfat Thickness in Qinchuan Cattle
Source: Animals (Basel). 2023 Mar 15;13(6):1060. doi: 10.3390/ani13061060 (PMC10044415; doi:10.3390/ani13061060)
Supplement: Supplementary file 1 [file animals-13-01060-s001.zip › Supplementary File S4 Supplementary Table S2.pdf]

Table S2. Statistics on mapping ratio of sequencing data against reference genome.

Sample	Total Reads	Mapped Reads	Uniq Mapped Reads	Multiple Map Reads	Reads Map to '+'
H1	51,585,452	47,581,154 (92.24%)	45,319,767 (87.85%)	2,261,387 (4.38%)	25,686,364 (49.79%)
H2	42,765,438	39,704,923 (92.84%)	37,888,277 (88.60%)	1,816,646 (4.25%)	21,468,023 (50.20%)
H3	41,499,272	38,278,028 (92.24%)	36,513,260 (87.99%)	1,764,768 (4.25%)	20,700,718 (49.88%)
H4	41,113,156	38,011,100 (92.45%)	36,179,461 (88.00%)	1,831,639 (4.46%)	20,639,341 (50.20%)
L1	46,984,026	43,996,358 (93.64%)	37,941,395 (80.75%)	6,054,963 (12.89%)	30,169,079 (64.21%)
L2	41,140,258	37,934,909 (92.21%)	35,173,762 (85.50%)	2,761,147 (6.71%)	22,184,419 (53.92%)
L3	41,697,748	38,271,451 (91.78%)	35,544,417 (85.24%)	2,727,034 (6.54%)	22,348,426 (53.60%)
L4	41,992,848	38,755,061 (92.29%)	35,959,156 (85.63%)	2,795,905 (6.66%)	22,521,988 (53.63%)

Reads Map to '-'
25,728,081 (49.87%)
21,464,144 (50.19%)
20,687,896 (49.85%)
20,675,260 (50.29%)
30,065,245 (63.99%)
22,137,193 (53.81%)
22,299,684 (53.48%)
22,478,318 (53.53%)
