# Supplementary material for: Integrative Analysis of Blood Transcriptomics and Metabolomics Reveals Molecular Regulation of Backfat Thickness in Qinchuan Cattle
Source: Animals (Basel). 2023 Mar 15;13(6):1060. doi: 10.3390/ani13061060 (PMC10044415; doi:10.3390/ani13061060)
Supplement: Supplementary file 1 [file animals-13-01060-s001.zip › Supplementary File S5 Supplementary Table S3.pdf]

Table S3. Differentially expressed genes (DEGs) in BFT.

#ID	gene_nam	H1_FPKM	H1_count	H2_FPKM	H2_count	H3_FPKM	H3_count	H4_FPKM
gene-CRA	CRABP2	8.143188	145	4.895172	73	4.539727	65	6.447078
gene-ZFP5	ZFP57	0.442817	123	0.309355	72	0.220943	49	0.242882
Bos_taurus	--	2.068774	163	2.021088	122	1.526224	110	1.05574
gene-LOC	LOC54032	3.53406	144	2.28963	78	1.662716	54	1.332272
gene-MTU	MTUS2	1.0109	175	0.295344	51	0.230778	54	0.135717
Bos_taurus	--	2.743102	117	1.408654	54	1.437804	47	1.330796
gene-LOC	LOC52993	7.309991	329	3.585061	135	5.00699	180	4.427381
Bos_taurus	--	8.901129	108	3.381177	34	6.516463	66	6.688237
gene-PAM	PAMR1	0.44939	52	0.694621	66	0.813974	75	1.179081
gene-HOX	HOXB6	0.730914	193	0.473287	104	0.607536	128	0.660127
gene-APC	APCDD1	1.046786	145	0.730503	84	0.293238	33	0.597729
gene-PRG	PRG3	10.54654	380	5.833452	175	6.762471	195	5.32208
gene-PRK	PRKCG	7.24272	908	5.895508	615	4.953426	496	3.98269
gene-OLI	OLIG2	1.765398	200	1.143508	105	0.869159	79	0.910845
gene-ASB	ASB2	46.26648	5038	29.57976	2681	27.88154	2424	26.47913
gene-LTBI	LTBP4	6.461422	1351	4.406244	781	3.626368	617	3.71201
gene-SPO	SPOCD1	0.435102	70	0.463517	62	0.426799	55	0.282208
gene-CEL	CELA2A	7.255178	270	6.944176	215	6.101418	182	4.29617
gene-SRM	SRMS	7.265142	482	4.928293	275	5.322034	281	5.504293
gene-ENH	ENHO	60.65575	2416	33.13696	1099	31.16266	991	33.92212
gene-LOC	LOC10190	2.062068	74	2.076234	62	2.566254	73	2.765573
gene-LOC	LOC10190	1.857801	335	1.343055	207	1.151833	173	0.982491
Bos_taurus	--	1.279437	38	2.288035	56	1.873969	44	2.116022
gene-RAB	RAB44	8.241726	1234	5.072481	632	6.71954	803	5.678891
gene-GHS	GHSR	0.349609	106	0.487948	117	0.374832	90	0.44579
gene-MGA	MGAT3	0.957965	201	0.666351	117	0.626139	109	0.311258
gene-LOC	LOC51527	10.07801	731	7.442693	457	7.209573	421	7.674706
gene-COL	COL7A1	2.22056	853	1.366922	437	1.395422	428	1.166399
gene-B3G	B3GAT1	2.163422	337	2.004617	263	1.099617	163	1.026816
gene-FBP1	FBP1	34.83762	1804	21.49076	927	21.13388	874	21.85921
gene-LOC	LOC11244	1.691026	121	2.216207	132	1.702011	98	1.634033
gene-CEB	CEBPE	165.4895	5785	103.7106	3018	94.71127	2643	108.6648
gene-PAD	PADI3	19.6062	2521	12.12178	1297	12.60965	1294	11.90826
Bos_taurus	--	6.036741	981	4.802721	553	3.390871	499	4.721807
gene-TDR	TDRD9	0.200571	51	0.232669	49	0.166839	34	0.37608
gene-PKN	PKNOX2	0.746647	114	0.405756	52	0.591693	72	0.557394
gene-ZBTI	ZBTB47	11.08142	2516	8.215124	1562	8.41762	1534	7.679233
gene-ALO	ALOX5	88.53973	9648	63.1903	5731	65.25858	5677	61.2306
gene-MPC	MPO	12.47671	1739	6.825712	1007	13.0269	1016	12.93997
gene-LOC	LOC50844	9.503612	953	6.625931	559	6.639309	536	5.839435
gene-LOC	LOC10029	890.754	33530	546.0069	17106	545.3704	16388	556.9105
Bos_taurus	--	3.558773	271	3.024923	192	2.891575	195	2.713862
gene-CRT	CRTAC1	28.86073	2730	18.89484	1493	18.37888	1387	18.48228
Bos_taurus	--	2.303848	320	1.52141	179	2.816902	259	2.224251
gene-LOC	LOC61854	1060.883	70105	780.0893	42825	791.2359	41780	744.9258
gene-SFXI	SFXN5	6.61597	1124	4.473849	632	3.447108	468	4.565683
gene-LOC	LOC50532	12.91774	492	7.667766	243	4.623733	141	7.968814
gene-CUE	CUEDC1	22.17838	2639	14.74081	1460	14.95899	1414	16.5575

Bos_taurus	--	2.620871	338	2.109092	210	1.994882	192	1.488902
gene-CAC	CACNB3	6.221005	759	5.569953	581	5.303737	499	4.083029
gene-GPR	GPR4	33.69633	3614	22.92021	2235	22.73375	2062	19.11516
gene-KCT	KCTD15	15.71229	1859	11.04409	1037	7.421486	849	8.639202
gene-C6H	C6H4orf19	2.9047	233	2.327263	155	2.041766	130	2.25033
gene-KRT4	KRT42	2.962562	217	1.389572	79	1.239574	112	2.501179
gene-LOC	LOC10496	121.6927	3928	87.4529	2349	78.63213	2026	96.19714
Bos_taurus	--	2.710043	217	1.401762	93	1.273239	82	2.970565
Bos_taurus	--	21.67274	451	4.671122	81	9.969015	166	9.18408
gene-LOC	LOC10190	0.207544	38	0.440506	68	0.440561	66	0.369897
gene-GSC	GSC2	3.63593	433	2.596272	257	2.460167	234	2.232955
gene-LOC	LOC10013	66.18333	4335	45.21218	2399	44.23902	2245	46.44888
gene-CD3	CD300LB	122.5615	4746	82.60318	2662	87.5498	2706	89.73905
Bos_taurus	--	0.439167	45	0.710436	64	0.812705	64	0.8955
gene-TM4	TM4SF5	12.77485	443	7.86008	227	7.752688	215	9.602764
gene-PLC	PLCXD1	1.669939	106	0.873296	47	3.084255	156	1.061514
Bos_taurus	--	7.320616	624	5.500146	383	4.019331	294	5.034172
gene-LOC	LOC51171	3.9384	257	2.717787	147	2.878462	151	2.938575
gene-LOC	LOC40714	0.636961	107	0.31558	44	0.610987	82	0.510983
gene-SCN	SCN2B	12.07742	1961	5.61527	756	5.610551	832	5.661379
gene-FBLL	FBLL1	0.861017	151	0.654246	96	0.827651	116	0.975603
gene-FAM	FAM107A	1.640837	221	1.333619	153	1.202728	143	1.085834
gene-CDK	CDKN2B	2.387549	264	1.824472	168	1.68343	149	1.485424
gene-JAG2	JAG2	0.930569	174	0.487472	76	0.864261	129	0.544983
gene-PAQ	PAQR7	1.523029	278	1.076909	167	0.717952	107	0.638935
Bos_taurus	--	1.008199	83	0.921495	63	1.541286	101	1.087557
Bos_taurus	--	0.474749	174	0.389195	119	0.420074	123	0.468047
gene-EFN	EFNA2	1.055521	87	1.11666	77	0.937078	62	0.752216
Bos_taurus	--	15.36714	239	7.515912	97	9.137752	114	6.206219
gene-FAD	FADS6	4.86881	270	2.265495	105	3.020817	134	2.788451
gene-LOC	LOC10033	8.55734	1157	7.993464	896	7.647176	837	7.348177
Bos_taurus	--	1.60183	111	3.114876	180	2.754318	153	3.250809
Bos_taurus	--	1.472029	101	0.753358	44	1.13812	63	1.349018
gene-TMI	TMIGD3	1.850238	109	1.039235	50	1.647989	78	1.516112
gene-ADC	ADORA3	21.95944	1524	15.38509	879	14.22629	779	14.85078
gene-MM	MMP15	1.105578	165	1.017489	126	0.952574	114	0.875841
gene-CAP	CAPN5	6.040041	1132	5.195328	802	5.500954	823	5.196912
Bos_taurus	--	0.493889	61	1.332366	123	0.836689	80	1.677861
gene-TME	TMEM205	42.3475	1344	25.6797	683	27.34687	695	24.64646
gene-CHA	CHAD	1.338574	86	1.21446	65	1.637425	86	0.966301
gene-LOC	LOC78612	29.5742	1188	21.30458	713	21.75142	698	24.00404
gene-ALO	ALOX15	65.36674	7862	58.28085	5842	58.62505	5618	55.84257
Bos_taurus	--	3.356473	144	3.697507	132	2.907985	99	2.064556
gene-LOC	LOC78973	4.430746	243	3.845239	176	3.098831	136	4.095742
gene-LOC	LOC10033	18.15335	3025	14.35659	1988	14.60116	1953	14.38878
gene-IL5R	IL5RA	3.925202	541	3.151735	363	3.608352	394	4.543293
gene-OCS	OCSTAMP	7.141545	555	8.858241	573	8.156718	508	7.326185
gene-RDH	RDH5	1.726024	97	1.54887	73	1.515684	69	1.280099
gene-TME	TMEM145	6.48772	594	6.225586	468	5.575883	406	6.57523
gene-SYN	SYNGR1	7.404634	508	3.774634	284	5.825453	289	6.698788
gene-PDZ	PDZD3	1.861387	279	1.35184	133	1.35773	117	1.199535

gene-PDE	PDE8A	0.477411	78	0.31257	43	0.460292	60	0.929502
gene-C11	C11H2orf5	0.603895	64	0.704276	62	1.0883	92	0.816374
Bos_taurus	--	1.187558	511	0.781943	280	0.663974	228	0.585401
gene-SLC	SLC7A11	0.346586	140	0.225421	76	0.269839	87	0.34349
gene-MGI	MGLL	11.08374	1427	8.393154	884	7.137046	755	6.234771
gene-HSP	HSPG2	2.012206	1240	2.18014	1118	1.8819	926	1.443606
gene-EEPI	EEPD1	2.794292	316	1.785221	168	1.920581	175	1.841596
gene-STA	STARD10	26.09903	1389	17.87204	782	20.05868	867	16.5877
gene-LOC	LOC51286	2.317396	1589	1.571249	897	1.573467	862	1.629635
Bos_taurus	--	6.389517	360	13.13141	595	12.53073	563	16.83732
gene-ADA	ADAMDEC	7.873536	715	7.060924	559	8.431878	623	7.319498
gene-ANK	ANKRD13I	48.30767	4611	36.8559	2901	36.06126	2557	33.62383
gene-BFSI	BFSP2	22.54038	1433	13.36506	710	14.29539	727	15.42406
gene-LOC	LOC11244	1.966909	489	2.578036	534	1.968583	391	1.786996
gene-LOC	LOC51813	32.96736	4220	27.43561	2919	24.3225	2489	28.0734
gene-RAB	RAB17	8.749096	669	8.445346	546	7.052551	437	7.305398
gene-TUB	TUBA8	0.922948	58	2.356057	123	1.328623	67	1.318135
gene-ME3	ME3	2.431551	197	2.760928	182	2.6253	175	2.632436
gene-KCT	KCTD14	1.789963	107	1.183094	59	1.245232	60	1.745393
gene-EBF	EBF4	1.148153	135	1.141263	112	1.004012	95	0.799231
Bos_taurus	--	0.59663	152	0.694015	147	0.46605	95	0.676617
gene-IGFE	IGFBP4	87.70918	7564	64.95502	4662	62.36468	4293	62.84294
gene-EDA	EDA	0.567302	119	0.747004	131	0.881413	147	0.515569
gene-SMF	SMPD3	7.500033	1652	5.628159	1022	5.757426	1016	4.52538
gene-LOC	LOC61878	0.61413	261	0.673814	238	0.38066	129	0.434532
gene-PAD	PADI4	120.4037	13281	91.65714	8423	89.01999	7821	84.046
gene-GFI1	GFI1B	19.70096	1903	15.49239	1230	15.07566	1149	14.85527
gene-LY6	LY6G5B	2.144208	112	2.795395	121	1.894922	79	1.540773
Bos_taurus	--	1.478827	193	1.141485	124	1.478151	130	0.99458
Bos_taurus	--	3.953061	554	3.045981	321	3.392928	353	3.480729
gene-CAN	CAMK1	28.15479	1655	18.23196	893	20.15055	948	20.48377
gene-CPT	CPT1B	0.798175	100	0.452839	47	0.874741	90	0.642056
gene-RBN	RBM41	1.323059	275	1.217179	228	1.832879	296	0.88286
Bos_taurus	--	0.564736	103	1.099507	166	1.523574	221	1.348006
gene-PTG	PTGDR2	56.8864	7300	38.91725	4153	42.52538	4344	39.91077
gene-VIPF	VIPR2	17.25241	1826	11.22321	989	9.699785	820	8.984639
gene-MG	MGC1370	3.742091	170	2.79175	106	2.399977	87	1.590274
gene-LRR	LRRC32	1.197529	330	1.428153	254	1.110911	306	1.479725
gene-GGT	GGT1	6.443556	633	7.332448	599	8.098586	587	7.777738
gene-HID	HID1	1.72032	244	2.004195	236	1.711672	194	2.92119
gene-MTC	MTCP1	2.553615	89	2.967387	86	2.203092	61	2.603416
gene-SAG	SAG	1.814653	127	1.557817	97	1.54043	74	1.560519
gene-HIC	HIC1	18.32513	3964	11.61618	2166	12.54496	2196	13.15038
gene-LOC	LOC10190	27.29948	1149	61.04567	2138	55.34258	1859	46.97184
Bos_taurus	--	2.550356	126	3.373325	142	4.732013	181	5.080219
gene-DSB	DSB	0.782232	111	1.086817	131	1.539114	178	1.519952
gene-VW	VWCE	1.607779	219	2.207516	249	1.722962	187	1.836103
gene-LOC	LOC78950	0.493109	452	0.449019	342	0.54966	402	0.366878
gene-LOC	LOC11244	7.650108	607	6.245983	412	6.309258	400	6.197264
gene-GZN	GZMB	170.6893	6179	109.2421	3291	117.9597	3409	118.1638
Bos_taurus	--	1.951831	227	1.198706	142	1.250695	122	1.190686

gene-TME	TMEM120	2.190228	411	1.548769	219	1.327858	127	1.818962
gene-C19	C19H17or	4.239878	494	3.099983	291	3.244182	304	3.572633
gene-CHC	CHCHD10	25.6712	721	10.90894	255	14.33331	322	11.06823
gene-DGK	DGKG	1.024203	246	1.501199	307	1.760147	346	1.300537
Bos_taurus	--	1.045714	229	0.885268	162	1.086039	190	0.939606
gene-OLF	OLFML3	2.193142	160	1.52704	93	2.198593	128	2.315439
gene-LOC	LOC10014	28.31352	1880	20.82665	1151	22.1669	1175	20.10716
gene-ITGA	ITGA9	1.188223	180	0.870217	110	1.026927	124	0.871621
gene-NKIF	NKIRAS1	7.50816	395	6.041732	284	4.61194	225	4.252506
gene-LOC	LOC61555	8.911063	1846	8.241649	1421	8.019629	1326	7.849082
gene-RHB	RHBDF1	0.377114	89	0.597295	122	0.372717	53	0.874916
Bos_taurus	--	1.10465	95	1.117484	80	1.145354	79	1.395001
gene-LOC	LOC61636	11.88911	731	7.85223	403	7.847972	383	8.631832
gene-SLC2	SLC2A1	24.65372	2577	16.19633	1409	15.80878	1319	13.84491
gene-LOC	LOC50988	4.39555	294	3.494426	195	3.314156	177	3.208231
Bos_taurus	--	13.77994	1581	9.100352	924	9.284558	876	9.924024
gene-LOC	LOC10030	1.378881	127	0.643602	50	1.183601	95	1.088207
gene-NEC	NECAB3	0.994809	79	0.851131	56	0.926569	56	1.052451
gene-MYH	MYH7	1.094036	276	0.630949	133	0.614754	124	0.554203
gene-ANC	ANO9	2.359405	278	3.059848	302	2.252838	212	2.016472
gene-FFAI	FFAR2	6.214943	617	4.071046	350	4.084273	342	3.810312
gene-CDC	CDC42EP1	2.860278	249	2.930484	212	2.488336	173	3.037075
gene-SNT	SNTA1	3.525998	295	5.74895	400	4.860509	324	4.326817
gene-ADA	ADAM11	4.416076	904	2.512539	430	2.601973	415	2.578143
Bos_taurus	--	3.35298	152	2.882872	126	2.516142	96	2.727885
gene-EEF2	EEF2KMT	8.893576	445	8.15448	344	10.91099	439	10.85328
Bos_taurus	--	117.3457	2935	76.592	1651	77.9687	1586	84.13701
Bos_taurus	--	0.720949	343	0.658988	261	0.743964	282	0.673849
gene-RAB	RAB11FIP5	20.80354	4513	19.28654	3351	14.97567	2914	17.14172
gene-ITGE	ITGB4	1.074737	267	0.90671	192	1.183328	240	0.843765
gene-TME	TMEM150	17.51103	897	11.39635	542	13.55303	585	12.37425
gene-ELAI	ELANE	29.44902	1133	19.01507	609	22.41001	689	20.26723
gene-SYTI	SYTL1	114.8746	9713	85.68384	6030	81.91607	5529	86.80799
gene-SEPT	5-9月	40.41647	3608	34.84744	2565	35.08307	2433	34.38017
gene-OPL	OPLAH	0.702119	120	0.505035	75	0.843713	112	0.403402
gene-HRH	HRH2	25.61371	4701	26.73227	4034	26.90037	3952	25.55239
gene-FHL3	FHL3	129.404	8597	113.9741	6305	111.8299	5932	106.9165
gene-NMU	NMUR1	0.882479	185	1.381802	241	1.935582	324	1.407548
gene-LOC	LOC50477	120.2704	3603	87.54614	2179	78.79905	1905	85.04553
Bos_taurus	--	1.111474	101	1.774961	135	1.260871	93	1.882487
gene-GAT	GATA1	60.81305	3724	39.56951	2016	42.1931	2071	40.94907
Bos_taurus	--	1.251081	186	1.333149	132	1.450339	114	1.095314
Bos_taurus	--	1.874027	237	1.723153	177	2.414074	199	1.777408
gene-ZFPI	ZFPM1	24.0709	3179	19.9212	2190	15.45031	1626	16.95976
gene-PAN	PANX2	4.040549	481	2.843285	286	2.700106	252	3.38008
gene-PRSS	PRSS53	0.431598	83	0.41802	65	0.441401	67	0.446656
Bos_taurus	--	1.550289	167	2.688301	242	2.709752	234	2.768655
gene-RNF	RNF223	0.844429	189	0.771232	144	1.013639	181	0.581277
gene-LPIN	LPIN1	4.004624	881	3.139074	577	3.639664	646	3.390721
gene-MBC	MBOAT7	293.7659	27922	255.7664	20253	232.219	17661	238.0877
gene-MYE	MYBL1	0.973313	159	1.726824	268	1.671907	257	1.201373

gene-KREI	KREMEN2	1.042724	95	1.202455	92	2.02405	148	0.961901
gene-GDF	GDPD3	6.530502	292	3.76699	142	4.680953	169	3.967305
gene-SSH	SSH3	29.7339	3320	29.06921	2749	26.62333	2425	24.32691
gene-NO)	NOXO1	1.923479	128	2.121062	113	1.115828	73	0.412601
gene-RAS	RASD1	24.28553	1512	18.92805	981	17.62665	876	15.80729
gene-DO(	DOC2G	15.14104	962	13.99709	739	13.08845	671	12.49461
gene-DDF	DDR1	1.376601	211	0.782542	100	1.049194	128	0.990792
gene-PTPI	PTPN5	8.794843	1186	6.568268	737	6.188237	666	7.374171
Bos_taurus	--	1.479649	76	1.406065	61	1.523179	61	1.839093
gene-FOX	FOXO6	1.620433	316	1.15454	158	2.161333	232	1.681494
gene-CDK	CDKN2A	17.49165	666	9.33069	293	11.95151	358	12.21344
gene-GFR	GFRA3	1.297446	110	1.438082	112	0.685906	68	1.521713
Bos_taurus	--	1.534568	159	1.962328	117	0.667778	81	1.099735
gene-GAS	GAS6	30.81937	3141	26.02435	2207	25.7486	2095	23.77647
gene-TME	TMEM132	4.812256	689	4.747711	566	4.353803	499	4.360619
gene-SGS	SGSM1	1.17003	264	1.757035	330	1.842288	332	1.842556
gene-FES	FES	119.8206	14102	99.72312	9770	104.0055	9760	102.5505
gene-NUF	NUP210L	0.449661	125	0.910867	211	0.735094	163	0.794091
gene-STX	STX3	9.896505	1151	10.01106	967	9.936432	919	7.990014
gene-C7H	C7H19orf	1.134736	128	0.818414	77	0.778512	70	0.735986
gene-DAC	DAGLA	4.360549	1042	3.334031	668	2.627554	494	3.18615
gene-PRS	PRSS50	4.081503	306	4.093174	255	3.044214	182	4.407913
Bos_taurus	--	2.342856	404	1.47632	232	1.747563	189	1.420212
gene-PHE	PHETA2	4.043977	431	3.313984	294	5.100685	434	3.742214
gene-ZDH	ZDHHC14	19.42753	2000	14.10581	1214	14.87708	1107	14.69024
Bos_taurus	--	4.506335	201	3.383772	166	4.71457	217	4.359329
gene-NLR	NLRX1	58.64875	9165	50.7913	6604	50.23808	6267	45.92811
gene-RAP	RAPGEF3	5.517784	874	3.910454	487	3.210768	429	4.001765
Bos_taurus	--	446.1678	21459	571.7568	22887	569.582	21868	573.4077
gene-WDI	WDR35	1.558575	291	1.398571	217	1.642259	245	1.134526
Bos_taurus	--	0.602944	100	0.717994	99	0.981993	129	0.748319
gene-PLD	PLD3	81.1208	7841	61.20574	5831	64.8352	5320	51.40687
gene-LOC	LOC10190	2.209857	357	2.720134	362	3.056758	392	1.661628
gene-NOS	NOS1AP	2.73979	191	2.735621	158	1.371629	76	2.748366
gene-DCB	DCBLD2	0.459987	106	1.170488	232	0.773978	148	0.89043
gene-POD	PODXL2	9.458947	788	5.954483	413	6.604508	439	6.313596
gene-TP5	TP53I13	5.723911	378	6.197491	341	6.112067	323	5.388499
gene-WDI	WDR86	4.410135	395	3.72776	254	0.827969	80	3.394116
gene-TTYI	TTYH1	1.747111	138	2.030098	139	1.718132	103	1.518907
gene-LOC	LOC11244	5.020163	239	2.652261	106	4.761227	181	4.357491
gene-LOC	LOC78863	19.77501	2981	17.41039	2185	17.90229	2155	16.54566
gene-KIA	KIAA1522	1.025883	231	0.635327	119	0.802504	147	0.865389
gene-HK3	HK3	278.6878	38629	225.0544	26044	230.6256	25464	228.1105
gene-TNF	TNFRSF18	6.929026	320	7.585608	278	6.132573	303	7.778656
gene-BTN	BTN3A3	5.538087	564	6.646973	563	8.38008	681	7.081282
gene-MY	MYADM	448.1376	41670	398.7504	29752	382.1389	27433	373.5825
gene-NEU	NEURL2	7.9146	525	6.032615	333	4.788107	254	5.661767
gene-CO(	COQ8A	16.35487	1848	12.62609	1248	12.76208	1195	13.49463
gene-PIGI	PIGR	1.087816	160	0.920763	113	0.925435	109	0.722528
gene-SPN	SPNS2	1.898478	343	1.527079	235	1.580314	209	1.541467
gene-C3H	C3H1orf2	3.920921	961	3.830788	797	4.230816	749	3.998524

gene-CPN	CPNE5	2.406523	254	2.933217	257	1.249135	103	1.364176
gene-ACE	ACE	2.11938	361	7.343877	1042	7.22399	983	7.518154
gene-C23	C23H6orf1	1.007488	240	0.993773	197	0.984347	187	1.170187
gene-LOC	LOC11244	693.0212	34822	636.4215	26688	655.7826	26358	757.5772
gene-IL9R	IL9R	4.766479	949	3.428682	553	4.157724	587	3.953105
gene-B3G	B3GNTL1	1.433219	176	2.062729	188	2.407936	202	2.423714
gene-CLB	CLBA1	3.052602	221	2.620813	158	3.406238	197	2.593234
gene-LOC	LOC61731	39.95678	1612	32.2898	1080	32.14192	1035	36.0303
Bos_taurus	--	6.458546	324	6.257624	267	7.733324	253	5.865386
gene-SDS	SDSL	93.75083	5005	73.46026	3423	79.18667	3494	74.58693
gene-HIST	HIST1H1C	62.34825	4264	56.50124	3216	57.81619	3157	48.71783
gene-CAC	CACNB1	1.254315	221	1.197624	149	1.15459	160	1.515646
Bos_taurus	--	2.115376	605	2.83296	562	2.305772	500	3.019771
gene-TPPI	TPPP3	62.01866	2657	63.07265	2247	70.44671	2403	68.33507
gene-PRR	PRR33	1.856813	180	1.514608	122	2.193305	169	2.286402
gene-HIST	HIST1H1E	4.398277	294	4.598246	256	4.32113	231	3.720472
gene-NKD	NKD2	5.045806	503	4.075633	338	3.744571	298	4.256703
gene-BAI	BAIAP2	1.298527	299	1.536156	287	2.417375	450	1.838344
gene-STA	STAB1	13.76624	4400	15.83569	4288	14.5465	3746	13.57536
gene-TKT	TKT	1426.97	121577	1180.621	83719	1154.343	78510	1160.665
gene-LOC	LOC10713	1.775809	585	2.412275	651	2.137517	561	2.780151
gene-ADA	ADAM8	269.1894	37370	222.7405	25773	217.8893	24088	206.4946
gene-CSR	CSRP1	212.0012	17569	147.3761	11733	157.8339	11995	155.7325
gene-ANK	ANKS3	3.453046	359	4.305178	368	3.911236	322	3.732922
gene-CDK	CDK3	4.236063	282	3.213939	177	2.278009	119	3.403386
gene-CD6	CD63	400.2534	14058	313.1016	9153	332.6371	9326	320.7277
gene-DST	DST	0.725571	696	1.305142	948	1.150889	867	0.956248
gene-LOC	LOC10496	0.265104	143	0.290887	127	0.32905	134	0.261573
gene-PCB	PCBP4	53.77619	4322	44.12967	2948	44.55726	2865	42.55241
gene-CBX	CBX7	60.01367	7516	50.49171	5281	50.20377	4919	47.7672
gene-BTBI	BTBD8	0.744809	221	0.854035	211	1.295042	305	0.908614
gene-AGE	AGER	6.455789	401	5.887707	315	6.099152	342	8.287109
gene-LOC	LOC61690	1.944807	446	1.876945	369	1.5847	298	1.425712
gene-SAP	SAP25	39.63014	1994	32.28704	1357	31.12852	1256	33.96223
gene-LOC	LOC78974	43.86872	3737	30.91777	2185	32.21219	2192	32.46072
Bos_taurus	--	1.782653	307	1.769567	270	1.048228	237	1.135675
gene-SCN	SCNN1D	2.427168	481	3.582282	413	2.669274	414	2.01704
gene-PGD	PGD	390.177	31405	329.4269	22069	340.5921	21884	338.0533
gene-ZNF	ZNF8	13.26707	2057	10.52711	1326	10.2628	1179	9.76318
gene-SLC	SLC25A29	41.70933	4975	31.58706	3271	31.43605	3176	33.89405
gene-TH	TH	6.628633	538	6.829617	467	5.860081	382	5.708713
gene-CER	CERS1	8.037763	488	5.136394	269	7.070457	338	5.751551
Bos_taurus	--	3.826662	221	3.979743	191	3.70207	171	3.099916
gene-RTN	RTN2	4.759762	362	2.975302	189	2.936412	179	2.928455
gene-ZNF	ZNF775	3.51558	361	3.549562	325	3.422432	281	3.540034
gene-PAC	PACSIN1	6.925627	1179	6.45452	914	5.909517	803	6.00179
gene-RAB	RABAC1	470.0854	15421	415.7623	11339	381.9981	9999	380.7262
gene-LOC	LOC53900	10.45335	600	11.2738	539	11.92204	546	11.86474
gene-MCA	MCAM	3.987427	514	4.205358	451	3.460264	356	5.309431
gene-ADA	ADAMTSL4	31.22681	5468	29.37458	4322	30.2388	4255	29.59464
gene-ZNF	ZNF177	5.99835	829	5.326732	619	5.753215	637	4.39504

gene-TNN	TNNI2	7.466596	223	7.929652	197	6.732307	160	7.231792
gene-LOC	LOC10084	1.880779	320	2.157271	305	2.007931	273	1.484579
gene-SLC4	SLC49A3	37.08255	4982	29.70803	3254	28.18379	3277	27.5889
gene-EPS8	EPS8L2	2.796379	412	3.069692	376	2.478375	291	2.399699
gene-TME	TMEM53	4.18233	268	4.696559	219	4.420771	189	4.241053
gene-ZBEI	ZBED6CL	3.586365	847	4.132053	812	3.474143	655	3.654244
gene-GAS	GAS7	17.83453	5659	14.98771	3825	15.26217	3748	16.26213
Bos_taurus	--	66.22622	3935	56.85105	2812	61.78783	2931	61.36215
Bos_taurus	--	116.7284	1452	81.8861	848	107.1476	1064	108.3585
gene-SULI	SULF2	1.385782	274	1.368426	226	1.002329	159	0.859227
gene-SGS	SGSM2	1.825233	354	1.4511	241	1.720733	275	1.472054
gene-ALC	ALCAM	0.64749	131	1.65462	280	2.074706	334	1.46312
gene-PALI	PALM	94.66653	11275	78.34601	7800	74.34843	7086	72.28411
gene-SCR	SCRN2	10.13553	642	8.203207	433	10.98695	556	9.152175
gene-DPM	DPM3	124.3593	2102	73.66178	1037	88.31345	1192	100.5747
gene-KCN	KCNT1	15.68682	3116	15.35459	2539	14.97818	2515	14.95844
Bos_taurus	--	8.266708	4159	9.597291	4122	8.95992	3569	10.10339
gene-LOC	LOC52481	1425.323	69670	1262.832	51376	1163.733	45409	1116.275
Bos_taurus	--	1681.067	84996	1418.33	59644	1308.709	52439	1311.476
gene-DBN	DBNDD1	3.170118	250	3.233427	206	3.480934	221	4.024885
gene-SDK	SDK1	1.663736	508	1.697061	431	1.399007	341	1.405659
gene-ZBTI	ZBTB48	14.10052	1443	12.25215	1089	14.16036	1151	12.524
gene-LOC	LOC52001	0.882189	214	1.252255	253	1.151551	223	0.834179
gene-TG	TG	1.746438	609	1.745928	507	1.479184	412	1.729576
gene-SPA	SPATA20	6.507997	697	6.335105	565	5.298513	453	6.365841
Bos_taurus	--	1.781089	556	3.686511	990	3.132	810	2.679598
gene-AMI	AMPD3	19.05148	3444	17.8888	2692	16.32896	2357	15.1474
gene-SPT	SPTY2D1C	1.447348	184	2.210056	234	1.740989	177	1.50405
gene-RFX	RFX2	1.70098	258	2.368501	326	2.684297	345	1.75247
gene-SYN	SYNE1	6.165555	5750	6.944438	6510	10.00533	6407	6.672803
gene-FCA	FCAR	34.14171	1576	39.11623	1576	34.82719	1428	43.23528
gene-ANG	ANG	45.83467	1372	40.25091	1003	50.44759	1205	46.96736
Bos_taurus	--	0.363297	104	0.485534	115	0.526161	120	0.678629
gene-EIF4	EIF4EBP1	166.0197	5564	151.5339	4227	167.1706	4472	174.1542
Bos_taurus	--	3.428561	284	4.297083	317	3.446861	246	3.856852
gene-LRS	LRSAM1	5.198814	676	6.261273	667	5.288422	527	5.444656
gene-USP	USP20	23.57962	4159	20.08936	2950	21.66214	3050	21.38958
gene-TUB	TUBA1C	18.54898	1403	11.08543	671	10.14262	587	12.30938
gene-SCA	SCAP	127.9968	22852	102.986	15361	101.4154	14330	95.49542
Bos_taurus	--	0.728587	401	0.576758	264	0.80387	335	0.79938
gene-GOI	GOLGB1	1.552963	724	2.897954	1120	2.709267	1040	2.402326
Bos_taurus	--	14.21579	3487	9.933476	2097	9.41834	2145	8.285002
gene-GCC	GCC2	0.506431	107	1.324241	232	1.325858	223	1.037964
gene-ICAI	ICAM3	610.4639	45731	485.192	30251	486.9311	29119	474.2491
gene-IFNI	IFNLR1	0.787053	141	0.683881	102	0.82283	117	0.830092
gene-C1H	C1H21orf2	4.677421	374	5.004489	337	5.769805	376	3.665449
Bos_taurus	--	1.348572	232	2.062366	323	0.961509	200	1.426154
gene-ALM	ALMS1	0.488646	272	0.624057	287	0.595553	263	0.686431
gene-ULB	ULBP13	2.608755	125	5.495594	217	4.532396	170	3.173412
gene-PGA	PGA5	52.06497	2941	57.01214	2681	56.37884	2543	58.07832
gene-ITGA	ITGAD	8.635437	1382	9.592678	1278	8.320988	1063	8.115909

Bos_taurus	--	4.665585	382	3.95328	285	4.191268	303	5.191879	
gene-CRA	CRACR2B	3.412213	238	3.740388	216	2.333292	131	2.725638	
gene-TNF	TNFRSF4	18.97167	861	18.0237	681	16.55808	600	20.967	
gene-PAX	PAXX	180.9514	6522	138.4278	4189	137.3068	4000	150.5645	
gene-CCL	CCL3	6.128491	90	8.159017	100	11.24532	132	8.629251	
gene-PLXI	PLXNB2	1.603756	431	1.854607	419	1.354708	267	1.554009	
Bos_taurus	--	8.361642	473	7.552143	356	5.77752	261	5.961739	
gene-SLC(	SLCO4A1	6.164559	861	6.9493	808	8.422599	896	5.629349	
gene-IL3R	IL3RA	5.017608	355	4.522053	266	3.627523	206	2.808695	
Bos_taurus	--	3.99132	444	4.756905	441	4.265192	379	4.320378	
gene-TEL(	TELO2	12.07658	2174	10.83358	1618	10.64019	1528	9.717037	
gene-RON	ROM1	19.25256	1471	16.77762	1094	16.94681	1072	15.95573	
gene-PPP	PPP1R26	2.74473	652	2.35811	468	2.093271	394	1.513741	
gene-TAZ	TAZ	12.66522	878	12.78376	795	11.92198	699	10.81895	
gene-NIN	NINL	0.85139	149	0.750751	109	0.830699	116	1.239048	
gene-NRA	NRADD	37.41927	3826	32.70973	3067	33.61535	2813	35.61579	
gene-HAC	HAGHL	2.761589	152	4.397981	202	3.610461	160	4.902488	
gene-CYB	CYB561A3	135.3658	12172	119.4762	9694	110.489	8608	105.6857	
gene-HON	HOMER3	31.28025	2052	27.52126	1501	29.26947	1533	27.73303	
gene-ACC	ACOT7	95.65947	5634	78.68503	3863	86.97831	4074	85.32921	
gene-SFI1	SFI1	5.568995	1033	5.066858	783	6.322329	937	5.559279	
gene-ARA	ARAP3	20.02055	4238	17.24474	3042	17.29954	2925	15.71682	
gene-NOT	NOTCH1	85.66109	33628	75.59598	24696	75.29991	23586	67.81091	
gene-GPA	GPAA1	153.8162	12709	144.7369	10002	140.6249	9359	131.4994	
gene-GPI	GPI	235.1962	19738	179.2798	12522	186.7794	12513	172.868	
gene-MAN	MAN2B1	442.8776	57334	388.042	41811	390.0503	40309	385.097	
gene-HS1	HS1BP3	3.727443	501	3.358397	376	3.388013	363	3.459507	
gene-DTX	DTX1	11.32216	1751	11.28266	1454	10.78686	1343	11.03879	
gene-SLC2	SLC22A18	7.72803	493	10.04228	512	8.309679	418	7.656215	
gene-LOC	LOC50864	10.25928	421	7.453664	255	6.873008	225	7.186364	
gene-MAS	MAST1	1.863001	379	1.28874	218	1.08331	176	1.297005	
gene-SLC2	SLC25A1	96.90742	6335	88.31329	4805	83.85611	4376	82.58782	
gene-LTBI	LTBP3	3.841591	884	3.816366	723	4.585195	861	3.536202	
gene-RNP	RNPEPL1	493.656	56888	450.2024	43180	437.5074	40247	413.0921	
gene-RFN	RFNG	75.83596	5906	65.34117	4239	60.5468	3765	60.82694	
gene-PLCI	PLCB2	25.54527	4534	24.82827	3716	25.22359	3505	22.72023	
gene-PLXI	PLXNA1	3.124724	1158	2.671888	836	2.244308	673	2.435901	
gene-LOC	LOC51079	13.58005	681	8.075285	337	8.084998	324	9.08131	
Bos_taurus	--	49.79645	2035	36.3785	1238	39.10148	1276	35.32071	
gene-LIPE	LIPE	24.1694	2907	21.6798	2169	21.55589	2069	19.78899	
gene-ZNF	ZNF469	0.685011	387	0.423886	196	0.523179	269	0.536104	
gene-AKA	AKAP9	1.456421	785	1.981094	889	2.210062	947	2.099582	
gene-CLIC	CLIC3	11.36386	1577	16.26285	1878	18.18095	2014	14.75294	
gene-PNP	PNPLA2	39.0898	3362	43.62173	3121	35.48327	2440	35.65654	
gene-PLXI	PLXNB1	1.725038	520	1.551547	390	0.972097	235	1.203473	
gene-WS(	WSCD1	2.082083	353	1.124635	243	1.031523	194	1.111715	
gene-TES(	TESC	39.75063	1624	33.72624	1132	36.50442	1189	39.31938	
gene-TCA	TCAP	17.41462	361	19.62467	339	18.72744	310	22.21612	
gene-C16I	C16H1orf1	13.78649	1584	10.20405	1090	12.073	1324	9.154143	
gene-PLEI	PLEKHG3	37.91057	7164	34.71235	5459	33.6531	5153	33.25094	
gene-APB	APBB3	6.669956	531	7.899827	537	7.571565	494	7.487933	

gene-CAM	CAMTA2	49.65543	8579	43.67052	6301	40.84661	5707	43.01572
gene-PWF	PWP1	3.862663	300	5.28282	341	5.300925	328	4.940345
gene-ADA	ADAL	1.400726	145	2.162106	190	2.063698	159	1.540315
gene-BTG	BTG1	99.60812	7277	170.1643	10347	169.6225	9892	171.2296
gene-PAF	PAFAH1B2	1.837343	166	7.90043	594	6.363153	459	5.867548
gene-GPN	GPN1	1.854817	204	3.176384	291	2.925741	257	2.091373
gene-EGLI	EGLN3	13.65917	1146	15.83806	1106	18.12361	1214	18.61771
Bos_taurus	--	0.939009	75	1.994311	133	2.82404	180	2.294346
gene-CAS	CASD1	0.799479	138	2.103085	296	1.883989	259	1.318598
gene-UBE	UBE2B	28.03335	781	57.92667	1343	53.10559	1181	55.22226
gene-HSP	HSPA5	12.12583	1235	28.54137	2430	26.17209	2124	27.32786
gene-CDC	CDC123	4.088358	255	6.616449	343	6.308036	314	7.53834
gene-DIM	DIMT1	2.022656	125	3.212543	165	4.358975	216	3.07579
gene-TMC	TMCO1	2.429744	122	6.787235	283	9.818047	393	7.824857
gene-GTF	GTF2B	5.303849	289	11.41612	520	9.95725	430	9.730639
gene-TME	TMEM167	1.315042	100	4.279849	270	3.255593	197	3.948802
gene-TM9	TM9SF2	12.04551	1373	26.80375	2543	27.71972	2522	24.70001
gene-NCB	NCBP2	6.717665	375	13.14505	656	11.86177	595	11.56344
gene-STT	STT3B	1.612493	295	5.789468	881	5.243666	765	5.505896
gene-LOC	LOC50815	10.33297	1547	11.37102	1463	10.35984	1346	9.249469
gene-RAP	RAP2A	0.714775	64	1.836453	137	1.755959	125	2.019629
gene-FAM	FAM210B	5.158331	617	14.82819	1475	12.32615	1176	13.41316
gene-SEC	SEC22B	2.153318	338	5.155275	654	5.641041	691	5.251552
gene-UTP	UTP18	2.281083	177	2.927595	189	3.713565	230	3.782165
gene-TTC	TTC1	3.968575	242	6.347695	321	9.717057	474	6.678432
gene-RPL	RPL36A	112.7146	1952	80.40366	1159	85.44041	1181	94.62557
gene-IGBF	IGBP1	4.693223	366	7.242083	469	8.17219	507	10.85203
gene-FCF	FCF1	2.205621	65	5.733951	140	4.932635	115	4.265874
gene-TME	TMEM30A	4.067423	281	12.78115	735	14.59422	805	14.74726
gene-MRF	MRPL32	2.186571	69	3.651738	96	3.633925	92	3.212197
gene-MAC	MAGT1	3.585134	484	12.33482	1385	12.42755	1339	11.05953
gene-RBM	RBM48	1.771809	98	3.235434	149	3.837877	170	3.327673
gene-PDC	PDCL	2.955669	304	7.207545	664	5.746245	530	5.700204
Bos_taurus	--	1.050517	54	1.86152	80	1.502053	62	1.578617
gene-HPR	HPRT1	5.344767	300	21.64233	1010	20.31605	909	17.94726
gene-PPID	PPID	2.075579	127	6.208858	317	6.370769	311	7.366666
gene-APC	APOPT1	1.868731	146	3.489574	198	3.819349	223	3.731299
gene-AOC	AOC3	1.552529	254	2.204529	300	1.754052	229	1.671795
gene-RAB	RAB33B	0.874388	113	3.236915	341	2.191207	225	3.584933
gene-DBI	DBI	11.83797	298	21.72029	455	18.88579	379	18.94986
gene-PLEI	PLEKHF2	3.208391	379	14.56953	1423	13.41122	1250	15.9504
gene-NSA	NSA2	3.486591	156	18.06594	672	17.06322	609	16.10399
gene-CDC	CDCA7	9.653773	994	16.4182	1408	18.74398	1537	18.3992
gene-LYPI	LYPLAL1	0.653695	70	1.224979	109	1.935474	164	1.651337
gene-MED	MED28	5.731934	188	6.691089	182	6.57417	172	6.973784
gene-C1D	C1D	0.520772	115	1.143446	159	1.056859	133	0.721523
gene-GMC	GMCL1	1.428261	215	6.086079	780	6.026181	747	5.597018
gene-RAB	RAB10	6.080483	573	20.30346	1590	22.05966	1657	24.04032
gene-NT5	NT5C3A	4.063884	293	10.69427	645	9.649158	557	11.48802
gene-DHX	DHX40	0.670392	99	3.261226	398	2.896874	339	2.213444
gene-SNR	SNRPD1	3.786498	128	6.147864	149	7.986035	190	7.365454

gene-S1PI	S1PR1	14.81033	1914	28.3608	3021	28.00904	2844	26.56265
gene-NAF	NARS2	0.386631	40	0.988359	83	1.0753	85	1.186361
gene-TRIN	TRIM37	0.694137	131	0.929225	150	0.815461	117	1.02089
gene-TLR2	TLR2	1.533133	256	6.317167	889	7.886584	990	6.641574
gene-SET	SET	13.76252	758	46.32013	2122	47.59735	2092	42.45086
gene-MRF	MRPS31	1.041141	56	3.085404	136	2.051818	87	3.087443
gene-LUZ	LUZP6	15.80769	479	33.74712	851	36.46392	882	30.48679
gene-SKA	SKA2	1.200311	88	2.070807	102	1.682466	98	1.954169
gene-NDU	NDUFV2	5.747013	262	10.55256	399	9.462609	343	10.20012
gene-LOC	LOC10713	1.318396	154	1.616483	157	2.307838	214	1.85533
gene-GHI	GHITM	10.09934	1089	19.30064	1683	20.25299	1700	20.15648
gene-VNN	VNN2	85.90025	6878	155.2552	10282	145.0621	9209	152.7969
gene-PDC	PDCD5	2.819273	66	3.992602	78	3.737974	70	3.23348
gene-POL	POLB	1.44358	78	4.514362	203	3.559547	153	3.399053
gene-MEX	MEX3C	1.141396	181	4.200385	554	3.855384	488	3.444725
gene-TFD	TFDP2	2.352033	707	4.245807	1053	4.020921	1018	4.176459
gene-PIGY	PIGY	4.728576	262	11.39097	525	10.16698	450	10.62554
gene-RPS2	RPS27A	702.3801	16347	761.6157	15004	835.2815	15926	780.6064
gene-UBX	UBXN2A	1.147669	209	3.841947	590	3.594471	514	3.379218
gene-ACT	ACTR3	16.63895	1723	57.6971	4973	62.02475	5127	58.50343
gene-CCT	CCT3	18.74633	1503	33.82839	2257	27.9281	1788	32.25571
gene-FBXI	FBXL4	0.748823	80	1.524312	135	1.457172	125	1.136804
gene-LNP	LNPK	0.159076	49	1.795849	220	1.811464	224	1.630978
gene-TM9	TM9SF3	2.064012	451	9.204884	1673	9.473465	1651	7.6936
gene-BLM	BLMH	2.414345	239	4.566926	376	4.876693	385	5.816102
gene-ARL	ARL2BP	3.289545	223	6.983316	394	8.950547	484	7.177138
gene-YIPF	YIPF5	4.456795	347	8.578087	613	7.418807	582	8.226827
gene-GIM	GIMAP4	23.89724	1790	83.29015	5188	83.60837	4995	86.1422
gene-MFF	MFF	7.376304	417	16.20163	865	16.25982	890	15.10947
gene-RPP3	RPP30	1.537112	88	2.230772	110	2.931902	139	2.91588
gene-RCH	RCHY1	2.247913	160	5.261862	311	5.586456	317	4.854179
gene-TUB	TUBA1D	1.811085	110	2.61604	132	1.970678	96	2.09727
gene-ZNF	ZNF32	0.639652	34	1.455339	62	1.514931	64	1.699758
gene-DOC	DOC2A	0.834523	85	0.945677	80	2.494239	188	1.445296
gene-AMI	AMIGO2	0.418818	52	1.290934	131	1.970951	192	2.040952
gene-LOC	LOC11244	2.886192	159	3.939992	180	2.631119	116	2.781642
gene-SH3	SH3GL3	0.861483	80	0.850247	63	1.571095	106	1.140749
gene-EMC	EMC3	17.39487	933	33.95128	1516	32.40558	1388	32.91346
gene-TBC	TBCCD1	0.786224	86	1.157234	105	1.272844	110	1.586137
gene-TME	TMEM14C	24.42275	908	27.18784	841	31.68114	940	23.78275
gene-FAN	FAM32A	8.281336	490	13.69975	674	16.2074	765	15.96661
gene-HDA	HDAC2	3.103277	247	7.373774	487	9.010859	571	6.221893
Bos_taurus	--	2.821648	122	5.510518	198	6.887169	237	6.396233
gene-RFX	RFXAP	0.542642	54	1.988789	163	2.379231	187	1.49455
gene-HSP	HSPD1	2.174257	237	11.48112	983	11.27736	891	10.98101
gene-DYR	DYRK3	1.116387	100	1.498935	112	1.193288	88	1.241226
gene-IL18	IL18	2.183737	73	3.463007	93	3.311634	84	3.277636
gene-ORC	ORC1	0.949087	117	1.039625	107	1.099206	108	0.986509
gene-MRF	MRPS21	1.937036	160	1.627615	114	2.073946	139	1.643796
gene-LOC	LOC54001	0.968087	100	1.492443	128	1.59346	131	1.763691
gene-RCN	RCN2	1.391895	108	3.584818	231	4.615427	285	3.990263

gene-RCN	RCN1	0.569136	54	1.8917	145	1.82722	134	1.737464
gene-SOD	SOD1	39.39542	1395	51.51533	1519	55.50055	1569	49.37688
gene-GLT	GLT8D1	0.593235	47	1.249818	74	1.867817	114	1.163001
gene-PPP	PPP2R1B	1.809293	312	5.550625	787	5.066732	696	4.562974
gene-GIM	GIMAP7	35.49055	2276	90.79945	4847	95.56236	4892	99.37109
gene-THA	THAP1	1.864469	135	3.003967	175	3.463592	203	3.341706
gene-RAL	RALB	7.522511	766	18.24766	1487	17.18592	1377	16.05386
gene-LOC	LOC10190	0.803352	39	1.389006	56	1.7762	69	1.354873
gene-OLR	OLR1	1.290172	104	4.55838	305	3.851622	249	3.835849
gene-HM(	HMGB1	14.04395	722	45.31556	1938	45.88459	1882	39.68338
gene-PDH	PDHB	4.688876	291	9.859746	509	11.93752	591	9.790473
gene-ACT	ACTL6A	1.10269	82	3.450764	212	4.122007	244	3.176482
gene-GN(	GNG10	10.35383	507	18.17377	741	18.29509	716	15.81126
gene-MYL	MYLIP	5.353978	678	10.02651	1057	10.68073	1080	7.925813
gene-UBA	UBA3	3.676105	236	8.542451	524	4.981059	387	9.080393
gene-TME	TMEM165	1.978836	161	4.092257	277	4.513106	293	3.389809
gene-PPP	PPP1CB	6.32821	1051	20.47623	2658	21.43055	2634	17.98396
gene-RPL	RPL17	108.1098	4459	263.9533	9060	275.9048	9083	265.4077
gene-ARP	ARPC3	67.04543	2280	148.0463	4191	150.5609	4088	159.2303
gene-TWS	TWSG1	0.523642	77	1.868207	229	1.926809	227	1.662378
gene-MRF	MRPL22	0.709065	79	1.380338	129	0.995223	119	0.986779
gene-XRC	XRCC5	2.126736	216	3.773702	319	4.454232	361	3.479162
gene-ARM	ARMCX2	2.34486	244	4.041119	349	3.721956	309	3.337712
gene-BCC	BCCIP	2.188092	111	5.710772	238	6.320954	253	5.209847
gene-GLR	GLRX3	10.38702	520	13.95371	582	16.36769	655	16.51665
gene-THE	THEM4	1.842828	98	2.225613	99	1.970285	84	1.777757
gene-FAM	FAM184B	0.655471	265	1.683442	566	1.588234	512	1.647707
gene-SAR	SARAF	10.26444	817	26.31977	1744	24.00562	1525	21.54156
gene-POT	POT1	0.520629	84	1.683831	222	1.516936	197	2.040707
gene-MKF	MKRN1	26.76099	2019	34.5661	2098	33.39679	2010	33.29457
gene-SELE	SELENOK	21.2974	759	23.54693	699	24.55052	699	24.07218
gene-RRM	RRM1	2.011681	250	4.900798	506	5.072807	502	4.771769
gene-SNX	SNX3	12.09779	648	34.27245	1527	33.98517	1452	29.97452
gene-ARP	ARPP19	2.487937	438	8.748911	916	8.40745	989	7.897506
gene-OST	OSTC	9.398746	402	10.28635	366	12.74964	435	11.8453
gene-MPL	MPLKIP	2.224096	101	3.66155	138	2.758072	100	3.318227
gene-GM	GM2A	14.73583	695	22.76122	894	24.61481	927	19.02985
gene-GDE	GDE1	1.61845	113	5.495052	296	6.38271	332	4.888329
gene-NPT	NPTN	5.442665	544	8.67979	723	9.389342	750	7.069337
gene-RBM	RBM7	1.063573	83	3.586954	232	3.592311	223	3.745829
gene-NMI	NMD3	0.982229	119	2.523196	253	2.859333	275	2.932961
gene-RAN	RAN	27.23992	1310	38.68483	1761	38.27317	1669	41.30247
gene-BOR	BORCS7	2.309728	117	3.651624	154	3.302849	134	4.269591
gene-TME	TMEM242	1.562584	103	1.880827	102	1.2065	63	1.762499
gene-RPL	RPL22	105.1159	2508	107.5898	2140	104.1528	1990	113.9534
gene-CCN	CCNG1	9.228805	884	35.75481	2848	36.78216	2810	34.43227
gene-GCS	GCSAML	0.313571	67	0.816536	195	0.86057	197	1.108296
gene-TATI	TATDN1	1.429714	61	3.299859	122	2.82675	102	3.685111
Bos_taurus	--	0.943634	61	2.730352	145	4.294412	219	3.125579
Bos_taurus	--	3.701199	90	3.299994	62	4.302614	91	4.116376
gene-VMA	VMA21	1.934912	348	6.144036	919	5.226911	750	5.40612

gene-CBX	CBX1	1.495967	74	4.221835	162	3.792581	148	2.141046
gene-C1R	C1R	3.881643	414	5.990141	528	5.633668	457	5.78413
gene-ATA	ATAD1	1.922729	242	6.235519	653	6.48897	652	4.764948
gene-ACP	ACP1	5.894475	328	13.94547	646	13.45639	597	11.17771
gene-ADS	ADSS	1.310304	139	5.599716	492	5.7883	488	4.463049
gene-CLIC	CLIC4	2.301185	154	6.358728	353	6.355388	338	5.719402
gene-TPC	TPC3	0.492494	62	0.387782	41	0.483798	49	0.867574
gene-BOL	BOLA	63.31142	13308	66.27035	11626	66.83216	11268	68.94807
gene-TME	TMEM70	2.608154	125	6.660727	264	5.909912	224	4.504364
gene-CHC	CHCHD3	6.932902	443	14.63122	776	18.82333	958	16.94201
gene-AAG	AAGAB	2.327472	223	3.159189	265	4.324885	346	3.395635
gene-DIP2	DIP2C	0.31933	112	0.758917	220	0.678668	189	0.400981
gene-DR1	DR1	2.668913	466	10.05495	1459	10.5077	1463	10.58753
gene-BET	BET1	1.365026	85	2.340984	121	3.043049	150	2.759214
gene-LOC	LOC10190	8.952024	172	7.024265	113	8.467293	130	8.594552
gene-CXC	CXCL12	4.600492	204	5.031552	157	3.058476	120	3.915103
gene-RID/	RIDA	12.60185	536	36.988	1308	40.53397	1375	34.87399
gene-TAF	TAF13	0.979573	60	2.591931	132	3.35452	164	1.029677
gene-SLC	SLC41A2	0.142671	32	0.398407	74	0.543657	74	0.520171
gene-DCL	DCLRE1B	0.343411	52	0.730968	91	0.761281	91	0.491574
gene-KRT	KRT72	1.431861	118	3.093516	212	2.89825	191	3.338768
gene-SLC	SLC28A3	1.456229	216	2.799035	336	2.042311	257	1.785089
gene-EIF4	EIF4A2	22.37512	1836	56.53982	3844	58.78782	3712	47.34484
gene-MET	METTL21A	0.892438	77	1.213392	102	1.270123	94	1.918231
gene-HNF	HNRNPH2	3.846787	375	8.895887	724	9.65913	753	8.383888
gene-PAB	PABPC4	5.676102	628	11.15626	909	8.643217	766	9.607272
Bos_taurus	--	10.10014	159	8.521112	112	9.95165	125	11.53264
gene-HSD	HSD17B12	1.304247	156	3.98707	396	4.467234	426	4.34619
gene-DST	DSTN	7.078671	215	12.76399	323	14.12481	343	20.64199
gene-ECD	ECD	0.578309	52	2.237228	164	2.779481	198	2.267858
gene-SMI	SMIM10L1	3.533301	358	10.77896	909	11.90143	963	11.4781
gene-RIO	RIOK2	1.002237	78	4.27081	275	4.510286	279	2.103466
gene-NCA	NCAPG2	0.489987	79	0.741294	100	0.617925	81	0.949474
gene-EIF2	EIF2S2	2.440159	144	9.520128	465	11.58394	543	9.873106
gene-C15	C15H11or	2.356727	281	8.270542	819	9.13185	867	9.263203
gene-ENP	ENPP5	1.165522	187	3.826467	510	4.106623	525	4.67431
gene-CLN	CLNS1A	5.444858	338	6.053146	319	7.793222	386	7.033759
gene-TIGI	TIGIT	0.419293	64	1.484756	180	1.64127	191	1.894637
gene-DAP	DAPL1	2.914409	64	4.603883	78	5.876827	93	4.77689
gene-PIN	PIN4	3.73316	72	3.746975	61	2.808655	44	5.607605
gene-VRK	VRK1	0.900237	52	3.203721	153	2.592666	119	2.257382
gene-RAD	RAD51	0.390066	52	0.390127	44	0.430291	46	0.485948
gene-CDC	CDC42SE2	9.159368	1259	24.05929	2737	23.84277	2602	23.27458
gene-CEM	CEMIP	0.691076	206	1.014617	258	0.779048	190	0.538426
gene-IFI6	IFI6	28.61611	754	52.02971	1143	50.3508	1045	47.94294
gene-CCN	CCNB2	1.346531	84	1.042847	54	1.329664	66	2.076942
gene-ARL	ARL14EP	3.573907	195	7.509348	339	6.947035	301	7.198432
gene-DYN	DYNLT1	164.3152	4970	226.2456	5696	238.6314	5762	260.205
gene-DCK	DCK	2.005758	233	4.268942	413	3.88881	361	4.099843
gene-RNF	RNF139	3.700536	406	8.201665	748	7.128264	624	7.429622
gene-MAF	MAFF	1.000926	92	0.662418	51	1.153122	85	0.87629

gene-SEM	SEM1	2.473885	48	5.834706	94	5.853261	90	6.222764
gene-OST	OSTF1	7.306541	382	24.27505	1068	24.24716	1042	22.34703
gene-MO(	MOCS2	2.615752	204	6.792307	429	7.120799	442	5.688636
gene-SERI	SERINC1	5.131263	597	19.84148	1921	23.19945	2155	17.95788
gene-PDC	PDCD10	1.744677	96	5.837873	334	9.925562	434	7.784148
gene-NDL	NDUFS1	1.551057	219	5.310709	648	4.436064	536	4.602492
gene-NAC	NACA	46.72584	1878	89.15968	2955	94.16079	2949	89.10428
gene-KIA/	KIAA1143	0.438889	41	1.273994	99	1.449253	108	1.057065
gene-ADA	ADARB1	0.332911	94	1.042858	240	1.237564	276	1.103167
gene-CO/	COA5	3.077893	99	3.05247	82	3.435532	88	4.908849
gene-FAN	FAM96A	8.614705	316	13.91423	425	13.01156	384	13.04254
gene-PTN	PTMA	49.91955	2785	78.91643	3654	90.33011	4015	103.0145
Bos_taurus	--	9.077774	317	10.45165	304	8.759365	245	9.148139
gene-AM(	AMOTL1	0.252742	88	0.431116	124	0.569138	157	0.414842
gene-B3G	B3GNT2	3.064396	363	6.951389	705	8.434894	798	6.475708
gene-TIMI	TIMM8A	0.767596	39	1.762589	74	1.696585	69	1.759797
gene-CDC	CDC40	0.529833	82	3.071266	374	2.922344	341	2.407147
gene-RNF	RNF14	1.784523	237	3.706462	406	4.015346	408	3.128392
gene-CISE	CISD2	3.417106	152	12.58618	464	10.27208	363	10.57192
gene-MCL	MCUB	2.823429	144	5.640672	240	4.592868	187	4.747284
gene-THC	THOC7	1.708335	75	3.403726	113	5.898357	183	5.274729
gene-KNS	KNSTRN	0.690257	50	1.972353	118	1.424154	82	1.767823
gene-DPY	DPYD	0.836593	153	3.404661	518	3.319472	485	3.198797
gene-NFU	NFU1	3.099381	114	6.712787	205	6.792817	198	8.053352
gene-MRF	MRPL50	3.574003	121	5.980618	168	4.966715	134	4.808968
gene-ZWI	ZWINT	1.789598	116	2.799913	151	1.329442	69	1.478613
gene-ICOS	ICOS	0.902018	96	2.355608	209	3.769074	320	2.135066
gene-LAP:	LAP3	6.145935	509	16.97247	1168	19.82435	1309	19.55533
gene-PM/	PMAIP1	1.980912	109	3.73884	170	3.954677	173	2.823212
gene-CEP	CEP76	0.515521	54	1.541361	134	2.145584	179	1.168289
gene-SOX	SOX4	0.62622	100	0.643743	85	0.699556	90	0.768305
gene-SDH	SDHD	14.1556	757	25.794	1151	28.44023	1216	22.0667
gene-FBXI	FBXL5	15.7881	1865	36.27773	3565	37.49068	3533	41.03078
gene-HM(	HMGN1	10.65298	530	30.3339	1256	28.91192	1149	24.36484
Bos_taurus	--	1.070349	80	1.174353	73	0.753614	45	0.961919
gene-GP9	GP9	22.17739	2048	31.96637	2456	30.53667	2251	28.2343
gene-PSN	PSMD14	2.388431	155	6.404811	343	6.506657	338	4.232495
gene-DAF	DARS	1.870342	141	9.573304	600	5.933816	357	7.54532
gene-E2F:	E2F3	0.639259	123	2.111398	312	1.16825	179	1.461691
gene-PRC	PRORSD1	7.965097	179	4.737189	89	3.584375	64	5.418004
gene-ADH	ADH5	5.595942	339	10.59627	535	12.76029	618	10.02316
gene-RPL	RPL4	94.26089	5410	380.232	18163	356.2928	16324	354.0196
gene-PAI(	PAICS	3.292839	322	11.89463	985	14.01259	1162	12.87808
gene-ORC	ORC6	1.288424	68	3.293287	156	3.864794	186	3.985639
gene-RSL:	RSL24D1	2.379126	121	5.264091	223	7.78535	316	5.156089
gene-EIF2	EIF2S1	1.308013	190	4.484595	255	4.791321	483	5.206892
gene-MRF	MRPL39	2.76035	123	3.944532	147	4.294598	153	2.759631
gene-CAS	CASP4	7.395072	629	24.34629	1695	24.28795	1614	22.50586
gene-UBE	UBE2N	8.177798	742	12.81485	967	14.00188	1014	11.75136
gene-UBE	UBE2E1	5.180271	300	10.10278	485	10.11459	465	9.123426
gene-LOC	LOC78726	0.805296	82	1.340886	114	1.918334	156	2.316969

gene-AIM	AIMP1	1.828166	83	9.875638	371	7.655602	276	7.939304
gene-TME	TMEM170.	1.289208	176	5.285606	592	4.051654	460	5.114584
gene-CAN	CAMLG	3.232938	182	3.678016	173	5.325072	239	5.369601
gene-UBE	UBE2D3	23.0568	2341	38.60074	3980	59.45385	4198	44.52144
gene-F2R	F2R	3.983128	568	16.49483	1973	15.06144	1733	14.47341
Bos_taurus	--	9.551548	279	13.57573	322	9.07891	207	14.43466
gene-ISCA	ISCA1	5.985505	534	14.81487	1094	15.48918	1105	14.38741
gene-NUF	NUPR1	3.91395	93	5.937699	118	4.507928	86	5.681783
gene-TME	TMEM126.	3.160478	100	6.21944	162	4.76855	119	4.446316
gene-STAI	STAU2	0.171841	31	1.121097	165	0.815635	115	0.705055
gene-SYT:	SYT7	0.309018	71	0.855881	163	0.608564	111	0.689759
gene-HAT	HAT1	0.887521	61	4.228051	237	4.666024	249	4.005468
gene-ACA	ACADM	0.816786	72	4.86686	356	5.172169	364	4.541581
gene-SRG	SRGN	200.6636	9668	577.0211	23138	573.364	22051	566.6191
gene-LOC	LOC11244	1.945776	63	8.394632	224	7.841921	200	8.473414
gene-CAT	CAT	6.772635	637	15.86439	1241	15.36852	1153	13.83508
gene-ZMA	ZMAT2	3.858956	242	11.40218	593	9.551946	477	10.78379
gene-ATP	ATP5PB	15.56005	741	31.56994	1251	33.28133	1265	32.54383
gene-ZCC	ZCCHC9	1.390318	79	2.823667	134	4.065086	185	2.171
gene-RAB	RAB2A	8.505264	770	17.16485	1275	15.82416	1142	18.92033
gene-IL15	IL15	2.922495	133	6.821117	262	7.042932	238	4.896012
gene-RDH	RDH14	3.117202	193	2.804877	145	3.853923	191	4.736722
gene-IPO5	IPO5	1.870899	156	6.443826	447	6.156667	410	6.253718
gene-TRM	TRMT5	0.573758	67	1.141578	93	1.381159	107	1.154525
gene-LOC	LOC50903	0.179462	53	1.216347	296	0.682612	159	0.962976
gene-GAR	GAR1	1.930916	91	3.136937	124	4.177879	158	2.658355
gene-CYP.	CYP2U1	0.298895	51	0.621253	95	0.40799	60	0.426919
gene-CON	COMMD1(	0.356737	26	1.694634	102	2.284346	132	2.281832
gene-NCA	NCALD	1.242913	171	3.691993	415	2.513998	283	2.808796
gene-CNE	CNEP1R1	1.259326	96	2.323232	146	3.372296	204	2.947498
gene-TBC.	TBCA	5.902552	145	10.97543	224	10.34555	203	15.03761
gene-CCS	CCSAP	0.495372	84	0.57791	93	0.679227	105	1.077104
gene-NAS	NASP	1.320267	178	4.026537	329	4.062733	324	4.243551
gene-VBP	VBP1	1.998317	72	4.401865	131	2.775057	79	4.191074
gene-AGP	AGPS	1.70335	273	8.04982	1071	7.712518	984	6.839065
Bos_taurus	--	0.297113	48	0.64686	49	0.478437	63	0.269092
Bos_taurus	--	0.385519	23	2.251523	104	1.888088	87	1.381768
gene-MCI	MCM6	2.50436	285	5.586607	528	5.535606	502	5.137009
gene-RWI	RWDD1	2.10095	93	8.595247	336	9.377964	334	11.96917
gene-RRN	RRM2	2.038245	261	3.595405	382	3.09936	316	3.627588
gene-LOC	LOC10190	6.9519	172	11.3097	232	9.866954	194	9.052782
gene-CRL	CRLS1	3.837738	307	6.193189	417	6.141746	394	7.078501
gene-NMI	NMNAT1	0.221859	33	0.724658	90	0.57026	69	0.685099
gene-PIK3	PIK3R3	0.316498	52	0.667329	79	0.505738	86	1.33229
gene-LOC	LOC78268	0.590453	38	1.100179	59	1.659362	86	1.714256
gene-UBE	UBE2G1	20.78823	2353	35.29714	3975	34.47424	3664	37.89784
gene-TES	TES	2.777688	175	6.768786	355	9.525533	478	8.32762
gene-MA(	MAGOH	4.878386	137	5.911561	138	7.403421	166	7.510436
gene-PAR	PARVB	11.82617	1624	21.10764	2230	22.46935	2012	20.13361
gene-CLE(	CLECL1	1.187333	52	4.184355	153	4.835257	171	4.53995
gene-HIGI	HIGD1A	2.189317	118	3.936086	176	4.256133	182	4.051627

gene-KLRI	KLRB1	0.824796	43	1.023009	42	0.697474	24	0.985937
gene-SMII	SMIM15	0.97253	103	5.378984	471	4.585486	385	3.190891
gene-TME	TMED2	10.76091	945	27.0454	1887	27.9765	1774	30.99598
gene-RPL!	RPL5	97.45542	4148	375.1775	13290	366.0901	12438	361.6811
gene-RAB	RABL3	0.49641	40	1.512182	102	2.18784	141	1.583141
gene-TRIF	TRIP13	0.777936	74	1.163071	93	1.224414	93	1.518524
gene-DN/	DNAJB9	0.955947	93	3.087039	255	3.740305	297	3.339182
gene-CAS	CASP6	6.572257	345	11.78099	515	11.7959	495	13.02555
gene-HIB/	HIBADH	0.843056	64	2.450101	154	2.828474	171	2.845661
gene-TM>	TMX1	2.075719	190	8.11032	617	7.056157	515	6.647573
gene-POL	POLE3	7.022573	553	10.74931	704	10.20795	642	10.01095
gene-CCD	CCDC43	0.725703	67	2.238809	174	2.846917	213	1.896394
gene-EIF3	EIF3H	21.59576	1084	53.06897	2216	59.42168	2380	54.03549
gene-UM/	UMAD1	0.906472	82	2.560726	191	2.259492	162	2.173593
Bos_taurus	--	2.593457	64	3.096228	64	5.523281	109	6.253182
gene-BPG	BPGM	1.44387	83	2.141955	102	1.648063	75	3.180527
gene-SFXI	SFXN1	0.84835	93	2.635373	229	2.72001	231	3.185869
gene-TME	TMEM128	3.741188	183	5.511484	223	7.728459	300	4.371361
gene-MAI	MAD2L1	2.810878	138	2.495019	102	3.262369	128	3.13286
gene-PSA	PSAT1	2.45411	228	4.725265	366	4.913685	365	3.89928
gene-SPR	SPRY2	0.633826	55	1.138338	86	1.365094	99	0.984327
gene-MEI	MED30	2.193453	93	2.965047	104	3.752017	126	3.205784
gene-SOR	SORBS1	0.144765	49	0.230461	67	0.179762	50	0.344928
gene-MU1	MUT	0.964059	112	2.164676	209	2.633121	244	2.768536
gene-FAN	FAM98B	0.566017	44	2.042706	131	1.392376	86	1.597463
gene-CYB	CYB5A	0.842765	74	1.872468	136	1.307496	91	1.593405
gene-MRF	MRPS22	0.978162	46	3.726041	144	3.071569	114	5.501203
gene-LSM	LSM3	4.347807	115	7.426417	164	6.706362	142	9.834174
gene-XPO	XPOT	1.915724	278	6.763569	804	6.727274	758	6.048341
gene-SLC	SLC24A3	3.377837	547	6.590298	888	6.030195	779	5.723329
gene-ASN	ASNSD1	3.152947	311	7.858737	645	8.537717	672	6.736197
gene-IPO	IPO11	1.725847	316	3.066248	466	2.637999	376	3.490836
gene-TRA	TRAF3IP1	0.208627	37	0.438744	65	0.315324	45	0.339896
gene-CISI	CISD1	1.487481	47	2.143792	56	1.805836	47	2.493005
gene-CEP	CEP78	0.29449	34	1.043604	99	0.935068	85	1.325641
gene-RPL	RPL22L1	7.661734	369	19.24713	770	21.91143	841	17.70325
gene-EIF4	EIF4E	1.90682	130	6.205863	350	5.236138	283	6.363914
gene-HSD	HSD17B11	7.615343	527	18.55432	1073	19.52761	1082	18.6139
gene-RPL(	RPL6	104.6575	4063	272.7026	9260	303.2633	9509	283.2928
gene-FRM	FRMD6	0.493603	100	1.995528	335	1.556868	245	1.853629
gene-HPS	HPSE	0.976538	68	3.377539	193	3.882763	213	3.382199
gene-CRIF	CRIPT	3.73345	155	10.12085	348	7.12491	235	6.831035
gene-UPR	UPRT	1.428306	67	2.155389	163	2.132373	154	2.195549
gene-TME	TMEM69	0.910539	47	1.183063	60	1.261179	52	2.791516
gene-MRF	MRPL3	4.86578	273	9.741427	455	9.843552	441	10.23684
gene-ETF/	ETFA	3.705451	212	6.070865	289	6.169221	282	5.130877
gene-HSP	HSP90AB1	48.00015	5010	125.1591	10873	119.5592	9962	120.6116
gene-NDI	NDUFS4	5.460389	151	5.163322	119	6.716419	148	6.185137
gene-EXO	EXOSC8	0.656928	26	3.237	106	2.768375	87	2.403777
Bos_taurus	--	0.487677	35	1.689213	80	2.169232	57	1.204326
gene-SLC(	SLC6A4	1.495995	183	3.780384	382	2.721493	263	4.08776

gene-ILDF	ILDR1	0.246153	72	0.373706	91	0.493268	113	0.232229
gene-HAU	HAUS2	0.418759	45	3.184272	280	1.81773	155	1.937752
gene-EPS	EPSTI1	4.469156	299	16.00973	892	16.75106	895	16.65068
gene-CNII	CNIH1	7.319224	480	10.35887	565	11.33727	593	11.57753
gene-LIMS	LIMS1	4.084054	614	10.80553	1371	12.54088	1489	11.20713
gene-TMC	TMCC2	6.372629	789	11.77435	1217	10.281	1025	11.2131
gene-ANX	ANXA4	1.888057	158	6.461112	450	5.852057	391	5.670157
gene-TXN	TXNL1	3.96908	232	9.582292	502	8.044736	488	9.638987
gene-LOC	LOC10029	6.824504	280	27.80942	948	30.13975	986	26.35572
gene-DNA	DNAJA1	4.551925	273	13.05888	651	12.82274	613	8.545985
gene-LOC	LOC52276	0.639768	119	1.798042	271	1.385571	228	1.473815
gene-MAF	MAPRE1	6.551748	684	18.38139	1556	19.13225	1515	16.21936
gene-CMF	CMPK1	2.247206	166	5.837509	357	4.49983	271	5.075754
gene-C29	C29H11or	0.244792	24	0.793599	65	0.672765	53	0.521611
gene-PCN	PCNA	5.085944	419	9.391653	643	9.244945	607	10.50336
gene-CD4	CD48	46.00028	1787	109.0642	3526	111.4513	3456	129.5347
gene-SYN	SYNPO2	0.154426	47	0.442821	100	0.309764	68	0.262598
gene-LLPH	LLPH	1.102937	44	3.39275	111	4.587772	144	4.436989
gene-CW(	CWC15	5.264191	258	12.05147	493	15.68627	615	11.72331
gene-RFK	RFK	0.693073	89	2.139824	229	1.742877	179	1.92398
gene-KIA/	KIAA0391	0.317534	67	0.568498	102	0.655758	108	0.624747
gene-SERI	SERBP1	10.76441	791	47.05107	2876	44.7705	2625	45.90275
gene-CCT	CCT4	8.03207	655	25.1811	1707	23.26102	1513	25.86066
gene-DER	DERA	2.787504	191	8.091618	455	7.644843	408	7.259598
gene-RPL	RPL9	219.2017	6295	354.0915	8463	391.6406	8978	402.7718
gene-MM	MMADHC	2.997959	162	8.689775	391	9.582722	414	11.36415
gene-AGP	AGPAT5	0.502697	79	1.239109	162	1.721101	215	0.869679
gene-MA(	MAGEH1	0.754416	45	1.900819	94	2.629821	125	1.762547
gene-WDI	WDR12	1.042069	86	4.267292	292	3.536962	233	3.238771
gene-NMI	NMI	4.141494	262	14.12114	732	12.17003	627	10.9311
gene-CYP	CYP20A1	1.78112	117	3.728713	204	2.861145	149	2.754504
gene-SCP	SCP2	3.773221	528	16.4219	1733	18.15215	1643	15.22412
gene-PPA	PPA1	5.467431	286	17.1899	747	13.61027	567	13.53652
gene-MRF	MRPS36	2.516348	45	3.077742	46	3.462499	49	5.008354
gene-BLVI	BLVRB	112.3238	3514	132.1603	3441	121.183	3026	129.5771
gene-CDC	CDC25A	0.125414	21	0.308024	42	0.464944	33	0.274805
gene-CN(	CNOT7	4.422746	480	16.32894	1475	15.06863	1293	15.01486
gene-DPY	DPY30	4.588769	125	7.119475	166	6.989136	157	6.359228
gene-SFR	SFR1	0.304664	29	1.861153	143	1.225502	90	2.222454
gene-UBD	UBD	135.7948	6004	224.1413	8248	234.1264	8263	210.6377
gene-GMI	GMNN	0.630165	30	1.766426	65	1.60771	57	1.04046
gene-GAL	GALK2	1.398571	112	3.069134	202	2.720782	172	2.016151
gene-PDF	PDHX	0.571429	54	1.836103	145	1.284139	97	1.742262
gene-RTR	RTRAF	11.67202	467	24.80035	825	24.21834	773	22.19768
gene-IFRD	IFRD1	1.498899	141	3.736091	290	4.438722	335	3.209445
gene-CXC	CXCL5	2.475844	153	2.982149	153	2.123355	105	2.310854
gene-CD3	CD3G	13.82942	644	44.11667	1709	44.24854	1644	39.973
gene-TTC	TTC27	0.481978	114	4.437018	442	2.469512	374	3.189402
gene-RFC	RFC3	1.469715	73	2.950306	122	2.928818	117	2.866954
gene-RPF	RPF2	0.293786	41	0.785289	72	1.276292	94	0.765041
gene-EIF2	EIF2S3	10.24545	757	44.97426	2766	43.33558	2556	39.46328

gene-AUH	AUH	0.473414	35	1.355377	83	1.49929	88	1.263101
gene-NIPS	NIPSNAP3	1.778041	118	2.683188	149	3.312465	176	2.932705
Bos_taurus	--	0.818396	18	4.514046	82	2.578769	45	4.468606
gene-ODC	ODC1	18.31796	1479	30.90038	2067	32.46251	2094	27.96153
gene-MRF	MRPS30	1.162986	67	2.457744	118	2.309139	106	1.502464
gene-ING:	ING2	0.543732	52	1.094143	87	1.332764	101	1.401939
gene-PIGI	PIGK	0.921988	55	4.096924	204	2.841115	136	4.002596
gene-LTC	LTC4S	14.78161	373	36.537	771	39.2248	793	33.46635
gene-MZT	MZT1	0.41121	35	1.741342	124	1.046452	72	0.987209
gene-IFI47	IFI47	12.32319	909	11.00321	678	9.512173	562	7.946701
gene-SCC	SCCPDH	1.393389	91	3.903722	211	3.83333	199	3.854886
gene-GTF:	GTF2H3	0.451267	42	1.576536	97	1.517339	103	2.115748
gene-DSN	DSN1	0.533477	51	1.099438	85	1.214533	93	0.808761
gene-CCT	CCT8	3.997825	310	10.95499	706	10.41511	643	9.160923
gene-MA(	MAGOHB	1.074005	53	1.104282	46	2.196537	87	1.932457
gene-AP3	AP3S1	3.677	199	6.196737	279	7.902989	341	5.416279
gene-SMF	SMPDL3A	1.481915	121	4.951171	334	4.536432	294	3.539617
gene-CCN	CCNA2	0.321716	25	1.590994	100	0.608816	37	0.827845
gene-SLC:	SLC16A1	0.509219	68	2.408426	263	3.080933	321	2.707381
gene-CAL	CALD1	0.449162	95	1.90742	332	2.156758	327	1.368594
gene-RPS:	RPS25	364.4714	7289	522.9305	8704	545.7822	8713	533.8256
gene-LMB	LMBR1	0.664528	97	1.217094	146	2.170759	224	1.947469
gene-IFIT2	IFIT2	13.8375	1792	44.32917	4777	45.25087	4677	45.72408
gene-NPN	NPM1	9.121039	487	31.02688	1386	33.14849	1423	28.63866
gene-SLC	SLC40A1	10.96688	1517	22.81107	2625	21.69366	2395	20.61248
gene-RAN	RAMP3	2.599556	155	3.0392	150	3.081391	146	3.012656
gene-MG!	MGST1	2.178518	205	5.403743	387	3.234669	328	4.130113
gene-CDK	CDK1	0.241443	34	1.517902	94	2.067897	117	0.668
gene-ZNF	ZNF330	1.770269	130	7.766688	487	6.480212	390	7.818521
gene-INT!	INTS7	1.876768	326	4.322743	625	3.947679	548	4.455043
gene-ZCC	ZCCHC10	0.506123	20	2.031237	67	1.099324	35	1.423097
gene-PSN	PSMC6	2.208912	146	6.078262	333	7.253007	381	5.500835
gene-HSP	HSPA8	45.43392	4232	136.3683	10571	137.7565	10242	135.0988
gene-UFN	UFM1	0.446869	45	2.769017	228	2.413458	190	1.774089
gene-EID1	EID1	1.873912	137	5.633614	341	5.501206	319	4.192835
gene-TUB	TUBA3E	0.677334	43	1.232153	65	0.66955	34	0.798413
gene-LMN	LMNB1	2.667152	319	9.076682	918	8.518797	813	6.622617
gene-IFI4	IFI44L	1.180434	125	3.146032	276	3.77411	318	2.647436
gene-BIR(	BIRC5	1.377146	95	1.688761	97	1.630368	90	1.383575
gene-BAR	BARD1	0.128155	29	0.208491	39	0.337248	60	0.294514
gene-PPIL	PPIL3	0.642759	42	0.862676	51	1.793577	78	2.97369
gene-ABH	ABHD5	0.59971	67	3.916714	293	3.790603	262	3.838267
gene-SH2	SH2D1A	4.079734	154	12.42369	389	12.07558	361	12.0356
gene-ZKS(	ZKSCAN4	0.33107	85	0.115859	25	0.086541	18	0.235333
gene-GPN	GPN3	0.602182	36	1.185648	58	0.634298	31	1.043015
gene-VDA	VDAC3	25.22007	1511	41.74376	1897	42.99511	1983	42.25156
gene-SUN	SUMO1	2.491538	119	11.01667	437	11.5016	438	12.41834
gene-SLC:	SLC25A17	4.722674	330	7.298663	424	6.499836	362	6.474743
gene-CCN	CCNB1	0.432201	27	0.895131	46	2.037306	99	1.450482
gene-PSN	PSMC2	4.827942	288	12.35705	612	11.00667	523	13.14635
gene-LOC	LOC10033	1.942145	326	3.317631	462	2.667521	358	2.498655

gene-TOB	TOB1	1.680683	157	4.298334	333	5.137207	382	4.895689
gene-RBP	RBPMS2	1.541445	135	1.46938	107	1.495598	105	1.442873
gene-MN/	MNAT1	0.905709	52	1.17446	57	1.334958	62	2.260828
gene-SNR	SNRPB2	1.099122	46	5.821559	202	5.649738	188	6.01014
gene-SERI	SERPINI1	0.122557	15	0.970769	97	0.852806	67	1.059742
gene-CW(	CWC27	0.21991	17	0.625062	41	1.057077	66	1.060393
gene-FAM	FAM213A	0.753631	49	2.047283	111	2.295743	120	2.858845
gene-C17I	C17H4orf4	0.37797	31	1.366175	92	1.375397	89	1.083199
gene-LOC	LOC11244	10.13073	393	18.43026	592	21.10913	652	22.40622
gene-REX(	REXO5	0.392955	48	1.043333	104	0.778161	75	0.506556
gene-LTBI	LTBP1	0.686494	169	0.728951	150	0.759171	148	1.309406
gene-MTU	MTURN	3.391552	808	7.294866	1446	7.729831	1470	7.858675
gene-RPL	RPL7	74.58872	3347	193.9738	7121	201.4376	7236	222.3363
gene-CCT	CCT2	5.291565	422	14.11982	937	16.3293	1039	13.02317
gene-FHL	FHL1	27.7018	2391	46.30969	3290	40.14848	2900	37.67894
gene-YBX	YBX1	32.23705	2042	72.81879	3840	74.95069	3790	78.37628
gene-PNC	PNO1	1.018872	50	2.912212	117	2.266492	88	1.741735
gene-GJA	GJA10	0.135853	55	0.323485	108	0.241898	77	0.316029
gene-RNF	RNFT1	0.884636	81	3.965949	302	3.659137	267	3.253581
gene-FAR	FARSB	1.191641	94	2.603645	171	2.476165	156	2.95182
gene-RSU	RSU1	20.32264	1190	37.23555	1814	34.76443	1624	32.85611
gene-SELI	SELP	3.904517	429	5.376663	491	6.40353	558	5.875622
gene-STRI	STRIP2	0.486296	97	1.171677	194	1.26216	200	1.279405
gene-PDS	PDSS1	0.339777	28	0.915983	59	0.407106	21	0.668278
gene-KIR3	KIR3DS1	0.641626	85	0.862183	93	0.808431	62	0.614903
gene-RPS	RPS3A	315.6536	11282	793.6573	23609	833.6061	23784	827.0344
gene-PDS	PDSS2	0.344028	30	0.768767	53	0.45733	31	0.928219
Bos_taurus	--	1.053386	115	1.288789	118	0.802064	68	1.106178
gene-CYC	CYCS	1.684841	76	6.117671	231	4.380918	161	4.194984
gene-PLS(	PLSCR2	2.782414	222	9.815516	651	8.976133	569	7.210156
gene-AQF	AQP9	0.279593	21	0.844406	55	1.077189	69	1.028348
gene-STM	STMP1	8.422963	706	18.81568	1312	19.03286	1273	18.99054
Bos_taurus	--	3.133084	240	3.037499	275	6.238668	349	5.366909
gene-APP	APP	2.109387	297	3.037643	351	2.337186	277	2.679132
gene-OLA	OLA1	0.377538	27	3.170189	183	2.847682	158	2.612797
gene-GG(	GGCT	0.339897	17	2.198781	92	1.960071	79	1.404999
gene-DD>	DDX10	0.116254	16	0.343439	39	0.613244	66	0.731384
gene-MRF	MRPS35	1.285111	93	2.794936	156	3.012539	170	3.005079
gene-TPT	TPT1	1860.546	64268	3400.268	97756	3385.602	93356	3520.33
gene-CCR	CCR4	1.038227	115	1.706461	162	2.990268	258	1.848798
gene-PLA(	PLAC8	0.421514	44	3.13995	126	5.996932	214	3.206454
Bos_taurus	--	1.330802	70	1.171411	52	1.237352	52	1.029216
Bos_taurus	--	1.070753	74	1.125392	65	1.122923	62	0.863575
gene-COL	COL14A1	0.218496	65	0.24979	62	0.320359	74	0.288317
Bos_taurus	--	1.183185	111	2.12948	158	2.709411	195	2.450049
gene-BCA	BCAS2	0.727748	28	1.376592	44	2.302979	70	2.375324
gene-AK1	AK1	0.476376	39	0.761814	52	0.83525	38	0.466665
gene-CD6	CD69	1.15079	81	5.603103	328	3.458703	194	4.528255
gene-RAB	RAB13	3.906078	250	3.039507	162	3.676919	188	2.906116
gene-PREI	PRELID3B	2.500158	237	6.644073	557	7.189999	482	7.820033
gene-CCN	CCNJ	0.102395	18	0.159336	24	0.199973	28	0.186538

gene-MYL	MYLK	0.202948	68	0.39508	114	0.661116	147	0.395377
gene-EIF3	EIF3E	15.77752	976	83.81277	4312	79.95387	3946	81.68618
gene-KCT	KCTD1	0.249011	38	0.176046	22	0.159045	19	0.706312
gene-MIO	MIOX	1.403025	60	1.623735	58	1.587478	55	1.717453
gene-PF4	PF4	540.3236	12614	822.5983	15984	841.0607	15674	858.9998
gene-IFIT3	IFIT3	14.23183	1396	43.9763	3533	44.04221	3460	43.58441
gene-IER3	IER3IP1	3.269964	188	8.586849	411	8.504196	390	8.048302
Bos_taurus	--	0.806298	32	0.445485	17	0.887648	27	1.037762
gene-LOC	LOC53330	0.808024	21	2.429723	53	0.791747	17	1.993057
gene-SLC4	SLC4A1	0.922041	132	0.895829	107	0.847483	97	0.759215
gene-OAS	OAS2	1.125932	158	3.703029	432	4.178385	467	3.860366
gene-END	ENDOD1	4.540901	855	9.189466	1440	9.596257	1443	8.433186
gene-EID3	EID3	0.819326	51	1.817088	93	1.918585	95	1.456047
gene-PDG	PDGFD	0.397159	31	1.055483	51	1.019302	69	0.347212
gene-TCN	TCN1	8.042979	484	24.69492	1235	22.75048	1092	23.8136
gene-LOC	LOC11244	1.56626	72	7.785127	294	8.608417	312	8.936733
gene-NAP	NAP1L1	14.86778	988	56.12973	3946	71.88929	4237	61.59763
gene-KLR	KLRG1	0.157821	12	1.107858	70	0.974046	59	0.916203
gene-TUB	TUBB1	79.15968	8569	154.0668	14275	154.8429	13685	158.2525
gene-NRIP	NRIP3	0.151678	24	0.856045	112	0.859858	107	0.876286
Bos_taurus	--	0.369171	35	0.617574	30	0.926418	46	1.760978
gene-GNG	GNG11	66.45496	2046	134.5683	3448	139.7216	3434	143.1508
gene-USP	USP15	1.136781	273	3.917878	695	5.44591	871	4.925567
gene-OAS	OAS1Z	6.77498	444	18.99288	1035	18.05424	944	16.19052
gene-PLXI	PLXDC1	0.181093	21	0.437651	41	0.491258	45	0.799299
gene-FBX	FBXO9	12.84727	1134	21.7438	1608	21.62752	1574	22.27796
gene-EIF1	EIF1B	42.51014	1689	78.9099	2612	82.8809	2635	75.15967
gene-LOC	LOC50530	5.765696	116	3.991865	67	4.415882	71	4.725456
gene-LOC	LOC10190	0.362164	16	0.74964	27	0.256013	9	1.021243
gene-ISG1	ISG15	212.6393	5193	230.6043	4687	236.6256	4613	251.1499
gene-CRE	CREG1	5.485193	448	14.26651	969	14.07489	914	14.05932
gene-TME	TMEM86B	3.320577	139	2.954676	103	3.258723	109	3.507191
gene-AHS	AHSP	1.802682	43	0.324868	19	0.5228	19	1.221974
gene-EIF3	EIF3M	13.70555	725	31.61892	1394	30.43348	1289	32.69848
gene-LOC	LOC51153	0.97986	93	3.545467	274	3.181942	256	4.791034
gene-TCE	TCEAL8	0.702291	29	3.275011	96	1.355816	45	2.214692
gene-CRC	CROT	0.234854	30	0.625237	65	0.454198	45	0.577983
gene-C16	C16H1orf2	0.764735	105	1.91248	217	2.037924	229	2.823034
gene-SUC	SUCLG2	3.7785	357	11.78845	933	11.02984	832	12.92814
gene-BZW	BZW2	1.179368	89	5.075773	315	4.551812	271	3.831474
gene-LOC	LOC52457	0.518493	82	1.68129	220	1.366116	171	1.47789
gene-CLE	CLEC6A	0.293326	22	0.953417	58	1.118333	65	0.624129
gene-SLFN	SLFN14	0.1902	45	0.321634	64	0.300781	57	0.278241
gene-ATP	ATP5F1E	61.29417	1457	45.43454	899	48.91991	928	60.87829
gene-LOC	LOC51365	0.536859	107	1.683169	239	1.357373	191	1.65139
Bos_taurus	--	0.440257	12	1.785479	38	1.003261	21	1.742062
gene-APC	APOLD1	0.339988	57	0.431628	64	0.378934	53	0.413246
gene-PGR	PGRMC1	4.921891	378	11.29684	722	11.93601	732	11.26555
gene-SKP1	SKP1	51.70694	2071	126.7058	4223	133.1725	4257	120.2435
gene-LOC	LOC78454	0.151518	17	0.460567	32	0.574489	41	0.513041
gene-MRF	MRPL1	0.350391	23	1.018707	54	0.937404	48	1.44282

gene-RHC	RHOBTB1	0.206354	36	0.108822	16	0.240646	33	0.504422
gene-YEA	YEATS4	0.157413	10	1.262855	63	0.754115	36	0.786021
Bos_taurus	--	0.519796	21	2.013628	63	1.138605	36	2.124453
Bos_taurus	--	0.588611	25	1.910765	54	0.292588	11	0.538832
gene-GAB	GABARAPI	17.10178	1354	24.74525	1630	26.52168	1676	28.13052
gene-LOC	LOC78129	0.596254	50	0.601056	43	1.123929	77	0.730963
gene-LOC	LOC10190	0.348686	22	1.749838	88	1.856	90	1.054836
gene-SEM	SEMA6B	0.097142	17	0.240584	34	0.183971	25	0.076908
gene-CAC	CACNG4	0.094451	13	0.172057	19	0.213195	22	0.3013
gene-TRA	TRAK2	1.505825	208	2.986942	344	2.98461	329	4.829865
Bos_taurus	--	2.566905	60	1.957222	38	1.781406	34	2.796464
gene-GPR	GPR19	0.077894	18	0.174151	27	0.201105	37	0.493673
gene-TME	TMEM119	1.393085	145	5.259387	455	4.770046	396	5.086151
gene-TEX	TEX30	0.856484	51	1.599551	66	2.53789	98	2.62542
gene-AOX	AOX1	0.508651	104	0.388072	66	0.377	62	0.306498
gene-ACB	ACBD6	0.491393	25	2.072294	82	0.969573	41	0.996692
gene-SLC	SLC16A9	0.129951	22	0.161914	23	0.143302	19	0.254354
Bos_taurus	--	0.265018	79	0.245132	61	0.356556	85	0.211214
gene-ITGE	ITGB3	7.681859	1854	16.67589	3341	17.009	3278	15.90799
gene-HMI	HMBS	15.74839	948	11.5601	583	13.2062	643	16.37101
gene-CSD	CSDC2	0.410513	47	0.22242	23	0.436431	39	0.35866
gene-HBN	HBM	75.9773	1617	78.82506	1397	82.01748	1394	93.08472
gene-ZDF	ZDHHC2	0.530808	80	2.587515	324	2.633123	317	2.044586
gene-HRC	HRCT1	0.169359	21	0.389872	39	0.525584	50	0.441591
gene-FBX	FBXO48	0.108985	14	0.233212	25	0.875916	37	0.204115
gene-XK	XK	0.375383	69	0.716784	109	1.108165	162	1.225227
gene-TEX	TEX12	0.673488	27	1.046786	43	0.353051	19	0.11331
gene-DSC	DSCC1	0.493106	32	1.741855	93	1.008255	52	1.426269
gene-MM	MMD	5.200922	515	9.773948	901	10.87309	942	9.89599
gene-LOC	LOC10084	0.045047	15	0.153899	43	0.074127	20	0.118236
gene-KLRI	KLRF1	0.378674	19	1.473041	58	1.304232	48	1.683509
gene-LOC	LOC78336	0.493027	19	0.508014	17	0.219111	7	1.267587
gene-LOC	LOC10190	0.447723	18	0.550408	19	0.890464	29	0.993625
Bos_taurus	--	3.64199	269	6.423924	389	6.445256	362	5.003565
gene-GPN	GPM6A	0.126238	17	1.039815	106	0.699664	71	0.930311
gene-APC	APOBEC3Z	3.041745	97	6.584364	173	5.922748	150	6.512877
gene-TME	TMEM267	0.165018	25	0.306464	38	0.562305	58	0.235
gene-RAB	RAB27A	9.999418	1205	23.50106	2449	26.59445	2675	24.34203
gene-LOC	LOC78634	0.070822	6	0.391603	25	0.431939	27	0.50663
gene-SIGL	SIGLEC1	0.486962	138	1.038261	245	1.156387	262	0.560592
gene-LGA	LGALSL	5.383432	789	9.385641	1145	10.35067	1211	8.078101
gene-PTG	PTGR1	3.67354	192	8.341478	363	9.145966	381	8.035165
gene-GAT	GATM	0.346124	35	2.468503	197	3.058343	235	3.727794
gene-ZNF	ZNF774	0.175702	21	0.322405	31	0.073591	7	0.353177
gene-RAN	RANBP10	3.869985	830	4.665679	833	4.75683	815	5.221817
gene-MES	MEST	0.161156	16	0.486859	40	0.304718	24	0.251738
Bos_taurus	--	0.27714	17	0.502414	26	0.96638	41	0.369461
Bos_taurus	--	0.123047	21	0.199721	28	0.37005	49	0.271473
gene-UCH	UCHL3	0.620231	32	2.994902	125	2.757898	111	3.774111
gene-LOC	LOC78171	0.781207	72	1.739695	132	1.922648	140	1.180229
gene-CXC	CXCL8	0.80358	50	3.520097	180	1.959521	96	2.003616

gene-TME	TMEM8A	16.22821	1664	20.04304	1730	18.07974	1499	19.10911
gene-PCL/	PCLAF	1.031354	58	3.525815	163	2.49553	111	1.191348
gene-VSTI	VSTM4	0.468416	43	0.360657	29	0.443459	34	0.463082
gene-LOC	LOC78679	1.644715	214	1.84046	198	1.839956	191	1.768247
gene-OAS	OAS1Y	20.38373	1411	39.96379	2302	47.28539	2613	46.22503
gene-LOC	LOC11244	0.281584	12	0.40375	28	1.715676	56	0.335967
gene-LOC	LOC61707	1.188689	119	1.458033	122	1.209966	97	1.178013
gene-FRM	FRMD4A	0.252187	47	0.545457	84	0.436529	65	0.335178
gene-LOC	LOC78698	0.946199	86	2.107641	159	2.171468	157	3.624166
gene-FAM	FAM171A1	0.033393	6	0.310713	46	0.404337	57	0.568729
gene-CDH	CDHR5	0.19107	19	0.350452	33	0.465051	42	0.644844
gene-RNF	RNF128	0.044489	6	0.161173	17	0.239275	23	0.078651
gene-LOC	LOC10713	0	0	0.396269	22	0.637314	34	0.735608
gene-SHC	SHCBP1	0.303344	31	1.25659	107	0.832561	68	1.228382
gene-RBM	RBM11	0.322293	27	0.52927	37	0.347789	23	0.380928
Bos_taurus	--	0.504443	57	0.967067	81	1.604028	124	1.214463
gene-QPC	QPCT	0.362337	32	0.304437	22	0.14404	10	0.18679
gene-TFF2	TFF2	31.82661	786	34.79563	715	33.34959	657	31.18446
gene-LOC	LOC51425	0.442123	116	0.86749	181	0.831967	162	0.791928
Bos_taurus	--	0.38966	14	0.86513	26	0.962703	28	0
gene-MX2	MX2	1.546035	239	12.87657	1852	13.736	1730	13.7276
Bos_taurus	--	0.524569	10	0.798026	12	1.831308	26	1.335216
gene-DMT	DMTN	0.56835	69	0.825152	82	0.910443	85	0.507286
gene-AQP	AQP1	0.543704	61	0.685908	64	0.379052	34	0.978658
gene-BBS1	BBS12	0.062728	7	0.414194	34	0.192368	16	0.165779
gene-LOC	LOC11244	0.853784	48	1.227342	57	0.645551	29	0.232127
gene-RAB	RAB3IL1	0.291814	35	0.11104	11	0.172198	17	0.586968
gene-TRIN	TRIM10	2.339254	197	1.842314	129	1.320711	89	1.397381
gene-FKBI	FKBP14	0.378549	49	0.258852	28	0.281446	29	0.445168
gene-PYG	PYGM	1.23049	145	1.817992	179	2.050431	193	1.544844
gene-ICAI	ICAM4	0.783498	67	0.134982	10	0.572511	39	1.046866
Bos_taurus	--	0.273428	54	0.184764	33	0.60913	95	0.137315
gene-SOD	SOD3	0.120666	12	0.748733	51	0.729515	46	0.834096
gene-TME	TMEM232	0	0	0.408148	37	0.229796	20	0.360893
gene-KLF1	KLF1	3.026953	205	2.338902	132	3.284587	177	1.551325
gene-MIN	MINDY3	7.704817	736	22.7101	1793	25.64298	1960	22.10348
gene-SRXI	SRXN1	5.067073	532	10.43899	912	10.97648	919	11.41514
gene-ALA	ALAS2	95.45297	7603	155.9518	10405	148.9929	9482	151.1116
gene-LOC	LOC10190	0.156461	23	0.265975	33	0.320333	37	0.440678
gene-HBB	HBB	20566.05	522603	24817.59	524877	26008.18	527574	25089.34
gene-CMF	CMPK2	2.180464	331	5.188792	654	5.200127	629	4.62231
gene-RHP	RHPN2	0.084155	11	0.119071	13	0.227258	24	0.019364
gene-HBA	HBA1	5743.814	140498	6934.287	141172	6964.034	135982	8076.004
gene-PLA2	PLA2G1B	1.407844	37	0.419147	9	0.485349	10	1.206999
gene-CRY	CRYGS	0.24755	9	4.645743	100	3.167256	82	4.756555
gene-MEIS	MEIS1	0.014758	3	0.122767	15	0.478792	43	0.091012
gene-ERM	ERMAP	0.04917	7	0.227428	24	0.19352	20	0.182892
gene-CPX	CPXM2	0.078215	12	1.07205	128	1.303182	146	1.017821
gene-RSA	RSAD2	0.537868	58	5.154739	456	4.366329	370	4.750841
gene-OAS	OAS1X	6.331606	326	12.8304	549	11.97183	492	9.127501
gene-HBA	HBA	5954.145	138754	7433.696	144181	7712.564	143475	8904.281

gene-LOC	LOC11244	0.62008	77	1.661449	164	1.6258	154	1.070064
gene-LOC	LOC10029	0.587185	69	1.683298	163	0.998967	93	0.997985
gene-CD1	CD1E	0.29767	23	0.832811	53	0.445858	27	0.461574
Bos_taurus	--	0.34687	24	0.040802	3	0.263962	17	0
gene-TRIN	TRIM58	1.946709	143	2.501923	152	2.299955	135	2.536752
gene-WN'	WNT5A	0.157511	25	0.049727	13	0.039456	10	0.031875
gene-STR/	STRADB	0.935914	64	0.779299	51	2.179434	116	1.826866
gene-ATP	ATP5IF1	616.8455	11546	458.1403	7138	485.6255	7257	511.9882
gene-CA2	CA2	8.903765	545	7.963872	406	8.050713	394	10.19873
gene-SVIF	SVIP	0.80006	57	1.353258	80	1.913442	108	1.57601
gene-LOC	LOC10190	0.596154	11	1.585459	24	1.324223	19	0.35055
gene-TAC	TAC3	7.774902	242	37.308	1007	35.84829	915	43.70284
gene-STE/	STEAP2	0.158686	14	0.191048	14	0.147548	10	0.36357
gene-CLIC	CLIC2	0.228776	26	0.467293	44	0.519643	47	0.685938
gene-LOC	LOC61657	0.273231	36	0.148315	16	0.36142	38	0.423338
gene-GYP	GYPB	5.153777	155	3.840597	93	4.00217	93	2.928293
gene-LOC	LOC78767	38.50292	707	40.64596	621	62.04149	909	32.22276
gene-LOC	LOC52582	0	0	0.17605	16	0.186688	16	0.220314
Bos_taurus	--	0.130372	15	0.064788	7	0.216456	20	0
gene-SMII	SMIM6	0.138588	9	0.125246	8	0.115517	6	0.107948
Bos_taurus	--	2.982808	61	0.839926	15	1.281191	21	3.383584
gene-ADD	ADD2	0.019094	7	0.062156	19	0.053714	16	0.061595
Bos_taurus	--	4.115924	98	4.017063	79	2.533412	48	6.686853
gene-REEI	REEP1	1.135187	191	1.068459	149	1.167017	156	1.428679
gene-MG(	MGC1271:	0.219385	11	0.113636	5	0.271626	11	0.101133
gene-SSX!	SSX5	1.268919	50	2.318515	86	2.479243	90	3.216719
gene-CHA	CHAC2	0.56412	51	1.696626	122	1.414274	118	1.291821
gene-LOC	LOC78780	3.556314	81	2.073405	39	1.402055	26	3.284544
gene-STU	STUM	0.071668	6	0.166263	10	0.09527	6	0.628624
gene-EPB	EPB42	0.038206	5	0.521895	48	0.259431	23	0.311945
gene-LOC	LOC10713	1.022359	23	0.927875	18	0	0	0.943525
gene-LOC	LOC10084	0.927029	40	1.058783	38	1.3401	46	1.020199
gene-LOC	LOC51244	0	0	0.018658	2	0.252948	22	0
Bos_taurus	--	0	0	0.008289	2	0.03457	9	0.055938
gene-ARG	ARG1	1.219349	82	2.076868	117	1.962776	97	1.926958
gene-HBQ	HBQ1	5.113055	182	3.990968	170	5.98628	174	7.147796
Bos_taurus	--	0.054965	2	0.464173	14	0.092546	3	0.135635
gene-ANK	ANKS1B	0	0	0	0	0	0	0

H4_count	L1_FPKM	L1_count	L2_FPKM	L2_count	L3_FPKM	L3_count	L4_FPKM	L4_count
91	0	0	0	0	0	0	0	0
54	0	0	0	0	0	0	0	0
123	0.023296	4	0	0	0	0	0	0
43	0	0	0.105613	4	0	0	0	0
33	0	0	0	0	0.019677	5	0	0
50	0	0	0	0	0.189986	8	0	0
158	0	0	0.203254	8	0.444709	17	0.23848	9
65	0	0	0.105751	2	0.121384	2	0.825697	8
107	0.097325	11	0	0	0	0	0.042885	5
138	0.041529	11	0	0	0.037995	9	0.055119	12
66	0	0	0.098285	11	0	0	0.085687	10
153	0.144295	15	0	0	0.229456	19	0.335079	29
396	0.26688	32	0.627236	63	0.39509	40	0.351861	37
83	0.031338	4	0.183511	17	0.136272	13	0	0
2287	1.114469	117	3.394404	296	3.163853	278	3.003371	268
627	0.145555	30	0.428988	74	0.436585	75	0.599385	105
36	0.072318	11	0	0	0.061847	8	0	0
127	0.721079	26	0	0	0.738984	23	0.718168	22
287	0	0	0.44223	26	0.762732	45	1.058913	61
1072	1.692968	65	4.83581	155	6.542685	211	4.127844	135
79	0	0	0.700764	20	0.320956	10	0	0
148	0.071207	13	0.185696	26	0.189365	26	0.187457	28
49	0.114054	4	0.652297	16	0	0	0	0
674	0.58001	84	1.088753	131	0.757601	92	0.621889	77
107	0.05398	16	0.093232	23	0.028651	7	0.008135	2
62	0.073865	18	0.127289	26	0.016949	3	0.045665	10
437	0.902381	60	0.570297	32	0.237921	17	2.311903	129
356	0.082555	31	0.203692	63	0.210647	66	0.30851	97
187	0.055894	12	0.140205	27	0.295153	57	0.152843	30
898	2.534372	126	4.087747	170	4.929984	206	2.504283	107
93	0	0	0.610477	35	0.269796	16	0.187014	11
3013	8.175012	274	18.37405	515	21.0747	594	22.82051	653
1214	0.872376	108	2.289782	236	2.975989	309	2.327528	245
480	0.684929	81	0.808462	104	0.698517	118	0.405484	50
75	0.03524	9	0.019516	4	0.038155	8	0.038827	8
67	0.073135	11	0.170291	21	0	0	0.079937	11
1379	0.758883	166	2.044004	376	1.17409	220	1.283147	243
5291	6.790074	708	11.57873	1010	11.68324	1027	11.63165	1037
1018	0.726244	124	1.730984	144	1.493353	214	2.765714	223
464	1.182862	112	0.489504	43	1.621082	128	1.055298	85
16625	51.86223	1868	109.6647	3304	121.1032	3676	126.0973	3881
172	0.169277	17	0.162839	10	0.291388	25	1.080323	73
1391	2.072408	189	3.479631	265	3.512888	269	4.560397	354
275	0.201958	31	0.286148	28	0.829701	61	0.327381	40
38981	13.01573	824	187.2726	9855	183.2503	9733	196.1639	10671
616	0.493834	81	0.850746	116	1.292893	178	0.563058	79
241	0.122846	5	1.850227	57	2.202831	68	2.024062	63
1546	1.969883	225	3.237356	309	3.039252	331	3.328924	328

161	0.240817	28	0.593729	65	0.36538	40	0.205895	22
454	0.385378	70	1.254369	119	0.629739	96	0.696229	107
1812	2.348458	276	6.92256	516	3.874398	362	5.829109	573
823	1.54262	203	1.930114	217	1.935247	192	1.543269	207
143	0.409697	34	0.127472	9	0.375873	27	0.669241	47
166	0.021843	4	0.788513	50	0.139581	22	0.565499	33
2462	8.680812	268	26.33178	681	22.43996	584	18.24754	482
188	0.134826	11	0.424341	28	0.490873	32	0.588271	39
152	3.067172	61	0.900934	15	2.813202	48	2.173136	37
55	0	0	0.146363	22	0.092682	14	0.049401	8
211	0.409922	47	0.623829	60	0.553945	54	0.580229	57
2368	4.46182	274	12.14768	636	12.76873	647	12.71132	669
2756	15.13623	561	23.58439	731	18.80862	588	20.68951	655
71	0	0	0.21121	13	0.247733	22	0.14652	14
265	1.926902	64	2.837849	79	0.73536	21	2.215944	63
54	0.049088	3	0.671209	35	0.477006	25	0.210114	11
324	1.097258	90	1.157272	88	1.350763	109	0.509653	40
152	0.412605	28	0.873222	48	0.508777	29	0.774712	42
68	0	0	0.150625	21	0.128527	18	0.189046	26
834	0.411476	74	1.890285	281	2.746284	398	1.336108	204
136	0.099364	17	0.110234	16	0.166176	24	0.331746	48
129	0.215025	28	0.582571	69	0.228064	25	0.130064	16
130	0.293095	31	0.383297	34	0.598406	54	0.364128	33
81	0.149442	27	0.166937	25	0.191441	29	0.108217	17
97	0.197447	36	0.394264	60	0.117491	18	0.158365	25
71	0.178698	14	0.057889	4	0.480448	32	0.270234	18
136	0.030429	11	0.110072	33	0.122467	37	0.139924	42
50	0.268896	22	0	0	0.163168	11	0.383103	26
77	2.034127	31	1.304255	17	3.194341	40	2.516747	32
123	0.200737	11	0.308991	14	1.029076	47	1.676835	77
789	2.10349	276	1.395425	153	2.609974	289	1.068642	120
179	0.65808	44	0.567417	32	0.557969	32	0.587086	34
55	0.132171	9	0.429261	24	0.21241	12	0.472235	18
71	0.227752	13	0.570942	27	0	0	0.712489	35
802	4.169887	273	3.378749	192	5.340489	297	3.687876	205
104	0.110831	16	0.295546	36	0.450128	54	0.158841	20
771	0.893074	161	1.397133	216	2.035933	313	1.192638	181
162	0.053861	8	0.225545	26	0.264505	31	0.343285	40
619	4.787303	152	7.820816	197	9.790731	255	8.923166	232
50	0.113072	7	0.261039	14	0.48519	28	0.421151	23
765	3.153836	122	7.3002	235	7.792387	253	7.48384	246
5323	13.9581	1609	16.3611	1574	16.92283	1645	13.35359	1322
70	0.554503	23	1.034319	36	0.917091	32	0.589449	21
178	1.03432	55	0.723981	32	1.437723	64	0.79176	36
1901	1.637378	262	5.239071	697	4.916315	664	5.236501	720
478	0.525008	71	1.163205	131	1.448718	149	1.051359	118
455	0.670633	51	2.540691	152	3.580274	217	2.130726	134
58	0.388209	21	0.665078	31	0.186651	9	0.340696	16
473	0.578432	53	2.165938	164	2.439967	184	1.543309	121
278	1.385672	61	2.10222	135	4.134158	113	0.452141	58
143	0.192696	28	0.402674	49	0.359971	44	0.492323	61

119	0.156272	25	0.115232	16	0.06657	9	0.212218	29
68	0.086302	9	0.187058	16	0.126739	11	0.479952	42
200	0.065177	27	0.278431	96	0.299604	104	0.32793	116
110	0.041316	16	0.057199	19	0.214053	70	0.030917	11
636	1.142467	140	1.987488	233	3.378799	348	3.057538	314
696	0.105824	63	0.496225	245	1.054752	524	0.590419	298
167	0.269511	30	0.512618	48	0.842651	79	0.798581	76
690	5.367337	277	5.629869	235	5.157658	229	6.443747	290
887	0.161442	106	0.609051	335	0.616731	341	0.793733	445
752	4.543863	245	2.454067	111	2.641167	116	3.212102	148
594	0.433977	78	1.683217	253	2.281381	173	1.398066	215
2632	5.710747	507	11.79862	874	14.236	1063	17.01253	1247
815	3.555766	227	5.916356	312	4.383498	236	5.423246	294
353	0.339925	81	0.670821	134	0.658066	132	0.787822	161
2856	7.850302	962	9.100191	936	7.573483	785	8.447498	887
449	1.354695	102	2.548353	161	2.607709	166	2.871607	180
66	0.432644	26	0.430108	22	0.13778	7	0.632623	33
174	0.83564	64	0.433927	29	1.123798	76	0.584682	40
83	0.265558	16	0.623698	30	0.424714	21	0.496171	25
75	0.362895	41	0.138303	13	0.261377	26	0.424759	41
137	0.06483	16	0.241492	50	0.349638	72	0.108382	23
4298	19.07124	1574	20.49165	1415	23.24208	1616	22.95137	1619
86	0.201001	42	0.183523	31	0.23781	40	0.171013	30
793	1.267635	267	2.251933	373	1.881144	338	2.180447	397
147	0.090367	37	0.112522	39	0.317535	109	0.154277	54
7353	18.51534	1959	35.00442	3098	34.33523	3060	36.89847	3336
1153	2.867684	249	5.041513	381	6.009625	489	7.281932	575
64	0.522278	26	1.195907	50	0.393557	17	0.496183	22
116	0.456029	25	0.323975	38	0.64997	66	0.394465	48
315	0.527954	78	1.555625	152	1.418552	166	1.161578	95
956	4.23925	239	7.089636	334	9.128821	434	8.457333	408
72	0.130605	16	0.169025	20	0.131281	16	0.38775	46
193	0.217958	43	0.474036	61	0.537783	106	0.801034	105
194	0.069834	13	0.46238	68	0.509713	75	0.415686	62
4059	14.09555	1731	14.41969	1482	16.87786	1747	12.44641	1306
754	3.180029	322	5.037652	427	4.271964	365	3.347872	290
58	0.298548	13	0.706018	26	1.09508	40	1.52126	57
319	0.174573	60	0.312476	90	0.363158	102	0.46198	135
558	0.662541	63	2.904216	225	2.909676	230	3.127755	251
320	0.328704	44	0.738252	84	0.68113	78	0.973155	113
72	0.79537	27	1.035331	29	0.368992	11	1.028822	30
86	0.282239	39	0.232438	22	0.36403	43	0.277327	18
2156	3.445931	663	5.364074	955	4.67154	856	4.899958	925
1567	14.05002	566	16.72438	564	15.2399	517	13.47676	464
192	0.34175	19	2.138411	87	0.920938	32	1.714985	70
177	0.520656	62	0.770345	39	0.411679	50	0.978943	37
198	0.331322	44	0.478187	52	0.778754	86	0.839193	93
267	0.044946	40	0.100685	74	0.211635	157	0.280165	210
390	0.468242	36	2.988101	190	2.766038	177	3.068501	199
3392	16.65017	577	56.55121	1639	51.12973	1493	57.46098	1701
121	0.432432	50	0.209383	25	0.265366	33	0.877099	90

254	0.644491	74	0.676213	68	0.688826	68	1.244141	122
319	0.773579	88	1.40146	125	1.361988	123	1.328107	128
247	3.946817	106	5.590428	126	6.122636	139	6.281871	145
254	0.175627	42	0.745508	148	0.470683	94	0.495258	100
164	0.110621	24	0.294372	52	0.311892	56	0.658568	118
134	0.401115	28	0.991265	58	0.893902	53	0.574477	35
1059	8.483179	539	6.807118	362	7.915746	424	7.651788	416
105	0.284773	42	0.132442	16	0.6925	85	0.251258	32
248	2.474762	111	3.215161	122	1.021741	93	1.557732	59
1290	1.90459	378	3.047469	506	2.905997	486	3.62452	614
114	0.108645	26	0.0647	13	0.186453	38	0.237641	49
95	0.539096	44	0.478442	33	0.223636	16	0.306767	22
420	2.484065	147	3.272626	160	3.702325	185	3.319721	167
1148	5.708289	571	6.788069	568	6.686298	564	5.667336	485
170	0.905953	58	0.675164	37	1.98459	107	1.514843	83
1025	2.540145	298	5.53724	453	3.224861	355	4.2124	406
87	0.121253	12	0.464424	38	0.683898	56	0.232929	20
66	0.400402	25	0.302593	20	0.346118	23	0.279571	19
111	0.126056	31	0.099901	21	0.400205	82	0.435863	90
188	0.494718	56	0.804043	75	0.949282	91	1.05473	113
303	0.612971	62	2.160583	183	1.78709	152	2.160268	171
210	0.314096	27	1.032107	72	0.95423	67	1.809517	129
287	1.217803	98	1.677054	112	2.192784	148	1.354625	93
416	0.840058	169	0.730647	123	1.626003	272	1.226196	200
103	0.55675	31	0.600618	30	0.487922	23	1.853851	82
428	2.058867	106	3.387275	141	3.330976	145	3.861658	189
1740	17.62632	494	30.24966	738	37.38234	891	29.03896	697
254	0.087465	40	0.411825	157	0.265855	102	0.280311	109
2855	3.294593	758	8.307103	1221	10.99687	1601	8.650635	1285
170	0.293499	72	0.289711	59	0.407737	83	0.435456	91
534	3.149161	144	6.112652	234	6.471168	320	4.536716	223
619	3.698555	137	10.43537	322	8.830811	274	11.85674	373
5821	25.09433	2029	37.25376	2521	38.02007	2591	37.08344	2563
2421	5.581077	517	15.77885	1187	16.55101	1175	15.61108	1111
57	0.074917	13	0.360801	50	0.258922	37	0.215082	32
3646	5.226423	944	12.00573	1715	9.893327	1505	11.41705	1686
5638	22.57649	1433	49.37321	2629	44.74178	2396	56.91327	3079
234	0.389392	78	0.695534	117	0.513012	87	0.378645	65
2064	38.9803	1111	29.85065	730	33.7958	836	30.62779	781
140	0.303527	28	0.295436	23	1.020413	76	0.541465	41
2010	14.79154	879	19.18546	965	14.04932	712	19.42372	969
114	0.278267	25	0.740914	70	0.423714	43	0.651577	61
181	0.293335	32	0.606364	64	0.993674	112	0.75206	82
1777	5.988507	757	7.624389	806	8.056852	858	6.875085	743
312	0.780546	90	1.11568	109	2.007082	196	1.059073	93
68	0.131941	25	0.181948	28	0.139827	22	0.16946	27
237	0.54824	57	1.134947	98	1.246848	109	0.604597	54
103	0.203303	44	0.455199	82	0.089337	17	0.43892	80
597	0.959637	215	1.615272	302	1.080614	204	1.381942	264
17938	57.31557	5207	102.8966	7840	114.7442	8790	117.5465	9134
200	0.222	44	0.980006	164	0.424827	72	0.259356	45

70	0.400405	35	0.273355	20	0.902395	67	0.337946	26
143	0.961038	43	3.30689	124	1.988875	73	1.061586	41
2186	5.460006	590	13.94405	1249	12.10305	1096	11.34916	1049
37	0.18998	21	0.654849	41	0.487785	44	0.265335	25
781	5.903954	352	8.381427	418	8.774935	441	6.544452	333
633	2.091282	131	5.54422	288	5.884914	312	7.578625	404
121	0.276456	41	0.608344	75	0.330085	41	0.430661	54
788	1.697758	219	3.27109	353	2.958765	322	3.439902	379
75	0.482107	22	0.638821	27	0.75588	32	0.486136	21
169	0.205676	28	0.793368	80	1.296335	134	0.91428	94
360	4.719869	182	4.042644	124	5.534155	164	5.119491	160
101	0.251312	38	0.107345	14	0.34463	33	0.591782	60
86	0.548029	24	0.703783	41	0.614844	57	2.007979	47
1922	9.038911	881	13.17777	1075	7.887453	648	11.03077	919
496	0.552726	77	2.720242	311	1.936123	223	2.195329	257
329	0.302422	67	0.755415	140	0.684597	128	0.74011	141
9520	21.51662	2429	48.18673	4545	47.96419	4556	52.78376	5080
175	0.149051	40	0.387247	87	0.308636	70	0.25596	59
737	1.789771	199	4.850825	452	3.32302	310	5.149592	491
66	0.245653	27	0.415148	38	0.516795	47	0.209282	20
554	0.689104	160	1.736575	335	1.305234	254	1.627167	324
262	1.095003	79	1.550299	93	1.682371	102	1.821913	112
212	0.535974	84	0.917489	68	0.49525	74	0.980965	176
316	1.279026	131	1.544959	132	1.69759	146	1.838119	160
1040	4.119395	373	6.592025	517	8.677789	608	8.744483	612
181	1.15098	48	1.401109	66	1.664611	75	3.574874	109
5695	11.2686	1685	22.47592	2821	23.00478	2897	27.37786	3500
518	1.122414	166	1.93887	266	1.214615	231	2.049972	247
21871	105.0195	4832	258.652	9957	276.9755	10741	231.5839	9107
168	0.277987	50	0.865921	130	0.453783	69	0.743397	114
98	0.230632	37	0.455166	60	0.374301	50	0.141357	19
5468	16.26832	2196	25.48546	2317	33.05069	2773	20.39723	2289
213	0.912817	141	0.907716	120	0.875651	114	1.019892	137
152	0.629176	42	1.60586	90	0.743146	42	0.962037	55
166	0.200077	46	0.516844	99	0.167425	33	0.386215	76
417	2.061427	165	3.668747	227	2.028418	137	4.296633	293
283	1.78892	113	2.844686	151	2.127547	114	2.732558	148
249	1.343441	123	1.659104	121	0.829594	85	0.570401	60
99	0.532701	43	0.659192	44	1.136592	77	0.385865	27
165	2.050587	94	2.195951	84	1.232663	48	1.186879	48
1978	9.960978	1437	6.743804	814	5.656539	688	5.636433	695
146	0.200088	45	0.477726	88	0.378685	70	0.315986	59
25031	60.01571	7925	117.5863	13099	110.5553	12323	118.8426	13437
280	2.601502	114	1.72494	78	2.55553	110	3.609854	169
572	1.428201	139	4.017375	327	3.165174	260	2.86863	239
26664	119.9952	10729	185.0547	13560	177.4261	13187	175.7308	13423
298	1.968917	125	3.69624	196	1.84464	99	2.839634	154
1259	4.409194	497	6.820884	636	6.121308	585	5.703739	541
85	0.321805	46	0.568983	67	0.299081	36	0.341175	41
175	0.397858	70	0.674653	104	0.932932	102	1.052301	119
801	0.812459	182	2.091642	399	2.561881	507	1.315081	284

172	0.715417	112	0.245152	32	0.613033	81	0.664122	89
1003	1.839958	302	2.364426	325	2.602319	360	2.636522	370
221	0.117007	27	0.292299	56	0.502383	97	0.878451	171
30288	256.2497	12362	274.293	11090	264.7079	10825	338.2647	13988
599	1.959245	354	1.892291	293	1.428457	276	0.888635	177
232	0.678253	86	0.868304	93	1.209165	108	0.366071	40
149	0.898782	63	1.723122	100	1.423945	83	0.923308	55
1151	10.72326	414	15.94084	515	19.0153	619	14.88783	488
245	1.99597	104	3.627202	158	4.668222	127	2.763393	64
3260	22.78018	1176	39.32847	1691	41.48756	1795	39.31948	1724
2642	19.50919	1277	30.25938	1657	21.80619	1203	25.08604	1403
195	0.413212	72	0.453321	67	0.539994	80	0.562112	84
540	0.906579	134	1.222018	258	0.966379	222	2.223413	316
2323	22.4963	923	28.10823	964	29.14213	1005	32.0576	1126
176	0.388855	36	0.868485	68	1.236008	97	0.942368	75
197	2.033822	130	1.457425	78	1.621149	88	2.007164	110
336	0.905277	87	2.388038	191	1.707576	137	2.663405	217
271	0.686084	143	0.682725	109	0.53644	102	1.249179	189
3526	3.411109	1054	7.162745	1897	7.445364	1969	7.039278	1891
78423	434.3444	35397	578.5063	39450	584.42	40146	564.6958	39336
723	0.597829	185	1.269038	336	0.800756	213	1.382944	340
22820	69.67291	9290	107.7036	11998	114.9652	12884	115.3769	13157
11462	58.09852	4163	80.576	5728	88.31655	5922	107.3275	6925
308	1.252437	125	1.246196	103	2.259821	191	1.88445	159
175	1.00001	65	1.641355	89	0.86963	47	2.244558	123
8934	113.1652	3802	167.8696	4719	159.4039	4514	167.1332	4800
758	0.248322	231	0.580754	448	0.41756	341	0.502879	376
105	0.096915	50	0.077669	34	0.106942	47	0.201173	85
2697	14.86813	1138	19.27248	1240	21.8574	1424	25.88598	1716
4870	17.08861	2038	23.18837	2245	27.82823	2672	26.89794	2760
214	0.249935	71	0.467516	111	0.546087	128	0.405029	99
411	1.55328	92	2.981832	157	3.683145	202	3.632552	187
260	0.414227	101	0.837035	184	0.837916	158	0.828045	152
1361	5.546029	269	17.93211	725	22.14144	906	17.74964	731
2194	11.29475	910	18.11075	1233	15.86797	1093	17.86441	1245
230	0.707824	84	1.044676	87	1.844202	188	0.348848	94
317	0.842992	104	0.705248	112	1.33564	210	1.840165	279
21579	131.0117	10087	166.8683	10750	161.4819	10480	160.3486	10553
1085	3.017736	412	5.409063	624	6.526594	778	5.61117	680
2988	11.22904	1162	17.5533	1604	16.96852	1676	21.51117	1899
357	1.710031	132	3.075533	205	3.598501	226	3.430851	203
276	1.783156	108	4.707665	226	3.207288	159	2.189734	115
142	1.888177	105	1.47658	69	1.121957	53	1.769304	84
177	1.320781	96	1.531709	91	0.961202	59	2.464759	154
291	1.071394	105	1.873019	174	1.191391	102	1.989577	173
810	1.839467	300	2.81567	384	3.242059	445	3.678096	512
9892	134.4397	4221	196.9955	5170	204.2577	5402	218.2321	5848
540	2.79474	154	4.748718	218	8.270475	383	4.97497	234
542	1.973835	243	1.140195	118	2.123998	221	2.158805	228
4185	7.9635	1361	13.01092	1846	17.8681	2525	16.48591	2379
483	1.872338	248	3.081153	347	2.241051	255	2.486591	286

171	3.527143	101	3.026059	73	3.44955	83	2.880398	71
200	0.672543	110	0.959274	131	0.818843	112	0.933938	131
3016	8.60176	1017	16.10178	1781	16.51444	1737	18.21841	1993
280	0.903047	128	1.395256	165	1.072769	127	1.501177	181
185	1.379185	78	3.529897	141	2.380604	106	1.033196	59
685	0.501608	114	1.665454	315	2.083546	397	2.735189	528
3990	4.833197	1513	6.759345	1768	8.728312	2267	10.32704	2171
2892	14.73924	838	31.23868	1486	37.42391	1793	32.0349	1557
1069	33.14892	395	57.83344	576	44.60768	448	55.93447	569
135	0.292021	56	0.316619	50	0.911002	146	0.654937	106
233	0.392369	75	0.890128	143	0.810604	132	0.901525	148
234	0.420817	82	0.692702	113	0.63738	105	0.794522	132
6847	21.94714	2506	41.19203	3956	40.74764	3936	46.60197	4558
460	4.713804	286	3.822276	194	4.275778	219	4.421688	229
1348	42.22636	683	43.85203	594	39.51639	539	53.30938	737
2321	4.292466	791	8.109076	1328	7.678335	1283	8.078712	1349
4069	1.24859	619	4.737358	1980	5.589539	2316	6.737182	2373
43272	445.3422	20822	585.3054	22899	663.497	26149	625.246	24988
52613	585.611	28206	660.795	26691	698.0146	27932	718.9137	29728
248	1.802106	137	1.605878	112	1.39879	83	1.12979	80
340	0.624677	183	1.10846	271	0.764418	188	0.367853	92
1052	3.598542	347	7.685992	598	8.204085	630	6.454385	594
161	0.298575	70	0.425523	83	0.55372	109	0.61939	123
479	0.533451	178	1.091995	305	0.759787	214	0.768249	219
541	0.972653	100	2.712529	233	3.823962	331	4.286209	380
705	0.515477	165	1.677678	438	1.686019	428	1.287483	363
2172	6.176952	1069	8.957692	1297	8.855616	1291	8.158308	1206
152	0.560837	69	0.981381	100	0.798636	82	0.832467	87
226	0.740215	128	0.66829	90	0.882632	137	1.086473	163
5769	1.914825	1090	4.111777	3349	4.066342	3791	6.219459	3069
1598	15.80541	702	15.53811	564	25.53901	915	10.72505	620
1115	16.96702	486	24.96359	598	21.30852	514	22.3234	546
153	0.13983	38	0.272957	63	0.347028	80	0.190541	45
4629	49.50211	1587	79.88624	2143	97.40856	2632	85.8295	2352
272	1.260916	100	1.583936	112	2.310087	166	1.973636	134
543	1.695472	208	2.456008	243	2.761006	291	3.629856	365
2992	6.489254	1095	11.57711	1635	11.28497	1605	12.15309	1753
681	6.55646	405	7.726378	401	6.116242	320	6.697129	418
13578	37.70976	6483	47.64859	6832	60.82285	8801	58.71382	8572
349	0.153207	81	0.325206	144	0.508723	226	0.405013	182
916	0.500091	230	1.337566	515	1.450449	562	1.175764	462
1997	4.094525	1131	4.906105	1029	5.022752	1219	5.200774	1157
173	0.335875	68	0.5589	94	0.405319	69	0.615763	106
28175	216.7986	15535	259.4012	15553	257.4219	15548	255.2651	15635
118	0.23039	40	0.481875	69	0.432547	63	0.358433	53
237	1.447917	113	2.539932	166	2.290138	151	2.838222	189
202	0.322561	58	0.792096	111	1.30934	197	0.506935	85
302	0.147167	78	0.328006	146	0.493593	214	0.203637	93
114	1.256427	56	2.263038	82	1.974631	73	2.052059	79
2602	26.3044	1422	30.35076	1373	24.48546	1116	23.29769	1076
1030	2.088014	320	5.470356	701	4.482699	579	4.978839	652

333	0.536521	51	2.527666	189	3.241047	211	2.455135	178
151	0.926971	63	1.641645	92	1.946352	110	1.498495	85
755	5.515656	240	10.29751	374	10.24106	375	10.52806	391
4345	57.18039	1968	77.71058	2224	74.39177	2143	92.20132	2713
101	2.305987	33	5.542816	66	4.310167	51	4.122541	50
333	0.415721	109	0.605806	132	1.422391	308	0.650688	145
268	2.15378	117	4.070985	185	3.19063	146	4.334925	201
610	1.710879	235	3.437973	387	3.295223	382	4.838124	512
157	1.098329	74	2.687307	152	1.781105	102	2.525052	146
381	1.397318	149	3.393905	285	2.315056	208	1.586674	145
1386	3.336177	575	6.220892	889	4.805396	700	7.162395	1054
1009	7.081572	491	9.83486	570	10.02118	613	8.721996	547
345	0.677702	155	0.802377	185	1.429554	302	1.088619	256
630	3.723573	255	5.843807	357	7.444098	450	6.319968	380
172	0.301228	51	0.369235	52	0.523954	74	0.59367	85
2828	6.364316	651	20.59256	1745	21.64251	1890	21.18059	1847
215	1.04748	56	2.139841	95	1.885596	83	2.570816	116
8202	26.11928	2234	70.71743	5116	73.62635	5734	80.42195	5866
1443	7.063126	443	18.87119	989	14.97481	791	17.98474	966
3955	28.69906	1646	45.07431	2128	47.39038	2247	51.57625	2479
818	2.097444	373	3.246202	482	2.460852	369	3.287827	499
2651	5.022124	1019	8.833134	1493	12.21509	2092	9.696562	1686
21100	18.70768	7029	44.40496	13951	46.31468	14652	46.49223	14927
8657	44.75888	3546	74.15322	4949	80.73616	5416	87.35187	5942
11505	79.9547	6418	101.6432	6828	112.3782	7604	94.97701	6517
39536	150.7269	18665	217.2172	22507	222.7022	23246	213.6899	22619
369	1.242386	160	2.441585	263	1.554187	169	1.732167	191
1376	4.199053	636	4.389143	564	6.169152	787	6.766777	881
384	1.720018	108	5.184889	256	4.356812	226	5.748319	294
234	2.946876	116	3.213214	106	4.375584	145	5.583519	188
209	0.527099	103	0.500217	82	0.999699	164	0.800497	133
4282	33.25231	2080	44.17031	2311	43.86861	2312	54.90788	2935
662	1.771853	404	1.93771	370	1.521114	283	2.297387	449
37753	152.4862	16808	237.7404	21928	249.2648	23160	267.8929	25241
3758	26.92941	2008	34.42943	2146	33.57214	2109	36.77901	2343
3234	8.572086	1408	15.05128	2040	14.26656	1981	13.21593	1905
726	0.565677	204	1.41839	427	1.682722	508	1.771167	544
361	2.362902	114	6.825904	274	6.115752	248	5.225464	215
1145	21.01937	822	21.27497	696	17.23107	568	20.09296	672
1889	9.380483	1084	9.915132	955	11.55114	1119	12.51181	1235
232	0.186495	98	0.172712	90	0.375403	197	0.283796	151
895	0.471256	243	1.162922	502	1.232598	536	1.008628	445
1623	3.035175	403	8.137503	904	11.06412	1238	8.364749	949
2432	11.596	967	22.30572	1537	22.6956	1574	21.24006	1498
303	0.619273	181	0.582563	143	0.531835	131	0.960565	249
185	0.316918	82	0.759254	162	0.407594	88	0.802277	150
1265	13.24103	517	23.16028	756	17.3097	573	21.44258	716
365	4.681413	93	11.10625	185	11.01972	184	12.81829	217
1125	3.734237	482	6.61207	653	9.178374	778	6.819044	622
4984	11.22024	2056	19.43956	2921	19.49981	2942	21.60448	3356
489	1.756076	138	4.82543	324	3.223517	220	5.030456	336

5914	13.93643	2309	24.6505	3421	26.87055	3779	26.05141	3692
304	9.662627	716	9.146165	567	11.58036	723	7.380755	468
123	3.45331	299	3.257824	277	3.249281	310	3.47243	315
9920	325.0076	22710	276.822	16186	286.5853	16880	272.7999	16294
421	8.241343	713	12.25186	886	11.05277	805	10.28811	760
183	4.163862	438	6.022821	530	5.461262	484	4.151526	373
1239	36.82443	2955	31.84338	2138	28.78982	1947	30.21918	2073
146	3.926828	300	4.530667	290	2.422983	156	4.322855	282
180	2.364975	382	3.360549	443	3.258288	452	3.125348	419
1220	96.4463	2570	101.5146	2263	87.84226	1973	84.90119	1934
2205	36.68765	3560	51.92593	4220	49.20544	4083	44.15528	3711
373	13.33457	793	11.34292	565	13.36702	671	9.000098	459
151	5.606946	331	5.401337	267	7.402493	368	6.222289	314
311	12.8	613	11.74961	471	14.89919	602	11.03138	452
418	17.52561	908	17.90596	774	17.64232	775	16.96341	748
237	5.131824	371	7.085006	429	5.550701	339	6.765906	419
2233	42.90201	4677	47.36914	4321	44.84002	4120	39.26417	3659
517	17.12494	1060	23.03424	1192	19.53033	989	17.48385	926
798	5.695982	996	10.05006	1470	11.33173	1669	8.176201	1222
1228	18.03358	2720	24.24872	2793	24.58286	2715	23.50297	2759
143	1.905086	163	3.233814	231	4.140087	298	3.15613	231
1271	22.7875	2604	24.04705	2300	21.22056	2044	18.34841	1792
609	7.203584	1053	10.09895	1238	9.868233	1201	7.800857	978
233	5.552543	412	7.16374	444	5.839756	365	6.383453	405
325	13.6304	789	13.41173	649	13.27518	648	11.30984	559
1300	265.259	4393	148.1791	2054	149.9802	2094	163.7137	2318
666	13.45023	1002	16.41425	1023	14.85684	932	15.4005	979
99	8.042016	225	8.513236	199	8.091068	191	8.21926	197
808	17.28951	1143	25.75877	1425	25.31564	1411	20.44846	1156
81	5.876689	177	7.073591	179	7.023096	179	4.921668	127
1184	17.99134	2322	20.50643	2215	19.45199	2116	16.75709	1849
146	6.250825	331	6.363967	282	5.682808	254	5.043856	228
519	8.329098	921	10.32915	937	11.05568	994	11.90689	1086
65	2.949911	145	2.390815	99	2.998932	124	3.347982	141
798	27.08156	1452	32.82665	1473	36.45419	1647	28.08585	1287
358	11.06209	648	10.01632	491	10.34738	511	10.08053	504
215	8.378914	523	6.924784	461	5.248186	300	5.208548	233
217	2.95997	463	3.68055	481	3.69391	486	4.075473	544
369	4.865956	595	5.131263	523	4.535228	474	3.97775	439
378	44.03325	1059	32.54717	655	29.94921	607	28.86055	593
1469	23.17072	2560	21.8597	2030	24.44939	2272	19.49494	1873
571	23.92377	1023	27.87368	997	26.17957	943	25.3021	924
1502	29.16102	2955	34.58688	2848	29.09065	2489	28.07596	2362
139	2.63848	268	2.660489	226	3.007557	257	2.211252	192
181	14.2549	446	11.17894	293	14.67847	387	10.98606	294
102	1.322184	244	1.855107	293	1.246811	222	1.569867	244
640	7.561678	1035	10.24411	1140	11.02775	1267	9.906317	1208
1794	32.95978	2967	38.2697	2882	38.72699	2938	28.08215	2161
660	12.04911	833	19.32669	1119	20.52543	1191	18.94313	1118
257	3.201788	449	5.692653	667	4.767542	563	3.843747	461
173	13.21623	352	18.20971	405	10.68558	240	11.02851	263

2733	51.82099	6318	46.80802	4733	50.69405	5158	40.73682	4301
95	1.308094	130	1.844909	146	2.31972	195	1.540584	129
157	1.163393	216	1.898972	274	1.440275	225	2.475964	392
911	8.266504	1347	11.29186	1509	11.90991	1692	10.15184	1433
1853	68.57734	3611	78.03506	3438	76.96488	3416	63.65357	2865
130	3.749639	190	3.815536	162	6.0923	260	4.48445	195
733	53.20116	1542	61.9799	1503	63.1412	1542	48.81313	1209
103	3.750631	244	5.079119	233	1.976316	117	3.277483	175
369	19.56296	850	19.24465	707	17.88087	654	13.03766	484
171	3.433458	382	3.408015	317	3.426797	321	3.789248	360
1689	33.3432	3331	37.11096	3118	32.2174	2730	34.46178	2979
9673	272.7853	20876	269.8291	17199	267.5125	17207	241.7844	15765
60	5.799315	129	7.290225	136	7.340548	138	7.572236	144
146	7.293538	375	6.957957	300	5.351797	233	5.066612	221
433	4.641	703	7.070206	896	6.456772	825	6.565199	850
1030	5.879828	1667	8.015776	1966	9.205534	2206	7.226065	1773
467	19.48695	1033	15.23779	676	20.24666	904	16.13271	731
14822	1885.551	41947	1483.759	28186	1387.215	26492	1358.826	26029
496	5.493992	925	5.229102	745	6.248953	908	6.934281	981
4805	81.81016	8103	109.9709	9114	102.9657	8596	83.24603	7048
2051	58.03821	4450	61.4665	3944	52.61977	3401	49.5123	3245
97	1.873101	190	2.775393	236	2.636054	226	2.562006	222
193	0.598855	253	3.048191	349	3.829608	399	3.183588	364
1332	11.16586	2331	14.3224	2502	16.89562	2973	12.88784	2300
456	8.918357	843	8.8953	704	9.668004	770	7.101902	574
385	13.08432	847	15.7898	855	10.86394	593	11.64347	644
511	16.62309	1263	14.00156	960	14.4557	1081	10.30545	749
5113	128.5255	9197	147.5739	8838	139.2863	8404	118.8957	7273
915	26.9781	1762	26.81328	1421	37.13115	1695	23.81768	1228
120	4.727086	233	4.645179	222	5.031935	227	4.673591	232
274	7.593745	516	9.923539	564	9.853008	564	8.144314	473
101	4.268556	247	4.670052	227	4.009697	196	4.167946	207
76	2.073634	100	3.118962	125	3.466722	139	2.622406	111
104	3.139269	281	1.976564	151	3.770002	270	2.710989	209
198	2.780629	324	3.578596	349	2.441139	240	2.262299	226
121	8.045815	422	5.95345	262	3.963363	176	6.242237	280
74	2.49464	191	1.966523	122	2.743888	169	2.450897	167
1400	64.05919	3287	57.23301	2457	55.19173	2387	51.6765	2266
137	1.746614	181	2.760423	240	3.312163	289	2.047056	182
701	64.65057	2298	54.47752	1620	47.09606	1411	47.37647	1439
749	28.19052	1594	26.61976	1259	27.21631	1297	24.86926	1202
392	11.99681	910	13.76865	874	16.16766	1034	8.87451	576
218	8.741475	360	13.22058	455	11.30508	392	10.10496	356
117	3.011502	284	3.212442	253	3.823373	304	2.42329	195
879	13.94309	1359	17.94461	1560	21.02543	1727	16.13865	1328
89	1.682981	145	2.411982	175	2.915515	213	3.532106	261
81	7.524433	235	4.580739	120	7.318938	184	4.712436	121
97	1.56315	184	2.112373	208	2.405485	238	2.446348	247
107	4.563515	360	3.118158	208	3.883621	255	3.393156	224
144	2.821272	278	4.002393	329	3.376815	280	1.549209	131
245	7.864202	580	7.554414	466	7.001423	435	3.915133	247

125	3.307109	291	2.109955	154	3.489331	254	2.863427	210
1387	118.3839	4010	90.53969	2567	94.46605	2697	85.65461	2480
66	2.352345	155	3.378188	190	2.166969	137	1.9526	125
623	7.855626	1293	8.85186	1219	10.04644	1394	6.670284	939
5054	155.8792	9562	178.7209	9173	153.931	7959	142.9261	7494
204	4.845378	421	5.649586	355	5.117147	401	4.258247	270
1290	28.12808	2696	29.52588	2397	31.09673	2546	27.321	2242
52	2.507207	116	3.12892	121	2.994244	117	2.08989	83
246	6.86876	524	7.915058	511	6.383514	415	5.450749	354
1617	57.6751	2834	80.35923	3304	73.9218	3062	76.21316	3201
482	17.50719	1038	19.57553	972	18.91864	946	16.06596	815
186	4.315668	306	4.915455	291	7.909657	472	6.53803	396
614	31.42825	1472	30.5837	1199	32.48462	1283	31.04444	1243
796	18.31032	2217	14.74544	1494	17.41374	1777	16.91674	1751
511	10.23583	849	10.86443	763	13.67757	898	13.99839	835
219	6.000569	466	7.618574	495	6.889522	451	7.689962	510
2253	38.11516	4675	37.43441	4337	39.15912	4600	30.44214	3640
8681	536.6931	21169	435.73	14381	407.6894	13555	405.1586	13141
4295	274.9662	8944	267.8185	7290	256.4214	7031	235.172	6539
194	2.174903	306	3.583306	422	3.560291	422	2.697274	324
117	2.144288	250	1.873488	224	2.431758	207	2.486552	216
280	7.436542	722	6.976174	567	6.378698	522	6.676155	555
276	7.125519	706	5.667126	474	7.286299	612	6.64813	586
209	8.354167	401	12.08845	485	8.845493	357	9.726563	398
656	28.8249	1380	29.92466	1199	33.35723	1346	24.06463	986
75	2.613428	133	6.514926	278	3.460221	149	4.063197	177
528	2.402491	928	2.90944	940	3.255478	1059	2.628348	867
1360	37.64828	2866	41.89293	2669	49.97379	3207	35.05005	2281
250	2.350122	349	2.628054	331	3.943375	498	2.786355	354
1905	78.60431	5450	57.68087	3456	58.14768	3554	61.01073	3787
680	60.01853	2045	43.63034	1244	44.42164	1276	40.38097	1176
469	6.86376	814	9.145993	907	9.825952	982	8.083726	819
1272	53.37689	2732	53.01514	2271	56.11782	2421	56.16589	2458
1024	10.15362	1615	16.36467	1625	15.954	1865	13.74365	1753
402	25.2224	1031	22.48271	769	23.4114	807	19.08853	667
120	7.104155	308	5.480983	199	6.512362	238	4.985321	185
712	29.07078	1311	49.75936	1878	44.539	1693	46.16082	1780
252	9.9624	619	7.577684	393	9.214037	482	9.379626	501
561	15.32218	1465	18.18837	1456	14.2876	1152	14.55707	1190
231	5.041192	375	7.696087	479	6.421478	403	4.900524	312
281	3.915416	452	5.270954	509	5.779523	562	3.836527	378
1795	71.22341	3783	79.21113	3588	68.85827	3097	72.68583	2889
172	7.305151	354	8.69243	352	5.517915	225	5.970499	247
92	3.575434	222	3.11357	162	2.336966	123	4.340826	231
2162	297.2752	6798	194.9869	3735	176.6689	3408	201.438	3940
2614	50.23726	4599	60.10587	4604	66.10867	5101	55.64043	4354
156	0.532955	143	2.194149	451	1.232209	274	1.975043	410
127	5.096194	208	6.274094	213	7.072669	243	5.258808	183
159	5.948036	363	5.735464	293	5.375021	277	4.797923	251
91	8.449812	244	5.962756	147	6.153535	168	5.259325	126
770	7.332841	1260	10.66268	1533	11.79573	1708	8.34879	1226

150	3.11889	199	7.974925	329	4.950162	351	5.647831	230
474	9.093427	907	11.49706	987	13.01532	1117	10.51358	872
475	8.342848	1004	10.91951	1099	9.607484	974	10.22763	1052
493	22.01607	1171	25.93795	1154	21.08509	945	20.76622	944
374	5.995198	606	9.921456	838	9.509696	810	9.378315	810
302	9.848945	628	11.18356	596	9.962965	535	10.67825	582
87	0.769803	93	1.337438	133	1.440909	146	1.27812	132
11538	123.0462	24865	145.7982	24661	148.1286	25184	142.8189	24629
171	9.94063	450	10.11716	386	11.66078	449	8.13637	317
857	26.1576	1595	29.51023	1508	33.07229	1699	27.54939	1435
282	5.841928	583	8.857285	684	7.530303	581	5.453462	462
111	1.027295	336	1.076154	293	1.117921	314	1.259598	350
1464	15.00351	2502	18.75346	2617	19.54036	2747	14.52079	2070
135	5.423661	321	2.826822	140	5.788972	288	5.051897	255
131	21.67667	398	19.90116	306	11.75052	182	15.32179	241
142	5.850334	536	4.399454	232	6.449521	296	2.248909	210
1175	46.80204	1902	76.53345	2602	67.89881	2326	64.97758	2257
50	4.086393	239	4.148338	203	4.55742	224	3.255706	163
92	0.691672	141	0.951038	169	0.791536	143	0.592983	109
59	1.095421	157	0.735856	88	1.695611	205	1.2592	154
218	3.622518	285	6.123107	403	7.519242	499	5.328086	359
216	2.82251	419	5.245235	657	4.028427	501	4.406101	562
3039	89.90034	6853	94.97155	6229	104.1475	6735	85.86668	5744
152	3.184274	274	3.055253	228	3.530973	248	1.578842	129
651	14.09547	1323	15.67834	1232	16.74778	1316	16.15121	1293
760	14.3388	1898	17.55104	1607	18.12042	1522	15.3706	1288
144	11.81592	177	27.18299	341	20.46889	259	29.09833	373
412	5.302986	606	7.786822	744	8.494001	818	7.395816	722
498	22.43181	652	34.40791	837	32.71618	802	23.97527	596
159	3.403883	289	2.504155	181	4.631498	321	5.241608	388
923	18.67323	1810	21.84635	1772	19.58084	1600	15.82606	1311
129	4.391294	325	7.941121	492	5.759702	360	6.241698	395
122	1.375964	211	1.474241	191	1.02341	134	1.958384	259
460	14.76	829	17.66961	830	21.06639	997	14.11836	678
874	13.77781	1568	16.77803	1597	13.84259	1328	14.07044	1368
592	6.211656	930	8.166498	1046	7.625708	984	6.143602	804
362	13.74008	835	13.46407	681	15.49871	794	11.73138	629
220	2.036101	283	4.294718	501	2.478452	291	2.484274	294
76	10.29331	195	11.27884	182	12.46585	202	4.151752	70
86	9.20814	170	8.324586	129	6.837969	107	9.129483	144
103	3.786014	208	6.703818	307	5.167232	239	2.884158	135
52	1.121717	122	0.84697	77	1.163424	125	0.754626	83
2512	42.06156	5517	39.30486	4296	43.47335	4790	37.68975	4210
130	1.972426	575	1.364289	333	1.40898	336	1.493953	372
1070	119.7764	3032	82.07012	1763	89.30291	1941	67.24411	1510
103	3.356371	200	3.08242	154	2.703515	136	3.029261	155
310	12.4645	646	13.35063	579	15.6856	686	10.75381	477
6242	416.3497	12045	507.7016	12291	479.2191	11686	470.4879	11635
378	7.853796	872	6.549239	609	7.483506	701	7.248592	688
646	12.15781	1275	15.33999	1346	14.88501	1316	12.64147	1133
64	1.74086	153	2.482331	182	2.369386	175	1.511988	113

96	12.04827	222	10.50896	162	7.577893	118	11.10038	175
943	38.66439	1919	42.52476	1775	41.70136	1771	38.80423	1656
347	11.38499	836	13.07749	827	11.77463	737	9.07861	557
1657	32.50261	3617	38.47626	3582	34.46385	3232	28.13913	2676
329	10.61557	561	17.05542	735	13.02881	560	14.37937	632
550	6.460583	879	9.226236	1082	8.665641	1104	8.866887	1024
2738	176.9055	6577	164.564	5160	180.7608	5631	142.16	4547
78	2.069616	184	2.25376	168	2.335077	175	2.016618	153
244	2.140397	567	1.910198	422	1.910778	425	1.563108	354
125	9.040831	277	8.815246	226	6.739101	174	5.805475	152
379	27.55179	965	24.10545	708	23.80683	702	25.63273	771
4556	199.2085	10643	165.5047	7403	138.7965	6233	154.2811	7011
254	23.00356	769	18.8511	527	20.61568	581	16.95686	485
114	0.709685	235	1.113319	307	0.807047	224	0.913287	258
626	14.33661	1658	11.11613	1076	14.01522	1365	11.28741	1091
71	3.102974	150	2.872131	116	3.783992	154	2.724514	112
279	4.019654	563	5.189271	608	4.886859	576	4.174962	499
326	7.119179	880	6.357998	660	6.757715	695	5.994864	646
371	17.80616	753	19.96568	707	19.3429	690	18.64808	675
191	13.8456	670	8.559052	349	9.002707	369	4.412623	185
164	6.264365	237	8.070916	251	8.634727	259	12.65811	390
101	2.842288	196	3.398445	196	2.802529	163	3.135234	184
464	4.706298	823	6.03861	883	6.200246	914	5.285856	790
234	14.79118	517	13.07084	382	11.6031	342	11.16347	333
129	12.02006	388	8.810753	238	11.52096	313	8.033711	222
76	3.897074	241	3.219471	167	5.522488	288	3.415994	181
181	4.376324	446	6.177448	526	3.432809	295	4.941187	430
1283	31.38912	2483	35.48824	2349	30.93662	2063	30.98224	2095
123	5.434576	284	6.597118	288	8.297809	365	6.476168	289
100	1.876418	189	3.347062	280	3.19182	276	2.878575	250
95	1.728613	276	1.49055	174	2.009191	233	0.90467	103
941	48.70209	2499	52.67349	2264	43.40792	1871	44.93783	1968
3836	68.2096	7706	69.44026	6563	73.89406	7027	59.96417	5784
962	55.75509	2654	50.67673	2018	47.83731	1919	38.65538	1573
57	1.537926	109	1.934481	116	2.82406	170	2.598489	159
2067	51.85888	4579	59.22155	4376	62.56818	4657	70.0155	5284
218	10.35156	633	10.4841	539	12.0489	628	8.056368	426
451	12.05857	868	13.23113	797	14.35581	871	11.7742	725
214	1.873559	343	2.980463	457	3.807745	571	2.578681	404
97	14.64966	314	11.11081	199	8.468617	153	14.11392	258
482	22.84805	1324	22.23972	1079	19.80679	968	16.20024	803
16113	580.7755	31882	652.9872	29996	630.5626	29178	578.8563	27162
934	19.4664	1852	19.80941	1962	21.01152	1723	22.12107	1669
174	4.872097	279	8.587847	393	6.06849	308	5.777496	270
208	9.289102	452	10.54851	429	13.01506	533	10.59924	441
282	7.43747	648	9.470553	786	7.179163	559	6.000491	598
98	6.302505	269	10.02442	358	7.218389	260	6.499166	237
1493	45.59881	3629	43.60689	2916	36.726	2469	35.59294	2429
845	30.5691	2651	24.61759	1787	22.10783	1616	20.24679	1501
417	18.78629	1035	18.23332	841	16.95574	787	18.83138	886
187	3.175834	308	3.205593	260	4.278725	350	2.917824	242

285	13.94102	602	14.95498	541	13.76239	501	13.53685	500
582	7.206427	926	10.22605	1101	8.475229	925	8.397215	926
240	9.414923	507	9.664335	436	9.704557	439	9.180425	422
3726	105.3995	9174	108.3681	7382	98.94972	6986	84.93793	6910
1651	21.61819	2972	29.28259	3376	29.95376	3474	24.58767	2884
328	34.95931	952	22.69531	524	20.2944	472	19.91978	471
1012	30.1218	2555	25.34486	1797	25.87517	1851	24.7022	1789
107	6.833775	156	9.847958	187	14.74535	282	13.59747	264
110	9.563975	295	8.077917	201	12.43042	311	9.876004	253
73	0.939488	149	1.504064	207	1.622986	210	2.029472	260
125	1.290534	245	1.703416	311	1.646266	263	1.065313	199
214	5.950153	386	7.574143	410	8.165395	443	7.266155	399
317	9.043244	761	8.271736	583	7.975068	565	6.17586	443
21649	982.1878	45262	1069.857	41255	1059.556	41157	932.8853	36747
215	11.63224	356	17.16027	439	15.57355	402	12.17813	319
1031	26.21365	2355	30.77551	2314	31.90532	2416	22.02121	1691
535	18.78667	1123	18.19504	910	21.05696	1061	17.13746	876
1229	67.71441	3083	55.27298	2106	58.67002	2252	56.20728	2188
98	5.444464	296	4.790246	218	7.70006	353	4.424211	206
1347	34.01974	2922	35.10219	2543	31.72472	2312	27.48053	2016
165	11.12529	481	9.80232	362	14.18037	539	9.082446	342
233	8.155405	483	10.27008	509	7.05172	352	6.413808	325
414	9.267564	739	11.22211	749	12.15353	817	11.46948	782
103	1.578404	175	2.713518	252	2.706355	232	1.647791	154
224	0.906425	254	2.179914	509	1.573849	371	1.946482	463
100	6.378838	288	8.281215	309	6.689239	253	4.620785	181
62	1.009036	174	1.292022	174	0.783791	106	0.913068	126
131	3.041479	211	4.445539	258	3.609005	211	2.855224	169
316	6.888325	880	5.470304	601	5.489379	552	4.507109	507
177	4.76344	344	8.253181	499	4.165016	254	4.315952	267
292	26.27962	615	22.6938	445	21.87922	432	19.00785	381
149	1.004816	185	1.619205	250	1.458101	226	1.871309	295
336	6.416831	584	8.225004	632	9.549295	744	7.642759	607
119	7.724747	264	6.706892	192	8.904594	256	5.60278	164
867	10.21266	1562	14.15407	1811	15.76857	2032	12.09555	1581
34	0.604495	104	0.64182	85	1.02731	152	0.596662	90
63	3.021337	162	3.259068	147	3.607476	158	2.848301	134
463	9.069198	984	11.88675	1080	12.85511	1176	7.359201	683
426	17.65906	771	15.47463	542	19.04242	667	16.64103	605
368	6.532728	794	6.55386	666	8.602845	883	5.651429	590
177	21.70594	512	23.22134	458	18.8733	375	17.84008	360
452	11.87091	912	13.74875	897	14.6661	968	10.65094	703
81	1.130257	146	1.466238	174	1.135721	136	1.369191	145
107	1.133542	205	1.564432	191	0.999114	180	0.756375	139
88	3.111639	192	2.769007	143	2.26868	118	2.64799	140
3759	87.48493	9948	65.91908	7106	65.15318	7123	51.53734	5846
415	14.30566	864	16.26274	818	13.57845	688	14.71393	756
167	14.9712	400	16.45477	368	15.97103	360	9.773776	224
2073	38.59426	3972	44.93066	4670	57.89211	4412	58.8202	4670
159	6.886564	293	6.581503	235	10.01827	354	8.16572	298
173	8.216291	421	9.772498	419	5.458128	236	8.137788	356

41	1.822498	90	1.968698	79	2.148316	88	1.841026	79
266	6.265013	630	8.662136	729	9.911514	840	5.79292	498
1950	48.225	4055	53.20501	3448	53.71402	3580	51.31974	3388
12207	656.8182	26738	657.7678	22406	666.7491	22879	585.2422	20364
101	2.095018	161	3.547076	229	3.942414	256	3.254803	214
115	2.437533	222	2.478472	189	2.438721	188	3.058236	238
260	5.4411	518	7.918579	630	5.39388	434	5.228971	424
543	22.38026	1124	27.31405	1148	23.98244	1015	22.1503	951
171	4.63688	335	5.309292	321	5.365291	327	4.227546	261
482	12.43431	1086	13.34788	976	11.81902	870	14.0434	1048
625	21.80259	1641	21.55186	1358	22.5427	1430	18.76674	1208
139	3.380838	300	6.014467	444	4.099288	304	3.643633	276
2150	106.6995	5120	109.1797	4384	110.1439	4455	82.90101	3400
155	3.885227	331	4.16848	299	4.014961	290	5.355656	392
123	9.746963	230	8.338717	165	9.290277	185	11.18216	226
144	7.094656	387	4.319799	197	3.647704	168	2.909574	136
215	3.602433	322	6.184942	525	5.339062	429	5.738265	456
169	13.91912	646	14.83893	583	9.301181	364	8.951208	355
122	5.565803	262	8.406234	330	8.427248	334	4.858449	195
288	9.380376	834	9.37828	697	9.03198	677	7.612977	578
68	2.515795	202	2.885201	190	1.581829	116	2.410188	180
107	6.816716	273	7.890993	268	6.259125	216	6.239342	214
82	0.729413	168	0.497622	139	0.426323	120	0.563149	131
254	4.00521	442	5.121333	475	5.632681	524	4.347266	412
98	2.981872	220	3.783563	233	3.477792	216	2.157216	136
110	4.214225	351	3.005336	210	2.225265	157	2.699252	192
203	7.756095	344	9.093576	338	4.518117	169	7.412982	281
206	18.23217	461	17.36038	367	12.39954	264	14.61256	316
683	8.473727	1159	14.20977	1607	15.93799	1836	9.672759	1126
735	11.68246	1808	11.79505	1528	11.08237	1446	14.11674	1868
527	13.58861	1281	16.98875	1340	15.24528	1212	12.72905	1026
509	6.971879	1145	5.845599	834	6.400596	937	5.749413	864
48	0.487048	83	1.028108	146	0.741488	106	0.782133	114
64	3.976258	120	5.517728	140	4.039366	114	4.619473	118
119	1.240976	135	2.075655	188	3.164845	289	1.787544	165
675	35.14642	1616	43.10146	1658	37.61397	1458	33.15691	1303
342	10.20386	661	11.78637	639	13.53136	739	8.636867	478
1026	31.47716	2090	37.69161	2095	37.99105	2127	37.93182	2155
9217	552.6824	21961	546.3515	17902	519.1028	17143	479.5684	15475
296	2.715293	518	3.639743	587	3.397905	552	3.519146	568
185	4.668388	307	9.358244	515	5.614775	311	6.737018	379
224	16.53457	653	15.89468	526	16.57191	552	13.31029	450
158	3.844977	332	5.606428	406	3.54578	259	3.310852	239
114	3.377292	194	3.188602	138	4.090531	167	3.034955	127
455	21.59471	1158	18.47525	829	19.78757	894	17.72369	812
233	11.71367	640	12.1641	556	12.63836	582	11.84749	553
9984	230.2832	22990	240.677	20106	242.6731	20421	213.1265	18187
137	16.30142	430	12.49921	276	12.18532	271	13.29597	300
75	4.178359	158	5.707207	180	5.433185	173	5.012315	162
51	2.357635	128	3.626363	166	5.896237	116	2.435707	103
380	4.773147	558	8.037036	810	6.831227	691	7.254326	744

54	0.801239	215	0.515393	120	0.781734	183	1.025213	239
164	3.244272	328	4.16206	351	4.796169	408	4.424462	381
884	33.70989	2157	27.56121	1476	34.73305	1874	22.61598	1237
601	23.00375	1441	23.7826	1246	24.21774	1278	22.76114	1218
1345	19.74392	2867	21.96841	2691	23.80608	2898	21.26095	2632
1108	26.64202	3181	21.67323	2164	21.08342	2111	19.71519	2015
376	10.19068	815	12.53788	839	11.45646	772	10.72645	733
534	20.67712	1148	20.8101	1031	20.22897	936	19.29702	920
856	55.93288	2191	49.79842	1633	54.39453	1796	40.85309	1368
406	18.01914	1032	25.99143	1246	23.45214	1133	22.0082	1078
238	0.985489	188	3.504293	512	4.882556	615	4.168554	707
1267	31.65319	3049	34.67029	2779	37.30419	3121	32.45914	2631
293	10.97712	770	10.54823	621	9.076669	537	9.639135	573
41	0.888543	83	1.950592	153	1.65465	131	0.768629	62
685	22.5754	1776	17.06285	1123	18.95609	1257	18.89771	1271
3990	262.4681	9751	224.3194	6974	207.859	6510	193.1507	6134
63	0.465547	135	0.506484	123	0.951157	226	0.724347	169
139	7.361826	279	8.157476	259	7.58693	243	7.289198	237
458	24.44758	1148	26.2367	1031	29.31489	1161	22.80501	914
196	3.233434	397	4.741507	487	4.415708	457	2.687832	282
107	0.949827	194	1.295212	206	1.298051	220	1.831116	286
2673	82.47907	5793	86.49262	5083	89.08157	5274	77.07767	4628
1671	48.27406	3761	45.23783	2949	52.49542	3447	40.94565	2727
397	13.11853	837	21.85975	1167	14.67493	795	10.94227	605
9173	1069.575	29379	727.8699	16730	632.1321	14636	649.3154	15245
487	17.6745	913	21.62783	935	20.36229	887	15.25376	674
108	1.702374	255	3.191969	400	3.299368	416	2.08105	267
83	3.324151	189	5.37161	256	4.738933	227	3.058899	149
212	5.788359	456	7.580273	499	7.958122	528	6.713065	452
558	21.83254	1364	25.23772	1286	25.55318	1323	21.4299	1139
143	6.709162	416	6.205628	323	7.255104	380	6.100557	325
1532	33.10294	3555	37.5365	3320	31.81487	2900	28.49225	2961
561	31.51333	1574	26.65981	1114	25.01819	1053	30.62377	1307
71	7.992014	136	11.13444	158	5.814202	83	8.756442	127
3215	455.52	13629	210.0603	5259	242.7019	6121	227.5794	5820
35	0.551146	71	0.412209	52	0.854227	91	0.751893	99
1292	27.05675	2803	29.24351	2523	32.77835	2868	28.75276	2551
140	16.99524	475	12.47387	287	14.8591	328	13.0374	301
163	3.103335	265	3.685494	272	3.36268	250	2.930663	221
7386	620.7382	26250	437.586	15485	420.3821	14985	383.5576	13865
37	3.288108	136	4.745514	169	2.259549	80	1.915008	67
129	4.976135	376	4.339307	276	6.944366	438	6.050525	392
131	2.16498	196	3.037853	230	4.567465	331	3.621441	280
704	51.72325	1977	45.40805	1453	50.4282	1625	48.36007	1580
237	4.628557	418	9.415914	705	8.366501	630	8.957896	691
113	6.47417	381	5.817871	287	6.126624	304	5.91647	298
1475	102.1487	4546	83.63286	3115	78.45506	2943	62.10102	2363
309	7.017353	795	5.145353	798	6.900764	663	7.137528	702
113	8.161055	388	4.350744	173	6.904185	277	4.251595	173
83	1.194371	156	3.565779	185	1.836321	203	3.346904	164
2313	75.95519	5368	87.6311	5182	85.20448	5076	75.02134	4532

74	2.73471	192	3.374895	199	2.127108	126	2.625585	158
155	5.910151	376	7.942389	422	5.90114	316	6.376566	346
78	5.281809	110	7.630831	133	8.827298	155	8.134619	145
1816	74.73576	5758	64.46855	4202	66.50728	4365	56.0546	3715
69	5.769372	316	5.658435	260	4.194117	194	2.003931	94
112	2.513875	229	3.049468	232	2.60669	200	2.538728	197
190	6.54307	373	8.739228	417	7.057869	340	5.839298	285
668	67.41037	1628	74.15452	1507	69.11386	1412	86.70032	1784
67	3.060909	249	2.550313	174	1.970461	136	2.184111	152
466	23.64718	1649	29.00206	1718	28.13507	1676	24.37565	1474
198	8.915979	553	8.589035	446	5.744702	300	7.359654	390
133	2.87762	213	3.408159	231	3.461599	213	3.54719	265
61	1.409522	122	2.657709	193	2.803132	205	2.727332	202
564	17.71466	1310	21.10874	1306	24.41274	1521	20.83583	1320
76	3.871321	183	3.446613	136	3.677689	147	4.460766	180
232	15.42726	785	13.38657	573	13.91544	606	13.70497	605
228	7.520219	583	11.51781	747	9.357217	612	6.87775	456
50	2.135691	153	1.412784	85	2.441738	148	2.059042	127
281	4.891281	611	5.98151	626	4.390428	463	5.394858	576
226	2.920079	515	4.854295	715	4.799352	704	3.290494	486
8467	1596.667	30542	1073.932	17190	977.7736	15766	1062.341	17370
219	3.565754	435	2.97278	326	5.241769	547	3.939063	397
4695	101.5485	12574	91.24309	9454	84.622	8833	76.426	8089
1213	59.33091	3037	63.66507	2722	64.55872	2792	58.64387	2577
2260	44.30979	5858	51.21212	5663	48.91061	5446	43.62912	4916
142	5.545778	315	8.471189	403	9.472806	453	6.929176	336
384	10.16892	817	13.56317	1083	8.388944	752	9.020547	616
74	2.308557	194	4.124918	214	2.755203	179	2.64113	207
369	18.96802	1082	17.1469	792	13.1608	795	11.44779	707
614	7.658203	1273	9.871958	1373	9.907933	1388	8.893785	1263
45	2.461194	93	4.041732	127	3.535996	112	2.676398	86
287	14.14735	889	15.06556	792	11.95865	634	9.801247	527
9979	267.7319	23850	295.1481	22001	294.0163	22078	253.2984	19288
139	3.057961	289	5.805056	458	5.104165	406	4.392949	354
242	9.237366	642	11.84183	689	10.27691	602	11.28196	670
40	2.974777	178	1.081886	55	2.080086	105	2.173	112
628	15.49551	1771	16.72738	1608	17.43285	1679	16.75093	1637
222	7.520678	758	7.049411	595	6.33239	538	5.236152	452
76	4.482947	294	5.17002	284	3.979942	220	1.923478	108
52	0.546057	115	0.746517	132	0.714936	127	0.467675	84
104	4.861373	208	3.98901	202	3.899597	149	3.491482	138
276	6.438823	546	9.857868	702	6.105687	423	8.108492	586
362	29.44009	1052	26.35789	786	18.42365	562	24.8595	758
48	0.428661	105	0.511618	105	0.656258	135	0.574593	120
49	1.276997	72	2.325888	113	3.32015	157	2.231477	109
1989	143.0358	6881	95.67898	3970	87.10118	3817	97.69771	3856
469	24.16729	1101	20.82697	794	24.95437	958	21.08616	821
359	17.66524	1179	17.05484	953	15.26766	859	13.34075	761
70	3.179052	185	1.653597	81	2.958872	145	4.02157	200
621	26.1141	1487	27.11848	1292	26.30841	1263	23.57759	1147
334	6.651523	1101	7.499133	1009	5.667032	788	6.29629	875

362	10.76255	950	9.474285	706	11.46591	836	8.647955	646
100	3.786885	316	3.746001	262	3.498382	246	4.671531	333
103	2.937343	162	4.226671	195	4.971708	231	2.699519	127
199	12.94882	517	11.54963	387	11.3664	381	9.288237	318
92	1.498115	172	2.26209	181	2.88694	226	1.159932	114
65	0.800348	60	2.142343	131	3.142927	196	1.762255	112
147	5.751209	358	5.090227	265	4.02656	207	4.969591	264
70	2.098895	162	3.362698	217	2.477898	161	2.892045	191
686	46.81107	1729	45.12737	1394	46.0545	1439	45.94377	1452
50	1.271002	145	2.290602	221	2.039828	195	1.654626	163
254	2.312062	517	3.071959	600	2.542796	497	1.454618	290
1485	14.31748	3261	17.17847	3274	19.18718	3684	17.64842	3436
7935	462.8476	19908	528.1193	19018	348.941	12653	406.0741	14939
824	31.39664	2394	30.3891	1939	32.0045	2057	30.28307	1974
2908	109.8276	9441	96.08071	6976	98.09913	7225	85.32806	6306
3937	199.7401	12092	161.4057	8181	146.8081	7490	142.8149	7391
67	5.236088	242	5.603574	217	5.700814	222	4.00476	158
100	0.625012	238	0.655302	209	0.522992	168	0.839495	274
236	6.031231	527	7.956424	581	9.282237	683	7.076762	528
185	5.993862	453	6.788044	429	5.746316	366	5.275356	341
1526	75.24425	4212	82.49105	3864	90.89159	4289	80.57496	3856
503	17.77767	1849	11.99694	1031	13.92644	1222	11.93554	1063
202	2.545201	483	3.552987	564	2.751486	436	2.108743	342
36	1.730505	111	1.220521	64	2.459993	129	1.390277	74
66	2.801581	260	3.944854	188	1.9259	196	1.453528	163
23442	2055.673	70276	1758.851	50315	1682.681	48489	1541.916	45057
64	1.402025	116	1.62713	111	2.098792	146	1.439068	102
93	1.402061	144	4.614957	392	3.469967	300	2.918578	250
153	11.16774	490	12.90358	464	10.72517	398	7.937581	287
455	23.12805	1749	18.70093	1188	15.58676	997	15.88671	1030
64	1.03884	79	2.544956	160	2.695405	169	2.474295	160
1262	50.83745	4073	42.98587	2882	41.15973	2780	34.48085	2362
296	56.78552	1354	20.12115	541	23.52095	636	17.77601	532
307	5.79496	807	8.058568	901	8.516294	947	6.452801	713
144	5.369534	356	6.413543	356	7.820537	437	3.940099	223
56	3.794562	182	4.269333	171	2.976314	120	4.307642	177
78	0.566562	73	1.334436	143	1.613091	174	1.460503	160
161	7.162249	475	6.195205	291	7.123839	412	7.798356	391
96436	10493.45	346704	7395.122	204455	7347.93	204641	6678.617	188616
162	5.400868	559	4.406551	385	5.699068	499	5.045448	448
136	15.67713	467	14.30393	337	10.69691	291	11.70936	298
43	4.135295	208	4.211885	177	1.840131	78	3.032295	130
48	4.342319	287	2.437585	135	2.177948	122	2.296835	130
67	0.956969	265	0.785856	181	0.79235	186	0.419989	101
166	8.090003	698	4.455831	318	5.709778	418	3.834128	264
71	4.843241	175	5.025757	152	4.276086	130	4.182347	129
31	1.76947	62	2.861952	79	3.754608	115	5.479783	194
253	8.37781	563	12.95013	729	9.866686	559	8.928359	513
148	15.94023	970	7.060563	360	5.956908	305	7.957155	414
522	19.33993	1562	16.63145	1224	16.23347	1141	16.36346	1049
26	0.413222	68	0.531537	75	0.541543	76	0.359373	52

109	1.062775	343	1.160112	278	2.370449	326	1.987733	278
4005	184.4873	10908	180.4684	8929	170.1892	8482	163.6351	8270
84	1.142802	160	0.701527	81	1.181051	143	0.63114	78
59	6.377003	261	4.94504	170	4.158993	144	2.047609	72
15904	2870.822	64105	1876.675	35066	1869.483	35188	1730.581	33032
3356	128.3566	11863	87.7845	6838	97.28508	7662	76.95115	6180
367	23.21431	1276	17.32097	797	18.42944	854	18.10467	851
34	1.516217	68	2.414859	104	1.873017	73	1.670348	74
42	3.873832	97	3.735155	78	6.482833	136	3.110051	66
87	4.168068	571	1.791873	206	1.682369	195	1.723994	202
429	13.78756	1847	5.715938	641	7.318001	826	6.580995	754
1259	21.90979	3946	22.46392	3385	23.79996	3613	20.84507	3209
71	3.82892	226	3.804955	188	4.004128	199	5.377963	271
24	1.528296	113	2.735524	148	2.353029	149	1.713427	95
1135	58.78569	3379	60.20561	2895	56.95702	2759	44.96278	2209
322	17.98334	781	18.91028	687	20.25612	741	17.47146	649
3766	138.6548	9670	188.4803	9997	139.0531	9190	125.8857	8163
55	2.498461	174	2.469728	150	2.604539	159	1.249844	78
13883	381.7604	39929	409.3198	36322	401.0262	36322	377.9125	33793
109	1.355245	202	1.387113	173	3.365086	423	1.792318	229
69	1.838729	125	3.058412	112	3.671963	188	1.760094	108
3495	344.0917	10132	359.9254	8868	339.1693	8418	344.4482	8669
729	14.60825	2850	13.45228	1584	9.192926	1582	7.51396	1295
841	54.34204	3402	38.73784	2030	43.1982	2280	30.65882	1641
72	1.435292	155	0.9256	84	2.13237	194	0.975401	90
1611	69.83154	6064	50.97685	3719	54.52143	4007	45.96626	3381
2370	193.1529	7344	212.0935	6751	214.3881	6878	194.9885	6343
76	22.97891	442	11.83064	191	10.3578	168	9.564681	158
35	1.271075	52	2.492694	85	2.355509	81	1.228668	43
4864	835.9283	19525	619.2099	12103	658.4261	12964	635.9927	12698
911	34.75419	2720	37.72013	2476	35.37101	2272	31.82571	2130
117	14.94901	598	6.997355	234	8.361843	282	7.865841	269
20	5.117612	138	4.658967	75	1.037964	42	2.798262	46
1375	97.43417	4940	75.96093	3221	76.97723	3291	61.93576	2685
363	8.225958	723	8.554327	635	12.90757	917	9.594572	688
71	5.85501	219	5.753983	173	5.1821	165	5.531259	158
57	1.660195	197	1.661417	165	1.033948	104	1.169892	119
309	7.160507	958	4.448535	497	6.258416	707	3.387101	390
967	27.65795	2500	33.78305	2557	31.36004	2393	23.62097	1828
227	10.01363	713	10.14387	610	12.01848	722	10.79649	657
182	1.797987	270	4.715137	588	4.394605	551	4.853644	610
36	1.716973	120	2.340278	137	2.956722	157	2.343431	140
52	1.086058	236	1.127521	188	0.598462	114	0.67405	123
1148	293.0089	6659	122.4179	2328	98.34278	1884	128.7864	2502
179	4.050013	702	3.44172	492	3.360564	557	4.419871	432
35	2.975004	72	2.259357	46	5.871891	119	4.38872	91
53	1.075258	166	1.536669	214	1.584288	225	0.820894	108
686	33.89694	2490	32.11444	1974	28.30433	1752	23.74754	1491
3818	379.2193	14523	308.8358	9897	319.1988	10304	283.6576	9286
43	1.658673	154	1.491456	99	0.912218	71	0.769056	82
73	2.513156	153	2.664945	136	2.880905	148	3.439275	179

68	0.376464	62	1.200515	160	1.109374	152	0.877527	122
37	2.316618	132	3.090687	147	1.79605	86	1.674879	82
60	1.521638	47	5.800141	172	7.482891	205	5.816649	151
14	1.677052	66	3.826034	119	2.490364	78	1.929584	64
1766	107.4134	8130	62.40505	3953	66.98784	4274	52.29953	3384
49	2.441036	196	2.439254	164	3.546431	240	1.334458	92
51	4.868598	282	3.374047	164	3.265073	160	3.278041	162
11	0.764176	114	0.301787	41	0.42839	59	0.377652	52
31	0.402173	50	0.667611	69	0.774956	81	0.688269	73
529	13.92833	1839	7.901342	873	8.594028	957	6.180526	698
53	8.152272	182	4.031969	78	8.798503	166	8.630138	165
84	0.892712	193	0.703397	129	0.347487	60	0.758857	141
419	14.38893	1430	11.9584	995	10.80647	904	13.22883	1124
101	6.304965	288	5.816422	226	7.860099	300	5.311266	207
50	0.882225	173	1.271671	208	1.698942	280	1.658185	277
42	4.0903	205	3.459877	145	1.876312	80	4.009048	171
34	0.611243	97	0.284737	38	0.774314	103	0.595343	81
51	0.573992	164	1.016041	241	0.970636	228	1.179382	285
3065	46.58991	10796	47.85416	9383	45.67477	8910	42.17158	8318
787	76.31571	4374	38.18972	1803	42.98105	2060	28.11641	1373
35	1.532699	149	1.22357	109	0.689074	69	1.737393	151
1571	452.5913	9212	210.7074	3589	203.301	3488	172.9875	3010
244	6.260368	901	7.08565	853	5.603824	680	5.791663	713
42	1.875169	214	0.584815	56	0.719594	70	1.534623	150
21	1.046633	115	0.726597	71	0.652679	68	1.306239	64
178	2.028545	355	3.102967	454	2.847811	420	3.540579	529
6	2.20819	86	2.799949	96	1.575305	66	1.320497	72
73	4.992687	306	2.311624	119	4.688131	242	3.020291	159
861	30.92514	3293	28.95495	2565	32.01995	2858	23.59032	2135
32	0.320628	102	0.303435	81	0.349051	94	0.34634	95
62	4.07914	180	4.664003	169	4.050376	149	3.726055	138
39	3.438415	125	2.548296	78	1.120316	35	1.268947	40
32	2.298062	88	2.342728	75	2.661955	86	2.745004	90
280	26.66174	1839	14.08871	793	14.78419	850	15.20634	886
91	2.798146	326	2.365803	231	2.087837	205	1.970827	197
164	30.27564	915	13.43898	340	14.1317	360	12.91259	334
28	0.866631	124	0.604988	73	1.367692	165	1.262344	154
2386	74.39225	8524	76.71901	7544	71.40014	7076	69.74728	6851
30	0.531535	38	2.015998	120	1.78051	107	0.776115	47
126	2.343681	637	2.915557	662	3.355465	767	2.739931	635
939	27.55469	3863	28.18955	3307	31.18097	3685	28.91597	3465
333	32.71695	1632	23.04189	962	25.45363	1071	17.09792	729
283	7.223444	661	9.629067	738	8.455564	653	7.396687	579
33	0.704181	79	0.909978	86	0.60415	57	1.111593	107
888	24.76951	5080	12.92342	2218	14.69381	2541	11.03553	1935
20	1.380237	124	0.908124	72	1.277279	101	0.697842	53
16	3.29818	174	1.589296	67	1.271375	63	1.097737	40
36	0.238822	38	1.038818	138	1.396132	187	0.991305	135
150	15.74161	551	6.833614	274	6.92243	280	8.85833	363
85	2.087563	182	6.814834	497	6.636248	486	5.665011	421
98	9.034524	529	6.399498	314	7.778423	384	5.694457	285

1552	72.47582	7189	118.4931	9901	38.9426	3262	39.41317	3338
53	6.437242	342	7.977723	355	6.143085	276	9.348285	425
35	1.991409	180	1.422	108	1.442736	110	1.688338	130
182	10.42711	1267	5.467142	566	5.725048	592	4.504556	473
2538	196.4829	13007	127.1489	7044	121.1603	6761	106.7146	6039
29	1.90004	126	1.09578	96	2.924453	152	1.95503	103
94	3.11622	298	6.588458	527	6.785297	547	4.087173	334
50	2.143584	380	1.173002	174	1.717754	257	0.82619	125
261	10.39063	878	8.235348	585	7.846713	573	7.45401	533
80	0.439464	76	1.649257	232	1.697575	241	1.479438	209
57	2.243355	237	0.975132	87	1.421151	127	1.504422	133
8	0.505135	59	0.345579	34	0.689535	68	0.516694	51
39	0.87281	55	1.832544	96	3.520366	174	1.228058	59
100	4.735431	462	2.141178	175	4.729471	389	2.180218	182
26	1.846358	147	1.680871	112	1.319687	89	1.612609	110
97	3.176312	264	3.769113	337	5.865024	480	5.332444	404
13	1.026083	85	1.542515	107	1.300677	91	0.539577	39
611	173.428	4092	112.0377	2212	123.7301	2461	125.0995	2523
157	3.430681	855	2.496152	517	2.94887	604	2.480572	528
0	2.458503	85	3.11937	90	2.050236	60	1.451676	43
1694	57.63043	8996	33.18432	4377	38.22871	4897	27.98034	3814
19	7.833232	134	4.376804	63	2.79003	40	2.250638	33
48	5.160357	586	1.894784	181	2.71197	260	1.3884	135
86	3.96724	419	2.947997	261	2.212991	197	1.631189	148
13	1.155916	110	1.009122	80	0.896553	72	0.320033	26
11	5.422389	288	2.158013	96	3.495589	157	1.461022	67
55	2.08469	195	0.786811	74	1.234149	117	1.52127	123
93	12.63048	1013	6.869464	461	5.389195	365	4.699507	322
46	0.876542	108	1.894284	194	1.655395	172	2.003297	211
145	10.64339	1199	6.969673	657	6.318501	600	4.970166	479
71	4.947114	401	1.996784	136	2.571547	176	2.22378	154
24	1.130683	207	1.36308	215	1.131357	172	2.480535	380
57	2.715562	205	2.484046	151	2.697682	177	3.696936	238
31	1.321642	136	1.162232	100	0.917237	80	0.995372	88
83	20.97733	1354	8.796003	475	10.20852	556	8.617107	476
1699	94.03546	8654	94.49659	7242	99.40019	7762	81.53427	6434
950	51.76318	5193	48.45322	4067	47.40318	4008	37.08028	3180
9568	1181.467	89019	467.329	29735	514.0658	32841	435.1854	28448
51	1.015237	138	1.720991	199	1.667271	187	1.863792	217
505604	212740.8	5170826	88310.24	1796100	89027.33	1823976	75957.43	1578086
555	25.50222	3692	20.25684	2454	20.95425	2557	18.61298	2304
3	0.443813	56	0.743292	78	0.404111	43	0.811163	87
156663	62628.99	1465315	24899.05	487471	25367.76	500293	23307.41	466124
25	8.933504	220	3.196285	66	3.836951	80	2.780236	59
98	27.94715	686	16.98442	350	10.8487	225	10.84214	228
11	0.854829	102	0.872667	89	0.964316	101	0.891239	94
18	0.986763	120	1.184717	120	0.761635	79	0.534734	56
117	2.859601	381	4.380693	489	4.510491	506	7.768854	884
400	30.40856	3088	17.09119	1453	16.79238	1438	12.71973	1104
373	84.93286	4176	43.16302	1776	53.05009	2199	38.53964	1620
164560	76874.53	1713545	29451.45	549326	29810.02	560096	26775.42	510153

101	11.25663	1275	5.439316	517	5.670111	542	5.412138	519
93	10.58273	1177	4.112026	383	5.699045	535	3.807864	362
28	3.707924	269	3.597425	218	1.881621	115	3.127539	194
0	1.884465	86	0.900413	38	2.324937	120	0.872901	45
148	24.80196	1731	13.20099	771	10.84727	637	8.255765	492
8	1.00814	131	0.222723	56	0.222995	57	0.457899	118
100	13.17071	965	7.253002	423	7.84903	470	4.063688	251
7600	6418.857	114919	2234.286	33472	2050.789	30949	2198.678	33647
495	92.64895	5423	45.36976	2223	48.44762	2391	35.91166	1797
88	15.15812	1021	7.574996	427	7.166325	407	5.539534	319
5	11.15515	188	3.502709	50	4.186056	60	5.747987	83
1098	230.8355	6923	200.6097	5086	196.0494	5072	195.1218	5023
24	1.217142	97	2.35747	156	1.794866	120	1.073748	73
48	5.894464	554	2.04441	183	2.733125	246	1.591855	144
44	3.835894	473	1.623687	168	2.043009	213	1.828024	193
62	63.4499	1737	20.83669	456	21.85439	501	22.18736	505
469	697.4302	12239	237.5799	3489	193.4607	2862	207.9567	3120
19	1.249057	127	0.71254	61	1.325175	114	1.359706	118
0	1.288467	138	0.838764	75	0.654678	59	0.863315	79
5	2.138296	98	1.01791	51	0.976611	49	0.697723	36
55	42.54662	829	12.56061	205	14.08342	232	11.50999	192
18	1.744543	310	0.419574	123	0.262448	78	0.286538	86
126	100.1176	2260	26.29717	497	21.34438	407	22.53868	435
190	26.84656	4285	8.221773	1098	6.815175	917	8.144308	1111
4	6.275031	298	0.861574	35	1.642608	66	1.399071	57
115	71.09981	3078	19.65292	699	15.3572	581	19.53935	704
109	31.16575	2875	18.62349	1448	17.68215	1330	12.2046	952
59	96.88731	2087	27.62846	498	19.14752	348	31.59594	582
35	3.993326	268	4.651704	261	4.636541	262	4.775069	274
27	9.02308	903	3.925809	322	3.890421	321	4.535967	380
17	41.14066	880	3.333551	60	3.840516	70	6.03762	111
35	52.10566	2138	15.68242	539	14.08823	488	14.39989	505
0	2.188196	270	2.681521	165	1.410398	147	0.566074	60
13	1.20987	210	0.712339	166	0.765677	179	0.786328	163
115	128.6773	7815	56.52027	2789	56.54651	2929	48.41281	2453
220	28.58173	787	161.1083	13485	141.3113	12598	146.3717	12829
4	31.78408	1029	4.286734	117	1.328995	37	7.406204	205
0	0.655033	80	0.491581	52	0.404117	48	0.265205	32

FDR	log2FC	regulated	COG_class	COG_class	GO_annot	KEGG_ann	KEGG_path	KOG_class
5.33E-13	8.982506	up	--	--	Molecular	K17289|1.3	--	[I]
1.40E-11	8.653221	up	--	--	Molecular	K09228|1.7	Herpes sin	[R]
1.19E-14	6.976294	up	--	--	--	--	--	--
1.38E-09	6.330529	up	[O]	Posttransla	Molecular	K01329|1.1	Renin-ang	[E]
1.15E-08	6.010503	up	--	--	Cellular Co	--	--	--
0.001315	5.124567	up	--	--	--	--	--	--
1.22E-16	4.593249	up	--	--	Biological	K10604|5.9	Ubiquitin r	[O]
4.21E-08	4.523754	up	--	--	--	--	--	--
7.86E-07	4.127866	up	[O]	Posttransla	Molecular	K05502|2.3	--	[E]
8.61E-13	4.106644	up	--	--	Molecular	K09306|7.5	--	[R]
0.004873	3.999614	up	--	--	Biological	--	--	--
0.000325	3.827783	up	--	--	Biological	K10786|5.0	Asthma (ko	[TV]
1.59E-31	3.8079	up	[T]	Signal tran	Molecular	K19663|0.0	EGFR tyros	[T]
2.68E-08	3.805616	up	--	--	Biological	K09085|1.1	--	[K]
5.38E-36	3.714414	up	--	--	Cellular Co	K10324|9.8	--	[R]
3.77E-25	3.585204	up	--	--	Molecular	K08023|0.0	--	[T]
0.000133	3.482465	up	--	--	Biological	K17596|0.0	--	[K]
0.000887	3.43201	up	[O]	Posttransla	Molecular	K01346|8.4	Pancreatic	[E]
0.004062	3.363094	up	[T]	Signal tran	Molecular	K08895|0.0	--	[T]
1.31E-22	3.328522	up	--	--	Molecular	--	--	--
0.00014	3.293272	up	[V]	Defense m	Molecular	K05673|1.5	Antifolate	[Q]
5.84E-15	3.217741	up	--	--	--	--	--	[S]
0.001137	3.201186	up	--	--	Cellular Co	K22869|2.2	--	--
6.06E-27	3.11742	up	[R]	General fu	Molecular	K17199|7.8	--	--
2.37E-07	3.089945	up	--	--	Molecular	K04284|3.5	cAMP sign	[R]
6.09E-07	3.076955	up	--	--	Cellular Co	K00737|0.0	N-Glycan	--
3.33E-05	3.071223	up	--	--	Molecular	K13334|3.7	--	[W]
1.98E-16	3.024782	up	[X]	Mobilome	Molecular	K16628|0.0	Protein dig	[W]
2.43E-11	2.927237	up	--	--	Cellular Co	K00735|4.9	Mannose t	[O]
5.14E-22	2.890556	up	[G]	Carbohydr	Biological	K03841|2.9	Glycolysis	[G]
2.07E-05	2.860384	up	[V]	Defense m	Molecular	K05673|4.0	Antifolate	[Q]
6.82E-23	2.842841	up	--	--	Molecular	K10051|9.7	Transcripti	[K]
4.56E-20	2.835949	up	--	--	Molecular	K01481|0.0	--	--
1.44E-17	2.829438	up	--	--	--	--	--	--
3.54E-05	2.813891	up	[J]	Translation	Molecular	K18408|0.0	--	[A]
0.000139	2.81213	up	--	--	Molecular	K15613|2.9	--	[K]
4.14E-25	2.800822	up	--	--	Molecular	K10518|1.3	--	[R]
1.49E-34	2.798898	up	--	--	Biological	K00461|0.0	Arachidon	--
3.55E-24	2.760578	up	[Q]	Secondary	Biological	K10789|0.0	Drug meta	[R]
1.53E-14	2.747053	up	--	--	Biological	K06473|6.1	Hematopo	--
8.11E-11	2.729207	up	--	--	Cellular Co	K05408|4.9	Cytokine-c	--
2.00E-07	2.718767	up	--	--	--	K15196|2.5	--	--
7.89E-23	2.702109	up	[O]	Posttransla	Molecular	K19511|5.1	--	--
1.69E-13	2.684814	up	--	--	--	K00308|1.3	Peroxisom	--
0.000785	2.67423	up	--	--	Biological	K22632|9.6	Cytokine-c	--
2.94E-15	2.655263	up	--	--	Molecular	K23503|2.8	--	[R]
0.002399	2.579176	up	[O]	Posttransla	Molecular	K01353|3.4	Apoptosis	[E]
2.60E-24	2.565084	up	--	--	Molecular	--	--	[O]

1.65E-09	2.541881	up	--	--	Cellular Cc	--	--	--
3.62E-21	2.539597	up	--	--	Molecular	K04864|2.(	MAPK sigr	[PT]
5.60E-20	2.493874	up	--	--	Molecular	K04312|1.6	--	--
1.44E-17	2.46894	up	--	--	Molecular	K21754|7.2	--	[R]
6.55E-07	2.466578	up	--	--	Cellular Cc	--	--	--
4.40E-05	2.433	up	--	--	Molecular	K07604|2.1	Estrogen s	--
1.26E-18	2.431566	up	--	--	--	K06856|1.5	Calcium si	--
2.84E-06	2.418725	up	--	--	--	--	--	--
3.54E-05	2.387814	up	--	--	--	--	--	--
0.002177	2.373097	up	--	--	Cellular Cc	K00286|1.6	Arginine a	[V]
7.92E-12	2.372469	up	--	--	Molecular	K09325|2.(	--	[R]
1.98E-16	2.365401	up	--	--	Cellular Cc	K06473|6.8	Hematopc	--
6.24E-23	2.336108	up	--	--	--	K20395|4.1	--	--
0.001595	2.325806	up	--	--	--	K08111|6.4	--	--
8.66E-08	2.321028	up	--	--	Cellular Cc	--	--	--
0.000711	2.319185	up	--	--	Biological	K22156|4.5	--	[T]
7.04E-10	2.300406	up	--	--	--	--	--	--
1.75E-08	2.260806	up	--	--	Molecular	K05631|2.8	Tight junct	[R]
0.002355	2.244608	up	--	--	Molecular	K06716|8.2	--	--
0.000758	2.239135	up	--	--	Cellular Cc	K04846|2.6	--	--
4.33E-06	2.237345	up	[J]	Translation	Cellular Cc	K14563|1.2	Ribosome	[A]
5.08E-06	2.221492	up	--	--	Cellular Cc	K08405|5.9	--	--
4.39E-08	2.220773	up	[R]	General fu	Biological	K04685|1.2	FoxO signa	[R]
6.85E-06	2.217558	up	--	--	Biological	K21635|0.(	Endocrine	[T]
1.99E-05	2.212167	up	--	--	Cellular Cc	K17387|6.9	MicroRNA	[RT]
0.000311	2.207542	up	--	--	--	--	--	--
1.82E-06	2.173472	up	--	--	--	K05647|1.5	ABC transp	--
0.005153	2.166003	up	--	--	Cellular Cc	K05462|1.5	MAPK sigr	[T]
4.29E-05	2.129654	up	--	--	Cellular Cc	K10785|2.5	Ras signali	--
0.000424	2.107575	up	[I]	Lipid trans	Biological	K12419|5.(	--	--
1.78E-11	2.098682	up	--	--	Molecular	K08444|0.(	--	[T]
4.49E-08	2.081293	up	--	--	Biological	K17261|4.2	--	[ZT]
0.000579	2.069532	up	--	--	--	K08111|2.4	--	--
0.008956	2.029112	up	--	--	Molecular	K04268|1.(	cGMP-PK(	--
6.77E-12	2.024497	up	--	--	Molecular	K04268|1.2	cGMP-PK(	[R]
2.07E-05	2.021336	up	[O]	Posttransla	Molecular	K07995|0.(	Parathyroi	[OW]
3.13E-15	2.01395	up	--	--	Molecular	K08574|0.(	--	[OT]
0.000247	2.0075	up	--	--	--	--	--	--
4.04E-11	2.003241	up	--	--	Cellular Cc	K07884|2.(	Pancreatic	[S]
0.000562	1.996291	up	--	--	Biological	K06248|3.8	PI3K-Akt s	[R]
2.49E-12	1.981568	up	[O]	Posttransla	Molecular	K01353|4.9	Apoptosis	[E]
2.13E-23	1.978948	up	--	--	Biological	K00460|0.(	Arachidon	--
1.84E-05	1.974317	up	--	--	--	--	--	--
1.40E-06	1.94568	up	--	--	Cellular Cc	K20395|1.5	--	--
1.02E-11	1.93153	up	--	--	Molecular	K05066|4.5	Cytokine-(	--
2.44E-11	1.921879	up	--	--	Molecular	K05067|2.(	Cytokine-(	--
1.01E-08	1.921125	up	--	--	Cellular Cc	--	--	--
0.000687	1.92101	up	[IQR]	Lipid trans	Biological	K00061|7.3	Retinol me	[QR]
1.09E-08	1.905993	up	--	--	Biological	K07188|4.7	Apelin sigr	[S]
2.87E-07	1.897767	up	--	--	Cellular Cc	K04403|2.8	MAPK sigr	[TU]
2.04E-05	1.893506	up	[O]	Posttransla	Cellular Cc	K12653|1.3	NOD-like	[S]

0.001904	1.887886	up	[TK]	Signal tran	Molecular	K18437|0.(	Purine me	[T]
0.00248	1.854418	up	--	--	--	--	--	--
3.43E-05	1.854166	up	--	--	--	K09228|9.8	Herpes sin	[R]
0.002259	1.851493	up	[E]	Amino acid	Biological	K13869|7.7	Ferroptosis	[E]
1.08E-08	1.848556	up	[I]	Lipid trans	Biological	K01054|4.3	Glycerolipi	[I]
0.004307	1.837275	up	[G]	Carbohydr	Molecular	K06255|0.(	ECM-recep	[O]
1.14E-05	1.836574	up	[L]	Replicatior	Molecular	K10260|1.2	Ubiquitin r	[K]
8.09E-11	1.833556	up	--	--	Molecular	K24142|2.7	--	[I]
4.97E-07	1.807149	up	--	--	Molecular	K22754|0.(	--	--
1.89E-07	1.797762	up	--	--	Molecular	--	--	--
5.52E-09	1.7964	up	--	--	Molecular	K08614|0.(	--	[O]
1.27E-10	1.789652	up	--	--	Biological	K21437|0.(	--	[R]
7.71E-10	1.782428	up	--	--	Molecular	K10379|4.1	--	--
1.00E-10	1.781966	up	--	--	Molecular	K07374|2.4	Phagosom	[Z]
3.35E-15	1.781949	up	--	--	Molecular	K08445|0.(	--	[T]
3.38E-11	1.778492	up	[R]	General fu	Biological	K07909|7.9	--	[U]
0.002637	1.769817	up	--	--	Molecular	K07374|1.5	Phagosom	[Z]
1.02E-05	1.763773	up	[C]	Energy pro	Molecular	K00029|0.(	Pyruvate n	[C]
0.000751	1.747011	up	--	--	Biological	K21917|6.6	--	[R]
0.00077	1.738402	up	--	--	Molecular	K09103|0.(	--	[K]
0.000473	1.732399	up	--	--	Biological	K06531|7.2	Cell adhes	--
1.91E-13	1.724733	up	--	--	Molecular	K23576|3.(	--	--
3.81E-05	1.713924	up	--	--	Molecular	K05480|5.6	Cytokine-(	--
2.29E-10	1.699161	up	--	--	Cellular Cc	K12352|0.(	Sphingolip	--
0.000135	1.69597	up	--	--	Molecular	--	--	--
1.25E-12	1.687146	up	--	--	Molecular	K01481|0.(	--	--
1.83E-09	1.684236	up	--	--	Biological	K09223|3.2	--	[R]
0.001138	1.683408	up	--	--	Cellular Cc	K20005|2.5	--	--
9.34E-05	1.672941	up	--	--	--	K09228|1.3	Herpes sin	[R]
7.81E-07	1.659451	up	--	--	Biological	K15621|0.(	Transcripti	[UT]
3.16E-09	1.658008	up	[T]	Signal tran	Molecular	K08794|6.4	Calcium si	[T]
0.004136	1.647382	up	--	--	Biological	K19523|0.(	Fatty acid	[I]
5.33E-06	1.647181	up	--	--	Molecular	K13157|4.4	--	--
0.000589	1.646274	up	--	--	--	--	--	--
4.32E-11	1.645332	up	--	--	Molecular	K06715|1.6	--	--
1.24E-07	1.64126	up	--	--	Molecular	K04590|5.6	cAMP sign	[T]
0.003255	1.641092	up	--	--	--	--	--	--
1.66E-07	1.633474	up	--	--	Cellular Cc	K05404|6.7	Toll-like re	[R]
1.38E-06	1.633133	up	[E]	Amino acid	Molecular	K18592|0.(	Taurine an	[E]
3.69E-06	1.630124	up	--	--	Cellular Cc	--	--	[R]
0.002084	1.629669	up	--	--	Biological	K16837|1.3	PI3K-Akt s	--
0.001189	1.624332	up	--	--	Biological	K19627|1.8	Phototrans	[T]
1.50E-10	1.623663	up	--	--	Molecular	K10495|1.9	--	[R]
1.69E-14	1.618217	up	--	--	Cellular Cc	K02155|1.2	Oxidative	[C]
0.000618	1.61715	up	--	--	--	--	--	--
5.13E-05	1.616948	up	--	--	Biological	K06752|5.4	Phagosom	--
3.53E-06	1.614934	up	--	--	Molecular	K23379|5.2	ECM-recep	[WV]
0.00019	1.609628	up	--	--	Cellular Cc	K13908|0.(	IL-17 signa	[WV]
9.34E-05	1.608058	up	--	--	--	K06512|8.1	Osteoclast	--
2.87E-07	1.607953	up	[O]	Posttransla	Molecular	K01353|1.4	Apoptosis	[E]
0.001151	1.601909	up	--	--	--	--	--	--

0.000168	1.599064	up	--	--	Cellular Cc	K14574|1.4	Ribosome	[R]
1.78E-07	1.59718	up	--	--	Molecular	--	--	--
5.23E-05	1.590747	up	--	--	Cellular Cc	K22759|1.0	Amyotropl	[S]
8.34E-06	1.576879	up	[IR]	Lipid trans	Molecular	K00901|0.0	Glycerolipi	[IT]
0.00052	1.575403	up	--	--	Molecular	K22869|1.2	--	--
0.000205	1.566839	up	--	--	--	K19909|4.9	--	[W]
1.08E-08	1.56632	up	--	--	Molecular	K07374|1.8	Phagosom	[Z]
0.00294	1.558056	up	[Q]	Secondary	Biological	K06585|0.0	PI3K-Akt s	[W]
1.25E-05	1.557342	up	--	--	Molecular	K17197|7.6	--	[T]
1.66E-12	1.555311	up	--	--	Cellular Cc	--	--	--
0.004002	1.553492	up	[O]	Posttransla	Molecular	K02857|1.7	--	[T]
0.002444	1.550619	up	--	--	--	--	--	--
3.11E-07	1.548766	up	--	--	Biological	K05408|6.0	Cytokine-c	--
5.11E-08	1.547422	up	[G]	Carbohydr	Molecular	K07299|9.3	HIF-1 sign	[G]
0.000163	1.54367	up	--	--	Cellular Cc	K20395|5.6	--	--
4.66E-09	1.539274	up	--	--	--	--	--	--
0.007526	1.537521	up	--	--	Molecular	K08443|0.0	--	[T]
0.003181	1.535662	up	--	--	Cellular Cc	--	--	--
0.004067	1.535288	up	--	--	Molecular	K17751|0.0	cGMP-PKC	[Z]
5.08E-06	1.529976	up	--	--	Molecular	K19503|0.0	--	[S]
4.25E-05	1.520759	up	--	--	Biological	K04328|2.4	cAMP sign	--
0.00039	1.516982	up	--	--	Biological	--	--	--
3.00E-08	1.504224	up	--	--	Biological	K12196|8.5	Endocytos	[W]
3.35E-05	1.502399	up	--	--	Molecular	K16067|0.0	--	[O]
0.002983	1.50027	up	--	--	--	K10434|3.2	--	--
2.36E-08	1.495705	up	--	--	Cellular Cc	K22696|6.6	--	[R]
1.56E-08	1.493359	up	--	--	--	--	--	--
6.77E-05	1.490377	up	--	--	Biological	--	--	--
6.24E-10	1.487468	up	--	--	Cellular Cc	K12484|0.0	Endocytos	--
1.21E-05	1.487326	up	[R]	General fu	Biological	K06525|0.0	PI3K-Akt s	[TW]
5.75E-07	1.479222	up	--	--	Cellular Cc	K21846|2.1	--	[S]
7.16E-06	1.474759	up	[O]	Posttransla	Biological	K01327|1.1	Transcripti	[E]
7.81E-11	1.474244	up	--	--	Cellular Cc	K17598|0.0	--	[TU]
1.22E-08	1.473206	up	--	--	Molecular	K04557|4.2	Parkinson	[DZU]
0.005376	1.473141	up	[EQ]	Amino acid	Molecular	K01469|0.0	Glutathion	[E]
2.85E-13	1.472937	up	--	--	Biological	K04150|5.8	Calcium si	[R]
5.92E-10	1.471104	up	--	--	Cellular Cc	K12827|1.2	Spliceosor	[TZR]
5.57E-06	1.471099	up	--	--	Molecular	K05052|9.5	Neuroactiv	[R]
1.51E-07	1.464981	up	--	--	Cellular Cc	K05408|8.3	Cytokine-c	--
0.001995	1.46446	up	--	--	--	K08111|6.7	--	--
4.26E-08	1.463783	up	--	--	Biological	K09182|2.7	--	[K]
0.000819	1.458849	up	--	--	--	--	--	--
0.00021	1.458543	up	--	--	--	--	--	--
4.60E-09	1.456868	up	--	--	Biological	K17441|0.0	--	[R]
5.26E-05	1.451674	up	--	--	Biological	K20857|0.0	--	--
0.002768	1.447563	up	[O]	Posttransla	Molecular	K09630|1.1	--	[E]
2.12E-05	1.447	up	--	--	Molecular	K22460|0.0	--	[S]
0.003706	1.446457	up	--	--	Cellular Cc	K22157|2.7	--	[O]
2.87E-08	1.440771	up	--	--	Molecular	K15728|0.0	Glycerolipi	[NI]
1.15E-10	1.432494	up	--	--	Cellular Cc	K13516|1.5	Glyceroph	[S]
0.001074	1.427802	up	--	--	Biological	K09421|0.0	--	--

0.005891	1.424686	up	--	--	Cellular Cc	K23091|9.6	--	[GO]
0.001787	1.423506	up	[I]	Lipid trans	Cellular Cc	K22387|5.6	Ether lipid	[C]
1.48E-09	1.420803	up	--	--	Molecular	K05766|0.0	Axon guid	--
0.009535	1.418524	up	--	--	Molecular	K17934|6.9	--	--
1.03E-07	1.416075	up	--	--	Molecular	K07843|2.7	Circadian e	[R]
6.13E-06	1.407705	up	--	--	Cellular Cc	K19918|8.0	--	[U]
0.001159	1.407224	up	[M]	Cell wall/m	Biological	K05124|0.0	--	[T]
2.59E-07	1.406727	up	[T]	Signal tran	Molecular	K18018|2.2	MAPK sigr	[T]
0.004368	1.404116	up	--	--	--	--	--	--
0.001746	1.403585	up	--	--	Molecular	K17847|4.8	FoxO signa	[K]
6.01E-05	1.395369	up	--	--	Biological	K06621|1.7	Endocrine	--
0.008122	1.392754	up	--	--	Molecular	K19895|6.2	--	--
0.00244	1.39259	up	--	--	--	--	--	--
1.82E-08	1.388293	up	--	--	Biological	K05464|0.0	EGFR tyros	--
7.06E-05	1.384025	up	--	--	Cellular Cc	K17599|0.0	--	[S]
2.07E-06	1.382449	up	--	--	Molecular	K21847|0.0	--	[R]
5.87E-09	1.379078	up	[M]	Cell wall/m	Biological	K07527|0.0	Axon guid	[T]
0.000137	1.373345	up	--	--	Cellular Cc	K14314|0.0	RNA trans	[YU]
1.52E-06	1.372822	up	--	--	Molecular	K08486|1.5	SNARE inte	[U]
0.007279	1.368289	up	--	--	Biological	--	--	--
1.92E-05	1.367333	up	--	--	Biological	K13806|0.0	Retrograde	[IOT]
1.49E-05	1.364644	up	[O]	Posttransla	Molecular	K09640|5.1	--	[E]
0.001328	1.354521	up	--	--	--	--	--	--
1.35E-06	1.352541	up	--	--	Biological	K23791|5.1	--	--
8.86E-07	1.345555	up	--	--	Cellular Cc	K17923|2.9	Salmonella	[R]
0.000151	1.344993	up	--	--	--	--	--	--
1.91E-08	1.344777	up	--	--	Molecular	K12653|0.0	NOD-like	[S]
1.03E-05	1.344529	up	[T]	Signal tran	Molecular	K08014|0.0	Rap1 signa	[T]
5.92E-11	1.339649	up	[J]	Translation	Biological	K02877|9.1	Ribosome	[J]
0.000403	1.339449	up	--	--	Cellular Cc	K19674|0.0	--	[R]
0.003695	1.337857	up	--	--	--	K09228|3.6	Herpes sin	[R]
1.71E-11	1.336628	up	[I]	Lipid trans	Cellular Cc	K16860|4.4	Glyceroph	[R]
1.26E-05	1.330275	up	--	--	--	K06511|1.0	--	--
0.003104	1.326052	up	--	--	Biological	K16513|9.2	Circadian e	[R]
0.002341	1.321759	up	--	--	Cellular Cc	K17253|1.6	--	--
0.000132	1.316397	up	--	--	Cellular Cc	K06818|0.0	--	--
1.35E-06	1.309887	up	--	--	Cellular Cc	--	--	--
0.006013	1.308997	up	--	--	Molecular	--	--	[R]
0.003104	1.307631	up	--	--	Molecular	K22641|9.5	--	[R]
0.002824	1.303418	up	--	--	--	K12464|1.7	Neurotrop	[S]
1.86E-05	1.300797	up	--	--	Cellular Cc	K06751|3.7	Endocytos	--
0.001076	1.298827	up	--	--	Biological	K19527|1.1	--	--
5.52E-09	1.297699	up	[G]	Carbohydr	Biological	K00844|0.0	Glycolysis	[G]
5.61E-05	1.294473	up	--	--	Molecular	K05154|7.9	Cytokine-c	--
6.77E-07	1.292809	up	--	--	Cellular Cc	K06712|0.0	--	--
1.43E-10	1.289966	up	--	--	Cellular Cc	K00286|3.4	Arginine a	[V]
0.000235	1.286643	up	--	--	Biological	K16782|5.2	--	[T]
1.56E-08	1.284624	up	[HT]	Coenzyme	Cellular Cc	K08869|0.0	--	[R]
0.003334	1.278643	up	--	--	Biological	K13073|0.0	Intestinal i	--
0.000229	1.278228	up	[G]	Carbohydr	Cellular Cc	K23677|0.0	--	[G]
8.34E-06	1.274211	up	--	--	Biological	K16513|1.4	Circadian e	--

0.006983	1.272116	up	--	--	Biological	K20493|4.(	--	[T]
2.24E-05	1.271689	up	[E]	Amino acio	Biological	K01283|0.(	Renin-ang	[E]
0.007468	1.270287	up	--	--	--	--	--	--
1.17E-10	1.263867	up	--	--	Cellular Cc	K19831|8.3	B cell rece	--
6.44E-05	1.260846	up	--	--	Biological	K05073|4.5	Cytokine-(	--
0.000759	1.257438	up	[M]	Cell wall/m	--	K14529|5.3	RNA trans	[R]
0.00044	1.255569	up	--	--	Cellular Cc	--	--	--
1.01E-07	1.25294	up	[O]	Posttransla	Molecular	K09616|2.4	--	[E]
0.000226	1.249082	up	--	--	--	--	--	--
9.88E-09	1.243727	up	[E]	Amino acio	Molecular	K17989|3.1	Glycine, se	[E]
1.05E-08	1.241653	up	--	--	Biological	K11275|7.1	--	[B]
0.00029	1.239304	up	--	--	Molecular	K04862|0.(	MAPK sign	[PT]
6.61E-06	1.237297	up	--	--	--	--	--	--
2.31E-12	1.236546	up	--	--	Biological	K16532|6.7	--	[RT]
0.001541	1.230762	up	--	--	Biological	--	--	--
0.000297	1.225027	up	--	--	Cellular Cc	K11275|6.7	--	[B]
0.00031	1.224442	up	--	--	Biological	K03213|9.8	Wnt signal	--
0.0002	1.224249	up	--	--	Biological	K05627|8.8	Adherens j	--
2.45E-09	1.219637	up	[R]	General fu	Molecular	K19020|0.(	--	[T]
2.64E-09	1.215665	up	[G]	Carbohydr	Molecular	K00615|0.(	Pentose pl	[G]
3.11E-06	1.210869	up	--	--	Cellular Cc	K06712|1.2	--	--
1.77E-08	1.209173	up	--	--	Cellular Cc	K06540|0.(	--	[O]
4.33E-08	1.207951	up	--	--	Cellular Cc	K09377|3.4	--	[TZ]
2.42E-05	1.206526	up	--	--	Cellular Cc	K18756|1.4	--	[R]
0.005293	1.206302	up	[R]	General fu	Molecular	K02088|6.7	--	[R]
1.29E-08	1.206261	up	--	--	Biological	K06497|9.4	Lysosome	[R]
8.66E-08	1.205345	up	--	--	Molecular	K10382|0.(	--	[Z]
0.004628	1.204967	up	--	--	Molecular	K10408|0.(	Amyotropl	[Z]
1.89E-07	1.204964	up	--	--	Molecular	K21444|3.7	--	[AR]
2.13E-08	1.204715	up	--	--	Biological	K11454|5.(	--	[B]
0.000101	1.200605	up	--	--	Cellular Cc	K10480|0.(	--	--
5.27E-05	1.199735	up	--	--	Molecular	K19722|1.1	AGE-RAGI	--
7.67E-05	1.198951	up	[P]	Inorganic i	Biological	K03926|3.6	--	[P]
0.000128	1.197069	up	--	--	Cellular Cc	K19193|1.8	--	--
1.24E-06	1.196277	up	--	--	Cellular Cc	K06549|1.6	--	--
0.000551	1.195005	up	--	--	--	K08111|7.3	--	--
0.000613	1.193841	up	--	--	Molecular	K04826|0.(	--	[PT]
1.48E-09	1.191868	up	[G]	Carbohydr	Molecular	K00033|1.6	Pentose pl	[G]
1.65E-05	1.181429	up	--	--	Biological	K09228|6.2	Herpes sin	[R]
5.85E-07	1.179124	up	--	--	Molecular	K15109|2.3	Thermoge	[C]
1.49E-05	1.177372	up	[E]	Amino acio	Biological	K00501|1.1	Tyrosine m	[E]
0.000645	1.176056	up	--	--	Cellular Cc	K04710|4.1	Sphingolip	[U]
0.002177	1.173953	up	--	--	--	--	--	--
0.005736	1.165209	up	--	--	Cellular Cc	K20722|4.7	--	[U]
0.000123	1.164929	up	--	--	Molecular	K09228|4.9	Herpes sin	--
2.22E-06	1.164642	up	--	--	Molecular	K20123|1.5	--	[TUZ]
2.53E-08	1.164178	up	--	--	Cellular Cc	K20359|7.9	--	[U]
3.28E-05	1.164172	up	--	--	Molecular	K16185|1.(	Autophagy	[T]
0.000241	1.163286	up	--	--	Cellular Cc	K06534|3.(	--	--
8.66E-08	1.161607	up	--	--	Molecular	K23369|0.(	--	[O]
8.00E-06	1.159692	up	--	--	Molecular	K09228|5.1	Herpes sin	[R]

0.000772	1.157997	up	--	--	Biological	K12043|2.(	--	[Z]
8.42E-05	1.155298	up	--	--	--	--	--	--
3.93E-06	1.155262	up	[G]	Carbohydr	Cellular Cc	K12306|0.(	--	[R]
6.03E-05	1.154841	up	--	--	Molecular	K17277|0.(	--	[T]
0.001385	1.154605	up	--	--	Cellular Cc	--	--	[S]
0.001106	1.150669	up	--	--	Molecular	--	--	--
1.83E-07	1.149704	up	--	--	Biological	K18618|7.3	--	[D]
1.44E-06	1.149207	up	--	--	--	--	--	--
4.60E-06	1.148751	up	--	--	--	K06554|1.9	Primary im	--
0.00802	1.14601	up	[P]	Inorganic i	Biological	K14607|0.(	--	[G]
0.000376	1.144953	up	--	--	Molecular	K21851|0.(	--	[R]
0.000963	1.140137	up	--	--	Cellular Cc	K06547|0.(	Cell adhes	--
1.59E-06	1.139554	up	--	--	Cellular Cc	K16519|5.4	--	--
5.72E-05	1.136303	up	[E]	Amino aci	Molecular	K14358|1.8	--	--
6.35E-05	1.135471	up	--	--	Biological	K09659|3.9	N-Glycan	[OT]
6.05E-08	1.135077	up	[P]	Inorganic i	Molecular	K04946|0.(	--	[P]
0.000225	1.133449	up	--	--	--	K08111|5.6	--	--
4.34E-08	1.129886	up	--	--	--	--	--	--
2.12E-07	1.12857	up	--	--	Molecular	K05863|2.6	Calcium si	[C]
0.000912	1.124576	up	--	--	Cellular Cc	K20189|2.4	--	--
0.001121	1.122629	up	[X]	Mobilome	Cellular Cc	K16353|0.(	--	[T]
1.67E-06	1.122239	up	--	--	Cellular Cc	K10519|1.2	--	[R]
0.000562	1.119484	up	[V]	Defense m	Molecular	K05673|0.(	Antifolate	[Q]
2.40E-05	1.119309	up	[I]	Lipid trans	Cellular Cc	K10809|0.(	Thyroid hc	--
0.001465	1.118949	up	[R]	General fu	Molecular	--	--	[R]
0.000111	1.118568	up	--	--	--	--	--	--
1.00E-07	1.116676	up	[F]	Nucleotide	Molecular	K01490|0.(	Purine me	[F]
0.000452	1.115803	up	--	--	Cellular Cc	--	--	--
0.000301	1.115361	up	--	--	Molecular	K09173|0.(	--	[K]
6.78E-05	1.114104	up	--	--	Cellular Cc	K19326|0.(	--	[Z]
1.66E-07	1.112072	up	--	--	Cellular Cc	K06513|7.7	Phagosom	--
8.66E-08	1.111266	up	--	--	Molecular	K16631|1.8	Amyotropl	--
0.005371	1.10881	up	--	--	--	--	--	--
2.20E-08	1.107774	up	--	--	Molecular	K07205|1.1	EGFR tyros	--
8.46E-05	1.1066	up	--	--	--	--	--	--
1.10E-05	1.104455	up	--	--	Molecular	K10641|0.(	--	[R]
2.47E-07	1.104054	up	[O]	Posttransla	Molecular	K11848|0.(	--	[O]
0.000718	1.102789	up	--	--	Molecular	K07374|7.6	Phagosom	[Z]
1.34E-06	1.096611	up	--	--	Cellular Cc	K16302|3.1	--	[I]
0.001059	1.096464	up	--	--	--	K09228|8.(	Herpes sin	--
3.01E-05	1.087602	up	--	--	Cellular Cc	K20478|0.(	--	--
1.10E-05	1.08414	up	--	--	--	--	--	--
0.002895	1.081547	up	--	--	Cellular Cc	K20282|0.(	Salmonella	--
7.42E-07	1.078858	up	--	--	Molecular	K06486|0.(	Cell adhes	--
0.005222	1.078852	up	--	--	Biological	K05140|4.4	Cytokine-(	--
0.000197	1.078476	up	--	--	Biological	K23456|7.7	--	[S]
0.005193	1.078022	up	--	--	--	--	--	--
0.001504	1.077205	up	--	--	Cellular Cc	K16741|0.(	--	--
0.002736	1.074544	up	--	--	Cellular Cc	K07986|1.3	Natural kil	--
1.15E-07	1.074363	up	--	--	Molecular	K06002|1.3	Protein dig	[O]
1.91E-05	1.072354	up	--	--	Biological	K06594|0.(	Regulatior	[W]

0.005032	1.064834	up	--	--	--	K06512|3.6	Osteoclast	--
0.003181	1.062539	up	--	--	Biological	K17199|1.8	--	--
1.22E-05	1.061818	up	--	--	Molecular	K05142|5.6	Cytokine-c	--
4.22E-06	1.061759	up	--	--	Cellular Cc	--	--	--
0.008888	1.061445	up	--	--	Biological	K05408|2.7	Cytokine-c	--
0.002886	1.060133	up	--	--	Cellular Cc	K06821|0.0	Axon guid	[T]
0.001008	1.056796	up	--	--	--	--	--	--
5.43E-05	1.052462	up	--	--	Cellular Cc	K14354|0.0	--	[Q]
0.005132	1.050391	up	--	--	Cellular Cc	K04737|2.3	Cytokine-c	--
0.000194	1.049339	up	--	--	Molecular	--	--	--
2.56E-05	1.049224	up	--	--	Cellular Cc	K11137|0.0	Fanconi ar	[S]
1.52E-06	1.048798	up	--	--	Biological	K17344|5.5	--	[R]
0.000863	1.04623	up	--	--	Molecular	K17565|0.0	--	--
9.80E-06	1.046041	up	[C]	Energy prc	Biological	K13511|6.2	Glyceroph	[I]
0.007636	1.04482	up	--	--	Molecular	K16477|0.0	--	--
0.000244	1.039182	up	--	--	Cellular Cc	K02583|7.3	MAPK sigr	--
0.002457	1.036833	up	[R]	General fu	Molecular	K01069|1.9	Pyruvate n	[R]
0.000115	1.034596	up	--	--	Cellular Cc	K16295|9.0	--	[C]
6.97E-05	1.034518	up	--	--	Cellular Cc	K15010|2.8	FoxO signa	--
1.20E-06	1.034382	up	[I]	Lipid trans	Molecular	K17360|2.7	Fatty acid	[I]
5.61E-06	1.031136	up	--	--	Cellular Cc	K16489|0.0	--	[D]
2.69E-05	1.03076	up	--	--	Molecular	K12490|0.0	Rap1 signa	[TZ]
3.15E-05	1.029536	up	[T]	Signal tran	Biological	K02599|0.0	Endocrine	[T]
9.73E-07	1.027181	up	--	--	Molecular	K05289|0.0	Glycosylph	[O]
5.43E-06	1.026824	up	[G]	Carbohydr	Biological	K01810|0.0	Glycolysis	[G]
1.23E-07	1.026199	up	[G]	Carbohydr	Molecular	K12311|0.0	Other glyc	[G]
0.000247	1.025764	up	--	--	Cellular Cc	--	--	--
5.10E-06	1.024679	up	--	--	Molecular	K06058|0.0	Notch sigr	--
0.000779	1.024541	up	[G]	Carbohydr	Cellular Cc	K08214|5.7	--	--
0.003676	1.022009	up	[O]	Posttransla	Molecular	K01353|5.5	Apoptosis	[E]
0.007316	1.021807	up	[T]	Signal tran	Molecular	K08789|0.0	--	[TR]
1.70E-06	1.020921	up	--	--	Molecular	K15100|2.3	--	[C]
5.43E-05	1.020386	up	--	--	Molecular	K08023|0.0	--	[T]
5.67E-07	1.019847	up	[E]	Amino acic	Molecular	K09605|0.0	--	[IOVE]
2.83E-06	1.019612	up	--	--	Biological	K05948|1.2	Other type	--
1.53E-07	1.018878	up	--	--	Molecular	K05858|0.0	Inositol ph	[I]
0.001008	1.018628	up	--	--	Cellular Cc	K06820|0.0	Axon guid	[T]
0.004437	1.018486	up	--	--	Cellular Cc	K04722|7.0	Cytokine-c	--
0.000202	1.017471	up	--	--	Cellular Cc	--	--	--
5.22E-06	1.016558	up	[I]	Lipid trans	Biological	K07188|0.0	cAMP sign	[I]
0.006321	1.016327	up	--	--	Molecular	K17441|6.8	--	[R]
2.38E-05	1.016039	up	--	--	Molecular	K16551|0.0	--	--
0.00016	1.015619	up	--	--	Molecular	K05023|2.1	--	[P]
1.34E-06	1.015305	up	[R]	General fu	Molecular	K16816|1.9	Glycerolipi	[U]
0.004659	1.014187	up	--	--	Cellular Cc	K06821|0.0	Axon guid	[T]
0.006827	1.011925	up	--	--	Molecular	K23091|7.8	--	[GO]
9.86E-06	1.011775	up	--	--	Molecular	K17612|3.7	--	[T]
0.000644	1.010638	up	--	--	Biological	K19879|1.1	--	--
9.56E-06	1.006928	up	--	--	Cellular Cc	--	--	--
1.94E-06	1.003881	up	--	--	Molecular	K16463|3.6	--	--
0.00052	1.000575	up	--	--	Molecular	K04530|3.5	--	--

4.91E-06	1.000538 up	--	--	Molecular	K21596|0.(	--	[S]
4.51E-05	-1.00004 down	--	--	Cellular Cc	K14791|1.9	--	[S]
0.000171	-1.00019 down	[F]	Nucleotide	Molecular	K16606|9.1	--	[F]
1.70E-06	-1.00094 down	--	--	Cellular Cc	K14443|3.8	RNA degra	[TR]
0.001218	-1.00119 down	[E]	Amino acid	Cellular Cc	K16795|1.8	Ether lipid	[TV]
6.22E-05	-1.00217 down	[R]	General fu	Molecular	K24104|1.7	--	[L]
1.00E-05	-1.00253 down	[JO]	Translation	Biological	K09592|2.3	HIF-1 sign	[T]
0.006253	-1.00311 down	--	--	--	K09228|8.7	Herpes sin	[R]
0.000239	-1.00323 down	--	--	Biological	K03377|0.(	--	[R]
6.07E-06	-1.0043 down	--	--	Biological	K10574|9.1	Ubiquitin r	[O]
9.89E-07	-1.00442 down	[O]	Posttransla	Molecular	K09490|0.(	Protein exp	[O]
0.000187	-1.00455 down	--	--	Cellular Cc	--	--	[S]
0.000363	-1.00457 down	[J]	Translation	Molecular	K14191|2.1	--	[A]
0.002365	-1.00458 down	--	--	Molecular	K21891|5.(	--	[S]
1.90E-05	-1.00493 down	[K]	Transcripti	Molecular	K03124|6.1	Basal trans	[K]
0.001206	-1.00519 down	--	--	Cellular Cc	K09532|1.3	--	[S]
4.17E-06	-1.00543 down	--	--	Cellular Cc	K17086|0.(	--	[U]
4.58E-06	-1.00545 down	[J]	Translation	Molecular	K12883|1.3	RNA trans	[A]
0.000599	-1.00575 down	[O]	Posttransla	Molecular	K07151|0.(	N-Glycan	[O]
1.02E-08	-1.0063 down	--	--	Cellular Cc	K00286|4.2	Arginine a	[V]
0.002829	-1.00643 down	[R]	General fu	Molecular	K07837|4.1	--	[R]
0.000128	-1.0065 down	--	--	Cellular Cc	K11415|9.9	Nicotinate	[S]
2.12E-05	-1.00668 down	--	--	Cellular Cc	K08517|3.5	SNARE inte	[U]
4.20E-05	-1.00768 down	--	--	Cellular Cc	K14553|2.5	Ribosome	[R]
0.000168	-1.00921 down	--	--	Cellular Cc	K09527|1.7	--	[R]
0.001865	-1.0093 down	[J]	Translation	Molecular	K02929|3.1	Ribosome	[J]
1.18E-05	-1.00964 down	--	--	Cellular Cc	K17606|2.3	Autophagy	[T]
0.002259	-1.0098 down	--	--	Biological	K14566|1.9	Ribosome	[R]
0.000467	-1.01002 down	--	--	Cellular Cc	K00972|3.9	Amino sug	[DKT]
0.002259	-1.01089 down	--	--	Molecular	K02911|6.6	Ribosome	[J]
0.000325	-1.01247 down	--	--	Cellular Cc	K19478|3.1	--	[O]
0.000912	-1.01258 down	--	--	Molecular	K08658|5.6	Terpenoid	--
2.08E-05	-1.01292 down	--	--	Cellular Cc	K00509|1.2	Arachidon	[T]
0.006842	-1.01297 down	[QR]	Secondary	Molecular	--	--	[R]
0.000729	-1.01352 down	[F]	Nucleotide	Molecular	K00760|7.1	Purine me	[F]
0.001493	-1.01417 down	[O]	Posttransla	Biological	K05864|1.(	Necroptos	[O]
0.002625	-1.01425 down	--	--	Biological	K23506|1.3	--	[S]
8.80E-06	-1.01443 down	[Q]	Secondary	Molecular	K00276|0.(	Glycine, se	[Q]
0.003036	-1.01536 down	[R]	General fu	Biological	K07920|1.3	Autophagy	[TU]
0.000389	-1.0157 down	[I]	Lipid trans	Molecular	K08762|4.5	PPAR signa	[I]
0.00217	-1.01572 down	--	--	Cellular Cc	K05724|4.7	--	[R]
0.003155	-1.01611 down	--	--	Biological	K14842|6.2	--	[R]
5.79E-07	-1.01621 down	--	--	Cellular Cc	K23408|7.8	--	--
0.003039	-1.01717 down	[R]	General fu	Cellular Cc	K06999|1.6	--	[I]
0.00038	-1.01721 down	--	--	Cellular Cc	K15141|1.6	--	--
0.000389	-1.01748 down	--	--	Biological	K12592|3.(	RNA degra	[L]
0.001207	-1.01773 down	--	--	Cellular Cc	K10485|6.(	--	[R]
0.000618	-1.01872 down	[R]	General fu	Molecular	K07903|1.8	Endocytos	[TU]
5.71E-05	-1.01964 down	--	--	Molecular	K24242|8.9	Purine me	[S]
0.003445	-1.0199 down	[J]	Translation	Molecular	K18711|0.(	--	[A]
0.000782	-1.0201 down	--	--	--	--	--	--

2.39E-06	-1.0201	down	--	--	Biological	K04288|1.(	FoxO signa	--
0.007364	-1.02194	down	[J]	Translatior	Molecular	K01893|3.3	Aminoacyl	[J]
0.00089	-1.02279	down	--	--	Molecular	K10608|0.(	Ubiquitin r	[O]
0.001496	-1.02342	down	--	--	Molecular	K10159|0.(	Phagosom	[T]
0.000202	-1.02426	down	--	--	Cellular Cc	K11290|3.4	--	[L]
0.004905	-1.02468	down	--	--	Molecular	K17410|1.4	--	--
5.43E-06	-1.02539	down	--	--	--	--	--	--
0.00481	-1.02671	down	--	--	Biological	--	--	--
0.000104	-1.02688	down	[C]	Energy prc	Molecular	K03943|3.(	Oxidative	[C]
0.000123	-1.02932	down	--	--	--	--	--	--
3.00E-07	-1.02961	down	--	--	Cellular Cc	K21890|2.7	--	[T]
1.02E-07	-1.03055	down	[R]	General fu	Biological	K08069|6.3	Pantothen	[E]
0.002215	-1.03101	down	[R]	General fu	Molecular	K06875|3.2	--	[D]
0.003793	-1.03107	down	[L]	Replicatior	Molecular	K02330|9.3	Base excisi	[L]
0.000417	-1.03196	down	--	--	Biological	K15686|0.(	--	[R]
1.33E-08	-1.03262	down	--	--	Biological	K09392|2.2	Cell cycle (	[K]
0.00014	-1.03279	down	--	--	Cellular Cc	K11001|6.9	Glycosylph	--
7.16E-06	-1.03294	down	[O]	Posttransla	Molecular	K02977|1.(	Ribosome	[J]
0.00038	-1.03446	down	--	--	Cellular Cc	K24349|6.(	Protein prc	[Y]
0.000202	-1.03514	down	--	--	Molecular	K18584|1.1	Tight junct	[Z]
5.57E-07	-1.03612	down	[O]	Posttransla	Cellular Cc	K09495|2.(	--	[O]
0.000431	-1.03754	down	--	--	Cellular Cc	K10270|0.(	--	[R]
0.008496	-1.03765	down	--	--	Cellular Cc	K23292|5.6	--	[S]
0.000844	-1.03842	down	--	--	Cellular Cc	K17087|0.(	--	[U]
7.44E-05	-1.03876	down	[E]	Amino aci(	Molecular	K01372|1.1	--	[E]
0.000182	-1.03883	down	--	--	Molecular	K16742|4.(	--	--
6.08E-05	-1.03897	down	--	--	Cellular Cc	K20363|1.4	--	[U]
0.000216	-1.03897	down	--	--	Molecular	K09228|1.4	Herpes sin	--
9.04E-05	-1.0391	down	--	--	Cellular Cc	K22076|9.2	--	--
0.00036	-1.03996	down	--	--	Cellular Cc	K03539|2.7	Ribosome	[J]
5.13E-05	-1.04015	down	--	--	Cellular Cc	K10144|1.4	p53 signal	[R]
0.000612	-1.04052	down	--	--	Molecular	K07374|1.3	Phagosom	[Z]
0.008221	-1.04265	down	--	--	Molecular	--	--	[R]
0.00774	-1.04532	down	--	--	Molecular	K19916|2.8	--	[U]
0.009188	-1.04664	down	--	--	Biological	K22529|6.4	--	--
0.004349	-1.04701	down	[G]	Carbohydr	Molecular	K01837|3.9	Glycolysis	[G]
0.004335	-1.04755	down	--	--	Cellular Cc	K11247|1.1	Endocytos	[IT]
4.60E-06	-1.04794	down	--	--	Cellular Cc	K23564|2.7	--	[S]
0.000785	-1.04882	down	--	--	Biological	K16810|0.(	--	[S]
3.34E-05	-1.04883	down	--	--	Cellular Cc	--	--	[S]
3.48E-06	-1.04974	down	--	--	Cellular Cc	K13120|4.5	--	[S]
0.000237	-1.04984	down	[BQ]	Chromatin	Biological	K06067|1.1	Cell cycle (	[B]
0.000104	-1.05024	down	--	--	Cellular Cc	K10802|1.1	Base excisi	[R]
0.005801	-1.05067	down	--	--	Molecular	K08063|3.(	Antigen pr	--
0.001515	-1.05113	down	[O]	Posttransla	Molecular	K04077|0.(	RNA degra	[O]
0.00109	-1.0517	down	[M]	Cell wall/m	Cellular Cc	K18669|0.(	--	[R]
0.006287	-1.05401	down	--	--	Biological	K05482|2.6	Cytokine-(	--
0.000265	-1.05422	down	[L]	Replicatior	Cellular Cc	K02603|0.(	Cell cycle (	[L]
0.002141	-1.05429	down	--	--	Molecular	K02970|2.4	Ribosome	--
0.00266	-1.05444	down	--	--	Molecular	K09228|2.2	Herpes sin	[R]
0.003402	-1.05619	down	--	--	Molecular	K12804|6.2	NOD-like	[TU]

0.006136	-1.05659	down	--	--	Biological	K18584|9.4	--	[TU]
6.24E-06	-1.05693	down	[P]	Inorganic i	Molecular	K04565|2.6	Peroxisom	[P]
0.006399	-1.05694	down	--	--	Cellular Co	--	--	--
0.000103	-1.05708	down	--	--	Molecular	K03456|3.9	mRNA sur	[T]
1.68E-05	-1.05739	down	--	--	Molecular	K09228|8.8	Herpes sin	--
0.0002	-1.05801	down	--	--	Molecular	K23203|7.6	--	--
1.55E-06	-1.05858	down	[R]	General fu	Molecular	K07835|3.0	Ras signali	[R]
0.008097	-1.05883	down	--	--	Cellular Co	K11290|3.9	--	[L]
0.001224	-1.05888	down	--	--	Molecular	K08763|2.6	PPAR signa	[TV]
4.20E-05	-1.05984	down	--	--	Molecular	K10802|2.0	Base excisi	[R]
1.61E-05	-1.06046	down	[C]	Energy pro	Molecular	K00162|9.3	Glycolysis	[C]
0.001669	-1.06059	down	--	--	Biological	K11340|1.1	Thermoge	[Z]
2.59E-07	-1.06108	down	--	--	Molecular	K04545|3.4	Ras signali	[T]
5.10E-06	-1.06127	down	--	--	Cellular Co	K10637|1.1	Cholestero	--
4.69E-05	-1.06177	down	[O]	Posttransla	Molecular	K10686|1.1	Ubiquitin r	[O]
2.21E-05	-1.06186	down	[R]	General fu	Cellular Co	K23541|9.1	--	[S]
4.40E-05	-1.0619	down	[T]	Signal tran	Cellular Co	K06269|2.9	mRNA sur	[TR]
5.87E-05	-1.06193	down	[J]	Translation	Molecular	K02880|1.9	Ribosome	[J]
4.37E-06	-1.06196	down	--	--	Molecular	K05756|7.3	Endocytos	[Z]
0.001362	-1.06294	down	--	--	Biological	K01810|5.7	Glycolysis	--
0.000447	-1.06375	down	[J]	Translation	Cellular Co	K02890|4.7	Ribosome	[J]
3.87E-05	-1.06402	down	--	--	Biological	K10885|0.0	Non-hom	[L]
1.77E-05	-1.0652	down	--	--	--	--	--	--
0.000266	-1.06525	down	--	--	Biological	K15262|1.6	--	[R]
5.03E-07	-1.06562	down	[O]	Posttransla	Molecular	K15216|9.7	--	[O]
0.002638	-1.06613	down	[Q]	Secondary	Cellular Co	K16339|1.7	Fatty acid	[S]
1.49E-05	-1.06634	down	--	--	--	--	--	--
4.06E-06	-1.06785	down	--	--	Cellular Co	--	--	--
0.001382	-1.06817	down	--	--	Cellular Co	K11109|0.0	--	[R]
2.74E-06	-1.06952	down	--	--	Biological	K15687|7.7	--	[O]
4.25E-05	-1.0702	down	--	--	Biological	--	--	--
8.98E-06	-1.07099	down	[F]	Nucleotide	Molecular	K10807|0.0	Purine me	[F]
1.97E-05	-1.07113	down	--	--	Molecular	K17918|6.9	Endocytos	[U]
1.84E-05	-1.07141	down	--	--	Molecular	K02539|2.3	Apoptosis	[TU]
1.14E-05	-1.07255	down	--	--	Biological	--	--	[S]
0.001136	-1.07296	down	--	--	Cellular Co	--	--	--
2.15E-09	-1.07342	down	--	--	Molecular	K12383|7.2	Lysosome	--
0.001219	-1.07473	down	[I]	Lipid trans	Molecular	K19179|2.3	--	[C]
3.62E-07	-1.07637	down	--	--	Cellular Co	K22653|1.5	--	--
0.001167	-1.0764	down	--	--	Molecular	K13188|1.4	--	[R]
0.000291	-1.07693	down	--	--	Cellular Co	K07562|3.5	Ribosome	[J]
2.48E-08	-1.07733	down	[R]	General fu	Biological	K07936|4.9	Ribosome	[U]
0.00051	-1.07754	down	--	--	Cellular Co	K20821|4.5	--	--
0.004592	-1.07996	down	--	--	Cellular Co	--	--	--
8.84E-05	-1.08007	down	--	--	Molecular	K02891|3.3	Ribosome	[J]
0.000183	-1.08194	down	--	--	Biological	K10145|3.5	p53 signal	[D]
0.007383	-1.08195	down	--	--	Biological	--	--	--
0.000969	-1.08227	down	[N]	Cell motilit	Molecular	K03424|4.7	--	[L]
0.003855	-1.08263	down	[J]	Translation	Molecular	K19788|1.1	--	[R]
0.005408	-1.08292	down	--	--	--	--	--	--
4.06E-05	-1.08322	down	--	--	Cellular Co	K23952|1.8	--	[S]

0.000876	-1.08404	down	--	--	Cellular Cc	K11585|1.3	--	[B]
4.66E-09	-1.08416	down	[O]	Posttransla	Molecular	K01330|0.0	Phagosom	[E]
9.72E-05	-1.08445	down	[O]	Posttransla	Biological	K22530|1.1	--	[O]
8.13E-06	-1.08448	down	[T]	Signal tran	Molecular	K14394|7.5	Thiamine r	[T]
0.000795	-1.08449	down	[F]	Nucleotide	Molecular	K01939|2.1	Purine me	[F]
8.76E-05	-1.08463	down	[O]	Posttransla	Biological	K05024|5.3	--	[P]
0.007906	-1.08477	down	--	--	Molecular	K16897|0.0	--	[PT]
3.16E-11	-1.08516	down	--	--	Biological	K06751|6.7	Endocytos	--
0.00021	-1.08574	down	--	--	Cellular Cc	K17966|3.7	--	[S]
5.31E-06	-1.08597	down	--	--	Molecular	K17563|9.0	--	[K]
7.17E-06	-1.08615	down	--	--	Cellular Cc	K23878|1.2	--	[S]
0.000364	-1.08616	down	[Q]	Secondary	Molecular	K13122|7.3	--	[R]
0.000276	-1.08769	down	--	--	Biological	K21751|4.8	--	[K]
0.002762	-1.08922	down	--	--	Cellular Cc	K08504|4.6	SNARE int	[U]
0.002561	-1.09025	down	--	--	Cellular Cc	K10881|4.4	Proteasom	[S]
0.005767	-1.09032	down	--	--	Molecular	K10031|1.3	Cytokine-c	--
1.97E-05	-1.09126	down	[V]	Defense m	Molecular	K09022|1.3	--	[J]
0.009016	-1.09147	down	--	--	Biological	K03127|4.2	Basal trans	[K]
0.007695	-1.0917	down	[P]	Inorganic i	Cellular Cc	K15122|0.0	--	[P]
0.006862	-1.09183	down	[J]	Translation	Cellular Cc	K15341|0.0	--	[L]
9.22E-05	-1.09257	down	--	--	Cellular Cc	K07605|3.9	--	--
5.89E-06	-1.09279	down	[F]	Nucleotide	Cellular Cc	K11536|0.0	--	[FP]
9.86E-07	-1.09391	down	[L]	Replication	Molecular	K03257|1.9	RNA trans	[J]
0.003598	-1.09403	down	[R]	General fu	Molecular	K21804|2.3	--	[A]
2.08E-06	-1.09454	down	--	--	Molecular	K12898|3.4	--	[A]
3.89E-07	-1.09499	down	[J]	Translation	Molecular	K13126|0.0	RNA trans	[AJ]
0.000653	-1.09586	down	--	--	Molecular	K02918|1.4	Ribosome	[J]
0.000163	-1.09781	down	[R]	General fu	Molecular	K10251|2.4	Fatty acid	[I]
5.71E-05	-1.09785	down	--	--	Biological	K10363|4.1	--	[Z]
0.006454	-1.09864	down	--	--	Cellular Cc	--	--	[K]
0.000121	-1.09919	down	--	--	--	--	--	--
0.003104	-1.09931	down	[T]	Signal tran	Molecular	K07179|0.0	Ribosome	[TR]
0.001992	-1.10109	down	--	--	Biological	K11492|0.0	--	[S]
0.000947	-1.10135	down	[J]	Translation	Biological	K03238|6.1	RNA trans	[J]
0.000282	-1.10235	down	--	--	--	--	--	--
0.000301	-1.10237	down	[R]	General fu	Molecular	K18398|3.3	--	[R]
1.20E-06	-1.10285	down	--	--	Biological	K05019|1.1	RNA trans	[P]
0.003078	-1.10347	down	--	--	Molecular	K16350|6.1	Cell adhes	--
0.008378	-1.104	down	--	--	Molecular	--	--	--
0.006586	-1.10414	down	[O]	Posttransla	Molecular	K09579|9.3	--	[O]
0.004486	-1.10511	down	[T]	Signal tran	Molecular	K08816|1.0	--	[T]
0.009402	-1.10551	down	[L]	Replication	Molecular	K04482|1.0	Homologo	[L]
6.24E-06	-1.10568	down	--	--	Molecular	--	--	--
0.000817	-1.10572	down	[S]	Function u	Molecular	K19031|0.0	--	--
4.58E-05	-1.10676	down	--	--	Biological	--	--	--
0.004162	-1.10685	down	--	--	Biological	K21770|9.9	FoxO signa	[D]
1.06E-05	-1.10719	down	--	--	Cellular Cc	--	--	[S]
4.34E-13	-1.10811	down	--	--	Biological	K10420|2.6	Salmonella	[N]
1.58E-05	-1.10857	down	[F]	Nucleotide	Molecular	K00893|1.9	Purine me	[F]
1.76E-07	-1.10996	down	--	--	Molecular	K15703|0.0	--	[O]
0.003582	-1.11032	down	--	--	Molecular	K09037|5.3	--	[K]

0.00438	-1.11039	down	--	--	Cellular Cc	K10881|4.4	Proteasom	[S]
6.77E-05	-1.11044	down	--	--	Biological	K05729|6.5	Regulatior	--
6.20E-05	-1.11136	down	[H]	Coenzyme	Cellular Cc	K03635|8.6	Folate bios	[H]
0.000302	-1.11192	down	--	--	Biological	K23544|4.2	--	[S]
0.001791	-1.11227	down	--	--	Biological	K18269|5.5	--	[S]
7.46E-05	-1.11269	down	[C]	Energy prc	Molecular	K03934|0.0	Oxidative	[C]
1.51E-07	-1.1127	down	--	--	--	K03626|0.0	Parathyroi	[K]
0.002776	-1.11568	down	--	--	--	--	--	--
0.00111	-1.11619	down	--	--	Molecular	K13194|0.0	--	[A]
0.002099	-1.11703	down	--	--	Cellular Cc	K18178|3.1	Thermoge	[O]
1.82E-06	-1.11743	down	--	--	Biological	K11660|2.5	--	[S]
5.40E-06	-1.11975	down	--	--	Cellular Cc	K13784|5.1	--	--
3.70E-05	-1.12182	down	--	--	--	--	--	--
0.00015	-1.12192	down	--	--	Biological	K06104|0.0	Tight junct	--
4.45E-05	-1.12193	down	--	--	Cellular Cc	K00741|3.3	Glycosami	[G]
0.003493	-1.12244	down	--	--	Cellular Cc	K17780|6.7	--	[U]
0.001995	-1.12255	down	--	--	Biological	K12816|0.0	Spliceosor	[S]
1.08E-05	-1.12294	down	--	--	Molecular	K11971|7.0	--	[O]
0.000163	-1.12452	down	--	--	Biological	K23882|1.3	--	[R]
0.004064	-1.12488	down	--	--	Cellular Cc	K22829|5.1	--	[R]
0.00118	-1.12556	down	--	--	Cellular Cc	K13176|7.2	RNA trans	[S]
0.001385	-1.12683	down	--	--	Biological	--	--	--
0.000322	-1.12754	down	[F]	Nucleotide	Molecular	K00207|0.0	Pyrimidine	[F]
0.000315	-1.12868	down	[O]	Posttransla	Molecular	K22074|4.4	--	[O]
0.000381	-1.12869	down	--	--	Cellular Cc	K17431|4.6	--	--
0.002593	-1.12929	down	--	--	Cellular Cc	K19943|1.3	--	--
0.001369	-1.13053	down	--	--	Cellular Cc	K06713|6.4	Cell adhes	--
3.03E-05	-1.13064	down	[E]	Amino aci	Molecular	K11142|1.8	Arginine a	[R]
3.54E-05	-1.13079	down	--	--	Biological	K10131|7.8	Platinum c	--
0.001846	-1.13102	down	--	--	Cellular Cc	K16457|0.0	--	--
0.004156	-1.13236	down	--	--	Molecular	K23581|3.1	MicroRNA	[K]
1.33E-07	-1.13453	down	--	--	Cellular Cc	K00237|4.9	Citrate cyc	[CU]
1.09E-06	-1.13469	down	--	--	Molecular	K10271|0.0	--	--
4.44E-05	-1.1353	down	--	--	Cellular Cc	K11299|1.9	--	--
0.003229	-1.13554	down	--	--	--	--	--	--
6.73E-13	-1.13566	down	--	--	Cellular Cc	K06263|4.4	ECM-rece	[R]
0.000124	-1.13598	down	[O]	Posttransla	Cellular Cc	K03030|5.8	Proteasom	[O]
0.000782	-1.13725	down	[J]	Translatior	Molecular	K22503|5.0	Aminoacyl	[J]
0.000144	-1.13748	down	--	--	Molecular	K06620|2.1	Endocrine	[K]
0.008141	-1.1383	down	[S]	Function u	Molecular	--	--	--
1.22E-05	-1.13845	down	[R]	General fu	Biological	K00121|1.3	Glycolysis	[Q]
7.71E-05	-1.13916	down	[J]	Translatior	Molecular	K02930|2.3	Ribosome	[A]
0.000194	-1.13952	down	[F]	Nucleotide	Molecular	K01587|0.0	Purine me	[F]
0.000657	-1.13955	down	--	--	Cellular Cc	K02608|1.7	Cell cycle (	[L]
0.000102	-1.14152	down	[J]	Translatior	Molecular	K02896|1.6	Ribosome	[J]
0.000124	-1.14212	down	[J]	Translatior	Molecular	K03237|2.4	RNA trans	[J]
9.05E-05	-1.14347	down	[J]	Translatior	Molecular	K17420|4.8	--	[J]
7.35E-05	-1.14421	down	--	--	Molecular	K04394|8.2	NOD-like	[D]
5.84E-06	-1.14429	down	--	--	Molecular	K10580|2.6	Ubiquitin r	[O]
8.58E-07	-1.14443	down	--	--	Molecular	K20217|1.4	Ubiquitin r	[O]
0.00054	-1.14639	down	--	--	Cellular Cc	K11252|9.9	Alcoholism	[B]

0.001482	-1.14672	down	[J]	Translatior	Molecular	K15437|1.8	--	[J]
0.000224	-1.1517	down	--	--	Cellular Cc	K09239|1.8	--	[S]
7.62E-06	-1.15243	down	--	--	Cellular Cc	K22385|1.4	--	--
1.51E-07	-1.15356	down	--	--	Cellular Cc	K06689|2.3	Ubiquitin r	[O]
7.71E-05	-1.15376	down	--	--	Molecular	K03914|5.8	Rap1 signa	--
0.000454	-1.15503	down	--	--	--	--	--	--
9.84E-06	-1.15849	down	[O]	Posttransla	Molecular	K22063|3.5	--	[P]
0.000266	-1.15871	down	--	--	Biological	K15626|4.4	Transcripti	[K]
0.00016	-1.15902	down	--	--	Cellular Cc	K18157|1.0	--	--
0.008699	-1.15938	down	--	--	Molecular	K17597|2.1	--	[UK]
0.000341	-1.15985	down	--	--	Molecular	K19907|0.0	--	[TU]
0.00141	-1.16023	down	--	--	Cellular Cc	K11303|7.2	Alcoholism	[B]
0.003913	-1.16059	down	[I]	Lipid trans	Biological	K00249|7.0	Fatty acid	[I]
2.20E-06	-1.16072	down	--	--	Cellular Cc	K06849|9.3	--	--
0.001087	-1.16109	down	--	--	--	--	--	--
4.32E-07	-1.16138	down	[P]	Inorganic i	Molecular	K03781|0.0	Tryptopha	[P]
2.07E-05	-1.16153	down	--	--	Biological	K12848|4.4	Spliceosor	[R]
1.34E-06	-1.16291	down	--	--	Cellular Cc	K02127|9.5	Oxidative p	[C]
0.000983	-1.16334	down	--	--	Molecular	K17578|1.6	--	[O]
3.51E-07	-1.16412	down	[R]	General fu	Molecular	K07877|1.0	AMPK sigr	[U]
9.34E-05	-1.16445	down	--	--	Molecular	K05433|3.6	Cytokine-c	--
8.99E-05	-1.16466	down	[IQR]	Lipid trans	Cellular Cc	K11162|1.4	--	[Q]
6.61E-05	-1.16469	down	--	--	Biological	K20222|2.6	--	[YU]
0.000325	-1.16485	down	[J]	Translatior	Cellular Cc	K15429|5.4	--	[A]
0.004636	-1.1695	down	--	--	Cellular Cc	K08220|9.7	--	[R]
0.00031	-1.16953	down	--	--	Biological	K11128|2.9	Ribosome	[J]
0.002762	-1.1701	down	[QV]	Secondary	Molecular	K07422|1.4	Arachidon	[Q]
0.008232	-1.17056	down	--	--	Cellular Cc	K22566|6.2	--	--
0.000183	-1.17244	down	--	--	Biological	K19695|2.2	Olfactory t	[T]
0.000513	-1.17252	down	--	--	Cellular Cc	--	--	[S]
0.000116	-1.17293	down	--	--	Cellular Cc	K17292|2.6	--	[O]
0.000239	-1.17341	down	--	--	Cellular Cc	K16454|2.1	--	--
1.49E-06	-1.17671	down	--	--	Cellular Cc	K11291|0.0	--	[BD]
0.000779	-1.17917	down	--	--	Cellular Cc	K20044|7.2	--	[O]
0.000197	-1.18091	down	[C]	Energy prc	Cellular Cc	K00803|0.0	Ether lipid	[R]
0.005722	-1.1823	down	--	--	--	--	--	--
0.007745	-1.18246	down	--	--	--	--	--	--
2.60E-06	-1.18476	down	[L]	Replicatior	Biological	K02542|0.0	DNA replic	[L]
0.001372	-1.18507	down	--	--	Biological	K17532|1.4	mTOR sigr	[S]
4.80E-07	-1.18661	down	[F]	Nucleotide	Molecular	K10808|1.3	Purine me	[F]
7.17E-06	-1.18699	down	--	--	Cellular Cc	K22526|1.4	--	--
4.42E-08	-1.1876	down	[I]	Lipid trans	Biological	K08744|6.2	Glyceroph	[I]
0.002244	-1.18776	down	[H]	Coenzyme	Molecular	K06210|5.5	Nicotinate	[H]
0.00093	-1.18807	down	--	--	Molecular	K02649|2.0	EGFR tyros	[T]
0.00394	-1.18843	down	--	--	Cellular Cc	K11290|1.6	--	[L]
6.16E-07	-1.18855	down	--	--	Molecular	K10575|1.3	Ubiquitin r	[O]
4.92E-05	-1.19014	down	--	--	Cellular Cc	K04511|2.6	Wnt signal	[TZR]
6.35E-05	-1.19283	down	--	--	Biological	K12877|1.7	RNA trans	[A]
2.18E-15	-1.19322	down	--	--	Molecular	K06275|1.1	Focal adhe	[Z]
0.001093	-1.19352	down	--	--	Cellular Cc	K17518|9.0	--	[TV]
9.28E-05	-1.19472	down	--	--	Cellular Cc	--	--	[R]

0.008326	-1.19504	down	--	--	Cellular Cc	K06543|3.(	Malaria (kc	[TV]
0.000988	-1.19579	down	--	--	Cellular Cc	--	--	--
2.51E-07	-1.19658	down	--	--	Biological	K20347|1.1	--	[U]
3.81E-05	-1.1993	down	[J]	Translatior	Molecular	K02932|4.6	Ribosome	[J]
0.001606	-1.20028	down	--	--	--	K07933|6.(	--	--
0.000143	-1.20032	down	[MDT]	Cell wall/m	Biological	K22399|4.7	--	[O]
0.000332	-1.20201	down	[O]	Posttransla	Cellular Cc	K09515|1.9	--	[O]
1.25E-08	-1.20281	down	--	--	Cellular Cc	K04396|1.4	Apoptosis	[D]
0.000348	-1.20368	down	[I]	Lipid trans	Biological	K00020|2.4	Valine, leu	[R]
7.10E-05	-1.20542	down	[O]	Posttransla	Cellular Cc	K16458|3.2	--	[OC]
1.96E-09	-1.20543	down	--	--	Cellular Cc	K02326|7.8	DNA replic	[K]
0.000579	-1.20573	down	--	--	Cellular Cc	--	--	--
1.45E-06	-1.20615	down	--	--	Biological	K03247|1.8	RNA trans	[J]
9.24E-05	-1.20656	down	--	--	--	--	--	--
0.001019	-1.20705	down	--	--	--	K06856|3.8	Calcium si	--
0.00552	-1.20923	down	[G]	Carbohydr	Molecular	K01837|3.9	Glycolysis	[G]
7.50E-05	-1.21026	down	--	--	Molecular	K23500|1.6	--	[R]
0.000156	-1.21189	down	--	--	Cellular Cc	--	--	--
7.17E-05	-1.21238	down	--	--	--	--	--	--
1.02E-06	-1.21396	down	[HE]	Coenzyme	Molecular	K00831|4.6	Glycine, se	[HE]
0.001053	-1.2149	down	--	--	Biological	K17383|1.(	MicroRNA	--
4.25E-05	-1.21737	down	--	--	Cellular Cc	K15143|8.8	Thyroid hc	--
0.001451	-1.2178	down	--	--	Cellular Cc	K06086|0.(	PPAR sign	[T]
1.85E-05	-1.21915	down	[I]	Lipid trans	Molecular	K01847|0.(	Valine, leu	--
0.001515	-1.22054	down	--	--	Molecular	K15434|6.2	--	[S]
0.001503	-1.22196	down	--	--	Cellular Cc	K23490|1.6	--	[C]
0.004368	-1.22253	down	--	--	Molecular	K17401|5.5	--	[J]
0.000127	-1.22318	down	[K]	Transcripti	Biological	K12622|6.8	RNA degra	[A]
2.88E-05	-1.22352	down	--	--	Molecular	K14288|0.(	RNA trans	[YUJ]
5.73E-09	-1.22445	down	[P]	Inorganic i	Cellular Cc	K13751|0.(	--	[PT]
7.31E-07	-1.22465	down	[E]	Amino aci	Molecular	K06825|6.(	TGF-beta	[E]
3.49E-07	-1.23254	down	--	--	Cellular Cc	K20224|2.1	--	[YU]
0.001995	-1.23292	down	--	--	Biological	K19680|7.7	--	[Z]
0.00082	-1.23423	down	--	--	Cellular Cc	K23885|5.(	--	[R]
0.002974	-1.2347	down	--	--	Cellular Cc	K16765|0.(	--	[S]
6.55E-07	-1.23763	down	--	--	Molecular	K02891|6.(	Ribosome	[J]
5.29E-05	-1.23924	down	--	--	Biological	K03259|2.3	EGFR tyros	[J]
3.70E-08	-1.23956	down	[R]	General fu	Cellular Cc	K15734|1.7	Retinol me	[Q]
1.50E-06	-1.23957	down	--	--	Molecular	K02934|2.4	Ribosome	[J]
9.32E-05	-1.24084	down	--	--	Biological	K16822|0.(	Hippo sigr	[T]
0.000376	-1.24133	down	--	--	Cellular Cc	K07964|3.6	Glycosami	--
1.28E-05	-1.24144	down	--	--	Molecular	--	--	[Z]
0.00031	-1.24187	down	[F]	Nucleotide	Cellular Cc	K00761|8.(	Pyrimidine	[R]
0.002951	-1.24288	down	--	--	--	K13168|2.3	--	--
7.77E-07	-1.24297	down	[J]	Translatior	Molecular	K02906|6.5	Ribosome	[J]
2.63E-08	-1.2435	down	[C]	Energy prc	Cellular Cc	K03522|1.3	--	[C]
5.26E-08	-1.24587	down	[O]	Posttransla	Biological	K04079|0.(	Protein prc	[O]
6.53E-05	-1.2495	down	--	--	Biological	K03937|2.8	Oxidative	[C]
0.00248	-1.24988	down	[J]	Translatior	Cellular Cc	K12586|1.1	RNA degra	[J]
0.001855	-1.24993	down	--	--	--	K10784|2.1	Ras signali	--
1.67E-06	-1.24994	down	[R]	General fu	Molecular	K05037|0.(	Synaptic v	[T]

0.001206	-1.24996	down	--	--	Cellular Cc	--	--	--	
0.002053	-1.25005	down	--	--	Cellular Cc	K16585|1.4	--	--	
0.000101	-1.2511	down	--	--	--	--	--	--	
7.44E-11	-1.25346	down	--	--	Cellular Cc	K20368|3.2	--	[OUT]	
3.63E-07	-1.25436	down	--	--	Cellular Cc	K23354|5.8	--	[TZ]	
1.29E-07	-1.2557	down	--	--	Cellular Cc	--	--	[S]	
1.26E-05	-1.25574	down	[NW]	Cell motilit	Molecular	K17093|1.9	--	[U]	
2.84E-06	-1.2563	down	--	--	Cellular Cc	K03671|3.8	--	[O]	
0.000126	-1.2567	down	[J]	Translatior	Molecular	K02932|4.3	Ribosome	[J]	
8.61E-07	-1.25713	down	[O]	Posttransla	Molecular	K09502|3.1	Protein prc	[O]	
0.000653	-1.25718	down	--	--	Cellular Cc	K00286|5.4	Arginine a	[V]	
1.20E-07	-1.25929	down	--	--	Cellular Cc	K10436|1.5	--	[DZ]	
5.88E-06	-1.26423	down	[F]	Nucleotide	Molecular	K13800|2.8	Pyrimidine	[F]	
0.007945	-1.26462	down	--	--	Cellular Cc	--	--	[S]	
3.94E-07	-1.26482	down	[L]	Replicatior	Biological	K04802|3.9	DNA replic	[L]	
1.67E-06	-1.268	down	--	--	Biological	K06479|4.7	Natural kil	--	
0.00075	-1.27045	down	--	--	Cellular Cc	K21112|1.2	Tight junct	--	
0.000637	-1.2716	down	--	--	Molecular	--	--	[S]	
8.55E-07	-1.27327	down	--	--	Biological	K12863|4.1	Spliceosor	[S]	
6.40E-05	-1.27434	down	[H]	Coenzyme	Molecular	K00861|1.4	Riboflavin	[H]	
4.05E-05	-1.275	down	--	--	Biological	K17655|0.0	--	--	
3.45E-05	-1.27635	down	--	--	Molecular	K13199|2.1	--	[R]	
2.97E-06	-1.27666	down	[O]	Posttransla	Cellular Cc	K09496|2.5	--	[O]	
1.25E-05	-1.27753	down	[F]	Nucleotide	Molecular	K01619|8.0	Pentose pl	[F]	
2.29E-06	-1.27835	down	[J]	Translatior	Biological	K02940|1.7	Ribosome	[J]	
2.67E-05	-1.2804	down	--	--	Biological	K22538|4.3	--	[S]	
0.000169	-1.28103	down	[I]	Lipid trans	Cellular Cc	K19007|1.8	Glycerolipi	[I]	
0.000549	-1.28411	down	--	--	Cellular Cc	K22823|2.2	--	[S]	
5.72E-05	-1.28675	down	--	--	Biological	K14863|1.8	--	[Z]	
2.23E-06	-1.28822	down	--	--	Molecular	--	--	--	
3.53E-06	-1.28841	down	[QV]	Secondary	Molecular	K07435|6.7	--	[QI]	
1.37E-05	-1.29198	down	[I]	Lipid trans	Molecular	K08764|6.8	Primary bil	[I]	
5.38E-06	-1.29363	down	[CP]	Energy prc	Molecular	K01507|1.9	Oxidative	[C]	
0.001731	-1.29386	down	--	--	Cellular Cc	K17414|1.1	--	--	
0.005017	-1.29824	down	[R]	General fu	Molecular	K05901|6.1	Riboflavin	--	
0.007383	-1.29994	down	--	--	Molecular	K06645|6.5	Cell cycle (	[D]	
3.20E-06	-1.3008	down	--	--	Biological	K12581|6.1	RNA degra	[A]	
1.69E-05	-1.3012	down	--	--	Cellular Cc	K14965|4.4	--	[K]	
0.002744	-1.304	down	--	--	Biological	--	--	--	
2.19E-07	-1.30731	down	--	--	Cellular Cc	K12157|1.4	--	[OR]	
0.006302	-1.31661	down	--	--	Molecular	K10749|3.6	--	--	
5.19E-06	-1.31682	down	[G]	Carbohydr	Molecular	K18674|3.1	--	[G]	
0.000157	-1.31715	down	[C]	Energy prc	Molecular	K13997|4.4	--	[C]	
5.87E-09	-1.31794	down	--	--	Molecular	K15433|2.3	--	[R]	
2.65E-06	-1.31796	down	--	--	Cellular Cc	K13855|2.6	Salivary se	[Z]	
1.07E-05	-1.31831	down	--	--	Cellular Cc	K05506|5.0	Cytokine-(	--	
1.44E-05	-1.32013	down	--	--	Molecular	K06452|1.1	Hematopc	--	
6.77E-05	-1.32033	down	--	--	Molecular	--	--	[R]	
0.000579	-1.32118	down	[L]	Replicatior	Molecular	K10756|5.4	DNA replic	[DL]	
0.000173	-1.32438	down	--	--	Biological	K14847|8.7	--	[J]	
1.10E-05	-1.32625	down	[J]	Translatior	Molecular	K03242|4.4	RNA trans	[J]	

0.000718	-1.32757	down	--	--	Molecular	K05607|1.1	Valine, leu	[I]
3.49E-07	-1.32804	down	--	--	Cellular Cc	--	--	[S]
0.004387	-1.32996	down	--	--	--	--	--	--
4.64E-10	-1.33085	down	[E]	Amino aci	Molecular	K01581|1.6	Arginine a	[E]
0.00189	-1.33087	down	--	--	Molecular	K17409|4.8	--	[J]
7.39E-05	-1.33118	down	--	--	Molecular	K19198|1.5	--	[B]
0.000301	-1.33128	down	--	--	Molecular	K05290|9.8	Glycosylph	[O]
2.55E-08	-1.33457	down	[S]	Function u	Biological	K00807|1.1	Arachidon	--
0.002036	-1.33604	down	--	--	Cellular Cc	K18633|8.6	--	--
5.18E-08	-1.33887	down	--	--	Molecular	K17072|2.1	TNF signal	--
6.06E-05	-1.34098	down	[S]	Function u	Cellular Cc	K17581|3.0	--	[S]
0.000239	-1.34235	down	--	--	Cellular Cc	K03143|4.4	Basal trans	[KL]
0.000101	-1.34392	down	--	--	Cellular Cc	K11544|1.0	--	--
1.36E-08	-1.34408	down	[O]	Posttransla	Cellular Cc	K09500|0.0	--	[O]
0.000389	-1.34433	down	--	--	Biological	K12877|2.0	RNA trans	[A]
1.89E-07	-1.34453	down	--	--	Biological	K12399|1.1	Lysosome	[U]
9.18E-06	-1.34643	down	--	--	Molecular	K01128|2.1	--	[I]
0.005685	-1.34685	down	--	--	Biological	K06627|4.6	Cell cycle (	[D]
0.000325	-1.34879	down	[P]	Inorganic i	Cellular Cc	K08179|1.3	--	[G]
5.51E-05	-1.35211	down	--	--	Molecular	K12327|1.1	Vascular sr	--
9.90E-08	-1.35223	down	--	--	Cellular Cc	K02975|1.3	Ribosome	[J]
2.49E-05	-1.35549	down	--	--	Cellular Cc	--	--	[V]
1.99E-06	-1.3581	down	--	--	Molecular	K14217|2.8	Hepatitis C	[R]
8.86E-07	-1.3634	down	--	--	Molecular	K11276|9.9	--	--
6.07E-13	-1.36378	down	--	--	Biological	K14685|0.0	Ferroptosis	[P]
1.90E-08	-1.366	down	--	--	Molecular	K08449|3.6	Vascular sr	--
2.11E-07	-1.3687	down	--	--	Molecular	K00799|8.1	Glutathion	--
0.000374	-1.36927	down	[R]	General fu	Molecular	K02087|4.3	Cell cycle (	[R]
5.29E-05	-1.36931	down	--	--	Cellular Cc	--	--	--
2.58E-10	-1.36944	down	--	--	Biological	K13144|0.0	--	[S]
0.00292	-1.37047	down	--	--	Molecular	K08337|8.5	Autophagy	[R]
9.77E-06	-1.37293	down	[O]	Posttransla	Molecular	K03064|4.5	Proteasom	[O]
3.53E-08	-1.37333	down	[O]	Posttransla	Cellular Cc	K03283|0.0	Spliceosor	[O]
0.000429	-1.37371	down	--	--	Biological	K12162|4.1	--	[S]
5.15E-07	-1.37832	down	--	--	Biological	K22825|1.2	--	--
0.005739	-1.37957	down	--	--	Molecular	K07374|5.3	Phagosom	[Z]
3.18E-07	-1.38217	down	--	--	Cellular Cc	K07611|2.4	Apoptosis	[DY]
1.10E-05	-1.38249	down	--	--	Biological	--	--	--
0.000353	-1.38358	down	--	--	Biological	K08731|1.8	Platinum c	[DR]
0.000988	-1.3846	down	--	--	Molecular	K10683|0.0	Homologc	--
0.000625	-1.3876	down	[O]	Posttransla	Molecular	K12734|3.9	--	[O]
0.000207	-1.38791	down	[HR]	Coenzyme	Molecular	K13699|8.1	Regulatior	[R]
1.31E-05	-1.39084	down	--	--	Cellular Cc	K07990|1.9	Natural kil	[U]
0.009478	-1.3918	down	--	--	Molecular	K09229|5.3	--	[R]
0.001128	-1.39268	down	[R]	General fu	Molecular	K24104|2.2	--	[K]
4.55E-08	-1.3936	down	--	--	Cellular Cc	K15041|1.1	Calcium si	[P]
6.36E-05	-1.39509	down	--	--	Cellular Cc	K12160|3.3	RNA trans	[O]
1.96E-09	-1.3982	down	--	--	Molecular	K13354|4.9	Peroxisom	[C]
0.003948	-1.39835	down	--	--	Cellular Cc	K05868|5.5	FoxO signa	[D]
2.87E-08	-1.39965	down	[O]	Posttransla	Cellular Cc	K03061|3.0	Proteasom	[O]
5.52E-10	-1.40023	down	[F]	Nucleotide	Cellular Cc	K14611|6.8	Vitamin di	[F]

1.57E-06	-1.40551	down	--	--	Molecular	K14443|2.4	RNA degra	[TR]
2.75E-06	-1.40571	down	--	--	Molecular	K16613|4.6	--	[R]
0.0002	-1.40743	down	--	--	Cellular Co	K10842|5.8	Basal trans	[O]
0.000432	-1.40896	down	--	--	Biological	K11094|2.4	Spliceosor	[A]
0.004207	-1.41	down	[O]	Posttransla	Molecular	K23412|1.1	--	[V]
0.005893	-1.41574	down	[O]	Posttransla	Biological	K12737|8.1	--	[O]
0.000169	-1.42274	down	--	--	Cellular Co	K15717|7.6	Arachidon	[S]
0.000202	-1.42288	down	--	--	--	--	--	--
1.67E-10	-1.42305	down	--	--	Biological	K14365|5.1	JAK-STAT	[TZR]
7.81E-05	-1.42428	down	--	--	Molecular	K14570|0.0	Ribosome	[L]
6.56E-06	-1.42937	down	--	--	Cellular Co	K19559|0.0	TGF-beta s	[T]
8.61E-13	-1.43359	down	--	--	Cellular Co	K08051|9.4	--	--
4.34E-08	-1.43568	down	[J]	Translation	Biological	K02937|1.1	Ribosome	[J]
1.75E-08	-1.43584	down	[O]	Posttransla	Cellular Co	K09494|8.2	--	[O]
8.61E-13	-1.43633	down	--	--	Biological	K14365|6.2	JAK-STAT	[TZR]
2.23E-08	-1.43725	down	[K]	Transcripti	Molecular	K09276|8.1	--	[J]
5.71E-05	-1.43915	down	[J]	Translation	Molecular	K11884|8.1	--	[O]
3.28E-05	-1.43939	down	--	--	Molecular	K07631|1.1	--	--
1.44E-05	-1.43995	down	--	--	Cellular Co	K22379|1.1	--	[S]
5.23E-07	-1.44176	down	[J]	Translation	Molecular	K01890|0.0	Aminoacyl	[J]
1.68E-17	-1.44283	down	--	--	Molecular	--	--	[T]
3.36E-08	-1.44286	down	--	--	Molecular	K06496|0.0	Cell adhes	[TV]
1.23E-06	-1.44471	down	--	--	Cellular Co	--	--	[S]
0.004234	-1.44567	down	--	--	--	--	--	--
3.87E-05	-1.44601	down	--	--	Cellular Co	K07980|1.2	Antigen pr	--
1.21E-08	-1.44948	down	[J]	Translation	Molecular	K02984|1.1	Ribosome	[J]
0.000641	-1.45308	down	[H]	Coenzyme	Molecular	K12505|7.6	Terpenoid	[H]
4.25E-05	-1.46409	down	--	--	Molecular	K20899|3.2	NOD-like	[R]
1.80E-05	-1.46455	down	[C]	Energy pro	Cellular Co	K08738|1.2	Platinum c	[C]
4.56E-06	-1.4677	down	[S]	Function u	Molecular	--	--	[M]
0.00099	-1.46797	down	[G]	Carbohydr	Molecular	K09877|1.3	Bile secreti	[G]
7.27E-09	-1.47511	down	--	--	--	--	--	--
2.46E-05	-1.48026	down	--	--	--	--	--	--
6.80E-14	-1.48169	down	--	--	Molecular	K04520|0.0	Serotonerg	[R]
0.000909	-1.48392	down	[J]	Translation	Molecular	K19788|9.9	--	[R]
0.001547	-1.48606	down	[P]	Inorganic i	Molecular	K00682|1.8	Glutathion	[S]
0.003104	-1.48699	down	[L]	Replication	Molecular	K14776|0.0	--	[A]
6.55E-07	-1.49187	down	--	--	Molecular	K17413|3.5	--	[J]
1.96E-09	-1.49336	down	--	--	Cellular Co	K01115|4.9	Glyceroph	[DZ]
5.93E-07	-1.49349	down	--	--	Biological	K04179|2.5	Cytokine-c	[R]
0.000269	-1.4949	down	--	--	--	--	--	--
0.0008	-1.49777	down	--	--	Cellular Co	K20818|3.7	--	[S]
0.000631	-1.50376	down	[J]	Translation	Molecular	K03231|1.1	RNA transp	[J]
0.000167	-1.50419	down	[G]	Carbohydr	Biological	K08133|0.0	Protein dig	[W]
4.20E-05	-1.50555	down	--	--	--	--	--	--
0.00029	-1.51215	down	--	--	Biological	K12861|2.3	Spliceosor	[S]
0.001631	-1.51356	down	[F]	Nucleotide	Cellular Co	K00939|1.1	Purine me	[F]
9.26E-06	-1.52074	down	--	--	Cellular Co	K06502|3.4	--	[TV]
0.000199	-1.52574	down	--	--	Molecular	K06109|8.7	Tight junct	[TU]
2.37E-08	-1.53282	down	--	--	Cellular Co	--	--	[U]
0.001958	-1.53339	down	--	--	Cellular Co	K06627|1.5	--	--

5.29E-07	-1.53461	down	[T]	Signal tran	Cellular Cc	K00907|0.(	Calcium si	[Z]
0.002585	-1.53775	down	--	--	Biological	K03250|6.2	RNA trans	[J]
0.007102	-1.53899	down	--	--	Biological	K21754|0.(	--	[R]
0.00072	-1.53967	down	--	--	Molecular	K00469|6.8	Ascorbate	[R]
1.25E-09	-1.54416	down	--	--	Cellular Cc	K05407|3.5	Cytokine-(	--
2.40E-07	-1.54794	down	--	--	Molecular	K14217|4.5	Hepatitis C	[R]
7.57E-08	-1.54812	down	--	--	Cellular Cc	K22939|1.1	--	[S]
0.001553	-1.54861	down	--	--	Molecular	--	--	--
0.002972	-1.54867	down	[O]	Posttransla	Molecular	K22068|4.7	--	[C]
0.000417	-1.55044	down	--	--	Molecular	K06573|0.(	Collecting	[P]
0.008895	-1.55267	down	--	--	Molecular	K14216|0.(	NOD-like	--
3.88E-15	-1.55343	down	--	--	Molecular	K15049|2.(	--	--
2.66E-06	-1.56231	down	--	--	Cellular Cc	K22826|7.(	--	[S]
0.000411	-1.56747	down	--	--	Molecular	K05450|4.8	EGFR tyros	--
4.63E-09	-1.57275	down	--	--	Cellular Cc	K14615|1.3	Vitamin di	--
1.39E-05	-1.57638	down	--	--	Cellular Cc	K11279|2.7	--	[BD]
0.001073	-1.57756	down	--	--	Cellular Cc	K11279|3.4	--	[BD]
0.002042	-1.58717	down	--	--	Cellular Cc	K10076|3.3	--	[TV]
9.83E-18	-1.58721	down	--	--	Biological	K07375|3.5	Phagosom	[Z]
0.000461	-1.58748	down	--	--	Molecular	K13215|4.4	--	[L]
0.000244	-1.58966	down	--	--	--	--	--	--
1.06E-15	-1.59059	down	--	--	Molecular	K04546|3.9	Ras signali	[T]
0.001729	-1.59284	down	--	--	Molecular	K21343|0.(	Mitophagy	[O]
6.62E-08	-1.59466	down	--	--	Molecular	K14216|1.8	NOD-like	--
0.001225	-1.59506	down	[O]	Posttransla	Biological	--	--	[W]
3.32E-11	-1.59793	down	--	--	Cellular Cc	K10295|2.3	--	[R]
1.26E-19	-1.60097	down	--	--	Molecular	K03113|2.6	RNA trans	[J]
0.000371	-1.60163	down	--	--	--	K10784|3.(	Ras signali	--
0.007061	-1.60659	down	--	--	Molecular	K05175|1.1	Neuroactiv	--
3.73E-13	-1.61518	down	--	--	Molecular	K12159|9.5	RIG-I-like	[OR]
8.09E-12	-1.62245	down	--	--	Cellular Cc	--	--	[K]
2.65E-05	-1.6278	down	[S]	Function u	Cellular Cc	K18575|1.4	Ether lipid	[R]
0.009552	-1.62847	down	--	--	Biological	--	--	--
5.92E-10	-1.63186	down	--	--	Biological	K15030|1.7	--	[R]
9.39E-07	-1.63525	down	--	--	Molecular	K20899|0.(	NOD-like	[R]
8.25E-05	-1.63539	down	--	--	Molecular	--	--	--
0.000115	-1.63562	down	--	--	Molecular	K05940|0.(	Peroxisom	[I]
9.36E-06	-1.63972	down	--	--	--	--	--	--
1.41E-09	-1.64067	down	[C]	Energy pro	Molecular	K01900|9.7	Citrate cyc	[C]
2.18E-07	-1.64437	down	--	--	Cellular Cc	K03240|8.2	RNA trans	[J]
3.93E-07	-1.65162	down	--	--	Cellular Cc	K14480|4.4	--	--
6.43E-05	-1.65963	down	--	--	Molecular	K17514|1.5	C-type lec	[TV]
4.06E-05	-1.66298	down	--	--	Molecular	--	--	--
0.006053	-1.66792	down	--	--	Cellular Cc	K02135|3.1	Oxidative	[C]
1.33E-08	-1.67285	down	--	--	Molecular	K20908|0.(	--	[R]
0.00235	-1.67412	down	--	--	Molecular	K20899|3.9	NOD-like	--
4.59E-06	-1.67652	down	--	--	Cellular Cc	K14480|5.4	--	--
4.68E-11	-1.67657	down	--	--	Cellular Cc	K17278|4.8	--	[R]
9.16E-12	-1.68206	down	--	--	Cellular Cc	K03094|6.5	Cell cycle (	[O]
0.000866	-1.68229	down	--	--	Cellular Cc	K04722|1.3	Cytokine-(	--
4.27E-05	-1.68479	down	[J]	Translation	Molecular	K02863|1.7	Ribosome	[J]

0.001121	-1.68671	down	--	--	Molecular	K07868|0.(	Ubiquitin r	[R]
0.001596	-1.68701	down	--	--	Biological	K11341|1.2	--	[K]
0.000912	-1.68707	down	--	--	--	--	--	--
0.004268	-1.69031	down	--	--	Molecular	--	--	[A]
3.70E-09	-1.69148	down	--	--	Cellular Cc	K08341|8.3	FoxO signa	[Z]
4.99E-05	-1.69457	down	--	--	Molecular	--	--	[I]
0.000217	-1.7002	down	--	--	Cellular Cc	K22939|1.1	--	[S]
0.005808	-1.70064	down	--	--	Cellular Cc	K06842|0.(	Axon guid	[T]
0.001101	-1.70731	down	--	--	Molecular	K04869|1.8	MAPK sigr	--
1.86E-06	-1.70873	down	--	--	Molecular	K15374|0.(	GABAergic	[S]
0.000124	-1.70941	down	--	--	Cellular Cc	--	--	--
0.002344	-1.72208	down	--	--	Molecular	K04316|5.3	--	[R]
5.11E-08	-1.7269	down	--	--	Biological	--	--	--
3.15E-07	-1.7357	down	[R]	General fu	Molecular	--	--	[R]
1.09E-06	-1.73992	down	[F]	Nucleotide	Molecular	K00157|0.(	Valine, leu	[F]
0.00026	-1.74313	down	[I]	Lipid trans	Molecular	K23151|1.5	--	[I]
0.001253	-1.7455	down	[P]	Inorganic i	Cellular Cc	K08186|6.9	--	[G]
1.24E-07	-1.74957	down	--	--	--	--	--	--
1.03E-17	-1.75195	down	--	--	Biological	K06493|0.(	Rap1 signa	[TW]
0.001065	-1.76082	down	[H]	Coenzyme	Molecular	K01749|3.6	Porphyrin	[H]
0.000202	-1.76829	down	--	--	Molecular	K15441|2.4	--	[J]
0.000785	-1.76855	down	--	--	Molecular	K13828|3.8	--	[C]
2.56E-07	-1.7739	down	--	--	Cellular Cc	K20028|7.5	--	[R]
0.001224	-1.78147	down	--	--	Cellular Cc	--	--	--
0.000811	-1.78279	down	--	--	Molecular	--	--	--
7.62E-09	-1.79646	down	--	--	Cellular Cc	K19522|1.2	--	--
0.002341	-1.80059	down	--	--	Biological	K19537|1.1	--	--
5.09E-05	-1.8018	down	--	--	Cellular Cc	K11271|3.9	--	[D]
2.27E-15	-1.80937	down	[U]	Intracellula	Cellular Cc	K11064|3.(	--	[V]
0.000182	-1.81428	down	[V]	Defense m	Molecular	K05673|0.(	Antifolate	[Q]
1.30E-05	-1.82291	down	--	--	Cellular Cc	K10075|1.4	--	[TV]
0.008507	-1.8273	down	--	--	--	--	--	--
0.000112	-1.82823	down	--	--	Cellular Cc	K13176|3.1	RNA trans	[S]
9.03E-09	-1.82972	down	--	--	--	--	--	--
7.45E-05	-1.83151	down	--	--	Biological	K17271|5.1	--	[X]
3.61E-06	-1.83211	down	--	--	Cellular Cc	K18750|3.2	Human im	--
6.23E-05	-1.83651	down	--	--	Cellular Cc	--	--	--
5.07E-16	-1.83993	down	[R]	General fu	Molecular	K07885|3.7	--	[U]
0.003057	-1.84401	down	[O]	Posttransla	Cellular Cc	K13963|7.7	Amoebiasi	[V]
2.11E-12	-1.85328	down	--	--	Cellular Cc	K06548|0.(	Cell adhes	[RP]
6.69E-25	-1.85721	down	--	--	Cellular Cc	K06832|3.6	--	[W]
6.89E-10	-1.86436	down	[QR]	Secondary	Cellular Cc	K13948|1.5	--	[R]
0.003259	-1.86675	down	[E]	Amino acid	Cellular Cc	K00613|1.7	Glycine, se	--
0.000977	-1.87569	down	--	--	Molecular	K16848|1.(	Adherens j	[R]
7.48E-10	-1.87787	down	--	--	Cellular Cc	K23334|0.(	--	[R]
0.000412	-1.87797	down	[HR]	Coenzyme	Molecular	K08726|6.6	Arachidon	[I]
0.002638	-1.88165	down	--	--	--	--	--	--
0.000276	-1.89458	down	--	--	--	K08111|1.9	--	--
1.02E-05	-1.89656	down	--	--	Molecular	K05609|1.2	--	[O]
2.21E-08	-1.9029	down	--	--	Molecular	K20897|1.4	NOD-like	[R]
4.78E-07	-1.91483	down	--	--	Molecular	K10030|6.3	Cytokine-(	--

2.89E-05	-1.9195	down	--	--	Cellular Cc	--	--	--
1.33E-07	-1.92142	down	--	--	Biological	--	--	--
4.99E-06	-1.94974	down	--	--	Cellular Cc	--	--	--
1.29E-08	-1.95468	down	--	--	Molecular	K06545|0.(	--	--
3.65E-12	-1.96668	down	--	--	Molecular	K14216|6.3	NOD-like	--
6.40E-05	-1.98389	down	--	--	--	--	--	--
6.73E-13	-1.99276	down	--	--	Molecular	K05673|3.(	Antifolate	[Q]
2.51E-06	-2.00467	down	--	--	Cellular Cc	K16684|4.9	Tight junct	[R]
1.48E-09	-2.0165	down	--	--	Biological	K12157|2.3	--	[OR]
0.00038	-2.02321	down	--	--	Cellular Cc	--	--	--
4.21E-05	-2.0282	down	--	--	Molecular	K16505|0.(	--	[S]
0.001964	-2.03315	down	--	--	Cellular Cc	K10629|7.4	--	[O]
0.006704	-2.04123	down	[O]	Posttransla	Cellular Cc	K13963|4.5	Amoebiasi	[V]
5.28E-06	-2.06103	down	--	--	Biological	--	--	--
2.16E-06	-2.07302	down	[J]	Translation	Biological	K05670|3.2	ABC transp	--
6.50E-12	-2.07394	down	--	--	--	--	--	--
0.000433	-2.07858	down	[O]	Posttransla	Molecular	K00683|5.(	--	[O]
1.06E-16	-2.08237	down	--	--	Molecular	K22453|5.6	--	--
1.86E-14	-2.08545	down	[F]	Nucleotide	Cellular Cc	K14611|0.(	Vitamin di	[F]
0.006265	-2.09472	down	--	--	--	K10784|7.9	Ras signali	--
0.004648	-2.10115	down	--	--	Biological	K14754|0.(	Hepatitis C	[UR]
0.001802	-2.10188	down	--	--	--	K10784|4.9	Ras signali	--
7.49E-06	-2.12432	down	--	--	Molecular	K07520|9.3	Axon guid	[TZ]
1.08E-06	-2.12644	down	[G]	Carbohydr	Biological	K09864|3.5	Renin secr	[G]
0.001334	-2.1278	down	--	--	Molecular	K19402|0.(	--	--
0.000292	-2.14895	down	--	--	Cellular Cc	K14480|3.7	--	--
0.000185	-2.15013	down	--	--	Molecular	K16779|1.4	--	[U]
4.27E-07	-2.15246	down	--	--	Cellular Cc	K12002|3.5	--	[O]
4.29E-08	-2.17302	down	[O]	Posttransla	Molecular	K09577|1.6	--	[O]
3.91E-12	-2.22107	down	[G]	Carbohydr	Biological	K00688|0.(	Starch and	[G]
6.92E-05	-2.26243	down	--	--	Cellular Cc	K06581|2.(	--	--
5.86E-07	-2.27187	down	--	--	--	--	--	--
2.34E-07	-2.27618	down	[P]	Inorganic i	Biological	K16627|6.3	--	[P]
0.001048	-2.2875	down	--	--	--	--	--	--
0.000116	-2.33224	down	--	--	Cellular Cc	K09204|8.2	--	[R]
3.77E-21	-2.34176	down	--	--	Cellular Cc	K22647|4.5	--	[S]
2.51E-25	-2.36981	down	--	--	Molecular	K12260|8.(	--	[L]
0.000168	-2.37507	down	[H]	Coenzyme	Biological	K00643|0.(	Glycine, se	[H]
4.94E-11	-2.38928	down	[V]	Defense m	Molecular	K05673|1.7	Antifolate	[Q]
0.000169	-2.4048	down	--	--	Molecular	K13823|1.1	African try	[C]
7.00E-23	-2.41078	down	--	--	Molecular	K13809|4.2	Pyrimidine	[F]
0.000241	-2.41157	down	--	--	Biological	K18040|6.4	--	[R]
0.000174	-2.43374	down	--	--	Molecular	K13822|4.7	African try	[C]
0.000225	-2.44635	down	--	--	Biological	K01047|5.6	Glyceroph	[I]
0.004028	-2.47713	down	--	--	Biological	K23483|7.9	--	--
8.82E-05	-2.48514	down	--	--	Molecular	K15613|2.(	Signaling p	[K]
4.17E-06	-2.50717	down	--	--	Cellular Cc	K06712|1.1	--	[O]
0.000911	-2.54152	down	[O]	Posttransla	Molecular	K08639|0.(	--	[R]
0.000687	-2.56983	down	[R]	General fu	Cellular Cc	K15045|1.5	Hepatitis C	--
4.92E-17	-2.5742	down	--	--	Molecular	K14216|2.(	NOD-like	--
0.000126	-2.58745	down	--	--	Molecular	K13822|4.7	African try	[C]

7.61E-13	-2.61934	down	--	--	Cellular Cc	K06731|9.4	Herpes sin	--
5.71E-11	-2.65705	down	--	--	Cellular Cc	K06731|3.5	Herpes sin	--
2.28E-10	-2.67413	down	--	--	Biological	K06448|5.9	Tight junct	--
0.001548	-2.71756	down	--	--	--	--	--	--
6.80E-14	-2.73314	down	--	--	Biological	K12027|4.0	--	[O]
6.54E-06	-2.73637	down	--	--	Biological	K00444|2.4	mTOR sigr	[T]
4.44E-11	-2.74757	down	[T]	Signal tran	Biological	K17532|5.9	mTOR sigr	[T]
0.000381	-2.74932	down	--	--	Biological	K22255|2.8	--	--
4.33E-08	-2.75355	down	[P]	Inorganic i	Biological	K18245|2.4	Nitrogen r	[R]
7.32E-13	-2.79596	down	--	--	Molecular	K14014|4.5	Protein prc	--
1.78E-05	-2.79932	down	--	--	Biological	K22562|3.5	--	--
2.11E-08	-2.83261	down	--	--	Biological	K05240|2.5	Neuroactiv	--
6.06E-09	-2.86537	down	[R]	General fu	Cellular Cc	K14738|1.2	Mineral ab	--
5.62E-10	-2.86619	down	[O]	Posttransla	Molecular	K05022|4.1	--	[P]
4.44E-11	-3.03162	down	--	--	Molecular	K24141|4.1	--	[I]
1.52E-05	-3.07055	down	--	--	Cellular Cc	K06575|5.4	Hematopc	--
8.00E-06	-3.10726	down	--	--	Molecular	K13823|2.7	African try	[C]
1.50E-06	-3.11501	down	[E]	Amino acic	Biological	K13865|0.0	--	[E]
1.90E-05	-3.12158	down	[G]	Carbohydr	Molecular	K08142|6.1	--	[G]
4.76E-06	-3.12669	down	--	--	Cellular Cc	--	--	--
0.000139	-3.33064	down	--	--	--	--	--	--
2.59E-09	-3.42204	down	[G]	Carbohydr	Molecular	K18622|0.0	--	[TZ]
4.23E-05	-3.46399	down	--	--	Molecular	--	--	--
4.27E-07	-3.53245	down	--	--	Cellular Cc	K17338|5.0	--	[V]
1.03E-07	-3.98852	down	[IQR]	Lipid trans	Biological	K00079|1.3	Arachidon	[Q]
2.08E-07	-4.01575	down	--	--	Molecular	K15624|1.3	Transcripti	--
1.57E-16	-4.13676	down	[P]	Inorganic i	Molecular	K07232|1.5	Glutathion	[P]
5.43E-07	-4.18525	down	[J]	Translatior	Molecular	K02973|5.9	Ribosome	[J]
5.60E-20	-4.25824	down	--	--	Cellular Cc	--	--	--
1.09E-08	-4.34573	down	--	--	Biological	K05622|7.3	--	--
0.00458	-4.40435	down	--	--	Molecular	K13823|2.1	African try	[C]
2.18E-11	-4.6357	down	--	--	Molecular	K15624|1.1	Transcripti	--
0.004465	-4.84362	down	--	--	Cellular Cc	K00286|1.2	Arginine a	[V]
2.27E-19	-4.95234	down	--	--	--	--	--	--
9.16E-26	-5.37442	down	[E]	Amino acic	Biological	K01476|1.2	Arginine b	[E]
3.73E-13	-5.71004	down	--	--	Molecular	K13827|6.4	--	[C]
2.44E-06	-6.1012	down	--	--	--	--	--	--
5.52E-10	-8.22904	down	--	--	Biological	K21413|0.0	--	[T]

KOG_class	Pfam_annc	Swiss_Prot	eggNOG_c	eggNOG_c	NR_annota	GO_second_level_annotation
Lipid trans	Lipocalin /	Cellular re	I	Lipid trans	Cellular re	molecular function: binding (GO
General fu	Zinc finger	Neurotrop	S	Function u	zinc finger	molecular function: binding (GO
--	--	--	--	--	hypothetic	--
Amino aci	Trypsin	Mast cell p	O	Posttransla	mast cell p	molecular function: catalytic acti
--	--	Microtubu	Z	Cytoskelet	microtubu	cellular component: cell (GO:000
--	--	--	--	--	--	--
Posttransla	--	--	S	Function u	E3 ubiquiti	biological process: metabolic pro
--	--	--	--	--	--	--
Amino aci	Trypsin	Inactive se	O	Posttransla	PREDICTEI	molecular function: catalytic acti
General fu	Homeodo	Homeobo:	K	Transcripti	PREDICTEI	molecular function: nucleic acid
--	Adenomat	--	S	Function u	protein AP	biological process: multicellular o
Signal tran	Lectin C-ty	--	S	Function u	proteoglyc	biological process: immune syste
Signal tran	Protein kir	Protein kir	T	Signal tran	protein kir	molecular function: catalytic acti
Transcripti	Helix-loop	--	K	Transcripti	oligodend	biological process: biological reg
General fu	Ankyrin re	Ankyrin re	S	Function u	TPA: ankyr	cellular component: cell (GO:000
Signal tran	Calcium-b	Latent-tra	O	Posttransla	latent-trar	molecular function: binding (GO
Transcripti	SPOC dom	--	S	Function u	SPOC dom	biological process: metabolic pro
Amino aci	Trypsin	Chymotryp	O	Posttransla	PREDICTEI	molecular function: catalytic acti
Signal tran	Protein tyr	Tyrosine-p	T	Signal tran	PREDICTEI	molecular function: catalytic acti
--	--	Adropin O	--	--	adropin pr	molecular function: binding (GO
Secondary	ABC transp	Multidrug	V	Defense m	multidrug	molecular function: binding (GO
Function u	--	--	S	Function u	maestro h	--
--	--	--	S	Function u	ATP-bindi	cellular component: cell (GO:000
--	Ras family	Ras-relate	S	Function u	LOW QUA	molecular function: catalytic acti
General fu	7 transmer	Growth hc	U	Intracellula	growth ho	molecular function: signal transd
--	Glycosyltra	--	S	Function u	beta-1,4-r	cellular component: cell (GO:000
Extracellula	Galactosid	--	S	Function u	placental p	molecular function: binding (GO
Extracellula	Collagen t	Tenascin C	S	Function u	collagen a	molecular function: molecular fu
Posttransla	Glycosyltra	Galactosyl	S	Function u	PREDICTEI	cellular component: cell (GO:000
Carbohydr	Fructose-1	Fructose-1	G	Carbohydr	Fructose-1	biological process: biological reg
Secondary	ABC transp	Multidrug	V	Defense m	multidrug	molecular function: binding (GO
Transcripti	Basic regic	CCAAT/en	K	Transcripti	CCAAT/en	molecular function: binding (GO
--	Protein-ar	Protein-ar	S	Function u	protein-ar	molecular function: catalytic acti
--	--	--	--	--	--	--
RNA proce	Helicase cc	ATP-depe	L	Replicatior	ATP-depe	molecular function: binding (GO
Transcripti	N-termina	Homeobo:	K	Transcripti	homeobox	molecular function: binding (GO
General fu	Zinc finger	--	S	Function u	zinc finger	molecular function: nucleic acid
--	Lipoxygen	Polyunsatu	S	Function u	polyunsatu	biological process: biological reg
General fu	Animal hae	Lactopero:	S	Function u	myeloperc	biological process: response to s
--	Immunogl	Sialic acid-	S	Function u	myeloid ce	biological process: cellular proce
--	Small cyto	C-C motif	O	Posttransla	C-C motif	cellular component: extracellular
--	--	--	--	--	hypothetic	--
--	Repeat do	--	S	Function u	cartilage a	molecular function: binding (GO
--	--	--	--	--	DTW dom.	--
--	Interleukin	--	--	--	uncharacte	biological process: immune syste
General fu	Siderofiexi	Siderofiexi	U	Intracellula	sideroflexi	molecular function: transporter a
Amino aci	Trypsin	Duodenas	O	Posttransla	mast cell p	molecular function: catalytic acti
Posttransla	CUE doma	--	S	Function u	CUE doma	molecular function: binding (GO

--	--	--	T	Signal tran	hypothetic	cellular component: membrane (
Inorganic i	Guanylate	Voltage-d	U	Intracellula	voltage-de	molecular function: transporter a
--	7 transmei	G-protein	U	Intracellula	G-protein	molecular function: signal transd
General fu	BTB/POZ c	BTB/POZ c	S	Function u	BTB/POZ c	molecular function: binding (GO
--	Domain of	--	S	Function u	uncharacte	cellular component: cell (GO:000
--	Intermedia	Keratin, tyI	Z	Cytoskelet	keratin, typ	molecular function: structural mo
--	Immunogl	Ig heavy cl	S	Function u	immunogl	--
--	--	--	--	--	hypothetic	--
--	--	--	--	--	--	--
Defense m	Membrane	Myeloid-a	S	Function u	myeloid-a	cellular component: membrane (
General fu	Homeodo	Homeobo:	K	Transcripti	homeobo›	molecular function: nucleic acid
--	Immunogl	Sialic acid-	S	Function u	CD33 antiç	cellular component: membrane (
--	Immunogl	--	--	--	CMRF35-li	--
--	--	--	--	--	hypothetic	--
--	L6 membr	Transmem	U	Intracellula	transmeml	cellular component: cell (GO:000
Signal tran	Phosphati	--	O	Posttransla	PI-PLC X c	biological process: metabolic pro
--	--	--	--	--	--	--
General fu	PDZ doma	--	O	Posttransla	PDZ doma	molecular function: binding (GO
--	NAD:argin	GPI-linked	O	Posttransla	LOW QUA	molecular function: catalytic acti
--	Immunogl	Sodium ch	U	Intracellula	sodium ch	cellular component: cell (GO:000
RNA proce	Fibrillarin	--	J	Translatior	rRNA/tRN	cellular component: cell (GO:000
--	Protein of	Actin-assc	S	Function u	protein FA	cellular component: cell (GO:000
General fu	Ankyrin re	Cyclin-dep	S	Function u	PREDICTEI	biological process: cellular proce
Signal tran	EGF-like d	--	S	Function u	protein jag	biological process: multicellular (
General fu	Haemolysi	Membrane	S	Function u	membrane	cellular component: membrane (
--	--	--	--	--	--	--
--	Reverse tr	--	S	Function u	hypothetic	--
Signal tran	Ephrin	Ephrin-A1	S	Function u	ephrin-A2	cellular component: cell (GO:000
--	Immunogl	--	S	Function u	Unknown (	cellular component: membrane (
--	Fatty acid	Fatty acid	I	Lipid trans	fatty acid (	biological process: metabolic pro
Signal tran	7 transmei	Adhesion (	S	Function u	adhesion (	molecular function: signal transd
Cytoskelet	Adenylate	Adenylyl c	--	--	TPA: CAP1	biological process: cellular proce
--	--	--	--	--	hypothetic	--
--	7 transmei	Adenosine	U	Intracellula	transmeml	molecular function: signal transd
General fu	7 transmei	Adenosine	U	Intracellula	adenosine	molecular function: signal transd
Posttransla	Matrixin	Matrix met	O	Posttransla	matrix met	molecular function: catalytic acti
Posttransla	Calpain fa	Calpain-3	O	Posttransla	calpain-5 |	molecular function: catalytic acti
--	--	--	--	--	--	--
Function u	Domain of	Transmem	S	Function u	transmeml	cellular component: membrane (
General fu	Leucine ric	Chondroa	S	Function u	chondroac	biological process: multicellular (
Amino acid	Trypsin	Duodenas	O	Posttransla	duodenase	molecular function: catalytic acti
--	Lipoxygen	Polyunsatu	S	Function u	arachidona	biological process: biological reg
--	--	--	--	--	--	--
--	Immunogl	--	S	Function u	CMRF35-li	cellular component: membrane (
--	IL-3 recep	--	S	Function u	granulocyt	molecular function: signal transd
--	Interleukin	--	U	Intracellula	interleukin	molecular function: signal transd
--	DC-STAM	--	S	Function u	osteoclast	cellular component: membrane (
Secondary	short chair	Retinol de	S	Function u	11-cis retii	biological process: metabolic pro
Function u	Rhodopsir	--	S	Function u	transmeml	biological process: cellular proce
Signal tran	Membrane	Synaptogy	U	Intracellula	synaptogy	cellular component: membrane (
Function u	PDZ doma	Na(+)/H(+	O	Posttransla	Na(+)/H(+	cellular component: cell (GO:000

Signal trar	3'5'-cyclic	High affini	T	Signal trar	high affinit	molecular function: catalytic acti
--	Normal lu	Uncharact	S	Function u	uncharacte	--
General fu	Reverse tr	LINE-1 rev	S	Function u	hypothetic	--
Amino aci	Amino aci	Cystine/gl	E	Amino aci	cystine/glu	biological process: metabolic pro
Lipid trans	Serine ami	--	I	Lipid trans	monoglyce	biological process: biological reg
Posttransl	Immunogl	Pikachurin	S	Function u	basement	molecular function: binding (GO
Transcripti	Helix-hair	Endonucle	L	Replicatior	endonucle	molecular function: binding (GO
Lipid trans	START dor	--	U	Intracellula	START dor	molecular function: binding (GO
--	ATPase far	--	S	Function u	E3 ubiquiti	molecular function: catalytic acti
--	--	--	--	--	hypothetic	molecular function: catalytic acti
Posttransl	Reprolysin	Disintegrir	S	Function u	ADAM DE	molecular function: catalytic acti
General fu	GPCR-cha	--	S	Function u	ankyrin re	biological process: biological reg
--	Intermedia	Phakinin C	Z	Cytoskelet	phakinin is	molecular function: structural mo
Cytoskelet	Tubulin/Ft	Tubulin al	Z	Cytoskelet	tubulin alp	molecular function: catalytic acti
Signal trar	7 transme	Adhesion	S	Function u	adhesion (	molecular function: signal transd
Intracellula	Ras family	Ras-relate	S	Function u	ras-relate	biological process: single-organi
Cytoskelet	Tubulin/Ft	Tubulin al	Z	Cytoskelet	tubulin alp	molecular function: catalytic acti
Energy prc	Malic enzy	NADP-de	C	Energy prc	NADP-de	molecular function: catalytic acti
General fu	BTB/POZ (	--	S	Function u	PREDICTE	biological process: cellular proce
Transcripti	Transcripti	Transcripti	K	Transcripti	transcripti	molecular function: nucleic acid
--	--	--	T	Signal trar	hypothetic	biological process: cellular proce
--	Thyroglob	Insulin-like	A	RNA proce	insulin-like	molecular function: binding (GO
--	TNF(Tumo	Ectodyspl	S	Function u	ectodyspla	molecular function: binding (GO
--	Endonucle	--	S	Function u	sphingomy	cellular component: membrane (
--	IQ calmod	IQ domain	S	Function u	uncharacte	molecular function: binding (GO
--	Protein-ar	Protein-ar	S	Function u	protein-ar	molecular function: catalytic acti
General fu	Zinc-finge	Zinc finger	S	Function u	zinc finger	biological process: biological reg
--	--	--	O	Posttransl	lymphocyt	cellular component: cell (GO:000
General fu	Reverse tr	--	S	Function u	Transposo	--
Intracellula	C2 domair	Protein kir	--	--	BAI1-asso	biological process: biological reg
Signal trar	Protein kir	Serine/thre	T	Signal trar	calcium/ca	molecular function: catalytic acti
Lipid trans	Choline/C	Carnitine (	S	Function u	carnitine C	biological process: reproduction
--	RNA recog	RNA-bind	S	Function u	RNA-bind	molecular function: binding (GO
--	--	--	--	--	uncharacte	--
--	7 transme	N-formyl	U	Intracellula	prostaglan	molecular function: signal transd
Signal trar	7 transme	Pituitary a	U	Intracellula	vasoactive	molecular function: signal transd
--	--	--	S	Function u	uncharacte	--
General fu	Leucine ric	Transform	S	Function u	transformi	cellular component: cell (GO:000
Amino aci	Gamma-g	Glutathion	E	Amino aci	gamma-gl	molecular function: catalytic acti
General fu	High-tem	--	S	Function u	protein HI	cellular component: cell (GO:000
--	TCL1/MTC	--	S	Function u	protein p1	biological process: biological reg
Signal trar	Arrestin (o	S-arrestin	O	Posttransl	S-arrestin	biological process: cellular proce
General fu	BTB/POZ (	Zinc finger	S	Function u	hypermeth	molecular function: nucleic acid
Energy prc	ATP synth	V-type prc	C	Energy prc	V-type prc	cellular component: cell (GO:000
--	--	--	--	--	--	--
--	Class II his	DLA class	U	Intracellula	MHC class	biological process: immune syste
Extracellul	von Willeb	--	S	Function u	von Willeb	molecular function: binding (GO
Extracellul	Mucin-2 p	von Willeb	S	Function u	mucin-5B	cellular component: extracellular
--	Immunogl	Leukocyte	U	Intracellula	leukocyte	--
Amino aci	Trypsin	Duodenas	O	Posttransl	PREDICTE	molecular function: catalytic acti
--	--	--	--	--	--	--

General fu	TMPIT-like	Transmem	S	Function u	transmeml	cellular component: cell (GO:000
--	--	--	S	Function u	transmeml	molecular function: binding (GO
Function u	CHCH dor	--	S	Function u	PREDICTEI	cellular component: cell (GO:000
Lipid trans	Diacylglyc	Diacylglyc	I	Lipid trans	diacylglyce	molecular function: catalytic activ
--	--	--	S	Function u	Trace amir	molecular function: signal transd
Extracellul	Olfactome	Olfactome	S	Function u	olfactome	--
Cytoskelet	Tubulin/Ft	Tubulin alp	Z	Cytoskelet	PREDICTEI	molecular function: catalytic activ
Extracellul	Integrin al	Integrin al	U	Intracellul	integrin al	biological process: biological adl
Signal trar	Ras family	NF-kappa	S	Function u	NF-kappa	molecular function: catalytic activ
--	Exocyst co	--	U	Intracellul	uncharacte	cellular component: cell (GO:000
Signal trar	Rhomboid	Inactive rh	S	Function u	inactive rh	molecular function: catalytic activ
--	--	--	--	--	--	--
--	Small cyto	C-C motif	O	Posttransl	C-C motif	biological process: immune syste
Carbohydr	Sugar (anc	Solute carr	G	Carbohydr	solute carr	molecular function: transporter a
--	Immunogl	--	S	Function u	CMRF35-li	cellular component: membrane (
--	--	--	--	--	--	--
Signal trar	Calcium-b	Adhesion (	S	Function u	adhesion (	molecular function: signal transd
--	EF hand	N-termina	S	Function u	N-termina	cellular component: cell (GO:000
Cytoskelet	Myosin he	Myosin-7	Z	Cytoskelet	myosin-7	molecular function: catalytic activ
Function u	Calcium-a	Anoctamir	U	Intracellul	anoctamin	molecular function: transporter a
--	7 transme	Proteinase	S	Function u	free fatty a	biological process: immune syste
--	Cdc42 effe	Cdc42 effe	S	Function u	cdc42 effe	biological process: biological reg
Extracellul	PH domair	Alpha-1-s	O	Posttransl	alpha-1-sy	biological process: biological reg
Posttransl	Reprolysin	Disintegrir	S	Function u	disintegrin	molecular function: catalytic activ
--	--	--	--	--	caudal typ	--
General fu	Family of u	Protein-lys	S	Function u	protein-lys	cellular component: cell (GO:000
--	--	--	--	--	--	--
--	--	--	--	--	hypothetic	biological process: multi-organis
--	C2 domair	--	S	Function u	rab11 fam	cellular component: cell (GO:000
Signal trar	Integrin be	Integrin be	U	Intracellul	integrin be	biological process: biological adl
Function u	Frag1/DRA	Modulator	S	Function u	modulator	cellular component: cell (GO:000
Amino acio	Trypsin	Duodenas	O	Posttransl	neutrophil	biological process: biological reg
Signal trar	C2 domair	Synaptota	S	Function u	synaptotag	cellular component: cell (GO:000
Cell cycle (	Septin	Septin-5 C	DZ	Cell cycle (	septin-5 is	molecular function: binding (GO
Amino acio	Hydantoin	5-oxoproli	EQ	Amino acio	5-oxoproli	molecular function: catalytic activ
General fu	7 transme	Histamine	U	Intracellul	histamine	biological process: multicellular (
Signal trar	LIM doma	Four and a	S	Function u	four and a	cellular component: cell (GO:000
General fu	7 transme	Neuromed	U	Intracellul	neuromed	molecular function: signal transd
--	Small cyto	Regakine-	S	Function u	regakine-1	cellular component: extracellular
--	--	--	--	--	hypothetic	--
Transcripti	GATA zinc	Trans-actir	K	Transcripti	erythroid t	biological process: biological reg
--	--	--	--	--	--	--
--	--	--	--	--	--	--
General fu	Zinc finger	--	S	Function u	zinc finger	biological process: biological reg
--	Innexin	--	U	Intracellul	pannexin-	biological process: response to s
Amino acio	Trypsin	Acrosin OS	O	Posttransl	serine prot	molecular function: catalytic activ
Function u	2OG-Fe(II)	--	S	Function u	prolyl 3-hy	molecular function: binding (GO
Posttransl	Ring finge	--	O	Posttransl	RING finge	cellular component: membrane (
Cell motilit	LNS2 (Lipi	--	S	Function u	phosphatic	molecular function: transcription
Function u	MBOAT, m	Lysophosp	I	Lipid trans	lysophosp	cellular component: membrane (
--	C-myb, C-	Transcripti	K	Transcripti	myb-relate	biological process: cellular proce

Carbohydr	Kringle do	Hyalurona	S	Function u	kremen pr	cellular component: cell (GO:000
Energy prc	Glyceroph	--	C	Energy prc	lysophosp	cellular component: cell (GO:000
--	Dual speci	Dual speci	T	Signal tran	protein ph	molecular function: binding (GO
--	SH3 doma	--	O	Posttransla	NADPH ox	molecular function: binding (GO
General fu	Ras family	Ras-relate	S	Function u	dexametha	molecular function: catalytic acti
Intracellula	C2 domair	Rabphilin-	S	Function u	double C2	cellular component: membrane (
Signal trar	Protein tyr	Epithelial c	T	Signal tran	epithelial c	biological process: biological reg
Signal trar	Protein-ty	Receptor-t	T	Signal tran	tyrosine-p	molecular function: binding (GO
--	--	--	--	--	hypothetic	--
Transcripti	Transactiva	Forkhead I	K	Transcripti	forkhead b	molecular function: nucleic acid
--	Cyclin-dep	--	S	Function u	tumor sup	biological process: cellular proce
--	GDNF/GAS	GDNF fam	U	Intracellula	GDNF fam	molecular function: signal transd
--	--	--	--	--	--	--
--	Laminin G	Vitamin K-	O	Posttransla	growth arr	biological process: cellular proce
Function u	Transmem	--	S	Function u	transmeml	cellular component: membrane (
General fu	RUN doma	--	S	Function u	small G pr	molecular function: molecular fu
Signal trar	Protein tyr	Tyrosine-p	T	Signal tran	tyrosine-p	biological process: cellular proce
Nuclear str	Bacterial Ig	--	U	Intracellula	nuclear pc	cellular component: cell (GO:000
Intracellula	Syntaxin	Syntaxin-1	U	Intracellula	syntaxin-3	molecular function: binding (GO
--	Domain of	--	S	Function u	uncharacte	biological process: multicellular (
Lipid trans	Lipase (cla	--	S	Function u	diacylglyce	biological process: cellular proce
Amino acid	Trypsin	Plasma kal	O	Posttransla	probable t	molecular function: catalytic acti
--	--	--	--	--	--	--
--	PH domair	Sesquiped	U	Intracellula	sesquipeda	biological process: metabolic pro
General fu	DHHC palı	Palmitoyltr	S	Function u	PREDICTEI	cellular component: membrane (
--	--	--	--	--	--	--
Function u	NACHT dc	NACHT, LF	S	Function u	NLR family	molecular function: binding (GO
Signal trar	RasGEF dc	Rap guani	T	Signal tran	rap guanir	molecular function: molecular fu
Translatior	Ribosomal	60S riboso	J	Translatior	TPA: ribos	biological process: metabolic pro
General fu	Anaphase-	--	S	Function u	WD repeat	cellular component: cell (GO:000
General fu	Reverse tra	--	S	Function u	Transposo	--
General fu	PLD-like d	5'-3' exon	I	Lipid trans	5'-3' exon	cellular component: cell (GO:000
--	Immunogl	--	S	Function u	SLAM fam	--
General fu	Phosphoty	--	S	Function u	carboxyl-t	biological process: biological reg
--	F5/8 type (	Coagulatic	U	Intracellula	discoidin, (	cellular component: membrane (
--	CD34/Pod	Podocalyx	U	Intracellula	podocalyx	cellular component: membrane (
--	--	--	S	Function u	tumor pro	cellular component: cell (GO:000
General fu	WD doma	--	S	Function u	WD repeat	molecular function: binding (GO
General fu	Tweety	Protein tw	U	Intracellula	protein tw	molecular function: transporter a
Function u	MAGE fam	Melanoma	S	Function u	melanoma	--
--	Class I Hist	BOLA class	U	Intracellula	LOW QUA	biological process: immune syste
--	--	--	S	Function u	uncharacte	biological process: cellular proce
Carbohydr	Hexokinas	Hexokinas	G	Carbohydr	hexokinase	biological process: cellular proce
--	--	--	U	Intracellula	tumor nec	molecular function: signal transd
--	Immunogl	Butyrophil	S	Function u	LOW QUA	cellular component: membrane (
Defense m	Membrane	Myeloid-a	S	Function u	myeloid-a	cellular component: cell (GO:000
Signal trar	Neuralizec	--	O	Posttransla	neuralized	biological process: cellular proce
General fu	ABC1 fami	Atypical ki	S	Function u	atypical kir	cellular component: cell (GO:000
--	Immunogl	Polymeric	U	Intracellula	polymeric	biological process: multicellular (
Carbohydr	Major Faci	Protein spi	G	Carbohydr	protein spi	cellular component: membrane (
--	Domain of	Uncharact	S	Function u	uncharacte	biological process: biological reg

Signal trar	Copine	Copine-3	S	Function u	copine-5 i	biological process: biological reg
Amino aci	Angiotens	Angiotens	S	Function u	angiotensi	biological process: multicellular
--	--	--	S	Function u	uncharacte	--
--	Interferon-	--	S	Function u	interferon-	cellular component: membrane
--	--	--	U	Intracellula	interleukin	biological process: biological reg
General fu	Glycosyl tr	--	M	Cell wall/m	UDP-GlcN	--
--	Clathrin-b	Uncharact	S	Function u	uncharacte	cellular component: cell (GO:000
Amino aci	Trypsin	Mast cell p	O	Posttransla	granzyme	molecular function: catalytic acti
--	--	--	S	Function u	reverse tra	--
Amino aci	Pyridoxal-	L-serine d	E	Amino aci	serine deh	molecular function: binding (GO
Chromatin	linker histo	Histone H	B	Chromatin	PREDICTEI	biological process: biological reg
Inorganic i	Guanylate	Voltage-d	U	Intracellula	PREDICTEI	molecular function: transporter a
--	--	--	--	--	--	--
General fu	p25-alpha	Tubulin pc	S	Function u	hypothetic	biological process: cellular proce
--	Domain of	--	S	Function u	basic proli	biological process: response to s
Chromatin	linker histo	Histone H	B	Chromatin	H1.12 linke	cellular component: cell (GO:000
--	--	--	O	Posttransla	protein na	biological process: biological reg
--	IRSp53/MI	Brain-spec	S	Function u	brain-spec	biological process: cellular proce
Signal trar	Fasciclin d	Transform	S	Function u	stabilin-1 i	molecular function: molecular tra
Carbohydr	Transketol	Transketol	G	Carbohydr	PREDICTEI	molecular function: catalytic acti
--	SPRY-assc	Butyrophil	S	Function u	butyrophil	cellular component: cell (GO:000
Posttransla	Reprolysin	Disintegrir	S	Function u	disintegrin	cellular component: cell (GO:000
Signal trar	LIM domai	Cysteine a	O	Posttransla	cysteine ar	cellular component: cell (GO:000
General fu	Ankyrin re	--	S	Function u	ankyrin rep	cellular component: cell (GO:000
General fu	Protein kir	Cyclin-dep	T	Signal trar	cyclin-dep	molecular function: catalytic acti
General fu	Tetraspani	CD63 antig	S	Function u	PREDICTEI	biological process: biological reg
Cytoskelet	Spectrin re	Alpha-acti	Z	Cytoskelet	dystonin is	molecular function: binding (GO
Cytoskelet	Hydrolytic	--	Z	Cytoskelet	dynein hea	molecular function: catalytic acti
RNA proce	KH domair	Poly(rC)-b	S	Function u	poly(rC)-b	molecular function: binding (GO
Chromatin	Chromo (C	--	S	Function u	PREDICTEI	biological process: biological reg
--	BTB/POZ c	--	S	Function u	BTB/POZ c	cellular component: cell (GO:000
--	CD80-like	Advanced	U	Intracellula	advanced	molecular function: binding (GO
Inorganic i	CutA1 diva	Protein Cu	P	Inorganic i	protein Cu	biological process: response to s
--	Histone de	--	K	Transcripti	histone de	cellular component: cell (GO:000
--	Immunogl	Sialic acid-	S	Function u	sialic acid-	cellular component: cell (GO:000
--	--	--	--	--	hypothetic	--
Inorganic i	Amiloride-	Amiloride-	S	Function u	amiloride-	molecular function: transporter a
Carbohydr	6-phosph	6-phosph	G	Carbohydr	6-phosph	molecular function: catalytic acti
General fu	Zinc-finge	Replicatior	S	Function u	zinc finger	biological process: biological reg
Energy prc	Mitochonc	Mitochonc	S	Function u	mitochonc	molecular function: transporter a
Amino aci	Biopterin-	Tyrosine 3	E	Amino aci	PREDICTEI	biological process: response to s
Intracellula	TLC doma	--	U	Intracellula	ceramide s	cellular component: membrane
--	--	--	--	--	--	--
Intracellula	Reticulon	Reticulon-	U	Intracellula	reticulon-2	cellular component: cell (GO:000
--	Zinc finger	--	S	Function u	zinc finger	molecular function: nucleic acid
Signal trar	Fes/CIP4, a	Protein kir	Z	Cytoskelet	protein kir	molecular function: binding (GO
Intracellula	PRA1 fami	Prenylated	U	Intracellula	prenylated	cellular component: cell (GO:000
Signal trar	Gtr1/RagA	Ras-relate	S	Function u	ras-relatec	molecular function: catalytic acti
--	CD80-like	--	U	Intracellula	cell surface	cellular component: membrane
Posttransla	Thrombos	A disintegi	S	Function u	ADAMTS-	molecular function: binding (GO
General fu	Zinc-finge	--	S	Function u	zinc finger	molecular function: binding (GO

Cytoskelet	Troponin	Troponin I	Z	Cytoskelet	troponin I,	biological process: multicellular (
--	--	--	--	--	uncharacte	--
General fu	Major Faci	--	U	Intracellula	solute carr	cellular component: membrane (
Signal trar	Phosphoty	Epidermal	T	Signal tran	epidermal	molecular function: binding (GO
Function u	Eukaryotic	Transmem	S	Function u	transmeml	cellular component: membrane (
--	--	--	S	Function u	LOW QUA	molecular function: binding (GO
Cell cycle (	Unstructur	--	Z	Cytoskelet	growth arr	biological process: cellular proce
--	--	--	--	--	--	--
--	Immunogl	--	T	Signal tran	immunogl	--
Carbohydr	Sulfatase	N-acetylgl	P	Inorganic i	extracellul	biological process: cellular proce
General fu	RUN doma	--	S	Function u	small G pr	molecular function: molecular fu
--	Immunogl	CD166 ant	U	Intracellula	CD166 ant	cellular component: cell (GO:000
--	Paralemmi	Paralemmi	Z	Cytoskelet	paralemmi	cellular component: cell (GO:000
--	Peptidase	Secernin-3	E	Amino aci	secernin-2	molecular function: catalytic acti
Posttransla	Dolichol-p	Dolichol-p	S	Function u	dolichol-p	biological process: metabolic pro
Inorganic i	Calcium-a	--	U	Intracellula	potassium	molecular function: transporter a
--	--	--	--	--	hypothetic	--
--	--	--	S	Function u	uncharacte	--
Energy pro	Mitochonc	ADP/ATP t	C	Energy pro	ADP/ATP t	molecular function: transporter a
--	Dysbindin	Dysbindin	S	Function u	dysbindin	cellular component: cell (GO:000
Signal trar	Fibronectir	Receptor-t	U	Intracellula	protein sid	cellular component: membrane (
General fu	Zinc-finge	Telomere z	S	Function u	PREDICTEI	cellular component: cell (GO:000
Secondary	ABC transp	Multidrug	V	Defense m	multidrug	molecular function: binding (GO
--	Carboxyles	Thyroglob	I	Lipid trans	thyroglobu	cellular component: extracellular
General fu	Protein of	--	O	Posttransla	PREDICTEI	molecular function: catalytic acti
--	--	--	--	--	--	--
Nucleotide	Adenosine	--	F	Nucleotide	PREDICTEI	molecular function: catalytic acti
--	--	--	S	Function u	SPTY2D1 c	cellular component: membrane (
Transcripti	RFX1 trans	DNA-bind	K	Transcripti	PREDICTEI	molecular function: binding (GO
Cytoskelet	Spectrin re	Dystrophir	Z	Cytoskelet	nesprin-1	cellular component: cell (GO:000
--	Immunogl	Natural cy	U	Intracellula	immunogl	cellular component: cell (GO:000
--	Pancreatic	Angiogeni	S	Function u	angiogenir	molecular function: binding (GO
--	--	--	--	--	--	--
--	Eukaryotic	Eukaryotic	J	Translatior	eukaryotic	molecular function: binding (GO
--	--	--	--	--	--	--
General fu	Leucine ric	--	S	Function u	E3 ubiquiti	molecular function: catalytic acti
Posttransla	Ubiquitin c	Ubiquitin c	O	Posttransla	ubiquitin c	molecular function: binding (GO
Cytoskelet	Tubulin/Ft	Tubulin alp	Z	Cytoskelet	tubulin alp	molecular function: catalytic acti
Lipid trans	Sterol-sen	Sterol regu	U	Intracellula	sterol regu	cellular component: cell (GO:000
--	Reverse tra	--	S	Function u	hypothetic	--
--	--	--	S	Function u	golgin sub	cellular component: cell (GO:000
--	--	--	--	--	--	--
--	Rab bindir	--	U	Intracellula	GRIP and c	cellular component: cell (GO:000
--	Intercellula	Intercellula	U	Intracellula	intercellula	molecular function: binding (GO
--	Tissue fact	--	U	Intracellula	interferon	biological process: immune syste
Function u	Leucine Ri	--	S	Function u	uncharacte	biological process: cellular proce
--	--	--	S	Function u	hypothetic	--
--	Alstrom sy	--	U	Intracellula	Alstrom sy	cellular component: cell (GO:000
--	Class I Hist	--	S	Function u	UL16 bind	cellular component: membrane (
Posttransla	Eukaryotic	Pepsin A C	O	Posttransla	pepsin A p	molecular function: catalytic acti
Extracellula	von Willeb	Integrin alp	U	Intracellula	TPA: integ	biological process: cellular proce

--	Immunogl	Leukocyte	T	Signal tran	leukocyte	--
--	EF-hand d	--	S	Function u	EF-hand c	biological process: localization (
--	TNFR/NGF	--	U	Intracellula	tumor nec	molecular function: signal transd
--	PAXX, PAr	--	S	Function u	protein PA	cellular component: cell (GO:000
--	Small cyto	C-C motif	O	Posttransla	TPA: C-C r	biological process: immune syste
Signal tran	Plexin cytc	--	U	Intracellula	plexin-B2	cellular component: membrane (
--	--	--	--	--	--	--
Secondary	Organic A	Solute carr	P	Inorganic i	solute carr	cellular component: cell (GO:000
--	IL-3 recep	--	U	Intracellula	interleukin	cellular component: membrane (
--	--	--	K	Transcripti	--	molecular function: nucleic acid
Function u	Telomere l	--	S	Function u	telomere l	cellular component: cell (GO:000
General fu	Tetraspani	Rod outer	S	Function u	rod outer s	biological process: multicellular c
--	Protein ph	--	T	Signal tran	protein ph	molecular function: molecular fu
Lipid trans	Acyltransfe	Tafazzin O	I	Lipid trans	tafazzin iso	biological process: metabolic pro
--	EF-hand d	--	S	Function u	ninein-like	molecular function: binding (GO
--	Tumor nec	--	U	Intracellula	death dom	cellular component: cell (GO:000
General fu	Hydroxyac	Hydroxyac	S	Function u	hydroxyac	molecular function: catalytic activ
Energy prc	Eukaryotic	Cytochron	O	Posttransla	cytochrom	cellular component: membrane (
--	WH1 dom	Homer prc	Z	Cytoskelet	homer prc	cellular component: cell (GO:000
Lipid trans	Thioestera	--	I	Lipid trans	Cytosolic a	molecular function: catalytic activ
Cell cycle c	--	Protein SF	U	Intracellula	protein SF	cellular component: cell (GO:000
Signal tran	RhoGAP d	Rho GTPas	U	Intracellula	arf-GAP w	molecular function: molecular fu
Signal tran	EGF-like d	--	S	Function u	neurogeni	biological process: biological reg
Posttransla	Gaa1-like,	--	S	Function u	glycosylph	molecular function: binding (GO
Carbohydr	Phosphog	Glucose-6	G	Carbohydr	PREDICTEI	biological process: multicellular c
Carbohydr	Glycosyl h	Lysosomal	G	Carbohydr	lysosomal	molecular function: catalytic activ
--	PX domair	--	S	Function u	HCLS1-bir	cellular component: cell (GO:000
--	Deltex C-t	--	O	Posttransla	E3 ubiquiti	molecular function: binding (GO
--	Major Faci	--	U	Intracellula	solute carr	cellular component: cell (GO:000
Amino acic	Trypsin	Duodenase	O	Posttransla	granzyme	molecular function: catalytic activ
Signal tran	Domain of	cAMP-dep	T	Signal tran	microtubu	molecular function: binding (GO
Energy prc	Mitochonc	Tricarboxy	S	Function u	tricarboxyl	molecular function: transporter a
Signal tran	Calcium-b	Latent-tra	A	RNA proce	latent-trar	molecular function: binding (GO
Lipid trans	Peptidase	Leukotrien	E	Amino acic	aminopep	molecular function: binding (GO
--	Fringe-like	Beta-1,3-N	O	Posttransla	beta-1,3-N	biological process: multicellular c
Lipid trans	PLC-beta (	1-phospha	S	Function u	1-phospha	molecular function: catalytic activ
Signal tran	Plexin cytc	Hepatocyt	S	Function u	plexin-A1	cellular component: macromolec
--	TNFR/NGF	--	S	Function u	tumor nec	cellular component: membrane (
--	--	--	--	--	hypothetic	cellular component: cell (GO:000
Lipid trans	Hormone-	Hormone-	I	Lipid trans	hormone-	biological process: reproduction
General fu	C2H2-type	--	S	Function u	zinc finger	molecular function: nucleic acid
--	Pericentrir	A-kinase a	S	Function u	A-kinase a	molecular function: binding (GO
Inorganic i	Glutathion	Chloride ir	U	Intracellula	chloride in	molecular function: transporter a
Intracellula	Patatin-lik	Patatin-lik	S	Function u	patatin-lik	molecular function: catalytic activ
Signal tran	Plexin cytc	Hepatocyt	S	Function u	plexin-B1	cellular component: macromolec
Carbohydr	WSC dom	--	S	Function u	WSC doma	molecular function: catalytic activ
Signal tran	EF-hand d	--	DTZ	Cell cycle c	calcineurir	molecular function: binding (GO
--	Telethonin	Telethonin	S	Function u	telethonin	biological process: multicellular c
--	Domain of	--	U	Intracellula	uncharacte	cellular component: membrane (
--	RhoGEF dc	Proto-onc	T	Signal tran	pleckstrin l	molecular function: molecular fu
--	Phosphoty	--	O	Posttransla	TPA: amylo	molecular function: binding (GO

Function u	CG-1 dom	--	K	Transcripti	calmodulir	molecular function: binding (GO
Function u	WD doma	Periodic tr	S	Function u	periodic tr	cellular component: cell (GO:000
Nucleotide	Adenosine	Adenosine	F	Nucleotide	adenosine	molecular function: catalytic acti
Signal trar	BTG family	Protein BT	S	Function u	protein BT	cellular component: cell (GO:000
Signal trar	GDSL-like	Platelet-ac	S	Function u	platelet-ac	cellular component: cell (GO:000
Replicatior	Conserved	GPN-loop	S	Function u	GPN-loop	molecular function: catalytic acti
Signal trar	2OG-Fe(II)	--	O	Posttransla	prolyl hydr	biological process: response to s
General fu	Reverse tra	--	S	Function u	hypothetic	--
General fu	10 TM Acy	--	S	Function u	N-acetylne	biological process: metabolic pro
Posttransla	Ubiquitin-	Ubiquitin-	O	Posttransla	ubiquitin-c	biological process: metabolic pro
Posttransla	Hsp70 pro	Endoplasm	O	Posttransla	endoplasm	molecular function: binding (GO
Function u	D123	Cell divisic	S	Function u	cell divisio	cellular component: cell (GO:000
RNA proce	Ribosomal	Probable c	J	Translatior	PREDICTEI	molecular function: catalytic acti
Function u	Integral m	Calcium lo	S	Function u	PREDICTEI	molecular function: transporter a
Transcripti	Transcripti	Transcripti	K	Transcripti	transcriptio	molecular function: binding (GO
Function u	Protein of	Protein kis	S	Function u	protein kis	cellular component: cell (GO:000
Intracellula	Endomem	Transmem	S	Function u	transmeml	cellular component: membrane (
RNA proce	RNA recog	Nuclear ca	A	RNA proce	nuclear ca	molecular function: binding (GO
Posttransla	Oligosacch	Dolichyl-d	S	Function u	dolichyl-d	molecular function: catalytic acti
Defense m	Membrane	Myeloid-a	S	Function u	uncharacte	cellular component: membrane (
General fu	Ras family	Ras-relate	S	Function u	ras-relatec	molecular function: binding (GO
Function u	Protein of	--	S	Function u	protein FA	cellular component: cell (GO:000
Intracellula	Regulated	Vesicle-tra	U	Intracellula	PREDICTEI	cellular component: cell (GO:000
General fu	Anaphase-	--	S	Function u	U3 small n	cellular component: cell (GO:000
General fu	Tetratricop	Tetratricop	S	Function u	tetratricop	cellular component: cell (GO:000
Translatior	Ribosomal	60S riboso	J	Translatior	60S riboso	molecular function: structural mo
Signal trar	TAP42-like	--	S	Function u	immunogl	cellular component: cell (GO:000
General fu	Fcf1	rRNA-proc	S	Function u	PREDICTEI	biological process: metabolic pro
Cell cycle c	LEM3 (liga	Cell cycle c	DKT	Cell cycle c	PREDICTEI	cellular component: cell (GO:000
Translatior	Ribosomal	39S riboso	J	Translatior	39S riboso	molecular function: structural mo
Posttransla	OST3 / OS	Magnesiur	L	Replicatior	magnesiur	cellular component: cell (GO:000
--	--	RNA-bind	S	Function u	RNA-bind	molecular function: binding (GO
Signal trar	Phosducin	Phosducin	S	Function u	phosducin	cellular component: cell (GO:000
General fu	Methyltrar	--	Q	Secondary	TPA: hypo	molecular function: catalytic acti
Nucleotide	Phosphori	Hypoxanth	F	Nucleotide	hypoxanth	molecular function: binding (GO
Posttransla	Cyclophilir	Peptidyl-p	O	Posttransla	peptidyl-p	biological process: biological reg
Function u	Apoptoge	Cytochron	O	Posttransla	apoptoger	biological process: response to s
Secondary	Copper an	Membrane	Q	Secondary	primary ar	molecular function: binding (GO
Signal trar	Ras family	Ras-relate	S	Function u	ras-relatec	biological process: metabolic pro
Lipid trans	Acyl CoA k	Acyl-CoA-	I	Lipid trans	acyl-CoA-	molecular function: binding (GO
General fu	FYVE zinc	Zinc finger	S	Function u	pleckstrin	cellular component: cell (GO:000
General fu	Ribosomal	Ribosome	J	Translatior	ribosome	biological process: metabolic pro
--	Zinc-finge	Cell divisic	S	Function u	cell divisio	cellular component: cell (GO:000
Lipid trans	Phospholip	Lysophosp	S	Function u	lysophosp	cellular component: cell (GO:000
--	Mediator c	Mediator c	K	Transcripti	PREDICTEI	cellular component: cell (GO:000
Replicatior	Sas10/Utp	Nuclear nu	S	Function u	Nuclear nu	biological process: metabolic pro
General fu	BTB/POZ c	--	S	Function u	germ cell-	cellular component: cell (GO:000
Signal trar	Ras family	Ras-relate	S	Function u	ras-relatec	molecular function: catalytic acti
Function u	Pyrimidine	--	F	Nucleotide	cytosolic 5	molecular function: binding (GO
RNA proce	Oligonucle	Probable A	L	Replicatior	probable A	molecular function: binding (GO
--	--	--	S	Function u	small nucle	--

--	7 transmer	Sphingosir	U	Intracellula	sphingosir	biological process: development
Translatior	tRNA syntl	Asparagin	J	Translatior	probable a	molecular function: binding (GO
Posttransla	B-box zinc	--	S	Function u	E3 ubiquiti	molecular function: binding (GO
Signal trar	Leucine ric	Toll-like re	S	Function u	toll-like re	molecular function: binding (GO
Replicatior	Nucleosom	Testis-spe	S	Function u	protein SE	cellular component: cell (GO:000
--	Mitochonc	28S riboso	S	Function u	28S riboso	molecular function: structural mo
--	--	--	--	--	leucine zip	--
--	Spindle an	Spindle an	S	Function u	spindle an	biological process: cellular proce
Energy prc	Thioredoxi	NADH deh	C	Energy prc	NADH deh	molecular function: catalytic activ
--	--	--	--	--	uncharacte	--
Signal trar	Inhibitor o	--	S	Function u	growth ho	cellular component: cell (GO:000
Amino acic	Vanin C-te	Pantetheir	S	Function u	TPA: vanin	biological process: metabolic prc
Cell cycle c	Double-st	Programm	S	Function u	programm	molecular function: binding (GO
Replicatior	DNA polyr	DNA polyr	L	Replicatior	DNA polyr	molecular function: binding (GO
General fu	KH domair	--	S	Function u	RNA-bind	biological process: cellular proce
Transcripti	Transcripti	Transcripti	K	Transcripti	transcriptic	biological process: cellular proce
--	Phosphatic	Phosphatic	S	Function u	phosphatic	cellular component: cell (GO:000
Translatior	Ubiquitin f	Ubiquitin-	O	Posttransla	ubiquitin-4	molecular function: structural mo
Nuclear str	SEP domai	NSFL1 cofa	O	Posttransla	UBX doma	cellular component: cell (GO:000
Cytoskelet	Actin	Actin-relat	Z	Cytoskelet	PREDICTEI	molecular function: binding (GO
Posttransla	TCP-1/cpr	T-complex	O	Posttransla	T-complex	cellular component: macromolec
General fu	F-box don	F-box/LRR	S	Function u	F-box/LRR	cellular component: cell (GO:000
Function u	Predicted i	Endoplasm	S	Function u	PREDICTEI	cellular component: membrane (
Intracellula	Endomem	Transmem	S	Function u	transmeml	cellular component: membrane (
Amino acic	Peptidase	Bleomycin	E	Amino acic	bleomycin	molecular function: catalytic activ
--	The ARF-li	ADP-ribos	S	Function u	ADP-ribos	molecular function: transcription
Intracellula	--	Protein YIF	U	Intracellula	protein YIF	cellular component: cell (GO:000
--	AIG1 famil	GTPase IM	S	Function u	GTPase IM	molecular function: binding (GO
--	Mitochonc	Mitochonc	U	Intracellula	mitochonc	cellular component: membrane (
Translatior	RNase P su	Ribonuclea	J	Translatior	ribonuclea	cellular component: cell (GO:000
General fu	Zinc-ribbc	--	S	Function u	RING finge	cellular component: cell (GO:000
Cytoskelet	Tubulin/Fts	Tubulin alp	Z	Cytoskelet	tubulin alp	molecular function: catalytic activ
General fu	Zinc-finge	--	S	Function u	zinc finger	molecular function: nucleic acid
Intracellula	C2 domair	Rabphilin-	S	Function u	double C2	molecular function: binding (GO
--	Leucine ric	Amphoter	S	Function u	amphoteri	biological process: multicellular c
Carbohydr	Histidine p	Bisphosph	G	Carbohydr	PREDICTEI	molecular function: catalytic activ
Lipid trans	BAR doma	Endophilin	U	Intracellula	endophilin	cellular component: cell (GO:000
Function u	Integral m	ER membr	S	Function u	ER membr	cellular component: cell (GO:000
Function u	Tubulin bir	TBCC dom	S	Function u	TBCC dom	biological process: cellular proce
Function u	Transmem	Transmem	S	Function u	transmeml	cellular component: cell (GO:000
Function u	Eukaryotic	Protein FA	S	Function u	protein FA	cellular component: cell (GO:000
Chromatin	Histone de	Histone de	BQ	Chromatin	histone de	biological process: biological reg
General fu	HMG (high	High mobi	K	Transcripti	TPA: high-	cellular component: cell (GO:000
--	Regulatory	--	K	Transcripti	TPA: regul	molecular function: binding (GO
Posttransla	TCP-1/cpr	60 kDa hea	O	Posttransla	60 kDa hea	molecular function: binding (GO
General fu	Protein kir	Dual speci	T	Signal trar	dual specif	cellular component: cell (GO:000
--	Interleukin	Interleukin	K	Transcripti	interleukin	biological process: metabolic prc
Replicatior	BAH doma	Origin recc	L	Replicatior	origin recc	cellular component: cell (GO:000
--	Ribosomal	28S riboso	J	Translatior	hypothetic	molecular function: structural mo
General fu	Zinc-finge	Zinc finger	S	Function u	zinc finger	molecular function: binding (GO
Signal trar	EF hand	Calumenir	S	Function u	reticulocal	molecular function: binding (GO

Signal trar	EF hand	Calumenir	S	Function u	reticulocal	biological process: multicellular (
Inorganic i	Copper/zi	Superoxid	P	Inorganic i	superoxide	molecular function: catalytic acti
--	Glycosyl tr	Glycosyltra	O	Posttransla	glycosyltra	cellular component: membrane (
Signal trar	HEAT repe	Serine/thre	T	Signal tran	PREDICTEI	molecular function: binding (GO
--	AIG1 famil	GTPase IM	S	Function u	GTPase, IN	molecular function: binding (GO
--	THAP dom	THAP dom	K	Transcripti	THAP dom	molecular function: binding (GO
General fu	Ras family	Ras-relate	S	Function u	ras-relatec	molecular function: catalytic acti
Replicatior	Nucleoson	Testis-spe	S	Function u	protein SE	cellular component: cell (GO:000
Signal trar	Lectin C-ty	Oxidized l	S	Function u	oxidized lc	molecular function: molecular tra
General fu	HMG (higl	High mobi	B	Chromatin	High mobi	molecular function: binding (GO
Energy prc	Transketol	Pyruvate d	C	Energy prc	pyruvate d	molecular function: catalytic acti
Cytoskelet	Actin	Actin-like	Z	Cytoskelet	actin-like	biological process: development
Signal trar	GGL doma	Guanine n	T	Signal tran	guanine nu	molecular function: catalytic acti
--	FERM N-te	--	O	Posttransla	E3 ubiquiti	cellular component: cell (GO:000
Posttransla	ThiF family	NEDD8-ac	H	Coenzyme	NEDD8-ac	molecular function: catalytic acti
Function u	Uncharact	--	S	Function u	transmeml	cellular component: cell (GO:000
Signal trar	Calcineurir	Serine/thre	T	Signal tran	serine/thre	cellular component: cell (GO:000
Translatior	Ribosomal	60S riboso	J	Translatior	hypothetic	molecular function: structural mc
Cytoskelet	ARP2/3 co	Actin-relat	S	Function u	actin-relat	molecular function: structural mc
--	Twisted ga	Twisted ga	S	Function u	twisted ga	biological process: multicellular (
Translatior	Ribosomal	39S riboso	J	Translatior	39S riboso	cellular component: cell (GO:000
Replicatior	Ku70/Ku8(	--	L	Replicatior	X-ray repa	biological process: metabolic prc
--	Armadillo-	Armadillo	S	Function u	armadillo r	--
General fu	p21-C-ter	BRCA2 anc	U	Intracellula	BRCA2 anc	biological process: biological reg
Posttransla	Glutaredo:	Glutaredo:	O	Posttransla	PREDICTEI	molecular function: electron carr
Function u	Thioestera	Acyl-coen	S	Function u	acyl-coenz	cellular component: cell (GO:000
--	Family witl	--	S	Function u	protein FA	--
--	SOCE-ass(	Store-ope	U	Intracellula	store-oper	cellular component: cell (GO:000
General fu	ssDNA-bir	Protection	S	Function u	protection	cellular component: cell (GO:000
Posttransla	E3 ubiquiti	E3 ubiquiti	S	Function u	E3 ubiquiti	biological process: metabolic prc
--	Selenopro	Selenopro	S	Function u	selenoprot	biological process: biological reg
Nucleotide	Ribonucle	Ribonucle	F	Nucleotide	PREDICTEI	molecular function: catalytic acti
Intracellula	PX domair	Sorting ne	U	Intracellula	hypothetic	molecular function: binding (GO
Signal trar	cAMP-reg	cAMP-reg	S	Function u	cAMP-reg	molecular function: molecular fu
Function u	OST3 / OS	Oligosacch	S	Function u	oligosacch	biological process: metabolic prc
--	M-phase-	--	O	Posttransla	M-phase-	cellular component: cell (GO:000
--	ML domair	Gangliosid	O	Posttransla	gangliosid	molecular function: catalytic acti
Energy prc	Glyceroph	Glyceroph	C	Energy prc	glyceroph(	molecular function: catalytic acti
--	Immunogl	Basigin OS	U	Intracellula	neuroplast	cellular component: cell (GO:000
General fu	--	RNA-bind	S	Function u	RNA-bind	molecular function: binding (GO
Translatior	NMD3 fam	60S riboso	J	Translatior	60S riboso	cellular component: cell (GO:000
Intracellula	Ras family	GTP-bindi	S	Function u	GTP-bindi	biological process: localization ((
--	BLOC-1-re	BLOC-1-re	S	Function u	BLOC-1-re	cellular component: cell (GO:000
--	Protein of	Transmem	S	Function u	transmeml	cellular component: membrane (
Translatior	Ribosomal	60S riboso	J	Translatior	PREDICTEI	molecular function: structural mc
Cell cycle (	Cyclin, N-t	Cyclin-G1	D	Cell cycle (	cyclin-G1	biological process: biological reg
--	Germinal c	--	S	Function u	germinal c	biological process: biological reg
Replicatior	TatD relate	Putative d	L	Replicatior	PREDICTEI	molecular function: catalytic acti
General fu	Protein of	Obg-like A	J	Translatior	Obg like A	molecular function: binding (GO
--	--	--	--	--	hypothetic	--
Function u	VMA21-lik	Vacuolar A	U	Intracellula	vacuolar A	cellular component: cell (GO:000

Chromatin	Chromo sl	Chromobc	S	Function u	chromobo	cellular component: cell (GO:000
Amino aci	Trypsin	Compleme	O	Posttransla	compleme	molecular function: catalytic acti
Posttransla	ATPase far	ATPase far	O	Posttransla	ATPase far	biological process: biological reg
Signal trar	Low molec	Low molec	T	Signal tran	low molec	molecular function: catalytic acti
Nucleotide	Adenylosu	Adenylosu	F	Nucleotide	RecName:	molecular function: binding (GO
Inorganic i	Glutathion	Chloride ir	U	Intracellula	chloride in	biological process: development
Inorganic i	Ion transp	Voltage-d	U	Intracellula	two pore c	molecular function: transporter a
--	Class I Hist	BOLA class	U	Intracellula	BOLA class	biological process: immune syste
Function u	Assembly,	Transmem	S	Function u	transmeml	cellular component: cell (GO:000
Transcripti	Protein of	MICOS coi	S	Function u	MICOS coi	molecular function: binding (GO
Function u	Alpha and	Alpha- an	S	Function u	alpha- anc	cellular component: cell (GO:000
General fu	AMP-bind	--	IQ	Lipid trans	disco-inte	molecular function: catalytic acti
Transcripti	Histone-lil	--	K	Transcripti	protein Dr	biological process: biological reg
Intracellula	--	--	U	Intracellula	BET1 hom	cellular component: cell (GO:000
Function u	DSS1/SEM	26S protea	O	Posttransla	26S protea	cellular component: cell (GO:000
--	Small cyto	Stromal ce	O	Posttransla	stromal ce	molecular function: binding (GO
Translatior	Endoribon	2-iminobu	J	Translatior	2-iminobu	molecular function: catalytic acti
Transcripti	Transcripti	Transcripti	K	Transcripti	transcriptic	biological process: metabolic pro
Inorganic i	Divalent ca	Solute carı	P	Inorganic i	solute carr	cellular component: membrane (
Replicatior	DNA repai	5' exonucl	J	Translatior	5' exonucl	cellular component: cell (GO:000
--	Intermedia	Keratin, ty	Z	Cytoskelet	keratin, typ	cellular component: cell (GO:000
Nucleotide	Na+ depe	Sodium/nu	F	Nucleotide	solute carr	cellular component: cell (GO:000
Translatior	DEAD/DEA	Eukaryotic	L	Replicatior	PREDICTEI	molecular function: binding (GO
RNA proce	Lysine met	Protein N-	S	Function u	protein N-	molecular function: catalytic acti
RNA proce	RNA recog	Heteroger	S	Function u	heterogen	molecular function: binding (GO
RNA proce	RNA recog	Polyadeny	S	Function u	polyadeny	molecular function: binding (GO
Translatior	Ribosomal	60S riboso	J	Translatior	60S riboso	molecular function: structural mo
Lipid trans	short chair	Very-long	S	Function u	very-long-	molecular function: binding (GO
Cytoskelet	Cofilin/trop	Destrin OS	S	Function u	destrin [Su	biological process: cellular proce
Transcripti	SGT1 prot	--	S	Function u	protein ec	cellular component: cell (GO:000
--	Domain of	--	S	Function u	small integ	--
Signal trar	RIO1 famil	--	T	Signal tran	serine/thre	molecular function: catalytic acti
Function u	Condensir	--	S	Function u	condensin	biological process: cellular proce
Translatior	Domain fo	Eukaryotic	J	Translatior	eukaryotic	biological process: multicellular o
--	Small acidi	Small acidi	S	Function u	small acidi	--
General fu	Type I phc	Bis(5'-ade	S	Function u	ectonuclec	molecular function: catalytic acti
Inorganic i	Regulator	Methyloso	U	Intracellula	methyloso	biological process: cellular proce
--	Immunogl	--	U	Intracellula	T-cell imm	molecular function: binding (GO
--	Death-ass	Death-ass	S	Function u	death-ass	molecular function: binding (GO
Posttransla	PPIC-type	Peptidyl-p	O	Posttransla	peptidyl-p	molecular function: binding (GO
Signal trar	Protein kir	Serine/thre	T	Signal tran	serine/thre	molecular function: catalytic acti
Replicatior	Rad51	DNA repai	L	Replicatior	DNA repai	molecular function: catalytic acti
--	P21-Rho-I	CDC42 sm	O	Posttransla	CDC42 sm	molecular function: binding (GO
--	Interleukin	--	S	Function u	cell migrat	molecular function: catalytic acti
--	Interferon-	Interferon	U	Intracellula	interferon	biological process: cellular proce
Cell cycle c	Cyclin, N-t	G2/mitotic	D	Cell cycle c	Cyclin B2 [	biological process: biological reg
Function u	ARF7 effec	ARL14 effe	S	Function u	ARL14 effe	cellular component: cell (GO:000
Cell motilit	Tctex-1 fa	Dynein ligl	S	Function u	dynein ligl	biological process: cellular proce
Nucleotide	Deoxynucl	Deoxycytic	F	Nucleotide	deoxycytic	molecular function: catalytic acti
Posttransla	TRC8 N-te	E3 ubiquiti	O	Posttransla	E3 ubiquiti	molecular function: binding (GO
Transcripti	bZIP Maf t	Transcripti	K	Transcripti	transcriptic	molecular function: nucleic acid

Function u	DSS1/SEM	26S protea	O	Posttransla	26S protea	cellular component: cell (GO:000
--	Ankyrin re	Osteoclast	S	Function u	osteoclast-	biological process: multicellular (
Coenzyme	MoaE prot	Molybdop	H	Coenzyme	molybdop	cellular component: cell (GO:000
Function u	Serine inco	Serine inco	S	Function u	serine inco	biological process: metabolic pro
Function u	Protein of	--	S	Function u	programm	biological process: response to s
Energy pro	Molybdop	NADH-ub	C	Energy pro	NADH-ub	molecular function: signal transd
Transcripti	NAC doma	Nascent p	K	Transcripti	nascent po	--
--	Domain of	Uncharact	S	Function u	uncharacte	--
RNA proce	Adenosine	Spermatid	S	Function u	double-str	molecular function: binding (GO
Posttransla	Cytochrom	Cytochrom	O	Posttransla	cytochrom	cellular component: cell (GO:000
Function u	Iron-sulfur	Cytosolic i	S	Function u	cytosolic ir	biological process: metabolic pro
--	Prothymos	Prothymos	--	--	prothymos	cellular component: cell (GO:000
--	--	--	--	--	--	--
--	Angiomoti	Angiomoti	Z	Cytoskelet	angiomoti	biological process: cellular proce
Carbohydr	Galactosyl	UDP-GlcN	S	Function u	N-acetylla	cellular component: cell (GO:000
Intracellula	Tim10/DD	Mitochonc	O	Posttransla	mitochonc	cellular component: cell (GO:000
Function u	WD doma	--	A	RNA proce	pre-mRNA	biological process: metabolic pro
Posttransla	RWD dom	--	S	Function u	E3 ubiquiti	molecular function: catalytic acti
General fu	Iron-conta	CDGSH irc	S	Function u	CDGSH irc	biological process: metabolic pro
General fu	Mitochonc	--	S	Function u	calcium ur	cellular component: cell (GO:000
Function u	Tho comp	THO comp	S	Function u	THO comp	cellular component: cell (GO:000
--	--	--	Z	Cytoskelet	small kinet	biological process: cellular proce
Nucleotide	Dihydroor	Dihydropy	F	Nucleotide	TPA: dihyd	molecular function: binding (GO
Posttransla	Scaffold pr	--	O	Posttransla	NFU1 iron	molecular function: binding (GO
--	Ribosomal	39S riboso	S	Function u	39S riboso	cellular component: cell (GO:000
--	ZW10 inte	ZW10 inte	Z	Cytoskelet	PREDICTED	cellular component: cell (GO:000
--	ICOS V-se	Inducible T	U	Intracellula	inducible T	cellular component: cell (GO:000
General fu	Cytosol an	Cytosol an	E	Amino acid	cytosol am	molecular function: catalytic acti
--	Phorbol-1	Phorbol-1	B	Chromatin	phorbol-1	biological process: cellular proce
--	CEP76 C2	Centrosom	S	Function u	PREDICTED	cellular component: cell (GO:000
Transcripti	HMG (high	Transcripti	B	Chromatin	transcriptio	molecular function: binding (GO
Energy pro	CybS, succ	Succinate	I	Lipid trans	succinate	cellular component: cell (GO:000
--	F-box-like	F-box/LRR	S	Function u	PREDICTED	molecular function: binding (GO
--	HMG14 ar	Non-histo	S	Function u	non-histor	cellular component: cell (GO:000
--	--	--	--	--	--	--
General fu	Leucine ric	--	S	Function u	platelet gly	cellular component: membrane (
Posttransla	JAB1/Mov3	--	S	Function u	26S protea	cellular component: cell (GO:000
Translation	tRNA synth	Aspartate-	J	Translation	TPA: aspar	molecular function: binding (GO
Transcripti	E2F transc	Transcripti	K	Transcripti	transcriptio	molecular function: binding (GO
--	Aminoacyl	Prolyl-tRN	S	Function u	prolyl-tRN	molecular function: catalytic acti
Secondary	Zinc-bindi	Alcohol de	C	Energy pro	alcohol de	biological process: metabolic pro
RNA proce	Ribosomal	60S riboso	J	Translation	60S riboso	molecular function: structural mo
Nucleotide	SAICAR sy	Multifunct	F	Nucleotide	multifuncti	molecular function: catalytic acti
Replication	Origin reco	Origin reco	L	Replication	origin reco	cellular component: cell (GO:000
Translation	Ribosomal	Probable r	J	Translation	PREDICTED	molecular function: structural mo
Translation	Eukaryotic	Eukaryotic	J	Translation	PREDICTED	molecular function: binding (GO
Translation	--	Threonine	J	Translation	39S riboso	molecular function: binding (GO
Cell cycle (	Caspase d	Caspase-4	O	Posttransla	caspase-4	molecular function: catalytic acti
Posttransla	Ubiquitin-	Ubiquitin-	O	Posttransla	ubiquitin-c	molecular function: binding (GO
Posttransla	Ubiquitin-	Ubiquitin-	O	Posttransla	PREDICTED	molecular function: binding (GO
Chromatin	Core histo	Histone H2	B	Chromatin	histone H2	cellular component: cell (GO:000

Translatior	Putative tF	Tyrosine--	J	Translatior	aminoacyl	molecular function: binding (GO
Function u	Putative tr	--	S	Function u	transmeml	cellular component: membrane (
--	Calcium si	--	S	Function u	calcium si	cellular component: membrane (
Posttransla	Ubiquitin-	Ubiquitin-	O	Posttransla	PREDICTEI	cellular component: cell (GO:000
--	7 transmeı	Proteinase	U	Intracellula	TPA: prote	molecular function: binding (GO
--	--	--	--	--	--	--
Inorganic i	Iron-sulph	Iron-sulfu	S	Function u	iron-sulfur	molecular function: structural mo
Transcripti	DNA-bind	--	K	Transcripti	nuclear pro	biological process: response to s
--	Transmem	Transmem	S	Function u	transmeml	cellular component: cell (GO:000
Intracellula	Double-st	Double-st	K	Transcripti	double-str	molecular function: binding (GO
Signal tran	C2 domair	Synaptota	S	Function u	synaptota	molecular function: binding (GO
Chromatin	Histone ac	--	B	Chromatin	histone ac	cellular component: cell (GO:000
Lipid trans	Acyl-CoA	Medium-c	I	Lipid trans	medium-c	biological process: multicellular o
--	Serglycin	--	O	Posttransla	serglycin p	cellular component: extracellular
--	--	--	--	--	PREDICTEI	--
Inorganic i	Catalase	Catalase C	P	Inorganic i	catalase [B	molecular function: binding (GO
General fu	Zinc-finge	--	S	Function u	zinc finger	biological process: metabolic pro
Energy prc	Mitochonc	ATP synth	Z	Cytoskelet	ATP synth	cellular component: cell (GO:000
Posttransla	Zinc knuck	--	OU	Posttransla	PREDICTEI	molecular function: binding (GO
Intracellula	Ras family	Ras-relate	S	Function u	ras-relate	molecular function: catalytic acti
--	Interleukin	Interleukin	O	Posttransla	interleukin	molecular function: binding (GO
Secondary	short chair	Retinol de	S	Function u	retinol deh	cellular component: cell (GO:000
Nuclear st	Importin r	--	U	Intracellula	RAN bindi	biological process: single-organi
RNA proce	Met-10+ l	tRNA (gua	S	Function u	tRNA (gua	cellular component: cell (GO:000
General fu	Major Faci	Feline leuk	U	Intracellula	feline leuk	cellular component: membrane (
Translatior	Gar1/Naf1	--	J	Translatior	H/ACA rib	biological process: metabolic pro
Secondary	Cytochron	Cytochron	Q	Secondary	cytochrom	molecular function: binding (GO
--	COMM do	--	S	Function u	COMM do	cellular component: cell (GO:000
Signal tran	EF-hand d	Neurocalci	DTZ	Cell cycle c	PREDICTEI	biological process: multicellular o
Function u	Transmem	Nuclear er	U	Intracellula	nuclear en	cellular component: cell (GO:000
Posttransla	Tubulin bi	Tubulin-sp	S	Function u	tubulin-sp	cellular component: cell (GO:000
--	Centriole,	--	S	Function u	centriole, c	cellular component: cell (GO:000
Chromatin	SHNi-TPR	Nuclear au	S	Function u	nuclear au	cellular component: cell (GO:000
Posttransla	Prefoldin s	Prefoldin s	S	Function u	prefoldin s	cellular component: cell (GO:000
General fu	FAD linkec	--	C	Energy prc	alkyldihydı	cellular component: cell (GO:000
--	--	--	--	--	--	--
--	--	--	--	--	--	--
Replicatior	MCM P-lo	DNA replic	L	Replicatior	DNA replic	biological process: metabolic pro
Function u	RWD dom	--	S	Function u	RWD dom	biological process: metabolic pro
Nucleotide	Ribonucle	Ribonucle	F	Nucleotide	ribonuclec	molecular function: catalytic acti
--	Domain of	Small inte	S	Function u	hypothetic	cellular component: cell (GO:000
Lipid trans	CDP-alcoh	--	I	Lipid trans	PREDICTEI	biological process: metabolic pro
Coenzyme	Cytidylyltr	Nicotinam	H	Coenzyme	nicotinami	molecular function: catalytic acti
Signal tran	Phosphati	Phosphati	T	Signal tran	PREDICTEI	molecular function: catalytic acti
Replicatior	Nucleoson	Testis-spe	S	Function u	LOW QUA	cellular component: cell (GO:000
Posttransla	Ubiquitin-	Ubiquitin-	O	Posttransla	ubiquitin-c	molecular function: binding (GO
Signal tran	PET Doma	Testin OS=	O	Posttransla	testin isofc	cellular component: cell (GO:000
RNA proce	Mago nasl	Protein ma	A	RNA proce	protein ma	biological process: metabolic pro
Cytoskelet	Calponin h	--	Z	Cytoskelet	PREDICTEI	molecular function: binding (GO
Signal tran	Lectin C-ty	C-type lec	S	Function u	C-type lec	cellular component: membrane (
General fu	Hypoxia in	HIG1 dom	S	Function u	HIG1 dom	cellular component: membrane (

Signal trar	Lectin C-ty	Natural kil	S	Function u	killer cell le	cellular component: membrane (
--	Uncharact	Small inte	S	Function u	hypothetic	cellular component: membrane (
Intracellula	emp24/gp	--	U	Intracellula	transmeml	biological process: multicellular c
Translatior	Ribosomal	60S riboso	J	Translatior	60S riboso	molecular function: structural mo
--	Ras of Cor	Rab-like p	S	Function u	rab-like pr	--
Posttransla	ATPase far	Pachytene	O	Posttransla	pachytene	biological process: reproduction
Posttransla	DnaJ dom	DnaJ hom	O	Posttransla	dnaJ homo	cellular component: cell (GO:000
Cell cycle (	Caspase d	Caspase-6	O	Posttransla	caspase-6	cellular component: cell (GO:000
General fu	NAD bindi	3-hydroxy	I	Lipid trans	3-hydroxy	biological process: metabolic pro
Posttransla	Thioredoxi	Thioredoxi	U	Intracellula	thioredoxi	cellular component: cell (GO:000
Transcripti	Histone-lik	DNA polyr	K	Transcripti	DNA polyr	cellular component: cell (GO:000
--	--	--	S	Function u	coiled-coil	cellular component: cell (GO:000
Translatior	JAB1/Mov	Eukaryotic	J	Translatior	eukaryotic	biological process: cellular proce
--	--	--	S	Function u	TPA: hypo	--
--	Immunogl	Ig heavy c	T	Signal trar	uncharact	--
Carbohydr	Histidine p	Bisphosph	G	Carbohydr	PREDICTEI	molecular function: catalytic acti
General fu	Sideroflexi	Sideroflexi	U	Intracellula	sideroflexi	molecular function: transporter a
--	--	Transmem	S	Function u	transmeml	cellular component: membrane (
--	--	--	S	Function u	--	--
Coenzyme	Aminotran	Phosphose	E	Amino aci	phosphose	molecular function: catalytic acti
--	Sprouty pr	Protein sp	S	Function u	protein sp	biological process: multicellular c
--	Mediator c	Mediator c	K	Transcripti	mediator c	cellular component: cell (GO:000
Signal trar	Variant SH	Sorbin anc	S	Function u	sorbin and	cellular component: cell (GO:000
--	Methylmal	Methylmal	I	Lipid trans	methylmal	molecular function: catalytic acti
Function u	Protein of	Protein FA	S	Function u	protein FA	molecular function: catalytic acti
Energy pro	Cytochron	Cytochron	C	Energy pro	cytochrom	cellular component: cell (GO:000
Translatior	Mitochonc	28S riboso	S	Function u	28S riboso	molecular function: structural mo
RNA proce	LSM doma	U6 snRNA	K	Transcripti	U6 snRNA	biological process: metabolic pro
Nuclear str	Exportin 1	Exportin-T	J	Translatior	exportin-T	molecular function: binding (GO
Inorganic i	Sodium/ca	Sodium/pc	P	Inorganic i	sodium/pc	cellular component: cell (GO:000
Amino aci	Asparagin	Asparagin	E	Amino aci	TPA: aspar	molecular function: catalytic acti
Nuclear str	Importin-k	--	O	Posttransla	importin-1	cellular component: cell (GO:000
Cytoskelet	Microtubu	--	S	Function u	TRAF3-int	biological process: development
General fu	Iron-conta	CDGSH irc	S	Function u	CDGSH irc	cellular component: membrane (
Function u	Leucine Ri	--	S	Function u	centrosom	cellular component: cell (GO:000
Translatior	Ribosomal	60S riboso	J	Translatior	PREDICTEI	molecular function: structural mo
Translatior	Eukaryotic	Eukaryotic	J	Translatior	eukaryotic	biological process: cellular proce
Secondary	short chair	Estradiol 1	S	Function u	estradiol 1	cellular component: membrane (
Translatior	Ribosomal	60S riboso	J	Translatior	PREDICTEI	molecular function: structural mo
Signal trar	FERM cent	Band 4.1-l	S	Function u	FERM dom	biological process: cellular proce
--	Glycosyl hy	Heparanas	G	Carbohydr	LOW QUA	cellular component: cell (GO:000
Cytoskelet	Microtubu	Cysteine-r	S	Function u	cysteine-ri	molecular function: binding (GO
General fu	Uracil pho	Uracil pho	F	Nucleotide	uracil phos	cellular component: cell (GO:000
--	Protein of	--	S	Function u	transmeml	--
Translatior	Ribosomal	39S riboso	J	Translatior	39S riboso	molecular function: structural mo
Energy pro	Electron tr	Electron tr	C	Energy pro	TPA: electr	cellular component: cell (GO:000
Posttransla	Hsp90 pro	Heat shocl	O	Posttransla	heat shock	biological process: reproduction
Energy pro	ETC comp	NADH deh	C	Energy pro	NADH deh	biological process: biological reg
Translatior	3' exoribo	Exosome c	J	Translatior	exosome c	cellular component: cell (GO:000
--	Immunogl	--	S	Function u	TPA: hypo	--
Signal trar	Sodium:ne	Sodium-d	P	Inorganic i	sodium-de	molecular function: transporter a

--	Lipolysis st	Immunogl	U	Intracellula	immunogl	cellular component: cell (GO:000
--	HAUS aug	HAUS aug	U	Intracellula	HAUS aug	cellular component: cell (GO:000
--	--	--	U	Intracellula	epithelial-s	--
Posttransla	Cornichon	Protein co	U	Intracellula	protein co	cellular component: membrane (
Signal trar	LIM domai	LIM and se	S	Function u	LIM and se	cellular component: cell (GO:000
Function u	Predicted	--	S	Function u	transmeml	cellular component: cell (GO:000
Intracellula	Annexin	Annexin A	S	Function u	annexin A4	molecular function: binding (GO
Posttransla	PITH doma	Thioredoxi	O	Posttransla	thioredoxi	cellular component: cell (GO:000
Translatior	Ribosomal	60S riboso	J	Translatior	LOW QUA	molecular function: structural mo
Posttransla	DnaJ C ter	DnaJ hom	O	Posttransla	dnaJ hom	molecular function: binding (GO
Defense m	Membrane	Myeloid-a	S	Function u	uncharacte	cellular component: cell (GO:000
Cell cycle (	EB1-like C	Microtubu	Z	Cytoskelet	microtubu	cellular component: cell (GO:000
Nucleotide	Adenylate	UMP-CMF	F	Nucleotide	UMP-CMF	molecular function: catalytic activ
Function u	Domain of	Ester hydr	S	Function u	ester hydr	cellular component: cell (GO:000
Replicatior	Proliferatin	Proliferatin	L	Replicatior	proliferatin	biological process: biological reg
--	Immunogl	--	U	Intracellula	CD48 antig	biological process: biological reg
--	PDZ doma	PDZ and L	O	Posttransla	synaptopo	cellular component: cell (GO:000
Function u	Learning-a	Uncharact	S	Function u	uncharacte	molecular function: binding (GO
Function u	Cwf15/Cw	Spliceosor	A	RNA proce	spliceoson	biological process: metabolic pro
Coenzyme	Riboflavin	--	H	Coenzyme	riboflavin k	molecular function: catalytic activ
--	Protein-or	Mitochonc	S	Function u	PREDICTEI	biological process: metabolic pro
General fu	Intracellula	--	S	Function u	plasminog	molecular function: binding (GO
Posttransla	TCP-1/cpr	T-complex	O	Posttransla	PREDICTEI	cellular component: macromolec
Nucleotide	DeoC/LacI	Deoxyribo	F	Nucleotide	deoxyribos	molecular function: catalytic activ
Translatior	Ribosomal	60S riboso	J	Translatior	60S riboso	biological process: metabolic pro
Function u	Methylmal	--	S	Function u	cobalamin	biological process: metabolic pro
Lipid trans	Acyltransfe	--	I	Lipid trans	1-acyl-sn-	cellular component: membrane (
Function u	MAGE fam	Melanoma	S	Function u	melanoma	cellular component: membrane (
Cytoskelet	WD doma	Ribosome	J	Translatior	ribosome l	biological process: metabolic pro
--	Nmi/IFP 35	N-myc-int	S	Function u	N-myc-int	molecular function: transcription
Secondary	Cytochron	Cytochron	Q	Secondary	cytochrom	molecular function: binding (GO
Lipid trans	SCP-2 ster	Non-speci	I	Lipid trans	TPA: non-s	molecular function: binding (GO
Energy prc	Inorganic	Inorganic	C	Energy prc	inorganic	molecular function: binding (GO
--	Ribosomal	28S riboso	J	Translatior	28S riboso	cellular component: cell (GO:000
--	NAD(P)H-	Flavin redu	S	Function u	flavin redu	molecular function: catalytic activ
Cell cycle (	M-phase i	M-phase i	D	Cell cycle (	M-phase i	molecular function: catalytic activ
RNA proce	CAF1 fami	CCR4-NO	K	Transcripti	CCR4-NO	biological process: metabolic pro
Transcripti	Dpy-30 m	Protein dp	S	Function u	protein dp	cellular component: cell (GO:000
--	Double-st	--	L	Replicatior	swi5-depe	biological process: metabolic pro
Posttransla	Ubiquitin f	Polyubiqui	O	Posttransla	TPA: ubiq	cellular component: cell (GO:000
--	Geminin	--	L	Replicatior	geminin is	molecular function: binding (GO
Carbohydr	Galactokin	N-acetylga	G	Carbohydr	N-acetylga	molecular function: catalytic activ
Energy prc	2-oxoacid	Pyruvate d	C	Energy prc	pyruvate d	molecular function: catalytic activ
General fu	RNA trans	RNA trans	S	Function u	RNA trans	molecular function: binding (GO
Cytoskelet	Interferon-	Interferon-	K	Transcripti	interferon-	cellular component: cell (GO:000
--	Small cyto	C-X-C mo	O	Posttransla	C-X-C mo	cellular component: extracellular
--	T-cell surf	T-cell surf	U	Intracellula	T-cell surf	molecular function: signal transd
General fu	Tetratricop	Tetratricop	S	Function u	tetratricop	molecular function: binding (GO
Cell cycle (	DNA polyr	Replicatior	L	Replicatior	replication	molecular function: catalytic activ
Translatior	Brix domai	Ribosome	S	Function u	ribosome	biological process: cellular proce
Translatior	Initiation fa	Eukaryotic	J	Translatior	eukaryotic	molecular function: catalytic activ

Lipid trans	Enoyl-CoA	--	I	Lipid trans	methylglut	molecular function: catalytic acti
Function u	NIPSNAP	Protein Ni	S	Function u	PREDICTEI	cellular component: cell (GO:000
--	--	--	--	--	--	--
Amino aci	Pyridoxal-	Ornithine	E	Amino aci	ornithine c	molecular function: catalytic acti
Translatior	Mitochonc	28S ribosc	J	Translatior	28S riboso	molecular function: structural mo
Chromatin	Inhibitor o	Inhibitor o	B	Chromatin	inhibitor o	molecular function: binding (GO
Posttransla	Peptidase	GPI-anchc	O	Posttransla	GPI-anchc	molecular function: catalytic acti
--	MAPEG fa	Leukotrien	I	Lipid trans	leukotriene	biological process: metabolic pro
--	Mitotic-sp	--	O	Posttransla	mitotic-sp	cellular component: cell (GO:000
--	Interferon-	Interferon-	S	Function u	interferon	molecular function: binding (GO
Function u	Saccharop	Saccharop	S	Function u	Putative sa	cellular component: cell (GO:000
Transcripti	Transcripti	General tra	K	Transcripti	general tra	cellular component: cell (GO:000
--	Mis12-Mtw	--	S	Function u	PREDICTEI	cellular component: cell (GO:000
Posttransla	TCP-1/cpr	T-complex	O	Posttransla	T-complex	cellular component: macromolec
RNA proce	Mago nasl	Protein ma	A	RNA proce	protein ma	biological process: metabolic pro
Intracellula	Clathrin ac	AP-3 com	U	Intracellula	AP-3 com	biological process: localization (
Lipid trans	Calcineurir	Acid sphin	S	Function u	PREDICTEI	molecular function: catalytic acti
Cell cycle c	N-termina	Cyclin-A2	D	Cell cycle c	cyclin-A2	biological process: biological reg
Carbohydr	Major Faci	Monocarb	P	Inorganic i	monocarb	cellular component: cell (GO:000
--	Caldesmor	Non-musc	Z	Cytoskelet	non-musc	molecular function: binding (GO
Translatior	S25 ribosc	40S ribosc	J	Translatior	hypothetic	cellular component: cell (GO:000
Defense m	LMBR1-lik	Protein LM	U	Intracellula	PREDICTEI	cellular component: membrane (
General fu	Tetratricor	Interferon-	S	Function u	interferon-	molecular function: binding (GO
--	Nucleopla	Nucleophc	S	Function u	nucleophc	molecular function: binding (GO
Inorganic i	Ferroportir	--	U	Intracellula	solute carr	biological process: immune syste
--	Receptor a	Receptor a	U	Intracellula	receptor a	molecular function: signal transd
--	MAPEG fa	Microsoma	S	Function u	microsoma	molecular function: catalytic acti
General fu	Protein kir	Cyclin-dep	T	Signal tran	cyclin-dep	molecular function: catalytic acti
--	NOA36 pr	Zinc finger	S	Function u	zinc finger	cellular component: cell (GO:000
Function u	--	Integrator	S	Function u	integrator	biological process: cellular proce
General fu	Zinc knuck	--	S	Function u	PREDICTEI	molecular function: binding (GO
Posttransla	ATPase fa	26S protea	O	Posttransla	26S protea	molecular function: binding (GO
Posttransla	Hsp70 pro	Heat shocl	O	Posttransla	heat shock	cellular component: macromolec
Function u	Ubiquitin f	Ubiquitin-	O	Posttransla	ubiquitin-f	biological process: metabolic pro
--	--	EP300-inte	K	Transcripti	EP300-inte	biological process: biological reg
Cytoskelet	Tubulin/Ft	Tubulin alp	Z	Cytoskelet	PREDICTEI	molecular function: catalytic acti
Cell cycle c	Intermedia	Prelamin-/	S	Function u	lamin-B1 i	cellular component: cell (GO:000
--	TLD	Interferon-	S	Function u	interferon-	biological process: immune syste
Cell cycle c	Inhibitor o	Baculovira	O	Posttransla	baculovira	biological process: cellular proce
--	zf-RING o	--	S	Function u	BRCA1-as	molecular function: binding (GO
Posttransla	Cyclophilir	Peptidylpr	O	Posttransla	peptidyl-p	molecular function: catalytic acti
General fu	alpha/beta	1-acylglyc	S	Function u	1-acylglyc	molecular function: catalytic acti
Intracellula	SH2 doma	SH2 doma	S	Function u	SH2 doma	cellular component: cell (GO:000
General fu	Zinc-finge	Zinc finger	S	Function u	zinc finger	molecular function: binding (GO
Transcripti	Conserved	GPN-loop	S	Function u	GPN-loop	molecular function: binding (GO
Inorganic i	Eukaryotic	Voltage-d	U	Intracellula	voltage-de	cellular component: cell (GO:000
Posttransla	Ubiquitin-	Small ubiq	O	Posttransla	small ubiq	cellular component: cell (GO:000
Energy prc	Mitochonc	Mitochonc	S	Function u	peroxisom	molecular function: transporter a
Cell cycle c	Cyclin, N-t	G2/mitotic	D	Cell cycle c	G2/mitotic	cellular component: cell (GO:000
Posttransla	ATPase fa	26S protea	O	Posttransla	PREDICTEI	cellular component: cell (GO:000
Nucleotide	Permease	--	F	Nucleotide	solute carr	cellular component: membrane (

Signal trar	BTG family	Protein BT	S	Function u	protein To	molecular function: transcription
General fu	RNA recog	--	S	Function u	RNA-bind	molecular function: binding (GO
Posttransla	CDK-activa	--	K	Transcripti	CDK-activa	cellular component: cell (GO:000
RNA proce	RNA recog	U1 small n	S	Function u	U2 small n	biological process: metabolic pro
Defense m	Serpin (ser	Plasminog	O	Posttransla	neuroserp	molecular function: molecular fu
Posttransla	Cyclophilin	Spliceosor	O	Posttransla	LOW QUA	biological process: metabolic pro
Function u	AhpC/TSA	Peroxiredo	O	Posttransla	redox-reg	cellular component: cell (GO:000
--	Renal canc	Uncharact	S	Function u	uncharacte	--
Signal trar	LIM domai	Four and a	O	Posttransla	four and a	biological process: multicellular o
Replicatior	RNA recog	RNA exonu	L	Replicatior	RNA exonu	molecular function: binding (GO
Signal trar	Calcium-b	Latent-tra	A	RNA proce	latent-trar	cellular component: extracellular
--	Domain of	Maturin O	S	Function u	maturin iso	cellular component: cell (GO:000
Translatior	Ribosomal	60S riboso	J	Translatior	60S riboso	biological process: metabolic pro
Posttransla	TCP-1/cpr	T-complex	O	Posttransla	T-complex	cellular component: macromolec
Signal trar	LIM domai	Four and a	O	Posttransla	four and a	biological process: multicellular o
Translatior	'Cold-shoc	Y-box-bin	K	Transcripti	Y-box-bin	molecular function: binding (GO
Posttransla	--	RNA-bind	S	Function u	RNA-bind	molecular function: binding (GO
--	Connexin	Gap juncti	D	Cell cycle c	gap junctio	molecular function: transporter a
Function u	Zinc finger	RING finge	O	Posttransla	E3 ubiquiti	cellular component: cell (GO:000
Translatior	Phenylalar	Phenylalar	J	Translatior	phenylalar	molecular function: binding (GO
Signal trar	Leucine ric	Ras suppre	S	Function u	ras suppre	molecular function: binding (GO
Signal trar	Sushi repe	P-selectin	U	Intracellula	P-selectin	molecular function: binding (GO
Function u	Domain of	Striatin-int	S	Function u	striatin-int	cellular component: cell (GO:000
--	Stage VI sp	--	H	Coenzyme	decapreny	--
--	Immunogl	Leukocyte	U	Intracellula	killer cell ir	cellular component: membrane (
Translatior	Ribosomal	40S riboso	J	Translatior	40S riboso	molecular function: structural mo
Coenzyme	Polyprenyl	--	H	Coenzyme	decapreny	molecular function: catalytic activ
General fu	Guanylate	Guanylate	S	Function u	PREDICTED	molecular function: catalytic activ
Energy pro	Cytochrom	Cytochrom	C	Energy pro	cytochrom	cellular component: cell (GO:000
Cell wall/m	Scramblas	Phospholip	S	Function u	PREDICTED	molecular function: transporter a
Carbohydr	Major intri	Aquaporin	G	Carbohydr	aquaporin	molecular function: transporter a
--	Domain of	--	S	Function u	PREDICTED	--
--	--	--	--	--	--	--
General fu	E2 domain	Amyloid-b	U	Intracellula	amyloid-b	molecular function: binding (GO
General fu	Protein of	Obg-like A	J	Translatior	obg-like A	molecular function: binding (GO
Function u	AIG2-like f	Gamma-g	S	Function u	gamma-gl	molecular function: catalytic activ
RNA proce	DEAD/DEA	ATP-depe	L	Replicatior	probable A	molecular function: binding (GO
Translatior	Mitochonc	28S riboso	S	Function u	28S riboso	molecular function: structural mo
Cell cycle c	Translatior	Translatior	S	Function u	hypothetic	cellular component: cell (GO:000
General fu	7 transmer	C-C chem	U	Intracellula	C-C chem	biological process: cellular proce
--	--	Placenta-s	S	Function u	placenta-s	--
Function u	Uncharact	KxDL moti	S	Function u	kxDL motif	cellular component: cell (GO:000
Translatior	Elongatior	Elongatior	J	Translatior	elongatior	molecular function: binding (GO
Extracellula	von Willeb	Collagen a	U	Intracellula	collagen a	biological process: biological reg
--	--	--	S	Function u	hypothetic	--
Function u	Breast carc	Pre-mRNA	A	RNA proce	hypothetic	biological process: metabolic pro
Nucleotide	Adenylate	Adenylate	F	Nucleotide	adenylate	cellular component: cell (GO:000
Signal trar	Lectin C-ty	--	S	Function u	PREDICTED	cellular component: membrane (
Signal trar	Ras family	Ras-relate	S	Function u	PREDICTED	molecular function: catalytic activ
Intracellula	PRELI-like	PRELI dom	S	Function u	PRELI dom	cellular component: cell (GO:000
--	Cyclin, N-t	G1/S-spec	D	Cell cycle c	cyclin-J [B	cellular component: other organ

Cytoskelet	Immunogl	Myosin lig	S	Function u	myosin lig	cellular component: cell (GO:000
Translatior	eIF3 subur	Eukaryotic	J	Translatior	eukaryotic	biological process: cellular proce
General fu	BTB/POZ c	BTB/POZ c	S	Function u	BTB/POZ c	biological process: cellular proce
General fu	Myo-inosi	Inositol ox	S	Function u	inositol ox	molecular function: binding (GO
--	Small cyto	Platelet fac	U	Intracellula	platelet fac	cellular component: extracellular
General fu	Tetratricop	Interferon-	S	Function u	interferon-	molecular function: binding (GO
Function u	Yos1-like	Immediate	U	Intracellula	immediate	cellular component: cell (GO:000
--	--	--	--	--	Zinc finger	molecular function: binding (GO
Energy prc	NifU-like N	--	C	Energy prc	iron-sulfur	molecular function: binding (GO
Inorganic i	HCO3- tra	Anion exch	U	Intracellula	band 3 ani	molecular function: transporter a
--	2'-5'-oligc	2'-5'-oligc	O	Posttransla	RecName:	molecular function: catalytic acti
--	--	--	F	Nucleotide	endonucle	molecular function: binding (GO
Function u	Nse4 C-te	EP300-inte	S	Function u	EP300-inte	cellular component: cell (GO:000
--	CUB doma	Platelet-de	A	RNA proce	platelet-de	molecular function: binding (GO
--	Eukaryotic	Transcoba	S	Function u	transcobal	cellular component: extracellular
Chromatin	Nucleosom	Nucleosom	S	Function u	nucleosom	cellular component: cell (GO:000
Chromatin	Nucleosom	Nucleosom	S	Function u	nucleosom	cellular component: cell (GO:000
Signal trar	Lectin C-ty	--	S	Function u	killer cell le	cellular component: membrane (
Cytoskelet	Tubulin/Fts	Tubulin be	Z	Cytoskelet	tubulin be	biological process: cellular proce
Replicatior	Aspartyl pı	Nuclear re	O	Posttransla	nuclear rec	molecular function: catalytic acti
--	--	--	--	--	--	--
Signal trar	GGL doma	Guanine n	T	Signal trar	guanine nu	molecular function: catalytic acti
Posttransla	Ubiquitin c	Ubiquitin c	O	Posttransla	ubiquitin c	molecular function: catalytic acti
--	2'-5'-oligc	2'-5'-oligc	O	Posttransla	TPA: 2',5'-	molecular function: catalytic acti
Extracellula	Plexin repe	--	S	Function u	plexin don	biological process: development
General fu	F-box-like	F-box only	S	Function u	PREDICTED	cellular component: cell (GO:000
Translatior	Translatior	Eukaryotic	J	Translatior	eukaryotic	molecular function: binding (GO
--	Immunogl	--	S	Function u	TPA: hypo	--
--	SOUL hem	Heme-bin	O	Posttransla	heme-bind	molecular function: binding (GO
Posttransla	Ubiquitin f	Ubiquitin-	O	Posttransla	ubiquitin-l	molecular function: binding (GO
Transcripti	Pyridoxam	--	S	Function u	PREDICTED	cellular component: extracellular
General fu	YhhN fami	Lysoplasm	S	Function u	lysoplasma	cellular component: cell (GO:000
--	Alpha-hae	Alpha-her	O	Posttransla	PREDICTED	biological process: cellular proce
General fu	PCI domai	Eukaryotic	J	Translatior	eukaryotic	biological process: cellular proce
General fu	Guanylate	Guanylate	S	Function u	guanylate-	molecular function: catalytic acti
--	Brain expr	Transcripti	K	Transcripti	transcriptic	molecular function: binding (GO
Lipid trans	Choline/Ca	Peroxisom	S	Function u	peroxisom	molecular function: catalytic acti
--	Domain of	--	S	Function u	uncharacte	--
Energy prc	ATP-grasp	Succinate-	C	Energy prc	succinate-	molecular function: binding (GO
Translatior	eIF4-gamr	Basic leuci	S	Function u	basic leuci	cellular component: cell (GO:000
--	Apolipopr	--	S	Function u	apolipopr	cellular component: extracellular
Signal trar	Lectin C-ty	C-type lec	U	Intracellula	C-type lec	molecular function: binding (GO
--	Putative D	Protein SLI	S	Function u	protein SL	molecular function: catalytic acti
Energy prc	Mitochonc	ATP syntha	C	Energy prc	ATP syntha	cellular component: cell (GO:000
General fu	Guanylate	Guanylate	S	Function u	guanylate-	molecular function: catalytic acti
--	--	--	S	Function u	guanylate-	molecular function: catalytic acti
--	Apolipopr	--	U	Intracellula	apolipopr	cellular component: extracellular
General fu	Cytochron	Membrane	S	Function u	PREDICTED	cellular component: membrane (
Posttransla	Skp1 famil	S-phase ki	O	Posttransla	hypothetic	cellular component: cell (GO:000
--	TNFR/NGF	--	S	Function u	tumor nec	cellular component: membrane (
Translatior	Ribosomal	39S riboso	J	Translatior	39S riboso	molecular function: binding (GO

General fu	BTB/POZ c	Ras-relate	S	Function u	rho-relate	molecular function: catalytic acti
Transcripti	YEATS fam	--	K	Transcripti	YEATS dor	biological process: biological reg
--	--	--	--	--	--	--
RNA proce	--	R3H doma	A	RNA proce	cAMP-reg	molecular function: binding (GO
Cytoskelet	Autophagy	Gamma-a	U	Intracellula	PREDICTEI	cellular component: cell (GO:000
Lipid trans	START dor	Phosphatic	S	Function u	phosphatic	molecular function: binding (GO
Function u	Yos1-like	Immediate	U	Intracellula	immediate	cellular component: cell (GO:000
Signal tran	Sema dom	Semaphor	S	Function u	semaphori	cellular component: membrane (
--	PMP-22/E	Voltage-d	U	Intracellula	voltage-de	molecular function: transporter a
Function u	HAP1 N-te	--	U	Intracellula	trafficking	molecular function: binding (GO
--	--	--	S	Function u	hypothetic	cellular component: cell (GO:000
General fu	7 transmer	Probable (	U	Intracellula	probable (	molecular function: signal transd
--	TMEM119	--	S	Function u	transmeml	biological process: multicellular o
General fu	Dienelacto	Testis-exp	S	Function u	testis-expr	molecular function: catalytic acti
Nucleotide	Molybdop	Aldehyde c	F	Nucleotide	aldehyde c	molecular function: catalytic acti
Lipid trans	Acyl CoA k	Acyl-CoA-	I	Lipid trans	acyl-CoA-	molecular function: binding (GO
Carbohydr	Major Faci	Monocarb	G	Carbohydr	monocarb	cellular component: membrane (
--	--	--	--	--	--	--
Signal tran	Integrin be	Integrin be	U	Intracellula	integrin be	biological process: biological reg
Coenzyme	Porphobili	Porphobili	H	Coenzyme	porphobili	molecular function: catalytic acti
Translatior	'Cold-shoc	--	K	Transcripti	cold shock	molecular function: binding (GO
Energy pro	Globin	Hemoglob	C	Energy pro	hemoglob	molecular function: transporter a
General fu	DHHC palı	Palmitoyltı	S	Function u	palmitoyltı	cellular component: cell (GO:000
--	Histidine-r	--	S	Function u	histidine-r	cellular component: membrane (
--	F-box-like	--	S	Function u	F-box only	molecular function: binding (GO
--	XK-relatec	Membrane	U	Intracellula	membrane	cellular component: membrane (
--	Testis-exp	--	S	Function u	testis-expr	biological process: reproduction
Cell cycle c	Sister chro	--	S	Function u	sister chro	cellular component: cell (GO:000
Defense m	Haemolysi	--	S	Function u	monocyte	cellular component: membrane (
Secondary	ABC transp	Multidrug	V	Defense m	multidrug	molecular function: binding (GO
Signal tran	Lectin C-ty	Killer cell le	S	Function u	killer cell le	cellular component: membrane (
--	--	--	--	--	uncharacte	--
Function u	Tho comp	THO comp	S	Function u	THO comp	cellular component: cell (GO:000
--	--	--	--	--	--	--
Mobilome	Myelin pro	Neuronal r	U	Intracellula	PREDICTEI	biological process: cellular proce
--	APOBEC2	DNA dC->	L	Replicatior	TPA: apolip	cellular component: cell (GO:000
--	--	Transmem	S	Function u	transmeml	cellular component: membrane (
Intracellula	Ras family	Ras-relate	S	Function u	ras-relatec	molecular function: catalytic acti
Defense m	Serpin (ser	Leukocyte	O	Posttransla	serpin B4-	cellular component: extracellular
General fu	Immunogl	Sialoadhes	U	Intracellula	sialoadhes	cellular component: cell (GO:000
Extracellula	Galactosid	Galectin-re	S	Function u	galectin-re	cellular component: cell (GO:000
General fu	N-termina	Prostaglan	S	Function u	TPA: prost	cellular component: cell (GO:000
--	Amidinotra	Glycine an	E	Amino acio	glycine am	cellular component: cell (GO:000
General fu	Zinc-finge	--	S	Function u	zinc finger	molecular function: nucleic acid
General fu	CTLH/CRA	Ran-bindiı	S	Function u	Ran-bindiı	cellular component: cell (GO:000
Lipid trans	alpha/beta	Mesoderm	S	Function u	mesoderm	molecular function: catalytic acti
--	--	--	--	--	--	--
--	--	--	--	--	hypothetic	--
Posttransla	Ubiquitin c	Ubiquitin c	O	Posttransla	ubiquitin c	molecular function: catalytic acti
General fu	Guanylate	Guanylate	S	Function u	guanylate-	molecular function: catalytic acti
--	Small cyto	Interleukin	O	Posttransla	interleukin	molecular function: binding (GO

--	Protein of	Transmem	S	Function u	post-GPI a	cellular component: membrane (
--	PCNA-ass	PCNA-ass	K	Transcripti	PCNA-ass	biological process: cellular proce
--	Immunogl	--	U	Intracellula	PREDICTEI	cellular component: membrane (
--	Scavenger	Antigen W	S	Function u	antigen W	molecular function: molecular tra
--	2'-5'-oligc	2'-5'-oligc	O	Posttransla	TPA: 2',5'-	molecular function: catalytic acti
--	--	--	--	--	uncharacte	--
Secondary	ABC transp	Cystic fibrc	V	Defense m	uncharacte	molecular function: binding (GO
General fu	Cytohesin	--	S	Function u	FERM dom	cellular component: cell (GO:000
Posttransla	Ubiquitin f	Protein C1	O	Posttransla	uncharacte	biological process: response to s
--	Uncharact	Protein FA	S	Function u	protein FA	cellular component: cell (GO:000
Function u	Cadherin c	Protocadh	U	Intracellula	cadherin-r	molecular function: binding (GO
Posttransla	Ring finge	E3 ubiquiti	O	Posttransla	E3 ubiquiti	cellular component: membrane (
Defense m	Serpin (ser	Leukocyte	O	Posttransla	serpin B3-	cellular component: extracellular
--	Right hanc	--	S	Function u	SHC SH2 c	biological process: cellular proce
--	RNA recog	RNA-bind	S	Function u	PREDICTEI	biological process: biological reg
--	--	--	--	--	--	--
Posttransla	Peptidase	Glutaminy	S	Function u	glutaminyl	molecular function: binding (GO
--	Trefoil (P-t	Trefoil fact	O	Posttransla	PREDICTEI	molecular function: binding (GO
Nucleotide	Permease	--	F	Nucleotide	uncharacte	cellular component: membrane (
--	Immunogl	--	S	Function u	hypothetic	--
Intracellula	Dynamin c	Interferon-	S	Function u	interferon-	biological process: cellular proce
--	Immunogl	--	S	Function u	TPA: TRD@	--
Signal trar	Putative ac	Dematin C	S	Function u	PREDICTEI	molecular function: binding (GO
Carbohydr	Major intri	Aquaporin	G	Carbohydr	Aquaporin	biological process: multicellular c
--	TCP-1/cpr	Bardet-Bie	O	Posttransla	Bardet-Bie	molecular function: binding (GO
--	Apolipopr	--	S	Function u	apolipopro	cellular component: extracellular
Intracellula	GDP/GTP (	Guanine n	S	Function u	guanine nu	molecular function: molecular fu
Posttransla	SPRY-assc	Tripartite r	S	Function u	TPA: tripar	cellular component: cell (GO:000
Posttransla	FKBP-type	Peptidyl-p	O	Posttransla	peptidyl-p	molecular function: catalytic acti
Carbohydr	Carbohydr	Glycogen p	G	Carbohydr	PREDICTEI	biological process: metabolic pro
--	Intercellula	Intercellula	U	Intracellula	intercellula	cellular component: membrane (
--	--	--	--	--	--	--
Inorganic i	Copper/zir	Extracellula	P	Inorganic i	extracellula	biological process: response to s
--	Transmem	--	S	Function u	transmeml	--
General fu	Zinc finger	Krueppel-	S	Function u	Krueppel-l	cellular component: cell (GO:000
Function u	Domain of	Ubiquitin c	S	Function u	PREDICTEI	cellular component: cell (GO:000
Replicatior	ParB-like r	--	S	Function u	sulfiredoxi	molecular function: catalytic acti
Coenzyme	Aminotran	5-aminole	H	Coenzyme	5-aminole	biological process: response to s
Secondary	ABC transp	Multidrug	V	Defense m	multidrug	molecular function: binding (GO
Energy prc	Globin	Hemoglob	C	Energy prc	hemoglob	molecular function: transporter a
Nucleotide	Thymidyla	--	F	Nucleotide	UMP-CMF	molecular function: catalytic acti
General fu	BRO1-like	Rhophilin-	S	Function u	rhophilin-2	biological process: cellular proce
Energy prc	Globin	Hemoglob	C	Energy prc	hemoglob	molecular function: transporter a
Lipid trans	Phospholip	Phospholip	I	Lipid trans	phospholip	biological process: immune syste
--	Beta/Gam	Gamma-cr	S	Function u	beta-cryst	biological process: development
Transcripti	N-termina	Homeobo	K	Transcripti	PREDICTEI	molecular function: binding (GO
Posttransla	SPRY-assc	Butyrophil	S	Function u	erythroid r	cellular component: membrane (
General fu	Zinc carbo	Carboxype	E	Amino acic	inactive ca	molecular function: catalytic acti
--	Radical SA	Radical S-a	O	Posttransla	radical S-a	cellular component: cell (GO:000
--	2'-5'-oligc	2'-5'-oligc	O	Posttransla	2',5'-oligo	molecular function: catalytic acti
Energy prc	Globin	Hemoglob	C	Energy prc	hemoglob	molecular function: transporter a

--	Bone marr	--	D	Cell cycle c	bone marr	cellular component: cell (GO:000
--	Bone marr	--	D	Cell cycle c	bone marr	cellular component: cell (GO:000
--	MHC-I fan	T-cell surfa	U	Intracellula	TPA: CD1E	biological process: biological reg
--	--	--	--	--	--	--
Posttransla	SPRY-assc	E3 ubiquiti	S	Function u	PREDICTEI	biological process: metabolic pro
Signal trar	wnt family	Protein Wr	O	Posttransla	hypothetic	biological process: development
Signal trar	Protein kir	STE20-rela	T	Signal trar	STE20-rela	biological process: cellular proce
--	Mitochonc	ATPase inh	K	Transcripti	ATPase inh	biological process: biological reg
General fu	Eukaryotic	Carbonic a	P	Inorganic i	carbonic a	biological process: development
--	Small VCP.	Small VCP.	U	Intracellula	small VCP/	molecular function: binding (GO
--	COMM do	COMM do	S	Function u	COMM do	biological process: biological reg
--	Neurokinir	Tachykinin	O	Posttransla	tachykinin	biological process: cellular proce
--	NADP oxic	Metallorec	S	Function u	metallorec	cellular component: cell (GO:000
Inorganic i	Glutathion	Chloride ir	U	Intracellula	chloride in	molecular function: catalytic activ
Lipid trans	START dor	Phosphatic	U	Intracellula	phosphatic	molecular function: binding (GO
--	--	--	--	--	glycophori	cellular component: membrane (
Energy prc	Globin	Hemoglob	C	Energy prc	hemoglob	molecular function: transporter a
Amino acic	Amino acic	Cationic ar	E	Amino acic	cationic ar	biological process: single-organi
Carbohydr	Sugar (anc	Solute carr	G	Carbohydr	TPA: solute	molecular function: transporter a
--	--	--	S	Function u	small integ	cellular component: membrane (
--	--	--	--	--	--	--
Signal trar	Class II Alc	Beta-addu	G	Carbohydr	beta-addu	molecular function: structural mc
--	--	--	--	--	hypothetic	molecular function: catalytic activ
Defense m	TB2/DP1, I	Receptor e	U	Intracellula	receptor e	cellular component: membrane (
Secondary	short chair	Carbonyl r	S	Function u	20-beta-h	biological process: reproduction
--	SSXRD mc	--	K	Transcripti	protein SS	molecular function: binding (GO
Inorganic i	ChaC-like	Putative gl	P	Inorganic i	PREDICTEI	molecular function: catalytic activ
Translatior	Ribosomal	40S riboso	J	Translatior	40S riboso	molecular function: structural mc
--	Ectoderma	--	S	Function u	PREDICTEI	cellular component: membrane (
--	Transgluta	Protein 4.2	S	Function u	erythrocyt	biological process: cellular proce
Energy prc	Globin	Hemoglob	C	Energy prc	hemoglob	molecular function: transporter a
--	SSXRD mc	--	K	Transcripti	protein SS	molecular function: binding (GO
Defense m	Membrane	Myeloid-a	S	Function u	uncharacte	cellular component: cell (GO:000
--	--	--	--	--	--	--
Amino acic	Arginase fa	Arginase-1	E	Amino acic	arginase-1	biological process: metabolic pro
Energy prc	Globin	Hemoglob	C	Energy prc	hemoglob	molecular function: transporter a
--	--	--	--	--	--	--
Signal trar	SAM doma	Phosphatic	S	Function u	ankyrin rep	biological process: localization (C

:0005488);; cellular component: cell (GO:0005623);; cellular component: organelle (GO:0043226);; c
:0005488);; biological process: metabolic process (GO:0008152);; biological process: cellular proces

vity (GO:0003824);; biological process: metabolic process (GO:0008152);; cellular component: extra
)5623);; cellular component: organelle (GO:0043226);; cellular component: cell part (GO:0044464);;

ocess (GO:0008152);; biological process: cellular process (GO:0009987);; molecular function: catalyt

vity (GO:0003824);; biological process: metabolic process (GO:0008152);; molecular function: bindi
binding transcription factor activity (GO:0001071);; biological process: biological regulation (GO:00
organismal process (GO:0032501);; biological process: developmental process (GO:0032502);; biolo
em process (GO:0002376);; biological process: response to stimulus (GO:0050896)
vity (GO:0003824);; biological process: metabolic process (GO:0008152);; biological process: cellula
julation (GO:0065007);; molecular function: nucleic acid binding transcription factor activity (GO:00
)5623);; cellular component: macromolecular complex (GO:0032991);; cellular component: cell part
:0005488);; biological process: cellular process (GO:0009987);; biological process: signaling (GO:00
ocess (GO:0008152);; biological process: cellular process (GO:0009987);; biological process: biologi
vity (GO:0003824);; biological process: metabolic process (GO:0008152)
vity (GO:0003824);; biological process: metabolic process (GO:0008152);; biological process: cellula
:0005488);; cellular component: cell (GO:0005623);; cellular component: membrane (GO:0016020);
:0005488);; cellular component: membrane (GO:0016020);; cellular component: membrane part (G

)5623);; cellular component: membrane (GO:0016020);; cellular component: organelle (GO:004322
vity (GO:0003824);; molecular function: binding (GO:0005488)
lucer activity (GO:0004871);; molecular function: molecular transducer activity (GO:0060089);; biolo
)5623);; cellular component: membrane (GO:0016020);; cellular component: organelle (GO:004322
:0005488);; biological process: biological regulation (GO:0065007)
nction regulator (GO:0098772);; biological process: biological regulation (GO:0065007);; cellular co
)5623);; cellular component: membrane (GO:0016020);; cellular component: organelle (GO:004322
julation (GO:0065007);; molecular function: binding (GO:0005488);; cellular component: cell (GO:00
:0005488);; cellular component: membrane (GO:0016020);; cellular component: membrane part (G
:0005488);; molecular function: nucleic acid binding transcription factor activity (GO:0001071);; bio
vity (GO:0003824);; molecular function: binding (GO:0005488);; cellular component: cell (GO:00056

:0005488);; molecular function: catalytic activity (GO:0003824);; cellular component: cell (GO:00056
:0005488);; cellular component: cell (GO:0005623);; cellular component: organelle (GO:0043226);; c
binding transcription factor activity (GO:0001071);; biological process: biological regulation (GO:00
julation (GO:0065007);; biological process: immune system process (GO:0002376);; biological proc
stimulus (GO:0050896);; biological process: multi-organism process (GO:0051704);; biological proc
ss (GO:0009987);; biological process: single-organism process (GO:0044699);; biological process: i
region (GO:0005576);; cellular component: extracellular region part (GO:0044421);; biological pro

:0005488)

em process (GO:0002376);; biological process: response to stimulus (GO:0050896)
activity (GO:0005215);; biological process: localization (GO:0051179);; cellular component: membra
vity (GO:0003824);; biological process: metabolic process (GO:0008152)
:0005488)

(GO:0016020);; cellular component: membrane part (GO:0044425)
ctivity (GO:0005215);; biological process: localization (GO:0051179);; biological process: biological
ucer activity (GO:0004871);; molecular function: molecular transducer activity (GO:0060089);; biolo
:0005488);; biological process: cellular process (GO:0009987);; biological process: cellular compon
5623);; cellular component: organelle (GO:0043226);; cellular component: organelle part (GO:004
lecule activity (GO:0005198);; cellular component: cell (GO:0005623);; cellular component: organe

(GO:0016020)
binding transcription factor activity (GO:0001071);; biological process: biological regulation (GO:0
(GO:0016020);; cellular component: membrane part (GO:0044425)

5623);; cellular component: membrane (GO:0016020);; cellular component: organelle (GO:004322
cess (GO:0008152);; biological process: single-organism process (GO:0044699);; molecular functi

:0005488)
vity (GO:0003824);; biological process: metabolic process (GO:0008152);; biological process: cellul
5623);; cellular component: membrane (GO:0016020);; cellular component: macromolecular comp
5623);; cellular component: membrane-enclosed lumen (GO:0031974);; cellular component: orga
5623);; cellular component: organelle (GO:0043226);; cellular component: organelle part (GO:004
ss (GO:0009987);; biological process: single-organism process (GO:0044699);; molecular function:
rganismal process (GO:0032501);; biological process: developmental process (GO:0032502);; biol
(GO:0016020);; cellular component: membrane part (GO:0044425)

5623);; cellular component: membrane (GO:0016020);; cellular component: cell part (GO:0044464)
(GO:0016020);; cellular component: membrane part (GO:0044425)
cess (GO:0008152);; biological process: single-organism process (GO:0044699)
ucer activity (GO:0004871);; molecular function: molecular transducer activity (GO:0060089);; biolo
ss (GO:0009987);; biological process: developmental process (GO:0032502);; biological process: si

ucer activity (GO:0004871);; molecular function: molecular transducer activity (GO:0060089);; biolo
ucer activity (GO:0004871);; molecular function: molecular transducer activity (GO:0060089);; biolo
vity (GO:0003824);; biological process: metabolic process (GO:0008152);; cellular component: extra
vity (GO:0003824);; biological process: metabolic process (GO:0008152);; cellular component: cell

(GO:0016020);; cellular component: membrane part (GO:0044425)
rganismal process (GO:0032501);; biological process: developmental process (GO:0032502);; biol
vity (GO:0003824);; biological process: metabolic process (GO:0008152)
ulation (GO:0065007);; molecular function: catalytic activity (GO:0003824);; biological process: me

(GO:0016020);; cellular component: membrane part (GO:0044425)
ucer activity (GO:0004871);; molecular function: molecular transducer activity (GO:0060089);; biolo
ucer activity (GO:0004871);; molecular function: molecular transducer activity (GO:0060089);; biolo
(GO:0016020);; cellular component: membrane part (GO:0044425);; biological process: biological
cess (GO:0008152);; biological process: cellular process (GO:0009987);; biological process: single-
ss (GO:0009987);; biological process: signaling (GO:0023052);; biological process: single-organism
(GO:0016020);; cellular component: membrane part (GO:0044425);; biological process: biological
5623);; cellular component: cell part (GO:0044464);; biological process: localization (GO:0051179);

vity (GO:0003824);; biological process: cellular process (GO:0009987);; biological process: biologica

ocess (GO:0008152);; biological process: cellular process (GO:0009987);; biological process: behavi
gulation (GO:0065007);; biological process: metabolic process (GO:0008152);; biological process: ce
:0005488)
:0005488);; biological process: metabolic process (GO:0008152);; biological process: cellular proces
:0005488)
vity (GO:0003824);; molecular function: binding (GO:0005488)
vity (GO:0003824);; biological process: metabolic process (GO:0008152);; biological process: cellula
vity (GO:0003824);; biological process: metabolic process (GO:0008152);; cellular component: mem
gulation (GO:0065007);; cellular component: cell (GO:0005623);; cellular component: cell part (GO:0
olecule activity (GO:0005198);; cellular component: cell (GO:0005623);; cellular component: cell par
vity (GO:0003824);; molecular function: structural molecule activity (GO:0005198);; molecular funct
lucer activity (GO:0004871);; molecular function: molecular transducer activity (GO:0060089);; biolo
ism process (GO:0044699);; biological process: localization (GO:0051179);; molecular function: cata
vity (GO:0003824);; molecular function: structural molecule activity (GO:0005198);; molecular funct
vity (GO:0003824);; biological process: metabolic process (GO:0008152);; biological process: single
ess (GO:0009987);; biological process: cellular component organization or biogenesis (GO:0071840
binding transcription factor activity (GO:0001071);; biological process: biological regulation (GO:00
ess (GO:0009987);; biological process: biological regulation (GO:0065007);; cellular component: me
:0005488);; cellular component: extracellular region (GO:0005576)
:0005488);; cellular component: cell (GO:0005623);; cellular component: membrane (GO:0016020);
(GO:0016020);; cellular component: membrane part (GO:0044425)
:0005488);; cellular component: cell (GO:0005623);; cellular component: cell part (GO:0044464);; ce
vity (GO:0003824);; molecular function: binding (GO:0005488);; cellular component: cell (GO:00056
gulation (GO:0065007)
05623);; cellular component: membrane (GO:0016020);; cellular component: membrane part (GO:0

gulation (GO:0065007);; molecular function: binding (GO:0005488);; cellular component: cell (GO:00
vity (GO:0003824);; biological process: metabolic process (GO:0008152);; biological process: cellula
(GO:0000003);; biological process: cellular process (GO:0009987);; biological process: reproductive
:0005488)

lucer activity (GO:0004871);; molecular function: molecular transducer activity (GO:0060089);; biolo
lucer activity (GO:0004871);; molecular function: molecular transducer activity (GO:0060089);; biolo

05623);; cellular component: organelle (GO:0043226);; cellular component: organelle part (GO:0044
vity (GO:0003824);; biological process: biological regulation (GO:0065007);; cellular component: ce
05623);; cellular component: organelle (GO:0043226);; cellular component: organelle part (GO:0044
gulation (GO:0065007);; molecular function: molecular function regulator (GO:0098772)
ess (GO:0009987);; biological process: biological regulation (GO:0065007)
binding transcription factor activity (GO:0001071);; biological process: biological regulation (GO:00
05623);; cellular component: membrane (GO:0016020);; cellular component: macromolecular comp

em process (GO:0002376);; biological process: response to stimulus (GO:0050896);; cellular compo
:0005488)
region (GO:0005576)

vity (GO:0003824);; biological process: metabolic process (GO:0008152)

)5623);; cellular component: membrane (GO:0016020);; cellular component: organelle (GO:004322(
:0005488);; biological process: cellular process (GO:0009987);; biological process: single-organism
)5623);; cellular component: organelle (GO:0043226);; cellular component: cell part (GO:0044464);;
vity (GO:0003824);; biological process: metabolic process (GO:0008152);; biological process: cellula
lucer activity (GO:0004871);; molecular function: molecular transducer activity (GO:0060089);; biolo

vity (GO:0003824);; molecular function: structural molecule activity (GO:0005198);; molecular funct
hesion (GO:0022610);; biological process: cellular process (GO:0009987);; biological process: signal
vity (GO:0003824);; molecular function: binding (GO:0005488);; biological process: cellular process
)5623);; cellular component: macromolecular complex (GO:0032991);; cellular component: cell part
vity (GO:0003824);; biological process: metabolic process (GO:0008152);; cellular component: mem

em process (GO:0002376);; biological process: cellular process (GO:0009987);; biological process: lo
activity (GO:0005215);; biological process: single-organism process (GO:0044699);; biological proce
(GO:0016020);; cellular component: membrane part (GO:0044425)

lucer activity (GO:0004871);; molecular function: molecular transducer activity (GO:0060089);; biolo
)5623);; cellular component: organelle (GO:0043226);; cellular component: organelle part (GO:0044
vity (GO:0003824);; molecular function: binding (GO:0005488);; cellular component: cell (GO:00056
activity (GO:0005215);; biological process: localization (GO:0051179);; cellular component: cell (GO:
em process (GO:0002376);; biological process: cellular process (GO:0009987);; biological process: lo
gulation (GO:0065007)
gulation (GO:0065007);; molecular function: structural molecule activity (GO:0005198);; molecular fu
vity (GO:0003824);; biological process: metabolic process (GO:0008152);; cellular component: mem

)5623);; cellular component: cell part (GO:0044464);; molecular function: catalytic activity (GO:0003

sm process (GO:0051704)
)5623);; cellular component: organelle (GO:0043226);; cellular component: cell part (GO:0044464);;
hesion (GO:0022610);; biological process: cellular process (GO:0009987);; biological process: signal
)5623);; cellular component: membrane (GO:0016020);; cellular component: membrane part (GO:0
gulation (GO:0065007);; biological process: response to stimulus (GO:0050896);; biological process:
)5623);; cellular component: membrane (GO:0016020);; cellular component: cell part (GO:0044464)
:0005488);; cellular component: cell (GO:0005623);; cellular component: organelle (GO:0043226);; c
vity (GO:0003824)
organismal process (GO:0032501);; biological process: single-organism process (GO:0044699);; bic
)5623);; cellular component: organelle (GO:0043226);; cellular component: organelle part (GO:0044
lucer activity (GO:0004871);; molecular function: molecular transducer activity (GO:0060089);; biolo
region (GO:0005576);; cellular component: extracellular region part (GO:0044421);; biological pro

gulation (GO:0065007);; molecular function: binding (GO:0005488);; molecular function: nucleic aci

gulation (GO:0065007);; molecular function: binding (GO:0005488);; molecular function: nucleic aci
stimulus (GO:0050896);; cellular component: cell (GO:0005623);; cellular component: cell part (GO:(
vity (GO:0003824);; biological process: metabolic process (GO:0008152)
:0005488);; cellular component: cell (GO:0005623);; cellular component: organelle (GO:0043226);; c
(GO:0016020);; cellular component: membrane part (GO:0044425)
factor activity, protein binding (GO:0000988);; biological process: biological regulation (GO:00650
(GO:0016020);; cellular component: membrane part (GO:0044425);; biological process: multicellula
ess (GO:0009987);; biological process: single-organism process (GO:0044699);; molecular function:

)5623);; cellular component: membrane (GO:0016020);; cellular component: cell part (GO:0044464)
)5623);; cellular component: membrane (GO:0016020);; cellular component: organelle (GO:004322
:0005488);; molecular function: catalytic activity (GO:0003824);; biological process: metabolic proce
:0005488)
vity (GO:0003824);; molecular function: binding (GO:0005488);; biological process: cellular process
(GO:0016020);; molecular function: catalytic activity (GO:0003824)
ulation (GO:0065007);; molecular function: binding (GO:0005488);; cellular component: cell (GO:0
:0005488);; molecular function: catalytic activity (GO:0003824);; biological process: metabolic proce

binding transcription factor activity (GO:0001071);; biological process: biological regulation (GO:0
ess (GO:0009987);; biological process: single-organism process (GO:0044699);; biological process: l
lucer activity (GO:0004871);; molecular function: molecular transducer activity (GO:0060089);; biolo

ess (GO:0009987);; biological process: locomotion (GO:0040011);; biological process: single-organi
(GO:0016020);; cellular component: membrane part (GO:0044425)
nction regulator (GO:0098772);; biological process: biological regulation (GO:0065007)
ess (GO:0009987);; biological process: cellular component organization or biogenesis (GO:0071840
)5623);; cellular component: macromolecular complex (GO:0032991);; cellular component: organel
:0005488);; biological process: localization (GO:0051179);; cellular component: membrane (GO:001
organismal process (GO:0032501);; biological process: developmental process (GO:0032502);; biolo
ess (GO:0009987);; biological process: signaling (GO:0023052);; biological process: single-organism
vity (GO:0003824);; biological process: metabolic process (GO:0008152)

ocess (GO:0008152);; biological process: cellular process (GO:0009987);; cellular component: cell (G
(GO:0016020);; cellular component: membrane part (GO:0044425);; molecular function: catalytic ac

:0005488);; cellular component: cell (GO:0005623);; cellular component: organelle (GO:0043226);; c
nction regulator (GO:0098772);; biological process: biological regulation (GO:0065007);; biological
ocess (GO:0008152);; biological process: cellular process (GO:0009987);; molecular function: bindin
)5623);; cellular component: organelle (GO:0043226);; cellular component: organelle part (GO:004

)5623);; cellular component: membrane (GO:0016020);; cellular component: organelle (GO:004322

ulation (GO:0065007);; cellular component: cell (GO:0005623);; cellular component: organelle (GO
(GO:0016020);; cellular component: membrane part (GO:0044425)
(GO:0016020);; cellular component: membrane part (GO:0044425);; biological process: biological a
)5623);; cellular component: cell part (GO:0044464);; cellular component: membrane (GO:0016020)
:0005488)
activity (GO:0005215);; biological process: localization (GO:0051179);; cellular component: cell (GO:

em process (GO:0002376);; biological process: response to stimulus (GO:0050896);; cellular compo
ess (GO:0009987);; biological process: developmental process (GO:0032502);; biological process: si
ess (GO:0009987);; biological process: single-organism process (GO:0044699);; biological process: l
lucer activity (GO:0004871);; molecular function: molecular transducer activity (GO:0060089);; biolo
(GO:0016020);; cellular component: membrane part (GO:0044425)
)5623);; cellular component: cell part (GO:0044464);; biological process: biological regulation (GO:0
ess (GO:0009987);; biological process: signaling (GO:0023052);; biological process: single-organism
)5623);; cellular component: organelle (GO:0043226);; cellular component: cell part (GO:0044464);;
organismal process (GO:0032501);; biological process: response to stimulus (GO:0050896);; molecu
(GO:0016020);; cellular component: membrane part (GO:0044425);; molecular function: transporte
ulation (GO:0065007);; molecular function: binding (GO:0005488);; cellular component: cell (GO:0

gulation (GO:0065007);; biological process: multicellular organismal process (GO:0032501);; biologi
organismal process (GO:0032501);; biological process: developmental process (GO:0032502);; biolo

(GO:0016020);; cellular component: membrane part (GO:0044425)
gulation (GO:0065007);; molecular function: signal transducer activity (GO:0004871);; molecular fun

05623);; cellular component: cell part (GO:0044464);; cellular component: membrane (GO:0016020)
vity (GO:0003824);; biological process: metabolic process (GO:0008152);; cellular component: cell (

:0005488)
gulation (GO:0065007);; cellular component: cell (GO:0005623);; cellular component: macromolecu
activity (GO:0005215);; biological process: localization (GO:0051179);; biological process: biological

ess (GO:0009987);; biological process: cellular component organization or biogenesis (GO:0071840
stimulus (GO:0050896)
05623);; cellular component: macromolecular complex (GO:0032991);; cellular component: organel
gulation (GO:0065007)
ess (GO:0009987);; biological process: single-organism process (GO:0044699);; biological process: c
ansducer activity (GO:0060089);; biological process: localization (GO:0051179);; molecular function
vity (GO:0003824);; cellular component: cell (GO:0005623);; cellular component: membrane-enclos
05623);; cellular component: cell part (GO:0044464);; biological process: biological regulation (GO:0
05623);; cellular component: organelle (GO:0043226);; cellular component: cell part (GO:0044464);;
05623);; cellular component: organelle (GO:0043226);; cellular component: cell part (GO:0044464);;
05623);; cellular component: cell part (GO:0044464);; cellular component: organelle (GO:0043226)
vity (GO:0003824);; biological process: metabolic process (GO:0008152);; biological process: cellula
gulation (GO:0065007);; cellular component: cell (GO:0005623);; cellular component: membrane (G
:0005488);; cellular component: cell (GO:0005623);; cellular component: organelle (GO:0043226);; c
vity (GO:0003824);; molecular function: binding (GO:0005488);; biological process: cellular process
:0005488);; cellular component: cell (GO:0005623);; cellular component: cell part (GO:0044464);; bi
gulation (GO:0065007);; cellular component: cell (GO:0005623);; cellular component: membrane-er
05623);; cellular component: membrane (GO:0016020);; cellular component: macromolecular comp
:0005488);; cellular component: cell (GO:0005623);; cellular component: membrane-enclosed lume
stimulus (GO:0050896);; cellular component: membrane (GO:0016020);; cellular component: memb
05623);; cellular component: organelle (GO:0043226);; cellular component: cell part (GO:0044464);;
05623);; cellular component: membrane (GO:0016020);; cellular component: cell part (GO:0044464)

activity (GO:0005215);; molecular function: molecular transducer activity (GO:0060089);; biological
vity (GO:0003824);; biological process: metabolic process (GO:0008152);; biological process: single
gulation (GO:0065007);; cellular component: cell (GO:0005623);; cellular component: organelle (GO
activity (GO:0005215);; biological process: single-organism process (GO:0044699);; biological proce
stimulus (GO:0050896);; biological process: cellular process (GO:0009987);; biological process: signa
(GO:0016020);; cellular component: membrane part (GO:0044425)

05623);; cellular component: membrane (GO:0016020);; cellular component: organelle (GO:004322
binding transcription factor activity (GO:0001071);; biological process: biological regulation (GO:00
:0005488);; biological process: cellular process (GO:0009987);; biological process: single-organism
05623);; cellular component: organelle (GO:0043226);; cellular component: cell part (GO:0044464);;
vity (GO:0003824);; molecular function: binding (GO:0005488);; cellular component: cell (GO:00056
(GO:0016020);; cellular component: membrane part (GO:0044425)
:0005488);; biological process: cellular process (GO:0009987);; biological process: developmental p
:0005488);; biological process: biological regulation (GO:0065007)

organismal process (GO:0032501);; molecular function: binding (GO:0005488);; cellular component

(GO:0016020);; cellular component: membrane part (GO:0044425);; molecular function: transporte
:0005488);; cellular component: cell (GO:0005623);; cellular component: cell part (GO:0044464);; bi
(GO:0016020);; cellular component: membrane part (GO:0044425)
:0005488)
ess (GO:0009987);; biological process: developmental process (GO:0032502);; biological process: si

ess (GO:0009987);; biological process: developmental process (GO:0032502);; biological process: si
nction regulator (GO:0098772);; biological process: biological regulation (GO:0065007);; molecular
)5623);; cellular component: membrane (GO:0016020);; cellular component: membrane part (GO:0
)5623);; cellular component: organelle (GO:0043226);; cellular component: organelle part (GO:004
vity (GO:0003824);; biological process: metabolic process (GO:0008152)
ocess (GO:0008152);; biological process: cellular process (GO:0009987);; biological process: single-
activity (GO:0005215);; biological process: cellular process (GO:0009987);; biological process: single

activity (GO:0005215);; biological process: single-organism process (GO:0044699);; biological proce
)5623);; cellular component: cell part (GO:0044464)
(GO:0016020);; cellular component: membrane part (GO:0044425);; biological process: behavior (G
)5623);; cellular component: organelle (GO:0043226);; cellular component: organelle part (GO:004
:0005488);; cellular component: membrane (GO:0016020);; cellular component: membrane part (G
region (GO:0005576);; cellular component: extracellular region part (GO:0044421);; biological pro
vity (GO:0003824);; cellular component: extracellular region (GO:0005576);; biological process: mul

vity (GO:0003824);; biological process: metabolic process (GO:0008152);; biological process: cellula
(GO:0016020);; cellular component: membrane part (GO:0044425)
:0005488);; molecular function: nucleic acid binding transcription factor activity (GO:0001071);; bio
)5623);; cellular component: macromolecular complex (GO:0032991);; cellular component: organel
)5623);; cellular component: membrane (GO:0016020);; cellular component: cell part (GO:0044464)
:0005488);; molecular function: catalytic activity (GO:0003824);; biological process: metabolic proce

:0005488);; biological process: biological regulation (GO:0065007)

vity (GO:0003824);; cellular component: cell (GO:0005623);; cellular component: cell part (GO:0044
:0005488);; molecular function: catalytic activity (GO:0003824);; biological process: metabolic proce
vity (GO:0003824);; molecular function: structural molecule activity (GO:0005198);; molecular funct
)5623);; cellular component: membrane (GO:0016020);; cellular component: organelle (GO:00432

)5623);; cellular component: organelle (GO:0043226);; cellular component: cell part (GO:0044464);;

)5623);; cellular component: organelle (GO:0043226);; cellular component: organelle part (GO:004
:0005488);; cellular component: cell (GO:0005623);; cellular component: membrane (GO:0016020);
em process (GO:0002376);; biological process: response to stimulus (GO:0050896);; molecular func
ess (GO:0009987);; biological process: cellular component organization or biogenesis (GO:0071840

)5623);; cellular component: organelle (GO:0043226);; cellular component: organelle part (GO:004
(GO:0016020);; cellular component: membrane part (GO:0044425)
vity (GO:0003824);; biological process: metabolic process (GO:0008152)
ess (GO:0009987);; biological process: signaling (GO:0023052);; biological process: single-organism

3O:0051179);; molecular function: binding (GO:0005488);; cellular component: cell (GO:0005623);;
lucer activity (GO:0004871);; molecular function: molecular transducer activity (GO:0060089);; biolc
)5623);; cellular component: organelle (GO:0043226);; cellular component: organelle part (GO:0044
em process (GO:0002376);; biological process: cellular process (GO:0009987);; biological process: lc
(GO:0016020);; cellular component: membrane part (GO:0044425);; molecular function: signal tran

)5623);; cellular component: membrane (GO:0016020);; cellular component: membrane part (GO:0
(GO:0016020);; cellular component: membrane part (GO:0044425)
binding transcription factor activity (GO:0001071);; biological process: biological regulation (GO:00
)5623);; cellular component: membrane-enclosed lumen (GO:0031974);; cellular component: orgar
organismal process (GO:0032501);; biological process: biological regulation (GO:0065007);; cellular
nction regulator (GO:0098772);; biological process: biological regulation (GO:0065007)
ocess (GO:0008152);; biological process: cellular process (GO:0009987);; biological process: single-
:0005488);; cellular component: cell (GO:0005623);; cellular component: organelle (GO:0043226);; c
)5623);; cellular component: membrane-enclosed lumen (GO:0031974);; cellular component: orgar
vity (GO:0003824);; biological process: metabolic process (GO:0008152);; biological process: cellula
(GO:0016020);; cellular component: membrane part (GO:0044425);; molecular function: catalytic ac
)5623);; cellular component: cell part (GO:0044464);; molecular function: binding (GO:0005488);; ce
vity (GO:0003824)
)5623);; cellular component: organelle (GO:0043226);; cellular component: organelle part (GO:0044
nction regulator (GO:0098772);; biological process: biological regulation (GO:0065007);; molecular
gulation (GO:0065007);; molecular function: binding (GO:0005488);; molecular function: nucleic acic
:0005488);; cellular component: cell (GO:0005623);; cellular component: membrane (GO:0016020);
organismal process (GO:0032501);; biological process: developmental process (GO:0032502);; biolc
vity (GO:0003824);; biological process: metabolic process (GO:0008152);; biological process: single
)5623);; cellular component: organelle (GO:0043226);; cellular component: cell part (GO:0044464);;
:0005488);; cellular component: cell (GO:0005623);; cellular component: organelle (GO:0043226);; c
)5623);; cellular component: organelle (GO:0043226);; cellular component: organelle part (GO:0044
vity (GO:0003824);; biological process: metabolic process (GO:0008152);; cellular component: cell (
:0005488);; molecular function: catalytic activity (GO:0003824);; biological process: metabolic proce
activity (GO:0005215);; biological process: single-organism process (GO:0044699);; biological proce
:0005488)
:0005488);; molecular function: catalytic activity (GO:0003824);; biological process: metabolic proce
organismal process (GO:0032501);; biological process: single-organism process (GO:0044699);; cel
vity (GO:0003824);; molecular function: binding (GO:0005488);; biological process: metabolic proce
cular complex (GO:0032991);; cellular component: cell (GO:0005623);; cellular component: organel
(GO:0016020);; cellular component: membrane part (GO:0044425)
)5623);; cellular component: organelle (GO:0043226);; cellular component: cell part (GO:0044464);;
(GO:0000003);; biological process: metabolic process (GO:0008152);; biological process: cellular p
binding transcription factor activity (GO:0001071);; biological process: biological regulation (GO:00
:0005488);; cellular component: cell (GO:0005623);; cellular component: organelle (GO:0043226);; c
activity (GO:0005215);; biological process: localization (GO:0051179);; cellular component: cell (GO:
vity (GO:0003824);; cellular component: cell (GO:0005623);; cellular component: organelle (GO:004
cular complex (GO:0032991);; cellular component: cell (GO:0005623);; cellular component: membra
vity (GO:0003824)
:0005488);; biological process: response to stimulus (GO:0050896);; cellular component: cell (GO:0
organismal process (GO:0032501);; biological process: developmental process (GO:0032502);; biolc
(GO:0016020);; cellular component: membrane part (GO:0044425)
nction regulator (GO:0098772);; biological process: biological regulation (GO:0065007)
:0005488)

:0005488)
)5623);; cellular component: organelle (GO:0043226);; cellular component: organelle part (GO:004
vity (GO:0003824);; biological process: metabolic process (GO:0008152);; biological process: cellula
)5623);; cellular component: organelle (GO:0043226);; cellular component: cell part (GO:0044464);;
)5623);; cellular component: membrane-enclosed lumen (GO:0031974);; cellular component: orgar
vity (GO:0003824);; molecular function: binding (GO:0005488);; cellular component: cell (GO:0005
stimulus (GO:0050896);; molecular function: binding (GO:0005488);; cellular component: cell (GO:0

ocess (GO:0008152);; cellular component: cell (GO:0005623);; cellular component: membrane (GO:
ocess (GO:0008152);; biological process: cellular process (GO:0009987);; cellular component: cell (G
:0005488);; cellular component: cell (GO:0005623);; cellular component: organelle (GO:0043226);; c
)5623);; cellular component: cell part (GO:0044464);; biological process: cellular process (GO:0009
vity (GO:0003824);; biological process: metabolic process (GO:0008152);; biological process: cellula
activity (GO:0005215);; biological process: localization (GO:0051179);; cellular component: cell (GO:
:0005488);; biological process: metabolic process (GO:0008152);; biological process: cellular proces
)5623);; cellular component: organelle (GO:0043226);; cellular component: cell part (GO:0044464);;
(GO:0016020);; cellular component: membrane part (GO:0044425)
:0005488)
vity (GO:0003824);; biological process: metabolic process (GO:0008152);; biological process: cellula
(GO:0016020);; cellular component: membrane part (GO:0044425)
:0005488);; molecular function: catalytic activity (GO:0003824);; cellular component: cell (GO:0005
)5623);; cellular component: membrane (GO:0016020);; cellular component: organelle (GO:004322
)5623);; cellular component: membrane (GO:0016020);; cellular component: organelle (GO:004322
)5623);; cellular component: organelle (GO:0043226);; cellular component: organelle part (GO:004
)5623);; cellular component: cell part (GO:0044464)
olecule activity (GO:0005198);; cellular component: cell (GO:0005623);; cellular component: macror
)5623);; cellular component: cell part (GO:0044464);; cellular component: organelle (GO:0043226);;
ocess (GO:0008152);; biological process: cellular process (GO:0009987);; biological process: cellular
)5623);; cellular component: organelle (GO:0043226);; cellular component: cell part (GO:0044464);;
olecule activity (GO:0005198);; cellular component: cell (GO:0005623);; cellular component: macror
)5623);; cellular component: membrane (GO:0016020);; cellular component: macromolecular comp
:0005488)
)5623);; cellular component: cell part (GO:0044464);; biological process: multicellular organismal pr
vity (GO:0003824);; cellular component: membrane (GO:0016020);; cellular component: membrane
:0005488);; molecular function: catalytic activity (GO:0003824);; cellular component: cell (GO:0005
gulation (GO:0065007);; molecular function: catalytic activity (GO:0003824);; cellular component: ce
stimulus (GO:0050896);; biological process: cellular process (GO:0009987);; biological process: cellu
:0005488);; cellular component: cell (GO:0005623);; cellular component: organelle (GO:0043226);; c
ocess (GO:0008152);; biological process: cellular process (GO:0009987);; biological process: cellular
:0005488)
)5623);; cellular component: organelle (GO:0043226);; cellular component: cell part (GO:0044464);;
ocess (GO:0008152);; biological process: cellular process (GO:0009987);; biological process: cellular
)5623);; cellular component: organelle (GO:0043226);; cellular component: organelle part (GO:004
)5623);; cellular component: cell part (GO:0044464);; molecular function: catalytic activity (GO:0003
)5623);; cellular component: cell part (GO:0044464);; cellular component: organelle (GO:0043226);;
ocess (GO:0008152);; biological process: cellular process (GO:0009987);; biological process: cellular
)5623);; cellular component: organelle (GO:0043226);; cellular component: cell part (GO:0044464);;
vity (GO:0003824);; molecular function: binding (GO:0005488)
:0005488);; cellular component: cell (GO:0005623);; cellular component: cell part (GO:0044464);; m
:0005488);; molecular function: catalytic activity (GO:0003824)

:al process (GO:0032502);; biological process: single-organism process (GO:0044699);; molecular fu
:0005488);; molecular function: catalytic activity (GO:0003824);; cellular component: cell (GO:00056
:0005488);; biological process: metabolic process (GO:0008152);; biological process: cellular proces
:0005488);; biological process: cellular process (GO:0009987);; biological process: single-organism
)5623);; cellular component: organelle (GO:0043226);; cellular component: cell part (GO:0044464);;
olecule activity (GO:0005198);; cellular component: cell (GO:0005623);; cellular component: organe

ess (GO:0009987);; biological process: single-organism process (GO:0044699);; cellular component:
vity (GO:0003824);; molecular function: binding (GO:0005488)

)5623);; cellular component: organelle (GO:0043226);; cellular component: cell part (GO:0044464);;
ocess (GO:0008152);; biological process: cellular process (GO:0009987);; biological process: single-
:0005488)
:0005488);; molecular function: catalytic activity (GO:0003824);; biological process: metabolic proce
ess (GO:0009987);; biological process: developmental process (GO:0032502);; biological process: gr
ess (GO:0009987);; biological process: single-organism process (GO:0044699);; molecular function:
)5623);; cellular component: membrane (GO:0016020);; cellular component: macromolecular comp
olecule activity (GO:0005198);; cellular component: cell (GO:0005623);; cellular component: macror
)5623);; cellular component: organelle (GO:0043226);; cellular component: cell part (GO:0044464);;
:0005488);; cellular component: cell (GO:0005623);; cellular component: macromolecular complex
cular complex (GO:0032991);; molecular function: binding (GO:0005488);; cellular component: cell
)5623);; cellular component: membrane-enclosed lumen (GO:0031974);; cellular component: orgar
(GO:0016020);; cellular component: membrane part (GO:0044425);; biological process: cellular pro
(GO:0016020);; cellular component: membrane part (GO:0044425)
vity (GO:0003824);; biological process: metabolic process (GO:0008152);; cellular component: cell (
factor activity, protein binding (GO:0000988);; biological process: biological regulation (GO:00650
)5623);; cellular component: organelle (GO:0043226);; cellular component: organelle part (GO:004
:0005488);; cellular component: cell (GO:0005623);; cellular component: cell part (GO:0044464)
(GO:0016020);; cellular component: membrane part (GO:0044425)
)5623);; cellular component: macromolecular complex (GO:0032991);; cellular component: cell part
)5623);; cellular component: macromolecular complex (GO:0032991);; cellular component: cell part
vity (GO:0003824);; molecular function: structural molecule activity (GO:0005198);; molecular funct
binding transcription factor activity (GO:0001071);; biological process: biological regulation (GO:00
:0005488);; cellular component: cell (GO:0005623);; cellular component: organelle (GO:0043226);; c
organismal process (GO:0032501);; biological process: developmental process (GO:0032502);; biolo
vity (GO:0003824);; biological process: metabolic process (GO:0008152);; biological process: cellula
)5623);; cellular component: cell part (GO:0044464);; biological process: localization (GO:0051179)
)5623);; cellular component: membrane (GO:0016020);; cellular component: macromolecular comp
ess (GO:0009987);; biological process: developmental process (GO:0032502);; biological process: sir
)5623);; cellular component: membrane (GO:0016020);; cellular component: organelle (GO:004322
)5623);; cellular component: cell part (GO:0044464);; cellular component: organelle (GO:0043226);;
gulation (GO:0065007);; molecular function: binding (GO:0005488);; cellular component: cell (GO:00
)5623);; cellular component: membrane-enclosed lumen (GO:0031974);; cellular component: orgar
:0005488);; cellular component: cell (GO:0005623);; cellular component: organelle (GO:0043226);; c
:0005488);; cellular component: cell (GO:0005623);; cellular component: membrane (GO:0016020);
)5623);; cellular component: organelle (GO:0043226);; cellular component: organelle part (GO:004
ocess (GO:0008152);; biological process: cellular process (GO:0009987);; biological process: signalir
)5623);; cellular component: membrane-enclosed lumen (GO:0031974);; cellular component: orgar
olecule activity (GO:0005198);; cellular component: cell (GO:0005623);; cellular component: macror
:0005488);; biological process: biological regulation (GO:0065007)
:0005488);; cellular component: cell (GO:0005623);; cellular component: organelle (GO:0043226);; c

organismal process (GO:0032501);; biological process: developmental process (GO:0032502);; biolo
vity (GO:0003824);; molecular function: antioxidant activity (GO:0016209);; biological process: meta
(GO:0016020);; cellular component: membrane part (GO:0044425);; molecular function: catalytic ac
:0005488);; biological process: cellular process (GO:0009987);; biological process: single-organism
:0005488);; cellular component: cell (GO:0005623);; cellular component: cell part (GO:0044464)
:0005488);; molecular function: nucleic acid binding transcription factor activity (GO:0001071);; bio
vity (GO:0003824);; molecular function: binding (GO:0005488);; cellular component: cell (GO:00056
)5623);; cellular component: organelle (GO:0043226);; cellular component: cell part (GO:0044464);;
ansducer activity (GO:0060089);; biological process: localization (GO:0051179);; cellular componen
:0005488);; cellular component: cell (GO:0005623);; cellular component: organelle (GO:0043226);; c
vity (GO:0003824);; biological process: metabolic process (GO:0008152);; biological process: single
al process (GO:0032502);; biological process: single-organism process (GO:0044699);; molecular fu
vity (GO:0003824);; cellular component: cell (GO:0005623);; cellular component: membrane (GO:00
)5623);; cellular component: organelle (GO:0043226);; cellular component: cell part (GO:0044464);;
vity (GO:0003824);; biological process: metabolic process (GO:0008152);; biological process: cellula
)5623);; cellular component: membrane (GO:0016020);; cellular component: organelle (GO:004322
)5623);; cellular component: membrane-enclosed lumen (GO:0031974);; cellular component: orgar
olecule activity (GO:0005198);; biological process: metabolic process (GO:0008152);; biological pro
olecule activity (GO:0005198);; cellular component: cell (GO:0005623);; cellular component: cell par
organismal process (GO:0032501);; biological process: single-organism process (GO:0044699);; bic
)5623);; cellular component: macromolecular complex (GO:0032991);; cellular component: organel
ocess (GO:0008152);; biological process: cellular process (GO:0009987);; biological process: biologi

gulation (GO:0065007);; cellular component: cell (GO:0005623);; cellular component: organelle (GO
rier activity (GO:0009055);; molecular function: catalytic activity (GO:0003824);; biological process: r
)5623);; cellular component: organelle (GO:0043226);; cellular component: cell part (GO:0044464);;

)5623);; cellular component: membrane (GO:0016020);; cellular component: organelle (GO:004322
)5623);; cellular component: membrane-enclosed lumen (GO:0031974);; cellular component: orgar
ocess (GO:0008152);; biological process: cellular process (GO:0009987);; molecular function: bindin
gulation (GO:0065007);; cellular component: cell (GO:0005623);; cellular component: membrane (G
vity (GO:0003824);; biological process: metabolic process (GO:0008152);; biological process: single
:0005488)
nction regulator (GO:0098772);; biological process: biological regulation (GO:0065007);; cellular cc
ocess (GO:0008152);; biological process: cellular process (GO:0009987);; biological process: single-
)5623);; cellular component: organelle (GO:0043226);; cellular component: organelle part (GO:0044
vity (GO:0003824);; biological process: metabolic process (GO:0008152);; biological process: cellula
vity (GO:0003824);; biological process: metabolic process (GO:0008152);; biological process: cellula
)5623);; cellular component: membrane (GO:0016020);; cellular component: cell part (GO:0044464)
:0005488);; cellular component: cell (GO:0005623);; cellular component: organelle (GO:0043226);; c
)5623);; cellular component: organelle (GO:0043226);; cellular component: cell part (GO:0044464);;
GO:0051179);; molecular function: catalytic activity (GO:0003824);; molecular function: binding (GC
)5623);; cellular component: cell part (GO:0044464);; cellular component: macromolecular complex
(GO:0016020);; cellular component: membrane part (GO:0044425)
olecule activity (GO:0005198);; cellular component: cell (GO:0005623);; cellular component: macror
gulation (GO:0065007)
gulation (GO:0065007)
vity (GO:0003824);; biological process: metabolic process (GO:0008152);; biological process: cellula
:0005488);; cellular component: cell (GO:0005623);; cellular component: organelle (GO:0043226);; c

)5623);; cellular component: organelle (GO:0043226);; cellular component: cell part (GO:0044464);;

)5623);; cellular component: organelle (GO:0043226);; cellular component: cell part (GO:0044464)
vity (GO:0003824);; biological process: metabolic process (GO:0008152);; molecular function: bindi
julation (GO:0065007);; molecular function: binding (GO:0005488);; biological process: behavior (G
vity (GO:0003824);; biological process: metabolic process (GO:0008152);; biological process: cellula
:0005488);; molecular function: catalytic activity (GO:0003824);; cellular component: cell (GO:00056
al process (GO:0032502);; biological process: single-organism process (GO:0044699);; biological p
activity (GO:0005215);; biological process: localization (GO:0051179);; cellular component: membra
em process (GO:0002376);; biological process: response to stimulus (GO:0050896);; cellular compo
)5623);; cellular component: organelle (GO:0043226);; cellular component: organelle part (GO:0044
:0005488);; cellular component: cell (GO:0005623);; cellular component: membrane (GO:0016020);
)5623);; cellular component: cell part (GO:0044464);; cellular component: organelle (GO:0043226);;
vity (GO:0003824)
julation (GO:0065007);; molecular function: transcription factor activity, protein binding (GO:00009
)5623);; cellular component: organelle (GO:0043226);; cellular component: organelle part (GO:0044
)5623);; cellular component: macromolecular complex (GO:0032991);; cellular component: cell part
:0005488);; cellular component: extracellular region (GO:0005576);; biological process: response tc
vity (GO:0003824);; biological process: metabolic process (GO:0008152);; biological process: cellula
ocess (GO:0008152);; biological process: cellular process (GO:0009987);; molecular function: bindin
(GO:0016020);; cellular component: membrane part (GO:0044425);; biological process: localization
)5623);; cellular component: membrane-enclosed lumen (GO:0031974);; cellular component: orgar
)5623);; cellular component: organelle (GO:0043226);; cellular component: organelle part (GO:0044
)5623);; cellular component: membrane (GO:0016020);; cellular component: membrane part (GO:0
:0005488);; molecular function: catalytic activity (GO:0003824)
vity (GO:0003824);; molecular function: binding (GO:0005488);; biological process: metabolic proce
:0005488)
:0005488);; cellular component: cell (GO:0005623);; cellular component: cell part (GO:0044464)
olecule activity (GO:0005198);; cellular component: cell (GO:0005623);; cellular component: macror
:0005488);; biological process: biological regulation (GO:0065007);; cellular component: membrane
ess (GO:0009987);; biological process: single-organism process (GO:0044699);; biological process: c
)5623);; cellular component: organelle (GO:0043226);; cellular component: organelle part (GO:0044

vity (GO:0003824);; biological process: metabolic process (GO:0008152);; biological process: cellula
ess (GO:0009987);; biological process: single-organism process (GO:0044699);; biological process: c
organismal process (GO:0032501);; biological process: developmental process (GO:0032502);; biolo

vity (GO:0003824);; cellular component: membrane (GO:0016020);; cellular component: membrane
ess (GO:0009987);; biological process: cellular component organization or biogenesis (GO:0071840
:0005488);; cellular component: membrane (GO:0016020);; cellular component: membrane part (G
:0005488);; biological process: cellular process (GO:0009987);; biological process: single-organism
:0005488);; molecular function: catalytic activity (GO:0003824);; biological process: metabolic proce
vity (GO:0003824);; biological process: metabolic process (GO:0008152);; biological process: cellula
vity (GO:0003824);; biological process: metabolic process (GO:0008152);; biological process: cellula
:0005488);; biological process: biological regulation (GO:0065007)
vity (GO:0003824);; molecular function: binding (GO:0005488);; cellular component: cell (GO:00056
ess (GO:0009987);; biological process: single-organism process (GO:0044699);; biological process: c
julation (GO:0065007);; cellular component: cell (GO:0005623);; cellular component: macromolecu
)5623);; cellular component: organelle (GO:0043226);; cellular component: organelle part (GO:0044
ess (GO:0009987);; biological process: single-organism process (GO:0044699);; biological process: l
vity (GO:0003824);; biological process: metabolic process (GO:0008152);; biological process: cellula
:0005488);; molecular function: catalytic activity (GO:0003824);; biological process: biological regul
binding transcription factor activity (GO:0001071);; biological process: biological regulation (GO:00

)5623);; cellular component: macromolecular complex (GO:0032991);; cellular component: cell part
organismal process (GO:0032501);; biological process: single-organism process (GO:0044699);; bic
)5623);; cellular component: cell part (GO:0044464);; biological process: metabolic process (GO:000
ocess (GO:0008152);; biological process: cellular process (GO:0009987);; biological process: single-
stimulus (GO:0050896);; biological process: biological regulation (GO:0065007);; biological process:
lucer activity (GO:0004871);; molecular function: molecular transducer activity (GO:0060089);; biolc

:0005488);; molecular function: catalytic activity (GO:0003824);; biological process: metabolic proce
)5623);; cellular component: cell part (GO:0044464);; cellular component: organelle (GO:0043226);;
ocess (GO:0008152)
)5623);; cellular component: organelle (GO:0043226);; cellular component: organelle part (GO:004

ess (GO:0009987);; biological process: locomotion (GO:0040011);; biological process: single-organi
)5623);; cellular component: membrane (GO:0016020);; cellular component: organelle (GO:004322
)5623);; cellular component: membrane (GO:0016020);; cellular component: organelle (GO:004322
ocess (GO:0008152);; biological process: cellular process (GO:0009987);; cellular component: cell (G
vity (GO:0003824);; cellular component: cell (GO:0005623);; cellular component: organelle (GO:004
ocess (GO:0008152);; biological process: cellular process (GO:0009987);; biological process: single-
)5623);; cellular component: organelle (GO:0043226);; cellular component: organelle part (GO:004
)5623);; cellular component: macromolecular complex (GO:0032991);; cellular component: organel
ess (GO:0009987);; biological process: single-organism process (GO:0044699);; biological process: c
:0005488);; cellular component: cell (GO:0005623);; cellular component: cell part (GO:0044464);; bi
:0005488);; biological process: metabolic process (GO:0008152);; biological process: cellular proces
)5623);; cellular component: macromolecular complex (GO:0032991);; cellular component: organel
)5623);; cellular component: macromolecular complex (GO:0032991);; cellular component: organel
)5623);; cellular component: membrane (GO:0016020);; cellular component: membrane part (GO:0
vity (GO:0003824);; biological process: metabolic process (GO:0008152);; cellular component: cell (
ess (GO:0009987);; biological process: single-organism process (GO:0044699);; biological process: c
)5623);; cellular component: organelle (GO:0043226);; cellular component: organelle part (GO:004
:0005488);; molecular function: nucleic acid binding transcription factor activity (GO:0001071);; bio
)5623);; cellular component: membrane (GO:0016020);; cellular component: organelle (GO:004322
:0005488);; biological process: metabolic process (GO:0008152);; biological process: cellular proces
)5623);; cellular component: organelle (GO:0043226);; cellular component: organelle part (GO:004

(GO:0016020);; cellular component: membrane part (GO:0044425)
)5623);; cellular component: macromolecular complex (GO:0032991);; cellular component: cell part
:0005488);; molecular function: catalytic activity (GO:0003824);; cellular component: cell (GO:00056
:0005488);; molecular function: nucleic acid binding transcription factor activity (GO:0001071);; bio
vity (GO:0003824)
ocess (GO:0008152);; biological process: single-organism process (GO:0044699);; molecular functic
olecule activity (GO:0005198);; cellular component: cell (GO:0005623);; cellular component: macror
vity (GO:0003824);; biological process: metabolic process (GO:0008152);; biological process: cellula
)5623);; cellular component: membrane-enclosed lumen (GO:0031974);; cellular component: orgar
olecule activity (GO:0005198);; cellular component: cell (GO:0005623);; cellular component: organe
:0005488);; biological process: metabolic process (GO:0008152);; biological process: cellular proces
:0005488);; cellular component: cell (GO:0005623);; cellular component: organelle (GO:0043226);; c
vity (GO:0003824);; biological process: metabolic process (GO:0008152);; biological process: biolog
:0005488);; molecular function: catalytic activity (GO:0003824)
:0005488);; molecular function: catalytic activity (GO:0003824)
)5623);; cellular component: macromolecular complex (GO:0032991);; cellular component: organel

:0005488);; biological process: biological regulation (GO:0065007);; cellular component: cell (GO:0(
(GO:0016020);; cellular component: membrane part (GO:0044425)
(GO:0016020)
)5623);; cellular component: macromolecular complex (GO:0032991);; cellular component: cell part
:0005488);; biological process: multicellular organismal process (GO:0032501);; biological process:

olecule activity (GO:0005198);; molecular function: binding (GO:0005488);; biological process: meta
stimulus (GO:0050896);; molecular function: binding (GO:0005488);; molecular function: transcriptic
)5623);; cellular component: organelle (GO:0043226);; cellular component: cell part (GO:0044464);;
:0005488)
:0005488);; biological process: cellular process (GO:0009987);; biological process: single-organism
)5623);; cellular component: organelle (GO:0043226);; cellular component: organelle part (GO:0044
organismal process (GO:0032501);; biological process: developmental process (GO:0032502);; biolo
region (GO:0005576);; cellular component: extracellular region part (GO:0044421);; cellular compo

:0005488);; biological process: response to stimulus (GO:0050896);; molecular function: catalytic ac
ocess (GO:0008152);; biological process: cellular process (GO:0009987);; molecular function: bindin
)5623);; cellular component: membrane (GO:0016020);; cellular component: macromolecular comp
:0005488);; cellular component: cell (GO:0005623);; cellular component: organelle (GO:0043226);; c
vity (GO:0003824);; molecular function: binding (GO:0005488);; cellular component: cell (GO:00056
:0005488);; cellular component: extracellular region (GO:0005576);; cellular component: extracellul
)5623);; cellular component: organelle (GO:0043226);; cellular component: cell part (GO:0044464);;
ism process (GO:0044699);; biological process: localization (GO:0051179);; molecular function: binc
)5623);; cellular component: organelle (GO:0043226);; cellular component: cell part (GO:0044464);;
(GO:0016020);; cellular component: membrane part (GO:0044425);; molecular function: transporte
ocess (GO:0008152);; biological process: cellular process (GO:0009987);; cellular component: cell (G
:0005488);; molecular function: catalytic activity (GO:0003824);; biological process: metabolic proce
)5623);; cellular component: organelle (GO:0043226);; cellular component: organelle part (GO:0044
organismal process (GO:0032501);; biological process: biological regulation (GO:0065007);; molecu
)5623);; cellular component: cell part (GO:0044464);; biological process: biological regulation (GO:0
)5623);; cellular component: organelle (GO:0043226);; cellular component: organelle part (GO:0044
)5623);; cellular component: organelle (GO:0043226);; cellular component: organelle part (GO:0044
)5623);; cellular component: membrane-enclosed lumen (GO:0031974);; cellular component: orgar
)5623);; cellular component: cell part (GO:0044464);; biological process: cellular process (GO:00099
)5623);; cellular component: organelle (GO:0043226);; cellular component: organelle part (GO:0044

ocess (GO:0008152);; biological process: cellular process (GO:0009987);; biological process: respon
ocess (GO:0008152);; biological process: cellular process (GO:0009987);; molecular function: bindin
vity (GO:0003824);; biological process: metabolic process (GO:0008152);; biological process: single
)5623);; cellular component: organelle (GO:0043226);; cellular component: cell part (GO:0044464);;
ocess (GO:0008152);; biological process: cellular process (GO:0009987);; biological process: single-
vity (GO:0003824);; molecular function: binding (GO:0005488);; biological process: metabolic proce
vity (GO:0003824)
)5623);; cellular component: organelle (GO:0043226);; cellular component: cell part (GO:0044464);;
:0005488);; biological process: metabolic process (GO:0008152);; biological process: cellular proces
)5623);; cellular component: cell part (GO:0044464);; cellular component: membrane (GO:0016020)
ocess (GO:0008152);; biological process: cellular process (GO:0009987);; biological process: biologi
:0005488);; cellular component: cell junction (GO:0030054);; biological process: biological adhesior
(GO:0016020);; cellular component: membrane part (GO:0044425);; molecular function: binding (G
(GO:0016020);; cellular component: membrane part (GO:0044425)

(GO:0016020);; cellular component: membrane part (GO:0044425);; molecular function: binding (G
(GO:0016020);; cellular component: membrane part (GO:0044425)
organismal process (GO:0032501);; biological process: developmental process (GO:0032502);; biolo
olecule activity (GO:0005198);; cellular component: cell (GO:0005623);; cellular component: macror

(GO:0000003);; biological process: cellular process (GO:0009987);; biological process: reproductive
)5623);; cellular component: organelle (GO:0043226);; cellular component: cell part (GO:0044464);;
)5623);; cellular component: organelle (GO:0043226);; cellular component: organelle part (GO:0044
ocess (GO:0008152);; biological process: cellular process (GO:0009987);; biological process: single-
)5623);; cellular component: membrane (GO:0016020);; cellular component: organelle (GO:004322
)5623);; cellular component: membrane-enclosed lumen (GO:0031974);; cellular component: macro
)5623);; cellular component: cell part (GO:0044464)
ess (GO:0009987);; biological process: cellular component organization or biogenesis (GO:0071840

vity (GO:0003824);; biological process: metabolic process (GO:0008152);; biological process: cellula
activity (GO:0005215);; biological process: localization (GO:0051179);; cellular component: cell (GO:
(GO:0016020);; cellular component: membrane part (GO:0044425)

vity (GO:0003824);; cellular component: cell (GO:0005623);; cellular component: cell part (GO:0044
organismal process (GO:0032501);; biological process: developmental process (GO:0032502);; biolo
)5623);; cellular component: macromolecular complex (GO:0032991);; cellular component: cell part
)5623);; cellular component: organelle (GO:0043226);; cellular component: organelle part (GO:0044
vity (GO:0003824);; biological process: multicellular organismal process (GO:0032501);; biological p
vity (GO:0003824);; molecular function: binding (GO:0005488);; cellular component: cell (GO:00056
)5623);; cellular component: cell part (GO:0044464);; cellular component: membrane (GO:0016020)
olecule activity (GO:0005198);; cellular component: cell (GO:0005623);; cellular component: macror
ocess (GO:0008152);; biological process: cellular process (GO:0009987);; cellular component: cell (G
:0005488);; cellular component: cell (GO:0005623);; cellular component: organelle (GO:0043226);; c
)5623);; cellular component: membrane (GO:0016020);; cellular component: cell part (GO:0044464)
vity (GO:0003824);; biological process: metabolic process (GO:0008152);; biological process: cellula
)5623);; cellular component: organelle (GO:0043226);; cellular component: organelle part (GO:0044
al process (GO:0032502);; biological process: single-organism process (GO:0044699);; biological p
(GO:0016020);; cellular component: membrane part (GO:0044425);; cellular component: cell (GO:0
)5623);; cellular component: organelle (GO:0043226);; cellular component: organelle part (GO:0044
olecule activity (GO:0005198);; cellular component: cell (GO:0005623);; cellular component: macror
ess (GO:0009987);; biological process: single-organism process (GO:0044699);; molecular function:
(GO:0016020);; cellular component: membrane part (GO:0044425)
olecule activity (GO:0005198);; cellular component: cell (GO:0005623);; cellular component: macror
ess (GO:0009987);; biological process: single-organism process (GO:0044699);; cellular component:
)5623);; cellular component: organelle (GO:0043226);; cellular component: organelle part (GO:0044
:0005488)
)5623);; cellular component: organelle (GO:0043226);; cellular component: organelle part (GO:0044

olecule activity (GO:0005198);; cellular component: cell (GO:0005623);; cellular component: macror
)5623);; cellular component: organelle (GO:0043226);; cellular component: cell part (GO:0044464);;
(GO:0000003);; biological process: reproductive process (GO:0022414);; biological process: multic
gulation (GO:0065007);; cellular component: cell (GO:0005623);; cellular component: membrane (G
)5623);; cellular component: macromolecular complex (GO:0032991);; cellular component: cell part

activity (GO:0005215);; biological process: single-organism process (GO:0044699);; biological proce

)5623);; cellular component: membrane (GO:0016020);; cellular component: cell part (GO:0044464)
)5623);; cellular component: organelle (GO:0043226);; cellular component: organelle part (GO:004

(GO:0016020);; cellular component: membrane part (GO:0044425);; biological process: localization
)5623);; cellular component: membrane (GO:0016020);; cellular component: cell part (GO:0044464)
)5623);; cellular component: organelle (GO:0043226);; cellular component: cell part (GO:0044464);;
:0005488);; cellular component: cell (GO:0005623);; cellular component: membrane (GO:0016020);
)5623);; cellular component: cell part (GO:0044464);; molecular function: catalytic activity (GO:0003
olecule activity (GO:0005198);; cellular component: cell (GO:0005623);; cellular component: macror
:0005488);; molecular function: molecular function regulator (GO:0098772);; biological process: bic
)5623);; cellular component: membrane (GO:0016020);; cellular component: cell part (GO:0044464)
)5623);; cellular component: organelle (GO:0043226);; cellular component: organelle part (GO:004
vity (GO:0003824);; biological process: metabolic process (GO:0008152);; biological process: cellula
)5623);; cellular component: organelle (GO:0043226);; cellular component: cell part (GO:0044464)
gulation (GO:0065007);; cellular component: cell (GO:0005623);; cellular component: macromolecu
gulation (GO:0065007);; molecular function: binding (GO:0005488);; molecular function: signal trans
)5623);; cellular component: organelle (GO:0043226);; cellular component: organelle part (GO:004
:0005488);; cellular component: cell (GO:0005623);; cellular component: organelle (GO:0043226);; c
ocess (GO:0008152);; biological process: cellular process (GO:0009987);; cellular component: cell (G
vity (GO:0003824);; biological process: metabolic process (GO:0008152);; biological process: cellula
ocess (GO:0008152);; biological process: cellular process (GO:0009987);; cellular component: cell (G
:0005488)
cular complex (GO:0032991);; molecular function: binding (GO:0005488);; cellular component: cell
vity (GO:0003824);; cellular component: cell (GO:0005623);; cellular component: cell part (GO:0044
ocess (GO:0008152);; biological process: cellular process (GO:0009987);; molecular function: bindin
ocess (GO:0008152);; biological process: cellular process (GO:0009987);; biological process: single-
(GO:0016020);; cellular component: membrane part (GO:0044425);; molecular function: catalytic ac
(GO:0016020);; cellular component: membrane part (GO:0044425)
ocess (GO:0008152);; biological process: cellular process (GO:0009987);; biological process: cellular
factor activity, protein binding (GO:0000988);; biological process: biological regulation (GO:00650
:0005488);; molecular function: catalytic activity (GO:0003824);; biological process: metabolic proce
:0005488);; cellular component: cell (GO:0005623);; cellular component: organelle (GO:0043226);; c
:0005488);; molecular function: catalytic activity (GO:0003824);; cellular component: cell (GO:00056
)5623);; cellular component: macromolecular complex (GO:0032991);; cellular component: organel
vity (GO:0003824);; biological process: metabolic process (GO:0008152);; biological process: single
vity (GO:0003824);; biological process: metabolic process (GO:0008152);; biological process: cellula
ocess (GO:0008152);; biological process: cellular process (GO:0009987);; biological process: biologi
)5623);; cellular component: membrane-enclosed lumen (GO:0031974);; cellular component: macr
ocess (GO:0008152);; biological process: cellular process (GO:0009987);; biological process: respon
)5623);; cellular component: organelle (GO:0043226);; cellular component: cell part (GO:0044464);;
:0005488);; molecular function: transcription factor activity, protein binding (GO:0000988);; biologi
vity (GO:0003824);; biological process: metabolic process (GO:0008152);; biological process: cellula
vity (GO:0003824);; biological process: metabolic process (GO:0008152);; biological process: single
:0005488);; cellular component: cell (GO:0005623);; cellular component: organelle (GO:0043226);; c
)5623);; cellular component: organelle (GO:0043226);; cellular component: cell part (GO:0044464);;
region (GO:0005576);; cellular component: extracellular region part (GO:0044421);; biological pro
ducer activity (GO:0004871);; molecular function: molecular transducer activity (GO:0060089);; biolo
:0005488)
vity (GO:0003824);; molecular function: binding (GO:0005488);; cellular component: cell (GO:00056
ess (GO:0009987);; biological process: cellular component organization or biogenesis (GO:0071840
vity (GO:0003824);; molecular function: binding (GO:0005488)

vity (GO:0003824);; cellular component: cell (GO:0005623);; cellular component: organelle (GO:004
)5623);; cellular component: cell part (GO:0044464);; cellular component: organelle (GO:0043226)

vity (GO:0003824);; biological process: metabolic process (GO:0008152);; biological process: cellula
olecule activity (GO:0005198);; cellular component: cell (GO:0005623);; cellular component: macror
:0005488);; cellular component: cell (GO:0005623);; cellular component: organelle (GO:0043226);; c
vity (GO:0003824);; biological process: metabolic process (GO:0008152);; biological process: cellula
ocess (GO:0008152);; biological process: cellular process (GO:0009987);; biological process: single-
)5623);; cellular component: organelle (GO:0043226);; cellular component: organelle part (GO:004
:0005488);; biological process: response to stimulus (GO:0050896);; cellular component: membran
)5623);; cellular component: organelle (GO:0043226);; cellular component: cell part (GO:0044464);;
)5623);; cellular component: membrane-enclosed lumen (GO:0031974);; cellular component: macr
)5623);; cellular component: macromolecular complex (GO:0032991);; cellular component: organel
cular complex (GO:0032991);; molecular function: binding (GO:0005488);; cellular component: cell
ocess (GO:0008152);; biological process: cellular process (GO:0009987);; cellular component: cell (G
GO:0051179);; cellular component: cell (GO:0005623);; cellular component: membrane (GO:001602
vity (GO:0003824);; cellular component: extracellular region (GO:0005576);; cellular component: ex
gulation (GO:0065007);; biological process: cellular process (GO:0009987);; biological process: singl
)5623);; cellular component: organelle (GO:0043226);; cellular component: organelle part (GO:004
:0005488);; cellular component: cell (GO:0005623);; cellular component: membrane (GO:0016020);
)5623);; cellular component: macromolecular complex (GO:0032991);; cellular component: organel
(GO:0016020);; cellular component: membrane part (GO:0044425)
:0005488);; cellular component: cell (GO:0005623);; cellular component: organelle (GO:0043226);; c
:0005488)
em process (GO:0002376);; biological process: biological regulation (GO:0065007);; biological proc
ducer activity (GO:0004871);; molecular function: molecular transducer activity (GO:0060089);; biolo
vity (GO:0003824);; molecular function: antioxidant activity (GO:0016209);; biological process: meta
vity (GO:0003824);; biological process: metabolic process (GO:0008152);; biological process: cellula
)5623);; cellular component: organelle (GO:0043226);; cellular component: cell part (GO:0044464);;
ess (GO:0009987);; biological process: response to stimulus (GO:0050896);; biological process: biolo
:0005488)
:0005488);; cellular component: cell (GO:0005623);; cellular component: cell part (GO:0044464);; ce
cular complex (GO:0032991);; molecular function: binding (GO:0005488);; cellular component: cell
ocess (GO:0008152);; biological process: cellular process (GO:0009987)
gulation (GO:0065007);; cellular component: cell (GO:0005623);; cellular component: organelle (GO
vity (GO:0003824);; molecular function: structural molecule activity (GO:0005198);; molecular funct
)5623);; cellular component: membrane (GO:0016020);; cellular component: organelle (GO:004322
em process (GO:0002376);; biological process: response to stimulus (GO:0050896);; biological proc
ess (GO:0009987);; biological process: cellular component organization or biogenesis (GO:0071840
:0005488);; molecular function: catalytic activity (GO:0003824);; cellular component: cell (GO:0005
vity (GO:0003824);; biological process: cellular process (GO:0009987)
vity (GO:0003824);; cellular component: cell (GO:0005623);; cellular component: organelle (GO:004
)5623);; cellular component: cell part (GO:0044464);; biological process: immune system process (G
:0005488);; molecular function: nucleic acid binding transcription factor activity (GO:0001071);; bio
:0005488);; molecular function: catalytic activity (GO:0003824);; cellular component: macromolecul
)5623);; cellular component: membrane (GO:0016020);; cellular component: organelle (GO:004322
)5623);; cellular component: organelle (GO:0043226);; cellular component: cell part (GO:0044464);;
activity (GO:0005215);; biological process: single-organism process (GO:0044699);; biological proce
)5623);; cellular component: organelle (GO:0043226);; cellular component: organelle part (GO:004
)5623);; cellular component: macromolecular complex (GO:0032991);; cellular component: cell part
(GO:0016020);; cellular component: membrane part (GO:0044425);; molecular function: transporte

factor activity, protein binding (GO:0000988);; biological process: biological regulation (GO:0065C
:0005488)
)5623);; cellular component: membrane-enclosed lumen (GO:0031974);; cellular component: macr
ocess (GO:0008152);; biological process: cellular process (GO:0009987);; cellular component: cell (G
nction regulator (GO:0098772);; biological process: biological regulation (GO:0065007);; cellular cc
ocess (GO:0008152);; biological process: cellular process (GO:0009987);; molecular function: catalyt
)5623);; cellular component: cell part (GO:0044464);; cellular component: membrane (GO:0016020)

organismal process (GO:0032501);; biological process: developmental process (GO:0032502);; biolo
:0005488)
region (GO:0005576);; cellular component: extracellular region part (GO:0044421);; cellular compo
)5623);; cellular component: cell part (GO:0044464);; biological process: biological regulation (GO:(
ocess (GO:0008152);; biological process: cellular process (GO:0009987);; biological process: cellular
cular complex (GO:0032991);; molecular function: binding (GO:0005488);; cellular component: cell
organismal process (GO:0032501);; biological process: developmental process (GO:0032502);; biolo
:0005488)
:0005488);; cellular component: cell (GO:0005623);; cellular component: organelle (GO:0043226);; c
activity (GO:0005215);; biological process: localization (GO:0051179);; cellular component: cell (GO:
)5623);; cellular component: organelle (GO:0043226);; cellular component: cell part (GO:0044464);;
:0005488);; molecular function: catalytic activity (GO:0003824);; cellular component: cell (GO:0005€
:0005488);; cellular component: cell (GO:0005623);; cellular component: cell part (GO:0044464);; ce
:0005488);; cellular component: extracellular region (GO:0005576);; cellular component: extracellul
)5623);; cellular component: cell part (GO:0044464);; biological process: cellular process (GO:00099

(GO:0016020);; cellular component: membrane part (GO:0044425)
olecule activity (GO:0005198);; cellular component: cell (GO:0005623);; cellular component: organe
vity (GO:0003824);; cellular component: cell (GO:0005623);; cellular component: cell part (GO:0044
vity (GO:0003824);; molecular function: binding (GO:0005488)
)5623);; cellular component: organelle (GO:0043226);; cellular component: cell part (GO:0044464);;
activity (GO:0005215);; biological process: single-organism process (GO:0044699);; biological proce
activity (GO:0005215);; biological process: localization (GO:0051179);; cellular component: membra

:0005488);; biological process: immune system process (GO:0002376);; biological process: cellular
:0005488);; cellular component: cell (GO:0005623);; cellular component: organelle (GO:0043226);; c
vity (GO:0003824)
:0005488);; molecular function: catalytic activity (GO:0003824);; cellular component: cell (GO:0005€
olecule activity (GO:0005198);; cellular component: cell (GO:0005623);; cellular component: organe
)5623);; cellular component: cell part (GO:0044464)
ess (GO:0009987);; biological process: locomotion (GO:0040011);; biological process: single-organi

)5623);; cellular component: cell part (GO:0044464);; biological process: localization (GO:0051179);
:0005488)
gulation (GO:0065007);; biological process: multicellular organismal process (GO:0032501);; biologi

ocess (GO:0008152);; biological process: cellular process (GO:0009987);; cellular component: macro
)5623);; cellular component: organelle (GO:0043226);; cellular component: organelle part (GO:0044
(GO:0016020);; cellular component: membrane part (GO:0044425);; molecular function: binding (G
vity (GO:0003824);; molecular function: binding (GO:0005488);; cellular component: cell (GO:0005€
)5623);; cellular component: membrane-enclosed lumen (GO:0031974);; cellular component: orgar
ism (GO:0044215);; cellular component: other organism part (GO:0044217)

)5623);; cellular component: organelle (GO:0043226);; cellular component: organelle part (GO:004
ess (GO:0009987);; biological process: cellular component organization or biogenesis (GO:0071840
ess (GO:0009987);; biological process: cellular component organization or biogenesis (GO:0071840
:0005488);; cellular component: cell (GO:0005623);; cellular component: cell part (GO:0044464);; bi
region (GO:0005576);; cellular component: extracellular region part (GO:0044421);; biological pro
:0005488);; cellular component: cell (GO:0005623);; cellular component: organelle (GO:0043226);;
)5623);; cellular component: membrane (GO:0016020);; cellular component: organelle (GO:004322
:0005488);; biological process: biological regulation (GO:0065007)
:0005488);; biological process: metabolic process (GO:0008152);; biological process: cellular proce
activity (GO:0005215);; biological process: localization (GO:0051179);; cellular component: membra
vity (GO:0003824);; molecular function: binding (GO:0005488);; cellular component: cell (GO:0005
:0005488);; cellular component: membrane (GO:0016020);; cellular component: membrane part (G
)5623);; cellular component: organelle (GO:0043226);; cellular component: cell part (GO:0044464);;
:0005488);; cellular component: extracellular region (GO:0005576);; cellular component: extracellul
region (GO:0005576);; cellular component: extracellular region part (GO:0044421);; biological pro
)5623);; cellular component: organelle (GO:0043226);; cellular component: cell part (GO:0044464);;
)5623);; cellular component: organelle (GO:0043226);; cellular component: cell part (GO:0044464);;
(GO:0016020);; cellular component: membrane part (GO:0044425);; molecular function: binding (G
ess (GO:0009987);; biological process: cellular component organization or biogenesis (GO:0071840
vity (GO:0003824);; biological process: metabolic process (GO:0008152)

vity (GO:0003824);; cellular component: cell (GO:0005623);; cellular component: membrane (GO:0
vity (GO:0003824);; biological process: metabolic process (GO:0008152);; molecular function: bindi
vity (GO:0003824);; molecular function: binding (GO:0005488);; cellular component: cell (GO:0005
al process (GO:0032502);; biological process: single-organism process (GO:0044699);; cellular com
)5623);; cellular component: macromolecular complex (GO:0032991);; cellular component: cell part
:0005488);; biological process: metabolic process (GO:0008152);; biological process: cellular proce

:0005488);; cellular component: extracellular region (GO:0005576);; cellular component: cell (GO:0
:0005488);; cellular component: extracellular region (GO:0005576);; biological process: cellular pro
region (GO:0005576);; cellular component: extracellular region part (GO:0044421);; cellular compo
)5623);; cellular component: cell part (GO:0044464);; cellular component: membrane (GO:0016020
ess (GO:0009987);; biological process: immune system process (GO:0002376);; biological process: n
ess (GO:0009987);; biological process: cellular component organization or biogenesis (GO:0071840
vity (GO:0003824);; molecular function: binding (GO:0005488);; cellular component: cell (GO:0005
:0005488);; cellular component: cell (GO:0005623);; cellular component: cell part (GO:0044464);; ce
vity (GO:0003824)

:0005488);; molecular function: catalytic activity (GO:0003824);; cellular component: cell (GO:0005
)5623);; cellular component: cell part (GO:0044464)
region (GO:0005576);; biological process: single-organism process (GO:0044699);; biological proc
:0005488);; cellular component: membrane (GO:0016020);; cellular component: membrane part (G
vity (GO:0003824);; biological process: metabolic process (GO:0008152);; biological process: cellula
)5623);; cellular component: membrane (GO:0016020);; cellular component: macromolecular comp
vity (GO:0003824);; molecular function: binding (GO:0005488);; cellular component: extracellular re
vity (GO:0003824);; molecular function: binding (GO:0005488)
region (GO:0005576);; cellular component: cell (GO:0005623);; cellular component: organelle (GO
(GO:0016020);; cellular component: membrane part (GO:0044425)
)5623);; cellular component: cell part (GO:0044464);; molecular function: binding (GO:0005488);; ce
(GO:0016020);; cellular component: membrane part (GO:0044425);; molecular function: binding (G
:0005488);; molecular function: structural molecule activity (GO:0005198);; cellular component: cell

vity (GO:0003824);; molecular function: binding (GO:0005488);; cellular component: cell (GO:0005€
julation (GO:0065007);; molecular function: binding (GO:0005488);; cellular component: cell (GO:0(

:0005488)
)5623);; cellular component: cell part (GO:0044464);; biological process: metabolic process (GO:000
:0005488)
)5623);; cellular component: membrane (GO:0016020);; cellular component: organelle (GO:004322
(GO:0016020);; cellular component: membrane part (GO:0044425);; molecular function: binding (G
activity (GO:0005215);; biological process: localization (GO:0051179);; biological process: biological
:0005488);; cellular component: cell (GO:0005623);; cellular component: cell part (GO:0044464);; ce
)5623);; cellular component: cell part (GO:0044464);; molecular function: binding (GO:0005488)
lucer activity (GO:0004871);; molecular function: molecular transducer activity (GO:0060089);; biolo
organismal process (GO:0032501);; biological process: single-organism process (GO:0044699);; cel
vity (GO:0003824)
vity (GO:0003824);; biological process: metabolic process (GO:0008152);; biological process: single
:0005488)
(GO:0016020);; cellular component: membrane part (GO:0044425);; molecular function: transporte

julation (GO:0065007);; molecular function: binding (GO:0005488);; cellular component: cell junctic
vity (GO:0003824);; biological process: metabolic process (GO:0008152);; biological process: cellula
:0005488)
activity (GO:0005215);; biological process: localization (GO:0051179);; cellular component: cell (GO:
)5623);; cellular component: membrane (GO:0016020);; cellular component: organelle (GO:004322
(GO:0016020);; cellular component: membrane part (GO:0044425)
:0005488)
(GO:0016020);; cellular component: membrane part (GO:0044425)
(GO:0000003);; cellular component: cell (GO:0005623);; cellular component: organelle (GO:004322
)5623);; cellular component: organelle (GO:0043226);; cellular component: organelle part (GO:0044
(GO:0016020);; cellular component: membrane part (GO:0044425);; biological process: cellular pro
:0005488);; cellular component: membrane (GO:0016020);; cellular component: membrane part (G
(GO:0016020);; cellular component: membrane part (GO:0044425);; molecular function: binding (G

)5623);; cellular component: macromolecular complex (GO:0032991);; cellular component: organel

ess (GO:0009987);; biological process: locomotion (GO:0040011);; biological process: single-organi
)5623);; cellular component: macromolecular complex (GO:0032991);; cellular component: organel
(GO:0016020);; cellular component: membrane part (GO:0044425)
vity (GO:0003824);; molecular function: binding (GO:0005488);; biological process: localization (GC
region (GO:0005576);; cellular component: extracellular region part (GO:0044421)
)5623);; cellular component: organelle (GO:0043226);; cellular component: cell part (GO:0044464);;
)5623);; cellular component: cell part (GO:0044464);; molecular function: binding (GO:0005488)
)5623);; cellular component: cell part (GO:0044464);; biological process: metabolic process (GO:000
)5623);; cellular component: membrane-enclosed lumen (GO:0031974);; cellular component: orgar
binding transcription factor activity (GO:0001071);; biological process: biological regulation (GO:00
)5623);; cellular component: cell part (GO:0044464);; biological process: cellular process (GO:00099
vity (GO:0003824);; cellular component: membrane (GO:0016020);; cellular component: membrane

vity (GO:0003824);; biological process: metabolic process (GO:0008152);; cellular component: cell (
vity (GO:0003824);; molecular function: binding (GO:0005488);; cellular component: cell (GO:0005€
:0005488);; cellular component: extracellular region (GO:0005576);; cellular component: extracellul

(GO:0016020);; cellular component: membrane part (GO:0044425)
ess (GO:0009987);; biological process: response to stimulus (GO:0050896);; biological process: biolo
(GO:0016020);; cellular component: membrane part (GO:0044425)
ansducer activity (GO:0060089);; biological process: localization (GO:0051179);; cellular componen
vity (GO:0003824);; molecular function: binding (GO:0005488);; cellular component: cell (GO:0005

:0005488);; cellular component: membrane (GO:0016020);; cellular component: membrane part (G
)5623);; cellular component: cell part (GO:0044464);; cellular component: organelle (GO:0043226);;
stimulus (GO:0050896);; molecular function: binding (GO:0005488)
)5623);; cellular component: membrane (GO:0016020);; cellular component: cell part (GO:0044464)
:0005488);; biological process: biological adhesion (GO:0022610);; cellular component: cell (GO:00
(GO:0016020);; cellular component: membrane part (GO:0044425)
region (GO:0005576);; cellular component: extracellular region part (GO:0044421)
ess (GO:0009987);; biological process: signaling (GO:0023052);; biological process: single-organism
gulation (GO:0065007);; molecular function: binding (GO:0005488);; cellular component: cell (GO:00

:0005488);; molecular function: catalytic activity (GO:0003824);; biological process: metabolic proce
:0005488);; biological process: biological regulation (GO:0065007);; molecular function: structural r
(GO:0016020);; cellular component: membrane part (GO:0044425);; molecular function: transporte

ess (GO:0009987);; biological process: cellular component organization or biogenesis (GO:0071840

:0005488);; biological process: cellular process (GO:0009987);; biological process: cellular compone
organismal process (GO:0032501);; molecular function: transporter activity (GO:0005215);; biologic
:0005488);; cellular component: cell (GO:0005623);; cellular component: organelle (GO:0043226);; c
region (GO:0005576);; biological process: single-organism process (GO:0044699);; biological proc
nction regulator (GO:0098772);; biological process: biological regulation (GO:0065007)
)5623);; cellular component: cell part (GO:0044464);; molecular function: binding (GO:0005488);; bi
vity (GO:0003824);; molecular function: binding (GO:0005488)
ocess (GO:0008152);; biological process: cellular process (GO:0009987);; biological process: single-
(GO:0016020);; cellular component: membrane part (GO:0044425);; biological process: biological a

stimulus (GO:0050896);; molecular function: catalytic activity (GO:0003824);; molecular function: an

)5623);; cellular component: membrane-enclosed lumen (GO:0031974);; cellular component: orgar
)5623);; cellular component: organelle (GO:0043226);; cellular component: organelle part (GO:0044
vity (GO:0003824);; molecular function: antioxidant activity (GO:0016209);; biological process: meta
stimulus (GO:0050896);; molecular function: catalytic activity (GO:0003824);; cellular component: ce
:0005488);; cellular component: membrane (GO:0016020);; cellular component: membrane part (G
activity (GO:0005215);; biological process: localization (GO:0051179);; cellular component: cell (GO:
vity (GO:0003824);; biological process: metabolic process (GO:0008152);; biological process: cellula
ess (GO:0009987);; biological process: biological regulation (GO:0065007)
activity (GO:0005215);; biological process: localization (GO:0051179);; molecular function: binding (
em process (GO:0002376);; biological process: response to stimulus (GO:0050896);; molecular func
al process (GO:0032502);; biological process: single-organism process (GO:0044699);; molecular fu
:0005488);; molecular function: nucleic acid binding transcription factor activity (GO:0001071);; bio
(GO:0016020);; cellular component: membrane part (GO:0044425)
vity (GO:0003824);; biological process: metabolic process (GO:0008152);; molecular function: bindi
)5623);; cellular component: membrane-enclosed lumen (GO:0031974);; cellular component: orgar
vity (GO:0003824);; molecular function: binding (GO:0005488);; biological process: immune system
activity (GO:0005215);; biological process: localization (GO:0051179);; molecular function: binding (

)5623);; cellular component: organelle (GO:0043226);; cellular component: cell part (GO:0044464);;
)5623);; cellular component: organelle (GO:0043226);; cellular component: cell part (GO:0044464);;
julation (GO:0065007);; cellular component: extracellular region (GO:0005576);; cellular componen

ocess (GO:0008152);; biological process: cellular process (GO:0009987);; biological process: biologi
:al process (GO:0032502);; biological process: single-organism process (GO:0044699);; biological p
:ss (GO:0009987);; biological process: developmental process (GO:0032502);; biological process: sir
julation (GO:0065007);; cellular component: cell (GO:0005623);; cellular component: organelle (GO
:al process (GO:0032502);; biological process: single-organism process (GO:0044699);; molecular fu
:0005488);; cellular component: cell (GO:0005623);; cellular component: cell part (GO:0044464);; ce
julation (GO:0065007);; molecular function: binding (GO:0005488)
:ss (GO:0009987);; biological process: signaling (GO:0023052);; biological process: single-organism
)5623);; cellular component: organelle (GO:0043226);; cellular component: cell part (GO:0044464);;
vity (GO:0003824);; molecular function: antioxidant activity (GO:0016209);; biological process: meta
:0005488)
(GO:0016020);; cellular component: membrane part (GO:0044425)
activity (GO:0005215);; biological process: localization (GO:0051179);; cellular component: cell (GO:
ism process (GO:0044699);; biological process: localization (GO:0051179);; cellular component: me
activity (GO:0005215);; biological process: single-organism process (GO:0044699);; biological proce
(GO:0016020);; cellular component: membrane part (GO:0044425)

olecule activity (GO:0005198);; biological process: cellular process (GO:0009987);; biological proces
vity (GO:0003824);; cellular component: cell (GO:0005623);; cellular component: membrane-enclos
(GO:0016020);; cellular component: membrane part (GO:0044425)
(GO:0000003);; biological process: metabolic process (GO:0008152);; biological process: cellular p
:0005488);; cellular component: cell (GO:0005623);; cellular component: organelle (GO:0043226);; c
vity (GO:0003824);; cellular component: cell (GO:0005623);; cellular component: cell part (GO:0044
olecule activity (GO:0005198);; biological process: metabolic process (GO:0008152);; biological pro
(GO:0016020);; cellular component: membrane part (GO:0044425)
:ss (GO:0009987);; biological process: developmental process (GO:0032502);; biological process: sir
activity (GO:0005215);; biological process: localization (GO:0051179);; cellular component: cell (GO:
:0005488);; cellular component: cell (GO:0005623);; cellular component: organelle (GO:0043226);; c
)5623);; cellular component: membrane (GO:0016020);; cellular component: cell part (GO:0044464)

ocess (GO:0008152);; biological process: cellular process (GO:0009987);; biological process: single-
activity (GO:0005215);; biological process: localization (GO:0051179);; molecular function: binding (

GO:0051179)

:ellular component: organelle part (GO:0044422);; cellular component: cell part (GO:0044464);; bic
ss (GO:0009987);; biological process: biological regulation (GO:0065007);; biological process: multi

acellular region (GO:0005576);; cellular component: extracellular region part (GO:0044421);; cellula
molecular function: binding (GO:0005488);; cellular component: extracellular region (GO:0005576)

tic activity (GO:0003824);; cellular component: cell (GO:0005623);; cellular component: cell part (GC

ng (GO:0005488)
)65007);; cellular component: cell (GO:0005623);; cellular component: organelle (GO:0043226);; cel
ogical process: single-organism process (GO:0044699);; cellular component: cell (GO:0005623);; ce

ır process (GO:0009987);; molecular function: binding (GO:0005488);; cellular component: cell (GO
)01071);; cellular component: cell (GO:0005623);; cellular component: organelle (GO:0043226);; cel
: (GO:0044464);; biological process: metabolic process (GO:0008152);; biological process: cellular p
23052);; biological process: single-organism process (GO:0044699);; biological process: response t
cal regulation (GO:0065007)

ır process (GO:0009987);; molecular function: binding (GO:0005488);; cellular component: cell (GO
; cellular component: cell part (GO:0044464);; cellular component: membrane part (GO:0044425);;
O:0044425);; molecular function: catalytic activity (GO:0003824);; molecular function: transporter a

6);; cellular component: organelle part (GO:0044422);; cellular component: cell part (GO:0044464);;

ogical process: cellular process (GO:0009987);; biological process: signaling (GO:0023052);; biologic
6);; cellular component: organelle part (GO:0044422);; cellular component: cell part (GO:0044464);;

omponent: macromolecular complex (GO:0032991)
6);; cellular component: organelle part (GO:0044422);; cellular component: cell part (GO:0044464);;
)05623);; cellular component: organelle (GO:0043226);; cellular component: cell part (GO:0044464)
O:0044425);; molecular function: catalytic activity (GO:0003824);; molecular function: transporter a
logical process: biological regulation (GO:0065007);; cellular component: cell (GO:0005623);; cellul
523);; cellular component: organelle (GO:0043226);; cellular component: cell part (GO:0044464);; bi

523);; cellular component: organelle (GO:0043226);; cellular component: cell part (GO:0044464);; bi
:ellular component: organelle part (GO:0044422);; cellular component: cell part (GO:0044464);; bic
)65007);; molecular function: binding (GO:0005488)
ess: cellular process (GO:0009987);; biological process: locomotion (GO:0040011);; biological proce
ess: metabolic process (GO:0008152);; biological process: cellular process (GO:0009987);; biologica
mmune system process (GO:0002376);; biological process: response to stimulus (GO:0050896);; bi
cess: immune system process (GO:0002376);; biological process: response to stimulus (GO:005089

ıne (GO:0016020);; cellular component: membrane part (GO:0044425);; cellular component: cell (G

regulation (GO:0065007);; cellular component: cell (GO:0005623);; cellular component: membrane
ogical process: cellular process (GO:0009987);; biological process: signaling (GO:0023052);; biologic
ent organization or biogenesis (GO:0071840)
4422);; cellular component: cell part (GO:0044464);; cellular component: cell junction (GO:0030054)
lle (GO:0043226);; cellular component: organelle part (GO:0044422);; cellular component: cell part

065007);; cellular component: cell (GO:0005623);; cellular component: organelle (GO:0043226);; cel

6);; cellular component: organelle part (GO:0044422);; cellular component: cell part (GO:0044464);;
on: catalytic activity (GO:0003824)

ar process (GO:0009987);; biological process: single-organism process (GO:0044699);; cellular com
plex (GO:0032991);; cellular component: membrane part (GO:0044425);; cellular component: cell pa
nelle (GO:0043226);; cellular component: organelle part (GO:0044422);; cellular component: cell pa
4422);; cellular component: cell part (GO:0044464);; biological process: cellular process (GO:000998
molecular function regulator (GO:0098772);; biological process: biological regulation (GO:006500
ogical process: single-organism process (GO:0044699);; molecular function: binding (GO:0005488)

);; biological process: cellular process (GO:0009987);; biological process: multicellular organismal p

ogical process: cellular process (GO:0009987);; biological process: signaling (GO:0023052);; biologic
ngle-organism process (GO:0044699);; biological process: cellular component organization or bio

ogical process: cellular process (GO:0009987);; biological process: signaling (GO:0023052);; biologic
ogical process: cellular process (GO:0009987);; biological process: signaling (GO:0023052);; biologic
acellular region (GO:0005576);; cellular component: extracellular region part (GO:0044421);; cellula
(GO:0005623);; cellular component: cell part (GO:0044464)

ogical process: single-organism process (GO:0044699);; biological process: biological regulation (G

tabolic process (GO:0008152);; biological process: single-organism process (GO:0044699);; molecu

ogical process: cellular process (GO:0009987);; biological process: signaling (GO:0023052);; biologic
ogical process: cellular process (GO:0009987);; biological process: signaling (GO:0023052);; biologic
egulation (GO:0065007);; biological process: cellular process (GO:0009987);; biological process: res
organism process (GO:0044699);; molecular function: catalytic activity (GO:0003824);; cellular com
process (GO:0044699);; biological process: response to stimulus (GO:0050896);; biological proces
egulation (GO:0065007);; biological process: cellular process (GO:0009987);; biological process: res
; biological process: cellular process (GO:0009987);; biological process: signaling (GO:0023052);; bi

al regulation (GO:0065007);; molecular function: binding (GO:0005488);; biological process: respon

or (GO:0007610);; biological process: multicellular organismal process (GO:0032501);; biological pr
ellular process (GO:0009987);; biological process: single-organism process (GO:0044699);; biologic

ss (GO:0009987);; biological process: response to stimulus (GO:0050896);; biological process: biolo

ar process (GO:0009987);; molecular function: binding (GO:0005488)
nbrane (GO:0016020);; cellular component: membrane part (GO:0044425)
0044464);; cellular component: membrane (GO:0016020);; molecular function: binding (GO:000548
rt (GO:0044464);; cellular component: organelle (GO:0043226);; cellular component: organelle part
ion: binding (GO:0005488);; cellular component: cell (GO:0005623);; cellular component: cell part (
ogical process: cellular process (GO:0009987);; biological process: signaling (GO:0023052);; biologic
alytic activity (GO:0003824);; molecular function: binding (GO:0005488);; cellular component: cell (G
ion: binding (GO:0005488);; cellular component: cell (GO:0005623);; cellular component: cell part (
-organism process (GO:0044699);; biological process: cellular process (GO:0009987);; molecular fu
)
065007);; molecular function: binding (GO:0005488);; cellular component: cell (GO:0005623);; cellul
mbrane (GO:0016020);; cellular component: membrane part (GO:0044425);; biological process: de

; cellular component: organelle (GO:0043226);; cellular component: organelle part (GO:0044422);;

ellular component: organelle (GO:0043226)
523);; cellular component: organelle (GO:0043226);; cellular component: cell part (GO:0044464);; bi

044425);; cellular component: cell part (GO:0044464)

005623);; cellular component: cell part (GO:0044464);; cellular component: membrane (GO:001602
ar process (GO:0009987);; molecular function: binding (GO:0005488)
e process (GO:0022414);; biological process: single-organism process (GO:0044699);; biological pr

ogical process: cellular process (GO:0009987);; biological process: signaling (GO:0023052);; biologic
ogical process: cellular process (GO:0009987);; biological process: signaling (GO:0023052);; biologic

4422);; cellular component: cell part (GO:0044464);; biological process: cellular process (GO:000998
ell (GO:0005623);; cellular component: membrane (GO:0016020);; cellular component: cell part (GO
4422);; cellular component: cell part (GO:0044464);; cellular component: supramolecular complex (

065007);; biological process: cellular process (GO:0009987);; biological process: signaling (GO:0023
plex (GO:0032991);; cellular component: organelle (GO:0043226);; cellular component: organelle pa

nent: membrane (GO:0016020);; cellular component: membrane part (GO:0044425);; cellular comp

6);; cellular component: organelle part (GO:0044422);; cellular component: cell part (GO:0044464);;
process (GO:0044699);; biological process: biological regulation (GO:0065007);; biological process
biological process: cellular process (GO:0009987);; biological process: cellular component organiz
ır process (GO:0009987);; molecular function: binding (GO:0005488);; cellular component: cell (GO
ɔgical process: cellular process (GO:0009987);; biological process: signaling (GO:0023052);; biologic

ion: binding (GO:0005488);; cellular component: cell (GO:0005623);; cellular component: cell part (
ling (GO:0023052);; biological process: single-organism process (GO:0044699);; biological process:
(GO:0009987);; biological process: signaling (GO:0023052);; biological process: single-organism p
: (GO:0044464);; biological process: cellular process (GO:0009987);; biological process: single-orga
nbrane (GO:0016020);; cellular component: membrane part (GO:0044425)

ocomotion (GO:0040011);; biological process: single-organism process (GO:0044699);; biological p
ess: localization (GO:0051179);; cellular component: membrane (GO:0016020);; cellular component

ɔgical process: cellular process (GO:0009987);; biological process: signaling (GO:0023052);; biologic
4422);; cellular component: cell part (GO:0044464);; molecular function: binding (GO:0005488);; cel
623);; cellular component: macromolecular complex (GO:0032991);; cellular component: organelle
:0005623);; cellular component: membrane (GO:0016020);; cellular component: cell part (GO:0044
ocomotion (GO:0040011);; biological process: single-organism process (GO:0044699);; biological p

unction: molecular function regulator (GO:0098772);; cellular component: macromolecular comple
nbrane (GO:0016020);; cellular component: membrane part (GO:0044425)

824);; biological process: metabolic process (GO:0008152)

molecular function: binding (GO:0005488);; biological process: cellular process (GO:0009987);; bio
ling (GO:0023052);; biological process: single-organism process (GO:0044699);; biological process:
044425);; cellular component: cell part (GO:0044464)
: multi-organism process (GO:0051704);; molecular function: binding (GO:0005488);; biological pro
);; biological process: localization (GO:0051179);; biological process: cellular process (GO:0009987)
cellular component: synapse part (GO:0044456);; cellular component: cell part (GO:0044464);; cellu

ological process: localization (GO:0051179);; biological process: cellular process (GO:0009987);; bio
4422);; cellular component: cell part (GO:0044464);; molecular function: binding (GO:0005488);; cel
ɔgical process: cellular process (GO:0009987);; biological process: signaling (GO:0023052);; biologic
cess: immune system process (GO:0002376);; biological process: response to stimulus (GO:005089

d binding transcription factor activity (GO:0001071);; biological process: multicellular organismal p

d binding transcription factor activity (GO:0001071);; biological process: developmental process (G
0044464);; cellular component: membrane (GO:0016020);; cellular component: cell junction (GO:00

cellular component: cell part (GO:0044464);; biological process: biological regulation (GO:0065007

007);; cellular component: cell (GO:0005623);; cellular component: organelle (GO:0043226);; cellular
r organismal process (GO:0032501);; biological process: developmental process (GO:0032502);; bi
binding (GO:0005488);; molecular function: nucleic acid binding transcription factor activity (GO:0

);; cellular component: membrane part (GO:0044425);; biological process: cellular process (GO:000[illegible]
6);; cellular component: organelle part (GO:0044422);; cellular component: membrane part (GO:00
ess (GO:0008152);; biological process: cellular process (GO:0009987);; cellular component: cell (GO

(GO:0009987);; biological process: biological regulation (GO:0065007);; cellular component: meml

)05623);; cellular component: membrane (GO:0016020);; cellular component: membrane part (GO
ess (GO:0008152);; biological process: cellular process (GO:0009987)

)65007);; cellular component: cell (GO:0005623);; cellular component: organelle (GO:0043226);; cel
biological regulation (GO:0065007)
gical process: cellular process (GO:0009987);; biological process: signaling (GO:0023052);; biologic

sm process (GO:0044699);; biological process: localization (GO:0051179);; molecular function: binc

);; molecular function: catalytic activity (GO:0003824);; biological process: metabolic process (GO:0
le (GO:0043226);; cellular component: organelle part (GO:0044422);; cellular component: cell part (
.6020);; cellular component: membrane part (GO:0044425);; biological process: cellular process (G
gical process: single-organism process (GO:0044699)
process (GO:0044699);; biological process: response to stimulus (GO:0050896);; biological proces

GO:0005623);; cellular component: organelle (GO:0043226);; cellular component: cell part (GO:004
ctivity (GO:0003824)

cellular component: cell part (GO:0044464);; cellular component: membrane (GO:0016020);; cellula
process: cellular process (GO:0009987);; biological process: signaling (GO:0023052);; biological pr
g (GO:0005488);; molecular function: structural molecule activity (GO:0005198);; cellular compone
4422);; cellular component: cell part (GO:0044464);; cellular component: macromolecular complex (

6);; cellular component: organelle part (GO:0044422);; cellular component: cell part (GO:0044464);;

:0043226);; cellular component: cell part (GO:0044464);; cellular component: membrane (GO:0016

dhesion (GO:0022610);; biological process: single-organism process (GO:0044699)
);; cellular component: membrane part (GO:0044425)

:0005623);; cellular component: membrane (GO:0016020);; cellular component: cell part (GO:004

nent: membrane (GO:0016020)
ngle-organism process (GO:0044699)
biological regulation (GO:0065007);; molecular function: catalytic activity (GO:0003824);; biological
gical process: cellular process (GO:0009987);; biological process: signaling (GO:0023052);; biologic

0065007);; cellular component: membrane (GO:0016020);; cellular component: cell junction (GO:00
process (GO:0044699);; biological process: response to stimulus (GO:0050896);; biological proces
biological process: metabolic process (GO:0008152);; biological process: cellular process (GO:000
ular function: signal transducer activity (GO:0004871);; molecular function: molecular transducer ac
r activity (GO:0005215);; biological process: localization (GO:0051179)
)05623);; cellular component: cell part (GO:0044464);; cellular component: organelle (GO:0043226

cal process: developmental process (GO:0032502);; biological process: single-organism process (G
ogical process: single-organism process (GO:0044699);; biological process: immune system proces

ıction: molecular transducer activity (GO:0060089);; biological process: cellular process (GO:00998

);; cellular component: macromolecular complex (GO:0032991);; cellular component: organelle (GC
(GO:0005623);; cellular component: cell part (GO:0044464);; biological process: cellular process (GC

lar complex (GO:0032991);; cellular component: organelle (GO:0043226);; cellular component: orga
regulation (GO:0065007);; cellular component: cell (GO:0005623);; cellular component: membrane

);; biological process: reproduction (GO:0000003);; biological process: reproductive process (GO:00

le (GO:0043226);; cellular component: organelle part (GO:0044422);; cellular component: cell part (

cellular component organization or biogenesis (GO:0071840);; molecular function: binding (GO:00
: binding (GO:0005488);; cellular component: cell (GO:0005623);; cellular component: membrane (
sed lumen (GO:0031974);; cellular component: organelle (GO:0043226);; cellular component: organ
0065007);; cellular component: membrane (GO:0016020);; cellular component: membrane part (GC
biological process: cellular process (GO:0009987);; biological process: developmental process (GC
molecular function: binding (GO:0005488)

ar process (GO:0009987);; molecular function: binding (GO:0005488)
O:0016020);; cellular component: organelle (GO:0043226);; cellular component: organelle part (GC
cellular component: cell part (GO:0044464)
(GO:0009987);; biological process: single-organism process (GO:0044699);; cellular component: ce
ological process: biological regulation (GO:0065007)
nclosed lumen (GO:0031974);; cellular component: organelle (GO:0043226);; cellular component: o
plex (GO:0032991);; cellular component: organelle (GO:0043226);; cellular component: organelle pa
en (GO:0031974);; cellular component: organelle (GO:0043226);; cellular component: organelle par
brane part (GO:0044425)
biological process: biological regulation (GO:0065007)
);; biological process: biological adhesion (GO:0022610);; molecular function: binding (GO:0005488

process: localization (GO:0051179);; cellular component: membrane (GO:0016020);; cellular compo
-organism process (GO:0044699);; biological process: cellular process (GO:0009987);; molecular fu
:0043226);; cellular component: cell part (GO:0044464);; biological process: cellular process (GO:00
ess: localization (GO:0051179);; cellular component: cell (GO:0005623);; cellular component: organ
aling (GO:0023052);; biological process: single-organism process (GO:0044699);; molecular functic

6);; cellular component: organelle part (GO:0044422);; cellular component: membrane part (GO:00
065007);; molecular function: binding (GO:0005488)
process (GO:0044699);; biological process: cellular component organization or biogenesis (GO:00
cellular component: membrane (GO:0016020);; cellular component: membrane part (GO:0044425
523);; cellular component: organelle (GO:0043226);; cellular component: cell part (GO:0044464);; bi

process (GO:0032502);; biological process: single-organism process (GO:0044699);; cellular compo

:: cell (GO:0005623);; cellular component: organelle (GO:0043226);; cellular component: cell part (G

r activity (GO:0005215);; biological process: localization (GO:0051179)
ological process: cellular process (GO:0009987);; biological process: signaling (GO:0023052);; biolc

ngle-organism process (GO:0044699);; biological process: cellular component organization or bio(

ngle-organism process (GO:0044699);; biological process: multicellular organismal process (GO:00
function: binding (GO:0005488);; biological process: localization (GO:0051179);; cellular compone
044425);; cellular component: cell part (GO:0044464);; biological process: biological adhesion (GO:
4422);; cellular component: cell part (GO:0044464);; cellular component: membrane (GO:0016020);;

organism process (GO:0044699);; molecular function: molecular function regulator (GO:0098772);;
-organism process (GO:0044699);; biological process: localization (GO:0051179);; cellular compon

ess: localization (GO:0051179);; cellular component: cell (GO:0005623);; cellular component: memb

O:0007610);; biological process: multicellular organismal process (GO:0032501);; biological proces
4422);; cellular component: cell part (GO:0044464);; molecular function: binding (GO:0005488);; bio
O:0044425);; molecular function: catalytic activity (GO:0003824);; molecular function: transporter a
cess: metabolic process (GO:0008152);; biological process: cellular process (GO:0009987);; biologic
lticellular organismal process (GO:0032501);; biological process: developmental process (GO:0032

ar process (GO:0009987);; biological process: single-organism process (GO:0044699);; molecular fu

logical process: biological regulation (GO:0065007);; cellular component: cell (GO:0005623);; cellul
le (GO:0043226);; cellular component: cell part (GO:0044464);; molecular function: binding (GO:00
)
ess (GO:0008152);; biological process: cellular process (GO:0009987)

464);; cellular component: membrane (GO:0016020);; biological process: biological regulation (GO
ess (GO:0008152);; cellular component: cell (GO:0005623);; cellular component: organelle (GO:004
ion: binding (GO:0005488);; cellular component: cell (GO:0005623);; cellular component: cell part (
6);; cellular component: organelle part (GO:0044422);; cellular component: cell part (GO:0044464);;

cellular component: membrane (GO:0016020);; cellular component: membrane part (GO:0044425

4422);; cellular component: cell part (GO:0044464);; biological process: localization (GO:0051179);;
; cellular component: membrane part (GO:0044425);; cellular component: cell part (GO:0044464);;
tion: signal transducer activity (GO:0004871);; molecular function: molecular transducer activity (G
);; biological process: signaling (GO:0023052);; biological process: single-organism process (GO:00

4422);; cellular component: cell part (GO:0044464);; molecular function: binding (GO:0005488);; bio

process (GO:0044699);; biological process: response to stimulus (GO:0050896);; biological proces

cellular component: cell part (GO:0044464);; biological process: biological regulation (GO:0065007
ɔgical process: cellular process (GO:0009987);; biological process: signaling (GO:0023052);; biologic
4422);; cellular component: cell part (GO:0044464);; biological process: metabolic process (GO:0008
ɔcomotion (GO:0040011);; biological process: single-organism process (GO:0044699);; biological p
sducer activity (GO:0004871);; molecular function: molecular transducer activity (GO:0060089);; bic

044425);; cellular component: cell part (GO:0044464);; molecular function: transporter activity (GO:

065007)
ıelle (GO:0043226);; cellular component: organelle part (GO:0044422);; cellular component: cell pa
component: membrane (GO:0016020);; cellular component: membrane part (GO:0044425);; cellul

organism process (GO:0044699);; molecular function: catalytic activity (GO:0003824)
cellular component: organelle part (GO:0044422);; cellular component: cell part (GO:0044464);; bic
ıelle (GO:0043226);; cellular component: organelle part (GO:0044422);; cellular component: cell pa
ır process (GO:0009987);; biological process: single-organism process (GO:0044699)
ctivity (GO:0003824)
ellular component: organelle (GO:0043226);; cellular component: synapse part (GO:0044456);; cellu

4422);; cellular component: cell part (GO:0044464)
function: binding (GO:0005488);; biological process: cellular process (GO:0009987)
d binding transcription factor activity (GO:0001071);; biological process: multicellular organismal p
; cellular component: macromolecular complex (GO:0032991);; cellular component: organelle (GO:
ɔgical process: single-organism process (GO:0044699);; molecular function: catalytic activity (GO:0
-organism process (GO:0044699);; biological process: behavior (GO:0007610);; biological process:
molecular function: binding (GO:0005488);; biological process: biological regulation (GO:0065007
cellular component: organelle part (GO:0044422);; cellular component: cell part (GO:0044464);; bic
4422);; cellular component: cell part (GO:0044464);; cellular component: membrane (GO:0016020);;
(GO:0005623);; cellular component: cell part (GO:0044464);; biological process: cellular process (GC
ess (GO:0008152);; biological process: cellular process (GO:0009987);; biological process: cellular cc
ess: localization (GO:0051179);; cellular component: membrane (GO:0016020);; cellular component

ess (GO:0008152)
lular component: cell (GO:0005623);; cellular component: membrane (GO:0016020);; cellular comp
ess (GO:0008152);; biological process: single-organism process (GO:0044699);; biological process:
le (GO:0043226);; cellular component: organelle part (GO:0044422);; cellular component: cell part (

molecular function: binding (GO:0005488)
rocess (GO:0009987);; biological process: single-organism process (GO:0044699);; molecular funct
065007);; molecular function: binding (GO:0005488)
cellular component: organelle part (GO:0044422);; cellular component: cell part (GO:0044464);; bic
:0005623);; cellular component: cell part (GO:0044464);; cellular component: membrane-enclosed
3226);; cellular component: organelle part (GO:0044422);; cellular component: cell part (GO:00444
ane (GO:0016020);; cellular component: membrane part (GO:0044425);; cellular component: cell pa

005623);; cellular component: cell part (GO:0044464);; biological process: biological regulation (GC
ɔgical process: single-organism process (GO:0044699);; molecular function: structural molecule ac

4422);; cellular component: cell part (GO:0044464);; biological process: biological regulation (GO:0(
ar process (GO:0009987);; biological process: single-organism process (GO:0044699)
biological process: biological regulation (GO:0065007);; molecular function: binding (GO:0005488)
nelle (GO:0043226);; cellular component: organelle part (GO:0044422);; cellular component: cell pa
523);; cellular component: organelle (GO:0043226);; cellular component: organelle part (GO:00442
005623);; cellular component: organelle (GO:0043226);; cellular component: cell part (GO:0044464

0016020);; cellular component: organelle (GO:0043226);; cellular component: organelle part (GO:0
GO:0005623);; cellular component: membrane-enclosed lumen (GO:0031974);; cellular component
cellular component: cell part (GO:0044464);; cellular component: membrane-enclosed lumen (GO:
987);; biological process: single-organism process (GO:0044699)
ar process (GO:0009987);; biological process: cellular component organization or biogenesis (GO:0
:0005623);; cellular component: membrane (GO:0016020);; cellular component: organelle (GO:004
ss (GO:0009987);; biological process: cellular component organization or biogenesis (GO:0071840)
cellular component: membrane (GO:0016020);; cellular component: membrane part (GO:0044425

ar process (GO:0009987);; biological process: response to stimulus (GO:0050896);; cellular compon

523);; cellular component: cell part (GO:0044464);; cellular component: membrane (GO:0016020);; l
6);; cellular component: organelle part (GO:0044422);; cellular component: cell part (GO:0044464);;
6);; cellular component: organelle part (GO:0044422);; cellular component: cell part (GO:0044464);;
4422);; cellular component: cell part (GO:0044464);; cellular component: membrane (GO:0016020)

molecular complex (GO:0032991);; cellular component: organelle (GO:0043226);; cellular compone
cellular component: organelle part (GO:0044422);; cellular component: supramolecular complex (
component organization or biogenesis (GO:0071840);; cellular component: cell (GO:0005623);; ce
cellular component: membrane (GO:0016020);; biological process: single-organism process (GO:0
molecular complex (GO:0032991);; cellular component: organelle (GO:0043226);; cellular compone
plex (GO:0032991);; cellular component: organelle (GO:0043226);; cellular component: organelle pa

rocess (GO:0032501);; biological process: biological regulation (GO:0065007);; biological process: c
e part (GO:0044425)
523);; cellular component: cell part (GO:0044464);; biological process: metabolic process (GO:0008
ll (GO:0005623);; cellular component: organelle (GO:0043226);; cellular component: organelle part
ular component organization or biogenesis (GO:0071840);; biological process: biological regulation
cellular component: cell part (GO:0044464);; cellular component: membrane (GO:0016020);; molec
component organization or biogenesis (GO:0071840);; molecular function: catalytic activity (GO:0

molecular function: binding (GO:0005488)
component organization or biogenesis (GO:0071840);; cellular component: cell (GO:0005623);; ce
4422);; cellular component: cell part (GO:0044464);; biological process: biological regulation (GO:0(
824)
cellular component: organelle part (GO:0044422);; cellular component: membrane-enclosed lume
component organization or biogenesis (GO:0071840);; molecular function: binding (GO:0005488)
cellular component: organelle part (GO:0044422);; biological process: biological regulation (GO:0(

olecular function: catalytic activity (GO:0003824);; biological process: metabolic process (GO:0008

unction: binding (GO:0005488);; biological process: multicellular organismal process (GO:0032501)
523);; cellular component: organelle (GO:0043226);; cellular component: organelle part (GO:00442
ss (GO:0009987)
process (GO:0044699);; biological process: immune system process (GO:0002376);; biological proc
biological process: cellular process (GO:0009987);; biological process: cellular component organiz
lle (GO:0043226);; cellular component: organelle part (GO:0044422);; cellular component: cell part

: cell (GO:0005623);; cellular component: macromolecular complex (GO:0032991);; cellular compor

cellular component: membrane (GO:0016020);; cellular component: membrane part (GO:0044425
organism process (GO:0044699);; cellular component: membrane (GO:0016020);; cellular compon

ess (GO:0008152);; biological process: cellular process (GO:0009987);; cellular component: cell (GO
owth (GO:0040007);; biological process: single-organism process (GO:0044699);; molecular functi
binding (GO:0005488);; molecular function: nucleic acid binding transcription factor activity (GO:0
olex (GO:0032991);; cellular component: organelle (GO:0043226);; cellular component: organelle pa
nolecular complex (GO:0032991);; cellular component: organelle (GO:0043226);; cellular compone
biological process: biological regulation (GO:0065007);; molecular function: binding (GO:0005488
(GO:0032991);; cellular component: organelle (GO:0043226);; cellular component: organelle part (
(GO:0005623);; cellular component: cell part (GO:0044464);; cellular component: organelle (GO:00
nelle (GO:0043226);; cellular component: organelle part (GO:0044422);; cellular component: cell pa
cess (GO:0009987);; biological process: cellular component organization or biogenesis (GO:00718

GO:0005623);; cellular component: cell part (GO:0044464)
007);; cellular component: cell (GO:0005623);; cellular component: organelle (GO:0043226);; cellular
4422);; cellular component: cell part (GO:0044464);; cellular component: membrane (GO:0016020);;

: (GO:0044464);; biological process: metabolic process (GO:0008152);; biological process: cellular p
: (GO:0044464);; molecular function: binding (GO:0005488);; cellular component: organelle (GO:00
ion: binding (GO:0005488);; cellular component: cell (GO:0005623);; cellular component: cell part (
065007);; molecular function: binding (GO:0005488)
cellular component: organelle part (GO:0044422);; cellular component: cell part (GO:0044464);; cel
ogical process: single-organism process (GO:0044699);; cellular component: membrane (GO:0016
ar process (GO:0009987);; biological process: single-organism process (GO:0044699)

olex (GO:0032991);; cellular component: organelle (GO:0043226);; cellular component: organelle pa
ngle-organism process (GO:0044699);; biological process: cellular component organization or biog
6);; cellular component: organelle part (GO:0044422);; cellular component: cell part (GO:0044464);;
cellular component: organelle part (GO:0044422);; cellular component: membrane-enclosed lume
005623);; cellular component: cell part (GO:0044464);; cellular component: membrane-enclosed lu
nelle (GO:0043226);; cellular component: organelle part (GO:0044422);; cellular component: cell pa
cellular component: cell part (GO:0044464);; cellular component: membrane-enclosed lumen (GO:
; cellular component: organelle (GO:0043226);; cellular component: organelle part (GO:0044422);;
4422);; cellular component: cell part (GO:0044464);; molecular function: binding (GO:0005488);; mo
ng (GO:0023052);; biological process: single-organism process (GO:0044699);; biological process: r
nelle (GO:0043226);; cellular component: organelle part (GO:0044422);; cellular component: cell pa
nolecular complex (GO:0032991);; cellular component: organelle (GO:0043226);; cellular compone

cellular component: organelle part (GO:0044422);; cellular component: cell part (GO:0044464)

ɔgical process: single-organism process (GO:0044699);; molecular function: binding (GO:0005488)
abolic process (GO:0008152);; biological process: cellular process (GO:0009987);; biological proces:
ctivity (GO:0003824)
process (GO:0044699);; biological process: developmental process (GO:0032502);; biological proc

logical process: biological regulation (GO:0065007);; cellular component: cell (GO:0005623);; cellul
523);; cellular component: membrane (GO:0016020);; cellular component: cell part (GO:0044464);; l
biological process: cellular process (GO:0009987);; biological process: cellular component organiz
t: cell (GO:0005623);; cellular component: organelle (GO:0043226);; cellular component: organelle
cellular component: cell part (GO:0044464)
-organism process (GO:0044699);; cellular component: cell (GO:0005623);; cellular component: or
unction: binding (GO:0005488);; cellular component: cell (GO:0005623);; cellular component: meml
)16020);; cellular component: macromolecular complex (GO:0032991);; cellular component: memb
biological process: metabolic process (GO:0008152);; biological process: cellular process (GO:000
ar process (GO:0009987)
6);; cellular component: organelle part (GO:0044422);; cellular component: cell part (GO:0044464);;
nelle (GO:0043226);; cellular component: organelle part (GO:0044422);; cellular component: cell pa
cess: cellular process (GO:0009987);; cellular component: cell (GO:0005623);; cellular component: r
t (GO:0044464);; cellular component: macromolecular complex (GO:0032991);; cellular componen
ological process: developmental process (GO:0032502);; biological process: biological regulation (G
le (GO:0043226);; cellular component: organelle part (GO:0044422);; cellular component: cell part (
cal regulation (GO:0065007);; biological process: cellular component organization or biogenesis (G

:0043226);; cellular component: organelle part (GO:0044422);; cellular component: cell part (GO:00
metabolic process (GO:0008152);; biological process: single-organism process (GO:0044699)
biological process: metabolic process (GO:0008152);; biological process: cellular process (GO:000

6);; cellular component: organelle part (GO:0044422);; cellular component: membrane part (GO:00
nelle (GO:0043226);; cellular component: organelle part (GO:0044422);; cellular component: cell pa
g (GO:0005488);; molecular function: catalytic activity (GO:0003824)
O:0016020);; cellular component: organelle (GO:0043226);; cellular component: organelle part (GC
-organism process (GO:0044699);; molecular function: binding (GO:0005488);; cellular component

omponent: cell (GO:0005623);; cellular component: cell part (GO:0044464);; biological process: cellu
organism process (GO:0044699);; cellular component: cell (GO:0005623);; cellular component: mer
4422);; cellular component: cell part (GO:0044464)
ar process (GO:0009987);; biological process: single-organism process (GO:0044699);; biological pr
ar process (GO:0009987);; biological process: single-organism process (GO:0044699);; cellular com
);; biological process: biological adhesion (GO:0022610);; biological process: cellular process (GO:0
cellular component: cell part (GO:0044464)
biological process: localization (GO:0051179);; molecular function: binding (GO:0005488)
):0005488);; cellular component: cell (GO:0005623);; cellular component: organelle (GO:0043226);;
: (GO:0032991)

molecular complex (GO:0032991);; cellular component: organelle (GO:0043226);; cellular compone

ar process (GO:0009987)
cellular component: organelle part (GO:0044422);; cellular component: cell part (GO:0044464);; mc

cellular component: membrane (GO:0016020);; cellular component: organelle part (GO:0044422);;

ng (GO:0005488);; biological process: immune system process (GO:0002376);; biological process: r
iO:0007610);; biological process: multicellular organismal process (GO:0032501);; biological proces
ar process (GO:0009987);; cellular component: cell (GO:0005623);; cellular component: cell part (GC
523);; cellular component: cell part (GO:0044464);; cellular component: membrane (GO:0016020);; b
rocess: cellular process (GO:0009987);; biological process: cellular component organization or biog
ine (GO:0016020);; cellular component: membrane part (GO:0044425)
nent: membrane (GO:0016020);; cellular component: membrane part (GO:0044425)
4422);; cellular component: cell part (GO:0044464);; cellular component: membrane (GO:0016020);;
; cellular component: macromolecular complex (GO:0032991);; cellular component: organelle (GO:
cellular component: organelle part (GO:0044422);; cellular component: membrane-enclosed lume

)88);; cellular component: cell (GO:0005623);; cellular component: organelle (GO:0043226);; cellular
4422);; cellular component: cell part (GO:0044464);; molecular function: binding (GO:0005488);; bio
: (GO:0044464);; biological process: metabolic process (GO:0008152);; biological process: cellular p
) stimulus (GO:0050896);; biological process: immune system process (GO:0002376);; biological pro
ar process (GO:0009987);; cellular component: cell (GO:0005623);; cellular component: cell part (GC
ig (GO:0005488)
(GO:0051179);; molecular function: transporter activity (GO:0005215)
nelle (GO:0043226);; cellular component: organelle part (GO:0044422);; cellular component: cell pa
4422);; cellular component: cell part (GO:0044464);; cellular component: supramolecular complex ((
044425);; cellular component: cell part (GO:0044464);; molecular function: transporter activity (GO:

ess (GO:0008152);; biological process: cellular process (GO:0009987);; cellular component: macrom

nolecular complex (GO:0032991);; cellular component: organelle (GO:0043226);; cellular compone
e (GO:0016020);; cellular component: membrane part (GO:0044425);; molecular function: catalytic
cellular component organization or biogenesis (GO:0071840);; biological process: biological regula
4422);; cellular component: cell part (GO:0044464);; molecular function: binding (GO:0005488);; bio

ar process (GO:0009987);; molecular function: binding (GO:0005488);; cellular component: cell (GO
cellular component organization or biogenesis (GO:0071840);; cellular component: cell (GO:00056
ogical process: single-organism process (GO:0044699);; biological process: reproduction (GO:0000

e part (GO:0044425)
);; cellular component: cell (GO:0005623);; cellular component: cell part (GO:0044464);; cellular cor
O:0044425);; biological process: biological regulation (GO:0065007)
process (GO:0044699);; biological process: response to stimulus (GO:0050896);; biological process
ess (GO:0008152);; biological process: cellular process (GO:0009987)
ar process (GO:0009987);; molecular function: binding (GO:0005488)
ar process (GO:0009987);; biological process: response to stimulus (GO:0050896);; molecular functi

523);; cellular component: organelle (GO:0043226);; cellular component: cell part (GO:0044464);; ce
cellular component organization or biogenesis (GO:0071840);; cellular component: cell (GO:00056
lar complex (GO:0032991);; cellular component: cell part (GO:0044464);; cellular component: orgar
4422);; cellular component: cell part (GO:0044464);; cellular component: membrane (GO:0016020);;
ocalization (GO:0051179);; biological process: cellular component organization or biogenesis (GO:
ar process (GO:0009987);; biological process: single-organism process (GO:0044699);; molecular fu
ation (GO:0065007);; cellular component: membrane (GO:0016020);; cellular component: membra
)65007);; biological process: multicellular organismal process (GO:0032501);; biological process: de

: (GO:0044464);; biological process: metabolic process (GO:0008152);; biological process: cellular p
ological process: cellular process (GO:0009987);; biological process: immune system process (GO:0
08152);; biological process: cellular process (GO:0009987);; cellular component: membrane-enclose
organism process (GO:0044699);; cellular component: membrane (GO:0016020);; cellular compon
: developmental process (GO:0032502);; biological process: single-organism process (GO:0044699
ogical process: cellular process (GO:0009987);; biological process: signaling (GO:0023052);; biologic

ess (GO:0008152);; biological process: cellular process (GO:0009987)
biological process: cellular process (GO:0009987);; biological process: cellular component organiz

4422);; cellular component: cell part (GO:0044464);; molecular function: binding (GO:0005488);; bio

sm process (GO:0044699);; biological process: localization (GO:0051179);; molecular function: binc
6);; cellular component: organelle part (GO:0044422);; cellular component: cell part (GO:0044464);;
6);; cellular component: organelle part (GO:0044422);; cellular component: cell part (GO:0044464);;
GO:0005623);; cellular component: membrane-enclosed lumen (GO:0031974);; cellular component
3226);; cellular component: cell part (GO:0044464);; biological process: biological regulation (GO:0
organism process (GO:0044699);; biological process: cellular component organization or biogenes
4422);; cellular component: cell part (GO:0044464);; molecular function: molecular function regulat
le (GO:0043226);; cellular component: organelle part (GO:0044422);; cellular component: cell part (
cellular component organization or biogenesis (GO:0071840);; cellular component: cell (GO:00056
ological process: metabolic process (GO:0008152);; biological process: cellular process (GO:00099
ss (GO:0009987);; biological process: cellular component organization or biogenesis (GO:0071840)
le (GO:0043226);; cellular component: organelle part (GO:0044422);; cellular component: cell part (
le (GO:0043226);; cellular component: organelle part (GO:0044422);; cellular component: cell part (
044425);; cellular component: cell part (GO:0044464);; biological process: immune system process
GO:0005623);; cellular component: organelle (GO:0043226);; cellular component: organelle part (G
cellular component organization or biogenesis (GO:0071840);; cellular component: cell (GO:00056
4422);; cellular component: cell part (GO:0044464);; cellular component: macromolecular complex (
logical process: biological regulation (GO:0065007);; biological process: multicellular organismal p
6);; cellular component: organelle part (GO:0044422);; cellular component: cell part (GO:0044464);;
ss (GO:0009987);; cellular component: cell (GO:0005623);; cellular component: macromolecular co
4422);; cellular component: cell part (GO:0044464);; molecular function: binding (GO:0005488)

: (GO:0044464);; biological process: metabolic process (GO:0008152);; biological process: cellular p
623);; cellular component: cell part (GO:0044464);; biological process: metabolic process (GO:0008
logical process: biological regulation (GO:0065007);; cellular component: cell (GO:0005623);; cellul

on: binding (GO:0005488);; molecular function: catalytic activity (GO:0003824)
molecular complex (GO:0032991);; cellular component: organelle (GO:0043226);; cellular compone
r process (GO:0009987);; biological process: single-organism process (GO:0044699)
nelle (GO:0043226);; cellular component: organelle part (GO:0044422);; cellular component: cell pa
lle (GO:0043226);; cellular component: organelle part (GO:0044422);; cellular component: cell part
ss (GO:0009987);; cellular component: cell (GO:0005623);; cellular component: organelle (GO:0043
cellular component: cell part (GO:0044464);; cellular component: macromolecular complex (GO:00
gical regulation (GO:0065007)

le (GO:0043226);; cellular component: organelle part (GO:0044422);; cellular component: cell part (

)05623);; cellular component: cell part (GO:0044464);; biological process: cellular process (GO:000

: (GO:0044464);; biological process: metabolic process (GO:0008152);; biological process: cellular p
single-organism process (GO:0044699);; molecular function: signal transducer activity (GO:000487

abolic process (GO:0008152)
on factor activity, protein binding (GO:0000988);; biological process: biological regulation (GO:006
cellular component: membrane (GO:0016020);; cellular component: membrane part (GO:0044425

process (GO:0044699);; biological process: cellular component organization or biogenesis (GO:00
4422);; cellular component: cell part (GO:0044464);; cellular component: membrane-enclosed lume
ogical process: single-organism process (GO:0044699);; cellular component: cell (GO:0005623);; ce
onent: cell (GO:0005623);; cellular component: organelle (GO:0043226);; cellular component: cell p

ctivity (GO:0003824);; molecular function: antioxidant activity (GO:0016209);; biological process: me
ng (GO:0005488);; cellular component: cell (GO:0005623);; cellular component: macromolecular co
plex (GO:0032991);; cellular component: organelle (GO:0043226);; cellular component: organelle pa
cellular component: organelle part (GO:0044422);; cellular component: cell part (GO:0044464);; bio
623);; cellular component: organelle (GO:0043226);; cellular component: cell part (GO:0044464);; bi
ar region part (GO:0044421);; biological process: immune system process (GO:0002376);; biologica
molecular function: catalytic activity (GO:0003824);; biological process: metabolic process (GO:000
ding (GO:0005488)
cellular component: membrane-enclosed lumen (GO:0031974);; cellular component: organelle pa
r activity (GO:0005215);; biological process: localization (GO:0051179)
GO:0005623);; cellular component: organelle (GO:0043226);; cellular component: cell part (GO:004
ess (GO:0008152);; biological process: single-organism process (GO:0044699);; biological process:
4422);; cellular component: cell part (GO:0044464)
ular function: binding (GO:0005488);; biological process: cellular process (GO:0009987);; biological
0065007);; cellular component: membrane (GO:0016020);; cellular component: membrane part (GO
4422);; cellular component: cell part (GO:0044464);; cellular component: supramolecular complex (
4422);; cellular component: cell part (GO:0044464);; molecular function: binding (GO:0005488);; bio
nelle (GO:0043226);; cellular component: organelle part (GO:0044422);; cellular component: cell pa
987);; cellular component: macromolecular complex (GO:0032991);; cellular component: organelle (
4422);; cellular component: cell part (GO:0044464);; molecular function: catalytic activity (GO:00038

se to stimulus (GO:0050896);; molecular function: catalytic activity (GO:0003824);; molecular functi
ng (GO:0005488);; cellular component: cell (GO:0005623);; cellular component: cell part (GO:00444
-organism process (GO:0044699);; cellular component: cell (GO:0005623);; cellular component: ce
cellular component: membrane (GO:0016020);; cellular component: membrane part (GO:0044425
organism process (GO:0044699);; cellular component: membrane (GO:0016020);; cellular compon
ess (GO:0008152);; biological process: cellular process (GO:0009987);; biological process: single-or

biological process: cellular process (GO:0009987);; biological process: cellular component organiz
ss (GO:0009987);; molecular function: catalytic activity (GO:0003824)
);; cellular component: cell junction (GO:0030054);; molecular function: binding (GO:0005488);; biol
cal regulation (GO:0065007);; cellular component: cell (GO:0005623);; cellular component: macrom
n (GO:0022610);; cellular component: cell (GO:0005623);; cellular component: cell part (GO:004446
O:0005488)

O:0005488)

ogical process: single-organism process (GO:0044699);; biological process: reproduction (GO:000C
nolecular complex (GO:0032991);; cellular component: organelle (GO:0043226);; cellular compone

e process (GO:0022414);; biological process: multicellular organismal process (GO:0032501);; biolo
biological process: metabolic process (GO:0008152);; biological process: cellular process (GO:000
4422);; cellular component: cell part (GO:0044464);; biological process: cellular process (GO:00098
organism process (GO:0044699);; molecular function: catalytic activity (GO:0003824);; molecular fu
6);; cellular component: organelle part (GO:0044422);; cellular component: membrane part (GO:00
omolecular complex (GO:0032991);; cellular component: organelle (GO:0043226);; cellular compor

);; molecular function: binding (GO:0005488);; biological process: metabolic process (GO:0008152)

ar process (GO:0009987);; biological process: single-organism process (GO:0044699)
:0005623);; cellular component: membrane (GO:0016020);; cellular component: organelle (GO:004

464);; biological process: metabolic process (GO:0008152);; biological process: cellular process (GC
ogical process: single-organism process (GO:0044699);; biological process: biological regulation (G
: (GO:0044464);; molecular function: transcription factor activity, protein binding (GO:0000988);; bi
4422);; cellular component: cell part (GO:0044464);; biological process: multicellular organismal pro
process: single-organism process (GO:0044699);; molecular function: binding (GO:0005488);; biolo
623);; cellular component: cell part (GO:0044464);; cellular component: organelle (GO:0043226);; ce
);; cellular component: membrane part (GO:0044425);; molecular function: binding (GO:0005488);;
nolecular complex (GO:0032991);; cellular component: organelle (GO:0043226);; cellular compone
GO:0005623);; cellular component: macromolecular complex (GO:0032991);; cellular component: o
cellular component: cell part (GO:0044464);; biological process: single-organism process (GO:0044
);; biological process: cellular process (GO:0009987);; biological process: single-organism process (
ar process (GO:0009987);; biological process: single-organism process (GO:0044699)
4422);; cellular component: cell part (GO:0044464);; biological process: single-organism process (G
rocess: multicellular organismal process (GO:0032501);; biological process: biological regulation (G
005623);; cellular component: organelle (GO:0043226);; cellular component: cell part (GO:0044464
4422);; cellular component: cell part (GO:0044464);; biological process: cellular process (GO:00098
nolecular complex (GO:0032991);; cellular component: organelle (GO:0043226);; cellular compone
binding (GO:0005488);; cellular component: cell (GO:0005623);; cellular component: macromolecu

nolecular complex (GO:0032991);; cellular component: organelle (GO:0043226);; cellular compone
: cell (GO:0005623);; cellular component: organelle (GO:0043226);; cellular component: cell part (G
4422);; cellular component: cell part (GO:0044464);; biological process: biological adhesion (GO:00

4422);; cellular component: cell part (GO:0044464);; biological process: metabolic process (GO:000

nolecular complex (GO:0032991);; cellular component: organelle (GO:0043226);; cellular compone
cellular component: membrane-enclosed lumen (GO:0031974);; cellular component: organelle pa
ellular organismal process (GO:0032501);; biological process: developmental process (GO:0032502
O:0016020);; cellular component: macromolecular complex (GO:0032991);; cellular component: or
: (GO:0044464);; cellular component: membrane-enclosed lumen (GO:0031974);; cellular compone

ess: localization (GO:0051179);; cellular component: cell (GO:0005623);; cellular component: memb

);; cellular component: membrane part (GO:0044425);; cellular component: macromolecular compl
4422);; cellular component: cell part (GO:0044464);; cellular component: supramolecular complex (

(GO:0051179)
);; cellular component: cell junction (GO:0030054);; molecular function: binding (GO:0005488)
cellular component: membrane (GO:0016020);; cellular component: membrane part (GO:0044425
; cellular component: cell part (GO:0044464);; biological process: biological regulation (GO:006500
824);; biological process: metabolic process (GO:0008152);; biological process: single-organism pr
nolecular complex (GO:0032991);; cellular component: organelle (GO:0043226);; cellular compone
ological regulation (GO:0065007);; cellular component: cell (GO:0005623);; cellular component: cell
);; cellular component: cell junction (GO:0030054);; cellular component: membrane part (GO:00444
4422);; cellular component: cell part (GO:0044464);; cellular component: supramolecular complex (
ar process (GO:0009987);; biological process: single-organism process (GO:0044699);; molecular fu

lar complex (GO:0032991);; cellular component: cell part (GO:0044464);; molecular function: cataly
sducer activity (GO:0004871);; molecular function: molecular transducer activity (GO:0060089);; bio
4422);; cellular component: cell part (GO:0044464);; molecular function: binding (GO:0005488);; cel
cellular component: cell part (GO:0044464);; cellular component: organelle part (GO:0044422);; bio
GO:0005623);; cellular component: macromolecular complex (GO:0032991);; cellular component: o
ar process (GO:0009987);; biological process: single-organism process (GO:0044699)
GO:0005623);; cellular component: nucleoid (GO:0009295);; cellular component: organelle (GO:004

(GO:0005623);; cellular component: organelle (GO:0043226);; cellular component: organelle part (
464);; biological process: metabolic process (GO:0008152);; biological process: cellular process (GO
g (GO:0005488);; molecular function: structural molecule activity (GO:0005198);; cellular compone
organism process (GO:0044699)
ctivity (GO:0003824)

component organization or biogenesis (GO:0071840);; cellular component: cell (GO:0005623);; ce
007);; cellular component: cell (GO:0005623);; cellular component: organelle (GO:0043226);; cellular
ess (GO:0008152);; biological process: single-organism process (GO:0044699)
cellular component: organelle part (GO:0044422);; cellular component: cell part (GO:0044464);; bio
523);; cellular component: cell part (GO:0044464);; biological process: metabolic process (GO:00081
le (GO:0043226);; cellular component: organelle part (GO:0044422);; cellular component: cell part (
-organism process (GO:0044699);; cellular component: cell (GO:0005623);; cellular component: or
ar process (GO:0009987);; cellular component: cell (GO:0005623);; cellular component: organelle (G
cal regulation (GO:0065007);; cellular component: cell (GO:0005623);; cellular component: macrom
omolecular complex (GO:0032991);; cellular component: organelle (GO:0043226);; cellular compor
se to stimulus (GO:0050896);; molecular function: transcription factor activity, protein binding (GO
biological process: metabolic process (GO:0008152);; biological process: cellular process (GO:000
cal process: biological regulation (GO:0065007);; cellular component: cell (GO:0005623);; cellular co
ar process (GO:0009987);; molecular function: binding (GO:0005488)
-organism process (GO:0044699);; cellular component: cell (GO:0005623);; cellular component: ma
cellular component: organelle part (GO:0044422);; cellular component: cell part (GO:0044464);; bio
biological process: developmental process (GO:0032502);; biological process: cellular process (GC
cess: response to stimulus (GO:0050896);; molecular function: binding (GO:0005488);; biological pr
ogical process: cellular process (GO:0009987);; biological process: signaling (GO:0023052);; biologic

523);; cellular component: macromolecular complex (GO:0032991);; cellular component: organelle
);; biological process: metabolic process (GO:0008152);; cellular component: cell (GO:0005623);; ce

-3226);; cellular component: cell part (GO:0044464);; biological process: metabolic process (GO:000

ar process (GO:0009987)
nolecular complex (GO:0032991);; cellular component: organelle (GO:0043226);; cellular compone
cellular component: cell part (GO:0044464);; biological process: cellular process (GO:0009987);; bio
ar process (GO:0009987);; cellular component: cell (GO:0005623);; cellular component: membrane
organism process (GO:0044699);; biological process: response to stimulus (GO:0050896);; molecul
4422);; cellular component: cell part (GO:0044464);; cellular component: macromolecular complex (
e (GO:0016020)
cellular component: membrane (GO:0016020);; biological process: metabolic process (GO:000815
omolecular complex (GO:0032991);; cellular component: organelle (GO:0043226);; cellular compor
le (GO:0043226);; cellular component: organelle part (GO:0044422);; cellular component: cell part (
(GO:0005623);; cellular component: organelle (GO:0043226);; cellular component: organelle part (
GO:0005623);; cellular component: macromolecular complex (GO:0032991);; cellular component: o
20);; cellular component: macromolecular complex (GO:0032991);; cellular component: membrane
tracellular region part (GO:0044421);; biological process: metabolic process (GO:0008152);; biolog
e-organism process (GO:0044699);; cellular component: cell (GO:0005623);; cellular component: n
4422);; cellular component: cell part (GO:0044464);; cellular component: membrane (GO:0016020);;
; cellular component: cell part (GO:0044464);; biological process: multicellular organismal process (
le (GO:0043226);; cellular component: cell part (GO:0044464)

cellular component: cell part (GO:0044464);; biological process: biological regulation (GO:0065007)

ess: developmental process (GO:0032502);; molecular function: transporter activity (GO:0005215);;
ogical process: cellular process (GO:0009987);; biological process: signaling (GO:0023052);; biologic
abolic process (GO:0008152);; biological process: cellular process (GO:0009987);; biological process
ar process (GO:0009987);; biological process: biological regulation (GO:0065007);; molecular functi
molecular function: binding (GO:0005488)
ogical regulation (GO:0065007);; cellular component: cell (GO:0005623);; cellular component: orgar

ellular component: macromolecular complex (GO:0032991);; molecular function: catalytic activity (C
(GO:0005623);; cellular component: organelle (GO:0043226);; cellular component: cell part (GO:004

:0043226);; cellular component: cell part (GO:0044464)
ion: binding (GO:0005488);; cellular component: cell (GO:0005623);; cellular component: cell part (
6);; cellular component: organelle part (GO:0044422);; cellular component: cell part (GO:0044464);;
ess: multi-organism process (GO:0051704)
);; cellular component: cell (GO:0005623);; cellular component: cell part (GO:0044464);; biological p
523);; cellular component: membrane-enclosed lumen (GO:0031974);; cellular component: organel

-3226);; cellular component: cell part (GO:0044464);; biological process: metabolic process (GO:000
GO:0002376);; biological process: response to stimulus (GO:0050896);; biological process: cellular p
logical process: biological regulation (GO:0065007);; cellular component: cell (GO:0005623);; cellul
ar complex (GO:0032991)
6);; cellular component: organelle part (GO:0044422);; cellular component: cell part (GO:0044464);;
molecular function: binding (GO:0005488);; biological process: metabolic process (GO:0008152);; l
ess: localization (GO:0051179);; cellular component: cell (GO:0005623);; cellular component: memb
4422);; cellular component: cell part (GO:0044464);; cellular component: membrane-enclosed lume
: (GO:0044464);; molecular function: binding (GO:0005488);; biological process: metabolic process
r activity (GO:0005215);; biological process: localization (GO:0051179)

)07);; cellular component: cell (GO:0005623);; cellular component: cell part (GO:0044464);; cellular c

omolecular complex (GO:0032991);; cellular component: organelle (GO:0043226);; cellular compor
GO:0005623);; cellular component: membrane-enclosed lumen (GO:0031974);; cellular component
omponent: extracellular region (GO:0005576);; cellular component: extracellular region part (GO:00
tic activity (GO:0003824);; cellular component: cell (GO:0005623);; cellular component: organelle (G
);; cellular component: membrane part (GO:0044425);; molecular function: antioxidant activity (GO:

ogical process: single-organism process (GO:0044699);; molecular function: binding (GO:0005488)

onent: supramolecular complex (GO:0099080);; biological process: multicellular organismal process
)065007)
component organization or biogenesis (GO:0071840);; molecular function: structural molecule ac
(GO:0005623);; cellular component: cell part (GO:0044464);; cellular component: organelle (GO:004
ogical process: single-organism process (GO:0044699);; molecular function: binding (GO:0005488)

cellular component: organelle part (GO:0044422);; cellular component: cell part (GO:0044464)
:0005623);; cellular component: membrane (GO:0016020);; cellular component: macromolecular co
cellular component: membrane (GO:0016020);; cellular component: membrane part (GO:0044425
523);; cellular component: cell part (GO:0044464);; biological process: metabolic process (GO:0008
ellular component: cell junction (GO:0030054);; biological process: cellular process (GO:0009987);; b
ar region part (GO:0044421);; cellular component: cell (GO:0005623);; cellular component: membra
)87);; biological process: cellular component organization or biogenesis (GO:0071840);; biological p

lle (GO:0043226);; cellular component: organelle part (GO:0044422);; cellular component: cell part
464);; biological process: metabolic process (GO:0008152);; biological process: cellular process (GC

molecular function: electron carrier activity (GO:0009055);; molecular function: binding (GO:00054
ess: localization (GO:0051179)
ne (GO:0016020);; cellular component: membrane part (GO:0044425)

process (GO:0009987);; biological process: single-organism process (GO:0044699);; biological proc
cellular component: organelle part (GO:0044422);; cellular component: cell part (GO:0044464);; mc

523);; cellular component: organelle (GO:0043226);; cellular component: cell part (GO:0044464);; bi
lle (GO:0043226);; cellular component: cell part (GO:0044464);; biological process: metabolic proce

sm process (GO:0044699);; biological process: localization (GO:0051179);; biological process: immu

; cellular component: macromolecular complex (GO:0032991)

cal process: developmental process (GO:0032502);; biological process: single-organism process (G

omolecular complex (GO:0032991);; cellular component: cell (GO:0005623);; cellular component: m
4422);; cellular component: cell part (GO:0044464);; cellular component: supramolecular complex (
O:0005488)
523);; cellular component: organelle (GO:0043226);; cellular component: cell part (GO:0044464);; ce
nelle (GO:0043226);; cellular component: organelle part (GO:0044422);; cellular component: cell pa

4422);; cellular component: cell part (GO:0044464);; molecular function: catalytic activity (GO:00038
);; molecular function: binding (GO:0005488);; biological process: metabolic process (GO:0008152)
)
ological process: metabolic process (GO:0008152);; biological process: cellular process (GO:00099
cess: response to stimulus (GO:0050896);; biological process: immune system process (GO:000237
cellular component: cell part (GO:0044464);; biological process: biological regulation (GO:0065007
6);; cellular component: organelle part (GO:0044422);; cellular component: cell part (GO:0044464);;

ss (GO:0009987);; biological process: cellular component organization or biogenesis (GO:0071840)
ne (GO:0016020);; cellular component: membrane part (GO:0044425);; biological process: cellular
623);; cellular component: cell part (GO:0044464);; biological process: immune system process (GO
O:0044425);; molecular function: catalytic activity (GO:0003824)
cellular component: organelle part (GO:0044422);; cellular component: macromolecular complex (
ar region part (GO:0044421);; biological process: biological regulation (GO:0065007);; cellular com
cess: single-organism process (GO:0044699);; biological process: localization (GO:0051179);; mole
biological process: cellular process (GO:0009987);; biological process: cellular component organiz
biological process: cellular process (GO:0009987);; biological process: cellular component organiz
O:0005488);; cellular component: cell (GO:0005623);; cellular component: organelle (GO:0043226)
);; biological process: single-organism process (GO:0044699);; molecular function: catalytic activity

016020);; cellular component: macromolecular complex (GO:0032991);; cellular component: memb
ng (GO:0005488);; cellular component: cell (GO:0005623);; cellular component: organelle (GO:004
623);; cellular component: organelle (GO:0043226);; cellular component: cell part (GO:0044464);; ce
nponent: cell (GO:0005623);; cellular component: cell part (GO:0044464);; cellular component: mem
(GO:0044464);; biological process: metabolic process (GO:0008152);; biological process: cellular p
ss (GO:0009987)

005623);; cellular component: cell part (GO:0044464);; biological process: metabolic process (GO:0
cess (GO:0009987);; biological process: signaling (GO:0023052);; biological process: single-organis
onent: cell (GO:0005623);; cellular component: macromolecular complex (GO:0032991);; cellular cc
);; cellular component: membrane part (GO:0044425);; biological process: metabolic process (GO:0
nulticellular organismal process (GO:0032501);; biological process: developmental process (GO:003
);; molecular function: binding (GO:0005488);; biological process: metabolic process (GO:0008152)
623);; cellular component: organelle (GO:0043226);; cellular component: cell part (GO:0044464);; bi
ellular component: organelle (GO:0043226);; biological process: biological regulation (GO:0065007

623);; cellular component: organelle (GO:0043226);; cellular component: cell part (GO:0044464);; bi

cess: localization (GO:0051179);; molecular function: binding (GO:0005488);; cellular component: m
O:0044425);; biological process: biological regulation (GO:0065007);; biological process: immune s
ar process (GO:0009987);; cellular component: cell (GO:0005623);; cellular component: organelle (G
plex (GO:0032991);; cellular component: organelle (GO:0043226);; cellular component: organelle pa
egion (GO:0005576);; cellular component: organelle (GO:0043226);; cellular component: other orga

:0043226);; cellular component: organelle part (GO:0044422);; cellular component: cell part (GO:00

ellular component: macromolecular complex (GO:0032991);; biological process: metabolic process
O:0005488)
(GO:0005623);; cellular component: organelle (GO:0043226);; cellular component: cell part (GO:00

523);; cellular component: cell part (GO:0044464);; biological process: cellular process (GO:0009987
)05623);; cellular component: membrane (GO:0016020);; cellular component: organelle (GO:00432

)8152);; biological process: cellular process (GO:0009987)

6);; cellular component: organelle part (GO:0044422);; cellular component: cell part (GO:0044464);;
O:0005488)
regulation (GO:0065007);; molecular function: molecular function regulator (GO:0098772);; biolog
ellular component: organelle (GO:0043226);; biological process: localization (GO:0051179);; biologi

ogical process: cellular process (GO:0009987);; biological process: signaling (GO:0023052);; biologic
lular component: cell (GO:0005623);; cellular component: membrane (GO:0016020);; cellular comp

-organism process (GO:0044699);; molecular function: binding (GO:0005488);; cellular component

r activity (GO:0005215);; biological process: localization (GO:0051179);; biological process: metabo

on (GO:0030054);; biological process: cellular process (GO:0009987);; biological process: single-org
ar process (GO:0009987)

:0005623);; cellular component: macromolecular complex (GO:0032991);; cellular component: cell p
6);; cellular component: organelle part (GO:0044422);; cellular component: membrane part (GO:00

26);; cellular component: organelle part (GO:0044422);; cellular component: cell part (GO:0044464)
4422);; cellular component: cell part (GO:0044464);; molecular function: catalytic activity (GO:00038
cess (GO:0009987)
O:0044425);; molecular function: catalytic activity (GO:0003824);; molecular function: transporter a
O:0005488)

le (GO:0043226);; cellular component: organelle part (GO:0044422);; cellular component: cell part (

sm process (GO:0044699);; biological process: localization (GO:0051179);; biological process: deve
le (GO:0043226);; cellular component: cell part (GO:0044464);; molecular function: binding (GO:00

):0051179);; cellular component: cell (GO:0005623);; cellular component: cell part (GO:0044464);; c

cellular component: membrane (GO:0016020);; cellular component: membrane part (GO:0044425

)8152);; biological process: cellular process (GO:0009987);; biological process: single-organism pro
nelle (GO:0043226);; cellular component: organelle part (GO:0044422);; cellular component: cell pa
)65007);; molecular function: binding (GO:0005488)
)87);; biological process: cellular component organization or biogenesis (GO:0071840);; biological p
e part (GO:0044425)

(GO:0005623);; cellular component: organelle (GO:0043226);; cellular component: organelle part (G
523);; cellular component: organelle (GO:0043226);; cellular component: organelle part (GO:00442
ar region part (GO:0044421);; biological process: response to stimulus (GO:0050896);; biological pr

ɔgical regulation (GO:0065007)

t: cell (GO:0005623);; cellular component: membrane (GO:0016020);; cellular component: membra
523);; cellular component: organelle (GO:0043226);; cellular component: cell part (GO:0044464);; ce

O:0044425);; molecular function: catalytic activity (GO:0003824);; molecular function: transporter a
cellular component: cell junction (GO:0030054);; molecular function: binding (GO:0005488);; biolo

);; biological process: biological regulation (GO:0065007);; cellular component: membrane part (GC
05623);; cellular component: membrane (GO:0016020);; cellular component: membrane part (GO:(

ı process (GO:0044699);; biological process: response to stimulus (GO:0050896);; biological proces
)05623);; cellular component: organelle (GO:0043226);; cellular component: cell part (GO:0044464)

ess (GO:0008152);; biological process: cellular process (GO:0009987)
nolecule activity (GO:0005198);; cellular component: extracellular region (GO:0005576);; cellular cc
r activity (GO:0005215);; biological process: localization (GO:0051179)

);; molecular function: catalytic activity (GO:0003824);; molecular function: binding (GO:0005488);;

ent organization or biogenesis (GO:0071840)
al process: localization (GO:0051179);; cellular component: cell (GO:0005623);; cellular component
cellular component: cell part (GO:0044464);; biological process: cellular process (GO:0009987);; bio
cess: localization (GO:0051179);; molecular function: binding (GO:0005488);; biological process: me

ological process: biological regulation (GO:0065007);; biological process: metabolic process (GO:0

organism process (GO:0044699);; molecular function: catalytic activity (GO:0003824);; molecular fu
ıdhesion (GO:0022610)

tioxidant activity (GO:0016209);; biological process: metabolic process (GO:0008152);; biological pı

nelle (GO:0043226);; cellular component: organelle part (GO:0044422);; cellular component: cell pa
4422);; cellular component: cell part (GO:0044464);; molecular function: catalytic activity (GO:00038
abolic process (GO:0008152);; biological process: cellular process (GO:0009987);; biological process
ell (GO:0005623);; cellular component: membrane (GO:0016020);; cellular component: organelle (G
O:0044425);; molecular function: catalytic activity (GO:0003824);; molecular function: transporter a
:0005623);; cellular component: macromolecular complex (GO:0032991);; cellular component: cell
ar process (GO:0009987);; biological process: single-organism process (GO:0044699);; cellular com

GO:0005488);; cellular component: cell (GO:0005623);; cellular component: macromolecular comp
tion: binding (GO:0005488);; cellular component: extracellular region (GO:0005576);; cellular comp
unction: structural molecule activity (GO:0005198)
logical process: biological regulation (GO:0065007);; biological process: developmental process (G

ng (GO:0005488)
nelle (GO:0043226);; cellular component: organelle part (GO:0044422);; cellular component: cell pa
ı process (GO:0002376);; biological process: response to stimulus (GO:0050896);; biological process
GO:0005488);; cellular component: cell (GO:0005623);; cellular component: macromolecular comp

molecular function: molecular function regulator (GO:0098772);; biological process: biological reg
molecular function: molecular function regulator (GO:0098772);; biological process: biological reg
t: extracellular region part (GO:0044421);; cellular component: cell (GO:0005623);; cellular compon

cal regulation (GO:0065007);; molecular function: catalytic activity (GO:0003824);; molecular functi
rocess: multicellular organismal process (GO:0032501);; biological process: biological regulation (G
ngle-organism process (GO:0044699);; biological process: cellular component organization or biog
:0043226);; cellular component: cell part (GO:0044464);; biological process: metabolic process (GC
unction: catalytic activity (GO:0003824);; cellular component: cell (GO:0005623);; cellular componer
ellular component: organelle (GO:0043226);; cellular component: membrane (GO:0016020);; cellula

process (GO:0044699);; biological process: response to stimulus (GO:0050896);; biological proces
cellular component: membrane (GO:0016020);; biological process: localization (GO:0051179);; mo
abolic process (GO:0008152);; biological process: cellular process (GO:0009987);; biological process

:0005623);; cellular component: macromolecular complex (GO:0032991);; cellular component: cell
mbrane (GO:0016020);; cellular component: membrane part (GO:0044425);; molecular function: tr
ess: localization (GO:0051179);; cellular component: membrane (GO:0016020);; cellular component

ss: single-organism process (GO:0044699);; biological process: cellular component organization or
sed lumen (GO:0031974);; cellular component: organelle (GO:0043226);; cellular component: orgar

rocess (GO:0009987);; biological process: single-organism process (GO:0044699);; biological proce
cellular component: cell part (GO:0044464);; biological process: biological regulation (GO:0065007
464);; biological process: metabolic process (GO:0008152);; biological process: cellular process (GC
cess: cellular process (GO:0009987);; cellular component: cell (GO:0005623);; cellular component: r

ngle-organism process (GO:0044699);; biological process: cellular component organization or biog
:0005623);; cellular component: macromolecular complex (GO:0032991);; cellular component: cell
cellular component: cell part (GO:0044464);; biological process: biological regulation (GO:0065007
);; cellular component: cell junction (GO:0030054);; cellular component: membrane part (GO:00444

organism process (GO:0044699);; molecular function: catalytic activity (GO:0003824);; cellular com
GO:0005488);; cellular component: cell (GO:0005623);; cellular component: macromolecular comp

ological process: multicellular organismal process (GO:0032501);; biological process: developmenta
cellular organismal process (GO:0032501);; biological process: developmental process (GO:00325C

r component: cell (GO:0005623);; cellular component: cell part (GO:0044464)
);; cellular component: extracellular region part (GO:0044421)

O:0044464);; cellular component: organelle (GO:0043226);; cellular component: membrane (GO:00[illegible]

lular component: cell part (GO:0044464);; biological process: multicellular organismal process (GO
ellular component: membrane (GO:0016020);; cellular component: cell part (GO:0044464);; cellular

:0005623);; cellular component: organelle (GO:0043226);; cellular component: cell part (GO:00444[illegible]
lular component: cell part (GO:0044464);; biological process: cellular process (GO:0009987);; biolo[illegible]
process (GO:0009987);; biological process: signaling (GO:0023052);; biological process: single-orga
to stimulus (GO:0050896);; biological process: biological regulation (GO:0065007);; biological proce

:0005623);; cellular component: cell part (GO:0044464)
biological process: biological regulation (GO:0065007)
ctivity (GO:0005215);; biological process: localization (GO:0051179)

; biological process: localization (GO:0051179);; cellular component: membrane part (GO:0044425)

cal process: single-organism process (GO:0044699);; biological process: response to stimulus (GO:[illegible]
; molecular function: catalytic activity (GO:0003824);; biological process: metabolic process (GO:00[illegible]

; biological process: metabolic process (GO:0008152);; biological process: cellular process (GO:000
);; biological process: metabolic process (GO:0008152);; biological process: cellular process (GO:00
ctivity (GO:0005215);; biological process: localization (GO:0051179)
ar component: organelle (GO:0043226);; cellular component: cell part (GO:0044464);; biological pr
ological process: metabolic process (GO:0008152);; biological process: cellular process (GO:00099[illegible]

ological process: reproduction (GO:0000003);; biological process: cellular process (GO:0009987);; b
ological process: biological regulation (GO:0065007);; cellular component: extracellular region (GO:

ess: single-organism process (GO:0044699);; biological process: response to stimulus (GO:005089[illegible]
l process: single-organism process (GO:0044699);; biological process: immune system process (G[illegible]
ological process: biological regulation (GO:0065007);; cellular component: cell (GO:0005623);; cellu
6);; molecular function: binding (GO:0005488)

O:0005623);; cellular component: organelle (GO:0043226);; cellular component: organelle part (GC

e (GO:0016020);; cellular component: macromolecular complex (GO:0032991);; cellular component
cal process: single-organism process (GO:0044699);; biological process: response to stimulus (GO:(

)
(GO:0044464);; cellular component: supramolecular complex (GO:0099080)

llular component: cell part (GO:0044464);; molecular function: binding (GO:0005488)

; cellular component: membrane part (GO:0044425);; molecular function: binding (GO:0005488);; b

ponent: cell (GO:0005623);; cellular component: cell part (GO:0044464);; cellular component: mem
art (GO:0044464);; molecular function: molecular function regulator (GO:0098772);; biological proc
rt (GO:0044464);; molecular function: binding (GO:0005488);; biological process: metabolic proces
37);; biological process: single-organism process (GO:0044699);; biological process: cellular compo
7);; cellular component: cell (GO:0005623);; cellular component: organelle (GO:0043226);; cellular c
;; cellular component: cell (GO:0005623);; cellular component: membrane (GO:0016020);; cellular c

rocess (GO:0032501);; biological process: developmental process (GO:0032502);; biological proces

cal process: single-organism process (GO:0044699);; biological process: response to stimulus (GO:(
genesis (GO:0071840);; molecular function: binding (GO:0005488)

cal process: single-organism process (GO:0044699);; biological process: response to stimulus (GO:(
cal process: single-organism process (GO:0044699);; biological process: response to stimulus (GO:(
r component: cell (GO:0005623);; cellular component: membrane (GO:0016020);; cellular compone

GO:0065007)

ular function: binding (GO:0005488);; cellular component: cell (GO:0005623);; cellular component: c

cal process: single-organism process (GO:0044699);; biological process: response to stimulus (GO:(
cal process: single-organism process (GO:0044699);; biological process: response to stimulus (GO:(
sponse to stimulus (GO:0050896);; biological process: immune system process (GO:0002376);; biolo
ponent: cell (GO:0005623);; cellular component: membrane-enclosed lumen (GO:0031974);; cellula
s: biological regulation (GO:0065007);; cellular component: membrane (GO:0016020);; cellular com
sponse to stimulus (GO:0050896)
ological process: single-organism process (GO:0044699);; biological process: response to stimulus

se to stimulus (GO:0050896)

ocess: single-organism process (GO:0044699);; biological process: response to stimulus (GO:0050
al process: multicellular organismal process (GO:0032501);; molecular function: catalytic activity (G

gical regulation (GO:0065007);; cellular component: cell (GO:0005623);; cellular component: memb

8)
(GO:0044422);; cellular component: supramolecular complex (GO:0099080);; biological process: ce
GO:0044464);; cellular component: organelle (GO:0043226);; cellular component: organelle part (G
cal process: single-organism process (GO:0044699);; biological process: response to stimulus (GO:
GO:0005623);; cellular component: cell part (GO:0044464);; cellular component: membrane (GO:00
GO:0044464);; cellular component: organelle (GO:0043226);; cellular component: organelle part (G
nction: binding (GO:0005488)

ar component: organelle (GO:0043226);; cellular component: cell part (GO:0044464);; biological pr
velopmental process (GO:0032502);; biological process: single-organism process (GO:0044699)

cellular component: membrane part (GO:0044425);; cellular component: cell part (GO:0044464);; b

ological process: cellular process (GO:0009987);; biological process: cellular component organizati

0);; biological process: cellular process (GO:0009987);; biological process: signaling (GO:0023052);;

ocess: cellular component organization or biogenesis (GO:0071840);; biological process: metabolic

cal process: single-organism process (GO:0044699);; biological process: response to stimulus (GO:
cal process: single-organism process (GO:0044699);; biological process: response to stimulus (GO:

37);; biological process: signaling (GO:0023052);; biological process: single-organism process (GO:
:0044464);; biological process: metabolic process (GO:0008152);; biological process: cellular proce
GO:0099080);; cellular component: membrane (GO:0016020);; cellular component: membrane part

052);; biological process: single-organism process (GO:0044699);; biological process: response to
rt (GO:0044422);; cellular component: membrane part (GO:0044425);; cellular component: cell par

onent: cell (GO:0005623);; cellular component: macromolecular complex (GO:0032991);; cellular c

; cellular component: membrane part (GO:0044425);; biological process: cellular process (GO:0009
;: signaling (GO:0023052);; biological process: response to stimulus (GO:0050896)
ation or biogenesis (GO:0071840)
:0005623);; cellular component: membrane (GO:0016020);; cellular component: cell part (GO:0044
cal process: single-organism process (GO:0044699);; biological process: response to stimulus (GO:(

GO:0044464);; cellular component: organelle (GO:0043226);; cellular component: organelle part (G
: response to stimulus (GO:0050896);; biological process: biological regulation (GO:0065007);; cellu
rocess (GO:0044699);; biological process: response to stimulus (GO:0050896);; biological process: I
nism process (GO:0044699);; biological process: localization (GO:0051179)

process: response to stimulus (GO:0050896);; biological process: localization (GO:0051179);; cellula
:: membrane part (GO:0044425)

cal process: single-organism process (GO:0044699);; biological process: response to stimulus (GO:(
lular component: membrane (GO:0016020);; cellular component: membrane part (GO:0044425);; b
(GO:0043226);; cellular component: organelle part (GO:0044422);; cellular component: cell part (G(
464);; cellular component: membrane part (GO:0044425);; biological process: single-organism proc
process: response to stimulus (GO:0050896);; biological process: localization (GO:0051179);; biolog

x (GO:0032991);; molecular function: binding (GO:0005488);; biological process: cellular process (G

logical process: single-organism process (GO:0044699);; biological process: localization (GO:0051
: response to stimulus (GO:0050896);; biological process: biological regulation (GO:0065007);; cellu

ocess: immune system process (GO:0002376);; biological process: metabolic process (GO:0008152)
;; biological process: single-organism process (GO:0044699);; molecular function: binding (GO:000
lar component: synapse (GO:0045202);; biological process: biological regulation (GO:0065007)

logical process: developmental process (GO:0032502);; biological process: cellular component org
lular component: supramolecular complex (GO:0099080);; biological process: cellular process (GO:
cal process: single-organism process (GO:0044699);; biological process: response to stimulus (GO:(
6);; molecular function: binding (GO:0005488)

rocess (GO:0032501);; biological process: developmental process (GO:0032502);; biological proces

O:0032502);; biological process: single-organism process (GO:0044699);; biological process: multi
030054);; biological process: localization (GO:0051179);; molecular function: transporter activity (GC

);; biological process: metabolic process (GO:0008152);; biological process: cellular process (GO:00

component: cell part (GO:0044464);; cellular component: membrane (GO:0016020);; cellular comp
ological process: single-organism process (GO:0044699);; cellular component: organelle (GO:0043
001071);; biological process: biological regulation (GO:0065007);; molecular function: transcription

9987);; biological process: signaling (GO:0023052);; biological process: single-organism process (G
44425);; cellular component: cell part (GO:0044464);; molecular function: catalytic activity (GO:000
:0005623);; cellular component: cell part (GO:0044464);; biological process: single-organism proce

orane (GO:0016020);; cellular component: cell (GO:0005623);; cellular component: organelle (GO:0

:0044425);; cellular component: cell part (GO:0044464);; biological process: reproduction (GO:000

lular component: cell part (GO:0044464);; molecular function: binding (GO:0005488)

cal process: single-organism process (GO:0044699);; biological process: response to stimulus (GO:

ding (GO:0005488);; biological process: biological regulation (GO:0065007);; biological process: mu

008152);; molecular function: binding (GO:0005488);; cellular component: cell junction (GO:0030
(GO:0044464);; biological process: reproduction (GO:0000003);; biological process: cellular process
D:0009987);; biological process: signaling (GO:0023052);; biological process: single-organism proc

s: biological regulation (GO:0065007);; biological process: metabolic process (GO:0008152);; cellul

4464);; cellular component: organelle part (GO:0044422);; biological process: cellular component o

r component: cell junction (GO:0030054);; biological process: biological regulation (GO:0065007);;
ocess: single-organism process (GO:0044699);; biological process: response to stimulus (GO:0050
nt: cell (GO:0005623);; cellular component: macromolecular complex (GO:0032991);; cellular comp
(GO:0032991);; biological process: cellular process (GO:0009987);; biological process: single-organ

; cellular component: membrane part (GO:0044425);; biological process: cellular process (GO:0009

020);; cellular component: membrane part (GO:0044425);; cellular component: organelle part (GO:

464);; cellular component: macromolecular complex (GO:0032991);; cellular component: membran

process: metabolic process (GO:0008152);; molecular function: binding (GO:0005488)
cal process: single-organism process (GO:0044699);; biological process: response to stimulus (GO:

30054);; cellular component: membrane part (GO:0044425);; biological process: cellular process (G
s: biological regulation (GO:0065007);; molecular function: catalytic activity (GO:0003824);; biologi
9987);; biological process: single-organism process (GO:0044699)
tivity (GO:0060089);; biological process: cellular process (GO:0009987);; biological process: signalir

);; cellular component: membrane (GO:0016020);; cellular component: organelle part (GO:004442

GO:0044699);; biological process: cellular process (GO:0009987)
ss (GO:0002376);; biological process: biological regulation (GO:0065007);; molecular function: catal

37);; biological process: signaling (GO:0023052);; biological process: single-organism process (GO:(

):0043226);; cellular component: organelle part (GO:0044422);; cellular component: membrane par
):0009987);; biological process: signaling (GO:0023052);; biological process: single-organism proce

anelle part (GO:0044422);; cellular component: cell part (GO:0044464);; cellular component: memb
e (GO:0016020);; cellular component: macromolecular complex (GO:0032991);; cellular component

)22414);; biological process: single-organism process (GO:0044699);; molecular function: binding (

(GO:0044464);; cellular component: membrane-enclosed lumen (GO:0031974);; molecular functior

05488);; biological process: biological regulation (GO:0065007)
GO:0016020);; cellular component: membrane part (GO:0044425);; cellular component: cell part (G
nelle part (GO:0044422);; cellular component: cell part (GO:0044464);; biological process: biologica
):0044425)
):0032502);; biological process: single-organism process (GO:0044699);; biological process: cellular

):0044422);; cellular component: cell part (GO:0044464);; biological process: biological adhesion (G

ell (GO:0005623);; cellular component: macromolecular complex (GO:0032991);; cellular componer

organelle part (GO:0044422);; cellular component: cell part (GO:0044464);; cellular component: mac
art (GO:0044422);; cellular component: membrane part (GO:0044425);; cellular component: cell par
t (GO:0044422);; cellular component: cell part (GO:0044464);; biological process: response to stimu

3)

onent: membrane part (GO:0044425)
unction: binding (GO:0005488)
)09987);; biological process: signaling (GO:0023052);; biological process: single-organism process
elle (GO:0043226);; cellular component: cell part (GO:0044464);; cellular component: membrane (G
on: catalytic activity (GO:0003824);; biological process: metabolic process (GO:0008152);; molecular

44425);; cellular component: cell part (GO:0044464);; biological process: biological regulation (GO:

71840);; cellular component: cell (GO:0005623);; cellular component: organelle (GO:0043226);; cellu
);; molecular function: binding (GO:0005488)
ological process: cellular process (GO:0009987);; biological process: response to stimulus (GO:005(

nent: extracellular region (GO:0005576);; cellular component: extracellular region part (GO:004442

3O:0044464);; cellular component: macromolecular complex (GO:0032991);; cellular component: o

ogical process: single-organism process (GO:0044699);; biological process: response to stimulus (G

genesis (GO:0071840);; cellular component: cell (GO:0005623);; cellular component: cell part (GO:0

32501);; molecular function: catalytic activity (GO:0003824);; molecular function: binding (GO:0005
nt: cell (GO:0005623);; cellular component: organelle (GO:0043226);; cellular component: cell part
:0022610);; biological process: cellular process (GO:0009987);; biological process: multicellular orga
; biological process: cellular process (GO:0009987);; biological process: cellular component organiz

biological process: biological regulation (GO:0065007);; cellular component: cell (GO:0005623);; c
ent: cell (GO:0005623);; cellular component: membrane (GO:0016020);; cellular component: cell pa

rane (GO:0016020);; cellular component: organelle (GO:0043226);; cellular component: organelle

s: single-organism process (GO:0044699);; biological process: cellular process (GO:0009987);; biol
logical process: metabolic process (GO:0008152);; biological process: cellular process (GO:000998
ctivity (GO:0005215);; biological process: localization (GO:0051179)
al process: biological regulation (GO:0065007);; biological process: localization (GO:0051179);; bio
02);; biological process: single-organism process (GO:0044699);; biological process: reproduction

nction: binding (GO:0005488)

ar component: organelle (GO:0043226);; cellular component: cell part (GO:0044464)
05488);; cellular component: organelle part (GO:0044422);; biological process: cellular process (GC

:0065007);; biological process: multi-organism process (GO:0051704);; biological process: metabo
3226);; cellular component: organelle part (GO:0044422);; cellular component: cell part (GO:00444
GO:0044464);; cellular component: organelle (GO:0043226);; cellular component: organelle part (G
; biological process: response to stimulus (GO:0050896);; cellular component: membrane part (GO

);; biological process: localization (GO:0051179)

biological process: cellular process (GO:0009987);; biological process: cellular component organiz
biological process: biological adhesion (GO:0022610)
O:0060089);; biological process: cellular process (GO:0009987);; biological process: signaling (GO:0
44699);; biological process: response to stimulus (GO:0050896);; biological process: biological reg

logical process: localization (GO:0051179);; biological process: biological regulation (GO:0065007)

s: biological regulation (GO:0065007);; cellular component: cell (GO:0005623);; cellular component

')
:al process: single-organism process (GO:0044699);; biological process: response to stimulus (GO:(
3152);; biological process: cellular process (GO:0009987);; biological process: response to stimulus
process: response to stimulus (GO:0050896);; biological process: localization (GO:0051179);; cellula
ological process: cellular process (GO:0009987);; biological process: signaling (GO:0023052);; biolog

:0005215);; biological process: localization (GO:0051179)

irt (GO:0044464);; biological process: metabolic process (GO:0008152);; biological process: cellular
lar component: cell (GO:0005623);; cellular component: organelle (GO:0043226);; cellular compone

ological process: cellular process (GO:0009987);; biological process: cellular component organizatic
irt (GO:0044464);; biological process: cellular process (GO:0009987);; biological process: biological

ilar component: synapse (GO:0045202);; biological process: biological regulation (GO:0065007)

rocess (GO:0032501);; biological process: developmental process (GO:0032502);; biological proces
:0043226);; cellular component: organelle part (GO:0044422);; cellular component: membrane part
003824);; biological process: metabolic process (GO:0008152);; biological process: cellular process
multicellular organismal process (GO:0032501);; molecular function: binding (GO:0005488)
)
ological process: cellular process (GO:0009987);; biological process: signaling (GO:0023052);; biolog
; cellular component: membrane part (GO:0044425);; molecular function: transporter activity (GO:(
):0009987);; biological process: signaling (GO:0023052);; biological process: single-organism proce
omponent organization or biogenesis (GO:0071840);; biological process: multicellular organismal p
:: membrane part (GO:0044425)

ponent: organelle (GO:0043226);; cellular component: organelle part (GO:0044422);; cellular compo
cellular process (GO:0009987);; biological process: signaling (GO:0023052);; biological process: res
(GO:0044464);; cellular component: membrane (GO:0016020);; cellular component: membrane pai

ion: binding (GO:0005488);; molecular function: catalytic activity (GO:0003824);; cellular componer

ological process: cellular process (GO:0009987);; biological process: cellular component organizatic
lumen (GO:0031974);; cellular component: organelle (GO:0043226);; cellular component: organelle
-64);; biological process: biological regulation (GO:0065007);; cellular component: membrane (GO:(
irt (GO:0044464);; biological process: biological regulation (GO:0065007);; molecular function: sigr

):0065007);; molecular function: molecular function regulator (GO:0098772);; cellular component: c
tivity (GO:0005198);; biological process: response to stimulus (GO:0050896);; cellular component: c

)65007);; biological process: metabolic process (GO:0008152);; biological process: cellular process

)
ırt (GO:0044464);; cellular component: membrane (GO:0016020);; biological process: biological reg
22);; cellular component: cell part (GO:0044464)
);; biological process: cellular process (GO:0009987);; biological process: single-organism process (

044422);; cellular component: membrane part (GO:0044425);; cellular component: cell part (GO:00
: organelle (GO:0043226);; cellular component: organelle part (GO:0044422);; cellular component:
0031974);; cellular component: organelle part (GO:0044422);; cellular component: membrane (GO

071840);; molecular function: binding (GO:0005488)
3226);; cellular component: organelle part (GO:0044422);; cellular component: membrane part (GC

);; biological process: cellular process (GO:0009987);; biological process: single-organism process (

ent: cell (GO:0005623);; cellular component: membrane (GO:0016020);; cellular component: macrc

biological process: cellular process (GO:0009987);; biological process: single-organism process (GC
; cellular component: membrane part (GO:0044425);; biological process: biological regulation (GO:
; cellular component: membrane part (GO:0044425);; biological process: localization (GO:0051179)

nt: cell part (GO:0044464);; biological process: metabolic process (GO:0008152);; biological proces
GO:0099080);; molecular function: binding (GO:0005488);; biological process: biological regulation
ellular component: macromolecular complex (GO:0032991);; cellular component: cell part (GO:004
0044699);; biological process: response to stimulus (GO:0050896);; biological process: localization (
nt: organelle part (GO:0044422);; cellular component: cell part (GO:0044464);; biological process: r
art (GO:0044422);; cellular component: membrane part (GO:0044425);; cellular component: cell par

cellular process (GO:0009987);; biological process: cellular component organization or biogenesis (

152);; biological process: cellular process (GO:0009987);; biological process: single-organism proce
: (GO:0044422);; cellular component: cell part (GO:0044464);; molecular function: binding (GO:000
ı (GO:0065007);; biological process: signaling (GO:0023052);; biological process: single-organism p
ular function: catalytic activity (GO:0003824);; biological process: metabolic process (GO:0008152);
003824);; molecular function: binding (GO:0005488);; cellular component: cell (GO:0005623);; cellu

ellular component: cell part (GO:0044464);; cellular component: organelle (GO:0043226);; cellular co
)65007)

en (GO:0031974);; cellular component: macromolecular complex (GO:0032991);; biological process
;; molecular function: transcription factor activity, protein binding (GO:0000988);; biological proces
)65007);; biological process: reproduction (GO:0000003);; biological process: cellular process (GO:(

152);; biological process: cellular process (GO:0009987);; biological process: single-organism proce

;; biological process: growth (GO:0040007);; cellular component: cell (GO:0005623);; cellular compo
22);; cellular component: cell part (GO:0044464);; biological process: metabolic process (GO:00815

cess: signaling (GO:0023052);; biological process: response to stimulus (GO:0050896);; biological p
ation or biogenesis (GO:0071840)
(GO:0044464);; cellular component: macromolecular complex (GO:0032991);; molecular function:

nent: organelle (GO:0043226);; cellular component: organelle part (GO:0044422);; cellular compone

)
ent: membrane part (GO:0044425);; molecular function: catalytic activity (GO:0003824)

:0005623);; cellular component: organelle (GO:0043226);; cellular component: cell part (GO:00444
on: binding (GO:0005488);; biological process: biological regulation (GO:0065007);; biological proc
001071);; biological process: biological regulation (GO:0065007);; molecular function: transcription
art (GO:0044422);; cellular component: membrane part (GO:0044425);; cellular component: cell par
nt: cell part (GO:0044464);; biological process: metabolic process (GO:0008152);; biological proces
);; biological process: cellular process (GO:0009987);; biological process: response to stimulus (GO:
GO:0044422);; cellular component: cell part (GO:0044464);; biological process: cellular process (GC
43226);; cellular component: organelle part (GO:0044422);; cellular component: supramolecular co
rt (GO:0044464);; biological process: metabolic process (GO:0008152);; biological process: cellular
40)

component: cell part (GO:0044464);; cellular component: membrane-enclosed lumen (GO:0031
; cellular component: membrane part (GO:0044425);; biological process: biological regulation (GO:

rocess (GO:0009987);; molecular function: catalytic activity (GO:0003824);; molecular function: bin
43226);; cellular component: organelle part (GO:0044422);; biological process: metabolic process (
GO:0044464);; cellular component: organelle (GO:0043226);; cellular component: organelle part (G

lular component: membrane (GO:0016020);; biological process: biological regulation (GO:0065007
020);; cellular component: membrane part (GO:0044425);; biological process: biological regulation

art (GO:0044422);; cellular component: membrane part (GO:0044425);; cellular component: cell par
genesis (GO:0071840);; biological process: biological regulation (GO:0065007);; cellular component
; biological process: localization (GO:0051179);; cellular component: membrane part (GO:0044425)
en (GO:0031974)
men (GO:0031974);; cellular component: macromolecular complex (GO:0032991);; cellular compo
rt (GO:0044464);; biological process: immune system process (GO:0002376);; biological process: b
0031974);; cellular component: organelle part (GO:0044422);; biological process: biological regulat
cellular component: cell part (GO:0044464);; cellular component: membrane-enclosed lumen (GO
lecular function: catalytic activity (GO:0003824);; biological process: metabolic process (GO:00815
response to stimulus (GO:0050896);; biological process: biological regulation (GO:0065007);; biolog
rt (GO:0044464);; molecular function: binding (GO:0005488);; cellular component: macromolecula
nt: organelle part (GO:0044422);; cellular component: cell part (GO:0044464);; biological process: r

s: single-organism process (GO:0044699);; biological process: response to stimulus (GO:0050896);;

ess: biological regulation (GO:0065007);; cellular component: membrane (GO:0016020);; cellular cc

ar component: membrane-enclosed lumen (GO:0031974);; cellular component: organelle (GO:004
biological process: cellular process (GO:0009987);; biological process: signaling (GO:0023052);; bio
ation or biogenesis (GO:0071840)
part (GO:0044422);; cellular component: cell part (GO:0044464);; cellular component: membrane (

ganelle (GO:0043226);; cellular component: organelle part (GO:0044422);; cellular component: cell
brane (GO:0016020);; cellular component: cell part (GO:0044464);; biological process: cellular proc
rane part (GO:0044425);; cellular component: cell part (GO:0044464);; biological process: cellular p
9987);; molecular function: binding (GO:0005488);; biological process: multicellular organismal pro

; biological process: metabolic process (GO:0008152);; biological process: cellular process (GO:000
rt (GO:0044464);; cellular component: membrane (GO:0016020);; molecular function: binding (GO
nacromolecular complex (GO:0032991);; cellular component: organelle (GO:0043226);; cellular cor
t: organelle (GO:0043226);; cellular component: organelle part (GO:0044422);; biological process: c
GO:0065007);; cellular component: extracellular region (GO:0005576);; cellular component: extracel
(GO:0044464);; molecular function: structural molecule activity (GO:0005198);; cellular component:
GO:0071840);; cellular component: cell (GO:0005623);; cellular component: membrane-enclosed lu

044464);; biological process: metabolic process (GO:0008152);; biological process: cellular process

9987);; biological process: single-organism process (GO:0044699);; molecular function: catalytic ac

44425);; cellular component: cell part (GO:0044464);; biological process: biological regulation (GO:
rt (GO:0044464);; biological process: metabolic process (GO:0008152);; biological process: cellular

):0044422);; cellular component: membrane part (GO:0044425);; cellular component: cell part (GO:
:: cell (GO:0005623);; cellular component: macromolecular complex (GO:0032991);; cellular compo

ular process (GO:0009987);; biological process: single-organism process (GO:0044699)
mbrane (GO:0016020);; cellular component: macromolecular complex (GO:0032991);; cellular com

rocess: behavior (GO:0007610);; biological process: multicellular organismal process (GO:0032501)
ponent: membrane (GO:0016020)
009987);; biological process: multicellular organismal process (GO:0032501);; biological process: de

cellular component: cell part (GO:0044464);; biological process: single-organism process (GO:004

nt: cell part (GO:0044464);; biological process: metabolic process (GO:0008152);; biological proces

lecular function: catalytic activity (GO:0003824)

; cellular component: membrane part (GO:0044425);; biological process: cellular process (GO:0009

response to stimulus (GO:0050896);; biological process: biological regulation (GO:0065007)
s: single-organism process (GO:0044699);; molecular function: catalytic activity (GO:0003824);; cell
):0044464)
biological process: metabolic process (GO:0008152);; biological process: cellular process (GO:0009
genesis (GO:0071840);; molecular function: transporter activity (GO:0005215);; biological process: l

; cellular component: membrane part (GO:0044425);; biological process: cellular process (GO:0009
:0043226);; cellular component: organelle part (GO:0044422);; cellular component: membrane part
en (GO:0031974)

component: cell part (GO:0044464);; cellular component: membrane-enclosed lumen (GO:00319
logical process: localization (GO:0051179);; cellular component: membrane (GO:0016020);; cellular
process (GO:0009987);; biological process: response to stimulus (GO:0050896);; biological process:
ocess: cellular process (GO:0009987);; biological process: cellular component organization or bioge
):0044464);; cellular component: organelle (GO:0043226);; cellular component: membrane-enclose

rt (GO:0044464);; molecular function: binding (GO:0005488);; biological process: metabolic proces
GO:0099080)
:0005215);; biological process: single-organism process (GO:0044699);; biological process: localiza

nolecular complex (GO:0032991)

nt: cell part (GO:0044464);; biological process: metabolic process (GO:0008152);; biological proces
activity (GO:0003824);; biological process: cellular process (GO:0009987);; biological process: singl
ation (GO:0065007);; cellular component: cell (GO:0005623);; cellular component: organelle (GO:00
logical process: biological regulation (GO:0065007)

:0005623);; cellular component: organelle (GO:0043226);; cellular component: cell part (GO:00444
23);; cellular component: macromolecular complex (GO:0032991);; cellular component: organelle (
003);; biological process: reproductive process (GO:0022414);; biological process: multi-organism

nponent: membrane (GO:0016020);; biological process: localization (GO:0051179);; biological proc

: biological regulation (GO:0065007);; biological process: signaling (GO:0023052)

on: binding (GO:0005488);; cellular component: cell (GO:0005623);; cellular component: organelle

llular component: membrane (GO:0016020);; biological process: multicellular organismal process (
23);; cellular component: membrane (GO:0016020);; cellular component: organelle (GO:0043226);;
nelle (GO:0043226);; cellular component: organelle part (GO:0044422);; molecular function: molecu
; cellular component: cell junction (GO:0030054)
:0071840);; biological process: immune system process (GO:0002376);; biological process: respons
nction: binding (GO:0005488);; cellular component: cell (GO:0005623);; cellular component: organ
ne part (GO:0044425);; biological process: metabolic process (GO:0008152);; biological process: ce
velopmental process (GO:0032502);; biological process: single-organism process (GO:0044699);; c

ırocess (GO:0009987);; biological process: response to stimulus (GO:0050896);; biological process:
002376);; biological process: response to stimulus (GO:0050896);; molecular function: binding (GO
ed lumen (GO:0031974);; cellular component: organelle (GO:0043226);; cellular component: organ
ent: membrane part (GO:0044425)
);; molecular function: binding (GO:0005488);; cellular component: cell (GO:0005623);; cellular com
cal process: single-organism process (GO:0044699);; biological process: response to stimulus (GO:(

ation or biogenesis (GO:0071840)

ological process: biological regulation (GO:0065007);; biological process: cellular process (GO:0009

ding (GO:0005488);; cellular component: cell (GO:0005623);; cellular component: cell part (GO:0044
; biological process: metabolic process (GO:0008152);; biological process: cellular process (GO:000
; cellular component: membrane-enclosed lumen (GO:0031974);; molecular function: binding (GO
: organelle (GO:0043226);; cellular component: organelle part (GO:0044422);; cellular component:
0065007);; molecular function: binding (GO:0005488)
sis (GO:0071840);; cellular component: cell (GO:0005623);; cellular component: membrane (GO:001
or (GO:0098772);; biological process: biological regulation (GO:0065007);; cellular component: mer
(GO:0044464);; biological process: metabolic process (GO:0008152);; biological process: cellular pr
23);; cellular component: organelle (GO:0043226);; cellular component: organelle part (GO:004442
37);; biological process: single-organism process (GO:0044699);; molecular function: catalytic activ

(GO:0044464)
(GO:0044464);; biological process: cellular process (GO:0009987);; biological process: single-organ
(GO:0002376);; biological process: biological regulation (GO:0065007)
GO:0044422);; cellular component: cell part (GO:0044464);; molecular function: binding (GO:00054
23);; cellular component: organelle (GO:0043226);; cellular component: cell part (GO:0044464);; bio
(GO:0032991);; biological process: biological regulation (GO:0065007)
rocess (GO:0032501);; biological process: developmental process (GO:0032502);; biological proces
; biological process: metabolic process (GO:0008152);; biological process: cellular process (GO:000
mplex (GO:0032991);; cellular component: cell part (GO:0044464);; biological process: biological re

ırocess (GO:0009987);; biological process: response to stimulus (GO:0050896);; biological process:
152);; biological process: cellular process (GO:0009987);; biological process: single-organism proce
ar component: organelle (GO:0043226);; cellular component: cell part (GO:0044464);; cellular com

nt: cell part (GO:0044464);; biological process: metabolic process (GO:0008152);; biological proces

ırt (GO:0044464);; molecular function: binding (GO:0005488);; cellular component: macromolecular
(GO:0044464);; cellular component: macromolecular complex (GO:0032991);; biological process: r
226);; cellular component: cell part (GO:0044464);; cellular component: macromolecular complex (
32991)

(GO:0044464);; biological process: immune system process (GO:0002376);; biological process: resp

9987);; biological process: signaling (GO:0023052);; biological process: single-organism process (G

rocess (GO:0009987);; molecular function: binding (GO:0005488);; cellular component: organelle (
'1);; molecular function: molecular transducer activity (GO:0060089);; biological process: cellular pr

5007);; cellular component: cell (GO:0005623);; cellular component: organelle (GO:0043226);; cellul
);; biological process: multicellular organismal process (GO:0032501);; biological process: developr

71840);; cellular component: cell (GO:0005623);; cellular component: organelle (GO:0043226);; cell
en (GO:0031974);; biological process: cellular process (GO:0009987);; biological process: cellular co
llular component: organelle (GO:0043226);; cellular component: cell part (GO:0044464);; biologica
art (GO:0044464);; biological process: cellular process (GO:0009987);; biological process: signaling

etabolic process (GO:0008152);; biological process: cellular process (GO:0009987);; biological proce
mplex (GO:0032991);; cellular component: organelle (GO:0043226);; cellular component: organelle
art (GO:0044422);; cellular component: membrane part (GO:0044425);; cellular component: cell par
ological process: biological regulation (GO:0065007)
ological process: cellular process (GO:0009987);; biological process: cellular component organizati
al process: response to stimulus (GO:0050896)
08152);; biological process: single-organism process (GO:0044699)

art (GO:0044422);; molecular function: catalytic activity (GO:0003824);; biological process: metaboli

4464);; cellular component: membrane (GO:0016020);; cellular component: membrane part (GO:00
cellular process (GO:0009987)

process: signaling (GO:0023052);; biological process: single-organism process (GO:0044699);; biol
O:0044425);; cellular component: organelle (GO:0043226);; cellular component: organelle part (GO:
GO:0099080);; biological process: cellular process (GO:0009987);; biological process: cellular comp
ological process: biological regulation (GO:0065007);; cellular component: supramolecular complex
art (GO:0044464);; biological process: cellular process (GO:0009987);; biological process: cellular co
(GO:0043226)
324);; biological process: metabolic process (GO:0008152);; biological process: cellular process (GO

on: binding (GO:0005488);; cellular component: cell (GO:0005623);; cellular component: organelle
64);; cellular component: macromolecular complex (GO:0032991);; biological process: biological re
ll part (GO:0044464);; cellular component: macromolecular complex (GO:0032991);; molecular func
);; biological process: cellular process (GO:0009987);; biological process: cellular component organ
ent: membrane part (GO:0044425);; molecular function: catalytic activity (GO:0003824)
ganism process (GO:0044699);; biological process: response to stimulus (GO:0050896);; cellular co

ation or biogenesis (GO:0071840)

logical process: biological regulation (GO:0065007);; cellular component: macromolecular complex
nolecular complex (GO:0032991);; cellular component: organelle (GO:0043226);; cellular componer
4);; biological process: cellular process (GO:0009987);; biological process: single-organism process

)003);; biological process: reproductive process (GO:0022414);; biological process: multi-organism
nt: cell part (GO:0044464);; biological process: metabolic process (GO:0008152);; biological proces

gical process: developmental process (GO:0032502);; biological process: single-organism process
9987);; biological process: single-organism process (GO:0044699);; biological process: response to
37);; biological process: single-organism process (GO:0044699);; biological process: developmental
ınction: binding (GO:0005488)
44425);; cellular component: cell part (GO:0044464);; molecular function: catalytic activity (GO:000
ıent: organelle part (GO:0044422);; cellular component: cell part (GO:0044464);; biological process

;; cellular component: cell (GO:0005623);; cellular component: macromolecular complex (GO:0032

3226);; cellular component: organelle part (GO:0044422);; cellular component: cell part (GO:00444

):0009987);; biological process: single-organism process (GO:0044699)
GO:0065007);; cellular component: cell (GO:0005623);; cellular component: organelle (GO:0043226)
ological process: biological regulation (GO:0065007);; molecular function: binding (GO:0005488);;
ocess (GO:0032501);; molecular function: binding (GO:0005488);; biological process: biological regu
gical process: biological regulation (GO:0065007);; biological process: metabolic process (GO:0008
llular component: organelle part (GO:0044422);; biological process: metabolic process (GO:00081
cellular component: organelle (GO:0043226)
nt: organelle part (GO:0044422);; cellular component: cell part (GO:0044464)
rganelle (GO:0043226);; cellular component: cell part (GO:0044464);; biological process: biological
699);; biological process: localization (GO:0051179)
GO:0044699);; biological process: biological regulation (GO:0065007);; molecular function: transpo

O:0044699);; biological process: localization (GO:0051179);; molecular function: binding (GO:0005
GO:0065007);; cellular component: cell (GO:0005623);; cellular component: organelle (GO:0043226)
);; molecular function: binding (GO:0005488)
37);; biological process: single-organism process (GO:0044699);; biological process: cellular compo
nt: cell part (GO:0044464);; biological process: metabolic process (GO:0008152);; biological proces
ılar complex (GO:0032991);; cellular component: organelle (GO:0043226);; cellular component: cell

nt: cell part (GO:0044464);; biological process: metabolic process (GO:0008152);; biological proces
O:0044464);; biological process: biological regulation (GO:0065007);; biological process: localizatic
22610);; biological process: biological regulation (GO:0065007);; biological process: metabolic proc

3152);; biological process: cellular process (GO:0009987);; biological process: single-organism proc

nt: organelle part (GO:0044422);; cellular component: cell part (GO:0044464);; biological process: r
ırt (GO:0044422);; molecular function: electron carrier activity (GO:0009055);; biological process: m
);; biological process: single-organism process (GO:0044699);; molecular function: binding (GO:00
ganelle (GO:0043226);; cellular component: organelle part (GO:0044422);; cellular component: me
ent: organelle (GO:0043226);; cellular component: organelle part (GO:0044422);; biological process

rane (GO:0016020);; cellular component: membrane part (GO:0044425);; cellular component: cell

ex (GO:0032991);; molecular function: binding (GO:0005488);; cellular component: cell junction (G(
GO:0099080);; biological process: cellular process (GO:0009987);; biological process: single-organi

);; biological process: metabolic process (GO:0008152)
7);; biological process: cellular process (GO:0009987);; biological process: signaling (GO:0023052);;
ocess (GO:0044699);; biological process: cellular process (GO:0009987);; biological process: biolog
nt: cell part (GO:0044464);; biological process: metabolic process (GO:0008152);; biological proces
part (GO:0044464);; biological process: cellular process (GO:0009987);; biological process: respons
25)
GO:0099080);; biological process: biological regulation (GO:0065007);; molecular function: binding
nction: binding (GO:0005488)

tic activity (GO:0003824);; cellular component: organelle (GO:0043226);; cellular component: orgar
ological process: cellular process (GO:0009987);; biological process: signaling (GO:0023052);; biolog
lular component: cell junction (GO:0030054);; cellular component: supramolecular complex (GO:00
ological process: cellular process (GO:0009987);; biological process: multicellular organismal proces
rganelle (GO:0043226);; cellular component: organelle part (GO:0044422);; cellular component: cel

3226);; cellular component: organelle part (GO:0044422);; cellular component: cell part (GO:00444

GO:0044422);; cellular component: cell part (GO:0044464);; cellular component: supramolecular co
):0009987);; biological process: single-organism process (GO:0044699)
nt: cell (GO:0005623);; cellular component: cell part (GO:0044464);; cellular component: macromol

llular component: organelle (GO:0043226);; cellular component: organelle part (GO:0044422);; cell
component: organelle part (GO:0044422);; cellular component: cell part (GO:0044464);; biologica

ological process: metabolic process (GO:0008152);; biological process: single-organism process (G(
152);; biological process: cellular process (GO:0009987)
(GO:0044464)
ganelle (GO:0043226);; cellular component: organelle part (GO:0044422);; cellular component: cell
GO:0043226);; cellular component: cell part (GO:0044464);; molecular function: binding (GO:00054
nolecular complex (GO:0032991);; cellular component: organelle (GO:0043226);; cellular componer
nent: organelle part (GO:0044422);; cellular component: cell part (GO:0044464)
:0000988);; biological process: biological regulation (GO:0065007);; cellular component: cell (GO:0
9987);; biological process: response to stimulus (GO:0050896);; biological process: biological regula
omponent: organelle (GO:0043226);; cellular component: organelle part (GO:0044422);; cellular co

acromolecular complex (GO:0032991);; cellular component: cell part (GO:0044464)
ological process: metabolic process (GO:0008152);; biological process: cellular process (GO:000998
):0009987);; biological process: multicellular organismal process (GO:0032501);; biological process:
rocess: immune system process (GO:0002376);; biological process: cellular process (GO:0009987);;
cal process: single-organism process (GO:0044699);; biological process: response to stimulus (GO:(

(GO:0043226);; cellular component: organelle part (GO:0044422);; cellular component: cell part (G(
llular component: organelle (GO:0043226);; cellular component: organelle part (GO:0044422);; cell

)8152);; biological process: cellular process (GO:0009987);; biological process: single-organism prc

nt: organelle part (GO:0044422);; cellular component: cell part (GO:0044464);; biological process: r
ological process: cellular component organization or biogenesis (GO:0071840);; biological process:
(GO:0016020);; cellular component: macromolecular complex (GO:0032991);; cellular component:
ar function: catalytic activity (GO:0003824);; molecular function: antioxidant activity (GO:0016209);;
(GO:0032991);; biological process: localization (GO:0051179)

2);; biological process: cellular process (GO:0009987);; biological process: single-organism process
nent: organelle part (GO:0044422);; cellular component: cell part (GO:0044464);; biological process
(GO:0044464);; cellular component: membrane-enclosed lumen (GO:0031974);; biological process
GO:0044422);; cellular component: cell part (GO:0044464);; cellular component: supramolecular co
rganelle (GO:0043226);; cellular component: organelle part (GO:0044422);; cellular component: cel
part (GO:0044425);; cellular component: cell part (GO:0044464)
ical process: cellular process (GO:0009987);; biological process: single-organism process (GO:0044
nacromolecular complex (GO:0032991);; cellular component: cell part (GO:0044464);; cellular comp
; cellular component: membrane part (GO:0044425);; biological process: metabolic process (GO:00
(GO:0032501);; cellular component: organelle (GO:0043226);; cellular component: organelle part ((

);; biological process: cellular process (GO:0009987);; biological process: response to stimulus (GO:

biological process: localization (GO:0051179);; cellular component: cell (GO:0005623);; cellular cor
cal process: single-organism process (GO:0044699);; biological process: response to stimulus (GO:(
s: single-organism process (GO:0044699);; biological process: response to stimulus (GO:0050896);;
on: binding (GO:0005488);; cellular component: cell (GO:0005623);; cellular component: organelle

nelle (GO:0043226);; cellular component: cell part (GO:0044464);; biological process: metabolic prc

GO:0003824);; biological process: metabolic process (GO:0008152);; biological process: cellular pro
44464);; molecular function: catalytic activity (GO:0003824);; biological process: biological regulatic

GO:0044464);; cellular component: organelle (GO:0043226);; cellular component: organelle part (G
; cellular component: supramolecular complex (GO:0099080);; molecular function: binding (GO:000

process: biological regulation (GO:0065007)
le (GO:0043226);; cellular component: organelle part (GO:0044422);; cellular component: cell part

)8152);; biological process: cellular process (GO:0009987);; biological process: single-organism prc
rocess (GO:0009987);; biological process: signaling (GO:0023052);; biological process: single-orga
ar component: organelle (GO:0043226);; cellular component: organelle part (GO:0044422);; cellula

; molecular function: transporter activity (GO:0005215);; biological process: localization (GO:00511
biological process: cellular process (GO:0009987)
brane (GO:0016020);; cellular component: organelle (GO:0043226);; cellular component: organelle p
en (GO:0031974);; cellular component: macromolecular complex (GO:0032991);; biological process
(GO:0008152);; biological process: cellular process (GO:0009987);; molecular function: catalytic act

component: macromolecular complex (GO:0032991);; molecular function: binding (GO:0005488)

nent: organelle part (GO:0044422);; cellular component: cell part (GO:0044464);; biological process
: organelle (GO:0043226);; cellular component: organelle part (GO:0044422);; cellular component:
044421);; cellular component: cell (GO:0005623);; cellular component: membrane-enclosed lumen
GO:0043226);; cellular component: organelle part (GO:0044422);; cellular component: cell part (GO
:0016209);; biological process: biological regulation (GO:0065007);; biological process: metabolic p

s (GO:0032501);; biological process: developmental process (GO:0032502);; biological process: sing

tivity (GO:0005198);; cellular component: cell (GO:0005623);; cellular component: macromolecular
43226);; cellular component: organelle part (GO:0044422);; cellular component: supramolecular co

omplex (GO:0032991);; cellular component: membrane part (GO:0044425);; cellular component: ce
);; molecular function: binding (GO:0005488);; biological process: metabolic process (GO:0008152)
152);; biological process: cellular process (GO:0009987);; biological process: single-organism proce
biological process: biological regulation (GO:0065007);; biological process: signaling (GO:0023052)
ane (GO:0016020);; cellular component: membrane part (GO:0044425);; cellular component: cell pa
process: biological regulation (GO:0065007);; biological process: locomotion (GO:0040011);; biolog

(GO:0044464);; biological process: metabolic process (GO:0008152);; biological process: cellular p
O:0009987);; biological process: single-organism process (GO:0044699);; molecular function: bindir

488);; cellular component: membrane (GO:0016020);; cellular component: membrane part (GO:004

cess: behavior (GO:0007610);; biological process: multi-organism process (GO:0051704);; biologica
olecular function: catalytic activity (GO:0003824)

iological process: metabolic process (GO:0008152);; biological process: cellular process (GO:00099
ess (GO:0008152);; biological process: cellular process (GO:0009987)

une system process (GO:0002376);; biological process: response to stimulus (GO:0050896);; cellula

GO:0044699);; molecular function: binding (GO:0005488);; cellular component: extracellular region

membrane-enclosed lumen (GO:0031974);; cellular component: organelle (GO:0043226);; cellular co
GO:0099080);; molecular function: catalytic activity (GO:0003824);; biological process: metabolic pr

ellular component: cell junction (GO:0030054);; biological process: localization (GO:0051179);; biolo
rt (GO:0044464)

324);; biological process: metabolic process (GO:0008152);; biological process: cellular process (GO
);; cellular component: cell (GO:0005623);; cellular component: macromolecular complex (GO:0032

37);; biological process: single-organism process (GO:0044699);; molecular function: catalytic activ
6);; molecular function: binding (GO:0005488);; cellular component: membrane (GO:0016020);; cel
);; biological process: cellular process (GO:0009987);; biological process: response to stimulus (GO:
; cellular component: membrane part (GO:0044425);; biological process: localization (GO:0051179)

)

process (GO:0009987);; biological process: single-organism process (GO:0044699);; biological pro
:0002376);; biological process: response to stimulus (GO:0050896);; biological process: multi-orgar

(GO:0032991);; cellular component: membrane-enclosed lumen (GO:0031974)
ponent: membrane (GO:0016020);; biological process: cellular process (GO:0009987);; biological p
cular function: binding (GO:0005488)
ation or biogenesis (GO:0071840)
ation or biogenesis (GO:0071840)
;; cellular component: cell part (GO:0044464)
' (GO:0003824);; molecular function: structural molecule activity (GO:0005198);; molecular function

rane part (GO:0044425);; cellular component: cell part (GO:0044464);; biological process: cellular p
3226);; cellular component: cell part (GO:0044464);; biological process: cellular process (GO:00099
ellular component: organelle part (GO:0044422);; cellular component: membrane (GO:0016020);; b
nbrane (GO:0016020);; cellular component: membrane part (GO:0044425);; cellular component: ma
rocess (GO:0009987);; biological process: immune system process (GO:0002376);; molecular funct

008152);; biological process: cellular process (GO:0009987);; biological process: biological regulatic
m process (GO:0044699);; biological process: response to stimulus (GO:0050896);; biological proce
omponent: cell part (GO:0044464);; biological process: biological regulation (GO:0065007);; molecu
008152);; biological process: cellular process (GO:0009987);; biological process: single-organism p
32502);; biological process: single-organism process (GO:0044699);; molecular function: binding (G
);; cellular component: cell (GO:0005623);; cellular component: macromolecular complex (GO:0032
ological process: immune system process (GO:0002376);; biological process: cellular process (GO:0
)

ological process: metabolic process (GO:0008152);; biological process: cellular process (GO:00099

nembrane (GO:0016020);; cellular component: membrane part (GO:0044425);; biological process: r
system process (GO:0002376);; biological process: response to stimulus (GO:0050896);; biological p
GO:0043226);; cellular component: cell part (GO:0044464);; biological process: biological regulation
art (GO:0044422);; cellular component: membrane part (GO:0044425);; cellular component: cell par
anism (GO:0044215);; cellular component: other organism part (GO:0044217);; cellular component:

044464);; biological process: single-organism process (GO:0044699);; biological process: localizatic

(GO:0008152);; biological process: cellular process (GO:0009987);; cellular component: organelle (

044464);; biological process: metabolic process (GO:0008152);; biological process: cellular process

');; biological process: signaling (GO:0023052);; biological process: single-organism process (GO:0(
!26);; cellular component: organelle part (GO:0044422);; cellular component: cell part (GO:0044464

; cellular component: membrane part (GO:0044425);; biological process: localization (GO:0051179)

jical process: signaling (GO:0023052);; biological process: multicellular organismal process (GO:003
cal process: cellular process (GO:0009987);; biological process: single-organism process (GO:0044(

cal process: single-organism process (GO:0044699);; biological process: response to stimulus (GO:(
ponent: cell part (GO:0044464);; cellular component: membrane part (GO:0044425);; biological pro

:: cell (GO:0005623);; cellular component: cell part (GO:0044464);; molecular function: electron carr

lic process (GO:0008152);; biological process: cellular process (GO:0009987);; biological process: si

ganism process (GO:0044699);; biological process: cellular component organization or biogenesis (

part (GO:0044464);; molecular function: binding (GO:0005488)
44425);; cellular component: cell part (GO:0044464);; biological process: metabolic process (GO:0C

;; biological process: cellular process (GO:0009987);; biological process: cellular component organi
324);; molecular function: binding (GO:0005488);; biological process: biological regulation (GO:006!

ictivity (GO:0005215);; biological process: localization (GO:0051179)

(GO:0044464);; biological process: metabolic process (GO:0008152);; biological process: cellular pr

lopmental process (GO:0032502);; biological process: response to stimulus (GO:0050896);; biologi
05488);; molecular function: catalytic activity (GO:0003824);; biological process: biological regulatic

ellular component: organelle (GO:0043226);; biological process: cellular process (GO:0009987);; bic

);; molecular function: binding (GO:0005488);; biological process: localization (GO:0051179);; biolo

ocess (GO:0044699);; molecular function: catalytic activity (GO:0003824)
irt (GO:0044464);; biological process: metabolic process (GO:0008152);; biological process: cellular

process: signaling (GO:0023052);; biological process: single-organism process (GO:0044699);; biolc

GO:0044422);; cellular component: cell part (GO:0044464);; biological process: cellular process (GO
22);; cellular component: cell part (GO:0044464);; cellular component: extracellular region (GO:000!
rocess: immune system process (GO:0002376);; biological process: cellular process (GO:0009987);;

ne part (GO:0044425);; cellular component: cell part (GO:0044464)
ellular component: organelle part (GO:0044422);; cellular component: membrane (GO:0016020);; b

ctivity (GO:0005215);; biological process: localization (GO:0051179)
gical process: biological regulation (GO:0065007);; biological process: cellular process (GO:000998

):0044425);; biological process: cellular process (GO:0009987);; biological process: single-organism
)044425);; cellular component: cell part (GO:0044464);; biological process: cellular process (GO:000

s: biological regulation (GO:0065007);; molecular function: binding (GO:0005488)
);; cellular component: membrane-enclosed lumen (GO:0031974);; cellular component: organelle p

omponent: extracellular region part (GO:0044421);; biological process: response to stimulus (GO:00

cellular component: cell (GO:0005623);; cellular component: organelle (GO:0043226);; cellular com

: membrane (GO:0016020);; cellular component: membrane part (GO:0044425);; cellular compone
ological process: single-organism process (GO:0044699);; biological process: localization (GO:0051
etabolic process (GO:0008152)

008152);; biological process: cellular process (GO:0009987);; biological process: immune system pr

unction: binding (GO:0005488)

rocess: cellular process (GO:0009987);; biological process: single-organism process (GO:0044699);;

rt (GO:0044464);; molecular function: binding (GO:0005488);; molecular function: nucleic acid binc
324);; biological process: metabolic process (GO:0008152);; cellular component: membrane (GO:00
s: single-organism process (GO:0044699);; biological process: response to stimulus (GO:0050896);;
O:0043226);; cellular component: organelle part (GO:0044422);; cellular component: cell part (GO:
ctivity (GO:0005215);; biological process: localization (GO:0051179)
part (GO:0044464);; molecular function: binding (GO:0005488)
ponent: cell (GO:0005623);; cellular component: cell part (GO:0044464);; cellular component: orga

lex (GO:0032991);; cellular component: cell part (GO:0044464)
oonent: extracellular region part (GO:0044421);; biological process: metabolic process (GO:000815

O:0032502);; biological process: single-organism process (GO:0044699);; biological process: multi

rt (GO:0044464);; molecular function: catalytic activity (GO:0003824);; cellular component: membra
s: multi-organism process (GO:0051704)
lex (GO:0032991);; cellular component: cell part (GO:0044464)

ulation (GO:0065007);; cellular component: membrane (GO:0016020);; cellular component: membr
ulation (GO:0065007);; cellular component: membrane (GO:0016020);; cellular component: membr
ient: membrane (GO:0016020);; cellular component: membrane part (GO:0044425);; cellular compo

on: binding (GO:0005488)
;O:0065007);; biological process: cellular process (GO:0009987);; biological process: signaling (GO:
genesis (GO:0071840);; molecular function: catalytic activity (GO:0003824);; biological process: met
):0008152);; biological process: cellular process (GO:0009987);; biological process: single-organism
nt: cell part (GO:0044464);; cellular component: membrane (GO:0016020);; biological process: meta
r component: organelle part (GO:0044422);; cellular component: membrane part (GO:0044425);; b

s: biological regulation (GO:0065007)
lecular function: catalytic activity (GO:0003824);; biological process: metabolic process (GO:000815
s: single-organism process (GO:0044699);; biological process: response to stimulus (GO:0050896);;

part (GO:0044464);; molecular function: binding (GO:0005488)
ansporter activity (GO:0005215)
:: membrane part (GO:0044425)

biogenesis (GO:0071840);; cellular component: cell (GO:0005623);; cellular component: macromol
ielle part (GO:0044422);; cellular component: cell part (GO:0044464);; cellular component: macrom

ess: reproductive process (GO:0022414);; biological process: multicellular organismal process (GO:(
)
):0009987)
nacromolecular complex (GO:0032991);; cellular component: organelle (GO:0043226);; cellular cor

genesis (GO:0071840);; molecular function: catalytic activity (GO:0003824);; cellular component: me
part (GO:0044464);; molecular function: binding (GO:0005488)
)
-25)

ponent: extracellular region (GO:0005576);; cellular component: extracellular region part (GO:0044
lex (GO:0032991);; cellular component: cell part (GO:0044464);; biological process: metabolic proc

l process (GO:0032502);; biological process: single-organism process (GO:0044699);; biological pr
)2);; biological process: single-organism process (GO:0044699)

16020);; biological process: localization (GO:0051179);; biological process: biological regulation (G

:0032501);; biological process: developmental process (GO:0032502);; biological process: single-or
component: membrane part (GO:0044425);; molecular function: binding (GO:0005488);; biological

54);; cellular component: cell junction (GO:0030054);; biological process: signaling (GO:0023052);; b
gical process: multicellular organismal process (GO:0032501);; biological process: developmental p
nism process (GO:0044699);; biological process: response to stimulus (GO:0050896);; biological pr
ess: localization (GO:0051179)

)

0050896);; biological process: biological regulation (GO:0065007);; biological process: cellular com
08152);; biological process: cellular process (GO:0009987);; biological process: single-organism pr

9987);; biological process: single-organism process (GO:0044699);; molecular function: catalytic ac
09987);; biological process: single-organism process (GO:0044699);; biological process: response t

ocess: single-organism process (GO:0044699);; biological process: localization (GO:0051179);; biol
37);; biological process: cellular component organization or biogenesis (GO:0071840)

biological process: reproductive process (GO:0022414);; biological process: multicellular organisma
0005576);; cellular component: extracellular region part (GO:0044421)

5);; biological process: localization (GO:0051179);; biological process: multicellular organismal proc
O:0002376);; molecular function: catalytic activity (GO:0003824);; molecular function: antioxidant a
ılar component: cell part (GO:0044464);; cellular component: organelle (GO:0043226);; cellular con

):0044422);; cellular component: cell part (GO:0044464)

:: membrane part (GO:0044425);; cellular component: cell part (GO:0044464);; biological process: ir
)050896);; biological process: biological regulation (GO:0065007);; cellular component: membrane

iological process: biological regulation (GO:0065007)

ıbrane (GO:0016020);; cellular component: membrane part (GO:0044425)
:ess: biological regulation (GO:0065007);; molecular function: transporter activity (GO:0005215);; bi
s (GO:0008152);; biological process: cellular process (GO:0009987);; molecular function: catalytic a
nent organization or biogenesis (GO:0071840);; biological process: biological regulation (GO:0065
:omponent: cell part (GO:0044464);; molecular function: binding (GO:0005488);; biological process:
:omponent: membrane part (GO:0044425);; cellular component: cell part (GO:0044464);; biological

s: locomotion (GO:0040011);; biological process: single-organism process (GO:0044699);; biologic

)050896);; biological process: biological regulation (GO:0065007);; molecular function: binding (GC

)050896);; biological process: biological regulation (GO:0065007);; cellular component: membrane
)050896);; biological process: biological regulation (GO:0065007);; cellular component: cell (GO:00
ent: membrane part (GO:0044425);; cellular component: cell part (GO:0044464);; molecular functior

organelle (GO:0043226);; cellular component: cell part (GO:0044464);; biological process: cellular p

)050896);; biological process: biological regulation (GO:0065007);; cellular component: membrane
)050896);; biological process: biological regulation (GO:0065007);; cellular component: membrane
ogical process: multicellular organismal process (GO:0032501);; biological process: developmental
ar component: organelle (GO:0043226);; cellular component: organelle part (GO:0044422);; cellular
ıponent: membrane part (GO:0044425)

(GO:0050896);; biological process: biological regulation (GO:0065007);; molecular function: bindir

896);; cellular component: cell (GO:0005623);; cellular component: cell part (GO:0044464);; cellular
O:0003824);; molecular function: binding (GO:0005488);; cellular component: cell (GO:0005623);; c

orane (GO:0016020);; cellular component: membrane part (GO:0044425);; cellular component: cell

ellular process (GO:0009987);; biological process: single-organism process (GO:0044699);; biologic
GO:0044422);; cellular component: supramolecular complex (GO:0099080);; biological process: cellu
0050896);; biological process: biological regulation (GO:0065007);; molecular function: binding (GC
16020);; cellular component: membrane part (GO:0044425);; cellular component: organelle (GO:00
GO:0044422);; cellular component: supramolecular complex (GO:0099080);; biological process: cellu

ocess: multicellular organismal process (GO:0032501);; biological process: developmental process

biological process: immune system process (GO:0002376);; biological process: response to stimulus

on or biogenesis (GO:0071840);; biological process: multicellular organismal process (GO:0032501

; biological process: single-organism process (GO:0044699);; biological process: response to stimu

c process (GO:0008152);; biological process: multicellular organismal process (GO:0032501);; biolog

0050896);; biological process: biological regulation (GO:0065007);; cellular component: cell (GO:00
0050896);; biological process: biological regulation (GO:0065007);; cellular component: membrane

0044699);; biological process: response to stimulus (GO:0050896);; biological process: biological re
ss (GO:0009987);; biological process: reproduction (GO:0000003);; biological process: reproductive
: (GO:0044425)

stimulus (GO:0050896);; molecular function: binding (GO:0005488)
t (GO:0044464);; molecular function: catalytic activity (GO:0003824);; molecular function: transport

omponent: cell part (GO:0044464)

987);; biological process: developmental process (GO:0032502);; biological process: single-organis

464);; biological process: signaling (GO:0023052);; biological process: single-organism process (GC
)050896);; biological process: biological regulation (GO:0065007);; cellular component: membrane

;O:0044422);; cellular component: supramolecular complex (GO:0099080);; biological process: cellu
ılar component: cell (GO:0005623);; cellular component: membrane (GO:0016020);; cellular compo
biological regulation (GO:0065007)

r component: extracellular region (GO:0005576);; cellular component: extracellular region part (GC

)050896);; biological process: biological regulation (GO:0065007);; molecular function: binding (GC
iological process: metabolic process (GO:0008152);; biological process: biological regulation (GO:(
O:0044464)
cess (GO:0044699);; biological process: cellular process (GO:0009987);; biological process: biologic
ical process: biological regulation (GO:0065007);; biological process: signaling (GO:0023052);; mol

;O:0009987);; biological process: multicellular organismal process (GO:0032501);; biological proces

179)
ılar component: cell (GO:0005623);; cellular component: membrane (GO:0016020);; cellular compo

;; biological process: cellular process (GO:0009987);; molecular function: transcription factor activit
5488);; cellular component: organelle (GO:0043226)

anization or biogenesis (GO:0071840);; molecular function: signal transducer activity (GO:000487
0009987);; biological process: single-organism process (GO:0044699);; biological process: cellular
)050896);; biological process: biological regulation (GO:0065007);; biological process: localization (

s: single-organism process (GO:0044699);; cellular component: cell (GO:0005623);; cellular compo

cellular organismal process (GO:0032501);; cellular component: cell (GO:0005623);; cellular compo
):0005215);; cellular component: membrane part (GO:0044425);; biological process: biological regu

09987);; molecular function: catalytic activity (GO:0003824);; biological process: single-organism p

ponent: organelle part (GO:0044422);; molecular function: catalytic activity (GO:0003824);; biologic
226);; cellular component: organelle part (GO:0044422);; biological process: metabolic process (GC
factor activity, protein binding (GO:0000988);; cellular component: cell (GO:0005623);; cellular cor

iO:0044699);; biological process: response to stimulus (GO:0050896);; biological process: biologica
3824);; biological process: metabolic process (GO:0008152);; biological process: cellular process (G
ess (GO:0044699);; biological process: cellular component organization or biogenesis (GO:0071840

043226);; cellular component: cell part (GO:0044464)

)003);; biological process: reproductive process (GO:0022414);; biological process: single-organism

)050896);; biological process: biological regulation (GO:0065007)

ilticellular organismal process (GO:0032501);; cellular component: extracellular region (GO:000557

54);; biological process: single-organism process (GO:0044699);; biological process: biological regu
s (GO:0009987);; biological process: reproductive process (GO:0022414);; biological process: multic
ess (GO:0044699);; biological process: presynaptic process involved in chemical synaptic transmiss

ar component: cell (GO:0005623);; cellular component: cell part (GO:0044464);; cellular component

rganization or biogenesis (GO:0071840);; biological process: localization (GO:0051179);; molecular

biological process: cellular process (GO:0009987);; biological process: signaling (GO:0023052);; bio
396)
onent: organelle (GO:0043226);; cellular component: organelle part (GO:0044422);; cellular compo
iism process (GO:0044699);; biological process: localization (GO:0051179);; biological process: cellu

987);; biological process: developmental process (GO:0032502);; biological process: single-organis

:0044422);; cellular component: supramolecular complex (GO:0099080);; molecular function: bindir

e part (GO:0044425)

)050896);; biological process: biological regulation (GO:0065007);; cellular component: cell (GO:00

iO:0009987);; biological process: single-organism process (GO:0044699);; biological process: cellul
cal process: metabolic process (GO:0008152)

ng (GO:0023052);; biological process: single-organism process (GO:0044699);; biological process: b

?);; cellular component: membrane part (GO:0044425);; biological process: localization (GO:005117

ytic activity (GO:0003824);; biological process: metabolic process (GO:0008152);; cellular compone

0044699);; biological process: response to stimulus (GO:0050896);; molecular function: binding (GC

rt (GO:0044425);; molecular function: binding (GO:0005488);; biological process: localization (GO:0
ess (GO:0044699);; biological process: response to stimulus (GO:0050896);; biological process: biol

orane-enclosed lumen (GO:0031974);; biological process: cellular process (GO:0009987);; biologica
:: membrane part (GO:0044425);; cellular component: cell part (GO:0044464);; biological process: c

(GO:0005488);; biological process: developmental process (GO:0032502);; cellular component: cell

n: binding (GO:0005488);; biological process: cellular process (GO:0009987);; biological process: ce

GO:0044464);; biological process: biological adhesion (GO:0022610);; biological process: cellular pro
l regulation (GO:0065007);; molecular function: binding (GO:0005488);; biological process: metabo

: component organization or biogenesis (GO:0071840);; biological process: response to stimulus (C

GO:0022610);; cellular component: membrane part (GO:0044425);; biological process: cellular proce

nt: organelle (GO:0043226);; cellular component: organelle part (GO:0044422);; cellular component

cromolecular complex (GO:0032991)
rt (GO:0044464);; cellular component: synapse part (GO:0044456);; cellular component: synapse (G
ulus (GO:0050896);; biological process: immune system process (GO:0002376);; biological process:

(GO:0044699);; biological process: response to stimulus (GO:0050896)
GO:0016020);; cellular component: membrane part (GO:0044425)
: function: binding (GO:0005488);; cellular component: cell (GO:0005623);; cellular component: org

:0065007);; biological process: localization (GO:0051179)

ular component: organelle part (GO:0044422);; cellular component: cell part (GO:0044464);; cellula

0896);; biological process: biological regulation (GO:0065007);; cellular component: membrane (GC

1);; molecular function: catalytic activity (GO:0003824);; biological process: cellular component org

rganelle part (GO:0044422);; cellular component: supramolecular complex (GO:0099080);; biologic

O:0050896);; biological process: biological regulation (GO:0065007);; biological process: multicellu

044464);; molecular function: nucleic acid binding transcription factor activity (GO:0001071);; biolc

488);; cellular component: cell (GO:0005623);; cellular component: organelle (GO:0043226);; cellula
(GO:0044464)
anismal process (GO:0032501);; biological process: developmental process (GO:0032502);; biologic
zation or biogenesis (GO:0071840);; biological process: signaling (GO:0023052);; biological process

ellular component: membrane (GO:0016020);; cellular component: organelle (GO:0043226);; cellula
art (GO:0044464);; biological process: biological regulation (GO:0065007);; cellular component: me

part (GO:0044422);; cellular component: cell part (GO:0044464);; cellular component: membrane p

ogical process: developmental process (GO:0032502);; biological process: biological regulation (G
7);; biological process: biological regulation (GO:0065007);; biological process: cellular component

logical process: multicellular organismal process (GO:0032501);; biological process: developmenta
(GO:0000003);; biological process: reproductive process (GO:0022414);; biological process: multi-

:0009987);; biological process: cellular component organization or biogenesis (GO:0071840);; cellu

lic process (GO:0008152);; biological process: cellular process (GO:0009987);; biological process: si
64);; biological process: cellular process (GO:0009987);; biological process: biological regulation (G
GO:0044422);; cellular component: supramolecular complex (GO:0099080);; biological process: cellu
:0044425);; biological process: immune system process (GO:0002376);; biological process: metabol

ation or biogenesis (GO:0071840);; molecular function: binding (GO:0005488);; biological process: l

0023052);; biological process: single-organism process (GO:0044699);; biological process: biologica
ulation (GO:0065007);; cellular component: cell (GO:0005623);; cellular component: organelle (GO:

: membrane (GO:0016020);; cellular component: macromolecular complex (GO:0032991);; cellular

)050896);; biological process: biological regulation (GO:0065007);; cellular component: membrane
(GO:0050896);; molecular function: binding (GO:0005488);; cellular component: macromolecular c
r component: extracellular region (GO:0005576);; cellular component: extracellular region part (GC
gical process: single-organism process (GO:0044699);; biological process: response to stimulus (GC

process (GO:0009987);; biological process: biological regulation (GO:0065007);; biological process
ent: organelle part (GO:0044422);; cellular component: cell part (GO:0044464);; biological process:

on or biogenesis (GO:0071840)
regulation (GO:0065007);; cellular component: membrane (GO:0016020);; cellular component: me

s: single-organism process (GO:0044699);; biological process: cellular process (GO:0009987);; cellu
: (GO:0044425);; cellular component: cell part (GO:0044464);; biological process: cellular process (G
(GO:0009987);; biological process: immune system process (GO:0002376);; biological process: bio

gical process: single-organism process (GO:0044699);; biological process: response to stimulus (GC
)005215);; biological process: localization (GO:0051179);; molecular function: binding (GO:0005488
ess (GO:0044699);; biological process: response to stimulus (GO:0050896);; biological process: biol
process (GO:0032501);; biological process: developmental process (GO:0032502);; biological proce

onent: membrane part (GO:0044425);; cellular component: cell part (GO:0044464);; biological proc
sponse to stimulus (GO:0050896);; biological process: biological regulation (GO:0065007);; biologic
rt (GO:0044425);; biological process: biological regulation (GO:0065007);; molecular function: signa

nt: extracellular region (GO:0005576);; cellular component: extracellular region part (GO:0044421);;

on or biogenesis (GO:0071840);; biological process: biological regulation (GO:0065007);; cellular co
e part (GO:0044422)
)016020);; biological process: metabolic process (GO:0008152);; biological process: cellular process
nal transducer activity (GO:0004871);; molecular function: molecular transducer activity (GO:06000[illegible]

organelle (GO:0043226);; cellular component: membrane (GO:0016020);; biological process: metab
cell (GO:0005623);; cellular component: organelle (GO:0043226);; cellular component: organelle pa

(GO:0009987);; biological process: cellular component organization or biogenesis (GO:0071840);; r

julation (GO:0065007);; molecular function: catalytic activity (GO:0003824)

(GO:0044699);; biological process: biological regulation (GO:0065007);; biological process: metabo

)44464);; molecular function: catalytic activity (GO:0003824)
cell part (GO:0044464);; biological process: multicellular organismal process (GO:0032501);; biolog
:0016020);; biological process: cellular process (GO:0009987);; biological process: signaling (GO:00

):0044425);; cellular component: cell part (GO:0044464);; biological process: cellular process (GO:0

(GO:0044699);; biological process: localization (GO:0051179)

omolecular complex (GO:0032991);; cellular component: organelle (GO:0043226);; cellular compon

):0044699);; biological process: cellular component organization or biogenesis (GO:0071840);; biol
:0065007);; biological process: cellular process (GO:0009987);; biological process: response to stim
);; biological process: biological regulation (GO:0065007)

s: cellular process (GO:0009987)
(GO:0065007)
4464)
GO:0051179);; biological process: cellular process (GO:0009987);; biological process: multicellular
metabolic process (GO:0008152);; biological process: cellular process (GO:0009987)
t (GO:0044464);; molecular function: transporter activity (GO:0005215);; biological process: localiz

GO:0071840)

ess (GO:0044699)
5488);; biological process: cellular process (GO:0009987);; biological process: cellular component o
process (GO:0044699);; cellular component: cell (GO:0005623);; cellular component: membrane (GC
; biological process: single-organism process (GO:0044699);; biological process: response to stimu
ılar component: membrane-enclosed lumen (GO:0031974);; cellular component: organelle (GO:00

omponent: organelle part (GO:0044422);; cellular component: macromolecular complex (GO:0032

: multicellular organismal process (GO:0032501);; biological process: developmental process (GO:0
ss: biological regulation (GO:0065007);; cellular component: cell (GO:0005623);; cellular componen
)009987);; biological process: reproductive process (GO:0022414);; biological process: multicellular

ess (GO:0044699)

onent: organelle (GO:0043226);; cellular component: organelle part (GO:0044422);; cellular compo
52);; biological process: cellular process (GO:0009987);; biological process: single-organism proces

rocess: biological regulation (GO:0065007);; biological process: multicellular organismal process (G

binding (GO:0005488)

ent: cell part (GO:0044464);; cellular component: supramolecular complex (GO:0099080);; molecula

54);; biological process: response to stimulus (GO:0050896)
cess: multicellular organismal process (GO:0032501)
factor activity, protein binding (GO:0000988);; cellular component: cell (GO:0005623);; cellular cor
rt (GO:0044464);; biological process: metabolic process (GO:0008152);; biological process: cellular
s: cellular process (GO:0009987)
0050896)
):0009987);; biological process: single-organism process (GO:0044699);; biological process: biologi
mplex (GO:0099080);; cellular component: membrane (GO:0016020);; biological process: cellular p
process (GO:0009987);; cellular component: macromolecular complex (GO:0032991)

74);; cellular component: organelle part (GO:0044422);; biological process: cellular process (GO:00
:0065007)

ding (GO:0005488)
GO:0008152);; biological process: cellular process (GO:0009987);; biological process: biological reg
GO:0044422);; cellular component: supramolecular complex (GO:0099080);; biological process: cellu

');; biological process: cellular process (GO:0009987);; biological process: signaling (GO:0023052);;
(GO:0065007)

rt (GO:0044464)
t: cell (GO:0005623);; cellular component: organelle (GO:0043226);; cellular component: organelle
);; biological process: immune system process (GO:0002376);; biological process: cellular process (

nent: organelle (GO:0043226);; cellular component: organelle part (GO:0044422);; molecular functi
iological regulation (GO:0065007);; biological process: metabolic process (GO:0008152);; biologica
tion (GO:0065007)
:0031974);; biological process: cellular process (GO:0009987);; biological process: cellular compone
52);; biological process: cellular process (GO:0009987);; cellular component: macromolecular comp
gical process: developmental process (GO:0032502);; molecular function: binding (GO:0005488);; c
r complex (GO:0032991);; biological process: metabolic process (GO:0008152);; biological process:
metabolic process (GO:0008152);; biological process: cellular process (GO:0009987)

; biological process: detoxification (GO:0098754);; molecular function: binding (GO:0005488)

omponent: membrane part (GO:0044425)

3226);; cellular component: organelle part (GO:0044422);; cellular component: cell part (GO:00444
logical process: single-organism process (GO:0044699);; biological process: response to stimulus (

GO:0016020);; biological process: response to stimulus (GO:0050896);; biological process: biologic

part (GO:0044464);; cellular component: macromolecular complex (GO:0032991)
ess (GO:0009987);; biological process: cellular component organization or biogenesis (GO:007184
process (GO:0009987);; biological process: signaling (GO:0023052);; biological process: single-orga
cess (GO:0032501);; biological process: developmental process (GO:0032502);; biological process:

9987);; biological process: single-organism process (GO:0044699);; biological process: biological re
:0005488);; biological process: biological regulation (GO:0065007);; molecular function: catalytic ac
nponent: organelle part (GO:0044422);; cellular component: cell part (GO:0044464)
cellular process (GO:0009987);; biological process: single-organism process (GO:0044699);; biologi
lular region part (GO:0044421);; biological process: cellular process (GO:0009987);; biological proc
membrane (GO:0016020);; cellular component: membrane-enclosed lumen (GO:0031974);; biolog
men (GO:0031974);; cellular component: organelle (GO:0043226);; cellular component: organelle p

(GO:0009987);; biological process: response to stimulus (GO:0050896);; molecular function: molecu

tivity (GO:0003824);; biological process: signaling (GO:0023052);; biological process: response to st

:0065007)
process (GO:0009987);; biological process: biological regulation (GO:0065007);; biological process

:0044464);; biological process: localization (GO:0051179);; biological process: response to stimulus
nent: cell part (GO:0044464);; biological process: cellular process (GO:0009987);; biological process

ponent: organelle (GO:0043226);; cellular component: organelle part (GO:0044422);; cellular comp

;; molecular function: molecular function regulator (GO:0098772);; biological process: biological re

evelopmental process (GO:0032502);; biological process: locomotion (GO:0040011);; biological pro

4699)

s: cellular process (GO:0009987);; molecular function: binding (GO:0005488);; molecular function: t

987);; biological process: cellular component organization or biogenesis (GO:0071840)

lular component: cell (GO:0005623);; cellular component: membrane (GO:0016020);; cellular comp

987);; biological process: single-organism process (GO:0044699)
ocalization (GO:0051179);; biological process: biological regulation (GO:0065007);; cellular compor

987);; biological process: cellular component organization or biogenesis (GO:0071840)
: (GO:0044425);; cellular component: cell part (GO:0044464)

74);; cellular component: macromolecular complex (GO:0032991);; cellular component: organelle p
r component: membrane part (GO:0044425);; cellular component: macromolecular complex (GO:0
localization (GO:0051179);; biological process: cellular component organization or biogenesis (GO
enesis (GO:0071840);; biological process: signaling (GO:0023052);; biological process: single-organ
ed lumen (GO:0031974);; cellular component: organelle part (GO:0044422);; biological process: bic

s (GO:0008152);; biological process: cellular process (GO:0009987);; biological process: response to

tion (GO:0051179)

s: cellular process (GO:0009987)
e-organism process (GO:0044699);; biological process: cellular component organization or biogen
43226);; cellular component: organelle part (GO:0044422);; cellular component: cell part (GO:0044

54);; biological process: biological regulation (GO:0065007);; biological process: cellular componen
GO:0043226);; cellular component: organelle part (GO:0044422);; cellular component: cell part (GC
process (GO:0051704);; molecular function: binding (GO:0005488);; biological process: metabolic p

cess: single-organism process (GO:0044699);; biological process: biological regulation (GO:006500

(GO:0043226);; cellular component: cell part (GO:0044464);; biological process: single-organism p

(GO:0032501);; biological process: biological regulation (GO:0065007);; biological process: metabo
cellular component: organelle part (GO:0044422);; cellular component: cell part (GO:0044464);; ce
ular function regulator (GO:0098772);; cellular component: other organism (GO:0044215);; cellular

e to stimulus (GO:0050896);; molecular function: binding (GO:0005488);; cellular component: extra
elle (GO:0043226);; cellular component: organelle part (GO:0044422);; cellular component: cell par
llular process (GO:0009987);; biological process: single-organism process (GO:0044699);; biologica
cellular component: cell (GO:0005623);; cellular component: organelle (GO:0043226);; cellular comp

localization (GO:0051179);; biological process: cellular component organization or biogenesis (GO
:0005488);; cellular component: cell (GO:0005623);; cellular component: cell part (GO:0044464);; bi
elle part (GO:0044422);; cellular component: macromolecular complex (GO:0032991);; molecular fu

ıponent: cell part (GO:0044464);; cellular component: organelle (GO:0043226);; biological process:
)0050896);; biological process: biological regulation (GO:0065007);; cellular component: cell (GO:00

987);; biological process: cellular component organization or biogenesis (GO:0071840)

4464);; cellular component: membrane (GO:0016020);; cellular component: cell junction (GO:00300
9987);; biological process: single-organism process (GO:0044699);; biological process: multicellula
:0005488);; biological process: single-organism process (GO:0044699);; biological process: localiza
cell part (GO:0044464);; cellular component: macromolecular complex (GO:0032991)

16020);; cellular component: organelle (GO:0043226);; cellular component: organelle part (GO:004
mbrane (GO:0016020);; cellular component: membrane part (GO:0044425);; biological process: loc
ocess (GO:0009987)
2);; cellular component: cell part (GO:0044464);; cellular component: macromolecular complex (GC
ity (GO:0003824)

ıism process (GO:0044699);; biological process: biological regulation (GO:0065007)

38)
ological process: biological regulation (GO:0065007);; biological process: response to stimulus (GO

s: single-organism process (GO:0044699);; biological process: immune system process (GO:00023
9987);; biological process: single-organism process (GO:0044699);; molecular function: catalytic ac
gulation (GO:0065007)

single-organism process (GO:0044699);; biological process: immune system process (GO:0002376
ss (GO:0044699);; cellular component: macromolecular complex (GO:0032991)
ponent: macromolecular complex (GO:0032991)

s: cellular process (GO:0009987)

r complex (GO:0032991);; biological process: metabolic process (GO:0008152);; biological process:
metabolic process (GO:0008152);; biological process: cellular process (GO:0009987)
GO:0032991);; biological process: response to stimulus (GO:0050896);; biological process: biologic

onse to stimulus (GO:0050896);; molecular function: binding (GO:0005488);; cellular component: e

O:0044699);; cellular component: macromolecular complex (GO:0032991);; biological process: imn

GO:0043226);; molecular function: catalytic activity (GO:0003824)
ocess (GO:0009987);; biological process: signaling (GO:0023052);; biological process: response to s

lar component: organelle part (GO:0044422);; cellular component: cell part (GO:0044464);; biologic
nental process (GO:0032502);; biological process: single-organism process (GO:0044699)

ular component: cell part (GO:0044464);; cellular component: synapse part (GO:0044456);; cellular
mponent organization or biogenesis (GO:0071840);; biological process: single-organism process (
l process: metabolic process (GO:0008152);; biological process: cellular process (GO:0009987);; bio
(GO:0023052);; biological process: single-organism process (GO:0044699);; biological process: res

ess: single-organism process (GO:0044699);; biological process: detoxification (GO:0098754);; cellu
part (GO:0044422);; cellular component: cell part (GO:0044464)
rt (GO:0044464);; molecular function: transporter activity (GO:0005215);; biological process: localiz

on or biogenesis (GO:0071840)

c process (GO:0008152);; biological process: cellular process (GO:0009987)

044425);; biological process: cellular component organization or biogenesis (GO:0071840);; biologi

ogical process: response to stimulus (GO:0050896)
:0044422);; biological process: localization (GO:0051179);; cellular component: macromolecular co
onent organization or biogenesis (GO:0071840);; molecular function: binding (GO:0005488)
(GO:0099080);; biological process: cellular process (GO:0009987);; biological process: single-orga
mponent organization or biogenesis (GO:0071840);; cellular component: macromolecular complex

:0009987);; biological process: single-organism process (GO:0044699);; molecular function: bindin

(GO:0043226);; cellular component: cell part (GO:0044464);; cellular component: organelle part (G
gulation (GO:0065007);; biological process: response to stimulus (GO:0050896);; biological process
ction: binding (GO:0005488);; biological process: cellular process (GO:0009987);; biological process
nization or biogenesis (GO:0071840)

mponent: cell (GO:0005623);; cellular component: membrane-enclosed lumen (GO:0031974);; cell

k (GO:0032991)
nt: organelle part (GO:0044422);; cellular component: cell part (GO:0044464)
s (GO:0044699);; biological process: cellular component organization or biogenesis (GO:0071840);;

process (GO:0051704);; cellular component: cell (GO:0005623);; cellular component: organelle (GC
s: cellular process (GO:0009987);; molecular function: binding (GO:0005488)

(GO:0044699);; cellular component: cell (GO:0005623);; cellular component: organelle (GO:004322
stimulus (GO:0050896);; molecular function: binding (GO:0005488)
l process (GO:0032502);; molecular function: binding (GO:0005488);; biological process: response t

3824);; biological process: metabolic process (GO:0008152);; biological process: single-organism p
: metabolic process (GO:0008152);; biological process: cellular process (GO:0009987);; biological p

991);; cellular component: cell part (GO:0044464)

64)

);; cellular component: cell part (GO:0044464);; cellular component: membrane (GO:0016020);; mol
cellular component: organelle (GO:0043226);; cellular component: organelle part (GO:0044422);; b
ulation (GO:0065007);; cellular component: membrane (GO:0016020);; cellular component: membr
3152);; biological process: cellular process (GO:0009987)
52);; biological process: cellular process (GO:0009987);; biological process: biological regulation (G

regulation (GO:0065007);; molecular function: binding (GO:0005488);; cellular component: organe

rter activity (GO:0005215);; biological process: localization (GO:0051179);; cellular component: me

488);; molecular function: transporter activity (GO:0005215)
);; cellular component: organelle part (GO:0044422);; cellular component: cell part (GO:0044464);; r

nent organization or biogenesis (GO:0071840)
s: cellular process (GO:0009987)
l part (GO:0044464);; biological process: behavior (GO:0007610);; biological process: multicellular c

s: cellular process (GO:0009987)
on (GO:0051179);; cellular component: cell junction (GO:0030054)
cess (GO:0008152);; biological process: cellular process (GO:0009987);; biological process: single-o

cess (GO:0044699)

metabolic process (GO:0008152);; biological process: cellular process (GO:0009987)
etabolic process (GO:0008152);; biological process: cellular process (GO:0009987);; biological proc
05488);; cellular component: extracellular region (GO:0005576);; cellular component: cell (GO:0005
mbrane part (GO:0044425);; cellular component: cell part (GO:0044464);; biological process: multic
: metabolic process (GO:0008152);; biological process: cellular process (GO:0009987);; molecular fu

part (GO:0044464);; cellular component: cell junction (GO:0030054);; biological process: response t

O:0030054);; molecular function: molecular transducer activity (GO:0060089);; biological process: lo
sm process (GO:0044699);; biological process: cellular component organization or biogenesis (GO

; biological process: single-organism process (GO:0044699);; biological process: response to stimu
ical regulation (GO:0065007)
s: cellular process (GO:0009987);; molecular function: binding (GO:0005488)
se to stimulus (GO:0050896);; cellular component: organelle (GO:0043226);; cellular component: m

(GO:0005488)

nelle part (GO:0044422);; molecular function: binding (GO:0005488);; biological process: metabolic
gical process: single-organism process (GO:0044699);; biological process: response to stimulus (GC
099080);; biological process: biological regulation (GO:0065007)
ss (GO:0032501);; biological process: developmental process (GO:0032502);; biological process: sin
ll part (GO:0044464)

64)

mplex (GO:0099080);; biological process: cellular process (GO:0009987);; biological process: repro

ecular complex (GO:0032991);; cellular component: organelle (GO:0043226);; cellular component:

lular component: cell part (GO:0044464);; biological process: signaling (GO:0023052);; biological pr
l process: metabolic process (GO:0008152);; biological process: cellular process (GO:0009987);; mc

O:0044699);; biological process: cellular process (GO:0009987);; biological process: cellular compor

part (GO:0044464);; cellular component: membrane (GO:0016020);; biological process: cellular pro
38);; biological process: response to stimulus (GO:0050896);; biological process: biological regulatio
nt: cell part (GO:0044464);; molecular function: binding (GO:0005488);; molecular function: transcri

005623);; cellular component: membrane-enclosed lumen (GO:0031974);; cellular component: ma
ation (GO:0065007);; molecular function: binding (GO:0005488)
mponent: cell part (GO:0044464);; biological process: developmental process (GO:0032502);; biolo

7);; biological process: biological regulation (GO:0065007);; cellular component: macromolecular c
single-organism process (GO:0044699);; biological process: biological regulation (GO:0065007);; b
biological process: locomotion (GO:0040011);; biological process: single-organism process (GO:00
0050896);; biological process: biological regulation (GO:0065007);; cellular component: cell (GO:00

O:0044464);; biological process: metabolic process (GO:0008152);; biological process: cellular proc
lular component: cell part (GO:0044464);; molecular function: binding (GO:0005488);; biological pr

ocess (GO:0044699)

metabolic process (GO:0008152);; biological process: cellular process (GO:0009987)
biological regulation (GO:0065007)
organelle (GO:0043226);; cellular component: organelle part (GO:0044422);; cellular component: m
; biological process: detoxification (GO:0098754);; molecular function: binding (GO:0005488);; cellu

s (GO:0044699);; cellular component: membrane part (GO:0044425);; molecular function: catalytic a
: metabolic process (GO:0008152);; biological process: cellular process (GO:0009987);; biological p
: cellular process (GO:0009987);; biological process: single-organism process (GO:0044699)
mplex (GO:0099080);; biological process: cellular process (GO:0009987);; biological process: repro
ll part (GO:0044464)

4699);; molecular function: binding (GO:0005488)
ponent: organelle (GO:0043226);; cellular component: organelle part (GO:0044422);; biological pro
008152);; biological process: single-organism process (GO:0044699);; biological process: cellular pr
GO:0044422)

0050896);; biological process: immune system process (GO:0002376);; biological process: multi-or

mponent: organelle (GO:0043226);; cellular component: organelle part (GO:0044422);; cellular com
0050896);; biological process: biological regulation (GO:0065007);; cellular component: cell (GO:00
; biological process: detoxification (GO:0098754);; cellular component: cell (GO:0005623);; cellular c
(GO:0043226);; cellular component: organelle part (GO:0044422);; cellular component: cell part (G

ocess (GO:0008152);; cellular component: membrane-enclosed lumen (GO:0031974);; cellular comp

cess (GO:0009987);; biological process: single-organism process (GO:0044699);; biological process
on (GO:0065007);; biological process: cellular process (GO:0009987);; biological process: multi-orga

GO:0044422);; cellular component: supramolecular complex (GO:0099080);; biological process: cellu
05488)

(GO:0044464);; cellular component: macromolecular complex (GO:0032991);; biological process: b

ocess (GO:0044699);; biological process: biological regulation (GO:0065007)
nism process (GO:0044699);; biological process: biological regulation (GO:0065007)
r component: cell part (GO:0044464)

79);; biological process: biological regulation (GO:0065007)

part (GO:0044422);; cellular component: membrane part (GO:0044425);; cellular component: cell p
: multicellular organismal process (GO:0032501);; biological process: developmental process (GO:0
tivity (GO:0003824);; cellular component: organelle (GO:0043226)

: metabolic process (GO:0008152);; biological process: cellular process (GO:0009987);; biological p
cell part (GO:0044464);; cellular component: macromolecular complex (GO:0032991);; molecular fu
(GO:0031974);; cellular component: organelle (GO:0043226);; cellular component: organelle part (
:0044464);; cellular component: macromolecular complex (GO:0032991)
process (GO:0008152);; biological process: single-organism process (GO:0044699)

gle-organism process (GO:0044699);; molecular function: binding (GO:0005488);; cellular compone

complex (GO:0032991);; cellular component: organelle (GO:0043226);; cellular component: organe
mplex (GO:0099080);; biological process: reproduction (GO:0000003);; biological process: cellular

ll part (GO:0044464);; biological process: cellular process (GO:0009987);; biological process: signali
;; biological process: cellular process (GO:0009987);; molecular function: catalytic activity (GO:0003
ess (GO:0044699)
);; biological process: response to stimulus (GO:0050896)
art (GO:0044464);; biological process: response to stimulus (GO:0050896);; biological process: biol
gical process: single-organism process (GO:0044699);; biological process: localization (GO:005117

rocess (GO:0009987);; cellular component: macromolecular complex (GO:0032991);; biological pro
ng (GO:0005488);; biological process: biological regulation (GO:0065007);; cellular component: ma

4425)

al process: multicellular organismal process (GO:0032501);; biological process: developmental proc

37);; biological process: developmental process (GO:0032502);; biological process: single-organism

r component: cell (GO:0005623);; cellular component: membrane (GO:0016020);; cellular compone

(GO:0005576);; cellular component: extracellular region part (GO:0044421);; biological process: cel

omponent: organelle part (GO:0044422);; cellular component: cell part (GO:0044464)
rocess (GO:0008152);; biological process: cellular process (GO:0009987);; biological process: single

ogical process: cellular process (GO:0009987);; biological process: multicellular organismal process

:0009987);; molecular function: binding (GO:0005488);; biological process: biological regulation (G
991);; cellular component: cell part (GO:0044464);; cellular component: membrane-enclosed lume

ity (GO:0003824)
lular component: membrane part (GO:0044425);; biological process: cellular process (GO:0009987
0050896);; biological process: immune system process (GO:0002376);; biological process: multi-or
);; biological process: biological regulation (GO:0065007)

cess: biological regulation (GO:0065007)
nism process (GO:0051704)

rocess: signaling (GO:0023052);; biological process: single-organism process (GO:0044699);; biolo

: binding (GO:0005488);; cellular component: cell (GO:0005623);; cellular component: cell part (GO

process (GO:0009987);; biological process: signaling (GO:0023052);; biological process: single-orga
37);; biological process: signaling (GO:0023052);; biological process: single-organism process (GO:
iological process: biological regulation (GO:0065007);; biological process: immune system process
acromolecular complex (GO:0032991)
ion: catalytic activity (GO:0003824);; molecular function: binding (GO:0005488);; biological process

on (GO:0065007);; biological process: signaling (GO:0023052);; biological process: single-organism
ess: biological regulation (GO:0065007);; biological process: metabolic process (GO:0008152);; mol
lar function: binding (GO:0005488)
process (GO:0044699);; molecular function: catalytic activity (GO:0003824)
GO:0005488);; biological process: biological regulation (GO:0065007)
991);; cellular component: cell part (GO:0044464)
0009987);; biological process: response to stimulus (GO:0050896)

37);; biological process: single-organism process (GO:0044699)

metabolic process (GO:0008152)
process: multi-organism process (GO:0051704)
n (GO:0065007);; biological process: developmental process (GO:0032502);; biological process: sing
t (GO:0044464);; biological process: metabolic process (GO:0008152);; biological process: cellular
extracellular region part (GO:0044421);; cellular component: organelle part (GO:0044422);; cellula

on (GO:0051179);; molecular function: binding (GO:0005488);; cellular component: membrane (GO

GO:0043226);; cellular component: organelle part (GO:0044422);; biological process: cellular comp

(GO:0009987);; cellular component: macromolecular complex (GO:0032991);; cellular component:

)44699);; biological process: response to stimulus (GO:0050896);; biological process: biological reg
4);; cellular component: membrane-enclosed lumen (GO:0031974);; cellular component: macromol

);; biological process: biological regulation (GO:0065007)

32501);; biological process: single-organism process (GO:0044699);; cellular component: cell (GO:0
699);; biological process: developmental process (GO:0032502);; biological process: cellular compo

0050896);; biological process: biological regulation (GO:0065007);; cellular component: cell (GO:00
cess: biological regulation (GO:0065007)

rier activity (GO:0009055);; biological process: cellular process (GO:0009987)

ngle-organism process (GO:0044699)

(GO:0071840);; biological process: biological adhesion (GO:0022610);; biological process: signaling

)08152);; biological process: cellular process (GO:0009987);; molecular function: catalytic activity (G

zation or biogenesis (GO:0071840);; biological process: metabolic process (GO:0008152);; biologic
5007);; biological process: cellular process (GO:0009987);; biological process: cellular component o

ocess (GO:0009987)

cal process: multi-organism process (GO:0051704);; cellular component: membrane (GO:0016020)
on (GO:0065007);; biological process: metabolic process (GO:0008152);; biological process: cellular

ological process: single-organism process (GO:0044699);; biological process: signaling (GO:002305

gical process: multi-organism process (GO:0051704)

process (GO:0009987);; biological process: single-organism process (GO:0044699);; biological pro

ogical process: response to stimulus (GO:0050896);; biological process: biological regulation (GO:0

:0009987);; molecular function: binding (GO:0005488)
5576);; cellular component: other organism (GO:0044215);; cellular component: other organism pa
biological process: single-organism process (GO:0044699);; biological process: biological regulatio

iological process: biological regulation (GO:0065007);; biological process: immune system process

7);; biological process: single-organism process (GO:0044699)

ı process (GO:0044699);; biological process: cellular component organization or biogenesis (GO:0(
)9987);; biological process: single-organism process (GO:0044699);; biological process: biological r

ɔart (GO:0044422);; biological process: cellular process (GO:0009987);; biological process: response

)50896);; biological process: cellular process (GO:0009987);; biological process: signaling (GO:0023

ıponent: cell part (GO:0044464);; cellular component: macromolecular complex (GO:0032991);; cell

ınt: cell part (GO:0044464);; cellular component: extracellular region (GO:0005576);; cellular compo
179);; biological process: behavior (GO:0007610);; biological process: multicellular organismal proc

ocess (GO:0002376);; biological process: multicellular organismal process (GO:0032501);; biologica

; biological process: detoxification (GO:0098754);; cellular component: extracellular region (GO:000

ding transcription factor activity (GO:0001071);; biological process: biological regulation (GO:0065C
16020)
; biological process: detoxification (GO:0098754)
0044464);; cellular component: membrane-enclosed lumen (GO:0031974);; biological process: me

nelle (GO:0043226);; cellular component: membrane (GO:0016020);; cellular component: membrar

2);; biological process: cellular process (GO:0009987);; biological process: single-organism process

cellular organismal process (GO:0032501);; cellular component: cell (GO:0005623);; cellular compo

ane (GO:0016020);; cellular component: membrane part (GO:0044425);; molecular function: bindin

rane part (GO:0044425);; biological process: immune system process (GO:0002376);; biological prc
rane part (GO:0044425);; biological process: immune system process (GO:0002376);; biological prc
onent: cell part (GO:0044464);; molecular function: binding (GO:0005488);; biological process: imm

0023052);; biological process: response to stimulus (GO:0050896);; molecular function: nucleic acic
abolic process (GO:0008152);; molecular function: binding (GO:0005488);; cellular component: cell
process (GO:0044699);; biological process: immune system process (GO:0002376);; biological pro
abolic process (GO:0008152);; biological process: cellular process (GO:0009987);; molecular functic
biological process: biological regulation (GO:0065007)

52);; biological process: single-organism process (GO:0044699);; biological process: response to sti
; biological process: detoxification (GO:0098754);; molecular function: transporter activity (GO:0005

ecular complex (GO:0032991);; cellular component: organelle (GO:0043226);; cellular component:
nolecular complex (GO:0032991)

0032501);; biological process: rhythmic process (GO:0048511);; biological process: multi-organism

nponent: organelle part (GO:0044422);; cellular component: cell part (GO:0044464)

embrane (GO:0016020);; biological process: metabolic process (GO:0008152);; cellular component:

421);; biological process: response to stimulus (GO:0050896);; biological process: multi-organism
ess (GO:0008152);; biological process: cellular process (GO:0009987);; biological process: response

ocess: metabolic process (GO:0008152);; biological process: cellular process (GO:0009987);; biolog

O:0065007)

rganism process (GO:0044699);; biological process: immune system process (GO:0002376);; molecu
process: biological regulation (GO:0065007);; biological process: cellular process (GO:0009987);; b

biological process: single-organism process (GO:0044699);; biological process: behavior (GO:0007
process (GO:0032502);; biological process: single-organism process (GO:0044699);; molecular funct
ocess: biological regulation (GO:0065007);; biological process: developmental process (GO:003250

ponent organization or biogenesis (GO:0071840);; biological process: behavior (GO:0007610);; bio
ocess (GO:0044699);; biological process: biological regulation (GO:0065007);; biological process: si

tivity (GO:0003824);; cellular component: membrane part (GO:0044425);; molecular function: bind
to stimulus (GO:0050896);; molecular function: catalytic activity (GO:0003824)

ogical process: immune system process (GO:0002376);; biological process: cellular process (GO:00

l process (GO:0032501);; biological process: single-organism process (GO:0044699);; biological pr

ess (GO:0032501);; molecular function: catalytic activity (GO:0003824);; biological process: metabo
ctivity (GO:0016209);; biological process: detoxification (GO:0098754);; cellular component: extrace
nponent: membrane (GO:0016020);; cellular component: membrane part (GO:0044425);; biologica

mmune system process (GO:0002376);; biological process: cellular process (GO:0009987);; biologic
(GO:0016020);; cellular component: membrane part (GO:0044425);; biological process: multicellul

ological process: localization (GO:0051179);; biological process: signaling (GO:0023052);; biologica
ctivity (GO:0003824)
007);; cellular component: synapse (GO:0045202)
: immune system process (GO:0002376);; biological process: multicellular organismal process (GO:
process: cellular process (GO:0009987);; biological process: signaling (GO:0023052);; biological pr

al process: response to stimulus (GO:0050896);; biological process: cellular component organizatic

):0005488);; cellular component: membrane (GO:0016020);; cellular component: membrane part (

(GO:0016020);; cellular component: membrane part (GO:0044425)
05623);; cellular component: membrane (GO:0016020);; cellular component: cell part (GO:004446
n: binding (GO:0005488);; biological process: cellular process (GO:0009987);; biological process: sir

rocess (GO:0009987);; biological process: multicellular organismal process (GO:0032501);; biologic

(GO:0016020);; cellular component: membrane part (GO:0044425)
(GO:0016020);; cellular component: membrane part (GO:0044425)
process (GO:0032502);; biological process: single-organism process (GO:0044699)
r component: cell part (GO:0044464);; cellular component: membrane (GO:0016020);; cellular com

ng (GO:0005488);; molecular function: molecular function regulator (GO:0098772);; cellular compor

component: membrane (GO:0016020);; cellular component: membrane part (GO:0044425);; biolo
cellular component: cell part (GO:0044464);; cellular component: organelle (GO:0043226);; cellular

part (GO:0044464)

cal process: cellular component organization or biogenesis (GO:0071840);; biological process: deve
ular process (GO:0009987)
0:0005488);; cellular component: membrane (GO:0016020);; cellular component: membrane part (
043226);; biological process: cellular process (GO:0009987);; biological process: signaling (GO:0023
ular process (GO:0009987)

(GO:0032502);; biological process: single-organism process (GO:0044699)

s (GO:0050896);; biological process: biological adhesion (GO:0022610);; biological process: metabo

);; biological process: developmental process (GO:0032502);; biological process: single-organism p

lus (GO:0050896);; cellular component: organelle (GO:0043226);; cellular component: organelle pa

gical process: multi-organism process (GO:0051704);; cellular component: membrane (GO:001602

05623);; cellular component: membrane (GO:0016020);; cellular component: membrane part (GO:
(GO:0016020);; cellular component: membrane part (GO:0044425)

gulation (GO:0065007);; cellular component: membrane (GO:0016020);; cellular component: mem
e process (GO:0022414);; biological process: multicellular organismal process (GO:0032501);; biolo

ter activity (GO:0005215);; biological process: localization (GO:0051179);; biological process: cellula

ɜm process (GO:0044699);; biological process: cellular component organization or biogenesis (GO:(

):0044699);; biological process: response to stimulus (GO:0050896);; biological process: biological
(GO:0016020);; cellular component: membrane part (GO:0044425)

ılar process (GO:0009987)
ɪnent: macromolecular complex (GO:0032991);; cellular component: membrane part (GO:0044425)

):0044421);; biological process: signaling (GO:0023052);; biological process: biological regulation ((

):0005488);; cellular component: cell (GO:0005623);; cellular component: membrane (GO:0016020)
)065007)

al regulation (GO:0065007);; biological process: cellular component organization or biogenesis (G(
ecular function: signal transducer activity (GO:0004871);; molecular function: molecular transducer

ss: single-organism process (GO:0044699)

ɪnent: macromolecular complex (GO:0032991);; cellular component: membrane part (GO:0044425)

y, protein binding (GO:0000988);; molecular function: catalytic activity (GO:0003824);; cellular com

);; molecular function: molecular transducer activity (GO:0060089);; biological process: signaling (G
component organization or biogenesis (GO:0071840)
(GO:0051179);; biological process: multicellular organismal process (GO:0032501);; cellular compor

nent: organelle (GO:0043226);; cellular component: organelle part (GO:0044422);; cellular compon

nent: organelle (GO:0043226);; cellular component: cell part (GO:0044464);; cellular component: m
ılation (GO:0065007)

rocess (GO:0044699);; biological process: multicellular organismal process (GO:0032501);; cellular (

:al process: metabolic process (GO:0008152);; biological process: cellular process (GO:0009987);; b
):0008152);; biological process: cellular process (GO:0009987);; molecular function: catalytic activity
nponent: organelle (GO:0043226);; cellular component: cell part (GO:0044464);; cellular componer

l regulation (GO:0065007);; biological process: multicellular organismal process (GO:0032501);; bic
O:0009987);; biological process: single-organism process (GO:0044699)
);; biological process: biological regulation (GO:0065007)

ı process (GO:0044699);; biological process: cellular process (GO:0009987);; biological process: loc

5);; cellular component: extracellular region part (GO:0044421);; cellular component: cell (GO:0005

ılation (GO:0065007);; biological process: multicellular organismal process (GO:0032501);; biologic
cellular organismal process (GO:0032501);; biological process: developmental process (GO:003250
ion (GO:0099531);; cellular component: cell (GO:0005623);; cellular component: synapse part (GO:

t: membrane (GO:0016020);; cellular component: membrane part (GO:0044425);; cellular compone

function: binding (GO:0005488)

ological process: single-organism process (GO:0044699);; biological process: response to stimulus

onent: cell part (GO:0044464)
ılar component organization or biogenesis (GO:0071840);; biological process: response to stimulus

sm process (GO:0044699);; cellular component: membrane-enclosed lumen (GO:0031974);; molecu

ng (GO:0005488);; biological process: cellular process (GO:0009987);; biological process: single-org

05623);; cellular component: membrane (GO:0016020);; cellular component: membrane part (GO:

ar component organization or biogenesis (GO:0071840);; cellular component: organelle (GO:0043

biological regulation (GO:0065007);; biological process: localization (GO:0051179);; cellular compo

9);; biological process: single-organism process (GO:0044699);; biological process: response to sti

nt: cell (GO:0005623);; cellular component: organelle (GO:0043226);; cellular component: cell part

):0005488);; cellular component: extracellular region (GO:0005576);; cellular component: extracellu

051179)
ogical regulation (GO:0065007)

l process: cellular component organization or biogenesis (GO:0071840);; molecular function: bindi
cellular process (GO:0009987);; biological process: response to stimulus (GO:0050896)

(GO:0005623);; cellular component: cell part (GO:0044464);; cellular component: organelle (GO:00

llular component organization or biogenesis (GO:0071840);; biological process: biological regulati

ocess (GO:0009987);; biological process: signaling (GO:0023052);; biological process: single-organ
olic process (GO:0008152);; biological process: cellular process (GO:0009987);; biological process: s

GO:0050896);; biological process: locomotion (GO:0040011);; biological process: localization (GO:0

ess (GO:0009987);; biological process: locomotion (GO:0040011);; biological process: single-organ

t: cell part (GO:0044464)

O:0045202)
cellular process (GO:0009987);; biological process: single-organism process (GO:0044699);; biolog

anelle (GO:0043226);; cellular component: cell part (GO:0044464);; biological process: multicellular

r component: membrane (GO:0016020);; cellular component: membrane part (GO:0044425);; biol

):0016020);; cellular component: macromolecular complex (GO:0032991);; cellular component: org

anization or biogenesis (GO:0071840);; biological process: biological regulation (GO:0065007)

al process: biological regulation (GO:0065007)

ılar organismal process (GO:0032501);; molecular function: molecular function regulator (GO:0098[illegible]

ɔgical process: biological regulation (GO:0065007);; molecular function: binding (GO:0005488);; cel

ar component: cell part (GO:0044464);; cellular component: membrane (GO:0016020);; biological p

:al process: locomotion (GO:0040011);; biological process: single-organism process (GO:0044699);
: single-organism process (GO:0044699);; biological process: response to stimulus (GO:0050896);;

ar component: organelle part (GO:0044422);; cellular component: membrane part (GO:0044425);; c
mbrane part (GO:0044425)

art (GO:0044425)

ɔ:0065007)
organization or biogenesis (GO:0071840)

ıl process (GO:0032502);; biological process: single-organism process (GO:0044699);; molecular fu
organism process (GO:0051704);; biological process: cellular process (GO:0009987)

ılar component: membrane (GO:0016020);; cellular component: membrane part (GO:0044425);; ce

ngle-organism process (GO:0044699);; biological process: localization (GO:0051179)
O:0065007)
ılar process (GO:0009987)
lic process (GO:0008152);; biological process: single-organism process (GO:0044699);; biological p

biological regulation (GO:0065007)

ıl regulation (GO:0065007);; cellular component: cell (GO:0005623);; cellular component: membrar
0043226);; cellular component: organelle part (GO:0044422);; cellular component: cell part (GO:00

component: membrane part (GO:0044425);; cellular component: cell part (GO:0044464);; biologica

(GO:0016020);; cellular component: membrane part (GO:0044425)
omplex (GO:0032991)
):0044421);; biological process: signaling (GO:0023052);; biological process: biological regulation ((
O:0050896);; biological process: biological regulation (GO:0065007)

;: cellular component organization or biogenesis (GO:0071840);; molecular function: binding (GO:0
cellular process (GO:0009987);; biological process: developmental process (GO:0032502);; biologic

mbrane part (GO:0044425)

ılar component: macromolecular complex (GO:0032991);; biological process: immune system proc
GO:0009987);; biological process: cellular component organization or biogenesis (GO:0071840)
logical regulation (GO:0065007);; cellular component: cell (GO:0005623);; cellular component: mer

):0050896);; biological process: biological regulation (GO:0065007);; biological process: developme
);; biological process: single-organism process (GO:0044699);; biological process: response to stim
ogical regulation (GO:0065007);; biological process: cell killing (GO:0001906);; biological process: i
ss: single-organism process (GO:0044699);; cellular component: membrane (GO:0016020);; biolog

ess: biological regulation (GO:0065007);; molecular function: catalytic activity (GO:0003824);; biolo
al process: localization (GO:0051179)
al transducer activity (GO:0004871);; molecular function: molecular transducer activity (GO:006008

cellular component: cell (GO:0005623);; cellular component: cell part (GO:0044464);; cellular comp

mponent: membrane (GO:0016020);; cellular component: macromolecular complex (GO:0032991)

s (GO:0009987);; biological process: single-organism process (GO:0044699);; biological process: lo
39);; biological process: cellular process (GO:0009987);; biological process: signaling (GO:0023052);

olic process (GO:0008152);; biological process: localization (GO:0051179);; biological process: cellu
rt (GO:0044422);; cellular component: cell part (GO:0044464);; cellular component: supramolecular

molecular function: binding (GO:0005488)

lic process (GO:0008152);; molecular function: catalytic activity (GO:0003824)

gical process: developmental process (GO:0032502);; biological process: single-organism process (G
23052);; biological process: single-organism process (GO:0044699);; biological process: response t

009987);; biological process: single-organism process (GO:0044699);; biological process: biologica

ent: organelle part (GO:0044422);; cellular component: membrane part (GO:0044425);; cellular con

logical process: biological regulation (GO:0065007);; biological process: signaling (GO:0023052);; b
ulus (GO:0050896)

organismal process (GO:0032501);; biological process: developmental process (GO:0032502);; biolo

ation (GO:0051179);; biological process: metabolic process (GO:0008152);; biological process: cellu

rganization or biogenesis (GO:0071840);; biological process: response to stimulus (GO:0050896)
O:0016020);; cellular component: organelle (GO:0043226);; cellular component: organelle part (GO
ulus (GO:0050896);; biological process: biological regulation (GO:0065007)
43226);; cellular component: organelle part (GO:0044422);; cellular component: cell part (GO:0044

991);; cellular component: membrane-enclosed lumen (GO:0031974)

0032502);; biological process: single-organism process (GO:0044699);; biological process: biologica
t: organelle (GO:0043226);; cellular component: organelle part (GO:0044422);; cellular component:
organismal process (GO:0032501);; biological process: developmental process (GO:0032502);; bio

nent: cell part (GO:0044464);; biological process: cellular process (GO:0009987);; biological process
s (GO:0044699)

GO:0032501);; biological process: localization (GO:0051179);; molecular function: signal transducer

ar function: binding (GO:0005488)

nponent: organelle (GO:0043226);; cellular component: cell part (GO:0044464);; cellular componer
process (GO:0009987);; biological process: single-organism process (GO:0044699)

cal regulation (GO:0065007);; biological process: cellular component organization or biogenesis (G
process (GO:0009987);; biological process: reproduction (GO:0000003);; biological process: reprodu

09987);; biological process: single-organism process (GO:0044699);; biological process: localization

gulation (GO:0065007);; molecular function: catalytic activity (GO:0003824)
ular process (GO:0009987)

biological process: single-organism process (GO:0044699);; biological process: localization (GO:00

part (GO:0044422);; cellular component: cell part (GO:0044464);; biological process: localization (G
GO:0009987);; biological process: multicellular organismal process (GO:0032501);; biological proces

on: catalytic activity (GO:0003824);; biological process: metabolic process (GO:0008152);; biologica
l process: cellular process (GO:0009987);; biological process: cellular component organization or b

ent organization or biogenesis (GO:0071840);; biological process: response to stimulus (GO:00508
plex (GO:0032991);; cellular component: membrane-enclosed lumen (GO:0031974);; biological pro
ellular component: extracellular region (GO:0005576);; cellular component: extracellular region par
cellular process (GO:0009987)

·64);; biological process: single-organism process (GO:0044699);; biological process: metabolic prc
GO:0050896);; biological process: biological regulation (GO:0065007)

al adhesion (GO:0022610);; biological process: single-organism process (GO:0044699);; biological

0);; biological process: multicellular organismal process (GO:0032501);; cellular component: macroı
ınism process (GO:0044699);; biological process: response to stimulus (GO:0050896);; biological pr
single-organism process (GO:0044699);; biological process: biological regulation (GO:0065007);; m

egulation (GO:0065007);; cellular component: membrane part (GO:0044425);; biological process: lc
:tivity (GO:0003824);; biological process: metabolic process (GO:0008152);; biological process: cellu

cal process: biological regulation (GO:0065007);; biological process: cellular component organizati
ess: signaling (GO:0023052);; biological process: response to stimulus (GO:0050896);; biological pr
gical process: metabolic process (GO:0008152);; biological process: cellular process (GO:0009987);;
part (GO:0044422);; cellular component: cell part (GO:0044464);; biological process: immune syster

ular function regulator (GO:0098772);; cellular component: macromolecular complex (GO:0032991

timulus (GO:0050896);; biological process: biological regulation (GO:0065007);; biological process:

: cellular component organization or biogenesis (GO:0071840);; molecular function: molecular fun

(GO:0050896);; biological process: cellular process (GO:0009987);; biological process: developmer
: cellular component organization or biogenesis (GO:0071840)

onent: membrane part (GO:0044425);; cellular component: cell part (GO:0044464)

gulation (GO:0065007);; biological process: localization (GO:0051179)

ocess: single-organism process (GO:0044699);; biological process: response to stimulus (GO:00508

ranslation regulator activity (GO:0045182);; biological process: immune system process (GO:00023

onent: membrane part (GO:0044425);; cellular component: synapse part (GO:0044456);; cellular cc

nent: cell (GO:0005623);; cellular component: organelle (GO:0043226);; cellular component: cell pa

part (GO:0044422);; molecular function: binding (GO:0005488)
032991);; biological process: cellular process (GO:0009987);; biological process: single-organism p
:0071840)
ism process (GO:0044699);; biological process: biological regulation (GO:0065007)
logical regulation (GO:0065007)

stimulus (GO:0050896);; biological process: single-organism process (GO:0044699);; biological p

esis (GO:0071840);; cellular component: extracellular region (GO:0005576);; cellular component: ex
464);; molecular function: binding (GO:0005488)

t organization or biogenesis (GO:0071840);; cellular component: macromolecular complex (GO:00
):0044464);; biological process: metabolic process (GO:0008152);; cellular component: membrane-
process (GO:0008152);; biological process: cellular process (GO:0009987);; cellular component: cell

7);; cellular component: macromolecular complex (GO:0032991)

rocess (GO:0044699)

lic process (GO:0008152);; biological process: cellular process (GO:0009987);; biological process: si
llular component: membrane part (GO:0044425);; biological process: biological regulation (GO:006
component: other organism part (GO:0044217);; biological process: cellular process (GO:0009987)

cellular region (GO:0005576);; cellular component: cell (GO:0005623);; cellular component: cell par
t (GO:0044464)
al process: response to stimulus (GO:0050896);; cellular component: cell (GO:0005623);; cellular co
ponent: organelle part (GO:0044422);; cellular component: cell part (GO:0044464);; biological proc

:0071840)
ological process: localization (GO:0051179);; biological process: biological regulation (GO:0065007
ınction: catalytic activity (GO:0003824)

cellular process (GO:0009987);; biological process: cellular component organization or biogenesis
05623);; cellular component: membrane (GO:0016020);; cellular component: macromolecular com

54);; biological process: biological regulation (GO:0065007);; cellular component: membrane part (
r organismal process (GO:0032501);; biological process: developmental process (GO:0032502);; bic
tion (GO:0051179)

4422);; cellular component: cell part (GO:0044464);; cellular component: membrane part (GO:0044
alization (GO:0051179);; biological process: cellular process (GO:0009987);; biological process: sing

):0032991);; cellular component: supramolecular complex (GO:0099080);; biological process: biolo

:0050896);; biological process: metabolic process (GO:0008152);; biological process: immune syste

76);; biological process: cellular process (GO:0009987);; molecular function: transcription factor act
tivity (GO:0003824);; cellular component: membrane part (GO:0044425);; molecular function: bind

);; molecular function: catalytic activity (GO:0003824);; molecular function: binding (GO:0005488);;

cellular process (GO:0009987);; biological process: biological regulation (GO:0065007)

al regulation (GO:0065007);; biological process: cellular component organization or biogenesis (G(

extracellular region (GO:0005576);; cellular component: extracellular region part (GO:0044421);; bic

nune system process (GO:0002376);; biological process: locomotion (GO:0040011);; biological proc

stimulus (GO:0050896);; biological process: biological regulation (GO:0065007);; cellular componer

cal process: metabolic process (GO:0008152);; biological process: cellular process (GO:0009987);; b

component: synapse (GO:0045202);; biological process: biological regulation (GO:0065007);; cellu
GO:0044699);; biological process: biological regulation (GO:0065007);; molecular function: catalyti
ological process: biological regulation (GO:0065007);; biological process: response to stimulus (GO:
sponse to stimulus (GO:0050896);; biological process: biological regulation (GO:0065007)

ılar component: cell (GO:0005623);; cellular component: membrane (GO:0016020);; cellular compo

ation (GO:0051179);; biological process: metabolic process (GO:0008152);; biological process: cellu

cal process: single-organism process (GO:0044699);; biological process: biological regulation (GO:

mplex (GO:0032991)

nism process (GO:0044699);; biological process: cellular component organization or biogenesis (G
x (GO:0032991);; molecular function: binding (GO:0005488)

g (GO:0005488)

O:0044422);; biological process: single-organism process (GO:0044699);; biological process: cellul
s: developmental process (GO:0032502);; biological process: single-organism process (GO:004469
s: cellular component organization or biogenesis (GO:0071840)

ular component: organelle (GO:0043226);; cellular component: organelle part (GO:0044422);; cellu

biological process: biological regulation (GO:0065007)

):0043226);; cellular component: cell part (GO:0044464);; biological process: localization (GO:0051

6);; cellular component: cell part (GO:0044464);; molecular function: binding (GO:0005488);; biolog

to stimulus (GO:0050896);; molecular function: catalytic activity (GO:0003824);; biological process: r

process (GO:0044699);; biological process: cellular process (GO:0009987);; biological process: respo
rocess: cellular component organization or biogenesis (GO:0071840);; molecular function: binding

ecular function: binding (GO:0005488);; biological process: cellular process (GO:0009987);; biologi
iological process: metabolic process (GO:0008152);; biological process: cellular process (GO:00099
ane part (GO:0044425);; cellular component: macromolecular complex (GO:0032991);; cellular com

O:0065007);; cellular component: membrane-enclosed lumen (GO:0031974);; cellular component:

lle part (GO:0044422);; biological process: cellular component organization or biogenesis (GO:007

mbrane part (GO:0044425)

molecular function: binding (GO:0005488);; cellular component: macromolecular complex (GO:003

organismal process (GO:0032501);; biological process: response to stimulus (GO:0050896);; biologi

rganism process (GO:0044699);; molecular function: catalytic activity (GO:0003824);; cellular comp

ess: single-organism process (GO:0044699);; molecular function: binding (GO:0005488)
5623);; cellular component: cell part (GO:0044464);; biological process: metabolic process (GO:0008
cellular organismal process (GO:0032501);; biological process: developmental process (GO:003250
unction: binding (GO:0005488)

to stimulus (GO:0050896);; biological process: biological regulation (GO:0065007);; biological proce

ocalization (GO:0051179);; biological process: biological regulation (GO:0065007);; biological proce
:0071840);; cellular component: macromolecular complex (GO:0032991)

lus (GO:0050896);; cellular component: organelle (GO:0043226);; cellular component: organelle pa

nembrane (GO:0016020);; biological process: localization (GO:0051179)

process (GO:0008152);; biological process: cellular process (GO:0009987);; biological process: resp
):0050896)

gle-organism process (GO:0044699);; biological process: biological regulation (GO:0065007);; biol

duction (GO:0000003);; biological process: reproductive process (GO:0022414);; biological process

organelle part (GO:0044422)

rocess: single-organism process (GO:0044699);; biological process: response to stimulus (GO:0050
lecular function: binding (GO:0005488);; biological process: signaling (GO:0023052);; biological pro

nent organization or biogenesis (GO:0071840);; biological process: localization (GO:0051179);; mol

ocess (GO:0009987)
on (GO:0065007)
ption factor activity, protein binding (GO:0000988);; molecular function: catalytic activity (GO:0003

cromolecular complex (GO:0032991);; cellular component: organelle (GO:0043226);; cellular compo

gical process: single-organism process (GO:0044699);; biological process: cellular process (GO:000

omplex (GO:0032991)
biological process: cellular component organization or biogenesis (GO:0071840);; biological proces
044699);; biological process: localization (GO:0051179);; biological process: multi-organism proces
05623);; cellular component: cell part (GO:0044464);; cellular component: membrane (GO:001602

ess (GO:0009987);; biological process: cellular component organization or biogenesis (GO:007184
ocess: biological regulation (GO:0065007);; biological process: localization (GO:0051179)

nembrane part (GO:0044425);; cellular component: cell part (GO:0044464)
ılar component: cell (GO:0005623);; cellular component: cell part (GO:0044464);; cellular componer

ıctivity (GO:0003824)
rocess: response to stimulus (GO:0050896);; biological process: biological regulation (GO:0065007

duction (GO:0000003);; biological process: reproductive process (GO:0022414);; biological process

cess: signaling (GO:0023052);; biological process: response to stimulus (GO:0050896);; molecular fı
ocess (GO:0009987);; biological process: cellular component organization or biogenesis (GO:0071[illegible]

ganism process (GO:0051704)

ıponent: cell part (GO:0044464);; biological process: cellular process (GO:0009987);; biological proc
05623);; cellular component: organelle (GO:0043226);; cellular component: cell part (GO:0044464);
component: organelle (GO:0043226);; cellular component: cell part (GO:0044464);; cellular compor
O:0044464);; cellular component: membrane-enclosed lumen (GO:0031974);; cellular component:

ponent: organelle part (GO:0044422);; cellular component: macromolecular complex (GO:0032991

s: response to stimulus (GO:0050896)
anism process (GO:0051704);; biological process: metabolic process (GO:0008152);; biological proc

ılar process (GO:0009987)

iological regulation (GO:0065007);; biological process: metabolic process (GO:0008152);; biologica

art (GO:0044464);; biological process: metabolic process (GO:0008152);; biological process: cellula
0032502);; biological process: single-organism process (GO:0044699);; molecular function: binding

rocess: response to stimulus (GO:0050896);; biological process: single-organism process (GO:0044
ınction: binding (GO:0005488)
GO:0044422);; cellular component: cell part (GO:0044464)

ent: macromolecular complex (GO:0032991);; biological process: localization (GO:0051179);; biolog

elle part (GO:0044422);; cellular component: cell part (GO:0044464)
process (GO:0009987);; biological process: reproductive process (GO:0022414);; biological process

ing (GO:0023052);; biological process: single-organism process (GO:0044699);; biological process:
8824);; biological process: biological regulation (GO:0065007)

ogical adhesion (GO:0022610);; biological process: biological regulation (GO:0065007);; cellular co
9)

ocess: developmental process (GO:0032502);; biological process: single-organism process (GO:004
cromolecular complex (GO:0032991)

ess (GO:0032502);; biological process: response to stimulus (GO:0050896);; molecular function: mo

ı process (GO:0044699)

ent: membrane part (GO:0044425);; cellular component: cell part (GO:0044464);; molecular functio

llular process (GO:0009987);; biological process: cellular component organization or biogenesis (G

-organism process (GO:0044699);; molecular function: binding (GO:0005488);; cellular component

(GO:0032501);; biological process: locomotion (GO:0040011);; biological process: single-organism

O:0065007);; biological process: single-organism process (GO:0044699);; biological process: cellula
n (GO:0031974);; cellular component: organelle (GO:0043226);; cellular component: organelle part

);; biological process: locomotion (GO:0040011);; biological process: single-organism process (GO:
ganism process (GO:0051704)

gical process: response to stimulus (GO:0050896)

:0044464);; cellular component: organelle (GO:0043226);; cellular component: organelle part (GO:

nism process (GO:0044699);; biological process: response to stimulus (GO:0050896);; biological pr
0044699);; biological process: response to stimulus (GO:0050896);; biological process: biological re
(GO:0002376);; biological process: response to stimulus (GO:0050896);; biological process: multi-

: response to stimulus (GO:0050896);; biological process: biological regulation (GO:0065007);; biol

process (GO:0044699);; biological process: response to stimulus (GO:0050896);; biological process
ecular function: protein tag (GO:0031386);; biological process: multi-organism process (GO:0051

gle-organism process (GO:0044699);; molecular function: binding (GO:0005488)
process (GO:0009987);; biological process: single-organism process (GO:0044699);; biological proc
r component: cell (GO:0005623);; cellular component: cell part (GO:0044464);; biological process:

:0016020);; cellular component: membrane part (GO:0044425);; biological process: metabolic proc

onent organization or biogenesis (GO:0071840);; biological process: single-organism process (GO

organelle part (GO:0044422)

ulation (GO:0065007)
ecular complex (GO:0032991);; biological process: metabolic process (GO:0008152);; biological pro

005623);; cellular component: membrane (GO:0016020);; cellular component: macromolecular cor
onent organization or biogenesis (GO:0071840)

05623);; cellular component: organelle (GO:0043226);; cellular component: cell part (GO:0044464);

(GO:0023052);; biological process: response to stimulus (GO:0050896);; cellular component: cell (

O:0003824);; biological process: biological regulation (GO:0065007);; molecular function: binding (

al process: reproductive process (GO:0022414);; biological process: single-organism process (GO:
rganization or biogenesis (GO:0071840);; cellular component: macromolecular complex (GO:0032

;; cellular component: membrane part (GO:0044425);; cellular component: cell (GO:0005623);; cellu
process (GO:0009987);; biological process: immune system process (GO:0002376);; biological proc

2);; biological process: response to stimulus (GO:0050896);; biological process: biological regulatic

cess: multicellular organismal process (GO:0032501);; biological process: developmental process (

065007);; molecular function: binding (GO:0005488)

rt (GO:0044217);; cellular component: extracellular region part (GO:0044421);; biological process: l
on (GO:0065007);; biological process: locomotion (GO:0040011);; biological process: localization (G

(GO:0002376);; biological process: response to stimulus (GO:0050896);; biological process: multi-[illegible]

[illegible]71840)
[illegible]egulation (GO:0065007);; biological process: cellular component organization or biogenesis (GO:0[illegible]

[illegible] to stimulus (GO:0050896)

[illegible]052);; biological process: single-organism process (GO:0044699);; biological process: multicellular

[illegible]lular component: organelle part (GO:0044422);; cellular component: membrane (GO:0016020);; cel[illegible]

[illegible]nent: organelle (GO:0043226);; cellular component: other organism (GO:0044215);; cellular compo[illegible]
[illegible]ess (GO:0032501);; biological process: biological regulation (GO:0065007);; biological process: cell[illegible]

[illegible]l process: developmental process (GO:0032502);; biological process: single-organism process (GC[illegible]

[illegible]5576);; cellular component: extracellular region part (GO:0044421);; cellular component: membran[illegible]

[illegible]007);; biological process: immune system process (GO:0002376);; biological process: cellular proces[illegible]

[illegible]tabolic process (GO:0008152);; biological process: cellular process (GO:0009987);; molecular functi[illegible]

[illegible]e part (GO:0044425);; biological process: response to stimulus (GO:0050896);; biological process:

(GO:0044699);; cellular component: cell (GO:0005623);; cellular component: cell part (GO:0044464[illegible]

[illegible]nent: organelle (GO:0043226);; cellular component: cell part (GO:0044464);; cellular component: m[illegible]

[illegible]g (GO:0005488);; biological process: biological regulation (GO:0065007);; biological process: immu[illegible]

ocess: response to stimulus (GO:0050896);; biological process: multi-organism process (GO:00517C
ocess: response to stimulus (GO:0050896);; biological process: multi-organism process (GO:00517C
iune system process (GO:0002376)

d binding transcription factor activity (GO:0001071);; molecular function: binding (GO:0005488);; ce
(GO:0005623);; cellular component: organelle (GO:0043226);; cellular component: cell part (GO:00
cess: multicellular organismal process (GO:0032501);; biological process: developmental process (
on: binding (GO:0005488);; biological process: localization (GO:0051179);; biological process: biolo

mulus (GO:0050896);; cellular component: organelle part (GO:0044422);; cellular component: mem
5215);; biological process: localization (GO:0051179);; cellular component: cell (GO:0005623);; cellu

organelle part (GO:0044422);; cellular component: cell part (GO:0044464);; cellular component: sy

process (GO:0051704);; biological process: response to stimulus (GO:0050896);; biological process

cell (GO:0005623);; cellular component: organelle (GO:0043226);; cellular component: cell part (G

process (GO:0051704);; biological process: biological regulation (GO:0065007);; molecular functior
to stimulus (GO:0050896);; biological process: detoxification (GO:0098754)

ical process: biological regulation (GO:0065007);; biological process: cellular component organizat

ular function: binding (GO:0005488)
biological process: locomotion (GO:0040011);; biological process: localization (GO:0051179)

610);; cellular component: synapse part (GO:0044456);; cellular component: synapse (GO:0045202)
tion: binding (GO:0005488)
02);; molecular function: catalytic activity (GO:0003824)

logical process: multicellular organismal process (GO:0032501);; cellular component: cell (GO:0005
gnaling (GO:0023052);; biological process: response to stimulus (GO:0050896);; cellular componen

ing (GO:0005488)

09987);; biological process: multicellular organismal process (GO:0032501);; biological process: de

ocess: multi-organism process (GO:0051704);; biological process: cellular component organization

lic process (GO:0008152);; molecular function: binding (GO:0005488);; cellular component: cell (GC
ellular region (GO:0005576);; cellular component: extracellular region part (GO:0044421);; cellular c
l process: localization (GO:0051179);; biological process: biological adhesion (GO:0022610);; biolog

:al process: signaling (GO:0023052);; biological process: single-organism process (GO:0044699);; b
ar organismal process (GO:0032501);; biological process: developmental process (GO:0032502)

l process: multicellular organismal process (GO:0032501);; biological process: single-organism pro

0032501);; biological process: developmental process (GO:0032502);; biological process: response
ocess: response to stimulus (GO:0050896);; biological process: biological regulation (GO:0065007)

on or biogenesis (GO:0071840);; biological process: immune system process (GO:0002376);; molecu

GO:0044425)

4);; cellular component: membrane part (GO:0044425)
ngle-organism process (GO:0044699);; biological process: cellular component organization or biog

al process: developmental process (GO:0032502);; biological process: cellular component organiza

ponent: membrane part (GO:0044425);; molecular function: binding (GO:0005488)

nent: cell junction (GO:0030054)

gical process: developmental process (GO:0032502);; molecular function: transporter activity (GO:0
component: organelle part (GO:0044422);; cellular component: membrane (GO:0016020);; cellular

elopmental process (GO:0032502)

GO:0044425)
052);; biological process: response to stimulus (GO:0050896);; biological process: biological regula

olic process (GO:0008152);; biological process: biological regulation (GO:0065007);; biological proc

process (GO:0044699);; cellular component: macromolecular complex (GO:0032991);; biological pro

rt (GO:0044422);; biological process: localization (GO:0051179);; cellular component: synapse part

0);; cellular component: membrane part (GO:0044425);; molecular function: catalytic activity (GO:0

0044425);; cellular component: cell part (GO:0044464);; molecular function: binding (GO:0005488)

brane part (GO:0044425);; molecular function: binding (GO:0005488)
gical process: single-organism process (GO:0044699);; biological process: multi-organism process

ir process (GO:0009987);; biological process: single-organism process (GO:0044699);; biological pr

)0071840)

regulation (GO:0065007)

;; cellular component: cell part (GO:0044464);; biological process: immune system process (GO:00[illegible]

GO:0065007);; molecular function: binding (GO:0005488)

);; cellular component: membrane part (GO:0044425);; cellular component: cell part (GO:0044464)

O:0071840)
activity (GO:0060089);; cellular component: cell (GO:0005623);; cellular component: membrane (G

;; cellular component: cell part (GO:0044464);; biological process: cellular component organization

ponent: extracellular region (GO:0005576);; cellular component: extracellular region part (GO:0044

O:0023052);; biological process: response to stimulus (GO:0050896);; biological process: biologica

nent: membrane (GO:0016020);; cellular component: membrane part (GO:0044425);; molecular fur

ent: cell part (GO:0044464);; biological process: metabolic process (GO:0008152);; biological proce

acromolecular complex (GO:0032991);; biological process: cellular process (GO:0009987);; biologi

component: macromolecular complex (GO:0032991)

iological process: single-organism process (GO:0044699);; biological process: response to stimulus
y (GO:0003824)
nt: organelle part (GO:0044422)

ological process: developmental process (GO:0032502)

omotion (GO:0040011);; biological process: localization (GO:0051179);; molecular function: catalyti

623);; cellular component: cell part (GO:0044464);; biological process: metabolic process (GO:0008

al process: developmental process (GO:0032502);; cellular component: cell (GO:0005623);; cellular
2);; biological process: single-organism process (GO:0044699);; biological process: multi-organism
0044456);; cellular component: cell part (GO:0044464);; cellular component: synapse (GO:0045202

ent: synapse part (GO:0044456);; cellular component: synapse (GO:0045202)

(GO:0050896)

s (GO:0050896)

ular function: catalytic activity (GO:0003824);; biological process: metabolic process (GO:0008152);;

ganism process (GO:0044699);; biological process: cellular component organization or biogenesis (

0044425);; cellular component: cell part (GO:0044464)

226);; cellular component: organelle part (GO:0044422);; biological process: developmental proces

nent: extracellular region (GO:0005576);; cellular component: extracellular region part (GO:004442

mulus (GO:0050896);; biological process: cellular process (GO:0009987);; biological process: signali

(GO:0044464);; cellular component: membrane (GO:0016020);; biological process: reproduction (G

ılar region part (GO:0044421);; cellular component: cell (GO:0005623);; cellular component: memb

ng (GO:0005488);; cellular component: other organism (GO:0044215);; cellular component: other c

043226);; cellular component: organelle part (GO:0044422)

on (GO:0065007);; biological process: reproduction (GO:0000003);; biological process: reproductive

ism process (GO:0044699);; biological process: biological regulation (GO:0065007);; biological proc
ingle-organism process (GO:0044699)

051179);; biological process: biological regulation (GO:0065007);; biological process: multicellular c

ism process (GO:0044699);; biological process: localization (GO:0051179);; cellular component: me

gical process: biological regulation (GO:0065007);; biological process: behavior (GO:0007610);; biol

organismal process (GO:0032501);; biological process: developmental process (GO:0032502);; bic

ogical process: biological regulation (GO:0065007);; biological process: localization (GO:0051179);;

ganelle part (GO:0044422);; cellular component: membrane part (GO:0044425)

772);; cellular component: organelle (GO:0043226);; cellular component: organelle part (GO:00442

llular component: organelle (GO:0043226);; cellular component: organelle part (GO:0044422);; cellu

process: response to stimulus (GO:0050896);; biological process: biological regulation (GO:0065007

; biological process: response to stimulus (GO:0050896);; biological process: cellular component or
biological process: biological regulation (GO:0065007);; cellular component: synapse part (GO:004

cellular component: cell part (GO:0044464);; cellular component: macromolecular complex (GO:00

nction: binding (GO:0005488)

ellular component: supramolecular complex (GO:0099080);; biological process: developmental proc

process: cellular process (GO:0009987);; biological process: signaling (GO:0023052);; biological proc

ne (GO:0016020);; cellular component: cell part (GO:0044464);; cellular component: membrane par
44464)

al process: biological adhesion (GO:0022610)

GO:0065007);; molecular function: binding (GO:0005488);; cellular component: membrane (GO:001

)005488);; cellular component: macromolecular complex (GO:0032991)
:al process: single-organism process (GO:0044699)

:ess (GO:0002376);; biological process: response to stimulus (GO:0050896);; biological process: gro

nbrane (GO:0016020);; cellular component: organelle (GO:0043226);; cellular component: organell

ental process (GO:0032502);; biological process: metabolic process (GO:0008152);; cellular compor
nulus (GO:0050896)
mmune system process (GO:0002376)
ical process: signaling (GO:0023052);; biological process: response to stimulus (GO:0050896);; biol

gical process: metabolic process (GO:0008152);; biological process: cellular process (GO:0009987);

9);; biological process: cellular process (GO:0009987);; biological process: signaling (GO:0023052);;

ponent: organelle (GO:0043226);; cellular component: membrane (GO:0016020);; cellular compone

;; cellular component: membrane part (GO:0044425);; molecular function: molecular function regu

calization (GO:0051179);; biological process: cellular component organization or biogenesis (GO:0
; biological process: single-organism process (GO:0044699);; biological process: response to stimu

ular process (GO:0009987);; biological process: single-organism process (GO:0044699);; biological
complex (GO:0099080);; biological process: cellular process (GO:0009987);; biological process: ce

GO:0044699);; molecular function: binding (GO:0005488);; biological process: response to stimulus
to stimulus (GO:0050896);; biological process: biological regulation (GO:0065007);; cellular compor

l regulation (GO:0065007)

nponent: cell part (GO:0044464);; biological process: single-organism process (GO:0044699)

iological process: response to stimulus (GO:0050896);; biological process: localization (GO:005117

ogical process: biological regulation (GO:0065007);; molecular function: transporter activity (GO:00

ilar process (GO:0009987);; biological process: single-organism process (GO:0044699)

:0044422);; cellular component: membrane part (GO:0044425);; cellular component: cell part (GO:(

464);; biological process: localization (GO:0051179);; biological process: signaling (GO:0023052);; b

al regulation (GO:0065007)
cell part (GO:0044464);; cellular component: macromolecular complex (GO:0032991)
ological process: single-organism process (GO:0044699)

s: signaling (GO:0023052);; biological process: response to stimulus (GO:0050896);; biological proc

activity (GO:0004871);; molecular function: molecular transducer activity (GO:0060089);; cellular co

nt: macromolecular complex (GO:0032991)

GO:0071840)
uctive process (GO:0022414);; biological process: single-organism process (GO:0044699);; biologica

n (GO:0051179)

51179);; biological process: presynaptic process involved in chemical synaptic transmission (GO:00

O:0051179)
ss: developmental process (GO:0032502);; biological process: single-organism process (GO:00446

al process: cellular process (GO:0009987);; biological process: cellular component organization or b
biogenesis (GO:0071840);; molecular function: binding (GO:0005488);; biological process: developm

96);; biological process: biological regulation (GO:0065007);; biological process: immune system pr
cess: immune system process (GO:0002376);; biological process: multicellular organismal process (
rt (GO:0044421);; biological process: immune system process (GO:0002376);; biological process: m

ocess (GO:0008152);; biological process: cellular process (GO:0009987)

process: cellular process (GO:0009987);; cellular component: membrane part (GO:0044425);; molec

molecular complex (GO:0032991);; cellular component: organelle (GO:0043226);; cellular compone
ocess: biological regulation (GO:0065007)
nolecular function: catalytic activity (GO:0003824)

ocalization (GO:0051179)
ılar process (GO:0009987);; cellular component: macromolecular complex (GO:0032991)

on or biogenesis (GO:0071840);; molecular function: binding (GO:0005488)
ocess: immune system process (GO:0002376);; molecular function: binding (GO:0005488)
; biological process: single-organism process (GO:0044699);; biological process: cellular componer
n process (GO:0002376);; molecular function: catalytic activity (GO:0003824);; molecular function: b

);; biological process: cellular component organization or biogenesis (GO:0071840);; biological prc

localization (GO:0051179);; biological process: cellular component organization or biogenesis (GO

ction regulator (GO:0098772);; molecular function: binding (GO:0005488);; cellular component: ma

ntal process (GO:0032502);; biological process: single-organism process (GO:0044699);; biological

96);; biological process: cellular component organization or biogenesis (GO:0071840);; cellular con

376);; biological process: multicellular organismal process (GO:0032501);; biological process: develo

ɔmponent: cell part (GO:0044464);; cellular component: synapse (GO:0045202)

rt (GO:0044464);; cellular component: organelle part (GO:0044422);; cellular component: membrar

rocess (GO:0044699);; biological process: cellular component organization or biogenesis (GO:0071

rocess: biological regulation (GO:0065007);; biological process: cellular component organization o

xtracellular region part (GO:0044421)

32991)
-enclosed lumen (GO:0031974);; biological process: immune system process (GO:0002376);; biolog
(GO:0005623);; cellular component: macromolecular complex (GO:0032991);; cellular component:

ingle-organism process (GO:0044699);; cellular component: organelle part (GO:0044422)
55007);; biological process: immune system process (GO:0002376);; biological process: response tc
;; biological process: single-organism process (GO:0044699)

t (GO:0044464);; cellular component: organelle (GO:0043226);; cellular component: macromolecul

mponent: macromolecular complex (GO:0032991);; cellular component: organelle (GO:0043226);;
ess: cellular process (GO:0009987);; cellular component: other organism (GO:0044215);; cellular co

');; biological process: signaling (GO:0023052);; cellular component: organelle (GO:0043226);; cellul

(GO:0071840);; biological process: signaling (GO:0023052);; biological process: locomotion (GO:00
plex (GO:0032991);; cellular component: organelle (GO:0043226);; cellular component: organelle p

(GO:0044425);; biological process: signaling (GO:0023052);; cellular component: organelle (GO:004
ological process: locomotion (GO:0040011);; biological process: response to stimulus (GO:0050896

425);; biological process: multicellular organismal process (GO:0032501);; biological process: devel
gle-organism process (GO:0044699);; cellular component: macromolecular complex (GO:0032991)

gical regulation (GO:0065007)

m process (GO:0002376);; biological process: multi-organism process (GO:0051704);; biological pr

ivity, protein binding (GO:0000988);; cellular component: cell (GO:0005623);; cellular component: c
ing (GO:0005488);; biological process: biological regulation (GO:0065007);; biological process: resp

cellular component: extracellular region (GO:0005576);; cellular component: organelle (GO:004322

O:0071840);; cellular component: synapse (GO:0045202)

ological process: multi-organism process (GO:0051704)

:ess: localization (GO:0051179)

nt: cell (GO:0005623);; cellular component: organelle (GO:0043226);; cellular component: cell part (

iological process: reproduction (GO:0000003);; biological process: reproductive process (GO:0022

lar component: membrane (GO:0016020);; cellular component: membrane part (GO:0044425);; bic
c activity (GO:0003824);; biological process: metabolic process (GO:0008152);; cellular component:
0050896);; molecular function: binding (GO:0005488);; molecular function: catalytic activity (GO:00

nent: organelle (GO:0043226);; cellular component: organelle part (GO:0044422);; cellular compon

ılar process (GO:0009987);; biological process: single-organism process (GO:0044699)

0065007)

O:0071840)

ar component organization or biogenesis (GO:0071840);; cellular component: macromolecular cor
9);; biological process: signaling (GO:0023052)

lar component: cell part (GO:0044464);; biological process: biological regulation (GO:0065007)

179);; biological process: cellular process (GO:0009987);; biological process: cellular component org

gical process: metabolic process (GO:0008152);; biological process: response to stimulus (GO:0050[illegible]

metabolic process (GO:0008152)

onse to stimulus (GO:0050896);; biological process: biological regulation (GO:0065007)
(GO:0005488)

cal process: response to stimulus (GO:0050896);; molecular function: molecular function regulator
87);; biological process: response to stimulus (GO:0050896);; biological process: signaling (GO:002
nponent: cell junction (GO:0030054);; biological process: response to stimulus (GO:0050896);; biolo

macromolecular complex (GO:0032991)

1840)

2991);; biological process: cellular process (GO:0009987);; biological process: localization (GO:005[illegible]

cal process: metabolic process (GO:0008152);; biological process: biological regulation (GO:00650[illegible]

onent: extracellular region (GO:0005576);; cellular component: extracellular region part (GO:00444

8152);; biological process: cellular process (GO:0009987);; biological process: biological regulation
2);; biological process: single-organism process (GO:0044699);; molecular function: catalytic activit

ess: behavior (GO:0007610);; biological process: multi-organism process (GO:0051704);; biological

ess: cellular process (GO:0009987);; biological process: response to stimulus (GO:0050896)

rt (GO:0044422);; biological process: developmental process (GO:0032502)

ponse to stimulus (GO:0050896);; cellular component: membrane-enclosed lumen (GO:0031974);;

ogical process: growth (GO:0040007);; biological process: cellular component organization or biog

: single-organism process (GO:0044699);; biological process: multi-organism process (GO:005170[illegible]

896);; biological process: biological regulation (GO:0065007);; cellular component: macromolecula
ocess: single-organism process (GO:0044699);; biological process: response to stimulus (GO:0050[illegible]

ecular function: catalytic activity (GO:0003824);; biological process: biological regulation (GO:0065

824);; cellular component: membrane-enclosed lumen (GO:0031974);; cellular component: organe

onent: organelle part (GO:0044422);; cellular component: cell part (GO:0044464)

)9987);; biological process: cellular component organization or biogenesis (GO:0071840)

ss: growth (GO:0040007);; biological process: response to stimulus (GO:0050896)
s (GO:0051704)
0);; cellular component: membrane part (GO:0044425);; biological process: localization (GO:005117

0);; biological process: response to stimulus (GO:0050896);; biological process: biological regulatio

nt: organelle (GO:0043226);; cellular component: organelle part (GO:0044422);; cellular componen

);; molecular function: catalytic activity (GO:0003824);; molecular function: binding (GO:0005488)

: single-organism process (GO:0044699);; biological process: multi-organism process (GO:00517

unction: molecular function regulator (GO:0098772);; biological process: metabolic process (GO:00
840);; molecular function: transporter activity (GO:0005215);; biological process: localization (GO:0

cess: single-organism process (GO:0044699);; cellular component: synapse part (GO:0044456);; cel
;; biological process: localization (GO:0051179);; biological process: metabolic process (GO:000815
nent: membrane (GO:0016020);; cellular component: membrane part (GO:0044425)
supramolecular complex (GO:0099080);; biological process: developmental process (GO:0032502)

)

cess: single-organism process (GO:0044699);; biological process: localization (GO:0051179);; cellul

l process: cellular process (GO:0009987)

r process (GO:0009987);; molecular function: binding (GO:0005488)
(GO:0005488);; biological process: cellular process (GO:0009987);; biological process: cellular com

I699);; biological process: multicellular organismal process (GO:0032501);; biological process: deve

jical process: biological regulation (GO:0065007)

: single-organism process (GO:0044699);; biological process: multi-organism process (GO:005170[illegible]

reproduction (GO:0000003);; biological process: reproductive process (GO:0022414);; biological pr

mponent: organelle (GO:0043226);; cellular component: organelle part (GO:0044422);; biological p

4699);; biological process: biological regulation (GO:0065007);; molecular function: binding (GO:00

olecular function regulator (GO:0098772);; biological process: biological regulation (GO:0065007);;

n: signal transducer activity (GO:0004871);; molecular function: molecular transducer activity (GO:0

O:0071840)

: membrane (GO:0016020);; biological process: biological regulation (GO:0065007)

process (GO:0044699);; biological process: response to stimulus (GO:0050896)

ar component organization or biogenesis (GO:0071840);; biological process: multicellular organism
(GO:0044422)

0044699);; biological process: localization (GO:0051179);; biological process: multi-organism proc

0044422);; cellular component: supramolecular complex (GO:0099080)

ocess: biological regulation (GO:0065007)
egulation (GO:0065007);; biological process: cellular component organization or biogenesis (GO:00
organism process (GO:0051704)

ogical process: developmental process (GO:0032502);; biological process: single-organism process

s: rhythmic process (GO:0048511)
04);; biological process: immune system process (GO:0002376);; biological process: multicellular or

cess: localization (GO:0051179);; molecular function: catalytic activity (GO:0003824);; molecular fun
response to stimulus (GO:0050896);; biological process: multi-organism process (GO:0051704);; bio

ess (GO:0008152)

:0044699);; biological process: localization (GO:0051179);; biological process: biological regulation

ocess: cellular process (GO:0009987);; biological process: cellular component organization or bioge

nplex (GO:0032991);; cellular component: membrane part (GO:0044425);; cellular component: cell

;; cellular component: membrane (GO:0016020);; cellular component: membrane part (GO:004442

GO:0005623);; cellular component: membrane (GO:0016020);; cellular component: macromolecula

(GO:0005488)

0044699);; biological process: response to stimulus (GO:0050896);; cellular component: membrane
991);; biological process: single-organism process (GO:0044699);; biological process: metabolic pro

ular component: cell part (GO:0044464);; biological process: cellular component organization or bi
cess: response to stimulus (GO:0050896);; biological process: multi-organism process (GO:005170

on (GO:0065007)

GO:0032502);; biological process: behavior (GO:0007610);; biological process: response to stimulus

ocalization (GO:0051179);; biological process: response to stimulus (GO:0050896);; biological proc
GO:0051179);; biological process: metabolic process (GO:0008152);; biological process: signaling (G

organism process (GO:0051704)

0071840)

organismal process (GO:0032501)

llular component: synapse part (GO:0044456);; cellular component: synapse (GO:0045202);; biolog

onent: other organism part (GO:0044217);; cellular component: extracellular region part (GO:00444
ular component organization or biogenesis (GO:0071840)

):0044699);; biological process: response to stimulus (GO:0050896);; molecular function: catalytic a

ne (GO:0016020);; cellular component: membrane part (GO:0044425);; molecular function: binding

s (GO:0009987);; biological process: multicellular organismal process (GO:0032501);; biological pro

on: binding (GO:0005488)

multi-organism process (GO:0051704)

);; biological process: multi-organism process (GO:0051704);; molecular function: catalytic activity

acromolecular complex (GO:0032991);; biological process: behavior (GO:0007610);; biological pro

ne system process (GO:0002376);; biological process: response to stimulus (GO:0050896);; biologi

)4)
)4)

ellular component: extracellular region (GO:0005576);; cellular component: extracellular region part
)44464);; biological process: localization (GO:0051179);; biological process: signaling (GO:0023052)
GO:0032502);; molecular function: molecular function regulator (GO:0098772);; molecular function
gical regulation (GO:0065007);; biological process: signaling (GO:0023052);; biological process: resp

nbrane part (GO:0044425);; biological process: cellular process (GO:0009987)
lar component: organelle (GO:0043226);; cellular component: cell part (GO:0044464);; biological p

napse part (GO:0044456);; cellular component: synapse (GO:0045202);; molecular function: binding

s: developmental process (GO:0032502);; molecular function: catalytic activity (GO:0003824);; cellul

O:0044464);; biological process: immune system process (GO:0002376);; biological process: multic

n: binding (GO:0005488)

:ion or biogenesis (GO:0071840)

;; biological process: biological regulation (GO:0065007);; biological process: response to stimulus

623);; cellular component: cell part (GO:0044464);; cellular component: membrane (GO:0016020);;
t: membrane part (GO:0044425)

velopmental process (GO:0032502);; biological process: metabolic process (GO:0008152);; biologic

n or biogenesis (GO:0071840);; biological process: biological regulation (GO:0065007);; biological p

):0005623);; cellular component: organelle (GO:0043226);; cellular component: organelle part (GO:
omponent: cell (GO:0005623);; cellular component: organelle (GO:0043226);; cellular component: c
gical process: signaling (GO:0023052);; cellular component: organelle part (GO:0044422);; molecula

iological process: response to stimulus (GO:0050896)

ocess (GO:0044699)

to stimulus (GO:0050896)
;; biological process: reproduction (GO:0000003);; biological process: reproductive process (GO:00

ular function: binding (GO:0005488);; biological process: signaling (GO:0023052);; biological proces

genesis (GO:0071840);; biological process: multicellular organismal process (GO:0032501);; biologic

ation or biogenesis (GO:0071840);; cellular component: membrane (GO:0016020);; cellular compor

)005215);; biological process: localization (GO:0051179);; biological process: biological regulation (
component: membrane part (GO:0044425);; biological process: response to stimulus (GO:005089

tion (GO:0065007);; biological process: cellular component organization or biogenesis (GO:00718

ess: multicellular organismal process (GO:0032501);; biological process: developmental process (G

ocess: metabolic process (GO:0008152)

(GO:0044456);; cellular component: synapse (GO:0045202);; biological process: developmental pr

003824)

(GO:0051704);; cellular component: membrane part (GO:0044425);; cellular component: organelle

ocess: biological regulation (GO:0065007);; biological process: cellular component organization or

)2376);; biological process: locomotion (GO:0040011);; biological process: localization (GO:005117

O:0016020);; cellular component: cell part (GO:0044464);; cellular component: membrane part (GC

ı or biogenesis (GO:0071840);; biological process: locomotion (GO:0040011)

421);; biological process: single-organism process (GO:0044699);; biological process: localization (

l regulation (GO:0065007);; biological process: behavior (GO:0007610);; cellular component: memb

ıction: binding (GO:0005488);; cellular component: cell (GO:0005623);; cellular component: organe

ess: cellular process (GO:0009987);; biological process: signaling (GO:0023052);; biological process:

cal process: immune system process (GO:0002376);; biological process: cellular component organi:

s (GO:0050896)

ic activity (GO:0003824);; molecular function: signal transducer activity (GO:0004871);; molecular fu

152);; biological process: signaling (GO:0023052);; biological process: response to stimulus (GO:00

component: organelle (GO:0043226);; cellular component: cell part (GO:0044464);; cellular compo
process (GO:0051704);; cellular component: membrane (GO:0016020);; cellular component: meml
)

biological process: biological regulation (GO:0065007)

(GO:0071840);; cellular component: synapse (GO:0045202)

ss (GO:0032502);; biological process: localization (GO:0051179)

1);; cellular component: cell (GO:0005623);; cellular component: membrane (GO:0016020);; cellular

ng (GO:0023052);; biological process: presynaptic process involved in chemical synaptic transmissi

GO:0000003);; biological process: reproductive process (GO:0022414);; biological process: multi-org

rane (GO:0016020);; cellular component: cell part (GO:0044464);; cellular component: membrane p

organism part (GO:0044217);; biological process: metabolic process (GO:0008152)

e process (GO:0022414);; biological process: multicellular organismal process (GO:0032501);; biolo

cess: response to stimulus (GO:0050896);; biological process: multi-organism process (GO:005170

organismal process (GO:0032501);; cellular component: macromolecular complex (GO:0032991);; c

mbrane-enclosed lumen (GO:0031974);; biological process: developmental process (GO:0032502)

ogical process: multicellular organismal process (GO:0032501);; biological process: signaling (GO:0

logical process: behavior (GO:0007610);; biological process: reproduction (GO:0000003);; biologica

biological process: developmental process (GO:0032502);; cellular component: synapse (GO:0045

22);; cellular component: membrane (GO:0016020);; cellular component: membrane part (GO:004[illegible]

ılar component: supramolecular complex (GO:0099080);; biological process: multicellular organism

7);; biological process: metabolic process (GO:0008152);; biological process: cellular component or[illegible]

rganization or biogenesis (GO:0071840);; cellular component: macromolecular complex (GO:0032[illegible]
14456);; cellular component: synapse (GO:0045202);; cellular component: membrane part (GO:004[illegible]

32991)

cess (GO:0032502);; biological process: single-organism process (GO:0044699);; cellular componer

cess: biological regulation (GO:0065007);; molecular function: binding (GO:0005488)

t (GO:0044425);; cellular component: macromolecular complex (GO:0032991);; biological process:

l6020);; cellular component: membrane part (GO:0044425);; biological process: multicellular organ

wth (GO:0040007);; biological process: signaling (GO:0023052);; biological process: locomotion (G

e part (GO:0044422);; cellular component: membrane part (GO:0044425);; cellular component: cell

nent: membrane-enclosed lumen (GO:0031974);; biological process: multicellular organismal proce

ogical process: biological regulation (GO:0065007);; cellular component: cell (GO:0005623);; cellula

; molecular function: binding (GO:0005488)

biological process: single-organism process (GO:0044699);; biological process: response to stimul

ent: membrane part (GO:0044425);; biological process: reproductive process (GO:0022414);; biolog

lator (GO:0098772);; biological process: single-organism process (GO:0044699);; biological process

071840)

ulus (GO:0050896);; molecular function: binding (GO:0005488);; biological process: multicellular org

process: cellular component organization or biogenesis (GO:0071840)

llular component organization or biogenesis (GO:0071840);; molecular function: binding (GO:0005

; (GO:0050896);; biological process: reproduction (GO:0000003);; biological process: reproductive p
nent: macromolecular complex (GO:0032991);; molecular function: catalytic activity (GO:0003824);;

9);; biological process: multicellular organismal process (GO:0032501);; biological process: develop

)05215);; cellular component: membrane part (GO:0044425)

)044464)

biological process: single-organism process (GO:0044699);; biological process: response to stimulu

ess: biological regulation (GO:0065007);; cellular component: membrane (GO:0016020);; cellular co

mponent: cell (GO:0005623);; cellular component: organelle (GO:0043226);; cellular component: c

al process: multi-organism process (GO:0051704);; biological process: biological regulation (GO:00

99531);; cellular component: synapse part (GO:0044456);; cellular component: synapse (GO:00452

99);; biological process: biological regulation (GO:0065007)

biogenesis (GO:0071840)
mental process (GO:0032502);; biological process: locomotion (GO:0040011);; biological process: si

ocess (GO:0002376);; biological process: single-organism process (GO:0044699);; biological proce
(GO:0032501);; biological process: developmental process (GO:0032502);; biological process: single
ulticellular organismal process (GO:0032501);; biological process: cell killing (GO:0001906)

cular function: binding (GO:0005488);; biological process: metabolic process (GO:0008152);; cellula

ent: organelle part (GO:0044422);; cellular component: membrane-enclosed lumen (GO:0031974);;

nt organization or biogenesis (GO:0071840)
binding (GO:0005488);; cellular component: membrane (GO:0016020);; biological process: respons

ocess: developmental process (GO:0032502);; biological process: single-organism process (GO:004

:0071840)

acromolecular complex (GO:0032991);; biological process: localization (GO:0051179)

process: metabolic process (GO:0008152);; molecular function: binding (GO:0005488);; biological p

nponent: membrane part (GO:0044425)

opmental process (GO:0032502);; biological process: single-organism process (GO:0044699);; cellu

ne (GO:0016020);; cellular component: cell junction (GO:0030054);; biological process: reproductio

1840)

r biogenesis (GO:0071840);; molecular function: catalytic activity (GO:0003824)

gical process: multicellular organismal process (GO:0032501);; biological process: developmental pr
cell part (GO:0044464)

stimulus (GO:0050896);; biological process: multi-organism process (GO:0051704);; biological pro

ar complex (GO:0032991);; cellular component: organelle part (GO:0044422);; cellular component:

cellular component: organelle part (GO:0044422);; cellular component: cell part (GO:0044464)
mponent: other organism part (GO:0044217);; molecular function: binding (GO:0005488)

lar component: membrane-enclosed lumen (GO:0031974);; cellular component: organelle part (G(

)40011);; biological process: localization (GO:0051179)
part (GO:0044422);; cellular component: membrane part (GO:0044425);; cellular component: cell pa

43226);; biological process: response to stimulus (GO:0050896)
);; biological process: cellular component organization or biogenesis (GO:0071840);; molecular fun

opmental process (GO:0032502);; biological process: biological regulation (GO:0065007);; cellular c

rocess: signaling (GO:0023052)

organelle (GO:0043226);; cellular component: cell part (GO:0044464);; cellular component: organell
ponse to stimulus (GO:0050896)

26);; cellular component: organelle part (GO:0044422);; biological process: localization (GO:005117

GO:0044464);; cellular component: membrane (GO:0016020);; cellular component: membrane par

414);; biological process: multicellular organismal process (GO:0032501);; biological process: devel

ological process: localization (GO:0051179);; biological process: signaling (GO:0023052);; biological
macromolecular complex (GO:0032991);; molecular function: binding (GO:0005488)
003824)

ent: cell part (GO:0044464);; biological process: biological regulation (GO:0065007);; cellular comp

nplex (GO:0032991)

ganization or biogenesis (GO:0071840);; biological process: biological regulation (GO:0065007);; ce

896);; biological process: biological regulation (GO:0065007);; biological process: cellular compone

(GO:0098772);; cellular component: organelle part (GO:0044422)
3052);; biological process: single-organism process (GO:0044699);; cellular component: membrane
ogical process: cellular process (GO:0009987);; biological process: cellular component organization

1179);; biological process: cellular component organization or biogenesis (GO:0071840)

07);; biological process: developmental process (GO:0032502);; cellular component: synapse part (

21);; cellular component: membrane (GO:0016020);; cellular component: membrane part (GO:004

(GO:0065007);; biological process: cellular component organization or biogenesis (GO:0071840);;
y (GO:0003824);; biological process: metabolic process (GO:0008152);; biological process: cellular

process: multicellular organismal process (GO:0032501);; biological process: developmental proce

molecular function: molecular function regulator (GO:0098772);; biological process: developmenta

jenesis (GO:0071840)

4);; biological process: biological regulation (GO:0065007);; biological process: localization (GO:005

r complex (GO:0032991);; molecular function: binding (GO:0005488);; cellular component: membra
396)

007);; cellular component: macromolecular complex (GO:0032991)

elle part (GO:0044422);; biological process: single-organism process (GO:0044699);; biological proc

'9);; biological process: immune system process (GO:0002376);; biological process: multicellular org

n (GO:0065007)

t: membrane (GO:0016020);; cellular component: membrane part (GO:0044425);; biological proces

4);; biological process: biological regulation (GO:0065007);; biological process: cellular component

)08152);; biological process: cellular component organization or biogenesis (GO:0071840);; molecu
)51179);; biological process: response to stimulus (GO:0050896);; biological process: biological reg

llular component: synapse (GO:0045202);; cellular component: membrane (GO:0016020);; cellular c
2);; biological process: cellular component organization or biogenesis (GO:0071840);; cellular com

;; biological process: single-organism process (GO:0044699);; biological process: localization (GO:(

ar component: extracellular region (GO:0005576);; cellular component: extracellular region part (G(

ponent organization or biogenesis (GO:0071840);; biological process: localization (GO:0051179);; b

lopmental process (GO:0032502);; molecular function: catalytic activity (GO:0003824);; biological p

4);; biological process: biological regulation (GO:0065007);; biological process: cellular component

rocess: multicellular organismal process (GO:0032501);; biological process: cellular component org

rocess: cellular process (GO:0009987);; biological process: signaling (GO:0023052);; biological proc

)05488)

cellular component: extracellular region (GO:0005576);; cellular component: extracellular region pa

)060089);; biological process: signaling (GO:0023052);; biological process: biological regulation (GO

ıal process (GO:0032501);; biological process: developmental process (GO:0032502);; biological pr

ess (GO:0051704);; biological process: signaling (GO:0023052);; biological process: biological regul

)71840)

s (GO:0044699)

ganismal process (GO:0032501);; biological process: localization (GO:0051179)

ction: transporter activity (GO:0005215)
ological process: immune system process (GO:0002376);; biological process: cellular process (GO:0

(GO:0065007);; molecular function: catalytic activity (GO:0003824)

enesis (GO:0071840)

part (GO:0044464);; cellular component: synapse (GO:0045202)

5)

r complex (GO:0032991);; cellular component: membrane part (GO:0044425);; cellular component:

-enclosed lumen (GO:0031974)
ocess (GO:0008152)

ogenesis (GO:0071840)
4);; biological process: single-organism process (GO:0044699)

s (GO:0050896);; molecular function: catalytic activity (GO:0003824)

ess: multi-organism process (GO:0051704);; biological process: immune system process (GO:0002
GO:0023052);; biological process: multicellular organismal process (GO:0032501);; biological proces

ical process: single-organism process (GO:0044699);; biological process: localization (GO:0051179

21);; biological process: developmental process (GO:0032502);; biological process: single-organism

ctivity (GO:0003824)

(GO:0005488)

ocess: developmental process (GO:0032502);; biological process: single-organism process (GO:004

(GO:0003824);; biological process: biological regulation (GO:0065007);; biological process: localiza

cess: immune system process (GO:0002376);; biological process: cellular process (GO:0009987)

cal process: multi-organism process (GO:0051704)

t (GO:0044421);; biological process: reproduction (GO:0000003);; biological process: reproductive [illegible]
);; biological process: response to stimulus (GO:0050896);; biological process: biological regulation
: binding (GO:0005488);; biological process: localization (GO:0051179);; biological process: cellular
ponse to stimulus (GO:0050896)

rocess: biological regulation (GO:0065007)

g (GO:0005488);; biological process: immune system process (GO:0002376);; biological process: mu

ar component: cell (GO:0005623);; cellular component: cell part (GO:0044464);; cellular componen

ellular organismal process (GO:0032501);; biological process: biological regulation (GO:0065007)

(GO:0050896);; biological process: multicellular organismal process (GO:0032501);; cellular compo

cellular component: membrane part (GO:0044425);; biological process: localization (GO:0051179)

:al process: response to stimulus (GO:0050896);; biological process: multi-organism process (GO:0

process: metabolic process (GO:0008152);; cellular component: macromolecular complex (GO:0032

:0044422);; cellular component: cell part (GO:0044464);; cellular component: membrane-enclosed
organelle part (GO:0044422);; cellular component: cell part (GO:0044464);; molecular function: bin
ar function: signal transducer activity (GO:0004871);; molecular function: molecular transducer activ

22414);; biological process: multi-organism process (GO:0051704);; biological process: immune sys

ss: biological regulation (GO:0065007)

cal process: developmental process (GO:0032502)

nent: membrane part (GO:0044425);; biological process: response to stimulus (GO:0050896);; biolo

GO:0065007);; biological process: biological adhesion (GO:0022610)
5);; biological process: developmental process (GO:0032502);; biological process: cellular compone

40);; biological process: multicellular organismal process (GO:0032501);; biological process: develop

O:0032502);; biological process: single-organism process (GO:0044699)

ocess (GO:0032502);; biological process: cellular component organization or biogenesis (GO:0071

(GO:0043226);; biological process: response to stimulus (GO:0050896)

biogenesis (GO:0071840)

9)

):0044425);; biological process: developmental process (GO:0032502)

(GO:0051179);; cellular component: cell (GO:0005623);; cellular component: cell part (GO:0044464)

brane (GO:0016020);; cellular component: membrane part (GO:0044425)

elle (GO:0043226);; cellular component: cell part (GO:0044464)

reproduction (GO:0000003);; biological process: reproductive process (GO:0022414);; cellular com

zation or biogenesis (GO:0071840)

ınction: molecular transducer activity (GO:0060089);; biological process: metabolic process (GO:00

50896);; biological process: immune system process (GO:0002376);; biological process: multi-orga

ɔnent: membrane (GO:0016020);; cellular component: membrane part (GO:0044425);; biological pr
brane part (GO:0044425)

component: cell part (GO:0044464);; cellular component: membrane part (GO:0044425);; cellular

ion (GO:0099531);; biological process: multicellular organismal process (GO:0032501);; biological p

ganism process (GO:0051704);; molecular function: binding (GO:0005488);; cellular component: me

part (GO:0044425)

gical process: single-organism process (GO:0044699);; biological process: multi-organism process

4)

cellular component: organelle part (GO:0044422);; biological process: immune system process (GO

;; biological process: response to stimulus (GO:0050896);; cellular component: extracellular region

0023052);; cellular component: membrane (GO:0016020);; cellular component: membrane part (GO

al process: reproductive process (GO:0022414);; biological process: biological regulation (GO:0065

202);; cellular component: synapse part (GO:0044456)

425);; cellular component: macromolecular complex (GO:0032991)

nal process (GO:0032501)

ganization or biogenesis (GO:0071840);; biological process: signaling (GO:0023052)

991);; molecular function: binding (GO:0005488)
425);; molecular function: binding (GO:0005488);; biological process: developmental process (GO:

nt: synapse part (GO:0044456);; cellular component: synapse (GO:0045202);; biological process: loc

multi-organism process (GO:0051704)

ismal process (GO:0032501);; biological process: developmental process (GO:0032502)

O:0040011);; biological process: localization (GO:0051179);; molecular function: molecular function

l part (GO:0044464)

ess (GO:0032501);; molecular function: catalytic activity (GO:0003824)

r component: cell part (GO:0044464)

us (GO:0050896);; biological process: multicellular organismal process (GO:0032501);; biological pr

gical process: multicellular organismal process (GO:0032501);; biological process: multi-organism p

s: localization (GO:0051179);; biological process: response to stimulus (GO:0050896);; biological pr

ganismal process (GO:0032501);; biological process: developmental process (GO:0032502);; biologi

5488)

›rocess (GO:0022414);; biological process: cellular component organization or biogenesis (GO:007
biological process: developmental process (GO:0032502);; cellular component: membrane part (G

›mental process (GO:0032502);; cellular component: organelle (GO:0043226);; cellular component:

s (GO:0050896);; biological process: biological regulation (GO:0065007);; biological process: multic

omponent: membrane part (GO:0044425);; biological process: cellular component organization or

ell part (GO:0044464);; cellular component: membrane (GO:0016020);; cellular component: membr

)65007);; biological process: cellular component organization or biogenesis (GO:0071840);; biologi

02)

ngle-organism process (GO:0044699);; biological process: response to stimulus (GO:0050896);; bic

ss: localization (GO:0051179)
e-organism process (GO:0044699);; biological process: cellular component organization or biogen

ır component: macromolecular complex (GO:0032991)

biological process: metabolic process (GO:0008152)

e to stimulus (GO:0050896);; biological process: single-organism process (GO:0044699);; cellular c

4699)

process: immune system process (GO:0002376);; biological process: signaling (GO:0023052)

lar component: synapse part (GO:0044456);; cellular component: synapse (GO:0045202);; biologica

n (GO:0000003);; biological process: reproductive process (GO:0022414);; cellular component: mac

rocess (GO:0032502);; molecular function: binding (GO:0005488);; biological process: biological reg

ocess: metabolic process (GO:0008152);; biological process: signaling (GO:0023052)

supramolecular complex (GO:0099080);; biological process: multicellular organismal process (GO:

D:0044422)

art (GO:0044464);; cellular component: membrane-enclosed lumen (GO:0031974);; molecular func

ıction: catalytic activity (GO:0003824);; cellular component: membrane part (GO:0044425)

component: macromolecular complex (GO:0032991);; molecular function: binding (GO:0005488)

le part (GO:0044422);; biological process: signaling (GO:0023052);; biological process: response to

9);; molecular function: molecular function regulator (GO:0098772);; biological process: biological

t (GO:0044425);; biological process: multi-organism process (GO:0051704);; cellular component: sy

opmental process (GO:0032502);; biological process: single-organism process (GO:0044699);; mol

process: presynaptic process involved in chemical synaptic transmission (GO:0099531)

ponent: macromolecular complex (GO:0032991)

ellular component: membrane (GO:0016020);; cellular component: membrane part (GO:0044425);;

ent organization or biogenesis (GO:0071840);; biological process: multi-organism process (GO:005

e-enclosed lumen (GO:0031974);; biological process: multicellular organismal process (GO:003250
or biogenesis (GO:0071840);; biological process: single-organism process (GO:0044699);; biologic

GO:0044456);; cellular component: synapse (GO:0045202)

4425);; molecular function: binding (GO:0005488);; biological process: multicellular organismal pro

cellular component: macromolecular complex (GO:0032991);; cellular component: organelle (GO:0
process (GO:0009987);; biological process: signaling (GO:0023052);; biological process: response t

ess (GO:0032502);; molecular function: binding (GO:0005488);; biological process: cellular process (

ıl process (GO:0032502);; biological process: single-organism process (GO:0044699);; biological pr

51179)

ane-enclosed lumen (GO:0031974)

cess: cellular component organization or biogenesis (GO:0071840);; biological process: immune sys

ganismal process (GO:0032501);; biological process: developmental process (GO:0032502);; cellula

ss: multi-organism process (GO:0051704)

organization or biogenesis (GO:0071840);; biological process: localization (GO:0051179)

ılar function: binding (GO:0005488);; cellular component: other organism (GO:0044215);; cellular co
ulation (GO:0065007);; cellular component: synapse (GO:0045202);; biological process: behavior (G

component: membrane part (GO:0044425);; molecular function: binding (GO:0005488);; biological
ponent: membrane (GO:0016020);; cellular component: macromolecular complex (GO:0032991);; c

0051179);; cellular component: macromolecular complex (GO:0032991)

O:0044421);; biological process: cellular component organization or biogenesis (GO:0071840);; cell

biological process: biological regulation (GO:0065007);; cellular component: membrane (GO:0016C

rocess: biological regulation (GO:0065007);; molecular function: binding (GO:0005488);; molecular

organization or biogenesis (GO:0071840);; biological process: localization (GO:0051179)

anization or biogenesis (GO:0071840);; biological process: response to stimulus (GO:0050896)

cess: single-organism process (GO:0044699)

art (GO:0044421);; cellular component: cell (GO:0005623);; cellular component: membrane-enclose

):0065007)

ocess: response to stimulus (GO:0050896)

ation (GO:0065007)

009987)

: cell part (GO:0044464);; biological process: locomotion (GO:0040011);; biological process: localiza

376);; biological process: cellular process (GO:0009987)
ss: developmental process (GO:0032502);; biological process: multi-organism process (GO:00517

);; biological process: metabolic process (GO:0008152);; biological process: response to stimulus (G

n process (GO:0044699);; biological process: biological regulation (GO:0065007);; biological proces

l4699)

ation (GO:0051179)

process (GO:0022414);; cellular component: cell (GO:0005623);; cellular component: cell part (GO:0
(GO:0065007)
component organization or biogenesis (GO:0071840);; biological process: response to stimulus (G

ılticellular organismal process (GO:0032501);; biological process: developmental process (GO:0032

t: organelle (GO:0043226);; cellular component: membrane (GO:0016020)

nent: membrane (GO:0016020);; cellular component: membrane part (GO:0044425)

;; biological process: reproduction (GO:0000003);; biological process: reproductive process (GO:00

051704)

991)

lumen (GO:0031974);; cellular component: membrane (GO:0016020)
ding (GO:0005488);; biological process: multicellular organismal process (GO:0032501);; biological
vity (GO:0060089)

stem process (GO:0002376

gical process: localization (GO:0051179)

nt organization or biogenesis (GO:0071840);; cellular component: membrane-enclosed lumen (GC

omental process (GO:0032502);; cellular component: organelle part (GO:0044422)

340)

);; cellular component: macromolecular complex (GO:0032991);; cellular component: organelle (GC

nponent: macromolecular complex (GO:0032991);; biological process: immune system process (GO

08152);; biological process: signaling (GO:0023052);; biological process: response to stimulus (GO:

nism process (GO:0051704);; biological process: cellular component organization or biogenesis (G

ocess: response to stimulus (GO:0050896)

component: macromolecular complex (GO:0032991)

process: developmental process (GO:0032502);; biological process: cellular component organization

embrane part (GO:0044425);; biological process: cellular process (GO:0009987);; biological process

; (GO:0051704);; cellular component: other organism (GO:0044215);; cellular component: other org

:0002376);; molecular function: catalytic activity (GO:0003824);; biological process: metabolic proce

(GO:0005576);; cellular component: extracellular region part (GO:0044421)

):0044425);; cellular component: cell junction (GO:0030054);; biological process: cellular componer

007);; cellular component: membrane (GO:0016020);; cellular component: membrane part (GO:004

0032502);; biological process: localization (GO:0051179)

calization (GO:0051179);; biological process: biological regulation (GO:0065007)

ı regulator (GO:0098772);; cellular component: cell (GO:0005623);; cellular component: organelle (

rocess: developmental process (GO:0032502);; biological process: locomotion (GO:0040011);; biolc

process (GO:0051704);; biological process: response to stimulus (GO:0050896)

ocess: signaling (GO:0023052);; biological process: multicellular organismal process (GO:0032501);

ical process: cellular component organization or biogenesis (GO:0071840)

1840);; biological process: biological regulation (GO:0065007);; cellular component: macromolecul
O:0044425);; biological process: localization (GO:0051179);; biological process: metabolic process

organelle part (GO:0044422

cellular organismal process (GO:0032501);; biological process: developmental process (GO:003250

biogenesis (GO:0071840);; biological process: immune system process (GO:0002376);; biological p

rane part (GO:0044425);; biological process: metabolic process (GO:0008152);; cellular component

cal process: localization (GO:0051179)

ological process: localization (GO:0051179)

esis (GO:0071840);; biological regulation (GO:0065007)

omponent: macromolecular complex (GO:0032991);; biological process: multicellular organismal p

al process: biological regulation (GO:0065007)

:romolecular complex (GO:0032991);; cellular component: membrane part (GO:0044425);; biologic

gulation (GO:0065007)

0032501);; biological process: developmental process (GO:0032502);; biological process: biologica

tion: catalytic activity (GO:0003824);; biological process: metabolic process (GO:0008152);; biologic

stimulus (GO:0050896)

regulation (GO:0065007);; cellular component: membrane-enclosed lumen (GO:0031974)

ynapse (GO:0045202);; biological process: cellular component organization or biogenesis (GO:007

ecular function: molecular function regulator (GO:0098772);; cellular component: macromolecular

biological process: growth (GO:0040007

1704

1);; biological process: developmental process (GO:0032502);; molecular function: signal transduce
cal process: biological adhesion (GO:0022610);; biological process: signaling (GO:0023052);; cellula

cess (GO:0032501);; biological process: developmental process (GO:0032502);; biological process:

043226);; cellular component: organelle part (GO:0044422);; biological process: biological adhesio
o stimulus (GO:0050896);; biological process: cellular component organization or biogenesis (GO:0

GO:0009987);; cellular component: synapse part (GO:0044456);; cellular component: synapse (GO:

ocess: cellular component organization or biogenesis (GO:0071840)

stem process (GO:0002376);; biological process: response to stimulus (GO:0050896);; biological pro

r component: macromolecular complex (GO:0032991);; molecular function: binding (GO:0005488)

omponent: other organism part (GO:0044217)
GO:0007610)

process: multicellular organismal process (GO:0032501)
cellular component: membrane part (GO:0044425)

lular component: synapse (GO:0045202);; cellular component: synapse part (GO:0044456);; cellular

020);; cellular component: other organism (GO:0044215);; cellular component: other organism part

function: molecular function regulator (GO:0098772)

ed lumen (GO:0031974);; cellular component: organelle (GO:0043226);; cellular component: organ

ation (GO:0051179);; cellular component: synapse (GO:0045202);; cellular component: synapse part

3O:0050896);; cellular component: membrane part (GO:0044425);; biological process: biological re[illegible]

ss: cellular process (GO:0009987);; biological process: cellular component organization or biogene[illegible]

)044464);; biological process: localization (GO:0051179);; biological process: locomotion (GO:0040[illegible]

GO:0050896)

2502);; biological process: biological regulation (GO:0065007);; cellular component: membrane (GC

22414);; biological process: developmental process (GO:0032502

process: biological regulation (GO:0065007);; biological process: cellular component organization

):0031974);; cellular component: synapse (GO:0045202)

):0043226);; biological process: locomotion (GO:0040011);; biological process: cell killing (GO:0001

):0002376);; biological process: biological adhesion (GO:0022610);; biological process: cellular com

0050896);; cellular component: macromolecular complex (GO:0032991);; biological process: multic

O:0071840);; molecular function: molecular function regulator (GO:0098772);; biological process: b

n or biogenesis (GO:0071840);; cellular component: supramolecular complex (GO:0099080)

;: localization (GO:0051179);; cellular component: extracellular region (GO:0005576);; cellular comp

ganism part (GO:0044217)

ess (GO:0008152);; molecular function: binding (GO:0005488);; cellular component: membrane (GC

nt organization or biogenesis (GO:0071840);; molecular function: signal transducer activity (GO:000

44425);; cellular component: organelle part (GO:0044422);; cellular component: synapse (GO:0045

GO:0043226);; cellular component: cell part (GO:0044464);; cellular component: cell junction (GO:C

ogical process: cellular component organization or biogenesis (GO:0071840);; biological process: g

;; cellular component: synapse (GO:0045202)

ar complex (GO:0032991);; biological process: localization (GO:0051179);; molecular function: cata
(GO:0008152)

2)

›rocess: locomotion (GO:0040011);; biological process: localization (GO:0051179);; molecular functi

: macromolecular complex (GO:0032991);; biological process: multi-organism process (GO:005170

rocess (GO:0032501);; biological process: developmental process (GO:0032502

al process: multicellular organismal process (GO:0032501);; biological process: growth (GO:00400

l regulation (GO:0065007);; biological process: multi-organism process (GO:0051704);; cellular con

:al process: cellular component organization or biogenesis (GO:0071840);; molecular function: elec

1840

complex (GO:0032991);; biological process: signaling (GO:0023052);; biological process: cellular cc

er activity (GO:0004871);; molecular function: molecular transducer activity (GO:0060089)
r component: membrane-enclosed lumen (GO:0031974)

response to stimulus (GO:0050896)

n (GO:0022610);; biological process: multi-organism process (GO:0051704);; molecular function: m
0071840)

0045202)

ɔcess: multi-organism process (GO:0051704);; cellular component: other organism (GO:0044215);;

- component: membrane (GO:0016020);; cellular component: organelle part (GO:0044422);; cellula

(GO:0044217);; molecular function: molecular function regulator (GO:0098772);; biological proces

elle part (GO:0044422);; cellular component: cell part (GO:0044464);; cellular component: membra

t (GO:0044456)

gulation (GO:0065007);; biological process: immune system process (GO:0002376);; biological proc

sis (GO:0071840);; biological process: response to stimulus (GO:0050896);; molecular function: bin

011);; biological process: cellular component organization or biogenesis (GO:0071840);; biological

):0016020);; cellular component: membrane part (GO:0044425);; biological process: biological adh

or biogenes

906

ponent organizatic

:ellular organisma

oiological adhe

ponent: extracellular

:0016020);; cellular co

)4871);; molecular function

202

)030054);; biolog

rowth (GO:0040007)

lytic activity (GO:00038[illegible]

ion: signal transducer ac

)4

)7);; biological process:

nponent: membrane-enclose

:tron carrier activity (GO

›mponent organiz

ıolecular function regı

cellular component: other

r component: membrane

s: metabolic process (GO:000815

ne (GO:0016020);; cellular

:cess: multi-organism process

:ding (GO:0005488);; b

process:

esion (GO:0022610)
