# Supplementary material for: Integrative Analysis of Blood Transcriptomics and Metabolomics Reveals Molecular Regulation of Backfat Thickness in Qinchuan Cattle
Source: Animals (Basel). 2023 Mar 15;13(6):1060. doi: 10.3390/ani13061060 (PMC10044415; doi:10.3390/ani13061060)
Supplement: Supplementary file 1 [file animals-13-01060-s001.zip › Supplementary File S6 Supplementary Table S4.pdf]

Table S4. GO enrichment analysis for DEGs.

#GO_classify1	GO_classify2	All	DE	DE_ID
#Total_gene		19889	996	--
cellular component	extracellular region	1534	61	gene-PLA2G1B;gene-
cellular component	cell	12899	642	gene-ACTR3;gene-C
cellular component	nucleoid	32	1	gene-KIAA0391
cellular component	membrane	8143	385	gene-GGT1;gene-HS
cellular component	cell junction	454	28	gene-ITGB3;gene-GJ
cellular component	membrane-enclosed lumer	3038	174	gene-CWC27;gene-T
cellular component	macromolecular complex	3864	230	gene-HAUS2;gene-L
cellular component	organelle	8888	480	gene-RPF2;gene-MA
cellular component	other organism	234	11	gene-HIST1H1C;gene
cellular component	other organism part	234	11	gene-CCNB2;gene-C
cellular component	extracellular region part	1066	49	gene-SRGN;gene-CD
cellular component	organelle part	5726	322	gene-USP20;gene-C
cellular component	membrane part	6886	323	gene-EMC3;gene-SL
cellular component	synapse part	432	23	gene-SLC6A4;gene-D
cellular component	cell part	12897	642	gene-NINL;gene-TO
cellular component	synapse	638	32	gene-F2R;gene-ITGB
cellular component	supramolecular complex	550	36	gene-LOC112443216
molecular function	transcription factor activity,	327	15	gene-NUPR1;gene-A
molecular function	nucleic acid binding transcr	954	28	gene-E2F3;gene-ZN
molecular function	catalytic activity	5374	312	gene-TRMT5;gene-C
molecular function	signal transducer activity	2183	39	gene-GPR4;gene-GF
molecular function	structural molecule activity	521	41	gene-TUBA3E;gene-l
molecular function	transporter activity	1143	63	gene-SLC4A1;gene-C
molecular function	binding	10564	551	gene-HBM;gene-LO
molecular function	electron carrier activity	54	5	gene-GLRX3;gene-E
molecular function	morphogen activity	3	0	--
molecular function	antioxidant activity	60	9	gene-MGST1;gene-F
molecular function	metallochaperone activity	4	0	--
molecular function	protein tag	5	1	gene-ISG15
molecular function	chemoattractant activity	15	1	gene-WNT5A
molecular function	translation regulator activity	26	1	gene-RPL22
molecular function	chemorepellent activity	13	0	--
molecular function	nutrient reservoir activity	1	0	--
molecular function	molecular transducer activit	2214	43	gene-LOC508441;ge
molecular function	molecular function regulato	970	40	gene-SERPINI1;gene-
biological process	reproduction	788	32	gene-CLIC4;gene-NU
biological process	cell killing	43	3	gene-ELANE;gene-IL
biological process	immune system process	1271	101	gene-SLC40A1;gene-
biological process	behavior	355	17	gene-AGER;gene-MA
biological process	metabolic process	6732	390	gene-AGPS;gene-HB
biological process	cellular process	11866	596	gene-NCAPG2;gene-
biological process	reproductive process	787	32	gene-CCT2;gene-TP
biological process	biological adhesion	521	27	gene-DDR1;gene-GA
biological process	signaling	4274	145	gene-SAG;gene-PLX
biological process	multicellular organismal pro	4956	168	gene-SOX4;gene-RC
biological process	developmental process	3290	162	gene-TBCCD1;gene-
biological process	growth	270	13	gene-IFRD1;gene-LL

biological process	locomotion	757	41	gene-CXCL8;gene-DI
biological process	single-organism process	10207	475	gene-CAT;gene-RFK;
biological process	rhythmic process	142	2	gene-MGC127133;ge
biological process	response to stimulus	6232	278	gene-TMX1;gene-DN
biological process	localization	3705	215	gene-MBOAT7;gene-
biological process	multi-organism process	1239	72	gene-GAS6;gene-GH
biological process	biological regulation	10167	456	gene-MEIS1;gene-TH
biological process	cellular component organiz	3579	211	gene-COL14A1;gene
biological process	cell aggregation	16	0	--
biological process	detoxification	71	10	gene-CLIC2;gene-LT
biological process	presynaptic process involve	54	4	gene-C3H1orf226;ge

-LOC524576;gene-TWSG1;gene-LOC513659;gene-PKNOX2;gene-CD63;gene-HSD17B12;gene-C
RACR2B;gene-ACE;gene-SAP25;gene-UCHL3;gene-ALOX15;gene-CDK1;gene-TMED2;gene-ICAN

PA8;gene-TMEM267;gene-CLEC6A;gene-IFI47;gene-GDPD3;gene-CACNB1;gene-GPR4;gene-FF
A10;gene-APP;gene-FRMD4A;gene-C6H4orf19;gene-NLRX1;gene-PRKCG;gene-NOTCH1;gene-F
ELO2;gene-DYRK3;gene-NDUFS1;gene-PNO1;gene-PALM;gene-PWP1;gene-NRADD;gene-MCN
OC787803;gene-MAGOHB;gene-PCNA;gene-AKAP9;gene-SKA2;gene-RRM1;gene-ITGB3;gene-E
GOH;gene-EXOSC8;gene-AGER;gene-ARPC3;gene-RPL5;gene-GCC2;gene-LPIN1;gene-CHCHD3
e-LOC781710;gene-CCNB2;gene-CCNJ;gene-LOC513659;gene-HIST1H1E;gene-CNOT7;gene-CC
CNJ;gene-LOC513659;gene-HIST1H1E;gene-HIST1H1C;gene-LOC781710;gene-CCNB1;gene-CN
)1E;gene-MPO;gene-LOC616364;gene-MMP15;gene-SMPDL3A;gene-LOC107131803;gene-PF4;g
K1;gene-UCHL3;gene-PDHB;gene-MRPL1;gene-ZKSCAN4;gene-CISD2;gene-APP;gene-ACTR3;g
C28A3;gene-SLC16A9;gene-AQP9;gene-TMCC2;gene-TMEM119;gene-SMPD3;gene-CNEP1R1;ge
OC2A;gene-SEPT5;gene-BTBD8;gene-MX2;gene-SYT7;Bos_taurus_newGene_9362;gene-HOMER
31;gene-GPN1;gene-MRPL3;gene-MRPL39;gene-ADORA3;gene-ECD;gene-CASD1;gene-CD3G;g
3;gene-EIF4E;gene-PACSIN1;gene-CACNG4;gene-RPL22;gene-PALM;gene-MGLL;gene-AKAP9;B
;gene-C3H1orf226;gene-CCT8;gene-NOS1AP;gene-TNNI2;gene-TUBA3E;gene-CCT4;gene-AK1;g
RL2BP;gene-ELANE;gene-C1D;gene-TOB1;gene-LPIN1;gene-TFDP2;gene-SFR1;gene-MED30;ger
774;gene-GATA1;gene-EBF4;gene-MYBL1;gene-MAFF;gene-ZNF32;Bos_taurus_newGene_9419;g
LIC2;gene-LOC104968411;gene-MGAT3;gene-LOC529930;gene-ACOT7;gene-SPATA20;gene-PF
RA3;gene-VIPR2;gene-PLXNB1;gene-S1PR1;gene-HRH2;gene-LOC518134;gene-LOC508441;gen
OC112443216;gene-LOC100299845;gene-MRPL1;gene-RPS27A;gene-TFF2;gene-RSL24D1;Bos_t
LIC3;gene-GJA10;gene-LOC787671;gene-SLC41A2;gene-TTYH1;gene-SLC2A1;gene-VDAC3;gen
533308;gene-MEX3C;gene-TCEAL8;gene-MED30;gene-AQP1;gene-ADARB1;gene-ACBD6;gene-
FA;gene-CYCS;gene-AOX1;gene-NDUFS1

AM213A;gene-CAT;gene-CLIC2;gene-LTC4S;gene-SRXN1;gene-MPO;gene-SOD1;gene-SOD3

ne-STAB1;gene-FFAR2;gene-S1PR1;gene-HRH2;gene-LOC518134;gene-GFRA3;gene-VIPR2;gene
-PPP1R26;gene-TESC;gene-DPM3;gene-LOC100298356;gene-LOC112441507;gene-MNAT1;gene
JPR1;gene-TPPP3;gene-CCT2;gene-TH;gene-SPATA20;gene-TMED2;gene-CPT1B;gene-TDRD9;g
18;gene-LOC508646
-SCAP;gene-IFIT3;gene-CACNB3;gene-LOC504773;gene-DSB;gene-APOBEC3Z1;gene-DYNLT1;g
AN2B1;gene-ATAD1;gene-GM2A;gene-TH;gene-SLC6A4;gene-GHSR;gene-SLC16A1;gene-SDK1;g
Q1;gene-FAM98B;gene-TEX12;gene-SCP2;gene-GAS6;gene-NECAB3;gene-PPA1;gene-FBXL4;ge
NPTN;gene-ITGA9;gene-TMEM86B;gene-ADD2;gene-DSN1;gene-LRSAM1;gene-METTL21A;gen
P3;gene-TH;gene-SPATA20;gene-CLIC4;gene-NUPR1;gene-CCT8;gene-TDRD9;gene-APP;gene-
S6;gene-STAB1;gene-LOC508441;gene-LOC789748;gene-CD63;gene-PODXL2;gene-ICAM3;gen
NB2;gene-SELENOK;gene-ISG15;gene-GATA1;gene-NDUFS4;gene-TLR2;gene-LRRC32;gene-THE
N1;gene-GGT1;gene-GPR4;gene-C3H1orf226;gene-TDRD9;gene-TRIM10;gene-TFF2;gene-SDK1;
PLD3;gene-TMEM126A;gene-CASP6;gene-TMEM14C;gene-RPL22;gene-PLXNA1;gene-ACTL6A;g
H;gene-PLXNA1;gene-APP;gene-NCAPG2;gene-MEX3C;gene-S1PR1;gene-NOTCH1;gene-HSP

DR1;gene-CCR4;gene-CCL3;Bos_taurus_newGene_5578;gene-GAS6;gene-CD63;gene-APCDD1;ge
gene-FES;gene-CACNB3;gene-TMEM145;gene-LOC504773;gene-DSCC1;gene-AOX1;gene-DYN
ene-LOC101905711

NAJB9;gene-ITGB4;gene-SULF2;gene-CXCL5;gene-GFRA3;gene-UBD;gene-RAP2A;gene-SLC7A11
-CD3G;gene-SLC49A3;gene-PLSCR2;gene-SLC6A4;gene-STRADB;gene-SYNE1;gene-STX3;gene-S
SR;gene-DDR1;gene-LOC513659;gene-PLA2G1B;gene-CCT3;gene-PMAIP1;gene-CEBPE;gene-G
HAP1;gene-ZNF8;gene-ITGB3;gene-NMNAT1;gene-ROM1;gene-RPL22;gene-TMEM14C;gene-PL
-TPPP3;gene-LOC782688;gene-TUBA1D;gene-RIOK2;gene-USP15;gene-PLSCR2;gene-FAM171A

C4S;gene-SRXN1;gene-SOD1;gene-MPO;gene-SOD3;gene-MGST1;gene-FAM213A;gene-HBQ1;g
ne-SYT7;gene-STX3;gene-DOC2A

:CL3;gene-CXCL8;gene-ADAMTSL4;gene-GAS6;gene-MTUS2;gene-PF4;gene-IGFBP4;gene-LOC1
M3;gene-HPSE;gene-RTN2;gene-SNRPB2;gene-RPL9;gene-ADSS;gene-CDC123;Bos_taurus_newG

AR2;gene-TIGIT;gene-SDK1;gene-LOC100337457;gene-CASD1;gene-ADORA3;gene-AMIGO2;ge
PANX2;gene-TES;gene-PDZD3;gene-ARL14EP;gene-PARVB;gene-RSU1;gene-AMOTL1;gene-SLC
M6;gene-ETFA;gene-INTS7;gene-POLE3;gene-GATA1;gene-RPS3A;gene-BCCIP;gene-GMNN;gen
BET1;gene-MEIS1;gene-HBB;gene-ACTL6A;gene-RPL36A-2;gene-CACNG4;gene-OSTC;gene-RPL
3;gene-SPRY2;gene-PPID;gene-DPY30;gene-THEM4;gene-MAFF;gene-PIGK;gene-POLE3;gene-T
:NA2;gene-MAFF;gene-AQP1;gene-CCNB1
IOT7;gene-CCNA2;gene-MAFF;gene-AQP1
gene-MTUS2;gene-GAS6;gene-ADAMTSL4;gene-CCL3;gene-CXCL8;gene-HSD17B12;gene-CD63
gene-RPL9;gene-SNRPB2;gene-RTN2;gene-HPSE;gene-HAUS2;gene-LOC787803;gene-PCNA;Bo
ene-HIGD1A;gene-LOC784541;gene-SLC40A1;gene-DSB;gene-SORBS1;gene-PAQR7;gene-NOT
R3;gene-ADD2;gene-WNT5A;gene-ATAD1;gene-DAGLA;gene-SYNE1;gene-STX3;gene-SLC40A1;
gene-NMI;gene-TPPP3;gene-TKT;gene-LOC782688;gene-ATP5F1E;gene-RFXAP;gene-CBX7;gene
os_taurus_newGene_9362;gene-DOC2A;gene-BTBD8;gene-WNT5A;gene-DAGLA;gene-ATAD1;ge
gene-TUBA8;gene-KRT42;gene-LTBP1;gene-HID1;gene-CDK1;gene-TUBA1D;gene-IGBP1;gene-
ne-CNOT7;gene-SOX4;gene-GMNN;gene-DR1;gene-MYBL1;gene-NMI
gene-ZFPM1;gene-GAS7;gene-WNT5A;gene-ZBTB47;gene-HOXB6;gene-THAP1;gene-HIC1;gene
PIL3;gene-OAS1Y;Bos_taurus_newGene_11955;gene-CMPK2;gene-MUT;gene-GATM;gene-ADAM
e-FFAR2;gene-CD3G;gene-CD48;gene-GPR19;gene-LOC100337081;gene-TNFRSF18;gene-ADO
taurus_newGene_7943;gene-RPL7;gene-KRT42;gene-MRPL3;gene-TUBA8;gene-MRPL32;gene-R
ne-HBB;gene-CACNG4;gene-SLC24A3;gene-SLCO4A1;gene-SCNN1D;gene-PANX2;gene-KCNT1;
-RAB27A;gene-PARVB;gene-LOC617079;gene-HSPA5;gene-KLRB1;gene-RBM11;gene-MMP15;g

e-PLXNB1;gene-GPR4;gene-RAMP3;gene-LOC100337081;gene-ADORA3;gene-TNFRSF18;gene-
e-DNAJA1;gene-CCNA2;gene-APP;gene-MTCP1;gene-SGSM2;gene-SNTA1;gene-RAB3IL1;gene-
ene-CCT8;gene-GGT1;gene-CCT4;gene-APP;gene-ACE;gene-GMCL1;gene-HSP90AB1;gene-WN

ene-ALOX5;gene-CNOT7;gene-IFIT2;gene-IFI6;gene-SH2D1A;gene-MX2;gene-CXCL12;gene-AH
gene-HRH2;gene-PRKCG;gene-SLC7A11;gene-APP;gene-MEIS1;gene-GATM;gene-EIF4E;gene-B
ene-IL18;gene-LOC407145;Bos_taurus_newGene_6876;gene-POLB;gene-MINDY3;gene-CDK3;gen
e-FRMD4A;gene-THOC7;gene-SKP1;gene-MPO;gene-SLC24A3;gene-UBE2B;gene-ORC6;gene-
-ACE;gene-CCT4;gene-GGT1;gene-TMED2;gene-CPT1B;gene-GATA1;gene-MGC127133;gene-H
e-ITGB4;gene-EDA;gene-SLC7A11;gene-ADAM8;gene-ITGB3;gene-ICAM4;gene-ITGAD;gene-A
M4;gene-RHOBTB1;gene-IL9R;gene-TNFRSF4;gene-WNT5A;gene-AGER;gene-LOC101905711;g
;gene-MYLIP;gene-AMIGO2;gene-EFNA2;gene-SLC6A4;gene-CD3G;gene-TPPP3;gene-COL14A1
gene-ROM1;gene-MEIS1;Bos_taurus_newGene_5578;gene-ADAMTSL4;gene-CCR4;gene-CRYGS;g
0AB1;gene-WNT5A;gene-TMED2;gene-DDR1;gene-CLIC4

ene-NOTCH1;gene-B3GNT2;gene-PLXNA1;gene-LOC616364;gene-UBE2B;gene-ITGB3;gene-PF4
LT1;gene-CD48;gene-ANXA4;gene-PDCD10;gene-GPM6A;gene-IFI6;gene-TH;gene-PNPLA2;gen

.;gene-WDR12;gene-LOC100297044;gene-HSPD1;gene-TESC;gene-CACNB3;gene-CDC25A;gene
SPNS2;gene-TCN1;gene-CLIC4;gene-ATP5IF1;gene-ATP5F1E;gene-PSMD14;gene-CACNB1;gene
JA10;gene-NUP210L;gene-F2R;gene-TRIP13;gene-PF4;gene-EIF2S2;gene-IL18;gene-OAS1Z;gene
D3;gene-RAPGEF3;gene-PCNA;gene-ADAMTSL4;gene-SPOCD1;gene-TXNL1;Bos_taurus_newGer
.1;gene-EFNA2;gene-PADI4;gene-GAS7;gene-STX3;gene-STRADB;gene-SYNE1;gene-EIF3E;gene-

gene-CAT

.07131803;gene-MMP15;gene-LOC616364;gene-SMPDL3A;gene-APOLD1;gene-MPO;gene-SRGI
Gene_13499;gene-OSTC;gene-TMEM14C;gene-ROM1;gene-THAP1;gene-ITGB3;gene-TXNL1;Bos_

ne-CD3G;gene-RASD1;gene-CLIC4;gene-BLVRB;gene-ATP5F1E;gene-CDHR5;gene-SYNE1;Bos_ta
6A4;gene-LOC522763;gene-FRMD6;gene-SORBS1;gene-RAB13;gene-FES;gene-LIMS1;gene-MY
e-PPID;gene-DPY30;gene-BCAS2;gene-LRRC32;gene-MAFF;gene-OLA1;gene-GCC2;gene-RTRAI
.17;gene-RPL22;gene-PLXNA1;gene-COL7A1;gene-ZFPM1;gene-GNG11;gene-MCUB;gene-RPL4
M4SF5;gene-NDUFS4;gene-SELENOK;gene-GATA1;gene-RPS3A;gene-LOC101906131;gene-STE

;gene-PKNOX2;gene-LOC513659;gene-TWSG1;gene-PLA2G1B;gene-LOC540321;gene-AQP1;ge
s_taurus_newGene_5578;gene-SKA2;gene-NMNAT1;gene-THAP1;gene-ROM1;gene-TMEM14C;g
CH1;gene-SEC22B;gene-LOC101906131;gene-GPI;gene-PIGY;gene-STEAP2;gene-MFF;gene-CCL
gene-PALM;gene-APP;gene-HSPA8;gene-RPL22;gene-PACSIN1;gene-EIF4E;gene-ITGB3;gene-PI
-STRADB;gene-PADI3;Bos_taurus_newGene_6763;gene-SFR1;gene-MRPL22;gene-MMP15;gene-
ene-ADD2;gene-HOMER3;gene-NOS1AP;gene-APP;gene-HSPA8;gene-SLC16A1;gene-PRKCG;ge
DYNLT1;gene-CCT2;gene-KRT72;gene-MAPRE1;gene-SYNE1;gene-GAS7;gene-LOC100141266;ge

-MEIS1;gene-RFX2;gene-FOXO6;gene-ZKSCAN4;gene-CEBPE;gene-SOX4;gene-ZNF469;gene-N
8;gene-OAS1X;gene-CHAC2;gene-HIBADH;gene-NDUFV2;gene-MYH7;gene-ENPP5;gene-PRKC
RA3;gene-RAMP3;gene-NDUFS1;gene-NMUR1;gene-TMIGD3;gene-F2R;gene-PLXNA1;gene-NO
PL6;gene-SNTA1;gene-TUBA1D;gene-RPL9;gene-TUBA1C;gene-MRPS22;gene-ISCA1;gene-LOC1
gene-LOC100848700;gene-SLC25A1;gene-HBQ1;gene-AQP1;gene-LOC101902462;gene-SLC25
ene-PADI3;Bos_taurus_newGene_6763;gene-ENDOD1;Bos_taurus_newGene_3076;gene-CCNB1;g

CD3G;gene-CD48;gene-GPR19;gene-SCNN1D;Bos_taurus_newGene_3867;gene-CCR4;gene-GHS
SGSM1;gene-ARAP3;gene-PLEKHG3;gene-PSMD14;gene-NUPR1;gene-ATP5IF1;gene-CCNB1;ge
IT5A;gene-HIST1H1E;gene-LIPE;gene-EIF2S2;gene-GATA1;gene-MGC127133;gene-NOTCH1;gen

ISP;gene-EDA;gene-LOC787269;gene-XRCC5;gene-S1PR1;gene-RSAD2;gene-RAB17;gene-KLF1;
BS12
ne-CAMK1;gene-B3GAT1;gene-SERINC1;gene-MRPS21;gene-RRM2;gene-MRPL32;gene-YEATS4;
CXCL8;gene-RHPN2;gene-ASNSD1;gene-KCTD1;gene-SLFN14;gene-SUMO1;gene-EFNA2;gene-
SP90AB1;gene-WNT5A;gene-GMCL1;gene-HIST1H1E;gene-LIPE;gene-EIF2S2;gene-TRIP13;gene-
DD2;gene-ALCAM;gene-CDHR5;gene-OLR1;gene-ITGA9;gene-HSP90AB1;gene-SORBS1;gene-NI
ene-ITGAD;gene-PF4;gene-NDUFS1;gene-HIC1;gene-F2R;gene-NLRX1;gene-PALM;gene-C19H1
;gene-MBOAT7;gene-ATP5IF1;gene-CLIC4;gene-CHAD;gene-PADI4;gene-LOC112445030;gene-
ene-PCNA;Bos_taurus_newGene_3602;gene-ZFPM1;gene-DTX1;gene-DAGLA;gene-ACE;gene-AI

;gene-ELANE;gene-ADD2;gene-ITGA9;gene-WNT5A;gene-RAB13;gene-NPTN;gene-SELP;gene-
ne-CXCL12;gene-FHL1;gene-ITGB4;gene-LTBP1;gene-SULF2;gene-RAN;gene-DNAJB9;gene-TMX

e-LOC504773;gene-TMEM145;gene-FES;gene-CAT;gene-IFIT2;gene-IFI6;gene-TH;gene-GPM6A;g
-WDR35;gene-C3H1orf226;gene-SIGLEC1;gene-HSPA8;gene-IPO5;gene-STRIP2;gene-LOC10033
e-GMCL1;gene-HSP90AB1;gene-WNT5A;gene-TLR2;gene-ISG15;gene-OAS2;gene-RSAD2;gene-
ne_5578;gene-ARPP19;gene-COL7A1;gene-ZFPM1;gene-DTX1;gene-IL5RA;gene-CASP4;gene-LC
-LOC101904963;gene-CLIC4;gene-FAM107A;gene-ATP5IF1;gene-CDHR5;gene-WDR35;gene-TD

N;gene-CD1E;gene-IL9R;gene-WNT5A;gene-HSP90AB1;gene-LOC789503;gene-LIPE;gene-IL18;g
_taurus_newGene_5578;gene-PCNA;gene-UPRT;gene-RAB13;gene-GOLGB1;gene-MAGT1;gene-I

aurus_newGene_12749;gene-MMP15;gene-MRPL22;gene-FCAR;gene-ENDOD1;gene-SYNGR1;ge
ADM;gene-SYNPO2;gene-CLIC4;gene-ILDR1;gene-AGER;gene-LOC512440
F;gene-C1D;gene-RPF2;gene-OLR1;gene-AGER;gene-EXOSC8;gene-FAM32A;gene-SERPINI1;ger
;gene-ELANE;gene-ILDR1;gene-LOC539009;gene-MRPL50;gene-GTF2H3;gene-MYH7;gene-RPL7
AP2;gene-CBX1;gene-UBE2D3;gene-GPI;gene-PIGY;gene-ACADM;gene-SEC22B;gene-NOTCH1;g

ne-TG;gene-SELP;gene-IL15;gene-CREG1;gene-PIGR;gene-LOC781710;gene-ELANE;gene-IL18;g
gene-OSTC;gene-PLD3;gene-TBCA;gene-DTX1;gene-LTC4S;gene-ARL14EP;gene-UPRT;gene-EID
L3;gene-ICAM4;gene-PF4;gene-NDUFS1;gene-NUP210L;gene-F2R;gene-ERMAP;gene-TMEM150
RKCG
CCNB1;gene-LOC615559;gene-HSPA5;gene-RBM11;gene-AQP1;gene-MED30;gene-TCEAL8;gen
ene-SYT7;gene-EIF2S1;gene-MX2;gene-SLC6A4;gene-TH;gene-SEPT5;gene-SLC40A1;gene-STX3
ene-SYNPO2;gene-TUBA1C;gene-TCAP;gene-CCT3;gene-TBCA;gene-LMNB1;gene-HAUS2;gene

IOTCH1;gene-ZNF775;gene-OLIG2;gene-TFDP2;gene-GSC2;gene-KLF1
G;gene-LOC508646;gene-RAB44;gene-GTF2H3;gene-LOC539009;gene-AGPAT5;gene-GM2A;ger
TCH1;Bos_taurus_newGene_3867;gene-GAS6;gene-CCR4;gene-DDR1;gene-GHSR;gene-PLXNB2;
.00141266;gene-MRPS30;gene-RPL22;gene-RPL17;gene-MRPL22;gene-TCAP;gene-RPL36A-2;ge
A29;gene-SFXN5;gene-LOC101904667;gene-IPO11;gene-TPC3;gene-LOC617079;gene-TMEM30
ene-RFXAP;gene-STRADB;gene-ARAP3;gene-ECD;gene-TKT;gene-TPPP3;gene-COL14A1;gene-(

R;gene-DDR1;gene-NOTCH1;gene-LOC786796;gene-PLXNA1;gene-NDUFS1;gene-TMIGD3;gene
ne-CACNG4;gene-RAPGEF3;gene-NOTCH1;gene-AKAP9;gene-CCNB2;gene-PCNA;gene-PDZD3
e-TEX12;gene-JAG2;gene-GHSR;gene-DDR1;gene-TRIP13;gene-NUP210L;gene-UBE2B;gene-GJ/

gene-EPB42;gene-LOC100297044;gene-HSPD1;gene-UBD;gene-CXCL5;gene-ICOS;gene-LOC10

gene-SULF2;gene-CPXM2;gene-EIF3H;gene-PPP1CB;gene-DNAJB9;gene-TMX1;gene-ACP1;gene
DPYD;gene-MBOAT7;gene-STT3B;gene-TUBA1D;gene-EIF3E;gene-GAS7;gene-RALB;gene-SOX4;
-UBE2B;gene-NUP210L;gene-GJA10;gene-CCT3;gene-NOTCH1;gene-JAG2;gene-TEX12;gene-DD
PTN;gene-HPSE;gene-GATA1;gene-SELP;gene-PARVB
7orf113;Bos_taurus_newGene_13402;gene-PMAIP1;gene-NRADD;gene-TWSG1;gene-NOTCH1;ge
GAS7;gene-NCALD;gene-MPO;gene-MMP15;gene-UBE2B;gene-CCNB1;gene-HOXB6;gene-CXC
P;gene-CISD2;gene-KREMEN2;gene-ZFP57;gene-ADAM8;gene-GATM;gene-ALOX15;gene-CDK

FFAR2;gene-PODXL2;gene-S1PR1;gene-ITGB4;gene-STRIP2;gene-APP;gene-CXCL5;gene-ADAM
(1;gene-CCT8;gene-HSPD1;gene-WDR12;gene-LOC100297044;gene-MED28;gene-TESC;gene-G

gene-PDCD10;gene-CXCL12;gene-DYNLT1;gene-ANXA4;gene-CD48;gene-TEX12;Bos_taurus_ne
37457;gene-PLCB2;gene-PODXL2;gene-LOC508441;gene-STAB1;gene-FFAR2;gene-LTBP4;gene-
LOC787269;gene-LOC100298356;gene-CCT4;gene-CXCL5;gene-CCT8;Bos_taurus_newGene_107
OC100336476;gene-ZFP57;gene-ZKSCAN4;gene-KREMEN2;gene-SAP25;gene-CISD2;gene-CRAC
RD9;gene-LOC112443216;gene-C3H1orf226;gene-COA5;gene-HSPA8;gene-STRIP2;gene-LSM3;

gene-LOC101905711;gene-LOC781710;gene-ELANE;gene-PIGR;gene-CREG1;gene-IL15;gene-ISC
JBD;gene-TUBA3E;gene-SLC7A11;gene-HSPD1;gene-CCT8;gene-EEF2KMT;gene-LOC10029984[illegible]

ne-CCNB1;gene-SLC41A2;gene-HSPA5;gene-JAG2;gene-KLRB1;gene-IL3RA;gene-MAST1;gene-

ne-DR1;gene-MNAT1;gene-PAXX;gene-C6H4orf19;gene-BARD1;gene-HAT1;gene-RSAD2;gene-
7;gene-CDK1;gene-RPS25;gene-BORCS7;gene-PDHB;gene-MRPL1;gene-HBA1;gene-ADAM8;ger
gene-PWP1;gene-PMAIP1;gene-CCT3;gene-CWC27;gene-TRIP13;gene-DYRK3;gene-F2R;gene-N

gene-LIPE;gene-WNT5A;gene-IL9R;gene-HSPA8;gene-ACE;gene-CXCL5;gene-APP;gene-PDGFD;
3;gene-MRPL32;gene-HID1;gene-YEATS4;gene-PPP1CB;gene-TMX1;gene-SFXN1;Bos_taurus_ne
B;gene-TNFRSF4;gene-ATAD1;gene-AGER;gene-TM4SF5;gene-PIGK;gene-PLXDC1;gene-CLECL1

e-MGC127133;gene-PAFAH1B2;gene-RAB27A;gene-PARVB;gene-FAM213A;gene-SFI1;gene-HB
;gene-SYNE1;gene-FAM107A
-TUBB1;gene-SKA2;gene-KNSTRN;gene-FHL3;gene-BFSP2;gene-SPRY2;gene-CCSAP

ne-FBLL1;gene-GNG11;gene-SCCPDH;gene-LIPE;gene-AUH;gene-LOC781710;gene-ELANE;gene
gene-TLR2;gene-IFNLR1;gene-MED30;gene-TNFRSF4;gene-IL9R;gene-LOC100300051;gene-AG
ne-MRPS31;gene-TUBB1;gene-LOC787803;gene-RPL22L1;gene-CRYGS;gene-MRPS21;gene-RP
A;gene-LOC509034;gene-LOC107131172;gene-LOC112449073;gene-TMCO1;gene-ATP5PB;gen
CSDC2;gene-CD3G;gene-NMI;gene-NINL;gene-MRPL39;gene-PLCB2;gene-TOB1;gene-GPN1;ge

-F2R;gene-NMUR1;gene-ILDR1;gene-AGER;gene-OLR1;gene-LOC100336476;gene-PIGR;gene-
3;gene-EPS8L2;gene-GAS6;gene-GM2A;gene-CDKN2B;gene-BCCIP;gene-SPRY2;gene-POT1;gen
A10;gene-CCT3

0298356;gene-WNT5A;gene-BOLA-2;gene-OAS1Z;gene-AGER;gene-IL18;gene-OAS2;gene-SEL

-WDR12;gene-CELA2A;gene-EEF2KMT;gene-LOC100299845;gene-TESC;gene-NARS2;gene-UB
gene-GNG10;gene-FBXL5;gene-TDRD9;gene-C3H1orf226;gene-SDHD;gene-TRIM10;gene-GTF2
R1;gene-GHSR

ene-DGKG;gene-DDR1;gene-GHSR;gene-CCL3;gene-SYT7;gene-GPR19;gene-LOC617313;gene-
L8;gene-EPS8L2;gene-JAG2;gene-HSPA5;gene-AQP1;gene-CPNE5;gene-NPTN;gene-MED30;ge
1;gene-PPP2R1B;gene-PRKCG;gene-TMED2;gene-HRH2;gene-HPSE;gene-OCSTAMP;gene-SPA

8;gene-LOC100297044;gene-ALCAM;gene-LOC504773;gene-EFNA2;gene-CXCL12;gene-PDCD1
FRA3;gene-NARS2;gene-CXCL5;gene-MAGT1;gene-SLC7A11;gene-RAP2A;gene-CCSAP;gene-SL

wGene_3867;gene-GAS6;gene-HBQ1;gene-GJA10;gene-SRGN;gene-NMUR1;gene-IL18;gene-OA
RAB27A;gene-LOC617079;gene-TG;gene-SH3GL3;gene-IPO11;gene-SLC25A29;gene-AQP1;gene
03;gene-IFIT3;gene-ARG1;gene-MX2;gene-CNOT7;gene-GPM6A;gene-IFI6;gene-IFIT2;gene-TH;
R2B;gene-ACTR3;gene-ACE;gene-APP;gene-USP20;gene-TMED2;gene-PPP2R1B;gene-CDK1;gen
gene-GTF2B;gene-NINL;gene-PARVB;gene-VMA21;gene-FHL3;gene-LLPH;gene-NCAPG2;gene-

G15;gene-SELP;gene-TG;gene-AQP1;gene-LOC540321;gene-LTBP1;gene-LOC787269;gene-TFF2
5;gene-TESC;Bos_taurus_newGene_7180;gene-EIF3H;gene-CYB5A;gene-SFXN1;gene-TMX1;gene-

MGC127133;gene-CAMLG;gene-IFNLR1;gene-AQP1;gene-PAFAH1B2;gene-LOC617079;gene-PI

YIPF5;gene-S1PR1;gene-XRCC5;gene-KLF1;gene-CDC40;gene-ALOX5;gene-TIMM8A;gene-NSA2
ne-RPS27A;gene-CISD2;gene-NASP;gene-APP;gene-CWC15;gene-CCNA2;gene-ACTR3;gene-RA
NDUFS1;gene-NUP210L;gene-RDH14;gene-TRAK2;gene-MZT1;gene-SLC40A1;gene-TIMM8A;gen

gene-SERPINI1;gene-LOC100297044;gene-TFF2;gene-LOC787269;gene-LTBP1;gene-HPSE;gene
wGene_7180;gene-CCT8;gene-HSPD1;gene-WDR12;gene-MED28;gene-RAP2A;gene-TUBA3E;ge
L;gene-NDUFS4;gene-SELENOK;gene-CHCHD3;gene-EEPD1;gene-PODXL2;gene-STAB1;gene-LC

M;gene-MOCS2;gene-ICOS;gene-COQ8A;gene-PAXX;gene-PTGR1;gene-CDC40;gene-PDSS2;ge

-RDH5;gene-TATDN1;gene-C1R;gene-SOD1;gene-CASP6;gene-RHBDF1;gene-GLT8D1;gene-CE
ER;gene-PIGR;gene-LOC100336476;gene-IL5RA;gene-PTGDR2
S3A;gene-BFSP2;Bos_taurus_newGene_6876;gene-ADD2;gene-RPL4;gene-MRPS35;gene-ARPC3;g
e-SLC22A18;gene-LOC520016;gene-HBM;gene-HBA;gene-SCN2B;Bos_taurus_newGene_6888;ge
ne-NCBP2;gene-IFI47;gene-OLR1;gene-HOMER3;Bos_taurus_newGene_4522;gene-LOC1124490[illegible]

PTGDR2;gene-IL5RA;gene-TNFRSF4;gene-LOC100300051;gene-IL9R;gene-TLR2;gene-IFNLR1;ger
e-COL7A1;gene-ARPP19;gene-HSP90AB1;gene-MCUB;gene-SCN2B

P;gene-SELENOK;gene-PRG3;gene-FBXO9;gene-ISG15;gene-GATA1;gene-TLR2;gene-OSTF1;gen

D;gene-MAGT1;gene-SLC7A11;gene-RFK;gene-CAT;gene-FES;gene-GABARAPL1;gene-CDC25A;g
2B;gene-TFF2;gene-MYLIP;gene-LSM3;gene-STRIP2;gene-THEM4;gene-DARS;gene-SRXN1;gene-

SH2D1A;gene-TNFRSF18;gene-SORBS1;gene-NEURL2;gene-SHCBP1;gene-PDE8A;gene-RANBP1
ne-NCAPG2;gene-MGC127133;gene-MAST1;gene-TG;gene-LLPH;gene-ADD2;gene-PIGR;gene-I
TA20;gene-TRAF3IP1;gene-NUPR1;gene-MUT;gene-ALCAM;gene-GJA10;gene-B3GNT2;gene-PA

0;gene-GPM6A;gene-AIMP1;gene-AMOTL1;gene-ALOX5;gene-MBOAT7
.C22A18;gene-IL18;gene-LOC407145;gene-ARL2BP;gene-BFSP2;gene-B3GAT1;gene-SELP;gene-

\S1Z;gene-SLC22A18;gene-BOLA-2;gene-POLB;gene-SELP;gene-NKIRAS1;gene-EGLN3;gene-AL
-HBA;gene-LRSAM1;gene-HBM;gene-CLBA1;gene-LOC520016;gene-ITGA9;gene-PIGR;gene-AD
gene-APOBEC3Z1;gene-CCT2;gene-DYNLT1;gene-CXCL8;gene-JAG2;gene-IFI44L;gene-MPO;ge
ne-EGLN3;gene-ALOX15;gene-AIMP1;gene-SNTA1;gene-AMOTL1;gene-EIF2S1;gene-RTN2;gene
MAST1;gene-AQP1;gene-NPTN;gene-LOC533308;gene-HIST1H1E;gene-ADD2;gene-ING2;gene-

;gene-LOC100297044;gene-LOC112446726;gene-SERPINI1;gene-PDGFD;gene-ACE;gene-APP;ge
-DNAJB9;gene-ITGB4;gene-YEATS4;gene-CCDC43;gene-MRPL32;gene-SULF2;gene-EID3;gene-IF

GR;gene-FAM213A;gene-MEST;gene-DNAJA1;gene-ICOS;gene-CLNS1A;gene-LOC100138951;ge

2;gene-ALAS2;gene-SORBS1;gene-CRABP2;gene-SLC40A1;gene-ZCCHC9;gene-UBE2B;gene-CCN
AMP3;Bos_taurus_newGene_13499;gene-RPP30;gene-NUPR1;gene-RPL9;gene-ALCAM;gene-SNR
ne-ALAS2;gene-KRT72;gene-SORBS1;gene-APOBEC3Z1;gene-IFRD1;gene-ZMAT2;gene-CEP76;ge

-SOD3;gene-COL14A1;gene-LOC504773;gene-LOC786348;gene-TCN1;gene-ARG1
ne-NARS2;gene-MAGT1;gene-CALD1;gene-CAT;gene-LOC100141266;gene-DSCC1;gene-SYNPO
OC508441;gene-KLRG1;gene-SDHD;gene-DPM3;gene-SIGLEC1;gene-C3H1orf226;gene-TMEM8A

ene-KLF1;gene-YIPF5;gene-XRCC5;gene-TNFRSF18;gene-SH2D1A;gene-ZNF330;gene-NSA2;gen

MIP;gene-ADAM11;gene-RCHY1;gene-CNOT7;gene-PSAT1;gene-GLRX3;gene-TUBA1C;gene-DO

gene-RPL5
ne-SLC7A11;gene-MAGT1;gene-HBA1;gene-CACNB1;gene-ANO9;gene-LOC101902555;gene-A
73;gene-KCTD15;gene-IL9R;gene-RTRAF;gene-C1D;gene-GMNN;gene-CDKN2B;gene-LOC10190

ne-MED30;gene-PLXNB2

ne-CDKN2B;gene-LOC788634;gene-PLA2G1B;gene-NOTCH1;gene-TWSG1;gene-LOC513659;gen

gene-DSCC1;gene-AOX1;gene-HDAC2;gene-LOC505326;gene-TH;gene-IFI6;gene-PNPLA2;gene
-POLE3;gene-SELENOK;gene-NDUFS4;gene-EXOSC8;gene-AGER;gene-ADAL;gene-WNT5A;gene

0;gene-S1PR1;gene-RAB27A;gene-RSU1;gene-MAST1;gene-IFNLR1;gene-MED30;gene-AQP1;ge
HIST1H1E;gene-MEX3C;gene-PDCL;gene-MNAT1;gene-TMEM119;gene-TNNI2;gene-KLF1;gene-
CSIN1;gene-EIF4E;gene-TCAP;gene-CD63;gene-GAS6;gene-OLIG2;gene-MGLL;gene-TMEM120E

NKIRAS1;gene-SERINC1;gene-RRM2;gene-AGPS;gene-HBQ1;gene-TFDP2;gene-TMEM120B;gen[illegible]

[illegible]OX15;gene-LOC786987;gene-APP;gene-KREMEN2;gene-PCLAF;gene-FBP1;gene-RAMP3;gene-[illegible]
[illegible]D2;gene-SLC24A3;gene-CCNB1;gene-XPOT;gene-LOC101907965;gene-SLC41A2;gene-UBE2B;[illegible]
ne-UBE2B;gene-LOC781710;gene-ELANE;gene-LIPE;gene-HIST1H1E;gene-LRSAM1;gene-IFNLR1
[illegible]-HPSE;gene-OCSTAMP;Bos_taurus_newGene_13499;gene-RAMP3;gene-GCSAML;gene-PCLAF;ge[illegible]
-UBE2B;gene-CCNB1;gene-LOC101907965;gene-MMP15;gene-MRPL22;gene-SKP1;gene-PADI3;

ene-CXCL5;gene-HSPA8;gene-ARG1;gene-TCN1;gene-LOC786348;gene-LOC504773;gene-PSMI
=I6;gene-TH;gene-PDCD10;gene-BIRC5;gene-PNPLA2;gene-DYNLT1;gene-GPAA1;gene-PTMA-2

ene-KLRF1;gene-XRCC5;gene-YIPF5;gene-S1PR1;gene-SPTY2D1OS;gene-RSAD2;gene-RNF139;g

NB1;gene-MRPL22;gene-LMNB1;gene-MPO;gene-SFR1;gene-RIDA;gene-RBM11;gene-ORC6;ger
PB2;gene-TRAF3IP1;gene-SNTA1;gene-AIMP1;gene-SEM1;gene-EIF2S1;gene-LOC104968411;ger
ene-NAP1L1;gene-ALOX5;gene-BARD1;gene-HAT1;gene-SSX5;gene-EMC3;gene-C6H4orf19;ger

D2;gene-HDAC2;gene-ANXA4;gene-PTMA-2;gene-GPAA1;gene-DYNLT1;gene-PNPLA2;gene-TH
;gene-GNG10;gene-STX3;gene-SPNS2;gene-LOC510798;gene-RABAC1;gene-MBOAT7;gene-BT

e-SYT7;gene-CCT2;gene-VBP1;gene-NT5C3A;gene-MGST1;gene-CISD1;gene-ETFA;gene-MCM6

CK;gene-CA2;gene-MIOX;gene-RAB33B;gene-MYLK;gene-RFNG;gene-RAB17;gene-MKRN1;gene

QP9;gene-SFXN1;gene-LOC100337457;gene-SLC16A1;gene-SLC16A9;gene-SLC28A3;Bos_taurus
)3064;gene-LOC513659;gene-PALM;gene-MCM6;gene-ETFA;gene-CISD1;gene-GIMAP7-3;gene-

ne-GAS6;gene-GPI;gene-CCL3;gene-DYRK3;gene-PF4;gene-CEBPE;gene-CD1E;gene-PMAIP1;ger

-FKBP14;gene-PCNA;gene-LOC524576;gene-PIK3R3;gene-LOC787803;gene-RRM1;gene-SPOCI
-PIN4;gene-GCC2;gene-RPL5;gene-LPIN1;gene-RHOBTB1;gene-PMAIP1;gene-CCT3;gene-HK3;

ene-LOC100300051;gene-DAPL1;gene-ITGA9;gene-PIGR;gene-TMIGD3;gene-RWDD1;gene-HSF
-XRCC5;gene-S1PR1;gene-AHSP;gene-SORBS1;gene-CRABP2;gene-IFRD1;gene-ALOX5;gene-C7
3;gene-APCDD1;Bos_taurus_newGene_9362;gene-BFSP2;gene-FBXO9;gene-TMEM30A;gene-IL18

e-APCDD1;gene-TEX12;gene-KCNT1;gene-SCP2;gene-GAS6;Bos_taurus_newGene_3867;gene-N

OCSTAMP;gene-HPSE;gene-EIF2S1;gene-AMOTL1;Bos_taurus_newGene_5578;gene-PCNA;gene-
gene-SKP1;gene-LOC100848700;gene-PANX2;gene-HSPA5;gene-SCNN1D;gene-LOC615559;gen
1;gene-AQP1;gene-MGC127133;gene-LTC4S;gene-CPT1B;gene-STAB1;gene-TMED2;gene-LOC1
ene-FBP1;gene-NMUR1;gene-SRGN;gene-SLC4A1;gene-ALMS1;gene-APCDD1;gene-TFDP2;gen
gene-MPO;gene-KCTD1;gene-HSD17B12;gene-CCT2;gene-SYT7;gene-IFRD1;gene-NAP1L1;gen

D14;gene-DYNLT1;gene-COL14A1;gene-SOD3;gene-HPSE;gene-CXCL12;gene-SPATA2(
2;gene-AOX1;gene-ANXA4;gene-HDAC2;gene-CACNB3;gene-DSCC1;gene-LOC100141266;gene

ene-TNFRSF18;gene-MX2;gene-SYT7;gene-GPR19;Bos_taurus_newGene_7753;gene-MGST1;gene

ne-HSPA5;gene-LLPH;gene-PAFAH1B2;gene-UTP18;gene-NCAPG2;gene-IPO11;gene-MED30;ge
ne-ORC1;gene-HBQ1;gene-TFDP2;gene-MRPS31;gene-PSMC6;gene-FAM98B;gene-SCP2;gene-
ne-DDX10;gene-CNEP1R1;gene-TMCC2;gene-SERPINI1;gene-UBXN2A;gene-ADD2;gene-DSN1;g

H;gene-IFI6;gene-ALMS1;gene-TMEM120B;gene-AGPS;gene-MRPS31;gene-NECAB3;gene-TEX12
N3A3;gene-TMEM242;gene-STT3B;gene-HRCT1;gene-SLC49A3;gene-SOD3;gene-FAM171A1;ge

5;gene-PALM;gene-LOC101904614;gene-BLMH;gene-PHETA2;gene-C29H11orf54;gene-GIMAP7

e-EPB42;gene-RNF14;gene-FBXO9;gene-LOC101902462;gene-SCRN2;gene-EIF4A2;gene-DCLRE1

_newGene_9777;gene-SLC6A4;gene-PLSCR2;gene-SLC49A3;gene-LOC525820;gene-CLIC2;gene-
-GPN3;gene-PHETA2;gene-NT5C3A;gene-LOC781298;gene-DIMT1;gene-ZNF330;gene-SYT7;ger

ne-OAS1Y;gene-ATP5IF1;gene-PSMD14;gene-NMI;gene-CD3G;gene-AIMP1;gene-OCSTAMP;ger

D1;Bos_taurus_newGene_5578;gene-TXNL1;gene-ADAMTSL4;gene-RPL36A-2;gene-VRK1;gene-N
gene-PF4;gene-DYRK3;gene-TRIP13;gene-KIAA1522;gene-F2R;gene-NUP210L;gene-NDUFS1;ge

PA5;gene-JAG2;gene-RHPN2;gene-EPS8L2;gene-CXCL8;gene-NMI;gene-CD3G;gene-USP15;gene
H19orf57;gene-TRAK2;gene-SLC40A1;gene-MAN2B1;Bos_taurus_newGene_13402;gene-PALM;ge
;gene-MYADM;gene-ASB2;gene-EIF2S2;gene-GMCL1;gene-MYLK;gene-PLXNB1;gene-SLC7A11;

MUR1;gene-LOC787671;gene-SLC4A1;gene-GJA10;gene-SRGN;gene-CDC123;gene-FBP1;gene-

RAPGEF3;gene-LOC511531;gene-ZNF8;gene-ITGB3;gene-NMNAT1;gene-IL5RA;gene-LOC10033
ne-CXCL8;gene-CCT2;gene-SYT7;gene-ALOX5;gene-TIMM8A;gene-MX2;gene-MZT1;Bos_taurus_
12441507;gene-CLEC6A;gene-GGT1;gene-HSPA8;gene-ACE;gene-OAS1X;gene-APP;gene-SIGLE
e-NECAB3;gene-GAS6;Bos_taurus_newGene_3867;gene-SCP2;gene-FAM98B;gene-KCNT1;gene-
e-NSA2;gene-MX2;gene-SORBS1;gene-CRABP2;gene-DIMT1;gene-TRAK2;gene-EIF3M;gene-FC

-CAT;gene-CALD1;gene-CLIC3;gene-GJA10;gene-SRGN;gene-NMUR1;gene-PPA1;gene-KCNT1;

e-SARAF;gene-NLRX1;gene-LOC101904614;gene-PALM;gene-CD1E;Bos_taurus_newGene_13402

ne-DSN1;gene-MOCS2;gene-HIST1H1E;gene-SOX4;gene-MRPL3;gene-GPN1;gene-RSL24D1;Bo
EIF4E;gene-LOC787671;gene-GJA10;gene-FBXL4;gene-MRPS36;gene-SCN2B;gene-EIF2S2;gene-
gene-CLBA1;gene-NCAPG2;gene-IPO11;gene-SLC25A29;gene-VMA21;gene-FHL3;gene-AOC3;g

2;gene-FAM98B;gene-SCP2;gene-CLIC3;gene-OAS1Z;gene-SLC22A18;gene-FBXL4;gene-CCSAP;
ne-SLC6A4;gene-LOC100848700;gene-HSD17B12;gene-PANX2;gene-EPS8L2;gene-SCNN1D;ge

-3;gene-SLCO4A1;gene-LOC513659;gene-CCNB2;gene-CDKN2B;gene-GMNN;gene-LOC101904

1B;gene-ZDHHC2;Bos_taurus_newGene_969;gene-ASB2;gene-RAB10;gene-SSH3;gene-B3GNT2;g

-ATP5F1E;gene-CACNB3;gene-SLC25A17;gene-CLIC4;gene-SLC40A1;gene-SPNS2;gene-LOC514
ne-CCT2;gene-KLF1;gene-PDSS2;gene-CDC42SE2;gene-XRCC5;gene-CAMTA2;gene-PAXX;gene-

ne-EFNA2;gene-LOC508646;gene-PODXL2;gene-TRIM10;gene-LOC508441;gene-FFAR2;gene-A

MNAT1;gene-MEIS1;gene-THAP1;gene-OSTC;gene-PLD3;gene-PLCXD1;gene-RPL22;gene-HPR
ne-CCL3;gene-STEAP2;gene-UBE2D3;gene-ACADM;gene-UFM1;gene-TWSG1;gene-SRMS;gene

e-ADORA3;gene-EFNA2;gene-NCALD;gene-STRADB;gene-STX3;gene-ARAP3;gene-RALB;gene-
ene-CEBPE;gene-F2R;gene-NUP210L;gene-TRIP13;gene-DYRK3;gene-GHSR;gene-CCL3;gene-D
gene-RAP2A;gene-MED28;gene-RAB33B;gene-EPB42;gene-FHL1;gene-LTBP1;gene-EDA;gene-

ADSS;gene-TMEM167B;gene-RAMP3;gene-VNN2;Bos_taurus_newGene_13499;gene-PGD;gene-[illegible]

[illegible]6476;gene-DTX1;gene-RAB13;gene-IL15;gene-LTC4S;gene-RSAD2;gene-XRCC5;gene-S1PR1;ge[illegible]
_newGene_7753;gene-SLC40A1;gene-TRAK2;gene-LOC112446726;gene-CLNS1A;gene-DNAJA1;[illegible]
[illegible]C1;gene-TDRD9;gene-CLIC4;gene-CMPK2;gene-OAS1Y;gene-SPATA20;gene-SLC6A4;gene-TPP[illegible]
EIF4EBP1;gene-SELP;gene-NKIRAS1;gene-CDC42EP1;gene-OAS1Z;gene-CCSAP;gene-ARL2BP;ge[illegible]
F1;gene-CLNS1A;gene-RANBP10;gene-CEP78;gene-DDX10;gene-PDCL;gene-TUBA8;gene-HAT1

gene-FAM98B;gene-TEX12;gene-MRPS31;gene-LYPLAL1;gene-TMEM120B;gene-ALMS1;gene-A

;gene-CISD1;gene-TMEM132A;gene-NRADD;gene-PGRMC1;gene-ITGAD;gene-SLCO4A1;gene-

s_taurus_newGene_10857;gene-NMI;gene-TKT;gene-ECD;gene-CBX7;gene-SYNE1;gene-LAP3;ge
ASB2;Bos_taurus_newGene_6876;gene-ATP5PB;gene-FBXO9;gene-KNSTRN;gene-ZWINT;gene-N
ene-LLPH;gene-E2F3;gene-UTP18;gene-ORC6;gene-EPS8L2;gene-RAD51;gene-SLFN14;gene-TH

gene-ARL2BP;Bos_taurus_newGene_6876;gene-B3GAT1;gene-SELP;gene-MINDY3;gene-BFSP2;ge
ne-REEP1;gene-SLC24A3;gene-TMIGD3;gene-LOC107131749;gene-LOC100300051;gene-CLBA1

4667;gene-BTBD8;gene-FAM32A;Bos_taurus_newGene_4522;gene-HOMER3;gene-OLR1;gene-RT

gene-RNFT1;gene-PLA2G1B;gene-MGLL;gene-TUBB1;gene-PSMC6;gene-ZDHHC14;gene-HMBS;

25
-LTBP3;Bos_taurus_newGene_3602;gene-NOXO1;gene-DMTN;gene-RAB13;gene-ZBED6CL;Bos_ta

DAM8;gene-SOX4;gene-ACE;gene-APP;gene-OAS1X;gene-LOC112441507;gene-CLEC6A;gene-Z

T1;gene-DTX1;gene-GZMB;gene-CASP4;gene-RPL4;gene-LTC4S;gene-UPRT;gene-USP20;gene-U
-ALAS2;gene-SORBS1;gene-NEURL2;gene-APOBEC3Z1;gene-NAP1L1;gene-TRAK2;gene-SLC40A

RASD1;gene-GPR4;gene-C3H1orf226;gene-SOX4;gene-GNG10;gene-PLCB2;gene-LOC508441;ge
DR1;gene-GPI;gene-NOTCH1;gene-TWSG1;gene-ACADM;gene-MAFF;gene-CDKN2B;gene-SPRY
RAB17;gene-RFNG;gene-SULF2;gene-PDCD10;gene-GPM6A;gene-TH;gene-EBF4;gene-DYNLT1;g

AMOTL1;gene-AIMP1;gene-SNTA1;gene-OCSTAMP;gene-HPSE;gene-DERA;gene-TMED2;gene-A

ne-DNAJA1;gene-MNAT1;gene-PAXX;gene-PDE8A;gene-IFIT3;gene-SHCBP1;gene-ARG1;gene-N
gene-CNEP1R1;gene-SLC28A3;gene-S1PR1;gene-SLC16A9;gene-AQP9;gene-LOC107131172;gen
P3
ene-IL18;gene-TESC;gene-WDR12;gene-HSPD1;gene-MED28;gene-CCT8;gene-RAP2A;gene-SLC
.;gene-S1PR1;gene-XRCC5;gene-POLE3;gene-RFC3;gene-NDUFS4;gene-GATA1;gene-BCCIP;gen

PCDD1;gene-RRM2;gene-SFXN5;gene-TMEM70;gene-POLB;gene-B3GAT1;gene-LOC407145;Bos

TMEM165;gene-TTYH1;gene-SLC2A1;gene-CRLS1;gene-ADAMDEC1;gene-MMD;gene-DGKG;gen

ene-BLVRB;gene-EIF3E;gene-RFXAP;gene-CLIC4;gene-PSMD14;gene-COMMD10;gene-CLIC3;gen
MRPS21;gene-POT1;gene-RRM2;gene-EID3;Bos_taurus_newGene_6570;gene-MRPL32;gene-YEAT
HOC7;gene-FRMD4A;gene-LMNB1;gene-SKP1;gene-MPO;gene-ZCCHC9;gene-UBE2B;gene-FOX

ene-TMEM70;gene-MRPS21;gene-SFXN5;gene-GPN1;gene-MRPL3;Bos_taurus_newGene_10857;g
;gene-LOC520016;gene-TMEM86B;gene-LOC112444847;gene-ITGA9;gene-ADD2;gene-VMA21;g

RAF;gene-C1D;gene-IL9R;gene-ACTR3;gene-ACE;gene-CRACR2B;gene-SAP25;gene-CDK1;gene-

gene-DPYD;gene-MBOAT7;gene-STT3B;gene-TUBA1D;gene-RIOK2;gene-SOD3;gene-PADI4;gen

aurus_newGene_5578;gene-PCNA;gene-LOC524576;gene-VRK1;gene-ITGB3;gene-THAP1;gene-F

ZFPM1;gene-ITGA9;gene-PIGR;gene-LOC781710;gene-ELANE;gene-ADD2;gene-IL15;gene-NCAF

UCHL3;gene-ALOX15;gene-EGLN3;gene-PDHB;gene-CDK1;gene-CPT1B;gene-PRSS53;gene-MRF
A1;gene-RANBP10;gene-DDX10;gene-UBXN2A;gene-AHSP;gene-HAT1;gene-SLC16A9;gene-CH(

ene-STAB1;gene-TFF2;gene-FFAR2;gene-LTBP4;Bos_taurus_newGene_9362;gene-SELP;gene-NKIF
2;gene-GMNN;gene-TLR2;gene-NDUFS4;gene-ISG15;gene-GATA1;gene-PLXDC1;gene-AGER;ge
gene-ANXA4;gene-CA2;gene-FES;gene-MMP15;gene-CCNB1;gene-HOXB6;gene-UBE2B;gene-JA

ALOX15;gene-EGLN3;gene-PDHB;gene-CDK1;gene-PPP2R1B;gene-CPT1B;gene-KREMEN2;gene-

MGST1;gene-MX2;gene-RNF139;gene-TNFRSF18;gene-SH2D1A;gene-SYT7;gene-GPR19;gene-D
ne-SELENOK;gene-ISG15;gene-TLR2;gene-FRMD6;gene-LOC101904667;gene-THEM4;Bos_taurus

7A11;gene-GFRA3;gene-UBD;gene-SULF2;gene-YEATS4;gene-ITGB4;gene-LTBP1;gene-TMX1;ge
ne-GMNN;gene-PPID;gene-FRMD6;gene-LOC101904667;gene-THEM4;gene-ARPC3;gene-KCTD1

s_taurus_newGene_6876;gene-NINL;gene-MRPL39;ger

ne-DDR1;gene-GHSR;gene-SLC25A1;gene-LOC101904667

ne-APOLD1;gene-AGPS;gene-MGLL;gene-ORC1;gene-SCP
'S4;gene-LOC787269;gene-ITGB4;gene-LTBP1;gene
O6;gene-RFX2;gene-HOXB6;gene-XPOT;gene-LOC10190496

gene-NINL;gene-LOC112443216;gene-GDPD3;gene-HS
gene-LOC522763;gene-SLC25A29;gene-NPTN;gene-EDA;gene-F

-UCHL3;gene-ALOX15;gene-ICAM3;gene-TMED2;gene-RTN2

ne-CAPN5;gene-RALB;gene-LAP3;gene-DHX40;gene-UBA3;gene

RPL9;Bos_taurus_newGene_11957;gene-SNRPB2;gene

PG2;gene-IFNLR1;gene-JAG2;Bos_taurus_newGer

PL1;gene-ZFP57;gene-CYB561A3;gene-ACE;gene-AF
CHD10;gene-C1H21orf2;gene-GM2A;gene-MYBL1;gene-LIPE;

RAS1;gene-DOC2A;gene-OSTF1;gene-SCN2B;gene-I
ene-ATAD1;gene-HSP90AB1;gene-WNT5A;gene-CISD2;gene
AG2;gene-HSPA5;gene-CXCL8;gene-RWDD1;gene-SLFN14;g

-ZFP57;gene-CYB561A3;gene-ACE;gene-ACTR3;gene-APF

GKG;gene-DDR1;gene-GHSR;gene-LOC513659;ge
_newGene_6888;gene-WNT5A;gene-GCC2;gene-/

ene-PPP1CB;gene-ANXA4;gene-HDAC2;gene-CD48;ge
L5;gene-HSP90AB1;gene-PIN4;gene-GCC2;gene-WNT5A
