# Supplementary material for: Integrative Analysis of Blood Transcriptomics and Metabolomics Reveals Molecular Regulation of Backfat Thickness in Qinchuan Cattle
Source: Animals (Basel). 2023 Mar 15;13(6):1060. doi: 10.3390/ani13061060 (PMC10044415; doi:10.3390/ani13061060)
Supplement: Supplementary file 1 [file animals-13-01060-s001.zip › Supplementary File S7 Supplementary Table S5.pdf]

Table S5. KEGG enrichment analysis for DEGs.

#Pathway	ko_ID	DEgene	gene	DEgene_al	gene_all
Pathways i	ko05200	29	575	533	10647
Herpes sin	ko05168	29	731	533	10647
Alzheimer	ko05010	27	398	533	10647
Epstein-Ba	ko05169	27	339	533	10647
Prion disea	ko05020	26	276	533	10647
Amyotropl	ko05014	26	388	533	10647
Parkinson	ko05012	26	256	533	10647
RNA trans	ko03013	25	203	533	10647
Huntingto	ko05016	24	315	533	10647
Cytokine-c	ko04060	23	364	533	10647
Transcripti	ko05202	23	307	533	10647
Ribosome	ko03010	22	183	533	10647
Apoptosis	ko04210	21	172	533	10647
Human T-	ko05166	21	334	533	10647
PI3K-Akt s	ko04151	21	400	533	10647
Human im	ko05170	20	338	533	10647
Phagosom	ko04145	20	217	533	10647
Rheumato	ko05323	19	204	533	10647
NOD-like	ko04621	19	234	533	10647
Measles	ko05162	18	253	533	10647
Human cy	ko05163	18	262	533	10647
Influenza A	ko05164	18	214	533	10647
Human pa	ko05165	17	389	533	10647
Cellular se	ko04218	17	181	533	10647
Cell adhes	ko04514	16	283	533	10647
Chagas dis	ko05142	16	214	533	10647
Calcium si	ko04020	16	333	533	10647
Rap1 signa	ko04015	16	314	533	10647
Malaria	ko05144	16	150	533	10647
Tight junct	ko04530	15	203	533	10647
Viral carcir	ko05203	15	254	533	10647
Regulation	ko04810	15	246	533	10647
Carbon me	ko01200	15	132	533	10647
Autoimmu	ko05320	15	173	533	10647
Cell cycle	ko04110	15	131	533	10647
Allograft r	ko05330	14	157	533	10647
Natural kil	ko04650	14	186	533	10647
Salmonella	ko05132	14	265	533	10647
Hematopo	ko04640	14	129	533	10647
Hepatitis C	ko05160	14	183	533	10647
Ras signali	ko04014	14	340	533	10647
cAMP sign	ko04024	14	271	533	10647
Graft-vers	ko05332	13	166	533	10647
Chemokin	ko04062	13	191	533	10647
Antigen pr	ko04612	13	193	533	10647
MicroRNA	ko05206	13	186	533	10647
Thermoge	ko04714	13	229	533	10647
Ubiquitin r	ko04120	13	176	533	10647

Type I diak	ko04940	13	155	533	10647
Intestinal i	ko04672	12	154	533	10647
Viral myoc	ko05416	12	177	533	10647
Staphyloc	ko05150	12	213	533	10647
Yersinia in	ko05135	12	244	533	10647
Spinocerel	ko05017	12	151	533	10647
Viral prote	ko04061	12	101	533	10647
Phospholi	ko04072	11	164	533	10647
Bile secreti	ko04976	11	145	533	10647
p53 signal	ko04115	11	97	533	10647
Protein pr	ko04141	11	195	533	10647
Endocytos	ko04144	11	314	533	10647
Spliceosor	ko03040	11	184	533	10647
Systemic l	ko05322	11	290	533	10647
Gap juncti	ko04540	11	108	533	10647
AMPK sigr	ko04152	11	124	533	10647
Amoebiasi	ko05146	11	136	533	10647
Hepatitis E	ko05161	11	182	533	10647
Axon guid	ko04360	11	204	533	10647
Necroptos	ko04217	11	211	533	10647
Kaposi sar	ko05167	10	230	533	10647
Ribosome	ko03008	10	104	533	10647
NF-kappa	ko04064	10	207	533	10647
mTOR sigr	ko04150	10	203	533	10647
MAPK sigr	ko04010	10	322	533	10647
African try	ko05143	10	49	533	10647
Focal adhe	ko04510	10	236	533	10647
Neuroacti	ko04080	10	407	533	10647
Insulin sigr	ko04910	10	155	533	10647
Arachidon	ko00590	10	114	533	10647
Purine me	ko00230	10	153	533	10647
JAK-STAT	ko04630	10	224	533	10647
Glutathion	ko00480	10	67	533	10647
FoxO signa	ko04068	9	140	533	10647
Th1 and Tl	ko04658	9	188	533	10647
Tuberculos	ko05152	9	219	533	10647
Dilated ca	ko05414	9	106	533	10647
Biosynthes	ko01230	9	108	533	10647
Serotoner	ko04726	9	129	533	10647
Thyroid hc	ko04919	9	122	533	10647
Proteoglyc	ko05205	9	225	533	10647
Arginine a	ko00330	9	82	533	10647
Retrograd	ko04723	9	147	533	10647
Toll-like re	ko04620	9	139	533	10647
Asthma	ko05310	9	137	533	10647
Autophag	ko04140	9	164	533	10647
Glyceroph	ko00564	9	112	533	10647
Pyrimidine	ko00240	8	65	533	10647
PD-L1 exp	ko05235	8	184	533	10647
Sphingolip	ko04071	8	122	533	10647
Th17 cell c	ko04659	8	205	533	10647

Adrenergic	ko04261	8	165	533	10647
Glycolysis	ko00010	8	74	533	10647
Non-alcoh	ko04932	8	156	533	10647
HIF-1 sign	ko04066	8	110	533	10647
ABC transp	ko02010	8	122	533	10647
Oxidative p	ko00190	8	137	533	10647
RNA degra	ko03018	8	83	533	10647
Hypertrop	ko05410	8	93	533	10647
T cell rece	ko04660	8	199	533	10647
Inflammat	ko05321	8	159	533	10647
Peroxisom	ko04146	8	134	533	10647
Apelin sigr	ko04371	7	150	533	10647
Cushing sy	ko04934	7	177	533	10647
Pancreatic	ko04972	7	110	533	10647
Hepatocel	ko05225	7	192	533	10647
ECM-rece	ko04512	7	124	533	10647
PPAR sign	ko03320	7	103	533	10647
Drug meta	ko00983	7	87	533	10647
Glycine, se	ko00260	7	47	533	10647
Progester	ko04914	7	95	533	10647
Vascular sı	ko04270	7	156	533	10647
Oxytocin s	ko04921	7	154	533	10647
Ether lipid	ko00565	7	63	533	10647
Legionello	ko05134	7	64	533	10647
cGMP-PK	ko04022	7	173	533	10647
Circadian	ko04713	7	108	533	10647
Wnt signal	ko04310	6	219	533	10647
Antifolate	ko01523	6	78	533	10647
mRNA sur	ko03015	6	122	533	10647
Gastric aci	ko04971	6	79	533	10647
Arrhythmo	ko05412	6	79	533	10647
B cell rece	ko04662	6	107	533	10647
Platelet ac	ko04611	6	135	533	10647
Glucagon	ko04922	6	104	533	10647
Platinum c	ko01524	6	80	533	10647
Pentose pl	ko00030	6	34	533	10647
EGFR tyros	ko01521	6	81	533	10647
Oocyte me	ko04114	6	130	533	10647
Dopamine	ko04728	6	134	533	10647
Alcoholism	ko05034	6	237	533	10647
Longevity	ko04213	5	65	533	10647
Proteasom	ko03050	5	48	533	10647
Nucleotide	ko03420	5	58	533	10647
Endocrine	ko01522	5	99	533	10647
Valine, leu	ko00280	5	60	533	10647
Primary im	ko05340	5	55	533	10647
Estrogen s	ko04915	5	143	533	10647
Acute mye	ko05221	5	71	533	10647
Pancreatic	ko05212	5	78	533	10647
Notch sigr	ko04330	5	70	533	10647
Toxoplasm	ko05145	5	116	533	10647

Fluid shear	ko05418	5	149	533	10647
Breast can	ko05224	5	170	533	10647
Regulation	ko04923	5	62	533	10647
Adherens	ko04520	5	94	533	10647
Glycerolipi	ko00561	5	73	533	10647
Colorectal	ko05210	5	91	533	10647
Hippo sigr	ko04390	5	181	533	10647
TGF-beta	ko04350	5	99	533	10647
Aldosteror	ko04925	5	97	533	10647
Lysosome	ko04142	5	175	533	10647
Glioma	ko05214	5	77	533	10647
Fc gamma	ko04666	5	101	533	10647
Base excisi	ko03410	5	48	533	10647
Choline m	ko05231	5	101	533	10647
Glutamate	ko04724	5	116	533	10647
Cholinergi	ko04725	5	117	533	10647
Non-small	ko05223	4	69	533	10647
Cardiac m	ko04260	4	101	533	10647
Phosphatic	ko04070	4	112	533	10647
Melanoma	ko05218	4	74	533	10647
Leishmania	ko05140	4	83	533	10647
Small cell l	ko05222	4	97	533	10647
Prostate ca	ko05215	4	102	533	10647
Starch and	ko00500	4	38	533	10647
Morphine	ko05032	4	98	533	10647
Retinol me	ko00830	4	83	533	10647
AGE-RAGE	ko04933	4	105	533	10647
Fatty acid	ko01212	4	68	533	10647
Parathyroi	ko04928	4	110	533	10647
Osteoclast	ko04380	4	156	533	10647
Apoptosis	ko04215	4	36	533	10647
Longevity	ko04211	4	91	533	10647
DNA replic	ko03030	4	41	533	10647
Leukocyte	ko04670	4	120	533	10647
Central ca	ko05230	4	71	533	10647
TNF signal	ko04668	4	131	533	10647
Thyroid hc	ko04918	4	77	533	10647
RIG-I-like	ko04622	4	121	533	10647
Fc epsilon	ko04664	4	79	533	10647
GABAergic	ko04727	4	92	533	10647
Basal trans	ko03022	4	49	533	10647
Salivary se	ko04970	4	119	533	10647
IL-17 signa	ko04657	4	111	533	10647
Relaxin sig	ko04926	4	136	533	10647
Homologo	ko03440	4	49	533	10647
Ferroptosis	ko04216	4	53	533	10647
Growth hc	ko04935	4	122	533	10647
Insulin resi	ko04931	4	112	533	10647
Chronic m	ko05220	4	80	533	10647
Protein dig	ko04974	4	139	533	10647
Amino sug	ko00520	4	62	533	10647

Gastric car	ko05226	4	180	533	10647
Tyrosine m	ko00350	4	50	533	10647
Aminoacyl	ko00970	3	54	533	10647
Collecting	ko04966	3	30	533	10647
Inflammat	ko04750	3	116	533	10647
Nicotinate	ko00760	3	50	533	10647
Neurotrop	ko04722	3	135	533	10647
Biosynthes	ko01040	3	37	533	10647
Long-term	ko04720	3	67	533	10647
Mitophagy	ko04137	3	79	533	10647
Autophagy	ko04136	3	37	533	10647
Metabolisr	ko00980	3	73	533	10647
Citrate cyc	ko00020	3	34	533	10647
Folate bios	ko00790	3	49	533	10647
Amphetan	ko05031	3	69	533	10647
Fatty acid	ko00071	3	48	533	10647
GnRH secr	ko04929	3	66	533	10647
SNARE int	ko04130	3	41	533	10647
Carbohydr	ko04973	3	47	533	10647
Glycosylpł	ko00563	3	28	533	10647
Pertussis	ko05133	3	83	533	10647
Chemical c	ko05204	3	87	533	10647
Synaptic v	ko04721	3	82	533	10647
Fatty acid	ko00062	3	31	533	10647
Renal cell	ko05211	3	70	533	10647
Pyruvate n	ko00620	3	49	533	10647
N-Glycan	ko00510	3	64	533	10647
Bladder ca	ko05219	3	44	533	10647
Melanoger	ko04916	3	127	533	10647
Vitamin di	ko04977	3	31	533	10647
Porphyrin	ko00860	3	49	533	10647
Renin secr	ko04924	3	77	533	10647
Drug meta	ko00982	3	68	533	10647
ErbB signa	ko04012	3	91	533	10647
Riboflavin	ko00740	3	9	533	10647
Insulin sec	ko04911	3	86	533	10647
Signaling p	ko04550	3	174	533	10647
Long-term	ko04730	3	66	533	10647
Proximal tu	ko04964	2	23	533	10647
Fanconi ar	ko03460	2	58	533	10647
Tryptopha	ko00380	2	55	533	10647
Renin-ang	ko04614	2	26	533	10647
Glyoxylate	ko00630	2	38	533	10647
Bacterial ir	ko05100	2	76	533	10647
Thiamine r	ko00730	2	20	533	10647
Mineral ab	ko04978	2	63	533	10647
Type II dia	ko04930	2	48	533	10647
Endocrine	ko04961	2	57	533	10647
Cysteine a	ko00270	2	57	533	10647
Cholesterc	ko04979	2	53	533	10647
C-type lec	ko04625	2	116	533	10647

Aldosteror	ko04960	2	39	533	10647
Prolactin s	ko04917	2	85	533	10647
Mismatch	ko03430	2	26	533	10647
Adipocyto	ko04920	2	81	533	10647
beta-Alani	ko00410	2	34	533	10647
Vitamin B6	ko00750	2	11	533	10647
Inositol ph	ko00562	2	81	533	10647
Compleme	ko04610	2	110	533	10647
Cortisol sy	ko04927	2	71	533	10647
Terpenoid	ko00900	2	26	533	10647
VEGF signa	ko04370	2	65	533	10647
Propanoat	ko00640	2	38	533	10647
Fructose a	ko00051	2	37	533	10647
Pantothen	ko00770	2	24	533	10647
Linoleic ac	ko00591	2	51	533	10647
Sphingolip	ko00600	2	63	533	10647
Cytosolic D	ko04623	1	76	533	10647
Ovarian st	ko04913	1	68	533	10647
Glycosami	ko00531	1	30	533	10647
Mannose t	ko00515	1	29	533	10647
Primary bil	ko00120	1	18	533	10647
Valine, leu	ko00290	1	5	533	10647
Nitrogen r	ko00910	1	21	533	10647
alpha-Linc	ko00592	1	36	533	10647
Arginine b	ko00220	1	22	533	10647
Steroid ho	ko00140	1	88	533	10647
Olfactory t	ko04740	1	1224	533	10647
Hippo sigr	ko04392	1	37	533	10647
Phototrans	ko04744	1	31	533	10647
Taste trans	ko04742	1	122	533	10647
Glycosphir	ko00601	1	37	533	10647
Other glyc	ko00511	1	33	533	10647
Protein ex	ko03060	1	24	533	10647
Alanine, as	ko00250	1	42	533	10647
Non-hom	ko03450	1	17	533	10647
Galactose	ko00052	1	39	533	10647
Neomycin	ko00524	1	6	533	10647
Sulfur rela	ko04122	1	14	533	10647
Endometri	ko05213	1	63	533	10647
Hedgehog	ko04340	1	57	533	10647
Other type	ko00514	1	57	533	10647
Phenylalar	ko00360	1	25	533	10647
GnRH sign	ko04912	1	105	533	10647
Various ty	ko00513	1	55	533	10647
Taurine an	ko00430	1	16	533	10647
Ascorbate	ko00053	1	27	533	10647
Fat digesti	ko04975	1	57	533	10647
Glycosami	ko00533	1	16	533	10647
Cocaine ac	ko05030	1	50	533	10647
Basal cell c	ko05217	1	80	533	10647

Gene_id
gene-BIRC5;gene-CCNA2;gene-CDKN2A;gene-CDKN2B;gene-CXCL12;gene-CXCL8;gene-
Bos_taurus_newGene_12809;Bos_taurus_newGene_3633;Bos_taurus_newGene_5402;Bos_ta
Bos_taurus_newGene_9777;gene-AGER;gene-APP;gene-ATP5F1E;gene-ATP5PB;gene-CY
Bos_taurus_newGene_11530;Bos_taurus_newGene_12749;Bos_taurus_newGene_2175;Bos_t
Bos_taurus_newGene_9777;gene-ATP5F1E;gene-ATP5PB;gene-CYCS;gene-EIF2S1;gene-H
gene-ANG;gene-ATP5F1E;gene-ATP5PB;gene-CAT;gene-CHCHD10;gene-CYCS;gene-EIF
Bos_taurus_newGene_9777;gene-ATP5F1E;gene-ATP5PB;gene-CYCS;gene-EIF2S1;gene-H
Bos_taurus_newGene_10660;gene-B3GNTL1;gene-BZW2;gene-CLNS1A;gene-EIF1B;gene-
Bos_taurus_newGene_9777;gene-ATP5F1E;gene-ATP5PB;gene-CYCS;gene-HDAC2;gene-L
gene-CCL3;gene-CCR4;gene-CXCL12;gene-CXCL5;gene-CXCL8;gene-EDA;gene-IFNLR1;
Bos_taurus_newGene_11530;Bos_taurus_newGene_12749;Bos_taurus_newGene_2175;Bos_t
Bos_taurus_newGene_6876;Bos_taurus_newGene_7943;gene-LOC100299845;gene-LOC78
gene-ARPP19;gene-BIRC5;gene-CASP6;gene-CYCS;gene-EIF2S1;gene-GZMB;gene-IL3RA
Bos_taurus_newGene_12749;Bos_taurus_newGene_2175;Bos_taurus_newGene_2236;Bos_ta
Bos_taurus_newGene_11530;gene-CHAD;gene-EFNA2;gene-EIF4E;gene-EIF4EBP1;gene-F
Bos_taurus_newGene_12749;Bos_taurus_newGene_2175;Bos_taurus_newGene_2236;Bos_ta
Bos_taurus_newGene_11530;gene-BOLA-2;gene-C1R;gene-DSB;gene-FCAR;gene-ITGB3;
Bos_taurus_newGene_11530;Bos_taurus_newGene_12749;Bos_taurus_newGene_2175;Bos_t
Bos_taurus_newGene_11955;Bos_taurus_newGene_11957;gene-CASP4;gene-CXCL8;gene-
Bos_taurus_newGene_12749;Bos_taurus_newGene_2175;Bos_taurus_newGene_2236;Bos_ta
gene-BOLA-2;gene-CCL3;gene-CDKN2A;gene-CXCL12;gene-CXCL8;gene-CYCS;gene-E2
Bos_taurus_newGene_9777;gene-CXCL8;gene-CYCS;gene-DSB;gene-EIF2S1;gene-IL18;ge
gene-BOLA-2;gene-CCNA2;gene-CHAD;gene-EIF4EBP1;gene-HDAC2;gene-ISG15;gene-
Bos_taurus_newGene_9777;gene-BOLA-2;gene-CCNA2;gene-CCNB1;gene-CCNB2;gene-
Bos_taurus_newGene_12749;Bos_taurus_newGene_13402;Bos_taurus_newGene_2175;Bos_t
Bos_taurus_newGene_12749;Bos_taurus_newGene_2175;Bos_taurus_newGene_2236;Bos_ta
Bos_taurus_newGene_11530;Bos_taurus_newGene_12749;Bos_taurus_newGene_2175;Bos_t
Bos_taurus_newGene_12749;Bos_taurus_newGene_2175;Bos_taurus_newGene_2236;Bos_ta
Bos_taurus_newGene_12749;Bos_taurus_newGene_2175;Bos_taurus_newGene_2236;Bos_ta
gene-ACTR3;gene-AMOTL1;gene-CD1E;gene-FRMD4A;gene-LOC100141266;gene-LOC1
gene-BOLA-2;gene-CCNA2;gene-CCR4;gene-CDK1;gene-CDKN2A;gene-CDKN2B;gene-
gene-ARPC3;gene-BAIAP2;gene-CXCL12;gene-F2R;gene-ITGA9;gene-ITGAD;gene-ITGB3
gene-ADH5;gene-CAT;gene-FBP1;gene-GPI;gene-HK3;gene-ME3;gene-MUT;gene-PDHB
Bos_taurus_newGene_11530;Bos_taurus_newGene_12749;Bos_taurus_newGene_2175;Bos_t
gene-CCNA2;gene-CCNB1;gene-CCNB2;gene-CDC25A;gene-CDK1;gene-CDKN2A;gene-
Bos_taurus_newGene_11530;Bos_taurus_newGene_12749;Bos_taurus_newGene_2175;Bos_t
Bos_taurus_newGene_11530;gene-CD48;gene-GZMB;gene-KIR3DS1;gene-LOC104968484
gene-ARPC3;gene-CASP4;gene-CXCL8;gene-CYCS;gene-DYNLT1;gene-GCC2;gene-HSP
Bos_taurus_newGene_11530;gene-CD1E;gene-CD3G;gene-DSB;gene-GP9;gene-GYPB;ge
gene-CYCS;gene-E2F3;gene-EIF2S1;gene-EIF3E;gene-IFIT2;gene-IFIT3;gene-MX2;gene-O
Bos_taurus_newGene_12749;Bos_taurus_newGene_2175;Bos_taurus_newGene_2236;Bos_ta
gene-ARAP3;gene-F2R;gene-FFAR2;gene-GHSR;gene-LIPE;gene-LOC100848700;gene-L
Bos_taurus_newGene_12749;Bos_taurus_newGene_2175;Bos_taurus_newGene_2236;Bos_ta
gene-CCL3;gene-CCR4;gene-CXCL12;gene-CXCL5;gene-CXCL8;gene-GNG10;gene-GNG
Bos_taurus_newGene_12749;Bos_taurus_newGene_2175;Bos_taurus_newGene_2236;Bos_ta
gene-CCNG1;gene-CDC25A;gene-CDKN2A;gene-E2F3;gene-EFNA2;gene-HDAC2;gene-I
gene-ACTL6A;gene-ATP5F1E;gene-ATP5PB;gene-COA5;gene-CPT1B;gene-LIPE;gene-M
gene-EEPD1;gene-LOC529930;gene-RCHY1;gene-RHOBTB1;gene-RPS27A;gene-SKP1;ge

Bos_taurus_newGene_12749;Bos_taurus_newGene_2175;Bos_taurus_newGene_2236;Bos_ta
Bos_taurus_newGene_11530;Bos_taurus_newGene_12749;Bos_taurus_newGene_2175;Bos_t
Bos_taurus_newGene_11530;Bos_taurus_newGene_12749;Bos_taurus_newGene_2175;Bos_t
Bos_taurus_newGene_11530;Bos_taurus_newGene_12749;Bos_taurus_newGene_2175;Bos_t
Bos_taurus_newGene_11530;Bos_taurus_newGene_12749;Bos_taurus_newGene_2175;Bos_t
Bos_taurus_newGene_9777;gene-CYCS;gene-GTF2B;gene-LOC101907965;gene-PIK3R3;ge
gene-CCL3;gene-CCR4;gene-CXCL12;gene-CXCL5;gene-CXCL8;gene-IL18;gene-LOC100
Bos_taurus_newGene_11530;gene-AGPAT5;gene-CXCL8;gene-DGKG;gene-F2R;gene-LOC
gene-AQP1;gene-AQP9;gene-CA2;gene-IFRD1;gene-LOC100848700;gene-LOC10190246
gene-CCNB1;gene-CCNB2;gene-CCNG1;gene-CDK1;gene-CDKN2A;gene-CYCS;gene-LO
gene-DNAJA1;gene-EIF2S1;gene-HSP90AB1;gene-HSPA5;gene-HSPA8;gene-SKP1;gene-
gene-ARAP3;gene-ARPC3;gene-BOLA-2;gene-HSPA8;gene-LOC788634;gene-RAB10;gen
gene-BCAS2;gene-CDC40;gene-CWC15;gene-FHL3;gene-HSPA8;gene-LSM3;gene-MAG
Bos_taurus_newGene_11530;Bos_taurus_newGene_12749;Bos_taurus_newGene_2175;Bos_t
gene-CDK1;gene-LOC100141266;gene-LOC112443216;gene-PDGFD;gene-PLCB2;gene-P
gene-CCNA2;gene-CPT1B;gene-EIF4EBP1;gene-FBP1;gene-LIPE;gene-PIK3R3;gene-PPP2
Bos_taurus_newGene_11530;gene-ARG1;gene-CD1E;gene-CXCL8;gene-LOC104968484;ge
gene-BIRC5;gene-CCNA2;gene-CXCL8;gene-CYCS;gene-E2F3;gene-HSPG2;gene-PCNA;g
gene-CXCL12;gene-DMTN;gene-EFNA2;gene-FES;gene-PIK3R3;gene-PLXNA1;gene-PLX
Bos_taurus_newGene_5578;Bos_taurus_newGene_9777;gene-ALOX15;gene-HMGB1;gene-
gene-BOLA-2;gene-CCR4;gene-CXCL8;gene-CYCS;gene-E2F3;gene-GNG10;gene-GNG1
gene-FBLL1;gene-FCF1;gene-GAR1;gene-NMD3;gene-RAN;gene-REXO5;gene-RIOK2;ge
Bos_taurus_newGene_11530;Bos_taurus_newGene_12749;Bos_taurus_newGene_2175;Bos_t
gene-EIF4E;gene-EIF4EBP1;gene-LOC539009;gene-LPIN1;gene-PIK3R3;gene-PRKCG;gene
gene-CACNB1;gene-CACNB3;gene-CACNG4;gene-EFNA2;gene-HSPA8;gene-NRADD;ge
Bos_taurus_newGene_11530;gene-HBA;gene-HBA1;gene-HBB;gene-IL18;gene-LOC10496
gene-CHAD;gene-ITGA9;gene-ITGB3;gene-ITGB4;gene-MYLK;gene-PARVB;gene-PDGFD;
gene-ADORA3;gene-F2R;gene-GHSR;gene-HRH2;gene-LOC101905711;gene-NMUR1;ge
gene-EIF4E;gene-EIF4EBP1;gene-FBP1;gene-HK3;gene-LIPE;gene-PIK3R3;gene-PPP1CB;g
gene-ALOX15;gene-ALOX5;gene-CYP2U1;gene-FAM213A;gene-GGT1;gene-LTC4S;gene-
gene-ADSS;gene-AK1;gene-AMPD3;gene-DCK;gene-HPRT1;gene-NT5C3A;gene-PAICS;g
gene-AOX1;gene-FHL1;gene-IFNLR1;gene-IL15;gene-IL3RA;gene-IL5RA;gene-IL9R;gene-
gene-CHAC2;gene-GGCT;gene-GGT1;gene-LAP3;gene-MGST1;gene-ODC1;gene-OPLAH
gene-CAT;gene-CCNB1;gene-CCNB2;gene-CDKN2B;gene-FOXO6;gene-GABARAPL1;gen
Bos_taurus_newGene_12749;Bos_taurus_newGene_2175;Bos_taurus_newGene_2236;Bos_ta
Bos_taurus_newGene_11530;gene-CYCS;gene-DSB;gene-HSPD1;gene-IL18;gene-LOC10
Bos_taurus_newGene_11530;gene-CACNB1;gene-CACNB3;gene-CACNG4;gene-ITGA9;ge
gene-ARG1;gene-LOC101906218;gene-LOC508153;gene-LOC512440;gene-LOC522763;g
gene-ALOX15;gene-ALOX5;gene-APP;gene-GNG10;gene-GNG11;gene-PLCB2;gene-PRK
gene-HDAC2;gene-ITGB3;gene-MED30;gene-MYH7;gene-NOTCH1;gene-PIK3R3;gene-P
gene-CD63;gene-HPSE;gene-HSPG2;gene-ITGB3;gene-PIK3R3;gene-PRKCG;gene-TLR2;g
gene-ARG1;gene-GATM;gene-LAP3;gene-LOC101906218;gene-LOC508153;gene-LOC51
gene-DAGLA;gene-GNG10;gene-GNG11;gene-MGLL;gene-NDUFS1;gene-NDUFS4;gene-
gene-CCL3;gene-CXCL8;gene-LOC100297044;gene-LOC504773;gene-LOC616364;gene-
Bos_taurus_newGene_11530;Bos_taurus_newGene_12749;Bos_taurus_newGene_2175;Bos_t
Bos_taurus_newGene_5578;gene-EIF2S1;gene-GABARAPL1;gene-HMGB1;gene-IGBP1;gen
gene-AGPAT5;gene-CRLS1;gene-DGKG;gene-LPIN1;gene-MBOAT7;gene-PLA2G1B;gene-
gene-CMPK1;gene-CMPK2;gene-DCK;gene-DPYD;gene-NT5C3A;gene-RRM1;gene-RRM
Bos_taurus_newGene_12749;Bos_taurus_newGene_2175;Bos_taurus_newGene_2236;Bos_ta
gene-ADORA3;gene-CERS1;gene-PIK3R3;gene-PLCB2;gene-PPP2R1B;gene-PRKCG;gene-
Bos_taurus_newGene_12749;Bos_taurus_newGene_2175;Bos_taurus_newGene_2236;Bos_ta

gene-CACNB1;gene-CACNB3;gene-CACNG4;gene-MYH7;gene-PLCB2;gene-PPP1CB;gen
gene-ADH5;gene-BPGM;gene-FBP1;gene-GPI;gene-HK3;gene-LOC112447087;gene-PDH
gene-CXCL8;gene-CYCS;gene-EIF2S1;gene-NDUFS1;gene-NDUFS4;gene-NDUFV2;gene-
gene-EGLN3;gene-EIF4E;gene-EIF4EBP1;gene-HK3;gene-PDHB;gene-PIK3R3;gene-PRKC
Bos_taurus_newGene_10702;gene-LOC100848700;gene-LOC101902462;gene-LOC101902
gene-ATP5F1E;gene-ATP5PB;gene-LOC101904667;gene-NDUFS1;gene-NDUFS4;gene-N
gene-BTG1;gene-C1D;gene-CNOT7;gene-EXOSC8;gene-HSPD1;gene-LSM3;gene-PABPC
gene-ACE;gene-CACNB1;gene-CACNB3;gene-CACNG4;gene-ITGA9;gene-ITGB3;gene-IT
Bos_taurus_newGene_12749;Bos_taurus_newGene_2175;Bos_taurus_newGene_2236;Bos_ta
Bos_taurus_newGene_12749;Bos_taurus_newGene_2175;Bos_taurus_newGene_2236;Bos_ta
Bos_taurus_newGene_4681;gene-AGPS;gene-CAT;gene-CROT;gene-MEST;gene-SCP2;gen
gene-GABARAPL1;gene-GNG10;gene-GNG11;gene-LIPE;gene-MYLK;gene-PLCB2;gene-T
gene-CDKN2A;gene-CDKN2B;gene-E2F3;gene-PDE8A;gene-PLCB2;gene-RASD1;gene-W
gene-CA2;gene-CELA2A;gene-IFRD1;gene-PLA2G1B;gene-PLCB2;gene-PRKCG;gene-TM
gene-ACTL6A;gene-CDKN2A;gene-E2F3;gene-MGST1;gene-PIK3R3;gene-PRKCG;gene-W
gene-CHAD;gene-GP9;gene-HSPG2;gene-ITGA9;gene-ITGB3;gene-ITGB4;gene-VWCE
gene-ACADM;gene-CPT1B;gene-DBI;gene-ME3;gene-OLR1;gene-SCP2;gene-SORBS1
gene-CMPK1;gene-DPYD;gene-HPRT1;gene-MGST1;gene-MPO;gene-RRM1;gene-RRM2
gene-ALAS2;gene-AOC3;gene-BPGM;gene-GATM;gene-LOC112447087;gene-PSAT1;gen
gene-CCNA2;gene-CCNB1;gene-CCNB2;gene-CDC25A;gene-CDK1;gene-HSP90AB1;gen
gene-CALD1;gene-MYLK;gene-PLA2G1B;gene-PLCB2;gene-PPP1CB;gene-PRKCG;gene-R
gene-CACNB1;gene-CACNB3;gene-CACNG4;gene-CAMK1;gene-MYLK;gene-PLCB2;gene
gene-AGPS;gene-GDPD3;gene-PAFAH1B2;gene-PLA2G1B;gene-PLD3;gene-TMEM86B;ge
gene-CXCL8;gene-CYCS;gene-HSPA8;gene-HSPD1;gene-IL18;gene-SEC22B;gene-TLR2
Bos_taurus_newGene_9777;gene-ADORA3;gene-MYH7;gene-MYLK;gene-PLCB2;gene-TM
gene-C3H1orf226;gene-GNG10;gene-GNG11;gene-NOS1AP;gene-PLCB2;gene-PRKCG;g
gene-NKD2;gene-PLCB2;gene-PRKCG;gene-SKP1;gene-TES;gene-WNT5A
gene-LOC100848700;gene-LOC101902462;gene-LOC101902555;gene-LOC112449073;ge
gene-MAGOH;gene-MAGOHB;gene-NCBP2;gene-PABPC4;gene-PPP1CB;gene-PPP2R1B
gene-CA2;gene-HRH2;gene-IFRD1;gene-MYLK;gene-PLCB2;gene-PRKCG
gene-CACNB1;gene-CACNB3;gene-CACNG4;gene-ITGA9;gene-ITGB3;gene-ITGB4
Bos_taurus_newGene_11530;Bos_taurus_newGene_6916;gene-LOC104968484;gene-LOC1
gene-F2R;gene-GP9;gene-ITGB3;gene-MYLK;gene-PIK3R3;gene-PLCB2
gene-CPT1B;gene-FBP1;gene-PDHB;gene-PLCB2;gene-PYGM;gene-SLC2A1
gene-BIRC5;gene-CDKN2A;gene-CYCS;gene-MGST1;gene-PIK3R3;gene-PMAIP1
gene-DERA;gene-FBP1;gene-GPI;gene-PGD;gene-TKT;gene-TWSG1
gene-EIF4E;gene-EIF4EBP1;gene-GAS6;gene-PDGFD;gene-PIK3R3;gene-PRKCG
gene-CCNB1;gene-CCNB2;gene-CDK1;gene-PPP1CB;gene-PPP2R1B;gene-SKP1
gene-GNG10;gene-GNG11;gene-PLCB2;gene-PPP2R1B;gene-PRKCG;gene-TH
gene-GNG10;gene-GNG11;gene-HAT1;gene-HDAC2;gene-LOC787269;gene-TH
gene-CAT;gene-HDAC2;gene-HSPA8;gene-PIK3R3;gene-SOD1
gene-LOC101907965;gene-PSMC2;gene-PSMC6;gene-PSMD14;gene-SEM1
gene-GTF2H3;gene-MNAT1;gene-PCNA;gene-POLE3;gene-RFC3
gene-CDKN2A;gene-E2F3;gene-JAG2;gene-NOTCH1;gene-PIK3R3
gene-ACADM;gene-AOX1;gene-AUH;gene-HIBADH;gene-MUT
Bos_taurus_newGene_11459;Bos_taurus_newGene_11530;gene-ICOS;gene-LOC104968484
gene-HSP90AB1;gene-HSPA8;gene-KRT42;gene-PIK3R3;gene-PLCB2
gene-CCNA2;gene-CEBPE;gene-EIF4EBP1;gene-MPO;gene-PIK3R3
gene-CDKN2A;gene-E2F3;gene-PIK3R3;gene-RAD51;gene-RALB
gene-DTX1;gene-HDAC2;gene-JAG2;gene-NOTCH1;gene-RFNG
gene-ALOX5;gene-CYCS;gene-DSB;gene-HSPA8;gene-TLR2

gene-HSP90AB1;gene-ITGB3;gene-MGST1;gene-PIK3R3;gene-SUMO1
gene-E2F3;gene-JAG2;gene-NOTCH1;gene-PIK3R3;gene-WNT5A
gene-ABHD5;gene-LIPE;gene-MGLL;gene-PIK3R3;gene-PNPLA2
Bos_taurus_newGene_13402;gene-ACP1;gene-BAIAP2;gene-SORBS1;gene-ZNF774
gene-AGPAT5;gene-DGKG;gene-LPIN1;gene-MGLL;gene-PNPLA2
gene-BIRC5;gene-CYCS;gene-PIK3R3;gene-PMAIP1;gene-RALB
gene-BIRC5;gene-FRMD6;gene-NKD2;gene-PPP2R1B;gene-WNT5A
gene-ASNSD1;gene-CDKN2B;gene-LTBP1;gene-PPP2R1B;gene-SKP1
gene-CAMK1;gene-DAGLA;gene-LIPE;gene-PLCB2;gene-PRKCG
gene-AP3S1;gene-CD63;gene-GM2A;gene-LOC101904667;gene-MAN2B1
gene-CAMK1;gene-CDKN2A;gene-E2F3;gene-PIK3R3;gene-PRKCG
Bos_taurus_newGene_11530;gene-ARPC3;gene-LOC104968484;gene-PIK3R3;gene-PRKCG
Bos_taurus_newGene_5578;gene-HMGB1;gene-PCNA;gene-POLB;gene-POLE3
gene-DGKG;gene-EIF4EBP1;gene-PDGFD;gene-PIK3R3;gene-PRKCG
gene-GNG10;gene-GNG11;gene-HOMER3;gene-PLCB2;gene-PRKCG
gene-GNG10;gene-GNG11;gene-PIK3R3;gene-PLCB2;gene-PRKCG
gene-CDKN2A;gene-E2F3;gene-PIK3R3;gene-PRKCG
gene-CACNB1;gene-CACNB3;gene-CACNG4;gene-MYH7
gene-DGKG;gene-PIK3R3;gene-PLCB2;gene-PRKCG
gene-CDKN2A;gene-E2F3;gene-PDGFD;gene-PIK3R3
Bos_taurus_newGene_11530;gene-DSB;gene-LOC104968484;gene-TLR2
gene-CDKN2B;gene-CYCS;gene-E2F3;gene-PIK3R3
gene-E2F3;gene-HSP90AB1;gene-PDGFD;gene-PIK3R3
gene-GPI;gene-HK3;gene-PYGM;gene-TWSG1
gene-GNG10;gene-GNG11;gene-PDE8A;gene-PRKCG
gene-ADH5;gene-AOX1;gene-HSD17B11;gene-RDH5
gene-AGER;gene-CXCL8;gene-PIK3R3;gene-PLCB2
gene-ACADM;gene-CPT1B;gene-HSD17B12;gene-SCP2
gene-MMP15;gene-NACA;gene-PLCB2;gene-PRKCG
Bos_taurus_newGene_6916;gene-ITGB3;gene-LOC112445615;gene-PIK3R3
gene-BIRC5;gene-CYCS;gene-NRADD;gene-PMAIP1
gene-CAT;gene-EIF4E;gene-EIF4EBP1;gene-PIK3R3
gene-MCM6;gene-PCNA;gene-POLE3;gene-RFC3
gene-CXCL12;gene-PIK3R3;gene-PRKCG;gene-RAPGEF3
gene-HK3;gene-PDHB;gene-PIK3R3;gene-SLC2A1
gene-CXCL5;gene-IFI47;gene-IL15;gene-PIK3R3
gene-HSPA5;gene-PLCB2;gene-PRKCG;gene-TG
gene-CXCL8;gene-ISG15;gene-NLRX1;gene-PDZD3
Bos_taurus_newGene_11530;gene-ALOX5;gene-LOC104968484;gene-PIK3R3
gene-GNG10;gene-GNG11;gene-PRKCG;gene-TRAK2
gene-GTF2B;gene-GTF2H3;gene-MNAT1;gene-TAF13
gene-IFRD1;gene-LOC789503;gene-PLCB2;gene-PRKCG
gene-CXCL5;gene-CXCL8;gene-HSP90AB1;gene-LOC789503
gene-GNG10;gene-GNG11;gene-PIK3R3;gene-PLCB2
gene-BARD1;gene-LOC101907965;gene-RAD51;gene-SEM1
gene-ALOX15;gene-SLC40A1;gene-SLC7A11;gene-VDAC3
gene-GHSR;gene-PIK3R3;gene-PLCB2;gene-PRKCG
gene-CPT1B;gene-PIK3R3;gene-PYGM;gene-SLC2A1
gene-CDKN2A;gene-E2F3;gene-HDAC2;gene-PIK3R3
gene-CELA2A;gene-COL14A1;gene-COL7A1;gene-PGA5
gene-GPI;gene-HK3;gene-TMEM30A;gene-TWSG1

gene-CDKN2B;gene-E2F3;gene-PIK3R3;gene-WNT5A
gene-ADH5;gene-AOC3;gene-AOX1;gene-TH
gene-DARS;gene-FARSB;gene-NARS2
gene-CA2;gene-LOC101904667;gene-SLC4A1
gene-PIK3R3;gene-PLCB2;gene-PRKCG
gene-AOX1;gene-FAM210B;gene-NMNAT1
gene-LOC112445044;gene-NRADD;gene-PIK3R3
gene-ACOT7;gene-HSD17B12;gene-SCP2
gene-PLCB2;gene-PRKCG;gene-RAPGEF3
gene-GABARAPL1;gene-RPS27A;gene-USP15
gene-GABARAPL1;gene-IGBP1;gene-ZCCHC10
gene-ADH5;gene-MGC127133;gene-MGST1
gene-PDHB;gene-SDHD;gene-SUCLG2
gene-MGC127133;gene-MOCS2;gene-TH
gene-HDAC2;gene-PRKCG;gene-TH
gene-ACADM;gene-ADH5;gene-CPT1B
gene-PIK3R3;gene-PLCB2;gene-PRKCG
gene-BET1;gene-SEC22B;gene-STX3
gene-HK3;gene-PIK3R3;gene-PLCB2
gene-GPAA1;gene-PIGK;gene-PIGY
gene-C1R;gene-CXCL5;gene-CXCL8
gene-ADH5;gene-MGC127133;gene-MGST1
gene-LOC101904667;gene-SLC6A4;gene-STX3
gene-ACOT7;gene-HSD17B12;gene-THEM4
gene-EGLN3;gene-PIK3R3;gene-SLC2A1
gene-HAGHL;gene-ME3;gene-PDHB
gene-DPM3;gene-MGAT3;gene-STT3B
gene-CDKN2A;gene-CXCL8;gene-E2F3
gene-PLCB2;gene-PRKCG;gene-WNT5A
gene-LOC100337457;gene-LOC514257;gene-TCN1
gene-ALAS2;gene-BLVRB;gene-HMBS
gene-ACE;gene-AQP1;gene-PLCB2
gene-ADH5;gene-AOX1;gene-MGST1
gene-EIF4EBP1;gene-PIK3R3;gene-PRKCG
gene-ACP1;gene-BLVRB;gene-RFK
gene-PLCB2;gene-PRKCG;gene-SLC2A1
gene-MEIS1;gene-PIK3R3;gene-WNT5A
gene-PLCB2;gene-PPP2R1B;gene-PRKCG
gene-AQP1;gene-CA2
gene-RAD51;gene-TELO2
gene-AOX1;gene-CAT
gene-ACE;gene-LOC540321
gene-CAT;gene-MUT
gene-ARPC3;gene-PIK3R3
gene-ACP1;gene-AK1
gene-SLC40A1;gene-STEAP2
gene-HK3;gene-PIK3R3
gene-PLCB2;gene-PRKCG
gene-PSAT1;gene-SDSL
gene-MYLIP;gene-VDAC3
gene-CLEC6A;gene-PIK3R3

gene-PIK3R3;gene-PRKCG
gene-PIK3R3;gene-TH
gene-PCNA;gene-RFC3
gene-CPT1B;gene-SLC2A1
gene-AOC3;gene-DPYD
gene-AOX1;gene-PSAT1
gene-MIOX;gene-PLCB2
gene-C1R;gene-F2R
gene-PDE8A;gene-PLCB2
gene-PDSS2;gene-RBM48
gene-PIK3R3;gene-PRKCG
gene-MUT;gene-SUCLG2
gene-FBP1;gene-HK3
gene-DPYD;gene-VNN2
gene-ALOX15;gene-PLA2G1B
gene-CERS1;gene-SMPD3
gene-IL18
gene-ALOX5
gene-HPSE
gene-B3GAT1
gene-SCP2
gene-SDSL
gene-CA2
gene-PLA2G1B
gene-ARG1
gene-HSD17B12
gene-NCALD
gene-FRMD6
gene-SAG
gene-PLCB2
gene-B3GNT2
gene-MAN2B1
gene-HSPA5
gene-ADSS
gene-XRCC5
gene-HK3
gene-HK3
gene-MOCS2
gene-PIK3R3
gene-LOC529930
gene-RFNG
gene-AOC3
gene-PLCB2
gene-STT3B
gene-GGT1
gene-MIOX
gene-PLA2G1B
gene-B3GNT2
gene-TH
gene-WNT5A

KOs
K08731+K06627+K06621+K04685+K10031+K10030+K08738+K06620+K09592+K03914+K04545+K04
K09228+K09228+K09228+K09228+K09228+K06751+K08738+K06752+K03237+K07205+K09228+K09
K05863+K19722+K04520+K02135+K02127+K08738+K03237+K07374+K10881+K07374+K03934+K03
K06856+K10785+K10784+K10784+K10784+K06751+K06627+K06452+K08738+K06752+K06620+K06
K05863+K02135+K02127+K08738+K03237+K09490+K03283+K07374+K10881+K07374+K03934+K03
K16631+K02135+K02127+K03781+K22759+K08738+K03237+K09490+K07374+K10881+K10408+K07
K05863+K02135+K02127+K08738+K03237+K09490+K07374+K10881+K07374+K03934+K03937+K03
K03231+K14529+K03240+K05019+K03113+K03237+K03238+K03242+K03250+K03247+K03257+K03
K05863+K02135+K02127+K08738+K06067+K07374+K10881+K10408+K07374+K03934+K03937+K03
K05408+K04179+K10031+K05506+K10030+K05480+K05140+K05433+K05482+K04737+K05067+K05
K06856+K10785+K10784+K10784+K10784+K15621+K06627+K10051+K10030+K01327+K01353+K06
K02877+K02918+K02932+K02973+K02863+K02890+K02906+K02911+K02970+K02880+K02891+K02
K02539+K08731+K04396+K08738+K03237+K01353+K04737+K07611+K07374+K07374+K01353+K01
K10785+K10784+K10784+K10784+K05863+K06751+K06627+K21770+K06452+K06621+K04685+K06
K06856+K06248+K05462+K03259+K07205+K03914+K04545+K04546+K04079+K04737+K06585+K06
K10785+K10784+K10784+K10784+K18750+K06751+K05868+K21770+K06452+K02087+K08738+K04
K06856+K06751+K01330+K06752+K06513+K06493+K07374+K02155+K06856+K07374+K06751+K10
K06856+K10785+K10784+K10784+K10784+K05408+K10031+K05506+K10030+K06752+K05433+K05
K20899+K20899+K04394+K10030+K08341+K04079+K05482+K20899+K20897+K12653+K14216+K14
K10785+K10784+K10784+K10784+K06452+K08738+K03237+K03247+K03283+K10784+K14754+K14
K06751+K05408+K06621+K10031+K10030+K08738+K06620+K07205+K04545+K04546+K06493+K05
K05863+K10030+K08738+K06752+K03237+K05482+K04722+K04722+K05404+K14754+K12653+K14
K06751+K06627+K06248+K07205+K06067+K12159+K06585+K06493+K06525+K02155+K06751+K14
K05863+K06751+K06627+K05868+K21770+K06645+K02087+K06621+K04685+K10030+K06620+K07
K10785+K06531+K10784+K10784+K10784+K06547+K06751+K06752+K06486+K06713+K06585+K10
K10785+K10784+K10784+K10784+K01283+K05408+K06452+K10030+K05408+K05408+K10784+K05
K06856+K10785+K10784+K10784+K10784+K05863+K08794+K03914+K04150+K06856+K10784+K00
K10785+K10784+K10784+K10784+K12490+K05462+K03914+K06493+K10784+K02583+K05450+K02
K10785+K10784+K10784+K10784+K10030+K06575+K13822+K13822+K13823+K05482+K06543+K13
K18584+K06104+K06448+K16684+K07374+K07374+K05631+K04802+K03456+K06109+K21112+K07
K06751+K06627+K04179+K02087+K06621+K04685+K03124+K03143+K06067+K11252+K06751+K02
K05756+K05627+K10031+K03914+K06585+K06594+K06493+K06525+K00907+K05729+K05450+K02
K00121+K03781+K03841+K01810+K00844+K00029+K01847+K00162+K00033+K00831+K00237+K17
K06856+K10785+K10784+K10784+K10784+K06751+K06752+K01353+K06856+K10784+K01353+K01
K06627+K05868+K21770+K06645+K02087+K06621+K04685+K06620+K06067+K02542+K02603+K02
K06856+K10785+K10784+K10784+K10784+K06751+K06752+K01353+K06856+K10784+K01353+K01
K06856+K06479+K01353+K07980+K06856+K01353+K01353+K04722+K04722+K01353+K02649+K19
K05756+K04394+K10030+K08738+K10420+K20282+K04079+K05482+K04722+K04722+K03094+K04
K06856+K06448+K06452+K06752+K06263+K06575+K04737+K05067+K05073+K06493+K06473+K05
K08738+K06620+K03237+K03250+K14217+K14217+K14754+K14216+K14216+K14216+K14216+K02
K10785+K10784+K10784+K10784+K05462+K04545+K04546+K10784+K02583+K05450+K02649+K01
K12490+K03914+K04328+K04284+K07188+K05673+K05673+K05673+K05673+K05673+K05673+K02
K10785+K10784+K10784+K10784+K06751+K06752+K01353+K07980+K10784+K01353+K01353+K01
K05408+K04179+K10031+K05506+K10030+K04545+K04546+K05408+K05408+K05408+K05407+K02
K10785+K10784+K10784+K10784+K06751+K06752+K04079+K09490+K03283+K07980+K10784+K06
K10145+K06645+K06621+K06620+K05462+K06067+K06493+K02599+K17387+K02649+K19663+K23
K11340+K02135+K02127+K18178+K19523+K07188+K01054+K03934+K03937+K03943+K16816+K00
K10260+K10604+K10144+K07868+K02977+K03094+K10608+K10686+K10574+K06689+K20217+K10

K10785+K10784+K10784+K10784+K06751+K06752+K01353+K04077+K10784+K01353+K01353+K01[illegible]
K06856+K10785+K10784+K10784+K10784+K10031+K06752+K06713+K05433+K06856+K10784+K13[illegible]
K06856+K10785+K10784+K10784+K10784+K06751+K08738+K06752+K06856+K10784+K06751+K17[illegible]
K06856+K10785+K10784+K10784+K10784+K01330+K06752+K06513+K07604+K06856+K10784+K06[illegible]
K06856+K10785+K10784+K10784+K10784+K18584+K05627+K10030+K05482+K06856+K10784+K02[illegible]
K05863+K08738+K03124+K10881+K02649+K05858+K19663+K03061+K03064+K03030+K10881+K15[illegible]
K05408+K04179+K10031+K05506+K10030+K05482+K05408+K05408+K04722+K05408+K04722+K05[illegible]
K06856+K19007+K10030+K00901+K03914+K06856+K05450+K02649+K05858+K07835+K08014
K09864+K09877+K18245+K13855+K05673+K05673+K05673+K05673+K05673+K05673+K07299
K05868+K21770+K10145+K02087+K06621+K08738+K04722+K04722+K10131+K10144+K10808
K09502+K03237+K04079+K09490+K03283+K03094+K07151+K14014+K06689+K10575+K24349
K12490+K05756+K06751+K03283+K06751+K07903+K12484+K11247+K12196+K17918+K01115
K12861+K12816+K12863+K12827+K03283+K12622+K12877+K12877+K12883+K11094+K12848
K06856+K10785+K10784+K10784+K10784+K01330+K06752+K01327+K06856+K10784+K11252
K02087+K07374+K07374+K05450+K05858+K19663+K07374+K07374+K07374+K07374+K07375
K06627+K19523+K07205+K03841+K07188+K02649+K03456+K07903+K07877+K17532+K17532
K06856+K01476+K06448+K10030+K06856+K13963+K13963+K02649+K05858+K19663+K10159
K08731+K06627+K10030+K08738+K06620+K06255+K04802+K02649+K19663+K10159+K15041
K10031+K07520+K05462+K07527+K02649+K06820+K06821+K06821+K06842+K05766+K00444
K10802+K05863+K00460+K10802+K04079+K04722+K04722+K05864+K00688+K12196+K15041
K06751+K04179+K10030+K08738+K06620+K04545+K04546+K06751+K02649+K02977
K14563+K14566+K11128+K07562+K07936+K14570+K07179+K03539+K14574+K14553
K06856+K10785+K10784+K10784+K10784+K10031+K10030+K05480+K06856+K10784
K03259+K07205+K16185+K15728+K02649+K19663+K17532+K17532+K11137+K00444
K04862+K04864+K04869+K05462+K03283+K02583+K05450+K19663+K18018+K04403
K06856+K13822+K13822+K13823+K05482+K06856+K13823+K13823+K05858+K19663
K06248+K06585+K06493+K06525+K00907+K06275+K05450+K02649+K06269+K19663
K04268+K03914+K04284+K04150+K05175+K05052+K04288+K05240+K04268+K04590
K03259+K07205+K03841+K00844+K07188+K02649+K06269+K00688+K06086+K07188
K00460+K00461+K07422+K15717+K18592+K00807+K08726+K00079+K00509+K01047
K01939+K00939+K01490+K00893+K00760+K24242+K01587+K18437+K10807+K10808
K00157+K14365+K05140+K05433+K04737+K05067+K05073+K05066+K14365+K02649
K07232+K00682+K18592+K11142+K00799+K01581+K01469+K00033+K10807+K10808
K03781+K05868+K21770+K04685+K17847+K08341+K15010+K02649+K04288
K10785+K10784+K10784+K10784+K06452+K06752+K21635+K10784+K02599
K06856+K08738+K06752+K04077+K05482+K02155+K06856+K08063+K10159
K06856+K04862+K04864+K04869+K06585+K06493+K06525+K06856+K17751
K01476+K00286+K00286+K00286+K00286+K00286+K00831+K17989+K00615
K00460+K00461+K04520+K04545+K04546+K05858+K19663+K08014+K05037
K06067+K06493+K15143+K17751+K02599+K02649+K05858+K19663+K07299
K06497+K07964+K06255+K06493+K02649+K19663+K10159+K00444+K16848
K01476+K00613+K11142+K00286+K00286+K00286+K00286+K00286+K01581
K13806+K04545+K04546+K01054+K03934+K03937+K03943+K05858+K19663
K05408+K10030+K05408+K05408+K05408+K05404+K02649+K04403+K10159
K06856+K10785+K10784+K10784+K10784+K06752+K06856+K10784+K10786
K10802+K03237+K08341+K10802+K17606+K16185+K02649+K07920+K08337
K19007+K08744+K00901+K15728+K13516+K01047+K16860+K13511+K01115
K13800+K13809+K00893+K00207+K24242+K10807+K10808+K00761
K10785+K10784+K10784+K10784+K06452+K10784+K02649+K10159
K04268+K04710+K02649+K05858+K03456+K19663+K04288+K04268
K10785+K10784+K10784+K10784+K06452+K06752+K04079+K10784

K04862+K04864+K04869+K17751+K05858+K06269+K03456+K08014
K00121+K01837+K03841+K01810+K00844+K01837+K00162+K01810
K10030+K08738+K03237+K03934+K03937+K03943+K02649+K00237
K09592+K03259+K07205+K00844+K00162+K02649+K19663+K07299
K05647+K05673+K05673+K05673+K05673+K05673+K05673+K05670
K02135+K02127+K02155+K03934+K03937+K03943+K01507+K00237
K14443+K12592+K12581+K12586+K04077+K12622+K13126+K14443
K01283+K04862+K04864+K04869+K06585+K06493+K06525+K17751
K10785+K10784+K10784+K10784+K06452+K06713+K10784+K02649
K10785+K10784+K10784+K10784+K06752+K05482+K10784+K10159
K00308+K00803+K03781+K05940+K08726+K08764+K13354+K04565
K08341+K04545+K04546+K07188+K00907+K05858+K07188
K06621+K04685+K06620+K18437+K05858+K07843+K00444
K18245+K01346+K13855+K01047+K05858+K19663+K07884
K11340+K06621+K06620+K00799+K02649+K19663+K00444
K06248+K06263+K06255+K06585+K06493+K06525+K23379
K00249+K19523+K08762+K00029+K08763+K08764+K06086
K13800+K00207+K00760+K00799+K10789+K10807+K10808
K00643+K00276+K01837+K00613+K01837+K00831+K17989
K06627+K05868+K21770+K06645+K02087+K04079+K02649
K12327+K00907+K01047+K05858+K06269+K19663+K08449
K04862+K04864+K04869+K08794+K00907+K05858+K19663
K00803+K22387+K16795+K01047+K16860+K18575+K01115
K10030+K08738+K03283+K04077+K05482+K08517+K10159
K05863+K04268+K17751+K00907+K05858+K04268+K15041
K16513+K04545+K04546+K16513+K05858+K19663+K07843
K03213+K05858+K19663+K03094+K04511+K00444
K05673+K05673+K05673+K05673+K05673+K05673
K12877+K12877+K12883+K13126+K06269+K03456
K18245+K04150+K13855+K00907+K05858+K19663
K04862+K04864+K04869+K06585+K06493+K06525
K06856+K06512+K06856+K19831+K06512+K02649
K03914+K06263+K06493+K00907+K02649+K05858
K19523+K03841+K00162+K05858+K00688+K07299
K08731+K06621+K08738+K00799+K02649+K10131
K01619+K03841+K01810+K00033+K00615+K01810
K03259+K07205+K05464+K05450+K02649+K19663
K05868+K21770+K02087+K06269+K03456+K03094
K04545+K04546+K05858+K03456+K19663+K00501
K04545+K04546+K11303+K06067+K11252+K00501
K03781+K06067+K03283+K02649+K04565
K10881+K03061+K03064+K03030+K10881
K03143+K10842+K04802+K02326+K10756
K06621+K06620+K21635+K02599+K02649
K00249+K00157+K05607+K00020+K01847
K06554+K06856+K06713+K06856+K08063
K04079+K03283+K07604+K02649+K05858
K06627+K10051+K07205+K10789+K02649
K06621+K06620+K02649+K04482+K07835
K06058+K06067+K21635+K02599+K05948
K00461+K08738+K06752+K03283+K10159

K04079+K06493+K00799+K02649+K12160
K06620+K21635+K02599+K02649+K00444
K13699+K07188+K01054+K02649+K16816
K06531+K14394+K05627+K06086+K16848
K19007+K00901+K15728+K01054+K16816
K08731+K08738+K02649+K10131+K07835
K08731+K16822+K03213+K03456+K00444
K06825+K04685+K19559+K03456+K03094
K08794+K13806+K07188+K05858+K19663
K12399+K06497+K12383+K02155+K12311
K08794+K06621+K06620+K02649+K19663
K06856+K05756+K06856+K02649+K19663
K10802+K10802+K04802+K02330+K02326
K00901+K07205+K05450+K02649+K19663
K04545+K04546+K15010+K05858+K19663
K04545+K04546+K02649+K05858+K19663
K06621+K06620+K02649+K19663
K04862+K04864+K04869+K17751
K00901+K02649+K05858+K19663
K06621+K06620+K05450+K02649
K06856+K06752+K06856+K10159
K04685+K08738+K06620+K02649
K06620+K04079+K05450+K02649
K01810+K00844+K00688+K01810
K04545+K04546+K18437+K19663
K00121+K00157+K15734+K00061
K19722+K10030+K02649+K05858
K00249+K19523+K10251+K08764
K07995+K03626+K05858+K19663
K06512+K06493+K06512+K02649
K08731+K08738+K02583+K10131
K03781+K03259+K07205+K02649
K02542+K04802+K02326+K10756
K10031+K02649+K19663+K08014
K00844+K00162+K02649+K07299
K05506+K17072+K05433+K02649
K09490+K05858+K19663+K10809
K10030+K12159+K12653+K12653
K06856+K00461+K06856+K02649
K04545+K04546+K19663+K15374
K03124+K03143+K10842+K03127
K13855+K13908+K05858+K19663
K05506+K10030+K04079+K13908
K04545+K04546+K02649+K05858
K10683+K10881+K04482+K10881
K00460+K14685+K13869+K15041
K04284+K02649+K05858+K19663
K19523+K02649+K00688+K07299
K06621+K06620+K06067+K02649
K01346+K08133+K16628+K06002
K01810+K00844+K00972+K01810

K04685+K06620+K02649+K00444
K00121+K00276+K00157+K00501
K22503+K01890+K01893
K18245+K02155+K06573
K02649+K05858+K19663
K00157+K11415+K06210
K12464+K02583+K02649
K17360+K10251+K08764
K05858+K19663+K08014
K08341+K02977+K21343
K08341+K17606+K08337
K00121+K00079+K00799
K00162+K00237+K01900
K00079+K03635+K00501
K06067+K19663+K00501
K00249+K00121+K19523
K02649+K05858+K19663
K08504+K08517+K08486
K00844+K02649+K05858
K05289+K05290+K11001
K01330+K05506+K10030
K00121+K00079+K00799
K02155+K05037+K08486
K17360+K10251+K16339
K09592+K02649+K07299
K01069+K00029+K00162
K09659+K00737+K07151
K06621+K10030+K06620
K05858+K19663+K00444
K14611+K14611+K14615
K00643+K05901+K01749
K01283+K09864+K05858
K00121+K00157+K00799
K07205+K02649+K19663
K14394+K05901+K00861
K05858+K19663+K07299
K15613+K02649+K00444
K05858+K03456+K19663
K09864+K18245
K04482+K11137
K00157+K03781
K01283+K01329
K03781+K01847
K05756+K02649
K14394+K00939
K14685+K14738
K00844+K02649
K05858+K19663
K00831+K17989
K10637+K15041
K17514+K02649

K02649+K19663
K02649+K00501
K04802+K10756
K19523+K07299
K00276+K00207
K00157+K00831
K00469+K05858
K01330+K03914
K18437+K05858
K12505+K08658
K02649+K19663
K01847+K01900
K03841+K00844
K00207+K08069
K00460+K01047
K04710+K12352
K05482
K00461
K07964
K00735
K08764
K17989
K18245
K01047
K01476
K10251
K19695
K16822
K19627
K05858
K00741
K12311
K09490
K01939
K10885
K00844
K00844
K03635
K02649
K10604
K05948
K00276
K05858
K07151
K18592
K00469
K01047
K00741
K00501
K00444

546+K06067+K04079+K05433+K04737+K05067+K21635+K05066+K00799+K02599+K02649+K0
228+K06493+K06731+K06731+K09228+K06751+K14216+K14216+K14216+K14216+K02649+K0
937+K03943+K02649+K05858+K05864+K03061+K03064+K03030+K00237+K10881+K07374+K0
067+K12159+K10881+K06856+K10784+K06751+K14216+K14216+K14216+K14216+K02649+K0
937+K03943+K02599+K02649+K03061+K03064+K03030+K00237+K10881+K04565+K07374+K0
374+K03934+K03937+K03943+K14314+K03061+K03064+K03030+K00237+K10881+K04565+K0
943+K03061+K03064+K03030+K02977+K00237+K10881+K04557+K00501+K07374+K07374+K0
259+K07205+K13176+K12877+K12877+K12883+K07562+K14314+K13126+K07936+K03539+K1
943+K05858+K03061+K03064+K03030+K00237+K10881+K04565+K07374+K07374+K07374+K0
073+K05408+K05066+K05408+K04722+K05408+K22632+K04722+K02583+K05407+K05154+K0
067+K15624+K06856+K10784+K01353+K01353+K01353+K15613+K10789+K02583+K15626+K1
891+K02929+K02930+K02932+K02934+K02937+K02940+K02975+K02977+K02984+K0289
353+K04722+K04722+K01353+K02649+K10131+K07374+K07374+K07374+K0737
752+K06620+K05433+K10784+K06751+K02649+K02330+K07936+K07299+K15041
493+K06525+K06856+K16837+K02583+K05450+K02649+K03456+K16339+K1015
545+K04546+K06731+K06731+K10784+K06751+K02649+K19663+K1015
789+K08763+K08517+K10159+K07374+K07374+K07374+K07374+K0737
482+K05408+K02155+K06856+K05408+K10784+K05408+K1015
216+K14216+K14216+K12653+K05858+K12804+K04403+K15041
216+K14216+K14216+K14216+K02649+K10144+K1015
408+K05408+K05408+K06751+K02649+K05858+K1966
216+K14216+K14216+K14216+K12653+K02649+K1504
754+K02599+K02649+K03456+K05948+K0044
205+K06751+K02649+K05864+K06269+K15041
784+K06751+K06496+K06548+K1635
408+K02649+K05858+K03456+K1015
907+K05450+K05858+K19663+K15041
649+K05858+K19663+K07835+K0801
823+K10784+K13823+K06496+K1015
374+K07374+K07374+K0737
649+K10131+K02330+K15041
649+K06269+K05766+K1684
989+K01900+K00615+K0181
353+K01353+K06751+K1080
608+K04802+K03094+K0939
353+K01353+K06751
663+K07990+K0798
403+K10159+K1792
066+K06856+K0647
649+K03456+K1504
047+K19663+K0783
649+K08014+K0459
353+K06751
649+K0585
751+K0806
581+K1738
237+K1510
575+K1058

353+K06751
073
751
496
649
041
407

5858+K10131+K19663+K04482+K07835+K07299+K0044[illegible]
6269+K04403+K10159+K09228+K09228+K09228+K0922[illegible]
7374+K07374+K07375+K15041+K0044[illegible]
3061+K03064+K03030+K10881+K1015[illegible]
7374+K07374+K07375+K1504[illegible]
7374+K07374+K07374+K0737[illegible]
7374+K07375+K10575+K1504[illegible]
2160+K13176+K1428[illegible]
7375+K1504[illegible]
514[illegible]
562[illegible]
