# Supplementary material for: Integrative Analysis of Blood Transcriptomics and Metabolomics Reveals Molecular Regulation of Backfat Thickness in Qinchuan Cattle
Source: Animals (Basel). 2023 Mar 15;13(6):1060. doi: 10.3390/ani13061060 (PMC10044415; doi:10.3390/ani13061060)
Supplement: Supplementary file 1 [file animals-13-01060-s001.zip › Supplementary File S8 Supplementary Table S6.pdf]

Table S6. Identified full metabolites.

#ID	name	H1	H2	H3	H4	L1	L2	L3
neg_1003	1H-1,2,4-T	914.921	572.7147	1043.132	688.6959	934.0937	734.8969	714.4156
neg_1005	Methyl 5-(	156.1932	225.8496	145.9965	219.6347	182.5723	278.3911	180.4958
neg_1012	Fagomine	139.4255	161.4321	116.0614	83.20446	146.5616	106.4735	82.94956
neg_1018	2,4-Dihydı	102.2246	132.3797	134.3318	99.93165	134.834	160.4813	113.5164
neg_1019	Fructosyl v	838.8038	1201.668	817.9929	715.1966	2130.483	949.7522	731.311
neg_1020	N-(2-Hydı	247.9466	429.2127	377.6872	354.2854	296.1479	476.6513	387.5232
neg_1021	Aspartyl-G	65.7032	114.0935	119.5879	102.7296	93.89532	128.1266	102.5398
neg_1028	UDP-N-ac	277.1601	282.0425	119.2347	274.2102	278.7503	298.8153	230.642
neg_1031	L-Cystine	656.9374	629.4978	408.0261	697.952	544.8765	662.5586	587.1188
neg_1033	UDP-alpha	292.247	304.0495	219.587	310.3264	249.3444	325.2321	302.2479
neg_1042	5-Fluorod	522.5826	496.0626	544.6362	545.7515	498.2891	570.7675	558.5878
neg_1045	Oxoglutari	338.6475	237.8364	444.2513	271.3357	674.8101	328.2662	288.4391
neg_1047	Sulfate	12592.97	11114.87	19645.88	13144.45	14687.86	14737.8	14857.49
neg_1049	L-Aspartat	23.63313	67.26138	268.1329	82.29709	58.0408	45.98663	169.7194
neg_1053	Semidehy	121.0508	101.2496	269.8946	212.5967	328.5443	241.7201	184.4267
neg_1058	Ascorbic a	900.2852	1661.015	2917.729	1329.834	1151.54	1532.537	1574.482
neg_1064	DL-Isocitri	158.4369	195.6778	261.247	193.6994	220.6644	183.3411	218.9905
neg_1065	(R)-2-(Pho	116.9992	145.0443	117.666	143.9076	102.6922	117.0739	137.7085
neg_1069	UDP-L-rha	588.1584	689.4325	398.9523	699.6587	470.6673	615.4547	659.8335
neg_1071	XTP	65.03529	55.85786	1.72E-06	70.66845	12.33361	65.9507	59.66146
neg_1079	6-Mercapt	3094.845	3580.303	3010.993	3430.393	2764.452	3252.906	3408.265
neg_1083	D-Ribose 1	299.8587	370.3567	194.2537	361.8646	218.1003	326.9318	348.3374
neg_1087	m7G(5')pp	1607.412	2032.515	977.6933	1957.967	831.647	1722.875	1716.612
neg_1089	dTDP-3-a	49.24851	45.744	7.206273	50.89009	12.97713	43.84476	49.67231
neg_1093	D-Arabinc	468.4891	604.152	645.135	612.8718	530.1191	520.1647	551.0889
neg_1094	3-Chlorob	175.5793	241.4191	270.356	223.7003	177.1081	213.4792	244.2984
neg_1095	Aconitic ac	4520.136	5820.704	5929.078	5250.627	4103.412	5447.014	5329.876
neg_1104	Cyanuric a	189.4552	238.2971	330.4619	364.5328	445.657	369.6874	247.8357
neg_1106	2-(3,4-Dic	430.4817	509.7054	419.5593	506.4587	399.2983	519.1544	469.5956
neg_1108	5-Fluorou	1477.042	1888.86	1517.538	1650.26	1199.167	1608.051	1594.505
neg_1110	dTDP-D-g	307.334	415.4715	315.0973	369.9988	277.9145	366.79	382.7239
neg_1111	Calicheam	4318.475	5811.782	3889.707	5376.733	2630.303	4628.054	4898.021
neg_1120	A,b-Dihyd	107.3963	146.8934	213.7877	140.0666	158.7867	154.9193	128.0407
neg_1121	5-(Ethylthi	130.7008	99.35937	172.543	129.6539	145.5465	107.4345	92.46348
neg_1122	2-n-Propy	39.09162	40.66986	42.71956	41.91651	86.85238	51.93444	38.68147
neg_1126	8-Hydroxy	56.98063	99.07806	169.9716	121.6022	67.21843	177.0572	106.4042
neg_1129	FMN	212.1518	199.0116	175.2313	198.5882	319.5369	255.3104	223.9942
neg_1130	Malvidin 3	241.9285	316.1298	255.3499	279.9098	337.8588	347.3858	271.274
neg_1131	Apigenin 7	614.1596	729.1647	437.837	689.6379	766.4518	715.5213	661.5338
neg_1133	Propanoyl	146.5788	197.6827	43.33327	214.1092	192.6137	153.5187	164.917
neg_1134	3-Oxoprop	95.12754	129.9013	32.79932	126.4121	87.68868	100.1256	115.5936
neg_1135	But-2-enc	662.679	849.054	818.1516	780.0135	622.8835	836.6067	780.4387
neg_1141	L-Norleuc	619.7439	899.0797	946.8657	767.9763	1595.092	723.9636	771.34
neg_1142	Deoxyribo	83.77306	101.3659	110.3975	174.193	108.5914	120.0622	102.3366
neg_1145	3-Hydroxy	52.93094	63.94876	68.26365	76.24752	67.74035	55.12076	55.18655
neg_1147	Hypoxanth	359.6672	364.4059	317.3315	1056.587	1364.438	985.7022	418.9934
neg_1148	3D-3,5/4-T	24.84743	24.36469	22.69554	37.61427	49.25817	39.3522	22.58462
neg_1154	Pantothen	495.1771	647.7491	759.4109	434.162	609.9874	554.6904	548.5548

neg_1156	1-[Ethyl-(6	494.4176	658.0315	677.1443	493.555	599.7504	561.3106	619.2706
neg_1157	O-Succiny	599.0556	718.7561	952.66	1104.516	1019.509	908.9753	857.2813
neg_1162	N-Acetyl-	102.5141	129.7287	105.3963	97.44319	231.1366	128.1047	94.98228
neg_1164	5-Hydroxy	471.82	917.6168	1091.706	599.863	587.253	800.4444	650.9426
neg_1165	tubercidin	2295.957	900.5203	1567.623	1356.292	2079.952	1582.034	2442.697
neg_1166	Articaine	218.5758	261.0128	283.5122	253.1741	293.9908	199.1189	253.2289
neg_1168	Deoxyguai	209.3558	291.9353	376.8405	249.9005	308.5518	230.2307	353.256
neg_1170	Quinoceto	185.441	258.0677	228.8127	272.0086	350.165	242.6281	225.7048
neg_1172	N-(1-Deox	142.9947	222.4094	76.27066	134.2545	552.8187	228.2196	133.478
neg_1173	Neosaxitox	235.6586	357.1837	233.8276	332.6599	543.1367	285.4533	310.1377
neg_1174	Glutathion	135.9132	201.2335	191.8069	239.1926	355.9442	192.2134	199.9667
neg_1176	3',5'-Cyclic	327.1039	325.3188	335.8279	406.6203	214.5852	285.859	347.4811
neg_1177	S-(2-Chlor	259.3805	262.0181	257.8824	309.9672	351.1234	311.2139	301.8146
neg_1178	para-hydr	491.7607	696.7672	569.439	757.9487	558.4692	609.5994	587.9152
neg_1179	Aloesone	7344.58	7694.921	6261.01	7242.875	14452.16	8318.489	7888.262
neg_1183	Methylsyri	33.88619	70.9848	48.65994	77.32003	155.8065	66.56421	45.40187
neg_1184	6-Thiogua	158.9156	196.1983	143.2723	212.5992	147.4162	190.7329	158.4664
neg_1186	Phosphog	94.42625	104.6631	75.18799	103.1176	149.7937	99.47139	98.17949
neg_1188	(7R)-7-(5-	247.5942	263.9696	209.8072	319.1554	352.9245	281.9152	273.7387
neg_1189	ent-Copal	34.08004	75.92707	31.20932	41.29993	243.9111	70.10042	34.64794
neg_1190	Bis-gamm	130.2075	161.8199	80.14822	167.656	162.0783	165.6156	165.7372
neg_1191	2-(alpha-H	498.8133	574.0329	407.2842	572.8338	509.2299	526.8388	485.4337
neg_1192	dTDP-2,6-	50.28535	66.73066	27.34042	75.0995	68.61175	71.89979	61.58132
neg_1198	Biochanin	102.345	174.1615	186.0493	127.7089	147.3213	154.6885	157.321
neg_1200	ADP-D-gly	68.82097	83.20065	23.80403	75.72553	51.43101	80.91126	63.64046
neg_1201	alpha-D-S	113.5551	131.4495	51.82881	140.6639	125.2416	135.1648	111.8392
neg_1204	Deamino-	65.39553	69.83876	17.10365	73.1738	56.33508	78.52142	76.38809
neg_1206	2'-Deoxyir	146.9343	139.1565	42.51717	143.2217	159.0826	179.7071	147.9316
neg_1208	dGMP	85.42551	86.01685	36.60248	66.22292	71.08796	70.81324	57.58835
neg_1212	dTDP-acai	36.26674	40.76114	4.343635	33.44349	53.16637	43.17483	40.28267
neg_1216	(S)-Lactate	655.7603	785.0165	1109.736	2050.336	3248.856	1752.086	986.1655
neg_1219	L-Sorbose	432.4969	498.1784	757.3224	2470.867	4401.938	1909.922	725.9798
neg_1220	Enprofyllin	1893.868	2248.696	2318.914	2201.449	2012.757	2019.511	2069.528
neg_1223	(R)-3-Hyd	518.8194	433.4596	635.2362	240.2714	137.5689	226.6005	381.4856
neg_1224	(S)-2-Hyd	103.4755	97.68379	108.3743	23.12716	0.393691	25.72788	84.24649
neg_1228	2-Hydroxy	256.3773	291.0593	292.157	618.2657	618.1923	506.9839	334.5491
neg_1230	ITP	282.356	259.248	152.9106	293.8317	177.3895	296.4564	323.0426
neg_1234	2-Thiourac	31.42987	38.21794	26.74136	82.29865	81.18894	68.42214	41.66263
neg_1247	3-Dehydro	56.2817	187.7601	117.5205	108.3582	95.08726	169.2646	151.1109
neg_1250	4-Methyle	97.54578	94.62523	34.90515	283.9285	299.8821	309.5472	96.47281
neg_1251	Parapyruva	71.44939	60.37951	1.72E-06	205.6133	210.9743	232.3442	50.11362
neg_1253	Norsolorin	66.10574	66.25783	26.38298	61.73464	99.46791	75.60145	63.27564
neg_1254	N-Methyl-	73.28009	112.1159	64.45388	89.83787	67.15797	99.8071	95.01284
neg_1256	3-Hydroxy	33.44255	64.88752	47.71423	68.39822	89.04795	77.96959	91.15137
neg_1271	L-Valine, N	590.9812	902.3153	553.688	587.7515	428.3116	683.7461	616.2571
neg_1272	N-Lactoyll	278.1246	382.3572	229.6506	378.2946	566.6491	365.8146	289.4493
neg_1278	Galactitol	82.75888	94.93244	151.2946	91.52617	107.3295	97.64324	98.08548
neg_1282	L-Phenylal	4489.264	4995.311	5088.323	3459.524	4320.163	4513.681	5159.865
neg_1283	3-Methoxy	539.0048	589.4344	390.0891	308.6305	377.6843	542.8963	606.4813
neg_1285	trans-Cinn	978.7553	828.5197	789.5195	560.6249	761.5388	851.9053	819.0003
neg_1287	Mannitol	62.89739	76.36797	110.0227	73.13929	87.42398	73.05199	82.57927

neg_1291	Guanidino	1009.951	299.4512	547.1651	569.0195	681.2307	737.8795	1190.048
neg_1298	2-chloro-3	361.8609	464.5464	331.8659	249.981	573.9598	547.1993	1398.869
neg_1303	Creatine g	53.99987	145.3678	84.57873	83.94001	58.91076	64.11926	128.622
neg_1305	5-(3'-Carb	33.65945	81.84387	193.25	392.435	60.83515	78.72731	114.3634
neg_1307	Arabinopy	20.34352	17.66809	1.72E-06	15.43147	6.387334	21.37285	15.03601
neg_1308	Zidovudin	107.7139	175.4378	161.2778	125.6779	121.2348	120.3724	183.8688
neg_1311	3-Hydroxy	350.4119	282.2545	101.1948	261.647	434.5412	325.9986	262.4567
neg_1313	gamma-L-	160.7727	185.951	99.54928	165.6942	121.2808	103.83	147.1147
neg_1314	(2S,3R)-3-	1247.494	1431.198	1543.484	1358.96	1570.181	1455.901	1294.948
neg_1320	N-Methac	365.9933	709.9423	1548.004	1298.697	348.913	639.0757	1671.566
neg_1321	4-ethylam	586.9439	1002.607	307.4214	732.1647	598.2039	739.5574	588.7369
neg_1324	D-Glucosa	528.7716	580.8618	641.6806	474.6539	467.4556	511.0055	566.2724
neg_1325	trans-Cinn	734.4832	837.6303	689.8774	622.897	606.9157	754.8025	783.9953
neg_1327	Desmethyl	61.72366	47.3568	20.08858	44.93499	25.71106	48.78559	41.14288
neg_1328	3-Methoxy	452.6646	480.0068	317.3487	406.313	313.9866	430.5146	441.8325
neg_1331	9,10-Anth	164.7206	182.212	167.6656	118.6164	119.0177	144.9312	167.1962
neg_1339	N,N''-Sulfo	181.1409	166.8691	126.1013	222.1372	126.5582	117.7355	95.18511
neg_1346	2-O-a-L-I	651.3652	10.21039	10.9326	32.7928	265.3856	120.5936	89.9539
neg_1354	Hydroxyat	375.6244	601.4124	256.8302	537.4566	467.1607	444.8186	385.3009
neg_1362	Lysylglutar	415.9441	321.8741	423.593	426.6134	319.7037	1017.324	483.2813
neg_1369	Methylmal	42.71481	61.5071	81.14247	62.13511	63.30264	50.45914	54.73623
neg_1392	Nona-4,6-	19.37266	19.89134	133.8673	56.75869	1.72E-06	29.36229	53.84682
neg_1396	PIP(20:2(1	18.30088	264.7985	203.6978	231.5923	258.6284	168.7175	346.9397
neg_1406	Arachidon	51.03326	518.268	1064.759	562.9877	31.8127	223.3416	496.9066
neg_1418	delta9-Tet	27.7318	723.5826	1459.554	810.4764	14.36321	337.0008	619.1686
neg_1421	Dextrorpha	9.176601	345.6805	1534.576	577.7412	6.154729	44.78648	246.9077
neg_1435	Zucchini fa	116.8723	814.0306	1595.559	831.7734	165.9869	395.7868	761.59
neg_1447	Olsalazine	6404.413	7843.825	12783.94	7362.577	8814.232	6889.052	8384.353
neg_1462	MG(a-21:0	515.6942	731.3989	619.714	820.7799	641.801	719.9406	828.2483
neg_1467	uric acid ci	6164.53	8054.928	12502.43	7462.524	8973.209	6777.099	8247.887
neg_1476	Cerivastati	18.62035	42.53786	233.6312	88.55787	21.0245	73.36195	150.4172
neg_148	Methylphc	2971.447	3384.692	4533.635	3205.315	3650.123	3431.576	3507.692
neg_1481	PIP(20:0/1	921.2571	1269.396	1477.168	1118.632	1669.817	1047.092	1172.905
neg_1484	Tetracosap	77.87194	82.51368	135.9034	113.7369	208.3763	176.4059	134.3553
neg_1488	Tetracosar	2445.794	3290.842	2570.661	3629.149	2952.649	3943.964	3100.549
neg_1507	Niveusin C	563.412	628.7375	1264.872	556.6691	868.2472	594.3702	565.3216
neg_1515	5S-HETE-c	2073.067	2041.441	1643.921	1196.112	2793.042	3478.521	2228.043
neg_1522	Colistin A	318.4119	2353.786	163.7335	485.2871	122.2572	334.9884	79.77158
neg_1529	Phenylalar	50.57875	18.93638	34.11484	1.162328	43.66108	128.6988	31.16666
neg_1556	Propylene	359.9395	200.1388	534.2377	295.9337	1853.327	1680.692	768.0436
neg_1560	3,4-Dimet	93.32671	17.88074	33.30934	33.49376	53.00333	101.1449	67.37941
neg_1569	Ginkgolic A	33.65385	9.410485	44.228	27.29808	6.285557	31.03097	29.62029
neg_1570	13,14-Dihy	14333.26	14452.35	5403.353	2783.329	9036.559	9739.97	12433.41
neg_1573	Diguanosii	5620.586	6892.373	10826.62	6463.193	8162.564	6192.909	7245.723
neg_1575	Zipeprol	1260.016	1279.844	282.0905	181.3832	811.0218	804.1593	1173.413
neg_1582	10-Oxo-1	61.92622	41.37415	26.86934	80.05506	42.04791	90.75646	40.40797
neg_1585	N-Palmito	66.06595	61.17158	107.484	79.3587	103.7777	59.33883	98.69475
neg_1601	2,22-Dide	1683.947	211.6583	5593.546	1103.323	440.3882	5167.203	3359.156
neg_1610	18-hydrox	389.8074	311.2853	132.8936	365.2677	299.6588	314.6961	310.5641
neg_1611	N-Undecy	6916.336	7334.862	15093.85	6986.834	11539.27	7119.51	7338.936
neg_1616	MG(0:0/22	635.2376	864.199	719.6757	978.0365	838.2257	1101.739	824.1426

neg_1625	alpha-Terp	90.10439	54.99703	95.83567	74.65917	31.79901	78.87366	68.76448
neg_1628	Furostanol	1325.445	1687.111	3213.089	1613.515	2183.515	1764.897	2373.773
neg_1637	L-Arginine	253.6208	272.8503	398.7687	244.3615	367.3507	269.9642	271.5838
neg_1638	DG(13:0/2(	615.6681	356.2703	248.3325	408.9069	260.9228	319.7018	326.4624
neg_1639	Furfural	344.5464	393.6372	636.666	395.8072	464.7478	392.5953	433.9756
neg_1643	4-Carboxy	199.4914	234.6149	367.0192	222.4288	271.7703	212.0553	244.6217
neg_1644	sn-Glycerc	41166.3	49115.12	77511.72	45523.82	57000.1	44176.32	52145.32
neg_1645	1-(3-Hydr	4049.31	4763.901	7465.575	4422.686	5420.188	4340.206	4995.637
neg_1646	thiol-male	38023.36	45091.92	71512.44	41131.74	52855.8	40283.57	47358.39
neg_1651	Adenosine	6129.244	7493.77	11571.92	6810.588	9011.05	6715.378	7947.134
neg_1660	Chelidonic	22305.23	26361.15	42018.4	24450.36	30977.77	23948.59	27970.96
neg_1661	D-Erythros	60610.87	71926.85	113808.8	66509.13	83865.85	64645.5	76266.12
neg_1662	Daucic acid	45042.21	53532.57	85230.78	49307.54	62561.34	48087.31	56982.93
neg_1663	dehydroas	11888.23	14197.64	22435.94	13040.39	16556.79	12660.79	15046.02
neg_1665	Phosphog	2480.404	2877.29	4637.641	2679.282	3494.564	2640.075	3141.841
neg_1666	(2-Carbam	2518.952	3023.919	4571.918	2734.896	3453.203	2760.472	3211.427
neg_1668	D-glycero	8836.298	10391.86	16607.95	9565.333	12327.36	9460.313	11136.26
neg_1671	6-Methyltl	2918.393	3611.493	5784.191	3323.682	4350.575	3375.119	3902.027
neg_1672	ZANOTER(	136.5253	382.3399	762.9908	435.3927	433.9051	328.1468	553.3132
neg_1678	Vignatic ac	900.1444	2010.573	311.1146	1257.582	6466.28	42.90083	4052.701
neg_1683	Cer(d18:2(	362.1463	779.6402	1629.713	854.6499	820.3765	615.0331	1001.577
neg_1685	TG(22:1(13	47.32519	139.6474	289.2895	135.6185	64.08013	210.8344	229.1357
neg_1691	2,3-Dihydı	65.62851	67.89931	131.4681	72.6971	81.53468	79.14071	89.87688
neg_1694	2-Hydroxy	70.71019	108.1282	156.5807	99.78486	113.6209	85.08779	101.7907
neg_1697	4-Hydroxy	5767.608	6816.938	10836.42	6325.929	7990.613	6235.522	7306.945
neg_1698	cis-2-Metl	50521.24	59753.55	94425.42	55272.14	69519.58	54003.99	63472.22
neg_1699	Mercaptos	9430.52	11246.27	17838.8	10307.82	13050.37	10053.3	11888.58
neg_1700	1,5-Isoqui	1616.723	1975.602	3162.892	1782.574	2320.539	1828.967	2160.601
neg_1701	5-Hydroxy	13272.71	15821.01	25066.15	14683.86	18161.95	14235.66	16801.2
neg_1702	D-Glycera	14318.42	17006.22	27165.51	15822.52	19758.41	15366.68	18281.08
neg_1703	Methazola	34783.78	41218.35	65199.53	37961.69	48482.99	37053.58	43505.42
neg_1704	Bergaptol	18267.06	21706.28	34376.03	19977.09	25422.43	19690.53	23069.69
neg_1705	4-Ketoniri	24957.03	30862.96	49342.56	28586.35	35506.27	29168.89	33539.47
neg_1711	1,7-Dipho	1212.238	1398.796	2227.283	1285.142	1753.475	1295.416	1500.226
neg_1715	Cysteic aci	2599.325	3161.866	5093.275	2937.57	3722.364	2789.027	3473.827
neg_1718	IDP	3041.486	3652.746	5869.344	3280.959	4304.664	3250.486	3872.046
neg_1719	Dabigatrar	109.4589	203.1345	554.9294	217.3767	218.5525	179.3302	347.2738
neg_1730	N-[2-(Dim	599.5367	721.2768	822.868	1688.765	796.3803	522.5498	1338.786
neg_1746	(1S,4Ar,5R	127.818	31.09184	81.59914	122.8065	23.35597	47.00273	76.26548
neg_1747	PE-NMe(1	2950.019	2326.558	5503.8	2736.213	2480.196	2255.294	2042.71
neg_1759	Tuberoside	1062.055	1302.381	1464.515	1147.992	1446.835	1528.463	1720.644
neg_1780	7a,12a-Dil	195.5332	207.979	310.1037	245.8503	292.6701	284.3373	263.8653
neg_1783	1-Cyclohe	7068.502	8413.848	15088.33	6653.975	11070	7471.867	7225.022
neg_1789	1-O-Isope	1489.526	1735.957	1515.294	2144.969	1808.944	2263.572	1541.688
neg_1791	Imidazotet	134.159	168.6692	235.7739	143.6203	181.6249	150.1573	161.8187
neg_1794	DG(14:0/P	71.40939	110.4415	232.036	71.62027	124.3816	88.72465	81.04999
neg_1799	Tsugaric a	170.8228	79.40382	52.98168	132.6435	170.9961	182.1418	128.5301
neg_1801	Barbituric	83.57563	91.93871	151.6173	87.4463	113.7356	79.1804	95.36891
neg_1808	CL(8:0/8:0/	493.8336	608.1214	946.4106	561.6015	803.4641	511.6625	633.2371
neg_1812	Vitamin D3	35.007	6.082422	7.78131	4.558326	21.17555	24.49946	20.88083
neg_1817	TG(8:0/8:0	2139.456	4072.43	2888.008	4644.54	5366.289	8191.842	4708.908

neg_1818	2-Hydroxy	785.4389	957.8887	1437.347	847.2131	1058.183	897.8886	978.495
neg_1821	Butylparab	235.8423	304.6457	454.6927	292.1974	344.5277	264.7291	312.1018
neg_1829	Hydroxyisc	939.5872	840.864	1518.168	667.8038	1026.87	697.9335	687.1802
neg_1835	PIP(20:1(1	6653.904	6732.941	12624.94	6200.275	8516.002	6798.265	7161.818
neg_1845	DG(13:0/2	575.6519	505.9425	244.1622	499.4801	719.3038	595.8003	410.2672
neg_1860	CDP-DG(1	1441.284	1675.826	2254.719	1259.609	1799.768	987.198	1378.789
neg_1866	2-Dodecyl	25931.79	26868	60247.77	26336.03	42996.8	27340.15	26671.89
neg_1875	CDP-DG(F	2898.053	3217.502	5716.707	2854.47	3662.833	2857.765	3300.144
neg_1887	Thiazole	16892	20043.72	31084.41	18769.11	22102.99	18502.64	20998.87
neg_1888	(1E)-4-Ox	10957.52	13039.22	20752.91	12093.4	15126.78	11649.97	13810.73
neg_1889	7-Hydroxy	19527.08	23411.89	37138.54	21490.67	27170.37	20850.08	24707.81
neg_1904	5,7-Dihydı	16501.79	19520.36	30888.58	18174.28	22703.8	17966.19	20907.04
neg_1909	PE(16:0/18	2106.319	2105.41	3362.808	1443.936	2295.447	1977.718	1823.929
neg_1913	Microcysti	12557.44	12700.22	18428.2	11537.25	15353.69	11123.48	12719.45
neg_1970	Phosphon	1516.26	1693.272	2695.876	1562.841	2025.627	1557.016	1760.269
neg_1973	5-(Methox	772.2779	954.8037	1499.927	849.8444	1163.891	849.6251	1026.798
neg_2012	LysoPI(18:2	162.6166	177.444	285.236	238.3235	230.6529	175.6441	217.7753
neg_2021	PG(18:1(11	16529.23	19100.25	30292.21	17100.71	23206.19	15123.31	18047.55
neg_2044	all-trans-H	2928.411	4472.319	4069.334	3256.174	4565.175	3810.459	3321.461
neg_2045	H-Phe-D-	4708.64	7049.126	8174.501	7583.287	10469.79	2976.119	8898.686
neg_2049	PI(16:0/22	90120.54	93668.73	170083	92841.72	112227.7	98827.8	117161.8
neg_2059	[5-(Amino	4389.795	5278.366	8223.545	4816.39	5951.542	4935.655	5360.419
neg_2064	1-Oleoyl L	138.5499	206.6709	253.9761	207.3214	124.2238	150.5174	138.9803
neg_2067	LysoPE(18:	80.60908	128.4407	147.8641	139.6961	83.96665	78.62338	143.9812
neg_2068	LysoPI(16:(	868.4941	1171.155	1444.229	1453.479	1160.131	977.8123	981.9941
neg_2127	Moracin P	241.77	1.669373	12.93116	23.50241	103.5796	51.52765	37.3598
neg_2128	(5R,6S)-3-	135.4939	6.260903	1.72E-06	1.291093	61.77782	22.02807	23.37273
neg_2130	1,7-Phena	384.5596	7.147732	4.714276	7.747925	93.46479	21.86584	18.57896
neg_2133	1-hexen-3	39.89495	54.13983	41.40232	43.88554	22.87334	39.27934	49.65404
neg_2135	N-Formyl-	76.41763	100.7884	71.68889	78.18415	40.49444	78.45042	80.3866
neg_2137	2,8-Quino	366.3768	233.9421	483.3127	144.4849	420.9555	266.6075	242.6365
neg_2138	2-Ethylhyc	1378.054	639.8814	1120.916	300.858	1098.535	836.9434	688.1358
neg_2144	[(1R,5R)-5	937.8748	1213.451	984.7189	785.8167	811.5573	1061.582	1126.471
neg_2148	Dehypoxa	62.45813	41.33899	59.5706	78.04775	65.76551	63.16125	58.85098
neg_2149	Ketotifen	711.6205	12.64165	6.39369	213.8248	218.6759	91.51329	16.70235
neg_2150	(2r,3r,4s,5r	392.2607	152.0774	223.5785	215.1233	519.119	598.3212	99.14607
neg_2151	5-Sulfosali	833.7048	345.5317	384.5293	501.459	573.6878	392.1093	322.1421
neg_2152	Isoprotere	56.68708	47.27299	29.04604	31.23688	46.06173	50.41202	46.42961
neg_2154	4-Hydroxy	23.11476	18.82773	23.67112	19.02381	23.4141	19.23178	13.20558
neg_2155	Solerol	560.1568	763.7125	768.1075	688.9309	498.0822	805.7009	643.6762
neg_2156	(R)-4-Deh	247.8553	390.8498	448.7045	437.1091	414.4559	626.4394	424.3826
neg_2158	N-lactoyl-	447.4982	768.1547	143.8361	365.7087	614.5599	867.108	234.3357
neg_2159	Tryptopha	1067.41	2059.974	432.1809	798.1012	1630.541	2342.407	644.3523
neg_2160	N1-(5-Pho	69.96064	166.1123	22.05453	38.17872	106.9509	190.8346	37.6176
neg_2163	Salicyluric	579.0717	755.1111	442.6745	11849.69	475.4779	981.4034	1075.979
neg_2169	Tryptamin	281.6349	418.974	230.9453	139.4966	243.9528	449.1807	205.8866
neg_2170	L-Tryptop	12569.32	18718.66	10820.78	5933.048	10715.48	19753.65	9606.19
neg_2171	Corchoion	188.1809	384.121	41.12257	17.10445	74.96346	422.5508	72.80783
neg_2172	Tetraceno	131.0279	170.5869	19.25016	209.744	58.72357	95.07968	46.08609
neg_2174	cis-3-Hex	753.4772	1366.641	293.3769	169.2294	356.2267	1587.311	374.9898
neg_2175	Cefmetazo	139.4463	199.1296	421.8366	106.65	169.3872	212.808	223.894

neg_2177	dTDP-D-f	137.9441	215.0445	70.08744	61.25036	268.0843	255.4935	68.82267
neg_2180	Imidazoox	186.9857	231.1641	127.9466	200.4501	215.4052	144.1487	135.397
neg_2181	2,5-Dihydı	61629.66	73661.25	41635.97	59553.38	68247	46314.03	41924.04
neg_2184	8-[(Amino	2635.76	3272.474	4991.918	3972.163	10007.01	4009.905	3076.952
neg_2187	N-(4-Hydı	3664.279	4548.125	6903.473	5931.465	15156.33	5481.517	4196.868
neg_2190	2'',3''-Di-(	707.9125	1199.469	1163.954	2154.525	12700.6	1895.928	721.4995
neg_2191	Pyrocatecl	5011.248	5977.76	3342.099	4925.008	5568.709	3816.045	3443.816
neg_2193	5-Isoxazol	6573.942	8434.007	11734.15	9555.143	25247.44	9923.812	7440.301
neg_2194	2-Amino-	906.3938	1109.505	1715.42	1409.362	3545.24	1359.119	1095.913
neg_2195	3',6'-Dihy	242.5629	289.1772	105.5963	277.5348	184.6082	141.9165	135.9152
neg_2197	D-Mannit	406.2758	473.6962	756.4073	609.4732	1436.307	563.4362	494.8566
neg_2200	2-Amino-	94.24988	95.53509	206.7805	168.3502	559.1846	196.8917	102.6813
neg_2201	(E)-4-Chlc	908.3783	1067.139	1217.573	1441.809	3173.165	1232.718	1058.212
neg_2205	FLOPROPI	741.7021	1682.196	2466.019	967.2692	1245.121	1354.662	1189.511
neg_2207	4-Acetami	86.80195	240.4081	206.2666	638.8257	92.21492	160.4332	195.537
neg_2209	Emtricitab	997.2555	1118.716	1760.6	1017.189	1244.713	1108.197	1300.075
neg_2210	Neuramini	66.13532	74.96134	143.9728	79.03372	251.6886	102.6634	94.7309
neg_2211	S-(Indolyl	317.5857	208.0167	324.3153	163.931	131.2321	125.7631	143.4239
neg_2212	O-feruloyl	37.18083	84.02164	82.97701	69.76902	46.56441	73.83673	47.55068
neg_2214	Glucoiber	97.25036	110.4037	416.8397	159.7808	513.7792	155.7446	150.0041
neg_2217	Hydroxym	30.09038	38.86589	82.52242	34.56806	55.16953	41.8777	47.84817
neg_2219	3-Methyld	5114.789	5867.38	12283.56	5276.76	8295.513	5992.271	6742.846
neg_2220	Theobrom	152.5911	206.5547	313.9636	265.0553	217.9193	201.3872	210.3337
neg_2221	Coumarin	7741.529	8855.69	18287.98	8046.764	12500.83	9086.945	10254.37
neg_2222	2-Chloro-	62331.44	71373.89	150581.1	64069.72	104584.1	71282.96	81500.44
neg_2225	2-Propena	21788.88	24647.93	52252.1	22252.02	36240.13	24573.9	28172.91
neg_2227	Pymetrozi	77.06242	219.3183	75.52331	189.1137	106.5719	137.0929	84.81297
neg_2228	3'-thiacyti	2437.678	2783.346	4031.283	2488.709	2940.499	2662.607	3194.191
neg_2229	3-Thiacyti	199.5691	256.6643	244.314	223.9032	228.745	198.2914	183.9503
neg_2232	4-Demeth	3436.274	3750.889	6130.629	3409.441	4187.733	3750.123	4506.335
neg_2237	GDP-D-m	931.3247	1057.073	2776.765	966.917	1556.101	1120.015	1354.219
neg_2239	Degalloylt	487.5348	518.1512	1797.518	473.8189	754.8365	571.575	747.1553
neg_2246	Glucococh	336.2196	332.3811	452.9393	660.9986	979.2772	413.7371	247.9337
neg_2247	2-Amino-	199.1457	213.0478	579.6933	222.518	344.7592	230.1577	302.7353
neg_2254	L-Pyridosi	87.83245	134.7423	69.14146	47.54251	60.96719	139.9486	41.28881
neg_2260	2'-Deoxy-	244.5748	257.4509	601.9108	238.923	352.5209	282.3159	364.9307
neg_2264	Isovalerylg	160.0509	130.7429	9.69271	12.49135	165.9865	62.64188	42.37484
neg_2268	L-Adrenali	8.836824	116.9397	356.3438	169.0869	54.66212	118.4928	238.0392
neg_2271	Methyl me	108.045	114.1562	191.3144	157.6993	126.4681	126.8763	118.6342
neg_2273	1-Phenylp	209.5817	250.4823	375.8093	229.6493	301.6449	171.7539	202.9715
neg_2274	2-(3-Meth	234.267	957.2333	17.28779	229.1852	226.2351	119.2948	235.1664
neg_2276	N-(2-(Met	249.2994	285.5741	497.7095	283.7581	321.7333	252.6803	295.1382
neg_2279	4-Methoxy	330.0956	155.8955	10.1887	187.9672	312.9654	144.2524	74.11825
neg_2284	4-(2-Amir	108.9827	189.5501	90.44268	603.6616	116.3468	309.6383	195.8344
neg_2285	4-Hydroxy	18784.47	8459.384	2741.118	8572.541	9313.12	4582.219	2803.193
neg_2300	p-coumar	833.8082	989.8642	1872.168	923.9895	1716.45	976.0109	951.6946
neg_2301	4-Hydroxy	318.2802	367.3651	706.1732	321.7935	605.8397	386.1025	383.8944
neg_2304	Tebupirim	150.3857	22.04115	60.80228	318.0782	168.9584	43.87988	45.62499
neg_2307	4-ACETO>	157.8587	79.52859	39.13722	117.8756	61.63961	148.3612	49.76445
neg_2308	Vanillin 4-	798.2279	366.5722	252.5389	650.2721	344.7912	747.3752	221.8523
neg_2311	Eudesmic	97.27548	377.8384	127.2921	123.1936	133.8208	202.3919	116.0079

neg_2313	2,8-Dihydı	297.7609	293.6045	104.6585	96.21471	320.9256	223.3121	244.2379
neg_2315	Ascorbate	197.7457	165.7082	2.494463	22.2443	187.0373	116.4881	126.2541
neg_2316	Genistein 4	539.6348	663.8348	178.5724	434.7081	363.0343	751.5836	476.862
neg_2317	4-Azidobe	163.941	96.99583	114.3165	101.7702	143.3364	197.722	88.9778
neg_2318	D-Lombric	533.1816	364.7301	21.63123	214.7647	325.0136	106.6901	133.9457
neg_2320	4-Aminop	11717.02	14141.11	21684.36	10780.3	17518.82	12061.94	13318.36
neg_2323	Guanosine	98.36044	91.73054	190.922	84.816	148.6957	94.76468	85.99192
neg_2324	6-Hydroxy	180.3289	316.0803	137.4687	127.7113	183.9858	276.2446	121.2838
neg_2326	Dextran-7	933.5523	1193.921	415.4744	1187.374	750.7369	767.733	685.4586
neg_2328	3-Quinolir	130.2707	134.7425	28.6626	121.4288	58.60095	42.73451	38.86908
neg_2329	2-Amino-(	33.16441	14.79269	34.00705	2337.564	13.00099	0.536191	19.10541
neg_2333	D-Erythro-	308.8276	200.522	356.4447	486.2033	268.4924	181.3201	227.5146
neg_2335	Indoxylsulf	6110.588	7466.333	6183.963	6638.683	4847.277	4619.954	4434.241
neg_2336	Stattic	85.13672	153.1096	13.552	1.72E-06	65.13627	88.65439	56.41458
neg_2338	3,3',5-Trih	82.76616	108.1437	243.7812	77.48105	264.8911	88.07313	96.15217
neg_2339	3-Formyl-	925.0208	583.5651	532.4789	337.5442	1039.334	804.5617	605.0432
neg_2340	2-[(3S)-3-	508.998	278.1083	47.27587	50.14465	89.06942	230.049	86.38856
neg_2341	Indoxyl glu	116.636	108.2867	49.83107	241.0008	212.3068	103.8316	100.1781
neg_2345	N6-methy	34.34366	16.80223	87.58519	25.13879	40.3481	32.01027	39.6883
neg_2347	3h-Adrena	624.6502	333.9344	778.607	281.0962	358.5469	528.4115	494.2313
neg_2350	6''-O-Ace	2250.085	1925.438	459.8108	1047.442	342.8423	1111.369	577.0938
neg_2353	Benzoquin	141.952	157.0779	172.23	616.7515	122.7043	145.6264	137.8811
neg_2354	Asparagin	135.3775	137.3496	323.6409	143.1002	219.8084	149.9325	146.8974
neg_2355	Nocardicir	162.6096	126.0309	24.18202	101.5424	75.38903	97.84172	59.56882
neg_2358	Erinapyror	27.27838	43.89719	28.17262	41.27401	26.55228	43.99327	23.32148
neg_2362	1-(2-Furar	167.0927	36.58929	30.73	65.79238	147.7819	131.2628	35.96119
neg_2365	1-Deoxy-I	90.54872	134.1749	135.4663	105.8926	56.75531	110.3728	111.1871
neg_2366	2,4-Dihydı	642.6622	167.9588	229.8749	384.1712	704.2344	606.9861	229.9483
neg_2368	Nipradilol	158.7725	35.72914	1.455359	89.3492	145.7805	48.49646	8.986121
neg_2369	Eurycomar	884.2775	464.2343	311.7229	534.8347	546.9617	505.0121	498.2691
neg_2370	D-Phenyla	1.72E-06	1.72E-06	1.72E-06	1.72E-06	1.72E-06	1.72E-06	1.72E-06
neg_2373	Benzonata	355.6039	175.9721	196.5393	465.8899	111.0423	226.639	456.7813
neg_2374	Zymonic a	66.14328	44.68282	43.53687	166.8929	115.8723	84.96667	44.07638
neg_2386	CTP	899.5324	990.8713	1194.882	881.3261	1099.247	952.3783	1039.775
neg_2387	L-Histidinc	53.67536	21.48144	6.595374	11.91516	66.73865	33.40987	7.99183
neg_2388	Thiophene	307.1711	327.4506	353.6352	341.2138	410.9563	371.4132	338.9829
neg_2392	Glisoxepid	262.1104	1.72E-06	1.72E-06	12.82103	141.8577	31.31856	1.72E-06
neg_2394	6-Methyl-	595.2182	742.833	124.2412	553.8602	522.5139	679.6034	169.902
neg_2395	AFMK	1916.04	172.0196	21.95066	337.6774	409.9646	199.7588	123.2053
neg_2397	L-2-Aminc	59.52055	21.48969	20.86345	27.7239	64.34198	27.95789	15.40007
neg_2399	12alpha-H	188.8779	189.6377	101.2674	157.8174	178.1	154.5856	155.7166
neg_240	L-Arginine	87.70786	253.0726	182.9557	143.5797	178.381	135.6861	128.8026
neg_2400	Benzylsucc	149.8699	187.6048	40.2313	146.7343	168.7187	136.839	115.6279
neg_2401	2,5-Dihydı	19654.43	19443.99	10682.74	17292.98	20644.54	17591.25	14455.01
neg_2402	(±)-2-Hyd	137850.1	141164.9	80436.45	123877	148491	125285.7	105916.6
neg_2404	2,4-Quino	318.354	307.5063	210.5011	281.3696	339.5403	289.3804	253.4587
neg_2413	4-[(2R,5R)	667.6979	703.7536	293.8857	624.7697	716.9989	576.0237	453.8615
neg_2415	Nogalavike	2178.574	2300.409	1311.955	2034.668	2360.903	2057.098	1805.407
neg_2417	dADP	1122.984	1148.496	757.6164	1049.852	1293.641	1079.731	933.8773
neg_2418	Blue pigm	146.4391	157.422	116.1672	148.6027	149.5365	177.0732	151.7511
neg_2420	dTDP-L-m	2258.5	2302.048	1093.649	2063.347	2327.805	2030.828	1662.603

neg_2421	UDP-2-ac	2898.763	2862.222	1301.239	2520.618	3264.661	2487.394	2007.145
neg_2422	UDP-2,6-c	1750.672	1796.492	792.0858	1583.683	1993.633	1535.576	1266.736
neg_2423	Islanditoxi	447.419	466.7482	240.7525	391.0435	562.4718	423.7063	367.9343
neg_2424	Coenzyme	148.7031	87.78013	290.6266	68.87979	169.382	86.86703	116.9577
neg_2425	Procyanidi	809.138	863.4871	356.1838	707.1095	957.4247	744.3031	632.6101
neg_2430	(x)-2-Hep	88.19983	67.24962	61.64342	127.9031	57.66117	174.4825	67.28519
neg_2445	3,6-Dihydı	136.2421	127.3238	124.7631	157.4026	87.52181	162.725	201.5832
neg_2446	p-Acetami	410.4324	210.9559	281.4989	33.8108	282.2461	241.0089	170.8742
neg_2447	7,8-Dihydı	2125.869	1048.389	1588.006	119.0879	1370.831	1335.343	843.2001
neg_2454	LEUCOGEI	3951.645	71.34941	3.26165	744.9974	555.5481	531.7655	6.749738
neg_2455	Clusin	531.9439	822.5125	305.9884	2466.618	483.1172	705.4919	591.5544
neg_2459	2-(Formar	291.4765	266.3559	785.4117	228.7222	382.6537	238.7595	330.7757
neg_2460	Tetranor-I	1110.498	940.8938	209.2339	2169.133	703.9461	649.7655	394.072
neg_2461	2-[4-(3-H	854.1831	917.707	330.8369	896.9354	575.7351	606.6003	522.403
neg_2463	5-amino-1	61.20029	73.98584	259.808	138.664	44.60753	60.91426	88.77096
neg_2473	3-[(3aS,4S	147.0644	346.4812	139.0599	247.5864	205.1978	211.4839	180.6734
neg_2475	(7,8-Dihyc	310.3467	344.1573	117.172	62.26798	138.6127	179.1052	187.9235
neg_2476	Gentisic ac	479.9548	649.878	284.6119	767.608	440.6773	347.8103	311.3118
neg_2479	(7R)-7-(4-	98.20415	99.36114	77.6655	58.87456	92.10061	131.5154	119.8411
neg_2482	3-Oxo-14	2262.073	180.2195	326.6795	505.546	1789.051	704.189	107.029
neg_2489	Fructosam	64.51539	77.32105	25.45606	84.98433	55.90386	50.59551	45.02865
neg_2491	4-Hydroxy	117.8994	178.1382	66.2111	177.5234	104.346	116.388	101.6268
neg_2492	Lactupicrir	975.051	992.262	319.2514	155.9102	634.489	717.7535	601.4604
neg_2495	4-Hydroxy	376.4633	380.0727	502.6223	596.254	609.8675	512.8581	450.4383
neg_2497	(2R,3S,4S,5	515.361	516.6109	244.5955	856.3248	326.3909	532.6007	275.8012
neg_2502	Valeric aci	1261.943	913.0252	266.6434	1621.168	619.8087	559.8076	359.4935
neg_2503	5-Acetami	99.88584	157.401	182.1098	138.2721	99.47499	127.4577	129.5273
neg_2504	O-methox	16593.68	13301.53	4111.192	19593.71	9988.497	8435.167	5406.786
neg_2508	4-Keto-an	214.3891	91.55322	8.6792	134.339	68.89563	72.24568	29.66357
neg_2510	6-Methyln	139.7626	66.50896	41.64604	75.60001	93.97979	70.28444	42.20835
neg_2518	Phenylace	16045.7	5793.82	4533.778	6475.92	8033.727	7242.832	5499.931
neg_2520	sn-Glycerc	881.7884	549.9361	471.8043	534.4124	622.1238	601.4309	519.5697
neg_2525	DOPA sulf	224.8566	131.4588	134.0747	137.3129	165.0975	144.6859	128.1834
neg_2530	5'-Butyrylp	220.1923	57.99076	47.15596	74.19791	92.47891	89.29333	61.48346
neg_2531	Crocin 5	183.7329	271.342	12.25457	304.3815	67.984	318.4216	110.0383
neg_2532	2-(Fluoror	82.23163	116.9941	1.72E-06	143.6323	14.68774	145.6172	32.31713
neg_2536	Cyanidin 3	390.0827	18.45184	1.72E-06	49.90609	69.87294	43.24233	16.30139
neg_2539	gamma-L-	167.3183	84.69675	1.72E-06	107.0489	73.6323	91.87053	43.02341
neg_2540	Luteolin 7-	181.3983	6.212093	1.72E-06	27.30574	29.75374	33.15043	7.198031
neg_2544	Phenylethy	205.6407	145.4793	53.738	181.2205	145.6424	138.8737	101.9918
neg_2545	Morphine-	128.6185	1.72E-06	1.72E-06	60.94072	23.61879	21.09997	1.72E-06
neg_2546	PI(TXB2/16	91.3808	935.088	200.9218	292.8144	451.6391	328.4811	488.4556
neg_2547	2-Ethylglu	259.8517	161.5181	86.39903	147.2324	154.4852	276.2388	152.2808
neg_2548	8-Amino-7	2458.57	2007.19	2039.071	1152.726	795.0577	1313.52	1769.816
neg_2554	Dansyl-L-a	662.7952	64.07983	85.66794	164.6171	241.6928	96.01321	33.16683
neg_2559	Deoxyloga	1797.126	1622.173	608.5104	2197.49	1115.058	775.0631	639.927
neg_2560	Belotecan	30.86616	69.91132	354.163	76.36051	21.25146	16.24455	126.8755
neg_2565	3-(1,2,5,6-	72.63661	78.92387	27.44216	103.9903	76.75662	78.79231	72.87242
neg_2567	4-Amino-.	12.40099	70.80172	92.99643	289.5466	76.87292	171.1334	164.1635
neg_2568	Candicine	7.369566	53.99605	41.22376	88.48031	54.35754	97.64129	50.38216
neg_2569	Hydroxyty	255.1617	601.2527	548.2762	708.4855	177.6621	302.1918	426.5085

neg_2572	Tetrahydrc	34.03439	11.19995	1.72E-06	9.562828	8.80683	5.931508	4.256464
neg_2574	2'-Deoxyc	112.7255	66.88781	36.20966	30.42536	82.09838	46.59065	45.53252
neg_2575	dTDP-5-d	153.0577	76.88196	22.67254	25.04596	96.64137	42.93814	42.02832
neg_2578	Trigoforin	3326.362	361.0107	1.72E-06	0.008509	211.1214	1.72E-06	1.72E-06
neg_2582	UDP-3-ke	360.6685	171.7661	42.00473	73.08777	211.15	88.61398	112.7089
neg_2583	UDP-glucu	828.0581	390.0943	141.8992	148.5891	467.3511	202.9644	226.3799
neg_2591	p-Tolyl Su	333167.7	202514.1	124401.8	97880.62	230545.7	128062.9	144345.5
neg_2596	(4As,5aS,6	816.9351	536.6357	447.5259	384.6919	674.6419	392.7967	447.265
neg_2597	Melledonc	78.55189	21.06848	1.72E-06	3.544694	31.84869	6.128204	5.933821
neg_2601	Maysin 3'-	235.0132	107.3658	20.83836	35.44751	155.2388	65.85419	73.38462
neg_2602	3-methyltl	117.5532	62.85515	53.17929	51.4243	84.33994	57.93051	54.81818
neg_2606	2'',3'',6''-T	310.2471	156.0178	41.32205	56.56806	183.8762	93.0468	102.2582
neg_2612	Methylimic	204.0003	302.6029	116.5478	107.6832	176.003	64.53526	233.8056
neg_2615	Valylserine	5.027089	54.13871	297.607	125.7527	25.98203	139.4962	139.4232
neg_2616	5-Sulfo-1,	350.0796	255.2898	231.2352	171.4964	313.5892	202.0595	215.5321
neg_2619	Psilocybine	3188.782	5012.317	2017.211	1593.064	2820.513	1044.966	3503.524
neg_2624	Tetracenoi	212.836	328.0571	102.0272	114.0291	189.5868	59.85923	216.6574
neg_2625	UDP-L-Ar	98.22067	50.0254	12.99888	19.10875	62.58211	28.26137	33.11936
neg_2634	Tricin 7-[p	80.16841	85.42941	94.72043	69.81247	104.7308	76.16572	96.97018
neg_2642	Quinaprila	278.6856	236.6038	105.1398	144.2578	199.1967	272.0106	211.3977
neg_2643	ISOPEONC	271.7232	147.0554	73.61948	199.3161	147.4487	193.0549	97.26857
neg_2645	3-[3-(Sulfc	1217.415	648.8235	331.564	801.0076	656.4657	915.1715	406.8436
neg_2649	Ethionamic	327.9752	78.68264	144.2221	106.6498	173.0087	394.4391	74.86788
neg_2653	Oxonol	60.72232	71.30415	114.2429	83.21114	138.0593	103.4651	93.81326
neg_2655	2-Hydroxy	37.45197	9.20131	16.29698	14.0474	50.84883	28.42559	12.37287
neg_2656	Ethyl nicot	85.4881	105.7882	48.72064	141.5302	94.7341	78.44366	70.027
neg_2657	Methylnor	1066.237	275.3771	447.7034	584.7363	350.4217	320.689	228.7558
neg_2659	Dopaquinc	205.8622	234.7449	117.8993	321.4087	220.071	164.817	136.3315
neg_2661	Octanoylg	443.5501	39.62265	21.18219	93.23246	228.364	157.654	33.10574
neg_2665	Suberic ac	1355.797	2204.928	1591.296	1784.983	1288.74	1669.512	1262.894
neg_2669	Isomaltotri	94.64334	34.7158	124.0818	141.5197	57.88557	82.23625	30.01065
neg_2670	Dihydrowy	232.364	20.8155	58.14012	169.4353	183.72	114.6887	26.72841
neg_2675	4-Hydroxy	28.58636	9.792815	9.432499	424.6611	8.418016	26.26605	4.918631
neg_2676	4-Methylb	7.379664	9.481527	2.528536	2431.783	10.31818	56.17622	7.226354
neg_2677	Dihydro-3	1887.67	346.8732	498.2953	8288.857	800.1633	746.7006	222.3884
neg_2679	Ecgonine r	477.5067	397.496	374.6087	236.7669	480.6578	1090.757	621.9637
neg_2682	BYSSOCHL	111.6161	134.5659	0.000684	8367.255	6.771544	82.44307	7.001534
neg_2685	2,4-Dipher	81.4177	66.36634	1.72E-06	13.24384	10.97708	63.44762	14.10423
neg_2696	(1'S,5'S)-5	500.8909	499.9582	101.7246	103.9162	254.6626	313.7846	222.9614
neg_2701	Picrocrocir	53.03681	68.11921	36.53738	63.90387	55.18613	31.07818	26.65681
neg_2702	2-Propylsu	336.9007	146.8598	76.45808	217.952	338.5269	268.9784	84.65345
neg_2703	Carbocron	864.8386	1.72E-06	1.72E-06	107.2541	107.3424	55.55383	1.72E-06
neg_2708	2-Heptene	8735.236	3565.705	4856.539	6016.975	5365.827	4836.334	3110.391
neg_2714	(1R,4E,6S,7	135.6639	15.86387	2.572548	69.54971	37.29188	56.59669	16.18258
neg_2716	Benzyl alcc	595.2226	225.2961	63.33745	354.3528	379.8311	314.6207	120.7907
neg_2718	Picroside I	10634.29	5057.215	1779.757	60697.22	4836.005	5946.069	2824.688
neg_2720	N-Acetyla	84.47617	155.6145	120.2274	112.415	145.3252	485.1929	97.72686
neg_2721	3-Hydroxy	71.81959	50.32628	15.05978	272.7515	62.42914	81.55359	30.56944
neg_2722	D-Clopros	256.0578	168.3293	14.34253	132.3738	90.41992	145.5788	40.84033
neg_2725	Erythronic	46.85151	23.24454	37.32587	171.3349	73.90748	27.66615	27.71969
neg_2728	Talastine	2327.795	2139.995	1538.005	2759.137	1050.149	2580.982	1539.369

neg_2729	Polyribopł	72.38564	49.44439	93.99404	222.5145	79.00738	97.11367	66.70307
neg_2732	Aprobarbi	56.78718	101.8061	55.80114	41.10228	32.65076	119.0537	27.6322
neg_2734	8-Methoxy	22.48626	34.39636	98.14209	52.89307	44.93825	37.90194	28.81038
neg_2735	6|A-Hydrc	4126.494	6186.508	1739.865	5708.685	2932.631	2598.991	2357.137
neg_2736	5-Hydroxy	56.9442	63.07469	199.9561	104.0646	91.98432	68.33063	63.45483
neg_2738	L-Prolinan	163.6371	39.48741	12.4291	157.8928	59.74168	92.51638	17.35109
neg_2739	Rhodamin	428.5064	398.1309	258.6873	268.919	377.8944	491.1876	274.4474
neg_2740	Taraxinic a	385.9218	386.0255	168.0241	290.631	283.4784	361.5181	263.1304
neg_2743	Cymorcin	767.0476	422.6911	132.2316	808.9416	384.4752	244.0642	176.9684
neg_2744	Oleandolic	337.6996	304.6512	220.4874	451.0465	205.1216	204.8394	239.5795
neg_2745	Sanazole	69.35964	65.88627	1.72E-06	16.9208	22.03577	51.95696	49.72571
neg_2747	5-Butyl-1,	40.74117	35.78231	14.33186	42.81791	38.76459	35.39452	22.78537
neg_2750	Dihydrone	76.84353	12.0586	1.72E-06	9.240983	3.48001	6.426534	1.56056
neg_2753	Methoxam	5528.495	215.5907	936.4525	788.3837	559.9186	281.2747	606.4169
neg_2758	Corchoion	1204.543	999.7304	382.7527	1840.548	851.2027	804.7684	518.3631
neg_2759	gamma-B	121.0891	154.5241	318.568	137.529	88.65898	142.5805	117.1024
neg_2760	Indolelacti	824.1586	1747.391	1962.749	642.9496	1136.007	1609.929	550.4684
neg_2765	Xanthuren	1181.444	1375.043	566.2427	641.8345	312.5422	143.9897	961.6639
neg_2766	4-Allylpyrc	704.2395	598.4277	395.5376	977.5867	631.678	767.7173	591.5862
neg_2769	Benzylsucc	141.0698	104.3541	51.02204	74.14555	101.1526	123.6223	55.51665
neg_2771	N-Acetyl-	48.07707	72.8197	61.15603	40.86049	105.2624	105.118	39.48796
neg_2773	(2S,4R)-4-	92.14046	94.98621	875.2864	191.8499	58.9058	47.55536	250.5911
neg_2776	Indolylmet	75.72145	53.11385	236.1341	51.5299	169.3945	84.40848	243.6926
neg_2778	Frenolicin	1131.049	1195.19	671.4806	913.5676	805.5074	1328.324	879.7388
neg_2783	Benzenesu	309.0247	71.86896	9.173454	652.1164	189.3583	89.86152	60.02418
neg_2785	1-Pyrimidi	58.28062	72.21448	29.38759	65.99733	50.71083	55.7466	38.08399
neg_2786	5-Hydroxy	29.53583	71.31029	11.30115	106.0739	9.698566	23.64436	14.49631
neg_2787	(2R,4S,5R)-	39.97859	39.50302	9.683899	204.3518	51.43068	8.101034	11.7321
neg_2788	Vignatic ac	120.8132	123.8117	215.8765	114.0884	142.4917	113.7676	120.7806
neg_2796	Ala-Abu-(	62.32286	79.18334	26.65544	288.9783	46.43439	110.1709	52.23605
neg_2797	MUCRONU	63.92759	67.71299	8.110522	82.84743	36.07694	106.3124	47.20896
neg_2798	Rivenprost	658.4245	773.0136	86.99507	1014.216	341.414	396.0416	325.2257
neg_2799	3-Heptene	101.1312	314.6595	227.9242	195.3661	422.2528	314.7751	290.3705
neg_2801	Methyl 7-e	303.6572	717.0974	124.5116	621.1687	247.2292	382.6999	321.8755
neg_2802	Isofluprede	32.78336	21.67041	1.72E-06	27.82515	6.35826	15.59439	3.641577
neg_2803	Abscisic ac	64.44394	56.23192	22.19859	68.40146	39.46852	42.05339	41.447
neg_2808	Formyl-5-	372.1502	323.7068	84.61973	800.2144	231.0519	222.29	188.495
neg_2810	1-Hexen-3	49.50566	36.42679	9.481521	35.77052	33.90577	33.49123	23.86024
neg_2811	Lentialexin	262.7967	204.5091	119.7222	207.6289	195.7351	180.5758	150.2217
neg_2813	3-Hydroxy	218.9711	209.2964	109.1338	149.8833	159.0871	176.1686	143.0116
neg_2817	4-Vinylphe	1247.951	1329.15	796.7837	1092.074	602.1856	610.5158	654.5745
neg_2818	BUTHIONI	102.1551	43.57041	20.24455	21.85509	55.08412	161.3872	53.62442
neg_2823	Pondaplin	73.63308	61.00143	51.98599	79.78677	34.4445	39.59212	48.49994
neg_2832	TILETAMIN	94.75958	67.32091	15.41446	166.3018	63.36206	88.85601	68.21984
neg_2834	7-Hydroxy	23.22784	10.85995	2.654712	1462.699	20.06078	17.29785	2.519099
neg_2835	LysoPA(22	49.61834	9.010214	238.0768	116.4803	16.36471	42.38594	116.1609
neg_2838	Bisbynin	121.8574	143.8985	1302.796	277.1372	63.93216	120.4082	352.3687
neg_2841	Ethyl galla	43.32508	45.79345	105.0472	74.20054	98.03845	58.094	43.81067
neg_2843	Polypodos	296.4919	1073.077	240.9167	137.0416	606.6374	308.8249	709.4652
neg_2846	3-(5-Meth	65.44827	53.029	28.62205	37.29889	52.92675	51.21212	36.66566
neg_2847	2-(4-Meth	8087.461	7190.559	3937.386	4118.379	7641.156	6410.144	4690.126

neg_2848	(S)-9-Hyd	74.24294	126.9686	82.57564	119.9341	102.4628	147.7493	79.32767
neg_2851	Flurandrer	223.0909	378.1083	74.08869	434.4616	198.9485	198.1224	122.4271
neg_2854	Etimizol	112.207	117.2985	1082.853	252.3035	90.3219	87.48584	302.5707
neg_2855	Deoxyguai	112.4854	122.9424	1105.607	229.3304	116.5789	64.17189	356.5095
neg_2857	Prunasin	13.72791	92.03661	121.1524	212.6231	18.93243	41.01944	184.6655
neg_2858	dCDP	122.4868	104.3752	211.1833	109.1295	170.7769	157.2316	176.2342
neg_2859	6-Acetyl-2	65.16698	22.53845	16.39731	74.0769	56.61501	19.72994	8.962839
neg_2863	Nicotine g	170.4735	211.7593	657.5535	335.2022	161.6661	162.3782	293.8784
neg_2865	(E)-2-Tride	182.7596	96.46647	42.32821	181.6558	80.10945	87.26227	70.89679
neg_2867	Amino (2S	274.2724	663.1037	714.2096	605.3966	796.3192	709.001	834.4612
neg_2868	Cinnamoyl	1060.325	2543.509	2828.807	2250.203	3063.299	2606.822	3129.937
neg_2869	(+)-Bottro	22.67387	31.91843	35.29475	98.87185	14.37778	13.07541	41.17858
neg_2870	Biopterin	61.53236	122.6048	145.3053	115.4355	160.727	127.4578	160.7675
neg_2876	Blumenol (	395.8893	711.1253	411.2337	299.4969	641.0367	1086.145	474.381
neg_2878	2-Methylir	17.45228	47.84579	48.87515	45.03139	58.97095	49.85035	61.43829
neg_2880	D-Galacta	124.6937	102.027	140.6214	111.184	155.1	138.2867	105.847
neg_2885	Capecitabi	20.06706	40.17662	65.67476	103.4626	9.056217	39.26788	81.95309
neg_2886	Sabine	1080.626	4780.052	160.4668	234.8921	54.28879	534.5863	932.2608
neg_2887	N-(4-Amir	404.1322	1.869572	1.72E-06	18.85343	119.8863	78.73018	1.397729
neg_2888	DL-Methic	93.44733	6.03611	318.11	41.04166	23.55162	436.428	519.2436
neg_2891	2,3-Dihydı	256.6433	87.00886	78.05753	181.8773	118.3427	83.71616	146.136
neg_2893	Asparagin	3527.132	1474.942	1420.929	2525.535	1786.293	1389.82	2083.127
neg_2894	Rutalinium	66.98002	1.838226	1.72E-06	1.867278	27.39519	2.477612	1.72E-06
neg_2896	Pantoyllac	223.1045	268.3611	67.00276	206.6257	267.8743	528.7938	264.6637
neg_2897	3-Acetyl-2	857.7024	150.9365	76.21979	165.3905	255.7964	273.1171	101.7869
neg_2898	Tyrosol 4-	18044.73	3549.595	2313.9	3902.015	5978.855	6260.066	2589.861
neg_2902	Pentoxifyll	2229.933	1210.351	2244.818	822.6975	1785.987	2788.395	1520.652
neg_2905	Salidroside	59.52951	31.03605	51.49834	12.41787	35.2655	100.0519	29.73073
neg_2909	Sepiapterii	14.65603	22.35142	19.07707	16.5656	58.48628	39.32532	12.58149
neg_2910	Celgosivir	211.112	178.3029	186.1109	155.408	246.3678	447.1168	245.9606
neg_2913	Obtustyrer	1038.635	1208.584	317.2452	336.6076	661.7291	950.3398	642.4817
neg_2914	(±)-3',4'-M	702.8777	911.3295	223.6956	261.7059	440.5029	675.6997	486.7915
neg_2915	6-Methyln	61.16043	221.8685	46.96682	310.3548	51.57892	100.6377	78.92236
neg_2917	Hexahydrc	24.89195	29.88693	4.685765	65.54792	52.49542	24.64274	8.632457
neg_2918	3-Hydroxy	42.47805	74.34936	223.413	251.3801	60.14228	61.95551	453.3488
neg_2919	(3xi,5Z)-1,!	622.5407	786.8508	389.2978	550.6786	576.4129	640.2529	550.7032
neg_2920	2,3-Dimet	228.0157	283.1108	130.9283	201.1837	212.1818	227.0314	200.8613
neg_2921	Azelaic aci	13016.1	16630.41	9315.149	11350.11	12357.79	13117.81	12144.73
neg_2931	Pentahom	57.35137	2.256876	199.0997	78.09901	10.03163	21.56419	129.5816
neg_2935	4-Amino-3	891.422	711.6163	922.0262	674.5742	866.001	838.0662	927.2009
neg_2938	Cinnavalin	82.32266	93.70122	143.6589	83.7581	111.8163	86.87493	97.46548
neg_2939	Moveltipril	212.8972	289.9079	30.51684	171.2148	156.5277	185.5093	173.7995
neg_2940	Ocfentanil	878.295	1136.899	661.2639	1171.179	1528.663	1379.016	630.6642
neg_2941	Benzoate	51.68793	57.04257	72.21132	43.4257	76.00246	46.79641	48.6092
neg_2946	Dibutyl ma	1018.163	453.8034	318.7919	811.7385	646.9475	807.0005	279.7268
neg_2948	Cycloprop	139.7514	271.0702	190.4283	318.3211	266.0789	388.5003	297.3483
neg_2949	2-(3-Carb	136.4175	178.1263	822.1945	255.4509	159.13	134.6971	286.628
neg_2950	3,4-Dimet	70.59232	74.6783	870.2355	157.1324	85.90328	38.54558	255.4632
neg_2951	(1R,3S,4S,6	982.1382	81.50021	54.42852	254.9816	731.8563	541.8633	75.38899
neg_2952	4'-Methyll	967.2767	689.9092	588.1005	507.0263	407.4235	847.1524	693.2839
neg_2953	(Z)-[(4-hyd	28.67139	46.24452	32.04887	43.91322	27.47607	41.84118	24.73262

neg_2954	Vanillyl oc	42.47332	104.7973	318.3552	191.903	40.259	64.75955	408.2182
neg_2957	4-Ethylphe	503.9436	192.1814	63.50028	53.04334	184.9834	131.7961	91.20893
neg_2958	2-((3-Ami	102.7596	148.8268	102.3839	129.5477	84.15408	130.1275	70.82548
neg_2963	4-Ethylphe	59559.02	25960.6	12007.52	7354.356	24709.53	17847.7	13330.94
neg_2969	D-Ribitol 5	296.7662	128.2749	196.648	120.9682	185.4402	384.237	125.2147
neg_2970	17alpha,21	172.9193	82.13173	69.65488	221.0533	142.7683	154.8698	77.56405
neg_2973	2,4-Dihydr	133.2471	22.27376	1.72E-06	1.72E-06	23.76533	13.4362	1.72E-06
neg_2976	Sulfite	30.59986	11.53339	3.678076	3.166214	8.249144	8.955998	5.306095
neg_2983	Uridine 2',3	95.23545	88.93125	149.124	93.04703	103.2713	93.42727	94.05697
neg_2986	Desonide	81.67308	44.09109	11.46119	49.51904	10.19771	49.82853	36.19351
neg_2988	Val-Gly-Va	650.082	60.03118	81.89519	223.7729	60.5286	19.55596	4.878596
neg_2996	Oxamyl	1112.774	1166.847	3055.798	1189.418	2748.661	1262.669	1224.278
neg_2997	5-[(Diamir	523.9893	550.7017	1331.104	546.5866	1164.291	589.4449	602.5924
neg_3000	Kiwiionosid	119.0636	66.25727	9.203198	134.0744	90.76725	61.33467	41.35181
neg_3002	p-CHLORO	63.18141	58.40106	145.0142	79.63973	168.0015	70.11666	101.7801
neg_3005	Angeloylse	547.042	706.3	118.1069	652.0216	522.5215	832.7424	282.0481
neg_3006	HoPhe-Ho	2073.731	1188.842	1200.214	1796.501	1873.548	2342.039	1557.121
neg_3012	5-Hydroxy	116.9401	101.7586	193.0377	152.0319	223.585	166.6515	287.8691
neg_3016	Vanilloyl g	512.0796	495.378	560.6851	615.9088	325.901	450.9279	453.8391
neg_3017	Allithiamin	575.5624	390.8681	922.0993	622.567	1142.405	733.6745	1418.549
neg_3018	Enterolacto	3253.573	1274.826	538.2818	18656.81	3496.957	1763.259	774.0375
neg_3020	Methionine	152.0931	46.85117	63.59015	275.8959	55.87697	74.9021	71.08608
neg_3025	(±)-Rollipy	84.16572	0.960224	0.265734	7.351873	50.49743	29.9898	1.72E-06
neg_3028	Butyl 3-hy	1171.478	2823.416	438.9416	2070.394	1226.022	961.5487	697.3562
neg_3030	N-[5-Meth	111.0042	12.70336	513.8145	143.2392	25.64251	52.4308	238.1835
neg_3031	Thymoctor	381.1484	1205.208	247.5374	120.4279	689.6966	344.0789	842.0197
neg_3034	6-Hydroxy	668.4886	727.2835	513.4776	329.1498	563.9365	106.3402	681.7809
neg_3035	2,6,7-Trihy	77.65257	83.1196	85.87693	52.7708	93.75626	59.23597	37.085
neg_3038	5-Hydroxy	102.5477	90.08727	137.9374	98.62583	118.6897	88.93269	89.86589
neg_3039	p-Coumar	340.6194	410.2671	3050.224	1000.07	271.1617	261.3437	822.6188
neg_3042	4-Nitrophe	335.027	325.4276	503.9857	469.2145	424.6267	365.485	418.6965
neg_3046	Nicotinate	128.8378	98.57764	11.57991	5.624642	78.59518	72.10256	96.24648
neg_3047	7-Hydroxy	112.045	101.7814	110.5155	328.8376	147.4514	167.1246	99.10397
neg_3053	15-Epi-lip	657.8496	939.4123	463.6413	3736.459	520.8329	633.6599	589.7118
neg_3055	Calicheam	11.45532	24.73376	147.8656	59.05011	3.945008	11.06467	46.26559
neg_3056	7-Epi-12-	295.4201	138.7639	85.13043	245.8641	123.6572	138.2445	159.7972
neg_3057	Corchoros	152.4632	120.6319	48.85174	8.928381	1.72E-06	4.563755	128.2855
neg_3061	2-(2-Furar	107.1623	139.2878	43.03351	125.8026	62.22146	96.023	49.73911
neg_3062	Aminocap	143.0547	164.1095	106.6503	208.875	191.7606	341.3059	154.5158
neg_3063	1-nitro-2-	745.3328	826.176	651.428	1058.2	1044.315	1652.102	826.0387
neg_3064	3-Phenylp	1572.266	2012.6	977.378	1861.282	1071.619	1483.345	871.5526
neg_3066	pyroglutar	258.1582	197.8821	47.38824	40.38478	169.6127	195.293	145.6252
neg_3068	3,7,8,15-Sc	96.0182	79.10055	212.6033	121.9331	126.3072	127.2295	158.1865
neg_3072	Pterostilbe	652.3357	888.9997	5859.738	2025.145	427.8386	512.6511	1622.326
neg_3073	Adhumulo	248.1736	158.0949	38.29274	65.55346	161.4397	234.9775	93.94865
neg_3074	20-Carbox	250.9074	403.5606	73.15945	660.6423	143.5819	113.1604	109.7766
neg_3076	L-Methion	253.8922	434.2074	809.8833	49.81886	354.696	640.2227	435.3139
neg_3079	(+/-)-(e)-4	78.53766	21.54886	0.365682	45.02589	35.02975	23.80066	30.51984
neg_3080	Orsellinic a	174.4379	159.5917	143.7046	168.2575	150.5654	165.2193	150.913
neg_3088	Garcinone	149.729	79.34009	11.90047	222.5253	88.42295	52.28364	35.39802
neg_3089	Avapritinib	136.1274	32.52099	70.18837	185.817	54.22797	43.39418	205.1115

neg_3090	Cortolone-	459.8543	50.68363	66.48029	491.7051	172.0252	48.12009	44.09526
neg_3091	(1R,6R)-1,4	43.02368	110.873	56.19993	90.27693	43.37534	92.17313	33.43536
neg_3093	Casticin	87.72401	41.97188	5.084158	44.48799	39.464	29.40201	39.04583
neg_3094	Traumatic	62.68186	149.2418	1.72E-06	39.16415	1.72E-06	14.36925	32.34107
neg_3096	3-Isobutyl	386.8089	339.5561	153.7879	12.02902	267.9462	330.9035	261.1838
neg_3098	2,3-Dihydr	172.38	130.4564	60.85369	12.71788	1.72E-06	4.64493	119.6099
neg_3099	2-Carboxy	705.6209	768.5177	1020.867	555.2379	1213.733	850.5184	1230.527
neg_3103	D-Glucono	916.3218	1219.478	815.7006	759.8747	788.3922	655.0029	624.0948
neg_3104	Sarcodon s	1620.656	4428.009	229.3784	225.7138	101.4631	635.7635	1269.478
neg_3105	xi-2,3-Dih	62.07119	61.34372	94.20481	340.6017	71.96802	58.29382	54.62386
neg_3106	1,3-Bis(4-r	1707.935	1025.1	2550.065	329.2168	1492.831	1059.762	636.2822
neg_3116	N-Acetylas	102.0537	105.0435	55.32675	27.22118	66.16091	126.4994	74.08656
neg_3117	Dihydroka	143.6962	151.9282	77.9476	100.6537	61.46411	96.80983	63.62966
neg_3118	2,3-Dinor-	167.0081	95.42798	40.43747	95.236	104.6801	147.2501	79.89164
neg_3123	l-lysyl-l-ly	409.062	385.0629	2117.397	506.9898	556.2124	609.7045	463.8461
neg_3126	2,6-Dihydr	195.7269	94.21282	44.1588	57.83239	34.21052	64.77312	83.52617
neg_3127	Glutaminy	101.7575	31.23375	5.19321	116.7191	9.268063	4.945211	1.72E-06
neg_3128	2-Hydroxy	701.1204	770.9454	1298.008	769.4989	924.7257	721.628	799.2332
neg_3131	Ancymidol	796.1962	869.5918	7051.372	2188.167	686.5503	591.4178	1865.843
neg_3132	2-(3-Carb	408.0097	577.6567	453.5516	675.484	645.3891	1174.81	646.4944
neg_3133	Phenylalar	1.72E-06	9.734687	273.1497	120.6563	1.209336	1.72E-06	81.13336
neg_3134	(E)-N-(3-(	2024.94	793.9987	622.1344	1759.185	1221.01	1411.631	792.1477
neg_3139	H-Gly-Arg	828.0019	1132.586	62.65164	36.2694	185.6231	112.1745	289.321
neg_3140	1-(11Z-do	143.143	341.2033	877.5048	127.3927	128.5132	192.0302	78.6005
neg_3143	(±)-Entero	23.69501	18.41823	42.26797	166.7051	35.32653	34.62542	32.38989
neg_3144	13,14-dihy	10196.9	25265.54	6181.161	27559.45	11822.46	9422.289	8970.766
neg_3146	11-beta-H	186.4416	27.65435	1.72E-06	52.66524	5.920995	13.71357	55.312
neg_3152	Delgocitini	231.818	243.2606	2653.901	705.8896	171.2164	146.8819	601.5104
neg_3153	Enterolacto	434.1655	356.4326	278.2237	7547.308	360.1793	310.0382	162.3241
neg_3154	(3S,5R,6S,7	159.885	86.3035	59.1564	171.7164	132.708	146.5805	85.71256
neg_3157	Melatonin	18.17689	21.38099	243.2035	63.17187	9.337214	10.7151	49.26979
neg_3158	Glutamate	87.33837	137.9982	134.8525	177.4539	151.2994	52.57549	135.2296
neg_3161	Glycocholi	106.3836	38.20492	7.219481	1.72E-06	2.026655	160.4434	2.539364
neg_3163	4-Allylphe	2150.149	3020.723	2186.922	2219.19	2908.007	2153.507	2069.052
neg_3164	Sebacic ac	133.935	146.4229	35.18499	181.8775	99.54461	85.86082	69.50658
neg_3169	TIC10	105.1235	73.74216	27.92667	133.4354	133.9486	178.852	185.857
neg_3172	DGAT-1 in	89.92897	331.3721	48.17986	469.0915	57.97925	73.38714	79.10673
neg_3178	PI(22:4(10Z	207.0281	505.4024	63.16827	30.70722	308.7433	109.767	330.091
neg_3180	Thr His Ph	1227.18	3229.9	29.08153	89.87175	29.96986	350.6661	559.2079
neg_3183	2H-1-Benz	75.89957	78.70901	285.7906	87.47865	178.003	79.23145	81.25921
neg_3189	Pisumiono	160.2214	74.56034	9.029035	202.4743	88.36002	115.5972	46.9558
neg_3190	Gln Leu Gl	543.7961	936.7414	84.24059	104.8015	38.01046	180.2506	371.9479
neg_3193	3-Hydroxy	218.0827	275.8859	311.1208	280.4902	337.9078	390.0957	425.1836
neg_3200	Heptylmal	7856.513	10359.48	5828.595	5582.863	6368.847	7582.283	6861.717
neg_3203	2-(4-Amin	254.5136	404.7271	93.08261	215.7329	157.1827	535.6098	224.2612
neg_3205	Pyroglutar	230.3148	27.44879	20.33375	41.55003	114.7544	56.07304	11.49438
neg_3207	Mevalonol	32.60451	53.00809	26.38219	77.98664	92.56818	49.84388	37.24422
neg_3210	Sagopilon	116.5514	90.7691	85.69359	23.14235	35.01009	129.1964	53.35731
neg_3225	Acetylshiko	140.718	231.7421	23.54623	2201.589	276.6186	717.1814	307.2011
neg_3230	Trihomom	292.0226	312.3672	518.6076	309.3155	335.9612	258.2267	271.0413
neg_3232	2,5-Dihydr	74.14039	88.34141	696.3758	182.5792	45.19707	55.25343	194.4366

neg_3237	Val-Cit	116.8667	246.4845	147.9581	216.3417	165.973	107.3225	91.78333
neg_3239	Sonchusid	113.0651	112.7636	5.017332	164.5904	34.84233	28.59104	7.963375
neg_3242	Dapitant	291.958	335.0489	1.72E-06	1.72E-06	1.72E-06	135.1848	35.86552
neg_3244	Tetrahydro	584.0238	90.19265	155.5253	7.413324	36.49015	225.8027	36.11477
neg_3248	Buprenorp	383.2295	309.1539	6.974862	1.821912	109.2537	160.1146	23.25896
neg_3250	Ascladiol	84.76461	91.37134	131.9254	66.59172	94.55466	89.38472	88.67221
neg_3255	2-Maleyla	803.7183	809.8348	1379.773	720.944	949.2214	819.3467	879.5509
neg_3257	1-(2-Furan	809.8906	550.5583	357.9409	983.4296	534.2117	523.3739	424.3078
neg_3260	2-Dehydro	18745.8	13412.85	9650.927	22942.05	12967.64	12780.53	10633.28
neg_3265	Colforsin c	6171.282	13603	1131.856	751.5125	265.1189	1914.978	3454.25
neg_3266	Fosinopril	120.3252	290.7074	1.72E-06	1.72E-06	1.72E-06	30.63509	64.37268
neg_3267	Ethyl 7-ep	475.9303	275.2594	234.4358	313.048	180.4476	383.9018	278.9025
neg_3268	Hypochoe	1098.407	660.0508	548.6793	1447.096	734.0561	1106.226	660.6497
neg_3269	Carindone	926.3	1389.571	419.166	537.3355	146.0861	251.2974	1215.192
neg_3271	Benzoic ac	195.9822	231.3847	299.5753	115.6962	254.4634	170.0186	203.387
neg_3272	4-Chloro-	14778.26	21884.05	7385.21	793.452	12900.82	7111.674	8831.318
neg_3276	Benzyl eth	93.85341	139.6214	33.63225	1.72E-06	74.11974	41.3742	47.86687
neg_3285	Sulfolithoc	5026.238	11096.13	3433.225	1349.845	461.7781	1692.257	2697.164
neg_3289	9,13-Dihyc	126.4037	121.6004	34.48827	166.9573	84.68001	188.7676	175.4699
neg_3290	5a,6a-Epo	83.21339	27.93992	7.807804	95.80421	54.9952	69.23962	12.99184
neg_3291	milbemyci	354.6471	426.054	30.60602	47.0431	3.983847	98.45599	75.43743
neg_3301	T2 Triol	486.5647	193.0454	157.2775	558.8422	321.7298	341.9042	210.7112
neg_3304	2-Heptanc	28.11758	64.92873	16.07678	7.321437	15.09817	16.5596	7.916866
neg_3305	N1-Methy	66.51455	78.49241	15.71182	89.43236	38.91163	33.44147	24.36824
neg_3306	Aminohipp	6484.189	13881.58	5665.684	2185.083	5328.711	4550.904	4168.915
neg_3311	1-(2,4-Dih	4.707222	1.219142	1.72E-06	2.935591	1.72E-06	10.76405	1.72E-06
neg_3313	Difructose	279.0135	78.77491	112.6493	299.9634	120.395	155.6409	149.487
neg_3315	N-Arachid	415.751	1175.672	69.79365	115.9386	75.40441	475.2725	640.534
neg_3322	(5Z)-7-[(1I	246.7066	470.1796	32.84342	93.43853	12.79357	57.92191	95.74024
neg_3335	2-[(2-Ami	141.0489	128.7122	307.9072	136.6345	213.1959	146.1502	120.272
neg_3336	Cinnzeylar	1216.956	571.2141	725.624	887.3976	659.5128	876.5116	797.3177
neg_3338	cis-3-Hexe	666.9422	144.2976	1194.998	859.8496	157.6601	408.4421	775.4375
neg_3339	((2R,3S)-3-	89.92452	1.72E-06	20.97912	13.69974	1.72E-06	6.735186	30.36391
neg_3342	3,5-Pyridir	51.43577	60.61475	26.43163	61.06495	69.77218	91.57307	50.57485
neg_3343	(3b,6b,8b,1	485.7752	353.5159	82.55913	139.9025	63.05628	318.5761	261.6331
neg_3357	(8-Methyl-	157.2698	45.23513	1.72E-06	1.72E-06	172.0891	8.539465	206.0498
neg_3361	3-Hydroxy	79.68706	30.33082	4.210821	44.11568	17.69179	47.82865	39.30678
neg_3364	Fexaramin	498.4877	267.3374	108.1815	398.3909	201.3455	239.2813	350.5975
neg_3365	4-hydroxy	490.7016	158.1648	252.9292	45.93834	282.0634	440.5367	370.0641
neg_3371	Dehydroas	650.9375	713.0315	1215.319	569.2273	832.0595	676.8059	765.3018
neg_3373	Myrigalon	2542.747	1925.919	1316.474	2085.865	2046.528	3424.516	2163.545
neg_3374	1-(2-Amir	680.8291	1323.672	185.3222	660.0145	889.2119	977.9795	655.8717
neg_3375	Mytilin A	82.91582	43.31365	11.20216	50.95032	49.2045	104.3356	51.84824
neg_3377	Glucosyl (2	38.46317	10.86687	5.148041	26.74635	25.52941	2.996192	1.477116
neg_3381	Phospho-a	108.4925	53.87163	13.8957	22.17361	70.28125	55.09709	63.22776
neg_3386	2,3-Dimet	171.2423	10.04416	3.939923	38.18904	37.56633	49.61577	15.16906
neg_3387	5-Megasti	439.7479	307.3741	67.07852	387.6343	229.3084	370.5067	197.2123
neg_3392	Salbutamc	262.8142	816.6029	1167.125	750.1352	859.4143	1800.887	773.3784
neg_3393	3-(2,4-Din	2398.545	1917.73	1678.01	2761.872	1013.641	1614.785	1056.997
neg_3395	Jasmolone	317.8272	134.7561	2.808464	359.2944	121.7771	77.68225	109.8445
neg_3396	N-Docosa	269.8706	1672.542	15.35157	13.10933	9.955975	140.0249	197.6255

neg_3404	Forskolin	2906.348	628.8364	494.2232	260.7182	664.6004	1248.073	460.7186
neg_3405	Phe Gly Pł	70.46734	43.74347	70.19532	54.39314	65.03268	116.7139	34.7684
neg_3406	(2S,3S,5S,8	3506.17	8294.773	1457.118	1574.498	841.9765	3597.531	6079.984
neg_341	Alginic aci	475.9103	384.1097	111.3824	405.2586	165.2748	422.806	345.6748
neg_3410	S-(2-(N,N-	386.3004	362.2994	56.37151	46.2346	58.29296	110.6697	308.9587
neg_3411	LysoPC(15	268.2841	122.5189	50.60522	121.4553	17.27086	47.08993	172.0487
neg_3412	Physagulir	117.733	131.3266	72.82502	118.1042	85.67467	176.4322	122.8191
neg_3414	(R)-Mevalo	790.0667	882.9075	986.3292	602.3678	999.6242	972.9925	758.8891
neg_3419	(2R,4S)-4-	246.7411	117.7783	123.712	153.2205	85.93573	116.6356	47.1351
neg_3423	Melanosta	24.77104	30.9853	78.961	29.38288	49.10444	91.85535	40.78088
neg_3432	Gentamicii	869.8865	475.5923	689.2586	2245.478	325.1223	387.1159	772.3725
neg_3433	isochorism	513.6327	663.8653	575.7917	351.5271	616.5668	610.3747	459.0907
neg_3436	Protease-/	152.3158	92.10695	91.33508	83.76311	31.45301	47.8064	72.74163
neg_3440	Undecane	2090.844	2663.195	2039.914	1664.513	1580.083	2206.651	1940.273
neg_3444	Taraxacoli	858.4046	247.0088	308.0294	475.198	409.9948	499.6704	196.231
neg_3447	11-Dehyd	2327.498	1480.823	1311.6	3392.667	1921.096	1913.345	1837.129
neg_3454	Thymoquir	47.86446	45.77123	14.07691	7.712383	49.21063	36.41268	37.21873
neg_3455	Methyl bet	579.5095	688.5683	591.9627	219.8398	638.5335	591.3958	666.9302
neg_3458	Lisinopril-1	887.1615	409.6101	108.7196	380.5526	233.5167	387.7099	325.2276
neg_3466	Cyclo(glyc	196.7856	73.79237	991.0562	237.6825	17.59316	45.63252	95.6867
neg_3476	4alpha-car	17253.65	21913.73	23573.62	9759.17	2915.388	9540.472	20500.58
neg_3483	(1(10)E,4a,	323.5413	84.67923	26.63053	138.6701	155.4326	180.9765	146.4408
neg_3484	Cyclopassi	31.06064	107.3246	1.72E-06	99.24845	11.48382	63.12116	78.50766
neg_3485	PG(i-14:0/	100.1567	109.5163	12.5958	36.8903	1.72E-06	56.796	66.97552
neg_3490	(6Z)-Oct-6	98.44271	75.82512	28.84056	84.2189	108.4213	100.7984	68.81007
neg_3491	7-hydroxy	313.9984	21.39947	1662.664	22.35213	72.1945	571.4717	2533.556
neg_3494	(ent-2b,4S	1624.251	59.534	1183.542	927.5543	152.528	368.3412	1870.651
neg_3501	CL(10:0/10	9541.129	89129.05	52.92203	40.23969	53.33714	2187.4	7328.987
neg_3502	Cyclolinop	297.2727	2928.923	1.72E-06	1.72E-06	1.72E-06	43.51492	217.2182
neg_3513	Ergocornir	3161.057	7686.551	703.299	490.206	559.8702	1618.962	3078.171
neg_3519	Chlorophy	418.8522	1225.612	54.13722	28.87841	39.00059	194.7445	365.9043
neg_3520	Matesapor	329.4673	1277.931	1.72E-06	1.72E-06	1.72E-06	52.31647	292.2092
neg_3524	Carmofur	84.09499	153.4613	5.815252	7.777184	14.01942	46.64381	106.3253
neg_3525	Apramycir	170.6923	292.9419	20.30698	18.40559	15.99432	82.83588	166.6559
neg_3530	1-(9Z-Nor	128.0122	86.84613	191.4866	28.60918	2.707414	28.34693	106.2297
neg_3534	Simulansir	611.7239	228.4096	37.0683	58.25792	37.51055	3.054379	115.325
neg_3544	Fusidic Aci	400.4204	246.1848	55.85986	3731.143	109.7227	147.7331	82.44998
neg_3546	20-Trihydr	413.5115	225.1444	75.68325	744.2719	205.8193	433.947	154.6419
neg_3550	1R-cis-3,3	1898.257	1626.27	772.5209	2665.739	1462.24	1922.089	1457.398
neg_3558	Chromafer	244.5275	160.3997	174.3048	292.408	152.9541	262.4648	175.7726
neg_3559	24-Fluoro	573.4252	79.34685	93.20622	339.5002	1011.006	62.07134	1514.542
neg_3562	MG(0:0/20	788.6262	268.6149	355.8797	224.3356	259.2095	184.7584	359.1648
neg_3565	alpha-Zea	102.0597	35.17775	29.03627	84.98847	45.87163	69.34372	32.77461
neg_3566	7,8-Dihydi	125.7235	95.45415	139.3537	196.1213	71.26415	121.0628	70.28101
neg_3572	5-(N-Meth	76.06832	44.79669	17.88303	68.20843	60.82926	74.39663	24.30187
neg_3580	Benzyl phe	524.879	160.2195	282.7528	303.4042	300.6157	318.0527	539.3194
neg_3582	Palmitoyl /	224.8269	354.2438	12.54249	42.80163	18.49611	128.8086	154.4666
neg_3583	N-Docosa	92.11481	78.11443	1.72E-06	371.933	21.33894	21.98219	29.29048
neg_3586	N-Arachid	6042.795	1735.436	1207.523	6186.696	32500.75	355.2959	20999.13
neg_3590	Sinapoylsp	820.1677	601.02	398.3467	225.3309	473.9958	658.4505	668.7294
neg_3593	1,3-Diacet	134.8658	53.62817	16.37432	4.715036	63.02714	124.0493	75.75118

neg_3596	Prostaglan	438.3465	553.0187	405.8382	565.713	478.8488	520.5477	513.2913
neg_3597	Dihydroxy	91.5756	111.1758	166.8822	101.9895	134.5405	97.74718	105.2178
neg_3601	3-Geranyl	147.2999	91.80368	38.99497	51.84486	23.97978	34.82782	64.6783
neg_3603	His Thr Lys	1636.338	504.1634	1915.425	814.3357	583.9826	1018.232	614.0909
neg_3605	Ethyl 3,4-c	169.1401	197.1453	197.299	178.6485	386.3544	247.9599	177.4848
neg_3609	Pteroside I	665.7212	617.5067	145.6217	588.031	570.6946	791.0374	419.1938
neg_3613	3-carboxy	1309.258	2478.208	1445.795	5693.843	2636.825	2451.708	1819.588
neg_3617	Prednisolo	147.2953	220.4355	2.814235	96.81226	219.5699	136.4168	120.6755
neg_3620	Mupirocin	4479.56	6501.189	639.0892	1359.665	569.1061	2204.04	2686.118
neg_3625	3-Oxoocta	8.310092	19.15235	18.6751	64.83186	15.70974	48.72114	20.91669
neg_3626	9,12,13-Tr	576.1735	573.8307	605.6602	625.8775	426.2909	558.1111	434.588
neg_3629	1-(2,6,6-T	31.78076	31.7369	7.501523	113.9778	59.50846	48.39458	10.6986
neg_3636	11-deoxy-	235.1806	141.5668	13.4687	95.0368	114.4607	149.8399	58.82849
neg_3639	PS(22:5(4Z	1.72E-06	5.37573	378.0848	403.2097	1.72E-06	41.57828	203.1945
neg_3644	3a,7b,21-T	5433.178	2880.33	3201.474	2419.842	2982.121	2235.883	5762.396
neg_3647	11-trans-L	14167.71	15833.57	58981.07	43882.85	9111.264	21146.84	35249.97
neg_3648	Austalide (	950.2102	959.4174	4246.329	3177.509	533.6967	1514.448	2492.122
neg_3650	Manumyci	166.1238	172.9672	553.7586	396.9891	238.3977	125.9695	458.902
neg_3656	PG(16:0/0:	1294.534	81.23862	504.7846	269.0037	156.7366	163.1263	325.1557
neg_3671	(S)-10,16-	3956.82	6509.491	1515.79	12939.99	5163.139	1952.214	2334.61
neg_3673	Auxin b	200.1011	351.4193	51.08697	687.5119	261.1949	100.3694	107.033
neg_3675	16,16-dim	925.95	807.5018	540.1958	1047.782	754.9452	997.8867	609.6551
neg_3677	DG(10:0/2	336.6156	110.9665	252.3859	139.7812	37.73778	47.71131	86.65123
neg_3679	(1S,2S,4R)-	350.0804	209.1295	86.34038	105.337	353.8382	225.7556	116.6344
neg_3680	13(S)-HPC	30.8804	40.87721	1.72E-06	80.53814	19.13789	13.5532	1.72E-06
neg_3685	Corticoste	1397.091	1002.875	672.5723	362.6913	573.4063	586.7515	1092.042
neg_3686	9'-Carboxy	9643.449	6568.777	2914.791	1698.243	2758.193	4420.793	7089.701
neg_3687	3-alpha-A	370.4448	264.9089	55.21681	58.36497	76.26074	161.4246	235.9798
neg_3688	Kanamycir	262.1692	240.2609	281.5626	297.5789	73.88662	158.053	372.9394
neg_3689	Mibefradil	1677.413	485.4981	407.1626	212.1076	148.3801	337.4373	754.9304
neg_3690	Cholic acic	228.8885	87.15565	214.8891	210.0755	294.6536	74.491	58.23294
neg_3694	5alpha-Pre	351.2434	364.3166	382.9241	359.1235	516.8012	238.4721	366.5349
neg_3695	N-Arachid	3156.656	381.1439	2812.337	379.6255	435.2584	1931.362	2167.702
neg_3696	Lucyoside	9.518942	14.42229	9.235619	96.18312	2.031836	20.14601	3.785133
neg_3697	DIHYDROJ	251.5709	228.0585	168.1675	150.7704	144.262	224.6674	192.013
neg_3699	Dodecane	13779.03	13003.56	9649.054	8690.86	9087.075	11483.34	10323.88
neg_3701	(S)-[8]-Gir	3867.611	6637.766	1536.928	2369.244	6788.968	4404.883	6267.762
neg_3703	2-Ethylsub	196.9605	79.55518	107.8439	146.2733	67.03784	64.50265	159.9638
neg_3709	Moxidectir	1051.065	290.9488	1.72E-06	31.72093	135.0917	191.5423	64.02515
neg_3717	N-Eicosap	656.6487	824.1386	192.4801	170.5053	254.7763	328.1086	774.8653
neg_3726	Taurolitho	666.2126	728.0933	581.652	283.5546	290.4094	449.9502	838.1334
neg_3731	Taurochen	84.23193	107.8375	79.50452	49.26684	56.50561	66.28432	131.7483
neg_3739	PC(20:3(5Z	60.76338	154.5131	1.72E-06	2.478311	13.9166	33.84612	111.4291
neg_3744	PG(i-24:0/	14346.43	17512.31	6080.861	2355.519	2305.746	7729.475	17484.3
neg_3745	26-Hydrox	115.4353	74.9499	5.330252	2.930747	1.72E-06	23.62376	29.71939
neg_3748	PC(22:4(7Z	391.9901	426.3094	202.5041	94.81229	75.95556	200.583	546.4205
neg_3758	Lefamulin	170.6477	86.9037	3.753239	87.92473	403.6038	2.20565	681.2559
neg_3759	Milbemyci	1295.979	1352.329	1162.418	726.6177	900.169	785.0244	1880.994
neg_3776	Adamanty	8.57779	7.182773	1.72E-06	11.11066	10.65881	7.977042	1.752799
neg_3777	(20-{[1-Ca	2492.345	693.9278	361.8745	332.373	454.5287	578.8882	1179.566
neg_3779	PI(22:5(4Z,	74.99546	59.0529	703.8206	299.8673	1.72E-06	8.810193	40.82804

neg_3781	PIP(22:5(4Z	409.2409	849.3922	10101.94	6462.367	87.24845	269.1187	493.3497
neg_3782	Urotensin-	25.68759	79.43828	351.6798	218.2708	1.72E-06	11.10136	35.00462
neg_3783	Arginine v.	38.21134	97.13667	811.9197	392.1811	1.72E-06	3.3013	35.7692
neg_3784	Glucurono	162.6055	236.5759	643.5723	522.6782	97.54395	150.1601	187.2748
neg_3788	15-Keto-1	4115.504	5664.381	17192.51	13089.77	2217.1	3679.523	4327.753
neg_3796	PA(2:0/18:	46492.72	78466.38	287958.9	208688.9	26403.32	41357.18	54807
neg_3799	Hygromyc	197.3459	267.2488	1044.264	500.0935	97.1614	120.7776	304.014
neg_3800	Netupitant	1804.427	2535.473	11485.35	6438.721	1416.843	1540.812	2140.909
neg_3801	Divinylpro	1570.515	1921.825	4927.777	2676.844	797.7525	1197.75	1926.135
neg_3807	Benzoyl-fv	75.93203	142.2947	617.1029	442.2793	14.42266	71.95047	105.4762
neg_3809	Norfurane	26.83028	38.05137	89.71046	78.94352	8.063201	19.37523	19.91351
neg_3810	Bambuterc	18.48417	96.52965	9.714173	60.47295	70.6254	62.92061	8.352589
neg_3811	19-hydrox	680.9067	381.1242	844.7033	597.5507	635.4709	512.9848	424.1192
neg_3813	Metkephai	60.71995	56.64327	498.2688	251.0564	63.66215	23.07446	47.95108
neg_3816	6-(6-(3r-H	9.764004	1.72E-06	1.72E-06	2.564386	0.853693	7.598242	1.72E-06
neg_3819	6'-Oxo-G4	1028.379	476.1254	393.7804	525.4737	404.3466	787.5677	849.6544
neg_3828	cis-p-Mer	1911.101	1156.986	464.2386	431.8483	720.3502	1097.926	920.6643
neg_3831	3-hydroxy	3887.178	3856.534	1419.208	2297.005	3074.654	1364.641	1582.809
neg_3834	DG(12:0/2	13.60567	262.4465	59.16355	47.40022	44.02886	81.27386	36.92993
neg_3841	9'-desmet	3.468439	2.629723	3.900023	135.8032	36.55897	120.0886	2.181124
neg_3849	Filipin II	47.60416	245.5907	28.55966	169.2716	72.21705	134.9919	55.70515
neg_3852	(1S,2S,4S,5	365.1859	525.0727	307.3925	352.062	225.0439	359.189	291.5058
neg_3857	(R)-Butapr	3711.248	3423.672	1695.412	784.738	1739.437	1984.651	2776.391
neg_3862	Methyl (2E	512.7048	183.2781	501.868	406.055	223.688	184.4614	102.1697
neg_3864	16-Hydrox	767.5417	1629.513	301.8415	1834.051	1381.821	420.2054	580.8668
neg_3868	1-Hydroxy	605.1433	590.1823	1219.443	568.2291	599.7825	575.2004	689.8143
neg_3874	Urocortiso	26.69687	1.72E-06	142.5339	102.2854	1.72E-06	1.72E-06	1.72E-06
neg_3875	Tuberose l	6.138555	1.72E-06	97.37481	24.46769	1.72E-06	1.72E-06	1.72E-06
neg_3885	Epothilone	93.61002	3.456804	443.9835	144.015	35.04287	13.64062	44.74818
neg_3889	Leukotrien	227.2014	32.42668	770.2433	307.1995	92.09831	22.87344	133.1475
neg_3893	2-Ethyl-5-	269.9847	67.23881	1206.926	428.2426	132.2523	29.18422	122.823
neg_3894	11-Hydrox	570.0729	19.88329	7502.494	1398.507	93.31947	12.11763	157.8669
neg_3895	Azaspiraci	122.7986	1.72E-06	1669.544	285.626	1.72E-06	1.72E-06	12.441
neg_3896	PGP(20:2(1	62.92361	4.765468	1294.316	251.7099	1.72E-06	1.72E-06	1.72E-06
neg_3898	2,3-Dihydı	1070.329	1179.317	2053.413	1082.768	1506.906	1013.536	1278.47
neg_3903	Tsugariosi	155.7463	651.5919	616.2963	1153.012	328.5809	201.1985	122.2274
neg_3906	3,4-Dihydı	3936.242	3427.9	6818.955	4075.048	4041.178	3254.949	3544.039
neg_391	Eicosanoyl	210.1569	356.2871	592.167	211.3603	417.4381	263.7814	266.0376
neg_3918	1-tetradec	161.4435	142.2186	343.4349	166.6086	294.746	190.8928	255.9208
neg_3921	Canesceol	193.4593	188.5224	218.3003	157.6627	220.2014	204.7913	183.0673
neg_3922	2,5-Di-ter	270.944	140.1092	80.84167	472.9337	212.9916	204.6274	122.5642
neg_3924	9-Hydroxy	815.3993	343.0354	143.7249	408.4901	593.151	264.6326	242.2216
neg_3928	Crustecdys	105.7162	3.127904	1.72E-06	3.472691	58.86681	4.528159	1.72E-06
neg_3932	Linalyl cinr	187.3993	128.2107	110.3398	68.96529	9.580331	205.4233	346.1333
neg_3937	3,3',5,5'-Te	935.8532	275.2035	167.094	67.23963	128.3448	111.7787	375.4308
neg_3938	Taurourso	34897.05	117425.9	7264.886	5987.621	9532.419	14965.13	25054.61
neg_3943	2-Amino-	50.95006	179.8504	1.72E-06	1.72E-06	1.72E-06	8.415922	30.08466
neg_3945	PI(22:3(10Z	626.6613	7047.062	568.309	179.2783	53.50017	824.7888	832.5244
neg_3946	3-Methylb	2730.039	2846.858	6100.767	2870.362	3829.549	2614.098	3354.542
neg_3962	Metoprolc	5518.437	1342.444	496.3772	1147.981	1248.992	1082.188	3827.28
neg_3972	Brassilexin	462.2396	507.1607	917.518	487.2957	621.2341	465.2576	565.8279

neg_398	Tetracoser	81.64468	93.61985	135.7991	117.0086	131.4859	81.09143	112.5033
neg_3986	Pregnanec	15229.26	5765.401	6307.527	6111.201	4525.146	9306.091	5660.541
neg_3999	1,11-Unde	1661.824	1428.423	1528.533	385.8005	898.6372	1495.862	1066.652
neg_4003	5,20-DiHE	694.3212	1644.11	321.4882	479.3671	567.7799	707.5831	1061.348
neg_4006	Neoporrig	3697.984	598.5639	3293.419	1704.919	1128.953	1202.792	2343.564
neg_4013	Talaromyc	1358.722	593.1457	265.7495	1300.588	392.2433	451.5671	320.962
neg_4015	5-Heptylte	1354.83	1370.598	2877.296	1444.221	1852.535	1370.45	1610.401
neg_4020	Metapro	128.6401	16.37711	29.69191	57.59753	66.71882	42.42531	77.54684
neg_4023	Isoplumba	443.4163	456.1788	774.7385	482.0988	551.8813	476.8251	501.9919
neg_4033	N-Docosa	194.1921	166.4021	39.60197	673.7666	257.5117	154.3731	164.0272
neg_4036	(3Z)-Phytc	52.52102	122.4516	13.5064	16.55201	11.39679	30.30992	53.75685
neg_4038	Lividomyci	223.9609	540.3477	76.05068	33.09715	65.48236	130.9774	321.3371
neg_4042	PS(22:2(13	2960.041	20510.93	92.63473	1.72E-06	131.8284	963.0006	3018.224
neg_4048	(24R)-5b,8	104.6006	322.9519	104.8235	360.6036	163.0877	346.0527	107.7422
neg_4057	MG(0:0/16	925.6288	428.6044	143.7834	538.8851	728.7956	390.566	432.3413
neg_4060	5b-Cyprin	387.3658	954.8329	2144.622	370.6973	64.3757	282.5473	1704.503
neg_4062	1-Stearoyl	29.19411	1.72E-06	1.72E-06	1.72E-06	1.72E-06	133.1215	4.309836
neg_4066	Cyclohexa	4122.045	106.6455	2271.041	563.1174	380.2595	523.5619	1884.462
neg_4068	Baccatin II	672.4995	850.0395	6691.495	2091.252	342.914	458.6377	1981.491
neg_407	[(2S)-6-Ar	702.1013	1088.685	581.6268	712.6443	678.8573	861.9564	714.7388
neg_4076	13,14-Dihy	459.2963	554.4585	179.1632	1882.054	507.1117	418.9547	400.3977
neg_408	Proclavam	65.47179	77.42331	59.29522	73.97614	77.09908	65.30934	70.48521
neg_4085	trans-2-tra	368.4213	322.5142	157.4376	120.4581	222.5033	148.7681	173.7893
neg_4092	(15S)-15-I	3318.346	1015.968	7659.641	4322.139	2338.66	3442.17	2818.925
neg_4099	Vomifoliol	920.406	353.2461	163.6071	244.3287	402.0628	364.191	387.9662
neg_4103	L-2-Aminc	100.7683	101.3547	207.1026	99.01941	144.4031	100.2311	103.5143
neg_4107	10-hydrox	353.1526	1395.359	1712.59	991.9651	236.6022	577.672	976.9574
neg_4111	1-acyl-PA	765.777	547.9092	193.0578	848.492	579.8948	997.9327	452.2844
neg_4116	Dethiobiot	90.46216	76.49168	20.74257	81.17792	90.15855	45.64408	33.39896
neg_412	Edetic Acic	11984.82	13226.81	10229.48	11764.25	10751.97	12036.95	11987.01
neg_4120	(14S)-14,1	2629.609	1472.656	1131.529	510.126	971.5128	1057.695	1165.431
neg_4126	Momordic	22.43449	71.01745	26.03744	178.0341	40.65497	105.8712	34.91885
neg_4127	Dioscoretiı	16.76407	135.7086	344.5079	263.369	73.77687	134.7211	427.5536
neg_4131	Glutathion	364.8887	459.1714	571.8097	1570.687	196.9252	280.7512	755.8133
neg_4139	Deoxyshik	61.18596	110.7681	53.80708	11.46875	0.093869	10.62717	3.901595
neg_414	Dillanol	1901.406	2017.72	1252.049	1694.262	1574.646	1786.334	1695.585
neg_4140	8,8a-Deox	291.0234	404.6728	242.5067	475.8544	256.7543	159.0612	206.3612
neg_4142	Digoxigen	201.4598	264.6233	256.3255	356.3296	271.1184	242.6848	230.1193
neg_4145	Dehydrotu	973.886	765.3226	812.6174	600.3751	925.6143	467.744	412.9822
neg_4154	Panaquinc	1266.236	820.1227	1921.742	1187.698	565.8936	321.5973	1047.822
neg_4155	Antibiotic	23.95145	1.72E-06	1.72E-06	18.02677	3.747382	1.72E-06	1.72E-06
neg_4156	PI(18:2(9Z,	401.4876	325.5649	792.9161	441.9855	671.7091	375.7907	464.9275
neg_4165	Cholic acic	1280876	304682.8	690982.4	438330.4	772432.8	386609.9	414974.3
neg_4172	N-desprop	320.0732	122.4213	227.7202	163.4545	274.963	168.4358	139.5567
neg_4175	L-Pyrrolysi	631.9773	241.9582	476.1755	326.0884	538.7558	297.3466	312.4984
neg_4181	SM(d18:1/	48103.21	11729.06	29633.19	18901.94	35737.83	16096.39	17359.68
neg_4184	PG(i-19:0/	689.9378	411.5426	959.61	564.8063	897.2883	469.9883	576.3053
neg_4185	PGP(20:1(1	254.464	96.99496	225.3801	137.8426	230.9624	126.0248	127.8239
neg_4186	PGP(20:1(1	1120.797	606.4924	1305.863	788.4971	1309.165	699.1415	782.7458
neg_4187	Tetradecar	5009.595	8516.45	4999.45	4591.857	2650.557	4895.784	4538.637
neg_4188	25-Acetylv	1991.487	1126.636	1430.218	1684.569	829.1373	1033.87	1316.804

neg_4190	trans-Gera	758.8874	1262.344	835.5344	745.8725	481.568	810.1125	743.612
neg_4192	Milbemyci	15.43313	50.46924	16.68723	14.866	2.395991	22.42973	4.31075
neg_4196	(Indol-3-y	86.16287	89.00167	6.625693	1.72E-06	32.2656	25.65644	15.94725
neg_4200	Cyclopassi	17.79362	54.73997	1.72E-06	31.29844	6.417801	21.52874	11.23223
neg_4201	HYDROCH	58.16814	64.05511	36.06042	48.41359	38.97189	58.09872	24.12373
neg_4205	Prostaglan	1214.941	913.895	1595.178	922.4881	1710.869	2467.832	2180.577
neg_4210	(10S)-Juve	384.3313	322.8993	142.5856	550.5064	384.9503	210.5113	224.6371
neg_4211	20-Cooh l	2107.04	799.6079	951.2345	487.5348	758.1384	1064.22	1369.92
neg_4218	CDP-DG(i-	94.51852	43.73037	1.72E-06	3.07922	129.7044	199.7765	59.77397
neg_4220	Annosqua	178.3534	345.2979	384.209	217.6804	135.0133	281.6189	409.2107
neg_4222	[16]-Ginge	158.6047	122.7935	213.4941	99.35044	60.14481	33.77892	124.9488
neg_4235	Glyuranoli	539.5458	613.8456	204.4569	159.5261	181.7366	249.0171	436.7928
neg_4236	1-Octadec	4935.207	6879.022	2402.991	1645.895	2361.874	2994.935	4973.815
neg_4242	11-Malein	85.5101	77.01079	134.3523	104.3115	27.80408	42.37411	21.78972
neg_4245	Didemnin	442.758	802.317	2.944442	8.385616	17.50925	136.7289	399.8722
neg_4246	Linaprazar	534.6285	97.25385	143.871	171.5857	272.1387	176.0776	129.8277
neg_4256	PA(22:6(4Z	282.8466	15.28531	17.12411	25.99652	46.6301	14.28991	27.8829
neg_4260	5'-Oxoave	96.15639	112.3614	92.02786	123.3639	77.49811	126.774	149.9734
neg_4262	Isosakuran	45102.21	50261.96	63969.66	46424.74	42007.5	51122.96	64393.27
neg_4265	N-(3-(3-H	1200.012	1458.192	818.0224	781.178	636.9781	1303.807	1408.947
neg_4266	4'-O-meth	1476.051	1619.692	2322.982	1570.92	1571.722	1680.895	2228.382
neg_4267	Rifaximin	154.1286	98.77869	69.59087	80.54959	503.0702	472.6646	170.2493
neg_4273	Androsten	317.5979	233.1513	297.9886	232.3255	233.4607	307.0728	219.5679
neg_4276	Andrograp	450.5091	363.4719	409.5282	393.9871	411.7463	470.8663	435.8506
neg_4281	Tempo	320.2888	343.198	562.4253	328.7376	267.2619	344.1082	347.3931
neg_4283	N'-(1,6-Di	3126.776	3355.658	5367.188	3236.243	2312.158	3299.576	3342.807
neg_4284	Eremopeta	618.5477	648.1235	1020.921	629.5273	447.791	646.1038	638.4653
neg_4285	4-Hydroxy	7040.325	7437.472	12119.61	7260.67	5336.868	7369.508	7498.817
neg_4286	L-Norvalir	16864.05	17905.26	29677.33	16935.15	13270.01	17587.38	17960.65
neg_4288	3,4-Dihydı	2256.475	2228.715	3822.709	2115.427	1666.06	2133.96	2270.477
neg_4289	9-F1-phyt	1687.301	1800.312	1569.237	918.4941	1031.219	1593.97	1193.234
neg_4290	Azastene	1550.124	1016.519	1435.393	1824.024	1704.651	1866.797	1448.624
neg_4291	(1'S)-Avera	233.127	215.4152	464.1105	227.8644	73.50549	233.6642	261.1205
neg_4304	Sorbitan la	620.8463	526.5191	688.7612	776.6795	540.4117	401.4937	610.8099
neg_4307	(R)-N1-((S	139.2291	131.8867	57.72755	64.8716	61.73873	109.8341	74.46747
neg_432	(2S)-Lactyl	313.8695	367.855	107.1627	301.8113	232.8334	325.8092	313.1433
neg_4321	2-Hexylbe	422.118	264.279	1047.476	483.5169	691.2551	307.6042	338.4088
neg_4325	Hydroxysir	1270.737	578.3868	520.8023	580.0833	714.4607	1376.71	970.4716
neg_4342	Cholylserir	8725.226	9994.097	8370.236	2694.637	5573.852	6224.123	10513.42
neg_435	Perfluoro t	714.0941	845.3925	622.179	708.1677	763.6062	758.8387	725.0051
neg_4352	(9Z,12Z,15	5491.686	6441.971	2250.261	301.2192	1256.786	2244.552	6515.79
neg_4355	PI(20:0/5-i	187.7292	226.808	69.77141	1.72E-06	48.57114	79.8234	269.2422
neg_4356	PI(20:0/PG	382.4035	429.2605	125.6774	6.547155	52.96606	130.4618	485.6123
neg_4357	Fungichroı	120.1381	65.64182	36.63251	127.22	14.15596	100.197	76.34756
neg_4372	16-Oxopa	252.4802	350.518	426.495	297.3734	385.901	779.6786	645.4043
neg_4379	9,10-Epox	953.9378	469.3948	519.6611	468.6723	613.9625	823.527	498.6832
neg_4383	Lysylserine	234.7583	178.0524	223.6268	184.7581	221.9101	207.7461	206.3531
neg_4385	L-Menthyl	310.9023	248.0816	287.5608	178.4848	178.2227	385.9941	265.2252
neg_4388	Muricataci	515.7883	402.3743	311.4886	452.9257	519.206	779.8058	466.1297
neg_4389	Corchorifa	4893.861	3693.427	3050.046	4059.669	4866.926	6711.184	4319.449
neg_439	dTDP-4-o	291.1101	346.5868	211.4716	274.7396	280.8753	280.1584	306.8509

neg_4397	Arg-Thr-L	64.54982	75.35465	35.86899	76.62967	90.16686	92.19874	29.7868
neg_4401	3alpha,21-	1357.603	264.4414	841.8136	322.5291	438.0714	523.3603	196.4891
neg_4411	7(14)-Bisa	418.9569	153.7738	216.1151	3149.976	230.4519	397.8847	293.579
neg_4416	Lofentanilu	47.14514	291.1879	243.1979	104.1907	37.71039	47.97992	268.5454
neg_4419	N-Jasmon	39.60821	18.88025	43.98155	34.27818	43.67203	39.2106	21.79274
neg_4433	Cholyl-L-c	1513.586	167.2331	1611.721	914.2896	248.2557	7152.516	4187.181
neg_4434	Remogliflc	76.46388	66.12729	112.6325	18.6311	132.4059	177.6313	61.44806
neg_4438	(4E,7E,10E	277.604	69.54808	244.4232	239.0381	443.3101	152.1992	340.6191
neg_4441	ORTHOTH	78.78534	114.0112	297.3012	117.2921	187.3244	314.7058	270.9531
neg_4445	4-Hydroxy	859.4386	761.0194	567.6619	544.5808	650.5037	1115.054	973.0682
neg_4448	N-Docosa	672.8855	652.7599	1480.769	1058.999	827.1851	1408.917	1722.867
neg_4449	PL	225.4763	198.3405	377.867	307.0981	237.5408	389.7186	504.1957
neg_4459	Becocalcid	172.0627	55.95714	717.8692	383.04	143.2468	66.13577	67.34185
neg_4461	Dslet	2272.557	1836.011	2203.871	3153.58	3864.156	1561.075	1903.749
neg_4468	Coagulin F	80078.67	64732.94	78161.76	103706.4	120551.9	56625.68	69394.83
neg_4471	10-Hydrox	14.25984	1.72E-06	112.3809	7.038448	15.45993	13.10825	36.1328
neg_4473	Nystatin A	7.873242	1.72E-06	131.3863	1.72E-06	1.72E-06	22.6941	50.44829
neg_4476	9'-Carboxy	250.7426	276.2445	491.7651	238.6701	344.9818	162.0294	282.4412
neg_4488	DEXAMETI	299.6844	113.1321	741.8177	277.2769	253.8525	322.001	433.8251
neg_4490	(3beta,17a	86.75044	97.82967	115.8264	73.24391	24.81162	79.48837	104.9179
neg_4492	Laniquidar	1187.772	1088.035	494.2216	1159.731	6950.927	52.10357	3112.082
neg_4493	Momorcha	1345.742	166.4889	4929.42	1074.131	600.9447	1376.641	2236.068
neg_4495	PA(18:2(9Z	1.72E-06	1.72E-06	16.2294	3.290861	1.72E-06	10.70608	6.527711
neg_4496	Iclaprim	16.5734	1.72E-06	158.4932	11.02659	3.096792	22.91218	57.1079
neg_4497	Ttatpn	102.0865	1.72E-06	525.1531	86.22532	42.69686	125.4663	213.2017
neg_4498	[Bala8]-Ne	149.9292	1.72E-06	467.693	122.2276	48.89274	147.5035	235.9294
neg_4500	Semax	57.87197	1.72E-06	261.8992	45.20584	25.11453	52.08018	122.6026
neg_4501	PI(22:5(4Z,	73.83785	19.81702	123.1275	150.7335	93.16685	76.22783	82.43344
neg_4504	Prolylpher	216.2779	200.264	448.9701	316.5656	178.8384	201.9772	284.3001
neg_4512	Pikromycir	1386.76	902.4849	403.4807	932.6789	3029.255	894.8072	1836.262
neg_4515	3-Oxotetra	349.1204	522.25	369.6099	326.7735	336.0096	684.0801	625.887
neg_4518	(13E)-11a-	650.1425	302.678	206.7691	338.1481	627.3421	686.0499	435.2213
neg_452	Cytidine 5'	3493.952	3802.769	1537.292	3343.931	2473.082	3315.148	3155.219
neg_4520	Lysocellin	273.6045	220.1498	123.9712	499.9247	369.7032	358.7495	276.2941
neg_4526	gamma-To	632.2539	405.0584	762.2333	633.6888	495.5365	941.4993	733.2784
neg_4535	Ciliatochol	274.7933	190.35	63.24319	173.769	157.817	208.0573	151.4693
neg_4538	Pregnan-2	704.7178	288.9043	726.2816	423.7716	476.3753	354.5279	493.4495
neg_4539	2,4-Decad	553.6291	324.5491	1334.078	1972.538	388.7639	298.7602	373.5145
neg_4541	hydroxyoc	526.1221	209.9802	149.5851	92.34687	222.6843	423.6264	276.2589
neg_4542	11beta,17a	272.889	155.4595	159.6577	122.1919	177.2907	264.3134	210.2458
neg_4543	17alpha,20	119.711	102.2556	82.51915	74.48401	91.68006	103.0951	55.54203
neg_4545	5-Tetradeo	747.079	896.2674	492.7074	449.3632	354.3618	639.1271	439.2306
neg_4546	3beta-7-D	810.0465	947.17	922.1047	1040.387	703.9505	1071.203	672.9607
neg_4548	Quadrone	138.9366	43.50951	46.43395	173.0643	24.02757	51.2422	42.0913
neg_4549	Gemfibroz	9046.728	3230.525	5470.337	11399.25	2876.497	4036.044	4241.813
neg_4551	4-Gingero	176.3511	129.1487	216.7842	239.1781	147.6648	129.4337	177.6525
neg_4553	Aspartame	39363.22	12735.66	23016.63	51023.42	11069.1	16723.73	17950.21
neg_4560	Heptyl 4-h	226.1286	228.5779	409.6272	213.9026	292.618	230.2313	247.7838
neg_4562	Leu-Leu-L	3753.772	3888.728	4583.527	4399.507	4843.933	5223.098	4636.147
neg_4564	LysoPE(0:0	243.5709	232.1879	254.9971	295.2642	292.3041	321.0248	307.4669
neg_4570	7b,12a-Dil	356.4734	195.217	35.44828	146.2413	206.3772	100.5891	181.6933

neg_4591	Monoketo	559.1213	393.9767	1496.502	197.4299	500.0161	1680.151	883.8548
neg_4592	Bardoxolo	1390.743	964.4998	206.0265	595.0252	1426.835	1621.174	655.4427
neg_4593	Cholylgluta	129.5352	139.2167	1.72E-06	63.82579	61.20611	79.80493	61.7731
neg_4598	10-Deoxyr	409.8343	390.4057	429.9181	611.0693	419.5435	489.514	326.4136
neg_4604	24(28)-De	144.974	88.66522	1.72E-06	67.3954	140.6285	222.7611	121.6446
neg_4605	[(3R,4S)-1,	8.973141	3.249403	1.72E-06	3.57126	13.99606	32.16687	15.9313
neg_4609	Hexosylspl	9697.412	9537.758	4355.96	8466.638	12391.06	11609.52	10435.97
neg_4610	Retinoyl-A	319.5082	334.1875	66.72675	254.0835	464.8183	372.5227	390.6487
neg_4614	His Leu Se	21349.73	30146.58	3648.089	6867.269	7207.537	9242.46	12549.77
neg_4617	(E)-2-hydr	88.11362	55.03907	46.84873	219.3516	77.29654	131.8129	90.92771
neg_4627	(±)α-CMB	246.4419	171.3393	218.3713	229.6117	102.1387	192.0408	134.5472
neg_4629	6-keto PG	88.65282	200.5822	361.2055	249.3296	126.1138	320.4159	54.08144
neg_4630	4-Hydroxy	214.661	192.0365	132.9061	195.6184	213.9635	274.9539	253.3439
neg_4633	N-Eicosap	5324.798	3599.506	2434.725	3193.141	3481.084	4359.168	3533.17
neg_4634	LysoPS(18:	269.2564	178.3286	100.6074	140.4698	151.8815	249.6961	175.3644
neg_4635	Cyclopent	97.71228	46.7473	27.00388	366.0619	145.9982	50.04873	67.94072
neg_4642	Fumarycar	155.8133	1.72E-06	266.7019	1.72E-06	1.771435	571.8783	267.9381
neg_4646	Mesoporp	20.10285	19.3934	2.868575	15.22182	11.1238	27.51941	3.027747
neg_4653	Chenodeo	202.6397	98.80294	83.03148	90.54852	330.9126	49.23288	293.4211
neg_4655	PE(LTE4/2:	275.5277	154.3456	25.73215	100.9743	101.6226	261.6965	105.4441
neg_4658	CDP-DG(a	34.00277	17.16169	1.72E-06	7.725831	21.34927	47.83294	21.57891
neg_4660	CDP-DG(a	87.66541	42.44833	1.72E-06	22.87256	27.26428	150.5633	37.69585
neg_4662	Ganoderic	135.669	161.1768	21.77266	132.668	78.24906	163.7344	88.6533
neg_4664	Desglucoc	40.11574	14.19966	1.72E-06	12.35005	18.54354	69.75406	15.99163
neg_4665	Calendasa	129.292	95.82253	13.54323	57.28524	103.5875	215.928	102.6641
neg_4670	9(S)-HpOI	289.6581	340.6904	498.237	359.1447	323.1461	335.6876	338.6981
neg_4671	12-Ketode	339.9938	23.12055	437.3854	139.1838	114.2817	41.90086	83.94837
neg_4672	1-(6Z,9Z,1	190.5903	164.1548	94.52068	153.5575	182.4896	264.9189	175.1618
neg_4673	VAPIPROS	2303.104	1791.24	909.6214	1683.501	2415.958	1780.617	1664.555
neg_4677	Destruxin	480.2261	387.0687	243.1566	303.5448	439.2443	516.4142	380.6477
neg_4682	Dihydrostr	464.8044	368.3061	255.7402	301.5154	436.2487	572.6955	439.6737
neg_4687	PE(PGF1al	69.28226	4.221349	39.02632	17.92102	22.53082	9.751572	12.83004
neg_4688	PC(LTE4/2	317.7368	108.2991	1.72E-06	62.90024	177.6725	284.2589	73.92467
neg_4689	4-Butyl-ga	30.25843	60.89039	47.51185	64.70699	43.55222	58.37438	59.04851
neg_469	Cyclic GMI	2554.792	2835.584	1152.321	2330.159	1891.03	2373.578	2186.355
neg_4690	Testostero	68.06251	53.32888	36.60666	63.57507	81.88387	85.19596	58.64809
neg_4693	Trypanoth	93.284	90.63279	66.92842	67.09692	119.924	105.9207	109.7916
neg_4695	CL(10:0/11	951.2701	557.669	101.6702	639.3615	1288.193	1806.975	585.809
neg_4696	Cucurbitac	170.0573	88.42588	2.233585	96.69927	205.596	309.0647	111.6246
neg_4697	Mepartrici	62.70946	16.19129	1.72E-06	25.4122	70.5528	130.1477	24.26653
neg_4699	Probucol	659.4357	727.0187	1074.901	657.9233	442.3169	700.944	1244.314
neg_470	UDP-N-ac	390.637	502.9129	105.1478	390.5929	205.5708	324.5347	316.6822
neg_4703	LysoPC(20	16659.11	13699.55	9864.14	17958.8	29074.65	24364.61	14988.59
neg_4704	Mavoglura	38.30748	20.19669	10.66145	47.00363	69.27243	57.43016	30.00913
neg_4705	N-((Hexah	1408.854	1206.388	1052.84	1756.981	2490.574	1732.757	1339.216
neg_4707	(5E)-7-{4,6	27.93385	5.215563	1.72E-06	1.72E-06	32.04535	58.22045	3.499617
neg_4709	Quinquen	57.43601	15.13226	4.540686	62.38536	128.7578	133.3193	21.5645
neg_4711	5,6-dehyd	73.65101	66.77948	33.08928	82.14307	118.5761	92.64555	56.00057
neg_4713	Ethyl abiet	486.3727	253.7288	221.4277	176.0487	228.5077	315.6764	213.3227
neg_4714	Homo-L-a	69.52795	20.39352	13.16546	62.65547	13.05601	35.13652	19.7586
neg_4717	Glucosylsp	14581.75	14201.47	6828.677	5050.176	20287.97	18706.15	9407.341

neg_4719	Galactocer	7944.135	5526.99	3250.667	6239.092	10586.08	8908.734	5429.88
neg_4728	Janthitrem	209.3535	113.6803	101.0647	215.0559	184.2729	207.6476	119.1528
neg_4745	LysoPE(0:0	1139.418	971.6869	427.1355	850.147	1014.115	1065.794	777.7341
neg_4750	4-Hydroxy	148.1452	78.13229	69.85143	395.4665	101.2399	81.23937	138.5451
neg_4754	13,14-dihy	3334.894	2170.88	723.8881	1330.04	4224.424	1803.52	2393.533
neg_4765	N-Lauroyl	1339.444	1025.073	2508.402	1135.858	1316.587	937.7372	1569.206
neg_4766	N-[(Ethoxy	210.4827	1256.604	2822.114	2489.575	826.6338	1023.176	4140.372
neg_4771	Galactosyl	5515.357	7476.075	3490.056	2887.822	8300.085	8395.575	5017.305
neg_4773	1-Nonade	98.50155	97.3093	11.79647	67.69844	112.2908	133.6166	72.97335
neg_4779	3alpha,7al	75.75412	1.72E-06	1.72E-06	16.56896	7.459314	1.72E-06	1.72E-06
neg_4781	3-Hydroxy	104.0745	73.61269	55.01572	60.99157	130.6069	201.1951	63.39917
neg_4783	PS(5-iso P	525.4925	213.1625	426.4693	445.5397	377.5027	230.8964	228.7726
neg_4788	24,25-Diac	154.2096	469.9334	92.08925	657.5937	112.189	119.6542	142.4091
neg_4789	(3Z)-Phycc	1.72E-06	40.77559	1.72E-06	89.59132	4.469365	3.396714	9.837475
neg_4792	16-hydrox	409.4402	525.3314	368.7013	520.3173	332.5568	295.7799	367.7109
neg_4796	Cholylglut	494.7704	602.7616	298.2807	604.2525	546.2612	508.5019	476.755
neg_4807	2-Nonene	478.0721	443.0838	532.89	576.9966	425.0467	549.9171	414.5067
neg_4811	2(R)-HPOT	3701.267	2940.514	3925.6	3037.996	2721.381	3340.483	3028.495
neg_4816	Hexadecar	3197.389	3650.936	2760.953	2073.973	1812.681	4547.725	2983.77
neg_4817	(Z)-7-Hexa	358.4214	356.4811	447.2934	423.334	297.8012	498.2907	275.3098
neg_4819	Erythronol	49.02293	22.69246	16.47819	37.99921	21.10409	10.45735	16.44937
neg_4822	Polysorbat	91.05316	46.841	85.96258	80.27638	8.809388	15.78239	49.78641
neg_4823	Gly-Pro-A	79.38472	64.9709	21.58356	49.90513	187.0387	96.84215	43.59789
neg_4828	LysoSM(d1	2699.424	1831.057	670.07	4672.986	2947.057	1557.361	1523.917
neg_4830	Fumonisin	1649.184	1196.622	945.7761	1725.156	1296.581	1534.853	1111.492
neg_4833	(8R,9S,10S	349.4762	322.7763	654.6079	329.1823	449.2906	398.0703	339.3884
neg_4837	Prostaglan	763.7174	585.5341	982.3597	683.9576	411.3793	510.5207	436.4473
neg_4844	Tanacetol	27.01998	12.22911	1.72E-06	30.45161	8.675868	40.6563	5.302831
neg_4848	Isocupress	1609.918	923.6869	1562.09	3032.26	1698.383	1270.078	1306.211
neg_4854	N-Lauroyl	58.95689	20.21588	51.13352	19.59459	14.95472	15.30313	12.57946
neg_4865	DG(22:5(4Z	1.72E-06	1.72E-06	4.028006	1.72E-06	3.488444	1.72E-06	1.715177
neg_4867	PC(16:1(9Z	177.1413	58.92754	187.6181	104.4665	106.0901	58.68194	67.96692
neg_4869	PGP(a-25:	54.66821	6.193065	36.4631	15.06836	32.87719	1.72E-06	21.95885
neg_4871	N-Lauroyl	155.6418	66.6795	97.8131	56.08105	55.29451	93.82031	63.9969
neg_4874	Sodium de	75103.93	12572.22	107510.8	31618.48	55758.96	18297.09	26400.81
neg_4876	2-[[3-Cycl	4660.396	1180.636	7872.479	2478.097	4058.651	1568.877	2232.367
neg_4878	Cholyllysin	270.2151	61.28189	360.3393	151.3336	257.8301	101.4547	112.8503
neg_4879	Deforolimu	152.441	23.21974	160.5049	59.40171	136.4146	35.31804	40.52433
neg_4880	PC(22:1(13	716.7727	556.3384	296.786	497.7987	728.5762	637.1727	510.1243
neg_4883	5beta-Cyp	26.68244	20.66647	18.60125	25.60763	14.29561	47.03647	20.04254
neg_4886	Trihexosylc	4443.937	4168.706	2906.412	4181.001	5014.686	7457.328	4648.793
neg_4888	Foliandrin	1358.107	1495.059	1130.752	1440.453	1627.475	2403.47	1674.288
neg_4893	1-(9Z-hex	267.7647	118.3989	222.0891	163.8044	237.5858	297.1317	230.3826
neg_4896	Fexofenad	2493.702	2369.781	1748.848	2114.015	2674.134	1798.716	2050.135
neg_4900	Cholylcyste	1259.923	1258.255	1255.309	1290.852	1488.997	1503.122	1350.245
neg_4908	20-Oxo-5	207.5763	205.1242	176.583	200.1552	226.4833	250.5537	212.5971
neg_4909	Streptomy	70.18309	61.79442	1.72E-06	33.72218	95.58731	77.07404	67.07276
neg_4915	Maytansin	518.6784	601.7896	668.6396	554.7293	813.6866	680.3134	699.2847
neg_4917	PC(20:1(11	1722.718	487.7254	1140.289	684.7525	1725.246	749.29	647.0811
neg_4918	PC(22:3(10	10466.72	1639.028	9706.94	4527.32	7641.059	3273.812	3498.321
neg_4922	PE(22:0/LT	151.5812	44.23326	72.32511	76.09778	110.3402	90.14057	34.22346

neg_4923	PC(22:2(13	506.3994	254.6869	268.0959	331.3706	347.6614	386.8797	244.8082
neg_4924	PC(22:0/LT	5029.323	3765.463	1739.506	2931.082	7330.868	7582.106	4018.031
neg_4926	Retapamu	41.3127	51.51219	1.72E-06	33.11706	124.519	50.32413	16.57159
neg_4929	Ganoderic	668.5143	602.7932	288.8775	448.2843	1099.944	1036.434	623.0696
neg_4934	Lithocholic	6045.894	7142.955	2281.536	6607.598	3640.026	3262.807	6504.234
neg_4938	HAMI3379	1203.568	1156.724	769.0198	887.3943	2027.547	1495.926	1215.892
neg_4941	10-Deoxyr	1182.962	1223.988	364.9328	1191.42	668.3844	741.1251	1134.401
neg_4942	Everolimus	64.58219	7.83857	1.72E-06	9.597552	58.67183	31.64528	2.786019
neg_4943	1H-Benzin	105.4192	111.3281	3.679227	73.78437	125.0091	77.01389	52.7729
neg_4946	Notoginse	165.6421	169.0179	1.72E-06	75.84607	364.905	249.2567	109.9532
neg_4948	Cucurbitac	490.8591	484.1679	157.7849	357.7459	705.6719	714.8654	310.2747
neg_4952	Dynorphin	20.7643	30.91157	9.617033	17.29315	42.3175	42.13021	6.951483
neg_4956	Bioadykini	10.86777	22.41537	3.3349	9.248673	49.7052	29.70896	1.72E-06
neg_4958	trans-16-(	245.8195	266.2899	185.7107	214.2836	404.111	305.8886	192.1078
neg_4963	Rhizoxin	1484.404	1689.205	1039.75	1196.711	2565.135	1990.452	1245.079
neg_4971	ROCCELLI(	202.0371	154.559	145.4914	128.3874	197.2654	216.3898	274.1903
neg_4973	8,9-DHET	616.5951	560.5058	462.4503	720.4663	548.9168	637.0632	713.7799
neg_4978	9-Acetoxy	339.8764	1.72E-06	325.7916	68.35862	1.72E-06	522.8678	351.8601
neg_4982	Docosanar	24.14385	17.87965	117.8583	149.6225	137.7747	13.61704	16.8387
neg_5004	Glycochen	48.93155	43.37366	14.07682	44.86533	50.84759	30.79104	36.20219
neg_5008	Acrimarine	220.1457	170.1078	131.1665	206.1027	115.6179	190.5815	162.0127
neg_5010	PA(8:0/20:	105674.2	92234.37	118518.6	132569.8	170004	82185.04	101325.7
neg_5013	N-[2-[5-[[	40.57263	38.38563	11.48725	26.77488	37.23325	56.32575	11.53628
neg_5014	PI(22:2(13	1404.994	745.5129	340.5377	563.6251	873.0931	1084.928	490.6856
neg_5015	PG(i-24:0/	372.5114	271.5523	98.53834	334.8236	226.2103	274.977	142.8471
neg_5016	((S)-2-((S)	260.3947	162.3272	38.9867	43.28866	154.5771	208.2169	80.80869
neg_5019	Cholylpher	216.0562	390.9038	149.6466	254.3138	515.2905	288.2687	214.7483
neg_5023	PGP(a-25:	695.8255	477.3733	426.3084	544.9789	531.1073	597.1624	479.5316
neg_5025	Chenodeo	713.1322	571.5473	202.0182	589.4919	733.4208	649.346	428.0965
neg_5027	Blumenol (	175.6257	149.2821	68.1573	152.7917	171.8634	166.9829	133.7198
neg_5028	Acrip	42.84255	53.94719	1.72E-06	50.71801	47.71691	56.99659	26.03096
neg_5032	10,11-Epo	13070.41	9538.753	5527.517	2842.29	13186.27	13627.15	5813.712
neg_5035	Hydroxype	38.47458	34.13689	1.72E-06	1.72E-06	45.9079	39.42863	10.75191
neg_5037	Penitrem [	2999.377	9446.579	4833.225	9217.422	13964.93	5841.865	4831.657
neg_5038	Peanut oil,	17122.61	19478.01	28220.52	18556.27	22223.28	18970.14	21338.36
neg_5039	(1alpha,3b	445.6291	4.269378	383.2561	189.7084	15.15609	560.629	421.3682
neg_5046	PE(24:1(15	21779.21	15859.94	9553.6	7893.442	21905.79	22743.77	11099.55
neg_5049	CL(10:0/10	31.28412	36.34685	11.32232	33.49113	48.21217	69.60721	36.27143
neg_5051	CDP-DG(a	5159.41	5634.202	5973.601	8392.752	9431.345	4956.702	5550.334
neg_5053	Stanolone	16.91874	12.54413	29.7305	167.1562	1.72E-06	6.616287	48.78632
neg_5060	Angiotens	3081.594	2731.857	1584.559	2350.929	2516.191	3143.096	2612.302
neg_5066	I-Urobilinc	1569.405	1669.403	2396.226	1629.971	1858.935	1600.441	1894.802
neg_5069	Linoleate	2198.671	2200.644	2617.595	2280.05	2495.142	2550.677	2477.241
neg_5070	LysoPA(0:(	1064.046	1120.983	1537.976	1112.382	1278.289	1202.51	1221.106
neg_5072	Cholylaspa	864.9924	941.1345	1275.222	923.9591	1005.527	903.694	1028.733
neg_5077	Stercobilin	452.5068	441.8841	438.1765	448.0768	488.286	461.8213	410.84
neg_5080	CL(11:0/11	44988.58	53721.81	47010.11	47859.75	102927.7	61533.05	43286.04
neg_5091	Cholylmetl	122.8619	67.76497	70.56646	75.95381	158.8831	134.883	81.29749
neg_5093	D-Urobilir	1779.688	2209.986	2443.653	1892.184	3388.858	2170.018	1914.487
neg_5096	(E)-3,7-Dir	438.496	472.3891	490.8396	415.9229	859.7506	506.7061	384.4636
neg_5100	Fluoroacet	784.1788	877.0067	1294.411	778.294	1011.259	863.1047	929.7828

neg_5101	CDP-DG(1	42.16119	58.0682	38.2617	66.92084	175.0297	76.29002	42.08941
neg_5104	CDP-DG(a	1028.397	1391.741	1313.675	1532.878	3172.762	1179.647	996.8963
neg_5106	(17alpha,2	1174.101	1379.506	1998.95	1916.355	993.7885	1171.67	1221.175
neg_5107	LysoPC(20	2286.671	2406.544	2509.793	1522.78	3818.216	2509.87	1869.689
neg_5109	N-Acetyl-	145.1115	177.8913	211.155	182.5489	270.0431	222.2451	196.9539
neg_5112	PC(2:0/PG	3584.327	4503.039	5724.778	3795.687	7789.305	4049.76	3898.459
neg_5113	Leukotrien	1879.479	2313.524	2941.242	1879.191	4121.067	2292.727	1986.306
neg_5114	3-Formyl I	270.9516	364.8364	503.5792	324.0316	714.1914	317.916	348.058
neg_5117	PC(24:0/2(	209.1926	168.8619	226.7791	76.59427	126.2897	90.65938	79.28074
neg_5128	Pangamic	544.3545	514.8672	374.685	913.3763	772.015	765.0388	802.0885
neg_5135	Levofloxac	744.5275	1014.847	1260.235	1531.74	1902.866	685.8751	794.14
neg_5136	16-Hydrox	795.5157	1307.995	1831.26	1409.125	1197.662	2284.523	1763.606
neg_5138	3b,5a,6b-(	645	633.1646	625.0733	825.4957	525.0974	586.8452	676.6733
neg_5141	Methymyc	299.1204	163.0542	88.16634	196.6095	125.5078	137.2653	146.8518
neg_5143	Valnemulir	36379.17	40264.75	81822.15	38147.59	45533.06	18678.32	38565.42
neg_5150	1-Hydroxy	213.8966	154.347	298.789	110.3286	439.8845	201.8712	249.0786
neg_5153	Deoxychol	334.863	243.0951	46.24961	118.6131	252.9629	325.5287	100.0071
neg_5158	Cyclohexa	203.7972	168.5482	117.4769	130.2953	118.8977	153.5134	99.05567
neg_5163	Gentamicir	68.99976	64.62562	24.91385	75.8154	49.69561	65.30748	41.6875
neg_5180	13(S)-HpC	16356.36	15254.02	23228.67	21292.3	13415.68	17558.93	13268.9
neg_5186	Secasteror	4288.569	5381.179	5025.094	4835.53	5927.716	5231.969	5652.284
neg_5188	1-Nonade	101261.6	122136.5	111572.2	105514.9	139661.1	126829.3	129901.5
neg_5192	Chenodeo	1014.36	662.1294	924.3916	773.4254	835.1854	760.9286	812.2955
neg_5198	Ponasteros	122.7311	141.3547	104.5735	119.0109	184.8416	154.6322	145.1907
neg_5204	PE(18:1(12	2947.841	3129.46	3771.575	3873.412	2904.389	3722.293	2789.755
neg_5207	Exametazir	107.2874	58.73493	51.80632	36.51258	60.80583	64.23716	64.99625
neg_5212	Nonadece	1049.399	815.66	995.2212	1156.105	944.8597	875.8462	883.9972
neg_5213	Decanoic a	886.5476	757.8797	642.6292	2249.588	887.8873	881.0069	981.8193
neg_5217	Octanoic a	113.9143	37.97002	66.70742	66.12246	33.58122	42.01279	52.42832
neg_5226	Aminovale	199.5533	115.4528	366.8347	272.1707	116.1085	113.5795	169.1144
neg_5227	Tetrofosm	187.5121	168.8202	312.5283	185.4185	93.20997	187.1421	239.5866
neg_5239	CL(11:0/12	603.3808	856.9392	70.94816	437.1507	1623.51	1484.636	267.1178
neg_5242	Cucurbitac	23.13845	11.86259	1.72E-06	4.338866	73.84256	75.66298	1.72E-06
neg_5243	Hydroxypr	383.9227	511.8351	523.4637	389.1907	206.1202	414.6429	457.1607
neg_5250	Cereulide	104.332	145.8203	3.792862	86.96778	308.0336	272.8362	63.40521
neg_5252	7Z, 10Z, 1:	215.9006	275.0134	145.3773	195.5552	394.5723	340.4034	190.32
neg_5254	Chenodeo	8448	9700.126	10092.36	8734.762	12700.85	10838.22	9146.641
neg_5258	Isepamicin	178.0436	1.72E-06	1.72E-06	45.15099	80.63427	69.90871	25.25468
neg_5260	N-Acetyl-:	108.7474	144.9088	80.8365	102.0486	214.2997	166.3907	95.18535
neg_5261	Hydroxyhc	65.40911	74.15417	34.62224	52.97358	137.3333	101.5816	44.67173
neg_5274	Phenmetra	209.6178	192.7234	557.1542	241.097	247.1445	314.3517	304.2187
neg_528	1,2-O-Isop	30.9455	40.4653	30.77838	30.20585	45.2177	23.15494	31.02112
neg_5280	3b-Allotet	856.288	850.4002	731.6014	848.14	867.3938	1109.287	873.285
neg_5281	7-[(1R,2R,:	1114.713	1013.919	1720.503	1430.627	2679.924	902.1472	1251.257
neg_5284	25-Hydrox	206.8121	193.0887	227.1667	284.1746	135.77	351.5491	247.7348
neg_5288	Oxyphency	116.7845	177.5115	55.23721	119.585	0.907974	129.7276	162.5223
neg_5311	Polyporust	751.5817	928.1095	331.4835	692.3144	941.9872	757.5292	476.2633
neg_5326	Chenodeo	1045.749	1029.69	1559.526	993.5527	1217.956	1046.495	1149.907
neg_5334	Didemnin	3330.365	3578.167	2831.106	2786.298	3500.331	3127.552	3696.892
neg_5345	(6Z,9Z,12Z	57.98082	39.85283	157.9002	93.79289	25.78982	33.00477	43.37944
neg_5347	PC(24:1(15	3222.616	1516.434	1031.224	903.6879	3287.02	3468.507	1630.229

neg_5352	Stearic aci	469.9887	607.0916	229.9093	377.488	776.1341	710.1449	336.6468
neg_5353	15(S)-Hyd	142.379	136.5382	176.5768	111.2569	88.32124	154.6309	130.123
neg_5356	Vertilmicin	95.42325	88.67748	10.2801	100.5032	60.61094	75.09028	38.96133
neg_5358	Gangliosid	6649.456	10042.83	1552.381	4778.68	15457.78	14590.68	3200.439
neg_5365	LysoPE(0:0	10554.01	4187.215	4075.086	1997.804	10445.33	10714.47	4538.872
neg_5366	LANEPITAI	3776.481	4609.784	3052.313	3393.174	5991.221	4951.725	3294.373
neg_5369	(1R,9S)-10	285.3029	361.305	183.132	253.1367	492.7135	422.464	243.4138
neg_5373	Actein	111.3203	56.00842	0.737232	31.28364	36.43638	67.27608	8.824417
neg_5374	Viomycin	16.75327	34.04136	16.52344	1.72E-06	17.22057	40.69519	12.71209
neg_5394	Deoxychol	1044.913	1100.449	1587.39	1126.888	1410.277	1244.974	1396.193
neg_5395	DG(12:0/2	2528.831	5482.811	3292.657	2614.617	2680.06	2429.497	2900.092
neg_5400	3-Deoxyes	28600	39010.46	32256.12	38628.22	31428.7	27112.41	28865.25
neg_5402	Docosadie	668.4058	1035.165	1512.417	1299.763	1260.446	3366.743	1984.685
neg_5403	N-Myristo	1201.704	1583.166	1346.294	1535.172	1336.166	1234.537	1257.113
neg_5407	1-Oleoyl-s	4651.492	5880.633	5267.858	5794.462	5436.78	4573.05	4876.739
neg_5412	3,4-dihydr	284.504	304.2952	291.2362	323.5619	334.8792	263.5858	295.6838
neg_5415	Lividamine	43.25261	88.97884	42.98735	64.27702	58.96017	33.63322	46.46101
neg_5422	(R)-3-Hyd	653.9138	270.0663	501.6533	339.4707	297.7778	356.8304	447.4008
neg_5423	7(S),17(S)-	110.3986	83.87251	159.4514	79.06844	56.5634	58.27212	53.54075
neg_544	6-Thioxan	10452.84	11864.79	14126.74	11292.04	11520.92	10439.2	12155.01
neg_5441	16-Hydrox	260.1982	979.3347	1193.958	787.6233	334.3255	1573.646	1178.214
neg_5442	Pristanic a	915.5109	567.0956	331.2751	332.1744	707.8116	745.7663	614.6961
neg_5444	Austroinul	496.6393	388.1756	288.9524	236.556	304.599	479.3891	308.9744
neg_5446	Janthitrem	7838.042	10153.69	3997.014	6755.449	25882.66	14004.15	7912.512
neg_5448	Bax inhibit	86.77505	76.8267	37.20589	75.12423	190.8407	121.4698	61.21656
neg_5451	Nummular	526.7563	697.7556	199.1357	473.8077	1959.132	944.2986	552.0403
neg_5454	(13R,14R)-	101.1863	145.6231	220.3237	153.5221	126.7795	352.334	183.8776
neg_5457	Capsaicin	135.0257	177.2435	213.141	159.6716	239.5978	135.8416	174.0707
neg_5462	MG(20:5(5	395.5261	327.4735	364.7128	364.0864	284.0543	346.5125	378.0303
neg_5465	1-heneico	66.92298	13.37095	8.013585	13.57359	46.82966	102.5912	14.20558
neg_5466	(4Z,7Z,11Z	73.69562	14.66725	24.52422	12.44636	58.40316	87.69019	20.29709
neg_5468	cis-9,10-E	1209.797	2666.179	2762.133	2464.537	1861.725	4725.79	2513.321
neg_547	Ribavirin 5	625.5102	769.127	1166.82	688.4602	1009.089	745.9089	813.6284
neg_5470	Epoxyeicos	248.6145	350.0742	427.319	346.5984	286.1905	588.1838	361.1732
neg_5471	LysoPE(P-1	5583.626	2369.63	1463.748	2021.899	4073.069	5887.677	2431.098
neg_5472	7-[(1R)-2-	15812.13	6860.577	4162.343	5693.112	11809.09	16457.05	6706.26
neg_5479	PE(18:2(9Z	53.44105	148.0282	769.4059	124.7234	273.8807	289.4556	402.2745
neg_5482	7-Methyl-	283.0969	149.0476	155.8714	387.4371	264.2257	295.4994	319.1833
neg_5486	(E)-8(9)-p	69.63459	60.11697	56.36358	75.24917	48.60265	72.54321	76.98765
neg_549	1,1,2,2-Tet	267.8723	307.8942	481.3237	273.1461	387.3964	280.7916	320.7157
neg_5498	beta-Phell	10.37333	7.623299	1.72E-06	1.72E-06	43.64716	32.43898	6.124771
neg_5500	Fumitremo	820.1431	800.8815	1255.956	785.9097	1077.819	920.5971	947.8703
neg_5504	Lotusine	50.79583	67.40233	189.29	61.59863	107.0305	86.98201	76.89826
neg_5517	PE(LTE4/2	1493.899	1514.938	1499.293	1564.895	1389.763	1513.325	1551.872
neg_5528	Indinavir	28405.81	31979.63	39769.52	32920.37	41102.16	32380.92	34231.41
neg_5544	CL(10:0/11	1314.791	1248.312	1057.243	1078.524	1582.101	1574.956	1100.295
neg_5552	DHAP(18:0	3009.076	3328.891	4389.792	2998.643	3535.986	3503.151	3684.857
neg_5554	Psychosine	124746.5	155998.3	145204.6	159470.6	160384.5	135946.4	135145.8
neg_5567	PG(i-24:0/	189.3679	44.27055	148.9029	91.30694	123.0137	104.1345	56.47768
neg_5568	6,8a-Seco	185.6694	153.3813	49.48477	80.66625	300.9071	246.4473	90.10643
neg_5572	LysoPC(P-	390.2273	303.249	315.1338	182.8971	428.2993	500.4009	335.8754

neg_5574	Methyl 3b,	1000.46	1107.608	1490.683	1621.57	1027.405	972.0228	1028.837
neg_5581	9,10-DiHC	26996.48	40270.01	55019.75	25790	19494.24	39425.47	39873.55
neg_5585	PA(P-16:0/	149.5114	286.1965	599.3892	99.49596	102.2399	292.1949	359.6213
neg_5588	9R,10S-Ep	74.66282	167.5715	239.4152	162.7	69.72996	327.7342	188.5759
neg_5589	13-Heptac	1219.222	2958.985	3303.514	2057.767	1378.513	4246.239	3056.373
neg_5591	PE(15:0/24	1674.413	2000.281	1739.359	928.2949	1340.363	2577.305	1721.109
neg_5600	trans-Dec-	62.06194	26.2305	63.82016	133.8211	72.1744	80.99058	83.12948
neg_5601	PC(16:0/0:	13558.25	11059.76	9287.214	8146.713	17539.42	14891.21	11027.97
neg_5602	Narbomyc	65609.9	55779.35	45493.56	39198.2	92532.7	76409.69	54724.61
neg_5622	Testostero	468.7424	415.1115	762.0408	319.6422	246.5713	269.2406	278.3091
neg_5626	Hovenidul	2505.386	2333.198	331.2346	1208.632	6558.001	5906.298	1780.942
neg_5627	Finasteride	169.9244	155.2567	242.3384	254.1349	200.8405	396.1103	340.8996
neg_5634	LysoPC(22	25408.27	27101.09	10667.88	11772	52449.12	40547.54	19095.21
neg_5637	Minaprine	209.3694	154.9624	76.90199	225.0777	330.2033	284.9175	260.8007
neg_5638	Mucronine	371.8159	338.0983	115.7114	171.4654	664.685	431.7503	259.8325
neg_5640	KOdiA-PC	2277.866	2671.846	1069.166	1295.104	4386.166	3285.351	1736.186
neg_5647	L-Fucono-	80.51388	102.6408	166.8088	95.01773	90.44822	115.0174	117.5811
neg_5649	Monacolin	252.082	495.6613	402.4378	559.2854	134.517	92.16558	168.6727
neg_5651	L-argininiu	78.16934	73.74849	109.5078	126.1152	64.35795	90.30789	96.40174
neg_5652	Docosa-2,	126.7557	101.462	169.2684	143.9201	69.64854	123.3249	144.7218
neg_5656	15-Dihydr	318.2097	367.6933	597.3235	460.2434	377.9522	644.0722	543.3466
neg_5658	N(6)-Meth	142.5994	173.9662	107.8272	132.3114	151.896	172.8557	150.0916
neg_5665	Tetraethyl	214.3087	163.7563	455.4277	349.5669	221.9343	569.1537	287.798
neg_5667	DG(15:0/2	228.3431	356.4151	464.5902	287.6239	345.7728	294.1451	318.2505
neg_5668	Ile-Ile-Ala	216.1106	160.2814	9.296041	93.12298	319.3803	337.7266	138.9353
neg_5682	LysoPC(16	19243.15	16518.6	11151.47	10882.49	26460.76	23384	14888.5
neg_5684	LysoPE(0:0	81874.97	68890.48	45779.25	43712.77	115379.6	102439.4	62711.13
neg_5689	BQ 123	210.277	151.2896	74.16904	149.3377	284.4335	248.2229	183.6197
neg_5696	PG(a-25:0/	90.50812	113.0844	133.0859	64.14234	105.4678	128.5645	92.06759
neg_5701	Gangliosid	121.1682	72.33887	1.72E-06	11.98349	232.5708	177.7852	26.08146
neg_5704	15(R)-HED	391.5865	153.0291	250.5267	127.2874	323.8196	526.5083	656.7195
neg_5705	Glycerylmo	154.1026	287.4538	331.634	256.828	301.032	679.0385	313.5614
neg_5708	35S-Methy	611.7145	445.5526	1.72E-06	163.2042	1126.962	817.7391	258.3176
neg_5709	CL(10:0/12	91.21662	74.68926	0.041286	40.59763	233.9583	186.9508	14.50398
neg_5710	13-Tetrad	20527.29	17108.6	29490.42	13621.45	19722.75	17835.8	15512.25
neg_5711	Linaloyl ox	74.80995	61.36469	0.972017	38.7769	100.1132	63.61992	59.35355
neg_5712	6-isobutyl	630.4808	510.5604	1185.126	548.351	669.0029	433.914	481.6911
neg_5716	LysoPC(20	22120.14	24108.61	11259.99	16026.58	34824.29	25354.44	17371.05
neg_5728	(±)12(13)-	225.5813	620.2246	836.2532	577.1304	263.6457	839.9125	699.2109
neg_5730	D8'-Merul	873.5971	531.2877	775.548	572.9667	512.9918	1028.851	666.7359
neg_5748	4-Trimethy	129.9993	96.23318	114.8011	89.67812	45.42567	107.1031	98.73881
neg_5754	N(4)-Oleyl	12848.71	8674.762	5170.268	6050.041	11518.99	12537.47	7161.822
neg_5755	Primaquin	69.48701	20.55067	1.72E-06	24.18823	68.15351	51.52341	16.26702
neg_5773	LTB4-d4	1111.137	1426.409	1823.589	1630.982	1178.388	1187.348	1122.69
neg_5774	Rotundine	229.4708	177.3254	100.3649	203.1607	170.356	228.6267	157.7825
neg_5804	Methyl 8-[	1528.704	1984.787	2932.581	3413.829	1869.337	2007.422	1803.952
neg_5805	Misoprost	908.2543	805.5423	944.1577	862.3612	688.0555	1278.059	898.1118
neg_5807	Lucidenic a	2589.322	1778.426	1781.204	1818.998	1093.938	1428.36	1216.126
neg_5809	(5Z)-7-[(1	83.08787	75.9599	60.78151	38.44558	66.00606	47.87946	67.51052
neg_5811	PS(22:0/22	335.8739	498.4013	667.1642	495.623	430.5212	491.5731	454.7323
neg_5825	25-Cinnan	215.8866	207.3888	102.0299	94.0995	317.9783	255.3519	187.1772

neg_5826	Eicosanedi	965.468	881.5667	950.4123	1099.94	871.8961	1096.117	927.0433
neg_5827	cis-p-Mer	192.7509	176.0204	246.2398	266.7313	161.2497	170.9024	170.2679
neg_5830	LysoPC(17	21937.27	23212.85	21544.06	16668.76	30079.84	24794.36	22200.17
neg_5831	LysoPC(18	4576.296	4828.097	4474.573	3593.32	6086.587	5257.588	4773.427
neg_5832	Cholylaspa	205.1331	305.8539	237.821	491.2921	336.363	139.494	167.3406
neg_5836	Ilmofosine	8525.979	8669.799	8157.294	6680.222	11758.73	9746.244	8567.455
neg_5837	Melperone	539.4824	546.1382	542.5857	451.9278	733.076	607.0636	589.2151
neg_5841	(17E,19E,2	257.487	258.0141	258.3575	191.433	445.4899	332.0456	250.0233
neg_5848	PC(24:0/22	131.1919	115.4883	119.7371	105.9418	75.00649	81.94973	88.73969
neg_5850	2-Nitroph	10346.31	12065	22218.83	11756.13	21737.41	13210.09	15603.27
neg_5853	Methyl 2-(	669.8998	522.6863	637.1542	642.1682	433.3901	681.9373	480.5291
neg_5857	Fumagillol	13.00869	3.10103	3.197633	8.614418	23.59543	9.658224	7.012852
neg_5860	Cyclopassi	1334.522	703.5098	208.4889	357.4736	1608.598	1603.759	666.8679
neg_5868	6-Ethylche	891.3643	583.2983	637.924	915.2784	527.681	587.9935	727.1538
neg_5882	Icosanoic a	238.0413	105.784	105.4059	256.5721	99.84236	120.7463	214.2805
neg_5890	METENEPI	4772.824	3348.518	4268.721	4873.954	2891.662	3645.921	4635.237
neg_5900	Rotundine	1020.596	1119.21	1851.964	1022.937	1467.045	1038.688	1219.185
neg_5901	9(S)-HETE	448.0647	416.3722	602.1528	527.1588	432.5237	538.4991	465.9883
neg_5903	MG(0:0/20	9957.674	7564.41	7498.881	7055.227	4695.387	6373.656	5033.623
neg_5904	6-Deoxoca	330.5088	148.5614	208.5709	497.5587	224.2816	211.5883	257.7732
neg_5909	DG(19:0/P	11583.23	14081.11	21344.56	12194.31	14184.41	6410.265	12803.15
neg_5916	Ganoderic	5338.295	5967.451	10883.5	6618.177	5977.42	5693.503	6739.808
neg_5918	PE(20:0/18	29110.5	22322.39	40361.11	30797.32	39960.17	15140.98	17631.78
neg_5926	CL(8:0/10:(	269.0014	289.5339	119.7315	145.5393	321.3313	155.4186	284.0697
neg_5931	1,2-Ethane	1163.631	1169.23	1282.983	1258.88	1093.382	1094.785	1072.76
neg_5948	Caspofung	5559.577	5962.698	4464.31	4050.994	6893.188	6073.298	5594.307
neg_5957	3-Oxoocta	258.335	443.721	593.615	521.5845	279.2528	710.1149	721.5126
neg_5958	3,7-Dihydı	1913.227	2188.639	2454.753	1862.312	2597.281	2006.414	2192.15
neg_5960	1-Stearoyl	737563.7	804830.8	892412.7	612390.5	1019032	813980.1	805730.3
neg_5961	Molindone	13.68854	17.24801	32.51387	13.95324	19.77146	17.59549	10.25829
neg_5963	Goyaglyco	167.8267	166.7501	225.7216	180.3936	171.9469	189.1951	279.0893
neg_5964	(1S,2S,3S,4	200.3208	229.9107	340.4946	217.7511	197.5971	228.4037	288.6627
neg_5969	Colistin	591.4257	651.9396	911.1845	551.6992	796.1315	695.2775	726.2379
neg_5972	1-heptade	138157.1	149655	168021.8	117512.6	190324.4	151853.2	149913.8
neg_5978	LysoPC(20	331.8586	392.0444	479.4956	334.634	509.3888	363.5052	409.0289
neg_5979	Cyclotricus	37.32335	149.4793	105.5573	157.1349	107.8094	60.29235	87.75137
neg_5980	CDP-DG(i-	2764.452	3563.464	6564.81	3565.669	5666.058	2917.327	3738.504
neg_5983	Leptomyci	1622.745	1717.964	2289.037	1467.189	2185.199	1814.015	1866.265
neg_5984	PC(20:4(5Z	598.3965	567.7841	512.7863	825.197	636.8718	663.8554	650.9426
neg_5988	CPA(18:0/(	508.4859	417.4023	656.6094	436.6227	506.1358	382.4426	525.7858
neg_5990	Avermectii	17425.42	21334.21	49124	23859.64	34218.1	17548.01	23645.66
neg_5994	Undecapre	27.81112	55.61152	30.1041	54.3778	10.74694	26.53566	57.0069
neg_5995	Fluoro 2,2-	1241.182	1439.393	2149.603	1306.79	1667.644	1347.398	1560.583
neg_6004	Palmitic ar	5.59892	11.84784	10.65713	8.25707	12.70235	20.66807	8.283569
neg_6005	MG(18:0/0	977.5265	548.168	765.4651	995.4784	512.51	629.1937	834.1007
neg_6008	Karpoxant	138.7706	102.6416	65.53967	144.1197	114.2083	130.4135	102.1119
neg_6011	8-Hydroxy	441.337	551.4415	804.4046	498.2069	795.4615	571.4663	607.5536
neg_6014	(3a,5b)-24	6563.602	7472.622	10920.77	6379.836	9545.681	6925.232	8246.87
neg_6022	PC(22:1(13	277.8411	396.4456	435.8962	426.5466	275.2169	409.4377	464.2665
neg_6025	Monacolin	5333.759	5840.764	2964.983	956.6044	2945.171	2949.5	5060.092
neg_6026	(-)-alpha-	150.6711	238.6149	213.3525	233.4237	133.8257	156.7684	141.4999

neg_6031	Decanal	43.45209	11.89654	9.730827	10.40237	24.6327	72.94201	24.75339
neg_6035	5-O-beta-	124.2981	79.45585	67.70941	88.43019	137.7336	151.8643	96.42794
neg_6038	SM(d18:2(	2171.316	1495.83	2950.927	2612.96	3580.133	2586.54	2545.702
neg_6042	11beta-Hy	21.34393	4.305108	6.110557	17.87373	12.48539	12.44084	2.486113
neg_6045	13-HODE	158.9251	158.2898	165.4976	135.3411	151.2746	167.3296	151.4834
neg_6046	Docosa-2,	889.2139	505.0318	879.5937	828.9527	599.687	648.9054	795.9202
neg_6049	Lansioside	1325.375	1391.576	1418.79	1231.963	2302.168	1714.571	1379.825
neg_6051	Linoleamic	16.06568	40.75793	11.24313	28.93179	4.473589	27.6198	34.48104
neg_6061	(R)-3-Hyd	685.6573	1933.318	1081.666	713.2568	729.388	1330.254	926.9622
neg_6062	DG(13:0/1	277.6372	190.5069	88.97763	213.7267	248.6801	228.2193	166.1856
neg_6064	DG(8:0/20	4.479274	49.38529	5.537614	13.33175	17.13359	2.314826	4.968384
neg_6065	MEDICA 1	3406.512	4077.582	5865.924	5255.243	4006.2	4869.524	3809.677
neg_6067	Armillatin	60.29608	55.04466	26.20669	56.14221	63.7839	89.23777	48.29911
neg_6071	DG(12:0/1	899.8875	885.7745	1267.338	960.0476	853.4339	694.8297	1166.9
neg_6078	SM(d16:1/	17713.24	22926.41	28068.49	20025.13	25438.02	11140.93	21479
neg_6082	hydroxytet	149.4947	184.8129	224.0651	328.0201	103.4546	149.9924	254.0705
neg_6084	2-Deoxye	6666.021	5590.142	5284.523	6315.96	4758.062	5019.713	5875.703
neg_6091	Sapacitabi	598.5744	1020.314	705.8026	861.0179	340.3002	674.0526	783.9455
neg_6094	2,8-Diben	373.4496	281.7943	717.7139	1235.055	362.6057	572.3497	652.475
neg_6095	PE(15:0/20	832.5418	961.5136	1837.717	813.7976	1082.31	808.1459	1119.251
neg_6097	20-Hydrox	154.2654	216.7215	191.2285	230.4291	61.65306	209.6033	203.4598
neg_6099	Tetrahydrc	560.2699	561.5776	623.2454	627.0326	510.2408	486.3352	658.8532
neg_6102	Calcidiol	2350.486	1765.819	1641.987	2001.76	1512.213	1555.659	1594.994
neg_6105	Juvocimen	1.72E-06	4.366028	1.72E-06	22.30052	1.72E-06	22.21176	1.72E-06
neg_6106	PC(24:0/P(	48.41449	12.57526	1.939656	2.016387	1.820468	76.71248	9.806133
neg_6108	DG(20:5(5Z	16.51038	1.72E-06	1.72E-06	1.72E-06	18.96556	104.8002	1.72E-06
neg_6112	Butanoyl P	48703.12	39382.39	30130.89	28359.81	55213.84	77362.58	43670.6
neg_6114	(2R,3S)-3-	230.9184	150.6667	78.87449	114.3836	234.1791	332.848	173.4318
neg_6126	Stearyl citr	2566.793	2286.729	2091.262	2706.177	1681.034	2163.218	2268.627
neg_6130	2-(3,7,11-	710.5315	816.9326	1338.644	703.4894	873.6864	678.1536	826.6428
neg_6131	PC(24:0/T)	27.34031	14.15373	21.70197	15.3062	10.86424	35.97391	7.083173
neg_6134	7-Hydroxy	132.717	81.37937	80.51797	111.6324	80.75396	77.33655	86.727
neg_6137	13-Azaprc	43.29785	41.26336	63.86078	40.95556	29.82218	58.78495	65.48073
neg_6140	2-Hexapre	207.1395	146.1175	33.46665	155.2923	192.5307	170.1229	123.8013
neg_6146	(8S,9S,10R	4517.701	3948.561	3488.912	4969.635	3645.007	3888.084	4159.171
neg_6148	gamma-L-	339.676	323.6047	256.8953	454.2633	248.3258	255.1516	274.6714
neg_6149	meso-Tart	129.3231	146.0175	236.3302	128.3056	178.5418	140.4817	154.0546
neg_6150	11-Oxahe:	183.2659	196.9293	294.4782	6143.286	119.3945	592.1818	595.8276
neg_6156	Docosatrie	336.0936	219.7173	431.5994	300.1541	225.5852	232.6749	247.0665
neg_6162	5-Choleste	1507.366	1406.448	1055.384	1376.587	1032.43	1056.364	1256.996
neg_6167	3b,15b,17a	72.83378	71.46501	83.6962	87.01099	106.1576	135.4386	88.20196
neg_6172	3-Heptade	649.5843	424.286	475.7743	239.2539	381.0571	374.5029	276.0063
neg_6174	(3beta,22R	318.997	224.5306	184.4724	264.6829	189.1992	224.4387	209.7696
neg_6175	PE(18:0/18	71052.39	24985.18	127256.9	31259.37	64471.38	19795.85	31442.03
neg_6176	PE(P-18:0/	176459.7	118829.6	295485.9	108710.4	140773	88550.78	95869.42
neg_6177	Prasterone	389.6661	326.5919	352.4573	478.7784	306.7317	341.3238	415
neg_6182	Oleoyl-est	83.75435	61.85856	39.51446	79.6592	75.93457	83.75797	65.26112
neg_6186	Digitoxige	1272.049	1318.009	1961.454	1232.717	1613.738	1319.169	1475.627
neg_6194	Procarbazi	149.2833	72.9655	82.76059	139.4297	65.28254	113.2393	127.7718
neg_6198	2-hydroxy	4213.992	5509.568	6757.352	5713.541	5060.748	5193.227	5940.41
neg_6200	Arachidon	1316.735	1601.924	2147.724	1676.929	1493.083	1603.965	1884.783

neg_6203	1-Stearoyl	2736.92	2485.766	2193.54	2337.134	3580.329	2808.781	2528.342
neg_6204	LysoPE(0:0	9336.498	9669.483	8620.524	8280.684	14507.54	10785.57	10069.07
neg_6209	5-Hydroxy	93.71602	92.0176	153.1285	115.067	90.98132	158.3264	134.7314
neg_6211	Androsterc	170.7011	83.99851	9.655238	150.1667	146.2057	272.5136	123.3695
neg_6217	5,6-Dihydı	634.9758	476.2513	313.4216	408.3595	566.926	521.8355	438.068
neg_6222	Phytosphir	125.6788	304.2634	507.0517	428.9869	127.6656	112.301	397.6367
neg_6224	Levorphan	59.91897	97.59638	84.26564	97.72466	57.8924	71.94293	119.0073
neg_6228	4Alpha-hy	4954.911	4324.178	3969.002	5185.381	3642.082	3785.428	4445.356
neg_6235	2-Oxo-2,3	194.7651	240.9657	379.2457	234.4432	269.3518	226.9423	260.941
neg_6236	2,2,2-Triflı	73.46405	95.43491	142.0395	86.42681	105.3273	83.14876	97.42794
neg_6237	L-Carnitin	3893.216	3548.194	4540.71	5751.569	4236.357	3024.436	3661.625
neg_6245	Avocaden	128.0954	168.2051	226.6834	122.8433	174.2057	154.8387	160.7349
neg_6246	5alpha-Pr	138.8706	168.9985	159.2483	184.5596	129.8918	136.8032	138.5094
neg_6249	Phoenicox	319.0921	229.4751	122.162	357.4497	241.6436	355.8147	257.5307
neg_6257	Tanacetol	342.7271	159.1894	113.8304	132.0818	85.7982	158.1346	128.0538
neg_6258	DG(13:0/P	3120.135	2596.836	4750.709	3766.121	4700.343	3504.572	4138.026
neg_6259	PE(20:0/20	296988.5	472603.9	1241439	477046.4	547528.6	464325.3	585870.5
neg_6260	PS(15:0/24	216428.8	442005.2	563357.7	379487.6	474327.4	358954.1	503359.4
neg_6265	PE(14:1(9Z	2565.325	2265.884	4311.178	2163.644	3757.473	2440.41	2511.569
neg_6273	(±)-Menth	1158.509	1116.632	834.9758	739.546	674.7228	807.2237	752.8875
neg_6277	4,6-Heneic	644.283	757.5374	1133.936	901.9459	723.8524	849.3973	552.0072
neg_6281	Methyl 3b	125.8038	88.73488	6.52105	136.0133	57.4893	48.15672	57.89861
neg_6282	PC(P-16:0/	64425.44	19259.49	122246.5	29941.96	41642.82	17087.04	29113.65
neg_6283	Obtusilact	121.6293	100.367	82.17063	118.0491	119.9297	129.734	89.93942
neg_6287	(S)-N-Met	838.1376	842.6543	495.3023	691.1852	811.6998	478.1166	1316.326
neg_6290	5-Hexyltet	586.943	1409.347	1288.909	1068.03	729.1982	793.3317	1030.952
neg_6293	Ile-Ile-Ile-	360.0693	721.4563	971.5386	794.9918	217.0588	398.1847	843.0269
neg_6294	Brassinolic	166.6419	129.4983	84.72378	177.26	86.28302	136.0432	147.6332
neg_6302	Docosahe›	670.4743	1658.568	1416.694	1766.519	1679.277	1548.352	1390.086
neg_6304	Digitoxige	64.50128	309.6178	200.0333	315.7993	261.7966	259.0657	238.7236
neg_6319	Physapube	109.857	17.27029	193.4436	51.64235	5.801772	12.2615	9.652676
neg_6335	3-O-Sulfo	142365.9	179449.4	383960.6	158135.5	201465.9	162551.1	189510.8
neg_6337	3-Oxohex	155.0047	869.2426	490.6737	5021.418	48.11186	370.113	1459.587
neg_6342	DG(17:0/2	3299.939	2785.044	5128.746	2400.435	4225.863	2785.837	3333.224
neg_6343	CDP-DG(2	2600.716	4168.931	8869.643	3092.737	4537.887	3535.085	4778.67
neg_6352	Menthyl et	73.78714	32.28009	1.72E-06	46.09437	22.39793	32.26215	34.50504
neg_6355	2-Hydroxy	77.41137	79.55734	130.4363	80.75422	87.66318	88.13063	102.2914
neg_6361	Momordol	21980.23	19584.83	16943.42	22460.93	15660.43	15901.86	18733.47
neg_6362	28-Homol	55.99883	64.91127	17.80284	66.62766	27.53834	18.34898	48.81073
neg_6363	Contignas	741.9475	714.0445	631.843	816.4326	576.9314	609.2363	710.6846
neg_6366	Cocaine	86.45848	92.00391	7.898098	126.2031	68.28952	83.08629	124.8781
neg_6370	12S-HHT	336.8056	180.4055	95.27264	251.8332	348.8246	180.2264	240.2198
neg_6372	2-Aminoe	89.94675	100.0055	67.91508	103.7457	59.35818	74.22834	117.6528
neg_6375	Arachidon	543.6835	530.1453	613.4106	415.3651	377.3767	534.3019	492.9775
neg_6377	4alpha-Me	438.1938	445.4942	306.0882	434.0829	266.7838	289.8056	367.0027
neg_6383	Valylargini	33.41228	11.2065	21.0835	11.75904	1.72E-06	1.72E-06	1.72E-06
neg_6386	8,11-eicos	1687.894	2234.458	3082.317	1830.337	2891.091	2900.684	2300.169
neg_6387	Tetrahydrc	310.6345	414.1467	558.2735	333.409	535.179	545.5419	462.8125
neg_6390	DG(22:6(5Z	829.6953	817.854	986.857	1298.591	2214.503	1242.815	835.0289
neg_6395	DG(14:1(9Z	717.6254	424.5581	201.9488	949.3006	592.2918	579.3554	495.7136
neg_6402	Eicosapent	102.1701	85.42967	74.37477	756.9032	108.1399	158.7576	194.5258

neg_6409	PA(15:0/18	14411.57	13156.96	24627.71	12371.88	15338.42	14695.06	16194.74
neg_6414	1,5-Dibuty	395.004	352.8777	810.1465	314.7307	392.3753	319.6912	728.1552
neg_6417	Docosane	3615.273	4753.637	4524.565	5012.846	4275.669	5787.973	4239.175
neg_6422	15-Hexad	1530.173	2524.411	3901.789	4389.106	7363.009	3805.449	2335.755
neg_6423	PA(2:0/PG	9.054271	7.010085	1.72E-06	11.35432	6.145969	34.37002	13.59222
neg_6433	PA(10:0/21	1347.668	1279.457	2036.069	1358.37	1808.564	1474.404	1495.718
neg_6442	(S)-Ureido	293.0536	383.2969	578.5806	354.9338	427.1108	321.3737	406.4325
neg_6445	Eicosadien	262.3257	320.4931	431.6758	308.7404	332.238	572.2748	381.8107
neg_6446	(1R,2S,3S,4	150.8536	185.0311	141.2208	138.0591	234.3955	154.2093	152.5405
neg_6447	11beta,21-	83.52659	113.7802	219.1545	101.8497	68.53451	98.53743	172.6563
neg_6454	1-[4-[2-[2	713.287	853.0869	1572.518	818.4211	997.2363	957.8038	1160.432
neg_6455	1,2,10-Trih	67.00102	72.80459	172.0165	56.3674	90.65607	146.4454	126.9773
neg_6456	beta-Bixin	1483.504	1656.305	3131.463	1562.654	2251.75	2832.778	2715.021
neg_6458	3beta,5bet	13411.48	2242.124	24561.99	3781.393	3713.833	36289.41	16000.41
neg_6460	1-(6-((3-M	55667.11	65329.81	87211.48	79661.54	83092.72	52018.46	65207.37
neg_6463	N-Docosa	567.2431	62.63757	648.2519	84.63371	53.86634	1579.238	515.7494
neg_6487	PI(22:5(4Z,	2174.002	3176.093	3651.492	2597.664	4138.925	1868.104	3220.52
neg_6489	Dehydropl	61.3399	28.00048	61.4628	33.02195	298.5898	321.6011	113.6279
neg_6490	1,4-Undec	26.22308	8.359632	12.74151	42.09097	5.915355	15.56407	12.22708
neg_6500	20-Hydrox	202.4364	489.2756	447.0375	217.3994	172.4003	364.7631	302.9708
neg_6501	PE(16:1(9Z	1385.224	671.435	1433.181	919.8444	1513.959	887.1383	1139.344
neg_6510	10,20-Dihy	877.6837	1053.79	730.903	716.4861	1753.341	830.6746	792.7979
neg_6515	(-)-Vesam	179.4818	88.80643	13.98273	117.9643	62.98399	110.7841	70.48132
neg_6516	DG(8:0/PG	61.38939	36.82299	0.421092	49.00435	5.456494	49.49233	26.54599
neg_6518	2-isopenty	55.82256	104.7246	79.18806	179.8301	101.7379	45.83721	47.4785
neg_6520	trans-Dod	758.0193	558.2214	634.8276	598.1298	696.857	530.2445	664.2227
neg_6522	3-O-alpha	11.17555	14.4194	1.72E-06	21.08588	1.72E-06	1.7994	6.441878
neg_6528	Filfiline	233.6456	163.5786	172.1942	182.0957	149.714	222.2052	172.2534
neg_6529	4-choleste	225.1118	160.9768	186.1454	200.8623	162.8482	198.8062	136.9688
neg_6536	medicager	85.59271	447.8058	1169.827	451.0425	92.43593	256.6102	70.00308
neg_6538	(7Z,10Z,13	26135.67	20000.03	39226.97	30799.74	32666.61	31962.79	21801.85
neg_6544	Sclareol	166.0329	279.6381	173.2571	221.0091	433.8507	458.9622	301.5837
neg_6545	1-Phenyl-	3253.009	3307.489	4825.161	4298.793	4279.513	3183.94	3484.178
neg_6569	Petasalbin	98.0615	84.9751	263.2879	72.48333	33.67101	87.39877	47.05318
neg_6582	DG(14:1n5	255.6468	103.6954	100.8282	133.7504	113.3352	224.429	146.4513
neg_6583	Phytol	62.36132	61.99146	79.5369	62.90754	128.9338	101.6292	83.2344
neg_6584	(Z)-3-(1,2-	1910.752	2854.014	2210.241	5117.677	2906.238	2828.071	2812.619
neg_6589	Hericenon	211.7418	157.2268	93.34883	202.9641	234.72	222.096	181.3256
neg_6593	Nervonoyl	116.9852	100.8373	117.0146	132.3944	81.45822	99.60664	143.8212
neg_6600	Methoxym	361.2877	450.2416	712.7284	434.109	520.9816	420.0949	474.0089
neg_6601	DL-2-hydr	2115.794	3161.12	4250.286	2936.99	2274.211	2556.336	3737.483
neg_6631	TOFA	31.63082	66.64248	125.7695	70.29128	83.0518	65.5698	98.46315
neg_6636	Ceftizoxim	6085.228	7278.77	11598.5	6719.585	8687.962	6467.58	7588.564
neg_6649	(S)-Argpyr	19.83095	65.98602	144.8795	191.9174	180.2739	25.24726	97.39955
neg_6656	3-methoxy	105.4005	143.5813	291.4169	180.8461	180.6807	170.9685	227.6084
neg_6659	Momordic	310.1398	544.4703	1248.011	701.4739	841.2938	520.1455	706.9726
neg_6700	(5Z,9Z)-No	310.1044	580.2477	819.1729	621.6636	325.1707	476.4198	476.8482
neg_6703	Guanosine	11994.34	14818.82	23492.65	13258.78	17297.02	12957.02	15561.22
neg_68	Methylpho	4553.672	5812.947	7385.836	4866.401	6377.16	5185.857	5797.45
neg_736	L-beta-Eth	59.02474	58.77538	102.436	62.88353	132.4443	52.25251	57.22426
neg_744	1,7-Dimet	2685.545	3750.38	4373.826	3686.136	3937.084	3548.684	3527.822

neg_823	6-Dimethy	23.26944	37.36516	62.19601	42.5463	43.90307	50.05012	37.29124
neg_828	Z-Ala-ON	83.87126	137.9827	185.1492	350.052	302.5061	187.0924	341.3919
neg_838	ppGpp	73.64729	78.12349	62.18423	81.27229	76.71753	88.84405	70.57729
neg_850	2',3'-Cyclic	8549.438	9717.858	12806.83	8603.798	11514.13	8771.393	9944.35
neg_854	dUDP	57.38193	65.54241	109.8145	61.60124	91.44556	73.42556	76.03669
neg_856	ascorbic a	179.7132	194.7916	280.2664	206.3418	251.104	217.7308	203.9625
neg_867	2'-Deoxyir	834.7893	859.4767	975.2578	800.7532	915.6678	813.9787	836.0941
neg_869	(6R,7R)-7-	180.3835	218.0801	343.1722	245.2668	132.8485	221.0222	220.0866
neg_886	L-Malyl-C	73.38876	68.89758	38.96254	95.20781	83.49173	99.86912	104.154
neg_893	N2-Acetyl	243.714	752.7666	278.4516	311.599	593.3932	635.4907	414.7991
neg_895	Artemidinc	259.2414	345.517	403.6229	354.2686	311.6638	373.4455	312.364
neg_897	Pinazepan	8095.108	8881.555	14092.53	7510.185	11891.24	8323.076	9311.454
neg_898	(3S,7R)-16	31539.98	36033.57	50577.64	32860.16	39505.82	32683.66	36826.04
neg_900	alpha-D-x	5378.887	5381.344	7343.762	5395.907	5941.379	5804.065	5934.138
neg_907	Delphinidi	365.3932	458.6066	1149.081	450.6263	498.4445	485.5104	512.0751
neg_910	L-3-Aminc	795.6548	1089.757	1114.755	1047.45	1206.651	1062.535	1148.598
neg_913	4-Hydroxy	66.37272	119.0549	50.06241	122.5216	101.3357	114.6776	85.2979
neg_922	Carvone	272.288	324.9824	576.0609	299.3795	437.365	338.9263	338.7469
neg_924	Alanylhydr	5784.782	6470.102	11271.62	5612.397	9059.072	6568.438	6948.512
neg_926	N1-Acetyl	361.1837	429.8483	687.2775	338.9518	593.1619	398.2857	425.6424
neg_927	Isoniazid a	596.9394	624.6645	1370.767	524.3527	996.6803	660.6548	700.8982
neg_931	Calcein	10345.1	11380.08	19819.52	9120.675	16857.69	10816.2	12263.05
neg_934	Amritoside	12493.87	14174.96	17344.74	12968.29	14591.14	12607.59	14378.88
neg_940	2-(alpha-H	317.337	314.6498	422.805	245.3609	356.7793	289.16	326.5245
neg_941	D-Glycerat	84.42623	124.0119	74.31946	118.089	72.1664	105.3914	78.80126
neg_944	D-Ribulose	320.9808	569.4426	373.3404	474.2696	312.0449	509.2327	696.6127
neg_946	Azetidinyl	411.7714	429.9266	908.8721	392.0633	634.9961	453.3072	484.4668
neg_950	Creatinine	83.95537	149.9479	59.39617	155.5448	62.43797	157.1764	93.71216
neg_951	(2S,3'S)-alp	70.09223	83.12944	118.7828	84.5493	100.3314	83.19088	76.41504
neg_952	5,6-Dihydr	1220.377	1794.226	980.3843	1941.348	666.1434	2648.909	1547.546
neg_953	Asparaginy	70.81644	104.8275	110.4375	95.50006	99.93542	127.8799	127.9264
neg_958	Chloroxurc	323.7236	447.2461	283.248	527.3794	258.3942	598.5221	445.6884
neg_959	2,8-bis-Tri	97755.29	109857.6	188460.9	106155.7	145849.9	111880.2	123467.4
neg_960	(E)-2-Amir	3439.887	2880.383	8061.93	3313.317	5730.799	4305.394	4088.86
neg_962	Cyanidin 3	68.07136	109.7237	32.18112	111.7836	78.66198	159.538	105.3471
neg_963	Anti-phos	31.85288	46.1696	41.50176	41.10742	49.21407	57.25322	48.39319
neg_964	Rutaretin 9	29714.41	32589.99	70040.04	27286.48	49970.73	32400.03	36426.55
neg_969	dTDP	315.2227	285.3355	241.7943	279.012	246.2726	294.5979	285.5196
neg_972	(8S)-8-am	4662.35	5114.24	9687.254	3949.1	8123.748	5086.187	5632.695
neg_974	5-Hydroxy	650.6698	823.6689	1527.046	788.1949	1171.588	817.6311	851.7214
neg_977	N-Acetyl-	22.7258	34.85335	34.45937	30.82841	19.67976	41.33823	35.8613
neg_984	N-Acetyl-	59.23854	94.61848	98.01	87.67611	81.48805	79.65586	62.08051
neg_985	Serylhydrc	125.9306	229.8102	193.709	174.7885	102.7466	261.2591	193.3426
neg_986	N5-Citryl-	183.7117	235.351	523.5446	176.7984	321.1388	199.9165	220.4185
neg_988	dTDP-mac	37.9523	59.88232	11.67431	54.99262	60.24267	54.24618	54.24464
neg_992	Acipimox	254.6967	400.2772	421.3946	332.2936	289.0825	408.0968	387.4875
neg_993	cis-Aconiti	161.0176	206.7316	219.7148	170.9432	171.8875	220.7534	194.3399
neg_994	Dehydroas	1290.468	1622.063	1841.871	1206.232	1320.009	1745.416	1578.232
neg_995	Uridine	1475.659	2225.043	2389.998	1909.804	1654.047	2353.062	2280.32
neg_997	AG 2034	22.46547	23.74859	21.22583	17.09217	8.900858	27.2645	19.41221
neg_998	dTDP-4-o	232.721	292.1137	244.9088	278.3321	220.314	344.79	296.3588

pos_1003	PE(22:2(13	9933.806	9111.548	11925.25	11294.59	12038.95	7012.986	6668.119
pos_1007	n-docosal	6285.223	7985.784	9651.217	9302.945	8167.549	12355.66	10587.42
pos_1009	Basilol	129.4856	162.6599	180.0046	190.9947	99.12904	165.5656	183.5376
pos_1012	MG(19:0/0	207.5281	240.8448	301.6787	349.6233	294.679	291.0302	269.5182
pos_1017	Glucose la	1.02878	2.170741	37.67188	2.948518	32.80604	15.70113	13.28877
pos_1021	3'-Sialyllac	105.9522	136.0679	596.5295	157.102	625.135	379.5817	310.054
pos_1022	Crocin 3	5048.089	6705.39	25684.75	8006.567	22566.86	16892.76	14183.02
pos_1024	Hordatine	173.2545	142.8292	600.5353	184.1263	825.4402	555.4582	336.5852
pos_1025	Acarbose	4.224705	8.866007	90.49076	6.4376	25.79136	43.54801	37.94761
pos_1028	L-threo-sp	957.2438	512.7215	1186.322	414.2439	393.3649	368.9576	558.004
pos_1031	(3b,16b,20	169.5843	540.3121	1203.539	387.8441	153.9092	160.603	222.1927
pos_1032	PI(18:1(11	463.8882	297.2799	592.5992	273.2673	475.1732	256.6488	384.7267
pos_1033	PC(22:2(13	166.7311	39.72917	78.13615	27.33849	0.357515	18.55151	34.92243
pos_1034	[(3S,4S,5S,	7.601134	11.14548	22.51388	9.102547	14.04306	8.882849	6.650675
pos_1035	Heptanoyl	56.46561	70.98719	94.44798	63.67757	66.49415	55.12581	54.9687
pos_1036	Ginsenosid	53.05417	86.59353	88.55123	82.0222	90.39097	80.97237	82.88254
pos_1040	Ergocalcife	99.90777	118.7152	124.2053	176.5002	153.5936	149.6321	120.852
pos_1041	3-Amino-	57.52993	162.9916	92.10167	39.89307	32.45833	72.57397	69.17489
pos_1042	3,4,3',4'-Te	34.77139	31.2879	48.51651	125.6351	37.35786	26.89328	30.85126
pos_1043	PA(14:0/18	113566	109260.1	94585.29	170165.8	154798.1	127941.6	152140.9
pos_1048	N-Lauroyl	120.7911	127.7229	140.1919	136.8035	170.6766	139.1552	139.6528
pos_1059	Notoginse	41553.64	30745.8	25604.6	74340.52	37690.2	30193.09	27767.9
pos_1060	DG(15:0/P	5219.961	3478.534	5752.295	5156.564	4332.754	2980.374	3313.92
pos_1067	Cyclospori	742.4375	995.4082	1575.757	773.7782	1012.354	512.5306	840.5426
pos_1073	7,21-Dihyd	120.4908	125.684	177.0966	136.0416	172.1943	147.8949	165.5324
pos_1077	12a-Hydro	12618.33	15035.37	17549.11	15441.04	15898.99	16070.16	16704.88
pos_1078	(20R,24R)-	1285.456	1019.803	1161.529	1056.052	1360.133	2304.168	1103.547
pos_1081	1-(O-alph	249.6176	222.6373	311.3747	226.5033	249.3501	214.837	238.8657
pos_1086	Azacyclotr	687.0097	705.0246	1167.245	759.145	850.9809	866.8992	935.9539
pos_1087	beta-Caro	370.8483	407.7534	727.7533	500.1608	539.0416	551.9142	588.7668
pos_1089	DG(17:0/P	410.8206	412.3541	388.7066	660.9219	436.1169	569.0977	574.9775
pos_1091	Angiotens	1532.784	1416.822	2033.863	1412.884	1767.337	1488.239	1156.406
pos_1094	(Trifluorom	66.8036	78.56709	59.83068	80.8928	77.19656	86.51444	77.62788
pos_1097	3-Aminop	110.1199	162.6519	123.0232	148.3238	153.8697	174.07	176.4575
pos_1100	6.alpha.-M	358.7979	351.2874	193.1756	126.5932	234.3846	306.974	294.2701
pos_1101	PE(16:1(9Z	976.6471	1129.554	997.922	1406.683	1353.405	985.599	958.6298
pos_1107	27-Norcho	283.7811	301.9862	355.6393	356.0382	401.6261	344.465	294.6873
pos_1113	Sorbitan o	309.7111	353.4923	389.147	387.4361	409.0591	395.4084	389.1622
pos_1119	α-Methylt	2338.82	2692.059	2917.032	2617.01	2948.47	2731.492	2701.843
pos_1122	Cer(d17:1/	1060.326	1245.68	1158.793	1216.91	1316.656	1268.358	1268.809
pos_1123	(±)9,10-Di	62.33314	104.5155	67.69814	77.19864	84.50784	110.1656	81.0313
pos_1130	Deoxychol	180.1272	214.7799	255.3275	267.6826	260.782	240.2944	242.8063
pos_1139	(3beta,5alp	572.5497	682.9184	702.0484	858.7012	971.1763	855.7993	792.2886
pos_1141	Pyridine-2	16.07452	24.0566	17.63069	32.44067	22.23635	22.30104	17.42158
pos_1144	2-Methyl-	50.76084	68.16238	68.8415	77.26253	80.24843	72.81425	77.34006
pos_1148	PGP(i-20:0	891.1346	888.9486	1025.533	915.9915	1071.466	888.3478	956.4421
pos_1151	(1R,6R)-6-	147.0162	178.2047	211.8688	182.4948	230.8671	215.3501	209.8153
pos_1156	Morronisid	100.0476	93.52693	158.3808	83.40956	120.9986	126.3055	109.1059
pos_1158	Tetraphylli	4.741966	19.81127	34.08364	6.005366	17.45584	59.3617	21.13466
pos_1160	3-Methylb	55.86	62.10266	72.28725	56.66305	65.56477	62.28065	65.21564
pos_1165	Dehydroep	291.9271	331.0985	373.877	416.7092	386.3059	383.3017	358.7304

pos_1167	Cer(d16:1/	323.7881	406.671	332.4049	549.9727	525.1559	498.1488	404.7337
pos_1173	N-Monom	469.7357	613.6672	681.1737	668.8429	629.7327	652.9692	721.54
pos_1174	22-Angelc	46.58773	74.95996	59.97995	94.92736	62.65005	102.4906	87.05781
pos_1176	Epomusen	457.7406	345.5353	754.6474	376.7656	256.5233	237.9395	282.0478
pos_1177	All-trans-1	440.8616	471.3922	548.8223	747.9795	440.7269	584.2988	637.4263
pos_1180	DG(20:3(6,	705.8282	1179.223	1005.395	1302.103	1107.318	1163.143	1591.354
pos_1182	Galactosyl	3269.378	3618.845	2758.823	4033.084	4263.369	3222.1	3707.903
pos_1184	PA(22:6(4Z	18870.01	14413.58	28467.96	13734.88	14669.01	12362.3	14234
pos_1186	SM(d16:1/	86790.85	107756.8	98680.15	63591.15	81233.82	50473.24	81860.76
pos_1187	PE(P-18:0/	11049.02	13434.79	16314.72	11138.55	12992.25	10208.68	12910.81
pos_1188	PE(P-18:0/	214216.9	333068	421984.2	341149.7	290582.4	221238.8	352718.2
pos_1189	DG(18:0/L	676120.7	643998.7	999128.5	521861.7	537097.5	367736.7	506985.4
pos_1191	DG(19:0/L	225158.1	206229.7	260100.9	196804	195316.8	203009.9	189568.1
pos_1192	PC(P-18:0/	685202.2	680072.6	768606.4	733483.3	748907.6	719213	627023.3
pos_1194	PS(17:2(9Z	23439.5	38241.84	37505.2	35076.13	28288.17	29497.2	32960.47
pos_1197	PC(17:0/P(	34135.69	25596.12	55499.03	23965.42	25067.8	22481.48	23631.34
pos_1198	PS(20:0/P(	3532.535	5471.301	2249.861	3473.83	4848.675	2765.149	4403.991
pos_1199	PG(20:1(11	4070.481	4649.66	8772.633	4881.776	4580.305	5872.395	7924.216
pos_1200	Dioleoylph	24835.63	35746.54	38639.74	36429.4	43604.93	44924.37	44492.04
pos_1204	PG(20:1(11	5941.884	3160.984	3057.042	3828.848	3195.118	1477.14	1444.289
pos_1205	PE(22:1(13	28733.56	27131.52	38945.47	28049.42	30566.13	35064.12	29911.61
pos_1206	N-[1-[[5-(	146.318	227.7697	229.7279	262.5193	246.6392	239.3053	258.557
pos_1211	Vitamin D2	92.20683	113.6106	103.1393	158.0859	139.8608	119.3597	121.8503
pos_1213	Carteolol	1.72E-06	0.912172	4.569863	9.023285	2.420733	3.943699	12.8293
pos_1214	PC(14:1(9Z	55982.98	70108.28	54045.46	25715.17	62007.61	16950.07	33810.07
pos_1217	(7S,8S)-Dil	28.26002	18.23053	10.93328	42.27871	25.27844	43.67386	27.62852
pos_1218	Asitribin	55.72507	76.66731	50.90848	68.43248	64.78664	73.7064	73.05738
pos_1219	Capparilos	1.72E-06	1.72E-06	22.90703	1.076389	96.97109	16.74661	14.28648
pos_1222	Somatotrc	154.8315	247.4429	281.4314	267.3434	310.8544	368.0499	328.1438
pos_1224	Alisporivir	2114.726	2237.607	3992.902	1796.036	1573.894	2233.741	1497.912
pos_1229	Ellagic acic	48.82089	132.4179	108.6539	132.1839	185.812	203.7859	188.5036
pos_1238	Antiarrhytl	70.3184	41.24273	124.6822	80.57871	101.1921	78.48182	138.1639
pos_1240	Iganidipine	228.1521	179.861	474.3028	275.8419	354.4838	272.8896	506.1055
pos_1242	LysoPI(20:4	8865.259	7621.176	17026.31	11930.24	14852.35	12097.35	19621.01
pos_1243	Scutianine	120.6264	94.47946	167.9268	196.6599	198.818	190.2945	289.8466
pos_1244	Amphibine	302.8756	285.1966	515.3837	421.2903	520.529	428.7755	651.702
pos_1245	Avermectin	3121.623	3428.886	4170.109	4411.564	4609.291	4797.896	5371.018
pos_1250	3-(Acetylo	54.70998	37.51539	47.37357	51.77453	154.1659	220.1025	60.62832
pos_1254	2-Acetyl-1	24.23695	93.76815	68.07662	60.20482	121.4176	111.4329	50.31677
pos_1257	4-Hydroxy	133.9516	194.5937	179.782	246.24	255.126	274.7155	279.137
pos_1259	benzoyl-l-	44.92631	56.30132	54.35265	59.42324	66.20837	59.72351	66.25782
pos_1261	Ustiloxin B	2271.369	1519.45	3283.217	1766.785	48222.42	1887.777	2001.486
pos_1265	Amphibine	98.10919	150.1094	162.0464	298.8618	288.6144	300.0697	218.7086
pos_1268	N-(1-Deo:	139.642	268.7665	138.0303	263.176	250.2675	293.7164	245.8789
pos_1270	L-Rhamnc	26.77679	41.15969	29.44183	41.30659	42.2884	52.82538	44.89623
pos_1271	(E)-2-metl	106.214	141.7048	133.5984	144.7189	140.4131	187.6837	157.3841
pos_1282	2-[4,6-Bis(	393.1801	329.0343	263.057	457.4069	487.8807	643.8839	412.6549
pos_1284	N-Palmito	5188.155	7314.177	4610.036	5772.571	6026.033	5893.044	4923.458
pos_1285	Cer(d20:1/	204.9977	327.2659	134.6908	473.4134	248.3516	378.3605	211.33
pos_1286	7-a,27-Dil	57.66119	68.83886	111.2141	115.9657	208.6747	110.1632	84.27172
pos_1288	9,10-Epox	81.50045	79.61397	41.16957	228.7851	144.622	73.43912	86.6545

pos_1291	Ectocarper	7.623197	5.240901	15.62593	7.849903	6.014801	7.659908	4.027734
pos_1293	Ethyl ment	29.66563	31.27827	56.67133	22.48768	28.17444	37.68288	28.13654
pos_1297	1,2,4-Trim	3.696115	3.775613	6.535503	1.988215	2.022188	4.842525	6.412729
pos_1305	PG(i-12:0/	237.7571	195.5005	109.7415	508.2625	232.2991	162.8432	178.8308
pos_1309	Cer(t18:0/I	1140.935	941.4555	1245.365	1506.348	1296.709	1446.666	987.7296
pos_1313	PC(16:0/18	83915.18	108654	58232.04	59466.88	76409.12	27762.71	66229.49
pos_1319	PC(14:0/2(	1986873	1257348	3062490	1017847	1115324	784784.6	949697
pos_1321	5-Amino-(	200.0853	169.7017	400.3211	332.4868	606.3793	290.8522	204.2688
pos_1324	PS(14:0/14	89.18368	106.3719	149.6364	132.0174	184.842	145.981	157.727
pos_1329	5S,8R-DiH	193.755	134.4388	71.95804	193.454	173.7168	260.3285	181.7997
pos_1330	Duloxetine	49.97143	37.43577	63.6667	34.94392	50.70462	47.60863	32.41489
pos_1331	(4r,5s,6s,7r	128.3098	658.5062	2095.585	453.7191	175.3491	233.3998	219.7556
pos_1333	beta-D-3-	343.6982	384.9142	442.4244	417.1354	424.9514	442.1019	441.4875
pos_1340	DG(8:0/20	164.3927	135.8055	94.7805	139.5526	157.8939	270.8519	154.0022
pos_1343	CDP-DG(F	467.0681	330.0306	708.4011	276.0026	342.5211	174.495	251.3144
pos_1348	3-(O-Gera	57.7841	64.10576	107.8272	93.06266	152.6977	119.0481	135.2783
pos_1354	Angiotens	182.3238	318.6491	523.4028	310.2175	282.8283	287.3549	393.9949
pos_1356	PGP(i-19:(	1637.1	1876.841	2260.92	1828.794	2292.509	2611.689	2436.352
pos_1359	p-coumar	6232.101	7662.856	8584.449	8591.818	10662.53	10849.86	9798.594
pos_1369	Oxybupro	70.14864	73.33517	83.83501	70.29216	78.28687	74.51711	82.63502
pos_1370	DG(2:0/PG	31.0877	32.06347	27.12588	45.05502	44.42046	47.53093	53.46056
pos_1374	Oleoyl Eth	2720.599	3067.291	3408.156	3123.013	3811.226	3493.674	3336.453
pos_1382	CDP-DG(a	433.2937	544.3133	711.5793	535.4696	572.0337	675.08	728.8797
pos_1386	5-ethyl-5-	11.8748	14.8933	16.35914	9.966534	19.13526	12.52946	18.40826
pos_1387	Methylitac	89.73941	112.6845	110.0554	127.9407	133.335	136.2544	135.7466
pos_1388	Methylisop	279.844	322.9352	381.1498	322.1305	359.9056	360.3538	367.1106
pos_1396	PI(18:2(9Z,	1238.619	1188.532	1541.538	1236.657	1454.116	1874.903	1575.666
pos_1398	4-(4-Fluor	9.757234	11.99986	10.72809	13.35156	13.0352	12.43008	12.76158
pos_1401	DG(20:0/2	522.3335	524.19	744.4431	529.8362	631.6617	631.0265	645.3618
pos_1405	Creatine	13.70505	13.48157	18.16954	18.41353	16.6838	15.50651	21.65627
pos_1406	Urocanate	155.8156	175.8114	191.3173	140.1865	191.3396	152.44	186.0704
pos_1411	2-Amino-	656.8428	793.9646	935.8614	784.406	861.4402	794.9908	785.0808
pos_1417	Lophopho	11.24014	6.662924	11.17094	23.12032	17.68995	17.87861	18.9639
pos_1450	DG(18:1(1	575.7703	660.9147	826.6059	623.1887	780.8372	704.7603	786.3721
pos_1464	4,6-Dihydı	6750.706	7873.044	8969.035	7747.15	8561.647	7719.199	7969.365
pos_1519	5-Chloro-	127.6156	148.5375	173.8561	155.6714	148.5966	151.0481	150.5913
pos_1525	DG(18:0/2	61.86934	6.643098	42.24987	54.28039	1.72E-06	37.45281	50.9101
pos_1537	Trifluoroac	102.7453	119.9818	121.0553	121.9017	116.4954	106.5669	118.5262
pos_1557	Carboxyim	107.1703	126.3336	141.3396	117.2212	136.5251	126.0199	126.7343
pos_1562	2-Fluoro-	100.7007	102.1735	126.4728	111.2793	114.9731	107.9327	114.6697
pos_1564	Chlorodiflu	49.44228	58.64394	69.07918	58.09083	62.3247	59.16939	64.09072
pos_1566	Methyl sul	98.35207	110.4459	128.0127	102.7948	123.7705	120.8981	117.5747
pos_1567	Kynuramir	79.43155	85.02965	41.29297	144.4578	74.21264	133.9458	74.23294
pos_1569	Morph	61.48104	80.27374	28.42363	137.4657	43.84132	89.43897	66.88681
pos_1570	2,4-Thiazo	92.18487	34.18779	73.68059	1.72E-06	62.75064	43.32029	35.10601
pos_1572	Samin	978.4355	1245.956	649.6307	819.4871	745.4115	1095.962	1057.837
pos_1573	dCMP	644.2319	95.13009	285.6198	1.72E-06	448.8825	229.723	119.7587
pos_1574	Glycylproli	4232.857	5213.055	2986.937	4029.093	2925.687	4529.971	3841.669
pos_1575	p-Butylam	1002.564	1303.895	558.9647	550.4092	814.4288	1537.559	1601.211
pos_1577	Isocolumb	46.41767	139.6881	12.42563	31.6582	76.01068	41.24698	68.59817
pos_1578	p-Tolualde	16.55829	0.883223	0.955722	3.611422	0.523483	1.070463	1.72E-06

pos_1580	3'-Formyl-	53.10237	44.25353	4.511232	49.40799	1.399951	32.17414	45.30584
pos_1581	6-O-Acety	93.25717	1.72E-06	1.72E-06	21.91467	12.69574	3.329817	1.72E-06
pos_1582	Dyphylline	173.4187	253.2057	270.2832	393.1873	1.72E-06	107.5063	207.4353
pos_1583	bk-MDEA	717.132	819.9951	764.7669	704.4454	791.1834	819.6017	753.6216
pos_1584	(R)-2-(4-(	539.7055	673.8428	425.2256	472.4449	389.3173	589.4104	583.0768
pos_1585	CYPMPO	100.0561	104.7479	116.6847	80.03731	95.73302	115.298	118.8942
pos_1586	Semilepidi	2846.647	5326.579	819.8839	2035.395	3974.18	6040.627	1443.185
pos_1587	2-Hydroxy	23.13271	31.36884	1.133756	702.6168	16.59774	30.02015	34.88594
pos_1589	Asparagin	28.52398	46.71272	1.72E-06	20.65349	27.29145	54.07655	15.83458
pos_1590	9-(trans-4	2623.21	3815.631	1319.047	1337.243	1635.032	3730.965	1564.212
pos_1591	Glycyl-Try	274.8257	358.3827	183.8228	243.3257	368.2305	658.3334	589.3619
pos_1592	Porphobili	41.78104	142.9547	1.72E-06	95.76081	2.4656	55.21405	69.67501
pos_1594	Serotonin	1.72E-06	1.72E-06	11.04326	25.6028	23.93975	1.72E-06	1.72E-06
pos_1595	L-Tryptop	41.08602	45.6267	49.73606	24.91262	68.29292	42.83422	42.61626
pos_1596	Asparagin	307.4287	516.7395	146.4317	328.5392	23.83289	203.8522	178.7837
pos_1597	2-Methox	434.2396	613.805	307.5337	250.5952	346.9074	628.3184	299.5659
pos_1598	3-Methyle	774.3468	1033.661	642.3685	723.747	999.7725	1041.921	635.8104
pos_1599	Benzenep	2693.116	3988.363	1428.934	1369.997	1971.69	4203.722	1742.09
pos_1600	5-[2H-Pyr	642.5101	916.4913	584.2836	441.3276	639.4209	940.5907	501.2017
pos_1601	6-Methylq	4268.815	6399.067	2400.958	2050.346	3203.374	6851.325	2777.92
pos_1603	Isoquinoli	10645.85	16019.05	6112.17	5298.436	8158.82	17120.63	7119.061
pos_1604	Indoleacry	36675.16	55171.5	21032.74	18615.1	27697.52	57399.51	24425.52
pos_1605	3',5'-Dide	128.1763	171.1215	123.1066	166.3096	52.29996	160.9585	144.1586
pos_1606	Trp Gly Ph	150.7485	264.1109	24.59825	10.06539	21.13618	218.8279	51.41796
pos_1607	(1-Methyl	211.1006	274.4839	261.7819	237.6466	315.5123	274.1422	216.6799
pos_1608	(1S,2R)-1-	50.99171	62.07829	106.7861	47.03907	60.58779	57.25619	53.11979
pos_1610	5-O-p-Co	117.2498	174.0942	18.61377	39.92712	40.30521	69.77844	31.32717
pos_1613	Hexahydro	1012.266	1283.234	1026.353	1783.34	3941.755	1413.304	940.4473
pos_1614	fenbendaz	378.2972	422.9408	314.6094	626.4998	1321.588	476.181	315.9985
pos_1615	Hippuryl-g	179.1572	232.2473	165.6487	317.4459	674.789	268.69	161.6336
pos_1616	Aminomet	37491.98	46815.55	37956.62	63175.55	136380.2	50813.63	34128.15
pos_1617	Asp Asp A	12655.43	15735.5	12458.59	21735.94	46809.02	17328.35	11367.3
pos_1618	4-Chloro-	395.0661	443.4345	281.906	405.6701	403.0711	259.0529	269.1882
pos_1619	Cystine-gl	269.4819	289.2748	176.992	258.3063	230.1074	159.8708	168.6019
pos_1620	GDP-L-fu	42.71378	59.00389	1.72E-06	33.72212	9.191734	7.621967	1.72E-06
pos_1621	1-(3-Fluor	24.80659	33.65498	1.72E-06	21.64597	1.820189	3.338969	1.575819
pos_1624	H-D-Arg(	116.2433	68.98662	200.9255	128.7007	113.9955	122.1326	47.55899
pos_1625	Lysyl-Gam	110.8409	133.214	84.96272	113.1432	101.1766	117.6139	116.5292
pos_1626	alpha-Hyc	32.55164	49.91114	21.44958	75.82831	14.00904	36.55426	31.19433
pos_1627	3-(3-Amir	496.9485	548.3017	917.4385	931.5207	1925.92	786.6394	653.3783
pos_1628	6-Thioura	42646.98	47726.22	58418.65	45323.82	56102.25	45957.83	46887.69
pos_1629	Dihydro-2	14223.63	16163.08	21009.92	15033.89	19661.61	16227.82	15306.93
pos_1638	Fluoromis	345589.3	391205.2	494527.7	373316.7	472912	383716.7	385310.1
pos_1645	L-quinate	121093.8	136314.5	177979.1	129917	166749.7	133354.6	133249.9
pos_1650	4-Fluoro-2	106.6944	106.7646	128.6137	89.08703	93.14385	97.94171	119.1102
pos_1652	2-Hydroxy	84.34672	101.6415	125.5982	107.3439	50.99144	86.11602	99.50834
pos_1656	dTDP-alph	183.3719	188.6	261.5133	187.5108	204.8029	184.303	201.227
pos_1659	GDP-valie	41.52673	43.62028	104.5195	46.26848	60.69324	43.26116	50.76476
pos_1666	Cyclohexa	201.6084	221.7404	319.0984	221.5064	291.9069	221.7389	213.9586
pos_1668	4,6-Hepta	747.9851	849.198	1065.741	787.4994	1025.096	850.1463	792.1082
pos_1673	L-alpha-A	503.6047	638.2313	584.8098	738.049	360.7681	685.4601	652.4451

pos_1674	Benzyl gly	424.8633	509.1668	262.4392	345.2415	283.3374	490.2717	438.0267
pos_1675	Chakanosi	29.09914	27.97172	17.70212	36.88529	20.32035	27.99808	24.72757
pos_1676	Plantagon	68.38804	44.2665	73.18043	34.94629	31.38425	36.8438	60.7929
pos_1677	2,3-Pentyl	43.6728	28.58881	57.28064	25.32239	24.64858	30.04402	41.14853
pos_1678	Methaphe	986.5483	1353.373	834.8625	1305.943	865.2541	1202.699	1153.626
pos_1679	Lamivudin	75.94984	103.5172	141.1637	133.5987	87.03683	85.01832	81.17077
pos_1680	Isopropyl I	161.1446	242.8079	85.98395	223.8534	88.61703	136.6295	174.4585
pos_1681	Asparagin	117.854	123.1696	102.5287	181.1314	43.22997	85.16349	68.93597
pos_1682	Sulfocipro	153.4953	152.9563	146.7272	315.4141	355.0012	165.0419	87.59709
pos_1683	5-(2-Amir	613.4646	749.207	750.3896	669.4058	730.4956	478.8731	576.6168
pos_1684	4-Dimethy	277.1251	398.1939	287.6955	331.1446	283.0875	253.8821	270.5705
pos_1685	Asn Gly Se	44.32494	70.05884	25.8146	18.70563	33.89935	66.10842	25.47668
pos_1686	caffeoylshi	153.1711	133.7301	419.0967	191.2997	292.1177	152.6518	174.6007
pos_1687	Dehydrogl	299.1242	187.9413	140.4297	249.7006	196.3176	760.5057	274.879
pos_1688	thienodiaz	69.8563	67.584	87.42857	51.0894	52.00807	66.24089	68.68405
pos_1689	L-Citrulline	191.164	198.149	266.6174	186.662	169.6778	182.8098	207.4498
pos_1690	4-Hydroxy	107.792	980.599	468.1638	1194.171	235.613	47.77818	422.9354
pos_1693	N-Acetylse	200.8716	553.7307	176.4696	341.9394	205.2479	337.7382	176.6528
pos_1694	2-Hydroxy	62.21191	69.74361	64.45767	35.19229	17.56476	37.77621	85.30586
pos_1695	(1aalpha,2	556.2235	962.9705	280.534	264.0773	341.5324	996.5445	355.3994
pos_1696	Honyudisi	439.4484	576.4304	339.6942	523.1187	393.0423	534.9609	512.4643
pos_1697	1-Hydroxy	164.8649	182.8429	135.5222	196.4036	113.5524	160.6882	157.9087
pos_1698	1-beta-D-	101.6071	107.4377	146.664	116.2091	143.5442	109.708	98.42749
pos_1699	Cysteinyl-(	1327.855	1465.985	2035.274	1478.436	2111.765	1472.2	1284.095
pos_1702	5-Formimi	97.99518	193.122	1.72E-06	20.56787	54.3144	63.80887	123.5131
pos_1705	Asparagin	34.645	48.53991	37.906	118.6392	43.47455	58.52137	32.91538
pos_1706	Adrenoste	273.7621	307.5152	249.5749	250.2126	200.6128	287.7437	258.7809
pos_1707	N-Ribosyl	169.148	182.9163	91.86719	162.2643	111.3612	177.9086	144.6054
pos_1709	5-(3-Pyrid	150.1086	182.6623	160.3164	158.379	158.5054	141.3404	146.4604
pos_1710	N-(gamma	230.4813	506.4423	281.6756	298.645	453.8791	747.0998	645.2257
pos_1712	N-Butyl-N	36.50825	18.00232	1.72E-06	90.05604	19.67552	45.23933	29.31122
pos_1714	D-Cathino	72.66395	38.73547	11.42108	177.7713	40.00961	83.07985	60.27129
pos_1715	Pretyrosin	43.53582	48.20889	43.15252	39.09963	56.5749	49.19917	35.56105
pos_1717	Valylprolin	511.0023	47.12069	43.94031	238.4524	89.26305	107.0192	67.66502
pos_1719	1-(Indol-3	1416.748	1690.103	2223.482	1494.841	2321.141	1695.453	1554.961
pos_1720	Islatravir	477.7797	644.7563	712.834	497.0966	756.759	671.7152	544.2544
pos_1721	6-Hydroxy	3971.01	4813.893	6447.887	4213.95	6120.318	4737.98	4225.472
pos_1722	gamma-G	13937.51	16592.31	20731.27	14270.54	20820.02	15859.76	14644.42
pos_1723	Epicatechi	70.14325	83.61299	1.72E-06	49.94573	21.76734	88.63808	39.96743
pos_1724	2-Naphthy	445.5503	690.3579	284.4137	310.3485	357.5248	609.3743	304.2055
pos_1726	Uracil anal	175.1394	315.2896	95.62985	117.0027	146.621	263.9681	113.7585
pos_1730	Dexelvucit	486.9993	590.8367	671.9395	541.964	863.782	732.3523	608.9258
pos_1731	Pseudouric	58.56842	80.11369	240.5328	180.0371	2.046213	28.25691	81.70804
pos_1734	5-Phenyl-	10.69838	35.185	4.268493	7.393875	9.065525	9.321522	23.1668
pos_1735	N-Nitroso	14.95818	75.84481	24.78681	74.16246	57.65723	149.5596	55.75736
pos_1736	Tridecanoi	26.15295	75.6042	7.03285	44.31293	19.68906	50.58136	26.50912
pos_1737	3-Feruloyl	28.98692	34.027	12.90484	35.22717	19.40028	30.128	30.27411
pos_1738	Indole-3-a	6.933735	9.209698	5.293486	311.0848	5.780769	10.89892	11.39312
pos_1739	4-Hydroxy	36.41848	36.59301	22.76212	40.1509	36.02111	24.86034	27.49132
pos_1740	SALSOLIDI	117.5493	464.896	199.1006	216.8286	354.6686	573.9989	639.5385
pos_1741	Fusarochr	90.59082	135.773	68.26998	68.88339	49.28712	93.25405	59.04582

pos_1742	Aflatoxin F	60.13132	74.24683	265.8452	105.5717	0.904101	20.21619	76.85855
pos_1744	Threonylis	543.6444	27.285	40.2269	231.0639	108.1733	83.47053	40.90849
pos_1746	8beta-Ang	57.98357	163.0552	1.72E-06	119.6661	1.72E-06	120.5158	27.75637
pos_1747	Phaseollini	82.56632	135.7427	27.55045	123.8467	42.78053	76.45529	64.4476
pos_1754	3''-Hydrox	114.6943	71.16879	27.11115	260.8185	59.17015	105.9765	86.05679
pos_1755	11beta,17l	184.9791	178.2961	172.1944	155.2177	122.5888	153.3731	145.9841
pos_1756	1-Methoxy	418.5451	142.2581	148.5092	264.5011	306.8969	195.9847	78.20286
pos_1757	Estetrol	232.4369	88.08318	38.86027	311.3106	42.66606	117.0041	253.4449
pos_1759	Trp Glu	90.09617	104.8721	41.85269	38.21095	50.98642	114.603	31.58897
pos_1763	5,2'-O-dir	62.92612	45.96542	14.00903	55.64311	53.19407	60.57321	40.73602
pos_1765	Pamidrona	553.1668	541.5402	293.7602	521.326	555.2237	527.3618	426.1332
pos_1766	Phenylacet	893.9658	169.9795	89.84057	256.6947	214.5629	173.3176	114.5444
pos_1768	3-O-Meth	1449.639	1451.183	469.9464	1376.633	1393.801	1295.034	890.8604
pos_1769	Peonidin 3	26.08753	1.72E-06	1.72E-06	1.72E-06	1.72E-06	1.72E-06	1.72E-06
pos_1771	2,5-Dimet	1923.349	1827.225	907.5586	1621.21	1669.714	1548.288	1200.651
pos_1774	3-Hydroxy	13745	14402.61	5749.951	12978.89	13057.05	12716.18	9762.178
pos_1775	Oxindole	183.9347	182.9256	85.10525	167.8394	182.2929	178.5078	138.1384
pos_1776	Umbellifer	2376.19	2464.825	1087.933	2274.424	2284.417	2142.196	1650.92
pos_1777	D-Xylonat	481.0294	339.8802	755.7385	336.6048	411.0527	316.5883	349.4991
pos_1778	2-Methyl-	76.35465	48.95149	160.1963	45.97519	53.30329	37.96584	46.1742
pos_1779	5-Hydroxy	1385.349	1283.402	1189.525	1158.929	1296.74	1083.52	923.6238
pos_1780	Zalcitabine	488.4368	513.6861	483.2828	526.9316	508.4167	537.7592	453.427
pos_1781	4-Hydroxy	206.7782	229.0871	128.9985	197.8444	199.3069	218.6563	177.2544
pos_1782	Fluoroazor	2856.874	1770.18	6599.905	1810.78	2281.099	1551.333	2291.985
pos_1783	Cysteinyl-'	462.8424	289.9741	1094.182	315.3584	371.5008	255.9581	389.7788
pos_1786	Tephcalost	539.0436	549.9536	221.8697	534.6431	475.1304	460.44	343.3789
pos_1788	1-(5'-Phos	23.36646	28.44635	6.360652	31.02493	30.95524	17.57472	15.80179
pos_1789	Desmethyl	1158.975	1100.785	260.6554	992.8421	1075.789	932.132	577.6237
pos_179	4-Guanidi	180.6465	362.9526	331.1598	389.1434	146.0221	215.4462	217.9075
pos_1791	Benzene	1826.096	1892.131	595.2593	1639.904	1670.129	1595.574	1199.509
pos_1792	Benzoyl ch	156.3178	118.6026	1.72E-06	95.45526	106.2333	88.71649	38.62221
pos_1794	Thymidine	6875.335	7639.013	9633.814	7159.212	9922.735	7834.109	6317.715
pos_1795	2'-Fluorotl	2341.771	2505.541	3204.414	2339.095	3293.916	2597.596	2047.265
pos_1796	15-keto-P	244.4022	152.9789	2701.99	1520.166	113.7074	124.1795	229.4848
pos_1797	2-Succinyl	125.8914	132.2185	105.7256	239.9733	258.114	143.5988	131.291
pos_1799	4-Methoxy	28.93091	27.40296	9.142544	23.49084	13.97355	23.47715	11.55027
pos_1800	Icofungipe	105.2403	65.85073	47.85634	14.96334	71.58452	64.30791	43.79859
pos_1801	cis-1,2-Dip	73.97137	26.44285	4.932642	132.5864	22.73464	60.55719	25.64624
pos_1803	2-Aminob	166.8132	292.8464	66.38386	250.5228	38.65829	124.0555	126.1471
pos_1804	Benzoyl gl	1169.628	1224.891	1828.866	1277.203	1031.877	768.2542	982.1849
pos_1807	S-Acetyldi	151.6477	182.2712	338.056	285.4138	131.8945	152.9306	166.1409
pos_1808	1,4-Benzo	233.0449	187.5793	611.1342	186.5055	293.6161	162.0486	239.0307
pos_1810	[[(3R,4R,5S	387.0982	730.5768	69.21886	1020.773	1.199887	440.3149	314.6296
pos_1811	(E)-indol-3	18.96077	15.26485	4.190978	11.75742	4.059158	22.59284	6.086023
pos_1812	3-Des(dim	186.5898	145.9141	46.47597	105.2448	124.0775	172.595	94.91452
pos_1813	Cidofovir	65.60953	159.935	30.64022	160.923	31.78347	50.09953	85.02301
pos_1814	Ribavirin m	63.3313	88.75823	247.771	200.6694	1.72E-06	35.07379	92.57533
pos_1815	3-keto-Di	305.6121	213.1283	279.1908	235.2975	193.7333	238.914	185.702
pos_1816	9-Hydroxy	494.1045	1.72E-06	79.10058	106.7877	29.1296	1.72E-06	27.40397
pos_1817	para-Nitro	298.9002	415.7752	314.5803	597.2916	321.5841	634.6889	474.9993
pos_1818	5-Acetylar	1105.308	1691.709	1303.232	2529.997	1372.113	2628.896	1950.769

pos_1822	Ethyl L-try	141.5021	141.8948	120.7073	173.8199	100.3947	138.9954	117.8186
pos_1823	Cornoside	215.0699	148.2248	105.6292	123.7296	30.50458	85.49879	44.82865
pos_1825	Tezacitabi	82.50659	80.15491	155.613	106.3534	233.9364	98.75229	87.17427
pos_1826	2-Amino-	604.002	575.4098	1091.193	654.5533	1558.645	643.9289	593.4016
pos_1827	Convicine	177.9025	182.1895	338.7093	209.8793	486.4073	196.473	171.4745
pos_1828	R-(-)-Mar	59.04072	78.65878	37.35887	75.96512	67.45851	50.53457	77.93701
pos_1829	trans-O-M	105.2533	85.8103	69.00368	128.4894	42.30731	81.42035	65.27658
pos_1830	Carbazole	53.20964	17.67365	24.40987	25.30863	14.30903	25.93339	13.25759
pos_1831	Indanone	137.4781	109.7971	124.2593	129.7459	107.6679	111.4813	101.0774
pos_1832	1-Benzaze	209.1128	106.0581	119.2385	107.501	81.24243	119.8228	102.4113
pos_1833	8-Isoquinc	739.016	359.8338	468.6531	440.7367	327.4073	411.7813	335.6725
pos_1837	Hexyl 2-fu	65.08054	61.62697	32.68223	74.07085	27.06627	73.74703	50.79885
pos_1838	Phenylalar	5.813219	13.23835	3.799425	6.878914	3.332683	13.55913	14.8883
pos_1839	Trimetazid	171.9295	237.5054	186.3703	183.3389	133.2264	137.9911	153.1169
pos_1840	Helenalin	30.01179	38.80171	1.05395	1.945602	14.832	17.19478	11.53888
pos_1841	Harman	110.5796	34.38192	34.3204	53.26595	46.99272	64.82085	34.81273
pos_1842	Harmalan	82.5482	35.08114	43.83876	42.77137	38.19864	47.13224	31.47861
pos_1843	Cyclic Ure	13.45049	17.14434	27.55109	18.4544	14.85547	20.97651	12.2608
pos_1844	Desmethyl	1.72E-06	3.553217	2.051719	46.76395	18.31569	34.42168	80.29642
pos_1846	Eremosulp	3.999582	18.0089	4.31377	122.1235	29.83677	80.62179	176.3242
pos_1848	4-Hydroxy	110.3211	91.04667	182.1225	119.3432	198.2413	100.0259	92.74781
pos_1849	Methyl hel	11.80491	21.51226	4.462912	4.930457	13.67835	10.47531	6.414732
pos_1850	ingenol	41.8224	64.72113	32.67807	16.47754	53.22559	8.551759	18.16294
pos_1857	6a,12b-Dil	420.9986	37.00033	5.143896	75.81501	99.45671	91.86776	50.89852
pos_1858	2-ETHOXY	193.6674	420.6759	21.63952	509.2436	18.53829	490.5368	88.93822
pos_1859	Phenethyl	83.06279	30.28954	12.68819	32.62086	30.65772	35.11908	19.41031
pos_1863	5-(3'-Carb	67.97637	15.17358	5.328198	17.89071	29.33662	26.10534	15.72593
pos_1865	xi-4-Hydro	1026.06	360.7972	182.3851	416.6142	407.88	448.1405	290.6538
pos_1866	C20914	45.28127	49.87231	68.44832	48.42385	49.23599	44.09147	45.27278
pos_1867	7-Hydroxy	20.75548	17.86951	82.87653	59.7159	7.616034	20.72057	42.92469
pos_1868	Pro Gly Se	146.8406	166.6924	728.4653	468.6921	61.84581	109.2744	314.6927
pos_1869	N-Docosa	107.3303	911.0599	216.5126	318.8336	395.6943	299.4136	441.5336
pos_1872	[(2S,3S,5R)	17.28971	103.6477	1.72E-06	38.95319	24.4381	157.0488	6.216806
pos_1874	2,3,6,7-Tet	271.5974	172.0218	63.22143	730.6887	189.7639	283.2537	202.7459
pos_1875	8-Amino-	168.0417	246.3508	145.2279	206.5571	89.87973	190.3244	192.4489
pos_1876	Panthenol	135.782	210.0583	145.3452	139.2547	61.18051	138.5477	166.5852
pos_1877	N-(3-Amir	454.0752	343.4266	175.8099	1271.981	383.8649	525.4849	403.2816
pos_1878	Isoleucylpr	1626.085	135.0784	178.5614	1338.796	283.4517	355.0545	250.0633
pos_1879	Carbazoch	99.14292	6.269062	14.20746	22.88062	32.52142	22.34754	10.79706
pos_1880	Dimethyl (	70.9927	1.72E-06	1.72E-06	5.740265	3.986768	1.72E-06	1.72E-06
pos_1881	S-(2-Hydr	139.395	115.4241	222.3422	115.1936	196.2348	107.8856	111.5895
pos_1882	Voriconaz	35.62028	35.74685	45.46328	40.42164	47.5041	42.96049	41.55468
pos_1883	Hastatosid	110.9846	130.3825	20.40586	176.9354	12.489	29.0991	53.31958
pos_1884	Fluridone	126.8309	110.2611	177.5466	124.7599	193.4807	116.7513	102.5041
pos_1885	Validamyc	26.6895	33.44727	156.5439	46.69238	2.830007	11.55177	70.91057
pos_1887	Nebramyc	14.32834	9.683985	1.72E-06	1.602963	35.74432	66.24134	2.766453
pos_1888	L-Glutami	50.22268	6.249757	1.994905	3.524024	4.555115	4.432414	3.619447
pos_1889	7-Ethyl-5,	149.1618	71.15508	13.19577	126.1824	44.54825	58.94999	52.54546
pos_1892	Forodesine	61.70564	13.67096	1.604514	38.5765	2.784102	2.567047	5.623104
pos_1893	6-Acetyl-2	34.65438	37.09706	69.38459	55.91397	20.58807	31.33339	39.77433
pos_1894	Armillane	85.41187	145.7568	113.9633	132.9481	115.1683	67.67357	84.72424

pos_1897	3-Mercapt	33.68286	15.67364	9.712412	19.15094	26.98023	19.85355	14.8306
pos_1898	Reduced p	31.214	48.40073	61.71131	46.87743	22.48842	61.30873	61.36972
pos_1903	Phenyllact	444.5686	658.2283	352.0664	773.5588	113.0008	267.7925	284.0132
pos_1904	3-Indolebu	42.13529	59.265	67.28929	81.50259	26.26883	80.04389	67.6703
pos_1906	5-Amino-	124.3821	389.54	708.1603	475.1136	221.9208	658.2577	602.2663
pos_1907	Austdiol	24.68997	23.34242	44.39071	25.41496	49.07829	25.40159	19.89675
pos_1908	4'-Azidocy	275.9339	344.1456	233.9436	222.8969	298.948	165.7868	197.473
pos_1909	Erosone	1104.762	2041.37	1116.725	2313.146	332.7614	809.1948	912.8861
pos_1910	3-O-fucop	1.72E-06	1.72E-06	307.4337	1.72E-06	1.72E-06	2.906571	1.72E-06
pos_1912	Harmanine	173.2463	162.56	174.5818	152.3321	162.011	205.2526	129.5919
pos_1913	Glutaminy	285.9155	305.0376	518.6851	390.7256	683.7403	340.4009	294.5218
pos_1914	Dihydroxy	65.92375	92.51153	26.18667	75.5656	68.60007	112.669	66.14474
pos_1915	Norophtha	87.19647	77.50296	113.7077	93.03555	125.4416	64.77607	75.10261
pos_1916	Dodecane	580.6129	575.7654	228.2298	484.3701	406.1726	426.6534	217.8764
pos_1919	Pentigetid	7.522388	33.93738	13.06906	3.082604	27.64623	15.06634	29.06846
pos_1920	xymedon	99.44863	77.36527	35.99629	155.3812	56.12265	53.10683	36.32927
pos_1921	Pro-leu	3150.684	153.2342	232.6732	2111.799	659.3942	520.8911	322.3381
pos_1924	(1R,6S)-6-	183.2327	200.5911	213.6347	242.5369	200.1116	196.8454	187.9572
pos_1927	3-Methyl-	400.6349	465.2989	188.4665	564.9371	356.6121	695.9597	363.2655
pos_1928	Nalidixic A	269.9169	337.3195	157.9431	442.602	256.7783	500.0241	267.0208
pos_1929	Dihyroxy-	60.93237	80.73846	37.64982	116.0893	18.86343	65.25565	41.98063
pos_1930	2,5-Dimet	39.50669	45.91824	43.11427	39.84727	51.18356	40.78387	37.94248
pos_1931	4'-Thiothy	388.6158	403.3668	647.9649	466.1986	657.333	403.9399	379.5018
pos_1932	Gly His Gly	148.8443	15.60215	5.700924	4.870366	23.59756	7.626509	1.72E-06
pos_1933	3-Methoxy	117.6992	32.48608	50.56689	270.8786	39.87025	70.64024	57.35869
pos_1934	Mofebutaz	14.19691	9.83073	8.441916	11.2516	7.438628	13.43256	8.961958
pos_1935	1-[6-(2-Ca	168.4307	112.87	108.2709	159.7845	161.509	157.7522	358.8177
pos_1938	2-Hydroxy	800.5008	1076.473	322.8373	2144.176	163.6078	768.3907	571.5887
pos_1939	3'-N'-Acet	123.1891	101.8037	388.3973	303.2574	41.26919	54.52109	155.0437
pos_1941	L-histidino	252.1301	291.8012	73.21223	713.1452	19.88072	195.4917	136.0129
pos_1944	Indole-3-	943.1962	253.2907	208.0268	280.9776	365.5368	335.2056	272.6516
pos_1946	(R)-1-O-[	33.46404	14.84599	3.981219	4.342514	1.72E-06	1.706868	1.72E-06
pos_1948	(2S)-2-(Ca	39.24248	21.49998	16.2063	14.757	28.64	81.93375	43.13423
pos_1951	L,L-Cyclo(	1746.646	226.8504	291.0927	1057.264	594.8341	399.8624	268.8582
pos_1953	Hexanoylg	168.6193	90.90633	85.20623	267.7509	132.9057	95.65625	91.43901
pos_1954	3-Furanm	166.7751	149.5464	138.6031	165.3546	160.0113	154.7023	139.4957
pos_1955	Deoxycytid	30.29494	1.72E-06	1.72E-06	27.66479	3.477899	4.22703	1.72E-06
pos_1956	Mannopin	219.497	213.8573	128.8477	348.6641	72.70266	288.535	141.9232
pos_1957	Diethylpro	238.4211	1.72E-06	1.72E-06	18.59312	1.72E-06	2.783845	1.72E-06
pos_1958	2-Isopropy	85.70956	26.68216	27.60665	52.63225	44.47617	44.1567	19.40523
pos_1959	N-Methyls	222.9737	297.1727	304.3592	292.3778	290.0804	251.3231	263.0646
pos_1960	1-Ipomea	299.2149	130.5494	97.35483	174.255	151.5944	162.9836	78.22184
pos_1963	Hydroxypr	104.6216	39.13566	1.72E-06	659.2663	22.57249	43.57277	9.388091
pos_1964	Morinidaz	390.6179	371.4439	209.2422	545.4207	175.6966	436.0312	239.2233
pos_1965	2-Hydroxy	1764.336	966.9806	811.0958	9324.368	829.0371	1056.696	740.076
pos_1968	Canavanin	82.80222	83.40528	30.9602	193.2101	85.18853	145.8766	109.4514
pos_1969	S-Adenosy	205.2221	302.5384	66.90169	449.3476	46.299	234.4757	106.8406
pos_1971	3b,6a-Dihy	56.73271	453.182	75.55853	141.811	174.767	137.913	201.6259
pos_1973	4-(Methyl	41.15134	39.09923	22.40646	27.71856	36.22103	46.57617	20.38844
pos_1974	Tyramine g	123.9575	133.8439	209.1809	100.7085	259.959	112.123	131.0258
pos_1976	5-(2-carbo	59.19442	36.39177	29.43311	91.62061	21.48859	47.73416	42.74616

pos_1977	N-trans-F	28.41908	33.65865	178.5493	116.5475	8.770649	18.29178	74.86982
pos_1978	Dihydroetl	832.3045	29.50609	1.72E-06	5.623626	1.72E-06	18.17541	1.72E-06
pos_1979	2-Hydroxy	211.8468	15.83003	11.82467	56.78784	29.2068	22.35023	22.58337
pos_1980	[(1S,2S,4S,	883.628	14.06788	13.5564	92.43838	55.10722	29.2374	60.57943
pos_1981	l-Tyrosino	24.61568	29.55003	32.58819	36.39396	24.93448	38.10698	19.8774
pos_1983	desethylet	34.30724	48.0259	32.24238	34.6351	28.43233	54.14422	25.35945
pos_1984	Indoleacet	78.93055	120.9286	81.50006	79.6257	78.42626	115.1116	62.69919
pos_1987	PGDM	45.30052	55.94713	22.77225	81.13172	9.881102	100.1133	39.3017
pos_1988	2,4(1H,3H)	41.48504	95.28003	1.72E-06	235.4184	1.72E-06	75.37852	13.7563
pos_1990	2,4-Dimet	148.0556	123.4588	93.88435	66.74623	93.01552	123.3375	56.17274
pos_1991	n2-(1-Car	70.83935	39.2733	14.12877	65.00867	91.51581	40.95391	16.13356
pos_1992	(2R,3R)-3-	44.58789	25.57359	37.25484	23.83659	10.23396	25.0904	12.07509
pos_1993	Licoagroch	224.4469	156.246	602.2146	260.1358	149.1557	158.9581	282.4966
pos_1994	D-Fructos	63.16106	72.64182	73.03748	64.10237	68.18667	66.44046	73.75088
pos_1995	Feruloyl-2	35.09234	1.72E-06	1.72E-06	4.626396	7.74183	4.128175	1.72E-06
pos_1996	Desmethyl	33.15842	35.0826	68.88465	38.60755	59.96422	32.18263	29.59015
pos_1997	cis,trans-5	6.253926	10.76349	25.25139	14.0873	11.18475	5.093968	9.287189
pos_1998	5'-S-Meth	45.53558	49.08847	57.32504	62.89749	50.01196	42.45111	41.72975
pos_1999	3-Hydroxy	152.9214	108.513	322.8957	174.3184	334.476	198.4184	488.7964
pos_2000	MM 42842	42.83446	23.94069	124.9146	43.11802	91.97691	47.50222	151.0507
pos_2001	Tyrosyl-Ar	451.9293	26.54451	1.72E-06	1.72E-06	1.72E-06	5.625776	1.72E-06
pos_2002	2-Naphtha	70.99738	99.35815	27.02361	64.36566	47.48139	117.9152	47.5916
pos_2003	5-Hydroxy	251.0636	105.7737	48.3801	83.8655	74.22364	148.0705	86.01483
pos_2005	Oxyapram	48.25919	38.65625	55.00273	39.03801	43.43039	40.60059	44.85099
pos_2007	Indole-3-e	220.906	250.1126	378.0637	354.3312	411.9131	226.8323	249.6395
pos_2011	oleandom	186.6261	39.9296	63.59929	37.77179	35.43227	47.70262	25.90357
pos_2012	Arkofix	201.6791	170.9471	204.6612	210.7684	200.1469	227.6014	285.5276
pos_2013	N-(3-oxo-	35.87184	16.4852	50.72662	46.1873	29.43308	30.26471	26.13097
pos_2014	5-Hydroxy	40.97341	44.96028	44.30739	56.70596	32.64819	45.99478	49.16151
pos_2015	Hydroxypr	5.149244	9.338298	20.05311	12.67581	10.18937	59.71609	32.6089
pos_2016	Dimethylsp	42.08346	24.1889	1.72E-06	8.972177	28.18365	38.36418	31.92934
pos_2017	cis-trans-N	31.40415	25.79905	7.963329	60.49873	9.058262	22.68865	21.99555
pos_2018	(R)-(Indol-	141.7761	58.22603	40.57693	36.74233	69.67174	239.8669	63.98519
pos_2020	beta-Amir	71.13817	86.70848	94.24698	86.42454	98.49439	308.4258	112.5767
pos_2021	3 Hydroxy	846.8923	960.8769	798.6857	1080.547	791.2941	1022.575	791.3044
pos_2022	10-Metho	56.99859	42.0613	12.33598	37.77399	38.49401	93.69188	59.89257
pos_2024	Ser Cys Al	67.01946	67.54284	130.965	96.55681	42.14925	31.16279	60.0555
pos_2025	7-Methylg	55.32886	52.32782	121.1537	450.1471	29.84318	39.78781	59.69655
pos_2026	3-Pyridine	12.02158	21.70524	14.39883	7.821459	23.90057	93.61592	39.4405
pos_2030	Pantetheir	206.2267	221.3657	1138.05	392.1353	141.1979	153.3515	444.0296
pos_2031	Rifampicin	41.97924	253.1034	8.403216	15.37889	117.8239	54.70117	133.4804
pos_2032	N4-Acetyl	13.90469	11.77152	12.48268	21.18172	11.73564	20.49389	31.80838
pos_2033	4-formyl I	33.15388	17.93078	26.72882	39.88896	20.2579	38.73293	49.16438
pos_2035	Val Gly His	61.27048	105.9232	4.925625	97.92515	32.60575	31.14521	14.93512
pos_2036	Glycyl-Arg	317.8516	314.7723	360.6815	366.8735	304.3867	340.1813	310.7606
pos_2038	(2S,3S,4R,5	440.8891	462.1034	532.8779	522.7812	508.7676	600.9935	586.3521
pos_2040	Asparagin	85.07644	463.7338	49.75973	115.2212	210.6722	127.5133	216.7494
pos_2042	Capillene	48.50449	51.12028	63.97966	51.21012	60.07584	51.06054	44.89729
pos_2043	4,4'-Diami	217.301	117.7119	226.3084	245.5409	167.8888	131.356	112.6382
pos_2045	6-Fluorom	672.4397	1020.308	738.5155	1045.045	1031.37	1070.402	1183.447
pos_2047	Eflornithin	112.7256	159.9881	134.2899	331.5163	127.9675	113.5718	164.2578

pos_2048	(-)-Jasmor	91.79206	133.9887	74.25993	174.3335	77.28721	147.1792	71.89802
pos_2049	Germacror	17.63996	32.09091	8.377448	63.43599	8.759239	46.6458	45.3706
pos_2052	Tiaprost	46.90773	47.58135	134.5617	77.65395	34.39701	40.55826	61.26273
pos_2053	7a-Hydrox	15.62009	50.13071	38.91787	140.1346	8.80451	14.80786	53.53078
pos_2054	Homomet	11.19744	12.55863	14.85501	13.18345	11.2506	22.50118	14.6145
pos_2055	3-Methylc	44.62266	50.45574	65.76596	88.63408	122.1763	85.2195	53.74271
pos_2056	Isocurcum	10.56088	19.56378	36.6129	17.32035	13.61237	73.54472	60.12246
pos_2057	1-(9Z,12Z,	5.986796	1.72E-06	1.72E-06	39.29555	1.72E-06	15.61585	1.72E-06
pos_2058	Cinnoline	536.5949	211.7836	145.9142	392.8785	191.3352	186.4999	285.3981
pos_2059	O-methylr	251.5707	123.1521	190.6423	202.8456	143.3507	120.9459	161.0299
pos_2063	Methyl 3-r	459.3964	489.9779	390.4587	650.1177	603.9977	567.8462	475.3783
pos_2064	6-(Hydrox	33.08668	29.53971	14.42227	46.91527	42.40555	31.94003	25.23111
pos_2066	δ-Valerola	840.4134	537.7833	78.95908	631.4996	261.9265	401.8491	304.632
pos_2069	2-Hydroxy	37.7637	59.98839	15.52951	92.6964	51.76545	40.45752	21.73257
pos_2071	5-Hydroxy	584.3677	640.3243	107.5723	204.3938	270.224	500.9557	298.8913
pos_2072	2'-Deoxy-	23.60251	39.90156	25.43131	13.34731	86.4076	78.23165	13.02958
pos_2073	Morpholin	35.88651	21.11442	1.72E-06	28.35369	12.09746	14.96025	14.98877
pos_2074	n6-[2-(4-/	33.09087	9.982188	123.1523	99.72858	1.72E-06	9.1957	42.33378
pos_2075	cis- and tr	122.4142	161.2635	33.21814	88.57609	73.15616	115.9688	80.98656
pos_2078	6-Thioinos	105.0686	133.1139	53.59489	94.63806	88.68118	111.0928	80.70797
pos_2086	(1R,6S)-1,6	101.3463	125.5818	129.8719	113.0908	109.7091	126.1294	116.1744
pos_2087	HARMOL	195.8955	290.433	152.1033	334.2632	273.3676	447.5712	273.3949
pos_2088	4-(Butoxyr	111.0999	113.8574	33.59356	119.3019	65.06595	95.64144	38.16228
pos_2089	Dodecano	3208.116	3542.713	2677.407	4065.795	4023.837	3733.925	4372.965
pos_2090	7-Methyl-	167.6314	223.9229	114.6504	238.6275	190.2401	330.5374	217.3373
pos_2091	5-Benzyla	807.5147	1293.314	564.7209	1470.674	1123.793	1982.502	1259.087
pos_2093	Pratenol A	49.58462	47.37548	569.2065	125.897	23.36416	29.76334	176.2607
pos_2094	5-Phospho	564.6749	647.5388	1023.464	626.4416	936.4491	627.1985	639.9339
pos_2095	Homovani	426.3486	480.1969	780.4501	462.3945	683.5687	483.2745	465.0668
pos_2098	2,6-Dimet	24.30629	61.86248	17.2261	57.39501	51.39167	55.2029	31.98058
pos_2101	Deoxypyri	251.1039	1.72E-06	1.72E-06	34.6536	15.4606	21.43102	1.72E-06
pos_2103	3-Methylir	140.3909	106.8248	73.72184	74.20946	173.6849	76.52535	92.52919
pos_2104	6-Hydroxy	574.7808	478.6364	492.28	709.4186	985.0769	805.5005	1232.832
pos_2105	3alpha,4,7	916.0145	1018.391	1046.897	1051.228	1031.78	967.449	976.9657
pos_2108	Arginyl-Va	11.39439	16.1061	29.77839	15.98156	21.26998	48.19253	17.48619
pos_2109	7-Acetylin	192.0735	110.6585	18.26797	153.2569	207.8085	202.5077	121.3422
pos_2110	2-Methylp	25.44621	25.93267	18.79785	31.51379	39.33585	35.91887	60.97277
pos_2111	Isoquinolir	419.0568	258.3541	119.687	386.6992	155.9364	304.0885	287.9674
pos_2112	Indole-3-a	190.58	112.0029	54.11693	172.2741	68.44183	144.1513	130.1986
pos_2113	S-adenosy	73.08125	55.70922	93.9632	99.37277	77.64429	50.70854	33.2762
pos_2114	1-Phenyl-	493.031	518.1572	948.9717	586.271	1069.745	572.493	505.2216
pos_2115	Methyl N-	44.65465	43.28669	33.2039	52.53989	53.08917	57.0908	37.49795
pos_2116	5-Methoxy	29.38864	15.52736	26.63305	29.34168	80.06192	3.39476	9.469858
pos_2117	6-Fluoroh	256.8126	274.2317	523.8134	313.5462	566.3085	292.5837	267.2683
pos_2118	L-Oxalylall	46.93591	45.22229	93.3756	52.81094	99.961	46.5914	53.86796
pos_2119	Leu-Leu-T	1.72E-06	39.76918	20.55972	128.7584	1.273274	1.72E-06	28.40022
pos_2120	(2S,5S)-tra	12.13196	11.82188	14.42552	15.03145	21.64258	18.39252	15.25435
pos_2121	2-Phenyl-	34.95993	25.95893	20.49859	41.34104	33.65089	41.43601	28.12353
pos_2125	6-hydroxy	166.032	85.64457	44.77299	1161.237	181.0343	105.5641	63.68758
pos_2126	Estradiol-1	678.6419	494.9945	735.9038	816.6194	1175.608	920.3104	1546.589
pos_2127	4-Hydroxy	234.2504	163.3314	230.1477	298.6925	361.3315	293.8441	504.2333

pos_2130	7C-aglyco	385.6589	228.6006	669.5775	1726.272	219.0374	179.7858	303.5809
pos_2131	1-Methoxy	103.2351	24.94368	14.07714	61.57255	3.069532	27.95439	46.50505
pos_2133	7-Aminom	43.74892	49.93538	45.20076	108.668	43.21836	45.58397	33.80145
pos_2134	N-[[(2S,3S	166.2154	168.3654	415.4309	260.4931	185.7537	211.1875	232.7525
pos_2135	Cotinine g	501.754	461.1661	642.9657	509.5108	454.6325	524.4666	413.2188
pos_2137	Ethyl 3-hy	2505.699	2688.143	1757.058	2970.24	2979.796	3034.385	3361.397
pos_2142	Metaxalon	15.70665	70.9005	1.72E-06	1.72E-06	28.14856	9.446125	34.10729
pos_2144	10-Hydrox	15.79313	17.27398	202.7733	82.74087	5.715129	14.56224	56.88946
pos_2145	Semilepidi	374.6736	380.8601	1560.773	737.6534	274.6152	303.9577	604.2249
pos_2147	Arbutin	90.57778	100.3318	340.9894	217.1573	198.4142	99.31877	183.0573
pos_2148	Loganin	4.994726	8.553861	139.9775	90.69846	1.72E-06	5.14536	34.5806
pos_2150	1,2,3,4-Tet	45.09922	16.78629	47.32941	25.9717	21.35911	16.52773	22.2453
pos_2151	7-Methylx	60.08869	63.87291	172.5243	122.8945	159.4212	60.53447	116.8633
pos_2154	3'-Deoxyth	22.6017	26.70414	151.5199	84.97408	11.39675	16.31055	48.22607
pos_2155	5-Acetylar	22.07584	22.35687	76.01507	55.74787	17.05234	20.89087	37.71204
pos_2156	Gly Asp Al	1003.557	1058.449	5915.53	2963.588	585.4644	705.2443	1942.883
pos_2158	Oxytocin	1.72E-06	123.0497	5.68013	40.80942	68.67334	144.644	1.72E-06
pos_2159	N-Acetylh	9.355524	7.720615	33.1905	18.82611	3.652455	4.505409	9.21033
pos_2160	N-Oxalyl-	57.18825	70.52711	73.79641	75.7757	63.93582	86.54186	65.62135
pos_2161	N-Hydroxy	27.43004	24.9285	40.18282	52.38552	27.99762	55.879	36.54815
pos_2164	xi-3-Hydro	42.54526	30.04834	31.69833	48.15337	43.70911	56.83765	50.7491
pos_2165	ribostamyc	6.246827	46.64127	1.72E-06	36.89696	30.97037	61.72086	2.370023
pos_2168	ethyl 2-cya	35.02962	39.708	212.8053	103.6681	15.89961	24.48557	67.59796
pos_2169	Decanoyl-	14.78872	43.89545	145.263	35.29751	33.56536	173.2394	101.9432
pos_2170	1-{2-[(3-E	667.9416	827.0338	4140.258	1980.151	407.0994	502.2046	1410.338
pos_2171	Grifola frar	59.6848	100.5221	51.0879	76.14075	82.43125	42.87098	59.20098
pos_2172	kainic acid	53.08833	64.78638	325.2711	174.1173	10.92963	44.61285	94.71181
pos_2173	Zanamivir	26.92281	38.43824	243.8704	122.9884	2.801184	17.04448	73.34806
pos_2174	Nevirapine	38.49092	13.17049	3.392896	113.5695	13.14851	21.4181	10.68599
pos_2175	Amyl 2-fur	67.85671	75.3905	54.28772	53.70511	61.3191	81.41184	64.14072
pos_2176	1,2-Benzis	299.1236	375.6361	244.9854	356.0457	354.8103	348.2458	339.071
pos_2177	Citrinin	18.55198	44.62305	2.099959	40.67192	1.72E-06	33.46324	4.22416
pos_2178	(S)-Mande	53.19939	111.4389	110.4443	116.4073	39.65614	96.09771	52.37481
pos_2179	Zolpidem	17.5905	35.20002	1.773155	28.39107	41.84592	43.59484	19.57398
pos_2181	(±)-threo-	165.4374	81.8369	73.16359	78.60861	101.6219	84.41415	67.55976
pos_2185	Methionyll	25.88846	28.21333	31.99363	33.41949	40.54916	22.53476	32.18374
pos_2186	5-Hydroxy	12.62355	1.72E-06	7.079712	5.042077	2.968991	3.175295	1.72E-06
pos_2187	Gentiobios	25.04104	21.08122	47.11088	27.83222	38.08181	26.22724	37.28958
pos_2190	N6-Acetyl	29.14676	28.59909	11.90897	20.77591	32.25106	19.75363	18.83921
pos_2191	Trihydroxy	118.4956	141.9457	83.1448	135.8701	115.9374	180.6009	170.4948
pos_2192	Hexenoylc	57.87751	39.37208	38.71381	22.17835	19.02553	42.65836	34.82279
pos_2193	15d PGD2	163.8636	98.17454	16.34339	87.11936	78.08059	188.104	69.8373
pos_2194	Allotetrahy	30.39539	69.72414	2.903116	97.00906	25.43538	20.1121	10.47243
pos_2195	Misoprosto	1434.27	1482.554	563.84	1145.624	757.3071	713.009	734.5817
pos_2196	Quillaic ac	3.810938	16.56209	1.72E-06	9.968543	45.16376	39.28966	1.72E-06
pos_2200	4,5-Dihydr	44.09446	102.9545	59.94578	88.13642	110.4726	250.5756	108.2376
pos_2201	Tryptophy	50.31092	113.359	254.1322	236.6518	30.46491	17.6162	84.87102
pos_2202	Aminoglut	74.52449	60.41987	386.8249	196.7337	44.69728	52.86615	125.7429
pos_2203	5-NITRO-:	572.4566	629.2708	3607.587	1661.675	440.1046	417.8539	1214.118
pos_2204	cis-3,4-Ph	1.572509	6.73827	101.4181	13.37075	1.706471	10.58107	9.269848
pos_2206	Met His Gl	1.72E-06	1.683741	752.2789	3.409309	1.72E-06	17.39199	3.15773

pos_2207	Cichoriosi	202.5074	289.8543	1.72E-06	1.72E-06	18.48368	15.80045	53.74369
pos_2209	Serylprolin	56.68677	75.32229	67.99154	75.86216	89.5882	75.76146	74.73789
pos_2210	5,6,7,8-Tet	29.47633	30.85507	106.4516	60.81552	14.96782	25.2662	44.01957
pos_2211	Lubiprosto	293.3798	706.2528	65.01533	896.9042	244.2601	250.1126	193.382
pos_2212	alpha-(Dir	36.38527	82.26524	34.43632	99.81307	34.25611	21.56043	7.287773
pos_2213	carbenicilli	41.41261	39.55111	30.33689	358.0825	44.62419	31.93976	36.26036
pos_2215	Irene	471.6665	541.8073	526.6922	548.4927	522.5994	507.7165	471.3832
pos_2218	(2R,3S,4R,5	25.91978	8.822407	1.72E-06	9.572632	4.86392	8.855611	1.72E-06
pos_2219	PHENACYI	22.73844	29.41863	21.02676	37.72224	25.4866	35.24453	26.91001
pos_2220	3,5,7-Trim	68.48295	267.3379	38.2742	371.5458	55.26148	63.39975	61.10693
pos_2221	(Z)-3-Oxo	221.2904	193.5772	215.0333	222.2081	159.2672	193.0493	193.5823
pos_2222	2-(Ethoxyr	1.72E-06	32.85025	27.40165	34.55128	28.36183	124.712	33.72862
pos_2223	5a,6a-Epo	94.29276	447.5984	30.57138	653.7997	62.82607	85.59562	69.38882
pos_2224	3,4-Dimet	24.63849	27.32713	7.067505	75.88252	37.16657	35.1134	26.13701
pos_2225	Thymidine	44.52787	47.53237	25.95849	33.56213	28.11634	50.67745	42.75587
pos_2226	Littorine	147.5954	168.0927	178.4277	183.9793	308.675	213.4688	170.6034
pos_2227	N,N-Dihyc	72.08256	45.37298	10.96628	5.582929	46.69	59.27603	36.58219
pos_2228	Harmalol	33.49132	35.26991	94.7916	41.79949	74.14538	27.75926	36.08581
pos_2229	5'-Methylt	27.13634	40.32261	112.1923	59.17682	17.80928	30.91688	56.06671
pos_2233	5-(3E-Pen	65.08971	182.4956	68.94561	234.9472	107.6052	71.23599	91.09953
pos_2234	5,8,12-Trik	101.2612	72.4593	3.139677	52.48832	73.44193	58.58151	81.14281
pos_2235	loganate	21.04311	38.91145	14.16227	55.02339	35.89413	25.8513	22.99939
pos_2237	L-4-Hydrc	201.9455	75.53378	66.66396	70.67318	130.3785	130.292	55.0506
pos_2238	Mopidrala	460.9686	155.9009	104.4454	138.2011	260.2128	304.1554	122.8397
pos_2239	3-Pyridyla	126.0815	163.1035	180.3295	174.2546	167.8016	172.2785	163.1823
pos_2240	Isosalsolid	29.84519	51.23892	54.24475	47.5528	50.45702	92.89525	69.45545
pos_2241	Harmine	61.55886	115.7046	108.7275	96.94497	99.95206	158.508	132.6151
pos_2242	Oxdralazir	318.5961	759.5378	501.9834	607.9075	607.5932	1334.48	968.653
pos_2243	5-ethyl-2-	13.67901	24.45679	21.76716	19.45298	17.71105	37.84182	27.52193
pos_2244	5-methoxy	81.32308	120.3324	50.56174	43.40009	44.5027	91.33778	82.81183
pos_2246	2-Hydroxy	94.28891	128.6346	26.50441	125.0127	139.7108	87.21785	50.74205
pos_2247	Ethylene b	43.08914	79.82701	112.4933	64.24678	12.73319	26.02867	72.06712
pos_2248	Tryptophy	64.15704	57.93428	45.48881	77.38931	70.21154	91.5637	138.0373
pos_2249	2-Phenyle	63.25464	76.49479	84.89254	64.22615	73.98337	65.20064	65.91948
pos_2253	Agar	70.48375	76.45389	105.3554	77.13881	80.62726	82.22299	79.42945
pos_2254	Kelampayo	9.872235	15.92075	132.4871	29.99467	1.72E-06	5.135148	47.74668
pos_2255	7-Amino-4	38.56684	21.97162	18.61851	31.96122	106.1972	16.1002	36.47532
pos_2256	Threonylhy	20.92969	37.56833	11.69032	34.04792	29.33783	20.87776	9.078426
pos_2257	4-Bis(2-hy	91.85602	86.68836	115.1425	103.0937	95.67184	95.5044	110.0355
pos_2258	N-(Dimeth	176.4156	399.6433	165.5176	326.7535	217.5783	186.4733	130.2148
pos_2259	1-Methylh	17.67999	32.04623	18.70954	8.053946	14.7715	13.38408	6.844879
pos_2263	Cinnamyl a	82.94524	74.58524	77.06341	73.99611	84.64608	100.6602	83.97936
pos_2264	Cyclocreat	29.46447	26.10568	26.65957	6.857169	29.00123	38.03708	27.42715
pos_2265	3-Indolepı	1326.783	558.4521	426.1612	302.4123	565.6752	2011.294	810.5469
pos_2266	2-Fluoroac	257.7647	203.6854	260.4392	209.0683	279.7712	325.5859	315.0527
pos_2267	Dihydrocu	23.93197	32.16781	16.781	11.75651	22.86776	18.52075	24.74401
pos_2269	Lovastatin	158.4164	203.954	23.40088	29.34434	22.9004	114.4485	210.0011
pos_2273	7-(Trifluor	37.41095	48.87098	68.14477	50.33549	147.22	31.34525	45.39241
pos_2274	2,3-Epoxyı	10.64321	5.551451	263.7586	32.29802	22.42159	1.72E-06	23.27288
pos_2275	L-alpha-A	17.21217	20.21243	1.72E-06	71.57579	1.693414	1.72E-06	1.983343
pos_2276	Val Cys Try	4.695175	50.52583	6.991941	53.53352	45.57125	72.43985	46.94057

pos_2277	O2'-4a-cy	81.21945	93.05095	87.5165	69.40302	92.42276	76.60387	102.3651
pos_2278	L-Fucose 1	47.609	42.43928	19.99097	31.66343	23.52939	43.48816	25.40731
pos_2279	Thr Arg Th	430.697	1098.131	129.6525	57.15569	1.72E-06	91.38846	178.5651
pos_2280	Cinnamalc	66.09148	61.74987	33.23299	54.49511	39.04595	71.1437	42.78559
pos_2284	2-[(L-Alan	113.0521	92.93053	128.8142	91.31488	128.5714	144.8161	144.4978
pos_2286	Pluviatolid	59.27774	13.11437	2.657775	5.557608	1.72E-06	26.02313	1.128672
pos_2289	Phenylalar	73.7027	196.8738	49.44935	98.38387	127.3802	395.3931	225.4573
pos_2290	Cyclohexyl	36.51527	21.27774	25.13878	20.0388	23.26424	44.88857	29.38557
pos_2291	Indoleacet	35.09951	19.59804	16.57454	9.611728	9.204112	1.72E-06	49.60917
pos_2292	Humulinic	28.84655	45.81968	61.87773	110.589	11.29628	17.94355	56.39427
pos_2294	tetranor-P	227.6128	514.2864	177.5792	316.9386	235.5175	193.1164	149.6869
pos_2295	Polysorbat	115.2212	113.9286	4.692102	70.63882	47.84203	31.4417	23.60616
pos_2296	sphingosir	209.4897	156.554	191.0795	111.5692	65.24099	174.0348	148.9464
pos_2297	Dodecatrie	19.37159	31.40872	27.10733	42.30142	29.66645	25.13735	15.68741
pos_2299	3alpha-Hy	69.81028	37.38779	1.72E-06	83.47551	241.3879	196.396	1.72E-06
pos_2300	Alfaprosto	41.34999	22.64527	22.76998	11.03848	2.545641	5.665345	21.3812
pos_2301	Thesinine	94.93637	67.46014	52.31966	87.07432	84.24268	62.56104	55.33192
pos_2302	2-Amino-4	64.36529	63.45657	54.00187	48.94372	58.29357	55.26042	48.27565
pos_2304	DROPROP	44.87083	40.76052	40.06644	47.31808	33.95123	40.61041	41.31368
pos_2306	(E)-2-(2,4-	712.7902	988.7557	480.6706	537.7827	631.9776	829.8063	871.0935
pos_2308	3-(2-Hydr	61.77771	51.6061	69.25597	39.20097	84.99141	73.0535	68.13785
pos_2309	Pantothen	3.686571	6.922566	25.52025	70.20289	1.72E-06	4.624223	41.1358
pos_2310	5alpha-Ar	465.4011	156.0063	101.1092	114.0694	167.5634	327.9603	238.5809
pos_2311	Indicine	65.29495	97.20722	230.0638	432.6869	30.68928	78.69058	317.4405
pos_2312	ISOSTEVIC	562.9357	227.1033	61.22562	125.3934	237.1277	412.0501	243.5248
pos_2313	5'-N-Ethyl	27.64035	32.15172	42.4134	27.51734	32.48113	32.40221	35.12753
pos_2314	17-phenyl	151.2664	84.41463	1.72E-06	1.72E-06	1.72E-06	43.94868	54.91043
pos_2315	2',5'-Dide	48.69004	41.95954	27.93313	49.82258	23.36973	54.28122	50.78905
pos_2316	Carbopros	13.09711	1.72E-06	1.72E-06	13.2942	1.72E-06	10.2346	3.46303
pos_2317	Cucurbic a	79.04421	73.42287	43.66566	56.2971	60.52165	82.38909	53.59972
pos_2319	cis-3-Hex	31.86663	37.4817	32.04364	18.47007	26.67879	23.76429	36.28268
pos_2320	all-trans-3	123.4464	26.48995	1.72E-06	1.72E-06	99.6229	3.372504	113.8544
pos_2321	Lysergic ac	473.3854	350.6232	166.2138	351.4126	349.045	698.5561	385.6618
pos_2322	Penicillani	18.71994	13.07207	61.74703	23.96954	87.83618	31.45523	28.50225
pos_2323	Sucrose 6'	12.55614	3.083332	40.65468	7.302728	42.00148	18.81236	15.60398
pos_2324	(-)-Epigall	30.33195	28.84063	63.18499	71.17941	141.9622	26.1608	32.90542
pos_2325	Cytidine 5'	12.70044	13.91269	26.69169	46.99734	85.96544	16.40304	16.851
pos_2326	2'-N-Acet	33.63815	24.62838	1.72E-06	4.792082	22.10123	50.49903	1.72E-06
pos_2329	2-O-(4-Hy	39.23844	50.14568	32.75891	84.90154	119.4664	43.66465	33.97188
pos_2330	Valtrate	21.26272	28.8844	12.73539	56.21362	102.4183	28.27027	22.19406
pos_2331	Glutaminy	124.0196	138.1449	178.3074	129.0744	144.6385	130.3106	135.5002
pos_2333	TETRAHYD	27.70195	24.11104	13.90962	5.716367	7.926328	14.85466	5.446719
pos_2334	1-Tert-bu	93.55759	402.4543	388.1923	314.6475	375.9038	942.5403	293.3886
pos_2335	Dynorphin	452.1148	865.6721	40.54196	70.21415	51.58375	396.6854	571.0378
pos_2336	Estradiol-1	1607.132	4040.085	1990.595	2424.499	547.0506	1747.238	2901.772
pos_2337	3-Oxo-4,6	32.6704	102.2436	1.844844	2.971293	1.72E-06	34.57371	46.68475
pos_2339	beta-Thuja	12.31375	34.64421	18.80355	15.83862	17.70524	12.0897	19.15522
pos_2342	Amprotrop	1302.01	2654.259	1496.437	1346.983	25.01214	1761.278	2578.675
pos_2343	Dihydromo	36309.48	72125.77	40249.5	39264.38	3134.099	45895.35	66950.01
pos_2346	Thiomorph	47.42591	119.7684	47.13293	75.79613	61.10081	74.67063	44.83256
pos_2347	3-hydroxy	693.8487	772.7477	983.0901	743.7165	863.0125	729.3391	781.8757

pos_2348	Prolylproli	140.1301	85.28756	58.4999	82.48594	77.40279	78.67546	103.5338
pos_2349	2-(1-Napł	17.57776	56.65038	1.383589	31.65638	13.32912	28.88799	4.217747
pos_2350	Etamicasta	279.6754	306.6618	459.8516	286.0081	543.5345	317.5658	300.0837
pos_2351	Pyridinolin	98.16971	338.7043	14.01702	179.3038	103.8058	204.337	56.84419
pos_2352	Asp Tyr As	39.71624	162.9806	1.72E-06	76.72004	42.61966	90.23511	13.3747
pos_2353	Helinorbisa	23.12008	18.43752	25.0066	11.06057	18.71985	18.17001	15.5357
pos_2354	3-Hydroxy	65.75581	72.32041	126.8495	70.70084	133.2908	83.73034	74.67666
pos_2357	α-Hydroxy	39.11621	249.4783	254.9477	545.1073	180.817	137.7601	747.9459
pos_2360	Momilacto	55.20447	49.12926	56.79203	52.67452	49.28944	62.62273	48.72925
pos_2361	Hercynine	68.44035	88.00075	56.91365	74.85026	47.42058	64.84956	70.46418
pos_2366	(S)-alpha-	157.5994	77.97614	38.28467	27.74981	52.95691	96.79725	74.02723
pos_2367	ent-8-iso	65.1299	50.48748	5.36584	44.3187	85.80394	126.7041	46.10769
pos_2368	LysoPA(0:0	84.51294	124.5379	1.72E-06	5.95614	1.72E-06	39.96506	62.28299
pos_2369	Leu Ser Ar	65.80395	33.98917	16.75405	103.3224	49.03833	30.41881	42.20632
pos_2370	6-octenoy	36.02574	15.98435	1.362365	70.29117	25.28106	29.48305	20.61229
pos_2376	(±)-Anisox	72.30708	70.80546	74.12989	73.57978	84.52821	81.42077	67.71304
pos_2377	Zerumbon	1900.983	1873.493	2063.38	1864.982	1946.142	1977.384	1843.453
pos_2379	alpha-hyd	49.40387	90.62546	55.0271	61.12559	45.81961	58.41677	131.2868
pos_2380	Apo-12'-z	233.8975	209.5357	301.9153	311.6115	711.1037	392.1714	250.07
pos_2381	N,N-Dihyc	10.79401	53.88959	19.9745	24.05978	5.897323	63.871	15.76128
pos_2382	Imidazolin	193.2071	276.4295	216.9285	181.5334	184.3115	186.4995	194.2868
pos_2383	17beta-Es	127.4321	73.13999	1.72E-06	97.57887	53.93554	108.4715	60.54553
pos_2386	2,6-Dihydı	74.56356	93.22873	63.23114	70.34588	32.64096	75.42674	83.70295
pos_2387	20-hydrox	335.0505	432.1786	320.0829	203.3414	67.51922	205.7776	392.766
pos_2388	N-Palmito	535.335	594.5775	421.8107	214.688	30.94878	222.7222	492.4021
pos_2392	8-iso Misc	251.2074	174.4367	156.5636	239.0175	327.0175	190.9616	72.4804
pos_2393	Mandelic a	72.05898	74.80018	64.03189	87.08032	84.51915	72.41285	70.5023
pos_2394	Santene hy	98.90651	102.5288	50.5359	87.27188	108.1378	128.5714	89.62903
pos_2399	Indicine-N	2758.522	2584.772	1009.935	2080.826	2514.242	3167.393	2296.849
pos_2400	(9Z)-Hexa	74.74892	49.87542	59.8889	72.28335	55.8208	68.32234	86.12427
pos_2401	MG(15:0/0	1162.382	1185.665	1129.217	1214.883	1335.538	1448.686	1693.062
pos_2402	1,4,8,11-Te	84.72852	156.6686	20.0306	84.87916	54.77034	58.14032	59.05466
pos_2408	2(3H)-Ben	126.8084	392.9392	57.50066	356.7241	276.7543	112.1187	333.4613
pos_2410	Pentobarb	95.98044	284.9631	37.75414	37.7918	23.67805	51.28103	85.26608
pos_2411	15H-11,12	472.9618	2088.596	48.82767	99.51964	72.10076	212.1203	406.6486
pos_2412	5,15-diHP	45.90686	42.12814	1.72E-06	5.032043	13.61019	81.60638	32.39438
pos_2413	Cytochalas	7736.271	32250.1	983.6148	798.8292	820.5232	3433.15	6624.378
pos_2414	Taurochola	14483.28	71273.79	1400.499	1313.816	1463.249	5848.73	12141.41
pos_2415	Hydroxyde	102.0538	19.03276	6.10886	46.28361	20.6018	36.21317	13.0774
pos_2418	MG(a-13:0	38.28098	45.77285	4.280903	12.15575	19.09392	31.40586	31.26594
pos_2422	Valyl-Hydı	18.65758	23.55724	5.489914	26.22161	6.954624	21.53441	10.50943
pos_2426	2,4-Dimet	72.38423	62.06902	43.27145	71.15855	73.73878	98.93041	68.95069
pos_2427	Rizatriptar	47.86156	26.62078	7.570161	42.27748	39.64232	53.06522	20.841
pos_2429	Alizapride	1628.081	1536.91	747.6182	1675.97	1466.605	2283.52	1427.243
pos_2430	L-Thyroxir	431.1433	409.276	293.3816	462.6787	306.3015	605.8336	397.7223
pos_2431	2-Pyrazinc	76.26881	77.56572	28.67412	97.70178	65.59309	81.92678	73.48877
pos_2437	Butyl (S)-3	104.6314	74.95712	6.686001	134.4693	49.40763	129.3779	69.88292
pos_2439	Sapidolide	103.7401	98.00093	28.1913	88.90086	43.45081	84.81889	48.49732
pos_2440	3-Piperidir	104.7925	93.25398	32.0076	83.40429	31.34861	78.52675	48.70427
pos_2442	1H-Purine	516.096	87.68712	1.72E-06	1.72E-06	16.58322	67.37138	1.72E-06
pos_2444	Dactimicin	116.5644	103.2962	18.59437	244.4361	148.3733	64.89667	30.79772

pos_2445	L-trans-5-	29.49873	28.18145	28.69913	19.61624	25.80702	19.64901	21.34374
pos_2446	Thr Ser His	1.72E-06	1.72E-06	1.72E-06	9.961611	1.72E-06	28.39559	4.58069
pos_2448	Arginylhyc	65.12423	67.30621	70.00474	56.5195	59.553	53.32672	64.33756
pos_2449	2-Aminoh	52.705	43.61089	31.81706	38.64418	53.79515	50.70159	44.88428
pos_2450	6-hydroxy	1133.065	1185.61	1795.513	1132.712	1337.416	1068.279	1192.516
pos_2451	Pterosin O	546.2268	374.5121	270.9388	325.2258	471.9945	480.1406	365.828
pos_2453	Illudin C2	261.9437	173.1812	90.56433	332.7252	221.2201	163.2926	147.0959
pos_2454	Cys Trp	62.03802	106.1537	85.17699	116.4632	130.4662	89.68942	90.66828
pos_2455	Valyl-Argi	128.1309	154.876	2.0617	77.97298	86.94691	118.9009	99.1787
pos_2458	Suberenor	1.72E-06	1.973411	1.72E-06	197.0449	0.377698	0.180182	0.031381
pos_2460	Deoxychol	21.97437	19.45474	21.32539	49.80039	60.89006	12.61373	63.40173
pos_2461	Asn Ala Va	295.8112	227.1408	5.24336	44.97772	86.94282	343.5045	227.8288
pos_2465	Etimicin	168.5583	67.43334	1.72E-06	2.89504	5.028291	24.60971	166.938
pos_2466	Achillin	96.82016	38.34875	37.80112	57.97999	31.92248	52.14581	31.75311
pos_2467	homosalat	131.9188	120.9564	27.12121	132.8732	117.6289	160.4049	78.0772
pos_2469	20-COOH	226.5103	186.1719	7.176525	119.8601	132.3295	259.9974	90.73725
pos_2470	2-Methyl-	39.20103	23.24272	44.03387	46.35188	21.36728	31.30121	25.75274
pos_2471	Alongside	60.91629	69.94331	53.35807	102.4169	70.05699	67.45211	46.81932
pos_2472	1-Cyclohe	202.298	342.1717	130.5661	769.3117	351.9665	352.6095	248.617
pos_2473	Kamahine	835.321	1279.771	494.9158	2697.373	1177.901	1324.833	917.605
pos_2474	Valproic a	21.34901	47.38246	4.074465	73.28934	34.21616	45.12366	23.60873
pos_2475	Cerulenin	1028.983	1679.358	101.8006	212.4429	90.06809	471.9462	491.4268
pos_2477	Guanidino	76.05378	35.61462	12.27232	44.91707	41.05651	66.65972	46.87726
pos_2478	Calligonine	676.9317	718.0022	993.6616	747.1302	878.868	638.4161	763.678
pos_2480	5-Formimi	187.2567	182.3155	318.853	188.6034	246.3535	160.5646	219.137
pos_2482	Nicotinate	35.59226	31.13216	50.23556	34.89314	41.37593	31.84255	36.43289
pos_2483	2-Methyl-	27.06852	26.20177	75.06625	84.48966	12.75907	33.09502	52.72522
pos_2484	2E,6E,8E,1(	74.45941	71.10316	163.9692	193.3323	54.97238	98.75013	137.149
pos_2485	Crispolide	135.4056	65.92704	75.63006	74.60696	134.3077	75.39034	68.41469
pos_2487	Asparagine	73.58395	88.17683	108.4893	79.7198	98.03966	73.15322	75.45904
pos_2488	6,7-dihydr	12.46265	6.233393	41.68921	66.26245	1.72E-06	16.07595	30.13956
pos_2490	12R-hydrc	71.59362	121.7618	3.319588	309.8623	67.55038	47.87249	63.83223
pos_2491	Thrombox	1037.608	572.2216	452.1649	247.1681	247.5946	510.6943	968.4652
pos_2493	1α-hydrox	195.3862	104.7091	98.25409	46.48507	24.55819	83.67412	197.6931
pos_2495	geneticin	58.40825	43.8213	285.5505	263.2569	15.17181	92.1851	199.4208
pos_2496	Prodigiosi	49.48142	50.09798	8.142325	106.12	27.4755	20.86013	31.76503
pos_2497	2-(1-Adar	114.3866	151.3614	135.7016	250.6812	141.7101	87.97635	121.0838
pos_2499	N1-(alpha	73.68088	40.46903	103.6337	48.31759	56.72637	42.13971	58.06355
pos_2500	PC(6:0/6:0	14.64782	50.00173	1.72E-06	1.72E-06	54.33607	16.52681	35.29473
pos_2503	Cannabidi	14.33975	7.467271	24.21438	11.94386	22.29796	7.022299	10.4358
pos_2505	Perindopri	117.8995	89.92787	1.72E-06	9.536317	19.31229	65.78626	98.34607
pos_2506	Dodecyl-b	38.46385	16.94295	2.642609	30.58336	42.60421	55.53699	14.86517
pos_2507	2-Methyl-	6.465497	1.606907	3.505234	18.41348	2.979472	8.058081	7.108246
pos_2508	N-Palmito	201.236	151.4228	104.4975	222.7799	132.4558	143.8798	188.1419
pos_2509	(2s)-7-Am	32.46054	38.28259	1.72E-06	29.28753	28.30887	50.04492	34.43532
pos_2511	Rigin	277.8359	349.2164	26.26122	11.64874	19.17927	78.29177	95.24991
pos_2515	N(6)-(Octa	51.26518	22.15349	22.92202	16.9684	6.165701	16.37742	52.67257
pos_2518	3-Hydroxy	214.8031	199.1553	28.31938	132.5161	159.9915	159.6304	147.6084
pos_2521	12-Oxo-9(	861.2998	1008.537	522.0197	506.8435	436.3732	580.4879	804.0272
pos_2522	Terbutryn	2.946342	63.89748	107.0969	137.626	0.224527	2.661916	81.0588
pos_2523	1-[(2R,4S,5	53.25774	53.70502	99.73582	54.24025	117.086	49.70724	53.49559

pos_2524	gamma-G	17.04179	24.2778	32.51578	22.24942	50.73982	15.83796	22.85173
pos_2525	o6-Carbo)	88.82005	86.06912	52.34683	38.36375	43.43887	56.55883	54.66682
pos_2533	Citrusin I	658.6323	151.5611	1.72E-06	4.583147	49.35032	105.1521	2.838948
pos_2536	2-Oxo-10	81.58267	121.0193	54.75563	153.576	64.33886	65.99296	95.16602
pos_2538	(S)-Coclau	49.19316	37.72991	14.30146	42.60204	29.64926	67.74352	51.29051
pos_2540	N-[[3-Hyc	52.67496	471.4471	207.1783	239.9798	1.72E-06	176.1419	94.83644
pos_2541	Antibiotic .	444.6692	204.1651	64.76616	70.77459	154.0565	241.2887	132.6663
pos_2542	(-)-Huperz	107.1451	155.0392	139.73	328.0861	58.63054	62.72685	114.2721
pos_2544	2-Hydroxy	59.46585	66.90792	36.77777	21.99654	16.55911	19.14268	78.06362
pos_2550	5-Hydroxy	94.51968	108.3345	81.29154	62.88572	61.93738	59.78139	101.79
pos_2557	3h-Phenyl	192.675	277.5464	91.15207	73.09767	46.95076	106.7367	219.896
pos_2558	o-Methylb	631.5005	863.9592	333.6732	266.2261	193.0782	383.8915	739.6572
pos_2560	Amobarbit	1757.485	2378.047	870.4587	602.9866	498.2718	1062.896	2017.071
pos_2562	Nitropolyz	355.3639	499.2905	136.0028	110.8664	54.05432	171.4937	424.2654
pos_2565	Polyethyle	45.05301	48.16038	21.22315	16.04826	8.963998	21.11722	55.78324
pos_2566	alpha-Am	570.8513	744.1078	310.7037	237.8767	110.8736	300.825	686.5313
pos_2567	Tryptopha	652.663	835.7007	508.2866	513.8539	257.6877	344.382	724.6627
pos_2569	8,11,14-O	905.2439	1183.281	402.8581	295.2515	224.0325	516.7068	1022.043
pos_2570	Colnelenic	398.3097	576.8907	105.6253	66.90604	53.56687	230.4457	456.6418
pos_2571	Carazolol	137.0133	158.9818	59.17614	23.92787	18.3093	40.00732	132.7943
pos_2572	16-Ketoes	126.6149	178.1891	30.76458	28.4607	22.2118	75.29242	141.4469
pos_2573	Exaprolol	238.5908	322.1974	63.82095	60.636	38.86445	122.8631	253.5307
pos_2574	Tetradecyl	12971.14	18927.19	3776.67	2577.84	2309.289	8076.89	14907.98
pos_2576	Megestrol	246.9247	338.9892	54.34522	33.76231	23.32176	123.5093	240.3453
pos_2577	Stearoylca	6.942731	14.88763	19.01208	22.48484	32.1516	14.61829	28.90694
pos_2578	Docosahe)	196.7839	323.368	60.69778	50.19602	41.39459	123.529	241.2172
pos_2579	Antibiotic	76549.31	112736.3	24468.49	16655.16	15429.69	50368.83	87666.45
pos_2583	3-Dehydro	543.4955	665.5668	250.4232	143.7779	111.2873	282.4294	736.6853
pos_2584	Glycocholi	255390.3	358160.2	79516.47	51072.56	49694.31	152309.6	299013.3
pos_2590	DG(10:0/2	274.161	339.0477	197.8268	73.47212	53.94998	81.19262	419.1819
pos_2591	Thr Cys Le	303.4333	302.3186	224.658	105.0219	101.8427	147.8262	357.6316
pos_2593	Prostaglan	166.1789	224.3946	29.40166	3.200293	1.72E-06	43.54785	220.4183
pos_2595	Torvoside	92.82644	142.669	1.72E-06	1.72E-06	1.72E-06	7.847973	130.8544
pos_2599	PG(a-21:0/	288.6572	335.2203	123.619	26.6827	59.9292	33.0069	390.7953
pos_2603	Guanidoac	24.41521	26.32978	56.31388	38.50807	27.51602	15.76395	25.74488
pos_2604	Policapran	74.80159	90.39377	99.99958	80.58529	5.519827	27.57079	89.29258
pos_2605	11-Dehyd	141.9318	137.7727	52.34284	105.5232	76.22274	90.90284	82.04165
pos_2606	Persicaxan	271.1796	68.25838	15.61448	24.82094	32.33716	44.56904	125.9595
pos_2608	4-Oxo-9-	164.6315	225.537	332.8649	239.9944	7.451867	39.03927	175.0233
pos_2613	alpha-Terp	129.8881	139.9168	207.5655	172.9025	97.92846	57.88607	120.3448
pos_2615	4-Dihydro	574.9278	913.356	2563.075	2206.261	222.4382	378.2101	623.0696
pos_2617	2'-Fluoro-	6.999333	1.484857	55.70833	41.06959	1.72E-06	1.72E-06	5.716573
pos_2619	20-carbox	268.1279	337.6347	942.6049	852.2274	76.64159	133.7373	276.9276
pos_2621	17-beta-E	12.58341	16.60942	115.5156	73.32122	1.72E-06	6.006579	10.27398
pos_2623	Nebramyc	699.2501	910.3171	6565.969	5204.605	320.6093	444.2843	800.6004
pos_2629	Trioxilin A	62.72656	78.66278	26.28108	4.971957	9.427066	23.42709	55.95781
pos_2632	Leukotrien	51.44369	7.767495	10.09345	23.36623	0.20203	35.91271	32.9821
pos_2634	Yucalexin I	142.783	685.1612	144.2295	186.6782	78.2894	123.3577	258.1393
pos_2635	Buspirone	84.01902	168.3847	44.08803	59.61853	131.701	60.62249	126.5748
pos_2637	9,10-Dihy	184.8188	111.3457	34.63727	47.92392	62.30985	94.02058	72.30098
pos_2638	N-(8-Amir	62.7137	302.6111	89.55053	149.5068	57.14665	86.83473	222.6762

pos_2639	Ala Asn Ile	905.5429	559.1024	99.00771	227.5894	322.0662	462.6603	490.881
pos_2644	Acutifolan	51.91731	67.90447	16.46775	94.96578	30.902	75.40282	37.78258
pos_2645	Sequiterpe	8.150905	17.0711	4.581922	119.1662	19.79698	12.876	5.111249
pos_2646	alatolide	30.77713	28.72456	20.76692	53.85601	13.48668	23.97573	25.48158
pos_2650	N-Eicosap	195.0635	159.6742	110.573	322.4668	280.3061	47.44661	122.2308
pos_2651	Glycolipids	408.3019	151.1493	3.04073	15.38585	1.72E-06	73.65378	451.7478
pos_2654	L-Glutama	42.39281	30.29205	17.39098	1.502167	39.63181	30.33749	34.08921
pos_2655	TEGASERC	160.6048	86.99909	24.32332	13.50206	127.2878	97.8894	108.6262
pos_2656	(9R,10S,12	29.97687	10.87886	0.588091	1.72E-06	1.72E-06	1.72E-06	1.72E-06
pos_2658	Alfentanil	154.8882	126.933	25.16887	31.08327	53.07152	69.30672	110.9591
pos_2659	Demethylc	45.89539	45.90718	77.94282	59.19227	18.19014	48.53124	91.82651
pos_2664	1-[(5-Ami	28.9804	32.72952	56.0344	22.58651	28.61393	32.95154	25.35085
pos_2665	Ascorbyl p	31.31339	11.3489	1.72E-06	25.68308	11.74185	3.634426	1.72E-06
pos_2670	Peramine	51.91625	41.83661	68.77603	78.44603	72.873	84.63243	68.60275
pos_2671	2-(3-(Diisc	43.53092	18.94251	12.04546	184.3499	73.74711	28.35817	61.50605
pos_2672	3-(3-Meth	86.38089	103.3255	208.0472	225.7213	62.90138	86.37404	67.15684
pos_2675	4-O-Meth	32.11526	67.33958	30.15016	69.21628	33.03661	97.73434	62.34731
pos_2676	Chamissor	336.404	377.1038	268.2881	582.8995	240.6588	314.3877	239.1034
pos_2681	Apronal	145.4124	77.48181	540.1461	244.1451	67.61611	38.59751	89.85535
pos_2683	Methionyl-	34.99709	26.42769	110.3382	53.9112	29.84113	23.00988	36.07967
pos_2687	[3-(2-Ami	47.70858	32.45111	105.6942	69.29775	32.48629	30.94805	38.96311
pos_2688	Argyrolobi	81.61147	0.976004	381.7276	142.3545	10.27308	2.400996	36.88574
pos_2689	3',4',5'-Tri	409.8884	452.9915	645.3052	785.0183	263.7876	376.8518	274.9572
pos_2690	3β-hydrox	765.9478	212.0225	3274.032	1268.385	306.6852	183.1081	411.4084
pos_2691	Cetamolol	13.72829	1.72E-06	81.91089	14.21029	4.569456	1.954786	7.347935
pos_2692	Eicosatetra	95.8085	11.58049	492.7605	189.6559	31.67082	15.15612	39.63484
pos_2694	(2E,4E)-5-	9352.938	1832.54	43515.17	16223.58	3355.175	1687.622	4865.132
pos_2695	8-Methyltl	64.71717	97.22419	28.63073	198.5965	82.23121	30.36692	40.18319
pos_2696	3,7-Dihydı	10156.13	1702.352	52772.51	17980.37	3192.305	1555.032	4941.497
pos_2697	(22E)-3α,7	232.3448	1.642118	553.593	205.7031	85.01234	15.44513	57.88393
pos_2699	PGD2 etha	18.71755	1.72E-06	326.1359	64.84989	1.72E-06	1.72E-06	1.72E-06
pos_2701	(5alpha,6b	11.03396	1.72E-06	101.0972	24.36846	3.051601	0.907037	1.431878
pos_2702	SM(d19:1/	3031.07	165.4384	13443.38	5417.147	501.4841	154.2657	1088.725
pos_2706	3-{[(2E)-4-	8.903099	1.72E-06	71.21253	22.58019	1.72E-06	1.72E-06	1.72E-06
pos_2708	Leucylhydı	364.7198	85.73367	1679.221	630.6728	131.4475	67.87221	176.9616
pos_2709	3'-Hydroxy	13.79861	1.72E-06	84.77846	36.32737	1.72E-06	1.72E-06	1.72E-06
pos_2712	3-Hydroxy	68.04195	8.338076	430.0368	111.2242	2.244852	1.72E-06	17.21318
pos_2713	Chitotriose	52.68046	1.72E-06	223.3773	78.86671	2.647172	2.207629	18.91824
pos_2714	Macrocin	32.77485	1.72E-06	522.0012	93.12993	1.72E-06	1.72E-06	1.72E-06
pos_2726	9-Octadec	10.36849	5.323055	104.4909	12.68216	32.62982	2.0964	23.4897
pos_2728	(1S)-3-[2-	5.962937	21.47618	51.59534	115.6097	13.75834	9.292399	16.02918
pos_2730	Homoarec	55.68771	47.97249	58.62823	44.70842	62.68726	60.53658	49.34569
pos_2732	alantolacto	222.3068	192.1402	226.968	150.09	199.4034	220.5656	170.8162
pos_2734	N-Linoleo	341.7068	86.92162	28.96121	4.361818	11.94876	15.70302	108.8556
pos_2735	Proliferin	472.2069	256.5415	28.41004	7.594523	2.177759	39.21486	160.6628
pos_2736	PC(O-16:1	390.3768	120.7386	37.62075	2.082807	23.05427	19.28809	135.9346
pos_2737	Taurodeox	3036.297	10923.72	393.0372	376.3609	578.3002	1015.179	1978.196
pos_2738	Ricinoleic a	75.93187	94.12917	79.51529	112.606	104.6237	133.0949	132.8711
pos_2751	3,4-Dimet	190.8226	190.4976	174.5333	198.7763	193.354	333.3088	337.6
pos_2752	Diisopropa	250.5953	294.4097	506.5958	264.7555	319.4443	254.0829	291.2538
pos_2757	Pregnanec	56.83779	34.26457	8.713465	16.54141	2.693567	24.66001	25.19166

pos_2762	3,8-Dihydı	2536.965	897.093	211.8412	1003.304	656.0277	413.2736	1851.834
pos_2764	Cetylmanı	188.125	170.8795	192.5299	174.1025	166.2052	331.5895	272.889
pos_2770	1-Isothioc	66.59601	209.1005	128.5787	183.3507	149.6323	182.3746	84.0463
pos_2771	12-amino-	2262.073	2119.428	3181.865	2527.227	3811.619	2781.514	3228.9
pos_2772	Aminopen	22317.9	20342.25	31849.45	23723.21	35871.61	27779.92	31602.87
pos_2775	Dicycloper	20.81803	25.77224	31.45025	24.25495	25.29095	22.68827	24.50506
pos_2776	Vulgarone	2967.235	2887.616	3986.402	3116.447	3158.502	2817.897	2862.682
pos_2778	C16 Sphin	1270.718	1292.935	2413.984	1522.774	2333.984	1624.475	1795.523
pos_2779	Ethyl penta	73.07941	86.85149	145.5922	84.66687	228.2101	65.78579	72.61828
pos_2780	N-Acetylsı	520.975	470.1685	717.6243	546.7065	778.1551	622.4881	699.7438
pos_2782	alpha-Toc	81.30453	66.64228	55.15119	62.76709	51.43159	132.666	100.7634
pos_2784	ent-16b,1!	125.0977	33.54569	99.01408	130.8026	16.13474	68.07051	65.70643
pos_2785	(3Z,6Z)-3,(	1210.68	1284.217	2129.116	1345.659	1587.515	1093.563	1363.942
pos_2786	4-Octylph	12.95209	68.37594	98.97887	132.8022	1.72E-06	7.196211	49.03813
pos_2789	Heliotron	136.0896	156.0394	31.73167	191.5863	85.04596	77.40493	171.0245
pos_2791	Antibiotic	65.6543	7.228878	11.60371	35.07556	3.680962	27.42966	11.995
pos_2792	Prolyl-Lysi	38.86401	215.5932	358.1344	457.1433	7.116237	20.24252	162.3518
pos_2794	(S)-4-(2-(4	171.9682	293.5442	795.9692	322.6367	39.62261	88.58342	364.8271
pos_2795	Globotriac	43.11754	6.494842	551.6076	239.3284	47.78183	544.5401	865.7325
pos_2796	Zingiberer	357.6454	345.9187	498.0844	359.2308	392.4203	320.7092	323.9233
pos_2798	3-O-Dem	17.1897	33.71217	1.72E-06	23.20092	21.99468	18.11021	13.49485
pos_2801	2,4,5,7alph	176.0623	130.0853	83.74838	165.4897	107.6856	165.3251	113.0887
pos_2803	7-oxo-11E	99.51796	91.20285	48.31502	117.3522	66.13993	80.2794	77.24301
pos_2804	Biotin sulfo	1.72E-06	13.13326	9.103469	2.87856	23.95974	27.11463	25.2403
pos_2805	Arg Pro Cy	81.27667	32.29621	14.71088	75.09695	51.93537	38.66227	31.35158
pos_2807	Pithedulos	1.72E-06	3.282071	1.72E-06	109.7225	1.72E-06	1.72E-06	3.624683
pos_2812	Androstan	194.2257	1.86091	122.6349	68.30083	1.72E-06	36.22915	93.88457
pos_2817	Flaviolin	97.22175	98.09389	133.6704	107.791	118.3598	120.0939	106.6359
pos_2818	(Z)-[3-(Me	99.56968	86.13707	113.9513	108.1336	104.974	101.2945	91.26449
pos_2819	Phantasmi	516.6884	501.266	661.3186	576.0376	582.2367	580.3537	518.3745
pos_2825	Phytosphir	18.25108	46.62286	1.72E-06	14.47003	29.68473	40.8817	35.01817
pos_2826	5β-CHOLA	8989.856	28572.87	2353.163	1222.202	2202.597	4681.84	9357.303
pos_2828	Gly Arg Ty	126.5089	297.1049	39.66529	22.42474	31.06847	64.11436	121.8595
pos_2829	L-Anticaps	17.43986	11.70899	17.0462	53.42446	17.41077	14.64176	10.04912
pos_2832	8-Acetylne	1.72E-06	1.72E-06	1.72E-06	273.116	9.730464	1.72E-06	0.399793
pos_2835	L-Histidin	53.14957	49.04128	108.9858	72.25527	86.14761	53.14424	83.87404
pos_2836	5-Megasti	43.7965	20.6153	13.54101	44.44636	20.24263	25.13982	16.91571
pos_2838	Homodihy	79.08183	48.64648	6.226434	47.68139	60.19294	29.7251	33.28892
pos_2841	(Z)-11-He	375.0229	315.0737	591.5081	412.7457	429.8505	344.8553	418.1851
pos_2843	Casbene	25.28455	23.62898	10.34222	22.40274	12.01366	5.331372	9.979462
pos_2844	14alpha-H	82.23515	63.45922	124.1013	83.34699	90.1666	73.42477	85.07478
pos_2846	Myricanol	661.6863	708.5543	5752.891	2248.765	310.6333	401.2661	1806.766
pos_2848	trans-trisn	43.57021	27.05142	16.6716	7.372089	16.29339	9.561383	6.578383
pos_2850	Phytantrio	124.1712	122.8983	103.2545	149.3202	135.5293	144.9204	149.4174
pos_2852	CerP(d18:1	266.7629	209.9984	345.6244	256.2213	302.3378	301.7704	340.8005
pos_2853	beta-L-Di	33.75474	23.84029	55.88675	36.84402	54.77486	35.7075	38.81073
pos_2855	3-Hydroxy	194.6435	228.0516	45.46079	664.0825	240.9346	150.7426	164.5312
pos_2859	3-Pentade	2.44988	45.47592	113.7793	137.8989	1.72E-06	1.72E-06	33.93749
pos_2861	C12:5n-1,3	57.11765	159.6996	176.8354	220.0062	110.3099	226.7861	494.8638
pos_2863	Strictosidir	289.0604	469.8025	217.0582	593.801	314.1376	762.1555	253.4049
pos_2865	3-Oxocho	197.0531	13.37719	19.16459	43.62856	30.90969	9.604179	30.61564

pos_2866	gamma-T	35.97713	33.88722	32.84995	46.26973	42.21666	51.90019	50.20913
pos_2871	Apigenin	73.12514	111.664	607.3618	198.1655	1.72E-06	39.51501	250.0402
pos_2873	12-trans-I	35.3895	19.63027	45.14856	42.71335	14.37166	31.69257	39.46628
pos_2874	His Leu Le	527.5851	323.9927	67.58292	591.1097	319.4968	669.3892	248.8153
pos_2878	6-Hydroxy	25.22138	7.448654	4.739798	186.6971	10.78029	18.99935	48.92654
pos_2879	7,8-Dihydı	21.87321	40.81957	44.26337	26.35617	15.37743	23.11521	37.16665
pos_2880	Ajulemic a	69.84711	42.22035	45.09222	174.7125	110.5615	30.83088	31.15378
pos_2881	Sorbitan p	190.3232	263.7752	210.976	1794.639	113.4528	99.85459	88.50457
pos_2884	CDP-DG(1	162.5734	248.0756	450.4627	89.32074	102.6972	159.2333	488.7418
pos_2887	7-Ketodec	224.0578	29.33217	129.4376	95.51319	96.18764	25.33281	49.96036
pos_2888	N-Linoleo	15.47338	19.05678	19.6004	73.09692	7.150496	3.306635	36.18311
pos_2890	6-Deoxyer	136.135	142.0455	110.5994	109.9218	394.0171	130.0794	106.9683
pos_2891	Tetracosal	79.05728	181.4141	69.6976	173.5394	72.72695	43.35103	70.68618
pos_2893	geldanamy	28.92858	8.740616	315.8743	17.072	451.0651	33.49883	118.071
pos_2902	6E,8E,12E,	3429.823	844.0625	1516.77	1284.433	1871.074	986.119	1064.41
pos_2903	Sophoranc	82.00653	21.11514	48.25948	34.55922	42.53611	20.23099	23.16563
pos_2904	Brefeldin A	248.592	142.885	194.8765	128.657	163.5363	136.4312	154.4716
pos_2905	DL-Estron	132.0333	22.01408	47.94587	45.25284	54.52937	31.92817	27.72964
pos_2906	9(S)-HpOT	316.2408	142.7783	129.2299	161.997	151.9818	153.5165	108.2326
pos_2916	L-Cysteine	255.3116	85.6389	151.7621	117.3866	154.3264	77.52823	94.80814
pos_2920	Carvyl ace	978.7812	287.1031	481.7891	424.0677	594.8364	323.9621	371.261
pos_2923	1,4,6-Trim	718.9382	415.0741	669.4015	474.3824	628.5522	396.6053	450.2733
pos_2924	(R)-2-Nitr	1191.747	369.6152	563.8383	437.3698	658.8197	359.7248	414.2131
pos_2925	Chamazul	647.7397	180.034	316.1762	242.1187	354.2184	200.9605	231.2162
pos_2926	(4E,7E,10E	1757.194	430.3626	777.7545	619.9966	950.4058	498.4307	550.3936
pos_2927	Isamoxole	1962.269	612.0037	1063.826	848.779	1166.34	706.19	746.613
pos_2931	(8R,9S,13S	1040.985	234.872	417.6081	365.4229	472.6478	284.1367	280.8745
pos_2932	Ginsenoyn	2357.859	619.5178	957.0415	811.7454	1190.45	664.8531	721.5001
pos_2933	Dehydroal	377.0384	105.2597	128.8254	156.597	184.886	113.8437	129.6572
pos_2934	16-Oxoan	676.3409	125.8119	285.4395	275.7455	359.1299	187.7256	231.8949
pos_2935	Methyl 4,7	263.6214	45.73982	93.73116	119.8738	122.1508	53.54867	52.06575
pos_2936	Norgestrel	1352.111	277.0664	525.96	492.7069	722.1188	355.887	391.4308
pos_2937	delta2-TH	230916.3	61701.17	110796.6	89232.31	134405.4	71845.58	78978.75
pos_2947	24,24-Dfh	448.0727	177.7213	279.8238	215.8033	315.5582	168.9983	197.5404
pos_2948	O-(17-Car	627.5168	745.3504	804.3222	651.8129	520.1287	439.1633	777.6672
pos_2949	Prostaglan	192.169	79.86414	180.9838	121.3064	183.5716	95.73629	113.5044
pos_2950	7-Sulfoch	1023.499	553.8178	1113.733	811.1446	1041.344	592.2601	765.8331
pos_2954	Lucyoside	631.8622	86.46708	282.5794	219.743	394.4501	146.258	162.6014
pos_2955	Avermecti	469.4585	59.18269	244.0939	186.0474	327.5025	110.0726	141.3817
pos_2956	SM(d19:1/	20764.66	1563.866	3861.138	3149.72	5943.135	2214.983	2557.988
pos_2958	PG(18:2(9Z	354457.1	36410.79	78975.67	66417.01	118973	51033.64	54344.72
pos_2966	PG(18:0/2(	1132.942	202.2984	530.2117	402.7726	685.5963	286.8797	314.8751
pos_2969	7-Ethyl-3,	391.9792	182.1121	226.0588	197.7491	246.8027	135.5653	175.6622
pos_2973	PA(19:2(1(	95.02707	246.2445	81.15747	108.2754	19.97765	121.0135	103.1515
pos_2976	2,6-Diamir	81.53557	110.8905	86.87663	150.2171	76.87148	43.29604	173.8248
pos_2977	(±)-(E)-13	68.78111	56.59414	46.19761	62.67933	64.3346	64.69364	69.62219
pos_2980	1,8-Octan	543.0588	261.1496	442.2764	332.5632	406.7872	245.7629	305.2994
pos_2981	C16 Sphin	143.0965	138.446	239.6424	162.3588	276.3506	176.2506	204.8581
pos_2982	Glucitol-ly	57.13893	79.93815	70.33806	69.71621	63.83895	84.63875	70.02868
pos_2984	PC(PGF1al	1703.67	405.6679	25.8562	53.06551	86.91759	169.1642	284.2848
pos_2986	Androsta-	139.1951	140.0523	116.0201	83.18517	67.45004	74.83531	109.4025

pos_2987	KIRENOL	1036.036	1414.696	287.4408	435.3041	370.837	450.5235	796.4441
pos_2988	24,24-Diflu	34.21305	54.4206	0.352002	9.822587	1.72E-06	19.45482	21.43497
pos_2989	Sodium gl	30921.37	47271.9	8151.992	6491.442	8392.513	14449.62	26440
pos_2991	Norlinolen	47.04942	32.18076	2.520253	21.68726	27.29558	23.36015	8.115197
pos_2992	Isopersin	945.2546	2172.663	1352.582	1439.773	736.0043	709.3588	2580.464
pos_2995	Pregnanet	142.2397	76.90817	22.20971	101.3972	135.2802	230.2523	111.487
pos_2996	PE(10:0/11	58.15087	58.22418	3.349138	26.58791	39.5519	74.29394	37.18907
pos_2999	sphinganir	213.4822	198.0006	336.5645	206.8907	554.051	165.2564	212.1265
pos_3	Triphenylp	1950.201	2101.334	2533.472	2086.76	2390.971	2096.508	2081.721
pos_3000	Pyranocya	234.0025	31.27424	241.1981	339.8818	360.0798	372.5847	867.3741
pos_3003	Gancaonir	962.7996	1051.567	1161.521	1033.252	792.5868	1068.778	1419.599
pos_3005	Eucaglobu	168.9554	200.6436	86.65208	104.0186	69.15188	162.9529	186.5173
pos_3007	Formonon	15.75139	16.93921	30.95515	20.53152	3.703444	14.95325	40.54819
pos_3008	7-Hydroxy	214.4037	224.0257	317.276	233.4439	213.5478	233.6823	323.4822
pos_3009	Carboxyde	23.72469	33.2449	46.3748	31.55298	20.02993	29.68064	51.18308
pos_3011	CDP-DG(i-	84.71578	48.46354	73.75658	35.89168	308.9149	283.7471	61.8627
pos_3012	Heptadeca	44.00669	6.326089	11.17439	9.226675	3.425707	16.27615	11.39261
pos_3019	N-Acetyl-l	685.6445	683.2047	1016.064	761.3788	464.2127	734.3773	695.3674
pos_3020	Hexazinon	234.1152	228.3698	266.765	184.2949	169.2053	242.7193	219.1067
pos_3021	3-(4-Meth	1781.124	1779.056	2514.799	1872.41	1311.899	1792.908	1788.179
pos_3022	(5alpha,17	251.5304	185.9569	236.7345	297.764	310.5077	336.8478	232.7929
pos_3023	Fesoterodi	165.3391	80.24728	49.35734	181.4321	69.75192	56.31839	84.24855
pos_3024	3a,6b,7b,1	70.49873	83.76549	15.23429	41.86427	104.8476	118.8693	66.92261
pos_3025	DG(2:0/18	43.10887	77.16224	1.881365	41.30858	22.68022	43.79312	41.8494
pos_3026	(1R,7S,13S	850.5162	860.793	590.3809	541.3844	716.1083	989.8645	884.5153
pos_3027	Asparenyc	32.47229	5.912542	22.36287	46.96599	44.50986	18.53321	21.8731
pos_3028	Sterebin A	91.4171	39.98904	9.686785	29.29093	40.09751	59.79709	49.12021
pos_3029	Octadecyl	82.82676	9.46924	39.72999	59.20409	12.16717	49.19743	60.30799
pos_3030	Pamatolol	117.9281	90.49328	35.45622	62.68165	65.09912	91.86317	48.55814
pos_3033	(±)16-HET	338.9976	93.56092	73.12446	70.59764	109.6209	202.6792	164.1153
pos_3034	Quinine	84.52647	60.69125	56.16738	144.3831	57.03776	81.18938	52.01625
pos_3035	(2R,5R)-2-	36.30455	23.09746	2.34671	49.99762	1.72E-06	36.504	17.12355
pos_3036	1,4-Ipome	19.12122	18.18278	27.50172	11.35602	28.5634	14.07234	21.57341
pos_3038	His Lys Me	1954.716	2093.688	3302.761	2216.477	3231.967	2476.762	2890.004
pos_3039	Sordarin	40.57458	30.67264	7.813928	44.1275	72.55677	75.11051	28.4885
pos_3040	2-Phenylp	158.8417	172.8364	250.9845	165.6457	180.48	143.811	169.6455
pos_3041	MG(0:0/18	134.3746	888.6257	2286.804	813.6322	201.009	160.7648	556.697
pos_3042	Lc3Cer	79.02053	111.3814	47.78962	10.85047	48.32934	81.02937	145.2398
pos_3043	10,11-Diflu	106.776	87.61619	92.11837	99.20589	100.3902	102.3298	105.3602
pos_3045	6-O-Acety	195.7067	110.9322	104.8812	128.7484	90.74834	126.4886	125.0654
pos_3047	13'-Carbo:	576.314	264.7388	99.45283	221.8437	228.7405	890.5574	329.4525
pos_3048	Thr Phe Ar	12.945	3.350896	1.72E-06	1.894815	1.72E-06	3.532119	4.210459
pos_3053	4-Hydroxy	101.8029	113.8699	64.3068	31.29804	46.26691	60.67746	106.5295
pos_3055	p-Mentha	24.52037	24.62253	14.69322	1.071667	2.785249	12.88715	20.98035
pos_3058	Estreptoqu	143.884	168.3507	78.05726	13.85941	49.71868	79.25922	161.2364
pos_3061	Methionyl-	60.85176	57.2087	46.2065	15.62697	34.64155	42.35184	69.31422
pos_3062	(2R,4S)-1-	72.83726	93.46416	48.27763	15.2411	26.89963	40.51227	89.81413
pos_3064	O-Formylc	236.7172	240.7316	182.8618	65.59988	110.4865	140.1301	253.5133
pos_3070	3b-Hydrox	35.11269	42.32294	31.14217	40.1591	16.05183	29.89429	33.2257
pos_3072	Deoxychol	100558.2	118239.9	53111.88	17374.17	37381.5	53891.66	115950
pos_3074	N-Eicosap	576.4005	707.5066	327.0456	120.7505	239.0436	352.6802	699.145

pos_3077	PC(22:4(7Z	297.829	410.7734	82.1672	1.72E-06	43.51102	90.14679	426.8741
pos_3078	PG(i-22:0/	132.873	198.5347	49.09606	4.624801	9.229888	64.54615	178.5346
pos_3082	Boviquinor	133.5539	73.11482	22.65919	92.45139	40.53589	61.10815	59.45452
pos_3083	Alpha-Tris	35.66903	31.95366	285.7535	77.51874	19.99544	7.85508	92.65018
pos_3084	Palmitalde	454.0028	600.3052	599.1015	533.3434	334.0059	483.4039	1256.654
pos_3086	Sphinganir	429.0143	465.6611	676.9907	502.0048	579.9151	499.1249	655.8728
pos_3092	Octadecar	191.417	239.8134	221.5904	213.6482	219.2459	194.8296	460.0663
pos_3094	12-oxo-5E	123.7135	122.2873	161.9248	133.6562	137.6023	116.5836	109.8349
pos_3096	Pantothen	33.00386	16.59671	10.21797	23.5659	6.823208	25.82807	17.10441
pos_3097	3-Mercapt	47.5822	42.0855	64.90963	61.61149	65.9125	54.75526	57.78989
pos_3098	Valdiate	1302.394	1255.187	1345.987	1219.484	1268.22	1207.458	1110.969
pos_3099	hydroxync	623.7074	308.7244	205.7059	358.4964	306.6995	563.2229	325.0565
pos_3100	QUININE E	48.83241	29.57135	46.0522	82.16309	54.59937	28.30926	38.7613
pos_3101	LysoPE(0:0	26.81634	17.14307	58.294	29.32438	40.02248	14.70364	30.5853
pos_3102	(4-Methylp	39.72017	30.17975	67.08256	45.90377	49.11072	35.3001	52.00569
pos_3103	8-Deoxy-1	34.03918	27.15499	56.42477	46.51181	52.1742	31.25796	43.88725
pos_3104	5'-N-Meth	22.76398	20.12085	39.29802	32.85379	28.67786	22.06162	35.93834
pos_3105	11Z-Eicos	76.1199	73.37623	100.6818	196.6393	92.37646	91.06926	124.6449
pos_3106	Docosahex	203.5372	86.48655	203.8515	69.73453	72.30058	68.11667	120.0052
pos_3109	Armillarin	3077.732	2927.34	4639.355	3735.604	4091.103	3439.186	4101.801
pos_3112	Austalide I	56.42044	38.14392	141.9017	77.56072	88.4048	32.78144	70.37206
pos_3113	[(2S,4R,5R,	291.1632	229.6852	668.9812	320.205	500.2074	195.0632	391.0947
pos_3115	O-Acetylca	38.61707	40.43777	54.76485	36.56264	48.13438	45.88586	44.03258
pos_3117	PA(10:0/LT	27.13324	15.99424	1.72E-06	20.79777	25.01959	50.56067	20.81374
pos_3119	Dihomo-g	105.5628	75.54343	10.89498	63.79951	66.77672	72.36144	45.16721
pos_3120	12-oxo-PI	1089.187	888.5571	681.4587	1197.815	936.2712	1253.631	846.5502
pos_3127	Verruculog	184.7224	197.468	176.844	174.0509	176.1512	186.6854	210.3607
pos_313	Butanoic a	144.9392	196.6767	217.6231	227.2211	309.1539	179.7329	143.8907
pos_3135	LysoPE(15:	52.55693	30.03498	98.20042	29.88189	47.0641	33.82336	48.43887
pos_3138	N-Nervon	40.71394	45.19005	44.49113	37.03359	35.54639	40.57501	60.99647
pos_3139	2-Phenyle	129.9848	154.9043	159.0442	154.2765	122.5472	152.4084	196.953
pos_3141	Gly His Lys	110.461	105.6453	89.40705	102.1565	105.0842	107.4025	131.6355
pos_3143	7alpha,12a	7.693906	8.073012	1.72E-06	1.72E-06	13.48221	2.152452	100.3899
pos_3144	DN-isobut	237.5933	273.9858	238.0488	274.5547	178.2392	295.4567	344.6012
pos_3145	17-Dimeth	9635.819	6655.616	7726.054	12910.59	14395.53	6055.173	7532.068
pos_3146	Nomilinic a	1058.189	685.6641	837.1181	1453.841	1582.965	640.1163	852.3719
pos_315	(2-Quinox	38.10635	39.02286	22.46562	44.89389	35.58818	37.37932	31.64602
pos_3151	6-Ketopro	39.79991	30.25889	40.94675	46.35859	32.50022	42.66461	25.90304
pos_3152	Cys Arg Ile	71.10151	40.14782	6.956974	49.3381	43.27603	57.40287	16.11294
pos_3153	LysoPC(16	39.48292	11.5997	1.72E-06	7.394491	9.245268	43.81846	4.16891
pos_3155	Ala Gly Me	6993.996	8110.038	6453.816	6985.015	4426.924	4563.382	6754.236
pos_3156	6-Keto-pr	22.24756	7.855669	22.95305	10.3482	10.52943	2.011586	26.31325
pos_3157	Neotame	246.0424	207.5596	130.0182	133.2205	176.636	174.5184	164.0471
pos_3158	Neamine	119.903	21.71419	4.686328	21.85197	19.67955	31.02215	17.65859
pos_3159	Neolinusta	474.2532	189.7443	178.2105	67.23916	296.666	287.9076	74.23098
pos_3162	Farnesoic a	124.9718	132.6007	116.8666	154.9368	166.3965	156.7669	143.4381
pos_3165	Tetracyclin	11.2259	1.812459	88.80351	59.63902	1.72E-06	1.72E-06	20.87438
pos_3166	3,4-Dihydr	275.6348	276.471	87.23025	169.7038	141.5614	458.5933	243.0475
pos_3170	Spergualin	91.57701	92.10307	155.9645	125.744	174.727	70.33637	84.79796
pos_3176	3-Oxodod	8.778533	67.07594	444.3289	189.0349	0.902323	12.32064	475.3459
pos_3177	Carthamos	108.9694	102.799	102.0878	93.25343	101.9794	160.3277	112.4393

pos_3179	1b,3a,7b-1	103.9009	86.78286	132.8423	133.6457	119.3318	101.6753	113.2363
pos_3180	Sterebin G	522.6325	386.5072	471.2012	507.3047	263.8786	481.937	431.3946
pos_3181	2-[2-[(1R,2	865.283	737.4386	1061.3	950.7528	1025.099	1033.883	991.3019
pos_3182	Antibiotic .	164.8986	133.8809	211.273	167.4991	201.0021	167.2126	190.4949
pos_3184	Phenylpro	22.95298	13.64392	26.84533	19.35395	26.81218	15.33362	20.02755
pos_3186	3-Phenylp	45.99894	28.12761	69.09767	44.2923	44.98302	40.31012	46.94097
pos_3188	2,3-Dihydı	86.52763	85.36783	148.4964	88.814	97.81342	75.12526	90.72072
pos_3189	Pinolenic a	593.5108	156.8976	1194.461	474.3471	376.0919	553.4167	755.4101
pos_3191	trans-Deh	960.671	279.5958	1619.786	755.9942	613.7825	856.4863	1015.418
pos_3192	Dehydropi	430.3828	206.6499	633.0516	399.6298	263.3378	369.5203	425.7537
pos_3193	N6-Acetyl	19.02687	2.943982	13.29777	22.52352	16.3786	18.02246	8.382395
pos_3194	Thr Cys Th	79.42294	18.64904	192.5598	84.45887	70.15778	74.40568	111.6986
pos_3195	1,2-Diocta	23.74508	3.229154	12.12261	16.5149	14.32564	52.13694	25.26291
pos_3197	2-Isopropy	28.4516	5.239774	38.73279	14.87464	12.47614	19.35689	19.34374
pos_3199	LysoPC(18	139.7327	28.16185	6.063824	52.66744	52.85377	118.8137	30.59637
pos_320	Ceftibuten	1540.774	1720.119	955.8234	1597.406	1619.056	1699.368	1349.5
pos_3200	2-Amino-3	18.37205	21.31036	15.29368	18.67532	9.273319	24.08168	25.35941
pos_3202	Neplanoci	30.85588	32.36672	47.27586	37.33373	47.60905	40.98437	47.16261
pos_3203	H-Tyr-gly-	79.45822	72.27327	117.2597	81.21305	96.62553	100.3011	112.4296
pos_3204	N-Palmito	1343.683	1546.598	2393.113	1338.779	1084.518	862.7275	1175.582
pos_3208	(S,E)-2-(5-	25.18573	16.40355	39.81827	32.573	39.72669	22.53501	26.59828
pos_3209	N2-(2-Car	383.7918	363.6361	392.5121	335.9613	368.4443	377.7328	419.7579
pos_3210	(+)-Eudesı	2738.978	2704.74	3281.987	2736.607	3160.214	3122.223	3461.861
pos_3212	PGP(20:5(7	186.7073	185.368	256.3642	196.9437	298.0646	290.352	332.4692
pos_3214	Citronellyl	79.21789	67.41349	110.4708	70.16972	106.7369	63.07755	86.90959
pos_3216	(S)-Batatic	166.9519	177.6134	240.4454	185.8833	203.2323	172.8657	182.2109
pos_3217	Dehydroor	659.032	685.9961	1083.477	703.2975	807.1086	600.3894	714.1787
pos_3218	Neryl gluc	68.81404	85.88014	7.048754	31.21043	72.13617	59.99993	125.9024
pos_3222	Ile His Leu	35.61411	34.79669	46.84539	37.9791	42.52649	37.56478	26.8017
pos_3223	Prostaglan	82.91896	49.69525	63.17972	23.48143	60.71846	13.78382	21.37991
pos_3224	Pro Arg Va	230.1	162.8524	33.47176	147.9948	167.6035	299.3435	141.0443
pos_3225	1-Heptade	87.24811	85.18079	11.60835	87.30258	85.77288	153.4153	71.99725
pos_3226	N-Myristo	483.8414	192.0177	451.312	246.2788	312.2883	191.2584	330.1078
pos_3227	Dexanabin	54.46067	42.89612	16.62535	43.72058	29.72943	58.01934	23.81843
pos_3229	5(S)-HEPE	784.0172	325.3973	247.263	142.7929	358.6023	640.1958	406.0514
pos_3231	Tetradeca-	26.60808	1.972032	30.31369	21.60157	14.07621	2.925604	6.827114
pos_3234	1α-hydrox	294.8516	124.3126	568.8351	433.0247	356.2089	219.6637	310.1666
pos_3235	Valyl-leucy	18.27665	1.818263	39.33802	47.33446	14.71297	6.20319	9.971661
pos_3236	5-Hexyl-2	72.42837	61.61153	52.6962	80.92368	43.31551	75.65505	38.04993
pos_3237	Ketosantal	179.3329	76.42617	121.0728	238.6757	71.49907	88.73701	91.91729
pos_3238	Epothilone	73.12507	57.8366	101.7053	70.08159	103.0373	72.83785	67.01704
pos_3241	o-Xylene	215.4147	217.7786	335.9523	226.1332	261.7537	235.0268	277.0515
pos_3242	alpha-Met	8625.593	8794.546	13263.06	9443.325	11614.24	10153.41	11692.01
pos_3244	3,4-Dimet	535.2	522.5716	789.5477	581.2649	709.0591	608.4105	707.0738
pos_3247	Dodecylbe	28.11309	26.61604	21.25794	33.1057	39.65203	40.74554	24.65747
pos_3249	Sphingosir	543.0288	595.3777	543.0096	729.1682	710.7265	800.166	647.8915
pos_3250	(Z)-5-Octe	135.5272	69.40026	43.58534	49.45164	33.02674	107.8602	58.31132
pos_3253	Dihydroca	13.59666	16.37418	38.67544	16.26589	18.02078	14.03289	24.65423
pos_3255	Sphingosir	1140.232	1030.511	854.3051	1434.683	1255.011	1251.379	1047.342
pos_3256	N-Palmito	115414.8	118747.6	183655	125789.1	155103.7	128988.5	152993.7
pos_3259	Eltrombop	6894.865	6717	10322.7	7127	8895.033	6764.109	8056.573

pos_3261	Imatinib	22.89217	16.1937	54.35096	19.28823	10.72739	18.26986	25.88178
pos_3262	Deflazacor	101.4088	57.7125	161.1355	93.81285	127.0589	58.64932	104.5763
pos_3263	Prednisolo	29.55875	19.63114	69.72879	30.58567	38.0527	42.29351	49.4622
pos_3265	Glu Cys Cy	50.08148	54.60462	120.8187	54.51186	66.18365	58.34091	78.68637
pos_3267	PS(5-iso P	155.0456	144.23	169.4864	192.3309	190.1781	239.6442	217.1836
pos_3269	Toluene-c	212.0366	215.9306	325.8231	232.5856	270.514	235.4779	288.6694
pos_3270	2-Aceto-2	50.91773	52.52143	78.37066	59.08028	64.51196	57.66368	68.71725
pos_3271	10,12-Tetr	53.64039	57.6809	100.3168	56.03458	67.54903	58.64757	69.14062
pos_3273	Atorvastat	133.2881	147.1941	251.1047	150.9554	174.4786	115.3373	157.7982
pos_3275	3-dehydro	59.95484	14.55477	2.202842	43.17264	51.49129	14.39777	24.36765
pos_3276	Tert-butyl	989.3481	976.1805	1711.724	1039.205	1366.474	921.282	1206.349
pos_3277	Lucidenic a	2052.024	2052.339	3519.821	2154.286	2791.709	1970.968	2488.346
pos_3279	Nebramine	39.43731	20.54104	22.96466	31.65685	27.46348	35.93193	15.67794
pos_3280	3-Dehydro	144.6117	153.2956	218.2999	164.0786	183.2552	140.9008	164.523
pos_3285	10-alpha-	126.4467	128.247	179.6733	151.89	153.496	114.4353	126.6047
pos_3286	3beta-Hyc	7.352085	23.39593	194.4649	97.23939	1.72E-06	1.133083	75.59812
pos_3287	2-Amino-:	14.12368	18.21335	21.00122	17.89661	20.93507	26.60209	20.56367
pos_3289	Vipadenar	126.5	104.8437	234.7306	137.2805	141.7943	128.2098	134.0594
pos_3290	17-Amino	98.40544	91.14889	111.0956	63.94269	68.90542	57.09594	71.0786
pos_3291	Davercin	29.2608	54.19025	66.83896	35.57761	46.18565	51.20657	54.89097
pos_3292	xi-5-Isopr	27.71777	23.42542	38.05065	18.80671	24.49325	28.05079	33.79408
pos_3296	PC(14:0/0:	9477.759	9243.838	2525.994	8405.574	11205.64	12011.72	9119.045
pos_3297	Postin	26.77268	22.08081	7.654059	12.45626	21.32279	22.64112	8.095571
pos_3298	9,10-DHO	41.17083	30.30605	30.48543	18.32425	9.867577	62.10173	30.5673
pos_3300	MG(0:0/20	207.729	662.9374	1255.973	811.7105	213.7102	243.6987	579.7018
pos_3302	Androstero	1787.578	2665.43	197.38	517.0523	262.5023	561.3675	840.4705
pos_3303	Lithocholic	197.9654	297.8172	17.25678	73.27714	28.50043	50.56656	96.18212
pos_3305	DG(2:0/PG	13.72433	14.27986	12.24948	21.46438	31.52769	46.97253	18.71912
pos_3308	Cervonoyl	44.65886	42.52221	10.54683	67.43732	33.62519	13.19316	19.07881
pos_3311	Ethyl salicy	735.8072	785.909	1154.614	804.5581	969.6822	695.0649	796.9655
pos_3312	Tetradecar	18.29638	23.30638	29.12654	21.16883	28.88432	36.71124	45.39321
pos_3314	Narbonolic	32.48401	9.035028	15.05208	23.8797	29.71083	39.23365	21.39852
pos_3315	1-(3-Carb	31.54851	72.29748	68.53085	41.58002	35.11022	37.04408	75.63048
pos_3316	Phenol	36.01069	45.13745	59.30937	43.55513	46.19333	38.49394	39.08705
pos_3317	Isoamyl sa	137.4849	282.3527	245.4112	278.4665	81.45684	134.3124	198.9301
pos_3318	DL-Kavain	4.905097	7.202705	18.62491	14.52247	1.326136	16.73487	4.779703
pos_3319	6-Keto-pr	29.74595	31.39135	46.43416	50.79934	107.1888	74.4486	13.88343
pos_3320	Cichoriosic	126.3842	150.9487	216.105	157.1348	169.828	136.7814	131.5826
pos_3324	6-Keto-pr	297.2144	257.4319	207.3081	310.226	235.7419	271.7567	189.1656
pos_3326	(Z)-3-Met	59.31151	57.11245	65.18635	100.6187	41.63043	37.60831	80.69524
pos_3328	(2S)-2-[[(2	113.459	100.7544	171.6068	145.7741	153.7806	126.0309	142.2159
pos_3331	1-(5Z,8Z,1	333.4844	218.5463	143.9275	107.7302	195.448	266.26	318.5455
pos_3333	MG(LTE4/(	7.398457	9.816667	28.65975	8.335944	14.75008	2.60155	10.93138
pos_3334	Hydrocinn	15.64368	13.36729	22.06403	20.65626	19.66737	17.7589	21.53663
pos_3335	Pantothen	18.25655	11.35106	1.310445	19.3744	12.82701	10.08723	6.727621
pos_3336	Myristoleic	11.75148	3.142508	13.98583	10.06552	6.294754	9.576185	5.632452
pos_3337	Tyr Asn	2.972784	0.633478	2.352524	1.220903	2.621449	0.663146	3.192724
pos_3341	Abt-510	88.96147	90.38324	1.72E-06	106.0807	26.58371	95.94282	35.38745
pos_3342	2-Amino-:	124.3384	104.4567	42.49361	87.50654	118.1476	179.2423	102.7972
pos_3343	CDP-DG(1	28.41867	12.26218	1.72E-06	17.41052	1.72E-06	71.40594	22.5198
pos_3344	12-Oxo-2	71.347	71.09376	97.87068	77.89976	98.35255	72.32946	77.27546

pos_3348	Indolylacry	45.01594	30.0416	4.402046	18.68388	33.35377	60.04823	31.63007
pos_3350	Arginylasp	40.1824	37.89991	11.37512	11.89665	37.49558	66.3521	33.49974
pos_3351	Nivalenol	150.2796	110.1256	286.9994	142.4282	175.9069	157.5819	273.8425
pos_3352	3-Methylb	18.43006	69.36766	24.33838	26.72723	13.14307	10.54321	137.7501
pos_3353	DG(2:0/18	228.1415	329.9863	152.9845	169.2912	279.2008	225.9896	198.8918
pos_3355	DG(5-iso F	410.7893	341.2654	134.4313	288.079	297.9765	516.0946	333.9553
pos_3357	LysoPC(18	103694.5	78903.67	38639.1	68817.07	87785.18	143156.7	79226.59
pos_3360	Diethylstilk	71.83747	46.30661	7.014848	26.88882	57.93378	85.78755	40.23708
pos_3363	(2R)-2-Ac	63.31247	28.16339	6.271426	25.63813	45.06354	85.39478	31.22504
pos_3365	Dihydroer	26.5045	20.31621	13.22462	15.73729	28.908	26.9494	27.48337
pos_3370	PC(20:0/18	106.8835	67.68194	12.81918	41.81433	51.66894	154.0927	95.92725
pos_3371	PGP(a-17:	61.89932	47.7066	2.617489	26.58354	50.92539	86.82646	50.01411
pos_3373	N-Arachid	50.70394	43.5707	56.06354	52.91032	47.70093	60.60016	38.75122
pos_3374	PS(18:1(11	4.742774	0.363626	1.72E-06	1.931669	1.72E-06	15.31196	1.72E-06
pos_3375	CDP-DG(a	27.76683	6.426077	1.72E-06	6.196431	23.15652	97.24972	9.135522
pos_3377	Spisulosine	221.0746	273.1773	442.9137	212.4139	198.4628	193.3466	435.4615
pos_3378	Octadecar	251.9811	255.0697	526.6679	267.3038	327.012	237.3233	374.7238
pos_3380	3-hydroxy	210.4497	537.5992	256.3168	221.6497	89.38394	142.4079	612.7231
pos_3383	Dipyridam	147.7078	71.01301	24.46472	113.8401	151.7862	168.3745	65.73935
pos_3384	PE(DiMe(1	119.8055	61.15016	1.72E-06	1.72E-06	72.32496	285.8437	13.51542
pos_3385	Epioxylubi	26.42619	23.80273	32.96949	31.41172	53.76705	28.62954	26.38941
pos_3389	PC(20:5(5Z	16533.94	12675.53	6354.489	17993.64	26562.18	24891.53	12580.45
pos_3395	2-cis-absc	9.468062	6.591503	1.72E-06	1.72E-06	28.73224	13.51733	6.722781
pos_3401	concanam	18.98614	16.14139	1.558457	1.72E-06	40.63418	38.19516	6.724394
pos_3405	Persenone	200.6286	138.5511	113.0178	84.48046	36.09058	122.877	101.8439
pos_3409	(Z,Z)-2,9,1	168.0481	118.7587	31.00009	126.4677	113.4283	155.5982	77.03647
pos_3411	Dihydroalp	71.12408	65.42181	78.66032	171.0373	84.05018	139.8943	93.8725
pos_3412	Bipindogu	56.29438	55.0795	93.93549	53.37865	66.39007	41.49252	48.18999
pos_3415	Norecasar	17.03711	4.035579	7.814406	35.82839	16.01757	11.28905	17.06133
pos_3416	2-Pentade	767.5459	425.2301	395.4638	2338.408	599.776	411.4225	699.4071
pos_3418	PGF1α Alc	490.4491	271.8885	287.4008	1671.502	414.0561	273.9068	463.824
pos_3420	DG(2:0/18	176.8209	269.3307	112.1744	119.1797	166.8451	136.6717	155.3674
pos_3421	PC(17:2(9Z	173.7281	116.6808	62.67263	161.5447	185.9491	160.382	112.2253
pos_3422	2-Phenyle	36.80434	27.83536	41.49261	48.02774	43.33284	32.04687	43.98704
pos_3424	5-Hydroxy	536.4646	483.1826	799.3865	559.0372	735.6874	634.8412	714.3843
pos_3427	Tutin	722.6249	611.5581	1068.867	794.5816	1083.204	918.4916	1043.274
pos_3435	Armillaripi	2628.747	2646.213	3564.908	2955.128	3465.313	3149.18	3580.084
pos_3436	1-Nitro-7-	1.72E-06	0.970736	6.715454	3.07059	5.484776	5.232837	6.737764
pos_3439	Cyclo(aspa	5.744331	1.72E-06	1.72E-06	40.40175	3.350343	1.162586	6.177762
pos_3442	Pregeijere	75.3892	58.47819	23.95857	63.58385	83.43283	43.91258	28.4798
pos_3444	Cortisol la	945.5255	736.7257	1349.12	992.266	1209.425	956.1615	1160.417
pos_3446	3-oxo-2-(	56.34272	36.6925	80.95072	41.2758	33.67125	27.00481	55.84455
pos_3449	N-Oleoyl p	5596.721	7377.98	2170.548	2790.719	6499.222	7684.221	4209.327
pos_3451	Lysyl-Thre	50.31761	3.066991	30.99304	18.64264	31.30342	15.2886	16.06729
pos_3452	Trimeprazi	26.49491	10.24376	9.832938	17.23389	17.3076	66.706	29.10406
pos_3453	5(S)-HETE	141.0691	42.02609	75.59494	68.43767	89.32348	61.11031	49.83911
pos_3454	12α-Hydro	7921.918	893.1857	2785.221	2530.033	3905.391	1210.951	1178.677
pos_3455	N-Stearoy	3.240803	44.6719	79.94178	58.9866	1.72E-06	4.348998	30.72212
pos_3456	DG(2:0/22	114.3419	51.59827	60.86202	146.9213	100.3966	64.55663	34.13251
pos_3457	(10E,12E,1	75.51725	11.15848	17.74694	15.96589	43.90303	15.8349	4.687889
pos_3460	PC(22:5(4Z	357.8786	278.8415	46.79829	489.4766	110.5801	129.9291	110.656

pos_3466	Siguazoda	48.80453	54.08802	42.47589	75.45879	40.90769	46.12744	36.44976
pos_3469	N-Acetyln	62.6853	35.25062	15.88275	22.22347	8.923738	45.35884	68.87004
pos_3478	(4Z,7Z,10Z	37.97667	42.79398	22.91227	33.22497	24.34322	17.89633	10.79689
pos_3479	4-Benzofu	98.92861	71.45326	112.115	75.87391	83.14223	49.6532	71.76228
pos_3483	10-OPDA	376.6593	236.4225	319.1953	311.6934	251.8381	357.6588	244.8706
pos_3486	L-Gizzeros	220.0158	227.1533	126.3348	335.4449	214.5581	275.2253	218.0278
pos_3487	Imidazo(1,	54.43058	54.64786	87.70473	54.16417	59.98773	41.59388	59.11447
pos_3489	cis-15-Oc	67.41563	44.47145	30.52166	31.69398	40.37668	52.945	45.2083
pos_3492	LysoPC(15	25239.9	12533.08	3680.431	9160.869	21896.34	22479.15	10005.41
pos_3493	CDP-DG(1	94.13017	30.54421	1.72E-06	5.821491	36.32376	85.90509	3.937325
pos_3496	Benzylami	108.9245	103.5381	155.9817	104.3822	134.1102	121.2606	134.713
pos_3497	Threonate	1.72E-06	1.72E-06	1.72E-06	1.72E-06	1.72E-06	5.345416	1.72E-06
pos_3498	N-Methyl-	157.6217	139.0122	227.0567	178.6082	223.6275	217.3321	220.7246
pos_3499	3-Phenylp	222.853	195.5626	329.1747	238.7541	303.3551	272.669	307.8861
pos_3500	cis-1,2-Dil	228.8713	181.8288	316.5174	229.4364	301.4234	249.4699	313.7275
pos_3503	[(2R,3R,4R)	294.7699	242.7221	446.3425	302.782	418.4822	366.0601	429.2119
pos_3505	Dapdiamic	65.41817	58.6546	107.2841	69.48392	63.27657	63.64703	58.36892
pos_3507	Campester	1121.909	1094.863	1806.965	1515.676	1586.417	1300.609	1808.736
pos_3511	Hordatine	29.51662	14.7076	29.75331	24.73249	45.71099	33.58349	32.29737
pos_3512	LysoPE(0:0	64.36223	36.36537	2.270699	17.89954	34.22047	81.68385	7.587773
pos_3513	PA(8:0/20:	435.4448	259.7951	185.0642	447.9372	281.509	354.6308	245.0529
pos_3517	L-Pipecola	21.28872	24.66639	32.31793	31.96102	23.66021	27.03534	32.96693
pos_3519	4,4-Dimet	277.2268	275.2269	514.5497	401.203	363.9357	292.8202	469.9242
pos_3521	(1'R)-Nepe	35.52768	45.92071	212.4717	95.47706	74.36076	8.301155	70.45066
pos_3522	Octocryler	119.1059	86.00325	106.9375	111.7506	116.5392	70.19749	71.3466
pos_3524	Iridal	55.6854	67.13311	101.2851	37.30023	35.12647	4.774495	22.27434
pos_3526	PGF2α Alc	882.4647	650.9038	603.2406	770.0874	764.9673	743.6764	629.2924
pos_3528	Palmitoylc	1385.323	2275.346	4410.511	4333.819	1123.782	1165.036	2699.791
pos_3531	(3Z,6Z)-3,6	161.6674	133.8415	188.0354	148.3633	127.2193	96.2038	158.0932
pos_3532	Tabtoxinin	23.96784	12.15786	37.80113	12.66473	21.31233	12.57058	17.37216
pos_3533	3,4-Dihydı	38.2686	1.14769	41.8839	14.34874	31.82729	1.875395	4.357126
pos_3534	Eticyclidine	36.32277	8.301398	46.61973	14.05365	23.42797	9.009552	11.68994
pos_3535	Mirogabal	29.8431	13.63344	28.72997	13.28849	20.63976	15.37545	17.59414
pos_3537	(-)-Fumiga	141.7069	84.84582	175.5049	104.0261	124.3427	77.33094	95.88917
pos_3538	3,5-Dihydı	209.2049	26.19667	250.6027	102.1374	138.2663	31.6055	71.53752
pos_3540	3,6,9,12,15	117.5827	29.41743	132.5258	65.59263	87.07586	30.98089	45.31838
pos_3541	(±)13-HpC	300.8135	407.9008	368.4284	442.8956	366.2739	260.7738	541.9037
pos_3543	Cortol	147.9315	82.00183	111.7213	144.1724	287.2413	157.7132	109.5165
pos_3544	Docosahex	17196.2	2994.753	19844.45	7732.765	12369.41	4105.006	5833.167
pos_3546	Enisoprost	268.6607	230.8571	223.7651	271.1535	301.4815	238.4663	210.2703
pos_3548	16-Hydrox	204.318	160.1352	53.25398	1.72E-06	36.40593	1.72E-06	87.18124
pos_3549	L-Olivosyl-	16.18203	8.21671	2.543984	8.043107	7.065319	6.263848	6.033987
pos_3550	Estradiol-1	280.8103	32.42098	396.6428	120.0871	193.0375	56.36448	88.08587
pos_3551	PS(17:1(9Z	325.9271	3.69356	397.1462	22.76768	109.1646	15.56939	17.73771
pos_3552	PE(22:2(13	164.7283	1.72E-06	27.87974	23.41397	94.91926	40.42535	25.87984
pos_3554	3-(2-Meth	29.41461	28.67051	56.73046	26.80822	31.03646	23.57875	27.31402
pos_3556	Propamoc	44.92383	6.119043	55.78639	20.18349	31.67966	8.169988	5.139471
pos_3557	(Z)-alpha-	73.24319	45.2771	101.314	58.84614	67.41231	49.09112	53.58317
pos_3558	alpha-Linc	293.0523	45.92649	322.1724	118.337	231.7328	82.85878	89.32437
pos_3560	PC(P-18:1(	691.2988	410.7064	539.7535	561.8166	679.7788	222.6637	287.6411
pos_3565	PG(i-20:0/	2206.158	353.4305	1910.284	1061.71	1583.452	548.561	769.219

pos_3566	Saponin D	43.12778	3.599889	34.90862	7.764079	11.2728	5.279367	7.934997
pos_3570	Temsirolim	252.782	99.60252	80.02133	88.24801	281.3373	192.0632	42.35245
pos_3573	CDP-DG(a	123.2859	80.47739	27.98082	141.9778	138.6972	108.2393	86.61865
pos_3577	5-Methylc	45.11306	50.20169	37.47503	38.79566	50.33666	55.08216	49.29262
pos_3581	Matricarin	33.14682	27.00991	16.56898	25.82251	31.42393	33.40729	32.62497
pos_3583	Cyclooctat	689.813	476.7969	458.9041	483.7152	550.6908	473.7861	479.5097
pos_3584	ELAIDYLPH	2437.737	2586.742	959.4476	2393.861	1154.232	1104.904	2234.069
pos_3585	HEPC	31709.25	24726.76	9394.201	18804.65	45434.77	36577.67	23342.56
pos_3589	1-Linoleoy	117300.4	106920	84869.56	111314.3	131459.8	145233.6	109911.1
pos_3594	Ouabain	171.8391	140.9848	120.8657	140.7855	186.2686	179.1615	134.7415
pos_3598	PS(15:0/18	77.29302	81.02181	60.37447	73.63968	83.16008	95.69219	73.15816
pos_3600	PGP(a-17:	332.8205	331.1095	336.8816	353.8259	440.1783	410.7171	481.4381
pos_3605	2-(3-Phen	43.20689	39.28304	82.51625	47.88866	88.38598	50.88661	56.11523
pos_3606	4-Acetoxy	12.3868	2.415593	28.42091	12.85326	43.8123	17.44624	15.62877
pos_3609	7-Amino-4	23.45858	16.86034	54.15402	16.4296	33.75386	19.23692	17.23771
pos_3612	Asparaginy	2.338106	1.72E-06	4.810099	1.784504	4.72225	2.136366	2.848824
pos_3614	Glutathion	267.327	215.3416	398.5237	263.5863	318.071	291.5779	357.1607
pos_3615	LysoPC(20	41274.64	41854.04	17923.83	31701.67	57466.05	55459.68	26982.04
pos_3617	Milbemyci	27.66426	28.12363	43.68058	29.8016	80.56422	39.04022	24.16673
pos_3620	3'-Deamir	27.64877	16.3869	8.008045	9.991446	44.62233	30.85832	11.88839
pos_3621	Gln Met Ar	61.39507	149.9932	59.82483	165.5102	206.6918	92.08867	71.50477
pos_3622	Spinorphir	41.6311	36.24267	9.314118	27.8409	89.17538	51.15602	44.83171
pos_3623	PS(18:1(12	76.17354	1.590421	1.72E-06	3.231167	120.8124	36.02729	4.989359
pos_3624	PI(PGE2/22	43.85573	56.2657	10.7022	34.83345	88.9768	92.27883	34.5984
pos_3628	PC(DiMe(1	290.1384	33.18556	7.596478	49.90612	494.7312	162.1696	27.98178
pos_3629	PGF2α 1,1	70.44623	30.82772	33.94103	39.15571	68.44939	39.50342	40.58392
pos_3630	12'-Apo-b	1636.489	245.8222	547.1767	572.3405	2344.738	668.4125	530.5138
pos_3632	Cholic Acid	2137.634	287.3266	784.6308	744.6527	2979.472	850.1161	650.1605
pos_3633	Volicitin	747.011	317.7651	346.8787	380.554	937.3611	477.9793	412.5846
pos_3638	5-(3-Meth	6423.567	5736.053	13528.45	7124.936	9790.381	7397.429	11129
pos_3639	Ser Arg As	65.2681	56.0087	82.49765	67.63282	74.56239	61.96213	72.81323
pos_3640	4-Fluoro-l	326.1804	303.4751	52.35141	151.2984	272.818	261.3378	185.4329
pos_3641	Obacunon	1253.408	938.278	1136.044	1550.936	1867.685	873.7946	1011.842
pos_3647	12,13-DH(	65.89828	59.81394	87.0034	64.37251	77.26816	115.8899	101.9386
pos_3648	Sphinganir	42.72378	36.10357	39.38271	42.9155	98.33447	37.55841	27.37171
pos_3653	N-Stearoy	705.7445	786.2792	472.8337	284.1885	865.4655	631.9667	524.6293
pos_3654	Cholesterc	46.42616	20.24199	15.01094	21.87236	9.826276	26.92542	16.1133
pos_3658	4alpha-car	45.05704	44.08424	96.43085	55.88042	108.4021	48.60116	64.53396
pos_3659	Val-Tyr-Le	58.84281	57.58868	60.92411	57.78544	35.56273	47.04388	55.23215
pos_3663	(9Z,12Z)-(	128.233	96.60054	118.0109	101.6397	91.70614	98.55877	90.84709
pos_3664	(2S,3R)-2-	107.0064	55.62507	43.30544	71.9312	46.03037	68.53757	46.75444
pos_3672	Val Trp Leu	6.222357	14.04606	3.538226	14.35455	15.6319	4.32015	11.56685
pos_3673	Glycochen	145.6775	452.1034	203.1868	500.3734	688.3101	266.0459	210.4254
pos_3676	Polypodos	37.87734	17.87434	21.14751	5.286074	43.82572	29.12293	24.2556
pos_3680	Surfactin A	809.756	580.9299	253.8203	162.848	769.9633	845.7099	310.5614
pos_3684	(3b,5a,22x	119.074	106.903	169.0427	119.4016	132.7251	68.15409	142.8085
pos_3686	TRIMOPRC	150.4638	116.1105	111.6232	127.543	178.1701	90.45997	103.8277
pos_3690	PI(PGJ2/20	360.7526	322.9445	859.2028	402.8306	454.7778	317.7513	626.2898
pos_3699	Glyceroph	187.0061	199.348	185.8804	206.2185	236.5862	214.4759	189.3801
pos_3702	2'-Deamir	226.7664	241.7919	292.8646	264.4021	276.5126	266.2044	260.5904
pos_3705	Adynerin	558.2663	572.5154	615.7463	643.1324	602.6104	648.1861	645.431

pos_3708	Janthitrem	3086.982	9776.256	3454.725	10370.06	14643.05	6131.613	4468.735
pos_3709	Glutaminy	31.08804	36.57742	64.37034	42.59539	55.89851	24.30925	31.17159
pos_3723	Phosphocl	1285.941	1267.808	1143.381	1280.353	1586.61	1502.581	1218.53
pos_3727	LysoPC(18	781597	760069.8	670185.8	798876.5	890859.2	949051.2	793627.8
pos_3732	SM(d16:2(4	551.418	508.7056	655.2186	532.4457	521.8308	563.4808	608.8522
pos_3742	Glyyunnan	265.1846	167.6885	37.077	106.5503	222.3623	399.1826	191.021
pos_3743	2,4-Bis(1-(	300.7312	387.9148	397.1056	380.1483	576.6651	343.605	314.8808
pos_3751	Pseudamir	10.85935	11.66267	20.60013	7.628411	30.73651	17.1412	5.976779
pos_3755	Milbemyci	2900.972	3478.018	3572.217	3185.648	5883.785	3358.873	2808.537
pos_3756	Gly Arg Ar	89075.09	105709.5	81750.07	93870.48	199339.1	114614.3	78270.94
pos_3764	Lyciumosic	46.63941	42.74716	47.70481	46.15567	70.77422	52.7724	43.46427
pos_3766	PS(15:0/22	73.57759	103.1244	125.1619	120.7213	264.9607	84.39831	53.78994
pos_3771	Biliverdin-	113.4495	135.7828	258.7237	164.8038	244.9854	108.4908	140.8175
pos_3775	PC(20:3(5Z	75.97489	46.12195	46.59591	22.75346	45.83685	24.02775	16.18206
pos_3777	N-Stearoy	1495.033	1198.971	2138.152	917.8705	1281.621	664.3949	713.0177
pos_3781	Galabiosyl	28.11019	13.22702	45.78859	35.3291	8.611002	2.336539	9.364669
pos_3782	Guanadrel	16.901	13.37367	32.18274	12.69125	16.72075	12.2304	15.9092
pos_3783	Rumenic a	72.24819	37.74831	30.89042	25.71657	17.34117	17.74841	45.35015
pos_3788	Phorone A	247.1251	262.947	286.4157	257.3418	301.2777	231.0763	214.3319
pos_3790	LysoPE(0:0	74.983	78.19013	55.73617	108.5627	84.94074	73.81244	68.51051
pos_3791	(3S,5R,6R,6	152.9218	171.2785	105.1969	152.898	292.3187	257.2483	131.7582
pos_3792	LysoPI(0:0/	1829.101	2118.29	4000.925	2225.924	2228.807	962.6178	2068.183
pos_3794	9,10-EOT	25.48368	22.69102	47.48215	83.71461	23.69747	26.86073	25.99686
pos_3796	3-[[(2S)-2,	58.39501	65.86905	72.45759	45.1722	59.94799	91.49189	85.27158
pos_3798	N-Arachid	79.12723	64.52669	85.09873	49.06278	71.52986	107.5617	103.551
pos_3799	Lys Lys Leu	24.87636	10.74627	29.36671	34.84421	49.88214	25.98099	30.39625
pos_3802	2-(2-Amir	9.294443	12.0128	1.72E-06	8.902892	1.72E-06	5.681842	1.72E-06
pos_3804	(3R,4R)-3-	28.32817	12.33946	23.71815	29.99441	25.1284	10.45984	8.81535
pos_3806	Glycocholi	9919.102	7750.417	3652.935	4234.445	8647.885	12476.5	5120.011
pos_3807	(3b,9R)-5-	554.0485	346.4613	234.868	437.99	408.3689	460.4498	305.9043
pos_3813	PA(PGE2/2	206.5827	89.16793	57.39619	134.3145	45.20238	86.72429	45.87861
pos_3816	Biolimus A	1901.612	2335.015	313.076	2163.128	1655.829	1927.092	1189.905
pos_3821	PG(a-25:0/	281.1546	302.5213	5.525319	258.2102	276.6392	318.8498	113.2026
pos_3826	Clavaminic	98.22504	146.0046	96.53963	126.3321	143.4133	146.2049	130.7143
pos_3828	Glutamylal	40.57253	51.76628	58.25413	48.58246	50.83684	54.49113	52.24467
pos_3838	Meproscill	170.1101	85.87816	16.25513	70.8797	103.4429	174.848	74.43134
pos_3842	Batatasin I	68.02133	90.13181	67.13793	77.81793	105.0985	70.90342	91.43373
pos_3843	9-OxoODE	1490.891	1392.495	1722.87	2076.786	1173.864	1611.074	1178.79
pos_3845	Cerebronic	1533.262	1562.638	4018.933	1638.82	1813.919	1391.853	2439.285
pos_3846	Stigmatelli	85.66331	75.90246	46.62731	65.99611	99.95682	91.0595	72.1802
pos_3855	1,3,5-Bisak	39.80376	44.73913	60.07285	47.35115	44.18566	44.99823	49.5078
pos_3856	5α-Andros	228.2806	41.42953	137.3556	104.1793	48.77588	69.59691	114.998
pos_3858	2,3-Dinor-	58.78728	72.09772	72.33252	91.53729	46.15644	81.83239	80.00887
pos_3860	2(R)-hydro	121.3582	355.8476	173.753	85.40291	60.97285	50.8361	293.4949
pos_3861	Glutamylal	30.834	12.18973	53.21731	34.5571	35.81615	24.02272	41.03704
pos_3862	24,25-Dihy	326.7639	324.9047	785.6887	358.1946	381.3727	324.841	563.0405
pos_3868	Glyceroph	30.27704	26.26075	6.685493	23.10403	27.69054	28.50689	35.78162
pos_3869	PE(20:4(5Z	209.3471	335.8655	1004.024	167.5939	149.305	540.3486	228.227
pos_3871	Mizoribine	73.662	82.79856	158.028	87.64265	113.6316	39.64128	88.14674
pos_3873	Lys Arg Ly:	321.8922	303.043	257.7076	449.6719	165.2135	196.468	285.2583
pos_3874	Pro Trp Le	551.4787	564.0704	275.0943	548.461	784.1886	530.7202	401.284

pos_3876	PC(P-18:1(	170.6644	134.5533	216.0531	167.8258	78.7967	158.0542	56.15629
pos_3885	PG(a-21:0/	871.7432	749.2964	1242.987	867.3615	842.5487	787.1154	968.7646
pos_3888	CL(10:0/11	125.2665	224.0299	1.72E-06	52.35343	495.9648	538.3646	17.18695
pos_3890	Macrolacti	78.41719	38.55146	52.8269	58.96459	75.2983	70.44136	45.38035
pos_3891	Simvastatii	435.2409	154.2416	204.8839	447.7434	172.7466	180.7886	268.2427
pos_3892	Gangliosid	666.5409	574.5199	5.635079	233.5393	1427.39	1889.541	262.0993
pos_3897	RepSox	24.57357	35.94884	15.54436	36.18832	50.03563	44.77708	26.33641
pos_3899	3-Carboxy	116.7874	141.6682	223.7239	156.5506	143.9899	121.4208	142.3297
pos_3904	5-Oxoavei	289.8284	281.6225	97.93148	245.9782	452.3993	360.0952	174.583
pos_3907	Etazolate	35.74181	23.18811	28.78337	80.77337	96.96196	38.30511	20.05578
pos_3909	Premithrar	67.80236	112.4071	47.43613	86.61606	151.4596	93.19549	64.5953
pos_3910	PS(20:5(5Z	49.58522	21.72144	42.70509	31.07876	24.37208	43.16084	30.02474
pos_3912	N-(2,3,4-T	64.71451	78.83543	55.80573	77.82114	54.66879	81.74248	76.4436
pos_3914	2-Amino-4	22.54913	19.31444	18.30749	17.48051	19.29715	17.51404	15.07946
pos_3917	(S)-Neroli(	1723.056	831.869	1506.948	1030.206	2927.811	1596.716	850.2773
pos_3919	2-Hydroxy	61.6827	61.89468	59.9765	57.27296	44.15194	76.89255	67.9956
pos_3921	16,16-Dim	236.7101	138.0518	203.4611	123.7773	177.6407	148.0203	169.0466
pos_3922	3-Dehydro	81.10672	74.35217	85.37658	97.2108	29.89542	77.94065	67.92025
pos_3923	DG(2:0/20	1245.547	1250.62	978.18	1341.258	1273.237	1189.733	1212.497
pos_3924	Sambutoxi	83.64625	69.65303	58.54163	99.27277	87.47346	71.31364	67.5428
pos_3925	Gymnodim	50.02186	37.61508	20.71361	53.88208	75.19044	58.62652	46.29789
pos_3926	LysoPE(0:0	147.1722	132.9969	241.9552	159.2085	188.1878	113.6432	166.7806
pos_3928	Piperdial	78.41363	22.76661	44.10589	19.25563	23.33316	65.63779	59.77756
pos_3929	Neohercul	15.96297	17.22358	51.30477	21.03845	76.76801	26.47363	26.14178
pos_3931	(23R,25R)-	136.1664	139.3409	142.3957	205.063	111.0552	177.9899	151.3708
pos_3932	Butirosina	192.7468	219.096	278.5317	235.3091	202.9541	146.011	219.2913
pos_3933	Hypaconit	56.65638	51.75431	87.33875	64.29933	86.05517	58.32735	58.79468
pos_3935	(3a,5b,7a,1	45.90531	43.91707	37.84784	107.1495	38.92915	17.86148	41.49112
pos_3939	Dehydrodi	53.18375	55.56144	78.35394	55.69235	97.25338	34.98529	55.29576
pos_3946	Choline	1095.511	1385.494	985.8341	1312.421	1499.311	1487.523	1332.86
pos_3949	Valyltrypto	187.8993	213.6641	235.4748	220.7348	233.6588	195.2725	243.3184
pos_3950	Glutamyl-(	205.0391	260.7319	246.7606	239.1069	262.0528	228.333	263.3572
pos_3952	Ethyl (S)-3	269.1527	327.4995	340.5989	311.5945	333.7512	314.3644	349.2857
pos_3954	Didemethy	206.5794	233.7765	315.9627	229.7807	254.4466	185.2184	251.8071
pos_3956	1-Palmitoy	241.8924	267.843	237.636	263.7155	257.2711	242.4564	249.8672
pos_3957	C20911	29.2567	34.13862	28.85999	22.74429	27.53637	21.64551	37.26448
pos_3959	N-Stearoy	366.0587	408.331	631.4935	193.4811	249.8504	108.1819	155.0194
pos_3960	(R)-1-O-[l	2245.645	2473.341	2887.563	2575.415	2788.652	2367.634	2767.425
pos_3970	Geranylhy(	436.7569	521.432	533.9369	529.9625	529.1824	487.1323	565.9249
pos_3981	Mabioside	190.9082	238.7573	225.4213	236.8537	228.7165	216.2808	273.1963
pos_3983	selamectin	113.1259	148.6137	179.7824	133.5409	138.1688	123.6305	174.1872
pos_3985	Kemptide	993.6749	1311.478	1748.622	1277.849	1359.489	1285.466	1541.665
pos_3987	PS(24:1(15	64.98979	58.17308	72.58518	16.78862	31.4929	10.39369	20.72707
pos_3991	7',8'-Dihyc	154.3919	193.6058	20.53316	152.8601	166.7758	190.5326	70.71293
pos_3994	LysoPC(18	49.77974	51.04355	11.12209	52.21965	82.13727	67.38125	42.22224
pos_4002	(24E)-15al	1033.388	553.9196	106.2961	270.006	1221.395	1625.474	218.4826
pos_4006	CDP-DG(5	6.141742	24.09072	1.72E-06	1.72E-06	54.56032	33.34189	1.72E-06
pos_4016	LysoPC(20	108572.1	138347.2	47152.49	92425.85	180017.3	171760.7	72395.15
pos_4021	ROSAMICI	74.92287	34.67219	8.756505	38.80697	53.63529	47.06333	49.41493
pos_4026	VIPROSTO	31.71809	47.47775	19.16513	9.447223	44.70431	51.12862	24.26785
pos_4031	Estrone	114.9744	111.5975	1.72E-06	30.5242	215.0483	145.4277	60.25896

pos_4035	MG(0:0/18	259.295	299.9741	299.0072	449.2578	193.7847	205.9614	263.7948
pos_4036	DL-Stearo	1006.645	2573.265	5727.815	4934.239	1278.06	1177.553	4419.896
pos_4042	Lysyl-Glyc	148.3665	139.9631	219.8685	148.8779	164.2306	161.969	171.0386
pos_4044	Dihydrosh	17.52407	26.34005	4.781595	22.67791	23.51437	13.8112	16.6784
pos_4045	Fasciculic a	158.5568	423.8939	206.0808	81.99036	83.56096	143.9846	142.4429
pos_4046	Surfactin	670.2788	1048.746	424.1765	1057.573	801.0421	852.4476	747.7137
pos_4048	Maraviroc	1259.391	2496.778	347.672	2695.4	816.8607	908.6923	742.2542
pos_4052	N2-gamm	86.64525	116.1576	82.00665	102.4114	85.53048	84.86112	85.48069
pos_4054	9(10)-EpO	112.4623	151.3071	168.5438	135.2493	144.8157	342.79	190.8348
pos_4056	Threoninyl	30.46345	52.35187	16.32696	54.15919	26.39129	14.32216	26.26439
pos_4057	n-methyl-	109.9711	156.0788	129.3268	147.7554	126.2991	105.5759	119.546
pos_4059	DG(2:0/18	684.2938	956.7781	664.0546	891.5189	741.9078	692.8851	706.9134
pos_4060	(1S,2R,3R,4	597.441	703.6052	484.8682	773.2465	639.0653	550.0607	540.9353
pos_4062	Corosin	488.9079	565.1878	323.616	590.5962	504.1161	459.5463	419.768
pos_4071	PA(16:0/18	25.56591	6.748537	80.14887	30.56949	65.10058	36.5541	57.99674
pos_4079	PS(TXB2/2	16.70849	33.81867	12.52019	46.67993	11.03172	8.821734	14.11419
pos_4080	L-(-)Sorbc	83.39889	84.91587	123.8105	91.8465	90.78003	83.20213	82.86163
pos_4081	(E,Z)-2,4-D	63.46837	84.35239	81.32418	23.35091	39.85026	75.51004	45.40968
pos_4082	Chalcone	2.51419	0.295351	9.966391	262.6383	2.246529	0.55624	3.234223
pos_4083	1,3,5,11-B	28.27765	22.60669	32.15261	5.995613	21.25847	24.72221	12.46862
pos_4084	Confertifol	2575.193	2830.683	3999.835	2762.132	3408.118	2897.765	2914.11
pos_4086	12(r)-Hydr	78.54475	29.64261	52.55258	40.07694	36.0055	50.01778	55.72622
pos_4087	8-Hydroxy	124.4394	47.96947	55.47366	54.91994	49.34884	63.89187	61.57837
pos_4090	(+-)-Zoap	125.1712	102.8489	180.8959	132.766	95.39071	137.9252	121.9359
pos_4093	1-hexadec	378.2771	275.7414	251.0542	308.9891	348.1691	379.0425	304.1351
pos_4095	LysoPC(22	671.784	889.6324	228.726	623.528	2491.843	1320.722	590.6261
pos_4098	IPA imine	58.65986	58.79423	103.4473	70.32851	105.4821	82.59576	81.56918
pos_4101	12-Hydrox	15.52858	179.6906	277.8511	310.2194	0.00634	11.36653	97.5761
pos_4102	N-(2,6-Die	99.79876	48.21285	76.42854	86.06407	68.21554	170.276	87.04177
pos_4106	Citrulline	60.12176	58.09762	90.22821	62.8375	66.96375	69.59707	70.07096
pos_4107	tetranor-1	500.552	560.7721	799.4471	545.6908	657.9801	529.0547	572.8613
pos_4113	2-Hydroxy	28.38031	24.23897	44.18581	25.07502	23.78625	32.62033	31.25889
pos_4114	9,10-12,13	72.52489	60.15299	82.31595	70.97351	67.83773	82.66158	82.58847
pos_4119	LysoPC(22	30.91127	20.25486	61.96529	26.15734	81.10331	30.2795	33.69235
pos_4126	Gamithron	314.0378	224.0178	365.4897	304.8319	365.0959	141.873	249.2738
pos_4130	1,2,4-Non	598.8136	722.2771	640.6343	930.2554	538.0774	561.9347	611.194
pos_4132	N-[2-(3,4-	344.2026	362.4851	328.8682	580.673	264.4276	241.635	305.4304
pos_4133	Cucurbitac	47.21137	24.2472	61.52859	30.63345	44.3712	34.10089	47.59227
pos_4137	Protoporp	378.7324	392.8377	474.3383	441.8349	573.9615	455.2015	410.3658
pos_4152	5-Oxoavei	84.45841	110.0384	150.9987	106.0861	141.7557	101.912	112.0588
pos_4154	Permetin A	20096.18	28930.84	20788.17	28774.64	27042.53	22355.66	21915.18
pos_4156	Glutethimi	164.7389	196.3594	162.0456	195.7414	197.5732	171.9006	162.2217
pos_4157	2-Propena	50.965	49.08963	45.28234	64.28857	65.58255	37.28531	46.36775
pos_4164	Prenol	983.1855	1223.199	763.3727	1284.381	1149.731	1137.694	939.2595
pos_4168	Phosphory	1240.584	1438.409	1143.185	1467.203	1488.238	1319.625	1155.925
pos_4171	PC(O-16:1	725345.6	948483.4	622823	1007538	819516	774191.2	712952.5
pos_4191	2-Methyl-	125.3594	26.68653	1.72E-06	15.17451	1.72E-06	228.0428	26.22992
pos_4204	Aliskiren	91.35966	165.9404	1087.161	67.2784	21.47195	189.9146	198.7205
pos_4205	5-Nonylte	52.49754	52.95787	80.53831	54.40557	61.05805	50.37657	61.0786
pos_4207	N-Undeca	34.47912	58.58555	65.54174	40.24223	10.40891	70.05064	55.09635
pos_4208	(±)12,13-D	1456.97	2338.919	2756.432	1500.492	1125	2433.909	2345.792

pos_4209	Clausarino	34.66346	34.1759	108.1065	42.07766	87.36249	34.7417	77.98325
pos_4210	MG(0:0/22	191.8958	121.1435	307.9853	220.131	180.0452	213.6787	170.8427
pos_4213	Lys Leu Le	1805.93	1637.225	1453.653	1082.778	1735.835	2563.799	1616.412
pos_4214	LysoPC(18	64218.34	79714.59	65941.43	83004	75909.15	74654	72081.68
pos_4216	Cys-Arg-C	433.4177	453.5314	573.617	563.9193	602.6297	462.7432	567.8972
pos_4217	PC(P-18:0/	257.8927	335.268	585.2688	331.4567	145.2486	336.8676	448.9336
pos_4220	Rehmanni	17.29995	142.6274	357.9273	68.32904	58.96065	156.0451	250.1324
pos_4223	12-Hydro>	67.84347	22.96609	28.96213	33.32058	49.02631	44.83515	21.57769
pos_4224	Dihydropic	6833.907	2372.443	1464.029	1646.916	5307.476	6663.223	1684.108
pos_4225	PE(P-16:0/	93.03792	94.9959	124.6442	96.94741	81.15677	50.0882	69.03616
pos_4226	PC(20:4(8Z	567.8428	556.01	744.0436	379.1843	480.5718	578.2206	510.4682
pos_4227	PE(20:0/PC	72.03132	51.81085	45.25623	12.54809	16.79016	71.84675	70.32366
pos_4228	(-)-Dihydr	39.9248	60.38257	87.2308	39.68294	34.70217	80.04372	80.13708
pos_4237	Phaseolic a	27.6336	29.0585	45.94235	26.74714	41.05851	30.38975	40.2639
pos_4259	Gangliosid	1267.053	1031.299	237.1311	555.0288	3436.85	3762.24	731.7122
pos_4260	Gangliosid	67.27343	67.68742	1.72E-06	44.58065	248.3319	233.3245	65.70888
pos_4263	Tyromycic	86.46165	93.27888	180.9603	126.2882	80.82032	72.44179	93.84887
pos_4270	Epothilone	171.6325	143.3507	44.54737	71.98675	227.8894	206.7596	103.0665
pos_4275	capsidiol	117.7156	138.4367	190.1861	148.8694	148.4568	129.4385	139.3121
pos_4276	(1S,2S,4R,8	183.3172	191.3284	327.7757	215.2728	224.4856	235.0768	233.0341
pos_4277	11-Deoxyc	22.24244	27.23665	34.83018	35.96706	23.6607	1.72E-06	16.22464
pos_4278	2-Amino-9	72.90238	44.45412	48.8478	43.68537	21.77032	114.7194	42.93466
pos_4279	17-Octade	22.15204	29.42355	30.76415	26.26797	29.9693	43.82649	22.59842
pos_4280	13,14-Dihy	13.25069	17.77685	21.45297	12.04754	6.456446	7.954056	29.33525
pos_4281	3alpha,17a	64.60407	40.22155	63.37382	54.38966	60.00874	55.78297	48.64881
pos_4284	Glycochola	22.66931	14.45077	17.50799	28.22155	5.33302	27.73946	20.96052
pos_4285	Cytochalas	64.63759	59.83201	36.03357	67.05399	66.5363	71.94096	52.13706
pos_4286	5-Hydroxy	4056.755	2398.357	2336.995	2320.495	1196.903	4751.43	1679.16
pos_4287	PA(13:0/18	3374.962	2096.971	2102.183	2044.921	1160.882	3858.816	1459.766
pos_4289	Prostaglan	64.75165	55.16881	172.4868	103.1551	78.94142	52.42259	95.50375
pos_4295	B-Octylglu	61.35511	38.33202	36.30231	37.05511	52.00702	81.29874	40.23307
pos_4296	Leupeptin	2893.449	1885.245	2076.364	1975.198	1249.92	3803.781	1732.616
pos_4297	Antcin K	3004.754	1937.031	1889.57	2005.35	973.682	3978.31	1569.124
pos_4298	Notoginse	665.3728	422.05	293.7843	390.6437	474.5514	841.7356	303.1121
pos_43	Isopropyl i	322.3987	275.2702	316.0302	321.2153	290.5171	284.5714	279.2833
pos_4305	1-(3-Hydr	30.52617	24.04032	14.84956	17.32243	40.73393	27.53954	21.83578
pos_4308	1-[5-(Thio	28.32384	31.54846	7.615169	21.48055	46.58025	28.98688	21.22065
pos_4309	DG(2:0/PG	794.3936	649.729	359.3366	492.3328	980.5684	978.3563	583.7313
pos_4315	Brincidofo	83.73809	76.53664	9.787843	47.90181	155.7006	106.322	67.11208
pos_4319	DG(2:0/22	266.1313	282.0208	317.6925	302.6982	144.0807	330.8692	342.6085
pos_4320	Difelikefali	41.29905	15.93504	11.46099	29.07036	46.59481	25.09223	38.0047
pos_4322	8(R)-HPOD	46.31166	140.2275	145.7183	118.9161	134.7126	348.8039	172.7349
pos_4324	LysoPC(P-	5272.717	7729.968	2759.967	5078.091	7676.57	10296.19	5133.987
pos_4326	Vitamin D3	408.6101	631.8425	133.644	420.2941	1449.773	734.3477	423.9263
pos_4331	Cholylvalir	7206.399	8974.275	5228.26	9550.036	8564.201	8266.076	7373.037
pos_4332	PC(O-18:2	16527.75	17365.56	5780.277	12115.29	24742.64	19262.98	11436.34
pos_4336	1α-hydrox	107.6124	89.1136	90.1949	134.6338	95.74872	94.10552	105.0231
pos_4338	Deoxyurid	30.06522	17.38741	20.4548	15.49744	1.72E-06	20.35587	31.17653
pos_4339	Lysylargini	86.41079	55.1886	68.80135	71.96537	150.7564	110.1031	83.72401
pos_4340	Prostaglan	145.4547	90.70739	144.5756	134.64	128.5245	121.6288	114.7774
pos_4343	(6E,8R,10Z	44.42973	17.94673	43.50327	49.60502	25.73821	19.3283	15.67885

pos_4345	N-Palmito	276.6779	194.2604	368.0434	216.988	248.9369	167.8577	159.6313
pos_4351	Mifentidin	142.8284	77.60163	86.76763	97.19888	123.9056	129.3883	100.8294
pos_4353	Morantel	69.67288	73.24264	111.3671	81.86057	94.89587	81.48924	85.7087
pos_4354	Tetrahydro	126.4951	120.8074	178.411	146.9714	204.3049	154.8005	151.0032
pos_4358	Cholestero	12360.33	7827.842	3619.072	5891.958	9745.884	12145.93	5978.879
pos_4359	Milbemyci	99.03159	135.9246	229.7101	289.77	324.2838	146.519	150.7782
pos_4360	Milbemyci	69.07573	14.38238	43.56341	51.70163	133.8573	73.99737	19.05795
pos_4361	4-Hydroxy	210.0137	228.6896	335.6352	235.1393	308.3499	255.3847	258.8824
pos_4362	Dopamine	61.39904	59.91622	89.28418	65.72859	88.98065	68.22413	64.17755
pos_4363	3-Hydrazi	2157.695	2325.165	3401.064	2399.372	3117.142	2453.676	2662.483
pos_4364	Anofinic a	1342.402	1408.877	2153.473	1452.467	1916.031	1432.173	1618.986
pos_4366	(2E)-2-do	52.96768	41.3049	81.57251	50.73969	62.61196	53.94842	54.36441
pos_4367	p-Salicylic	38.33066	40.45919	62.94512	29.5563	33.55636	32.56445	47.49063
pos_4368	N1-(5-Pho	28.13551	26.43545	51.83618	29.04934	39.61373	26.33366	36.46294
pos_4372	6-Methylt	98.1691	99.37208	139.3698	85.11604	105.8999	109.8119	117.8225
pos_4373	[(2S,3S,4S,	240.0818	242.27	364.4774	226.466	248.6926	270.0524	255.8032
pos_4375	Desidustat	207.9429	221.5634	320.0271	189.3549	221.7697	250.8386	251.3673
pos_4378	Esmolol	138.8308	73.8284	59.28622	86.70158	170.2181	129.8699	65.5662
pos_4379	Oxidized c	36.06749	37.97219	73.47107	39.91927	80.33306	46.32296	49.16915
pos_4383	N2-Ethyl-:	181.4314	175.8891	269.6979	204.6012	351.878	220.0833	257.4123
pos_4387	Latanopro	192.9378	190.0724	285.6982	209.4229	248.6761	212.5249	212.4458
pos_4388	12-[Methy	219.5643	177.9871	248.7838	250.1117	278.0577	209.8969	206.4827
pos_4393	1,25-Dihy	35.12941	19.52295	37.30031	30.40082	16.43426	24.8535	15.20744
pos_4396	Physapruir	197.7535	153.6356	227.9298	249.7691	356.8558	188.656	149.0224
pos_4397	[8]-Parado	66.02244	39.51545	95.66527	60.98754	37.93876	60.1997	42.02177
pos_4398	Avenester	49.01252	7.373673	17.57369	29.25805	60.11203	64.15275	13.43268
pos_4403	Gentamici	200.6257	157.6218	150.4685	210.4165	174.3712	201.7103	152.689
pos_4404	PGP(i-22:(	64.02217	45.23888	4.683941	24.32011	26.96742	32.4313	18.72728
pos_4405	12-OPDA	949.7014	682.4082	576.5016	754.2523	523.9198	602.0782	480.1839
pos_4406	dl-alpha-T	1703.409	1633.247	2598.924	1817.451	3320.273	2150.359	1877.579
pos_4408	DG(10:0/P	466.0623	477.0268	971.8136	521.9986	975.8094	520.343	682.3185
pos_4409	Asparagos	164.6961	107.8245	126.4986	144.9778	169.5399	160.8513	119.7531
pos_4414	Imazodan	104.5406	123.5492	93.91207	86.94443	164.4061	125.0582	113.8864
pos_4417	PG(22:6(5Z	83.34705	87.18278	67.23791	66.56236	126.3476	99.47172	83.09998
pos_4428	Ribalinium	180.6822	173.6458	193.3706	167.2706	215.2074	192.2391	180.3847
pos_4429	Nepetasid	58.02612	51.517	35.80822	45.74405	69.42077	47.36168	57.60408
pos_4439	N-[(Z)-1,3	203.3201	254.849	204.5002	191.8731	306.8444	238.4682	233.4522
pos_4441	Tragopogo	914.729	911.5006	1469.566	1224.677	1072.437	912.6116	935.4209
pos_4447	Sirolimus	56.88037	41.49562	55.75079	56.805	58.8865	63.84336	48.48198
pos_4452	PC(24:0/P(	25.96905	13.63541	17.80953	0.611484	3.32919	24.31785	8.839247
pos_4453	Pterosin N	15.49874	22.25225	33.50253	36.44593	46.72946	25.59092	33.15399
pos_4454	Tylactone	87.00241	106.3163	162.0595	105.6011	102.7327	76.32948	84.52067
pos_4455	PC(22:5(7Z	71.58841	77.33427	98.01978	71.42598	68.17286	79.30697	82.78067
pos_4456	Isopropyl (	258.1963	198.0149	202.0035	295.0411	204.8857	249.7913	211.3081
pos_4457	4-(4-Amir	123.7615	77.15887	53.33432	73.0228	153.0069	133.0719	67.81344
pos_4458	PC(19:1(9Z	19203.72	12916.93	8060.999	11299.32	22933.97	22090.76	10988.06
pos_4459	6,8a-Seco	26.07546	12.43506	15.80513	10.59656	46.80684	72.22307	8.152065
pos_4460	1-Heneico	58.9912	17.52773	61.84045	38.72549	37.40291	33.85307	39.73399
pos_4465	3beta-(3-r	251.7412	143.4581	149.0932	310.5893	143.952	168.8256	218.9496
pos_4466	Ganoderic	222.9499	174.0959	176.13	251.1557	165.9574	210.1957	151.9502
pos_4467	DG(13:0/2(	296.2501	240.5739	425.2147	270.8469	300.2233	271.3169	292.1523

pos_4468	Nystatin	53.36903	119.6501	175.4572	106.9418	139.5835	73.15657	120.7621
pos_4470	(±)16-HDo	91.78855	64.66616	126.1494	102.87	132.9624	111.129	87.75177
pos_4472	DG(17:0/T.	1237.567	934.4092	1133.224	1442.31	1186.916	1066.546	785.7397
pos_4473	PA(15:0/2(	1991.941	1734.95	2124.67	2429.546	2560.622	2156.515	1821.936
pos_4474	2,5-Dimet	26.93027	22.24594	31.89016	30.886	22.78564	21.67599	39.72817
pos_4478	Lubiminol	69.93304	76.47913	100.3667	75.92554	75.15475	64.69806	78.36587
pos_4479	3-Phenylp	55.73483	46.80988	93.87515	63.32446	58.57342	53.70388	63.31736
pos_4480	(9Z)-Octa(	27.87263	21.4498	29.35346	25.95126	37.89994	18.73032	15.83705
pos_4486	Capryloylc	66.45082	44.01815	61.14589	61.4528	35.65419	52.4022	39.44853
pos_4489	Neomycin	398.3244	410.6601	669.0512	563.5697	475.9119	420.6843	415.5872
pos_4500	(9E)-7-Hy(	74.82622	57.72214	170.6579	108.3782	66.33584	115.5377	131.3853
pos_4502	Ascorbic a	199.7056	210.8832	229.7451	277.9781	267.4618	161.2931	196.5421
pos_4504	5alpha-Dil	52.93126	55.77152	64.69189	65.74236	48.88079	63.83962	55.00655
pos_4511	Cer(d18:2(	262.6115	192.973	398.3561	228.4613	301.4181	156.3473	206.3893
pos_4512	Lopinavir	46.00179	23.84163	45.72443	85.36275	218.1491	60.83442	36.04227
pos_4513	Momordic	50.89152	41.8121	57.18257	70.32224	91.18372	50.5078	26.2616
pos_4514	PS(O-20:0	3059.129	3491.814	3595.15	1671.146	2831.217	924.464	2147.036
pos_4515	Roxithrom	300.2321	301.3356	333.658	425.016	478.774	202.6461	245.5391
pos_4520	gamma-A	84.05734	108.8093	112.9784	99.84739	90.33868	81.45139	110.6338
pos_4521	1-Stearoyl	343.5205	331.38	321.9215	298.8436	394.6322	312.7344	373.7578
pos_4522	O-Arachid	608.7497	571.9082	987.6382	768.925	768.2372	587.295	694.8369
pos_4524	Frangulani	1183.788	1263.376	1182.383	1152.815	1197.439	959.203	1272.92
pos_4528	PE(20:0/22	34966.46	55161.51	77694.41	54318.39	65557.3	67435.71	79298.62
pos_4529	N-Palmito	425.1471	413.0282	454.7794	539.3425	390.6479	345.3924	377.8487
pos_4531	norerythro	62.19243	63.4146	116.3717	78.11114	62.62984	59.21676	52.95723
pos_4532	PC(P-18:0/	43.565	24.48972	13.81651	11.94678	12.73317	17.4202	3.725076
pos_4533	22-Deoxo	389.8014	419.8151	218.719	228.8519	381.9361	303.5202	374.6442
pos_4535	Gangliosid	38.4099	24.48335	55.96386	35.95097	19.91813	18.85189	39.61131
pos_4536	CDP-DG(a	226.331	230.5941	426.5159	299.3529	374.8965	195.4109	266.3352
pos_4538	Dynorphin	121.0155	252.8366	412.5762	309.719	362.3257	180.438	213.5412
pos_4540	D-Pipecoli	27.16842	32.26569	39.79375	36.11917	16.95545	28.5068	33.57267
pos_4542	Avermecti	13.3992	16.98042	37.40769	24.93459	13.25849	5.760602	21.53256
pos_4544	PE(24:1(15	78.25969	68.6628	116.1404	118.2409	47.33926	48.3815	89.87215
pos_4549	2,3-bis (4-	44.83857	45.86852	45.72343	46.34531	50.22951	51.33365	52.69906
pos_4550	Isoleucyl-I	739.0781	801.8876	895.5315	770.2409	913.3733	843.8705	816.5118
pos_4559	Choline pl	1873.815	1975.282	2039.262	1773.503	2322.139	2029.669	1871.607
pos_4561	Ilicifolinosi	49.62917	50.09621	50.62766	38.7257	65.95875	46.1769	34.71558
pos_4563	24,25-Epo	5732.019	5892.789	6053.089	5656.613	7229.334	6131.179	5984.73
pos_4578	6beta-Hyc	186.9675	209.2339	252.6703	206.4659	254.4617	193.2992	223.4766
pos_4580	Neurospo	69.09068	59.68867	26.74357	49.29261	66.53595	70.18363	59.25884
pos_4586	CL(8:0/11:(	278.99	306.393	394.5152	322.0351	357.7313	290.1838	353.1323
pos_4588	Gramicidir	39.19911	29.34186	48.79812	22.93811	34.93395	13.52093	34.71248
pos_4592	Aurachin D	16.45977	7.482277	16.893	42.36868	26.00523	26.36077	25.00664
pos_4594	5-O-beta-	1828.285	1925.192	1932.497	1867.694	2181.502	1985.859	1918.73
pos_4595	3'-N-Acet	928.8022	962.7837	1060.958	998.0749	1047.845	866.3873	849.4618
pos_4598	Sandoricin	177.0992	216.5854	257.8947	180.4776	228.3729	202.3297	212.4198
pos_4605	PS(22:0/22	416.4195	407.7217	750.0541	407.1515	381.2828	348.3221	413.005
pos_4611	1,2-Di-(9Z	40.72034	47.35897	55.52366	116.0677	70.70617	36.07808	70.17032
pos_4613	Cholylargi	70.24547	71.49371	123.124	57.30049	114.9154	70.53075	102.2727
pos_4614	DG(13:0/2.	259.6867	313.1267	485.2318	352.9208	413.8526	319.3393	316.0724
pos_4618	MG(0:0/14	95.02065	89.88503	168.6687	97.88035	127.0256	104.1217	128.4724

pos_4619	4-[(1R,2S)-	174.0246	143.5591	387.198	279.8148	194.2378	329.2331	329.7738
pos_4620	N-Stearoy	86.99585	117.784	144.4236	157.6046	101.9956	100.8515	129.5055
pos_4621	Goshonosi	45.71098	23.64633	51.93919	35.55743	51.16999	50.53615	51.30927
pos_4628	Saringoste	21.69012	8.299634	2.524999	8.661629	9.191955	27.67962	12.61399
pos_4629	Nonacosa	648.6571	390.7725	538.846	484.625	761.7177	708.1705	444.5773
pos_4630	8-[(1R,2R)	647.7431	406.33	355.8973	570.9098	490.5197	703.7554	472.9591
pos_4631	MG(18:1(1	593.9203	239.9431	578.542	428.9541	1142.482	414.0245	390.5034
pos_4634	Cer(d18:0/	163.5225	185.2306	267.4795	249.0698	294.0969	225.9554	187.3602
pos_4638	1-O-Myris	346.0052	179.1991	143.4859	214.0783	260.8126	343.0014	186.3772
pos_4639	13,14-Dihy	288.2686	270.5238	331.2649	421.1404	257.5753	382.5455	350.1565
pos_4642	(2R)-2-[[(2	80.817	31.66788	54.57844	19.07238	54.66536	92.78173	43.6678
pos_4643	Isoleucyl-A	54.61119	63.6503	78.10112	52.19803	63.84434	57.79353	68.45994
pos_4644	Mytilin B	11.20703	11.8051	21.23869	15.53664	28.56122	8.71388	18.06597
pos_4649	N-Acetyln	14.59007	14.58677	30.12191	8.483343	19.63916	21.62849	12.90322
pos_4650	Cer(d18:0/	509.7189	454.3174	538.0337	442.7509	335.8818	497.0029	617.5922
pos_4656	(3b,5a,22E	24.09131	4.951987	23.04968	14.45065	2.749395	16.76509	13.36657
pos_4658	delta-Toco	839.0611	801.1158	1110.197	1163.043	853.5808	788.2884	850.2002
pos_4666	Salmeterol	937.9515	799.9341	859.0358	1096.364	809.1406	748.5366	794.7335
pos_4671	L-Oleandr	1124.303	1757.223	1365.986	1927.317	1471.418	1287.17	1077.137
pos_4674	[(2R,3S,4S,	262.1445	186.0429	157.0542	265.1249	227.6258	215.0511	200.6202
pos_4676	Oxyglutino	147.0015	105.0329	119.8728	114.3999	71.53099	121.7371	122.7434
pos_4677	N-Lauroyl	167.6309	154.8316	91.51627	182.2302	100.9096	122.4787	129.6525
pos_4680	5,8-Epoxy	1423.309	1468.868	1039.055	1512.442	1417.454	1341.727	1621.499
pos_4681	Medicager	264.2208	267.1017	335.4768	344.2403	281.6659	227.4938	233.6347
pos_4683	Donhexoc	42.40515	20.66686	30.13188	13.79128	18.79335	20.17411	11.9135
pos_4685	2,5-Furano	126.5337	135.4523	190.4825	137.8602	141.7802	128.8397	138.9847
pos_4687	Danielone	347.5706	368.5223	518.0506	384.5959	402.3214	357.0693	377.4302
pos_4688	D-Glucuro	275.0042	296.1377	409.1471	325.1421	321.6827	286.7575	308.0008
pos_4689	(6S)-Tetral	1059.28	1150.839	1540.125	1223.513	1277.622	1138.617	1178.801
pos_4690	Osmundal	1058.987	1133.122	1637.763	1227.853	1303.212	1122.256	1188.343
pos_4691	Cromakali	398.9126	435.1897	614.9046	454.1684	487.7362	401.6656	452.2198
pos_4694	1-(beta-D	4491.009	5046.181	6994.301	5150.878	5595.701	4724.844	5299.038
pos_4695	5-Heptyl-3	1352.424	1382.726	2055.228	1538.408	1620.081	1376.004	1476.91
pos_4696	Tetrameth	260.4753	211.5584	343.6823	340.5841	302.0487	333.0171	306.0685
pos_4697	Arg Asp Le	27812.62	31446.07	41248.91	31949.27	34986.16	30140.86	34011.46
pos_4701	N-Arachid	153.4336	109.8939	134.0861	151.378	131.8701	114.0414	111.6961
pos_4703	Proscillaric	17.33849	22.47399	46.08022	28.05232	32.34097	25.61533	42.10012
pos_4704	DG(14:0/2	23948.34	26680.64	31935.52	28114.66	31286.95	26682.98	27850.32
pos_4705	LacCer(d18	19.18007	31.62158	47.72085	28.59545	26.4421	16.81568	29.56525
pos_4706	beta-Toco	74.07126	76.7384	86.52266	99.95014	112.8772	72.08294	72.91607
pos_4710	DG(PGF2a	1641.539	1770.739	2125.405	2008.137	1979.665	1756.616	1794.889
pos_4717	N-Myristo	151.8745	149.1499	194.5772	197.814	182.4597	275.3984	297.1613
pos_4718	Trilostane	391.5419	358.7354	432.8345	408.1647	424.0121	381.0056	358.7245
pos_4720	Homodolic	631.739	462.3014	297.9	382.5709	653.5307	990.1194	492.5413
pos_4728	(3b,20R,22	33.36668	20.20437	9.930768	21.39352	42.31971	54.6756	29.08906
pos_4732	Heptaethy	522.9827	600.059	743.3917	663.3015	683.3621	592.304	578.0223
pos_4734	Rocuroniu	26004.08	29548.25	36433.65	30610.1	33589.32	28636.27	30806.04
pos_4735	3-Hydroxy	430.4285	490.441	570.1521	512.3508	553.6744	500.6898	485.0848
pos_4736	6-deoxote	1774.883	1864.667	2258.87	2076.568	2145.703	1825.185	1902.593
pos_4737	DG(18:3(6Z	468.562	52.10403	114.6049	164.1282	132.8686	118.9659	133.9409
pos_4739	(S)-2-Ami	13.46572	10.94273	28.77394	6.134016	9.536249	7.523301	13.25887

pos_4741	2,4-Undec	34.30188	32.8997	55.92743	36.80478	37.27457	27.68447	35.00616
pos_4742	Papa nonc	247.3757	275.7412	360.7814	283.6738	302.5673	273.1828	282.3248
pos_4744	Farnesyl ac	3418.433	2547.462	6809.701	2339.825	2970.771	1413.076	2910.16
pos_4745	Pregnanol	1035.691	1207.02	1376.651	1177.515	1283.124	861.2524	1136.647
pos_4748	7-Dehydrc	3432.583	3723.328	5003.853	4024.589	4319.755	3557.063	3943.701
pos_4750	Gallopamil	246.1922	190.0242	323.5943	297.65	305.713	219.257	222.4644
pos_4755	Secalcifero	368.5641	283.416	257.9886	478.2025	340.6608	308.4158	294.0884
pos_4756	Deoxychol	1023.076	662.2436	425.8008	788.4417	1061.506	982.2617	900.6362
pos_4757	PC(O-17:0	3529.987	4392.239	2352.567	3307.748	5493.635	3921.031	3212.04
pos_4759	Docosahex	318.7801	243.6054	293.3427	395.4853	261.0662	319.5919	312.6632
pos_4761	PAz-PC	403.0804	264.826	413.9885	365.0141	703.7837	431.5854	287.1787
pos_4767	6,8,10,12-|	147.1637	179.5853	213.4118	183.5296	195.7389	172.9678	176.1685
pos_4770	12,13 diHC	426.7368	453.9633	595.5016	439.9628	556.8906	458.2994	472.0547
pos_4771	Soyasapog	924.7024	942.8707	768.8428	1050.55	800.8291	732.2873	849.4769
pos_4772	DG(2:0/PG	234.2489	211.892	100.8432	315.0372	182.9718	185.2076	169.3617
pos_4776	8-[5]-ladd	438.7262	462.5909	277.3653	490.6641	442.2714	532.5358	426.47
pos_4777	Eicosapent	2242.28	2332.171	2906.012	2434.234	2846.488	2393.36	2413.491
pos_4778	3Alpha,7A	4949.373	4902.468	4475.548	5600.862	4517.937	4238.884	4638.242
pos_4779	27-Nor-5l	1651.694	1484.578	1370.725	2014.549	1418.636	1405.989	1543.913
pos_4782	Bafilomyci	979.2645	889.3032	1059.552	1086.705	2648.149	989.9639	803.7304
pos_4783	Isoleucyl-(	179.1319	168.3522	161.9131	179.9974	140.2165	194.8407	172.3952
pos_4789	Glutaminy	70.15683	89.73902	133.8487	80.97775	37.82033	114.08	117.2333
pos_4790	N-Acetylm	90.82112	100.0915	153.8874	93.07021	56.95812	104.655	115.7654
pos_4791	DG(8:0/20	10208.67	10745.62	7348.709	10134.91	14956.75	12656.84	10433.05
pos_4797	2',3'-Dide	194.2782	209.7186	269.9226	218.8418	222.4718	220.4241	236.5763
pos_4800	Sitosterol l	285.271	322.3962	370.2917	441.372	230.5563	248.0953	280.2539
pos_4801	Saponin H	59.3635	27.56039	41.13327	25.43547	34.74383	37.00397	48.9707
pos_4809	N-Myristo	44.00688	30.63228	44.84902	44.92166	37.42564	28.71665	26.11461
pos_4810	5-Amino-	59.43061	92.80477	137.2292	105.4665	60.86441	86.01902	105.0556
pos_4811	Monocrota	24.79492	76.54388	98.50684	81.1018	26.53892	50.71746	76.40116
pos_4812	N-Acetyl-	829.253	1025.414	794.3655	1877.85	1006.022	727.4114	1059.084
pos_4813	DG(2:0/20	69.21087	49.31771	65.09581	161.4997	77.57853	76.26766	72.06366
pos_4816	2-Octapre	10084.81	13516.77	11432.47	7850.001	11451.47	5934.732	10163.49
pos_4817	PC(18:1(9Z	117614.9	109993.8	160470.1	138400.7	119833.7	94570.67	81912.9
pos_4820	Osmaronir	129.1454	180.255	148.5589	140.1011	121.458	173.1615	140.9663
pos_4822	(1R,3S)-3-	82.2309	60.01533	109.8157	40.55323	40.76608	86.94675	80.47124
pos_4824	Lansic acic	577.3079	568.5126	656.3643	656.3293	562.2722	561.6061	597.9345
pos_4826	9,9-Dimet	130.6288	98.38333	119.4272	150.0352	86.94284	104.6333	96.72212
pos_4828	2-Methylb	53.18648	53.1959	62.13028	49.79599	54.76121	61.97686	52.52575
pos_4831	N-Oleoyl /	197.4065	93.37086	139.4871	120.0429	129.2826	133.8094	137.693
pos_4837	Cer(d17:1/	389.0283	261.9398	298.8281	430.0522	448.1098	463.4091	281.1289
pos_4839	Leucyl-Try	87.9524	91.99048	103.0279	97.88358	91.0144	117.1897	116.4637
pos_4841	3-Carboxy	435.568	442.0039	578.0565	415.7449	445.3501	463.474	453.7322
pos_4844	Digalactur	3124.259	3805.853	4533.65	3276.12	1824.566	2252.607	1648.777
pos_4847	(Z)-3-(1-F	208.3891	224.1608	267.8772	215.8341	206.9314	242.4588	240.404
pos_4849	Deoxythio	38.462	43.66157	65.80657	37.50473	39.20822	45.17626	43.23465
pos_4855	N,N'-diace	86.07858	95.38919	110.6973	85.86077	93.86726	108.2184	110.7397
pos_4858	Spirolide E	2569.649	2314.135	2780.936	3487.076	2984.798	2534.216	2503.156
pos_4860	Methyltrie	38.55072	57.32838	74.75217	49.60387	40.54031	63.905	46.42506
pos_4861	PC(18:0/20	72615.94	76601.86	136683.5	69982.84	79178.22	72941.53	66044.48
pos_4863	N2-Succin	77.38243	87.51321	102.78	99.59769	98.3196	108.6682	91.67324

pos_4864	vamorolor	332.9007	323.5943	435.2743	411.8045	408.1031	469.9339	355.79
pos_4868	Ciprostene	15770.28	18238.08	26033.86	18336.57	21260.29	18299.01	18366.58
pos_4872	(4Z,7Z)-9-	459.6045	571.4731	574.8518	695.4061	598.7385	617.7064	538.9751
pos_4874	(x)-1,2-Prc	292.301	123.0045	365.2073	1.72E-06	1.72E-06	89.1043	178.4542
pos_4880	4-Hydroxy	518.6435	473.031	333.2822	525.9591	331.1038	401.5593	421.6666
pos_4881	Arginyl-Isc	68.29791	60.71335	33.59738	65.78279	41.80008	97.0224	55.40403
pos_4886	Lithocholic	1206.403	1235.041	1419.685	1530.302	1269.421	1291.754	1283.348
pos_4887	DG(13:0/2	66942	75127.91	91176.15	74683	84168.36	73551.7	76175.31
pos_4890	N-Arachid	657.4882	327.8016	383.6225	376.5661	334.1763	424.0675	349.0008
pos_4891	Cer(d18:2(	62380.54	70573.48	85583.68	69627.45	78767.95	69348.21	70575.01
pos_4892	3,6-Epoxy	9117.421	10654.21	13453.09	13616.02	10933.17	9847	10591.69
pos_4893	Bentazone	19.74369	49.81215	51.05302	73.01121	24.26022	27.59393	49.44638
pos_4895	Lysyltryptc	1.72E-06	19.3862	12.74959	133.1836	1.72E-06	1.72E-06	31.56908
pos_4896	Val Val Gly	1067.339	1299.739	1452.751	1291.872	1445.517	1325.913	1290.213
pos_4897	MG(PGJ2/(	1264.537	1500.933	1350.725	2283.919	1494.424	1181.103	1523.729
pos_4899	epsilon,ga	6027.193	7795.562	10339.01	8497.936	7443.254	6896.812	7510.379
pos_4905	Behenoyl-	803.6592	287.3658	317.8485	765.3318	954.0999	635.8652	915.3906
pos_4906	DG(PGJ2/1	2122.888	1669.369	1372.461	3010.227	2412.521	2447.551	2007.135
pos_4909	Tetraethyle	6416.159	7410.905	7788.384	7087.167	8217.811	7747.99	7691.273
pos_4910	Pentaethyl	5586.023	6396.131	7320.805	6274.242	6964.26	6501.38	6425.096
pos_4911	Hexaethyle	2116.802	2376.89	2847.464	2384.551	2740.829	2412.576	2402.125
pos_4914	3-hydroxy	2080.873	2267.814	2703.725	2179.785	2478.222	2355.689	2387.679
pos_4916	3alpha,7al	96163.01	110577.1	129462.8	110457.1	123532	110063.5	115766.5
pos_4917	Castastero	81133.75	93913	109023.8	100269.6	101779.2	88581.23	95687.71
pos_4918	Septacidin	72.34231	27.66618	80.49115	453.8057	364.7655	77.78967	68.84597
pos_4920	Polyporust	699.6478	732.5784	552.8986	933.8686	579.7888	702.1976	739.2352
pos_4922	PS(20:1(11	592.2345	738.2185	711.3728	591.8604	1004.149	619.528	769.7325
pos_4926	N-Formylr	68.89454	94.43025	93.79068	83.0076	95.21698	97.62997	90.84762
pos_4928	Zymosterc	220.8887	210.558	260.4466	270.5452	172.5648	236.3433	201.7748
pos_4929	Sphingosir	90.6229	91.1346	115.0246	125.2183	162.2184	191.7884	130.1103
pos_4937	Oleoyl Oxa	8906.595	5678.496	9195.324	7663.648	7413.262	10154.42	8093.256
pos_4939	N-Stearoy	34.55651	29.54722	37.07395	58.06406	58.07818	102.4374	45.44425
pos_4943	Ganoderic	29.13586	63.54982	79.55242	36.73138	45.45351	105.2061	100.1279
pos_4949	DG(14:0/2	603.7567	576.3826	409.4967	819.5539	1089.358	1084.87	887.696
pos_4951	arachidyl a	121940.4	39050.55	177579.3	71734.75	94984.49	27972.61	29285.31
pos_4953	PC(18:1(9Z	800273.8	1510720	1854669	1502977	1711963	1813311	1938398
pos_4954	PGP(a-21:	144.9339	168.7673	207.5853	183.4685	187.9811	161.0224	162.7244
pos_4955	Gangliosid	73.69903	57.41412	118.8017	87.45067	73.02538	63.49867	57.11423
pos_4958	(1R,4R,5S)-	229.6338	246.4835	273.7064	295.0934	339.3045	331.5884	263.9301
pos_4960	Cer(d16:1/	318.4001	206.1846	243.4025	239.1807	358.4103	381.6995	378.6951
pos_4961	(9Z,11E)-(	98.64238	84.39045	40.5683	113.0817	124.7381	118.1357	74.60486
pos_4964	Ser Asn Al	132.3835	141.2619	199.27	149.9754	169.5603	158.2778	153.6178
pos_4965	Galactose-	40.28602	38.8924	59.84303	41.13058	52.44116	45.80561	63.8833
pos_4968	PIP(6 keto	941.7491	1066.977	1519.724	1081.459	1528.446	1249.941	1503.614
pos_4970	beta-Meth	330.6806	362.2359	598.6927	344.2164	598.2089	422.0883	569.4297
pos_4974	Cytidine	15.83926	7.519451	26.82851	12.07306	5.999966	12.04771	21.1046
pos_4975	5,6-Dihydı	108.4768	113.8731	215.6343	131.9089	196.4029	161.6601	215.8723
pos_4977	8-Methoxy	1733.85	2044.436	2673.209	1977.184	2077.827	2403.614	2583.49
pos_4978	Macropho	522.826	601.9562	1035.998	627.0701	1002.553	744.7699	1061.019
pos_4981	Myotoxin /	11061.16	12530.25	20939.87	12817.19	21760.6	15541.01	21552.61
pos_4982	Etheno ad	1.72E-06	1.72E-06	1.72E-06	1.72E-06	1.72E-06	1.72E-06	27.36706

pos_4983	Ile Arg His	116.6453	136.7064	187.3171	145.8218	272.2396	180.9369	246.653
pos_4984	Fosteabine	690.4187	763.8404	1092.182	765.9482	1310.529	997.4442	1216.1
pos_4985	PA(8:0/22:	728.4592	908.3245	1150.136	927.1072	1779.822	1278.611	1520.594
pos_4989	CDP-DG(2	676.5925	728.8984	1003.139	761.872	908.6207	809.2813	908.109
pos_4990	2-[(Tetrah	16.30817	13.64513	33.97055	6.092296	6.376947	2.949759	18.61011
pos_4992	Undecylen	83.98617	41.88068	139.3187	34.7072	44.05247	13.7575	49.48068
pos_4993	3,5,5-Trim	39.29378	27.30871	72.30539	14.59246	20.50167	6.089731	29.56652
pos_4994	5-Ethyl-3-	23.88199	11.70601	40.57782	9.976279	9.033219	1.72E-06	17.9799
pos_4999	9,12,15-O	37078.55	29498.33	63876.6	24388.48	29173.31	16712.55	32392.94
pos_5004	N-Myristo	54.60745	46.18694	129.1933	52.30831	37.29412	17.22731	68.00968
pos_5005	PS(22:1(13	3548.878	2762.524	6314.829	2557.892	4246.793	2318.65	4288.987
pos_5006	CDP-DG(F	74.83222	52.22255	98.08599	48.79277	99.57637	121.892	108.7914
pos_5007	CDP-DG(2	16.64725	18.82912	20.1709	43.60557	26.74882	70.40838	83.14383
pos_5010	Lycaconitir	8843.633	8425.054	11874.09	8086.491	9483.611	9250.8	13417.04
pos_5011	Aypgkf	1056.828	965.9356	1395.52	931.7355	1587.652	1296.271	1295.305
pos_5012	PA(12:0/2(	1349.904	1234.308	1628.268	1295.086	2134.956	1990.243	2130.317
pos_5013	Isoachifoli	109.5369	138.4709	164.0666	145.9207	256.178	248.2965	397.5724
pos_5016	CDP-DG(F	109.6791	84.67965	186.9304	112.9409	170.2413	145.3837	201.0968
pos_5017	CDP-DG(F	146.3539	130.1899	201.1603	197.0481	276.6755	228.5882	246.2254
pos_5020	FUSARENC	52.23152	30.70257	98.36813	43.96697	116.9253	67.53009	102.8689
pos_5021	2-(6'-metl	97.62901	53.79013	168.6459	57.12191	197.122	115.5929	203.6236
pos_5022	Chaetoglo	1770.077	1028.363	2865.815	1204.96	3442.732	2223.17	3157.27
pos_5029	PIP(PGF2a	206.3811	203.7314	364.3363	220.5465	531.4739	396.3249	609.212
pos_5032	Yucalexin I	274.6331	388.7855	255.8156	289.1774	390.5514	580.2699	427.5939
pos_5035	Captopril-	322.6446	380.761	498.4334	343.5011	383.0226	375.8705	422.7973
pos_5036	MG(16:0/0	1246.129	1136.462	2122.02	1241.828	1595.533	1491.592	1618.934
pos_5038	Pinolidoxir	69.41704	137.3305	156.6356	119.2932	35.87955	124.3492	149.3312
pos_5039	3-[2-(3-G	44.81797	54.21034	108.1088	42.52003	73.16403	66.68359	72.98879
pos_504	o-(pentafl	2108.556	2280.183	1739.085	2355.573	1997.548	2088.195	2096.562
pos_5044	Arginylleu	141.2797	155.4773	229.3923	250.42	189.5912	268.631	196.2142
pos_5048	Canavanin	157.5526	223.1324	179.1107	258.4263	175.0302	192.3332	192.1099
pos_5049	Ergosta-5,	40.5172	52.5393	57.93221	49.5597	65.6743	55.18906	61.23865
pos_5050	N-Linoleo	630.6808	538.0541	611.6011	1051.14	943.9239	684.9823	709.0666
pos_5051	4-Megasti	70.52452	123.1309	100.8132	185.827	173.8326	133.2456	132.6836
pos_5052	Hovenidul	34.17685	85.62364	53.91284	109.3014	39.36877	90.91799	81.82954
pos_5054	Cohibin C	103231.3	125943.3	144901.5	123893.4	138692.8	137650.3	143040.4
pos_5058	3-Hydroxy	75.46793	73.80972	70.58757	86.76675	77.01322	93.63292	97.39023
pos_506	Iodohistan	203.4895	245.8313	182.5943	290.1907	216.184	251.1808	225.7997
pos_5061	AS 1-5	58339.97	55979.44	72435.2	28433.42	50725.35	25769.58	38083.18
pos_5064	Isopropyl I	4.308995	16.24566	6.583391	19.19176	2.455564	6.573878	10.24948
pos_5071	PGP(i-19:(	476.5878	443.3954	693.8841	328.6024	679.3638	781.1827	709.7373
pos_5078	8-Heptade	2255.627	2681.394	3265.31	2657.239	2851.276	2756.697	2878.744
pos_5080	LysoPE(P-:	485.6383	494.0761	549.6822	584.0315	569.0756	534.9495	496.01
pos_5087	Nigroxantl	1231.48	1374.859	2519.154	1379.684	1453.446	762.4141	915.9619
pos_5094	9-deoxy-9	1070.562	1572.477	642.2389	806.5103	1053.536	387.6337	625.444
pos_5095	1-Myristoy	102.7807	122.5033	95.26342	101.8816	102.8246	99.03347	85.99045
pos_5098	Cer(d18:0/	5266.596	6130.604	5952.325	5469.668	7418.276	7603.012	7144.53
pos_5099	DG(14:0/2.	495.4806	279.0052	194.6491	334.1118	552.4823	603.1957	334.9307
pos_5102	Isopentyl 3	183.8853	225.4006	285.2338	209.1051	280.3145	342.3071	268.1785
pos_5104	Octaethyle	450.8512	506.4396	582.0335	517.6375	609.7317	535.0517	517.42
pos_5109	Neoxanthi	536.1663	488.6752	474.4056	615.3441	585.1919	710.2679	564.869

pos_5111	Lidocaine	126.82	93.90375	93.56751	125.1511	89.02027	112.9475	103.5481
pos_5112	PIP(20:1(1	226.931	362.2675	371.8708	332.0047	325.6654	346.7294	411.1116
pos_5113	Ala Ala Va	71.63554	68.44894	71.84967	161.2765	182.0765	191.5736	139.1151
pos_5115	4-Tiglylcar	45.97284	103.1536	132.5129	134.8222	48.98018	99.00661	126.7705
pos_5117	3-Heptylp	74.30888	108.829	119.7277	93.23737	73.14854	126.3228	124.846
pos_5120	DG(16:0/2	34487.9	38306.55	44326.57	37903.75	43807.23	38547.02	38293.05
pos_5122	Mucocin	224.169	252.8415	296.3862	291.6081	233.9742	270.3283	265.7464
pos_5123	DG(16:0/P	590.0641	327.5943	470.3039	455.2205	414.3012	423.6802	503.7789
pos_5124	DG(14:1(9	66041.87	71210.78	85023.3	70699.89	80978.64	75881.06	75663.18
pos_5125	LysoPC(26	8606.055	9655.287	11429.6	10124.68	9848.255	9587.848	9727.206
pos_5133	11beta,21-	1329.678	1535.837	1749.129	1733.405	1668.748	1747.556	1567.537
pos_5135	MG(22:6(5	9.071449	24.97111	9.139139	29.21789	21.8396	42.90979	20.33587
pos_5139	L-Proparg	5.501742	3.542432	3.577043	4.810381	3.110785	4.919344	5.053253
pos_5142	Isoleucyl-T	28.33916	29.3177	35.49248	29.10037	23.93345	34.41365	39.13564
pos_5143	Methyl jas	16.88242	10.15703	12.88398	14.13398	7.061713	14.2813	10.24684
pos_5147	Paraldehy	778.1706	920.1361	1020.341	922.326	952.2208	956.6662	941.8219
pos_5149	7-[(1R,2R,3	84623.08	96630.49	114961.8	95960.78	106785.1	95960.32	98185.62
pos_5152	TRIETHYLE	2444.017	3391.467	3163.77	3032.168	3281.113	3974.815	3780.309
pos_5153	L-Metanep	11747.18	13372.14	15067.96	12874.46	14450.84	13572.15	13439.99
pos_5154	Argininosu	216.6269	232.274	277.2301	249.7432	258.2448	264.8486	249.9482
pos_5156	N-Oleoyl-	22140.29	26160.35	31043.71	26272.87	28197.04	26859.63	27596.99
pos_5158	3alpha,7al	91685.94	105649.8	125757.4	105369.3	115843	107216.9	109697.8
pos_5162	Ethyl oleat	168.4604	138.7313	161.2775	164.2174	199.08	246.0764	442.507
pos_5165	Hexadecar	34.58547	64.35911	79.29505	128.5015	102.2159	390.1223	214.4649
pos_5171	Palmitoyl g	239.4189	279.1863	346.5059	318.5132	391.8316	502.4557	372.5677
pos_5172	Daminozic	16.73759	26.57373	9.134851	11.9804	24.85672	52.45006	25.73658
pos_5174	PGP(i-22:0	1609.824	1592.111	2111.201	1792.129	3304.982	2020.638	2014.737
pos_5176	1,3,4-Pent	45.73806	47.07033	54.74614	43.52539	52.44903	46.63542	44.55221
pos_5179	(1R,2R,4R,5	451.939	517.5557	593.2274	500.4985	542.2649	551.6328	554.6412
pos_5187	3-Hydroxy	6977.62	3034.731	9763.933	3308.71	3135.681	3441.938	4629.868
pos_5199	Annonisin	49.12361	31.48265	33.26258	72.0209	50.28725	45.0701	50.62725
pos_5200	1-Stearoyl	34.16206	60.33091	96.71218	115.9714	35.36632	171.4326	124.3854
pos_5202	L-gamma-	215.2042	237.0812	291.3062	221.0094	271.1586	266.3175	277.0869
pos_5212	THIARABIN	36.79773	17.03298	37.79763	18.3598	42.28119	44.10432	11.57849
pos_5214	Glacin B	344.9368	354.563	463.1907	534.9593	314.383	422.9001	405.175
pos_5219	Erythromy	43944.35	44489.54	37652.92	39324.97	46570.91	29396.39	42821.04
pos_5222	PE(P-18:0/	26195.2	31205.83	54456.58	31260.65	29490.04	25651.06	28682.93
pos_5223	1-Octadec	187507.3	383656.7	436157.3	423768.2	347960.7	249392.3	310861
pos_5225	1-Octadec	15973.22	12661.55	21849.5	13655.29	12487.11	8273.552	11533.19
pos_5226	PC(16:0/20	472966.7	603424.4	680779.7	510647.4	513559.3	393150.9	550225.3
pos_5229	PE(18:0/20	41016.69	37259.57	76414.26	45256.03	44896.18	30447.42	39992.16
pos_5231	CE(LTE4)	725809.1	772023.8	1042400	813891.6	805758.4	716369.7	747316.1
pos_5232	PC(P-18:1(	175419.5	191733.6	263690.5	228575.9	191399.6	122583.6	144399.1
pos_5233	11-((2-Dir	2483.603	7850.409	2220.309	3788.201	4797.636	2111.516	4976.154
pos_5234	PS(22:0/18	11612.32	8722.017	9119.907	11399.71	10894.45	7157.806	8457.454
pos_5235	DG(22:0/L	6554.727	9138.322	13668.19	9128.003	8641.927	12800.65	14542.36
pos_5236	Muroctasir	1650.913	1271.167	2297.146	1637.668	2140.828	1564.173	1622.957
pos_5237	PS(20:0/20	10036.74	9957.929	11983.96	11601.87	12581.69	7262.174	7359.459
pos_5238	PC(18:1(11	14921.53	16869.9	17121.23	18885.68	16937.03	12554.32	14376.48
pos_5240	PE(DiMe(1	43832.71	54006.08	67542.58	51258.36	57216.51	50756.19	56218.37
pos_5241	PE(DiMe(1	3073.321	3573.992	4619.301	3487.634	4005.482	3830.357	3883.492

pos_5246	PC(P-18:0/	8408.395	8558.953	8652.743	9403.7	9386.911	7655.295	8108.99
pos_5247	PC(16:0/T>	5144.499	7317.805	6085.049	4979.631	7801.109	4958.922	7513.115
pos_5249	PI(22:5(4Z,	1032.339	1098.054	1309.859	1116.457	1249.988	1151.399	1143.12
pos_5254	N-Oleoyl (	48.90187	39.90446	66.92263	97.41028	116.561	138.7718	101.7597
pos_5257	PG(12:0/1:	189.4332	309.0634	191.0334	454.6107	334.7789	368.2616	333.0856
pos_5267	(9R,10S)-d	1021.01	947.1182	946.3997	900.6232	799.5107	1024.226	900.1114
pos_5273	PE(22:4(7Z	2145.078	2396.202	2938.487	2376.154	2996.535	2560.674	2641.143
pos_54	Iodoacetic	227.1848	260.8045	216.5611	207.7503	196.4011	220.2049	231.7342
pos_558	Glabrin A	230.3836	335.5745	172.4955	513.021	325.2381	384.5918	245.8314
pos_584	8-Nitrogu:	1600.71	1885.898	1020.617	1870.902	1475.01	1814.496	1634.82
pos_585	Homovani	217.9184	259.8016	158.4242	239.819	218.6204	310.9716	251.5355
pos_602	Betaine	8515.255	13699.59	8919.548	13761.25	15434.36	12987.01	10170.83
pos_604	Pyroglutar	158.1975	200.639	101.8524	171.4944	124.3347	144.6048	136.9372
pos_606	(2R)-2-Am	11369.69	13711.23	10774.55	14254.45	12684.9	13601.09	12076.39
pos_608	2-oxo-6-r	82.30658	86.46572	68.9595	88.38208	107.9951	74.49876	64.96552
pos_609	N,N-Dides	86.69477	157.1354	67.42798	150.8123	230.5243	227.9602	135.1955
pos_613	Ccc(C(O)=	61.61536	75.64202	81.51659	78.50402	80.41078	68.40507	68.52179
pos_614	Maltitol	1157.896	1331.015	730.1584	1733.181	1302.738	1378.973	1139.431
pos_618	4-Amino-	145.8804	125.2497	141.7179	154.2642	198.7635	127.346	136.2876
pos_622	Keratan	118.1026	109.8241	6.587371	121.8908	1.72E-06	66.68537	69.27936
pos_626	3-Acetami	127.1003	179.0278	179.7902	198.9591	184.9445	222.5957	154.3131
pos_627	Pentenylbe	231.0136	181.4677	128.5526	109.1983	147.7434	205.5287	171.4792
pos_630	alpha-D-G	116.3657	166.4616	54.76178	177.63	130.4043	206.3977	120.4226
pos_631	2-Nitroph	71.84908	71.2224	44.3458	150.1525	56.66292	75.66818	79.60482
pos_632	(+)-Bornyl	149349.6	161936.6	154266.5	151966.7	168130	161026.4	152765.4
pos_633	Distemona	7959.803	8214.888	6086.55	8248.016	5679.448	7000.609	7578.104
pos_635	Nicotinate	3971.4	3907.99	2433.168	3710.109	2734.348	3250.511	3415.964
pos_637	Persicarin	1094.373	914.1155	464.6266	1027.025	461.8141	780.2179	776.7651
pos_644	ADP-5-etl	42.3887	67.57674	7.36633	51.37688	29.88594	66.64467	77.08693
pos_645	8-oxo-GTI	46.9088	61.27981	9.732786	48.79778	15.22038	35.15525	59.99114
pos_646	UDP-L-idu	104.5589	128.8025	37.33446	134.1895	43.8758	91.39393	128.9179
pos_650	Adenylate	1787.004	1856.593	1132.277	1746.636	1380.379	1551.638	1580.979
pos_652	4-Aminob	25.52293	31.05249	32.30163	30.85817	31.05945	39.15286	31.90821
pos_659	UDP	168.412	174.5546	67.83516	201.576	97.75744	168.5334	148.1891
pos_661	P1,P4-Bis(	102.9494	122.7774	11.22069	123.943	24.32626	89.15411	106.4488
pos_662	Malonyl-C	49.81357	36.97715	7.238293	28.82076	2.846813	26.00217	34.42523
pos_663	GDP	42.08054	40.23607	1.72E-06	59.01145	1.990064	18.16444	25.61834
pos_667	L-beta-as|	504.6205	636.4888	584.421	639.7706	620.136	614.1097	558.3695
pos_673	2-Aminois	102.3457	110.6717	155.4135	88.21479	137.5929	120.3202	107.0939
pos_677	sinapoyl-C	28.65281	49.29947	11.48201	24.48897	36.93522	31.07898	43.91585
pos_679	4-hydroxy	78.94714	40.41224	32.30729	57.2714	48.19568	56.49373	51.38751
pos_680	5,3',4'-Trih	80.54263	62.0624	42.55942	60.90323	45.11229	79.05657	56.43493
pos_685	(R)-2,3-Di	583.3575	367.6821	244.2185	470.8205	680.0921	529.344	281.0984
pos_686	Aspartylhy	2315.871	2634.38	3548.656	2533.706	3813.466	2884.903	2535.403
pos_687	Creatinine	78.54062	116.2058	43.59823	167.4036	131.4673	173.1173	76.71502
pos_688	edetate	163763.4	182139	216461.5	168981.7	231572.2	186455.7	171968.4
pos_691	6''-Acetyla	7746.37	8611.991	10196.94	7391.446	10732.15	8460.873	7943.918
pos_693	3-Hydroxy	561.1267	676.2271	809.5541	604.4862	805.8756	693.3336	631.2245
pos_697	Indole-3-(	213.3093	293.6279	242.5235	365.7368	272.4508	291.0583	255.137
pos_698	2-n-Propy	4451.023	5266.614	1070.466	1491.708	2294.951	3334.617	6223.242
pos_700	l-Felinine	120.6979	153.6061	138.3464	183.1773	137.934	163.7799	124.9997

pos_701	Linamarin	214.8337	371.5604	238.1392	353.9777	176.1414	274.5474	230.1775
pos_704	Genistein 4	328.4468	393.4755	376.8005	272.4226	457.8055	375.5067	311.9188
pos_707	nona-4,6-	52.75111	50.81122	159.1655	33.94316	141.5434	65.68749	51.70805
pos_708	S-2-Octer	28.04415	46.91962	202.3095	31.11299	193.3062	37.96191	45.7835
pos_709	N-Nitroso	2.850508	0.868831	5.603579	6.983579	1.077963	1.960396	0.914001
pos_710	Glycyl-Glu	68.38172	114.4744	72.61277	129.7969	60.90576	95.44152	85.27319
pos_711	n2-Acetyl,	16.65483	16.27943	18.67192	18.15348	11.16888	11.7199	6.632969
pos_713	Phenylalar	17.77787	21.34423	26.89376	24.68688	3.924362	21.77454	11.72154
pos_714	Arabinosyl	397.0245	568.7566	245.3567	742.6553	405.5954	740.4776	392.654
pos_715	Uralenneo	76.62022	133.0209	75.01739	134.7784	46.65389	158.7406	106.9182
pos_716	(2R,4S)-6-	126.6489	197.4622	105.2134	203.4584	76.89909	213.635	170.6463
pos_719	8-Hydroxy	40.8556	54.84451	30.21662	65.15079	35.47544	41.34088	40.71245
pos_722	1-Methylp	14.38951	21.56342	19.2702	13.31911	28.7066	59.9592	64.74693
pos_723	Xylitol	1865	2501.806	2028.914	1735.651	2745.068	5543.794	5663.363
pos_724	2-Propeny	848.0881	1313.407	206.9353	328.4487	707.9275	479.1813	565.8684
pos_725	N-Acetyl-	1903.857	2495.068	2736.272	2669.122	1520.267	2534.879	1888.067
pos_727	6-[(2R,4S,5	374.6887	477.2225	615.356	528.9768	649.1735	730.4763	443.8111
pos_729	3-Methylc	456.9214	460.5768	459.7731	861.1954	731.5919	575.1082	467.0802
pos_730	1,2-Dihydı	85.02751	27.44172	38.33931	42.83982	59.84277	44.03161	29.34859
pos_735	N-(1-Deo:	11716.49	15314.03	8496.371	11562.95	25797.93	14671.93	9085.355
pos_736	Oxindanac	359.0643	497.2336	182.0005	490.3151	387.6819	390.6787	280.0157
pos_737	5-O-Caffe	45.07057	58.37614	6.143925	59.7068	69.16401	60.43773	27.07566
pos_739	6-Thiogua	50.60923	70.28605	9.217742	47.85591	92.86018	56.10719	23.34938
pos_741	Uric acid	801.0428	856.7759	640.2757	975.5343	771.3443	996.3627	667.5164
pos_743	UDP-2-ac	208.8435	228.3472	492.6097	221.3764	436.9285	241.3231	225.3361
pos_744	7-Methyltl	396.8912	440.8192	244.9972	384.5499	1134.482	473.4147	368.3099
pos_746	L-Dopachı	2041.206	2574.097	2951.157	2412.8	2935.687	2455.132	2208.54
pos_747	L-2-Aminc	76.04281	72.99737	80.43876	94.7922	76.1912	87.14686	69.3624
pos_749	N-alpha-a	36.17589	36.14533	41.21032	64.05744	53.66418	44.83163	52.18314
pos_750	Galantami	1033.017	1405.38	425.2543	1063.784	1223.911	1140.669	745.1285
pos_751	Glutathion	45.96652	52.70678	1.72E-06	31.31927	0.082994	41.36186	12.02435
pos_752	Phenacety	2703.036	5798.763	3345.751	3604.533	2362.963	3565.448	3790.469
pos_755	dTDP-3,4-	134.4353	130.3487	46.239	163.8579	40.77846	115.1405	108.1745
pos_765	Erdosteine	26.20286	37.1262	63.5624	35.04181	52.48846	36.68576	37.95954
pos_774	L-Homocy	48.05662	51.6946	102.6819	16.75282	137.7687	57.68097	51.02829
pos_777	Oxonal	44.4204	37.78264	31.92608	93.36817	72.12595	51.13307	36.27513
pos_780	Genipic ac	351.6676	324.8683	340.3949	356.8079	311.5252	439.1621	327.6877
pos_784	Norvaline	44.98601	35.26375	16.16493	47.36129	47.14014	48.38931	38.38214
pos_786	p-cresol	907.1261	1013.167	676.0788	637.1526	889.2139	1091.554	855.6374
pos_790	L-Isoleucir	964.3883	1050.956	1172.934	949.419	982.547	922.2807	1067.929
pos_792	(E)-5-(3,4,	166.8177	183.3632	146.0522	161.5151	148.1401	183.0119	138.2379
pos_793	alpha-Ace	131.9472	173.9526	142.6413	167.5919	166.6181	136.6589	105.6457
pos_794	Formylisog	907.6021	1125.192	879.2756	889.2028	938.2045	1038.982	862.8587
pos_796	4-Fluoro-l	1547.428	2016.031	1209.879	1302.903	1509.032	1997.541	1992.522
pos_799	N-Acetyl-	8479.881	9466.028	5674.738	8999.916	13222.54	10227.67	8317.922
pos_800	Nornefopa	463.4146	561.051	449.4012	466.8283	437.8877	533.244	453.3822
pos_803	2-(7'-Metl	283.4422	449.9418	249.3318	443.5836	200.4752	332.1491	291.7495
pos_804	Heliannuo	638.4191	800.6027	518.2846	796.295	1174.166	761.3017	630.4438
pos_805	Lagociclov	165.0995	166.2436	151.6107	168.8808	160.2229	192.441	146.5694
pos_806	(2S,2'S)-Py	15486.98	21536.61	12354.4	22434.28	33747.78	19834.51	15764.04
pos_810	Gln Phe	3893.022	4567.768	2904.737	5027.666	7551.894	4342.872	3502.802

pos_813	p-HPEA-E	1045.394	1336.749	868.1339	1559.946	785.5904	1357.378	1148.722
pos_814	Penilloic a	264.342	327.7941	172.3632	336.1332	416.9706	337.9415	223.7546
pos_817	8-Demeth	103.89	173.6847	58.17012	179.0483	183.7015	239.5833	94.6164
pos_818	De-O-met	3083.686	3999.677	2179.251	3164.404	4980.976	4545.762	2654.413
pos_821	Minoxidil (	63.02218	132.2783	71.00502	116.5367	82.07385	151.232	78.66066
pos_822	Hetacillin	87.52207	193.66	170.3558	218.8474	165.9075	374.1901	179.0253
pos_823	6-Hydroxy	179.6014	176.6046	48.11623	191.4667	224.3125	200.9913	163.7512
pos_824	Ononin	36.92797	14.16953	3.558282	15.51096	2.155699	26.85872	15.80233
pos_825	Phenethyl	76.16082	145.9704	105.3539	120.1763	59.61662	127.0803	126.212
pos_826	3-Hydroxy	41.71032	56.64878	40.04547	48.39649	27.47969	65.69808	39.84881
pos_827	5-O-[(3S)-	62.82718	95.68413	35.07935	189.2491	85.6242	115.1394	72.18545
pos_828	dTDP-3-m	183.6315	207.8239	187.5086	128.4484	176.5373	209.2424	162.3221
pos_829	Histamine-	2.780609	13.41727	9.729937	137.5302	46.55234	80.70107	6.473698
pos_830	ISOPRENE	1751.799	1969.729	2095.366	1546.216	1710.39	1760.92	1763.727
pos_836	2-Pyrrolidi	41.92349	33.95652	62.55881	21.95744	21.28384	33.80613	45.24712
pos_837	7-Deoxylo	79.82235	86.28097	58.97039	74.1362	75.18251	71.07767	82.90263
pos_838	Methyl (3x	48.99611	69.86663	38.07813	69.48326	30.84258	52.81542	57.23852
pos_839	Adipate se	303.3288	294.2771	245.171	353.0779	248.9657	314.9615	250.7173
pos_840	Tuliposide	507.5716	571.1969	456.4081	506.3399	518.0131	514.2257	467.6339
pos_844	D-Fructos	73.40872	91.59526	81.29115	66.09691	82.0982	75.04169	64.2249
pos_846	Mertiatide	2599.936	2881.725	3151.118	2893.285	3329.769	3022.658	2867.874
pos_849	1-Methyln	52.08325	57.44882	57.15894	46.72587	52.72249	57.36507	49.69051
pos_851	L-Histidine	22.89312	27.07476	9.838533	11.43233	15.53907	32.14285	24.29164
pos_853	2-OH-ber	48.14194	53.2811	29.74993	27.49631	27.27546	47.4417	39.09238
pos_855	Glutamylv	105.1867	148.8359	234.3175	351.7129	641.6538	354.9546	198.6547
pos_856	Tranylcypr	1918.472	2559.563	1014.53	2538.224	4413.178	2725.898	1787.668
pos_857	Asp Gly Hi	328.018	453.1446	194.7556	448.2928	964.1353	539.9331	327.217
pos_858	D-Threitol	90.81592	104.6091	112.0203	105.1349	98.25059	108.0946	93.98087
pos_859	Piperidine	427.1281	527.9554	346.9931	407.4264	340.9359	429.6036	420.1509
pos_861	4-[(2,4-Dil	1198.804	1754.641	1097.455	1278.34	848.6238	1360.238	1191.004
pos_866	3-Carbam	975.0974	759.7057	1032.441	1015.219	1949.621	1462.279	2574.152
pos_868	Cys Ala Tr	55.72707	88.57859	92.46136	40.88692	100.0309	121.2209	61.97097
pos_869	Aminoethy	84.99947	60.00746	85.16222	74.25196	133.5925	118.2894	181.3638
pos_871	Serylalanir	142.0067	89.64167	84.78811	101.5945	207.7806	73.0267	61.18572
pos_874	Benzyl alc	218.0223	226.2478	168.6092	166.4102	146.0098	229.1773	222.0367
pos_875	beta-Nitro	700.9967	758.7864	298.1458	505.8023	330.9234	709.5906	680.2773
pos_876	Edoxudine	12.99512	20.61439	7.490572	2.095111	6.503519	23.22223	15.48173
pos_878	Castanosp	135.567	149.0551	83.91582	188.3399	100.9161	142.2528	115.1321
pos_879	(3-Phenylp	255.6207	281.7327	305.1006	283.241	287.709	287.1024	252.4048
pos_880	(±)-Ribalir	313.8092	562.6954	161.0763	221.0814	49.2362	122.5721	115.066
pos_881	PI-103	2.923683	41.26581	21.19101	43.13565	33.36764	109.6028	32.21356
pos_882	2-(Difluoro	45.89155	22.23864	2.118445	15.75596	259.8243	27.00383	20.83519
pos_883	(2S)-1-[2-	302.3505	396.6359	284.4996	305.7298	225.9529	334.6283	325.527
pos_884	Galactosyl	61.79424	95.36627	125.7694	158.8719	1.72E-06	33.75685	80.1378
pos_885	4-(3-Benz	96.15213	113.936	87.79736	158.6302	89.48832	106.7097	104.1825
pos_886	Tyramine-	4.179722	5.05854	1.72E-06	1.72E-06	1.72E-06	1.72E-06	1.72E-06
pos_887	4-Quinolir	47.49508	29.36084	37.24486	43.3341	66.15844	60.40523	115.409
pos_888	Hydroxyph	1754.488	1485.447	1332.68	2149.961	2980.03	2620.444	4047.979
pos_889	5-Methoxy	413.1887	430.9942	293.4865	399.1634	257.8651	436.8986	390.5953
pos_892	Imidazolop	42.66042	33.98566	24.76834	56.54146	54.96117	59.38907	88.28852
pos_893	Tabun	87.21084	90.90468	89.72075	86.67659	76.52772	75.13902	89.82741

pos_896	4-Amino-	560.4734	729.4175	621.591	573.044	551.7539	647.4755	607.4334
pos_897	Amino (2S	39.68462	36.79375	21.52867	77.14724	15.92181	49.82968	38.5075
pos_898	Phenylace	5208.828	5971.82	3356.784	4510.944	3714.18	5280.131	5303.815
pos_900	3-Phenoxy	6643.426	7432.551	4193.203	5889.942	4764.689	6481.267	6582.448
pos_901	PRIMA-1	92.88641	93.48597	20.24089	96.20765	22.9031	98.36235	66.12517
pos_903	Toluene	244.2466	257.7127	139.7507	197.7236	153.8717	270.6501	337.313
pos_905	2,3,4,5-Tet	308.8301	499.0323	149.0873	382.8129	337.3288	415.5415	281.4838
pos_907	7-Deaza-2	49.91536	50.41384	42.05393	16.44735	50.05992	40.92012	96.8103
pos_908	Pro Pro As	1688.79	2170.702	761.6247	2145.365	3352.449	2180.424	1451.221
pos_909	5-Hydroxy	252.9595	517.9933	161.7846	163.4016	39.51148	105.6394	123.2666
pos_911	Flunixin	151.6231	1.72E-06	1.72E-06	2.576762	38.71414	8.413296	9.39293
pos_912	Feruloylpu	887.5259	16.78004	4.890974	52.98388	274.7154	115.4475	89.15299
pos_913	3-Hydroxy	89.15679	90.38194	48.37796	82.26227	88.50752	90.48141	79.32158
pos_916	(+/-)-(E)-E	16.6004	16.7131	20.90566	37.26162	17.97569	20.32886	29.33384
pos_919	Mofegiline	387.4263	339.2128	101.6586	334.063	267.7789	287.9357	195.8067
pos_920	Dibenzo[a	8.28691	40.22169	21.54119	20.22116	29.16352	74.01828	33.81244
pos_921	Azimexon	472.1451	533.0475	327.7001	501.617	351.7803	533.8622	474.2564
pos_922	3-Ethyl-5-	9.577762	58.7903	1.72E-06	1.651116	2.314519	21.15547	175.0359
pos_923	Xanthoang	140.5302	70.2278	42.11483	109.3662	57.01953	274.9173	93.53898
pos_924	(S)-2-(4-M	57.35378	71.8134	29.69802	48.45782	52.32081	52.56158	49.73899
pos_925	L-Arabino	75.36128	0.167095	3.49137	1.72E-06	26.90723	3.798051	3.473488
pos_927	N(alpha)-t	2734.978	1109.95	1346.036	941.3817	476.3859	673.5602	1009.036
pos_931	PE(15:0/20	13949.56	15830.75	18591.74	16159.14	18325.42	16890.64	17351.7
pos_933	2R-Hydrox	76.91685	57.92927	51.67689	49.55688	97.28408	81.80532	79.52696
pos_934	CL(8:0/8:0/	170.6749	197.1705	208.0081	146.2946	212.6503	65.16423	156.2462
pos_936	Carboxyno	350.167	386.1691	401.5152	323.0977	468.8227	401.7085	369.506
pos_939	Docosanal	532.1428	363.3112	241.331	1020.474	758.2925	468.1424	350.3214
pos_942	N-(L-Argir	172.9229	184.0741	204.1307	183.3143	206.799	198.2978	182.0753
pos_947	(2,5-Dihyc	132.7105	157.488	209.6099	151.785	162.565	162.0858	179.4188
pos_949	Pimonidaz	114.9416	207.0258	282.8285	353.8464	67.10363	225.0729	221.9751
pos_950	Cinncassio	3734.316	4524.864	6121.488	5093.154	4473.164	4573.215	5308.562
pos_953	PG(i-12:0/	351295.1	431521.9	492254.8	449674.1	419251	425020.7	460538.6
pos_956	Ginsenosic	18.40203	84.79866	100.1873	82.02368	39.23699	69.98912	112.0593
pos_957	D-Glucosa	271.6352	457.5379	575.8251	481.9887	320.9807	412.1471	472.474
pos_958	2-Methoxy	59.76544	167.988	289.9271	163.2827	109.8333	125.173	119.9748
pos_959	2,3-Dinor-	1262.046	1328.663	1705.418	1406.055	1501.372	1599.329	1535.403
pos_965	Lisdexamfe	57.73241	210.6977	158.9449	119.2872	23.0692	77.84677	108.6119
pos_966	DG(16:0/1	39772.84	55306.89	62582.55	58474.69	55988.2	59282.69	63083.04
pos_967	cis-Goniot	241.5287	394.7855	573.7591	621.929	184.29	381.8071	400.212
pos_968	PG(a-13:0/	21795.59	25412.03	31778.7	28010.76	25854.46	25974.87	28832.72
pos_970	PA(13:0/5-	40.67459	103.8102	150.4774	122.3587	53.67592	89.50579	115.1345
pos_974	PE(P-16:0/	2130.831	2857.766	3747.404	2619.215	2023.827	2345.946	2557.782
pos_975	Lupeoside	51142.37	59443.05	75629.08	58187.18	61398.47	57317.12	62293.84
pos_976	Cer(d18:0/	24458.9	37499.88	35438.51	65430.84	32352.34	34761.12	51203.09
pos_978	PC(20:5(5Z	906008.2	286492.3	1483161	472630.2	395516.8	259714.1	335135.2
pos_979	PE-NMe(1	43438.18	55489.28	114779.8	58895.74	33806.15	43776.29	42273.02
pos_981	PE(20:5(5Z	57682.55	129513.2	134852	132942.1	72513.93	59023.3	98248.01
pos_982	PE(18:0/20	201110.7	351102.3	607371.9	406043.7	258261.4	190220.5	335525.1
pos_983	PE(22:6(4Z	28978.91	37687.82	46694.69	29258.71	31945.56	24620.63	32683.59
pos_984	PS(16:1(9Z	9591.292	18698.18	21606.98	15854.42	9338.625	6572.9	14156.49
pos_985	PC(P-16:0/	169777.4	292136	306128.7	250995.5	200469.9	145353.2	259013.4

pos_987	PC(P-16:0/	481694.6	751817.3	977929.6	769047.3	672136.2	512903.1	700068.5
pos_989	LacCer(d18	3391.433	14683.3	6097.264	8501.55	6574.885	4725.003	9222.228
pos_990	DG(20:0/LT	349117.4	365262.9	642999.5	404930.4	327041.7	337639.5	336146.2
pos_991	PC(15:0/PC	6068.724	12178.37	17806.51	12054.17	6330.57	10447.1	15172.56
pos_992	PE(20:0/PC	9345.022	11801.01	17125.41	11489.28	10024.59	10192.8	11932.37
pos_993	PG(20:1(11	2244.669	4239.328	2024.14	4401.401	1828.677	1011.423	1073.021
pos_995	PE(22:1(13	14398.46	24542.27	41784.46	24628.91	20661.84	19038.81	25982.75
pos_996	PE(22:0/20	5367.736	8611.504	8686.571	9120.448	8432.372	9033.728	9781.393
pos_997	PC(22:3(10	448.3287	470.9617	639.4928	499.3855	691.9663	775.007	810.0707
pos_999	8-Propanc	1445.819	1626.666	2176.97	1941.254	1869.125	1856.146	2025.961

L4	L_vs_H_log	L_vs_H_Pva	L_vs_H_VIF	L_vs_H_(FC	Mass Error	Formula	m/z	Retention
843.3043	-0.00324	0.988451	0.002118	unchanged	-1.0846	C3H4N4O	133.0131	0.724867
183.9624	-0.14272	0.563829	0.737357	unchanged	3.71388	C11H12O2	207.0493	0.717733
129.5745	0.10332	0.705029	0.459944	unchanged	-10.5516	C6H13NO	146.0807	0.732017
158.984	-0.27624	0.141454	1.615671	unchanged	-19.8981	C12H16O4	245.0751	0.732017
1085.873	-0.45462	0.374995	0.974018	unchanged	-3.77303	C11H21N(	278.1235	0.732017
381.2909	-0.12963	0.555399	0.760715	unchanged	8.056644	C8H9F3N4	281.0526	0.732017
116.645	-0.13385	0.524414	0.820503	unchanged	-13.4566	C9H14N2(	283.0512	0.732017
259.9035	-0.16505	0.53389	0.72303	unchanged	30.39723	C23H36N4	795.1608	0.732017
519.3593	0.048132	0.797876	0.287531	unchanged	6.945938	C6H12N2(	525.0492	0.73915
282.2159	-0.04145	0.767478	0.374271	unchanged	10.55028	C15H22N2	585.02	0.73915
507.7755	-0.01794	0.771253	0.40151	unchanged	35.11263	C9H14FN2	530.9795	0.746283
309.5363	-0.30933	0.489646	0.746729	unchanged	-7.9309	C5H6O5	145.0131	0.760567
11272.97	0.024259	0.915088	0.104375	unchanged	-7.55636	H2O4S	96.95936	0.760567
59.0174	0.40734	0.678193	0.479916	unchanged	-9.62551	C4H7NO3	116.0342	0.774833
93.14643	-0.26659	0.593661	0.606774	unchanged	-5.80405	C6H8O6	197.0057	0.7677
687.4259	0.461156	0.383922	0.956399	unchanged	-5.2936	C6H8O9S	254.9803	0.7677
142.9723	0.078964	0.715935	0.435563	unchanged	-6.07471	C6H8O7	213.0005	0.781983
94.62918	0.211858	0.197183	1.38741	unchanged	-2.82102	C5H9O8P	248.9775	0.781983
556.0569	0.045762	0.827905	0.248595	unchanged	-3.57141	C15H24N2	571.0328	0.781983
38.79247	0.116194	0.861383	0.179588	unchanged	40.06519	C10H15N4	1107.005	0.789117
3037.389	0.073734	0.432844	0.851853	unchanged	-1.47401	C10H14N4	464.9596	0.789117
274.1398	0.070918	0.77942	0.310554	unchanged	-22.0419	C5H12O11	678.9712	0.789117
1455.729	0.199375	0.531178	0.675834	unchanged	-19.4979	C22H29N1	833.0463	0.789117
36.90277	0.094355	0.86041	0.194689	unchanged	-20.0918	C16H25N3	1057.144	0.8034
503.3712	0.147086	0.24587	1.318442	unchanged	-7.93367	C5H8O5	147.0287	0.8034
201.9505	0.122592	0.476896	0.767291	unchanged	43.12999	C7H5ClO2	154.9973	0.79625
4789.249	0.129751	0.339053	0.981899	unchanged	-6.14334	C6H6O6	173.0081	0.79625
213.566	-0.18544	0.589836	0.632213	unchanged	-4.15181	C3H3N3O	303.032	0.8034
410.2532	0.053473	0.661442	0.4347	unchanged	-7.57907	C12H14O1	387.0153	0.79625
1556.894	0.132922	0.326555	1.026414	unchanged	35.50067	C9H13FN2	421.0005	0.79625
325.6579	0.057293	0.70521	0.397052	unchanged	27.40366	C16H24N2	613.0403	0.79625
4115.545	0.253426	0.292202	1.115072	unchanged	-48.1115	C24H28N2	619.037	0.79625
104.4396	0.15502	0.573923	0.606931	unchanged	-7.86547	C4H8O4	119.034	0.810533
84.30434	0.30863	0.253179	1.243902	unchanged	-2.3798	C3H6N4S	151.0057	0.810533
44.62175	-0.43396	0.273769	1.254168	unchanged	2.549209	C7H13NO	220.0653	0.810533
84.30668	0.041344	0.928347	0.005111	unchanged	6.321458	C10H13N5	320.0631	0.810533
177.0154	-0.31401	0.209302	1.400171	unchanged	14.62177	C17H21N4	491.0807	0.810533
271.0909	-0.16714	0.252275	1.279663	unchanged	-18.2854	C25H27O1	556.11	0.810533
655.2292	-0.1798	0.30475	1.145115	unchanged	-2.02332	C27H28O1	607.1292	0.810533
179.0008	-0.19765	0.611088	0.564902	unchanged	11.89392	C24H40N7	822.1439	0.810533
120.7649	-0.14264	0.697296	0.440478	unchanged	1.357162	C24H38N7	836.1145	0.810533
707.6469	0.077338	0.53538	0.615206	unchanged	-13.3612	C4H6O2	85.02835	0.810533
771.444	-0.25612	0.522183	0.687322	unchanged	-9.31811	C6H13NO	130.0861	0.817667
89.33264	0.160332	0.586386	0.599403	unchanged	-10.0665	C5H10O4	133.0493	0.817667
62.73593	0.118472	0.411212	0.92572	unchanged	7.569071	C6H8O4	143.0361	0.8248
943.0021	-0.82324	0.176657	1.441727	unchanged	-2.14339	C5H4N4O	157.0129	0.817667
26.814	-0.33354	0.356703	1.042542	unchanged	-15.791	C6H8O5	159.0274	0.8248
409.7731	0.13824	0.559286	0.667927	unchanged	-4.90682	C9H17NO	218.1023	0.817667

504.9355	0.023719	0.873675	0.198004	unchanged	-0.58486	C9H17N5(	232.1179	0.8248
700.9744	-0.047	0.840605	0.24758	unchanged	-4.33371	C8H13NO	240.048	0.8248
118.8391	-0.3974	0.338806	1.074427	unchanged	-6.81577	C11H13N(	252.0863	0.817667
673.6625	0.183883	0.573755	0.592785	unchanged	1.985786	C11H12N2	255.0546	0.817667
2445.064	-0.48226	0.143866	1.485102	unchanged	-8.88403	C11H14N4	265.0919	0.8248
264.3456	0.007958	0.95562	0.13356	unchanged	-2.20216	C13H20N2	305.0935	0.817667
220.659	0.019747	0.938886	0.131824	unchanged	-8.14217	C10H13N5	312.0928	0.817667
250.5789	-0.179	0.398986	0.913952	unchanged	-1.2993	C18H14N2	327.0747	0.817667
245.9933	-1.01079	0.208588	1.380672	unchanged	-0.73773	C15H21N(	342.1192	0.817667
308.5212	-0.32002	0.347133	0.994937	unchanged	-2.45866	C10H17N7	350.0977	0.8248
199.6865	-0.30322	0.368477	0.96689	unchanged	6.483874	C10H17N3	352.084	0.8248
340.0415	0.231637	0.213169	1.332	unchanged	11.5932	C10H12N5	364.0257	0.817667
273.7201	-0.18453	0.120542	1.585099	unchanged	-34.1059	C12H20Cl	390.0707	0.8248
612.0478	0.087395	0.587315	0.603896	unchanged	-6.42562	C18H19N3	372.1	0.817667
6649.215	-0.38633	0.299464	1.153126	unchanged	-12.3552	C19H22O9	393.1142	0.817667
56.78072	-0.49149	0.438309	0.855263	unchanged	-12.1676	C18H26O9	407.1277	0.817667
162.157	0.110039	0.513941	0.68633	unchanged	-27.2035	C10H14N5	424.023	0.8248
91.03121	-0.21642	0.364644	0.961381	unchanged	0.142278	C3H8N3O	453.0542	0.817667
249.0833	-0.1539	0.392931	0.9057	unchanged	-4.81018	C16H18N2	459.0695	0.8248
49.70343	-1.12606	0.351375	1.040988	unchanged	-7.81912	C20H36O7	495.1883	0.817667
161.8274	-0.27955	0.245468	1.351095	unchanged	2.761367	C16H26N4	519.0851	0.817667
539.3958	-0.00556	0.964166	0.06314	unchanged	33.49444	C15H25N4	528.1012	0.817667
53.61577	-0.22057	0.468028	0.839211	unchanged	-22.9338	C16H27N3	530.0825	0.817667
172.1914	-0.09747	0.642657	0.585692	unchanged	1.334812	C26H26O1	591.1363	0.817667
64.86482	-0.05235	0.881405	0.198138	unchanged	6.150543	C17H27N5	618.0893	0.817667
144.1337	-0.23916	0.407852	0.965164	unchanged	-3.35485	C7H15O1(	639.0925	0.817667
59.55925	-0.26405	0.473757	0.822611	unchanged	11.40763	C21H27N6	710.1068	0.817667
150.3922	-0.43328	0.20026	1.454285	unchanged	21.31265	C10H13N4	723.1324	0.817667
81.13684	-0.03307	0.905446	0.123715	unchanged	-49.8351	C10H14N5	739.0898	0.8248
57.70612	-0.7592	0.091196	1.752762	unchanged	7.669131	C23H38N3	784.1198	0.817667
791.106	-0.559	0.43643	0.865455	unchanged	-13.1076	C3H6O3	89.02324	0.817667
518.9386	-0.86158	0.443313	0.849531	unchanged	-5.01789	C6H12O6	201.0372	0.83195
2126.413	0.074276	0.335795	1.082385	unchanged	-0.13104	C8H10N4(	215.055	0.83195
560.6586	0.484596	0.336601	1.049403	unchanged	-11.2299	C4H8O3	103.0389	0.839083
81.38288	0.794818	0.269331	1.211003	unchanged	-3.27651	C3H9O4P	160.9981	0.839083
280.5355	-0.25545	0.562818	0.661634	unchanged	-9.3059	C2H7O4P	146.9817	0.846217
264.3342	-0.10264	0.700657	0.45006	unchanged	-14.4523	C10H15N4	552.9706	0.846217
38.01755	-0.35974	0.473283	0.81379	unchanged	0.85084	C4H4N2O	148.9792	0.8605
77.35116	-0.06863	0.875106	0.224857	unchanged	-5.47551	C7H10O6	189.0394	0.988983
270.3726	-0.93395	0.165122	1.501187	unchanged	-11.1271	C6H6O5	157.0125	1.003267
228.2898	-1.0968	0.169588	1.49106	unchanged	-7.95609	C6H8O6	175.0234	1.003267
59.07564	-0.43185	0.196594	1.332154	unchanged	-18.6728	C20H18O7	415.0965	1.03825
90.99683	-0.05536	0.804166	0.326141	unchanged	-17.2683	C7H15NO	198.0717	1.059667
191.3499	-1.06779	0.109681	1.773876	unchanged	-9.5628	C5H9NO3	130.0497	1.109633
481.244	0.2539	0.335786	1.031782	unchanged	-6.2446	C9H17NO	232.1179	1.188167
350.967	-0.31037	0.332437	1.044854	unchanged	-6.91895	C9H17NO	202.1071	1.1953
85.52694	0.113917	0.652153	0.511409	unchanged	-7.06391	C6H14O6	181.0705	1.2524
4244.054	-0.01634	0.909386	0.11378	unchanged	-7.44548	C9H11NO	164.0705	1.295233
490.9913	-0.14336	0.579311	0.624956	unchanged	-4.49776	C10H13N(	232.0582	1.295233
775.065	-0.02271	0.896063	0.14506	unchanged	6.600737	C9H8O2	147.0461	1.309517
70.14754	0.041877	0.844199	0.233497	unchanged	-8.20625	C6H14O6	181.0703	1.330933

1093.609	-0.61027	0.153335	1.463161	unchanged	-0.46607	C9H16N4(	265.0917	1.4016
384.0852	-1.04419	0.19965	1.421816	unchanged	23.69865	C11H13ClI	299.0624	1.52295
58.02497	0.248496	0.592098	0.636873	unchanged	4.167251	C9H16N4(	297.0828	1.572917
74.87615	1.092583	0.328403	1.126529	unchanged	16.26897	C10H7NO	274.001	1.594333
87.21543	-1.28257	0.381663	1.048975	unchanged	-3.84651	C10H18O9	303.0687	1.630033
110.203	0.089865	0.720852	0.438516	unchanged	-1.39922	C10H13N5	312.0946	1.637167
1954.677	-1.58068	0.309235	1.208032	unchanged	-5.72006	C8H14O5	235.0812	1.658583
116.097	0.32562	0.204603	1.460727	unchanged	-4.93751	C10H14N2	241.0818	1.68
992.4713	0.070896	0.655466	0.51252	unchanged	-9.6009	C5H10O3	117.0546	1.687133
535.8797	0.295812	0.666621	0.490223	unchanged	-4.83934	C9H13NO	214.0711	1.729267
369.0339	0.195761	0.634586	0.545049	unchanged	-0.23726	C8H15N5(	218.1023	1.729267
538.0689	0.095907	0.428523	0.880077	unchanged	-30.85	C6H14NO	310.0015	1.729267
691.41	0.024086	0.848302	0.203396	unchanged	-6.79049	C9H8O2	147.0441	1.7364
43.66802	0.128135	0.726509	0.388933	unchanged	-1.71786	C6H9N3O	222.0284	1.7364
410.4868	0.052791	0.757978	0.341312	unchanged	-3.95722	C10H13N(	232.0583	1.7364
159.9299	0.099353	0.568535	0.644487	unchanged	-2.17629	C22H18O7	429.0738	1.7364
173.072	0.441907	0.125996	1.580659	unchanged	0.537227	C2H6N4O	216.9805	1.843483
688.5989	-0.72344	0.604736	0.563185	unchanged	-3.2258	C12H22O1	347.0949	1.850617
262.7369	0.183266	0.585723	0.637525	unchanged	-0.22742	C8H15N5(	218.1023	1.936283
647.6596	-0.63609	0.237476	1.48443	unchanged	0.360913	C11H22N4	295.1389	1.98625
49.1872	0.185182	0.428895	0.907303	unchanged	3.908427	C4H6O4	139.0017	10.00723
33.71543	0.975366	0.387265	0.953376	unchanged	-4.23581	C16H27N(	639.3837	10.03578
175.634	-0.40304	0.43748	0.832964	unchanged	11.48841	C52H89N(	1170.534	10.05007
270.4431	1.10346	0.264149	1.221011	unchanged	-10.1244	C20H32O2	349.2354	10.05007
455.6195	1.083063	0.278549	1.18198	unchanged	-3.61222	C25H34O5	449.1627	10.05007
134.7846	2.511642	0.218273	1.400016	unchanged	2.165146	C23H31N(	468.1804	10.05007
529.3816	0.858039	0.313588	1.113605	unchanged	7.219799	C44H57N(	662.4263	10.05007
6624.039	0.163401	0.58006	0.649404	unchanged	4.563558	C14H10N2	381.0052	10.0572
920.9781	-0.21105	0.285552	1.184929	unchanged	-3.39409	C24H48O4	399.3466	10.07077
6809.034	0.15007	0.599621	0.618049	unchanged	7.235527	C11H6N4(	767.0358	10.07792
58.90945	0.335943	0.73436	0.364396	unchanged	-2.41834	C26H32FN	440.2232	10.1136
3186.891	0.033006	0.837371	0.225276	unchanged	-14.7782	CH5O3P	130.9656	0.539983
930.1516	-0.01007	0.968189	0.000445	unchanged	-8.21902	C47H90O1	1025.527	10.12073
192.3292	-0.79508	0.011922	2.245991	down	13.49158	C24H38O2	393.2614	10.13502
3453.358	-0.17229	0.335753	1.144667	unchanged	-1.32963	C24H46O4	397.3318	10.16357
512.228	0.246608	0.562932	0.66462	unchanged	1.181936	C20H26O7	377.161	10.21353
2086.118	-0.60609	0.059805	1.92283	unchanged	30.2805	C20H24D8	327.288	10.22067
76.0659	2.437558	0.278341	1.268618	unchanged	12.7502	C53H100N	1189.755	10.24923
68.18743	-1.37456	0.152647	1.640896	unchanged	2.676742	C13H18N2	531.2475	10.24923
787.2019	-1.87211	0.045029	2.136523	down	-9.52722	C21H42O3	341.3029	10.27778
66.70905	-0.69529	0.218462	1.372383	unchanged	2.802815	C26H46O3	427.3205	10.2992
27.63565	0.276994	0.612339	0.517551	unchanged	-3.28353	C22H34O3	345.2424	10.32775
8413.691	-0.09992	0.844874	0.184268	unchanged	6.814614	C20H32O5	351.2201	10.33488
5698.664	0.126553	0.648929	0.536731	unchanged	3.073595	C20H28N1	867.0335	10.33488
688.5147	-0.21132	0.730017	0.348566	unchanged	-0.83351	C23H32N2	419.2104	10.34202
98.3539	-0.36937	0.460906	0.903871	unchanged	-2.38275	C18H30O3	587.4303	10.36343
91.59571	-0.1702	0.520644	0.66428	unchanged	6.930618	C22H44N2	383.3306	10.38485
245.3943	-0.10046	0.929615	0.172267	unchanged	-15.3029	C27H42O4	451.2764	10.42698
401	-0.14486	0.639593	0.52633	unchanged	-1.00761	C18H33O3	593.4781	10.4484
6667.219	0.153493	0.707956	0.44045	unchanged	-2.40491	C17H28O3	311.1679	10.45553
714.2634	-0.12163	0.553384	0.742266	unchanged	-1.18492	C25H48O4	411.3475	10.48408

93.40845	0.209995	0.532172	0.639489	unchange(	-3.69876	C15H26O2	535.3986	10.5055
1788.775	-0.04917	0.887947	0.161671	unchange(	3.06155	C27H46O2	423.3257	10.53405
258.4015	0.002841	0.990035	0.030881	unchange(	0.305132	C6H14N4(	209.0811	10.57688
363.1858	0.359006	0.333631	1.14481	unchange(	0.278878	C36H60O(	587.4319	10.58402
345.0706	0.113769	0.660142	0.509746	unchange(	-2.95463	C5H4O2	116.9955	10.59115
197.2269	0.145011	0.58427	0.640995	unchange(	1.209583	C4H4N2O	146.9968	10.61257
40771.08	0.136252	0.62116	0.581932	unchange(	22.4562	C3H9O6P	216.9942	10.61257
3914.925	0.148938	0.580403	0.646105	unchange(	-5.95326	C7H8O2S	176.9982	10.60543
37704.66	0.135565	0.627825	0.572116	unchange(	-1.44226	C8H5NO2	357.0004	10.61257
6381.709	0.090703	0.737073	0.403316	unchange(	-6.98825	C10H15N5	899.0511	10.60543
22292.55	0.130332	0.637854	0.554734	unchange(	2.778771	C7H4O6	218.9925	10.6197
60398.93	0.133642	0.627599	0.572101	unchange(	36.88872	C4H9O7P	234.9976	10.6197
44821.76	0.133884	0.632275	0.564726	unchange(	-22.4159	C7H8O7	224.9971	10.6197
11743.67	0.136431	0.624814	0.577097	unchange(	0.431489	C6H8O7	226.9965	10.6197
2555.027	0.099308	0.717125	0.43336	unchange(	-0.14334	C6H13O1(	296.9993	10.6197
2543.881	0.102432	0.686833	0.477263	unchange(	-8.23634	C7H11N3(	300.0041	10.6197
8670.5	0.126348	0.651711	0.533506	unchange(	12.39563	C7H16O1:	369.0039	10.6197
3048.885	0.091515	0.748836	0.379104	unchange(	-5.09152	C11H15N4	413.0074	10.6197
330.2999	0.061428	0.904103	0.140587	unchange(	-4.85022	C23H32N2	451.1807	10.6197
379.4236	-1.2884	0.375318	0.930421	unchange(	4.066545	C30H39N3	598.2793	10.6197
614.2324	0.249053	0.636855	0.548191	unchange(	2.67192	C38H65N(	666.4523	10.6197
64.48111	0.10601	0.877727	0.122832	unchange(	15.95537	C61H102C	975.7807	10.6197
61.18138	0.115398	0.720678	0.40415	unchange(	-2.2334	C5H4O3	132.9905	10.62685
90.41114	0.154853	0.591887	0.627607	unchange(	-19.6871	C5H6O3	148.9989	10.63398
5763.67	0.12401	0.651443	0.532902	unchange(	-1.16091	C8H6O3	185.0009	10.63398
50179.54	0.132404	0.628481	0.570248	unchange(	-5.85969	C7H8O6	232.9942	10.62685
9387.701	0.137664	0.622453	0.580413	unchange(	-4.26896	C4H6O4S	194.9962	10.63398
1680.175	0.095617	0.736943	0.39847	unchange(	-14.0407	C9H7NO2	196.0148	10.62685
13201.99	0.141761	0.608181	0.602537	unchange(	-6.30285	C5H4N4O	204.9968	10.63398
14272.63	0.134904	0.628827	0.569741	unchange(	-6.47228	C3H7O6P	214.9951	10.62685
34589.93	0.130821	0.635615	0.559392	unchange(	4.811595	C5H8N4O	270.999	10.62685
18164.3	0.127517	0.644491	0.544283	unchange(	-8.74797	C11H6O4	236.9942	10.62685
26859.12	0.096748	0.72795	0.407149	unchange(	2.086736	C6H4N4O	248.9705	10.62685
1251.144	0.078229	0.771813	0.350659	unchange(	20.59779	C7H14O11	335.0008	10.63398
2682.984	0.122624	0.670823	0.506369	unchange(	-1.90752	C3H7NO5	383.0065	10.63398
3029.995	0.132198	0.650145	0.53686	unchange(	4.77075	C10H14N4	427.0082	10.62685
161.724	0.258577	0.696257	0.452013	unchange(	4.420377	C25H25N7	470.1967	10.62685
446.731	0.303929	0.590648	0.650666	unchange(	3.914971	C31H35F3	669.2791	10.62685
79.3255	0.685221	0.246152	1.279718	unchange(	-3.14203	C15H26O2	535.3989	10.66253
1587.586	0.692158	0.170909	1.535192	unchange(	-2.95017	C42H78N(	754.537	10.6554
1415.95	-0.29636	0.047002	1.941581	down	1.329077	C34H56O8	591.391	10.72678
272.5011	-0.21463	0.233124	1.411238	unchange(	16.57723	C27H46O3	439.3263	10.82602
6333.463	0.213669	0.591778	0.619141	unchange(	-1.32102	C23H28N2	353.1995	10.84028
1272.907	-0.00029	0.998999	0.064337	unchange(	-4.48927	C26H50O4	425.3617	10.88317
130.474	0.128523	0.594684	0.617771	unchange(	5.260399	C3H2N6	157.0041	10.8903
41.34697	0.533166	0.417032	0.919056	unchange(	9.038338	C37H66O8	683.4797	10.8903
141.1972	-0.51508	0.180707	1.469819	unchange(	-1.70046	C33H52O5	527.3733	10.91885
77.46808	0.180774	0.534359	0.730722	unchange(	-2.01422	C4H4N2O	148.9966	10.926
451.2715	0.121216	0.694631	0.478039	unchange(	-24.3255	C56H104C	1109.641	10.96168
9.369645	-0.50696	0.517377	0.718588	unchange(	0.212273	C27H44O	429.3375	10.99737
8096.521	-0.9397	0.031633	2.14243	down	-0.28562	C36H68O(	595.4941	11.05448

788.5108	0.113528	0.656766	0.515954	unchanged	-23.9911	C4H8O3	125.0195	11.06162
249.3951	0.136998	0.600491	0.614183	unchanged	-4.93988	C11H14O3	193.0861	11.07518
677.5739	0.360438	0.337839	1.095424	unchanged	1.344273	C20H26O6	361.1661	11.12517
6140.565	0.170746	0.608872	0.595348	unchanged	5.190101	C52H91N(	1126.537	11.14657
855.3306	-0.49968	0.167763	1.457673	unchanged	-0.56576	C36H62O6	589.447	11.14657
1142.416	0.321107	0.283096	1.218423	unchanged	9.475428	C50H85N3	1098.515	11.18227
23027.07	0.215595	0.636502	0.546682	unchanged	-2.84236	C18H30O3	325.1834	11.17513
2525.089	0.250489	0.470299	0.839342	unchanged	5.813076	C50H83N3	1104.524	11.20368
16802.59	0.146532	0.573806	0.65476	unchanged	-5.30039	C3H3NS	168.9891	11.21795
10824.2	0.144888	0.60526	0.608114	unchanged	-23.3657	C7H6O7	200.9994	11.21795
19457.39	0.139835	0.618918	0.586133	unchanged	16.54666	C10H5F3C	250.9976	11.21795
16496.09	0.124079	0.647208	0.53856	unchanged	-12.7481	C9H6O4	212.9937	11.2251
1684.917	0.21274	0.508171	0.764662	unchanged	-1.03131	C39H76N(	716.5228	11.2251
9926.137	0.168881	0.466953	0.849117	unchanged	14.88631	C49H74N1	1015.538	11.25365
1479.706	0.130445	0.623213	0.578925	unchanged	-12.9822	C2H5O5P	174.955	11.26078
825.9152	0.076529	0.786554	0.330006	unchanged	-4.10353	C7H6O4S	206.9726	11.26078
128.9943	0.197619	0.478512	0.814652	unchanged	-3.07011	C27H49O1	595.2871	11.26078
13749.95	0.24353	0.439063	0.894646	unchanged	3.526518	C47H82N(	976.5259	11.26078
2099.683	0.094058	0.725774	0.413536	unchanged	-13.3338	C30H52O7	621.3046	11.26792
4707.599	0.024502	0.954491	0.164758	unchanged	-5.86719	C29H42N8	597.2942	11.26792
84892.51	0.112826	0.707741	0.43682	unchanged	-0.22463	C47H83O1	885.5497	11.26792
4485.412	0.131276	0.622516	0.571696	unchanged	38.24615	C6H11NO	285.9997	11.27507
183.6372	0.43311	0.115073	1.619798	unchanged	-14.7149	C21H41O7	435.2453	11.27507
78.21929	0.36804	0.250146	1.263026	unchanged	-2.92337	C23H46N(	524.298	11.27507
849.7584	0.314711	0.183946	1.454234	unchanged	-1.06961	C25H49O1	571.2883	11.27507
233.1217	-0.60469	0.635256	0.514948	unchanged	15.15724	C19H18O5	347.095	2.000517
125.3757	-0.70109	0.608053	0.548786	unchanged	15.75042	C11H14N2	379.0328	2.000517
312.5243	-0.14349	0.931308	0.071274	unchanged	2.571001	C12H8N2	419.1523	2.000517
36.20633	0.276833	0.276701	1.190321	unchanged	-13.4675	C6H10O	143.07	2.0148
65.59958	0.304022	0.22153	1.284469	unchanged	-0.68186	C7H10N4(	187.06	2.007667
315.624	-0.02065	0.9603	0.038254	unchanged	-5.21312	C9H7NO5	261.9779	2.0148
1243.381	-0.16893	0.712099	0.411232	unchanged	-9.81943	C5H10O3	117.0546	2.021933
866.3033	0.020729	0.908396	0.116588	unchanged	0.554842	C12H15N5	266.1025	2.04265
65.48484	-0.06912	0.723961	0.430907	unchanged	8.350839	C14H16O7	341.0903	2.085483
386.9581	0.403901	0.768947	0.370739	unchanged	5.045447	C19H19N(	354.1185	2.1069
309.5168	-0.63453	0.32994	1.151781	unchanged	3.123726	C6H12O5S	217.0158	2.121167
1023.405	-0.16243	0.761599	0.33491	unchanged	0.033926	C7H6O6S	216.9812	2.149733
38.91491	-0.14667	0.567527	0.637983	unchanged	14.77516	C11H17N(	246.0934	2.149733
26.87591	0.032931	0.888879	0.143772	unchanged	-9.04737	C7H6O3	137.0232	2.156867
604.8061	0.123778	0.504431	0.647882	unchanged	-7.43914	C6H10O4	145.0495	2.164
549.9797	-0.40261	0.125941	1.684487	unchanged	-9.46267	C6H10O4	337.1148	2.164
416.0772	-0.3055	0.607036	0.629677	unchanged	-3.1719	C14H16N2	275.1029	2.164
1074.585	-0.38535	0.534484	0.748656	unchanged	-1.74878	C17H22N2	365.1348	2.164
54.80352	-0.39715	0.636448	0.590256	unchanged	-2.60996	C14H19N2	403.0903	2.164
935.1756	1.974229	0.433663	0.916163	unchanged	-5.51019	C9H9NO4	194.0448	2.185417
166.3031	0.007736	0.987238	0.039707	unchanged	-8.30915	C10H12N2	159.0914	2.235383
7288.978	0.02049	0.965893	0.006985	unchanged	-5.08823	C11H12N2	203.0816	2.235383
67.80421	-0.01728	0.988058	0.082003	unchanged	1.038986	C19H30O8	407.1691	2.242533
90.4985	0.869668	0.242489	1.325175	unchanged	39.15458	C21H14O8	415.059	2.242533
285.7349	-0.01198	0.99005	0.081932	unchanged	-1.72351	C17H30O1	429.1526	2.235383
106.6898	0.282681	0.638793	0.520884	unchanged	-2.53023	C15H17N7	492.0188	2.242533

83.92292	-0.48173	0.488662	0.797496	unchanged	26.94763	C18H31N3	588.1511	2.235383
183.1657	0.138699	0.57025	0.691007	unchanged	2.393168	C4H3N3O	108.0206	2.26395
58529.81	0.137283	0.569074	0.691957	unchanged	-4.75891	C6H6O5S	188.9854	2.26395
3950.655	-0.50082	0.414303	0.909297	unchanged	-18.5498	C9H19NO	272.0507	2.26395
5770.462	-0.54014	0.421271	0.895748	unchanged	7.033707	C18H17N(	362.0824	2.26395
2262.028	-1.7502	0.349514	1.050987	unchanged	4.06259	C39H32O1	723.1749	2.26395
4799.805	0.127417	0.596515	0.647165	unchanged	-10.2302	C6H6O2	109.0284	2.271083
9790.759	-0.52977	0.403445	0.934713	unchanged	-3.78616	C12H13N(	270.053	2.271083
1412.465	-0.52805	0.399818	0.936069	unchanged	15.88584	C9H9N5O	312.0628	2.271083
267.6197	0.325554	0.411819	0.937761	unchanged	-10.5617	C21H11N(	410.0064	2.271083
621.8001	-0.47262	0.408195	0.913496	unchanged	-5.10701	C6H15O9I	583.1019	2.271083
190.4327	-0.89317	0.322574	1.113753	unchanged	16.05178	C9H15N5(	342.0719	2.278217
1485.36	-0.58436	0.323166	1.103234	unchanged	12.73853	C23H18ClI	428.0723	2.278217
889.6006	0.32404	0.511073	0.736195	unchanged	3.37409	C9H10O4	181.0512	2.313917
135.5717	1.005905	0.308801	1.169504	unchanged	-15.2475	C8H10N2(	249.0074	2.306767
962.5085	0.084458	0.74066	0.387721	unchanged	-2.34411	C8H10FN3	268.0168	2.313917
113.8979	-0.62874	0.290046	1.149565	unchanged	-15.886	C9H17NO	302.0606	2.313917
186.7626	0.787963	0.070295	1.936427	unchanged	-14.4877	C13H15N3	338.0774	2.306767
42.34901	0.38145	0.276072	1.121038	unchanged	-0.61988	C17H19O9	402.0721	2.313917
148.0591	-0.30303	0.710519	0.393196	unchanged	8.296452	C11H21N(	813.0752	2.313917
30.84955	0.082182	0.854504	0.212134	unchanged	2.379539	C5H5NO3	126.02	2.32105
5159.569	0.124084	0.766706	0.347549	unchanged	-6.87319	C9H9NO2	162.0549	2.32105
201.8137	0.174205	0.503105	0.764457	unchanged	-19.9978	C7H8N4O	179.0538	2.32105
7764.548	0.116308	0.77553	0.334492	unchanged	-7.68869	C9H6O2	181.0051	2.32105
62131.33	0.124752	0.770483	0.344906	unchanged	-4.48214	C9H10ClN	198.0318	2.32105
21708.52	0.127706	0.764914	0.353746	unchanged	-21.7351	C9H9NO3	200.029	2.32105
68.23536	0.49995	0.366618	1.00957	unchanged	3.338924	C10H11N5	238.0718	2.32105
2417.439	0.066162	0.763404	0.356413	unchanged	-9.86017	C8H11N3(	266.0193	2.32105
189.252	0.208166	0.102825	1.673367	unchanged	-21.7163	C9H12N2(	305.0392	2.32105
3313.114	0.086178	0.748177	0.376982	unchanged	-6.88981	C20H16O9	421.0513	2.32105
1006.482	0.186546	0.730476	0.400834	unchanged	5.613793	C16H23N5	654.0293	2.32105
428.1746	0.389453	0.600053	0.608102	unchanged	-8.30191	C36H32O2	819.1116	2.32105
624.2567	-0.34571	0.526704	0.70406	unchanged	15.76226	C11H21N(	374.0644	2.328183
235.5042	0.125593	0.807087	0.288528	unchanged	-34.2978	C4H8NO7	485.0069	2.328183
53.95894	0.195992	0.723898	0.328914	unchanged	3.883665	C12H18N2	275.1023	2.342467
210.7314	0.149707	0.74717	0.377972	unchanged	-4.52055	C10H18N3	541.9961	2.335333
197.9906	-0.58351	0.502888	0.709891	unchanged	2.993589	C11H18O8	299.0757	2.3496
95.32384	0.362503	0.681259	0.447631	unchanged	-0.66997	C9H13NO	228.0876	2.35675
108.5039	0.24955	0.333715	1.090324	unchanged	10.07397	C5H8O2	121.0281	2.406017
179.2926	0.316447	0.317785	1.126333	unchanged	-6.94524	C10H14N2	207.1128	2.406017
1232.007	-0.33411	0.787905	0.305507	unchanged	9.935408	C11H14N4	285.0607	2.41315
290.3085	0.182582	0.547094	0.703254	unchanged	1.193318	C12H15N3	300.0582	2.420283
426.3092	-0.48518	0.534061	0.672886	unchanged	-5.83487	C10H10N2	235.0713	2.434567
224.3101	0.230388	0.788458	0.267383	unchanged	4.25061	C8H10FN(	387.1388	2.47025
6430.073	0.737334	0.346123	1.123429	unchanged	-6.01306	C6H6O4S	172.9904	2.484533
945.4637	0.009466	0.981208	0.042213	unchanged	1.799889	C15H11O5	270.0539	2.541633
347.4616	-0.00813	0.983332	0.014556	unchanged	-14.4809	C12H13N(	272.0504	2.541633
211.9718	0.228861	0.806953	0.298125	unchanged	-1.75175	C13H23N2	353.0855	2.555917
65.96343	0.275989	0.632149	0.492831	unchanged	-7.98359	C8H8O3	151.0389	2.5916
308.0354	0.350143	0.540335	0.638168	unchanged	0.22642	C8H8O6S	230.9969	2.5916
104.7172	0.381658	0.578966	0.621863	unchanged	-4.14238	C10H12O5	211.0603	2.613017

431.0653	-0.62233	0.19744	1.338766	unchanged	-4.99199	C15H15N(	358.0528	2.620167
323.6052	-0.95661	0.232485	1.253477	unchanged	-6.8103	C6H8O6	175.0236	2.6273
527.5246	-0.22203	0.587404	0.665216	unchanged	1.872203	C21H20O	437.0862	2.634433
137.2858	-0.25011	0.439352	0.936956	unchanged	-0.8799	C6H6N4O	218.9956	2.641583
756.3069	-0.22086	0.809436	0.246226	unchanged	-0.35654	C6H15N4(	269.0655	2.641583
12733.69	0.068124	0.818496	0.281466	unchanged	29.01214	C12H15N(	300.0635	2.641583
99.31307	0.119612	0.76044	0.355167	unchanged	-47.6943	C10H14N!	384.0154	2.648717
165.6876	0.027515	0.949496	0.021105	unchanged	5.719238	C10H14N2	215.0813	2.663
1206.503	0.129346	0.728533	0.397889	unchanged	1.931745	C17H32O1	417.175	2.663
101.4057	0.780793	0.197606	1.42709	unchanged	1.601954	C17H17F2	394.1226	2.670133
72.51264	4.524133	0.390331	1.007765	unchanged	-0.58076	C5H7N3S	341.0858	2.6844
239.068	0.561051	0.162254	1.582558	unchanged	7.298189	C6H11N2(	273.0066	2.69155
5009.439	0.481295	0.005245	2.464746	up	-4.96158	C8H7NO4	212.0012	2.7194
35.46079	0.03557	0.969205	0.053832	unchanged	-4.72726	C8H5NO4	255.9911	2.7194
81.57396	-0.05124	0.940073	0.050752	unchanged	-2.54714	C23H20O1	519.0767	2.7194
741.1375	-0.42347	0.234799	1.25438	unchanged	-7.04592	C9H7NO2	160.0393	2.733667
265.3482	0.398904	0.678122	0.48089	unchanged	-8.89883	C24H26N2	489.1394	2.726533
940.2944	-1.39525	0.377662	1.04685	unchanged	-2.35461	C14H15N(	308.0768	2.740817
13.32737	0.386312	0.605156	0.59849	unchanged	-11.1741	C11H15N!	316.0787	2.74795
471.0876	0.123832	0.756749	0.332975	unchanged	-5.67213	C9H15NO	230.1023	2.762233
972.0624	0.920015	0.205596	1.40182	unchanged	-1.70802	C23H22O1	503.1187	2.769367
142.7917	0.986807	0.326277	1.143242	unchanged	-6.2758	C8H6O4	165.0183	2.783633
108.8414	0.241525	0.608515	0.588788	unchanged	-1.0118	C9H17N3(	284.0684	2.783633
196.2222	-0.05015	0.933976	0.102033	unchanged	-8.51901	C23H26N4	507.1456	2.783633
43.18474	0.037103	0.902078	0.045748	unchanged	-5.14514	C7H10O3	141.055	2.790783
182.1858	-0.72786	0.310755	1.115886	unchanged	-8.73165	C8H8O3	151.0387	2.797917
86.32292	0.35412	0.185667	1.348018	unchanged	6.907399	C6H14O5	187.0599	2.8122
745.0347	-0.68233	0.222484	1.31922	unchanged	-0.69613	C8H8O6S	230.9967	2.80505
358.4951	-0.97744	0.46235	0.83747	unchanged	-14.0421	C15H22N2	361.1126	2.80505
799.4815	-0.09823	0.794713	0.271791	unchanged	-1.65245	C20H26O	455.1552	2.80505
6480.675	-29.8134	0.391002	1.037925	unchanged	10.58077	C27H36N8	587.2561	2.8122
154.6379	0.331176	0.574849	0.659129	unchanged	17.28779	C30H53N(	624.347	2.819333
101.3814	-0.10829	0.858429	0.218085	unchanged	-8.66324	C6H6O5	157.0129	2.826467
744.337	0.048403	0.767612	0.36552	unchanged	-6.83194	C9H16N3(	481.9739	2.847883
42.61484	-0.68659	0.409593	0.883073	unchanged	3.590684	C6H12N3(	256.0267	2.862167
322.3146	-0.11889	0.256304	1.263896	unchanged	0.431874	C14H12O4	320.9822	2.862167
937.274	-2.014	0.424006	0.93603	unchanged	-18.3668	C20H27N!	494.1632	2.862167
462.362	0.136311	0.799028	0.258362	unchanged	-0.26987	C6H6N4S	187.0059	2.8693
879.6522	0.602048	0.6813	0.51308	unchanged	-3.54879	C13H16N2	263.1028	2.8693
166.5458	-1.08143	0.374517	1.033555	unchanged	2.491764	C16H23N6	525.092	2.8693
144.4546	0.010773	0.959666	0.118621	unchanged	-6.65081	C20H16O7	781.1725	2.8693
149.3113	0.172332	0.636399	0.552599	unchanged	13.10444	C6H15N4(	275.056	0.5614
139.7986	-0.09718	0.79966	0.26329	unchanged	20.01493	C32H46N7	992.1668	2.8693
22490.93	-0.16463	0.488331	0.758544	unchanged	12.05758	C6H11NO	134.0601	2.883583
150583	-0.13374	0.529722	0.68406	unchanged	-8.69845	C8H7NO	178.05	2.876433
354.226	-0.14581	0.409867	0.889851	unchanged	-6.82149	C9H7NO2	160.0393	2.883583
793.079	-0.14939	0.624024	0.536038	unchanged	0.231369	C13H18N6	357.1084	2.883583
2229.425	-0.11123	0.563149	0.632183	unchanged	-33.8209	C21H16O8	441.0595	2.883583
1080.483	-0.10528	0.532841	0.664942	unchanged	-16.1646	C10H15N!	410.0206	2.883583
132.5392	-0.10344	0.442153	0.887744	unchanged	-18.6156	C10H6N2(	545.0341	2.876433
2258.615	-0.10146	0.681007	0.449484	unchanged	19.13248	C18H31N3	580.131	2.883583

3334.811	-0.21126	0.472741	0.77492	unchanged	10.47513	C17H27N3	590.0856	2.883583
1815.187	-0.15859	0.568691	0.611768	unchanged	-26.2728	C17H28N4	589.0799	2.883583
421.6334	-0.19992	0.418365	0.853341	unchanged	2.070583	C24H31Cl2	606.1306	2.883583
112.6081	0.294881	0.633314	0.573508	unchanged	-2.62697	C24H29N4	679.1044	2.883583
800.085	-0.19617	0.486811	0.748784	unchanged	-5.54482	C36H36O1	739.1839	2.883583
164.0069	-0.42579	0.434458	0.994681	unchanged	-3.13274	C13H26O6	323.1703	2.890717
162.3284	-0.17042	0.535249	0.752587	unchanged	-8.78596	C7H6O3	137.0232	2.940683
215.6872	0.042009	0.939055	0.110025	unchanged	-7.39185	C9H9NO2	162.0548	2.940683
908.0909	0.131057	0.826465	0.266293	unchanged	21.4114	C10H7NO	242.0118	2.940683
1550.276	0.851461	0.621398	0.599066	unchanged	-1.85794	C18H25NO	382.15	2.969233
901.7821	0.621836	0.517563	0.74447	unchanged	12.27019	C22H26O7	401.1655	2.969233
295.5259	0.333282	0.586444	0.641481	unchanged	-0.57943	C8H16N3O	312.06	2.976383
1126.609	0.623971	0.420964	0.924696	unchanged	-2.56305	C16H24O7	373.1496	2.983517
1048.391	0.123728	0.751191	0.361672	unchanged	-3.61094	C19H30O1	417.1751	2.976383
100.2818	0.857283	0.283451	1.202563	unchanged	10.7902	C8H14N3O	340.0583	2.99065
216.3516	0.113309	0.758035	0.355143	unchanged	-1.09275	C13H18O4	273.0897	3.0192
373.8143	-0.07666	0.900901	0.134172	unchanged	-23.449	C27H26N2	477.1483	3.0192
562.3092	0.392671	0.32919	1.103119	unchanged	-8.01237	C7H6O4	153.0181	3.02635
134.5219	-0.51664	0.038591	2.005172	down	25.07551	C15H18N2	407.0627	3.039917
5749.992	-1.35054	0.405157	0.959139	unchanged	-14.2918	C19H18N2	327.1071	3.047067
96.79855	0.022769	0.957379	0.062969	unchanged	1.900974	C6H13NO	417.1733	3.089883
179.6806	0.104544	0.783119	0.302272	unchanged	-7.36582	C9H10N2O	415.1597	3.1113
1171.32	-0.35552	0.533315	0.70191	unchanged	-28.3845	C25H28O8	477.1401	3.1113
525.5669	-0.17778	0.373778	1.012864	unchanged	-9.61522	C7H6O2	121.0283	3.132717
517.7896	0.368089	0.438311	0.837922	unchanged	-1.85504	C17H24O9	371.1341	3.125583
764.6942	0.818449	0.226802	1.390146	unchanged	11.69445	C5H10O2	123.0439	3.161267
114.491	0.294666	0.227538	1.30688	unchanged	-6.89372	C7H13NO	158.0812	3.161267
10924.93	0.625	0.260704	1.291979	unchanged	-4.8174	C7H8O5S	203.001	3.161267
129.3398	0.58093	0.474751	0.832552	unchanged	5.239259	C20H15NO	418.0565	3.168417
97.11066	0.091752	0.846705	0.25141	unchanged	-1.21851	C6H6N4S	187.0058	3.182683
12057.01	0.00069	0.999005	0.023349	unchanged	-5.35392	C10H11NO	192.0656	3.189833
768.7825	-0.04312	0.868632	0.172913	unchanged	-5.70825	C5H14NO	260.0526	3.189833
201.6461	-0.02712	0.918172	0.095192	unchanged	-0.54083	C9H11NO	322.0237	3.189833
166.0466	-0.03484	0.96008	0.041396	unchanged	-15.3058	C14H19N4	439.0572	3.189833
260.9272	0.02706	0.969031	0.02758	unchanged	0.051579	C22H26O9	455.1324	3.189833
113.7469	0.162341	0.843922	0.147304	unchanged	-6.38609	C10H12FN	457.1399	3.189833
249.6268	0.274372	0.859693	0.228049	unchanged	-5.0791	C27H31O1	632.1328	3.189833
148.3298	0.008899	0.989795	0.014197	unchanged	-2.13637	C17H28N5	461.1576	3.2041
90.06678	0.424179	0.782914	0.340164	unchanged	0.943909	C27H30O1	631.1286	3.196967
245.1406	-0.10803	0.809631	0.272152	unchanged	0.868865	C19H28O1	415.1613	3.218383
86.82201	0.527138	0.703202	0.448455	unchanged	-2.58026	C23H27NO	460.1601	3.218383
351.5799	-0.09187	0.9048	0.11803	unchanged	6.393721	C45H81O1	959.4965	3.218383
187.2368	-0.23381	0.556718	0.709274	unchanged	-6.14967	C7H12O4	159.0653	3.225517
1538.937	0.499302	0.15833	1.499331	unchanged	-6.35067	C9H17NO	186.1124	3.225517
444.6998	0.260781	0.819443	0.291958	unchanged	1.070679	C18H25N5	428.1378	3.2398
2050.559	0.442604	0.409862	0.926496	unchanged	-3.33132	C17H26O9	373.1492	3.254067
82.18593	1.107605	0.421768	0.916269	unchanged	-3.55258	C25H27N3	478.1968	3.254067
117.7325	-0.29065	0.444232	0.86567	unchanged	8.31934	C12H13N3	429.208	3.261217
105.7581	-0.15321	0.849611	0.266745	unchanged	-1.56277	C6H10N4	159.065	3.289767
51.82801	-0.41192	0.469481	0.898125	unchanged	-7.33906	C11H18NO	201.1122	3.289767
644.7979	0.446065	0.351117	0.992759	unchanged	-15.6451	C14H20O8	351.0803	3.289767

13.12288	0.770734	0.496637	0.835291	unchanged	9.922064	C5H10O2	123.0438	3.2969
65.65869	0.037799	0.942346	0.147561	unchanged	-2.42008	C9H15N3(	439.9867	3.2969
109.9871	-0.07066	0.925451	0.054319	unchanged	38.20242	C17H28N2	597.0874	3.30405
983.59	1.625934	0.504448	0.816473	unchanged	-1.15856	C12H12O2	187.0762	3.311183
243.5359	-0.01877	0.980231	0.031693	unchanged	-4.35387	C15H22N2	585.0116	3.311183
520.2861	0.090428	0.904487	0.196617	unchanged	-45.8136	C15H22N2	600.9824	3.311183
231203.7	0.04604	0.924654	0.172659	unchanged	-5.01311	C7H8O4S	187.0061	3.318317
600.8897	0.047092	0.885096	0.241073	unchanged	-17.7282	C20H19N(	420.0787	3.318317
35.01799	0.386333	0.774896	0.386958	unchanged	26.44561	C23H30O8	455.1802	3.318317
177.9966	-0.24506	0.758002	0.298511	unchanged	-23.5464	C28H30O1	625.1191	3.318317
87.00609	0.004651	0.990256	0.064681	unchanged	-7.47719	C11H21N(	653.1499	3.318317
199.4387	-0.03652	0.959861	0.001075	unchanged	-23.5452	C35H30O2	807.0841	3.318317
895.9241	-0.90684	0.462816	0.85489	unchanged	-32.0572	C6H8N2O	175.0235	3.3326
54.99801	0.423011	0.684388	0.412757	unchanged	1.922772	C8H16N2(	239.0808	3.325467
292.6838	-0.02238	0.935189	0.026928	unchanged	20.47005	C8H6O7S	266.9631	3.325467
12906.38	-0.77955	0.493412	0.792825	unchanged	-13.8054	C12H17N2	283.0814	3.3326
808.4372	-0.75171	0.504816	0.766562	unchanged	5.270944	C23H20O1	493.0777	3.3326
76.17128	-0.15014	0.835969	0.182525	unchanged	2.690331	C14H23N3	580.0601	3.325467
84.23008	-0.13334	0.370694	0.910075	unchanged	5.013936	C38H36O2	863.1485	3.339733
431.8985	-0.54346	0.242487	1.286714	unchanged	2.304581	C23H26N2	445.1545	3.36115
171.1253	0.183977	0.677942	0.467289	unchanged	-7.40093	C9H10O3	165.0545	3.381867
773.3893	0.123978	0.78413	0.294748	unchanged	-5.07849	C9H10O6S	245.0113	3.381867
186.0088	-0.33314	0.64369	0.595957	unchanged	-22.1927	C8H10N2(	217.0167	3.396133
67.23825	-0.28907	0.367151	0.991431	unchanged	-9.32159	C8H8O2	135.0439	3.410417
54.86167	-0.9281	0.198905	1.377589	unchanged	-11.0818	C7H8O2	123.0438	3.4247
116.6455	0.084389	0.815663	0.274963	unchanged	-8.67953	C8H9NO2	150.0547	3.4247
988.2102	0.330435	0.63508	0.540893	unchanged	-7.70613	C9H13NO	182.0809	3.41755
267.3987	0.158037	0.672605	0.490412	unchanged	-5.03725	C9H9NO4	194.0449	3.4247
326.9112	-0.32009	0.763671	0.327949	unchanged	4.716653	C14H24O8	341.1233	3.4247
1788.786	0.206964	0.343409	0.984276	unchanged	-5.88116	C8H14O4	173.0809	3.431833
148.8026	0.308446	0.602104	0.530452	unchanged	14.53791	C18H32O1	525.151	3.460383
434.6139	-0.66023	0.52	0.741424	unchanged	27.93749	C15H18O4	297.0972	3.467517
4.202566	3.431055	0.371884	1.043147	unchanged	-10.3985	C8H8O	119.049	3.503217
13.16341	4.818235	0.401568	0.982043	unchanged	-9.38274	C8H10O	121.0647	3.503217
791.467	2.105725	0.342045	1.113932	unchanged	-6.93784	C9H10O3	165.0546	3.503217
496.414	-0.85569	0.123194	1.767523	unchanged	-4.11558	C10H17N(	244.1182	3.496083
181.8173	4.953259	0.388575	1.010473	unchanged	-0.05884	C18H20O6	353.0956	3.503217
67.05875	0.049583	0.95854	0.02346	unchanged	0.829407	C15H11N(	473.1511	3.496083
889.2429	-0.4782	0.567309	0.654824	unchanged	-2.01875	C20H20O8	433.1132	3.517483
86.89935	0.149236	0.741546	0.393999	unchanged	-1.13109	C16H26O7	375.1657	3.531767
477.4252	-0.58784	0.365046	1.016516	unchanged	-2.56771	C7H12O4	379.1601	3.531767
295.5833	1.084237	0.591891	0.657938	unchanged	18.7245	C20H27N(	396.1651	3.531767
9125.709	0.046575	0.916333	0.114208	unchanged	-5.47597	C9H15NO	184.0969	3.54605
69.89654	0.313505	0.75392	0.36667	unchanged	4.8234	C18H25N(	372.1445	3.553183
782.6592	-0.36792	0.633614	0.542289	unchanged	-0.83489	C19H28O1	415.1606	3.553183
7885.708	1.862756	0.381695	1.024376	unchanged	-1.80704	C24H28O1	491.1553	3.560317
105.872	-0.81922	0.404124	1.088947	unchanged	4.867279	C8H13NO	240.0323	3.56745
81.75908	0.677576	0.5592	0.66725	unchanged	-6.32162	C14H22N2	271.1412	3.5746
404.6882	-0.25502	0.783491	0.336225	unchanged	-2.13068	C22H29Cl(	459.1338	3.56745
60.27974	0.556253	0.573619	0.665166	unchanged	5.235443	C4H8O5	157.0126	3.588867
1500.309	0.393881	0.252802	1.169181	unchanged	-16.2115	C19H21N3	328.1382	3.588867

78.5728	0.44769	0.50774	0.749728	unchanged	-4.36399	C10H19O1	383.0345	3.588867
39.81962	0.221345	0.73387	0.295023	unchanged	2.364152	C10H14N2	231.0756	3.596017
37.07861	0.483325	0.442255	0.866074	unchanged	0.362218	C11H9NO	254.0226	3.60315
4787.812	0.486593	0.320456	1.119243	unchanged	1.894879	C23H32O4	403.1956	3.596017
79.80451	0.482149	0.432249	0.89701	unchanged	-5.03605	C10H9NO	210.0318	3.610283
142.3813	0.2594	0.758192	0.331678	unchanged	1.310082	C15H24N4	345.1548	3.610283
374.991	-0.16518	0.53349	0.749134	unchanged	1.772788	C20H14N2	365.0704	3.610283
488.9975	-0.1831	0.587846	0.634497	unchanged	11.72123	C21H28O9	445.153	3.610283
678.3069	0.52216	0.440089	0.884695	unchanged	-0.75554	C16H24O7	373.1502	3.617433
524.4727	0.16239	0.716462	0.410996	unchanged	-14.1727	C20H34O7	431.2232	3.617433
97.95529	-0.54278	0.48754	0.782993	unchanged	4.243687	C7H11N5O	457.1569	3.624567
35.65947	0.011587	0.972794	0.046632	unchanged	-9.89213	C8H14O3	157.0855	3.63885
40.03247	0.930327	0.584956	0.648586	unchanged	-11.6853	C9H14N5O	356.0338	3.63885
1465.761	1.35821	0.426723	0.969524	unchanged	-4.40636	C11H17NO	256.1181	3.645983
1333.232	0.336046	0.53587	0.701207	unchanged	-3.36843	C19H28O9	399.1647	3.681667
142.6444	0.57559	0.283483	1.182317	unchanged	-5.04246	C4H6O2	171.0654	3.688817
665.8622	0.385859	0.487472	0.718278	unchanged	-5.00402	C11H11NO	204.0656	3.688817
802.1658	0.761688	0.214945	1.388505	unchanged	23.97839	C10H7NO	226.0171	3.69595
663.2656	0.011663	0.96841	0.02074	unchanged	-5.3926	C9H10O5S	229.0164	3.69595
197.7563	-0.36732	0.482257	0.822547	unchanged	2.55451	C11H12O4	475.162	3.69595
27.64096	-0.31605	0.571085	0.691783	unchanged	5.848177	C13H14N2	245.0946	3.716667
155.6211	1.290728	0.403696	0.958938	unchanged	0.183925	C15H16N2	317.1143	3.716667
54.58879	-0.40657	0.602236	0.542671	unchanged	34.13438	C16H20N2	389.0914	3.730933
1280.201	-0.1346	0.613256	0.637578	unchanged	-2.21859	C18H16O6	327.0867	3.738083
272.2052	0.769305	0.524964	0.7495	unchanged	3.1333	C17H26N4	419.1383	3.745217
67.89651	0.088515	0.777787	0.306399	unchanged	1.748624	C8H12N4	185.0812	3.773767
40.93059	1.29765	0.228483	1.350546	unchanged	-5.50778	C9H12N2O	359.1705	3.766633
155.2944	0.373563	0.77561	0.322294	unchanged	-13.3726	C20H26N2	363.1644	3.773767
132.4367	0.173517	0.555894	0.684398	unchanged	-5.43616	C27H41N3	554.261	3.766633
91.39587	0.606533	0.562241	0.644135	unchanged	9.389886	C12H14N2	281.0806	3.78805
80.17385	-0.2773	0.62463	0.626435	unchanged	1.288702	C17H18O5	301.1085	3.78805
793.5218	0.448292	0.489197	0.785571	unchanged	8.2661	C24H34O6	471.186	3.78805
311.204	-0.67385	0.063155	1.856244	unchanged	7.605732	C9H15NO	206.0813	3.802317
394.4842	0.391853	0.507341	0.763976	unchanged	-3.12185	C19H30O9	401.1805	3.809467
38.75597	0.354578	0.691678	0.43123	unchanged	9.122401	C23H29FO	419.1914	3.809467
68.52179	0.141854	0.709862	0.435955	unchanged	-2.06983	C21H30O9	425.1808	3.809467
513.9726	0.451652	0.556003	0.67077	unchanged	-2.13407	C10H12N2	415.1614	3.823733
47.99527	-0.08611	0.844498	0.214688	unchanged	9.233626	C6H12O	121.0644	3.830883
249.5507	0.034121	0.902338	0.158845	unchanged	-10.8239	C8H8O	165.0544	3.830883
188.5146	0.043693	0.862612	0.209215	unchanged	4.427409	C16H18O6	327.0864	3.830883
534.4192	0.894917	0.0194	2.362471	up	-5.11332	C8H8O4S	199.006	3.84515
97.77775	-0.96982	0.209365	1.417721	unchanged	-9.08745	C8H18N2O	243.0765	3.852283
93.06792	0.305246	0.435411	0.88625	unchanged	12.3147	C14H14O3	459.187	3.84515
217.248	-0.34834	0.643001	0.54936	unchanged	-0.63515	C12H17NO	445.1986	3.866567
23.75838	4.558436	0.395239	0.997273	unchanged	-3.1728	C18H20O5	315.1228	3.887983
24.86526	1.048401	0.384745	0.975155	unchanged	6.030883	C25H41O7	483.2546	3.887983
183.3315	1.35801	0.396699	0.969786	unchanged	-5.5921	C15H22O5	317.1145	3.895117
206.2628	-0.59801	0.433153	0.925506	unchanged	3.080118	C15H18O1	409.0555	3.902267
273.1057	-0.11919	0.882493	0.112429	unchanged	-5.28866	C40H64O1	803.3949	3.895117
64.91469	-0.15785	0.616288	0.562648	unchanged	-8.25062	C9H12O2	151.0752	3.923667
7408.057	-0.16436	0.598387	0.575886	unchanged	-4.10038	C9H12O5S	231.0323	3.923667

150.8038	-0.25071	0.418343	0.993011	unchanged	-10.3594	C11H20O3	245.1374	3.923667
337.7496	0.37245	0.528424	0.709026	unchanged	8.892886	C24H33FC	457.2047	3.916533
189.0499	1.22485	0.411453	0.943682	unchanged	5.18935	C9H14N4(	255.111	3.930817
142.6549	1.207673	0.427245	0.916359	unchanged	21.18682	C9H16N4(	273.1253	3.930817
49.14317	0.581354	0.537355	0.704196	unchanged	-2.00427	C14H17N(	294.0977	3.93795
148.4014	-0.25429	0.375539	1.038354	unchanged	24.56795	C9H15N3(	422.0022	3.93795
122.9593	-0.2251	0.810108	0.270321	unchanged	2.080324	C13H14O2	449.1978	3.93795
285.2911	0.60628	0.372753	1.007309	unchanged	5.962346	C16H22N2	359.1245	3.952233
168.1824	0.30808	0.580648	0.633583	unchanged	0.3069	C13H14O	417.2072	3.952233
704.8698	-0.43188	0.139331	1.646795	unchanged	18.28492	C5H11N3(	160.0757	3.959367
2570.21	-0.38903	0.184061	1.481077	unchanged	-4.7116	C11H11N(	204.0657	3.959367
37.3434	0.832818	0.335304	1.09813	unchanged	-8.80746	C10H16O2	213.1118	3.959367
122.478	-0.36117	0.186328	1.41584	unchanged	-11.4217	C9H11N5(	272.0529	3.959367
971.2909	-0.80363	0.098899	1.789676	unchanged	20.24648	C19H32O7	407.1917	3.959367
54.75474	-0.49913	0.112141	1.739669	unchanged	-4.69517	C9H11N	132.0812	3.9665
132.717	-0.15269	0.353156	1.048495	unchanged	15.178	C6H8O7	226.9993	3.973633
28.18432	0.533614	0.483231	0.771249	unchanged	-43.0275	C15H22FN	380.1085	3.9665
107.4176	1.941658	0.369792	1.067464	unchanged	-20.4797	C27H45N(	530.2789	3.9665
219.0065	0.01995	0.989958	0.037656	unchanged	-9.46069	C14H20N4	321.1542	3.980783
22.21291	-1.12665	0.410456	0.969313	unchanged	-4.76979	C16H22N4	379.143	3.980783
180.1997	0.191946	0.708453	0.485777	unchanged	-5.80447	C12H13N(	232.0968	3.99505
2501.426	0.205471	0.617442	0.628941	unchanged	11.73798	C9H15N3(	276.087	3.99505
155.0577	-1.3875	0.516319	0.743302	unchanged	-3.93523	C16H20N(	311.1128	4.0022
371.1142	-0.90477	0.075042	1.867216	unchanged	2.852992	C12H20O8	327.0861	4.009333
241.4843	0.51951	0.645204	0.567301	unchanged	-8.58271	C8H10O2	137.0596	4.016467
5385.813	0.460219	0.649246	0.559754	unchanged	-4.32566	C8H10O5S	217.0167	4.016467
3608.75	-0.57638	0.235071	1.344845	unchanged	-9.92678	C13H18N4	277.1279	4.016467
116.1359	-0.86408	0.261103	1.337096	unchanged	-2.61356	C14H20O7	345.1183	4.016467
21.4977	-0.86031	0.241455	1.365944	unchanged	16.27378	C9H11N5(	236.0828	4.03075
281.7256	-0.74045	0.080406	1.99767	unchanged	-3.42112	C12H21N(	258.1338	4.03075
1273.637	-0.28234	0.593972	0.62124	unchanged	-3.62811	C16H16O2	285.1124	4.03075
948.1213	-0.28101	0.602949	0.607676	unchanged	-2.77159	C18H18O6	329.1021	4.03075
121.814	0.859386	0.346409	1.074952	unchanged	-5.88043	C8H10N2(	359.1707	4.03075
9.47501	0.392348	0.66451	0.515806	unchanged	-9.2219	C9H12O3	167.0698	4.044317
156.6258	-0.30731	0.75602	0.324376	unchanged	-4.77906	C15H27N(	300.1802	4.044317
694.5443	-0.06751	0.767067	0.351762	unchanged	-8.99551	C8H14O	125.0961	4.051467
240.7341	-0.06289	0.790906	0.30675	unchanged	-9.47761	C8H12O	169.0858	4.051467
13685.76	-0.02823	0.884697	0.166052	unchanged	-5.0735	C9H16O4	187.0966	4.051467
12.78046	0.953184	0.454682	0.847099	unchanged	1.79353	C10H21N(	483.2576	4.051467
1046.199	-0.2008	0.179151	1.421986	unchanged	1.242106	C11H22N2	297.1226	4.0586
96.36214	0.039595	0.86823	0.200537	unchanged	-12.5764	C14H8N2(	321.0091	4.0586
231.4082	-0.08491	0.861035	0.192671	unchanged	1.669982	C19H30N2	419.166	4.0586
1840.75	-0.48339	0.244017	1.334259	unchanged	6.549079	C22H27FN	405.1775	4.0586
62.49486	-0.06005	0.802609	0.248987	unchanged	-7.77811	C7H6O2	121.0286	4.072867
925.8744	-0.03129	0.948938	0.104887	unchanged	-4.35548	C12H22O5	245.1384	4.101433
306.2237	-0.45227	0.134729	1.646174	unchanged	-3.92755	C13H10N2	239.0818	4.108567
198.4029	0.837923	0.412213	0.945006	unchanged	-9.98087	C10H16N4	255.1073	4.108567
94.33207	1.306057	0.438722	0.895526	unchanged	-3.43201	C14H22O3	273.1255	4.108567
1122.179	-0.84788	0.406425	0.919375	unchanged	-2.79073	C16H26O8	345.1545	4.108567
847.293	-0.02228	0.943233	0.125397	unchanged	-1.99409	C22H24O8	461.1445	4.108567
31.59606	0.264019	0.313946	1.021039	unchanged	-5.27454	C8H9NO2	150.0553	4.12285

82.7757	0.141712	0.889387	0.183074	unchange(	24.89525	C16H25N(	314.1598	4.1157
162.3005	0.510974	0.608466	0.647918	unchange(	-9.73606	C8H10O	121.0647	4.129983
83.07169	0.393163	0.146638	1.44532	unchange(	-29.3148	C7H9N5S	194.0449	4.129983
21598.1	0.436748	0.60654	0.650987	unchange(	-5.21876	C8H10O4S	201.0216	4.129983
171.0977	-0.22165	0.678245	0.551577	unchange(	10.09451	C5H13O8I	231.0299	4.129983
185.8631	-0.0399	0.932431	0.123791	unchange(	-38.8885	C21H30O5	383.1699	4.129983
18.28594	1.486874	0.492614	0.848731	unchange(	5.761329	C16H21N(	448.112	4.129983
10.29533	0.578136	0.575787	0.691814	unchange(	-16.854	H2O3S	80.96381	4.129983
82.43155	0.192098	0.428056	0.911334	unchange(	10.61215	C9H11N2(	327.0032	4.158533
108.7145	-0.1341	0.864124	0.222238	unchange(	-9.96552	C24H32O(	415.2085	4.158533
200.2672	1.83239	0.279985	1.265797	unchange(	2.86371	C22H38N(	543.2798	4.158533
1004.702	0.064324	0.912684	0.144368	unchange(	-0.32482	C7H13N3(	240.0424	4.17995
467.612	0.064169	0.902889	0.158449	unchange(	-2.44784	C7H15N3(	242.0398	4.17995
153.895	-0.08006	0.904655	0.125276	unchange(	1.89708	C19H34O(	427.1957	4.17995
77.91021	-0.27108	0.57176	0.59041	unchange(	-3.00122	C9H10ClN	198.0321	4.187083
413.6544	-0.01947	0.970425	0.092033	unchange(	-3.29854	C17H20O4	309.1099	4.194233
2607.925	-0.42106	0.151554	1.542369	unchange(	20.34352	C25H24N2	447.1653	4.187083
112.018	-0.48698	0.248675	1.226266	unchange(	-4.60513	C10H9NO	190.0501	4.215633
207.4044	0.602871	0.042269	2.035421	up	10.05249	C14H18O(	365.0678	4.222783
490.8544	-0.59216	0.237364	1.245648	unchange(	5.950427	C15H22N4	389.0899	4.215633
2813.77	1.422889	0.45065	0.888143	unchange(	-2.73797	C24H26O1	473.144	4.215633
31.71535	1.20484	0.242855	1.356317	unchange(	-3.79153	C5H11NO	216.0096	4.229917
203.9526	-1.6168	0.388777	0.989428	unchange(	-5.36459	C16H20N2	323.1152	4.2442
1519.038	0.562575	0.397559	0.985209	unchange(	-1.75619	C9H16O3	389.2175	4.2442
24.24146	1.197234	0.412367	0.929702	unchange(	7.773758	C27H36N4	483.2567	4.251333
185.6707	-0.077	0.929456	0.03836	unchange(	0.646837	C43H67N(	916.4792	4.251333
670.6414	0.146186	0.752798	0.465018	unchange(	-4.58754	C9H9NO4	226.0169	4.27275
73.30633	0.185005	0.550988	0.693462	unchange(	2.251683	C14H10O5	257.0461	4.27275
100.7478	0.10802	0.565164	0.669631	unchange(	-0.95277	C5H6N4O	223.0083	4.287017
528.8879	1.349581	0.336234	1.102507	unchange(	4.451259	C13H18N2	255.1125	4.287017
325.5212	0.090495	0.651782	0.527355	unchange(	-7.98814	C6H5NO3	138.0186	4.3013
164.3474	-0.74962	0.314493	1.081184	unchange(	-1.03519	C11H14N(	301.08	4.3013
105.2185	0.332027	0.595374	0.592616	unchange(	-19.0868	C12H16N4	349.0703	4.3013
893.8995	1.135895	0.380559	1.022987	unchange(	13.29464	C20H30O5	385.1834	4.322717
28.36261	1.439398	0.305892	1.155684	unchange(	-7.49228	C18H17N(	457.9931	4.315583
369.5388	-0.04831	0.933856	0.089574	unchange(	-1.29804	C12H18O4	511.2543	4.322717
40.1336	0.935658	0.407074	0.984528	unchange(	5.931668	C29H42O(	533.2788	4.315583
63.28531	0.614382	0.196201	1.404952	unchange(	-7.25144	C9H10O2	149.0597	4.351267
259.7223	-0.60532	0.14628	1.658039	unchange(	10.26645	C6H13NO	152.0706	4.351267
1261.389	-0.54397	0.122135	1.746995	unchange(	-7.4399	C8H9NO2	196.0604	4.351267
992.0471	0.539788	0.118587	1.619246	unchange(	-7.14373	C9H10O5S	229.016	4.351267
368.5412	-0.69287	0.30342	1.123914	unchange(	0.595287	C13H17N5	358.0926	4.351267
110.0989	-0.03404	0.927837	0.121638	unchange(	-6.9266	C15H22O(	297.1323	4.3584
1044.939	1.385578	0.317066	1.142452	unchange(	40.20892	C16H16O3	255.113	4.36555
165.652	-0.36291	0.541959	0.714821	unchange(	7.65829	C21H30O5	397.1815	4.36555
359.8815	0.934451	0.292429	1.180808	unchange(	-1.59322	C20H30O(	411.2019	4.36555
191.5231	-0.06734	0.924985	0.160047	unchange(	-5.72142	C5H11NO	200.0144	4.379117
126.7715	-0.57104	0.574767	0.625294	unchange(	-0.72331	C14H18N4	327.1073	4.379117
89.26776	0.21652	0.285059	1.222161	unchange(	-0.04662	C14H18O(	365.0645	4.379117
151.3637	0.501198	0.544235	0.704698	unchange(	3.914617	C23H26O7	459.1677	4.386267
140.1821	-0.06075	0.93189	0.049665	unchange(	10.33082	C26H27FN	497.2383	4.386267

424.4192	0.634025	0.551825	0.690342	unchanged	-1.84594	C27H42O1	541.2644	4.386267
76.28639	0.292386	0.531954	0.617303	unchanged	-5.20266	C14H14N2	295.0686	4.400533
98.85439	-0.20588	0.777007	0.294141	unchanged	1.171898	C19H18O8	373.0933	4.400533
37.62482	1.573986	0.282095	1.259727	unchanged	-6.81348	C12H20O4	515.2831	4.400533
578.427	-0.68912	0.277794	1.186413	unchanged	1.055624	C10H14N4	257.0813	4.407667
50.93996	1.103338	0.309923	1.173574	unchanged	8.145417	C28H40O7	533.2796	4.414817
748.7071	-0.40668	0.168252	1.399417	unchanged	4.779082	C15H20N2	329.1134	4.42195
694.5274	0.426232	0.09912	1.818549	unchanged	15.87787	C6H10O6	213.02	4.429083
290.8266	1.501189	0.371371	1.063331	unchanged	14.2328	C23H39N3	530.2788	4.429083
53.87782	1.225255	0.321864	1.157996	unchanged	4.931667	C7H10O4	179.0334	4.436233
1880.25	0.146859	0.813858	0.25946	unchanged	8.223896	C13H10N4	337.037	4.436233
148.5805	-0.51996	0.297449	1.184419	unchanged	-3.84481	C11H16N2	325.0642	4.464783
86.67338	0.619943	0.096435	1.741504	unchanged	13.07929	C16H14O6	347.0812	4.45765
205.0251	-0.43135	0.393574	0.972076	unchanged	2.444226	C18H32O5	349.2004	4.464783
650.074	0.584438	0.548959	0.672515	unchanged	-23.8716	C18H38N6	803.5644	4.464783
78.24894	0.587884	0.418787	0.969461	unchanged	0.563767	C10H12N2	383.1727	4.471917
92.5883	1.255018	0.330371	1.075647	unchanged	1.006758	C10H19N3	549.2895	4.471917
802.8981	0.123808	0.645023	0.538152	unchanged	-5.87219	C6H6O5	192.99	4.479067
1180.963	1.334337	0.349083	1.076101	unchanged	-3.22611	C15H16N2	255.1131	4.4862
757.1826	-0.60834	0.112585	1.789646	unchanged	2.022041	C11H19N4	270.1339	4.4862
27.71758	1.87442	0.338611	1.089002	unchanged	0.683711	C14H18N2	299.1015	4.493333
731.1987	0.323392	0.532641	0.719016	unchanged	-7.32753	C17H17FN	351.0894	4.493333
323.4705	1.177429	0.376593	1.080698	unchanged	6.549278	C22H37N9	586.2629	4.507617
284.5961	1.12306	0.338223	1.069754	unchanged	-13.3461	C25H49O7	527.2844	4.521883
19.40491	1.0443	0.42489	0.93068	unchanged	7.69582	C18H18O4	297.1155	4.529033
19366.1	0.481031	0.447957	0.868667	unchanged	6.815444	C20H32F2	389.2172	4.529033
28.49451	1.36674	0.402138	1.02439	unchanged	-0.32003	C25H38O9	481.2442	4.529033
342.3489	1.603514	0.347478	1.080634	unchanged	0.801617	C16H18N6	331.1291	4.5433
224.2391	3.027364	0.370466	1.050426	unchanged	-5.80025	C18H18O7	377.0679	4.536167
293.7788	-0.46562	0.428425	0.918518	unchanged	-0.82511	C19H34O8	411.1997	4.5433
29.94476	1.80111	0.332406	1.112837	unchanged	-0.67393	C13H16N2	231.1137	4.550433
96.35934	0.304096	0.409111	0.994018	unchanged	12.60186	C11H14N2	301.0715	4.550433
46.46482	-0.47823	0.750875	0.448513	unchanged	4.093218	C26H43NO	580.2375	4.550433
2418.158	0.004263	0.980828	0.069164	unchanged	-13.713	C9H10O4S	213.0198	4.564717
135.4786	0.349547	0.478666	0.825687	unchanged	-6.55	C10H18O4	223.0939	4.564717
244.908	-1.12796	0.02004	2.135129	down	8.261082	C24H26N4	407.1885	4.57185
131.0515	1.458479	0.232715	1.380971	unchanged	7.668159	C24H28N4	455.1888	4.579
18.28313	0.072318	0.943346	0.156139	unchanged	7.646038	C51H85O1	1029.563	4.614683
31.88654	2.23547	0.316231	1.193914	unchanged	8.380291	C25H37N7	530.2777	4.614683
68.12951	0.376512	0.62272	0.57041	unchanged	-7.20921	C10H9NO	210.0315	4.621817
406.8589	-0.55962	0.595663	0.6338	unchanged	4.390343	C19H32O9	425.1811	4.628967
147.8472	1.177683	0.343424	1.119076	unchanged	4.944531	C22H40N6	515.286	4.628967
479.8606	-0.5891	0.011404	2.297232	down	-0.48178	C10H18O5	253.0971	4.643233
7485.374	0.066223	0.788336	0.312968	unchanged	-5.06993	C10H18O4	201.1122	4.657517
330.8425	-0.36634	0.530292	0.792029	unchanged	-1.88691	C16H16N6	353.1362	4.66465
170.1087	-0.14086	0.898144	0.120353	unchanged	-0.41497	C19H24N4	393.1543	4.657517
63.84269	-0.35806	0.451942	0.815923	unchanged	9.035383	C6H10O3	151.0388	4.678933
102.8059	-0.0191	0.972612	0.1164	unchanged	-25.5509	C30H41NO	578.221	4.678933
644.5819	0.416974	0.777464	0.29819	unchanged	-3.35426	C18H18O6	329.102	4.72105
244.0969	0.368663	0.235088	1.33887	unchanged	-3.92996	C8H17NO	236.0955	4.7282
144.9413	1.243563	0.387309	0.988123	unchanged	-11.9795	C22H37NO	494.193	4.7282

117.9167	0.591237	0.136519	1.606357	unchanged	-0.47279	C11H22N4	309.1334	4.742467
46.59116	1.744807	0.128597	1.716848	unchanged	-0.55257	C21H32O8	457.2077	4.735333
165.1123	0.899322	0.5029	0.765159	unchanged	-13.7291	C37H39N(	582.2549	4.735333
60.22921	1.222971	0.433163	0.899987	unchanged	4.890602	C27H40O1	585.2579	4.749617
47.67567	1.042966	0.442051	0.909913	unchanged	-3.79051	C35H49N(	642.3259	4.763883
93.44106	0.033504	0.886408	0.152176	unchanged	-2.6418	C7H8O4	191.0112	4.771033
821.4194	0.098335	0.717278	0.422193	unchanged	-1.88752	C6H6O5	192.9906	4.778167
521.6537	0.431375	0.29748	1.225655	unchanged	-8.12799	C9H12O2	151.0752	4.7853
12455.24	0.406951	0.267875	1.297835	unchanged	23.1824	C6H12O7	231.0322	4.7853
553.289	1.807417	0.29004	1.248874	unchanged	9.444224	C27H43N(	530.2783	4.799583
1.72E-06	2.113136	0.337117	1.144562	unchanged	-5.50787	C30H46N(	598.2675	4.799583
729.7608	-0.27649	0.626903	0.601802	unchanged	4.446048	C20H32O9	415.1992	4.806717
1711.497	-0.16613	0.73048	0.4385	unchanged	-2.50615	C21H32O9	427.1963	4.806717
577.2735	0.579506	0.437901	0.91823	unchanged	6.103254	C31H44O6	547.2863	4.806717
191.941	0.039624	0.898685	0.181832	unchanged	-11.2204	C7H6O2	121.0281	4.828133
14461.12	0.050286	0.941147	0.129345	unchanged	7.203745	C6H13ClN	215.0373	4.828133
91.84811	0.065739	0.933141	0.137015	unchanged	-8.49781	C9H12O	135.0804	4.835267
1011.407	1.834264	0.169916	1.577488	unchanged	-2.17527	C26H43N(	512.2676	4.84955
166.9891	-0.45455	0.298647	1.170936	unchanged	5.494974	C19H32O8	409.1865	4.863833
150.592	-0.4224	0.629581	0.580265	unchanged	2.196126	C19H32O8	433.2088	4.863833
47.05696	1.932063	0.220689	1.432162	unchanged	1.833857	C31H42O5	515.2788	4.856683
1199.134	-0.57103	0.534418	0.737328	unchanged	-1.71683	C20H30O7	427.1967	4.8781
18.68852	0.998987	0.336523	1.130637	unchanged	9.187714	C7H14O	135.0802	4.899517
42.95621	0.8407	0.188498	1.514876	unchanged	36.85165	C7H8N2O	197.0624	4.899517
4877.62	0.57616	0.41544	0.979722	unchanged	-33.8073	C9H10N2(	215.0372	4.899517
3.279154	-0.66417	0.661052	0.614459	unchanged	1.324647	C25H28O5	861.3866	4.899517
66.21973	0.647706	0.314505	1.162326	unchanged	1.327046	C12H20O1	359.0755	4.9138
118.4273	0.440401	0.705183	0.445457	unchanged	-2.39799	C25H39N(	478.28	4.9138
52.6585	1.944137	0.207771	1.478793	unchanged	-2.38477	C27H43N(	498.2826	4.928067
117.1833	0.259285	0.574785	0.637069	unchanged	25.76815	C8H14N2(	255.0659	4.978033
1998.401	-0.34891	0.52774	0.749472	unchanged	-3.73106	C20H32O7	429.2116	4.978033
713.6354	0.479821	0.47351	0.788832	unchanged	20.22206	C13H17N(	483.2589	4.978033
30.74253	0.8771	0.547603	0.732591	unchanged	-0.18862	C11H19N3	527.2834	4.978033
100.2597	-0.64565	0.092248	1.773288	unchanged	-0.04749	C14H21N(	312.1452	4.99945
293.3518	0.180916	0.788163	0.298473	unchanged	2.111955	C22H34O6	415.211	4.99945
127.9349	-1.34553	0.220292	1.208119	unchanged	8.937605	C18H25N(	605.365	5.028
43.93174	0.090089	0.895224	0.152568	unchanged	6.864519	C13H25N(	296.1498	5.03515
538.8273	-0.06393	0.903092	0.116925	unchanged	-11.9174	C32H36N2	495.2594	5.03515
495.451	-0.74476	0.197131	1.433627	unchanged	-1.90902	C10H17N(	236.09	5.048717
664.0159	0.099747	0.747921	0.37982	unchanged	2.173425	C6H6O6	208.9862	5.055867
3253.352	-0.46811	0.142227	1.609542	unchanged	-2.84961	C17H18O4	285.1124	5.055867
609.1948	-0.13632	0.79224	0.306924	unchanged	4.396676	C13H16N4	337.1414	5.055867
93.91808	-0.66796	0.224033	1.354482	unchanged	4.050827	C13H20N2	353.098	5.055867
39.75283	0.219606	0.818827	0.298896	unchanged	3.209432	C21H36O9	453.212	5.055867
99.02523	-0.53557	0.394047	0.930201	unchanged	16.92308	C22H37N(	447.2228	5.077267
84.78809	0.255617	0.839316	0.250286	unchanged	-2.00879	C19H28O6	397.1861	5.09155
669.96	-0.28762	0.643788	0.555516	unchanged	1.215277	C19H30O7	415.1978	5.09155
910.3415	-0.53567	0.312336	1.220657	unchanged	-2.6548	C13H21N(	284.1497	5.105833
683.7483	1.002938	0.013319	2.194299	up	-3.24207	C18H18N2	345.1001	5.105833
130.6242	0.888977	0.34148	1.154005	unchanged	3.881745	C17H26O7	387.1674	5.105833
27.94063	2.39177	0.390964	1.025211	unchanged	-2.23002	C28H39N3	500.2675	5.112967

545.6781	0.555511	0.625066	0.581188	unchanged	6.141756	C22H34O7	409.2257	5.12725
153.2658	-0.63087	0.306395	1.268706	unchanged	-2.94711	C22H26N4	425.1818	5.12725
905.3829	0.376589	0.690308	0.472215	unchanged	15.85268	C27H43NO	480.2957	5.12725
368.1821	0.080513	0.855211	0.183719	unchanged	8.010255	C12H16O1	448.9844	0.5828
139.126	0.464127	0.611882	0.63455	unchanged	-3.57541	C9H21N3S	465.3036	5.141517
148.6964	0.54753	0.483155	0.834446	unchanged	-1.32058	C23H48NO	516.2856	5.141517
314.1161	-0.66791	0.289424	1.276296	unchanged	9.866252	C30H38O9	577.2263	5.141517
809.6654	-0.11862	0.516971	0.760482	unchanged	-35.8292	C6H13O7P	263.0011	5.14865
195.1813	0.5279	0.299776	1.110977	unchanged	0.454251	C21H36N4	507.2229	5.1558
161.6273	-1.06518	0.21104	1.456289	unchanged	1.202058	C13H24N4	305.1599	5.177217
1227.281	0.658384	0.428366	0.894895	unchanged	-11.411	C19H38N4	481.246	5.198633
487.4336	-0.0463	0.833575	0.260303	unchanged	-2.30948	C10H10O6	261.0166	5.2129
89.10692	0.799063	0.073944	1.809648	unchanged	5.321302	C33H46N8	701.3219	5.205767
1898.35	0.149591	0.430038	0.847207	unchanged	-5.07198	C11H20O4	215.1278	5.234317
1334.142	-0.36956	0.650428	0.543843	unchanged	-1.85573	C21H32O9	427.1966	5.227183
3366.642	-0.08644	0.835142	0.247902	unchanged	-3.93823	C20H32O6	413.2166	5.24145
45.85452	-0.54748	0.297846	1.151599	unchanged	5.400404	C10H12O2	185.0593	5.255733
666.3916	-0.30147	0.326948	1.125033	unchanged	2.746671	C7H14O6	229.049	5.255733
539.4881	0.265389	0.68909	0.480698	unchanged	0.409152	C27H34N4	493.2458	5.255733
347.6425	1.565515	0.328846	1.087289	unchanged	1.516555	C27H46N6	611.3419	5.291433
7994.839	0.824076	0.154727	1.503766	unchanged	-12.2794	C28H43O3	462.2854	5.319983
41.51576	0.129273	0.87085	0.221268	unchanged	-1.39168	C17H28O4	651.4105	5.33425
58.91578	0.164482	0.839491	0.21835	unchanged	-15.4154	C36H58O1	701.357	5.327117
48.16234	0.591984	0.470801	0.820701	unchanged	-4.95913	C38H69O1	777.4523	5.33425
103.3097	-0.40838	0.239239	1.283911	unchanged	-0.40516	C15H25NO	336.1427	5.3414
18.03628	-0.66128	0.695427	0.427646	unchanged	-0.2229	C17H20N4	349.1104	5.3414
505.5157	0.389477	0.675521	0.520361	unchanged	15.51181	C26H40O6	483.2588	5.348533
1.72E-06	3.367426	0.378214	1.056421	unchanged	-27.6605	C50H96O1	1029.576	5.36995
1.72E-06	3.629188	0.374036	1.066019	unchanged	14.48768	C57H85N9	1074.631	5.36995
245.7811	1.129735	0.414662	0.966032	unchanged	1.884862	C31H39N5	582.2708	5.36995
8.226139	1.506821	0.397527	1.005533	unchanged	-47.7272	C35H34Mg	659.2068	5.36995
1.72E-06	2.222044	0.377039	1.059355	unchanged	-9.62818	C53H86O2	1119.538	5.383517
1.031465	0.579909	0.643897	0.566008	unchanged	-5.34379	C11H16FN	573.2462	5.383517
5.178735	0.892176	0.482018	0.831956	unchanged	-15.9193	C21H41N5	574.2411	5.383517
24.4857	1.426921	0.155244	1.523386	unchanged	-23.6342	C24H48NO	528.2746	5.390667
50.04909	2.183459	0.264885	1.351416	unchanged	27.14593	C20H25NO	713.3985	5.404933
201.8327	3.032812	0.348272	1.100393	unchanged	12.49947	C31H48O6	537.3262	5.42635
561.4199	0.105421	0.887577	0.117184	unchanged	-4.23682	C20H32O7	429.2114	5.440633
2427.357	-0.06211	0.872752	0.212208	unchanged	-4.4006	C14H23NO	298.1649	5.4549
485.1062	-0.30427	0.565971	0.716872	unchanged	-0.23177	C24H30N2	415.2002	5.48345
310.1485	-1.41661	0.271202	1.147064	unchanged	12.46587	C27H43FO	469.2944	5.476317
471.5355	0.361335	0.559376	0.719953	unchanged	-7.40436	C23H38O4	423.2724	5.4906
125.5937	-0.12279	0.845248	0.25212	unchanged	1.489467	C18H24O5	365.1611	5.504867
159.5965	0.398836	0.307763	1.04605	unchanged	4.259494	C19H34O8	411.2017	5.504867
119.4047	-0.43059	0.478969	0.834766	unchanged	15.75487	C10H12N2	397.1937	5.51915
339.6196	-0.23641	0.571401	0.58928	unchanged	3.743937	C13H12O	367.1717	5.533433
49.96694	0.850924	0.460467	0.86749	unchanged	-29.8453	C25H43N3	480.2935	5.526283
87.74465	1.757444	0.327668	1.141176	unchanged	9.46547	C28H44N4	519.3153	5.526283
810.0057	-1.84917	0.30189	1.104874	unchanged	-4.09006	C26H44N2	467.3028	5.540567
833.1941	-0.36545	0.366979	1.009573	unchanged	3.248564	C21H36N4	407.2677	5.554833
128.4055	-0.9005	0.239577	1.313371	unchanged	0.342362	C18H20O4	299.129	5.561983

542.0627	-0.06596	0.61926	0.603362	unchanged	-1.28921	C20H32O6	403.1888	5.561983
98.86771	0.112072	0.662794	0.512477	unchanged	0.950157	C3H9O4PS	170.9888	5.569117
115.5189	0.465179	0.502577	0.782397	unchanged	-3.32586	C28H39N	424.2764	5.569117
2251.864	0.124315	0.851518	0.170834	unchanged	-20.9723	C22H40N8	511.2891	5.569117
166.5197	-0.39843	0.328682	1.121756	unchanged	-5.45296	C9H10O4	203.0316	5.57625
1087.304	-0.50803	0.303771	1.161307	unchanged	-1.12469	C21H30O8	455.1918	5.5834
1752.919	0.335299	0.622444	0.566896	unchanged	-3.83025	C14H20O5	267.1228	5.590533
34.06195	-0.12802	0.861469	0.145844	unchanged	2.363358	C21H28O5	381.1692	5.590533
768.7568	1.05939	0.315656	1.165265	unchanged	-0.31016	C26H44O9	499.2911	5.604817
42.97198	-0.20958	0.783704	0.398928	unchanged	-1.9984	C8H14O3	315.1807	5.626217
418.4467	0.374201	0.019824	2.211479	up	-3.25367	C18H34O5	329.2323	5.626217
62.1387	0.033583	0.969287	0.023749	unchanged	1.849151	C16H24O	267.1525	5.6405
93.31618	0.22061	0.748429	0.379541	unchanged	-4.1764	C20H32O4	335.2214	5.647633
126.9097	1.081688	0.439776	0.867892	unchanged	12.49288	C51H81N2	1013.507	5.661917
3643.3	-0.06961	0.870518	0.102163	unchanged	-3.30598	C24H40O6	423.2738	5.661917
28037.02	0.506229	0.464498	0.799061	unchanged	11.7054	C25H40N2	495.2592	5.661917
1948.189	0.524538	0.480256	0.769151	unchanged	-0.39902	C28H38O9	563.2464	5.661917
254.9785	0.258502	0.667678	0.514105	unchanged	-11.1059	C31H38N2	585.2312	5.661917
350.3552	1.110731	0.361353	1.101604	unchanged	-32.0607	C22H45O9	483.2573	5.66905
4148.525	0.873979	0.339765	1.120827	unchanged	-3.74498	C16H32O4	287.2217	5.718317
217.5635	0.910886	0.355226	1.087065	unchanged	-11.8999	C18H30O4	355.2089	5.718317
1297.424	-0.14	0.665929	0.544492	unchanged	-2.85713	C22H36O6	417.2125	5.718317
139.3225	1.431084	0.079174	1.862905	unchanged	13.39499	C33H54O8	613.359	5.718317
381.5257	-0.52136	0.378898	0.948389	unchanged	-1.683	C16H28O7	331.1757	5.72545
27.77581	1.332658	0.266963	1.261428	unchanged	-7.92034	C18H30O4	345.1814	5.72545
1082.699	0.042764	0.928391	0.155086	unchanged	29.99078	C21H30O4	381.1942	5.754017
4851.804	0.123215	0.841862	0.27396	unchanged	-2.44457	C23H36O4	421.2586	5.754017
182.3839	0.191038	0.798268	0.332323	unchanged	4.653453	C25H40O8	489.2492	5.754017
82.63581	0.653667	0.252825	1.356895	unchanged	23.63151	C18H37N5	528.2637	5.754017
191.5707	0.957864	0.401434	1.006843	unchanged	9.666717	C29H38FN	530.2639	5.754017
228.0449	0.177065	0.762004	0.391085	unchanged	-1.4413	C30H48O1	583.3115	5.754017
551.3929	-0.19901	0.510004	0.715312	unchanged	23.85921	C21H36O5	435.2073	5.782567
548.232	0.405002	0.659994	0.495414	unchanged	-9.24575	C31H42N2	511.2897	5.782567
24.04673	1.371111	0.427025	0.891904	unchanged	-1.51043	C36H58O1	649.3947	5.775417
217.7322	0.036393	0.874518	0.148952	unchanged	-4.47104	C12H20O3	211.133	5.7897
10740.15	0.11607	0.551565	0.684029	unchanged	-3.90492	C12H22O4	229.1436	5.7897
7560.378	-0.79597	0.099123	1.669442	unchanged	5.724267	C19H30O4	367.2145	5.7897
127.413	0.341049	0.450462	0.896008	unchanged	8.222295	C10H18O4	403.2371	5.803983
49.91139	1.640658	0.41224	0.999856	unchanged	-3.81379	C37H53NO	684.3729	5.803983
113.1432	0.325968	0.684472	0.519718	unchanged	-3.9999	C25H39NO	446.2903	5.832533
194.2301	0.350044	0.510394	0.782711	unchanged	24.80331	C26H45NO	562.2654	5.832533
27.98948	0.183465	0.718653	0.466437	unchanged	8.946224	C26H45NO	614.2282	5.832533
24.76698	0.243322	0.850123	0.259996	unchanged	-1.06236	C44H80NO	832.5256	5.8254
1490.951	0.474031	0.599924	0.632808	unchanged	-2.07214	C50H91O1	951.5925	5.832533
7.94617	1.696494	0.304566	1.221719	unchanged	-18.6181	C27H44O7	959.5922	5.832533
42.51294	0.366281	0.671585	0.532528	unchanged	-7.7587	C53H89N2	1013.559	5.839667
18.80607	-1.66294	0.334277	1.023521	unchanged	-23.0291	C28H45NO	506.2829	5.839667
401.7909	0.193444	0.700491	0.517829	unchanged	-10.1298	C31H44O7	563.2256	5.839667
10.82621	-0.21617	0.74522	0.373781	unchanged	-28.9406	C18H30N4	371.1963	5.861083
702.6227	0.412454	0.679687	0.535726	unchanged	-1.801	C27H45NO	478.3165	5.861083
346.5498	1.521909	0.332564	1.048404	unchanged	-25.5725	C51H85O1	1005.507	5.868233

2854.592	2.266459	0.229166	1.338818	unchanged	-5.93952	C51H80O1	1041.469	5.868233
95.54983	2.252661	0.165353	1.528605	unchanged	3.562969	C49H70N1	1057.445	5.868233
142.3548	2.884192	0.200075	1.443721	unchanged	4.358334	C44H61N1	1072.403	5.868233
365.5542	0.967528	0.203431	1.357395	unchanged	-11.4878	C6H8O6	175.0228	5.868233
8887.082	1.067803	0.195364	1.387669	unchanged	-2.79725	C20H30O4	333.2062	5.868233
132622.1	1.284433	0.208231	1.364003	unchanged	-26.2362	C23H43O1	509.2387	5.868233
244.2335	1.390676	0.204379	1.427569	unchanged	9.42731	C20H37N3	572.2358	5.868233
4232.282	1.254631	0.244031	1.29795	unchanged	-25.45	C30H32F6	577.226	5.868233
1621.216	1.001464	0.162133	1.53791	unchanged	-15.2476	C35H30Mg	609.1901	5.868233
259.032	1.502627	0.207734	1.365624	unchanged	-5.43659	C33H40N8	689.3018	5.868233
53.33634	1.213747	0.125942	1.56594	unchanged	-12.0641	C5H6O3	113.023	5.875367
31.04694	0.098774	0.905845	0.115935	unchanged	3.143361	C18H29N3	412.2101	5.875367
815.4463	0.068583	0.828518	0.229046	unchanged	-2.68571	C20H32O6	413.2171	5.8825
130.6509	1.707679	0.246149	1.313559	unchanged	15.99617	C29H40N6	621.2573	5.875367
5.457883	-0.17412	0.897688	0.197684	unchanged	-18.7127	C22H35NO	382.2296	5.889633
1289.343	-0.45867	0.366317	1.012877	unchanged	2.533814	C20H38N4	493.2528	5.896783
864.9097	0.137481	0.816172	0.293807	unchanged	-5.69803	C10H20O3	421.2792	5.932467
2298.586	0.461823	0.325209	1.162402	unchanged	-2.82704	C19H38O3	359.2794	5.932467
101.6464	0.536021	0.642519	0.521007	unchanged	-1.47569	C35H58O8	651.4105	5.932467
38.36614	-0.43562	0.768137	0.418653	unchanged	-8.88681	C17H22N4	595.3461	5.961017
125.3497	0.338762	0.665829	0.464398	unchanged	-4.85633	C35H58O1	637.3926	5.9753
391.4343	0.290388	0.287835	1.120289	unchanged	-6.00589	C10H18O3	185.1172	6.00385
3005.856	0.016408	0.973251	0.075802	unchanged	-2.8583	C23H38O5	393.2635	5.996717
374.4034	0.858293	0.112229	1.62843	unchanged	8.015334	C26H35NO	454.2632	6.018133
1033.181	0.40811	0.537692	0.744695	unchanged	-4.82294	C16H30O4	285.2058	6.0324
480.1997	0.347177	0.393635	0.983239	unchanged	-4.05277	C15H22O3	285.1253	6.03955
38.58361	2.814978	0.175387	1.498263	unchanged	-49.2088	C21H34O5	731.4379	6.053117
21.57847	2.568265	0.32409	1.11229	unchanged	-7.32756	C12H18O2	387.2512	6.06025
162.4137	1.420969	0.352555	1.043073	unchanged	-1.80428	C27H41NO	490.2624	6.06025
299.0129	1.289115	0.3052	1.152687	unchanged	-39.368	C25H40N2	541.2394	6.06025
420.5524	1.484636	0.300118	1.164162	unchanged	-47.9945	C19H21N	585.3234	6.06025
2185.513	1.954469	0.392539	0.959199	unchanged	-29.6349	C23H38O5	833.5187	6.06025
420.8068	2.26191	0.372529	1.004031	unchanged	-2.70396	C46H69NO	864.4493	6.06025
294.0847	2.456082	0.358334	1.036185	unchanged	-2.95727	C44H78O1	913.4581	6.06025
1115.177	0.132244	0.672888	0.508245	unchanged	-4.95264	C12H14O3	251.0915	6.074533
450.7755	1.224347	0.16844	1.51449	unchanged	11.72384	C37H60O7	651.4105	6.0674
3023.63	0.397218	0.248876	1.318505	unchanged	-5.03836	C12H16O3	207.1016	6.088817
267.0777	0.173979	0.710602	0.431802	unchanged	47.9279	C41H74N7	1060.451	0.58995
155.1372	-0.14011	0.727899	0.371553	unchanged	1.676443	C17H35O7	417.1821	6.09595
146.7448	0.005989	0.970352	0.049657	unchanged	-17.4975	C29H44O1	1195.571	6.1245
297.7167	0.203495	0.75254	0.357291	unchanged	-3.77056	C14H22O2	267.1593	6.1245
691.124	-0.06633	0.915429	0.07174	unchanged	-3.46123	C10H20O3	375.2739	6.1245
28.52761	0.289082	0.868933	0.251869	unchanged	-1.10176	C27H44O7	525.3064	6.1245
28.36039	-0.2523	0.792232	0.30285	unchanged	9.835725	C19H24O2	627.3747	6.131633
68.15067	1.080013	0.415196	0.999482	unchanged	2.406397	C16H20N2	479.3192	6.138783
4109.961	1.625513	0.366037	1.083522	unchanged	-1.71553	C26H45NO	498.2886	6.138783
1.72E-06	2.583694	0.341499	1.139676	unchanged	-20.7919	C26H35N5	558.2463	6.138783
1.72E-06	2.299362	0.385679	1.026174	unchanged	21.12644	C52H87O1	997.587	6.138783
2806.431	0.206871	0.607565	0.606937	unchanged	7.70278	C9H16O3	193.0859	6.145917
758.2472	0.298266	0.779768	0.393033	unchanged	-2.32093	C15H25NO	312.181	6.210167
433.6722	0.186716	0.571147	0.665465	unchanged	-0.297	C9H6N2S	208.9945	6.238717

116.1604	-0.04371	0.844411	0.181674	unchanged	-21.7039	C45H80N7	1136.405	0.58995
8519.649	0.254411	0.623555	0.545473	unchanged	-1.59243	C27H44O8	495.2956	6.288683
1017.218	0.160276	0.701445	0.413929	unchanged	-3.93371	C13H24O4	243.1592	6.317233
1486.688	-0.28442	0.653997	0.500215	unchanged	-0.48251	C20H32O4	381.2281	6.317233
1108.972	0.684299	0.322244	1.151997	unchanged	-15.7795	C27H42O5	467.2708	6.317233
1175.93	0.587898	0.414939	0.913439	unchanged	-2.13705	C12H22O4	519.3165	6.331517
1403.562	0.176158	0.633267	0.560033	unchanged	6.982428	C12H20O4	249.1124	6.33865
117.1618	-0.38734	0.570518	0.600553	unchanged	4.36036	C22H36N2	423.2519	6.33865
398.3413	0.160764	0.541493	0.701196	unchanged	5.303815	C11H8O3	223.0177	6.345783
293.2518	0.305244	0.742301	0.394933	unchanged	-6.42288	C28H44N2	477.3069	6.360067
10.809	0.948073	0.41743	0.957464	unchanged	24.66514	C33H36N4	583.2706	6.360067
13.20777	0.718011	0.549355	0.719522	unchanged	-8.06388	C23H45N5	634.266	6.360067
1.72E-06	2.518279	0.397555	1.015314	unchanged	26.06068	C51H87N2	997.5854	6.360067
251.1285	0.040914	0.944917	0.002069	unchanged	-5.26123	C34H54O8	635.377	6.3672
1468.332	-0.56819	0.44505	0.837121	unchanged	-2.99636	C19H38O4	375.2742	6.39505
353.5617	0.681643	0.540123	0.700439	unchanged	-2.74716	C27H48O8	531.2983	6.39505
1.72E-06	-2.23496	0.476336	0.936088	unchanged	-17.9657	C24H48N(	560.2666	6.409333
257.9553	1.213221	0.36711	1.069388	unchanged	-1.27199	C25H34N4	467.2658	6.423617
1311.149	1.331734	0.356108	1.051462	unchanged	2.146549	C31H38O1	631.2409	6.416467
639.751	0.091583	0.712499	0.405302	unchanged	-6.23793	C8H16N2(	225.0898	0.58995
634.4181	0.64907	0.518163	0.761166	unchanged	-4.17101	C20H38O5	357.2632	6.445033
63.8833	-0.00319	0.9771	0.034965	unchanged	-8.38586	C8H14N2(	223.0683	0.58995
266.9223	0.254796	0.585112	0.684129	unchanged	-4.23556	C7H12O	269.1749	6.473583
6379.577	0.123322	0.847294	0.168966	unchanged	4.830571	C20H32O3	365.2349	6.48785
392.6478	0.120474	0.856667	0.262917	unchanged	2.789987	C13H20O3	493.2819	6.495
103.1277	0.171513	0.646238	0.534226	unchanged	6.724743	C5H13N2(	227.0454	6.509267
327.231	1.071782	0.146792	1.533138	unchanged	-5.98625	C10H20O3	187.1328	6.52355
1145.188	-0.43102	0.389293	1.010668	unchanged	-2.4024	C26H52N(	582.34	6.516417
126.0493	-0.13501	0.812211	0.244999	unchanged	-4.48048	C10H18N2	427.2543	6.530683
11953.46	0.014621	0.870463	0.180297	unchanged	-2.97296	C10H16N2	313.0645	0.58995
999.9892	0.453493	0.448933	0.934725	unchanged	3.877991	C20H32O4	381.2296	6.544967
71.26438	0.235524	0.791637	0.243662	unchanged	-6.14471	C36H56O1	647.3761	6.559233
108.5913	0.030114	0.972371	0.044897	unchanged	-1.39302	C13H23N(	240.1602	6.566383
207.7362	1.041493	0.281288	1.213507	unchanged	9.098519	C20H41N7	512.2681	6.566383
2.67112	3.777962	0.0728	1.991839	unchanged	19.55726	C16H16O4	293.0848	6.5878
1903.121	-0.01967	0.903459	0.140712	unchanged	-22.0822	C18H12O5	329.0363	0.58995
376.7226	0.501429	0.193578	1.400226	unchanged	-1.76695	C20H36O6	371.2433	6.5878
207.3597	0.181399	0.410989	0.935675	unchanged	7.330743	C29H44O8	519.3002	6.594933
1114.757	0.109849	0.774004	0.365864	unchanged	6.164536	C31H48O4	505.3329	6.602067
1135.861	0.758556	0.128971	1.591971	unchanged	-2.14902	C17H24O3	597.3421	6.609217
12.92169	1.332467	0.406179	0.968417	unchanged	25.29814	C33H58O1	659.4167	6.609217
393.7421	0.041615	0.914608	0.149675	unchanged	3.136186	C47H81O1	977.467	6.609217
672145.9	0.273421	0.645055	0.582729	unchanged	-1.84833	C24H40O5	407.2795	6.61635
219.7911	0.05453	0.888069	0.201282	unchanged	-12.4489	C13H18N2	435.2711	6.61635
452.0526	0.066533	0.862152	0.250767	unchanged	3.274024	C12H21N3	569.3321	6.623483
28783.75	0.145406	0.78941	0.360165	unchanged	14.89714	C43H81N2	837.5497	6.61635
687.1878	-0.00267	0.993698	0.031152	unchanged	-13.0343	C45H83O1	899.5153	6.61635
159.6065	0.149305	0.707017	0.476905	unchanged	-7.0197	C44H84O1	913.5148	6.61635
926.9747	0.039658	0.904988	0.183523	unchanged	1.413643	C44H82O1	917.4939	6.61635
5863.077	0.365149	0.303113	1.098399	unchanged	-3.50383	C14H26O4	257.1749	6.630617
1831.02	0.314856	0.325418	1.08318	unchanged	-14.9311	C27H42O7	523.2841	6.630617

1083.22	0.208196	0.512182	0.691944	unchanged	7.562886	C45H62O4	665.4626	6.630617
37.81182	0.541698	0.550672	0.618565	unchanged	20.09184	C39H58O1	721.3862	6.630617
73.76132	0.300283	0.769914	0.361336	unchanged	-1.15302	C10H10N2	347.1509	6.637767
39.57597	0.398813	0.665541	0.46915	unchanged	12.48398	C37H62O1	703.4124	6.637767
83.42699	0.014563	0.972473	0.016585	unchanged	13.25433	C42H60N2	709.4289	6.637767
3289.956	-1.05427	0.024187	2.224618	down	-3.01809	C20H30O4	333.2061	6.652033
369.6631	0.235086	0.609112	0.626389	unchanged	-3.53089	C15H26O4	269.1749	6.659167
1317.808	-0.05366	0.91913	0.091961	unchanged	4.527292	C21H32O6	379.2143	6.659167
4.362162	-1.47774	0.250182	1.304615	unchanged	3.858505	C51H91N3	1116.556	6.680583
203.4114	0.129019	0.764992	0.320637	unchanged	-0.46546	C22H34O4	397.2149	6.680583
165.3279	0.629193	0.227879	1.319702	unchanged	-5.14477	C27H46O4	479.3356	6.680583
194.0212	0.515381	0.423739	0.955321	unchanged	-13.607	C31H44O6	533.2874	6.687733
1958.722	0.368268	0.545639	0.734197	unchanged	14.96788	C23H48N(	516.2934	6.687733
63.74062	1.365418	0.009516	2.24037	up	-14.9008	C15H23N(	621.3309	6.694867
18.74196	1.133067	0.464071	0.876957	unchanged	21.94175	C49H78N6	987.587	6.687733
252.4649	0.189885	0.797395	0.340188	unchanged	0.446345	C21H26N4	411.2039	6.702
106.4373	0.805589	0.627736	0.608643	unchanged	-24.1943	C45H71O1	855.4228	6.722717
128.0407	-0.18613	0.434765	0.923228	unchanged	9.18019	C20H18O8	431.1019	6.72985
49476.58	-0.00868	0.962611	0.067437	unchanged	9.184148	C22H24O1	447.1338	6.72985
1206.03	-0.09772	0.763146	0.364407	unchanged	2.517966	C23H28N2	479.1602	6.72985
1650.793	-0.02905	0.889938	0.158169	unchanged	5.372847	C23H26O1	515.1197	6.72985
76.51132	-1.60081	0.150785	1.652352	unchanged	2.887867	C43H51N3	784.3474	6.72985
219.6303	0.141992	0.436894	0.805518	unchanged	4.03376	C19H30O2	311.2004	6.744133
609.1915	-0.25308	0.179671	1.524547	unchanged	-0.43847	C20H30O5	395.2074	6.751267
299.4361	0.305229	0.298489	1.149677	unchanged	-10.5265	C9H19NO	192.1144	6.7584
3005.263	0.335004	0.249911	1.240385	unchanged	1.381502	C12H19N3	220.1458	6.7584
566.6402	0.343538	0.220288	1.309758	unchanged	-3.94325	C14H20O3	235.133	6.7584
6596.84	0.337158	0.25238	1.244349	unchanged	2.362635	C10H13N3	236.1045	6.7584
15537.67	0.338638	0.271811	1.211381	unchanged	13.83378	C5H11NO	293.175	6.7584
1884.327	0.389914	0.229672	1.331598	unchanged	-4.10196	C19H24O4	361.1627	6.7584
855.5514	0.354372	0.24762	1.221681	unchanged	-3.70992	C18H32O5	327.2165	6.7584
1395.611	-0.13908	0.495017	0.81097	unchanged	1.204616	C23H33N(	376.2262	6.7584
144.0527	0.679044	0.200833	1.331085	unchanged	13.72679	C20H20O7	393.1007	6.7584
398.7425	0.421048	0.068709	1.852955	unchanged	13.06224	C18H34O6	367.2147	6.7941
84.32975	0.253068	0.540403	0.688586	unchanged	0.606557	C15H29N3	314.2087	6.801233
335.2743	-0.14625	0.661611	0.511655	unchanged	49.11691	C13H19N5	536.0454	0.58995
269.9844	0.464266	0.474555	0.823354	unchanged	-5.83012	C13H17NS	240.0816	6.836933
885.8009	-0.4202	0.316195	1.137727	unchanged	11.1552	C31H42O3	461.3113	6.844067
4340.398	0.160315	0.723062	0.447711	unchanged	-1.3043	C27H45N(	516.2936	6.851217
745.8214	-0.05074	0.617074	0.579752	unchanged	1.873867	C10F20	544.9672	0.58995
993.1554	0.395722	0.666566	0.542388	unchanged	-27.1967	C25H43N(	919.6027	6.851217
22.97616	0.203434	0.842251	0.285182	unchanged	17.84245	C47H85O1	981.5724	6.851217
45.84074	0.400914	0.707324	0.482901	unchanged	-8.85406	C49H89O1	999.5497	6.851217
110.8082	0.213639	0.708909	0.379361	unchanged	-26.4269	C35H58O1	715.3733	6.858367
658.1147	-0.89596	0.032818	2.191466	down	2.274204	C16H30O3	315.2183	6.901183
575.7011	-0.05873	0.861874	0.226539	unchanged	-13.0118	C18H32O4	311.2187	6.9226
265.8751	-0.13522	0.347061	1.032329	unchanged	1.39822	C9H19N3(	525.2896	6.9226
216.2446	-0.02879	0.927015	0.184996	unchanged	-4.0168	C14H26O3	241.1799	6.92975
832.915	-0.62676	0.082995	1.885295	unchanged	-5.09999	C17H32O3	283.2264	6.944017
7339.517	-0.56594	0.074376	1.898256	unchanged	-2.84163	C18H32O5	327.2168	6.944017
287.6473	-0.04003	0.797905	0.261597	unchanged	-7.48687	C16H24N2	581.0305	0.58995

110.0474	-0.35223	0.423817	0.951763	unchanged	-8.30017	C22H45N1	580.3255	6.9583
254.4505	0.980276	0.275982	1.253289	unchanged	6.108676	C21H32O4	347.2249	6.972567
184.742	1.831555	0.400515	0.98294	unchanged	-3.34368	C15H28O4	271.1906	7.001117
452.1826	-0.23391	0.802982	0.289246	unchanged	-6.83055	C25H32N2	429.2132	7.001117
33.54259	-0.01542	0.961183	0.086992	unchanged	-5.6213	C18H29NO	368.206	7.008267
149.144	-1.48027	0.34947	1.168481	unchanged	24.42674	C33H49NO	632.3584	7.029683
80.35038	-0.72239	0.225748	1.371377	unchanged	-4.36485	C23H34N2	959.4831	7.029683
246.9917	-0.51035	0.305092	1.037497	unchanged	-2.50626	C23H39NO	392.2796	7.036817
117.0264	-0.5512	0.326298	1.130237	unchanged	-5.7857	C11H14O3	193.0859	7.057533
1818.227	-0.73771	0.160265	1.61211	unchanged	-7.92594	C9H18O2	315.2516	7.064667
1399.15	-0.4711	0.216437	1.365462	unchanged	3.627807	C31H41NO	510.2798	7.057533
364.167	-0.43177	0.20993	1.378228	unchanged	-15.1256	C28H46O1	577.2703	7.057533
259.1812	1.310213	0.270214	1.236212	unchanged	2.625424	C23H36O2	389.2706	7.078933
2830.455	-0.10199	0.780621	0.25993	unchanged	-11.0285	C33H46N6	721.2894	7.078933
100122.7	-0.08579	0.7763	0.266997	unchanged	10.74364	C34H48O1	653.3011	7.086083
403.3019	-1.80774	0.453682	0.899856	unchanged	12.83592	C24H42O1	1039.582	7.114633
295.7947	-1.4056	0.488629	0.825796	unchanged	-7.56681	C53H85NO	1100.557	7.114633
276.8733	0.237821	0.529146	0.752914	unchanged	16.87866	C23H32O4	417.2345	7.1075
472.6149	-0.04989	0.934523	0.124406	unchanged	4.798216	C24H31FO	469.182	7.121767
126.4054	0.154846	0.70874	0.394369	unchanged	-3.95024	C29H42O5	515.2996	7.121767
198.0546	-1.39197	0.397736	0.900015	unchanged	-20.3495	C37H36N4	583.2596	7.121767
5899.096	-0.42818	0.694153	0.481815	unchanged	-1.71093	C36H62O1	689.4026	7.121767
20.64242	-0.95632	0.459306	0.902322	unchanged	-14.6587	C39H67O1	763.4059	7.121767
161.5947	-0.39506	0.785485	0.343708	unchanged	-8.59859	C19H22N4	767.3461	7.121767
565.4143	-0.40819	0.735147	0.421503	unchanged	-1.20002	C43H46N8	769.3458	7.121767
474.7715	-0.29402	0.767397	0.373188	unchanged	-5	C35H56N8	779.3728	7.121767
294.0057	-0.43613	0.714433	0.444421	unchanged	4.808637	C37H51N9	812.3446	7.121767
294.6486	-0.57235	0.492839	0.806658	unchanged	-6.19068	C53H81O1	1009.5	7.1289
415.5018	0.129469	0.757157	0.322057	unchanged	0.009943	C14H18N2	523.2562	7.1289
704.5178	-0.83447	0.282674	1.124546	unchanged	-1.54573	C28H47NO	560.2988	7.13605
303.4343	-0.31434	0.422102	0.962255	unchanged	-3.91699	C14H26O3	241.18	7.150317
986.1812	-0.86865	0.084884	1.754412	unchanged	-1.92135	C20H32O5	397.2225	7.150317
3481.536	-0.02897	0.91698	0.124948	unchanged	3.815727	C20H31N4	649.119	0.58995
397.5964	-0.32737	0.448571	0.892168	unchanged	0.758225	C34H60O1	649.3938	7.150317
636.7454	-0.20618	0.465489	0.908049	unchanged	1.927391	C28H42O2	445.2887	7.1646
192.0183	-0.01473	0.970554	0.040234	unchanged	44.88329	C26H46NO	536.299	7.178883
1225.683	-0.25043	0.672915	0.487976	unchanged	-0.07166	C24H38O4	389.2697	7.186017
866.8412	1.118142	0.235266	1.32306	unchanged	2.267006	C27H36N2	455.248	7.19315
230.4962	-0.23752	0.703435	0.451414	unchanged	1.503176	C18H32O3	317.2103	7.200283
165.047	-0.20193	0.529546	0.730738	unchanged	-10.1268	C21H32O5	385.196	7.200283
92.98279	0.142613	0.562468	0.620177	unchanged	12.50351	C21H32O3	367.2087	7.207433
464.4838	0.446523	0.219912	1.316716	unchanged	-3.98668	C14H26O2	271.1906	7.2217
860.6898	0.16888	0.36778	0.898818	unchanged	-3.83741	C15H26O2	283.1906	7.22885
38.60415	1.365772	0.156677	1.619614	unchanged	-2.41413	C15H20O3	247.1334	7.235983
2871.203	1.055281	0.127708	1.721669	unchanged	-4.02036	C15H22O3	249.1486	7.243117
109.2598	0.43305	0.141662	1.583049	unchanged	6.310924	C15H22O4	265.1462	7.243117
11415.65	1.141969	0.133864	1.695909	unchanged	49.96498	C14H18N2	329.1057	7.243117
217.81	0.125443	0.675829	0.490294	unchanged	-4.39168	C14H20O3	235.1329	7.25025
4749.758	-0.22659	0.029613	2.144018	down	11.4535	C18H35N3	378.2415	7.25025
281.0409	-0.22818	0.042009	2.028129	down	2.319513	C19H40NO	446.2299	7.25025
261.6331	-0.03289	0.957279	0.008817	unchanged	10.59683	C24H40O4	427.2662	7.2574

535.2724	-0.44334	0.571235	0.724052	unchangec	-2.6936	C24H38O6	421.2584	7.307367
977.9797	-0.56872	0.298519	1.148604	unchangec	-3.36376	C31H41N(	536.3001	7.307367
75.41612	0.257568	0.704707	0.470507	unchangec	28.96475	C29H48N2	581.3599	7.307367
494.9226	0.089568	0.682623	0.393854	unchangec	-6.29527	C17H28O4	295.1896	7.321633
197.406	-1.18077	0.048818	1.953238	down	-0.40859	C28H44O7	1029.615	7.328783
33.6054	-2.59915	0.024914	2.283197	down	0.482846	C26H44F2	1097.599	7.328783
12940.56	-0.56352	0.047021	2.014145	down	-1.53538	C24H47N(	512.2988	7.335917
428.0538	-0.765	0.063815	1.929698	unchangec	2.922796	C30H39N5	584.2307	7.335917
6492.16	0.805049	0.367946	1.088519	unchangec	41.42721	C21H37N7	482.2933	7.350183
209.0703	-0.31462	0.635721	0.582851	unchangec	-9.64216	C19H21N(	649.3226	7.350183
251.711	0.347511	0.269808	1.169308	unchangec	-0.81767	C19H28O4	319.1912	7.39945
128.9237	0.51527	0.432389	0.766085	unchangec	-1.1003	C20H32O6	367.2122	7.39945
190.2013	-0.34287	0.107068	1.658149	unchangec	-6.54947	C9H18O2	315.252	7.413733
2927.563	0.02512	0.930472	0.109843	unchangec	23.65568	C26H37N3	474.2633	7.413733
146.7206	-0.07152	0.84708	0.240437	unchangec	0.590244	C24H44N(	542.2503	7.413733
139.047	0.415429	0.706345	0.456687	unchangec	-2.32817	C17H25N(	581.3583	7.4066
1.72E-06	-0.99411	0.522648	0.81663	unchangec	20.09498	C11H17N(	258.1035	7.428017
34.77892	-0.40878	0.596921	0.674879	unchangec	25.24899	C34H38N4	1191.621	7.442283
38.82944	-0.58468	0.515154	0.638998	unchangec	0.447806	C27H45N(	494.2948	7.442283
166.3446	-0.19041	0.77217	0.371033	unchangec	20.16257	C50H89N2	991.5812	7.43515
25.78045	-0.98474	0.187709	1.440261	unchangec	-1.99673	C51H95N3	1096.62	7.449417
100.7078	-1.04758	0.286484	1.253189	unchangec	-8.08092	C55H99N3	1118.634	7.449417
184.0967	-0.18978	0.711274	0.467579	unchangec	8.546565	C30H44O8	1123.63	7.449417
43.37432	-1.1473	0.235928	1.365535	unchangec	-10.5658	C29H44O1	1163.589	7.449417
145.0878	-0.93871	0.112483	1.680339	unchangec	13.4358	C54H86O2	1179.559	7.449417
293.9081	0.204133	0.355239	1.045306	unchangec	-15.0819	C18H32O4	311.2181	7.449417
475.5267	0.392906	0.697413	0.435216	unchangec	-5.48298	C24H38O4	389.2676	7.449417
203.2247	-0.45405	0.100596	1.719591	unchangec	2.742411	C21H37O7	431.2216	7.449417
1936.282	-0.22154	0.442781	0.810603	unchangec	6.260108	C30H39N(	498.2656	7.449417
392.8578	-0.2903	0.247306	1.281641	unchangec	-15.4087	C29H47N5	592.326	7.449417
413.4149	-0.42141	0.090895	1.74194	unchangec	2.39456	C21H42N7	662.242	7.449417
96.24156	-0.11581	0.916842	0.115872	unchangec	17.54607	C47H86N(	906.5785	7.449417
218.6504	-0.62589	0.452904	0.870389	unchangec	-8.13712	C53H95N2	1043.629	7.456567
62.77843	-0.13782	0.592269	0.667136	unchangec	-0.45382	C8H14O2	283.1914	7.4637
2608.83	-0.03008	0.912628	0.129426	unchangec	-20.0797	C20H24N1	709.0558	0.58995
95.92373	-0.53772	0.055191	1.898692	unchangec	23.16672	C19H28O2	323.185	7.456567
94.54432	-0.43618	0.02258	2.079273	down	-12.3438	C27H47N9	766.278	7.456567
1635.769	-1.24064	0.061765	1.923514	unchangec	-11.1247	C52H100C	1103.631	7.470833
282.6346	-1.34655	0.051769	1.951571	unchangec	-3.28947	C32H44O8	1171.617	7.470833
115.5972	-1.70701	0.087055	1.839642	unchangec	1.500769	C60H88N2	1175.569	7.470833
620.1739	0.052528	0.894511	0.160256	unchangec	-5.68884	C31H48O2	515.2994	7.470833
417.9764	0.13548	0.758955	0.352932	unchangec	-0.13326	C20H29N3	712.0564	0.58995
29103.55	-0.7453	0.051905	1.970552	unchangec	1.192313	C28H48N(	586.3148	7.477983
60.53184	-0.90309	0.077138	1.773401	unchangec	2.056832	C19H23N(	625.3296	7.477983
2270.406	-0.52992	0.104583	1.68634	unchangec	-4.13316	C34H45N5	654.3038	7.477983
57.8683	-2.19354	0.105118	1.77418	unchangec	-4.04596	C27H45N(	1067.623	7.485117
176.8382	-1.72293	0.088194	1.846312	unchangec	4.718162	C56H94O2	1195.617	7.485117
125.3278	-0.61863	0.128791	1.594502	unchangec	-0.17263	C20H30O2	301.2173	7.485117
272.5053	0.143304	0.732684	0.397282	unchangec	-7.64774	C22H34O2	365.2228	7.485117
31.80324	0.732491	0.344559	1.072502	unchangec	-2.38467	C7H16N4(	375.2465	7.49225
13729.2	-0.61162	0.175487	1.431747	unchangec	-1.45817	C24H47N(	496.304	7.485117

10296.76	-0.61728	0.094047	1.701634	unchanged	-2.12791	C25H47N(	526.2987	7.485117
174.4457	-0.10103	0.758658	0.381162	unchanged	-1.49629	C37H49N(	638.3245	7.49225
911.8952	-0.15379	0.593921	0.609519	unchanged	-1.46901	C25H48N(	550.3143	7.506533
353.6039	0.035835	0.967185	0.042643	unchanged	-6.54385	C19H38O2	297.278	7.535083
5401.222	-0.87064	0.176019	1.39812	unchanged	14.59077	C20H32D4	359.2794	7.535083
834.537	0.36734	0.421988	0.935362	unchanged	-3.66617	C14H27N(	256.1909	7.5565
880.2634	-0.01938	0.982625	0.004059	unchanged	-3.11553	C15H27N(	268.191	7.563633
6231.71	-0.5288	0.160471	1.491953	unchanged	-1.74127	C24H47N(	496.3039	7.5565
84.02799	-0.54942	0.24709	1.274784	unchanged	2.093304	C25H50N(	560.2981	7.563633
31.96562	1.227583	0.534931	0.749158	unchanged	3.149034	C24H40O5	429.2635	7.570767
88.90937	-0.72102	0.21537	1.461929	unchanged	0.469275	C23H43N(	448.2837	7.570767
676.0945	0.08999	0.852593	0.220015	unchanged	-1.97279	C48H88N(	952.5669	7.570767
502.1517	0.648531	0.478886	0.784812	unchanged	-2.64727	C29H44O8	1099.618	7.577917
45.27745	1.049589	0.510892	0.743797	unchanged	3.853225	C33H38N4	1171.556	7.577917
487.6501	0.297742	0.188931	1.409072	unchanged	-3.93346	C16H32O3	271.2268	7.577917
611.9664	-0.09991	0.668385	0.486172	unchanged	21.91226	C29H47N(	582.3401	7.577917
356.2991	0.218357	0.210812	1.276595	unchanged	-3.77258	C9H18O2	315.2529	7.58505
2883.723	0.184262	0.204807	1.344338	unchanged	-9.18213	C18H30O4	309.2043	7.599333
2299.132	0.004941	0.988951	0.06325	unchanged	-3.16648	C16H30O4	285.2062	7.6136
414.5877	0.093541	0.68422	0.34045	unchanged	-3.39152	C16H28O2	297.2063	7.606467
32.50074	0.648362	0.246775	1.311967	unchanged	-27.6446	C21H38O7	401.2434	7.606467
56.114	1.22074	0.032317	2.035135	up	-0.28653	C26H50O1	521.333	7.6136
64.53275	-0.8609	0.266196	1.222403	unchanged	9.430354	C24H43N9	574.3135	7.6136
3195.012	0.098277	0.872061	0.212049	unchanged	-4.10898	C23H51N2	487.3263	7.620733
1244.081	0.088913	0.707706	0.411403	unchanged	-2.13497	C30H53N(	638.33	7.620733
299.6972	0.155872	0.651128	0.504602	unchanged	1.763608	C19H29FC	353.2139	7.627883
721.9985	0.535607	0.079149	1.724875	unchanged	-3.93627	C20H30O4	333.2058	7.64215
69.82935	-0.83649	0.45633	0.917773	unchanged	-3.8914	C17H28O4	637.3934	7.663567
3816.891	-0.18293	0.760434	0.363543	unchanged	-21.5205	C20H32O3	341.2029	7.67785
30.98514	1.021878	0.162137	1.542793	unchanged	-17.1612	C21H33N(	753.494	7.684983
12.56406	-2.14112	0.316272	1.151384	unchanged	-2.91896	C41H68O5	661.4795	7.692117
172.1918	0.383282	0.471298	0.81577	unchanged	8.140631	C44H80N(	890.5469	7.692117
34.99287	0.323297	0.69243	0.520798	unchanged	-7.80385	C51H92O1	1067.576	7.699267
84.11661	0.339987	0.45865	0.836596	unchanged	1.654987	C18H36N2	363.2425	7.699267
76307.79	0.359625	0.641101	0.544169	unchanged	-2.01608	C24H40O4	391.2846	7.699267
4669.816	0.369893	0.603159	0.607678	unchanged	3.197846	C21H38N6	459.2715	7.699267
283.7956	0.157569	0.797896	0.309756	unchanged	-11.9037	C30H52N2	571.3456	7.699267
147.7035	0.136085	0.851524	0.24274	unchanged	7.250711	C53H84N(	1010.545	7.7064
530.5944	-0.21889	0.438938	0.839487	unchanged	2.249049	C53H95N2	1019.616	7.713533
27.95199	-0.25589	0.585506	0.75439	unchanged	-15.2806	C27H48O8	1063.59	7.7064
4387.428	-0.45412	0.132198	1.683828	unchanged	10.11023	C54H99N(	1084.666	7.713533
1315.976	-0.37226	0.182248	1.558557	unchanged	-2.25074	C32H48O9	1151.65	7.713533
422.9683	-0.62184	0.113385	1.674708	unchanged	-11.9651	C19H37O7	429.1975	7.713533
1849.756	0.059678	0.745698	0.471357	unchanged	-0.24184	C32H39N(	500.2805	7.713533
1285.59	-0.15224	0.075312	1.987743	unchanged	16.70502	C27H45N(	556.3035	7.713533
177.3612	-0.1352	0.310865	1.165521	unchanged	-35.7392	C31H51N(	616.305	7.713533
51.20948	-0.81217	0.151081	1.494585	unchanged	-31.4094	C21H39N7	626.2456	7.713533
514.1314	-0.20804	0.254483	1.23541	unchanged	0.129838	C34H46ClI	712.2619	7.713533
1770.071	-0.27759	0.619993	0.523703	unchanged	-12.1846	C46H86N(	872.5921	7.713533
11157.76	0.04275	0.94801	0.092159	unchanged	-0.01699	C49H88N(	910.6179	7.7064
102.7861	0.028557	0.955205	0.067402	unchanged	-16.8715	C50H91N2	957.5847	7.7064

303.5125	0.084827	0.779661	0.31624	unchanged	-7.7139	C53H93N2	995.6088	7.7064
4697.682	-0.81129	0.070637	1.82215	unchanged	-6.46431	C53H97N2	1045.647	7.727117
47.13471	-0.92153	0.326854	1.065076	unchanged	-23.1498	C30H47N(	1093.635	7.73425
658.8355	-0.76718	0.063207	1.861726	unchanged	-0.78956	C30H46O8	1113.636	7.727117
3653.645	0.37193	0.388065	1.026304	unchanged	-2.01465	C26H43N(	432.3111	7.73425
1158.72	-0.55423	0.095798	1.712933	unchanged	-0.25133	C34H45N(	594.3071	7.727117
739.149	0.271663	0.504034	0.813619	unchanged	28.3801	C25H43N(	951.642	7.727117
70.34391	-0.99481	0.372799	0.967641	unchanged	20.98796	C53H83N(	1002.6	7.741383
88.58399	-0.22295	0.688655	0.415057	unchanged	-11.3669	C27H41N5	1043.619	7.741383
169.9597	-1.12299	0.131733	1.554958	unchanged	25.70619	C54H92O2	1137.634	7.741383
524.8747	-0.59771	0.17282	1.461622	unchanged	-3.50871	C32H46O8	1175.648	7.741383
27.40194	-0.5962	0.340284	1.109152	unchanged	-18.1215	C26H43N1	640.2982	7.741383
25.01092	-1.18695	0.254673	1.289671	unchanged	3.990992	C27H40N8	1189.617	7.748517
266.8126	-0.35791	0.249291	1.28658	unchanged	3.011257	C18H34O2	303.2314	7.748517
1758.557	-0.48259	0.149007	1.545069	unchanged	-14.898	C35H47N(	660.2611	7.748517
225.967	-0.53546	0.020722	2.086711	down	-0.66506	C17H32O4	299.2226	7.755667
786.9291	-0.18703	0.313766	1.107256	unchanged	-3.54594	C20H34O4	383.2427	7.755667
1.72E-06	-0.253	0.831702	0.30117	unchanged	-19.8509	C17H24O4	643.3372	7.7628
16.82783	0.74198	0.516223	0.753573	unchanged	21.83246	C22H45N(	374.3269	7.769933
38.64169	-0.04909	0.891209	0.082911	unchanged	6.056464	C26H43N(	494.315	7.812767
93.62373	0.372844	0.212326	1.316667	unchanged	1.482681	C35H33N(	616.1962	7.812767
138120.1	-0.13088	0.642998	0.475222	unchanged	-9.68019	C31H53O1	653.3011	7.805633
12.79597	-0.00823	0.989918	0.058757	unchanged	-20.518	C28H38N4	969.5782	7.805633
720.0907	-0.05292	0.917593	0.12514	unchanged	11.05045	C49H87O1	975.5918	7.812767
240.3778	0.284797	0.507989	0.752367	unchanged	5.460903	C50H93O1	983.6049	7.812767
139.3541	-0.20711	0.757071	0.364753	unchanged	24.5554	C52H74N1	1009.588	7.8199
328.6258	-0.41401	0.345836	1.003725	unchanged	0.190824	C33H49N(	576.3308	7.8199
381.9265	0.108061	0.621265	0.566209	unchanged	-3.5325	C51H98O1	1063.599	7.82705
616.7237	-0.22558	0.523171	0.710412	unchanged	-23.9618	C28H46N2	1071.661	7.834183
137.3207	-0.16002	0.563911	0.646158	unchanged	-4.30108	C25H42O1	1095.555	7.82705
18.98206	-0.02154	0.972513	0.047712	unchanged	-0.12122	C57H80N1	1111.586	7.82705
10953.87	-0.49241	0.316883	1.110977	unchanged	-7.52779	C28H39N(	450.2983	7.82705
37.2409	-0.87672	0.29306	1.148355	unchanged	-20.4304	C15H24O3	549.333	7.82705
6451.435	-0.23064	0.679529	0.433089	unchanged	-5.35186	C37H45N(	612.33	7.834183
16170.92	0.083244	0.699985	0.452434	unchanged	-17.367	C30H45N9	632.3184	7.834183
1.72E-06	0.036726	0.97187	0.008034	unchanged	-16.064	C34H52O1	641.3208	7.82705
18183.29	-0.42452	0.299386	1.146051	unchanged	19.62707	C52H93N2	1029.652	7.834183
22.16113	-0.64843	0.228468	1.361761	unchanged	7.261682	C52H100C	1057.644	7.841317
8146.131	-0.15864	0.59194	0.567776	unchanged	3.42792	C55H101N	1172.634	7.841317
38.79929	1.264724	0.448411	0.877348	unchanged	-8.42026	C26H42O3	447.3082	7.841317
2506.151	-0.14474	0.506377	0.771263	unchanged	2.429209	C26H36N6	1083.566	7.848467
1383.969	0.108612	0.58908	0.633968	unchanged	-45.9326	C33H44N4	1183.59	7.848467
2106.639	-0.05073	0.579857	0.649243	unchanged	-3.29906	C18H32O2	279.232	7.848467
1029.093	0.031487	0.841291	0.222451	unchanged	0.930938	C21H39O7	433.2365	7.848467
836.6901	0.085572	0.603304	0.60818	unchanged	6.39648	C28H46N2	557.3032	7.848467
375.6767	0.036114	0.693952	0.476617	unchanged	-7.07897	C33H48N4	595.3459	7.848467
66557.58	-0.50285	0.204584	1.44431	unchanged	12.69594	C54H104C	1107.663	7.862733
89.18537	-0.46152	0.215126	1.329514	unchanged	-12.1645	C29H49N(	1137.657	7.869867
2223.485	-0.21998	0.394288	0.940052	unchanged	-14.2952	C33H42N4	1179.597	7.869867
557.6664	-0.34494	0.312477	1.151234	unchanged	-3.87598	C10H16O	303.2318	7.869867
766.1122	0.064651	0.773538	0.338104	unchanged	43.45944	C2H3FO2	112.9845	7.877017

106.1577	-0.95992	0.184007	1.48857	unchanged	8.572008	C55H99N3	1148.663	7.877017
2049.977	-0.49049	0.365544	1.015278	unchanged	5.418965	C57H101N	1196.636	7.877017
765.6553	0.639616	0.056504	1.91823	unchanged	-25.0824	C29H44O5	517.3052	7.877017
2523.448	-0.29711	0.336982	1.051005	unchanged	2.82054	C28H50NO	578.3034	7.877017
190.5663	-0.29581	0.124455	1.598966	unchanged	15.2015	C33H43N5	618.3384	7.877017
4730.043	-0.21712	0.521005	0.693324	unchanged	-1.73	C30H54NO	656.317	7.88415
2535.264	-0.27885	0.416566	0.89006	unchanged	-17.0176	C30H47N3	660.2621	7.877017
381.2354	-0.2674	0.511351	0.701879	unchanged	11.45849	C38H47NO	724.3058	7.88415
106.9255	0.757225	0.126968	1.70882	unchanged	17.17708	C52H98NO	962.6782	7.88415
1904.155	-0.85419	0.194172	1.461638	unchanged	3.44049	C20H40N2	457.2546	7.898433
1352.858	-0.0573	0.893584	0.112634	unchanged	12.77963	C18H20FN	721.2895	7.905567
1240.678	-0.27954	0.424542	0.980906	unchanged	-4.84969	C16H32O3	271.2265	7.919833
704.4458	0.130313	0.388234	0.947034	unchanged	-4.93619	C27H48O3	455.3277	7.919833
196.988	0.300236	0.495225	0.821567	unchanged	3.92981	C25H43NO	983.6021	7.926983
28505.16	0.582695	0.249428	1.303453	unchanged	-17.056	C31H52N2	599.3195	7.934117
275.9626	-0.5859	0.192405	1.337814	unchanged	-6.20551	C9H18O2	315.2521	7.94125
295.7772	-0.39132	0.505677	0.772999	unchanged	-12.8643	C27H45NO	1017.651	7.9484
135.035	0.291974	0.267915	1.203534	unchanged	-6.08268	C17H32O2	267.2313	7.9484
50.4436	0.178128	0.613835	0.565678	unchanged	-1.32733	C20H41N5	971.5982	7.9484
14551.83	0.372789	0.107452	1.634809	unchanged	-3.43058	C18H32O4	311.2217	7.962667
5422.059	-0.18705	0.054348	1.882878	unchanged	7.235292	C28H46O4	481.3122	7.962667
124860.2	-0.24289	0.01318	2.205848	down	-1.09175	C24H50NO	540.3302	7.962667
676.9187	0.129167	0.445434	0.914116	unchanged	-15.5171	C29H49NO	544.2997	7.955533
153.3805	-0.38775	0.017723	2.12312	down	-4.78036	C33H54O1	647.3383	7.962667
3253.506	0.115111	0.430778	0.767902	unchanged	0.283424	C41H80NO	806.5555	7.962667
70.8237	-0.03653	0.922294	0.083598	unchanged	0.580266	C13H28N4	307.1908	7.969817
1349.609	-0.01356	0.946138	0.089082	unchanged	-9.72217	C19H36O2	341.2669	7.97695
1110.763	0.232473	0.685093	0.47654	unchanged	-30.887	C10H20O2	343.2747	7.97695
130.8031	0.137535	0.821689	0.256892	unchanged	-13.0351	C8H16O2	347.2402	7.991217
364.19	0.322338	0.571464	0.613801	unchanged	-3.22217	C8H17NO	377.2647	8.0055
200.0765	0.24668	0.488327	0.736655	unchanged	2.94047	C18H40O4	403.216	8.0055
779.6911	-1.0778	0.192421	1.447289	unchanged	-13.7958	C56H108O	1159.692	8.026917
34.10042	-2.22255	0.137627	1.697254	unchanged	-5.85064	C32H46O9	1193.62	8.03405
464.6582	0.229376	0.396331	0.898229	unchanged	2.722442	C8H14N2O	239.0655	8.026917
220.0009	-1.34209	0.091281	1.782023	unchanged	9.497895	C57H96N6	1173.664	8.041183
302.572	-0.56177	0.109041	1.683787	unchanged	-3.20097	C22H34O2	329.2475	8.041183
9483.676	-0.18964	0.215713	1.370307	unchanged	-2.97837	C29H48N2	555.3191	8.041183
30.63266	0.112647	0.929666	0.131426	unchanged	0.786271	C22H43N5	614.2895	8.041183
145.7558	-0.50994	0.164788	1.501083	unchanged	-14.7604	C15H23N3	631.3374	8.041183
86.36722	-0.70364	0.161977	1.531028	unchanged	-15.9636	C31H53N5	644.3541	8.041183
213.4096	0.153885	0.753985	0.328073	unchanged	9.887391	C11H15NO	353.227	8.069033
29.37873	0.040024	0.870933	0.284799	unchanged	-4.43186	C9H16O6	241.0684	0.597083
1327.452	-0.34609	0.133576	1.737196	unchanged	-2.25596	C21H34O5	365.2325	8.076183
1421.33	-0.24446	0.592274	0.555574	unchanged	6.500511	C22H36O5	415.2281	8.076183
289.4881	-0.16907	0.598084	0.721404	unchanged	-0.73243	C27H40O4	427.2851	8.083317
111.6604	0.212677	0.722921	0.381148	unchanged	-1.43721	C20H28N2	687.4117	8.076183
506.7196	0.011245	0.975946	0.058412	unchanged	-0.21722	C28H44O6	997.6256	8.104733
983.9011	0.073618	0.711016	0.440191	unchanged	-26.4724	C30H51N3	570.3379	8.111867
3377.695	-0.12952	0.251886	1.178686	unchanged	5.603563	C52H82N6	1035.569	8.119
70.28124	1.019181	0.18982	1.43454	unchanged	3.370591	C18H30O2	323.2237	8.133283
2375.24	-0.6892	0.188219	1.408873	unchanged	14.24348	C55H99N2	1047.66	8.140417

426.3954	-0.41719	0.332615	1.076982	unchanged	3.442635	C18H36O2	305.2472	8.140417
146.8271	0.124474	0.579564	0.542592	unchanged	-7.85447	C20H34O3	367.2465	8.140417
76.43313	0.231911	0.659037	0.505657	unchanged	5.725563	C22H43N5	1023.636	8.1547
5550.512	-0.75294	0.32391	1.120412	unchanged	-7.10375	C56H102N	1135.692	8.147567
6298.006	-0.62036	0.290753	1.159508	unchanged	-1.2254	C25H50NO	552.3301	8.147567
3560.514	-0.26301	0.350171	1.042698	unchanged	-9.13728	C33H45N5	580.3218	8.147567
246.0372	-0.37532	0.323031	1.098875	unchanged	-4.80142	C18H23NO	629.3569	8.147567
0.003735	0.824855	0.470841	0.812386	unchanged	1.833638	C37H56O1	697.3582	8.1547
11.92241	-0.29428	0.708598	0.489954	unchanged	17.73148	C25H43N1	720.3071	8.1547
1118.645	-0.08934	0.61236	0.571959	unchanged	-6.25334	C29H49NO	544.3045	8.18325
2905.067	0.350772	0.357336	1.093829	unchanged	-4.27721	C37H60O6	635.4058	8.176117
35079.13	0.177221	0.246122	1.271376	unchanged	1.7917	C18H22O	567.349	8.190417
1892.546	-0.91325	0.106463	1.83898	unchanged	18.99568	C22H40O2	371.2786	8.190417
722.596	0.316419	0.151881	1.544523	unchanged	-23.8681	C20H40N4	429.2991	8.190417
5274.252	0.099106	0.342668	1.081146	unchanged	2.803165	C26H52NO	556.319	8.190417
310.9082	-0.00175	0.983954	0.057764	unchanged	-12.8438	C37H53O4	596.3566	8.190417
53.88378	0.311862	0.389747	1.019715	unchanged	-31.019	C12H25N3	673.3435	8.190417
409.3049	0.22395	0.52913	0.748787	unchanged	1.976201	C18H36O3	321.2417	8.2047
72.90185	0.842973	0.077152	1.916317	unchanged	-3.06574	C22H34O4	361.2373	8.19755
10094.22	0.110738	0.382144	1.001298	unchanged	-3.97913	C10H13N4	414.9871	0.597083
591.1776	-0.19111	0.752971	0.450696	unchanged	-4.2648	C16H32O3	271.2267	8.23325
621.2436	-0.32566	0.402051	0.956687	unchanged	-8.63803	C19H38O2	343.2828	8.23325
347.2722	-0.03028	0.919324	0.157131	unchanged	-8.38265	C20H34O3	367.2463	8.2261
9386.755	-0.99239	0.179393	1.480207	unchanged	-5.70454	C37H47NO	614.3455	8.2261
81.76897	-0.72249	0.21882	1.3589	unchanged	-10.5742	C27H50N6	631.3433	8.2261
661.2345	-1.11742	0.182605	1.461954	unchanged	-7.78239	C36H49N5	682.3326	8.2261
182.1375	-0.44538	0.357574	1.157036	unchanged	-4.55741	C20H36O3	369.2632	8.240383
171.6721	-0.07409	0.751633	0.302123	unchanged	13.36579	C18H27NO	669.4202	8.240383
443.9003	-0.00069	0.996365	0.030288	unchanged	13.24931	C23H36O4	421.2645	8.254667
77.90745	-1.24534	0.195118	1.453255	unchanged	-3.73689	C24H49O7	1019.653	8.2618
84.60886	-1.00191	0.189315	1.438791	unchanged	5.919568	C29H45NO	1033.679	8.2618
2627.312	-0.36562	0.404281	1.058397	unchanged	-3.50095	C18H34O3	297.2425	8.2618
640.0171	0.018439	0.94575	0.092843	unchanged	4.289285	C8H15N4O	482.9746	0.597083
398.5406	-0.25157	0.419244	1.036501	unchanged	-7.15333	C20H32O3	365.2311	8.2618
4079.484	-0.52601	0.324511	1.117776	unchanged	-2.25155	C23H48NO	464.3136	8.2618
11310.17	-0.50878	0.339072	1.085636	unchanged	25.46495	C27H45NO	524.3351	8.2618
215.3208	-0.10821	0.90783	0.159756	unchanged	-7.12009	C41H78NO	726.5391	8.268933
696.141	-0.69125	0.256997	1.268525	unchanged	-1.46934	C25H42O5	443.2773	8.283217
159.6567	-0.45305	0.395472	1.028781	unchanged	-0.3426	C10H18O2	399.2751	8.29035
258.7104	0.092511	0.736055	0.406414	unchanged	-21.9312	C10H5F17	484.9714	0.597083
11.5655	-2.3815	0.117442	1.733931	unchanged	-3.97508	C10H16	331.2632	8.304633
772.6159	-0.02189	0.91853	0.113869	unchanged	19.90989	C32H41N3	624.3042	8.304633
59.24322	0.160821	0.790649	0.289913	unchanged	-7.72833	C19H24NO	673.3446	8.304633
1480.136	0.033144	0.414788	0.891333	unchanged	14.79093	C50H91N2	957.615	8.304633
30177.64	-0.0513	0.731422	0.362342	unchanged	-6.12471	C36H47N5	634.3337	8.311767
1166.406	-0.20698	0.271084	1.248045	unchanged	-0.39278	C53H102O	1117.657	8.333183
2829.722	0.018265	0.914057	0.115993	unchanged	2.154937	C21H41O7	435.2527	8.333183
147623.5	0.015659	0.87775	0.191068	unchanged	-19.106	C24H47NO	506.3246	8.32605
87.09211	0.354103	0.499437	0.779658	unchanged	3.675627	C50H95O1	969.6239	8.340317
213.1712	-0.85833	0.137286	1.562499	unchanged	-10.8548	C34H48O7	1181.666	8.34745
299.8947	-0.39289	0.186083	1.426414	unchanged	-1.78885	C24H50NO	514.3061	8.34745

1018.536	0.367356	0.143232	1.66233	unchanged	-4.51944	C31H48O4	519.3225	8.34745
22091.39	0.292709	0.468426	0.764315	unchanged	-3.41109	C18H34O4	313.2374	8.361733
80.91901	0.44237	0.59493	0.575583	unchanged	-17.6266	C37H69O8	671.4539	8.361733
131.9817	-0.15618	0.786915	0.423281	unchanged	-3.86465	C18H32O3	295.2267	8.368867
1804.946	-0.13649	0.778289	0.414576	unchanged	-5.19906	C17H32O	297.2422	8.368867
1227.286	-0.11447	0.74478	0.440705	unchanged	3.429958	C44H86N(	822.5812	8.368867
74.70184	-0.12122	0.799678	0.319505	unchanged	1.974554	C10H18O2	385.2602	8.38315
13546.84	-0.43893	0.083706	1.726677	unchanged	-1.46946	C24H50N(	494.3245	8.38315
64208.71	-0.48223	0.092442	1.706415	unchanged	25.30499	C28H47N(	554.3451	8.38315
224.6866	0.948042	0.088008	1.89534	unchanged	4.813223	C23H34O3	379.2272	8.411
2835.491	-1.42109	0.100473	1.768628	unchanged	-0.42855	C32H50O7	1137.709	8.418133
283.8486	-0.57229	0.095953	1.805146	unchanged	17.58926	C23H36N2	371.277	8.418133
24208.3	-0.8628	0.144705	1.550713	unchanged	-0.54886	C30H54N(	616.3617	8.425267
149.4297	-0.62185	0.129533	1.53997	unchanged	-22.2031	C17H22N4	655.3593	8.425267
363.33	-0.78627	0.144496	1.502624	unchanged	-21.6442	C37H51N5	660.3623	8.425267
1992.551	-0.64034	0.217183	1.325755	unchanged	-0.93503	C32H58N(	684.3488	8.425267
97.57852	0.081209	0.779165	0.279642	unchanged	-11.3172	C6H10O5	161.0437	8.453833
144.3103	1.663408	0.019006	2.335371	up	-9.80112	C19H30O4	343.1859	8.453833
164.6266	-0.10117	0.788422	0.356828	unchanged	-0.97504	C6H15N4(	349.2314	8.453833
187.7681	0.043121	0.894068	0.104884	unchanged	-9.86534	C22H34O2	375.2508	8.453833
655.5979	-0.34923	0.22768	1.38507	unchanged	-8.12599	C20H34O4	337.2357	8.460967
188.4924	-0.25283	0.163952	1.508021	unchanged	2.168343	C7H16N2(	319.2358	8.4681
398.8166	-0.32083	0.492439	0.888035	unchanged	0.989292	C20H42O5	383.2783	8.475233
203.0297	0.203355	0.493016	0.777622	unchanged	-1.70264	C38H66O7	669.4492	8.482383
248.7154	-1.12564	0.067008	1.827596	unchanged	-17.3193	C26H48N6	1143.68	8.489517
17034.34	-0.50057	0.131269	1.56611	unchanged	-1.25905	C24H50N(	494.3246	8.49665
72014.54	-0.55322	0.123909	1.59501	unchanged	-0.86781	C25H52N(	554.3459	8.49665
174.5634	-0.60655	0.093368	1.676654	unchanged	-48.3055	C31H42N6	655.2802	8.5038
105.6304	-0.10717	0.664478	0.565975	unchanged	14.10022	C53H93O1	971.6282	8.49665
104.1848	-1.39555	0.174035	1.458797	unchanged	10.56393	C55H100N	1169.692	8.518067
271.2734	-0.947	0.101269	1.686303	unchanged	-4.8546	C20H36O3	323.2576	8.518067
434.3727	-0.74644	0.140176	1.71942	unchanged	-1.69471	C21H38O5	369.264	8.518067
625.0753	-1.21239	0.132012	1.560855	unchanged	0.627556	C61H100C	1101.71	8.525217
139.6534	-1.47727	0.145995	1.605298	unchanged	4.66075	C56H108C	1113.704	8.525217
12391.8	0.302751	0.363431	1.025933	unchanged	11.01768	C14H20O2	265.147	8.53235
66.29718	-0.71804	0.19135	1.362875	unchanged	-6.43837	C10H18O	307.2623	8.53235
357.9575	0.565357	0.243903	1.315277	unchanged	4.751249	C9H11O3-	333.1359	8.53235
24218.56	-0.46917	0.180263	1.394421	unchanged	-2.23181	C28H54N(	592.3608	8.53235
374.803	0.053085	0.915804	0.037849	unchanged	-3.06902	C18H32O3	295.227	8.546617
685.5147	-0.0719	0.805295	0.371114	unchanged	-8.9582	C24H38O4	435.2717	8.553767
105.5249	0.271638	0.334495	1.020993	unchanged	2.137536	C7H16NO	351.2507	8.596583
10801.95	-0.35986	0.313627	1.12328	unchanged	-3.40246	C27H47N3	538.3503	8.603733
48.60385	-0.6921	0.375003	0.968599	unchanged	9.75629	C15H21N3	577.3558	8.596583
1176.643	0.361167	0.117063	1.746205	unchanged	-0.10483	C20H28D4	339.2479	8.62515
341.8382	-0.33921	0.391527	0.992371	unchanged	-4.74735	C15H23N(	511.3519	8.62515
2374.218	0.291701	0.379034	0.972341	unchanged	-4.38075	C19H36O4	327.2526	8.696517
750.3587	-0.03813	0.872216	0.294805	unchanged	-4.04853	C21H36O5	367.2475	8.696517
2304.262	0.399019	0.209148	1.336084	unchanged	2.320952	C27H38O6	457.2606	8.696517
37.44264	0.239039	0.45478	0.917698	unchanged	-8.12878	C27H45N(	1003.64	8.7108
429.4951	0.144825	0.536331	0.670616	unchanged	-11.5238	C50H86N(	958.5475	8.7108
199.1342	-0.63161	0.104794	1.629153	unchanged	-10.3791	C34H46O7	1191.651	8.731517

1053.858	-0.01895	0.859523	0.302451	unchanged	-7.89322	C20H38O4	341.267	8.731517
192.5941	0.343315	0.115128	1.706968	unchanged	-26.1297	C10H18O	353.2617	8.731517
21188.23	-0.23724	0.184456	1.38686	unchanged	-1.1141	C25H52NO	508.3403	8.731517
4381.686	-0.23051	0.151892	1.48385	unchanged	-0.27743	C26H54NO	558.333	8.731517
290.8832	0.408837	0.376977	1.008423	unchanged	-1.02919	C28H45NO	568.3122	8.731517
8589.93	-0.27136	0.118199	1.615625	unchanged	9.782463	C26H56NO	570.365	8.731517
523.1841	-0.2376	0.124578	1.599538	unchanged	1.132907	C16H22FN	585.3515	8.731517
284.893	-0.44323	0.132392	1.630149	unchanged	-16.6974	C35H58O1	675.3617	8.731517
109.9075	0.409615	0.022379	2.132675	up	-6.83171	C54H96NO	968.6453	8.731517
13148.87	-0.17594	0.611564	0.550692	unchanged	-3.76819	C14H21NO	250.1439	8.73865
756.9334	0.071253	0.742036	0.290124	unchanged	9.826703	C25H40O3	433.2998	8.73865
18.95581	-1.08475	0.147434	1.502769	unchanged	16.17469	C16H26O4	609.3735	8.73865
1008.681	-0.90849	0.14599	1.520092	unchanged	0.745668	C31H52O8	1103.726	8.745783
857.4856	0.165174	0.495838	0.775061	unchanged	28.67695	C26H44O4	455.3054	8.77435
269.248	0.003451	0.994359	0.027643	unchanged	-0.35683	C20H40O2	333.2774	8.8029
5701.229	0.032962	0.895261	0.142086	unchanged	-2.65566	C23H38O4	377.2687	8.810033
1036.273	0.074843	0.790859	0.325645	unchanged	-2.32217	C15H21NO	276.16	8.817167
481.2204	0.055707	0.707152	0.344145	unchanged	-2.63292	C20H32O3	365.2661	8.824317
10977.06	0.24429	0.474251	0.791872	unchanged	0.170581	C23H38O4	423.2753	8.824317
276.0072	0.289594	0.538305	0.736697	unchanged	-16.1324	C28H50O4	471.3383	8.817167
8036.71	0.514842	0.180241	1.46826	unchanged	14.63681	C42H72O7	733.5481	8.817167
3647.417	0.385129	0.292482	1.167822	unchanged	0.731323	C32H46O9	595.2893	8.83145
19729.31	0.406921	0.316895	1.157052	unchanged	2.33707	C43H84NO	826.5598	8.83145
178.6293	-0.18951	0.640197	0.421317	unchanged	4.016025	C53H102O	1071.656	8.852867
1255.451	0.110155	0.142459	1.504799	unchanged	-2.58768	C20H38O4	341.2688	8.845717
4956.188	-0.23099	0.202485	1.321047	unchanged	-6.33928	C52H88N1	1091.629	8.874283
307.8043	-0.15165	0.73734	0.446438	unchanged	-2.51086	C18H34O3	297.2428	8.867133
1928.005	-0.05133	0.719857	0.358749	unchanged	-0.7406	C31H50O4	521.34	8.874283
707129.8	-0.1349	0.429981	0.843851	unchanged	0.098406	C26H54NO	568.3616	8.867133
9.983336	0.426118	0.380586	0.955147	unchanged	-10.2264	C16H24N2	611.3758	8.874283
150.8577	-0.09497	0.70744	0.408594	unchanged	8.150762	C38H62O9	683.4195	8.867133
222.8483	0.076371	0.745508	0.355684	unchanged	18.1095	C15H28N4	701.4322	8.867133
586.8113	-0.05143	0.8008	0.286326	unchanged	-19.9708	C52H98N1	1175.702	8.881417
133573.1	-0.12598	0.445784	0.816511	unchanged	-0.88081	C25H52NO	508.3404	8.881417
350.8388	-0.08623	0.65181	0.466844	unchanged	6.651646	C28H58NO	572.3734	8.881417
163.1484	0.101348	0.835408	0.238528	unchanged	-1.63382	C31H52O7	1117.739	8.88855
2735.876	0.128316	0.755839	0.384887	unchanged	-0.53768	C55H103N	1142.655	8.88855
1695.223	-0.09132	0.601278	0.582938	unchanged	15.92305	C33H48O6	585.3519	8.88855
934.8023	-0.20498	0.371657	1.008679	unchanged	-19.1564	C52H96NO	930.6395	8.88855
404.3852	0.15078	0.474843	0.855916	unchanged	22.5264	C21H41O6	419.2663	8.895683
17796.42	0.261659	0.597288	0.622279	unchanged	-26.8195	C34H48O8	619.2886	8.902833
57.22236	0.14821	0.778121	0.323517	unchanged	-25.639	C60H100N	1012.668	8.909967
1219.973	0.082568	0.730735	0.410734	unchanged	-9.10944	C2HF3O2	112.9845	8.909967
5.905882	-0.38735	0.471469	0.911256	unchanged	26.13006	C16H33NO	300.2611	8.9171
1318.584	-0.0034	0.992889	0.017897	unchanged	2.586437	C21H42O4	379.2839	8.92425
171.9287	-0.20144	0.504559	0.769541	unchanged	-1.02804	C40H58O4	623.4076	8.92425
495.0625	-0.10551	0.686232	0.440616	unchanged	3.018968	C16H24N2	675.3629	8.9171
6244.555	0.017345	0.944557	0.110427	unchanged	7.7809	C32H53NO	704.3373	8.9171
606.7341	-0.19215	0.515327	0.795491	unchanged	13.38159	C52H92NO	934.6662	8.931383
3248.862	0.087918	0.86541	0.251018	unchanged	8.631695	C19H30O5	337.205	8.938517
197.2936	0.409661	0.086401	1.705365	unchanged	-12.3982	C10H18O	353.2659	8.938517

77.14438	-1.40199	0.126115	1.690144	unchanged	-7.15937	C10H20O	311.2933	8.94565
162.053	-0.60681	0.048211	1.946937	down	-2.35863	C31H53N(	588.3504	8.94565
2678.368	-0.3033	0.227312	1.285898	unchanged	-1.31333	C37H73N2	717.5179	8.9528
13.33473	0.284613	0.672829	0.472914	unchanged	-7.34728	C19H26O3	603.3647	8.959933
128.1331	0.047054	0.651733	0.484164	unchanged	-3.14504	C18H32O3	295.2269	8.98135
998.73	0.027958	0.91109	0.121828	unchanged	-1.27611	C22H36O2	377.2693	8.98135
1290.335	-0.31703	0.246336	1.322219	unchanged	-8.9031	C38H61N(	680.4085	8.98135
25.32119	0.077967	0.895106	0.124129	unchanged	4.550892	C18H33N(	324.2557	8.988483
646.2445	0.280952	0.580667	0.5918	unchanged	-3.97055	C16H32O3	271.2268	9.0099
262.8115	-0.2329	0.485751	0.779344	unchanged	-25.1447	C34H64O7	605.4252	9.0099
36.6355	0.252583	0.832375	0.255639	unchanged	2.920087	C31H52O7	1117.744	9.024183
4494.91	0.114955	0.587705	0.540297	unchanged	-2.58185	C20H38O4	341.2688	9.024183
29.68636	-0.2247	0.598504	0.636251	unchanged	-15.302	C38H58O6	631.3887	9.017033
988.0732	0.115912	0.58557	0.658428	unchanged	-18.7859	C33H62O7	591.4135	9.031317
14273.75	0.294847	0.345366	1.124336	unchanged	-3.1467	C38H77N2	733.548	9.03845
308.3623	0.11959	0.781852	0.298775	unchanged	-1.31048	C21H39N(	368.2801	9.052733
6120.303	0.131799	0.29855	1.161924	unchanged	-7.69472	C27H44O5	447.3081	9.052733
822.0334	0.281865	0.362369	0.969158	unchanged	0.038817	C26H42N4	489.3083	9.07345
240.9427	0.512391	0.453391	0.836425	unchanged	1.433611	C22H26O	671.4115	9.07345
699.9438	0.261085	0.525558	0.744228	unchanged	-2.58891	C40H80N(	754.5349	9.07345
215.3097	0.200023	0.560737	0.595936	unchanged	-6.80245	C20H36O3	323.257	9.080583
704.4468	0.007469	0.959974	0.082299	unchanged	-3.43482	C23H44O4	405.2973	9.080583
1988.093	0.222504	0.201443	1.390444	unchanged	14.87374	C27H44O2	435.3095	9.087717
12.20805	-0.36821	0.806073	0.381418	unchanged	0.609335	C20H26O	609.3953	9.087717
104.4905	-1.57002	0.307765	1.225009	unchanged	21.17193	C52H98N(	988.7059	9.09485
169.5697	-4.15111	0.176639	1.635563	unchanged	-21.9786	C33H54O6	1137.758	9.102
81635.41	-0.81506	0.045885	2.056448	down	-0.71244	C28H58N(	596.3929	9.102
292.9277	-0.84614	0.053098	1.927345	unchanged	-21.9797	C14H23N5	613.3822	9.102
3072.5	0.071333	0.733193	0.368232	unchanged	-29.1837	C24H44O7	443.2885	9.109133
597.7563	0.262271	0.416444	0.941332	unchanged	1.638121	C22H30O2	775.4083	9.109133
119.3917	-1.14257	0.432755	0.975544	unchanged	6.593426	C52H98N(	994.6584	9.109133
105.5206	0.213608	0.373899	1.038719	unchanged	2.893653	C10H20O2	403.3075	9.13055
51.55919	-0.11891	0.685323	0.518503	unchanged	3.930828	C19H37N(	356.2819	9.137683
187.0959	-0.31345	0.454077	0.837489	unchanged	1.137469	C38H56O3	605.4218	9.144817
4827.721	0.034927	0.814697	0.270658	unchanged	-6.99503	C27H48O5	451.3397	9.1591
428.7496	0.187539	0.505879	0.739947	unchanged	4.439002	C9H19N3(	433.2799	9.166233
126.5988	0.093833	0.737259	0.396068	unchanged	-5.40265	C4H6O6	170.9903	9.180517
186.1229	2.190617	0.435315	0.909058	unchanged	-3.14085	C15H28O3	255.1958	9.180517
359.3119	0.274281	0.345602	1.021982	unchanged	-2.81671	C22H38O2	379.2844	9.18765
1343.964	0.188889	0.236705	1.316808	unchanged	0.837494	C27H46O5	527.2991	9.201933
133.5395	-0.55668	0.041025	2.18216	down	4.785816	C21H32O4	369.2064	9.209067
239.1549	0.493424	0.229692	1.361633	unchanged	0.594548	C20H32O3	365.2335	9.2162
317.2387	0.077681	0.757945	0.345013	unchanged	-5.56711	C29H50O4	483.343	9.2162
13306.11	0.980428	0.291381	1.206875	unchanged	-0.25338	C41H78N(	758.534	9.22335
61546.62	0.854931	0.166425	1.543642	unchanged	0.954276	C43H80N(	800.5455	9.2162
487.6735	-0.00301	0.988161	0.022905	unchanged	-1.27514	C26H40O3	445.2954	9.230483
94.81067	-0.27218	0.298748	1.16489	unchanged	1.631852	C36H54O3	579.4064	9.237617
1192.135	0.046525	0.824627	0.272823	unchanged	3.417237	C35H54O1	655.3485	9.237617
169.8132	-0.0993	0.794141	0.315214	unchanged	-2.69973	C12H19N3	501.3183	9.259033
3667.282	0.160212	0.440166	0.854809	unchanged	-3.98856	C16H32O3	271.2268	9.273317
1210.24	0.123035	0.558196	0.650611	unchanged	10.28458	C20H32O2	339.2128	9.273317

3084.097	-0.29925	0.082438	1.773929	unchanged	-2.92174	C26H54N(	522.355	9.266167
11142.94	-0.37312	0.069276	1.90319	unchanged	-1.28259	C27H56N(	582.3772	9.266167
126.6419	-0.16996	0.503101	0.846755	unchanged	-3.38348	C20H40O2	357.3	9.28045
176.0116	-0.79274	0.172848	1.490573	unchanged	26.09004	C19H30O2	625.4625	9.28045
474.9736	-0.12709	0.595571	0.580181	unchanged	-3.51641	C40H60O2	607.4267	9.301867
240.4747	0.637516	0.296288	1.148427	unchanged	4.645143	C18H39N(	338.2691	9.323283
96.17146	-0.02322	0.935241	0.085746	unchanged	8.384788	C17H23N(	559.3584	9.323283
4910.377	0.135306	0.350273	1.047963	unchanged	9.570957	C28H48O2	451.3388	9.330417
196.694	0.137638	0.613085	0.586378	unchanged	21.64334	C6H6O4	176.9991	9.33755
71.88565	0.151354	0.582219	0.641739	unchanged	0.901121	C4H2F6	162.9989	9.3447
2765.685	0.37357	0.142247	1.580049	unchanged	27.88171	C7H15NO	321.2121	9.33755
131.2536	0.056479	0.821165	0.259537	unchanged	-7.16645	C17H34O3	285.2415	9.358967
99.2978	0.369296	0.031965	2.01431	up	-3.33079	C21H34O2	353.2242	9.358967
303.3259	-0.17194	0.604215	0.632549	unchanged	23.98738	C40H52O3	579.3983	9.358967
240.502	0.288025	0.609004	0.584797	unchanged	-0.29549	C17H26O4	587.3588	9.36615
2879.762	-0.0969	0.698892	0.410353	unchanged	-3.87332	C36H66O8	671.4715	9.3733
486092.6	0.255803	0.66586	0.49248	unchanged	-1.46968	C45H84N(	828.5748	9.3733
317934.7	-0.04724	0.881405	0.152303	unchanged	-2.19598	C45H86N(	830.5898	9.3733
1960.761	0.083504	0.810309	0.297259	unchanged	-9.60268	C34H66N(	692.4446	9.387567
889.955	0.300973	0.181603	1.478301	unchanged	-2.47783	C12H22O2	455.3368	9.408283
814.0324	0.225977	0.363313	0.932368	unchanged	-3.62776	C21H40O2	369.2999	9.422567
116.9848	0.348067	0.593145	0.638082	unchanged	6.599318	C33H52O4	547.3593	9.415417
9819.293	1.272132	0.23559	1.364236	unchanged	2.324912	C42H82N(	796.5492	9.415417
87.92629	-0.01804	0.927179	0.126555	unchanged	4.65469	C19H32O3	353.2348	9.4297
740.9397	-0.22322	0.566783	0.554444	unchanged	-5.66302	C18H21N(	597.2936	9.4297
678.497	0.429668	0.227602	1.339962	unchanged	-3.4205	C18H34O3	297.2425	9.443967
553.4125	0.501575	0.300706	1.117849	unchanged	-5.37862	C23H42N4	499.3113	9.443967
190.0899	-0.00497	0.98768	0.032139	unchanged	-5.82024	C28H48O6	501.317	9.436833
1101.398	-0.05315	0.859896	0.22521	unchanged	3.870559	C22H32O2	327.2342	9.45825
166.3987	-0.05726	0.8936	0.176509	unchanged	4.416346	C23H34O4	395.222	9.45825
53.59769	2.19456	0.154242	1.581831	unchanged	-8.01115	C30H42O8	551.2584	9.465383
181917.4	0.232265	0.611599	0.587564	unchanged	-6.21744	C44H85N(	870.5485	9.479667
451.3785	1.488654	0.430386	0.917638	unchanged	-2.64757	C16H30O3	269.2115	9.493933
2203.541	0.117597	0.732883	0.424348	unchanged	-7.36143	C40H72O7	685.4976	9.4868
3675.964	0.180629	0.730068	0.400452	unchanged	6.690559	C52H81N3	1142.505	9.501083
45.46607	0.176592	0.799896	0.32305	unchanged	14.35548	C13H24O4	547.3558	9.508217
65.25435	0.100694	0.695259	0.444636	unchanged	15.61882	C5H6O5	166.9985	9.5225
20288.72	0.198026	0.176812	1.455614	unchanged	-1.73333	C26H48O5	439.3421	9.51535
41.4217	0.593143	0.251223	1.317564	unchanged	10.43194	C29H50O6	515.3406	9.5225
711.8934	0.154818	0.202483	1.378204	unchanged	1.178832	C29H48O7	529.3153	9.51535
125.8271	-0.36334	0.476031	0.786907	unchanged	-6.03699	C17H21N(	665.3043	9.51535
179.3773	-0.13431	0.757525	0.263041	unchanged	-1.07383	C17H28O3	619.4209	9.529667
135.3757	-0.09645	0.765162	0.33465	unchanged	-1.74145	C20H39N(	370.2957	9.536817
550.3671	0.104992	0.539013	0.628537	unchanged	5.348266	C25H44N(	411.314	9.536817
441.3844	0.25055	0.259661	1.242374	unchanged	-0.44882	C28H46O	433.3241	9.54395
16.35046	2.244144	0.062726	1.8525	unchanged	-11.533	C11H23N5	545.3466	9.551083
2675.63	-0.28539	0.229606	1.39052	unchanged	-3.15596	C20H32O2	303.232	9.558233
476.0878	-0.32124	0.170209	1.570464	unchanged	-1.72504	C21H34O4	371.2198	9.558233
910.0952	-0.40356	0.402428	0.940929	unchanged	-5.44315	C38H62O7	651.3903	9.558233
834.827	-0.12568	0.785079	0.315441	unchanged	0.272699	C35H58O5	603.4268	9.5725
120.8743	0.807152	0.562455	0.676625	unchanged	15.40551	C20H30O2	663.4723	9.579633

12051.8	0.147821	0.630574	0.555088	unchanged	-11.045	C36H65O8	701.4922	9.586783
404.6674	0.021631	0.963748	0.081373	unchanged	-2.36322	C15H26O8	713.3222	9.6082
4859.465	-0.0978	0.531611	0.821671	unchanged	-2.47885	C22H42O4	369.3001	9.615333
10799.52	-0.9772	0.215691	1.411128	unchanged	-2.14134	C16H30O2	567.4619	9.622467
19.97514	-1.43399	0.146279	1.683669	unchanged	-0.58654	C25H43O1	595.2522	9.615333
1174.487	0.016479	0.940792	0.095595	unchanged	-1.17371	C34H67O8	669.426	9.6296
322.9014	0.123471	0.64979	0.540283	unchanged	0.286705	C3H6N2O4	155.0075	9.643883
403.8772	-0.35313	0.203314	1.479273	unchanged	4.628305	C20H36O2	329.2476	9.643883
197.7133	-0.26433	0.229033	1.272246	unchanged	-4.21437	C10H20O2	343.2839	9.643883
81.05265	0.300749	0.550335	0.667465	unchanged	-7.66104	C21H32O4	347.2201	9.63675
805.5233	0.013301	0.967644	0.037063	unchanged	3.114667	C22H30FN	412.1918	9.63675
90.97687	-0.30559	0.508096	0.815894	unchanged	1.693899	C16H30O1	427.1827	9.643883
2138.585	-0.34324	0.28496	1.253712	unchanged	0.081389	C25H30O4	429.1838	9.643883
2395.319	-0.40854	0.716674	0.501492	unchanged	29.42025	C27H44O4	467.3061	9.63675
69299.04	0.094502	0.649593	0.553513	unchanged	7.432952	C29H40N2	485.282	9.63675
123.0765	-0.73738	0.585562	0.724187	unchanged	7.164443	C33H42N2	535.2979	9.63675
2289.021	0.010321	0.973865	0.118875	unchanged	1.588312	C51H81O1	1025.526	9.6653
111.4758	-2.20112	0.06126	2.073861	unchanged	13.85401	C18H37NO	314.2744	9.672433
20.89534	0.71157	0.349472	1.043353	unchanged	-5.85259	C11H20	363.3251	9.6653
171.9164	0.422234	0.378757	0.934404	unchanged	-10.7714	C20H34O3	367.2455	9.686717
865.5827	0.001198	0.997066	0.068153	unchanged	-9.02988	C39H76NO	716.5171	9.69385
1148.137	-0.42136	0.295157	1.136141	unchanged	-2.7969	C20H40O4	343.2844	9.7224
130.471	0.095034	0.874226	0.177986	unchanged	-4.09377	C17H25NO	563.3833	9.715267
67.81328	-0.01623	0.98309	0.064983	unchanged	1.071319	C31H56O8	601.3963	9.715267
70.68909	0.658865	0.262797	1.263839	unchanged	-0.52666	C11H18N2	355.2865	9.735983
587.0766	0.040634	0.767953	0.440849	unchanged	-6.95534	C12H22O2	455.3351	9.743117
8.194514	1.505989	0.188867	1.468806	unchanged	-17.273	C28H50O1	545.3237	9.743117
214.5502	-0.01377	0.941006	0.150172	unchanged	-18.7177	C26H47NO	410.3331	9.75025
176.8849	0.194675	0.23904	1.209441	unchanged	-7.81013	C27H44O4	431.3133	9.75025
403.1424	1.389651	0.243392	1.268998	unchanged	2.22605	C30H44O6	499.3076	9.764533
29801.3	-0.00087	0.997202	0.060938	unchanged	10.29474	C22H34O2	719.5328	9.771667
246.0913	-0.7782	0.053596	1.984554	unchanged	4.307485	C20H36O2	329.2475	9.7788
2760.079	0.194348	0.363581	1.025599	unchanged	5.644483	C23H38N2	425.2598	9.7788
169.9014	0.618073	0.441239	0.808482	unchanged	-2.96008	C15H22O2	527.3364	9.828767
213.0737	-0.23149	0.589907	0.651835	unchanged	-3.65794	C35H58O5	603.4246	9.87875
78.14334	-0.55489	0.066025	1.933711	unchanged	-4.82647	C20H40O	341.3047	9.885883
2980.19	0.069102	0.85804	0.198804	unchanged	-1.6152	C23H44O4	383.3161	9.885883
237.1777	-0.39584	0.150252	1.555113	unchanged	-17.0822	C37H54O6	639.3801	9.90015
109.3692	0.105594	0.599951	0.62253	unchanged	-25.8351	C27H50O2	427.3452	9.921567
383.1301	0.123085	0.653761	0.52261	unchanged	8.26717	C5H6O5	166.9974	9.942983
1959.017	0.243689	0.441124	0.859108	unchanged	-3.0982	C18H36O3	299.2582	9.942983
68.57221	-0.1009	0.81135	0.278966	unchanged	1.783574	C19H32O4	323.2234	9.950117
6150.21	0.132882	0.638025	0.555957	unchanged	-3.65039	C13H13N5	404.0091	9.950117
112.5656	0.024539	0.972794	0.079069	unchanged	-4.36062	C11H18N4	553.2718	9.950117
98.71714	0.089258	0.830775	0.241998	unchanged	16.71115	C38H55O4	610.3891	9.950117
529.0058	0.110457	0.821161	0.266215	unchanged	2.335159	C36H58O9	655.3842	9.950117
540.2995	0.358128	0.323714	1.059877	unchanged	-27.512	C26H47NO	482.3367	9.985817
12351.11	0.128038	0.658982	0.523335	unchanged	-20.955	C20H27N1	793.0353	9.99295
4891.643	0.023584	0.90373	0.156051	unchanged	-16.2464	CH5O4P	146.9601	0.532833
59.85832	-0.09208	0.839621	0.183535	unchanged	-6.75153	C5H7NO3	128.0344	0.632783
3360.532	0.01217	0.938636	0.070801	unchanged	-13.7193	C7H8N4O	217.0292	0.632783

48.35235	-0.119	0.700136	0.526813	unchanged	4.855843	C7H9N5	198.056	0.639917
215.6392	-0.46728	0.334532	1.036929	unchanged	-4.62206	C17H16N2	379.0832	0.639917
69.22	-0.04868	0.693555	0.501898	unchanged	-0.85686	C10H12N5	618.892	0.639917
9652.614	-0.00742	0.966369	0.021993	unchanged	-10.8606	C9H11N2O	351.0202	0.654183
68.89721	-0.07388	0.783357	0.323795	unchanged	-11.2927	C9H14N2O	386.9956	0.64705
178.5069	0.016528	0.930927	0.089423	unchanged	1.89762	C12H10O1	396.9822	0.654183
798.2047	0.044896	0.586712	0.646025	unchanged	2.09078	C10H15N4	512.9605	0.654183
175.6666	0.396741	0.205133	1.31932	unchanged	-4.17174	C16H17N9	524.0756	0.654183
84.76513	-0.42933	0.129422	1.639348	unchanged	-10.7539	C25H40N7	904.0913	0.654183
356.0028	-0.3339	0.487726	0.807285	unchanged	-5.47886	C7H14N2O	173.0922	0.661333
314.649	0.054513	0.724342	0.317698	unchanged	-6.17666	C13H12O3	237.052	0.661333
8922.483	0.004912	0.985452	0.039743	unchanged	-26.7285	C18H13ClI	329.0381	0.661333
32200.5	0.096753	0.631183	0.56781	unchanged	-23.0905	C17H14O6	335.0465	0.661333
5270.119	0.034179	0.803808	0.275824	unchanged	-0.58853	C5H9O8P-	454.9995	0.661333
494.0382	0.284397	0.593971	0.604451	unchanged	17.75359	C32H39O2	780.1801	0.661333
957.1902	-0.1122	0.407435	0.908426	unchanged	-2.85521	C25H43N8	851.1583	0.661333
102.7026	-0.1744	0.587156	0.665676	unchanged	-14.0586	C28H40N7	908.0985	0.661333
342.0901	0.015346	0.960851	0.048327	unchanged	-19.7508	C10H14O	185.0918	0.668467
6585.915	-0.00114	0.997052	0.001282	unchanged	-6.05671	C8H14N2O	247.0926	0.668467
420.6478	-0.01617	0.95781	0.046321	unchanged	-4.13953	C9H14N2O	229.082	0.668467
682.2715	0.035719	0.933268	0.10086	unchanged	-11.3301	C11H11N3	286.0415	0.668467
11870.65	-0.03216	0.922246	0.093241	unchanged	-12.0024	C30H26N2	643.1107	0.668467
12659.66	0.071219	0.600471	0.625422	unchanged	-14.8409	C26H26O1	671.1008	0.668467
290.8641	0.041452	0.828938	0.281358	unchanged	-29.6976	C14H23N4	997.1284	0.668467
101.035	0.165535	0.492799	0.714134	unchanged	-9.65068	C3H6O4	105.0183	0.68275
398.9381	-0.14127	0.671532	0.489429	unchanged	-7.56417	C5H10O5	149.0444	0.68275
479.2438	0.062345	0.87214	0.187108	unchanged	-10.0941	C3H7N	159.1127	0.6756
101.4312	0.113946	0.793167	0.225324	unchanged	-7.0828	C7H11N3O	200.0663	0.68275
89.30526	0.02989	0.881594	0.151733	unchanged	-2.17771	C9H14N2O	251.0644	0.6756
1325.161	-0.05984	0.89948	0.242782	unchanged	0.586103	C8H14N4O	267.0712	0.68275
93.79283	-0.23644	0.225917	1.345413	unchanged	-2.38882	C10H15N5	290.0864	0.68275
417.2374	-0.12089	0.713222	0.50444	unchanged	0.188944	C15H15ClI	325.0517	0.6756
97543.88	0.0691	0.813769	0.279647	unchanged	6.741309	C12H5F6N	343.9939	0.68275
3406.539	0.013427	0.976614	0.025991	unchanged	-6.75499	C5H10NO	389.0494	0.6756
81.14468	-0.40043	0.372351	1.044899	unchanged	4.883766	C23H23O1	536.1196	0.6756
44.24187	-0.30975	0.055443	1.928709	unchanged	-3.79068	C9H12NO	567.0767	0.6756
33544.38	0.067429	0.875277	0.189341	unchanged	-15.0998	C26H34O1	605.1556	0.6756
288.6725	0.008131	0.935717	0.075338	unchanged	-37.7871	C10H16N2	803.0082	0.68275
5511.401	-0.05685	0.879795	0.15952	unchanged	-18.0944	C30H51N8	957.1841	0.6756
744.3851	0.079933	0.825723	0.25652	unchanged	-5.33021	C31H44N7	994.1218	0.68275
38.02955	-0.13489	0.613292	0.677175	unchanged	-12.6338	C5H9NO3	130.0493	0.70345
84.37217	0.142555	0.468706	0.765978	unchanged	-0.70343	C7H11NO	234.0618	0.70345
204.8549	-0.07371	0.818424	0.369306	unchanged	-5.69864	C8H14N2O	263.0872	0.70345
234.2542	0.198182	0.701327	0.446808	unchanged	-3.0665	C11H18N2	305.0981	0.70345
57.12035	-0.45729	0.253904	1.311954	unchanged	26.46398	C16H27N3	568.086	0.696317
324.0236	#######	0.999884	0.071798	unchanged	-8.30671	C6H6N2O	153.0293	0.7106
189.6777	-0.03431	0.801592	0.378028	unchanged	-4.07004	C6H4O5	154.998	0.7106
1392.571	-0.01818	0.918534	0.186156	unchanged	-6.43015	C6H6O6	173.008	0.7106
1840.698	-0.02283	0.907513	0.202968	unchanged	-2.93873	C9H12N2O	243.0615	0.7106
21.49856	0.133214	0.673673	0.397193	unchanged	9.783745	C18H21N5	502.0673	0.7106
266.0164	-0.10536	0.535161	0.799518	unchanged	18.70804	C16H24N2	549.0544	0.7106

9324.726	0.270271	0.258657	1.289213	unchanged	9.355413	C49H82N(	898.5439	10.05017
11335.45	-0.35335	0.098276	1.772708	unchanged	-3.87887	C24H37N(	413.3148	10.0573
126.7186	0.205886	0.384097	0.921904	unchanged	1.885716	C37H52O4	543.3843	10.0573
255.9635	-0.01503	0.935082	0.133926	unchanged	3.095239	C22H44O4	411.303	10.07088
3.453545	-0.57438	0.639819	0.531474	unchanged	-6.19327	C17H25N(	541.1481	10.07802
180.9791	-0.58716	0.435407	0.870538	unchanged	1.665931	C23H40N2	597.2148	10.07802
8286.737	-0.44651	0.4988	0.779961	unchanged	-5.0869	C32H44O1	653.2771	10.07802
223.1095	-0.81802	0.268771	1.205886	unchanged	-19.5233	C34H48N8	711.3318	10.07802
8.322798	-0.07151	0.953416	0.109232	unchanged	-3.85749	C25H44N(	743.2454	10.07802
470.8862	0.777551	0.177987	1.547014	unchanged	-26.5037	C18H40N(	285.2946	10.08515
149.8885	1.744909	0.167504	1.57219	unchanged	-4.2102	C27H44O8	535.2647	10.08515
274.027	0.226563	0.542261	0.746853	unchanged	-1.99437	C47H85O1	936.5551	10.08515
55.9395	1.506749	0.210546	1.425261	unchanged	3.089426	C54H102N	946.7264	10.08515
6.28006	0.490125	0.391143	0.959636	unchanged	7.79952	C24H44O7	462.346	10.09943
60.23214	0.270091	0.239136	1.327583	unchanged	-16.4465	C12H26N(	471.3488	10.09943
73.61549	-0.07979	0.648651	0.538914	unchanged	-31.8224	C53H90O2	1043.544	10.10657
125.9968	-0.08298	0.694973	0.490035	unchanged	-22.3484	C28H44O	419.3196	10.10657
43.7518	0.693633	0.31302	1.135519	unchanged	2.418843	C16H25N(	549.3676	10.10657
40.82871	0.821426	0.325634	1.146971	unchanged	19.14301	C42H64O2	639.4653	10.10657
114647.7	-0.17256	0.458103	0.797073	unchanged	15.30476	C35H67O1	701.5498	10.10657
161.3351	-0.21703	0.068478	1.846503	unchanged	-27.657	C17H31N(	371.2449	10.12085
36462.69	0.382676	0.431216	0.930472	unchanged	14.06533	C36H60O1	675.4756	10.17795
2403.65	0.58948	0.043306	1.980456	up	-6.76167	C38H68O8	691.4502	10.17795
673.0443	0.427831	0.291892	1.202937	unchanged	-17.4773	C62H111N	1184.817	10.20652
134.1497	-0.14808	0.371196	0.980592	unchanged	-0.22277	C24H34O5	403.2478	10.22793
14427.86	-0.05732	0.610421	0.613745	unchanged	2.741458	C24H34O4	425.2099	10.2422
1827.098	-0.54413	0.143931	1.749554	unchanged	-0.82592	C27H41FC	455.3129	10.23507
179.2216	0.195246	0.26374	1.243373	unchanged	-33.5801	C32H64O8	577.448	10.23507
720.1968	-0.02397	0.915009	0.144979	unchanged	4.031756	C23H36N2	341.2965	10.24933
524.4707	-0.13556	0.584011	0.681076	unchanged	1.841907	C30H40O	381.2948	10.24933
348.5959	-0.0425	0.874252	0.201696	unchanged	-0.80247	C40H68O7	625.4821	10.24933
1394.391	0.139612	0.477075	0.779367	unchanged	4.452218	C49H71N1	966.5353	10.26362
69.46006	-0.11949	0.352548	1.061726	unchanged	0.664634	C7H11F3	152.0808	10.29932
137.0043	-0.23731	0.161233	1.494475	unchanged	-10.0894	C3H6N2	141.1121	10.31358
283.9421	-0.12051	0.730833	0.394523	unchanged	1.498728	C22H30O5	397.1991	10.30645
1030.372	0.059683	0.746365	0.403092	unchanged	-0.71373	C39H70N(	694.4801	10.31358
322.5091	-0.07142	0.5954	0.616772	unchanged	20.64313	C26H46O6	419.325	10.32787
326.3138	-0.07816	0.47296	0.81444	unchanged	-4.63096	C24H44O6	470.3547	10.35642
2415.74	-0.03142	0.731702	0.389991	unchanged	43.28354	C11H14N2	175.1305	10.39212
1116.749	-0.08638	0.271995	1.182189	unchanged	-11.4376	C37H67N(	660.4529	10.41997
118.125	-0.3372	0.169872	1.51014	unchanged	-19.0186	C18H30D4	319.272	10.4271
190.3801	-0.02546	0.876039	0.185121	unchanged	2.146161	C29H49N(	491.3615	10.44138
689.7498	-0.23264	0.189748	1.390339	unchanged	27.71877	C28H46O4	447.3593	10.49135
24.85125	0.055302	0.843669	0.19484	unchanged	-5.27157	C13H14N4	227.1279	10.51277
61.90463	-0.14135	0.36262	1.003265	unchanged	8.475721	C14H15N3	259.1574	10.5199
814.5733	-0.00357	0.972443	0.016844	unchanged	14.1782	C44H86O1	955.5203	10.5199
166.0744	-0.19216	0.230543	1.294621	unchanged	4.077961	C11H12O6	223.0611	10.54847
99.16228	-0.06545	0.792908	0.344137	unchanged	9.588532	C17H26O1	445.1146	10.54847
4.361026	-0.66244	0.522846	0.819423	unchanged	1.831745	C12H17N(	613.1652	10.54847
54.42526	-0.00335	0.976197	0.029076	unchanged	-7.87259	C5H10O2	103.0746	10.5556
341.836	-0.0566	0.65118	0.564747	unchanged	-1.15619	C19H28O2	577.4245	10.58425

480.5853	-0.24293	0.268346	1.261588	unchanged	-12.7956	C36H61N(	642.4053	10.58425
603.8771	-0.10002	0.464209	0.855017	unchanged	19.93286	C3H10NO	301.0744	10.61282
75.98996	-0.24748	0.37243	1.060749	unchanged	-1.38451	C35H56O7	553.3879	10.60567
312.0611	0.829665	0.106729	1.801547	unchanged	1.985687	C37H66O3	597.4655	10.61282
428.0736	0.079564	0.744601	0.342346	unchanged	3.00162	C20H32O	599.4816	10.61282
1069.144	-0.23404	0.337726	1.039241	unchanged	13.14628	C38H68O6	603.5065	10.61282
2990.587	-0.05218	0.757971	0.2756	unchanged	4.706325	C38H73N(	710.4999	10.61282
12754.92	0.482717	0.212162	1.44688	unchanged	1.545394	C41H69O8	721.4814	10.61282
54954.98	0.410148	0.13387	1.62561	unchanged	3.983142	C41H73N2	736.5179	10.61282
9416.787	0.189994	0.342672	1.085902	unchanged	-5.68804	C43H76N(	748.5232	10.61282
271563.4	0.205935	0.428659	0.9149	unchanged	-15.9327	C43H78N(	806.5598	10.61282
415857	0.636444	0.083215	1.871533	unchanged	1.449903	C44H77N(	780.5454	10.61282
180083.2	0.20997	0.117296	1.696972	unchanged	2.25253	C45H79N(	794.5617	10.61282
597807.4	0.090537	0.346392	1.03017	unchanged	-11.1768	C46H82N(	808.5761	10.61282
25627.59	0.206296	0.298549	1.15919	unchanged	7.358808	C45H82N(	792.5599	10.61282
20808.95	0.597578	0.201993	1.473631	unchanged	-5.0631	C45H80N(	848.537	10.61282
3413.681	-0.06736	0.837136	0.145773	unchanged	0.530535	C46H84N(	854.5546	10.61282
5639.523	-0.10216	0.7612	0.37122	unchanged	-0.50742	C46H81O1	858.585	10.61282
28516.02	-0.25196	0.248033	1.266002	unchanged	-28.3574	C48H84N3	876.5608	10.61282
2818.382	0.83953	0.078398	1.835102	unchanged	2.454216	C44H79O1	856.5718	10.61995
25033.83	0.027076	0.874115	0.126171	unchanged	7.788031	C49H84N(	900.5582	10.61995
222.7149	-0.15891	0.391031	1.009922	unchanged	2.084168	C21H40N6	441.3193	10.62708
106.8857	-0.0632	0.758817	0.34363	unchanged	25.63558	C28H44O	419.3386	10.64137
6.956516	-0.85024	0.381756	0.958329	unchanged	2.188066	C16H24N2	623.3218	10.6485
16928.82	0.666466	0.227194	1.373979	unchanged	-11.6516	C40H76N(	714.5349	10.6485
38.32828	-0.43629	0.324087	1.150291	unchanged	-20.8023	C18H32O4	295.2203	10.6842
76.29138	-0.19338	0.22987	1.366074	unchanged	2.263773	C37H66O7	661.4454	10.67705
3.01233	-2.44964	0.307601	1.131922	unchanged	13.79432	C16H18N2	669.2495	10.67705
211.0078	-0.35699	0.180431	1.460108	unchanged	-1.4571	C41H66N1	976.5197	10.69133
1679.816	0.537831	0.211387	1.358151	unchanged	-28.2723	C63H113N	1198.819	10.69847
139.9578	-0.7666	0.025091	2.12933	down	5.056766	C20H16O1	431.0632	10.7406
83.27427	-0.34033	0.376493	0.947446	unchanged	7.707409	C19H30N6	509.1793	10.74775
302.5344	-0.31024	0.435403	0.839764	unchanged	3.833061	C28H38N4	565.2443	10.74775
12165.19	-0.37018	0.271954	1.165087	unchanged	1.531833	C29H49O1	621.3044	10.74775
200.7722	-0.60177	0.0623	1.814893	unchanged	-3.41392	C38H45N5	652.3471	10.74775
438.5874	-0.41971	0.135491	1.517095	unchanged	8.476724	C39H47N5	666.3706	10.74775
4043.231	-0.31476	0.065447	1.828356	unchanged	2.19658	C40H58O1	679.3856	10.74775
99.16317	-1.48061	0.089129	1.969962	unchanged	12.8793	C23H44O5	401.3313	10.79057
80.74549	-0.56325	0.222115	1.350594	unchanged	-0.1711	C9H13NO	399.1761	10.79772
224.7319	-0.45411	0.048983	1.99771	down	-8.70861	C8H6O4	149.0219	10.82627
60.03652	-0.23036	0.051464	1.92754	unchanged	2.155719	C13H18N4	261.1352	10.8334
1738.896	-2.60671	0.403192	0.92631	unchanged	-0.68651	C26H39N5	684.1943	10.8334
193.4614	-0.49712	0.206192	1.409437	unchanged	4.620982	C33H43N5	647.358	10.85482
290.361	-0.41602	0.162264	1.595679	unchanged	-1.00541	C12H23N(	587.3016	10.8691
48.58899	-0.44351	0.038927	2.076275	down	-2.6071	C6H12O5	129.0542	10.88338
144.9213	-0.26056	0.109799	1.723907	unchanged	-0.936	C16H22N2	259.1802	10.87623
517.8467	-0.51548	0.051995	1.954046	unchanged	15.06396	C33H39N3	551.3457	10.9048
4827.659	0.078686	0.666861	0.494821	unchanged	-5.54094	C22H43N(	369.3216	10.91193
379.4191	-0.09438	0.832199	0.303765	unchanged	-16.5285	C40H69N(	682.4701	10.91907
92.20458	-0.4859	0.32914	1.075184	unchanged	-28.5989	C27H46O3	401.3294	10.92622
99.08444	0.094278	0.885202	0.196444	unchanged	6.317082	C18H34O4	667.4585	10.93335

10.21551	0.380363	0.457982	0.780203	unchanged	-23.6076	C11H16	149.129	10.95477
46.24847	-0.00144	0.996943	0.069387	unchanged	-21.4876	C13H25N(	212.1964	10.95477
6.909159	-0.33574	0.497022	0.798791	unchanged	-29.5482	C9H12	121.0976	10.9619
187.074	0.466064	0.463823	0.881226	unchanged	11.25943	C33H65O1	653.4462	10.9619
1402.481	-0.08672	0.649822	0.592426	unchanged	-11.2333	C38H67N(	675.5235	11.01198
33600.16	0.604936	0.167071	1.543747	unchanged	-8.37181	C42H82N(	756.5472	11.01912
868077.3	0.97826	0.143491	1.674923	unchanged	-8.23725	C42H80N(	758.5632	11.02625
228.536	-0.27056	0.621441	0.543982	unchanged	0.112562	C9H15N4(	355.065	11.04053
136.5208	-0.3894	0.075359	1.772888	unchanged	21.03119	C34H64N(	642.4272	11.04767
247.3486	-0.54018	0.118136	1.661022	unchanged	-19.5348	C18H32O4	295.2207	11.08267
32.79709	0.185928	0.519341	0.705618	unchanged	3.129331	C18H19N(	339.1535	11.08267
117.0666	2.161753	0.232853	1.366046	unchanged	7.595191	C35H38N2	549.2791	11.07552
383.0545	-0.09102	0.354675	1.067355	unchanged	-4.70058	C10H12N4	301.0765	11.11835
163.8877	-0.48213	0.161211	1.593484	unchanged	-2.72859	C31H50O8	551.3563	11.13977
240.0711	0.821024	0.136897	1.661499	unchanged	-21.0655	C48H83N3	1052.5	11.15405
97.92387	-0.64559	0.033916	1.98809	down	-9.83402	C23H41O6	427.2564	11.14692
274.2037	0.107944	0.76762	0.32962	unchanged	1.202145	C50H71N1	1010.522	11.16118
1700.394	-0.24978	0.187684	1.449621	unchanged	-23.48	C45H84O1	944.5406	11.16118
8741.451	-0.36631	0.023076	2.128536	down	-3.03284	C44H80O2	623.6106	11.16842
76.24052	-0.06664	0.385603	0.959477	unchanged	0.632462	C17H28N2	347.1733	11.21125
36.32852	-0.42538	0.070628	1.786553	unchanged	13.75508	C25H44O8	490.3439	11.21125
3091.281	-0.15672	0.138077	1.546418	unchanged	-11.3159	C20H39N(	310.3069	11.2184
414.3056	-0.10361	0.662322	0.518335	unchanged	-5.62887	C44H81N3	976.4981	11.2184
16.16493	-0.31911	0.163779	1.39591	unchanged	1.177624	C6H9NO3	144.0657	11.22553
116.6485	-0.24513	0.077128	1.831309	unchanged	-8.96005	C6H8O4	162.0748	11.22553
304.452	-0.09175	0.434802	0.894823	unchanged	-2.09489	C9H17NO	178.1199	11.23267
1196.024	-0.22898	0.229258	1.380866	unchanged	-0.20799	C47H81O1	931.5177	11.22553
9.333731	-0.05326	0.723581	0.388492	unchanged	1.76628	C11H14FN	160.0924	11.23982
559.9766	-0.08873	0.562941	0.694554	unchanged	-22.0755	C47H92O5	719.6749	11.23982
17.76944	-0.16741	0.342432	1.017224	unchanged	-1.79651	C4H9N3O	132.0765	11.24695
160.6158	-0.05828	0.658634	0.436118	unchanged	-1.38807	C6H6N2O	139.0498	11.25408
697.727	0.014557	0.909109	0.119074	unchanged	6.599114	C4H6N2O	184.9791	11.24695
15.19627	-0.41786	0.304508	1.199902	unchanged	1.284705	C13H17N(	236.1284	11.24695
652.2635	-0.12234	0.392075	0.950174	unchanged	0.632275	C45H84O5	705.6396	11.24695
7022.966	0.003076	0.97707	0.03992	unchanged	20.95928	C4H4N2O	182.9655	11.26123
122.995	0.079441	0.517589	0.705452	unchanged	0.817488	C6H3ClO3	180.9664	11.26837
4.49E-05	0.901326	0.325027	1.071566	unchanged	-2.24138	C41H72O6	625.5176	11.26837
97.35139	0.085328	0.356556	1.050658	unchanged	-0.72655	C2H2F3N(	77.99491	11.29692
110.3705	-0.02207	0.84147	0.228572	unchanged	-5.88325	C2H2N2O4	156.9639	11.59603
96.54031	0.021476	0.832205	0.251114	unchanged	0.825584	C3H2FN3(	169.9764	11.74523
51.39225	-0.01051	0.933252	0.094802	unchanged	-12.1144	C2HClF2O	112.9585	11.9451
103.9034	-0.08457	0.436134	0.893363	unchanged	1.733028	CH4O4S	111.9826	11.95938
73.84085	-0.02459	0.955996	0.12502	unchanged	-10.7417	C9H12N2(	165.1005	2.0148
91.46338	0.077121	0.88198	0.128255	unchanged	-15.5759	C10H16N2	211.1042	2.0148
86.21267	-0.18478	0.783907	0.303424	unchanged	1.922413	C6H9NO4	214.0148	2.007667
933.8449	-0.0535	0.824505	0.271752	unchanged	-2.05289	C13H14O5	268.1174	2.007667
531.5203	-0.3757	0.674058	0.455691	unchanged	-29.4133	C9H14N3(	290.0446	2.007667
2634.785	0.24072	0.353884	0.996917	unchanged	3.988931	C7H12N2(	173.128	2.035517
1161.136	-0.58231	0.152035	1.512918	unchanged	-1.35652	C11H15N(	194.1173	2.049783
38.31086	0.038251	0.962346	0.107927	unchanged	18.71313	C20H22O6	400.1822	2.056933
8.503862	1.124028	0.516777	0.758305	unchanged	7.109739	C8H8O	121.0656	2.092617

50.90887	0.221009	0.745189	0.367506	unchanged	-5.30157	C17H14O6	315.0846	2.09975
51.39349	0.77256	0.655665	0.540448	unchanged	4.849554	C14H18O8	356.1355	2.09975
222.4859	1.020312	0.091766	1.683903	unchanged	-1.47162	C10H14N4	272.135	2.1069
734.7481	-0.04387	0.503219	0.794092	unchanged	-3.7981	C12H15N(	222.1116	2.121167
498.4496	0.035254	0.864495	0.170067	unchanged	9.94663	C11H19N(	268.118	2.121167
96.44283	-0.08661	0.549783	0.701288	unchanged	-1.14279	C10H18N(	248.1043	2.142583
2613.809	-0.35157	0.599199	0.644412	unchanged	-2.462	C17H22N2	367.1493	2.149717
49.13101	2.537138	0.426967	0.931432	unchanged	6.90941	C7H8O4	121.0295	2.164
13.79654	-0.21109	0.787462	0.352146	unchanged	6.662532	C7H13N3(	168.0781	2.156867
1515.087	0.106947	0.846958	0.166619	unchanged	5.799695	C9H11N5(	205.0969	2.164
451.4492	-0.96325	0.0223	2.209075	down	-29.2799	C13H15N3	226.0898	2.178283
66.98764	0.529383	0.567742	0.645726	unchanged	-14.1553	C10H14N2	244.126	2.185417
65.45713	-1.28657	0.472097	0.836902	unchanged	-7.61386	C10H12N2	218.1274	2.199683
40.00858	-0.26392	0.383579	0.917942	unchanged	-47.5515	C11H13N3	242.0594	2.2211
297.1148	0.884762	0.170799	1.432802	unchanged	-9.2944	C9H15N3(	271.138	2.2211
267.6937	0.058371	0.894666	0.095424	unchanged	1.428939	C9H10O2	115.0544	2.22825
690.9999	-0.08575	0.730078	0.421804	unchanged	4.929831	C9H7N	130.0658	2.22825
1550.398	0.001905	0.997266	0.051833	unchanged	5.007676	C9H9N	170.0605	2.22825
446.8451	0.031918	0.927408	0.049032	unchanged	3.472348	C10H11N(	142.0657	2.22825
2380.73	-0.00896	0.987446	0.074661	unchanged	-1.51397	C10H9N	144.0806	2.22825
6139.984	-0.01744	0.974921	0.092683	unchanged	-2.55452	C9H7NO	146.0597	2.22825
21651.41	0.003521	0.994817	0.04803	unchanged	-2.899	C11H9NO	188.0701	2.22825
126.5603	0.282628	0.381473	0.92081	unchanged	-18.7465	C10H14N2	228.1303	2.22825
36.45894	0.455399	0.701221	0.390994	unchanged	-2.10538	C22H24N4	409.1862	2.22825
212.2528	-0.04835	0.779989	0.331114	unchanged	9.494906	C5H10N2(	127.034	2.235383
45.34833	0.303159	0.430136	0.901292	unchanged	-3.68563	C11H14N(	288.0621	2.242517
57.40192	0.81547	0.37353	1.041625	unchanged	4.27006	C20H23N(	404.1358	2.235383
1742.284	-0.65483	0.3561	1.028696	unchanged	-25.6448	C13H15N(	298.0845	2.2568
605.2732	-0.64207	0.35887	1.017383	unchanged	4.521348	C15H13N3	300.0815	2.2568
304.9997	-0.65666	0.338736	1.066492	unchanged	10.03046	C13H15N3	316.0933	2.263933
62871.59	-0.61592	0.359121	1.023416	unchanged	-7.26447	C21H15N(	344.0891	2.2568
21346.42	-0.62994	0.358475	1.023799	unchanged	-3.92505	C12H17N3	346.0867	2.2568
394.9794	0.20243	0.376803	1.031722	unchanged	-1.12619	C9H10ClN	399.0868	2.2568
252.1344	0.294132	0.221149	1.356982	unchanged	9.702869	C11H17N3	401.0832	2.2568
40.73915	1.234692	0.25622	1.245569	unchanged	2.012036	C16H25N5	589.0828	2.2568
17.89934	1.701269	0.153912	1.545597	unchanged	2.436967	C10H13FN	591.0805	2.263933
155.5016	0.229328	0.613301	0.509342	unchanged	-4.34161	C6H13N5(	202.0925	2.278217
137.2313	-0.0959	0.562778	0.67	unchanged	-3.51962	C11H22N4	239.1493	2.278217
48.33187	0.466412	0.411136	0.888124	unchanged	-4.92225	C12H13N(	258.0516	2.28535
833.0482	-0.53687	0.362425	1.00533	unchanged	5.541941	C13H19N3	384.0823	2.292483
44818	0.002603	0.984543	0.039901	unchanged	25.28752	C5H4N4O	202.044	2.299633
13797.41	0.031545	0.861134	0.203516	unchanged	-15.8651	C5H8OS	117.0572	2.306767
352361.7	0.009325	0.952542	0.082358	unchanged	0.70445	C6H8FN3(	154.0412	2.306767
122507.3	0.024302	0.886822	0.175741	unchanged	-16.2606	C7H11O6-	156.0386	2.306767
94.55032	0.091205	0.539651	0.708839	unchanged	3.296451	C9H6F4O2	222.0305	2.306767
112.5145	0.262945	0.316902	1.05722	unchanged	1.372975	C8H6N2O	315.0856	2.3139
178.9334	0.093892	0.552715	0.695098	unchanged	-16.818	C16H24N2	545.0474	2.3139
38.64915	0.287038	0.545094	0.70414	unchanged	20.25624	C17H25N5	624.0836	2.306767
203.9598	0.049309	0.81587	0.27467	unchanged	8.410833	C6H4	77.03922	2.306767
737.2455	0.01929	0.907427	0.136394	unchanged	4.355853	C7H8O2	89.03912	2.306767
582.981	0.111329	0.624484	0.481734	unchanged	1.296068	C10H16N2	197.1287	2.34245

320.281	0.009194	0.973941	0.010627	unchanged	2.725177	C9H11NO	207.1133	2.34245
29.24268	0.126444	0.621102	0.541148	unchanged	-6.36192	C14H18O7	281.1001	2.335317
78.34743	0.090422	0.822573	0.263206	unchanged	-7.53513	C10H11N(	178.0849	2.356733
43.98786	0.147344	0.682554	0.461777	unchanged	2.339811	C13H17N(	236.1287	2.356733
1108.307	0.049404	0.806571	0.245313	unchanged	-2.55635	C15H20N2	261.1413	2.3703
88.59179	0.410194	0.156154	1.597426	unchanged	-8.75528	C8H11N3(	212.0468	2.37745
121.9385	0.452435	0.286251	1.203192	unchanged	2.763976	C9H18O6	245.1002	2.37745
138.4124	0.644096	0.125785	1.544442	unchanged	-5.01186	C7H13N3(	258.0507	2.37745
273.8112	-0.19766	0.709703	0.411192	unchanged	6.080873	C17H18FN	376.0787	2.37745
491.7453	0.288767	0.12004	1.704015	unchanged	-0.48318	C10H15N(	148.112	2.391717
268.6917	0.266026	0.140261	1.68917	unchanged	0.800771	C11H16N2	209.1286	2.391717
20.83785	0.119006	0.844484	0.178216	unchanged	6.413743	C9H16N4(	277.116	2.39885
97.75239	0.323367	0.587098	0.631177	unchanged	-0.49639	C16H15O8	300.0627	2.406
652.9697	-1.10334	0.16607	1.664217	unchanged	7.050894	C22H24O5	333.1511	2.413133
63.38122	0.14071	0.479232	0.777537	unchanged	-6.86723	C7H6N2S	168.058	2.427417
201.5524	0.14601	0.380254	0.976656	unchanged	4.13136	C6H13N3(	214.0596	2.43455
593.9239	1.081023	0.249914	1.289932	unchanged	-5.55564	C15H27N(	316.1736	2.427417
70.96979	0.687209	0.291531	1.138459	unchanged	13.06171	C12H14N2	219.1157	2.448833
46.10024	0.310584	0.521115	0.760268	unchanged	-9.27389	C12H23N(	244.1519	2.455967
299.9382	0.050065	0.942315	0.020889	unchanged	-15.3612	C20H18O3	307.1282	2.470233
543.7905	-0.07887	0.689073	0.492592	unchanged	-17.9538	C19H20O4	295.1273	2.4988
154.0829	0.213285	0.224862	1.270761	unchanged	1.247027	C6H13NO	114.0915	2.52735
88.14105	0.10162	0.627216	0.560366	unchanged	-0.75069	C9H12FN3	226.062	2.534483
1265.59	0.040334	0.869353	0.202877	unchanged	2.795447	C8H15N3(	272.0682	2.52735
33.32831	0.180843	0.857051	0.255079	unchanged	5.011811	C20H24N8	490.2181	2.52735
68.05665	0.240159	0.688506	0.421443	unchanged	22.57576	C8H15N3(	216.1031	2.548767
227.6659	0.14927	0.301644	1.083467	unchanged	15.48236	C19H24O3	283.1739	2.5559
136.1897	0.088658	0.727353	0.366277	unchanged	23.1619	C11H17N3	305.1522	2.5559
147.1322	0.134592	0.129955	1.654271	unchanged	4.046686	C9H11NO	130.0658	2.577317
564.3488	-0.87184	0.01995	2.168088	down	14.59436	C7H14N2(	208.132	2.58445
27.4525	0.248659	0.792934	0.283725	unchanged	0.739231	C8H18N2(	139.1231	2.598733
52.35935	0.350731	0.6919	0.444903	unchanged	-0.71093	C9H11NO	167.1178	2.605867
39.00879	-0.05169	0.7738	0.337813	unchanged	-18.8776	C10H13N(	192.0612	2.605867
148.0345	1.028694	0.404307	1.006265	unchanged	-2.30106	C10H18N2	197.128	2.634417
1407.046	-0.03207	0.892107	0.139917	unchanged	-0.06965	C11H14N(	256.0733	2.634417
482.8829	-0.07423	0.726973	0.412063	unchanged	-19.4255	C12H12FN	258.0729	2.634417
3746.4	0.046482	0.845509	0.226377	unchanged	-15.8491	C15H17N(	304.0762	2.634417
14078.19	0.002848	0.98872	0.028787	unchanged	-6.72878	C11H19N3	302.0782	2.634417
59.34031	-0.04196	0.950683	0.121484	unchanged	1.92624	C23H20O1	439.1032	2.627283
338.8932	0.104272	0.804486	0.234047	unchanged	-6.57401	C10H9N	144.0798	2.6487
153.0923	0.053558	0.917995	0.070525	unchanged	-2.8987	C12H16N2	217.0964	2.6487
533.2657	-0.25685	0.237936	1.30447	unchanged	1.65337	C9H10FN3	210.0677	2.655833
93.20655	1.446343	0.13129	1.592252	unchanged	18.06899	C9H13N2(	342.0755	2.662967
18.04296	-0.05053	0.95085	0.036449	unchanged	-5.34869	C10H9NO	214.0464	2.67725
70.2629	-0.81243	0.244274	1.359604	unchanged	7.54241	C12H15F3	243.1123	2.684383
58.40415	-0.01948	0.977157	0.079287	unchanged	12.90644	C13H26O2	253.1592	2.684383
37.49261	-0.07769	0.817545	0.282237	unchanged	-6.66007	C17H18O8	315.084	2.67725
6.579946	3.262406	0.399394	0.988088	unchanged	2.330068	C10H10N2	191.0819	2.691533
98.32658	-0.45791	0.523011	0.755801	unchanged	11.52917	C8H7NO	134.0616	2.712233
519.8868	-1.06453	0.031707	2.053871	down	-0.62357	C12H17N(	208.1331	2.712233
79.73772	0.369787	0.321421	1.05696	unchanged	9.58984	C15H20N2	275.1418	2.712233

121.6574	1.203437	0.25075	1.238687	unchanged	-4.13124	C16H10O6	340.0803	2.7051
167.4816	1.074075	0.430613	0.952319	unchanged	-0.46725	C10H20N2	197.1283	2.719383
92.0901	0.503312	0.600549	0.528881	unchanged	9.834968	C22H32O8	389.2	2.719383
63.96034	0.578113	0.305173	1.173767	unchanged	-3.34455	C20H20O4	289.1212	2.73365
74.6914	0.53985	0.522501	0.740107	unchanged	-35.5688	C16H22O3	227.1337	2.790767
163.338	0.238896	0.053539	1.868859	unchanged	23.10737	C19H28O3	327.2001	2.790767
1192.672	-0.86509	0.495236	0.812013	unchanged	2.215666	C21H20O5	317.118	2.812183
99.49831	0.387777	0.629382	0.575915	unchanged	-8.90487	C18H24O4	626.3633	2.812183
55.99348	0.119482	0.831555	0.179178	unchanged	-1.36462	C16H19N3	334.1393	2.819317
72.20381	-0.34455	0.38487	0.985599	unchanged	37.89521	C11H16N2	295.1004	2.847867
582.3534	-0.13083	0.549922	0.674575	unchanged	-22.747	C3H11NO	236.0012	2.855
486.1803	0.51272	0.62689	0.589612	unchanged	-4.31671	C13H16N2	287.0985	2.86215
1744.035	-0.1653	0.646292	0.520185	unchanged	-15.5667	C19H18O8	397.0706	2.86215
241.55	-3.21089	0.439129	0.933175	unchanged	-12.0421	C24H25O1	528.1177	2.855
2214.889	-0.07917	0.785789	0.304329	unchanged	0.289449	C6H8O2	77.03861	2.869283
16120.2	-0.14006	0.638424	0.53439	unchanged	-1.64849	C7H6O2	105.0333	2.869283
208.892	-0.19159	0.463304	0.826998	unchanged	7.76355	C8H7NO	134.0611	2.869283
2822.853	-0.11765	0.681973	0.463492	unchanged	0.113857	C9H6O3	180.0655	2.869283
496.5668	0.281861	0.468947	0.830011	unchanged	-30.7802	C5H10O6	166.042	2.869283
63.78679	0.720065	0.311519	1.170219	unchanged	-4.47643	C6H7N3O	168.0395	2.869283
1540.628	0.050533	0.778631	0.335327	unchanged	-12.6568	C10H9NO	383.0829	2.869283
472.5517	0.0291	0.660339	0.434117	unchanged	-5.41388	C9H13N3O	194.0913	2.869283
224.3421	-0.10372	0.584013	0.645453	unchanged	18.89798	C9H8N2O	231.0203	2.869283
2619.498	0.576343	0.419798	0.94188	unchanged	3.025348	C8H10FN3	270.0891	2.869283
422.7025	0.586596	0.411659	0.958564	unchanged	3.077041	C14H17N3	272.0862	2.869283
633.0927	-0.05109	0.873325	0.182824	unchanged	-7.33849	C21H16O7	381.0941	2.869283
27.15143	-0.03649	0.934765	0.052195	unchanged	-20.545	C13H19N4	419.0505	2.869283
1387.705	-0.17751	0.683472	0.457248	unchanged	-10.7044	C26H27N5	576.1301	2.869283
252.284	0.603819	0.099891	1.705322	unchanged	1.59282	C5H11N3O	168.0746	0.55425
2177.144	-0.15798	0.654378	0.505655	unchanged	-0.16084	C6H6	79.05421	2.869283
134.1417	0.010407	0.987021	0.035446	unchanged	3.335536	C7H5ClO	104.9895	2.876417
5098.334	0.101874	0.678456	0.481221	unchanged	-29.9346	C10H16N2	241.0736	2.883567
1666.241	0.11345	0.652184	0.522607	unchanged	-24.4956	C10H13FN	243.0712	2.883567
83.9903	3.066677	0.190075	1.496329	unchanged	-15.0203	C20H30O5	315.1902	2.904967
334.9247	-0.52348	0.300446	1.13807	unchanged	13.25978	C14H16O9	329.0911	2.904967
32.18522	0.13204	0.776012	0.289254	unchanged	7.00354	C11H10N2	169.0773	2.912117
97.26616	-0.24371	0.643303	0.518253	unchanged	1.239253	C7H11NO	164.0684	2.91925
68.16573	0.425962	0.645752	0.503065	unchanged	-0.51989	C16H16	209.1324	2.926383
213.7579	0.627644	0.311977	1.097588	unchanged	2.130681	C15H24N2	319.1424	2.940667
1442.341	0.380751	0.176064	1.426287	unchanged	-26.992	C13H14O8	340.0878	2.95495
238.392	0.473851	0.237821	1.261812	unchanged	-10.4373	C10H19NO	272.0723	2.962083
191.8896	0.458496	0.486707	0.818702	unchanged	-1.44988	C12H12N2	314.0801	2.962083
445.4243	0.877603	0.332463	1.058215	unchanged	-1.47199	C15H21N3	373.1713	2.969217
15.56144	0.054933	0.933148	0.016921	unchanged	22.86336	C10H10N2	197.0725	2.97635
179.5866	-0.23825	0.571736	0.666341	unchanged	-0.85658	C14H14N2	257.0918	2.97635
93.60333	0.679085	0.339359	1.090472	unchanged	-16.0213	C8H14N3O	297.0914	2.9835
105.501	1.364977	0.132777	1.572739	unchanged	-11.669	C8H13N4O	342.0771	2.9835
198.4435	0.339118	0.076981	1.78666	unchanged	13.72913	C23H32O5	371.227	2.97635
66.07496	2.47146	0.296625	1.261314	unchanged	-3.05444	C19H32O7	390.2475	2.9835
605.6818	-0.3246	0.340662	1.131037	unchanged	3.151292	C12H10N2	181.0766	2.990633
2561.672	-0.36068	0.317313	1.176791	unchanged	19.46081	C8H10N4O	227.0817	2.990633

165.6658	0.144415	0.471305	0.750053	unchanged	19.53903	C13H16N2	215.1224	3.0399
101.0308	1.178378	0.03374	2.023927	up	-27.2666	C14H20O8	281.0933	3.0399
102.324	-0.29837	0.560438	0.631155	unchanged	4.549988	C10H12FN	240.0791	3.061317
531.3193	-0.18583	0.728617	0.358473	unchanged	-2.36477	C11H19N3	286.0848	3.061317
153.6767	-0.1497	0.789051	0.269696	unchanged	0.454624	C10H15N3	288.0828	3.061317
58.97701	-0.02215	0.934145	0.019067	unchanged	12.24701	C8H8O3	135.0459	3.06845
61.95458	0.630674	0.07149	1.800862	unchanged	-26.2302	C16H18O6	329.0915	3.06845
20.55485	0.703585	0.241589	1.312144	unchanged	-0.0863	C12H9N	168.0808	3.082733
120.019	0.18731	0.080036	1.761936	unchanged	12.29176	C9H8O	155.0762	3.089867
97.58663	0.434225	0.253626	1.305202	unchanged	3.275958	C10H9N	143.0734	3.089867
311.1486	0.534994	0.153712	1.611489	unchanged	3.625409	C11H11N	158.097	3.089867
66.43073	0.098567	0.787569	0.239795	unchanged	-2.59324	C11H16O3	197.1167	3.111283
11.41816	-0.53906	0.34837	1.071868	unchanged	15.05461	C14H18N2	245.1324	3.111283
137.1925	0.472535	0.028588	2.226948	up	2.562784	C14H22N2	267.171	3.111283
48.65313	-0.36081	0.707378	0.425038	unchanged	5.468924	C15H18O4	285.1112	3.111283
62.54396	0.15285	0.778258	0.322764	unchanged	4.670092	C12H10N2	183.0925	3.1327
30.84253	0.468061	0.284132	1.225763	unchanged	-1.52105	C12H12N2	185.107	3.1327
11.7914	0.355175	0.302161	1.056951	unchanged	-10.0147	C5H10N2O	188.1015	3.1327
45.03896	-1.76568	0.120162	1.594293	unchanged	-11.8607	C14H19F3	631.2617	3.1327
63.58376	-1.23893	0.279429	1.16913	unchanged	5.31987	C24H32O7	946.4125	3.1327
81.77766	0.088873	0.830127	0.279087	unchanged	-18.312	C11H19NO	316.0949	3.146967
9.123187	0.105759	0.868284	0.217079	unchanged	4.296717	C17H22O8	319.1191	3.146967
18.87773	0.655915	0.350478	1.100813	unchanged	-6.32066	C20H28O5	371.1807	3.154117
275.4896	0.058021	0.963097	0.074597	unchanged	-8.96888	C22H16O1	441.0752	3.168383
380.3616	0.227174	0.800961	0.20301	unchanged	-0.11719	C24H24O9	457.1493	3.168383
69.97355	0.032188	0.964252	0.057721	unchanged	5.985217	C9H9NO	148.0766	3.175533
44.95759	-0.12661	0.880952	0.151428	unchanged	18.60685	C10H9NO	294.0058	3.175533
760.5762	0.058267	0.929039	0.115838	unchanged	3.664466	C7H10O2	91.05469	3.175533
50.3399	0.16631	0.357167	1.050483	unchanged	-16.9896	C7H14N2O	155.0783	3.19695
34.92968	0.77106	0.340941	1.045824	unchanged	12.18598	C18H18O5	315.1265	3.19695
277.0184	0.985772	0.282141	1.188293	unchanged	10.53471	C13H22N4	347.1598	3.19695
352.3543	0.061402	0.934469	0.117436	unchanged	3.738166	C26H38N2	481.248	3.204083
1.72E-06	-0.23137	0.879174	0.261591	unchanged	-0.24236	C16H24N6	783.3507	3.204083
196.9538	0.503877	0.580496	0.63957	unchanged	-5.9203	C10H13NO	181.1326	3.211217
178.079	0.235615	0.416502	0.861698	unchanged	3.96352	C9H17NO	210.1108	3.211217
148.8432	0.291348	0.364004	0.977646	unchanged	-2.12777	C9H19NO	170.1171	3.21835
360.6033	0.424264	0.60079	0.604456	unchanged	-13.8497	C9H20N2O	227.1389	3.21835
488.5693	1.251371	0.308038	1.212313	unchanged	-1.4932	C11H20N2	211.1438	3.232633
72.27533	0.046908	0.965754	0.06907	unchanged	-5.37143	C10H12N4	254.1235	3.2255
35.88985	0.944303	0.657036	0.540293	unchanged	-5.74607	C20H20O8	430.1474	3.232633
102.145	0.193914	0.602064	0.620788	unchanged	2.162863	C12H21N3	316.0969	3.239767
38.01695	-0.11276	0.336235	1.062208	unchanged	-1.87412	C16H14F3	332.1111	3.239767
147.9202	0.853328	0.314926	1.101363	unchanged	-0.70031	C17H24O1	427.1208	3.239767
112.6561	0.037957	0.896073	0.178991	unchanged	3.640712	C19H14F3	330.1112	3.246917
48.26671	0.97963	0.393353	0.95443	unchanged	10.80084	C20H35NO	480.2129	3.246917
18.86498	-2.2708	0.167826	1.635188	unchanged	18.09888	C19H38N6	527.2767	3.268317
20.71595	0.895552	0.593982	0.650877	unchanged	2.960535	C5H9NO4	130.0503	3.2826
88.8943	0.554358	0.424082	0.944226	unchanged	0.349971	C14H18	209.1301	3.275467
38.33433	1.228702	0.345464	1.070464	unchanged	6.134773	C11H14N4	266.1025	3.275467
58.0318	0.396222	0.339686	1.006663	unchanged	4.730792	C8H11NO	361.1386	3.275467
84.34132	0.442057	0.10688	1.698334	unchanged	-8.46082	C23H32O7	385.1974	3.2826

32.05612	-0.26083	0.566426	0.621434	unchanged	-5.76511	C4H10OS	107.0519	3.304017
105.6143	-0.41414	0.438523	0.958765	unchanged	-12.0026	C13H12N2	213.0997	3.304017
533.5665	0.894946	0.095293	1.671317	unchanged	19.63673	C9H10O3	355.0999	3.31115
63.90331	0.072764	0.836489	0.140986	unchanged	1.449085	C12H13NO	168.0811	3.31115
529.1022	-0.24515	0.630813	0.630779	unchanged	17.60769	C9H16N4O	241.098	3.31115
21.54885	0.023608	0.956799	0.085084	unchanged	-14.77	C12H12O5	254.0988	3.31115
686.6314	-0.32481	0.615573	0.587085	unchanged	5.233466	C9H12N6O	302.1006	3.31115
1518.808	0.879813	0.110171	1.621209	unchanged	0.32936	C20H16O6	353.1021	3.31115
3.306908	5.628729	0.399454	0.969306	unchanged	-0.38064	C14H25NO	409.1815	3.31115
178.9718	-0.02825	0.854763	0.323846	unchanged	8.328887	C12H10N2	199.0882	3.3183
263	-0.07613	0.862335	0.16824	unchanged	5.941883	C10H19N3	300.1005	3.3183
153.3088	-0.62305	0.216138	1.389113	unchanged	3.408743	C15H14N2	271.1086	3.325433
64.74991	0.170367	0.558742	0.730578	unchanged	23.16904	C10H17N3	298.1253	3.332567
604.113	0.175579	0.656156	0.492487	unchanged	1.22326	C19H35NO	412.2101	3.332567
33.44955	-0.86913	0.193707	1.373939	unchanged	1.769455	C22H36N8	606.2852	3.332567
113.3608	0.507952	0.402365	0.930846	unchanged	-24.4385	C8H12N2O	133.0719	3.34685
1001.429	1.173575	0.36615	1.083169	unchanged	1.006064	C11H20N2	211.1443	3.34685
198.6316	0.100364	0.344392	1.070231	unchanged	20.44434	C7H9NO3	155.0776	3.381833
528.5217	-0.26389	0.499924	0.835802	unchanged	3.59285	C9H9NO	130.0657	3.396117
388.3295	-0.22554	0.56	0.740359	unchanged	3.872451	C12H12N2	233.093	3.396117
108.5771	0.332043	0.571406	0.586758	unchanged	-8.43023	C14H15NO	367.1109	3.40325
51.16808	-0.10483	0.44677	0.857839	unchanged	-3.32141	C8H12O3	179.0673	3.410383
367.8025	0.075804	0.797842	0.315103	unchanged	3.928727	C10H14N2	300.1023	3.410383
76.01481	0.706673	0.685132	0.499251	unchanged	2.332155	C15H23N7	398.1792	3.410383
68.00451	0.999643	0.357376	1.071572	unchanged	-0.79386	C9H13NO	209.1283	3.417533
11.61032	0.077187	0.764809	0.275303	unchanged	1.13197	C13H16N2	197.1076	3.4318
103.5831	-0.5088	0.382734	0.952747	unchanged	-2.63989	C16H24N2	323.1593	3.4318
850.536	0.88383	0.297858	1.153203	unchanged	-3.45448	C14H11NO	441.1183	3.446083
139.9471	1.230006	0.156438	1.541068	unchanged	-3.59107	C17H22N2	299.1378	3.446083
249.5747	1.146398	0.27828	1.209172	unchanged	-4.49717	C6H12N3O	443.1184	3.4675
466.4167	0.22729	0.752365	0.404456	unchanged	4.291006	C9H7NO	146.0607	3.481767
1.093428	4.338009	0.146479	1.701904	unchanged	-0.21416	C20H38O1	453.2329	3.481767
34.13579	-1.03445	0.140433	1.666467	unchanged	2.574786	C9H18N4O	246.1328	3.488917
744.0076	0.726543	0.43563	0.936638	unchanged	0.356015	C11H18N2	211.1442	3.503183
122.9856	0.467406	0.398801	1.002223	unchanged	-4.76526	C11H20N2	209.1273	3.510333
113.403	0.128012	0.336832	1.089181	unchanged	4.947139	C11H16O7	225.077	3.517467
11.53979	1.590588	0.337746	1.119294	unchanged	-4.96282	C9H13N3O	245.1233	3.517467
175.4709	0.42461	0.398593	0.858867	unchanged	8.021892	C11H22N2	352.1591	3.5246
59.12993	2.053516	0.469804	0.876807	unchanged	1.48406	C19H27NO	398.1815	3.517467
96.06208	-0.08344	0.897293	0.157461	unchanged	10.32421	C7H10O	111.0816	3.531733
288.7643	0.030879	0.792915	0.301653	unchanged	31.53897	C11H14N2	155.1028	3.531733
313.5405	-0.01018	0.985664	0.030418	unchanged	2.887146	C9H12O3	186.113	3.531733
60.1328	2.565381	0.359225	1.07297	unchanged	3.809459	C10H18N2	263.107	3.55315
313.3433	0.381503	0.359959	0.947938	unchanged	-1.86824	C11H18N4	312.1457	3.55315
1367.47	1.688005	0.358022	1.075194	unchanged	8.002172	C18H20O5	299.1303	3.55315
73.93682	-0.08634	0.880424	0.20764	unchanged	8.626164	C9H16N4O	257.0906	3.5603
304.6919	0.564745	0.44049	0.815379	unchanged	0.084693	C14H20N6	367.1183	3.5603
97.86657	0.248581	0.779746	0.353362	unchanged	11.58182	C24H40O1	538.2918	3.5603
40.57978	-0.14104	0.65851	0.555999	unchanged	-27.31	C10H13N3	188.0757	3.567433
106.1827	-0.10202	0.819068	0.208501	unchanged	-23.2131	C14H19NO	278.095	3.574567
40.37339	0.507983	0.348548	1.046078	unchanged	6.897702	C6H8N2O	330.143	3.595983

76.88933	0.998108	0.324494	1.086188	unchanged	2.827234	C18H19N(	347.1611	3.603133
1.72E-06	5.576694	0.377044	1.08094	unchanged	-5.85659	C21H21N3	648.3772	3.610267
70.5353	1.034184	0.485314	0.851773	unchanged	8.040232	C5H6O4	148.0615	3.631683
212.5595	1.489366	0.504788	0.822798	unchanged	-5.00191	C13H17N5	258.1336	3.631683
32.54609	0.092937	0.702027	0.316317	unchanged	9.43289	C9H13NO	132.0824	3.638817
29.62508	0.117278	0.717273	0.319847	unchanged	17.84297	C12H12N2	217.101	3.6531
73.7501	0.129529	0.630667	0.466877	unchanged	-19.7199	C10H9NO	160.0726	3.674517
61.83233	-0.04143	0.949709	0.174953	unchanged	-1.23116	C16H24O7	311.1485	3.667367
117.7917	0.846895	0.51215	0.704412	unchanged	8.558019	C8H9FN2(	423.1121	3.674517
85.81287	0.270192	0.443554	0.825563	unchanged	8.760037	C7H9N	130.0637	3.68165
23.47728	0.13721	0.848059	0.267226	unchanged	16.46896	C13H17N5	381.1573	3.688783
5.872967	1.300889	0.023943	2.067643	up	-16.6526	C12H26N4	566.4257	3.695917
234.4511	0.591303	0.379469	1.002021	unchanged	0.924065	C21H20O4	301.1226	3.7095
52.41391	0.065699	0.589553	0.647118	unchanged	-27.3002	C6H13O9I	243.0193	3.723767
8.981438	0.929672	0.621646	0.61493	unchanged	-3.43754	C14H20N2	245.1275	3.723767
38.31368	0.134858	0.730996	0.40367	unchanged	-1.17418	C14H12FN	290.0932	3.716633
7.309415	0.777567	0.247099	1.320186	unchanged	-1.09254	C13H10N2	292.0925	3.716633
36.66605	0.330502	0.066974	1.841617	unchanged	7.917489	C11H14N4	299.0832	3.723767
111.8888	-0.57938	0.369601	0.950816	unchanged	-12.7536	C16H20N2	391.1065	3.716633
30.77028	-0.45244	0.560828	0.606102	unchanged	-8.42345	C13H22N4	393.104	3.716633
1.72E-06	6.410243	0.364812	1.108844	unchanged	39.77971	C15H23N5	692.4107	3.723767
108.5017	-0.29661	0.560728	0.735699	unchanged	-8.52887	C15H18O5	311.0921	3.730917
115.2306	0.207582	0.748255	0.370779	unchanged	-3.24049	C15H25N(	332.1695	3.730917
64.05584	-0.0925	0.669176	0.494846	unchanged	-8.35897	C21H41N5	556.2778	3.752333
307.346	0.00924	0.973971	0.065937	unchanged	4.325619	C10H11N(	200.0479	3.7666
29.79671	1.240001	0.274252	1.299406	unchanged	12.14663	C35H61N(	652.4139	3.780883
178.731	-0.17876	0.355145	1.038481	unchanged	-6.12335	C5H10N2(	179.0652	3.788017
51.25484	0.122878	0.760706	0.309268	unchanged	-9.45	C12H19N(	242.1364	3.79515
37.20458	0.180084	0.327506	1.038128	unchanged	3.629482	C12H25N3	256.1666	3.816567
27.46316	-1.4609	0.13494	1.759937	unchanged	13.80391	C11H20N2	262.1795	3.823717
14.6259	-0.58798	0.41176	0.907781	unchanged	3.467115	C20H37N(	320.2596	3.816567
29.59869	0.592485	0.418756	0.915275	unchanged	-18.689	C10H16O2	186.1457	3.83085
112.6863	-0.81002	0.324609	1.178889	unchanged	3.30093	C11H11N(	170.0607	3.837983
73.23474	-0.80815	0.324753	1.259826	unchanged	28.84548	C11H14N2	155.1022	3.845117
641.2157	0.183612	0.318227	1.043499	unchanged	2.493729	C9H6O3	163.0394	3.852267
15.39766	-0.47599	0.480871	0.83914	unchanged	-1.59495	C21H18N2	396.1548	3.852267
46.51203	1.009293	0.050176	2.02568	unchanged	-4.64469	C12H22N4	351.1317	3.8594
67.98971	1.782804	0.293962	1.218978	unchanged	8.077895	C11H16N5	281.1143	3.873683
36.46521	-1.78962	0.11086	1.878768	unchanged	1.864453	C9H11NO	130.0654	3.880817
287.4668	0.932121	0.376617	1.006909	unchanged	5.687526	C11H22N2	301.1208	3.88795
20.3838	-0.03365	0.977904	0.019357	unchanged	9.200627	C43H58N4	805.4094	3.88795
11.9471	-0.3567	0.466665	0.825825	unchanged	6.041789	C6H11NO	94.06591	3.88795
17.18216	-0.09067	0.839337	0.23885	unchanged	11.35389	C9H7NO	146.0617	3.8951
79.15326	0.77474	0.344736	1.054267	unchanged	3.004414	C17H28N6	413.2155	3.902233
341.3427	0.068984	0.385926	0.877897	unchanged	2.99788	C8H17N5(	273.1677	3.909367
477.1917	-0.15003	0.205672	1.410074	unchanged	2.590919	C13H16N6	321.1314	3.92365
88.2134	0.15035	0.870602	0.230973	unchanged	-16.4492	C10H20N6	594.3336	3.930783
41.04154	0.124345	0.445281	0.851393	unchanged	-21.4632	C12H10	155.0822	3.937917
259.4979	0.265191	0.465311	0.803022	unchanged	7.212628	C12H12N2	201.103	3.937917
1155.884	-0.35336	0.080298	1.874237	unchanged	-4.10565	C6H9FO3	131.0497	3.945067
170.8764	0.356882	0.484695	0.818039	unchanged	-5.1067	C7H14F2N	179.098	3.9522

153.3454	0.077031	0.850528	0.134724	unchanged	-36.0275	C12H18O3	252.1518	3.9522
27.23474	-0.07478	0.917954	0.161112	unchanged	-3.79291	C15H20O2	271.1086	3.9522
71.16881	0.564526	0.327968	1.083656	unchanged	-16.1528	C20H28O6	361.1404	3.945067
48.24887	0.965177	0.369468	1.020967	unchanged	3.22934	C15H20N4	397.0826	3.9522
21.40179	-0.42977	0.195304	1.580019	unchanged	-8.83582	C6H13NO	146.062	3.959333
52.60603	-0.33068	0.440145	0.872083	unchanged	-14.9596	C9H9NO	148.0735	3.966467
52.22956	-1.247	0.107066	1.81009	unchanged	-2.87043	C15H22O2	276.1951	3.966467
58.38695	-0.70863	0.684349	0.514206	unchanged	5.120655	C21H37O7	474.2637	3.973617
360.5063	0.330355	0.535871	0.750594	unchanged	-14.4649	C8H6N2	278.1032	3.98075
201.772	0.29281	0.312546	1.149691	unchanged	2.723627	C11H19N(	300.0851	3.98075
380.4611	-0.0271	0.903252	0.137204	unchanged	20.13451	C6H12S2	149.0483	4.0093
18.64595	0.068414	0.87006	0.21757	unchanged	4.716528	C7H6N4O	177.0416	4.0093
1069.992	0.035138	0.961304	0.050105	unchanged	2.059071	C5H9NO	100.0759	4.01645
20.00297	0.620704	0.374468	1.013681	unchanged	2.725331	C9H16O5	169.0865	4.023583
693.6263	-0.19881	0.745274	0.391585	unchanged	4.583412	C18H18O6	295.098	4.01645
26.9335	-1.00026	0.260037	1.319515	unchanged	6.063451	C9H13N2(	342.0539	4.023583
42.81034	0.008439	0.990999	0.021173	unchanged	10.63459	C4H9NO	88.07662	4.01645
79.63147	1.019838	0.342759	1.026843	unchanged	-5.48401	C18H22N6	351.1543	4.030717
128.6605	0.024038	0.958306	0.044167	unchanged	3.783678	C9H16O4	211.0948	4.0443
100.3076	0.021159	0.94076	0.068034	unchanged	2.353231	C10H12N4	284.058	4.0443
110.7012	0.022204	0.819918	0.183591	unchanged	-1.20466	C7H8O4	179.0642	4.094267
345.5165	-0.46201	0.16932	1.524473	unchanged	3.302526	C12H10N2	181.0767	4.094267
129.1808	0.203906	0.675163	0.43998	unchanged	3.253672	C12H18O3	211.1336	4.087117
3554.254	-0.21706	0.170456	1.428494	unchanged	-0.63829	C12H24O2	218.2113	4.087117
262.9757	-0.42659	0.176218	1.504399	unchanged	3.778184	C13H10N2	209.0718	4.094267
1513.86	-0.50732	0.174566	1.50643	unchanged	0.613459	C14H16N2	241.0973	4.094267
92.82809	1.297587	0.424422	0.913998	unchanged	4.132396	C14H12O5	302.1034	4.094267
562.1449	0.049425	0.862905	0.215582	unchanged	-10.3704	C9H14N3(	381.0771	4.087117
425.2701	0.063259	0.826366	0.259387	unchanged	1.840539	C15H20O9	383.0745	4.094267
40.52501	-0.15559	0.732072	0.425242	unchanged	4.718717	C9H12O3	169.0867	4.115683
117.1907	0.89109	0.643594	0.556511	unchanged	3.967824	C18H28N4	412.1968	4.115683
120.4309	-0.22915	0.54794	0.591423	unchanged	2.364857	C9H9N	132.0811	4.1371
511.1338	-0.64832	0.123104	1.64065	unchanged	3.97147	C9H9NO2	146.0607	4.144233
963.9844	0.033424	0.545581	0.695451	unchanged	0.195282	C8H9NO3	185.1151	4.144233
21.60417	-0.56729	0.32954	1.2037	unchanged	14.47852	C11H23N5	238.1702	4.144233
116.3049	-0.45024	0.376133	0.976098	unchanged	0.364478	C17H27N(	342.1895	4.1371
21.54746	-0.63368	0.184135	1.442399	unchanged	10.58996	C6H7N	94.06611	4.144233
435.4154	0.000475	0.999164	0.014332	unchanged	3.031428	C9H7N	130.0655	4.151367
191.4551	-0.01431	0.974842	0.056536	unchanged	3.579393	C10H9NO	176.0712	4.151367
140.7073	0.091473	0.856021	0.190031	unchanged	-10.5451	C14H24N6	379.1485	4.151367
445.6621	-0.02621	0.949982	0.046486	unchanged	8.120139	C11H6O	154.0425	4.16565
47.58941	-0.16898	0.389239	0.998422	unchanged	1.432732	C9H11NO	166.0865	4.172783
71.87857	-0.70797	0.488915	0.76737	unchanged	11.2335	C11H11N(	188.0729	4.172783
235.7063	0.006908	0.987264	0.043763	unchanged	0.457123	C9H9FO4	200.048	4.16565
45.85846	-0.04724	0.912114	0.087053	unchanged	-0.60415	C6H9N3O	202.0457	4.16565
43.87552	1.362274	0.396652	0.968707	unchanged	13.2029	C21H33N3	372.2335	4.16565
22.09524	-0.53492	0.024223	2.189096	down	21.10956	C7H11NO	138.0586	4.179917
45.00331	-0.27186	0.330972	1.115554	unchanged	0.84579	C11H12O	161.0962	4.179917
162.2741	1.507887	0.441932	0.90497	unchanged	2.021639	C18H16O3	281.1166	4.201333
578.2526	-0.63063	0.164651	1.498181	unchanged	24.00038	C18H24O5	391.1061	4.201333
181.1863	-0.53313	0.229152	1.292418	unchanged	-28.9765	C17H22O9	393.1049	4.201333

372.4334	1.485697	0.247071	1.340291	unchanged	20.24611	C18H18O4	299.1338	4.208467
16.08712	1.122527	0.278976	1.243695	unchanged	0.310536	C16H22O7	344.1705	4.208467
56.60901	0.466064	0.361815	1.029715	unchanged	41.03068	C7H9N5O	144.0742	4.215617
237.4485	0.220736	0.587815	0.599532	unchanged	-17.084	C13H18N6	321.1248	4.22275
523.7227	0.1428	0.344235	0.968625	unchanged	-9.18163	C16H20N2	335.1205	4.215617
3048.221	-0.32453	0.090153	1.814921	unchanged	0.063784	C14H28O3	262.2377	4.229883
1.72E-06	0.272473	0.850499	0.275952	unchanged	-9.54226	C12H15N(	460.24	4.23705
45.98086	1.371272	0.35276	1.052486	unchanged	-3.32865	C15H14N2	255.112	4.27275
534.9644	0.830151	0.322822	1.119878	unchanged	6.675185	C16H20N2	301.1205	4.27275
139.9357	0.271117	0.637687	0.557305	unchanged	5.45938	C12H16O7	314.1249	4.279883
20.56679	2.018152	0.258744	1.279539	unchanged	-21.8653	C17H26O1	355.1302	4.279883
33.94499	0.523037	0.277246	1.224566	unchanged	3.908875	C10H11N(	200.0689	4.287017
79.28979	0.011299	0.981911	0.068858	unchanged	6.775547	C11H15N4	282.0979	4.287017
38.79233	1.316816	0.255496	1.279903	unchanged	7.952073	C10H14N2	191.0833	4.308433
39.81889	0.609609	0.352063	1.032527	unchanged	-20.4876	C7H10N4(	216.1051	4.308433
1402.06	1.238917	0.268386	1.252647	unchanged	14.32672	C12H20N4	333.1452	4.308433
639.1061	-2.32995	0.326284	1.187533	unchanged	-14.7752	C43H66N1	1048.455	4.315567
11.11395	1.278477	0.180812	1.484227	unchanged	2.885101	C8H11N3(	215.1144	4.315567
82.71303	-0.10786	0.482711	0.910503	unchanged	0.03534	C5H7NO5	179.0663	4.336983
37.56101	-0.12447	0.718472	0.513799	unchanged	5.256936	C9H11NO4	198.0771	4.336983
56.6093	-0.44763	0.044759	1.996799	down	-2.13394	C17H24O8	321.1325	4.344133
303.4572	-2.1501	0.346637	1.14254	unchanged	-4.0627	C17H34N4	947.4145	4.344133
52.69798	1.283747	0.261583	1.2647	unchanged	0.54015	C14H12N2	241.0973	4.351267
60.37016	-0.6256	0.470179	0.90154	unchanged	1.663528	C17H33N(	280.2276	4.351267
1041.483	1.179973	0.28031	1.219965	unchanged	4.310771	C16H16N2	301.1196	4.351267
47.71549	0.307754	0.363805	1.074288	unchanged	1.394451	C6H11FO5	365.1259	4.351267
90.49679	1.358343	0.236008	1.317829	unchanged	-3.61594	C10H15N(	255.1126	4.3584
51.8173	1.575603	0.251065	1.285345	unchanged	12.54342	C12H20N4	355.1266	4.3584
32.12572	1.123807	0.430956	0.913519	unchanged	-0.11294	C15H14N4	267.124	4.365533
57.46099	-0.07329	0.677809	0.517477	unchanged	3.226207	C10H14O3	200.1287	4.379117
354.5771	-0.13063	0.382274	1.022232	unchanged	4.879262	C7H5NOS	152.0172	4.393383
33.45165	0.574628	0.542532	0.617613	unchanged	-9.23757	C13H14O5	251.0891	4.38625
75.66182	0.569583	0.154132	1.404887	unchanged	-20.2152	C14H18O8	297.0905	4.38625
32.42964	-0.72845	0.190026	1.423027	unchanged	34.41645	C19H21N3	324.1818	4.407667
103.1029	0.161851	0.676564	0.52102	unchanged	-22.9608	C9H12O4	185.0766	4.42195
61.67389	-0.39304	0.346245	1.081419	unchanged	-4.76299	C10H18N2	227.0836	4.429083
12.52833	0.406233	0.701106	0.421801	unchanged	6.96539	C10H14N2	276.1208	4.429083
50.43575	-0.32861	0.349172	1.024929	unchanged	13.2321	C13H24O1	321.1227	4.429083
54.80598	-0.47452	0.393689	0.952287	unchanged	-2.57831	C8H16N2(	205.1178	4.436217
181.5466	-0.43588	0.085388	1.777189	unchanged	0.520898	C18H36O5	350.2903	4.436217
51.61535	0.094432	0.811492	0.236922	unchanged	-4.44019	C13H23N(	299.1954	4.464783
98.49577	-0.24954	0.686017	0.510697	unchanged	-5.61133	C20H30O4	335.2191	4.457633
57.49638	0.817329	0.399492	0.951651	unchanged	19.88233	C21H34O3	373.2206	4.464783
852.5034	0.597549	0.159533	1.648516	unchanged	0.802972	C22H38O5	400.3061	4.464783
176.4566	-3.10418	0.23045	1.429947	unchanged	16.23559	C42H64O1	863.396	4.457633
148.8461	-1.06656	0.088872	1.912567	unchanged	-2.39026	C14H14N4	272.15	4.471917
117.1513	1.387767	0.131448	1.609833	unchanged	-27.7446	C16H20N4	355.1285	4.471917
100.4693	1.15	0.285646	1.211943	unchanged	15.39483	C13H16N2	255.114	4.47905
864.9492	1.139631	0.305171	1.167345	unchanged	2.660033	C16H16N2	301.1193	4.47905
11.60028	1.892411	0.412966	0.930305	unchanged	-0.47713	C15H12O4	257.0807	4.486183
16.23165	4.363955	0.407903	0.950051	unchanged	11.2669	C13H21N5	344.1426	4.486183

69.84598	1.640947	0.338641	1.156725	unchanged	26.78275	C27H38O1	588.2803	4.500467
90.45246	-0.26087	0.069159	1.80089	unchanged	-9.295	C8H14N2O	220.1273	4.51475
31.51277	0.975275	0.221751	1.35734	unchanged	-22.6486	C9H15N5O	299.1404	4.51475
604.913	0.601644	0.470515	0.815433	unchanged	6.742109	C20H32F2	413.2158	4.51475
74.50176	0.878022	0.237045	1.25899	unchanged	-0.45811	C18H23N3	355.2127	4.521883
22.70599	1.792151	0.375163	1.044402	unchanged	5.942396	C17H18N2	379.0981	4.521883
529.1105	0.040522	0.531859	0.66294	unchanged	-18.4317	C14H20	171.1497	4.5433
46.63727	-0.44573	0.752638	0.380703	unchanged	1.842229	C15H22O1	404.1558	4.550433
29.46664	-0.0785	0.737401	0.455136	unchanged	-18.2275	C8H9NO	136.0732	4.564717
105.9117	1.384081	0.246247	1.345185	unchanged	-5.85001	C13H20	177.1627	4.564717
181.3745	0.22854	0.025337	2.051105	up	-9.20575	C12H16O3	226.1419	4.557567
18.2407	-1.11292	0.35405	1.173131	unchanged	-4.79133	C13H14N4	284.1494	4.557567
173.445	1.648084	0.253101	1.321092	unchanged	-2.32658	C19H32O8	406.2426	4.564717
28.7513	0.085319	0.904854	0.135913	unchanged	4.138125	C10H12O3	181.0867	4.57185
31.23769	-0.01144	0.968015	0.078947	unchanged	14.38686	C10H14N2	260.1276	4.578983
173.1965	-0.35279	0.243074	1.322512	unchanged	26.34636	C17H23NO	331.2092	4.578983
91.65836	-0.8055	0.252633	1.248161	unchanged	29.47093	C11H12N2	237.0939	4.593267
32.73724	0.266404	0.650113	0.540378	unchanged	14.01962	C12H12N2	239.0609	4.6004
48.6271	0.638486	0.355404	1.019446	unchanged	28.09942	C11H15N5	315.1317	4.6004
120.7163	0.497401	0.417628	0.955975	unchanged	16.19762	C10H14O4	221.0816	4.60755
133.3616	-0.59543	0.31	1.076542	unchanged	1.105255	C18H34O5	348.2748	4.614683
25.46311	0.22871	0.651804	0.541455	unchanged	-8.17597	C16H23O1	393.1599	4.60755
145.4238	-0.15275	0.776277	0.32761	unchanged	4.639741	C5H9NO4	148.0611	4.621817
334.4664	-0.24934	0.688312	0.467581	unchanged	-1.87324	C14H19N5	274.1657	4.621817
169.3099	-0.06315	0.597687	0.684211	unchanged	-18.2379	C7H7NO2	179.0657	4.62895
81.94606	-0.6886	0.047125	2.055502	down	-5.17238	C12H13NO	168.0797	4.62895
157.5042	-0.5186	0.064906	1.903332	unchanged	9.574132	C13H12N2	195.0937	4.62895
1066.379	-0.86209	0.052413	2.011557	unchanged	24.42767	C8H15N5O	255.1132	4.62895
30.71207	-0.51992	0.135057	1.69187	unchanged	-3.12587	C14H12N2	241.0964	4.62895
78.81083	-0.00898	0.982791	0.048175	unchanged	-2.85058	C10H14N2	297.0685	4.643233
107.8583	-0.0421	0.929844	0.076321	unchanged	2.990152	C10H16O4	239.0908	4.66465
30.98551	1.079303	0.089326	1.716287	unchanged	0.459893	C15H26O4	288.2171	4.66465
52.91953	-0.52597	0.244596	1.290319	unchanged	21.97698	C14H17N3	290.1025	4.657517
75.57701	0.041482	0.741895	0.374506	unchanged	15.44172	C11H14O2	143.0883	4.678933
76.97585	0.045268	0.767465	0.309342	unchanged	8.734125	C14H24O9	301.1311	4.70035
30.02115	1.183344	0.442865	0.868745	unchanged	13.48596	C20H30O1	496.2089	4.713917
69.94141	-1.04145	0.234461	1.295587	unchanged	3.602985	C10H9NO	176.0712	4.72105
24.84056	0.309086	0.524205	0.720734	unchanged	20.65924	C9H16N2O	233.118	4.72105
90.62262	0.018098	0.876663	0.182978	unchanged	-0.46757	C13H20N2	291.1314	4.7282
215.26	0.511306	0.265823	1.260782	unchanged	6.179646	C12H18N6	333.1333	4.7282
18.04631	0.528001	0.339879	1.070952	unchanged	0.784281	C7H11N3O	356.2043	4.7282
87.11444	-0.20781	0.047051	2.061953	down	-7.64446	C9H10O	135.0794	4.75675
31.75171	-0.50262	0.175422	1.523481	unchanged	-7.85279	C5H9N3O	144.0756	4.75675
1184.024	-0.80653	0.261634	1.297287	unchanged	5.609384	C11H11NO	190.0867	4.75675
289.2315	-0.37779	0.011831	2.274355	down	2.176938	C10H12FN	303.1218	4.75675
24.82557	-0.10391	0.754026	0.313939	unchanged	-4.16833	C17H24O5	326.1949	4.75675
32.28462	0.128901	0.892792	0.184752	unchanged	-0.91889	C24H38O6	464.3003	4.763883
43.07425	-0.38306	0.610141	0.532559	unchanged	-15.514	C9H6F3NO	435.0707	4.792433
14.32298	2.379253	0.385683	1.006372	unchanged	-2.94715	C21H24O3	347.1608	4.799583
22.23695	2.072546	0.277042	1.238155	unchanged	-18.7336	C21H27NO	325.1975	4.81385
40.88089	-0.8305	0.20208	1.444989	unchanged	-3.43907	C19H26N4	407.1734	4.806717

96.4225	-0.15132	0.267767	1.130946	unchanged	-0.23208	C9H13N5(	281.1356	4.821
50.58763	-0.01327	0.972369	0.086342	unchanged	-12.5119	C6H13O8F	262.0656	4.842417
36.95623	2.482857	0.234759	1.399841	unchanged	19.9435	C20H35N9	514.2835	4.842417
64.61872	-0.01349	0.964069	0.116899	unchanged	17.47095	C9H8O	133.0671	4.84955
137.8059	-0.38305	0.028242	2.193329	down	-26.0462	C11H20N4	303.1216	4.863833
25.24617	0.621403	0.661112	0.484497	unchanged	-12.5744	C20H20O6	395.0847	4.856683
139.0148	-1.08442	0.160055	1.59206	unchanged	1.92879	C15H23N5	286.1669	4.8781
35.31433	-0.3676	0.256977	1.300755	unchanged	-2.27116	C16H23N5	314.1604	4.8781
15.69771	0.118398	0.900909	0.23439	unchanged	-2.51179	C15H17N3	326.1104	4.8781
44.04236	0.930371	0.213836	1.34977	unchanged	2.836614	C15H22O4	231.1387	4.892383
225.4652	0.621282	0.24295	1.349761	unchanged	-0.14393	C16H22O6	333.1327	4.885233
141.4818	0.317274	0.703668	0.449124	unchanged	-5.97286	C22H42O8	399.2715	4.892383
136.256	0.35046	0.299647	1.082213	unchanged	-3.56047	C18H34N(	409.2448	4.885233
25.85933	0.318941	0.338222	1.037503	unchanged	-3.73617	C12H18O2	212.1638	4.90665
199.8668	-1.74166	0.128295	1.705571	unchanged	9.508221	C19H32O5	363.2174	4.928067
33.43942	0.633814	0.397957	0.964973	unchanged	-5.61516	C24H38O5	371.2558	4.928067
91.98317	0.037148	0.887386	0.1956	unchanged	11.28419	C17H21N(	252.1415	4.94235
60.15103	0.056006	0.649073	0.529703	unchanged	3.042572	C7H14N2(	155.0821	4.956617
61.34491	-0.03464	0.873948	0.218667	unchanged	-2.5482	C13H20N2	237.1592	4.9709
882.465	-0.24137	0.383438	0.989888	unchanged	-3.19786	C14H14O2	467.1605	4.9709
59.46601	-0.36472	0.108769	1.629687	unchanged	2.093432	C15H20O5	303.1209	4.978033
11.51475	0.892608	0.523895	0.73075	unchanged	8.683868	C9H18N2(	236.1624	4.985183
224.4861	-0.1964	0.758387	0.362531	unchanged	-7.5522	C19H28O	273.2217	4.992317
118.7706	0.597017	0.530378	0.712547	unchanged	-2.30278	C15H25N(	282.1693	4.985183
279.7428	-0.2636	0.702615	0.449296	unchanged	2.32973	C20H30O3	319.2275	4.992317
38.92783	-0.09902	0.578066	0.669508	unchanged	-11.7165	C12H16N6	326.1535	4.992317
1.72E-06	1.25339	0.4353	0.928835	unchanged	-0.1212	C26H37N(	428.2795	4.992317
26.41701	0.121001	0.735372	0.356536	unchanged	8.962249	C10H13N5	253.1429	4.99945
1.72E-06	0.946136	0.512549	0.710644	unchanged	-5.30191	C22H38O5	400.3037	4.99945
72.73817	-0.09306	0.698291	0.486117	unchanged	-14.0436	C12H20O3	177.1244	5.006583
30.74103	0.029122	0.906763	0.189898	unchanged	-1.11665	C9H16O3	214.1436	5.020867
72.8363	-0.95014	0.397152	0.834919	unchanged	-16.4444	C20H28O	607.3818	5.020867
611.7982	-0.60815	0.153048	1.572224	unchanged	-10.9552	C16H16N2	269.118	5.03515
29.41123	-0.59265	0.446455	0.822704	unchanged	-0.68812	C8H11NO	403.0989	5.03515
13.37678	-0.49767	0.565986	0.635185	unchanged	42.06266	C12H23O1	405.097	5.03515
32.03122	-0.26809	0.758939	0.300922	unchanged	-4.14215	C21H22O1	447.0902	5.03515
12.56411	-0.39382	0.705061	0.38649	unchanged	13.41999	C11H19N3	449.0891	5.03515
10.84909	-0.40421	0.719544	0.466867	unchanged	-2.31238	C14H27N3	731.3652	5.03515
49.24974	-0.25078	0.684039	0.420605	unchanged	-8.8022	C22H22O1	461.1036	5.05585
34.06484	-0.65051	0.459428	0.792515	unchanged	6.633126	C10H12O5	463.1029	5.05585
137.7485	0.055116	0.699362	0.446359	unchanged	25.59158	C11H22N4	257.1678	5.070133
13.37869	0.779906	0.241713	1.296186	unchanged	21.76311	C9H16N2(	213.0924	5.09155
421.2705	-0.76203	0.266247	1.354352	unchanged	2.594261	C14H23N(	286.1656	5.09155
84.91486	0.371515	0.739206	0.397846	unchanged	-19.5269	C20H39N5	410.2664	5.112967
2324.137	0.420119	0.420098	0.888106	unchanged	1.502756	C18H24O2	290.2119	5.1201
10.2168	0.611189	0.664317	0.510041	unchanged	0.608716	C24H34O3	335.2372	5.1201
18.24622	0.280188	0.529116	0.764344	unchanged	-4.60863	C10H12O2	129.0691	5.134383
1724.913	0.159056	0.788147	0.283039	unchanged	-0.51101	C18H29N(	308.2219	5.127233
51636.43	0.165183	0.764307	0.318576	unchanged	36.03691	C19H32O4	325.249	5.127233
33.83404	0.436106	0.377005	0.977057	unchanged	-4.78925	C5H9NO2	130.0314	5.14865
684.8415	0.062002	0.670582	0.503663	unchanged	-3.04037	C7H6O6S	182.974	5.14865

88.71945	0.072972	0.817842	0.328514	unchanged	-25.7991	C10H16N2	195.1073	5.14865
1.72E-06	1.207941	0.309745	1.111417	unchanged	18.14305	C12H11N(	203.1212	5.14865
219.7187	-0.0518	0.887102	0.128966	unchanged	10.94679	C14H15F2	294.0905	5.14865
41.03985	0.634222	0.509246	0.724953	unchanged	9.020552	C18H28N4	467.1577	5.14865
1.72E-06	0.934185	0.445802	0.836604	unchanged	-0.33483	C19H24N4	469.1564	5.14865
20.93008	0.081609	0.763429	0.34313	unchanged	4.588295	C14H18O4	233.1184	5.162933
47.56879	-0.01556	0.969721	0.02452	unchanged	-3.0502	C6H5NO3	296.0869	5.1558
297.9572	-0.32581	0.706962	0.391381	unchanged	1.451024	C15H25N(	284.186	5.18435
69.0531	-0.10345	0.499073	0.880262	unchanged	-4.47824	C20H26O4	295.1678	5.2129
70.04258	0.189231	0.335875	1.044862	unchanged	35.16647	C9H16N3(	199.1385	5.220033
40.01324	0.193269	0.782077	0.334742	unchanged	14.62394	C16H28O6	317.2111	5.234317
49.93449	-0.9004	0.172494	1.499046	unchanged	0.322674	C20H25D9	346.2939	5.227183
1.72E-06	1.07231	0.451074	0.884123	unchanged	1.881408	C19H39O7	428.2779	5.227183
106.3295	-0.05234	0.939052	0.074448	unchanged	6.346912	C17H33N7	432.2592	5.227183
60.31755	-0.13394	0.869795	0.203637	unchanged	20.66468	C10H17N(	437.2131	5.234317
93.3904	-0.16938	0.187269	1.56487	unchanged	2.530317	C14H18O	203.1436	5.27
2462.892	-0.09548	0.42138	0.974769	unchanged	1.052538	C15H22O	219.1746	5.27
68.14783	-0.24534	0.601766	0.561727	unchanged	2.794158	C14H28O3	262.2384	5.27715
255.936	-0.6065	0.293733	1.193933	unchanged	8.36328	C25H34O2	389.2521	5.284283
14.2282	0.124088	0.894554	0.054179	unchanged	18.01741	C5H11NO4	321.1322	5.291417
171.6442	0.2367	0.218442	1.424801	unchanged	-5.85224	C3H6N2	179.0685	5.298567
144.7511	-0.3025	0.634302	0.567135	unchanged	4.173929	C18H22O3	309.1473	5.298567
60.24173	0.258038	0.385597	0.948468	unchanged	26.47229	C10H14N2	252.1398	5.3057
174.6057	0.618493	0.2266	1.327212	unchanged	47.98329	C20H32O5	335.2386	5.3057
184.3252	0.924901	0.152824	1.519943	unchanged	-2.72391	C21H41N(	410.2689	5.3057
259.6011	-0.04979	0.908759	0.120029	unchanged	-7.57826	C22H38O5	383.2763	5.319983
68.73216	0.008765	0.942355	0.121151	unchanged	8.341499	C8H8O3	135.0453	5.327117
119.8187	-0.39523	0.12143	1.643829	unchanged	-3.88811	C9H16O	141.1268	5.327117
2883.505	-0.36499	0.232625	1.342266	unchanged	1.322732	C15H25N(	316.1759	5.327117
80.68134	-0.18014	0.374793	0.9748	unchanged	-0.33667	C16H30O2	272.2583	5.3414
1540.624	-0.35901	0.018958	2.325899	down	1.636294	C18H36O4	334.2957	5.3414
82.76156	0.4431	0.477349	0.851316	unchanged	-0.3375	C10H24N4	239.1632	5.348533
100.256	0.183206	0.794498	0.366243	unchanged	4.842503	C7H5NS2	167.9944	5.3628
21.39478	1.329659	0.32958	1.152017	unchanged	8.907893	C11H18N2	209.1305	5.3628
69.45313	1.833558	0.386601	1.036877	unchanged	38.09573	C20H32O4	337.2525	5.3628
47.35685	-0.91075	0.318313	1.161072	unchanged	-11.6931	C20H32O6	369.2229	5.3628
480.0447	1.878705	0.38525	1.037714	unchanged	9.036794	C29H37N(	462.2682	5.3628
595.71	2.141673	0.382341	1.04596	unchanged	2.380776	C26H45N(	498.2896	5.3628
33.43295	0.747567	0.476715	0.842106	unchanged	4.19464	C30H51N5	592.373	5.3628
24.96701	-0.08694	0.88927	0.146828	unchanged	-0.82798	C16H32O4	311.219	5.36995
22.24826	0.271456	0.617667	0.509345	unchanged	-34.0448	C10H18N2	231.1261	5.39065
88.87203	-0.40915	0.078494	1.813962	unchanged	-8.20486	C9H16O	141.1262	5.419217
57.38415	-0.45925	0.377248	1.014106	unchanged	-4.21526	C15H19N5	270.1702	5.419217
2188.319	-0.39834	0.209796	1.39812	unchanged	-2.03061	C16H21N5	316.1762	5.419217
483.4634	-0.16774	0.534852	0.801617	unchanged	5.723775	C15H11I4N	777.6984	5.419217
96.714	-0.18126	0.589747	0.635395	unchanged	3.703996	C6H7N5O	148.0624	5.433483
132.4224	-0.24871	0.677996	0.524127	unchanged	2.177031	C14H26O8	322.1629	5.440633
66.09153	0.392686	0.385208	0.954676	unchanged	12.41362	C14H18O5	231.1049	5.46205
71.96199	0.443247	0.331343	1.04695	unchanged	-14.4262	C19H24N2	335.144	5.46205
1.397166	2.822537	0.37207	1.079015	unchanged	-0.37184	C17H21N5	672.3726	5.469183
210.1926	0.088179	0.911636	0.135714	unchanged	-5.77724	C18H36N6	397.2533	5.476317

40.86759	-0.02258	0.941255	0.074669	unchanged	-14.4313	C6H11NO	145.0712	5.4906
1.72E-06	-1.72698	0.473853	0.932183	unchanged	0.896626	C18H28N6	441.2096	5.4906
53.74651	0.165033	0.124949	1.644395	unchanged	3.517544	C11H21N5	287.1598	5.497733
74.4553	-0.42452	0.123421	1.609228	unchanged	-10.7443	C7H15NO	187.1425	5.51915
1186.22	0.133118	0.538685	0.722666	unchanged	6.411462	C7H9N5O	213.1107	5.51915
700.7998	-0.41234	0.223661	1.31364	unchanged	1.927625	C15H20O2	233.1541	5.51915
273.8164	0.091924	0.834854	0.261039	unchanged	1.69766	C15H20O3	249.1489	5.533433
72.17464	-0.05047	0.854863	0.177172	unchanged	-1.30156	C14H17N3	308.1059	5.526283
129.9695	-0.26087	0.6376	0.546318	unchanged	7.236116	C11H23N5	312.1452	5.533433
0.01402	8.365858	0.386773	1.015398	unchanged	11.65788	C14H12O4	209.0626	5.540567
42.07628	-0.66918	0.281961	1.09584	unchanged	-15.3831	C24H40O4	415.2758	5.540567
250.2628	-0.66458	0.37968	1.008514	unchanged	0.750139	C18H34N6	431.2616	5.540567
103.4029	-0.32853	0.788019	0.248152	unchanged	-10.1727	C21H43N5	442.2975	5.561983
94.31432	0.136259	0.805485	0.260873	unchanged	2.925495	C15H18O3	247.1336	5.569117
248.639	-0.55065	0.328058	1.118991	unchanged	0.148976	C16H22O3	263.1642	5.569117
144.5592	-0.21769	0.728002	0.436749	unchanged	-4.13528	C20H30O6	367.21	5.569117
28.66759	0.513115	0.112229	1.673634	unchanged	9.939377	C10H10O	147.0819	5.5834
70.56874	0.169297	0.546906	0.662896	unchanged	23.65453	C12H16O4	207.1069	5.5834
277.6438	0.230779	0.736472	0.383783	unchanged	3.230773	C12H16O3	209.1179	5.57625
1039.903	0.250878	0.694346	0.445893	unchanged	17.17979	C14H20O5	251.1324	5.57625
43.55426	-0.00402	0.995235	0.039316	unchanged	23.11685	C14H24O8	321.1618	5.590533
119.4131	1.365759	0.306014	1.197368	unchanged	21.18597	C12H17N(	464.285	5.590533
72.05936	-0.42468	0.387296	0.991387	unchanged	-0.46759	C7H17N7(	249.1781	5.633367
708.9835	0.06868	0.693287	0.487726	unchanged	22.46806	C12H14N2	187.1272	5.6405
202.0331	0.082839	0.760178	0.384255	unchanged	-1.7067	C20H24N8	437.1672	5.6405
38.53256	0.035288	0.852945	0.234006	unchanged	32.32627	C6H5NO2	88.02215	5.6405
48.32181	0.53483	0.400932	0.911082	unchanged	-7.0322	C11H16O	147.1157	5.654783
119.9674	0.291596	0.550663	0.645429	unchanged	5.61637	C12H16O2	193.1234	5.654783
114.537	-0.15943	0.66336	0.438408	unchanged	3.279951	C15H20O5	245.1181	5.647633
91.3207	0.050324	0.768765	0.347715	unchanged	-0.43932	C13H15N3	290.077	5.647633
25.78459	0.81475	0.421858	0.884597	unchanged	-0.32545	C20H34O4	303.2317	5.654783
101.3254	0.852254	0.45596	0.883411	unchanged	-3.80233	C20H34O3	305.2463	5.647633
606.7846	-0.01515	0.979212	0.008349	unchanged	38.00956	C20H34O6	353.2459	5.647633
116.7978	0.073555	0.911346	0.163419	unchanged	0.571733	C24H36O4	389.2689	5.654783
118.3364	0.614889	0.486431	0.762252	unchanged	-14.2522	C20H40N4	514.3012	5.654783
77.11228	0.443824	0.578039	0.640672	unchanged	3.148026	C20H25N3	341.2346	5.661917
171.1806	0.321248	0.395341	0.972286	unchanged	-13.4803	C12H18O2	177.1248	5.683333
42.50293	0.416074	0.333335	1.136681	unchanged	18.48524	C14H18N2	243.1179	5.6762
244.2831	-2.43846	0.270717	1.278695	unchanged	-0.30831	C20H40N(	454.2563	5.690467
26.59063	-0.19484	0.733873	0.365335	unchanged	-9.98365	C21H30O2	297.2182	5.697617
21.81973	0.082628	0.934129	0.128988	unchanged	1.234064	C19H32N2	410.2654	5.70475
52.2989	-0.89922	0.16671	1.500491	unchanged	-17.2904	C24H46O1	552.329	5.70475
10.28082	0.077293	0.928127	0.071023	unchanged	4.501377	C10H12O3	181.0867	5.718317
172.0294	0.095223	0.729005	0.435066	unchanged	7.005311	C14H22O4	237.1503	5.718317
63.13577	-0.81452	0.153498	1.526313	unchanged	-8.65123	C21H35N2	409.2214	5.718317
48.45306	1.463198	0.309177	1.213087	unchanged	27.08742	C18H32N8	498.2907	5.7326
26.91173	0.149893	0.832852	0.29188	unchanged	10.23978	C14H28N2	255.2095	5.76115
220.5733	-0.25895	0.569049	0.636186	unchanged	5.524756	C15H27N(	351.2144	5.768283
503.8736	0.318323	0.377282	1.023683	unchanged	-17.4226	C12H20O3	213.1505	5.775433
24.732	1.519491	0.202608	1.378283	unchanged	6.986393	C10H19N5	259.1716	5.782567
52.48136	-0.06397	0.887602	0.118878	unchanged	1.024558	C9H10F2N	282.0899	5.782567

25.87475	-0.26307	0.591384	0.55742	unchanged	-4.77898	C11H15N3	284.0863	5.782567
63.5106	0.283767	0.423255	0.939823	unchanged	-0.22416	C12H15N5	325.1016	5.775433
8.698507	2.294875	0.375749	1.069688	unchanged	3.795218	C34H53N7	686.3899	5.796833
41.44642	0.622367	0.206641	1.413435	unchanged	-3.02874	C11H20O3	233.1199	5.811117
49.81466	-0.46479	0.255923	1.275484	unchanged	2.851273	C17H19NO	286.1446	5.811117
280.6083	0.8163	0.361029	0.963206	unchanged	2.279216	C18H31NO	348.2153	5.811117
317.8838	-0.10894	0.88302	0.170267	unchanged	10.63226	C20H41N5	496.303	5.811117
77.41995	1.221507	0.123687	1.720055	unchanged	12.69154	C15H18N2	207.1311	5.81825
9.875381	0.582525	0.451935	0.912515	unchanged	-7.08323	C13H12N4	282.1332	5.81825
50.36403	0.341561	0.268149	1.263395	unchanged	2.440012	C10H14O2	131.0859	5.8254
43.04775	0.606784	0.420968	0.933463	unchanged	-3.39396	C9H13NO	185.1279	5.8254
177.5158	0.48788	0.459971	0.863334	unchanged	0.146637	C14H14O	199.1512	5.8254
423.5268	0.487101	0.492437	0.806575	unchanged	23.67999	C11H18N2	227.141	5.8254
45.45321	0.663862	0.457756	0.870678	unchanged	3.879878	C13H22N2	277.1936	5.8254
7.91734	0.476502	0.534506	0.752118	unchanged	23.7553	C12H20O5	245.1441	5.8254
127.1135	0.604859	0.404709	0.964644	unchanged	-0.64862	C16H22O2	493.2604	5.8254
231.0774	0.688458	0.141683	1.595055	unchanged	11.16147	C15H20N2	225.1415	5.8254
208.7967	0.499172	0.498896	0.796987	unchanged	-8.47214	C18H24O2	295.2143	5.8254
46.0681	0.544859	0.583499	0.654127	unchanged	-21.6726	C18H28O3	293.2258	5.8254
13.49734	0.889711	0.342956	1.094656	unchanged	0.346414	C18H22N2	281.1903	5.8254
14.85115	0.52035	0.581421	0.658276	unchanged	7.667989	C18H22O3	304.1929	5.832533
34.12578	0.608671	0.50551	0.787407	unchanged	0.571182	C18H29NO	309.2538	5.8254
2177.954	0.477599	0.604777	0.618879	unchanged	-23.7619	C20H33NO	337.2536	5.8254
35.3655	0.6737	0.51322	0.77259	unchanged	4.824273	C21H28O3	370.2393	5.8254
17.0974	-0.5509	0.229186	1.234915	unchanged	-29.399	C25H49NO	392.3397	5.8254
36.29121	0.512289	0.580435	0.660015	unchanged	6.934729	C24H37NO	394.2742	5.8254
14431.38	0.456628	0.605389	0.615868	unchanged	2.93084	C24H34O3	412.2857	5.8254
68.87539	0.418846	0.624969	0.601871	unchanged	-3.6475	C27H42O6	485.2857	5.8254
46546.16	0.442547	0.621004	0.596368	unchanged	2.623169	C26H43NO	430.2964	5.8254
17.0436	0.630454	0.505466	0.815687	unchanged	-7.97941	C33H54O6	511.3738	5.8254
42.98858	0.524551	0.42703	0.942209	unchanged	0.929498	C24H35N5	522.2386	5.8254
1.72E-06	0.680903	0.613753	0.624496	unchanged	-13.1928	C20H30O5	718.4432	5.8254
1.72E-06	0.763707	0.631306	0.606312	unchanged	9.324464	C38H62O1	726.4252	5.832533
1.72E-06	0.678461	0.554713	0.740946	unchanged	-0.35479	C50H90NO	975.5895	5.8254
29.26037	0.566636	0.210762	1.399778	unchanged	-23.8501	C3H7N3O	135.0849	5.85395
54.02121	0.970969	0.096436	1.830213	unchanged	4.223322	C6H13NO	253.1896	5.85395
67.97115	0.464403	0.24284	1.371477	unchanged	-1.0608	C18H28O6	323.1849	5.85395
60.17765	0.530218	0.670568	0.552241	unchanged	2.98337	C25H36O3	426.3014	5.846817
59.7166	1.775822	0.014913	2.181606	up	3.164386	C26H34O9	998.5519	5.846817
117.7656	0.723125	0.031739	2.051816	up	-1.3998	C11H18O2	205.1182	5.861083
1169.594	1.386606	0.13937	1.5787	unchanged	4.5462	C19H28O2	289.2179	5.861083
16.46439	2.246592	0.213488	1.386385	unchanged	2.347374	C10H11FN	296.1159	5.861083
408.385	1.422318	0.115452	1.671135	unchanged	2.423612	C20H30O4	335.2225	5.861083
44.45471	1.843918	0.213524	1.352911	unchanged	-2.23324	C24H32O8	471.1979	5.861083
2144.208	1.850718	0.20469	1.410091	unchanged	-34.0472	C19H38N6	528.2814	5.861083
8.612643	0.825429	0.391343	1.00025	unchanged	41.15361	C20H34O5	372.289	5.8825
66.49151	-0.54905	0.549899	0.706368	unchanged	-2.2092	C20H30O3	301.2155	5.889633
138.1485	0.954636	0.373516	1.048335	unchanged	2.238165	C20H26O4	348.2177	5.896783
71.19584	-0.1315	0.808143	0.194916	unchanged	4.743464	C21H31N5	408.24	5.896783
72.88816	0.328901	0.617554	0.610892	unchanged	3.518096	C18H34O5	313.2385	5.91105
194.6258	0.106731	0.878137	0.191224	unchanged	-3.11161	C17H31N3	375.2591	5.91105

363.4228	0.128118	0.849205	0.260273	unchanged	2.194208	C19H36N6	445.2779	5.91105
101.3882	-0.08609	0.883687	0.226051	unchanged	4.853189	C16H22O3	263.1654	5.932467
26.78596	1.206083	0.500642	0.783439	unchanged	7.327521	C15H20O4	265.1454	5.932467
31.32994	0.508643	0.274804	1.195896	unchanged	8.156762	C19H26O6	351.1831	5.932467
226.1569	0.220465	0.700382	0.504651	unchanged	10.25765	C24H36N2	381.2579	5.953883
55.88371	-0.00848	0.995337	0.057222	unchanged	-1.1373	C24H50N2	494.3474	5.961017
43.84008	-0.69154	0.209044	1.377875	unchanged	9.439248	C5H9NO4	148.0618	5.96815
159.2966	-0.78875	0.22876	1.311326	unchanged	-2.01108	C16H23N5	302.1969	5.96815
3.725153	3.475783	0.271165	1.324046	unchanged	-3.73837	C18H32O5	293.2099	5.982433
123.3054	-0.07714	0.905749	0.097702	unchanged	2.896926	C21H32N6	417.2621	5.982433
47.15179	0.154415	0.747704	0.365022	unchanged	8.243147	C24H30O1	565.1361	5.9753
32.53327	0.23243	0.531913	0.685778	unchanged	2.261279	C12H24N2	273.1452	6.00385
16.8253	1.085712	0.316276	1.15144	unchanged	-6.18608	C22H38O7	437.2484	6.00385
104.3648	-0.45565	0.099164	1.732852	unchanged	-0.01545	C12H17N5	265.1771	6.018117
16.33447	0.524659	0.669764	0.526933	unchanged	1.083538	C22H31N0	367.2747	6.018117
137.5023	0.816849	0.157784	1.490172	unchanged	-4.8028	C13H14O2	203.1057	6.025267
35.43009	-0.20103	0.703373	0.512317	unchanged	-6.12953	C13H24O1	321.1158	6.0324
243.2118	0.592963	0.145083	1.613047	unchanged	-3.15944	C19H24O6	349.1635	6.0324
226.0133	1.254733	0.255432	1.257875	unchanged	-26.3644	C9H16N20	223.1195	6.039533
52.11896	0.678038	0.352326	1.050767	unchanged	0.107743	C10H20N2	213.1056	6.039533
59.60974	0.655297	0.247114	1.274697	unchanged	-28.2477	C11H17N0	237.1542	6.039533
151.2023	1.595424	0.317056	1.108592	unchanged	-4.38259	C15H22N2	285.1848	6.039533
364.4934	0.841119	0.056263	1.964657	unchanged	-2.60158	C14H18O5	231.1009	6.039533
1461.587	1.224279	0.338135	1.059396	unchanged	-3.83108	C18H22O2	271.1694	6.039533
21.01362	1.654816	0.388735	0.982991	unchanged	1.879339	C16H26N2	275.176	6.039533
200.134	1.46248	0.329898	1.079283	unchanged	2.508815	C20H24O2	297.1856	6.039533
18509.89	1.319483	0.341491	1.054585	unchanged	1.451627	C24H32O2	353.2476	6.039533
76.17184	0.765343	0.363701	1.065045	unchanged	-5.41045	C9H19NO	379.2427	6.039533
20549.45	1.449964	0.339376	1.062885	unchanged	1.089034	C24H38O5	389.2688	6.039533
294.5093	1.133169	0.349379	1.035585	unchanged	-1.34103	C24H38O5	407.2787	6.039533
82.25502	2.316404	0.364949	1.018479	unchanged	10.45325	C22H37N0	434.2345	6.039533
22.61413	2.285158	0.324185	1.116789	unchanged	0.682128	C28H42O8	545.2515	6.039533
6662.988	1.391496	0.344185	1.033377	unchanged	-25.7428	C44H81N2	835.5363	6.039533
20.02139	2.358763	0.289252	1.182308	unchanged	-13.3783	C7H11N30	202.0795	6.053117
699.1585	1.359923	0.326287	1.087972	unchanged	10.98575	C11H20N2	245.1533	6.053117
36.19238	1.89818	0.293714	1.142577	unchanged	0.334849	C17H26N2	291.2068	6.053117
122.7199	2.119071	0.302172	1.156655	unchanged	-1.26117	C21H41N0	426.2611	6.053117
79.68163	1.778513	0.288858	1.177969	unchanged	3.137765	C18H35N3	502.2258	6.053117
119.6627	2.436811	0.361259	1.030214	unchanged	-23.2009	C45H75N0	866.4687	6.053117
9.715919	0.967797	0.552891	0.699223	unchanged	29.84306	C18H34O	555.527	6.0888
18.7793	1.75022	0.253313	1.321184	unchanged	-29.827	C27H44O3	416.316	6.09595
56.54512	-0.14646	0.25391	1.25949	unchanged	-13.0679	C9H15NO	187.1419	6.11735
246.3995	-0.08095	0.64955	0.564695	unchanged	0.867969	C15H20O2	233.1538	6.1245
5.686292	1.699884	0.386173	1.057925	unchanged	-3.1223	C27H41N0	426.2989	6.131633
23.3113	1.762719	0.312079	1.210748	unchanged	-2.04011	C27H40O5	462.3205	6.131633
7.00197	1.571879	0.384867	1.060725	unchanged	-31.8351	C24H50N0	480.3296	6.131633
230.8733	1.953661	0.354853	1.109572	unchanged	-0.61455	C26H45N0	464.2826	6.131633
129.1265	-0.46439	0.019862	2.170682	down	-0.24069	C18H34O3	316.2845	6.138767
235.4981	-0.54335	0.094848	1.910331	unchanged	22.19601	C24H42O3	378.3212	6.160183
273.7631	0.20936	0.517084	0.757268	unchanged	-1.82251	C6H15NO	175.1439	6.174467
13.26967	0.822075	0.344598	1.07958	unchanged	-1.8499	C21H36O2	285.2571	6.1816

647.3138	0.381686	0.664641	0.574842	unchanged	-0.7111	C16H24O5	314.196	6.203017
252.9484	-0.49638	0.1166	1.853173	unchanged	-3.2917	C22H44O6	422.3463	6.203017
149.5693	0.055058	0.889612	0.090816	unchanged	-1.51506	C10H19NS	235.1294	6.217283
2530.992	-0.29185	0.175311	1.393003	unchanged	-0.05084	C18H37NO	300.2897	6.217283
22983.6	-0.26742	0.228974	1.258425	unchanged	-2.31251	C22H47NO	406.3518	6.2387
24.15744	0.082024	0.575142	0.651985	unchanged	-4.01835	C10H12	155.0826	6.252983
3003.069	0.129879	0.358746	1.053466	unchanged	-1.70618	C15H22O	219.174	6.24585
1510.25	-0.16028	0.581101	0.610046	unchanged	-1.04543	C16H33NO	272.2581	6.252983
63.35154	-0.14004	0.830355	0.194821	unchanged	-5.19912	C17H34O2	288.2883	6.24585
554.0922	-0.235	0.217196	1.29063	unchanged	-5.06776	C20H41NO	344.3142	6.24585
110.6498	-0.57302	0.15422	1.666182	unchanged	0.360153	C29H44O2	466.3681	6.24585
67.86049	0.834947	0.160346	1.481559	unchanged	2.334729	C22H36O3	371.2565	6.260117
1209.981	0.183961	0.492933	0.805395	unchanged	-1.75854	C9H16O	158.1537	6.2744
29.23555	1.873177	0.107926	1.713546	unchanged	5.462522	C14H22O	207.1716	6.2744
136.8637	0.132123	0.793457	0.35068	unchanged	-0.49982	C16H27NO	336.178	6.26725
33.20446	0.647819	0.507804	0.741604	unchanged	19.79859	C20H40N4	514.3181	6.2744
48.852	2.164814	0.102142	1.75494	unchanged	6.582777	C11H21N3	285.1937	6.281533
233.5592	1.124462	0.231982	1.305736	unchanged	3.167659	C21H27N7	464.182	6.281533
6.367949	-0.80093	0.547001	0.709619	unchanged	4.620191	C36H67NO	786.4518	6.281533
320.7979	0.201034	0.269734	1.238882	unchanged	-2.19858	C15H26O	240.2317	6.288667
38.72719	-0.31722	0.629733	0.544821	unchanged	-1.62186	C16H33N5	391.2418	6.288667
152.8289	0.043397	0.876733	0.134698	unchanged	-3.89646	C13H20O	193.1579	6.295817
104.4232	0.119377	0.690948	0.453279	unchanged	-1.80125	C14H22O3	239.1637	6.30295
40.60341	-2.21886	0.003827	2.411866	down	-5.16588	C10H16N2	241.0627	6.30295
34.07755	0.382389	0.526944	0.776918	unchanged	-12.9821	C14H26N6	375.176	6.30295
1.72E-06	4.962381	0.388401	1.013639	unchanged	8.726052	C30H48O5	527.3176	6.30295
22.91215	1.338641	0.262065	1.272472	unchanged	-3.92013	C19H32O2	257.2252	6.317217
74.3321	0.058495	0.760884	0.324099	unchanged	18.51295	C10H6O5	207.0326	6.3315
70.37736	0.148478	0.352068	1.020959	unchanged	3.850489	C7H12OS3	209.0131	6.3315
351.0291	0.15043	0.430481	0.870642	unchanged	2.857378	C11H11ClI	223.0639	6.3315
48.94245	-0.96167	0.149979	1.590066	unchanged	1.137143	C18H40NO	420.249	6.345783
835.4524	1.268404	0.420347	0.962216	unchanged	-2.02858	C26H45NO	464.2819	6.345783
4.971397	1.129428	0.386093	1.02234	unchanged	-18.877	C23H38N8	523.2888	6.345783
19.44671	0.694708	0.398086	0.975806	unchanged	-16.6759	C9H13NO4	217.115	6.36005
1.72E-06	4.75277	0.406722	0.975614	unchanged	1.070764	C21H28O9	425.1811	6.36005
56.01399	0.021807	0.950391	0.10582	unchanged	2.162117	C6H11N3O	321.1442	6.374333
50.19701	0.121731	0.828728	0.233528	unchanged	11.59108	C19H30O7	335.1896	6.3672
109.044	-0.35463	0.612424	0.541595	unchanged	2.208598	C12H20O	399.2668	6.374333
296.5948	0.185918	0.484116	0.81335	unchanged	-1.37411	C16H30O	256.2632	6.3879
33.82831	0.417184	0.510633	0.774889	unchanged	-3.94633	C20H32	311.2125	6.39505
61.02812	0.189406	0.486751	0.80812	unchanged	-16.4743	C27H42O3	415.3138	6.39505
1275.16	1.304688	0.333833	1.096639	unchanged	0.666032	C32H44O1	633.2546	6.409317
37.16017	0.443887	0.568255	0.666207	unchanged	-10.7681	C17H18O3	271.13	6.4236
192.3458	-0.31651	0.103388	1.688812	unchanged	-5.33293	C20H42O3	372.3455	6.4236
250.6521	-0.14852	0.423872	0.893607	unchanged	-13.5961	C34H68NO	582.4562	6.4236
33.35387	-0.11365	0.723408	0.38148	unchanged	-17.3714	C8H11N3O	231.1051	6.430733
249.3923	0.491041	0.586513	0.651327	unchanged	18.15379	C21H35NO	381.2578	6.430733
11.88183	2.709029	0.131761	1.67309	unchanged	0.358976	C21H36O	343.2399	6.45215
107.2015	-0.61394	0.452091	0.854499	unchanged	-1.33786	C12H14O2	191.1064	6.459283
362.7107	-0.10857	0.838444	0.332382	unchanged	30.89785	C21H24N2	333.1711	6.459283
96.50174	0.70479	0.606159	0.636138	unchanged	-19.1474	C24H38O5	429.2604	6.466417

68.01309	-0.51121	0.053897	1.97455	unchanged	14.68844	C28H48O2	416.3709	6.466417
187.8608	1.052643	0.396497	0.946654	unchanged	-5.66102	C15H10O5	541.1099	6.494983
42.28508	0.160757	0.6737	0.436134	unchanged	-4.82718	C16H26O4	265.1785	6.50925
796.5227	-0.42967	0.489305	0.833145	unchanged	5.655968	C24H43N9	538.349	6.50925
102.679	0.305131	0.835727	0.231211	unchanged	-5.30524	C22H43NO	440.2752	6.516383
27.64681	0.367885	0.332255	1.055078	unchanged	-12.8775	C10H15N5	292.0982	6.530667
47.90223	0.590186	0.480022	0.831851	unchanged	-2.8521	C25H36O4	365.2464	6.530667
93.77347	2.636429	0.281109	1.259921	unchanged	-12.4599	C22H42O6	385.2898	6.5378
158.5723	0.063916	0.93318	0.116322	unchanged	6.1019	C48H83N3	1069.555	6.559217
117.3471	0.727829	0.352107	1.090661	unchanged	-7.12566	C24H38O5	371.2552	6.559217
11.53119	1.129028	0.324518	1.125862	unchanged	-0.31145	C24H43NO	416.3134	6.559217
207.0396	-0.74895	0.284111	1.200024	unchanged	13.24155	C21H38O6	369.2687	6.566367
162.0815	0.53	0.365536	1.007643	unchanged	4.386459	C24H36O2	395.2363	6.5735
123.5771	-0.97047	0.482801	0.739904	unchanged	-16.0013	C29H40N2	583.2536	6.5735
1735.781	0.322613	0.595509	0.666227	unchanged	18.38695	C16H20O2	227.1475	6.60205
40.23426	0.559506	0.352756	1.100795	unchanged	-5.71756	C15H24N2	265.1895	6.60205
211.5355	0.102497	0.714905	0.452059	unchanged	10.35327	C16H24O4	281.1776	6.60205
68.38031	0.437518	0.567703	0.697534	unchanged	-1.78872	C19H24O2	285.1844	6.60205
200.5861	0.28838	0.511666	0.771429	unchanged	8.72093	C18H30O4	293.2138	6.60205
136.7561	0.396727	0.417236	0.97833	unchanged	4.358271	C3H7NO2	163.053	6.609183
545.3658	0.242738	0.635775	0.603285	unchanged	-2.82434	C12H18O2	159.1163	6.609183
502.6743	0.20352	0.435883	0.930803	unchanged	-16.7109	C13H14	171.1325	6.609183
584.7066	0.345049	0.536162	0.772287	unchanged	25.95673	C10H15NO	199.1488	6.609183
323.434	0.320661	0.567889	0.716643	unchanged	-9.78257	C14H16	185.1307	6.609183
905.5967	0.303645	0.621608	0.623575	unchanged	-2.7228	C16H24O2	213.1631	6.609183
1059.582	0.286506	0.558699	0.726942	unchanged	-23.1924	C12H20N2	247.1683	6.609183
511.381	0.410491	0.541331	0.752685	unchanged	-2.68316	C18H24O2	273.1842	6.609183
1237.814	0.315224	0.613368	0.635843	unchanged	-1.25157	C17H24O3	241.1888	6.609183
197.4025	0.294904	0.62127	0.627738	unchanged	-6.47457	C20H28O2	301.2143	6.609183
357.1929	0.263252	0.675195	0.53929	unchanged	-4.45604	C19H28O3	287.1992	6.609183
126.5257	0.561783	0.455395	0.899741	unchanged	-18.1912	C23H36O2	309.2514	6.609183
671.942	0.306278	0.645557	0.585667	unchanged	-1.66311	C21H28O2	313.2157	6.609183
121694.6	0.275792	0.623708	0.620899	unchanged	-3.08143	C24H34O2	355.2617	6.609183
222.3189	0.31027	0.462255	0.900126	unchanged	3.354278	C27H42F2	454.3506	6.609183
583.6673	0.285779	0.189441	1.476508	unchanged	-18.1987	C25H47NO	458.3462	6.609183
135.1257	0.121495	0.734295	0.442617	unchanged	-24.8367	C23H38O7	465.2143	6.609183
815.153	0.123624	0.660799	0.556135	unchanged	-29.957	C24H40O8	506.2407	6.609183
336.1793	0.231777	0.746447	0.425503	unchanged	12.48639	C36H56O9	632.3997	6.609183
272.2449	0.171703	0.799342	0.348015	unchanged	7.31561	C35H50O8	640.3888	6.609183
6470.086	0.771588	0.5544	0.73937	unchanged	-28.9475	C44H85N2	799.5723	6.609183
121425.8	0.633091	0.572891	0.707125	unchanged	-14.6002	C45H85O1	817.5834	6.609183
570.9008	0.287617	0.667603	0.548358	unchanged	-9.93362	C44H81O1	871.5015	6.609183
253.1206	0.298924	0.444432	0.926894	unchanged	-22.4539	C14H18	187.1439	6.616333
149.6356	0.430525	0.499142	0.724158	unchanged	7.309939	C40H69O9	689.4594	6.623467
72.50088	0.228938	0.648895	0.595098	unchanged	13.98375	C10H14N6	268.1551	6.637733
82.79621	-0.2648	0.11844	1.589869	unchanged	-8.5544	C18H32O4	335.2166	6.637733
361.3601	0.259381	0.404695	0.997636	unchanged	-7.87349	C8H18S2	179.0909	6.6663
158.657	-0.25574	0.380349	0.931748	unchanged	-2.00269	C16H35NO	274.2735	6.673433
84.06983	-0.12673	0.39778	1.034418	unchanged	4.253103	C12H26N2	333.1645	6.6663
187.8377	1.587369	0.424604	0.985734	unchanged	20.23081	C46H84NO	880.5884	6.673433
63.87554	0.600446	0.055482	1.922464	unchanged	12.14476	C19H22O2	265.1621	6.680567

333.2261	0.701828	0.342218	1.128825	unchanged	34.53958	C20H34O4	339.2645	6.680567
1.72E-06	1.272891	0.341557	1.087889	unchanged	1.620407	C27H42F2	435.3076	6.680567
7910.24	0.698873	0.44951	0.905155	unchanged	-4.66049	C26H43N(	414.2982	6.680567
27.403	0.263439	0.698239	0.469662	unchanged	-13.1717	C17H28O2	287.1947	6.69485
1800.429	0.020655	0.969416	0.087023	unchanged	-42.2292	C23H40O4	381.2839	6.69485
108.4867	-0.77251	0.162556	1.513917	unchanged	21.44095	C27H44O9	530.3433	6.69485
66.75967	-0.57392	0.320452	1.134054	unchanged	-5.79492	C26H52N(	538.3472	6.69485
247.5792	-0.3041	0.584604	0.572192	unchanged	5.805773	C20H44N(	369.3023	6.702017
1950.094	0.025592	0.817004	0.271639	unchanged	-17.8219	C18H15OI	279.0884	0.026717
266.8666	-1.14131	0.159786	1.49303	unchanged	-4.03723	C30H33O1	616.1761	6.702017
965.989	-0.0129	0.949208	0.077925	unchanged	-24.7802	C20H18O7	393.0853	6.70915
148.0531	-0.0164	0.966879	0.055537	unchanged	6.221066	C23H30O1	481.1735	6.70915
13.1045	0.219248	0.747278	0.385228	unchanged	-15.2565	C29H26O1	589.1023	6.70915
209.7588	0.012713	0.953446	0.076646	unchanged	6.579147	C22H24O1	471.1291	6.722717
29.3376	0.050787	0.890701	0.156345	unchanged	-0.92738	C19H32O1	587.1215	6.722717
22.73299	-1.47977	0.238555	1.391593	unchanged	4.352073	C40H73N3	915.4894	6.722717
11.55546	0.729857	0.494065	0.801976	unchanged	1.071153	C17H28O	271.2035	6.729867
620.9256	0.323161	0.164425	1.406895	unchanged	27.22151	C8H16N2(	262.1431	6.751267
218.0734	0.105533	0.508948	0.672235	unchanged	-2.9563	C12H20N4	235.1546	6.751267
1483.47	0.317726	0.122405	1.568647	unchanged	0.726101	C18H22O	277.1798	6.751267
278.9086	-0.25394	0.195603	1.429721	unchanged	-2.92362	C25H31N(	378.2375	6.751267
129.2787	0.488274	0.38968	1.004586	unchanged	-7.28962	C26H37N(	412.2816	6.751267
105.0953	-0.90481	0.054551	1.916454	unchanged	-3.54007	C24H40O6	424.2804	6.751267
45.03499	0.092046	0.884706	0.169299	unchanged	7.6455	C23H40O6	454.3195	6.758417
714.591	-0.21723	0.327867	1.095051	unchanged	-11.2492	C16H24O4	263.161	6.772683
30.84484	-0.10395	0.853934	0.169712	unchanged	11.22266	C18H16O3	322.1469	6.772683
47.68911	-0.20724	0.735446	0.382808	unchanged	-0.52228	C18H30O4	349.1774	6.772683
196.1534	-0.73293	0.504979	0.794735	unchanged	-16.5249	C22H40O4	410.2653	6.772683
65.43784	0.178095	0.675619	0.471841	unchanged	17.4805	C16H26N2	275.1808	6.779833
154.4776	-0.13062	0.851788	0.212497	unchanged	-8.71877	C20H32O3	303.2291	6.80125
91.6724	0.294541	0.513346	0.710976	unchanged	27.28252	C20H24N2	325.1988	6.80125
60.22894	-0.02699	0.975516	0.089109	unchanged	3.525936	C16H24N6	347.1839	6.80125
24.62529	-0.22205	0.508959	0.682859	unchanged	-4.75972	C9H14O3	135.0796	6.808383
2205.412	-0.17535	0.4523	0.81906	unchanged	-4.88373	C17H30N6	437.1921	6.808383
52.10677	-0.88982	0.104574	1.696017	unchanged	14.21888	C27H40O8	534.3131	6.808383
144.1218	0.229944	0.302996	1.180333	unchanged	-30.1167	C11H14O2	179.1013	6.815517
208.6024	1.871266	0.19567	1.468641	unchanged	1.862903	C21H36O4	370.2958	6.82265
34.52564	-0.3118	0.662612	0.459472	unchanged	-5.36641	C40H72N2	886.5072	6.82265
85.28658	-0.02833	0.76561	0.305858	unchanged	-13.2331	C12H20F2	219.1526	6.8298
274.332	-0.19074	0.696726	0.465036	unchanged	-5.88214	C22H36O4	329.2454	6.8298
377.5326	-0.65187	0.394956	1.023452	unchanged	6.397936	C28H46O4	485.3056	6.8298
8.612792	0.153438	0.897368	0.161738	unchanged	19.57977	C25H41N7	536.3296	6.8298
44.62977	0.270252	0.596168	0.647606	unchanged	1.356339	C9H10O2	115.0544	6.844083
4.663191	0.651693	0.431325	0.901449	unchanged	-12.1617	C10H14	157.0971	6.836933
38.09904	0.299822	0.685548	0.506032	unchanged	3.469449	C11H19N(	215.1761	6.844083
15.981	0.148585	0.78046	0.364048	unchanged	0.874912	C11H22N2	245.1321	6.836933
24.06055	0.342233	0.612667	0.619306	unchanged	8.367667	C16H21N(	256.1356	6.836933
101.4669	0.261434	0.59669	0.644727	unchanged	-6.67788	C15H20O4	265.1417	6.836933
5.845583	0.806933	0.081101	1.854767	unchanged	-2.16742	C24H38O3	397.2705	6.844083
31514.51	0.277061	0.688452	0.506253	unchanged	-3.17359	C26H43N(	414.2988	6.844083
213.3361	0.203191	0.752334	0.406496	unchanged	-11.5576	C29H39N(	488.2718	6.836933

16.4682	0.454686	0.704902	0.489043	unchanged	6.083942	C52H84N(	955.5567	6.844083
3.851648	0.588283	0.606153	0.623033	unchanged	13.65993	C48H89O1	943.6008	6.844083
79.79054	0.417703	0.456671	0.880577	unchanged	-11.2507	C26H36O4	454.2905	6.851217
15.98962	1.658537	0.316021	1.145493	unchanged	7.551175	C20H37N(	480.2114	6.85835
555.5907	-0.26608	0.629409	0.533509	unchanged	-3.6404	C16H32O	258.2783	6.872633
552.4425	-0.14149	0.442034	0.847622	unchanged	-2.81844	C18H39N(	284.2939	6.879767
170.6654	-0.27002	0.555744	0.632932	unchanged	-5.19948	C18H36O3	318.2987	6.89405
143.0643	0.094951	0.506886	0.729471	unchanged	2.347177	C12H16O3	209.1177	6.901183
30.6309	0.052823	0.920062	0.070107	unchanged	6.944448	C9H17NO	261.146	6.901183
56.28117	-0.11876	0.48201	0.801203	unchanged	-3.27039	C6H12OS	133.0677	6.908317
1279.465	0.074235	0.229204	1.284652	unchanged	7.123017	C17H26O5	333.1678	6.908317
472.1213	-0.15562	0.708711	0.46725	unchanged	-10.5736	C9H16O2	335.216	6.908317
31.60569	0.430845	0.338391	1.114283	unchanged	-0.41791	C23H28N2	397.212	6.908317
12.24781	0.431566	0.473239	0.845377	unchanged	-4.57602	C27H46N(	569.3326	6.908317
30.72261	0.129897	0.691539	0.484682	unchanged	-2.50458	C9H10O	135.0801	6.915467
34.9139	0.016775	0.954969	0.105661	unchanged	-7.01976	C15H20O5	281.1364	6.915467
23.66449	0.060107	0.83718	0.266306	unchanged	4.973272	C11H14N6	295.1164	6.915467
100.3728	0.129478	0.767734	0.34623	unchanged	0.732049	C20H38O2	328.3212	6.9226
221.6614	0.225412	0.70327	0.439761	unchanged	-3.39477	C23H34O2	343.262	6.9226
3293.159	-0.05369	0.772235	0.316491	unchanged	-4.40969	C24H30O6	437.1916	6.915467
34.6522	0.473222	0.444568	0.893719	unchanged	0.637033	C25H32O6	446.254	6.915467
176.3628	0.258038	0.642584	0.570718	unchanged	-0.74673	C26H34O6	460.269	6.915467
42.9996	-0.08763	0.572329	0.685	unchanged	2.858068	C9H18NO	187.1209	6.929733
64.17611	-1.32875	0.100965	1.772667	unchanged	5.298834	C36H62N(	765.4159	6.929733
84.48249	-0.07145	0.8867	0.155478	unchanged	-11.0233	C20H34O2	271.2387	6.944017
1420.112	-0.20845	0.428197	0.940723	unchanged	-8.34779	C18H28O3	293.1973	6.936867
207.8923	-0.09151	0.275288	1.184774	unchanged	-0.86929	C27H33N3	534.209	6.95115
142.5352	0.020595	0.951785	0.097605	unchanged	4.633543	C9H19O6P	219.0792	0.575667
45.67858	0.267618	0.622253	0.587152	unchanged	-10.3408	C20H42N(	457.2992	6.965433
40.92743	-0.0887	0.680018	0.446002	unchanged	0.067824	C33H55N(	478.4044	6.9797
226.0024	-0.22239	0.364144	1.07268	unchanged	0.769244	C14H20O6	267.1229	6.993983
164.0257	-0.31785	0.163838	1.546921	unchanged	-0.61133	C14H24N6	323.1824	6.993983
35.91807	-3.26855	0.218744	1.370492	unchanged	-13.9066	C27H46O3	401.3356	6.98685
399.518	-0.24982	0.383936	1.056923	unchanged	-3.78415	C20H32N6	419.2385	6.993983
11782.12	-0.10678	0.774787	0.268454	unchanged	7.552576	C32H48N4	655.315	6.993983
1425.251	-0.15765	0.693527	0.394224	unchanged	23.52389	C34H48O1	677.2971	6.98685
43.90415	-0.03968	0.860849	0.213144	unchanged	-2.74932	C11H10N4	269.0429	0.5828
46.13485	0.096303	0.67428	0.391227	unchanged	-0.12106	C20H32O6	369.2271	7.001117
104.1292	-0.39899	0.580604	0.655058	unchanged	-3.17046	C15H30N6	391.211	7.001117
11.83102	-0.24005	0.838859	0.289961	unchanged	-4.16073	C24H48N(	516.304	7.001117
6342.238	0.369947	0.069281	1.886996	unchanged	13.38218	C10H19N3	278.1198	7.00825
36.05488	-0.24056	0.753461	0.321394	unchanged	-0.76091	C20H34O6	371.2425	7.00825
356.8267	-0.28272	0.508115	0.750155	unchanged	-1.78573	C20H30N2	396.2486	7.00825
53.56714	0.463773	0.698528	0.47396	unchanged	-25.7635	C12H26N4	364.2108	7.0154
353.7829	-0.15498	0.817331	0.269752	unchanged	0.049835	C17H29N(	388.1602	7.0154
168.4465	-0.26257	0.04258	1.985443	down	5.561124	C15H24O2	259.1682	7.036817
27.78513	1.730569	0.269001	1.236437	unchanged	3.615315	C22H24N2	444.1543	7.04395
233.7358	-0.41265	0.445479	0.929889	unchanged	-2.94268	C16H26O5	263.1633	7.064667
55.24435	0.273181	0.54659	0.737775	unchanged	-28.6253	C17H37N7	421.313	7.064667
7.00585	0.517127	0.737505	0.4045	unchanged	-4.22758	C12H22O3	256.1898	7.086083
131.8138	-0.31532	0.14613	1.734714	unchanged	4.168427	C19H26O6	333.1711	7.086083

113.7341	0.02931	0.859171	0.215439	unchange(	-4.68339	C24H40O(	424.2799	7.093217
638.686	0.055906	0.839702	0.172801	unchange(	-9.88948	C20H34O!	337.2338	7.10035
1096.563	-0.19811	0.145448	1.660269	unchange(	-0.69407	C24H34N2	453.1759	7.10035
149.0262	-0.06288	0.716559	0.383294	unchange(	-6.6599	C19H39N!	446.2577	7.10035
28.47539	-0.13072	0.652577	0.47008	unchange(	7.336638	C9H10O2	115.0553	7.114633
47.33228	0.0625	0.830814	0.246751	unchange(	8.156004	C12H16O2	193.1239	7.114633
76.53135	0.266483	0.355004	1.072448	unchange(	-2.82705	C11H22O4	241.1404	7.114633
1346.59	-0.3255	0.631137	0.574918	unchange(	-2.77668	C18H30O2	243.21	7.107483
1906.831	-0.28063	0.641353	0.561443	unchange(	-1.27042	C19H28O2	289.2113	7.107483
771.0208	-0.13195	0.785703	0.347141	unchange(	-9.40516	C20H30O4	335.2192	7.107483
17.49993	-0.06089	0.903071	0.177578	unchange(	6.435664	C8H16N2(	431.2139	7.107483
185.9977	-0.23765	0.723046	0.429683	unchange(	5.277807	C20H30N4	471.1933	7.107483
31.25523	-1.14497	0.126024	1.712888	unchange(	19.45569	C24H48N(	510.3289	7.107483
39.92545	-0.06152	0.923459	0.143333	unchange(	2.482661	C9H16	147.1147	7.121767
83.64514	-0.33524	0.688963	0.494386	unchange(	5.427165	C26H46N(	538.2932	7.121767
1647.291	-0.11927	0.538247	0.720051	unchange(	-26.4543	C15H14N4	375.0108	0.5828
13.61089	0.026213	0.940312	0.049658	unchange(	0.193911	C9H17NO	189.1598	7.1289
37.24081	-0.22679	0.216012	1.32349	unchange(	-4.9114	C11H13N!	281.1344	7.1289
81.913	-0.15996	0.427098	0.903171	unchange(	5.094407	C13H17N3	295.1177	7.1289
1003.614	0.682406	0.08473	1.888603	unchange(	-4.41656	C21H39N(	371.3253	7.1289
32.59396	-0.09162	0.776176	0.300058	unchange(	8.206022	C13H14O3	183.0822	7.13605
431.1007	-0.1138	0.180348	1.411281	unchange(	15.3559	C12H20N4	331.1302	7.143183
3214.995	-0.17709	0.06856	1.862984	unchange(	30.3743	C22H26O(	387.1919	7.143183
288.3106	-0.55091	0.004755	2.399954	down	0.845161	C38H66O1	823.38	7.13605
67.0376	0.015558	0.952382	0.132335	unchange(	2.165216	C22H40O1	445.2442	7.150317
178.9594	0.064343	0.659042	0.515734	unchange(	-7.30014	C10H12O4	179.0688	7.15745
685.9734	0.15763	0.498964	0.794567	unchange(	-5.17802	C14H18O3	235.1317	7.15745
351.1658	-1.65867	0.223723	1.391201	unchange(	2.445189	C16H28O(	339.1786	7.1646
26.5584	0.218142	0.309648	1.098578	unchange(	-2.45314	C29H41N7	568.3228	7.1646
45.29544	0.635232	0.28254	1.230334	unchange(	-12.8185	C20H30O4	352.244	7.171733
177.8439	-0.45212	0.365284	1.045194	unchange(	0.309212	C21H39N7	492.3087	7.171733
164.7202	-0.81057	0.141604	1.592607	unchange(	-7.37801	C22H46N(	490.287	7.171733
854.8983	-0.29798	0.655009	0.508607	unchange(	6.279685	C18H34N2	343.2652	7.178867
104.4492	-0.45393	0.507016	0.812718	unchange(	7.056825	C25H38O3	425.248	7.178867
330.1384	-0.21047	0.726728	0.415075	unchange(	-3.76264	C20H30O3	301.215	7.186017
53.4617	0.058612	0.954185	0.061775	unchange(	-6.63776	C21H33N(	413.262	7.186017
675.1231	-0.13569	0.807235	0.289604	unchange(	-13.8554	C25H38O4	403.2738	7.214567
58.42408	0.257547	0.793685	0.276975	unchange(	-6.04351	C23H38N(	443.2736	7.214567
62.73344	0.284511	0.309201	1.036373	unchange(	-8.69788	C16H26O3	249.1826	7.2217
72.15001	0.924433	0.129791	1.723117	unchange(	-18.0111	C15H22O3	251.1597	7.2217
46.55212	0.064834	0.831298	0.266903	unchange(	19.36968	C27H41N(	530.2645	7.2217
206.7264	0.021497	0.915908	0.131883	unchange(	-12.4255	C8H10	107.0842	7.243117
9177.239	-0.08755	0.638049	0.528242	unchange(	-5.83849	C9H10	119.0848	7.243117
559.9812	-0.08978	0.613493	0.559466	unchange(	3.923784	C9H10O2	133.0654	7.243117
42.11526	-0.43193	0.102527	1.746524	unchange(	-1.64934	C18H30	247.2416	7.235983
684.9791	-0.23842	0.098767	1.751176	unchange(	-2.64104	C18H37N(	264.2678	7.243117
67.88307	0.157861	0.778951	0.282907	unchange(	13.62233	C8H16O	279.2329	7.243117
13.01606	0.284317	0.584717	0.638869	unchange(	-7.50456	C15H18O1	376.1211	7.243117
1372.385	-0.14349	0.442973	0.889774	unchange(	-0.56731	C18H38N(	380.2536	7.243117
119395.8	-0.03377	0.868038	0.17459	unchange(	-12.3278	C21H34O(	415.2098	7.243117
4972.065	0.114694	0.641163	0.561329	unchange(	-19.1273	C25H22N4	460.2677	7.243117

7.807783	0.846574	0.264132	1.23422	unchanged	14.72391	C29H31N7	458.2524	7.243117
75.6952	0.17811	0.665015	0.544911	unchanged	-3.21694	C25H31NC	483.2475	7.243117
42.02108	-0.20079	0.653033	0.557959	unchanged	-18.4773	C25H32O8	499.1644	7.235983
37.4629	0.218433	0.630167	0.555896	unchanged	-12.1487	C20H28N4	517.1359	7.235983
196.3529	-0.35129	0.024774	2.133987	down	-0.26548	C42H72NC	794.4601	7.243117
219.3672	-0.03989	0.83323	0.224162	unchanged	0.467359	C7H10O2	91.05429	7.243117
56.04145	-0.03575	0.838214	0.228308	unchanged	0.773031	C6H10O4	147.0653	7.25025
59.72531	0.069619	0.799714	0.295543	unchanged	-11.9281	C14H16O2	217.1197	7.25025
115.0226	0.278715	0.380842	1.011686	unchanged	-13.0255	C33H35FN	576.2796	7.25025
37.18234	-0.08816	0.907389	0.060601	unchanged	-2.47989	C18H38NC	339.2527	7.264533
832.5979	0.124436	0.67095	0.517737	unchanged	0.503907	C23H34N4	446.2525	7.2574
1860.448	0.101925	0.708388	0.457121	unchanged	20.7252	C27H36O7	473.2632	7.2574
38.29184	-0.0344	0.921167	0.168287	unchanged	-17.7072	C12H26N4	289.1816	7.271667
178.7209	0.027589	0.87299	0.200523	unchanged	-4.19836	C18H37NC	299.3041	7.278817
111.8878	0.211188	0.253423	1.264508	unchanged	17.63502	C17H22N2	251.1593	7.307367
11.60884	1.867946	0.274702	1.222102	unchanged	17.38983	C29H46O3	442.3518	7.307367
20.57915	-0.31603	0.077112	1.852085	unchanged	-8.94332	C4H11NO	106.0853	7.321633
111.4531	0.226988	0.505816	0.767063	unchanged	-5.28822	C16H15N7	344.1213	7.321633
55.30747	0.530645	0.060056	1.992772	unchanged	0.800053	C28H39N3	546.2814	7.321633
38.16206	-0.0351	0.908338	0.160179	unchanged	19.9458	C38H65NC	724.4418	7.321633
26.7873	-0.06688	0.791695	0.312716	unchanged	-1.72028	C7H10O2	91.05401	7.321633
13879.09	-0.64019	0.084344	1.785807	unchanged	-3.87912	C22H46NC	468.3067	7.328783
22.02071	-0.10325	0.826943	0.26197	unchanged	15.25143	C22H40N8	538.3536	7.328783
23.23864	-0.06437	0.914601	0.216398	unchanged	-2.13445	C18H34O4	279.2312	7.34305
276.6914	1.161258	0.156915	1.548701	unchanged	-4.47408	C23H40O4	398.3248	7.335917
398.6805	1.324692	0.269813	1.309414	unchanged	-47.1891	C25H38O8	466.297	7.335917
35.15079	1.478546	0.233726	1.404822	unchanged	-5.7714	C26H45NC	484.3063	7.335917
22.90846	-0.96081	0.097114	1.878799	unchanged	11.7049	C25H42O8	512.3273	7.3502
34.06071	0.724518	0.269969	1.2789	unchanged	2.799165	C24H36O3	372.2669	7.364467
738.0218	0.121503	0.561894	0.685605	unchanged	-5.2189	C9H10O3	167.0701	7.3716
33.7037	-0.65488	0.023421	2.142808	down	-0.1402	C14H28O2	270.2427	7.3716
17.52931	-0.42315	0.367507	1.024439	unchanged	15.95043	C20H32O5	375.2198	7.3716
23.99902	0.316726	0.510822	0.765131	unchanged	-2.75687	C9H10FN3	519.1267	7.3716
47.68017	0.101979	0.590455	0.603485	unchanged	10.66106	C6H6O	95.05014	7.3716
146.4125	0.750063	0.065553	1.826701	unchanged	-2.26191	C12H16O3	191.1062	7.37875
1.72E-06	0.986476	0.303548	1.032691	unchanged	5.3048	C14H14O3	231.1028	7.37875
42.76927	-0.58941	0.399889	1.002007	unchanged	16.21932	C20H32O6	391.2151	7.37875
144.3071	0.159454	0.458104	0.838526	unchanged	-1.43252	C22H28O1	470.2014	7.37875
236.1789	0.200841	0.274166	1.159877	unchanged	9.034588	C20H34O6	335.225	7.4066
27.75926	0.58849	0.1777	1.462235	unchanged	-6.8693	C11H20O2	386.324	7.413733
101.9979	0.02069	0.926393	0.121344	unchanged	-19.3968	C21H29N5	454.1703	7.413733
170.2807	-0.2421	0.56734	0.616368	unchanged	-0.6883	C23H37O7	495.1905	7.413733
6.970894	0.620798	0.447175	0.893123	unchanged	3.878936	C26H43NC	555.3118	7.4066
12.71176	0.001139	0.996128	0.023601	unchanged	12.59785	C9H10O2	115.0561	7.420867
29.80844	-0.24134	0.741182	0.375963	unchanged	-0.29911	C9H19NO	247.1652	7.420867
17.89344	-0.01663	0.976442	0.086983	unchanged	-18.4639	C14H26O2	249.1783	7.420867
1.72E-06	0.148524	0.857302	0.298837	unchanged	-7.22143	C13H17N3	296.122	7.420867
175.2257	-0.22301	0.786636	0.35766	unchanged	-25.1165	C46H83N1	1011.642	7.43515
133.0814	-0.5717	0.1194	1.649192	unchanged	4.428349	C5H13NO	104.1074	7.442283
25.09823	-1.03486	0.398895	1.062194	unchanged	5.744028	C50H85N3	1135.565	7.43515
76.27641	-0.02705	0.866963	0.158281	unchanged	-2.57313	C16H24O3	265.1791	7.43515

35.86663	-0.71319	0.202654	1.399365	unchanged	-2.22662	C13H12N2	286.1181	7.442283
41.64604	-0.8205	0.123708	1.634431	unchanged	-27.4608	C10H20N6	288.1218	7.442283
188.231	-0.20573	0.59632	0.585741	unchanged	9.386981	C15H20O7	330.1764	7.43515
35.57409	-0.50461	0.675897	0.42749	unchanged	-5.34499	C11H22O2	390.3558	7.442283
280.3358	-0.16111	0.590153	0.594688	unchanged	-3.47362	C23H38O6	410.2648	7.442283
394.3374	-0.39302	0.27207	1.240117	unchanged	26.51799	C23H38O8	481.2316	7.442283
92714.21	-0.47404	0.20286	1.409048	unchanged	-1.95924	C26H48N(	518.3231	7.442283
73.46515	-0.75962	0.177141	1.468209	unchanged	-27.1796	C18H20O2	537.2854	7.442283
63.20447	-0.86603	0.179163	1.469681	unchanged	4.479742	C22H35N5	547.2746	7.442283
22.79086	-0.48592	0.074	1.829988	unchanged	-0.55933	C31H41N5	602.2736	7.43515
100.6289	-0.81173	0.185755	1.449976	unchanged	1.211364	C46H88N(	847.6545	7.43515
64.37758	-0.86117	0.124944	1.638726	unchanged	-0.24697	C43H78O1	954.5101	7.43515
56.87663	-0.00482	0.976903	0.145998	unchanged	7.892938	C26H39N3	442.3099	7.449433
6.488508	-1.63111	0.391313	1.103445	unchanged	4.262576	C48H92N(	891.6834	7.449433
74.4265	-2.3363	0.142782	1.728422	unchanged	-0.36295	C51H91N3	1081.621	7.456567
218.5112	0.136524	0.754179	0.384991	unchanged	-2.47036	C18H39N(	286.3097	7.4637
278.249	0.095951	0.789592	0.329212	unchanged	-2.57112	C18H38O	312.3254	7.456567
329.8321	0.062118	0.93066	0.141402	unchanged	-4.77855	C20H40O3	346.33	7.4637
206.3517	-0.73018	0.191998	1.413075	unchanged	2.583384	C24H40N8	505.2441	7.4637
128.7818	-1.46764	0.282399	1.289278	unchanged	12.58121	C49H86N(	969.6292	7.4637
47.39687	-0.4465	0.226482	1.348783	unchanged	2.256656	C15H24O3	291.1363	7.470833
30492.46	-0.81963	0.076653	1.81977	unchanged	-2.67848	C28H48N(	542.3227	7.470833
5.338814	-1.75782	0.175918	1.448595	unchanged	-1.52318	C15H19O4	246.1246	7.477983
26.6018	-1.6122	0.093502	1.751846	unchanged	-3.74463	C46H75N(	888.5047	7.477983
26.85844	0.899642	0.119965	1.592524	unchanged	-10.5336	C23H42O4	424.3381	7.49225
131.3784	-0.10387	0.813222	0.295428	unchanged	4.28096	C17H22O	507.3254	7.4994
160.986	-0.30992	0.486041	0.854365	unchanged	18.68886	C15H25N(	251.1926	7.513667
19.06075	0.56276	0.18151	1.437587	unchanged	15.53936	C29H44O1	594.3359	7.506533
38.01477	-0.34823	0.650569	0.512359	unchanged	-13.3028	C11H16O2	181.1199	7.520817
1902.479	0.120067	0.895699	0.146248	unchanged	-5.91198	C19H34O	279.2671	7.520817
1294.03	0.153948	0.871646	0.180097	unchanged	-4.20501	C20H38O4	325.2723	7.520817
265.6747	-0.09687	0.808731	0.255521	unchanged	-5.63518	C23H38O6	410.2639	7.520817
186.7983	-0.32657	0.332137	1.06393	unchanged	13.05784	C25H48N(	528.3127	7.520817
39.38211	-0.04232	0.829217	0.196109	unchanged	3.184985	C8H10O	105.0703	7.52795
552.7132	-0.14945	0.463165	0.817624	unchanged	23.85956	C4H7N3O	281.1379	7.52795
811.1053	-0.27013	0.212107	1.33835	unchanged	1.791553	C15H18O6	295.1174	7.52795
2763.211	-0.13564	0.348454	1.015265	unchanged	-1.43279	C24H30O6	453.1668	7.52795
1.72E-06	-0.69843	0.456838	0.834112	unchanged	-47.3603	C20H24N4	514.1367	7.52795
52.47644	-0.45297	0.795305	0.311638	unchanged	-8.35117	C39H53N7	813.4075	7.52795
67.01659	-0.0093	0.983335	0.022032	unchanged	-1.40432	C12H18	363.2444	7.542217
968.7215	-0.09407	0.657452	0.484765	unchanged	-3.26661	C24H34O8	473.2629	7.535083
35.62647	0.500625	0.236781	1.321362	unchanged	21.78017	C18H29O3	258.2042	7.549367
6440.819	-0.46944	0.27955	1.217115	unchanged	-5.75788	C27H43N(	452.311	7.549367
46.6711	-0.08577	0.903584	0.114786	unchanged	39.68117	C10H21N3	247.1624	7.563633
9.739179	-0.94523	0.333974	1.188999	unchanged	15.87735	C18H22N2	316.1889	7.5565
139.3696	-0.05416	0.917619	0.101685	unchanged	11.1315	C20H32O3	321.246	7.563633
6126.693	0.185934	0.83289	0.276761	unchanged	-1.18811	C24H38O3	357.2784	7.5565
5.676083	2.197039	0.105813	1.72321	unchanged	14.19858	C23H44N2	412.3354	7.563633
141.6087	0.133493	0.807046	0.27783	unchanged	-22.2427	C27H40O7	441.253	7.563633
75.44593	-0.21641	0.831667	0.218433	unchanged	-11.0132	C25H41N(	490.2516	7.5565
712.954	0.140537	0.882877	0.15971	unchanged	-9.4104	C50H86N(	930.5113	7.563633

53.10184	0.322541	0.232742	1.289963	unchangec	1.672028	C14H16N(	267.1357	7.570783
37.73887	-0.24203	0.713891	0.40448	unchangec	-11.2639	C11H20N(	407.1018	7.570783
23.8325	0.832733	0.032511	2.051955	up	5.385828	C22H32O2	311.2387	7.577917
128.5281	0.105559	0.755799	0.382309	unchangec	6.312418	C24H32N2	379.2405	7.577917
349.3002	0.047515	0.817927	0.186193	unchangec	-9.40205	C18H28O3	275.1978	7.592183
429.6149	-0.3235	0.421411	0.931172	unchangec	-2.85004	C11H20N4	498.3497	7.592183
52.32407	0.236395	0.362055	1.059421	unchangec	-9.99132	C13H11N3	264.0511	7.599333
56.01317	-0.16015	0.611138	0.590328	unchangec	-10.5803	C18H34O2	305.2421	7.599333
19261.43	-0.54099	0.335361	1.072043	unchangec	-3.4572	C23H48N(	482.3225	7.599333
81.03631	-0.66704	0.533754	0.743015	unchangec	9.467644	C44H81N3	918.5095	7.599333
108.9116	-0.07772	0.663954	0.493276	unchangec	19.79747	C7H9N	107.075	7.606467
3.801305	-20.3448	0.190634	1.631899	unchangec	7.009467	C4H8O5	119.0348	7.6136
180.7541	-0.26249	0.169159	1.505356	unchangec	6.667862	C2H6N4O	120.0887	7.6136
247.334	-0.19775	0.318984	1.115153	unchangec	1.421773	C9H10O	135.0806	7.606467
224.969	-0.18772	0.383206	0.936127	unchangec	10.84626	C8H12O2	281.1371	7.6136
319.0105	-0.25255	0.279372	1.183907	unchangec	18.93536	C8H15NO	295.117	7.606467
64.65971	0.267348	0.32921	1.111464	unchangec	28.11181	C13H22N4	332.2017	7.606467
1556.645	-0.17468	0.413169	0.898304	unchangec	15.15393	C28H48O	400.376	7.6136
22.99707	-0.44729	0.179173	1.396102	unchangec	-0.68817	C28H38N8	531.2823	7.606467
38.7956	-0.42477	0.629219	0.606749	unchangec	-1.89671	C27H44N(	567.3184	7.6136
283.4818	0.189591	0.587839	0.61833	unchangec	-27.4866	C31H51O9	640.3444	7.6136
31.4492	-0.06246	0.737256	0.413706	unchangec	-2.52327	C6H11NO	259.1646	7.635017
393.2182	-0.04992	0.856674	0.189415	unchangec	5.392053	C30H52O2	444.3985	7.635017
49.91085	0.939596	0.344857	1.091748	unchangec	-5.29957	C10H16O3	149.0951	7.6493
124.7134	0.146796	0.557112	0.713308	unchangec	0.941366	C24H27N(	379.2383	7.6493
26.92961	1.552704	0.040984	2.07281	up	-26.9044	C30H50O3	423.3498	7.656433
585.6418	0.093877	0.573074	0.660864	unchangec	-17.9423	C21H38O4	337.2674	7.670717
1397.528	0.957906	0.142605	1.577553	unchangec	8.603147	C23H45N(	400.3456	7.670717
135.716	0.288902	0.147724	1.567673	unchangec	-26.6053	C9H14O	277.2048	7.684983
26.40209	0.157107	0.754725	0.372119	unchangec	31.29498	C7H12N2(	153.0717	7.684983
39.92083	0.294633	0.757842	0.377573	unchangec	-15.9146	C15H20	201.1606	7.684983
33.41911	0.441337	0.544355	0.700765	unchangec	6.494908	C14H21N	203.1681	7.684983
32.94709	-0.0178	0.966331	0.03692	unchangec	-15.0406	C12H19N(	209.1378	7.684983
138.4877	0.214881	0.503632	0.778984	unchangec	-26.0169	C16H20N2	221.137	7.684983
200.1006	0.413718	0.584233	0.642477	unchangec	-20.3868	C13H20O3	247.1678	7.684983
126.0581	0.253862	0.679321	0.489379	unchangec	-3.0217	C18H26O2	275.1997	7.684983
214.5357	0.135799	0.686993	0.542524	unchangec	-6.31719	C18H32O4	335.2162	7.684983
204.4417	-0.64349	0.17068	1.487664	unchangec	-24.9538	C21H36O5	351.2438	7.67785
17650.01	0.25758	0.712194	0.438466	unchangec	-2.69198	C24H36O2	357.2778	7.684983
298.6013	-0.07682	0.62199	0.542948	unchangec	-17.1834	C22H36O5	363.2465	7.67785
128.5951	0.728025	0.485553	0.84725	unchangec	11.33077	C23H45N(	438.3237	7.684983
10.66629	0.220393	0.702021	0.482711	unchangec	9.255626	C26H44O1	481.2844	7.67785
300.2955	0.379977	0.644872	0.534688	unchangec	4.360205	C24H32O8	490.2455	7.684983
330.3913	0.664573	0.603964	0.589066	unchangec	-2.67691	C45H78N(	865.5309	7.684983
146.4824	-0.51038	0.640741	0.501677	unchangec	-5.72452	C47H84N(	908.5898	7.684983
43.48614	0.17535	0.647706	0.509579	unchangec	-3.48526	C12H14O2	155.0849	7.692133
53.72869	0.363591	0.674747	0.480344	unchangec	-4.96142	C9H20N2(	189.1588	7.692133
77.66108	0.16974	0.597544	0.608031	unchangec	-39.858	C15H22O2	235.1599	7.692133
290.9446	0.165804	0.811882	0.290946	unchangec	-7.31446	C18H30O2	243.2087	7.692133
364.8298	0.503012	0.225368	1.403163	unchangec	-0.89604	C46H82N(	804.553	7.692133
2256.582	0.100933	0.876153	0.205792	unchangec	15.68504	C46H85O1	934.6155	7.699267

30.2581	0.707546	0.480023	0.813945	unchange	-8.31188	C48H78O	968.55	7.699267
259.0696	-0.57354	0.387018	0.956627	unchange	-17.5318	C56H87N	1047.618	7.713533
174.4443	-0.44286	0.333525	1.054822	unchange	-27.6447	C57H107	1174.65	7.713533
49.51065	-0.25121	0.062764	1.940701	unchange	5.137155	C10H15N	240.0992	7.7064
21.26066	-0.21122	0.399312	0.903352	unchange	-2.62967	C17H20O	287.127	7.7064
585.9104	0.013284	0.940248	0.136488	unchange	-8.27942	C20H34O	305.2448	7.713533
1365.692	0.51594	0.227983	1.36569	unchange	-34.5552	C23H48N	416.3138	7.713533
29203.17	-0.66891	0.112537	1.605143	unchange	34.90079	C23H48N	482.3232	7.713533
118468.1	-0.26471	0.090017	1.745188	unchange	-2.2346	C26H50N	520.3386	7.7064
128.7643	-0.13067	0.485349	0.780279	unchange	32.51615	C29H44O	584.2591	7.7064
67.69523	-0.12915	0.406804	0.962243	unchange	-0.80775	C39H74N	802.4835	7.7064
368.2342	-0.32811	0.033203	2.17324	down	6.377437	C43H80O	956.5318	7.713533
40.0451	-0.14518	0.708567	0.39314	unchange	1.583256	C14H15N	198.128	7.73425
10.04545	-0.6325	0.439271	0.83437	unchange	3.997731	C12H22O	214.1572	7.73425
20.68124	0.286786	0.633612	0.552573	unchange	-12.1134	C10H9NO	281.0562	7.73425
0.890103	-0.24656	0.755826	0.322839	unchange	-8.34455	C13H17N	296.1216	7.727117
256.8416	-0.09612	0.678512	0.465392	unchange	-18.1093	C17H34N	417.22	7.727117
44076.85	-0.47083	0.204827	1.372265	unchange	-3.55467	C28H50N	544.3378	7.73425
38.37871	-0.49474	0.364729	1.023026	unchange	-2.88283	C32H48O	562.3178	7.73425
18.1288	-0.76606	0.257378	1.225867	unchange	-2.91688	C15H19N	572.295	7.73425
117.3523	-0.15909	0.766885	0.304184	unchange	0.933795	C22H43N	590.3197	7.727117
46.46403	-1.00981	0.067142	1.792731	unchange	5.092442	C45H64N	918.5128	7.727117
46.05283	-1.35986	0.346008	0.99294	unchange	7.78902	C48H90N	926.5952	7.73425
82.47238	-1.03432	0.065344	1.872701	unchange	-0.33474	C51H81O	980.5253	7.73425
189.8322	-1.19968	0.342824	1.006998	unchange	-2.72092	C52H88N	988.6251	7.7414
55.37142	-0.22576	0.543097	0.622742	unchange	-1.70588	C20H32O	319.2262	7.748533
945.191	-0.58051	0.500704	0.693257	unchange	-4.10065	C25H36O	369.2773	7.748533
1134.741	-0.50575	0.558573	0.597085	unchange	-5.60138	C25H42O	387.287	7.748533
537.6459	-0.40045	0.391836	0.887901	unchange	-4.9374	C23H38N	445.2652	7.748533
7170.841	-0.11305	0.757171	0.336602	unchange	18.4323	C26H25F3	437.1919	7.7628
54.997	0.038093	0.813907	0.307623	unchange	-2.94129	C18H32N	473.2453	7.7628
215.5872	-0.16639	0.729084	0.378936	unchange	-11.4497	C31H45F	556.3355	7.755667
1544.32	-0.11886	0.7098	0.370327	unchange	6.180514	C32H42O	599.2526	7.7628
78.83759	-0.43244	0.080438	1.847482	unchange	-1.63208	C18H34O	332.279	7.784217
61.47624	-0.48008	0.388107	0.974104	unchange	-24.2775	C18H40N	382.2624	7.777083
705.646	-0.27838	0.414522	0.880978	unchange	-2.58105	C22H43N	424.2814	7.791367
31.00402	0.304138	0.58724	0.593065	unchange	7.255984	C27H46O	449.3118	7.791367
36.90203	-0.09809	0.838626	0.186562	unchange	9.800075	C31H49O	434.3589	7.7985
61.32317	0.239584	0.204788	1.448107	unchange	4.86353	C26H43N	514.3163	7.7985
105.3705	0.201729	0.142301	1.577887	unchange	-8.72954	C18H32O	277.2135	7.812783
55.78786	0.355972	0.364322	1.032666	unchange	3.049022	C18H37N	306.2776	7.805633
14.32687	-0.26468	0.62523	0.481865	unchange	15.46604	C28H39N	554.3171	7.805633
302.4606	-0.17311	0.777801	0.284621	unchange	-20.9179	C32H51N	590.3193	7.812783
32.86231	-0.6623	0.189945	1.377825	unchange	2.503282	C45H72O	902.513	7.812783
664.8946	-0.5197	0.345376	1.056822	unchange	-8.36032	C51H89N	1007.643	7.819917
68.72108	0.318875	0.341006	1.130391	unchange	-2.78801	C52H88O	1122.602	7.819917
133.9377	-0.00187	0.994222	0.079683	unchange	-15.2041	C23H38O	361.268	7.819917
296.9016	0.198413	0.687715	0.484419	unchange	-10.2204	C49H79O	956.5399	7.819917
201.4345	-0.113	0.22564	1.34217	unchange	2.522656	C8H20NO	240.1002	7.834183
224.0472	-0.00215	0.9841	0.039554	unchange	11.61925	C12H22N	287.1275	7.82705
502.9418	-0.00573	0.95403	0.08697	unchange	13.71435	C30H44O	539.305	7.834183

7029.194	-0.27412	0.657673	0.469015	unchanged	-5.05071	C37H47N(	568.3392	7.82705
27.10712	0.334566	0.412789	0.94419	unchanged	-29.3321	C14H19N3	604.2917	7.82705
1293.228	-0.17026	0.17033	1.530226	unchanged	1.183132	C5H14NO4	184.0735	7.841333
773592.2	-0.17844	0.102038	1.71715	unchanged	-3.12827	C26H50N(	520.3381	7.841333
437.6229	0.076443	0.573308	0.632981	unchanged	-4.26638	C43H73N2	799.4755	7.841333
287.5706	-0.93229	0.097699	1.727389	unchanged	6.536603	C30H48O5	1015.67	7.86275
390.5078	-0.14924	0.561309	0.636314	unchanged	15.10051	C62H88N6	1073.649	7.86275
15.08658	-0.44194	0.472518	0.817652	unchanged	15.68818	C13H22N2	299.129	7.86275
3798.245	-0.27081	0.391231	0.966178	unchanged	-0.90055	C32H46O7	1085.655	7.86275
132743.2	-0.50312	0.22554	1.372929	unchanged	-6.42816	C20H41N1	544.3391	7.8556
49.1344	-0.23821	0.258449	1.290469	unchanged	5.894997	C38H64O1	818.4578	7.8556
131.3232	-0.33887	0.597016	0.583041	unchanged	-12.8912	C43H72N(	832.4631	7.86275
138.4288	0.088519	0.826601	0.284998	unchanged	-40.4049	C33H34N4	600.2581	7.869883
32.15148	0.695732	0.208145	1.413269	unchanged	-6.04239	C52H98N(	918.6868	7.869883
829.9672	0.720754	0.12043	1.671342	unchanged	-7.35928	C23H43N(	399.3553	7.88415
10.73903	1.979528	0.039846	2.144148	up	-14.1792	C50H95N(	940.6566	7.877017
10.95901	0.428982	0.374759	1.035737	unchanged	22.01134	C10H19N3	196.1491	7.8913
36.67802	0.508459	0.369898	1.056964	unchanged	-6.7421	C18H32O2	263.2351	7.8913
270.4731	0.051097	0.688621	0.487526	unchanged	-6.43581	C25H34O3	424.2794	7.912717
63.5929	0.126322	0.604043	0.620481	unchanged	-5.06524	C21H44N(	454.2905	7.912717
229.5566	-0.64551	0.092454	1.818866	unchanged	15.49415	C39H48O7	593.3359	7.912717
1422.889	0.606462	0.189865	1.450908	unchanged	-4.35838	C27H53O1	583.3216	7.905567
26.90985	0.793809	0.270911	1.269034	unchanged	-7.26841	C18H28O3	315.1909	7.91985
75.62499	-0.36873	0.100115	1.73362	unchanged	-14.0909	C9H17NO	237.1414	7.926983
121.7933	-0.54178	0.058034	1.916954	unchanged	5.913835	C24H38N2	401.2823	7.926983
26.24824	-0.40848	0.328135	1.025843	unchanged	13.6091	C24H44N8	525.3579	7.926983
2.202921	1.937893	0.122328	1.626564	unchanged	-19.0566	C11H16N2	274.1349	7.941267
12.41483	0.732126	0.135542	1.585362	unchanged	1.379123	C5H10N2(	283.138	7.941267
9596.168	-0.48788	0.271337	1.239321	unchanged	22.18691	C26H43N(	466.3266	7.941267
392.8695	0.005305	0.985505	0.014605	unchanged	-22.4456	C24H42O1	471.2475	7.941267
99.13248	0.815726	0.208083	1.39718	unchanged	0.129159	C45H77O1	842.5543	7.941267
2650.371	-0.14512	0.761569	0.362886	unchanged	26.01712	C55H87N(	1003.672	7.9484
344.4622	-0.31358	0.576163	0.655471	unchanged	-12.8472	C54H98N(	1049.65	7.9484
142.7578	-0.26963	0.134907	1.688468	unchanged	4.1344	C8H10N2(	240.0987	7.9484
49.49699	-0.05608	0.636777	0.612161	unchanged	3.217387	C11H21N5	268.1414	7.9484
86.28954	-0.35553	0.563663	0.681157	unchanged	12.56293	C31H44O8	562.3443	7.9484
110.1562	-0.31699	0.130293	1.542974	unchanged	-3.03621	C16H18O4	297.1391	7.955533
1312.857	0.3409	0.110404	1.587121	unchanged	-5.05215	C18H30O3	277.2147	7.955533
1508.613	0.291201	0.574957	0.657795	unchanged	-5.44351	C24H48O3	402.3921	7.962683
94.78084	-0.3847	0.092799	1.706016	unchanged	2.118295	C30H42O7	515.3014	7.955533
38.01016	0.11954	0.475976	0.796392	unchanged	9.97889	C15H22O	219.1765	7.9841
232.7144	0.133421	0.848614	0.223539	unchanged	-7.29762	C19H30O	257.2244	7.9841
56.46834	0.156433	0.521974	0.660491	unchanged	-7.7255	C18H30O5	291.193	7.9841
203.1855	0.275183	0.716274	0.454018	unchanged	-8.16151	C22H44O3	374.36	7.97695
26.14376	0.04229	0.923824	0.138109	unchanged	4.270077	C8H14N2(	437.1897	7.97695
355.7255	0.144006	0.748298	0.379847	unchanged	-41.7909	C30H52O	446.4177	7.9841
31.5248	-0.51667	0.17082	1.487406	unchanged	-2.80573	C21H44N(	454.2915	7.991233
131.626	0.710036	0.48133	0.737361	unchanged	-13.1041	C43H78N(	750.5332	7.991233
67.39325	0.380933	0.387114	1.007983	unchanged	0.414149	C9H13N3(	301.1144	7.998367
275.8917	0.529796	0.094566	1.722507	unchanged	0.239816	C18H38N8	413.2984	7.998367
516.029	-0.20309	0.518909	0.683274	unchanged	-13.1575	C28H41N5	528.3111	7.998367

103.2835	0.798147	0.040195	1.901986	up	-15.1233	C46H84N(	880.5337	7.998367
745.346	0.158235	0.454631	0.86611	unchange(	2.57955	C45H85O1	797.5712	8.005517
228.6878	-1.67236	0.171089	1.557304	unchange(	-11.8116	C56H108C	1115.7	8.019783
49.0183	-0.07003	0.807586	0.279004	unchange(	3.720523	C24H34O5	420.2759	8.019783
369.1631	0.325922	0.512884	0.761962	unchange(	15.69514	C25H36O7	449.2604	8.019783
1245.716	-1.70463	0.087343	1.861004	unchange(	-16.5135	C54H100N	1065.687	8.026917
40.6333	-0.52727	0.132345	1.575032	unchange(	-0.36854	C17H13N5	305.1508	8.026917
112.5212	0.295969	0.292698	1.200016	unchange(	11.78167	C15H16N2	327.0776	8.026917
356.6585	-0.55384	0.197731	1.375316	unchange(	-48.31	C33H44O8	533.2623	8.026917
34.11274	-0.16907	0.815954	0.235946	unchange(	-22.7554	C14H19N5	596.3284	8.034067
100.7947	-0.38382	0.33598	1.033823	unchange(	9.195427	C33H38O1	692.2611	8.034067
38.2182	0.095726	0.766834	0.279933	unchange(	-13.5779	C48H84N(	904.5554	8.034067
48.7557	0.083386	0.706213	0.397714	unchange(	-2.82152	C9H19NO	239.1595	8.0412
19.347	0.124375	0.325907	1.095582	unchange(	1.293791	C6H14N4(	349.2311	8.048333
1415.255	-0.41517	0.428735	0.875784	unchange(	-6.66624	C27H46O1	548.3394	8.061917
58.62259	-0.04038	0.824151	0.34903	unchange(	-4.83807	C15H24O2	259.1657	8.06905
187.3732	0.041529	0.868718	0.234199	unchange(	-0.52522	C22H38O5	365.2684	8.06905
92.67377	0.332674	0.292704	1.1275	unchange(	-13.535	C27H42O5	411.2833	8.06905
936.6255	0.062296	0.65418	0.55495	unchange(	-3.43789	C25H40O6	454.2914	8.06905
54.62787	0.147089	0.524396	0.75763	unchange(	0.528927	C28H39N(	476.2774	8.06905
36.90579	-0.41977	0.26565	1.174529	unchange(	-9.76803	C32H45N(	1037.649	8.083333
101.7918	0.256377	0.421407	0.938449	unchange(	8.236501	C27H48N(	1081.632	8.083333
57.96915	-0.32921	0.551938	0.700902	unchange(	-1.6674	C15H22O3	233.1532	8.076183
20.42515	-0.50547	0.509574	0.710204	unchange(	2.499866	C16H25N(	270.1835	8.083333
183.92	-0.00317	0.988793	0.098255	unchange(	-7.84444	C27H40O4	429.2966	8.076183
162.4231	0.34128	0.097185	1.727402	unchange(	-9.27985	C21H41N5	578.2592	8.083333
41.52376	0.087762	0.762591	0.370849	unchange(	0.286933	C33H45N(	580.2907	8.083333
66.61214	0.510016	0.401288	0.962425	unchange(	12.13774	C32H51N(	642.3562	8.076183
31.60595	0.147862	0.734189	0.473254	unchange(	-8.62431	C57H87N7	1074.603	8.090467
1413.208	-0.26248	0.076893	1.881287	unchange(	0.722378	C5H13NO	104.1071	8.104733
207.9246	-0.03719	0.720631	0.344734	unchange(	-5.43745	C16H21N3	268.1428	8.104733
231.8648	-0.0506	0.596176	0.549528	unchange(	6.474115	C10H17N3	275.1281	8.0976
282.0342	-0.03491	0.731206	0.369595	unchange(	4.49698	C12H22O8	277.1295	8.0976
210.6501	0.12841	0.502219	0.80039	unchange(	0.874305	C18H17FN	297.14	8.104733
239.309	0.032005	0.550138	0.73203	unchange(	-3.81875	C19H38O4	313.2725	8.104733
31.87483	-0.04108	0.845059	0.142222	unchange(	5.890331	C10H13N(	314.0289	8.104733
206.6647	1.151998	0.087636	1.857409	unchange(	-4.45502	C23H45N(	425.3721	8.0976
2541.929	-0.03964	0.686247	0.418511	unchange(	-18.8562	C21H40O9	459.2482	8.104733
522.6268	-0.05788	0.493164	0.743462	unchange(	-16.1428	C16H22O2	515.3052	8.104733
241.008	-0.10489	0.347941	0.997116	unchange(	15.83834	C42H70O1	763.4754	8.104733
137.3896	0.004238	0.98183	0.05234	unchange(	26.3093	C43H63N(	770.4676	8.104733
1316.011	-0.04555	0.810168	0.288303	unchange(	-9.41471	C32H61N1	772.4639	8.104733
31.06226	1.181962	0.092427	1.833757	unchange(	-3.97963	C54H102N	920.7065	8.0976
95.35742	-0.00549	0.991987	0.026338	unchange(	-2.4859	C33H44O3	999.6449	8.0976
52.55514	-0.57348	0.182308	1.399029	unchange(	3.639386	C26H48N(	1035.645	8.119017
560.1332	-0.88467	0.319538	1.142319	unchange(	14.77182	C32H46O6	1053.682	8.1333
1.72E-06	-1.5398	0.377696	1.001537	unchange(	-26.4347	C55H97N3	1191.628	8.1333
97248.92	-0.43199	0.348807	1.053129	unchange(	-2.42261	C28H52N(	546.3541	8.1333
64.16102	-0.44724	0.37753	0.985532	unchange(	-22.1879	C31H51N(	582.3508	8.12615
16.19363	-0.33826	0.563682	0.665356	unchange(	-7.60208	C23H36O5	823.4697	8.1333
76.84267	-0.95261	0.238577	1.246072	unchange(	-13.903	C18H22O2	558.3503	8.140433

262.5109	0.497684	0.103468	1.738399	unchanged	-7.55326	C21H40O4	339.2867	8.1547
1492.139	0.767254	0.317242	1.107411	unchanged	-3.6147	C25H49NO	428.3719	8.147567
150.4043	0.020863	0.908996	0.117493	unchanged	14.23728	C8H17N3O	204.1372	8.16185
38.11907	-0.36918	0.497005	0.737691	unchanged	-35.2643	C16H20O3	538.298	8.169
186.1269	0.646496	0.369035	1.031963	unchanged	-9.45667	C36H60O9	654.4515	8.169
1241.237	-0.18648	0.586054	0.635609	unchanged	-7.13957	C53H93N7	1018.672	8.176133
2406.831	0.480079	0.508146	0.737374	unchanged	16.29	C29H41F2	1044.706	8.183267
101.0308	0.117624	0.429635	0.897322	unchanged	3.678464	C10H17N3	240.0989	8.183267
184.6231	-0.60469	0.188451	1.599307	unchanged	-2.61868	C18H32O3	279.2311	8.183267
33.13264	0.614779	0.249296	1.328958	unchanged	-1.30831	C15H19N3	288.1339	8.183267
127.0561	0.182854	0.223997	1.370973	unchanged	0.442618	C15H16N2	290.1323	8.183267
441.7186	0.307274	0.178906	1.442534	unchanged	11.77851	C23H42O7	431.3095	8.183267
691.2769	0.079866	0.656324	0.528307	unchanged	0.694853	C24H39NO	485.2622	8.183267
508.2823	0.057263	0.779737	0.344327	unchanged	-2.89943	C30H46O7	560.3081	8.183267
31.27601	-0.41668	0.531045	0.696682	unchanged	1.808067	C37H67O8	670.458	8.176133
105.6123	-0.34717	0.780477	0.344276	unchanged	3.016877	C46H84NO	923.5995	8.190417
70.34035	0.230897	0.239836	1.302751	unchanged	-19.1608	C7H14O5	179.088	8.204683
57.33009	0.211269	0.617444	0.51286	unchanged	8.491845	C12H22	189.1628	8.19755
3.153502	4.905317	0.378857	1.032108	unchanged	-1.39347	C15H12O	209.0958	8.204683
22.59963	0.13554	0.768555	0.308122	unchanged	-13.0573	C15H20O	217.1559	8.19755
2643.74	0.036515	0.842694	0.228314	unchanged	-9.6652	C15H22O2	235.167	8.204683
54.81668	0.030865	0.930645	0.107034	unchanged	-14.3756	C20H34O3	305.2429	8.19755
69.23833	0.212574	0.6331	0.580393	unchanged	-3.26187	C23H43NO	468.2917	8.19755
125.6526	0.171696	0.456513	0.773203	unchanged	-7.69653	C20H34O4	321.2398	8.211817
305.3499	-0.13883	0.394372	0.96336	unchanged	-8.65315	C19H41O6	438.2945	8.218967
920.7454	-1.14126	0.177987	1.485168	unchanged	-4.48607	C30H52NO	592.3344	8.218967
70.98198	-0.22604	0.377432	0.981122	unchanged	17.32725	C11H10N2	220.1116	8.2261
40.82634	2.386746	0.092012	1.813895	unchanged	-11.5994	C27H53NO	454.3836	8.2261
64.09207	-0.32747	0.504693	0.856451	unchanged	2.510268	C13H19N3	473.28	8.2261
50.40967	0.077809	0.704351	0.414828	unchanged	-16.578	C6H13N3O	193.1266	8.240383
509.1206	0.084846	0.668135	0.505047	unchanged	-4.14701	C16H26O3	249.1838	8.240383
21.14238	0.16368	0.574351	0.598751	unchanged	7.539308	C15H24O5	267.1612	8.25465
70.51151	-0.08632	0.490595	0.821663	unchanged	-21.5942	C18H32O4	277.2095	8.25465
35.45213	-0.37414	0.524855	0.67488	unchanged	-20.199	C30H58NO	540.3696	8.247517
145.5429	0.422214	0.263497	1.315921	unchanged	5.304673	C40H76N2	818.5778	8.268933
616.6251	0.313071	0.150036	1.613466	unchanged	-1.31564	C19H40O3	339.2865	8.2832
375.1213	0.44578	0.173354	1.49042	unchanged	-4.84235	C28H41NO	462.2957	8.2832
18.45435	0.179098	0.670824	0.519835	unchanged	29.9869	C32H50O8	580.4013	8.276067
355.0149	-0.08852	0.629803	0.538924	unchanged	-37.35	C34H34N4	604.2708	8.29035
80.58308	0.049635	0.846077	0.245036	unchanged	-12.5103	C34H46O8	621.2751	8.297483
27281.12	#######	0.999689	0.011095	unchanged	3.850504	C54H92N1	1065.686	8.311767
173.7306	0.027267	0.79017	0.327077	unchanged	2.742724	C13H15NO	240.1001	8.311767
57.32092	0.021278	0.921778	0.186395	unchanged	4.047766	C16H20N2	273.1609	8.311767
1231.496	-0.06759	0.721627	0.428845	unchanged	0.623744	C5H10O	104.107	8.3189
1359.326	-0.00917	0.937993	0.083719	unchanged	-0.07241	C5H15NO4	184.0733	8.3189
898530.5	0.043887	0.814765	0.268155	unchanged	-4.46862	C26H52NO	522.3531	8.3189
138.6825	-1.23261	0.392526	1.056133	unchanged	14.42686	C16H27N4	480.1064	8.333167
52.88739	1.608407	0.408394	0.936251	unchanged	-15.569	C30H53N3	1120.804	8.354583
51.7693	0.100115	0.615415	0.599901	unchanged	-11.885	C14H24O4	221.1506	8.34745
22.07318	0.335137	0.544551	0.616718	unchanged	2.462969	C13H25NO	261.2179	8.354583
1169.163	0.186994	0.629506	0.492347	unchanged	-3.52896	C18H34O4	297.2413	8.354583

34.4767	-0.0989	0.870093	0.142659	unchanged	1.376027	C24H30O6	397.2015	8.354583
145.7536	0.243903	0.473359	0.783894	unchanged	-8.51929	C25H40O4	443.256	8.34745
1481.647	-0.30703	0.273458	1.266985	unchanged	-0.28412	C23H43N5	502.3234	8.34745
71241.55	-0.00496	0.961799	0.066426	unchanged	-3.02696	C26H52NO	544.3358	8.34745
421.8383	-0.02166	0.896108	0.097991	unchanged	4.905721	C23H43N9	644.2617	8.354583
189.2506	0.430554	0.366864	0.963802	unchanged	2.590185	C46H84NO	880.5486	8.354583
26.72998	0.253081	0.804211	0.260829	unchanged	19.99406	C35H52O5	1122.819	8.361733
37.86854	-0.00203	0.996524	0.001827	unchanged	9.384045	C24H42O1	455.2685	8.361733
4631.997	-0.57012	0.400688	0.958905	unchanged	19.59274	C28H49NO	492.3423	8.361733
56.57514	0.673344	0.009461	2.288165	up	15.38752	C39H70NO	660.4858	8.361733
373.1516	0.210202	0.41752	0.888859	unchanged	2.136064	C48H84NO	846.5662	8.361733
13.73522	0.072901	0.916113	0.104805	unchanged	-9.40107	C45H84NO	868.5595	8.361733
46.31144	-0.0861	0.836465	0.295981	unchanged	-20.3141	C10H18O	177.1219	8.368867
32.20429	-0.1536	0.525028	0.688314	unchanged	7.368768	C12H22O6	262.143	8.383133
1445.064	-1.60111	0.121665	1.745992	unchanged	-17.2337	C56H104N	1093.717	8.411
79.01024	-1.80271	0.102087	1.80369	unchanged	6.313241	C55H102N	1165.668	8.411
84.95983	0.552398	0.169647	1.554575	unchanged	-16.0178	C30H44O3	417.3079	8.411
153.2346	-0.67916	0.163896	1.458649	unchanged	-4.26363	C26H39NO	535.2815	8.418133
137.5281	0.101593	0.560942	0.672555	unchanged	-7.98386	C15H24O2	219.1725	8.43955
142.9817	0.135239	0.62979	0.527045	unchanged	-2.85769	C10H20O4	227.1248	8.43955
25.868	0.871217	0.101173	1.768857	unchanged	5.638824	C21H30O4	364.2502	8.43955
21.09644	0.065879	0.924889	0.021207	unchanged	4.888092	C10H15N5	271.1526	8.453817
43.1159	-0.36124	0.23832	1.43786	unchanged	-15.9136	C18H32O2	303.225	8.453817
15.1131	0.132667	0.81448	0.313878	unchanged	-17.3875	C22H38O5	347.2514	8.446683
47.68632	0.069456	0.699059	0.447395	unchanged	-2.03377	C21H32O4	349.2366	8.446683
14.61045	0.271373	0.556496	0.596232	unchanged	9.184388	C26H43NO	430.2995	8.453817
43.88916	-0.04338	0.862474	0.203837	unchanged	3.751439	C28H37NO	469.3078	8.453817
824.6806	0.394802	0.537297	0.636979	unchanged	4.900775	C39H54O5	620.4339	8.453817
740.2301	0.413954	0.478121	0.743192	unchanged	6.865213	C34H63O9	664.4592	8.446683
49.19759	0.518896	0.358687	1.055047	unchanged	-2.04502	C20H34O5	396.2737	8.460967
16.97367	-0.13874	0.780309	0.365653	unchanged	4.443798	C14H28O6	315.1791	8.4681
1260.863	0.133972	0.779005	0.240298	unchanged	-1.96716	C20H38N6	449.2952	8.4681
968.0663	0.2387	0.683061	0.391936	unchanged	7.798915	C29H44O6	488.3545	8.4681
352.5192	-0.15434	0.743954	0.438238	unchanged	-3.67303	C30H50O9	537.3402	8.4681
263.2793	0.143941	0.074191	1.856061	unchanged	22.11431	C4H7NS	66.0183	0.518567
27.72151	-0.44197	0.196045	1.32903	unchanged	-5.61752	C16H21NO	275.15	8.489517
28.77357	-0.49703	0.271459	1.148176	unchanged	-1.09885	C16H18N2	304.1475	8.489517
752.8179	-0.52149	0.113271	1.630828	unchanged	27.15712	C25H38O7	473.2632	8.489517
81.44217	-0.91356	0.111122	1.60341	unchanged	5.769233	C27H52N3	600.3207	8.489517
268.02	0.106247	0.685042	0.409153	unchanged	27.1161	C27H40O6	460.3047	8.503783
38.51619	-0.60023	0.177245	1.368556	unchanged	14.15501	C36H53N7	662.4121	8.49665
197.4939	-0.92012	0.120452	1.766899	unchanged	-11.5685	C18H32O4	295.2232	8.510933
6322.376	-0.49784	0.203567	1.404867	unchanged	-7.18783	C26H52NO	506.3569	8.510933
1062.394	-1.20295	0.095412	1.745059	unchanged	-10.6561	C33H52O7	560.3647	8.518067
11478.26	-0.20482	0.405562	0.934877	unchanged	16.17173	C29H49NO	508.3715	8.532333
18165.95	-0.50722	0.20193	1.34209	unchanged	-7.40468	C28H54NO	548.367	8.5252
133.2028	-0.02216	0.910892	0.127037	unchanged	4.887243	C26H43O3	435.3044	8.539483
18.50485	0.252009	0.666021	0.488547	unchanged	2.542489	C9H12N2O	479.1396	8.546617
94.52982	-0.63703	0.069291	1.88009	unchanged	-11.3529	C12H26N6	267.1894	8.5609
169.9432	-0.05357	0.794815	0.313831	unchanged	0.285721	C21H34O5	349.2374	8.5609
59.75473	0.367739	0.508023	0.731864	unchanged	5.781281	C16H26O4	265.1815	8.582317

186.6355	0.468697	0.161186	1.530059	unchanged	-14.1648	C21H39N(	427.3809	8.582317
136.5864	-0.2791	0.250467	1.280331	unchanged	-3.28425	C13H16N4	457.2808	8.58945
69.6516	0.018999	0.923244	0.111992	unchanged	-10.1229	C12H16N2	185.0873	8.603717
143.8053	-0.19136	0.32574	1.059797	unchanged	7.116321	C9H15N5(	259.153	8.603717
10277.83	-0.3612	0.390602	0.979954	unchanged	-14.1444	C32H51N(	494.3554	8.596583
196.5407	-0.11692	0.800046	0.26658	unchanged	13.55734	C33H48O7	598.3814	8.603717
60.84041	-0.6871	0.355078	1.033901	unchanged	-29.7398	C33H50O7	600.3729	8.596583
212.9505	-0.03681	0.856397	0.203213	unchanged	-0.43342	C7H6O3	121.0283	8.618
58.69264	-0.01943	0.924827	0.095925	unchanged	4.554186	C8H9NO2	134.0607	8.618
2118.266	-0.00955	0.962867	0.034856	unchanged	-2.36298	C4H6N4	149.0221	8.618
1229.496	0.036899	0.872548	0.209195	unchanged	-6.89803	C12H12O3	205.0845	8.618
45.39526	0.066884	0.797868	0.295519	unchanged	-8.98826	C12H24O	386.3959	8.618
34.64631	0.208344	0.504484	0.775852	unchanged	-1.02571	C7H6O3	139.0388	8.625133
20.32619	0.142265	0.687223	0.491049	unchanged	-5.77366	C14H19N2	381.0801	8.625133
68.63768	0.069523	0.767407	0.337238	unchanged	15.66328	C11H16N5	411.0908	8.639417
217.7402	0.113215	0.586816	0.598428	unchanged	-0.99858	C7H14O7S	281.0511	8.64655
179.6201	0.055276	0.804493	0.25804	unchanged	-17.6224	C16H16N2	297.0811	8.64655
77.32323	-0.30467	0.508571	0.726194	unchanged	5.32507	C16H25N(	608.3937	8.6537
45.72977	-0.24131	0.510905	0.711693	unchanged	-6.07597	C4H8O2S2	153.0029	8.660833
208.7424	-0.31997	0.240421	1.231205	unchanged	6.845651	C12H17N5	313.1639	8.660833
189.6155	0.024638	0.890513	0.158219	unchanged	-0.72261	C23H30D4	395.2727	8.667967
263.6284	-0.09591	0.559739	0.640143	unchanged	8.311751	C25H40N4	441.29	8.667967
20.94084	0.659978	0.059173	1.866525	unchanged	28.81797	C27H40O5	409.2865	8.68225
274.1234	-0.22446	0.528167	0.700829	unchanged	5.374169	C34H50O9	620.3825	8.68225
54.06918	0.432855	0.248632	1.229163	unchanged	-2.37227	C19H30O3	630.4714	8.68225
34.68262	-0.7399	0.291263	1.187159	unchanged	-2.28694	C37H52O6	634.4089	8.68225
191.5735	-0.00243	0.987523	0.068228	unchanged	3.699704	C19H39N5	491.3204	8.689383
44.75091	0.170223	0.796305	0.314491	unchanged	0.559338	C48H92O1	1004.62	8.703667
886.6161	0.249226	0.36806	0.982794	unchanged	-11.0133	C18H28O3	293.208	8.696517
2045.783	-0.27698	0.346969	1.024117	unchanged	32.04292	C35H53N(	558.4089	8.703667
501.6473	-0.13725	0.723158	0.363866	unchanged	0.620653	C33H60O8	602.463	8.696517
100.0307	-0.01629	0.942803	0.078291	unchanged	11.59214	C33H56O9	638.4332	8.703667
106.3911	-0.31786	0.157607	1.468367	unchanged	-8.70448	C13H12N4	240.0984	8.7108
59.51038	-0.27575	0.34885	1.036606	unchanged	-5.65216	C49H85O1	938.6066	8.7108
141.073	-0.02785	0.843547	0.206127	unchanged	-2.54593	C16H20N(	291.1371	8.717933
50.41345	-0.23435	0.261594	1.117685	unchanged	20.9307	C16H26O8	311.1562	8.717933
192.6169	-0.18489	0.338227	1.02089	unchanged	11.47114	C30H49N5	614.338	8.717933
879.6147	0.250442	0.27904	1.229908	unchanged	14.03977	C36H56O1	666.4303	8.717933
53.08141	-0.08861	0.531889	0.76131	unchanged	2.691993	C51H79N(	931.5914	8.717933
27.06503	-0.13124	0.866149	0.258465	unchanged	12.65968	C52H96N(	980.6472	8.731517
24.79175	-0.27445	0.4541	0.795505	unchanged	-6.73178	C14H18O3	252.1578	8.73865
62.1831	0.500866	0.13029	1.623749	unchanged	-13.6033	C23H38O5	395.2738	8.73865
81.75424	0.029083	0.83338	0.193888	unchanged	19.57632	C54H98N(	958.6842	8.73865
312.7005	-0.03798	0.858025	0.249629	unchanged	8.725681	C15H32FC	277.2117	8.752933
93.30271	-0.45039	0.266463	1.205032	unchanged	-0.34451	C16H18N2	499.2831	8.745783
18987.49	-0.54286	0.153714	1.507356	unchanged	-6.10423	C27H54N(	536.3678	8.745783
11.20375	-1.09213	0.319193	1.222165	unchanged	5.6357	C33H46O7	596.3613	8.752933
13.5022	0.508386	0.321674	1.124712	unchanged	3.400803	C27H54N(	568.3628	8.760067
212.4584	0.200063	0.567028	0.675111	unchanged	4.903747	C25H42O6	439.3076	8.7672
201.4732	0.176165	0.353545	0.970337	unchanged	-13.303	C30H44O4	491.307	8.774333
211.9881	0.196789	0.430252	0.902666	unchanged	-0.20872	C36H60O6	630.4727	8.7672

103.7275	0.058801	0.880874	0.199827	unchanged	-12.3549	C47H75N(	948.4813	8.7672
108.4945	-0.19198	0.420723	0.921644	unchanged	3.745757	C22H32O3	327.2331	8.781483
981.7654	0.239629	0.23053	1.279379	unchanged	4.362934	C40H72O9	661.5068	8.781483
2141.291	-0.06793	0.650245	0.519643	unchanged	-7.62456	C38H69O9	683.4593	8.781483
23.70079	0.053316	0.841806	0.281397	unchanged	2.082459	C9H10O	135.0807	8.788617
78.67463	0.120269	0.433498	0.904711	unchanged	-4.8471	C15H26O3	219.1731	8.8029
43.76087	0.243821	0.412503	0.937775	unchanged	-1.27567	C9H13N	271.2165	8.79575
22.39188	0.141398	0.665171	0.555607	unchanged	2.274172	C18H34O2	305.2457	8.79575
46.04334	0.425412	0.05468	1.842188	unchanged	-13.428	C13H28N(	499.381	8.79575
276.7019	0.361689	0.202248	1.368059	unchanged	-13.7972	C23H46N6	579.29	8.79575
72.90598	0.091972	0.838192	0.191479	unchanged	2.712452	C19H35N(	357.2519	8.810033
150.0818	0.244083	0.30795	1.188012	unchanged	-9.94905	C22H38O7	846.5491	8.810033
62.85108	0.052582	0.668985	0.375666	unchanged	16.6637	C21H32O3	297.2268	8.817167
210.8076	0.306944	0.379832	1.016115	unchanged	7.729834	C38H67N(	614.4829	8.8243
55.91423	-0.88449	0.395647	0.939156	unchanged	6.514759	C37H48N4	646.4004	8.8243
48.41595	0.025375	0.951281	0.086136	unchanged	-11.3163	C37H60O9	648.4158	8.8243
1095.507	0.755832	0.105282	1.721513	unchanged	-2.74783	C40H78N(	712.5255	8.8243
272.8493	0.181059	0.584865	0.696284	unchanged	4.887901	C41H76N2	854.5625	8.8243
74.30665	0.185551	0.274725	1.228355	unchanged	-4.44012	C20H41N(	310.309	8.83145
245.2255	-0.03377	0.838458	0.152051	unchanged	-18.4373	C21H42O4	341.2984	8.83145
584.1932	0.156889	0.507335	0.775002	unchanged	-16.2585	C23H36O3	361.2679	8.83145
842.5763	0.162765	0.296965	1.300367	unchanged	-13.8225	C28H44N4	483.3261	8.83145
42762.92	-0.19933	0.505195	0.755934	unchanged	20.26186	C47H84N(	786.5962	8.83145
403.8495	0.27173	0.063019	1.943505	unchanged	-4.90513	C25H41N(	419.3009	8.838583
49.80106	0.511088	0.15422	1.588492	unchanged	2.944172	C35H63N(	670.4182	8.838583
79.87886	-0.27802	0.804279	0.306931	unchanged	-2.50902	C49H89N2	970.6291	8.838583
249.1653	-0.05856	0.840482	0.154877	unchanged	19.15792	C30H46O6	1005.685	8.845717
15.54182	0.720928	0.124689	1.611243	unchanged	-16.2841	C58H104N	1129.68	8.845717
175.1775	0.225246	0.534785	0.738258	unchanged	7.486146	C57H103N	1144.666	8.845717
190.6688	0.211045	0.634866	0.55544	unchanged	6.692395	C53H85N1	1166.651	8.845717
28.27206	0.334919	0.167851	1.415358	unchanged	5.125904	C6H11NO	259.1666	8.845717
12.03626	0.818179	0.168682	1.49759	unchanged	2.272142	C34H48O8	623.2994	8.845717
38.19441	0.768813	0.062654	1.849237	unchanged	9.886441	C49H90N(	938.5972	8.845717
47.73451	-0.14426	0.015579	2.346111	down	-5.81138	C15H13N(	240.1005	8.86
693.6902	-0.02706	0.799561	0.285094	unchanged	-10.5849	C12H20N4	291.1399	8.86
1822.418	-0.07055	0.486739	0.773161	unchanged	-2.35033	C5H15NO4	184.0728	8.867133
47.79595	-0.04187	0.85305	0.189596	unchanged	6.542411	C11H20O7	282.1565	8.867133
5514.827	-0.09137	0.374929	0.991161	unchanged	17.99603	C28H38O7	487.2778	8.867133
178.4776	0.009515	0.950801	0.12161	unchanged	-17.9613	C18H24O3	311.1566	8.881417
68.55855	-0.36914	0.199818	1.450257	unchanged	10.06865	C35H46O2	1019.699	8.88855
292.4748	0.009351	0.94805	0.113577	unchanged	-8.7557	C58H108O	1103.682	8.895683
31.50803	0.290725	0.434085	0.945307	unchanged	-7.0012	C60H92N1	1123.695	8.895683
24.76314	-0.29577	0.573504	0.686096	unchanged	-18.0503	C25H33N(	381.2835	8.902833
1700.828	-0.04388	0.603985	0.581373	unchanged	-20.7214	C31H51N(	562.3251	8.902833
720.4636	0.181269	0.185098	1.500213	unchanged	-4.1097	C33H52N2	614.3398	8.902833
164.9958	0.042116	0.805799	0.292497	unchanged	-3.30479	C31H40O1	606.2889	8.902833
436.7695	0.327124	0.325036	1.132408	unchanged	-23.2965	C50H86N(	925.6065	8.902833
70.12141	0.071728	0.877771	0.211912	unchanged	-9.14061	C51H84O1	954.6063	8.895683
69.25828	-0.14804	0.65601	0.457991	unchanged	-9.03825	C30H52N4	582.4174	8.909967
345.5034	0.016658	0.942758	0.073926	unchanged	6.176712	C38H62O7	669.4166	8.909967
101.4885	-0.03052	0.910055	0.122092	unchanged	-8.60362	C17H34O4	285.2398	8.92425

216.1609	-0.1192	0.760997	0.408295	unchanged	0.816191	C22H29N(	357.2539	8.92425
131.2076	0.128683	0.57051	0.634326	unchanged	-3.69912	C23H43N(	413.312	8.92425
40.85878	-0.3057	0.239131	1.321925	unchanged	5.091779	C32H54O1	653.354	8.9171
29.16538	-0.93365	0.20352	1.416525	unchanged	-2.39259	C35H58O7	555.403	8.931383
601.0527	-0.28618	0.250023	1.267032	unchanged	-4.0647	C36H71N(	620.421	8.931383
580.4185	-0.18228	0.471747	0.860792	unchanged	-11.0872	C18H30O3	277.2129	8.938517
498.7892	-0.40953	0.48289	0.747154	unchanged	19.42323	C21H40O6	371.2867	8.938517
206.7727	-0.07928	0.732062	0.401261	unchanged	4.916221	C38H71N(	692.4894	8.938517
204.3186	-0.17195	0.639491	0.558918	unchanged	-8.11727	C19H36O5	309.2396	8.94565
256.0921	0.073154	0.740028	0.317274	unchanged	-6.18205	C20H36O5	321.2402	8.9528
54.56818	-0.40044	0.425111	0.942833	unchanged	20.21017	C34H51N5	642.3991	8.9528
53.41148	0.029621	0.859004	0.226131	unchanged	-0.84984	C12H25N5	326.1587	8.959933
19.72207	-0.32826	0.452949	0.780557	unchanged	-14.6512	C14H22N2	369.1218	8.959933
12.35902	0.026901	0.954464	0.021594	unchanged	-18.7088	C11H20N(	390.0723	8.967067
468.1219	0.019584	0.921136	0.101614	unchanged	-9.71869	C36H69N(	580.5243	8.967067
12.67173	0.546761	0.371815	0.948817	unchanged	2.146582	C35H56O6	595.3981	8.988483
790.129	0.253766	0.185578	1.511999	unchanged	27.21595	C27H40O2	419.3028	8.995617
929.3404	0.170439	0.228979	1.32624	unchanged	3.632214	C25H37N(	433.3076	9.0099
1886.873	0.109729	0.668188	0.461117	unchanged	-11.0323	C27H46O1	572.3668	9.024183
249.0228	-0.03594	0.86	0.189189	unchanged	-8.3022	C28H47N(	494.3258	9.03845
132.7809	0.115819	0.592864	0.569103	unchanged	-3.92118	C14H20O3	237.1476	9.052733
159.3631	0.218536	0.411136	0.929752	unchanged	-0.17959	C16H30N2	315.2278	9.052733
1303.291	-0.06232	0.662573	0.43203	unchanged	-16.32	C30H40O3	466.3243	9.052733
256.2233	0.277664	0.087952	1.775021	unchanged	1.372247	C30H46O6	485.3268	9.052733
18.06432	0.634022	0.224514	1.387794	unchanged	-2.00977	C35H66O8	653.4377	9.0456
121.9552	0.151287	0.394568	0.981775	unchanged	-3.46573	C6H4O5	157.0126	9.07345
334.8143	0.137451	0.424113	0.918991	unchanged	-4.94563	C10H12O5	213.0747	9.07345
272.3743	0.135002	0.408365	0.949549	unchanged	7.360798	C6H10O7	217.0333	9.07345
1093.572	0.085175	0.55907	0.672504	unchanged	8.060971	C9H15N5(	259.1533	9.07345
1051.937	0.11638	0.521887	0.738654	unchanged	-6.21308	C12H18O8	273.0951	9.07345
396.0956	0.131215	0.472906	0.834512	unchanged	28.37203	C16H18N2	269.1366	9.07345
4467.849	0.110229	0.541932	0.710644	unchanged	1.806313	C14H26O7	329.1576	9.07345
1270.985	0.139878	0.457953	0.853876	unchanged	20.11749	C20H34O3	361.2204	9.07345
256.3527	-0.05049	0.787523	0.356898	unchanged	-9.46246	C20H30O7	383.2808	9.07345
27556.01	0.064169	0.684788	0.480806	unchanged	-0.96096	C16H30N6	425.2115	9.07345
116.3996	0.21135	0.163318	1.555914	unchanged	17.23692	C24H37N(	461.3082	9.07345
24.03738	-0.12309	0.748004	0.350849	unchanged	0.27565	C30H42O8	569.2513	9.0663
25126.68	-0.00349	0.975852	0.017399	unchanged	-3.22895	C37H62O6	644.4865	9.0663
18.41497	0.478461	0.243579	1.310668	unchanged	6.999714	C54H101N	989.7679	9.07345
76.85072	0.010973	0.957541	0.108888	unchanged	7.307563	C28H42O2	375.3076	9.080583
1699.396	0.061569	0.559792	0.669039	unchanged	23.73174	C33H58O8	605.4191	9.080583
196.9747	-0.45723	0.103872	1.770041	unchanged	1.409933	C19H35N(	357.2514	9.09485
305.9946	0.114627	0.343046	1.069091	unchanged	-12.5347	C20H27N(	371.2288	9.087717
1036.119	-0.83811	0.070974	1.913134	unchanged	-10.7284	C29H48O5	515.3082	9.087717
67.5301	-1.18943	0.03733	2.077075	down	2.102217	C34H50O1	634.336	9.09485
553.4547	0.071664	0.599895	0.591884	unchanged	0.119687	C14H30O8	327.2014	9.102
26276.9	0.039216	0.769428	0.352513	unchanged	25.78115	C32H53N2	512.4109	9.102
447.9709	0.011534	0.916247	0.112535	unchanged	14.07269	C19H24O3	283.1735	9.109133
1717.915	0.071117	0.52443	0.73701	unchanged	-4.63522	C28H50O3	473.3371	9.109133
83.38595	0.768832	0.439104	0.954035	unchanged	-5.36692	C39H62O5	633.4457	9.109133
14.64625	0.399639	0.528305	0.733167	unchanged	20.73582	C4H9NO2	121.0993	9.123417

27.83455	0.32359	0.242575	1.322012	unchanged	19.92884	C11H18O	167.1464	9.123417
251.1542	0.073954	0.610394	0.58392	unchanged	0.752196	C6H16N4(	177.1144	9.116267
2521.36	0.622907	0.29867	1.207449	unchanged	-8.7948	C18H30O	280.261	9.123417
946.1743	0.182394	0.276304	1.257104	unchanged	14.72718	C21H34O2	336.2944	9.123417
3240.656	0.103765	0.525985	0.736531	unchanged	3.699225	C27H42O	424.3588	9.116267
216.7729	0.133189	0.549615	0.697386	unchanged	4.822923	C28H40N2	485.3033	9.13055
394.5304	0.053436	0.827744	0.258704	unchanged	-33.2195	C27H44O3	417.3225	9.144817
819.1842	-0.37627	0.184723	1.435925	unchanged	-11.3321	C30H51N3	532.3683	9.137683
6074.335	-0.46136	0.164587	1.471114	unchanged	-6.83405	C27H56N(	538.383	9.137683
350.3798	0.008688	0.961229	0.015557	unchanged	-9.39622	C24H37N(	389.3128	9.151967
338.5837	-0.28353	0.473368	0.80449	unchanged	0.884667	C33H64N(	666.4346	9.151967
155.8781	0.046466	0.732099	0.391633	unchanged	-9.70806	C15H22O	219.1722	9.18765
385.0595	0.033407	0.84218	0.247304	unchanged	9.750543	C18H30D4	319.2812	9.194783
874.6552	0.17878	0.169199	1.532397	unchanged	-28.7675	C30H50O4	439.3434	9.194783
234.7701	0.158541	0.656862	0.525602	unchanged	0.391349	C25H42O8	493.2736	9.194783
626.8366	-0.28086	0.226513	1.333375	unchanged	-12.1258	C20H30O2	303.2291	9.201933
2136.968	0.018215	0.886321	0.182907	unchanged	12.7601	C22H35N(	363.305	9.201933
4875.635	0.125283	0.184586	1.471305	unchanged	-23.3545	C26H44O4	421.3309	9.201933
1757.39	0.090286	0.570046	0.658344	unchanged	-15.218	C26H46O5	461.3171	9.201933
1394.428	-0.53971	0.353525	1.044746	unchanged	-5.79807	C35H58O9	622.4039	9.230483
185.5228	-0.00747	0.947401	0.169651	unchanged	-17.1102	C11H21N3	301.1826	9.24475
78.73833	0.107265	0.783093	0.245591	unchanged	-18.4983	C16H20N4	333.1496	9.259033
101.5023	0.208759	0.484473	0.728815	unchanged	4.365861	C11H19N(	335.1462	9.259033
12844.92	-0.4049	0.0423	1.942236	down	-5.91498	C31H54O7	538.3832	9.259033
228.2181	-0.02393	0.837042	0.26763	unchanged	11.22611	C9H12N2(	251.0452	9.294733
330.838	0.381222	0.094141	1.701767	unchanged	21.02631	C35H62O6	596.5006	9.287583
22.70086	0.09793	0.800645	0.33504	unchanged	2.449007	C36H58O1	668.4384	9.287583
23.82204	0.502191	0.039894	1.987787	up	20.38905	C19H36N2	357.282	9.31615
67.94452	0.304054	0.366364	0.974894	unchanged	-8.33767	C16H21N5	365.1903	9.323283
51.78376	0.451574	0.361745	0.984591	unchanged	0.705086	C16H23N(	367.1866	9.323283
1102.006	0.217071	0.58914	0.652193	unchanged	-1.52155	C21H19N(	375.1698	9.323283
63.61694	0.253418	0.624273	0.571891	unchanged	10.15068	C25H40O6	401.2731	9.323283
5758.392	0.364569	0.251838	1.322662	unchanged	-32.0799	C48H72O4	712.5196	9.323283
69105.45	0.526812	0.042472	1.947824	up	0.572667	C41H78N(	744.5542	9.323283
162.8492	-0.0009	0.995502	0.099988	unchanged	-0.39438	C11H17N(	301.1393	9.330417
69.83515	0.07382	0.847366	0.168116	unchanged	3.325093	C10H13N5	237.1466	9.3447
569.3265	0.101722	0.182705	1.482934	unchanged	-0.94126	C30H46O4	470.3386	9.3447
102.5177	0.351031	0.08281	1.832457	unchanged	-3.41247	C12H25N(	485.319	9.3447
51.84154	-0.01836	0.849015	0.299826	unchanged	-10.3822	C5H10O2	103.0743	9.351833
117.2775	0.087112	0.742316	0.416052	unchanged	20.46073	C22H40N2	379.3036	9.351833
388.4544	-0.19642	0.409923	0.956549	unchanged	-2.47844	C37H63N(	656.4272	9.351833
89.08547	-0.11953	0.383126	1.050451	unchanged	21.56663	C17H23N3	318.1881	9.358967
414.8309	0.074339	0.58038	0.616887	unchanged	9.508786	C18H24Cl2	371.0989	9.358967
6403.969	0.281155	0.612458	0.553972	unchanged	-2.87632	C12H18O1	758.1812	9.358967
208.5655	0.028466	0.796235	0.238391	unchanged	23.3042	C9H12O5	223.0624	9.366117
42.09848	0.127776	0.598253	0.576281	unchanged	-11.615	C10H13N5	248.0568	9.366117
72.64276	-0.02813	0.866101	0.213874	unchanged	-14.8701	C16H28N2	463.1262	9.366117
2477.62	0.086915	0.588536	0.63433	unchanged	0.324287	C42H63N(	676.4574	9.380483
39.9224	0.207038	0.468077	0.729188	unchanged	-4.30608	C19H24O2	307.1656	9.387617
65197.58	0.328762	0.341272	1.088477	unchanged	-0.81158	C46H84N(	790.5738	9.387617
82.86611	-0.05493	0.670736	0.554813	unchanged	10.81796	C9H16N2(	233.1157	9.4012

312.39	-0.04035	0.817029	0.337245	unchanged	-8.3769	C22H28O4	398.2296	9.4012
16984.22	0.065303	0.736604	0.382246	unchanged	5.188274	C22H36O4	746.5603	9.408333
496.599	0.031252	0.833609	0.19913	unchanged	-0.92499	C29H45N(	484.3053	9.415467
98.94149	1.090609	0.313266	1.141138	unchanged	20.46781	C9H18O7	256.144	9.42975
493.9731	0.167258	0.402153	0.934397	unchanged	-9.13945	C23H43N(	463.3337	9.42975
62.18332	-0.16694	0.642109	0.613745	unchanged	48.16211	C12H25N5	305.2434	9.444017
1215.038	0.091656	0.363962	1.029515	unchanged	-3.07721	C24H40O3	415.2597	9.451167
68223.68	0.027481	0.820329	0.27065	unchanged	8.339928	C38H62O6	614.4794	9.451167
536.3601	0.086759	0.783588	0.306339	unchanged	26.63954	C25H40N2	433.3176	9.4583
63154.53	0.03199	0.796961	0.300476	unchanged	2.751062	C36H63N(	570.4534	9.4583
9895.991	0.182745	0.297516	1.197852	unchanged	-1.39675	C40H58O5	619.4348	9.4583
50.64241	0.349699	0.45809	0.823953	unchanged	-9.38733	C10H12N2	263.0438	9.465433
5.693315	2.149463	0.380865	1.023969	unchanged	4.00795	C17H24N4	315.1829	9.472583
1194.376	-0.04017	0.718717	0.425369	unchanged	2.36214	C17H30N4	371.2298	9.465433
1591.835	0.144262	0.576717	0.670132	unchanged	-14.6838	C23H36O6	431.2344	9.465433
6943.033	0.181769	0.360055	1.055074	unchanged	-23.7036	C40H56O	575.4092	9.465433
1943.775	-1.03304	0.142691	1.571507	unchanged	-1.72214	C31H55N3	566.4154	9.479717
2347.462	-0.17272	0.527779	0.752806	unchanged	-2.74236	C37H62O7	641.4371	9.479717
6655.199	-0.07872	0.39571	0.939124	unchanged	-1.64742	C8H18O5	195.1211	9.48685
5743.628	-0.00322	0.975058	0.044309	unchanged	-7.86022	C10H22O6	239.147	9.493983
2171.654	-0.00022	0.998531	0.001621	unchanged	-6.73864	C12H26O7	283.1732	9.48685
2182.824	-0.02666	0.788583	0.321328	unchanged	6.791586	C20H39N(	356.2821	9.493983
99672.34	-0.00765	0.946648	0.065136	unchanged	-48.8639	C27H48O3	443.3294	9.493983
86125.14	0.046409	0.676118	0.494511	unchanged	36.21365	C28H48O5	482.4008	9.48685
97.26425	0.059528	0.959962	0.086678	unchanged	4.730504	C30H51N7	639.4218	9.48685
755.3346	0.072174	0.704242	0.42389	unchanged	-20.0712	C28H46O5	463.3325	9.501133
486.6554	-0.12902	0.629871	0.486154	unchanged	-11.104	C44H82N(	870.5373	9.501133
80.98757	-0.10058	0.421986	0.941186	unchanged	25.04407	C5H5NO4	161.0593	9.522633
321.4096	0.046221	0.840323	0.160314	unchanged	-18.1074	C27H42O	383.3235	9.529767
135.6385	-0.55446	0.030986	2.138992	down	-31.2638	C18H38N(	397.2707	9.529767
8606.795	-0.12406	0.504857	0.828864	unchanged	-11.6504	C24H36N2	385.2805	9.536917
60.04087	-0.74021	0.120281	1.751322	unchanged	-28.0472	C22H41N(	417.3211	9.54405
79.51836	-0.66051	0.142588	1.594611	unchanged	-26.3834	C33H52O6	1106.758	9.551183
1150.13	-0.80598	0.006004	2.370052	down	-3.02613	C39H64O5	635.4627	9.551183
22863.35	1.22847	0.15476	1.549755	unchanged	-12.68	C44H79N(	740.5501	9.551183
1096483	-0.21073	0.471797	0.82486	unchanged	-9.40865	C44H84N(	786.5933	9.551183
153.3872	0.083518	0.542795	0.693546	unchanged	-1.35715	C47H88O1	970.5528	9.551183
71.64436	0.346782	0.263714	1.25906	unchanged	15.75917	C53H96N2	1138.702	9.558317
268.9428	-0.20417	0.163842	1.517031	unchanged	-1.67469	C12H19N(	259.1648	9.558317
566.9341	-0.74308	0.031506	2.129944	down	10.61905	C36H61N(	613.5	9.558317
102.5798	-0.3192	0.3243	1.094992	unchanged	-35.6405	C18H32O4	647.4271	9.558317
142.6573	-0.00283	0.985679	0.044134	unchanged	-4.46455	C10H18N4	291.1286	9.5726
40.32099	-0.16836	0.461655	0.786057	unchanged	1.164146	C11H20O1	295.1027	9.5726
1073.446	-0.21627	0.306949	1.091049	unchanged	11.55174	C49H90O2	1078.596	9.579733
385.5465	-0.27203	0.345429	1.001382	unchanged	-8.35368	C9H10	119.0845	9.579733
11.94231	0.285142	0.609877	0.57027	unchanged	-8.31311	C9H13N3(	261.1173	9.579733
127.3136	-0.29924	0.341569	1.028875	unchanged	-8.44452	C15H18O4	263.1256	9.579733
2168.875	-0.13162	0.424406	0.942928	unchanged	-25.9073	C20H24O4	679.4187	9.579733
655.6896	-0.3133	0.307695	1.082486	unchanged	-3.36108	C22H30O5	397.1973	9.579733
13780.31	-0.3409	0.254843	1.192431	unchanged	21.6054	C29H38O9	531.2703	9.579733
1.72E-06	-21.9259	0.391002	0.958412	unchanged	11.31311	C12H16N5	531.0012	9.579733

144.1746	-0.52514	0.115557	1.625457	unchanged	-11.7615	C24H39N1	562.3142	9.579733
782.7485	-0.37875	0.147903	1.483655	unchanged	-8.17726	C27H50N3	576.3361	9.579733
1097.379	-0.61199	0.036893	2.007422	down	-7.48566	C33H53O9	589.3242	9.579733
761.6287	-0.09557	0.536627	0.699901	unchanged	23.27428	C52H81N3	1083.568	9.594017
13.26352	0.765032	0.34293	1.080641	unchanged	22.56242	C7H14O3	111.0837	9.586883
51.25877	0.919514	0.243316	1.341707	unchanged	-8.5098	C11H20O2	149.1309	9.586883
27.66189	0.872879	0.264699	1.27755	unchanged	-10.227	C9H14O	156.1369	9.594017
16.01031	1.001596	0.245472	1.307583	unchanged	-17.8596	C12H20	165.1608	9.586883
27958.35	0.543508	0.268665	1.276109	unchanged	-9.83827	C18H32O	282.2764	9.586883
40.30298	0.793806	0.241849	1.31325	unchanged	6.842093	C18H33NO	382.2014	9.586883
2941.631	0.138307	0.742261	0.421921	unchanged	17.34507	C48H82NO	918.5622	9.594017
58.63301	-0.50555	0.15922	1.501543	unchanged	-12.6531	C46H81N3	1043.52	9.60115
64.7766	-1.30406	0.049455	2.016225	down	1.586206	C52H87N3	1145.582	9.60115
9373.957	-0.15756	0.452461	0.806405	unchanged	-16.7713	C36H48N2	669.327	9.60115
931.2781	-0.23244	0.311398	1.087678	unchanged	17.42528	C34H47N7	682.3678	9.60115
1511.34	-0.49592	0.02234	2.14526	down	-1.8618	C35H61O9	695.3673	9.60115
212.8851	-0.99863	0.037247	2.130129	down	0.887949	C20H24O5	727.2885	9.60115
135.0282	-0.39914	0.194961	1.373744	unchanged	12.03484	C53H95N3	1124.644	9.6083
199.8415	-0.49559	0.028646	2.032073	down	-1.55079	C57H99N3	1140.624	9.6083
52.92089	-0.59492	0.219134	1.268614	unchanged	-29.5923	C17H22O8	393.0841	9.6083
86.47918	-0.67644	0.205189	1.297829	unchanged	-9.19939	C11H18O5	525.1774	9.6083
1660.05	-0.60987	0.173289	1.391468	unchanged	-4.31921	C33H38N2	581.2389	9.6083
285.6798	-0.87331	0.056016	1.894945	unchanged	-7.3401	C47H80O1	975.4556	9.6083
634.0577	-0.75012	0.030475	2.176342	down	-13.8625	C20H32O2	269.2222	9.615433
328.185	0.033495	0.848586	0.216512	unchanged	26.6122	C12H20N2	301.0765	9.6297
1321.161	-0.06882	0.78605	0.329764	unchanged	-9.66381	C19H38O4	313.2705	9.6297
73.33026	0.334125	0.462341	0.776905	unchanged	-11.5872	C18H26O6	339.1626	9.6297
58.96424	-0.1226	0.747352	0.395612	unchanged	27.72828	C19H24N4	358.2332	9.6297
2041.587	0.044821	0.671471	0.512148	unchanged	-1.25407	C15H4ClF9	460.9592	0.597083
396.6227	-0.43666	0.271324	1.290344	unchanged	2.284834	C12H25N5	592.4266	9.6297
197.0943	0.113023	0.54809	0.678915	unchanged	7.355458	C5H12N4O	375.1737	9.643983
58.782	-0.26439	0.064801	1.852421	unchanged	-26.6959	C28H42O	417.3023	9.63685
666.8312	-0.08572	0.758591	0.327048	unchanged	-2.0978	C29H42N2	449.3153	9.63685
118.5694	-0.2172	0.509483	0.747874	unchanged	1.57057	C24H42O1	471.2597	9.63685
82.18438	-0.05641	0.8945	0.218066	unchanged	3.957588	C32H48O7	567.3314	9.63685
120328.5	-0.11613	0.340643	1.081705	unchanged	6.621416	C37H68O4	559.5123	9.63685
74.69998	-0.16059	0.238266	1.310302	unchanged	8.758248	C17H26O5	275.1669	9.651117
220.7176	0.012924	0.939009	0.053948	unchanged	-17.2006	C5H8IN3	496.9337	0.597083
21492.69	0.66124	0.136203	1.611124	unchanged	-19.7037	C40H77NO	738.535	9.658267
6.805637	0.828745	0.268333	1.232963	unchanged	-10.6213	C19H38O2	340.3178	9.6654
468.0401	-0.44173	0.138995	1.562885	unchanged	-3.66767	C43H82O1	926.5486	9.69395
2550.361	-0.02339	0.851081	0.237899	unchanged	-10.8544	C17H32O	270.2764	9.708233
451.4471	0.042918	0.669105	0.474975	unchanged	15.09135	C21H44NO	455.331	9.708233
1067.472	0.631442	0.153361	1.557203	unchanged	20.38915	C40H54O2	1171.811	9.7225
479.8413	0.684241	0.179536	1.501715	unchanged	19.82796	C23H38O4	343.2707	9.7225
103.8481	0.108972	0.329772	1.070511	unchanged	-8.60624	C22H46NO	485.331	9.7225
6098.282	-0.30872	0.018301	2.211789	down	-14.1912	C38H77NO	595.5813	9.715367
380.6688	-0.52191	0.169009	1.463189	unchanged	4.332042	C39H68O5	655.4725	9.715367
235.7603	-0.31813	0.122432	1.660229	unchanged	11.23058	C10H18O2	171.1399	9.736083
469.7234	-0.05164	0.652909	0.505219	unchanged	8.923506	C16H34O9	371.2309	9.743217
596.6804	-0.21652	0.109441	1.705646	unchanged	8.44648	C40H56O4	642.4568	9.743217

113.9903	0.066982	0.668455	0.458272	unchanged	1.359425	C14H22N2	491.3363	9.75035
297.5033	-0.09492	0.613487	0.583088	unchanged	-18.2528	C49H86O1	1026.549	9.764633
181.3561	-0.8952	0.029568	2.155287	down	15.86513	C17H33N7	416.2682	9.7575
95.08542	0.171272	0.673135	0.432077	unchanged	-7.8007	C12H21NO	509.2795	9.764633
83.2866	-0.04129	0.871768	0.25079	unchanged	0.56022	C12H19N	372.3375	9.771767
36004.71	-0.01506	0.882261	0.162223	unchanged	-0.92332	C39H66O6	672.5192	9.778917
289.6234	0.007242	0.950822	0.005139	unchanged	12.46774	C37H66O8	603.4699	9.78605
524.3637	-0.01785	0.928503	0.07914	unchanged	5.861661	C39H66O7	611.4708	9.793183
66608.48	-0.03	0.772645	0.335442	unchanged	2.980691	C39H62O5	628.4954	9.793183
9078.384	0.058188	0.558371	0.661127	unchanged	-6.72096	C34H68NO	633.4514	9.793183
1496.832	-0.02983	0.78161	0.384405	unchanged	8.837852	C21H30O5	327.1987	9.8146
26.25577	-0.62093	0.235547	1.358664	unchanged	-6.19503	C25H38O5	383.2555	9.8146
5.242243	-0.07216	0.75722	0.378186	unchanged	7.26149	C5H7NO2	131.0823	9.821733
36.7661	-0.13508	0.463557	0.884886	unchanged	-5.47369	C10H20N2	250.1749	9.821733
18.80383	0.10125	0.765778	0.279766	unchanged	-28.7582	C13H20O3	266.1686	9.821733
823.0208	-0.01292	0.895277	0.181589	unchanged	-10.21	C6H12O3	133.0846	9.828883
88116.35	0.011556	0.919489	0.118495	unchanged	9.265251	C27H51NO	540.4415	9.828883
2940.309	-0.2162	0.169191	1.495412	unchanged	-8.72658	C6H14O4	151.0952	9.84315
12287.43	-0.0186	0.841966	0.238301	unchanged	37.48456	C10H15NO	239.1464	9.836017
213.5106	-0.0157	0.882621	0.206632	unchanged	-8.43631	C10H18N4	291.1275	9.84315
24442.02	-0.02006	0.861905	0.217262	unchanged	16.85635	C21H39NO	369.2935	9.84315
96202.79	-0.00168	0.988389	0.021758	unchanged	32.44552	C27H46O4	457.3436	9.836017
135.2883	-0.69318	0.237434	1.344806	unchanged	-14.904	C20H38O2	328.3164	9.864567
182.4133	-1.53551	0.091568	1.922097	unchanged	4.137618	C16H32O2	279.2305	9.871717
412.7345	-0.5049	0.016393	2.248517	down	4.7917	C22H42O7	419.3023	9.87885
28.38836	-1.02859	0.080837	1.869795	unchanged	1.070738	C6H12N2O	161.0922	9.885983
1670.407	-0.34276	0.283813	1.180689	unchanged	20.51776	C46H90O1	962.6287	9.893117
43.85578	0.027344	0.783996	0.320853	unchanged	-9.96154	C5H12O3	103.0742	9.9074
469.9119	-0.03811	0.712643	0.445987	unchanged	-5.43288	C9H18O3	175.1319	9.914533
2482.936	0.753788	0.242077	1.343311	unchanged	14.63948	C24H47NO	430.359	9.914533
79.11142	-0.27609	0.452003	0.840215	unchanged	1.948149	C35H62O8	611.4529	9.928817
49.22348	-0.30848	0.642197	0.620598	unchanged	-5.29608	C24H49O9	1063.581	9.93595
248.3743	-0.14005	0.257194	1.300157	unchanged	-36.9415	C10H14N2	281.0496	9.93595
25.53889	-0.1672	0.736272	0.431471	unchanged	-0.35471	C9H13N3O	519.1324	9.93595
414.6934	0.124629	0.531955	0.646099	unchanged	-12.5709	C35H64O7	597.465	9.9431
28494.41	0.167473	0.411291	1.062343	unchanged	15.91288	C37H67NO	700.4745	9.93595
21438.86	0.443211	0.2344	1.349291	unchanged	11.1589	C43H76NO	748.5312	9.9431
385169.1	0.145964	0.620261	0.566235	unchanged	-13.3051	C43H78NO	806.5622	9.9431
7370.497	0.693372	0.05223	1.966709	unchanged	-43.2634	C45H82NO	778.5401	9.9431
472171.6	0.233372	0.197318	1.424914	unchanged	-7.45313	C44H78NO	780.548	9.9431
28143.69	0.47877	0.220795	1.376831	unchanged	27.41505	C43H78NO	800.5624	9.9431
640770.9	0.20481	0.223491	1.347179	unchanged	-15.969	C50H81NO	808.5779	9.9431
150219.3	0.497864	0.045884	1.966638	up	-7.31695	C46H80NO	804.5478	9.9431
3718.602	0.066723	0.904739	0.202854	unchanged	-15.126	C43H81N3	828.5449	9.9431
7913.16	0.247109	0.19574	1.454214	unchanged	-17.7901	C46H88NO	842.5749	9.9431
8990.907	-0.2247	0.463147	0.860611	unchanged	-3.14167	C48H85NO	858.5862	9.9431
953.3758	0.126484	0.672206	0.507933	unchanged	-15.0844	C43H78N6	869.546	9.9431
8520.361	0.286802	0.220515	1.415441	unchanged	1.852486	C46H82NO	872.5362	9.9431
18410.07	0.122529	0.410271	0.957425	unchanged	4.43484	C46H84NO	874.548	9.9431
46494.64	0.040205	0.80046	0.298718	unchanged	-2.07565	C47H82NO	848.5418	9.9431
3454.024	-0.04041	0.780283	0.346518	unchanged	-15.0545	C49H84NO	916.5332	9.9431

7016.743	0.122712	0.260516	1.324294	unchanged	-17.2631	C46H82N(	846.5477	9.950233
3676.822	-0.02571	0.92973	0.018161	unchanged	-10.4825	C44H82N(	870.5378	9.950233
1057.349	-0.01422	0.880556	0.176709	unchanged	-4.24149	C51H83O1	1040.566	9.9645
107.089	-0.87476	0.016671	2.249619	down	4.379366	C23H41N(	411.2997	9.957367
341.6903	-0.26812	0.422196	0.974295	unchanged	-9.87122	C31H61O1	625.4013	9.957367
929.5348	0.062507	0.479119	0.722832	unchanged	1.482659	C18H35O4	631.4674	9.993067
2282.887	-0.08875	0.510608	0.723359	unchanged	-8.56836	C47H80N(	920.4974	9.985917
227.2173	0.059307	0.539911	0.691084	unchanged	-22.177	C2H3IO2	186.9209	0.532833
392.2906	-0.10714	0.782897	0.364271	unchanged	-22.0542	C7H13NO	140.0667	0.611367
1709.056	-0.05661	0.781827	0.343869	unchanged	-4.2762	C5H4N6O	219.0229	0.6185
268.5343	-0.26098	0.187785	1.455289	unchanged	2.564882	C9H10O4	221.0215	0.6185
14426.67	-0.23993	0.315764	1.113306	unchanged	-17.9815	C5H11NO	118.0841	0.632767
128.0927	0.243586	0.32423	1.154494	unchanged	-13.641	C5H7NO3	152.0212	0.625633
11900.92	-0.00441	0.969289	0.079247	unchanged	2.075465	C7H12N2(	203.0489	0.625633
76.22416	0.010792	0.955763	0.116066	unchanged	-1.83079	C7H11O3	214.0057	0.632767
213.7117	-0.80516	0.035266	2.024646	down	4.921818	C14H21N(	274.0848	0.625633
71.49565	0.041576	0.70281	0.440038	unchanged	-21.6116	C4H7O4P	322.9991	0.632767
1159.558	-0.00826	0.975501	0.046221	unchanged	7.329407	C12H24O1	383.109	0.625633
116.1496	-0.0288	0.891115	0.08668	unchanged	4.319098	C2H4N4	207.0124	0.625633
73.51075	0.766736	0.311653	1.113973	unchanged	1.500814	C28H48N2	1075.098	0.654183
182.7524	-0.12063	0.500584	0.85456	unchanged	-10.6052	C5H9NO2	116.0694	0.64705
130.1861	-0.0104	0.972073	0.042255	unchanged	5.791286	C11H14	146.1098	0.64705
204.5305	-0.36111	0.354347	1.083446	unchanged	25.67739	C6H14NO	277.0862	0.64705
124.6438	0.004237	0.993036	0.010923	unchanged	4.4065	C12H15N(	266.0672	0.64705
159252.8	-0.05423	0.206333	1.356152	unchanged	-2.97512	C10H20O7	315.0751	0.654183
7273.138	0.148174	0.307768	1.117643	unchanged	-5.23217	C17H10O9	359.0379	0.654183
3712.591	0.096718	0.609327	0.596257	unchanged	1.37104	C11H15N(	375.0121	0.654183
870.7924	0.276548	0.403904	0.931269	unchanged	-2.85069	C16H12O1	793.0353	0.64705
71.57718	-0.53939	0.294765	1.157503	unchanged	1.048992	C17H22N6	561.0359	0.64705
53.87288	0.021618	0.968435	0.062837	unchanged	16.63181	C10H12N5	576.997	0.64705
102.452	0.143151	0.748987	0.372327	unchanged	13.79198	C15H22N2	619.0054	0.64705
1769.01	0.054202	0.761163	0.362625	unchanged	39.33966	C20H26N1	689.0795	0.654183
38.31686	-0.23008	0.097599	1.798517	unchanged	-6.01185	C4H9NO	70.0646	0.64705
160.9538	0.089772	0.79386	0.277481	unchanged	16.04592	C9H14N2(	809.0246	0.654183
102.9767	0.160447	0.783707	0.307504	unchanged	2.028355	C20H26N8	853.0046	0.654183
35.26731	0.318092	0.622727	0.563465	unchanged	-6.87146	C24H38N7	876.0781	0.654183
38.3241	0.748923	0.373578	0.998953	unchanged	29.18775	C10H15N5	909.0637	0.654183
593.9426	-0.01291	0.884816	0.213173	unchanged	-18.0695	C8H14N2(	257.0702	0.661333
108.8871	-0.05349	0.801314	0.305478	unchanged	7.917279	C4H9NO2	86.06086	0.661333
50.59778	-0.51262	0.235588	1.271264	unchanged	1.79573	C32H42N7	992.1328	0.661333
87.79661	-0.22306	0.549242	0.677445	unchanged	-11.1813	C32H48N7	1022.136	0.668467
82.38319	-0.09594	0.733851	0.442644	unchanged	-26.9033	C23H20O1	1079.106	0.668467
274.4415	-0.08319	0.847762	0.198194	unchanged	-23.0188	C6H12O4	190.104	0.668467
2853.558	-0.13172	0.521001	0.719302	unchanged	-13.1653	C9H14N2(	247.0892	0.668467
190.1846	-0.49413	0.301241	1.187091	unchanged	-7.0919	C8H10N4(	260.0972	0.668467
186541.6	-0.0865	0.54373	0.675616	unchanged	-14.5328	C10H16N2	293.0937	0.668467
9070.686	-0.09302	0.540114	0.677088	unchanged	-6.20187	C28H30O1	629.1439	0.668467
705.8038	-0.09723	0.509391	0.762396	unchanged	7.170023	C28H46N7	943.2136	0.668467
334.1722	-0.04787	0.812956	0.320332	unchanged	-21.1617	C9H9NO	112.0514	0.6756
3320.749	-0.30527	0.611638	0.528618	unchanged	-17.8683	C8H14O2	160.1307	0.68275
144.1515	0.061746	0.704147	0.356115	unchanged	28.71784	C8H17NO	172.085	0.6756

253.1267	0.335481	0.238094	1.252282	unchangec	17.56946	C10H17N(	270.0991	0.6756
393.7724	-0.16661	0.340529	1.043547	unchangec	6.414445	C27H26O1	623.1283	0.6756
68.00524	-0.14018	0.837125	0.22724	unchangec	-21.0973	C30H48N7	921.2188	0.6756
39.7626	-0.0389	0.971546	0.014126	unchangec	3.62727	C29H48N7	930.1704	0.6756
0.983658	1.724027	0.12854	1.687552	unchangec	0.020339	C4H8N2O	139.0478	0.696317
96.99026	0.186227	0.534133	0.659635	unchangec	-22.5165	C7H13N3(	168.0722	0.70345
25.61672	0.339334	0.440609	0.869732	unchangec	-4.06897	C9H18N2(	203.1382	0.70345
12.56892	0.859525	0.062089	1.814841	unchangec	12.09633	C11H14N2	261.0663	0.70345
559.2966	-0.10275	0.79862	0.367869	unchangec	-1.72084	C10H12N4	291.0695	0.70345
119.9656	-0.04351	0.914824	0.217385	unchangec	6.067698	C12H14O8	287.0779	0.70345
154.5155	0.039493	0.913841	0.050879	unchangec	-2.28462	C12H11FN	289.038	0.70345
53.71647	0.158019	0.592467	0.600114	unchangec	2.393661	C19H14O4	329.0792	0.70345
18.58092	-1.32729	0.106533	1.833641	unchangec	3.967903	C5H10N+	107.0709	0.7106
1844.539	-0.95806	0.142415	1.695367	unchangec	3.546853	C5H12O5	152.0684	0.7106
538.2374	0.235178	0.71988	0.474023	unchangec	-12.4054	C10H11N(	178.0841	0.7106
2552.891	0.206616	0.344561	0.939643	unchangec	40.04392	C8H15NO	186.0849	0.7106
563.7706	-0.25805	0.265526	1.294556	unchangec	28.04771	C11H15N3	218.0995	0.7106
645.6512	-0.11216	0.711383	0.432306	unchangec	-0.24849	C7H14S	130.081	0.724867
100.2724	-0.26995	0.633936	0.533574	unchangec	15.35076	C8H10O4	135.0467	0.724867
14740.56	-0.44931	0.318521	1.103733	unchangec	-12.4992	C11H21N(	262.125	0.724867
426.2036	0.042169	0.897585	0.140688	unchangec	14.3094	C17H14O4	324.096	0.724867
53.58156	-0.31261	0.53436	0.707077	unchangec	33.12949	C16H16O8	319.0924	0.724867
70.04606	-0.44554	0.437935	0.845014	unchangec	10.20128	C10H13N5	616.1776	0.724867
722.929	0.05181	0.783092	0.244806	unchangec	-9.30038	C5H4N4O	169.0341	0.732
231.85	0.019861	0.964858	0.062417	unchangec	-23.5343	C17H26N4	638.0961	0.732
363.0489	-0.67293	0.326756	1.086306	unchangec	41.10125	C8H17NO	158.107	0.73915
2429.427	-0.00714	0.961086	0.067586	unchangec	18.88401	C9H7NO4	235.0901	0.73915
99.04277	-0.03287	0.825755	0.332264	unchangec	-13.6933	C6H11NO	128.0686	0.746283
73.81721	-0.33815	0.245364	1.254542	unchangec	-0.5268	C9H19N2(	242.1026	0.746283
1343.162	-0.18115	0.60949	0.586099	unchangec	10.86433	C17H21N(	310.1445	0.753417
45.28922	0.396453	0.645188	0.473839	unchangec	10.18429	C13H23N3	332.131	0.753417
3297.628	0.247459	0.455472	0.831317	unchangec	9.629141	C15H22N(	245.1437	0.7677
105.0293	0.363465	0.425437	0.871128	unchangec	9.728501	C16H22N2	551.049	0.774833
30.45423	0.039242	0.911747	0.131199	unchangec	-9.21601	C8H11NO	287.9738	0.781967
9.280389	-0.22263	0.787374	0.271651	unchangec	-6.82642	C4H9NO2	100.0206	0.789117
50.97121	-0.02076	0.964245	0.04769	unchangec	-25.1301	C9H8O2	113.0349	0.789117
351.9945	-0.05828	0.658636	0.641493	unchangec	-10.1651	C9H12O4	202.1055	0.789117
87.18282	-0.62084	0.194718	1.411717	unchangec	-9.88732	C5H11NO	100.0745	0.803383
853.54	-0.19049	0.334604	1.10301	unchangec	-7.98469	C7H8O	91.05336	0.803383
1006.258	0.056417	0.535323	0.731895	unchangec	-20.5345	C6H13NO	132.0992	0.803383
161.1917	0.060852	0.603498	0.509352	unchangec	4.749796	C11H13N(	156.0817	0.803383
125.1326	0.206253	0.254895	1.2355	unchangec	-14.4994	C8H16N2(	171.1101	0.803383
874.2894	0.033379	0.771568	0.290206	unchangec	-10.1395	C6H10N2(	174.1124	0.79625
1619.959	-0.22851	0.286065	1.182623	unchangec	15.43589	C9H10FN(	166.0691	0.803383
7898.523	-0.28215	0.284326	1.141123	unchangec	-7.71397	C8H11N3(	395.1253	0.79625
387.4299	0.099035	0.448137	0.801926	unchangec	-3.54133	C16H17N(	204.1163	0.803383
378.9379	0.245266	0.425739	0.84617	unchangec	-1.87668	C12H22O5	243.1044	0.803383
857.5636	-0.31414	0.26951	1.184895	unchangec	-0.17154	C15H20O3	248.1406	0.803383
198.6802	-0.09854	0.435541	0.997846	unchangec	-1.0709	C10H12FN	252.0888	0.803383
22157.53	-0.3496	0.328648	1.050521	unchangec	8.266002	C11H18N2	276.1407	0.803383
4979.93	-0.31388	0.363684	0.982719	unchangec	17.29274	C14H19N3	294.1499	0.803383

1370.11	0.045216	0.862574	0.133347	unchanged	-0.18329	C17H20O5	269.1172	0.803383
377.9615	-0.30169	0.29948	1.134093	unchanged	-7.65713	C15H20N2	308.1165	0.803383
218.6909	-0.51687	0.247383	1.3105	unchanged	-7.07682	C17H21N5	356.1326	0.803383
4548.512	-0.42893	0.14841	1.543897	unchanged	-10.2602	C15H23N(	326.1197	0.79625
156.3956	-0.29087	0.462809	0.91297	unchanged	-3.4524	C15H23N5	386.1657	0.803383
203.7386	-0.46112	0.313937	1.233594	unchanged	1.351824	C19H23N3	390.1487	0.803383
164.0162	-0.33798	0.344895	1.049315	unchanged	-23.2764	C19H22O9	433.0804	0.803383
46.53137	-0.38059	0.667897	0.525629	unchanged	3.586492	C22H22O9	448.1618	0.803383
88.01065	0.159095	0.612707	0.517426	unchanged	8.123316	C21H24O1	454.1743	0.803383
64.16746	-0.07811	0.81001	0.386756	unchanged	-2.53577	C21H30O1	481.1669	0.803383
163.5463	-0.18923	0.746198	0.413348	unchanged	6.389245	C27H29N3	456.1949	0.803383
161.5323	-0.00452	0.979216	0.074349	unchanged	-12.0684	C17H26N2	527.0761	0.803383
55.70192	-0.21274	0.864277	0.25862	unchanged	-3.90911	C14H16N4	626.2658	0.803383
1502.458	0.128103	0.308076	1.136602	unchanged	-5.7355	C5H8	86.09534	0.803383
49.762	0.095725	0.817714	0.241988	unchanged	4.197285	C4H7NO	86.0604	0.810533
83.64573	-0.06412	0.629651	0.497301	unchanged	-1.24253	C11H16O4	251.0678	0.817667
84.0373	0.009527	0.978898	0.001437	unchanged	12.22079	C19H24O9	361.133	0.817667
295.0859	0.107833	0.466535	0.756	unchanged	-3.56151	C6H10O3	172.0964	0.83195
585.4167	-0.03061	0.75693	0.369022	unchanged	-4.31606	C11H18O9	295.1011	0.83195
62.97446	0.135744	0.363761	1.006169	unchanged	-7.63302	C6H12O6	145.0482	0.846217
2564.701	-0.03205	0.752536	0.358707	unchanged	-13.0255	C8H13N3(	302.0173	0.8605
57.15946	-0.02361	0.789277	0.376263	unchanged	-29.0974	C7H9N2O	102.0536	0.910467
23.85471	-0.42779	0.302823	1.168608	unchanged	-13.5872	C6H9N3O	120.0535	0.9176
38.92891	0.054959	0.854557	0.181912	unchanged	5.66599	C7H8O	91.05484	0.924733
415.2477	-0.93897	0.13238	1.594258	unchanged	-11.5071	C10H18N2	211.1049	0.988983
3177.768	-0.59193	0.178353	1.418814	unchanged	-7.46241	C15H19N(	310.1262	1.017533
660.7238	-0.80715	0.138548	1.584872	unchanged	34.0139	C12H17N5	328.1363	1.017533
96.95897	0.0545	0.508208	0.653426	unchanged	9.691038	C4H10O4	145.0483	1.045383
402.0587	0.102058	0.526133	0.710266	unchanged	-4.80341	C5H11N	86.09602	1.059667
1025.044	0.268282	0.264587	1.174794	unchanged	-11.8364	C10H19N(	234.1308	1.109633
839.9842	-0.85172	0.12902	1.663905	unchanged	-6.30951	C10H11N(	192.0642	1.202433
240.0557	-0.91429	0.21033	1.46503	unchanged	4.807523	C17H22N4	379.1453	1.202433
70.7921	-0.72746	0.113421	1.713274	unchanged	19.99461	C5H13N3(	163.0806	1.209567
203.224	-0.38322	0.496084	0.748019	unchanged	6.659798	C6H12N2(	194.1147	1.22385
175.0901	0.012972	0.947454	0.046568	unchanged	-1.3153	C7H8O	91.05408	1.245267
537.1582	0.003689	0.99185	0.0057	unchanged	-12.1768	C3H5NO4	120.0277	1.259533
6.046557	-0.24679	0.735541	0.426876	unchanged	7.092496	C11H16N2	221.0939	1.259533
166.5071	0.085571	0.769821	0.296712	unchanged	-0.68252	C8H15NO	172.0967	1.295233
260.7141	0.04923	0.513502	0.675018	unchanged	-11.5511	C12H15N(	222.1099	1.3095
169.4477	1.463767	0.104772	1.795022	unchanged	-0.98488	C15H17N(	240.1016	1.3095
69.49198	-1.17296	0.166223	1.610101	unchanged	-16.4178	C19H16N4	390.1504	1.37305
18.58256	-1.92347	0.388578	0.947378	unchanged	11.91346	C7H14F2N	189.0973	1.408733
285.5191	0.13798	0.437104	0.825658	unchanged	23.145	C12H19N3	234.1299	1.46585
91.69822	1.103609	0.093866	1.673774	unchanged	4.003214	C9H18O8	272.135	1.572917
139.6987	0.052901	0.837168	0.203842	unchanged	8.660795	C14H17N(	247.1224	1.580067
94.22706	-3.35045	0.433837	0.947563	unchanged	16.66994	C8H11NO	259.0783	1.5872
32.29207	-0.80081	0.189288	1.447387	unchanged	2.588603	C10H7NO	174.0554	1.6443
1605.574	-0.74336	0.106248	1.71027	unchanged	-3.13925	C10H11N(	192.0649	1.6443
365.7089	0.082845	0.677367	0.423213	unchanged	25.3681	C12H14N2	217.1031	1.6443
32.60546	-0.57464	0.207205	1.335372	unchanged	-6.15534	C5H8N4	147.0634	1.65145
75.08236	0.163284	0.072476	1.990098	unchanged	5.08136	C5H11N2(	127.0428	1.672867

480.6906	0.119291	0.386429	0.929695	unchanged	-22.6575	C12H17N3	234.118	1.687133
52.43096	0.160714	0.761348	0.294814	unchanged	12.1002	C9H12N2O	180.0915	1.714983
4769.596	-0.00146	0.994494	0.0176	unchanged	-2.83076	C8H8O	103.0539	1.722117
5979.248	0.021143	0.91764	0.11596	unchanged	-6.52457	C9H10O3	166.0855	1.722117
77.10291	0.195233	0.708252	0.378827	unchanged	-3.82809	C9H15NO	186.1118	1.722117
190.9855	-0.18279	0.586925	0.611345	unchanged	-0.94694	C7H8	93.06979	1.722117
212.1465	0.104094	0.795154	0.286995	unchanged	12.66042	C16H17NO	220.1153	1.750683
53.75916	-0.60483	0.219404	1.267501	unchanged	-15.868	C11H14N4	267.1046	1.750683
2608.533	-0.50352	0.221862	1.290473	unchanged	-37.9581	C14H21N3	310.1273	1.750683
152.1329	1.38208	0.135957	1.671621	unchanged	-1.20086	C15H13NO	240.1016	1.757817
181.2928	-0.62503	0.721194	0.388043	unchanged	-1.04885	C14H11F3	335.0401	1.779233
942.586	-0.56344	0.709687	0.403237	unchanged	-21.6911	C14H20N2	303.1048	1.786367
75.42447	-0.1056	0.607203	0.57983	unchanged	12.50919	C10H12O2	203.0489	1.807783
17.87553	0.097309	0.801312	0.294041	unchanged	-14.8668	C8H13N3O	180.0736	1.872033
264.9281	0.193519	0.619173	0.592481	unchanged	0.202425	C11H13F2	220.1163	1.922
33.10272	-0.91402	0.169177	1.564646	unchanged	4.17517	C16H16	208.1255	1.929133
460.0534	0.011494	0.952996	0.022867	unchanged	-22.2587	C9H14N4O	217.1038	1.936283
25.87095	-1.6801	0.417142	0.888529	unchanged	-1.17246	C8H13NO	197.1283	1.964833
220.7345	-0.83506	0.272675	1.331208	unchanged	-0.47848	C21H22O5	319.1327	1.971967
66.94893	-0.09588	0.729782	0.386658	unchanged	10.80362	C10H12O4	238.1095	1.9791
107.1031	-0.83829	0.632296	0.527551	unchanged	12.2743	C5H8O5	335.0411	1.9791
1215.238	0.861885	0.19403	1.473347	unchanged	-1.99391	C11H21NO	232.1539	1.993383
14733.33	-0.06063	0.59141	0.606031	unchanged	3.804702	C40H74NO	708.4991	10.0002
87.60544	-0.55242	0.013122	2.217459	down	25.70162	C5H11NO	100.0787	10.00733
108.3091	0.413018	0.262189	1.356383	unchanged	21.27185	C63H118O	1173.796	10.00733
353.2651	-0.12511	0.332967	1.053524	unchanged	-5.82201	C7H17N3O	175.1305	10.00733
359.9694	0.15558	0.790258	0.32971	unchanged	-20.2778	C22H44O	366.3665	10.00733
168.1432	-0.02092	0.810067	0.29529	unchanged	-11.4978	C10H18N4	291.1266	10.02162
199.4667	-0.11065	0.520269	0.764027	unchanged	-2.59681	C14H19N3	383.1375	10.02875
246.2709	0.334191	0.481455	0.719378	unchanged	-6.22389	C11H18N4	509.2799	10.02875
4885.657	0.017382	0.918857	0.093913	unchanged	4.823256	C26H42O1	495.2614	10.02875
408645.4	0.009475	0.933247	0.074525	unchanged	-16.4507	C35H69O1	663.4484	10.02875
102.9741	-0.1841	0.706378	0.460971	unchanged	-31.4929	C54H92O2	1091.565	10.04303
379.4728	0.172978	0.513695	0.705539	unchanged	-17.5667	C6H14NO	301.075	10.04303
81.17323	0.642738	0.286222	1.203163	unchanged	-11.0662	C19H26O4	357.1427	10.04303
1363.961	-0.07346	0.530958	0.767058	unchanged	8.482062	C18H30O6	360.241	10.03588
48.41164	1.083617	0.11275	1.65008	unchanged	-8.26228	C15H25N3	549.3844	10.03588
56084.51	-0.11726	0.437244	0.937223	unchanged	2.781648	C37H70O5	559.5101	10.04303
596.2033	0.229554	0.598279	0.532045	unchanged	-4.4148	C35H64O7	635.4494	10.04303
25951.32	0.005183	0.967874	0.025073	unchanged	-16.3909	C31H61O1	607.3867	10.03588
125.2047	0.121852	0.776019	0.275822	unchanged	-2.54416	C34H61O1	641.3796	10.03588
1555.715	0.420663	0.133255	1.562458	unchanged	2.720476	C43H74NO	712.5085	10.04303
58723.55	0.027826	0.838735	0.229988	unchanged	4.865504	C41H68O1	721.492	10.04303
33018.59	0.105603	0.782663	0.323445	unchanged	-6.43201	C41H74N2	723.5294	10.04303
91088.2	1.541597	0.145579	1.644247	unchanged	16.20174	C44H78NO	728.5501	10.04303
37213.5	0.795403	0.166202	1.555782	unchanged	-0.38571	C42H76NO	792.5593	10.04303
86384.66	0.525134	0.163487	1.52838	unchanged	11.50914	C43H76NO	804.5464	10.04303
287365.9	0.547281	0.242853	1.318536	unchanged	-14.2979	C43H78NO	806.5606	10.04303
19961.51	0.385056	0.163298	1.504795	unchanged	13.97326	C45H76NO	738.5329	10.04303
8052.036	0.78646	0.07163	1.844989	unchanged	-0.07716	C44H80NO	778.5381	10.04303
197771.4	0.34444	0.212489	1.373345	unchanged	-8.94693	C44H78NO	780.5468	10.04303

699560.8	0.20557	0.422401	0.923495	unchanged	-8.90279	C44H82N(	782.5623	10.04303
7627.296	0.215018	0.685154	0.500588	unchanged	-2.24392	C42H79N(	828.5463	10.04303
297325.3	0.441008	0.1885	1.492893	unchanged	1.732305	C46H81N(	808.577	10.04303
10563.63	0.178337	0.658872	0.470457	unchanged	-27.5812	C43H82N(	858.5866	10.04303
9983.323	0.240054	0.338157	1.090446	unchanged	-2.14239	C45H80N(	848.5394	10.04303
2745.941	0.955046	0.091201	1.747248	unchanged	4.427714	C44H79O1	856.5734	10.04303
28618.98	0.159881	0.674932	0.465888	unchanged	-10.4966	C47H82N(	874.5479	10.04303
6466.333	-0.08494	0.682715	0.481614	unchanged	11.79609	C47H84N(	876.5614	10.04303
532.4869	-0.44897	0.05162	1.93453	unchanged	-5.06747	C51H86N(	926.5627	10.03588
1997.869	-0.1079	0.460907	0.891241	unchanged	7.283709	C22H30O9	439.1995	10.05017

KEGG_ann	KEGG_path	LM_ID(lipic	CATEGORY	MAIN_CLA	SUB_CLAS	HMDB_ID	CAS	kingdom(H
--	--	--	--	--	--	HMDB024	--	Organic cc
--	--	--	--	--	--	HMDB003	67901-30-	Organic cc
--	--	--	--	--	--	--	--	--
--	--	--	--	--	--	--	--	--
--	--	--	--	--	--	HMDB025	--	Organic cc
--	--	--	--	--	--	HMDB025	--	Organic cc
--	--	--	--	--	--	HMDB002	--	Organic cc
--	--	--	--	--	--	--	--	--
--	--	--	--	--	--	--	--	--
--	--	--	--	--	--	--	--	--
--	--	--	--	--	--	--	--	--
C00026	Citrate cyc	--	--	--	--	HMDB000	328-50-7	Organic cc
C00059	Purine me	--	--	--	--	HMDB000	14808-79-	Inorganic c
C00441	Glycine, se	--	--	--	--	HMDB001	2338/3/6	Organic cc
--	--	--	--	--	--	--	--	--
--	--	--	--	--	--	HMDB024	22430-27-	Organic cc
--	--	--	--	--	--	--	--	--
--	--	--	--	--	--	--	--	--
C02199	--	--	--	--	--	HMDB001	1955-26-6	Organic cc
C00700	Purine me	--	--	--	--	HMDB000	6253-56-1	Organic cc
C16616	Drug meta	--	--	--	--	HMDB006	--	Organic cc
--	--	--	--	--	--	--	--	--
--	--	--	--	--	--	--	--	--
--	--	--	--	--	--	--	--	--
C00652	Ascorbate	--	--	--	--	HMDB030	--	Organic cc
--	--	--	--	--	--	--	--	--
--	--	--	--	--	--	HMDB024	--	Organic cc
C06554	Atrazine d	--	--	--	--	HMDB004	108-80-5	Organic cc
--	--	--	--	--	--	HMDB025	--	Organic cc
--	--	--	--	--	--	--	--	--
--	--	--	--	--	--	--	--	--
--	--	--	--	--	--	--	--	--
--	--	LMFA0105	Fatty Acyls	Fatty Acids	Hydroxy fa	HMDB000	21620-60-	Organic cc
--	--	--	--	--	--	HMDB024	--	Organic cc
--	--	--	--	--	--	HMDB024	--	Organic cc
--	--	--	--	--	--	HMDB000	3868-31-3	Organic cc
C00061	Oxidative		--	--	--	--	HMDB000	146-17-8	Organic cc
--	--	--	--	--	--	HMDB030	75-07-0	Organic cc
--	--	--	--	--	--	HMDB030	--	Organic cc
C00100	Valine, leu	LMFA0705	Fatty Acyls	Fatty ester	Fatty acyl (	HMDB000	317-66-8	Organic cc
C05989	Propanoat	LMFA0705	Fatty Acyls	Fatty ester	Fatty acyl (	HMDB000	--	--
C01771	--	LMFA0103	Fatty Acyls	Fatty Acids	Unsaturate	HMDB001	3724-65-0	Organic cc
C01933	--	LMFA0110	Fatty Acyls	Fatty Acids	Amino fatt	HMDB000	327-57-1	Organic cc
C01801	--	--	--	--	--	HMDB000	533-67-5	Organic cc
--	--	--	--	--	--	HMDB002	60551-20-	Organic cc
C00262	Purine me	--	--	--	--	HMDB000	68-94-0	Organic cc
--	--	--	--	--	--	--	--	--
--	--	--	--	--	--	--	--	--

--	--	--	--	--	--	HMDB024	--	Organic cc
--	--	--	--	--	--	--	--	--
C03519	Phenylalar	--	--	--	--	HMDB000	2018-61-3	Organic cc
C00643	Tryptopha	--	--	--	--	HMDB000	4350/9/8	Organic cc
--	--	--	--	--	--	HMDB025	69-33-0	Organic cc
--	--	--	--	--	--	HMDB024	23964-58-	Organic cc
C00330	Purine me	--	--	--	--	HMDB000	961-07-9	Organic cc
--	--	--	--	--	--	HMDB025	--	Organic cc
--	--	--	--	--	--	HMDB003	34393-22-	Organic cc
C17208	--	--	--	--	--	HMDB002	64296-20-	Organic cc
--	--	--	--	--	--	HMDB006	--	Organic cc
C00575	Purine me	--	--	--	--	HMDB000	60-92-4	Organic cc
--	--	--	--	--	--	--	--	--
--	--	--	--	--	--	HMDB006	--	Organic cc
--	--	--	--	--	--	HMDB003	128701-0	Organic cc
--	--	--	--	--	--	HMDB003	139742-2	Organic cc
C16619	Drug meta	--	--	--	--	HMDB006	--	Organic cc
C03166	Arginine a	--	--	--	--	HMDB000	5115-19-5	Organic cc
C04712	Penicillin a	--	--	--	--	HMDB006	--	Organic cc
C06089	Diterpenoi	LMPR0104	Prenol Lipi	Isoprenoid	C20 isopre	HMDB030	--	Organic cc
--	--	--	--	--	--	HMDB003	23052-19-	Organic cc
--	--	--	--	--	--	--	--	--
--	--	--	--	--	--	--	--	--
--	--	--	--	--	--	HMDB003	34232-19-	Organic cc
--	--	--	--	--	--	--	--	--
--	--	--	--	--	--	--	--	--
C00857	Nicotinate	--	--	--	--	HMDB000	6450-77-7	Organic cc
C06196	Purine me	--	--	--	--	HMDB000	3393-18-8	Organic cc
C00362	Purine me	--	--	--	--	HMDB000	902-04-5	Organic cc
--	--	--	--	--	--	--	--	--
--	--	--	--	--	--	--	--	--
C08356	--	--	--	--	--	HMDB000	470-15-5	Organic cc
--	--	--	--	--	--	HMDB001	41078-02-	Organic cc
C01089	Butanoate	LMFA0105	Fatty Acyls	Fatty Acids	Hydroxy fa	HMDB000	625-72-9	Organic cc
--	--	--	--	--	--	--	--	--
--	--	--	--	--	--	--	--	--
C00081	Purine me	--	--	--	--	HMDB000	132-06-9	Organic cc
C19304	--	--	--	--	--	HMDB024	141-90-2	Organic cc
C00944	Phenylalar	--	--	--	--	HMDB001	10534-44-	Organic cc
--	--	--	--	--	--	--	--	--
--	--	--	--	--	--	--	--	--
C20452	Aflatoxin b	--	--	--	--	--	--	--
--	--	--	--	--	--	--	--	--
C04397	--	--	--	--	--	HMDB000	4298/8/2	Organic cc
--	--	--	--	--	--	HMDB025	--	Organic cc
--	--	--	--	--	--	HMDB006	210769-8	Organic cc
C01697	Galactose	--	--	--	--	HMDB000	608-66-2	Organic cc
C02057	--	--	--	--	--	HMDB025	63-91-2	Organic cc
--	--	--	--	--	--	HMDB006	--	Organic cc
C10438	Ubiquinon	--	--	--	--	HMDB000	140-10-3	Organic cc
C00392	Fructose a	--	--	--	--	HMDB000	69-65-8	Organic cc

--	--	--	--	--	--	--	--	--
--	--	--	--	--	--	--	--	--
--	--	--	--	--	--	HMDB025	--	Organic cc
--	--	--	--	--	--	--	--	--
--	--	--	--	--	--	HMDB002	20546-04-	Organic cc
C07210	Bile secret	--	--	--	--	HMDB001	30516-87-	Organic cc
--	--	LMFA0117	Fatty Acyls	Fatty Acids	Dicarboxyl	HMDB000	73141-47-	Organic cc
--	--	--	--	--	--	--	--	--
--	--	LMFA0105	Fatty Acyls	Fatty Acids	Hydroxy fa	HMDB000	71526-30-	Organic cc
--	--	--	--	--	--	HMDB025	--	Organic cc
C06552	Atrazine d	--	--	--	--	HMDB006	2163-68-(	Organic cc
--	--	--	--	--	--	--	--	--
C10438	--	--	--	--	--	HMDB000	102-94-3	Organic cc
--	--	--	--	--	--	HMDB024	--	Organic cc
--	--	--	--	--	--	--	--	--
--	--	--	--	--	--	HMDB024	93513-59-	Organic cc
--	--	--	--	--	--	HMDB025	--	Organic cc
--	--	--	--	--	--	HMDB000	24656-24-	Organic cc
C06552	Atrazine d	--	--	--	--	HMDB006	2163-68-(	Organic cc
--	--	--	--	--	--	HMDB002	92352-82-	Organic cc
--	--	--	--	--	--	--	--	--
--	--	--	--	--	--	HMDB024	--	Organic cc
--	--	--	--	--	--	HMDB027	--	_
--	--	--	--	--	--	--	--	--
--	--	--	--	--	--	HMDB025	--	Organic cc
--	--	--	--	--	--	HMDB001	--	Organic cc
--	--	--	--	--	--	HMDB003	246248-1(	Organic cc
--	--	--	--	--	--	HMDB006	--	Organic cc
--	--	--	--	--	--	HMDB007	--	Organic cc
--	--	--	--	--	--	HMDB025	--	Organic cc
--	--	--	--	--	--	HMDB024	--	Organic cc
C20396	Phosphon	--	--	--	--	HMDB024	993-13-5	Organic cc
--	--	--	--	--	--	HMDB027	--	_
--	--	LMFA0103	Fatty Acyls	Fatty Acids	Unsaturate	HMDB000	--	Organic cc
--	--	LMFA0117	Fatty Acyls	Fatty Acids	Dicarboxyl	HMDB030	--	Organic cc
--	--	--	--	--	--	--	--	--
C04805	--	LMFA0306	Fatty Acyls	Eicosanoid	Hydroxy/h	HMDB111	--	--
--	--	--	--	--	--	HMDB025	--	Organic cc
--	--	--	--	--	--	HMDB002	51352-44-	Organic cc
--	--	LMFA0701	Fatty Acyls	Fatty ester	Wax monc	HMDB002	1323-39-3	Organic cc
--	--	LMFA0115	Fatty Acyls	Fatty Acids	Heterocycl	HMDB011	116627-42	Organic cc
--	--	--	--	--	--	--	--	--
C04671	--	LMFA0301	Fatty Acyls	Eicosanoid	Prostaglan	HMDB000	363-23-5	Organic cc
C01261	Purine me	--	--	--	--	HMDB000	4130-19-2	Organic cc
--	--	--	--	--	--	HMDB026	34758-83-	Organic cc
--	--	--	--	--	--	HMDB002	--	Organic cc
--	--	--	--	--	--	HMDB024	--	Organic cc
--	--	--	--	--	--	--	--	--
--	--	--	--	--	--	HMDB030	--	Organic cc
--	--	--	--	--	--	HMDB003	50854-94-	Organic cc
--	--	--	--	--	--	HMDB001	--	Organic cc

--	--	--	--	--	--	HMDB003	14481-55-	Organic cc
--	--	--	--	--	--	HMDB025	24744-50-	Organic cc
--	--	--	--	--	--	--	--	--
--	--	--	--	--	--	HMDB029	--	_
C14279	Furfural de	--	--	--	--	HMDB003	1998/1/1	Organic cc
--	--	--	--	--	--	HMDB006	37718-11-	Organic cc
C00093	Glycerolipi	--	--	--	--	HMDB000	1957/3/4	Organic cc
--	--	--	--	--	--	HMDB003	133860-42	Organic cc
--	--	--	--	--	--	HMDB025	--	Organic cc
C00054	Purine me	--	--	--	--	HMDB000	1053-73-2	Organic cc
C08476	--	--	--	--	--	HMDB024	99-32-1	Organic cc
--	--	--	--	--	--	--	--	--
--	--	--	--	--	--	HMDB003	34098-52-	Organic cc
--	--	--	--	--	--	HMDB030	--	Organic cc
--	--	--	--	--	--	HMDB025	--	Organic cc
--	--	--	--	--	--	HMDB024	--	Organic cc
--	--	--	--	--	--	--	--	--
--	--	--	--	--	--	HMDB024	--	Organic cc
--	--	--	--	--	--	HMDB025	--	Organic cc
--	--	--	--	--	--	HMDB003	181485-19	Organic cc
--	--	--	--	--	--	HMDB029	--	_
--	--	--	--	--	--	HMDB005	--	Organic cc
--	--	--	--	--	--	HMDB003	124902-00	Organic cc
--	--	--	--	--	--	--	--	--
--	--	--	--	--	--	HMDB003	13161-32-	Organic cc
C04225	Propanoat	--	--	--	--	HMDB000	6061-93-4	Organic cc
--	--	--	--	--	--	HMDB025	70-49-5	Organic cc
--	--	--	--	--	--	HMDB024	--	Organic cc
--	--	--	--	--	--	--	--	--
C00118	Glycolysis	--	--	--	--	HMDB000	591-57-1	Organic cc
C07764	--	--	--	--	--	HMDB001	554-57-4	Organic cc
C00758	Biosynthes	--	--	--	--	HMDB001	486-60-2	Organic cc
--	--	--	--	--	--	HMDB024	--	Organic cc
--	--	--	--	--	--	--	--	--
C00506	--	--	--	--	--	HMDB000	498-40-8	Organic cc
C00104	--	--	--	--	--	HMDB000	1986/4/4	Organic cc
C21556	--	--	--	--	--	HMDB025	--	Organic cc
--	--	--	--	--	--	HMDB026	--	Organic cc
--	--	--	--	--	--	HMDB025	--	Organic cc
--	--	--	--	--	--	HMDB011	--	Organic cc
--	--	--	--	--	--	HMDB003	140447-21	Organic cc
C05458	--	--	--	--	--	HMDB000	--	Organic cc
--	--	--	--	--	--	HMDB024	--	Organic cc
--	--	--	--	--	--	HMDB030	--	Organic cc
--	--	--	--	--	--	HMDB025	--	Organic cc
--	--	--	--	--	--	HMDB029	--	_
--	--	--	--	--	--	HMDB003	201045-20	Organic cc
C00813	Pyrimidine	--	--	--	--	HMDB004	67-52-7	Organic cc
--	--	--	--	--	--	HMDB011	--	Organic cc
--	--	--	--	--	--	HMDB024	--	Organic cc
--	--	--	--	--	--	HMDB007	--	Organic cc

C05984	Propanoat	LMFA0105	Fatty Acyls	Fatty Acids	Hydroxy fa	HMDB000	3347-90-8	Organic co
--	--	--	--	--	--	HMDB003	94-26-8	Organic co
--	--	--	--	--	--	HMDB003	--	Organic co
--	--	--	--	--	--	HMDB027	--	_
--	--	--	--	--	--	HMDB029	--	_
--	--	--	--	--	--	HMDB029	--	_
--	--	--	--	--	--	HMDB003	--	Organic co
--	--	--	--	--	--	HMDB029	--	_
C02740	--	--	--	--	--	HMDB002	288-47-1	Organic co
--	--	--	--	--	--	--	--	--
--	--	--	--	--	--	HMDB024	--	Organic co
C09001	--	--	--	--	--	HMDB003	31721-94-	Organic co
--	--	LMGP0201	Glyceroph	Glyceroph	Diacylglyce	HMDB000	--	Organic co
--	--	--	--	--	--	--	--	--
C05682	Phosphona	--	--	--	--	HMDB000	4408-78-0	Organic co
--	--	--	--	--	--	HMDB024	--	Organic co
--	--	LMGP0605	Glyceroph	Glyceroph	Monoacylg	HMDB024	1425501-1	Organic co
--	--	--	--	--	--	HMDB026	--	_
C01230	Terpenoid	LMPR0303	Prenol Lipi	Polypreno	Bactopren	HMDB001	207513-95	Organic co
--	--	--	--	--	--	HMDB025	--	Organic co
--	--	--	--	--	--	HMDB000	--	Organic co
--	--	--	--	--	--	--	--	--
--	--	--	--	--	--	--	--	--
--	--	LMGP0205	Glyceroph	Glyceroph	Monoacylg	HMDB001	--	Organic co
--	--	LMGP0605	Glyceroph	Glyceroph	Monoacylg	HMDB006	1425501-1	Organic co
--	--	--	--	--	--	HMDB004	102841-46	Organic co
--	--	--	--	--	--	HMDB024	--	Organic co
--	--	--	--	--	--	HMDB024	230-46-6	Organic co
--	--	--	--	--	--	HMDB030	1629-60-3	Organic co
--	--	--	--	--	--	--	--	--
--	--	--	--	--	--	HMDB024	--	Organic co
--	--	LMFA0105	Fatty Acyls	Fatty Acids	Hydroxy fa	HMDB000	4374-62-3	Organic co
--	--	--	--	--	--	HMDB024	--	Organic co
--	--	--	--	--	--	--	--	--
--	--	--	--	--	--	HMDB001	34580-14-	Organic co
--	--	--	--	--	--	HMDB024	--	Organic co
C16199	Naphthale	--	--	--	--	HMDB001	--	Organic co
C07056	Adrenergi	--	--	--	--	HMDB001	7683-59-2	Organic co
C00156	Ubiquinon	--	--	--	--	HMDB000	99-96-7	Organic co
--	--	--	--	--	--	HMDB000	27610-27-	Organic co
C01053	--	LMFA0105	Fatty Acyls	Fatty Acids	Hydroxy fa	--	--	--
--	--	--	--	--	--	HMDB006	--	Organic co
--	--	--	--	--	--	HMDB024	180509-18	Organic co
C04778	Porphyrin	--	--	--	--	HMDB000	--	Organic co
C07588	--	--	--	--	--	HMDB000	487-54-7	Organic co
C00398	Tryptopha	--	--	--	--	HMDB000	61-54-1	Organic co
C00078	Glycine, se	--	--	--	--	HMDB000	73-22-3	Organic co
--	--	--	--	--	--	HMDB002	--	Organic co
C12369	Biosynthes	--	--	--	--	--	--	--
--	--	--	--	--	--	HMDB003	132278-37	Organic co
C08103	--	--	--	--	--	HMDB001	56796-20-	Organic co

--	--	--	--	--	--	--	--	--
--	--	--	--	--	--	HMDB025	--	Organic cc
--	--	--	--	--	--	HMDB024	--	Organic cc
--	--	--	--	--	--	HMDB001	--	Organic cc
--	--	--	--	--	--	HMDB003	77201-66-	Organic cc
--	--	--	--	--	--	HMDB003	--	Organic cc
C15571	Chlorocycl	--	--	--	--	HMDB000	120-80-9	Organic cc
--	--	--	--	--	--	HMDB025	--	Organic cc
C05255	--	--	--	--	--	HMDB000	--	Organic cc
--	--	--	--	--	--	HMDB024	--	Organic cc
--	--	--	--	--	--	--	--	--
C05252	--	--	--	--	--	HMDB001	--	Organic cc
--	--	--	--	--	--	HMDB024	--	Organic cc
--	--	--	--	--	--	--	--	--
--	--	--	--	--	--	HMDB024	--	Organic cc
C12599	--	--	--	--	--	HMDB001	143491-57	Organic cc
C06469	--	--	--	--	--	HMDB000	114-04-5	Organic cc
--	--	--	--	--	--	--	--	--
--	--	--	--	--	--	HMDB030	--	Organic cc
--	--	--	--	--	--	--	--	--
--	--	--	--	--	--	HMDB003	--	Organic cc
C05834	Tryptopha	--	--	--	--	HMDB000	3040-34-4	Organic cc
C07480	Caffeine m	--	--	--	--	HMDB000	83-67-0	Organic cc
C05851	Biosynthes	--	--	--	--	HMDB000	91-64-5	Organic cc
--	--	--	--	--	--	--	--	--
--	--	--	--	--	--	HMDB024	--	Organic cc
--	--	--	--	--	--	HMDB025	--	Organic cc
--	--	--	--	--	--	HMDB024	--	Organic cc
--	--	--	--	--	--	HMDB024	--	Organic cc
--	--	--	--	--	--	--	--	--
--	--	--	--	--	--	--	--	--
--	--	--	--	--	--	HMDB004	143833-95	Organic cc
C08407	Glucosinol	--	--	--	--	HMDB030	--	Organic cc
--	--	--	--	--	--	--	--	--
--	--	--	--	--	--	HMDB002	31489-08-	Organic cc
--	--	--	--	--	--	--	--	--
--	--	--	--	--	--	HMDB000	88070-93-	Organic cc
C00788	Tyrosine m	--	--	--	--	HMDB000	51-43-4	Organic cc
C08279	--	LMFA0102	Fatty Acyls	Fatty Acids	Branched f	HMDB000	80-59-1	Organic cc
--	--	--	--	--	--	HMDB024	92-54-6	Organic cc
--	--	--	--	--	--	HMDB024	--	Organic cc
--	--	--	--	--	--	HMDB024	--	Organic cc
--	--	--	--	--	--	--	--	--
--	--	--	--	--	--	HMDB024	71144-39-	Organic cc
--	--	--	--	--	--	--	--	--
--	--	--	--	--	--	HMDB030	--	Organic cc
C02564	--	--	--	--	--	HMDB005	--	Organic cc
C18692	--	--	--	--	--	HMDB025	96182-53-	Organic cc
--	--	--	--	--	--	--	--	--
--	--	--	--	--	--	HMDB004	--	Organic cc
--	--	--	--	--	--	HMDB003	118-41-2	Organic cc

--	--	--	--	--	--	HMDB001	--	Organic cc
--	--	--	--	--	--	--	--	--
--	--	--	--	--	--	HMDB003	--	Organic cc
--	--	--	--	--	--	HMDB024	--	Organic cc
--	--	--	--	--	--	--	--	--
--	--	--	--	--	--	--	--	--
C06193	Purine me	--	--	--	--	HMDB024	117-68-0	Organic cc
C01297	Nicotinate	--	--	--	--	HMDB024	7424-35-3	Organic cc
--	--	--	--	--	--	HMDB025	--	Organic cc
--	--	--	--	--	--	HMDB025	--	Organic cc
--	--	--	--	--	--	HMDB024	6307-44-4	Organic cc
C04666	Histidine n	--	--	--	--	HMDB001	36244-87-	Organic cc
--	--	--	--	--	--	HMDB000	487-94-5	Organic cc
--	--	--	--	--	--	HMDB025	--	Organic cc
--	--	--	--	--	--	HMDB003	--	Organic cc
--	--	--	--	--	--	HMDB003	192184-71	Organic cc
--	--	--	--	--	--	HMDB025	--	Organic cc
--	--	--	--	--	--	HMDB001	35804-66-	Organic cc
--	--	--	--	--	--	--	--	--
--	--	--	--	--	--	HMDB024	--	Organic cc
--	--	--	--	--	--	HMDB003	71385-83-	Organic cc
--	--	--	--	--	--	HMDB000	10275-07-	Organic cc
--	--	--	--	--	--	HMDB002	--	Organic cc
--	--	--	--	--	--	--	--	--
--	--	--	--	--	--	HMDB004	146064-66	Organic cc
--	--	--	--	--	--	HMDB004	25790-35-	Organic cc
--	--	--	--	--	--	--	--	--
--	--	--	--	--	--	HMDB004	--	Organic cc
--	--	--	--	--	--	HMDB025	1000144-2	Organic cc
--	--	--	--	--	--	HMDB025	--	Organic cc
--	--	--	--	--	--	HMDB025	--	Organic cc
--	--	--	--	--	--	HMDB001	104-31-4	Organic cc
--	--	--	--	--	--	HMDB003	24891-71-	Organic cc
C00063	Pyrimidine	--	--	--	--	HMDB000	65-47-4	Organic cc
--	--	--	--	--	--	--	--	--
--	--	--	--	--	--	HMDB006	--	Organic cc
--	--	--	--	--	--	HMDB001	25046-79-	Organic cc
--	--	--	--	--	--	HMDB024	--	Organic cc
C05642	Tryptopha	--	--	--	--	HMDB000	52450-38-	Organic cc
C05560	--	--	--	--	--	HMDB000	--	Organic cc
--	--	LMPK1206	Polyketide	Flavonoids	Rotenoid f	HMDB003	66322-32-	Organic cc
C05945	--	--	--	--	--	HMDB002	1189-11-3	Organic cc
--	--	--	--	--	--	--	--	--
--	--	--	--	--	--	HMDB003	22694-96-	Organic cc
--	--	--	--	--	--	HMDB003	613-88-7	Organic cc
C16716	Biosynthes	--	--	--	--	HMDB024	86-95-3	Organic cc
--	--	--	--	--	--	HMDB025	--	Organic cc
--	--	--	--	--	--	--	--	--
C00206	Purine me	--	--	--	--	HMDB000	2793/6/8	Organic cc
--	--	--	--	--	--	--	--	--
--	--	--	--	--	--	--	--	--

--	--	--	--	--	--	--	--	--
--	--	--	--	--	--	--	--	--
--	--	--	--	--	--	HMDB003	10089-09-	Organic cc
--	--	--	--	--	--	--	--	--
--	--	--	--	--	--	HMDB003	105330-57	Organic cc
--	--	--	--	--	--	HMDB003	--	Organic cc
--	--	--	--	--	--	HMDB026	--	Organic cc
--	--	--	--	--	--	--	--	--
--	--	--	--	--	--	--	--	--
--	--	--	--	--	--	HMDB025	--	Organic cc
--	--	--	--	--	--	HMDB002	86992-94-	Organic cc
C04640	Purine me	--	--	--	--	HMDB000	6157-85-3	Organic cc
--	--	--	--	--	--	HMDB025	--	Organic cc
--	--	--	--	--	--	HMDB004	68340-35-	Organic cc
--	--	--	--	--	--	HMDB006	--	Organic cc
--	--	--	--	--	--	--	--	--
--	--	--	--	--	--	HMDB025	--	Organic cc
--	--	--	--	--	--	--	--	--
--	--	--	--	--	--	--	--	--
--	--	--	--	--	--	HMDB003	139955-87	Organic cc
--	--	--	--	--	--	HMDB000	4429/4/3	Organic cc
--	--	--	--	--	--	--	--	--
--	--	--	--	--	--	HMDB030	--	Organic cc
C00633	Bisphenol	--	--	--	--	HMDB001	123-08-0	Organic cc
--	--	--	--	--	--	HMDB024	--	Organic cc
C00803	--	LMFA0101	Fatty Acyls	Fatty Acids	Straight ch	HMDB000	109-52-4	Organic cc
C03087	Lysine deg	LMFA0103	Fatty Acyls	Fatty Acids	Unsaturate	HMDB001	--	Organic cc
--	--	--	--	--	--	HMDB006	--	Organic cc
--	--	--	--	--	--	--	--	--
C16614	Drug meta	--	--	--	--	HMDB006	50-66-8	Organic cc
C05598	Phenylalar	--	--	--	--	HMDB000	500-98-1	Organic cc
C01233	--	--	--	--	--	HMDB000	1190-00-7	Organic cc
--	--	--	--	--	--	HMDB000	96253-55-	Organic cc
--	--	--	--	--	--	--	--	--
--	--	--	--	--	--	HMDB025	--	Organic cc
--	--	--	--	--	--	HMDB024	--	Organic cc
--	--	--	--	--	--	HMDB003	142562-01	Organic cc
--	--	--	--	--	--	--	--	--
--	--	--	--	--	--	--	--	--
--	--	--	--	--	--	HMDB004	129932-48	Organic cc
C16578	Drug meta	--	--	--	--	HMDB004	20290-10-	Organic cc
--	--	--	--	--	--	HMDB027	--	_
--	--	--	--	--	--	HMDB005	--	Organic cc
C01092	--	LMFA0110	Fatty Acyls	Fatty Acids	Amino fatt	HMDB024	682799-71	Organic cc
--	--	--	--	--	--	HMDB025	28217-22-	Organic cc
--	--	--	--	--	--	HMDB025	--	Organic cc
--	--	--	--	--	--	HMDB024	--	Organic cc
--	--	--	--	--	--	HMDB025	--	Organic cc
--	--	--	--	--	--	--	--	--
C10575	--	--	--	--	--	HMDB030	--	Organic cc
--	--	--	--	--	--	HMDB004	76873-99-	Organic cc

--	--	--	--	--	--	HMDB003	97-99-4	Organic cc
--	--	--	--	--	--	--	--	--
--	--	--	--	--	--	--	--	--
--	--	--	--	--	--	HMDB002	14002-93-	Organic cc
--	--	--	--	--	--	--	--	--
--	--	--	--	--	--	--	--	--
--	--	--	--	--	--	--	--	--
--	--	--	--	--	--	HMDB025	--	Organic cc
--	--	--	--	--	--	--	--	--
--	--	--	--	--	--	HMDB003	74158-05-	Organic cc
--	--	--	--	--	--	HMDB030	--	Organic cc
--	--	--	--	--	--	HMDB004	152041-22	Organic cc
C05828	Histidine n	--	--	--	--	HMDB000	2625-49-2	Organic cc
--	--	--	--	--	--	HMDB002	13588-94-	Organic cc
--	--	--	--	--	--	HMDB003	22326-31-	Organic cc
C07576	Indole alka	--	--	--	--	HMDB025	520-52-5	Organic cc
--	--	--	--	--	--	--	--	--
--	--	--	--	--	--	--	--	--
--	--	--	--	--	--	--	--	--
C21540	--	--	--	--	--	HMDB004	82768-85-	Organic cc
--	--	--	--	--	--	--	--	--
--	--	--	--	--	--	HMDB009	--	Organic cc
--	--	--	--	--	--	HMDB006	--	Organic cc
--	--	--	--	--	--	HMDB025	--	_
C02323	--	--	--	--	--	HMDB005	1990/1/7	Organic cc
--	--	--	--	--	--	HMDB005	614-18-6	Organic cc
C17925	--	--	--	--	--	HMDB000	6539-57-7	Organic cc
C00822	Tyrosine m	--	--	--	--	HMDB000	4430-97-1	Organic cc
--	--	--	--	--	--	HMDB001	99910-55-	Organic cc
C08278	--	LMFA0117	Fatty Acyls	Fatty Acids	Dicarboxyl	HMDB000	505-48-6	Organic cc
--	--	--	--	--	--	HMDB002	32590-17-	Organic cc
--	--	--	--	--	--	--	--	--
C05627	Phenylpro	--	--	--	--	HMDB000	2628-17-3	Organic cc
C06757	Xylene deg	--	--	--	--	HMDB004	589-18-4	Organic cc
C11457	Phenylalar	--	--	--	--	HMDB000	621-54-5	Organic cc
C12448	Tropane, p	--	--	--	--	HMDB000	7143/9/1	Organic cc
--	--	--	--	--	--	--	--	--
--	--	--	--	--	--	HMDB024	--	Organic cc
--	--	--	--	--	--	--	--	--
--	--	--	--	--	--	--	--	--
--	--	--	--	--	--	--	--	--
--	--	--	--	--	--	HMDB024	--	Organic cc
--	--	--	--	--	--	HMDB009	--	Organic cc
--	--	--	--	--	--	--	--	--
--	--	--	--	--	--	HMDB030	--	Organic cc
--	--	--	--	--	--	--	--	--
--	--	--	--	--	--	HMDB024	--	Organic cc
C16560	Drug meta	--	--	--	--	HMDB006	--	Organic cc
--	--	--	--	--	--	HMDB024	--	Organic cc
--	--	--	--	--	--	--	--	--
--	--	--	--	--	--	HMDB024	16188-61-	Organic cc

--	--	--	--	--	--	HMDB025	--	Organic cc
C07826	--	--	--	--	--	HMDB001	1977/2/1	Organic cc
C05830	Tryptopha	--	--	--	--	HMDB006	--	Organic cc
--	--	--	--	--	--	HMDB024	--	Organic cc
C05634	Tryptopha	--	--	--	--	HMDB000	1892-21-3	Organic cc
--	--	--	--	--	--	HMDB025	--	Organic cc
--	--	--	--	--	--	HMDB025	--	Organic cc
--	--	--	--	--	--	HMDB003	75911-14-	Organic cc
--	--	--	--	--	--	HMDB002	76859-62-	Organic cc
C11990	Biosynthes	LMPK0400	Polyketide	Macrolides	-	--	--	--
--	--	--	--	--	--	HMDB025	--	Organic cc
--	--	--	--	--	--	HMDB005	--	Organic cc
C05925	Folate bios	--	--	--	--	HMDB000	19622-42-	Organic cc
C07513	--	--	--	--	--	HMDB001	390-28-3	Organic cc
--	--	--	--	--	--	--	--	--
C01770	--	LMFA0704	Fatty Acyls	Fatty ester	Lactones [	HMDB000	96-48-0	Organic cc
C02043	--	--	--	--	--	--	--	--
C02470	Tryptopha	--	--	--	--	HMDB000	59-00-7	Organic cc
--	--	--	--	--	--	HMDB030	--	Organic cc
--	--	--	--	--	--	--	--	--
--	--	--	--	--	--	--	--	--
--	--	--	--	--	--	HMDB003	220345-69	Organic cc
--	--	--	--	--	--	--	--	--
--	--	--	--	--	--	--	--	--
--	--	--	--	--	--	HMDB024	--	Organic cc
--	--	--	--	--	--	HMDB006	20980-22-	Organic cc
C05638	Tryptopha	--	--	--	--	HMDB000	708-23-6	Organic cc
--	--	--	--	--	--	HMDB024	--	Organic cc
--	--	--	--	--	--	HMDB003	181485-20	Organic cc
--	--	--	--	--	--	--	--	--
--	--	--	--	--	--	--	--	--
--	--	--	--	--	--	HMDB025	--	Organic cc
--	--	--	--	--	--	HMDB009	--	Organic cc
--	--	--	--	--	--	HMDB003	142465-56	Organic cc
--	--	--	--	--	--	HMDB025	--	Organic cc
--	--	--	--	--	--	--	--	--
C05647	Tryptopha	--	--	--	--	HMDB001	958733-17	Organic cc
--	--	--	--	--	--	HMDB003	4798-44-1	Organic cc
--	--	LMFA0500	Fatty Acyls	Fatty alcoh	-	HMDB003	114020-45	Organic cc
--	--	--	--	--	--	--	--	--
--	--	--	--	--	--	HMDB006	131351-90	Organic cc
C04543	--	--	--	--	--	HMDB024	--	Organic cc
--	--	--	--	--	--	HMDB003	--	Organic cc
--	--	--	--	--	--	HMDB025	--	Organic cc
--	--	--	--	--	--	HMDB003	122577-99	Organic cc
--	--	--	--	--	--	HMDB011	--	Organic cc
--	--	--	--	--	--	HMDB004	163365-14	Organic cc
--	--	--	--	--	--	HMDB016	1456546-8	Organic cc
--	--	--	--	--	--	HMDB004	120015-17	Organic cc
--	--	--	--	--	--	HMDB003	31704-80-	Organic cc
--	--	--	--	--	--	HMDB030	--	Organic cc

--	--	--	--	--	--	HMDB003	--	Organic cc
--	--	--	--	--	--	HMDB001	1524-88-5	Organic cc
--	--	--	--	--	--	HMDB025	--	Organic cc
--	--	--	--	--	--	--	--	--
--	--	--	--	--	--	HMDB003	138-53-4	Organic cc
C00705	Pyrimidine	--	--	--	--	HMDB000	800-73-7	Organic cc
--	--	--	--	--	--	HMDB003	19013-07-	Organic cc
--	--	--	--	--	--	HMDB000	152306-59	Organic cc
--	--	--	--	--	--	HMDB003	--	Organic cc
--	--	--	--	--	--	HMDB025	--	Organic cc
--	--	--	--	--	--	HMDB001	16534-24-	Organic cc
--	--	--	--	--	--	--	--	--
--	--	--	--	--	--	HMDB000	13039-82-	Organic cc
--	--	--	--	--	--	--	--	--
--	--	--	--	--	--	--	--	--
--	--	--	--	--	--	--	--	--
C12650	Drug meta	--	--	--	--	HMDB001	154361-50	Organic cc
--	--	--	--	--	--	HMDB025	--	Organic cc
--	--	--	--	--	--	HMDB025	--	Organic cc
--	--	--	--	--	--	HMDB025	--	Organic cc
--	--	--	--	--	--	HMDB004	--	Organic cc
--	--	--	--	--	--	HMDB024	--	Organic cc
--	--	--	--	--	--	HMDB003	27539-40-	Organic cc
--	--	--	--	--	--	HMDB004	--	Organic cc
--	--	--	--	--	--	HMDB002	10599-70-	Organic cc
--	--	--	--	--	--	HMDB004	--	Organic cc
C07424	--	--	--	--	--	HMDB001	6493/5/6	Organic cc
--	--	--	--	--	--	HMDB025	--	Organic cc
C00835	Folate bios	--	--	--	--	HMDB000	17094-01-	Organic cc
--	--	--	--	--	--	HMDB024	--	Organic cc
--	--	--	--	--	--	HMDB003	21148-31-	Organic cc
--	--	--	--	--	--	HMDB003	87562-72-	Organic cc
--	--	--	--	--	--	HMDB001	6960-22-1	Organic cc
--	--	--	--	--	--	HMDB024	--	Organic cc
--	--	LMFA0707	Fatty Acyls	Fatty ester	Fatty acyl	HMDB024	--	Organic cc
--	--	LMFA0500	Fatty Acyls	Fatty alcoh	-	HMDB003	50306-18-	Organic cc
--	--	--	--	--	--	HMDB003	1122-20-9	Organic cc
C08261	--	LMFA0117	Fatty Acyls	Fatty Acids	Dicarboxyl	HMDB000	123-99-9	Organic cc
--	--	--	--	--	--	--	--	--
--	--	--	--	--	--	HMDB024	--	Organic cc
C05640	Tryptopha	--	--	--	--	HMDB000	--	Organic cc
--	--	--	--	--	--	HMDB025	--	Organic cc
--	--	--	--	--	--	HMDB025	--	Organic cc
--	--	--	--	--	--	--	--	--
--	--	--	--	--	--	--	--	--
--	--	--	--	--	--	HMDB025	--	Organic cc
C04441	--	--	--	--	--	HMDB001	--	Organic cc
--	--	LMFA0115	Fatty Acyls	Fatty Acids	Heterocycl	HMDB011	116627-39	Organic cc
--	--	--	--	--	--	HMDB003	217960-73	Organic cc
--	--	--	--	--	--	HMDB004	83697-43-	Organic cc
C04353	--	--	--	--	--	HMDB030	--	Organic cc

--	--	--	--	--	--	HMDB030	--	Organic cc
C13637	Bisphenol	--	--	--	--	HMDB002	123-07-9	Organic cc
--	--	--	--	--	--	HMDB024	--	Organic cc
--	--	--	--	--	--	HMDB006	--	_
--	--	--	--	--	--	--	--	--
C05469	Steroid ho	LMST0203	Sterol Lipi	Steroids [S	C21 steroi	HMDB000	68-54-2	Organic cc
--	--	--	--	--	--	HMDB003	40246-08-	Organic cc
C00094	Cysteine a	--	--	--	--	HMDB000	14265-45-	Inorganic
C02355	Pyrimidine	--	--	--	--	HMDB001	40632-52-	Organic cc
--	--	--	--	--	--	HMDB001	638-94-8	Organic cc
--	--	--	--	--	--	HMDB025	--	Organic cc
--	--	--	--	--	--	HMDB025	--	Organic cc
--	--	--	--	--	--	HMDB024	--	Organic cc
--	--	--	--	--	--	HMDB003	141897-12	Organic cc
--	--	--	--	--	--	--	--	--
--	--	--	--	--	--	--	--	--
--	--	--	--	--	--	--	--	--
C05635	Tryptopha	--	--	--	--	HMDB000	54-16-0	Organic cc
C20470	--	--	--	--	--	HMDB030	--	Organic cc
--	--	--	--	--	--	HMDB003	554-44-9	Organic cc
--	--	--	--	--	--	HMDB024	2188234-7	Organic cc
--	--	--	--	--	--	HMDB006	7314-32-1	Organic cc
--	--	--	--	--	--	HMDB003	--	Organic cc
--	--	--	--	--	--	HMDB004	22141-40-	Organic cc
--	--	--	--	--	--	HMDB025	--	Organic cc
--	--	--	--	--	--	HMDB025	--	Organic cc
--	--	--	--	--	--	HMDB030	--	_
--	--	--	--	--	--	--	--	--
C12248	--	--	--	--	--	HMDB005	1320343-6	Organic cc
--	--	--	--	--	--	--	--	--
--	--	--	--	--	--	--	--	--
--	--	--	--	--	--	--	--	--
--	--	--	--	--	--	--	--	--
--	--	LMFA0302	Fatty Acyls	Eicosanoic	Leukotrien	HMDB001	--	Organic cc
--	--	--	--	--	--	--	--	--
--	--	LMFA0202	Fatty Acyls	Octadecar	Jasmonic a	HMDB030	--	Organic cc
--	--	--	--	--	--	HMDB003	508-76-9	Organic cc
--	--	--	--	--	--	HMDB004	31681-28-	Organic cc
C02378	Caprolacta	LMFA011C	Fatty Acyls	Fatty Acids	Amino fatt	HMDB000	60-32-2	Organic cc
--	--	--	--	--	--	HMDB030	--	Organic cc
--	--	--	--	--	--	HMDB024	2177304-6	Organic cc
--	--	--	--	--	--	HMDB025	--	Organic cc
--	--	--	--	--	--	HMDB003	128534-67	Organic cc
--	--	--	--	--	--	HMDB030	--	Organic cc
--	--	--	--	--	--	--	--	--
C05950	Arachidon	LMFA0302	Fatty Acyls	Eicosanoic	Leukotrien	HMDB000	80434-82-	Organic cc
C02989	Cysteine a	--	--	--	--	HMDB000	3226-65-1	Organic cc
--	--	--	--	--	--	HMDB024	--	Organic cc
--	--	--	--	--	--	HMDB030	--	Organic cc
--	--	--	--	--	--	HMDB002	76996-27-	Organic cc
--	--	--	--	--	--	HMDB030	--	Organic cc

--	--	--	--	--	--	HMDB001	56162-46-	Organic cc
--	--	--	--	--	--	--	--	--
--	--	LMPK1211	Polyketide	Flavonoids	Flavones a	HMDB003	479-91-4	Organic cc
C16308	alpha-Linc	LMFA0117	Fatty Acyls	Fatty Acids	Dicarboxyl	HMDB000	6402-36-4	Organic cc
C13708	--	--	--	--	--	HMDB024	--	Organic cc
--	--	--	--	--	--	HMDB003	38253-76-	Organic cc
--	--	--	--	--	--	HMDB003	214150-43	Organic cc
C00198	Pentose pl	--	--	--	--	HMDB000	90-80-2	Organic cc
--	--	--	--	--	--	HMDB004	--	Organic cc
--	--	--	--	--	--	HMDB003	--	Organic cc
--	--	--	--	--	--	HMDB024	587-90-6	Organic cc
C12270	--	--	--	--	--	HMDB025	--	Organic cc
--	--	--	--	--	--	--	--	--
--	--	--	--	--	--	--	--	--
--	--	--	--	--	--	HMDB025	--	Organic cc
--	--	--	--	--	--	--	--	--
--	--	--	--	--	--	HMDB002	31189-73-	Organic cc
--	--	--	--	--	--	--	--	--
C18774	--	--	--	--	--	HMDB024	12771-68-	Organic cc
C04692	--	--	--	--	--	HMDB001	--	Organic cc
--	--	--	--	--	--	HMDB001	90965-82-	Organic cc
--	--	--	--	--	--	HMDB024	--	Organic cc
--	--	--	--	--	--	HMDB025	--	Organic cc
--	--	LMGP1005	Glyceroph	Glyceroph	Monoacylg	HMDB006	--	Organic cc
--	--	--	--	--	--	--	--	--
--	--	--	--	--	--	--	--	--
C05643	--	LMST0501	Sterol Lipi	Steroid co	Glucuronic	HMDB001	--	Organic cc
--	--	--	--	--	--	HMDB025	--	Organic cc
--	--	--	--	--	--	HMDB024	--	Organic cc
--	--	LMFA1301	Fatty Acyls	Fatty acyl g	Fatty acyl g	HMDB004	--	Organic cc
C01598	Tryptopha	--	--	--	--	HMDB000	73-31-4	Organic cc
--	--	--	--	--	--	HMDB025	--	Organic cc
--	--	--	--	--	--	HMDB024	--	Organic cc
--	--	--	--	--	--	HMDB017	1823179-0	Organic cc
C08277	--	LMFA0117	Fatty Acyls	Fatty Acids	Dicarboxyl	HMDB000	111-20-6	Organic cc
--	--	--	--	--	--	HMDB025	--	Organic cc
--	--	--	--	--	--	HMDB025	--	Organic cc
--	--	--	--	--	--	HMDB027	--	_
--	--	--	--	--	--	--	--	--
--	--	--	--	--	--	HMDB024	--	Organic cc
--	--	--	--	--	--	HMDB003	371113-06	Organic cc
--	--	--	--	--	--	--	--	--
--	--	LMFA0117	Fatty Acyls	Fatty Acids	Dicarboxyl	HMDB000	--	--
--	--	--	--	--	--	HMDB005	--	Organic cc
--	--	--	--	--	--	HMDB025	--	Organic cc
--	--	--	--	--	--	HMDB025	--	Organic cc
--	--	--	--	--	--	HMDB000	503-48-0	Organic cc
--	--	--	--	--	--	HMDB025	--	Organic cc
C17413	--	--	--	--	--	HMDB024	24502-78-	Organic cc
--	--	--	--	--	--	--	--	--
--	--	--	--	--	--	HMDB025	--	Organic cc

--	--	--	--	--	--	HMDB025	--	Organic cc
--	--	--	--	--	--	HMDB030	--	Organic cc
--	--	--	--	--	--	HMDB025	--	Organic cc
--	--	--	--	--	--	HMDB001	20605-81-	Organic cc
--	--	--	--	--	--	HMDB006	--	Organic cc
--	--	--	--	--	--	--	--	--
C02222	Chlorocycl	--	--	--	--	HMDB006	--	Organic cc
--	--	--	--	--	--	HMDB003	3194-17-(	Organic cc
C00257	Pentose pl	--	--	--	--	HMDB000	526-95-4	Organic cc
--	--	--	--	--	--	HMDB025	--	Organic cc
C07016	--	--	--	--	--	HMDB001	98048-97-	Organic cc
--	--	--	--	--	--	HMDB003	--	Organic cc
--	--	--	--	--	--	HMDB030	--	Organic cc
--	--	--	--	--	--	--	--	--
--	--	--	--	--	--	--	--	--
--	--	--	--	--	--	--	--	--
--	--	--	--	--	--	HMDB003	539-30-0	Organic cc
--	--	--	--	--	--	HMDB006	--	Organic cc
--	--	--	--	--	--	HMDB003	188305-07	Organic cc
--	--	--	--	--	--	HMDB003	189351-14	Organic cc
--	--	--	--	--	--	HMDB025	--	Organic cc
--	--	--	--	--	--	HMDB003	34114-98-	Organic cc
C08380	--	LMFA1200	Fatty Acyls	Oxygenate	-	HMDB000	110-43-0	Organic cc
C05843	Nicotinate	--	--	--	--	HMDB000	769-49-3	Organic cc
D06890	--	--	--	--	--	HMDB000	61-78-9	Organic cc
--	--	--	--	--	--	--	--	--
--	--	--	--	--	--	HMDB025	--	Organic cc
--	--	--	--	--	--	HMDB024	--	Organic cc
--	--	--	--	--	--	HMDB024	--	Organic cc
--	--	--	--	--	--	--	--	--
--	--	--	--	--	--	HMDB003	62394-04-	Organic cc
--	--	--	--	--	--	HMDB003	65405-76-	Organic cc
--	--	--	--	--	--	HMDB024	--	Organic cc
--	--	--	--	--	--	HMDB024	632-93-9	Organic cc
--	--	--	--	--	--	HMDB003	--	Organic cc
--	--	--	--	--	--	HMDB025	--	Organic cc
--	--	--	--	--	--	HMDB001	1469900-9	Organic cc
--	--	--	--	--	--	HMDB025	--	Organic cc
--	--	--	--	--	--	HMDB009	--	Organic cc
C05422	Ascorbate	--	--	--	--	HMDB000	490-83-5	Organic cc
--	--	LMPK1212	Polyketide	Flavonoids	Chalcones	HMDB004	143502-0(	Organic cc
--	--	--	--	--	--	HMDB024	--	Organic cc
--	--	--	--	--	--	HMDB003	73112-73-	Organic cc
--	--	--	--	--	--	HMDB003	204927-92	Organic cc
--	--	--	--	--	--	--	--	--
--	--	--	--	--	--	HMDB025	--	Organic cc
--	--	--	--	--	--	HMDB003	62512-26-	Organic cc
C11770	--	--	--	--	--	HMDB000	18559-94-	Organic cc
--	--	--	--	--	--	HMDB025	--	Organic cc
--	--	--	--	--	--	HMDB003	--	Organic cc
--	--	--	--	--	--	HMDB024	--	Organic cc

--	--	--	--	--	--	--	--	--
--	--	--	--	--	--	--	--	--
--	--	--	--	--	--	HMDB026	--	Organic cc
--	--	--	--	--	--	--	--	--
--	--	--	--	--	--	HMDB024	--	Organic cc
C04230	--	LMGP0105	Glyceroph	Glyceroph	Monoacylg	HMDB001	--	Organic cc
--	--	--	--	--	--	HMDB003	136997-67	Organic cc
--	--	--	--	--	--	--	--	--
--	--	--	--	--	--	HMDB025	--	Organic cc
--	--	--	--	--	--	HMDB025	--	Organic cc
C17702	Neomycin	--	--	--	--	--	--	--
C00885	Ubiquinon	--	--	--	--	HMDB030	--	Organic cc
--	--	--	--	--	--	HMDB025	--	Organic cc
--	--	LMFA0117	Fatty Acyls	Fatty Acids	Dicarboxyl	HMDB000	1852-04-6	Organic cc
--	--	--	--	--	--	HMDB003	75911-12-	Organic cc
C05964	--	LMFA0303	Fatty Acyls	Eicosanoic	Thrombox	HMDB000	67910-12-	Organic cc
--	--	--	--	--	--	HMDB003	490-91-5	Organic cc
--	--	--	--	--	--	HMDB024	--	Organic cc
--	--	--	--	--	--	HMDB024	--	Organic cc
--	--	--	--	--	--	HMDB030	--	Organic cc
--	--	--	--	--	--	HMDB030	--	Organic cc
--	--	--	--	--	--	HMDB003	143305-08	Organic cc
--	--	--	--	--	--	HMDB003	292167-43	Organic cc
--	--	--	--	--	--	HMDB027	--	_
--	--	--	--	--	--	HMDB024	--	Organic cc
--	--	--	--	--	--	HMDB006	--	Organic cc
--	--	--	--	--	--	HMDB004	80489-86-	Organic cc
--	--	--	--	--	--	HMDB012	--	Organic cc
--	--	--	--	--	--	--	--	--
--	--	--	--	--	--	--	--	--
--	--	--	--	--	--	--	--	--
--	--	--	--	--	--	HMDB004	164178-28	Organic cc
C13252	Drug meta	--	--	--	--	HMDB024	--	Organic cc
--	--	--	--	--	--	HMDB024	--	Organic cc
--	--	--	--	--	--	HMDB024	--	Organic cc
--	--	--	--	--	--	HMDB004	176520-66	Organic cc
C06694	--	LMPR0106	Prenol Lipi	Isoprenoid	C30 isopre	HMDB001	6990/6/3	Organic cc
--	--	LMFA0302	Fatty Acyls	Eicosanoid	Leukotrien	HMDB001	--	Organic cc
--	--	--	--	--	--	HMDB024	--	Organic cc
C18515	--	--	--	--	--	HMDB025	--	Organic cc
--	--	--	--	--	--	HMDB024	--	Organic cc
C13856	Neuroactiv	LMGL0101	Glycerolipi	Monorady	Monoacylg	HMDB000	53847-30-	Organic cc
C14750	--	--	--	--	--	HMDB004	36455-72-	Organic cc
--	--	--	--	--	--	HMDB004	177261-75	Organic cc
--	--	--	--	--	--	--	--	--
--	--	--	--	--	--	HMDB024	946-80-5	Organic cc
--	--	--	--	--	--	HMDB025	--	Organic cc
--	--	--	--	--	--	HMDB024	--	_
--	--	--	--	--	--	HMDB024	--	_
--	--	--	--	--	--	HMDB003	70185-65-	Organic cc
--	--	--	--	--	--	--	--	--

C05956	Arachidon	LMFA0301	Fatty Acyls	Eicosanoic	Prostaglan	HMDB000	51982-36-	Organic cc
--	--	--	--	--	--	HMDB025	--	Organic cc
--	--	--	--	--	--	--	--	--
--	--	--	--	--	--	--	--	--
--	--	--	--	--	--	HMDB025	3943-89-3	Organic cc
--	--	--	--	--	--	--	--	--
--	--	LMFA0115	Fatty Acyls	Fatty Acids	Heterocycl	HMDB006	--	Organic cc
--	--	--	--	--	--	HMDB024	50-24-8	Organic cc
C11758	--	--	--	--	--	HMDB001	12650-69-	Organic cc
--	--	LMFA0106	Fatty Acyls	Fatty Acids	Oxo fatty a	HMDB001	--	Organic cc
C14833	Linoleic ac	LMFA0200	Fatty Acyls	Octadecar	Other Octa	HMDB000	--	Organic cc
--	--	--	--	--	--	HMDB002	79-78-7	Organic cc
--	--	LMFA0301	Fatty Acyls	Eicosanoic	Prostaglan	--	--	--
--	--	--	--	--	--	HMDB028	--	_
--	--	--	--	--	--	HMDB001	--	Organic cc
--	--	LMFA0302	Fatty Acyls	Eicosanoic	Leukotrien	--	--	--
--	--	--	--	--	--	HMDB003	96817-09-	Organic cc
--	--	--	--	--	--	HMDB024	--	Organic cc
--	--	--	--	--	--	--	--	--
C08285	Cutin, sub	LMFA0105	Fatty Acyls	Fatty Acids	Hydroxy fa	HMDB003	69232-67-	Organic cc
--	--	--	--	--	--	HMDB003	--	Organic cc
--	--	--	--	--	--	--	--	--
--	--	--	--	--	--	HMDB029	--	_
--	--	--	--	--	--	HMDB003	155836-26	Organic cc
C04785	alpha-Linc	LMFA0200	Fatty Acyls	Octadecar	Other Octa	HMDB030	67597-26-	Organic cc
--	--	--	--	--	--	--	--	--
--	--	--	--	--	--	HMDB001	1215088-6	Organic cc
--	--	--	--	--	--	HMDB001	--	Organic cc
C00825	Neomycin	--	--	--	--	--	--	--
--	--	--	--	--	--	HMDB025	--	Organic cc
--	--	LMST0501	Sterol Lipi	Steroid co	Glucuronic	HMDB000	76060-22-	Organic cc
--	--	--	--	--	--	HMDB024	103674-15	Organic cc
--	--	--	--	--	--	HMDB024	--	_
--	--	--	--	--	--	HMDB004	152845-77	Organic cc
--	--	--	--	--	--	--	--	--
--	--	--	--	--	--	--	--	--
C17495	--	--	--	--	--	HMDB003	23513-08-	Organic cc
--	--	--	--	--	--	HMDB005	3971-33-3	Organic cc
--	--	--	--	--	--	HMDB024	--	Organic cc
--	--	--	--	--	--	HMDB024	--	_
C03642	Bile secreti	LMST0502	Sterol Lipi	Steroid co	Sulfates [S	HMDB000	15324-65-	Organic cc
--	--	LMST0502	Sterol Lipi	Steroid co	Sulfates [S	HMDB000	66874-12-	Organic cc
--	--	--	--	--	--	HMDB028	--	_
--	--	--	--	--	--	HMDB027	--	_
--	--	--	--	--	--	--	--	--
--	--	--	--	--	--	HMDB028	--	_
--	--	--	--	--	--	HMDB025	--	Organic cc
--	--	--	--	--	--	HMDB025	--	Organic cc
--	--	--	--	--	--	HMDB024	--	Organic cc
--	--	--	--	--	--	--	--	--
--	--	--	--	--	--	HMDB027	--	_

--	--	--	--	--	--	HMDB028	--	_
--	--	--	--	--	--	HMDB025	--	Organic co
--	--	--	--	--	--	HMDB001	37552-33-	Organic co
--	--	--	--	--	--	HMDB024	32449-92-	Organic co
--	--	LMFA0301	Fatty Acyls	Eicosanoic	Prostaglan	HMDB000	74872-89-	Organic co
--	--	--	--	--	--	HMDB026	--	_
C01925	--	--	--	--	--	HMDB003	31282-04-	Organic co
--	--	--	--	--	--	HMDB025	--	Organic co
--	--	--	--	--	--	--	--	--
--	--	--	--	--	--	HMDB024	--	Organic co
--	--	--	--	--	--	HMDB003	19322-27-	Organic co
--	--	--	--	--	--	HMDB001	81732-46-	Organic co
--	--	--	--	--	--	HMDB006	--	Organic co
--	--	--	--	--	--	HMDB025	--	Organic co
--	--	--	--	--	--	HMDB024	--	Organic co
--	--	--	--	--	--	--	--	--
--	--	--	--	--	--	HMDB003	154843-7	Organic co
--	--	--	--	--	--	HMDB006	--	Organic co
--	--	--	--	--	--	HMDB029	--	_
--	--	--	--	--	--	HMDB006	--	Organic co
--	--	--	--	--	--	--	--	--
--	--	--	--	--	--	HMDB003	219546-8	Organic co
--	--	--	--	--	--	--	--	--
--	--	--	--	--	--	HMDB025	--	Organic co
--	--	--	--	--	--	HMDB004	53833-25-	Organic co
--	--	--	--	--	--	HMDB003	185154-9	Organic co
C05465	--	--	--	--	--	HMDB000	1953/2/1	Organic co
--	--	--	--	--	--	HMDB030	--	Organic co
--	--	--	--	--	--	--	--	--
--	--	--	--	--	--	HMDB024	--	Organic co
C16660	Drug meta	--	--	--	--	HMDB006	--	Organic co
--	--	--	--	--	--	HMDB001	--	Organic co
--	--	--	--	--	--	HMDB003	--	Organic co
--	--	--	--	--	--	HMDB027	--	_
--	--	--	--	--	--	HMDB004	13229-59-	Organic co
--	--	--	--	--	--	HMDB003	267649-9	Organic co
--	--	--	--	--	--	HMDB004	65383-71-	Organic co
C02041	Biosynthes	LMFA0705	Fatty Acyls	Fatty ester	Fatty acyl (	HMDB000	15895-27-	Organic co
--	--	LMGP1005	Glyceroph	Glyceroph	Monoacylg	HMDB006	--	Organic co
--	--	--	--	--	--	--	--	--
--	--	--	--	--	--	HMDB024	--	Organic co
--	--	LMFA0105	Fatty Acyls	Fatty Acids	Hydroxy fa	HMDB003	1422-27-1	Organic co
--	--	--	--	--	--	HMDB024	--	Organic co
--	--	--	--	--	--	HMDB003	78-37-5	Organic co
--	--	--	--	--	--	HMDB024	--	Organic co
--	--	--	--	--	--	HMDB000	14605-22-	Organic co
--	--	--	--	--	--	HMDB024	--	Organic co
--	--	--	--	--	--	HMDB027	--	_
--	--	--	--	--	--	HMDB003	2308-18-1	Organic co
C07202	--	--	--	--	--	HMDB000	37350-58-	Organic co
--	--	--	--	--	--	HMDB003	119752-7	Organic co

C16532	Biosynthes	LMFA0705	Fatty Acyls	Fatty ester	Fatty acyl (	HMDB006	--	Organic cc
C03033	--	--	--	--	--	HMDB001	1852-49-9	Organic cc
--	--	LMFA0117	Fatty Acyls	Fatty Acids	Dicarboxyl	HMDB000	505-52-2	Organic cc
--	--	--	--	--	--	HMDB006	--	Organic cc
--	--	--	--	--	--	HMDB003	196607-74	Organic cc
--	--	--	--	--	--	HMDB003	83720-10-	Organic cc
--	--	--	--	--	--	HMDB003	--	Organic cc
--	--	--	--	--	--	HMDB025	--	Organic cc
--	--	--	--	--	--	HMDB003	14777-17-	Organic cc
--	--	--	--	--	--	HMDB024	--	_
--	--	--	--	--	--	--	--	--
--	--	--	--	--	--	--	--	--
--	--	--	--	--	--	HMDB028	--	_
--	--	--	--	--	--	HMDB003	348144-81	Organic cc
--	--	LMGL0101	Glycerolipi	Monorady	Monoacylg	HMDB001	23470-00-	Organic cc
C05468	--	--	--	--	--	HMDB000	--	Organic cc
--	--	--	--	--	--	HMDB006	--	Organic cc
--	--	--	--	--	--	HMDB025	--	Organic cc
--	--	--	--	--	--	HMDB024	--	Organic cc
--	--	--	--	--	--	HMDB025	--	Organic cc
--	--	--	--	--	--	HMDB000	--	Organic cc
--	--	--	--	--	--	--	--	--
--	--	--	--	--	--	HMDB003	33467-79-	Organic cc
C04742	Arachidon	LMFA0306	Fatty Acyls	Eicosanoic	Hydroxy/h	HMDB000	54845-95-	Organic cc
C04166	--	--	--	--	--	HMDB030	--	Organic cc
C03872	--	--	--	--	--	HMDB003	1186-34-1	Organic cc
C02774	--	LMFA0105	Fatty Acyls	Fatty Acids	Hydroxy fa	HMDB024	--	Organic cc
--	--	--	--	--	--	HMDB024	--	Organic cc
--	--	--	--	--	--	--	--	--
C00284	--	--	--	--	--	HMDB001	62-33-9	Organic cc
--	--	--	--	--	--	--	--	--
--	--	--	--	--	--	HMDB030	--	Organic cc
--	--	--	--	--	--	HMDB003	128637-87	Organic cc
--	--	--	--	--	--	--	--	--
--	--	--	--	--	--	--	--	--
--	--	--	--	--	--	HMDB030	--	Organic cc
C11989	Biosynthes	LMPK0400	Polyketide	Macrolides	-	--	--	--
--	--	--	--	--	--	HMDB006	--	Organic cc
--	--	--	--	--	--	--	--	--
--	--	LMFA0500	Fatty Acyls	Fatty alcoh	-	HMDB003	133921-58	Organic cc
--	--	--	--	--	--	--	--	--
--	--	--	--	--	--	HMDB027	--	_
--	--	--	--	--	--	HMDB024	81-25-4	Organic cc
--	--	--	--	--	--	HMDB006	--	Organic cc
--	--	--	--	--	--	--	--	--
--	--	--	--	--	--	HMDB029	--	_
--	--	--	--	--	--	HMDB027	--	_
--	--	--	--	--	--	HMDB027	--	_
--	--	--	--	--	--	HMDB027	--	_
C11002	--	LMFA0117	Fatty Acyls	Fatty Acids	Dicarboxyl	HMDB000	821-38-5	Organic cc
--	--	--	--	--	--	--	--	--

--	--	--	--	--	--	HMDB003	247030-35	Organic cc
--	--	--	--	--	--	HMDB025	--	Organic cc
--	--	--	--	--	--	--	--	--
--	--	--	--	--	--	HMDB003	292167-39	Organic cc
--	--	--	--	--	--	HMDB025	--	Organic cc
--	--	--	--	--	--	HMDB024	--	Organic cc
--	--	--	--	--	--	--	--	--
--	--	--	--	--	--	HMDB024	--	Organic cc
--	--	--	--	--	--	HMDB029	--	_
--	--	--	--	--	--	--	--	--
--	--	--	--	--	--	--	--	--
--	--	--	--	--	--	--	--	--
C21484	Ferroptosis	LMGP0205	Glyceroph	Glyceroph	Monoacylg	HMDB001	69747-55-	Organic cc
--	--	--	--	--	--	HMDB024	--	Organic cc
--	--	--	--	--	--	HMDB025	--	Organic cc
--	--	--	--	--	--	HMDB025	--	Organic cc
--	--	--	--	--	--	HMDB026	--	_
--	--	--	--	--	--	--	--	--
--	--	--	--	--	--	--	--	--
--	--	--	--	--	--	HMDB025	--	Organic cc
--	--	--	--	--	--	HMDB002	--	Organic cc
--	--	--	--	--	--	HMDB001	80621-81-	Organic cc
--	--	--	--	--	--	HMDB024	521-17-5	Organic cc
--	--	--	--	--	--	--	--	--
--	--	--	--	--	--	--	--	--
--	--	--	--	--	--	HMDB024	--	Organic cc
--	--	--	--	--	--	HMDB004	163135-94	Organic cc
--	--	--	--	--	--	HMDB000	59333-79-	Organic cc
--	--	--	--	--	--	--	--	--
--	--	--	--	--	--	--	--	--
--	--	--	--	--	--	HMDB030	--	Organic cc
--	--	--	--	--	--	HMDB024	--	Organic cc
--	--	--	--	--	--	--	--	--
--	--	--	--	--	--	--	--	--
--	--	--	--	--	--	HMDB025	--	Organic cc
--	--	--	--	--	--	--	--	--
--	--	--	--	--	--	HMDB004	65718-88-	Organic cc
--	--	--	--	--	--	HMDB003	--	Organic cc
--	--	--	--	--	--	HMDB024	--	Organic cc
--	--	--	--	--	--	HMDB025	--	Organic cc
--	--	LMFA0707	Fatty Acyls	Fatty ester	Fatty acyl (	HMDB024	--	Organic cc
--	--	--	--	--	--	HMDB027	--	_
--	--	--	--	--	--	HMDB027	--	_
--	--	--	--	--	--	--	--	--
--	--	--	--	--	--	--	--	--
--	--	--	--	--	--	--	--	--
--	--	--	--	--	--	HMDB002	6665-19-6	Organic cc
--	--	--	--	--	--	HMDB003	108766-16	Organic cc
--	--	--	--	--	--	--	--	--
--	--	--	--	--	--	HMDB003	95341-44-	Organic cc
C11907	Streptomy	--	--	--	--	HMDB000	16752-71-	Organic cc

--	--	--	--	--	--	HMDB030	--	Organic cc
C05478	--	LMST0203	Sterol Lipi	Steroids [S	C21 steroi	HMDB000	566-03-0	Organic cc
--	--	--	--	--	--	HMDB003	122470-42	Organic cc
--	--	--	--	--	--	HMDB025	1000380-(	Organic cc
C18699	Plant horm	LMFA0202	Fatty Acyls	Octadecar	Jasmonic a	HMDB002	120330-93	Organic cc
--	--	--	--	--	--	HMDB024	--	_
--	--	--	--	--	--	HMDB025	--	Organic cc
--	--	--	--	--	--	HMDB024	--	Organic cc
--	--	--	--	--	--	--	--	--
--	--	--	--	--	--	HMDB024	--	Organic cc
--	--	LMFA0802	Fatty Acyls	Fatty amid	N-acyl am	HMDB006	--	Organic cc
--	--	--	--	--	--	HMDB030	--	Organic cc
--	--	--	--	--	--	HMDB024	--	Organic cc
--	--	--	--	--	--	HMDB025	--	Organic cc
--	--	--	--	--	--	HMDB003	221695-66	Organic cc
--	--	--	--	--	--	HMDB006	--	Organic cc
--	--	--	--	--	--	HMDB024	--	Organic cc
--	--	--	--	--	--	HMDB001	17-15-2	Organic cc
--	--	--	--	--	--	--	--	--
--	--	--	--	--	--	HMDB003	--	Organic cc
--	--	--	--	--	--	HMDB025	--	Organic cc
--	--	--	--	--	--	--	--	--
--	--	--	--	--	--	HMDB026	--	_
--	--	--	--	--	--	HMDB025	--	Organic cc
--	--	--	--	--	--	HMDB025	--	Organic cc
--	--	--	--	--	--	HMDB024	--	Organic cc
--	--	--	--	--	--	HMDB025	--	Organic cc
--	--	--	--	--	--	HMDB027	--	_
--	--	--	--	--	--	HMDB001	13589-02-	Organic cc
--	--	--	--	--	--	--	--	--
--	--	LMFA0106	Fatty Acyls	Fatty Acids	Oxo fatty a	HMDB001	--	Organic cc
C04654	--	LMFA0301	Fatty Acyls	Eicosanoic	Prostaglan	HMDB000	22973-19-	Organic cc
C00128	Amino sug	--	--	--	--	HMDB000	3063-71-6	Organic cc
--	--	--	--	--	--	--	--	--
--	--	--	--	--	--	--	--	--
--	--	--	--	--	--	--	--	--
--	--	--	--	--	--	--	--	--
--	--	--	--	--	--	HMDB024	--	Organic cc
--	--	--	--	--	--	HMDB025	--	Organic cc
C05489	--	--	--	--	--	HMDB000	--	Organic cc
C04518	Steroid ho	LMST0203	Sterol Lipi	Steroids [S	C21 steroi	HMDB001	652-69-7	Organic cc
--	--	LMFA0103	Fatty Acyls	Fatty Acids	Unsaturate	HMDB000	5684-69-5	Organic cc
--	--	--	--	--	--	HMDB003	--	Organic cc
--	--	--	--	--	--	--	--	--
C07020	Chemical (	--	--	--	--	HMDB001	25812-30-	Organic cc
--	--	--	--	--	--	--	--	--
C11045	Taste trans	--	--	--	--	HMDB000	22839-47-	Organic cc
C14718	--	--	--	--	--	HMDB003	1085-12-7	Organic cc
--	--	--	--	--	--	HMDB009	--	Organic cc
--	--	LMGP0205	Glyceroph	Glyceroph	Monoacylg	HMDB001	--	Organic cc
--	--	LMST0401	Sterol Lipi	Bile acids a	C24 bile ac	HMDB000	84413-81-	Organic cc

--	--	--	--	--	--	HMDB025	--	Organic cc
--	--	--	--	--	--	--	--	--
--	--	--	--	--	--	HMDB024	--	Organic cc
C11993	Biosynthes	LMPK0400	Polyketide	Macrolides	-	--	--	--
--	--	--	--	--	--	HMDB030	--	Organic cc
--	--	--	--	--	--	HMDB025	--	Organic cc
--	--	--	--	--	--	HMDB025	--	Organic cc
--	--	--	--	--	--	HMDB025	--	Organic cc
--	--	--	--	--	--	--	--	--
--	--	--	--	--	--	HMDB006	--	Organic cc
--	--	--	--	--	--	--	--	--
C05962	Arachidon	LMFA0301	Fatty Acyls	Eicosanoic	Prostaglan	HMDB000	67786-53-	Organic cc
--	--	--	--	--	--	HMDB024	--	Organic cc
--	--	--	--	--	--	HMDB024	--	_
--	--	LMGP0305	Glyceroph	Glyceroph	Monoacylg	HMDB024	1218913-8	Organic cc
C06932	--	--	--	--	--	HMDB001	512-15-2	Organic cc
--	--	LMFA0707	Fatty Acyls	Fatty ester	Fatty acyl (	HMDB001	--	Organic cc
--	--	--	--	--	--	HMDB000	493-90-3	Organic cc
--	--	--	--	--	--	HMDB024	--	Organic cc
--	--	--	--	--	--	HMDB028	--	_
--	--	--	--	--	--	HMDB011	--	Organic cc
--	--	--	--	--	--	HMDB029	--	_
--	--	--	--	--	--	--	--	--
--	--	--	--	--	--	HMDB003	29336-13-	Organic cc
--	--	--	--	--	--	HMDB003	358732-35	Organic cc
--	--	--	--	--	--	--	--	--
--	--	LMST0401	Sterol Lipid	Bile acids a	C24 bile ac	HMDB000	5130-29-0	Organic cc
--	--	LMGP1005	Glyceroph	Glyceroph	Monoacylg	HMDB006	--	Organic cc
--	--	--	--	--	--	HMDB025	--	Organic cc
--	--	--	--	--	--	HMDB025	--	Organic cc
--	--	--	--	--	--	--	--	--
--	--	--	--	--	--	HMDB028	--	_
--	--	--	--	--	--	HMDB028	--	_
--	--	LMFA0704	Fatty Acyls	Fatty ester	Lactones [	HMDB003	104-50-7	Organic cc
--	--	--	--	--	--	HMDB006	--	Organic cc
--	--	--	--	--	--	--	--	--
C03170	Glutathion	--	--	--	--	HMDB006	--	Organic cc
--	--	--	--	--	--	HMDB018	--	Organic cc
--	--	--	--	--	--	--	--	--
--	--	--	--	--	--	HMDB025	--	Organic cc
C07373	--	--	--	--	--	HMDB001	23288-49-	Organic cc
--	--	--	--	--	--	--	--	--
C04230	--	LMGP0105	Glyceroph	Glyceroph	Monoacylg	HMDB001	--	Organic cc
--	--	--	--	--	--	HMDB025	--	Organic cc
--	--	--	--	--	--	HMDB025	--	Organic cc
--	--	--	--	--	--	HMDB024	--	Organic cc
--	--	--	--	--	--	--	--	--
--	--	--	--	--	--	--	--	--
--	--	--	--	--	--	HMDB003	631-71-0	Organic cc
C01924	--	--	--	--	--	HMDB000	156-86-5	Organic cc
--	--	--	--	--	--	HMDB024	--	Organic cc

--	--	--	--	--	--	HMDB024	--	Organic co
--	--	--	--	--	--	--	--	--
--	--	LMGP0205	Glyceroph	Glyceroph	Monoacylg	HMDB001	--	Organic co
--	--	--	--	--	--	HMDB003	--	Organic co
--	--	--	--	--	--	--	--	--
--	--	LMFA0802	Fatty Acyls	Fatty amid	N-acyl am	HMDB001	--	Organic co
--	--	--	--	--	--	HMDB003	68489-14-	Organic co
--	--	--	--	--	--	HMDB024	--	Organic co
--	--	--	--	--	--	HMDB024	--	Organic co
--	--	LMST0401	Sterol Lipi	Bile acids a	C24 bile ac	HMDB000	71883-64-	Organic co
--	--	--	--	--	--	HMDB001	--	Organic co
--	--	--	--	--	--	HMDB028	--	_
--	--	--	--	--	--	--	--	--
--	--	--	--	--	--	--	--	--
--	--	--	--	--	--	--	--	--
--	--	--	--	--	--	HMDB024	--	_
--	--	--	--	--	--	HMDB024	--	Organic co
--	--	--	--	--	--	--	--	--
--	--	--	--	--	--	--	--	--
--	--	--	--	--	--	HMDB003	123-69-3	Organic co
--	--	--	--	--	--	--	--	--
--	--	--	--	--	--	HMDB030	--	Organic co
--	--	--	--	--	--	HMDB025	--	Organic co
--	--	LMSP0106	Sphingolip	Sphingoid	Lysosphing	HMDB001	--	Organic co
--	--	--	--	--	--	HMDB003	170591-49	Organic co
--	--	--	--	--	--	HMDB026	--	Organic co
--	--	--	--	--	--	HMDB030	13345-50-	Organic co
--	--	--	--	--	--	--	--	--
--	--	--	--	--	--	HMDB025	--	Organic co
--	--	--	--	--	--	HMDB024	--	Organic co
--	--	--	--	--	--	HMDB000	--	Organic co
--	--	--	--	--	--	HMDB028	--	_
--	--	--	--	--	--	HMDB027	--	_
--	--	--	--	--	--	HMDB024	--	Organic co
C04483	Secondary	LMST0401	Sterol Lipi	Bile acids a	C24 bile ac	HMDB000	83-44-3	Organic co
--	--	--	--	--	--	HMDB024	--	Organic co
--	--	--	--	--	--	HMDB024	--	Organic co
--	--	--	--	--	--	HMDB024	--	Organic co
--	--	--	--	--	--	HMDB028	--	_
C05468	--	--	--	--	--	HMDB000	--	Organic co
C04737	--	--	--	--	--	HMDB000	--	Organic co
--	--	--	--	--	--	HMDB025	--	Organic co
--	--	LMGP1005	Glyceroph	Glyceroph	Monoacylg	HMDB006	--	Organic co
C06999	Bile secreti	--	--	--	--	HMDB000	83799-24-	Organic co
--	--	--	--	--	--	HMDB024	--	Organic co
--	--	--	--	--	--	--	--	--
C00413	Streptomy	--	--	--	--	HMDB001	57-92-1	Organic co
--	--	--	--	--	--	--	--	--
--	--	--	--	--	--	HMDB028	--	_
--	--	--	--	--	--	HMDB028	--	_
--	--	--	--	--	--	HMDB028	--	_

--	--	--	--	--	--	HMDB028	--	_
--	--	--	--	--	--	HMDB028	--	_
--	--	--	--	--	--	HMDB001	224452-6	Organic cc
--	--	--	--	--	--	HMDB003	102607-2	Organic cc
C15557	--	LMST0503	Sterol Lipi	Steroid co	Glycine co	HMDB000	474-74-8	Organic cc
--	--	--	--	--	--	--	--	--
C11994	Biosynthes	LMPK040	Polyketide	Macrolides	-	--	--	--
--	--	--	--	--	--	HMDB001	159351-6	Organic cc
--	--	--	--	--	--	HMDB025	--	Organic cc
--	--	--	--	--	--	HMDB003	193977-0	Organic cc
--	--	--	--	--	--	--	--	--
--	--	--	--	--	--	HMDB001	--	Organic cc
--	--	--	--	--	--	HMDB024	--	Organic cc
--	--	LMFA0103	Fatty Acyls	Fatty Acids	Unsaturate	HMDB030	--	Organic cc
--	--	--	--	--	--	HMDB025	--	Organic cc
--	--	--	--	--	--	--	--	--
C14773	Arachidon	LMFA0305	Fatty Acyls	Eicosanoic	Hydroxy/h	HMDB000	192461-9	Organic cc
--	--	--	--	--	--	--	--	--
--	--	LMFA0801	Fatty Acyls	Fatty amid	Primary an	HMDB000	3061-75-	Organic cc
C05466	Primary bil	LMST0503	Sterol Lipi	Steroid co	Glycine co	HMDB000	640-79-9	Organic cc
--	--	--	--	--	--	HMDB004	147395-9	Organic cc
--	--	--	--	--	--	HMDB026	--	_
--	--	--	--	--	--	HMDB026	--	Organic cc
--	--	--	--	--	--	HMDB027	--	_
--	--	--	--	--	--	HMDB027	--	_
--	--	--	--	--	--	HMDB024	--	Organic cc
--	--	--	--	--	--	HMDB024	--	_
--	--	--	--	--	--	HMDB027	--	_
--	--	--	--	--	--	HMDB024	--	Organic cc
--	--	--	--	--	--	--	--	--
--	--	--	--	--	--	HMDB024	--	Organic cc
--	--	--	--	--	--	--	--	--
--	--	--	--	--	--	HMDB003	17909-94-	Organic cc
--	--	--	--	--	--	--	--	--
--	--	--	--	--	--	HMDB025	--	Organic cc
--	--	--	--	--	--	HMDB003	100217-9	Organic cc
--	--	--	--	--	--	HMDB028	--	_
--	--	--	--	--	--	HMDB012	--	Organic cc
--	--	--	--	--	--	HMDB029	--	_
--	--	--	--	--	--	HMDB025	--	Organic cc
--	--	--	--	--	--	--	--	--
--	--	--	--	--	--	--	--	--
--	--	--	--	--	--	HMDB024	--	Organic cc
--	--	--	--	--	--	HMDB000	--	Organic cc
--	--	--	--	--	--	HMDB024	--	Organic cc
C05789	--	--	--	--	--	HMDB000	17095-63-	Organic cc
--	--	--	--	--	--	HMDB019	--	Organic cc
--	--	--	--	--	--	HMDB024	--	Organic cc
C05791	Porphyrin	--	--	--	--	HMDB000	17208-65-	Organic cc
--	--	--	--	--	--	HMDB003	29171-20-	Organic cc
C06108	Drug meta	--	--	--	--	HMDB025	144-49-0	Organic cc

--	--	--	--	--	--	HMDB011	--	Organic cc
--	--	--	--	--	--	HMDB029	--	_
--	--	--	--	--	--	HMDB003	--	Organic cc
C04230	--	LMGP0105	Glyceroph	Glyceroph	Monoacylg	HMDB001	60701-99-	Organic cc
--	--	--	--	--	--	HMDB025	--	Organic cc
--	--	--	--	--	--	HMDB028	--	_
--	--	LMFA0302	Fatty Acyls	Eicosanoic	Leukotrien	HMDB000	74841-69-	Organic cc
--	--	--	--	--	--	HMDB024	--	Organic cc
--	--	--	--	--	--	HMDB028	--	_
--	--	--	--	--	--	HMDB002	11006-56-	Organic cc
C07660	Bile secreti	--	--	--	--	HMDB000	100986-85	Organic cc
C18218	Cutin, sub	LMFA0105	Fatty Acyls	Fatty Acids	Hydroxy fa	HMDB000	506-13-8	Organic cc
C05425	--	LMST0101	Sterol Lipi	Sterols [ST	Cholesterc	HMDB000	1253-84-5	Organic cc
C11996	Biosynthes	LMPK0400	Polyketide	Macrolides	-	--	--	--
--	--	--	--	--	--	HMDB025	--	Organic cc
--	--	LMFA0500	Fatty Acyls	Fatty alcoh	-	HMDB003	67801-46-	Organic cc
--	--	--	--	--	--	HMDB024	--	_
C12100	--	LMFA0114	Fatty Acyls	Fatty Acids	Carbocycli	HMDB003	--	Organic cc
--	--	--	--	--	--	HMDB025	25876-11-	Organic cc
--	--	--	--	--	--	--	--	--
--	--	--	--	--	--	--	--	--
--	--	--	--	--	--	HMDB024	--	Organic cc
--	--	--	--	--	--	HMDB024	--	Organic cc
--	--	--	--	--	--	HMDB003	20117-33-	Organic cc
--	--	--	--	--	--	HMDB028	--	_
--	--	--	--	--	--	HMDB025	--	Organic cc
--	--	--	--	--	--	HMDB030	--	Organic cc
--	--	--	--	--	--	--	--	--
--	--	--	--	--	--	--	--	--
--	--	--	--	--	--	HMDB024	--	Organic cc
--	--	--	--	--	--	HMDB025	--	Organic cc
--	--	--	--	--	--	HMDB019	--	_
--	--	--	--	--	--	--	--	--
--	--	--	--	--	--	HMDB002	--	Organic cc
--	--	--	--	--	--	HMDB024	--	Organic cc
--	--	--	--	--	--	--	--	--
--	--	--	--	--	--	HMDB024	--	Organic cc
--	--	--	--	--	--	HMDB004	58152-03-	Organic cc
--	--	--	--	--	--	HMDB006	--	Organic cc
--	--	--	--	--	--	HMDB003	--	Organic cc
C07432	--	--	--	--	--	HMDB001	134-49-6	Organic cc
--	--	--	--	--	--	HMDB024	--	Organic cc
--	--	LMST0203	Sterol Lipi	Steroids [S	C21 steroi	HMDB000	651-43-4	Organic cc
--	--	--	--	--	--	HMDB024	--	Organic cc
--	--	--	--	--	--	HMDB006	--	Organic cc
C07851	--	--	--	--	--	HMDB001	125-53-1	Organic cc
--	--	--	--	--	--	HMDB003	141360-89	Organic cc
--	--	--	--	--	--	HMDB024	--	_
--	--	--	--	--	--	HMDB025	--	Organic cc
C06426	Linoleic ac	LMFA0103	Fatty Acyls	Fatty Acids	Unsaturate	HMDB000	506-26-3	Organic cc
--	--	--	--	--	--	HMDB028	--	_

--	--	--	--	--	--	--	--	--
--	--	LMFA0305	Fatty Acyls	Eicosanoic	Hydroxy/h	HMDB000	13-16-1	Organic cc
--	--	--	--	--	--	HMDB024	--	Organic cc
C06135	--	--	--	--	--	HMDB000	--	Organic cc
--	--	LMGP0205	Glyceroph	Glyceroph	Monoacylg	HMDB001	--	Organic cc
--	--	--	--	--	--	HMDB025	--	Organic cc
--	--	--	--	--	--	HMDB024	--	Organic cc
--	--	--	--	--	--	HMDB024	--	Organic cc
--	--	--	--	--	--	--	--	--
--	--	--	--	--	--	--	--	--
--	--	--	--	--	--	HMDB029	--	_
--	--	--	--	--	--	HMDB024	--	Organic cc
C16533	Biosynthes	LMFA0103	Fatty Acyls	Fatty Acids	Unsaturate	HMDB006	17735-98-	Organic cc
--	--	--	--	--	--	HMDB024	--	Organic cc
--	--	LMGP0105	Glyceroph	Glyceroph	Monoacylg	HMDB000	19420-56-	Organic cc
--	--	--	--	--	--	HMDB030	--	Organic cc
C21255	Neomycin	--	--	--	--	--	--	--
--	--	LMFA0200	Fatty Acyls	Octadecar	Other Octa	HMDB001	--	Organic cc
--	--	--	--	--	--	--	--	--
C16618	Drug meta	--	--	--	--	HMDB006	--	Organic cc
C18218	Cutin, sub	LMFA0105	Fatty Acyls	Fatty Acids	Hydroxy fa	HMDB000	506-13-8	Organic cc
--	--	LMPR0104	Prenol Lipi	Isoprenoid	C20 isopre	HMDB000	1189-37-3	Organic cc
--	--	--	--	--	--	--	--	--
--	--	--	--	--	--	HMDB004	73561-91-	Organic cc
--	--	--	--	--	--	HMDB024	--	Organic cc
--	--	--	--	--	--	--	--	--
--	--	--	--	--	--	HMDB003	--	Organic cc
C06866	Biosynthes	LMFA0802	Fatty Acyls	Fatty amid	N-acyl am	HMDB000	404-86-4	Organic cc
--	--	--	--	--	--	HMDB001	--	Organic cc
--	--	LMGP1005	Glyceroph	Glyceroph	Monoacylg	HMDB006	--	Organic cc
--	--	--	--	--	--	HMDB024	--	Organic cc
C19418	Cutin, sub	LMFA0200	Fatty Acyls	Octadecar	Other Octa	HMDB024	13980-07-	Organic cc
--	--	--	--	--	--	HMDB025	--	Organic cc
--	--	--	--	--	--	HMDB025	--	Organic cc
--	--	LMGP0207	Glyceroph	Glyceroph	1Z-alkenyl	HMDB024	174062-73	Organic cc
--	--	--	--	--	--	HMDB024	--	Organic cc
--	--	--	--	--	--	HMDB000	--	Organic cc
--	--	--	--	--	--	HMDB024	--	Organic cc
--	--	--	--	--	--	HMDB030	--	Organic cc
--	--	--	--	--	--	HMDB024	678-39-7	Organic cc
C19818	--	--	--	--	--	HMDB003	555-10-2	Organic cc
--	--	--	--	--	--	--	--	--
C17567	--	--	--	--	--	HMDB003	6871-67-6	Organic cc
--	--	--	--	--	--	HMDB028	--	_
C07051	Bile secret	--	--	--	--	HMDB001	150378-17	Organic cc
--	--	--	--	--	--	HMDB018	--	Organic cc
C03805	--	--	--	--	--	HMDB001	--	Organic cc
--	--	--	--	--	--	HMDB024	--	Organic cc
--	--	--	--	--	--	HMDB027	--	_
--	--	--	--	--	--	--	--	--
--	--	LMGP0107	Glyceroph	Glyceroph	1Z-alkenyl	HMDB001	--	Organic cc

--	--	--	--	--	--	HMDB003	98063-17-	Organic cc
--	--	--	--	--	--	HMDB000	125356-8(	Organic cc
--	--	--	--	--	--	HMDB026	--	_
C14825	Linoleic ac	LMFA020(	Fatty Acyls	Octadecar	Other Octa	HMDB047	--	--
--	--	--	--	--	--	HMDB001	56554-77-	Organic cc
--	--	--	--	--	--	HMDB000	--	Organic cc
--	--	LMFA0103	Fatty Acyls	Fatty Acids	Unsaturate	HMDB001	3913-85-7	Organic cc
--	--	--	--	--	--	--	--	--
C11998	Biosynthes	--	--	--	--	--	--	--
--	--	--	--	--	--	HMDB025	1000335-(	Organic cc
--	--	--	--	--	--	HMDB004	182173-5(	Organic cc
--	--	--	--	--	--	HMDB000	98319-26-	Organic cc
C04230	--	LMGP0105	Glyceroph	Glyceroph	Monoacylg	HMDB001	--	Organic cc
--	--	--	--	--	--	HMDB001	25905-77-	Organic cc
--	--	--	--	--	--	--	--	--
--	--	--	--	--	--	--	--	--
--	--	--	--	--	--	--	--	--
--	--	--	--	--	--	--	--	--
--	--	--	--	--	--	HMDB006	--	Organic cc
--	--	--	--	--	--	HMDB025	--	Organic cc
--	--	--	--	--	--	HMDB024	--	Organic cc
C02728	--	--	--	--	--	HMDB000	1188-07-4	Organic cc
--	--	--	--	--	--	HMDB024	5274-68-(	Organic cc
--	--	--	--	--	--	HMDB029	--	_
--	--	--	--	--	--	HMDB025	--	Organic cc
C04230	--	LMGP0105	Glyceroph	Glyceroph	Monoacylg	HMDB001	17364-16-	Organic cc
--	--	LMGP0205	Glyceroph	Glyceroph	Monoacylg	HMDB001	--	Organic cc
--	--	--	--	--	--	--	--	--
--	--	--	--	--	--	HMDB027	--	_
--	--	--	--	--	--	HMDB001	--	Organic cc
--	--	--	--	--	--	--	--	--
--	--	--	--	--	--	HMDB025	--	Organic cc
--	--	--	--	--	--	HMDB003	--	Organic cc
--	--	--	--	--	--	HMDB018	--	Organic cc
--	--	LMFA0500	Fatty Acyls	Fatty alcoh	-	HMDB003	122855-4(	Organic cc
--	--	--	--	--	--	HMDB003	7392-19-(	Organic cc
--	--	--	--	--	--	HMDB030	--	Organic cc
C04230	--	LMGP0105	Glyceroph	Glyceroph	Monoacylg	HMDB001	--	Organic cc
--	--	--	--	--	--	--	--	--
--	--	--	--	--	--	HMDB004	69506-65-	Organic cc
--	--	--	--	--	--	--	--	--
--	--	--	--	--	--	HMDB025	--	Organic cc
C07627	--	--	--	--	--	HMDB001	90-34-6	Organic cc
C02165	--	LMFA0302	Fatty Acyls	Eicosanoic	Leukotrien	HMDB010	--	--
--	--	--	--	--	--	HMDB003	--	Organic cc
--	--	--	--	--	--	HMDB024	--	Organic cc
--	--	--	--	--	--	--	--	--
--	--	--	--	--	--	HMDB003	95311-94-	Organic cc
--	--	--	--	--	--	HMDB024	--	Organic cc
--	--	--	--	--	--	HMDB028	--	_
--	--	--	--	--	--	HMDB004	172616-9(	Organic cc

--	--	LMFA0117	Fatty Acyls	Fatty Acids	Dicarboxyl	HMDB024	2424-92-2	Organic co
--	--	--	--	--	--	HMDB030	--	Organic co
C04230	--	LMGP0105	Glyceroph	Glyceroph	Monoacylg	HMDB001	50930-23-	Organic co
--	--	--	--	--	--	--	--	--
--	--	--	--	--	--	HMDB024	--	Organic co
--	--	--	--	--	--	HMDB025	--	Organic co
--	--	--	--	--	--	HMDB025	--	Organic co
--	--	--	--	--	--	HMDB024	--	Organic co
--	--	--	--	--	--	HMDB028	--	_
--	--	--	--	--	--	HMDB024	37682-29-	Organic co
--	--	--	--	--	--	HMDB003	111047-3	Organic co
--	--	--	--	--	--	HMDB025	--	Organic co
--	--	--	--	--	--	HMDB003	301540-7	Organic co
--	--	--	--	--	--	HMDB024	--	Organic co
C06425	Biosynthes	LMFA0101	Fatty Acyls	Fatty Acids	Straight ch	HMDB000	506-30-9	Organic co
--	--	--	--	--	--	HMDB025	--	Organic co
--	--	--	--	--	--	HMDB003	--	Organic co
--	--	--	--	--	--	--	--	--
--	--	--	--	--	--	HMDB001	--	Organic co
--	--	--	--	--	--	--	--	--
--	--	--	--	--	--	HMDB029	--	_
--	--	--	--	--	--	HMDB003	220181-8	Organic co
--	--	--	--	--	--	HMDB026	--	_
--	--	--	--	--	--	HMDB011	--	Organic co
--	--	--	--	--	--	HMDB025	--	Organic co
--	--	--	--	--	--	HMDB001	179463-1	Organic co
--	--	LMFA0200	Fatty Acyls	Octadecan	Other Octa	HMDB001	--	Organic co
--	--	--	--	--	--	HMDB003	--	Organic co
--	--	--	--	--	--	--	--	--
C07230	--	--	--	--	--	HMDB001	7416-34-4	Organic co
--	--	--	--	--	--	HMDB003	333332-4	Organic co
--	--	--	--	--	--	HMDB024	--	Organic co
C13768	--	--	--	--	--	HMDB001	1264-72-8	Organic co
C04230	--	LMGP0105	Glyceroph	Glyceroph	Monoacylg	HMDB001	50930-23-	Organic co
C04230	--	LMGP0105	Glyceroph	Glyceroph	Monoacylg	HMDB001	108341-8	Organic co
--	--	--	--	--	--	--	--	--
--	--	--	--	--	--	HMDB011	--	Organic co
--	--	--	--	--	--	HMDB025	--	Organic co
--	--	--	--	--	--	HMDB028	--	_
--	--	--	--	--	--	HMDB000	--	Organic co
--	--	--	--	--	--	--	--	--
C16157	--	LMPR0302	Prenol Lipi	Polypreno	Bactopren	--	--	--
--	--	--	--	--	--	HMDB025	--	Organic co
--	--	LMFA0801	Fatty Acyls	Fatty amid	Primary an	HMDB001	629-54-9	Organic co
--	--	--	--	--	--	HMDB001	22610-61-	Organic co
--	--	--	--	--	--	HMDB003	99664-48-	Organic co
--	--	--	--	--	--	HMDB006	--	Organic co
--	--	--	--	--	--	HMDB000	99794-82-	Organic co
--	--	--	--	--	--	HMDB000	--	Organic co
--	--	--	--	--	--	--	--	--
--	--	--	--	--	--	--	--	--

--	--	--	--	--	--	--	--	--
--	--	--	--	--	--	--	--	--
--	--	LMSP0301	Sphingolip	Phosphosp	Ceramide	HMDB024	2292199-2	Organic co
C07432	Steroid ho	LMST0202	Sterol Lipi	Steroids [S	C19 steroi	HMDB000	382-44-5	Organic co
--	--	--	--	--	--	HMDB024	--	Organic co
--	--	--	--	--	--	HMDB025	--	Organic co
--	--	--	--	--	--	HMDB003	82537-86-	Organic co
--	--	LMFA0801	Fatty Acyls	Fatty amid	Primary an	HMDB006	3072-13-7	Organic co
--	--	LMFA0105	Fatty Acyls	Fatty Acids	Hydroxy fa	HMDB001	--	Organic co
--	--	--	--	--	--	HMDB029	--	_
--	--	--	--	--	--	HMDB029	--	_
--	--	--	--	--	--	--	--	--
--	--	--	--	--	--	--	--	--
--	--	--	--	--	--	HMDB029	--	_
--	--	LMSP0301	Sphingolip	Phosphosp	Ceramide	HMDB024	121999-56	Organic co
--	--	--	--	--	--	HMDB025	--	Organic co
--	--	--	--	--	--	--	--	--
--	--	--	--	--	--	HMDB025	--	Organic co
--	--	--	--	--	--	HMDB024	--	Organic co
--	--	LMGP0201	Glyceroph	Glyceroph	Diacylglyce	HMDB000	--	Organic co
--	--	--	--	--	--	HMDB024	--	Organic co
--	--	LMFA0500	Fatty Acyls	Fatty alcoh	-	HMDB003	163955-67	Organic co
C01561	Steroid bio	LMST0302	Sterol Lipi	Secosteroi	Vitamin D3	HMDB000	19356-17-	Organic co
--	--	--	--	--	--	HMDB004	75539-64-	Organic co
--	--	--	--	--	--	HMDB028	--	_
--	--	--	--	--	--	HMDB029	--	_
--	--	--	--	--	--	--	--	--
--	--	--	--	--	--	HMDB024	--	Organic co
--	--	--	--	--	--	HMDB003	1337-33-3	Organic co
--	--	--	--	--	--	HMDB024	--	Organic co
--	--	--	--	--	--	HMDB028	--	_
--	--	--	--	--	--	HMDB003	107-75-5	Organic co
--	--	--	--	--	--	HMDB024	--	Organic co
C05804	--	--	--	--	--	HMDB000	--	Organic co
--	--	--	--	--	--	HMDB026	--	Organic co
C15699	Arginine a	--	--	--	--	HMDB001	--	Organic co
--	--	--	--	--	--	--	--	--
--	--	--	--	--	--	HMDB030	--	Organic co
--	--	LMFA0103	Fatty Acyls	Fatty Acids	Unsaturate	HMDB000	28845-86-	Organic co
--	--	--	--	--	--	HMDB025	--	Organic co
--	--	--	--	--	--	HMDB000	80380-40-	Organic co
--	--	--	--	--	--	HMDB025	--	Organic co
--	--	--	--	--	--	HMDB003	90524-90-	Organic co
--	--	--	--	--	--	HMDB026	--	_
--	--	--	--	--	--	HMDB028	--	_
--	--	--	--	--	--	HMDB025	--	Organic co
--	--	--	--	--	--	HMDB025	--	Organic co
--	--	--	--	--	--	HMDB025	--	Organic co
C07402	--	--	--	--	--	HMDB001	671-16-9	Organic co
--	--	--	--	--	--	--	--	--
C00219	Arachidon	LMFA0103	Fatty Acyls	Fatty Acids	Unsaturate	HMDB000	506-32-1	Organic co

--	--	--	--	--	--	HMDB025	--	Organic cc
--	--	LMGP0205	Glyceroph	Glyceroph	Monoacylg	HMDB001	--	Organic cc
--	--	--	--	--	--	HMDB003	--	Organic cc
--	--	--	--	--	--	HMDB024	53-41-8	Organic cc
--	--	--	--	--	--	HMDB000	66803-17-	Organic cc
--	--	--	--	--	--	--	--	--
C08014	--	--	--	--	--	HMDB001	1977/7/6	Organic cc
--	--	--	--	--	--	HMDB001	--	Organic cc
--	--	--	--	--	--	--	--	--
--	--	--	--	--	--	HMDB024	--	Organic cc
--	--	--	--	--	--	--	--	--
--	--	LMFA050C	Fatty Acyls	Fatty alcoh	-	HMDB003	--	--
C18041	Steroid ho	--	--	--	--	HMDB006	--	Organic cc
--	--	--	--	--	--	HMDB024	--	Organic cc
--	--	--	--	--	--	HMDB003	86778-06-	Organic cc
--	--	--	--	--	--	HMDB029	--	_
--	--	--	--	--	--	HMDB026	--	_
--	--	--	--	--	--	HMDB011	--	Organic cc
--	--	LMGP0201	Glyceroph	Glyceroph	Diacylglyce	HMDB000	--	Organic cc
C09870	--	--	--	--	--	HMDB004	89-48-5	Organic cc
--	--	--	--	--	--	HMDB003	--	Organic cc
--	--	--	--	--	--	HMDB003	--	Organic cc
--	--	--	--	--	--	HMDB028	--	_
--	--	--	--	--	--	--	--	--
C05176	Isoquinolir	--	--	--	--	HMDB006	--	Organic cc
--	--	--	--	--	--	HMDB003	61781-98-	Organic cc
--	--	--	--	--	--	HMDB030	--	Organic cc
--	--	--	--	--	--	--	--	--
--	--	--	--	--	--	HMDB024	--	Organic cc
--	--	--	--	--	--	HMDB025	143-62-4	Organic cc
--	--	--	--	--	--	HMDB003	74747-52-	Organic cc
C06125	--	--	--	--	--	HMDB001	265096-81	Organic cc
--	--	LMFA0106	Fatty Acyls	Fatty Acids	Oxo fatty a	HMDB001	--	Organic cc
--	--	--	--	--	--	HMDB029	--	_
--	--	--	--	--	--	HMDB029	--	_
--	--	--	--	--	--	HMDB003	156324-78	Organic cc
--	--	--	--	--	--	--	--	--
--	--	--	--	--	--	HMDB002	189156-42	Organic cc
--	--	--	--	--	--	--	--	--
--	--	--	--	--	--	--	--	--
--	--	--	--	--	--	--	--	--
C20388	--	LMFA0305	Fatty Acyls	Eicosanoic	Hydroxy/h	HMDB001	54397-84-	Organic cc
--	--	--	--	--	--	HMDB024	--	Organic cc
--	--	--	--	--	--	HMDB024	57718-75-	Organic cc
--	--	--	--	--	--	--	--	--
--	--	--	--	--	--	HMDB030	--	Organic cc
--	--	--	--	--	--	--	--	--
--	--	LMST0203	Sterol Lipi	Steroids [S	C21 steroi	HMDB000	600-63-5	Organic cc
--	--	--	--	--	--	HMDB029	--	_
--	--	--	--	--	--	HMDB000	--	Organic cc
C06428	Biosynthes	LMFA0103	Fatty Acyls	Fatty Acids	Unsaturate	HMDB000	10417-94-	Organic cc

--	--	LMGP1001	Glyceroph	Glyceroph	Diacylglyc	HMDB011	--	Organic cc
--	--	--	--	--	--	HMDB004	--	Organic cc
--	--	--	--	--	--	--	--	--
--	--	--	--	--	--	HMDB024	--	Organic cc
--	--	--	--	--	--	HMDB026	--	_
--	--	--	--	--	--	HMDB011	--	Organic cc
--	--	--	--	--	--	--	--	--
C16525	Biosynthes	LMFA0103	Fatty Acyls	Fatty Acids	Unsaturate	HMDB000	5598-38-9	Organic cc
--	--	--	--	--	--	HMDB003	51745-21-	Organic cc
C05475	--	LMST0203	Sterol Lipi	Steroids [S	C21 steroi	HMDB000	566-01-8	Organic cc
--	--	--	--	--	--	HMDB025	--	Organic cc
--	--	--	--	--	--	HMDB003	219814-37	Organic cc
--	--	LMPR0107	Prenol Lipi	Isoprenoid	C40 isopre	HMDB003	39937-23-	Organic cc
--	--	--	--	--	--	--	--	--
--	--	--	--	--	--	HMDB024	--	Organic cc
--	--	--	--	--	--	HMDB024	--	_
--	--	--	--	--	--	HMDB027	--	_
--	--	--	--	--	--	HMDB003	3687-54-5	Organic cc
--	--	--	--	--	--	HMDB003	53786-93-	Organic cc
--	--	--	--	--	--	HMDB024	--	Organic cc
--	--	LMGP0201	Glyceroph	Glyceroph	Diacylglyc	HMDB000	--	Organic cc
--	--	--	--	--	--	HMDB003	120727-26	Organic cc
--	--	--	--	--	--	HMDB024	--	Organic cc
--	--	--	--	--	--	HMDB029	--	_
--	--	--	--	--	--	HMDB030	18433-98-	Organic cc
--	--	LMFA0103	Fatty Acyls	Fatty Acids	Unsaturate	HMDB001	--	Organic cc
--	--	--	--	--	--	--	--	--
--	--	--	--	--	--	--	--	--
--	--	LMST0403	Sterol Lipi	Bile acids a	C27 bile ac	HMDB006	--	Organic cc
--	--	--	--	--	--	--	--	--
--	--	--	--	--	--	--	--	--
--	--	--	--	--	--	--	--	--
--	--	--	--	--	--	HMDB024	--	Organic cc
--	--	--	--	--	--	HMDB003	#######	Organic cc
--	--	--	--	--	--	HMDB005	--	Organic cc
--	--	--	--	--	--	--	--	--
--	--	--	--	--	--	HMDB025	--	Organic cc
--	--	--	--	--	--	HMDB003	141973-37	Organic cc
--	--	--	--	--	--	HMDB003	--	Organic cc
--	--	--	--	--	--	HMDB030	142160-72	Organic cc
--	--	LMFA0200	Fatty Acyls	Octadecar	Other Octa	HMDB024	1330-70-7	Organic cc
--	--	--	--	--	--	--	--	--
C06890	Bile secreti	--	--	--	--	HMDB001	68401-81-	Organic cc
--	--	--	--	--	--	HMDB003	--	Organic cc
--	--	--	--	--	--	HMDB030	--	Organic cc
--	--	--	--	--	--	HMDB003	91590-75-	Organic cc
--	--	--	--	--	--	HMDB024	--	Organic cc
--	--	--	--	--	--	HMDB000	10527-47-	Organic cc
--	--	--	--	--	--	HMDB006	812-00-0	Organic cc
--	--	--	--	--	--	--	--	--
C16356	Caffeine m	--	--	--	--	HMDB001	33868-03-	Organic cc

--	--	--	--	--	--	HMDB000	938-55-6	Organic co
--	--	--	--	--	--	HMDB025	--	Organic co
--	--	--	--	--	--	--	--	--
--	--	--	--	--	--	--	--	--
C01346	Pyrimidine	--	--	--	--	HMDB000	4208-67-7	Organic co
--	--	--	--	--	--	HMDB024	--	Organic co
C01345	Purine me	--	--	--	--	HMDB000	16595-02-	Organic co
--	--	--	--	--	--	HMDB024	--	Organic co
C04348	--	LMFA0705	Fatty Acyls	Fatty ester	Fatty acyl (	HMDB000	2043-93-8	Organic co
C00437	Arginine b	--	--	--	--	HMDB000	6205/8/9	Organic co
--	--	--	--	--	--	--	--	--
--	--	--	--	--	--	HMDB004	52463-83-	Organic co
--	--	--	--	--	--	HMDB024	29611-03-	Organic co
--	--	--	--	--	--	--	--	--
--	--	--	--	--	--	HMDB003	--	Organic co
C05117	--	--	--	--	--	HMDB001	--	Organic co
C02949	Ubiquinon	--	--	--	--	HMDB006	--	Organic co
--	--	--	--	--	--	--	--	--
--	--	--	--	--	--	HMDB002	76400-25-	Organic co
--	--	--	--	--	--	--	--	--
C16625	Drug meta	--	--	--	--	HMDB006	--	Organic co
--	--	--	--	--	--	HMDB024	--	Organic co
--	--	--	--	--	--	--	--	--
C05125	Glycolysis	--	--	--	--	--	--	--
--	--	--	--	--	--	--	--	--
C00309	--	--	--	--	--	HMDB000	131064-70	Organic co
--	--	--	--	--	--	--	--	--
--	--	--	--	--	--	HMDB025	--	Organic co
--	--	--	--	--	--	HMDB003	--	Organic co
--	--	--	--	--	--	HMDB024	--	Organic co
--	--	--	--	--	--	HMDB002	224638-52	Organic co
--	--	--	--	--	--	HMDB025	1982-47-4	Organic co
--	--	--	--	--	--	HMDB006	--	Organic co
--	--	--	--	--	--	HMDB024	--	Organic co
--	--	--	--	--	--	--	--	--
--	--	--	--	--	--	HMDB024	--	Organic co
--	--	--	--	--	--	HMDB003	160845-05	Organic co
C00363	Pyrimidine	--	--	--	--	HMDB000	491-97-4	Organic co
--	--	--	--	--	--	HMDB030	--	Organic co
--	--	--	--	--	--	--	--	--
--	--	--	--	--	--	HMDB025	97-69-8	Organic co
--	--	--	--	--	--	--	--	--
--	--	--	--	--	--	HMDB002	90965-81-	Organic co
--	--	--	--	--	--	--	--	--
--	--	--	--	--	--	--	--	--
--	--	--	--	--	--	HMDB024	--	Organic co
--	--	--	--	--	--	HMDB025	6318-55-4	Organic co
C05422	Ascorbate	--	--	--	--	HMDB000	490-83-5	Organic co
--	--	--	--	--	--	--	--	--
--	--	--	--	--	--	--	--	--
--	--	--	--	--	--	--	--	--

--	--	--	--	--	--	HMDB028	--	_
--	--	--	--	--	--	HMDB025	--	Organic cc
--	--	--	--	--	--	--	--	--
--	--	--	--	--	--	HMDB007	--	Organic cc
--	--	--	--	--	--	HMDB025	--	Organic cc
--	--	--	--	--	--	HMDB000	126151-6(	Organic cc
--	--	--	--	--	--	HMDB003	55750-85-	Organic cc
--	--	--	--	--	--	HMDB003	--	Organic cc
--	--	--	--	--	--	--	--	--
--	--	--	--	--	--	HMDB030	--	Organic cc
--	--	--	--	--	--	--	--	--
--	--	--	--	--	--	HMDB027	--	_
--	--	--	--	--	--	HMDB000	--	Organic cc
--	--	--	--	--	--	HMDB026	--	Organic cc
--	--	--	--	--	--	HMDB001	--	Organic cc
--	--	--	--	--	--	--	--	--
--	--	--	--	--	--	--	--	--
--	--	--	--	--	--	HMDB024	--	Organic cc
C15888	Carotenoi	LMPR0107	Prenol Lipi	Isoprenoid	C40 isopre	--	--	--
--	--	--	--	--	--	HMDB026	--	_
--	--	--	--	--	--	HMDB024	--	Organic cc
--	--	--	--	--	--	HMDB003	343962-53	Organic cc
--	--	--	--	--	--	HMDB029	--	_
--	--	--	--	--	--	--	--	--
--	--	--	--	--	--	--	--	--
--	--	--	--	--	--	HMDB000	13535-96-	Organic cc
--	--	--	--	--	--	--	--	--
--	--	LMFA1301	Fatty Acyls	Fatty acyl (	Fatty acyl (	--	--	--
--	--	--	--	--	--	HMDB025	--	Organic cc
--	--	--	--	--	--	HMDB003	1107-26-2	Organic cc
--	--	--	--	--	--	HMDB029	--	_
--	--	--	--	--	--	HMDB024	--	Organic cc
--	--	--	--	--	--	HMDB024	--	Organic cc
C05670	beta-Alani	--	--	--	--	HMDB000	151-18-8	Organic cc
--	--	--	--	--	--	--	--	--
--	--	LMGP0201	Glyceroph	Glyceroph	Diacylglyc	HMDB000	--	Organic cc
--	--	LMST0402	Sterol Lipi	Bile acids a	C26 bile ac	HMDB000	91999-66-	Organic cc
--	--	--	--	--	--	HMDB024	--	Organic cc
--	--	--	--	--	--	--	--	--
--	--	--	--	--	--	HMDB028	--	_
--	--	--	--	--	--	--	--	--
--	--	--	--	--	--	HMDB024	--	_
--	--	--	--	--	--	HMDB003	200942-15	Organic cc
--	--	--	--	--	--	--	--	--
--	--	--	--	--	--	HMDB025	--	Organic cc
--	--	--	--	--	--	HMDB027	--	_
C05817	Ubiquinon	--	--	--	--	HMDB030	--	Organic cc
--	--	--	--	--	--	--	--	--
--	--	--	--	--	--	HMDB002	34323-07-	Organic cc
C08262	Biosynthes	LMFA0102	Fatty Acyls	Fatty Acids	Branched f	HMDB000	503-74-2	Organic cc
--	--	LMST0202	Sterol Lipi	Steroids [S	C19 steroi	HMDB000	2283-82-1	Organic cc

--	--	--	--	--	--	HMDB028	--	_
--	--	--	--	--	--	--	--	--
--	--	--	--	--	--	HMDB003	200492-4	Organic co
--	--	--	--	--	--	--	--	--
C15492	Retinol me	LMPR010	Prenol Lipi	Isoprenoid	Retinoids [	HMDB001	115797-1	Organic co
--	--	--	--	--	--	HMDB029	--	_
C02686	--	--	--	--	--	HMDB001	148347-4	Organic co
--	--	LMGP1001	Glyceroph	Glyceroph	Diacylglyce	HMDB011	--	Organic co
--	--	--	--	--	--	HMDB026	--	_
--	--	--	--	--	--	HMDB028	--	_
--	--	--	--	--	--	HMDB028	--	_
--	--	--	--	--	--	HMDB029	--	_
--	--	--	--	--	--	HMDB029	--	_
--	--	--	--	--	--	HMDB028	--	_
--	--	LMGP0301	Glyceroph	Glyceroph	Diacylglyce	--	--	--
--	--	--	--	--	--	HMDB028	--	_
--	--	--	--	--	--	HMDB028	--	_
--	--	--	--	--	--	HMDB026	--	_
--	--	--	--	--	--	HMDB025	--	Organic co
--	--	--	--	--	--	HMDB026	--	_
--	--	--	--	--	--	HMDB028	--	_
--	--	--	--	--	--	HMDB024	--	Organic co
--	--	--	--	--	--	HMDB024	--	Organic co
C06874	--	--	--	--	--	HMDB001	51781-06-	Organic co
--	--	--	--	--	--	HMDB000	--	Organic co
--	--	--	--	--	--	--	--	--
--	--	--	--	--	--	--	--	--
--	--	--	--	--	--	HMDB003	229483-4	Organic co
--	--	--	--	--	--	HMDB025	--	Organic co
--	--	--	--	--	--	HMDB024	--	Organic co
--	--	--	--	--	--	HMDB030	--	Organic co
--	--	--	--	--	--	HMDB024	--	Organic co
--	--	--	--	--	--	HMDB025	--	Organic co
--	--	LMGP0605	Glyceroph	Glyceroph	Monoacylg	HMDB006	1246430-	Organic co
--	--	--	--	--	--	--	--	--
--	--	--	--	--	--	--	--	--
--	--	--	--	--	--	--	--	--
--	--	--	--	--	--	HMDB005	--	Organic co
--	--	--	--	--	--	HMDB003	--	Organic co
--	--	--	--	--	--	--	--	--
--	--	--	--	--	--	HMDB024	--	Organic co
--	--	--	--	--	--	--	--	--
--	--	--	--	--	--	--	--	--
--	--	--	--	--	--	HMDB003	34393-18-	Organic co
C00507	Fructose a	--	--	--	--	HMDB000	3615-41-	Organic co
--	--	--	--	--	--	--	--	--
--	--	--	--	--	--	HMDB003	2725-22-	Organic co
--	--	--	--	--	--	HMDB024	--	Organic co
--	--	--	--	--	--	HMDB029	--	_
C06341	Primary bil	LMST0403	Sterol Lipi	Bile acids a	C27 bile ac	HMDB000	4725-24-	Organic co
--	--	--	--	--	--	--	--	--

--	--	--	--	--	--	--	--	--
--	--	--	--	--	--	HMDB003	39711-79-	Organic cc
C14533	--	--	--	--	--	HMDB001	95-63-6	Organic cc
--	--	--	--	--	--	HMDB011	--	Organic cc
--	--	--	--	--	--	HMDB029	--	_
--	--	--	--	--	--	HMDB028	--	_
--	--	--	--	--	--	--	--	--
C01268	Riboflavin	--	--	--	--	--	--	--
--	--	LMGP0301	Glyceroph	Glyceroph	Diacylglyce	HMDB001	--	Organic cc
C20186	Linoleic ac	LMFA0200	Fatty Acyls	Octadecar	Other Octa	--	--	--
--	--	--	--	--	--	HMDB001	136434-34	Organic cc
--	--	--	--	--	--	HMDB025	--	Organic cc
C05513	--	--	--	--	--	HMDB002	2124-54-1	Organic cc
--	--	--	--	--	--	HMDB029	--	_
--	--	--	--	--	--	HMDB029	--	_
--	--	--	--	--	--	--	--	--
C02135	Renin-ang	--	--	--	--	HMDB000	11128-99-	Organic cc
--	--	--	--	--	--	HMDB027	--	_
--	--	--	--	--	--	HMDB030	--	Organic cc
--	--	--	--	--	--	HMDB001	99-43-4	Organic cc
--	--	--	--	--	--	HMDB029	--	_
--	--	--	--	--	--	--	--	--
--	--	--	--	--	--	HMDB011	--	Organic cc
--	--	--	--	--	--	HMDB006	--	Organic cc
--	--	--	--	--	--	--	--	--
C06184	Tropane, p	--	--	--	--	HMDB003	18747-42-	Organic cc
--	--	--	--	--	--	HMDB027	--	_
--	--	--	--	--	--	HMDB024	--	Organic cc
--	--	--	--	--	--	HMDB000	--	Organic cc
C00300	Glycine, se	--	--	--	--	HMDB000	57-00-1	Organic cc
--	--	--	--	--	--	HMDB006	--	Organic cc
--	--	--	--	--	--	HMDB024	2150-55-2	Organic cc
--	--	--	--	--	--	--	--	--
--	--	--	--	--	--	HMDB000	--	Organic cc
--	--	--	--	--	--	HMDB024	--	Organic cc
--	--	--	--	--	--	--	--	--
--	--	--	--	--	--	HMDB029	--	_
--	--	--	--	--	--	HMDB024	354-38-1	Organic cc
--	--	--	--	--	--	HMDB025	--	Organic cc
--	--	--	--	--	--	HMDB025	--	Organic cc
--	--	--	--	--	--	HMDB025	76-04-0	Organic cc
C02704	--	--	--	--	--	HMDB025	--	Organic cc
--	--	--	--	--	--	HMDB001	363-36-0	Organic cc
--	--	--	--	--	--	HMDB025	--	Organic cc
--	--	--	--	--	--	HMDB024	--	Organic cc
--	--	--	--	--	--	HMDB003	--	Organic cc
C00239	Pyrimidine	--	--	--	--	HMDB000	1032-65-1	Organic cc
--	--	--	--	--	--	HMDB000	704-15-4	Organic cc
--	--	--	--	--	--	--	--	--
--	--	--	--	--	--	--	--	--
--	--	--	--	--	--	--	--	--

--	--	LMPK1212	Polyketide	Flavonoids	Chalcones	--	--	--
--	--	--	--	--	--	--	--	--
C07819	--	--	--	--	--	HMDB001	479-18-5	Organic cc
--	--	--	--	--	--	--	--	--
--	--	--	--	--	--	HMDB024	--	Organic cc
--	--	--	--	--	--	--	--	--
--	--	--	--	--	--	--	--	--
--	--	--	--	--	--	--	--	--
--	--	--	--	--	--	HMDB002	20917-57-	Organic cc
--	--	--	--	--	--	HMDB025	--	Organic cc
--	--	--	--	--	--	HMDB002	2390-74-1	Organic cc
C00931	Porphyrin	--	--	--	--	HMDB000	487-90-1	Organic cc
C00780	Tryptopha	--	--	--	--	HMDB000	50-67-9	Organic cc
--	--	--	--	--	--	--	--	--
--	--	--	--	--	--	HMDB002	--	Organic cc
C17883	--	--	--	--	--	HMDB001	7786-61-(	Organic cc
--	--	--	--	--	--	HMDB001	40642-83-	Organic cc
--	--	--	--	--	--	HMDB003	645-59-0	Organic cc
--	--	--	--	--	--	HMDB004	--	Organic cc
--	--	--	--	--	--	HMDB003	91-62-3	Organic cc
--	--	--	--	--	--	--	--	--
C21283	--	--	--	--	--	HMDB000	29953-71-	Organic cc
--	--	--	--	--	--	HMDB024	--	Organic cc
--	--	--	--	--	--	--	--	--
--	--	--	--	--	--	HMDB024	--	Organic cc
C03506	Phenylalar	--	--	--	--	HMDB030	--	Organic cc
--	--	--	--	--	--	HMDB003	--	Organic cc
--	--	--	--	--	--	HMDB003	396714-67	Organic cc
--	--	--	--	--	--	--	--	--
--	--	--	--	--	--	HMDB025	--	Organic cc
--	--	--	--	--	--	HMDB024	--	Organic cc
--	--	--	--	--	--	--	--	--
--	--	--	--	--	--	HMDB024	--	Organic cc
--	--	--	--	--	--	HMDB025	--	Organic cc
--	--	--	--	--	--	--	--	--
--	--	--	--	--	--	HMDB024	--	Organic cc
--	--	--	--	--	--	HMDB024	--	Organic cc
--	--	--	--	--	--	HMDB002	--	Organic cc
--	--	--	--	--	--	HMDB003	--	Organic cc
--	--	--	--	--	--	HMDB024	--	Organic cc
C16613	Drug meta	--	--	--	--	HMDB006	--	Organic cc
--	--	--	--	--	--	HMDB004	65936-86-	Organic cc
--	--	--	--	--	--	HMDB025	--	Organic cc
--	--	--	--	--	--	HMDB030	--	_
--	--	--	--	--	--	HMDB024	--	Organic cc
--	--	--	--	--	--	HMDB024	1196-57-2	Organic cc
--	--	--	--	--	--	--	--	--
--	--	--	--	--	--	--	--	--
--	--	--	--	--	--	HMDB024	--	Organic cc
--	--	--	--	--	--	HMDB003	--	Organic cc
--	--	--	--	--	--	HMDB004	156539-32	Organic cc

--	--	--	--	--	--	HMDB005	2462-31-9	Organic co
--	--	--	--	--	--	HMDB030	--	Organic co
--	--	--	--	--	--	HMDB003	21857-97-	Organic co
--	--	--	--	--	--	--	--	--
--	--	--	--	--	--	--	--	--
C07065	Bile secreti	--	--	--	--	HMDB001	134678-17	Organic co
--	--	--	--	--	--	HMDB003	5391-17-3	Organic co
--	--	--	--	--	--	HMDB002	71190-89-	Organic co
--	--	--	--	--	--	HMDB024	--	Organic co
--	--	--	--	--	--	HMDB024	21618-99-	Organic co
--	--	--	--	--	--	--	--	--
--	--	--	--	--	--	--	--	--
--	--	--	--	--	--	HMDB030	--	Organic co
--	--	--	--	--	--	HMDB025	--	Organic co
--	--	--	--	--	--	HMDB025	--	Organic co
C00327	Arginine b	--	--	--	--	HMDB000	372-75-8	Organic co
--	--	--	--	--	--	HMDB024	--	Organic co
C00978	Tryptopha	--	--	--	--	HMDB000	1210-83-9	Organic co
--	--	--	--	--	--	HMDB024	--	Organic co
C19559	Metabolism	--	--	--	--	HMDB006	86941-58-	Organic co
--	--	--	--	--	--	HMDB003	114542-45	Organic co
--	--	--	--	--	--	HMDB025	--	Organic co
--	--	--	--	--	--	HMDB024	--	Organic co
--	--	--	--	--	--	HMDB002	--	Organic co
C00664	--	--	--	--	--	HMDB000	2311-81-1	Organic co
--	--	--	--	--	--	HMDB002	--	Organic co
C05285	Steroid ho	--	--	--	--	HMDB000	382-45-6	Organic co
--	--	--	--	--	--	HMDB000	98379-91-	Organic co
C19578	Metabolism	--	--	--	--	HMDB006	53798-73-	Organic co
--	--	--	--	--	--	HMDB003	2650-74-0	Organic co
--	--	--	--	--	--	HMDB025	#######	Organic co
--	--	--	--	--	--	--	--	--
C00826	Phenylalan	--	--	--	--	HMDB030	--	Organic co
--	--	--	--	--	--	HMDB002	20488-27-	Organic co
--	--	--	--	--	--	--	--	--
--	--	--	--	--	--	HMDB025	--	Organic co
--	--	--	--	--	--	HMDB001	77463-72-	Organic co
--	--	--	--	--	--	HMDB003	144331-30	Organic co
--	--	--	--	--	--	--	--	--
C02227	--	--	--	--	--	HMDB004	91-59-8	Organic co
--	--	--	--	--	--	HMDB025	--	Organic co
--	--	--	--	--	--	HMDB025	--	Organic co
C01168	Pyrimidine	--	--	--	--	HMDB000	1157-60-4	Organic co
C16596	Drug meta	--	--	--	--	HMDB006	--	Organic co
--	--	--	--	--	--	HMDB025	--	Organic co
C17076	--	LMFA0101	Fatty Acyls	Fatty Acids	Straight ch	HMDB000	638-53-9	Organic co
--	--	--	--	--	--	HMDB002	--	Organic co
--	--	--	--	--	--	--	--	--
--	--	--	--	--	--	--	--	--
--	--	--	--	--	--	--	--	--
--	--	--	--	--	--	--	--	--

--	--	--	--	--	--	HMDB003	32215-02-	Organic cc
--	--	--	--	--	--	HMDB002	129050-49	Organic cc
--	--	--	--	--	--	HMDB003	--	Organic cc
C10515	--	--	--	--	--	HMDB003	40323-57-	Organic cc
--	--	--	--	--	--	--	--	--
C18075	Steroid ho	--	--	--	--	HMDB006	--	Organic cc
--	--	--	--	--	--	--	--	--
--	--	--	--	--	--	HMDB030	--	Organic cc
--	--	--	--	--	--	--	--	--
--	--	--	--	--	--	--	--	--
C07395	--	--	--	--	--	HMDB001	40391-99-	Organic cc
--	--	--	--	--	--	HMDB025	--	Organic cc
--	--	--	--	--	--	HMDB004	--	Organic cc
--	--	--	--	--	--	HMDB030	75-07-0	Organic cc
--	--	--	--	--	--	HMDB003	14400-67-	Organic cc
--	--	--	--	--	--	--	--	--
--	--	--	--	--	--	HMDB024	--	Organic cc
C09315	Biosynthes	--	--	--	--	HMDB002	93-35-6	Organic cc
--	--	--	--	--	--	--	--	--
--	--	--	--	--	--	HMDB024	--	Organic cc
C05635	Tryptopha	--	--	--	--	HMDB000	54-16-0	Organic cc
C07207	Bile secreti	--	--	--	--	HMDB001	7481-89-2	Organic cc
--	--	--	--	--	--	HMDB024	--	Organic cc
--	--	--	--	--	--	HMDB025	--	Organic cc
--	--	--	--	--	--	HMDB002	--	Organic cc
--	--	--	--	--	--	--	--	--
--	--	--	--	--	--	--	--	--
--	--	--	--	--	--	HMDB025	--	Organic cc
C01035	Arginine a	--	--	--	--	HMDB000	463-00-3	Organic cc
C01407	Chlorocycl	--	--	--	--	HMDB000	71-43-2	Organic cc
C19168	--	--	--	--	--	HMDB024	98-88-4	Organic cc
--	--	--	--	--	--	HMDB004	32645-65-	Organic cc
--	--	--	--	--	--	HMDB024	--	Organic cc
C04707	--	LMFA0301	Fatty Acyls	Eicosanoic	Prostaglan	HMDB000	26441-05-	Organic cc
--	--	--	--	--	--	--	--	--
--	--	--	--	--	--	HMDB030	--	Organic cc
--	--	--	--	--	--	HMDB025	--	Organic cc
C14467	--	--	--	--	--	HMDB003	3018-21-1	Organic cc
--	--	--	--	--	--	HMDB024	--	Organic cc
--	--	--	--	--	--	HMDB001	19237-53-	Organic cc
C16255	--	LMFA0802	Fatty Acyls	Fatty amid	N-acyl am	HMDB000	--	Organic cc
--	--	--	--	--	--	HMDB001	--	Organic cc
--	--	--	--	--	--	HMDB024	--	Organic cc
--	--	--	--	--	--	HMDB030	--	Organic cc
--	--	--	--	--	--	HMDB025	--	Organic cc
C06909	--	--	--	--	--	HMDB001	113852-37	Organic cc
--	--	--	--	--	--	HMDB025	40925-28-	Organic cc
C07359	--	--	--	--	--	HMDB006	--	Organic cc
--	--	--	--	--	--	HMDB004	--	Organic cc
--	--	--	--	--	--	--	--	--
C16365	Caffeine m	--	--	--	--	HMDB001	--	Organic cc

--	--	--	--	--	--	HMDB025	--	Organic cc
--	--	--	--	--	--	--	--	--
--	--	--	--	--	--	HMDB025	--	Organic cc
--	--	--	--	--	--	HMDB024	--	Organic cc
--	--	--	--	--	--	HMDB025	--	Organic cc
--	--	--	--	--	--	--	--	--
--	--	--	--	--	--	--	--	--
C08060	Dioxin deg	--	--	--	--	HMDB024	86-74-8	Organic cc
--	--	--	--	--	--	--	--	--
--	--	--	--	--	--	HMDB024	--	Organic cc
--	--	--	--	--	--	--	--	--
--	--	--	--	--	--	HMDB003	39251-86-	Organic cc
--	--	--	--	--	--	HMDB001	7669-65-(	Organic cc
--	--	--	--	--	--	HMDB025	5011-34-7	Organic cc
--	--	--	--	--	--	--	--	--
C09209	Indole alka	--	--	--	--	HMDB003	486-84-0	Organic cc
--	--	--	--	--	--	HMDB002	525-41-7	Organic cc
--	--	--	--	--	--	HMDB025	--	Organic cc
--	--	--	--	--	--	HMDB025	--	Organic cc
--	--	--	--	--	--	HMDB004	182127-79	Organic cc
--	--	--	--	--	--	HMDB002	80185-44-	Organic cc
--	--	--	--	--	--	HMDB004	--	Organic cc
--	--	--	--	--	--	HMDB025	--	Organic cc
--	--	--	--	--	--	--	--	--
--	--	--	--	--	--	--	--	--
--	--	--	--	--	--	HMDB025	--	Organic cc
--	--	--	--	--	--	--	--	--
--	--	--	--	--	--	HMDB003	--	Organic cc
--	--	--	--	--	--	--	--	--
--	--	--	--	--	--	HMDB004	--	Organic cc
--	--	--	--	--	--	--	--	--
--	--	--	--	--	--	HMDB024	--	_
--	--	--	--	--	--	HMDB025	--	Organic cc
--	--	--	--	--	--	HMDB003	97826-67-	Organic cc
C01092	--	LMFA011C	Fatty Acyls	Fatty Acids	Amino fatt	HMDB024	682799-71	Organic cc
--	--	--	--	--	--	HMDB030	--	Organic cc
--	--	--	--	--	--	HMDB024	--	Organic cc
--	--	--	--	--	--	HMDB001	37462-92-	Organic cc
--	--	--	--	--	--	HMDB024	--	Organic cc
--	--	--	--	--	--	HMDB003	191280-2(	Organic cc
C14875	Metabolisr	--	--	--	--	HMDB006	28747-20-	Organic cc
C07622	--	--	--	--	--	HMDB001	137234-62	Organic cc
--	--	--	--	--	--	--	--	--
C18857	--	--	--	--	--	HMDB025	59756-60-	Organic cc
C12112	--	--	--	--	--	HMDB003	37248-47-	Organic cc
--	--	--	--	--	--	--	--	--
C00302	Cyanoamii	--	--	--	--	HMDB006	138-16-9	Organic cc
--	--	--	--	--	--	HMDB003	18454-89-	Organic cc
--	--	--	--	--	--	HMDB025	--	Organic cc
--	--	--	--	--	--	HMDB003	214218-63	Organic cc
--	--	--	--	--	--	--	--	--

--	--	--	--	--	--	HMDB004	54812-86-	Organic cc
--	--	--	--	--	--	--	--	--
C01479	--	--	--	--	--	HMDB000	828-01-3	Organic cc
C11284	--	--	--	--	--	HMDB000	133-32-4	Organic cc
C04732	Methane r	--	--	--	--	HMDB001	17014-74-	Organic cc
--	--	--	--	--	--	HMDB003	53043-28-	Organic cc
--	--	--	--	--	--	HMDB024	--	Organic cc
--	--	LMPK1206	Polyketide	Flavonoids	Rotenoid f	--	--	--
--	--	--	--	--	--	HMDB000	52630-68-	Organic cc
--	--	--	--	--	--	HMDB003	2506/9/4	Organic cc
--	--	--	--	--	--	HMDB002	114659-59	Organic cc
--	--	--	--	--	--	--	--	--
--	--	--	--	--	--	HMDB000	1116-21-8	Organic cc
--	--	--	--	--	--	HMDB001	--	Organic cc
--	--	--	--	--	--	HMDB003	62087-72-	Organic cc
--	--	--	--	--	--	HMDB025	14716-32-	Organic cc
--	--	--	--	--	--	HMDB025	--	Organic cc
--	--	--	--	--	--	--	--	--
C02366	--	--	--	--	--	HMDB030	--	Organic cc
C05079	--	--	--	--	--	HMDB001	389-08-2	Organic cc
--	--	--	--	--	--	HMDB005	--	Organic cc
--	--	--	--	--	--	HMDB003	65330-49-	Organic cc
--	--	--	--	--	--	HMDB024	--	Organic cc
--	--	--	--	--	--	--	--	--
C05587	Tyrosine m	--	--	--	--	HMDB000	554-52-9	Organic cc
--	--	--	--	--	--	HMDB025	--	Organic cc
--	--	--	--	--	--	HMDB025	--	Organic cc
C16604	Drug meta	LMPK1309	Polyketide	Aromatic p	Diphenyl e	HMDB006	--	Organic cc
--	--	--	--	--	--	HMDB003	120976-97	Organic cc
C01100	Histidine n	--	--	--	--	HMDB030	--	Organic cc
C08493	--	--	--	--	--	HMDB002	487-89-8	Organic cc
--	--	--	--	--	--	--	--	--
--	--	--	--	--	--	HMDB026	--	Organic cc
--	--	--	--	--	--	HMDB003	2873-36-1	Organic cc
--	--	--	--	--	--	HMDB024	--	Organic cc
--	--	--	--	--	--	HMDB003	86425-28-	Organic cc
--	--	--	--	--	--	HMDB025	--	Organic cc
--	--	--	--	--	--	--	--	--
--	--	--	--	--	--	--	--	--
--	--	--	--	--	--	HMDB004	10599-59-	Organic cc
C06212	Tryptopha	--	--	--	--	HMDB000	1134-01-6	Organic cc
--	--	--	--	--	--	HMDB003	34435-70-	Organic cc
--	--	--	--	--	--	HMDB002	--	Organic cc
--	--	--	--	--	--	HMDB025	--	Organic cc
--	--	LMPK1212	Polyketide	Flavonoids	Chalcones	--	--	--
--	--	--	--	--	--	HMDB001	56073-32-	Organic cc
C00021	Cysteine a	--	--	--	--	HMDB000	979-92-0	Organic cc
--	--	--	--	--	--	--	--	--
C19602	Metabolisr	--	--	--	--	HMDB006	76014-82-	Organic cc
C03033	--	--	--	--	--	HMDB001	27972-85-	Organic cc
--	--	--	--	--	--	HMDB006	--	Organic cc

--	--	--	--	--	--	HMDB003	66648-44-	Organic cc
--	--	--	--	--	--	HMDB025	--	Organic cc
--	--	--	--	--	--	HMDB005	4344-84-7	Organic cc
--	--	--	--	--	--	HMDB026	--	Organic cc
--	--	--	--	--	--	HMDB025	1000131-2	Organic cc
--	--	--	--	--	--	--	--	--
C00637	Tryptopha	--	--	--	--	HMDB000	2591-98-2	Organic cc
--	--	--	--	--	--	--	--	--
C12673	Drug meta	--	--	--	--	HMDB024	--	Organic cc
--	--	--	--	--	--	HMDB003	108-47-4	Organic cc
--	--	--	--	--	--	HMDB024	--	Organic cc
--	--	--	--	--	--	--	--	--
--	--	LMPK1212	Polyketide	Flavonoids	Chalcones	HMDB003	--	Organic cc
C00085	--	--	--	--	--	HMDB000	643-13-0	Organic cc
--	--	--	--	--	--	HMDB003	--	Organic cc
--	--	--	--	--	--	--	--	--
--	--	--	--	--	--	HMDB001	--	Organic cc
--	--	--	--	--	--	--	--	--
--	--	--	--	--	--	HMDB024	--	Organic cc
--	--	--	--	--	--	--	--	--
--	--	--	--	--	--	HMDB002	--	Organic cc
--	--	--	--	--	--	--	--	--
--	--	--	--	--	--	HMDB024	--	Organic cc
--	--	--	--	--	--	--	--	--
C00955	Tryptopha	--	--	--	--	HMDB000	526-55-6	Organic cc
--	--	--	--	--	--	HMDB025	--	Organic cc
--	--	--	--	--	--	HMDB024	--	Organic cc
C11841	Quorum s	LMFA0803	Fatty Acyls	Fatty amid	Fatty acyl	--	--	--
--	--	--	--	--	--	HMDB024	--	Organic cc
--	--	--	--	--	--	HMDB002	--	Organic cc
--	--	--	--	--	--	HMDB025	--	Organic cc
--	--	--	--	--	--	--	--	--
--	--	--	--	--	--	--	--	--
--	--	--	--	--	--	HMDB024	--	Organic cc
--	--	--	--	--	--	HMDB000	939-19-5	Organic cc
--	--	--	--	--	--	HMDB024	19685-10-	Organic cc
--	--	--	--	--	--	--	--	--
C20674	--	--	--	--	--	HMDB000	20244-86-	Organic cc
--	--	--	--	--	--	HMDB000	477251-67	Organic cc
C00831	--	--	--	--	--	HMDB000	496-65-1	Organic cc
--	--	--	--	--	--	HMDB025	--	Organic cc
C05936	Arginine a	--	--	--	--	HMDB000	--	Organic cc
--	--	--	--	--	--	--	--	--
--	--	--	--	--	--	--	--	--
--	--	--	--	--	--	HMDB002	--	Organic cc
--	--	--	--	--	--	HMDB024	--	Organic cc
--	--	--	--	--	--	HMDB002	2478/1/5	Organic cc
C16927	--	--	--	--	--	HMDB003	520-74-1	Organic cc
--	--	--	--	--	--	--	--	--
--	--	--	--	--	--	HMDB024	--	Organic cc
--	--	--	--	--	--	--	--	--

C08491	alpha-Linc	LMFA0202	Fatty Acyls	Octadecar	Jasmonic a	HMDB003	59366-47-	Organic cc
--	--	--	--	--	--	HMDB003	129673-85	Organic cc
--	--	--	--	--	--	HMDB025	--	Organic cc
--	--	--	--	--	--	--	--	--
C17213	Glucosinol	--	--	--	--	HMDB003	25148-30-	Organic cc
C02366	--	--	--	--	--	HMDB030	--	Organic cc
--	--	--	--	--	--	--	--	--
--	--	LMGP1005	Glyceroph	Glyceroph	Monoacylg	HMDB006	--	Organic cc
--	--	--	--	--	--	HMDB025	253-66-7	Organic cc
--	--	--	--	--	--	HMDB006	--	Organic cc
--	--	--	--	--	--	HMDB003	--	Organic cc
--	--	--	--	--	--	HMDB003	10129-99-	Organic cc
--	--	--	--	--	--	--	--	--
--	--	--	--	--	--	HMDB030	--	Organic cc
--	--	--	--	--	--	HMDB003	93078-82-	Organic cc
--	--	--	--	--	--	HMDB025	--	Organic cc
C14452	--	--	--	--	--	HMDB003	110-91-8	Organic cc
--	--	--	--	--	--	HMDB024	--	Organic cc
--	--	LMFA0701	Fatty Acyls	Fatty ester	Wax monc	HMDB003	6290-17-1	Organic cc
--	--	--	--	--	--	HMDB025	574-25-4	Organic cc
--	--	--	--	--	--	--	--	--
--	--	--	--	--	--	--	--	--
--	--	--	--	--	--	HMDB003	82654-98-	Organic cc
--	--	--	--	--	--	--	--	--
--	--	--	--	--	--	HMDB024	--	Organic cc
--	--	--	--	--	--	HMDB024	--	Organic cc
--	--	--	--	--	--	HMDB004	147838-41	Organic cc
--	--	--	--	--	--	HMDB006	--	Organic cc
--	--	--	--	--	--	HMDB024	--	Organic cc
--	--	--	--	--	--	HMDB002	6638/5/7	Organic cc
--	--	--	--	--	--	HMDB000	83462-55-	Organic cc
C08313	Tryptopha	--	--	--	--	HMDB000	83-34-1	Organic cc
--	--	--	--	--	--	HMDB024	--	Organic cc
--	--	--	--	--	--	HMDB003	--	Organic cc
--	--	--	--	--	--	--	--	--
C16750	--	--	--	--	--	HMDB030	--	Organic cc
C14447	--	--	--	--	--	HMDB006	109-06-8	Organic cc
C06323	--	--	--	--	--	HMDB003	119-65-3	Organic cc
--	--	--	--	--	--	--	--	--
--	--	--	--	--	--	--	--	--
--	--	--	--	--	--	HMDB003	29743-36-	Organic cc
--	--	--	--	--	--	HMDB003	85-91-6	Organic cc
C05660	Tryptopha	--	--	--	--	HMDB000	3471-31-6	Organic cc
--	--	--	--	--	--	HMDB024	--	Organic cc
--	--	--	--	--	--	HMDB003	99694-81-	Organic cc
C11331	--	--	--	--	--	HMDB030	--	Organic cc
--	--	--	--	--	--	--	--	--
--	--	--	--	--	--	HMDB003	24401-36-	Organic cc
--	--	LMFA0103	Fatty Acyls	Fatty Acids	Unsaturate	--	--	--
C08357	Steroid ho	LMST0502	Sterol Lipi	Steroid co	Sulfates [S	HMDB000	481-96-9	Organic cc
--	--	--	--	--	--	HMDB005	--	Organic cc

--	--	--	--	--	--	HMDB000	51732-61-	Organic cc
--	--	--	--	--	--	HMDB003	--	Organic cc
C16675	Folate bios	--	--	--	--	HMDB001	--	Organic cc
--	--	--	--	--	--	HMDB025	--	Organic cc
C00190	--	--	--	--	--	HMDB000	139427-57	Organic cc
--	--	--	--	--	--	HMDB005	--	Organic cc
C07934	--	--	--	--	--	HMDB001	1665-48-1	Organic cc
C07493	Drug meta	--	--	--	--	HMDB006	--	Organic cc
--	--	--	--	--	--	--	--	--
--	--	--	--	--	--	--	--	--
--	--	--	--	--	--	--	--	--
--	--	--	--	--	--	HMDB024	35186-99-	Organic cc
--	--	--	--	--	--	--	--	--
--	--	--	--	--	--	HMDB024	--	Organic cc
C16366	Caffeine m	--	--	--	--	HMDB000	19893-78-	Organic cc
--	--	--	--	--	--	--	--	--
C00746	--	--	--	--	--	HMDB000	50-56-6	Organic cc
C02997	--	--	--	--	--	HMDB003	2497/2/1	Organic cc
--	--	--	--	--	--	HMDB025	--	Organic cc
--	--	--	--	--	--	HMDB003	64448-49-	Organic cc
--	--	--	--	--	--	HMDB003	--	Organic cc
--	--	--	--	--	--	HMDB025	--	Organic cc
--	--	--	--	--	--	--	--	--
C03299	--	LMFA0707	Fatty Acyls	Fatty ester	Fatty acyl c	HMDB000	1492-27-9	Organic cc
--	--	--	--	--	--	HMDB024	--	Organic cc
--	--	--	--	--	--	HMDB030	--	Organic cc
--	--	--	--	--	--	HMDB025	--	Organic cc
C08095	--	--	--	--	--	HMDB001	139110-80	Organic cc
C07263	--	--	--	--	--	HMDB001	129618-40	Organic cc
--	--	--	--	--	--	HMDB002	4996-48-9	Organic cc
--	--	--	--	--	--	HMDB003	2634-33-5	Organic cc
C16765	--	--	--	--	--	HMDB004	518-75-2	Organic cc
--	--	--	--	--	--	HMDB003	--	Organic cc
--	--	--	--	--	--	--	--	--
--	--	--	--	--	--	HMDB004	25743-67-	Organic cc
--	--	--	--	--	--	HMDB002	148305-57	Organic cc
--	--	--	--	--	--	HMDB024	--	Organic cc
--	--	--	--	--	--	HMDB025	--	Organic cc
--	--	--	--	--	--	HMDB003	--	Organic cc
--	--	--	--	--	--	HMDB030	--	Organic cc
--	--	--	--	--	--	HMDB025	--	Organic cc
--	--	--	--	--	--	HMDB006	--	Organic cc
C13713	Steroid ho	LMST0503	Sterol Lipic	Steroid co	Glycine co	HMDB000	567-03-3	Organic cc
--	--	--	--	--	--	HMDB001	59122-46-	Organic cc
--	--	--	--	--	--	--	--	--
--	--	--	--	--	--	HMDB025	--	Organic cc
--	--	--	--	--	--	HMDB002	--	Organic cc
C07617	--	--	--	--	--	HMDB001	125-84-8	Organic cc
--	--	--	--	--	--	--	--	--
--	--	--	--	--	--	--	--	--
--	--	--	--	--	--	--	--	--

--	--	--	--	--	--	HMDB030	--	Organic cc
--	--	--	--	--	--	HMDB002	23827-93-	Organic cc
--	--	--	--	--	--	--	--	--
C13707	--	--	--	--	--	HMDB001	136790-76	Organic cc
--	--	--	--	--	--	HMDB024	--	Organic cc
--	--	--	--	--	--	--	--	--
--	--	--	--	--	--	HMDB025	--	Organic cc
--	--	--	--	--	--	HMDB024	--	Organic cc
--	--	--	--	--	--	--	--	--
--	--	LMFA110C	Fatty Acyls	Hydrocarb	-	--	--	--
--	--	--	--	--	--	HMDB003	120282-76	Organic cc
--	--	--	--	--	--	HMDB024	--	Organic cc
--	--	--	--	--	--	HMDB003	--	Organic cc
--	--	--	--	--	--	HMDB030	1131-62-(	Organic cc
C00214	Pyrimidine	--	--	--	--	HMDB000	50-89-5	Organic cc
--	--	--	--	--	--	--	--	--
--	--	--	--	--	--	--	--	--
C06537	--	--	--	--	--	HMDB002	525-57-5	Organic cc
C00170	Cysteine a	--	--	--	--	HMDB000	2457-80-9	Organic cc
--	--	--	--	--	--	HMDB003	--	Organic cc
--	--	LMFA020C	Fatty Acyls	Octadecar	Other Octa	HMDB003	--	Organic cc
--	--	--	--	--	--	HMDB030	--	Organic cc
C05938	Arginine a	--	--	--	--	HMDB000	--	Organic cc
--	--	--	--	--	--	HMDB025	--	Organic cc
--	--	--	--	--	--	HMDB000	501-81-5	Organic cc
--	--	--	--	--	--	HMDB004	4594/2/9	Organic cc
C06538	Indole alka	--	--	--	--	HMDB003	442-51-3	Organic cc
--	--	--	--	--	--	HMDB025	--	Organic cc
--	--	--	--	--	--	--	--	--
--	--	--	--	--	--	--	--	--
--	--	--	--	--	--	--	--	--
--	--	--	--	--	--	HMDB004	105-95-3	Organic cc
--	--	--	--	--	--	HMDB002	--	Organic cc
--	--	--	--	--	--	HMDB003	122-70-3	Organic cc
--	--	--	--	--	--	HMDB024	--	Organic cc
--	--	--	--	--	--	--	--	--
C01386	--	--	--	--	--	HMDB024	26093-31-	Organic cc
--	--	--	--	--	--	HMDB002	844641-04	Organic cc
--	--	--	--	--	--	HMDB024	--	Organic cc
--	--	--	--	--	--	HMDB024	--	Organic cc
--	--	--	--	--	--	--	--	--
C02394	--	--	--	--	--	HMDB002	104-54-1	Organic cc
--	--	--	--	--	--	HMDB025	--	Organic cc
C22236	--	--	--	--	--	HMDB000	830-96-6	Organic cc
--	--	--	--	--	--	HMDB024	--	Organic cc
--	--	--	--	--	--	--	--	--
--	--	--	--	--	--	HMDB025	--	Organic cc
--	--	--	--	--	--	HMDB024	--	Organic cc
C20265	Ubiquinon	--	--	--	--	HMDB006	--	Organic cc
--	--	--	--	--	--	--	--	--
--	--	--	--	--	--	--	--	--

--	--	--	--	--	--	HMDB001	--	Organic cc
C02985	Fructose a	--	--	--	--	HMDB000	16562-59-	Organic cc
--	--	--	--	--	--	--	--	--
--	--	--	--	--	--	--	--	--
--	--	--	--	--	--	--	--	--
--	--	--	--	--	--	--	--	--
--	--	--	--	--	--	HMDB002	1238-09-1	Organic cc
--	--	--	--	--	--	HMDB025	--	Organic cc
--	--	--	--	--	--	HMDB001	6899/4/3	Organic cc
--	--	--	--	--	--	HMDB003	24184-12-	Organic cc
--	--	--	--	--	--	--	--	--
--	--	--	--	--	--	HMDB003	9005-67-8	Organic cc
--	--	--	--	--	--	HMDB025	--	Organic cc
--	--	--	--	--	--	HMDB030	--	Organic cc
--	--	--	--	--	--	--	--	--
--	--	--	--	--	--	HMDB024	--	Organic cc
--	--	--	--	--	--	HMDB030	--	Organic cc
--	--	--	--	--	--	HMDB003	38710-35-	Organic cc
--	--	--	--	--	--	--	--	--
--	--	--	--	--	--	HMDB002	3306-40-9	Organic cc
--	--	--	--	--	--	--	--	--
--	--	--	--	--	--	HMDB004	7757-97-3	Organic cc
C02941	--	--	--	--	--	HMDB024	3248-10-0	Organic cc
--	--	--	--	--	--	--	--	--
--	--	--	--	--	--	--	--	--
--	--	--	--	--	--	HMDB025	--	Organic cc
--	--	--	--	--	--	--	--	--
--	--	--	--	--	--	HMDB024	--	Organic cc
--	--	--	--	--	--	HMDB024	--	Organic cc
--	--	--	--	--	--	--	--	--
--	--	--	--	--	--	HMDB003	61931-81-	Organic cc
C21797	Retinol me	LMPR0109	Prenol Lipi	Isoprenoid	Retinoids [	HMDB001	79-80-1	Organic cc
--	--	--	--	--	--	--	--	--
--	--	--	--	--	--	HMDB025	--	Organic cc
--	--	--	--	--	--	--	--	--
--	--	--	--	--	--	HMDB004	--	Organic cc
--	--	--	--	--	--	--	--	--
--	--	--	--	--	--	--	--	--
--	--	--	--	--	--	HMDB003	94356-18-	Organic cc
C09801	--	--	--	--	--	HMDB003	18296-44-	Organic cc
--	--	--	--	--	--	HMDB002	45234-03-	Organic cc
--	--	--	--	--	--	HMDB025	--	Organic cc
--	--	--	--	--	--	HMDB024	--	Organic cc
--	--	--	--	--	--	HMDB001	--	Organic cc
--	--	--	--	--	--	--	--	--
--	--	LMST0401	Sterol Lipi	Bile acids a	C24 bile ac	HMDB000	88179-71-	Organic cc
C09904	--	--	--	--	--	HMDB003	499-44-5	Organic cc
--	--	--	--	--	--	--	--	--
--	--	--	--	--	--	--	--	--
C03901	--	--	--	--	--	HMDB005	--	Organic cc
--	--	--	--	--	--	HMDB005	--	Organic cc

--	--	--	--	--	--	HMDB001	20488-28-	Organic co
C18533	--	--	--	--	--	HMDB003	86-86-2	Organic co
--	--	--	--	--	--	HMDB025	--	Organic co
--	--	--	--	--	--	HMDB000	63800-01-	Organic co
--	--	--	--	--	--	--	--	--
--	--	--	--	--	--	HMDB003	201288-95	Organic co
C18620	--	--	--	--	--	HMDB001	874-24-8	Organic co
--	--	--	--	--	--	--	--	--
--	--	--	--	--	--	--	--	--
--	--	--	--	--	--	--	--	--
--	--	--	--	--	--	HMDB002	114673-99	Organic co
--	--	--	--	--	--	--	--	--
--	--	--	--	--	--	HMDB000	--	Organic co
--	--	--	--	--	--	--	--	--
--	--	--	--	--	--	HMDB009	--	Organic co
--	--	--	--	--	--	HMDB003	--	Organic co
--	--	--	--	--	--	--	--	--
C13790	--	LMFA0105	Fatty Acyls	Fatty Acids	Hydroxy fa	HMDB022	--	--
--	--	--	--	--	--	--	--	--
--	--	--	--	--	--	--	--	--
--	--	--	--	--	--	HMDB024	--	Organic co
--	--	--	--	--	--	HMDB006	--	Organic co
--	--	--	--	--	--	--	--	--
C04853	Arachidon	LMFA0302	Fatty Acyls	Eicosanoic	Leukotrien	HMDB000	79516-82-	Organic co
--	--	--	--	--	--	HMDB024	--	Organic co
--	--	--	--	--	--	--	--	--
C01984	Aminoben	--	--	--	--	HMDB000	90-64-2	Organic co
--	--	--	--	--	--	HMDB003	59432-92-	Organic co
--	--	--	--	--	--	HMDB025	41708-76-	Organic co
C08362	Fatty acid	LMFA0103	Fatty Acyls	Fatty Acids	Unsaturate	HMDB000	373-49-9	Organic co
--	--	--	--	--	--	HMDB001	--	Organic co
--	--	--	--	--	--	HMDB024	--	Organic co
C14437	--	--	--	--	--	HMDB003	149-30-4	Organic co
C07422	--	--	--	--	--	HMDB001	76-74-4	Organic co
C14781	Arachidon	LMFA0308	Fatty Acyls	Eicosanoic	Epoxyeicos	HMDB000	877878-78	Organic co
--	--	LMFA0306	Fatty Acyls	Eicosanoic	Hydroxy/h	--	--	--
--	--	--	--	--	--	HMDB025	--	Organic co
--	--	--	--	--	--	HMDB025	--	Organic co
--	--	--	--	--	--	HMDB004	--	Organic co
--	--	--	--	--	--	HMDB007	--	Organic co
--	--	--	--	--	--	--	--	--
--	--	LMFA0500	Fatty Acyls	Fatty alcoh	-	--	--	--
--	--	--	--	--	--	HMDB001	145202-66	Organic co
--	--	--	--	--	--	HMDB001	59338-93-	Organic co
C01829	Tyrosine m	--	--	--	--	HMDB000	51-48-9	Organic co
--	--	--	--	--	--	HMDB024	--	Organic co
--	--	--	--	--	--	HMDB003	--	Organic co
--	--	--	--	--	--	HMDB004	182191-87	Organic co
--	--	--	--	--	--	HMDB025	--	Organic co
--	--	--	--	--	--	HMDB024	--	Organic co
--	--	--	--	--	--	HMDB025	--	Organic co

--	--	--	--	--	--	HMDB002	50439-45-	Organic co
--	--	--	--	--	--	--	--	--
--	--	--	--	--	--	HMDB002	182002-02	Organic co
--	--	LMFA0110	Fatty Acyls	Fatty Acids	Amino fatt	HMDB024	--	Organic co
C01300	Folate bios	--	--	--	--	HMDB030	--	Organic co
--	--	--	--	--	--	HMDB003	54854-89-	Organic co
--	--	--	--	--	--	--	--	--
--	--	--	--	--	--	--	--	--
--	--	--	--	--	--	HMDB030	--	Organic co
--	--	--	--	--	--	HMDB003	35897-95-	Organic co
--	--	--	--	--	--	HMDB024	83-44-3	Organic co
--	--	--	--	--	--	--	--	--
--	--	--	--	--	--	HMDB025	--	Organic co
--	--	--	--	--	--	--	--	--
--	--	--	--	--	--	--	--	--
--	--	--	--	--	--	--	--	--
--	--	--	--	--	--	--	--	--
--	--	--	--	--	--	HMDB024	--	Organic co
--	--	--	--	--	--	--	--	--
--	--	--	--	--	--	HMDB003	144841-1	Organic co
--	--	--	--	--	--	HMDB000	60113-83-	Organic co
C12058	--	--	--	--	--	HMDB001	17397-89-	Organic co
--	--	--	--	--	--	HMDB025	--	Organic co
--	--	--	--	--	--	--	--	--
C00664	--	--	--	--	--	HMDB000	2311-81-1	Organic co
--	--	--	--	--	--	--	--	--
--	--	--	--	--	--	HMDB003	11050-62-	Organic co
--	--	LMFA0103	Fatty Acyls	Fatty Acids	Unsaturate	--	--	--
--	--	--	--	--	--	--	--	--
--	--	--	--	--	--	HMDB030	--	Organic co
--	--	LMFA0302	Fatty Acyls	Eicosanoic	Leukotrien	HMDB001	--	Organic co
--	--	LMFA0305	Fatty Acyls	Eicosanoic	Hydroxy/h	HMDB006	--	Organic co
C05963	--	LMFA0303	Fatty Acyls	Eicosanoic	Thrombox	HMDB000	54397-85-	Organic co
--	--	--	--	--	--	--	--	--
--	--	--	--	--	--	HMDB024	--	Organic co
--	--	--	--	--	--	HMDB025	--	Organic co
--	--	--	--	--	--	HMDB025	--	Organic co
--	--	--	--	--	--	HMDB001	--	Organic co
--	--	LMGP0101	Glyceroph	Glyceroph	Diacylglyce	--	--	--
--	--	--	--	--	--	--	--	--
C07706	--	--	--	--	--	HMDB001	107133-36	Organic co
--	--	--	--	--	--	HMDB024	--	Organic co
--	--	--	--	--	--	--	--	--
--	--	--	--	--	--	--	--	--
--	--	--	--	--	--	HMDB024	--	Organic co
--	--	--	--	--	--	HMDB000	77727-17-	Organic co
--	--	--	--	--	--	HMDB001	--	Organic co
--	--	--	--	--	--	HMDB024	--	Organic co
--	--	--	--	--	--	--	--	--
C18811	--	--	--	--	--	HMDB025	--	Organic co
--	--	--	--	--	--	HMDB025	--	Organic co

--	--	--	--	--	--	HMDB004	142115-2	Organic co
--	--	--	--	--	--	HMDB025	--	Organic co
--	--	--	--	--	--	--	--	--
--	--	--	--	--	--	--	--	--
--	--	--	--	--	--	--	--	--
--	--	--	--	--	--	HMDB004	162854-9	Organic co
--	--	--	--	--	--	--	--	--
--	--	--	--	--	--	HMDB024	--	Organic co
C20286	Chemical	--	--	--	--	HMDB025	--	Organic co
--	--	--	--	--	--	HMDB003	56423-48-	Organic co
--	--	--	--	--	--	HMDB024	--	Organic co
--	--	--	--	--	--	--	--	--
C07536	--	--	--	--	--	HMDB001	57-43-2	Organic co
--	--	--	--	--	--	--	--	--
--	--	--	--	--	--	HMDB003	68441-17-	Organic co
--	--	--	--	--	--	HMDB003	7493-78-	Organic co
--	--	--	--	--	--	HMDB025	--	Organic co
--	--	LMFA0103	Fatty Acyls	Fatty Acids	Unsaturate	--	--	--
C16320	alpha-Linc	LMFA1000	Fatty Acyls	Fatty ether	-	HMDB003	52591-16-	Organic co
--	--	--	--	--	--	HMDB024	57775-29-	Organic co
C14383	--	LMST0201	Sterol Lipi	Steroids [S	C18 steroi	HMDB000	566-75-6	Organic co
--	--	--	--	--	--	HMDB025	--	Organic co
--	--	--	--	--	--	HMDB025	273203-6	Organic co
C07120	--	LMST0203	Sterol Lipi	Steroids [S	C21 steroi	HMDB001	3562-63-8	Organic co
--	--	--	--	--	--	--	--	--
--	--	LMFA0804	Fatty Acyls	Fatty amid	N-acyl eth	HMDB001	162758-9	Organic co
--	--	--	--	--	--	--	--	--
C02513	--	LMST0101	Sterol Lipi	Sterols [ST	Cholestero	--	--	--
--	--	--	--	--	--	--	--	--
--	--	--	--	--	--	HMDB029	--	_
--	--	--	--	--	--	--	--	--
C06439	--	LMFA0301	Fatty Acyls	Eicosanoid	Prostaglan	HMDB000	802-31-3	Organic co
--	--	--	--	--	--	HMDB002	--	Organic co
--	--	--	--	--	--	HMDB027	--	_
C00581	Glycine, se	--	--	--	--	HMDB000	352-97-6	Organic co
--	--	--	--	--	--	--	--	--
--	--	--	--	--	--	HMDB024	--	Organic co
--	--	--	--	--	--	HMDB003	80952-82-	Organic co
--	--	--	--	--	--	HMDB006	--	Organic co
--	--	--	--	--	--	HMDB003	2153-26-6	Organic co
--	--	--	--	--	--	HMDB000	10529-96-	Organic co
--	--	--	--	--	--	HMDB024	--	Organic co
--	--	--	--	--	--	--	--	--
--	--	--	--	--	--	HMDB001	--	Organic co
--	--	--	--	--	--	--	--	--
C14809	Arachidon	LMFA0309	Fatty Acyls	Eicosanoid	Hepoxilins	HMDB000	68860-46-	Organic co
C00909	Arachidon	LMFA0302	Fatty Acyls	Eicosanoid	Leukotrien	HMDB000	72059-45-	Organic co
--	--	--	--	--	--	HMDB003	119626-5	Organic co
C06861	--	--	--	--	--	HMDB001	36505-84-	Organic co
--	--	--	--	--	--	--	--	--
--	--	--	--	--	--	HMDB024	--	Organic co

--	--	--	--	--	--	--	--	--
--	--	LMPR0103	Prenol Lipi	Isoprenoid	C15 isopre	--	--	--
--	--	--	--	--	--	--	--	--
C09290	--	LMPR0103	Prenol Lipi	Isoprenoid	C15 isopre	--	--	--
--	--	--	--	--	--	HMDB024	--	_
--	--	--	--	--	--	HMDB030	--	Organic c
C00302	Cyanoami	--	--	--	--	HMDB006	138-16-9	Organic c
--	--	--	--	--	--	--	--	--
--	--	--	--	--	--	HMDB004	142036-1	Organic c
C08005	--	--	--	--	--	HMDB001	71195-58-	Organic c
--	--	--	--	--	--	--	--	--
--	--	--	--	--	--	HMDB003	21291-40-	Organic c
--	--	LMFA0701	Fatty Acyls	Fatty ester	Wax monc	HMDB003	137-66-6	Organic c
--	--	--	--	--	--	HMDB025	--	Organic c
--	--	--	--	--	--	HMDB024	--	Organic c
--	--	--	--	--	--	HMDB003	--	Organic c
--	--	--	--	--	--	HMDB003	75589-40-	Organic c
--	--	--	--	--	--	--	--	--
--	--	--	--	--	--	HMDB024	528-92-7	Organic c
--	--	--	--	--	--	HMDB002	--	Organic c
--	--	--	--	--	--	HMDB025	--	Organic c
--	--	--	--	--	--	--	--	--
--	--	--	--	--	--	HMDB004	87200-84-	Organic c
--	--	--	--	--	--	--	--	--
--	--	--	--	--	--	HMDB024	--	Organic c
--	--	--	--	--	--	--	--	--
--	--	--	--	--	--	HMDB024	--	Organic c
C17251	Glucosinol	--	--	--	--	--	--	--
--	--	LMST0401	Sterol Lipi	Bile acids a	C24 bile ac	HMDB000	2458/8/4	Organic c
--	--	--	--	--	--	--	--	--
--	--	LMFA0301	Fatty Acyls	Eicosanoic	Prostaglan	HMDB001	398138-2	Organic c
--	--	--	--	--	--	HMDB003	222609-9	Organic c
--	--	--	--	--	--	HMDB029	--	_
--	--	--	--	--	--	--	--	--
--	--	--	--	--	--	HMDB002	63912-01-	Organic c
--	--	--	--	--	--	HMDB025	--	Organic c
--	--	LMFA0707	Fatty Acyls	Fatty ester	Fatty acyl	HMDB006	282523-6	Organic c
--	--	--	--	--	--	HMDB000	41708-93-	Organic c
--	--	--	--	--	--	--	--	--
--	--	LMFA0600	Fatty Acyls	Fatty aldeh	-	HMDB003	5090-41-5	Organic c
--	--	--	--	--	--	HMDB026	--	Organic c
--	--	--	--	--	--	HMDB003	28125-84-	Organic c
C09289	--	LMPR0103	Prenol Lipi	Isoprenoid	C15 isopre	--	--	--
--	--	--	--	--	--	HMDB024	--	Organic c
--	--	--	--	--	--	HMDB025	--	Organic c
--	--	--	--	--	--	--	--	--
C05463	--	LMST0504	Sterol Lipi	Steroid co	Taurine co	HMDB000	516-50-7	Organic c
--	--	--	--	--	--	--	--	--
--	--	LMFA0115	Fatty Acyls	Fatty Acids	Heterocycl	HMDB006	57818-43-	Organic c
--	--	--	--	--	--	HMDB025	110-97-4	Organic c
--	--	--	--	--	--	HMDB024	566-58-5	Organic c

--	--	--	--	--	--	HMDB004	--	Organic cc
--	--	--	--	--	--	HMDB024	--	Organic cc
--	--	--	--	--	--	HMDB003	4430-41-5	Organic cc
--	--	LMFA011C	Fatty Acyls	Fatty Acids	Amino fatt	--	--	--
--	--	--	--	--	--	HMDB024	--	Organic cc
C14411	--	--	--	--	--	HMDB025	77-73-6	Organic cc
--	--	--	--	--	--	--	--	--
--	--	LMSP0104	Sphingolip	Sphingoid	Sphingoid	HMDB024	--	Organic cc
--	--	--	--	--	--	HMDB005	41114-00-	Organic cc
--	--	--	--	--	--	HMDB024	--	Organic cc
--	--	--	--	--	--	--	--	--
--	--	--	--	--	--	HMDB003	30320-70-	Organic cc
--	--	LMFA050C	Fatty Acyls	Fatty alcoh	-	HMDB003	53046-97-	Organic cc
C14132	--	--	--	--	--	HMDB024	--	Organic cc
--	--	--	--	--	--	HMDB025	303-33-3	Organic cc
--	--	--	--	--	--	--	--	--
--	--	--	--	--	--	HMDB002	--	Organic cc
--	--	--	--	--	--	HMDB024	--	Organic cc
--	--	--	--	--	--	HMDB025	--	Organic cc
--	--	--	--	--	--	--	--	--
--	--	--	--	--	--	HMDB024	--	Organic cc
--	--	--	--	--	--	HMDB003	99901-23-	Organic cc
--	--	LMFA0106	Fatty Acyls	Fatty Acids	Oxo fatty a	--	--	--
C20387	Biotin met	--	--	--	--	HMDB000	40720-05-	Organic cc
--	--	--	--	--	--	--	--	--
--	--	--	--	--	--	HMDB003	200127-56	Organic cc
C07645	--	--	--	--	--	HMDB006	--	Organic cc
--	--	--	--	--	--	--	--	--
--	--	--	--	--	--	HMDB003	104324-40	Organic cc
--	--	--	--	--	--	--	--	--
--	--	--	--	--	--	HMDB001	--	Organic cc
--	--	--	--	--	--	--	--	--
--	--	--	--	--	--	--	--	--
--	--	--	--	--	--	--	--	--
--	--	--	--	--	--	HMDB024	--	Organic cc
--	--	--	--	--	--	--	--	--
--	--	--	--	--	--	HMDB003	62512-27-	Organic cc
--	--	--	--	--	--	HMDB003	4868-24-(	Organic cc
--	--	LMFA060C	Fatty Acyls	Fatty aldeh	-	HMDB024	--	Organic cc
C01414	Diterpenoi	LMPR0104	Prenol Lipi	Isoprenoid	C20 isopre	--	--	--
--	--	--	--	--	--	--	--	--
--	--	--	--	--	--	--	--	--
--	--	--	--	--	--	--	--	--
--	--	--	--	--	--	HMDB025	--	Organic cc
--	--	LMSP0205	Sphingolip	Ceramides	Ceramide	HMDB001	--	Organic cc
--	--	--	--	--	--	HMDB024	--	Organic cc
--	--	--	--	--	--	HMDB024	--	Organic cc
--	--	LMPK1501	Polyketide	Phenolic li	Alkyl phen	HMDB003	501-24-6	Organic cc
--	--	LMFA0103	Fatty Acyls	Fatty Acids	Unsaturate	--	--	--
--	--	--	--	--	--	--	--	--
--	--	LMST0401	Sterol Lipi	Bile acids a	C24 bile ac	HMDB000	2304-89-4	Organic cc

--	--	--	--	--	--	--	--	--
C01477	Flavonoid	LMPK1211	Polyketide	Flavonoids	Flavones a	HMDB000	520-36-5	Organic cc
--	--	--	--	--	--	--	--	--
--	--	--	--	--	--	--	--	--
--	--	--	--	--	--	HMDB024	--	Organic cc
--	--	--	--	--	--	HMDB024	--	Organic cc
--	--	--	--	--	--	HMDB024	--	Organic cc
--	--	LMFA0701	Fatty Acyls	Fatty ester	Wax monc	HMDB002	26266-57-	Organic cc
--	--	--	--	--	--	HMDB029	--	_
C04643	--	LMST0401	Sterol Lipi	Bile acids a	C24 bile ac	HMDB000	911-40-0	Organic cc
--	--	--	--	--	--	HMDB024	--	_
--	--	--	--	--	--	--	--	--
--	--	--	--	--	--	HMDB025	--	Organic cc
--	--	--	--	--	--	HMDB025	--	Organic cc
--	--	LMFA0103	Fatty Acyls	Fatty Acids	Unsaturate	--	--	--
--	--	--	--	--	--	--	--	--
--	--	--	--	--	--	--	--	--
--	--	--	--	--	--	--	--	--
--	--	--	--	--	--	--	--	--
--	--	--	--	--	--	--	--	--
--	--	--	--	--	--	--	--	--
--	--	--	--	--	--	--	--	--
--	--	--	--	--	--	HMDB003	--	Organic cc
C09633	--	LMPR0103	Prenol Lipi	Isoprenoid	C15 isopre	HMDB003	529-05-5	Organic cc
--	--	--	--	--	--	HMDB025	--	Organic cc
--	--	--	--	--	--	HMDB025	--	Organic cc
--	--	--	--	--	--	HMDB026	--	Organic cc
--	--	LMFA0500	Fatty Acyls	Fatty alcoh	-	HMDB004	141947-42	Organic cc
--	--	--	--	--	--	--	--	--
--	--	--	--	--	--	HMDB000	1159-66-6	Organic cc
--	--	--	--	--	--	HMDB025	--	Organic cc
--	--	--	--	--	--	HMDB024	6533-00-2	Organic cc
--	--	LMFA0103	Fatty Acyls	Fatty Acids	Unsaturate	--	--	--
--	--	--	--	--	--	HMDB024	--	Organic cc
--	--	LMFA0707	Fatty Acyls	Fatty ester	Fatty acyl (	HMDB024	--	Organic cc
--	--	--	--	--	--	HMDB001	--	Organic cc
--	--	LMST0502	Sterol Lipi	Steroid co	Sulfates [S	HMDB000	60320-05-	Organic cc
--	--	--	--	--	--	HMDB004	32448-19-	Organic cc
--	--	--	--	--	--	--	--	--
--	--	--	--	--	--	HMDB029	--	_
--	--	LMGP0401	Glyceroph	Glyceroph	Diacylglyce	--	--	--
--	--	--	--	--	--	HMDB026	--	_
--	--	--	--	--	--	HMDB003	18454-88-	Organic cc
--	--	--	--	--	--	HMDB026	--	_
--	--	--	--	--	--	HMDB025	--	Organic cc
--	--	LMFA0200	Fatty Acyls	Octadecar	Other Octa	HMDB004	28979-44-	Organic cc
--	--	--	--	--	--	HMDB004	1191-62-4	Organic cc
C13915	--	LMSP0104	Sphingolip	Sphingoid	Sphingoid	--	--	--
--	--	--	--	--	--	HMDB025	--	Organic cc
--	--	--	--	--	--	HMDB028	--	_
--	--	--	--	--	--	HMDB024	633-35-2	Organic cc

--	--	--	--	--	--	--	--	--
--	--	--	--	--	--	HMDB024	--	Organic cc
--	--	--	--	--	--	--	--	--
C16344	alpha-Linc	LMFA0103	Fatty Acyls	Fatty Acids	Unsaturate	--	--	--
--	--	LMFA0500	Fatty Acyls	Fatty alcoh	-	HMDB003	--	Organic cc
--	--	--	--	--	--	HMDB024	--	Organic cc
--	--	--	--	--	--	--	--	--
--	--	--	--	--	--	--	--	--
--	--	--	--	--	--	--	--	--
--	--	--	--	--	--	HMDB003	--	Organic cc
--	--	LMPK1211	Polyketide	Flavonoids	Flavones a	HMDB003	129145-54	Organic cc
--	--	--	--	--	--	--	--	--
--	--	--	--	--	--	HMDB003	122130-27	Organic cc
--	--	--	--	--	--	HMDB003	--	Organic cc
--	--	--	--	--	--	HMDB024	--	Organic cc
--	--	--	--	--	--	HMDB011	--	Organic cc
C16343	--	--	--	--	--	HMDB004	56797-44-	Organic cc
C01239	--	--	--	--	--	HMDB000	--	Organic cc
C10926	--	--	--	--	--	HMDB025	51235-04-	Organic cc
--	--	--	--	--	--	HMDB024	--	Organic cc
--	--	--	--	--	--	HMDB025	--	Organic cc
--	--	--	--	--	--	HMDB001	286930-03	Organic cc
--	--	LMST0401	Sterol Lipi	Bile acids a	C24 bile ac	HMDB000	75110-48-	Organic cc
--	--	--	--	--	--	HMDB029	--	_
--	--	--	--	--	--	HMDB024	--	Organic cc
--	--	--	--	--	--	--	--	--
--	--	--	--	--	--	--	--	--
--	--	LMFA0701	Fatty Acyls	Fatty ester	Wax mono	HMDB003	1741-93-1	Organic cc
--	--	--	--	--	--	HMDB025	--	Organic cc
--	--	--	--	--	--	--	--	--
--	--	--	--	--	--	--	--	--
--	--	--	--	--	--	HMDB025	--	Organic cc
--	--	--	--	--	--	HMDB003	53011-73-	Organic cc
--	--	--	--	--	--	--	--	--
--	--	--	--	--	--	HMDB024	--	Organic cc
--	--	--	--	--	--	--	--	--
--	--	--	--	--	--	HMDB001	--	Organic cc
--	--	--	--	--	--	HMDB006	--	Organic cc
--	--	LMFA0500	Fatty Acyls	Fatty alcoh	-	--	--	--
--	--	--	--	--	--	HMDB003	75207-46-	Organic cc
--	--	--	--	--	--	HMDB001	--	Organic cc
--	--	--	--	--	--	--	--	--
--	--	--	--	--	--	HMDB024	--	Organic cc
--	--	--	--	--	--	HMDB003	18368-95-	Organic cc
--	--	--	--	--	--	HMDB025	--	Organic cc
--	--	--	--	--	--	HMDB002	--	Organic cc
--	--	--	--	--	--	HMDB024	--	Organic cc
--	--	--	--	--	--	HMDB003	--	Organic cc
--	--	LMST0401	Sterol Lipi	Bile acids a	C24 bile ac	HMDB000	5255-17-4	Organic cc
C05464	--	--	--	--	--	HMDB000	360-65-6	Organic cc
--	--	--	--	--	--	HMDB024	--	_

--	--	--	--	--	--	HMDB028	--	_
--	--	--	--	--	--	HMDB027	--	_
--	--	--	--	--	--	HMDB003	28129-52-	Organic cc
--	--	--	--	--	--	HMDB000	49777-13-	Organic cc
C00517	Fatty acid	LMFA060	Fatty Acyls	Fatty aldeh	-	HMDB000	629-80-1	Organic cc
--	--	--	--	--	--	--	--	--
--	--	--	--	--	--	HMDB025	--	Organic cc
--	--	LMFA010	Fatty Acyls	Fatty Acids	Oxo fatty a	--	--	--
--	--	--	--	--	--	--	--	--
--	--	--	--	--	--	HMDB003	227456-2	Organic cc
--	--	--	--	--	--	HMDB004	162232-3	Organic cc
--	--	--	--	--	--	HMDB025	--	Organic cc
--	--	--	--	--	--	--	--	--
--	--	LMGP020	Glyceroph	Glyceroph	Monoacylg	HMDB001	--	Organic cc
--	--	--	--	--	--	HMDB002	104-09-6	Organic cc
--	--	--	--	--	--	HMDB004	83551-03-	Organic cc
--	--	--	--	--	--	HMDB024	--	Organic cc
C16526	Biosynthes	LMFA010	Fatty Acyls	Fatty Acids	Unsaturate	HMDB000	5561-99-	Organic cc
--	--	--	--	--	--	--	--	--
--	--	--	--	--	--	HMDB003	83329-14-	Organic cc
--	--	--	--	--	--	--	--	--
--	--	--	--	--	--	HMDB024	--	Organic cc
C02571	Insulin resi	--	--	--	--	HMDB000	3040-38-	Organic cc
--	--	--	--	--	--	HMDB026	--	_
C03242	Linoleic ac	LMFA010	Fatty Acyls	Fatty Acids	Unsaturate	HMDB000	1783-84-	Organic cc
--	--	--	--	--	--	HMDB030	--	Organic cc
C20045	Staurospo	--	--	--	--	--	--	--
--	--	--	--	--	--	HMDB024	--	Organic cc
--	--	LMGP020	Glyceroph	Glyceroph	Monoacylg	HMDB001	--	Organic cc
--	--	--	--	--	--	HMDB024	--	_
--	--	--	--	--	--	HMDB002	18997-54-	Organic cc
--	--	--	--	--	--	--	--	--
--	--	--	--	--	--	HMDB000	--	Organic cc
--	--	--	--	--	--	HMDB025	--	Organic cc
--	--	--	--	--	--	HMDB024	--	Organic cc
--	--	--	--	--	--	HMDB003	113301-6	Organic cc
--	--	--	--	--	--	HMDB024	--	Organic cc
C05962	Arachidon	LMFA0301	Fatty Acyls	Eicosanoid	Prostaglan	HMDB000	67786-53-	Organic cc
--	--	--	--	--	--	--	--	--
C04230	--	LMGP010	Glyceroph	Glyceroph	Monoacylg	HMDB001	76790-27-	Organic cc
--	--	--	--	--	--	--	--	--
C05961	Arachidon	LMFA0301	Fatty Acyls	Eicosanoid	Prostaglan	HMDB000	58962-34-	Organic cc
--	--	--	--	--	--	HMDB003	165450-1	Organic cc
--	--	--	--	--	--	HMDB025	--	Organic cc
--	--	--	--	--	--	--	--	--
--	--	--	--	--	--	--	--	--
C06570	Tetracyclin	--	--	--	--	HMDB001	60-54-8	Organic cc
--	--	--	--	--	--	HMDB024	--	Organic cc
--	--	--	--	--	--	HMDB025	--	Organic cc
C02367	--	LMFA010	Fatty Acyls	Fatty Acids	Oxo fatty a	HMDB001	--	Organic cc
--	--	--	--	--	--	HMDB030	--	Organic cc

--	--	--	--	--	--	HMDB001	--	Organic cc
--	--	--	--	--	--	HMDB003	114437-32	Organic cc
--	--	--	--	--	--	HMDB025	--	Organic cc
--	--	--	--	--	--	--	--	--
C05629	Phenylalar	--	--	--	--	HMDB000	501-52-0	Organic cc
--	--	--	--	--	--	HMDB003	122-74-7	Organic cc
--	--	--	--	--	--	HMDB025	--	Organic cc
--	--	--	--	--	--	HMDB025	--	Organic cc
C01227	Steroid ho	LMST0202	Sterol Lipi	Steroids [S	C19 steroi	HMDB000	53-43-0	Organic cc
--	--	--	--	--	--	HMDB003	33123-07-	Organic cc
--	--	--	--	--	--	--	--	--
--	--	--	--	--	--	--	--	--
--	--	--	--	--	--	--	--	--
--	--	--	--	--	--	HMDB003	--	Organic cc
--	--	LMGP0105	Glyceroph	Glyceroph	Monoacylg	HMDB001	--	Organic cc
C08117	--	--	--	--	--	HMDB001	97519-39-	Organic cc
--	--	--	--	--	--	HMDB024	--	Organic cc
--	--	--	--	--	--	HMDB025	--	Organic cc
--	--	--	--	--	--	HMDB024	--	Organic cc
--	--	--	--	--	--	HMDB024	--	Organic cc
--	--	--	--	--	--	HMDB025	--	Organic cc
--	--	--	--	--	--	HMDB003	87605-91-	Organic cc
--	--	--	--	--	--	--	--	--
--	--	--	--	--	--	HMDB027	--	_
--	--	--	--	--	--	--	--	--
--	--	--	--	--	--	HMDB003	--	Organic cc
--	--	--	--	--	--	--	--	--
--	--	--	--	--	--	HMDB002	22850-13-	Organic cc
--	--	--	--	--	--	--	--	--
C05954	--	--	--	--	--	HMDB000	13367-85-	Organic cc
--	--	--	--	--	--	--	--	--
--	--	LMGP0205	Glyceroph	Glyceroph	Monoacylg	HMDB006	--	Organic cc
--	--	--	--	--	--	HMDB024	--	Organic cc
--	--	--	--	--	--	HMDB025	112830-95	Organic cc
--	--	--	--	--	--	--	--	--
--	--	--	--	--	--	HMDB024	--	Organic cc
--	--	--	--	--	--	--	--	--
--	--	--	--	--	--	HMDB025	--	Organic cc
--	--	--	--	--	--	HMDB011	--	Organic cc
--	--	--	--	--	--	HMDB003	--	Organic cc
--	--	--	--	--	--	HMDB025	--	Organic cc
C07212	Xylene deg	--	--	--	--	HMDB005	95-47-6	Organic cc
C14395	--	--	--	--	--	HMDB005	98-83-9	Organic cc
--	--	--	--	--	--	HMDB000	619-04-5	Organic cc
--	--	--	--	--	--	HMDB025	--	Organic cc
--	--	--	--	--	--	--	--	--
--	--	LMFA0500	Fatty Acyls	Fatty alcoh	-	HMDB003	64275-73-	Organic cc
--	--	--	--	--	--	HMDB004	--	Organic cc
C06124	Sphingolip	LMSP0105	Sphingolip	Sphingoid	Sphingoid	HMDB000	26993-30-	Organic cc
--	--	--	--	--	--	--	--	--
--	--	--	--	--	--	HMDB025	--	Organic cc

--	--	--	--	--	--	HMDB001	152459-9	Organic cc
--	--	--	--	--	--	HMDB025	--	Organic cc
--	--	--	--	--	--	HMDB024	2920-86-7	Organic cc
--	--	--	--	--	--	--	--	--
--	--	--	--	--	--	HMDB028	--	_
--	--	--	--	--	--	--	--	--
C06006	Valine, leu	LMFA0105	Fatty Acyls	Fatty Acids	Hydroxy fa	HMDB000	113919-07	Organic cc
--	--	LMFA0103	Fatty Acyls	Fatty Acids	Unsaturate	--	--	--
C06834	--	--	--	--	--	HMDB000	134523-00	Organic cc
--	--	--	--	--	--	HMDB006	--	Organic cc
--	--	--	--	--	--	HMDB024	--	Organic cc
--	--	--	--	--	--	HMDB003	--	Organic cc
--	--	--	--	--	--	--	--	--
C02934	Sphingolip	LMSP0102	Sphingolip	Sphingoid	Sphinganin	HMDB000	16105-69-	Organic cc
--	--	--	--	--	--	HMDB006	--	Organic cc
--	--	--	--	--	--	--	--	--
C11260	--	--	--	--	--	HMDB024	115-69-5	Organic cc
--	--	--	--	--	--	HMDB025	--	Organic cc
--	--	--	--	--	--	HMDB025	--	Organic cc
--	--	--	--	--	--	HMDB024	--	Organic cc
--	--	--	--	--	--	HMDB004	100019-33	Organic cc
--	--	LMGP0105	Glyceroph	Glyceroph	Monoacylg	HMDB001	20559-16-	Organic cc
--	--	--	--	--	--	HMDB000	103745-46	Organic cc
--	--	--	--	--	--	HMDB000	125356-86	Organic cc
--	--	--	--	--	--	HMDB001	--	Organic cc
C11135	Steroid ho	LMST0501	Sterol Lipi	Steroid co	Glucuronic	HMDB000	1852-43-3	Organic cc
C02592	--	LMST0504	Sterol Lipi	Steroid co	Taurine co	HMDB000	516-90-5	Organic cc
--	--	--	--	--	--	HMDB029	--	_
C13828	--	--	--	--	--	HMDB001	--	Organic cc
--	--	--	--	--	--	HMDB002	118-61-6	Organic cc
C06424	Fatty acid	LMFA0101	Fatty Acyls	Fatty Acids	Straight ch	HMDB000	544-63-8	Organic cc
--	--	--	--	--	--	--	--	--
--	--	--	--	--	--	HMDB024	--	Organic cc
--	--	--	--	--	--	--	--	--
--	--	--	--	--	--	HMDB004	87-20-7	Organic cc
C09947	--	--	--	--	--	HMDB003	500-64-1	Organic cc
--	--	--	--	--	--	--	--	--
--	--	--	--	--	--	HMDB030	--	Organic cc
C05961	Arachidon	LMFA0301	Fatty Acyls	Eicosanoic	Prostaglan	HMDB000	58962-34-	Organic cc
--	--	--	--	--	--	HMDB003	22882-87-	Organic cc
--	--	--	--	--	--	HMDB025	--	Organic cc
--	--	--	--	--	--	HMDB006	--	Organic cc
--	--	--	--	--	--	HMDB026	--	_
C05629	Phenylalar	--	--	--	--	HMDB000	501-52-0	Organic cc
--	--	--	--	--	--	HMDB030	--	Organic cc
--	--	LMFA0103	Fatty Acyls	Fatty Acids	Unsaturate	HMDB006	--	Organic cc
--	--	--	--	--	--	--	--	--
--	--	--	--	--	--	HMDB024	--	Organic cc
--	--	--	--	--	--	HMDB024	4276/9/9	Organic cc
--	--	--	--	--	--	HMDB029	--	_
--	--	--	--	--	--	HMDB003	197247-23	Organic cc

--	--	--	--	--	--	HMDB000	3475-68-1	Organic cc
--	--	--	--	--	--	HMDB002	68040-95-	Organic cc
--	--	--	--	--	--	--	--	--
--	--	LMFA0701	Fatty Acyls	Fatty ester	Wax monc	HMDB003	2198-61-(	Organic cc
--	--	--	--	--	--	HMDB029	--	_
--	--	--	--	--	--	HMDB029	--	_
C04230	--	LMGP0105	Glyceroph	Glyceroph	Monoacylç	HMDB001	--	Organic cc
C07620	--	--	--	--	--	HMDB001	56-53-1	Organic cc
--	--	--	--	--	--	HMDB026	--	Organic cc
--	--	--	--	--	--	HMDB025	25447-65-	Organic cc
--	--	--	--	--	--	HMDB028	--	_
--	--	--	--	--	--	HMDB027	--	_
--	--	--	--	--	--	HMDB024	--	Organic cc
--	--	--	--	--	--	HMDB011	--	Organic cc
--	--	--	--	--	--	HMDB029	--	_
--	--	--	--	--	--	HMDB025	--	Organic cc
D01924	--	LMFA0500	Fatty Acyls	Fatty alcoh	-	HMDB000	112-92-5	Organic cc
--	--	LMFA0105	Fatty Acyls	Fatty Acids	Hydroxy fa	HMDB006	--	Organic cc
--	--	--	--	--	--	HMDB001	58-32-2	Organic cc
--	--	--	--	--	--	HMDB028	--	_
--	--	--	--	--	--	HMDB003	69350-60-	Organic cc
C04230	--	LMGP0105	Glyceroph	Glyceroph	Monoacylç	HMDB001	--	Organic cc
--	--	--	--	--	--	HMDB030	--	Organic cc
--	--	--	--	--	--	HMDB025	--	Organic cc
--	--	--	--	--	--	HMDB003	--	Organic cc
--	--	--	--	--	--	HMDB003	13894-72-	Organic cc
--	--	--	--	--	--	HMDB025	--	Organic cc
--	--	--	--	--	--	--	--	--
--	--	--	--	--	--	HMDB003	59300-52-	Organic cc
--	--	--	--	--	--	HMDB003	59958-20-	Organic cc
--	--	--	--	--	--	--	--	--
--	--	--	--	--	--	HMDB029	--	_
--	--	--	--	--	--	HMDB025	--	Organic cc
--	--	--	--	--	--	--	--	--
--	--	--	--	--	--	HMDB024	--	Organic cc
--	--	--	--	--	--	--	--	--
--	--	--	--	--	--	--	--	--
C14803	Metabolisr	--	--	--	--	HMDB006	--	Organic cc
--	--	--	--	--	--	HMDB030	--	Organic cc
--	--	--	--	--	--	--	--	--
--	--	--	--	--	--	HMDB025	--	Organic cc
--	--	--	--	--	--	HMDB030	--	Organic cc
--	--	LMFA0802	Fatty Acyls	Fatty amid	N-acyl am	HMDB006	--	Organic cc
--	--	--	--	--	--	--	--	--
C07172	--	--	--	--	--	HMDB001	84-96-8	Organic cc
C04805	Arachidon	LMFA0306	Fatty Acyls	Eicosanoic	Hydroxy/h	HMDB001	70608-72-	Organic cc
--	--	--	--	--	--	--	--	--
--	--	--	--	--	--	HMDB024	--	Organic cc
--	--	--	--	--	--	HMDB029	--	_
--	--	--	--	--	--	HMDB024	--	Organic cc
--	--	--	--	--	--	HMDB028	--	_

--	--	--	--	--	--	HMDB025	--	Organic cc
C06241	--	--	--	--	--	HMDB000	--	Organic cc
--	--	--	--	--	--	HMDB025	--	Organic cc
--	--	--	--	--	--	HMDB024	--	Organic cc
--	--	--	--	--	--	--	--	--
--	--	--	--	--	--	HMDB003	89238-78-	Organic cc
--	--	--	--	--	--	HMDB025	--	Organic cc
--	--	--	--	--	--	HMDB030	--	Organic cc
C04230	--	LMGP0105	Glyceroph	Glyceroph	Monoacylg	HMDB001	--	Organic cc
--	--	--	--	--	--	HMDB000	--	Organic cc
C15562	--	--	--	--	--	HMDB003	100-46-9	Organic cc
C01620	Ascorbate	--	--	--	--	HMDB000	7306-96-9	Organic cc
--	--	--	--	--	--	HMDB025	--	Organic cc
--	--	--	--	--	--	HMDB003	104-53-0	Organic cc
--	--	--	--	--	--	--	--	--
--	--	--	--	--	--	HMDB026	--	Organic cc
C20963	Biosynthes	--	--	--	--	--	--	--
C01789	--	LMST0103	Sterol Lipi	Sterols [ST	Ergosterols	HMDB003	4651-51-8	Organic cc
--	--	--	--	--	--	--	--	--
--	--	LMGP0205	Glyceroph	Glyceroph	Monoacylg	HMDB001	--	Organic cc
--	--	--	--	--	--	HMDB026	--	_
C00408	Lysine deg	--	--	--	--	HMDB000	3105-95-1	Organic cc
--	--	--	--	--	--	HMDB001	--	Organic cc
--	--	--	--	--	--	HMDB003	21651-54-	Organic cc
--	--	--	--	--	--	HMDB025	6197-30-4	Organic cc
--	--	--	--	--	--	HMDB025	--	Organic cc
--	--	--	--	--	--	--	--	--
--	--	LMFA0707	Fatty Acyls	Fatty ester	Fatty acyl c	HMDB024	--	Organic cc
C16323	alpha-Linc	--	--	--	--	HMDB003	21944-83-	Organic cc
--	--	--	--	--	--	--	--	--
--	--	--	--	--	--	HMDB003	21391-99-	Organic cc
--	--	--	--	--	--	HMDB025	--	Organic cc
--	--	--	--	--	--	HMDB025	--	Organic cc
--	--	--	--	--	--	HMDB003	6879-93-2	Organic cc
--	--	--	--	--	--	HMDB030	--	Organic cc
--	--	LMFA0103	Fatty Acyls	Fatty Acids	Unsaturate	--	--	--
--	--	--	--	--	--	--	--	--
C05482	Steroid ho	LMST0203	Sterol Lipi	Steroids [S	C21 steroi	HMDB000	516-38-1	Organic cc
--	--	--	--	--	--	--	--	--
--	--	--	--	--	--	HMDB025	--	Organic cc
--	--	--	--	--	--	HMDB024	--	Organic cc
C11991	Biosynthes	LMPK0400	Polyketide	Macrolides	-	--	--	--
C05503	Steroid ho	LMST0501	Sterol Lipi	Steroid co	Glucuronic	HMDB000	15270-30-	Organic cc
--	--	LMGP0301	Glyceroph	Glyceroph	Diacylglyce	--	--	--
--	--	--	--	--	--	HMDB028	--	_
--	--	--	--	--	--	HMDB003	66983-88-	Organic cc
--	--	--	--	--	--	--	--	--
--	--	--	--	--	--	HMDB003	124439-27	Organic cc
C06427	alpha-Linc	LMFA0103	Fatty Acyls	Fatty Acids	Unsaturate	HMDB000	463-40-1	Organic cc
--	--	--	--	--	--	HMDB028	--	_
--	--	--	--	--	--	HMDB027	--	_

--	--	--	--	--	--	HMDB004	79190-14-	Organic cc
C15182	--	--	--	--	--	HMDB001	162635-04	Organic cc
--	--	--	--	--	--	HMDB011	--	Organic cc
--	--	--	--	--	--	HMDB000	2140-61-6	Organic cc
--	--	--	--	--	--	HMDB003	5989-43-5	Organic cc
--	--	--	--	--	--	--	--	--
--	--	--	--	--	--	--	--	--
--	--	--	--	--	--	--	--	--
--	--	LMGP0105	Glyceroph	Glyceroph	Monoacylg	HMDB001	22252-07-	Organic cc
--	--	--	--	--	--	--	--	--
--	--	--	--	--	--	HMDB028	--	_
--	--	--	--	--	--	HMDB027	--	_
--	--	--	--	--	--	HMDB003	2110-18-1	Organic cc
--	--	--	--	--	--	HMDB003	10039-39-	Organic cc
--	--	--	--	--	--	HMDB024	--	Organic cc
--	--	--	--	--	--	HMDB002	--	Organic cc
--	--	--	--	--	--	--	--	--
--	--	LMGP0105	Glyceroph	Glyceroph	Monoacylg	HMDB006	67341-29-	Organic cc
--	--	--	--	--	--	HMDB025	--	Organic cc
--	--	--	--	--	--	HMDB004	162287-96	Organic cc
--	--	--	--	--	--	--	--	--
--	--	--	--	--	--	HMDB005	--	Organic cc
--	--	--	--	--	--	HMDB028	--	_
--	--	--	--	--	--	HMDB027	--	_
--	--	--	--	--	--	HMDB028	--	_
--	--	--	--	--	--	--	--	--
--	--	--	--	--	--	HMDB003	120021-87	Organic cc
--	--	--	--	--	--	--	--	--
--	--	LMFA0802	Fatty Acyls	Fatty amid	N-acyl am	--	--	--
--	--	--	--	--	--	HMDB026	--	Organic cc
--	--	--	--	--	--	--	--	--
--	--	--	--	--	--	HMDB024	--	Organic cc
--	--	--	--	--	--	HMDB030	--	Organic cc
C14829	--	LMFA0200	Fatty Acyls	Octadecar	Other Octa	HMDB000	7293-40-5	Organic cc
--	--	--	--	--	--	HMDB024	--	Organic cc
--	--	--	--	--	--	HMDB024	--	Organic cc
C18043	Steroid ho	LMST0502	Sterol Lipi	Steroid co	Sulfates [S	HMDB000	1256-86-6	Organic cc
--	--	--	--	--	--	HMDB030	--	Organic cc
--	--	--	--	--	--	HMDB030	--	Organic cc
C07338	Linoleic ac	LMFA0200	Fatty Acyls	Octadecar	Other Octa	HMDB006	--	Organic cc
--	--	--	--	--	--	HMDB024	--	Organic cc
--	--	--	--	--	--	--	--	--
C03033	--	--	--	--	--	HMDB000	79254-98-	Organic cc
--	--	--	--	--	--	HMDB003	119784-25	Organic cc
--	--	--	--	--	--	HMDB025	--	Organic cc
--	--	--	--	--	--	--	--	--
--	--	--	--	--	--	HMDB025	--	Organic cc
--	--	--	--	--	--	HMDB027	--	_
--	--	--	--	--	--	HMDB025	--	Organic cc
--	--	--	--	--	--	--	--	--
--	--	--	--	--	--	--	--	--

--	--	--	--	--	--	--	--	--
--	--	--	--	--	--	HMDB002	92327-75-	Organic co
--	--	--	--	--	--	--	--	--
--	--	LMGP0105	Glyceroph	Glyceroph	Monoacylg	HMDB001	22252-07-	Organic co
--	--	--	--	--	--	HMDB029	--	_
--	--	--	--	--	--	HMDB003	20528-70-	Organic co
--	--	--	--	--	--	HMDB024	--	_
--	--	--	--	--	--	--	--	--
--	--	--	--	--	--	HMDB025	--	Organic co
--	--	--	--	--	--	--	--	--
--	--	--	--	--	--	HMDB003	212773-3	Organic co
--	--	--	--	--	--	HMDB028	--	_
--	--	--	--	--	--	--	--	--
--	--	--	--	--	--	HMDB000	--	Organic co
--	--	--	--	--	--	HMDB024	--	Organic co
C06126	--	--	--	--	--	HMDB000	--	Organic co
C07035	--	--	--	--	--	HMDB001	40580-59-	Organic co
--	--	--	--	--	--	HMDB024	--	Organic co
--	--	--	--	--	--	HMDB025	--	Organic co
--	--	LMGP0205	Glyceroph	Glyceroph	Monoacylg	HMDB001	--	Organic co
--	--	--	--	--	--	HMDB003	256505-5	Organic co
--	--	--	--	--	--	HMDB006	1913287-8	Organic co
--	--	--	--	--	--	--	--	--
--	--	--	--	--	--	HMDB025	--	Organic co
--	--	--	--	--	--	HMDB024	--	_
--	--	--	--	--	--	--	--	--
--	--	--	--	--	--	HMDB025	--	Organic co
--	--	--	--	--	--	HMDB024	--	Organic co
--	--	--	--	--	--	--	--	--
--	--	LMFA1301	Fatty Acyls	Fatty acyl g	Fatty acyl g	HMDB003	347852-04	Organic co
--	--	--	--	--	--	HMDB026	--	_
--	--	--	--	--	--	HMDB024	--	Organic co
--	--	--	--	--	--	HMDB027	--	_
--	--	--	--	--	--	--	--	--
--	--	--	--	--	--	HMDB002	7219-59-2	Organic co
--	--	--	--	--	--	HMDB025	--	Organic co
--	--	--	--	--	--	HMDB004	39354-56-	Organic co
C14766	Linoleic ac	LMFA0200	Fatty Acyls	Octadecar	Other Octa	HMDB000	--	Organic co
C17873	--	LMFA0105	Fatty Acyls	Fatty Acids	Hydroxy fa	HMDB003	544-57-0	Organic co
--	--	--	--	--	--	--	--	--
--	--	--	--	--	--	HMDB003	76760-40-	Organic co
--	--	--	--	--	--	--	--	--
--	--	--	--	--	--	--	--	--
--	--	LMFA0105	Fatty Acyls	Fatty Acids	Hydroxy fa	HMDB006	--	Organic co
--	--	--	--	--	--	HMDB000	21064-18-	Organic co
C05109	Steroid bio	LMST0101	Sterol Lipid	Sterols [ST	Cholestero	HMDB000	79-62-9	Organic co
--	--	--	--	--	--	--	--	--
--	--	--	--	--	--	HMDB028	--	_
--	--	--	--	--	--	HMDB004	50924-49-	Organic co
--	--	--	--	--	--	--	--	--
--	--	--	--	--	--	--	--	--

--	--	--	--	--	--	HMDB028	--	_
--	--	--	--	--	--	HMDB027	--	_
--	--	--	--	--	--	HMDB018	--	Organic cc
--	--	--	--	--	--	HMDB025	--	Organic cc
--	--	--	--	--	--	--	--	--
--	--	--	--	--	--	HMDB000	--	Organic cc
--	--	--	--	--	--	HMDB025	--	Organic cc
--	--	--	--	--	--	HMDB003	--	Organic cc
--	--	--	--	--	--	--	--	--
--	--	--	--	--	--	HMDB025	--	Organic cc
--	--	--	--	--	--	--	--	--
--	--	--	--	--	--	HMDB028	--	_
--	--	--	--	--	--	HMDB025	--	Organic cc
--	--	--	--	--	--	HMDB025	--	Organic cc
--	--	--	--	--	--	--	--	--
--	--	--	--	--	--	HMDB003	185154-94	Organic cc
--	--	--	--	--	--	HMDB024	--	Organic cc
--	--	--	--	--	--	--	--	--
--	--	--	--	--	--	HMDB029	--	_
--	--	--	--	--	--	HMDB004	160047-56	Organic cc
--	--	--	--	--	--	HMDB004	173792-58	Organic cc
--	--	LMGP0205	Glyceroph	Glyceroph	Monoacylg	HMDB001	--	Organic cc
--	--	--	--	--	--	--	--	--
--	--	--	--	--	--	HMDB003	504-97-2	Organic cc
--	--	LMST0302	Sterol Lipi	Secosteroi	Vitamin D3	--	--	--
--	--	--	--	--	--	HMDB024	--	Organic cc
--	--	--	--	--	--	--	--	--
--	--	--	--	--	--	HMDB000	99794-78-	Organic cc
--	--	--	--	--	--	HMDB025	--	Organic cc
--	--	--	--	--	--	--	--	--
--	--	--	--	--	--	HMDB002	24587-37-	Organic cc
--	--	--	--	--	--	HMDB002	--	Organic cc
--	--	--	--	--	--	HMDB003	--	Organic cc
C16609	Drug meta	--	--	--	--	HMDB006	--	Organic cc
--	--	LMGL0101	Glycerolipi	Monorady	Monoacylg	HMDB024	542-44-9	Organic cc
--	--	--	--	--	--	--	--	--
--	--	--	--	--	--	HMDB024	--	Organic cc
--	--	--	--	--	--	--	--	--
C10793	Ubiquinon	--	--	--	--	--	--	--
--	--	--	--	--	--	HMDB004	156980-55	Organic cc
--	--	--	--	--	--	HMDB024	--	Organic cc
--	--	--	--	--	--	HMDB025	--	Organic cc
--	--	--	--	--	--	HMDB011	--	Organic cc
--	--	--	--	--	--	HMDB003	15446-86-	Organic cc
C04230	--	LMGP0105	Glyceroph	Glyceroph	Monoacylg	HMDB001	--	Organic cc
--	--	--	--	--	--	HMDB003	117383-35	Organic cc
--	--	--	--	--	--	HMDB029	--	_
C04230	--	LMGP0105	Glyceroph	Glyceroph	Monoacylg	HMDB001	1199257-4	Organic cc
--	--	--	--	--	--	HMDB025	--	Organic cc
--	--	--	--	--	--	HMDB025	--	Organic cc
--	--	--	--	--	--	--	--	--

--	--	LMGL0101	Glycerolipi	Monorady	Monoacylg	HMDB001	3443-84-3	Organic cc
--	--	--	--	--	--	--	--	--
--	--	--	--	--	--	HMDB030	--	Organic cc
--	--	--	--	--	--	--	--	--
--	--	--	--	--	--	HMDB003	126882-5	Organic cc
--	--	--	--	--	--	HMDB025	--	Organic cc
--	--	--	--	--	--	HMDB001	376348-6	Organic cc
C05283	--	--	--	--	--	HMDB001	10148-81-	Organic cc
C14825	--	LMFA020C	Fatty Acyls	Octadecar	Other Octa	HMDB000	--	Organic cc
--	--	--	--	--	--	HMDB030	--	Organic cc
--	--	--	--	--	--	HMDB025	--	Organic cc
--	--	--	--	--	--	HMDB029	--	_
--	--	--	--	--	--	HMDB024	--	Organic cc
--	--	--	--	--	--	HMDB003	53527-49-	Organic cc
--	--	LMGP1001	Glyceroph	Glyceroph	Diacylglyc	HMDB011	--	Organic cc
--	--	--	--	--	--	HMDB028	--	_
--	--	--	--	--	--	--	--	--
--	--	--	--	--	--	HMDB006	74685-27-	Organic cc
C15589	--	--	--	--	--	HMDB000	94-41-7	Organic cc
--	--	--	--	--	--	HMDB003	76760-39-	Organic cc
--	--	--	--	--	--	--	--	--
--	--	--	--	--	--	HMDB025	--	Organic cc
--	--	--	--	--	--	HMDB024	--	Organic cc
--	--	--	--	--	--	HMDB024	--	Organic cc
--	--	LMGP1006	Glyceroph	Glyceroph	Monoalkyl	HMDB006	--	Organic cc
C04230	--	LMGP0105	Glyceroph	Glyceroph	Monoacylg	HMDB001	--	Organic cc
--	--	--	--	--	--	--	--	--
--	--	--	--	--	--	HMDB024	--	Organic cc
--	--	--	--	--	--	HMDB025	--	Organic cc
C00327	Arginine b	--	--	--	--	HMDB000	372-75-8	Organic cc
--	--	--	--	--	--	--	--	--
--	--	--	--	--	--	HMDB025	--	Organic cc
--	--	--	--	--	--	--	--	--
C04230	--	LMGP0105	Glyceroph	Glyceroph	Monoacylg	HMDB001	--	Organic cc
--	--	--	--	--	--	HMDB025	--	Organic cc
--	--	LMFA050C	Fatty Acyls	Fatty alcoh	-	HMDB003	--	Organic cc
--	--	--	--	--	--	HMDB024	--	Organic cc
--	--	--	--	--	--	HMDB024	--	Organic cc
--	--	--	--	--	--	--	--	--
--	--	--	--	--	--	--	--	--
--	--	--	--	--	--	--	--	--
C07489	--	--	--	--	--	HMDB001	77-21-4	Organic cc
--	--	--	--	--	--	HMDB025	--	Organic cc
C01390	Riboflavin	LMFA050C	Fatty Acyls	Fatty alcoh	-	HMDB003	556-82-1	Organic cc
C00588	Teichoic ac	--	--	--	--	HMDB000	3616/4/4	Organic cc
--	--	LMGP0102	Glyceroph	Glyceroph	1-alkyl,2-a	--	--	--
C15976	Valine, leu	--	--	--	--	HMDB000	--	Organic cc
--	--	--	--	--	--	HMDB001	173334-5	Organic cc
--	--	--	--	--	--	HMDB003	--	Organic cc
--	--	--	--	--	--	HMDB001	--	Organic cc
--	--	--	--	--	--	--	--	--

--	--	--	--	--	--	--	--	--
--	--	--	--	--	--	HMDB001	--	Organic cc
--	--	--	--	--	--	--	--	--
--	--	LMGP0105	Glyceroph	Glyceroph	Monoacylg	HMDB000	19420-56-	Organic cc
--	--	--	--	--	--	HMDB025	--	Organic cc
--	--	--	--	--	--	HMDB028	--	_
--	--	--	--	--	--	HMDB025	--	Organic cc
--	--	--	--	--	--	HMDB006	--	Organic cc
--	--	--	--	--	--	--	--	--
--	--	LMGP0203	Glyceroph	Glyceroph	1-(1Z-alke	HMDB001	--	Organic cc
--	--	--	--	--	--	HMDB028	--	_
--	--	--	--	--	--	HMDB026	--	_
C11396	--	--	--	--	--	HMDB003	20549-47-	Organic cc
--	--	--	--	--	--	--	--	--
--	--	--	--	--	--	HMDB000	--	Organic cc
--	--	--	--	--	--	HMDB001	--	Organic cc
--	--	--	--	--	--	--	--	--
--	--	--	--	--	--	HMDB025	--	Organic cc
C09627	Sesquiterp	LMPR0103	Prenol Lipi	Isoprenoid	C15 isopre	HMDB000	37208-05-	Organic cc
--	--	--	--	--	--	HMDB003	402593-4(	Organic cc
--	--	--	--	--	--	HMDB025	152-58-9	Organic cc
--	--	--	--	--	--	HMDB024	--	Organic cc
--	--	LMFA0103	Fatty Acyls	Fatty Acids	Unsaturate	HMDB024	34450-18-	Organic cc
--	--	--	--	--	--	HMDB024	--	Organic cc
--	--	--	--	--	--	--	--	--
--	--	--	--	--	--	--	--	--
--	--	--	--	--	--	--	--	--
--	--	--	--	--	--	HMDB030	--	Organic cc
--	--	--	--	--	--	HMDB026	--	_
C05959	Arachidon	LMFA0301	Fatty Acyls	Eicosanoic	Prostaglan	HMDB001	--	Organic cc
--	--	--	--	--	--	HMDB024	29836-26-	Organic cc
--	--	--	--	--	--	HMDB025	--	Organic cc
--	--	--	--	--	--	HMDB024	--	Organic cc
--	--	--	--	--	--	--	--	--
--	--	--	--	--	--	HMDB030	2253-73-8	Organic cc
--	--	--	--	--	--	HMDB025	--	Organic cc
--	--	--	--	--	--	HMDB024	--	Organic cc
--	--	--	--	--	--	HMDB029	--	_
--	--	--	--	--	--	HMDB024	--	Organic cc
--	--	--	--	--	--	HMDB029	--	_
--	--	--	--	--	--	HMDB030	--	Organic cc
C14831	Linoleic ac	LMFA0200	Fatty Acyls	Octadecar	Other Octa	HMDB000	143343-95	Organic cc
C04230	--	LMGP0107	Glyceroph	Glyceroph	1Z-alkenyl	HMDB001	--	Organic cc
--	--	--	--	--	--	HMDB025	--	Organic cc
--	--	--	--	--	--	HMDB024	--	Organic cc
--	--	LMGP0102	Glyceroph	Glyceroph	1-alkyl,2-a	--	--	--
--	--	--	--	--	--	--	--	--
C00526	Pyrimidine	--	--	--	--	HMDB000	951-78-0	Organic cc
--	--	--	--	--	--	HMDB002	29586-66-	Organic cc
--	--	--	--	--	--	--	--	--
--	--	--	--	--	--	HMDB006	2190485-6	Organic cc

--	--	--	--	--	--	HMDB024	--	Organic cc
--	--	--	--	--	--	HMDB025	--	Organic cc
--	--	--	--	--	--	HMDB025	--	Organic cc
--	--	--	--	--	--	--	--	--
--	--	--	--	--	--	HMDB025	--	Organic cc
--	--	--	--	--	--	HMDB025	--	Organic cc
--	--	--	--	--	--	HMDB025	--	Organic cc
--	--	--	--	--	--	--	--	--
C17755	Betalain bi	--	--	--	--	HMDB001	50673-96-	Organic cc
--	--	--	--	--	--	HMDB024	--	Organic cc
--	--	--	--	--	--	HMDB003	34818-56-	Organic cc
--	--	--	--	--	--	HMDB030	69064-37-	Organic cc
C00156	Ubiquinon	--	--	--	--	HMDB000	99-96-7	Organic cc
C04778	Porphyrin	--	--	--	--	HMDB000	--	Organic cc
C16620	Drug meta	--	--	--	--	HMDB006	--	Organic cc
--	--	--	--	--	--	HMDB025	--	Organic cc
--	--	--	--	--	--	HMDB025	--	Organic cc
C06980	--	--	--	--	--	HMDB001	103598-0	Organic cc
C01119	--	--	--	--	--	HMDB005	--	Organic cc
--	--	--	--	--	--	HMDB025	--	Organic cc
--	--	--	--	--	--	--	--	--
--	--	--	--	--	--	--	--	--
--	--	LMST0302	Sterol Lipi	Secosteroi	Vitamin D	HMDB000	81203-50-	Organic cc
--	--	--	--	--	--	HMDB004	--	Organic cc
--	--	--	--	--	--	HMDB004	27113-23-	Organic cc
--	--	--	--	--	--	HMDB003	90578-32-	Organic cc
--	--	--	--	--	--	--	--	--
--	--	--	--	--	--	HMDB027	--	_
C01226	alpha-Linc	LMFA0201	Fatty Acyls	Octadecar	12-oxophy	HMDB030	85551-10-	Organic cc
--	--	--	--	--	--	--	--	--
--	--	--	--	--	--	HMDB029	--	_
--	--	--	--	--	--	--	--	--
--	--	--	--	--	--	HMDB025	--	Organic cc
--	--	--	--	--	--	HMDB027	--	_
--	--	--	--	--	--	--	--	--
--	--	--	--	--	--	HMDB003	114076-5	Organic cc
--	--	--	--	--	--	HMDB024	--	Organic cc
--	--	--	--	--	--	HMDB003	134361-6	Organic cc
C07909	Type I poly	--	--	--	--	HMDB001	53123-88-	Organic cc
--	--	--	--	--	--	HMDB028	--	_
--	--	--	--	--	--	--	--	--
--	--	--	--	--	--	--	--	--
--	--	--	--	--	--	HMDB000	--	Organic cc
--	--	--	--	--	--	HMDB025	--	Organic cc
--	--	--	--	--	--	HMDB025	--	Organic cc
--	--	LMGP0105	Glyceroph	Glyceroph	Monoacylg	--	--	--
--	--	--	--	--	--	--	--	--
--	--	--	--	--	--	HMDB024	--	Organic cc
--	--	LMPR0104	Prenol Lipi	Isoprenoid	C20 isopre	--	--	--
--	--	--	--	--	--	--	--	--
--	--	--	--	--	--	HMDB029	--	_

--	--	--	--	--	--	HMDB024	--	Organic cc
--	--	--	--	--	--	--	--	--
--	--	--	--	--	--	HMDB029	--	_
--	--	--	--	--	--	HMDB026	--	_
--	--	--	--	--	--	--	--	--
--	--	--	--	--	--	HMDB002	55784-92-	Organic cc
--	--	--	--	--	--	HMDB024	2038-57-	Organic cc
--	--	--	--	--	--	--	--	--
--	--	--	--	--	--	HMDB001	--	Organic cc
--	--	--	--	--	--	--	--	--
--	--	--	--	--	--	HMDB024	--	Organic cc
--	--	--	--	--	--	--	--	--
C18040	Steroid ho	LMST0203	Sterol Lipi	Steroids [S	C21 steroi	HMDB006	--	Organic cc
--	--	--	--	--	--	HMDB029	--	_
C12871	--	--	--	--	--	HMDB001	192725-17	Organic cc
--	--	--	--	--	--	--	--	--
--	--	--	--	--	--	HMDB025	--	Organic cc
C13173	--	--	--	--	--	HMDB001	80214-83-	Organic cc
--	--	--	--	--	--	HMDB024	--	Organic cc
--	--	--	--	--	--	HMDB001	22610-61-	Organic cc
--	--	--	--	--	--	--	--	--
--	--	--	--	--	--	--	--	--
--	--	--	--	--	--	HMDB000	--	Organic cc
--	--	LMFA0802	Fatty Acyls	Fatty amid	N-acyl am	HMDB006	--	Organic cc
--	--	--	--	--	--	HMDB006	--	Organic cc
--	--	--	--	--	--	HMDB028	--	_
--	--	--	--	--	--	HMDB003	15371-77-	Organic cc
C04730	--	--	--	--	--	HMDB000	--	Organic cc
--	--	--	--	--	--	HMDB029	--	_
--	--	--	--	--	--	HMDB001	--	Organic cc
--	--	--	--	--	--	HMDB000	1723-00-	Organic cc
--	--	--	--	--	--	--	--	--
--	--	--	--	--	--	HMDB028	--	_
--	--	--	--	--	--	--	--	--
--	--	--	--	--	--	HMDB002	--	Organic cc
C00588	Teichoic ac	--	--	--	--	HMDB000	3616/4/4	Organic cc
--	--	--	--	--	--	HMDB004	--	Organic cc
--	--	--	--	--	--	HMDB003	--	Organic cc
--	--	--	--	--	--	--	--	--
C08607	Carotenoi	LMPR0107	Prenol Lipi	Isoprenoid	C40 isopre	--	--	--
--	--	--	--	--	--	HMDB011	--	Organic cc
--	--	--	--	--	--	HMDB025	--	Organic cc
C10641	Biosynthes	--	--	--	--	--	--	--
--	--	--	--	--	--	--	--	--
--	--	--	--	--	--	HMDB003	136762-4	Organic cc
--	--	--	--	--	--	HMDB003	133585-5	Organic cc
--	--	--	--	--	--	HMDB028	--	_
--	--	--	--	--	--	HMDB001	--	Organic cc
--	--	--	--	--	--	HMDB024	--	Organic cc
--	--	--	--	--	--	HMDB029	--	_
--	--	--	--	--	--	HMDB001	3443-83-	Organic cc

--	--	--	--	--	--	HMDB025	--	Organic cc
--	--	--	--	--	--	HMDB024	--	Organic cc
--	--	--	--	--	--	HMDB003	90851-27-	Organic cc
--	--	--	--	--	--	HMDB002	--	Organic cc
--	--	--	--	--	--	HMDB024	--	Organic cc
--	--	--	--	--	--	--	--	--
--	--	--	--	--	--	HMDB026	--	_
--	--	--	--	--	--	HMDB028	--	_
--	--	--	--	--	--	HMDB024	--	Organic cc
--	--	LMFA0301	Fatty Acyls	Eicosanoic	Prostaglan	HMDB000	19313-28-	Organic cc
--	--	--	--	--	--	HMDB026	--	Organic cc
--	--	--	--	--	--	HMDB002	--	Organic cc
--	--	--	--	--	--	HMDB003	70579-26-	Organic cc
C06241	Amino sug	--	--	--	--	HMDB000	37992-17-	Organic cc
--	--	--	--	--	--	HMDB028	--	_
--	--	--	--	--	--	HMDB003	99782-87-	Organic cc
--	--	--	--	--	--	--	--	--
C07241	--	--	--	--	--	HMDB001	89365-50-	Organic cc
C11992	Biosynthes	LMPK0400	Polyketide	Macrolides	-	--	--	--
--	--	--	--	--	--	HMDB025	--	Organic cc
--	--	--	--	--	--	HMDB030	--	Organic cc
--	--	--	--	--	--	HMDB024	--	Organic cc
--	--	--	--	--	--	HMDB003	54899-35-	Organic cc
--	--	--	--	--	--	--	--	--
--	--	--	--	--	--	HMDB003	--	Organic cc
C20450	Furfural de	--	--	--	--	HMDB000	3238-40-2	Organic cc
C10674	--	--	--	--	--	HMDB003	90426-22-	Organic cc
--	--	--	--	--	--	--	--	--
--	--	--	--	--	--	HMDB025	--	Organic cc
--	--	--	--	--	--	--	--	--
C11819	--	--	--	--	--	HMDB025	150871-62	Organic cc
--	--	--	--	--	--	HMDB003	194919-40	Organic cc
--	--	--	--	--	--	HMDB011	116643-35	Organic cc
--	--	--	--	--	--	HMDB025	--	Organic cc
--	--	--	--	--	--	--	--	--
--	--	--	--	--	--	HMDB024	--	Organic cc
--	--	--	--	--	--	HMDB025	466-06-8	Organic cc
--	--	--	--	--	--	HMDB029	--	_
--	--	LMSP0501	Sphingolip	Neutral gly	Simple Glc	HMDB000	483370-78	Organic cc
C14154	--	--	--	--	--	HMDB003	490-23-3	Organic cc
--	--	--	--	--	--	HMDB029	--	_
--	--	--	--	--	--	HMDB024	--	Organic cc
C12580	--	--	--	--	--	HMDB001	13647-35-	Organic cc
--	--	--	--	--	--	HMDB003	85797-14-	Organic cc
--	--	--	--	--	--	HMDB003	227179-09	Organic cc
--	--	--	--	--	--	HMDB006	5617-32-3	Organic cc
C07556	--	--	--	--	--	HMDB001	119302-91	Organic cc
--	--	--	--	--	--	--	--	--
--	--	--	--	--	--	HMDB030	--	Organic cc
--	--	--	--	--	--	HMDB000	--	Organic cc
C02356	Cysteine a	LMFA0110	Fatty Acyls	Fatty Acids	Amino fatt	HMDB000	1492-24-6	Organic cc

--	--	--	--	--	--	HMDB003	13162-46-	Organic cc
--	--	--	--	--	--	HMDB025	--	Organic cc
--	--	--	--	--	--	--	--	--
--	--	--	--	--	--	HMDB024	4406-35-3	Organic cc
C05107	Steroid bio	LMST0101	Sterol Lipi	Sterols [ST	Cholestero	HMDB000	1715-86-2	Organic cc
C13764	--	--	--	--	--	HMDB025	16662-47-	Organic cc
--	--	--	--	--	--	HMDB006	--	Organic cc
--	--	--	--	--	--	HMDB024	--	_
--	--	LMGP0102	Glyceroph	Glyceroph	1-alkyl,2-a	--	--	--
--	--	--	--	--	--	HMDB025	--	Organic cc
--	--	--	--	--	--	--	--	--
--	--	LMFA0600	Fatty Acyls	Fatty aldeh	-	--	--	--
C14829	--	LMFA0200	Fatty Acyls	Octadecar	Other Octa	HMDB047	--	--
C17419	--	--	--	--	--	--	--	--
--	--	--	--	--	--	HMDB029	--	_
--	--	LMFA0114	Fatty Acyls	Fatty Acids	Carbocycli	--	--	--
--	--	LMFA0804	Fatty Acyls	Fatty amid	N-acyl eth	HMDB001	109001-03	Organic cc
--	--	LMST0402	Sterol Lipi	Bile acids a	C26 bile ac	--	--	--
--	--	LMST0101	Sterol Lipi	Sterols [ST	Cholestero	HMDB000	78648-95-	Organic cc
--	--	--	--	--	--	HMDB024	--	Organic cc
--	--	--	--	--	--	HMDB002	--	Organic cc
--	--	--	--	--	--	HMDB002	66851-83-	Organic cc
C02713	Amino sug	--	--	--	--	HMDB006	--	Organic cc
--	--	--	--	--	--	HMDB029	--	_
--	--	--	--	--	--	HMDB024	--	Organic cc
--	--	--	--	--	--	HMDB030	--	Organic cc
--	--	--	--	--	--	HMDB003	85202-26-	Organic cc
--	--	--	--	--	--	HMDB024	--	Organic cc
--	--	--	--	--	--	HMDB025	--	Organic cc
--	--	--	--	--	--	--	--	--
C11341	--	--	--	--	--	--	--	--
--	--	--	--	--	--	HMDB029	--	_
--	--	--	--	--	--	--	--	--
--	--	LMGP0101	Glyceroph	Glyceroph	Diacylglyce	--	--	--
--	--	--	--	--	--	HMDB003	160551-60	Organic cc
--	--	--	--	--	--	HMDB024	--	Organic cc
--	--	--	--	--	--	--	--	--
--	--	--	--	--	--	HMDB024	--	Organic cc
C18319	Protein dig	LMFA0102	Fatty Acyls	Fatty Acids	Branched f	HMDB000	116-53-0	Organic cc
--	--	--	--	--	--	HMDB024	--	Organic cc
--	--	--	--	--	--	HMDB028	--	_
--	--	--	--	--	--	HMDB002	--	Organic cc
--	--	--	--	--	--	HMDB025	--	Organic cc
C02273	--	--	--	--	--	HMDB003	5894-59-7	Organic cc
--	--	--	--	--	--	HMDB003	--	Organic cc
--	--	--	--	--	--	HMDB025	--	Organic cc
--	--	--	--	--	--	HMDB025	--	Organic cc
--	--	--	--	--	--	HMDB003	170713-72	Organic cc
--	--	--	--	--	--	HMDB025	--	Organic cc
--	--	--	--	--	--	HMDB028	--	_
C03415	Arginine a	--	--	--	--	HMDB000	899816-95	Organic cc

--	--	--	--	--	--	HMDB025	--	Organic cc
--	--	--	--	--	--	HMDB025	--	Organic cc
--	--	--	--	--	--	HMDB024	--	Organic cc
--	--	--	--	--	--	HMDB003	--	Organic cc
--	--	--	--	--	--	HMDB024	--	Organic cc
--	--	--	--	--	--	--	--	--
--	--	--	--	--	--	--	--	--
--	--	--	--	--	--	HMDB029	--	_
--	--	--	--	--	--	HMDB024	--	Organic cc
--	--	--	--	--	--	HMDB029	--	_
--	--	--	--	--	--	HMDB002	181046-8	Organic cc
C10965	--	--	--	--	--	HMDB024	--	Organic cc
--	--	--	--	--	--	HMDB002	50674-18-	Organic cc
--	--	--	--	--	--	--	--	--
--	--	--	--	--	--	HMDB026	--	_
--	--	LMPR0107	Prenol Lipi	Isoprenoid	C40 isopre	HMDB003	75324-12-	Organic cc
--	--	--	--	--	--	HMDB024	--	Organic cc
--	--	--	--	--	--	HMDB029	--	_
--	--	--	--	--	--	HMDB009	--	Organic cc
--	--	--	--	--	--	HMDB025	--	Organic cc
--	--	--	--	--	--	HMDB006	2615-15-8	Organic cc
--	--	--	--	--	--	HMDB006	--	Organic cc
C05454	Primary bil	LMST0403	Sterol Lipi	Bile acids a	C27 bile ac	HMDB000	547-96-6	Organic cc
--	--	--	--	--	--	--	--	--
C15751	--	--	--	--	--	HMDB025	62362-59-	Organic cc
--	--	--	--	--	--	--	--	--
--	--	--	--	--	--	HMDB028	--	_
--	--	--	--	--	--	--	--	--
C22136	Steroid bic	LMST0101	Sterol Lipi	Sterols [ST	Cholestero	HMDB030	--	Organic cc
C06124	Sphingolip	LMSP0105	Sphingolip	Sphingoid	Sphingoid	HMDB000	26993-30-	Organic cc
--	--	--	--	--	--	--	--	--
--	--	--	--	--	--	HMDB024	--	Organic cc
--	--	--	--	--	--	HMDB003	110024-16	Organic cc
--	--	LMGL0201	Glycerolipi	Diradylglyc	Diacylglyce	HMDB000	--	Organic cc
--	--	--	--	--	--	HMDB024	--	Organic cc
--	--	LMGP0101	Glyceroph	Glyceroph	Diacylglyce	HMDB000	4235-95-4	Organic cc
--	--	--	--	--	--	HMDB027	--	_
C04730	--	--	--	--	--	HMDB000	--	Organic cc
--	--	--	--	--	--	HMDB024	--	Organic cc
--	--	--	--	--	--	HMDB028	--	_
C04717	Linoleic ac	LMFA0200	Fatty Acyls	Octadecan	Other Octa	HMDB000	33964-75-	Organic cc
--	--	--	--	--	--	--	--	--
--	--	--	--	--	--	HMDB001	--	Organic cc
--	--	--	--	--	--	HMDB027	--	_
--	--	--	--	--	--	HMDB024	--	Organic cc
--	--	--	--	--	--	--	--	--
--	--	--	--	--	--	HMDB003	49776-58-	Organic cc
--	--	--	--	--	--	HMDB003	30430-91-	Organic cc
--	--	--	--	--	--	HMDB003	85764-12-	Organic cc
--	--	--	--	--	--	HMDB025	--	Organic cc
--	--	--	--	--	--	HMDB025	--	Organic cc

--	--	--	--	--	--	--	--	--
--	--	--	--	--	--	HMDB025	--	Organic cc
--	--	--	--	--	--	HMDB026	--	_
--	--	--	--	--	--	HMDB029	--	_
--	--	--	--	--	--	HMDB025	--	Organic cc
C13910	--	LMFA0103	Fatty Acyls	Fatty Acids	Unsaturate	HMDB003	112-38-9	Organic cc
C14743	--	--	--	--	--	HMDB003	78-59-1	Organic cc
--	--	LMFA110C	Fatty Acyls	Hydrocarb	-	--	--	--
--	--	LMFA050C	Fatty Acyls	Fatty alcoh	-	--	--	--
--	--	--	--	--	--	HMDB024	--	Organic cc
--	--	--	--	--	--	HMDB028	--	_
--	--	--	--	--	--	HMDB029	--	_
--	--	--	--	--	--	HMDB029	--	_
--	--	--	--	--	--	--	--	--
--	--	--	--	--	--	HMDB024	--	Organic cc
--	--	--	--	--	--	HMDB026	--	_
--	--	--	--	--	--	HMDB003	--	Organic cc
--	--	--	--	--	--	HMDB029	--	_
--	--	--	--	--	--	HMDB029	--	_
--	--	--	--	--	--	HMDB025	23255-69-	Organic cc
--	--	--	--	--	--	--	--	--
--	--	--	--	--	--	--	--	--
--	--	--	--	--	--	HMDB027	--	_
--	--	--	--	--	--	HMDB003	119626-54	Organic cc
--	--	--	--	--	--	HMDB006	--	Organic cc
--	--	LMGL0101	Glycerolipi	Monorady	Monoacylg	HMDB001	32899-41-	Organic cc
--	--	--	--	--	--	--	--	--
--	--	--	--	--	--	HMDB024	--	Organic cc
--	--	--	--	--	--	HMDB025	--	Organic cc
--	--	--	--	--	--	HMDB002	1188-24-5	Organic cc
C00308	--	--	--	--	--	HMDB000	543-38-4	Organic cc
--	--	--	--	--	--	--	--	--
--	--	--	--	--	--	HMDB024	--	_
--	--	--	--	--	--	--	--	--
--	--	--	--	--	--	HMDB004	171499-81	Organic cc
--	--	--	--	--	--	--	--	--
--	--	--	--	--	--	HMDB004	162613-65	Organic cc
--	--	--	--	--	--	HMDB025	--	Organic cc
--	--	--	--	--	--	--	--	--
--	--	LMFA0701	Fatty Acyls	Fatty ester	Wax monc	HMDB003	142-91-6	Organic cc
--	--	--	--	--	--	HMDB027	--	_
--	--	LMFA060C	Fatty Acyls	Fatty aldeh	-	HMDB004	56797-41-	Organic cc
--	--	LMGP0207	Glyceroph	Glyceroph	1Z-alkenyl	HMDB001	174062-72	Organic cc
--	--	LMPR0107	Prenol Lipi	Isoprenoid	C40 isopre	HMDB003	158312-69	Organic cc
--	--	LMFA0301	Fatty Acyls	Eicosanoic	Prostaglan	--	--	--
--	--	LMGP0105	Glyceroph	Glyceroph	Monoacylg	HMDB001	20559-16-	Organic cc
--	--	LMSP0202	Sphingolip	Ceramides	N-acylsph	HMDB001	--	Organic cc
--	--	LMGL0201	Glycerolipi	Diradylglyc	Diacylglyce	HMDB000	--	Organic cc
--	--	LMFA0701	Fatty Acyls	Fatty ester	Wax monc	HMDB004	56922-73-	Organic cc
--	--	--	--	--	--	HMDB009	5117-19-1	Organic cc
C13431	Carotenoic	--	--	--	--	HMDB030	--	Organic cc

C07073	Drug meta	--	--	--	--	HMDB001	137-58-6	Organic cc
--	--	--	--	--	--	HMDB027	--	_
--	--	--	--	--	--	--	--	--
--	--	--	--	--	--	HMDB024	--	Organic cc
--	--	--	--	--	--	HMDB030	--	Organic cc
--	--	--	--	--	--	HMDB029	--	_
--	--	--	--	--	--	--	--	--
--	--	--	--	--	--	HMDB029	--	_
--	--	LMGL0201	Glycerolipi	Diradylgly	Diacylglyce	HMDB000	--	Organic cc
--	--	--	--	--	--	HMDB002	--	Organic cc
--	--	--	--	--	--	--	--	--
--	--	--	--	--	--	HMDB026	--	_
--	--	--	--	--	--	--	--	--
--	--	--	--	--	--	HMDB002	--	Organic cc
--	--	--	--	--	--	--	--	--
C07834	--	--	--	--	--	HMDB003	123-63-7	Organic cc
--	--	--	--	--	--	HMDB024	--	Organic cc
--	--	--	--	--	--	HMDB025	112-27-6	Organic cc
C05588	Tyrosine m	--	--	--	--	HMDB006	--	Organic cc
C03406	Arginine b	--	--	--	--	HMDB000	2387-71-5	Organic cc
--	--	LMFA0802	Fatty Acyls	Fatty amid	N-acyl am	HMDB024	--	Organic cc
C01301	Primary bil	LMST0403	Sterol Lipi	Bile acids a	C27 bile ac	HMDB000	3836/1/9	Organic cc
C03425	--	LMFA0701	Fatty Acyls	Fatty ester	Wax monc	HMDB003	111-62-6	Organic cc
--	--	--	--	--	--	--	--	--
C03033	--	--	--	--	--	HMDB001	--	Organic cc
C10996	--	--	--	--	--	HMDB025	1596-84-5	Organic cc
--	--	--	--	--	--	HMDB027	--	_
--	--	--	--	--	--	HMDB024	--	Organic cc
--	--	--	--	--	--	--	--	--
--	--	--	--	--	--	HMDB024	--	Organic cc
--	--	--	--	--	--	--	--	--
--	--	--	--	--	--	HMDB006	--	Organic cc
--	--	--	--	--	--	--	--	--
--	--	--	--	--	--	HMDB025	--	Organic cc
--	--	--	--	--	--	HMDB003	--	Organic cc
C06653	Biosynthes	LMPK040C	Polyketide	Macrolides	-	--	--	--
--	--	--	--	--	--	HMDB028	--	_
C21480	Ferroptosi	LMGP0201	Glyceroph	Glyceroph	Diacylglyce	HMDB000	61216-62-	Organic cc
C21481	Ferroptosi	LMGP0201	Glyceroph	Glyceroph	Diacylglyce	HMDB000	--	Organic cc
--	--	--	--	--	--	--	--	--
--	--	--	--	--	--	HMDB026	--	_
--	--	--	--	--	--	HMDB029	--	_
--	--	--	--	--	--	HMDB028	--	_
--	--	--	--	--	--	HMDB025	--	Organic cc
--	--	--	--	--	--	HMDB028	--	_
--	--	--	--	--	--	HMDB029	--	_
--	--	--	--	--	--	HMDB025	--	Organic cc
--	--	--	--	--	--	HMDB028	--	_
--	--	--	--	--	--	HMDB028	--	_
--	--	--	--	--	--	HMDB028	--	_
--	--	--	--	--	--	HMDB028	--	_

--	--	--	--	--	--	HMDB028	--	_
--	--	--	--	--	--	HMDB028	--	_
--	--	--	--	--	--	HMDB027	--	_
--	--	--	--	--	--	HMDB024	--	Organic cc
--	--	LMGP0401	Glyceroph	Glyceroph	Diacylglyce	--	--	--
--	--	--	--	--	--	HMDB030	--	Organic cc
--	--	--	--	--	--	HMDB028	--	_
--	--	--	--	--	--	--	--	--
--	--	--	--	--	--	HMDB030	162157-0(	Organic cc
--	--	--	--	--	--	HMDB024	--	Organic cc
C05582	Tyrosine m	--	--	--	--	HMDB000	306-08-1	Organic cc
--	--	--	--	--	--	--	--	--
C01879	Glutathion	--	--	--	--	HMDB000	98-79-3	Organic cc
--	--	--	--	--	--	HMDB024	--	Organic cc
--	--	--	--	--	--	HMDB030	--	Organic cc
--	--	--	--	--	--	HMDB006	931115-27	Organic cc
--	--	--	--	--	--	HMDB025	--	Organic cc
--	--	--	--	--	--	HMDB000	585-88-6	Organic cc
--	--	--	--	--	--	HMDB024	584-13-4	Organic cc
C00573	--	--	--	--	--	HMDB000	69992-87-	Organic cc
C18170	--	--	--	--	--	HMDB001	--	Organic cc
--	--	--	--	--	--	HMDB030	826-18-6	Organic cc
--	--	--	--	--	--	HMDB000	--	Organic cc
--	--	--	--	--	--	HMDB024	369-07-3	Organic cc
C03190	Monoterp	LMPR0102	Prenol Lipi	Isoprenoid	C10 isopre	--	--	--
--	--	LMPK1211	Polyketide	Flavonoids	Flavones a	--	--	--
C01185	Nicotinate	--	--	--	--	HMDB000	321-02-8	Organic cc
--	--	LMPK1211	Polyketide	Flavonoids	Flavones a	HMDB030	--	Organic cc
--	--	--	--	--	--	--	--	--
--	--	--	--	--	--	--	--	--
C02330	O-Antigen	--	--	--	--	HMDB001	--	Organic cc
C19848	Folate bios	--	--	--	--	HMDB005	220456-6(	Organic cc
C02903	Arginine a	--	--	--	--	HMDB000	4390-05-(	Organic cc
C00015	Pyrimidine	--	--	--	--	HMDB000	58-98-0	Organic cc
C04392	Purine me	--	--	--	--	HMDB000	--	Organic cc
C05989	Propanoat	LMFA0705	Fatty Acyls	Fatty ester	Fatty acyl (	HMDB000	--	--
C00035	Purine me	--	--	--	--	HMDB000	146-91-8	Organic cc
--	--	--	--	--	--	HMDB001	--	Organic cc
C03665	--	LMFA0110	Fatty Acyls	Fatty Acids	Amino fatt	HMDB000	62-57-7	Organic cc
--	--	--	--	--	--	--	--	--
--	--	--	--	--	--	HMDB030	--	_
--	--	--	--	--	--	--	--	--
--	--	--	--	--	--	--	--	--
--	--	--	--	--	--	HMDB001	106414-0	Organic cc
--	--	--	--	--	--	HMDB025	--	Organic cc
--	--	--	--	--	--	--	--	--
--	--	--	--	--	--	HMDB003	--	Organic cc
C06714	--	--	--	--	--	HMDB001	--	Organic cc
--	--	--	--	--	--	HMDB000	700-06-1	Organic cc
--	--	--	--	--	--	--	--	--
--	--	--	--	--	--	HMDB024	--	Organic cc

--	--	--	--	--	--	--	--	--
--	--	--	--	--	--	HMDB004	--	Organic cc
--	--	--	--	--	--	HMDB030	--	_
--	--	--	--	--	--	HMDB000	71629-68-	Organic cc
--	--	--	--	--	--	HMDB006	--	Organic cc
--	--	--	--	--	--	HMDB002	--	Organic cc
--	--	--	--	--	--	HMDB024	--	Organic cc
--	--	--	--	--	--	HMDB030	--	Organic cc
--	--	--	--	--	--	HMDB000	7013-16-3	Organic cc
--	--	--	--	--	--	HMDB004	143986-3(	Organic cc
--	--	--	--	--	--	HMDB024	--	Organic cc
--	--	--	--	--	--	HMDB025	--	Organic cc
C06178	Nicotinate	--	--	--	--	HMDB006	--	Organic cc
C00379	--	--	--	--	--	HMDB024	--	Organic cc
--	--	--	--	--	--	HMDB003	7493-63-2	Organic cc
C00140	Amino suç	--	--	--	--	HMDB000	7512-17-6	Organic cc
--	--	--	--	--	--	HMDB026	--	Organic cc
--	--	--	--	--	--	HMDB003	38699-50-	Organic cc
--	--	--	--	--	--	--	--	--
--	--	--	--	--	--	HMDB003	--	Organic cc
--	--	--	--	--	--	HMDB025	--	Organic cc
C10434	Phenylpro	--	--	--	--	HMDB003	73263-62-	Organic cc
--	--	--	--	--	--	HMDB024	85-31-4	Organic cc
C00366	Purine me	--	--	--	--	HMDB000	69-93-2	Organic cc
--	--	--	--	--	--	--	--	--
--	--	--	--	--	--	--	--	--
C01693	Tyrosine m	--	--	--	--	HMDB000	89762-39-	Organic cc
C04076	Lysine bios	--	--	--	--	HMDB000	#######	Organic cc
--	--	--	--	--	--	--	--	--
C08526	Isoquinolir	--	--	--	--	HMDB001	357-70-0	Organic cc
--	--	--	--	--	--	HMDB025	--	Organic cc
--	--	--	--	--	--	HMDB024	--	Organic cc
--	--	--	--	--	--	--	--	--
--	--	--	--	--	--	HMDB025	--	Organic cc
--	--	--	--	--	--	--	--	--
--	--	--	--	--	--	HMDB025	--	_
--	--	--	--	--	--	--	--	--
C01799	--	--	--	--	--	HMDB001	760-78-1	Organic cc
--	--	--	--	--	--	--	--	--
C21096	--	--	--	--	--	HMDB000	1509-34-8	Organic cc
--	--	--	--	--	--	HMDB003	--	Organic cc
--	--	--	--	--	--	HMDB024	--	Organic cc
--	--	--	--	--	--	--	--	--
C11217	--	--	--	--	--	HMDB024	51-65-0	Organic cc
--	--	--	--	--	--	--	--	--
--	--	--	--	--	--	HMDB025	--	Organic cc
--	--	--	--	--	--	--	--	--
--	--	--	--	--	--	HMDB003	161730-07	Organic cc
--	--	--	--	--	--	HMDB025	--	Organic cc
--	--	--	--	--	--	HMDB003	38495-84-	Organic cc
--	--	--	--	--	--	--	--	--

--	--	--	--	--	--	HMDB002	--	Organic co
--	--	--	--	--	--	HMDB025	--	Organic co
--	--	--	--	--	--	--	--	--
--	--	--	--	--	--	HMDB004	135105-75	Organic co
--	--	--	--	--	--	HMDB025	--	Organic co
C11729	--	--	--	--	--	HMDB001	3511-16-8	Organic co
--	--	--	--	--	--	HMDB003	23566-96-	Organic co
C10509	--	--	--	--	--	HMDB003	486-62-4	Organic co
--	--	--	--	--	--	HMDB003	--	Organic co
--	--	--	--	--	--	--	--	--
--	--	--	--	--	--	HMDB026	--	Organic co
--	--	--	--	--	--	--	--	--
--	--	--	--	--	--	HMDB030	--	Organic co
C16521	Terpenoid	--	--	--	--	HMDB025	78-79-5	Organic co
C11118	--	--	--	--	--	HMDB000	616-45-5	Organic co
--	--	--	--	--	--	--	--	--
--	--	--	--	--	--	HMDB004	142449-85	Organic co
C06102	Caprolacta	--	--	--	--	HMDB001	928-81-4	Organic co
--	--	--	--	--	--	--	--	--
C02336	Amino sug	--	--	--	--	HMDB000	53188-23-	Organic co
--	--	--	--	--	--	HMDB025	--	Organic co
C02918	Nicotinate	--	--	--	--	HMDB000	3106-60-3	Organic co
C00135	Histidine n	--	--	--	--	HMDB000	71-00-1	Organic co
--	--	--	--	--	--	--	--	--
--	--	--	--	--	--	HMDB002	5879/6/1	Organic co
--	--	--	--	--	--	--	--	--
--	--	--	--	--	--	--	--	--
--	--	--	--	--	--	--	--	--
C01746	Tropane, p	--	--	--	--	HMDB003	110-89-4	Organic co
--	--	--	--	--	--	HMDB024	--	Organic co
C16591	Drug meta	--	--	--	--	HMDB006	--	Organic co
--	--	--	--	--	--	--	--	--
--	--	--	--	--	--	HMDB024	--	Organic co
--	--	--	--	--	--	HMDB002	6403-17-4	Organic co
C03485	Toluene de	--	--	--	--	HMDB000	100-51-6	Organic co
C05669	beta-Alani	--	--	--	--	HMDB003	504-88-1	Organic co
--	--	--	--	--	--	HMDB025	15176-29-	Organic co
--	--	--	--	--	--	--	--	--
--	--	--	--	--	--	--	--	--
--	--	--	--	--	--	HMDB003	41234-32-	Organic co
--	--	--	--	--	--	HMDB025	--	Organic co
--	--	--	--	--	--	HMDB024	--	Organic co
--	--	--	--	--	--	HMDB026	--	Organic co
C05401	--	--	--	--	--	HMDB000	--	Organic co
--	--	--	--	--	--	HMDB024	--	Organic co
--	--	--	--	--	--	HMDB000	30223-92-	Organic co
C06414	--	--	--	--	--	HMDB025	--	Organic co
C05596	Tyrosine m	--	--	--	--	HMDB000	28116-23-	Organic co
--	--	--	--	--	--	HMDB000	2504-22-5	Organic co
--	--	--	--	--	--	HMDB025	--	Organic co
--	--	--	--	--	--	HMDB025	77-81-6	Organic co

--	--	--	--	--	--	HMDB025	--	Organic co
--	--	--	--	--	--	HMDB026	--	Organic co
--	--	--	--	--	--	--	--	--
--	--	--	--	--	--	HMDB000	7170-38-	Organic co
--	--	--	--	--	--	--	--	--
C01455	Toluene d	--	--	--	--	HMDB003	108-88-3	Organic co
--	--	--	--	--	--	HMDB025	--	Organic co
--	--	--	--	--	--	HMDB024	--	Organic co
--	--	--	--	--	--	--	--	--
--	--	--	--	--	--	--	--	--
--	--	--	--	--	--	HMDB025	--	Organic co
C10497	--	--	--	--	--	HMDB003	501-13-3	Organic co
--	--	--	--	--	--	HMDB003	5355-63-	Organic co
--	--	--	--	--	--	HMDB024	--	Organic co
--	--	--	--	--	--	HMDB025	--	Organic co
--	--	--	--	--	--	HMDB024	--	Organic co
--	--	--	--	--	--	HMDB024	--	Organic co
--	--	--	--	--	--	HMDB025	--	Organic co
--	--	LMPK1212	Polyketide	Flavonoids	Chalcones	HMDB030	--	Organic co
--	--	--	--	--	--	HMDB004	4276-74-	Organic co
--	--	--	--	--	--	--	--	--
--	--	--	--	--	--	--	--	--
--	--	--	--	--	--	HMDB026	--	_
--	--	--	--	--	--	HMDB030	--	Organic co
--	--	--	--	--	--	HMDB011	--	Organic co
--	--	--	--	--	--	--	--	--
--	--	--	--	--	--	HMDB025	--	Organic co
C03406	Arginine b	--	--	--	--	HMDB000	2387-71-	Organic co
--	--	--	--	--	--	HMDB024	--	Organic co
--	--	--	--	--	--	HMDB025	--	Organic co
--	--	--	--	--	--	HMDB003	--	Organic co
--	--	--	--	--	--	HMDB011	--	Organic co
--	--	--	--	--	--	HMDB003	41753-43-	Organic co
--	--	--	--	--	--	--	--	--
--	--	--	--	--	--	HMDB024	1236-72-	Organic co
--	--	--	--	--	--	HMDB000	--	Organic co
--	--	--	--	--	--	HMDB001	608137-3	Organic co
C00165	--	LMGL0201	Glycerolipi	Diradylgly	Diacylglyc	HMDB000	--	Organic co
--	--	--	--	--	--	--	--	--
--	--	--	--	--	--	HMDB011	--	Organic co
--	--	--	--	--	--	HMDB026	--	_
C00350	--	LMGP020	Glyceroph	Glyceroph	1-(1Z-alke	HMDB000	206059-9	Organic co
--	--	--	--	--	--	--	--	--
--	--	--	--	--	--	HMDB028	--	_
--	--	--	--	--	--	HMDB000	--	Organic co
--	--	--	--	--	--	--	--	--
--	--	LMGP0201	Glyceroph	Glyceroph	Diacylglyc	HMDB000	--	Organic co
--	--	--	--	--	--	HMDB000	--	Organic co
--	--	--	--	--	--	HMDB000	--	Organic co
--	--	LMGP0301	Glyceroph	Glyceroph	Diacylglyc	HMDB011	--	Organic co
--	--	--	--	--	--	HMDB028	--	_

--	--	--	--	--	--	HMDB028	--	_
--	--	LMSP0501	Sphingolip	Neutral gly	Simple Glc	HMDB000	474943-8(	_
--	--	--	--	--	--	HMDB029	--	_
--	--	--	--	--	--	HMDB028	--	_
--	--	--	--	--	--	HMDB026	--	_
--	--	--	--	--	--	HMDB026	--	_
--	--	--	--	--	--	HMDB028	--	_
--	--	--	--	--	--	HMDB028	--	_
--	--	--	--	--	--	HMDB028	--	_
--	--	--	--	--	--	HMDB003	111112-47	Organic cc

super_clas	HMDB_tax	sub_class(I	species
Organic ac	Carboxylic	Carboxylic	Animals
Lipids and	Fatty Acyls	Fatty acid	Animals
--	--	--	--
--	--	--	--
Organic ac	Carboxylic	Amino aci	Animals
Organic 1,	Allyl-type	Organic ni	Animals
Organic ac	Carboxylic	Amino aci	Animals
--	--	--	--
--	--	--	--
--	--	--	--
--	--	--	--
Organic ac	Keto acids	Gamma-k	Microbe,Animals,Plants
Homogen	Non-meta	Non-meta	Animals,Plants
Organic ac	Carboxylic	Amino aci	Microbe,Animals
--	--	--	--
Organohe	Dihydrofu	Furanones	Animals
--	--	--	--
--	--	--	--
Nucleoside	Pyrimidine	Pyrimidine	Animals
Nucleoside	Purine nuc	Purine ribo	Animals
Nucleoside	Purine nuc	Purine ribo	Animals
--	--	--	--
--	--	--	--
--	--	--	--
Organic ox	Organooxy	Carbohydr	Microbe,Animals
--	--	--	--
Organic ac	Carboxylic	Tricarboxy	Animals
Organohe	Triazines	1,3,5-triaz	Animals
Organic ac	Carboxylic	Hexacarbo	Animals
--	--	--	--
--	--	--	--
--	--	--	--
Organic ac	Hydroxy a	Beta hydro	Animals
Organosul	Thioethers	Aryl thioet	Animals
Organic ac	Carboxylic	Amino aci	Animals
Nucleoside	Purine nuc	_	Animals
Nucleoside	Flavin nucl	_	Microbe,Animals
Phenylpro	Flavonoids	Flavonoid	Animals
Phenylpro	Flavonoids	Flavonoid	Animals
Lipids and	Fatty Acyls	Fatty acyl t	Microbe,Animals
--	--	--	--
Lipids and	Fatty Acyls	Fatty acids	Animals
Organic ac	Carboxylic	Amino aci	Microbe,Animals,Plants
Organic ox	Organooxy	Carbohydr	Animals
Organohe	Lactones	Gamma bu	Animals
Organohe	Imidazopy	Purines an	Microbe,Animals,Plants
--	--	--	--
--	--	--	--

Organic ni	onitrogen	Amines	Animals
--	--	--	--
Organic ac	Carboxylic	Amino aci	Microbe,Animals
Organohe	Indoles an	Tryptamin	Animals
Nucleoside	Pyrrolopyr	_	Animals
Organohe	Thiophene	Thiophene	Animals
Nucleoside	Purine nuc	Purine 2'-(	Microbe,Animals,Plants
Phenylpro	Cinnamic a	_	Animals
Organic ac	Carboxylic	Amino aci	Animals
Phenylpro	Saxitoxins,	_	Animals
Organic ac	Carboxylic	Amino aci	Animals
Nucleoside	Purine nuc	Cyclic puri	Microbe,Animals
--	--	--	--
Benzenoid	Phenol eth	_	Animals
Organic ox	Organooxy	Carbohydr	Animals
Organic ox	Organooxy	Carbohydr	Animals
Nucleoside	Purine nuc	Purine ribo	Animals
Organic ac	Carboxylic	Amino aci	Animals
Organohe	Lactams	Beta lactar	Animals
Lipids and	Prenol lipi	Diterpenoi	Animals
Organic ac	Carboxylic	Amino aci	Animals
--	--	--	--
--	--	--	--
Phenylpro	Isoflavono	Isoflavono	Animals
--	--	--	--
--	--	--	--
Nucleoside	(5'-5')-din	_	Microbe,Animals
Nucleoside	Purine nuc	Purine dec	Animals
Nucleoside	Purine nuc	Purine dec	Microbe,Animals
--	--	--	--
--	--	--	--
Organic ox	Organooxy	Carbohydr	Microbe,Animals
Organohe	Imidazopy	Purines an	Animals
Organic ac	Hydroxy a	Beta hydro	Animals
--	--	--	--
--	--	--	--
Nucleoside	Purine nuc	Purine ribo	Microbe,Animals
Organohe	Diazines	Pyrimidine	Animals
Organic ac	Hydroxy a	Alpha hyd	Microbe,Animals
--	--	--	--
--	--	--	--
--	--	--	--
--	--	--	--
Organic ac	Carboxylic	Amino aci	Animals
Organic ac	Carboxylic	Amino aci	Animals
Organic ac	Carboxylic	Amino aci	Animals
Organic ox	Organooxy	Carbohydr	Animals
Organic ac	Carboxylic	Amino aci	Animals
Organic ac	Carboxylic	Amino aci	Animals
Phenylpro	Cinnamic a	Cinnamic a	Microbe,Animals,Plants
Organic ox	Organooxy	Carbohydr	Microbe,Animals

--	--	--	--
--	--	--	--
Organic ac	Carboxylic	Amino aci	Animals
--	--	--	--
Organic o	Organooxy	Carbohydr	Animals
Nucleoside	Pyrimidine	Pyrimidine	Animals
Organic ac	Hydroxy a	Medium-c	Animals
--	--	--	--
Lipids and	Fatty Acyls	Fatty acids	Animals
Organic ac	Carboxylic	Amino aci	Animals
Organohe	Triazines	1,3,5-triaz	Animals
--	--	--	--
Phenylpro	Cinnamic a	Cinnamic a	Animals,Plants
c 1,3-dipo	Allyl-type	Organic ni	Animals
--	--	--	--
Benzenoid	Anthracen	Anthraqui	Animals
Organic ac	Organic su	_	Animals
Lipids and	Fatty Acyls	Fatty acyl	Animals
Organohe	Triazines	1,3,5-triaz	Animals
Organic ac	Carboxylic	Amino aci	Animals
--	--	--	--
Lipids and	Fatty Acyls	Fatty acid	Animals
_	_	_	Animals
--	--	--	--
Organohe	Benzopyra	1-benzopy	Animals
Alkaloids a	Morphinar	_	Animals
Lipids and	Prenol lipi	Triterpeno	Animals
Organic ac	Organic su	Arylsulfate	Animals
Lipids and	Glycerolipi	Monorady	Animals
Organohe	Imidazopy	Purines an	Animals
Organohe	Pyridines a	Phenylpyri	Animals
Organic ac	Organic ph	Organic ph	Animals
_	_	_	Animals
Lipids and	Fatty Acyls	Fatty acids	Animals
Lipids and	Fatty Acyls	Fatty acids	Animals
--	--	--	--
--	--	--	--
Organic Po	Polypeptic	_	Animals
Organic ac	Carboxylic	Amino aci	Animals
Lipids and	Fatty Acyls	Fatty acid	Animals
Lipids and	Fatty Acyls	Fatty acids	Animals
--	--	--	--
Lipids and	Fatty Acyls	Eicosanoic	Animals
Nucleoside	(5'-5')-din	_	Microbe,Animals
Benzenoid	Benzene a	Benzylethe	Animals
Phenylpro	Macrolides	_	Animals
Organic ac	Carboxylic	Amino aci	Animals
--	--	--	--
Lipids and	Fatty Acyls	Fatty acids	Animals
Benzenoid	Benzene a	Benzenesu	Animals
Lipids and	Glycerolipi	Monorady	Animals

Lipids and	Prenol lipi	Monoterp	Animals
Lipids and	Steroids a	Furostanes	Animals
--	--	--	--
_	_	_	Animals
Organic o	Organooxy	Carbonyl c	Microbe,Animals,Plants
Organohe	Azoles	Pyrazoles	Animals
Lipids and	Glyceroph	Glyceroph	Microbe,Animals
Organic o	Organooxy	Carbonyl c	Animals
Organohe	Pyrrolidine	Pyrrolidon	Animals
Nucleoside	Purine nuc	Purine rib	Microbe,Animals
Organohe	Pyrans	Pyranones	Animals,Plants
--	--	--	--
Organic ac	Hydroxy a	Beta hydro	Animals
Organohe	Furofurans	Isosorbide	Animals
Organic o	Organooxy	Carbohydr	Animals
Organic ac	Carboxylic	Amino aci	Animals
--	--	--	--
Nucleoside	Purine nuc	Purine rib	Animals
Lipids and	Steroids a	Estrane ste	Animals
Organic ac	Carboxylic	Amino aci	Animals
_	_	_	Animals
Lipids and	Glycerolipi	Triradylcgl	Animals
Organic ac	Vinylogous	_	Animals,Plants
--	--	--	--
Organohe	Isocoumar	Isobenzofu	Animals
Organic ac	Carboxylic	Tricarboxy	Microbe,Animals
Lipids and	Fatty Acyls	Fatty acids	Animals
Organohe	Isoquinolir	_	Animals
--	--	--	--
Organic o	Organooxy	Carbohydr	Animals
Organohe	Azoles	Thiadiazol	Animals
Phenylpro	Coumarins	Furanocou	Animals,Plants
Organohe	Azolidines	Imidazolid	Animals
--	--	--	--
Organic ac	Carboxylic	Amino aci	Animals
Nucleoside	Purine nuc	Purine rib	Microbe,Animals
Organohe	Benzimida	_	Animals
Benzenoid	Benzene a	N-phenylu	Animals
Lipids and	Prenol lipi	Sesquiterp	Animals
Lipids and	Glyceroph	Glyceroph	Animals
Lipids and	Steroids a	Steroidal g	Animals
Lipids and	Steroids a	Cholestane	Animals
Organohe	Pyrroles	Substitute	Animals
Lipids and	Glycerolipi	Diradylgly	Animals
Organohe	Tetrazines	_	Animals
_	_	_	Animals
Lipids and	Prenol lipi	Monoterp	Animals
Organohe	Diazines	Pyrimidine	Animals
Lipids and	Glyceroph	Glyceroph	Animals
Lipids and	Steroids a	Vitamin D	Animals
Lipids and	Glycerolipi	Triradylcgl	Animals

Organic ac	Hydroxy ac	Alpha hyd	Animals
Benzenoid	Benzene a	Benzoic ac	Animals
Lipids and	Prenol lipi	Terpene la	Animals
_	_	_	Animals
_	_	_	Animals
_	_	_	Animals
Benzenoid	Benzene a	Benzenesu	Animals
_	_	_	Animals
Organohe	Azoles	Thiazoles	Animals
--	--	--	--
Phenylpro	Coumarins	Hydroxycc	Animals
Organohe	Benzopyra	1-benzopy	Animals,Plants
Lipids and	Glyceroph	Glyceroph	Animals
--	--	--	--
Organic ac	Organic pl	Organic pl	Animals
Organohe	Thiophene	Thiophene	Animals
Lipids and	Glyceroph	Glyceroph	Animals
_	_	_	Animals
Lipids and	Prenol lipi	Polypreno	Microbe,Animals
Organic ac	Carboxylic	Amino aci	Animals
Lipids and	Glyceroph	Glyceroph	Animals
--	--	--	--
--	--	--	--
Lipids and	Glyceroph	Glyceroph	Animals
Lipids and	Glyceroph	Glyceroph	Animals
Phenylpro	2-arylbenz	_	Animals
Organic ac	Carboxylic	Amino aci	Animals
Organohe	Phenanthr	_	Animals
Organic ox	Organooxy	Carbonyl c	Animals
--	--	--	--
Organohe	Quinolines	Quinolone	Animals
Lipids and	Fatty Acyls	Fatty acids	Animals
Organohe	Imidazopy	Purines an	Animals
--	--	--	--
Organohe	Cyclohepta	_	Animals
Organic ox	Organooxy	Carbohydr	Animals
Benzenoid	Benzene a	Benzenesu	Animals
Benzenoid	Phenols	Benzenedi	Animals
Benzenoid	Benzene a	Benzoic ac	Microbe,Animals,Plants
Organohe	Lactones	_	Animals
--	--	--	--
Organic ac	Carboxylic	Amino aci	Animals
Organohe	Indoles an	Indolyl car	Animals
Nucleoside	Benzimida	_	Animals
Benzenoid	Benzene a	Benzoic ac	Microbe,Animals
Organohe	Indoles an	Tryptamin	Animals,Plants,Microbe
Organohe	Indoles an	Indolyl car	Microbe,Animals
Lipids and	Fatty Acyls	Fatty acyl g	Animals
--	--	--	--
Lipids and	Fatty Acyls	Fatty acyl g	Animals
Organic ac	Carboxylic	Amino aci	Animals

--	--	--	--
Organohe	Azolines	Oxazolines	Animals
Benzenoid	Benzene a	Benzenesu	Animals
Lipids and	Fatty Acyls	Fatty acids	Animals
Organic ac	Carboxylic	Amino aci	Animals
Phenylpro	Flavonoids	Flavonoid	Animals
Benzenoid	Phenols	Benzenedi	Animals,Plants
Benzenoid	Phenols	1-hydroxy	Animals
Organohe	Pteridines	Pterins and	Animals
Organohe	Benzopyra	1-benzopy	Animals
--	--	--	--
Organohe	Pteridines	Pterins and	Animals
Phenylpro	Linear 1,3-	Chalcones	Animals
--	--	--	--
Benzenoid	Benzene a	Benzenesu	Animals
Nucleoside	Nucleoside	3'-thia pyr	Animals
Lipids and	Fatty Acyls	Fatty acid	Animals
--	--	--	--
Organic ox	Organooxy	Alcohols a	Animals
--	--	--	--
Organohe	Pyrroles	Pyrrole ca	Animals
Organohe	Indoles an	Indolines	Animals,Plants
Organohe	Imidazopy	Purines an	Animals,Plants
Phenylpro	Coumarins	_	Animals,Plants
--	--	--	--
Phenylpro	Cinnamic a	Hydroxycir	Animals
Organohe	Triazines	1,2,4-triaz	Animals
Nucleoside	Nucleoside	3'-thia pyr	Animals
Organic ox	Organooxy	Carbohydr	Animals
--	--	--	--
--	--	--	--
Phenylpro	Tannins	Complex t	Animals
Organic ox	Organooxy	Carbohydr	Animals
--	--	--	--
Organic ac	Carboxylic	Amino aci	Animals
--	--	--	--
Organic ox	Organooxy	Carbohydr	Animals
Benzenoid	Phenols	Benzenedi	Animals
Lipids and	Fatty Acyls	Fatty acids	Animals,Plants
Organohe	Diazinanes	Piperazine	Animals
Organic ni	Organonit	Amines	Animals
Organohe	Isoquinolir	_	Animals
--	--	--	--
Benzenoid	Phenols	Benzenedi	Animals
--	--	--	--
Phenylpro	Cinnamic a	Hydroxycir	Animals
Organic ac	Carboxylic	Amino aci	Animals
Organic ac	Organic th	Thiophosp	Animals
--	--	--	--
Organic ac	Organic su	Arylsulfate	Animals
Benzenoid	Benzene a	Benzoic ac	Microbe,Animals,Plants

Organic o›	Organooxy	Carbohydr	Animals
--	--	--	--
Phenylpro	Isoflavono	Isoflavono	Animals
Benzenoid	Benzene a	Benzenesu	Animals
--	--	--	--
--	--	--	--
Nucleoside	Ribonucle	_	Animals
Organic o›	Organooxy	Carbonyl c	Animals
Organic o›	Organooxy	Carbohydr	Animals
Organohe	Quinolines	Quinoline	Animals
Organohe	Diazines	Pyrimidine	Animals
Organic ac	Organic pl	Phosphate	Microbe,Animals
Organic ac	Organic su	Arylsulfate	Animals
Organic 1,	Allyl-type	Organic ni	Animals
Phenylpro	Flavonoids	Flavonoid	Animals
Organohe	Indoles an	Hydroxyin	Microbe,Animals
Organic ac	Peptidomi	Depsipept	Animals
Organic o›	Organooxy	Carbohydr	Animals
--	--	--	--
Organic o›	Organooxy	Alcohols a	Animals
Phenylpro	Isoflavono	Isoflavono	Animals
Organic o›	Organooxy	Carbonyl c	Animals
Organic ac	Carboxylic	Amino aci	Animals
--	--	--	--
Organohe	Pyrans	Pyranones	Animals
Organic o›	Organooxy	Carbonyl c	Animals
--	--	--	--
Organic o›	Organooxy	Carbonyl c	Animals
Organohe	Benzopyra	1-benzopy	Animals
Lipids and	Prenol lipi	Terpene la	Animals
Organic ac	Carboxylic	Amino aci	Animals
Benzenoid	Benzene a	Benzoic ac	Animals
Organohe	Dihydrofu	Furanones	Animals
Organic o›	Organooxy	Carbohydr	Microbe,Animals
--	--	--	--
Phenylpro	Phenylpro	_	Animals
Benzenoid	Benzene a	Benzenesu	Animals
Organohe	Imidazopy	Purines an	Animals
Organic o›	Organooxy	Carbonyl c	Animals
Nucleoside	Purine nuc	Purine ribo	Animals
Phenylpro	Isoflavono	Rotenoids	Animals
Organic ac	Carboxylic	Amino aci	Animals
--	--	--	--
Organohe	Azolines	Oxazolines	Animals
Benzenoid	Benzene a	_	Microbe,Animals
Organohe	Quinolines	Quinolone	Animals,Plants,Microbe
Nucleoside	5'-deoxyri	_	Animals
--	--	--	--
Nucleoside	Purine nuc	Purine dec	Animals
--	--	--	--
--	--	--	--

--	--	--	--
--	--	--	--
Organic ac	Peptidomi	Hybrid pe	Animals,Microbe
--	--	--	--
Phenylpro	Flavonoids	Biflavonoi	Animals
Lipids and	Fatty Acyls	Fatty acyl	Animals
Hydrocarb	Tropones	_	Animals
--	--	--	--
--	--	--	--
Organohe	Oxazines	1,3-oxazin	Animals
Lignans, n	Furanoid li	Tetrahydr	Animals
Organic o	Organooxy	Carbohydr	Animals
Organic ac	Keto acids	Medium-c	Animals
Organic o	Organooxy	Carbohydr	Animals
Organic o	Organooxy	Carbohydr	Animals
--	--	--	--
Benzenoid	Benzene a	Phenoxyac	Animals
--	--	--	--
--	--	--	--
Benzenoid	_	_	Animals,Plants
Organic o	Organooxy	Carbohydr	Animals
--	--	--	--
Lipids and	Prenol lipi	Sesquiterp	Animals
Organic o	Organooxy	Carbonyl c	Animals,Plants,Microbe
Organic o	Organooxy	Carbohydr	Animals
Lipids and	Fatty Acyls	Fatty acids	Microbe,Animals,Plants
Lipids and	Fatty Acyls	Fatty acids	Animals
Organic ac	Organic su	Arylsulfate	Animals
--	--	--	--
Organohe	Imidazopy	Purines an	Animals
Organic ac	Carboxylic	Amino aci	Microbe,Animals
Lipids and	Glyceroph	Glyceroph	Microbe,Animals,Plants
Organic ac	Carboxylic	Amino aci	Animals
--	--	--	--
Lipids and	Fatty Acyls	Fatty acyl	Animals
Phenylpro	Phenylpro	_	Animals
Phenylpro	Flavonoids	Flavonoid	Animals
--	--	--	--
--	--	--	--
Organic o	Organooxy	Carbohydr	Animals
Alkaloids a	Morphinar	_	Animals
_	_	_	Animals
Lipids and	Fatty Acyls	Fatty acids	Animals
Lipids and	Fatty Acyls	Fatty acids	Animals
Benzenoid	Naphthale	Naphthale	Animals
Lipids and	Prenol lipi	Terpene g	Animals
Alkaloids a	Camptoth	_	Animals
Organohe	Pyrrolopyr	_	Animals
--	--	--	--
Benzenoid	Benzene a	Phenethyl	Animals,Plants
Organic o	Organooxy	Carbohydr	Animals

Organohe	Tetrahydro	_	Animals
--	--	--	--
--	--	--	--
Phenylpro	Coumarins	_	Animals
--	--	--	--
--	--	--	--
--	--	--	--
Phenylpro	Tetracyclin	_	Animals
--	--	--	--
Phenylpro	Flavonoids	Flavonoid	Animals
Organic ox	Organooxy	Carbohydr	Animals
Organic ox	Organooxy	Carbohydr	Animals
Organohe	Azoles	Imidazoles	Animals
Organic ac	Carboxylic	Amino acid	Animals
Benzenoid	Benzene a	Benzenesu	Animals
Organohe	Indoles an	Tryptamine	Animals
--	--	--	--
--	--	--	--
--	--	--	--
Organic ac	Carboxylic	Amino acid	Animals
--	--	--	--
Organic ac	Organic su	Arylsulfate	Animals
Organohe	Pyridines a	_	Animals
_	_	_	Animals
Benzenoid	Benzene a	Benzyl alco	Animals
Organohe	Pyridines a	Pyridineca	Animals,Plants
Benzenoid	Benzene a	Phenylpro	Animals
Organic ac	Carboxylic	Amino acid	Animals,Plants
Organic ox	Organooxy	Carbohydr	Animals
Lipids and	Fatty Acyls	Fatty acids	Animals,Plants
Organic ox	Organooxy	Carbohydr	Animals
--	--	--	--
Benzenoid	Benzene a	Styrenes	Microbe,Animals,Plants
Benzenoid	Benzene a	Benzyl alco	Animals
Phenylpro	Phenylpro	_	Animals
Alkaloids a	Tropane a	_	Animals
--	--	--	--
Organic ac	Carboxylic	Amino acid	Animals
--	--	--	--
--	--	--	--
--	--	--	--
Phenylpro	Coumarins	_	Animals
Organic ac	Carboxylic	Amino acid	Animals
--	--	--	--
Organic ox	Organooxy	Carbohydr	Animals
--	--	--	--
Organic ac	Carboxylic	Amino acid	Animals
Organic ac	Carboxylic	Amino acid	Animals
Lipids and	Fatty Acyls	Eicosanoid	Animals
--	--	--	--
Organohe	Diazanaph	Benzodiaz	Animals

Organic o:	Organooxy	Carbohydr	Animals
Organohe	Diazines	Pyrimidine	Animals
Organohe	Quinolines	Quinoline	Animals
Lipids and	Steroids ar	Steroid lac	Animals
Organohe	Indoles an	Hydroxyin	Animals
Organic ac	Carboxylic	Amino aci	Animals
Organohe	Benzopyra	1-benzopy	Animals
Lipids and	Prenol lipi	Terpene la	Animals
Organic o:	Organooxy	Carbohydr	Animals
--	--	--	--
Organic 1,	Allyl-type	Organic ni	Animals
Organohe	Dioxanes	1,4-dioxar	Animals
Organohe	Pteridines	Pterins anc	Microbe,Animals
Benzenoid	Benzene a	Methoxyb	Animals
--	--	--	--
Organohe	Lactones	Gamma bu	Microbe,Animals
--	--	--	--
Organohe	Quinolines	Quinoline	Microbe,Animals
Organic ac	Organic su	Arylsulfate	Animals
--	--	--	--
--	--	--	--
Alkaloids a	Harmala a	_	Animals
--	--	--	--
--	--	--	--
Benzenoid	Benzene a	Benzenesu	Animals
Organohe	Diazinanes	Piperazine	Animals
Organic o:	Organooxy	Carbonyl c	Animals
Alkaloids a	Cinchona a	_	Animals
Organic ac	Carboxylic	Amino aci	Animals
--	--	--	--
--	--	--	--
Benzenoid	Benzene a	Benzylethe	Animals
Organic ac	Carboxylic	Amino aci	Animals
Lipids and	Fatty Acyls	Fatty acyl g	Animals
Lipids and	Steroids ar	Pregnane s	Animals
--	--	--	--
Organic o:	Organooxy	Carbonyl c	Animals
Organic o:	Organooxy	Alcohols a	Animals
Lipids and	Fatty Acyls	Fatty alcoh	Animals,Microbe
--	--	--	--
Organic ac	Organic su	Arylsulfate	Animals
Organic ac	Carboxylic	Amino aci	Animals
Organic o:	Organooxy	Ethers	Animals
Organic ni	Organonit	Amines	Animals
Phenylpro	Isoflavono	O-methyla	Animals
Lipids and	Glyceroph	Glyceroph	Animals
Organohe	Pyrans	_	Animals
Phenylpro	Tannins	Hydrolyzal	Animals
Lipids and	Steroids ar	Steroidal g	Animals
Organohe	Heteroaro	_	Animals
Benzenoid	Phenols	Tyrosols ar	Animals

Organic ac	Hydroxy a	Medium-c	Animals
Lipids and	Steroids ar	Hydroxyst	Animals
Organic ac	Carboxylic	Carboxylic	Animals
--	--	--	--
Organic o	Organoox	Carbohydr	Animals
Organic o	Organic o	Organic p	Microbe,Animals
Organohe	Benzopyra	1-benzopy	Animals,Plants
Nucleoside	Purine nuc	Purine ribo	Animals
Lipids and	Fatty Acyls	Fatty alcoh	Animals
Organic ac	Carboxylic	Amino acid	Animals
Organic ac	Carboxylic	Amino acid	Animals
--	--	--	--
Organohe	Pteridines	Pterins and	Animals
--	--	--	--
--	--	--	--
--	--	--	--
Nucleoside	5'-deoxyri	_	Animals
Lipids and	Steroids ar	Steroidal a	Animals,Plants
Organohe	Diazanaph	Benzodiaz	Animals
Organic ac	Carboxylic	Amino acid	Animals
Organohe	Pyrrolizine	_	Animals
Organic ac	Carboxylic	Amino acid	Animals
Organohe	Quinolines	Quinolone	Animals,Plants
Lipids and	Fatty Acyls	Fatty acyl g	Animals
Organic o	Organoox	Carbonyl c	Animals
Organic ac	Organic su	Arylsulfate	Animals
Organohe	Imidazopy	Purines an	Animals
Organic o	Organoox	Carbohydr	Animals
Organohe	Pteridines	Pterins and	Animals
Organohe	Indolizidin	_	Animals
Phenylpro	Linear 1,3-	Cinnamylp	Animals,Plants
Phenylpro	Flavonoids	Flavans	Animals
Organohe	Pyridines a	Pyridineca	Animals
Organohe	Isobenzofu	_	Animals
Lipids and	Fatty Acyls	Fatty acid	Animals
Lipids and	Fatty Acyls	Fatty alcoh	Animals,Plants
Organic o	Organoox	Carbonyl c	Animals
Lipids and	Fatty Acyls	Fatty acids	Animals,Plants
--	--	--	--
Lipids and	Fatty Acyls	Fatty acid	Animals
Organohe	Benzoxazi	Phenoxazi	Animals
Organic ac	Carboxylic	Amino acid	Animals
Benzenoid	Benzene a	Anilides	Animals
--	--	--	--
--	--	--	--
Organohe	Indoles an	Pyrroloind	Animals
Organic ac	Carboxylic	Amino acid	Microbe,Animals
Lipids and	Fatty Acyls	Fatty acids	Animals
Lipids and	Prenol lipi	Terpene g	Animals
Phenylpro	Flavonoids	Flavonoid	Animals
Benzenoid	Phenols	1-hydroxy	Microbe,Animals

Benzenoid	Phenols	Methoxypl	Animals
Benzenoid	Phenols	1-hydroxy	Microbe,Animals,Plants
Organohe	Pyridines a	Aminopyri	Animals
_	_	_	Animals
--	--	--	--
Lipids and	Steroids ar	Hydroxyst	Animals
Organic o	Organooxy	Carbohydr	Animals
Homogen	Non-meta	Non-meta	Animals
Nucleosid	Pyrimidine	Cyclic pyri	Animals
Lipids and	Steroids ar	Hydroxyst	Animals
Organic ac	Carboxylic	Amino aci	Animals
Organic ac	Carboxylic	Carboxylic	Animals
Organic ni	Organonit	Guanidine	Animals
Lipids and	Prenol lipi	Terpene g	Animals
--	--	--	--
--	--	--	--
--	--	--	--
Organohe	Indoles an	Indolyl car	Animals,Plants
Phenylpro	Tannins	Hydrolyzal	Animals
Organohe	Diazines	Pyrimidine	Animals,Plants
Lignans, n	Lignan gly	_	Animals
Organic ac	Carboxylic	Amino aci	Animals
Organohe	Pyrrolines	_	Animals
Organic ac	Hydroxy a	Beta hydro	Animals
Organohe	Oxazinane	Morpholin	Animals
Organic ac	Carboxylic	Amino aci	Animals
_	_	_	Animals
--	--	--	--
Organic ac	Carboxylic	Amino aci	Animals
--	--	--	--
--	--	--	--
--	--	--	--
--	--	--	--
Lipids and	Fatty Acyls	Eicosanoic	Animals
--	--	--	--
Lipids and	Fatty Acyls	Lineolic ac	Animals
Lipids and	Steroids ar	Steroid lac	Animals
Organohe	Heteroaro	_	Animals
Lipids and	Fatty Acyls	Fatty acids	Animals
Benzenoid	Benzene a	_	Animals,Plants
Benzenoid	Benzene a	_	Animals
Organic ac	Carboxylic	Amino aci	Animals
Lipids and	Prenol lipi	Sesquiterp	Animals
Phenylpro	Stilbenes	_	Animals
--	--	--	--
Lipids and	Fatty Acyls	Fatty acids	Animals
Organic ac	Carboxylic	Amino aci	Animals
Organohe	Pyridines a	Pyridineca	Animals
Organic o	Organooxy	Carbohydr	Animals
Organohe	Benzopyra	1-benzopy	Animals,Plants
Organohe	Diazinanes	Piperazine	Animals

Lipids and	Steroids ar	Steroidal g	Animals
--	--	--	--
Phenylpro	Flavonoids	O-methyla	Animals,Plants
Lipids and	Fatty Acyls	Fatty acids	Animals
Organohe	Imidazopy	Purines an	Animals
Lipids and	Steroids ar	Steroid lac	Animals
Organic ac	Carboxylic	Amino aci	Animals
Organic ox	Organooxy	Carbohydr	Animals
Organic ac	Peptidomi	Depsipept	Animals
Organohe	Pyrans	Pyranones	Animals
Benzenoid	Benzene a	N-phenylu	Animals
Organic ac	Carboxylic	Amino aci	Animals
--	--	--	--
--	--	--	--
Organic ac	Carboxylic	Amino aci	Animals
--	--	--	--
Organic ac	Carboxylic	Amino aci	Animals
--	--	--	--
Benzenoid	Phenol eth	Anisoles	Animals
Organic ac	Carboxylic	Amino aci	Microbe,Animals
Organic ac	Carboxylic	Amino aci	Animals
Benzenoid	Benzene a	Diphenyle	Animals
Organic ac	Carboxylic	Amino aci	Animals
Lipids and	Glyceroph	Glyceroph	Animals
--	--	--	--
--	--	--	--
Organohe	Indoles an	Hydroxyin	Animals
Organohe	Pyrrolopyr	Pyrrolo[2,3	Animals
Lignans, n	Furanoid li	Tetrahydro	Animals
Lipids and	Fatty Acyls	Fatty acyl g	Animals
Organohe	Indoles an	Indoles	Animals
Organic ac	Carboxylic	Tetracarbo	Animals
Lipids and	Steroids ar	Bile acids,	Animals
Organic ac	Organic su	Arylsulfate	Animals
Lipids and	Fatty Acyls	Fatty acids	Animals,Plants
Organohe	Imidazopy	_	Animals
Organohe	Diazines	Pyrimidine	Animals
_	_	_	Animals
--	--	--	--
Phenylpro	Coumarins	_	Animals
Lipids and	Prenol lipi	Terpene g	Animals
--	--	--	--
--	--	--	Animals
Lipids and	Fatty Acyls	Fatty acids	Animals
Organohe	Indoles an	Hydroxyin	Animals
Organic ac	Carboxylic	Amino aci	Animals
Organohe	Lactones	Delta valer	Animals
Phenylpro	Macrolides	_	Animals
Benzenoid	Naphthale	Naphthoq	Animals
--	--	--	--
Organic ac	Organic su	Organosul	Animals

Organic ac	Carboxylic	Amino aci	Animals
Lipids and	Prenol lipi	Terpene la	Animals
Benzenoid	Benzene a	Diphenylm	Animals
Lipids and	Steroids ar	Steroidal g	Animals
Organohe	Pyridines a	Bipyridines	Animals
--	--	--	--
Organic ac	Keto acids	Medium-c	Microbe,Animals
Organic ox	Organooxy	Carbonyl c	Animals
Organic ox	Organooxy	Carbohydr	Microbe,Animals
Lipids and	Prenol lipi	Triterpeno	Animals
Organic ac	Carboxylic	Amino aci	Animals
Lipids and	Fatty Acyls	Fatty acyl g	Animals
Lipids and	Prenol lipi	Terpene la	Animals
--	--	--	--
--	--	--	--
--	--	--	--
Benzenoid	Benzene a	Benzylethe	Animals
Lipids and	Steroids ar	Bile acids,	Animals
Lipids and	Fatty Acyls	Fatty acyl g	Animals
Organic ox	Organooxy	Carbohydr	Animals
Phenylpro	Macrolides	Milbemyci	Animals
Lipids and	Prenol lipi	Sesquiterp	Animals
Organic ox	Organooxy	Carbonyl c	Microbe,Animals,Plants
Organohe	Pyridines a	Pyridineca	Animals
Benzenoid	Benzene a	Benzoic ac	Animals
--	--	--	--
Organic ox	Organooxy	Carbohydr	Animals
Organic ac	Carboxylic	Amino aci	Animals
Lipids and	Fatty Acyls	Eicosanoic	Animals
--	--	--	--
Lipids and	Prenol lipi	Diterpenoi	Animals
Benzenoid	Benzene a	Benzoic ac	Animals
Organic ac	Carboxylic	Amino aci	Animals
Organohe	Pyridines a	Hydropyri	Animals
Organohe	Naphthofu	_	Animals
Alkaloids a	Tropane a	_	Animals
Lipids and	Fatty Acyls	Fatty acid	Animals
Benzenoid	Benzene a	Biphenyls a	Animals
Organic ac	Carboxylic	Amino aci	Animals
Organohe	Lactones	Gamma bu	Animals,Plants
Phenylpro	Linear 1,3-	Chalcones	Animals,Plants
Organic ac	Carboxylic	Amino aci	Animals
Organic ac	Carboxylic	Amino aci	Animals
Lipids and	Prenol lipi	Terpene g	Animals
--	--	--	--
Lipids and	Prenol lipi	Quinone a	Animals
Lipids and	Fatty Acyls	Fatty acyl g	Animals
Benzenoid	Benzene a	Benzyl alco	Animals
Organohe	Indoles an	Indolines	Animals
Organic ox	Organooxy	Carbohydr	Animals
Organic ac	Carboxylic	Amino aci	Animals

--	--	--	--
--	--	--	--
Lipids and	Steroids ar	Pregnane :	Animals
--	--	--	--
Organosul	Isothiourea	_	Animals
Lipids and	Glyceroph	Glyceroph	Animals
Lipids and	Steroids ar	Steroid lac	Animals
--	--	--	--
Lipids and	Fatty Acyls	Fatty acid	Animals
Organic ac	Carboxylic	Amino aci	Animals
--	--	--	--
Organic ac	Hydroxy a	Beta hydro	Animals
Organic ac	Carboxylic	Amino aci	Animals
Lipids and	Fatty Acyls	Fatty acids	Animals
Lipids and	Prenol lipi	Terpene la	Animals
Lipids and	Fatty Acyls	Eicosanoic	Animals
Organic ox	Organooxy	Carbonyl c	Animals,Plants
Organic ox	Organooxy	Carbohydr	Animals
Organic ac	Carboxylic	Amino aci	Animals
Organic ac	Carboxylic	Amino aci	Animals
Lipids and	Steroids ar	Cholestane	Animals
Lipids and	Prenol lipi	Sesquiterp	Animals
Lipids and	Prenol lipi	Terpene g	Animals
_	_	_	Animals
Lipids and	Fatty Acyls	Fatty acid	Animals
Benzenoid	Phenol eth	_	Animals
Lipids and	Prenol lipi	Sesquiterp	Animals
Lipids and	Glyceroph	Glyceroph	Animals
--	--	--	--
--	--	--	--
--	--	--	--
Lipids and	Prenol lipi	Terpene g	Animals
Organohe	Diazines	Pyrimidine	Animals
Organic ox	Organooxy	Carbohydr	Animals
Lipids and	Glyceroph	Glyceroph	Animals
Organohe	Quinolines	Quinolone	Animals,Plants
Lipids and	Steroids ar	Steroid est	Animals
Lipids and	Fatty Acyls	Eicosanoic	Animals
Organic ac	Carboxylic	Amino aci	Animals
Organohe	Benzopyra	1-benzopy	Animals
Lipids and	Steroids ar	Vitamin D	Animals
Lipids and	Endocanna	_	Animals
Phenylpro	Macrolides	_	Animals
Lipids and	Fatty Acyls	Fatty acyl g	Animals
--	--	--	--
Benzenoid	Phenol eth	_	Animals
Nucleoside	Pyrimidine	_	Animals
_	_	_	Animals
_	_	_	Animals
Phenylpro	Cinnamic a	Hydroxycir	Animals
--	--	--	--

Lipids and	Fatty Acyls	Eicosanoic	Animals
Organic ac	Organic th	Thiophosp	Animals
--	--	--	--
--	--	--	--
Benzenoid	Benzene a	Benzoic ac	Animals,Plants
--	--	--	--
Lipids and	Fatty Acyls	Fatty acids	Animals
Lipids and	Steroids ar	Hydroxyste	Animals
Lipids and	Fatty Acyls	Fatty acids	Microbe,Animals
Organic ac	Keto acids	Medium-c	Animals
Lipids and	Fatty Acyls	Fatty acids	Animals
Lipids and	Prenol lipi	Sesquiterp	Animals
--	--	--	--
_	_	_	Animals
Lipids and	Steroids ar	Androstan	Animals
--	--	--	--
Organohe	Benzopyra	1-benzopy	Animals,Microbe
Organic ox	Organooxy	Carbonyl c	Animals
--	--	--	--
Lipids and	Fatty Acyls	Fatty acids	Animals
Lipids and	Prenol lipi	Monoterpe	Animals
--	--	--	--
_	_	_	Animals
Organic ox	Organooxy	Carbohydr	Animals
Lipids and	Fatty Acyls	Lineolic ac	Animals
--	--	--	--
Organohe	Benzopyra	1-benzopy	Animals
Alkaloids a	Morphinar	_	Animals
--	--	--	--
Benzenoid	Tetralins	_	Animals
Lipids and	Steroids ar	Steroidal g	Animals
Lipids and	Steroids ar	Sulfated st	Animals
_	_	_	Animals
Lipids and	Prenol lipi	Terpene g	Animals
--	--	--	--
--	--	--	--
Benzenoid	Phenols	Methoxypl	Animals
Lipids and	Fatty Acyls	Fatty acids	Animals
Phenylpro	Macrolides	Milbemyci	Animals
_	_	_	Animals
Lipids and	Steroids ar	Bile acids,	Animals
Lipids and	Steroids ar	Bile acids,	Animals
_	_	_	Animals
_	_	_	Animals
--	--	--	--
_	_	_	Animals
Lipids and	Prenol lipi	Diterpenoi	Animals
Phenylpro	Macrolides	Milbemyci	Animals
Organic ac	Carboxylic	Amino aci	Animals
--	--	--	--
_	_	_	Animals

_	_	_	Animals
Organic ac	Carboxylic	Amino acid	Animals
Lipids and	Fatty Acyls	Fatty acyl t	Animals
Organohe	Lactones	Gamma bu	Animals
Lipids and	Fatty Acyls	Eicosanoic	Animals
_	_	_	Animals
Organic ox	Organooxy	Carbohydr	Animals
Phenylpro	Flavonoids	Flavonoid	Animals
--	--	--	--
Organic ac	Carboxylic	Amino acid	Animals
Organohe	Dihydrofur	Furanones	Animals
Benzenoid	Benzene a	Phenoxy c	Animals
Lipids and	Fatty Acyls	Eicosanoic	Animals
Organic ac	Carboxylic	Amino acid	Animals
Organohe	Pyridines a	_	Animals
--	--	--	--
Lipids and	Prenol lipid	Monoterpe	Animals
Lipids and	Prenol lipid	Diterpenoi	Animals
_	_	_	Animals
Organohe	Benzopyra	Indazoles	Animals
--	--	--	--
Organohe	Oxanes	_	Animals
--	--	--	--
Lipids and	Steroids ar	Pregnane s	Animals
Lipids and	Fatty Acyls	Fatty acids	Animals
Organic ox	Organooxy	Carbonyl c	Animals
Lipids and	Steroids ar	Bile acids,	Animals
Organohe	Lactones	Gamma bu	Animals
--	--	--	--
Lipids and	Fatty Acyls	Eicosanoic	Animals
Benzenoid	Benzene a	Diphenylm	Animals
Lipids and	Endocanna	_	Animals
Organohe	Azaspirode	_	Animals
_	_	_	Animals
Organohe	Benzopyra	1-benzopy	Animals,Microbe
Lipids and	Prenol lipid	Triterpeno	Animals
Organohe	Benzopyra	1-benzopy	Animals,Microbe
Lipids and	Fatty Acyls	Fatty acyl t	Animals
Lipids and	Glyceroph	Glyceroph	Microbe,Animals
--	--	--	--
Benzenoid	Benzene a	Phenylpro	Animals
Organic ac	Hydroxy ac	Medium-c	Animals
Lipids and	Steroids ar	Bile acids,	Animals
Phenylpro	Cinnamic a	Cinnamic a	Animals
Benzenoid	Benzene a	Biphenyls a	Animals
Lipids and	Steroids ar	Bile acids,	Animals
Organic ac	Carboxylic	Amino acid	Animals
_	_	_	Animals
Organic ac	Keto acids	Beta-keto	Animals
Benzenoid	Phenols	Tyrosols ar	Animals
Organohe	Indoles an	Indoles	Animals,Plants

Lipids and	Fatty Acyls	Fatty acyl t	Animals
Lipids and	Steroids ar	Steroidal g	Animals
Lipids and	Fatty Acyls	Fatty acids	Animals
Lipids and	Fatty Acyls	Eicosanoic	Animals
Lipids and	Prenol lipi	Triterpeno	Animals
Organic ox	Organooxy	Ethers	Animals
Organohet	Lactones	Gamma bu	Animals
Organic ac	Carboxylic	Amino aci	Animals
Benzenoid	Naphthale	Naphthoq	Animals
_	_	_	Animals
--	--	--	--
--	--	--	--
_	_	_	Animals
Lipids and	Steroids ar	Steroidal g	Animals
Lipids and	Glycerolipi	Monorady	Animals,Plants
Lipids and	Steroids ar	Bile acids,	Animals
Lipids and	Glyceroph	Glyceroph	Animals
Organohet	Diazinanes	Piperazine	Animals
Lipids and	Prenol lipi	Diterpenoi	Animals
Organic ac	Carboxylic	Amino aci	Animals
Lipids and	Fatty Acyls	Eicosanoic	Animals
--	--	--	--
Lipids and	Fatty Acyls	Fatty alcoh	Animals
Lipids and	Fatty Acyls	Eicosanoic	Animals
Lipids and	Prenol lipi	Sesquiterp	Animals
Organic ac	Carboxylic	Amino aci	Animals
Organic ac	Hydroxy ac	Medium-c	Animals
Lipids and	Glyceroph	Glyceroph	Animals
--	--	--	--
Organic ac	Carboxylic	Tetracarbo	Animals
--	--	--	--
pids and li	Steroids ar	Cucurbitac	Animals
Lipids and	Fatty Acyls	Fatty acids	Animals
--	--	--	--
--	--	--	--
Phenylpro	Isoflavono	Isoflav-2-e	Animals
--	--	--	--
Organic ox	Organooxy	Ethers	Animals
--	--	--	--
Lipids and	Fatty Acyls	Fatty alcoh	Animals
--	--	--	--
_	_	_	Animals
Lipids and	Steroids ar	Bile acids,	Animals
Organohet	Indoles an	Indolines	Animals
--	--	--	--
_	_	_	Animals
_	_	_	Animals
_	_	_	Animals
_	_	_	Animals
Lipids and	Fatty Acyls	Fatty acids	Animals,Plants
--	--	--	--

Lipids and	Prenol lipi	Diterpenoi	Animals
Phenylpro	Macrolides	Milbemyci	Animals
--	--	--	--
Lipids and	Prenol lipi	Terpene g	Animals
Lipids and	Prenol lipi	Triterpeno	Animals
Lipids and	Fatty Acyls	Eicosanoic	Animals
--	--	--	--
Lipids and	Fatty Acyls	Fatty acids	Animals
_	_	_	Animals
--	--	--	--
--	--	--	--
--	--	--	--
Lipids and	Glyceroph	Glyceroph	Microbe,Animals
Lipids and	Fatty Acyls	Fatty acids	Animals
Organic ac	Peptidomi	Depsipept	Animals
Organohe	Imidazopy	_	Animals
_	_	_	Animals
--	--	--	--
--	--	--	--
Organic ac	Carboxylic	Amino aci	Animals
Phenylpro	Flavonoids	Flavonoid	Animals
Phenylpro	Macrolacta	_	Animals
Lipids and	Steroids ar	Androstan	Animals
--	--	--	--
--	--	--	--
Organohe	Pyridines a	Hydropyri	Animals
Lipids and	Prenol lipi	Sesquiterp	Animals
Organohe	Tetrahydro	_	Animals
--	--	--	--
--	--	--	--
Lipids and	Fatty Acyls	Eicosanoic	Animals
Lipids and	Steroids ar	Estrane ste	Animals
--	--	--	--
--	--	--	--
Organic ac	Carboxylic	Amino aci	Animals
--	--	--	--
Organohe	Benzothiaz	_	Animals
Lipids and	Prenol lipi	Triterpeno	Animals
Lipids and	Steroids ar	Bile acids,	Animals
Organoha	Alkyl halid	Cyclohexyl	Animals
Lipids and	Fatty Acyls	Fatty acid	Animals
_	_	_	Animals
_	_	_	Animals
--	--	--	--
--	--	--	--
--	--	--	--
Organic ac	Carboxylic	Amino aci	Animals
Lipids and	Prenol lipi	Monoterp	Animals
--	--	--	--
Lipids and	Fatty Acyls	Lineolic ac	Animals
Nucleoside	Pyrimidine	Pyrimidine	Animals

Organic ac	Carboxylic	Amino acic	Animals
Lipids and	Steroids ar	Hydroxyste	Animals
Lipids and	Prenol lipic	Sesquiterp	Animals,Microbe
Organohe	Piperidines	Fentanyls	Animals
Organic ac	Carboxylic	Amino acic	Animals,Plants
_	_	_	Animals
Organic ox	Organooxy	Carbohydr	Animals
Lipids and	Fatty Acyls	Fatty acid	Animals
--	--	--	--
Lipids and	Fatty Acyls	Fatty alcoh	Animals
Organic ac	Carboxylic	Amino acic	Animals
Phenylpro	Macrolides	_	Animals
Lipids and	Steroids ar	Vitamin D	Animals
Organic ac	Carboxylic	Amino acic	Animals
Lipids and	Steroids ar	Steroid lac	Animals
Lipids and	Saccharoli	_	Animals
Organic ox	Organooxy	Carbohydr	Animals
Lipids and	Prenol lipic	Monoterpe	Animals
--	--	--	--
Lipids and	Steroids ar	Oxosteroic	Animals
Organohe	Benzazepi	_	Animals
--	--	--	--
_	_	_	Animals
Organohe	Benzopyra	1-benzopy	Animals
Organic ac	Carboxylic	Amino acic	Animals
Organic ac	Carboxylic	Amino acic	Animals
Organic ac	Carboxylic	Amino acic	Animals
_	_	_	Animals
Organic ac	Carboxylic	Amino acic	Animals
--	--	--	--
Lipids and	Fatty Acyls	Fatty acids	Animals
Lipids and	Fatty Acyls	Eicosanoic	Animals
Nucleoside	Pyrimidine	Pyrimidine	Animals
--	--	--	--
--	--	--	--
--	--	--	--
--	--	--	--
Benzenoid	Phenol eth	Anisoles	Animals
Lipids and	Fatty Acyls	Lineolic ac	Animals
Lipids and	Steroids ar	Hydroxyste	Animals
Lipids and	Steroids ar	Pregnane :	Animals
Lipids and	Fatty Acyls	Fatty acids	Animals
Organic ox	Organooxy	Alcohols a	Animals
--	--	--	--
Benzenoid	Phenol eth	_	Animals
--	--	--	--
Organic ac	Carboxylic	Amino acic	Animals
Benzenoid	Benzene a	Benzoic ac	Animals
Organic ac	Carboxylic	Amino acic	Animals
Lipids and	Glyceroph	Glyceroph	Animals
Lipids and	Glyceroph	Glyceroph	Animals

Lipids and	Steroids ar	Bile acids,	Animals
--	--	--	--
Lipids and	Steroids ar	Bile acids,	Animals
--	--	--	--
Lipids and	Steroids ar	Bile acids,	Animals
Lipids and	Fatty Acyls	Fatty amid	Animals
Lipids and	Sphingolip	Glycosphir	Animals
Lipids and	Prenol lipi	Retinoids	Animals
--	--	--	--
Organohe	Benzoxepi	Dibenzoxe	Animals
--	--	--	--
Lipids and	Fatty Acyls	Eicosanoic	Animals
Lipids and	Fatty Acyls	Fatty alcoh	Animals
_	_	_	Animals
Lipids and	Glyceroph	Glyceroph	Animals
Benzenoid	Benzene a	_	Animals
Lipids and	Fatty Acyls	Fatty acid	Animals
Organohe	Tetrapyrro	Porphyrins	Animals
Lipids and	Steroids ar	Bile acids,	Animals
_	_	_	Animals
Lipids and	Glyceroph	CDP-glyce	Animals
_	_	_	Animals
--	--	--	--
Lipids and	Steroids ar	Steroid lac	Animals
Lipids and	Prenol lipi	Terpene g	Animals
--	--	--	--
Lipids and	Steroids ar	Bile acids,	Animals
Lipids and	Glyceroph	Glyceroph	Animals
Benzenoid	Benzene a	Biphenyls a	Animals
Organic ac	Peptidomi	Depsipept	Animals
--	--	--	--
_	_	_	Animals
_	_	_	Animals
Organohe	Lactones	Gamma bu	Animals
Organic ox	Organooxy	Carbohydr	Animals
--	--	--	--
Organic ac	Carboxylic	Amino aci	Animals
Lipids and	Glyceroph	Glyceroph	Animals
--	--	--	--
Organic ox	Organooxy	Carbohydr	Animals
Benzenoid	Benzene a	Phenylpro	Animals
--	--	--	--
Lipids and	Glyceroph	Glyceroph	Animals
Organohe	Indoles an	_	Animals
Organic ac	Carboxylic	Amino aci	Animals
Lipids and	Fatty Acyls	Eicosanoic	Animals
--	--	--	--
--	--	--	--
Lipids and	Prenol lipi	Diterpenoi	Animals
Organic ac	Carboxylic	Amino aci	Animals
Lipids and	Sphingolip	Glycosphir	Animals

Lipids and	Fatty Acyls	Fatty acyl g	Animals
--	--	--	--
Lipids and	Glyceroph	Glyceroph	Animals
Lipids and	Fatty Acyls	Fatty alcoh	Animals
--	--	--	--
Organic ac	Carboxylic	Amino acid	Animals
Organic ac	Carboxylic	Amino acid	Animals
Lipids and	Sphingolip	Glycosphin	Animals
Lipids and	Glyceroph	Glyceroph	Animals
Lipids and	Steroids ar	Bile acids,	Animals
Lipids and	Fatty Acyls	Fatty acid	Animals
_	_	_	Animals
--	--	--	--
--	--	--	--
--	--	--	--
_	_	_	Animals
Lipids and	Fatty Acyls	Fatty alcoh	Animals
--	--	--	--
--	--	--	--
Phenylpro	Macrolides	_	Animals,Plants
--	--	--	--
Lipids and	Fatty Acyls	Fatty acid	Animals
Organic ac	Carboxylic	Amino acid	Animals
Lipids and	Sphingolip	Phosphosp	Animals
Organic ac	Carboxylic	Tricarboxy	Animals
Lipids and	Steroids ar	Androstan	Animals
Lipids and	Fatty Acyls	Eicosanoic	Animals
--	--	--	--
Lipids and	Prenol lipid	Diterpenoi	Animals
Organic ac	Carboxylic	Amino acid	Animals
Lipids and	Glycerolipi	Diradylglyc	Animals
_	_	_	Animals
_	_	_	Animals
Organic ac	Carboxylic	Amino acid	Animals
Lipids and	Steroids ar	Bile acids,	Animals
Organic ac	Carboxylic	Amino acid	Animals
Lipids and	Steroids ar	Bile acids,	Animals
Phenylpro	Macrolide	_	Animals
_	_	_	Animals
Lipids and	Steroids ar	Bile acids,	Animals
Lipids and	Sphingolip	Glycosphin	Animals
Lipids and	Steroids ar	Steroid lac	Animals
Lipids and	Glyceroph	Glyceroph	Microbe,Animals
Benzenoid	Benzene a	Diphenylm	Animals
Lipids and	Steroids ar	Bile acids,	Animals
--	--	--	--
Benzenoid	Benzene a	Diphenylac	Microbe,Animals
--	--	--	--
_	_	_	Animals
_	_	_	Animals
_	_	_	Animals

_	_	_	Animals
_	_	_	Animals
Lipids and	Prenol lipi	Diterpenoi	Animals
Phenylpro	Isoflavono	O-methyla	Animals
Lipids and	Steroids ar	Bile acids,	Animals
--	--	--	--
--	--	--	--
Phenylpro	Macrolide	_	Animals
Organic ac	Carboxylic	Amino acid	Animals
Lipids and	Prenol lipi	Triterpeno	Animals
--	--	--	--
Organic ac	Carboxylic	Amino acid	Animals
Organic ac	Carboxylic	Amino acid	Animals
Lipids and	Fatty Acyls	Fatty acids	Animals
Lipids and	Prenol lipi	Terpene la	Animals
--	--	--	--
Lipids and	Fatty Acyls	Eicosanoic	Animals
--	--	--	--
Lipids and	Fatty Acyls	Fatty amid	Animals
Lipids and	Steroids ar	Bile acids,	Animals
Organohet	Quinolines	Benzoquin	Animals,Plants
_	_	_	Animals
Organohet	Indoles an	Tryptamin	Animals
_	_	_	Animals
_	_	_	Animals
Organic ac	Carboxylic	Amino acid	Animals
_	_	_	Animals
_	_	_	Animals
Lipids and	Steroids ar	Bile acids,	Animals
--	--	--	--
Organic ac	Peptidomi	Hybrid pep	Animals
--	--	--	--
Lipids and	Prenol lipi	Terpene la	Animals
--	--	--	--
Organic ac	Carboxylic	Amino acid	Animals
Lipids and	Prenol lipi	Sesquiterp	Animals
_	_	_	Animals
Lipids and	Glyceroph	Glyceroph	Animals
_	_	_	Animals
Lipids and	Steroids ar	Steroid est	Animals
--	--	--	--
--	--	--	--
Lipids and	Fatty Acyls	Lineolic ac	Animals
Lipids and	Glyceroph	Glyceroph	Animals
Lipids and	Steroids ar	Bile acids,	Animals
Organohet	Tetrapyrro	Bilirubins	Animals
Lipids and	Glyceroph	Glyceroph	Animals
Lipids and	Steroids ar	Bile acids,	Animals
Organohet	Tetrapyrro	Bilirubins	Animals
Organic ox	Organooxy	Carbonyl c	Animals,Plants
Organic ac	Carboxylic	Alpha-halo	Microbe,Animals,Plants

Lipids and	Glyceroph	CDP-glyce	Animals
_	_	_	Animals
Lipids and	Prenol lipi	Triterpeno	Animals
Lipids and	Glyceroph	Glyceroph	Animals
Organohe	Indoles an	Tryptamin	Animals
_	_	_	Animals
Organic ac	Carboxylic	Amino aci	Animals
Organic ac	Carboxylic	Amino aci	Animals
_	_	_	Animals
Organic ac	Carboxylic	Amino aci	Animals
Organohe	Quinolines	Quinoline	Animals
Lipids and	Fatty Acyls	Fatty acids	Animals,Plants
Lipids and	Glyceroph	Glyceroph	Animals
--	--	--	--
Lipids and	Prenol lipi	Diterpenoi	Animals
Lipids and	Fatty Acyls	Fatty alcoh	Animals
_	_	_	Animals
Lipids and	Fatty Acyls	Fatty acids	Animals,Microbe
Organic ox	Organooxy	Carbohydr	Animals
--	--	--	--
--	--	--	--
Lipids and	Glyceroph	Glyceroph	Animals
Lipids and	Steroids ar	Bile acids,	Animals
Lipids and	Steroids ar	Steroidal g	Animals
_	_	_	Animals
Organic ni	Organonit	Oximes	Animals
Lipids and	Fatty Acyls	Fatty acids	Animals
--	--	--	--
--	--	--	--
Lipids and	Fatty Acyls	Fatty acids	Animals
Organoph	Organic ph	_	Animals
_	_	_	Animals
--	--	--	--
Organic ac	Carboxylic	Amino aci	Animals
Organic ac	Peptidomi	Depsipept	Animals
--	--	--	--
Lipids and	Steroids ar	Bile acids,	Animals
Organic ox	Organooxy	Carbohydr	Animals
Benzenoid	Benzene a	Benzoic ac	Animals
Organic ac	Peptidomi	Depsipept	Animals
Organohe	Oxazinane	Morpholin	Animals
Organic ox	Organooxy	Ethers	Animals
Lipids and	Steroids ar	Hydroxyste	Animals
Lipids and	Fatty Acyls	Fatty acids	Animals
Lipids and	Steroids ar	Vitamin D	Animals
Organohe	Diazines	Pyrimidine	Animals
Lipids and	Steroids ar	Bile acids,	Animals
_	_	_	Animals
Organic ac	Peptidomi	Depsipept	Animals
Lipids and	Fatty Acyls	Lineolic ac	Animals,Plants
_	_	_	Animals

--	--	--	--
Lipids and	Fatty Acyls	Eicosanoic	Animals
Organic ox	Organooxy	Carbohydr	Animals
Lipids and	Sphingolip	Glycosphin	Animals
Lipids and	Glyceroph	Glyceroph	Animals
Organohet	Indoles an	Tryptamin	Animals
Alkaloids a	6,7-benzo	2,6-dimetl	Animals
Lipids and	Steroids ar	Cycloartan	Animals
--	--	--	--
--	--	--	--
_	_	_	Animals
Lipids and	Steroids ar	Estrane ste	Animals
Lipids and	Fatty Acyls	Fatty acids	Animals
Organic ac	Carboxylic	Amino acid	Animals
Lipids and	Glyceroph	Glyceroph	Microbe,Animals
Lipids and	Prenol lipi	Sesterterp	Animals
--	--	--	--
Lipids and	Fatty Acyls	Fatty acids	Animals
--	--	--	--
Organohet	Diazines	Pyrimidine	Animals
Lipids and	Fatty Acyls	Fatty acids	Animals,Plants
Lipids and	Prenol lipi	Diterpenoi	Animals
--	--	--	--
Organic ac	Peptidomi	Depsipept	Animals
Phenylpro	Flavonoids	Flavans	Animals
--	--	--	--
Lipids and	Prenol lipi	Diterpenoi	Animals
Benzenoid	Phenols	Methoxypl	Animals,Plants
Lipids and	Glycerolipi	Monorady	Animals
Lipids and	Glyceroph	Glyceroph	Animals
Lipids and	Fatty Acyls	Fatty acid	Animals
Lipids and	Fatty Acyls	Lineolic ac	Animals
Nucleoside	Triazole rib	_	Animals
Lipids and	Fatty Acyls	Lineolic ac	Animals
Lipids and	Glyceroph	Glyceroph	Animals
Lipids and	Fatty Acyls	Eicosanoic	Animals
Lipids and	Glyceroph	Glyceroph	Animals
Lipids and	Steroids ar	Bile acids,	Animals
Lipids and	Prenol lipi	Monoterp	Animals
Organoha	Alkyl halid	Alkyl fluori	Animals
Lipids and	Prenol lipi	Monoterp	Animals
--	--	--	--
Organohet	Isoquinolir	Benzylisoq	Animals
_	_	_	Animals
Organic ac	Carboxylic	Amino acid	Animals
Lipids and	Glyceroph	Glyceroph	Animals
Organic ox	Organooxy	Carbonyl c	Animals
Lipids and	Sphingolip	Glycosphin	Animals
_	_	_	Animals
--	--	--	--
Lipids and	Glyceroph	Glyceroph	Animals

Lipids and	Prenol lipi	Triterpeno	Animals
Lipids and	Fatty Acyls	Fatty acids	Animals
_	_	_	Animals
--	--	--	Plants
Lipids and	Fatty Acyls	Fatty alcoh	Animals
Lipids and	Glyceroph	Glyceroph	Animals
Lipids and	Fatty Acyls	Fatty acids	Microbe,Animals
--	--	--	--
--	--	--	--
Lipids and	Steroids ar	Steroid est	Animals
Lipids and	Prenol lipi	Terpene la	Animals
Lipids and	Steroids ar	Androstan	Animals
Lipids and	Glyceroph	Glyceroph	Animals
Organohe	Diazines	Pyridazine	Animals
--	--	--	--
--	--	--	--
--	--	--	--
--	--	--	--
Organic ac	Carboxylic	Amino aci	Animals
Lipids and	Fatty Acyls	Fatty acids	Animals
Lipids and	Fatty Acyls	Eicosanoic	Animals
Organic ac	Carboxylic	Amino aci	Animals
Organic ox	Organooxy	Ethers	Animals
_	_	_	Animals
Organic ac	Carboxylic	Amino aci	Animals
Lipids and	Glyceroph	Glyceroph	Microbe,Animals
Lipids and	Glyceroph	Glyceroph	Animals
--	--	--	--
_	_	_	Animals
Lipids and	Sphingolip	Glycosphir	Animals
--	--	--	--
Organic ox	Organooxy	Carbohydr	Animals
Lipids and	Fatty Acyls	Fatty acid	Animals
Lipids and	Glyceroph	Glyceroph	Animals
Lipids and	Fatty Acyls	Fatty alcoh	Animals
Organohe	Oxanes	_	Animals
Organohe	Pyrans	Pyranones	Animals
Lipids and	Glyceroph	Glyceroph	Animals
--	--	--	--
Benzenoid	Benzene a	Benzoic ac	Animals
--	--	--	--
Nucleoside	Pyrimidine	_	Animals
Organohe	Quinolines	Aminoquir	Animals
--	--	--	--
Organohe	Pyridines a	Methylpyri	Animals
Lipids and	Fatty Acyls	Fatty alcoh	Animals
--	--	--	--
Lipids and	Prenol lipi	Sesquiterp	Animals
Lipids and	Fatty Acyls	Eicosanoic	Animals
_	_	_	Animals
Lipids and	Prenol lipi	Sesterterp	Animals

Lipids and	Fatty Acyls	Fatty acids	Microbe,Animals
Lipids and	Prenol lipi	Monoterp	Animals
Lipids and	Glyceroph	Glyceroph	Animals
--	--	--	--
Lipids and	Steroids ar	Bile acids,	Animals
Organic ni	Organonit	Quaternar	Animals
Organic o	Organooxy	Carbonyl c	Animals
Phenylpro	Macrolides	_	Animals
_	_	_	Animals
Benzenoid	Benzene a	Nitrobenz	Animals
Benzenoid	Benzene a	Benzoic ac	Animals
Organic o	Organooxy	Alcohols a	Animals
Lipids and	Steroids ar	Cycloartan	Animals
Lipids and	Steroids ar	Bile acids,	Animals
Lipids and	Fatty Acyls	Fatty acids	Animals,Plants
Lipids and	Fatty Acyls	Fatty acids	Animals
Organohe	Pyridines a	Methylpyri	Animals
--	--	--	--
Lipids and	Endocanna	_	Animals
--	--	--	--
_	_	_	Animals
Lipids and	Prenol lipi	Triterpeno	Animals
_	_	_	Animals
Lipids and	Glyceroph	Glyceroph	Animals
Lipids and	Fatty Acyls	Fatty alcoh	Animals
Organic ac	Carboxylic	Amino aci	Animals
Lipids and	Fatty Acyls	Fatty acids	Animals
Lipids and	Steroids ar	Cucurbitac	Animals
--	--	--	--
Organohe	Indoles an	_	Animals
Lipids and	Prenol lipi	Terpene g	Animals
Organic ac	Carboxylic	Amino aci	Animals
Organic Po	Polypeptic	_	Animals
Lipids and	Glyceroph	Glyceroph	Animals
Lipids and	Glyceroph	Glyceroph	Animals
--	--	--	--
Lipids and	Glyceroph	CDP-glyce	Animals
Lipids and	Fatty Acyls	Fatty acids	Animals
_	_	_	Animals
Lipids and	Glyceroph	Glyceroph	Animals
--	--	--	--
--	--	--	--
Organic ac	Carboxylic	Alpha-halo	Animals
Lipids and	Fatty Acyls	Fatty amid	Animals,Plants
Lipids and	Glycerolipi	Monorady	Animals
Lipids and	Prenol lipi	Tetraterpe	Animals
Organohe	Quinolines	Quinolone	Animals
Lipids and	Steroids ar	Bile acids,	Animals
Lipids and	Glyceroph	Glyceroph	Animals
--	--	--	--
--	--	--	--

--	--	--	--
--	--	--	--
Lipids and	Sphingolip	Phosphosp	Animals
Organohe	Oxazinane	Morpholin	Animals
Lipids and	Fatty Acyls	Lineolic ac	Animals
Lipids and	Fatty Acyls	Fatty acids	Animals
Lipids and	Prenol lipi	Terpene g	Animals
Lipids and	Fatty Acyls	Fatty amid	Animals,Plants
Lipids and	Fatty Acyls	Fatty acids	Animals
_	_	_	Animals
_	_	_	Animals
--	--	--	--
--	--	--	--
_	_	_	Animals
Lipids and	Sphingolip	Phosphosp	Animals
Lipids and	Fatty Acyls	Fatty acids	Animals
--	--	--	--
Nucleoside	Pyrimidine	Pyrimidine	Animals
Benzenoid	Benzene a	_	Animals
Lipids and	Glyceroph	Glyceroph	Animals
Lipids and	Fatty Acyls	Fatty acids	Animals
Lipids and	Fatty Acyls	Fatty alcoh	Animals
Lipids and	Steroids ar	Vitamin D	Animals
Lipids and	Prenol lipi	Monoterpe	Animals
_	_	_	Animals
_	_	_	Animals
--	--	--	--
Organohe	Imidazopy	Purines an	Animals
Organic ac	Carboxylic	Tricarboxy	Animals
Lipids and	Prenol lipi	Sesquiterp	Animals
_	_	_	Animals
Organic ox	Organooxy	Carbonyl c	Animals
Lipids and	Fatty Acyls	Fatty acids	Animals
Lipids and	Prenol lipi	Quinone a	Microbe,Animals
Lipids and	Steroids ar	Cholestane	Animals
Organic ac	Carboxylic	Amino aci	Animals
--	--	--	--
Phenylpro	Macrolides	_	Animals
Lipids and	Fatty Acyls	Fatty acids	Animals
Lipids and	Steroids ar	Cholestane	Animals
Lipids and	Fatty Acyls	Fatty acids	Animals
Organohe	Oxirenes	_	Animals
Lipids and	Steroids ar	Stigmastar	Animals
_	_	_	Animals
_	_	_	Animals
Lipids and	Steroids ar	Steroid est	Animals
Lipids and	Steroids ar	Steroid est	Animals
Lipids and	Steroids ar	Steroid lac	Animals
Benzenoid	Benzene a	Benzoic ac	Animals
--	--	--	--
Lipids and	Fatty Acyls	Fatty acids	Animals,Microbe

Lipids and	Glyceroph	Glyceroph	Animals
Lipids and	Glyceroph	Glyceroph	Animals
Lipids and	Fatty Acyls	Fatty alcoh	Animals
Lipids and	Steroids ar	Androstan	Animals
Lipids and	Prenol lipi	Tetraterpe	Animals
--	--	--	--
Alkaloids a	Morphinar	_	Animals
Lipids and	Steroids ar	Cholestane	Animals
--	--	--	--
Organoha	Organoflu	_	Animals
--	--	--	--
--	--	--	Plants
Lipids and	Steroids ar	Pregnane	Animals
Lipids and	Prenol lipi	Tetraterpe	Animals
Lipids and	Prenol lipi	Sesquiterp	Animals
_	_	_	Animals
_	_	_	Animals
Lipids and	Glyceroph	Glyceroph	Animals
Lipids and	Glyceroph	Glyceroph	Animals
Lipids and	Prenol lipi	Monoterpe	Animals
Organic ox	Organooxy	Carbonyl c	Animals
Lipids and	Prenol lipi	Triterpeno	Animals
_	_	_	Animals
--	--	--	--
Organohet	Isoquinolir	Benzylisoq	Animals
Lipids and	Fatty Acyls	Eicosanoic	Animals
Organic ac	Carboxylic	Amino acid	Animals
--	--	--	--
Lipids and	Fatty Acyls	Fatty acids	Animals
Lipids and	Steroids ar	Steroid lac	Animals
Lipids and	Fatty Acyls	Fatty acids	Animals
Lipids and	Sphingolip	Glycosphir	Animals
Lipids and	Fatty Acyls	Fatty acids	Animals
_	_	_	Animals
_	_	_	Animals
Lipids and	Prenol lipi	Monoterpe	Animals
--	--	--	--
Lipids and	Fatty Acyls	Fatty alcoh	Animals
--	--	--	--
--	--	--	--
--	--	--	--
Lipids and	Fatty Acyls	Fatty acids	Animals
Lipids and	Fatty Acyls	Fatty acid	Animals
Organic ni	Organonit	Quaternar	Animals
--	--	--	--
Organic ac	Carboxylic	Amino acid	Animals
--	--	--	--
Lipids and	Steroids ar	Hydroxyste	Animals
_	_	_	Animals
Lipids and	Fatty Acyls	Lineolic ac	Animals
Lipids and	Fatty Acyls	Fatty acids	Animals

Lipids and	Glyceroph	Glyceroph	Animals
Organic ac	Carboxylic	Tricarboxy	Animals
--	--	--	--
Lipids and	Fatty Acyls	Fatty acids	Animals
_	_	_	Animals
Lipids and	Glyceroph	Glyceroph	Animals
--	--	--	--
Lipids and	Fatty Acyls	Fatty acids	Animals
Lipids and	Prenol lipi	Monoterpe	Animals
Lipids and	Steroids ar	Hydroxyste	Animals
Benzenoid	Phenol eth	_	Animals
Organic ox	Organooxy	Carbohydr	Animals
Lipids and	Prenol lipi	Diterpenoi	Animals,Plants
--	--	--	--
Lipids and	Steroids ar	Azasteroid	Animals
_	_	_	Animals
_	_	_	Animals
Organic ni	Organonit	Amines	Animals
Hydrocarb	Unsaturate	Olefins	Animals
Lipids and	Fatty Acyls	Fatty acids	Animals
Lipids and	Glyceroph	Glyceroph	Animals
Lipids and	Fatty Acyls	Fatty acids	Animals,Plants
Organohe	Piperidines	Phenylpipe	Animals
_	_	_	Animals
Organohe	Diazines	Pyrazines	Microbe,Animals
Lipids and	Fatty Acyls	Fatty acids	Animals
--	--	--	--
--	--	--	--
Lipids and	Steroids ar	Bile acids,	Animals
--	--	--	--
--	--	--	--
--	--	--	--
Organic ni	Organonit	Amines	Animals
Lipids and	Prenol lipi	Sesquiterp	Animals
Lipids and	Fatty Acyls	Lineolic ac	Animals
--	--	--	--
Lipids and	Fatty Acyls	Fatty alcoh	Animals
Lipids and	Fatty Acyls	Lineolic ac	Animals
Organic ox	Organooxy	Carbonyl c	Animals
Organic ac	Carboxylic	Dicarboxyl	Animals
Lipids and	Fatty Acyls	Fatty acids	Animals
--	--	--	--
Organohe	Lactams	Beta lactar	Animals
Organic ac	Carboxylic	Amino acid	Animals
Lipids and	Prenol lipi	Sesterterpe	Animals
Lipids and	Steroids ar	Steroidal g	Animals
Lipids and	Fatty Acyls	Fatty acid	Animals
Nucleoside	(5'-5')-din	_	Animals
Organic ac	Organic pl	Phosphate	Animals
--	--	--	--
Organohe	Imidazopy	Purines an	Animals

Organohe	Imidazopy	Purines an	Animals,Plants
Organic ac	Carboxylic	Amino aci	Animals
--	--	--	--
--	--	--	--
Nucleoside	Pyrimidine	Pyrimidine	Microbe,Animals
Organic ac	Carboxylic	Tetracarbo	Animals
Nucleoside	Purine nuc	Purine dec	Animals
Organohe	Lactams	Beta lactar	Animals
Lipids and	Fatty Acyls	Fatty acyl t	Animals
Organic ac	Carboxylic	Amino aci	Microbe,Animals
--	--	--	--
Organohe	Benzodiaz	1,4-benzo	Animals
Phenylpro	Coumarins	Furanocou	Animals
--	--	--	--
Phenylpro	Flavonoids	Flavonoid	Animals
Lipids and	Fatty Acyls	Fatty acyl t	Animals
Lipids and	Fatty Acyls	Fatty acyl t	Microbe,Animals
--	--	--	--
Organic ac	Carboxylic	Amino aci	Animals
--	--	--	--
Organohe	Pyridines a	Pyridineca	Animals
Organic ac	Carboxylic	Pentacarb	Animals
--	--	--	--
--	--	--	--
--	--	--	--
Organic ox	Organooxy	Carbohydr	Animals
--	--	--	--
Organic ac	Carboxylic	Amino aci	Animals
Organic ac	Carboxylic	Amino aci	Animals
Organic ox	Organooxy	Carbohydr	Animals
Organic ac	Carboxylic	Amino aci	Animals
Benzenoid	Benzene a	Diphenyle	Animals
Organohe	Quinolines	Quinoline	Animals
Organic ac	Carboxylic	Amino aci	Animals
--	--	--	--
Organic ac	Carboxylic	Amino aci	Animals
Phenylpro	Coumarins	Coumarin	Animals
Nucleoside	Pyrimidine	Pyrimidine	Microbe,Animals
Lipids and	Fatty Acyls	Fatty acyl t	Animals
--	--	--	--
Organic ac	Carboxylic	Amino aci	Animals
--	--	--	--
Organic ac	Carboxylic	Amino aci	Animals
--	--	--	--
--	--	--	--
Organohe	Diazines	Pyrazines	Animals
Organic ac	Carboxylic	Tricarboxy	Animals
Organohe	Lactones	Gamma bu	Animals,Plants
--	--	--	--
--	--	--	--
--	--	--	--

_	_	_	Animals
Organic ni	Organonit	Amines	Animals
--	--	--	--
Lipids and	Glycerolipi	Monorady	Animals
Organic ac	Carboxylic	Tetracarbo	Animals
Organic ox	Organooxy	Carbohydr	Animals
Lipids and	Prenol lipi	Diterpenoi	Animals
Phenylpro	2-arylbenz	_	Animals
--	--	--	--
Organic ni	Organonit	Amines	Animals
--	--	--	--
_	_	_	Animals
Lipids and	Glyceroph	Glyceroph	Animals
Lipids and	Fatty Acyls	Fatty acyl g	Animals
Organic ni	Organonit	Quaternar	Animals
--	--	--	--
--	--	--	--
Benzenoid	Phenol eth	_	Animals
--	--	--	--
_	_	_	Animals
Organic ac	Carboxylic	Amino acid	Animals
Lipids and	Prenol lipi	Triterpeno	Animals
_	_	_	Animals
--	--	--	--
--	--	--	--
Lipids and	Steroids ar	Bile acids,	Animals
--	--	--	--
--	--	--	--
Benzenoid	Benzene a	_	Animals
Lipids and	Prenol lipi	Triterpeno	Animals
_	_	_	Animals
Organic ac	Carboxylic	Amino acid	Animals
Organoha	Organoflu	_	Animals
Organic ni	Organonit	Organic cy	Animals,Plants
--	--	--	--
Lipids and	Glyceroph	Glyceroph	Animals
Lipids and	Steroids ar	Bile acids,	Animals
Lipids and	Fatty Acyls	Fatty acid	Animals
--	--	--	--
_	_	_	Animals
--	--	--	--
_	_	_	Animals
Lipids and	Steroids ar	Ergostane	Animals
--	--	--	--
Organohe	Pyrazolopy	_	Animals
_	_	_	Animals
Organic ac	Keto acids	Gamma-ke	Animals
--	--	--	--
Organic ox	Organooxy	Carbohydr	Animals
Lipids and	Fatty Acyls	Fatty acids	Microbe,Animals,Plants
Lipids and	Steroids ar	Androstan	Animals

_	_	_	Animals
--	--	--	--
Lipids and	Prenol lipi	Triterpeno	Animals
--	--	--	--
Lipids and	Prenol lipi	Retinoids	Animals
_	_	_	Animals
Lipids and	Sphingolip	Glycosphir	Animals
Lipids and	Glyceroph	Glyceroph	Animals
_	_	_	Animals
_	_	_	Animals
_	_	_	Animals
_	_	_	Animals
_	_	_	Animals
_	_	_	Animals
--	--	--	--
_	_	_	Animals
_	_	_	Animals
_	_	_	Animals
Lipids and	Glyceroph	_	Animals
_	_	_	Animals
_	_	_	Animals
Organic ac	Carboxylic	Amino aci	Animals
Lipids and	Steroids ar	Vitamin D	Animals
Organohe	Quinolines	Hydroquin	Animals
Lipids and	Glyceroph	Glyceroph	Animals
--	--	--	--
--	--	--	--
Organic ox	Organooxy	Carbohydr	Animals
Organic ac	Carboxylic	Amino aci	Animals
Organic ac	Carboxylic	Amino aci	Animals
Phenylpro	Tannins	Hydrolyzal	Animals
Organic ac	Carboxylic	Amino aci	Animals
Organohe	Pyridines a	Hydropyri	Animals
Lipids and	Glyceroph	Glyceroph	Animals
--	--	--	--
--	--	--	--
--	--	--	--
Lipids and	Glycerolipi	Diradylgly	Animals
Organohe	Azepines	_	Animals
--	--	--	--
Organic ox	Organooxy	Carbonyl c	Animals
--	--	--	--
--	--	--	--
Organic ac	Carboxylic	Amino aci	Animals
Organic ox	Organooxy	Carbohydr	Animals
--	--	--	--
Benzenoid	Phenol eth	_	Animals
Organic ac	Carboxylic	Amino aci	Animals
_	_	_	Animals
Lipids and	Steroids ar	Bile acids,	Animals
--	--	--	--

--	--	--	--
Lipids and	Prenol lipi	Monoterp	Animals
Benzenoid	Benzene a	_	Animals
Lipids and	Glyceroph	Glyceroph	Animals
_	_	_	Animals
_	_	_	Animals
--	--	--	--
--	--	--	--
Lipids and	Glyceroph	Glyceroph	Animals
--	--	--	--
Benzenoid	Naphthale	_	Animals
Benzenoid	Benzene a	Benzyl alc	Animals
Organohe	Imidazopy	Purines an	Animals
_	_	_	Animals
_	_	_	Animals
--	--	--	--
Organic ac	Carboxylic	Amino aci	Animals
_	_	_	Animals
Lipids and	Steroids ar	Bile acids,	Animals
Benzenoid	Benzene a	Benzoic ac	Animals
_	_	_	Animals
--	--	--	--
Lipids and	Glyceroph	CDP-glyce	Animals
Organohe	Azolidines	Oxazolidin	Animals
--	--	--	--
Organohe	Piperidines	_	Animals
_	_	_	Animals
Organohe	Piperidines	Phenylpipe	Animals
Lipids and	Glycerolipi	Diradylgly	Animals
Organic ac	Carboxylic	Amino aci	Animals,Plants
Organohe	Azoles	Imidazoles	Animals
Organic ac	Carboxylic	Amino aci	Animals
--	--	--	--
Lipids and	Glycerolipi	Diradylgly	Animals
Organohe	Diazines	Pyrimidine	Animals
--	--	--	--
_	_	_	Animals
Organic ac	Carboxylic	Carboxylic	Animals
Organic ni	Organonit	Azo comp	Animals
Organoha	Aryl halide	Aryl fluoric	Animals
Organic ac	Carboxylic	Alpha-hal	Animals
Organic ac	Organic su	Sulfuric ac	Animals
Organic ox	Organooxy	Carbonyl c	Animals
Organohe	Oxazinane	Morpholin	Animals
Organic ac	Carboxylic	Amino aci	Animals
Organohe	Benzodiox	_	Animals
Nucleoside	Pyrimidine	Pyrimidine	Microbe,Animals
Organic ac	Carboxylic	Amino aci	Animals
--	--	--	--
--	--	--	--
--	--	--	--

--	--	--	--
--	--	--	--
Organohe	Imidazopy	Purines an	Animals
--	--	--	--
Organohe	Oxazinane	Morpholin	Animals
--	--	--	--
--	--	--	--
--	--	--	--
Organic ac	Carboxylic	Amino aci	Animals
Organohe	Imidazopy	Purines an	Animals
Organic ac	Carboxylic	Amino aci	Animals
Organic ni	Organonit	Amines	Microbe,Animals
Organohe	Indoles an	Tryptamin	Animals,Plants,Microbe
--	--	--	--
Organic ac	Carboxylic	Amino aci	Animals
Benzenoid	Phenols	Methoxypl	Microbe,Animals,Plants
Organohe	Indoles an	_	Animals
Benzenoid	Benzene a	_	Animals,Plants
Organohe	Heteroaro	_	Animals
Organohe	Quinolines	_	Animals
--	--	--	--
Organohe	Indoles an	Indoles	Animals
Organohe	Diazines	Pyrimidine	Animals
--	--	--	--
Organic ox	Organooxy	Ketene ace	Animals
Organohe	Indoles an	Indoles	Microbe,Animals
Phenylpro	Tannins	_	Animals
Benzenoid	Phenols	1-hydroxy	Animals
--	--	--	--
Organic ac	Carboxylic	Amino aci	Animals
Organohe	Benzopyra	1-benzopy	Animals
--	--	--	--
Organic ac	Carboxylic	Amino aci	Animals
Organic ac	Carboxylic	Tetracarbo	Animals
--	--	--	--
Nucleosid	2',5'-didec	_	Animals
Organic ac	Carboxylic	Amino aci	Animals
Organic ac	Carboxylic	Amino aci	Animals
Organohe	Indoles an	Indolyl car	Animals
Nucleosid	Pyrimidine	_	Animals
Organohe	Imidazopy	Purines an	Animals
Organohe	Dihydrofur	_	Animals
Organic 1,	Allyl-type	Organic ni	Animals
_	_	_	Animals
Benzenoid	Benzene a	Trifluorom	Animals
Organohe	Diazanaph	Benzodiaz	Animals
--	--	--	--
--	--	--	--
Allenes	Cyclic aller	_	Animals
Lipids and	Fatty Acyls	Fatty alcoh	Animals
Organic ac	Carboxylic	Amino aci	Animals

Organic ac	Carboxylic	Amino aci	Animals
Organic ox	Organooxy	Carbonyl c	Animals
Organohe	Pyridines a	Pyridineca	Animals
--	--	--	--
--	--	--	--
Nucleoside	Nucleoside	3'-thia pyr	Animals
Organic ox	Organooxy	Carbohydr	Animals
Organic ac	Carboxylic	Amino aci	Animals
Organohe	Quinolines	Quinoline	Animals
Benzenoid	Benzene a	Phenethyla	Animals
--	--	--	--
--	--	--	--
Phenylpro	Cinnamic a	Hydroxycir	Animals
Phenylpro	Isoflavono	O-methyla	Animals
Organohe	Thienodiaz	_	Animals
Organic ac	Carboxylic	Amino aci	Microbe,Animals
Lipids and	Fatty Acyls	Fatty acid	Animals
Organohe	Indoles an	Hydroxyin	Animals
Lipids and	Fatty Acyls	Fatty acid	Animals
Benzenoid	Phenanthr	Phenanthr	Animals
Phenylpro	Coumarins	Pyranocou	Animals,Plants
Organohe	Pyrrolidine	_	Animals
Nucleoside	Pyrimidine	_	Animals
Organic ac	Carboxylic	Amino aci	Animals
Organohe	Pteridines	Pterins and	Animals
Organic ac	Carboxylic	Amino aci	Animals
Lipids and	Steroids ar	Androstan	Animals
Organic ac	Carboxylic	Amino aci	Animals
Organohe	Pyridines a	_	Animals
Organic ac	Carboxylic	Amino aci	Animals
Organic ni	Organonit	Organic ni	Animals
--	--	--	--
Organic ac	Carboxylic	Amino aci	Animals
Organic ac	Carboxylic	Amino aci	Animals
--	--	--	--
Organohe	Imidazopy	Purines an	Animals
Organic ox	Organooxy	Carbohydr	Animals
Organic ac	Carboxylic	Amino aci	Animals
--	--	--	--
Benzenoid	Naphthale	_	Animals
Nucleoside	Nucleoside	Cyclopenty	Animals
Nucleoside	Nucleoside	_	Animals
Organic ox	Organooxy	Carbohydr	Microbe,Animals
Benzenoid	Benzene a	_	Animals
Benzenoid	Benzene a	Phenethyla	Animals
Lipids and	Fatty Acyls	Fatty acids	Animals,Plants
Phenylpro	Cinnamic a	Hydroxycir	Animals
--	--	--	--
--	--	--	--
--	--	--	--
--	--	--	--

Lipids and	Prenol lipi	Sesquiterp	Animals
Organic ac	Carboxylic	Amino aci	Animals
Lipids and	Prenol lipi	Terpene la	Animals
Phenylpro	Isoflavono	Pyranoisof	Animals
--	--	--	--
Lipids and	Steroids ar	Androstan	Animals
--	--	--	--
Lipids and	Steroids ar	Estrane ste	Animals
--	--	--	--
--	--	--	--
Organic ac	Organic pl	Bisphosph	Animals
Organic ac	Carboxylic	Amino aci	Animals
Phenylpro	Cinnamic a	Hydroxycir	Animals
Phenylpro	Flavonoids	Flavonoid	Animals
Organohe	Dihydrofur	Furanones	Animals
--	--	--	--
Organohe	Indoles an	Hydroxyin	Animals
Phenylpro	Coumarins	Hydroxycc	Animals,Plants
--	--	--	--
Organic ac	Carboxylic	nd analog	Animals
Organohe	Indoles an	Indolyl car	Animals,Plants
Nucleoside	Pyrimidine	Pyrimidine	Animals
Organohe	Pyrans	Pyranones	Animals
Organic ox	Organooxy	Carbohydr	Animals
Organic ac	Carboxylic	Amino aci	Animals
--	--	--	--
--	--	--	--
Organohe	Diazines	Pyrimidine	Animals
Organic ac	Carboxylic	Amino aci	Microbe,Animals,Plants
Benzenoid	Benzene a	_	Animals
Benzenoid	Benzene a	Benzoic ac	Animals
Nucleoside	Pyrimidine	Pyrimidine	Animals
Nucleoside	Pyrimidine	Pyrimidine	Animals
Lipids and	Fatty Acyls	Eicosanoic	Animals
--	--	--	--
Organohe	Indoles an	Indoles	Animals,Plants
Organic ac	Carboxylic	Amino aci	Animals
Phenylpro	Stilbenes	_	Animals
Organic ac	Carboxylic	Amino aci	Animals
Organic ox	Organooxy	Carbohydr	Animals
Lipids and	Fatty Acyls	Fatty amid	Animals
Organohe	Benzothiaz	_	Animals
Organic ox	Organooxy	Carbohydr	Animals
Organohe	Indoles an	Indoles	Animals
Organohe	Indoles an	Indolyl car	Animals
Organohe	Diazines	Pyrimidine	Animals
Nucleoside	Triazole rib	_	Animals
Organohe	Benzodiaz	1,4-benzo	Animals
Lipids and	Fatty Acyls	Fatty acyl	Animals
--	--	--	--
Organohe	Diazines	Pyrimidine	Animals

Organic ac	Carboxylic	Amino aci	Animals
--	--	--	--
Organohe	Diazines	Pyrimidine	Animals
Organic ac	Carboxylic	Amino aci	Animals
Organic o	Organooxy	Carbohydr	Animals
--	--	--	--
--	--	--	--
Organohe	Indoles an	Carbazoles	Animals,Plants
--	--	--	--
Organohe	Benzazepi	_	Animals
--	--	--	--
Organohe	Furans	Furoic acid	Animals
Organic ac	Carboxylic	Amino aci	Animals
Benzenoid	Benzene a	Phenylmet	Animals
--	--	--	--
Alkaloids a	Harmala a	_	Animals,Plants
Alkaloids a	Harmala a	_	Animals,Plants
Organohe	Diazepane	1,3-diazep	Animals
Benzenoid	Benzene a	Trifluorom	Animals
Lipids and	Prenol lipi	Terpene la	Animals
Lipids and	Fatty Acyls	Fatty alcoh	Animals
Lipids and	Fatty Acyls	Fatty acyl	Animals
Lipids and	Prenol lipi	Diterpenoi	Animals
--	--	--	--
--	--	--	--
Benzenoid	Benzene a	_	Animals
--	--	--	--
Organic o	Organooxy	Carbonyl c	Animals
--	--	--	--
Lignans, n	Furanoid li	Tetrahydro	Animals
--	--	--	--
_	_	_	Animals
Nucleoside	Pyrimidine	Pyrimidine	Animals
Organohe	Azepines	_	Animals
Lipids and	Fatty Acyls	Fatty acids	Animals
Lipids and	Fatty Acyls	Fatty amid	Animals
Organic ac	Carboxylic	Amino aci	Animals
Organic ac	Carboxylic	Amino aci	Animals
Organohe	Indoles an	_	Animals
Benzenoid	Indanes	_	Animals
Organic ac	Carboxylic	Amino aci	Animals
Benzenoid	Benzene a	Phenylpro	Animals
--	--	--	--
Organohe	Pyridines a	Phenylpyri	Animals
Organic o	Organooxy	Carbohydr	Animals
--	--	--	--
Organic ac	Carboxylic	Amino aci	Animals
Hydrocarb	Unsaturate	Branched	Animals
Organohe	Pyrrolopyr	_	Animals
Organohe	Pyridines a	Hydropyri	Animals
--	--	--	--

Organic o:	Organooxy	Alcohols a	Animals
--	--	--	--
Phenylpro	Phenylpro	_	Animals
Organohe	Indoles an	Indoles	Animals,Plants
Organic o:	Organooxy	Carbohydr	Microbe,Animals
Organohe	Azaphilon	_	Animals
Organic o:	Organooxy	Carbohydr	Animals
--	--	--	--
Lipids and	Fatty Acyls	Fatty acyl	Animals
Organic o:	Organooxy	Alcohols a	Animals
Organic ac	Carboxylic	Amino aci	Animals
--	--	--	--
Organic ac	Carboxylic	Amino aci	Animals
Organic ac	Carboxylic	Tricarboxy	Animals
Phenylpro	Isoflavono	Hydroxyis	Animals
Organohe	Diazines	Pyrimidine	Animals
Organic ac	Carboxylic	Amino aci	Animals
--	--	--	--
Organohe	Indoles an	Indolines	Animals
Organohe	Diazanaph	Naphthyri	Animals
Organic o:	Organooxy	Carbohydr	Animals
Organohe	Dihydrofu	Furanones	Animals
Nucleosid	Nucleosid	Thionucle	Animals
--	--	--	--
Benzenoid	Phenols	Methoxypl	Animals,Plants
Benzenoid	Benzene a	_	Animals
Organic ac	Carboxylic	Amino aci	Animals
Organohe	Benzazepi	Dibenzaze	Animals
Organohe	Benzopyra	1-benzopy	Animals,Microbe
Organic ac	Organic pl	Phosphate	Microbe,Animals
Organohe	Indoles an	Indoles	Animals,Plants,Microbe
--	--	--	--
Organic ac	Carboxylic	Amino aci	Animals
Organic ac	Carboxylic	Amino aci	Animals
Organic ac	Carboxylic	Amino aci	Animals
Organic o:	Organooxy	Carbohydr	Animals
Nucleosid	Pyrimidine	Pyrimidine	Animals
--	--	--	--
--	--	--	--
Organohe	Heteroaro	_	Microbe,Animals,Plants
Organohe	Indoles an	Tryptamin	Animals,Plants
Organohe	Heteroaro	_	Animals
Organic ac	Carboxylic	Amino aci	Animals
Organohe	Azoles	Imidazoles	Animals
--	--	--	--
Organic ac	Carboxylic	Amino aci	Animals
Organohe	Lactones	Gamma bu	Microbe,Animals
--	--	--	--
Organic o:	Organooxy	Carbonyl c	Animals
Organic o:	Organooxy	Carbohydr	Animals
Organohe	Azoles	Imidazoles	Animals

Phenylpro	Cinnamic a	Hydroxycir	Animals
Organohe	Quinolines	Phenylquir	Animals
Organohe	Lactones	Gamma bu	Animals
Nucleoside	Nucleoside	Cyclopenty	Animals
Benzenoid	Benzene a	Phenethyla	Animals
--	--	--	--
Organohe	Indoles an	Indoles	Microbe,Animals,Plants
--	--	--	--
Organohe	Diazines	Pyrimidine	Animals
Organohe	Pyridines a	Methylpyri	Animals
Nucleoside	Purine nuc	Purine 2'-(	Animals
--	--	--	--
Phenylpro	Linear 1,3-	Chalcones	Animals,Plants
Organic ox	Organooxy	Carbohydr	Microbe,Animals
Phenylpro	Cinnamic a	Hydroxycir	Animals
--	--	--	--
Organohe	Isoindoles	Isoindoline	Animals
--	--	--	--
Organic ox	Organooxy	Carbohydr	Animals
--	--	--	--
Organic ac	Carboxylic	Amino acid	Animals
--	--	--	--
Lipids and	Fatty Acyls	Fatty acid	Animals
--	--	--	--
Organohe	Indoles an	Indoles	Microbe,Animals,Plants
Organic ox	Organooxy	Carbohydr	Animals
Organohe	Azolidines	Imidazolid	Animals
--	--	--	--
Organic ac	Carboxylic	Amino acid	Animals
Organic ac	Carboxylic	Amino acid	Microbe,Animals
Organic ox	Organooxy	Alcohols a	Animals
--	--	--	--
--	--	--	--
Organohe	Indoles an	Indoles	Animals
Phenylpro	Coumarins	Hydroxycc	Animals,Plants
Alkaloids a	Camptothe	_	Animals
--	--	--	--
Nucleoside	Purine nuc	_	Animals
Organohe	Pyridines a	_	Animals
Organic ac	Carboxylic	Amino acid	Animals
Phenylpro	Macrolacta	_	Animals
Organic ox	Organooxy	Carbonyl c	Microbe,Animals
--	--	--	--
--	--	--	--
Organic ac	Carboxylic	Amino acid	Animals
Nucleoside	Purine nuc	_	Animals
Organic ac	Carboxylic	Amino acid	Animals
Benzenoid	Benzene a	_	Animals,Plants
--	--	--	--
Organohe	Lactones	Delta valer	Animals
--	--	--	--

Lipids and	Fatty Acyls	Lineolic ac	Animals
Lipids and	Prenol lipi	Sesquiterp	Animals,Plants
Lipids and	Fatty Acyls	Eicosanoic	Animals
--	--	--	--
Organic ac	Carboxylic	Amino aci	Animals
Organohe	Indoles an	Indolines	Animals
--	--	--	--
Lipids and	Glyceroph	Glyceroph	Animals
Organohe	Diazanaph	Benzodiaz	Animals
Lipids and	Fatty Acyls	Fatty acid	Animals
Organosul	Organic di	_	Animals
Organohe	Pteridines	_	Animals,Plants
--	--	--	--
Lipids and	Fatty Acyls	Fatty acid	Animals
Phenylpro	Homoisofl	Homoisofl	Animals
Nucleoside	Pyrimidine	Pyrimidine	Animals
Organohe	Oxazinane	Morpholin	Animals
Nucleoside	Purine nuc	_	Animals
Lipids and	Fatty Acyls	Fatty acid	Animals
Nucleoside	Purine nuc	_	Animals
--	--	--	--
--	--	--	--
Benzenoid	Phenols	Methoxypl	Animals
--	--	--	--
Organohe	Diazanaph	Naphthyri	Animals
Organohe	Diazines	Pyrimidine	Animals
Organohe	Benzopyra	1-benzopy	Animals
Organic ox	Organooxy	Carbohydr	Animals
Organic ox	Organooxy	Carbohydr	Animals
Benzenoid	Phenols	Methoxypl	Animals
Organic ac	Carboxylic	Amino aci	Animals
Organohe	Indoles an	Indoles	Animals,Plants
Organohe	Quinolines	Quinolone	Animals
Organohe	Isoindoles	Isoindoline	Animals
--	--	--	--
Alkaloids a	_	_	Animals
Organohe	Pyridines a	Methylpyri	Microbe,Animals
Organohe	Isoquinolir	_	Animals,Plants
--	--	--	--
--	--	--	--
Benzenoid	Benzene a	Benzoyl de	Animals,Plants
Benzenoid	Benzene a	Benzoic ac	Animals
Organohe	Indoles an	Indolyl car	Animals
Benzenoid	Phenols	Methoxypl	Animals
Organic ac	Carboxylic	Amino aci	Animals
Organic ac	Carboxylic	Amino aci	Animals
--	--	--	--
Benzenoid	Benzene a	Phenylace	Animals
--	--	--	--
Lipids and	Steroids ar	Sulfated st	Animals
Organic ox	Organooxy	Carbohydr	Animals

Lipids and	Prenol lipi(	Quinone a	Animals
Organic o›	Organoox¹	Carbohydr	Animals
Organohe¹	Pyrrolopyr	Pyrrolo[2,3	Animals
Nucleoside	Purine nuc	_	Animals
Nucleoside	Pyrimidine	Pyrimidine	Animals
Lipids and	Fatty Acyls	Fatty alcoh	Animals
Benzenoid	Phenol eth	_	Animals
Organohe¹	Benzazepi	Dibenzaze	Animals
--	--	--	--
--	--	--	--
--	--	--	--
Organohe¹	Tetrahydrc	_	Animals
--	--	--	--
Nucleoside	Pyrimidine	Pyrimidine	Animals
Organic ni	Organonit	N-arylami(	Animals
--	--	--	--
Organic ac	Carboxylic	Amino aci(	Animals
Organic ac	Carboxylic	Amino aci(	Animals
Organic ac	Carboxylic	Amino aci(	Animals
Organic ac	Carboxylic	Amino aci(	Animals
Lipids and	Fatty Acyls	Fatty acyl (	Animals
Organic o›	Organoox¹	Carbohydr	Animals
--	--	--	--
Lipids and	Fatty Acyls	Fatty acid	Animals
Organohe¹	Pyridines a	Pyridineca	Animals
Organic o›	Organoox¹	Carbohydr	Animals
Organic ac	Carboxylic	Amino aci(	Animals
Organic ac	Carboxylic	Carboxylic	Animals
Organic ni	Organonit	Amines	Animals
Organohe¹	Furans	Furoic acic	Animals
Organohe¹	Benzothia:	_	Animals
Organohe¹	Benzopyra	_	Animals
Organic o›	Organoox¹	Carbohydr	Animals
--	--	--	--
Benzenoid	Phenols	1-hydroxy	Animals
Organic ac	Carboxylic	Amino aci(	Animals
Nucleoside	Pyrimidine	Pyrimidine	Animals
Organic o›	Organoox¹	Carbohydr	Animals
Organic ac	Carboxylic	Amino aci(	Animals
Lipids and	Fatty Acyls	Fatty acids	Animals
Organic ac	Keto acids	Medium-c	Animals
Lipids and	Fatty Acyls	Eicosanoic	Animals
Lipids and	Steroids ar	Hydroxyst(	Animals
Lipids and	Fatty Acyls	Eicosanoic	Animals
--	--	--	--
Organohe¹	Azoles	Imidazoles	Animals
Organic ac	Carboxylic	Amino aci(	Animals
Benzenoid	Benzene a	Aniline anc	Animals
--	--	--	--
--	--	--	--
--	--	--	--

Organic o›	Organooxy	Carbohydr	Animals
Organic ac	Carboxylic	Amino aci	Animals
--	--	--	--
Lipids and	Fatty Acyls	Eicosanoic	Animals
Organohe	Indoles an	Indoles	Animals
--	--	--	--
Benzenoid	Tetralins	_	Animals
Organic o›	Organooxy	Carbohydr	Animals
--	--	--	--
--	--	--	--
Organic o›	Organooxy	Carbonyl c	Animals
Organohe	Quinolines	Imidazoqu	Animals
Lipids and	Fatty Acyls	Fatty acyl (	Animals
Organic o›	Organooxy	Carbonyl c	Animals
Nucleoside	Pyrimidine	Pyrimidine	Microbe,Animals
--	--	--	--
--	--	--	--
Alkaloids a	Harmala a	_	Animals
Nucleoside	5'-deoxyri	5'-deoxy-!	Microbe,Animals
Organohe	Lactones	Gamma bu	Animals
Lipids and	Fatty Acyls	Fatty acids	Animals
Lipids and	Prenol lipi	Terpene g	Animals
Organic ac	Carboxylic	Amino aci	Microbe,Animals
Organic ni	Organonit	Amines	Animals
Organohe	Pyridines a	_	Animals
Organohe	Isoquinolir	_	Animals,Plants
Alkaloids a	Harmala a	_	Animals,Plants
Organic ni	Organonit	Amines	Animals
--	--	--	--
--	--	--	--
--	--	--	--
Phenylpro	Macrolides	_	Animals
Organic ac	Carboxylic	Amino aci	Animals
Benzenoid	Benzene a	_	Animals
Organic o›	Organooxy	Carbohydr	Animals
--	--	--	--
Phenylpro	Coumarins	_	Animals,Microbe
Organic ac	Carboxylic	Amino aci	Animals
Organic ac	Carboxylic	Amino aci	Animals
Organohe	Imidazopy	nes and pu	Animals
--	--	--	--
Phenylpro	Cinnamyl a	_	Animals
Organic ac	Carboxylic	Amino aci	Animals
Organohe	Indoles an	Indolyl car	Animals,Plants
Nucleoside	Purine nuc	_	Animals
--	--	--	--
Organic ac	Hydroxy a	Medium-c	Animals
Organohe	Indoles an	Indolines	Animals
Lipids and	Prenol lipi	Quinone a	Animals
--	--	--	--
--	--	--	--

Organohe	Pteridines	Pterins and	Animals
Organic o:	Organooxy	Carbohydr	Animals
--	--	--	--
--	--	--	--
--	--	--	--
--	--	--	--
Organic ac	Carboxylic	Amino aci	Animals
Nucleoside	Purine nuc	_	Animals
Organic ac	Carboxylic	Amino aci	Animals
Organic ac	Vinylogous	_	Animals
--	--	--	--
Lipids and	Fatty Acyls	Fatty acid	Animals
Organic ac	Organic pl	_	Animals
Lipids and	Fatty Acyls	Fatty acids	Animals
--	--	--	--
Lipids and	Fatty Acyls	Fatty acid	Animals
Phenylpro	Cinnamic a	Hydroxycir	Animals
Organic ac	Carboxylic	Amino aci	Animals
--	--	--	--
Organic o:	Organooxy	Ethers	Animals
--	--	--	--
Organic ac	Carboxylic	Amino aci	Animals
Lipids and	Steroids ar	Androstan	Animals
--	--	--	--
--	--	--	--
Nucleoside	Purine nuc	_	Animals
--	--	--	--
Nucleoside	2',5'-didec	_	Animals
Lipids and	Fatty Acyls	Eicosanoic	Animals
--	--	--	--
Organic ac	Carboxylic	Carboxylic	Animals
Lipids and	Prenol lipi	Retinoids	Animals
--	--	--	--
Organic ac	Carboxylic	Amino aci	Animals
--	--	--	--
Phenylpro	Flavonoids	Flavonoid	Animals
--	--	--	--
--	--	--	--
Phenylpro	Tannins	_	Animals
Lipids and	Prenol lipi	Monoterp	Animals
Organic ac	Carboxylic	Amino aci	Animals
Nucleoside	Pyrimidine	_	Animals
Organohe	Azepanes	_	Animals
Organic ac	Carboxylic	Amino aci	Animals
--	--	--	--
Lipids and	Steroids ar	Bile acids,	Animals
Hydrocarb	Tropones	Tropolone	Animals,Plants
--	--	--	--
--	--	--	--
Organic ac	Carboxylic	Amino aci	Animals
Organic ac	Organic su	Arylsulfate	Animals

Organic ac	Carboxylic	Amino aci	Animals
Benzenoid	Naphthale	_	Animals
Organohe	Benzopyra	1-benzopy	Animals
Organic ac	Carboxylic	Amino aci	Animals
--	--	--	--
Lipids and	Prenol lipi	Monoterp	Animals
Organohe	Pyridines a	Pyridineca	Animals
--	--	--	--
--	--	--	--
--	--	--	--
Organic ox	Organooxy	Carbohydr	Animals
--	--	--	--
Lipids and	Glyceroph	Glyceroph	Animals
--	--	--	--
Organic ac	Carboxylic	Amino aci	Animals
Organohe	Coumaran	_	Animals
--	--	--	--
--	--	--	--
--	--	--	--
--	--	--	--
Organohe	Azolines	Imidazolin	Animals
Lipids and	Steroids ar	Oxosteroic	Animals
--	--	--	--
Lipids and	Fatty Acyls	Eicosanoic	Animals
Organic ac	Carboxylic	Amino aci	Animals
--	--	--	--
Benzenoid	Benzene a	_	Animals
Lipids and	Prenol lipi	Monoterp	Animals
Alkaloids a	_	_	Animals
Lipids and	Fatty Acyls	Fatty acids	Microbe,Animals,Plants
Lipids and	Glycerolipi	Monorady	Animals
Organic ni	Organonit	Amines	Animals
Organohe	Benzothiaz	_	Animals
Organohe	Diazines	Pyrimidine	Animals
Organic ac	Hydroxy a	Medium-c	Animals
--	--	--	--
Alkaloids a	Cytochalas	Cytochalas	Animals
Lipids and	Steroids ar	Bile acids,	Animals
Organic ac	Peptidomi	Depsipept	Animals
Lipids and	Glycerolipi	Monorady	Animals
--	--	--	--
--	--	--	--
Organohe	Indoles an	Tryptamin	Animals
Organohe	Benzotriaz	_	Animals
Organic ac	Carboxylic	Amino aci	Animals
Organohe	Diazines	Pyrazines	Animals
Lipids and	Fatty Acyls	Fatty acyl (	Animals
Organohe	Lactones	Gamma bu	Animals
Organohe	Piperidines	Benzylpipe	Animals
Organohe	Imidazopy	Purines an	Animals
Organic ox	Organooxy	Carbohydr	Animals

Organic ac	Carboxylic	Amino aci	Animals
--	--	--	--
Organic ac	Carboxylic	Amino aci	Animals
Organic ac	Carboxylic	Amino aci	Animals
Organohe	Pteridines	Pterins an	Microbe,Animals,Plants
Benzenoid	Indanes	Indanones	Animals
--	--	--	--
--	--	--	--
Organic ac	Carboxylic	Amino aci	Animals
Phenylpro	Coumarins	_	Animals,Plants
Lipids and	Steroids a	Bile acids,	Animals
--	--	--	--
Organic o	Organoox	Carbohydr	Animals
--	--	--	--
--	--	--	--
--	--	--	--
--	--	--	--
Phenylpro	Macrolides	_	Animals
--	--	--	--
Organic o	Organoox	Ethers	Animals
Organic o	Organoox	Carbohydr	Animals
Organohe	Epoxides	Oxirane ca	Animals
Organic ac	Carboxylic	Amino aci	Animals
--	--	--	--
Organohe	Pteridines	Pterins an	Animals
--	--	--	--
Organic o	Organoox	Carbonyl c	Animals
--	--	--	--
--	--	--	--
Organic ac	Carboxylic	Amino aci	Animals,Plants
Lipids and	Fatty Acyls	Eicosanoic	Animals
Lipids and	Fatty Acyls	Eicosanoic	Animals
Lipids and	Fatty Acyls	Eicosanoic	Animals
--	--	--	--
Organic o	Organoox	Carbohydr	Animals
Organohe	Pyrroles	Substitute	Animals
Organohe	Dioxetanes	_	Animals
Nucleoside	Benzimida	_	Animals
--	--	--	--
--	--	--	--
Organic ac	Carboxylic	Amino aci	Animals
Lipids and	Fatty Acyls	Fatty acyl	Animals
--	--	--	--
--	--	--	--
Lipids and	Fatty Acyls	Fatty acids	Animals
Organic ac	Carboxylic	Amino aci	Animals
Organic ac	Carboxylic	Amino aci	Animals
Lipids and	Fatty Acyls	Fatty acid	Animals
--	--	--	--
Organohe	Triazines	1,3,5-triaz	Animals
Organohe	Diazines	Pyrimidine	Animals

Organic ac	Carboxylic	Amino aci	Animals
Nucleoside	Purine nuc	Purine 2'-(	Animals
--	--	--	--
--	--	--	--
--	--	--	--
Organic ac	Carboxylic	Amino aci	Animals
--	--	--	--
Organohe	Quinolines	Quinolone	Microbe,Animals
Organohe	Pyridines a	Phenylpyri	Animals
Lipids and	Prenol lipi	Monoterp	Animals
Organic ac	Carboxylic	Amino aci	Animals
--	--	--	--
Organohe	Diazines	Pyrimidine	Animals
--	--	--	--
rganic acic	Keto acids	Medium-c	Animals
Benzenoid	Benzene a	_	Animals
Organic ac	Carboxylic	Amino aci	Animals
--	--	--	--
Lipids and	Fatty Acyls	Fatty acids	Animals,Plants
Organohe	Indoles an	Carbazoles	Animals
Lipids and	Steroids ar	Estrane ste	Animals
Benzenoid	Phenol eth	_	Animals
Organohe	Pyridines a	Pyridineca	Animals
Lipids and	Steroids ar	Pregnane	Animals
--	--	--	--
Organic ni	Organonit	Amines	Animals
--	--	--	--
--	--	--	--
--	--	--	--
_	_	_	Animals
--	--	--	--
Lipids and	Fatty Acyls	Eicosanoic	Animals
Lipids and	Steroids ar	Steroidal g	Animals
_	_	_	Animals
Organic ac	Carboxylic	Amino aci	Animals
--	--	--	--
Organic ac	Hydroxy a	Medium-c	Animals
Lipids and	Prenol lipi	Diterpenoi	Animals
Lipids and	Prenol lipi	Terpene g	Animals
Lipids and	Prenol lipi	Monoterp	Animals
Lipids and	Steroids ar	Androstan	Animals
Nucleoside	Purine nuc	Purine 2',3	Animals
--	--	--	--
Lipids and	Steroids ar	Steroidal g	Animals
--	--	--	--
Lipids and	Fatty Acyls	Eicosanoic	Animals
Lipids and	Fatty Acyls	Fatty acids	Microbe,Animals
Lipids and	Prenol lipi	Diterpenoi	Animals
Organohe	Diazinanes	Piperazine	Animals
--	--	--	--
Nucleoside	Purine nuc	_	Animals

--	--	--	--
--	--	--	--
--	--	--	--
--	--	--	--
_	_	_	Animals
Lipids and	Sphingolip	Phosphosp	Animals
Organic ac	Carboxylic	Amino aci	Animals
--	--	--	--
Lipids and	Fatty Acyls	Lineolic ac	Animals
Benzenoid	Benzene a	Anilides	Animals
--	--	--	--
Organic ox	Organooxy	Carbohydr	Animals
Lipids and	Fatty Acyls	Fatty acid	Animals
Organohe	Pyrrolopyr	_	Animals,Microbe
Benzenoid	Benzene a	Diphenylm	Animals
Organohe	Isocoumar	Isobenzofu	Animals
Organic ox	Organooxy	Carbohydr	Animals
--	--	--	--
Organic ac	Organic ca	Ureas	Animals
Organic ac	Carboxylic	Amino aci	Animals
Organic ox	Organooxy	Alcohols a	Animals
--	--	--	--
Benzenoid	Phenol eth	Anisoles	Animals
--	--	--	--
Benzenoid	Phenol eth	_	Animals
--	--	--	--
Benzenoid	Tetralins	_	Animals
--	--	--	--
Lipids and	Steroids ar	Bile acids,	Animals
--	--	--	--
Lipids and	Fatty Acyls	Eicosanoic	Animals
Lipids and	Steroids ar	Steroid lac	Animals
_	_	_	Animals
--	--	--	--
Organic ac	Carboxylic	Amino aci	Animals
Organic ac	Carboxylic	Amino aci	Animals
Lipids and	Fatty Acyls	Fatty acid	Animals
Organic ox	Organooxy	Carbohydr	Animals
--	--	--	--
Lipids and	Fatty Acyls	Fatty aldeh	Animals
Lipids and	Steroids ar	Vitamin D	Animals
Alkaloids a	_	_	Animals,Plants
--	--	--	--
Organic ac	Carboxylic	Amino aci	Animals
Organic ac	Carboxylic	Carboxylic	Animals
--	--	--	--
Lipids and	Steroids ar	Bile acids,	Animals
--	--	--	--
Lipids and	Fatty Acyls	Fatty acids	Animals
Organic ni	Organonit	Amines	Animals
Lipids and	Steroids ar	Pregnane s	Animals

Lipids and	Prenol lipi	Terpene la	Animals
Lipids and	Fatty Acyls	Fatty acyl	Animals
Organosul	Isothiocya	_	Animals
--	--	--	--
Organic ni	Organonit	Amines	Animals
Hydrocarb	Polycyclic	_	Animals
--	--	--	--
Organic ni	Organonit	Amines	Animals
Lipids and	Fatty Acyls	Fatty acid	Animals
Lipids and	Sphingolip	Ceramides	Animals
--	--	--	--
Lipids and	Prenol lipi	Diterpenoi	Animals
Lipids and	Fatty Acyls	Fatty alcoh	Animals
Benzenoid	Phenols	1-hydroxy	Animals
Alkaloids a	_	_	Animals
--	--	--	--
Organic ac	Carboxylic	Amino aci	Animals
Organohe	Imidazopy	Imidazo-[4	Animals
Lipids and	Sphingolip	Glycosphin	Animals
--	--	--	--
Organic ox	Organooxy	Carbohydr	Animals
Organic ox	Organooxy	Alcohols a	Animals
--	--	--	--
Organohe	Thienoimid	_	Animals
--	--	--	--
Lipids and	Prenol lipi	Triterpeno	Animals
Organic ni	Organonit	Nitrogen r	Animals
--	--	--	--
Organosul	Sulfoxides	_	Animals
--	--	--	--
Lipids and	Sphingolip	Phosphosp	Animals
--	--	--	--
--	--	--	--
--	--	--	--
Lipids and	Prenol lipi	Sesquiterp	Animals
--	--	--	--
Organic ox	Organooxy	Carbohydr	Animals
Organic ox	Organooxy	Carbonyl c	Animals
Lipids and	Fatty Acyls	Fatty aldeh	Animals
--	--	--	--
--	--	--	--
--	--	--	--
--	--	--	--
Lipids and	Prenol lipi	Diterpenoi	Animals
Lipids and	Sphingolip	Phosphosp	Animals
Nucleoside	Nucleoside	_	Animals
Lipids and	Fatty Acyls	Fatty acid	Animals
Benzenoid	Phenols	1-hydroxy	Animals,Plants
--	--	--	--
--	--	--	--
Lipids and	Steroids ar	Bile acids,	Animals

--	--	--	--
Phenylpro	Flavonoids	Flavones	Animals,Plants
--	--	--	--
--	--	--	--
Lipids and	Fatty Acyls	Fatty acid	Animals
Nucleoside	Purine nuc	Purine 2'-(	Animals
Organohe	Benzopyra	1-benzopy	Animals
Lipids and	Fatty Acyls	Fatty acid	Animals
_	_	_	Animals
Lipids and	Steroids ar	Bile acids,	Animals
_	_	_	Animals
--	--	--	--
Lipids and	Fatty Acyls	Fatty acids	Animals
Phenylpro	Macrolacta	_	Animals
--	--	--	--
--	--	--	--
--	--	--	--
--	--	--	--
--	--	--	--
--	--	--	--
--	--	--	--
--	--	--	--
Lipids and	Prenol lipi	Monoterp	Animals
Lipids and	Prenol lipi	Sesquiterp	Animals,Plants
Lipids and	Fatty Acyls	Fatty acids	Animals
Organohe	Azoles	Oxazoles	Animals
Lipids and	Steroids ar	Oxosteroid	Animals
Lipids and	Fatty Acyls	Fatty alcoh	Animals
--	--	--	--
Lipids and	Steroids ar	Androstan	Animals
Lipids and	Fatty Acyls	Fatty acid	Animals
Lipids and	Steroids ar	Estrane ste	Animals
--	--	--	--
Lipids and	Steroids ar	Vitamin D	Animals
Lipids and	Fatty Acyls	Fatty acid	Animals
Lipids and	Fatty Acyls	Eicosanoic	Animals
Lipids and	Steroids ar	Bile acids,	Animals
Lipids and	Prenol lipi	Terpene g	Animals
--	--	--	--
_	_	_	Animals
--	--	--	--
_	_	_	Animals
Hydrocarb	Unsaturate	Branched	Animals
_	_	_	Animals
Nucleoside	Purine nuc	Purine 2',3	Animals
Lipids and	Fatty Acyls	Lineolic ac	Animals
Organosul	Thiols	Alkylthiols	Animals
--	--	--	--
Organic ac	Carboxylic	Amino aci	Animals
_	_	_	Animals
Lipids and	Steroids ar	Androstan	Animals

--	--	--	--
Lipids and	Steroids ar	Vitamin D	Animals
--	--	--	--
--	--	--	--
Lipids and	Fatty Acyls	Fatty alcoh	Animals
Lipids and	Steroids ar	Steroidal g	Animals
--	--	--	--
--	--	--	--
--	--	--	--
Phenylpro	Flavonoids	Flavonoid	Animals
Phenylpro	Flavonoids	Flavones	Animals,Plants
--	--	--	--
Phenylpro	Isoflavono	Isoflavono	Animals
Benzenoid	Anthracen	_	Animals
Lipids and	Fatty Acyls	Fatty acyl g	Animals
Lipids and	Glyceroph	CDP-glyce	Animals
Lipids and	Fatty Acyls	Fatty aldeh	Animals
Organic ox	Organooxy	Carbohydr	Animals
Organic ni	Organonit	Amines	Animals
Lipids and	Prenol lipi	Monoterpe	Animals
Lipids and	Steroids ar	Androstan	Animals
Benzenoid	Benzene a	Diphenylm	Animals
Lipids and	Sphingolip	Glycosphin	Animals
_	_	_	Animals
Phenylpro	Macrolides	_	Animals
--	--	--	--
--	--	--	--
Lipids and	Fatty Acyls	Fatty acid	Animals
Benzenoid	Phenol eth	_	Animals
--	--	--	--
--	--	--	--
Nucleoside	Purine nuc	_	Animals
Organohe	Heteroaro	_	Animals
--	--	--	--
Lipids and	Prenol lipi	Terpene g	Animals
--	--	--	--
Lipids and	Fatty Acyls	Lineolic ac	Animals
Lipids and	Sphingolip	Glycosphin	Animals
--	--	--	--
Lipids and	Prenol lipi	Diterpenoi	Animals
Lipids and	Prenol lipi	Quinone a	Animals
--	--	--	--
Organic ox	Organooxy	Alcohols a	Animals
Lipids and	Prenol lipi	Monoterpe	Animals,Plants
Organic ac	Carboxylic	Amino aci	Animals
Organic ac	Carboxylic	Amino aci	Animals
Organic ac	Carboxylic	Amino aci	Animals
Organohe	Naphthofu	_	Animals
Lipids and	Steroids ar	Bile acids,	Animals
Lipids and	Steroids ar	Bile acids,	Animals
_	_	_	Animals

_	_	_	Animals
_	_	_	Animals
Lipids and	Prenol lipi	Diterpenoi	Animals,Microbe
Organic o	Organooxy	Carbohydr	Animals
Lipids and	Fatty Acyls	Fatty aldel	Microbe,Animals,Plants
--	--	--	--
Organic ac	Carboxylic	Peroxycarl	Animals
--	--	--	--
--	--	--	--
Organosul	Thiols	Alkylthiols	Animals
Lipids and	Prenol lipi	Monoterp	Animals
Organic o	Organooxy	Carbonyl c	Animals
--	--	--	--
Lipids and	Glyceroph	Glyceroph	Animals
Benzenoid	Benzene a	Phenylace	Animals
Lipids and	Prenol lipi	Terpene la	Animals,Plants
Nucleoside	Purine nuc	_	Animals
Lipids and	Fatty Acyls	Fatty acids	Animals,Plants
--	--	--	--
Organic o	Organooxy	Carbohydr	Animals
--	--	--	--
Lipids and	Steroids ar	Steroid est	Animals
Lipids and	Fatty Acyls	Fatty acid	Microbe,Animals
_	_	_	Animals
Lipids and	Fatty Acyls	Fatty acids	Animals
Lipids and	Fatty Acyls	Eicosanoic	Animals
--	--	--	--
Organic ac	Organic pl	Phosphate	Animals
Lipids and	Glyceroph	Glyceroph	Animals
_	_	_	Animals
Organic o	Organooxy	Carbohydr	Animals
--	--	--	--
Lipids and	Glycerolipi	Diradylgly	Animals
Organic ac	Carboxylic	Amino aci	Animals
Phenylpro	Macrolacta	_	Animals
Lipids and	Saccharoli	_	Animals
Organohe	Diazanaph	Benzodiaz	Animals
Lipids and	Fatty Acyls	Eicosanoic	Animals
--	--	--	--
Lipids and	Glyceroph	Glyceroph	Microbe,Animals
--	--	--	--
Lipids and	Fatty Acyls	Eicosanoic	Animals
Organic ac	Carboxylic	Amino aci	Animals
Organic o	Organooxy	Carbohydr	Animals
--	--	--	--
--	--	--	--
Phenylpro	Tetracyclin	_	Animals
Organic o	Organooxy	Alcohols a	Animals
Organic ac	Carboxylic	Amino aci	Animals
Organic ac	Keto acids	Medium-c	Animals
Lipids and	Fatty Acyls	Fatty acyl	Animals

Lipids and	Glyceroph	Glyceroph	Animals
Lipids and	Prenol lipi	Diterpenoi	Animals
Benzenoid	Benzene a	Diphenylm	Animals
--	--	--	--
Phenylpro	Phenylpro	_	Animals,Plants
Benzenoid	Benzene a	_	Animals
Lipids and	Glycerolipi	Monorady	Animals
Lipids and	Fatty Acyls	Lineolic ac	Animals
Lipids and	Steroids ar	Androstan	Animals
Lipids and	Prenol lipi	Diterpenoi	Animals
--	--	--	--
--	--	--	--
--	--	--	--
Hydrocarb	Unsaturate	Branched	Animals
Lipids and	Glyceroph	Glyceroph	Animals
Organohe	Lactams	Beta lactar	Animals
Organic ac	Carboxylic	Amino aci	Animals
Nucleoside	Nucleoside	Cyclopenty	Animals
Organic ac	Carboxylic	Amino aci	Animals
Organic ac	Carboxylic	Amino aci	Animals
Benzenoid	_	_	Animals
Organic ac	Carboxylic	Amino aci	Animals
--	--	--	--
_	_	_	Animals
--	--	--	--
Lipids and	Fatty Acyls	Fatty acids	Animals
--	--	--	--
Lipids and	Prenol lipi	Terpene g	Animals
--	--	--	--
Lipids and	Fatty Acyls	Eicosanoic	Animals
--	--	--	--
Lipids and	Glyceroph	Glyceroph	Animals
Organic ac	Carboxylic	Amino aci	Animals
Organohe	Benzopyra	1-benzopy	Animals
--	--	--	--
Lipids and	Fatty Acyls	Fatty acid	Animals
--	--	--	--
Organic ac	Carboxylic	Amino aci	Animals
Lipids and	Fatty Acyls	Fatty acids	Animals
Lipids and	Prenol lipi	Sesquiterp	Animals
Phenylpro	Macrolides	Epothilone	Animals
Benzenoid	Benzene a	Xylenes	Animals,Plants
Benzenoid	Benzene a	Phenylpro	Animals
Benzenoid	Benzene a	Benzoic ac	Animals
Benzenoid	Benzene a	_	Animals
--	--	--	--
Lipids and	Fatty Acyls	Fatty alcoh	Animals
Organic ox	Organooxy	Carbohydr	Animals
Lipids and	Sphingolip	Phosphosp	Animals
--	--	--	--
Benzenoid	Benzene a	Biphenyls	Animals

Organohe	Azolidines	Oxazolidin	Animals
Lipids and	Steroids ar	Pregnane :	Animals
Lipids and	Steroids ar	Pregnane :	Animals
--	--	--	--
_	_	_	Animals
--	--	--	--
Organic ac	Keto acids	Short-chai	Microbe,Animals
--	--	--	--
Organohe	Pyrroles	Substitute	Animals
Organic o:	Organooxy	Carbonyl c	Animals
Benzenoid	Phenol eth	_	Animals
Lipids and	Prenol lipi	Triterpeno	Animals
--	--	--	--
Organic o:	Organooxy	Carbonyl c	Microbe,Animals
Phenylpro	Linear 1,3-	Cinnamylp	Animals
--	--	--	--
Organic ni	Organonit	Amines	Microbe,Animals
Organohe	Triazolopy	_	Animals
Phenylpro	Macrolact	_	Animals
Organic o:	Organooxy	Carbohydr	Animals
Organohe	Dihydrofu	Furanones	Animals
Lipids and	Glyceroph	Glyceroph	Microbe,Animals
Organic ac	Carboxylic	Amino aci	Animals
Lipids and	Fatty Acyls	Fatty acids	Animals
Lipids and	Endocann	_	Animals
Lipids and	Steroids ar	Steroidal g	Animals
Lipids and	Steroids ar	Bile acids,	Animals
_	_	_	Animals
Lipids and	Fatty Acyls	Fatty acid	Animals
Benzenoid	Benzene a	Benzoic ac	Animals
Lipids and	Fatty Acyls	Fatty acids	Microbe,Animals,Plants
--	--	--	--
Organic ac	Carboxylic	Amino aci	Animals
--	--	--	--
Benzenoid	Benzene a	Benzoic ac	Animals
Phenylpro	Kavalactor	_	Animals
--	--	--	--
Organic o:	Organooxy	Carbohydr	Animals
Lipids and	Fatty Acyls	Eicosanoic	Animals
Lipids and	Fatty Acyls	Fatty acids	Animals
Organic ac	Carboxylic	Amino aci	Animals
Lipids and	Glyceroph	Glyceroph	Animals
_	_	_	Animals
Phenylpro	Phenylpro	_	Animals,Plants
Lipids and	Fatty Acyls	Fatty amid	Animals
Lipids and	Fatty Acyls	Fatty acids	Animals,Plants
--	--	--	--
Organohe	Diazinanes	Piperazine	Animals
Organic ni	Organonit	Amines	Animals
_	_	_	Animals
Lipids and	Fatty Acyls	Eicosanoic	Animals

Organic ac	Carboxylic	Amino acio	Animals
Organic ac	Carboxylic	Amino acio	Animals
--	--	--	--
Lipids and	Fatty Acyls	Fatty acid	Microbe,Animals
_	_	_	Animals
_	_	_	Animals
Lipids and	Glyceroph	Glyceroph	Animals
Phenylpro	Stilbenes	_	Animals
Organic ac	Peptidomi	Hybrid pep	Animals
Alkaloids a	Ergoline ar	Lysergic ac	Animals
_	_	_	Animals
_	_	_	Animals
Organic ac	Carboxylic	Amino acio	Animals
Lipids and	Glyceroph	Glyceroph	Animals
_	_	_	Animals
Organic ni	Organonit	Amines	Animals
Lipids and	Fatty Acyls	Fatty alcoh	Animals,Plants
Lipids and	Fatty Acyls	Fatty acids	Animals
Organic ni	Organonit	Amines	Animals,Plants
_	_	_	Animals
Lipids and	Prenol lipic	Sesquiterp	Animals
Lipids and	Glyceroph	Glyceroph	Animals
Lipids and	Prenol lipic	Sesquiterp	Animals
Phenylpro	Macrolides	_	Animals
Lipids and	Fatty Acyls	Fatty alcoh	Animals
Lipids and	Fatty Acyls	Fatty alcoh	Animals
Benzenoid	Benzene a	Phenylpro	Animals
--	--	--	--
Lipids and	Prenol lipic	Monoterp	Animals
Organohe	Heteroaro	_	Animals
--	--	--	--
_	_	_	Animals
Lipids and	Glyceroph	Glyceroph	Animals
--	--	--	--
Organic ac	Carboxylic	Amino acio	Animals
--	--	--	--
--	--	--	--
Organic ac	Carboxylic	Amino acio	Animals
Organic ac	Carboxylic	Amino acio	Animals
--	--	--	--
Lipids and	Steroids ar	Hydroxyste	Animals
Lipids and	Fatty Acyls	Eicosanoic	Animals
Organic ac	Carboxylic	Amino acio	Animals
--	--	--	--
Organohe	Benzothiaz	Phenothia:	Animals
Lipids and	Fatty Acyls	Eicosanoic	Animals
--	--	--	--
Organic ac	Carboxylic	Amino acio	Animals
_	_	_	Animals
Lipids and	Fatty Acyls	Fatty acid	Animals
_	_	_	Animals

Organohe	Diazines	Pyridazine	Animals
Organic o›	Organooxy	Carbohydr	Animals
Lipids and	Fatty Acyls	Fatty acids	Animals
Organohe	Benzofura	_	Animals
--	--	--	--
Organic ac	Carboxylic	Amino aci	Animals
Organohe	Imidazopy	_	Animals
Lipids and	Fatty Acyls	Fatty acids	Animals
Lipids and	Glyceroph	Glyceroph	Animals
Lipids and	Glyceroph	CDP-glyce	Animals
Benzenoid	Benzene a	Phenylmet	Animals,Plants
Organic o›	Organooxy	Carbohydr	Animals,Plants
Organic ni	Organonit	Organic ni	Animals
Benzenoid	Benzene a	_	Animals
--	--	--	--
Organic ac	Carboxylic	Amino aci	Animals
--	--	--	--
Lipids and	Steroids ar	Ergostane	Animals
--	--	--	--
Lipids and	Glyceroph	Glyceroph	Animals
_	_	_	Animals
Organic ac	Carboxylic	Amino aci	Animals
Lipids and	Prenol lipi	Triterpeno	Animals
Lipids and	Prenol lipi	Monoterp	Animals
Benzenoid	Benzene a	Diphenylm	Animals
Lipids and	Prenol lipi	Sesterterp	Animals
--	--	--	--
Lipids and	Fatty Acyls	Fatty acid	Animals
Organic o›	Organooxy	Carbonyl c	Animals
--	--	--	--
Lipids and	Prenol lipi	Sesquiterp	Animals
Organic ni	Organonit	Amines	Animals
Organic ac	Carboxylic	Amino aci	Animals
Organic ac	Carboxylic	Amino aci	Animals
Lipids and	Prenol lipi	Sesquiterp	Animals
--	--	--	--
--	--	--	--
Lipids and	Steroids ar	Hydroxyst	Animals
--	--	--	--
Lipids and	Fatty Acyls	Fatty acid	Animals
Lipids and	Fatty Acyls	Fatty acid	Animals
--	--	--	--
Lipids and	Steroids ar	Steroidal g	Animals
--	--	--	--
_	_	_	Animals
Organohe	Isobenzofu	_	Animals
--	--	--	--
Lipids and	Prenol lipi	Monoterp	Animals
Lipids and	Fatty Acyls	Lineolic ac	Animals,Plants,Microbe
_	_	_	Animals
_	_	_	Animals

Lipids and	Prenol lipi	Monoterp	Animals
Phenylpro	Macrolide	_	Animals
Lipids and	Glyceroph	CDP-glyce	Animals
Nucleosid	Pyrimidine	_	Animals
Organohe	Lactones	Gamma bu	Animals
--	--	--	--
--	--	--	--
--	--	--	--
Lipids and	Glyceroph	Glyceroph	Animals
--	--	--	--
_	_	_	Animals
_	_	_	Animals
Organohe	Pyridines a	_	Animals
Organohe	Tetrahydro	_	Animals
Benzenoid	Naphthale	Naphthale	Animals
Organic ac	Carboxylic	Amino aci	Animals
--	--	--	--
Lipids and	Glyceroph	Glyceroph	Animals
Phenylpro	Macrolides	Milbemyci	Animals
Organohe	Benzopyra	1-benzopy	Microbe,Animals
--	--	--	--
Organic ac	Carboxylic	Amino aci	Animals
_	_	_	Animals
_	_	_	Animals
_	_	_	Animals
--	--	--	--
Lipids and	Prenol lipi	Diterpenoi	Animals
--	--	--	--
--	--	--	--
Organohe	Pyrrolidine	Phenylpyr	Animals
--	--	--	--
Organic ac	Carboxylic	Amino aci	Animals
Lipids and	Prenol lipi	Triterpeno	Animals
Lipids and	Fatty Acyls	Fatty acids	Animals
Lipids and	Sphingolip	Phosphosp	Animals
Organic ac	Carboxylic	Amino aci	Animals
Lipids and	Steroids a	Cholestane	Animals,Plants
Lipids and	Steroids a	Cycloartan	Animals
Lipids and	Fatty Acyls	Fatty acyl	Animals
Lipids and	Fatty Acyls	Lineolic ac	Animals
Organic ox	unds	Alcohols a	Animals
--	--	--	--
Lipids and	Steroids a	Steroidal g	Animals
Lipids and	Steroids a	Steroidal g	Animals
Organic ac	Peptidomi	Depsipept	Animals
--	--	--	--
Lipids and	Fatty Acyls	Eicosanoic	Animals
_	_	_	Animals
Lipids and	Glyceroph	Glyceroph	Animals,Plants
--	--	--	--
--	--	--	--

--	--	--	--
Organic ac	Carboxylic	Amino aci	Animals
--	--	--	--
Lipids and	Glyceroph	Glyceroph	Animals
_	_	_	Animals
Lipids and	Prenol lipi	Triterpeno	Animals
_	_	_	Animals
--	--	--	--
Phenylpro	Macrolides	Milbemyci	Animals
--	--	--	--
Lipids and	Prenol lipi	Terpene g	Animals
_	_	_	Animals
--	--	--	--
Lipids and	Glyceroph	Glyceroph	Animals
Organic ac	Carboxylic	Amino aci	Animals
Lipids and	Sphingolip	Glycosphin	Animals
Organic ox	Organooxy	Ethers	Animals
Lipids and	Fatty Acyls	Lineolic ac	Animals
Benzenoid	Phenols	1-hydroxy	Animals
Lipids and	Glyceroph	Glyceroph	Animals
Lipids and	Prenol lipi	Terpene la	Animals
Lipids and	Glyceroph	Glyceroph	Animals
--	--	--	--
Organic ac	Carboxylic	Amino aci	Animals
_	_	_	Animals
--	--	--	--
Organic ac	Carboxylic	Amino aci	Animals
Organic ac	Carboxylic	Amino aci	Animals
--	--	--	--
Lipids and	Fatty Acyls	Fatty acyl	Animals
_	_	_	Animals
Phenylpro	Macrolide	_	Animals
_	_	_	Animals
--	--	--	--
Organic ac	Carboxylic	Amino aci	Animals
Lipids and	Steroids ar	Steroid lac	Animals
Phenylpro	Stilbenes	_	Animals,Plants
Lipids and	Fatty Acyls	Lineolic ac	Animals
Lipids and	Fatty Acyls	Fatty acids	Animals
--	--	--	--
Lipids and	Prenol lipi	Sesquiterp	Animals
--	--	--	--
--	--	--	--
Lipids and	Fatty Acyls	Fatty acids	Animals
Organic ac	Carboxylic	Amino aci	Animals
Lipids and	Prenol lipi	Triterpeno	Microbe,Animals
--	--	--	--
_	_	_	Animals
Nucleoside	Imidazole	1-ribosyl-i	Animals
--	--	--	--
--	--	--	--

_	_	_	Animals
_	_	_	Animals
Lipids and	Glyceroph	Glyceroph	Animals
Phenylpro	Macrolides	_	Animals
--	--	--	--
Lipids and	Sphingolip	Glycosphin	Animals
Organohe	Pyridines a	Pyrazolylp	Animals
Alkaloids a	Harmala a	_	Microbe,Animals
--	--	--	--
Organohe	Pyrazolopy	_	Animals
--	--	--	--
_	_	_	Animals
Organic ac	Carboxylic	Amino acid	Animals
Organic ac	Carboxylic	Amino acid	Animals
--	--	--	--
Organic ox	Organooxy	Carbonyl c	Animals
Lipids and	Fatty Acyls	Eicosanoic	Animals
--	--	--	--
_	_	_	Animals
Lipids and	Prenol lipid	Terpene g	Animals
Organohe	Pyridines a	Hydropyrid	Animals
Lipids and	Glyceroph	Glyceroph	Animals
--	--	--	--
Organic ac	Carboximi	Carboximi	Animals
--	--	--	--
Organic ox	Organooxy	Carbohydr	Animals
--	--	--	--
Organic ni	Organonit	Oximes	Animals
Organic ac	Peptidomi	Depsipept	Animals
--	--	--	--
Organic ac	Carboxylic	Amino acid	Animals
Organic ac	Carboxylic	Amino acid	Animals
Lipids and	Fatty Acyls	Fatty acyl g	Animals
Benzenoid	Benzene a	Phenylbuty	Animals
Lipids and	Glycerolipi	Monorady	Animals
--	--	--	--
Organic ac	Carboxylic	Amino acid	Animals
--	--	--	--
--	--	--	--
Lipids and	Prenol lipid	Triterpeno	Animals
Phenylpro	Macrolides	Milbemyci	Animals
Organic ac	Carboxylic	Amino acid	Animals
Lipids and	Glyceroph	Glyceroph	Animals
Lipids and	Prenol lipid	Triterpeno	Animals
Lipids and	Glyceroph	Glyceroph	Animals
Lipids and	Fatty Acyls	Fatty alcoh	Animals
_	_	_	Animals
Lipids and	Glyceroph	Glyceroph	Animals
Organic ox	Organooxy	Carbohydr	Animals
Lipids and	Fatty Acyls	Eicosanoic	Animals
--	--	--	--

Lipids and	Glycerolipi	Monorady	Animals,Plants
--	--	--	--
Organic ac	Carboxylic	Amino acic	Animals
--	--	--	--
Lipids and	Prenol lipic	Triterpeno	Animals
Organic ac	Peptidomi	Depsipept	Animals
Alkaloids a	Tropane a	_	Animals
Organic ac	Carboxylic	Amino acic	Animals
Lipids and	Fatty Acyls	Fatty acids	Animals
Organic ac	Carboxylic	Amino acic	Animals
Organohe	Benzothiaz	_	Animals
_	_	_	Animals
Lipids and	Prenol lipic	Diterpenoi	Animals
Lipids and	Prenol lipic	Triterpeno	Animals
Lipids and	Glyceroph	Glyceroph	Animals
_	_	_	Animals
--	--	--	--
Hydrocarb	Unsaturate	Olefins	Animals
Phenylpro	Linear 1,3-	Chalcones	Animals
Lipids and	Prenol lipic	Sesquiterp	Animals
--	--	--	--
Lipids and	Fatty Acyls	Eicosanoic	Animals
Lipids and	Fatty Acyls	Fatty acid	Animals
Lipids and	Prenol lipic	Monoterpe	Animals
Lipids and	Glyceroph	Glyceroph	Animals
Lipids and	Glyceroph	Glyceroph	Animals
--	--	--	--
Lipids and	Fatty Acyls	Fatty acid	Animals
Benzenoid	Benzene a	Aniline anc	Animals
Organic ac	Carboxylic	Amino acic	Microbe,Animals
--	--	--	--
Lipids and	Fatty Acyls	Fatty alcoh	Animals
--	--	--	--
Lipids and	Glyceroph	Glyceroph	Animals
Organic ox	Organooxy	Carbohydr	Animals
Lipids and	Fatty Acyls	Fatty alcoh	Animals
Benzenoid	Phenols	Benzenedi	Animals
Lipids and	Steroids ar	Cucurbitac	Animals
--	--	--	--
--	--	--	--
--	--	--	--
Organohe	Piperidines	Phenylpipe	Animals
Phenylpro	Cinnamic a	Hydroxycir	Animals
Organic ox	Organooxy	Alcohols a	Animals,Plants
Organic ni	Organonit	Quaternar	Microbe,Animals,Plants
--	--	--	--
Organohe	Diazines	Pyrimidine	Animals
Organic ac	Carboxylic	Amino acic	Animals
Organohe	Lactones	Gamma bu	Animals
Organic ac	Carboxylic	Amino acic	Animals
--	--	--	--

--	--	--	--
Lipids and	Glycerolipi	Monorady	Animals
--	--	--	--
Lipids and	Glyceroph	Glyceroph	Microbe,Animals
Organic ac	Carboxylic	Amino aci	Animals
_	_	_	Animals
Lipids and	Prenol lipi	Triterpeno	Animals
Lipids and	Saccharoli	_	Animals
--	--	--	--
Lipids and	Glyceroph	Glyceroph	Animals
_	_	_	Animals
_	_	_	Animals
Lipids and	Prenol lipi	Monoterpe	Animals
--	--	--	--
Lipids and	Sphingolip	Glycosphin	Animals
Lipids and	Sphingolip	Glycosphin	Animals
--	--	--	--
Phenylpro	Macrolides	Epothilone	Animals
Lipids and	Prenol lipi	Sesquiterp	Animals
Lipids and	Prenol lipi	Monoterpe	Animals
Lipids and	Steroids an	Hydroxyste	Animals
Organohe	Imidazopy	Purines an	Animals
Lipids and	Fatty Acyls	Fatty acids	Animals
Lipids and	Fatty Acyls	Eicosanoic	Animals
--	--	--	--
--	--	--	--
--	--	--	--
Lipids and	Prenol lipi	Triterpeno	Animals
_	_	_	Animals
Lipids and	Fatty Acyls	Eicosanoic	Animals
Lipids and	Fatty Acyls	Fatty acyl	Animals
Organic ac	Carboxylic	Amino aci	Microbe,Animals
Lipids and	Steroids an	Bile acids,	Animals
--	--	--	--
Organosul	Isothiocyan	_	Animals,Plants
Organohe	Benzopyra	1-benzopy	Animals
Organohe	Indoles an	Tryptamin	Animals
_	_	_	Animals
Organohe	Diazines	Pyrimidine	Animals
_	_	_	Animals
Organic ac	Carboxylic	Amino aci	Animals
Lipids and	Fatty Acyls	Lineolic ac	Animals
Lipids and	Glyceroph	Glyceroph	Animals
Lipids and	Steroids an	Vitamin D	Animals
Lipids and	Steroids an	Bile acids,	Animals
--	--	--	--
--	--	--	--
Nucleoside	Pyrimidine	Pyrimidine	Animals
Organic ac	Carboxylic	Amino aci	Animals
--	--	--	--
Lipids and	Fatty Acyls	Fatty acids	Animals

Organic ac	Carboxylic	Amino aci	Animals
Organohe	Azoles	Imidazoles	Animals
Organohe	Diazines	Pyrimidine	Animals
--	--	--	--
Lipids and	Steroids ar	Cholestane	Animals
Phenylpro	Macrolides	Milbemyci	Animals
Phenylpro	Macrolides	Milbemyci	Animals
--	--	--	--
Organic ox	Organooxy	Carbonyl c	Animals
Organohe	Diazines	Pyridazine	Animals
Organohe	Benzopyra	1-benzopy	Microbe,Animals,Plants
Lipids and	Fatty Acyls	Fatty alcoh	Animals
Benzenoid	Benzene a	Benzoic ac	Microbe,Animals,Plants
Nucleoside	Benzimida	_	Animals
Nucleoside	Purine nuc	Purine ribo	Animals
Organohe	Oxanes	_	Animals
Organic ac	Carboxylic	Amino aci	Animals
Benzenoid	Phenol eth	_	Animals
Organohe	Dithianes	_	Animals
Nucleoside	Purine nuc	Purine 2'-c	Animals
--	--	--	--
--	--	--	--
Lipids and	Steroids ar	Vitamin D	Animals
Organohe	Benzopyra	1-benzopy	Animals
Benzenoid	Phenols	Methoxypl	Animals,Plants
Lipids and	Prenol lipi	Triterpeno	Animals
--	--	--	--
_	_	_	Animals
Lipids and	Fatty Acyls	Eicosanoic	Animals
--	--	--	--
_	_	_	Animals
--	--	--	--
Organohe	Azoles	Imidazoles	Animals
_	_	_	Animals
--	--	--	--
Lipids and	Prenol lipi	Terpene g	Animals
Lipids and	Sphingolip	Ceramides	Animals
Lipids and	Prenol lipi	Terpene g	Animals
Benzenoid	Benzene a	Benzoic ac	Microbe,Animals
_	_	_	Animals
--	--	--	--
--	--	--	--
Lipids and	Glyceroph	Glyceroph	Animals
Organic ac	Organic pl	Phosphoni	Animals
Benzenoid	Tetralins	_	Animals
--	--	--	--
--	--	--	--
Lipids and	Glyceroph	Glyceroph	Animals
--	--	--	--
--	--	--	--
_	_	_	Animals

Organic ox	Organooxy	Carbohydr	Animals
--	--	--	--
_	_	_	Animals
_	_	_	Animals
--	--	--	--
Lipids and	Prenol lipi	Sesquiterp	Animals
Benzenoid	Benzene a	Phenylpro	Animals
--	--	--	--
Organic ni	Organonit	Quaternar	Animals
--	--	--	--
Lipids and	Fatty Acyls	Fatty acid	Animals
--	--	--	--
Lipids and	Steroids ar	Hydroxyste	Animals
_	_	_	Animals
Organic ac	Carboxylic	Amino aci	Animals
--	--	--	--
Lipids and	Glyceroph	Glyceroph	Animals
Benzenoid	Benzene a	_	Animals
Organic ac	Carboxylic	Amino aci	Animals
Lipids and	Glycerolipi	Monorady	Animals
--	--	--	--
--	--	--	--
Lipids and	Glyceroph	Glyceroph	Animals
Organic ac	Carboxylic	Amino aci	Animals
Lipids and	Steroids ar	Estrane ste	Animals
_	_	_	Animals
Lipids and	Steroids ar	Cucurbitac	Animals
Lipids and	Sphingolip	Glycosphir	Animals
_	_	_	Animals
Lipids and	Steroids ar	Oxosteroic	Animals
Organic ac	Carboxylic	Amino aci	Animals
--	--	--	--
_	_	_	Animals
--	--	--	--
Organic ac	Carboxylic	Amino aci	Animals
Organic ni	Organonit	Quaternar	Microbe,Animals,Plants
Lipids and	Fatty Acyls	Fatty acyl (	Animals
Lipids and	Steroids ar	Steroid lac	Animals
--	--	--	--
--	--	--	--
Lipids and	Glyceroph	Glyceroph	Animals
Organic ac	Carboxylic	Amino aci	Animals
--	--	--	--
--	--	--	--
Organohe	Benzopyra	1-benzopy	Animals,Microbe
Organic ac	Carboxylic	Tetracarbo	Animals
_	_	_	Animals
Lipids and	Glycerolipi	Glycosylgly	Animals
Lipids and	Steroids ar	Bile acids,	Animals
_	_	_	Animals
Lipids and	Glycerolipi	Monorady	Animals

Organohe	Piperidines	Benzylpipe	Animals
Organic ac	Carboxylic	Amino aci	Animals
Lipids and	Prenol lipi	Terpene g	Animals
Lipids and	Glycerolipi	Glycosylgl	Animals
Lipids and	Fatty Acyls	Fatty acid	Animals
--	--	--	--
_	_	_	Animals
_	_	_	Animals
Lipids and	Glycerolipi	Diradylgly	Animals
Lipids and	Fatty Acyls	Eicosanoic	Animals
Organic ac	Carboxylic	Amino aci	Animals
Organic ac	Carboxylic	Amino aci	Animals
Organic ac	Carboxylic	Amino aci	Animals
Organic ox	Organooxy	Carbohydr	Animals
_	_	_	Animals
Lipids and	Steroids ar	Stigmastar	Animals
--	--	--	--
Benzenoid	Benzene a	Benzyl alc	Animals
--	--	--	--
Lipids and	Prenol lipi	Diterpenoi	Animals
Organic ox	Organooxy	Carbonyl c	Animals
Organic ac	Carboxylic	Amino aci	Animals
Lipids and	Prenol lipi	Triterpeno	Animals
--	--	--	--
Lipids and	Fatty Acyls	Fatty alcoh	Animals
Organohe	Furans	Furoic acic	Animals
Organic ox	Organooxy	Carbonyl c	Animals,Plants
--	--	--	--
Organohe	Pteridines	Pterins anc	Animals
--	--	--	--
Organohe	Benzopyra	1-benzopy	Animals
Lipids and	Fatty Acyls	Fatty acyl g	Animals
Lipids and	Fatty Acyls	Fatty acids	Animals
Organic ox	Organooxy	Carbohydr	Animals
--	--	--	--
Organic ac	Carboxylic	Amino aci	Animals
Lipids and	Steroids ar	Steroid lac	Animals
_	_	_	Animals
Lipids and	Sphingolip	Glycosphir	Animals
Lipids and	Prenol lipi	Quinone a	Animals
_	_	_	Animals
Organic ac	Carboxylic	Amino aci	Animals
Lipids and	Steroids ar	Estrane ste	Animals
Lipids and	Steroids ar	Stigmastar	Animals
Lipids and	Prenol lipi	Terpene g	Animals
Organic ox	Organooxy	Ethers	Animals
Lipids and	Steroids ar	Steroid est	Animals
--	--	--	--
Lipids and	Steroids ar	Bile acids,	Animals
Lipids and	Fatty Acyls	Lineolic ac	Animals
Organic ac	Carboxylic	Amino aci	Microbe,Animals

Organic o›	Organooxy	Carbonyl c	Animals
Organic 1,	Allyl-type	_	Animals
--	--	--	--
Lipids and	Steroids ar	Pregnane s	Animals
Lipids and	Steroids ar	Cholestane	Animals,Microbe
Benzenoid	Benzene a	Phenylbuty	Animals
Lipids and	Steroids ar	Vitamin D	Animals
_	_	_	Animals
--	--	--	--
Organic o›	Organooxy	Carbohydr	Animals
--	--	--	--
--	--	--	--
--	--	--	--
--	--	--	--
_	_	_	Animals
--	--	--	--
Organic ni	Organonit	Amines	Animals
--	--	--	--
Lipids and	Steroids ar	Bile acids,	Animals
Phenylpro	Macrolides	_	Animals
Organic ac	Carboxylic	Amino acid	Animals
Organic ac	Carboxylic	Amino acid	Animals
Organic o›	Organooxy	Carbohydr	Animals
_	_	_	Animals
Nucleoside	Pyrimidine	Pyrimidine	Animals
Lipids and	Steroids ar	Stigmastar	Animals
Lipids and	Prenol lipid	Terpene g	Animals
Organic ac	Carboxylic	Amino acid	Animals
Organic o›	Organooxy	Carbohydr	Animals
--	--	--	--
--	--	--	--
_	_	_	Animals
--	--	--	--
--	--	--	--
Lipids and	Fatty Acyls	Fatty acyl g	Animals
Nucleoside	Nucleoside	Cyclopenty	Animals
--	--	--	--
Organic ni	Organonit	_	Animals
Lipids and	Fatty Acyls	Fatty acids	Microbe,Animals
Organic ac	Carboxylic	Amino acid	Animals
_	_	_	Animals
Organic ac	Carboxylic	Amino acid	Animals
Lipids and	Fatty Acyls	Fatty acids	Animals
Organic o›	Organooxy	Carbohydr	Animals
Lipids and	Fatty Acyls	Fatty acids	Animals
Nucleoside	Purine nuc	Purine 2',3	Animals
Organic o›	Organooxy	Carbohydr	Animals
Organohe	Azepines	_	Animals
Lipids and	Steroids ar	Estrane ste	Animals
_	_	_	Animals
Organic ac	Carboxylic	Amino acid	Animals

Lipids and	Steroids ar	Hydroxyste	Animals
Lipids and	Fatty Acyls	Eicosanoic	Animals
Lipids and	Fatty Acyls	Fatty acid	Animals
Organic ox	Organooxy	Carbohydr	Animals
Lipids and	Fatty Acyls	Fatty acid	Animals
--	--	--	--
--	--	--	--
_	_	_	Animals
Organic ac	Carboxylic	Amino acic	Animals
_	_	_	Animals
Lipids and	Prenol lipic	Tetraterpe	Animals
Benzenoid	_	_	Animals
Organic ac	Carboxylic	Amino acic	Animals
--	--	--	--
_	_	_	Animals
Lipids and	Prenol lipic	Tetraterpe	Animals
Nucleoside	Pyrimidine	_	Animals
_	_	_	Animals
Organic ox	Organooxy	Ethers	Animals
Organic ox	Organooxy	Ethers	Animals
Organic ox	Organooxy	Ethers	Animals
Lipids and	Fatty Acyls	Fatty acid	Animals
Lipids and	Steroids ar	Cholestane	Animals
--	--	--	--
Organic ac	Carboxylic	Amino acic	Animals
--	--	--	--
_	_	_	Animals
--	--	--	--
Lipids and	Steroids ar	Cholestane	Microbe,Animals
Lipids and	Sphingolip	Phosphosp	Animals
--	--	--	--
Organic ac	Carboxylic	Amino acic	Animals
Lipids and	Prenol lipic	Triterpeno	Animals
Lipids and	Glycerolipi	Diradylglyc	Animals
Lipids and	Steroids ar	Bile acids,	Animals
Lipids and	Glyceroph	Glyceroph	Microbe,Animals
_	_	_	Animals
Lipids and	Sphingolip	Glycosphir	Animals
Organic ac	Carboxylic	Amino acic	Animals
_	_	_	Animals
Lipids and	Fatty Acyls	Lineolic ac	Animals
--	--	--	--
Organic ox	Organooxy	Carbohydr	Animals
_	_	_	Animals
Benzenoid	Benzene a	Styrenes	Animals
--	--	--	--
Phenylpro	Kavalactor	_	Animals
Phenylpro	Coumarins	Hydroxycc	Animals
Organic ox	Organooxy	Carbonyl c	Animals
Lipids and	Prenol lipic	Sesquiterp	Animals
Nucleoside	Purine nuc	Purine ribc	Animals

--	--	--	--
Nucleosid	Pyrimidine	Pyrimidine	Animals
_	_	_	Animals
_	_	_	Animals
Organohe	Tetrahydro	_	Animals
Lipids and	Fatty Acyls	Fatty acids	Animals
Organic o	Organooxy	Carbonyl c	Animals,Plants
--	--	--	--
--	--	--	--
Organic ac	Carboxylic	Amino aci	Animals
_	_	_	Animals
_	_	_	Animals
_	_	_	Animals
--	--	--	--
Organic ac	Carboxylic	Amino aci	Animals
_	_	_	Animals
Lipids and	Fatty Acyls	Fatty acid	Animals
_	_	_	Animals
_	_	_	Animals
Lipids and	Prenol lipi	Sesquiterp	Animals
--	--	--	--
--	--	--	--
_	_	_	Animals
Lipids and	Prenol lipi	Diterpenoi	Animals
Organic ac	Carboxylic	Amino aci	Animals
Lipids and	Glycerolipi	Monorady	Animals
--	--	--	--
Phenylpro	Cinnamic a	_	Animals
Benzenoid	Benzene a	Benzyloxy	Animals
Organic ac	Carboxylic	Amino aci	Animals
Organic ac	Carboxylic	Amino aci	Animals
--	--	--	--
_	_	_	Animals
--	--	--	--
Lipids and	Prenol lipi	Terpene la	Animals
--	--	--	--
Lipids and	Prenol lipi	Terpene la	Animals
Organohe	Azoles	Imidazoles	Animals
--	--	--	--
Lipids and	Fatty Acyls	Fatty acid	Animals
_	_	_	Animals
Lipids and	Fatty Acyls	Fatty aldeh	Animals
Lipids and	Glyceroph	Glyceroph	Animals
Lipids and	Prenol lipi	Tetraterpe	Animals
--	--	--	--
Lipids and	Glyceroph	Glyceroph	Microbe,Animals
Lipids and	Sphingolip	Ceramides	Animals
Lipids and	Glycerolipi	Diradylgly	Animals
Lipids and	Fatty Acyls	Fatty acid	Animals,Plants
Organic o	Organooxy	Ethers	Animals
Lipids and	Prenol lipi	Tetraterpe	Animals

Benzenoid	Benzene a	Xylenes	Animals
_	_	_	Animals
--	--	--	--
Lipids and	Fatty Acyls	Fatty acid	Animals
Organohe	Pyridines a	_	Animals
_	_	_	Animals
--	--	--	--
_	_	_	Animals
Lipids and	Glycerolipi	Diradylgly	Animals
Lipids and	Glyceroph	Glyceroph	Animals
--	--	--	--
_	_	_	Animals
--	--	--	--
Organic ac	Carboxylic	Amino aci	Animals
--	--	--	--
Organohe	Trioxanes	_	Animals
Lipids and	Fatty Acyls	Eicosanoic	Animals
Organic ox	Organooxy	Ethers	Animals
Benzenoid	Phenols	Methoxypl	Animals
Organic ac	Carboxylic	Amino aci	Animals
Organic ac	Carboxylic	Amino aci	Animals
Lipids and	Steroids ar	Bile acids,	Animals
Lipids and	Fatty Acyls	Fatty acid	Animals
--	--	--	--
Lipids and	Fatty Acyls	Fatty acyl g	Animals
Lipids and	Fatty Acyls	Fatty acids	Animals
_	_	_	Animals
Organic ox	Organooxy	Alcohols a	Animals
--	--	--	--
Lipids and	Fatty Acyls	Fatty acid	Animals
--	--	--	--
Lipids and	Glyceroph	Glyceroph	Animals
--	--	--	--
Nucleoside	Nucleoside	Thionucleo	Animals
Lipids and	Fatty Acyls	Fatty alcoh	Animals
--	--	--	--
_	_	_	Animals
Lipids and	Glyceroph	Glyceroph	Animals
Lipids and	Glyceroph	Glyceroph	Animals
--	--	--	--
_	_	_	Animals
_	_	_	Animals
_	_	_	Animals
Organic ox	Organooxy	Carbohydr	Animals
_	_	_	Animals
_	_	_	Animals
Organohe	Azoles	Imidazoles	Animals
_	_	_	Animals
_	_	_	Animals
_	_	_	Animals
_	_	_	Animals

_	_	_	Animals
_	_	_	Animals
_	_	_	Animals
Organic ac	Carboxylic	Amino aci	Animals
--	--	--	--
Lipids and	Fatty Acyls	Fatty acids	Animals
_	_	_	Animals
--	--	--	--
Organic ac	Carboxylic	Amino aci	Animals
Organohe	Imidazopy	Purines an	Animals
Benzenoid	Phenols	Methoxypl	Microbe,Animals,Plants
--	--	--	--
Organic ac	Carboxylic	Amino aci	Microbe,Animals
Organic ac	Carboxylic	Amino aci	Animals
Organic ac	Keto acids	Medium-c	Animals
Benzenoid	Phenol eth	Anisoles	Animals
Lipids and	Fatty Acyls	Fatty acids	Animals
Lipids and	Fatty Acyls	Fatty acyl g	Animals
Organohe	Azoles	Triazoles	Animals
Organic ox	Organooxy	Carbohydr	Animals
Organic ox	Organooxy	Carbonyl c	Microbe,Animals
Benzenoid	Benzene a	Styrenes	Animals
Organic ox	Organooxy	Carbohydr	Animals
Organic ox	Organooxy	Carbohydr	Animals
--	--	--	--
--	--	--	--
Nucleoside	Pyridine nu	Nicotinic a	Microbe,Animals
Phenylpro	Flavonoids	Sulfated fla	Animals,Plants
--	--	--	--
--	--	--	--
Nucleoside	Pyrimidine	Pyrimidine	Animals
Nucleoside	Molybdop	_	Animals
Organic ox	Organooxy	Carbonyl c	Microbe,Animals,Plants
Nucleoside	Pyrimidine	Pyrimidine	Microbe,Animals
Nucleoside	(5'-5')-din	_	Animals
--	--	--	--
Nucleoside	Purine nuc	Purine ribo	Microbe,Animals
Organic ac	Peptidomi	Hybrid pep	Animals
Organic ac	Carboxylic	Amino aci	Animals
--	--	--	--
_	_	_	Animals
--	--	--	--
--	--	--	--
Organic ac	Carboxylic	Amino aci	Animals
Organic ac	Carboxylic	Amino aci	Animals
--	--	--	--
Phenylpro	Flavonoids	Flavonoid	Animals
Lipids and	Fatty Acyls	Fatty acyl t	Animals
Organohe	Indoles an	Indoles	Animals,Plants
--	--	--	--
Organic ac	Carboxylic	Amino aci	Animals

--	--	--	--
Phenylpro	Isoflavono	Isoflavono	Animals
_	_	_	Animals
Lipids and	Fatty Acyls	Fatty acyl t	Animals
Organohe	Pyrrolidine	_	Animals
Organic ac	Carboxylic	Amino aci	Animals
Organic ac	Carboxylic	Amino aci	Animals
Organic ac	Carboxylic	Amino aci	Animals
Nucleoside	Purine nuc	_	Animals
Benzenoid	Benzene a	Benzoic ac	Animals
Organohe	Azolidines	Imidazolid	Animals
Phenylpro	Angucyclin	_	Animals
Organohe	Pyrrolines	_	Animals,Plants
Organic ox	Organooxy	Carbohydr	Animals
Benzenoid	Benzene a	Benzoic ac	Animals
Organic ox	Organooxy	Carbohydr	Animals
Organic ox	Organooxy	Carbohydr	Animals
Organosul	Thiols	Alkylthiols	Animals
--	--	--	--
Organic ac	Carboxylic	Amino aci	Animals
Benzenoid	Benzene a	Benzophen	Animals
Phenylpro	Cinnamic a	Hydroxycin	Animals
Nucleoside	Purine nuc	_	Animals
Organohe	Imidazopy	Purines an	Microbe,Animals,Plants
--	--	--	--
--	--	--	--
Organic ac	Carboxylic	Amino aci	Animals
Organic ac	Carboxylic	Amino aci	Microbe,Animals
--	--	--	--
Alkaloids a	Amaryllida	Galantham	Animals
Organic ac	Carboxylic	Amino aci	Animals
Organic ni	Organonit	Quaternary	Animals
--	--	--	--
Organic ac	Carboxylic	Amino aci	Animals
--	--	--	--
_	_	_	Animals
--	--	--	--
Organic ac	Carboxylic	Amino aci	Microbe,Animals
--	--	--	--
Organic ac	Carboxylic	Amino aci	Animals
Organohe	Pyridines a	Hydropyrid	Animals
Organic ac	Carboxylic	Amino aci	Animals
--	--	--	--
Organic ac	Carboxylic	Amino aci	Animals
--	--	--	--
Organic ni	Organonit	Amines	Animals
--	--	--	--
Organohe	Benzoxepi	_	Animals
Nucleoside	Purine nuc	Purine 2',3	Animals
Organic ac	Carboxylic	Amino aci	Animals
--	--	--	--

Benzenoid	Phenols	Tyrosols ar	Animals
Organic ac	Carboxylic	Amino acic	Animals
--	--	--	--
Organic ox	Organooxy	Carbohydr	Animals
Organic ox	Organooxy	Carbohydr	Animals
Organohe	Lactams	Beta lactar	Animals
Organic ox	Organooxy	Carbohydr	Animals
Phenylpro	Isoflavono	Isoflavono	Animals
Benzenoid	Benzene a	Benzoic ac	Animals
--	--	--	--
Organohe	Pyridines a	Hydropyric	Animals
--	--	--	--
Organic ac	Carboxylic	Amino acic	Animals
Hydrocarb	Unsaturate	Branched u	Animals,Plants,Microbe
Organohe	Pyrrolidine	Pyrrolidon	Animals,Plants,Microbe
--	--	--	--
Lipids and	Fatty Acyls	Fatty acyl g	Animals
Lipids and	Fatty Acyls	Fatty acids	Animals
--	--	--	--
Organic ox	Organooxy	Carbohydr	Animals
Organic ac	Carboxylic	Amino acic	Animals
Organohe	Pyridines a	Pyridineca	Microbe,Animals
Organic ac	Carboxylic	Amino acic	Microbe,Animals
--	--	--	--
Organic ac	Carboxylic	Amino acic	Animals
--	--	--	--
--	--	--	--
--	--	--	--
Organohe	Piperidines	_	Microbe,Animals
Organic ac	Carboxylic	Amino acic	Animals
Benzenoid	Benzene a	_	Animals
--	--	--	--
Organic ac	Carboxylic	Amino acic	Animals
Organic ac	Carboxylic	Amino acic	Animals
Benzenoid	Benzene a	Benzyl alco	Microbe,Animals,Plants
Organic 1,	Allyl-type	Organic ni	Microbe,Animals
Nucleoside	Pyrimidine	Pyrimidine	Animals
--	--	--	--
--	--	--	--
Organohe	Quinolines	Dihydrofur	Animals
Organohe	Furopyridi	_	Animals
Organic ac	Carboxylic	Amino acic	Animals
Organic ac	Carboxylic	Amino acic	Animals
Lipids and	Glycerolipi	Glycosylgly	Animals
Organic ox	Organooxy	Carbonyl c	Animals
Organic ac	Organic su	Arylsulfate	Animals
Organohe	Quinolines	Quinoline	Microbe,Animals
Organic ac	Carboxylic	Amino acic	Animals
Organohe	Indoles an	Tryptamine	Animals
Organohe	Imidolacta	_	Animals
Organic ac	Organic pl	Phosphoni	Animals

Nucleoside	Nucleoside	Cyclopenty	Animals
Organic ac	Carboxylic	Amino acio	Animals
--	--	--	--
Benzenoid	Benzene a	3-phenoxy	Animals
--	--	--	--
Benzenoid	Benzene a	Toluenes	Animals,Plants
Organohe	Benzazepi	_	Animals
Organohe	Pyrrolopyr	Pyrrolo[2,3	Animals
--	--	--	--
--	--	--	--
Benzenoid	Benzene a	Trifluorom	Animals
Phenylpro	Cinnamic a	Hydroxycir	Animals
Benzenoid	Benzene a	_	Animals
Organic ni	Organonit	Oximes	Animals
Benzenoid	Benzene a	Phenylbuty	Animals
Benzenoid	_	_	Animals
Organic ac	Carboxylic	Amino acio	Animals
Organohe	Pyrrolines	_	Animals
Phenylpro	Cinnamic a	Hydroxycir	Animals
Benzenoid	Benzene a	2-phenoxy	Animals
--	--	--	--
--	--	--	--
_	_	_	Animals
Organohe	Pyrrolidine	_	Animals
Lipids and	Glyceroph	Glyceroph	Animals
--	--	--	--
Lipids and	Fatty Acyls	Fatty aldeh	Animals,Plants
Organic ac	Carboxylic	Amino acio	Animals
Organohe	Biotin and	_	Animals
Organic 1,	Allyl-type	Organic ni	Animals
Lipids and	Prenol lipio	Terpene g	Animals
Lipids and	Glyceroph	Glyceroph	Animals
Lipids and	Prenol lipio	Triterpeno	Animals
--	--	--	--
Lipids and	Steroids ar	Estrane ste	Animals
Lipids and	Fatty Acyls	Eicosanoic	Animals
Organic ac	Carboxylic	Amino acio	Animals
Lipids and	Glycerolipi	Diradylglyc	Animals
--	--	--	--
Lipids and	Glyceroph	Glyceroph	Animals
_	_	_	Animals
Lipids and	Glyceroph	Glyceroph	Animals
--	--	--	--
_	_	_	Animals
Lipids and	Glyceroph	Glyceroph	Animals
--	--	--	--
Lipids and	Glyceroph	Glyceroph	Animals
Lipids and	Glyceroph	Glyceroph	Animals
Lipids and	Glyceroph	Glyceroph	Animals
Lipids and	Glyceroph	Glyceroph	Animals
_	_	_	Animals

_	_	_	Animals
_	_	_	Animals
_	_	_	Animals
_	_	_	Animals
_	_	_	Animals
_	_	_	Animals
_	_	_	Animals
_	_	_	Animals
_	_	_	Animals
Organohe	Isoquinolir	Benzylisoq	Animals
