# Supplementary material for: Integrative Analysis of Blood Transcriptomics and Metabolomics Reveals Molecular Regulation of Backfat Thickness in Qinchuan Cattle
Source: Animals (Basel). 2023 Mar 15;13(6):1060. doi: 10.3390/ani13061060 (PMC10044415; doi:10.3390/ani13061060)
Supplement: Supplementary file 1 [file animals-13-01060-s001.zip › Supplementary File S9 Supplementary Table S7.pdf]

Table S7. Differentially expressed metabolites (DEMs) in BFT.

#ID	name	L1	L2	L3	L4	H1	H2	H3
pos_3781	Galabiosylc	8.611002	2.336539	9.364669	10.73903	28.11019	13.22702	45.78859
pos_2608	4-Oxo-9-cis	7.451867	39.03927	175.0233	59.7166	164.6315	225.537	332.8649
neg_5649	Monacolin	134.517	92.16558	168.6727	144.3103	252.082	495.6613	402.4378
pos_3524	Iridal	35.12647	4.774495	22.27434	26.92961	55.6854	67.13311	101.2851
neg_4242	11-Maleim	27.80408	42.37411	21.78972	63.74062	85.5101	77.01079	134.3523
pos_1992	(2R,3R)-3-M	10.23396	25.0904	12.07509	5.872967	44.58789	25.57359	37.25484
neg_4822	Polysorbate	8.809388	15.78239	49.78641	56.114	91.05316	46.841	85.96258
pos_1823	Cornoside	30.50458	85.49879	44.82865	101.0308	215.0699	148.2248	105.6292
neg_3393	3-(2,4-Dime	1013.641	1614.785	1056.997	683.7483	2398.545	1917.73	1678.01
neg_2817	4-Vinylphe	602.1856	610.5158	654.5745	534.4192	1247.951	1329.15	796.7837
pos_3478	(4Z,7Z,10Z,	24.34322	17.89633	10.79689	23.8325	37.97667	42.79398	22.91227
pos_3876	PC(P-18:1(1	78.7967	158.0542	56.15629	103.2835	170.6644	134.5533	216.0531
pos_2613	alpha-Terp	97.92846	57.88607	120.3448	117.7656	129.8881	139.9168	207.5655
pos_4225	PE(P-16:0/1	81.15677	50.0882	69.03616	56.57514	93.03792	94.9959	124.6442
neg_3016	Vanilloyl gl	325.901	450.9279	453.8391	207.4044	512.0796	495.378	560.6851
pos_1060	DG(15:0/P(	4332.754	2980.374	3313.92	2403.65	5219.961	3478.534	5752.295
pos_4817	PC(18:1(9Z	119833.7	94570.67	81912.9	69105.45	117614.9	109993.8	160470.1
pos_4809	N-Myristoy	37.42564	28.71665	26.11461	23.82204	44.00688	30.63228	44.84902
pos_5232	PC(P-18:1(1	191399.6	122583.6	144399.1	150219.3	175419.5	191733.6	263690.5
neg_2335	Indoxylsulf	4847.277	4619.954	4434.241	5009.439	6110.588	7466.333	6183.963
pos_1839	Trimetazidi	133.2264	137.9911	153.1169	137.1925	171.9295	237.5054	186.3703
neg_5848	PC(24:0/22	75.00649	81.94973	88.73969	109.9075	131.1919	115.4883	119.7371
neg_3626	9,12,13-Tri	426.2909	558.1111	434.588	418.4467	576.1735	573.8307	605.6602
neg_6246	5alpha-Pre	129.8918	136.8032	138.5094	99.2978	138.8706	168.9985	159.2483
pos_2221	(Z)-3-Oxo-2	159.2672	193.0493	193.5823	181.3745	221.2904	193.5772	215.0333
pos_4549	2,3-bis (4-H	50.22951	51.33365	52.69906	47.73451	44.83857	45.86852	45.72343
pos_2263	Cinnamyl a	84.64608	100.6602	83.97936	87.11444	82.94524	74.58524	77.06341
neg_4562	Leu-Leu-Le	4843.933	5223.098	4636.147	4749.758	3753.772	3888.728	4583.527
neg_4564	LysoPE(0:0,	292.3041	321.0248	307.4669	281.0409	243.5709	232.1879	254.9971
neg_5188	1-Nonadec	139661.1	126829.3	129901.5	124860.2	101261.6	122136.5	111572.2
pos_3162	Farnesoic a	166.3965	156.7669	143.4381	168.4465	124.9718	132.6007	116.8666
neg_1759	Tuberoside	1446.835	1528.463	1720.644	1415.95	1062.055	1302.381	1464.515
pos_5098	Cer(d18:0/	7418.276	7603.012	7144.53	6098.282	5266.596	6130.604	5952.325
pos_3600	PGP(a-17:0	440.1783	410.7171	481.4381	368.2342	332.8205	331.1095	336.8816
pos_3267	PS(5-iso PG	190.1781	239.6442	217.1836	196.3529	155.0456	144.23	169.4864
pos_2401	MG(15:0/0	1335.538	1448.686	1693.062	1540.624	1162.382	1185.665	1129.217
pos_1359	p-coumaro	10662.53	10849.86	9798.594	8741.451	6232.101	7662.856	8584.449
pos_2266	2-Fluoroad	279.7712	325.5859	315.0527	289.2315	257.7647	203.6854	260.4392
pos_2284	2-[(L-Alanir	128.5714	144.8161	144.4978	137.8059	113.0521	92.93053	128.8142
neg_5198	Ponasteros	184.8416	154.6322	145.1907	153.3805	122.7311	141.3547	104.5735
pos_4791	DG(8:0/20:	14956.75	12656.84	10433.05	12844.92	10208.67	10745.62	7348.709
neg_4693	Trypanothi	119.924	105.9207	109.7916	94.54432	93.284	90.63279	66.92842
pos_1270	L-Rhamnos	42.2884	52.82538	44.89623	48.58899	26.77679	41.15969	29.44183
pos_2164	xi-3-Hydro:	43.70911	56.83765	50.7491	56.6093	42.54526	30.04834	31.69833
pos_1257	4-Hydroxyp	255.126	274.7155	279.137	224.7319	133.9516	194.5937	179.782
pos_2738	Ricinoleic a	104.6237	133.0949	132.8711	129.1265	75.93187	94.12917	79.51529
pos_5017	CDP-DG(PG	276.6755	228.5882	246.2254	199.8415	146.3539	130.1899	201.1603
pos_5012	PA(12:0/20	2134.956	1990.243	2130.317	1511.34	1349.904	1234.308	1628.268

pos_5171	Palmitoyl g	391.8316	502.4557	372.5677	412.7345	239.4189	279.1863	346.5059
neg_2479	(7R)-7-(4-C	92.10061	131.5154	119.8411	134.5219	98.20415	99.36114	77.6655
pos_2120	(2S,5S)-trai	21.64258	18.39252	15.25435	22.09524	12.13196	11.82188	14.42552
neg_4971	ROCCELLIC	197.2654	216.3898	274.1903	225.967	202.0371	154.559	145.4914
pos_3212	PGP(20:5(7	298.0646	290.352	332.4692	288.3106	186.7073	185.368	256.3642
pos_933	2R-Hydroxy	97.28408	81.80532	79.52696	87.60544	76.91685	57.92927	51.67689
pos_4929	Sphingosin	162.2184	191.7884	130.1103	135.6385	90.6229	91.1346	115.0246
neg_6167	3b,15b,17a	106.1576	135.4386	88.20196	133.5395	72.83378	71.46501	83.6962
neg_4609	Hexosylsph	12391.06	11609.52	10435.97	12940.56	9697.412	9537.758	4355.96
neg_3193	3-Hydroxys	337.9078	390.0957	425.1836	479.8606	218.0827	275.8859	311.1208
neg_6035	5-O-beta-D	137.7336	151.8643	96.42794	162.053	124.2981	79.45585	67.70941
pos_4985	PA(8:0/22:	1779.822	1278.611	1520.594	1097.379	728.4592	908.3245	1150.136
pos_1348	3-(O-Geran	152.6977	119.0481	135.2783	97.92387	57.7841	64.10576	107.8272
pos_3312	Tetradecar	28.88432	36.71124	45.39321	33.7037	18.29638	23.30638	29.12654
pos_2240	Isosalsolidi	50.45702	92.89525	69.45545	81.94606	29.84519	51.23892	54.24475
pos_4960	Cer(d16:1/	358.4103	381.6995	378.6951	566.9341	318.4001	206.1846	243.4025
pos_5032	Yucalexin F	390.5514	580.2699	427.5939	634.0577	274.6331	388.7855	255.8156
pos_1229	Ellagic acid	185.812	203.7859	188.5036	139.9578	48.82089	132.4179	108.6539
neg_1484	Tetracosap	208.3763	176.4059	134.3553	192.3292	77.87194	82.51368	135.9034
pos_609	N,N-Didesr	230.5243	227.9602	135.1955	213.7117	86.69477	157.1354	67.42798
pos_4949	DG(14:0/2:	1089.358	1084.87	887.696	1150.13	603.7567	576.3826	409.4967
neg_6112	Butanoyl P.	55213.84	77362.58	43670.6	81635.41	48703.12	39382.39	30130.89
pos_1710	N-(gamma-	453.8791	747.0998	645.2257	564.3488	230.4813	506.4423	281.6756
pos_5254	N-Oleoyl G	116.561	138.7718	101.7597	107.089	48.90187	39.90446	66.92263
pos_5113	Ala Ala Val	182.0765	191.5736	139.1151	181.3561	71.63554	68.44894	71.84967
neg_4372	16-Oxopalr	385.901	779.6786	645.4043	658.1147	252.4802	350.518	426.495
neg_1817	TG(8:0/8:0,	5366.289	8191.842	4708.908	8096.521	2139.456	4072.43	2888.008
pos_1591	Glycyl-Tryp	368.2305	658.3334	589.3619	451.4492	274.8257	358.3827	183.8228
pos_5013	Isoachifolic	256.178	248.2965	397.5724	212.8851	109.5369	138.4709	164.0666
neg_4205	Prostaglan	1710.869	2467.832	2180.577	3289.956	1214.941	913.895	1595.178
pos_1740	SALSOLIDII	354.6686	573.9989	639.5385	519.8868	117.5493	464.896	199.1006
neg_3169	TIC10	133.9486	178.852	185.857	244.908	105.1235	73.74216	27.92667
neg_4604	24(28)-Deh	140.6285	222.7611	121.6446	197.406	144.974	88.66522	1.72E-06
pos_4728	(3b,20R,22	42.31971	54.6756	29.08906	67.5301	33.36668	20.20437	9.930768
pos_5007	CDP-DG(20	26.74882	70.40838	83.14383	64.7766	16.64725	18.82912	20.1709
neg_1556	Propylene	1853.327	1680.692	768.0436	787.2019	359.9395	200.1388	534.2377
pos_2804	Biotin sulfc	23.95974	27.11463	25.2403	40.60341	1.72E-06	13.13326	9.103469
neg_4605	[(3R,4S)-1,:	13.99606	32.16687	15.9313	33.6054	8.973141	3.249403	1.72E-06

H4	L_Mean	H_Mean	Fold_chang	log2FC	Pvalue	VIP	regulated
35.3291	7.76281	30.61372	3.94364	1.979528	0.039846	2.144148	up
239.9944	70.30775	240.7569	3.42433	1.775822	0.014913	2.181606	up
559.2854	134.9164	427.3666	3.16764	1.663408	0.019006	2.335371	up
37.30023	22.27623	65.35097	2.933664	1.552704	0.040984	2.07281	up
104.3115	38.92713	100.2962	2.57651	1.365418	0.009516	2.24037	up
23.83659	13.31811	32.81323	2.463806	1.300889	0.023943	2.067643	up
80.27638	32.62305	76.03328	2.330662	1.22074	0.032317	2.035135	up
123.7296	65.46571	148.1634	2.263221	1.178378	0.03374	2.023927	up
2761.872	1092.293	2189.039	2.004077	1.002938	0.013319	2.194299	up
1092.074	600.4238	1116.49	1.859503	0.894917	0.0194	2.362471	up
33.22497	19.21723	34.22697	1.781056	0.832733	0.032511	2.051955	up
167.8258	99.07268	172.2742	1.738867	0.798147	0.040195	1.901986	up
172.9025	98.48123	162.5682	1.650753	0.723125	0.031739	2.051816	up
96.94741	64.21407	102.4064	1.594765	0.673344	0.009461	2.288165	up
615.9088	359.5181	546.0129	1.518736	0.602871	0.042269	2.035421	up
5156.564	3257.675	4901.838	1.504705	0.58948	0.043306	1.980456	up
138400.7	91355.68	131619.9	1.440742	0.526812	0.042472	1.947824	up
44.92166	29.01973	41.10246	1.416363	0.502191	0.039894	1.987787	up
228575.9	152150.4	214854.9	1.412121	0.497864	0.045884	1.966638	up
6638.683	4727.728	6599.892	1.395997	0.481295	0.005245	2.464746	up
183.3389	140.3817	194.786	1.387545	0.472535	0.028588	2.226948	up
105.9418	88.90085	118.0898	1.328331	0.409615	0.022379	2.132675	up
625.8775	459.3592	595.3855	1.296122	0.374201	0.019824	2.211479	up
184.5596	126.1256	162.9192	1.291723	0.369296	0.031965	2.01431	up
222.2081	181.8183	213.0273	1.171649	0.22854	0.025337	2.051105	up
46.34531	50.49918	45.69396	0.904845	-0.14426	0.015579	2.346111	down
73.99611	89.10002	77.1475	0.865853	-0.20781	0.047051	2.061953	down
4399.507	4863.234	4156.383	0.854654	-0.22659	0.029613	2.144018	down
295.2642	300.4592	256.505	0.85371	-0.22818	0.042009	2.028129	down
105514.9	130313	110121.3	0.845052	-0.24289	0.01318	2.205848	down
154.9368	158.762	132.344	0.8336	-0.26257	0.04258	1.985443	down
1147.992	1527.973	1244.235	0.814305	-0.29636	0.047002	1.941581	down
5469.668	7066.025	5704.798	0.807356	-0.30872	0.018301	2.211789	down
353.8259	425.1419	338.6594	0.79658	-0.32811	0.033203	2.17324	down
192.3309	210.8397	165.2732	0.783881	-0.35129	0.024774	2.133987	down
1214.883	1504.478	1173.037	0.779697	-0.35901	0.018958	2.325899	down
8591.818	10013.11	7767.806	0.775764	-0.36631	0.023076	2.128536	down
209.0683	302.4103	232.7394	0.769615	-0.37779	0.011831	2.274355	down
91.31488	138.9228	106.5279	0.766814	-0.38305	0.028242	2.193329	down
119.0109	159.5112	121.9175	0.764319	-0.38775	0.017723	2.12312	down
10134.91	12722.89	9609.476	0.75529	-0.4049	0.0423	1.942236	down
67.09692	107.5451	79.48554	0.73909	-0.43618	0.02258	2.079273	down
41.30659	47.14975	34.67122	0.735343	-0.44351	0.038927	2.076275	down
48.15337	51.97629	38.11132	0.733244	-0.44763	0.044759	1.996799	down
246.24	258.4276	188.6418	0.72996	-0.45411	0.048983	1.99771	down
112.606	124.929	90.54557	0.724776	-0.46439	0.019862	2.170682	down
197.0481	237.8327	168.6881	0.709272	-0.49559	0.028646	2.032073	down
1295.086	1941.714	1376.891	0.709111	-0.49592	0.02234	2.14526	down

318.5132	419.8974	295.9061	0.70471	-0.5049	0.016393	2.248517	down
58.87456	119.4948	83.52634	0.698996	-0.51664	0.038591	2.005172	down
15.03145	19.34617	13.3527	0.690199	-0.53492	0.024223	2.189096	down
128.3874	228.4531	157.6187	0.689939	-0.53546	0.020722	2.086711	down
196.9437	302.2991	206.3458	0.682588	-0.55091	0.004755	2.399954	down
49.55688	86.55545	59.01997	0.681875	-0.55242	0.013122	2.217459	down
125.2183	154.9389	105.5001	0.680914	-0.55446	0.030986	2.138992	down
87.01099	115.8344	78.75149	0.679863	-0.55668	0.041025	2.18216	down
8466.638	11844.28	8014.442	0.676651	-0.56352	0.047021	2.014145	down
280.4902	408.2619	271.3949	0.664757	-0.5891	0.011404	2.297232	down
88.43019	137.0197	89.97339	0.656646	-0.60681	0.048211	1.946937	down
927.1072	1419.101	928.5067	0.654292	-0.61199	0.036893	2.007422	down
93.06266	126.237	80.69492	0.639233	-0.64559	0.033916	1.98809	down
21.16883	36.17312	22.97453	0.635127	-0.65488	0.023421	2.142808	down
47.5528	73.68844	45.72041	0.620456	-0.6886	0.047125	2.055502	down
239.1807	421.4347	251.792	0.597464	-0.74308	0.031506	2.129944	down
289.1774	508.1182	302.1029	0.594552	-0.75012	0.030475	2.176342	down
132.1839	179.5148	105.5192	0.587802	-0.7666	0.025091	2.12933	down
113.7369	177.8667	102.5065	0.576311	-0.79508	0.011922	2.245991	down
150.8123	201.8479	115.5176	0.5723	-0.80516	0.035266	2.024646	down
819.5539	1053.013	602.2975	0.571975	-0.80598	0.006004	2.370052	down
28359.81	64470.61	36644.05	0.568384	-0.81506	0.045885	2.056448	down
298.645	602.6384	329.311	0.546449	-0.87184	0.01995	2.168088	down
97.41028	116.0454	63.28481	0.545345	-0.87476	0.016671	2.249619	down
161.2765	173.5303	93.30267	0.537674	-0.8952	0.029568	2.155287	down
297.3734	617.2747	331.7167	0.537389	-0.89596	0.032818	2.191466	down
4644.54	6590.89	3436.108	0.521342	-0.9397	0.031633	2.14243	down
243.3257	516.8438	265.0892	0.5129	-0.96325	0.0223	2.209075	down
145.9207	278.733	139.4988	0.500475	-0.99863	0.037247	2.130129	down
922.4881	2412.308	1161.625	0.481541	-1.05427	0.024187	2.224618	down
216.8286	522.0232	249.5936	0.478127	-1.06453	0.031707	2.053871	down
133.4354	185.8914	85.05693	0.457563	-1.12796	0.02004	2.135129	down
67.3954	170.61	75.25864	0.441115	-1.18077	0.048818	1.953238	down
21.39352	48.40362	21.22384	0.438476	-1.18943	0.03733	2.077075	down
43.60557	61.26941	24.81321	0.404985	-1.30406	0.049455	2.016225	down
295.9337	1272.316	347.5624	0.273173	-1.87211	0.045029	2.136523	down
2.87856	29.22952	6.278823	0.214811	-2.21886	0.003827	2.411866	down
3.57126	23.92491	3.948451	0.165035	-2.59915	0.024914	2.283197	down
